# Estimating the reduction in US mortality if cigarettes were largely replaced by e-cigarettes

**DOI:** 10.1007/s00204-021-03180-3

**Published:** 2021-10-22

**Authors:** Peter N. Lee, John S. Fry, Stanley Gilliland, Preston Campbell, Andrew R. Joyce

**Affiliations:** 1 P N Lee Statistics and Computing Ltd, 17 Cedar Road, Sutton, SM2 5DA Surrey UK; 2 RoeLee Statistics Ltd, 17 Cedar Road, Sutton, SM2 5DA Surrey UK; 3 Consilium Sciences, LLC, 7400 Beaufont Springs Drive, Suite 300, N. Chesterfield, 23325 VA USA

**Keywords:** Smoking-related disease, Loss of life, Public health impact modelling, e-cigarettes, US, Vaping

## Abstract

**Background:**

Recent estimates indicated substantially replacing cigarettes by e-cigarettes would, during 2016–2100, reduce US deaths and life-years lost (millions) by 6.6 and 86.7 (Optimistic Scenario) and 1.6 and 20.8 (Pessimistic). To provide additional insight we use alternative modelling based on a shorter period (1991–2040), four main smoking-associated diseases, deaths aged 30–79 years, and a full product history. We consider variations in: assumed effective dose of e-cigarettes versus cigarettes (F); their relative quitting rate (Q); proportions smoking after 10 years (X); and initiation rate (I) of vaping, relative to smoking.

**Methods:**

We set F = 0.05, X = 5%, Q = 1.0 and I = 1.0 (Main Scenario) and F = 0.4, X = 10%, Q = 0.5 and I = 1.5 (Pessimistic Scenario). Sensitivity Analyses varied Main Scenario parameters singly; F from 0 to 0.4, X 0.01% to 15%, and Q and I 0.5 to 1.5. To allow comparison with prior work, individuals cannot be dual users, re-initiate, or switch except from cigarettes to e-cigarettes.

**Results:**

Main Scenario reductions were 2.52 and 26.23 million deaths and life-years lost; Pessimistic Scenario reductions were 0.76 and 8.31 million. These were less than previously, due to the more limited age-range and follow-up, and restriction to four diseases. Reductions in deaths (millions) varied most for X, from 3.22 (X = 0.01%) to 1.31 (X = 15%), and F, 2.74 (F = 0) to 1.35 (F = 0.4). Varying Q or I had little effect.

**Conclusions:**

Substantial reductions in deaths and life-years lost were observed even under pessimistic assumptions. Estimates varied most for X and F. These findings supplement literature indicating e-cigarettes can importantly impact health challenges from smoking.

**Supplementary Information:**

The online version contains supplementary material available at 10.1007/s00204-021-03180-3.

## Introduction

In 2018, Levy et al. (2018) (subsequently referred to as Levy) estimated the effects on US mortality that would occur over 2016 to 2100 if e-cigarette use (“vaping”) largely replaced cigarette smoking (“smoking”) over the first 10 years. Their methodology involved forward projection of smoking rates based on age- and sex-specific initiation and cessation rates using an age-period-cohort model. Forward projection of all-cause life tables were sub-classified by smoking status using projected data on smoking prevalence and relative risks by age, sex and smoking from the first two American Cancer Society Prevention Studies (Garfinkel 1985). Levy considered an “Optimistic Scenario” and a “Pessimistic Scenario,” differing regarding the relative harm of e-cigarettes versus cigarettes and their impact on initiation, cessation and switching. Compared to where e-cigarettes were not introduced, they estimated that substantially replacing smoking by vaping would reduce premature deaths by 6.6 million and life-years lost by 86.7 million in their Optimistic Scenario, and by, respectively, 1.6 million and 20.8 million in their Pessimistic Scenario. Levy concluded, “a strategy of replacing cigarette smoking with vaping (i.e., e-cigarette use) would yield substantial life year gains, even under pessimistic assumptions regarding cessation, initiation and relative harm.”

While Levy’s findings are certainly interesting, they are somewhat limited. First, the Scenarios which Levy compare differ on a number of parameters, and one cannot clearly determine how varying individual parameters affects the estimated life year gains. Second, Levy projects to 2100, ignoring possible effects on future mortality of factors such as medical advances, infections, global warming and wars. Third, Levy derived estimates of relative mortality rates by smoking status from only two studies. Fourth, Levy only considered overall mortality. Fifth, Levy estimated risks at a year based only on the product use distribution at that year.

Here, we approach the same problem using a different framework, first described by Weitkunat et al. (2015), known as the Population Health Impact Model (PHIM). This approach has been used to estimate reductions in mortality associated with uptake of reduced risk tobacco products in the US (Lee et al. 2017), Japan (Lee et al. 2018) and Sweden (Djurdjevic et al. 2019). The partial hindcasting approach of PHIM limits uncertainty regarding the future. We also rely on more precise mortality rates from published meta-analyses and consider the four major smoking-related diseases—lung cancer (LC), chronic obstructive pulmonary disease (COPD), ischaemic heart disease (IHD) and stroke, rather than overall mortality. Furthermore, risks are derived based on the full product history over time, and sensitivity analyses are used to determine the impact and relative importance of the key parameters: F (relative excess risk of e-cigarettes to cigarettes), X (proportion of population currently smoking after 10 years), I (relative initiation rate of vaping to smoking), and Q (relative quitting rate of vaping to smoking) on the estimated reductions in deaths and years of life lost (YLL).

## Materials and Methods

### PHIM methodology

The modelling begins with individuals of a given sex and age with a defined smoking distribution. In the prevalence component, the simulated population is followed over discrete time intervals under a “Null Scenario” and various “Alternative Scenarios” based on estimated transition probabilities (TPs) between different product use groups. In the Null Scenario, the new product (here e-cigarettes) is never introduced, and each individual’s smoking status (never, current, former) is updated at each interval. In each Alternative Scenario, the new product is introduced, and the TPs allow switching between five groups (never users of either product, current exclusive smokers, current exclusive new product users, current dual users of both products, and former users of either product not current users of either). At the end of follow-up, each individual then has a complete history of use over the follow-up period under each Scenario.

The epidemiologic component then uses the histories to estimate, for each individual and for each of the four diseases considered (LC, COPD, IHD and stroke), their relative risk (RR), compared to never users, for each follow-up year and Scenario where the individual is aged 30 to 79 years. The estimation involves a negative exponential model, allowing for multiple changes in product use, described and justified elsewhere (Lee et al. 2015, 2017). For each individual, this model estimates the excess risk (RR-1) of the given disease at each year of age from the excess risk at the previous year, together with knowledge of the product smoked during that year. The model requires estimates for each disease of the RR for continued smoking and the quitting half-life (H): the time taken from quitting for the excess risk (RR–1) to become half that for continued smoking. It also requires estimates of the excess risk for exclusive new product use relative to exclusive smoking (F).

For each Scenario, the average RR for each disease for individuals of a given sex and age group is then calculated at each year, thus deriving the proportion of product-related deaths and population counts using national mortality estimates by sex, age group and year. Differences in estimates between the Null and Alternative Scenarios then quantify the effect of introducing the new product. For a given Scenario, YLL before age 75 years are estimated using the method of Gardner and Sanborn (1990), assuming that deaths occurring in a given age group have a mean age of death at the midpoint of the corresponding range. Thus, someone dying in the age group 50 to 54 years would have an estimated YLL of 75–52.5 = 22.5 years.

Initial estimates of between-Scenario differences in deaths and YLL assume that the populations at risk in each Scenario remain the same during follow-up. However, a correction for differences in survival is available (Weitkunat et al. 2015).

### Similarities and differences between the Levy model and PHIM

A comparison between the Levy model and our application of PHIM is shown in Table 1.

**Table 1:** Similarities and differences between the Levy model and our application of PHIM

Characteristic	Similarity
New product	E-cigarettes
Country	USA
Sex	Both sexes considered, results presented by sex, TPs sex-specific
Groups—null scenario	Never, current and former smokers
Groups—alternative scenarios	Never users, current exclusive smokers, current exclusive vapers and former users, but not dual users
Stability of population in follow-up	Only initial population studied, neither model allowing for immigration or emigration
TPs considered	Quitters cannot re-initiate. Switching to cigarettes not allowed, but switching to e-cigarettes is. Initiation and quitting rates derived from national distributions for same birth cohort in successive five-year periods

Characteristic	Difference
Time period	1990–2040 vs. Levy 2016–2100, to allow some hindcasting, and avoid extrapolation to years where rates very uncertain
Diseases considered	Lung cancer (LC), chronic obstructive pulmonary disease (COPD), ischaemic heart disease (IHD) and stroke vs. Levy all causes combined. These form about two thirds of smoking-related mortality (Weitkunat et al. 2015)
Age range	10–79 years (30–79 years for estimating mortality) vs. Levy 15–99 years. As death certification unreliable at older years, individuals not considered after age 79. PHIM includes those aged 10–14 years as product use may start then
Years quit	Considered only by PHIM, to estimate risk more precisely
Age of initiation	To age 35 years vs. Levy to age 25, to reflect US patterns of initiation
Period over which smoking declines to X	First 11 years vs. Levy first 10 years. This reflects periods for which TPs estimated in PHIM (1986–1990, 1991–1995 and 1996–2000)
Former users	Former users of each product combined, rather than separately. Distinction not relevant as re-initiation disallowed, and PHIM retains full product history
Relative risks	Derived from meta-analyses vs. Levy from two CPS studies
Estimating mortality	Full product history used, while Levy uses smoking status at the time

Another major difference is that while Levy uses only the Optimistic and Pessimistic Scenarios, PHIM uses a Main Scenario (similar to Levy’s Optimistic Scenario), a Pessimistic Scenario (similar to Levy’s Pessimistic Scenario) and various Sensitivity Scenarios where the value of one parameter (F, X, I or Q) is varied, with the remainder held at their respective Main Scenario values (Table 2).

**Table 2:** Values of the four key parameters in the PHIM modelling

Parameter	Main scenario	Pessimistic scenario	Sensitivity scenarios
F (Excess risk of vaping relative to smoking)	0.05	0.4	0, 0.1, 0.2, 0.3
X^†^ (Residual prevalence of smoking after 10 years)	5%	10%	0.01%, 2.5%, 7.5%, 15%
I (Initiation rate for vaping relative to smoking)	1.0	1.5	0.5, 0.75, 1.25
Q (Quitting rate for vaping relative to smoking)	1.0	0.5	0.75, 1.25, 1.5

^†^Values for F, I, and Q are exact, but those for X are approximate as discussed in the methods section.

Note that the initiation rates for cigarettes and e-cigarettes in the Main Scenario are set equal to half that for smoking in the Null Scenario, so the combined initiation rate of cigarettes (conventional and e-) remains equal to the Null Scenario rate. Changing I only affects the initiation rate for vaping. In the Main Scenario, the quitting rates of smoking and vaping are each assumed to be the same as in the Null Scenario. Changing Q only affects the quitting rate for vaping.

### Common features of each PHIM simulation

Each simulation starts in 1990 with 100,000 individuals of a given sex aged 10–79 years, each individual then being randomly allocated first to a year of age, then a smoking group (never, current, former), and then, for former smokers, to an age of quitting using the data described below. Population estimates by sex and 5-year age groups were downloaded from the United Nations website on April 15, 2020, for the years 1990 to 2040, using the “medium variant projections” for 2018 to 2040 (Online Resource 1). Sex- and age-specific distributions for annual current and former smoking prevalence from 1986 to 2018 and for five-year periods from 1986–1990 to 2016–2020 were estimated from the National Survey on Drug Use and Health (NSDUH) and the National Health Interview Survey (NHIS). For 2021–2040, 5-year estimates were derived from the data for 2016–2018, later data being unavailable (Online Resource 2). As previously described (Lee et al. 2017), data on age of quitting were based on NHIS data for 2006 (Online Resource 3).

As each simulation starts with the same random number, the initial distribution of age, sex and smoking is always the same. Each individual is then followed at yearly intervals from 1990 under two Alternative Scenarios, with each individual’s product use status being estimated at each year of follow-up until the year 2040 (or age 79, if earlier). Under the Null and Alternative Scenarios, each individual’s smoking status may change annually according to the TPs described in the following two subsections.

### Estimating transition probabilities in the Null Scenario

Initiation and quitting rates were derived from data on the distribution of smoking habits for a given 5-year age group and for the same birth cohort five years later when they are 5 years older, as described in Online Resource 4, which also details the derived initiation and quitting rates.

### Estimating transition probabilities in the Alternative Scenarios

Only five TPs are relevant to any Alternative Scenario, since re-initiation, dual use, or switching from e-cigarettes to cigarettes was not allowed in order to allow for direct comparison with Levy. The derivation of the initiation, quitting and switching rates is also described in Online Resource 4.

### Mortality

Annual number of deaths for 1966–2017 from LC, COPD, IHD, and stroke by sex and age group were downloaded from the WHO database (Online Resource 5). Numbers of deaths and in the population, were then used to estimate death rates, both for single years and five-year periods from 1966–1970 to 2011–2015.

The age-period-cohort model (Osmond and Gardner 1982; Osmond et al. 1982) was then fitted to the rates by 5-year periods and used to estimate rates for later 5-year periods up to 2036–2040, with single year estimates obtained and numbers of deaths derived from the rates using the population data. The Osmond and Gardner approach describes mortality from major smoking-related diseases well and provides a reasonable prediction of future mortality trends (e.g., Barker and Osmond 1986; Lee et al. 2014a)). The methodology produced estimates for the period to 2011–2015, but estimates were also required up to 2036–2040. Based on our previous experience (Forey 1989a,b), we used the age effects fitted to the period up to 2015. The ratio of the fitted period values for 2011–2015 to 2006–2010 was used to extrapolate period values from 2016 to 2020 onwards, with period values for single years obtained using log-linear interpolation. Cohort values for later born cohorts were estimated by log-linear extrapolation from the values fitted for earlier cohorts, using as weights powers of two decreasing into the past. Fuller details are given in Online Resource 6.

Online Resource 6 gives further details on methods and for both sexes, the four diseases and two selected age groups (50–54 and 70–74 years), gives the observed single-year rates, the fitted five-year period rates, the fitted rates for 2016 and 2017, where observed rates are also available and the estimated rates for 2018 to 2040. The fit to the observed data is good, and the predicted future rates seem plausible. Online Resource 6 also gives details of the fitted and extrapolated age, period and cohort values, and also goodness-of-fit statistics and observed and fitted rates for all age groups.

When estimating mortality based on product use history, an individual may each year be a non-smoker (never or former smoker), a current smoker or a current vaper. Associated with each status is an effective dose, or excess risk, for vaping relative to smoking, taken as 0 for a non-smoker, 1 for a current smoker and F for a current vaper, with F varying as in Table 2. Estimating the risk of a disease (relative to a never user of either product) by age at each year of follow-up for an individual is described elsewhere (Lee et al. 2017) and depends not only on the individual’s history of product use and on the assumed F value, but also on RR estimates for continued smoking and quitting half-life (H) estimates. The estimates used (see Online Resource 4 Table S4.2) are well justified and used in previous applications of PHIM in the US (Lee et al. 2017). Note that age-dependent estimates are used only where there is strong evidence of variation by age and that the estimates are assumed applicable to each sex, there being little evidence of variation by sex.

## Results

### Introduction

Online Resource 7 gives the full output from all runs of PHIM as well as an explanation of the results. Online Resource 4 gives tables of results not included in the main paper (Tables S4.3 to S4.6), while Online Resource 8 gives figures presenting some results (Figures S8.1 to S8.4).

### Main Scenario

Predicted trends in never and former user prevalence are indistinguishable between the Null and Main Scenarios, whereas the switch from current smoking to current vaping in the Main Scenario is clear (Figures S8.1 and S8.2). Since the population, aged 10–79 years in 1990, is not replaced during follow-up, there are no 30- to 34-year-olds after 2010, nor 50-to 54-year-olds after 2030. Also, as prevalences for successive age groups relate to different birth cohorts, the prevalence of never smokers for an age group can fall over time.

Over the entire follow-up, the switch to e-cigarettes is associated with 2,524,975 less deaths from the four diseases combined, 1,524,711 in males and 1,000,264 in females.

Declines in mortality associated with switching to e-cigarettes by sex, disease, period and age are reported in Table 3 and cumulative drops over the entire follow-up displayed in Figure S8.3. In males, drops are highest for IHD, 38.2% of the total, with drops in LC, COPD and stroke accounting for, respectively, 34.2%, 22.2% and 5.3%. In females, IHD constitutes a smaller percentage of the total, 19.9%, while LC, COPD and stroke constitute 43.7%, 31.0% and 5.3%. In both sexes, the drops per 5-year interval for IHD and stroke rise over time until about 2010 and then fall, while those for LC and COPD rise over a longer period. This difference in time trend can be explained by LC and COPD having longer half-lives (see Table S4.2), so effects of switching to e-cigarettes take longer to appear. The drops in death rise sharply with age for LC and COPD. For IHD and stroke they rise initially, but fall at higher ages, reflecting the decline in RR with age (see Table S4.2) for these conditions.

**Table 3:** Drops in deaths (D, hundreds) comparing Main with Null Scenario by sex, age and cause

Year	Age	LC D (%)	IHD D (%)	Stroke D (%)	COPD D (%)	Total D (%)
Males						
1990–2039	30–79	5220 (17.0)	5832 (9.2)	804 (5.4)	3392 (15.2)	15,247 (11.6)
2000	30–79	21 (2.8)	97 (6.1)	12 (3.5)	9 (2.5)	139 (4.6)
2010	30–79	81 (12.1)	163 (13.5)	23 (8.4)	35 (9.4)	301 (12.0)
2020	30–79	167 (26.3)	162 (13.4)	23 (7.9)	85 (17.5)	437 (16.7)
2030	30–79	197 (40.1)	112 (10.7)	16 (5.5)	139(24.2)	464 (19.3)
2039	30–79	156 (48.1)	75 (8.7)	10 (4.1)	159 (28.0)	399 (19.9)
1990–2039	45–49	140 (19.5)	603 (25.0)	69 (15.4)	41 (18.7)	854 (22.4)
1990–2039	55–59	602 (20.5)	977 (15.0)	144 (13.0)	259 (20.1)	1982 (16.7)
1990–2039	65–69	1082 (17.3)	707 (6.5)	94 (4.0)	675 (16.4)	2558 (10.8)
1990–2039	75–79	995 (14.3)	269 (1.7)	18 (0.4)	939 (12.6)	2222 (6.4)
Females						
1990–2039	30–79	4371 (18.8)	1993 (6.1)	535 (4.0)	3104 (14.4)	10,003 (11.0)
2000	30–79	16 (3.3)	32 (3.5)	9 (2.7)	9 (2.8)	67 (3.2)
2010	30–79	63 (12.5)	53 (9.2)	15 (6.2)	33 (9.1)	166 (9.7)
2020	30–79	133 (25.8)	55 (9.9)	15 (6.0)	77 (16.0)	280 (15.5)
2030	30–79	167 (38.5)	41 (8.3)	10 (4.2)	126 (22.2)	345 (19.8)
2039	30–79	139 (44.6)	26 (6.2)	5 (2.8)	138 (24.8)	309 (20.8)
1990–2039	45–49	113 (20.3)	159 (22.3)	49 (13.2)	36 (16.3)	357 (19.2)
1990–2039	55–59	448 (21.6)	289 (12.4)	86 (10.5)	197 (17.2)	1020 (16.0)
1990–2039	65–69	884 (19.3)	279 (5.2)	61 (3.2)	582 (15.3)	1807 (11.5)
1990–2039	75–79	946 (16.5)	145 (1.4)	17 (0.3)	976 (12.7)	2085 (7.1)

Associated with the drops in deaths are reductions in YLL before age 75, i.e., years of life saved (YLS). For the diseases combined, YLS was estimated (in millions) to be 17.25 for males and 8.98 for females, or 26.23 combined. Per death saved, these represent an average of 11.31 years in males and 8.97 years in females. For the sexes combined, the drops were 8.46 years per death saved for LC, 15.35 for IHD, 15.58 for stroke, 6.17 for COPD and 10.39 for the diseases combined.

The results cited above are unadjusted for differential mortality between the Null and Main Scenarios. Compared to the unadjusted drops in deaths of 1,524,711 in males and 1,000,264 in females, the adjusted drops were 1,506,814 in males and 991,427 in females, representing 98.8% and 99.1% of the unadjusted drops. Because of these small differences, only unadjusted results are considered further.

### Pessimistic Scenario

Figure S8.4 in Online Resource 8 shows the prevalence of tobacco use by sex and time of follow-up for three age groups (30–34, 50–54, 70–74) as predicted in the Pessimistic Scenario where, compared to the Main Scenario, e-cigarettes are assumed to have a less reduced risk, with the rate of switching to e-cigarettes lower, of initiating e-cigarettes higher and of quitting e-cigarettes lower.. A shallower initial decline in current smoking relative to the Main Scenario is apparent, although prevalence again drops to virtually zero by 2010.

For the entire follow-up and age range, Table 4 reports drops in deaths by cause and overall, expressed as percentages of both the total number of predicted deaths for that cause and the drops in the Main Scenario, respectively. Overall, the drops total 759,056 (472,306 for males and 286,750 for females). As a percentage of the drops in the Main Scenario, the drops in the Pessimistic Scenario are 31.0% in males and 28.7% in females and are, in both sexes, somewhat lower for LC and COPD than for IHD and stroke.

**Table 4:** Overall drops in deaths (hundreds) in the Pessimistic vs. Null Scenario by sex and cause

Sex	Statistic	LC	IHD	Stroke	COPD	Four diseases
Males	Drop in Pessimistic Scenario	1308	2044	289	1082	4723
	% of all predicted deaths from cause^a^	4.3	3.2	1.9	4.8	3.6
	% of drop in deaths in Main Scenario	25.0	35.0	36.0	31.9	31.0
Females	Drop in Pessimistic Scenario	1065	664	182	957	2867
	% of all predicted deaths from cause^a^	4.6	2.0	1.3	4.4	3.2
	% of drop in deaths in Main Scenario	24.4	33.3	34.1	30.8	28.7

^a^Overall death rates were based on WHO statistics up to 2015 and predicted by extrapolations subsequently as described in the Mortality section of the methods.

As for the results for drops in deaths, the estimates of YLS (in millions) were also substantially less in the Pessimistic Scenario, being 2.06 for LC, 4.16 for IHD, 0.76 for stroke and 1.32 for COPD, totalling 8.31.

### Sensitivity Scenarios

Table S4.3 in Online Resource 4 shows the distribution of product use at 10, 25 and 50 years follow-up for the Null Scenario and also the Main, Pessimistic and Sensitivity Scenarios. At baseline, all models for the same sex start with the same distribution of smoking habits (see footnote to Table S4.3). In all the models where e-cigarettes are introduced, current smoking prevalence declines with time, to almost zero after 50 years, and current vaping prevalence also declines except in the Pessimistic Scenario. Prevalence of former use increases with time. In the sensitivity analyses, increasing X slows the decline in current smoking, while increasing I slows the decline in current vaping. Increasing Q reduces the decline in current vaping and accelerates the increase in former product use. Note that some of the trends over time are due to the ageing population. Thus, by year 25, the population was aged 35–79 years, with no further initiation.

Drops in death for the Alternative Scenarios compared to those in the Main Scenario are shown by sex for the four causes of death combined in Table S4.4, and by cause for the sexes combined in Table S4.5. Varying X most affects drops in death, with varying F also having a pronounced effect, but varying Q has less effect, and varying I very little effect. Thus, for the sexes and causes combined, the difference in drops in death between extreme values of the parameters tested is 1.91 million varying X, 1.39 million varying F, 110 thousand varying Q and only 13.5 thousand varying I. These differences are evident for both sexes and all causes. This is unsurprising since, while varying X materially affects the prevalence of current smoking and increasing F reduces the apparent benefit of vaping, varying Q and I has almost no effect on current smoking prevalence (see Table S4.3), which accounts for most of the increased death risk.

To understand better the interdependence of effects of varying individual parameter values, the last entries of Tables S4.4 and S4.5 compare difference in drops relative to the Main Scenario for a Pessimistic Scenario with all four parameters varied simultaneously against the sum of the differences for Scenarios where single parameters are set individually to the Pessimistic Scenario values. For sexes and causes combined, the Pessimistic Scenario resulted in an estimated increase of 17,659 deaths compared to the Main Scenario, while summing the individual values produced a quite similar estimate of 18.469 deaths. Thus, an approximate idea of the drop associated with scenarios where two or more parameters vary from their Main Scenario values could be derived from the drops from varying individual parameter values.

Table S4.6 in Online Resource 4 shows the estimated YLS for the different scenarios for the two sexes and four diseases combined. These results follow those for drops in deaths with values varying considerably more when X and F are varied than when I and Q are varied.

## Discussion

Comparing our Main Scenario with the Null Scenario with no e-cigarette introduction, we estimated the total deaths saved over the 50-year follow-up to be 2.52 million, corresponding to a reduction in YLL of 26.23 million. These differences were less than Levy’s 6.6 million deaths and 86.7 million YLL, as we considered a narrower age range (30–79 vs 15–99 years), used a shorter follow-up (50 vs 85 years), used lower RR estimates, and restricted attention to four diseases forming about two-thirds of smoking-related mortality (Weitkunat et al. 2015). For the Pessimistic Scenario, our estimated reductions were 0.76 million deaths and 8.31 million YLL, again less than Levy’s 1.6 and 20.8 million. The reductions were evident in each sex and disease, age-dependent, and for LC and COPD were greater in later years. The unadjusted estimates presented were little affected by adjusting for mortality differences between the Scenarios.

Various issues concerning applying PHIM to estimate effects of introducing reduced risk products into the US seem unlikely to materially affect our conclusions, as discussed earlier (Lee et al. 2017; Weitkunat et al. 2015). These include ignoring pipe/cigar smokers, ignoring environmental tobacco smoke exposure, not accounting for risk factors other than smoking affecting the diseases studied and using a methodology that involves simulation, with inherent sampling errors in the estimates.

Our modelling involves projection into the future. While uncertainty about future mortality trends is inevitable, extrapolating to 2040 involves less uncertainty than Levy’s extrapolation to 2100. Our mortality predictions derived from age-period-cohort modelling appear plausible, but one cannot be sure. A cancer cure might become available by 2040, for example.

Four aspects of our modelling could be considered implausible, but were included to align with Levy’s approach. One is disallowing dual use, when many switching to exclusive vaping go through an intermediate stage of dual use, while some smokers become dual users but revert to exclusive smoking. Another is disallowing reverting from exclusive vaping to exclusive smoking. The third is disallowing re-initiation, so an individual no longer using either product never subsequently uses them. Clearly some quitters do later return to smoking, and all forms of re-initiation seem possible. PHIM does allow for all the forms of initiating, quitting, re-initiation and switching (Lee et al. 2017), though difficulties in obtaining satisfactory estimates for all the relevant TPs increases. As we aimed to give greater insight into Levy’s approach, extending the set of TPs was not pursued here.

A major conclusion is that estimated drops in deaths and YLL vary most with X (the percentage of smokers after 11 years) and with F (the excess risk for e-cigarettes relative to smoking). In contrast, varying Q or I (the quitting or initiation rates of e-cigarettes relative to cigarettes) has little effect.

The relative importance of these factors is predictable. Varying Q would be expected to have little effect, especially where e-cigarettes have a low effective dose, while varying I should also have little effect, again assuming a much lower risk for e-cigarettes than cigarettes. Variations in F and X are much more important than variations in Q and I, as they directly relate to the risk in smokers.

However, the relative importance of F and X depends on the range of values considered. Thus, if we let F exceed 0.4, an assumption that runs contrary to evidence that e-cigarettes involve a considerable reduction in risk of the major diseases associated with smoking (Abrams et al. 2018; Nutt et al. 2014), the estimated drop in deaths could become lower than that for the highest value of X we considered.

We could have carried out further analyses by modifying the parameters considered or widening their range, or varying combinations of parameters. However, such analyses would only confirm our main conclusion that a large reduction in deaths and in YLL should result providing the effective dose of harmful constituents from e-cigarettes is substantially less than that from cigarettes, as an expert panel concluded (Nutt et al. 2014), and provided that many smokers switch to e-cigarettes.

Our analyses assume that, compared to smoking, vaping involves much less risk of the four considered diseases. The US National Academy of Sciences (National Academies of Sciences Engineering and Medicine 2018) stated that “There is *conclusive evidence* that completely substituting e-cigarettes for combustible tobacco cigarettes reduces exposure to numerous toxicants and carcinogens present in combustible tobacco cigarettes” and that “There is *substantial evidence* that completely switching from regular use of combustible tobacco cigarettes to e-cigarettes results in reduced short-term adverse health outcomes in several organ systems.” However, reliable evidence on mortality risks of exclusive vapers or of dual users, as compared to exclusive cigarette use is lacking due primarily to the relatively limited time that e-cigarettes have been available to consumers.

Such evidence might be obtained from a large cohort study where a history of vaping and smoking is recorded at baseline in the disease-free and is related to subsequent disease onset. Substantial reductions in excess risk of IHD and stroke occur quickly in those quitting (Lee et al. 2012, 2014c), and, if vaping has a small effective dose (i.e., F), substantial reductions should also occur in those switching to e-cigarettes. While the decline in excess risk after quitting is slower for LC and COPD (Fry et al. 2013; Lee et al. 2014b), one might also see an advantage in switchers in 10 years.

However, no such study has been conducted, and care must be taken in existing studies to account for reverse causation, where smokers switch to e-cigarettes due to prior disease. Thus, having a myocardial infarction (MI) might lead some smokers who are advised to quit to switch to a hopefully less dangerous source of nicotine. Analyses based on annual large surveys (Osei et al. 2019), for example, are limited by their cross-sectional design, with some endpoints starting prior to vaping. Some analyses based on the PATH study, a cohort study with regular follow-up, have also ignored reverse causation. One analysis claiming vaping substantially increased risk of MI (Bhatta and Glantz 2019) was severely biased by ignoring the sequencing of exposure and disease (Rodu and Plurphanswat 2020), the original odds ratio (OR) of 2.25 [95% confidence interval (CI): 1.23–4.11] reducing to 0.69 (95% CI 0.22–2.12) after taking account of it. While the same group (Bhatta and Glantz 2020) recently reported vaping increased risk of respiratory disease, the adjusted OR for current vaping of 1.29 (95% CI 1.03–1.61) was much less than for current smoking of 2.56 (95% CI 1.92–3.41). Note that respiratory diseases are considered linked to long-term smoking, making a true effect of short-term vaping somewhat implausible, and that the combined analysis included many asthma cases, a disease hardly related to smoking.

Our analyses assume that smoking prevalence will decline rapidly initially and then continue to decline. While the rapid decline is only intended as illustrative, predictions of reduced mortality risk could be much affected if introducing e-cigarettes adversely affected the decline in smoking. As noted elsewhere (Lee et al. 2019), there are various possible adverse and beneficial effects of vaping on smoking prevalence.

One benefit of vaping is helping smokers quit. Evidence from trials where nicotine e-cigarette users are compared with a placebo or alternative nicotine replacement therapy generally show higher quitting in the e-cigarette group (Baldassarri et al. 2018; Hajek et al. 2019), though not always so (Halpern et al. 2018). The epidemiological evidence, though better reflecting general population use, is difficult to interpret, partly due to the low quality of the research (Malas et al. 2016). While many cohort studies find no clear difference in quit rates by vaping (e.g. Comiford et al. 2020; Lozano et al. 2019)), others report higher quit rates in vapers (e.g. Jackson et al. 2019; Piper et al. 2019)), and studies reporting lower rates are rare (Al Delaimy et al. 2015; Kalkhoran and Glantz 2016), with the latter widely criticized (West et al. 2016).

While the quitting evidence does not suggest vaping might slow the declining trend in smoking, much attention has been given to vaping possibly encouraging young people to start smoking, the so-called “gateway effect.” A 2017 review (Soneji et al. 2017) considered nine cohort studies relating baseline vaping in youth never smokers to subsequent smoking, an unadjusted overall OR of 5.12 (95% CI 4.41–5.95) reducing to 3.62 (85% CI 2.42–5.41) after adjustment for smoking predictors. Considering these and six other studies, a review (Lee et al. 2019) noted the range of predictors considered was usually limited, that no study adjusted for residual confounding arising from inaccuracies in the predictors, and considered better adjustment may substantially reduce the gateway effect. Later, the same authors (Lee and Fry 2020) cited detailed results from PATH adjusting for many smoking predictors and concluded, “much of the unadjusted gateway effect results from confounding … though doubts still remain about the completeness of the adjustment.” They also referred to various other recent analyses generally consistent with most of the observed gateway effect being explained by factors linked to general susceptibility to tobacco use.

In interpreting this effect’s importance, if real, one must recognize that, in PATH, in both adults and youths, initiating e-cigarettes is far more common in current smokers than never smokers. Based on Waves 1 + 2, we estimate that among Wave 1 current cigarette-only smokers, 71/114 (weighted 64.16%) of youths and 740/5561 (weighted 12.65%) of adults vaped by Wave 2, far greater than among current never users of either product, 716/7414 (9.73%) of youths and 166/8410 (0.72%) of adults.

Evidence from trends in smoking prevalence over a period where vaping increased, though limited by the difficulty of accounting for other factors affecting smoking prevalence, such as changes in smoking restrictions, suggest no material adverse effect due to vaping. Indeed, most publications suggest some benefit of e-cigarette introduction in the US and UK (Beard et al. 2019; Lee et al. 2019).

The evidence strongly suggests introducing e-cigarettes has benefitted public health and reduced smoking prevalence. A rapid switch to e-cigarettes should therefore produce a substantial mortality reduction.

Our findings are consistent with recent modelling by Levy et al. (2021), which considers a more gradual switch to e-cigarettes using current rather than optimistic patterns of switching to e-cigarettes. As we do, they estimate substantial potential benefits of e-cigarettes, while emphasising the dependence of these estimates on the assumed risk of e-cigarettes relative to cigarettes and the rate of decline in smoking.

Our findings agree with Levy’s conclusion that rapidly replacing smoking by vaping would substantially reduce deaths and YLL from smoking. Over a 50 year period from 1990, the estimated reduction in deaths in our Main Scenario would be 2.52 million, 11.4% of the total number of deaths from the diseases considered. The reduction in YLL would be 26.23 million. These estimates depend most on the assumed smoking prevalence after 10 years and the effective dose of harmful constituents from e-cigarettes compared to cigarettes. Even where less optimistic assumptions are made about parameter values, substantial reductions in deaths and YLL are still seen. Our findings support literature indicating e-cigarettes can have an important effect on the health challenges created by smoking (Mendez and Warner 2021).

## Supplementary Information

Below is the link to the electronic supplementary material.

Supplementary file1 (PDF 258 KB)

Supplementary file2 (PDF 238 KB)

Supplementary file3 (PDF 142 KB)

Supplementary file4 (PDF 435 KB)

Supplementary file5 (PDF 551 KB)

Supplementary file6 (PDF 5432 KB)

Supplementary file7 (DOCX 8210 KB)

Supplementary file8 (PDF 1777 KB)

Supplementary file9 (PDF 48 KB)

## Data Availability

All data generated or analysed during this study are included in this published article and its additional files.

## Code availability

Available from John S. Fry at RoeLee Statistics Ltd.

## References

[CR1] Abrams DB, Glasser AM, Pearson JL, Villanti AC, Collins LK, Niaura RS (2018). Harm minimization and tobacco control: reframing societal views of nicotine use to rapidly save lives. Annu Rev Public Health.

[CR2] Al Delaimy WK, Myers MG, Leas EC, Strong DR, Hofstetter CR (2015). E-cigarette use in the past and quitting behavior in the future: a population-based study. Am J Public Health.

[CR3] Baldassarri SR, Bernstein SL, Chupp GL, Slade MD, Fucito LM, Toll BA (2018). Electronic cigarettes for adults with tobacco dependence enrolled in a tobacco treatment program: a pilot study. Addict Behav.

[CR4] Barker DJP, Osmond C (1986). Childhood respiratory infection and adult chronic bronchitis in England and Wales. Br Med J.

[CR5] Beard E, West R, Michie S, Brown J (2019). Association of prevalence of electronic cigarette use with smoking cessation and cigarette consumption in England: a time-series analysis between 2006 and 2017. Addiction.

[CR6] Bhatta DN, Glantz SA (2019). Electronic cigarette use and myocardial infarction among adults in the US population assessment of tobacco and health—retracted by journal. J Am Heart Assoc.

[CR7] Bhatta DN, Glantz SA (2020). Association of e-cigarette use with respiratory disease among adults: a longitudinal analysis. Am J Prev Med.

[CR8] Comiford AL, Rhoades DA, Spicer P (2020). Impact of e-cigarette use among a cohort of American Indian cigarette smokers: associations with cigarette smoking cessation and cigarette consumption. Tob Control.

[CR9] Djurdjevic S, Pecze L, Weitkunat R, Luedicke F, Fry J, Lee P (2019). Using data on snus use in Sweden to compare different modelling approaches to estimate the population health impact of introducing a smoke-free tobacco product. BMC Public Health.

[CR10] Forey BA (1989a) Prediction of lung cancer rates 1986–2005 in 24 countries (https://file:\\\x:\refscan\FOREY1989.pdf). P N Lee Statistics and Computing Ltd, Sutton, Surrey

[CR11] Forey BA (1989b) Prediction of lung cancer rates 1986–2005. Comparison of several methods of prediction all based on O&G analysis, applied to males in 6 countries (https://file:\\\x:\refscan\FOREY1989A.pdf). P N Lee Statistics and Computing Ltd, Sutton, Surrey

[CR12] Fry JS, Lee PN, Forey BA, Coombs KJ (2013). How rapidly does the excess risk of lung cancer decline following quitting smoking? A quantitative review using the negative exponential model. Regul Toxicol Pharmacol.

[CR13] Gardner JW, Sanborn JS (1990). Years of potential life lost (YPLL)—what does it measure?. Epidemiology.

[CR14] Garfinkel L (1985). Selection, follow-up, and analysis in the American Cancer Society prospective studies. Natl Cancer Inst Monogr.

[CR15] Hajek P, Phillips-Waller A, Przulj D (2019). A randomized trial of e-cigarettes versus nicotine-replacement therapy. N Engl J Med.

[CR16] Halpern SD, Harhay MO, Saulsgiver K, Brophy C, Troxel AB, Volpp KG (2018). A pragmatic trial of e-cigarettes, incentives, and drugs for smoking cessation. N Engl J Med.

[CR17] Jackson SE, Kotz D, West R, Brown J (2019). Moderators of real-world effectiveness of smoking cessation aids: a population study. Addiction.

[CR18] Kalkhoran S, Glantz SA (2016). E-cigarettes and smoking cessation in real-world and clinical settings: a systematic review and meta-analysis. Lancet Respir Med.

[CR19] Lee P, Fry J (2020). Further investigation of gateway effects using the PATH study [version 2; peer review: 2 approved with reservations]. F1000Research.

[CR20] Lee PN, Fry JS, Hamling JS (2012). Using the negative exponential distribution to quantitatively review the evidence on how rapidly the excess risk of ischaemic heart disease declines following quitting smoking. Regul Toxicol Pharmacol.

[CR21] Lee PN, Fry J, Hamling J (2014a) Using the age-period-cohort model to describe and predict mortality from major smoking-related diseases (https://file:\\\x:\refscan\LEE2014L.pdf). P N Lee Statistics and Computing Ltd, Sutton, Surrey

[CR22] Lee PN, Fry JS, Forey BA (2014). Estimating the decline in excess risk of chronic obstructive pulmonary disease following quitting smoking—a systematic review based on the negative exponential model. Regul Toxicol Pharmacol.

[CR23] Lee PN, Fry JS, Thornton A (2014). Estimating the decline in excess risk of cerebrovascular disease following quitting smoking—a systematic review based on the negative exponential model. Regul Toxicol Pharmacol.

[CR24] Lee PN, Hamling J, Fry J, Forey B (2015). Using the negative exponential model to describe changes in risk of smoking-related diseases following changes in exposure to tobacco. Adv Epidemiol.

[CR25] Lee PN, Fry JS, Hamling JF, Sponsiello-Wang Z, Baker G, Weitkunat R (2017). Estimating the effect of differing assumptions on the population health impact of introducing a reduced risk tobacco product in the USA. Regul Toxicol Pharmacol.

[CR26] Lee PN, Djurdjevic S, Weitkunat R, Baker G (2018). Estimating the population health impact of introducing a reduced-risk tobacco product into Japan. The effect of differing assumptions, and some comparisons with the U.S. Regul Toxicol Pharmacol.

[CR27] Lee PN, Coombs KJ, Afolalu EF (2019). Considerations related to vaping as a possible gateway into cigarette smoking: an analytical review [version 3; peer review: 2 approved]. F1000Research.

[CR28] Levy DT, Borland R, Lindblom EN (2018). Potential deaths averted in USA by replacing cigarettes with e-cigarettes. Tob Control.

[CR29] Levy DT, Tam J, Sanchez-Romero LM (2021). Public health implications of vaping in the USA: the smoking and vaping simulation model. Popul Health Metr.

[CR30] Lozano P, Arillo-Santillán E, Barrientos-Gutiérrez I, Zavala-Arciniega L, Reynales-Shigematsu LM, Thrasher JF (2019). E-cigarette use and its association with smoking reduction and cessation intentions among Mexican smokers (uso de cigarros electrónicos y su asociación con la reducción en el consumo de cigarros convencionales y la intencion de dejar de fumar entre fumadores mexicanos). Salud Publica Mex.

[CR31] Malas M, van der Tempel J, Schwartz R (2016). Electronic cigarettes for smoking cessation: a systematic review. Nicotine Tob Res.

[CR32] Mendez D, Warner KE (2021). A magic bullet? The potential impact of e-cigarettes on the toll of cigarette smoking. Nicotine Tob Res.

[CR33] National Academies of Sciences Engineering and Medicine (2018) Public health consequences of e-cigarettes (https://file:\\\x:\refscan\NATION2018.pdf). The National Academies Press, Washington DC

[CR34] Nutt DJ, Phillips LD, Balfour D (2014). Estimating the harms of nicotine-containing products using the MCDA approach. Eur Addict Res.

[CR35] Osei AD, Mirbolouk M, Orimoloye OA (2019). Association between e-cigarette use and chronic obstructive pulmonary disease by smoking status: behavioral risk factor surveillance system 2016 and 2017. Am J Prev Med.

[CR36] Osmond C, Gardner MJ (1982). Age, period and cohort models applied to cancer mortality rates. Stat Med.

[CR37] Osmond C, Gardner MJ, Acheson ED (1982). Analysis of trends in cancer mortality in England and Wales during 1951–80 separating changes associated with period of birth and period of death. Br Med J.

[CR38] Piper ME, Baker TB, Benowitz NL, Jorenby DE (2019). Changes in use patterns over 1 year among smokers and dual users of combustible and electronic cigarettes. Nicotine Tob Res.

[CR39] Rodu B, Plurphanswat N (2020). A re-analysis of e-cigarette use and heart attacks in PATH wave 1 data. Addiction.

[CR40] Soneji S, Barrington-Trimis JL, Wills TA (2017). Association between initial use of e-cigarettes and subsequent cigarette smoking among adolescents and young adults: a systematic review and meta-analysis. JAMA Pediatr.

[CR41] Weitkunat R, Lee PN, Baker G, Sponsiello-Wang Z, González-Zuloeta Ladd AM, Lüdicke F (2015). A novel approach to assess the population health impact of introducing a modified risk tobacco product. Regul Toxicol Pharmacol.

[CR42] West R, Shahab L, Brown J (2016). Estimating the population impact of e-cigarettes on smoking cessation in England. Addiction.

