# Supplementary material for: Estimating the reduction in US mortality if cigarettes were largely replaced by e-cigarettes
Source: Arch Toxicol. 2021 Oct 22;96(1):167–76. doi: 10.1007/s00204-021-03180-3 (PMC8748352; doi:10.1007/s00204-021-03180-3)
Supplement: Supplementary file 1 — Supplementary file1 (PDF 258 KB) [file 204_2021_3180_MOESM1_ESM.pdf]

# Estimating the reduction in US mortality if cigarettes were largely replaced by e-cigarettes

Published in: Archives of Toxicology

Peter N Lee[1*], John S Fry[2], Stanley Gilliland III[3], Preston Campbell[3], Andrew R. Joyce[3]

[1]P N Lee Statistics and Computing Ltd, 17 Cedar Road, Sutton, Surrey, SM2 5DA

[2]RoeLee Statistics Ltd., 17 Cedar Road, Sutton, Surrey SM2 5DA

[3]Consilium Sciences, LLC, 7400 Beaufont Springs Drive, Suite 300, N. Chesterfield, VA 23325

[*] Author for correspondence

E-mail: Peterlee@pnlee.co.uk

## ONLINE RESOURCE 1

### Population estimates

Population estimates (in thousands) by sex and five year age groups (10-14, 15-19 … 75-79 were downloaded from the United Nations website (http://population.un.org/wpp/Download/Standard/Population/) on April 15, 2020 for the years 1990 to 2040, those for 2018 to 2040 being what are termed medium variant projections. The data are shown in the pages that follow.

| | 1990 | 1991 | 1992 | 1993 | 1994 | 1995 | 1996 | 1997 | 1998 | 1999 | 2000 | 2001 | 2002 |
|---|---|---|---|---|---|---|---|---|---|---|---|---|---|
| | | | | | | | | | | | | | |
| **Male** | | | | | | | | | | | | | |
| 10-14 | 89031.79 | 90093.6 | 91490.97 | 93079.16 | 94770 | 96512.57 | 98371.88 | 100412.4 | 102464.7 | 104379.3 | 106077.3 | 107756.3 | 109118.3 |
| 15-19 | 92222.14 | 91302.19 | 90966.03 | 91303.71 | 92257.42 | 93654.31 | 94834.36 | 96333.3 | 98162.73 | 100192 | 102236.4 | 104176.7 | 105968.2 |
| 20-24 | 100348.9 | 98390.93 | 96510.26 | 94886.76 | 93679.01 | 93010.96 | 93359.15 | 94094.85 | 95031.09 | 96016.26 | 97058.99 | 98121.35 | 99481.11 |
| 25-29 | 109467.9 | 108580.8 | 107180 | 105248.4 | 103031.1 | 100852 | 99475.17 | 98598.51 | 98093.31 | 97798.89 | 97619.52 | 97313.25 | 97023.56 |
| 30-34 | 111409.7 | 112212.1 | 112772.2 | 113189.8 | 113410.1 | 113222.2 | 111782.9 | 110062.4 | 108257 | 106592.9 | 105173 | 103578.7 | 102067.8 |
| 35-39 | 101184.6 | 103486 | 105908.9 | 108279.6 | 110431.5 | 112330 | 113473.9 | 114420.3 | 115013.1 | 115051.2 | 114480.8 | 113408.1 | 111647.3 |
| 40-44 | 87675.59 | 90529.83 | 92984.73 | 95313.84 | 97826.52 | 100621.5 | 103279.7 | 105936.2 | 108441.5 | 110556.4 | 112133.2 | 113472.3 | 114268.8 |
| 45-49 | 66913.26 | 70105.78 | 73880.27 | 77872.73 | 81678.24 | 85148.38 | 88745.03 | 92068.86 | 95059.86 | 97798.85 | 100379.2 | 102949.8 | 105314.7 |
| 50-54 | 57242.9 | 58289.97 | 59405.76 | 60841.8 | 62890.22 | 65623.29 | 68687.82 | 72544.65 | 76959.04 | 81408.23 | 85447.29 | 88866.89 | 91652.95 |
| 55-59 | 50454.24 | 50659.52 | 51337.86 | 52352.31 | 53528.8 | 54809.16 | 56134.65 | 57539.83 | 59116.67 | 61117.44 | 63750.46 | 67108.47 | 71067.43 |
| 60-64 | 49829.53 | 49093.72 | 48320.34 | 47659.28 | 47330.28 | 47453.14 | 47776.08 | 48636.01 | 49881.02 | 51264.2 | 52625.59 | 53915.79 | 55109.19 |
| 65-69 | 45494.37 | 45463.44 | 45364.87 | 45251.86 | 45139.98 | 45018.38 | 44477.92 | 43949.16 | 43563.66 | 43482.99 | 43785.58 | 44327.13 | 45212.58 |
| 70-74 | 35396.16 | 35964.08 | 36641.51 | 37307.79 | 37873.18 | 38342.31 | 38539.7 | 38730.51 | 38858.67 | 38873.49 | 38789.13 | 38599.61 | 38329.11 |
| 75-79 | 25225.57 | 25533.44 | 25803.59 | 26193.56 | 26788.88 | 27551.98 | 28127.37 | 28752.86 | 29408.69 | 30022.8 | 30533.84 | 30729.04 | 30864.57 |
| | | | | | | | | | | | | | |

| | 2003 | 2004 | 2005 | 2006 | 2007 | 2008 | 2009 | 2010 | 2011 | 2012 | 2013 | 2014 | 2015 |
|---|---|---|---|---|---|---|---|---|---|---|---|---|---|
| | | | | | | | | | | | | | |
| **Male** | | | | | | | | | | | | | |
| 10-14 | 110140.4 | 110688.1 | 110548.6 | 111533.1 | 108775.3 | 107784.4 | 107080.9 | 106920.5 | 106475.8 | 106257.1 | 106161.8 | 106089.4 | 106047.7 |
| 15-19 | 107647.5 | 109309.5 | 110959.2 | 106705.2 | 112301.3 | 113121.9 | 113728.3 | 113973.9 | 113924.8 | 113157.8 | 111881.9 | 110510.9 | 109331.8 |
| 20-24 | 101208.4 | 103239.7 | 105433 | 98729.93 | 107848 | 108925.5 | 109986.1 | 111059.9 | 112938.4 | 114840.8 | 116538.3 | 117693.7 | 118085.6 |
| 25-29 | 96809.07 | 96881.61 | 97461.26 | 98196.82 | 100360.4 | 102056 | 103557 | 104813.5 | 106739.5 | 108751.6 | 110797.3 | 112835.9 | 114818.5 |
| 30-34 | 100759.6 | 99719.31 | 98923.82 | 103896 | 97861.38 | 97978.12 | 98524.19 | 99371.16 | 100462.4 | 101907.8 | 103621.4 | 105495.7 | 107455.9 |
| 35-39 | 109513 | 107424.9 | 105629.1 | 112467.7 | 102485.9 | 101353.1 | 100433.3 | 99734.77 | 99324.74 | 99246.69 | 99487.85 | 100043.1 | 100908.5 |
| 40-44 | 114543.9 | 114335.8 | 113684.9 | 111962.6 | 110882.8 | 109085.4 | 107281 | 105611.2 | 104061.5 | 102762.6 | 101706.6 | 100876.6 | 100277.1 |
| 45-49 | 107446.9 | 109300.7 | 110828.7 | 100974.6 | 112845.7 | 113341.8 | 113317 | 112749.8 | 111588.6 | 110082.9 | 108360 | 106630.4 | 105045.4 |
| 50-54 | 94012.93 | 96311.53 | 98746.95 | 86313.21 | 103347.8 | 105738.2 | 107896.9 | 109630.6 | 110553.2 | 111172.7 | 111433.2 | 111279.1 | 110697.3 |
| 55-59 | 75344.81 | 79503.46 | 83261.19 | 64101.69 | 88975.02 | 91370.69 | 93732.36 | 96204.21 | 98332.62 | 100546.5 | 102695.8 | 104563.1 | 106015.4 |
| 60-64 | 56452.26 | 58344.95 | 61008.35 | 50052.68 | 67943.39 | 72169.73 | 76249.04 | 79866.26 | 82659.8 | 85036.34 | 87130.21 | 89193.64 | 91370.31 |
| 65-69 | 46370.93 | 47670.51 | 49031.47 | 39075.52 | 51152.93 | 52540.84 | 54507.44 | 57180.19 | 60057.39 | 63548.89 | 67338.56 | 70969.12 | 74169.14 |
| 70-74 | 38113.16 | 38166.45 | 38620.85 | 31439.05 | 39921.58 | 41051.87 | 42299.41 | 43591.46 | 44675.65 | 45772.21 | 47062.35 | 48822.71 | 51187.63 |
| 75-79 | 31021.66 | 31222.12 | 31446.04 | 111533.1 | 31336.98 | 31261.2 | 31389.37 | 31831.6 | 32397 | 33272.08 | 34357.3 | 35518.64 | 36701.31 |

| | | | | | | | | | | | | | |
|---|---|---|---|---|---|---|---|---|---|---|---|---|---|

| | 2016 | 2017 | 2018 | 2019 | 2020 | 2021 | 2022 | 2023 | 2024 | 2025 | 2026 | 2027 | 2028 |
|---|---|---|---|---|---|---|---|---|---|---|---|---|---|
| | | | | | | | | | | | | | |
| **Male** | | | | | | | | | | | | | |
| 10-14 | 106662.4 | 107281.3 | 107820.2 | 108058.2 | 107775.1 | 107441.8 | 106584.5 | 105339.4 | 104004.7 | 102858.8 | 102347.1 | 101967.3 | 101686.2 |
| 15-19 | 108871.9 | 108585.4 | 108437 | 108371.9 | 108343.2 | 108844.9 | 109515 | 110116.3 | 110337.6 | 110010.6 | 109660.7 | 108831.4 | 107648.3 |
| 20-24 | 117866.1 | 116915.1 | 115549.4 | 114235.5 | 113227.3 | 112480.5 | 112060.2 | 111946.1 | 112037.4 | 112220.6 | 112555.5 | 113161.8 | 113837.9 |
| 25-29 | 116664.5 | 118445.2 | 119991.4 | 121043 | 121444.6 | 121074.8 | 120072.5 | 118718.7 | 117445.5 | 116529.3 | 115783.2 | 115395.3 | 115330.5 |
| 30-34 | 109396.5 | 111313 | 113212.6 | 115120.7 | 117025.1 | 118757.7 | 120480.8 | 122003 | 123037.1 | 123431.2 | 123099.8 | 122150.7 | 120849.6 |
| 35-39 | 102029.4 | 103397.3 | 104980.4 | 106727.8 | 108588.7 | 110434 | 112312.9 | 114204.9 | 116096.6 | 117963.2 | 119699.6 | 121440.5 | 122984.9 |
| 40-44 | 99919.43 | 99793.01 | 99936.3 | 100395.6 | 101185.8 | 102208.8 | 103543.2 | 105128.9 | 106876.4 | 108719.3 | 110549.1 | 112434.2 | 114340.8 |
| 45-49 | 103581.6 | 102272.1 | 101165.7 | 100298.9 | 99690.99 | 99242.93 | 99093.21 | 99254.35 | 99737.28 | 100542.9 | 101546.3 | 102882.9 | 104481.4 |
| 50-54 | 109627.2 | 108137.6 | 106413 | 104711.9 | 103195.4 | 101687.2 | 100399 | 99352.7 | 98551.39 | 98004.48 | 97565.92 | 97442.82 | 97640.03 |
| 55-59 | 106941.1 | 107502 | 107703.7 | 107543 | 107020.7 | 105928.8 | 104506.2 | 102905 | 101336.9 | 99943.84 | 98515.25 | 97312.38 | 96350.73 |
| 60-64 | 93413.07 | 95463.36 | 97419.45 | 99129.39 | 100498.9 | 101325.9 | 101894.7 | 102171.6 | 102106.2 | 101686.4 | 100706.9 | 99431.26 | 97989.85 |
| 65-69 | 76738.31 | 78871.94 | 80741.69 | 82621.82 | 84652.74 | 86461.99 | 88359.04 | 90225.39 | 91890.97 | 93257.36 | 94090.33 | 94720.98 | 95097.64 |
| 70-74 | 53807.54 | 56941.82 | 60322.41 | 63567.12 | 66455.19 | 68627.23 | 70495.96 | 72213.7 | 73996.64 | 75946.51 | 77642.7 | 79455.67 | 81271.07 |
| 75-79 | 37638.95 | 38552.76 | 39637.64 | 41167.73 | 43269.86 | 45476.76 | 48148.98 | 51047.2 | 53839.87 | 56355.46 | 58243.73 | 59927.44 | 61548.21 |
| | | | | | | | | | | | | | |

| | 2029 | 2030 | 2031 | 2032 | 2033 | 2034 | 2035 | 2036 | 2037 | 2038 | 2039 | 2040 |
|---|---|---|---|---|---|---|---|---|---|---|---|---|
| | | | | | | | | | | | | |
| **Male** | | | | | | | | | | | | |
| 10-14 | 101498.6 | 101470.6 | 102114.8 | 102877.4 | 103681 | 104401.5 | 104955.6 | 105877.4 | 106570.9 | 107039 | 107313.7 | 107433.9 |
| 15-19 | 106394.3 | 105276.7 | 104802.1 | 104470.8 | 104248.2 | 104119.5 | 104083.9 | 104723.3 | 105537.1 | 106382.5 | 107105.1 | 107619.1 |
| 20-24 | 114259.7 | 114195.9 | 113718.7 | 112837.2 | 111736 | 110690.4 | 109833.5 | 109162.2 | 108713.5 | 108513.4 | 108545.6 | 108746.9 |
| 25-29 | 115497.6 | 115822.8 | 116202.2 | 116849.2 | 117569.1 | 118056.4 | 118118.8 | 117643.5 | 116765.5 | 115670.6 | 114651.2 | 113890.4 |
| 30-34 | 119629.2 | 118778.2 | 118134.8 | 117811 | 117782.7 | 117975 | 118328.8 | 118772.5 | 119447 | 120162.2 | 120630.5 | 120687.7 |
| 35-39 | 124045.5 | 124469.9 | 124226.7 | 123342.5 | 122091.5 | 120908.7 | 120074.1 | 119494.5 | 119205.3 | 119187.7 | 119374.6 | 119711.4 |
| 40-44 | 116250.1 | 118133.6 | 119919.8 | 121692.8 | 123260.6 | 124341.2 | 124775.8 | 124577.7 | 123731.6 | 122510.5 | 121348.4 | 120525.3 |
| 45-49 | 106248.9 | 108117 | 109976.9 | 111882.2 | 113806.8 | 115734.3 | 117628.6 | 119421 | 121196.2 | 122766.3 | 123855.7 | 124310.2 |
| 50-54 | 98167.01 | 99023.15 | 100068.6 | 101429.1 | 103046.3 | 104833.8 | 106719.4 | 108574 | 110468.9 | 112385.7 | 114312.8 | 116218 |
| 55-59 | 95635.8 | 95179.27 | 94853.58 | 94810.04 | 95066.3 | 95640.25 | 96531.34 | 97604.83 | 98971.23 | 100579.3 | 102352.2 | 104228.7 |
| 60-64 | 96579.59 | 95336.98 | 94127.23 | 93103.03 | 92285.06 | 91686.74 | 91322.81 | 91095.36 | 91124.32 | 91426.53 | 92024.97 | 92928.27 |
| 65-69 | 95160.86 | 94892.86 | 94152.8 | 93125.67 | 91934.49 | 90764.25 | 89736.52 | 88691 | 87817.73 | 87132.51 | 86653 | 86398.47 |
| 70-74 | 82935.72 | 84358.3 | 85296.91 | 86070.81 | 86633.91 | 86924.62 | 86911.54 | 86311.54 | 85470.66 | 84501.56 | 83576.13 | 82803.34 |

| | | | | | | | | | | | | |
|---|---|---|---|---|---|---|---|---|---|---|---|---|
| 75-79 | 63282.21 | 65200.04 | 66882.79 | 68676.42 | 70497.44 | 72222.45 | 73769.11 | 74675.1 | 75475.6 | 76138.72 | 76605.74 | 76840.03 |
| | | | | | | | | | | | | |

| | 1990 | 1991 | 1992 | 1993 | 1994 | 1995 | 1996 | 1997 | 1998 | 1999 | 2000 | 2001 | 2002 |
|---|---|---|---|---|---|---|---|---|---|---|---|---|---|
| | | | | | | | | | | | | | |
| **Female** | | | | | | | | | | | | | |
| 10-14 | 84910.03 | 85944.58 | 87265.62 | 88808.99 | 90491.35 | 92211.44 | 93889.98 | 95724.85 | 97637.54 | 99500.59 | 101190.6 | 102598 | 103729.3 |
| 15-19 | 87224.15 | 86822.22 | 86728.4 | 87022.06 | 87748.19 | 88880.47 | 90166.24 | 91724.36 | 93491.76 | 95322.42 | 97103.57 | 98936.56 | 100560.8 |
| 20-24 | 94131.08 | 92713.06 | 91465.69 | 90472.86 | 89779.73 | 89421.36 | 89906.59 | 90649.98 | 91499.67 | 92374.36 | 93299.33 | 94545.33 | 95928.76 |
| 25-29 | 104689.8 | 103476.3 | 101947.2 | 100118.4 | 98212.14 | 96484.87 | 95339.86 | 94819.52 | 94797.76 | 95009.68 | 95233.99 | 95257.29 | 95272.78 |
| 30-34 | 110174.6 | 110451 | 110295.1 | 109920.5 | 109454.5 | 108824.8 | 107328.2 | 105623.1 | 103850.1 | 102240.1 | 100963.4 | 99909.59 | 99108.39 |
| 35-39 | 101477.5 | 103611.5 | 105871.4 | 108006 | 109770.9 | 111119.3 | 111833.9 | 112290.5 | 112415.4 | 112090.2 | 111269.9 | 110038.2 | 108236.8 |
| 40-44 | 90433.92 | 93002.17 | 95086.57 | 97008.3 | 99128.25 | 101557.1 | 103939.5 | 106389.5 | 108712.6 | 110601.1 | 111876.7 | 112864.9 | 113272.8 |
| 45-49 | 70860.05 | 74345.33 | 78125.63 | 81900.98 | 85392.85 | 88540.37 | 91820.56 | 94795.24 | 97434.74 | 99846.43 | 102134.3 | 104512.1 | 106731.8 |
| 50-54 | 58832.07 | 60293.89 | 62114.08 | 64376.66 | 67128.52 | 70297.49 | 73622.11 | 77485.06 | 81701.82 | 85859.7 | 89594.31 | 92791.07 | 95334.33 |
| 55-59 | 54785.26 | 54545.06 | 54677.37 | 55188.34 | 56090.79 | 57415.46 | 59112.73 | 61197.47 | 63611.79 | 66349.33 | 69442.67 | 73052.67 | 77024.9 |
| 60-64 | 57015.65 | 56036.42 | 54991.69 | 53985.82 | 53219.87 | 52842.51 | 52729.96 | 53106.95 | 53907.3 | 55038.38 | 56437.68 | 58079.47 | 59941.12 |
| 65-69 | 55534.24 | 55333.81 | 54985.49 | 54600.51 | 54227.73 | 53837 | 52939.2 | 52045.5 | 51297.24 | 50834.88 | 50713.24 | 50748.87 | 51072.26 |
| 70-74 | 46425.14 | 47104.21 | 47898.12 | 48618.93 | 49151.76 | 49526.41 | 49663.11 | 49754.4 | 49702.86 | 49447.6 | 49025.78 | 48422.99 | 47768.47 |
| 75-79 | 37272.45 | 37322.75 | 37521.94 | 37999.44 | 38778.48 | 39753.32 | 40370.95 | 41109.91 | 41914.66 | 42667.05 | 43257.29 | 43092.66 | 42868.5 |
| | | | | | | | | | | | | | |

| | 2003 | 2004 | 2005 | 2006 | 2007 | 2008 | 2009 | 2010 | 2011 | 2012 | 2013 | 2014 | 2015 |
|---|---|---|---|---|---|---|---|---|---|---|---|---|---|
| | | | | | | | | | | | | | |
| **Female** | | | | | | | | | | | | | |
| 10-14 | 104625 | 105168.8 | 105125.7 | 104421.5 | 103536.3 | 102653.8 | 102070.2 | 101989.2 | 101766.1 | 101653.3 | 101576.3 | 101485.3 | 101423.5 |
| 15-19 | 102016.3 | 103394.4 | 104735.9 | 105559.5 | 106480.9 | 107335.4 | 107871.8 | 107995.7 | 108176.6 | 107724.9 | 106796.6 | 105730.7 | 104768.8 |
| 20-24 | 97433.94 | 99036.61 | 100697.6 | 101985.5 | 103233.3 | 104383.4 | 105414.2 | 106345.7 | 107937.8 | 109619.6 | 111185.3 | 112317.7 | 112795.3 |
| 25-29 | 95412.95 | 95860.83 | 96698.1 | 97596.19 | 98820.66 | 100240.1 | 101658.2 | 102937.8 | 104432.5 | 105952.1 | 107492.2 | 109078.2 | 110704.6 |
| 30-34 | 98554.57 | 98201.91 | 98019.89 | 97816.06 | 97849.09 | 98097.1 | 98564.17 | 99250.61 | 100147.6 | 101368.4 | 102822 | 104383.8 | 105974.6 |
| 35-39 | 106199.1 | 104363.4 | 102970.8 | 101638.2 | 100824.7 | 100431.7 | 100247.8 | 100128.8 | 99959.44 | 99908.46 | 100019.2 | 100384.4 | 101062.2 |
| 40-44 | 113171.9 | 112665.4 | 111832.5 | 110422.1 | 108696.9 | 106839.8 | 105108.9 | 103687.9 | 102561.9 | 101845.9 | 101439.2 | 101172.5 | 100951.4 |
| 45-49 | 108725.4 | 110390.6 | 111659.3 | 112512.4 | 113028.1 | 113106.8 | 112671.4 | 111741.2 | 110339.4 | 108615.8 | 106748.6 | 105015.7 | 103603 |
| 50-54 | 97442.99 | 99497.07 | 101704.3 | 103846.7 | 106149.2 | 108426.2 | 110368.1 | 111758.9 | 112334.8 | 112506.6 | 112264.8 | 111623.2 | 110617.4 |
| 55-59 | 81147.81 | 85101.16 | 88668.75 | 91578.69 | 94105.81 | 96382.59 | 98629.37 | 100961.8 | 102984 | 105047.9 | 106984.1 | 108552.6 | 109612.8 |
| 60-64 | 62101.78 | 64711.39 | 67844.02 | 71236.17 | 75167.37 | 79327.14 | 83283.91 | 86787.92 | 89568.23 | 91915.53 | 93957.27 | 95934.66 | 97981.03 |
| 65-69 | 51728.93 | 52755.14 | 54157.06 | 55532.91 | 57306.17 | 59517.87 | 62212.32 | 65369.38 | 68570.38 | 72199.86 | 76007.45 | 79633.4 | 82854.48 |

| | | | | | | | | | | | | | |
|---|---|---|---|---|---|---|---|---|---|---|---|---|---|
| 70-74 | 47162.18 | 46791.56 | 46790.99 | 46711.59 | 47018.06 | 47706.27 | 48746.16 | 50124.71 | 51568.91 | 53310.08 | 55376.99 | 57837.21 | 60711.32 |
| 75-79 | 42692.25 | 42602.36 | 42565.51 | 42187.39 | 41723.49 | 41251.46 | 40941.18 | 40932.41 | 40975.05 | 41373.68 | 42101.1 | 43135.96 | 44475.6 |
| | | | | | | | | | | | | | |

| | 2016 | 2017 | 2018 | 2019 | 2020 | 2021 | 2022 | 2023 | 2024 | 2025 | 2026 | 2027 | 2028 |
|---|---|---|---|---|---|---|---|---|---|---|---|---|---|
| | | | | | | | | | | | | | |
| **Female** | | | | | | | | | | | | | |
| 10-14 | 102048.4 | 102668.1 | 103196.5 | 103416.5 | 103119.7 | 102838.6 | 102041.8 | 100854.3 | 99561.97 | 98437.76 | 98019.03 | 97707.32 | 97463.3 |
| 15-19 | 104490.8 | 104310.5 | 104200.4 | 104134.1 | 104085.9 | 104593.7 | 105258.8 | 105846.2 | 106048.8 | 105699.1 | 105411 | 104647.8 | 103524.1 |
| 20-24 | 112754.5 | 112105.8 | 111103.8 | 110124.2 | 109360.1 | 108770 | 108425.4 | 108322.6 | 108395.5 | 108550 | 108894 | 109496.4 | 110158.4 |
| 25-29 | 112260.4 | 113851.2 | 115313.9 | 116388 | 116908.8 | 116732.9 | 116025.6 | 115011.7 | 114041.8 | 113339.6 | 112753.7 | 112440.8 | 112383.4 |
| 30-34 | 107497.8 | 108962 | 110408.6 | 111913.5 | 113499.7 | 114988.6 | 116553.3 | 118007.1 | 119063.4 | 119562.5 | 119436.4 | 118779.2 | 117804.6 |
| 35-39 | 101999.3 | 103179.7 | 104553.8 | 106038.7 | 107569.2 | 109034.1 | 110485.7 | 111939.6 | 113436.5 | 114986.8 | 116509.2 | 118107.6 | 119585 |
| 40-44 | 100826.1 | 100749.5 | 100804.8 | 101120.1 | 101760.2 | 102630.7 | 103794.4 | 105177.6 | 106662.9 | 108171.1 | 109650.2 | 111123.9 | 112595.5 |
| 45-49 | 102532 | 101803.7 | 101360.1 | 101069.8 | 100847 | 100659.6 | 100575.9 | 100653.9 | 100986.7 | 101625.5 | 102500.8 | 103677.9 | 105073 |
| 50-54 | 109268.3 | 107545.3 | 105669.8 | 103952.8 | 102582.7 | 101478.7 | 100767.1 | 100364.9 | 100110.8 | 99910.25 | 99735.09 | 99674.37 | 99776.49 |
| 55-59 | 110181.6 | 110305 | 110017.5 | 109369.2 | 108402.1 | 107034.4 | 105367.2 | 103586.7 | 101959.8 | 100659.3 | 99601.26 | 98936.25 | 98571.78 |
| 60-64 | 99941.81 | 101888.1 | 103692.9 | 105167.1 | 106192.6 | 106670 | 106802.2 | 106586.9 | 106027.6 | 105148.8 | 103844 | 102267.9 | 100585.3 |
| 65-69 | 85495.31 | 87701.52 | 89628.99 | 91532.64 | 93537.53 | 95311.43 | 97151.03 | 98914.02 | 100389.8 | 101451.9 | 101911.2 | 102082.3 | 101945.4 |
| 70-74 | 63688.32 | 67061.37 | 70611.49 | 74021.46 | 77093.44 | 79442.78 | 81480.14 | 83335.8 | 85212.39 | 87206.59 | 88867.16 | 90635.93 | 92365.82 |
| 75-79 | 45747.25 | 47272.23 | 49103.09 | 51336.03 | 54007.48 | 56601.46 | 59618.26 | 62846.26 | 65980.48 | 68843.41 | 70916.17 | 72776.1 | 74545.1 |
| | | | | | | | | | | | | | |

| | 2029 | 2030 | 2031 | 2032 | 2033 | 2034 | 2035 | 2036 | 2037 | 2038 | 2039 | 2040 |
|---|---|---|---|---|---|---|---|---|---|---|---|---|
| | | | | | | | | | | | | |
| **Female** | | | | | | | | | | | | |
| 10-14 | 97277.15 | 97213.74 | 97897.69 | 98659.12 | 99422 | 100073.8 | 100545.7 | 101474.2 | 102160.1 | 102601.7 | 102829.1 | 102884.6 |
| 15-19 | 102309.3 | 101201.4 | 100810.5 | 100543.2 | 100360 | 100239.4 | 100174.8 | 100832.7 | 101636 | 102445.8 | 103113.7 | 103554.7 |
| 20-24 | 110560.6 | 110472.5 | 110043.6 | 109215.5 | 108165.5 | 107158.7 | 106321.3 | 105709.1 | 105313.1 | 105152.8 | 105201 | 105384.1 |
| 25-29 | 112526.1 | 112817.5 | 113197.9 | 113832.5 | 114532.9 | 115000.7 | 115047.4 | 114600.1 | 113762 | 112710.9 | 111728.5 | 110989.3 |
| 30-34 | 116869.6 | 116215.1 | 115722 | 115461.7 | 115432.3 | 115598 | 115923.7 | 116352.7 | 117007.5 | 117704.8 | 118160.6 | 118208.2 |
| 35-39 | 120656 | 121163.9 | 121121.1 | 120513.4 | 119568.1 | 118651.5 | 118001.6 | 117559.6 | 117326 | 117302.7 | 117459.2 | 117763.4 |
| 40-44 | 114101.7 | 115652.2 | 117230.5 | 118857.3 | 120347.8 | 121425 | 121928.4 | 121919.3 | 121336.5 | 120407.5 | 119497.3 | 118841.1 |
| 45-49 | 106566.6 | 108079.3 | 109596.5 | 111088 | 112568.4 | 114080.5 | 115628.4 | 117216 | 118846.1 | 120337.2 | 121414.6 | 121918.3 |
| 50-54 | 100131 | 100788.2 | 101703 | 102896.1 | 104296.7 | 105794.2 | 107306.8 | 108824.8 | 110313.1 | 111790.4 | 113300.6 | 114847.4 |
| 55-59 | 98349.26 | 98179.66 | 98068.83 | 98049.73 | 98181.04 | 98557.22 | 99225.88 | 100154.4 | 101350.3 | 102745.4 | 104232.7 | 105735.3 |
| 60-64 | 99052.38 | 97837.8 | 96887.58 | 96301.1 | 95993.99 | 95818.8 | 95689.54 | 95610.22 | 95623.12 | 95783.17 | 96181.29 | 96864.11 |

| | | | | | | | | | | | | |
|---|---|---|---|---|---|---|---|---|---|---|---|---|
| 65-69 | 101493.2 | 100741.8 | 99562.46 | 98120.48 | 96581.42 | 95196.79 | 94122.43 | 93233.27 | 92707.19 | 92457.99 | 92344.12 | 92283.66 |
| 70-74 | 93857.76 | 94993.39 | 95491.46 | 95744.27 | 95736.57 | 95458.32 | 94912.65 | 93804.05 | 92481.96 | 91099.81 | 89896.79 | 89017.71 |
| 75-79 | 76397.46 | 78406 | 79998.44 | 81713.29 | 83427.8 | 84971.38 | 86235.81 | 86675.68 | 86953.29 | 87056.02 | 86967.53 | 86679.86 |
| | | | | | | | | | | | | |
