# Supplementary material for: Estimating the reduction in US mortality if cigarettes were largely replaced by e-cigarettes
Source: Arch Toxicol. 2021 Oct 22;96(1):167–76. doi: 10.1007/s00204-021-03180-3 (PMC8748352; doi:10.1007/s00204-021-03180-3)
Supplement: Supplementary file 2 — Supplementary file2 (PDF 238 KB) [file 204_2021_3180_MOESM2_ESM.pdf]

Estimating the reduction in US mortality if cigarettes were largely replaced by e-cigarettes

Published in: Archives of Toxicology

Peter N Lee[1*], John S Fry[2], Stanley Gilliland III[3], Preston Campbell[3], Andrew R. Joyce[3]

[1]P N Lee Statistics and Computing Ltd, 17 Cedar Road, Sutton, Surrey, SM2 5DA

[2]RoeLee Statistics Ltd., 17 Cedar Road, Sutton, Surrey SM2 5DA

[3]Consilium Sciences, LLC, 7400 Beaufont Springs Drive, Suite 300, N. Chesterfield, VA 23325

[*] Author for correspondence

E-mail: Peterlee@pnlee.co.uk

ONLINE RESOURCE 2

Prevalence of smoking

Sex- and age-specific distributions of current and former smoking prevalence for individual years from 1986 to 2018 and for the five-year periods from 1986-1990 to 2016-2020 were estimated from annual data from two national US surveys, the National Survey of Drug Use and Health (NSDUH) and the National Health Interview Survey (NHIS). For the final period, the estimates were derived from data for 2016-2018 only, later data being currently unavailable. The data and more complete details of their derivation are given in the pages that follow.

1 NSDUH

On-line queries were carried out using the facility available via the NSDUH websites (see https://pdas.samhsa.gov/#/). Data were available for current and former smoking prevalence up to 2018.

There are three variables available that are relevant to current smoking prevalence:-

CIGMON (Smoked cigarettes in the last month)
DCIGMON (Smoked cigarettes daily in the last month)
TOBMON (Smoked tobacco products in the last month)

The worksheets are labelled accordingly e.g. [2018 CIGMON] with prevalence data formatted by age groups in cells G4:Q5.

Former smoking prevalence, EXCIGLF, was calculated using the variable CIG100LF (Smoked at least 100 cigarettes in lifetime) and CIGMON (Used cigarettes in past month, 0=No 1=Yes) using the following formula:-

```
If (CIG100LF eq Yes)
   EXCIGLF=1- CIGMON
elseif (CIG100LF eq No or CIG100LF eq Never used cigs)
  EXCIGLF=0
endif
```

Former smoking prevalence data (weighted and unweighted) are held in worksheets [2018 EXCIGLF], [2017 EXCIGLF] etc. We used the weighted data in Rows 20 (Male) and 32 (Female).

2 NHIS

IPUMS on-line queries were carried out (see https://nhis.ipums.org/nhis/). These queries involved generating a variable for cigarette smoking status (current daily, current occasional, former and never) and a variable for age groups 15-19, 20-24, 25-29 … 80-84, 85+; and then running analyses using these variables to calculate prevalence estimates. Note that the surveys asked smoking habit questions of participants aged 18 and over (20 and over in some years) so no data were available for people aged 15-17.

3 Producing age-standardized prevalence data

The process of age standardisation involves estimating prevalence for those age groups without data (usually for young people or the highest age group) and then apportioning the prevalence values (which may have been provided using wide or narrow age ranges) into the necessary five-year age groups. This was necessary for the NSDUH data because the on-line analyses made use of the age groups provided. It was done separately for current and former smoking prevalence estimates. For the NHIS data the estimation of prevalence for ages 15-17 was necessary but apportioning the prevalence estimates into five-year age groups was not needed because the on-line analyses had been made to report these age groups.

4 Combining age standardized prevalence data into five-year periods

The age-standardized data were then combined to give five-year period data.

The process of estimating prevalence in five-year periods involved selecting a single set of results for each combination of survey series (NSDUH or NHIS), year and sex; and then averaging the selected results over five-year periods. The selection of results was based on the smoking frequency reported and on the tobacco product reported. Note that a set of results consists of the prevalence estimates for each of the age groups.

4.1 Selecting results by smoking frequency

For selecting results by smoking frequency, we chose from the available results in the order:-

A All smoking, whether daily or occasional
U Unspecified frequency of smoking
R Regular (daily) smoking

This was not relevant for former smoking prevalence because neither survey series reported more than one former smoking frequency.

4.2 Selecting results by tobacco product reported

Prevalence data are recorded using the tobacco product classifications:-

MC Manufactured Cigarettes
TC Total Cigarettes

UC	Unspecified cigarettes
U	Unspecified
A	All tobacco products

Only smoking products MC, TC and UC were considered for inclusion in each five-year period, results for other smoking products being rejected. Once this had been done, no further exclusions were needed.

5 The data

Note that the age group 10-14 years is assumed to have prevalence zero throughout.

5.1 Current smoking prevalence (%), males

	**Age group (years)**												
	15-19	20-24	25-29	30-34	35-39	40-44	45-49	50-54	55-59	60-64	65-69	70-74	75-79
**1976-1980**	18.5	41.9	44.8	45.8	47.7	44.4	45.9	41.6	38.7	35.0	28.3	22.0	18.4
**1981-1985**	20.5	37.6	42.6	42.1	42.6	41.8	41.4	36.9	36.0	32.4	27.7	25.7	19.5
**1986-1990**	16.9	32.7	36.2	37.3	35.5	36.0	34.8	32.5	30.0	26.1	21.8	19.6	14.8
**1991-1995**	18.4	33.1	32.2	33.8	35.4	34.2	33.1	30.8	26.8	24.2	21.2	17.0	14.0
**1996-2000**	22.3	41.2	32.6	32.6	32.5	32.1	30.6	28.2	25.4	21.1	18.4	14.3	11.9
**2001-2005**	20.9	38.6	32.7	31.7	30.1	30.1	29.2	25.7	23.1	20.0	15.4	12.9	10.2
**2006-2010**	17.6	35.5	33.9	32.2	27.1	26.6	27.0	26.8	23.1	21.4	14.1	10.8	8.3
**2011-2015**	12.7	30.2	31.0	30.3	24.8	23.9	22.9	23.5	22.1	18.1	13.9	10.9	8.2
**2016-2020**	7.3	20.9	25.0	24.9	23.1	22.1	20.4	21.5	20.2	17.7	12.7	10.5	8.2

5.2 Current smoking prevalence (%), females

	Age group (years)												
	15-19	20-24	25-29	30-34	35-39	40-44	45-49	50-54	55-59	60-64	65-69	70-74	75-79
**1976-1980**	20.2	35.3	35.3	36.1	38.5	37.6	38.3	34.0	31.0	27.4	20.9	14.6	10.2
**1981-1985**	21.1	37.5	36.0	35.2	34.4	34.8	34.5	30.5	27.9	24.3	19.6	15.6	11.5
**1986-1990**	16.4	29.1	32.8	31.5	27.6	28.7	29.5	27.5	23.9	22.2	17.9	14.4	10.2
**1991-1995**	17.0	28.8	28.8	30.2	29.4	28.4	27.3	26.6	23.9	20.9	16.8	14.5	11.1
**1996-2000**	21.8	32.2	27.1	27.2	28.9	28.1	25.9	25.2	22.1	20.2	16.8	14.1	10.6
**2001-2005**	19.2	30.3	25.5	24.1	25.5	26.4	24.8	20.6	18.8	16.8	13.1	11.0	8.6
**2006-2010**	15.0	26.4	25.6	24.3	22.8	23.7	23.9	22.0	18.1	16.6	12.4	10.5	7.6
**2011-2015**	10.6	21.1	21.7	22.7	20.0	20.5	20.8	21.1	17.9	15.2	10.8	9.2	7.2
**2016-2020**	5.8	15.1	17.9	18.8	17.2	17.1	17.0	18.4	17.1	15.0	11.0	9.7	7.2

5.3 Former smoking prevalence (%), males

	Age group (years)												
	15-19	20-24	25-29	30-34	35-39	40-44	45-49	50-54	55-59	60-64	65-69	70-74	75-79
**1976-1980**	3.1	11.9	17.1	21.9	24.8	27.6	31.2	35.3	38.6	39.7	45.0	46.9	44.7
**1981-1985**	5.6	9.8	15.5	20.4	24.2	29.9	33.9	38.2	43.7	48.9	50.8	48.8	56.4
**1986-1990**	4.2	9.0	14.1	19.1	24.8	29.3	35.7	38.3	43.3	49.4	53.8	53.2	54.6
**1991-1995**	2.7	7.5	10.8	15.3	21.0	26.6	31.3	38.1	42.2	45.6	50.7	53.2	50.6
**1996-2000**	3.7	7.0	10.4	13.6	17.7	22.1	28.3	35.9	42.4	47.8	52.7	60.6	59.1
**2001-2005**	2.4	7.7	11.1	14.1	16.1	19.0	25.2	31.2	38.6	45.5	49.6	55.8	57.7
**2006-2010**	1.7	6.6	12.6	16.2	17.3	18.7	22.9	28.9	35.0	40.9	46.9	53.0	58.1
**2011-2015**	0.8	6.3	12.2	17.0	19.0	19.6	22.5	26.0	30.6	37.4	44.0	50.1	54.4
**2016-2020**	0.9	6.8	13.2	18.3	21.4	22.0	22.9	22.9	27.3	32.8	40.6	47.5	50.6

5.4 Former smoking prevalence (%), females

	Age group (years)											
	15-19	20-24	25-29	30-34	35-39	40-44	45-49	50-54	55-59	60-64	65-69	70-74	75-79
**1976-1980**	3.8	9.9	12.2	15.3	15.1	15.7	14.1	15.4	15.9	17.3	16.4	14.5	10.5
**1981-1985**	5.0	9.3	12.9	14.0	16.8	19.6	20.0	18.0	20.8	21.1	23.2	22.7	19.2
**1986-1990**	5.5	10.0	14.0	17.4	19.9	20.3	22.9	23.8	23.4	21.8	24.5	23.6	20.7
**1991-1995**	3.7	7.8	11.3	15.4	17.8	19.0	20.4	23.4	24.2	25.3	26.2	26.4	22.8
**1996-2000**	3.7	7.4	9.9	12.7	15.8	19.1	20.7	24.9	28.0	28.1	28.3	30.2	26.4
**2001-2005**	2.5	7.7	10.6	12.4	14.8	17.6	20.1	22.5	26.2	28.2	29.3	30.2	29.7
**2006-2010**	1.3	6.6	11.6	12.9	14.2	15.8	19.2	21.3	24.0	27.3	30.2	30.6	31.9
**2011-2015**	0.5	5.7	10.0	14.1	14.9	15.2	17.4	21.2	24.9	26.5	30.2	32.3	32.2
**2016-2020**	0.2	5.1	9.0	13.2	14.7	16.1	17.7	19.9	23.4	25.3	27.1	30.6	32.5
