# Supplementary material for: Estimating the reduction in US mortality if cigarettes were largely replaced by e-cigarettes
Source: Arch Toxicol. 2021 Oct 22;96(1):167–76. doi: 10.1007/s00204-021-03180-3 (PMC8748352; doi:10.1007/s00204-021-03180-3)
Supplement: Supplementary file 3 — Supplementary file3 (PDF 142 KB) [file 204_2021_3180_MOESM3_ESM.pdf]

Estimating the reduction in US mortality if cigarettes were largely replaced by e-cigarettes

Published in: Archives of Toxicology

Peter N Lee[1*], John S Fry[2], Stanley Gilliland III[3], Preston Campbell[3], Andrew R. Joyce[3]

[1]P N Lee Statistics and Computing Ltd, 17 Cedar Road, Sutton, Surrey, SM2 5DA

[2]RoeLee Statistics Ltd., 17 Cedar Road, Sutton, Surrey SM2 5DA

[3]Consilium Sciences, LLC, 7400 Beaufont Springs Drive, Suite 300, N. Chesterfield, VA 23325

[*] Author for correspondence

E-mail: Peterlee@pnlee.co.uk

ONLINE RESOURCE 3

Age of quitting

The data on age of quitting were based on NHIS data for 2006 (www.cdc.gov/nchs/nhis.htm) and are shown on the following page.

		Percentage quitting by year quit										
SEX	AGE	QTD <1	1	2	3-5	6-10	11-15	16-20	21-30	21-40	41-50	51+
Male	10-14	99.9	0.1	0	0	0	0	0	0	0	0	0
	15-19	69.4	30.6	0	0	0	0	0	0	0	0	0
	20-24	30.2	22.5	17.8	29.5	0	0	0	0	0	0	0
	25-29	27.3	14	16.2	21.1	21.4	0	0	0	0	0	0
	30-34	20.3	8	7.4	36.7	23.7	3.9	0	0	0	0	0
	35-39	10.2	8.2	3.1	18.5	29.6	13.8	16.6	0	0	0	0
	40-44	8.8	3.1	2	13.5	15.9	28	28.7	0	0	0	0
	45-49	7.2	2.5	3.8	10.6	17.5	14.6	21.2	22.6	0	0	0
	50-54	8.6	4.2	2.6	11	8.7	12.5	20.9	31.5	0	0	0
	55-59	7.7	2	4.5	12.9	17.4	10.8	11.4	22	11.3	0	0
	60-64	2.5	2.3	1.3	7.7	13	16.9	14	24.5	17.8	0	0
	65-69	1.8	2.9	1.7	8.7	12.9	10.4	17.9	20.9	17.2	5.6	0
	70-74	0.6	0.7	0.8	14.8	8.7	11.8	15.4	19.9	20.6	6.7	0
	75-79	1.8	2.6	2.3	2.8	6.3	18.4	11.3	24	17.4	13.1	0
Female	10-14	99.9	0.1	0	0	0	0	0	0	0	0	0
	15-19	60.2	39.8	0	0	0	0	0	0	0	0	0
	20-24	34.1	13	21	31.9	0	0	0	0	0	0	0
	25-29	17.3	9	10.9	31.1	31.7	0	0	0	0	0	0
	30-34	20.1	7.9	14.9	19.7	26.7	10.7	0	0	0	0	0
	35-39	10	6.7	5.9	20.3	24.2	18.5	14.4	0	0	0	0
	40-44	10.7	6.7	10	19.9	20.6	19.9	12.2	0	0	0	0
	45-49	9.1	4.3	3.5	11.1	20.2	13.1	19.8	19	0	0	0
	50-54	5.6	4.8	5	9.9	20.9	12.2	14	27.6	0	0	0
	55-59	4.5	2.3	3.3	8.4	11.2	11	17.9	32.8	8.6	0	0
	60-64	2.7	2.4	2.6	9.1	11.6	15.4	17.8	23.5	14.9	0	0

	65-69	4.1	1.5	1.7	7.9	12.8	8.7	13.8	26	19.1	4.4	0
	70-74	0.5	1.6	1.8	2.6	11.8	11.5	14.3	19.8	23.4	12.7	0
	75-79	1.2	1.6	0.3	3.1	6.3	11.5	16.8	23.6	21.1	14.5	0
