# Supplementary material for: Estimating the reduction in US mortality if cigarettes were largely replaced by e-cigarettes
Source: Arch Toxicol. 2021 Oct 22;96(1):167–76. doi: 10.1007/s00204-021-03180-3 (PMC8748352; doi:10.1007/s00204-021-03180-3)
Supplement: Supplementary file 4 — Supplementary file4 (PDF 435 KB) [file 204_2021_3180_MOESM4_ESM.pdf]

Estimating the reduction in US mortality if cigarettes were largely replaced by e-cigarettes

Published in: Archives of Toxicology

Peter N Lee[1*], John S Fry[2], Stanley Gilliland III[3], Preston Campbell[3], Andrew R. Joyce[3]

[1]P N Lee Statistics and Computing Ltd, 17 Cedar Road, Sutton, Surrey, SM2 5DA

[2]RoeLee Statistics Ltd., 17 Cedar Road, Sutton, Surrey SM2 5DA

[3]Consilium Sciences, LLC, 7400 Beaufont Springs Drive, Suite 300, N. Chesterfield, VA 23325

[*] Author for correspondence
E-mail: Peterlee@pnlee.co.uk

ONLINE RESOURCE 4

Methods of estimating transition probabilities and Supplemental Tables

Estimating transition probabilities in the Null Scenario

Initiation and quitting rates were derived from data on the distribution of smoking habits for a given five-year age group, and for the same birth cohort five years later when they are five years older (see Online Resource 2). Defining the proportions of never, current and former smokers as N_O, C_O and F_O at baseline, and as N_5, C_5 and F_5 five years later, and ignoring mortality, immigration and emigration, and assuming that re-initiation rates are zero, five-year rates of initiation (S) and quitting (Q) can then be derived from the formulae

$$N_5 = N_0(1 - S) \quad (1)$$

and

$$C_5 = N_0 S + C_0(1 - Q) \quad (2)$$

After rearranging and substituting terms, these equations yield five-year rates of initiation as

$$S = (N_0 - N_5)/N_0 \quad (3)$$

and five-year rates of quitting as

$$Q = (N_0 - N_5 + C_0 - C_5)/C_0 \quad (4)$$

One-year rates of initiation (S_1) and quitting (Q_1) can then be estimated, assuming that they do not change within the 5 year period. Given the recurrence relationships between the proportions of never, current and former smokers at successive years:

$$N_j = N_{j-1}(1 - S_1) \quad (5)$$

$$C_j = N_{j-1}S_1 + C_{j-1}(1 - Q_1) \quad (6)$$

$$F_j = C_{j-1}Q_1 \quad (7)$$

it is clear that

$$N_5 = N_0(1 - S_1)^5 \quad (8)$$

and

$$S_1 = 1 - (1 - S)^{0.2} \quad (9)$$

Estimating Q_1 is more difficult, since using the recurrence relationship to express N_5, C_5 and F_5 in terms of N_0, C_0, F_0, S and Q, gives a non-linear equation with no direct solution. However, it can be readily solved by numerical methods. In this process, it should be noted that zeros in the 10-14 age group were replaced by 0.5 for current smoking and 0.1 for former smoking, that initiation was not allowed above the age of 35, that if the percentage of never smokers increased over a five-year period, the previous initiation rate was halved, and that initiation and quitting rates for periods from 2016-2020 to 2036-2040 were assumed, in the absence of available data, to be the same as for 2011-2015. The derived initiation and quitting rates are shown below in Table S4.1.

Estimating transition probabilities in the Alternative Scenarios

Only five TPs are relevant to any Alternative Scenario, since the methodology of Levy, which our PHIM-based approach attempts to match, does not consider re-initiation, dual use, or switching from e-cigarettes to cigarettes.

For quitting cigarettes, the TPs are identical to those in the Null Scenario, while for quitting from e-cigarettes the TPs are the multiples, Q, of those in the Null Scenario relating to cigarettes.

In the Main Scenario, the TPs for initiating cigarettes for the periods 1, 2-6, 7-11 and for the three periods for 12+ years follow-up were set to equal, respectively, 50%, 40%, 20% and 5% of those in the Null Scenario, while the TPs for initiating e-cigarettes were set as 50%, 60%, 80% and 95% of those in the Null Scenario. This preserved the same overall initiation rate as that in the Null Scenario, while reflecting the increasing usage of e-cigarettes during follow-up. Compared to the TPs in the Main Scenario, the TPs in the other Alternative Scenarios were identical for initiating cigarettes, but those for initiating e-cigarettes were multiplied by the relevant I factor.

Following Levy, switching rates from e-cigarettes to cigarettes were set to zero. Although Levy allows the major switch from cigarettes to e-cigarettes to be in the first 10 years of follow-up, the PHIM modelling allowed this to be in the first 11 years of follow-up, to be consistent with the periods considered for the Null Scenario, which were 1, 2-6, 7-11, 12-16, 17-21 and 22+ years from baseline. In Levy's Optimistic Scenario, the proportion of smokers (CS) in the population was reduced after 10 years to 5% (X = 5%). To achieve this, we set age specific switching rates from smoking to vaping such that the resulting prevalence at 10 years was around 5% for each 5-year age group (a). To find these switching rates a simplified prevalence model was implemented whereby for each 5-year age group *a* and year *t*:

$$\text{Prev\_CS}(t+1) = \text{Prev\_CS}(t) \times (1 - \text{P\_CS\_Ex}(t)) \times (1 - \text{P\_CS\_EC})) + \text{Prev\_NS}(t) \times \text{P\_I\_CS}(t) \qquad (10)$$

where Prev_CS(t) is the prevalence of current smoking at year *t*, and initial value Prev_CS(0) is the starting prevalence in 1990. Prev_NS(t) is the prevalence of non-smoking in 1990 for age group *a* where $t < 5$, and for age group $a + 1$ where $t \geq 5$. P_CS_Ex(t) is the probability of quitting cigarettes in 1990 for age group a where $t < 5$, and for age group $a + 1$ where $t \geq 5$. P_I_CS(t) is the probability of initiating cigarettes at year *t* for age group *a* where $t < 5$ and for age group $a + 1$ for $t \geq 5$. P_CS_EC is the probability of switching from cigarettes to e-cigarettes and assumed to be constant over *t*. This value can then be found by using the generalized reduced gradient non-linear method in the Excel Solver function to solve Prev_CS(10) = 5%.

In practice we looked at the prevalence values predicted by PHIM at 10 years and altered the solver target value to ensure the final prevalence was indeed around 5%. The same approach was used to determine switching probabilities for the pessimistic scenario where X = 10% and for alternative scenarios where X has values 0.01%, 2.5%, 7.5% and 15%, respectively.

The resulting switching probabilities used and 10-year smoking prevalences are documented in the detailed results presented in Online Resource 7.

Table S4.1 Annual initiation and quitting rates (per million) in Null Scenario by sex, age and period

		1980		1991-1995		1996-2000	
Sex	Age	Initiation	Quitting	Initiation	Quitting	Initiation	Quitting
**Males**	10-14	3842.1	8741.6	4906.0	8741.6	4311.5	8741.6
	15-19	4720.3	3614.2	6988.6	4426.4	5329.9	3289.3
	20-24	375.8	943.2	687.1	1527.0	2455.8	3843.6
	25-29	186.8	1142.7	962.5	1515.0	839.2	2005.8
	30-34	93.2	871.0	364.1	1226.8	414.4	1328.9
	35-39	0.0	866.9	0.0	1629.6	0.0	1277.8
	40-44	0.0	1398.8	0.0	1852.0	0.0	1691.2
	45-49	0.0	2033.0	0.0	2666.6	0.0	2904.3
	50-54	0.0	3208.8	0.0	3207.6	0.0	3319.3
	55-59	0.0	3574.3	0.0	3977.5	0.0	3975.7
	60-64	0.0	3459.6	0.0	5771.2	0.0	5234.7
	65-69	0.0	4136.4	0.0	10431.8	0.0	5901.2
	70-74	0.0	5592.2	0.0	7079.3	0.0	5615.4
	75-79	0.0	5592.2	0.0	7079.3	0.0	5615.4
**Females**	10-14	3758.2	8741.6	4794.4	8741.6	3968.8	8741.6
	15-19	3469.4	2515.3	4527.2	4082.5	3056.4	3372.8
	20-24	275.9	761.4	105.5	1261.1	1459.3	3880.6
	25-29	137.4	1375.5	52.7	952.2	713.9	1953.5
	30-34	68.6	1149.2	26.3	733.1	111.3	1338.3
	35-39	0.0	991.2	0.0	753.5	0.0	1506.8
	40-44	0.0	833.2	0.0	1534.6	0.0	2079.9
	45-49	0.0	1723.2	0.0	2997.6	0.0	3808.6
	50-54	0.0	2335.7	0.0	3159.5	0.0	4871.2
	55-59	0.0	2233.0	0.0	2964.7	0.0	4559.6
	60-64	0.0	4634.5	0.0	3632.9	0.0	7191.9
	65-69	0.0	3504.7	0.0	4522.0	0.0	7033.2
	70-74	0.0	4328.7	0.0	5208.0	0.0	8206.4

	75-79	0.0	4328.7	0.0	5208.0	0.0	8206.4

		1991-2005		2006-2010		2011 onwards	
Sex	Age	Initiation	Quitting	Initiation	Quitting	Initiation	Quitting
**Males**	10-14	3467.5	8741.6	2314.1	8741.6	1324.8	8741.6
	15-19	4675.4	3732.0	3987.0	5036.4	2984.2	10601.6
	20-24	62.2	2260.0	319.6	2857.1	452.2	4313.8
	25-29	1422.2	2822.0	251.1	2314.4	224.6	3645.3
	30-34	696.2	2609.6	125.1	4342.6	111.9	4511.7
	35-39	0.0	2058.1	0.0	2092.1	0.0	2146.6
	40-44	0.0	2310.1	0.0	2565.9	0.0	2635.6
	45-49	0.0	2255.6	0.0	2311.3	0.0	1050.8
	50-54	0.0	2663.2	0.0	3208.6	0.0	2518.8
	55-59	0.0	1746.5	0.0	4057.1	0.0	3693.4
	60-64	0.0	5809.0	0.0	7165.9	0.0	5887.8
	65-69	0.0	5896.2	0.0	4281.0	0.0	4823.0
	70-74	0.0	7322.6	0.0	4579.7	0.0	4732.6
	75-79	0.0	7322.6	0.0	4579.7	0.0	4732.6
**Females**	10-14	2861.1	8741.6	1858.9	8741.6	930.5	8741.6
	15-19	2594.2	3995.6	2231.6	5769.9	1798.2	9440.1
	20-24	1247.5	2805.3	1079.0	3262.2	22.8	2830.6
	25-29	289.4	1574.2	530.9	2001.8	73.4	2655.5
	30-34	131.7	1292.9	263.4	3240.5	36.6	4613.6
	35-39	0.0	1219.3	0.0	1770.7	0.0	2607.5
	40-44	0.0	1656.7	0.0	2173.0	0.0	3115.3
	45-49	0.0	1994.7	0.0	2074.6	0.0	2131.9
	50-54	0.0	2154.0	0.0	3431.5	0.0	3497.1
	55-59	0.0	2072.1	0.0	2906.0	0.0	2941.5
	60-64	0.0	5048.6	0.0	7138.7	0.0	5375.5
	65-69	0.0	3680.5	0.0	4962.5	0.0	1788.7
	70-74	0.0	6143.5	0.0	6268.5	0.0	4077.0
	75-79	0.0	6143.5	0.0	6268.5	0.0	4077.0

Table S4.2 Assumed relative risks for continued cigarette smoking and quitting half-lives for the four diseases

		Disease			
	Age	Lung cancer	IHD	Stroke	COPD
Relative risks	Any	11.68			4.56
	to 54		3.38	2.48	
	55-64		2.32	2.13	
	65-74		1.70	1.39	
	75-79		1.27	1.06	
Half-life	Any			4.78	13.32
	to 49	6.98	1.47		
	50-59	10.39	5.32		
	60-69	10.60	7.48		
	70-79	12.99	13.77		

These estimates are derived from meta-analyses of published data as described in Lee et al. (2017).

Table S4.3 Distribution of product use by year in the Null, Main, Pessimistic and Sensitivity Scenarios

Parameter		Current cigarette smokers			Current e-cigarette users			Former users		
varied	Value	10	25	50	10	25	50	10	25	50
	**Males**									
	Null Scenario	27.11	18.27	7.88	0.00	0.00	0.00	31.41	38.46	41.43
X	0.01	0.06	0.00	0.00	27.24	18.05	7.52	31.06	38.42	41.29
	2.5	2.65	0.06	0.00	24.57	18.04	7.49	31.15	38.37	41.32
	5 M	5.15	0.36	0.00	22.03	17.67	7.48	31.19	38.43	41.33
	7.5	7.55	0.96	0.03	19.67	17.14	7.49	31.15	38.37	41.29
	10 P	10.22	1.97	0.12	16.93	16.03	7.36	31.21	38.47	41.33
	15	14.49	4.83	0.71	12.58	13.13	6.74	31.29	38.50	41.36
Q	0.5 P	5.15	0.36	0.00	24.12	24.31	16.75	29.10	31.79	32.05
	0.75	5.15	0.36	0.00	23.03	20.74	11.20	30.18	35.37	37.60
	1 M	5.15	0.36	0.00	22.03	17.67	7.48	31.19	38.43	41.33
	1.25	5.15	0.36	0.00	21.11	15.21	5.17	32.10	40.89	43.63
	1.5	5.15	0.36	0.00	20.28	13.10	3.52	32.94	43.00	45.29
I	0.5	5.21	0.37	0.00	20.42	16.28	6.43	30.81	37.16	36.86
	0.75	5.18	0.36	0.00	21.26	17.00	6.97	31.02	37.84	39.22
	1 M	5.15	0.36	0.00	22.03	17.67	7.48	31.19	38.43	41.33
	1.25	5.11	0.36	0.00	22.78	18.34	8.05	31.36	38.99	43.27
	1.5 P	5.09	0.35	0.00	23.45	18.90	8.53	31.52	39.53	45.06
All four in combination	As P	10.14	1.95	0.12	19.94	23.16	18.29	29.98	33.67	35.18

	**Females**									
	Null Scenario	21.95	15.15	7.00	0.00	0.00	0.00	23.06	30.61	34.19
X	0.01	0.06	0.00	0.00	22.07	15.49	7.04	23.01	30.42	34.48
	2.5	2.64	0.09	0.00	19.45	15.44	7.12	23.06	30.39	34.40
	5 M	5.00	0.45	0.01	17.02	15.09	7.10	23.12	30.37	34.40
	7.5	7.38	1.19	0.05	14.68	14.40	7.06	23.07	30.32	34.41
	10 P	9.91	2.54	0.24	12.09	12.98	6.90	23.14	30.39	34.38
	15	13.81	5.71	1.26	8.18	9.81	5.76	23.16	30.39	34.50
Q	0.5 P	5.00	0.45	0.01	18.69	20.13	15.13	21.45	25.33	26.37
	0.75	5.00	0.45	0.01	17.84	17.39	10.37	22.30	28.07	31.13
	1 M	5.00	0.45	0.01	17.02	15.09	7.10	23.12	30.37	34.40
	1.25	5.00	0.45	0.01	16.27	13.07	4.93	23.87	32.39	36.57
	1.5	5.00	0.45	0.01	15.59	11.33	3.39	24.55	34.13	38.12
I	0.5	5.03	0.45	0.01	15.95	14.10	6.32	22.87	29.52	31.22
	0.75	5.01	0.45	0.01	16.49	14.61	6.73	22.99	29.96	32.86
	1 M	5.00	0.45	0.01	17.02	15.09	7.10	23.12	30.37	34.40
	1.25	4.99	0.45	0.01	17.47	15.50	7.43	23.25	30.74	35.73
	1.5 P	4.97	0.45	0.01	17.92	15.86	7.75	23.37	31.14	37.07
All four in combination	As P	9.87	2.54	0.24	14.14	18.06	15.70	22.25	26.86	28.90

X percentage of cigarette smokers after 10 years
Q TP for quitting e-cigarettes relative to that for quitting cigarettes in the Null Scenario
I TP for initiating e-cigarettes relative to that for initiating cigarettes in the Main Scenario
M = Main Scenario
P = Pessimistic Scenario

In all Scenarios at baseline, the prevalence of current cigarette smoking was 31.90% in males and 26.14% in females, and that of former cigarette smoking was 28.33% in males and 18.95% in females, with no current e-cigarette users

Table S4.4 Drops in deaths (hundreds) and differences vs. Main Scenario by sex for entire follow-up

Parameter		Males		Females		Combined	
varied	Value	Drop	Difference	Drop	Difference	Drop	Difference
F	0	16528	+1281	10854	+851	27381	+2131
	0.05 M	15247	0	10003	0	25250	0
	0.1	14053	-1194	9208	-795	23262	-1988
	0.2	11880	-3367	7762	-2241	19642	-5608
	0.3	9936	-5311	6468	-3535	16404	-8846
	0.4 P	8173	-7074	5297	-4706	13470	-11780
X	0.01	19196	+3949	13003	+3000	32199	+6949
	2.5	16703	+1456	11106	+1103	27809	+2559
	5 M	15247	0	10003	0	25250	0
	7.5	13812	-1435	8753	-1250	22565	-2685
	10 P	12045	-3202	7230	-2773	19275	-5975
	15	8646	-6601	4470	-5533	13116	-12134
Q	0.5 P	14849	-398	9748	-255	24598	-652
	0.75	15064	-183	9888	-115	24952	-298
	1 M	15247	0	10003	0	25250	0
	1.25	15392	+145	10099	+96	25491	+241
	1.5	15513	+266	10180	+177	25694	+444
I	0.5	15298	+51	10025	+22	25323	+73
	0.75	15272	+25	10013	+10	25285	+35
	1 M	15247	0	10003	0	25250	0
	1.25	15223	-24	9993	-10	25217	-33
	1.5 P	15203	-44	9985	-18	25188	-62
All four in combination	As P	4723	-10524	2867	-7136	7591	-17659

Summation of individual reductions			-10718		-7752		-18469

F effective dose for e-cigarettes compared to cigarettes
X percentage of cigarette smokers after 10 years
Q TP for quitting e-cigarettes relative to that for quitting cigarettes in the Null Scenario
I TP for initiating e-cigarettes relative to that for initiating cigarettes in the Main Scenario
M = Main Scenario
P = Pessimistic Scenario

Table S4.5 Drops in deaths (hundreds) and differences vs. Main Scenario by cause for entire follow-up

Parameter		LC		IHD		Stroke		COPD	
varied	Value	Drop	Difference	Drop	Difference	Drop	Difference	Drop	Difference
F	0	10650	1059	8345	+520	1421	+82	6966	+471
	0.05 M	9591	0	7825	0	1339	0	6495	0
	0.1	8643	-948	7320	-505	1258	-81	6042	-453
	0.2	7010	-2581	6348	-1477	1100	-239	5184	-1311
	0.3	5649	-3942	5425	-2400	947	-392	4383	-2112
	0.4 P	4491	-5100	4546	-3279	799	-540	3634	-2861
X	0.01	12730	3139	9734	+1909	1665	+326	8070	+1575
	2.5	10745	1154	8528	+703	1457	+118	7078	+583
	5 M	9591	0	7825	0	1339	0	6495	0
	7.5	8382	-1209	7115	-710	1216	-123	5852	-643
	10 P	6931	-2660	6247	-1578	1065	-274	5032	-1463
	15	4359	-5232	4564	-3261	771	-568	3422	-3073
Q	0.5 P	9266	-325	7680	-145	1315	-24	6337	-158
	0.75	9442	-149	7759	-66	1328	-11	6424	-71
	1 M	9591	0	7825	0	1339	0	6495	0
	1.25	9712	121	7880	+55	1348	+9	6552	+57
	1.5	9813	222	7926	+101	1355	+16	6599	+104
	0.5	9626	35	7848	+23	1342	+3	6507	+12
I	0.75	9608	17	7836	+11	1340	+1	6501	+6
	1 M	9591	0	7825	0	1339	0	6495	0
	1.25	9575	-16	7815	-10	1337	-2	6490	-5
	1.5 P	9561	-30	7806	-19	1336	-3	6485	-10
All four in combination	As P	2372	-7219	2707	-5118	472	-867	2039	-4456

Summation of individual reductions			-8115		-5021		-841		-4492

F effective dose for e-cigarettes compared to cigarettes
X percentage of cigarette smokers after 10 years
Q TP for quitting e-cigarettes relative to that for quitting cigarettes in the Null Scenario
I TP for initiating e-cigarettes relative to that for initiating cigarettes in the Main Scenario
M = Main Scenario
P = Pessimistic Scenario

Table S4.6 Overall years of life saved (millions) by sex for the four causes combined

Parameter varied	Value	Males	Females	Combined
F	0	18.72	9.77	28.49
	0.05 M	17.25	8.98	26.23
	0.1	15.88	8.24	24.13
	0.2	13.40	6.92	20.32
	0.3	11.20	5.74	16.94
	0.4 P	9.20	4.68	13.88
X	0.01	21.55	11.61	33.16
	2.5	18.79	9.91	28.70
	5 M	17.25	8.98	26.23
	7.5	15.79	8.00	23.79
	10 P	14.07	6.82	20.89
	15	10.80	4.62	15.42
Q	0.5 P	16.87	8.78	25.65
	0.75	17.07	8.89	25.96
	1 M	17.25	8.98	26.23
	1.25	17.39	9.05	26.44
	1.5	17.52	9.12	26.63
I	0.5	17.34	9.02	26.36
	0.75	17.29	9.00	26.29
	1 M	17.25	8.98	26.23
	1.25	17.21	8.96	26.16
	1.5 P	17.17	8.94	26.11
All four in combination	As P	5.59	2.71	8.31

F effective dose for e-cigarettes compared to cigarettes

X percentage of cigarette smokers after 10 years
Q TP for quitting e-cigarettes relative to that for quitting cigarettes in the Null Scenario
I TP for initiating e-cigarettes relative to that for initiating cigarettes in the Main Scenario
M = Main Scenario
P = Pessimistic Scenario

References

Lee PN, Fry JS, Hamling JF, Sponsiello-Wang Z, Baker G, Weitkunat R (2017) Estimating the effect of differing assumptions on the population health impact of introducing a Reduced Risk Tobacco Product in the USA. Regul Toxicol Pharmacol 88:192-213 doi:10.1016/j.yrtph.2017.06.009
