# Supplementary material for: Estimating the reduction in US mortality if cigarettes were largely replaced by e-cigarettes
Source: Arch Toxicol. 2021 Oct 22;96(1):167–76. doi: 10.1007/s00204-021-03180-3 (PMC8748352; doi:10.1007/s00204-021-03180-3)
Supplement: Supplementary file 5 — Supplementary file5 (PDF 551 KB) [file 204_2021_3180_MOESM5_ESM.pdf]

Estimating the reduction in US mortality if cigarettes were largely replaced by e-cigarettes

Published in: Archives of Toxicology

Peter N Lee[1*], John S Fry[2], Stanley Gilliland III[3], Preston Campbell[3], Andrew R. Joyce[3]

[1]P N Lee Statistics and Computing Ltd, 17 Cedar Road, Sutton, Surrey, SM2 5DA

[2]RoeLee Statistics Ltd., 17 Cedar Road, Sutton, Surrey SM2 5DA

[3]Consilium Sciences, LLC, 7400 Beaufont Springs Drive, Suite 300, N. Chesterfield, VA 23325

[*] Author for correspondence

E-mail: Peterlee@pnlee.co.uk

ONLINE RESOURCE 5

Numbers of deaths

Numbers of deaths by sex and the same five year age groups for LC, COPD, IHD and stroke were downloaded from the WHO website (https://www.who.int/data/data-collection-tools/who-mortality-database) on April 20, 2020 for the years 1966 to 2017 and are shown in the following pages. Results are shown first for males, then for females. Within each sex, results are then shown, successively, for the periods 1966-1978, 1979-1991, 1992-2004 and 2005-2017. Within each period, results are shown for COPD, IHD, lung cancer and stroke in turn, with numbers of deaths given by 5-year age group from 10-14 to 75-79.

**COPD**	1966	1967	1968	1969	1970	1971	1972	1973	1974	1975	1976	1977	1978
**Male**													
10-14	28	36	72	63	68	56	66	67	47	85	49	63	53
15-19	42	50	96	111	117	141	124	80	112	121	68	78	81
20-24	49	45	96	115	132	114	144	125	127	110	104	108	106
25-29	46	53	112	99	94	100	144	114	98	113	104	96	91
30-34	82	70	115	107	130	127	128	86	104	165	105	96	107
35-39	154	151	204	197	209	179	172	137	151	280	135	128	155
40-44	311	298	424	423	379	354	314	310	279	615	260	244	216
45-49	664	668	824	767	748	793	714	682	629	1361	611	558	531
50-54	1290	1272	1609	1520	1523	1493	1510	1502	1454	2615	1341	1260	1238
55-59	2510	2467	3039	2870	2854	2773	2860	2803	2633	4634	2644	2552	2504
60-64	3919	4022	4778	4511	4640	4680	4834	4859	4636	6726	4653	4370	4575
65-69	5011	5050	6028	5814	6006	6084	6384	6585	6536	7494	6860	6784	7204
70-74	5480	5800	6624	6322	6496	6586	7078	7316	7266	6857	7773	7871	8257
75-79	4579	4796	5691	5471	5806	6025	6446	6620	6540	85	7079	7144	7657

**IHD**	1966	1967	1968	1969	1970	1971	1972	1973	1974	1975	1976	1977	1978
**Male**													
10-14	27	25	7	10	7	16	8	13	8	9	6	5	5
15-19	67	74	33	39	29	52	32	41	33	31	27	29	23
20-24	136	114	87	100	115	113	120	98	79	92	66	74	75
25-29	289	302	262	274	307	287	270	268	245	270	284	265	280
30-34	970	891	855	886	847	903	804	830	844	811	829	844	827
35-39	3087	3027	2932	2838	2674	2652	2432	2396	2194	2178	2136	2156	2199
40-44	7667	7764	7695	7406	7092	6846	6600	6317	5844	5631	5416	5109	4868
45-49	14577	14663	15114	14883	14437	14101	14012	13605	13016	11997	11529	10837	10304
50-54	24131	23813	24094	23801	23416	23376	23602	23069	22293	21558	20624	19978	18965
55-59	34753	34259	35581	34906	34546	34198	33430	33855	31781	30688	30147	29562	29224
60-64	43349	43494	46008	45288	44993	45182	46360	45332	43487	42101	41587	40083	39697
65-69	51259	50400	53227	52811	52620	52087	53236	53107	52155	50874	50590	49652	48819
70-74	57487	56629	59497	57856	56664	56264	56808	56507	55157	53091	52846	53119	53542
75-79	54651	54439	58423	57289	56650	57178	57992	56191	53133	51432	51609	50744	50871

**Lung cancer**	1966	1967	1968	1969	1970	1971	1972	1973	1974	1975	1976	1977	1978
**Male**													
10-14	1	1	3	6	2	9	6	5	1	0	0	1	4
15-19	6	7	8	6	7	6	10	11	6	6	9	5	4
20-24	17	11	14	14	20	20	10	12	11	14	17	12	10
25-29	30	21	35	26	37	33	26	29	27	35	27	41	29
30-34	144	128	123	113	125	130	104	112	117	128	116	130	130
35-39	429	454	447	453	426	435	428	483	423	389	381	408	399
40-44	1178	1197	1283	1354	1377	1273	1280	1172	1216	1168	1163	1102	1087
45-49	2321	2398	2633	2681	2769	2863	2998	2972	3014	2958	2949	2881	2749
50-54	4167	4316	4536	4641	4789	4793	5068	5224	5506	5555	5587	5719	5889
55-59	6044	6305	6771	7083	7452	7397	7736	7755	7958	7952	8355	8514	9013
60-64	7707	7940	8641	8874	9226	9348	9994	10325	10753	10936	11171	11371	11790
65-69	7884	8274	8699	9155	9483	10141	10524	10958	11309	11878	12286	12856	13241
70-74	6657	6960	7694	7726	7960	8372	8900	9185	9546	10158	10641	11371	12005
75-79	4184	4629	4922	5119	5471	5952	6298	6329	6713	6813	7375	7876	7933

**Stroke**	1966	1967	1968	1969	1970	1971	1972	1973	1974	1975	1976	1977	1978
**Male**													
10-14	92	85	89	82	98	71	86	74	88	59	73	52	69
15-19	128	95	123	137	131	148	160	118	137	135	111	94	100
20-24	133	121	167	125	177	198	146	153	150	175	170	156	145
25-29	181	186	217	247	214	208	222	201	209	212	228	212	180
30-34	311	311	341	320	318	344	338	345	294	298	327	312	263
35-39	625	615	628	616	581	573	554	535	513	449	464	434	444
40-44	1209	1120	1252	1167	1184	1054	1050	998	959	837	807	767	761
45-49	1862	1922	2002	1934	1954	1978	1834	1741	1638	1434	1350	1299	1203
50-54	3254	3128	3264	3106	3019	3088	3074	2954	2714	2453	2454	2179	2014
55-59	4888	4810	4996	4787	4816	4711	4830	4396	4156	3920	3641	3500	3258
60-64	7202	7019	7386	7216	7376	7247	7334	7062	6635	6167	5751	5420	5087
65-69	10742	10404	10841	10678	10492	10321	10384	10484	10024	9359	8835	8288	7921
70-74	14983	14643	15082	14452	13946	13974	14320	13956	13536	12537	11804	11377	10903

75-79	17608	17264	17828	17178	17076	17295	17096	16850	15794	14832	14050	13554	12851

**COPD**	1979	1980	1981	1982	1983	1984	1985	1986	1987	1988	1989	1990	1991
**Male**													
10-14	23	30	36	23	37	43	34	43	59	39	43	47	52
15-19	30	34	40	54	49	35	37	60	60	42	49	43	59
20-24	37	44	48	66	44	55	54	54	57	57	47	48	58
25-29	43	49	59	50	56	54	50	64	60	70	67	70	77
30-34	49	57	54	74	60	51	68	55	79	87	102	85	97
35-39	72	70	74	92	92	92	106	107	127	124	128	120	125
40-44	148	144	146	132	143	161	151	183	206	205	206	202	232
45-49	364	385	347	306	338	325	345	370	307	378	340	401	411
50-54	926	945	957	896	883	874	886	849	789	824	801	752	754
55-59	1989	2127	2132	2029	2203	2145	2097	2042	1913	1946	1999	1859	1770
60-64	3776	3958	3981	3819	4040	4168	4213	4157	4213	4217	3998	3973	3939
65-69	5869	6346	6359	6202	6537	6397	6806	6747	6594	6931	6797	6899	6905
70-74	7208	7842	8182	8036	8635	8874	9256	9390	9072	9429	8965	9233	9330
75-79	6520	7421	7727	7956	8733	8927	9499	9742	9870	10093	10137	10469	10529

**IHD**	1979	1980	1981	1982	1983	1984	1985	1986	1987	1988	1989	1990	1991
**Male**													
10-14	7	2	6	7	6	3	5	9	3	6	5	9	3
15-19	26	29	17	23	25	20	21	24	22	21	17	16	26
20-24	71	85	81	71	52	77	73	57	71	76	58	62	62
25-29	256	229	250	314	239	237	265	249	226	218	206	202	224
30-34	792	738	737	746	757	699	735	747	715	681	663	611	595
35-39	1960	1920	1980	1963	1934	1941	1993	2025	1837	1789	1636	1598	1668
40-44	4198	4201	4150	4090	4037	4024	4058	4033	3964	3719	3681	3671	3728
45-49	8743	8292	8125	7605	7203	7048	6812	6298	6324	6246	6247	6112	6135
50-54	16192	15626	14753	14164	13295	12372	11810	10864	10344	9561	9384	9031	8888
55-59	25616	25306	24159	23093	22100	20787	19981	18335	17048	15986	15123	14423	13854
60-64	34559	33686	33218	32797	31810	31155	29849	28295	26664	25310	24153	22952	21933
65-69	42934	43480	42133	41620	40458	38510	37880	36374	35349	34813	33698	32367	31433

70-74	47509	48104	47529	47233	47227	45409	45119	43185	42001	41203	39233	38677	38374
75-79	45047	45950	45190	45860	46182	45654	45296	44089	43460	43514	42626	42042	41436
**Lung cancer**	1979	1980	1981	1982	1983	1984	1985	1986	1987	1988	1989	1990	1991
**Male**													
10-14	1	3	0	2	2	3	2	0	0	0	2	2	2
15-19	5	3	5	3	3	3	4	6	4	6	8	4	6
20-24	9	15	13	12	15	13	9	16	9	7	7	11	8
25-29	35	38	29	31	30	43	41	38	39	25	30	38	28
30-34	113	108	114	119	99	97	112	122	139	140	111	154	113
35-39	369	397	421	409	410	392	412	351	407	408	412	425	420
40-44	1118	1099	1106	1025	1098	1085	1148	1143	1085	1158	1088	1151	1123
45-49	2702	2670	2617	2559	2437	2471	2433	2397	2508	2466	2555	2573	2573
50-54	5733	5696	5703	5474	5227	5099	4933	4928	4887	4871	4820	4733	4625
55-59	9079	9361	9408	9329	9433	9622	9424	9131	9064	8747	8531	8458	8128
60-64	12104	12323	12381	13054	12869	13433	13789	13762	13828	13856	13634	13736	13363
65-69	13782	14227	14565	14908	14993	15219	15536	15863	16406	16526	16813	17295	17380
70-74	12200	13062	13240	13893	14386	14825	15007	15294	15954	15963	16361	16736	17019
75-79	8520	9184	9431	10119	10547	10995	11464	11903	12240	12649	13007	13561	13718

**Stroke**	1979	1980	1981	1982	1983	1984	1985	1986	1987	1988	1989	1990	1991
**Male**													
10-14	33	24	22	40	18	31	24	23	20	20	33	18	28
15-19	62	79	57	63	61	57	58	43	36	45	32	45	37
20-24	136	161	130	89	115	118	92	99	84	99	83	83	68
25-29	202	189	193	197	190	186	171	176	180	174	159	155	126
30-34	287	293	318	290	280	280	296	314	309	347	299	300	261
35-39	460	418	463	438	457	467	480	533	529	523	531	503	536
40-44	706	677	650	636	611	713	675	729	744	789	741	757	839
45-49	1150	1048	1067	1050	1073	1046	992	961	954	1082	1029	1086	1100
50-54	1996	1952	1822	1709	1576	1631	1539	1441	1506	1388	1397	1419	1454
55-59	3161	2963	2872	2781	2664	2573	2559	2365	2352	2291	2118	2117	2024
60-64	4815	4623	4467	4167	4207	4091	4036	3898	3832	3827	3586	3294	3213

65-69	7329	7208	6844	6542	6214	6116	5940	5774	5631	5783	5545	5493	5426
70-74	10462	10306	9859	9450	9098	8934	8843	8373	8300	8241	7758	7698	7715
75-79	12339	12488	11694	11415	11371	10790	11033	10526	10578	10752	10380	10195	10051

**COPD**	1992	1993	1994	1995	1996	1997	1998	1999	2000	2001	2002	2003	2004
**Male**													
10-14	35	56	56	69	61	41	62	64	53	37	50	49	44
15-19	51	56	75	69	64	67	56	60	58	43	68	50	55
20-24	55	72	68	64	67	68	93	63	62	56	52	65	55
25-29	61	62	82	74	76	79	87	69	72	55	70	67	64
30-34	97	74	106	117	84	111	89	85	93	91	95	89	88
35-39	134	132	151	157	143	150	169	143	145	171	159	143	132
40-44	224	223	221	210	258	265	259	268	292	287	338	287	300
45-49	377	436	446	464	484	495	467	547	618	591	617	682	613
50-54	792	892	907	897	929	948	913	1006	1045	1111	1181	1146	1193
55-59	1836	1828	1824	1766	1790	1770	1827	2169	2072	2160	2142	2252	2156
60-64	3746	3943	3753	3546	3512	3477	3530	3790	3494	3545	3643	3994	3879
65-69	6788	7111	6866	6613	6528	6656	6458	6645	6151	6050	5843	6002	5847
70-74	9441	10054	9881	9809	9506	9987	10139	10839	10063	9835	9506	9336	8663
75-79	10507	11220	10919	11090	11348	11408	11824	13024	12310	12440	12459	12274	11740

**IHD**	1992	1993	1994	1995	1996	1997	1998	1999	2000	2001	2002	2003	2004
**Male**													
10-14	6	4	5	3	10	7	7	7	4	11	12	6	5
15-19	11	31	12	26	33	27	33	36	21	24	18	21	20
20-24	58	60	60	57	50	60	48	57	60	95	74	86	76
25-29	197	198	178	181	170	172	170	169	181	211	191	197	206
30-34	626	632	582	610	601	557	488	570	523	619	550	616	616
35-39	1634	1642	1747	1715	1698	1560	1585	1801	1665	1667	1670	1630	1467
40-44	3740	3707	3537	3694	3508	3443	3452	4169	4122	4045	4170	4049	3899
45-49	6403	6394	6489	6588	6696	6466	6155	7447	7601	7323	7582	7661	7442
50-54	8988	9285	9450	9733	9450	9663	9387	11614	11548	11818	11969	11820	11544
55-59	13303	13068	12859	12673	12360	12445	12329	15021	15154	14794	15350	15342	15004

60-64	20911	20472	19424	18723	18483	17277	16587	19102	18641	18212	18306	18425	17864
65-69	30125	30124	28517	27591	26324	25093	23564	26184	24225	23181	22573	21547	20392
70-74	37923	37717	37301	36681	35408	33790	32762	36003	34329	31891	30563	28452	25881
75-79	40895	41321	39960	40075	40347	39676	39098	44041	42452	40894	39154	37191	34272
**Lung cancer**	1992	1993	1994	1995	1996	1997	1998	1999	2000	2001	2002	2003	2004
**Male**													
10-14	1	1	0	3	2	1	0	2	0	0	2	1	2
15-19	2	6	3	8	0	3	3	5	3	2	2	4	4
20-24	12	8	8	9	7	6	18	8	11	10	14	10	10
25-29	17	23	25	22	22	27	24	24	28	17	19	9	30
30-34	116	136	119	133	126	99	87	73	74	60	71	69	57
35-39	428	452	428	401	440	381	356	323	370	315	290	258	236
40-44	1137	1111	1106	1092	1127	1163	1180	1151	1172	1189	1096	1086	1044
45-49	2577	2524	2400	2447	2525	2373	2362	2346	2540	2487	2471	2659	2566
50-54	4740	4693	4760	4694	4726	4659	4540	4505	4583	4703	4723	4654	4751
55-59	7811	7893	7523	7388	7331	7202	7453	7465	7546	7523	7769	7758	7663
60-64	12779	12650	12194	11434	11112	10845	10743	10548	10391	10350	10646	10589	10777
65-69	17285	17246	16872	16435	16104	15580	14945	14186	14189	13798	13209	13218	13243
70-74	17098	17674	17827	18349	17973	17829	18191	17101	17193	16901	16334	15717	15210
75-79	14016	14163	14208	14531	14768	15342	15524	15461	15726	15968	15833	15934	15754

**Stroke**	1992	1993	1994	1995	1996	1997	1998	1999	2000	2001	2002	2003	2004
**Male**													
10-14	21	27	29	26	22	27	31	17	21	31	31	22	22
15-19	36	37	29	33	32	41	45	36	38	34	34	51	41
20-24	82	57	69	64	64	65	64	59	68	69	69	65	75
25-29	129	146	111	115	107	103	107	102	111	100	100	101	112
30-34	271	274	267	263	244	232	220	197	197	184	184	213	172
35-39	523	541	515	566	515	498	485	449	430	389	389	411	389
40-44	843	824	952	919	930	921	891	859	867	789	789	872	851
45-49	1162	1214	1321	1379	1452	1383	1366	1282	1401	1402	1402	1389	1394
50-54	1427	1528	1619	1624	1710	1777	1755	1714	1834	1876	1876	1961	2015

55-59	1999	2045	2068	2170	2190	2214	2234	2256	2294	2373	2373	2394	2395
60-64	3271	3171	3171	3192	3143	3128	3111	3049	3202	3083	3083	3084	3128
65-69	5040	5157	5141	5029	5037	4914	4568	4522	4513	4305	4305	4138	4180
70-74	7616	7809	7795	7975	7845	7749	7449	7698	7529	6902	6902	6530	6027
75-79	9948	10333	10474	10452	10804	10700	10716	11349	11192	10283	10283	9993	9333

**COPD**	2005	2006	2007	2008	2009	2010	2011	2012	2013	2014	2015	2016	2017
**Male**													
10-14	35	45	42	30	39	46	43	31	50	44	59	47	48
15-19	43	39	43	40	44	36	48	35	38	38	36	50	44
20-24	52	54	53	53	64	60	69	63	63	78	94	78	71
25-29	58	66	76	64	75	74	68	79	78	100	73	84	94
30-34	72	71	74	71	69	97	89	91	82	113	90	110	99
35-39	124	105	108	137	117	121	92	102	143	130	130	142	125
40-44	302	292	284	253	238	214	248	240	224	230	205	224	200
45-49	707	617	703	656	684	591	612	577	557	527	492	483	469
50-54	1331	1343	1423	1556	1591	1465	1565	1563	1591	1479	1518	1454	1369
55-59	2399	2430	2480	2737	2758	2765	2976	2988	3174	3322	3338	3365	3351
60-64	4193	3957	4260	4636	4600	4661	4920	4903	5126	5241	5677	5714	6091
65-69	6275	5801	6210	6891	6831	7036	7184	7457	7599	7834	8347	8634	8601
70-74	9145	8359	8473	9256	8898	9190	9326	9462	10346	10275	10808	11086	11609
75-79	12278	11375	11311	12055	11116	11094	11081	11121	11448	11188	11788	12002	12292

**IHD**	2005	2006	2007	2008	2009	2010	2011	2012	2013	2014	2015	2016	2017
**Male**													
10-14	5	5	6	11	7	3	5	7	2	5	4	6	5
15-19	23	30	27	23	24	14	21	17	12	26	19	14	12
20-24	98	76	80	91	65	69	88	82	87	74	79	63	63
25-29	229	224	246	226	216	234	226	218	206	212	211	173	179
30-34	545	606	563	531	498	554	564	545	537	531	498	539	529
35-39	1439	1411	1340	1293	1271	1250	1182	1192	1095	1059	1151	1117	1137
40-44	3749	3665	3395	3151	2924	2783	2785	2648	2620	2516	2444	2400	2262
45-49	7355	7249	7053	6784	6491	6353	6140	5673	5408	5088	4881	4990	4705

50-54	11878	11723	11480	11459	11167	10974	10831	10694	10485	10220	9931	9464	9161
55-59	15484	15921	15450	15274	15104	15148	15094	15263	15475	15617	15439	15584	15338
60-64	18064	17673	18031	18414	18372	18580	19096	19345	19382	19541	19847	20257	20855
65-69	19607	19362	18883	19576	19639	19252	19505	20706	21390	21839	22620	23890	23886
70-74	24890	23487	21904	21756	21268	20740	20726	21038	22010	22484	23265	24033	25617
75-79	32870	30825	28403	27621	25694	24903	24298	23823	24246	24334	24196	24483	25304
**Lung cancer**	2005	2006	2007	2008	2009	2010	2011	2012	2013	2014	2015	2016	2017
**Male**													
10-14	0	0	3	1	2	2	2	2	2	3	1	0	2
15-19	4	4	5	6	5	5	6	3	2	6	5	5	5
20-24	5	6	12	16	12	10	11	15	8	9	5	12	7
25-29	19	26	25	32	27	19	23	27	17	30	24	18	17
30-34	58	59	54	53	58	71	39	74	47	46	48	58	53
35-39	235	216	177	172	183	167	140	138	134	151	146	121	128
40-44	962	818	734	662	583	496	492	447	427	435	372	361	332
45-49	2535	2432	2298	2251	2148	1929	1746	1610	1480	1280	1142	1033	905
50-54	4792	4764	4641	4800	4704	4717	4449	4428	4106	3825	3573	3154	2759
55-59	7668	7812	7516	7510	7535	7558	7674	7616	7855	7553	7519	7049	6508
60-64	10995	10702	10810	10786	10886	10900	11150	10667	10513	10572	10632	10189	10010
65-69	13007	13182	13331	13458	13653	13666	13357	13838	13549	13497	13593	13238	12625
70-74	15200	14513	14237	14214	13894	14094	14178	14174	14297	14844	14401	14021	13959
75-79	15512	15160	14767	14320	13865	13598	13281	13163	12925	12830	12767	12452	12671

**Stroke**	2005	2006	2007	2008	2009	2010	2011	2012	2013	2014	2015	2016	2017
**Male**													
10-14	28	30	20	25	27	27	27	26	25	27	26	31	33
15-19	34	36	40	42	41	53	39	35	29	30	32	37	37
20-24	60	79	73	68	68	54	65	73	56	72	57	55	46
25-29	109	106	90	99	107	88	92	100	101	132	104	118	116
30-34	192	184	162	197	191	177	192	197	178	199	216	213	223
35-39	355	394	401	368	380	343	285	334	306	363	333	398	398
40-44	793	769	743	715	667	686	621	628	632	638	657	662	636

45-49	1345	1353	1410	1307	1330	1278	1222	1186	1129	1078	1027	1087	1110
50-54	2101	2130	2097	2039	2058	2024	1979	1915	1938	1917	1873	1891	1900
55-59	2535	2757	2757	2648	2683	2692	2735	2810	2869	2952	2973	2981	2981
60-64	3101	3141	3236	3204	3300	3421	3670	3600	3782	3844	3997	4179	4351
65-69	3996	3963	3978	4083	4046	4123	4039	4288	4458	4702	4990	5293	5298
70-74	5692	5400	5373	5191	5242	5165	5189	5312	5575	5802	6130	6292	6734
75-79	8598	8097	7869	7376	7171	7002	7027	6787	7037	7297	7580	7730	8008

**COPD**	1966	1967	1968	1969	1970	1971	1972	1973	1974	1975	1976	1977	1978
**Female**													
10-14	20	31	51	53	53	53	54	38	50	39	33	33	35
15-19	29	41	79	67	73	75	58	62	80	55	55	62	68
20-24	38	22	92	91	108	100	78	89	89	83	76	51	70
25-29	39	46	106	118	113	72	92	79	82	93	76	63	81
30-34	60	55	114	115	132	137	126	102	82	99	94	78	84
35-39	117	92	175	162	193	171	184	141	145	145	126	121	105
40-44	196	183	330	300	301	279	270	300	226	247	219	194	226
45-49	318	292	468	519	505	511	498	480	436	449	409	395	415
50-54	431	447	722	690	714	760	716	734	748	772	791	701	803
55-59	578	608	951	950	1050	1041	1068	1182	1159	1210	1251	1209	1422
60-64	670	781	1192	1118	1257	1246	1406	1497	1634	1731	1835	1854	2089
65-69	826	899	1301	1208	1408	1528	1756	1815	1981	2014	2303	2455	2707
70-74	1002	949	1336	1344	1407	1557	1794	1903	2015	2165	2313	2574	3035
75-79	956	1023	1354	1312	1331	1500	1542	1720	1902	1946	2309	2404	2702

**IHD**	1966	1967	1968	1969	1970	1971	1972	1973	1974	1975	1976	1977	1978
**Female**													
10-14	13	13	4	6	6	14	10	9	10	1	1	1	5
15-19	26	32	11	22	20	29	22	23	21	15	12	12	10
20-24	73	54	45	53	46	52	64	41	34	32	24	24	30
25-29	129	119	94	107	109	117	98	107	73	68	90	90	83
30-34	280	324	302	255	299	263	226	253	278	224	214	214	205
35-39	735	747	744	748	706	687	700	701	550	579	477	477	488

40-44	1707	1680	2006	1859	1740	1739	1690	1561	1465	1332	1248	1248	1180
45-49	3287	3394	3743	3579	3571	3602	3378	3328	3102	2882	2836	2836	2558
50-54	6112	5964	6601	6429	6525	6461	6254	6398	6028	5822	5565	5565	5200
55-59	10248	10240	11622	11052	11121	10879	10656	10688	10125	9924	9681	9681	9411
60-64	16426	16394	18364	17741	18180	18281	18336	17927	17277	16459	16329	16329	15682
65-69	26455	26114	28921	28238	27919	27108	27892	27073	26341	25608	24996	24996	24791
70-74	38708	38045	41282	40440	40522	40152	40498	39546	38365	36306	35562	35562	35385
75-79	46978	47085	52434	51712	51655	52302	53252	52230	49529	47881	47967	47967	47044
**Lung cancer**	1966	1967	1968	1969	1970	1971	1972	1973	1974	1975	1976	1977	1978
**Female**													
10-14	0	4	4	3	2	1	0	2	3	1	3	1	1
15-19	5	2	5	5	3	6	2	5	2	2	3	5	4
20-24	7	9	6	15	8	10	6	6	8	6	9	4	7
25-29	14	13	14	21	15	13	16	21	17	21	28	25	22
30-34	48	47	47	64	52	70	62	73	71	58	76	75	81
35-39	174	164	192	196	229	188	210	209	196	215	217	240	227
40-44	427	429	504	484	497	548	594	530	557	599	568	643	619
45-49	691	828	819	938	1009	1095	1086	1229	1235	1276	1294	1315	1368
50-54	926	1043	1243	1293	1511	1672	1908	1745	1865	2012	2197	2330	2465
55-59	1100	1231	1405	1685	1818	1944	2198	2304	2553	2732	2899	3085	3377
60-64	1084	1180	1429	1578	1783	1974	2196	2472	2820	3061	3371	3631	3908
65-69	1109	1177	1423	1536	1627	1853	2080	2275	2565	2842	3228	3542	4024
70-74	1003	1124	1236	1376	1389	1617	1776	1830	2040	2216	2527	2787	3154
75-79	823	849	1049	1042	1174	1286	1290	1456	1578	1614	1913	2038	2288

**Stroke**	1966	1967	1968	1969	1970	1971	1972	1973	1974	1975	1976	1977	1978
**Female**													
10-14	70	66	80	70	66	69	52	71	65	53	52	35	40
15-19	88	104	119	108	100	113	88	119	104	97	107	81	91
20-24	143	141	169	180	145	182	138	149	144	141	118	148	137
25-29	191	240	225	218	229	242	238	246	235	237	220	183	195
30-34	385	373	372	423	370	386	358	406	317	332	320	309	264

35-39	695	719	726	698	685	682	580	607	558	511	507	487	465
40-44	1218	1212	1289	1210	1162	1114	1136	1037	999	868	864	735	714
45-49	1862	1970	2035	1938	1975	1916	1882	1871	1567	1419	1374	1260	1136
50-54	2690	2811	2830	2653	2716	2673	2780	2659	2494	2355	2243	1968	1995
55-59	3745	3817	3942	3746	3790	3563	3666	3585	3334	3248	3044	2856	2765
60-64	5582	5602	5690	5556	5547	5688	5526	5564	5302	4793	4773	4444	4212
65-69	9512	9111	9377	9091	9052	8521	8904	8645	8293	7768	7458	7080	6770
70-74	14733	14025	14576	14370	14273	13852	13808	13853	13420	12392	11655	11151	10796
75-79	20110	19923	20795	20274	20153	20399	20698	20978	20033	53	17700	16938	16087

**COPD**	1979	1980	1981	1982	1983	1984	1985	1986	1987	1988	1989	1990	1991
**Female**													
10-14	10	30	25	29	24	15	44	34	29	33	27	34	26
15-19	32	29	24	38	47	29	36	35	49	46	37	39	44
20-24	33	34	37	43	44	41	53	42	43	67	43	48	48
25-29	44	42	43	44	57	52	48	52	49	76	60	63	62
30-34	40	54	57	55	63	70	67	76	76	112	89	82	95
35-39	79	72	93	77	91	88	94	98	116	189	115	102	125
40-44	126	125	127	146	159	152	143	154	184	356	183	184	205
45-49	233	293	303	299	318	293	334	301	347	701	373	352	368
50-54	613	614	639	627	688	633	729	715	702	1552	769	772	804
55-59	1058	1203	1262	1297	1374	1461	1545	1490	1489	2944	1644	1492	1527
60-64	1803	1989	2116	2080	2368	2497	2752	2796	2833	4589	3107	3004	3196
65-69	2311	2775	3042	3146	3605	3758	4038	4228	4337	6236	4751	4931	5235
70-74	2558	3147	3501	3801	4288	4639	5049	5501	5704	6525	6552	6455	7091
75-79	2282	2686	3081	3343	3935	4494	5202	5547	5824	33	7030	7364	7916

**IHD**	1979	1980	1981	1982	1983	1984	1985	1986	1987	1988	1989	1990	1991
**Female**													
10-14	1	4	4	7	5	2	3	0	4	3	5	5	6
15-19	12	9	16	10	11	12	11	11	7	5	11	9	8
20-24	37	22	30	27	28	37	33	27	30	23	25	26	30
25-29	54	69	69	70	77	71	88	73	79	89	68	69	66

30-34	187	167	182	165	168	160	188	203	192	177	204	181	212
35-39	450	385	409	435	381	431	429	432	417	397	367	395	424
40-44	960	966	923	968	950	922	933	928	906	888	869	811	895
45-49	2006	1978	1979	1840	1752	1765	1660	1643	1632	1630	1542	1590	1638
50-54	4270	4178	4091	3888	3784	3440	3370	3081	3106	2943	2828	2724	2762
55-59	8058	8165	7864	7625	7523	7207	6833	6245	6077	5860	5331	5141	5106
60-64	13489	13670	13709	13489	13379	12967	12500	12161	11560	11111	10516	9965	9628
65-69	21087	21862	21456	20850	20558	20117	19764	19204	18819	18203	17656	17011	16262
70-74	30589	31552	30727	31067	30802	30360	29232	28348	27442	26893	25528	25084	24661
75-79	40125	41277	39551	40371	40476	40095	39597	38680	37895	37684	36923	36014	34815
**Lung Cancer**	1979	1980	1981	1982	1983	1984	1985	1986	1987	1988	1989	1990	1991
**Female**													
10-14	0	0	1	3	2	1	3	0	1	2	0	1	2
15-19	5	2	4	4	0	4	1	3	2	2	5	4	2
20-24	13	2	5	6	9	6	8	5	5	9	6	2	5
25-29	20	20	17	23	20	17	27	22	25	23	27	27	26
30-34	75	69	71	77	80	78	91	79	76	88	84	85	103
35-39	235	234	274	262	274	272	257	269	246	267	258	296	301
40-44	606	621	591	636	692	619	643	691	752	704	710	692	743
45-49	1360	1349	1346	1365	1356	1443	1402	1384	1444	1588	1518	1606	1570
50-54	2466	2622	2543	2583	2651	2597	2658	2623	2674	2657	2740	2823	2758
55-59	3524	3762	4032	4198	4449	4448	4456	4479	4452	4520	4503	4514	4514
60-64	4176	4552	4812	5356	5608	5851	6369	6311	6639	6975	7083	7207	7028
65-69	4352	4924	5270	5703	6136	6523	6935	7304	7810	8090	8721	9035	9313
70-74	3551	4136	4457	4920	5571	5841	6562	7059	7327	7888	8618	8973	9299
75-79	2490	2908	3076	3385	3785	4215	4635	5124	5778	6081	6997	7459	8063

**Stroke**	1979	1980	1981	1982	1983	1984	1985	1986	1987	1988	1989	1990	1991
**Female**													
10-14	27	27	28	28	26	28	27	25	22	20	18	22	24
15-19	58	62	59	44	53	56	38	38	38	33	34	29	41
20-24	123	116	123	99	107	98	119	83	86	89	83	77	73

25-29	170	201	196	165	174	175	159	186	181	168	151	179	149
30-34	288	282	307	291	234	282	276	284	297	280	304	303	277
35-39	430	443	453	428	437	435	426	451	453	441	417	454	445
40-44	681	651	665	655	647	679	691	638	684	670	665	701	710
45-49	1142	1122	1035	974	992	919	893	908	909	941	899	979	954
50-54	1773	1628	1676	1576	1460	1486	1350	1338	1303	1219	1258	1206	1212
55-59	2706	2649	2561	2348	2262	2179	2103	2076	1959	1894	1845	1757	1683
60-64	3928	3924	3899	3728	3684	3599	3438	3441	3345	3184	2992	2956	2824
65-69	6503	6448	6055	5819	5695	5418	5427	5459	5267	5343	5070	4989	4719
70-74	10513	10232	10041	9435	9061	9155	8919	8838	8569	8327	7933	7874	7663
75-79	15486	15613	14396	14314	13941	13896	13605	13268	13164	13126	12471	12285	12018

**COPD**	1992	1993	1994	1995	1996	1997	1998	1999	2000	2001	2002	2003	2004
**Female**													
10-14	39	39	30	36	49	38	36	26	35	25	45	28	29
15-19	44	35	45	63	47	29	42	47	28	31	25	34	30
20-24	42	43	44	50	59	37	48	39	42	41	47	43	39
25-29	78	64	76	67	86	77	68	77	53	47	46	51	49
30-34	135	96	100	116	105	90	84	102	78	98	90	75	55
35-39	208	151	153	155	164	165	168	160	165	184	181	159	145
40-44	373	227	212	309	290	306	288	320	328	330	332	363	311
45-49	732	401	453	468	486	473	473	535	559	536	609	595	622
50-54	1507	765	870	927	902	922	975	1027	1035	1087	1073	1118	1090
55-59	3009	1644	1622	1673	1685	1671	1685	2011	1915	2019	2083	2096	2052
60-64	5231	3256	3136	3003	3059	3191	3120	3331	3262	3447	3421	3748	3676
65-69	7245	5843	5788	5548	5536	5556	5662	5799	5686	5797	5574	5804	5483
70-74	8310	8260	8119	8148	8601	8370	8843	9371	9266	9083	8885	8786	8412
75-79	39	9131	9351	9408	10072	10384	10798	11960	12005	12011	12043	12330	11666

**IHD**	1992	1993	1994	1995	1996	1997	1998	1999	2000	2001	2002	2003	2004
**Female**													
10-14	1	3	3	3	4	5	7	2	4	5	4	6	4
15-19	11	2	11	8	9	12	8	16	12	7	12	11	4

20-24	25	28	29	29	21	24	22	28	30	37	29	42	25
25-29	80	65	80	82	64	77	64	76	55	68	60	77	86
30-34	196	226	208	210	202	200	214	199	176	218	195	192	184
35-39	434	480	476	522	532	482	477	586	568	545	541	522	510
40-44	981	951	961	961	1012	941	1021	1195	1164	1277	1302	1346	1256
45-49	1639	1660	1748	1740	1912	1748	1730	2077	2037	2138	2310	2328	2289
50-54	2790	2858	2931	2937	3087	3024	2893	3637	3704	3794	3714	3552	3648
55-59	4981	4823	4859	4709	4668	4686	4573	5554	5570	5476	5647	5565	5433
60-64	8974	8871	8425	8240	8090	7672	7479	8700	8372	8092	7959	8045	7701
65-69	15651	15556	14787	14594	13729	13018	12368	13896	13032	12321	11763	11101	10522
70-74	24444	24347	23579	23203	22747	21401	20931	22757	21299	20070	18968	17780	16307
75-79	33631	33907	32858	32582	32307	31605	30831	35338	34117	32188	30615	28871	26011
**Lung Cancer**	1992	1993	1994	1995	1996	1997	1998	1999	2000	2001	2002	2003	2004
**Female**													
10-14	2	0	1	2	1	0	3	0	1	1	0	1	1
15-19	1	2	4	4	3	4	5	1	0	4	2	3	2
20-24	13	7	5	5	4	4	3	6	8	4	3	8	7
25-29	25	14	15	18	18	26	17	19	9	19	16	20	14
30-34	93	97	99	100	98	79	93	63	86	61	59	56	46
35-39	326	282	311	345	337	354	330	345	294	325	293	262	234
40-44	745	700	682	725	778	851	868	905	920	967	1019	872	912
45-49	1622	1682	1619	1682	1621	1561	1603	1570	1680	1768	1870	1958	1998
50-54	2798	2841	2909	2987	3040	3053	2995	3013	3112	3083	3086	3103	3133
55-59	4497	4513	4564	4549	4461	4635	4699	4700	4907	5072	5161	5162	5183
60-64	7147	7022	6783	6826	6726	6619	6892	6631	6877	6832	7108	7447	7463
65-69	9544	9735	9938	9688	9774	9618	9479	9053	9249	9052	9149	9196	9309
70-74	10211	10568	10839	11257	11425	11681	11962	11508	11627	11474	11564	11255	11267
75-79	8440	8851	9273	9681	9984	10584	10765	11095	11579	11755	12136	12097	11854

**Stroke**	1992	1993	1994	1995	1996	1997	1998	1999	2000	2001	2002	2003	2004
**Female**													
10-14	16	19	17	16	20	24	16	22	30	21	27	18	21

15-19	31	37	34	21	29	30	20	31	29	32	19	37	28
20-24	48	77	51	54	42	52	49	56	64	61	49	68	67
25-29	124	101	124	89	113	100	126	100	98	94	83	87	96
30-34	272	276	300	253	256	243	217	181	196	196	200	182	187
35-39	473	452	504	532	514	483	494	467	450	405	408	350	330
40-44	752	702	746	755	763	885	780	799	852	819	839	827	791
45-49	962	1032	1021	1069	1197	1094	1073	1173	1224	1153	1213	1188	1228
50-54	1240	1283	1394	1401	1433	1441	1515	1394	1552	1552	1564	1589	1544
55-59	1714	1724	1664	1768	1749	1755	1829	1896	1851	1842	1920	1998	1973
60-64	2725	2685	2674	2605	2594	2579	2479	2451	2609	2489	2521	2470	2470
65-69	4571	4462	4503	4620	4524	4342	4218	4172	4158	3825	3721	3627	3626
70-74	7745	7883	7947	8110	7900	7939	7677	7700	7449	7124	7064	6413	6068
75-79	12106	12269	12252	12617	12820	12938	12992	13658	13486	13101	12628	11924	11171

**COPD**	2005	2006	2007	2008	2009	2010	2011	2012	2013	2014	2015	2016	2017
**Female**													
10-14	20	18	22	34	20	27	29	25	30	27	34	28	28
15-19	17	22	28	31	42	24	21	19	22	17	27	31	21
20-24	36	36	25	40	37	29	32	26	32	45	45	48	52
25-29	42	47	51	50	55	41	35	53	52	68	57	56	59
30-34	86	71	62	67	85	61	76	66	80	86	71	103	81
35-39	137	118	122	115	125	135	118	120	140	124	108	104	126
40-44	327	329	284	310	263	240	277	282	256	272	262	226	264
45-49	748	704	697	767	787	773	754	692	698	625	615	603	554
50-54	1199	1265	1336	1418	1611	1631	1708	1710	1781	1781	1729	1777	1598
55-59	2277	2274	2165	2329	2482	2536	2695	2912	3075	3292	3464	3570	3624
60-64	3886	3721	3885	4357	4334	4296	4466	4430	4590	4665	4998	5194	5639
65-69	5746	5806	5773	6675	6701	6649	6860	7186	7517	7268	7672	7897	7953
70-74	8757	8288	8220	9167	8904	8914	9086	9499	10173	10268	10867	10834	11588
75-79	12229	11405	11456	12220	11534	11553	11596	11368	11802	11703	12485	12401	13018

**IHD**	2005	2006	2007	2008	2009	2010	2011	2012	2013	2014	2015	2016	2017
**Female**													

10-14	2	5	3	3	3	1	2	3	2	3	5	5	4
15-19	6	8	13	6	5	9	8	9	10	8	10	7	16
20-24	24	24	31	24	19	22	23	27	31	18	25	24	18
25-29	54	62	83	98	63	73	72	78	90	57	76	68	69
30-34	186	163	156	190	181	164	171	184	177	171	192	174	162
35-39	479	462	447	430	411	380	379	401	345	422	408	389	401
40-44	1193	1162	1041	1078	980	920	920	940	931	923	863	836	811
45-49	2298	2378	2230	2238	2088	2138	1949	1995	1765	1736	1795	1656	1616
50-54	3779	3640	3634	3878	3564	3574	3610	3553	3588	3624	3512	3391	3215
55-59	5495	5603	5288	5272	5201	5145	5260	5434	5314	5540	5647	5757	5696
60-64	7756	7511	7401	7705	7380	7452	7711	7671	7426	7627	7958	7992	8078
65-69	10326	9596	9213	9361	9363	9224	9097	9470	9847	9929	10246	10703	10592
70-74	15298	14242	13039	12930	12088	11759	11760	11588	11842	12132	12487	12579	13087
75-79	24839	22794	21054	20089	18491	17252	16511	16175	15832	15434	15479	15355	15508
**Lung Cancer**	2005	2006	2007	2008	2009	2010	2011	2012	2013	2014	2015	2016	2017
**Female**													
10-14	0	1	0	1	0	2	1	2	1	1	3	1	2
15-19	0	7	1	1	3	7	1	0	2	4	4	4	2
20-24	7	10	7	6	5	9	12	7	10	3	12	7	3
25-29	20	17	15	16	12	18	17	13	23	11	11	14	11
30-34	36	53	41	44	49	54	43	60	37	43	50	37	31
35-39	206	210	175	167	172	155	117	118	127	117	98	125	100
40-44	920	779	766	603	580	533	510	471	458	407	399	278	283
45-49	2061	2112	2056	1990	1970	1775	1680	1527	1354	1211	1042	984	879
50-54	3236	3300	3486	3491	3666	3675	3619	3545	3499	3424	3080	2766	2446
55-59	5139	5272	5111	4903	4962	5104	5241	5597	5692	5779	5875	5654	5488
60-64	7561	7531	7779	7597	7584	7587	7708	7580	7373	7541	7387	7473	7499
65-69	9150	9323	9635	9875	9912	10024	9852	10209	10287	10196	10198	9738	9578
70-74	11033	10864	10954	10746	11079	10823	10791	11201	11445	11462	11663	11081	11492
75-79	12079	11911	11836	11560	11219	11123	10948	10937	10797	11071	10701	10755	10560

**Stroke**	2005	2006	2007	2008	2009	2010	2011	2012	2013	2014	2015	2016	2017

Female													
10-14	15	20	25	31	15	16	20	25	23	16	16	19	23
15-19	42	37	31	30	20	33	30	28	27	28	29	25	29
20-24	60	58	51	49	65	51	52	47	41	47	48	31	43
25-29	83	77	90	98	94	84	81	85	78	96	88	80	100
30-34	162	160	164	145	145	169	165	153	151	152	159	164	154
35-39	338	323	332	318	287	273	283	251	244	253	306	301	287
40-44	774	735	658	634	586	602	529	517	505	491	492	490	490
45-49	1284	1204	1206	1177	1173	1019	1010	1004	837	855	875	902	836
50-54	1651	1655	1673	1589	1605	1594	1494	1549	1521	1499	1532	1473	1352
55-59	1945	2054	1988	2070	1921	1917	1961	1986	2055	2114	2180	2210	2262
60-64	2447	2567	2520	2537	2628	2670	2839	2674	2658	2817	2966	2940	3114
65-69	3477	3371	3483	3484	3252	3412	3439	3548	3705	3867	4012	4344	4403
70-74	5674	5490	5173	5139	5047	5042	4917	5020	4984	5292	5661	5839	6255
75-79	10202	9642	9105	8905	8396	8142	8155	7798	7791	7956	8292	8236	8617
