# Supplementary material for: Estimating the reduction in US mortality if cigarettes were largely replaced by e-cigarettes
Source: Arch Toxicol. 2021 Oct 22;96(1):167–76. doi: 10.1007/s00204-021-03180-3 (PMC8748352; doi:10.1007/s00204-021-03180-3)
Supplement: Supplementary file 6 — Supplementary file6 (PDF 5432 KB) [file 204_2021_3180_MOESM6_ESM.pdf]

Estimating the reduction in US mortality if cigarettes were largely replaced by e-cigarettes

Published in: Archives of Toxicology

Peter N Lee[1*], John S Fry[2], Stanley Gilliland III[3], Preston Campbell[3], Andrew R. Joyce[3]

[1]P N Lee Statistics and Computing Ltd, 17 Cedar Road, Sutton, Surrey, SM2 5DA

[2]RoeLee Statistics Ltd., 17 Cedar Road, Sutton, Surrey SM2 5DA

[3]Consilium Sciences, LLC, 7400 Beaufont Springs Drive, Suite 300, N. Chesterfield, VA 23325

[*] Author for correspondence

E-mail: Peterlee@pnlee.co.uk

ONLINE RESOURCE 6

Projecting mortality rates

This file gives full details of the age, period and cohort values fitted to the mortality rate data for 1966 to 2015 and extrapolated to the years 2016 to 2040, as well as goodness-of-fit statistics and the observed and fitted mortality rates for all the age groups.

Methodology

Modelling disease rate patterns from data by period and age using the Osmond and Gardner method

Mortality trends are often portrayed as a matrix in which rows are age groups and columns time periods, data being rates based on numbers of deaths and person-years at risk. Where age groups and time periods are equal, diagonals of increasing age and period relate to "cohorts" born around the same year. Interest often centres on whether trends result from period or cohort effects. However, since knowledge of any two of age, period and cohort defines the other, there is no unique best fitting solution to the model $r_{ijk} = a_i p_j c_k$ where r is rate and a_i, p_j and c_k refer to effects due to age group i, period j and cohort k respectively.

In 1982, Osmond et al. and Osmond and Gardner recommended an approach based upon the relative success of the three two-variable submodels.

The method involves fitting a model in which the logged rates are expressed as linear functions of the logs of individual age, period and cohort parameters. This involves minimizing a weighted function of the squares of the residuals while constraining the period and cohort values, so that their averages are kept at unity. The basic problem is one of identifiability since the logical dependence between age, period and cohort values leads to an infinite set of possible solutions or minima. Given any one particular solution, another can be obtained by adding or subtracting successive multiples of a shift quantity to each individual logged age, period and cohort value. Thus, by shifting the cohort values in one direction and the period values in another, a new solution is produced which fits the data identically. The various shapes or rotations induced by the shifts will lead to different interpretations being placed upon the alternative solutions.

Osmond and Gardner (1982) approached the problem by first finding solutions to each of the two-parameter submodels (age-period, age-cohort and period-cohort) and then obtaining a full solution by minimizing a function of the three weighted squared-differences between each two-factor submodel and the full three-factor model. The resulting full solution thus gives more weight to those two-parameter models which produce better fits to the data. The method is unique in its approach to the problem of lack of identifiability, and it is difficult to envisage an alternative approach which does not involve rather arbitrary assumptions to overcome the pattern of dependence between the three variables. The method produces results that look sensible, and the period and cohort values broadly resemble those obtained by calculating period or cohort based SMRs (standardised mortality ratios).

Applying the method to England and Wales lung cancer data, Osmond and Gardner (1982) showed a sharply rising age effect, little period effect and an inverted V cohort pattern peaking around 1900-05 for men and 1925 for women. The cohort pattern was attributed to successive generations smoking differently, though Lee (1987) noted that it was difficult to explain the recent cohort decline for females in terms of changes in cigarette consumption.

Extrapolating death rates fitted by the Osmond and Gardner model to future years

As well as fitting the O&G model to observed rates we are also interested in predicting future rates. Some previous unpublished work at P.N. Lee Statistics and Computing Ltd. on lung cancer in England & Wales had suggested four methods of prediction based on O&G analysis. These were: 1) A-P-C model with linear extrapolation of P and C values; 2) A-P-C model with log-linear extrapolation of P and C values; 3) A-C model with log-linear extrapolation of C values; and 4) A-C model with log-linear extrapolation and residual correction.

Over the 1941 to 1980 period there was a distinct peak in lung cancer rates. Extrapolation of cohort values seemed to work best where a weighting of 0 was used for values before the peak and a weighting of 1 was used at the peak and after. Extrapolation of the period values in methods 1 and 2 and of residuals in method 4 used weights decreasing exponentially into the past, to allow more recent values to have more influence than those in the distant past.

From the then available 1941-80 data, various (4, 5 or 6) initial periods were used to predict the remaining periods and age-specific numbers of deaths were compared. It was found that predictions improved if more points were used, except that pre-1950 data did not improve predictions. The A-P-C model produced better results for males, but the A-C was better for females. Log-linear extrapolation gave poorer results than linear, but linear gave the possibility of negative results. In 1989, these four methods were then used to predict rates and numbers of deaths over the 20-year period 1981-2000 for a number of countries (Forey). From this work the following decisions were made. First, data below age 40 should be used where possible, there seeming to be no good reason to omit such data when available, as it did appear to be useful in indicating most recent trends. Second, linear extrapolation should be abandoned. Third, the full A-P-C model should be used for extrapolation, since the A-C model, though appealing as it did not suffer from the redundancy problem of the full model, gave a considerably less good fit than the full model for most countries. Fourth, weighting based on 'past the peak' was judged to be too arbitrary.

It was proposed that new cohort values be estimated by log-linear extrapolation using as weights powers of 2 decreasing into the past. The last cohort value from the fitted model should be excluded from this extrapolation procedure and replaced by an estimated value.

It was also proposed that new period values be estimated by applying to the succeeding periods the percentage change found between the last two period values.

We have extrapolated using this proposed method, as well as by the more standard method using weighted averages for the estimated period effects.

Applying the methodology

Extrapolating period parameters

As mentioned above, it seems that extrapolation of the period parameters is best carried out based on the last two periods rather than using weightings based on all the previous periods, or from periods where peaks have occurred. This method is selected by setting the Period Extrapolation Method parameter to 1 (see below).

Extracting year and age-group specific data on population size and deaths

The data used comes from two sources:

- UN population data – estimates, and projected values to 2100 (http://population.un.org/wpp/Download/Standard/Population/; see also Online Resource 1)
- WHO mortality and population data (https://www.who.int/data/data-collection-tools/who-mortality-database; see also Online Resource 5)

UN population data for 14 countries (Austria, Canada, France, Germany, Hungary, Italy, Japan, Poland, South Korea, Russia, Sweden, Switzerland, United Kingdom, United States) were downloaded to an Excel file, and then exported to a PHIM fixed file, POP.CSV, from which they can be read by the O&G Modelling System.

The latest version was downloaded on 15th April 2020 into two files:

WPP2019_POP_F15_2_ANNUAL_POPULATION_BY_AGE_MALE.xlsx

WPP2019_POP_F15_3_ANNUAL_POPULATION_BY_AGE_FEMALE.xlsx

The files contain population estimates up to 2017, and forecasts (Medium Variant) up to 2100. Values from 1950 to 2100 have been put into POP.CSV, with the source value set as “UN4”.

WHO data for each country, consisting of numbers of deaths from different causes up to 2017, were downloaded on 15th April 2020, with deaths from 1950 to 2015 for the chosen countries for the four individual PHIM cause-of-death categories (Ischaemic heart disease (IHD), Lung cancer (LC), Stroke (STR) and COPD) then added to the PHIM fixed file, MORT.CSV, This new version of the data was given the source value “Apr-20”.

Running the O&G modelling within PHIM

The program is run via new messages allowed in the output control file (OUTC.CSV).

Output type	User choice	Possible Values
OG MODEL	OUTPUT FILENAME	Filename for results.<br>This file will be put into the PLOT_RESULTS directory of the particular PHIM run.<br>If left blank the filename for the PHIM results will be used
OG MODEL	PERIODS FIRST YEAR	Integer >= 0 (Year such as 1991) BLANK if to be same as first year of particular PHIM run.
OG MODEL	NUMBER PERIODS	Integer >= 0. Number of 5 year periods to be used for O&G modelling. Set blank if the value is to be same as set by the particular PHIM run.
OG MODEL	SAVED MODEL	Integer 1-5 or BLANK for model 4, the default value.<br>(1=AGE-PERIOD, 2=AGE_COHORT, 3=PERIOD-COHORT, 4=FULL AGE-PERIOD-COHORT, 5=AGE ONLY).<br>Specifies the O&G Model to be used for extrapolations. This will usually be model 4, Full age-period-cohort.
OG MODEL	GOODNESS OF FIT	Integer 0, 1 or 2, or BLANK for default value of 1.<br>0= Show no goodness of fit statistics<br>1= Print output FOR chosen model to save (usually the AGE-PERIOD-COHORT MODEL) (Default)<br>2= Print output for all models
OG MODEL	CSV TEST RESULTS	Y or N. Y if results from the O&G modelling process are to be sent to the Output Filename set above (default value). This file of results can be used for plotting the results or for using in future runs of the PHIM system.
OG MODEL	NUMBER PERIODS IN FUTURE	Integer >0
OG MODEL	PERIOD EXTRAPOLATION METHOD	Integer -2, -1, 0, 1, 2 or BLANK for default value of 1.<br>If this line is not included or set to “0” the O&G modelling will not be run. Mortality will be computed by “last rate brought forwards”.<br>If the value is set to “-1” the old method of “Last value for number of deaths brought forwards” will be used.<br>1=Use percentage difference between the last two periods<br>2= Use weighted linear regression<br>If the value is set to “-2” the PHIM system will attempt to use the fitted results from a previous run of the system. The file with the results should be in the FIXED folder, with the name as specified in “OUTPUT FILENAME” as specified above.

OG MODEL	PERIOD WEIGHTS	When using period extrapolation method 2, weighted linear regression, this option defines the weights to be be used. Enter D or BLANK for default values of 1, 10, 100, 1000, … or a list of values delimited by the character "|".
OG MODEL	COHORT WEIGHTS	Enter D for the values of 1, 2, 4, 8, 16, . … or a list of values delimited by the character "|". Special values of D1 and D2 can be entered which set either the last or the last 2 parameters to be "-1" which means they are calculated by extrapolation from the other parameter values. The default value is D2.
OG MODEL	SHOW MATRIX	Integer 0 to 3 or BLANK for default (1)<br>Show matrix of 1:rates, 2:deaths 3:both, 0: none
OG MODEL	SHOW FITX	Integer 0 to 3 or BLANK for default (1)<br>Show matrix of 1:rates, 2:deaths 3:both, 0: none
OG MODEL	DISPLAY NUMBER DEATHS	Y or N. Y if number of deaths to be displayed in output (default value).
OG MODEL	SHOW FIT	Integer 0 to 3 or BLANK for default (1)<br>Show matrix of 1:rates, 2:deaths 3:both, 0: none
OG MODEL	SHOW PARAMETERS	Integer 0, 1, 2 or BLANK for default value of 1.<br>Show O&G parameters by 5 years, or by 5 and 1 year:<br>0) None 1) 5 Year 2)5 and 1 Year
OG MODEL	SHOW PROJECTIONS	Integer 0 to 3 or BLANK for default (1)<br>Show matrix of 1:rates, 2:deaths 3:both, 0: none
OG MODEL	SHOW 5YR PROJECTIONS	Integer 0 to 3 or BLANK for default (1)<br>Show matrix of 1:rates, 2:deaths 3:both, 0: none
OG MODEL	SHOW 1YR PROJECTIONS	Integer 0 to 3 or BLANK for default (1)<br>Show matrix of 1:rates, 2:deaths 3:both, 0: none
OG MODEL	SHOW PROJECTION TOTALS	Integer 0 to 3 or BLANK for default (1)<br>Show matrix of 1:rates, 2:deaths 3:both, 0: none

First year (all 4 digits)

This gives the first year for the first period of interest, such as "1986" for the grouped period 1986 to 1990. Note that it is currently assumed that ages and periods will be grouped into 5 year periods. For this study the start year of modelling was taken as 1966.

Last year

This gives the very last year for the period of interest, such as "2015" for the grouped period 2011 to 2015. For this study the last year for modelling was taken as 2015.

Model Type to Save

This is the model that will be used to do extrapolations, where required. It should be chosen from the following:

1. AGE-PERIOD
2. AGE-COHORT
3. PERIOD-COHORT

4. FULL AGE-PERIOD-COHORT (Default)
5. AGE-ONLY

Here, the value 4 was used.

Goodness of Fit Required

This defines whether all goodness of fit statistics are to be shown, or only particular ones, or even none. Choose from:

0. Show no goodness of fit statistics
1. Print output for chosen model to save (usually the AGE-PERIOD-COHORT MODEL) (Default)
2. Print output for all models

Here, the value 1 was used.

Number of Periods into the future to Predict

Note that this is periods, not years. Thus, as years will be grouped in 5 year periods, the value "2" will refer to two 5 year periods. Here the value 5 was used to predict deaths from 2016 to 2040.

Period Extrapolation Method

As mentioned above, it seems that extrapolation of the period parameters might best be carried out based on the last two periods rather than using weightings based on all the previous periods, or from periods where peaks have occurred. This method (extrapolation method 1) is the method used here.

If the value is set to "−2" the PHIM system will use the fitted results from a previous run of the system. As the periods of interest were to be the same for all the modelling the results

from the first run of the O&G modelling were put into the FIXED folder and then used without repeating the O&G modelling for the rest of the analyses.

Weights for period

If users have decided to use period extrapolation method 2, that is weighted linear regression to estimate periods into the future, then they can use this option to either choose the default values based on powers of 10 or enter their own set of weights, separated by the character "|". Use D to ask for the default values, 1, 10, 100, … or something like 1|2|4|8|16|32|64 … if powers of 2 are wanted.

Here we have used the default.

Weights for cohort

The O&G method uses weighted linear regression to estimate parameter values when extrapolating outside the periods considered. By default (using the option value of D) values based on increasing powers of 2 will be used, that is 1, 2, 4, 8, 16, ….. Alternatively a list of integers delimited by the character "|" can be entered. Special values of D1 and D2 can also be entered which set either the last or the last 2 parameters to be "-1" which means they are also calculated by linear extrapolation from the other parameter values. Here, the default value of D2 has been used.

Show Matrix for Rates, Deaths, Both or None

0) None 1) Rates 2) Deaths 3) Both

This refers to a simple table that shows the simple matrix of data that will be analysed. Here the default value of 1 has been used.

Show Results for Rates, Deaths, Both or None

0) None 1) Rates 2) Deaths 3) Both

This refers to the detailed tables of the fit of the different O&G models. Here the default value of 1 has been used.

Show O&G parameters by 5 years, or by 5 and 1 year

0) None 1) 5 Years 2) 5 and 1 Years

Where projections are requested users can select whether or not to see the extrapolated parameters. Here the default value of 1 has been used.

Show Projections for Rates, Deaths, Both or None

0) None 1) Rates 2) Deaths 3) Both

This allows users control over the detailed results shown for the projections. Here the default value of 1 has been used.

Show 5 year projections

0) None 1) Rates 2) Deaths 3) Both

This allows users to produce an extra table where just the 5 year projections are shown. Here the default value of 1 has been used.

Show 1 year projections

0) None 1) Rates 2) Deaths 3) Both

This allows users to produce an extra table where the 1 year projections are shown.

Note that currently one year projections are based on the same technique as used for the 5 year corrections. So that where users have used only the last 2 periods to predict into the future, the 1 year corrections are based on the same form of extrapolation.

Here the default value of 1 has been used.

Show Totals for projections:

0) None 1) First to Last 2) 40-74 3) Both

Here the default value of 1 has been used.

When showing results for projections users have the option of showing totals over particular age groups. The options are to show the totals over all the age groups, totals for just ages 40 to 74 or both. Note that when rates are being combined, the European standardizing population (1976) is used:

Age Groups	All	0-4	5-9	10-14	15-19	20-24	25-29	30-34	35-39	40-44
Males	100000	8000	7000	7000	7000	7000	7000	7000	7000	7000
Females	100000	8000	7000	7000	7000	7000	7000	7000	7000	7000

Age Groups	45-49	50-54	55-59	60-64	65-69	70-74	75-79	80-84	85+
Males	7000	7000	6000	5000	4000	3000	2000	1000	1000
Females	7000	7000	6000	5000	4000	3000	2000	1000	1000

Negative estimates

When using simple linear extrapolation of parameter estimates it is possible to get negative values. Where this would happen the last positive estimate is used instead.

Incomplete periods

It may happen when setting up the 5 year periods for mortality that, due to missing data, there are less than the complete 5 years of data present. The system will factor up the number of deaths as a simple ratio of the years present to the 5 years required. That is, if we have 2 years of data and the combined number of deaths in those two years is n, the number of deaths in the 5 year period will be assumed to be 5*n/2.

Disease Forecasts for the USA using Osmond and Gardner

Methodology

Content of the output

The analyses concern fitting the APC model to data by five year period (1966-70, 1971-75, ….. 2011-15) and age (30-34, 35-39, ….. 75-79) and then using the model to predict numbers and rates for five further five year periods (2016-2020, 2021-2025, 2026-2030, 2031-2035, 2036-2040), comparing predicted with known results.

Figure 1 shows in both sexes, for four diseases and two selected age groups (50-54 and 70-74 years), the observed rates for single years (in green), the fitted rates for the five year periods (in purple), the fitted rates for 2016 and 2017, where observed rates are also available (in red) and the estimated rates for 2018 to 2040 (in blue). As can be seen, the fit to the observed data is always very good, and the predicted future rates seem plausible (though this cannot be tested until data for later years become available).

The detailed outputs are given below in "Results for PHIM for Run 1 (Basic_US_OG_T1.RTF)" and "Results for PHIM for Run 2 (Basic_US_OG_T1.RTF)". Run 1 gives the results for the males and Run 2 the results for the females.

The output consists of 212 pages, the first 2 pages giving a table of contents, then pages 15 to 119 relating to males and pages 120 to 224 to females. For each sex, the 105 pages of APC model results are divided into a control page followed by 20 pages for all causes and then four sections of 21 pages, for in turn lung cancer, IHD, stroke and COPD.

We will first describe the structure of the 20 pages of output, referring to the results on pages 16-36 for all-cause mortality in males.

Page 16 shows the input values of the variable parameters and the known data used for 1966-2015. The output for the variable parameters is also self-explanatory. Note that, to minimize

output, it was decided only to give detailed prediction results for the full APC model, and not for any of the sub-models. The output of the matrices of numbers of deaths, mortality rates and log-rates is also self-explanatory. Note that numbers of deaths and numbers in the population were actually available by single years and have been combined as appropriate.

Page 17 shows the results of fitting the APC model and the various sub-models. The fitted age, period and cohort values are shown for the full APC model, while various statistics are shown for the different sub-models Age only (A), Age-Period (AP), Age-Cohort (AC) and Period-Cohort (PC). While the full APC model is not a perfect fit, with a chisquared value of 28726.6 on 90 degrees of freedom, it fits much better than the other models. Notably, compared to the simple A model, with no period or cohort effects, the residual sum of squares is much lower (5,414 vs 382,049, so that the period and cohort effects explains 98.6% of the variation.

Pages 18 to 20 still concern results for 1966-2015, comparing observed and expected (fitted) numbers and rates by five year period and age. Despite the chisquared value of 28726.6 seeming to be large, this is because there are very large numbers of deaths, differences between observed and fitted values being generally proportionately quite small.

The extrapolation output (for 2016-2040) starts on page 21. Here the user has specified that the method of projection of period values is to be based on the last two fitted period values in the earlier fit. Thus, as they were 0.8430 and 0.8243, a ratio of 0.9779, those for the five extrapolated periods would be 0.8061, 0.7883, 0.7707, 0.7538 and 0.7372, with the output also showing the extrapolation by single years. The output also shows how the cohort values were extrapolated using weighted regression and ignoring results for the 1956 and 1961 cohort in the original fit, which were based on minimal data.

Page 23 also shows the 1976 European Standard Population by age used in some of the subsequent output.

Page 24 shows the observed rates (including the total over ages standardized to the European Standard Population) and those expected for the five extrapolation periods, an extension of the rates shown on page 16. Note that the output also shows the observed and predicted drops in rates between the last observed rate (2011-2015) and the last predicted rate (2016-2020) where an observed rate is available.

Pages 25-26 show a matrix of observed and predicted numbers of deaths, by age and all nine periods, also an extension of the rates shown on page 16.

Pages 27-30 show observed and predicted rates (with residuals) and numbers (with chisquared values) just for the prediction period, by five year periods and then by single years.

Pages 31-35 show the list of values (estimated number of deaths) obtained from the O&G modelling process and used by PHIM. Note that the values start from 2018 as there were data available in the latest mortality download for the US for the years 2016 and 2017.

For diseases other than "all causes" an extra page is added at this point showing the value for RR used by the PHIM for values outside the range specified by the fixed RR file (using last value brought forwards).

References

Forey BA (1989) Prediction of lung cancer rates 1986-2005. Comparison of several methods of prediction all based on O&G analysis, applied to males in 6 countries (file:\\\x:\refscan\FOREY1989A.pdf). P N Lee Statistics and Computing Ltd, Sutton, Surrey

Lee PN (1987) Childhood respiratory infection and adult chronic bronchitis in England and Wales. Br Med J 294:118

Osmond C, Gardner MJ (1982) Age, period and cohort models applied to cancer mortality rates. Statistics In Medicine 1:245-259
Osmond C, Gardner MJ, Acheson ED (1982) Analysis of trends in cancer mortality in England and Wales during 1951-80 separating changes associated with period of birth and period of death. Br Med J 284:1005-1008

Results for PHIM for Run 1 (Basic_US_OG_T1.RTF)

System Date and Time: 20200429T103141
User: JohnS
SAS Version: 9.4
Project: Population Health Impact Model 7.0

The System is running in Test Mode

Maximum Upper Age for P Component = 79
Upper Age for ELL Calculations = 75
Description: Basic Analysis Using OG Modelling T1 on US
Assumption Files:
Options Control File: C:\VeneBio\OG_Test\\US_Control_OG_T1.CSV
TTP Test File: C:\VeneBio\OG_Test\\TTP_Test_SSA1.csv
TTP Reference File: C:\VeneBio\OG_Test\\TTP_Reference_SSA1.csv
TTP Factor Test File: C:\VeneBio\OG_Test\\TTP_FACTOR_SSA1.csv
TTP Factor Reference File: C:\VeneBio\OG_Test\\TTP_FACTOR_SSA1.csv
TP Socioeconomic Groups: Not Used
Output Choices:C:\VeneBio\OG_Test\\OUTC_OG_US_T1.csv

Main Results will be sent to the folder C:\VeneBio\Main_Results\
Plots will be sent to the folder C:\VeneBio\Plot_Results\Plots_20200429T103141
Saved P, E and/or A Component results will be sent to the folder C:\VeneBio\SAVED\Saved_20200429T103141\

Table of Contents

Control File Details for Run: 1 18
Osmond and Gardner Modeling of Death Rates for COD: All Causes 19
Osmond and Gardner Extrapolating Death Rates for COD: All Causes 24
List of values created by O and G modelling, using percentage change in last two period parameters for Fixed File MORT 34
Osmond and Gardner Modeling of Death Rates for COD: LUNG CANCER 39
Osmond and Gardner Extrapolating Death Rates for COD: LUNG CANCER 44
List of values created by O and G modelling, using percentage change in last two period parameters for Fixed File MORT 54
List of values created by last value brought forwards for Fixed File perm.Fixed_File_RR_LC 59
Osmond and Gardner Modeling of Death Rates for COD: IHD 60
Osmond and Gardner Extrapolating Death Rates for COD: IHD 65
List of values created by O and G modelling, using percentage change in last two period parameters for Fixed File MORT 75
List of values created by last value brought forwards for Fixed File perm.Fixed_File_RR_IHD 80
Osmond and Gardner Modeling of Death Rates for COD: STROKE 81
Osmond and Gardner Extrapolating Death Rates for COD: STROKE 86
List of values created by O and G modelling, using percentage change in last two period parameters for Fixed File MORT 96
List of values created by last value brought forwards for Fixed File perm.Fixed_File_RR_STR 101
Osmond and Gardner Modeling of Death Rates for COD: COPD 102
Osmond and Gardner Extrapolating Death Rates for COD: COPD 107
List of values created by O and G modelling, using percentage change in last two period parameters for Fixed File MORT 117
List of values created by last value brought forwards for Fixed File perm.Fixed_File_RR_COPD 122
Control File Details for Run: 2 123
Osmond and Gardner Modeling of Death Rates for COD: All Causes 124
Osmond and Gardner Extrapolating Death Rates for COD: All Causes 129
List of values created by O and G modelling, using percentage change in last two period parameters for Fixed File MORT 139
Osmond and Gardner Modeling of Death Rates for COD: LUNG CANCER 144
Osmond and Gardner Extrapolating Death Rates for COD: LUNG CANCER 149
List of values created by O and G modelling, using percentage change in last two period parameters for Fixed File MORT 159
List of values created by last value brought forwards for Fixed File perm.Fixed_File_RR_LC 164
Osmond and Gardner Modeling of Death Rates for COD: IHD 165
Osmond and Gardner Extrapolating Death Rates for COD: IHD 170
List of values created by O and G modelling, using percentage change in last two period parameters for Fixed File MORT 180
List of values created by last value brought forwards for Fixed File perm.Fixed_File_RR_IHD 185
Osmond and Gardner Modeling of Death Rates for COD: STROKE 186

Results for PHIM for Run 1 (Basic_US_OG_T1.RTF)

Osmond and Gardner Extrapolating Death Rates for COD: STROKE 191
List of values created by O and G modelling, using percentage change in last two period parameters for Fixed File MORT 201
List of values created by last value brought forwards for Fixed File perm.Fixed_File_RR_STR 206
Osmond and Gardner Modeling of Death Rates for COD: COPD 207
Osmond and Gardner Extrapolating Death Rates for COD: COPD 212
List of values created by O and G modelling, using percentage change in last two period parameters for Fixed File MORT 222
List of values created by last value brought forwards for Fixed File perm.Fixed_File_RR_COPD 227

Results for PHIM for Run 1 (Basic_US_OG_T1.RTF)

Control File Details for Run: 1

Variable Parameter	Value
1. Name for Reference Scenario	NULL
2. Name for Test Scenario	MRTP
3. Name for Product 1	CC
4. Name for Product 2	MRTP
5. Name for Product 3	-
6. Name for Product 4	Dual
7. Country	US (United States)
8. Sex	M (Males)
9. Year of start of process	1990
10. Number of months of follow-up	600
11. Follow-up interval length (in months)	12
12. Lower age for risk estimation	10
13. Upper age for risk estimation	79
14. The effective dose for Product 1 (F1) - Ref	1
15. The effective dose for Product 2 (F2) - Ref	-
16. The effective dose for Product 3 (F3) - Ref	-
17. The effective dose for Product 4 (F4) - Ref	-
18. The effective dose for Product 1 (F1) - Test	1
19. The effective dose for Product 2 (F2) - Test	0.2
20. The effective dose for Product 3 (F3) - Test	-
21. The effective dose for Product 4 (F4) - Test	0.6
22. Number in population to be simulated	10000
23. Number of MC simulations	1
24. The random number seed for the first simulation	15975263
25. Source for the population file (POP)	UN4
26. Source for the socioeconomic prevalence file (SEP)	-
27. Source for the current smoking prevalence file (CSP)	ISS2
28. Source for the former smoking prevalence file (FSP)	ISS2
29. Source for the quit-time distribution file (QTD)	NHIS2006
30. Source for the death file (MORT)	APR-20
31. Source for the relative risk file (RR)	PNLEST
32. Source for the half-life file (H)	PNLEST
33. Assumption Set for TTP Factor Reference	F1
34. Assumption Set for TTP Factor Test	F1
35. Assumption Set for TTP Reference	PNLNULL1
36. Assumption Set for TTP Test	PNLMRTP9
37. Assumption Set for TP for socioeconomic group	-
38. Source for Output Choice file (OUTC)	BASIC
39. Output for DTP P1	N
40. Output for DTP P2	N
41. Output for Main P Component	N
42. Output for DTP E1	N
43. Output for DTP E2	N
44. Output for DTP E3	N
45. Output for Main E Component	N
46. Output file name	Basic_US_OG_T1
47. Run number for P-Component	-
48. Results file for E-Component	-
49. Components required	PE
50. Results folder for P to be used in E-Component	-
51. Fixed age of starting product use	16
52. Output absolute risk file?	N

Results for PHIM for Run 1 (Basic_US_OG_T1.RTF)

****

Osmond and Gardner Modeling of Death Rates for COD: All Causes

Variable Parameter	Value
1. Country	US (United States)
2. Sex	M (Males)
3. Year of start of OG modelling	1966
4. Number 5-year periods for OG modelling	10
5. Age Range of interest	10 - 79
6. Scaling Factor for Rates	10000
7. Model Type to Save	4 (Full Age-Period-Cohort)
8. Goodness of Fit Required	1 (Analysis Printed For Age-Period-Cohort Model)
9. Cause of Death	All Causes
Note:	Death rates are per million population

Matrix of Numbers of Deaths

Age	1966-	1971-	1976-	1981-	1986-	1991-	1996-	2001-	2006-	2011-
10-14	26560	26099	20262	15955	14594	14836	13248	12098	9621	8755
15-19	70802	80511	76794	59105	57660	55780	50337	48880	44514	35615
20-24	78092	97616	102600	91635	83974	77417	65490	74265	75102	72091
25-29	61018	77239	87840	90638	98389	88487	66635	66799	74374	77458
30-34	61189	69073	76494	89432	115766	126810	86523	76938	73796	83349
35-39	87116	81285	79903	91163	126851	153726	124851	108590	95469	90955
40-44	142836	125693	106090	106866	134645	175091	169148	170588	144631	127624
45-49	218621	207798	169385	146767	156895	191902	214634	241200	235687	203520
50-54	316294	311085	278357	235659	211693	225226	254608	307687	336048	337324
55-59	438370	424808	400129	373924	324993	294093	306919	359207	418498	468032
60-64	548112	561927	529444	523418	495363	437607	399306	417036	477350	565709
65-69	632614	654522	653544	649802	651869	617477	552107	499882	521451	622044
70-74	694556	687884	700127	743175	752608	762390	742612	655839	596181	666060
75-79	675757	676012	662582	726917	783718	806011	856793	827712	739436	733939

Matrix of Age- and Period-Specific Mortality Rates

Age	1966-	1971-	1976-	1981-	1986-	1991-	1996-	2001-	2006-	2011-
10-14	506.744	489.132	404.530	346.786	328.843	318.406	258.899	220.665	178.067	164.868
15-19	1503.886	1533.059	1402.858	1139.496	1220.395	1213.971	1023.612	908.447	788.335	637.340
20-24	1999.636	2089.898	1942.710	1650.658	1609.435	1624.776	1377.112	1463.397	1379.221	1242.741
25-29	1854.844	1927.386	1843.972	1691.677	1770.221	1685.812	1355.512	1375.912	1459.697	1398.303
30-34	2068.943	2062.708	1891.765	1855.128	2142.419	2245.194	1596.754	1523.376	1500.127	1606.130
35-39	2927.725	2762.200	2472.195	2295.320	2635.782	2844.482	2181.035	1982.936	1879.670	1822.705
40-44	4562.385	4249.806	3669.732	3350.215	3391.690	3668.545	3130.359	2991.168	2652.184	2503.981
45-49	7198.101	6786.014	6005.066	5208.792	4985.903	4937.206	4527.649	4501.338	4177.242	3757.011
50-54	11565.44	10683.20	9598.566	8743.495	7701.962	7335.132	6612.387	6552.230	6369.514	6076.426
55-59	17983.44	16652.29	14749.74	13718.02	12652.66	11195.54	10311.09	9546.133	9165.618	9138.511
60-64	26541.39	25775.85	22646.65	20974.98	19613.40	18244.51	15960.56	14641.55	13247.57	12993.15
65-69	39062.19	37156.08	33801.97	31068.22	29350.36	27293.18	25180.55	21489.89	19645.22	18508.64
70-74	59637.80	54465.84	49158.16	47015.19	43635.97	40960.33	38320.15	34188.70	28949.28	28042.21
75-79	83587.17	81538.26	69325.19	67220.12	65911.28	61120.96	58346.54	53303.30	47020.51	42609.85

Matrix of Log-Rates

Age	1966-	1971-	1976-	1981-	1986-	1991-	1996-	2001-	2006-	2011-
10-14	2.705	2.689	2.607	2.540	2.517	2.503	2.413	2.344	2.251	2.217
15-19	3.177	3.186	3.147	3.057	3.087	3.084	3.010	2.958	2.897	2.804
20-24	3.301	3.320	3.288	3.218	3.207	3.211	3.139	3.165	3.140	3.094
25-29	3.268	3.285	3.266	3.228	3.248	3.227	3.132	3.139	3.164	3.146
30-34	3.316	3.314	3.277	3.268	3.331	3.351	3.203	3.183	3.176	3.206
35-39	3.467	3.441	3.393	3.361	3.421	3.454	3.339	3.297	3.274	3.261
40-44	3.659	3.628	3.565	3.525	3.530	3.564	3.496	3.476	3.424	3.399
45-49	3.857	3.832	3.779	3.717	3.698	3.693	3.656	3.653	3.621	3.575
50-54	4.063	4.029	3.982	3.942	3.887	3.865	3.820	3.816	3.804	3.784
55-59	4.255	4.221	4.169	4.137	4.102	4.049	4.013	3.980	3.962	3.961
60-64	4.424	4.411	4.355	4.322	4.293	4.261	4.203	4.166	4.122	4.114
65-69	4.592	4.570	4.529	4.492	4.468	4.436	4.401	4.332	4.293	4.267
70-74	4.776	4.736	4.692	4.672	4.640	4.612	4.583	4.534	4.462	4.448
75-79	4.922	4.911	4.841	4.827	4.819	4.786	4.766	4.727	4.672	4.630

Results for PHIM for Run 1 (Basic_US_OG_T1.RTF)

Fitting the Age, Period, Cohort Models

Model	RSS	MRSS	DF	Factor	%Account	ChiSq	P
Age Only	382049.190	3008.261	127	P, C	98.5828	2042847.98	0.0000
Age-Period	19291.140	164.882	117	Cohort	71.9330	102419.136	0.0000
Age-Cohort	7812.477	75.120	104	Period	30.6950	41455.723	0.0000
Period-Cohort	6582.909	60.953	108	Age	17.7501	34932.574	0.0000
Full Age-Period-Cohort	5414.435	56.400	96			28726.636	0.0000

**Key to terms:**		
	RSS =	residual sum of squares
	MRSS =	mean RSS (MRSS/DF)
	DF =	degrees of freedom
	Factor =	Factors not included in the model
	% Account =	1- (RSS for full model)/(RSS for model in question)
	Chisq =	chi-squared value for model
	P =	probability value based on Chisq and DF.

Age	Value	Log10 Value
10-	413.171205	2.616130
15-	1389.96388	3.143004
20-	1946.80406	3.289322
25-	1925.13456	3.284461
30-	2175.90732	3.337640
35-	2743.29059	3.438272
40-	3795.72795	3.579295
45-	5581.65748	3.746763
50-	8418.57608	3.925239
55-	12580.6688	4.099704
60-	18658.6871	4.270881
65-	26881.1765	4.429448
70-	38904.9981	4.590005
75-	56268.3997	4.750265

Period	Value	Log10 Value
1966	1.221487	0.086889
1971	1.185388	0.073861
1976	1.084477	0.035220
1981	1.035284	0.015060
1986	1.024055	0.010323
1991	1.009476	0.004096
1996	0.943378	-0.025314
2001	0.897015	-0.047201
2006	0.842968	-0.074189
2011	0.824333	-0.083897

Cohort	Value	Log10 Value
1891	1.216147	0.084986
1896	1.238822	0.093009
1901	1.168520	0.067636
1906	1.162125	0.065253
1911	1.159945	0.064437
1916	1.103531	0.042785
1921	1.072947	0.030578
1926	1.035896	0.015316
1931	0.981194	-0.008245
1936	0.894138	-0.048595
1941	0.864810	-0.063079
1946	0.841661	-0.074863
1951	0.872092	-0.059437
1956	0.907361	-0.042220
1961	0.891477	-0.049890
1966	0.817728	-0.087391
1971	0.802772	-0.095408
1976	0.803809	-0.094847
1981	0.854626	-0.068224
1986	0.815619	-0.088513
1991	0.719626	-0.142893
1996	0.546356	-0.262525
2001	0.484064	-0.315097

Results for PHIM for Run 1 (Basic_US_OG_T1.RTF)

Model: Full Age-Period-Cohort

Basic Analysis Using OG Modelling T1 on US
Fitting the Full Age-Period-Cohort Model
Matrix of observed, expected, and residual rates

**Age**		**1966-**	**1971-**	**1976-**	**1981-**	**1986-**	**1991-**	**1996-**	**2001-**	**2006-**	**2011-**
10-	Observed	506.744	489.132	404.530	346.786	328.843	318.406	258.899	220.665	178.067	164.868
	Expected	457.930	436.617	366.403	343.385	340.099	356.453	317.909	266.708	190.290	164.868
	Residual	-48.814	-52.515	-38.126	-3.401	11.256	38.047	59.010	46.043	12.224	-0.000
15-	Observed	1503.886	1533.059	1402.858	1139.496	1220.395	1213.971	1023.612	908.447	788.335	637.340
	Expected	1480.659	1495.010	1343.799	1176.718	1142.665	1127.852	1120.638	1016.928	843.183	626.011
	Residual	-23.227	-38.049	-59.060	37.222	-77.730	-86.119	97.027	108.481	54.848	-11.329
20-	Observed	1999.636	2089.898	1942.710	1650.658	1609.435	1624.776	1377.112	1463.397	1379.221	1242.741
	Expected	2001.466	2012.544	1915.679	1796.769	1630.251	1577.649	1476.253	1492.443	1338.508	1154.867
	Residual	1.830	-77.355	-27.031	146.111	20.816	-47.127	99.141	29.046	-40.713	-87.874
25-	Observed	1854.844	1927.386	1843.972	1691.677	1770.221	1685.812	1355.512	1375.912	1459.697	1398.303
	Expected	2033.625	1920.696	1820.724	1808.426	1757.497	1589.155	1457.939	1388.076	1386.910	1294.349
	Residual	178.782	-6.690	-23.248	116.749	-12.724	-96.657	102.426	12.164	-72.786	-103.955
30-	Observed	2068.943	2062.708	1891.765	1855.128	2142.419	2245.194	1596.754	1523.376	1500.127	1606.130
	Expected	2376.478	2230.601	1986.085	1964.548	2021.825	1958.154	1678.555	1566.867	1474.363	1532.919
	Residual	307.535	167.893	94.320	109.419	-120.594	-287.041	81.801	43.491	-25.764	-73.211
35-	Observed	2927.725	2762.200	2472.195	2295.320	2635.782	2844.482	2181.035	1982.936	1879.670	1822.705
	Expected	3287.876	2907.615	2572.842	2390.389	2449.952	2512.742	2307.109	2012.243	1856.416	1817.721
	Residual	360.151	145.415	100.648	95.069	-185.830	-331.740	126.074	29.307	-23.254	-4.984
40-	Observed	4562.385	4249.806	3669.732	3350.215	3391.690	3668.545	3130.359	2991.168	2652.184	2503.981
	Expected	4802.861	4414.793	3680.612	3398.409	3271.563	3341.594	3249.085	3035.323	2616.468	2511.829
	Residual	240.476	164.987	10.880	48.194	-120.127	-326.951	118.726	44.155	-35.715	7.848
45-	Observed	7198.101	6786.014	6005.066	5208.792	4985.903	4937.206	4527.649	4501.338	4177.242	3757.011
	Expected	7315.271	6853.931	5939.341	5166.869	4943.190	4742.379	4592.103	4542.999	4194.544	3762.488
	Residual	117.170	67.917	-65.725	-41.923	-42.713	-194.827	64.454	41.662	17.302	5.477
50-	Observed	11565.440	10683.202	9598.566	8743.495	7701.962	7335.132	6612.387	6552.230	6369.514	6076.426
	Expected	11347.815	10707.240	9457.472	8551.711	7708.438	7349.463	6684.387	6585.680	6439.171	6186.596
	Residual	-217.625	24.038	-141.094	-191.784	6.476	14.330	72.000	33.450	69.657	110.170
55-	Observed	17983.439	16652.288	14749.735	13718.019	12652.664	11195.540	10311.093	9546.133	9165.618	9138.511
	Expected	17825.017	16456.936	14638.699	13492.096	12641.006	11355.449	10263.857	9498.177	9248.633	9409.933
	Residual	-158.422	-195.353	-111.037	-225.923	-11.658	159.910	-47.236	-47.956	83.014	271.422
60-	Observed	26541.395	25775.846	22646.650	20974.983	19613.403	18244.514	15960.563	14641.548	13247.575	12993.147
	Expected	26486.383	25655.410	22329.869	20726.172	19793.395	18481.271	15738.798	14474.431	13238.214	13413.633
	Residual	-55.012	-120.436	-316.780	-248.811	179.993	236.757	-221.765	-167.117	-9.361	420.486
65-	Observed	39062.187	37156.082	33801.972	31068.218	29350.363	27293.185	25180.550	21489.892	19645.217	18508.637
	Expected	38368.360	37030.663	33814.736	30710.907	29535.872	28109.967	24882.208	21560.177	19596.589	18650.398
	Residual	-693.827	-125.419	12.764	-357.311	185.510	816.782	-298.342	70.285	-48.628	141.761
70-	Observed	59637.804	54465.840	49158.165	47015.188	43635.971	40960.330	38320.153	34188.699	28949.280	28042.205
	Expected	58871.240	53889.235	49031.872	46719.953	43965.629	42138.568	38019.584	34242.037	29323.867	27735.061
	Residual	-766.564	-576.605	-126.293	-295.234	329.658	1178.237	-300.570	53.339	374.587	-307.144
75-	Observed	83587.174	81538.257	69325.188	67220.117	65911.279	61120.963	58346.538	53303.305	47020.505	42609.848
	Expected	83587.174	82629.287	71305.173	67698.167	66838.252	62682.354	56954.604	52285.357	46540.448	41473.618
	Residual	0.000	1091.030	1979.985	478.050	926.973	1561.391	-1391.934	-1017.948	-480.057	-1136.230

Results for PHIM for Run 1 (Basic_US_OG_T1.RTF)

Fitting the Full Age-Period-Cohort Model

Matrix of observed and expected deaths and (O-E)2/E Values**

**Age**		**1966-**	**1971-**	**1976-**	**1981-**	**1986-**	**1991-**	**1996-**	**2001-**	**2006-**	**2011-**	**Total**
10-	Observed	26560.0	26099.0	20262.0	15955.0	14594.0	14836.0	13248.0	12098.0	9621.0	8755.0	162028.0
	Expected	24001.5	23296.9	18352.3	15798.5	15093.5	16608.8	16267.6	14622.3	10281.5	8755.0	163078.0
	Difference	2558.5	2802.1	1909.7	156.5	-499.5	-1772.8	-3019.6	-2524.3	-660.5	0.0	-1050.0
	Chi-Sq	272.7	337.0	198.7	1.5	16.5	189.2	560.5	435.8	42.4	0.0	2054.5
15-	Observed	70802.0	80511.0	76794.0	59105.0	57660.0	55780.0	50337.0	48880.0	44514.0	35615.0	579998.0
	Expected	69708.5	78512.8	73561.0	61035.7	53987.5	51823.0	55108.4	54717.0	47611.0	34981.9	581046.7
	Difference	1093.5	1998.2	3233.0	-1930.7	3672.5	3957.0	-4771.4	-5837.0	-3097.0	633.1	-1048.7
	Chi-Sq	17.2	50.9	142.1	61.1	249.8	302.1	413.1	622.7	201.5	11.5	2071.8
20-	Observed	78092.0	97616.0	102600.0	91635.0	83974.0	77417.0	65490.0	74265.0	75102.0	72091.0	818282.0
	Expected	78163.5	94002.9	101172.4	99746.3	85060.1	75171.5	70204.7	75739.0	72885.1	66993.5	819138.9
	Difference	-71.5	3613.1	1427.6	-8111.3	-1086.1	2245.5	-4714.7	-1474.0	2216.9	5097.5	-856.9
	Chi-Sq	0.1	138.9	20.1	659.6	13.9	67.1	316.6	28.7	67.4	387.9	1700.2
25-	Observed	61018.0	77239.0	87840.0	90638.0	98389.0	88487.0	66635.0	66799.0	74374.0	77458.0	788877.0
	Expected	66899.3	76970.9	86732.5	96893.3	97681.8	83413.5	71670.1	67389.5	70665.4	71699.5	790015.9
	Difference	-5881.3	268.1	1107.5	-6255.3	707.2	5073.5	-5035.1	-590.5	3708.6	5758.5	-1138.9
	Chi-Sq	517.0	0.9	14.1	403.8	5.1	308.6	353.7	5.2	194.6	462.5	2265.7
30-	Observed	61189.0	69073.0	76494.0	89432.0	115766.0	126810.0	86523.0	76938.0	73796.0	83349.0	859370.0
	Expected	70284.4	74695.2	80307.8	94706.9	109249.7	110597.8	90955.5	79134.5	72528.6	79549.8	862010.1
	Difference	-9095.4	-5622.2	-3813.8	-5274.9	6516.3	16212.2	-4432.5	-2196.5	1267.4	3799.2	-2640.1
	Chi-Sq	1177.0	423.2	181.1	293.8	388.7	2376.5	216.0	61.0	22.1	181.4	5320.9
35-	Observed	87116.0	81285.0	79903.0	91163.0	126851.0	153726.0	124851.0	108590.0	95469.0	90955.0	1039909.0
	Expected	97832.5	85564.2	83156.0	94938.8	117907.6	135797.6	132068.0	110194.9	94287.9	90706.3	1042453.9
	Difference	-10716.5	-4279.2	-3253.0	-3775.8	8943.4	17928.4	-7217.0	-1604.9	1181.1	248.7	-2544.9
	Chi-Sq	1173.9	214.0	127.3	150.2	678.4	2367.0	394.4	23.4	14.8	0.7	5143.9
40-	Observed	142836.0	125693.0	106090.0	106866.0	134645.0	175091.0	169148.0	170588.0	144631.0	127624.0	1403212.0
	Expected	150364.6	130572.7	106404.5	108403.3	129876.1	159486.4	175563.3	173106.2	142683.3	128024.0	1404484.5
	Difference	-7528.6	-4879.7	-314.5	-1537.3	4768.9	15604.6	-6415.3	-2518.2	1947.7	-400.0	-1272.5
	Chi-Sq	377.0	182.4	0.9	21.8	175.1	1526.8	234.4	36.6	26.6	1.2	2582.8
45-	Observed	218621.0	207798.0	169385.0	146767.0	156895.0	191902.0	214634.0	241200.0	235687.0	203520.0	1986409.0
	Expected	222179.7	209877.7	167531.1	145585.7	155550.9	184329.3	217689.5	243432.4	236663.2	203816.7	1986656.3
	Difference	-3558.7	-2079.7	1853.9	1181.3	1344.1	7572.7	-3055.5	-2232.4	-976.2	-296.7	-247.3
	Chi-Sq	57.0	20.6	20.5	9.6	11.6	311.1	42.9	20.5	4.0	0.4	498.2
50-	Observed	316294.0	311085.0	278357.0	235659.0	211693.0	225226.0	254608.0	307687.0	336048.0	337324.0	2813981.0
	Expected	310342.3	311785.0	274265.3	230490.0	211871.0	225666.0	257380.3	309257.8	339723.0	343439.9	2814220.6
	Difference	5951.7	-700.0	4091.7	5169.0	-178.0	-440.0	-2772.3	-1570.8	-3675.0	-6115.9	-239.6
	Chi-Sq	114.1	1.6	61.0	115.9	0.1	0.9	29.9	8.0	39.8	108.9	480.2
55-	Observed	438370.0	424808.0	400129.0	373924.0	324993.0	294093.0	306919.0	359207.0	418498.0	468032.0	3808973.0
	Expected	434508.2	419824.5	397116.8	367765.8	324693.6	298293.6	305513.0	357402.5	422288.4	481933.0	3809339.4
	Difference	3861.8	4983.5	3012.2	6158.2	299.4	-4200.6	1406.0	1804.5	-3790.4	-13901.0	-366.4
	Chi-Sq	34.3	59.2	22.8	103.1	0.3	59.2	6.5	9.1	34.0	401.0	729.4
60-	Observed	548112.0	561927.0	529444.0	523418.0	495363.0	437607.0	399306.0	417036.0	477350.0	565709.0	4955272.0
	Expected	546975.9	559301.4	522038.2	517209.1	499909.0	443285.8	393757.8	412276.0	477012.7	584016.6	4955782.4
	Difference	1136.1	2625.6	7405.8	6208.9	-4546.0	-5678.8	5548.2	4760.0	337.3	-18307.6	-510.4
	Chi-Sq	2.4	12.3	105.1	74.5	41.3	72.7	78.2	55.0	0.2	573.9	1015.6
65-	Observed	632614.0	654522.0	653544.0	649802.0	651869.0	617477.0	552107.0	499882.0	521451.0	622044.0	6055312.0
	Expected	621377.4	652312.7	653790.8	642328.7	655989.2	635955.8	545565.6	501516.9	520160.3	626808.4	6055805.6
	Difference	11236.6	2209.3	-246.8	7473.3	-4120.2	-18478.8	6541.4	-1634.9	1290.7	-4764.4	-493.6
	Chi-Sq	203.2	7.5	0.1	86.9	25.9	536.9	78.4	5.3	3.2	36.2	983.7

Results for PHIM for Run 1 (Basic_US_OG_T1.RTF)

70-	Observed	694556.0	687884.0	700127.0	743175.0	752608.0	762390.0	742612.0	655839.0	596181.0	666060.0	7001432.0
	Expected	685628.4	680601.7	698328.3	738508.2	758293.8	784320.4	736787.2	656862.2	603895.2	658764.7	7001990.1
	Difference	8927.6	7282.3	1798.7	4666.8	-5685.8	-21930.4	5824.8	-1023.2	-7714.2	7295.3	-558.1
	Chi-Sq	116.2	77.9	4.6	29.5	42.6	613.2	46.0	1.6	98.5	80.8	1111.1
75-	Observed	675757.0	676012.0	662582.0	726917.0	783718.0	806011.0	856793.0	827712.0	739436.0	733939.0	7488877.0
	Expected	675757.0	685057.4	681505.9	732086.6	794740.2	826601.3	836353.1	811905.0	731886.7	714367.8	7490261.0
	Difference	-0.0	-9045.4	-18923.9	-5169.6	-11022.2	-20590.3	20439.9	15807.0	7549.3	19571.2	-1384.0
	Chi-Sq	0.0	119.4	525.5	36.5	152.9	512.9	499.5	307.7	77.9	536.2	2768.5
Total over ages	Observed	4051937.0	4081552.0	3943551.0	3944456.0	4009018.0	4026853.0	3903211.0	3866721.0	3842158.0	4092475.0	39761932.0
	Expected	4054023.3	4082375.9	3944263.0	3945496.9	4009903.9	4031350.7	3904884.1	3867556.2	3842572.3	4093857.0	39776283.4
	Difference	-2086.3	-823.9	-712.0	-1040.9	-885.9	-4497.7	-1673.1	-835.2	-414.3	-1382.0	-14351.4
	Chi-Sq	4062.1	1645.7	1424.1	2047.9	1802.2	9244.2	3270.2	1620.5	827.1	2782.6	28726.6

Results for PHIM for Run 1 (Basic_US_OG_T1.RTF)

****

Osmond and Gardner Extrapolating Death Rates for COD: All Causes

Variable Parameter	Value
1. Country	US (United States)
2. Sex	M (Males)
3. Year of start of OG modelling	1966
4. Number 5-year periods for OG modelling	10
5. Age Range of interest	10 - 79
6. Scaling Factor for Rates	10000
7. Model Type to Save	4 (Full Age-Period-Cohort)
8. Goodness of Fit Required	1 (Analysis Printed For Age-Period-Cohort Model)
9. Cause of Death	All Causes
Note:	Death rates are per million population
10. Number of Periods into the future to Predict	5
11. Earliest projected year	2016
12. Extrapolate Period using (1: last 2 points 2: linear regression)	1
13. Ratio of last two period values	0.97789

Predictions of rates for future years from model:	Full Age-Period-Cohort
Effects for extending model to project rates for:	2016-2040

Extrapolating Model: Full Age-Period-Cohort
Log Transform Parameters

Model	ChiSq	MChiSq	DF	Factor	%Account	P
Age Only	666879.602	47634.257	14	P, C	93.7430	0.0000
Age-Period	78117.347	5579.810	14	Cohort	46.5847	0.0000
Age-Cohort	49204.726	3514.623	14	Period	15.1979	0.0000
Period-Cohort	54044.177	3860.298	14	Age	22.7916	0.0000
Full Age-Period-Cohort	41726.630	2980.474	14			0.0000

Key to terms:		
	Chisq =	chi-squared value for model
	MChisq =	mean Chi-squared (Chisq/DF)
	DF =	degrees of freedom
	Factor =	Factors not included in the model
	% Account =	1- (Chisq for full model)/(Chisq for model in question)
	P =	probability value based on Chisq and DF.

AGE	EFFECT
10	413.171205
15	1389.96388
20	1946.80406
25	1925.13456
30	2175.90732
35	2743.29059
40	3795.72795
45	5581.65748
50	8418.57608
55	12580.6688
60	18658.6871
65	26881.1765
70	38904.9981
75	56268.3997

PERIOD	EFFECT	
Period Change =	0.977893	
1966	1.221487	
1971	1.185388	
1976	1.084477	
1981	1.035284	
1986	1.024055	
1991	1.009476	
1996	0.943378	
2001	0.897015	
2006	0.842968	
2011	0.824333	
2016	0.806110	
2021	0.788290	
2026	0.770863	
2031	0.753822	
2036	0.737158	
2016	0.813350	Extrapolated
2017	0.809722	Extrapolated
2018	0.806110	Extrapolated
2019	0.802514	Extrapolated

Results for PHIM for Run 1 (Basic_US_OG_T1.RTF)

2020	0.798934	Extrapolated
2021	0.795370	Extrapolated
2022	0.791822	Extrapolated
2023	0.788290	Extrapolated
2024	0.784773	Extrapolated
2025	0.781272	Extrapolated
2026	0.777787	Extrapolated
2027	0.774317	Extrapolated
2028	0.770863	Extrapolated
2029	0.767424	Extrapolated
2030	0.764001	Extrapolated
2031	0.760593	Extrapolated
2032	0.757200	Extrapolated
2033	0.753822	Extrapolated
2034	0.750459	Extrapolated
2035	0.747112	Extrapolated
2036	0.743779	Extrapolated
2037	0.740461	Extrapolated
2038	0.737158	Extrapolated
2039	0.733869	Extrapolated
2040	0.730595	Extrapolated

Results for PHIM for Run 1 (Basic_US_OG_T1.RTF)

COHORT	EFFECT	WEIGHT	ORIGINAL
1891	1.216147	1.000	
1896	1.238822	2.000	
1901	1.168520	4.000	
1906	1.162125	8.000	
1911	1.159945	16.000	
1916	1.103531	32.000	
1921	1.072947	64.000	
1926	1.035896	128.000	
1931	0.981194	256.000	
1936	0.894138	512.000	
1941	0.864810	1024.000	
1946	0.841661	2048.000	
1951	0.872092	4096.000	
1956	0.907361	8192.000	
1961	0.891477	16384.000	
1966	0.817728	32768.000	
1971	0.802772	65536.000	
1976	0.803809	131072.000	
1981	0.854626	262144.000	
1986	0.815619	524288.000	
1991	0.719626	1048576.000	
1996	0.715936	Extrapolated	0.546356
2001	0.690069	Extrapolated	0.484064
2006	0.665136	Extrapolated	
2011	0.641103	Extrapolated	
2016	0.617940	Extrapolated	
2021	0.595613	Extrapolated	
2026	0.574093	Extrapolated	

Standardizing Population: The 1976 European Standard Population

Age Range	Population, Males
All	100000
0	0
1	0
2	0
3	0
0-4	8000
5-9	7000
10-14	7000
15-19	7000
20-24	7000
25-29	7000
30-34	7000
35-39	7000
40-44	7000
45-49	7000
50-54	7000
55-59	6000
60-64	5000
65-69	4000
70-74	3000
75-79	2000
80-84	1000
85+	1000

Results for PHIM for Run 1 (Basic_US_OG_T1.RTF)

Matrix of observed and expected rates including predictions

Total over ages standardized using: The 1976 European Standard Population

		1966-	1971-	1976-	1981-	1986-	1991-	1996-	2001-	2006-	2011-	2016-	2021-	2026-	2031-	2036-
10	OBS	506.7	489.1	404.5	346.8	328.8	318.4	258.9	220.7	178.1	164.9	173.6	.	.	.	.
	EXP											221.5	208.8	196.8	185.5	174.9
15	OBS	1503.9	1533.1	1402.9	1139.5	1220.4	1214.0	1023.6	908.4	788.3	637.3	717.0	.	.	.	.
	EXP											773.2	728.8	686.9	647.5	610.3
20	OBS	1999.6	2089.9	1942.7	1650.7	1609.4	1624.8	1377.1	1463.4	1379.2	1242.7	1382.6	.	.	.	.
	EXP											1123.5	1059.0	998.2	940.8	886.8
25	OBS	1854.8	1927.4	1844.0	1691.7	1770.2	1685.8	1355.5	1375.9	1459.7	1398.3	1669.0	.	.	.	.
	EXP											1116.8	1086.5	1024.1	965.3	909.8
30	OBS	2068.9	2062.7	1891.8	1855.1	2142.4	2245.2	1596.8	1523.4	1500.1	1606.1	1877.4	.	.	.	.
	EXP											1430.6	1234.3	1200.9	1131.9	1066.9
35	OBS	2927.7	2762.2	2472.2	2295.3	2635.8	2844.5	2181.0	1982.9	1879.7	1822.7	2230.9	.	.	.	.
	EXP											1889.9	1763.8	1521.8	1480.5	1395.5
40	OBS	4562.4	4249.8	3669.7	3350.2	3391.7	3668.5	3130.4	2991.2	2652.2	2504.0	2648.1	.	.	.	.
	EXP											2459.5	2557.2	2386.5	2059.1	2003.2
45	OBS	7198.1	6786.0	6005.1	5208.8	4985.9	4937.2	4527.6	4501.3	4177.2	3757.0	3944.3	.	.	.	.
	EXP											3612.0	3536.7	3677.2	3431.8	2960.9
50	OBS	11565.4	10683.2	9598.6	8743.5	7702.0	7335.1	6612.4	6552.2	6369.5	6076.4	6056.3	.	.	.	.
	EXP											5549.3	5327.4	5216.4	5423.5	5061.6
55	OBS	17983.4	16652.3	14749.7	13718.0	12652.7	11195.5	10311.1	9546.1	9165.6	9138.5	9178.0	.	.	.	.
	EXP											9040.8	8109.6	7785.3	7623.0	7925.7
60	OBS	26541.4	25775.8	22646.6	20975.0	19613.4	18244.5	15960.6	14641.5	13247.6	12993.1	12897.2	.	.	.	.
	EXP											13647.6	13112.3	11761.6	11291.3	11055.9
65	OBS	39062.2	37156.1	33802.0	31068.2	29350.4	27293.2	25180.5	21489.9	19645.2	18508.6	17940.3	.	.	.	.
	EXP											18897.5	19227.1	18472.9	16570.1	15907.5
70	OBS	59637.8	54465.8	49158.2	47015.2	43636.0	40960.3	38320.2	34188.7	28949.3	28042.2	25492.5	.	.	.	.
	EXP											26395.9	26745.7	27212.1	26144.8	23451.7
75	OBS	83587.2	81538.3	69325.2	67220.1	65911.3	61121.0	58346.5	53303.3	47020.5	42609.8	39991.1	.	.	.	.
	EXP											39226.5	37332.5	37827.2	38486.9	36977.3
10-79	OBS	11834.4	11228.6	9972.1	9288.1	8850.9	8359.1	7572.1	6942.8	6305.9	5995.7	5935.8	.	.	.	.
	EXP	11835.8	11240.0	9976.2	9267.0	8860.1	8393.3	7565.8	6942.8	6312.2	6018.0	5818.7*	5645.1*	5482.7*	5274.0*	5015.2*

Drop in overall standardized Observed and Predicted rates

comparing the last observed rate during the model fitting period to the last observed and predicted rates where an observed rate is available (2016)

Observed and Predicted %Drop = 1.000% and 2.953%

Results for PHIM for Run 1 (Basic_US_OG_T1.RTF)

Matrix of observed and expected deaths including predictions

		1966-	1971-	1976-	1981-	1986-	1991-	1996-	2001-	2006-	2011-	2016-	2021-	2026-	2031-	2036-
10-	OBS	26560.0	26099.0	20262.0	15955.0	14594.0	14836.0	13248.0	12098.0	9621.0	8755.0	9335.0*	.	.	.	.
	EXP	24001.5	23296.9	18352.3	15798.5	15093.5	16608.8	16267.6	14622.3	13472.7	12480.9	11909.4*	10988.0*	10017.2*	9609.9*	9341.2*
	ChiSq	272.730	337.025	198.710	1.550	16.533	189.224	560.500	435.786	1101.142	1112.283	556.514*	.	.	.	.
15-	OBS	70802.0	80511.0	76794.0	59105.0	57660.0	55780.0	50337.0	48880.0	44514.0	35615.0	38905.0*	.	.	.	.
	EXP	69708.5	78512.8	73561.0	61035.7	53987.5	51823.0	55108.4	54717.0	47611.0	45839.8	41954.4*	39997.5*	36943.6*	33780.0*	32428.2*
	ChiSq	17.154	50.856	142.089	61.071	249.825	302.146	413.112	622.660	201.458	2280.691	221.640*	.	.	.	.
20-	OBS	78092.0	97616.0	102600.0	91635.0	83974.0	77417.0	65490.0	74265.0	75102.0	72091.0	79885.0*	.	.	.	.
	EXP	78163.5	94002.9	101172.4	99746.3	85060.1	75171.5	70204.7	75739.0	72885.1	66993.5	64917.7*	59383.5*	56697.8*	52576.0*	48214.0*
	ChiSq	0.065	138.875	20.144	659.599	13.868	67.076	316.629	28.687	67.433	387.871	3450.808*	.	.	.	.
25-	OBS	61018.0	77239.0	87840.0	90638.0	98389.0	88487.0	66635.0	66799.0	74374.0	77458.0	99740.0*	.	.	.	.
	EXP	66899.3	76970.9	86732.5	96893.3	97681.8	83413.5	71670.1	67389.5	70665.4	71699.5	66736.7*	64519.5*	59173.9*	56640.5*	52643.4*
	ChiSq	517.041	0.934	14.141	403.832	5.120	308.585	353.739	5.175	194.631	462.491	16321.138*	.	.	.	.
30-	OBS	61189.0	69073.0	76494.0	89432.0	115766.0	126810.0	86523.0	76938.0	73796.0	83349.0	106275.0*	.	.	.	.
	EXP	70284.4	74695.2	80307.8	94706.9	109249.7	110597.8	90955.5	79134.5	72528.6	79549.8	80982.4*	75011.8*	72592.8*	66784.7*	63980.1*
	ChiSq	1177.011	423.171	181.120	293.796	388.671	2376.509	216.011	60.967	22.148	181.447	7899.442*	.	.	.	.
35-	OBS	87116.0	81285.0	79903.0	91163.0	126851.0	153726.0	124851.0	108590.0	95469.0	90955.0	117285.0*	.	.	.	.
	EXP	97832.5	85564.2	83156.0	94938.8	117907.6	135797.6	132068.0	110194.9	94287.9	90706.3	99357.2*	100714.0*	93231.3*	90407.1*	83306.6*
	ChiSq	1173.874	214.010	127.255	150.170	678.358	2366.967	394.378	23.375	14.794	0.682	3234.840*	.	.	.	.
40-	OBS	142836.0	125693.0	106090.0	106866.0	134645.0	175091.0	169148.0	170588.0	144631.0	127624.0	132732.5*	.	.	.	.
	EXP	150364.6	130572.7	106404.5	108403.3	129876.1	159486.4	175563.3	173106.2	142683.3	128024.0	123276.2*	134628.2*	136437.5*	126424.8*	122736.3*
	ChiSq	376.954	182.361	0.930	21.801	175.105	1526.802	234.423	36.632	26.586	1.250	725.374*	.	.	.	.
45-	OBS	218621.0	207798.0	169385.0	146767.0	156895.0	191902.0	214634.0	241200.0	235687.0	203520.0	199980.0*	.	.	.	.
	EXP	222179.7	209877.7	167531.1	145585.7	155550.9	184329.3	217689.5	243432.4	236663.2	203816.7	183132.5*	176083.3*	192418.9*	195278.2*	181076.5*
	ChiSq	57.000	20.609	20.515	9.585	11.614	311.102	42.886	20.472	4.027	0.432	1549.897*	.	.	.	.
50-	OBS	316294.0	311085.0	278357.0	235659.0	211693.0	225226.0	254608.0	307687.0	336048.0	337324.0	322245.0*	.	.	.	.
	EXP	310342.3	311785.0	274265.3	230490.0	211871.0	225666.0	257380.3	309257.8	339723.0	343439.9	295272.6*	265302.6*	255518.3*	279907.8*	284440.4*
	ChiSq	114.139	1.571	61.043	115.923	0.150	0.858	29.862	7.978	39.755	108.911	2463.860*	.	.	.	.
55-	OBS	438370.0	424808.0	400129.0	373924.0	324993.0	294093.0	306919.0	359207.0	418498.0	468032.0	492592.5*	.	.	.	.
	EXP	434508.2	419824.5	397116.8	367765.8	324693.6	298293.6	305513.0	357402.5	422288.4	481933.0	485230.9*	417336.1*	376023.1*	363541.5*	399248.5*
	ChiSq	34.322	59.157	22.848	103.117	0.276	59.154	6.471	9.111	34.022	400.963	111.687*	.	.	.	.
60-	OBS	548112.0	561927.0	529444.0	523418.0	495363.0	437607.0	399306.0	417036.0	477350.0	565709.0	626705.0*	.	.	.	.
	EXP	546975.9	559301.4	522038.2	517209.1	499909.0	443285.8	393757.8	412276.0	477012.7	584016.6	663168.5*	667655.8*	576372.4*	522248.5*	507023.0*
	ChiSq	2.360	12.325	105.062	74.536	41.339	72.749	78.176	54.958	0.238	573.899	2004.897*	.	.	.	.
65-	OBS	632614.0	654522.0	653544.0	649802.0	651869.0	617477.0	552107.0	499882.0	521451.0	622044.0	724117.5*	.	.	.	.
	EXP	621377.4	652312.7	653790.8	642328.7	655989.2	635955.8	545565.6	501516.9	520160.3	626808.4	762754.5*	865594.6*	875548.3*	761752.2*	694667.2*
	ChiSq	203.194	7.483	0.093	86.949	25.878	536.931	78.433	5.330	3.203	36.214	1957.142*	.	.	.	.

Results for PHIM for Run 1 (Basic_US_OG_T1.RTF)

70-	OBS	694556.0	687884.0	700127.0	743175.0	752608.0	762390.0	742612.0	655839.0	596181.0	666060.0	767565.0*	.	.	.	.
	EXP	685628.4	680601.7	698328.3	738508.2	758293.8	784320.4	736787.2	656862.2	603895.2	658764.7	794765.4*	966268.3*	1103897.2*	1129029.4*	991218.5*
	ChiSq	116.246	77.919	4.633	29.491	42.632	613.196	46.049	1.594	98.543	80.790	930.920*	.	.	.	.
75-	OBS	675757.0	676012.0	662582.0	726917.0	783718.0	806011.0	856793.0	827712.0	739436.0	733939.0	800890.0*	.	.	.	.
	EXP	675757.0	685057.4	681505.9	732086.6	794740.2	826601.3	836353.1	811905.0	731886.7	714367.8	785577.6*	951487.7*	1165841.1*	1354926.0*	1404158.5*
	ChiSq	.	119.435	525.474	36.505	152.865	512.895	499.539	307.749	77.870	536.180	298.470*	.	.	.	.
Total Deaths		4051937.0	4081552.0	3943551.0	3944456.0	4009018.0	4026853.0	3903211.0	3866721.0	3842158.0	4092475.0	4518252.5*	.	.	.	.
Expected		4054023.3	4082375.9	3944263.0	3945496.9	4009903.9	4031350.7	3904884.1	3867556.2	3845763.6	4108440.7	4459036.1*	4794970.8*	5010713.4*	5042906.7*	4874482.4*
Obs/Exp		0.999	1.000	1.000	1.000	1.000	0.999	1.000	1.000	0.999	0.996	1.013*	.	.	.	.

Chi Squared (Log) = **41726.6 on 14 D.F.** **P = 0.0000**

Results for PHIM for Run 1 (Basic_US_OG_T1.RTF)

****

Observed and predicted rates (5 year periods)

		10-	15-	20-	25-	30-	35-	40-	45-	50-	55-	60-	65-	70-	75-
2016-	OBS	173.6	717.0	1382.6	1669.0	1877.4	2230.9	2648.1	3944.3	6056.3	9178.0	12897.2	17940.3	25492.5	39991.1
	PRE	221.5	773.2	1123.5	1116.8	1430.6	1889.9	2459.5	3612.0	5549.3	9040.8	13647.6	18897.5	26395.9	39226.5
	RES	-47.888	-56.199	259.042	552.275	446.812	341.011	188.662	332.291	506.919	137.162	-750.394	-957.247	-903.386	764.602
2021-	PRE	208.8	728.8	1059.0	1086.5	1234.3	1763.8	2557.2	3536.7	5327.4	8109.6	13112.3	19227.1	26745.7	37332.5
2026-	PRE	196.8	686.9	998.2	1024.1	1200.9	1521.8	2386.5	3677.2	5216.4	7785.3	11761.6	18472.9	27212.1	37827.2
2031-	PRE	185.5	647.5	940.8	965.3	1131.9	1480.5	2059.1	3431.8	5423.5	7623.0	11291.3	16570.1	26144.8	38486.9
2036-	PRE	174.9	610.3	886.8	909.8	1066.9	1395.5	2003.2	2960.9	5061.6	7925.7	11055.9	15907.5	23451.7	36977.3

Results for PHIM for Run 1 (Basic_US_OG_T1.RTF)

****

Observed and predicted deaths (5 year periods)

		10-	15-	20-	25-	30-	35-	40-	45-	50-	55-	60-	65-	70-	75-
2016-	OBS	9335.0	38905.0	79885.0	99740.0	106275.0	117285.0	132732.5	199980.0	322245.0	492592.5	626705.0	724117.5	767565.0	800890.0
	PRE	11909.4	41954.4	64917.7	66736.7	80982.4	99357.2	123276.2	183132.5	295272.6	485230.9	663168.5	762754.5	794765.4	785577.6
	CHI	556.514	221.640	3450.808	16321.138	7899.442	3234.840	725.374	1549.897	2463.860	111.687	2004.897	1957.142	930.920	298.470
2021-	PRE	10988.0	39997.5	59383.5	64519.5	75011.8	100714.0	134628.2	176083.3	265302.6	417336.1	667655.8	865594.6	966268.3	951487.7
2026-	PRE	10017.2	36943.6	56697.8	59173.9	72592.8	93231.3	136437.5	192418.9	255518.3	376023.1	576372.4	875548.3	1103897.2	1165841.1
2031-	PRE	9609.9	33780.0	52576.0	56640.5	66784.7	90407.1	126424.8	195278.2	279907.8	363541.5	522248.5	761752.2	1129029.4	1354926.0
2036-	PRE	9341.2	32428.2	48214.0	52643.4	63980.1	83306.6	122736.3	181076.5	284440.4	399248.5	507023.0	694667.2	991218.5	1404158.5

Results for PHIM for Run 1 (Basic_US_OG_T1.RTF)

****

Observed and predicted rates (single years)

		10-	15-	20-	25-	30-	35-	40-	45-	50-	55-	60-	65-	70-	75-
2016	OBS	165.4	708.4	1382.9	1672.4	1896.4	2235.5	2642.7	3839.4	5958.1	9224.7	13245.9	18815.8	27571.0	41672.5
	PRE	223.5	780.1	1133.6	1126.8	1443.5	1906.9	2481.6	3644.5	5599.2	9122.0	13770.2	19067.3	26633.0	39578.9
	RES	-58.139	-71.694	249.287	545.606	452.941	328.643	161.165	194.931	358.907	102.673	-524.256	-251.505	938.040	2093.670
2017	OBS	183.6	722.8	1338.9	1721.0	1955.2	2331.3	2674.2	3932.9	5879.6	9152.1	13298.1	18416.9	27865.8	42410.7
	PRE	222.5	776.7	1128.6	1121.8	1437.0	1898.4	2470.5	3628.2	5574.2	9081.3	13708.7	18982.2	26514.2	39402.3
	RES	-38.894	-53.820	210.340	599.278	518.184	432.916	203.742	304.739	305.422	70.764	-410.640	-565.269	1351.616	3008.419
2018	PRE	221.5	773.2	1123.5	1116.8	1430.6	1889.9	2459.5	3612.0	5549.3	9040.8	13647.6	18897.5	26395.9	39226.5
2019	PRE	220.5	769.7	1118.5	1111.8	1424.2	1881.5	2448.5	3595.9	5524.6	9000.5	13586.7	18813.2	26278.2	39051.5
2020	PRE	219.6	766.3	1113.5	1106.8	1417.9	1873.1	2437.6	3579.9	5499.9	8960.4	13526.1	18729.3	26160.9	38877.3
2021	PRE	210.7	735.3	1068.5	1096.2	1245.4	1779.6	2580.1	3568.5	5375.3	8182.4	13230.0	19399.8	26985.9	37667.8
2022	PRE	209.7	732.1	1063.8	1091.3	1239.9	1771.7	2568.6	3552.6	5351.3	8145.9	13171.0	19313.3	26865.5	37499.8
2023	PRE	208.8	728.8	1059.0	1086.5	1234.3	1763.8	2557.2	3536.7	5327.4	8109.6	13112.3	19227.1	26745.7	37332.5
2024	PRE	207.9	725.5	1054.3	1081.6	1228.8	1755.9	2545.7	3521.0	5303.7	8073.4	13053.8	19141.3	26626.4	37166.0
2025	PRE	206.9	722.3	1049.6	1076.8	1223.3	1748.1	2534.4	3505.2	5280.0	8037.4	12995.5	19056.0	26507.6	37000.2
2026	PRE	198.6	693.1	1007.1	1033.3	1211.6	1535.5	2407.9	3710.2	5263.2	7855.2	11867.3	18638.9	27456.6	38167.0
2027	PRE	197.7	690.0	1002.7	1028.7	1206.2	1528.6	2397.2	3693.7	5239.7	7820.1	11814.3	18555.7	27334.1	37996.7
2028	PRE	196.8	686.9	998.2	1024.1	1200.9	1521.8	2386.5	3677.2	5216.4	7785.3	11761.6	18472.9	27212.1	37827.2
2029	PRE	195.9	683.9	993.7	1019.5	1195.5	1515.0	2375.8	3660.8	5193.1	7750.5	11709.2	18390.5	27090.8	37658.5
2030	PRE	195.1	680.8	989.3	1015.0	1190.2	1508.2	2365.2	3644.5	5169.9	7716.0	11656.9	18308.5	26969.9	37490.5
2031	PRE	187.2	653.3	949.3	973.9	1142.0	1493.8	2077.6	3462.6	5472.3	7691.5	11392.7	16719.0	26379.6	38832.6
2032	PRE	186.3	650.4	945.1	969.6	1137.0	1487.2	2068.3	3447.2	5447.9	7657.1	11341.8	16644.4	26261.9	38659.4
2033	PRE	185.5	647.5	940.8	965.3	1131.9	1480.5	2059.1	3431.8	5423.5	7623.0	11291.3	16570.1	26144.8	38486.9
2034	PRE	184.7	644.6	936.7	960.9	1126.8	1473.9	2049.9	3416.5	5399.4	7589.0	11240.9	16496.2	26028.1	38315.3
2035	PRE	183.9	641.7	932.5	956.7	1121.8	1467.3	2040.7	3401.2	5375.3	7555.1	11190.7	16422.6	25912.0	38144.3
2036	PRE	176.4	615.8	894.8	918.0	1076.5	1408.0	2021.2	2987.5	5107.0	7996.9	11155.2	16050.3	23662.4	37309.4
2037	PRE	175.6	613.0	890.8	913.9	1071.6	1401.7	2012.2	2974.2	5084.3	7961.3	11105.4	15978.7	23556.8	37143.0
2038	PRE	174.9	610.3	886.8	909.8	1066.9	1395.5	2003.2	2960.9	5061.6	7925.7	11055.9	15907.5	23451.7	36977.3
2039	PRE	174.1	607.6	882.9	905.7	1062.1	1389.3	1994.3	2947.7	5039.0	7890.4	11006.6	15836.5	23347.1	36812.4
2040	PRE	173.3	604.8	878.9	901.7	1057.4	1383.1	1985.4	2934.6	5016.5	7855.2	10957.5	15765.9	23243.0	36648.1

Results for PHIM for Run 1 (Basic_US_OG_T1.RTF)

****

Observed and predicted deaths (single years)

		10-	15-	20-	25-	30-	35-	40-	45-	50-	55-	60-	65-	70-	75-
2016	OBS	1764.0	7713.0	16300.0	19511.0	20746.0	22809.0	26406.0	39769.0	65317.0	98650.0	123734.0	144389.0	148353.0	156851.0
	PRE	2384.1	8493.6	13361.7	13145.7	15791.0	19455.9	24795.6	37749.9	61382.4	97552.0	128631.2	146319.0	143305.6	148970.6
	CHI	161.299	71.732	646.123	3082.133	1554.827	577.895	104.585	107.997	252.206	12.358	186.447	25.458	177.773	416.861
2017	OBS	1970.0	7849.0	15654.0	20385.0	21764.0	24105.0	26687.0	40223.0	63581.0	98387.0	126948.0	145258.0	158673.0	163505.0
	PRE	2387.3	8433.4	13194.8	13286.8	15995.9	19628.8	24653.8	37106.4	60278.2	97626.3	130868.1	149716.4	150976.7	151906.7
	CHI	72.932	40.498	458.333	3792.017	2079.934	1020.782	167.678	261.771	180.965	5.928	117.425	132.766	392.338	885.545
2018	PRE	2388.6	8384.3	12982.5	13400.2	16196.3	19840.4	24579.1	36541.2	59052.3	97373.1	132953.9	152581.9	159226.5	155484.7
2019	PRE	2383.1	8341.9	12777.6	13457.4	16395.8	20080.7	24581.9	36066.5	57849.0	96794.1	134684.0	155438.3	167042.7	160766.3
2020	PRE	2366.3	8302.5	12608.4	13441.8	16592.7	20339.6	24664.8	35688.0	56756.9	95894.2	135935.6	158548.7	173853.0	168221.7
2021	PRE	2263.6	8003.7	12018.8	13272.7	14790.3	19653.1	26371.1	35414.8	54659.6	86675.4	134054.4	167734.6	185196.9	171301.2
2022	PRE	2235.5	8017.1	11920.5	13104.1	14938.0	19898.3	26596.2	35203.6	53726.4	85129.9	134205.5	170650.2	189391.2	180557.8
2023	PRE	2199.6	8025.1	11855.2	12898.5	15059.3	20143.3	26883.1	35103.6	52929.3	83451.7	133969.9	173477.4	193140.5	190572.1
2024	PRE	2162.0	8005.4	11812.0	12703.3	15119.2	20385.6	27208.0	35117.0	52268.2	81813.4	133287.0	175891.7	197026.2	200101.2
2025	PRE	2128.6	7946.0	11778.5	12548.0	15099.9	20620.9	27553.7	35242.7	51746.3	80328.8	132146.8	177710.8	201315.9	208516.3
2026	PRE	2032.4	7600.5	11336.0	11963.5	14915.3	18379.4	26619.4	37675.9	51351.2	77385.6	119511.6	175373.6	213180.2	222298.7
2027	PRE	2015.8	7509.4	11346.2	11870.3	14734.3	18563.6	26952.5	38001.6	51057.6	76099.7	117471.4	175761.5	217184.8	227704.6
2028	PRE	2001.3	7394.6	11363.1	11810.7	14512.3	18715.8	27287.3	38419.8	50932.7	75011.6	115252.0	175673.3	221156.0	232819.8
2029	PRE	1988.7	7275.9	11354.3	11775.0	14301.7	18793.0	27619.2	38895.5	50979.1	74122.8	113086.6	175005.9	224679.1	238311.2
2030	PRE	1979.3	7167.3	11297.4	11755.5	14136.6	18773.2	27941.5	39402.8	51194.3	73439.9	111133.6	173734.5	227513.5	244438.1
2031	PRE	1911.3	6846.6	10795.3	11317.2	13491.6	18557.2	24914.1	38080.7	54760.2	72956.2	107236.1	157413.8	225009.7	259723.5
2032	PRE	1917.0	6794.5	10663.8	11329.4	13394.6	18343.0	25169.7	38567.5	55257.1	72597.4	105596.0	155002.0	226038.4	265498.9
2033	PRE	1923.4	6749.7	10512.7	11348.4	13331.6	18075.9	25380.2	39056.0	55887.6	72468.9	104201.4	152336.8	226502.2	271323.1
2034	PRE	1928.1	6711.3	10367.8	11344.6	13293.8	17821.0	25488.5	39540.3	56603.5	72581.2	103064.0	149726.7	226248.5	276722.2
2035	PRE	1929.7	6679.1	10241.7	11299.9	13274.2	17619.0	25463.5	40008.2	57364.5	72930.7	102197.0	147371.0	225205.3	281387.4
2036	PRE	1867.9	6448.4	9767.5	10799.4	12785.3	16825.0	25179.9	35677.5	55449.2	78053.9	101618.7	142352.1	204233.6	278608.6
2037	PRE	1871.8	6469.6	9684.0	10671.0	12800.5	16709.4	24897.3	36046.3	56165.3	78793.6	101197.6	140321.7	201341.7	280339.0
2038	PRE	1871.6	6492.3	9623.0	10523.8	12819.7	16632.4	24541.6	36350.4	56884.9	79716.6	101080.3	138605.7	198170.8	281540.5
2039	PRE	1868.0	6507.2	9582.9	10384.5	12812.3	16584.2	24200.4	36509.4	57602.3	80759.9	101288.0	137228.0	195126.1	282003.8
2040	PRE	1861.8	6509.3	9557.9	10269.6	12761.2	16556.8	23929.0	36479.9	58301.1	81873.7	101826.0	136214.6	192459.5	281604.4

Results for PHIM for Run 1 (Basic_US_OG_T1.RTF)

****
List of values created by O and G modelling, using percentage change in last two period parameters for Fixed File MORT

1. Country US (United States)
2. Sex M (Males)
3. Disease ALL (All Causes)

* Value comes from O and G Modelling.

Age	Years	Value	Death Rate	Population
10-14	2018	2388.5523	221.531021	107820.22 *
10-14	2019	2383.1450	220.542784	108058.17 *
10-14	2020	2366.2995	219.558955	107775.13 *
10-14	2021	2263.6047	210.681983	107441.78 *
10-14	2022	2235.5253	209.742143	106584.46 *
10-14	2023	2199.5553	208.806496	105339.41 *
10-14	2024	2161.9986	207.875022	104004.73 *
10-14	2025	2128.6386	206.947704	102858.77 *
10-14	2026	2032.4144	198.580617	102347.07 *
10-14	2027	2015.8399	197.694760	101967.29 *
10-14	2028	2001.3142	196.812855	101686.15 *
10-14	2029	1988.7107	195.934884	101498.55 *
10-14	2030	1979.2945	195.060830	101470.63 *
10-14	2031	1911.3276	187.174340	102114.83 *
10-14	2032	1917.0115	186.339367	102877.43 *
10-14	2033	1923.3669	185.508117	103681.01 *
10-14	2034	1928.0935	184.680577	104401.53 *
10-14	2035	1929.6789	183.856727	104955.58 *
10-14	2036	1867.9233	176.423230	105877.40 *
10-14	2037	1871.7703	175.636216	106570.86 *
10-14	2038	1871.6063	174.852713	107039.02 *
10-14	2039	1868.0390	174.072705	107313.72 *
15-19	2018	8384.3162	773.196850	108437.02 *
15-19	2019	8341.9048	769.747664	108371.94 *
15-19	2020	8302.4904	766.313864	108343.21 *
15-19	2021	8003.7016	735.331086	108844.87 *
15-19	2022	8017.0552	732.050816	109515.01 *
15-19	2023	8025.1128	728.785180	110116.30 *
15-19	2024	8005.3671	725.534112	110337.57 *
15-19	2025	7946.0408	722.297547	110010.63 *
15-19	2026	7600.5188	693.094388	109660.66 *
15-19	2027	7509.3969	690.002535	108831.44 *
15-19	2028	7394.6238	686.924473	107648.28 *
15-19	2029	7275.8801	683.860143	106394.27 *
15-19	2030	7167.3355	680.809483	105276.67 *
15-19	2031	6846.5519	653.283725	104802.12 *
15-19	2032	6794.4618	650.369465	104470.80 *
15-19	2033	6749.7382	647.468204	104248.18 *
15-19	2034	6711.3323	644.579886	104119.48 *
15-19	2035	6679.1102	641.704453	104083.90 *
15-19	2036	6448.4374	615.759748	104723.27 *
15-19	2037	6469.5620	613.012880	105537.13 *
15-19	2038	6492.2940	610.278265	106382.52 *
15-19	2039	6507.2318	607.555849	107105.08 *
20-24	2018	12982.5047	1123.546025	115549.38 *
20-24	2019	12777.6252	1118.533952	114235.47 *
20-24	2020	12608.3630	1113.544237	113227.32 *
20-24	2021	12018.7915	1068.522613	112480.46 *
20-24	2022	11920.4720	1063.755995	112060.21 *
20-24	2023	11855.2111	1059.010642	111946.10 *
20-24	2024	11811.9524	1054.286457	112037.41 *
20-24	2025	11778.4883	1049.583346	112220.61 *
20-24	2026	11335.9995	1007.147720	112555.48 *
20-24	2027	11346.2273	1002.654893	113161.84 *
20-24	2028	11363.0945	998.182108	113837.89 *
20-24	2029	11354.3219	993.729275	114259.71 *
20-24	2030	11297.3582	989.296307	114195.90 *
20-24	2031	10795.2960	949.298142	113718.71 *
20-24	2032	10663.8315	945.063378	112837.21 *
20-24	2033	10512.6537	940.847505	111736.00 *
20-24	2034	10367.8174	936.650439	110690.36 *
20-24	2035	10241.6702	932.472096	109833.53 *
20-24	2036	9767.5177	894.771386	109162.16 *
20-24	2037	9683.9797	890.779863	108713.50 *
20-24	2038	9623.0323	886.806146	108513.37 *
20-24	2039	9582.9500	882.850155	108545.60 *
25-29	2018	13400.2343	1116.766321	119991.39 *
25-29	2019	13457.3741	1111.784491	121043.01 *

Results for PHIM for Run 1 (Basic_US_OG_T1.RTF)

25-29	2020	13441.7850	1106.824885	121444.55 *
25-29	2021	13272.6759	1096.237421	121074.83 *
25-29	2022	13104.0794	1091.347170	120072.51 *
25-29	2023	12898.5386	1086.478734	118718.74 *
25-29	2024	12703.2791	1081.632015	117445.48 *
25-29	2025	12547.9535	1076.806918	116529.28 *
25-29	2026	11963.5358	1033.270617	115783.18 *
25-29	2027	11870.2623	1028.661257	115395.25 *
25-29	2028	11810.6758	1024.072459	115330.47 *
25-29	2029	11775.0291	1019.504132	115497.61 *
25-29	2030	11755.5067	1014.956183	115822.80 *
25-29	2031	11317.1742	973.920565	116202.23 *
25-29	2032	11329.4156	969.575962	116849.18 *
25-29	2033	11348.3680	965.250740	117569.12 *
25-29	2034	11344.5714	960.944813	118056.43 *
25-29	2035	11299.9277	956.658094	118118.77 *
25-29	2036	10799.4351	917.979522	117643.53 *
25-29	2037	10671.0140	913.884468	116765.46 *
25-29	2038	10523.7955	909.807683	115670.55 *
25-29	2039	10384.5228	905.749084	114651.21 *
30-34	2018	16196.3306	1430.612648	113212.55 *
30-34	2019	16395.8415	1424.230768	115120.68 *
30-34	2020	16592.7296	1417.877358	117025.14 *
30-34	2021	14790.3436	1245.421967	118757.69 *
30-34	2022	14938.0061	1239.866212	120480.79 *
30-34	2023	15059.2639	1234.335241	122003.03 *
30-34	2024	15119.1537	1228.828943	123037.09 *
30-34	2025	15099.9165	1223.347209	123431.16 *
30-34	2026	14915.3238	1211.645137	123099.77 *
30-34	2027	14734.3007	1206.240058	122150.65 *
30-34	2028	14512.3281	1200.859092	120849.55 *
30-34	2029	14301.6975	1195.502129	119629.21 *
30-34	2030	14136.6175	1190.169064	118778.23 *
30-34	2031	13491.5781	1142.049427	118134.80 *
30-34	2032	13394.5783	1136.954811	117811.00 *
30-34	2033	13331.6170	1131.882922	117782.65 *
30-34	2034	13293.8223	1126.833658	117975.02 *
30-34	2035	13274.2089	1121.806918	118328.82 *
30-34	2036	12785.2803	1076.451226	118772.50 *
30-34	2037	12800.5308	1071.649240	119447.02 *
30-34	2038	12819.7234	1066.868675	120162.15 *
30-34	2039	12812.2803	1062.109436	120630.51 *
35-39	2018	19840.3873	1889.914375	104980.35 *
35-39	2019	20080.6528	1881.483577	106727.76 *
35-39	2020	20339.6469	1873.090388	108588.71 *
35-39	2021	19653.1042	1779.624567	110433.99 *
35-39	2022	19898.3148	1771.685765	112312.89 *
35-39	2023	20143.2625	1763.782378	114204.92 *
35-39	2024	20385.5674	1755.914247	116096.60 *
35-39	2025	20620.9184	1748.081216	117963.16 *
35-39	2026	18379.4382	1535.463370	119699.62 *
35-39	2027	18563.5665	1528.613758	121440.53 *
35-39	2028	18715.7723	1521.794701	122984.87 *
35-39	2029	18792.9715	1515.006063	124045.52 *
35-39	2030	18773.1502	1508.247709	124469.94 *
35-39	2031	18557.2302	1493.820388	124226.65 *
35-39	2032	18342.9561	1487.156542	123342.47 *
35-39	2033	18075.9248	1480.522423	122091.53 *
35-39	2034	17820.9527	1473.917899	120908.72 *
35-39	2035	17618.9871	1467.342837	120074.10 *
35-39	2036	16825.0194	1408.016808	119494.45 *
35-39	2037	16709.4328	1401.735727	119205.30 *
35-39	2038	16632.4355	1395.482665	119187.69 *
35-39	2039	16584.2030	1389.257498	119374.58 *
40-44	2018	24579.0645	2459.473130	99936.30 *
40-44	2019	24581.8808	2448.501563	100395.61 *
40-44	2020	24664.8424	2437.578940	101185.82 *
40-44	2021	26371.1081	2580.122361	102208.75 *
40-44	2022	26596.2264	2568.612585	103543.16 *
40-44	2023	26883.0854	2557.154153	105128.92 *
40-44	2024	27208.0206	2545.746838	106876.38 *
40-44	2025	27553.7202	2534.390409	108719.32 *
40-44	2026	26619.4099	2407.926207	110549.11 *
40-44	2027	26952.5436	2397.184588	112434.16 *
40-44	2028	27287.3325	2386.490887	114340.82 *
40-44	2029	27619.2159	2375.844890	116250.08 *
40-44	2030	27941.5070	2365.246384	118133.60 *
40-44	2031	24914.0858	2077.563188	119919.75 *

Results for PHIM for Run 1 (Basic_US_OG_T1.RTF)

40-44	2032	25169.6605	2068.295300	121692.78 *
40-44	2033	25380.1947	2059.068755	123260.55 *
40-44	2034	25488.4917	2049.883369	124341.18 *
40-44	2035	25463.4836	2040.738959	124775.80 *
40-44	2036	25179.8693	2021.218029	124577.70 *
40-44	2037	24897.2890	2012.201493	123731.59 *
40-44	2038	24541.6118	2003.225180	122510.50 *
40-44	2039	24200.3848	1994.288909	121348.44 *
45-49	2018	36541.2228	3612.016282	101165.72 *
45-49	2019	36066.4964	3595.903288	100298.85 *
45-49	2020	35688.0004	3579.862172	99690.99 *
45-49	2021	35414.7903	3568.495034	99242.93 *
45-49	2022	35203.6178	3552.576185	99093.21 *
45-49	2023	35103.5673	3536.728350	99254.35 *
45-49	2024	35117.0097	3520.951210	99737.28 *
45-49	2025	35242.7337	3505.244452	100542.87 *
45-49	2026	37675.9377	3710.222237	101546.31 *
45-49	2027	38001.5563	3693.671152	102882.89 *
45-49	2028	38419.8477	3677.193900	104481.43 *
45-49	2029	38895.4890	3660.790151	106248.89 *
45-49	2030	39402.8000	3644.459580	108116.99 *
45-49	2031	38080.6530	3462.603749	109976.93 *
45-49	2032	38567.5436	3447.157275	111882.17 *
45-49	2033	39056.0004	3431.779707	113806.84 *
45-49	2034	39540.2986	3416.470737	115734.34 *
45-49	2035	40008.1998	3401.230060	117628.62 *
45-49	2036	35677.5155	2987.540924	119421.01 *
45-49	2037	36046.3336	2974.213678	121196.18 *
45-49	2038	36350.4311	2960.945884	122766.28 *
45-49	2039	36509.4182	2947.737276	123855.74 *
50-54	2018	59052.3048	5549.349579	106413.02 *
50-54	2019	57849.0485	5524.594254	104711.85 *
50-54	2020	56756.9199	5499.949360	103195.35 *
50-54	2021	54659.5777	5375.267337	101687.18 *
50-54	2022	53726.4022	5351.288582	100399.00 *
50-54	2023	52929.3243	5327.416795	99352.70 *
50-54	2024	52268.2227	5303.651498	98551.39 *
50-54	2025	51746.2892	5279.992217	98004.48 *
50-54	2026	51351.1551	5263.226655	97565.92 *
50-54	2027	51057.5793	5239.747707	97442.82 *
50-54	2028	50932.6865	5216.373498	97640.03 *
50-54	2029	50979.1449	5193.103559	98167.01 *
50-54	2030	51194.3489	5169.937426	99023.15 *
50-54	2031	54760.1565	5472.262225	100068.59 *
50-54	2032	55257.0711	5447.850782	101429.12 *
50-54	2033	55887.6307	5423.548236	103046.25 *
50-54	2034	56603.4592	5399.354103	104833.76 *
50-54	2035	57364.5365	5375.267899	106719.40 *
50-54	2036	55449.2414	5107.046016	108574.00 *
50-54	2037	56165.2976	5084.263781	110468.89 *
50-54	2038	56884.9467	5061.583176	112385.68 *
50-54	2039	57602.2527	5039.003748	114312.78 *
55-59	2018	97373.0994	9040.831407	107703.70 *
55-59	2019	96794.1036	9000.500784	107543.02 *
55-59	2020	95894.2489	8960.350074	107020.65 *
55-59	2021	86675.4230	8182.425910	105928.76 *
55-59	2022	85129.9216	8145.924584	104506.15 *
55-59	2023	83451.7281	8109.586088	102905.04 *
55-59	2024	81813.4230	8073.409697	101336.89 *
55-59	2025	80328.8088	8037.394686	99943.84 *
55-59	2026	77385.5969	7855.189620	98515.25 *
55-59	2027	76099.7221	7820.148076	97312.38 *
55-59	2028	75011.5759	7785.262851	96350.73 *
55-59	2029	74122.8447	7750.533246	95635.80 *
55-59	2030	73439.9304	7715.958568	95179.27 *
55-59	2031	72956.2334	7691.458081	94853.58 *
55-59	2032	72597.4407	7657.146934	94810.04 *
55-59	2033	72468.9345	7622.988847	95066.30 *
55-59	2034	72581.2244	7588.983137	95640.25 *
55-59	2035	72930.6738	7555.129124	96531.34 *
55-59	2036	78053.9371	7996.933871	97604.83 *
55-59	2037	78793.5696	7961.260014	98971.23 *
55-59	2038	79716.5597	7925.745297	100579.26 *
55-59	2039	80759.8753	7890.389008	102352.21 *
60-64	2018	132953.8985	13647.572276	97419.45 *
60-64	2019	134684.0416	13586.691250	99129.39 *
60-64	2020	135935.5802	13526.081811	100498.86 *
60-64	2021	134054.3570	13230.024106	101325.86 *

Results for PHIM for Run 1 (Basic_US_OG_T1.RTF)

60-64	2022	134205.5415	13171.005738	101894.68 *
60-64	2023	133969.9104	13112.250648	102171.56 *
60-64	2024	133286.9852	13053.757661	102106.22 *
60-64	2025	132146.8475	12995.525607	101686.42 *
60-64	2026	119511.6331	11867.273552	100706.90 *
60-64	2027	117471.4149	11814.334335	99431.26 *
60-64	2028	115252.0485	11761.631277	97989.85 *
60-64	2029	113086.6193	11709.163324	96579.59 *
60-64	2030	111133.6448	11656.929427	95336.98 *
60-64	2031	107236.0536	11392.670712	94127.23 *
60-64	2032	105596.0477	11341.848671	93103.03 *
60-64	2033	104201.3992	11291.253344	92285.06 *
60-64	2034	103063.9983	11240.883720	91686.74 *
60-64	2035	102196.9712	11190.738791	91322.81 *
60-64	2036	101618.7400	11155.204832	91095.36 *
60-64	2037	101197.5861	11105.442113	91124.32 *
60-64	2038	101080.2699	11055.901381	91426.53 *
60-64	2039	101288.0346	11006.581649	92024.97 *
65-69	2018	152581.8755	18897.533050	80741.69 *
65-69	2019	155438.3489	18813.232255	82621.82 *
65-69	2020	158548.7200	18729.307520	84652.74 *
65-69	2021	167734.6281	19399.811190	86461.99 *
65-69	2022	170650.1975	19313.269761	88359.04 *
65-69	2023	173477.3894	19227.114388	90225.39 *
65-69	2024	175891.6607	19141.343349	91890.97 *
65-69	2025	177710.8049	19055.954930	93257.36 *
65-69	2026	175373.6473	18638.859843	94090.33 *
65-69	2027	175761.5317	18555.712974	94720.98 *
65-69	2028	175673.2714	18472.937018	95097.64 *
65-69	2029	175005.8681	18390.530320	95160.86 *
65-69	2030	173734.5096	18308.491235	94892.86 *
65-69	2031	157413.8353	16718.975466	94152.80 *
65-69	2032	155002.0258	16644.393089	93125.67 *
65-69	2033	152336.7685	16570.143420	91934.49 *
65-69	2034	149726.7488	16496.224974	90764.25 *
65-69	2035	147371.0228	16422.636274	89736.52 *
65-69	2036	142352.0755	16050.340569	88691.00 *
65-69	2037	140321.6757	15978.740934	87817.73 *
65-69	2038	138605.6979	15907.460700	87132.51 *
65-69	2039	137228.0100	15836.498443	86653.00 *
70-74	2018	159226.5291	26395.916398	60322.41 *
70-74	2019	167042.7316	26278.165751	63567.12 *
70-74	2020	173853.0264	26160.940382	66455.19 *
70-74	2021	185196.8600	26985.915066	68627.23 *
70-74	2022	189391.1502	26865.532469	70495.96 *
70-74	2023	193140.5009	26745.686891	72213.70 *
70-74	2024	197026.2355	26626.375938	73996.64 *
70-74	2025	201315.9498	26507.597224	75946.51 *
70-74	2026	213180.1557	27456.561365	77642.70 *
70-74	2027	217184.7580	27334.079242	79455.67 *
70-74	2028	221156.0020	27212.143505	81271.07 *
70-74	2029	224679.0999	27090.751716	82935.72 *
70-74	2030	227513.5037	26969.901448	84358.30 *
70-74	2031	225009.7223	26379.586587	85296.91 *
70-74	2032	226038.3761	26261.908786	86070.81 *
70-74	2033	226502.2433	26144.755939	86633.91 *
70-74	2034	226248.4936	26028.125703	86924.62 *
70-74	2035	225205.3193	25912.015749	86911.54 *
70-74	2036	204233.5886	23662.373378	86311.54 *
70-74	2037	201341.6689	23556.816907	85470.66 *
70-74	2038	198170.7881	23451.731318	84501.56 *
70-74	2039	195126.1477	23347.114508	83576.13 *
75-79	2018	155484.6759	39226.522044	39637.64 *
75-79	2019	160766.3041	39051.534812	41167.73 *
75-79	2020	168221.6548	38877.328187	43269.86 *
75-79	2021	171301.1726	37667.848952	45476.76 *
75-79	2022	180557.7836	37499.814869	48148.98 *
75-79	2023	190572.1145	37332.530376	51047.20 *
75-79	2024	200101.2185	37165.992130	53839.87 *
75-79	2025	208516.3111	37000.196802	56355.46 *
75-79	2026	222298.7456	38166.983056	58243.73 *
75-79	2027	227704.6300	37996.722365	59927.44 *
75-79	2028	232819.7754	37827.221197	61548.21 *
75-79	2029	238311.1597	37658.476164	63282.21 *
75-79	2030	244438.1049	37490.483893	65200.04 *
75-79	2031	259723.4780	38832.632128	66882.79 *
75-79	2032	265498.9330	38659.402016	68676.42 *
75-79	2033	271323.1073	38486.944672	70497.44 *

Results for PHIM for Run 1 (Basic_US_OG_T1.RTF)

75-79	2034	276722.1708	38315.256651	72222.45 *
75-79	2035	281387.3609	38144.334520	73769.11 *
75-79	2036	278608.5763	37309.434638	74675.10 *
75-79	2037	280339.0167	37142.999421	75475.60 *
75-79	2038	281540.4798	36977.306661	76138.72 *
75-79	2039	282003.7546	36812.353047	76605.74 *

Results for PHIM for Run 1 (Basic_US_OG_T1.RTF)

****

Osmond and Gardner Modeling of Death Rates for COD: LUNG CANCER

Variable Parameter	Value
1. Country	US (United States)
2. Sex	M (Males)
3. Year of start of OG modelling	1966
4. Number 5-year periods for OG modelling	10
5. Age Range of interest	10 - 79
6. Scaling Factor for Rates	10000
7. Model Type to Save	4 (Full Age-Period-Cohort)
8. Goodness of Fit Required	1 (Analysis Printed For Age-Period-Cohort Model)
9. Cause of Death	LUNG CANCER
Note:	Death rates are per million population

Matrix of Numbers of Deaths

Age	1966-	1971-	1976-	1981-	1986-	1991-	1996-	2001-	2006-	2011-
10-14	13	22	10	10	6	8	6	6	9	10
15-19	34	39	26	18	28	25	15	16	25	22
20-24	76	67	63	62	50	45	50	49	56	48
25-29	149	150	170	174	170	115	125	94	129	121
30-34	633	591	597	541	666	617	459	315	295	254
35-39	2209	2158	1954	2044	2003	2129	1870	1334	915	709
40-44	6389	6109	5569	5462	5625	5569	5793	5377	3293	2173
45-49	12802	14805	13951	12517	12499	12521	12146	12718	11058	7258
50-54	22449	26146	28624	26436	24239	23512	23013	23623	23626	20381
55-59	33655	38798	44322	47216	43931	38743	36997	38381	37931	38217
60-64	42388	51356	58759	65526	68816	62420	53639	53357	54084	53534
65-69	43495	54810	66392	75221	82903	85218	75004	66475	67290	67834
70-74	36997	46161	59279	71351	80308	87967	88287	79362	70952	71894
75-79	24325	32105	40888	52556	63360	70636	76821	79001	71710	64966

Matrix of Age- and Period-Specific Mortality Rates

Age	1966-	1971-	1976-	1981-	1986-	1991-	1996-	2001-	2006-	2011-
10-14	0.248	0.403	0.190	0.206	0.124	0.161	0.117	0.109	0.157	0.188
15-19	0.722	0.743	0.475	0.347	0.593	0.544	0.295	0.297	0.443	0.394
20-24	1.946	1.434	1.193	1.117	0.958	0.944	1.051	0.966	1.028	0.827
25-29	4.529	3.743	3.569	3.248	3.059	2.191	2.543	1.936	2.532	2.184
30-34	21.403	17.649	14.764	11.222	12.325	10.924	8.471	6.237	5.997	4.895
35-39	74.238	73.332	60.457	51.464	41.619	39.394	32.667	24.360	18.015	14.208
40-44	204.074	206.551	192.636	171.232	141.693	116.683	107.209	94.283	60.386	42.634
45-49	421.506	483.484	494.593	444.231	397.201	322.137	256.217	237.347	195.989	133.984
50-54	820.858	897.899	987.040	980.837	881.880	765.736	597.667	503.055	447.811	367.136
55-59	1380.643	1520.865	1633.817	1732.197	1710.327	1474.869	1242.932	1019.997	830.735	746.202
60-64	2052.567	2355.723	2513.381	2625.830	2724.701	2602.387	2143.991	1873.289	1500.957	1229.563
65-69	2685.698	3111.469	3433.863	3596.453	3732.703	3766.732	3420.790	2857.756	2535.093	2018.370
70-74	3176.734	3654.973	4162.169	4513.850	4656.232	4726.134	4555.773	4137.118	3445.278	3026.854
75-79	3008.860	3872.395	4278.064	4860.005	5328.624	5356.429	5231.415	5087.536	4560.017	3771.691

Matrix of Log-Rates

Age	1966-	1971-	1976-	1981-	1986-	1991-	1996-	2001-	2006-	2011-
10-14	-0.605	-0.395	-0.722	-0.685	-0.907	-0.793	-0.931	-0.961	-0.803	-0.725
15-19	-0.141	-0.129	-0.323	-0.460	-0.227	-0.264	-0.530	-0.527	-0.354	-0.405
20-24	0.289	0.157	0.077	0.048	-0.019	-0.025	0.022	-0.015	0.012	-0.082
25-29	0.656	0.573	0.553	0.512	0.486	0.341	0.405	0.287	0.403	0.339
30-34	1.330	1.247	1.169	1.050	1.091	1.038	0.928	0.795	0.778	0.690
35-39	1.871	1.865	1.781	1.712	1.619	1.595	1.514	1.387	1.256	1.153
40-44	2.310	2.315	2.285	2.234	2.151	2.067	2.030	1.974	1.781	1.630
45-49	2.625	2.684	2.694	2.648	2.599	2.508	2.409	2.375	2.292	2.127
50-54	2.914	2.953	2.994	2.992	2.945	2.884	2.776	2.702	2.651	2.565
55-59	3.140	3.182	3.213	3.239	3.233	3.169	3.094	3.009	2.919	2.873
60-64	3.312	3.372	3.400	3.419	3.435	3.415	3.331	3.273	3.176	3.090
65-69	3.429	3.493	3.536	3.556	3.572	3.576	3.534	3.456	3.404	3.305
70-74	3.502	3.563	3.619	3.655	3.668	3.675	3.659	3.617	3.537	3.481
75-79	3.478	3.588	3.631	3.687	3.727	3.729	3.719	3.707	3.659	3.577

Results for PHIM for Run 1 (Basic_US_OG_T1.RTF)

Fitting the Age, Period, Cohort Models

Model	RSS	MRSS	DF	Factor	%Account	ChiSq	P
Age Only	34327.554	270.296	127	P, C	99.6158	185213.223	0.0000
Age-Period	11318.506	96.739	117	Cohort	98.8347	60450.977	0.0000
Age-Cohort	1726.386	16.600	104	Period	92.3599	9162.873	0.0000
Period-Cohort	704.869	6.527	108	Age	81.2877	3748.207	0.0000
Full Age-Period-Cohort	131.898	1.374	96			700.539	0.0000

Key to terms:

RSS =	residual sum of squares
MRSS =	mean RSS (MRSS/DF)
DF =	degrees of freedom
Factor =	Factors not included in the model
% Account =	1- (RSS for full model)/(RSS for model in question)
Chisq =	chi-squared value for model
P =	probability value based on Chisq and DF.

Age	Value	Log10 Value
10-	0.320230	-0.494538
15-	0.790868	-0.101896
20-	1.744712	0.241724
25-	4.236138	0.626970
30-	14.814930	1.170700
35-	50.673088	1.704777
40-	145.657367	2.163332
45-	345.572817	2.538540
50-	709.819441	2.851148
55-	1279.33735	3.106985
60-	2078.27159	3.317702
65-	3040.27603	3.482913
70-	4036.85716	3.606043
75-	4869.49747	3.687484

Period	Value	Log10 Value
1966	1.137322	0.055884
1971	1.170231	0.068272
1976	1.171735	0.068829
1981	1.152364	0.061590
1986	1.122440	0.050163
1991	1.059496	0.025099
1996	0.957005	-0.019086
2001	0.878878	-0.056072
2006	0.801556	-0.096066
2011	0.731831	-0.135589

Cohort	Value	Log10 Value
1891	0.543293	-0.264966
1896	0.686145	-0.163584
1901	0.767118	-0.115138
1906	0.872678	-0.059146
1911	0.966994	-0.014576
1916	1.028264	0.012105
1921	1.100250	0.041492
1926	1.178549	0.071348
1931	1.179505	0.071700
1936	1.076378	0.031965
1941	1.026555	0.011382
1946	0.899065	-0.046209
1951	0.801232	-0.096241
1956	0.787224	-0.103902
1961	0.708623	-0.149584
1966	0.529740	-0.275937
1971	0.427004	-0.369568
1976	0.434266	-0.362244
1981	0.537569	-0.269566
1986	0.672588	-0.172251
1991	0.638332	-0.194953
1996	0.660743	-0.179968
2001	0.803539	-0.094993

Results for PHIM for Run 1 (Basic_US_OG_T1.RTF)

Model: Full Age-Period-Cohort

Basic Analysis Using OG Modelling T1 on US
Fitting the Full Age-Period-Cohort Model
Matrix of observed, expected, and residual rates

Age		1966-	1971-	1976-	1981-	1986-	1991-	1996-	2001-	2006-	2011-
10-	Observed	0.248	0.403	0.190	0.206	0.124	0.161	0.117	0.109	0.157	0.188
	Expected	0.287	0.266	0.199	0.158	0.156	0.182	0.206	0.180	0.170	0.188
	Residual	0.039	-0.137	0.009	-0.049	0.032	0.021	0.089	0.070	0.012	0.000
15-	Observed	0.722	0.743	0.475	0.347	0.593	0.544	0.295	0.297	0.443	0.394
	Expected	0.721	0.729	0.657	0.483	0.379	0.364	0.407	0.467	0.405	0.382
	Residual	-0.001	-0.014	0.182	0.136	-0.214	-0.180	0.112	0.170	-0.038	-0.011
20-	Observed	1.946	1.434	1.193	1.117	0.958	0.944	1.051	0.966	1.028	0.827
	Expected	1.784	1.636	1.609	1.425	1.037	0.789	0.725	0.824	0.941	0.815
	Residual	-0.162	0.201	0.416	0.308	0.079	-0.155	-0.326	-0.141	-0.088	-0.012
25-	Observed	4.529	3.743	3.569	3.248	3.059	2.191	2.543	1.936	2.532	2.184
	Expected	4.946	4.457	3.977	3.843	3.369	2.378	1.731	1.617	1.825	2.085
	Residual	0.416	0.714	0.408	0.595	0.311	0.187	-0.812	-0.319	-0.706	-0.099
30-	Observed	21.403	17.649	14.764	11.222	12.325	10.924	8.471	6.237	5.997	4.895
	Expected	18.136	17.797	15.607	13.679	13.091	11.123	7.511	5.560	5.157	5.828
	Residual	-3.267	0.148	0.843	2.457	0.765	0.199	-0.960	-0.677	-0.840	0.934
35-	Observed	74.238	73.332	60.457	51.464	41.619	39.394	32.667	24.360	18.015	14.208
	Expected	67.977	63.828	60.952	52.500	45.572	42.264	34.364	23.592	17.344	16.104
	Residual	-6.262	-9.504	0.496	1.036	3.953	2.870	1.697	-0.768	-0.672	1.896
40-	Observed	204.074	206.551	192.636	171.232	141.693	116.683	107.209	94.283	60.386	42.634
	Expected	195.238	201.050	183.707	172.308	146.990	123.649	109.735	90.714	61.849	45.517
	Residual	-8.836	-5.502	-8.928	1.076	5.297	6.966	2.526	-3.568	1.463	2.883
45-	Observed	421.506	483.484	494.593	444.231	397.201	322.137	256.217	237.347	195.989	133.984
	Expected	432.429	476.605	477.605	428.641	398.185	329.177	264.979	239.093	196.286	133.972
	Residual	10.923	-6.879	-16.988	-15.590	0.984	7.040	8.763	1.746	0.297	-0.012
50-	Observed	820.858	897.899	987.040	980.837	881.880	765.736	597.667	503.055	447.811	367.136
	Expected	830.111	913.926	980.223	964.800	857.582	772.021	610.735	499.844	447.899	368.107
	Residual	9.252	16.027	-6.817	-16.037	-24.298	6.286	13.068	-3.210	0.087	0.972
55-	Observed	1380.643	1520.865	1633.817	1732.197	1710.327	1474.869	1242.932	1019.997	830.735	746.202
	Expected	1406.995	1539.434	1649.324	1737.489	1693.744	1458.979	1256.844	1010.892	821.632	737.045
	Residual	26.351	18.570	15.507	5.292	-16.582	-15.890	13.912	-9.106	-9.103	-9.157
60-	Observed	2052.567	2355.723	2513.381	2625.830	2724.701	2602.387	2143.991	1873.289	1500.957	1229.563
	Expected	2062.718	2351.786	2504.012	2635.017	2749.242	2597.175	2140.825	1875.051	1497.708	1218.629
	Residual	10.151	-3.937	-9.369	9.187	24.541	-5.212	-3.166	1.761	-3.249	-10.934
65-	Observed	2685.698	3111.469	3433.863	3596.453	3732.703	3766.732	3420.790	2857.756	2535.093	2018.370
	Expected	2652.519	3104.836	3444.819	3602.526	3754.635	3796.293	3431.838	2876.115	2501.665	2000.391
	Residual	-33.179	-6.633	10.956	6.073	21.932	29.561	11.048	18.360	-33.428	-17.979
70-	Observed	3176.734	3654.973	4162.169	4513.850	4656.232	4726.134	4555.773	4137.118	3445.278	3026.854
	Expected	3150.236	3623.906	4127.878	4498.387	4659.197	4705.807	4553.077	4184.769	3482.909	3032.749
	Residual	-26.498	-31.068	-34.291	-15.463	2.965	-20.327	-2.696	47.651	37.631	5.895
75-	Observed	3008.860	3872.395	4278.064	4860.005	5328.624	5356.429	5231.415	5087.536	4560.017	3771.691
	Expected	3008.860	3909.956	4376.990	4896.973	5285.319	5305.031	5127.312	5043.826	4603.813	3835.834
	Residual	-0.000	37.560	98.926	36.968	-43.305	-51.398	-104.103	-43.710	43.796	64.143

Fitting the Full Age-Period-Cohort Model

Results for PHIM for Run 1 (Basic_US_OG_T1.RTF)

Matrix of observed and expected deaths and (O-E)2/E Values**

Age		1966-	1971-	1976-	1981-	1986-	1991-	1996-	2001-	2006-	2011-	Total
10-	Observed	13.0	21.5	9.5	9.5	5.5	7.5	6.0	6.0	8.5	10.0	97.0
	Expected	15.0	14.2	10.0	7.2	6.9	8.5	10.5	9.8	9.2	10.0	101.4
	Difference	-2.0	7.3	-0.5	2.3	-1.4	-1.0	-4.5	-3.8	-0.7	-0.0	-4.4
	Chi-Sq	0.3	3.8	0.0	0.7	0.3	0.1	2.0	1.5	0.0	0.0	8.7
15-	Observed	34.0	39.0	26.0	18.0	28.0	25.0	14.5	16.0	25.0	22.0	247.5
	Expected	33.9	38.3	35.9	25.0	17.9	16.7	20.0	25.2	22.8	21.4	257.2
	Difference	0.1	0.7	-9.9	-7.0	10.1	8.3	-5.5	-9.2	2.2	0.6	-9.7
	Chi-Sq	0.0	0.0	2.8	2.0	5.7	4.1	1.5	3.3	0.2	0.0	19.6
20-	Observed	76.0	67.0	63.0	62.0	50.0	45.0	50.0	49.0	56.0	48.0	566.0
	Expected	69.7	76.4	85.0	79.1	54.1	37.6	34.5	41.8	51.2	47.3	576.7
	Difference	6.3	-9.4	-22.0	-17.1	-4.1	7.4	15.5	7.2	4.8	0.7	-10.7
	Chi-Sq	0.6	1.2	5.7	3.7	0.3	1.5	7.0	1.2	0.4	0.0	21.6
25-	Observed	149.0	150.0	170.0	174.0	170.0	115.0	125.0	94.0	129.0	121.0	1397.0
	Expected	162.7	178.6	189.5	205.9	187.3	124.8	85.1	78.5	93.0	115.5	1420.8
	Difference	-13.7	-28.6	-19.5	-31.9	-17.3	-9.8	39.9	15.5	36.0	5.5	-23.8
	Chi-Sq	1.2	4.6	2.0	4.9	1.6	0.8	18.7	3.1	13.9	0.3	51.0
30-	Observed	633.0	591.0	597.0	541.0	666.0	617.0	459.0	315.0	295.0	254.0	4968.0
	Expected	536.4	596.0	631.1	659.4	707.4	628.2	407.0	280.8	253.7	302.5	5002.3
	Difference	96.6	-5.0	-34.1	-118.4	-41.4	-11.2	52.0	34.2	41.3	-48.5	-34.3
	Chi-Sq	17.4	0.0	1.8	21.3	2.4	0.2	6.6	4.2	6.7	7.8	68.5
35-	Observed	2209.0	2158.0	1954.0	2044.0	2003.0	2129.0	1870.0	1334.0	915.0	709.0	17325.0
	Expected	2022.7	1878.3	1970.0	2085.1	2193.2	2284.1	1967.1	1292.0	880.9	803.6	17377.1
	Difference	186.3	279.7	-16.0	-41.1	-190.2	-155.1	-97.1	42.0	34.1	-94.6	-52.1
	Chi-Sq	17.2	41.6	0.1	0.8	16.5	10.5	4.8	1.4	1.3	11.1	105.4
40-	Observed	6389.0	6109.0	5569.0	5462.0	5625.0	5569.0	5793.0	5377.0	3293.0	2173.0	51359.0
	Expected	6112.4	5946.3	5310.9	5496.3	5835.3	5901.5	5929.5	5173.5	3372.8	2319.9	51398.3
	Difference	276.6	162.7	258.1	-34.3	-210.3	-332.5	-136.5	203.5	-79.8	-146.9	-39.3
	Chi-Sq	12.5	4.5	12.5	0.2	7.6	18.7	3.1	8.0	1.9	9.3	78.4
45-	Observed	12802.0	14805.0	13951.0	12517.0	12499.0	12521.0	12146.0	12718.0	11058.0	7258.0	122275.0
	Expected	13133.7	14594.4	13471.8	12077.7	12530.0	12794.6	12561.4	12811.6	11074.8	7257.3	122307.3
	Difference	-331.7	210.6	479.2	439.3	-31.0	-273.6	-415.4	-93.6	-16.8	0.7	-32.3
	Chi-Sq	8.4	3.0	17.0	16.0	0.1	5.9	13.7	0.7	0.0	0.0	64.8
50-	Observed	22449.0	26146.0	28624.0	26436.0	24239.0	23512.0	23013.0	23623.0	23626.0	20381.0	242049.0
	Expected	22702.0	26612.7	28426.3	26003.8	23571.2	23705.0	23516.2	23472.3	23630.6	20434.9	242074.9
	Difference	-253.0	-466.7	197.7	432.2	667.8	-193.0	-503.2	150.7	-4.6	-53.9	-25.9
	Chi-Sq	2.8	8.2	1.4	7.2	18.9	1.6	10.8	1.0	0.0	0.1	51.9
55-	Observed	33655.0	38798.0	44322.0	47216.0	43931.0	38743.0	36997.0	38381.0	37931.0	38217.0	398191.0
	Expected	34297.4	39271.7	44742.7	47360.3	43505.1	38325.6	37411.1	38038.4	37515.4	37748.0	398215.5
	Difference	-642.4	-473.7	-420.7	-144.3	425.9	417.4	-414.1	342.6	415.6	469.0	-24.5
	Chi-Sq	12.0	5.7	4.0	0.4	4.2	4.5	4.6	3.1	4.6	5.8	49.0
60-	Observed	42388.0	51356.0	58759.0	65526.0	68816.0	62420.0	53639.0	53357.0	54084.0	53534.0	563879.0
	Expected	42597.6	51270.2	58540.0	65755.2	69435.8	62295.0	53559.8	53407.2	53966.9	53057.9	563885.6
	Difference	-209.6	85.8	219.0	-229.2	-619.8	125.0	79.2	-50.2	117.1	476.1	-6.6
	Chi-Sq	1.0	0.1	0.8	0.8	5.5	0.3	0.1	0.0	0.3	4.3	13.3
65-	Observed	43495.0	54810.0	66392.0	75221.0	82903.0	85218.0	75004.0	66475.0	67290.0	67834.0	684642.0
	Expected	42957.7	54693.2	66603.8	75348.0	83390.1	85886.8	75246.2	66902.1	66402.7	67229.8	684660.3
	Difference	537.3	116.8	-211.8	-127.0	-487.1	-668.8	-242.2	-427.1	887.3	604.2	-18.3
	Chi-Sq	6.7	0.2	0.7	0.2	2.8	5.2	0.8	2.7	11.9	5.4	36.7
70-	Observed	36997.0	46161.0	59279.0	71351.0	80308.0	87967.0	88287.0	79362.0	70952.0	71894.0	692558.0
	Expected	36688.4	45768.6	58790.6	71106.6	80359.1	87588.6	88234.8	80276.1	71727.0	72034.0	692573.8

Results for PHIM for Run 1 (Basic_US_OG_T1.RTF)

	Difference	308.6	392.4	488.4	244.4	-51.1	378.4	52.2	-914.1	-775.0	-140.0	-15.8
	Chi-Sq	2.6	3.4	4.1	0.8	0.0	1.6	0.0	10.4	8.4	0.3	31.6
75-	Observed	24325.0	32105.0	40888.0	52556.0	63360.0	70636.0	76821.0	79001.0	71710.0	64966.0	576368.0
	Expected	24325.0	32416.4	41833.5	52955.8	62845.1	69958.2	75292.3	78322.3	72398.7	66070.8	576418.1
	Difference	0.0	-311.4	-945.5	-399.8	514.9	677.8	1528.7	678.7	-688.7	-1104.8	-50.1
	Chi-Sq	0.0	3.0	21.4	3.0	4.2	6.6	31.0	5.9	6.6	18.5	100.1
Total over ages	Observed	225614.0	273316.5	320603.5	359133.5	384603.5	389524.5	374224.5	360108.0	341372.5	327421.0	3355921.5
	Expected	225654.6	273355.1	320641.0	359165.5	384638.4	389555.3	374275.5	360131.4	341399.7	327453.0	3356269.5
	Difference	-40.6	-38.6	-37.5	-32.0	-34.9	-30.8	-51.0	-23.4	-27.2	-32.0	-348.0
	Chi-Sq	82.7	79.4	74.3	62.1	70.2	61.5	104.8	46.5	56.2	62.9	700.5

Results for PHIM for Run 1 (Basic_US_OG_T1.RTF)

****

Osmond and Gardner Extrapolating Death Rates for COD: LUNG CANCER

Variable Parameter	Value
1. Country	US (United States)
2. Sex	M (Males)
3. Year of start of OG modelling	1966
4. Number 5-year periods for OG modelling	10
5. Age Range of interest	10 - 79
6. Scaling Factor for Rates	10000
7. Model Type to Save	4 (Full Age-Period-Cohort)
8. Goodness of Fit Required	1 (Analysis Printed For Age-Period-Cohort Model)
9. Cause of Death	LUNG CANCER
Note:	Death rates are per million population
10. Number of Periods into the future to Predict	5
11. Earliest projected year	2016
12. Extrapolate Period using (1: last 2 points 2: linear regression)	1
13. Ratio of last two period values	0.91301

Predictions of rates for future years from model:	Full Age-Period-Cohort
Effects for extending model to project rates for:	2016-2040

Extrapolating Model: Full Age-Period-Cohort
Log Transform Parameters

Model	ChiSq	MChiSq	DF	Factor	%Account	P
Age Only	135107.044	9650.503	14	P, C	99.2924	0.0000
Age-Period	8350.504	596.465	14	Cohort	88.5516	0.0000
Age-Cohort	4498.548	321.325	14	Period	78.7487	0.0000
Period-Cohort	989.643	70.689	14	Age	3.3996	0.0000
Full Age-Period-Cohort	955.999	68.286	14			0.0000

Key to terms:		
	Chisq =	chi-squared value for model
	MChisq =	mean Chi-squared (Chisq/DF)
	DF =	degrees of freedom
	Factor =	Factors not included in the model
	% Account =	1- (Chisq for full model)/(Chisq for model in question)
	P =	probability value based on Chisq and DF.

AGE	EFFECT
10	0.320230
15	0.790868
20	1.744712
25	4.236138
30	14.814930
35	50.673088
40	145.657367
45	345.572817
50	709.819441
55	1279.33735
60	2078.27159
65	3040.27603
70	4036.85716
75	4869.49747

PERIOD	EFFECT	
Period Change =	0.913013	
1966	1.137322	
1971	1.170231	
1976	1.171735	
1981	1.152364	
1986	1.122440	
1991	1.059496	
1996	0.957005	
2001	0.878878	
2006	0.801556	
2011	0.731831	
2016	0.668171	
2021	0.610049	
2026	0.556983	
2031	0.508533	
2036	0.464297	
2016	0.692942	Extrapolated
2017	0.680444	Extrapolated
2018	0.668171	Extrapolated
2019	0.656120	Extrapolated

Results for PHIM for Run 1 (Basic_US_OG_T1.RTF)

2020	0.644286	Extrapolated
2021	0.632665	Extrapolated
2022	0.621254	Extrapolated
2023	0.610049	Extrapolated
2024	0.599046	Extrapolated
2025	0.588241	Extrapolated
2026	0.577632	Extrapolated
2027	0.567213	Extrapolated
2028	0.556983	Extrapolated
2029	0.546937	Extrapolated
2030	0.537072	Extrapolated
2031	0.527385	Extrapolated
2032	0.517873	Extrapolated
2033	0.508533	Extrapolated
2034	0.499360	Extrapolated
2035	0.490354	Extrapolated
2036	0.481510	Extrapolated
2037	0.472825	Extrapolated
2038	0.464297	Extrapolated
2039	0.455923	Extrapolated
2040	0.447699	Extrapolated

Results for PHIM for Run 1 (Basic_US_OG_T1.RTF)

COHORT	EFFECT	WEIGHT	ORIGINAL
1891	0.543293	1.000	
1896	0.686145	2.000	
1901	0.767118	4.000	
1906	0.872678	8.000	
1911	0.966994	16.000	
1916	1.028264	32.000	
1921	1.100250	64.000	
1926	1.178549	128.000	
1931	1.179505	256.000	
1936	1.076378	512.000	
1941	1.026555	1024.000	
1946	0.899065	2048.000	
1951	0.801232	4096.000	
1956	0.787224	8192.000	
1961	0.708623	16384.000	
1966	0.529740	32768.000	
1971	0.427004	65536.000	
1976	0.434266	131072.000	
1981	0.537569	262144.000	
1986	0.672588	524288.000	
1991	0.638332	1048576.000	
1996	0.673839	Extrapolated	0.660743
2001	0.708097	Extrapolated	0.803539
2006	0.744097	Extrapolated	
2011	0.781926	Extrapolated	
2016	0.821679	Extrapolated	
2021	0.863453	Extrapolated	
2026	0.907351	Extrapolated	

Standardizing Population: The 1976 European Standard Population

Age Range	Population, Males
All	100000
0	0
1	0
2	0
3	0
0-4	8000
5-9	7000
10-14	7000
15-19	7000
20-24	7000
25-29	7000
30-34	7000
35-39	7000
40-44	7000
45-49	7000
50-54	7000
55-59	6000
60-64	5000
65-69	4000
70-74	3000
75-79	2000
80-84	1000
85+	1000

Results for PHIM for Run 1 (Basic_US_OG_T1.RTF)

Matrix of observed and expected rates including predictions
Total over ages standardized using: The 1976 European Standard Population

		1966-	1971-	1976-	1981-	1986-	1991-	1996-	2001-	2006-	2011-	2016-	2021-	2026-	2031-	2036-
10	OBS	0.2	0.4	0.2	0.2	0.1	0.2	0.1	0.1	0.2	0.2	0.1	.	.	.	.
	EXP											0.2	0.2	0.1	0.1	0.1
15	OBS	0.7	0.7	0.5	0.3	0.6	0.5	0.3	0.3	0.4	0.4	0.5	.	.	.	.
	EXP											0.4	0.4	0.3	0.3	0.3
20	OBS	1.9	1.4	1.2	1.1	1.0	0.9	1.1	1.0	1.0	0.8	0.8	.	.	.	.
	EXP											0.8	0.8	0.7	0.7	0.7
25	OBS	4.5	3.7	3.6	3.2	3.1	2.2	2.5	1.9	2.5	2.2	1.5	.	.	.	.
	EXP											1.8	1.7	1.7	1.6	1.5
30	OBS	21.4	17.6	14.8	11.2	12.3	10.9	8.5	6.2	6.0	4.9	4.9	.	.	.	.
	EXP											6.7	5.8	5.6	5.3	5.1
35	OBS	74.2	73.3	60.5	51.5	41.6	39.4	32.7	24.4	18.0	14.2	11.8	.	.	.	.
	EXP											18.2	20.8	18.0	17.4	16.7
40	OBS	204.1	206.6	192.6	171.2	141.7	116.7	107.2	94.3	60.4	42.6	34.6	.	.	.	.
	EXP											42.3	47.8	54.6	47.3	45.6
45	OBS	421.5	483.5	494.6	444.2	397.2	322.1	256.2	237.3	196.0	134.0	95.6	.	.	.	.
	EXP											98.6	91.6	103.5	118.2	102.4
50	OBS	820.9	897.9	987.0	980.8	881.9	765.7	597.7	503.1	447.8	367.1	277.8	.	.	.	.
	EXP											251.2	184.9	171.7	194.0	221.7
55	OBS	1380.6	1520.9	1633.8	1732.2	1710.3	1474.9	1242.9	1020.0	830.7	746.2	631.5	.	.	.	.
	EXP											605.7	413.4	304.3	282.5	319.3
60	OBS	2052.6	2355.7	2513.4	2625.8	2724.7	2602.4	2144.0	1873.3	1501.0	1229.6	1039.2	.	.	.	.
	EXP											1093.2	898.4	613.2	451.3	419.0
65	OBS	2685.7	3111.5	3433.9	3596.5	3732.7	3766.7	3420.8	2857.8	2535.1	2018.4	1601.9	.	.	.	.
	EXP											1627.6	1460.1	1200.0	819.0	602.8
70	OBS	3176.7	3655.0	4162.2	4513.9	4656.2	4726.1	4555.8	4137.1	3445.3	3026.9	2323.2	.	.	.	.
	EXP											2425.1	1973.2	1770.0	1454.7	992.9
75	OBS	3008.9	3872.4	4278.1	4860.0	5328.6	5356.4	5231.4	5087.5	4560.0	3771.7	3136.2	.	.	.	.
	EXP											3340.1	2670.8	2173.1	1949.4	1602.1
10-79	OBS	670.9	769.4	836.5	877.3	889.1	851.0	759.5	669.7	568.8	473.3	381.1	.	.	.	.
	EXP	671.0	769.5	836.2	876.7	888.4	850.9	760.2	670.1	568.7	473.5	391.6*	319.9*	263.1*	219.1*	185.1*

Drop in overall standardized Observed and Predicted rates
comparing the last observed rate during the model fitting period to the last observed and predicted rates where an observed rate is available (2016)
Observed and Predicted %Drop = 19.498% and 17.259%

Results for PHIM for Run 1 (Basic_US_OG_T1.RTF)

Matrix of observed and expected deaths including predictions

		1966-	1971-	1976-	1981-	1986-	1991-	1996-	2001-	2006-	2011-	2016-	2021-	2026-	2031-	2036-
10-	OBS	13.0	21.5	9.5	9.5	5.5	7.5	6.0	6.0	8.5	10.0	6.3*	.	.	.	.
	EXP	15.0	14.2	10.0	7.2	6.9	8.5	10.5	9.8	9.3	8.8	8.6*	8.0*	7.5*	7.3*	7.2*
	ChiSq	0.274	3.793	0.021	0.699	0.294	0.117	1.961	1.505	0.076	0.160	0.623*	.	.	.	.
15-	OBS	34.0	39.0	26.0	18.0	28.0	25.0	14.5	16.0	25.0	22.0	25.0*	.	.	.	.
	EXP	33.9	38.3	35.9	25.0	17.9	16.7	20.0	25.2	22.8	21.8	20.3*	19.7*	18.5*	17.2*	16.8*
	ChiSq	.	0.014	2.752	1.980	5.686	4.101	1.516	3.332	0.202	0.002	1.086*	.	.	.	.
20-	OBS	76.0	67.0	63.0	62.0	50.0	45.0	50.0	49.0	56.0	48.0	47.5*	.	.	.	.
	EXP	69.7	76.4	85.0	79.1	54.1	37.6	34.5	41.8	51.2	47.3	45.4*	42.3*	41.1*	38.8*	36.2*
	ChiSq	0.575	1.159	5.692	3.694	0.315	1.452	6.983	1.228	0.446	0.011	0.098*	.	.	.	.
25-	OBS	149.0	150.0	170.0	174.0	170.0	115.0	125.0	94.0	129.0	121.0	87.5*	.	.	.	.
	EXP	162.7	178.6	189.5	205.9	187.3	124.8	85.1	78.5	93.0	115.5	108.0*	103.4*	96.5*	94.1*	89.0*
	ChiSq	1.154	4.582	1.997	4.941	1.593	0.769	18.711	3.063	13.933	0.262	3.881*	.	.	.	.
30-	OBS	633.0	591.0	597.0	541.0	666.0	617.0	459.0	315.0	295.0	254.0	277.5*	.	.	.	.
	EXP	536.4	596.0	631.1	659.4	707.4	628.2	407.0	280.8	253.7	302.5	376.9*	350.6*	336.1*	314.8*	306.9*
	ChiSq	17.404	0.041	1.840	21.268	2.418	0.200	6.650	4.166	6.729	7.764	26.206*	.	.	.	.
35-	OBS	2209.0	2158.0	1954.0	2044.0	2003.0	2129.0	1870.0	1334.0	915.0	709.0	622.5*	.	.	.	.
	EXP	2022.7	1878.3	1970.0	2085.1	2193.2	2284.1	1967.1	1292.0	880.9	803.6	956.9*	1187.2*	1103.8*	1060.3*	994.5*
	ChiSq	17.162	41.645	0.130	0.811	16.499	10.535	4.797	1.368	1.320	11.142	116.848*	.	.	.	.
40-	OBS	6389.0	6109.0	5569.0	5462.0	5625.0	5569.0	5793.0	5377.0	3293.0	2173.0	1732.5*	.	.	.	.
	EXP	6112.4	5946.3	5310.9	5496.3	5835.3	5901.5	5929.5	5173.5	3372.8	2319.9	2118.4*	2514.8*	3119.6*	2903.1*	2792.1*
	ChiSq	12.520	4.453	12.545	0.214	7.577	18.730	3.142	8.005	1.887	9.307	70.307*	.	.	.	.
45-	OBS	12802.0	14805.0	13951.0	12517.0	12499.0	12521.0	12146.0	12718.0	11058.0	7258.0	4845.0*	.	.	.	.
	EXP	13133.7	14594.4	13471.8	12077.7	12530.0	12794.6	12561.4	12811.6	11074.8	7257.3	4998.9*	4558.0*	5414.4*	6725.8*	6263.4*
	ChiSq	8.380	3.040	17.045	15.976	0.077	5.852	13.737	0.683	0.025	.	4.738*	.	.	.	.
50-	OBS	22449.0	26146.0	28624.0	26436.0	24239.0	23512.0	23013.0	23623.0	23626.0	20381.0	14782.5*	.	.	.	.
	EXP	22702.0	26612.7	28426.3	26003.8	23571.2	23705.0	23516.2	23472.3	23630.6	20434.9	13368.4*	9208.1*	8410.1*	10014.6*	12456.5*
	ChiSq	2.820	8.184	1.375	7.185	18.922	1.571	10.767	0.968	.	0.142	149.580*	.	.	.	.
55-	OBS	33655.0	38798.0	44322.0	47216.0	43931.0	38743.0	36997.0	38381.0	37931.0	38217.0	33892.5*	.	.	.	.
	EXP	34297.4	39271.7	44742.7	47360.3	43505.1	38325.6	37411.1	38038.4	37515.4	37748.0	32510.9*	21276.5*	14696.0*	13473.8*	16084.9*
	ChiSq	12.031	5.714	3.955	0.439	4.170	4.546	4.584	3.086	4.605	5.827	58.717*	.	.	.	.
60-	OBS	42388.0	51356.0	58759.0	65526.0	68816.0	62420.0	53639.0	53357.0	54084.0	53534.0	50497.5*	.	.	.	.
	EXP	42597.6	51270.2	58540.0	65755.2	69435.8	62295.0	53559.8	53407.2	53966.9	53057.9	53119.8*	45746.5*	30049.9*	20873.1*	19217.1*
	ChiSq	1.032	0.144	0.820	0.799	5.533	0.251	0.117	0.047	0.254	4.271	129.456*	.	.	.	.
65-	OBS	43495.0	54810.0	66392.0	75221.0	82903.0	85218.0	75004.0	66475.0	67290.0	67834.0	64657.5*	.	.	.	.
	EXP	42957.7	54693.2	66603.8	75348.0	83390.1	85886.8	75246.2	66902.1	66402.7	67229.8	65696.0*	65731.9*	56874.1*	37651.5*	26321.8*
	ChiSq	6.721	0.250	0.674	0.214	2.845	5.208	0.780	2.726	11.856	5.431	16.417*	.	.	.	.

Results for PHIM for Run 1 (Basic_US_OG_T1.RTF)

70-	OBS	36997.0	46161.0	59279.0	71351.0	80308.0	87967.0	88287.0	79362.0	70952.0	71894.0	69950.0*	.	.	.	.
	EXP	36688.4	45768.6	58790.6	71106.6	80359.1	87588.6	88234.8	80276.1	71727.0	72034.0	73017.1*	71287.1*	71804.1*	62820.1*	41965.9*
	ChiSq	2.596	3.364	4.057	0.840	0.033	1.634	0.031	10.408	8.373	0.272	128.832*	.	.	.	.
75-	OBS	24325.0	32105.0	40888.0	52556.0	63360.0	70636.0	76821.0	79001.0	71710.0	64966.0	62807.5*	.	.	.	.
	EXP	24325.0	32416.4	41833.5	52955.8	62845.1	69958.2	75292.3	78322.3	72398.7	66070.8	66890.4*	68070.0*	66976.0*	68628.3*	60838.2*
	ChiSq	.	2.991	21.369	3.018	4.219	6.567	31.038	5.882	6.552	18.475	249.209*	.	.	.	.
Total Deaths		225614.0	273316.5	320603.5	359133.5	384603.5	389524.5	374224.5	360108.0	341372.5	327421.0	304231.3*	.	.	.	.
Expected		225654.6	273355.1	320641.0	359165.5	384638.4	389555.3	374275.5	360131.4	341399.9	327452.3	313235.9*	290104.2*	258947.6*	224622.6*	187390.7*
Obs/Exp		1.000	1.000	1.000	1.000	1.000	1.000	1.000	1.000	1.000	1.000	0.971*	.	.	.	.

Chi Squared (Log) = **956.0 on 14 D.F.** **P = 0.0000**

Results for PHIM for Run 1 (Basic_US_OG_T1.RTF)

****

Observed and predicted rates (5 year periods)

		10-	15-	20-	25-	30-	35-	40-	45-	50-	55-	60-	65-	70-	75-
2016-	OBS	0.1	0.5	0.8	1.5	4.9	11.8	34.6	95.6	277.8	631.5	1039.2	1601.9	2323.2	3136.2
	PRE	0.2	0.4	0.8	1.8	6.7	18.2	42.3	98.6	251.2	605.7	1093.2	1627.6	2425.1	3340.1
	RES	-0.043	0.087	0.037	-0.343	-1.756	-6.360	-7.700	-3.036	26.576	25.743	-53.966	-25.730	-101.864	-203.871
2021-	PRE	0.2	0.4	0.8	1.7	5.8	20.8	47.8	91.6	184.9	413.4	898.4	1460.1	1973.2	2670.8
2026-	PRE	0.1	0.3	0.7	1.7	5.6	18.0	54.6	103.5	171.7	304.3	613.2	1200.0	1770.0	2173.1
2031-	PRE	0.1	0.3	0.7	1.6	5.3	17.4	47.3	118.2	194.0	282.5	451.3	819.0	1454.7	1949.4
2036-	PRE	0.1	0.3	0.7	1.5	5.1	16.7	45.6	102.4	221.7	319.3	419.0	602.8	992.9	1602.1

Results for PHIM for Run 1 (Basic_US_OG_T1.RTF)

****

Observed and predicted deaths (5 year periods)

		10-	15-	20-	25-	30-	35-	40-	45-	50-	55-	60-	65-	70-	75-
2016-	OBS	6.3	25.0	47.5	87.5	277.5	622.5	1732.5	4845.0	14782.5	33892.5	50497.5	64657.5	69950.0	62807.5
	PRE	8.6	20.3	45.4	108.0	376.9	956.9	2118.4	4998.9	13368.4	32510.9	53119.8	65696.0	73017.1	66890.4
	CHI	0.623	1.086	0.098	3.881	26.206	116.848	70.307	4.738	149.580	58.717	129.456	16.417	128.832	249.209
2021-	PRE	8.0	19.7	42.3	103.4	350.6	1187.2	2514.8	4558.0	9208.1	21276.5	45746.5	65731.9	71287.1	68070.0
2026-	PRE	7.5	18.5	41.1	96.5	336.1	1103.8	3119.6	5414.4	8410.1	14696.0	30049.9	56874.1	71804.1	66976.0
2031-	PRE	7.3	17.2	38.8	94.1	314.8	1060.3	2903.1	6725.8	10014.6	13473.8	20873.1	37651.5	62820.1	68628.3
2036-	PRE	7.2	16.8	36.2	89.0	306.9	994.5	2792.1	6263.4	12456.5	16084.9	19217.1	26321.8	41965.9	60838.2

Results for PHIM for Run 1 (Basic_US_OG_T1.RTF)

****

Observed and predicted rates (single years)

		10-	15-	20-	25-	30-	35-	40-	45-	50-	55-	60-	65-	70-	75-
2016	OBS	.	0.5	1.0	1.5	5.3	11.9	36.1	99.7	287.7	659.1	1090.7	1725.1	2605.8	3308.3
	PRE	0.2	0.4	0.8	1.9	6.9	18.9	43.8	102.3	260.6	628.2	1133.7	1688.0	2515.0	3463.9
	RES	.	0.071	0.203	-0.331	-1.603	-7.017	-7.702	-2.523	27.142	30.949	-42.952	37.099	90.807	-155.610
2017	OBS	0.2	0.5	0.6	1.4	4.8	12.4	33.3	88.5	255.1	605.4	1048.6	1600.7	2451.4	3286.7
	PRE	0.2	0.4	0.8	1.8	6.8	18.5	43.0	100.4	255.9	616.9	1113.3	1657.5	2469.6	3401.4
	RES	0.024	0.079	-0.201	-0.405	-2.019	-6.156	-9.772	-11.917	-0.723	-11.485	-64.681	-56.844	-18.152	-114.744
2018	PRE	0.2	0.4	0.8	1.8	6.7	18.2	42.3	98.6	251.2	605.7	1093.2	1627.6	2425.1	3340.1
2019	PRE	0.2	0.4	0.8	1.8	6.5	17.9	41.5	96.8	246.7	594.8	1073.5	1598.3	2381.3	3279.8
2020	PRE	0.2	0.4	0.8	1.7	6.4	17.6	40.8	95.1	242.3	584.1	1054.1	1569.5	2338.4	3220.7
2021	PRE	0.2	0.4	0.8	1.8	6.0	21.6	49.5	94.9	191.8	428.8	931.7	1514.2	2046.3	2769.8
2022	PRE	0.2	0.4	0.8	1.8	5.9	21.2	48.6	93.2	188.3	421.0	914.9	1486.9	2009.4	2719.8
2023	PRE	0.2	0.4	0.8	1.7	5.8	20.8	47.8	91.6	184.9	413.4	898.4	1460.1	1973.2	2670.8
2024	PRE	0.1	0.4	0.7	1.7	5.7	20.4	46.9	89.9	181.6	406.0	882.2	1433.7	1937.6	2622.6
2025	PRE	0.1	0.3	0.7	1.7	5.6	20.0	46.1	88.3	178.3	398.7	866.3	1407.9	1902.6	2575.3
2026	PRE	0.2	0.4	0.7	1.7	5.8	18.7	56.6	107.3	178.1	315.5	635.9	1244.5	1835.7	2253.7
2027	PRE	0.1	0.4	0.7	1.7	5.7	18.3	55.6	105.4	174.8	309.9	624.5	1222.0	1802.6	2213.0
2028	PRE	0.1	0.3	0.7	1.7	5.6	18.0	54.6	103.5	171.7	304.3	613.2	1200.0	1770.0	2173.1
2029	PRE	0.1	0.3	0.7	1.6	5.5	17.7	53.6	101.6	168.6	298.8	602.1	1178.3	1738.1	2133.9
2030	PRE	0.1	0.3	0.7	1.6	5.4	17.4	52.6	99.8	165.6	293.4	591.3	1157.1	1706.8	2095.4
2031	PRE	0.1	0.3	0.7	1.7	5.5	18.0	49.0	122.6	201.2	293.0	468.0	849.4	1508.6	2021.7
2032	PRE	0.1	0.3	0.7	1.6	5.4	17.7	48.2	120.4	197.6	287.7	459.6	834.1	1481.4	1985.2
2033	PRE	0.1	0.3	0.7	1.6	5.3	17.4	47.3	118.2	194.0	282.5	451.3	819.0	1454.7	1949.4
2034	PRE	0.1	0.3	0.7	1.6	5.2	17.1	46.4	116.1	190.5	277.4	443.1	804.2	1428.5	1914.2
2035	PRE	0.1	0.3	0.7	1.5	5.1	16.7	45.6	114.0	187.1	272.4	435.2	789.7	1402.7	1879.7
2036	PRE	0.1	0.3	0.7	1.6	5.3	17.3	47.3	106.2	229.9	331.1	434.6	625.1	1029.7	1661.5
2037	PRE	0.1	0.3	0.7	1.6	5.2	17.0	46.4	104.3	225.7	325.2	426.7	613.8	1011.1	1631.5
2038	PRE	0.1	0.3	0.7	1.5	5.1	16.7	45.6	102.4	221.7	319.3	419.0	602.8	992.9	1602.1
2039	PRE	0.1	0.3	0.7	1.5	5.0	16.4	44.7	100.6	217.7	313.6	411.5	591.9	975.0	1573.2
2040	PRE	0.1	0.3	0.6	1.5	4.9	16.1	43.9	98.8	213.7	307.9	404.1	581.2	957.4	1544.8

Results for PHIM for Run 1 (Basic_US_OG_T1.RTF)

****

Observed and predicted deaths (single years)

		10-	15-	20-	25-	30-	35-	40-	45-	50-	55-	60-	65-	70-	75-
2016	OBS	.	5.0	12.0	18.0	58.0	121.0	361.0	1033.0	3154.0	7049.0	10189.0	13238.0	14021.0	12452.0
	PRE	1.8	4.2	9.6	21.9	75.5	192.6	438.0	1059.1	2856.4	6718.0	10590.2	12953.3	13532.4	13037.7
	CHI	.	0.142	0.599	0.682	4.071	26.612	13.524	0.645	30.996	16.305	15.201	6.257	17.642	26.312
2017	OBS	2.0	5.0	7.0	17.0	53.0	128.0	332.0	905.0	2759.0	6508.0	10010.0	12625.0	13959.0	12671.0
	PRE	1.7	4.1	9.4	21.8	75.5	191.7	429.5	1026.9	2766.8	6631.5	10627.5	13073.3	14062.4	13113.4
	CHI	0.039	0.180	0.592	1.054	6.691	21.140	22.140	14.466	0.022	2.299	35.875	15.375	0.760	14.923
2018	PRE	1.7	4.1	9.1	21.7	75.4	191.1	422.4	997.5	2673.6	6524.1	10649.6	13141.9	14628.5	13239.2
2019	PRE	1.7	4.0	8.8	21.5	75.3	190.8	416.7	971.1	2583.4	6396.8	10641.1	13205.3	15137.4	13502.3
2020	PRE	1.7	3.9	8.6	21.2	75.1	190.6	412.4	947.8	2500.1	6251.0	10593.5	13285.9	15539.7	13935.8
2021	PRE	1.7	4.1	8.8	21.9	71.1	238.1	506.3	942.3	1949.9	4541.9	9440.9	13092.1	14043.4	12596.2
2022	PRE	1.7	4.0	8.6	21.3	70.8	237.8	503.7	923.9	1890.5	4400.1	9322.6	13138.1	14165.6	13095.8
2023	PRE	1.6	4.0	8.4	20.7	70.4	237.5	502.2	908.7	1837.1	4254.5	9179.4	13173.6	14249.1	13633.6
2024	PRE	1.6	3.9	8.3	20.1	69.7	237.0	501.3	896.6	1789.4	4114.1	9008.0	13174.8	14337.5	14120.2
2025	PRE	1.5	3.8	8.2	19.6	68.7	236.5	500.8	887.6	1747.4	3984.4	8809.2	13129.6	14449.9	14513.3
2026	PRE	1.6	3.9	8.4	20.1	71.0	223.6	625.6	1089.7	1737.2	3108.6	6404.4	11709.1	14252.6	13126.3
2027	PRE	1.5	3.8	8.3	19.6	69.2	222.8	624.8	1084.1	1703.7	3015.3	6209.2	11575.0	14322.3	13262.2
2028	PRE	1.5	3.7	8.2	19.3	67.2	221.6	623.9	1081.1	1676.4	2931.7	6008.8	11411.4	14385.3	13375.2
2029	PRE	1.5	3.6	8.1	18.9	65.3	219.5	622.9	1079.5	1655.0	2857.4	5815.5	11213.1	14415.2	13504.0
2030	PRE	1.4	3.5	8.0	18.7	63.7	216.2	621.6	1078.7	1639.4	2792.5	5637.1	10979.8	14398.0	13662.3
2031	PRE	1.5	3.6	8.2	19.3	65.4	223.7	588.0	1348.1	2013.8	2779.2	4405.3	7997.2	12868.3	13521.5
2032	PRE	1.5	3.5	8.0	19.1	64.0	218.1	586.0	1346.7	2004.3	2727.8	4278.8	7767.3	12750.8	13633.7
2033	PRE	1.5	3.4	7.8	18.8	62.8	212.0	582.8	1345.2	1999.6	2685.9	4164.7	7529.6	12602.8	13742.8
2034	PRE	1.4	3.4	7.5	18.6	61.8	206.2	577.3	1343.3	1997.5	2653.4	4063.1	7299.7	12417.0	13825.1
2035	PRE	1.4	3.3	7.3	18.3	60.9	201.0	568.9	1340.6	1996.8	2629.8	3974.0	7086.9	12191.2	13866.5
2036	PRE	1.5	3.4	7.5	18.8	63.0	206.5	588.8	1268.4	2495.9	3232.2	3958.8	5544.1	8887.5	12407.4
2037	PRE	1.5	3.4	7.4	18.3	62.3	202.2	574.2	1264.1	2493.7	3218.3	3888.6	5390.5	8642.2	12314.2
2038	PRE	1.4	3.4	7.2	17.8	61.5	198.6	558.3	1257.4	2491.2	3211.6	3831.1	5252.0	8390.1	12198.3
2039	PRE	1.4	3.3	7.1	17.3	60.6	195.3	543.0	1245.6	2488.2	3209.3	3786.7	5128.8	8148.5	12051.8
2040	PRE	1.4	3.3	7.0	16.9	59.6	192.3	529.6	1227.7	2484.0	3209.2	3754.9	5021.5	7927.6	11870.6

Results for PHIM for Run 1 (Basic_US_OG_T1.RTF)

****

List of values created by O and G modelling, using percentage change in last two period parameters for Fixed File MORT

1. Country	US (United States)
2. Sex	M (Males)
3. Disease	LC (LUNG CANCER)
* Value comes from O and G Modelling.	

Age	Years	Value	Death Rate	Population
10-14	2018	1.7166	0.159213	107820.22 *
10-14	2019	1.6894	0.156341	108058.17 *
10-14	2020	1.6546	0.153522	107775.13 *
10-14	2021	1.7021	0.158417	107441.78 *
10-14	2022	1.6580	0.155560	106584.46 *
10-14	2023	1.6091	0.152754	105339.41 *
10-14	2024	1.5601	0.149999	104004.73 *
10-14	2025	1.5150	0.147293	102858.77 *
10-14	2026	1.5556	0.151990	102347.07 *
10-14	2027	1.5218	0.149249	101967.29 *
10-14	2028	1.4903	0.146557	101686.15 *
10-14	2029	1.4607	0.143913	101498.55 *
10-14	2030	1.4340	0.141318	101470.63 *
10-14	2031	1.4891	0.145824	102114.83 *
10-14	2032	1.4731	0.143194	102877.43 *
10-14	2033	1.4579	0.140611	103681.01 *
10-14	2034	1.4415	0.138075	104401.53 *
10-14	2035	1.4230	0.135584	104955.58 *
10-14	2036	1.4813	0.139908	105877.40 *
10-14	2037	1.4641	0.137384	106570.86 *
10-14	2038	1.4440	0.134906	107039.02 *
10-14	2039	1.4216	0.132473	107313.72 *
15-19	2018	4.0575	0.374183	108437.02 *
15-19	2019	3.9820	0.367435	108371.94 *
15-19	2020	3.9091	0.360807	108343.21 *
15-19	2021	4.0524	0.372312	108844.87 *
15-19	2022	4.0038	0.365597	109515.01 *
15-19	2023	3.9532	0.359003	110116.30 *
15-19	2024	3.8897	0.352528	110337.57 *
15-19	2025	3.8082	0.346170	110010.63 *
15-19	2026	3.9172	0.357208	109660.66 *
15-19	2027	3.8174	0.350765	108831.44 *
15-19	2028	3.7078	0.344438	107648.28 *
15-19	2029	3.5985	0.338226	106394.27 *
15-19	2030	3.4965	0.332126	105276.67 *
15-19	2031	3.5917	0.342716	104802.12 *
15-19	2032	3.5158	0.336535	104470.80 *
15-19	2033	3.4450	0.330465	104248.18 *
15-19	2034	3.3787	0.324504	104119.48 *
15-19	2035	3.3166	0.318651	104083.90 *
15-19	2036	3.4434	0.328812	104723.27 *
15-19	2037	3.4076	0.322882	105537.13 *
15-19	2038	3.3729	0.317058	106382.52 *
15-19	2039	3.3346	0.311339	107105.08 *
20-24	2018	9.0769	0.785539	115549.38 *
20-24	2019	8.8118	0.771371	114235.47 *
20-24	2020	8.5765	0.757458	113227.32 *
20-24	2021	8.7916	0.781611	112480.46 *
20-24	2022	8.6008	0.767514	112060.21 *
20-24	2023	8.4370	0.753670	111946.10 *
20-24	2024	8.2916	0.740077	112037.41 *
20-24	2025	8.1554	0.726729	112220.61 *
20-24	2026	8.4406	0.749901	112555.48 *
20-24	2027	8.3330	0.736376	113161.84 *
20-24	2028	8.2316	0.723094	113837.89 *
20-24	2029	8.1130	0.710052	114259.71 *
20-24	2030	7.9623	0.697245	114195.90 *
20-24	2031	8.1818	0.719478	113718.71 *
20-24	2032	7.9720	0.706501	112837.21 *
20-24	2033	7.7518	0.693759	111736.00 *
20-24	2034	7.5407	0.681246	110690.36 *
20-24	2035	7.3474	0.668959	109833.53 *
20-24	2036	7.5353	0.690289	109162.16 *
20-24	2037	7.3690	0.677839	108713.50 *
20-24	2038	7.2228	0.665613	108513.37 *
20-24	2039	7.0946	0.653608	108545.60 *
25-29	2018	21.6798	1.806777	119991.39 *
25-29	2019	21.4753	1.774189	121043.01 *

Results for PHIM for Run 1 (Basic_US_OG_T1.RTF)

25-29	2020	21.1579	1.742189	121444.55 *
25-29	2021	21.8652	1.805928	121074.83 *
25-29	2022	21.2931	1.773355	120072.51 *
25-29	2023	20.6733	1.741370	118718.74 *
25-29	2024	20.0827	1.709962	117445.48 *
25-29	2025	19.5667	1.679121	116529.28 *
25-29	2026	20.0613	1.732662	115783.18 *
25-29	2027	19.6335	1.701411	115395.25 *
25-29	2028	19.2685	1.670724	115330.47 *
25-29	2029	18.9484	1.640590	115497.61 *
25-29	2030	18.6590	1.611000	115822.80 *
25-29	2031	19.3171	1.662369	116202.23 *
25-29	2032	19.0743	1.632386	116849.18 *
25-29	2033	18.8457	1.602943	117569.12 *
25-29	2034	18.5825	1.574032	118056.43 *
25-29	2035	18.2569	1.545642	118118.77 *
25-29	2036	18.7633	1.594927	117643.53 *
25-29	2037	18.2873	1.566160	116765.46 *
25-29	2038	17.7891	1.537913	115670.55 *
25-29	2039	17.3143	1.510174	114651.21 *
30-34	2018	75.3756	6.657886	113212.55 *
30-34	2019	75.2636	6.537802	115120.68 *
30-34	2020	75.1288	6.419883	117025.14 *
30-34	2021	71.0529	5.983017	118757.69 *
30-34	2022	70.7837	5.875105	120480.79 *
30-34	2023	70.3853	5.769140	122003.03 *
30-34	2024	69.7016	5.665085	123037.09 *
30-34	2025	68.6636	5.562908	123431.16 *
30-34	2026	70.9846	5.766428	123099.77 *
30-34	2027	69.1669	5.662423	122150.65 *
30-34	2028	67.1959	5.560293	120849.55 *
30-34	2029	65.3176	5.460005	119629.21 *
30-34	2030	63.6833	5.361527	118778.23 *
30-34	2031	65.3579	5.532487	118134.80 *
30-34	2032	64.0032	5.432701	117811.00 *
30-34	2033	62.8337	5.334714	117782.65 *
30-34	2034	61.8012	5.238495	117975.02 *
30-34	2035	60.8685	5.144012	118328.82 *
30-34	2036	63.0449	5.308036	118772.50 *
30-34	2037	62.2594	5.212298	119447.02 *
30-34	2038	61.5024	5.118287	120162.15 *
30-34	2039	60.6286	5.025972	120630.51 *
35-39	2018	191.0766	18.201178	104980.35 *
35-39	2019	190.7534	17.872895	106727.76 *
35-39	2020	190.5790	17.550532	108588.71 *
35-39	2021	238.1239	21.562559	110433.99 *
35-39	2022	237.8074	21.173648	112312.89 *
35-39	2023	237.4520	20.791752	114204.92 *
35-39	2024	237.0315	20.416744	116096.60 *
35-39	2025	236.4984	20.048500	117963.16 *
35-39	2026	223.6494	18.684222	119699.62 *
35-39	2027	222.8097	18.347226	121440.53 *
35-39	2028	221.5733	18.016308	122984.87 *
35-39	2029	219.4534	17.691359	124045.52 *
35-39	2030	216.2326	17.372271	124469.94 *
35-39	2031	223.7054	18.007840	124226.65 *
35-39	2032	218.1070	17.683044	123342.47 *
35-39	2033	212.0010	17.364105	122091.53 *
35-39	2034	206.1605	17.050920	120908.72 *
35-39	2035	201.0447	16.743383	120074.10 *
35-39	2036	206.4538	17.277270	119494.45 *
35-39	2037	202.2395	16.965650	119205.30 *
35-39	2038	198.5625	16.659651	119187.69 *
35-39	2039	195.2869	16.359172	119374.58 *
40-44	2018	422.3765	42.264569	99936.30 *
40-44	2019	416.6646	41.502269	100395.61 *
40-44	2020	412.3698	40.753718	101185.82 *
40-44	2021	506.3244	49.538262	102208.75 *
40-44	2022	503.6833	48.644771	103543.16 *
40-44	2023	502.1735	47.767395	105128.92 *
40-44	2024	501.3127	46.905844	106876.38 *
40-44	2025	500.7594	46.059832	108719.32 *
40-44	2026	625.5868	56.589044	110549.11 *
40-44	2027	624.7784	55.568382	112434.16 *
40-44	2028	623.9136	54.566129	114340.82 *
40-44	2029	622.8906	53.581953	116250.08 *
40-44	2030	621.5662	52.615529	118133.60 *
40-44	2031	588.0277	49.035100	119919.75 *

Results for PHIM for Run 1 (Basic_US_OG_T1.RTF)

40-44	2032	585.9591	48.150684	121692.78 *
40-44	2033	582.8032	47.282220	123260.55 *
40-44	2034	577.3089	46.429420	124341.18 *
40-44	2035	568.8778	45.592001	124775.80 *
40-44	2036	588.7542	47.259996	124577.70 *
40-44	2037	574.2086	46.407597	123731.59 *
40-44	2038	558.2873	45.570571	122510.50 *
40-44	2039	543.0178	44.748643	121348.44 *
45-49	2018	997.4531	98.595953	101165.72 *
45-49	2019	971.0698	96.817639	100298.85 *
45-49	2020	947.7762	95.071398	99690.99 *
45-49	2021	942.2569	94.944487	99242.93 *
45-49	2022	923.8661	93.232032	99093.21 *
45-49	2023	908.6782	91.550463	99254.35 *
45-49	2024	896.6304	89.899224	99737.28 *
45-49	2025	887.5700	88.277767	100542.87 *
45-49	2026	1089.6550	107.306212	101546.31 *
45-49	2027	1084.0852	105.370796	102882.89 *
45-49	2028	1081.0724	103.470288	104481.43 *
45-49	2029	1079.5318	101.604058	106248.89 *
45-49	2030	1078.6993	99.771488	108116.99 *
45-49	2031	1348.0874	122.579107	109976.93 *
45-49	2032	1346.7058	120.368223	111882.17 *
45-49	2033	1345.1652	118.197215	113806.84 *
45-49	2034	1343.2748	116.065364	115734.34 *
45-49	2035	1340.6365	113.971965	117628.62 *
45-49	2036	1268.4458	106.216299	119421.01 *
45-49	2037	1264.0827	104.300541	121196.18 *
45-49	2038	1257.3641	102.419336	122766.28 *
45-49	2039	1245.6427	100.572062	123855.74 *
50-54	2018	2673.5818	251.245736	106413.02 *
50-54	2019	2583.3897	246.714171	104711.85 *
50-54	2020	2500.0553	242.264339	103195.35 *
50-54	2021	1949.9335	191.758045	101687.18 *
50-54	2022	1890.5074	188.299423	100399.00 *
50-54	2023	1837.0630	184.903182	99352.70 *
50-54	2024	1789.3798	181.568197	98551.39 *
50-54	2025	1747.3548	178.293363	98004.48 *
50-54	2026	1737.2135	178.055359	97565.92 *
50-54	2027	1703.7281	174.843884	97442.82 *
50-54	2028	1676.3849	171.690332	97640.03 *
50-54	2029	1655.0335	168.593659	98167.01 *
50-54	2030	1639.3564	165.552839	99023.15 *
50-54	2031	2013.7611	201.238078	100068.59 *
50-54	2032	2004.3253	197.608470	101429.12 *
50-54	2033	1999.5540	194.044328	103046.25 *
50-54	2034	1997.5493	190.544469	104833.76 *
50-54	2035	1996.8025	187.107736	106719.40 *
50-54	2036	2495.9023	229.880296	108574.00 *
50-54	2037	2493.6594	225.734086	110468.89 *
50-54	2038	2491.1709	221.662659	112385.68 *
50-54	2039	2488.1853	217.664665	114312.78 *
55-59	2018	6524.0763	605.743009	107703.70 *
55-59	2019	6396.8480	594.817594	107543.02 *
55-59	2020	6250.9610	584.089235	107020.65 *
55-59	2021	4541.8832	428.767714	105928.76 *
55-59	2022	4400.0673	421.034294	104506.15 *
55-59	2023	4254.5096	413.440357	102905.04 *
55-59	2024	4114.1094	405.983387	101336.89 *
55-59	2025	3984.3703	398.660914	99943.84 *
55-59	2026	3108.6457	315.549692	98515.25 *
55-59	2027	3015.3050	309.858316	97312.38 *
55-59	2028	2931.6597	304.269592	96350.73 *
55-59	2029	2857.4224	298.781668	95635.80 *
55-59	2030	2792.4906	293.392726	95179.27 *
55-59	2031	2779.2201	293.001076	94853.58 *
55-59	2032	2727.8403	287.716396	94810.04 *
55-59	2033	2685.8800	282.527032	95066.30 *
55-59	2034	2653.3596	277.431265	95640.25 *
55-59	2035	2629.7783	272.427408	96531.34 *
55-59	2036	3232.1807	331.149670	97604.83 *
55-59	2037	3218.3160	325.176927	98971.23 *
55-59	2038	3211.6156	319.311910	100579.26 *
55-59	2039	3209.2809	313.552678	102352.21 *
60-64	2018	10649.6168	1093.171512	97419.45 *
60-64	2019	10641.0905	1073.454649	99129.39 *
60-64	2020	10593.5186	1054.093407	100498.86 *
60-64	2021	9440.8722	931.733736	101325.86 *

Results for PHIM for Run 1 (Basic_US_OG_T1.RTF)

60-64	2022	9322.6360	914.928627	101894.68 *
60-64	2023	9179.3649	898.426621	102171.56 *
60-64	2024	9008.0379	882.222252	102106.22 *
60-64	2025	8809.1978	866.310151	101686.42 *
60-64	2026	6404.3566	635.940197	100706.90 *
60-64	2027	6209.1852	624.470134	99431.26 *
60-64	2028	6008.8057	613.206950	97989.85 *
60-64	2029	5815.5102	602.146913	96579.59 *
60-64	2030	5637.1456	591.286359	95336.98 *
60-64	2031	4405.3178	468.017359	94127.23 *
60-64	2032	4278.7920	459.576017	93103.03 *
60-64	2033	4164.7041	451.286927	92285.06 *
60-64	2034	4063.0735	443.147342	91686.74 *
60-64	2035	3973.9538	435.154565	91322.81 *
60-64	2036	3958.7646	434.573677	91095.36 *
60-64	2037	3888.5986	426.735539	91124.32 *
60-64	2038	3831.1261	419.038772	91426.53 *
60-64	2039	3786.6511	411.480827	92024.97 *
65-69	2018	13141.8712	1627.643816	80741.69 *
65-69	2019	13205.3381	1598.287004	82621.82 *
65-69	2020	13285.9062	1569.459683	84652.74 *
65-69	2021	13092.1337	1514.206848	86461.99 *
65-69	2022	13138.0706	1486.896029	88359.04 *
65-69	2023	13173.6089	1460.077799	90225.39 *
65-69	2024	13174.8060	1433.743272	91890.97 *
65-69	2025	13129.5519	1407.883726	93257.36 *
65-69	2026	11709.1267	1244.455904	94090.33 *
65-69	2027	11575.0024	1222.010417	94720.98 *
65-69	2028	11411.4293	1199.969765	95097.64 *
65-69	2029	11213.0577	1178.326648	95160.86 *
65-69	2030	10979.8051	1157.073894	94892.86 *
65-69	2031	7997.1862	849.383791	94152.80 *
65-69	2032	7767.2767	834.063977	93125.67 *
65-69	2033	7529.6230	819.020477	91934.49 *
65-69	2034	7299.6994	804.248308	90764.25 *
65-69	2035	7086.8750	789.742575	89736.52 *
65-69	2036	5544.0764	625.100222	88691.00 *
65-69	2037	5390.4777	613.825673	87817.73 *
65-69	2038	5251.9510	602.754477	87132.51 *
65-69	2039	5128.8435	591.882964	86653.00 *
70-74	2018	14628.5373	2425.058503	60322.41 *
70-74	2019	15137.3604	2381.319212	63567.12 *
70-74	2020	15539.6744	2338.368820	66455.19 *
70-74	2021	14043.4042	2046.331206	68627.23 *
70-74	2022	14165.6188	2009.422787	70495.96 *
70-74	2023	14249.0633	1973.180062	72213.70 *
70-74	2024	14337.5226	1937.591025	73996.64 *
70-74	2025	14449.9163	1902.643886	75946.51 *
70-74	2026	14252.5700	1835.661300	77642.70 *
70-74	2027	14322.3025	1802.552605	79455.67 *
70-74	2028	14385.3132	1770.041071	81271.07 *
70-74	2029	14415.1896	1738.115927	82935.72 *
70-74	2030	14397.9929	1706.766597	84358.30 *
70-74	2031	12868.2698	1508.644308	85296.91 *
70-74	2032	12750.8209	1481.433817	86070.81 *
70-74	2033	12602.7571	1454.714105	86633.91 *
70-74	2034	12416.9761	1428.476320	86924.62 *
70-74	2035	12191.1840	1402.711770	86911.54 *
70-74	2036	8887.5116	1029.701428	86311.54 *
70-74	2037	8642.1892	1011.129336	85470.66 *
70-74	2038	8390.0941	992.892217	84501.56 *
70-74	2039	8148.5392	974.984030	83576.13 *
75-79	2018	13239.2082	3340.059655	39637.64 *
75-79	2019	13502.2623	3279.817051	41167.73 *
75-79	2020	13935.7551	3220.661005	43269.86 *
75-79	2021	12596.1750	2769.804832	45476.76 *
75-79	2022	13095.7886	2719.847563	48148.98 *
75-79	2023	13633.6420	2670.791343	51047.20 *
75-79	2024	14120.1516	2622.619920	53839.87 *
75-79	2025	14513.3193	2575.317335	56355.46 *
75-79	2026	13126.3158	2253.687350	58243.73 *
75-79	2027	13262.1760	2213.038975	59927.44 *
75-79	2028	13375.1877	2173.123750	61548.21 *
75-79	2029	13503.9708	2133.928452	63282.21 *
75-79	2030	13662.2778	2095.440095	65200.04 *
75-79	2031	13521.4938	2021.670118	66882.79 *
75-79	2032	13633.6875	1985.206496	68676.42 *
75-79	2033	13742.7748	1949.400546	70497.44 *

Results for PHIM for Run 1 (Basic_US_OG_T1.RTF)

75-79	2034	13825.1132	1914.240405	72222.45 *
75-79	2035	13866.4860	1879.714427	73769.11 *
75-79	2036	12407.3894	1661.516269	74675.10 *
75-79	2037	12314.2104	1631.548521	75475.60 *
75-79	2038	12198.3464	1602.121282	76138.72 *
75-79	2039	12051.8050	1573.224805	76605.74 *

Results for PHIM for Run 1 (Basic_US_OG_T1.RTF)

****
List of values created by last value brought forwards for Fixed File perm.Fixed_File_RR_LC

1. Country	US (United States)
2. Sex	M (Males)
3. Disease	LC (LC)

Age	Years	Value
10-14	2013-2039	11.6800
15-19	2013-2039	11.6800
20-24	2013-2039	11.6800
25-29	2013-2039	11.6800
30-34	2013-2039	11.6800
35-39	2013-2039	11.6800
40-44	2013-2039	11.6800
45-49	2013-2039	11.6800
50-54	2013-2039	11.6800
55-59	2013-2039	11.6800
60-64	2013-2039	11.6800
65-69	2013-2039	11.6800
70-74	2013-2039	11.6800
75-79	2013-2039	11.6800

Results for PHIM for Run 1 (Basic_US_OG_T1.RTF)

****

Osmond and Gardner Modeling of Death Rates for COD: IHD

Variable Parameter	Value
1. Country	US (United States)
2. Sex	M (Males)
3. Year of start of OG modelling	1966
4. Number 5-year periods for OG modelling	10
5. Age Range of interest	10 - 79
6. Scaling Factor for Rates	10000
7. Model Type to Save	4 (Full Age-Period-Cohort)
8. Goodness of Fit Required	1 (Analysis Printed For Age-Period-Cohort Model)
9. Cause of Death	IHD
Note:	Death rates are per million population

Matrix of Numbers of Deaths

Age	1966-	1971-	1976-	1981-	1986-	1991-	1996-	2001-	2006-	2011-
10-14	76	54	25	27	32	21	35	39	32	23
15-19	242	189	134	106	100	106	150	106	118	95
20-24	552	502	371	354	324	297	275	429	381	410
25-29	1434	1340	1314	1305	1101	978	862	1034	1146	1073
30-34	4449	4192	4030	3674	3417	3045	2739	2946	2752	2675
35-39	14558	11852	10371	9811	8885	8406	8309	7873	6565	5679
40-44	37624	31238	23792	20359	19068	18406	18694	19912	15918	13013
45-49	73674	66731	49705	36793	31227	32009	34365	37363	33930	27190
50-54	119255	113898	91385	66394	49184	46344	51662	59029	56803	52161
55-59	174045	163952	139855	110120	80915	65757	67309	75974	76897	76888
60-64	223132	222462	189612	158829	127374	101463	90090	90871	91070	97211
65-69	260317	261459	235475	200601	172601	147790	125390	107300	96712	106060
70-74	288133	277827	255120	232517	204299	187996	172292	141677	109155	109523
75-79	281452	275926	244221	228182	215731	203687	205614	184381	137446	120897

Matrix of Age- and Period-Specific Mortality Rates

Age	1966-	1971-	1976-	1981-	1986-	1991-	1996-	2001-	2006-	2011-
10-14	1.450	1.012	0.499	0.587	0.721	0.451	0.684	0.711	0.592	0.433
15-19	5.140	3.599	2.448	2.044	2.117	2.307	3.050	1.970	2.090	1.700
20-24	14.135	10.748	7.025	6.377	6.210	6.233	5.783	8.453	6.997	7.068
25-29	43.591	33.438	27.584	24.357	19.809	18.632	17.535	21.298	22.492	19.370
30-34	150.431	125.185	99.666	76.211	63.237	53.912	50.547	58.331	55.943	51.547
35-39	489.254	402.751	320.878	247.023	184.618	155.541	145.151	143.767	129.257	113.805
40-44	1201.764	1056.188	822.983	638.248	480.320	385.647	345.963	349.146	291.898	255.315
45-49	2425.718	2179.220	1762.150	1305.791	992.350	823.519	724.921	697.278	601.365	501.932
50-54	4360.615	3911.456	3151.223	2463.371	1789.447	1509.326	1341.706	1257.029	1076.654	939.608
55-59	7139.922	6426.847	5155.398	4039.934	3150.192	2503.239	2261.278	2019.053	1684.138	1501.269
60-64	10804.79	10204.43	8110.540	6364.771	5043.246	4230.150	3600.966	3190.353	2527.405	2232.732
65-69	16073.86	14842.58	12179.01	9591.099	7771.350	6532.486	5718.799	4612.819	3643.541	3155.767
70-74	24740.44	21998.01	17912.79	14709.63	11845.19	10100.31	8890.586	7385.581	5300.334	4611.096
75-79	34813.96	33281.25	25552.56	21100.65	18143.14	15445.88	14002.06	11873.84	8740.148	7018.843

Matrix of Log-Rates

Age	1966-	1971-	1976-	1981-	1986-	1991-	1996-	2001-	2006-	2011-
10-14	0.161	0.005	-0.302	-0.231	-0.142	-0.346	-0.165	-0.148	-0.227	-0.363
15-19	0.711	0.556	0.389	0.310	0.326	0.363	0.484	0.294	0.320	0.230
20-24	1.150	1.031	0.847	0.805	0.793	0.795	0.762	0.927	0.845	0.849
25-29	1.639	1.524	1.441	1.387	1.297	1.270	1.244	1.328	1.352	1.287
30-34	2.177	2.098	1.999	1.882	1.801	1.732	1.704	1.766	1.748	1.712
35-39	2.690	2.605	2.506	2.393	2.266	2.192	2.162	2.158	2.111	2.056
40-44	3.080	3.024	2.915	2.805	2.682	2.586	2.539	2.543	2.465	2.407
45-49	3.385	3.338	3.246	3.116	2.997	2.916	2.860	2.843	2.779	2.701
50-54	3.640	3.592	3.498	3.392	3.253	3.179	3.128	3.099	3.032	2.973
55-59	3.854	3.808	3.712	3.606	3.498	3.399	3.354	3.305	3.226	3.176
60-64	4.034	4.009	3.909	3.804	3.703	3.626	3.556	3.504	3.403	3.349
65-69	4.206	4.172	4.086	3.982	3.890	3.815	3.757	3.664	3.562	3.499
70-74	4.393	4.342	4.253	4.168	4.074	4.004	3.949	3.868	3.724	3.664
75-79	4.542	4.522	4.407	4.324	4.259	4.189	4.146	4.075	3.942	3.846

Results for PHIM for Run 1 (Basic_US_OG_T1.RTF)

Fitting the Age, Period, Cohort Models

**Model**	**RSS**	**MRSS**	**DF**	**Factor**	**%Account**	**ChiSq**	**P**
Age Only	491270.586	3868.272	127	P, C	99.8175	2719030.72	0.0000
Age-Period	4176.733	35.699	117	Cohort	78.5350	22233.748	0.0000
Age-Cohort	4045.733	38.901	104	Period	77.8400	21462.202	0.0000
Period-Cohort	1664.950	15.416	108	Age	46.1524	8835.442	0.0000
Full Age-Period-Cohort	896.535	9.339	96			4756.283	0.0000

**Key to terms:**		
	RSS =	residual sum of squares
	MRSS =	mean RSS (MRSS/DF)
	DF =	degrees of freedom
	Factor =	Factors not included in the model
	% Account =	1- (RSS for full model)/(RSS for model in question)
	Chisq =	chi-squared value for model
	P =	probability value based on Chisq and DF.

**Age**	**Value**	**Log10 Value**
10-	0.970387	-0.013055
15-	3.574221	0.553181
20-	10.886575	1.036891
25-	35.269829	1.547403
30-	111.570043	2.047548
35-	322.049812	2.507923
40-	768.629498	2.885717
45-	1503.69882	3.177161
50-	2586.83377	3.412769
55-	4018.46684	3.604060
60-	5983.37821	3.776946
65-	8500.49177	3.929444
70-	12197.3060	4.086264
75-	17483.7056	4.242633

**Period**	**Value**	**Log10 Value**
1966	1.562287	0.193761
1971	1.484369	0.171542
1976	1.245347	0.095290
1981	1.040268	0.017145
1986	0.883616	-0.053736
1991	0.792453	-0.101027
1996	0.749892	-0.125001
2001	0.693398	-0.159018
2006	0.576831	-0.238951
2011	0.519862	-0.284112

**Cohort**	**Value**	**Log10 Value**
1891	1.274557	0.105359
1896	1.290510	0.110761
1901	1.200387	0.079321
1906	1.168526	0.067638
1911	1.154651	0.062451
1916	1.091922	0.038192
1921	1.038574	0.016438
1926	0.972010	-0.012329
1931	0.892142	-0.049566
1936	0.786052	-0.104549
1941	0.748572	-0.125766
1946	0.715537	-0.145368
1951	0.699037	-0.155500
1956	0.691572	-0.160163
1961	0.678884	-0.168204
1966	0.645165	-0.190329
1971	0.669666	-0.174141
1976	0.749908	-0.124992
1981	0.967989	-0.014130
1986	1.047234	0.020044
1991	1.182129	0.072665
1996	0.949069	-0.022702
2001	0.858567	-0.066226

Results for PHIM for Run 1 (Basic_US_OG_T1.RTF)

Model: Full Age-Period-Cohort

Basic Analysis Using OG Modelling T1 on US
Fitting the Full Age-Period-Cohort Model
Matrix of observed, expected, and residual rates

Age		1966-	1971-	1976-	1981-	1986-	1991-	1996-	2001-	2006-	2011-
10-	Observed	1.450	1.012	0.499	0.587	0.721	0.451	0.684	0.711	0.592	0.433
	Expected	1.048	0.978	0.780	0.676	0.643	0.744	0.762	0.795	0.531	0.433
	Residual	-0.402	-0.034	0.281	0.089	-0.078	0.294	0.078	0.084	-0.061	0.000
15-	Observed	5.140	3.599	2.448	2.044	2.117	2.307	3.050	1.970	2.090	1.700
	Expected	3.903	3.669	3.022	2.399	2.115	2.124	2.594	2.595	2.437	1.763
	Residual	-1.237	0.070	0.574	0.355	-0.002	-0.183	-0.456	0.625	0.347	0.063
20-	Observed	14.135	10.748	7.025	6.377	6.210	6.233	5.783	8.453	6.997	7.068
	Expected	12.170	11.296	9.376	7.688	6.206	5.777	6.122	7.307	6.576	6.690
	Residual	-1.965	0.549	2.351	1.312	-0.004	-0.456	0.339	-1.146	-0.421	-0.378
25-	Observed	43.591	33.438	27.584	24.357	19.809	18.632	17.535	21.298	22.492	19.370
	Expected	41.248	37.461	30.704	25.374	21.157	18.032	17.712	18.340	19.693	19.202
	Residual	-2.344	4.023	3.120	1.017	1.348	-0.600	0.177	-2.958	-2.798	-0.169
30-	Observed	150.431	125.185	99.666	76.211	63.237	53.912	50.547	58.331	55.943	51.547
	Expected	137.012	123.972	99.419	81.132	68.179	60.023	53.978	51.807	48.262	56.144
	Residual	-13.419	-1.213	-0.246	4.921	4.942	6.111	3.431	-6.524	-7.681	4.597
35-	Observed	489.254	402.751	320.878	247.023	184.618	155.541	145.151	143.767	129.257	113.805
	Expected	448.867	375.765	300.225	239.718	198.924	176.496	163.952	144.071	124.403	125.551
	Residual	-40.387	-26.986	-20.653	-7.305	14.306	20.954	18.801	0.304	-4.854	11.746
40-	Observed	1201.764	1056.188	822.983	638.248	480.320	385.647	345.963	349.146	291.898	255.315
	Expected	1167.209	1017.871	752.417	598.544	485.974	425.785	398.614	361.822	286.047	267.586
	Residual	-34.555	-38.317	-70.566	-39.704	5.654	40.138	52.652	12.676	-5.851	12.271
45-	Observed	2425.718	2179.220	1762.150	1305.791	992.350	823.519	724.921	697.278	601.365	501.932
	Expected	2439.828	2169.568	1670.648	1229.581	994.623	852.641	788.242	721.075	588.851	504.336
	Residual	14.110	-9.651	-91.503	-76.211	2.273	29.122	63.321	23.797	-12.514	2.404
50-	Observed	4360.615	3911.456	3151.223	2463.371	1789.447	1509.326	1341.706	1257.029	1076.654	939.608
	Expected	4412.868	3987.933	3131.334	2400.752	1796.733	1534.531	1388.032	1253.865	1031.941	912.961
	Residual	52.253	76.477	-19.889	-62.619	7.286	25.205	46.326	-3.164	-44.713	-26.647
55-	Observed	7139.922	6426.847	5155.398	4039.934	3150.192	2503.239	2261.278	2019.053	1684.138	1501.269
	Expected	7248.900	6513.190	5197.424	4063.274	3167.801	2503.138	2255.759	1993.770	1620.352	1444.727
	Residual	108.977	86.343	42.026	23.340	17.610	-0.101	-5.519	-25.283	-63.787	-56.542
60-	Observed	10804.789	10204.433	8110.540	6364.771	5043.246	4230.150	3600.966	3190.353	2527.405	2232.732
	Expected	10923.091	10255.081	8136.325	6464.412	5139.027	4230.128	3526.925	3105.722	2469.606	2174.375
	Residual	118.302	50.649	25.785	99.641	95.781	-0.022	-74.041	-84.631	-57.798	-58.357
65-	Observed	16073.864	14842.575	12179.011	9591.099	7771.350	6532.486	5718.799	4612.819	3643.541	3155.767
	Expected	15941.391	14744.298	12223.207	9655.632	7800.911	6547.688	5686.910	4633.163	3670.513	3162.019
	Residual	-132.473	-98.277	44.196	64.533	29.562	15.203	-31.889	20.344	26.972	6.252
70-	Observed	24740.438	21998.012	17912.794	14709.632	11845.191	10100.314	8890.586	7385.581	5300.334	4611.096
	Expected	24591.568	21733.369	17749.758	14650.749	11768.451	10038.636	8890.643	7545.361	5530.495	4746.635
	Residual	-148.869	-264.644	-163.037	-58.882	-76.740	-61.678	0.057	159.780	230.161	135.539
75-	Observed	34813.960	33281.251	25552.561	21100.649	18143.141	15445.876	14002.058	11873.836	8740.148	7018.843
	Expected	34813.960	33491.666	26136.358	21252.833	17838.081	15128.586	13616.628	11783.832	8997.383	7144.515
	Residual	0.000	210.415	583.797	152.183	-305.060	-317.289	-385.430	-90.004	257.234	125.671

Fitting the Full Age-Period-Cohort Model

Results for PHIM for Run 1 (Basic_US_OG_T1.RTF)

Matrix of observed and expected deaths and (O-E)2/E Values**

**Age**		**1966-**	**1971-**	**1976-**	**1981-**	**1986-**	**1991-**	**1996-**	**2001-**	**2006-**	**2011-**	**Total**
10-	Observed	76.0	54.0	25.0	27.0	32.0	21.0	35.0	39.0	32.0	23.0	364.0
	Expected	55.0	52.2	39.1	31.1	28.5	34.7	39.0	43.6	28.7	23.0	374.8
	Difference	21.0	1.8	-14.1	-4.1	3.5	-13.7	-4.0	-4.6	3.3	-0.0	-10.8
	Chi-Sq	8.1	0.1	5.1	0.5	0.4	5.4	0.4	0.5	0.4	0.0	20.8
15-	Observed	242.0	189.0	134.0	106.0	100.0	106.0	150.0	106.0	118.0	95.0	1346.0
	Expected	183.8	192.7	165.4	124.4	99.9	97.6	127.6	139.6	137.6	98.5	1367.2
	Difference	58.2	-3.7	-31.4	-18.4	0.1	8.4	22.4	-33.6	-19.6	-3.5	-21.2
	Chi-Sq	18.5	0.1	6.0	2.7	0.0	0.7	3.9	8.1	2.8	0.1	42.9
20-	Observed	552.0	502.0	371.0	354.0	324.0	297.0	275.0	429.0	381.0	410.0	3895.0
	Expected	475.3	527.6	495.2	426.8	323.8	275.3	291.1	370.8	358.1	388.1	3932.1
	Difference	76.7	-25.6	-124.2	-72.8	0.2	21.7	-16.1	58.2	22.9	21.9	-37.1
	Chi-Sq	12.4	1.2	31.1	12.4	0.0	1.7	0.9	9.1	1.5	1.2	71.6
25-	Observed	1434.0	1340.0	1314.0	1305.0	1101.0	978.0	862.0	1034.0	1146.0	1073.0	11587.0
	Expected	1356.9	1501.2	1462.6	1359.5	1175.9	946.5	870.7	890.4	1003.4	1063.7	11630.8
	Difference	77.1	-161.2	-148.6	-54.5	-74.9	31.5	-8.7	143.6	142.6	9.3	-43.8
	Chi-Sq	4.4	17.3	15.1	2.2	4.8	1.0	0.1	23.2	20.3	0.1	88.4
30-	Observed	4449.0	4192.0	4030.0	3674.0	3417.0	3045.0	2739.0	2946.0	2752.0	2675.0	33919.0
	Expected	4052.1	4151.4	4020.0	3911.2	3684.0	3390.1	2924.9	2616.5	2374.2	2913.6	34038.1
	Difference	396.9	40.6	10.0	-237.2	-267.0	-345.1	-185.9	329.5	377.8	-238.6	-119.1
	Chi-Sq	38.9	0.4	0.0	14.4	19.4	35.1	11.8	41.5	60.1	19.5	241.1
35-	Observed	14558.0	11852.0	10371.0	9811.0	8885.0	8406.0	8309.0	7873.0	6565.0	5679.0	92309.0
	Expected	13356.3	11057.9	9703.5	9520.9	9573.5	9538.5	9385.3	7889.6	6318.5	6265.1	92608.9
	Difference	1201.7	794.1	667.5	290.1	-688.5	-1132.5	-1076.3	-16.6	246.5	-586.1	-299.9
	Chi-Sq	108.1	57.0	45.9	8.8	49.5	134.5	123.4	0.0	9.6	54.8	591.8
40-	Observed	37624.0	31238.0	23792.0	20359.0	19068.0	18406.0	18694.0	19912.0	15918.0	13013.0	218024.0
	Expected	36542.2	30104.7	21752.0	19092.5	19292.4	20321.7	21539.0	20634.9	15598.9	13638.4	218516.8
	Difference	1081.8	1133.3	2040.0	1266.5	-224.4	-1915.7	-2845.0	-722.9	319.1	-625.4	-492.8
	Chi-Sq	32.0	42.7	191.3	84.0	2.6	180.6	375.8	25.3	6.5	28.7	969.6
45-	Observed	73674.0	66731.0	49705.0	36793.0	31227.0	32009.0	34365.0	37363.0	33930.0	27190.0	422987.0
	Expected	74102.5	66435.5	47124.0	34645.6	31298.5	33140.9	37366.7	38638.1	33224.0	27320.2	423296.1
	Difference	-428.5	295.5	2581.0	2147.4	-71.5	-1131.9	-3001.7	-1275.1	706.0	-130.2	-309.1
	Chi-Sq	2.5	1.3	141.4	133.1	0.2	38.7	241.1	42.1	15.0	0.6	615.9
50-	Observed	119255.0	113898.0	91385.0	66394.0	49184.0	46344.0	51662.0	59029.0	56803.0	52161.0	706115.0
	Expected	120684.0	116124.9	90808.2	64706.3	49384.3	47117.9	53445.8	58880.4	54444.0	50681.7	706277.5
	Difference	-1429.0	-2226.9	576.8	1687.7	-200.3	-773.9	-1783.8	148.6	2359.0	1479.3	-162.5
	Chi-Sq	16.9	42.7	3.7	44.0	0.8	12.7	59.5	0.4	102.2	43.2	326.1
55-	Observed	174045.0	163952.0	139855.0	110120.0	80915.0	65757.0	67309.0	75974.0	76897.0	76888.0	1031712.0
	Expected	176701.5	166154.7	140995.1	110756.2	81367.3	65754.3	67144.7	75022.7	73984.5	73992.2	1031873.2
	Difference	-2656.5	-2202.7	-1140.1	-636.2	-452.3	2.7	164.3	951.3	2912.5	2895.8	-161.2
	Chi-Sq	39.9	29.2	9.2	3.7	2.5	0.0	0.4	12.1	114.7	113.3	325.0
60-	Observed	223132.0	222462.0	189612.0	158829.0	127374.0	101463.0	90090.0	90871.0	91070.0	97211.0	1392114.0
	Expected	225575.1	223566.2	190214.8	161315.5	129793.1	101462.5	88237.6	88460.5	88987.4	94670.2	1392282.7
	Difference	-2443.1	-1104.2	-602.8	-2486.5	-2419.1	0.5	1852.4	2410.5	2082.6	2540.8	-168.7
	Chi-Sq	26.5	5.5	1.9	38.3	45.1	0.0	38.9	65.7	48.7	68.2	338.7
65-	Observed	260317.0	261459.0	235475.0	200601.0	172601.0	147790.0	125390.0	107300.0	96712.0	106060.0	1713705.0
	Expected	258171.6	259727.8	236329.5	201950.7	173257.6	148133.9	124690.8	107773.2	97427.9	106270.1	1713733.2
	Difference	2145.4	1731.2	-854.5	-1349.7	-656.6	-343.9	699.2	-473.2	-715.9	-210.1	-28.2
	Chi-Sq	17.8	11.5	3.1	9.0	2.5	0.8	3.9	2.1	5.3	0.4	56.4
70-	Observed	288133.0	277827.0	255120.0	232517.0	204299.0	187996.0	172292.0	141677.0	109155.0	109523.0	1978539.0
	Expected	286399.2	274484.6	252798.0	231586.2	202975.4	186848.0	172293.1	144742.1	113894.9	112742.3	1978763.9

Results for PHIM for Run 1 (Basic_US_OG_T1.RTF)

	Difference	1733.8	3342.4	2322.0	930.8	1323.6	1148.0	-1.1	-3065.1	-4739.9	-3219.3	-224.9
	Chi-Sq	10.5	40.7	21.3	3.7	8.6	7.1	0.0	64.9	197.3	91.9	446.0
75-	Observed	281452.0	275926.0	244221.0	228182.0	215731.0	203687.0	205614.0	184381.0	137446.0	120897.0	2097537.0
	Expected	281452.0	277670.5	249800.7	229827.7	212103.7	199502.9	199954.1	182983.4	141491.2	123061.6	2097847.8
	Difference	-0.0	-1744.5	-5579.7	-1645.7	3627.3	4184.1	5659.9	1397.6	-4045.2	-2164.6	-310.8
	Chi-Sq	0.0	11.0	124.6	11.8	62.0	87.8	160.2	10.7	115.7	38.1	621.8
Total over ages	Observed	1478943.0	1431622.0	1245410.0	1069072.0	914258.0	816305.0	777786.0	728934.0	628925.0	612898.0	9704153.0
	Expected	1479107.4	1431751.9	1245708.0	1069254.7	914358.1	816564.8	778310.4	729085.9	629273.3	613128.8	9706543.3
	Difference	-164.4	-129.9	-298.0	-182.7	-100.1	-259.8	-524.4	-151.9	-348.3	-230.8	-2390.3
	Chi-Sq	336.4	260.7	599.7	368.8	198.4	506.0	1020.4	305.6	700.0	460.2	4756.3

Results for PHIM for Run 1 (Basic_US_OG_T1.RTF)

****

Osmond and Gardner Extrapolating Death Rates for COD: IHD

Variable Parameter	Value
1. Country	US (United States)
2. Sex	M (Males)
3. Year of start of OG modelling	1966
4. Number 5-year periods for OG modelling	10
5. Age Range of interest	10 - 79
6. Scaling Factor for Rates	10000
7. Model Type to Save	4 (Full Age-Period-Cohort)
8. Goodness of Fit Required	1 (Analysis Printed For Age-Period-Cohort Model)
9. Cause of Death	IHD
Note:	Death rates are per million population
10. Number of Periods into the future to Predict	5
11. Earliest projected year	2016
12. Extrapolate Period using (1: last 2 points 2: linear regression)	1
13. Ratio of last two period values	0.90124

Predictions of rates for future years from model:	Full Age-Period-Cohort
Effects for extending model to project rates for:	2016-2040

Extrapolating Model: Full Age-Period-Cohort
Log Transform Parameters

Model	ChiSq	MChiSq	DF	Factor	%Account	P
Age Only	792755.822	56625.416	14	P, C	99.5027	0.0000
Age-Period	9079.408	648.529	14	Cohort	56.5775	0.0000
Age-Cohort	6348.210	453.444	14	Period	37.8958	0.0000
Period-Cohort	2950.418	210.744	14	Age	-33.6253	0.0000
Full Age-Period-Cohort	3942.505	281.608	14			0.0000

**Key to terms:**		
	Chisq =	chi-squared value for model
	MChisq =	mean Chi-squared (Chisq/DF)
	DF =	degrees of freedom
	Factor =	Factors not included in the model
	% Account =	1- (Chisq for full model)/(Chisq for model in question)
	P =	probability value based on Chisq and DF.

AGE	EFFECT
10	0.970387
15	3.574221
20	10.886575
25	35.269829
30	111.570043
35	322.049812
40	768.629498
45	1503.69882
50	2586.83377
55	4018.46684
60	5983.37821
65	8500.49177
70	12197.3060
75	17483.7056

PERIOD	EFFECT	
Period Change =	0.901237	
1966	1.562287	
1971	1.484369	
1976	1.245347	
1981	1.040268	
1986	0.883616	
1991	0.792453	
1996	0.749892	
2001	0.693398	
2006	0.576831	
2011	0.519862	
2016	0.468519	
2021	0.422247	
2026	0.380545	
2031	0.342961	
2036	0.309089	
2016	0.488418	Extrapolated
2017	0.478365	Extrapolated
2018	0.468519	Extrapolated
2019	0.458876	Extrapolated

Results for PHIM for Run 1 (Basic_US_OG_T1.RTF)

2020	0.449431	Extrapolated
2021	0.440180	Extrapolated
2022	0.431120	Extrapolated
2023	0.422247	Extrapolated
2024	0.413556	Extrapolated
2025	0.405044	Extrapolated
2026	0.396707	Extrapolated
2027	0.388542	Extrapolated
2028	0.380545	Extrapolated
2029	0.372712	Extrapolated
2030	0.365041	Extrapolated
2031	0.357527	Extrapolated
2032	0.350168	Extrapolated
2033	0.342961	Extrapolated
2034	0.335902	Extrapolated
2035	0.328988	Extrapolated
2036	0.322217	Extrapolated
2037	0.315585	Extrapolated
2038	0.309089	Extrapolated
2039	0.302727	Extrapolated
2040	0.296496	Extrapolated

Results for PHIM for Run 1 (Basic_US_OG_T1.RTF)

COHORT	EFFECT	WEIGHT	ORIGINAL
1891	1.274557	1.000	
1896	1.290510	2.000	
1901	1.200387	4.000	
1906	1.168526	8.000	
1911	1.154651	16.000	
1916	1.091922	32.000	
1921	1.038574	64.000	
1926	0.972010	128.000	
1931	0.892142	256.000	
1936	0.786052	512.000	
1941	0.748572	1024.000	
1946	0.715537	2048.000	
1951	0.699037	4096.000	
1956	0.691572	8192.000	
1961	0.678884	16384.000	
1966	0.645165	32768.000	
1971	0.669666	65536.000	
1976	0.749908	131072.000	
1981	0.967989	262144.000	
1986	1.047234	524288.000	
1991	1.182129	1048576.000	
1996	1.309978	Extrapolated	0.949069
2001	1.463726	Extrapolated	0.858567
2006	1.635520	Extrapolated	
2011	1.827477	Extrapolated	
2016	2.041963	Extrapolated	
2021	2.281624	Extrapolated	
2026	2.549412	Extrapolated	

Standardizing Population: The 1976 European Standard Population

Age Range	Population, Males
All	100000
0	0
1	0
2	0
3	0
0-4	8000
5-9	7000
10-14	7000
15-19	7000
20-24	7000
25-29	7000
30-34	7000
35-39	7000
40-44	7000
45-49	7000
50-54	7000
55-59	6000
60-64	5000
65-69	4000
70-74	3000
75-79	2000
80-84	1000
85+	1000

Results for PHIM for Run 1 (Basic_US_OG_T1.RTF)

Matrix of observed and expected rates including predictions

Total over ages standardized using: The 1976 European Standard Population

		1966-	1971-	1976-	1981-	1986-	1991-	1996-	2001-	2006-	2011-	2016-	2021-	2026-	2031-	2036-
10	OBS	1.5	1.0	0.5	0.6	0.7	0.5	0.7	0.7	0.6	0.4	0.5	.	.	.	.
	EXP											0.7	0.7	0.8	0.8	0.8
15	OBS	5.1	3.6	2.4	2.0	2.1	2.3	3.1	2.0	2.1	1.7	1.2	.	.	.	.
	EXP											2.5	2.5	2.5	2.5	2.5
20	OBS	14.1	10.7	7.0	6.4	6.2	6.2	5.8	8.5	7.0	7.1	5.5	.	.	.	.
	EXP											6.7	6.7	6.8	6.8	6.9
25	OBS	43.6	33.4	27.6	24.4	19.8	18.6	17.5	21.3	22.5	19.4	14.7	.	.	.	.
	EXP											19.5	19.5	19.6	19.8	19.9
30	OBS	150.4	125.2	99.7	76.2	63.2	53.9	50.5	58.3	55.9	51.5	47.2	.	.	.	.
	EXP											54.7	55.7	55.6	56.0	56.4
35	OBS	489.3	402.8	320.9	247.0	184.6	155.5	145.2	143.8	129.3	113.8	107.2	.	.	.	.
	EXP											146.1	142.4	144.9	144.7	145.7
40	OBS	1201.8	1056.2	823.0	638.2	480.3	385.6	346.0	349.1	291.9	255.3	232.5	.	.	.	.
	EXP											270.1	314.2	306.3	311.6	311.2
45	OBS	2425.7	2179.2	1762.2	1305.8	992.4	823.5	724.9	697.3	601.4	501.9	478.0	.	.	.	.
	EXP											471.8	476.1	553.9	540.1	549.4
50	OBS	4360.6	3911.5	3151.2	2463.4	1789.4	1509.3	1341.7	1257.0	1076.7	939.6	875.1	.	.	.	.
	EXP											781.9	731.5	738.2	858.8	837.3
55	OBS	7139.9	6426.8	5155.4	4039.9	3150.2	2503.2	2261.3	2019.1	1684.1	1501.3	1440.3	.	.	.	.
	EXP											1278.2	1094.7	1024.1	1033.5	1202.3
60	OBS	10804.8	10204.4	8110.5	6364.8	5043.2	4230.1	3601.0	3190.4	2527.4	2232.7	2115.1	.	.	.	.
	EXP											1938.7	1715.2	1469.0	1374.2	1386.9
65	OBS	16073.9	14842.6	12179.0	9591.1	7771.3	6532.5	5718.8	4612.8	3643.5	3155.8	2959.2	.	.	.	.
	EXP											2784.0	2482.3	2196.1	1880.9	1759.5
70	OBS	24740.4	21998.0	17912.8	14709.6	11845.2	10100.3	8890.6	7385.6	5300.3	4611.1	4122.5	.	.	.	.
	EXP											4089.1	3600.2	3210.0	2839.9	2432.3
75	OBS	34814.0	33281.3	25552.6	21100.6	18143.1	15445.9	14002.1	11873.8	8740.1	7018.8	6215.1	.	.	.	.
	EXP											6131.9	5282.4	4650.9	4146.8	3668.7
10-79	OBS	4407.9	4043.1	3233.8	2579.6	2069.8	1737.1	1537.0	1327.6	1036.2	890.4	821.5	.	.	.	.
	EXP	4408.7	4043.6	3232.1	2576.9	2071.2	1738.1	1536.9	1327.2	1037.4	891.4	786.8*	707.0*	650.7*	614.4*	594.4*

Drop in overall standardized Observed and Predicted rates

comparing the last observed rate during the model fitting period to the last observed and predicted rates where an observed rate is available (2016)

Observed and Predicted %Drop = 7.734% and 11.628%

Results for PHIM for Run 1 (Basic_US_OG_T1.RTF)

Matrix of observed and expected deaths including predictions

		1966-	1971-	1976-	1981-	1986-	1991-	1996-	2001-	2006-	2011-	2016-	2021-	2026-	2031-	2036-
10-	OBS	76.0	54.0	25.0	27.0	32.0	21.0	35.0	39.0	32.0	23.0	27.5*	.	.	.	.
	EXP	55.0	52.2	39.1	31.1	28.5	34.7	39.0	43.6	39.6	39.2	40.0*	39.4*	38.4*	39.3*	40.9*
	ChiSq	8.062	0.064	5.056	0.541	0.420	5.399	0.409	0.487	1.465	6.702	3.893*	.	.	.	.
15-	OBS	242.0	189.0	134.0	106.0	100.0	106.0	150.0	106.0	118.0	95.0	65.0*	.	.	.	.
	EXP	183.8	192.7	165.4	124.4	99.9	97.6	127.6	139.6	137.6	136.0	133.0*	135.5*	133.7*	130.6*	133.9*
	ChiSq	18.451	0.071	5.967	2.728	.	0.724	3.938	8.108	2.797	12.369	34.768*	.	.	.	.
20-	OBS	552.0	502.0	371.0	354.0	324.0	297.0	275.0	429.0	381.0	410.0	315.0*	.	.	.	.
	EXP	475.3	527.6	495.2	426.8	323.8	275.3	291.1	370.8	358.1	388.1	386.1*	377.3*	384.9*	381.3*	373.6*
	ChiSq	12.388	1.245	31.139	12.421	.	1.715	0.895	9.127	1.465	1.236	13.080*	.	.	.	.
25-	OBS	1434.0	1340.0	1314.0	1305.0	1101.0	978.0	862.0	1034.0	1146.0	1073.0	880.0*	.	.	.	.
	EXP	1356.9	1501.2	1462.6	1359.5	1175.9	946.5	870.7	890.4	1003.4	1063.7	1167.3*	1158.5*	1135.2*	1160.9*	1152.7*
	ChiSq	4.381	17.315	15.102	2.185	4.775	1.049	0.087	23.168	20.261	0.082	70.729*	.	.	.	.
30-	OBS	4449.0	4192.0	4030.0	3674.0	3417.0	3045.0	2739.0	2946.0	2752.0	2675.0	2670.0*	.	.	.	.
	EXP	4052.1	4151.4	4020.0	3911.2	3684.0	3390.1	2924.9	2616.5	2374.2	2913.6	3098.8*	3384.3*	3362.2*	3304.7*	3382.4*
	ChiSq	38.868	0.397	0.025	14.387	19.358	35.135	11.815	41.492	60.134	19.535	59.324*	.	.	.	.
35-	OBS	14558.0	11852.0	10371.0	9811.0	8885.0	8406.0	8309.0	7873.0	6565.0	5679.0	5635.0*	.	.	.	.
	EXP	13356.3	11057.9	9703.5	9520.9	9573.5	9538.5	9385.3	7889.6	6318.5	6265.1	7678.5*	8131.6*	8875.6*	8835.3*	8698.0*
	ChiSq	108.125	57.032	45.920	8.842	49.517	134.450	123.422	0.035	9.620	54.832	543.854*	.	.	.	.
40-	OBS	37624.0	31238.0	23792.0	20359.0	19068.0	18406.0	18694.0	19912.0	15918.0	13013.0	11655.0*	.	.	.	.
	EXP	36542.2	30104.7	21752.0	19092.5	19292.4	20321.7	21539.0	20634.9	15598.9	13638.4	13536.0*	16539.9*	17512.2*	19133.2*	19068.1*
	ChiSq	32.028	42.662	191.327	84.013	2.611	180.592	375.787	25.327	6.527	28.683	261.380*	.	.	.	.
45-	OBS	73674.0	66731.0	49705.0	36793.0	31227.0	32009.0	34365.0	37363.0	33930.0	27190.0	24237.5*	.	.	.	.
	EXP	74102.5	66435.5	47124.0	34645.6	31298.5	33140.9	37366.7	38638.1	33224.0	27320.2	23920.1*	23705.6*	28984.6*	30731.5*	33600.1*
	ChiSq	2.478	1.315	141.364	133.096	0.163	38.661	241.135	42.083	15.004	0.621	4.212*	.	.	.	.
50-	OBS	119255.0	113898.0	91385.0	66394.0	49184.0	46344.0	51662.0	59029.0	56803.0	52161.0	46562.5*	.	.	.	.
	EXP	120684.0	116124.9	90808.2	64706.3	49384.3	47117.9	53445.8	58880.4	54444.0	50681.7	41605.2*	36426.6*	36160.6*	44321.5*	47054.5*
	ChiSq	16.921	42.706	3.663	44.021	0.812	12.712	59.533	0.375	102.215	43.178	590.671*	.	.	.	.
55-	OBS	174045.0	163952.0	139855.0	110120.0	80915.0	65757.0	67309.0	75974.0	76897.0	76888.0	77305.0*	.	.	.	.
	EXP	176701.5	166154.7	140995.1	110756.2	81367.3	65754.3	67144.7	75022.7	73984.5	73992.2	68599.9*	56335.8*	49461.3*	49288.0*	60564.4*
	ChiSq	39.936	29.200	9.219	3.655	2.514	.	0.402	12.064	114.652	113.332	1104.658*	.	.	.	.
60-	OBS	223132.0	222462.0	189612.0	158829.0	127374.0	101463.0	90090.0	90871.0	91070.0	97211.0	102780.0*	.	.	.	.
	EXP	225575.1	223566.2	190214.8	161315.5	129793.1	101462.5	88237.6	88460.5	88987.4	94670.2	94206.2*	87334.1*	71987.7*	63560.1*	63602.1*
	ChiSq	26.460	5.453	1.910	38.326	45.087	.	38.887	65.687	48.742	68.192	780.311*	.	.	.	.
65-	OBS	260317.0	261459.0	235475.0	200601.0	172601.0	147790.0	125390.0	107300.0	96712.0	106060.0	119440.0*	.	.	.	.
	EXP	258171.6	259727.8	236329.5	201950.7	173257.6	148133.9	124690.8	107773.2	97427.9	106270.1	112370.1*	111750.2*	104085.3*	86466.3*	76835.6*
	ChiSq	17.828	11.539	3.090	9.021	2.488	0.799	3.921	2.078	5.261	0.415	444.807*	.	.	.	.

Results for PHIM for Run 1 (Basic_US_OG_T1.RTF)

70-	OBS	288133.0	277827.0	255120.0	232517.0	204299.0	187996.0	172292.0	141677.0	109155.0	109523.0	124125.0*	.	.	.	.
	EXP	286399.2	274484.6	252798.0	231586.2	202975.4	186848.0	172293.1	144742.1	113894.9	112742.3	123119.2*	130069.1*	130218.5*	122637.9*	102804.7*
	ChiSq	10.496	40.699	21.328	3.741	8.631	7.053	.	64.905	197.260	91.927	8.217*	.	.	.	.
75-	OBS	281452.0	275926.0	244221.0	228182.0	215731.0	203687.0	205614.0	184381.0	137446.0	120897.0	124467.5*	.	.	.	.
	EXP	281452.0	277670.5	249800.7	229827.7	212103.7	199502.9	199954.1	182983.4	141491.2	123061.6	122801.5*	134631.9*	143342.1*	145988.1*	139313.6*
	ChiSq	.	10.960	124.631	11.784	62.033	87.753	160.207	10.675	115.652	38.076	22.601*	.	.	.	.
Total Deaths		1478943.0	1431622.0	1245410.0	1069072.0	914258.0	816305.0	777786.0	728934.0	628925.0	612898.0	640165.0*	.	.	.	.
Expected		1479107.4	1431751.9	1245708.0	1069254.7	914358.1	816564.8	778310.4	729085.9	629284.2	613182.5	612661.8*	610019.8*	595682.1*	575978.7*	556624.6*
Obs/Exp		1.000	1.000	1.000	1.000	1.000	1.000	0.999	1.000	0.999	1.000	1.045*	.	.	.	.

Chi Squared (Log) = 3942.5 on 14 D.F. P = 0.0000

Results for PHIM for Run 1 (Basic_US_OG_T1.RTF)

****

Observed and predicted rates (5 year periods)

		10-	15-	20-	25-	30-	35-	40-	45-	50-	55-	60-	65-	70-	75-
2016-	OBS	0.5	1.2	5.5	14.7	47.2	107.2	232.5	478.0	875.1	1440.3	2115.1	2959.2	4122.5	6215.1
	PRE	0.7	2.5	6.7	19.5	54.7	146.1	270.1	471.8	781.9	1278.2	1938.7	2784.0	4089.1	6131.9
	RES	-0.232	-1.253	-1.230	-4.808	-7.574	-38.871	-37.527	6.261	93.168	162.194	176.443	175.159	33.406	83.188
2021-	PRE	0.7	2.5	6.7	19.5	55.7	142.4	314.2	476.1	731.5	1094.7	1715.2	2482.3	3600.2	5282.4
2026-	PRE	0.8	2.5	6.8	19.6	55.6	144.9	306.3	553.9	738.2	1024.1	1469.0	2196.1	3210.0	4650.9
2031-	PRE	0.8	2.5	6.8	19.8	56.0	144.7	311.6	540.1	858.8	1033.5	1374.2	1880.9	2839.9	4146.8
2036-	PRE	0.8	2.5	6.9	19.9	56.4	145.7	311.2	549.4	837.3	1202.3	1386.9	1759.5	2432.3	3668.7

Results for PHIM for Run 1 (Basic_US_OG_T1.RTF)

****

Observed and predicted deaths (5 year periods)

		10-	15-	20-	25-	30-	35-	40-	45-	50-	55-	60-	65-	70-	75-
2016-	OBS	27.5	65.0	315.0	880.0	2670.0	5635.0	11655.0	24237.5	46562.5	77305.0	102780.0	119440.0	124125.0	124467.5
	PRE	40.0	133.0	386.1	1167.3	3098.8	7678.5	13536.0	23920.1	41605.2	68599.9	94206.2	112370.1	123119.2	122801.5
	CHI	3.893	34.768	13.080	70.729	59.324	543.854	261.380	4.212	590.671	1104.658	780.311	444.807	8.217	22.601
2021-	PRE	39.4	135.5	377.3	1158.5	3384.3	8131.6	16539.9	23705.6	36426.6	56335.8	87334.1	111750.2	130069.1	134631.9
2026-	PRE	38.4	133.7	384.9	1135.2	3362.2	8875.6	17512.2	28984.6	36160.6	49461.3	71987.7	104085.3	130218.5	143342.1
2031-	PRE	39.3	130.6	381.3	1160.9	3304.7	8835.3	19133.2	30731.5	44321.5	49288.0	63560.1	86466.3	122637.9	145988.1
2036-	PRE	40.9	133.9	373.6	1152.7	3382.4	8698.0	19068.1	33600.1	47054.5	60564.4	63602.1	76835.6	102804.7	139313.6

Results for PHIM for Run 1 (Basic_US_OG_T1.RTF)

****

Observed and predicted rates (single years)

		10-	15-	20-	25-	30-	35-	40-	45-	50-	55-	60-	65-	70-	75-
2016	OBS	0.6	1.3	5.3	14.8	49.3	109.5	240.2	481.7	863.3	1457.3	2168.5	3113.2	4466.5	6504.7
	PRE	0.8	2.6	7.0	20.4	57.1	152.3	281.5	491.8	815.1	1332.4	2021.0	2902.3	4262.7	6392.3
	RES	-0.213	-1.269	-1.620	-5.535	-7.796	-42.781	-41.331	-10.079	48.152	124.812	147.498	210.923	203.745	112.373
2017	OBS	0.5	1.1	5.4	15.1	47.5	110.0	226.7	460.0	847.2	1426.8	2184.6	3028.5	4498.8	6563.5
	PRE	0.8	2.5	6.8	19.9	55.9	149.1	275.7	481.7	798.4	1305.0	1979.4	2842.5	4175.0	6260.8
	RES	-0.293	-1.398	-1.434	-4.832	-8.369	-39.162	-49.061	-21.655	48.802	121.749	205.164	185.934	323.811	302.718
2018	PRE	0.7	2.5	6.7	19.5	54.7	146.1	270.1	471.8	781.9	1278.2	1938.7	2784.0	4089.1	6131.9
2019	PRE	0.7	2.4	6.5	19.1	53.6	143.1	264.5	462.1	765.8	1251.8	1898.8	2726.7	4004.9	6005.7
2020	PRE	0.7	2.4	6.4	18.7	52.5	140.1	259.1	452.6	750.1	1226.1	1859.7	2670.6	3922.5	5882.1
2021	PRE	0.8	2.6	7.0	20.3	58.1	148.5	327.5	496.4	762.5	1141.2	1788.0	2587.7	3753.1	5506.8
2022	PRE	0.8	2.5	6.9	19.9	56.9	145.4	320.8	486.1	746.8	1117.7	1751.2	2534.4	3675.9	5393.4
2023	PRE	0.7	2.5	6.7	19.5	55.7	142.4	314.2	476.1	731.5	1094.7	1715.2	2482.3	3600.2	5282.4
2024	PRE	0.7	2.4	6.6	19.1	54.5	139.5	307.7	466.3	716.4	1072.2	1679.9	2431.2	3526.1	5173.7
2025	PRE	0.7	2.4	6.5	18.7	53.4	136.6	301.4	456.7	701.7	1050.1	1645.3	2381.1	3453.6	5067.2
2026	PRE	0.8	2.6	7.1	20.5	58.0	151.0	319.3	577.4	769.6	1067.6	1531.4	2289.3	3346.3	4848.5
2027	PRE	0.8	2.5	6.9	20.1	56.8	147.9	312.8	565.5	753.7	1045.6	1499.9	2242.2	3277.5	4748.7
2028	PRE	0.8	2.5	6.8	19.6	55.6	144.9	306.3	553.9	738.2	1024.1	1469.0	2196.1	3210.0	4650.9
2029	PRE	0.7	2.4	6.6	19.2	54.5	141.9	300.0	542.5	723.0	1003.0	1438.8	2150.9	3143.9	4555.2
2030	PRE	0.7	2.4	6.5	18.8	53.4	139.0	293.8	531.3	708.1	982.3	1409.2	2106.6	3079.2	4461.4
2031	PRE	0.8	2.6	7.1	20.6	58.4	150.8	324.9	563.0	895.3	1077.4	1432.6	1960.8	2960.5	4322.9
2032	PRE	0.8	2.6	7.0	20.2	57.2	147.7	318.2	551.4	876.8	1055.2	1403.1	1920.4	2899.6	4234.0
2033	PRE	0.8	2.5	6.8	19.8	56.0	144.7	311.6	540.1	858.8	1033.5	1374.2	1880.9	2839.9	4146.8
2034	PRE	0.7	2.5	6.7	19.4	54.9	141.7	305.2	529.0	841.1	1012.2	1345.9	1842.2	2781.5	4061.5
2035	PRE	0.7	2.4	6.5	19.0	53.7	138.8	298.9	518.1	823.8	991.4	1318.2	1804.2	2724.2	3977.9
2036	PRE	0.8	2.6	7.2	20.8	58.8	151.9	324.4	572.8	872.9	1253.4	1445.8	1834.2	2535.6	3824.5
2037	PRE	0.8	2.6	7.0	20.3	57.6	148.8	317.8	561.0	854.9	1227.6	1416.0	1796.5	2483.4	3745.8
2038	PRE	0.8	2.5	6.9	19.9	56.4	145.7	311.2	549.4	837.3	1202.3	1386.9	1759.5	2432.3	3668.7
2039	PRE	0.7	2.5	6.7	19.5	55.2	142.7	304.8	538.1	820.1	1177.6	1358.3	1723.3	2382.2	3593.2
2040	PRE	0.7	2.4	6.6	19.1	54.1	139.8	298.5	527.0	803.2	1153.3	1330.4	1687.8	2333.2	3519.2

Results for PHIM for Run 1 (Basic_US_OG_T1.RTF)

****

Observed and predicted deaths (single years)

		10-	15-	20-	25-	30-	35-	40-	45-	50-	55-	60-	65-	70-	75-
2016	OBS	6.0	14.0	63.0	173.0	539.0	1117.0	2400.0	4990.0	9464.0	15584.0	20257.0	23890.0	24033.0	24483.0
	PRE	8.3	27.8	82.1	237.6	624.3	1553.5	2813.0	5094.4	8936.1	14249.3	18879.2	22271.4	22936.7	24060.0
	CHI	0.622	6.865	4.443	17.551	11.652	122.645	60.630	2.140	31.183	125.028	100.555	117.631	52.400	7.435
2017	OBS	5.0	12.0	63.0	179.0	529.0	1137.0	2262.0	4705.0	9161.0	15338.0	20855.0	23886.0	25617.0	25304.0
	PRE	8.1	27.2	79.8	236.2	622.2	1541.9	2751.6	4926.5	8633.3	14029.2	18896.4	22419.5	23773.2	24136.9
	CHI	1.214	8.474	3.522	13.867	13.947	106.335	87.114	9.956	32.259	122.105	203.000	95.926	143.007	56.430
2018	PRE	8.0	26.6	77.2	234.4	619.7	1533.3	2698.8	4772.9	8320.7	13766.2	18886.7	22478.6	24666.2	24305.4
2019	PRE	7.9	26.0	74.8	231.6	617.2	1526.7	2655.4	4634.6	8019.2	13462.7	18822.7	22528.6	25458.0	24724.0
2020	PRE	7.7	25.5	72.6	227.6	614.5	1521.4	2621.2	4511.7	7740.4	13121.6	18689.9	22607.3	26066.8	25451.6
2021	PRE	8.4	28.0	78.9	246.2	689.5	1639.5	3347.4	4926.1	7754.0	12088.6	18117.3	22373.7	25756.7	25043.0
2022	PRE	8.1	27.6	77.0	239.2	685.1	1633.0	3321.3	4817.4	7498.2	11680.8	17844.0	22394.0	25913.5	25968.8
2023	PRE	7.9	27.2	75.3	231.6	679.4	1626.4	3302.8	4725.9	7267.3	11265.1	17524.2	22396.3	25998.6	26965.2
2024	PRE	7.6	26.7	73.8	224.4	671.1	1619.3	3288.5	4651.2	7060.3	10865.1	17152.5	22340.3	26092.2	27855.1
2025	PRE	7.4	26.0	72.4	218.1	659.4	1611.4	3276.4	4592.2	6876.6	10495.2	16730.4	22205.8	26228.5	28556.4
2026	PRE	8.0	28.4	79.5	237.1	713.7	1807.8	3530.1	5863.6	7508.3	10517.0	15422.2	21540.4	25981.9	28239.2
2027	PRE	7.9	27.6	78.3	231.5	693.7	1796.3	3516.4	5818.5	7344.5	10174.8	14913.4	21238.5	26041.4	28457.5
2028	PRE	7.7	26.8	77.1	226.6	672.1	1781.7	3502.4	5787.3	7207.9	9866.9	14394.7	20884.1	26088.1	28625.6
2029	PRE	7.5	25.9	75.8	222.2	651.7	1760.1	3487.6	5764.1	7097.7	9592.1	13895.6	20467.8	26074.5	28826.3
2030	PRE	7.3	25.1	74.2	218.3	633.7	1729.8	3471.2	5744.7	7012.2	9349.8	13434.4	19990.1	25975.9	29088.6
2031	PRE	8.1	27.3	80.9	239.7	689.8	1873.7	3895.7	6191.8	8958.7	10219.5	13484.3	18461.1	25252.3	28913.1
2032	PRE	8.0	26.7	78.6	236.0	673.7	1822.1	3871.9	6169.4	8893.6	10004.6	13063.1	17883.8	24957.0	29077.4
2033	PRE	7.9	26.1	76.2	232.6	659.7	1766.5	3841.1	6146.4	8849.4	9825.2	12681.8	17291.7	24603.2	29234.0
2034	PRE	7.8	25.5	74.0	228.8	647.2	1713.4	3795.0	6121.8	8817.6	9681.0	12340.2	16720.2	24177.7	29332.9
2035	PRE	7.6	25.0	71.9	224.2	635.7	1666.5	3729.9	6093.9	8791.5	9570.1	12038.3	16190.6	23676.5	29344.4
2036	PRE	8.4	27.5	78.2	244.3	698.3	1815.0	4041.7	6840.0	9477.3	12233.5	13170.4	16267.9	21885.3	28559.7
2037	PRE	8.3	27.2	76.3	237.5	687.9	1773.3	3931.7	6798.8	9444.3	12149.4	12903.4	15776.1	21226.0	28271.7
2038	PRE	8.2	26.8	74.6	230.4	677.7	1736.6	3812.7	6745.1	9410.4	12092.7	12679.7	15330.9	20553.4	27933.1
2039	PRE	8.0	26.4	73.0	223.7	666.4	1703.5	3698.8	6664.9	9374.7	12052.6	12500.0	14932.7	19909.9	27525.9
2040	PRE	7.9	26.0	71.7	217.7	653.0	1673.2	3598.1	6551.7	9334.8	12020.9	12362.9	14582.4	19319.8	27041.8

Results for PHIM for Run 1 (Basic_US_OG_T1.RTF)

****
List of values created by O and G modelling, using percentage change in last two period parameters for Fixed File MORT

1. Country	US (United States)
2. Sex	M (Males)
3. Disease	IHD (IHD)
* Value comes from O and G Modelling.	

Age	Years	Value	Death Rate	Population
10-14	2018	8.0173	0.743581	107820.22 *
10-14	2019	7.8696	0.728276	108058.17 *
10-14	2020	7.6875	0.713286	107775.13 *
10-14	2021	8.3869	0.780598	107441.78 *
10-14	2022	8.1487	0.764532	106584.46 *
10-14	2023	7.8878	0.748796	105339.41 *
10-14	2024	7.6275	0.733384	104004.73 *
10-14	2025	7.3882	0.718289	102858.77 *
10-14	2026	8.0452	0.786073	102347.07 *
10-14	2027	7.8504	0.769894	101967.29 *
10-14	2028	7.6676	0.754047	101686.15 *
10-14	2029	7.4959	0.738527	101498.55 *
10-14	2030	7.3396	0.723326	101470.63 *
10-14	2031	8.0833	0.791586	102114.83 *
10-14	2032	7.9760	0.775293	102877.43 *
10-14	2033	7.8729	0.759335	103681.01 *
10-14	2034	7.7644	0.743706	104401.53 *
10-14	2035	7.6450	0.728399	104955.58 *
10-14	2036	8.4399	0.797137	105877.40 *
10-14	2037	8.3203	0.780730	106570.86 *
10-14	2038	8.1849	0.764661	107039.02 *
10-14	2039	8.0370	0.748922	107313.72 *
15-19	2018	26.5795	2.451142	108437.02 *
15-19	2019	26.0168	2.400691	108371.94 *
15-19	2020	25.4745	2.351279	108343.21 *
15-19	2021	28.0076	2.573167	108844.87 *
15-19	2022	27.6000	2.520205	109515.01 *
15-19	2023	27.1804	2.468332	110116.30 *
15-19	2024	26.6744	2.417528	110337.57 *
15-19	2025	26.0480	2.367769	110010.63 *
15-19	2026	28.4154	2.591213	109660.66 *
15-19	2027	27.6201	2.537879	108831.44 *
15-19	2028	26.7575	2.485643	107648.28 *
15-19	2029	25.9015	2.434482	106394.27 *
15-19	2030	25.1019	2.384374	105276.67 *
15-19	2031	27.3469	2.609386	104802.12 *
15-19	2032	26.6994	2.555678	104470.80 *
15-19	2033	26.0941	2.503076	104248.18 *
15-19	2034	25.5255	2.451556	104119.48 *
15-19	2035	24.9915	2.401096	104083.90 *
15-19	2036	27.5180	2.627686	104723.27 *
15-19	2037	27.1611	2.573601	105537.13 *
15-19	2038	26.8151	2.520630	106382.52 *
15-19	2039	26.4416	2.468749	107105.08 *
20-24	2018	77.2058	6.681629	115549.38 *
20-24	2019	74.7569	6.544104	114235.47 *
20-24	2020	72.5720	6.409409	113227.32 *
20-24	2021	78.8967	7.014260	112480.46 *
20-24	2022	76.9841	6.869888	112060.21 *
20-24	2023	75.3228	6.728488	111946.10 *
20-24	2024	73.8326	6.589999	112037.41 *
20-24	2025	72.4312	6.454359	112220.61 *
20-24	2026	79.5030	7.063452	112555.48 *
20-24	2027	78.2861	6.918068	113161.84 *
20-24	2028	77.1329	6.775676	113837.89 *
20-24	2029	75.8252	6.636215	114259.71 *
20-24	2030	74.2231	6.499625	114195.90 *
20-24	2031	80.8880	7.112989	113718.71 *
20-24	2032	78.6090	6.966586	112837.21 *
20-24	2033	76.2397	6.823195	111736.00 *
20-24	2034	73.9717	6.682756	110690.36 *
20-24	2035	71.8883	6.545208	109833.53 *
20-24	2036	78.1915	7.162874	109162.16 *
20-24	2037	76.2673	7.015443	108713.50 *
20-24	2038	74.5601	6.871047	108513.37 *
20-24	2039	73.0471	6.729624	108545.60 *
25-29	2018	234.3934	19.534187	119991.39 *
25-29	2019	231.5810	19.132123	121043.01 *

Results for PHIM for Run 1 (Basic_US_OG_T1.RTF)

25-29	2020	227.5669	18.738335	121444.55 *
25-29	2021	246.2361	20.337516	121074.83 *
25-29	2022	239.1714	19.918918	120072.51 *
25-29	2023	231.6076	19.508935	118718.74 *
25-29	2024	224.4077	19.107391	117445.48 *
25-29	2025	218.0742	18.714112	116529.28 *
25-29	2026	237.1257	20.480147	115783.18 *
25-29	2027	231.4669	20.058612	115395.25 *
25-29	2028	226.5754	19.645755	115330.47 *
25-29	2029	222.2335	19.241394	115497.61 *
25-29	2030	218.2722	18.845357	115822.80 *
25-29	2031	239.6529	20.623777	116202.23 *
25-29	2032	236.0270	20.199287	116849.18 *
25-29	2033	232.5933	19.783534	117569.12 *
25-29	2034	228.7501	19.376337	118056.43 *
25-29	2035	224.1602	18.977522	118118.77 *
25-29	2036	244.3270	20.768415	117643.53 *
25-29	2037	237.5120	20.340948	116765.46 *
25-29	2038	230.4421	19.922279	115670.55 *
25-29	2039	223.7100	19.512227	114651.21 *
30-34	2018	619.7454	54.741756	113212.55 *
30-34	2019	617.2199	53.615030	115120.68 *
30-34	2020	614.5165	52.511495	117025.14 *
30-34	2021	689.4532	58.055459	118757.69 *
30-34	2022	685.0601	56.860529	120480.79 *
30-34	2023	679.4372	55.690192	122003.03 *
30-34	2024	671.0928	54.543945	123037.09 *
30-34	2025	659.3852	53.421290	123431.16 *
30-34	2026	713.7375	57.980411	123099.77 *
30-34	2027	693.6572	56.787025	122150.65 *
30-34	2028	672.1435	55.618202	120849.55 *
30-34	2029	651.6614	54.473436	119629.21 *
30-34	2030	633.7084	53.352232	118778.23 *
30-34	2031	689.7541	58.387038	118134.80 *
30-34	2032	673.7055	57.185282	117811.00 *
30-34	2033	659.6801	56.008261	117782.65 *
30-34	2034	647.1575	54.855467	117975.02 *
30-34	2035	635.7382	53.726400	118328.82 *
30-34	2036	698.3409	58.796516	118772.50 *
30-34	2037	687.8516	57.586332	119447.02 *
30-34	2038	677.7272	56.401057	120162.15 *
30-34	2039	666.3651	55.240178	120630.51 *
35-39	2018	1533.3049	146.056368	104980.35 *
35-39	2019	1526.7422	143.050152	106727.76 *
35-39	2020	1521.3909	140.105811	108588.71 *
35-39	2021	1639.4585	148.455966	110433.99 *
35-39	2022	1633.0335	145.400359	112312.89 *
35-39	2023	1626.3654	142.407645	114204.92 *
35-39	2024	1619.2751	139.476529	116096.60 *
35-39	2025	1611.4445	136.605742	117963.16 *
35-39	2026	1807.8001	151.028060	119699.62 *
35-39	2027	1796.3424	147.919513	121440.53 *
35-39	2028	1781.7427	144.874948	122984.87 *
35-39	2029	1760.1197	141.893049	124045.52 *
35-39	2030	1729.7902	138.972524	124469.94 *
35-39	2031	1873.7457	150.832826	124226.65 *
35-39	2032	1822.1173	147.728298	123342.47 *
35-39	2033	1766.5139	144.687669	122091.53 *
35-39	2034	1713.3929	141.709624	120908.72 *
35-39	2035	1666.5429	138.792874	120074.10 *
35-39	2036	1815.0089	151.890642	119494.45 *
35-39	2037	1773.3498	148.764341	119205.30 *
35-39	2038	1736.5931	145.702388	119187.69 *
35-39	2039	1703.5165	142.703457	119374.58 *
40-44	2018	2698.8288	270.054903	99936.30 *
40-44	2019	2655.4285	264.496477	100395.61 *
40-44	2020	2621.2435	259.052458	101185.82 *
40-44	2021	3347.3880	327.505028	102208.75 *
40-44	2022	3321.2932	320.764130	103543.16 *
40-44	2023	3302.7509	314.161978	105128.92 *
40-44	2024	3288.5404	307.695715	106876.38 *
40-44	2025	3276.3931	301.362544	108719.32 *
40-44	2026	3530.0920	319.323425	110549.11 *
40-44	2027	3516.3888	312.750926	112434.16 *
40-44	2028	3502.4160	306.313706	114340.82 *
40-44	2029	3487.6068	300.008980	116250.08 *
40-44	2030	3471.1671	293.834022	118133.60 *
40-44	2031	3895.6639	324.855907	119919.75 *

Results for PHIM for Run 1 (Basic_US_OG_T1.RTF)

40-44	2032	3871.8935	318.169535	121692.78 *
40-44	2033	3841.0549	311.620786	123260.55 *
40-44	2034	3794.9777	305.206827	124341.18 *
40-44	2035	3729.8592	298.924884	124775.80 *
40-44	2036	4041.7486	324.435966	124577.70 *
40-44	2037	3931.6732	317.758237	123731.59 *
40-44	2038	3812.7467	311.217954	122510.50 *
40-44	2039	3698.8495	304.812286	121348.44 *
45-49	2018	4772.8738	471.787661	101165.72 *
45-49	2019	4634.5797	462.077055	100298.85 *
45-49	2020	4511.6784	452.566319	99690.99 *
45-49	2021	4926.0513	496.362946	99242.93 *
45-49	2022	4817.3819	486.146518	99093.21 *
45-49	2023	4725.9003	476.140370	99254.35 *
45-49	2024	4651.1500	466.340174	99737.28 *
45-49	2025	4592.2120	456.741691	100542.87 *
45-49	2026	5863.6097	577.432082	101546.31 *
45-49	2027	5818.5114	565.547042	102882.89 *
45-49	2028	5787.2956	553.906626	104481.43 *
45-49	2029	5764.0639	542.505800	106248.89 *
45-49	2030	5744.6842	531.339633	108116.99 *
45-49	2031	6191.7770	563.006898	109976.93 *
45-49	2032	6169.3928	551.418765	111882.17 *
45-49	2033	6146.3563	540.069146	113806.84 *
45-49	2034	6121.8042	528.953131	115734.34 *
45-49	2035	6093.9378	518.065913	117628.62 *
45-49	2036	6839.9739	572.761352	119421.01 *
45-49	2037	6798.7718	560.972447	121196.18 *
45-49	2038	6745.1009	549.426189	122766.28 *
45-49	2039	6664.8951	538.117582	123855.74 *
50-54	2018	8320.7249	781.927331	106413.02 *
50-54	2019	8019.1817	765.833252	104711.85 *
50-54	2020	7740.3781	750.070430	103195.35 *
50-54	2021	7753.9665	762.531375	101687.18 *
50-54	2022	7498.1639	746.836514	100399.00 *
50-54	2023	7267.2992	731.464694	99352.70 *
50-54	2024	7060.3129	716.409266	98551.39 *
50-54	2025	6876.6188	701.663717	98004.48 *
50-54	2026	7508.3462	769.566482	97565.92 *
50-54	2027	7344.5267	753.726820	97442.82 *
50-54	2028	7207.9157	738.213180	97640.03 *
50-54	2029	7097.6599	723.018850	98167.01 *
50-54	2030	7012.1982	708.137259	99023.15 *
50-54	2031	8958.7100	895.256947	100068.59 *
50-54	2032	8893.6121	876.830252	101429.12 *
50-54	2033	8849.4350	858.782825	103046.25 *
50-54	2034	8817.6395	841.106862	104833.76 *
50-54	2035	8791.4878	823.794715	106719.40 *
50-54	2036	9477.3374	872.891986	108574.00 *
50-54	2037	9444.2684	854.925619	110468.89 *
50-54	2038	9410.3794	837.329047	112385.68 *
50-54	2039	9374.7300	820.094657	114312.78 *
55-59	2018	13766.1925	1278.154098	107703.70 *
55-59	2019	13462.7342	1251.846392	107543.02 *
55-59	2020	13121.5896	1226.080167	107020.65 *
55-59	2021	12088.5894	1141.199934	105928.76 *
55-59	2022	11680.7684	1117.711098	104506.15 *
55-59	2023	11265.0736	1094.705723	102905.04 *
55-59	2024	10865.0764	1072.173858	101336.89 *
55-59	2025	10495.1602	1050.105757	99943.84 *
55-59	2026	10517.0073	1067.551199	98515.25 *
55-59	2027	10174.7707	1045.578244	97312.38 *
55-59	2028	9866.8692	1024.057549	96350.73 *
55-59	2029	9592.0776	1002.979806	95635.80 *
55-59	2030	9349.8014	982.335897	95179.27 *
55-59	2031	10219.5287	1077.400416	94853.58 *
55-59	2032	10004.5900	1055.224739	94810.04 *
55-59	2033	9825.1543	1033.505494	95066.30 *
55-59	2034	9681.0245	1012.233288	95640.25 *
55-59	2035	9570.1066	991.398918	96531.34 *
55-59	2036	12233.4797	1253.368266	97604.83 *
55-59	2037	12149.4184	1227.570717	98971.23 *
55-59	2038	12092.6861	1202.304148	100579.26 *
55-59	2039	12052.5626	1177.557630	102352.21 *
60-64	2018	18886.7261	1938.701774	97419.45 *
60-64	2019	18822.6717	1898.798295	99129.39 *
60-64	2020	18689.9351	1859.716132	100498.86 *
60-64	2021	18117.2825	1788.021592	101325.86 *

Results for PHIM for Run 1 (Basic_US_OG_T1.RTF)

60-64	2022	17843.9950	1751.219499	101894.68 *
60-64	2023	17524.2094	1715.174889	102171.56 *
60-64	2024	17152.5397	1679.872169	102106.22 *
60-64	2025	16730.4267	1645.296071	101686.42 *
60-64	2026	15422.1939	1531.393963	100706.90 *
60-64	2027	14913.4355	1499.873929	99431.26 *
60-64	2028	14394.7350	1469.002658	97989.85 *
60-64	2029	13895.5507	1438.766797	96579.59 *
60-64	2030	13434.4417	1409.153269	95336.98 *
60-64	2031	13484.3242	1432.563580	94127.23 *
60-64	2032	13063.0788	1403.077729	93103.03 *
60-64	2033	12681.8016	1374.198774	92285.06 *
60-64	2034	12340.2487	1345.914221	91686.74 *
60-64	2035	12038.2809	1318.211838	91322.81 *
60-64	2036	13170.3886	1445.780397	91095.36 *
60-64	2037	12903.4088	1416.022510	91124.32 *
60-64	2038	12679.7362	1386.877118	91426.53 *
60-64	2039	12500.0426	1358.331612	92024.97 *
65-69	2018	22478.5899	2784.012812	80741.69 *
65-69	2019	22528.5797	2726.710654	82621.82 *
65-69	2020	22607.2585	2670.587921	84652.74 *
65-69	2021	22373.6740	2587.688999	86461.99 *
65-69	2022	22393.9598	2534.427690	88359.04 *
65-69	2023	22396.3114	2482.262637	90225.39 *
65-69	2024	22340.2687	2431.171275	91890.97 *
65-69	2025	22205.8038	2381.131504	93257.36 *
65-69	2026	21540.4352	2289.335704	94090.33 *
65-69	2027	21238.4828	2242.215276	94720.98 *
65-69	2028	20884.0571	2196.064707	95097.64 *
65-69	2029	20467.8071	2150.864035	95160.86 *
65-69	2030	19990.0702	2106.593710	94892.86 *
65-69	2031	18461.0710	1960.756455	94152.80 *
65-69	2032	17883.8447	1920.399034	93125.67 *
65-69	2033	17291.7033	1880.872273	91934.49 *
65-69	2034	16720.2187	1842.159074	90764.25 *
65-69	2035	16190.6460	1804.242693	89736.52 *
65-69	2036	16267.8508	1834.216638	88691.00 *
65-69	2037	15776.1367	1796.463732	87817.73 *
65-69	2038	15330.8595	1759.487879	87132.51 *
65-69	2039	14932.6783	1723.273084	86653.00 *
70-74	2018	24666.1934	4089.059674	60322.41 *
70-74	2019	25457.9723	4004.896287	63567.12 *
70-74	2020	26066.8170	3922.465200	66455.19 *
70-74	2021	25756.7489	3753.138347	68627.23 *
70-74	2022	25913.5330	3675.889087	70495.96 *
70-74	2023	25998.5916	3600.229817	72213.70 *
70-74	2024	26092.1610	3526.127808	73996.64 *
70-74	2025	26228.5146	3453.551011	75946.51 *
70-74	2026	25981.9477	3346.347778	77642.70 *
70-74	2027	26041.3679	3277.471317	79455.67 *
70-74	2028	26088.1151	3210.012511	81271.07 *
70-74	2029	26074.5108	3143.942181	82935.72 *
70-74	2030	25975.8756	3079.231748	84358.30 *
70-74	2031	25252.3485	2960.523251	85296.91 *
70-74	2032	24956.9892	2899.588053	86070.81 *
70-74	2033	24603.2252	2839.907058	86633.91 *
70-74	2034	24177.6871	2781.454452	86924.62 *
70-74	2035	23676.4848	2724.204951	86911.54 *
70-74	2036	21885.2501	2535.611123	86311.54 *
70-74	2037	21225.9693	2483.421711	85470.66 *
70-74	2038	20553.3693	2432.306492	84501.56 *
70-74	2039	19909.8680	2382.243356	83576.13 *
75-79	2018	24305.3733	6131.892125	39637.64 *
75-79	2019	24724.0294	6005.681982	41167.73 *
75-79	2020	25451.6327	5882.069569	43269.86 *
75-79	2021	25042.9800	5506.764324	45476.76 *
75-79	2022	25968.7716	5393.420923	48148.98 *
75-79	2023	26965.2261	5282.410420	51047.20 *
75-79	2024	27855.0517	5173.684800	53839.87 *
75-79	2025	28556.4220	5067.197033	56355.46 *
75-79	2026	28239.2037	4848.453849	58243.73 *
75-79	2027	28457.5048	4748.660173	59927.44 *
75-79	2028	28625.5832	4650.920509	61548.21 *
75-79	2029	28826.2653	4555.192579	63282.21 *
75-79	2030	29088.5739	4461.434976	65200.04 *
75-79	2031	28913.0660	4322.945563	66882.79 *
75-79	2032	29077.3779	4233.968203	68676.42 *
75-79	2033	29234.0351	4146.822226	70497.44 *

Results for PHIM for Run 1 (Basic_US_OG_T1.RTF)

75-79	2034	29332.9309	4061.469937	72222.45 *
75-79	2035	29344.4256	3977.874419	73769.11 *
75-79	2036	28559.6566	3824.522047	74675.10 *
75-79	2037	28271.6769	3745.803527	75475.60 *
75-79	2038	27933.0521	3668.705237	76138.72 *
75-79	2039	27525.9272	3593.193828	76605.74 *

Results for PHIM for Run 1 (Basic_US_OG_T1.RTF)

****
List of values created by last value brought forwards for Fixed File perm.Fixed_File_RR_IHD

1.	Country	US (United States)
2.	Sex	M (Males)
3.	Disease	IHD (IHD)

**Age**	**Years**	**Value**
10-14	2013-2039	3.3800
15-19	2013-2039	3.3800
20-24	2013-2039	3.3800
25-29	2013-2039	3.3800
30-34	2013-2039	3.3800
35-39	2013-2039	3.3800
40-44	2013-2039	3.3800
45-49	2013-2039	3.3800
50-54	2013-2039	3.3800
55-59	2013-2039	2.3200
60-64	2013-2039	2.3200
65-69	2013-2039	1.7000
70-74	2013-2039	1.7000
75-79	2013-2039	1.2700

Results for PHIM for Run 1 (Basic_US_OG_T1.RTF)

****

Osmond and Gardner Modeling of Death Rates for COD: STROKE

Variable Parameter	Value
1. Country	US (United States)
2. Sex	M (Males)
3. Year of start of OG modelling	1966
4. Number 5-year periods for OG modelling	10
5. Age Range of interest	10 - 79
6. Scaling Factor for Rates	10000
7. Model Type to Save	4 (Full Age-Period-Cohort)
8. Goodness of Fit Required	1 (Analysis Printed For Age-Period-Cohort Model)
9. Cause of Death	STROKE
Note:	Death rates are per million population

Matrix of Numbers of Deaths

Age	1966-	1971-	1976-	1981-	1986-	1991-	1996-	2001-	2006-	2011-
10-14	446	378	251	135	114	131	118	124	129	131
15-19	614	698	446	296	201	172	192	196	212	165
20-24	723	822	768	544	448	340	320	336	342	323
25-29	1045	1052	1011	937	844	627	530	527	490	529
30-34	1601	1619	1482	1464	1569	1336	1090	967	911	982
35-39	3065	2624	2220	2305	2619	2681	2377	1979	1886	1621
40-44	5932	4898	3718	3285	3760	4377	4468	4137	3580	3176
45-49	9674	8625	6050	5228	5112	6176	6884	6884	6678	5642
50-54	15771	14283	10595	8277	7151	7652	8790	9804	10348	9622
55-59	24297	22013	16523	13449	11243	10306	11188	11948	13537	14339
60-64	36199	34445	25696	20968	18437	16018	15633	15422	16302	18893
65-69	53157	50572	39581	31656	28226	25793	23554	20964	20193	22477
70-74	73106	68323	54852	46184	40370	38910	38270	32455	26371	28008
75-79	86954	81867	65282	56303	52431	51258	54761	48878	37515	35728

Matrix of Age- and Period-Specific Mortality Rates

Age	1966-	1971-	1976-	1981-	1986-	1991-	1996-	2001-	2006-	2011-
10-14	8.509	7.084	5.011	2.934	2.569	2.811	2.306	2.262	2.388	2.467
15-19	13.042	13.291	8.147	5.707	4.254	3.743	3.904	3.643	3.754	2.953
20-24	18.513	17.599	14.542	9.799	8.586	7.136	6.729	6.621	6.281	5.568
25-29	31.766	26.251	21.223	17.488	15.185	11.945	10.781	10.855	9.617	9.550
30-34	54.134	48.348	36.651	30.368	29.037	23.654	20.116	19.147	18.519	18.923
35-39	103.006	89.168	68.687	58.036	54.419	49.608	41.524	36.138	37.133	32.484
40-44	189.477	165.606	128.608	102.984	94.714	91.708	82.688	72.540	65.649	62.313
45-49	318.517	281.665	214.486	185.543	162.452	158.895	145.216	128.471	118.359	104.152
50-54	576.674	490.503	365.347	307.096	260.173	249.209	228.284	208.777	196.138	173.327
55-59	996.746	862.900	609.078	493.399	437.714	392.329	375.866	317.525	296.477	279.975
60-64	1752.875	1580.008	1099.131	840.253	729.995	667.815	624.863	541.445	452.418	433.932
65-69	3282.300	2870.885	2047.170	1513.531	1270.874	1140.080	1074.253	901.241	760.754	668.793
70-74	6277.221	5409.734	3851.335	2921.720	2340.640	2090.487	1974.803	1691.870	1280.520	1179.182
75-79	10755.70	9874.518	6830.380	5206.501	4409.487	3886.967	3729.156	3147.664	2385.567	2074.239

Matrix of Log-Rates

Age	1966-	1971-	1976-	1981-	1986-	1991-	1996-	2001-	2006-	2011-
10-14	0.930	0.850	0.700	0.467	0.410	0.449	0.363	0.354	0.378	0.392
15-19	1.115	1.124	0.911	0.756	0.629	0.573	0.592	0.561	0.575	0.470
20-24	1.267	1.245	1.163	0.991	0.934	0.853	0.828	0.821	0.798	0.746
25-29	1.502	1.419	1.327	1.243	1.181	1.077	1.033	1.036	0.983	0.980
30-34	1.733	1.684	1.564	1.482	1.463	1.374	1.304	1.282	1.268	1.277
35-39	2.013	1.950	1.837	1.764	1.736	1.696	1.618	1.558	1.570	1.512
40-44	2.278	2.219	2.109	2.013	1.976	1.962	1.917	1.861	1.817	1.795
45-49	2.503	2.450	2.331	2.268	2.211	2.201	2.162	2.109	2.073	2.018
50-54	2.761	2.691	2.563	2.487	2.415	2.397	2.358	2.320	2.293	2.239
55-59	2.999	2.936	2.785	2.693	2.641	2.594	2.575	2.502	2.472	2.447
60-64	3.244	3.199	3.041	2.924	2.863	2.825	2.796	2.734	2.656	2.637
65-69	3.516	3.458	3.311	3.180	3.104	3.057	3.031	2.955	2.881	2.825
70-74	3.798	3.733	3.586	3.466	3.369	3.320	3.296	3.228	3.107	3.072
75-79	4.032	3.995	3.834	3.717	3.644	3.590	3.572	3.498	3.378	3.317

Results for PHIM for Run 1 (Basic_US_OG_T1.RTF)

Fitting the Age, Period, Cohort Models

**Model**	**RSS**	**MRSS**	**DF**	**Factor**	**%Account**	**ChiSq**	**P**
Age Only	97079.024	764.402	127	P, C	99.6835	534935.578	0.0000
Age-Period	1650.763	14.109	117	Cohort	81.3880	8768.728	0.0000
Age-Cohort	1847.699	17.766	104	Period	83.3718	9808.595	0.0000
Period-Cohort	591.859	5.480	108	Age	48.0891	3150.733	0.0000
Full Age-Period-Cohort	307.240	3.200	96			1630.681	0.0000

**Key to terms:**		
	RSS =	residual sum of squares
	MRSS =	mean RSS (MRSS/DF)
	DF =	degrees of freedom
	Factor =	Factors not included in the model
	% Account =	1- (RSS for full model)/(RSS for model in question)
	Chisq =	chi-squared value for model
	P =	probability value based on Chisq and DF.

**Age**	**Value**	**Log10 Value**
10-	4.919072	0.691883
15-	8.010732	0.903672
20-	12.806062	1.107416
25-	20.679707	1.315544
30-	37.474240	1.573733
35-	71.081192	1.851755
40-	130.078780	2.114206
45-	221.449482	2.345275
50-	362.150452	2.558889
55-	577.394538	2.761473
60-	951.277717	2.978307
65-	1601.85730	3.204624
70-	2805.51996	3.448013
75-	4817.35652	3.682809

**Period**	**Value**	**Log10 Value**
1966	1.642625	0.215538
1971	1.537616	0.186848
1976	1.172443	0.069092
1981	0.962431	-0.016631
1986	0.862775	-0.064102
1991	0.820189	-0.086086
1996	0.803171	-0.095192
2001	0.705982	-0.151206
2006	0.590620	-0.228691
2011	0.537468	-0.269647

**Cohort**	**Value**	**Log10 Value**
1891	1.359225	0.133291
1896	1.346706	0.129273
1901	1.236369	0.092148
1906	1.146682	0.059443
1911	1.073783	0.030917
1916	0.977397	-0.009929
1921	0.922644	-0.034966
1926	0.887994	-0.051590
1931	0.848935	-0.071125
1936	0.805519	-0.093924
1941	0.806758	-0.093256
1946	0.800118	-0.096846
1951	0.848859	-0.071165
1956	0.881513	-0.054771
1961	0.852256	-0.069430
1966	0.814074	-0.089336
1971	0.820400	-0.085974
1976	0.788416	-0.103245
1981	0.818698	-0.086876
1986	0.780550	-0.107599
1991	0.771834	-0.112476
1996	0.742452	-0.129331
2001	0.933071	-0.030085

Results for PHIM for Run 1 (Basic_US_OG_T1.RTF)

Model: Full Age-Period-Cohort

Basic Analysis Using OG Modelling T1 on US
Fitting the Full Age-Period-Cohort Model
Matrix of observed, expected, and residual rates

**Age**		**1966-**	**1971-**	**1976-**	**1981-**	**1986-**	**1991-**	**1996-**	**2001-**	**2006-**	**2011-**
10-	Observed	8.509	7.084	5.011	2.934	2.569	2.811	2.306	2.262	2.388	2.467
	Expected	7.123	6.446	4.695	3.884	3.346	3.303	3.084	2.680	2.157	2.467
	Residual	-1.387	-0.638	-0.316	0.950	0.777	0.492	0.778	0.419	-0.230	-0.000
15-	Observed	13.042	13.291	8.147	5.707	4.254	3.743	3.904	3.643	3.754	2.953
	Expected	11.170	10.858	8.004	6.276	5.670	5.180	5.267	4.414	3.652	3.197
	Residual	-1.872	-2.433	-0.143	0.570	1.416	1.437	1.363	0.772	-0.103	0.244
20-	Observed	18.513	17.599	14.542	9.799	8.586	7.136	6.729	6.621	6.281	5.568
	Expected	16.831	16.715	13.235	10.504	8.994	8.617	8.109	7.402	5.904	5.312
	Residual	-1.682	-0.884	-1.307	0.705	0.408	1.481	1.380	0.781	-0.377	-0.256
25-	Observed	31.766	26.251	21.223	17.488	15.185	11.945	10.781	10.855	9.617	9.550
	Expected	27.405	25.442	20.581	17.545	15.206	13.808	13.626	11.510	9.999	8.676
	Residual	-4.361	-0.809	-0.642	0.056	0.021	1.862	2.845	0.655	0.383	-0.874
30-	Observed	54.134	48.348	36.651	30.368	29.037	23.654	20.116	19.147	18.519	18.923
	Expected	49.585	46.486	35.154	30.615	28.501	26.195	24.502	21.705	17.450	16.490
	Residual	-4.549	-1.862	-1.497	0.247	-0.536	2.541	4.387	2.558	-1.069	-2.434
35-	Observed	103.006	89.168	68.687	58.036	54.419	49.608	41.524	36.138	37.133	32.484
	Expected	99.122	88.040	67.234	54.737	52.058	51.392	48.656	40.852	34.442	30.121
	Residual	-3.885	-1.128	-1.453	-3.299	-2.361	1.784	7.132	4.714	-2.691	-2.364
40-	Observed	189.477	165.606	128.608	102.984	94.714	91.708	82.688	72.540	65.649	62.313
	Expected	189.738	169.797	122.850	101.000	89.796	90.564	92.096	78.265	62.543	57.357
	Residual	0.262	4.190	-5.759	-1.984	-4.918	-1.144	9.409	5.725	-3.106	-4.956
45-	Observed	318.517	281.665	214.486	185.543	162.452	158.895	145.216	128.471	118.359	104.152
	Expected	335.620	302.366	220.415	171.680	154.140	145.326	150.980	137.815	111.469	96.893
	Residual	17.103	20.701	5.929	-13.863	-8.312	-13.569	5.763	9.344	-6.890	-7.259
50-	Observed	576.674	490.503	365.347	307.096	260.173	249.209	228.284	208.777	196.138	173.327
	Expected	581.431	513.773	377.043	295.892	251.688	239.633	232.729	217.029	188.550	165.887
	Residual	4.757	23.270	11.696	-11.204	-8.485	-9.577	4.445	8.252	-7.588	-7.440
55-	Observed	996.746	862.900	609.078	493.399	437.714	392.329	375.866	317.525	296.477	279.975
	Expected	1018.422	867.744	624.595	493.460	422.907	381.472	374.131	326.152	289.479	273.561
	Residual	21.676	4.844	15.516	0.061	-14.807	-10.857	-1.735	8.627	-6.998	-6.414
60-	Observed	1752.875	1580.008	1099.131	840.253	729.995	667.815	624.863	541.445	452.418	433.932
	Expected	1791.798	1570.623	1090.109	844.716	728.811	662.363	615.447	541.807	449.541	434.006
	Residual	38.923	-9.385	-9.022	4.463	-1.183	-5.453	-9.415	0.362	-2.877	0.073
65-	Observed	3282.300	2870.885	2047.170	1513.531	1270.874	1140.080	1074.253	901.241	760.754	668.793
	Expected	3253.196	2824.327	2016.657	1506.830	1275.133	1166.669	1092.211	910.947	763.266	688.859
	Residual	-29.103	-46.558	-30.513	-6.701	4.259	26.589	17.958	9.706	2.512	20.066
70-	Observed	6277.221	5409.734	3851.335	2921.720	2340.640	2090.487	1974.803	1691.870	1280.520	1179.182
	Expected	6206.186	5333.462	3771.796	2899.342	2365.821	2123.055	2000.928	1681.441	1334.743	1216.493
	Residual	-71.035	-76.272	-79.540	-22.379	25.181	32.568	26.125	-10.429	54.223	37.311
75-	Observed	10755.699	9874.518	6830.380	5206.501	4409.487	3886.967	3729.156	3147.664	2385.567	2074.239
	Expected	10755.699	9975.383	6983.101	5316.446	4462.960	3861.833	3569.857	3020.038	2415.416	2085.630
	Residual	-0.000	100.866	152.721	109.944	53.473	-25.134	-159.299	-127.625	29.849	11.391

Fitting the Full Age-Period-Cohort Model

Results for PHIM for Run 1 (Basic_US_OG_T1.RTF)

Matrix of observed and expected deaths and (O-E)2/E Values**

Age		1966-	1971-	1976-	1981-	1986-	1991-	1996-	2001-	2006-	2011-	Total
10-	Observed	446.0	378.0	251.0	135.0	114.0	131.0	118.0	124.0	129.0	131.0	1957.0
	Expected	373.3	344.0	235.2	178.7	148.5	153.9	157.8	147.0	116.5	131.0	1985.8
	Difference	72.7	34.0	15.8	-43.7	-34.5	-22.9	-39.8	-23.0	12.5	0.0	-28.8
	Chi-Sq	14.1	3.4	1.1	10.7	8.0	3.4	10.0	3.6	1.3	0.0	55.6
15-	Observed	614.0	698.0	446.0	296.0	201.0	172.0	192.0	196.0	212.0	165.0	3192.0
	Expected	525.9	570.2	438.2	325.5	267.9	238.0	259.0	237.5	206.2	178.6	3247.1
	Difference	88.1	127.8	7.8	-29.5	-66.9	-66.0	-67.0	-41.5	5.8	-13.6	-55.1
	Chi-Sq	14.8	28.6	0.1	2.7	16.7	18.3	17.3	7.3	0.2	1.0	107.0
20-	Observed	723.0	822.0	768.0	544.0	448.0	340.0	320.0	336.0	342.0	323.0	4966.0
	Expected	657.3	780.7	699.0	583.1	469.3	410.6	385.6	375.6	321.5	308.2	4990.9
	Difference	65.7	41.3	69.0	-39.1	-21.3	-70.6	-65.6	-39.6	20.5	14.8	-24.9
	Chi-Sq	6.6	2.2	6.8	2.6	1.0	12.1	11.2	4.2	1.3	0.7	48.7
25-	Observed	1045.0	1052.0	1011.0	937.0	844.0	627.0	530.0	527.0	490.0	529.0	7592.0
	Expected	901.5	1019.6	980.4	940.0	845.1	724.8	669.8	558.8	509.5	480.6	7630.2
	Difference	143.5	32.4	30.6	-3.0	-1.1	-97.8	-139.8	-31.8	-19.5	48.4	-38.2
	Chi-Sq	22.8	1.0	1.0	0.0	0.0	13.2	29.2	1.8	0.7	4.9	74.7
30-	Observed	1601.0	1619.0	1482.0	1464.0	1569.0	1336.0	1090.0	967.0	911.0	982.0	13021.0
	Expected	1466.5	1556.7	1421.5	1475.9	1540.1	1479.5	1327.7	1096.2	858.4	855.7	13078.1
	Difference	134.5	62.3	60.5	-11.9	28.9	-143.5	-237.7	-129.2	52.6	126.3	-57.1
	Chi-Sq	12.3	2.5	2.6	0.1	0.5	13.9	42.6	15.2	3.2	18.6	111.6
35-	Observed	3065.0	2624.0	2220.0	2305.0	2619.0	2681.0	2377.0	1979.0	1886.0	1621.0	23377.0
	Expected	2949.4	2590.8	2173.1	2174.0	2505.4	2777.4	2785.2	2237.1	1749.3	1503.0	23444.8
	Difference	115.6	33.2	46.9	131.0	113.6	-96.4	-408.2	-258.1	136.7	118.0	-67.8
	Chi-Sq	4.5	0.4	1.0	7.9	5.2	3.3	59.8	29.8	10.7	9.3	131.9
40-	Observed	5932.0	4898.0	3718.0	3285.0	3760.0	4377.0	4468.0	4137.0	3580.0	3176.0	41331.0
	Expected	5940.2	5021.9	3551.5	3221.7	3564.8	4322.4	4976.4	4463.5	3410.6	2923.4	41396.5
	Difference	-8.2	-123.9	166.5	63.3	195.2	54.6	-508.4	-326.5	169.4	252.6	-65.5
	Chi-Sq	0.0	3.1	7.8	1.2	10.7	0.7	51.9	23.9	8.4	21.8	129.6
45-	Observed	9674.0	8625.0	6050.0	5228.0	5112.0	6176.0	6884.0	6884.0	6678.0	5642.0	66953.0
	Expected	10193.4	9258.9	6217.2	4837.4	4850.4	5648.6	7157.2	7384.7	6289.3	5248.7	67085.9
	Difference	-519.4	-633.9	-167.2	390.6	261.6	527.4	-273.2	-500.7	388.7	393.3	-132.9
	Chi-Sq	26.5	43.4	4.5	31.5	14.1	49.2	10.4	33.9	24.0	29.5	267.1
50-	Observed	15771.0	14283.0	10595.0	8277.0	7151.0	7652.0	8790.0	9804.0	10348.0	9622.0	102293.0
	Expected	15901.1	14960.6	10934.2	7975.0	6917.8	7358.0	8961.2	10191.5	9947.7	9209.0	102355.9
	Difference	-130.1	-677.6	-339.2	302.0	233.2	294.0	-171.2	-387.5	400.3	413.0	-62.9
	Chi-Sq	1.1	30.7	10.5	11.4	7.9	11.8	3.3	14.7	16.1	18.5	126.0
55-	Observed	24297.0	22013.0	16523.0	13449.0	11243.0	10306.0	11188.0	11948.0	13537.0	14339.0	148843.0
	Expected	24825.4	22136.6	16943.9	13450.7	10862.7	10020.8	11136.4	12272.6	13217.5	14010.5	148877.0
	Difference	-528.4	-123.6	-420.9	-1.7	380.3	285.2	51.6	-324.6	319.5	328.5	-34.0
	Chi-Sq	11.2	0.7	10.5	0.0	13.3	8.1	0.2	8.6	7.7	7.7	68.1
60-	Observed	36199.0	34445.0	25696.0	20968.0	18437.0	16018.0	15633.0	15422.0	16302.0	18893.0	218013.0
	Expected	37002.8	34240.4	25485.1	21079.4	18407.1	15887.2	15397.4	15432.3	16198.3	18896.2	218026.3
	Difference	-803.8	204.6	210.9	-111.4	29.9	130.8	235.6	-10.3	103.7	-3.2	-13.3
	Chi-Sq	17.5	1.2	1.7	0.6	0.0	1.1	3.6	0.0	0.7	0.0	26.4
65-	Observed	53157.0	50572.0	39581.0	31656.0	28226.0	25793.0	23554.0	20964.0	20193.0	22477.0	316173.0
	Expected	52685.7	49751.9	38991.0	31515.8	28320.6	26394.6	23947.7	21189.8	20259.7	23151.4	316208.1
	Difference	471.3	820.1	590.0	140.2	-94.6	-601.6	-393.7	-225.8	-66.7	-674.4	-35.1
	Chi-Sq	4.2	13.5	8.9	0.6	0.3	13.7	6.5	2.4	0.2	19.6	70.1
70-	Observed	73106.0	68323.0	54852.0	46184.0	40370.0	38910.0	38270.0	32455.0	26371.0	28008.0	446849.0
	Expected	72278.7	67359.7	53719.2	45830.3	40804.3	39516.2	38776.3	32254.9	27487.7	28894.2	446921.5

Results for PHIM for Run 1 (Basic_US_OG_T1.RTF)

	Difference	827.3	963.3	1132.8	353.7	-434.3	-606.2	-506.3	200.1	-1116.7	-886.2	-72.5
	Chi-Sq	9.5	13.8	23.9	2.7	4.6	9.3	6.6	1.2	45.4	27.2	144.2
75-	Observed	86954.0	81867.0	65282.0	56303.0	52431.0	51258.0	54761.0	48878.0	37515.0	35728.0	570977.0
	Expected	86954.0	82703.3	66741.6	57491.9	53066.8	50926.6	52421.8	46896.2	37984.4	35924.2	571110.8
	Difference	0.0	-836.3	-1459.6	-1188.9	-635.8	331.4	2339.2	1981.8	-469.4	-196.2	-133.8
	Chi-Sq	0.0	8.5	31.9	24.6	7.6	2.2	104.4	83.8	5.8	1.1	269.7
Total over ages	Observed	312584.0	292219.0	228475.0	191031.0	172525.0	165777.0	168175.0	154621.0	138494.0	141636.0	1965537.0
	Expected	312655.2	292295.1	228531.1	191079.5	172570.8	165858.4	168359.6	154737.8	138556.6	141714.8	1966358.9
	Difference	-71.2	-76.1	-56.1	-48.5	-45.8	-81.4	-184.6	-116.8	-62.6	-78.8	-821.9
	Chi-Sq	145.1	152.9	112.3	96.7	90.0	160.3	357.1	230.4	125.8	159.9	1630.7

Results for PHIM for Run 1 (Basic_US_OG_T1.RTF)

****

Osmond and Gardner Extrapolating Death Rates for COD: STROKE

Variable Parameter	Value
1. Country	US (United States)
2. Sex	M (Males)
3. Year of start of OG modelling	1966
4. Number 5-year periods for OG modelling	10
5. Age Range of interest	10 - 79
6. Scaling Factor for Rates	10000
7. Model Type to Save	4 (Full Age-Period-Cohort)
8. Goodness of Fit Required	1 (Analysis Printed For Age-Period-Cohort Model)
9. Cause of Death	STROKE
Note:	Death rates are per million population
10. Number of Periods into the future to Predict	5
11. Earliest projected year	2016
12. Extrapolate Period using (1: last 2 points 2: linear regression)	1
13. Ratio of last two period values	0.91001

Predictions of rates for future years from model:	Full Age-Period-Cohort
Effects for extending model to project rates for:	2016-2040

Extrapolating Model: Full Age-Period-Cohort
Log Transform Parameters

Model	ChiSq	MChiSq	DF	Factor	%Account	P
Age Only	124510.295	8893.592	14	P, C	98.6741	0.0000
Age-Period	5250.848	375.061	14	Cohort	68.5587	0.0000
Age-Cohort	2447.674	174.834	14	Period	32.5509	0.0000
Period-Cohort	2919.954	208.568	14	Age	43.4603	0.0000
Full Age-Period-Cohort	1650.935	117.924	14			0.0000

**Key to terms:**	Chisq =	chi-squared value for model
	MChisq =	mean Chi-squared (Chisq/DF)
	DF =	degrees of freedom
	Factor =	Factors not included in the model
	% Account =	1- (Chisq for full model)/(Chisq for model in question)
	P =	probability value based on Chisq and DF.

AGE	EFFECT
10	4.919072
15	8.010732
20	12.806062
25	20.679707
30	37.474240
35	71.081192
40	130.078780
45	221.449482
50	362.150452
55	577.394538
60	951.277717
65	1601.85730
70	2805.51996
75	4817.35652

PERIOD	EFFECT	
Period Change =	0.910006	
1966	1.642625	
1971	1.537616	
1976	1.172443	
1981	0.962431	
1986	0.862775	
1991	0.820189	
1996	0.803171	
2001	0.705982	
2006	0.590620	
2011	0.537468	
2016	0.489099	
2021	0.445083	
2026	0.405029	
2031	0.368578	
2036	0.335409	
2016	0.507901	Extrapolated
2017	0.498412	Extrapolated
2018	0.489099	Extrapolated
2019	0.479961	Extrapolated

Results for PHIM for Run 1 (Basic_US_OG_T1.RTF)

2020	0.470993	Extrapolated
2021	0.462193	Extrapolated
2022	0.453558	Extrapolated
2023	0.445083	Extrapolated
2024	0.436767	Extrapolated
2025	0.428607	Extrapolated
2026	0.420599	Extrapolated
2027	0.412740	Extrapolated
2028	0.405029	Extrapolated
2029	0.397461	Extrapolated
2030	0.390035	Extrapolated
2031	0.382747	Extrapolated
2032	0.375596	Extrapolated
2033	0.368578	Extrapolated
2034	0.361692	Extrapolated
2035	0.354934	Extrapolated
2036	0.348302	Extrapolated
2037	0.341795	Extrapolated
2038	0.335409	Extrapolated
2039	0.329142	Extrapolated
2040	0.322992	Extrapolated

Results for PHIM for Run 1 (Basic_US_OG_T1.RTF)

COHORT	EFFECT	WEIGHT	ORIGINAL
1891	1.359225	1.000	
1896	1.346706	2.000	
1901	1.236369	4.000	
1906	1.146682	8.000	
1911	1.073783	16.000	
1916	0.977397	32.000	
1921	0.922644	64.000	
1926	0.887994	128.000	
1931	0.848935	256.000	
1936	0.805519	512.000	
1941	0.806758	1024.000	
1946	0.800118	2048.000	
1951	0.848859	4096.000	
1956	0.881513	8192.000	
1961	0.852256	16384.000	
1966	0.814074	32768.000	
1971	0.820400	65536.000	
1976	0.788416	131072.000	
1981	0.818698	262144.000	
1986	0.780550	524288.000	
1991	0.771834	1048576.000	
1996	0.762197	Extrapolated	0.742452
2001	0.751454	Extrapolated	0.933071
2006	0.740862	Extrapolated	
2011	0.730420	Extrapolated	
2016	0.720125	Extrapolated	
2021	0.709975	Extrapolated	
2026	0.699969	Extrapolated	

Standardizing Population: The 1976 European Standard Population

Age Range	Population, Males
All	100000
0	0
1	0
2	0
3	0
0-4	8000
5-9	7000
10-14	7000
15-19	7000
20-24	7000
25-29	7000
30-34	7000
35-39	7000
40-44	7000
45-49	7000
50-54	7000
55-59	6000
60-64	5000
65-69	4000
70-74	3000
75-79	2000
80-84	1000
85+	1000

Results for PHIM for Run 1 (Basic_US_OG_T1.RTF)

Matrix of observed and expected rates including predictions

Total over ages standardized using: The 1976 European Standard Population

		1966-	1971-	1976-	1981-	1986-	1991-	1996-	2001-	2006-	2011-	2016-	2021-	2026-	2031-	2036-
10	OBS	8.5	7.1	5.0	2.9	2.6	2.8	2.3	2.3	2.4	2.5	3.0	.	.	.	.
	EXP											1.8	1.6	1.4	1.3	1.2
15	OBS	13.0	13.3	8.1	5.7	4.3	3.7	3.9	3.6	3.8	3.0	3.4	.	.	.	.
	EXP											2.9	2.6	2.4	2.1	1.9
20	OBS	18.5	17.6	14.5	9.8	8.6	7.1	6.7	6.6	6.3	5.6	4.4	.	.	.	.
	EXP											4.8	4.3	3.8	3.4	3.1
25	OBS	31.8	26.3	21.2	17.5	15.2	11.9	10.8	10.9	9.6	9.5	9.8	.	.	.	.
	EXP											7.8	7.0	6.3	5.6	5.1
30	OBS	54.1	48.3	36.7	30.4	29.0	23.7	20.1	19.1	18.5	18.9	19.3	.	.	.	.
	EXP											14.3	12.9	11.6	10.4	9.3
35	OBS	103.0	89.2	68.7	58.0	54.4	49.6	41.5	36.1	37.1	32.5	37.9	.	.	.	.
	EXP											28.5	24.7	22.2	20.0	17.9
40	OBS	189.5	165.6	128.6	103.0	94.7	91.7	82.7	72.5	65.6	62.3	64.7	.	.	.	.
	EXP											50.2	47.4	41.1	37.0	33.3
45	OBS	318.5	281.7	214.5	185.5	162.5	158.9	145.2	128.5	118.4	104.2	108.3	.	.	.	.
	EXP											88.9	77.7	73.4	63.7	57.3
50	OBS	576.7	490.5	365.3	307.1	260.2	249.2	228.3	208.8	196.1	173.3	178.1	.	.	.	.
	EXP											144.2	132.2	115.6	109.3	94.8
55	OBS	996.7	862.9	609.1	493.4	437.7	392.3	375.9	317.5	296.5	280.0	277.7	.	.	.	.
	EXP											240.7	209.2	191.9	167.8	158.6
60	OBS	1752.9	1580.0	1099.1	840.3	730.0	667.8	624.9	541.4	452.4	433.9	438.9	.	.	.	.
	EXP											410.1	360.8	313.7	287.6	251.6
65	OBS	3282.3	2870.9	2047.2	1513.5	1270.9	1140.1	1074.3	901.2	760.8	668.8	656.0	.	.	.	.
	EXP											665.1	628.5	552.9	480.6	440.8
70	OBS	6277.2	5409.7	3851.3	2921.7	2340.6	2090.5	1974.8	1691.9	1280.5	1179.2	1081.6	.	.	.	.
	EXP											1097.9	1060.0	1001.7	881.3	766.0
75	OBS	10755.7	9874.5	6830.4	5206.5	4409.5	3887.0	3729.2	3147.7	2385.6	2074.2	1964.6	.	.	.	.
	EXP											1900.9	1715.6	1656.3	1565.2	1377.1
10-79	OBS	932.7	825.5	585.5	451.0	381.0	343.2	323.5	277.2	227.7	205.9	200.7	.	.	.	.
	EXP	933.0	826.1	585.9	450.5	380.4	342.8	323.9	277.7	228.0	205.9	188.6*	173.0*	159.0*	143.5*	127.6*

Drop in overall standardized Observed and Predicted rates

comparing the last observed rate during the model fitting period to the last observed and predicted rates where an observed rate is available (2016)

Observed and Predicted %Drop = 2.530% and 8.421%

Results for PHIM for Run 1 (Basic_US_OG_T1.RTF)

Matrix of observed and expected deaths including predictions

		1966-	1971-	1976-	1981-	1986-	1991-	1996-	2001-	2006-	2011-	2016-	2021-	2026-	2031-	2036-
10-	OBS	446.0	378.0	251.0	135.0	114.0	131.0	118.0	124.0	129.0	131.0	160.0*	.	.	.	.
	EXP	373.3	344.0	235.2	178.7	148.5	153.9	157.8	147.0	119.6	105.5	95.8*	84.2*	73.0*	66.7*	61.7*
	ChiSq	14.147	3.370	1.066	10.685	8.014	3.409	10.039	3.585	0.731	6.163	42.980*	.	.	.	.
15-	OBS	614.0	698.0	446.0	296.0	201.0	172.0	192.0	196.0	212.0	165.0	185.0*	.	.	.	.
	EXP	525.9	570.2	438.2	325.5	267.9	238.0	259.0	237.5	206.2	183.4	159.8*	145.0*	127.5*	110.9*	101.4*
	ChiSq	14.770	28.632	0.140	2.682	16.705	18.312	17.347	7.258	0.163	1.842	3.989*	.	.	.	.
20-	OBS	723.0	822.0	768.0	544.0	448.0	340.0	320.0	336.0	342.0	323.0	252.5*	.	.	.	.
	EXP	657.3	780.7	699.0	583.1	469.3	410.6	385.6	375.6	321.5	308.2	275.8*	240.2*	218.3*	192.7*	168.2*
	ChiSq	6.567	2.183	6.812	2.625	0.967	12.133	11.173	4.180	1.311	0.714	1.974*	.	.	.	.
25-	OBS	1045.0	1052.0	1011.0	937.0	844.0	627.0	530.0	527.0	490.0	529.0	585.0*	.	.	.	.
	EXP	901.5	1019.6	980.4	940.0	845.1	724.8	669.8	558.8	509.5	480.6	466.5*	416.6*	363.7*	331.4*	293.1*
	ChiSq	22.834	1.032	0.954	0.010	0.002	13.186	29.197	1.812	0.746	4.879	30.092*	.	.	.	.
30-	OBS	1601.0	1619.0	1482.0	1464.0	1569.0	1336.0	1090.0	967.0	911.0	982.0	1090.0*	.	.	.	.
	EXP	1466.5	1556.7	1421.5	1475.9	1540.1	1479.5	1327.7	1096.2	858.4	855.7	809.8*	782.3*	699.3*	612.4*	558.4*
	ChiSq	12.342	2.496	2.577	0.096	0.544	13.919	42.554	15.226	3.220	18.637	96.920*	.	.	.	.
35-	OBS	3065.0	2624.0	2220.0	2305.0	2619.0	2681.0	2377.0	1979.0	1886.0	1621.0	1990.0*	.	.	.	.
	EXP	2949.4	2590.8	2173.1	2174.0	2505.4	2777.4	2785.2	2237.1	1749.3	1503.0	1496.3*	1410.1*	1361.3*	1219.4*	1069.5*
	ChiSq	4.530	0.426	1.014	7.898	5.153	3.347	59.836	29.786	10.679	9.256	162.858*	.	.	.	.
40-	OBS	5932.0	4898.0	3718.0	3285.0	3760.0	4377.0	4468.0	4137.0	3580.0	3176.0	3245.0*	.	.	.	.
	EXP	5940.2	5021.9	3551.5	3221.7	3564.8	4322.4	4976.4	4463.5	3410.6	2923.4	2514.2*	2495.5*	2351.1*	2272.1*	2037.5*
	ChiSq	0.011	3.059	7.804	1.243	10.692	0.690	51.940	23.887	8.409	21.828	212.436*	.	.	.	.
45-	OBS	9674.0	8625.0	6050.0	5228.0	5112.0	6176.0	6884.0	6884.0	6678.0	5642.0	5492.5*	.	.	.	.
	EXP	10193.4	9258.9	6217.2	4837.4	4850.4	5648.6	7157.2	7384.7	6289.3	5248.7	4505.2*	3868.9*	3842.5*	3625.3*	3505.9*
	ChiSq	26.471	43.399	4.499	31.541	14.105	49.243	10.429	33.948	24.029	29.463	216.367*	.	.	.	.
50-	OBS	15771.0	14283.0	10595.0	8277.0	7151.0	7652.0	8790.0	9804.0	10348.0	9622.0	9477.5*	.	.	.	.
	EXP	15901.1	14960.6	10934.2	7975.0	6917.8	7358.0	8961.2	10191.5	9947.7	9209.0	7672.4*	6585.4*	5664.8*	5639.9*	5328.1*
	ChiSq	1.065	30.689	10.522	11.434	7.862	11.751	3.270	14.733	16.111	18.525	424.692*	.	.	.	.
55-	OBS	24297.0	22013.0	16523.0	13449.0	11243.0	10306.0	11188.0	11948.0	13537.0	14339.0	14905.0*	.	.	.	.
	EXP	24825.4	22136.6	16943.9	13450.7	10862.7	10020.8	11136.4	12272.6	13217.5	14010.5	12917.5*	10766.3*	9266.7*	8001.8*	7986.8*
	ChiSq	11.246	0.690	10.457	.	13.316	8.118	0.239	8.587	7.725	7.701	305.785*	.	.	.	.
60-	OBS	36199.0	34445.0	25696.0	20968.0	18437.0	16018.0	15633.0	15422.0	16302.0	18893.0	21325.0*	.	.	.	.
	EXP	37002.8	34240.4	25485.1	21079.4	18407.1	15887.2	15397.4	15432.3	16198.3	18896.2	19929.7*	18373.6*	15370.7*	13304.5*	11536.4*
	ChiSq	17.461	1.223	1.746	0.589	0.049	1.077	3.604	0.007	0.663	.	97.682*	.	.	.	.
65-	OBS	53157.0	50572.0	39581.0	31656.0	28226.0	25793.0	23554.0	20964.0	20193.0	22477.0	26477.5*	.	.	.	.
	EXP	52685.7	49751.9	38991.0	31515.8	28320.6	26394.6	23947.7	21189.8	20259.7	23151.4	26843.3*	28294.0*	26207.4*	22095.6*	19248.6*
	ChiSq	4.217	13.520	8.926	0.623	0.316	13.710	6.474	2.406	0.219	19.645	4.985*	.	.	.	.

Results for PHIM for Run 1 (Basic_US_OG_T1.RTF)

70-	OBS	73106.0	68323.0	54852.0	46184.0	40370.0	38910.0	38270.0	32455.0	26371.0	28008.0	32565.0*	.	.	.	.
	EXP	72278.7	67359.7	53719.2	45830.3	40804.3	39516.2	38776.3	32254.9	27487.7	28894.2	33057.2*	38294.3*	40634.4*	38057.0*	32377.7*
	ChiSq	9.469	13.776	23.889	2.730	4.623	9.299	6.610	1.241	45.364	27.181	7.330*	.	.	.	.
75-	OBS	86954.0	81867.0	65282.0	56303.0	52431.0	51258.0	54761.0	48878.0	37515.0	35728.0	39345.0*	.	.	.	.
	EXP	86954.0	82703.3	66741.6	57491.9	53066.8	50926.6	52421.8	46896.2	37984.4	35924.2	38067.9*	43724.0*	51046.4*	55102.3*	52291.8*
	ChiSq	.	8.456	31.923	24.587	7.618	2.157	104.385	83.750	5.801	1.072	42.846*	.	.	.	.
Total Deaths		312584.0	292219.0	228475.0	191031.0	172525.0	165777.0	168175.0	154621.0	138494.0	141636.0	157095.0*	.	.	.	.
Expected		312655.2	292295.1	228531.1	191079.5	172570.8	165858.4	168359.6	154737.8	138559.7	141694.0	148811.6*	155480.2*	157227.0*	150631.7*	136565.2*
Obs/Exp		1.000	1.000	1.000	1.000	1.000	1.000	0.999	0.999	1.000	1.000	1.056*	.	.	.	.

Chi Squared (Log) = 1650.9 on 14 D.F. P = 0.0000

Results for PHIM for Run 1 (Basic_US_OG_T1.RTF)

****

Observed and predicted rates (5 year periods)

		10-	15-	20-	25-	30-	35-	40-	45-	50-	55-	60-	65-	70-	75-
2016-	OBS	3.0	3.4	4.4	9.8	19.3	37.9	64.7	108.3	178.1	277.7	438.9	656.0	1081.6	1964.6
	PRE	1.8	2.9	4.8	7.8	14.3	28.5	50.2	88.9	144.2	240.7	410.1	665.1	1097.9	1900.9
	RES	1.194	0.465	-0.404	1.983	4.949	9.390	14.581	19.473	33.925	37.030	28.714	-9.063	-16.348	63.771
2021-	PRE	1.6	2.6	4.3	7.0	12.9	24.7	47.4	77.7	132.2	209.2	360.8	628.5	1060.0	1715.6
2026-	PRE	1.4	2.4	3.8	6.3	11.6	22.2	41.1	73.4	115.6	191.9	313.7	552.9	1001.7	1656.3
2031-	PRE	1.3	2.1	3.4	5.6	10.4	20.0	37.0	63.7	109.3	167.8	287.6	480.6	881.3	1565.2
2036-	PRE	1.2	1.9	3.1	5.1	9.3	17.9	33.3	57.3	94.8	158.6	251.6	440.8	766.0	1377.1

Results for PHIM for Run 1 (Basic_US_OG_T1.RTF)

****

Observed and predicted deaths (5 year periods)

		10-	15-	20-	25-	30-	35-	40-	45-	50-	55-	60-	65-	70-	75-
2016-	OBS	160.0	185.0	252.5	585.0	1090.0	1990.0	3245.0	5492.5	9477.5	14905.0	21325.0	26477.5	32565.0	39345.0
	PRE	95.8	159.8	275.8	466.5	809.8	1496.3	2514.2	4505.2	7672.4	12917.5	19929.7	26843.3	33057.2	38067.9
	CHI	42.980	3.989	1.974	30.092	96.920	162.858	212.436	216.367	424.692	305.785	97.682	4.985	7.330	42.846
2021-	PRE	84.2	145.0	240.2	416.6	782.3	1410.1	2495.5	3868.9	6585.4	10766.3	18373.6	28294.0	38294.3	43724.0
2026-	PRE	73.0	127.5	218.3	363.7	699.3	1361.3	2351.1	3842.5	5664.8	9266.7	15370.7	26207.4	40634.4	51046.4
2031-	PRE	66.7	110.9	192.7	331.4	612.4	1219.4	2272.1	3625.3	5639.9	8001.8	13304.5	22095.6	38057.0	55102.3
2036-	PRE	61.7	101.4	168.2	293.1	558.4	1069.5	2037.5	3505.9	5328.1	7986.8	11536.4	19248.6	32377.7	52291.8

Results for PHIM for Run 1 (Basic_US_OG_T1.RTF)

****

Observed and predicted rates (single years)

		10-	15-	20-	25-	30-	35-	40-	45-	50-	55-	60-	65-	70-	75-
2016	OBS	2.9	3.4	4.7	10.1	19.5	39.0	66.3	104.9	172.5	278.8	447.4	689.7	1169.4	2053.7
	PRE	1.9	3.1	5.0	8.1	14.9	29.6	52.1	92.3	149.7	249.9	425.9	690.6	1140.1	1973.9
	RES	1.055	0.341	-0.291	2.008	4.614	9.452	14.165	12.667	22.756	28.820	21.460	-0.872	29.243	79.794
2017	OBS	3.1	3.4	3.9	9.8	20.0	38.5	63.7	108.5	175.7	277.3	455.8	671.7	1182.6	2077.2
	PRE	1.8	3.0	4.9	8.0	14.6	29.0	51.1	90.6	146.9	245.3	417.9	677.7	1118.8	1937.0
	RES	1.260	0.407	-0.930	1.838	5.455	9.488	12.617	17.984	28.762	32.035	37.827	-5.994	63.803	140.105
2018	PRE	1.8	2.9	4.8	7.8	14.3	28.5	50.2	88.9	144.2	240.7	410.1	665.1	1097.9	1900.9
2019	PRE	1.7	2.9	4.7	7.7	14.0	27.9	49.2	87.2	141.5	236.2	402.5	652.6	1077.4	1865.3
2020	PRE	1.7	2.8	4.6	7.5	13.8	27.4	48.3	85.6	138.9	231.8	395.0	640.4	1057.3	1830.5
2021	PRE	1.7	2.7	4.4	7.3	13.4	25.6	49.2	80.7	137.3	217.3	374.7	652.6	1100.7	1781.5
2022	PRE	1.6	2.7	4.4	7.1	13.1	25.2	48.3	79.2	134.8	213.2	367.7	640.4	1080.1	1748.2
2023	PRE	1.6	2.6	4.3	7.0	12.9	24.7	47.4	77.7	132.2	209.2	360.8	628.5	1060.0	1715.6
2024	PRE	1.6	2.6	4.2	6.9	12.6	24.2	46.5	76.3	129.8	205.3	354.1	616.7	1040.2	1683.5
2025	PRE	1.5	2.5	4.1	6.8	12.4	23.8	45.6	74.8	127.3	201.5	347.5	605.2	1020.7	1652.0
2026	PRE	1.5	2.5	4.0	6.5	12.0	23.1	42.7	76.3	120.1	199.2	325.7	574.2	1040.2	1719.9
2027	PRE	1.5	2.4	3.9	6.4	11.8	22.6	41.9	74.8	117.8	195.5	319.6	563.5	1020.7	1687.8
2028	PRE	1.4	2.4	3.8	6.3	11.6	22.2	41.1	73.4	115.6	191.9	313.7	552.9	1001.7	1656.3
2029	PRE	1.4	2.3	3.8	6.2	11.4	21.8	40.4	72.1	113.5	188.3	307.8	542.6	983.0	1625.3
2030	PRE	1.4	2.3	3.7	6.1	11.1	21.4	39.6	70.7	111.4	184.8	302.0	532.5	964.6	1595.0
2031	PRE	1.3	2.2	3.6	5.9	10.8	20.7	38.4	66.2	113.5	174.2	298.7	499.1	915.2	1625.4
2032	PRE	1.3	2.2	3.5	5.8	10.6	20.3	37.7	64.9	111.4	171.0	293.1	489.8	898.1	1595.0
2033	PRE	1.3	2.1	3.4	5.6	10.4	20.0	37.0	63.7	109.3	167.8	287.6	480.6	881.3	1565.2
2034	PRE	1.3	2.1	3.4	5.5	10.2	19.6	36.3	62.5	107.2	164.7	282.3	471.7	864.8	1535.9
2035	PRE	1.2	2.0	3.3	5.4	10.0	19.2	35.6	61.4	105.2	161.6	277.0	462.8	848.7	1507.2
2036	PRE	1.2	2.0	3.2	5.3	9.7	18.6	34.5	59.5	98.5	164.6	261.2	457.7	795.5	1430.0
2037	PRE	1.2	1.9	3.2	5.2	9.5	18.3	33.9	58.4	96.6	161.6	256.3	449.2	780.6	1403.3
2038	PRE	1.2	1.9	3.1	5.1	9.3	17.9	33.3	57.3	94.8	158.6	251.6	440.8	766.0	1377.1
2039	PRE	1.1	1.9	3.0	5.0	9.1	17.6	32.6	56.3	93.0	155.6	246.9	432.5	751.7	1351.3
2040	PRE	1.1	1.8	3.0	4.9	9.0	17.3	32.0	55.2	91.3	152.7	242.2	424.5	737.7	1326.1

Results for PHIM for Run 1 (Basic_US_OG_T1.RTF)

****

Observed and predicted deaths (single years)

		10-	15-	20-	25-	30-	35-	40-	45-	50-	55-	60-	65-	70-	75-
2016	OBS	31.0	37.0	55.0	118.0	213.0	398.0	662.0	1087.0	1891.0	2981.0	4179.0	5293.0	6292.0	7730.0
	PRE	19.7	33.3	58.4	94.6	162.5	301.6	520.5	955.8	1641.5	2672.8	3978.5	5299.7	6134.6	7429.7
	CHI	6.419	0.414	0.202	5.801	15.677	30.837	38.489	18.013	37.911	35.538	10.101	0.008	4.036	12.141
2017	OBS	33.0	37.0	46.0	116.0	223.0	398.0	636.0	1110.0	1900.0	2981.0	4351.0	5298.0	6734.0	8008.0
	PRE	19.5	32.6	56.9	94.2	162.3	299.9	510.1	926.1	1589.0	2636.6	3989.9	5345.3	6370.7	7467.9
	CHI	9.371	0.600	2.080	5.031	22.719	32.090	31.077	36.529	60.879	44.981	32.683	0.418	20.718	39.068
2018	PRE	19.2	31.9	55.2	93.7	162.0	298.8	501.3	898.9	1534.4	2592.2	3995.6	5369.8	6622.8	7534.5
2019	PRE	18.9	31.3	53.5	92.7	161.6	298.1	494.2	874.6	1481.7	2540.0	3989.7	5392.1	6848.7	7679.2
2020	PRE	18.5	30.7	52.1	91.3	161.2	297.6	488.8	853.0	1432.9	2480.4	3969.3	5421.4	7026.0	7920.5
2021	PRE	17.8	29.9	50.0	88.2	158.8	283.2	503.1	800.9	1396.4	2301.3	3796.8	5642.9	7553.9	8101.7
2022	PRE	17.4	29.5	48.9	85.8	158.1	282.6	500.1	784.7	1352.9	2228.0	3746.8	5659.0	7614.6	8417.5
2023	PRE	16.8	29.1	47.9	83.3	157.1	282.0	498.3	771.3	1313.8	2152.9	3686.8	5670.5	7654.4	8757.4
2024	PRE	16.3	28.6	47.1	80.9	155.4	281.3	497.1	760.6	1278.9	2080.4	3615.6	5667.3	7696.8	9063.9
2025	PRE	15.8	28.0	46.3	78.7	153.0	280.5	496.2	752.4	1248.0	2013.5	3533.5	5644.1	7752.0	9310.2
2026	PRE	15.2	27.0	44.9	75.7	147.9	276.2	472.1	774.3	1171.7	1962.8	3280.2	5402.7	8076.3	10017.5
2027	PRE	14.9	26.3	44.3	74.0	144.0	275.0	471.2	769.9	1148.3	1902.6	3178.1	5337.2	8110.4	10114.6
2028	PRE	14.6	25.5	43.7	72.6	139.8	273.3	470.2	767.2	1129.2	1848.6	3073.5	5258.3	8140.7	10194.0
2029	PRE	14.3	24.7	43.1	71.3	135.8	270.5	469.1	765.6	1114.0	1800.6	2972.7	5163.5	8152.3	10285.4
2030	PRE	14.0	24.0	42.3	70.2	132.3	266.3	467.8	764.5	1102.8	1758.5	2879.6	5052.8	8137.2	10399.1
2031	PRE	13.6	23.1	40.7	68.1	127.3	257.6	460.8	727.6	1135.6	1652.7	2811.6	4699.3	7806.0	10870.9
2032	PRE	13.5	22.6	39.6	67.2	124.6	251.0	458.9	726.4	1129.5	1621.1	2729.1	4561.2	7729.7	10953.8
2033	PRE	13.3	22.2	38.5	66.4	122.2	243.8	456.1	725.1	1126.1	1595.1	2654.6	4418.7	7634.9	11034.2
2034	PRE	13.2	21.7	37.4	65.4	120.2	236.9	451.5	723.6	1124.2	1574.7	2588.1	4281.0	7517.4	11093.0
2035	PRE	13.0	21.3	36.5	64.2	118.3	230.9	444.6	721.7	1123.1	1559.7	2529.6	4153.4	7375.8	11118.8
2036	PRE	12.7	20.7	35.1	61.9	114.9	222.3	430.2	710.9	1069.0	1607.0	2379.7	4059.6	6866.0	10678.5
2037	PRE	12.5	20.5	34.3	60.3	113.3	217.6	419.3	708.0	1067.3	1599.1	2335.9	3944.5	6672.1	10591.3
2038	PRE	12.4	20.3	33.6	58.6	111.9	213.5	407.4	703.8	1065.6	1594.7	2299.9	3840.6	6473.2	10484.8
2039	PRE	12.2	20.0	32.9	57.0	110.2	209.9	396.0	696.8	1063.6	1592.5	2271.7	3748.1	6282.6	10352.0
2040	PRE	11.9	19.8	32.4	55.6	108.2	206.5	386.0	686.3	1061.1	1591.4	2251.1	3667.3	6108.3	10189.6

Results for PHIM for Run 1 (Basic_US_OG_T1.RTF)

****
List of values created by O and G modelling, using percentage change in last two period parameters for Fixed File MORT

1. Country	US (United States)
2. Sex	M (Males)
3. Disease	STR (STROKE)
* Value comes from O and G Modelling.	

Age	Years	Value	Death Rate	Population
10-14	2018	19.2184	1.782452	107820.22 *
10-14	2019	18.9010	1.749148	108058.17 *
10-14	2020	18.4992	1.716467	107775.13 *
10-14	2021	17.8424	1.660656	107441.78 *
10-14	2022	17.3693	1.629628	106584.46 *
10-14	2023	16.8457	1.599180	105339.41 *
10-14	2024	16.3215	1.569301	104004.73 *
10-14	2025	15.8400	1.539980	102858.77 *
10-14	2026	15.2488	1.489907	102347.07 *
10-14	2027	14.9083	1.462069	101967.29 *
10-14	2028	14.5894	1.434752	101686.15 *
10-14	2029	14.2904	1.407945	101498.55 *
10-14	2030	14.0196	1.381639	101470.63 *
10-14	2031	13.6498	1.336714	102114.83 *
10-14	2032	13.4948	1.311739	102877.43 *
10-14	2033	13.3461	1.287231	103681.01 *
10-14	2034	13.1878	1.263180	104401.53 *
10-14	2035	13.0101	1.239579	104955.58 *
10-14	2036	12.6976	1.199273	105877.40 *
10-14	2037	12.5420	1.176866	106570.86 *
10-14	2038	12.3617	1.154877	107039.02 *
10-14	2039	12.1619	1.133300	107313.72 *
15-19	2018	31.9263	2.944228	108437.02 *
15-19	2019	31.3110	2.889218	108371.94 *
15-19	2020	30.7179	2.835236	108343.21 *
15-19	2021	29.8567	2.743048	108844.87 *
15-19	2022	29.4792	2.691796	109515.01 *
15-19	2023	29.0872	2.641503	110116.30 *
15-19	2024	28.6011	2.592149	110337.57 *
15-19	2025	27.9836	2.543717	110010.63 *
15-19	2026	26.9876	2.461007	109660.66 *
15-19	2027	26.2831	2.415025	108831.44 *
15-19	2028	25.5116	2.369903	107648.28 *
15-19	2029	24.7433	2.325624	106394.27 *
15-19	2030	24.0259	2.282172	105276.67 *
15-19	2031	23.1400	2.207966	104802.12 *
15-19	2032	22.6358	2.166712	104470.80 *
15-19	2033	22.1656	2.126229	104248.18 *
15-19	2034	21.7246	2.086503	104119.48 *
15-19	2035	21.3114	2.047518	104083.90 *
15-19	2036	20.7451	1.980943	104723.27 *
15-19	2037	20.5157	1.943931	105537.13 *
15-19	2038	20.2936	1.907610	106382.52 *
15-19	2039	20.0497	1.871968	107105.08 *
20-24	2018	55.1629	4.773970	115549.38 *
20-24	2019	53.5167	4.684773	114235.47 *
20-24	2020	52.0533	4.597242	113227.32 *
20-24	2021	50.0286	4.447761	112480.46 *
20-24	2022	48.9105	4.364659	112060.21 *
20-24	2023	47.9477	4.283110	111946.10 *
20-24	2024	47.0903	4.203084	112037.41 *
20-24	2025	46.2860	4.124553	112220.61 *
20-24	2026	44.9146	3.990442	112555.48 *
20-24	2027	44.3129	3.915885	113161.84 *
20-24	2028	43.7447	3.842720	113837.89 *
20-24	2029	43.0864	3.770922	114259.71 *
20-24	2030	42.2578	3.700466	114195.90 *
20-24	2031	40.7129	3.580144	113718.71 *
20-24	2032	39.6426	3.513253	112837.21 *
20-24	2033	38.5222	3.447611	111736.00 *
20-24	2034	37.4487	3.383196	110690.36 *
20-24	2035	36.4646	3.319984	109833.53 *
20-24	2036	35.0633	3.212034	109162.16 *
20-24	2037	34.2667	3.152020	108713.50 *
20-24	2038	33.5646	3.093127	108513.37 *
20-24	2039	32.9472	3.035335	108545.60 *
25-29	2018	93.6732	7.806657	119991.39 *
25-29	2019	92.7286	7.660797	121043.01 *

Results for PHIM for Run 1 (Basic_US_OG_T1.RTF)

25-29	2020	91.2979	7.517662	121444.55 *
25-29	2021	88.2041	7.285091	121074.83 *
25-29	2022	85.8396	7.148976	120072.51 *
25-29	2023	83.2860	7.015404	118718.74 *
25-29	2024	80.8533	6.884328	117445.48 *
25-29	2025	78.7237	6.755701	116529.28 *
25-29	2026	75.6763	6.536037	115783.18 *
25-29	2027	74.0136	6.413918	115395.25 *
25-29	2028	72.5899	6.294080	115330.47 *
25-29	2029	71.3369	6.176481	115497.61 *
25-29	2030	70.2011	6.061079	115822.80 *
25-29	2031	68.1410	5.864001	116202.23 *
25-29	2032	67.2401	5.754438	116849.18 *
25-29	2033	66.3904	5.646922	117569.12 *
25-29	2034	65.4200	5.541414	118056.43 *
25-29	2035	64.2315	5.437878	118118.77 *
25-29	2036	61.8930	5.261064	117643.53 *
25-29	2037	60.2833	5.162766	116765.46 *
25-29	2038	58.6022	5.066305	115670.55 *
25-29	2039	57.0005	4.971646	114651.21 *
30-34	2018	161.9665	14.306408	113212.55 *
30-34	2019	161.6191	14.039106	115120.68 *
30-34	2020	161.2232	13.776799	117025.14 *
30-34	2021	158.7603	13.368421	118757.69 *
30-34	2022	158.0545	13.118645	120480.79 *
30-34	2023	157.0610	12.873535	122003.03 *
30-34	2024	155.4328	12.633006	123037.09 *
30-34	2025	153.0172	12.396970	123431.16 *
30-34	2026	147.8853	12.013450	123099.77 *
30-34	2027	144.0033	11.788990	122150.65 *
30-34	2028	139.8075	11.568723	120849.55 *
30-34	2029	135.8099	11.352573	119629.21 *
30-34	2030	132.3244	11.140461	118778.23 *
30-34	2031	127.3284	10.778225	118134.80 *
30-34	2032	124.6069	10.576845	117811.00 *
30-34	2033	122.2493	10.379226	117782.65 *
30-34	2034	120.1611	10.185300	117975.02 *
30-34	2035	118.2696	9.994997	118328.82 *
30-34	2036	114.8531	9.670007	118772.50 *
30-34	2037	113.3472	9.489332	119447.02 *
30-34	2038	111.8954	9.312033	120162.15 *
30-34	2039	110.2327	9.138046	120630.51 *
35-39	2018	298.8020	28.462657	104980.35 *
35-39	2019	298.0998	27.930859	106727.76 *
35-39	2020	297.6308	27.408997	108588.71 *
35-39	2021	283.1925	25.643603	110433.99 *
35-39	2022	282.6295	25.164476	112312.89 *
35-39	2023	282.0211	24.694301	114204.92 *
35-39	2024	281.3359	24.232912	116096.60 *
35-39	2025	280.5181	23.780143	117963.16 *
35-39	2026	276.2098	23.075242	119699.62 *
35-39	2027	274.9912	22.644103	121440.53 *
35-39	2028	273.2849	22.221019	122984.87 *
35-39	2029	270.4917	21.805840	124045.52 *
35-39	2030	266.3460	21.398419	124469.94 *
35-39	2031	257.6017	20.736424	124226.65 *
35-39	2032	250.9894	20.348984	123342.47 *
35-39	2033	243.8019	19.968782	122091.53 *
35-39	2034	236.9289	19.595684	120908.72 *
35-39	2035	230.8972	19.229557	120074.10 *
35-39	2036	222.3111	18.604303	119494.45 *
35-39	2037	217.6295	18.256699	119205.30 *
35-39	2038	213.5318	17.915590	119187.69 *
35-39	2039	209.8707	17.580854	119374.58 *
40-44	2018	501.2819	50.160139	99936.30 *
40-44	2019	494.1767	49.222944	100395.61 *
40-44	2020	488.7605	48.303260	101185.82 *
40-44	2021	503.0855	49.221376	102208.75 *
40-44	2022	500.1313	48.301721	103543.16 *
40-44	2023	498.3032	47.399249	105128.92 *
40-44	2024	497.1209	46.513639	106876.38 *
40-44	2025	496.2447	45.644575	108719.32 *
40-44	2026	472.0960	42.704640	110549.11 *
40-44	2027	471.1750	41.906744	112434.16 *
40-44	2028	470.2124	41.123756	114340.82 *
40-44	2029	469.1318	40.355397	116250.08 *
40-44	2030	467.8255	39.601394	118133.60 *
40-44	2031	460.8218	38.427514	119919.75 *

Results for PHIM for Run 1 (Basic_US_OG_T1.RTF)

40-44	2032	458.8978	37.709532	121692.78 *
40-44	2033	456.1252	37.004965	123260.55 *
40-44	2034	451.5271	36.313562	124341.18 *
40-44	2035	444.6395	35.635077	124775.80 *
40-44	2036	430.1998	34.532649	124577.70 *
40-44	2037	419.2947	33.887439	123731.59 *
40-44	2038	407.3999	33.254284	122510.50 *
40-44	2039	395.9959	32.632959	121348.44 *
45-49	2018	898.9405	88.858209	101165.72 *
45-49	2019	874.5857	87.197977	100298.85 *
45-49	2020	853.0435	85.568765	99690.99 *
45-49	2021	800.8535	80.696279	99242.93 *
45-49	2022	784.7047	79.188545	99093.21 *
45-49	2023	771.2954	77.708981	99254.35 *
45-49	2024	760.5672	76.257062	99737.28 *
45-49	2025	752.3851	74.832271	100542.87 *
45-49	2026	774.3377	76.254634	101546.31 *
45-49	2027	769.8715	74.829888	102882.89 *
45-49	2028	767.2255	73.431762	104481.43 *
45-49	2029	765.6269	72.059758	106248.89 *
45-49	2030	764.5319	70.713390	108116.99 *
45-49	2031	727.5941	66.158789	109976.93 *
45-49	2032	726.3690	64.922674	111882.17 *
45-49	2033	725.0595	63.709655	113806.84 *
45-49	2034	723.5630	62.519301	115734.34 *
45-49	2035	721.6655	61.351186	117628.62 *
45-49	2036	710.9442	59.532590	119421.01 *
45-49	2037	708.0315	58.420279	121196.18 *
45-49	2038	703.8038	57.328751	122766.28 *
45-49	2039	696.7829	56.257618	123855.74 *
50-54	2018	1534.4212	144.194876	106413.02 *
50-54	2019	1481.6803	141.500730	104711.85 *
50-54	2020	1432.9389	138.856923	103195.35 *
50-54	2021	1396.3834	137.321482	101687.18 *
50-54	2022	1352.9343	134.755759	100399.00 *
50-54	2023	1313.8200	132.237975	99352.70 *
50-54	2024	1278.8741	129.767233	98551.39 *
50-54	2025	1248.0151	127.342654	98004.48 *
50-54	2026	1171.6834	120.091465	97565.92 *
50-54	2027	1148.3409	117.847669	97442.82 *
50-54	2028	1129.1659	115.645796	97640.03 *
50-54	2029	1114.0489	113.485063	98167.01 *
50-54	2030	1102.7684	111.364702	99023.15 *
50-54	2031	1135.5929	113.481449	100068.59 *
50-54	2032	1129.5264	111.361155	101429.12 *
50-54	2033	1126.0943	109.280477	103046.25 *
50-54	2034	1124.2233	107.238674	104833.76 *
50-54	2035	1123.0618	105.235021	106719.40 *
50-54	2036	1068.9860	98.456906	108574.00 *
50-54	2037	1067.3209	96.617331	110468.89 *
50-54	2038	1065.5525	94.812127	112385.68 *
50-54	2039	1063.5736	93.040652	114312.78 *
55-59	2018	2592.2118	240.679925	107703.70 *
55-59	2019	2539.9838	236.183047	107543.02 *
55-59	2020	2480.4196	231.770190	107020.65 *
55-59	2021	2301.3036	217.250123	105928.76 *
55-59	2022	2227.9772	213.191009	104506.15 *
55-59	2023	2152.8530	209.207736	102905.04 *
55-59	2024	2080.4351	205.298886	101336.89 *
55-59	2025	2013.4993	201.463070	99943.84 *
55-59	2026	1962.7720	199.235348	98515.25 *
55-59	2027	1902.5818	195.512823	97312.38 *
55-59	2028	1848.5837	191.859850	96350.73 *
55-59	2029	1800.5843	188.275130	95635.80 *
55-59	2030	1758.5073	184.757386	95179.27 *
55-59	2031	1652.6991	174.236867	94853.58 *
55-59	2032	1621.0755	170.981415	94810.04 *
55-59	2033	1595.0869	167.786789	95066.30 *
55-59	2034	1574.7344	164.651850	95640.25 *
55-59	2035	1559.7098	161.575486	96531.34 *
55-59	2036	1607.0304	164.646607	97604.83 *
55-59	2037	1599.0815	161.570340	98971.23 *
55-59	2038	1594.6998	158.551551	100579.26 *
55-59	2039	1592.4895	155.589164	102352.21 *
60-64	2018	3995.5692	410.140813	97419.45 *
60-64	2019	3989.7371	402.477719	99129.39 *
60-64	2020	3969.2809	394.957802	100498.86 *
60-64	2021	3796.8320	374.715004	101325.86 *

Results for PHIM for Run 1 (Basic_US_OG_T1.RTF)

60-64	2022	3746.8081	367.713808	101894.68 *
60-64	2023	3686.7935	360.843422	102171.56 *
60-64	2024	3615.5956	354.101402	102106.22 *
60-64	2025	3533.4541	347.485351	101686.42 *
60-64	2026	3280.1838	325.715896	100706.90 *
60-64	2027	3178.1234	319.630202	99431.26 *
60-64	2028	3073.5321	313.658213	97989.85 *
60-64	2029	2972.6986	307.797805	96579.59 *
60-64	2030	2879.6239	302.046893	95336.98 *
60-64	2031	2811.6457	298.706943	94127.23 *
60-64	2032	2729.0908	293.125885	93103.03 *
60-64	2033	2654.5715	287.649104	92285.06 *
60-64	2034	2588.0843	282.274651	91686.74 *
60-64	2035	2529.6475	277.000616	91322.81 *
60-64	2036	2379.6618	261.227550	91095.36 *
60-64	2037	2335.9424	256.346760	91124.32 *
60-64	2038	2299.8998	251.557162	91426.53 *
60-64	2039	2271.7013	246.857053	92024.97 *
65-69	2018	5369.7516	665.053161	80741.69 *
65-69	2019	5392.1253	652.627269	82621.82 *
65-69	2020	5421.4454	640.433544	84652.74 *
65-69	2021	5642.8860	652.643551	86461.99 *
65-69	2022	5658.9505	640.449521	88359.04 *
65-69	2023	5670.5153	628.483325	90225.39 *
65-69	2024	5667.2902	616.740707	91890.97 *
65-69	2025	5644.0985	605.217488	93257.36 *
65-69	2026	5402.6502	574.198237	94090.33 *
65-69	2027	5337.2420	563.469884	94720.98 *
65-69	2028	5258.3477	552.941980	95097.64 *
65-69	2029	5163.5309	542.610780	95160.86 *
65-69	2030	5052.7849	532.472609	94892.86 *
65-69	2031	4699.2978	499.113970	94152.80 *
65-69	2032	4561.1882	489.788496	93125.67 *
65-69	2033	4418.7141	480.637260	91934.49 *
65-69	2034	4280.9594	471.657005	90764.25 *
65-69	2035	4153.4058	462.844539	89736.52 *
65-69	2036	4059.6224	457.726533	88691.00 *
65-69	2037	3944.5471	449.174344	87817.73 *
65-69	2038	3840.6437	440.781945	87132.51 *
65-69	2039	3748.1439	432.546350	86653.00 *
70-74	2018	6622.8208	1097.903880	60322.41 *
70-74	2019	6848.6616	1077.390581	63567.12 *
70-74	2020	7026.0451	1057.260553	66455.19 *
70-74	2021	7553.8601	1100.708867	68627.23 *
70-74	2022	7614.5729	1080.143159	70495.96 *
70-74	2023	7654.3756	1059.961702	72213.70 *
70-74	2024	7696.8146	1040.157316	73996.64 *
70-74	2025	7752.0346	1020.722957	75946.51 *
70-74	2026	8076.2637	1040.183266	77642.70 *
70-74	2027	8110.4250	1020.748422	79455.67 *
70-74	2028	8140.7337	1001.676699	81271.07 *
70-74	2029	8152.2604	982.961314	82935.72 *
70-74	2030	8137.1646	964.595608	84358.30 *
70-74	2031	7806.0075	915.157128	85296.91 *
70-74	2032	7729.6604	898.058279	86070.81 *
70-74	2033	7634.8637	881.278906	86633.91 *
70-74	2034	7517.3545	864.813040	86924.62 *
70-74	2035	7375.7898	848.654824	86911.54 *
70-74	2036	6865.9779	795.487826	86311.54 *
70-74	2037	6672.0524	780.624886	85470.66 *
70-74	2038	6473.1545	766.039645	84501.56 *
70-74	2039	6282.6426	751.726916	83576.13 *
75-79	2018	7534.5469	1900.856584	39637.64 *
75-79	2019	7679.1849	1865.340870	41167.73 *
75-79	2020	7920.4991	1830.488734	43269.86 *
75-79	2021	8101.6938	1781.501975	45476.76 *
75-79	2022	8417.4831	1748.216289	48148.98 *
75-79	2023	8757.4152	1715.552515	51047.20 *
75-79	2024	9063.9369	1683.499033	53839.87 *
75-79	2025	9310.1724	1652.044440	56355.46 *
75-79	2026	10017.5459	1719.935506	58243.73 *
75-79	2027	10114.5541	1687.800131	59927.44 *
75-79	2028	10194.0157	1656.265176	61548.21 *
75-79	2029	10285.3805	1625.319422	63282.21 *
75-79	2030	10399.0925	1594.951860	65200.04 *
75-79	2031	10870.8610	1625.359971	66882.79 *
75-79	2032	10953.8317	1594.991651	68676.42 *
75-79	2033	11034.1940	1565.190735	70497.44 *

Results for PHIM for Run 1 (Basic_US_OG_T1.RTF)

75-79	2034	11092.9828	1535.946621	72222.45 *
75-79	2035	11118.8410	1507.248906	73769.11 *
75-79	2036	10678.5227	1429.997781	74675.10 *
75-79	2037	10591.3371	1403.279620	75475.60 *
75-79	2038	10484.7636	1377.060663	76138.72 *
75-79	2039	10351.9756	1351.331582	76605.74 *

Results for PHIM for Run 1 (Basic_US_OG_T1.RTF)

****
List of values created by last value brought forwards for Fixed File perm.Fixed_File_RR_STR

1. Country	US (United States)
2. Sex	M (Males)
3. Disease	STR (STR)

Age	Years	Value
10-14	2013-2039	2.4800
15-19	2013-2039	2.4800
20-24	2013-2039	2.4800
25-29	2013-2039	2.4800
30-34	2013-2039	2.4800
35-39	2013-2039	2.4800
40-44	2013-2039	2.4800
45-49	2013-2039	2.4800
50-54	2013-2039	2.4800
55-59	2013-2039	2.1300
60-64	2013-2039	2.1300
65-69	2013-2039	1.3900
70-74	2013-2039	1.3900
75-79	2013-2039	1.0600

Results for PHIM for Run 1 (Basic_US_OG_T1.RTF)

****

Osmond and Gardner Modeling of Death Rates for COD: COPD

Variable Parameter	Value
1. Country	US (United States)
2. Sex	M (Males)
3. Year of start of OG modelling	1966
4. Number 5-year periods for OG modelling	10
5. Age Range of interest	10 - 79
6. Scaling Factor for Rates	10000
7. Model Type to Save	4 (Full Age-Period-Cohort)
8. Goodness of Fit Required	1 (Analysis Printed For Age-Period-Cohort Model)
9. Cause of Death	COPD
Note:	Death rates are per million population

Matrix of Numbers of Deaths

Age	1966-	1971-	1976-	1981-	1986-	1991-	1996-	2001-	2006-	2011-
10-14	267	287	218	173	231	268	281	215	202	227
15-19	416	542	291	215	254	310	305	259	202	195
20-24	437	631	399	267	263	317	353	280	284	367
25-29	404	566	383	269	331	356	383	314	355	398
30-34	504	558	414	307	408	491	462	435	382	465
35-39	915	804	560	456	606	699	750	729	588	597
40-44	1835	1537	1012	733	1002	1110	1342	1514	1281	1147
45-49	3671	3433	2449	1661	1796	2134	2611	3210	3251	2765
50-54	7214	7320	5710	4496	4015	4242	4841	5962	7378	7716
55-59	13740	13684	11816	10606	9759	9024	9628	11109	13170	15798
60-64	21870	23643	21332	20221	20558	18927	17803	19254	22114	25867
65-69	27909	32315	33063	32301	33968	34283	32438	30017	32769	38421
70-74	30722	35740	38951	42983	46089	48515	50534	46485	44176	50217
75-79	26343	32488	35821	42842	50311	54265	59914	61191	56951	56626

Matrix of Age- and Period-Specific Mortality Rates

Age	1966-	1971-	1976-	1981-	1986-	1991-	1996-	2001-	2006-	2011-
10-14	5.094	5.379	4.352	3.760	5.205	5.752	5.491	3.922	3.739	4.275
15-19	8.836	10.321	5.316	4.145	5.376	6.747	6.202	4.814	3.577	3.490
20-24	11.190	13.509	7.555	4.810	5.041	6.653	7.423	5.517	5.216	6.327
25-29	12.281	14.124	8.040	5.021	5.955	6.782	7.791	6.468	6.967	7.185
30-34	17.041	16.663	10.239	6.368	7.551	8.693	8.526	8.613	7.765	8.961
35-39	30.751	27.321	17.326	11.481	12.592	12.934	13.102	13.312	11.577	11.964
40-44	58.613	51.968	35.006	22.979	25.240	23.257	24.836	26.547	23.490	22.504
45-49	120.868	112.111	86.822	58.949	57.074	54.903	55.078	59.906	57.620	51.042
50-54	263.783	251.382	196.898	166.812	146.077	138.153	125.725	126.961	139.844	138.993
55-59	563.662	536.407	435.567	389.099	379.938	343.526	323.457	295.228	288.439	308.462
60-64	1059.018	1084.515	912.464	810.318	813.973	789.096	711.599	675.981	613.715	594.111
65-69	1723.305	1834.467	1710.053	1544.370	1529.407	1515.348	1479.435	1290.429	1234.544	1143.199
70-74	2637.934	2829.851	2734.875	2719.217	2672.225	2606.527	2607.648	2423.250	2145.092	2114.217
75-79	3258.474	3918.592	3747.910	3961.724	4231.193	4114.992	4080.069	3940.601	3621.496	3287.501

Matrix of Log-Rates

Age	1966-	1971-	1976-	1981-	1986-	1991-	1996-	2001-	2006-	2011-
10-14	0.707	0.731	0.639	0.575	0.716	0.760	0.740	0.593	0.573	0.631
15-19	0.946	1.014	0.726	0.618	0.730	0.829	0.793	0.682	0.554	0.543
20-24	1.049	1.131	0.878	0.682	0.702	0.823	0.871	0.742	0.717	0.801
25-29	1.089	1.150	0.905	0.701	0.775	0.831	0.892	0.811	0.843	0.856
30-34	1.232	1.222	1.010	0.804	0.878	0.939	0.931	0.935	0.890	0.952
35-39	1.488	1.437	1.239	1.060	1.100	1.112	1.117	1.124	1.064	1.078
40-44	1.768	1.716	1.544	1.361	1.402	1.367	1.395	1.424	1.371	1.352
45-49	2.082	2.050	1.939	1.770	1.756	1.740	1.741	1.777	1.761	1.708
50-54	2.421	2.400	2.294	2.222	2.165	2.140	2.099	2.104	2.146	2.143
55-59	2.751	2.729	2.639	2.590	2.580	2.536	2.510	2.470	2.460	2.489
60-64	3.025	3.035	2.960	2.909	2.911	2.897	2.852	2.830	2.788	2.774
65-69	3.236	3.264	3.233	3.189	3.185	3.181	3.170	3.111	3.092	3.058
70-74	3.421	3.452	3.437	3.434	3.427	3.416	3.416	3.384	3.331	3.325
75-79	3.513	3.593	3.574	3.598	3.626	3.614	3.611	3.596	3.559	3.517

Results for PHIM for Run 1 (Basic_US_OG_T1.RTF)

Fitting the Age, Period, Cohort Models

**Model**	**RSS**	**MRSS**	**DF**	**Factor**	**%Account**	**ChiSq**	**P**
Age Only	7884.413	62.082	127	P, C	96.3998	42140.547	0.0000
Age-Period	3114.209	26.617	117	Cohort	90.8852	16604.161	0.0000
Age-Cohort	1247.837	11.998	104	Period	77.2523	6641.523	0.0000
Period-Cohort	405.389	3.754	108	Age	29.9799	2161.586	0.0000
Full Age-Period-Cohort	283.854	2.957	96			1510.971	0.0000

**Key to terms:**	RSS =	residual sum of squares
	MRSS =	mean RSS (MRSS/DF)
	DF =	degrees of freedom
	Factor =	Factors not included in the model
	% Account =	1- (RSS for full model)/(RSS for model in question)
	Chisq =	chi-squared value for model
	P =	probability value based on Chisq and DF.

**Age**	**Value**	**Log10 Value**
10-	5.824736	0.765276
15-	7.235710	0.859481
20-	8.822176	0.945576
25-	9.352002	0.970905
30-	11.263621	1.051678
35-	17.594330	1.245373
40-	33.243499	1.521707
45-	73.358044	1.865448
50-	168.720617	2.227168
55-	374.865700	2.573876
60-	776.076978	2.889905
65-	1450.47739	3.161511
70-	2503.37134	3.398525
75-	3901.65444	3.591249

**Period**	**Value**	**Log10 Value**
1966	1.356373	0.132379
1971	1.278244	0.106614
1976	1.063219	0.026623
1981	0.959791	-0.017824
1986	0.953915	-0.020490
1991	0.943870	-0.025088
1996	0.946464	-0.023896
2001	0.920809	-0.035830
2006	0.900375	-0.045576
2011	0.905857	-0.042940

**Cohort**	**Value**	**Log10 Value**
1891	0.615724	-0.210614
1896	0.781415	-0.107118
1901	0.888804	-0.051194
1906	1.024254	0.010408
1911	1.121068	0.049632
1916	1.116506	0.047861
1921	1.106274	0.043863
1926	1.102656	0.042440
1931	1.055639	0.023516
1936	0.949213	-0.022636
1941	0.931652	-0.030746
1946	0.862958	-0.064010
1951	0.839860	-0.075793
1956	0.892401	-0.049440
1961	0.865239	-0.062864
1966	0.762808	-0.117585
1971	0.772407	-0.112154
1976	0.823944	-0.084102
1981	0.859102	-0.065955
1986	0.801539	-0.096075
1991	0.704906	-0.151869
1996	0.617647	-0.209259
2001	0.810158	-0.091431

Results for PHIM for Run 1 (Basic_US_OG_T1.RTF)

Model: Full Age-Period-Cohort

Basic Analysis Using OG Modelling T1 on US
Fitting the Full Age-Period-Cohort Model
Matrix of observed, expected, and residual rates

**Age**		**1966-**	**1971-**	**1976-**	**1981-**	**1986-**	**1991-**	**1996-**	**2001-**	**2006-**	**2011-**
10-	Observed	5.094	5.379	4.352	3.760	5.205	5.752	5.491	3.922	3.739	4.275
	Expected	7.050	6.442	4.724	4.318	4.578	4.723	4.419	3.781	3.239	4.275
	Residual	1.956	1.063	0.372	0.558	-0.627	-1.029	-1.073	-0.141	-0.499	-0.000
15-	Observed	8.836	10.321	5.316	4.145	5.376	6.747	6.202	4.814	3.577	3.490
	Expected	8.243	8.254	6.656	5.298	5.331	5.627	5.883	5.340	4.592	4.048
	Residual	-0.593	-2.067	1.340	1.153	-0.045	-1.120	-0.319	0.527	1.015	0.559
20-	Observed	11.190	13.509	7.555	4.810	5.041	6.653	7.423	5.517	5.216	6.327
	Expected	10.326	9.471	8.371	7.326	6.419	6.432	6.880	6.979	6.367	5.633
	Residual	-0.864	-4.038	0.816	2.517	1.379	-0.221	-0.543	1.462	1.151	-0.693
25-	Observed	12.281	14.124	8.040	5.021	5.955	6.782	7.791	6.468	6.967	7.185
	Expected	11.818	10.316	8.351	8.010	7.719	6.733	6.837	7.095	7.234	6.790
	Residual	-0.463	-3.808	0.311	2.990	1.763	-0.049	-0.954	0.628	0.267	-0.395
30-	Observed	17.041	16.663	10.239	6.368	7.551	8.693	8.526	8.613	7.765	8.961
	Expected	14.502	13.414	10.335	9.079	9.588	9.199	8.132	8.011	8.356	8.766
	Residual	-2.540	-3.250	0.096	2.711	2.038	0.505	-0.394	-0.602	0.591	-0.195
35-	Observed	30.751	27.321	17.326	11.481	12.592	12.934	13.102	13.312	11.577	11.964
	Expected	25.192	21.348	17.428	14.573	14.096	14.820	14.408	12.358	12.236	13.132
	Residual	-5.558	-5.974	0.102	3.091	1.504	1.886	1.306	-0.954	0.659	1.168
40-	Observed	58.613	51.968	35.006	22.979	25.240	23.257	24.836	26.547	23.490	22.504
	Expected	49.719	44.858	33.550	29.726	27.366	26.353	28.078	26.486	22.832	23.260
	Residual	-8.893	-7.110	-1.456	6.747	2.125	3.096	3.242	-0.061	-0.658	0.756
45-	Observed	120.868	112.111	86.822	58.949	57.074	54.903	55.078	59.906	57.620	51.042
	Expected	110.075	103.395	82.335	66.833	65.194	59.752	58.312	60.281	57.149	50.690
	Residual	-10.793	-8.715	-4.487	7.883	8.120	4.849	3.234	0.375	-0.471	-0.352
50-	Observed	263.783	251.382	196.898	166.812	146.077	138.153	125.725	126.961	139.844	138.993
	Expected	255.510	238.586	197.802	170.947	152.771	148.366	137.804	130.480	135.566	132.240
	Residual	-8.273	-12.796	0.904	4.134	6.695	10.213	12.079	3.519	-4.278	-6.753
55-	Observed	563.662	536.407	435.567	389.099	379.938	343.526	323.457	295.228	288.439	308.462
	Expected	570.016	534.996	440.921	396.727	377.486	335.855	330.547	297.876	283.470	303.037
	Residual	6.354	-1.411	5.354	7.629	-2.452	-7.671	7.090	2.648	-4.970	-5.425
60-	Observed	1059.018	1084.515	912.464	810.318	813.973	789.096	711.599	675.981	613.715	594.111
	Expected	1078.181	1112.117	921.273	824.032	816.308	773.273	697.224	665.776	603.001	590.434
	Residual	19.164	27.602	8.809	13.714	2.335	-15.823	-14.375	-10.205	-10.714	-3.676
65-	Observed	1723.305	1834.467	1710.053	1544.370	1529.407	1515.348	1479.435	1290.429	1234.544	1143.199
	Expected	1748.623	1899.033	1728.882	1554.348	1530.676	1509.605	1449.208	1267.781	1216.713	1133.862
	Residual	25.318	64.566	18.830	9.979	1.268	-5.743	-30.228	-22.648	-17.831	-9.337
70-	Observed	2637.934	2829.851	2734.875	2719.217	2672.225	2606.527	2607.648	2423.250	2145.092	2114.217
	Expected	2653.301	2844.101	2726.187	2693.603	2666.218	2613.968	2612.578	2433.383	2139.501	2112.703
	Residual	15.367	14.251	-8.687	-25.614	-6.007	7.441	4.930	10.134	-5.592	-1.514
75-	Observed	3258.474	3918.592	3747.910	3961.724	4231.193	4114.992	4080.069	3940.601	3621.496	3287.501
	Expected	3258.474	3897.127	3687.036	3835.598	4172.441	4111.706	4085.221	3961.489	3708.412	3354.842
	Residual	-0.000	-21.465	-60.873	-126.125	-58.752	-3.286	5.152	20.888	86.915	67.341

Fitting the Full Age-Period-Cohort Model

Results for PHIM for Run 1 (Basic_US_OG_T1.RTF)

Matrix of observed and expected deaths and (O-E)2/E Values**

Age		1966-	1971-	1976-	1981-	1986-	1991-	1996-	2001-	2006-	2011-	Total
10-	Observed	267.0	287.0	218.0	173.0	231.0	268.0	281.0	215.0	202.0	227.0	2369.0
	Expected	369.5	343.7	236.6	198.7	203.2	220.1	226.1	207.3	175.0	227.0	2407.2
	Difference	-102.5	-56.7	-18.6	-25.7	27.8	47.9	54.9	7.7	27.0	0.0	-38.2
	Chi-Sq	28.5	9.4	1.5	3.3	3.8	10.4	13.3	0.3	4.2	0.0	74.6
15-	Observed	416.0	542.0	291.0	215.0	254.0	310.0	305.0	259.0	202.0	195.0	2989.0
	Expected	388.1	433.5	364.4	274.8	251.9	258.6	289.3	287.3	259.3	226.2	3033.3
	Difference	27.9	108.5	-73.4	-59.8	2.1	51.4	15.7	-28.3	-57.3	-31.2	-44.3
	Chi-Sq	2.0	27.2	14.8	13.0	0.0	10.2	0.8	2.8	12.7	4.3	87.8
20-	Observed	437.0	631.0	399.0	267.0	263.0	317.0	353.0	280.0	284.0	367.0	3598.0
	Expected	403.3	442.4	442.1	406.7	334.9	306.5	327.2	354.2	346.7	326.8	3690.7
	Difference	33.7	188.6	-43.1	-139.7	-71.9	10.5	25.8	-74.2	-62.7	40.2	-92.7
	Chi-Sq	2.8	80.4	4.2	48.0	15.5	0.4	2.0	15.5	11.3	4.9	185.1
25-	Observed	404.0	566.0	383.0	269.0	331.0	356.0	383.0	314.0	355.0	398.0	3759.0
	Expected	388.8	413.4	397.8	429.2	429.0	353.4	336.1	344.5	368.6	376.1	3836.9
	Difference	15.2	152.6	-14.8	-160.2	-98.0	2.6	46.9	-30.5	-13.6	21.9	-77.9
	Chi-Sq	0.6	56.3	0.6	59.8	22.4	0.0	6.5	2.7	0.5	1.3	150.7
30-	Observed	504.0	558.0	414.0	307.0	408.0	491.0	462.0	435.0	382.0	465.0	4426.0
	Expected	428.9	449.2	417.9	437.7	518.1	519.5	440.6	404.6	411.1	454.9	4482.5
	Difference	75.1	108.8	-3.9	-130.7	-110.1	-28.5	21.4	30.4	-29.1	10.1	-56.5
	Chi-Sq	13.2	26.4	0.0	39.0	23.4	1.6	1.0	2.3	2.1	0.2	109.2
35-	Observed	915.0	804.0	560.0	456.0	606.0	699.0	750.0	729.0	588.0	597.0	6704.0
	Expected	749.6	628.2	563.3	578.8	678.4	800.9	824.8	676.8	621.5	655.3	6777.5
	Difference	165.4	175.8	-3.3	-122.8	-72.4	-101.9	-74.8	52.2	-33.5	-58.3	-73.5
	Chi-Sq	36.5	49.2	0.0	26.0	7.7	13.0	6.8	4.0	1.8	5.2	150.2
40-	Observed	1835.0	1537.0	1012.0	733.0	1002.0	1110.0	1342.0	1514.0	1281.0	1147.0	12513.0
	Expected	1556.6	1326.7	969.9	948.2	1086.4	1257.8	1517.2	1510.5	1245.1	1185.5	12603.9
	Difference	278.4	210.3	42.1	-215.2	-84.4	-147.8	-175.2	3.5	35.9	-38.5	-90.9
	Chi-Sq	49.8	33.3	1.8	48.8	6.6	17.4	20.2	0.0	1.0	1.3	180.2
45-	Observed	3671.0	3433.0	2449.0	1661.0	1796.0	2134.0	2611.0	3210.0	3251.0	2765.0	26981.0
	Expected	3343.2	3166.1	2322.4	1883.1	2051.5	2322.5	2764.3	3230.1	3224.4	2745.9	27053.6
	Difference	327.8	266.9	126.6	-222.1	-255.5	-188.5	-153.3	-20.1	26.6	19.1	-72.6
	Chi-Sq	32.1	22.5	6.9	26.2	31.8	15.3	8.5	0.1	0.2	0.1	143.8
50-	Observed	7214.0	7320.0	5710.0	4496.0	4015.0	4242.0	4841.0	5962.0	7378.0	7716.0	58894.0
	Expected	6987.7	6947.4	5736.2	4607.4	4199.0	4555.6	5306.1	6127.2	7152.3	7341.1	58960.2
	Difference	226.3	372.6	-26.2	-111.4	-184.0	-313.6	-465.1	-165.2	225.7	374.9	-66.2
	Chi-Sq	7.3	20.0	0.1	2.7	8.1	21.6	40.8	4.5	7.1	19.1	131.3
55-	Observed	13740.0	13684.0	11816.0	10606.0	9759.0	9024.0	9628.0	11109.0	13170.0	15798.0	118334.0
	Expected	13894.9	13648.0	11961.3	10813.9	9696.0	8822.5	9839.0	11208.6	12943.1	15520.1	118347.5
	Difference	-154.9	36.0	-145.3	-207.9	63.0	201.5	-211.0	-99.6	226.9	277.9	-13.5
	Chi-Sq	1.7	0.1	1.8	4.0	0.4	4.6	4.5	0.9	4.0	5.0	27.0
60-	Observed	21870.0	23643.0	21332.0	20221.0	20558.0	18927.0	17803.0	19254.0	22114.0	25867.0	211589.0
	Expected	22265.8	24244.7	21538.0	20563.2	20617.0	18547.5	17443.4	18963.3	21727.9	25706.9	211617.7
	Difference	-395.8	-601.7	-206.0	-342.2	-59.0	379.5	359.6	290.7	386.1	160.1	-28.7
	Chi-Sq	7.0	14.9	2.0	5.7	0.2	7.8	7.4	4.5	6.9	1.0	57.3
65-	Observed	27909.0	32315.0	33063.0	32301.0	33968.0	34283.0	32438.0	30017.0	32769.0	38421.0	327484.0
	Expected	28319.0	33452.4	33427.1	32509.7	33996.2	34153.1	31775.2	29490.2	32295.7	38107.2	327525.7
	Difference	-410.0	-1137.4	-364.1	-208.7	-28.2	129.9	662.8	526.8	473.3	313.8	-41.7
	Chi-Sq	5.9	38.7	4.0	1.3	0.0	0.5	13.8	9.4	6.9	2.6	83.2
70-	Observed	30722.0	35740.0	38951.0	42983.0	46089.0	48515.0	50534.0	46485.0	44176.0	50217.0	434412.0
	Expected	30901.0	35920.0	38827.3	42578.1	45985.4	48653.5	50629.5	46679.4	44060.8	50181.0	434416.1

Results for PHIM for Run 1 (Basic_US_OG_T1.RTF)

	Difference	-179.0	-180.0	123.7	404.9	103.6	-138.5	-95.5	-194.4	115.2	36.0	-4.1
	Chi-Sq	1.0	0.9	0.4	3.8	0.2	0.4	0.2	0.8	0.3	0.0	8.1
75-	Observed	26343.0	32488.0	35821.0	42842.0	50311.0	54265.0	59914.0	61191.0	56951.0	56626.0	476752.0
	Expected	26343.0	32310.0	35239.2	41478.1	49612.4	54221.7	59989.7	61515.4	58317.8	57785.9	476813.1
	Difference	0.0	178.0	581.8	1363.9	698.6	43.3	-75.7	-324.4	-1366.8	-1159.9	-61.1
	Chi-Sq	0.0	1.0	9.6	44.8	9.8	0.0	0.1	1.7	32.0	23.3	122.4
Total over ages	Observed	136247.0	153548.0	152419.0	157530.0	169591.0	174941.0	181645.0	180974.0	183103.0	200806.0	1690804.0
	Expected	136339.3	153725.7	152443.4	157707.7	169659.4	174993.0	181708.6	180999.3	183149.4	200840.2	1691565.9
	Difference	-92.3	-177.7	-24.4	-177.7	-68.4	-52.0	-63.6	-25.3	-46.4	-34.2	-761.9
	Chi-Sq	188.5	380.2	47.6	326.6	129.9	103.1	126.1	49.5	91.0	68.3	1511.0

Results for PHIM for Run 1 (Basic_US_OG_T1.RTF)

****

Osmond and Gardner Extrapolating Death Rates for COD: COPD

Variable Parameter	Value
1. Country	US (United States)
2. Sex	M (Males)
3. Year of start of OG modelling	1966
4. Number 5-year periods for OG modelling	10
5. Age Range of interest	10 - 79
6. Scaling Factor for Rates	10000
7. Model Type to Save	4 (Full Age-Period-Cohort)
8. Goodness of Fit Required	1 (Analysis Printed For Age-Period-Cohort Model)
9. Cause of Death	COPD
Note:	Death rates are per million population
10. Number of Periods into the future to Predict	5
11. Earliest projected year	2016
12. Extrapolate Period using (1: last 2 points 2: linear regression)	1
13. Ratio of last two period values	1.00609

Predictions of rates for future years from model:	Full Age-Period-Cohort
Effects for extending model to project rates for:	2016-2040

Extrapolating Model: Full Age-Period-Cohort
Log Transform Parameters

Model	ChiSq	MChiSq	DF	Factor	%Account	P
Age Only	16476.412	1176.887	14	P, C	94.1815	0.0000
Age-Period	466.129	33.295	14	Cohort	-105.6690	0.0000
Age-Cohort	853.636	60.974	14	Period	-12.3057	0.0000
Period-Cohort	693.826	49.559	14	Age	-38.1733	0.0000
Full Age-Period-Cohort	958.682	68.477	14			0.0000

**Key to terms:**	Chisq =	chi-squared value for model
	MChisq =	mean Chi-squared (Chisq/DF)
	DF =	degrees of freedom
	Factor =	Factors not included in the model
	% Account =	1- (Chisq for full model)/(Chisq for model in question)
	P =	probability value based on Chisq and DF.

AGE	EFFECT
10	5.824736
15	7.235710
20	8.822176
25	9.352002
30	11.263621
35	17.594330
40	33.243499
45	73.358044
50	168.720617
55	374.865700
60	776.076978
65	1450.47739
70	2503.37134
75	3901.65444

PERIOD	EFFECT	
Period Change =	1.006088	
1966	1.356373	
1971	1.278244	
1976	1.063219	
1981	0.959791	
1986	0.953915	
1991	0.943870	
1996	0.946464	
2001	0.920809	
2006	0.900375	
2011	0.905857	
2016	0.911372	
2021	0.916921	
2026	0.922503	
2031	0.928119	
2036	0.933770	
2016	0.909162	Extrapolated
2017	0.910266	Extrapolated
2018	0.911372	Extrapolated
2019	0.912479	Extrapolated

Results for PHIM for Run 1 (Basic_US_OG_T1.RTF)

2020	0.913587	Extrapolated
2021	0.914697	Extrapolated
2022	0.915808	Extrapolated
2023	0.916921	Extrapolated
2024	0.918034	Extrapolated
2025	0.919149	Extrapolated
2026	0.920266	Extrapolated
2027	0.921384	Extrapolated
2028	0.922503	Extrapolated
2029	0.923623	Extrapolated
2030	0.924745	Extrapolated
2031	0.925869	Extrapolated
2032	0.926993	Extrapolated
2033	0.928119	Extrapolated
2034	0.929247	Extrapolated
2035	0.930375	Extrapolated
2036	0.931506	Extrapolated
2037	0.932637	Extrapolated
2038	0.933770	Extrapolated
2039	0.934904	Extrapolated
2040	0.936040	Extrapolated

Results for PHIM for Run 1 (Basic_US_OG_T1.RTF)

COHORT	EFFECT	WEIGHT	ORIGINAL
1891	0.615724	1.000	
1896	0.781415	2.000	
1901	0.888804	4.000	
1906	1.024254	8.000	
1911	1.121068	16.000	
1916	1.116506	32.000	
1921	1.106274	64.000	
1926	1.102656	128.000	
1931	1.055639	256.000	
1936	0.949213	512.000	
1941	0.931652	1024.000	
1946	0.862958	2048.000	
1951	0.839860	4096.000	
1956	0.892401	8192.000	
1961	0.865239	16384.000	
1966	0.762808	32768.000	
1971	0.772407	65536.000	
1976	0.823944	131072.000	
1981	0.859102	262144.000	
1986	0.801539	524288.000	
1991	0.704906	1048576.000	
1996	0.701827	Extrapolated	0.617647
2001	0.674834	Extrapolated	0.810158
2006	0.648879	Extrapolated	
2011	0.623922	Extrapolated	
2016	0.599926	Extrapolated	
2021	0.576852	Extrapolated	
2026	0.554665	Extrapolated	

Standardizing Population: The 1976 European Standard Population

Age Range	Population, Males
All	100000
0	0
1	0
2	0
3	0
0-4	8000
5-9	7000
10-14	7000
15-19	7000
20-24	7000
25-29	7000
30-34	7000
35-39	7000
40-44	7000
45-49	7000
50-54	7000
55-59	6000
60-64	5000
65-69	4000
70-74	3000
75-79	2000
80-84	1000
85+	1000

Results for PHIM for Run 1 (Basic_US_OG_T1.RTF)

Matrix of observed and expected rates including predictions
Total over ages standardized using: The 1976 European Standard Population

		1966-	1971-	1976-	1981-	1986-	1991-	1996-	2001-	2006-	2011-	2016-	2021-	2026-	2031-	2036-
10	OBS	5.1	5.4	4.4	3.8	5.2	5.8	5.5	3.9	3.7	4.3	4.4	.	.	.	.
	EXP											3.4	3.3	3.2	3.1	3.0
15	OBS	8.8	10.3	5.3	4.1	5.4	6.7	6.2	4.8	3.6	3.5	4.3	.	.	.	.
	EXP											4.5	4.3	4.2	4.0	3.9
20	OBS	11.2	13.5	7.6	4.8	5.0	6.7	7.4	5.5	5.2	6.3	6.4	.	.	.	.
	EXP											5.6	5.5	5.3	5.1	4.9
25	OBS	12.3	14.1	8.0	5.0	6.0	6.8	7.8	6.5	7.0	7.2	7.4	.	.	.	.
	EXP											6.0	6.0	5.8	5.6	5.4
30	OBS	17.0	16.7	10.2	6.4	7.6	8.7	8.5	8.6	7.8	9.0	9.2	.	.	.	.
	EXP											8.2	7.3	7.3	7.1	6.8
35	OBS	30.8	27.3	17.3	11.5	12.6	12.9	13.1	13.3	11.6	12.0	12.7	.	.	.	.
	EXP											13.8	12.9	11.4	11.5	11.1
40	OBS	58.6	52.0	35.0	23.0	25.2	23.3	24.8	26.5	23.5	22.5	21.1	.	.	.	.
	EXP											25.0	26.2	24.6	21.7	21.8
45	OBS	120.9	112.1	86.8	58.9	57.1	54.9	55.1	59.9	57.6	51.0	46.9	.	.	.	.
	EXP											51.6	55.4	58.1	54.6	48.3
50	OBS	263.8	251.4	196.9	166.8	146.1	138.2	125.7	127.0	139.8	139.0	132.6	.	.	.	.
	EXP											117.3	119.5	128.2	134.5	126.3
55	OBS	563.7	536.4	435.6	389.1	379.9	343.5	323.5	295.2	288.4	308.5	312.8	.	.	.	.
	EXP											295.6	262.2	267.1	286.7	300.7
60	OBS	1059.0	1084.5	912.5	810.3	814.0	789.1	711.6	676.0	613.7	594.1	607.3	.	.	.	.
	EXP											631.2	615.7	546.1	556.4	597.1
65	OBS	1723.3	1834.5	1710.1	1544.4	1529.4	1515.3	1479.4	1290.4	1234.5	1143.2	1067.5	.	.	.	.
	EXP											1110.2	1186.9	1157.7	1026.9	1046.2
70	OBS	2637.9	2829.9	2734.9	2719.2	2672.2	2606.5	2607.6	2423.2	2145.1	2114.2	1884.4	.	.	.	.
	EXP											1968.8	1927.8	2060.9	2010.3	1783.1
75	OBS	3258.5	3918.6	3747.9	3961.7	4231.2	4115.0	4080.1	3940.6	3621.5	3287.5	3032.7	.	.	.	.
	EXP											3312.8	3087.2	3022.9	3231.6	3152.3
10-79	OBS	406.0	431.6	389.4	369.1	371.5	361.0	351.6	328.4	304.0	290.3	272.5	.	.	.	.
	EXP	406.4	432.4	389.2	369.7	371.9	361.0	351.4	328.1	303.9	290.3	283.7*	277.6*	276.3*	275.1*	268.1*

Drop in overall standardized Observed and Predicted rates
comparing the last observed rate during the model fitting period to the last observed and predicted rates where an observed rate is available (2016)
Observed and Predicted %Drop = 6.124% and 2.258%

Results for PHIM for Run 1 (Basic_US_OG_T1.RTF)

Matrix of observed and expected deaths including predictions

		1966-	1971-	1976-	1981-	1986-	1991-	1996-	2001-	2006-	2011-	2016-	2021-	2026-	2031-	2036-
10-	OBS	267.0	287.0	218.0	173.0	231.0	268.0	281.0	215.0	202.0	227.0	237.5*	.	.	.	.
	EXP	369.5	343.7	236.6	198.7	203.2	220.1	226.1	207.3	198.9	189.1	185.2*	175.4*	164.1*	161.5*	161.2*
	ChiSq	28.450	9.364	1.465	3.317	3.811	10.437	13.323	0.288	0.049	7.603	14.783*	.	.	.	.
15-	OBS	416.0	542.0	291.0	215.0	254.0	310.0	305.0	259.0	202.0	195.0	235.0*	.	.	.	.
	EXP	388.1	433.5	364.4	274.8	251.9	258.6	289.3	287.3	259.3	257.1	241.5*	236.3*	224.0*	210.2*	207.1*
	ChiSq	2.012	27.178	14.777	13.005	0.018	10.234	0.850	2.797	12.667	14.982	0.173*	.	.	.	.
20-	OBS	437.0	631.0	399.0	267.0	263.0	317.0	353.0	280.0	284.0	367.0	372.5*	.	.	.	.
	EXP	403.3	442.4	442.1	406.7	334.9	306.5	327.2	354.2	346.7	326.8	326.0*	306.1*	300.0*	285.5*	268.7*
	ChiSq	2.821	80.426	4.197	47.996	15.453	0.362	2.038	15.533	11.336	4.948	6.620*	.	.	.	.
25-	OBS	404.0	566.0	383.0	269.0	331.0	356.0	383.0	314.0	355.0	398.0	445.0*	.	.	.	.
	EXP	388.8	413.4	397.8	429.2	429.0	353.4	336.1	344.5	368.6	376.1	359.0*	357.4*	336.4*	330.5*	315.3*
	ChiSq	0.597	56.325	0.551	59.780	22.392	0.019	6.548	2.695	0.500	1.270	20.584*	.	.	.	.
30-	OBS	504.0	558.0	414.0	307.0	408.0	491.0	462.0	435.0	382.0	465.0	522.5*	.	.	.	.
	EXP	428.9	449.2	417.9	437.7	518.1	519.5	440.6	404.6	411.1	454.9	465.8*	442.4*	440.8*	416.3*	409.3*
	ChiSq	13.154	26.366	0.036	39.030	23.402	1.569	1.035	2.284	2.054	0.225	6.911*	.	.	.	.
35-	OBS	915.0	804.0	560.0	456.0	606.0	699.0	750.0	729.0	588.0	597.0	667.5*	.	.	.	.
	EXP	749.6	628.2	563.3	578.8	678.4	800.9	824.8	676.8	621.5	655.3	724.2*	738.4*	700.9*	699.8*	661.9*
	ChiSq	36.491	49.190	0.019	26.046	7.723	12.970	6.781	4.031	1.803	5.187	4.442*	.	.	.	.
40-	OBS	1835.0	1537.0	1012.0	733.0	1002.0	1110.0	1342.0	1514.0	1281.0	1147.0	1060.0*	.	.	.	.
	EXP	1556.6	1326.7	969.9	948.2	1086.4	1257.8	1517.2	1510.5	1245.1	1185.5	1251.2*	1378.7*	1405.3*	1335.4*	1334.8*
	ChiSq	49.800	33.330	1.826	48.844	6.553	17.358	20.232	0.008	1.035	1.252	29.227*	.	.	.	.
45-	OBS	3671.0	3433.0	2449.0	1661.0	1796.0	2134.0	2611.0	3210.0	3251.0	2765.0	2380.0*	.	.	.	.
	EXP	3343.2	3166.1	2322.4	1883.1	2051.5	2322.5	2764.3	3230.1	3224.4	2745.9	2618.2*	2759.3*	3042.2*	3105.3*	2952.9*
	ChiSq	32.139	22.495	6.898	26.201	31.826	15.293	8.501	0.125	0.219	0.133	21.674*	.	.	.	.
50-	OBS	7214.0	7320.0	5710.0	4496.0	4015.0	4242.0	4841.0	5962.0	7378.0	7716.0	7057.5*	.	.	.	.
	EXP	6987.7	6947.4	5736.2	4607.4	4199.0	4555.6	5306.1	6127.2	7152.3	7341.1	6241.1*	5950.7*	6281.8*	6943.0*	7096.4*
	ChiSq	7.326	19.983	0.120	2.695	8.063	21.587	40.768	4.456	7.121	19.143	106.797*	.	.	.	.
55-	OBS	13740.0	13684.0	11816.0	10606.0	9759.0	9024.0	9628.0	11109.0	13170.0	15798.0	16790.0*	.	.	.	.
	EXP	13894.9	13648.0	11961.3	10813.9	9696.0	8822.5	9839.0	11208.6	12943.1	15520.1	15865.3*	13493.0*	12901.2*	13671.2*	15148.3*
	ChiSq	1.726	0.095	1.764	3.999	0.409	4.602	4.526	0.886	3.978	4.975	53.900*	.	.	.	.
60-	OBS	21870.0	23643.0	21332.0	20221.0	20558.0	18927.0	17803.0	19254.0	22114.0	25867.0	29512.5*	.	.	.	.
	EXP	22265.8	24244.7	21538.0	20563.2	20617.0	18547.5	17443.4	18963.3	21727.9	25706.9	30671.1*	31350.7*	26762.3*	25733.0*	27382.7*
	ChiSq	7.034	14.934	1.969	5.695	0.169	7.766	7.415	4.455	6.859	0.997	43.764*	.	.	.	.
65-	OBS	27909.0	32315.0	33063.0	32301.0	33968.0	34283.0	32438.0	30017.0	32769.0	38421.0	43087.5*	.	.	.	.
	EXP	28319.0	33452.4	33427.1	32509.7	33996.2	34153.1	31775.2	29490.2	32295.7	38107.2	44811.9*	53432.2*	54873.0*	47208.2*	45684.9*
	ChiSq	5.937	38.670	3.965	1.340	0.023	0.494	13.824	9.411	6.936	2.584	66.357*	.	.	.	.

Results for PHIM for Run 1 (Basic_US_OG_T1.RTF)

70-	OBS	30722.0	35740.0	38951.0	42983.0	46089.0	48515.0	50534.0	46485.0	44176.0	50217.0	56737.5*	.	.	.	.
	EXP	30901.0	35920.0	38827.3	42578.1	45985.4	48653.5	50629.5	46679.4	44060.8	50181.0	59280.6*	69647.9*	83602.5*	86813.2*	75365.9*
	ChiSq	1.037	0.902	0.394	3.850	0.233	0.394	0.180	0.810	0.301	0.026	109.101*	.	.	.	.
75-	OBS	26343.0	32488.0	35821.0	42842.0	50311.0	54265.0	59914.0	61191.0	56951.0	56626.0	60735.0*	.	.	.	.
	EXP	26343.0	32310.0	35239.2	41478.1	49612.4	54221.7	59989.7	61515.4	58317.8	57785.9	66344.9*	78683.9*	93166.3*	113766.6*	119703.1*
	ChiSq	.	0.980	9.606	44.850	9.837	0.035	0.095	1.710	32.034	23.283	474.350*	.	.	.	.
Total Deaths		136247.0	153548.0	152419.0	157530.0	169591.0	174941.0	181645.0	180974.0	183103.0	200806.0	219840.0*	.	.	.	.
Expected		136339.3	153725.7	152443.4	157707.7	169659.4	174993.0	181708.6	180999.3	183173.2	200833.1	229386.0*	258952.4*	284200.8*	300679.7*	296692.4*
Obs/Exp		0.999	0.999	1.000	0.999	1.000	1.000	1.000	1.000	1.000	1.000	0.958*	.	.	.	.

Chi Squared (Log) = **958.7 on 14 D.F.** **P = 0.0000**

Results for PHIM for Run 1 (Basic_US_OG_T1.RTF)

****

Observed and predicted rates (5 year periods)

		10-	15-	20-	25-	30-	35-	40-	45-	50-	55-	60-	65-	70-	75-
2016-	OBS	4.4	4.3	6.4	7.4	9.2	12.7	21.1	46.9	132.6	312.8	607.3	1067.5	1884.4	3032.7
	PRE	3.4	4.5	5.6	6.0	8.2	13.8	25.0	51.6	117.3	295.6	631.2	1110.2	1968.8	3312.8
	RES	0.973	-0.119	0.804	1.439	1.002	-1.079	-3.815	-4.698	15.344	17.230	-23.843	-42.723	-84.463	-280.120
2021-	PRE	3.3	4.3	5.5	6.0	7.3	12.9	26.2	55.4	119.5	262.2	615.7	1186.9	1927.8	3087.2
2026-	PRE	3.2	4.2	5.3	5.8	7.3	11.4	24.6	58.1	128.2	267.1	546.1	1157.7	2060.9	3022.9
2031-	PRE	3.1	4.0	5.1	5.6	7.1	11.5	21.7	54.6	134.5	286.7	556.4	1026.9	2010.3	3231.6
2036-	PRE	3.0	3.9	4.9	5.4	6.8	11.1	21.8	48.3	126.3	300.7	597.1	1046.2	1783.1	3152.3

Results for PHIM for Run 1 (Basic_US_OG_T1.RTF)

****

Observed and predicted deaths (5 year periods)

		10-	15-	20-	25-	30-	35-	40-	45-	50-	55-	60-	65-	70-	75-
2016-	OBS	237.5	235.0	372.5	445.0	522.5	667.5	1060.0	2380.0	7057.5	16790.0	29512.5	43087.5	56737.5	60735.0
	PRE	185.2	241.5	326.0	359.0	465.8	724.2	1251.2	2618.2	6241.1	15865.3	30671.1	44811.9	59280.6	66344.9
	CHI	14.783	0.173	6.620	20.584	6.911	4.442	29.227	21.674	106.797	53.900	43.764	66.357	109.101	474.350
2021-	PRE	175.4	236.3	306.1	357.4	442.4	738.4	1378.7	2759.3	5950.7	13493.0	31350.7	53432.2	69647.9	78683.9
2026-	PRE	164.1	224.0	300.0	336.4	440.8	700.9	1405.3	3042.2	6281.8	12901.2	26762.3	54873.0	83602.5	93166.3
2031-	PRE	161.5	210.2	285.5	330.5	416.3	699.8	1335.4	3105.3	6943.0	13671.2	25733.0	47208.2	86813.2	113766.6
2036-	PRE	161.2	207.1	268.7	315.3	409.3	661.9	1334.8	2952.9	7096.4	15148.3	27382.7	45684.9	75365.9	119703.1

Results for PHIM for Run 1 (Basic_US_OG_T1.RTF)

****

Observed and predicted rates (single years)

		10-	15-	20-	25-	30-	35-	40-	45-	50-	55-	60-	65-	70-	75-
2016	OBS	4.4	4.6	6.6	7.2	10.1	13.9	22.4	46.6	132.6	314.7	611.7	1125.1	2060.3	3188.7
	PRE	3.4	4.4	5.6	6.0	8.2	13.7	24.9	51.5	117.0	294.9	629.7	1107.5	1964.1	3304.8
	RES	0.970	0.153	0.988	1.207	1.847	0.175	-2.485	-4.885	15.621	19.774	-17.968	17.583	96.239	-116.071
2017	OBS	4.5	4.1	6.1	7.9	8.9	12.1	20.0	45.9	126.6	311.7	638.0	1090.5	2038.7	3188.4
	PRE	3.4	4.4	5.6	6.0	8.2	13.8	24.9	51.6	117.2	295.2	630.4	1108.9	1966.5	3308.8
	RES	1.034	-0.393	0.437	1.935	0.676	-1.670	-4.891	-5.720	9.445	16.472	7.621	-18.383	72.295	-120.445
2018	PRE	3.4	4.5	5.6	6.0	8.2	13.8	25.0	51.6	117.3	295.6	631.2	1110.2	1968.8	3312.8
2019	PRE	3.4	4.5	5.6	6.0	8.2	13.8	25.0	51.7	117.4	296.0	632.0	1111.6	1971.2	3316.8
2020	PRE	3.5	4.5	5.7	6.0	8.2	13.8	25.0	51.8	117.6	296.3	632.7	1112.9	1973.6	3320.9
2021	PRE	3.3	4.3	5.4	6.0	7.3	12.9	26.1	55.3	119.2	261.6	614.2	1184.0	1923.1	3079.8
2022	PRE	3.3	4.3	5.5	6.0	7.3	12.9	26.2	55.4	119.3	261.9	615.0	1185.4	1925.5	3083.5
2023	PRE	3.3	4.3	5.5	6.0	7.3	12.9	26.2	55.4	119.5	262.2	615.7	1186.9	1927.8	3087.2
2024	PRE	3.3	4.3	5.5	6.0	7.3	12.9	26.2	55.5	119.6	262.5	616.5	1188.3	1930.2	3091.0
2025	PRE	3.3	4.3	5.5	6.0	7.3	13.0	26.3	55.6	119.8	262.8	617.2	1189.8	1932.5	3094.7
2026	PRE	3.2	4.2	5.3	5.8	7.3	11.4	24.5	58.0	127.9	266.5	544.8	1154.9	2055.9	3015.6
2027	PRE	3.2	4.2	5.3	5.8	7.3	11.4	24.6	58.1	128.1	266.8	545.5	1156.3	2058.4	3019.2
2028	PRE	3.2	4.2	5.3	5.8	7.3	11.4	24.6	58.1	128.2	267.1	546.1	1157.7	2060.9	3022.9
2029	PRE	3.2	4.2	5.3	5.8	7.3	11.5	24.6	58.2	128.4	267.4	546.8	1159.2	2063.4	3026.6
2030	PRE	3.2	4.2	5.3	5.8	7.3	11.5	24.6	58.3	128.6	267.8	547.4	1160.6	2065.9	3030.2
2031	PRE	3.1	4.0	5.1	5.6	7.0	11.4	21.7	54.4	134.2	286.0	555.0	1024.4	2005.4	3223.7
2032	PRE	3.1	4.0	5.1	5.6	7.0	11.4	21.7	54.5	134.4	286.3	555.7	1025.7	2007.9	3227.6
2033	PRE	3.1	4.0	5.1	5.6	7.1	11.5	21.7	54.6	134.5	286.7	556.4	1026.9	2010.3	3231.6
2034	PRE	3.1	4.0	5.1	5.6	7.1	11.5	21.8	54.6	134.7	287.0	557.0	1028.2	2012.8	3235.5
2035	PRE	3.1	4.0	5.1	5.6	7.1	11.5	21.8	54.7	134.9	287.4	557.7	1029.4	2015.2	3239.4
2036	PRE	3.0	3.9	4.9	5.4	6.8	11.1	21.7	48.2	126.0	300.0	595.6	1043.6	1778.8	3144.6
2037	PRE	3.0	3.9	4.9	5.4	6.8	11.1	21.8	48.2	126.1	300.4	596.4	1044.9	1781.0	3148.5
2038	PRE	3.0	3.9	4.9	5.4	6.8	11.1	21.8	48.3	126.3	300.7	597.1	1046.2	1783.1	3152.3
2039	PRE	3.0	3.9	4.9	5.5	6.8	11.1	21.8	48.3	126.4	301.1	597.8	1047.4	1785.3	3156.1
2040	PRE	3.0	3.9	5.0	5.5	6.8	11.1	21.8	48.4	126.6	301.4	598.5	1048.7	1787.5	3159.9

Results for PHIM for Run 1 (Basic_US_OG_T1.RTF)

****

Observed and predicted deaths (single years)

		10-	15-	20-	25-	30-	35-	40-	45-	50-	55-	60-	65-	70-	75-
2016	OBS	47.0	50.0	78.0	84.0	110.0	142.0	224.0	483.0	1454.0	3365.0	5714.0	8634.0	11086.0	12002.0
	PRE	36.7	48.3	66.3	69.9	89.8	140.2	248.8	533.6	1282.8	3153.5	5881.8	8499.1	10568.2	12438.9
	CHI	2.922	0.058	2.046	2.834	4.547	0.023	2.477	4.799	22.861	14.180	4.790	2.142	25.374	15.344
2017	OBS	48.0	44.0	71.0	94.0	99.0	125.0	200.0	469.0	1369.0	3351.0	6091.0	8601.0	11609.0	12292.0
	PRE	36.9	48.3	65.9	71.1	91.5	142.3	248.8	527.5	1266.9	3173.9	6018.2	8746.0	11197.3	12756.3
	CHI	3.333	0.377	0.396	7.394	0.618	2.095	9.576	6.487	8.235	9.879	0.879	2.404	15.134	16.903
2018	PRE	37.1	48.3	65.2	72.1	93.2	144.6	249.5	522.4	1248.2	3183.7	6149.0	8964.2	11876.5	13131.2
2019	PRE	37.3	48.3	64.5	72.8	94.8	147.2	250.9	518.6	1229.7	3182.9	6264.6	9184.1	12530.6	13654.7
2020	PRE	37.2	48.3	64.0	73.1	96.5	150.0	253.2	516.1	1213.4	3171.2	6358.8	9421.3	13115.8	14369.4
2021	PRE	35.7	46.7	61.3	72.7	86.2	142.5	267.0	548.7	1212.2	2770.7	6223.6	10237.0	13197.9	14005.7
2022	PRE	35.5	47.1	61.1	72.2	87.6	145.1	270.8	548.5	1198.3	2736.8	6266.1	10474.3	13573.8	14846.7
2023	PRE	35.1	47.4	61.1	71.4	88.8	147.7	275.3	550.1	1187.2	2698.1	6290.8	10708.6	13921.4	15759.5
2024	PRE	34.7	47.6	61.2	70.8	89.7	150.3	280.2	553.4	1179.1	2660.2	6294.4	10919.5	14282.5	16641.8
2025	PRE	34.4	47.5	61.4	70.3	90.1	152.9	285.4	558.6	1173.9	2626.8	6276.1	11095.3	14676.6	17440.6
2026	PRE	32.9	45.6	59.3	67.2	89.6	136.6	271.1	588.9	1248.2	2625.1	5486.5	10866.9	15962.4	17563.8
2027	PRE	32.8	45.3	59.7	67.1	89.0	138.8	276.0	597.4	1248.1	2596.2	5423.5	10953.0	16355.0	18093.5
2028	PRE	32.8	44.8	60.1	67.1	88.1	140.7	281.1	607.4	1252.2	2573.6	5351.4	11009.9	16749.0	18605.4
2029	PRE	32.8	44.4	60.4	67.3	87.3	142.1	286.1	618.5	1260.5	2557.6	5280.8	11030.6	17112.8	19152.8
2030	PRE	32.8	44.0	60.5	67.6	86.8	142.8	291.1	630.1	1273.0	2548.5	5219.2	11012.9	17427.5	19757.2
2031	PRE	31.8	42.1	58.0	65.3	83.1	142.0	260.2	598.7	1343.0	2712.5	5224.1	9645.1	17105.8	21561.2
2032	PRE	32.0	42.0	57.6	65.7	83.0	141.2	264.3	609.8	1362.9	2714.6	5173.6	9551.5	17282.0	22166.3
2033	PRE	32.3	42.0	57.1	66.2	83.1	139.9	268.1	621.1	1386.3	2725.2	5134.4	9440.8	17416.2	22781.7
2034	PRE	32.6	42.0	56.6	66.6	83.3	138.7	270.8	632.4	1412.0	2745.0	5107.3	9331.9	17495.8	23367.5
2035	PRE	32.8	42.0	56.2	66.7	83.7	137.9	272.0	643.5	1439.2	2774.0	5093.2	9237.5	17514.5	23896.9
2036	PRE	31.9	40.7	53.8	63.9	80.9	132.2	270.7	575.2	1367.7	2928.0	5426.1	9256.0	15353.1	23482.6
2037	PRE	32.1	41.1	53.7	63.5	81.4	132.0	269.2	584.5	1393.3	2972.6	5434.4	9176.0	15221.9	23763.1
2038	PRE	32.3	41.5	53.6	63.0	82.0	132.1	266.9	592.8	1419.2	3024.6	5459.0	9115.4	15067.6	24001.0
2039	PRE	32.4	41.8	53.7	62.5	82.4	132.5	264.7	598.8	1445.3	3081.7	5501.4	9076.3	14920.7	24177.6
2040	PRE	32.5	42.0	53.9	62.2	82.6	133.0	263.2	601.7	1471.2	3142.0	5562.2	9060.6	14800.7	24281.0

Results for PHIM for Run 1 (Basic_US_OG_T1.RTF)

****
List of values created by O and G modelling, using percentage change in last two period parameters for Fixed File MORT

1.	Country	US (United States)
2.	Sex	M (Males)
3.	Disease	COPD (COPD)
*	Value comes from O and G Modelling.	

Age	Years	Value	Death Rate	Population
10-14	2018	37.1395	3.444574	107820.22 *
10-14	2019	37.2667	3.448759	108058.17 *
10-14	2020	37.2142	3.452948	107775.13 *
10-14	2021	35.7155	3.324176	107441.78 *
10-14	2022	35.4736	3.328214	106584.46 *
10-14	2023	35.1018	3.332257	105339.41 *
10-14	2024	34.6991	3.336305	104004.73 *
10-14	2025	34.3585	3.340357	102858.77 *
10-14	2026	32.9126	3.215785	102347.07 *
10-14	2027	32.8303	3.219691	101967.29 *
10-14	2028	32.7796	3.223602	101686.15 *
10-14	2029	32.7588	3.227517	101498.55 *
10-14	2030	32.7896	3.231438	101470.63 *
10-14	2031	31.7672	3.110927	102114.83 *
10-14	2032	32.0433	3.114706	102877.43 *
10-14	2033	32.3328	3.118490	103681.01 *
10-14	2034	32.5971	3.122277	104401.53 *
10-14	2035	32.8098	3.126070	104955.58 *
10-14	2036	31.8637	3.009489	105877.40 *
10-14	2037	32.1113	3.013145	106570.86 *
10-14	2038	32.2916	3.016805	107039.02 *
10-14	2039	32.4138	3.020469	107313.72 *
15-19	2018	48.2560	4.450139	108437.02 *
15-19	2019	48.2856	4.455545	108371.94 *
15-19	2020	48.3314	4.460957	108343.21 *
15-19	2021	46.7445	4.294594	108844.87 *
15-19	2022	47.0894	4.299811	109515.01 *
15-19	2023	47.4054	4.305033	110116.30 *
15-19	2024	47.5584	4.310263	110337.57 *
15-19	2025	47.4751	4.315498	110010.63 *
15-19	2026	45.5592	4.154560	109660.66 *
15-19	2027	45.2696	4.159606	108831.44 *
15-19	2028	44.8318	4.164659	107648.28 *
15-19	2029	44.3634	4.169718	106394.27 *
15-19	2030	43.9507	4.174783	105276.67 *
15-19	2031	42.1209	4.019092	104802.12 *
15-19	2032	42.0388	4.023974	104470.80 *
15-19	2033	42.0001	4.028862	104248.18 *
15-19	2034	41.9993	4.033755	104119.48 *
15-19	2035	42.0359	4.038655	104083.90 *
15-19	2036	40.7168	3.888041	104723.27 *
15-19	2037	41.0831	3.892764	105537.13 *
15-19	2038	41.4625	3.897492	106382.52 *
15-19	2039	41.7948	3.902226	107105.08 *
20-24	2018	65.2032	5.642887	115549.38 *
20-24	2019	64.5401	5.649741	114235.47 *
20-24	2020	64.0482	5.656604	113227.32 *
20-24	2021	61.2529	5.445651	112480.46 *
20-24	2022	61.0982	5.452266	112060.21 *
20-24	2023	61.1101	5.458889	111946.10 *
20-24	2024	61.2343	5.465519	112037.41 *
20-24	2025	61.4089	5.472158	112220.61 *
20-24	2026	59.2952	5.268084	112555.48 *
20-24	2027	59.6870	5.274483	113161.84 *
20-24	2028	60.1165	5.280890	113837.89 *
20-24	2029	60.4126	5.287305	114259.71 *
20-24	2030	60.4522	5.293727	114195.90 *
20-24	2031	57.9546	5.096308	113718.71 *
20-24	2032	57.5752	5.102498	112837.21 *
20-24	2033	57.0825	5.108696	111736.00 *
20-24	2034	56.6170	5.114901	110690.36 *
20-24	2035	56.2470	5.121114	109833.53 *
20-24	2036	53.8184	4.930132	109162.16 *
20-24	2037	53.6623	4.936120	108713.50 *
20-24	2038	53.6286	4.942116	108513.37 *
20-24	2039	53.7097	4.948119	108545.60 *
25-29	2018	72.0911	6.008020	119991.39 *
25-29	2019	72.8112	6.015318	121043.01 *

Results for PHIM for Run 1 (Basic_US_OG_T1.RTF)

25-29	2020	73.1415	6.022625	121444.55 *
25-29	2021	72.6885	6.003602	121074.83 *
25-29	2022	72.1743	6.010894	120072.51 *
25-29	2023	71.4473	6.018196	118718.74 *
25-29	2024	70.7668	6.025506	117445.48 *
25-29	2025	70.3001	6.032825	116529.28 *
25-29	2026	67.2450	5.807842	115783.18 *
25-29	2027	67.1011	5.814897	115395.25 *
25-29	2028	67.1449	5.821960	115330.47 *
25-29	2029	67.3239	5.829032	115497.61 *
25-29	2030	67.5955	5.836112	115822.80 *
25-29	2031	65.2878	5.618465	116202.23 *
25-29	2032	65.7310	5.625290	116849.18 *
25-29	2033	66.2164	5.632123	117569.12 *
25-29	2034	66.5716	5.638964	118056.43 *
25-29	2035	66.6877	5.645813	118118.77 *
25-29	2036	63.9424	5.435263	117643.53 *
25-29	2037	63.5422	5.441865	116765.46 *
25-29	2038	63.0228	5.448476	115670.55 *
25-29	2039	62.5433	5.455094	114651.21 *
30-34	2018	93.1522	8.228077	113212.55 *
30-34	2019	94.8372	8.238072	115120.68 *
30-34	2020	96.5233	8.248078	117025.14 *
30-34	2021	86.2478	7.262505	118757.69 *
30-34	2022	87.6055	7.271326	120480.79 *
30-34	2023	88.8201	7.280159	122003.03 *
30-34	2024	89.6818	7.289002	123037.09 *
30-34	2025	90.0783	7.297856	123431.16 *
30-34	2026	89.5527	7.274805	123099.77 *
30-34	2027	88.9702	7.283641	122150.65 *
30-34	2028	88.1294	7.292489	120849.55 *
30-34	2029	87.3454	7.301347	119629.21 *
30-34	2030	86.8294	7.310216	118778.23 *
30-34	2031	83.1385	7.037595	118134.80 *
30-34	2032	83.0113	7.046143	117811.00 *
30-34	2033	83.0921	7.054702	117782.65 *
30-34	2034	83.3290	7.063271	117975.02 *
30-34	2035	83.6804	7.071851	118328.82 *
30-34	2036	80.8617	6.808119	118772.50 *
30-34	2037	81.4197	6.816389	119447.02 *
30-34	2038	82.0067	6.824669	120162.15 *
30-34	2039	82.4263	6.832958	120630.51 *
35-39	2018	144.6176	13.775681	104980.35 *
35-39	2019	147.2033	13.792414	106727.76 *
35-39	2020	149.9520	13.809167	108588.71 *
35-39	2021	142.4549	12.899554	110433.99 *
35-39	2022	145.0546	12.915223	112312.89 *
35-39	2023	147.6774	12.930911	114204.92 *
35-39	2024	150.3058	12.946618	116096.60 *
35-39	2025	152.9079	12.962344	117963.16 *
35-39	2026	136.6186	11.413456	119699.62 *
35-39	2027	138.7740	11.427320	121440.53 *
35-39	2028	140.7095	11.441201	122984.87 *
35-39	2029	142.0954	11.455098	124045.52 *
35-39	2030	142.7547	11.469012	124469.94 *
35-39	2031	142.0257	11.432787	124226.65 *
35-39	2032	141.1861	11.446674	123342.47 *
35-39	2033	139.9240	11.460578	122091.53 *
35-39	2034	138.7367	11.474499	120908.72 *
35-39	2035	137.9464	11.488437	120074.10 *
35-39	2036	132.1608	11.059997	119494.45 *
35-39	2037	132.0012	11.073431	119205.30 *
35-39	2038	132.1420	11.086882	119187.69 *
35-39	2039	132.5100	11.100349	119374.58 *
40-44	2018	249.4729	24.963192	99936.30 *
40-44	2019	250.9239	24.993514	100395.61 *
40-44	2020	253.2061	25.023873	101185.82 *
40-44	2021	267.0034	26.123340	102208.75 *
40-44	2022	270.8179	26.155071	103543.16 *
40-44	2023	275.2994	26.186841	105128.92 *
40-44	2024	280.2154	26.218650	106876.38 *
40-44	2025	285.3936	26.250497	108719.32 *
40-44	2026	271.0816	24.521371	110549.11 *
40-44	2027	276.0389	24.551157	112434.16 *
40-44	2028	281.0609	24.580979	114340.82 *
40-44	2029	286.1012	24.610837	116250.08 *
40-44	2030	291.0898	24.640731	118133.60 *
40-44	2031	260.1824	21.696378	119919.75 *

Results for PHIM for Run 1 (Basic_US_OG_T1.RTF)

40-44	2032	264.3500	21.722732	121692.78 *
40-44	2033	268.0808	21.749118	123260.55 *
40-44	2034	270.7596	21.775536	124341.18 *
40-44	2035	272.0360	21.801987	124775.80 *
40-44	2036	270.7463	21.733124	124577.70 *
40-44	2037	269.2340	21.759523	123731.59 *
40-44	2038	266.9008	21.785954	122510.50 *
40-44	2039	264.6903	21.812417	121348.44 *
45-49	2018	522.4236	51.640377	101165.72 *
45-49	2019	518.5762	51.703104	100298.85 *
45-49	2020	516.0595	51.765907	99690.99 *
45-49	2021	548.6840	55.286965	99242.93 *
45-49	2022	548.5218	55.354121	99093.21 *
45-49	2023	550.0811	55.421359	99254.35 *
45-49	2024	553.4290	55.488678	99737.28 *
45-49	2025	558.5768	55.556080	100542.87 *
45-49	2026	588.9384	57.997031	101546.31 *
45-49	2027	597.4150	58.067479	102882.89 *
45-49	2028	607.4343	58.138012	104481.43 *
45-49	2029	618.4602	58.208631	106248.89 *
45-49	2030	630.0986	58.279337	108116.99 *
45-49	2031	598.7195	54.440463	109976.93 *
45-49	2032	609.8316	54.506591	111882.17 *
45-49	2033	621.0758	54.572799	113806.84 *
45-49	2034	632.3619	54.639088	115734.34 *
45-49	2035	643.4927	54.705457	117628.62 *
45-49	2036	575.2346	48.168630	119421.01 *
45-49	2037	584.4945	48.227139	121196.18 *
45-49	2038	592.7858	48.285720	122766.28 *
45-49	2039	598.7728	48.344372	123855.74 *
50-54	2018	1248.1703	117.294889	106413.02 *
50-54	2019	1229.7084	117.437366	104711.85 *
50-54	2020	1213.3711	117.580015	103195.35 *
50-54	2021	1212.1536	119.204170	101687.18 *
50-54	2022	1198.2517	119.348966	100399.00 *
50-54	2023	1187.2045	119.493937	99352.70 *
50-54	2024	1179.0598	119.639084	98551.39 *
50-54	2025	1173.9409	119.784408	98004.48 *
50-54	2026	1248.1804	127.932007	97565.92 *
50-54	2027	1248.1198	128.087404	97442.82 *
50-54	2028	1252.1649	128.242989	97640.03 *
50-54	2029	1260.4523	128.398764	98167.01 *
50-54	2030	1272.9894	128.554728	99023.15 *
50-54	2031	1342.9505	134.202999	100068.59 *
50-54	2032	1362.8626	134.366013	101429.12 *
50-54	2033	1386.2732	134.529225	103046.25 *
50-54	2034	1412.0335	134.692635	104833.76 *
50-54	2035	1439.1777	134.856244	106719.40 *
50-54	2036	1367.7418	125.973231	108574.00 *
50-54	2037	1393.3027	126.126249	110468.89 *
50-54	2038	1419.2002	126.279452	112385.68 *
50-54	2039	1445.2890	126.432842	114312.78 *
55-59	2018	3183.7424	295.601953	107703.70 *
55-59	2019	3182.8541	295.961015	107543.02 *
55-59	2020	3171.2414	296.320514	107020.65 *
55-59	2021	2770.6531	261.558159	105928.76 *
55-59	2022	2736.7639	261.875869	104506.15 *
55-59	2023	2698.1080	262.193965	102905.04 *
55-59	2024	2660.2195	262.512448	101336.89 *
55-59	2025	2626.8371	262.831317	99943.84 *
55-59	2026	2625.0556	266.461857	98515.25 *
55-59	2027	2596.1534	266.785524	97312.38 *
55-59	2028	2573.6203	267.109584	96350.73 *
55-59	2029	2557.6268	267.434037	95635.80 *
55-59	2030	2548.5095	267.758885	95179.27 *
55-59	2031	2712.5424	285.971540	94853.58 *
55-59	2032	2714.5907	286.318905	94810.04 *
55-59	2033	2725.2342	286.666691	95066.30 *
55-59	2034	2745.0177	287.014901	95640.25 *
55-59	2035	2773.9587	287.363533	96531.34 *
55-59	2036	2928.0408	299.989339	97604.83 *
55-59	2037	2972.6378	300.353731	98971.23 *
55-59	2038	3024.6051	300.718566	100579.26 *
55-59	2039	3081.6597	301.083843	102352.21 *
60-64	2018	6149.0246	631.190654	97419.45 *
60-64	2019	6264.5547	631.957351	99129.39 *
60-64	2020	6358.8139	632.724979	100498.86 *
60-64	2021	6223.5520	614.211614	101325.86 *

Results for PHIM for Run 1 (Basic_US_OG_T1.RTF)

60-64	2022	6266.0917	614.957687	101894.68 *
60-64	2023	6290.7506	615.704665	102171.56 *
60-64	2024	6294.3640	616.452552	102106.22 *
60-64	2025	6276.0995	617.201346	101686.42 *
60-64	2026	5486.4654	544.795382	100706.90 *
60-64	2027	5423.5490	545.457135	99431.26 *
60-64	2028	5351.4187	546.119693	97989.85 *
60-64	2029	5280.8083	546.783055	96579.59 *
60-64	2030	5219.1965	547.447224	95336.98 *
60-64	2031	5224.1480	555.009218	94127.23 *
60-64	2032	5173.5806	555.683378	93103.03 *
60-64	2033	5134.3564	556.358357	92285.06 *
60-64	2034	5107.2646	557.034156	91686.74 *
60-64	2035	5093.1715	557.710776	91322.81 *
60-64	2036	5426.0553	595.645630	91095.36 *
60-64	2037	5434.3733	596.369151	91124.32 *
60-64	2038	5459.0191	597.093550	91426.53 *
60-64	2039	5501.4260	597.818830	92024.97 *
65-69	2018	8964.2011	1110.232043	80741.69 *
65-69	2019	9184.0814	1111.580623	82621.82 *
65-69	2020	9421.2645	1112.930841	84652.74 *
65-69	2021	10237.0207	1183.990869	86461.99 *
65-69	2022	10474.3372	1185.429043	88359.04 *
65-69	2023	10708.5715	1186.868963	90225.39 *
65-69	2024	10919.5017	1188.310633	91890.97 *
65-69	2025	11095.3322	1189.754054	93257.36 *
65-69	2026	10866.8890	1154.942167	94090.33 *
65-69	2027	10953.0137	1156.345056	94720.98 *
65-69	2028	11009.9259	1157.749648	95097.64 *
65-69	2029	11030.6277	1159.155947	95160.86 *
65-69	2030	11012.9233	1160.563955	94892.86 *
65-69	2031	9645.1474	1024.414296	94152.80 *
65-69	2032	9551.5148	1025.658635	93125.67 *
65-69	2033	9440.7940	1026.904485	91934.49 *
65-69	2034	9331.9431	1028.151849	90764.25 *
65-69	2035	9237.4839	1029.400727	89736.52 *
65-69	2036	9255.9705	1043.620038	88691.00 *
65-69	2037	9175.9666	1044.887706	87817.73 *
65-69	2038	9115.4278	1046.156914	87132.51 *
65-69	2039	9076.2749	1047.427663	86653.00 *
70-74	2018	11876.5242	1968.841137	60322.41 *
70-74	2019	12530.5583	1971.232655	63567.12 *
70-74	2020	13115.7762	1973.627078	66455.19 *
70-74	2021	13197.9406	1923.134683	68627.23 *
70-74	2022	13573.7904	1925.470682	70495.96 *
70-74	2023	13921.4258	1927.809519	72213.70 *
70-74	2024	14282.4703	1930.151197	73996.64 *
70-74	2025	14676.6305	1932.495719	75946.51 *
70-74	2026	15962.4425	2055.884516	77642.70 *
70-74	2027	16355.0102	2058.381765	79455.67 *
70-74	2028	16749.0089	2060.882046	81271.07 *
70-74	2029	17112.8351	2063.385365	82935.72 *
70-74	2030	17427.5114	2065.891724	84358.30 *
70-74	2031	17105.8200	2005.444282	85296.91 *
70-74	2032	17281.9881	2007.880262	86070.81 *
70-74	2033	17416.1813	2010.319200	86633.91 *
70-74	2034	17495.8494	2012.761101	86924.62 *
70-74	2035	17514.4654	2015.205968	86911.54 *
70-74	2036	15353.0569	1778.795382	86311.54 *
70-74	2037	15221.9489	1780.956055	85470.66 *
70-74	2038	15067.6367	1783.119352	84501.56 *
70-74	2039	14920.7234	1785.285277	83576.13 *
75-79	2018	13131.2447	3312.822023	39637.64 *
75-79	2019	13654.7023	3316.846052	41167.73 *
75-79	2020	14369.3795	3320.874969	43269.86 *
75-79	2021	14005.7170	3079.752605	45476.76 *
75-79	2022	14846.7068	3083.493529	48148.98 *
75-79	2023	15759.4906	3087.238996	51047.20 *
75-79	2024	16641.8447	3090.989013	53839.87 *
75-79	2025	17440.5698	3094.743586	56355.46 *
75-79	2026	17563.7989	3015.569045	58243.73 *
75-79	2027	18093.4845	3019.232006	59927.44 *
75-79	2028	18605.4048	3022.899416	61548.21 *
75-79	2029	19152.8119	3026.571281	63282.21 *
75-79	2030	19757.2265	3030.247606	65200.04 *
75-79	2031	21561.1874	3223.727263	66882.79 *
75-79	2032	22166.2971	3227.643071	68676.42 *
75-79	2033	22781.6963	3231.563634	70497.44 *

Results for PHIM for Run 1 (Basic_US_OG_T1.RTF)

75-79	2034	23367.4940	3235.488960	72222.45 *
75-79	2035	23896.9061	3239.419054	73769.11 *
75-79	2036	23482.5897	3144.634514	74675.10 *
75-79	2037	23763.1474	3148.454249	75475.60 *
75-79	2038	24001.0459	3152.278623	76138.72 *
75-79	2039	24177.5962	3156.107643	76605.74 *

Results for PHIM for Run 1 (Basic_US_OG_T1.RTF)

****
List of values created by last value brought forwards for Fixed File perm.Fixed_File_RR_COPD

1. Country	US (United States)
2. Sex	M (Males)
3. Disease	COPD (COPD)

**Age**	**Years**	**Value**
10-14	2013-2039	4.5600
15-19	2013-2039	4.5600
20-24	2013-2039	4.5600
25-29	2013-2039	4.5600
30-34	2013-2039	4.5600
35-39	2013-2039	4.5600
40-44	2013-2039	4.5600
45-49	2013-2039	4.5600
50-54	2013-2039	4.5600
55-59	2013-2039	4.5600
60-64	2013-2039	4.5600
65-69	2013-2039	4.5600
70-74	2013-2039	4.5600
75-79	2013-2039	4.5600

Results for PHIM for Run 2 (Basic_US_OG_T1.RTF)

Control File Details for Run: 2

Variable Parameter	Value
1. Name for Reference Scenario	NULL
2. Name for Test Scenario	MRTP
3. Name for Product 1	CC
4. Name for Product 2	MRTP
5. Name for Product 3	-
6. Name for Product 4	Dual
7. Country	US (United States)
8. Sex	F (Females)
9. Year of start of process	1990
10. Number of months of follow-up	600
11. Follow-up interval length (in months)	12
12. Lower age for risk estimation	10
13. Upper age for risk estimation	79
14. The effective dose for Product 1 (F1) - Ref	1
15. The effective dose for Product 2 (F2) - Ref	-
16. The effective dose for Product 3 (F3) - Ref	-
17. The effective dose for Product 4 (F4) - Ref	-
18. The effective dose for Product 1 (F1) - Test	1
19. The effective dose for Product 2 (F2) - Test	0.2
20. The effective dose for Product 3 (F3) - Test	-
21. The effective dose for Product 4 (F4) - Test	0.6
22. Number in population to be simulated	10000
23. Number of MC simulations	1
24. The random number seed for the first simulation	15975263
25. Source for the population file (POP)	UN4
26. Source for the socioeconomic prevalence file (SEP)	-
27. Source for the current smoking prevalence file (CSP)	ISS2
28. Source for the former smoking prevalence file (FSP)	ISS2
29. Source for the quit-time distribution file (QTD)	NHIS2006
30. Source for the death file (MORT)	APR-20
31. Source for the relative risk file (RR)	PNLEST
32. Source for the half-life file (H)	PNLEST
33. Assumption Set for TTP Factor Reference	F1
34. Assumption Set for TTP Factor Test	F1
35. Assumption Set for TTP Reference	PNLNULL1
36. Assumption Set for TTP Test	PNLMRTP9
37. Assumption Set for TP for socioeconomic group	-
38. Source for Output Choice file (OUTC)	BASIC
39. Output for DTP P1	N
40. Output for DTP P2	N
41. Output for Main P Component	N
42. Output for DTP E1	N
43. Output for DTP E2	N
44. Output for DTP E3	N
45. Output for Main E Component	N
46. Output file name	Basic_US_OG_T1
47. Run number for P-Component	-
48. Results file for E-Component	-
49. Components required	PE
50. Results folder for P to be used in E-Component	-
51. Fixed age of starting product use	16
52. Output absolute risk file?	N

Results for PHIM for Run 2 (Basic_US_OG_T1.RTF)

****

Osmond and Gardner Modeling of Death Rates for COD: All Causes

Variable Parameter	Value
1. Country	US (United States)
2. Sex	F (Females)
3. Year of start of OG modelling	1966
4. Number 5-year periods for OG modelling	10
5. Age Range of interest	10 - 79
6. Scaling Factor for Rates	10000
7. Model Type to Save	4 (Full Age-Period-Cohort)
8. Goodness of Fit Required	1 (Analysis Printed For Age-Period-Cohort Model)
9. Cause of Death	All Causes
Note:	Death rates are per million population

Matrix of Numbers of Deaths

Age	1966-	1971-	1976-	1981-	1986-	1991-	1996-	2001-	2006-	2011-
10-14	14979	14617	11213	9148	8164	8444	8260	7803	6486	5876
15-19	27374	29708	28228	22419	21491	19314	19727	19491	17394	14305
20-24	28587	32504	32691	29755	26215	23457	21023	23885	24162	24609
25-29	27618	31475	32574	32920	34903	31452	26902	25960	29221	30985
30-34	34525	35478	35457	38135	44925	48023	40658	37434	35370	40704
35-39	53951	47307	43129	46317	55118	65511	66000	61071	54406	52458
40-44	87729	76208	61766	60217	69177	82895	93362	102173	89435	81675
45-49	130147	121967	98320	85704	89530	105668	121850	144032	148875	132859
50-54	174493	175362	158914	137932	127309	135622	157913	185539	206759	217142
55-59	230110	231473	226882	221548	197615	185913	200217	233737	259586	294958
60-64	293085	308481	304275	320908	316885	285668	272825	290018	323744	368942
65-69	393085	396349	402929	423911	444824	434169	400928	372978	384761	449746
70-74	507440	505646	501057	547176	570059	594894	595312	536769	486983	530534
75-79	605146	615847	601792	652467	711435	736185	795741	779924	683129	660064

Matrix of Age- and Period-Specific Mortality Rates

Age	1966-	1971-	1976-	1981-	1986-	1991-	1996-	2001-	2006-	2011-
10-14	299.179	286.918	239.478	213.918	194.353	189.871	169.282	149.699	126.022	115.691
15-19	595.555	587.461	544.238	467.138	485.956	441.764	421.690	382.444	324.974	268.287
20-24	719.407	697.949	634.172	562.157	535.345	516.842	459.288	489.806	463.440	444.321
25-29	853.998	782.953	686.475	626.362	651.628	628.740	566.119	542.527	582.959	576.294
30-34	1199.825	1081.243	863.718	787.510	839.543	874.822	781.877	758.089	719.521	790.835
35-39	1834.235	1641.518	1307.388	1123.648	1133.348	1216.819	1178.782	1148.365	1081.048	1046.369
40-44	2773.812	2602.107	2136.281	1817.602	1671.283	1706.422	1724.075	1812.196	1672.447	1607.868
45-49	4190.976	3909.457	3371.733	2971.631	2728.432	2587.966	2507.040	2657.323	2644.035	2486.495
50-54	6137.605	5784.908	5154.872	4771.097	4467.220	4183.145	3867.923	3811.638	3824.982	3882.064
55-59	8700.018	8432.275	7652.602	7345.403	7014.562	6689.515	6262.378	5771.351	5389.423	5532.040
60-64	12378.80	12345.21	11537.54	11232.48	10931.47	10538.29	10059.17	9275.299	8179.433	7860.588
65-69	19853.09	17962.04	16985.85	16884.05	16469.65	15904.53	15550.09	14319.85	12827.99	11858.34
70-74	32626.49	29350.29	25448.09	25686.29	25280.65	24552.02	24043.90	22654.58	20265.05	19028.89
75-79	52812.96	47857.83	41519.63	38692.27	39227.33	38468.00	38015.55	36475.51	32995.67	31126.08

Matrix of Log-Rates

Age	1966-	1971-	1976-	1981-	1986-	1991-	1996-	2001-	2006-	2011-
10-14	2.476	2.458	2.379	2.330	2.289	2.278	2.229	2.175	2.100	2.063
15-19	2.775	2.769	2.736	2.669	2.687	2.645	2.625	2.583	2.512	2.429
20-24	2.857	2.844	2.802	2.750	2.729	2.713	2.662	2.690	2.666	2.648
25-29	2.931	2.894	2.837	2.797	2.814	2.798	2.753	2.734	2.766	2.761
30-34	3.079	3.034	2.936	2.896	2.924	2.942	2.893	2.880	2.857	2.898
35-39	3.263	3.215	3.116	3.051	3.054	3.085	3.071	3.060	3.034	3.020
40-44	3.443	3.415	3.330	3.259	3.223	3.232	3.237	3.258	3.223	3.206
45-49	3.622	3.592	3.528	3.473	3.436	3.413	3.399	3.424	3.422	3.396
50-54	3.788	3.762	3.712	3.679	3.650	3.622	3.587	3.581	3.583	3.589
55-59	3.940	3.926	3.884	3.866	3.846	3.825	3.797	3.761	3.732	3.743
60-64	4.093	4.091	4.062	4.050	4.039	4.023	4.003	3.967	3.913	3.895
65-69	4.298	4.254	4.230	4.227	4.217	4.202	4.192	4.156	4.108	4.074
70-74	4.514	4.468	4.406	4.410	4.403	4.390	4.381	4.355	4.307	4.279
75-79	4.723	4.680	4.618	4.588	4.594	4.585	4.580	4.562	4.518	4.493

Results for PHIM for Run 2 (Basic_US_OG_T1.RTF)

Fitting the Age, Period, Cohort Models

Model	RSS	MRSS	DF	Factor	%Account	ChiSq	P
Age Only	124576.173	980.915	127	P, C	98.3843	663921.737	0.0000
Age-Period	9395.745	80.306	117	Cohort	78.5774	49859.306	0.0000
Age-Cohort	4831.260	46.454	104	Period	58.3378	25626.850	0.0000
Period-Cohort	2144.601	19.857	108	Age	6.1452	11373.488	0.0000
Full Age-Period-Cohort	2012.811	20.967	96			10674.488	0.0000

Key to terms:		
	RSS =	residual sum of squares
	MRSS =	mean RSS (MRSS/DF)
	DF =	degrees of freedom
	Factor =	Factors not included in the model
	% Account =	1- (RSS for full model)/(RSS for model in question)
	Chisq =	chi-squared value for model
	P =	probability value based on Chisq and DF.

Age	Value	Log10 Value
10-	231.656134	2.364844
15-	510.416171	2.707924
20-	609.364503	2.784877
25-	703.679243	2.847375
30-	927.241125	2.967193
35-	1336.96236	3.126119
40-	2016.63729	3.304628
45-	3065.80341	3.486544
50-	4625.68732	3.665176
55-	6892.88243	3.838401
60-	10416.3270	4.017715
65-	15609.9630	4.193402
70-	24003.7647	4.380279
75-	37614.0888	4.575351

Period	Value	Log10 Value
1966	1.253522	0.098132
1971	1.183831	0.073290
1976	1.066225	0.027849
1981	1.023155	0.009941
1986	0.996169	-0.001667
1991	0.971753	-0.012444
1996	0.954113	-0.020400
2001	0.928126	-0.032393
2006	0.872740	-0.059115
2011	0.857018	-0.067010

Cohort	Value	Log10 Value
1891	1.120103	0.049258
1896	1.079071	0.033050
1901	1.029008	0.012419
1906	0.985861	-0.006184
1911	1.031054	0.013281
1916	1.051272	0.021715
1921	1.056901	0.024034
1926	1.050364	0.021340
1931	1.025037	0.010739
1936	0.980179	-0.008695
1941	0.935945	-0.028749
1946	0.886507	-0.052318
1951	0.883101	-0.053990
1956	0.934556	-0.029394
1961	0.969705	-0.013360
1966	0.929363	-0.031815
1971	0.908260	-0.041790
1976	0.871458	-0.059754
1981	0.925593	-0.033580
1986	0.874599	-0.058191
1991	0.781384	-0.107136
1996	0.616424	-0.210121
2001	0.582728	-0.234534

Results for PHIM for Run 2 (Basic_US_OG_T1.RTF)

Model: Full Age-Period-Cohort

Basic Analysis Using OG Modelling T1 on US
Fitting the Full Age-Period-Cohort Model
Matrix of observed, expected, and residual rates

**Age**		**1966-**	**1971-**	**1976-**	**1981-**	**1986-**	**1991-**	**1996-**	**2001-**	**2006-**	**2011-**
10-	Observed	299.179	286.918	239.478	213.918	194.353	189.871	169.282	149.699	126.022	115.691
	Expected	271.382	265.934	229.550	215.276	201.105	208.363	193.309	168.002	124.626	115.691
	Residual	-27.797	-20.985	-9.928	1.358	6.753	18.491	24.027	18.304	-1.396	-0.000
15-	Observed	595.555	587.461	544.238	467.138	485.956	441.764	421.690	382.444	324.974	268.287
	Expected	565.024	564.703	527.731	485.346	461.815	432.242	450.759	414.324	348.076	269.646
	Residual	-30.531	-22.758	-16.507	18.208	-24.142	-9.523	29.069	31.881	23.102	1.359
20-	Observed	719.407	697.949	634.172	562.157	535.345	516.842	459.288	489.806	463.440	444.321
	Expected	677.160	637.056	607.200	604.586	564.152	537.828	506.668	523.485	465.126	408.067
	Residual	-42.247	-60.893	-26.972	42.429	28.807	20.986	47.380	33.679	1.686	-36.254
25-	Observed	853.998	782.953	686.475	626.362	651.628	628.740	566.119	542.527	582.959	576.294
	Expected	825.577	738.494	662.573	672.855	679.747	635.501	609.796	569.152	568.433	527.441
	Residual	-28.421	-44.459	-23.902	46.493	28.119	6.761	43.677	26.625	-14.526	-48.853
30-	Observed	1199.825	1081.243	863.718	787.510	839.543	874.822	781.877	758.089	719.521	790.835
	Expected	1139.279	1027.385	876.443	837.808	863.240	873.752	822.201	781.645	705.219	735.534
	Residual	-60.546	-53.859	12.725	50.298	23.697	-1.070	40.323	23.557	-14.302	-55.301
35-	Observed	1834.235	1641.518	1307.388	1123.648	1133.348	1216.819	1178.782	1148.365	1081.048	1046.369
	Expected	1717.872	1551.366	1334.193	1212.671	1176.150	1214.174	1236.969	1153.219	1059.776	998.517
	Residual	-116.363	-90.151	26.805	89.023	42.802	-2.646	58.186	4.854	-21.272	-47.852
40-	Observed	2773.812	2602.107	2136.281	1817.602	1671.283	1706.422	1724.075	1812.196	1672.447	1607.868
	Expected	2655.214	2447.130	2107.570	1931.166	1780.916	1730.590	1798.180	1814.991	1635.678	1569.740
	Residual	-118.598	-154.976	-28.711	113.564	109.632	24.168	74.105	2.795	-36.768	-38.127
45-	Observed	4190.976	3909.457	3371.733	2971.631	2728.432	2587.966	2507.040	2657.323	2644.035	2486.495
	Expected	4061.726	3812.184	3350.677	3074.616	2858.433	2641.087	2583.179	2659.236	2594.590	2441.854
	Residual	-129.250	-97.274	-21.056	102.985	130.001	53.121	76.139	1.913	-49.445	-44.640
50-	Observed	6137.605	5784.908	5154.872	4771.097	4467.220	4183.145	3867.923	3811.638	3824.982	3882.064
	Expected	6095.699	5787.625	5180.417	4851.287	4516.633	4207.100	3912.537	3791.349	3772.824	3844.200
	Residual	-41.906	2.717	25.545	80.190	49.413	23.956	44.613	-20.289	-52.158	-37.864
55-	Observed	8700.018	8432.275	7652.602	7345.403	7014.562	6689.515	6262.378	5771.351	5389.423	5532.040
	Expected	8908.701	8578.393	7767.548	7407.673	7038.392	6565.416	6155.328	5671.400	5312.464	5520.729
	Residual	208.683	146.118	114.945	62.270	23.831	-124.098	-107.050	-99.951	-76.959	-11.311
60-	Observed	12378.796	12345.214	11537.544	11232.476	10931.467	10538.287	10059.167	9275.299	8179.433	7860.588
	Expected	12872.491	12714.108	11675.584	11263.934	10899.020	10375.525	9741.364	9048.409	8059.010	7883.427
	Residual	493.695	368.894	138.040	31.458	-32.447	-162.763	-317.803	-226.890	-120.423	22.838
65-	Observed	19853.086	17962.036	16985.853	16884.049	16469.653	15904.527	15550.088	14319.848	12827.990	11858.340
	Expected	20135.044	18218.291	17160.586	16790.296	16434.984	15933.000	15266.557	14200.849	12750.791	11859.715
	Residual	281.958	256.255	174.733	-93.754	-34.669	28.473	-283.530	-118.999	-77.199	1.375
70-	Observed	32626.486	29350.291	25448.092	25686.291	25280.646	24552.018	24043.903	22654.581	20265.054	19028.889
	Expected	32468.453	29240.705	25231.560	25322.237	25137.827	24652.991	24055.746	22836.306	20533.805	19253.954
	Residual	-158.034	-109.586	-216.532	-364.054	-142.819	100.973	11.844	181.726	268.752	225.066
75-	Observed	52812.960	47857.834	41519.630	38692.267	39227.327	38468.004	38015.552	36475.509	32995.674	31126.081
	Expected	52812.960	48049.686	41268.435	37940.909	38633.598	38425.699	37930.152	36668.849	33649.195	31597.006
	Residual	-0.000	191.852	-251.196	-751.357	-593.729	-42.305	-85.400	193.340	653.522	470.925

Fitting the Full Age-Period-Cohort Model

Results for PHIM for Run 2 (Basic_US_OG_T1.RTF)

Matrix of observed and expected deaths and (O-E)2/E Values**

Age		1966-	1971-	1976-	1981-	1986-	1991-	1996-	2001-	2006-	2011-	Total
10-	Observed	14979.0	14617.0	11213.0	9148.0	8164.0	8444.0	8260.0	7803.0	6486.0	5876.0	94990.0
	Expected	13587.3	13547.9	10748.2	9206.1	8447.7	9266.3	9432.4	8757.1	6414.1	5876.0	95283.1
	Difference	1391.7	1069.1	464.8	-58.1	-283.7	-822.3	-1172.4	-954.1	71.9	0.0	-293.1
	Chi-Sq	142.5	84.4	20.1	0.4	9.5	73.0	145.7	103.9	0.8	0.0	580.4
15-	Observed	27374.0	29708.0	28228.0	22419.0	21491.0	19314.0	19727.0	19491.0	17394.0	14305.0	219451.0
	Expected	25970.7	28557.1	27371.9	23292.8	20423.4	18897.7	21086.9	21115.8	18630.5	14377.5	219724.1
	Difference	1403.3	1150.9	856.1	-873.8	1067.6	416.3	-1359.9	-1624.8	-1236.5	-72.5	-273.1
	Chi-Sq	75.8	46.4	26.8	32.8	55.8	9.2	87.7	125.0	82.1	0.4	541.9
20-	Observed	28587.0	32504.0	32691.0	29755.0	26215.0	23457.0	21023.0	23885.0	24162.0	24609.0	266888.0
	Expected	26908.2	29668.2	31300.6	32000.8	27625.6	24409.5	23191.7	25527.3	24249.9	22601.0	267482.9
	Difference	1678.8	2835.8	1390.4	-2245.8	-1410.6	-952.5	-2168.7	-1642.3	-87.9	2008.0	-594.9
	Chi-Sq	104.7	271.1	61.8	157.6	72.0	37.2	202.8	105.7	0.3	178.4	1191.5
25-	Observed	27618.0	31475.0	32574.0	32920.0	34903.0	31452.0	26902.0	25960.0	29221.0	30985.0	304010.0
	Expected	26698.9	29687.7	31439.8	35363.5	36409.1	31790.2	28977.6	27234.0	28492.9	28358.4	304452.2
	Difference	919.1	1787.3	1134.2	-2443.5	-1506.1	-338.2	-2075.6	-1274.0	728.1	2626.6	-442.2
	Chi-Sq	31.6	107.6	40.9	168.8	62.3	3.6	148.7	59.6	18.6	243.3	885.1
30-	Observed	34525.0	35478.0	35457.0	38135.0	44925.0	48023.0	40658.0	37434.0	35370.0	40704.0	390709.0
	Expected	32782.8	33710.8	35979.4	40570.7	46193.0	47964.3	42754.8	38597.2	34666.9	37857.7	391077.5
	Difference	1742.2	1767.2	-522.4	-2435.7	-1268.0	58.7	-2096.8	-1163.2	703.1	2846.3	-368.5
	Chi-Sq	92.6	92.6	7.6	146.2	34.8	0.1	102.8	35.1	14.3	214.0	740.1
35-	Observed	53951.0	47307.0	43129.0	46317.0	55118.0	65511.0	66000.0	61071.0	54406.0	52458.0	545268.0
	Expected	50528.4	44708.9	44013.2	49986.5	57199.6	65368.6	69257.8	61329.1	53335.4	50059.0	545786.7
	Difference	3422.6	2598.1	-884.2	-3669.5	-2081.6	142.4	-3257.8	-258.1	1070.6	2399.0	-518.7
	Chi-Sq	231.8	151.0	17.8	269.4	75.8	0.3	153.2	1.1	21.5	115.0	1036.8
40-	Observed	87729.0	76208.0	61766.0	60217.0	69177.0	82895.0	93362.0	102173.0	89435.0	81675.0	804637.0
	Expected	83978.0	71669.2	60935.9	63979.4	73714.9	84069.0	97374.9	102330.6	87468.8	79738.3	805258.9
	Difference	3751.0	4538.8	830.1	-3762.4	-4537.9	-1174.0	-4012.9	-157.6	1966.2	1936.7	-621.9
	Chi-Sq	167.5	287.4	11.3	221.3	279.3	16.4	165.4	0.2	44.2	47.0	1240.1
45-	Observed	130147.0	121967.0	98320.0	85704.0	89530.0	105668.0	121850.0	144032.0	148875.0	132859.0	1178952.0
	Expected	126133.3	118932.3	97706.0	88674.2	93795.8	107836.9	125550.6	144135.7	146090.9	130473.8	1179329.4
	Difference	4013.7	3034.7	614.0	-2970.2	-4265.8	-2168.9	-3700.6	-103.7	2784.1	2385.2	-377.4
	Chi-Sq	127.7	77.4	3.9	99.5	194.0	43.6	109.1	0.1	53.1	43.6	751.9
50-	Observed	174493.0	175362.0	158914.0	137932.0	127309.0	135622.0	157913.0	185539.0	206759.0	217142.0	1676985.0
	Expected	173301.6	175444.4	159701.5	140250.3	128717.2	136398.7	159734.4	184551.4	203939.6	215024.1	1677063.1
	Difference	1191.4	-82.4	-787.5	-2318.3	-1408.2	-776.7	-1821.4	987.6	2819.4	2117.9	-78.1
	Chi-Sq	8.2	0.0	3.9	38.3	15.4	4.4	20.8	5.3	39.0	20.9	156.2
55-	Observed	230110.0	231473.0	226882.0	221548.0	197615.0	185913.0	200217.0	233737.0	259586.0	294958.0	2282039.0
	Expected	235629.5	235484.1	230289.9	223426.1	198286.4	182464.1	196794.5	229689.0	255879.2	294354.9	2282297.7
	Difference	-5519.5	-4011.1	-3407.9	-1878.1	-671.4	3448.9	3422.5	4048.0	3706.8	603.1	-258.7
	Chi-Sq	129.3	68.3	50.4	15.8	2.3	65.2	59.5	71.3	53.7	1.2	517.1
60-	Observed	293085.0	308481.0	304275.0	320908.0	316885.0	285668.0	272825.0	290018.0	323744.0	368942.0	3084831.0
	Expected	304773.9	317698.9	307915.5	321806.8	315944.4	281255.9	264205.5	282923.6	318977.6	370013.9	3085516.1
	Difference	-11688.9	-9217.9	-3640.5	-898.8	940.6	4412.1	8619.5	7094.4	4766.4	-1071.9	-685.1
	Chi-Sq	448.3	267.5	43.0	2.5	2.8	69.2	281.2	177.9	71.2	3.1	1366.7
65-	Observed	393085.0	396349.0	402929.0	423911.0	444824.0	434169.0	400928.0	372978.0	384761.0	449746.0	4103680.0
	Expected	398667.7	402003.5	407073.9	421557.1	443887.6	434946.3	393617.7	369878.5	382445.5	449798.2	4103876.1
	Difference	-5582.7	-5654.5	-4144.9	2353.9	936.4	-777.3	7310.3	3099.5	2315.5	-52.2	-196.1
	Chi-Sq	78.2	79.5	42.2	13.1	2.0	1.4	135.8	26.0	14.0	0.0	392.2
70-	Observed	507440.0	505646.0	501057.0	547176.0	570059.0	594894.0	595312.0	536769.0	486983.0	530534.0	5375870.0
	Expected	504982.1	503758.1	496793.6	539420.8	566838.5	597340.6	595605.2	541074.7	493441.3	536808.9	5376063.9

Results for PHIM for Run 2 (Basic_US_OG_T1.RTF)

	Difference	2457.9	1887.9	4263.4	7755.2	3220.5	-2446.6	-293.2	-4305.7	-6458.3	-6274.9	-193.9
	Chi-Sq	12.0	7.1	36.6	111.5	18.3	10.0	0.1	34.3	84.5	73.3	387.7
75-	Observed	605146.0	615847.0	601792.0	652467.0	711435.0	736185.0	795741.0	779924.0	683129.0	660064.0	6841730.0
	Expected	605146.0	618315.8	598151.1	639796.9	700667.0	735375.4	793953.4	784058.0	696659.2	670050.5	6842173.4
	Difference	0.0	-2468.8	3640.9	12670.1	10768.0	809.6	1787.6	-4134.0	-13530.2	-9986.5	-443.4
	Chi-Sq	0.0	9.9	22.2	250.9	165.5	0.9	4.0	21.8	262.8	148.8	886.7
Total over ages	Observed	2608269.0	2622422.0	2539227.0	2628557.0	2717650.0	2757215.0	2820718.0	2820814.0	2750311.0	2904857.0	27170040.0
	Expected	2609088.3	2623186.8	2539420.5	2629332.0	2718150.2	2757383.4	2821537.5	2821202.2	2750692.1	2905392.1	27175385.0
	Difference	-819.3	-764.8	-193.5	-775.0	-500.2	-168.4	-819.5	-388.2	-381.1	-535.1	-5345.0
	Chi-Sq	1650.4	1550.2	388.4	1528.1	989.8	334.4	1616.8	767.2	760.0	1089.1	10674.5

Results for PHIM for Run 2 (Basic_US_OG_T1.RTF)

****

Osmond and Gardner Extrapolating Death Rates for COD: All Causes

Variable Parameter	Value
1. Country	US (United States)
2. Sex	F (Females)
3. Year of start of OG modelling	1966
4. Number 5-year periods for OG modelling	10
5. Age Range of interest	10 - 79
6. Scaling Factor for Rates	10000
7. Model Type to Save	4 (Full Age-Period-Cohort)
8. Goodness of Fit Required	1 (Analysis Printed For Age-Period-Cohort Model)
9. Cause of Death	All Causes
Note:	Death rates are per million population
10. Number of Periods into the future to Predict	5
11. Earliest projected year	2016
12. Extrapolate Period using (1: last 2 points 2: linear regression)	1
13. Ratio of last two period values	0.98199

Predictions of rates for future years from model:	Full Age-Period-Cohort
Effects for extending model to project rates for:	2016-2040

Extrapolating Model: Full Age-Period-Cohort
Log Transform Parameters

Model	ChiSq	MChiSq	DF	Factor	%Account	P
Age Only	250006.823	17857.630	14	P, C	92.7335	0.0000
Age-Period	32018.213	2287.015	14	Cohort	43.2612	0.0000
Age-Cohort	20972.888	1498.063	14	Period	13.3798	0.0000
Period-Cohort	19888.774	1420.627	14	Age	8.6583	0.0000
Full Age-Period-Cohort	18166.754	1297.625	14			0.0000

**Key to terms:**	Chisq =	chi-squared value for model
	MChisq =	mean Chi-squared (Chisq/DF)
	DF =	degrees of freedom
	Factor =	Factors not included in the model
	% Account =	1- (Chisq for full model)/(Chisq for model in question)
	P =	probability value based on Chisq and DF.

AGE	EFFECT
10	231.656134
15	510.416171
20	609.364503
25	703.679243
30	927.241125
35	1336.96236
40	2016.63729
45	3065.80341
50	4625.68732
55	6892.88243
60	10416.3270
65	15609.9630
70	24003.7647
75	37614.0888

PERIOD	EFFECT	
Period Change =	0.981986	
1966	1.253522	
1971	1.183831	
1976	1.066225	
1981	1.023155	
1986	0.996169	
1991	0.971753	
1996	0.954113	
2001	0.928126	
2006	0.872740	
2011	0.857018	
2016	0.841580	
2021	0.826420	
2026	0.811533	
2031	0.796914	
2036	0.782558	
2016	0.847722	Extrapolated
2017	0.844645	Extrapolated
2018	0.841580	Extrapolated
2019	0.838526	Extrapolated

Results for PHIM for Run 2 (Basic_US_OG_T1.RTF)

2020	0.835483	Extrapolated
2021	0.832451	Extrapolated
2022	0.829430	Extrapolated
2023	0.826420	Extrapolated
2024	0.823421	Extrapolated
2025	0.820432	Extrapolated
2026	0.817455	Extrapolated
2027	0.814488	Extrapolated
2028	0.811533	Extrapolated
2029	0.808588	Extrapolated
2030	0.805653	Extrapolated
2031	0.802729	Extrapolated
2032	0.799816	Extrapolated
2033	0.796914	Extrapolated
2034	0.794022	Extrapolated
2035	0.791140	Extrapolated
2036	0.788269	Extrapolated
2037	0.785408	Extrapolated
2038	0.782558	Extrapolated
2039	0.779718	Extrapolated
2040	0.776889	Extrapolated

Results for PHIM for Run 2 (Basic_US_OG_T1.RTF)

COHORT	EFFECT	WEIGHT	ORIGINAL
1891	1.120103	1.000	
1896	1.079071	2.000	
1901	1.029008	4.000	
1906	0.985861	8.000	
1911	1.031054	16.000	
1916	1.051272	32.000	
1921	1.056901	64.000	
1926	1.050364	128.000	
1931	1.025037	256.000	
1936	0.980179	512.000	
1941	0.935945	1024.000	
1946	0.886507	2048.000	
1951	0.883101	4096.000	
1956	0.934556	8192.000	
1961	0.969705	16384.000	
1966	0.929363	32768.000	
1971	0.908260	65536.000	
1976	0.871458	131072.000	
1981	0.925593	262144.000	
1986	0.874599	524288.000	
1991	0.781384	1048576.000	
1996	0.773076	Extrapolated	0.616424
2001	0.743794	Extrapolated	0.582728
2006	0.715621	Extrapolated	
2011	0.688515	Extrapolated	
2016	0.662436	Extrapolated	
2021	0.637345	Extrapolated	
2026	0.613204	Extrapolated	

Standardizing Population: The 1976 European Standard Population

Age Range	Population, Females
All	100000
0	0
1	0
2	0
3	0
0-4	8000
5-9	7000
10-14	7000
15-19	7000
20-24	7000
25-29	7000
30-34	7000
35-39	7000
40-44	7000
45-49	7000
50-54	7000
55-59	6000
60-64	5000
65-69	4000
70-74	3000
75-79	2000
80-84	1000
85+	1000

Results for PHIM for Run 2 (Basic_US_OG_T1.RTF)

Matrix of observed and expected rates including predictions

Total over ages standardized using: The 1976 European Standard Population

		1966-	1971-	1976-	1981-	1986-	1991-	1996-	2001-	2006-	2011-	2016-	2021-	2026-	2031-	2036-
10	OBS	299.2	286.9	239.5	213.9	194.4	189.9	169.3	149.7	126.0	115.7	121.3	.	.	.	.
	EXP											139.5	131.8	124.5	117.7	111.2
15	OBS	595.6	587.5	544.2	467.1	486.0	441.8	421.7	382.4	325.0	268.3	294.3	.	.	.	.
	EXP											319.5	301.9	285.2	269.5	254.6
20	OBS	719.4	697.9	634.2	562.2	535.3	516.8	459.3	489.8	463.4	444.3	492.8	.	.	.	.
	EXP											396.5	374.6	353.9	334.4	315.9
25	OBS	854.0	783.0	686.5	626.4	651.6	628.7	566.1	542.5	583.0	576.3	671.3	.	.	.	.
	EXP											462.7	449.6	424.7	401.3	379.1
30	OBS	1199.8	1081.2	863.7	787.5	839.5	874.8	781.9	758.1	719.5	790.8	905.0	.	.	.	.
	EXP											682.5	598.8	581.7	549.6	519.3
35	OBS	1834.2	1641.5	1307.4	1123.6	1133.3	1216.8	1178.8	1148.4	1081.0	1046.4	1206.9	.	.	.	.
	EXP											1041.4	966.3	847.8	823.7	778.2
40	OBS	2773.8	2602.1	2136.3	1817.6	1671.3	1706.4	1724.1	1812.2	1672.4	1607.9	1599.0	.	.	.	.
	EXP											1479.0	1542.6	1431.3	1255.8	1220.0
45	OBS	4191.0	3909.5	3371.7	2971.6	2728.4	2588.0	2507.0	2657.3	2644.0	2486.5	2526.1	.	.	.	.
	EXP											2343.4	2208.0	2302.9	2136.8	1874.7
50	OBS	6137.6	5784.9	5154.9	4771.1	4467.2	4183.1	3867.9	3811.6	3825.0	3882.1	3944.9	.	.	.	.
	EXP											3617.9	3472.1	3271.4	3412.0	3165.9
55	OBS	8700.0	8432.3	7652.6	7345.4	7014.6	6689.5	6262.4	5771.4	5389.4	5532.0	5800.3	.	.	.	.
	EXP											5625.2	5294.0	5080.6	4786.9	4992.7
60	OBS	12378.8	12345.2	11537.5	11232.5	10931.5	10538.3	10059.2	9275.3	8179.4	7860.6	7909.2	.	.	.	.
	EXP											8192.5	8347.5	7856.1	7539.4	7103.6
65	OBS	19853.1	17962.0	16985.9	16884.0	16469.7	15904.5	15550.1	14319.8	12828.0	11858.3	11447.7	.	.	.	.
	EXP											11601.3	12056.1	12284.2	11561.1	11095.0
70	OBS	32626.5	29350.3	25448.1	25686.3	25280.6	24552.0	24043.9	22654.6	20265.1	19028.9	17158.8	.	.	.	.
	EXP											17908.4	17518.2	18205.0	18549.4	17457.5
75	OBS	52813.0	47857.8	41519.6	38692.3	39227.3	38468.0	38015.6	36475.5	32995.7	31126.1	28492.5	.	.	.	.
	EXP											29627.6	27557.1	26956.7	28013.5	28543.5
10-79	OBS	6352.3	5898.3	5247.0	5007.3	4890.0	4740.5	4577.9	4355.0	3992.9	3828.9	3746.2	.	.	.	.
	EXP	6354.8	5898.3	5252.9	5023.8	4901.9	4737.1	4572.6	4350.0	3990.5	3831.5	3704.5*	3611.0*	3551.7*	3487.3*	3369.2*

Drop in overall standardized Observed and Predicted rates

comparing the last observed rate during the model fitting period to the last observed and predicted rates where an observed rate is available (2016)

Observed and Predicted %Drop = 2.160% and 3.247%

Results for PHIM for Run 2 (Basic_US_OG_T1.RTF)

Matrix of observed and expected deaths including predictions

		1966-	1971-	1976-	1981-	1986-	1991-	1996-	2001-	2006-	2011-	2016-	2021-	2026-	2031-	2036-
10-	OBS	14979.0	14617.0	11213.0	9148.0	8164.0	8444.0	8260.0	7803.0	6486.0	5876.0	6240.0*	.	.	.	.
	EXP	13587.3	13547.9	10748.2	9206.1	8447.7	9266.3	9432.4	8757.1	8044.2	7500.1	7177.4*	6639.9*	6073.4*	5843.0*	5691.0*
	ChiSq	142.549	84.358	20.103	0.366	9.525	72.979	145.724	103.944	301.815	351.699	122.419*	.	.	.	.
15-	OBS	27374.0	29708.0	28228.0	22419.0	21491.0	19314.0	19727.0	19491.0	17394.0	14305.0	15340.0*	.	.	.	.
	EXP	25970.7	28557.1	27371.9	23292.8	20423.4	18897.7	21086.9	21115.8	18630.5	18031.2	16653.1*	15921.6*	14747.3*	13529.9*	13023.6*
	ChiSq	75.827	46.383	26.779	32.782	55.812	9.172	87.697	125.022	82.068	770.034	103.537*	.	.	.	.
20-	OBS	28587.0	32504.0	32691.0	29755.0	26215.0	23457.0	21023.0	23885.0	24162.0	24609.0	27370.0*	.	.	.	.
	EXP	26908.2	29668.2	31300.6	32000.8	27625.6	24409.5	23191.7	25527.3	24249.9	22601.0	22021.1*	20318.9*	19449.1*	18085.2*	16639.9*
	ChiSq	104.736	271.064	61.761	157.608	72.030	37.165	202.800	105.662	0.319	178.396	1299.257*	.	.	.	.
25-	OBS	27618.0	31475.0	32574.0	32920.0	34903.0	31452.0	26902.0	25960.0	29221.0	30985.0	38582.5*	.	.	.	.
	EXP	26698.9	29687.7	31439.8	35363.5	36409.1	31790.2	28977.6	27234.0	28492.9	28358.4	26594.5*	25857.1*	23910.1*	22938.8*	21375.8*
	ChiSq	31.642	107.598	40.915	168.844	62.305	3.599	148.664	59.600	18.607	243.287	5403.789*	.	.	.	.
30-	OBS	34525.0	35478.0	35457.0	38135.0	44925.0	48023.0	40658.0	37434.0	35370.0	40704.0	49980.0*	.	.	.	.
	EXP	32782.8	33710.8	35979.4	40570.7	46193.0	47964.3	42754.8	38597.2	34666.9	37857.7	37692.7*	35218.0*	34270.0*	31775.2*	30503.6*
	ChiSq	92.589	92.644	7.584	146.224	34.809	0.072	102.836	35.056	14.259	214.004	4005.469*	.	.	.	.
35-	OBS	53951.0	47307.0	43129.0	46317.0	55118.0	65511.0	66000.0	61071.0	54406.0	52458.0	63160.0*	.	.	.	.
	EXP	50528.4	44708.9	44013.2	49986.5	57199.6	65368.6	69257.8	61329.1	53335.4	50059.0	54502.8*	54103.6*	50530.3*	49243.5*	45712.0*
	ChiSq	231.839	150.977	17.765	269.381	75.753	0.310	153.247	1.086	21.488	114.965	1375.097*	.	.	.	.
40-	OBS	87729.0	76208.0	61766.0	60217.0	69177.0	82895.0	93362.0	102173.0	89435.0	81675.0	80792.5*	.	.	.	.
	EXP	83978.0	71669.2	60935.9	63979.4	73714.9	84069.0	97374.9	102330.6	87468.8	79738.3	74728.3*	81207.2*	80602.1*	75318.5*	73445.3*
	ChiSq	167.542	287.442	11.308	221.251	279.349	16.395	165.377	0.243	44.198	47.041	492.112*	.	.	.	.
45-	OBS	130147.0	121967.0	98320.0	85704.0	89530.0	105668.0	121850.0	144032.0	148875.0	132859.0	128227.5*	.	.	.	.
	EXP	126133.3	118932.3	97706.0	88674.2	93795.8	107836.9	125550.6	144135.7	146090.9	130473.8	118954.8*	111392.0*	121107.6*	120293.9*	112430.1*
	ChiSq	127.723	77.436	3.859	99.487	194.009	43.624	109.074	0.075	53.056	43.605	722.817*	.	.	.	.
50-	OBS	174493.0	175362.0	158914.0	137932.0	127309.0	135622.0	157913.0	185539.0	206759.0	217142.0	208692.5*	.	.	.	.
	EXP	173301.6	175444.4	159701.5	140250.3	128717.2	136398.7	159734.4	184551.4	203939.6	215024.1	191393.9*	174516.6*	163602.5*	178104.7*	176999.9*
	ChiSq	8.190	0.039	3.883	38.320	15.406	4.422	20.769	5.285	38.977	20.861	1563.478*	.	.	.	.
55-	OBS	230110.0	231473.0	226882.0	221548.0	197615.0	185913.0	200217.0	233737.0	259586.0	294958.0	318017.5*	.	.	.	.
	EXP	235629.5	235484.1	230289.9	223426.1	198286.4	182464.1	196794.5	229689.0	255879.2	294354.9	308414.3*	274552.7*	250798.9*	235557.3*	256734.8*
	ChiSq	129.293	68.321	50.430	15.788	2.273	65.191	59.523	71.340	53.699	1.236	299.016*	.	.	.	.
60-	OBS	293085.0	308481.0	304275.0	320908.0	316885.0	285668.0	272825.0	290018.0	323744.0	368942.0	408812.5*	.	.	.	.
	EXP	304773.9	317698.9	307915.5	321806.8	315944.4	281255.9	264205.5	282923.6	318977.6	370013.9	423455.1*	443447.3*	395622.3*	362411.4*	341016.1*
	ChiSq	448.302	267.453	43.041	2.510	2.800	69.213	281.202	177.892	71.222	3.105	506.326*	.	.	.	.
65-	OBS	393085.0	396349.0	402929.0	423911.0	444824.0	434169.0	400928.0	372978.0	384761.0	449746.0	512737.5*	.	.	.	.
	EXP	398667.7	402003.5	407073.9	421557.1	443887.6	434946.3	393617.7	369878.5	382445.5	449798.2	519618.7*	594630.5*	624251.8*	559075.0*	513729.0*
	ChiSq	78.176	79.535	42.205	13.144	1.975	1.389	135.766	25.973	14.019	0.006	91.127*	.	.	.	.

Results for PHIM for Run 2 (Basic_US_OG_T1.RTF)

70-	OBS	507440.0	505646.0	501057.0	547176.0	570059.0	594894.0	595312.0	536769.0	486983.0	530534.0	604805.0*	.	.	.	.
	EXP	504982.1	503758.1	496793.6	539420.8	566838.5	597340.6	595605.2	541074.7	493441.3	536808.9	631228.7*	729945.9*	838741.2*	885444.0*	796585.0*
	ChiSq	11.963	7.076	36.588	111.495	18.297	10.021	0.144	34.264	84.528	73.350	1106.112*	.	.	.	.
75-	OBS	605146.0	615847.0	601792.0	652467.0	711435.0	736185.0	795741.0	779924.0	683129.0	660064.0	705092.5*	.	.	.	.
	EXP	605146.0	618315.8	598151.1	639796.9	700667.0	735375.4	793953.4	784058.0	696659.2	670050.5	733182.5*	864989.5*	1005595.4*	1166332.9*	1239735.3*
	ChiSq	.	9.857	22.162	250.911	165.485	0.891	4.025	21.797	262.779	148.840	1076.198*	.	.	.	.
Total Deaths		2608269.0	2622422.0	2539227.0	2628557.0	2717650.0	2757215.0	2820718.0	2820814.0	2750311.0	2904857.0	3167850.0*	.	.	.	.
Expected		2609088.3	2623186.8	2539420.5	2629332.0	2718150.2	2757383.4	2821537.5	2821202.2	2752322.1	2910670.0	3165618.0*	3432740.6*	3629301.9*	3723953.2*	3643621.5*
Obs/Exp		1.000	1.000	1.000	1.000	1.000	1.000	1.000	1.000	0.999	0.998	1.001*	.	.	.	.

Chi Squared (Log) = **18166.8 on 14 D.F.** **P = 0.0000**

Results for PHIM for Run 2 (Basic_US_OG_T1.RTF)

****

Observed and predicted rates (5 year periods)

		10-	15-	20-	25-	30-	35-	40-	45-	50-	55-	60-	65-	70-	75-
2016-	OBS	121.3	294.3	492.8	671.3	905.0	1206.9	1599.0	2526.1	3944.9	5800.3	7909.2	11447.7	17158.8	28492.5
	PRE	139.5	319.5	396.5	462.7	682.5	1041.4	1479.0	2343.4	3617.9	5625.2	8192.5	11601.3	17908.4	29627.6
	RES	-18.221	-25.193	96.299	208.587	222.482	165.421	120.021	182.672	326.993	175.152	-283.287	-153.634	-749.658	-1135.106
2021-	PRE	131.8	301.9	374.6	449.6	598.8	966.3	1542.6	2208.0	3472.1	5294.0	8347.5	12056.1	17518.2	27557.1
2026-	PRE	124.5	285.2	353.9	424.7	581.7	847.8	1431.3	2302.9	3271.4	5080.6	7856.1	12284.2	18205.0	26956.7
2031-	PRE	117.7	269.5	334.4	401.3	549.6	823.7	1255.8	2136.8	3412.0	4786.9	7539.4	11561.1	18549.4	28013.5
2036-	PRE	111.2	254.6	315.9	379.1	519.3	778.2	1220.0	1874.7	3165.9	4992.7	7103.6	11095.0	17457.5	28543.5

Results for PHIM for Run 2 (Basic_US_OG_T1.RTF)

****

Observed and predicted deaths (5 year periods)

		10-	15-	20-	25-	30-	35-	40-	45-	50-	55-	60-	65-	70-	75-
2016-	OBS	6240.0	15340.0	27370.0	38582.5	49980.0	63160.0	80792.5	128227.5	208692.5	318017.5	408812.5	512737.5	604805.0	705092.5
	PRE	7177.4	16653.1	22021.1	26594.5	37692.7	54502.8	74728.3	118954.8	191393.9	308414.3	423455.1	519618.7	631228.7	733182.5
	CHI	122.419	103.537	1299.257	5403.789	4005.469	1375.097	492.112	722.817	1563.478	299.016	506.326	91.127	1106.112	1076.198
2021-	PRE	6639.9	15921.6	20318.9	25857.1	35218.0	54103.6	81207.2	111392.0	174516.6	274552.7	443447.3	594630.5	729945.9	864989.5
2026-	PRE	6073.4	14747.3	19449.1	23910.1	34270.0	50530.3	80602.1	121107.6	163602.5	250798.9	395622.3	624251.8	838741.2	1005595.4
2031-	PRE	5843.0	13529.9	18085.2	22938.8	31775.2	49243.5	75318.5	120293.9	178104.7	235557.3	362411.4	559075.0	885444.0	1166332.9
2036-	PRE	5691.0	13023.6	16639.9	21375.8	30503.6	45712.0	73445.3	112430.1	176999.9	256734.8	341016.1	513729.0	796585.0	1239735.3

Results for PHIM for Run 2 (Basic_US_OG_T1.RTF)

****

Observed and predicted rates (single years)

		10-	15-	20-	25-	30-	35-	40-	45-	50-	55-	60-	65-	70-	75-
2016	OBS	122.4	296.6	484.5	671.8	913.2	1222.4	1597.7	2517.8	3899.9	5762.9	8061.2	11961.6	18382.0	30175.4
	PRE	140.5	321.8	399.3	466.1	687.5	1049.0	1489.8	2360.5	3644.3	5666.2	8252.3	11686.0	18039.1	29843.8
	RES	-18.141	-25.252	85.155	205.717	225.756	173.320	107.904	157.330	255.635	96.628	-191.078	275.602	342.918	331.553
2017	OBS	121.5	291.1	489.3	693.1	933.8	1240.2	1608.7	2502.4	3799.6	5775.9	8142.3	11724.9	18617.3	30460.4
	PRE	140.0	320.7	397.9	464.4	685.0	1045.2	1484.4	2352.0	3631.1	5645.7	8222.3	11643.6	17973.6	29735.5
	RES	-18.564	-29.515	91.370	228.675	248.835	194.933	124.351	150.411	168.528	130.234	-80.058	81.302	643.637	724.870
2018	PRE	139.5	319.5	396.5	462.7	682.5	1041.4	1479.0	2343.4	3617.9	5625.2	8192.5	11601.3	17908.4	29627.6
2019	PRE	139.0	318.3	395.0	461.1	680.0	1037.7	1473.6	2334.9	3604.8	5604.8	8162.8	11559.2	17843.4	29520.1
2020	PRE	138.5	317.2	393.6	459.4	677.5	1033.9	1468.3	2326.4	3591.7	5584.4	8133.1	11517.3	17778.7	29412.9
2021	PRE	132.8	304.1	377.3	452.9	603.1	973.4	1553.8	2224.1	3497.4	5332.7	8408.4	12144.1	17646.1	27758.2
2022	PRE	132.3	303.0	375.9	451.2	600.9	969.9	1548.2	2216.0	3484.7	5313.3	8377.9	12100.0	17582.0	27657.5
2023	PRE	131.8	301.9	374.6	449.6	598.8	966.3	1542.6	2208.0	3472.1	5294.0	8347.5	12056.1	17518.2	27557.1
2024	PRE	131.3	300.8	373.2	447.9	596.6	962.8	1537.0	2199.9	3459.5	5274.8	8317.2	12012.4	17454.7	27457.1
2025	PRE	130.9	299.7	371.9	446.3	594.4	959.3	1531.4	2192.0	3446.9	5255.7	8287.0	11968.8	17391.3	27357.5
2026	PRE	125.4	287.3	356.5	427.8	586.0	854.0	1441.8	2319.7	3295.2	5117.7	7913.4	12373.9	18337.9	27153.4
2027	PRE	125.0	286.2	355.2	426.3	583.8	850.9	1436.6	2311.3	3283.3	5099.1	7884.7	12329.0	18271.3	27054.9
2028	PRE	124.5	285.2	353.9	424.7	581.7	847.8	1431.3	2302.9	3271.4	5080.6	7856.1	12284.2	18205.0	26956.7
2029	PRE	124.1	284.2	352.6	423.2	579.6	844.7	1426.1	2294.5	3259.5	5062.2	7827.6	12239.6	18138.9	26858.9
2030	PRE	123.6	283.1	351.3	421.7	577.5	841.7	1421.0	2286.2	3247.7	5043.8	7799.2	12195.2	18073.1	26761.4
2031	PRE	118.5	271.4	336.8	404.2	553.6	829.7	1264.9	2152.4	3436.9	4821.9	7594.4	11645.5	18684.8	28217.9
2032	PRE	118.1	270.4	335.6	402.8	551.6	826.7	1260.3	2144.6	3424.4	4804.4	7566.8	11603.2	18617.0	28115.5
2033	PRE	117.7	269.5	334.4	401.3	549.6	823.7	1255.8	2136.8	3412.0	4786.9	7539.4	11561.1	18549.4	28013.5
2034	PRE	117.2	268.5	333.1	399.8	547.6	820.7	1251.2	2129.0	3399.6	4769.6	7512.0	11519.1	18482.1	27911.8
2035	PRE	116.8	267.5	331.9	398.4	545.6	817.7	1246.7	2121.3	3387.3	4752.3	7484.8	11477.3	18415.0	27810.5
2036	PRE	112.0	256.4	318.2	381.9	523.1	783.9	1228.9	1888.4	3189.0	5029.2	7155.4	11176.0	17584.9	28751.8
2037	PRE	111.6	255.5	317.0	380.5	521.2	781.0	1224.5	1881.5	3177.5	5010.9	7129.5	11135.4	17521.1	28647.4
2038	PRE	111.2	254.6	315.9	379.1	519.3	778.2	1220.0	1874.7	3165.9	4992.7	7103.6	11095.0	17457.5	28543.5
2039	PRE	110.8	253.7	314.7	377.8	517.4	775.4	1215.6	1867.9	3154.4	4974.6	7077.8	11054.8	17394.1	28439.9
2040	PRE	110.4	252.7	313.6	376.4	515.5	772.6	1211.2	1861.1	3143.0	4956.6	7052.1	11014.6	17331.0	28336.7

Results for PHIM for Run 2 (Basic_US_OG_T1.RTF)

****

Observed and predicted deaths (single years)

		10-	15-	20-	25-	30-	35-	40-	45-	50-	55-	60-	65-	70-	75-
2016	OBS	1249.0	3099.0	5463.0	7542.0	9817.0	12468.0	16109.0	25816.0	42614.0	63496.0	80565.0	102266.0	117072.0	138044.0
	PRE	1434.1	3362.9	4502.8	5232.6	7390.2	10700.1	15021.0	24202.9	39820.7	62431.3	82474.7	99909.7	114888.0	136527.2
	CHI	23.897	20.703	204.740	1019.234	796.938	292.081	78.799	107.516	195.939	18.156	44.218	55.570	41.517	16.851
2017	OBS	1247.0	3037.0	5485.0	7891.0	10175.0	12796.0	16208.0	25475.0	40863.0	63711.0	82960.0	102829.0	124850.0	143993.0
	PRE	1437.6	3344.9	4460.7	5287.5	7463.6	10784.7	14955.2	23943.8	39050.6	62274.5	83775.7	102116.0	120533.7	140566.4
	CHI	25.269	28.337	235.214	1281.920	984.965	375.104	104.953	97.925	84.120	33.138	7.942	4.979	154.568	83.532
2018	PRE	1439.7	3329.2	4404.8	5336.0	7535.3	10888.7	14909.1	23752.9	38230.3	61886.7	84950.2	103981.5	126454.0	145480.7
2019	PRE	1437.6	3315.0	4350.1	5366.2	7610.3	11003.2	14901.4	23598.9	37472.6	61298.8	85845.3	105804.6	132079.6	151544.4
2020	PRE	1428.3	3301.5	4304.2	5370.6	7690.1	11121.5	14941.3	23461.4	36844.6	60536.2	86367.8	107729.7	137061.9	158851.9
2021	PRE	1365.4	3180.3	4103.9	5286.3	6935.4	10613.3	15947.2	22387.4	35491.1	57077.9	89692.2	115747.3	140185.4	157115.5
2022	PRE	1349.9	3188.9	4076.1	5235.2	7004.2	10715.5	16069.5	22287.6	35114.3	55985.0	89477.6	117553.2	143258.7	164889.0
2023	PRE	1329.4	3195.1	4057.4	5170.6	7065.9	10817.1	16224.5	22224.0	34847.3	54839.2	88973.1	119252.1	145989.6	173186.1
2024	PRE	1307.6	3189.6	4045.4	5108.4	7103.3	10922.0	16393.9	22216.5	34632.9	53782.0	88185.0	120592.1	148735.4	181163.2
2025	PRE	1288.1	3167.5	4036.5	5058.5	7107.1	11031.1	16565.4	22276.0	34438.1	52903.3	87136.7	121425.6	151663.8	188338.0
2026	PRE	1229.6	3028.2	3881.8	4824.2	6998.7	9949.6	15809.2	23776.9	32865.1	50972.9	82176.0	126103.5	162963.4	192561.8
2027	PRE	1221.2	2995.4	3889.1	4793.3	6934.9	10049.5	15963.5	23962.7	32725.9	50448.8	80635.1	125856.9	165603.8	196895.0
2028	PRE	1213.8	2952.5	3898.4	4773.5	6853.0	10138.3	16116.2	24197.0	32640.5	50080.6	79020.6	125232.0	168152.0	200949.1
2029	PRE	1207.1	2907.2	3898.4	4762.2	6774.0	10192.0	16272.6	24451.9	32637.6	49786.2	77534.0	124224.0	170248.0	205195.1
2030	PRE	1201.9	2865.3	3881.2	4757.2	6711.6	10197.8	16433.8	24709.0	32732.6	49520.0	76305.3	122856.8	171682.6	209825.5
2031	PRE	1160.3	2736.2	3706.2	4575.8	6406.6	10049.2	14828.7	23589.5	34954.2	47287.6	73580.4	115945.0	178423.8	225739.1
2032	PRE	1165.1	2719.0	3664.9	4584.7	6369.0	9962.5	14979.9	23823.8	35235.9	47106.8	72869.5	113851.1	178246.9	229741.3
2033	PRE	1169.8	2704.2	3616.5	4596.2	6344.3	9848.5	15112.7	24053.7	35585.9	46998.7	72373.6	111658.6	177585.8	233710.5
2034	PRE	1173.2	2691.2	3569.9	4598.2	6330.4	9737.5	15192.6	24288.3	35965.9	47007.6	71979.3	109658.4	176427.0	237170.7
2035	PRE	1174.5	2679.7	3529.1	4583.4	6325.2	9649.0	15200.2	24528.5	36347.7	47154.8	71621.3	108027.4	174781.9	239826.5
2036	PRE	1136.3	2585.7	3363.6	4376.7	6085.9	9215.2	14982.9	22134.5	34704.6	50369.2	68413.2	104197.5	164953.2	249208.0
2037	PRE	1139.8	2596.8	3338.9	4328.9	6098.0	9163.5	14857.2	22360.9	35051.6	50785.7	68174.1	103233.5	162038.2	249098.9
2038	PRE	1140.6	2608.0	3321.7	4273.4	6112.0	9128.4	14689.9	22559.3	35392.1	51297.9	68040.4	102582.4	159037.2	248488.1
2039	PRE	1138.9	2615.5	3311.1	4220.8	6113.4	9107.4	14526.0	22678.6	35740.0	51851.6	68075.2	102084.3	156367.5	247334.7
2040	PRE	1135.4	2617.1	3304.9	4177.6	6093.7	9097.9	14393.8	22690.1	36096.5	52408.2	68309.7	101647.2	154276.5	245621.9

Results for PHIM for Run 2 (Basic_US_OG_T1.RTF)

****

List of values created by O and G modelling, using percentage change in last two period parameters for Fixed File MORT

1. Country	US (United States)
2. Sex	F (Females)
3. Disease	ALL (All Causes)
* Value comes from O and G Modelling.	

Age	Years	Value	Death Rate	Population
10-14	2018	1439.7497	139.515416	103196.46 *
10-14	2019	1437.5838	139.009109	103416.52 *
10-14	2020	1428.2562	138.504640	103119.74 *
10-14	2021	1365.4383	132.774846	102838.63 *
10-14	2022	1349.9413	132.293001	102041.78 *
10-14	2023	1329.3900	131.812904	100854.31 *
10-14	2024	1307.5927	131.334550	99561.97 *
10-14	2025	1288.1362	130.857932	98437.76 *
10-14	2026	1229.5946	125.444474	98019.03 *
10-14	2027	1221.2363	124.989232	97707.32 *
10-14	2028	1213.7655	124.535641	97463.30 *
10-14	2029	1207.0508	124.083696	97277.15 *
10-14	2030	1201.8864	123.633392	97213.74 *
10-14	2031	1160.2717	118.518806	97897.69 *
10-14	2032	1165.0527	118.088696	98659.12 *
10-14	2033	1169.8007	117.660148	99422.00 *
10-14	2034	1173.1963	117.233155	100073.76 *
10-14	2035	1174.4513	116.807711	100545.70 *
10-14	2036	1136.2619	111.975497	101474.16 *
10-14	2037	1139.7915	111.569133	102160.11 *
10-14	2038	1140.5639	111.164245	102601.69 *
10-14	2039	1138.9436	110.760825	102829.10 *
15-19	2018	3329.2139	319.501130	104200.38 *
15-19	2019	3315.0215	318.341648	104134.08 *
15-19	2020	3301.4620	317.186374	104085.87 *
15-19	2021	3180.3261	304.064701	104593.73 *
15-19	2022	3188.9345	302.961238	105258.83 *
15-19	2023	3195.0925	301.861780	105846.21 *
15-19	2024	3189.5900	300.766312	106048.78 *
15-19	2025	3167.5362	299.674819	105699.11 *
15-19	2026	3028.2220	287.277581	105411.01 *
15-19	2027	2995.3879	286.235040	104647.84 *
15-19	2028	2952.4677	285.196282	103524.06 *
15-19	2029	2907.2343	284.161294	102309.30 *
15-19	2030	2865.3156	283.130061	101201.39 *
15-19	2031	2736.1702	271.417263	100810.47 *
15-19	2032	2719.0127	270.432279	100543.20 *
15-19	2033	2704.2097	269.450870	100360.03 *
15-19	2034	2691.1583	268.473022	100239.43 *
15-19	2035	2679.6631	267.498723	100174.80 *
15-19	2036	2585.6779	256.432577	100832.66 *
15-19	2037	2596.8196	255.501973	101635.99 *
15-19	2038	2608.0106	254.574747	102445.77 *
15-19	2039	2615.4886	253.650885	103113.72 *
20-24	2018	4404.7750	396.455734	111103.83 *
20-24	2019	4350.0929	395.016981	110124.20 *
20-24	2020	4304.2337	393.583449	109360.13 *
20-24	2021	4103.9063	377.301307	108770.00 *
20-24	2022	4076.0573	375.932066	108425.37 *
20-24	2023	4057.4142	374.567794	108322.56 *
20-24	2024	4045.4119	373.208473	108395.50 *
20-24	2025	4036.4765	371.854086	108550.01 *
20-24	2026	3881.7542	356.470866	108894.01 *
20-24	2027	3889.0616	355.177219	109496.37 *
20-24	2028	3898.3769	353.888267	110158.41 *
20-24	2029	3898.4098	352.603993	110560.57 *
20-24	2030	3881.1679	351.324380	110472.49 *
20-24	2031	3706.1634	336.790452	110043.60 *
20-24	2032	3664.9255	335.568227	109215.51 *
20-24	2033	3616.5172	334.350437	108165.47 *
20-24	2034	3569.8548	333.137066	107158.74 *
20-24	2035	3529.1027	331.928099	106321.30 *
20-24	2036	3363.6270	318.196576	105709.09 *
20-24	2037	3338.8667	317.041828	105313.13 *
20-24	2038	3321.6858	315.891271	105152.82 *
20-24	2039	3311.1461	314.744890	105200.95 *
25-29	2018	5336.0020	462.737262	115313.86 *
25-29	2019	5366.1601	461.057971	116387.97 *

Results for PHIM for Run 2 (Basic_US_OG_T1.RTF)

25-29	2020	5370.6100	459.384774	116908.75 *
25-29	2021	5286.2624	452.851152	116732.89 *
25-29	2022	5235.1658	451.207738	116025.62 *
25-29	2023	5170.5843	449.570288	115011.70 *
25-29	2024	5108.3754	447.938780	114041.82 *
25-29	2025	5058.4954	446.313193	113339.59 *
25-29	2026	4824.1643	427.849676	112753.72 *
25-29	2027	4793.3171	426.296993	112440.79 *
25-29	2028	4773.4830	424.749945	112383.37 *
25-29	2029	4762.2012	423.208511	112526.12 *
25-29	2030	4757.2065	421.672671	112817.52 *
25-29	2031	4575.7810	404.228508	113197.88 *
25-29	2032	4584.7346	402.761547	113832.48 *
25-29	2033	4596.2026	401.299910	114532.86 *
25-29	2034	4598.2303	399.843577	115000.73 *
25-29	2035	4583.4041	398.392529	115047.44 *
25-29	2036	4376.7086	381.911441	114600.09 *
25-29	2037	4328.9319	380.525470	113761.95 *
25-29	2038	4273.3732	379.144528	112710.93 *
25-29	2039	4220.7504	377.768598	111728.46 *
30-34	2018	7535.2868	682.491175	110408.56 *
30-34	2019	7610.2811	680.014388	111913.53 *
30-34	2020	7690.1301	677.546589	113499.65 *
30-34	2021	6935.3802	603.136488	114988.57 *
30-34	2022	7004.2417	600.947682	116553.27 *
30-34	2023	7065.8754	598.766820	118007.13 *
30-34	2024	7103.2519	596.593872	119063.44 *
30-34	2025	7107.1412	594.428810	119562.53 *
30-34	2026	6998.6705	585.974518	119436.43 *
30-34	2027	6934.9009	583.847994	118779.22 *
30-34	2028	6853.0345	581.729187	117804.55 *
30-34	2029	6773.9715	579.618070	116869.57 *
30-34	2030	6711.5936	577.514613	116215.13 *
30-34	2031	6406.6405	553.623430	115721.99 *
30-34	2032	6369.0337	551.614309	115461.72 *
30-34	2033	6344.3038	549.612479	115432.31 *
30-34	2034	6330.3541	547.617914	115598.01 *
30-34	2035	6325.1511	545.630588	115923.69 *
30-34	2036	6085.9248	523.058413	116352.68 *
30-34	2037	6097.9654	521.160214	117007.50 *
30-34	2038	6112.0448	519.268904	117704.81 *
30-34	2039	6113.4453	517.384457	118160.59 *
35-39	2018	10888.6553	1041.440516	104553.79 *
35-39	2019	11003.2254	1037.661088	106038.72 *
35-39	2020	11121.5298	1033.895376	107569.20 *
35-39	2021	10613.2644	973.389643	109034.08 *
35-39	2022	10715.5320	969.857174	110485.67 *
35-39	2023	10817.1475	966.337525	111939.64 *
35-39	2024	10922.0100	962.830649	113436.46 *
35-39	2025	11031.1034	959.336499	114986.80 *
35-39	2026	9949.6468	853.979425	116509.21 *
35-39	2027	10049.5413	850.880301	118107.58 *
35-39	2028	10138.3274	847.792424	119585.02 *
35-39	2029	10191.9990	844.715752	120655.96 *
35-39	2030	10197.7626	841.650246	121163.90 *
35-39	2031	10049.1743	829.679836	121121.11 *
35-39	2032	9962.4655	826.668896	120513.37 *
35-39	2033	9848.4515	823.668883	119568.09 *
35-39	2034	9737.4909	820.679756	118651.53 *
35-39	2035	9649.0091	817.701478	118001.61 *
35-39	2036	9215.1884	783.874011	117559.56 *
35-39	2037	9163.5044	781.029302	117326.00 *
35-39	2038	9128.4396	778.194917	117302.74 *
35-39	2039	9107.4451	775.370818	117459.22 *
40-44	2018	14909.0807	1479.004593	100804.83 *
40-44	2019	14901.4373	1473.637228	101120.12 *
40-44	2020	14941.3373	1468.289342	101760.17 *
40-44	2021	15947.1707	1553.839897	102630.72 *
40-44	2022	16069.4542	1548.200953	103794.37 *
40-44	2023	16224.5060	1542.582471	105177.56 *
40-44	2024	16393.9181	1536.984380	106662.88 *
40-44	2025	16565.3876	1531.406605	108171.06 *
40-44	2026	15809.1993	1441.785467	109650.15 *
40-44	2027	15963.5377	1436.553172	111123.89 *
40-44	2028	16116.2371	1431.339865	112595.46 *
40-44	2029	16272.5680	1426.145478	114101.74 *
40-44	2030	16433.8357	1420.969941	115652.24 *
40-44	2031	14828.6601	1264.914964	117230.49 *

Results for PHIM for Run 2 (Basic_US_OG_T1.RTF)

40-44	2032	14979.8721	1260.324539	118857.26 *
40-44	2033	15112.6843	1255.750772	120347.80 *
40-44	2034	15192.6171	1251.193604	121424.99 *
40-44	2035	15200.2365	1246.652974	121928.37 *
40-44	2036	14982.9395	1228.922394	121919.33 *
40-44	2037	14857.1968	1224.462587	121336.47 *
40-44	2038	14689.9458	1220.018965	120407.52 *
40-44	2039	14525.9899	1215.591470	119497.30 *
45-49	2018	23752.8961	2343.417489	101360.07 *
45-49	2019	23598.9250	2334.913137	101069.82 *
45-49	2020	23461.4436	2326.439647	100846.99 *
45-49	2021	22387.4435	2224.073916	100659.62 *
45-49	2022	22287.6396	2216.002666	100575.87 *
45-49	2023	22223.9834	2207.960706	100653.89 *
45-49	2024	22216.5372	2199.947932	100986.65 *
45-49	2025	22275.9505	2191.964236	101625.52 *
45-49	2026	23776.8991	2319.680043	102500.77 *
45-49	2027	23962.6866	2311.261835	103677.94 *
45-49	2028	24196.9829	2302.874176	105072.97 *
45-49	2029	24451.8802	2294.516957	106566.57 *
45-49	2030	24708.9868	2286.190067	108079.32 *
45-49	2031	23589.5245	2152.397413	109596.51 *
45-49	2032	23823.7779	2144.586279	111087.99 *
45-49	2033	24053.6593	2136.803493	112568.42 *
45-49	2034	24288.2948	2129.048951	114080.49 *
45-49	2035	24528.5175	2121.322550	115628.42 *
45-49	2036	22134.5092	1888.352850	117215.96 *
45-49	2037	22360.8855	1881.499944	118846.06 *
45-49	2038	22559.2731	1874.671908	120337.18 *
45-49	2039	22678.6469	1867.868652	121414.57 *
50-54	2018	38230.3077	3617.903936	105669.77 *
50-54	2019	37472.6359	3604.774423	103952.79 *
50-54	2020	36844.5592	3591.692557	102582.72 *
50-54	2021	35491.1315	3497.396102	101478.73 *
50-54	2022	35114.3334	3484.703917	100767.05 *
50-54	2023	34847.2664	3472.057792	100364.88 *
50-54	2024	34632.9064	3459.457560	100110.80 *
50-54	2025	34438.0946	3446.903055	99910.25 *
50-54	2026	32865.0638	3295.235785	99735.09 *
50-54	2027	32725.8591	3283.277247	99674.37 *
50-54	2028	32640.5029	3271.362107	99776.49 *
50-54	2029	32637.6112	3259.490208	100131.03 *
50-54	2030	32732.5978	3247.661392	100788.21 *
50-54	2031	34954.1808	3436.887881	101703.00 *
50-54	2032	35235.8840	3424.415282	102896.06 *
50-54	2033	35585.9186	3411.987947	104296.73 *
50-54	2034	35965.8669	3399.605711	105794.23 *
50-54	2035	36347.7036	3387.268411	107306.83 *
50-54	2036	34704.6331	3189.038335	108824.76 *
50-54	2037	35051.5940	3177.465192	110313.07 *
50-54	2038	35392.1160	3165.934048	111790.44 *
50-54	2039	35740.0388	3154.444751	113300.57 *
55-59	2018	61886.7497	5625.172727	110017.51 *
55-59	2019	61298.7982	5604.758758	109369.20 *
55-59	2020	60536.2454	5584.418872	108402.05 *
55-59	2021	57077.9220	5332.671300	107034.39 *
55-59	2022	55984.9687	5313.318830	105367.23 *
55-59	2023	54839.1833	5294.036591	103586.71 *
55-59	2024	53782.0034	5274.824328	101959.80 *
55-59	2025	52903.3355	5255.681787	100659.32 *
55-59	2026	50972.9257	5117.698885	99601.26 *
55-59	2027	50448.8460	5099.126559	98936.25 *
55-59	2028	50080.5918	5080.621631	98571.78 *
55-59	2029	49786.2037	5062.183859	98349.26 *
55-59	2030	49519.9845	5043.812999	98179.66 *
55-59	2031	47287.6135	4821.880053	98068.83 *
55-59	2032	47106.8286	4804.381264	98049.73 *
55-59	2033	46998.7335	4786.945979	98181.04 *
55-59	2034	47007.5951	4769.573967	98557.22 *
55-59	2035	47154.7677	4752.264999	99225.88 *
55-59	2036	50369.2243	5029.157911	100154.39 *
55-59	2037	50785.7118	5010.906903	101350.34 *
55-59	2038	51297.9282	4992.722129	102745.41 *
55-59	2039	51851.6090	4974.603347	104232.65 *
60-64	2018	84950.2380	8192.481992	103692.92 *
60-64	2019	85845.3112	8162.751159	105167.13 *
60-64	2020	86367.7788	8133.128220	106192.57 *
60-64	2021	89692.2494	8408.388773	106669.96 *

Results for PHIM for Run 2 (Basic_US_OG_T1.RTF)

60-64	2022	89477.5753	8377.874406	106802.24 *
60-64	2023	88973.1116	8347.470776	106586.91 *
60-64	2024	88185.0201	8317.177483	106027.58 *
60-64	2025	87136.7405	8286.994125	105148.79 *
60-64	2026	82176.0275	7913.413507	103843.97 *
60-64	2027	80635.1085	7884.695424	102267.88 *
60-64	2028	79020.6321	7856.081560	100585.30 *
60-64	2029	77533.9590	7827.571537	99052.38 *
60-64	2030	76305.3143	7799.164978	97837.80 *
60-64	2031	73580.3557	7594.405356	96887.58 *
60-64	2032	72869.5494	7566.844966	96301.10 *
60-64	2033	72373.5609	7539.384594	95993.99 *
60-64	2034	71979.3113	7512.023876	95818.80 *
60-64	2035	71621.3476	7484.762451	95689.54 *
60-64	2036	68413.1780	7155.425225	95610.22 *
60-64	2037	68174.1009	7129.457911	95623.12 *
60-64	2038	68040.3874	7103.584833	95783.17 *
60-64	2039	68075.2478	7077.805649	96181.29 *
65-69	2018	103981.5082	11601.325444	89628.99 *
65-69	2019	105804.6269	11559.223786	91532.64 *
65-69	2020	107729.7448	11517.274917	93537.53 *
65-69	2021	115747.3169	12144.117126	95311.43 *
65-69	2022	117553.1899	12100.045656	97151.03 *
65-69	2023	119252.0692	12056.134124	98914.02 *
65-69	2024	120592.0982	12012.381948	100389.83 *
65-69	2025	121425.6219	11968.788550	101451.89 *
65-69	2026	126103.4894	12373.864588	101911.16 *
65-69	2027	125856.8898	12328.959356	102082.33 *
65-69	2028	125231.9670	12284.217087	101945.42 *
65-69	2029	124223.9700	12239.637189	101493.18 *
65-69	2030	122856.8077	12195.219073	100741.78 *
65-69	2031	115945.0078	11645.454297	99562.46 *
65-69	2032	113851.0817	11603.192494	98120.48 *
65-69	2033	111658.5915	11561.084061	96581.42 *
65-69	2034	109658.4051	11519.128440	95196.79 *
65-69	2035	108027.3726	11477.325079	94122.43 *
65-69	2036	104197.4921	11175.998879	93233.27 *
65-69	2037	103233.5421	11135.440748	92707.19 *
65-69	2038	102582.4155	11095.029804	92457.99 *
65-69	2039	102084.2593	11054.765513	92344.12 *
70-74	2018	126453.9588	17908.411059	70611.49 *
70-74	2019	132079.6055	17843.420744	74021.46 *
70-74	2020	137061.8542	17778.666281	77093.44 *
70-74	2021	140185.3779	17646.081607	79442.78 *
70-74	2022	143258.7349	17582.043296	81480.14 *
70-74	2023	145989.6327	17518.237383	83335.80 *
70-74	2024	148735.3553	17454.663023	85212.39 *
70-74	2025	151663.7658	17391.319377	87206.59 *
70-74	2026	162963.3882	18337.863863	88867.16 *
70-74	2027	165603.7632	18271.315048	90635.93 *
70-74	2028	168152.0468	18205.007740	92365.82 *
70-74	2029	170248.0377	18138.941064	93857.76 *
70-74	2030	171682.6381	18073.114147	94993.39 *
70-74	2031	178423.7610	18684.787204	95491.46 *
70-74	2032	178246.9101	18616.979390	95744.27 *
70-74	2033	177585.7622	18549.417653	95736.57 *
70-74	2034	176427.0321	18482.101101	95458.32 *
70-74	2035	174781.9187	18415.028843	94912.65 *
70-74	2036	164953.2292	17584.872849	93804.05 *
70-74	2037	162038.1662	17521.056667	92481.96 *
70-74	2038	159037.2389	17457.472076	91099.81 *
70-74	2039	156367.5394	17394.118236	89896.79 *
75-79	2018	145480.6568	29627.597125	49103.09 *
75-79	2019	151544.3581	29520.077431	51336.03 *
75-79	2020	158851.9197	29412.947931	54007.48 *
75-79	2021	157115.5094	27758.207900	56601.46 *
75-79	2022	164889.0374	27657.472293	59618.26 *
75-79	2023	173186.0813	27557.102258	62846.26 *
75-79	2024	181163.2405	27457.096471	65980.48 *
75-79	2025	188338.0395	27357.453608	68843.41 *
75-79	2026	192561.7611	27153.434982	70916.17 *
75-79	2027	196894.9680	27054.894118	72776.10 *
75-79	2028	200949.0707	26956.710863	74545.10 *
75-79	2029	205195.0510	26858.883919	76397.46 *
75-79	2030	209825.5269	26761.411993	78406.00 *
75-79	2031	225739.0827	28217.935584	79998.44 *
75-79	2032	229741.2588	28115.531607	81713.29 *
75-79	2033	233710.4613	28013.499258	83427.80 *

Results for PHIM for Run 2 (Basic_US_OG_T1.RTF)

75-79	2034	237170.7324	27911.837188	84971.38 *
75-79	2035	239826.4793	27810.544053	86235.81 *
75-79	2036	249207.9583	28751.774234	86675.68 *
75-79	2037	249098.8544	28647.432936	86953.29 *
75-79	2038	248488.0921	28543.470296	87056.02 *
75-79	2039	247334.6547	28439.884941	86967.53 *

Results for PHIM for Run 2 (Basic_US_OG_T1.RTF)

****
Osmond and Gardner Modeling of Death Rates for COD: LUNG CANCER

Variable Parameter	Value
1. Country	US (United States)
2. Sex	F (Females)
3. Year of start of OG modelling	1966
4. Number 5-year periods for OG modelling	10
5. Age Range of interest	10 - 79
6. Scaling Factor for Rates	10000
7. Model Type to Save	4 (Full Age-Period-Cohort)
8. Goodness of Fit Required	1 (Analysis Printed For Age-Period-Cohort Model)
9. Cause of Death	LUNG CANCER
Note:	Death rates are per million population

Matrix of Numbers of Deaths

Age	1966-	1971-	1976-	1981-	1986-	1991-	1996-	2001-	2006-	2011-
10-14	14	8	6	10	5	8	6	4	5	8
15-19	20	17	19	14	16	13	14	12	19	12
20-24	45	36	35	34	27	35	25	29	37	44
25-29	77	88	115	104	124	98	89	89	78	75
30-34	258	334	376	397	412	492	419	258	241	233
35-39	955	1018	1153	1339	1336	1565	1660	1320	879	577
40-44	2341	2828	3057	3181	3549	3595	4322	4690	3261	2245
45-49	4285	5921	6686	6912	7540	8175	8035	9655	9903	6814
50-54	6016	9202	12080	13032	13517	14293	15213	15641	17618	17167
55-59	7239	11731	16647	21583	22468	22637	23402	25717	25352	28184
60-64	7054	12523	19638	27996	34215	34806	33745	36411	38078	37589
65-69	6872	11615	20070	30567	40960	48218	47173	45856	48769	50742
70-74	6128	9479	16155	27351	39865	52174	58203	56593	54466	56562
75-79	4937	7224	11637	19096	31439	44308	54007	59921	57649	54454

Matrix of Age- and Period-Specific Mortality Rates

Age	1966-	1971-	1976-	1981-	1986-	1991-	1996-	2001-	2006-	2011-
10-14	0.270	0.147	0.128	0.234	0.119	0.169	0.123	0.077	0.097	0.158
15-19	0.435	0.336	0.366	0.281	0.362	0.297	0.289	0.226	0.355	0.216
20-24	1.132	0.773	0.679	0.642	0.551	0.771	0.546	0.595	0.710	0.794
25-29	2.381	2.189	2.424	1.979	2.315	1.959	1.873	1.860	1.556	1.395
30-34	8.966	10.179	9.159	8.198	7.699	8.963	8.058	5.225	4.903	4.527
35-39	32.468	35.324	34.951	32.484	27.471	29.069	29.648	24.821	17.466	11.509
40-44	74.018	96.561	105.731	96.016	85.742	74.004	79.812	83.184	60.981	44.195
45-49	137.985	189.788	229.286	239.661	229.782	200.218	165.319	178.130	175.878	127.526
50-54	211.606	303.559	391.853	450.780	474.306	440.855	372.627	321.322	325.928	306.912
55-59	273.693	427.346	561.494	715.582	797.526	814.524	731.967	634.995	526.348	528.601
60-64	297.934	501.163	744.637	979.921	1180.302	1283.993	1244.192	1164.490	962.045	800.862
65-69	347.076	526.377	846.070	1217.460	1516.548	1766.327	1829.616	1760.562	1625.966	1337.902
70-74	394.007	550.210	820.493	1283.948	1767.910	2153.286	2350.746	2388.533	2266.519	2028.733
75-79	430.867	561.381	802.875	1132.421	1733.493	2315.234	2580.118	2802.387	2784.493	2567.841

Matrix of Log-Rates

Age	1966-	1971-	1976-	1981-	1986-	1991-	1996-	2001-	2006-	2011-
10-14	-0.569	-0.832	-0.892	-0.631	-0.924	-0.773	-0.910	-1.115	-1.013	-0.803
15-19	-0.361	-0.473	-0.436	-0.551	-0.442	-0.527	-0.540	-0.647	-0.450	-0.666
20-24	0.054	-0.112	-0.168	-0.192	-0.259	-0.113	-0.263	-0.226	-0.149	-0.100
25-29	0.377	0.340	0.384	0.296	0.365	0.292	0.273	0.270	0.192	0.145
30-34	0.953	1.008	0.962	0.914	0.886	0.952	0.906	0.718	0.690	0.656
35-39	1.511	1.548	1.543	1.512	1.439	1.463	1.472	1.395	1.242	1.061
40-44	1.869	1.985	2.024	1.982	1.933	1.869	1.902	1.920	1.785	1.645
45-49	2.140	2.278	2.360	2.380	2.361	2.302	2.218	2.251	2.245	2.106
50-54	2.326	2.482	2.593	2.654	2.676	2.644	2.571	2.507	2.513	2.487
55-59	2.437	2.631	2.749	2.855	2.902	2.911	2.864	2.803	2.721	2.723
60-64	2.474	2.700	2.872	2.991	3.072	3.109	3.095	3.066	2.983	2.904
65-69	2.540	2.721	2.927	3.085	3.181	3.247	3.262	3.246	3.211	3.126
70-74	2.596	2.741	2.914	3.109	3.247	3.333	3.371	3.378	3.355	3.307
75-79	2.634	2.749	2.905	3.054	3.239	3.365	3.412	3.448	3.445	3.410

Results for PHIM for Run 2 (Basic_US_OG_T1.RTF)

Fitting the Age, Period, Cohort Models

Model	RSS	MRSS	DF	Factor	%Account	ChiSq	P
Age Only	37699.973	296.850	127	P, C	99.5516	218055.760	0.0000
Age-Period	15063.146	128.745	117	Cohort	98.8776	81325.905	0.0000
Age-Cohort	2131.243	20.493	104	Period	92.0674	11334.212	0.0000
Period-Cohort	588.236	5.447	108	Age	71.2594	3131.942	0.0000
Full Age-Period-Cohort	169.063	1.761	96			897.737	0.0000

**Key to terms:**		
	RSS =	residual sum of squares
	MRSS =	mean RSS (MRSS/DF)
	DF =	degrees of freedom
	Factor =	Factors not included in the model
	% Account =	1- (RSS for full model)/(RSS for model in question)
	Chisq =	chi-squared value for model
	P =	probability value based on Chisq and DF.

Age	Value	Log10 Value
10-	0.224538	-0.648711
15-	0.432142	-0.364374
20-	0.927456	-0.032707
25-	2.423241	0.384397
30-	8.492446	0.929033
35-	28.607987	1.456487
40-	78.293396	1.893725
45-	177.011521	2.248002
50-	338.859777	2.530020
55-	584.835285	2.767034
60-	938.460154	2.972416
65-	1394.24532	3.144339
70-	1919.98135	3.283297
75-	2417.55878	3.383377

Period	Value	Log10 Value
1966	0.759082	-0.119711
1971	0.880165	-0.055436
1976	0.980155	-0.008705
1981	1.057436	0.024254
1986	1.100273	0.041500
1991	1.111005	0.045716
1996	1.056278	0.023778
2001	1.013230	0.005708
2006	0.952657	-0.021063
2011	0.882895	-0.054091

Cohort	Value	Log10 Value
1891	0.234789	-0.629322
1896	0.266798	-0.573817
1901	0.331614	-0.479367
1906	0.434544	-0.361966
1911	0.631222	-0.199818
1916	0.837165	-0.077189
1921	1.000767	0.000333
1926	1.152389	0.061599
1931	1.234678	0.091554
1936	1.241299	0.093876
1941	1.204831	0.080926
1946	1.069977	0.029375
1951	0.941646	-0.026112
1956	1.005794	0.002509
1961	1.029269	0.012529
1966	0.821613	-0.085333
1971	0.641783	-0.192612
1976	0.518796	-0.285003
1981	0.623174	-0.205391
1986	0.670574	-0.173553
1991	0.880922	-0.055063
1996	0.529015	-0.276532
2001	0.794529	-0.099890

Results for PHIM for Run 2 (Basic_US_OG_T1.RTF)

Model: Full Age-Period-Cohort

Basic Analysis Using OG Modelling T1 on US
Fitting the Full Age-Period-Cohort Model
Matrix of observed, expected, and residual rates

**Age**		**1966-**	**1971-**	**1976-**	**1981-**	**1986-**	**1991-**	**1996-**	**2001-**	**2006-**	**2011-**
10-	Observed	0.270	0.147	0.128	0.234	0.119	0.169	0.123	0.077	0.097	0.158
	Expected	0.171	0.203	0.181	0.152	0.128	0.155	0.159	0.200	0.113	0.158
	Residual	-0.098	0.056	0.053	-0.081	0.009	-0.013	0.036	0.124	0.016	-0.000
15-	Observed	0.435	0.336	0.366	0.281	0.362	0.297	0.289	0.226	0.355	0.216
	Expected	0.309	0.383	0.436	0.375	0.305	0.249	0.284	0.294	0.363	0.202
	Residual	-0.126	0.046	0.070	0.094	-0.057	-0.048	-0.004	0.068	0.008	-0.014
20-	Observed	1.132	0.773	0.679	0.642	0.551	0.771	0.546	0.595	0.710	0.794
	Expected	0.753	0.769	0.914	1.009	0.838	0.661	0.508	0.586	0.592	0.721
	Residual	-0.379	-0.004	0.235	0.367	0.287	-0.110	-0.038	-0.009	-0.117	-0.073
25-	Observed	2.381	2.189	2.424	1.979	2.315	1.959	1.873	1.860	1.556	1.395
	Expected	2.216	2.282	2.237	2.577	2.744	2.212	1.643	1.274	1.439	1.435
	Residual	-0.165	0.093	-0.187	0.598	0.429	0.253	-0.230	-0.586	-0.117	0.040
30-	Observed	8.966	10.179	9.159	8.198	7.699	8.963	8.058	5.225	4.903	4.527
	Expected	8.002	9.006	8.906	8.456	9.398	9.711	7.370	5.522	4.197	4.673
	Residual	-0.964	-1.173	-0.253	0.258	1.699	0.749	-0.687	0.298	-0.705	0.146
35-	Observed	32.468	35.324	34.951	32.484	27.471	29.069	29.648	24.821	17.466	11.509
	Expected	26.812	31.256	33.784	32.368	29.640	31.968	31.102	23.816	17.491	13.104
	Residual	-5.656	-4.068	-1.168	-0.116	2.169	2.899	1.454	-1.005	0.025	1.594
40-	Observed	74.018	96.561	105.731	96.016	85.742	74.004	79.812	83.184	60.981	44.195
	Expected	68.488	85.083	95.257	99.748	92.172	81.908	83.179	81.651	61.281	44.363
	Residual	-5.530	-11.478	-10.475	3.732	6.430	7.904	3.366	-1.533	0.300	0.168
45-	Observed	137.985	189.788	229.286	239.661	229.782	200.218	165.319	178.130	175.878	127.526
	Expected	134.469	179.541	214.215	232.344	234.654	210.423	176.063	180.393	173.567	128.404
	Residual	-3.516	-10.247	-15.071	-7.317	4.872	10.205	10.744	2.262	-2.311	0.878
50-	Observed	211.606	303.559	391.853	450.780	474.306	440.855	372.627	321.322	325.928	306.912
	Expected	215.338	298.481	382.749	442.413	462.804	453.589	382.977	323.307	324.688	307.934
	Residual	3.731	-5.078	-9.104	-8.367	-11.502	12.733	10.350	1.985	-1.240	1.023
55-	Observed	273.693	427.346	561.494	715.582	797.526	814.524	731.967	634.995	526.348	528.601
	Expected	280.223	430.932	573.669	712.667	794.489	806.540	744.283	634.040	524.636	519.340
	Residual	6.531	3.586	12.175	-2.915	-3.037	-7.984	12.316	-0.955	-1.713	-9.261
60-	Observed	297.934	501.163	744.637	979.921	1180.302	1283.993	1244.192	1164.490	962.045	800.862
	Expected	309.555	521.389	770.055	993.123	1189.913	1287.318	1230.469	1145.645	956.593	780.211
	Residual	11.621	20.226	25.418	13.202	9.611	3.325	-13.723	-18.845	-5.453	-20.651
65-	Observed	347.076	526.377	846.070	1217.460	1516.548	1766.327	1829.616	1760.562	1625.966	1337.902
	Expected	350.962	533.258	862.613	1234.254	1535.228	1785.065	1818.325	1753.573	1600.301	1317.112
	Residual	3.886	6.880	16.543	16.794	18.680	18.738	-11.291	-6.990	-25.664	-20.789
70-	Observed	394.007	550.210	820.493	1283.948	1767.910	2153.286	2350.746	2388.533	2266.519	2028.733
	Expected	388.838	560.394	817.760	1281.543	1768.515	2134.746	2337.084	2401.923	2270.440	2042.358
	Residual	-5.170	10.184	-2.734	-2.405	0.605	-18.540	-13.662	13.390	3.920	13.625
75-	Observed	430.867	561.381	802.875	1132.421	1733.493	2315.234	2580.118	2802.387	2784.493	2567.841
	Expected	430.867	567.706	785.786	1110.875	1679.034	2248.559	2555.574	2822.826	2843.594	2649.490
	Residual	-0.000	6.325	-17.089	-21.547	-54.459	-66.675	-24.544	20.439	59.101	81.649

Fitting the Full Age-Period-Cohort Model

Results for PHIM for Run 2 (Basic_US_OG_T1.RTF)

Matrix of observed and expected deaths and (O-E)2/E Values**

Age		1966-	1971-	1976-	1981-	1986-	1991-	1996-	2001-	2006-	2011-	Total
10-	Observed	13.5	7.5	6.0	10.0	5.0	7.5	6.0	4.0	5.0	8.0	72.5
	Expected	8.6	10.4	8.5	6.5	5.4	6.9	7.8	10.4	5.8	8.0	78.3
	Difference	4.9	-2.9	-2.5	3.5	-0.4	0.6	-1.8	-6.4	-0.8	0.0	-5.8
	Chi-Sq	2.8	0.8	0.7	1.9	0.0	0.0	0.4	4.0	0.1	0.0	10.8
15-	Observed	20.0	17.0	19.0	13.5	16.0	13.0	13.5	11.5	19.0	11.5	154.0
	Expected	14.2	19.3	22.6	18.0	13.5	10.9	13.3	15.0	19.4	10.8	157.0
	Difference	5.8	-2.3	-3.6	-4.5	2.5	2.1	0.2	-3.5	-0.4	0.7	-3.0
	Chi-Sq	2.4	0.3	0.6	1.1	0.5	0.4	0.0	0.8	0.0	0.1	6.1
20-	Observed	45.0	36.0	35.0	34.0	27.0	35.0	25.0	29.0	37.0	44.0	347.0
	Expected	29.9	35.8	47.1	53.4	41.1	30.0	23.3	28.6	30.9	40.0	360.0
	Difference	15.1	0.2	-12.1	-19.4	-14.1	5.0	1.7	0.4	6.1	4.0	-13.0
	Chi-Sq	7.6	0.0	3.1	7.1	4.8	0.8	0.1	0.0	1.2	0.4	25.2
25-	Observed	77.0	88.0	115.0	104.0	124.0	98.0	89.0	89.0	78.0	75.0	937.0
	Expected	71.7	91.7	106.1	135.5	147.0	110.7	78.1	61.0	72.1	77.1	950.9
	Difference	5.3	-3.7	8.9	-31.5	-23.0	-12.7	10.9	28.0	5.9	-2.1	-13.9
	Chi-Sq	0.4	0.2	0.7	7.3	3.6	1.4	1.5	12.9	0.5	0.1	28.6
30-	Observed	258.0	334.0	376.0	397.0	412.0	492.0	419.0	258.0	241.0	233.0	3420.0
	Expected	230.3	295.5	365.6	409.5	502.9	533.1	383.3	272.7	206.3	240.5	3439.6
	Difference	27.7	38.5	10.4	-12.5	-90.9	-41.1	35.7	-14.7	34.7	-7.5	-19.6
	Chi-Sq	3.3	5.0	0.3	0.4	16.4	3.2	3.3	0.8	5.8	0.2	38.8
35-	Observed	955.0	1018.0	1153.0	1339.0	1336.0	1565.0	1660.0	1320.0	879.0	577.0	11802.0
	Expected	788.6	900.8	1114.5	1334.2	1441.5	1721.1	1741.4	1266.5	880.3	656.9	11845.8
	Difference	166.4	117.2	38.5	4.8	-105.5	-156.1	-81.4	53.5	-1.3	-79.9	-43.8
	Chi-Sq	35.1	15.3	1.3	0.0	7.7	14.2	3.8	2.3	0.0	9.7	89.4
40-	Observed	2341.0	2828.0	3057.0	3181.0	3549.0	3595.0	4322.0	4690.0	3261.0	2245.0	33069.0
	Expected	2166.1	2491.8	2754.1	3304.7	3815.2	3979.0	4504.3	4603.6	3277.1	2253.5	33149.3
	Difference	174.9	336.2	302.9	-123.7	-266.2	-384.0	-182.3	86.4	-16.1	-8.5	-80.3
	Chi-Sq	14.1	45.4	33.3	4.6	18.6	37.1	7.4	1.6	0.1	0.0	162.1
45-	Observed	4285.0	5921.0	6686.0	6912.0	7540.0	8175.0	8035.0	9655.0	9903.0	6814.0	73926.0
	Expected	4175.8	5601.3	6246.5	6701.0	7699.9	8591.7	8557.2	9777.6	9772.9	6860.9	73984.8
	Difference	109.2	319.7	439.5	211.0	-159.9	-416.7	-522.2	-122.6	130.1	-46.9	-58.8
	Chi-Sq	2.9	18.2	30.9	6.6	3.3	20.2	31.9	1.5	1.7	0.3	117.6
50-	Observed	6016.0	9202.0	12080.0	13032.0	13517.0	14293.0	15213.0	15641.0	17618.0	17167.0	133779.0
	Expected	6122.1	9048.1	11799.3	12790.1	13189.2	14705.8	15635.5	15737.6	17551.0	17224.2	133803.0
	Difference	-106.1	153.9	280.7	241.9	327.8	-412.8	-422.5	-96.6	67.0	-57.2	-24.0
	Chi-Sq	1.8	2.6	6.7	4.6	8.1	11.6	11.4	0.6	0.3	0.2	47.9
55-	Observed	7239.0	11731.0	16647.0	21583.0	22468.0	22637.0	23402.0	25717.0	25352.0	28184.0	204960.0
	Expected	7411.7	11829.4	17008.0	21495.1	22382.4	22415.1	23795.8	25678.3	25269.5	27690.2	204975.6
	Difference	-172.7	-98.4	-361.0	87.9	85.6	221.9	-393.8	38.7	82.5	493.8	-15.6
	Chi-Sq	4.0	0.8	7.7	0.4	0.3	2.2	6.5	0.1	0.3	8.8	31.0
60-	Observed	7054.0	12523.0	19638.0	27996.0	34215.0	34806.0	33745.0	36411.0	38078.0	37589.0	282055.0
	Expected	7329.1	13028.4	20308.3	28373.2	34493.6	34896.1	33372.8	35821.8	37862.2	36619.7	282105.3
	Difference	-275.1	-505.4	-670.3	-377.2	-278.6	-90.1	372.2	589.2	215.8	969.3	-50.3
	Chi-Sq	10.3	19.6	22.1	5.0	2.3	0.2	4.2	9.7	1.2	25.7	100.3
65-	Observed	6872.0	11615.0	20070.0	30567.0	40960.0	48218.0	47173.0	45856.0	48769.0	50742.0	350842.0
	Expected	6948.9	11766.8	20462.4	30988.6	41464.5	48729.5	46881.9	45673.9	47999.2	49953.5	350869.5
	Difference	-76.9	-151.8	-392.4	-421.6	-504.5	-511.5	291.1	182.1	769.8	788.5	-27.5
	Chi-Sq	0.9	2.0	7.5	5.7	6.1	5.4	1.8	0.7	12.3	12.4	54.9
70-	Observed	6128.0	9479.0	16155.0	27351.0	39865.0	52174.0	58203.0	56593.0	54466.0	56562.0	376976.0
	Expected	6047.6	9654.5	16101.2	27299.8	39878.6	51724.8	57864.7	56910.2	54560.2	56941.9	376983.4

Results for PHIM for Run 2 (Basic_US_OG_T1.RTF)

	Difference	80.4	-175.5	53.8	51.2	-13.6	449.2	338.3	-317.2	-94.2	-379.9	-7.4
	Chi-Sq	1.1	3.2	0.2	0.1	0.0	3.9	2.0	1.8	0.2	2.5	14.9
75-	Observed	4937.0	7224.0	11637.0	19096.0	31439.0	44308.0	54007.0	59921.0	57649.0	54454.0	344672.0
	Expected	4937.0	7305.4	11389.3	18732.7	30451.3	43032.0	53493.2	60358.0	58872.6	56185.5	344757.0
	Difference	0.0	-81.4	247.7	363.3	987.7	1276.0	513.8	-437.0	-1223.6	-1731.5	-85.0
	Chi-Sq	0.0	0.9	5.4	7.0	32.0	37.8	4.9	3.2	25.4	53.4	170.1
Total over ages	Observed	46240.5	72023.5	107674.0	151615.5	195473.0	230416.5	246312.5	256195.5	256355.0	254705.5	1817011.5
	Expected	46281.7	72079.3	107733.7	151642.2	195526.0	230486.6	246352.5	256215.3	256379.4	254762.7	1817459.4
	Difference	-41.2	-55.8	-59.7	-26.7	-53.0	-70.1	-40.0	-19.8	-24.4	-57.2	-447.9
	Chi-Sq	86.7	114.2	120.6	51.9	103.8	138.4	79.3	39.9	49.1	113.8	897.7

Results for PHIM for Run 2 (Basic_US_OG_T1.RTF)

****

Osmond and Gardner Extrapolating Death Rates for COD: LUNG CANCER

Variable Parameter	Value
1. Country	US (United States)
2. Sex	F (Females)
3. Year of start of OG modelling	1966
4. Number 5-year periods for OG modelling	10
5. Age Range of interest	10 - 79
6. Scaling Factor for Rates	10000
7. Model Type to Save	4 (Full Age-Period-Cohort)
8. Goodness of Fit Required	1 (Analysis Printed For Age-Period-Cohort Model)
9. Cause of Death	LUNG CANCER
Note:	Death rates are per million population
10. Number of Periods into the future to Predict	5
11. Earliest projected year	2016
12. Extrapolate Period using (1: last 2 points 2: linear regression)	1
13. Ratio of last two period values	0.92677

Predictions of rates for future years from model:	Full Age-Period-Cohort
Effects for extending model to project rates for:	2016-2040

Extrapolating Model: Full Age-Period-Cohort
Log Transform Parameters

Model	ChiSq	MChiSq	DF	Factor	%Account	P
Age Only	16550.213	1182.158	14	P, C	93.3976	0.0000
Age-Period	8926.623	637.616	14	Cohort	87.7590	0.0000
Age-Cohort	8422.562	601.612	14	Period	87.0264	0.0000
Period-Cohort	862.687	61.621	14	Age	-26.6636	0.0000
Full Age-Period-Cohort	1092.711	78.051	14			0.0000

Key to terms:		
	Chisq =	chi-squared value for model
	MChisq =	mean Chi-squared (Chisq/DF)
	DF =	degrees of freedom
	Factor =	Factors not included in the model
	% Account =	1- (Chisq for full model)/(Chisq for model in question)
	P =	probability value based on Chisq and DF.

AGE	EFFECT
10	0.224538
15	0.432142
20	0.927456
25	2.423241
30	8.492446
35	28.607987
40	78.293396
45	177.011521
50	338.859777
55	584.835285
60	938.460154
65	1394.24532
70	1919.98135
75	2417.55878

PERIOD	EFFECT	
Period Change =	0.926771	
1966	0.759082	
1971	0.880165	
1976	0.980155	
1981	1.057436	
1986	1.100273	
1991	1.111005	
1996	1.056278	
2001	1.013230	
2006	0.952657	
2011	0.882895	
2016	0.818241	
2021	0.758322	
2026	0.702790	
2031	0.651326	
2036	0.603629	
2016	0.843514	Extrapolated
2017	0.830781	Extrapolated
2018	0.818241	Extrapolated
2019	0.805890	Extrapolated

Results for PHIM for Run 2 (Basic_US_OG_T1.RTF)

2020	0.793725	Extrapolated
2021	0.781744	Extrapolated
2022	0.769944	Extrapolated
2023	0.758322	Extrapolated
2024	0.746875	Extrapolated
2025	0.735601	Extrapolated
2026	0.724498	Extrapolated
2027	0.713561	Extrapolated
2028	0.702790	Extrapolated
2029	0.692182	Extrapolated
2030	0.681734	Extrapolated
2031	0.671443	Extrapolated
2032	0.661308	Extrapolated
2033	0.651326	Extrapolated
2034	0.641494	Extrapolated
2035	0.631811	Extrapolated
2036	0.622274	Extrapolated
2037	0.612881	Extrapolated
2038	0.603629	Extrapolated
2039	0.594518	Extrapolated
2040	0.585544	Extrapolated

Results for PHIM for Run 2 (Basic_US_OG_T1.RTF)

COHORT	EFFECT	WEIGHT	ORIGINAL
1891	0.234789	1.000	
1896	0.266798	2.000	
1901	0.331614	4.000	
1906	0.434544	8.000	
1911	0.631222	16.000	
1916	0.837165	32.000	
1921	1.000767	64.000	
1926	1.152389	128.000	
1931	1.234678	256.000	
1936	1.241299	512.000	
1941	1.204831	1024.000	
1946	1.069977	2048.000	
1951	0.941646	4096.000	
1956	1.005794	8192.000	
1961	1.029269	16384.000	
1966	0.821613	32768.000	
1971	0.641783	65536.000	
1976	0.518796	131072.000	
1981	0.623174	262144.000	
1986	0.670574	524288.000	
1991	0.880922	1048576.000	
1996	0.859968	Extrapolated	0.529015
2001	0.917328	Extrapolated	0.794529
2006	0.978514	Extrapolated	
2011	1.043780	Extrapolated	
2016	1.113400	Extrapolated	
2021	1.187664	Extrapolated	
2026	1.266881	Extrapolated	

Standardizing Population: The 1976 European Standard Population

Age Range	Population, Females
All	100000
0	0
1	0
2	0
3	0
0-4	8000
5-9	7000
10-14	7000
15-19	7000
20-24	7000
25-29	7000
30-34	7000
35-39	7000
40-44	7000
45-49	7000
50-54	7000
55-59	6000
60-64	5000
65-69	4000
70-74	3000
75-79	2000
80-84	1000
85+	1000

Results for PHIM for Run 2 (Basic_US_OG_T1.RTF)

Matrix of observed and expected rates including predictions

Total over ages standardized using: The 1976 European Standard Population

		1966-	1971-	1976-	1981-	1986-	1991-	1996-	2001-	2006-	2011-	2016-	2021-	2026-	2031-	2036-
10	OBS	0.3	0.1	0.1	0.2	0.1	0.2	0.1	0.1	0.1	0.2	0.1	.	.	.	.
	EXP											0.2	0.2	0.2	0.2	0.2
15	OBS	0.4	0.3	0.4	0.3	0.4	0.3	0.3	0.2	0.4	0.2	0.3	.	.	.	.
	EXP											0.3	0.3	0.3	0.3	0.3
20	OBS	1.1	0.8	0.7	0.6	0.6	0.8	0.5	0.6	0.7	0.8	0.5	.	.	.	.
	EXP											0.7	0.6	0.6	0.6	0.6
25	OBS	2.4	2.2	2.4	2.0	2.3	2.0	1.9	1.9	1.6	1.4	1.1	.	.	.	.
	EXP											1.7	1.6	1.6	1.5	1.5
30	OBS	9.0	10.2	9.2	8.2	7.7	9.0	8.1	5.2	4.9	4.5	3.1	.	.	.	.
	EXP											4.7	5.7	5.1	5.1	5.0
35	OBS	32.5	35.3	35.0	32.5	27.5	29.1	29.6	24.8	17.5	11.5	10.7	.	.	.	.
	EXP											14.6	14.5	17.7	16.0	15.8
40	OBS	74.0	96.6	105.7	96.0	85.7	74.0	79.8	83.2	61.0	44.2	27.8	.	.	.	.
	EXP											33.2	37.0	36.9	44.9	40.6
45	OBS	138.0	189.8	229.3	239.7	229.8	200.2	165.3	178.1	175.9	127.5	91.8	.	.	.	.
	EXP											93.0	69.6	77.5	77.3	94.1
50	OBS	211.6	303.6	391.9	450.8	474.3	440.9	372.6	321.3	325.9	306.9	246.3	.	.	.	.
	EXP											227.8	164.9	123.5	137.5	137.2
55	OBS	273.7	427.3	561.5	715.6	797.5	814.5	732.0	635.0	526.3	528.6	508.0	.	.	.	.
	EXP											492.5	364.4	263.8	197.6	220.0
60	OBS	297.9	501.2	744.6	979.9	1180.3	1284.0	1244.2	1164.5	962.0	800.9	724.1	.	.	.	.
	EXP											772.3	732.5	541.9	392.3	293.9
65	OBS	347.1	526.4	846.1	1217.5	1516.5	1766.3	1829.6	1760.6	1626.0	1337.9	1078.2	.	.	.	.
	EXP											1074.3	1063.4	1008.5	746.1	540.1
70	OBS	394.0	550.2	820.5	1283.9	1767.9	2153.3	2350.7	2388.5	2266.5	2028.7	1601.0	.	.	.	.
	EXP											1680.9	1371.0	1357.2	1287.1	952.2
75	OBS	430.9	561.4	802.9	1132.4	1733.5	2315.2	2580.1	2802.4	2784.5	2567.8	2153.3	.	.	.	.
	EXP											2383.3	1961.6	1599.9	1583.7	1502.0
10-79	OBS	118.7	173.7	240.6	313.2	377.4	418.8	418.7	406.7	373.0	328.1	274.2	.	.	.	.
	EXP	118.8	173.4	240.2	313.0	377.7	419.9	419.2	406.2	372.5	327.9	283.8*	243.4*	210.1*	182.5*	155.2*

Drop in overall standardized Observed and Predicted rates

comparing the last observed rate during the model fitting period to the last observed and predicted rates where an observed rate is available (2016)

Observed and Predicted %Drop = 16.406% and 13.491%

Results for PHIM for Run 2 (Basic_US_OG_T1.RTF)

Matrix of observed and expected deaths including predictions

		1966-	1971-	1976-	1981-	1986-	1991-	1996-	2001-	2006-	2011-	2016-	2021-	2026-	2031-	2036-
10-	OBS	13.5	7.5	6.0	10.0	5.0	7.5	6.0	4.0	5.0	8.0	7.5*	.	.	.	.
	EXP	8.6	10.4	8.5	6.5	5.4	6.9	7.8	10.4	9.5	9.2	9.2*	9.0*	8.6*	8.6*	8.8*
	ChiSq	2.817	0.791	0.719	1.862	0.027	0.050	0.399	3.978	2.108	0.166	0.331*	.	.	.	.
15-	OBS	20.0	17.0	19.0	13.5	16.0	13.0	13.5	11.5	19.0	11.5	15.0*	.	.	.	.
	EXP	14.2	19.3	22.6	18.0	13.5	10.9	13.3	15.0	19.4	17.5	16.9*	16.9*	16.4*	15.7*	15.8*
	ChiSq	2.371	0.285	0.577	1.133	0.465	0.409	0.003	0.802	0.009	2.054	0.215*	.	.	.	.
20-	OBS	45.0	36.0	35.0	34.0	27.0	35.0	25.0	29.0	37.0	44.0	25.0*	.	.	.	.
	EXP	29.9	35.8	47.1	53.4	41.1	30.0	23.3	28.6	30.9	40.0	36.2*	35.0*	35.1*	34.1*	32.8*
	ChiSq	7.584	0.001	3.123	7.065	4.812	0.829	0.130	0.007	1.209	0.410	3.491*	.	.	.	.
25-	OBS	77.0	88.0	115.0	104.0	124.0	98.0	89.0	89.0	78.0	75.0	62.5*	.	.	.	.
	EXP	71.7	91.7	106.1	135.5	147.0	110.7	78.1	61.0	72.1	77.1	100.4*	90.9*	87.9*	88.3*	86.1*
	ChiSq	0.396	0.153	0.742	7.304	3.596	1.447	1.533	12.907	0.481	0.059	14.298*	.	.	.	.
30-	OBS	258.0	334.0	376.0	397.0	412.0	492.0	419.0	258.0	241.0	233.0	170.0*	.	.	.	.
	EXP	230.3	295.5	365.6	409.5	502.9	533.1	383.3	272.7	206.3	240.5	257.3*	333.7*	302.4*	293.4*	294.7*
	ChiSq	3.343	5.016	0.295	0.381	16.433	3.168	3.334	0.792	5.826	0.233	29.647*	.	.	.	.
35-	OBS	955.0	1018.0	1153.0	1339.0	1336.0	1565.0	1660.0	1320.0	879.0	577.0	562.5*	.	.	.	.
	EXP	788.6	900.8	1114.5	1334.2	1441.5	1721.1	1741.4	1266.5	880.3	656.9	763.4*	814.5*	1055.6*	958.0*	930.5*
	ChiSq	35.097	15.261	1.331	0.017	7.717	14.154	3.807	2.257	0.002	9.726	52.878*	.	.	.	.
40-	OBS	2341.0	2828.0	3057.0	3181.0	3549.0	3595.0	4322.0	4690.0	3261.0	2245.0	1402.5*	.	.	.	.
	EXP	2166.1	2491.8	2754.1	3304.7	3815.2	3979.0	4504.3	4603.6	3277.1	2253.5	1679.3*	1947.8*	2077.8*	2694.4*	2446.7*
	ChiSq	14.122	45.352	33.302	4.627	18.567	37.053	7.378	1.623	0.079	0.032	45.614*	.	.	.	.
45-	OBS	4285.0	5921.0	6686.0	6912.0	7540.0	8175.0	8035.0	9655.0	9903.0	6814.0	4657.5*	.	.	.	.
	EXP	4175.8	5601.3	6246.5	6701.0	7699.9	8591.7	8557.2	9777.6	9772.9	6860.9	4718.5*	3513.3*	4077.0*	4352.4*	5645.0*
	ChiSq	2.854	18.245	30.919	6.645	3.319	20.206	31.867	1.538	1.733	0.321	0.788*	.	.	.	.
50-	OBS	6016.0	9202.0	12080.0	13032.0	13517.0	14293.0	15213.0	15641.0	17618.0	17167.0	13030.0*	.	.	.	.
	EXP	6122.1	9048.1	11799.3	12790.1	13189.2	14705.8	15635.5	15737.6	17551.0	17224.2	12051.5*	8289.2*	6178.8*	7179.5*	7668.5*
	ChiSq	1.838	2.619	6.676	4.574	8.147	11.589	11.419	0.593	0.256	0.190	79.454*	.	.	.	.
55-	OBS	7239.0	11731.0	16647.0	21583.0	22468.0	22637.0	23402.0	25717.0	25352.0	28184.0	27855.0*	.	.	.	.
	EXP	7411.7	11829.4	17008.0	21495.1	22382.4	22415.1	23795.8	25678.3	25269.5	27690.2	27004.9*	18897.0*	13021.4*	9724.5*	11312.6*
	ChiSq	4.025	0.819	7.661	0.360	0.327	2.196	6.516	0.058	0.269	8.805	26.761*	.	.	.	.
60-	OBS	7054.0	12523.0	19638.0	27996.0	34215.0	34806.0	33745.0	36411.0	38078.0	37589.0	37430.0*	.	.	.	.
	EXP	7329.1	13028.4	20308.3	28373.2	34493.6	34896.1	33372.8	35821.8	37862.2	36619.7	39920.7*	38912.2*	27288.8*	18856.8*	14108.5*
	ChiSq	10.330	19.607	22.127	5.014	2.250	0.233	4.151	9.692	1.230	25.654	155.398*	.	.	.	.
65-	OBS	6872.0	11615.0	20070.0	30567.0	40960.0	48218.0	47173.0	45856.0	48769.0	50742.0	48290.0*	.	.	.	.
	EXP	6948.9	11766.8	20462.4	30988.6	41464.5	48729.5	46881.9	45673.9	47999.2	49953.5	48115.5*	52449.5*	51251.5*	36080.8*	25009.4*
	ChiSq	0.852	1.959	7.526	5.737	6.139	5.370	1.808	0.726	12.345	12.445	0.633*	.	.	.	.

Results for PHIM for Run 2 (Basic_US_OG_T1.RTF)

70-	OBS	6128.0	9479.0	16155.0	27351.0	39865.0	52174.0	58203.0	56593.0	54466.0	56562.0	56432.5*	.	.	.	.
	EXP	6047.6	9654.5	16101.2	27299.8	39878.6	51724.8	57864.7	56910.2	54560.2	56941.9	59249.2*	57126.6*	62527.2*	61440.5*	43449.6*
	ChiSq	1.069	3.189	0.180	0.096	0.005	3.902	1.977	1.768	0.163	2.534	133.906*	.	.	.	.
75-	OBS	4937.0	7224.0	11637.0	19096.0	31439.0	44308.0	54007.0	59921.0	57649.0	54454.0	53287.5*	.	.	.	.
	EXP	4937.0	7305.4	11389.3	18732.7	30451.3	43032.0	53493.2	60358.0	58872.6	56185.5	58979.3*	61571.9*	59682.5*	65938.6*	65237.7*
	ChiSq	.	0.907	5.387	7.047	32.035	37.836	4.934	3.164	25.431	53.358	549.296*	.	.	.	.
Total Deaths		46240.5	72023.5	107674.0	151615.5	195473.0	230416.5	246312.5	256195.5	256355.0	254705.5	243227.5*	.	.	.	.
Expected		46281.7	72079.3	107733.7	151642.2	195526.0	230486.6	246352.5	256215.3	256383.1	254770.7	252902.4*	244007.3*	227610.8*	207665.5*	176246.6*
Obs/Exp		0.999	0.999	0.999	1.000	1.000	1.000	1.000	1.000	1.000	1.000	0.962*	.	.	.	.

Chi Squared (Log) = **1092.7 on 14 D.F.** **P = 0.0000**

Results for PHIM for Run 2 (Basic_US_OG_T1.RTF)

****

Observed and predicted rates (5 year periods)

		10-	15-	20-	25-	30-	35-	40-	45-	50-	55-	60-	65-	70-	75-
2016-	OBS	0.1	0.3	0.5	1.1	3.1	10.7	27.8	91.8	246.3	508.0	724.1	1078.2	1601.0	2153.3
	PRE	0.2	0.3	0.7	1.7	4.7	14.6	33.2	93.0	227.8	492.5	772.3	1074.3	1680.9	2383.3
	RES	-0.034	-0.037	-0.203	-0.659	-1.582	-3.839	-5.478	-1.202	18.497	15.505	-48.187	3.896	-79.912	-230.005
2021-	PRE	0.2	0.3	0.6	1.6	5.7	14.5	37.0	69.6	164.9	364.4	732.5	1063.4	1371.0	1961.6
2026-	PRE	0.2	0.3	0.6	1.6	5.1	17.7	36.9	77.5	123.5	263.8	541.9	1008.5	1357.2	1599.9
2031-	PRE	0.2	0.3	0.6	1.5	5.1	16.0	44.9	77.3	137.5	197.6	392.3	746.1	1287.1	1583.7
2036-	PRE	0.2	0.3	0.6	1.5	5.0	15.8	40.6	94.1	137.2	220.0	293.9	540.1	952.2	1502.0

Results for PHIM for Run 2 (Basic_US_OG_T1.RTF)

****

Observed and predicted deaths (5 year periods)

		10-	15-	20-	25-	30-	35-	40-	45-	50-	55-	60-	65-	70-	75-
2016-	OBS	7.5	15.0	25.0	62.5	170.0	562.5	1402.5	4657.5	13030.0	27855.0	37430.0	48290.0	56432.5	53287.5
	PRE	9.2	16.9	36.2	100.4	257.3	763.4	1679.3	4718.5	12051.5	27004.9	39920.7	48115.5	59249.2	58979.3
	CHI	0.331	0.215	3.491	14.298	29.647	52.878	45.614	0.788	79.454	26.761	155.398	0.633	133.906	549.296
2021-	PRE	9.0	16.9	35.0	90.9	333.7	814.5	1947.8	3513.3	8289.2	18897.0	38912.2	52449.5	57126.6	61571.9
2026-	PRE	8.6	16.4	35.1	87.9	302.4	1055.6	2077.8	4077.0	6178.8	13021.4	27288.8	51251.5	62527.2	59682.5
2031-	PRE	8.6	15.7	34.1	88.3	293.4	958.0	2694.4	4352.4	7179.5	9724.5	18856.8	36080.8	61440.5	65938.6
2036-	PRE	8.8	15.8	32.8	86.1	294.7	930.5	2446.7	5645.0	7668.5	11312.6	14108.5	25009.4	43449.6	65237.7

Results for PHIM for Run 2 (Basic_US_OG_T1.RTF)

****

Observed and predicted rates (single years)

		10-	15-	20-	25-	30-	35-	40-	45-	50-	55-	60-	65-	70-	75-
2016	OBS	0.1	0.4	0.6	1.2	3.4	12.3	27.6	96.0	253.1	513.2	747.7	1139.0	1739.9	2351.0
	PRE	0.2	0.3	0.7	1.8	4.8	15.0	34.3	95.8	234.8	507.8	796.2	1107.4	1732.9	2456.9
	RES	-0.087	0.048	-0.052	-0.554	-1.362	-2.783	-6.690	0.144	18.294	5.397	-48.456	31.573	7.018	-105.984
2017	OBS	0.2	0.2	0.3	1.0	2.8	9.7	28.1	86.3	227.4	497.5	736.0	1092.1	1713.7	2233.9
	PRE	0.2	0.3	0.7	1.8	4.7	14.8	33.7	94.4	231.3	500.1	784.2	1090.7	1706.7	2419.9
	RES	0.012	-0.138	-0.395	-0.807	-1.886	-5.119	-5.655	-8.037	-3.860	-2.562	-48.170	1.393	6.949	-185.988
2018	PRE	0.2	0.3	0.7	1.7	4.7	14.6	33.2	93.0	227.8	492.5	772.3	1074.3	1680.9	2383.3
2019	PRE	0.2	0.3	0.6	1.7	4.6	14.4	32.7	91.6	224.4	485.1	760.7	1058.0	1655.6	2347.4
2020	PRE	0.2	0.3	0.6	1.7	4.5	14.2	32.2	90.2	221.0	477.8	749.2	1042.1	1630.6	2311.9
2021	PRE	0.2	0.3	0.7	1.6	5.8	15.0	38.1	71.8	170.0	375.6	755.1	1096.3	1413.3	2022.2
2022	PRE	0.2	0.3	0.7	1.6	5.8	14.8	37.6	70.7	167.4	370.0	743.7	1079.7	1392.0	1991.6
2023	PRE	0.2	0.3	0.6	1.6	5.7	14.5	37.0	69.6	164.9	364.4	732.5	1063.4	1371.0	1961.6
2024	PRE	0.2	0.3	0.6	1.6	5.6	14.3	36.4	68.6	162.4	358.9	721.4	1047.4	1350.3	1932.0
2025	PRE	0.2	0.3	0.6	1.5	5.5	14.1	35.9	67.6	160.0	353.5	710.5	1031.6	1329.9	1902.8
2026	PRE	0.2	0.3	0.7	1.6	5.3	18.3	38.0	79.9	127.4	271.9	558.6	1039.7	1399.1	1649.3
2027	PRE	0.2	0.3	0.6	1.6	5.2	18.0	37.5	78.7	125.4	267.8	550.2	1024.0	1378.0	1624.4
2028	PRE	0.2	0.3	0.6	1.6	5.1	17.7	36.9	77.5	123.5	263.8	541.9	1008.5	1357.2	1599.9
2029	PRE	0.2	0.3	0.6	1.5	5.1	17.4	36.3	76.4	121.7	259.8	533.7	993.3	1336.7	1575.7
2030	PRE	0.2	0.3	0.6	1.5	5.0	17.2	35.8	75.2	119.8	255.9	525.7	978.3	1316.5	1552.0
2031	PRE	0.2	0.3	0.6	1.6	5.2	16.5	46.3	79.7	141.8	203.7	404.4	769.2	1326.9	1632.7
2032	PRE	0.2	0.3	0.6	1.6	5.2	16.3	45.6	78.5	139.6	200.6	398.3	757.5	1306.9	1608.0
2033	PRE	0.2	0.3	0.6	1.5	5.1	16.0	44.9	77.3	137.5	197.6	392.3	746.1	1287.1	1583.7
2034	PRE	0.2	0.3	0.6	1.5	5.0	15.8	44.2	76.1	135.5	194.6	386.4	734.9	1267.7	1559.8
2035	PRE	0.2	0.3	0.6	1.5	4.9	15.5	43.6	75.0	133.4	191.7	380.5	723.8	1248.6	1536.3
2036	PRE	0.2	0.3	0.6	1.6	5.2	16.3	41.9	97.0	141.4	226.8	303.0	556.8	981.6	1548.4
2037	PRE	0.2	0.3	0.6	1.6	5.1	16.1	41.3	95.6	139.3	223.4	298.4	548.4	966.8	1525.0
2038	PRE	0.2	0.3	0.6	1.5	5.0	15.8	40.6	94.1	137.2	220.0	293.9	540.1	952.2	1502.0
2039	PRE	0.2	0.3	0.6	1.5	4.9	15.6	40.0	92.7	135.1	216.7	289.5	532.0	937.8	1479.3
2040	PRE	0.2	0.3	0.6	1.5	4.9	15.4	39.4	91.3	133.1	213.4	285.1	523.9	923.7	1457.0

Results for PHIM for Run 2 (Basic_US_OG_T1.RTF)

****

Observed and predicted deaths (single years)

		10-	15-	20-	25-	30-	35-	40-	45-	50-	55-	60-	65-	70-	75-
2016	OBS	1.0	4.0	7.0	14.0	37.0	125.0	278.0	984.0	2766.0	5654.0	7473.0	9738.0	11081.0	10755.0
	PRE	1.9	3.5	7.6	20.2	51.6	153.4	345.5	982.5	2566.1	5594.5	7957.3	9468.1	11036.3	11239.8
	CHI	0.420	0.073	0.045	1.910	4.150	5.253	13.170	0.002	15.572	0.632	29.473	7.696	0.181	20.914
2017	OBS	2.0	2.0	3.0	11.0	31.0	100.0	283.0	879.0	2446.0	5488.0	7499.0	9578.0	11492.0	10560.0
	PRE	1.9	3.4	7.4	20.2	51.6	152.8	340.0	960.8	2487.5	5516.3	7989.8	9565.8	11445.4	11439.2
	CHI	0.008	0.600	2.640	4.184	8.193	18.256	9.549	6.967	0.693	0.145	30.148	0.016	0.190	67.575
2018	PRE	1.9	3.4	7.3	20.1	51.4	152.5	335.0	942.2	2407.2	5418.8	8008.6	9628.5	11869.4	11702.9
2019	PRE	1.8	3.3	7.1	20.0	51.4	152.3	331.0	925.3	2332.4	5305.6	7999.8	9684.5	12254.8	12050.4
2020	PRE	1.8	3.3	6.9	19.8	51.3	152.2	328.1	909.3	2266.9	5179.3	7955.9	9747.3	12570.7	12486.1
2021	PRE	1.9	3.5	7.2	19.0	67.2	163.5	391.5	722.6	1725.2	4020.6	8054.7	10448.6	11228.0	11445.7
2022	PRE	1.8	3.4	7.1	18.6	67.1	163.2	389.9	711.1	1687.3	3898.2	7943.0	10489.5	11342.1	11873.8
2023	PRE	1.8	3.4	7.0	18.2	66.9	162.8	389.1	700.9	1655.2	3774.5	7807.3	10518.6	11425.4	12327.8
2024	PRE	1.7	3.3	6.9	17.7	66.5	162.5	388.7	692.6	1626.1	3659.1	7649.1	10514.4	11506.3	12747.2
2025	PRE	1.7	3.3	6.8	17.4	65.8	162.3	388.2	686.5	1598.3	3557.9	7471.2	10465.3	11597.8	13099.6
2026	PRE	1.8	3.4	7.2	18.2	63.2	212.7	417.1	819.2	1270.3	2708.5	5801.0	10595.6	12433.2	11696.3
2027	PRE	1.7	3.4	7.1	17.8	61.9	212.4	416.3	816.1	1250.4	2649.8	5626.7	10453.2	12489.3	11821.8
2028	PRE	1.7	3.3	7.0	17.6	60.5	211.8	415.4	814.6	1232.7	2600.2	5450.6	10281.6	12535.5	11926.4
2029	PRE	1.7	3.2	6.9	17.3	59.1	210.5	414.7	813.7	1218.4	2555.1	5286.5	10081.5	12545.8	12038.3
2030	PRE	1.7	3.1	6.8	17.1	57.9	208.2	413.9	812.8	1207.9	2512.2	5142.9	9855.8	12505.9	12168.3
2031	PRE	1.8	3.3	7.2	18.0	60.5	200.1	542.9	873.5	1442.0	1997.9	3918.2	7657.9	12670.7	13061.0
2032	PRE	1.7	3.2	7.0	17.8	59.5	196.1	542.1	872.0	1436.9	1967.3	3835.6	7433.1	12512.5	13139.6
2033	PRE	1.7	3.1	6.8	17.7	58.6	191.6	540.6	870.3	1434.5	1940.2	3765.7	7206.1	12322.6	13212.8
2034	PRE	1.7	3.1	6.7	17.5	57.8	187.3	537.2	868.7	1433.1	1918.3	3702.1	6995.5	12101.3	13254.1
2035	PRE	1.7	3.0	6.5	17.2	57.1	183.4	531.3	867.2	1431.7	1902.1	3641.3	6812.2	11850.5	13248.3
2036	PRE	1.8	3.2	6.8	18.0	60.2	192.0	510.8	1137.4	1538.8	2271.4	2896.7	5191.3	9208.0	13421.0
2037	PRE	1.8	3.2	6.7	17.6	59.6	188.7	500.7	1135.8	1536.3	2263.8	2853.3	5084.1	8941.2	13260.7
2038	PRE	1.8	3.2	6.6	17.2	59.0	185.8	489.4	1132.7	1533.4	2260.4	2815.0	4993.9	8674.7	13076.0
2039	PRE	1.7	3.1	6.5	16.8	58.4	183.3	478.3	1125.6	1530.6	2258.5	2784.0	4912.5	8430.9	12865.5
2040	PRE	1.7	3.1	6.4	16.4	57.5	181.0	468.5	1113.2	1528.1	2256.4	2761.4	4835.2	8222.4	12629.4

Results for PHIM for Run 2 (Basic_US_OG_T1.RTF)

****
List of values created by O and G modelling, using percentage change in last two period parameters for Fixed File MORT

1. Country	US (United States)
2. Sex	F (Females)
3. Disease	LC (LUNG CANCER)
* Value comes from O and G Modelling.	

Age	Years	Value	Death Rate	Population
10-14	2018	1.8552	0.179778	103196.46 *
10-14	2019	1.8311	0.177065	103416.52 *
10-14	2020	1.7983	0.174392	103119.74 *
10-14	2021	1.8842	0.183216	102838.63 *
10-14	2022	1.8413	0.180450	102041.78 *
10-14	2023	1.7924	0.177726	100854.31 *
10-14	2024	1.7428	0.175044	99561.97 *
10-14	2025	1.6971	0.172401	98437.76 *
10-14	2026	1.7754	0.181125	98019.03 *
10-14	2027	1.7430	0.178391	97707.32 *
10-14	2028	1.7124	0.175698	97463.30 *
10-14	2029	1.6833	0.173046	97277.15 *
10-14	2030	1.6568	0.170434	97213.74 *
10-14	2031	1.7529	0.179057	97897.69 *
10-14	2032	1.7399	0.176354	98659.12 *
10-14	2033	1.7269	0.173692	99422.00 *
10-14	2034	1.7120	0.171071	100073.76 *
10-14	2035	1.6941	0.168488	100545.70 *
10-14	2036	1.7962	0.177014	101474.16 *
10-14	2037	1.7811	0.174342	102160.11 *
10-14	2038	1.7618	0.171710	102601.69 *
10-14	2039	1.7390	0.169118	102829.10 *
15-19	2018	3.3799	0.324364	104200.38 *
15-19	2019	3.3267	0.319467	104134.08 *
15-19	2020	3.2750	0.314645	104085.87 *
15-19	2021	3.4575	0.330566	104593.73 *
15-19	2022	3.4270	0.325576	105258.83 *
15-19	2023	3.3941	0.320661	105846.21 *
15-19	2024	3.3492	0.315821	106048.78 *
15-19	2025	3.2878	0.311054	105699.11 *
15-19	2026	3.4448	0.326793	105411.01 *
15-19	2027	3.3682	0.321860	104647.84 *
15-19	2028	3.2817	0.317001	103524.06 *
15-19	2029	3.1943	0.312216	102309.30 *
15-19	2030	3.1120	0.307504	101201.39 *
15-19	2031	3.2568	0.323063	100810.47 *
15-19	2032	3.1991	0.318186	100543.20 *
15-19	2033	3.1451	0.313383	100360.03 *
15-19	2034	3.0939	0.308653	100239.43 *
15-19	2035	3.0453	0.303994	100174.80 *
15-19	2036	3.2203	0.319375	100832.66 *
15-19	2037	3.1970	0.314554	101635.99 *
15-19	2038	3.1738	0.309806	102445.77 *
15-19	2039	3.1463	0.305130	103113.72 *
20-24	2018	7.2508	0.652615	111103.83 *
20-24	2019	7.0784	0.642764	110124.20 *
20-24	2020	6.9232	0.633061	109360.13 *
20-24	2021	7.2342	0.665093	108770.00 *
20-24	2022	7.1024	0.655054	108425.37 *
20-24	2023	6.9886	0.645166	108322.56 *
20-24	2024	6.8877	0.635427	108395.50 *
20-24	2025	6.7934	0.625836	108550.01 *
20-24	2026	7.1598	0.657502	108894.01 *
20-24	2027	7.0907	0.647577	109496.37 *
20-24	2028	7.0259	0.637802	110158.41 *
20-24	2029	6.9451	0.628175	110560.57 *
20-24	2030	6.8348	0.618692	110472.49 *
20-24	2031	7.1528	0.649997	110043.60 *
20-24	2032	6.9918	0.640186	109215.51 *
20-24	2033	6.8201	0.630522	108165.47 *
20-24	2034	6.6546	0.621005	107158.74 *
20-24	2035	6.5029	0.611631	106321.30 *
20-24	2036	6.7926	0.642578	105709.09 *
20-24	2037	6.6650	0.632879	105313.13 *
20-24	2038	6.5544	0.623326	105152.82 *
20-24	2039	6.4585	0.613917	105200.95 *
25-29	2018	20.1417	1.746687	115313.86 *
25-29	2019	20.0225	1.720321	116387.97 *

Results for PHIM for Run 2 (Basic_US_OG_T1.RTF)

25-29	2020	19.8085	1.694353	116908.75 *
25-29	2021	19.0168	1.629084	116732.89 *
25-29	2022	18.6162	1.604494	116025.62 *
25-29	2023	18.1750	1.580274	115011.70 *
25-29	2024	17.7497	1.556420	114041.82 *
25-29	2025	17.3741	1.532927	113339.59 *
25-29	2026	18.1589	1.610490	112753.72 *
25-29	2027	17.8351	1.586180	112440.79 *
25-29	2028	17.5569	1.562237	112383.37 *
25-29	2029	17.3139	1.538656	112526.12 *
25-29	2030	17.0967	1.515430	112817.52 *
25-29	2031	18.0223	1.592108	113197.88 *
25-29	2032	17.8498	1.568076	113832.48 *
25-29	2033	17.6885	1.544406	114532.86 *
25-29	2034	17.4927	1.521094	115000.73 *
25-29	2035	17.2356	1.498133	115047.44 *
25-29	2036	18.0373	1.573936	114600.09 *
25-29	2037	17.6351	1.550178	113761.95 *
25-29	2038	17.2085	1.526779	112710.93 *
25-29	2039	16.8010	1.503732	111728.46 *
30-34	2018	51.4474	4.659728	110408.56 *
30-34	2019	51.3615	4.589390	111913.53 *
30-34	2020	51.3031	4.520115	113499.65 *
30-34	2021	67.2495	5.848368	114988.57 *
30-34	2022	67.1357	5.760088	116553.27 *
30-34	2023	66.9471	5.673141	118007.13 *
30-34	2024	66.5268	5.587507	119063.44 *
30-34	2025	65.7972	5.503165	119562.53 *
30-34	2026	63.1959	5.291174	119436.43 *
30-34	2027	61.8995	5.211305	118779.22 *
30-34	2028	60.4649	5.132642	117804.55 *
30-34	2029	59.0795	5.055166	116869.57 *
30-34	2030	57.8619	4.978860	116215.13 *
30-34	2031	60.5316	5.230782	115721.99 *
30-34	2032	59.4839	5.151825	115461.72 *
30-34	2033	58.5710	5.074059	115432.31 *
30-34	2034	57.7697	4.997468	115598.01 *
30-34	2035	57.0580	4.922032	115923.69 *
30-34	2036	60.1669	5.171079	116352.68 *
30-34	2037	59.5922	5.093023	117007.50 *
30-34	2038	59.0424	5.016145	117704.81 *
30-34	2039	58.3764	4.940428	118160.59 *
35-39	2018	152.5168	14.587403	104553.79 *
35-39	2019	152.3481	14.367210	106038.72 *
35-39	2020	152.2141	14.150341	107569.20 *
35-39	2021	163.5162	14.996794	109034.08 *
35-39	2022	163.1920	14.770422	110485.67 *
35-39	2023	162.8438	14.547466	111939.64 *
35-39	2024	162.5304	14.327876	113436.46 *
35-39	2025	162.2648	14.111601	114986.80 *
35-39	2026	212.7266	18.258349	116509.21 *
35-39	2027	212.3898	17.982744	118107.58 *
35-39	2028	211.8006	17.711299	119585.02 *
35-39	2029	210.4717	17.443952	120655.96 *
35-39	2030	208.1673	17.180640	121163.90 *
35-39	2031	200.0777	16.518815	121121.11 *
35-39	2032	196.0688	16.269468	120513.37 *
35-39	2033	191.5945	16.023885	119568.09 *
35-39	2034	187.2559	15.782008	118651.53 *
35-39	2035	183.4191	15.543783	118001.61 *
35-39	2036	191.9780	16.330272	117559.56 *
35-39	2037	188.7045	16.083771	117326.00 *
35-39	2038	185.8192	15.840991	117302.74 *
35-39	2039	183.2584	15.601876	117459.22 *
40-44	2018	335.0307	33.235578	100804.83 *
40-44	2019	331.0055	32.733896	101120.12 *
40-44	2020	328.0726	32.239786	101760.17 *
40-44	2021	391.4502	38.141625	102630.72 *
40-44	2022	389.9128	37.565888	103794.37 *
40-44	2023	389.1448	36.998841	105177.56 *
40-44	2024	388.6833	36.440353	106662.88 *
40-44	2025	388.2291	35.890296	108171.06 *
40-44	2026	417.0785	38.037203	109650.15 *
40-44	2027	416.3039	37.463042	111123.89 *
40-44	2028	415.4496	36.897547	112595.46 *
40-44	2029	414.6524	36.340589	114101.74 *
40-44	2030	413.9429	35.792038	115652.24 *
40-44	2031	542.8905	46.309664	117230.49 *

Results for PHIM for Run 2 (Basic_US_OG_T1.RTF)

40-44	2032	542.1155	45.610632	118857.26 *
40-44	2033	540.6282	44.922152	120347.80 *
40-44	2034	537.2335	44.244064	121424.99 *
40-44	2035	531.3177	43.576212	121928.37 *
40-44	2036	510.8126	41.897588	121919.33 *
40-44	2037	500.6968	41.265155	121336.47 *
40-44	2038	489.3635	40.642269	120407.52 *
40-44	2039	478.3332	40.028785	119497.30 *
45-49	2018	942.1884	92.954591	101360.07 *
45-49	2019	925.3090	91.551466	101069.82 *
45-49	2020	909.3325	90.169522	100846.99 *
45-49	2021	722.6339	71.789846	100659.62 *
45-49	2022	711.1337	70.706198	100575.87 *
45-49	2023	700.9427	69.638907	100653.89 *
45-49	2024	692.6445	68.587726	100986.65 *
45-49	2025	686.5049	67.552413	101625.52 *
45-49	2026	819.1719	79.918607	102500.77 *
45-49	2027	816.0725	78.712258	103677.94 *
45-49	2028	814.5689	77.524117	105072.97 *
45-49	2029	813.6774	76.353912	106566.57 *
45-49	2030	812.7713	75.201370	108079.32 *
45-49	2031	873.4821	79.699810	109596.51 *
45-49	2032	872.0048	78.496763	111087.99 *
45-49	2033	870.2876	77.311876	112568.42 *
45-49	2034	868.6645	76.144874	114080.49 *
45-49	2035	867.1610	74.995488	115628.42 *
45-49	2036	1137.3839	97.033197	117215.96 *
45-49	2037	1135.7941	95.568508	118846.06 *
45-49	2038	1132.6849	94.125927	120337.18 *
45-49	2039	1125.5752	92.705121	121414.57 *
50-54	2018	2407.2397	227.807793	105669.77 *
50-54	2019	2332.3794	224.369096	103952.79 *
50-54	2020	2266.8966	220.982305	102582.72 *
50-54	2021	1725.2329	170.009314	101478.73 *
50-54	2022	1687.2744	167.443069	100767.05 *
50-54	2023	1655.1731	164.915562	100364.88 *
50-54	2024	1626.0617	162.426206	100110.80 *
50-54	2025	1598.3085	159.974426	99910.25 *
50-54	2026	1270.2868	127.366090	99735.09 *
50-54	2027	1250.3505	125.443533	99674.37 *
50-54	2028	1232.7385	123.549997	99776.49 *
50-54	2029	1218.4449	121.685043	100131.03 *
50-54	2030	1207.9290	119.848240	100788.21 *
50-54	2031	1442.0239	141.787747	101703.00 *
50-54	2032	1436.9178	139.647500	102896.06 *
50-54	2033	1434.4926	137.539558	104296.73 *
50-54	2034	1433.1250	135.463436	105794.23 *
50-54	2035	1431.6733	133.418652	107306.83 *
50-54	2036	1538.7774	141.399568	108824.76 *
50-54	2037	1536.2770	139.265180	110313.07 *
50-54	2038	1533.3513	137.163010	111790.44 *
50-54	2039	1530.6065	135.092571	113300.57 *
55-59	2018	5418.8302	492.542523	110017.51 *
55-59	2019	5305.5844	485.107727	109369.20 *
55-59	2020	5179.2891	477.785157	108402.05 *
55-59	2021	4020.5813	375.634530	107034.39 *
55-59	2022	3898.2127	369.964428	105367.23 *
55-59	2023	3774.4917	364.379916	103586.71 *
55-59	2024	3659.1302	358.879699	101959.80 *
55-59	2025	3557.9296	353.462507	100659.32 *
55-59	2026	2708.4661	271.930905	99601.26 *
55-59	2027	2649.7718	267.826182	98936.25 *
55-59	2028	2600.1601	263.783418	98571.78 *
55-59	2029	2555.1303	259.801679	98349.26 *
55-59	2030	2512.2216	255.880043	98179.66 *
55-59	2031	1997.8858	203.722816	98068.83 *
55-59	2032	1967.3450	200.647675	98049.73 *
55-59	2033	1940.2434	197.618953	98181.04 *
55-59	2034	1918.2778	194.635948	98557.22 *
55-59	2035	1902.1400	191.697971	99225.88 *
55-59	2036	2271.4048	226.790342	100154.39 *
55-59	2037	2263.8322	223.367003	101350.34 *
55-59	2038	2260.3511	219.995339	102745.41 *
55-59	2039	2258.4564	216.674569	104232.65 *
60-64	2018	8008.5772	772.335972	103692.92 *
60-64	2019	7999.8298	760.677770	105167.13 *
60-64	2020	7955.9000	749.195544	106192.57 *
60-64	2021	8054.7399	755.108547	106669.96 *

Results for PHIM for Run 2 (Basic_US_OG_T1.RTF)

60-64	2022	7942.9935	743.710388	106802.24 *
60-64	2023	7807.3236	732.484281	106586.91 *
60-64	2024	7649.1226	721.427629	106027.58 *
60-64	2025	7471.2198	710.537874	105148.79 *
60-64	2026	5800.9800	558.624638	103843.97 *
60-64	2027	5626.7005	550.192350	102267.88 *
60-64	2028	5450.5901	541.887345	100585.30 *
60-64	2029	5286.5018	533.707702	99052.38 *
60-64	2030	5142.8589	525.651529	97837.80 *
60-64	2031	3918.1518	404.401862	96887.58 *
60-64	2032	3835.6490	398.297526	96301.10 *
60-64	2033	3765.7034	392.285332	95993.99 *
60-64	2034	3702.0924	386.363891	95818.80 *
60-64	2035	3641.2916	380.531833	95689.54 *
60-64	2036	2896.6670	302.966248	95610.22 *
60-64	2037	2853.3275	298.393054	95623.12 *
60-64	2038	2814.9610	293.888892	95783.17 *
60-64	2039	2783.9936	289.452719	96181.29 *
65-69	2018	9628.4517	1074.256413	89628.99 *
65-69	2019	9684.5268	1058.040803	91532.64 *
65-69	2020	9747.2650	1042.069963	93537.53 *
65-69	2021	10448.5972	1096.258575	95311.43 *
65-69	2022	10489.5021	1079.710849	97151.03 *
65-69	2023	10518.6445	1063.412905	98914.02 *
65-69	2024	10514.4390	1047.360975	100389.83 *
65-69	2025	10465.2834	1031.551345	101451.89 *
65-69	2026	10595.6303	1039.692832	101911.16 *
65-69	2027	10453.2199	1023.998950	102082.33 *
65-69	2028	10281.6234	1008.541963	101945.42 *
65-69	2029	10081.5032	993.318295	101493.18 *
65-69	2030	9855.8144	978.324424	100741.78 *
65-69	2031	7657.9296	769.158333	99562.46 *
65-69	2032	7433.0983	757.548096	98120.48 *
65-69	2033	7206.0664	746.113113	96581.42 *
65-69	2034	6995.5431	734.850737	95196.79 *
65-69	2035	6812.1896	723.758364	94122.43 *
65-69	2036	5191.3430	556.812288	93233.27 *
65-69	2037	5084.1305	548.407357	92707.19 *
65-69	2038	4993.9269	540.129296	92457.99 *
65-69	2039	4912.4873	531.976190	92344.12 *
70-74	2018	11869.3858	1680.942546	70611.49 *
70-74	2019	12254.7647	1655.569173	74021.46 *
70-74	2020	12570.6929	1630.578804	77093.44 *
70-74	2021	11228.0306	1413.348150	79442.78 *
70-74	2022	11342.1499	1392.014042	81480.14 *
70-74	2023	11425.3546	1371.001966	83335.80 *
70-74	2024	11506.2892	1350.307062	85212.39 *
70-74	2025	11597.8184	1329.924542	87206.59 *
70-74	2026	12433.2432	1399.081862	88867.16 *
70-74	2027	12489.2967	1377.963099	90635.93 *
70-74	2028	12535.5484	1357.163119	92365.82 *
70-74	2029	12545.7519	1336.677109	93857.76 *
70-74	2030	12505.8829	1316.500329	94993.39 *
70-74	2031	12670.6737	1326.890768	95491.46 *
70-74	2032	12512.4520	1306.861711	95744.27 *
70-74	2033	12322.5889	1287.134987	95736.57 *
70-74	2034	12101.3088	1267.706032	95458.32 *
70-74	2035	11850.5121	1248.570353	94912.65 *
70-74	2036	9208.0456	981.625592	93804.05 *
70-74	2037	8941.2319	966.808219	92481.96 *
70-74	2038	8674.6561	952.214511	91099.81 *
70-74	2039	8430.8904	937.841090	89896.79 *
75-79	2018	11702.8893	2383.330512	49103.09 *
75-79	2019	12050.3876	2347.354783	51336.03 *
75-79	2020	12486.1086	2311.922097	54007.48 *
75-79	2021	11445.7412	2022.163594	56601.46 *
75-79	2022	11873.8086	1991.639582	59618.26 *
75-79	2023	12327.7735	1961.576321	62846.26 *
75-79	2024	12747.2101	1931.966857	65980.48 *
75-79	2025	13099.5539	1902.804340	68843.41 *
75-79	2026	11696.2534	1649.306974	70916.17 *
75-79	2027	11821.8307	1624.411131	72776.10 *
75-79	2028	11926.4041	1599.891085	74545.10 *
75-79	2029	12038.2622	1575.741162	76397.46 *
75-79	2030	12168.2644	1551.955775	78406.00 *
75-79	2031	13061.0167	1632.658926	79998.44 *
75-79	2032	13139.6145	1608.014381	81713.29 *
75-79	2033	13212.8097	1583.741839	83427.80 *

Results for PHIM for Run 2 (Basic_US_OG_T1.RTF)

75-79	2034	13254.1391	1559.835684	84971.38 *
75-79	2035	13248.3246	1536.290386	86235.81 *
75-79	2036	13420.9967	1548.415511	86675.68 *
75-79	2037	13260.7471	1525.042598	86953.29 *
75-79	2038	13076.0100	1502.022492	87056.02 *
75-79	2039	12865.5404	1479.349869	86967.53 *

Results for PHIM for Run 2 (Basic_US_OG_T1.RTF)

****

List of values created by last value brought forwards for Fixed File perm.Fixed_File_RR_LC

1. Country	US (United States)
2. Sex	F (Females)
3. Disease	LC (LC)

Age	Years	Value
10-14	2013-2039	11.6800
15-19	2013-2039	11.6800
20-24	2013-2039	11.6800
25-29	2013-2039	11.6800
30-34	2013-2039	11.6800
35-39	2013-2039	11.6800
40-44	2013-2039	11.6800
45-49	2013-2039	11.6800
50-54	2013-2039	11.6800
55-59	2013-2039	11.6800
60-64	2013-2039	11.6800
65-69	2013-2039	11.6800
70-74	2013-2039	11.6800
75-79	2013-2039	11.6800

Results for PHIM for Run 2 (Basic_US_OG_T1.RTF)

****

Osmond and Gardner Modeling of Death Rates for COD: IHD

Variable Parameter	Value
1. Country	US (United States)
2. Sex	F (Females)
3. Year of start of OG modelling	1966
4. Number 5-year periods for OG modelling	10
5. Age Range of interest	10 - 79
6. Scaling Factor for Rates	10000
7. Model Type to Save	4 (Full Age-Period-Cohort)
8. Goodness of Fit Required	1 (Analysis Printed For Age-Period-Cohort Model)
9. Cause of Death	IHD
Note:	Death rates are per million population

Matrix of Numbers of Deaths

Age	1966-	1971-	1976-	1981-	1986-	1991-	1996-	2001-	2006-	2011-
10-14	42	44	15	21	18	16	22	21	15	15
15-19	111	110	52	60	43	40	57	40	41	45
20-24	271	223	152	155	131	141	125	157	120	124
25-29	558	463	375	375	378	373	336	345	379	373
30-34	1460	1244	984	863	957	1052	991	975	854	895
35-39	3680	3217	2322	2085	2008	2336	2645	2597	2130	1955
40-44	8992	7787	5593	4696	4402	4749	5333	6374	5181	4577
45-49	17574	16292	12003	8996	8037	8425	9504	11363	11072	9240
50-54	31631	30963	24657	18573	14682	14278	16345	18487	18290	17887
55-59	54283	52272	44862	37052	28654	24478	25051	27616	26509	27195
60-64	87105	88280	75123	66044	55313	44138	40313	39553	37449	38393
65-69	137647	134022	117588	102745	90893	76850	66043	56033	46757	48589
70-74	198997	194867	168269	152188	133295	120234	109135	88423	64058	59809
75-79	249864	255194	223091	200090	187196	167793	164198	142524	99680	79431

Matrix of Age- and Period-Specific Mortality Rates

Age	1966-	1971-	1976-	1981-	1986-	1991-	1996-	2001-	2006-	2011-
10-14	0.839	0.864	0.320	0.491	0.417	0.360	0.451	0.403	0.291	0.295
15-19	2.415	2.175	1.003	1.250	0.972	0.915	1.218	0.785	0.766	0.844
20-24	6.820	4.788	2.949	2.928	2.675	3.107	2.731	3.220	2.302	2.239
25-29	17.254	11.517	7.903	7.135	7.057	7.456	7.071	7.210	7.561	6.937
30-34	50.738	37.913	23.970	17.821	17.884	19.164	19.058	19.745	17.373	17.389
35-39	125.113	111.628	70.388	50.582	41.289	43.390	47.241	48.833	42.323	38.996
40-44	284.309	265.886	193.443	141.745	106.350	97.760	98.482	113.053	96.885	90.104
45-49	565.916	522.214	411.624	311.920	244.928	206.341	195.543	209.642	196.640	172.929
50-54	1112.587	1021.419	799.827	642.444	515.185	440.393	400.355	379.789	338.360	319.784
55-59	2052.336	1904.204	1513.170	1228.456	1017.105	880.766	783.544	681.884	550.369	510.052
60-64	3678.984	3532.910	2848.525	2311.683	1908.112	1628.250	1486.356	1264.976	946.154	817.992
65-69	6951.976	6073.708	4957.033	4092.254	3365.322	2815.178	2561.493	2151.291	1558.885	1281.134
70-74	12794.76	11311.08	8546.183	7144.219	5911.289	4962.207	4407.825	3731.933	2665.676	2145.195
75-79	21806.40	19831.28	15391.79	11865.64	10321.67	8767.717	7844.358	6665.567	4814.623	3745.661

Matrix of Log-Rates

Age	1966-	1971-	1976-	1981-	1986-	1991-	1996-	2001-	2006-	2011-
10-14	-0.076	-0.064	-0.494	-0.309	-0.380	-0.444	-0.346	-0.395	-0.535	-0.530
15-19	0.383	0.337	0.001	0.097	-0.012	-0.039	0.086	-0.105	-0.116	-0.074
20-24	0.834	0.680	0.470	0.467	0.427	0.492	0.436	0.508	0.362	0.350
25-29	1.237	1.061	0.898	0.853	0.849	0.873	0.849	0.858	0.879	0.841
30-34	1.705	1.579	1.380	1.251	1.252	1.282	1.280	1.295	1.240	1.240
35-39	2.097	2.048	1.847	1.704	1.616	1.637	1.674	1.689	1.627	1.591
40-44	2.454	2.425	2.287	2.152	2.027	1.990	1.993	2.053	1.986	1.955
45-49	2.753	2.718	2.615	2.494	2.389	2.315	2.291	2.321	2.294	2.238
50-54	3.046	3.009	2.903	2.808	2.712	2.644	2.602	2.580	2.529	2.505
55-59	3.312	3.280	3.180	3.089	3.007	2.945	2.894	2.834	2.741	2.708
60-64	3.566	3.548	3.455	3.364	3.281	3.212	3.172	3.102	2.976	2.913
65-69	3.842	3.783	3.695	3.612	3.527	3.450	3.408	3.333	3.193	3.108
70-74	4.107	4.054	3.932	3.854	3.772	3.696	3.644	3.572	3.426	3.331
75-79	4.339	4.297	4.187	4.074	4.014	3.943	3.895	3.824	3.683	3.574

Results for PHIM for Run 2 (Basic_US_OG_T1.RTF)

Fitting the Age, Period, Cohort Models

**Model**	**RSS**	**MRSS**	**DF**	**Factor**	**%Account**	**ChiSq**	**P**
Age Only	248330.536	1955.359	127	P, C	99.7449	1379158.04	0.0000
Age-Period	3461.499	29.585	117	Cohort	81.7006	18469.716	0.0000
Age-Cohort	2411.663	23.189	104	Period	73.7346	12799.875	0.0000
Period-Cohort	973.252	9.012	108	Age	34.9157	5190.108	0.0000
Full Age-Period-Cohort	633.434	6.598	96			3368.502	0.0000

**Key to terms:**		
	RSS =	residual sum of squares
	MRSS =	mean RSS (MRSS/DF)
	DF =	degrees of freedom
	Factor =	Factors not included in the model
	% Account =	1- (RSS for full model)/(RSS for model in question)
	Chisq =	chi-squared value for model
	P =	probability value based on Chisq and DF.

**Age**	**Value**	**Log10 Value**
10-	0.587316	-0.231128
15-	1.604655	0.205382
20-	4.436719	0.647062
25-	11.620384	1.065220
30-	32.538051	1.512392
35-	84.370443	1.926190
40-	201.319941	2.303887
45-	404.809982	2.607251
50-	769.041842	2.885950
55-	1365.73808	3.135367
60-	2377.95952	3.376204
65-	3875.10956	3.588284
70-	6348.78274	3.802690
75-	10288.8139	4.012365

**Period**	**Value**	**Log10 Value**
1966	1.444418	0.159693
1971	1.411349	0.149634
1976	1.199437	0.078977
1981	1.041271	0.017564
1986	0.922967	-0.034814
1991	0.835735	-0.077932
1996	0.805342	-0.094020
2001	0.739894	-0.130831
2006	0.582583	-0.234642
2011	0.500020	-0.301013

**Cohort**	**Value**	**Log10 Value**
1891	1.467323	0.166526
1896	1.378557	0.139425
1901	1.251215	0.097332
1906	1.106931	0.044121
1911	1.071964	0.030180
1916	1.009276	0.004010
1921	0.939924	-0.026907
1926	0.872445	-0.059262
1931	0.812382	-0.090240
1936	0.745510	-0.127547
1941	0.693664	-0.158851
1946	0.660681	-0.180008
1951	0.666036	-0.176502
1956	0.720268	-0.142506
1961	0.803853	-0.094823
1966	0.824749	-0.083678
1971	0.868321	-0.061320
1976	0.902442	-0.044581
1981	1.061712	0.026007
1986	1.064322	0.027073
1991	0.954710	-0.020129
1996	0.997814	-0.000950
2001	1.005658	0.002450

Results for PHIM for Run 2 (Basic_US_OG_T1.RTF)

Model: Full Age-Period-Cohort

Basic Analysis Using OG Modelling T1 on US
Fitting the Full Age-Period-Cohort Model
Matrix of observed, expected, and residual rates

Age		1966-	1971-	1976-	1981-	1986-	1991-	1996-	2001-	2006-	2011-
10-	Observed	0.839	0.864	0.320	0.491	0.417	0.360	0.451	0.403	0.291	0.295
	Expected	0.611	0.666	0.581	0.531	0.489	0.521	0.503	0.415	0.341	0.295
	Residual	-0.228	-0.197	0.261	0.040	0.073	0.161	0.053	0.012	0.050	0.000
15-	Observed	2.415	2.175	1.003	1.250	0.972	0.915	1.218	0.785	0.766	0.844
	Expected	1.544	1.631	1.547	1.378	1.286	1.210	1.372	1.264	0.893	0.801
	Residual	-0.871	-0.544	0.545	0.128	0.314	0.295	0.154	0.479	0.126	-0.043
20-	Observed	6.820	4.788	2.949	2.928	2.675	3.107	2.731	3.220	2.302	2.239
	Expected	4.234	4.171	3.833	3.714	3.377	3.220	3.224	3.485	2.751	2.118
	Residual	-2.586	-0.618	0.884	0.785	0.702	0.113	0.494	0.266	0.449	-0.121
25-	Observed	17.254	11.517	7.903	7.135	7.057	7.456	7.071	7.210	7.561	6.937
	Expected	11.643	10.835	9.283	8.715	8.622	8.010	8.126	7.759	7.188	6.184
	Residual	-5.611	-0.682	1.380	1.580	1.564	0.553	1.055	0.549	-0.373	-0.753
30-	Observed	50.738	37.913	23.970	17.821	17.884	19.164	19.058	19.745	17.373	17.389
	Expected	35.038	31.855	25.785	22.566	21.631	21.859	21.612	20.905	17.107	17.274
	Residual	-15.701	-6.058	1.815	4.744	3.747	2.695	2.554	1.160	-0.266	-0.115
35-	Observed	125.113	111.628	70.388	50.582	41.289	43.390	47.241	48.833	42.323	38.996
	Expected	99.002	88.772	70.197	58.042	51.865	50.787	54.619	51.485	42.680	38.071
	Residual	-26.111	-22.855	-0.191	7.461	10.576	7.398	7.379	2.652	0.357	-0.925
40-	Observed	284.309	265.886	193.443	141.745	106.350	97.760	98.482	113.053	96.885	90.104
	Expected	253.698	230.824	180.019	145.412	122.762	112.061	116.778	119.738	96.731	87.409
	Residual	-30.610	-35.061	-13.425	3.667	16.412	14.301	18.296	6.686	-0.154	-2.695
45-	Observed	565.916	522.214	411.624	311.920	244.928	206.341	195.543	209.642	196.640	172.929
	Expected	549.587	498.452	394.447	314.245	259.171	223.518	217.135	215.732	189.577	166.940
	Residual	-16.328	-23.762	-17.177	2.325	14.243	17.177	21.592	6.090	-7.063	-5.990
50-	Observed	1112.587	1021.419	799.827	642.444	515.185	440.393	400.355	379.789	338.360	319.784
	Expected	1121.122	1020.181	804.758	650.540	529.163	445.828	409.188	378.981	322.702	309.111
	Residual	8.536	-1.239	4.931	8.096	13.978	5.436	8.833	-0.809	-15.658	-10.673
55-	Observed	2052.336	1904.204	1513.170	1228.456	1017.105	880.766	783.544	681.884	550.369	510.052
	Expected	2114.659	1945.414	1539.705	1240.706	1024.032	850.921	762.952	667.619	529.935	491.868
	Residual	62.323	41.209	26.535	12.251	6.927	-29.845	-20.592	-14.265	-20.434	-18.184
60-	Observed	3678.984	3532.910	2848.525	2311.683	1908.112	1628.250	1486.356	1264.976	946.154	817.992
	Expected	3802.053	3597.650	2878.671	2327.345	1914.821	1614.481	1427.705	1220.458	915.280	791.934
	Residual	123.069	64.741	30.146	15.662	6.709	-13.769	-58.652	-44.518	-30.874	-26.057
65-	Observed	6951.976	6073.708	4957.033	4092.254	3365.322	2815.178	2561.493	2151.291	1558.885	1281.134
	Expected	7003.401	6053.954	4982.433	4072.469	3361.728	2825.467	2535.272	2137.503	1565.997	1280.156
	Residual	51.424	-19.754	25.400	-19.786	-3.594	10.289	-26.221	-13.787	7.111	-0.978
70-	Observed	12794.760	11311.082	8546.183	7144.219	5911.289	4962.207	4407.825	3731.933	2665.676	2145.195
	Expected	12641.774	11211.325	8429.244	7086.540	5914.071	4987.139	4460.759	3816.101	2757.411	2202.048
	Residual	-152.985	-99.757	-116.940	-57.679	2.782	24.932	52.934	84.168	91.735	56.853
75-	Observed	21806.403	19831.276	15391.790	11865.636	10321.672	8767.717	7844.358	6665.567	4814.623	3745.661
	Expected	21806.403	20018.169	15440.977	11859.045	10179.615	8678.482	7788.224	6641.596	4869.485	3835.357
	Residual	0.000	186.893	49.188	-6.591	-142.057	-89.235	-56.134	-23.971	54.862	89.696

Fitting the Full Age-Period-Cohort Model

Results for PHIM for Run 2 (Basic_US_OG_T1.RTF)

Matrix of observed and expected deaths and (O-E)2/E Values**

Age		1966-	1971-	1976-	1981-	1986-	1991-	1996-	2001-	2006-	2011-	Total
10-	Observed	42.0	44.0	15.0	21.0	17.5	16.0	22.0	21.0	15.0	15.0	228.5
	Expected	30.6	33.9	27.2	22.7	20.5	23.2	24.6	21.6	17.6	15.0	236.9
	Difference	11.4	10.1	-12.2	-1.7	-3.0	-7.2	-2.6	-0.6	-2.6	-0.0	-8.4
	Chi-Sq	4.3	3.0	5.5	0.1	0.5	2.2	0.3	0.0	0.4	0.0	16.2
15-	Observed	111.0	110.0	52.0	60.0	43.0	40.0	57.0	40.0	41.0	45.0	599.0
	Expected	71.0	82.5	80.2	66.1	56.9	52.9	64.2	64.4	47.8	42.7	628.7
	Difference	40.0	27.5	-28.2	-6.1	-13.9	-12.9	-7.2	-24.4	-6.8	2.3	-29.7
	Chi-Sq	22.6	9.2	9.9	0.6	3.4	3.2	0.8	9.2	1.0	0.1	60.0
20-	Observed	271.0	223.0	152.0	155.0	131.0	141.0	125.0	157.0	120.0	124.0	1599.0
	Expected	168.2	194.2	197.6	196.6	165.4	146.1	147.6	170.0	143.4	117.3	1646.4
	Difference	102.8	28.8	-45.6	-41.6	-34.4	-5.1	-22.6	-13.0	-23.4	6.7	-47.4
	Chi-Sq	62.8	4.3	10.5	8.8	7.1	0.2	3.5	1.0	3.8	0.4	102.3
25-	Observed	558.0	463.0	375.0	375.0	378.0	373.0	336.0	345.0	379.0	373.0	3955.0
	Expected	376.5	435.6	440.5	458.0	461.8	400.7	386.2	371.3	360.3	332.5	4023.3
	Difference	181.5	27.4	-65.5	-83.0	-83.8	-27.7	-50.2	-26.3	18.7	40.5	-68.3
	Chi-Sq	87.5	1.7	9.7	15.1	15.2	1.9	6.5	1.9	1.0	4.9	145.4
30-	Observed	1460.0	1244.0	984.0	863.0	957.0	1052.0	991.0	975.0	854.0	895.0	10275.0
	Expected	1008.2	1045.2	1058.5	1092.7	1157.5	1200.0	1123.8	1032.3	840.9	889.1	10448.2
	Difference	451.8	198.8	-74.5	-229.7	-200.5	-148.0	-132.8	-57.3	13.1	5.9	-173.2
	Chi-Sq	202.4	37.8	5.2	48.3	34.7	18.2	15.7	3.2	0.2	0.0	365.9
35-	Observed	3680.0	3217.0	2322.0	2085.0	2008.0	2336.0	2645.0	2597.0	2130.0	1955.0	24975.0
	Expected	2912.0	2558.3	2315.7	2392.5	2522.3	2734.3	3058.1	2738.0	2148.0	1908.6	25287.9
	Difference	768.0	658.7	6.3	-307.5	-514.3	-398.3	-413.1	-141.0	-18.0	46.4	-312.9
	Chi-Sq	202.6	169.6	0.0	39.5	104.9	58.0	55.8	7.3	0.2	1.1	638.9
40-	Observed	8992.0	7787.0	5593.0	4696.0	4402.0	4749.0	5333.0	6374.0	5181.0	4577.0	57684.0
	Expected	8023.9	6760.2	5204.9	4817.5	5081.3	5443.7	6323.8	6750.9	5172.7	4440.1	58018.9
	Difference	968.1	1026.8	388.1	-121.5	-679.3	-694.7	-990.8	-376.9	8.3	136.9	-334.9
	Chi-Sq	116.8	156.0	28.9	3.1	90.8	88.7	155.2	21.0	0.0	4.2	664.8
45-	Observed	17574.0	16292.0	12003.0	8996.0	8037.0	8425.0	9504.0	11363.0	11072.0	9240.0	112506.0
	Expected	17066.9	15550.7	11502.1	9063.1	8504.4	9126.3	10553.4	11693.1	10674.3	8920.0	112654.3
	Difference	507.1	741.3	500.9	-67.1	-467.4	-701.3	-1049.4	-330.1	397.7	320.0	-148.3
	Chi-Sq	15.1	35.3	21.8	0.5	25.7	53.9	104.4	9.3	14.8	11.5	292.3
50-	Observed	31631.0	30963.0	24657.0	18573.0	14682.0	14278.0	16345.0	18487.0	18290.0	17887.0	205793.0
	Expected	31873.7	30925.5	24809.0	18807.0	15080.3	14454.2	16705.6	18447.6	17443.6	17290.0	205836.6
	Difference	-242.7	37.5	-152.0	-234.0	-398.3	-176.2	-360.6	39.4	846.4	597.0	-43.6
	Chi-Sq	1.8	0.0	0.9	2.9	10.5	2.1	7.8	0.1	41.1	20.6	88.0
55-	Observed	54283.0	52272.0	44862.0	37052.0	28654.0	24478.0	25051.0	27616.0	26509.0	27195.0	347972.0
	Expected	55931.4	53403.2	45648.7	37421.5	28849.1	23648.5	24392.6	27038.3	25524.8	26225.5	348083.7
	Difference	-1648.4	-1131.2	-786.7	-369.5	-195.1	829.5	658.4	577.7	984.2	969.5	-111.7
	Chi-Sq	48.6	24.0	13.6	3.6	1.3	29.1	17.8	12.3	38.0	35.8	224.1
60-	Observed	87105.0	88280.0	75123.0	66044.0	55313.0	44138.0	40313.0	39553.0	37449.0	38393.0	571711.0
	Expected	90018.8	89897.7	75918.0	66491.5	55507.5	43764.8	38722.2	38161.0	36227.0	37170.0	571878.5
	Difference	-2913.8	-1617.7	-795.0	-447.5	-194.5	373.2	1590.8	1392.0	1222.0	1223.0	-167.5
	Chi-Sq	94.3	29.1	8.3	3.0	0.7	3.2	65.3	50.8	41.2	40.2	336.2
65-	Observed	137647.0	134022.0	117588.0	102745.0	90893.0	76850.0	66043.0	56033.0	46757.0	48589.0	877167.0
	Expected	138665.2	133586.1	118190.5	102248.2	90795.9	77130.9	65366.9	55673.9	46970.3	48551.9	877179.9
	Difference	-1018.2	435.9	-602.5	496.8	97.1	-280.9	676.1	359.1	-213.3	37.1	-12.9
	Chi-Sq	7.5	1.4	3.1	2.4	0.1	1.0	7.0	2.3	1.0	0.0	25.8
70-	Observed	198997.0	194867.0	168269.0	152188.0	133295.0	120234.0	109135.0	88423.0	64058.0	59809.0	1289275.0
	Expected	196617.6	193148.4	165966.5	150959.3	133357.7	120838.1	110445.6	90417.3	66262.5	61394.1	1289407.1

Results for PHIM for Run 2 (Basic_US_OG_T1.RTF)

	Difference	2379.4	1718.6	2302.5	1228.7	-62.7	-604.1	-1310.6	-1994.3	-2204.5	-1585.1	-132.1
	Chi-Sq	28.8	15.3	31.9	10.0	0.0	3.0	15.6	44.0	73.3	40.9	262.9
75-	Observed	249864.0	255194.0	223091.0	200090.0	187196.0	167793.0	164198.0	142524.0	99680.0	79431.0	1769061.0
	Expected	249864.0	257599.0	223803.9	199978.9	184619.6	166085.3	163023.0	142011.4	100815.8	81333.1	1769134.1
	Difference	-0.0	-2405.0	-712.9	111.1	2576.4	1707.7	1175.0	512.6	-1135.8	-1902.1	-73.1
	Chi-Sq	0.0	22.5	2.3	0.1	36.0	17.6	8.5	1.8	12.8	44.5	145.9
Total over ages	Observed	792215.0	784978.0	675086.0	593943.0	526006.5	464903.0	440098.0	394508.0	312535.0	288528.0	5272800.5
	Expected	792628.0	785220.6	675163.4	594015.7	526180.4	465048.9	440337.7	394591.1	312649.0	288629.8	5274464.6
	Difference	-413.0	-242.6	-77.4	-72.7	-173.9	-145.9	-239.7	-83.1	-114.0	-101.8	-1664.1
	Chi-Sq	895.0	509.1	151.8	138.0	330.9	282.3	464.1	164.3	228.7	204.4	3368.5

Results for PHIM for Run 2 (Basic_US_OG_T1.RTF)

****

Osmond and Gardner Extrapolating Death Rates for COD: IHD

Variable Parameter	Value
1. Country	US (United States)
2. Sex	F (Females)
3. Year of start of OG modelling	1966
4. Number 5-year periods for OG modelling	10
5. Age Range of interest	10 - 79
6. Scaling Factor for Rates	10000
7. Model Type to Save	4 (Full Age-Period-Cohort)
8. Goodness of Fit Required	1 (Analysis Printed For Age-Period-Cohort Model)
9. Cause of Death	IHD
Note:	Death rates are per million population
10. Number of Periods into the future to Predict	5
11. Earliest projected year	2016
12. Extrapolate Period using (1: last 2 points 2: linear regression)	1
13. Ratio of last two period values	0.85828

Predictions of rates for future years from model:	Full Age-Period-Cohort
Effects for extending model to project rates for:	2016-2040

Extrapolating Model: Full Age-Period-Cohort
Log Transform Parameters

Model	ChiSq	MChiSq	DF	Factor	%Account	P
Age Only	431159.947	30797.139	14	P, C	99.7411	0.0000
Age-Period	15427.938	1101.996	14	Cohort	92.7643	0.0000
Age-Cohort	1276.071	91.148	14	Period	12.5195	0.0000
Period-Cohort	1901.729	135.838	14	Age	41.3001	0.0000
Full Age-Period-Cohort	1116.313	79.737	14			0.0000

**Key to terms:**		
	Chisq =	chi-squared value for model
	MChisq =	mean Chi-squared (Chisq/DF)
	DF =	degrees of freedom
	Factor =	Factors not included in the model
	% Account =	1- (Chisq for full model)/(Chisq for model in question)
	P =	probability value based on Chisq and DF.

AGE	EFFECT
10	0.587316
15	1.604655
20	4.436719
25	11.620384
30	32.538051
35	84.370443
40	201.319941
45	404.809982
50	769.041842
55	1365.73808
60	2377.95952
65	3875.10956
70	6348.78274
75	10288.8139

PERIOD	EFFECT	
Period Change =	0.858281	
1966	1.444418	
1971	1.411349	
1976	1.199437	
1981	1.041271	
1986	0.922967	
1991	0.835735	
1996	0.805342	
2001	0.739894	
2006	0.582583	
2011	0.500020	
2016	0.429157	
2021	0.368337	
2026	0.316137	
2031	0.271334	
2036	0.232881	
2016	0.456210	Extrapolated
2017	0.442477	Extrapolated
2018	0.429157	Extrapolated
2019	0.416239	Extrapolated

Results for PHIM for Run 2 (Basic_US_OG_T1.RTF)

2020	0.403709	Extrapolated
2021	0.391556	Extrapolated
2022	0.379769	Extrapolated
2023	0.368337	Extrapolated
2024	0.357250	Extrapolated
2025	0.346496	Extrapolated
2026	0.336065	Extrapolated
2027	0.325949	Extrapolated
2028	0.316137	Extrapolated
2029	0.306620	Extrapolated
2030	0.297390	Extrapolated
2031	0.288438	Extrapolated
2032	0.279756	Extrapolated
2033	0.271334	Extrapolated
2034	0.263166	Extrapolated
2035	0.255245	Extrapolated
2036	0.247561	Extrapolated
2037	0.240109	Extrapolated
2038	0.232881	Extrapolated
2039	0.225871	Extrapolated
2040	0.219071	Extrapolated

Results for PHIM for Run 2 (Basic_US_OG_T1.RTF)

COHORT	EFFECT	WEIGHT	ORIGINAL
1891	1.467323	1.000	
1896	1.378557	2.000	
1901	1.251215	4.000	
1906	1.106931	8.000	
1911	1.071964	16.000	
1916	1.009276	32.000	
1921	0.939924	64.000	
1926	0.872445	128.000	
1931	0.812382	256.000	
1936	0.745510	512.000	
1941	0.693664	1024.000	
1946	0.660681	2048.000	
1951	0.666036	4096.000	
1956	0.720268	8192.000	
1961	0.803853	16384.000	
1966	0.824749	32768.000	
1971	0.868321	65536.000	
1976	0.902442	131072.000	
1981	1.061712	262144.000	
1986	1.064322	524288.000	
1991	0.954710	1048576.000	
1996	1.016893	Extrapolated	0.997814
2001	1.034931	Extrapolated	1.005658
2006	1.053288	Extrapolated	
2011	1.071971	Extrapolated	
2016	1.090986	Extrapolated	
2021	1.110337	Extrapolated	
2026	1.130032	Extrapolated	

Standardizing Population: The 1976 European Standard Population

Age Range	Population, Females
All	100000
0	0
1	0
2	0
3	0
0-4	8000
5-9	7000
10-14	7000
15-19	7000
20-24	7000
25-29	7000
30-34	7000
35-39	7000
40-44	7000
45-49	7000
50-54	7000
55-59	6000
60-64	5000
65-69	4000
70-74	3000
75-79	2000
80-84	1000
85+	1000

Results for PHIM for Run 2 (Basic_US_OG_T1.RTF)

Matrix of observed and expected rates including predictions

Total over ages standardized using: The 1976 European Standard Population

		1966-	1971-	1976-	1981-	1986-	1991-	1996-	2001-	2006-	2011-	2016-	2021-	2026-	2031-	2036-
10	OBS	0.8	0.9	0.3	0.5	0.4	0.4	0.5	0.4	0.3	0.3	0.4	.	.	.	.
	EXP											0.3	0.2	0.2	0.2	0.2
15	OBS	2.4	2.2	1.0	1.3	1.0	0.9	1.2	0.8	0.8	0.8	1.1	.	.	.	.
	EXP											0.7	0.6	0.5	0.5	0.4
20	OBS	6.8	4.8	2.9	2.9	2.7	3.1	2.7	3.2	2.3	2.2	1.9	.	.	.	.
	EXP											1.9	1.7	1.5	1.3	1.1
25	OBS	17.3	11.5	7.9	7.1	7.1	7.5	7.1	7.2	7.6	6.9	6.0	.	.	.	.
	EXP											4.8	4.4	3.8	3.3	2.9
30	OBS	50.7	37.9	24.0	17.8	17.9	19.2	19.1	19.7	17.4	17.4	15.2	.	.	.	.
	EXP											14.9	11.4	10.5	9.1	8.0
35	OBS	125.1	111.6	70.4	50.6	41.3	43.4	47.2	48.8	42.3	39.0	37.7	.	.	.	.
	EXP											38.4	33.1	25.5	23.3	20.3
40	OBS	284.3	265.9	193.4	141.7	106.4	97.8	98.5	113.1	96.9	90.1	81.5	.	.	.	.
	EXP											78.0	78.7	67.7	52.2	47.7
45	OBS	565.9	522.2	411.6	311.9	244.9	206.3	195.5	209.6	196.6	172.9	161.1	.	.	.	.
	EXP											150.9	134.6	135.9	116.9	90.0
50	OBS	1112.6	1021.4	799.8	642.4	515.2	440.4	400.4	379.8	338.4	319.8	312.2	.	.	.	.
	EXP											272.2	246.0	219.4	221.5	190.6
55	OBS	2052.3	1904.2	1513.2	1228.5	1017.1	880.8	783.5	681.9	550.4	510.1	522.2	.	.	.	.
	EXP											471.2	414.9	374.9	334.4	337.7
60	OBS	3679.0	3532.9	2848.5	2311.7	1908.1	1628.2	1486.4	1265.0	946.2	818.0	777.3	.	.	.	.
	EXP											735.0	704.1	620.0	560.3	499.8
65	OBS	6952.0	6073.7	4957.0	4092.3	3365.3	2815.2	2561.5	2151.3	1558.9	1281.1	1188.6	.	.	.	.
	EXP											1107.6	1028.1	984.8	867.2	783.6
70	OBS	12794.8	11311.1	8546.2	7144.2	5911.3	4962.2	4407.8	3731.9	2665.7	2145.2	1820.4	.	.	.	.
	EXP											1800.1	1557.5	1445.6	1384.8	1219.4
75	OBS	21806.4	19831.3	15391.8	11865.6	10321.7	8767.7	7844.4	6665.6	4814.6	3745.7	3117.9	.	.	.	.
	EXP											3062.9	2503.8	2166.4	2010.8	1926.1
10-79	OBS	1875.6	1696.7	1327.1	1068.6	892.0	757.1	683.1	590.7	443.5	370.5	334.8	.	.	.	.
	EXP	1876.9	1695.9	1327.3	1069.7	894.6	757.4	682.5	590.2	443.3	370.0	318.0*	281.7*	255.6*	234.3*	213.2*

Drop in overall standardized Observed and Predicted rates

comparing the last observed rate during the model fitting period to the last observed and predicted rates where an observed rate is available (2016)

Observed and Predicted %Drop = 9.617% and 14.165%

Results for PHIM for Run 2 (Basic_US_OG_T1.RTF)

Matrix of observed and expected deaths including predictions

		1966-	1971-	1976-	1981-	1986-	1991-	1996-	2001-	2006-	2011-	2016-	2021-	2026-	2031-	2036-
10-	OBS	42.0	44.0	15.0	21.0	17.5	16.0	22.0	21.0	15.0	15.0	22.5*	.	.	.	.
	EXP	30.6	33.9	27.2	22.7	20.5	23.2	24.6	21.6	17.9	15.4	13.7*	11.7*	9.9*	8.8*	7.9*
	ChiSq	4.254	2.978	5.475	0.129	0.452	2.222	0.268	0.018	0.472	0.012	5.725*	.	.	.	.
15-	OBS	111.0	110.0	52.0	60.0	43.0	40.0	57.0	40.0	41.0	45.0	57.5*	.	.	.	.
	EXP	71.0	82.5	80.2	66.1	56.9	52.9	64.2	64.4	47.8	43.5	37.1*	32.8*	28.1*	23.9*	21.2*
	ChiSq	22.599	9.174	9.943	0.569	3.384	3.151	0.804	9.245	0.960	0.051	11.151*	.	.	.	.
20-	OBS	271.0	223.0	152.0	155.0	131.0	141.0	125.0	157.0	120.0	124.0	105.0*	.	.	.	.
	EXP	168.2	194.2	197.6	196.6	165.4	146.1	147.6	170.0	143.4	117.3	107.5*	91.7*	81.2*	69.8*	59.4*
	ChiSq	62.758	4.263	10.517	8.789	7.148	0.180	3.459	0.988	3.827	0.382	0.060*	.	.	.	.
25-	OBS	558.0	463.0	375.0	375.0	378.0	373.0	336.0	345.0	379.0	373.0	342.5*	.	.	.	.
	EXP	376.5	435.6	440.5	458.0	461.8	400.7	386.2	371.3	360.3	332.5	273.6*	250.3*	214.0*	189.8*	163.6*
	ChiSq	87.462	1.725	9.738	15.058	15.204	1.911	6.514	1.859	0.973	4.934	17.333*	.	.	.	.
30-	OBS	1460.0	1244.0	984.0	863.0	957.0	1052.0	991.0	975.0	854.0	895.0	840.0*	.	.	.	.
	EXP	1008.2	1045.2	1058.5	1092.7	1157.5	1200.0	1123.8	1032.3	840.9	889.1	820.8*	673.0*	616.2*	528.2*	468.8*
	ChiSq	202.445	37.801	5.243	48.305	34.727	18.244	15.700	3.176	0.203	0.040	0.449*	.	.	.	.
35-	OBS	3680.0	3217.0	2322.0	2085.0	2008.0	2336.0	2645.0	2597.0	2130.0	1955.0	1975.0*	.	.	.	.
	EXP	2912.0	2558.3	2315.7	2392.5	2522.3	2734.3	3058.1	2738.0	2148.0	1908.6	2011.9*	1851.9*	1517.7*	1391.8*	1194.5*
	ChiSq	202.563	169.577	0.017	39.528	104.882	58.011	55.814	7.263	0.151	1.126	0.675*	.	.	.	.
40-	OBS	8992.0	7787.0	5593.0	4696.0	4402.0	4749.0	5333.0	6374.0	5181.0	4577.0	4117.5*	.	.	.	.
	EXP	8023.9	6760.2	5204.9	4817.5	5081.3	5443.7	6323.8	6750.9	5172.7	4440.1	3939.5*	4144.6*	3814.5*	3128.0*	2870.1*
	ChiSq	116.812	155.973	28.945	3.063	90.818	88.656	155.225	21.046	0.013	4.221	8.045*	.	.	.	.
45-	OBS	17574.0	16292.0	12003.0	8996.0	8037.0	8425.0	9504.0	11363.0	11072.0	9240.0	8180.0*	.	.	.	.
	EXP	17066.9	15550.7	11502.1	9063.1	8504.4	9126.3	10553.4	11693.1	10674.3	8920.0	7657.4*	6788.6*	7145.5*	6581.2*	5397.8*
	ChiSq	15.064	35.340	21.813	0.496	25.685	53.896	104.355	9.318	14.816	11.482	35.669*	.	.	.	.
50-	OBS	31631.0	30963.0	24657.0	18573.0	14682.0	14278.0	16345.0	18487.0	18290.0	17887.0	16515.0*	.	.	.	.
	EXP	31873.7	30925.5	24809.0	18807.0	15080.3	14454.2	16705.6	18447.6	17443.6	17290.0	14399.9*	12363.1*	10972.5*	11564.6*	10656.8*
	ChiSq	1.848	0.046	0.931	2.913	10.522	2.149	7.784	0.084	41.067	20.614	310.676*	.	.	.	.
55-	OBS	54283.0	52272.0	44862.0	37052.0	28654.0	24478.0	25051.0	27616.0	26509.0	27195.0	28632.5*	.	.	.	.
	EXP	55931.4	53403.2	45648.7	37421.5	28849.1	23648.5	24392.6	27038.3	25524.8	26225.5	25832.1*	21516.6*	18506.8*	16456.2*	17364.2*
	ChiSq	48.582	23.963	13.558	3.648	1.320	29.093	17.769	12.345	37.952	35.842	303.589*	.	.	.	.
60-	OBS	87105.0	88280.0	75123.0	66044.0	55313.0	44138.0	40313.0	39553.0	37449.0	38393.0	40175.0*	.	.	.	.
	EXP	90018.8	89897.7	75918.0	66491.5	55507.5	43764.8	38722.2	38161.0	36227.0	37170.0	37993.3*	37403.7*	31223.1*	26931.2*	23991.4*
	ChiSq	94.317	29.112	8.326	3.011	0.681	3.183	65.350	50.774	41.219	40.242	125.282*	.	.	.	.
65-	OBS	137647.0	134022.0	117588.0	102745.0	90893.0	76850.0	66043.0	56033.0	46757.0	48589.0	53237.5*	.	.	.	.
	EXP	138665.2	133586.1	118190.5	102248.2	90795.9	77130.9	65366.9	55673.9	46970.3	48551.9	49610.7*	50706.4*	50043.6*	41935.5*	36283.1*
	ChiSq	7.476	1.422	3.072	2.413	0.104	1.023	6.992	2.316	0.969	0.028	265.138*	.	.	.	.

Results for PHIM for Run 2 (Basic_US_OG_T1.RTF)

70-	OBS	198997.0	194867.0	168269.0	152188.0	133295.0	120234.0	109135.0	88423.0	64058.0	59809.0	64165.0*	.	.	.	.
	EXP	196617.6	193148.4	165966.5	150959.3	133357.7	120838.1	110445.6	90417.3	66262.5	61394.1	63449.5*	64898.5*	66603.5*	66100.2*	55641.3*
	ChiSq	28.794	15.292	31.942	10.001	0.030	3.020	15.552	43.985	73.339	40.924	8.067*	.	.	.	.
75-	OBS	249864.0	255194.0	223091.0	200090.0	187196.0	167793.0	164198.0	142524.0	99680.0	79431.0	77157.5*	.	.	.	.
	EXP	249864.0	257599.0	223803.9	199978.9	184619.6	166085.3	163023.0	142011.4	100815.8	81333.1	75796.1*	78592.4*	80815.5*	83718.1*	83656.3*
	ChiSq	.	22.453	2.271	0.062	35.953	17.560	8.469	1.850	12.797	44.484	24.454*	.	.	.	.
Total Deaths		792215.0	784978.0	675086.0	593943.0	526006.5	464903.0	440098.0	394508.0	312535.0	288528.0	295522.5*	.	.	.	.
Expected		792628.0	785220.6	675163.4	594015.7	526180.4	465048.9	440337.7	394591.1	312649.4	288631.1	281943.1*	279325.3*	271592.2*	258627.2*	237776.3*
Obs/Exp		0.999	1.000	1.000	1.000	1.000	1.000	0.999	1.000	1.000	1.000	1.048*	.	.	.	.

Chi Squared (Log) = 1116.3 on 14 D.F. P = 0.0000

Results for PHIM for Run 2 (Basic_US_OG_T1.RTF)

****

Observed and predicted rates (5 year periods)

		10-	15-	20-	25-	30-	35-	40-	45-	50-	55-	60-	65-	70-	75-
2016-	OBS	0.4	1.1	1.9	6.0	15.2	37.7	81.5	161.1	312.2	522.2	777.3	1188.6	1820.4	3117.9
	PRE	0.3	0.7	1.9	4.8	14.9	38.4	78.0	150.9	272.2	471.2	735.0	1107.6	1800.1	3062.9
	RES	0.172	0.390	-0.046	1.198	0.348	-0.704	3.523	10.296	39.982	51.077	42.209	80.974	20.298	55.015
2021-	PRE	0.2	0.6	1.7	4.4	11.4	33.1	78.7	134.6	246.0	414.9	704.1	1028.1	1557.5	2503.8
2026-	PRE	0.2	0.5	1.5	3.8	10.5	25.5	67.7	135.9	219.4	374.9	620.0	984.8	1445.6	2166.4
2031-	PRE	0.2	0.5	1.3	3.3	9.1	23.3	52.2	116.9	221.5	334.4	560.3	867.2	1384.8	2010.8
2036-	PRE	0.2	0.4	1.1	2.9	8.0	20.3	47.7	90.0	190.6	337.7	499.8	783.6	1219.4	1926.1

Results for PHIM for Run 2 (Basic_US_OG_T1.RTF)

****

Observed and predicted deaths (5 year periods)

		10-	15-	20-	25-	30-	35-	40-	45-	50-	55-	60-	65-	70-	75-
2016-	OBS	22.5	57.5	105.0	342.5	840.0	1975.0	4117.5	8180.0	16515.0	28632.5	40175.0	53237.5	64165.0	77157.5
	PRE	13.7	37.1	107.5	273.6	820.8	2011.9	3939.5	7657.4	14399.9	25832.1	37993.3	49610.7	63449.5	75796.1
	CHI	5.725	11.151	0.060	17.333	0.449	0.675	8.045	35.669	310.676	303.589	125.282	265.138	8.067	24.454
2021-	PRE	11.7	32.8	91.7	250.3	673.0	1851.9	4144.6	6788.6	12363.1	21516.6	37403.7	50706.4	64898.5	78592.4
2026-	PRE	9.9	28.1	81.2	214.0	616.2	1517.7	3814.5	7145.5	10972.5	18506.8	31223.1	50043.6	66603.5	80815.5
2031-	PRE	8.8	23.9	69.8	189.8	528.2	1391.8	3128.0	6581.2	11564.6	16456.2	26931.2	41935.5	66100.2	83718.1
2036-	PRE	7.9	21.2	59.4	163.6	468.8	1194.5	2870.1	5397.8	10656.8	17364.2	23991.4	36283.1	55641.3	83656.3

Results for PHIM for Run 2 (Basic_US_OG_T1.RTF)

****

Observed and predicted rates (single years)

		10-	15-	20-	25-	30-	35-	40-	45-	50-	55-	60-	65-	70-	75-
2016	OBS	0.5	0.7	2.1	6.1	16.2	38.1	82.9	161.5	310.3	522.5	799.7	1251.9	1975.1	3356.5
	PRE	0.3	0.8	2.1	5.1	15.8	40.9	82.9	160.4	289.4	500.9	781.4	1177.5	1913.6	3256.0
	RES	0.208	-0.088	0.070	0.996	0.387	-2.728	0.031	1.151	20.979	21.650	18.284	74.421	61.505	100.525
2017	OBS	0.4	1.5	1.6	6.1	14.9	38.9	80.5	158.7	298.9	516.4	792.8	1207.7	1951.5	3280.6
	PRE	0.3	0.7	2.0	4.9	15.3	39.6	80.4	155.5	280.6	485.8	757.9	1142.0	1856.0	3157.9
	RES	0.116	0.799	-0.391	1.152	-0.456	-0.772	0.108	3.204	18.296	30.612	34.970	65.716	95.517	122.624
2018	PRE	0.3	0.7	1.9	4.8	14.9	38.4	78.0	150.9	272.2	471.2	735.0	1107.6	1800.1	3062.9
2019	PRE	0.3	0.7	1.9	4.6	14.4	37.3	75.6	146.3	264.0	457.0	712.9	1074.3	1745.9	2970.7
2020	PRE	0.2	0.7	1.8	4.5	14.0	36.2	73.3	141.9	256.1	443.2	691.5	1042.0	1693.4	2881.3
2021	PRE	0.2	0.7	1.8	4.6	12.2	35.2	83.7	143.0	261.5	441.0	748.5	1092.9	1655.7	2661.7
2022	PRE	0.2	0.6	1.7	4.5	11.8	34.1	81.2	138.7	253.6	427.8	725.9	1060.0	1605.9	2581.5
2023	PRE	0.2	0.6	1.7	4.4	11.4	33.1	78.7	134.6	246.0	414.9	704.1	1028.1	1557.5	2503.8
2024	PRE	0.2	0.6	1.6	4.2	11.1	32.1	76.4	130.5	238.6	402.4	682.9	997.1	1510.6	2428.4
2025	PRE	0.2	0.6	1.6	4.1	10.8	31.1	74.1	126.6	231.4	390.3	662.3	967.1	1465.2	2355.3
2026	PRE	0.2	0.6	1.6	4.0	11.1	27.1	72.0	144.4	233.2	398.5	659.1	1046.8	1536.8	2303.0
2027	PRE	0.2	0.6	1.5	3.9	10.8	26.3	69.8	140.1	226.2	386.5	639.3	1015.3	1490.5	2233.6
2028	PRE	0.2	0.5	1.5	3.8	10.5	25.5	67.7	135.9	219.4	374.9	620.0	984.8	1445.6	2166.4
2029	PRE	0.2	0.5	1.4	3.7	10.1	24.7	65.7	131.8	212.8	363.6	601.3	955.1	1402.1	2101.2
2030	PRE	0.2	0.5	1.4	3.6	9.8	24.0	63.7	127.8	206.4	352.7	583.2	926.4	1359.9	2037.9
2031	PRE	0.2	0.5	1.4	3.5	9.7	24.7	55.4	124.3	235.5	355.5	595.6	921.8	1472.0	2137.5
2032	PRE	0.2	0.5	1.3	3.4	9.4	24.0	53.8	120.5	228.4	344.8	577.6	894.1	1427.7	2073.2
2033	PRE	0.2	0.5	1.3	3.3	9.1	23.3	52.2	116.9	221.5	334.4	560.3	867.2	1384.8	2010.8
2034	PRE	0.2	0.5	1.3	3.2	8.9	22.6	50.6	113.4	214.9	324.4	543.4	841.1	1343.1	1950.2
2035	PRE	0.2	0.4	1.2	3.1	8.6	21.9	49.1	110.0	208.4	314.6	527.0	815.8	1302.6	1891.5
2036	PRE	0.2	0.4	1.2	3.1	8.5	21.6	50.7	95.7	202.6	359.0	531.3	833.0	1296.3	2047.5
2037	PRE	0.2	0.4	1.2	3.0	8.2	21.0	49.2	92.8	196.5	348.2	515.3	807.9	1257.2	1985.9
2038	PRE	0.2	0.4	1.1	2.9	8.0	20.3	47.7	90.0	190.6	337.7	499.8	783.6	1219.4	1926.1
2039	PRE	0.1	0.4	1.1	2.8	7.7	19.7	46.2	87.3	184.9	327.5	484.7	760.0	1182.7	1868.1
2040	PRE	0.1	0.4	1.1	2.7	7.5	19.1	44.8	84.7	179.3	317.7	470.1	737.1	1147.1	1811.9

Results for PHIM for Run 2 (Basic_US_OG_T1.RTF)

****

Observed and predicted deaths (single years)

		10-	15-	20-	25-	30-	35-	40-	45-	50-	55-	60-	65-	70-	75-
2016	OBS	5.0	7.0	24.0	68.0	174.0	389.0	836.0	1656.0	3391.0	5757.0	7992.0	10703.0	12579.0	15355.0
	PRE	2.9	7.9	23.2	56.8	169.8	416.8	835.7	1644.2	3161.8	5518.5	7809.3	10066.7	12187.3	14895.1
	CHI	1.561	0.106	0.027	2.201	0.102	1.858	0.000	0.085	16.619	10.311	4.276	40.215	12.590	14.198
2017	OBS	4.0	16.0	18.0	69.0	162.0	401.0	811.0	1616.0	3215.0	5696.0	8078.0	10592.0	13087.0	15508.0
	PRE	2.8	7.7	22.4	55.9	167.0	409.0	809.9	1583.4	3018.2	5358.3	7721.7	10015.7	12446.4	14928.3
	CHI	0.504	9.064	0.857	3.076	0.148	0.155	0.001	0.672	12.827	21.278	16.441	33.165	32.966	22.509
2018	PRE	2.7	7.4	21.5	54.9	164.1	401.9	786.0	1529.0	2876.3	5183.5	7621.9	9927.7	12710.8	15039.7
2019	PRE	2.7	7.2	20.7	53.7	161.3	395.4	764.7	1478.8	2744.4	4997.8	7497.6	9833.3	12923.6	15250.3
2020	PRE	2.6	7.0	19.9	52.4	158.7	389.0	746.4	1431.1	2626.7	4804.5	7342.8	9746.2	13054.7	15561.0
2021	PRE	2.5	6.9	19.6	54.0	139.9	383.4	858.9	1439.9	2653.4	4720.7	7983.9	10416.4	13153.4	15065.3
2022	PRE	2.4	6.8	18.9	52.1	137.5	376.8	842.5	1395.4	2555.5	4507.3	7753.2	10297.8	13084.6	15390.6
2023	PRE	2.3	6.6	18.3	50.1	135.0	370.2	828.1	1354.4	2468.6	4297.7	7504.7	10169.1	12979.7	15735.6
2024	PRE	2.2	6.4	17.8	48.1	132.1	363.9	814.5	1318.0	2388.3	4102.9	7240.6	10010.1	12872.5	16023.0
2025	PRE	2.1	6.2	17.3	46.4	128.7	357.8	801.1	1286.4	2311.7	3928.6	6964.4	9811.5	12777.2	16215.0
2026	PRE	2.1	6.1	17.1	45.6	132.8	315.4	789.6	1480.5	2326.2	3969.5	6844.3	10668.6	13656.8	16331.7
2027	PRE	2.0	5.9	16.7	44.1	128.1	310.1	776.1	1452.4	2254.8	3824.3	6537.5	10364.8	13509.3	16255.5
2028	PRE	2.0	5.6	16.3	42.7	123.2	304.5	762.7	1427.7	2189.1	3695.5	6236.4	10039.3	13352.8	16149.4
2029	PRE	1.9	5.4	15.8	41.5	118.6	298.0	749.6	1404.4	2130.8	3576.2	5956.5	9693.9	13160.0	16052.5
2030	PRE	1.9	5.2	15.4	40.3	114.4	290.2	737.0	1381.4	2080.2	3462.6	5706.4	9332.5	12918.3	15978.6
2031	PRE	1.8	5.1	15.1	40.0	112.4	299.7	649.9	1362.0	2395.2	3486.3	5770.4	9178.1	14056.7	17099.9
2032	PRE	1.8	4.9	14.5	39.0	108.8	289.3	639.1	1339.0	2350.4	3380.7	5562.8	8772.9	13669.7	16940.7
2033	PRE	1.8	4.8	14.0	38.0	105.5	278.3	627.6	1316.0	2310.6	3283.4	5378.2	8375.4	13257.1	16775.5
2034	PRE	1.7	4.6	13.4	37.0	102.4	267.9	614.2	1293.5	2273.3	3196.7	5206.7	8006.8	12820.7	16571.5
2035	PRE	1.7	4.5	12.9	35.9	99.6	258.4	598.2	1271.6	2236.4	3121.5	5043.2	7678.1	12363.7	16311.9
2036	PRE	1.7	4.4	12.7	35.3	98.7	254.1	617.9	1121.5	2205.1	3595.2	5079.4	7766.4	12159.5	17746.9
2037	PRE	1.6	4.3	12.2	34.0	96.3	246.0	596.4	1102.8	2168.0	3528.6	4927.1	7490.1	11627.3	17267.8
2038	PRE	1.6	4.3	11.9	32.7	93.9	238.5	574.1	1083.1	2130.9	3469.5	4786.8	7245.1	11108.7	16767.8
2039	PRE	1.5	4.1	11.5	31.4	91.5	231.7	552.6	1059.9	2094.7	3413.8	4662.0	7018.3	10632.0	16246.5
2040	PRE	1.5	4.0	11.2	30.3	88.8	225.3	533.0	1032.2	2059.3	3358.8	4553.8	6802.6	10211.1	15705.3

Results for PHIM for Run 2 (Basic_US_OG_T1.RTF)

****
List of values created by O and G modelling, using percentage change in last two period parameters for Fixed File MORT

1. Country	US (United States)
2. Sex	F (Females)
3. Disease	IHD (IHD)
* Value comes from O and G Modelling.	

Age	Years	Value	Death Rate	Population
10-14	2018	2.7397	0.265482	103196.46 *
10-14	2019	2.6629	0.257491	103416.52 *
10-14	2020	2.5753	0.249740	103119.74 *
10-14	2021	2.5352	0.246518	102838.63 *
10-14	2022	2.4398	0.239098	102041.78 *
10-14	2023	2.3388	0.231900	100854.31 *
10-14	2024	2.2393	0.224919	99561.97 *
10-14	2025	2.1474	0.218149	98437.76 *
10-14	2026	2.1107	0.215335	98019.03 *
10-14	2027	2.0406	0.208853	97707.32 *
10-14	2028	1.9743	0.202566	97463.30 *
10-14	2029	1.9112	0.196468	97277.15 *
10-14	2030	1.8524	0.190554	97213.74 *
10-14	2031	1.8414	0.188096	97897.69 *
10-14	2032	1.7999	0.182434	98659.12 *
10-14	2033	1.7592	0.176942	99422.00 *
10-14	2034	1.7174	0.171616	100073.76 *
10-14	2035	1.6736	0.166450	100545.70 *
10-14	2036	1.6672	0.164303	101474.16 *
10-14	2037	1.6280	0.159357	102160.11 *
10-14	2038	1.5858	0.154560	102601.69 *
10-14	2039	1.5415	0.149907	102829.10 *
15-19	2018	7.4264	0.712704	104200.38 *
15-19	2019	7.1983	0.691250	104134.08 *
15-19	2020	6.9784	0.670442	104085.87 *
15-19	2021	6.9220	0.661794	104593.73 *
15-19	2022	6.7563	0.641873	105258.83 *
15-19	2023	6.5895	0.622551	105846.21 *
15-19	2024	6.4033	0.603810	106048.78 *
15-19	2025	6.1901	0.585634	105699.11 *
15-19	2026	6.0936	0.578080	105411.01 *
15-19	2027	5.8674	0.560679	104647.84 *
15-19	2028	5.6296	0.543801	103524.06 *
15-19	2029	5.3961	0.527431	102309.30 *
15-19	2030	5.1770	0.511554	101201.39 *
15-19	2031	5.0905	0.504956	100810.47 *
15-19	2032	4.9242	0.489756	100543.20 *
15-19	2033	4.7672	0.475013	100360.03 *
15-19	2034	4.6182	0.460714	100239.43 *
15-19	2035	4.4763	0.446845	100174.80 *
15-19	2036	4.4475	0.441082	100832.66 *
15-19	2037	4.3480	0.427804	101635.99 *
15-19	2038	4.2507	0.414926	102445.77 *
15-19	2039	4.1497	0.402436	103113.72 *
20-24	2018	21.5121	1.936215	111103.83 *
20-24	2019	20.6806	1.877931	110124.20 *
20-24	2020	19.9189	1.821400	109360.13 *
20-24	2021	19.5558	1.797907	108770.00 *
20-24	2022	18.9071	1.743786	108425.37 *
20-24	2023	18.3205	1.691294	108322.56 *
20-24	2024	17.7810	1.640382	108395.50 *
20-24	2025	17.2703	1.591002	108550.01 *
20-24	2026	17.1016	1.570481	108894.01 *
20-24	2027	16.6785	1.523205	109496.37 *
20-24	2028	16.2743	1.477353	110158.41 *
20-24	2029	15.8420	1.432881	110560.57 *
20-24	2030	15.3529	1.389748	110472.49 *
20-24	2031	15.0960	1.371823	110043.60 *
20-24	2032	14.5314	1.330527	109215.51 *
20-24	2033	13.9585	1.290475	108165.47 *
20-24	2034	13.4123	1.251629	107158.74 *
20-24	2035	12.9069	1.213952	106321.30 *
20-24	2036	12.6671	1.198294	105709.09 *
20-24	2037	12.2397	1.162222	105313.13 *
20-24	2038	11.8532	1.127237	105152.82 *
20-24	2039	11.5017	1.093304	105200.95 *
25-29	2018	54.9022	4.761111	115313.86 *
25-29	2019	53.7455	4.617790	116387.97 *

Results for PHIM for Run 2 (Basic_US_OG_T1.RTF)

25-29	2020	52.3609	4.478784	116908.75 *
25-29	2021	54.0111	4.626897	116732.89 *
25-29	2022	52.0678	4.487616	116025.62 *
25-29	2023	50.0592	4.352528	115011.70 *
25-29	2024	48.1428	4.221507	114041.82 *
25-29	2025	46.4061	4.094429	113339.59 *
25-29	2026	45.5707	4.041617	112753.72 *
25-29	2027	44.0763	3.919955	112440.79 *
25-29	2028	42.7276	3.801955	112383.37 *
25-29	2029	41.4941	3.687507	112526.12 *
25-29	2030	40.3492	3.576504	112817.52 *
25-29	2031	39.9631	3.530372	113197.88 *
25-29	2032	38.9774	3.424100	113832.48 *
25-29	2033	38.0367	3.321026	114532.86 *
25-29	2034	37.0424	3.221055	115000.73 *
25-29	2035	35.9419	3.124094	115047.44 *
25-29	2036	35.3403	3.083798	114600.09 *
25-29	2037	34.0258	2.990968	113761.95 *
25-29	2038	32.6967	2.900933	112710.93 *
25-29	2039	31.4360	2.813608	111728.46 *
30-34	2018	164.0906	14.862127	110408.56 *
30-34	2019	161.3205	14.414742	111913.53 *
30-34	2020	158.6819	13.980823	113499.65 *
30-34	2021	139.8659	12.163457	114988.57 *
30-34	2022	137.5015	11.797308	116553.27 *
30-34	2023	135.0259	11.442181	118007.13 *
30-34	2024	132.1336	11.097744	119063.44 *
30-34	2025	128.6932	10.763675	119562.53 *
30-34	2026	132.8089	11.119630	119436.43 *
30-34	2027	128.1022	10.784902	118779.22 *
30-34	2028	123.2265	10.460251	117804.55 *
30-34	2029	118.5685	10.145372	116869.57 *
30-34	2030	114.3554	9.839972	116215.13 *
30-34	2031	112.4014	9.713051	115721.99 *
30-34	2032	108.7726	9.420665	115461.72 *
30-34	2033	105.4714	9.137080	115432.31 *
30-34	2034	102.4433	8.862032	115598.01 *
30-34	2035	99.6395	8.595264	115923.69 *
30-34	2036	98.7182	8.484398	116352.68 *
30-34	2037	96.2854	8.228997	117007.50 *
30-34	2038	93.9436	7.981285	117704.81 *
30-34	2039	91.4685	7.741029	118160.59 *
35-39	2018	401.9326	38.442662	104553.79 *
35-39	2019	395.3701	37.285446	106038.72 *
35-39	2020	389.0032	36.163064	107569.20 *
35-39	2021	383.3714	35.160693	109034.08 *
35-39	2022	376.7812	34.102272	110485.67 *
35-39	2023	370.2483	33.075711	111939.64 *
35-39	2024	363.9048	32.080053	113436.46 *
35-39	2025	357.7741	31.114367	114986.80 *
35-39	2026	315.3882	27.069813	116509.21 *
35-39	2027	310.0908	26.254946	118107.58 *
35-39	2028	304.5186	25.464609	119585.02 *
35-39	2029	297.9969	24.698063	120655.96 *
35-39	2030	290.2432	23.954592	121163.90 *
35-39	2031	299.7356	24.746771	121121.11 *
35-39	2032	289.2542	24.001834	120513.37 *
35-39	2033	278.3464	23.279321	119568.09 *
35-39	2034	267.8980	22.578558	118651.53 *
35-39	2035	258.4104	21.898889	118001.61 *
35-39	2036	254.1218	21.616426	117559.56 *
35-39	2037	245.9824	20.965720	117326.00 *
35-39	2038	238.5304	20.334601	117302.74 *
35-39	2039	231.6587	19.722481	117459.22 *
40-44	2018	785.9665	77.969131	100804.83 *
40-44	2019	764.6913	75.622073	101120.12 *
40-44	2020	746.3668	73.345667	101760.17 *
40-44	2021	858.9442	83.692699	102630.72 *
40-44	2022	842.5336	81.173348	103794.37 *
40-44	2023	828.0612	78.729835	105177.56 *
40-44	2024	814.4764	76.359878	106662.88 *
40-44	2025	801.1285	74.061262	108171.06 *
40-44	2026	789.5735	72.008425	109650.15 *
40-44	2027	776.0981	69.840799	111123.89 *
40-44	2028	762.7039	67.738423	112595.46 *
40-44	2029	749.6408	65.699334	114101.74 *
40-44	2030	736.9549	63.721626	115652.24 *
40-44	2031	649.9077	55.438457	117230.49 *

Results for PHIM for Run 2 (Basic_US_OG_T1.RTF)

40-44	2032	639.0910	53.769626	118857.26 *
40-44	2033	627.6262	52.151032	120347.80 *
40-44	2034	614.1817	50.581160	121424.99 *
40-44	2035	598.1629	49.058546	121928.37 *
40-44	2036	617.8983	50.680913	121919.33 *
40-44	2037	596.4330	49.155295	121336.47 *
40-44	2038	574.0501	47.675603	120407.52 *
40-44	2039	552.5609	46.240453	119497.30 *
45-49	2018	1529.0258	150.850903	101360.07 *
45-49	2019	1478.7518	146.309929	101069.82 *
45-49	2020	1431.0758	141.905649	100846.99 *
45-49	2021	1439.8593	143.042393	100659.62 *
45-49	2022	1395.3542	138.736473	100575.87 *
45-49	2023	1354.4005	134.560172	100653.89 *
45-49	2024	1317.9726	130.509588	100986.65 *
45-49	2025	1286.3853	126.580936	101625.52 *
45-49	2026	1480.5004	144.437982	102500.77 *
45-49	2027	1452.4248	140.090052	103677.94 *
45-49	2028	1427.6580	135.873005	105072.97 *
45-49	2029	1404.3652	131.782902	106566.57 *
45-49	2030	1381.4258	127.815920	108079.32 *
45-49	2031	1361.9899	124.273106	109596.51 *
45-49	2032	1338.9678	120.532187	111087.99 *
45-49	2033	1315.9685	116.903879	112568.42 *
45-49	2034	1293.4993	113.384791	114080.49 *
45-49	2035	1271.5847	109.971637	115628.42 *
45-49	2036	1121.4805	95.676433	117215.96 *
45-49	2037	1102.8480	92.796343	118846.06 *
45-49	2038	1083.0701	90.002950	120337.18 *
45-49	2039	1059.8720	87.293645	121414.57 *
50-54	2018	2876.3298	272.199876	105669.77 *
50-54	2019	2744.4161	264.006006	103952.79 *
50-54	2020	2626.7207	256.058792	102582.72 *
50-54	2021	2653.3794	261.471480	101478.73 *
50-54	2022	2555.4580	253.600561	100767.05 *
50-54	2023	2468.6406	245.966575	100364.88 *
50-54	2024	2388.2672	238.562391	100110.80 *
50-54	2025	2311.7343	231.381090	99910.25 *
50-54	2026	2326.1672	233.234583	99735.09 *
50-54	2027	2254.7704	226.213662	99674.37 *
50-54	2028	2189.1370	219.404088	99776.49 *
50-54	2029	2130.7833	212.799498	100131.03 *
50-54	2030	2080.2054	206.393722	100788.21 *
50-54	2031	2395.2087	235.510130	101703.00 *
50-54	2032	2350.3591	228.420709	102896.06 *
50-54	2033	2310.6388	221.544698	104296.73 *
50-54	2034	2273.2606	214.875671	105794.23 *
50-54	2035	2236.3537	208.407397	107306.83 *
50-54	2036	2205.1242	202.630740	108824.76 *
50-54	2037	2167.9946	196.531069	110313.07 *
50-54	2038	2130.8936	190.615012	111790.44 *
50-54	2039	2094.6674	184.877042	113300.57 *
55-59	2018	5183.4933	471.151666	110017.51 *
55-59	2019	4997.8320	456.968870	109369.20 *
55-59	2020	4804.5199	443.213010	108402.05 *
55-59	2021	4720.6996	441.045125	107034.39 *
55-59	2022	4507.2793	427.768608	105367.23 *
55-59	2023	4297.7271	414.891746	103586.71 *
55-59	2024	4102.8879	402.402509	101959.80 *
55-59	2025	3928.6248	390.289227	100659.32 *
55-59	2026	3969.5020	398.539340	99601.26 *
55-59	2027	3824.3051	386.542349	98936.25 *
55-59	2028	3695.5201	374.906497	98571.78 *
55-59	2029	3576.1848	363.620911	98349.26 *
55-59	2030	3462.5516	352.675049	98179.66 *
55-59	2031	3486.3486	355.500174	98068.83 *
55-59	2032	3380.7426	344.798765	98049.73 *
55-59	2033	3283.3654	334.419495	98181.04 *
55-59	2034	3196.7297	324.352665	98557.22 *
55-59	2035	3121.5358	314.588872	99225.88 *
55-59	2036	3595.2281	358.968602	100154.39 *
55-59	2037	3528.6417	348.162785	101350.34 *
55-59	2038	3469.5301	337.682250	102745.41 *
55-59	2039	3413.7986	327.517204	104232.65 *
60-64	2018	7621.9146	735.046771	103692.92 *
60-64	2019	7497.5761	712.920099	105167.13 *
60-64	2020	7342.7861	691.459493	106192.57 *
60-64	2021	7983.9453	748.471766	106669.96 *

Results for PHIM for Run 2 (Basic_US_OG_T1.RTF)

60-64	2022	7753.2122	725.940969	106802.24 *
60-64	2023	7504.6607	704.088403	106586.91 *
60-64	2024	7240.5561	682.893652	106027.58 *
60-64	2025	6964.3925	662.336914	105148.79 *
60-64	2026	6844.3273	659.097230	103843.97 *
60-64	2027	6537.5440	639.256821	102267.88 *
60-64	2028	6236.4260	620.013657	100585.30 *
60-64	2029	5956.5125	601.349757	99052.38 *
60-64	2030	5706.3670	583.247686	97837.80 *
60-64	2031	5770.3980	595.576644	96887.58 *
60-64	2032	5562.8172	577.648357	96301.10 *
60-64	2033	5378.1569	560.259755	95993.99 *
60-64	2034	5206.7418	543.394592	95818.80 *
60-64	2035	5043.1939	527.037110	95689.54 *
60-64	2036	5079.3787	531.258972	95610.22 *
60-64	2037	4927.1419	515.266802	95623.12 *
60-64	2038	4786.8217	499.756035	95783.17 *
60-64	2039	4662.0243	484.712178	96181.29 *
65-69	2018	9927.6553	1107.638862	89628.99 *
65-69	2019	9833.3174	1074.296274	91532.64 *
65-69	2020	9746.2119	1041.957377	93537.53 *
65-69	2021	10416.3878	1092.879188	95311.43 *
65-69	2022	10297.8236	1059.980901	97151.03 *
65-69	2023	10169.0827	1028.072932	98914.02 *
65-69	2024	10010.1256	997.125469	100389.83 *
65-69	2025	9811.5097	967.109599	101451.89 *
65-69	2026	10668.5677	1046.849796	101911.16 *
65-69	2027	10364.7977	1015.337105	102082.33 *
65-69	2028	10039.3099	984.773021	101945.42 *
65-69	2029	9693.9078	955.128989	101493.18 *
65-69	2030	9332.4900	926.377314	100741.78 *
65-69	2031	9178.1268	921.846131	99562.46 *
65-69	2032	8772.9163	894.096349	98120.48 *
65-69	2033	8375.3660	867.181903	96581.42 *
65-69	2034	8006.7892	841.077646	95196.79 *
65-69	2035	7678.1237	815.759190	94122.43 *
65-69	2036	7766.3601	833.003083	93233.27 *
65-69	2037	7490.0706	807.927690	92707.19 *
65-69	2038	7245.0740	783.607128	92457.99 *
65-69	2039	7018.3255	760.018672	92344.12 *
70-74	2018	12710.8405	1800.109368	70611.49 *
70-74	2019	12923.5677	1745.921755	74021.46 *
70-74	2020	13054.7358	1693.365319	77093.44 *
70-74	2021	13153.3614	1655.702554	79442.78 *
70-74	2022	13084.5855	1605.861933	81480.14 *
70-74	2023	12979.7311	1557.521634	83335.80 *
70-74	2024	12872.4946	1510.636495	85212.39 *
70-74	2025	12777.1844	1465.162710	87206.59 *
70-74	2026	13656.8129	1536.767115	88867.16 *
70-74	2027	13509.3464	1490.506736	90635.93 *
70-74	2028	13352.7623	1445.638906	92365.82 *
70-74	2029	13160.0002	1402.121705	93857.76 *
70-74	2030	12918.2886	1359.914476	94993.39 *
70-74	2031	14056.7464	1472.042252	95491.46 *
70-74	2032	13669.6990	1427.730247	95744.27 *
70-74	2033	13257.1420	1384.752140	95736.57 *
70-74	2034	12820.6994	1343.067776	95458.32 *
70-74	2035	12363.6845	1302.638212	94912.65 *
70-74	2036	12159.5059	1296.266627	93804.05 *
70-74	2037	11627.2564	1257.245890	92481.96 *
70-74	2038	11108.7087	1219.399771	91099.81 *
70-74	2039	10632.0296	1182.692911	89896.79 *
75-79	2018	15039.7218	3062.887039	49103.09 *
75-79	2019	15250.3266	2970.686788	51336.03 *
75-79	2020	15560.9699	2881.261984	54007.48 *
75-79	2021	15065.3431	2661.652733	56601.46 *
75-79	2022	15390.6362	2581.530597	59618.26 *
75-79	2023	15735.5743	2503.820330	62846.26 *
75-79	2024	16023.0252	2428.449328	65980.48 *
75-79	2025	16215.0131	2355.347174	68843.41 *
75-79	2026	16331.7176	2302.961025	70916.17 *
75-79	2027	16255.5344	2233.636371	72776.10 *
75-79	2028	16149.4397	2166.398554	74545.10 *
75-79	2029	16052.5178	2101.184758	76397.46 *
75-79	2030	15978.6257	2037.934053	78406.00 *
75-79	2031	17099.9108	2137.530538	79998.44 *
75-79	2032	16940.6827	2073.185738	81713.29 *
75-79	2033	16775.4774	2010.777870	83427.80 *

Results for PHIM for Run 2 (Basic_US_OG_T1.RTF)

75-79	2034	16571.5317	1950.248630	84971.38 *
75-79	2035	16311.8610	1891.541465	86235.81 *
75-79	2036	17746.8714	2047.502992	86675.68 *
75-79	2037	17267.7777	1985.868237	86953.29 *
75-79	2038	16767.7628	1926.088837	87056.02 *
75-79	2039	16246.4820	1868.108939	86967.53 *

Results for PHIM for Run 2 (Basic_US_OG_T1.RTF)

****

List of values created by last value brought forwards for Fixed File perm.Fixed_File_RR_IHD

1. Country	US (United States)
2. Sex	F (Females)
3. Disease	IHD (IHD)

Age	Years	Value
10-14	2013-2039	3.3800
15-19	2013-2039	3.3800
20-24	2013-2039	3.3800
25-29	2013-2039	3.3800
30-34	2013-2039	3.3800
35-39	2013-2039	3.3800
40-44	2013-2039	3.3800
45-49	2013-2039	3.3800
50-54	2013-2039	3.3800
55-59	2013-2039	2.3200
60-64	2013-2039	2.3200
65-69	2013-2039	1.7000
70-74	2013-2039	1.7000
75-79	2013-2039	1.2700

Results for PHIM for Run 2 (Basic_US_OG_T1.RTF)

****

Osmond and Gardner Modeling of Death Rates for COD: STROKE

Variable Parameter	Value
1. Country	US (United States)
2. Sex	F (Females)
3. Year of start of OG modelling	1966
4. Number 5-year periods for OG modelling	10
5. Age Range of interest	10 - 79
6. Scaling Factor for Rates	10000
7. Model Type to Save	4 (Full Age-Period-Cohort)
8. Goodness of Fit Required	1 (Analysis Printed For Age-Period-Cohort Model)
9. Cause of Death	STROKE
Note:	Death rates are per million population

Matrix of Numbers of Deaths

Age	1966-	1971-	1976-	1981-	1986-	1991-	1996-	2001-	2006-	2011-
10-14	352	310	181	137	107	92	112	102	107	100
15-19	519	521	399	250	172	164	139	158	151	142
20-24	778	754	642	546	418	303	263	305	274	235
25-29	1103	1198	969	869	865	587	537	443	443	428
30-34	1923	1799	1463	1390	1468	1378	1093	927	783	780
35-39	3523	2938	2332	2179	2216	2406	2408	1831	1533	1337
40-44	6091	5154	3645	3337	3358	3665	4079	4050	3215	2534
45-49	9780	8655	6034	4813	4636	5038	5761	6066	5779	4581
50-54	13700	12961	9607	7548	6324	6530	7335	7900	8116	7595
55-59	19040	17396	14020	11453	9531	8553	9080	9678	9950	10296
60-64	27977	26873	21281	18348	15918	13513	12712	12397	12922	13954
65-69	46143	42131	34259	28414	26128	22875	21414	18276	17002	18571
70-74	71977	67325	54347	46611	41541	39348	38665	32343	25891	25874
75-79	101255	100493	81824	70152	64314	61262	65894	59026	44190	39992

Matrix of Age- and Period-Specific Mortality Rates

Age	1966-	1971-	1976-	1981-	1986-	1991-	1996-	2001-	2006-	2011-
10-14	7.031	6.085	3.866	3.204	2.547	2.069	2.295	1.957	2.079	1.969
15-19	11.291	10.303	7.693	5.209	3.889	3.751	2.971	3.100	2.821	2.663
20-24	19.579	16.190	12.454	10.315	8.536	6.676	5.746	6.255	5.255	4.243
25-29	34.107	29.801	20.421	16.534	16.149	11.734	11.300	9.258	8.838	7.960
30-34	66.829	54.827	35.638	28.704	27.433	25.103	21.019	18.773	15.928	15.155
35-39	119.776	101.946	70.691	52.862	45.566	44.690	43.008	34.430	30.461	26.669
40-44	192.585	175.982	126.068	100.725	81.128	75.445	75.325	71.833	60.121	49.885
45-49	314.934	277.422	206.927	166.882	141.282	123.388	118.531	111.915	102.636	85.735
50-54	481.883	427.562	311.633	261.087	221.907	201.412	179.664	162.294	150.144	135.783
55-59	719.866	633.715	472.887	379.723	338.313	307.754	284.004	238.966	206.578	193.105
60-64	1181.642	1075.440	806.936	642.220	549.117	498.494	468.697	396.478	326.476	297.301
65-69	2330.491	1909.324	1444.221	1131.708	967.392	837.960	830.547	701.676	566.849	489.657
70-74	4627.851	3907.889	2760.220	2188.078	1842.236	1623.941	1561.631	1365.051	1077.414	928.034
75-79	8836.837	7809.370	5645.310	4160.118	3546.166	3201.134	3148.005	2760.530	2134.412	1885.869

Matrix of Log-Rates

Age	1966-	1971-	1976-	1981-	1986-	1991-	1996-	2001-	2006-	2011-
10-14	0.847	0.784	0.587	0.506	0.406	0.316	0.361	0.292	0.318	0.294
15-19	1.053	1.013	0.886	0.717	0.590	0.574	0.473	0.491	0.450	0.425
20-24	1.292	1.209	1.095	1.013	0.931	0.825	0.759	0.796	0.721	0.628
25-29	1.533	1.474	1.310	1.218	1.208	1.069	1.053	0.967	0.946	0.901
30-34	1.825	1.739	1.552	1.458	1.438	1.400	1.323	1.274	1.202	1.181
35-39	2.078	2.008	1.849	1.723	1.659	1.650	1.634	1.537	1.484	1.426
40-44	2.285	2.245	2.101	2.003	1.909	1.878	1.877	1.856	1.779	1.698
45-49	2.498	2.443	2.316	2.222	2.150	2.091	2.074	2.049	2.011	1.933
50-54	2.683	2.631	2.494	2.417	2.346	2.304	2.254	2.210	2.177	2.133
55-59	2.857	2.802	2.675	2.579	2.529	2.488	2.453	2.378	2.315	2.286
60-64	3.072	3.032	2.907	2.808	2.740	2.698	2.671	2.598	2.514	2.473
65-69	3.367	3.281	3.160	3.054	2.986	2.923	2.919	2.846	2.753	2.690
70-74	3.665	3.592	3.441	3.340	3.265	3.211	3.194	3.135	3.032	2.968
75-79	3.946	3.893	3.752	3.619	3.550	3.505	3.498	3.441	3.329	3.276

Results for PHIM for Run 2 (Basic_US_OG_T1.RTF)

Fitting the Age, Period, Cohort Models

Model	RSS	MRSS	DF	Factor	%Account	ChiSq	P
Age Only	87245.383	686.972	127	P, C	99.8140	479069.724	0.0000
Age-Period	518.694	4.433	117	Cohort	68.7071	2751.979	0.0000
Age-Cohort	1601.507	15.399	104	Period	89.8649	8499.981	0.0000
Period-Cohort	272.157	2.520	108	Age	40.3599	1444.622	0.0000
Full Age-Period-Cohort	162.314	1.691	96			861.011	0.0000

**Key to terms:**		
	RSS =	residual sum of squares
	MRSS =	mean RSS (MRSS/DF)
	DF =	degrees of freedom
	Factor =	Factors not included in the model
	% Account =	1- (RSS for full model)/(RSS for model in question)
	Chisq =	chi-squared value for model
	P =	probability value based on Chisq and DF.

Age	Value	Log10 Value
10-	3.922554	0.593569
15-	6.441639	0.808996
20-	11.401377	1.056957
25-	19.608677	1.292448
30-	35.806112	1.553957
35-	64.923694	1.812403
40-	114.357767	2.058266
45-	184.960962	2.267080
50-	280.773859	2.448357
55-	414.054773	2.617058
60-	675.024984	2.829320
65-	1170.46202	3.068357
70-	2196.17794	3.341668
75-	4204.79965	3.623745

Period	Value	Log10 Value
1966	1.776385	0.249537
1971	1.589726	0.201322
1976	1.212067	0.083527
1981	0.978761	-0.009324
1986	0.847730	-0.071743
1991	0.769910	-0.113560
1996	0.755667	-0.121670
2001	0.670359	-0.173693
2006	0.550778	-0.259023
2011	0.492519	-0.307577

Cohort	Value	Log10 Value
1891	1.183081	0.073014
1896	1.175747	0.070314
1901	1.114791	0.047193
1906	1.017643	0.007596
1911	1.003965	0.001719
1916	0.984329	-0.006860
1921	0.972551	-0.012088
1926	0.952840	-0.020980
1931	0.937685	-0.027943
1936	0.913745	-0.039175
1941	0.884652	-0.053228
1946	0.859449	-0.065780
1951	0.877257	-0.056873
1956	0.928278	-0.032322
1961	0.962710	-0.016505
1966	0.889919	-0.050650
1971	0.837023	-0.077262
1976	0.785924	-0.104619
1981	0.809551	-0.091756
1986	0.803628	-0.094945
1991	0.765186	-0.116233
1996	0.890175	-0.050525
2001	1.019122	0.008226

Results for PHIM for Run 2 (Basic_US_OG_T1.RTF)

Model: Full Age-Period-Cohort

Basic Analysis Using OG Modelling T1 on US
Fitting the Full Age-Period-Cohort Model
Matrix of observed, expected, and residual rates

**Age**		**1966-**	**1971-**	**1976-**	**1981-**	**1986-**	**1991-**	**1996-**	**2001-**	**2006-**	**2011-**
10-	Observed	7.031	6.085	3.866	3.204	2.547	2.069	2.295	1.957	2.079	1.969
	Expected	6.468	6.003	4.231	3.214	2.613	2.445	2.382	2.012	1.923	1.969
	Residual	-0.562	-0.082	0.365	0.010	0.066	0.376	0.087	0.055	-0.156	0.000
15-	Observed	11.291	10.303	7.693	5.209	3.889	3.751	2.971	3.100	2.821	2.663
	Expected	10.038	9.506	7.517	5.611	4.571	3.898	3.941	3.470	2.715	2.824
	Residual	-1.253	-0.797	-0.176	0.402	0.682	0.147	0.969	0.370	-0.106	0.161
20-	Observed	19.579	16.190	12.454	10.315	8.536	6.676	5.746	6.255	5.255	4.243
	Expected	17.407	15.900	12.828	10.743	8.601	7.347	6.771	6.187	5.046	4.297
	Residual	-2.172	-0.290	0.374	0.428	0.065	0.671	1.025	-0.067	-0.209	0.054
25-	Observed	34.107	29.801	20.421	16.534	16.149	11.734	11.300	9.258	8.838	7.960
	Expected	30.815	26.791	20.850	17.816	16.003	13.435	12.403	10.331	8.743	7.761
	Residual	-3.292	-3.010	0.429	1.281	-0.146	1.701	1.102	1.073	-0.095	-0.199
30-	Observed	66.829	54.827	35.638	28.704	27.433	25.103	21.019	18.773	15.928	15.155
	Expected	58.119	50.356	37.300	30.744	28.177	26.540	24.079	20.091	15.499	14.277
	Residual	-8.710	-4.471	1.661	2.040	0.743	1.437	3.060	1.318	-0.429	-0.878
35-	Observed	119.776	101.946	70.691	52.862	45.566	44.690	43.008	34.430	30.461	26.669
	Expected	108.143	94.309	69.615	54.613	48.282	46.400	47.231	38.731	29.931	25.131
	Residual	-11.633	-7.638	-1.076	1.751	2.716	1.711	4.224	4.301	-0.530	-1.538
40-	Observed	192.585	175.982	126.068	100.725	81.128	75.445	75.325	71.833	60.121	49.885
	Expected	193.563	170.469	126.654	99.018	83.319	77.238	80.218	73.802	56.052	47.144
	Residual	0.978	-5.513	0.585	-1.707	2.191	1.793	4.893	1.969	-4.069	-2.741
45-	Observed	314.934	277.422	206.927	166.882	141.282	123.388	118.531	111.915	102.636	85.735
	Expected	319.543	280.171	210.215	165.418	138.711	122.388	122.613	115.097	98.074	81.069
	Residual	4.609	2.748	3.288	-1.464	-2.572	-1.000	4.082	3.183	-4.562	-4.666
50-	Observed	481.883	427.562	311.633	261.087	221.907	201.412	179.664	162.294	150.144	135.783
	Expected	490.946	434.102	324.268	257.686	217.490	191.236	182.351	165.117	143.553	133.130
	Residual	9.064	6.539	12.634	-3.401	-4.416	-10.176	2.687	2.822	-6.591	-2.654
55-	Observed	719.866	633.715	472.887	379.723	338.313	307.754	284.004	238.966	206.578	193.105
	Expected	738.437	647.919	488.087	386.148	329.134	291.288	276.796	238.553	200.060	189.304
	Residual	18.571	14.204	15.200	6.425	-9.180	-16.465	-7.207	-0.413	-6.518	-3.802
60-	Observed	1181.642	1075.440	806.936	642.220	549.117	498.494	468.697	396.478	326.476	297.301
	Expected	1220.260	1077.360	805.354	642.553	545.252	487.323	466.096	400.313	319.533	291.655
	Residual	38.618	1.920	-1.582	0.333	-3.865	-11.171	-2.600	3.834	-6.942	-5.645
65-	Observed	2330.491	1909.324	1444.221	1131.708	967.392	837.960	830.547	701.676	566.849	489.657
	Expected	2317.863	1893.543	1424.304	1127.650	965.000	858.653	829.363	716.952	570.304	495.450
	Residual	-12.627	-15.781	-19.916	-4.058	-2.392	20.693	-1.184	15.276	3.454	5.794
70-	Observed	4627.851	3907.889	2760.220	2188.078	1842.236	1623.941	1561.631	1365.051	1077.414	928.034
	Expected	4586.890	3892.095	2708.880	2158.056	1832.591	1644.448	1581.314	1380.485	1105.272	956.891
	Residual	-40.961	-15.794	-51.340	-30.022	-9.646	20.507	19.683	15.434	27.858	28.858
75-	Observed	8836.837	7809.370	5645.310	4160.118	3546.166	3201.134	3148.005	2760.530	2134.412	1885.869
	Expected	8836.837	7859.256	5681.533	4188.102	3578.669	3186.589	3090.212	2685.794	2171.595	1892.315
	Residual	-0.000	49.886	36.223	27.984	32.503	-14.545	-57.794	-74.736	37.183	6.446

Fitting the Full Age-Period-Cohort Model

Results for PHIM for Run 2 (Basic_US_OG_T1.RTF)

Matrix of observed and expected deaths and (O-E)2/E Values**

**Age**		**1966-**	**1971-**	**1976-**	**1981-**	**1986-**	**1991-**	**1996-**	**2001-**	**2006-**	**2011-**	**Total**
10-	Observed	352.0	310.0	181.0	137.0	107.0	92.0	112.0	102.0	107.0	100.0	1600.0
	Expected	323.8	305.8	198.1	137.4	109.8	108.7	116.2	104.9	99.0	100.0	1603.8
	Difference	28.2	4.2	-17.1	-0.4	-2.8	-16.7	-4.2	-2.9	8.0	-0.0	-3.8
	Chi-Sq	2.4	0.1	1.5	0.0	0.1	2.6	0.2	0.1	0.6	0.0	7.5
15-	Observed	519.0	521.0	399.0	250.0	172.0	164.0	139.0	158.0	151.0	142.0	2615.0
	Expected	461.4	480.7	389.9	269.3	202.1	170.4	184.3	176.9	145.3	150.6	2630.9
	Difference	57.6	40.3	9.1	-19.3	-30.1	-6.4	-45.3	-18.9	5.7	-8.6	-15.9
	Chi-Sq	7.2	3.4	0.2	1.4	4.5	0.2	11.2	2.0	0.2	0.5	30.8
20-	Observed	778.0	754.0	642.0	546.0	418.0	303.0	263.0	305.0	274.0	235.0	4518.0
	Expected	691.7	740.5	661.3	568.6	421.2	333.5	309.9	301.7	263.1	238.0	4529.5
	Difference	86.3	13.5	-19.3	-22.6	-3.2	-30.5	-46.9	3.3	10.9	-3.0	-11.5
	Chi-Sq	10.8	0.2	0.6	0.9	0.0	2.8	7.1	0.0	0.5	0.0	22.9
25-	Observed	1103.0	1198.0	969.0	869.0	865.0	587.0	537.0	443.0	443.0	428.0	7442.0
	Expected	996.5	1077.0	989.3	936.3	857.2	672.1	589.4	494.3	438.3	417.3	7467.7
	Difference	106.5	121.0	-20.3	-67.3	7.8	-85.1	-52.4	-51.3	4.7	10.7	-25.7
	Chi-Sq	11.4	13.6	0.4	4.8	0.1	10.8	4.7	5.3	0.1	0.3	51.4
30-	Observed	1923.0	1799.0	1463.0	1390.0	1468.0	1378.0	1093.0	927.0	783.0	780.0	13004.0
	Expected	1672.4	1652.3	1531.2	1488.8	1507.8	1456.9	1252.1	992.1	761.9	734.8	13050.2
	Difference	250.6	146.7	-68.2	-98.8	-39.8	-78.9	-159.1	-65.1	21.1	45.2	-46.2
	Chi-Sq	37.6	13.0	3.0	6.6	1.0	4.3	20.2	4.3	0.6	2.8	93.3
35-	Observed	3523.0	2938.0	2332.0	2179.0	2216.0	2406.0	2408.0	1831.0	1533.0	1337.0	22703.0
	Expected	3180.8	2717.9	2296.5	2251.2	2348.1	2498.1	2644.5	2059.8	1506.3	1259.9	22763.1
	Difference	342.2	220.1	35.5	-72.2	-132.1	-92.1	-236.5	-228.8	26.7	77.1	-60.1
	Chi-Sq	36.8	17.8	0.5	2.3	7.4	3.4	21.1	25.4	0.5	4.7	120.1
40-	Observed	6091.0	5154.0	3645.0	3337.0	3358.0	3665.0	4079.0	4050.0	3215.0	2534.0	39128.0
	Expected	6121.9	4992.5	3661.9	3280.5	3448.7	3752.1	4344.0	4161.0	2997.4	2394.8	39154.8
	Difference	-30.9	161.5	-16.9	56.5	-90.7	-87.1	-265.0	-111.0	217.6	139.2	-26.8
	Chi-Sq	0.2	5.2	0.1	1.0	2.4	2.0	16.2	3.0	15.8	8.1	53.9
45-	Observed	9780.0	8655.0	6034.0	4813.0	4636.0	5038.0	5761.0	6066.0	5779.0	4581.0	61143.0
	Expected	9923.1	8740.7	6129.9	4770.8	4551.6	4997.2	5959.4	6238.5	5522.1	4331.7	61165.0
	Difference	-143.1	-85.7	-95.9	42.2	84.4	40.8	-198.4	-172.5	256.9	249.3	-22.0
	Chi-Sq	2.1	0.8	1.5	0.4	1.6	0.3	6.6	4.8	11.9	14.3	44.3
50-	Observed	13700.0	12961.0	9607.0	7548.0	6324.0	6530.0	7335.0	7900.0	8116.0	7595.0	87616.0
	Expected	13957.7	13159.2	9996.5	7449.7	6198.1	6200.1	7444.7	8037.4	7759.7	7446.6	87649.6
	Difference	-257.7	-198.2	-389.5	98.3	125.9	329.9	-109.7	-137.4	356.3	148.4	-33.6
	Chi-Sq	4.8	3.0	15.2	1.3	2.6	17.6	1.6	2.3	16.4	3.0	67.6
55-	Observed	19040.0	17396.0	14020.0	11453.0	9531.0	8553.0	9080.0	9678.0	9950.0	10296.0	118997.0
	Expected	19531.2	17785.9	14470.6	11646.8	9272.4	8095.4	8849.6	9661.3	9636.1	10093.3	119042.6
	Difference	-491.2	-389.9	-450.6	-193.8	258.6	457.6	230.4	16.7	313.9	202.7	-45.6
	Chi-Sq	12.4	8.5	14.0	3.2	7.2	25.9	6.0	0.0	10.2	4.1	91.6
60-	Observed	27977.0	26873.0	21281.0	18348.0	15918.0	13513.0	12712.0	12397.0	12922.0	13954.0	175895.0
	Expected	28891.3	26921.0	21239.3	18357.5	15806.0	13210.2	12641.5	12516.9	12647.2	13689.0	175919.8
	Difference	-914.3	-48.0	41.7	-9.5	112.0	302.8	70.5	-119.9	274.8	265.0	-24.8
	Chi-Sq	28.9	0.1	0.1	0.0	0.8	6.9	0.4	1.1	6.0	5.1	49.5
65-	Observed	46143.0	42131.0	34259.0	28414.0	26128.0	22875.0	21414.0	18276.0	17002.0	18571.0	275213.0
	Expected	45893.0	41782.8	33786.6	28312.1	26063.4	23439.9	21383.5	18673.9	17105.6	18790.7	275231.4
	Difference	250.0	348.2	472.4	101.9	64.6	-564.9	30.5	-397.9	-103.6	-219.7	-18.4
	Chi-Sq	1.4	2.9	6.6	0.4	0.2	13.6	0.0	8.5	0.6	2.6	36.7
70-	Observed	71977.0	67325.0	54347.0	46611.0	41541.0	39348.0	38665.0	32343.0	25891.0	25874.0	443922.0
	Expected	71339.9	67052.9	53336.2	45971.5	41323.5	39844.9	39152.3	32708.7	26560.4	26678.6	443968.9

Results for PHIM for Run 2 (Basic_US_OG_T1.RTF)

	Difference	637.1	272.1	1010.8	639.5	217.5	-496.9	-487.3	-365.7	-669.4	-804.6	-46.9
	Chi-Sq	5.7	1.1	19.2	8.9	1.1	6.2	6.1	4.1	16.9	24.3	93.5
75-	Observed	101255.0	100493.0	81824.0	70152.0	64314.0	61262.0	65894.0	59026.0	44190.0	39992.0	688402.0
	Expected	101255.0	101134.9	82349.0	70623.9	64903.5	60983.6	64684.3	57428.0	44959.8	40128.7	688450.8
	Difference	0.0	-641.9	-525.0	-471.9	-589.5	278.4	1209.7	1598.0	-769.8	-136.7	-48.8
	Chi-Sq	0.0	4.1	3.3	3.2	5.4	1.3	22.6	44.5	13.2	0.5	97.9
Total over ages	Observed	304161.0	288508.0	231003.0	196047.0	176996.0	165714.0	169492.0	153502.0	130356.0	126419.0	1942198.0
	Expected	304239.9	288544.3	231036.3	196064.3	177013.3	165763.0	169555.7	153555.3	130402.3	126453.9	1942628.2
	Difference	-78.9	-36.3	-33.3	-17.3	-17.3	-49.0	-63.7	-53.3	-46.3	-34.9	-430.2
	Chi-Sq	161.5	73.9	66.2	34.3	34.3	97.8	124.0	105.4	93.4	70.2	861.0

Results for PHIM for Run 2 (Basic_US_OG_T1.RTF)

****

Osmond and Gardner Extrapolating Death Rates for COD: STROKE

Variable Parameter	Value
1. Country	US (United States)
2. Sex	F (Females)
3. Year of start of OG modelling	1966
4. Number 5-year periods for OG modelling	10
5. Age Range of interest	10 - 79
6. Scaling Factor for Rates	10000
7. Model Type to Save	4 (Full Age-Period-Cohort)
8. Goodness of Fit Required	1 (Analysis Printed For Age-Period-Cohort Model)
9. Cause of Death	STROKE
Note:	Death rates are per million population
10. Number of Periods into the future to Predict	5
11. Earliest projected year	2016
12. Extrapolate Period using (1: last 2 points 2: linear regression)	1
13. Ratio of last two period values	0.89422

Predictions of rates for future years from model:	Full Age-Period-Cohort
Effects for extending model to project rates for:	2016-2040

Extrapolating Model: Full Age-Period-Cohort
Log Transform Parameters

Model	ChiSq	MChiSq	DF	Factor	%Account	P
Age Only	112363.718	8025.980	14	P, C	98.9026	0.0000
Age-Period	2441.737	174.410	14	Cohort	49.4996	0.0000
Age-Cohort	2505.158	178.940	14	Period	50.7781	0.0000
Period-Cohort	1607.128	114.795	14	Age	23.2738	0.0000
Full Age-Period-Cohort	1233.087	88.078	14			0.0000

**Key to terms:**		
	Chisq =	chi-squared value for model
	MChisq =	mean Chi-squared (Chisq/DF)
	DF =	degrees of freedom
	Factor =	Factors not included in the model
	% Account =	1- (Chisq for full model)/(Chisq for model in question)
	P =	probability value based on Chisq and DF.

AGE	EFFECT
10	3.922554
15	6.441639
20	11.401377
25	19.608677
30	35.806112
35	64.923694
40	114.357767
45	184.960962
50	280.773859
55	414.054773
60	675.024984
65	1170.46202
70	2196.17794
75	4204.79965

PERIOD	EFFECT	
Period Change =	0.894224	
1966	1.776385	
1971	1.589726	
1976	1.212067	
1981	0.978761	
1986	0.847730	
1991	0.769910	
1996	0.755667	
2001	0.670359	
2006	0.550778	
2011	0.492519	
2016	0.440422	
2021	0.393836	
2026	0.352178	
2031	0.314926	
2036	0.281614	
2016	0.460565	Extrapolated
2017	0.450381	Extrapolated
2018	0.440422	Extrapolated
2019	0.430684	Extrapolated

Results for PHIM for Run 2 (Basic_US_OG_T1.RTF)

2020	0.421161	Extrapolated
2021	0.411848	Extrapolated
2022	0.402741	Extrapolated
2023	0.393836	Extrapolated
2024	0.385128	Extrapolated
2025	0.376612	Extrapolated
2026	0.368285	Extrapolated
2027	0.360141	Extrapolated
2028	0.352178	Extrapolated
2029	0.344391	Extrapolated
2030	0.336776	Extrapolated
2031	0.329329	Extrapolated
2032	0.322047	Extrapolated
2033	0.314926	Extrapolated
2034	0.307962	Extrapolated
2035	0.301153	Extrapolated
2036	0.294494	Extrapolated
2037	0.287982	Extrapolated
2038	0.281614	Extrapolated
2039	0.275387	Extrapolated
2040	0.269298	Extrapolated

Results for PHIM for Run 2 (Basic_US_OG_T1.RTF)

COHORT	EFFECT	WEIGHT	ORIGINAL
1891	1.183081	1.000	
1896	1.175747	2.000	
1901	1.114791	4.000	
1906	1.017643	8.000	
1911	1.003965	16.000	
1916	0.984329	32.000	
1921	0.972551	64.000	
1926	0.952840	128.000	
1931	0.937685	256.000	
1936	0.913745	512.000	
1941	0.884652	1024.000	
1946	0.859449	2048.000	
1951	0.877257	4096.000	
1956	0.928278	8192.000	
1961	0.962710	16384.000	
1966	0.889919	32768.000	
1971	0.837023	65536.000	
1976	0.785924	131072.000	
1981	0.809551	262144.000	
1986	0.803628	524288.000	
1991	0.765186	1048576.000	
1996	0.752278	Extrapolated	0.890175
2001	0.735084	Extrapolated	1.019122
2006	0.718282	Extrapolated	
2011	0.701865	Extrapolated	
2016	0.685822	Extrapolated	
2021	0.670147	Extrapolated	
2026	0.654829	Extrapolated	

Standardizing Population: The 1976 European Standard Population

Age Range	Population, Females
All	100000
0	0
1	0
2	0
3	0
0-4	8000
5-9	7000
10-14	7000
15-19	7000
20-24	7000
25-29	7000
30-34	7000
35-39	7000
40-44	7000
45-49	7000
50-54	7000
55-59	6000
60-64	5000
65-69	4000
70-74	3000
75-79	2000
80-84	1000
85+	1000

Results for PHIM for Run 2 (Basic_US_OG_T1.RTF)

Matrix of observed and expected rates including predictions

Total over ages standardized using: The 1976 European Standard Population

		1966-	1971-	1976-	1981-	1986-	1991-	1996-	2001-	2006-	2011-	2016-	2021-	2026-	2031-	2036-
10	OBS	7.0	6.1	3.9	3.2	2.5	2.1	2.3	2.0	2.1	2.0	2.0	.	.	.	.
	EXP											1.2	1.1	0.9	0.8	0.7
15	OBS	11.3	10.3	7.7	5.2	3.9	3.8	3.0	3.1	2.8	2.7	2.6	.	.	.	.
	EXP											2.1	1.8	1.6	1.4	1.2
20	OBS	19.6	16.2	12.5	10.3	8.5	6.7	5.7	6.3	5.3	4.2	3.3	.	.	.	.
	EXP											3.8	3.3	2.9	2.5	2.2
25	OBS	34.1	29.8	20.4	16.5	16.1	11.7	11.3	9.3	8.8	8.0	7.8	.	.	.	.
	EXP											6.6	5.8	5.1	4.4	3.9
30	OBS	66.8	54.8	35.6	28.7	27.4	25.1	21.0	18.8	15.9	15.2	14.4	.	.	.	.
	EXP											12.7	10.8	9.5	8.3	7.2
35	OBS	119.8	101.9	70.7	52.9	45.6	44.7	43.0	34.4	30.5	26.7	28.1	.	.	.	.
	EXP											23.1	20.5	17.5	15.4	13.4
40	OBS	192.6	176.0	126.1	100.7	81.1	75.4	75.3	71.8	60.1	49.9	48.5	.	.	.	.
	EXP											39.6	36.5	32.4	27.6	24.2
45	OBS	314.9	277.4	206.9	166.9	141.3	123.4	118.5	111.9	102.6	85.7	85.6	.	.	.	.
	EXP											68.2	57.3	52.7	46.8	39.9
50	OBS	481.9	427.6	311.6	261.1	221.9	201.4	179.7	162.3	150.1	135.8	133.5	.	.	.	.
	EXP											110.0	92.6	77.7	71.6	63.5
55	OBS	719.9	633.7	472.9	379.7	338.3	307.8	284.0	239.0	206.6	193.1	203.9	.	.	.	.
	EXP											175.6	145.1	122.1	102.5	94.4
60	OBS	1181.6	1075.4	806.9	642.2	549.1	498.5	468.7	396.5	326.5	297.3	292.8	.	.	.	.
	EXP											276.0	255.9	211.6	177.9	149.4
65	OBS	2330.5	1909.3	1444.2	1131.7	967.4	838.0	830.5	701.7	566.8	489.7	488.2	.	.	.	.
	EXP											452.2	427.9	396.8	328.0	275.9
70	OBS	4627.9	3907.9	2760.2	2188.1	1842.2	1623.9	1561.6	1365.1	1077.4	928.0	857.8	.	.	.	.
	EXP											831.3	758.8	718.0	665.8	550.4
75	OBS	8836.8	7809.4	5645.3	4160.1	3546.2	3201.1	3148.0	2760.5	2134.4	1885.9	1702.6	.	.	.	.
	EXP											1638.3	1423.2	1299.1	1229.2	1140.0
10-79	OBS	721.0	624.8	455.3	354.5	302.4	270.2	259.9	226.2	184.2	162.3	155.4	.	.	.	.
	EXP	721.5	624.8	455.9	354.3	301.8	269.4	260.3	227.2	183.9	162.0	143.2*	127.6*	114.8*	102.7*	89.7*

Drop in overall standardized Observed and Predicted rates

comparing the last observed rate during the model fitting period to the last observed and predicted rates where an observed rate is available (2016)

Observed and Predicted %Drop = 4.232% and 11.775%

Results for PHIM for Run 2 (Basic_US_OG_T1.RTF)

Matrix of observed and expected deaths including predictions

		1966-	1971-	1976-	1981-	1986-	1991-	1996-	2001-	2006-	2011-	2016-	2021-	2026-	2031-	2036-
10-	OBS	352.0	310.0	181.0	137.0	107.0	92.0	112.0	102.0	107.0	100.0	105.0*	.	.	.	.
	EXP	323.8	305.8	198.1	137.4	109.8	108.7	116.2	104.9	83.6	72.1	63.8*	54.6*	46.2*	41.1*	37.0*
	ChiSq	2.448	0.057	1.477	0.001	0.070	2.574	0.154	0.079	6.519	10.769	26.542*	.	.	.	.
15-	OBS	519.0	521.0	399.0	250.0	172.0	164.0	139.0	158.0	151.0	142.0	135.0*	.	.	.	.
	EXP	461.4	480.7	389.9	269.3	202.1	170.4	184.3	176.9	145.3	127.3	108.7*	96.1*	82.3*	69.9*	62.2*
	ChiSq	7.191	3.375	0.214	1.380	4.494	0.241	11.155	2.011	0.223	1.708	6.364*	.	.	.	.
20-	OBS	778.0	754.0	642.0	546.0	418.0	303.0	263.0	305.0	274.0	235.0	185.0*	.	.	.	.
	EXP	691.7	740.5	661.3	568.6	421.2	333.5	309.9	301.7	263.1	238.0	209.8*	179.1*	158.5*	136.3*	116.0*
	ChiSq	10.771	0.246	0.562	0.901	0.024	2.783	7.109	0.036	0.451	0.037	2.936*	.	.	.	.
25-	OBS	1103.0	1198.0	969.0	869.0	865.0	587.0	537.0	443.0	443.0	428.0	450.0*	.	.	.	.
	EXP	996.5	1077.0	989.3	936.3	857.2	672.1	589.4	494.3	438.3	417.3	379.8*	334.1*	285.8*	253.5*	218.5*
	ChiSq	11.374	13.591	0.418	4.844	0.072	10.769	4.655	5.331	0.051	0.275	12.980*	.	.	.	.
30-	OBS	1923.0	1799.0	1463.0	1390.0	1468.0	1378.0	1093.0	927.0	783.0	780.0	795.0*	.	.	.	.
	EXP	1672.4	1652.3	1531.2	1488.8	1507.8	1456.9	1252.1	992.1	761.9	734.8	699.9*	634.7*	558.8*	479.2*	425.5*
	ChiSq	37.557	13.026	3.038	6.553	1.050	4.270	20.221	4.270	0.584	2.779	12.919*	.	.	.	.
35-	OBS	3523.0	2938.0	2332.0	2179.0	2216.0	2406.0	2408.0	1831.0	1533.0	1337.0	1470.0*	.	.	.	.
	EXP	3180.8	2717.9	2296.5	2251.2	2348.1	2498.1	2644.5	2059.8	1506.3	1259.9	1211.4*	1150.5*	1042.8*	919.6*	789.5*
	ChiSq	36.806	17.827	0.549	2.314	7.432	3.395	21.146	25.406	0.472	4.719	55.186*	.	.	.	.
40-	OBS	6091.0	5154.0	3645.0	3337.0	3358.0	3665.0	4079.0	4050.0	3215.0	2534.0	2450.0*	.	.	.	.
	EXP	6121.9	4992.5	3661.9	3280.5	3448.7	3752.1	4344.0	4161.0	2997.4	2394.8	2000.0*	1919.4*	1822.6*	1652.9*	1458.5*
	ChiSq	0.156	5.222	0.078	0.974	2.385	2.022	16.164	2.962	15.794	8.094	101.247*	.	.	.	.
45-	OBS	9780.0	8655.0	6034.0	4813.0	4636.0	5038.0	5761.0	6066.0	5779.0	4581.0	4345.0*	.	.	.	.
	EXP	9923.1	8740.7	6129.9	4770.8	4551.6	4997.2	5959.4	6238.5	5522.1	4331.7	3461.1*	2888.3*	2773.2*	2635.3*	2390.3*
	ChiSq	2.064	0.841	1.500	0.374	1.565	0.333	6.604	4.770	11.949	14.350	225.708*	.	.	.	.
50-	OBS	13700.0	12961.0	9607.0	7548.0	6324.0	6530.0	7335.0	7900.0	8116.0	7595.0	7062.5*	.	.	.	.
	EXP	13957.7	13159.2	9996.5	7449.7	6198.1	6200.1	7444.7	8037.4	7759.7	7446.6	5821.7*	4652.2*	3886.5*	3736.6*	3552.5*
	ChiSq	4.757	2.986	15.176	1.298	2.556	17.557	1.616	2.348	16.358	2.959	264.471*	.	.	.	.
55-	OBS	19040.0	17396.0	14020.0	11453.0	9531.0	8553.0	9080.0	9678.0	9950.0	10296.0	11180.0*	.	.	.	.
	EXP	19531.2	17785.9	14470.6	11646.8	9272.4	8095.4	8849.6	9661.3	9636.1	10093.3	9625.5*	7526.0*	6025.1*	5043.0*	4854.0*
	ChiSq	12.353	8.548	14.034	3.225	7.213	25.866	6.000	0.029	10.227	4.070	251.066*	.	.	.	.
60-	OBS	27977.0	26873.0	21281.0	18348.0	15918.0	13513.0	12712.0	12397.0	12922.0	13954.0	15135.0*	.	.	.	.
	EXP	28891.3	26921.0	21239.3	18357.5	15806.0	13210.2	12641.5	12516.9	12647.2	13689.0	14264.6*	13596.2*	10653.9*	8553.3*	7172.2*
	ChiSq	28.936	0.085	0.082	0.005	0.794	6.942	0.393	1.148	5.970	5.129	53.112*	.	.	.	.
65-	OBS	46143.0	42131.0	34259.0	28414.0	26128.0	22875.0	21414.0	18276.0	17002.0	18571.0	21867.5*	.	.	.	.
	EXP	45893.0	41782.8	33786.6	28312.1	26063.4	23439.9	21383.5	18673.9	17105.6	18790.7	20254.9*	21105.2*	20166.3*	15863.1*	12774.8*
	ChiSq	1.362	2.902	6.606	0.367	0.160	13.614	0.044	8.478	0.628	2.569	128.385*	.	.	.	.

Results for PHIM for Run 2 (Basic_US_OG_T1.RTF)

70-	OBS	71977.0	67325.0	54347.0	46611.0	41541.0	39348.0	38665.0	32343.0	25891.0	25874.0	30235.0*	.	.	.	.
	EXP	71339.9	67052.9	53336.2	45971.5	41323.5	39844.9	39152.3	32708.7	26560.4	26678.6	29301.3*	31616.2*	33078.4*	31783.5*	25114.4*
	ChiSq	5.689	1.104	19.158	8.897	1.145	6.196	6.066	4.089	16.873	24.264	29.755*	.	.	.	.
75-	OBS	101255.0	100493.0	81824.0	70152.0	64314.0	61262.0	65894.0	59026.0	44190.0	39992.0	42132.5*	.	.	.	.
	EXP	101255.0	101134.9	82349.0	70623.9	64903.5	60983.6	64684.3	57428.0	44959.8	40128.7	40541.7*	44674.3*	48460.8*	51178.4*	49512.8*
	ChiSq	.	4.075	3.347	3.153	5.354	1.271	22.625	44.467	13.181	0.466	62.417*	.	.	.	.
Total Deaths		304161.0	288508.0	231003.0	196047.0	176996.0	165714.0	169492.0	153502.0	130356.0	126419.0	137547.5*	.	.	.	.
Expected		304239.9	288544.3	231036.3	196064.3	177013.3	165763.0	169555.7	153555.3	130387.0	126402.7	127944.3*	130427.0*	129041.3*	122345.6*	108478.3*
Obs/Exp		1.000	1.000	1.000	1.000	1.000	1.000	1.000	1.000	1.000	1.000	1.075*	.	.	.	.

Chi Squared (Log) = **1233.1 on 14 D.F.** **P = 0.0000**

Results for PHIM for Run 2 (Basic_US_OG_T1.RTF)

****

Observed and predicted rates (5 year periods)

		10-	15-	20-	25-	30-	35-	40-	45-	50-	55-	60-	65-	70-	75-
2016-	OBS	2.0	2.6	3.3	7.8	14.4	28.1	48.5	85.6	133.5	203.9	292.8	488.2	857.8	1702.6
	PRE	1.2	2.1	3.8	6.6	12.7	23.1	39.6	68.2	110.0	175.6	276.0	452.2	831.3	1638.3
	RES	0.800	0.505	-0.447	1.222	1.722	4.941	8.906	17.412	23.455	28.353	16.840	36.003	26.491	64.282
2021-	PRE	1.1	1.8	3.3	5.8	10.8	20.5	36.5	57.3	92.6	145.1	255.9	427.9	758.8	1423.2
2026-	PRE	0.9	1.6	2.9	5.1	9.5	17.5	32.4	52.7	77.7	122.1	211.6	396.8	718.0	1299.1
2031-	PRE	0.8	1.4	2.5	4.4	8.3	15.4	27.6	46.8	71.6	102.5	177.9	328.0	665.8	1229.2
2036-	PRE	0.7	1.2	2.2	3.9	7.2	13.4	24.2	39.9	63.5	94.4	149.4	275.9	550.4	1140.0

Results for PHIM for Run 2 (Basic_US_OG_T1.RTF)

****

Observed and predicted deaths (5 year periods)

		10-	15-	20-	25-	30-	35-	40-	45-	50-	55-	60-	65-	70-	75-
2016-	OBS	105.0	135.0	185.0	450.0	795.0	1470.0	2450.0	4345.0	7062.5	11180.0	15135.0	21867.5	30235.0	42132.5
	PRE	63.8	108.7	209.8	379.8	699.9	1211.4	2000.0	3461.1	5821.7	9625.5	14264.6	20254.9	29301.3	40541.7
	CHI	26.542	6.364	2.936	12.980	12.919	55.186	101.247	225.708	264.471	251.066	53.112	128.385	29.755	62.417
2021-	PRE	54.6	96.1	179.1	334.1	634.7	1150.5	1919.4	2888.3	4652.2	7526.0	13596.2	21105.2	31616.2	44674.3
2026-	PRE	46.2	82.3	158.5	285.8	558.8	1042.8	1822.6	2773.2	3886.5	6025.1	10653.9	20166.3	33078.4	48460.8
2031-	PRE	41.1	69.9	136.3	253.5	479.2	919.6	1652.9	2635.3	3736.6	5043.0	8553.3	15863.1	31783.5	51178.4
2036-	PRE	37.0	62.2	116.0	218.5	425.5	789.5	1458.5	2390.3	3552.5	4854.0	7172.2	12774.8	25114.4	49512.8

Results for PHIM for Run 2 (Basic_US_OG_T1.RTF)

****

Observed and predicted rates (single years)

		10-	15-	20-	25-	30-	35-	40-	45-	50-	55-	60-	65-	70-	75-
2016	OBS	1.9	2.4	2.7	7.1	15.3	29.5	48.6	88.0	134.8	200.6	294.2	508.1	916.8	1800.3
	PRE	1.3	2.2	4.0	6.9	13.3	24.2	41.4	71.3	115.1	183.6	288.6	472.9	869.3	1713.2
	RES	0.564	0.212	-1.201	0.216	2.003	5.303	7.205	16.669	19.726	16.990	5.576	35.192	47.491	87.126
2017	OBS	2.2	2.8	3.8	8.8	14.1	27.8	48.6	82.1	125.7	205.1	305.6	502.0	932.7	1822.8
	PRE	1.3	2.1	3.9	6.8	13.0	23.7	40.5	69.7	112.5	179.5	282.2	462.4	850.1	1675.3
	RES	0.971	0.648	-0.027	2.026	1.174	4.144	8.157	12.392	13.180	25.539	23.416	39.595	82.633	147.527
2018	PRE	1.2	2.1	3.8	6.6	12.7	23.1	39.6	68.2	110.0	175.6	276.0	452.2	831.3	1638.3
2019	PRE	1.2	2.0	3.7	6.5	12.4	22.6	38.7	66.7	107.6	171.7	269.9	442.2	812.9	1602.1
2020	PRE	1.2	2.0	3.6	6.3	12.1	22.1	37.9	65.2	105.2	167.9	263.9	432.4	794.9	1566.6
2021	PRE	1.1	1.9	3.5	6.1	11.3	21.5	38.1	59.9	96.8	151.8	267.6	447.5	793.5	1488.3
2022	PRE	1.1	1.9	3.4	5.9	11.0	21.0	37.3	58.5	94.6	148.4	261.7	437.6	775.9	1455.4
2023	PRE	1.1	1.8	3.3	5.8	10.8	20.5	36.5	57.3	92.6	145.1	255.9	427.9	758.8	1423.2
2024	PRE	1.1	1.8	3.2	5.7	10.6	20.1	35.7	56.0	90.5	141.9	250.3	418.4	742.0	1391.8
2025	PRE	1.0	1.7	3.2	5.6	10.3	19.6	34.9	54.7	88.5	138.8	244.7	409.2	725.6	1361.0
2026	PRE	1.0	1.7	3.0	5.3	9.9	18.3	33.8	55.1	81.3	127.6	221.2	415.0	750.8	1358.5
2027	PRE	1.0	1.6	2.9	5.2	9.7	17.9	33.1	53.9	79.5	124.8	216.3	405.8	734.2	1328.4
2028	PRE	0.9	1.6	2.9	5.1	9.5	17.5	32.4	52.7	77.7	122.1	211.6	396.8	718.0	1299.1
2029	PRE	0.9	1.6	2.8	5.0	9.3	17.1	31.6	51.6	76.0	119.4	206.9	388.1	702.1	1270.3
2030	PRE	0.9	1.5	2.8	4.9	9.1	16.7	31.0	50.4	74.3	116.7	202.3	379.5	686.6	1242.3
2031	PRE	0.9	1.5	2.6	4.6	8.7	16.1	28.8	49.0	74.9	107.2	186.1	343.0	696.3	1285.4
2032	PRE	0.8	1.4	2.6	4.5	8.5	15.7	28.2	47.9	73.2	104.8	182.0	335.4	680.9	1257.0
2033	PRE	0.8	1.4	2.5	4.4	8.3	15.4	27.6	46.8	71.6	102.5	177.9	328.0	665.8	1229.2
2034	PRE	0.8	1.4	2.5	4.3	8.1	15.0	26.9	45.8	70.0	100.2	174.0	320.8	651.1	1202.0
2035	PRE	0.8	1.3	2.4	4.2	7.9	14.7	26.4	44.8	68.5	98.0	170.2	313.7	636.7	1175.5
2036	PRE	0.8	1.3	2.3	4.1	7.6	14.1	25.3	41.7	66.4	98.7	156.2	288.5	575.6	1192.1
2037	PRE	0.7	1.2	2.3	4.0	7.4	13.7	24.8	40.8	65.0	96.5	152.8	282.1	562.8	1165.8
2038	PRE	0.7	1.2	2.2	3.9	7.2	13.4	24.2	39.9	63.5	94.4	149.4	275.9	550.4	1140.0
2039	PRE	0.7	1.2	2.2	3.8	7.1	13.1	23.7	39.0	62.1	92.3	146.1	269.8	538.2	1114.8
2040	PRE	0.7	1.2	2.1	3.7	6.9	12.9	23.2	38.1	60.8	90.3	142.9	263.8	526.3	1090.1

Results for PHIM for Run 2 (Basic_US_OG_T1.RTF)

****

Observed and predicted deaths (single years)

		10-	15-	20-	25-	30-	35-	40-	45-	50-	55-	60-	65-	70-	75-
2016	OBS	19.0	25.0	31.0	80.0	164.0	301.0	490.0	902.0	1473.0	2210.0	2940.0	4344.0	5839.0	8236.0
	PRE	13.2	22.8	44.5	77.6	142.5	246.9	417.4	731.1	1257.5	2022.8	2884.3	4043.1	5536.5	7837.4
	CHI	2.503	0.215	4.117	0.076	3.256	11.850	12.643	39.957	36.948	17.325	1.077	22.390	16.524	20.270
2017	OBS	23.0	29.0	43.0	100.0	154.0	287.0	490.0	836.0	1352.0	2262.0	3114.0	4403.0	6255.0	8617.0
	PRE	13.0	22.2	43.3	76.9	141.2	244.2	407.8	709.8	1210.3	1980.3	2875.4	4055.8	5700.9	7919.6
	CHI	7.633	2.051	0.002	6.914	1.158	7.485	16.560	22.422	16.600	40.076	19.795	29.731	53.865	61.412
2018	PRE	12.8	21.7	42.0	76.2	139.9	242.0	399.0	691.1	1162.9	1931.5	2861.6	4053.2	5869.9	8044.4
2019	PRE	12.5	21.2	40.7	75.2	138.7	240.0	391.4	673.9	1118.7	1877.6	2838.2	4047.8	6017.3	8224.3
2020	PRE	12.2	20.8	39.5	73.9	137.5	238.1	385.2	657.5	1079.5	1819.9	2802.5	4045.0	6128.5	8461.0
2021	PRE	11.7	19.9	37.5	70.9	129.8	234.3	391.3	602.6	982.2	1624.3	2854.9	4265.0	6303.6	8424.2
2022	PRE	11.3	19.6	36.6	68.9	128.6	232.2	387.0	588.8	953.8	1563.7	2795.3	4251.2	6322.3	8677.0
2023	PRE	10.9	19.3	35.8	66.8	127.3	230.0	383.5	576.2	928.9	1503.2	2727.9	4232.6	6323.3	8944.6
2024	PRE	10.6	18.9	35.0	64.8	125.6	227.9	380.3	565.4	906.1	1446.9	2653.6	4200.8	6322.7	9183.0
2025	PRE	10.2	18.4	34.3	63.0	123.4	225.9	377.2	556.4	884.3	1396.9	2573.4	4151.4	6327.6	9369.6
2026	PRE	9.7	17.6	32.8	59.9	118.5	213.2	371.1	565.2	810.5	1271.3	2297.4	4229.2	6672.2	9633.9
2027	PRE	9.5	17.0	32.3	58.4	115.2	211.3	367.8	559.1	792.1	1234.9	2212.5	4142.6	6654.6	9667.9
2028	PRE	9.2	16.5	31.8	57.0	111.8	209.2	364.4	554.1	775.4	1203.1	2128.0	4045.6	6631.6	9684.0
2029	PRE	9.0	15.9	31.2	55.9	108.4	206.4	361.1	549.5	761.0	1173.9	2049.2	3938.6	6589.7	9705.1
2030	PRE	8.8	15.4	30.5	54.8	105.4	202.7	357.9	545.0	749.0	1145.9	1979.3	3823.0	6522.0	9740.1
2031	PRE	8.5	14.7	29.0	52.5	100.3	194.8	337.8	536.5	761.3	1051.0	1802.8	3415.3	6649.0	10283.4
2032	PRE	8.4	14.3	28.1	51.6	97.9	189.6	334.9	531.8	753.2	1027.6	1752.3	3291.4	6519.2	10271.5
2033	PRE	8.2	14.0	27.3	50.8	95.7	183.9	331.6	526.9	746.6	1006.2	1708.1	3168.2	6374.5	10255.2
2034	PRE	8.1	13.6	26.4	49.9	93.7	178.5	327.2	522.2	740.6	987.7	1667.3	3053.7	6215.5	10213.9
2035	PRE	8.0	13.3	25.6	48.8	91.9	173.6	321.3	517.6	734.5	972.4	1628.2	2952.5	6043.3	10136.7
2036	PRE	7.7	12.8	24.3	46.4	88.1	165.2	308.9	488.6	723.1	988.7	1493.8	2689.9	5399.0	10332.7
2037	PRE	7.6	12.6	23.7	45.1	86.7	161.3	300.6	484.4	716.8	978.3	1460.9	2615.6	5205.2	10136.6
2038	PRE	7.4	12.5	23.2	43.7	85.3	157.7	291.7	479.6	710.3	969.9	1431.0	2550.9	5014.1	9924.2
2039	PRE	7.3	12.3	22.7	42.3	83.7	154.4	283.1	473.2	704.0	962.2	1405.2	2491.4	4838.4	9694.9
2040	PRE	7.1	12.0	22.2	41.1	81.9	151.4	275.3	464.7	697.9	954.5	1383.9	2434.7	4685.2	9449.1

Results for PHIM for Run 2 (Basic_US_OG_T1.RTF)

****
List of values created by O and G modelling, using percentage change in last two period parameters for Fixed File MORT

1. Country	US (United States)
2. Sex	F (Females)
3. Disease	STR (STROKE)
* Value comes from O and G Modelling.	

Age	Years	Value	Death Rate	Population
10-14	2018	12.8055	1.240890	103196.46 *
10-14	2019	12.5491	1.213452	103416.52 *
10-14	2020	12.2364	1.186620	103119.74 *
10-14	2021	11.6605	1.133860	102838.63 *
10-14	2022	11.3143	1.108788	102041.78 *
10-14	2023	10.9353	1.084271	100854.31 *
10-14	2024	10.5565	1.060296	99561.97 *
10-14	2025	10.2065	1.036851	98437.76 *
10-14	2026	9.7112	0.990750	98019.03 *
10-14	2027	9.4663	0.968843	97707.32 *
10-14	2028	9.2339	0.947420	97463.30 *
10-14	2029	9.0124	0.926471	97277.15 *
10-14	2030	8.8074	0.905985	97213.74 *
10-14	2031	8.4750	0.865702	97897.69 *
10-14	2032	8.3521	0.846560	98659.12 *
10-14	2033	8.2306	0.827841	99422.00 *
10-14	2034	8.1013	0.809536	100073.76 *
10-14	2035	7.9596	0.791636	100545.70 *
10-14	2036	7.6759	0.756438	101474.16 *
10-14	2037	7.5569	0.739712	102160.11 *
10-14	2038	7.4217	0.723355	102601.69 *
10-14	2039	7.2737	0.707361	102829.10 *
15-19	2018	21.7306	2.085463	104200.38 *
15-19	2019	21.2366	2.039350	104134.08 *
15-19	2020	20.7574	1.994257	104085.87 *
15-19	2021	19.9312	1.905586	104593.73 *
15-19	2022	19.6145	1.863451	105258.83 *
15-19	2023	19.2878	1.822247	105846.21 *
15-19	2024	18.8974	1.781954	106048.78 *
15-19	2025	18.4186	1.742552	105699.11 *
15-19	2026	17.5517	1.665073	105411.01 *
15-19	2027	17.0393	1.628255	104647.84 *
15-19	2028	16.4836	1.592252	103524.06 *
15-19	2029	15.9300	1.557045	102309.30 *
15-19	2030	15.4091	1.522616	101201.39 *
15-19	2031	14.6671	1.454916	100810.47 *
15-19	2032	14.3047	1.422745	100543.20 *
15-19	2033	13.9629	1.391286	100360.03 *
15-19	2034	13.6378	1.360522	100239.43 *
15-19	2035	13.3276	1.330439	100174.80 *
15-19	2036	12.8187	1.271284	100832.66 *
15-19	2037	12.6351	1.243173	101635.99 *
15-19	2038	12.4542	1.215685	102445.77 *
15-19	2039	12.2582	1.188804	103113.72 *
20-24	2018	41.9695	3.777506	111103.83 *
20-24	2019	40.6797	3.693979	110124.20 *
20-24	2020	39.5042	3.612299	109360.13 *
20-24	2021	37.5440	3.451686	108770.00 *
20-24	2022	36.5975	3.375363	108425.37 *
20-24	2023	35.7543	3.300729	108322.56 *
20-24	2024	34.9873	3.227744	108395.50 *
20-24	2025	34.2624	3.156373	108550.01 *
20-24	2026	32.8428	3.016032	108894.01 *
20-24	2027	32.2942	2.949342	109496.37 *
20-24	2028	31.7711	2.884127	110158.41 *
20-24	2029	31.1820	2.820355	110560.57 *
20-24	2030	30.4682	2.757992	110472.49 *
20-24	2031	29.0005	2.635363	110043.60 *
20-24	2032	28.1458	2.577091	109215.51 *
20-24	2033	27.2589	2.520107	108165.47 *
20-24	2034	26.4080	2.464384	107158.74 *
20-24	2035	25.6223	2.409892	106321.30 *
20-24	2036	24.3421	2.302741	105709.09 *
20-24	2037	23.7147	2.251824	105313.13 *
20-24	2038	23.1550	2.202032	105152.82 *
20-24	2039	22.6534	2.153342	105200.95 *
25-29	2018	76.2019	6.608217	115313.86 *
25-29	2019	75.2111	6.462099	116387.97 *

Results for PHIM for Run 2 (Basic_US_OG_T1.RTF)

25-29	2020	73.8771	6.319211	116908.75 *
25-29	2021	70.9181	6.075248	116732.89 *
25-29	2022	68.9298	5.940914	116025.62 *
25-29	2023	66.8166	5.809551	115011.70 *
25-29	2024	64.7882	5.681092	114041.82 *
25-29	2025	62.9655	5.555474	113339.59 *
25-29	2026	59.8549	5.308461	112753.72 *
25-29	2027	58.3689	5.191082	112440.79 *
25-29	2028	57.0492	5.076299	112383.37 *
25-29	2029	55.8586	4.964054	112526.12 *
25-29	2030	54.7649	4.854290	112817.52 *
25-29	2031	52.5063	4.638454	113197.88 *
25-29	2032	51.6332	4.535890	113832.48 *
25-29	2033	50.8021	4.435594	114532.86 *
25-29	2034	49.8817	4.337516	115000.73 *
25-29	2035	48.7986	4.241606	115047.44 *
25-29	2036	46.4476	4.053012	114600.09 *
25-29	2037	45.0883	3.963393	113761.95 *
25-29	2038	43.6840	3.875756	112710.93 *
25-29	2039	42.3457	3.790057	111728.46 *
30-34	2018	139.9214	12.673058	110408.56 *
30-34	2019	138.6926	12.392836	111913.53 *
30-34	2020	137.5481	12.118810	113499.65 *
30-34	2021	129.7525	11.283946	114988.57 *
30-34	2022	128.6100	11.034439	116553.27 *
30-34	2023	127.3350	10.790450	118007.13 *
30-34	2024	125.6340	10.551855	119063.44 *
30-34	2025	123.3710	10.318536	119562.53 *
30-34	2026	118.4830	9.920173	119436.43 *
30-34	2027	115.2256	9.700822	118779.22 *
30-34	2028	111.7532	9.486321	117804.55 *
30-34	2029	108.4148	9.276562	116869.57 *
30-34	2030	105.4239	9.071442	116215.13 *
30-34	2031	100.3090	8.668099	115721.99 *
30-34	2032	97.8704	8.476433	115461.72 *
30-34	2033	95.6819	8.289005	115432.31 *
30-34	2034	93.7005	8.105722	115598.01 *
30-34	2035	91.8868	7.926491	115923.69 *
30-34	2036	88.1262	7.574056	116352.68 *
30-34	2037	86.6625	7.406581	117007.50 *
30-34	2038	85.2513	7.242809	117704.81 *
30-34	2039	83.6891	7.082659	118160.59 *
35-39	2018	242.0229	23.148169	104553.79 *
35-39	2019	240.0327	22.636325	106038.72 *
35-39	2020	238.1130	22.135798	107569.20 *
35-39	2021	234.2921	21.487966	109034.08 *
35-39	2022	232.1617	21.012831	110485.67 *
35-39	2023	230.0158	20.548203	111939.64 *
35-39	2024	227.9375	20.093848	113436.46 *
35-39	2025	225.9438	19.649540	114986.80 *
35-39	2026	213.1639	18.295885	116509.21 *
35-39	2027	211.3102	17.891333	118107.58 *
35-39	2028	209.2227	17.495726	119585.02 *
35-39	2029	206.4287	17.108866	120655.96 *
35-39	2030	202.7140	16.730561	121163.90 *
35-39	2031	194.8191	16.084652	121121.11 *
35-39	2032	189.5554	15.728994	120513.37 *
35-39	2033	183.9101	15.381200	119568.09 *
35-39	2034	178.4649	15.041096	118651.53 *
35-39	2035	173.5628	14.708513	118001.61 *
35-39	2036	165.2244	14.054529	117559.56 *
35-39	2037	161.2500	13.743760	117326.00 *
35-39	2038	157.6533	13.439863	117302.74 *
35-39	2039	154.3730	13.142685	117459.22 *
40-44	2018	399.0221	39.583629	100804.83 *
40-44	2019	391.4195	38.708369	101120.12 *
40-44	2020	385.1873	37.852463	101760.17 *
40-44	2021	391.3130	38.128253	102630.72 *
40-44	2022	386.9991	37.285174	103794.37 *
40-44	2023	383.4851	36.460737	105177.56 *
40-44	2024	380.3015	35.654530	106662.88 *
40-44	2025	377.1508	34.866150	108171.06 *
40-44	2026	371.1191	33.845747	109650.15 *
40-44	2027	367.7908	33.097362	111123.89 *
40-44	2028	364.4211	32.365525	112595.46 *
40-44	2029	361.1305	31.649870	114101.74 *
40-44	2030	357.9441	30.950039	115652.24 *
40-44	2031	337.8336	28.817893	117230.49 *

Results for PHIM for Run 2 (Basic_US_OG_T1.RTF)

40-44	2032	334.9479	28.180682	118857.26 *
40-44	2033	331.6492	27.557561	120347.80 *
40-44	2034	327.2187	26.948217	121424.99 *
40-44	2035	321.3099	26.352348	121928.37 *
40-44	2036	308.8823	25.334975	121919.33 *
40-44	2037	300.6084	24.774777	121336.47 *
40-44	2038	291.7109	24.226966	120407.52 *
40-44	2039	283.1043	23.691268	119497.30 *
45-49	2018	691.1206	68.184699	101360.07 *
45-49	2019	673.9035	66.677023	101069.82 *
45-49	2020	657.5494	65.202684	100846.99 *
45-49	2021	602.6333	59.868425	100659.62 *
45-49	2022	588.8178	58.544635	100575.87 *
45-49	2023	576.2447	57.250117	100653.89 *
45-49	2024	565.3659	55.984222	100986.65 *
45-49	2025	556.3623	54.746319	101625.52 *
45-49	2026	565.2425	55.145195	102500.77 *
45-49	2027	559.0920	53.925844	103677.94 *
45-49	2028	554.0861	52.733455	105072.97 *
45-49	2029	549.5364	51.567431	106566.57 *
45-49	2030	545.0136	50.427190	108079.32 *
45-49	2031	536.4900	48.951374	109596.51 *
45-49	2032	531.7669	47.868978	111087.99 *
45-49	2033	526.9386	46.810516	112568.42 *
45-49	2034	522.2087	45.775459	114080.49 *
45-49	2035	517.5908	44.763288	115628.42 *
45-49	2036	488.5508	41.679548	117215.96 *
45-49	2037	484.3921	40.757944	118846.06 *
45-49	2038	479.6245	39.856719	120337.18 *
45-49	2039	473.2184	38.975421	121414.57 *
50-54	2018	1162.8591	110.046521	105669.77 *
50-54	2019	1118.6694	107.613211	103952.79 *
50-54	2020	1079.5160	105.233706	102582.72 *
50-54	2021	982.2145	96.790189	101478.73 *
50-54	2022	953.7601	94.649999	100767.05 *
50-54	2023	928.9485	92.557131	100364.88 *
50-54	2024	906.1083	90.510541	100110.80 *
50-54	2025	884.2977	88.509203	99910.25 *
50-54	2026	810.5294	81.268228	99735.09 *
50-54	2027	792.1247	79.471254	99674.37 *
50-54	2028	775.4031	77.714013	99776.49 *
50-54	2029	760.9521	75.995628	100131.03 *
50-54	2030	749.0100	74.315240	100788.21 *
50-54	2031	761.3150	74.856693	101703.00 *
50-54	2032	753.2145	73.201488	102896.06 *
50-54	2033	746.5861	71.582883	104296.73 *
50-54	2034	740.5603	70.000067	105794.23 *
50-54	2035	734.5394	68.452250	107306.83 *
50-54	2036	723.1286	66.448908	108824.76 *
50-54	2037	716.8101	64.979613	110313.07 *
50-54	2038	710.3478	63.542806	111790.44 *
50-54	2039	704.0245	62.137770	113300.57 *
55-59	2018	1931.4534	175.558729	110017.51 *
55-59	2019	1877.6158	171.676836	109369.20 *
55-59	2020	1819.8620	167.880778	108402.05 *
55-59	2021	1624.3088	151.755787	107034.39 *
55-59	2022	1563.6520	148.400217	105367.23 *
55-59	2023	1503.2384	145.118843	103586.71 *
55-59	2024	1446.9118	141.910026	101959.80 *
55-59	2025	1396.8711	138.772162	100659.32 *
55-59	2026	1271.2872	127.637659	99601.26 *
55-59	2027	1234.8766	124.815380	98936.25 *
55-59	2028	1203.1229	122.055506	98571.78 *
55-59	2029	1173.8639	119.356658	98349.26 *
55-59	2030	1145.9283	116.717485	98179.66 *
55-59	2031	1050.9917	107.168779	98068.83 *
55-59	2032	1027.5524	104.799100	98049.73 *
55-59	2033	1006.1772	102.481820	98181.04 *
55-59	2034	987.6988	100.215778	98557.22 *
55-59	2035	972.4121	97.999842	99225.88 *
55-59	2036	988.6626	98.713858	100154.39 *
55-59	2037	978.3463	96.531132	101350.34 *
55-59	2038	969.8825	94.396670	102745.41 *
55-59	2039	962.1654	92.309404	104232.65 *
60-64	2018	2861.6485	275.973373	103692.92 *
60-64	2019	2838.1574	269.871146	105167.13 *
60-64	2020	2802.4628	263.903851	106192.57 *
60-64	2021	2854.9232	267.640787	106669.96 *

Results for PHIM for Run 2 (Basic_US_OG_T1.RTF)

60-64	2022	2795.2582	261.722808	106802.24 *
60-64	2023	2727.9394	255.935686	106586.91 *
60-64	2024	2653.6214	250.276526	106027.58 *
60-64	2025	2573.4378	244.742499	105148.79 *
60-64	2026	2297.3912	221.234921	103843.97 *
60-64	2027	2212.4945	216.343052	102267.88 *
60-64	2028	2127.9761	211.559351	100585.30 *
60-64	2029	2049.2097	206.881425	99052.38 *
60-64	2030	1979.3265	202.306935	97837.80 *
60-64	2031	1802.8324	186.074666	96887.58 *
60-64	2032	1752.2972	181.960248	96301.10 *
60-64	2033	1708.0864	177.936807	95993.99 *
60-64	2034	1667.2695	174.002331	95818.80 *
60-64	2035	1628.2040	170.154853	95689.54 *
60-64	2036	1493.7607	156.234413	95610.22 *
60-64	2037	1460.9282	152.779812	95623.12 *
60-64	2038	1431.0159	149.401599	95783.17 *
60-64	2039	1405.1902	146.098083	96181.29 *
65-69	2018	4053.2355	452.223714	89628.99 *
65-69	2019	4047.7958	442.224302	91532.64 *
65-69	2020	4044.9930	432.445994	93537.53 *
65-69	2021	4264.9846	447.478815	95311.43 *
65-69	2022	4251.1767	437.584320	97151.03 *
65-69	2023	4232.6161	427.908610	98914.02 *
65-69	2024	4200.7808	418.446845	100389.83 *
65-69	2025	4151.3535	409.194296	101451.89 *
65-69	2026	4229.1967	414.988577	101911.16 *
65-69	2027	4142.6285	405.812496	102082.33 *
65-69	2028	4045.5950	396.839313	101945.42 *
65-69	2029	3938.5904	388.064542	101493.18 *
65-69	2030	3822.9873	379.483795	100741.78 *
65-69	2031	3415.3337	343.034281	99562.46 *
65-69	2032	3291.4439	335.449228	98120.48 *
65-69	2033	3168.1786	328.031892	96581.42 *
65-69	2034	3053.7090	320.778567	95196.79 *
65-69	2035	2952.4853	313.685624	94122.43 *
65-69	2036	2689.9364	288.516790	93233.27 *
65-69	2037	2615.6148	282.137208	92707.19 *
65-69	2038	2550.9038	275.898690	92457.99 *
65-69	2039	2491.4269	269.798115	92344.12 *
70-74	2018	5869.9196	831.298081	70611.49 *
70-74	2019	6017.3282	812.916710	74021.46 *
70-74	2020	6128.4797	794.941782	77093.44 *
70-74	2021	6303.5584	793.471531	79442.78 *
70-74	2022	6322.2605	775.926568	81480.14 *
70-74	2023	6323.2668	758.769552	83335.80 *
70-74	2024	6322.6904	741.991907	85212.39 *
70-74	2025	6327.5815	725.585243	87206.59 *
70-74	2026	6672.2198	750.808260	88867.16 *
70-74	2027	6654.5503	734.206652	90635.93 *
70-74	2028	6631.6085	717.972133	92365.82 *
70-74	2029	6589.7213	702.096586	93857.76 *
70-74	2030	6521.9809	686.572073	94993.39 *
70-74	2031	6649.0139	696.294085	95491.46 *
70-74	2032	6519.2070	680.897875	95744.27 *
70-74	2033	6374.5439	665.842101	95736.57 *
70-74	2034	6215.4748	651.119235	95458.32 *
70-74	2035	6043.2964	636.721915	94912.65 *
70-74	2036	5399.0292	575.564615	93804.05 *
70-74	2037	5205.2355	562.837933	92481.96 *
70-74	2038	5014.0667	550.392659	91099.81 *
70-74	2039	4838.4481	538.222570	89896.79 *
75-79	2018	8044.4365	1638.275015	49103.09 *
75-79	2019	8224.2890	1602.050054	51336.03 *
75-79	2020	8460.9527	1566.626087	54007.48 *
75-79	2021	8424.2261	1488.340776	56601.46 *
75-79	2022	8677.0270	1455.431108	59618.26 *
75-79	2023	8944.5885	1423.249128	62846.26 *
75-79	2024	9183.0230	1391.778744	65980.48 *
75-79	2025	9369.6172	1361.004222	68843.41 *
75-79	2026	9633.8697	1358.487034	70916.17 *
75-79	2027	9667.9312	1328.448647	72776.10 *
75-79	2028	9683.9635	1299.074458	74545.10 *
75-79	2029	9705.1497	1270.349781	76397.46 *
75-79	2030	9740.0657	1242.260254	78406.00 *
75-79	2031	10283.3522	1285.444086	79998.44 *
75-79	2032	10271.5305	1257.020799	81713.29 *
75-79	2033	10255.1621	1229.225997	83427.80 *

Results for PHIM for Run 2 (Basic_US_OG_T1.RTF)

75-79	2034	10213.9489	1202.045785	84971.38 *
75-79	2035	10136.7312	1175.466571	86235.81 *
75-79	2036	10332.7069	1192.111437	86675.68 *
75-79	2037	10136.5962	1165.751888	86953.29 *
75-79	2038	9924.1703	1139.975193	87056.02 *
75-79	2039	9694.8660	1114.768462	86967.53 *

Results for PHIM for Run 2 (Basic_US_OG_T1.RTF)

****
List of values created by last value brought forwards for Fixed File perm.Fixed_File_RR_STR

1. Country	US (United States)
2. Sex	F (Females)
3. Disease	STR (STR)

Age	Years	Value
10-14	2013-2039	2.4800
15-19	2013-2039	2.4800
20-24	2013-2039	2.4800
25-29	2013-2039	2.4800
30-34	2013-2039	2.4800
35-39	2013-2039	2.4800
40-44	2013-2039	2.4800
45-49	2013-2039	2.4800
50-54	2013-2039	2.4800
55-59	2013-2039	2.1300
60-64	2013-2039	2.1300
65-69	2013-2039	1.3900
70-74	2013-2039	1.3900
75-79	2013-2039	1.0600

Results for PHIM for Run 2 (Basic_US_OG_T1.RTF)

****

Osmond and Gardner Modeling of Death Rates for COD: COPD

Variable Parameter	Value
1. Country	US (United States)
2. Sex	F (Females)
3. Year of start of OG modelling	1966
4. Number 5-year periods for OG modelling	10
5. Age Range of interest	10 - 79
6. Scaling Factor for Rates	10000
7. Model Type to Save	4 (Full Age-Period-Cohort)
8. Goodness of Fit Required	1 (Analysis Printed For Age-Period-Cohort Model)
9. Cause of Death	COPD
Note:	Death rates are per million population

Matrix of Numbers of Deaths

Age	1966-	1971-	1976-	1981-	1986-	1991-	1996-	2001-	2006-	2011-
10-14	208	234	141	137	156	158	184	147	121	145
15-19	289	330	246	174	193	226	193	137	147	106
20-24	351	439	264	218	222	229	225	206	167	180
25-29	422	418	306	244	291	311	361	235	244	265
30-34	476	546	350	312	399	485	459	404	346	379
35-39	739	786	503	443	543	719	822	806	615	610
40-44	1310	1322	890	727	894	1161	1532	1663	1426	1349
45-49	2102	2374	1745	1547	1729	2063	2526	3110	3728	3384
50-54	3004	3730	3522	3316	3659	4098	4861	5567	7261	8709
55-59	4137	5660	6143	6939	7667	7973	8967	10527	11786	15438
60-64	5018	7514	9570	11813	14684	15600	15963	18178	20593	23149
65-69	5642	9094	12551	17589	22836	27645	28239	28404	31604	36503
70-74	6038	9434	13627	21278	30448	38863	44451	43923	43493	49893
75-79	5976	8610	12383	20055	32290	44116	55219	60279	58168	58954

Matrix of Age- and Period-Specific Mortality Rates

Age	1966-	1971-	1976-	1981-	1986-	1991-	1996-	2001-	2006-	2011-
10-14	4.154	4.593	3.011	3.204	3.714	3.553	3.771	2.820	2.351	2.855
15-19	6.288	6.526	4.743	3.626	4.364	5.169	4.126	2.688	2.746	1.988
20-24	8.833	9.427	5.121	4.119	4.534	5.046	4.916	4.224	3.203	3.250
25-29	13.049	10.398	6.449	4.643	5.433	6.217	7.597	4.911	4.868	4.929
30-34	16.542	16.640	8.526	6.443	7.456	8.835	8.827	8.182	7.039	7.364
35-39	25.125	27.274	15.248	10.747	11.165	13.355	14.681	15.156	12.220	12.168
40-44	41.420	45.139	30.782	21.944	21.599	23.900	28.291	29.496	26.666	26.557
45-49	67.688	76.095	59.842	53.639	52.691	50.526	51.972	57.378	66.210	63.333
50-54	105.662	123.047	114.247	114.701	128.393	126.399	119.065	114.366	134.326	155.699
55-59	156.412	206.187	207.200	230.062	272.149	286.884	280.469	259.929	244.696	289.545
60-64	211.941	300.706	362.877	413.481	506.549	575.484	588.562	581.365	520.285	493.207
65-69	284.954	412.129	529.099	700.556	845.505	1012.695	1095.256	1090.523	1053.682	962.465
70-74	388.221	547.598	692.099	998.861	1350.290	1603.925	1795.320	1853.790	1809.895	1789.533
75-79	521.544	669.088	854.344	1189.291	1780.416	2305.201	2638.020	2819.130	2809.561	2780.044

Matrix of Log-Rates

Age	1966-	1971-	1976-	1981-	1986-	1991-	1996-	2001-	2006-	2011-
10-14	0.619	0.662	0.479	0.506	0.570	0.551	0.576	0.450	0.371	0.456
15-19	0.798	0.815	0.676	0.559	0.640	0.713	0.615	0.429	0.439	0.298
20-24	0.946	0.974	0.709	0.615	0.656	0.703	0.692	0.626	0.506	0.512
25-29	1.116	1.017	0.809	0.667	0.735	0.794	0.881	0.691	0.687	0.693
30-34	1.219	1.221	0.931	0.809	0.873	0.946	0.946	0.913	0.847	0.867
35-39	1.400	1.436	1.183	1.031	1.048	1.126	1.167	1.181	1.087	1.085
40-44	1.617	1.655	1.488	1.341	1.334	1.378	1.452	1.470	1.426	1.424
45-49	1.831	1.881	1.777	1.729	1.722	1.704	1.716	1.759	1.821	1.802
50-54	2.024	2.090	2.058	2.060	2.109	2.102	2.076	2.058	2.128	2.192
55-59	2.194	2.314	2.316	2.362	2.435	2.458	2.448	2.415	2.389	2.462
60-64	2.326	2.478	2.560	2.616	2.705	2.760	2.770	2.764	2.716	2.693
65-69	2.455	2.615	2.724	2.845	2.927	3.005	3.040	3.038	3.023	2.983
70-74	2.589	2.738	2.840	3.000	3.130	3.205	3.254	3.268	3.258	3.253
75-79	2.717	2.825	2.932	3.075	3.251	3.363	3.421	3.450	3.449	3.444

Results for PHIM for Run 2 (Basic_US_OG_T1.RTF)

Fitting the Age, Period, Cohort Models

**Model**	**RSS**	**MRSS**	**DF**	**Factor**	**%Account**	**ChiSq**	**P**
Age Only	24747.841	194.865	127	P, C	97.4030	140866.699	0.0000
Age-Period	7787.613	66.561	117	Cohort	91.7470	42603.450	0.0000
Age-Cohort	945.798	9.094	104	Period	32.0454	5045.375	0.0000
Period-Cohort	873.751	8.090	108	Age	26.4420	4695.950	0.0000
Full Age-Period-Cohort	642.714	6.695	96			3428.026	0.0000

**Key to terms:**		
	RSS =	residual sum of squares
	MRSS =	mean RSS (MRSS/DF)
	DF =	degrees of freedom
	Factor =	Factors not included in the model
	% Account =	1- (RSS for full model)/(RSS for model in question)
	Chisq =	chi-squared value for model
	P =	probability value based on Chisq and DF.

**Age**	**Value**	**Log10 Value**
10-	4.260117	0.629422
15-	5.052684	0.703522
20-	5.947052	0.774302
25-	7.072766	0.849589
30-	9.147724	0.961313
35-	14.330545	1.156263
40-	26.475717	1.422848
45-	53.699364	1.729969
50-	112.927047	2.052798
55-	232.146594	2.365762
60-	460.500378	2.663230
65-	858.571698	2.933777
70-	1498.42577	3.175635
75-	2400.46289	3.380295

**Period**	**Value**	**Log10 Value**
1966	0.989537	-0.004568
1971	1.003624	0.001571
1976	0.880373	-0.055333
1981	0.891049	-0.050098
1986	0.967917	-0.014162
1991	1.024402	0.010470
1996	1.047092	0.019985
2001	1.033143	0.014161
2006	1.008939	0.003865
2011	1.017319	0.007457

**Cohort**	**Value**	**Log10 Value**
1891	0.219565	-0.658436
1896	0.271058	-0.566938
1901	0.375321	-0.425597
1906	0.521571	-0.282687
1911	0.733841	-0.134398
1916	0.925924	-0.033425
1921	1.041798	0.017783
1926	1.147204	0.059641
1931	1.195258	0.077462
1936	1.187150	0.074505
1941	1.188380	0.074955
1946	1.090496	0.037624
1951	1.029424	0.012594
1956	1.158468	0.063884
1961	1.229270	0.089647
1966	1.045778	0.019440
1971	0.923981	-0.034337
1976	0.815251	-0.088709
1981	0.749825	-0.125040
1986	0.647818	-0.188547
1991	0.567879	-0.245744
1996	0.465240	-0.332323
2001	0.658729	-0.181293

Results for PHIM for Run 2 (Basic_US_OG_T1.RTF)

Model: Full Age-Period-Cohort

Basic Analysis Using OG Modelling T1 on US
Fitting the Full Age-Period-Cohort Model
Matrix of observed, expected, and residual rates

**Age**		**1966-**	**1971-**	**1976-**	**1981-**	**1986-**	**1991-**	**1996-**	**2001-**	**2006-**	**2011-**	
10-	Observed	4.154	4.593	3.011	3.204	3.714	3.553	3.771	2.820	2.351	2.855	
	Expected	4.884	5.256	3.922	3.507	3.362	3.272	2.890	2.499	2.000	2.855	
	Residual	0.729	0.663	0.911	0.304	-0.352	-0.280	-0.881	-0.321		-0.351	0.000
15-	Observed	6.288	6.526	4.743	3.626	4.364	5.169	4.126	2.688	2.746	1.988	
	Expected	5.147	5.875	5.468	4.708	4.519	4.220	3.967	3.382	2.895	2.391	
	Residual	-1.141	-0.651	0.725	1.083	0.155	-0.950	-0.159	0.694	0.149	0.403	
20-	Observed	8.833	9.427	5.121	4.119	4.534	5.046	4.916	4.224	3.203	3.250	
	Expected	6.417	6.144	6.065	6.514	6.020	5.629	5.077	4.607	3.887	3.436	
	Residual	-2.416	-3.282	0.944	2.395	1.486	0.583	0.161	0.383	0.684	0.186	
25-	Observed	13.049	10.398	6.449	4.643	5.433	6.217	7.597	4.911	4.868	4.929	
	Expected	8.317	7.741	6.410	7.301	8.415	7.577	6.843	5.957	5.351	4.661	
	Residual	-4.732	-2.657	-0.039	2.658	2.983	1.360	-0.754	1.046	0.483	-0.268	
30-	Observed	16.542	16.640	8.526	6.443	7.456	8.835	8.827	8.182	7.039	7.364	
	Expected	10.746	10.910	8.782	8.391	10.257	11.519	10.017	8.732	7.524	6.978	
	Residual	-5.796	-5.730	0.256	1.948	2.801	2.684	1.190	0.551	0.486	-0.386	
35-	Observed	25.125	27.274	15.248	10.747	11.165	13.355	14.681	15.156		12.220	12.168
	Expected	16.949	17.074	14.993	13.925	14.279	17.007	18.446	15.483		13.360	11.885
	Residual	-8.175	-10.199	-0.255	3.178	3.114	3.652	3.764	0.327	1.139	-0.282	
40-	Observed	41.420	45.139	30.782	21.944	21.599	23.900	28.291	29.496		26.666	26.557
	Expected	30.055	31.760	27.671	28.035	27.945	27.920	32.116	33.624		27.935	24.887
	Residual	-11.364	-13.379	-3.111	6.091	6.347	4.020	3.825	4.129	1.269	-1.670	
45-	Observed	67.688	76.095	59.842	53.639	52.691	50.526	51.972	57.378		66.210	63.333
	Expected	55.359	61.827	56.506	56.804	61.768	59.988	57.883	64.271		66.601	57.130
	Residual	-12.330	-14.267	-3.336	3.164	9.076	9.462	5.911	6.893	0.391	-6.202	
50-	Observed	105.662	123.047	114.247	114.701	128.393	126.399	119.065	114.366		134.326	155.699
	Expected	103.468	118.074	114.053	120.271	129.760	137.475	128.946	120.103		131.992	141.222
	Residual	-2.195	-4.973	-0.194	5.570	1.367	11.076	9.880	5.736	-2.335	-14.477	
55-	Observed	156.412	206.187	207.200	230.062	272.149	286.884	280.469	259.929		244.696	289.545
	Expected	168.576	215.729	212.918	237.304	268.573	282.318	288.870	261.545		241.113	273.592
	Residual	12.164	9.542	5.718	7.242	-3.576	-4.567	8.400	1.616	-3.583	-15.953	
60-	Observed	211.941	300.706	362.877	413.481	506.549	575.484	588.562	581.365		520.285	493.207
	Expected	237.671	339.159	375.381	427.479	511.339	563.848	572.427	565.387		506.663	482.260
	Residual	25.729	38.453	12.504	13.999	4.790	-11.636	-16.135	-15.978		-13.622	-10.947
65-	Observed	284.954	412.129	529.099	700.556	845.505	1012.695	1095.256	1090.523		1053.682	962.465
	Expected	318.869	449.429	554.683	708.359	865.761	1008.992	1074.541	1053.034		1029.430	952.485
	Residual	33.915	37.300	25.584	7.803	20.256	-3.703	-20.715	-37.488		-24.252	-9.981
70-	Observed	388.221	547.598	692.099	998.861	1350.290	1603.925	1795.320	1853.790		1809.895	1789.533
	Expected	401.911	564.429	688.042	979.803	1342.915	1599.149	1799.951	1850.365		1794.757	1811.539
	Residual	13.690	16.831	-4.057	-19.058	-7.375	-4.775	4.631	-3.425		-15.137	22.006
75-	Observed	521.544	669.088	854.344	1189.291	1780.416	2305.201	2638.020	2819.130		2809.561	2780.044
	Expected	521.544	653.023	793.167	1115.604	1705.040	2276.883	2618.565	2845.091		2894.822	2899.064
	Residual	0.000	-16.065	-61.177	-73.688	-75.376	-28.318	-19.456	25.961		85.261	119.020

Results for PHIM for Run 2 (Basic_US_OG_T1.RTF)

Fitting the Full Age-Period-Cohort Model

Matrix of observed and expected deaths and (O-E)2/E Values**

Age		1966-	1971-	1976-	1981-	1986-	1991-	1996-	2001-	2006-	2011-	Total
10-	Observed	208.0	234.0	141.0	137.0	156.0	158.0	184.0	147.0	121.0	145.0	1631.0
	Expected	244.5	267.8	183.6	150.0	141.2	145.5	141.0	130.3	102.9	145.0	1651.8
	Difference	-36.5	-33.8	-42.6	-13.0	14.8	12.5	43.0	16.7	18.1	-0.0	-20.8
	Chi-Sq	5.5	4.3	9.9	1.1	1.5	1.1	13.1	2.1	3.2	0.0	41.8
15-	Observed	289.0	330.0	246.0	174.0	193.0	226.0	193.0	137.0	147.0	106.0	2041.0
	Expected	236.6	297.1	283.6	226.0	199.8	184.5	185.6	172.3	155.0	127.5	2067.9
	Difference	52.4	32.9	-37.6	-52.0	-6.8	41.5	7.4	-35.3	-8.0	-21.5	-26.9
	Chi-Sq	11.6	3.6	5.0	11.9	0.2	9.3	0.3	7.2	0.4	3.6	53.4
20-	Observed	351.0	439.0	264.0	218.0	222.0	229.0	225.0	206.0	167.0	180.0	2501.0
	Expected	255.0	286.1	312.7	344.8	294.8	255.5	232.4	224.7	202.7	190.3	2598.8
	Difference	96.0	152.9	-48.7	-126.8	-72.8	-26.5	-7.4	-18.7	-35.7	-10.3	-97.8
	Chi-Sq	36.1	81.7	7.6	46.6	18.0	2.7	0.2	1.5	6.3	0.6	201.3
25-	Observed	422.0	418.0	306.0	244.0	291.0	311.0	361.0	235.0	244.0	265.0	3097.0
	Expected	269.0	311.2	304.2	383.7	450.8	379.0	325.2	285.1	268.2	250.6	3226.9
	Difference	153.0	106.8	1.8	-139.7	-159.8	-68.0	35.8	-50.1	-24.2	14.4	-129.9
	Chi-Sq	87.1	36.7	0.0	50.9	56.6	12.2	3.9	8.8	2.2	0.8	259.2
30-	Observed	476.0	546.0	350.0	312.0	399.0	485.0	459.0	404.0	346.0	379.0	4156.0
	Expected	309.2	358.0	360.5	406.3	548.9	632.4	520.9	431.2	369.9	359.2	4296.4
	Difference	166.8	188.0	-10.5	-94.3	-149.9	-147.4	-61.9	-27.2	-23.9	19.8	-140.4
	Chi-Sq	90.0	98.7	0.3	21.9	40.9	34.3	7.4	1.7	1.5	1.1	297.9
35-	Observed	739.0	786.0	503.0	443.0	543.0	719.0	822.0	806.0	615.0	610.0	6586.0
	Expected	498.5	492.1	494.6	574.0	694.4	915.6	1032.8	823.4	672.3	595.9	6793.6
	Difference	240.5	293.9	8.4	-131.0	-151.4	-196.6	-210.8	-17.4	-57.3	14.1	-207.6
	Chi-Sq	116.0	175.6	0.1	29.9	33.0	42.2	43.0	0.4	4.9	0.3	445.4
40-	Observed	1310.0	1322.0	890.0	727.0	894.0	1161.0	1532.0	1663.0	1426.0	1349.0	12274.0
	Expected	950.6	930.2	800.0	928.8	1156.7	1356.3	1739.1	1895.8	1493.9	1264.2	12515.5
	Difference	359.4	391.8	90.0	-201.8	-262.7	-195.3	-207.1	-232.8	-67.9	84.8	-241.5
	Chi-Sq	135.9	165.1	10.1	43.8	59.7	28.1	24.7	28.6	3.1	5.7	504.7
45-	Observed	2102.0	2374.0	1745.0	1547.0	1729.0	2063.0	2526.0	3110.0	3728.0	3384.0	24308.0
	Expected	1719.1	1928.9	1647.7	1638.3	2026.8	2449.3	2813.3	3483.6	3750.0	3052.6	24509.7
	Difference	382.9	445.1	97.3	-91.3	-297.8	-386.3	-287.3	-373.6	-22.0	331.4	-201.7
	Chi-Sq	85.3	102.7	5.7	5.1	43.8	60.9	29.3	40.1	0.1	36.0	409.0
50-	Observed	3004.0	3730.0	3522.0	3316.0	3659.0	4098.0	4861.0	5567.0	7261.0	8709.0	47727.0
	Expected	2941.6	3579.2	3516.0	3477.0	3698.0	4457.1	5264.4	5846.2	7134.8	7899.2	47813.6
	Difference	62.4	150.8	6.0	-161.0	-39.0	-359.1	-403.4	-279.2	126.2	809.8	-86.6
	Chi-Sq	1.3	6.3	0.0	7.5	0.4	28.9	30.9	13.3	2.2	83.0	174.0
55-	Observed	4137.0	5660.0	6143.0	6939.0	7667.0	7973.0	8967.0	10527.0	11786.0	15438.0	85237.0
	Expected	4458.7	5921.9	6312.5	7157.4	7566.3	7846.1	9235.6	10592.5	11613.4	14587.4	85291.9
	Difference	-321.7	-261.9	-169.5	-218.4	100.7	126.9	-268.6	-65.5	172.6	850.6	-54.9
	Chi-Sq	23.2	11.6	4.6	6.7	1.3	2.1	7.8	0.4	2.6	49.6	109.8
60-	Observed	5018.0	7514.0	9570.0	11813.0	14684.0	15600.0	15963.0	18178.0	20593.0	23149.0	142082.0
	Expected	5627.2	8474.9	9899.8	12212.9	14822.9	15284.6	15525.4	17678.4	20053.8	22635.2	142215.0
	Difference	-609.2	-960.9	-329.8	-399.9	-138.9	315.4	437.6	499.6	539.2	513.8	-133.0
	Chi-Sq	65.9	108.9	11.0	13.1	1.3	6.5	12.3	14.1	14.5	11.7	259.4
65-	Observed	5642.0	9094.0	12551.0	17589.0	22836.0	27645.0	28239.0	28404.0	31604.0	36503.0	220107.0
	Expected	6313.5	9917.1	13157.9	17784.9	23383.1	27543.9	27704.9	27427.6	30876.6	36124.5	220233.9
	Difference	-671.5	-823.1	-606.9	-195.9	-547.1	101.1	534.1	976.4	727.4	378.5	-126.9
	Chi-Sq	71.4	68.3	28.0	2.2	12.8	0.4	10.3	34.8	17.1	4.0	249.2

Results for PHIM for Run 2 (Basic_US_OG_T1.RTF)

70-	Observed	6038.0	9434.0	13627.0	21278.0	30448.0	38863.0	44451.0	43923.0	43493.0	49893.0	301448.0
	Expected	6250.9	9724.0	13547.1	20872.0	30281.7	38747.3	44565.7	43841.9	43129.2	50506.5	301466.3
	Difference	-212.9	-290.0	79.9	406.0	166.3	115.7	-114.7	81.1	363.8	-613.5	-18.3
	Chi-Sq	7.3	8.6	0.5	7.9	0.9	0.3	0.3	0.2	3.1	7.5	36.5
75-	Observed	5976.0	8610.0	12383.0	20055.0	32290.0	44116.0	55219.0	60279.0	58168.0	58954.0	356050.0
	Expected	5976.0	8403.3	11496.3	18812.4	30923.0	43574.1	54811.8	60834.1	59933.2	61478.0	356242.0
	Difference	-0.0	206.7	886.7	1242.6	1367.0	541.9	407.2	-555.1	-1765.2	-2524.0	-192.0
	Chi-Sq	0.0	5.1	68.4	82.1	60.4	6.7	3.0	5.1	52.0	103.6	386.4
Total over ages	Observed	35712.0	50491.0	62241.0	84792.0	116011.0	143647.0	164002.0	173586.0	179699.0	199064.0	1209245.0
	Expected	36050.5	50891.6	62316.6	84968.6	116188.2	143771.1	164097.9	173666.9	179756.0	199216.0	1210923.4
	Difference	-338.5	-400.6	-75.6	-176.6	-177.2	-124.1	-95.9	-80.9	-57.0	-152.0	-1678.4
	Chi-Sq	736.5	877.3	151.2	330.6	330.9	235.9	186.6	158.3	113.2	307.4	3428.0

Results for PHIM for Run 2 (Basic_US_OG_T1.RTF)

****

Osmond and Gardner Extrapolating Death Rates for COD: COPD

Variable Parameter	Value
1. Country	US (United States)
2. Sex	F (Females)
3. Year of start of OG modelling	1966
4. Number 5-year periods for OG modelling	10
5. Age Range of interest	10 - 79
6. Scaling Factor for Rates	10000
7. Model Type to Save	4 (Full Age-Period-Cohort)
8. Goodness of Fit Required	1 (Analysis Printed For Age-Period-Cohort Model)
9. Cause of Death	COPD
Note:	Death rates are per million population
10. Number of Periods into the future to Predict	5
11. Earliest projected year	2016
12. Extrapolate Period using (1: last 2 points 2: linear regression)	1
13. Ratio of last two period values	1.00831

Predictions of rates for future years from model:	Full Age-Period-Cohort
Effects for extending model to project rates for:	2016-2040

Extrapolating Model: Full Age-Period-Cohort
Log Transform Parameters

Model	ChiSq	MChiSq	DF	Factor	%Account	P
Age Only	3651.845	260.846	14	P, C	35.5096	0.0000
Age-Period	2604.224	186.016	14	Cohort	9.5665	0.0000
Age-Cohort	2712.586	193.756	14	Period	13.1791	0.0000
Period-Cohort	1228.435	87.745	14	Age	-91.7147	0.0000
Full Age-Period-Cohort	2355.091	168.221	14			0.0000

**Key to terms:**		
	Chisq =	chi-squared value for model
	MChisq =	mean Chi-squared (Chisq/DF)
	DF =	degrees of freedom
	Factor =	Factors not included in the model
	% Account =	1- (Chisq for full model)/(Chisq for model in question)
	P =	probability value based on Chisq and DF.

AGE	EFFECT
10	4.260117
15	5.052684
20	5.947052
25	7.072766
30	9.147724
35	14.330545
40	26.475717
45	53.699364
50	112.927047
55	232.146594
60	460.500378
65	858.571698
70	1498.42577
75	2400.46289

PERIOD	EFFECT	
Period Change =	1.008306	
1966	0.989537	
1971	1.003624	
1976	0.880373	
1981	0.891049	
1986	0.967917	
1991	1.024402	
1996	1.047092	
2001	1.033143	
2006	1.008939	
2011	1.017319	
2016	1.025769	
2021	1.034289	
2026	1.042880	
2031	1.051542	
2036	1.060276	
2016	1.022381	Extrapolated
2017	1.024074	Extrapolated
2018	1.025769	Extrapolated
2019	1.027468	Extrapolated

Results for PHIM for Run 2 (Basic_US_OG_T1.RTF)

2020	1.029169	Extrapolated
2021	1.030873	Extrapolated
2022	1.032580	Extrapolated
2023	1.034289	Extrapolated
2024	1.036002	Extrapolated
2025	1.037717	Extrapolated
2026	1.039435	Extrapolated
2027	1.041156	Extrapolated
2028	1.042880	Extrapolated
2029	1.044607	Extrapolated
2030	1.046336	Extrapolated
2031	1.048069	Extrapolated
2032	1.049804	Extrapolated
2033	1.051542	Extrapolated
2034	1.053283	Extrapolated
2035	1.055027	Extrapolated
2036	1.056774	Extrapolated
2037	1.058523	Extrapolated
2038	1.060276	Extrapolated
2039	1.062032	Extrapolated
2040	1.063790	Extrapolated

Results for PHIM for Run 2 (Basic_US_OG_T1.RTF)

COHORT	EFFECT	WEIGHT	ORIGINAL
1891	0.219565	1.000	
1896	0.271058	2.000	
1901	0.375321	4.000	
1906	0.521571	8.000	
1911	0.733841	16.000	
1916	0.925924	32.000	
1921	1.041798	64.000	
1926	1.147204	128.000	
1931	1.195258	256.000	
1936	1.187150	512.000	
1941	1.188380	1024.000	
1946	1.090496	2048.000	
1951	1.029424	4096.000	
1956	1.158468	8192.000	
1961	1.229270	16384.000	
1966	1.045778	32768.000	
1971	0.923981	65536.000	
1976	0.815251	131072.000	
1981	0.749825	262144.000	
1986	0.647818	524288.000	
1991	0.567879	1048576.000	
1996	0.511568	Extrapolated	0.465240
2001	0.455820	Extrapolated	0.658729
2006	0.406147	Extrapolated	
2011	0.361888	Extrapolated	
2016	0.322451	Extrapolated	
2021	0.287312	Extrapolated	
2026	0.256002	Extrapolated	

Standardizing Population: The 1976 European Standard Population

Age Range	Population, Females
All	100000
0	0
1	0
2	0
3	0
0-4	8000
5-9	7000
10-14	7000
15-19	7000
20-24	7000
25-29	7000
30-34	7000
35-39	7000
40-44	7000
45-49	7000
50-54	7000
55-59	6000
60-64	5000
65-69	4000
70-74	3000
75-79	2000
80-84	1000
85+	1000

Results for PHIM for Run 2 (Basic_US_OG_T1.RTF)

Matrix of observed and expected rates including predictions

Total over ages standardized using: The 1976 European Standard Population

		1966-	1971-	1976-	1981-	1986-	1991-	1996-	2001-	2006-	2011-	2016-	2021-	2026-	2031-	2036-
10	OBS	4.2	4.6	3.0	3.2	3.7	3.6	3.8	2.8	2.4	2.9	2.7	.	.	.	.
	EXP											1.8	1.6	1.4	1.3	1.2
15	OBS	6.3	6.5	4.7	3.6	4.4	5.2	4.1	2.7	2.7	2.0	2.5	.	.	.	.
	EXP											2.4	2.1	1.9	1.7	1.5
20	OBS	8.8	9.4	5.1	4.1	4.5	5.0	4.9	4.2	3.2	3.2	4.5	.	.	.	.
	EXP											3.1	2.8	2.5	2.3	2.0
25	OBS	13.0	10.4	6.4	4.6	5.4	6.2	7.6	4.9	4.9	4.9	5.0	.	.	.	.
	EXP											4.1	3.7	3.4	3.0	2.7
30	OBS	16.5	16.6	8.5	6.4	7.5	8.8	8.8	8.2	7.0	7.4	8.3	.	.	.	.
	EXP											6.1	5.4	4.9	4.4	3.9
35	OBS	25.1	27.3	15.2	10.7	11.2	13.4	14.7	15.2	12.2	12.2	11.0	.	.	.	.
	EXP											11.0	9.6	8.5	7.7	6.9
40	OBS	41.4	45.1	30.8	21.9	21.6	23.9	28.3	29.5	26.7	26.6	24.2	.	.	.	.
	EXP											22.1	20.5	17.9	15.8	14.4
45	OBS	67.7	76.1	59.8	53.6	52.7	50.5	52.0	57.4	66.2	63.3	57.0	.	.	.	.
	EXP											50.9	45.3	42.0	36.6	32.3
50	OBS	105.7	123.0	114.2	114.7	128.4	126.4	119.1	114.4	134.3	155.7	159.5	.	.	.	.
	EXP											121.1	107.9	96.0	89.0	77.6
55	OBS	156.4	206.2	207.2	230.1	272.1	286.9	280.5	259.9	244.7	289.5	328.0	.	.	.	.
	EXP											292.7	251.1	223.7	199.0	184.6
60	OBS	211.9	300.7	362.9	413.5	506.5	575.5	588.6	581.4	520.3	493.2	524.0	.	.	.	.
	EXP											547.2	585.5	502.2	447.4	398.1
65	OBS	285.0	412.1	529.1	700.6	845.5	1012.7	1095.3	1090.5	1053.7	962.5	884.7	.	.	.	.
	EXP											906.6	1028.7	1100.7	944.2	841.1
70	OBS	388.2	547.6	692.1	998.9	1350.3	1603.9	1795.3	1853.8	1809.9	1789.5	1590.3	.	.	.	.
	EXP											1676.1	1595.4	1810.3	1936.9	1661.5
75	OBS	521.5	669.1	854.3	1189.3	1780.4	2305.2	2638.0	2819.1	2809.6	2780.0	2567.9	.	.	.	.
	EXP											2926.2	2707.5	2577.1	2924.2	3128.7
10-79	OBS	88.8	115.7	128.9	158.9	202.8	238.2	257.5	261.5	254.8	252.2	240.4	.	.	.	.
	EXP	89.3	116.1	129.3	160.4	204.0	238.8	257.8	261.0	254.3	251.6	247.7*	242.7*	242.1*	241.0*	225.3*

Drop in overall standardized Observed and Predicted rates

comparing the last observed rate during the model fitting period to the last observed and predicted rates where an observed rate is available (2016)

Observed and Predicted %Drop = 4.644% and 1.771%

Results for PHIM for Run 2 (Basic_US_OG_T1.RTF)

Matrix of observed and expected deaths including predictions

		1966-	1971-	1976-	1981-	1986-	1991-	1996-	2001-	2006-	2011-	2016-	2021-	2026-	2031-	2036-
10-	OBS	208.0	234.0	141.0	137.0	156.0	158.0	184.0	147.0	121.0	145.0	140.0*	.	.	.	.
	EXP	244.5	267.8	183.6	150.0	141.2	145.5	141.0	130.3	113.2	100.3	91.3*	80.3*	69.9*	63.9*	59.2*
	ChiSq	5.451	4.256	9.904	1.125	1.549	1.069	13.111	2.146	0.542	19.882	25.969*	.	.	.	.
15-	OBS	289.0	330.0	246.0	174.0	193.0	226.0	193.0	137.0	147.0	106.0	130.0*	.	.	.	.
	EXP	236.6	297.1	283.6	226.0	199.8	184.5	185.6	172.3	155.0	140.2	123.1*	112.0*	98.6*	86.0*	78.7*
	ChiSq	11.618	3.648	4.988	11.949	0.234	9.341	0.297	7.249	0.408	8.346	0.383*	.	.	.	.
20-	OBS	351.0	439.0	264.0	218.0	222.0	229.0	225.0	206.0	167.0	180.0	250.0*	.	.	.	.
	EXP	255.0	286.1	312.7	344.8	294.8	255.5	232.4	224.7	202.7	190.3	173.3*	152.1*	138.4*	122.4*	107.1*
	ChiSq	36.135	81.658	7.573	46.624	17.968	2.744	0.234	1.550	6.273	0.556	33.903*	.	.	.	.
25-	OBS	422.0	418.0	306.0	244.0	291.0	311.0	361.0	235.0	244.0	265.0	287.5*	.	.	.	.
	EXP	269.0	311.2	304.2	383.7	450.8	379.0	325.2	285.1	268.2	250.6	236.8*	215.2*	189.3*	172.7*	153.0*
	ChiSq	87.058	36.667	0.011	50.872	56.618	12.211	3.947	8.789	2.185	0.826	10.863*	.	.	.	.
30-	OBS	476.0	546.0	350.0	312.0	399.0	485.0	459.0	404.0	346.0	379.0	460.0*	.	.	.	.
	EXP	309.2	358.0	360.5	406.3	548.9	632.4	520.9	431.2	369.9	359.2	335.7*	316.0*	287.5*	253.5*	231.4*
	ChiSq	89.955	98.735	0.307	21.898	40.929	34.337	7.353	1.716	1.542	1.097	46.008*	.	.	.	.
35-	OBS	739.0	786.0	503.0	443.0	543.0	719.0	822.0	806.0	615.0	610.0	575.0*	.	.	.	.
	EXP	498.5	492.1	494.6	574.0	694.4	915.6	1032.8	823.4	672.3	595.9	576.8*	537.6*	505.8*	460.9*	406.8*
	ChiSq	115.979	175.588	0.143	29.890	33.019	42.214	43.016	0.368	4.891	0.336	0.006*	.	.	.	.
40-	OBS	1310.0	1322.0	890.0	727.0	894.0	1161.0	1532.0	1663.0	1426.0	1349.0	1225.0*	.	.	.	.
	EXP	950.6	930.2	800.0	928.8	1156.7	1356.3	1739.1	1895.8	1493.9	1264.2	1118.7*	1080.9*	1007.3*	948.3*	864.5*
	ChiSq	135.903	165.070	10.116	43.847	59.663	28.121	24.668	28.581	3.082	5.692	10.106*	.	.	.	.
45-	OBS	2102.0	2374.0	1745.0	1547.0	1729.0	2063.0	2526.0	3110.0	3728.0	3384.0	2892.5*	.	.	.	.
	EXP	1719.1	1928.9	1647.7	1638.3	2026.8	2449.3	2813.3	3483.6	3750.0	3052.6	2583.5*	2284.4*	2208.3*	2059.3*	1939.1*
	ChiSq	85.279	102.715	5.742	5.084	43.765	60.937	29.335	40.067	0.130	35.978	36.949*	.	.	.	.
50-	OBS	3004.0	3730.0	3522.0	3316.0	3659.0	4098.0	4861.0	5567.0	7261.0	8709.0	8437.5*	.	.	.	.
	EXP	2941.6	3579.2	3516.0	3477.0	3698.0	4457.1	5264.4	5846.2	7134.8	7899.2	6408.5*	5424.4*	4801.6*	4647.9*	4336.5*
	ChiSq	1.323	6.350	0.010	7.458	0.411	28.929	30.908	13.337	2.232	83.016	642.381*	.	.	.	.
55-	OBS	4137.0	5660.0	6143.0	6939.0	7667.0	7973.0	8967.0	10527.0	11786.0	15438.0	17985.0*	.	.	.	.
	EXP	4458.7	5921.9	6312.5	7157.4	7566.3	7846.1	9235.6	10592.5	11613.4	14587.4	16049.4*	13022.2*	11042.5*	9793.1*	9490.5*
	ChiSq	23.216	11.587	4.553	6.666	1.341	2.053	7.810	0.405	2.564	49.596	233.448*	.	.	.	.
60-	OBS	5018.0	7514.0	9570.0	11813.0	14684.0	15600.0	15963.0	18178.0	20593.0	23149.0	27082.5*	.	.	.	.
	EXP	5627.2	8474.9	9899.8	12212.9	14822.9	15284.6	15525.4	17678.4	20053.8	22635.2	28285.0*	31103.3*	25291.7*	21507.3*	19109.0*
	ChiSq	65.947	108.941	10.985	13.097	1.301	6.509	12.335	14.119	14.495	11.662	51.120*	.	.	.	.
65-	OBS	5642.0	9094.0	12551.0	17589.0	22836.0	27645.0	28239.0	28404.0	31604.0	36503.0	39625.0*	.	.	.	.
	EXP	6313.5	9917.1	13157.9	17784.9	23383.1	27543.9	27704.9	27427.6	30876.6	36124.5	40606.7*	50739.0*	55933.3*	45657.7*	38946.1*
	ChiSq	71.421	68.310	27.992	2.158	12.800	0.371	10.296	34.761	17.137	3.966	23.733*	.	.	.	.

Results for PHIM for Run 2 (Basic_US_OG_T1.RTF)

70-	OBS	6038.0	9434.0	13627.0	21278.0	30448.0	38863.0	44451.0	43923.0	43493.0	49893.0	56055.0*	.	.	.	.
	EXP	6250.9	9724.0	13547.1	20872.0	30281.7	38747.3	44565.7	43841.9	43129.2	50506.5	59079.8*	66477.0*	83404.8*	92457.0*	75813.2*
	ChiSq	7.253	8.647	0.471	7.897	0.913	0.345	0.295	0.150	3.068	7.453	154.862*	.	.	.	.
75-	OBS	5976.0	8610.0	12383.0	20055.0	32290.0	44116.0	55219.0	60279.0	58168.0	58954.0	63547.5*	.	.	.	.
	EXP	5976.0	8403.3	11496.3	18812.4	30923.0	43574.1	54811.8	60834.1	59933.2	61478.0	72412.8*	84984.3*	96134.6*	121747.7*	135888.7*
	ChiSq	.	5.086	68.392	82.076	60.433	6.740	3.026	5.065	51.991	103.620	1085.363*	.	.	.	.
Total Deaths		35712.0	50491.0	62241.0	84792.0	116011.0	143647.0	164002.0	173586.0	179699.0	199064.0	218692.5*	.	.	.	.
Expected		36050.5	50891.6	62316.6	84968.6	116188.2	143771.1	164097.9	173666.9	179766.2	199184.0	228081.5*	256528.6*	281113.6*	299977.7*	287423.8*
Obs/Exp		0.991	0.992	0.999	0.998	0.998	0.999	0.999	1.000	1.000	0.999	0.959*	.	.	.	.

Chi Squared (Log) = **2355.1 on 14 D.F.** **P = 0.0000**

Results for PHIM for Run 2 (Basic_US_OG_T1.RTF)

****

Observed and predicted rates (5 year periods)

		10-	15-	20-	25-	30-	35-	40-	45-	50-	55-	60-	65-	70-	75-
2016-	OBS	2.7	2.5	4.5	5.0	8.3	11.0	24.2	57.0	159.5	328.0	524.0	884.7	1590.3	2567.9
	PRE	1.8	2.4	3.1	4.1	6.1	11.0	22.1	50.9	121.1	292.7	547.2	906.6	1676.1	2926.2
	RES	0.947	0.132	1.380	0.882	2.250	-0.035	2.104	6.087	38.353	35.304	-23.264	-21.918	-85.815	-358.245
2021-	PRE	1.6	2.1	2.8	3.7	5.4	9.6	20.5	45.3	107.9	251.1	585.5	1028.7	1595.4	2707.5
2026-	PRE	1.4	1.9	2.5	3.4	4.9	8.5	17.9	42.0	96.0	223.7	502.2	1100.7	1810.3	2577.1
2031-	PRE	1.3	1.7	2.3	3.0	4.4	7.7	15.8	36.6	89.0	199.0	447.4	944.2	1936.9	2924.2
2036-	PRE	1.2	1.5	2.0	2.7	3.9	6.9	14.4	32.3	77.6	184.6	398.1	841.1	1661.5	3128.7

Results for PHIM for Run 2 (Basic_US_OG_T1.RTF)

****

Observed and predicted deaths (5 year periods)

		10-	15-	20-	25-	30-	35-	40-	45-	50-	55-	60-	65-	70-	75-
2016-	OBS	140.0	130.0	250.0	287.5	460.0	575.0	1225.0	2892.5	8437.5	17985.0	27082.5	39625.0	56055.0	63547.5
	PRE	91.3	123.1	173.3	236.8	335.7	576.8	1118.7	2583.5	6408.5	16049.4	28285.0	40606.7	59079.8	72412.8
	CHI	25.969	0.383	33.903	10.863	46.008	0.006	10.106	36.949	642.381	233.448	51.120	23.733	154.862	1085.363
2021-	PRE	80.3	112.0	152.1	215.2	316.0	537.6	1080.9	2284.4	5424.4	13022.2	31103.3	50739.0	66477.0	84984.3
2026-	PRE	69.9	98.6	138.4	189.3	287.5	505.8	1007.3	2208.3	4801.6	11042.5	25291.7	55933.3	83404.8	96134.6
2031-	PRE	63.9	86.0	122.4	172.7	253.5	460.9	948.3	2059.3	4647.9	9793.1	21507.3	45657.7	92457.0	121747.7
2036-	PRE	59.2	78.7	107.1	153.0	231.4	406.8	864.5	1939.1	4336.5	9490.5	19109.0	38946.1	75813.2	135888.7

Results for PHIM for Run 2 (Basic_US_OG_T1.RTF)

****

Observed and predicted rates (single years)

		10-	15-	20-	25-	30-	35-	40-	45-	50-	55-	60-	65-	70-	75-
2016	OBS	2.7	3.0	4.3	5.0	9.6	10.2	22.4	58.8	162.6	324.0	519.7	923.7	1701.1	2710.8
	PRE	1.8	2.4	3.1	4.1	6.1	11.0	22.1	50.7	120.7	291.8	545.4	903.6	1670.6	2916.5
	RES	0.975	0.612	1.147	0.882	3.523	-0.790	0.347	8.083	41.887	32.253	-25.712	20.061	30.498	-205.743
2017	OBS	2.7	2.0	4.6	5.2	7.4	12.2	26.2	54.4	148.6	328.5	553.5	906.8	1728.0	2753.8
	PRE	1.8	2.4	3.1	4.1	6.1	11.0	22.1	50.8	120.9	292.2	546.3	905.1	1673.4	2921.3
	RES	0.955	-0.345	1.523	1.069	1.365	1.208	4.100	3.607	27.649	36.303	7.132	1.715	54.605	-167.499
2018	PRE	1.8	2.4	3.1	4.1	6.1	11.0	22.1	50.9	121.1	292.7	547.2	906.6	1676.1	2926.2
2019	PRE	1.8	2.4	3.1	4.1	6.1	11.0	22.2	51.0	121.3	293.2	548.1	908.1	1678.9	2931.0
2020	PRE	1.8	2.4	3.1	4.1	6.1	11.1	22.2	51.1	121.5	293.7	549.0	909.6	1681.7	2935.9
2021	PRE	1.6	2.1	2.8	3.7	5.4	9.6	20.5	45.1	107.6	250.3	583.6	1025.3	1590.1	2698.5
2022	PRE	1.6	2.1	2.8	3.7	5.4	9.6	20.5	45.2	107.7	250.7	584.5	1027.0	1592.8	2703.0
2023	PRE	1.6	2.1	2.8	3.7	5.4	9.6	20.5	45.3	107.9	251.1	585.5	1028.7	1595.4	2707.5
2024	PRE	1.6	2.1	2.8	3.7	5.4	9.6	20.6	45.4	108.1	251.5	586.5	1030.4	1598.0	2711.9
2025	PRE	1.6	2.1	2.8	3.8	5.4	9.6	20.6	45.4	108.3	251.9	587.4	1032.1	1600.7	2716.4
2026	PRE	1.4	1.9	2.5	3.4	4.9	8.5	17.8	41.9	95.7	223.0	500.6	1097.0	1804.3	2568.5
2027	PRE	1.4	1.9	2.5	3.4	4.9	8.5	17.9	41.9	95.9	223.3	501.4	1098.9	1807.3	2572.8
2028	PRE	1.4	1.9	2.5	3.4	4.9	8.5	17.9	42.0	96.0	223.7	502.2	1100.7	1810.3	2577.1
2029	PRE	1.4	1.9	2.5	3.4	4.9	8.5	17.9	42.1	96.2	224.1	503.1	1102.5	1813.3	2581.3
2030	PRE	1.4	1.9	2.5	3.4	4.9	8.5	17.9	42.1	96.3	224.4	503.9	1104.3	1816.3	2585.6
2031	PRE	1.3	1.7	2.3	3.0	4.4	7.7	15.8	36.5	88.7	198.4	445.9	941.0	1930.5	2914.5
2032	PRE	1.3	1.7	2.3	3.0	4.4	7.7	15.8	36.5	88.9	198.7	446.7	942.6	1933.7	2919.4
2033	PRE	1.3	1.7	2.3	3.0	4.4	7.7	15.8	36.6	89.0	199.0	447.4	944.2	1936.9	2924.2
2034	PRE	1.3	1.7	2.3	3.0	4.4	7.7	15.8	36.6	89.2	199.3	448.2	945.7	1940.1	2929.0
2035	PRE	1.3	1.7	2.3	3.0	4.4	7.7	15.9	36.7	89.3	199.7	448.9	947.3	1943.3	2933.9
2036	PRE	1.2	1.5	2.0	2.7	3.9	6.9	14.3	32.2	77.3	184.0	396.7	838.3	1656.0	3118.3
2037	PRE	1.2	1.5	2.0	2.7	3.9	6.9	14.3	32.3	77.4	184.3	397.4	839.7	1658.7	3123.5
2038	PRE	1.2	1.5	2.0	2.7	3.9	6.9	14.4	32.3	77.6	184.6	398.1	841.1	1661.5	3128.7
2039	PRE	1.2	1.5	2.0	2.7	3.9	6.9	14.4	32.4	77.7	184.9	398.7	842.5	1664.2	3133.9
2040	PRE	1.2	1.5	2.0	2.7	4.0	6.9	14.4	32.4	77.8	185.2	399.4	843.9	1667.0	3139.0

Results for PHIM for Run 2 (Basic_US_OG_T1.RTF)

****

Observed and predicted deaths (single years)

		10-	15-	20-	25-	30-	35-	40-	45-	50-	55-	60-	65-	70-	75-
2016	OBS	28.0	31.0	48.0	56.0	103.0	104.0	226.0	603.0	1777.0	3570.0	5194.0	7897.0	10834.0	12401.0
	PRE	18.1	24.6	35.1	46.1	65.1	112.1	222.5	520.1	1319.3	3214.6	5451.0	7725.5	10639.8	13342.2
	CHI	5.482	1.663	4.766	2.127	22.020	0.579	0.055	13.206	158.786	39.285	12.114	3.808	3.546	66.397
2017	OBS	28.0	21.0	52.0	59.0	81.0	126.0	264.0	554.0	1598.0	3624.0	5639.0	7953.0	11588.0	13018.0
	PRE	18.2	24.6	34.9	46.8	66.1	113.5	222.7	517.3	1300.6	3223.6	5566.3	7938.0	11221.8	13809.8
	CHI	5.288	0.527	8.345	3.163	3.346	1.367	7.661	2.606	67.980	49.744	0.949	0.028	11.949	45.399
2018	PRE	18.3	24.6	34.7	47.5	67.1	115.2	223.2	515.9	1280.1	3220.5	5674.3	8125.9	11835.4	14368.4
2019	PRE	18.4	24.6	34.4	48.0	68.1	117.1	224.3	515.3	1261.4	3206.8	5764.5	8312.2	12427.5	15046.7
2020	PRE	18.4	24.7	34.2	48.3	69.2	119.0	226.0	515.0	1246.8	3183.7	5830.4	8508.3	12964.7	15855.9
2021	PRE	16.3	22.1	30.4	43.5	61.6	104.3	210.0	454.3	1091.5	2678.7	6224.8	9772.6	12632.5	15274.0
2022	PRE	16.2	22.3	30.3	43.3	62.5	105.9	212.8	454.7	1085.7	2641.4	6242.8	9977.7	12977.9	16114.7
2023	PRE	16.1	22.5	30.4	43.0	63.4	107.5	216.0	455.8	1083.1	2601.0	6240.6	10175.6	13295.4	17015.3
2024	PRE	15.9	22.5	30.4	42.7	64.1	109.1	219.4	458.0	1082.2	2564.4	6218.1	10344.5	13617.3	17893.5
2025	PRE	15.7	22.5	30.5	42.6	64.5	110.8	222.8	461.7	1081.8	2535.9	6176.8	10471.3	13959.1	18700.8
2026	PRE	14.0	20.0	27.3	37.8	58.1	98.6	195.5	429.0	954.4	2220.7	5198.1	11180.0	16034.6	18215.1
2027	PRE	14.0	19.9	27.5	37.7	57.9	100.1	198.4	434.6	955.4	2209.5	5127.7	11217.3	16380.8	18723.8
2028	PRE	14.0	19.7	27.7	37.8	57.5	101.5	201.4	441.2	958.0	2205.0	5051.7	11220.8	16721.1	19210.7
2029	PRE	14.0	19.5	27.9	37.9	57.1	102.6	204.4	448.2	963.0	2203.7	4983.0	11189.6	17019.3	19720.6
2030	PRE	14.0	19.4	27.9	38.1	56.9	103.2	207.6	455.3	970.9	2203.5	4930.0	11125.1	17253.8	20272.6
2031	PRE	12.6	17.2	24.8	34.1	50.6	93.1	184.7	399.6	902.6	1945.2	4320.7	9369.2	18434.7	23315.8
2032	PRE	12.7	17.2	24.7	34.3	50.5	92.7	187.6	405.7	914.7	1948.1	4301.6	9248.8	18514.1	23855.0
2033	PRE	12.8	17.2	24.5	34.6	50.6	92.2	190.3	411.8	928.7	1953.9	4295.0	9118.8	18543.3	24395.9
2034	PRE	12.9	17.2	24.3	34.8	50.8	91.6	192.3	418.0	943.6	1964.7	4294.3	9002.9	18520.0	24888.4
2035	PRE	13.0	17.2	24.1	34.9	51.0	91.3	193.4	424.4	958.6	1981.3	4295.6	8916.1	18444.6	25300.6
2036	PRE	11.7	15.5	21.4	31.0	45.7	81.2	174.5	377.7	841.3	1842.4	3793.2	7816.1	15533.8	27028.5
2037	PRE	11.8	15.6	21.4	30.8	46.0	81.1	174.0	383.6	854.2	1867.4	3800.0	7784.9	15340.2	27159.9
2038	PRE	11.9	15.8	21.4	30.6	46.4	81.2	172.9	389.1	867.1	1896.3	3812.7	7776.8	15136.0	27237.0
2039	PRE	11.9	15.9	21.4	30.4	46.6	81.5	171.9	393.2	880.3	1926.9	3834.9	7780.1	14960.9	27254.4
2040	PRE	11.9	16.0	21.5	30.2	46.7	81.8	171.2	395.5	893.8	1957.9	3868.5	7787.9	14839.1	27209.2

Results for PHIM for Run 2 (Basic_US_OG_T1.RTF)

****
List of values created by O and G modelling, using percentage change in last two period parameters for Fixed File MORT

1. Country	US (United States)
2. Sex	F (Females)
3. Disease	COPD (COPD)
* Value comes from O and G Modelling.	

Age	Years	Value	Death Rate	Population
10-14	2018	18.3155	1.774823	103196.46 *
10-14	2019	18.3850	1.777761	103416.52 *
10-14	2020	18.3626	1.780705	103119.74 *
10-14	2021	16.3439	1.589280	102838.63 *
10-14	2022	16.2441	1.591911	102041.78 *
10-14	2023	16.0817	1.594547	100854.31 *
10-14	2024	15.9019	1.597187	99561.97 *
10-14	2025	15.7484	1.599831	98437.76 *
10-14	2026	13.9957	1.427851	98019.03 *
10-14	2027	13.9742	1.430215	97707.32 *
10-14	2028	13.9624	1.432583	97463.30 *
10-14	2029	13.9588	1.434955	97277.15 *
10-14	2030	13.9728	1.437330	97213.74 *
10-14	2031	12.5585	1.282818	97897.69 *
10-14	2032	12.6771	1.284942	98659.12 *
10-14	2033	12.7963	1.287070	99422.00 *
10-14	2034	12.9015	1.289201	100073.76 *
10-14	2035	12.9838	1.291335	100545.70 *
10-14	2036	11.6951	1.152517	101474.16 *
10-14	2037	11.7936	1.154426	102160.11 *
10-14	2038	11.8642	1.156337	102601.69 *
10-14	2039	11.9102	1.158251	102829.10 *
15-19	2018	24.6170	2.362466	104200.38 *
15-19	2019	24.6421	2.366377	104134.08 *
15-19	2020	24.6714	2.370295	104085.87 *
15-19	2021	22.1267	2.115490	104593.73 *
15-19	2022	22.3043	2.118993	105258.83 *
15-19	2023	22.4659	2.122501	105846.21 *
15-19	2024	22.5461	2.126015	106048.78 *
15-19	2025	22.5090	2.129535	105699.11 *
15-19	2026	20.0345	1.900611	105411.01 *
15-19	2027	19.9224	1.903758	104647.84 *
15-19	2028	19.7411	1.906910	103524.06 *
15-19	2029	19.5418	1.910067	102309.30 *
15-19	2030	19.3622	1.913230	101201.39 *
15-19	2031	17.2140	1.707559	100810.47 *
15-19	2032	17.1968	1.710386	100543.20 *
15-19	2033	17.1939	1.713218	100360.03 *
15-19	2034	17.2016	1.716054	100239.43 *
15-19	2035	17.2190	1.718896	100174.80 *
15-19	2036	15.4689	1.534115	100832.66 *
15-19	2037	15.6180	1.536655	101635.99 *
15-19	2038	15.7684	1.539200	102445.77 *
15-19	2039	15.8975	1.541748	103113.72 *
20-24	2018	34.6724	3.120722	111103.83 *
20-24	2019	34.4236	3.125889	110124.20 *
20-24	2020	34.2414	3.131065	109360.13 *
20-24	2021	30.3955	2.794477	108770.00 *
20-24	2022	30.3494	2.799104	108425.37 *
20-24	2023	30.3708	2.803738	108322.56 *
20-24	2024	30.4416	2.808381	108395.50 *
20-24	2025	30.5354	2.813030	108550.01 *
20-24	2026	27.3393	2.510631	108894.01 *
20-24	2027	27.5360	2.514788	109496.37 *
20-24	2028	27.7484	2.518952	110158.41 *
20-24	2029	27.8958	2.523122	110560.57 *
20-24	2030	27.9197	2.527300	110472.49 *
20-24	2031	24.8216	2.255617	110043.60 *
20-24	2032	24.6756	2.259351	109215.51 *
20-24	2033	24.4788	2.263092	108165.47 *
20-24	2034	24.2912	2.266839	107158.74 *
20-24	2035	24.1412	2.270592	106321.30 *
20-24	2036	21.4220	2.026505	105709.09 *
20-24	2037	21.3771	2.029860	105313.13 *
20-24	2038	21.3799	2.033221	105152.82 *
20-24	2039	21.4251	2.036587	105200.95 *
25-29	2018	47.5091	4.119980	115313.86 *
25-29	2019	48.0310	4.126801	116387.97 *

Results for PHIM for Run 2 (Basic_US_OG_T1.RTF)

25-29	2020	48.3258	4.133634	116908.75 *
25-29	2021	43.5403	3.729907	116732.89 *
25-29	2022	43.3481	3.736083	116025.62 *
25-29	2023	43.0405	3.742269	115011.70 *
25-29	2024	42.7482	3.748465	114041.82 *
25-29	2025	42.5553	3.754671	113339.59 *
25-29	2026	37.7843	3.351046	112753.72 *
25-29	2027	37.7418	3.356594	112440.79 *
25-29	2028	37.7850	3.362152	112383.37 *
25-29	2029	37.8956	3.367718	112526.12 *
25-29	2030	38.0567	3.373294	112817.52 *
25-29	2031	34.0801	3.010667	113197.88 *
25-29	2032	34.3279	3.015652	113832.48 *
25-29	2033	34.5963	3.020645	114532.86 *
25-29	2034	34.7952	3.025646	115000.73 *
25-29	2035	34.8669	3.030656	115047.44 *
25-29	2036	30.9977	2.704862	114600.09 *
25-29	2037	30.8220	2.709340	113761.95 *
25-29	2038	30.5878	2.713826	112710.93 *
25-29	2039	30.3714	2.718319	111728.46 *
30-34	2018	67.1148	6.078772	110408.56 *
30-34	2019	68.1423	6.088837	111913.53 *
30-34	2020	69.2225	6.098918	113499.65 *
30-34	2021	61.5785	5.355183	114988.57 *
30-34	2022	62.5197	5.364049	116553.27 *
30-34	2023	63.4044	5.372930	118007.13 *
30-34	2024	64.0779	5.381826	119063.44 *
30-34	2025	64.4530	5.390737	119562.53 *
30-34	2026	58.0966	4.864230	119436.43 *
30-34	2027	57.8726	4.872284	118779.22 *
30-34	2028	57.4928	4.880351	117804.55 *
30-34	2029	57.1309	4.888431	116869.57 *
30-34	2030	56.9050	4.896525	116215.13 *
30-34	2031	50.5723	4.370152	115721.99 *
30-34	2032	50.5421	4.377387	115461.72 *
30-34	2033	50.6129	4.384635	115432.31 *
30-34	2034	50.7694	4.391894	115598.01 *
30-34	2035	50.9968	4.399166	115923.69 *
30-34	2036	45.6831	3.926258	116352.68 *
30-34	2037	46.0162	3.932759	117007.50 *
30-34	2038	46.3671	3.939270	117704.81 *
30-34	2039	46.6237	3.945793	118160.59 *
35-39	2018	115.2424	11.022305	104553.79 *
35-39	2019	117.0726	11.040555	106038.72 *
35-39	2020	118.9590	11.058834	107569.20 *
35-39	2021	104.3478	9.570197	109034.08 *
35-39	2022	105.9120	9.586043	110485.67 *
35-39	2023	107.4835	9.601914	111939.64 *
35-39	2024	109.1011	9.617812	113436.46 *
35-39	2025	110.7752	9.633736	114986.80 *
35-39	2026	98.5545	8.458946	116509.21 *
35-39	2027	100.0720	8.472951	118107.58 *
35-39	2028	101.4916	8.486980	119585.02 *
35-39	2029	102.5700	8.501031	120655.96 *
35-39	2030	103.1724	8.515107	121163.90 *
35-39	2031	93.0628	7.683447	121121.11 *
35-39	2032	92.7491	7.696168	120513.37 *
35-39	2033	92.1740	7.708911	119568.09 *
35-39	2034	91.6188	7.721674	118651.53 *
35-39	2035	91.2679	7.734459	118001.61 *
35-39	2036	81.1515	6.903009	117559.56 *
35-39	2037	81.1243	6.914438	117326.00 *
35-39	2038	81.2425	6.925886	117302.74 *
35-39	2039	81.4856	6.937354	117459.22 *
40-44	2018	223.1875	22.140558	100804.83 *
40-44	2019	224.2563	22.177216	101120.12 *
40-44	2020	226.0494	22.213935	101760.17 *
40-44	2021	210.0343	20.465052	102630.72 *
40-44	2022	212.7674	20.498935	103794.37 *
40-44	2023	215.9598	20.532875	105177.56 *
40-44	2024	219.3722	20.566872	106662.88 *
40-44	2025	222.8424	20.600924	108171.06 *
40-44	2026	195.4823	17.827820	109650.15 *
40-44	2027	198.4377	17.857337	111123.89 *
40-44	2028	201.3984	17.886904	112595.46 *
40-44	2029	204.4306	17.916519	114101.74 *
40-44	2030	207.5516	17.946183	115652.24 *
40-44	2031	184.7286	15.757727	117230.49 *

Results for PHIM for Run 2 (Basic_US_OG_T1.RTF)

40-44	2032	187.6021	15.783817	118857.26 *
40-44	2033	190.2693	15.809950	120347.80 *
40-44	2034	192.2902	15.836127	121424.99 *
40-44	2035	193.4070	15.862347	121928.37 *
40-44	2036	174.5042	14.313091	121919.33 *
40-44	2037	173.9575	14.336789	121336.47 *
40-44	2038	172.9115	14.360526	120407.52 *
40-44	2039	171.8885	14.384303	119497.30 *
45-49	2018	515.8801	50.895792	101360.07 *
45-49	2019	515.2545	50.980060	101069.82 *
45-49	2020	514.9698	51.064467	100846.99 *
45-49	2021	454.2769	45.130004	100659.62 *
45-49	2022	454.6505	45.204726	100575.87 *
45-49	2023	455.7565	45.279571	100653.89 *
45-49	2024	458.0203	45.354540	100986.65 *
45-49	2025	461.6810	45.429633	101625.52 *
45-49	2026	428.9965	41.852998	102500.77 *
45-49	2027	434.6417	41.922294	103677.94 *
45-49	2028	441.2193	41.991704	105072.97 *
45-49	2029	448.2321	42.061230	106566.57 *
45-49	2030	455.3476	42.130870	108079.32 *
45-49	2031	399.5846	36.459606	109596.51 *
45-49	2032	405.6930	36.519972	111087.99 *
45-49	2033	411.7802	36.580438	112568.42 *
45-49	2034	418.0024	36.641003	114080.49 *
45-49	2035	424.3756	36.701670	115628.42 *
45-49	2036	377.7409	32.226067	117215.96 *
45-49	2037	383.6282	32.279424	118846.06 *
45-49	2038	389.0846	32.332869	120337.18 *
45-49	2039	393.2181	32.386402	121414.57 *
50-54	2018	1280.0829	121.139931	105669.77 *
50-54	2019	1261.3684	121.340501	103952.79 *
50-54	2020	1246.8048	121.541404	102582.72 *
50-54	2021	1091.5437	107.563789	101478.73 *
50-54	2022	1085.6832	107.741882	100767.05 *
50-54	2023	1083.1405	107.920269	100364.88 *
50-54	2024	1082.1873	108.098952	100110.80 *
50-54	2025	1081.8075	108.277930	99910.25 *
50-54	2026	954.4089	95.694399	99735.09 *
50-54	2027	955.4071	95.852839	99674.37 *
50-54	2028	957.9695	96.011542	99776.49 *
50-54	2029	962.9652	96.170507	100131.03 *
50-54	2030	970.8902	96.329736	100788.21 *
50-54	2031	902.5712	88.745782	101703.00 *
50-54	2032	914.6710	88.892717	102896.06 *
50-54	2033	928.6570	89.039896	104296.73 *
50-54	2034	943.5504	89.187319	105794.23 *
50-54	2035	958.6254	89.334986	107306.83 *
50-54	2036	841.3193	77.309544	108824.76 *
50-54	2037	854.2373	77.437545	110313.07 *
50-54	2038	867.1110	77.565758	111790.44 *
50-54	2039	880.2795	77.694183	113300.57 *
55-59	2018	3220.4825	292.724540	110017.51 *
55-59	2019	3206.8056	293.209202	109369.20 *
55-59	2020	3183.7104	293.694666	108402.05 *
55-59	2021	2678.7390	250.268999	107034.39 *
55-59	2022	2641.3812	250.683368	105367.23 *
55-59	2023	2601.0459	251.098422	103586.71 *
55-59	2024	2564.4334	251.514164	101959.80 *
55-59	2025	2535.9162	251.930594	100659.32 *
55-59	2026	2220.6882	222.957844	99601.26 *
55-59	2027	2209.5135	223.326993	98936.25 *
55-59	2028	2205.0187	223.696754	98571.78 *
55-59	2029	2203.6836	224.067127	98349.26 *
55-59	2030	2203.5258	224.438113	98179.66 *
55-59	2031	1945.2444	198.355013	98068.83 *
55-59	2032	1948.0856	198.683427	98049.73 *
55-59	2033	1953.9243	199.012386	98181.04 *
55-59	2034	1964.6582	199.341889	98557.22 *
55-59	2035	1981.2624	199.671938	99225.88 *
55-59	2036	1842.3595	183.951944	100154.39 *
55-59	2037	1867.4460	184.256512	101350.34 *
55-59	2038	1896.2856	184.561584	102745.41 *
55-59	2039	1926.9194	184.867161	104232.65 *
60-64	2018	5674.3080	547.222315	103692.92 *
60-64	2019	5764.5085	548.128347	105167.13 *
60-64	2020	5830.3531	549.035879	106192.57 *
60-64	2021	6224.7846	583.555541	106669.96 *

Results for PHIM for Run 2 (Basic_US_OG_T1.RTF)

60-64	2022	6242.8230	584.521730	106802.24 *
60-64	2023	6240.5519	585.489518	106586.91 *
60-64	2024	6218.0819	586.458908	106027.58 *
60-64	2025	6176.7544	587.429903	105148.79 *
60-64	2026	5198.1443	500.572571	103843.97 *
60-64	2027	5127.7255	501.401365	102267.88 *
60-64	2028	5051.7109	502.231531	100585.30 *
60-64	2029	4982.9595	503.063072	99052.38 *
60-64	2030	4930.0075	503.895990	97837.80 *
60-64	2031	4320.6676	445.946488	96887.58 *
60-64	2032	4301.6241	446.684838	96301.10 *
60-64	2033	4295.0054	447.424410	95993.99 *
60-64	2034	4294.2652	448.165207	95818.80 *
60-64	2035	4295.5726	448.907231	95689.54 *
60-64	2036	3793.2153	396.737426	95610.22 *
60-64	2037	3800.0083	397.394301	95623.12 *
60-64	2038	3812.6708	398.052264	95783.17 *
60-64	2039	3834.8569	398.711316	96181.29 *
65-69	2018	8125.8507	906.609645	89628.99 *
65-69	2019	8312.1771	908.110712	91532.64 *
65-69	2020	8508.3071	909.614263	93537.53 *
65-69	2021	9772.6141	1025.334962	95311.43 *
65-69	2022	9977.7275	1027.032601	97151.03 *
65-69	2023	10175.6122	1028.733050	98914.02 *
65-69	2024	10344.5327	1030.436315	100389.83 *
65-69	2025	10471.2797	1032.142401	101451.89 *
65-69	2026	11180.0266	1097.036534	101911.16 *
65-69	2027	11217.3463	1098.852888	102082.33 *
65-69	2028	11220.8495	1100.672250	101945.42 *
65-69	2029	11189.5685	1102.494625	101493.18 *
65-69	2030	11125.1164	1104.320016	100741.78 *
65-69	2031	9369.1795	941.035359	99562.46 *
65-69	2032	9248.7719	942.593424	98120.48 *
65-69	2033	9118.7741	944.154068	96581.42 *
65-69	2034	9002.9251	945.717296	95196.79 *
65-69	2035	8916.0589	947.283113	94122.43 *
65-69	2036	7816.1441	838.342804	93233.27 *
65-69	2037	7784.9087	839.730842	92707.19 *
65-69	2038	7776.8373	841.121177	92457.99 *
65-69	2039	7780.1197	842.513815	92344.12 *
70-74	2018	11835.4416	1676.135372	70611.49 *
70-74	2019	12427.5409	1678.910536	74021.46 *
70-74	2020	12964.7290	1681.690295	77093.44 *
70-74	2021	12632.4860	1590.136446	79442.78 *
70-74	2022	12977.9059	1592.769222	81480.14 *
70-74	2023	13295.4465	1595.406358	83335.80 *
70-74	2024	13617.3477	1598.047859	85212.39 *
70-74	2025	13959.1042	1600.693734	87206.59 *
70-74	2026	16034.5971	1804.333238	88867.16 *
70-74	2027	16380.8189	1807.320658	90635.93 *
70-74	2028	16721.1047	1810.313024	92365.82 *
70-74	2029	17019.3247	1813.310345	93857.76 *
70-74	2030	17253.7694	1816.312629	94993.39 *
70-74	2031	18434.7226	1930.510082	95491.46 *
70-74	2032	18514.1309	1933.706412	95744.27 *
70-74	2033	18543.2932	1936.908035	95736.57 *
70-74	2034	18520.0114	1940.114958	95458.32 *
70-74	2035	18444.6333	1943.327191	94912.65 *
70-74	2036	15533.8288	1655.987008	93804.05 *
70-74	2037	15340.2492	1658.728813	92481.96 *
70-74	2038	15136.0071	1661.475157	91099.81 *
70-74	2039	14960.8580	1664.226048	89896.79 *
75-79	2018	14368.4098	2926.172216	49103.09 *
75-79	2019	15046.6780	2931.017056	51336.03 *
75-79	2020	15855.8936	2935.869918	54007.48 *
75-79	2021	15273.9680	2698.511310	56601.46 *
75-79	2022	16114.6918	2702.979214	59618.26 *
75-79	2023	17015.3390	2707.454515	62846.26 *
75-79	2024	17893.4920	2711.937226	65980.48 *
75-79	2025	18700.8122	2716.427359	68843.41 *
75-79	2026	18215.1076	2568.540807	70916.17 *
75-79	2027	18723.7878	2572.793519	72776.10 *
75-79	2028	19210.6694	2577.053273	74545.10 *
75-79	2029	19720.6298	2581.320080	76397.46 *
75-79	2030	20272.6079	2585.593951	78406.00 *
75-79	2031	23315.8013	2914.531996	79998.44 *
75-79	2032	23855.0311	2919.357563	81713.29 *
75-79	2033	24395.8832	2924.191120	83427.80 *

Results for PHIM for Run 2 (Basic_US_OG_T1.RTF)

75-79	2034	24888.3949	2929.032680	84971.38 *
75-79	2035	25300.5713	2933.882256	86235.81 *
75-79	2036	27028.4667	3118.344929	86675.68 *
75-79	2037	27159.9292	3123.507948	86953.29 *
75-79	2038	27237.0386	3128.679515	87056.02 *
75-79	2039	27254.4033	3133.859645	86967.53 *

Results for PHIM for Run 2 (Basic_US_OG_T1.RTF)

****
List of values created by last value brought forwards for Fixed File perm.Fixed_File_RR_COPD

1. Country	US (United States)
2. Sex	F (Females)
3. Disease	COPD (COPD)

**Age**	**Years**	**Value**
10-14	2013-2039	4.5600
15-19	2013-2039	4.5600
20-24	2013-2039	4.5600
25-29	2013-2039	4.5600
30-34	2013-2039	4.5600
35-39	2013-2039	4.5600
40-44	2013-2039	4.5600
45-49	2013-2039	4.5600
50-54	2013-2039	4.5600
55-59	2013-2039	4.5600
60-64	2013-2039	4.5600
65-69	2013-2039	4.5600
70-74	2013-2039	4.5600
75-79	2013-2039	4.5600
