# Supplementary material for: Estimating the reduction in US mortality if cigarettes were largely replaced by e-cigarettes
Source: Arch Toxicol. 2021 Oct 22;96(1):167–76. doi: 10.1007/s00204-021-03180-3 (PMC8748352; doi:10.1007/s00204-021-03180-3)

Estimating the reduction in US mortality if cigarettes were largely replaced by e-cigarettes

Published in: Archives of Toxicology

Peter N Lee[1*], John S Fry[2], Stanley Gilliland III[3], Preston Campbell[3], Andrew R. Joyce[3]

[1]P N Lee Statistics and Computing Ltd, 17 Cedar Road, Sutton, Surrey, SM2 5DA

[2]RoeLee Statistics Ltd., 17 Cedar Road, Sutton, Surrey SM2 5DA

[3]Consilium Sciences, LLC, 7400 Beaufont Springs Drive, Suite 300, N. Chesterfield, VA 23325

[*] Author for correspondence

E-mail: Peterlee@pnlee.co.uk

ONLINE RESOURCE 8

Supplemental Figures

Description of Figures

Fig. S8.1 PHIM-predicted prevalence of tobacco use in males in the Null and Main Scenarios

Prevalences are shown by time of follow-up for three selected age groups (30-34, 50-54 and 70-74).

Fig. S8.2 PHIM-predicted prevalence of tobacco use in females in the Null and Main Scenarios

Again, prevalences are shown by time of follow-up for the three selected age groups (30-34, 50-54 and 70-74).

Fig. S8.3 Drops in deaths (hundreds)

Cumulative drops in deaths comparing the Main and Null Scenarios are shown by sex and disease over the whole follow-up period.

Fig. S8.4 PHIM-predicted prevalence of tobacco use in the Pessimistic Scenario

Prevalences are shown by sex and time of follow-up for the three selected age groups (30-34, 50-54 and 70-74).

Figures

Fig. S8.1 *PHIM-predicted prevalence of tobacco use in males in the Null and Main Scenarios*

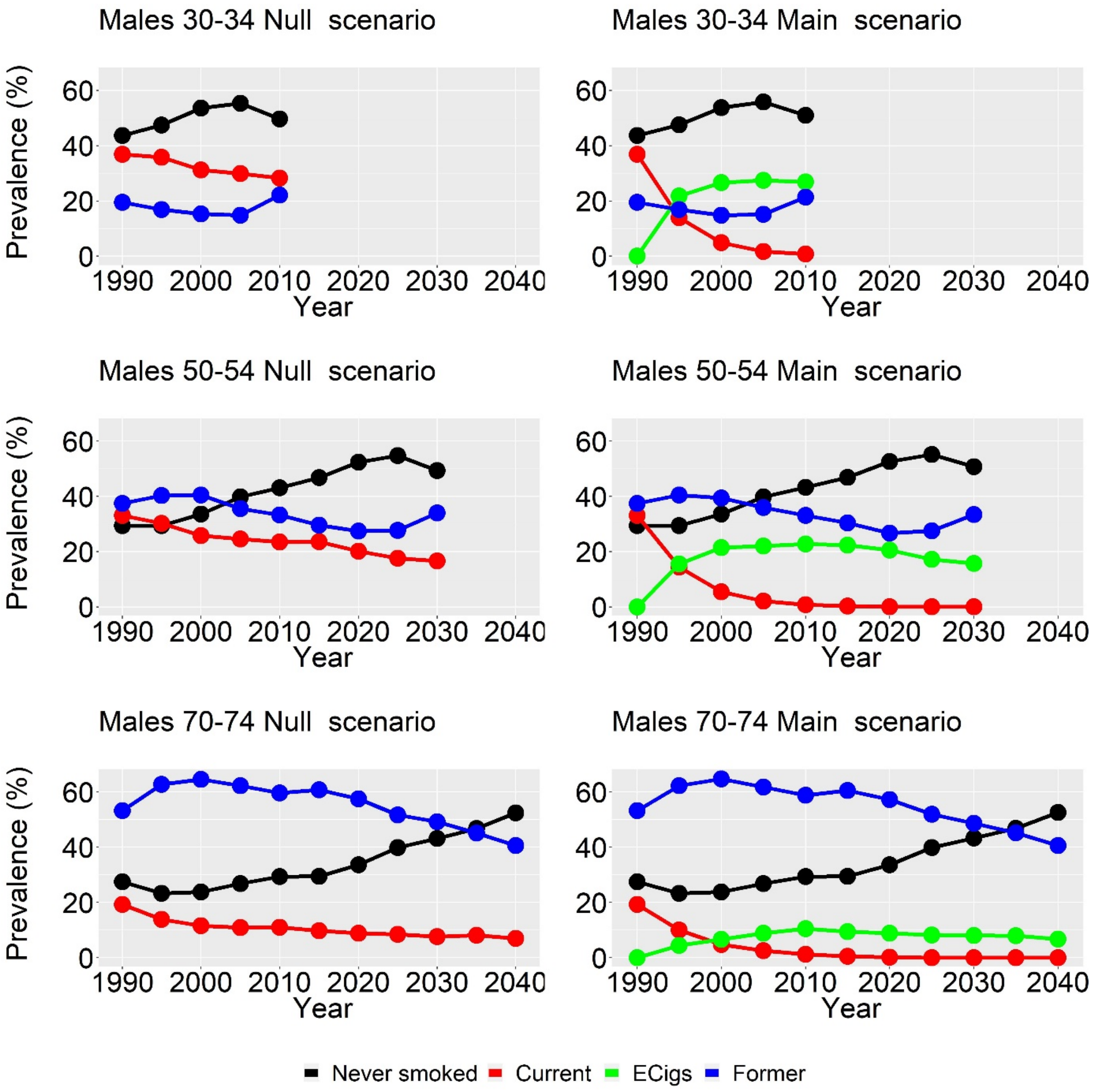

Fig. S8.2 *PHIM-predicted prevalence of tobacco use in females in the Null and Main Scenarios*

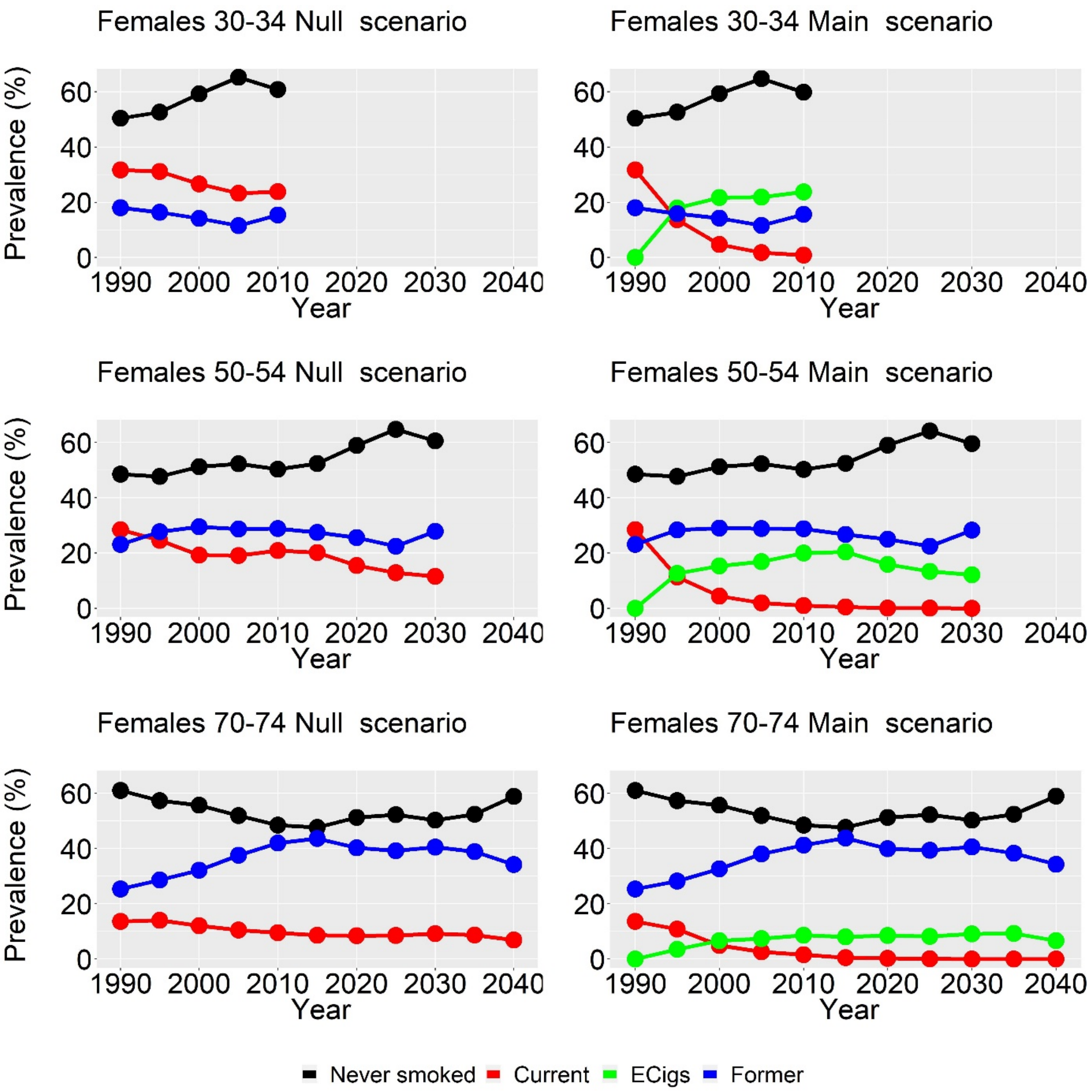

Fig. S8.3 *Drops in deaths (hundreds)*

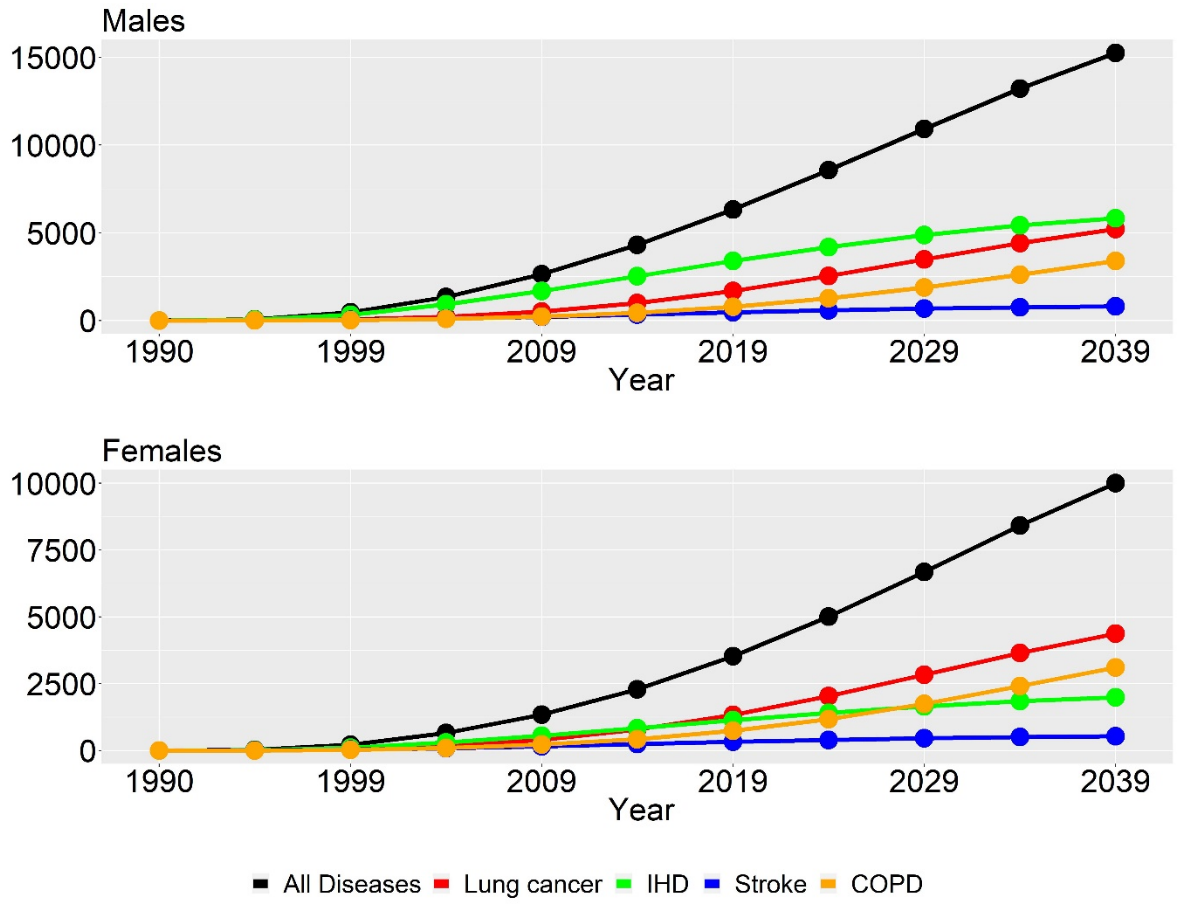

Fig. S8.4 *PHIM-predicted prevalence of tobacco use in the Pessimistic Scenario*

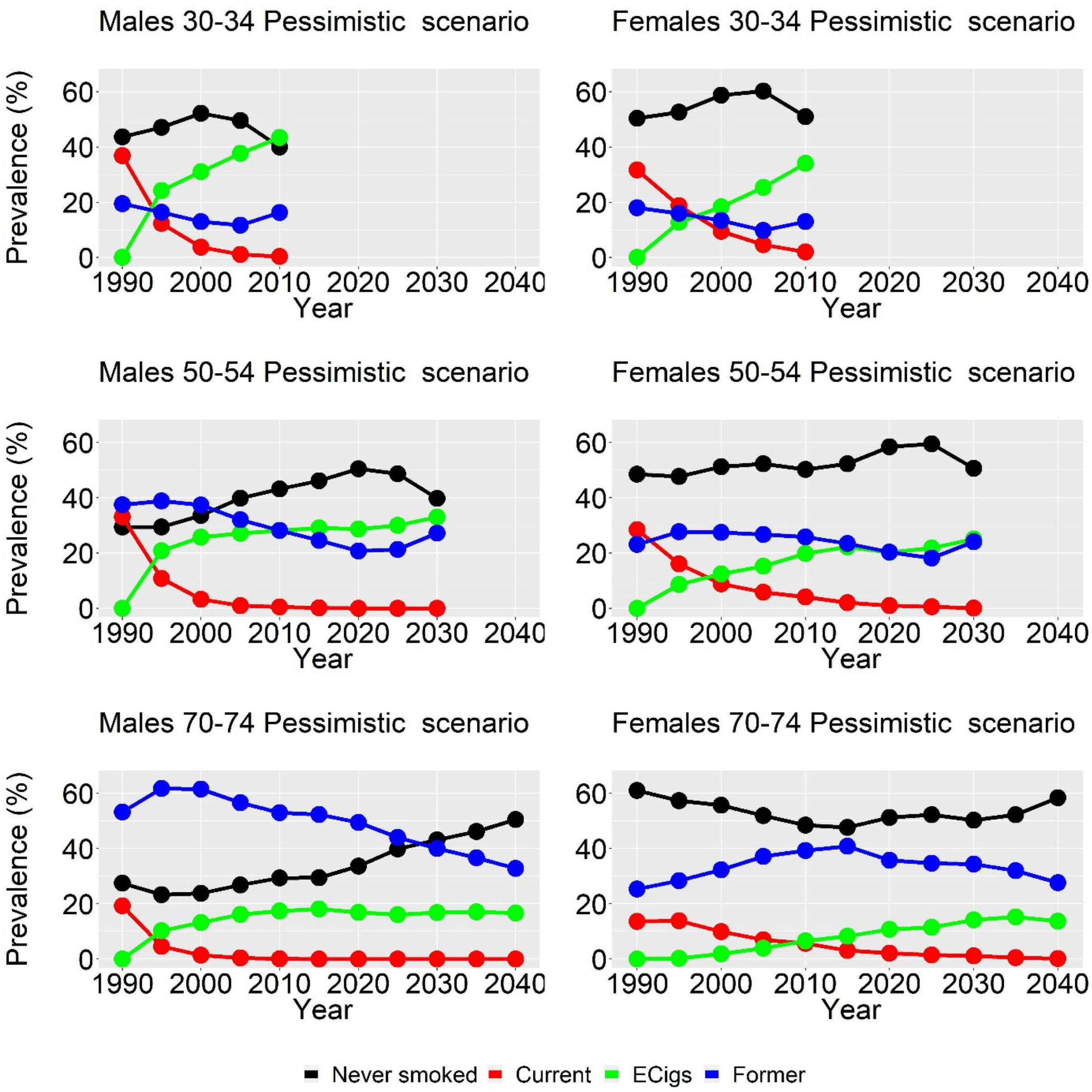

Supplement: Supplementary file 8 — Supplementary file8 (PDF 1777 KB) [file 204_2021_3180_MOESM8_ESM.pdf]
