# Supplementary material for: Estimating the reduction in US mortality if cigarettes were largely replaced by e-cigarettes
Source: Arch Toxicol. 2021 Oct 22;96(1):167–76. doi: 10.1007/s00204-021-03180-3 (PMC8748352; doi:10.1007/s00204-021-03180-3)
Supplement: Supplementary file 9 — Supplementary file9 (PDF 48 KB) [file 204_2021_3180_MOESM9_ESM.pdf]

Online Resources

Online Resource 1.pdf gives the population data used.

Online Resource 2.pdf gives the data on current and former prevalence of smoking.

Online Resource 3.pdf gives the data on age of quitting.

Online Resource 4.pdf gives tables outlining the annual initiation and quitting rates (per million) in Null Scenario by sex, age and period; assumed relative risks for continued cigarette smoking and quitting half-lives for the four diseases; distribution of product use by year in the Null, Main, Pessimistic and Sensitivity Scenarios; drops in deaths (hundreds) and differences vs. Main Scenario by sex for entire follow-up; drops in deaths (hundreds) and differences vs. Main Scenario by cause for entire follow-up; and overall years of life saved (millions) by sex for the four causes combined

Online Resource 5.pdf gives the mortality data for 1966-2017

Online Resource 6.pdf gives details of the estimation of mortality data for 2016-2040 using the age-period-cohort model.

Online Resource 7.pdf gives the full output from the runs of PHIM.

Online Resource 8.pdf gives figures presenting PHIM-predicted prevalence of tobacco use in males and females in the Null and Main Scenarios, drops in death, and PHIM-predicted prevalence of tobacco use in the Pessimistic Scenario.
