# Supplementary material for: Estimating the reduction in US mortality if cigarettes were largely replaced by e-cigarettes
Source: Arch Toxicol. 2021 Oct 22;96(1):167–76. doi: 10.1007/s00204-021-03180-3 (PMC8748352; doi:10.1007/s00204-021-03180-3)
Supplement: Supplementary file 7 — Supplementary file7 (DOCX 8210 KB) [file 204_2021_3180_MOESM7_ESM.pdf]

# Estimating the reduction in US mortality if cigarettes were largely replaced by e-cigarettes

Published in: Archives of Toxicology

Peter N Lee[1*], John S Fry[2], Stanley Gilliland III[3], Preston Campbell[3], Andrew R. Joyce[3]

[1]P N Lee Statistics and Computing Ltd, 17 Cedar Road, Sutton, Surrey, SM2 5DA

[2]RoeLee Statistics Ltd., 17 Cedar Road, Sutton, Surrey SM2 5DA

[3]Consilium Sciences, LLC, 7400 Beaufont Springs Drive, Suite 300, N. Chesterfield, VA 23325

[*] Author for correspondence

E-mail: Peterlee@pnlee.co.uk

## ONLINE RESOURCE 7

## Full output from PHIM

This gives the results from all the runs of PHIM as well as an explanation of it.

This file gives the full output from the Main Analysis, the Pessimistic Analysis and the four Sensitivity Analyses described in the paper.

## Introduction

This file provides the full computer output for all the simulations. There are six sets of detailed output, the main analysis (MA), the pessimistic analysis (PA) and then four sets of sensitivity analyses. For the MA and PA there are two runs, one for males then one for females. The four sensitivity analyses start with the values for the MA and then vary one factor. In sensitivity analysis 1 the effective dose (F) is varied from the MA value of 0.05, using values 0.4 (as in the PA), 0, 0.1, 0.2 or 0.3. This produces 10 runs, with the results for females following those for males. In sensitivity analysis 2 the switching rates (X) are varied from the MA value of 5% to 0.01%, 2.5%, 10% (as in the PA), 7.5% or 15% giving 10 runs. In sensitivity analysis 3 the MA quitting rates (Q) are multiplied by 0.5 (as in the PA), 0.75, 1.25 or 1.5 giving 8 runs. In sensitivity analysis 4 the MA initialisation rates (I) are multiplied by 0.5, 0.75, 1.25 or 1.5 (as in the PA).

For each set of output, the first page (see pages numbered 8, 123, 214, 667, 1120 and 1483 for the six sets of output) starts by giving details of when the simulations were run, the person who ran them, the version of the PHIM software that was used, and the input and output files used, followed by a table of contents. Within each set, each run starts with a page giving the control file details, which is then followed by the transition probabilities, the tobacco history output generated from the P-component, and the results for the E-component, firstly for drop in deaths and then for years of life saved. Full details of the NULL smoking prevalences are only given for the MA results, for which each run has 47 pages with a final 20 pages at the end giving details of years of life saved. For all other analyses each run has 35 pages. For the MA, the total output consists of 115 pages.

Further explanation of the output is given below, followed by the full computer output. Before that, it should be noted that the terminology on the output varies somewhat from that in the main paper. Thus:

| Terminology in paper | Terminology on output |
|---|---|
| Null Scenario | Reference Scenario or NULL Scenario |
| Alternative Scenario | Test Scenario or MRTP Scenario |
| TP | TTP |
| CC | Product 1, PR1 or CC |
| ECig | Product 2, PR2 or Ecig |
| Dual | Product 4, PR4 or Dual |

Below we use the terminology on the output.

**Control File Details**

When running the software, there are 52 variable parameters, not all relevant to all runs. Explanation of some parameters which may not be self-evident is given below.

12-13 Age limits for risk estimation. These are the limits for the baseline population. As individuals exceed 79 years of age they drop out of the analysis. The results concerning deaths or years of life lost only relate to those aged 30-79 years.

14-17 Effective doses in the Reference scenario. Only product 1 is relevant here. Current CC smokers have a dose of 1 unit.

18-21 Effective doses in the Test scenario. Current CC smokers have a dose of 1 unit, current ECig users have a dose of F units, and dual users have the dose "FROMF2" which implies the average of the doses for products 1 and 2, i.e. (1+F)/2.

23 Number of MC simulations. MC stands for Monte Carlo. Here we only have one simulation for each sex.

25-32 Files used. Parameter 26 is not used.

33-37 Assumption sets used. See section 3 below. Parameter 37 is not used.

38 Output choice file. The file determining which outputs are to be produced.

39-46 Further details of output. The user has the option to include or not include various parts of the output.

47-48 Run number for P-component and Results file for E-component. This is only relevant if results are only to be output for one of the components, so is not relevant here.

49 Components required. PE implies that both P-component and E-component results are to be produced.

50 Results folder for P to be used in E-component. This is not relevant here.

51 Fixed age of starting product use. This is the assumed age of starting for individuals whose product use history starts as a current or former user.

52 Output absolute risk file? This is an option for additional output, which is not used here.

**Transition probabilities (TPs)**

For the MA, pages 10 and 11 show the monthly TPs for the NULL Scenario and pages 13 to 16 show the monthly TTPs for the Alternative (ECig) Scenario. On pages 12 and 17 the values of special factors are shown. These factors are applied in special cases, such as Factor 1a which doubles the probability of the transition from current ECigs (PR2) to current cigaretters (PR1) when the individual has smoked current cigarettes in the past. The same factors have been applied through the analysis, but have only been shown for the MA.

For the NULL Scenario there are three TPs (initiation, cessation and re-initiation), while for the ECig Scenario there are 15 TPs, three relating each to initiation, cessation and re-initiation and six to switching. The TPs may vary by grouped age and by time period since baseline. Note that, in order to match the Levy results, TPs relating to re-initiation and dual usage have been set to zero in these simulations.

**Tobacco history output from the P-Component**

For the MA, the tobacco history output from the P-Component is then shown by time of follow-up from baseline in 1990 by five year period (0, 5, 10 … 50), and by age group (18-19, 20-24, 25-29 … 75-79, and for all ages combined).

The output on pages 18 to 29 for the first run for the NULL scenario gives results for three smoking groups: Never Any, CC (i.e. current conventional cigarettes) and former (i.e. former conventional cigarettes). The output on pages 30 to 45 for the first run for the ECig Scenario gives results for five smoking groups: never any, three current use groups (cigarettes [CC], ECig, Dual) and former (i.e. not a current user, but formerly used one or both products).

For each group, the output shows the total numbers and percentages in each group. For current CC smokers it also shows the number and percentage who have previously quit, while for former smokers it also shows the mean years quit, and the number and percentage of short term quitters.

Note that for scenarios other than the MA, when showing the prevalences for the NULL scenario only the results for the final year are shown. This is because the prevalence for the NULL scenario varies only by sex, but not by set or sensitivity analysis.

**<u>Results for drops in death from the E-Component</u>**

The results for drops in death from the E-Component are shown for all four diseases combined and then for the four diseases separately, with estimates unadjusted for differential mortality in the ECig Scenario shown on the first five pages and adjusted estimates shown on the next five pages. Each of the 10 pages for a given run (pages 46-55 and 93-102 for females in the MA) is laid out similarly, with results for all years combined shown first and then results for individual years. The results relate to the age groups 30-79 combined. The output shows the total number of deaths from the disease (or four diseases combined), followed by the number attributed to tobacco in the NULL and ECig Scenarios. The output also shows the drop in deaths associated with the ECig Scenario (compared to the NULL Scenario), expressed as a number, and as a percentage, both of the total deaths and of the attributable deaths in the NULL Scenario. Finally, the output shows the total death rate from the disease (or four diseases combined), the rate of attributable deaths in each scenario and the drop in rates. Rates are expressed per million of population.

**Results for years of life lost from the E-Component**

The final 20 pages (pages 103 to 122 for the MA) provide the full computer output for years of life lost for males and females. Each page gives data on the estimated cumulative years of life lost in the Reference (Null) Scenario and in the particular ECig Scenario of interest, as well as the difference, the cumulative years of life saved. The results are shown for the four diseases separately and combined, separately for males and females. Tables 1, 3 and 5 relate to results unadjusted for deaths in the Test Scenario, while Tables 2, 4 and 6 give the corresponding adjusted results. Tables 1 and 2 give overall results for the whole follow-up period and for ages 30-79 combined. Tables 3 and 4 give results by year of follow-up for ages 30-79 years combined. Tables 5 and 6 give results for the whole follow-up period by 5 year age group.

These 20 pages of output are repeated for every separate pair of runs (male and female). Thus for sensitivity analyses 1 and 2 there are five sets for results taking up 100 pages, while for sensitivity analyses 3 and 4 there are are four sets of results taking up 80 pages of output.

## Results for PHIM for Run 1 (Main_Analysis.RTF)

---

System Date and Time: 20210223T100622
User: JohnS
SAS Version: 9.4
Project: Population Health Impact Model 7.3

The System is running in Test Mode

Maximum Upper Age for P Component = 79
Upper Age for ELL Calculations = 75
Report in E-component: Social Classes A and B combined
Description: Main Analysis for VeneBio on US
Assumption Files:
Options Control File: C:\VeneBio\OG_Test\\US_Control_V_MA.CSV
TTP Test File: C:\VeneBio\OG_Test\\TTP_Test_MA.csv
TTP Reference File: C:\VeneBio\OG_Test\\TTP_Reference_V.csv
TTP Factor Test File: C:\VeneBio\OG_Test\\TTP_FACTOR_Test.csv
TTP Factor Reference File: C:\VeneBio\OG_Test\\TTP_FACTOR_Test.csv
TP Socioeconomic Groups: Not Used
Output Choices:C:\VeneBio\OG_Test\\OUTC_MA.csv

Main Results will be sent to the folder C:\VeneBio\Main_Results\
Plots will be sent to the folder C:\VeneBio\Plot_Results\Plots_20210223T100622
Saved P, E and/or A Component results will be sent to the folder C:\VeneBio\SAVED\Saved_20210223T100622\

# Table of Contents


**Control File Details for Run: 1** ........ 9
**TTP values for Run: 1, Scenario: NULL** ........ 10
**TTP values for Run: 1, Scenario: Ecig** ........ 13
**Main Output for the Tobacco History for Run: 1, Simulation: 1, Scenario: NULL** ........ 18
**Main Output for the Tobacco History for Run: 1, Simulation: 1, Scenario: Ecig** ........ 30
**Main Results for E-Component for Run: 1, Simulation: 1, COD: Four Diseases Combined** ........ 46
**Main Results for E-Component for Run: 1, Simulation: 1, COD: LUNG CANCER** ........ 47
**Main Results for E-Component for Run: 1, Simulation: 1, COD: IHD** ........ 48
**Main Results for E-Component for Run: 1, Simulation: 1, COD: STROKE** ........ 49
**Main Results for E-Component for Run: 1, Simulation: 1, COD: COPD** ........ 50
**Main Results for E-Component for Run: 1, Simulation: 1, COD: Four Diseases Combined, Adjusted** ........ 51
**Main Results for E-Component for Run: 1, Simulation: 1, COD: LUNG CANCER, Adjusted** ........ 52
**Main Results for E-Component for Run: 1, Simulation: 1, COD: IHD, Adjusted** ........ 53
**Main Results for E-Component for Run: 1, Simulation: 1, COD: STROKE, Adjusted** ........ 54
**Main Results for E-Component for Run: 1, Simulation: 1, COD: COPD, Adjusted** ........ 55
**Control File Details for Run: 2** ........ 56
**TTP values for Run: 2, Scenario: NULL** ........ 57
**TTP values for Run: 2, Scenario: Ecig** ........ 60
**Main Output for the Tobacco History for Run: 2, Simulation: 1, Scenario: NULL** ........ 65
**Main Output for the Tobacco History for Run: 2, Simulation: 1, Scenario: Ecig** ........ 77
**Main Results for E-Component for Run: 2, Simulation: 1, COD: Four Diseases Combined** ........ 93
**Main Results for E-Component for Run: 2, Simulation: 1, COD: LUNG CANCER** ........ 94
**Main Results for E-Component for Run: 2, Simulation: 1, COD: IHD** ........ 95
**Main Results for E-Component for Run: 2, Simulation: 1, COD: STROKE** ........ 96
**Main Results for E-Component for Run: 2, Simulation: 1, COD: COPD** ........ 97
**Main Results for E-Component for Run: 2, Simulation: 1, COD: Four Diseases Combined, Adjusted** ........ 98
**Main Results for E-Component for Run: 2, Simulation: 1, COD: LUNG CANCER, Adjusted** ........ 99
**Main Results for E-Component for Run: 2, Simulation: 1, COD: IHD, Adjusted** ........ 100
**Main Results for E-Component for Run: 2, Simulation: 1, COD: STROKE, Adjusted** ........ 101
**Main Results for E-Component for Run: 2, Simulation: 1, COD: COPD, Adjusted** ........ 102
**Estimated Loss of Life from the E Component for Run: 2, Simulation: 1** ........ 103
**Table 1: Estimated Loss of Life, at the end of follow-up, all age groups combined, Population unadjusted for deaths in Ecig scenario** ........ 103
**Table 2: Estimated Loss of Life, at the end of follow-up, all age groups combined, Population and Number deaths adjusted for Ecig scenario** ........ 104
**Table 3: Estimated Loss of Life, All age groups combined, Population unadjusted for deaths in Ecig scenario, cumulative over years** ........ 105
**Table 4: Estimated Loss of Life, All age groups combined, Population and Number deaths adjusted for Ecig scenario, cumulative over years** ........ 112
**Table 5: Estimated Loss of Life, at the end of follow-up, Population unadjusted for deaths in Ecig scenario** ........ 119
**Table 6: Estimated Loss of Life, at the end of follow-up, Population and Number deaths adjusted for Ecig scenario** ........ 121

# Results for PHIM for Run 1 (Main_Analysis.RTF)

---

------------------------------------------------------------------------------------------

**Control File Details for Run: 1**

| Variable Parameter | Value |
|---|---|
| 1. Name for Reference Scenario | NULL |
| 2. Name for Test Scenario | Ecig |
| 3. Name for Product 1 | CC |
| 4. Name for Product 2 | Ecig |
| 5. Name for Product 3 | - |
| 6. Name for Product 4 | Dual |
| 7. Country | US (United States) |
| 8. Sex | M (Males) |
| 9. Year of start of process | 1990 |
| 10. Number of months of follow-up | 600 |
| 11. Follow-up interval length (in months) | 12 |
| 12. Lower age for risk estimation | 10 |
| 13. Upper age for risk estimation | 79 |
| 14. The effective dose for Product 1 (F1) - Ref | 1 |
| 15. The effective dose for Product 2 (F2) - Ref | - |
| 16. The effective dose for Product 3 (F3) - Ref | - |
| 17. The effective dose for Product 4 (F4) - Ref | - |
| 18. The effective dose for Product 1 (F1) - Test | 1 |
| 19. The effective dose for Product 2 (F2) - Test | 0.05 |
| 20. The effective dose for Product 3 (F3) - Test | - |
| 21. The effective dose for Product 4 (F4) - Test | FROMF2 |
| 22. Number in population to be simulated | 100000 |
| 23. Number of MC simulations | 1 |
| 24. The random number seed for the first simulation | 15975263 |
| 25. Source for the population file (POP) | UN4 |
| 26. Source for the socioeconomic prevalence file (SEP) | - |
| 27. Source for the current smoking prevalence file (CSP) | ISS3 |
| 28. Source for the former smoking prevalence file (FSP) | ISS3 |
| 29. Source for the quit-time distribution file (QTD) | NHIS2006 |
| 30. Source for the death file (MORT) | APR-20 |
| 31. Source for the relative risk file (RR) | PNLEST |
| 32. Source for the half-life file (H) | PNLEST |
| 33. Assumption Set for TTP Factor Reference | F1 |
| 34. Assumption Set for TTP Factor Test | F1 |
| 35. Assumption Set for TTP Reference | ISS3 |
| 36. Assumption Set for TTP Test | ISS3 |
| 37. Assumption Set for TP for socioeconomic group | - |
| 38. Source for Output Choice file (OUTC) | BASIC |
| 39. Output for DTP P1 | N |
| 40. Output for DTP P2 | N |
| 41. Output for Main P Component | Y |
| 42. Output for DTP E1 | N |
| 43. Output for DTP E2 | N |
| 44. Output for DTP E3 | N |
| 45. Output for Main E Component | Y |
| 46. Output file name | Main_Analysis |
| 47. Run number for P-Component | - |
| 48. Results file for E-Component | - |
| 49. Components required | PE |
| 50. Results folder for P to be used in E-Component | - |
| 51. Fixed age of starting product use | 16 |
| 52. Output absolute risk file? | N |

# Results for PHIM for Run 1 (Main_Analysis.RTF)

************************************************************************************

**TTP values for Run: 1, Scenario: NULL**

| **Variable Parameter** | **Value** |
|---|---|
| 1. Country | US (United States) |
| 2. Sex | M (Males) |
| 3. Assumption Set | ISS3 |
| Note: | Period is in Months |

| Age | Period | **Initiation** Never CC | **Cessation** CC Former | **Re-initiation** Former CC |
|---|---|---|---|---|
| 10-14 | 1-12 | 0.003842 | 0.008742 | 0.000000 |
| 15-19 | 1-12 | 0.004720 | 0.003614 | 0.000000 |
| 20-24 | 1-12 | 0.000376 | 0.000943 | 0.000000 |
| 25-29 | 1-12 | 0.000187 | 0.001143 | 0.000000 |
| 30-34 | 1-12 | 0.000093 | 0.000871 | 0.000000 |
| 35-39 | 1-12 | 0.000000 | 0.000867 | 0.000000 |
| 40-44 | 1-12 | 0.000000 | 0.001399 | 0.000000 |
| 45-49 | 1-12 | 0.000000 | 0.002033 | 0.000000 |
| 50-54 | 1-12 | 0.000000 | 0.003209 | 0.000000 |
| 55-59 | 1-12 | 0.000000 | 0.003574 | 0.000000 |
| 60-64 | 1-12 | 0.000000 | 0.003460 | 0.000000 |
| 65-69 | 1-12 | 0.000000 | 0.004136 | 0.000000 |
| 70-74 | 1-12 | 0.000000 | 0.005592 | 0.000000 |
| 75-79 | 1-12 | 0.000000 | 0.005592 | 0.000000 |
| 10-14 | 13-72 | 0.004906 | 0.008742 | 0.000000 |
| 15-19 | 13-72 | 0.006989 | 0.004426 | 0.000000 |
| 20-24 | 13-72 | 0.000687 | 0.001527 | 0.000000 |
| 25-29 | 13-72 | 0.000962 | 0.001515 | 0.000000 |
| 30-34 | 13-72 | 0.000364 | 0.001227 | 0.000000 |
| 35-39 | 13-72 | 0.000000 | 0.001630 | 0.000000 |
| 40-44 | 13-72 | 0.000000 | 0.001852 | 0.000000 |
| 45-49 | 13-72 | 0.000000 | 0.002667 | 0.000000 |
| 50-54 | 13-72 | 0.000000 | 0.003208 | 0.000000 |
| 55-59 | 13-72 | 0.000000 | 0.003978 | 0.000000 |
| 60-64 | 13-72 | 0.000000 | 0.005771 | 0.000000 |
| 65-69 | 13-72 | 0.000000 | 0.010432 | 0.000000 |
| 70-74 | 13-72 | 0.000000 | 0.007079 | 0.000000 |
| 75-79 | 13-72 | 0.000000 | 0.007079 | 0.000000 |
| 10-14 | 73-132 | 0.004312 | 0.008742 | 0.000000 |
| 15-19 | 73-132 | 0.005330 | 0.003289 | 0.000000 |
| 20-24 | 73-132 | 0.002456 | 0.003844 | 0.000000 |
| 25-29 | 73-132 | 0.000839 | 0.002006 | 0.000000 |
| 30-34 | 73-132 | 0.000414 | 0.001329 | 0.000000 |
| 35-39 | 73-132 | 0.000000 | 0.001278 | 0.000000 |
| 40-44 | 73-132 | 0.000000 | 0.001691 | 0.000000 |
| 45-49 | 73-132 | 0.000000 | 0.002904 | 0.000000 |
| 50-54 | 73-132 | 0.000000 | 0.003319 | 0.000000 |
| 55-59 | 73-132 | 0.000000 | 0.003976 | 0.000000 |
| 60-64 | 73-132 | 0.000000 | 0.005235 | 0.000000 |
| 65-69 | 73-132 | 0.000000 | 0.005901 | 0.000000 |
| 70-74 | 73-132 | 0.000000 | 0.005615 | 0.000000 |
| 75-79 | 73-132 | 0.000000 | 0.005615 | 0.000000 |
| 10-14 | 133-192 | 0.003468 | 0.008742 | 0.000000 |
| 15-19 | 133-192 | 0.004675 | 0.003732 | 0.000000 |
| 20-24 | 133-192 | 0.000062 | 0.002260 | 0.000000 |
| 25-29 | 133-192 | 0.001422 | 0.002822 | 0.000000 |
| 30-34 | 133-192 | 0.000696 | 0.002610 | 0.000000 |
| 35-39 | 133-192 | 0.000000 | 0.002058 | 0.000000 |
| 40-44 | 133-192 | 0.000000 | 0.002310 | 0.000000 |
| 45-49 | 133-192 | 0.000000 | 0.002256 | 0.000000 |
| 50-54 | 133-192 | 0.000000 | 0.002663 | 0.000000 |
| 55-59 | 133-192 | 0.000000 | 0.001746 | 0.000000 |
| 60-64 | 133-192 | 0.000000 | 0.005809 | 0.000000 |
| 65-69 | 133-192 | 0.000000 | 0.005896 | 0.000000 |
| 70-74 | 133-192 | 0.000000 | 0.007323 | 0.000000 |
| 75-79 | 133-192 | 0.000000 | 0.007323 | 0.000000 |
| 10-14 | 193-252 | 0.002314 | 0.008742 | 0.000000 |
| 15-19 | 193-252 | 0.003987 | 0.005036 | 0.000000 |
| 20-24 | 193-252 | 0.000320 | 0.002857 | 0.000000 |
| 25-29 | 193-252 | 0.000251 | 0.002314 | 0.000000 |
| 30-34 | 193-252 | 0.000125 | 0.004343 | 0.000000 |
| 35-39 | 193-252 | 0.000000 | 0.002092 | 0.000000 |
| 40-44 | 193-252 | 0.000000 | 0.002566 | 0.000000 |
| 45-49 | 193-252 | 0.000000 | 0.002311 | 0.000000 |
| 50-54 | 193-252 | 0.000000 | 0.003209 | 0.000000 |

## Results for PHIM for Run 1 (Main_Analysis.RTF)

| | | | | |
|---|---|---|---|---|
| 55-59 | 193-252 | 0.000000 | 0.004057 | 0.000000 |
| 60-64 | 193-252 | 0.000000 | 0.007166 | 0.000000 |
| 65-69 | 193-252 | 0.000000 | 0.004281 | 0.000000 |
| 70-74 | 193-252 | 0.000000 | 0.004580 | 0.000000 |
| 75-79 | 193-252 | 0.000000 | 0.004580 | 0.000000 |
| 10-14 | 253+ | 0.001325 | 0.008742 | 0.000000 |
| 15-19 | 253+ | 0.002984 | 0.010602 | 0.000000 |
| 20-24 | 253+ | 0.000452 | 0.004314 | 0.000000 |
| 25-29 | 253+ | 0.000225 | 0.003645 | 0.000000 |
| 30-34 | 253+ | 0.000112 | 0.004512 | 0.000000 |
| 35-39 | 253+ | 0.000000 | 0.002147 | 0.000000 |
| 40-44 | 253+ | 0.000000 | 0.002636 | 0.000000 |
| 45-49 | 253+ | 0.000000 | 0.001051 | 0.000000 |
| 50-54 | 253+ | 0.000000 | 0.002519 | 0.000000 |
| 55-59 | 253+ | 0.000000 | 0.003693 | 0.000000 |
| 60-64 | 253+ | 0.000000 | 0.005888 | 0.000000 |
| 65-69 | 253+ | 0.000000 | 0.004823 | 0.000000 |
| 70-74 | 253+ | 0.000000 | 0.004733 | 0.000000 |
| 75-79 | 253+ | 0.000000 | 0.004733 | 0.000000 |

## Results for PHIM for Run 1 (Main_Analysis.RTF)

*******************************************************************************************

**TTP Factors**

| **Factor** | **TTP Affected** | **Relates to** | **Value** | **< or >** | **Years** |
|---|---|---|---|---|---|
| 1a | Current PR2 to Current PR1 | Previous PR1 use | 2 | - | - |
| 1b | Current PR3 to Current PR1 | Previous PR1 use | 2 | - | - |
| 1c | Current PR4 to Current PR1 | Previous PR1 use | 2 | - | - |
| 2a | Current PR1 to Former | Previously quit | 2 | - | - |
| 2b | Current PR2 to Former | Previously quit | 2 | - | - |
| 2c | Current PR3 to Former | Previously quit | 2 | - | - |
| 2d | Current PR4 to Former | Previously quit | 2 | - | - |
| 3a | Former to Current PR1 | Time quit | 2 | < | 2 |
| 3b | Former to Current PR2 | Time quit | 2 | < | 2 |
| 3c | Former to Current PR3 | Time quit | 2 | < | 2 |
| 3d | Former to Current PR4 | Time quit | 2 | < | 2 |
| 4a | Current PR2 to Current PR1 | Time PR2 use | 0 | > | 2 |
| 4b | Current PR2 to Current PR3 | Time PR2 use | 0 | > | 2 |
| 4c | Current PR2 to Current PR4 | Time PR2 use | 0 | > | 2 |
| 5a | Current PR3 to Current PR1 | Time PR3 use | 0 | > | 2 |
| 5b | Current PR3 to Current PR2 | Time PR3 use | 0 | > | 2 |
| 5c | Current PR3 to Current PR4 | Time PR3 use | 0 | > | 2 |
| 6a | Current PR4 to Current PR1 | Time PR4 use | - | - | - |
| 6b | Current PR4 to Current PR2 | Time PR4 use | - | - | - |
| 6c | Current PR4 to Current PR3 | Time PR4 use | - | - | - |
| 7a | Current PR1 to Current PR2 | Time PR1 use | - | - | - |
| 7b | Current PR1 to Current PR3 | Time PR1 use | - | - | - |
| 7c | Current PR1 to Current PR4 | Time PR1 use | - | - | - |

# Results for PHIM for Run 1 (Main_Analysis.RTF)

************************************************************************************
**TTP values for Run: 1, Scenario: Ecig**

| **Variable Parameter** | **Value** |
|---|---|
| 1. Country | US (United States) |
| 2. Sex | M (Males) |
| 3. Assumption Set | ISS3 |
| Note: | Period is in Months |

| | | **Initiation** | | | **Cessation** | | | **Re-initiation** | | |
|---|---|---|---|---|---|---|---|---|---|---|
| Age | Period | Never CC | Never Ecig | Never Dual | CC Former | Ecig Former | Dual Former | Former CC | Former Ecig | Former Dual |
| 10-14 | 1-12 | 0.001921 | 0.001921 | 0.000000 | 0.008742 | 0.008742 | 0.000000 | 0.000000 | 0.000000 | 0.000000 |
| 15-19 | 1-12 | 0.002360 | 0.002360 | 0.000000 | 0.003614 | 0.003614 | 0.000000 | 0.000000 | 0.000000 | 0.000000 |
| 20-24 | 1-12 | 0.000188 | 0.000188 | 0.000000 | 0.000943 | 0.000943 | 0.000000 | 0.000000 | 0.000000 | 0.000000 |
| 25-29 | 1-12 | 0.000093 | 0.000093 | 0.000000 | 0.001143 | 0.001143 | 0.000000 | 0.000000 | 0.000000 | 0.000000 |
| 30-34 | 1-12 | 0.000047 | 0.000047 | 0.000000 | 0.000871 | 0.000871 | 0.000000 | 0.000000 | 0.000000 | 0.000000 |
| 35-39 | 1-12 | 0.000000 | 0.000000 | 0.000000 | 0.000867 | 0.000867 | 0.000000 | 0.000000 | 0.000000 | 0.000000 |
| 40-44 | 1-12 | 0.000000 | 0.000000 | 0.000000 | 0.001399 | 0.001399 | 0.000000 | 0.000000 | 0.000000 | 0.000000 |
| 45-49 | 1-12 | 0.000000 | 0.000000 | 0.000000 | 0.002033 | 0.002033 | 0.000000 | 0.000000 | 0.000000 | 0.000000 |
| 50-54 | 1-12 | 0.000000 | 0.000000 | 0.000000 | 0.003209 | 0.003209 | 0.000000 | 0.000000 | 0.000000 | 0.000000 |
| 55-59 | 1-12 | 0.000000 | 0.000000 | 0.000000 | 0.003574 | 0.003574 | 0.000000 | 0.000000 | 0.000000 | 0.000000 |
| 60-64 | 1-12 | 0.000000 | 0.000000 | 0.000000 | 0.003460 | 0.003460 | 0.000000 | 0.000000 | 0.000000 | 0.000000 |
| 65-69 | 1-12 | 0.000000 | 0.000000 | 0.000000 | 0.004136 | 0.004136 | 0.000000 | 0.000000 | 0.000000 | 0.000000 |
| 70-74 | 1-12 | 0.000000 | 0.000000 | 0.000000 | 0.005592 | 0.005592 | 0.000000 | 0.000000 | 0.000000 | 0.000000 |
| 75-79 | 1-12 | 0.000000 | 0.000000 | 0.000000 | 0.005592 | 0.005592 | 0.000000 | 0.000000 | 0.000000 | 0.000000 |
| 10-14 | 13-72 | 0.001962 | 0.002944 | 0.000000 | 0.008742 | 0.008742 | 0.000000 | 0.000000 | 0.000000 | 0.000000 |
| 15-19 | 13-72 | 0.002796 | 0.004193 | 0.000000 | 0.004426 | 0.004426 | 0.000000 | 0.000000 | 0.000000 | 0.000000 |
| 20-24 | 13-72 | 0.000275 | 0.000412 | 0.000000 | 0.001527 | 0.001527 | 0.000000 | 0.000000 | 0.000000 | 0.000000 |
| 25-29 | 13-72 | 0.000385 | 0.000578 | 0.000000 | 0.001515 | 0.001515 | 0.000000 | 0.000000 | 0.000000 | 0.000000 |
| 30-34 | 13-72 | 0.000146 | 0.000218 | 0.000000 | 0.001227 | 0.001227 | 0.000000 | 0.000000 | 0.000000 | 0.000000 |
| 35-39 | 13-72 | 0.000000 | 0.000000 | 0.000000 | 0.001630 | 0.001630 | 0.000000 | 0.000000 | 0.000000 | 0.000000 |
| 40-44 | 13-72 | 0.000000 | 0.000000 | 0.000000 | 0.001852 | 0.001852 | 0.000000 | 0.000000 | 0.000000 | 0.000000 |
| 45-49 | 13-72 | 0.000000 | 0.000000 | 0.000000 | 0.002667 | 0.002667 | 0.000000 | 0.000000 | 0.000000 | 0.000000 |
| 50-54 | 13-72 | 0.000000 | 0.000000 | 0.000000 | 0.003208 | 0.003208 | 0.000000 | 0.000000 | 0.000000 | 0.000000 |
| 55-59 | 13-72 | 0.000000 | 0.000000 | 0.000000 | 0.003978 | 0.003978 | 0.000000 | 0.000000 | 0.000000 | 0.000000 |
| 60-64 | 13-72 | 0.000000 | 0.000000 | 0.000000 | 0.005771 | 0.005771 | 0.000000 | 0.000000 | 0.000000 | 0.000000 |
| 65-69 | 13-72 | 0.000000 | 0.000000 | 0.000000 | 0.010432 | 0.010432 | 0.000000 | 0.000000 | 0.000000 | 0.000000 |
| 70-74 | 13-72 | 0.000000 | 0.000000 | 0.000000 | 0.007079 | 0.007079 | 0.000000 | 0.000000 | 0.000000 | 0.000000 |
| 75-79 | 13-72 | 0.000000 | 0.000000 | 0.000000 | 0.007079 | 0.007079 | 0.000000 | 0.000000 | 0.000000 | 0.000000 |
| 10-14 | 73-132 | 0.000862 | 0.003449 | 0.000000 | 0.008742 | 0.008742 | 0.000000 | 0.000000 | 0.000000 | 0.000000 |
| 15-19 | 73-132 | 0.001066 | 0.004264 | 0.000000 | 0.003289 | 0.003289 | 0.000000 | 0.000000 | 0.000000 | 0.000000 |
| 20-24 | 73-132 | 0.000491 | 0.001965 | 0.000000 | 0.003844 | 0.003844 | 0.000000 | 0.000000 | 0.000000 | 0.000000 |
| 25-29 | 73-132 | 0.000168 | 0.000671 | 0.000000 | 0.002006 | 0.002006 | 0.000000 | 0.000000 | 0.000000 | 0.000000 |
| 30-34 | 73-132 | 0.000083 | 0.000332 | 0.000000 | 0.001329 | 0.001329 | 0.000000 | 0.000000 | 0.000000 | 0.000000 |
| 35-39 | 73-132 | 0.000000 | 0.000000 | 0.000000 | 0.001278 | 0.001278 | 0.000000 | 0.000000 | 0.000000 | 0.000000 |
| 40-44 | 73-132 | 0.000000 | 0.000000 | 0.000000 | 0.001691 | 0.001691 | 0.000000 | 0.000000 | 0.000000 | 0.000000 |
| 45-49 | 73-132 | 0.000000 | 0.000000 | 0.000000 | 0.002904 | 0.002904 | 0.000000 | 0.000000 | 0.000000 | 0.000000 |
| 50-54 | 73-132 | 0.000000 | 0.000000 | 0.000000 | 0.003319 | 0.003319 | 0.000000 | 0.000000 | 0.000000 | 0.000000 |
| 55-59 | 73-132 | 0.000000 | 0.000000 | 0.000000 | 0.003976 | 0.003976 | 0.000000 | 0.000000 | 0.000000 | 0.000000 |
| 60-64 | 73-132 | 0.000000 | 0.000000 | 0.000000 | 0.005235 | 0.005235 | 0.000000 | 0.000000 | 0.000000 | 0.000000 |
| 65-69 | 73-132 | 0.000000 | 0.000000 | 0.000000 | 0.005901 | 0.005901 | 0.000000 | 0.000000 | 0.000000 | 0.000000 |
| 70-74 | 73-132 | 0.000000 | 0.000000 | 0.000000 | 0.005615 | 0.005615 | 0.000000 | 0.000000 | 0.000000 | 0.000000 |
| 75-79 | 73-132 | 0.000000 | 0.000000 | 0.000000 | 0.005615 | 0.005615 | 0.000000 | 0.000000 | 0.000000 | 0.000000 |
| 10-14 | 133-192 | 0.000173 | 0.003294 | 0.000000 | 0.008742 | 0.008742 | 0.000000 | 0.000000 | 0.000000 | 0.000000 |
| 15-19 | 133-192 | 0.000234 | 0.004442 | 0.000000 | 0.003732 | 0.003732 | 0.000000 | 0.000000 | 0.000000 | 0.000000 |
| 20-24 | 133-192 | 0.000003 | 0.000059 | 0.000000 | 0.002260 | 0.002260 | 0.000000 | 0.000000 | 0.000000 | 0.000000 |
| 25-29 | 133-192 | 0.000071 | 0.001351 | 0.000000 | 0.002822 | 0.002822 | 0.000000 | 0.000000 | 0.000000 | 0.000000 |
| 30-34 | 133-192 | 0.000035 | 0.000661 | 0.000000 | 0.002610 | 0.002610 | 0.000000 | 0.000000 | 0.000000 | 0.000000 |
| 35-39 | 133-192 | 0.000000 | 0.000000 | 0.000000 | 0.002058 | 0.002058 | 0.000000 | 0.000000 | 0.000000 | 0.000000 |
| 40-44 | 133-192 | 0.000000 | 0.000000 | 0.000000 | 0.002310 | 0.002310 | 0.000000 | 0.000000 | 0.000000 | 0.000000 |
| 45-49 | 133-192 | 0.000000 | 0.000000 | 0.000000 | 0.002256 | 0.002256 | 0.000000 | 0.000000 | 0.000000 | 0.000000 |
| 50-54 | 133-192 | 0.000000 | 0.000000 | 0.000000 | 0.002663 | 0.002663 | 0.000000 | 0.000000 | 0.000000 | 0.000000 |
| 55-59 | 133-192 | 0.000000 | 0.000000 | 0.000000 | 0.001747 | 0.001747 | 0.000000 | 0.000000 | 0.000000 | 0.000000 |
| 60-64 | 133-192 | 0.000000 | 0.000000 | 0.000000 | 0.005809 | 0.005809 | 0.000000 | 0.000000 | 0.000000 | 0.000000 |
| 65-69 | 133-192 | 0.000000 | 0.000000 | 0.000000 | 0.005896 | 0.005896 | 0.000000 | 0.000000 | 0.000000 | 0.000000 |
| 70-74 | 133-192 | 0.000000 | 0.000000 | 0.000000 | 0.007323 | 0.007323 | 0.000000 | 0.000000 | 0.000000 | 0.000000 |
| 75-79 | 133-192 | 0.000000 | 0.000000 | 0.000000 | 0.007323 | 0.007323 | 0.000000 | 0.000000 | 0.000000 | 0.000000 |
| 10-14 | 193-252 | 0.000116 | 0.002198 | 0.000000 | 0.008742 | 0.008742 | 0.000000 | 0.000000 | 0.000000 | 0.000000 |
| 15-19 | 193-252 | 0.000199 | 0.003788 | 0.000000 | 0.005036 | 0.005036 | 0.000000 | 0.000000 | 0.000000 | 0.000000 |
| 20-24 | 193-252 | 0.000016 | 0.000304 | 0.000000 | 0.002857 | 0.002857 | 0.000000 | 0.000000 | 0.000000 | 0.000000 |
| 25-29 | 193-252 | 0.000013 | 0.000239 | 0.000000 | 0.002314 | 0.002314 | 0.000000 | 0.000000 | 0.000000 | 0.000000 |
| 30-34 | 193-252 | 0.000006 | 0.000119 | 0.000000 | 0.004343 | 0.004343 | 0.000000 | 0.000000 | 0.000000 | 0.000000 |
| 35-39 | 193-252 | 0.000000 | 0.000000 | 0.000000 | 0.002092 | 0.002092 | 0.000000 | 0.000000 | 0.000000 | 0.000000 |
| 40-44 | 193-252 | 0.000000 | 0.000000 | 0.000000 | 0.002566 | 0.002566 | 0.000000 | 0.000000 | 0.000000 | 0.000000 |
| 45-49 | 193-252 | 0.000000 | 0.000000 | 0.000000 | 0.002311 | 0.002311 | 0.000000 | 0.000000 | 0.000000 | 0.000000 |
| 50-54 | 193-252 | 0.000000 | 0.000000 | 0.000000 | 0.003209 | 0.003209 | 0.000000 | 0.000000 | 0.000000 | 0.000000 |

# Results for PHIM for Run 1 (Main_Analysis.RTF)

| | | | | | | | | | | |
|---|---|---|---|---|---|---|---|---|---|---|
| 55-59 | 193-252 | 0.000000 | 0.000000 | 0.000000 | 0.004057 | 0.004057 | 0.000000 | 0.000000 | 0.000000 | 0.000000 |
| 60-64 | 193-252 | 0.000000 | 0.000000 | 0.000000 | 0.007166 | 0.007166 | 0.000000 | 0.000000 | 0.000000 | 0.000000 |
| 65-69 | 193-252 | 0.000000 | 0.000000 | 0.000000 | 0.004281 | 0.004281 | 0.000000 | 0.000000 | 0.000000 | 0.000000 |
| 70-74 | 193-252 | 0.000000 | 0.000000 | 0.000000 | 0.004580 | 0.004580 | 0.000000 | 0.000000 | 0.000000 | 0.000000 |
| 75-79 | 193-252 | 0.000000 | 0.000000 | 0.000000 | 0.004580 | 0.004580 | 0.000000 | 0.000000 | 0.000000 | 0.000000 |
| 10-14 | 253+ | 0.000066 | 0.001259 | 0.000000 | 0.008742 | 0.008742 | 0.000000 | 0.000000 | 0.000000 | 0.000000 |
| 15-19 | 253+ | 0.000149 | 0.002835 | 0.000000 | 0.010602 | 0.010602 | 0.000000 | 0.000000 | 0.000000 | 0.000000 |
| 20-24 | 253+ | 0.000023 | 0.000430 | 0.000000 | 0.004314 | 0.004314 | 0.000000 | 0.000000 | 0.000000 | 0.000000 |
| 25-29 | 253+ | 0.000011 | 0.000213 | 0.000000 | 0.003645 | 0.003645 | 0.000000 | 0.000000 | 0.000000 | 0.000000 |
| 30-34 | 253+ | 0.000006 | 0.000106 | 0.000000 | 0.004512 | 0.004512 | 0.000000 | 0.000000 | 0.000000 | 0.000000 |
| 35-39 | 253+ | 0.000000 | 0.000000 | 0.000000 | 0.002147 | 0.002147 | 0.000000 | 0.000000 | 0.000000 | 0.000000 |
| 40-44 | 253+ | 0.000000 | 0.000000 | 0.000000 | 0.002636 | 0.002636 | 0.000000 | 0.000000 | 0.000000 | 0.000000 |
| 45-49 | 253+ | 0.000000 | 0.000000 | 0.000000 | 0.001051 | 0.001051 | 0.000000 | 0.000000 | 0.000000 | 0.000000 |
| 50-54 | 253+ | 0.000000 | 0.000000 | 0.000000 | 0.002519 | 0.002519 | 0.000000 | 0.000000 | 0.000000 | 0.000000 |
| 55-59 | 253+ | 0.000000 | 0.000000 | 0.000000 | 0.003693 | 0.003693 | 0.000000 | 0.000000 | 0.000000 | 0.000000 |
| 60-64 | 253+ | 0.000000 | 0.000000 | 0.000000 | 0.005888 | 0.005888 | 0.000000 | 0.000000 | 0.000000 | 0.000000 |
| 65-69 | 253+ | 0.000000 | 0.000000 | 0.000000 | 0.004823 | 0.004823 | 0.000000 | 0.000000 | 0.000000 | 0.000000 |
| 70-74 | 253+ | 0.000000 | 0.000000 | 0.000000 | 0.004733 | 0.004733 | 0.000000 | 0.000000 | 0.000000 | 0.000000 |
| 75-79 | 253+ | 0.000000 | 0.000000 | 0.000000 | 0.004733 | 0.004733 | 0.000000 | 0.000000 | 0.000000 | 0.000000 |

## Results for PHIM for Run 1 (Main_Analysis.RTF)

*******************************************************************************

| | | Switch from CC | | Switch from Ecig | | Switch from Dual | |
|---|---|---|---|---|---|---|---|
| | | **CC** | **CC** | **Ecig** | **Ecig** | **Dual** | **Dual** |
| Age | Period | Ecig | Dual | CC | Dual | CC | Ecig |
| 10-14 | 1-12 | 0.009549 | 0.000000 | 0.000000 | 0.000000 | 0.000000 | 0.000000 |
| 15-19 | 1-12 | 0.012695 | 0.000000 | 0.000000 | 0.000000 | 0.000000 | 0.000000 |
| 20-24 | 1-12 | 0.014490 | 0.000000 | 0.000000 | 0.000000 | 0.000000 | 0.000000 |
| 25-29 | 1-12 | 0.015212 | 0.000000 | 0.000000 | 0.000000 | 0.000000 | 0.000000 |
| 30-34 | 1-12 | 0.015460 | 0.000000 | 0.000000 | 0.000000 | 0.000000 | 0.000000 |
| 35-39 | 1-12 | 0.014711 | 0.000000 | 0.000000 | 0.000000 | 0.000000 | 0.000000 |
| 40-44 | 1-12 | 0.014241 | 0.000000 | 0.000000 | 0.000000 | 0.000000 | 0.000000 |
| 45-49 | 1-12 | 0.013014 | 0.000000 | 0.000000 | 0.000000 | 0.000000 | 0.000000 |
| 50-54 | 1-12 | 0.011768 | 0.000000 | 0.000000 | 0.000000 | 0.000000 | 0.000000 |
| 55-59 | 1-12 | 0.011032 | 0.000000 | 0.000000 | 0.000000 | 0.000000 | 0.000000 |
| 60-64 | 1-12 | 0.009525 | 0.000000 | 0.000000 | 0.000000 | 0.000000 | 0.000000 |
| 65-69 | 1-12 | 0.006897 | 0.000000 | 0.000000 | 0.000000 | 0.000000 | 0.000000 |
| 70-74 | 1-12 | 0.005435 | 0.000000 | 0.000000 | 0.000000 | 0.000000 | 0.000000 |
| 75-79 | 1-12 | 0.005435 | 0.000000 | 0.000000 | 0.000000 | 0.000000 | 0.000000 |
| 10-14 | 13-72 | 0.009549 | 0.000000 | 0.000000 | 0.000000 | 0.000000 | 0.000000 |
| 15-19 | 13-72 | 0.012695 | 0.000000 | 0.000000 | 0.000000 | 0.000000 | 0.000000 |
| 20-24 | 13-72 | 0.014490 | 0.000000 | 0.000000 | 0.000000 | 0.000000 | 0.000000 |
| 25-29 | 13-72 | 0.015212 | 0.000000 | 0.000000 | 0.000000 | 0.000000 | 0.000000 |
| 30-34 | 13-72 | 0.015460 | 0.000000 | 0.000000 | 0.000000 | 0.000000 | 0.000000 |
| 35-39 | 13-72 | 0.014711 | 0.000000 | 0.000000 | 0.000000 | 0.000000 | 0.000000 |
| 40-44 | 13-72 | 0.014241 | 0.000000 | 0.000000 | 0.000000 | 0.000000 | 0.000000 |
| 45-49 | 13-72 | 0.013014 | 0.000000 | 0.000000 | 0.000000 | 0.000000 | 0.000000 |
| 50-54 | 13-72 | 0.011768 | 0.000000 | 0.000000 | 0.000000 | 0.000000 | 0.000000 |
| 55-59 | 13-72 | 0.011032 | 0.000000 | 0.000000 | 0.000000 | 0.000000 | 0.000000 |
| 60-64 | 13-72 | 0.009525 | 0.000000 | 0.000000 | 0.000000 | 0.000000 | 0.000000 |
| 65-69 | 13-72 | 0.006897 | 0.000000 | 0.000000 | 0.000000 | 0.000000 | 0.000000 |
| 70-74 | 13-72 | 0.005435 | 0.000000 | 0.000000 | 0.000000 | 0.000000 | 0.000000 |
| 75-79 | 13-72 | 0.005435 | 0.000000 | 0.000000 | 0.000000 | 0.000000 | 0.000000 |
| 10-14 | 73-132 | 0.009549 | 0.000000 | 0.000000 | 0.000000 | 0.000000 | 0.000000 |
| 15-19 | 73-132 | 0.012695 | 0.000000 | 0.000000 | 0.000000 | 0.000000 | 0.000000 |
| 20-24 | 73-132 | 0.014490 | 0.000000 | 0.000000 | 0.000000 | 0.000000 | 0.000000 |
| 25-29 | 73-132 | 0.015212 | 0.000000 | 0.000000 | 0.000000 | 0.000000 | 0.000000 |
| 30-34 | 73-132 | 0.015460 | 0.000000 | 0.000000 | 0.000000 | 0.000000 | 0.000000 |
| 35-39 | 73-132 | 0.014711 | 0.000000 | 0.000000 | 0.000000 | 0.000000 | 0.000000 |
| 40-44 | 73-132 | 0.014241 | 0.000000 | 0.000000 | 0.000000 | 0.000000 | 0.000000 |
| 45-49 | 73-132 | 0.013014 | 0.000000 | 0.000000 | 0.000000 | 0.000000 | 0.000000 |
| 50-54 | 73-132 | 0.011768 | 0.000000 | 0.000000 | 0.000000 | 0.000000 | 0.000000 |
| 55-59 | 73-132 | 0.011032 | 0.000000 | 0.000000 | 0.000000 | 0.000000 | 0.000000 |
| 60-64 | 73-132 | 0.009525 | 0.000000 | 0.000000 | 0.000000 | 0.000000 | 0.000000 |
| 65-69 | 73-132 | 0.006897 | 0.000000 | 0.000000 | 0.000000 | 0.000000 | 0.000000 |
| 70-74 | 73-132 | 0.005435 | 0.000000 | 0.000000 | 0.000000 | 0.000000 | 0.000000 |
| 75-79 | 73-132 | 0.005435 | 0.000000 | 0.000000 | 0.000000 | 0.000000 | 0.000000 |
| 10-14 | 133-192 | 0.009549 | 0.000000 | 0.000000 | 0.000000 | 0.000000 | 0.000000 |
| 15-19 | 133-192 | 0.012695 | 0.000000 | 0.000000 | 0.000000 | 0.000000 | 0.000000 |
| 20-24 | 133-192 | 0.014490 | 0.000000 | 0.000000 | 0.000000 | 0.000000 | 0.000000 |
| 25-29 | 133-192 | 0.015212 | 0.000000 | 0.000000 | 0.000000 | 0.000000 | 0.000000 |
| 30-34 | 133-192 | 0.015460 | 0.000000 | 0.000000 | 0.000000 | 0.000000 | 0.000000 |
| 35-39 | 133-192 | 0.014711 | 0.000000 | 0.000000 | 0.000000 | 0.000000 | 0.000000 |
| 40-44 | 133-192 | 0.014241 | 0.000000 | 0.000000 | 0.000000 | 0.000000 | 0.000000 |
| 45-49 | 133-192 | 0.013014 | 0.000000 | 0.000000 | 0.000000 | 0.000000 | 0.000000 |
| 50-54 | 133-192 | 0.011768 | 0.000000 | 0.000000 | 0.000000 | 0.000000 | 0.000000 |
| 55-59 | 133-192 | 0.011032 | 0.000000 | 0.000000 | 0.000000 | 0.000000 | 0.000000 |
| 60-64 | 133-192 | 0.009525 | 0.000000 | 0.000000 | 0.000000 | 0.000000 | 0.000000 |
| 65-69 | 133-192 | 0.006897 | 0.000000 | 0.000000 | 0.000000 | 0.000000 | 0.000000 |
| 70-74 | 133-192 | 0.005435 | 0.000000 | 0.000000 | 0.000000 | 0.000000 | 0.000000 |
| 75-79 | 133-192 | 0.005435 | 0.000000 | 0.000000 | 0.000000 | 0.000000 | 0.000000 |
| 10-14 | 193-252 | 0.009549 | 0.000000 | 0.000000 | 0.000000 | 0.000000 | 0.000000 |
| 15-19 | 193-252 | 0.012695 | 0.000000 | 0.000000 | 0.000000 | 0.000000 | 0.000000 |
| 20-24 | 193-252 | 0.014490 | 0.000000 | 0.000000 | 0.000000 | 0.000000 | 0.000000 |
| 25-29 | 193-252 | 0.015212 | 0.000000 | 0.000000 | 0.000000 | 0.000000 | 0.000000 |
| 30-34 | 193-252 | 0.015460 | 0.000000 | 0.000000 | 0.000000 | 0.000000 | 0.000000 |
| 35-39 | 193-252 | 0.014711 | 0.000000 | 0.000000 | 0.000000 | 0.000000 | 0.000000 |
| 40-44 | 193-252 | 0.014241 | 0.000000 | 0.000000 | 0.000000 | 0.000000 | 0.000000 |
| 45-49 | 193-252 | 0.013014 | 0.000000 | 0.000000 | 0.000000 | 0.000000 | 0.000000 |
| 50-54 | 193-252 | 0.011768 | 0.000000 | 0.000000 | 0.000000 | 0.000000 | 0.000000 |
| 55-59 | 193-252 | 0.011032 | 0.000000 | 0.000000 | 0.000000 | 0.000000 | 0.000000 |
| 60-64 | 193-252 | 0.009525 | 0.000000 | 0.000000 | 0.000000 | 0.000000 | 0.000000 |
| 65-69 | 193-252 | 0.006897 | 0.000000 | 0.000000 | 0.000000 | 0.000000 | 0.000000 |
| 70-74 | 193-252 | 0.005435 | 0.000000 | 0.000000 | 0.000000 | 0.000000 | 0.000000 |
| 75-79 | 193-252 | 0.005435 | 0.000000 | 0.000000 | 0.000000 | 0.000000 | 0.000000 |
| 10-14 | 253+ | 0.009549 | 0.000000 | 0.000000 | 0.000000 | 0.000000 | 0.000000 |
| 15-19 | 253+ | 0.012695 | 0.000000 | 0.000000 | 0.000000 | 0.000000 | 0.000000 |
| 20-24 | 253+ | 0.014490 | 0.000000 | 0.000000 | 0.000000 | 0.000000 | 0.000000 |

## Results for PHIM for Run 1 (Main_Analysis.RTF)

| | | | | | | | |
|---|---|---|---|---|---|---|---|
| 25-29 | 253+ | 0.015212 | 0.000000 | 0.000000 | 0.000000 | 0.000000 | 0.000000 |
| 30-34 | 253+ | 0.015460 | 0.000000 | 0.000000 | 0.000000 | 0.000000 | 0.000000 |
| 35-39 | 253+ | 0.014711 | 0.000000 | 0.000000 | 0.000000 | 0.000000 | 0.000000 |
| 40-44 | 253+ | 0.014241 | 0.000000 | 0.000000 | 0.000000 | 0.000000 | 0.000000 |
| 45-49 | 253+ | 0.013014 | 0.000000 | 0.000000 | 0.000000 | 0.000000 | 0.000000 |
| 50-54 | 253+ | 0.011768 | 0.000000 | 0.000000 | 0.000000 | 0.000000 | 0.000000 |
| 55-59 | 253+ | 0.011032 | 0.000000 | 0.000000 | 0.000000 | 0.000000 | 0.000000 |
| 60-64 | 253+ | 0.009525 | 0.000000 | 0.000000 | 0.000000 | 0.000000 | 0.000000 |
| 65-69 | 253+ | 0.006897 | 0.000000 | 0.000000 | 0.000000 | 0.000000 | 0.000000 |
| 70-74 | 253+ | 0.005435 | 0.000000 | 0.000000 | 0.000000 | 0.000000 | 0.000000 |
| 75-79 | 253+ | 0.005435 | 0.000000 | 0.000000 | 0.000000 | 0.000000 | 0.000000 |

# Results for PHIM for Run 1 (Main_Analysis.RTF)

************************************************************************************

**TTP Factors**

| **Factor** | **TTP Affected** | **Relates to** | **Value** | **< or >** | **Years** |
|---|---|---|---|---|---|
| 1a | Current PR2 to Current PR1 | Previous PR1 use | 2 | - | - |
| 1b | Current PR3 to Current PR1 | Previous PR1 use | 2 | - | - |
| 1c | Current PR4 to Current PR1 | Previous PR1 use | 2 | - | - |
| 2a | Current PR1 to Former | Previously quit | 2 | - | - |
| 2b | Current PR2 to Former | Previously quit | 2 | - | - |
| 2c | Current PR3 to Former | Previously quit | 2 | - | - |
| 2d | Current PR4 to Former | Previously quit | 2 | - | - |
| 3a | Former to Current PR1 | Time quit | 2 | < | 2 |
| 3b | Former to Current PR2 | Time quit | 2 | < | 2 |
| 3c | Former to Current PR3 | Time quit | 2 | < | 2 |
| 3d | Former to Current PR4 | Time quit | 2 | < | 2 |
| 4a | Current PR2 to Current PR1 | Time PR2 use | 0 | > | 2 |
| 4b | Current PR2 to Current PR3 | Time PR2 use | 0 | > | 2 |
| 4c | Current PR2 to Current PR4 | Time PR2 use | 0 | > | 2 |
| 5a | Current PR3 to Current PR1 | Time PR3 use | 0 | > | 2 |
| 5b | Current PR3 to Current PR2 | Time PR3 use | 0 | > | 2 |
| 5c | Current PR3 to Current PR4 | Time PR3 use | 0 | > | 2 |
| 6a | Current PR4 to Current PR1 | Time PR4 use | - | - | - |
| 6b | Current PR4 to Current PR2 | Time PR4 use | - | - | - |
| 6c | Current PR4 to Current PR3 | Time PR4 use | - | - | - |
| 7a | Current PR1 to Current PR2 | Time PR1 use | - | - | - |
| 7b | Current PR1 to Current PR3 | Time PR1 use | - | - | - |
| 7c | Current PR1 to Current PR4 | Time PR1 use | - | - | - |

# Results for PHIM for Run 1 (Main_Analysis.RTF)

*************************************************************************************
**Main Output for the Tobacco History for Run: 1, Simulation: 1, Scenario: NULL**

| Variable Parameter | Value |
|---|---|
| 1. Country | US (United States) |
| 2. Sex | M (Males) |
| 3. Year of start of process | 1990 |

Year of Follow-up: 1990, Age Group: All Ages

| Period | Age Group | Smoking Group | N | % | Ever Quit (N) | Ever Quit % | Mean Yrs Quit | Short Term (n) | Short Term % |
|---|---|---|---|---|---|---|---|---|---|
| 0 | All Ages | Never Any | 34075 | 39.78 | | | | | |
| 0 | All Ages | CC | 27323 | 31.90 | 0 | 0.00 | | | |
| 0 | All Ages | Former | 24266 | 28.33 | | | 13.08 | 3623 | 14.93 |

Year of Follow-up: 1990, Age Group: 18-19

| Period | Age Group | Smoking Group | N | % | Ever Quit (N) | Ever Quit % | Mean Yrs Quit | Short Term (n) | Short Term % |
|---|---|---|---|---|---|---|---|---|---|
| 0 | 18-19 | Never Any | 2783 | 78.42 | | | | | |
| 0 | 18-19 | CC | 634 | 17.86 | 0 | 0.00 | | | |
| 0 | 18-19 | Former | 132 | 3.72 | | | 0.11 | 132 | 100.00 |

Year of Follow-up: 1990, Age Group: 20-24

| Period | Age Group | Smoking Group | N | % | Ever Quit (N) | Ever Quit % | Mean Yrs Quit | Short Term (n) | Short Term % |
|---|---|---|---|---|---|---|---|---|---|
| 0 | 20-24 | Never Any | 5722 | 58.29 | | | | | |
| 0 | 20-24 | CC | 3199 | 32.59 | 0 | 0.00 | | | |
| 0 | 20-24 | Former | 895 | 9.12 | | | 1.46 | 506 | 56.54 |

Year of Follow-up: 1990, Age Group: 25-29

| Period | Age Group | Smoking Group | N | % | Ever Quit (N) | Ever Quit % | Mean Yrs Quit | Short Term (n) | Short Term % |
|---|---|---|---|---|---|---|---|---|---|
| 0 | 25-29 | Never Any | 5232 | 49.06 | | | | | |
| 0 | 25-29 | CC | 3914 | 36.70 | 0 | 0.00 | | | |
| 0 | 25-29 | Former | 1518 | 14.23 | | | 2.87 | 643 | 42.36 |

Year of Follow-up: 1990, Age Group: 30-34

| Period | Age Group | Smoking Group | N | % | Ever Quit (N) | Ever Quit % | Mean Yrs Quit | Short Term (n) | Short Term % |
|---|---|---|---|---|---|---|---|---|---|
| 0 | 30-34 | Never Any | 4765 | 43.65 | | | | | |
| 0 | 30-34 | CC | 4023 | 36.85 | 0 | 0.00 | | | |
| 0 | 30-34 | Former | 2128 | 19.49 | | | 4.06 | 592 | 27.82 |

Year of Follow-up: 1990, Age Group: 35-39

| Period | Age Group | Smoking Group | N | % | Ever Quit (N) | Ever Quit % | Mean Yrs Quit | Short Term (n) | Short Term % |
|---|---|---|---|---|---|---|---|---|---|
| 0 | 35-39 | Never Any | 3941 | 39.85 | | | | | |
| 0 | 35-39 | CC | 3474 | 35.13 | 0 | 0.00 | | | |
| 0 | 35-39 | Former | 2474 | 25.02 | | | 8.31 | 425 | 17.18 |

Year of Follow-up: 1990, Age Group: 40-44

| Period | Age Group | Smoking Group | N | % | Ever Quit (N) | Ever Quit % | Mean Yrs Quit | Short Term (n) | Short Term % |
|---|---|---|---|---|---|---|---|---|---|
| 0 | 40-44 | Never Any | 2912 | 33.65 | | | | | |
| 0 | 40-44 | CC | 3113 | 35.97 | 0 | 0.00 | | | |
| 0 | 40-44 | Former | 2630 | 30.39 | | | 10.69 | 317 | 12.05 |

Year of Follow-up: 1990, Age Group: 45-49

| Period | Age Group | Smoking Group | N | % | Ever Quit (N) | Ever Quit % | Mean Yrs Quit | Short Term (n) | Short Term % |
|---|---|---|---|---|---|---|---|---|---|
| 0 | 45-49 | Never Any | 1919 | 29.46 | | | | | |
| 0 | 45-49 | CC | 2318 | 35.58 | 0 | 0.00 | | | |
| 0 | 45-49 | Former | 2278 | 34.97 | | | 13.26 | 221 | 9.70 |

Year of Follow-up: 1990, Age Group: 50-54

| Period | Age Group | Smoking Group | N | % | Ever Quit (N) | Ever Quit % | Mean Yrs Quit | Short Term (n) | Short Term % |
|---|---|---|---|---|---|---|---|---|---|
| 0 | 50-54 | Never Any | 1611 | 29.41 | | | | | |
| 0 | 50-54 | CC | 1816 | 33.16 | 0 | 0.00 | | | |
| 0 | 50-54 | Former | 2050 | 37.43 | | | 14.55 | 261 | 12.73 |

Year of Follow-up: 1990, Age Group: 55-59

| Period | Age Group | Smoking Group | N | % | Ever Quit (N) | Ever Quit % | Mean Yrs Quit | Short Term (n) | Short Term % |
|---|---|---|---|---|---|---|---|---|---|
| 0 | 55-59 | Never Any | 1337 | 26.83 | | | | | |
| 0 | 55-59 | CC | 1520 | 30.50 | 0 | 0.00 | | | |
| 0 | 55-59 | Former | 2126 | 42.67 | | | 14.80 | 216 | 10.16 |

Year of Follow-up: 1990, Age Group: 60-64

| Period | Age Group | Smoking Group | N | % | Ever Quit (N) | Ever Quit % | Mean Yrs Quit | Short Term (n) | Short Term % |
|---|---|---|---|---|---|---|---|---|---|
| 0 | 60-64 | Never Any | 1169 | 23.83 | | | | | |
| 0 | 60-64 | CC | 1287 | 26.24 | 0 | 0.00 | | | |
| 0 | 60-64 | Former | 2449 | 49.93 | | | 18.87 | 106 | 4.33 |

Year of Follow-up: 1990, Age Group: 65-69

| Period | Age Group | Smoking Group | N | % | Ever Quit (N) | Ever Quit % | Mean Yrs Quit | Short Term (n) | Short Term % |
|---|---|---|---|---|---|---|---|---|---|

# Results for PHIM for Run 1 (Main_Analysis.RTF)

| | | | | | | | | | |
|---|---|---|---|---|---|---|---|---|---|
| 0 | 65-69 | Never Any | 1023 | 23.32 | | | | | |
| 0 | 65-69 | CC | 979 | 22.32 | 0 | 0.00 | | | |
| 0 | 65-69 | Former | 2385 | 54.37 | | | 19.91 | 113 | 4.74 |

Year of Follow-up: 1990, Age Group: 70-74

| Period | Age Group | Smoking Group | N | % | Ever Quit (N) | Ever Quit % | Mean Yrs Quit | Short Term (n) | Short Term % |
|---|---|---|---|---|---|---|---|---|---|
| 0 | 70-74 | Never Any | 961 | 27.46 | | | | | |
| 0 | 70-74 | CC | 675 | 19.29 | 0 | 0.00 | | | |
| 0 | 70-74 | Former | 1863 | 53.24 | | | 20.69 | 23 | 1.23 |

Year of Follow-up: 1990, Age Group: 75-79

| Period | Age Group | Smoking Group | N | % | Ever Quit (N) | Ever Quit % | Mean Yrs Quit | Short Term (n) | Short Term % |
|---|---|---|---|---|---|---|---|---|---|
| 0 | 75-79 | Never Any | 700 | 29.06 | | | | | |
| 0 | 75-79 | CC | 371 | 15.40 | 0 | 0.00 | | | |
| 0 | 75-79 | Former | 1338 | 55.54 | | | 22.88 | 68 | 5.08 |

Year of Follow-up: 1995, Age Group: All Ages

| Period | Age Group | Smoking Group | N | % | Ever Quit (N) | Ever Quit % | Mean Yrs Quit | Short Term (n) | Short Term % |
|---|---|---|---|---|---|---|---|---|---|
| 5 | All Ages | Never Any | 38379 | 41.59 | | | | | |
| 5 | All Ages | CC | 26792 | 29.03 | 0 | 0.00 | | | |
| 5 | All Ages | Former | 27119 | 29.38 | | | 15.09 | 1786 | 6.59 |

Year of Follow-up: 1995, Age Group: 18-19

| Period | Age Group | Smoking Group | N | % | Ever Quit (N) | Ever Quit % | Mean Yrs Quit | Short Term (n) | Short Term % |
|---|---|---|---|---|---|---|---|---|---|
| 5 | 18-19 | Never Any | 2456 | 69.30 | | | | | |
| 5 | 18-19 | CC | 989 | 27.91 | 0 | 0.00 | | | |
| 5 | 18-19 | Former | 99 | 2.79 | | | 0.96 | 70 | 70.71 |

Year of Follow-up: 1995, Age Group: 20-24

| Period | Age Group | Smoking Group | N | % | Ever Quit (N) | Ever Quit % | Mean Yrs Quit | Short Term (n) | Short Term % |
|---|---|---|---|---|---|---|---|---|---|
| 5 | 20-24 | Never Any | 5811 | 64.28 | | | | | |
| 5 | 20-24 | CC | 2706 | 29.93 | 0 | 0.00 | | | |
| 5 | 20-24 | Former | 523 | 5.79 | | | 2.70 | 162 | 30.98 |

Year of Follow-up: 1995, Age Group: 25-29

| Period | Age Group | Smoking Group | N | % | Ever Quit (N) | Ever Quit % | Mean Yrs Quit | Short Term (n) | Short Term % |
|---|---|---|---|---|---|---|---|---|---|
| 5 | 25-29 | Never Any | 5473 | 55.76 | | | | | |
| 5 | 25-29 | CC | 3144 | 32.03 | 0 | 0.00 | | | |
| 5 | 25-29 | Former | 1199 | 12.21 | | | 5.30 | 123 | 10.26 |

Year of Follow-up: 1995, Age Group: 30-34

| Period | Age Group | Smoking Group | N | % | Ever Quit (N) | Ever Quit % | Mean Yrs Quit | Short Term (n) | Short Term % |
|---|---|---|---|---|---|---|---|---|---|
| 5 | 30-34 | Never Any | 5055 | 47.40 | | | | | |
| 5 | 30-34 | CC | 3816 | 35.78 | 0 | 0.00 | | | |
| 5 | 30-34 | Former | 1793 | 16.81 | | | 6.95 | 112 | 6.25 |

Year of Follow-up: 1995, Age Group: 35-39

| Period | Age Group | Smoking Group | N | % | Ever Quit (N) | Ever Quit % | Mean Yrs Quit | Short Term (n) | Short Term % |
|---|---|---|---|---|---|---|---|---|---|
| 5 | 35-39 | Never Any | 4711 | 43.16 | | | | | |
| 5 | 35-39 | CC | 3760 | 34.44 | 0 | 0.00 | | | |
| 5 | 35-39 | Former | 2445 | 22.40 | | | 8.12 | 150 | 6.13 |

Year of Follow-up: 1995, Age Group: 40-44

| Period | Age Group | Smoking Group | N | % | Ever Quit (N) | Ever Quit % | Mean Yrs Quit | Short Term (n) | Short Term % |
|---|---|---|---|---|---|---|---|---|---|
| 5 | 40-44 | Never Any | 3941 | 39.85 | | | | | |
| 5 | 40-44 | CC | 3197 | 32.33 | 0 | 0.00 | | | |
| 5 | 40-44 | Former | 2751 | 27.82 | | | 12.14 | 128 | 4.65 |

Year of Follow-up: 1995, Age Group: 45-49

| Period | Age Group | Smoking Group | N | % | Ever Quit (N) | Ever Quit % | Mean Yrs Quit | Short Term (n) | Short Term % |
|---|---|---|---|---|---|---|---|---|---|
| 5 | 45-49 | Never Any | 2912 | 33.65 | | | | | |
| 5 | 45-49 | CC | 2701 | 31.21 | 0 | 0.00 | | | |
| 5 | 45-49 | Former | 3042 | 35.15 | | | 13.81 | 165 | 5.42 |

Year of Follow-up: 1995, Age Group: 50-54

| Period | Age Group | Smoking Group | N | % | Ever Quit (N) | Ever Quit % | Mean Yrs Quit | Short Term (n) | Short Term % |
|---|---|---|---|---|---|---|---|---|---|
| 5 | 50-54 | Never Any | 1919 | 29.46 | | | | | |
| 5 | 50-54 | CC | 1968 | 30.21 | 0 | 0.00 | | | |
| 5 | 50-54 | Former | 2628 | 40.34 | | | 16.08 | 147 | 5.59 |

Year of Follow-up: 1995, Age Group: 55-59

| Period | Age Group | Smoking Group | N | % | Ever Quit (N) | Ever Quit % | Mean Yrs Quit | Short Term (n) | Short Term % |
|---|---|---|---|---|---|---|---|---|---|
| 5 | 55-59 | Never Any | 1611 | 29.41 | | | | | |
| 5 | 55-59 | CC | 1466 | 26.77 | 0 | 0.00 | | | |
| 5 | 55-59 | Former | 2400 | 43.82 | | | 16.98 | 153 | 6.38 |

Year of Follow-up: 1995, Age Group: 60-64

| Period | Age Group | Smoking Group | N | % | Ever Quit (N) | Ever Quit % | Mean Yrs Quit | Short Term (n) | Short Term % |
|---|---|---|---|---|---|---|---|---|---|

# Results for PHIM for Run 1 (Main_Analysis.RTF)

| | | | | | | | | | |
|---|---|---|---|---|---|---|---|---|---|
| 5 | 60-64 | Never Any | 1337 | 26.83 | | | | | |
| 5 | 60-64 | CC | 1155 | 23.18 | 0 | 0.00 | | | |
| 5 | 60-64 | Former | 2491 | 49.99 | | | 17.18 | 144 | 5.78 |

Year of Follow-up: 1995, Age Group: 65-69

| Period | Age Group | Smoking Group | N | % | Ever Quit (N) | Ever Quit % | Mean Yrs Quit | Short Term (n) | Short Term % |
|---|---|---|---|---|---|---|---|---|---|
| 5 | 65-69 | Never Any | 1169 | 23.83 | | | | | |
| 5 | 65-69 | CC | 832 | 16.96 | 0 | 0.00 | | | |
| 5 | 65-69 | Former | 2904 | 59.20 | | | 20.40 | 208 | 7.16 |

Year of Follow-up: 1995, Age Group: 70-74

| Period | Age Group | Smoking Group | N | % | Ever Quit (N) | Ever Quit % | Mean Yrs Quit | Short Term (n) | Short Term % |
|---|---|---|---|---|---|---|---|---|---|
| 5 | 70-74 | Never Any | 1023 | 23.32 | | | | | |
| 5 | 70-74 | CC | 610 | 13.90 | 0 | 0.00 | | | |
| 5 | 70-74 | Former | 2754 | 62.78 | | | 21.83 | 137 | 4.97 |

Year of Follow-up: 1995, Age Group: 75-79

| Period | Age Group | Smoking Group | N | % | Ever Quit (N) | Ever Quit % | Mean Yrs Quit | Short Term (n) | Short Term % |
|---|---|---|---|---|---|---|---|---|---|
| 5 | 75-79 | Never Any | 961 | 27.46 | | | | | |
| 5 | 75-79 | CC | 448 | 12.80 | 0 | 0.00 | | | |
| 5 | 75-79 | Former | 2090 | 59.73 | | | 23.12 | 87 | 4.16 |

Year of Follow-up: 2000, Age Group: All Ages

| Period | Age Group | Smoking Group | N | % | Ever Quit (N) | Ever Quit % | Mean Yrs Quit | Short Term (n) | Short Term % |
|---|---|---|---|---|---|---|---|---|---|
| 10 | All Ages | Never Any | 39029 | 41.48 | | | | | |
| 10 | All Ages | CC | 25505 | 27.11 | 0 | 0.00 | | | |
| 10 | All Ages | Former | 29558 | 31.41 | | | 16.78 | 1706 | 5.77 |

Year of Follow-up: 2000, Age Group: 18-19

| Period | Age Group | Smoking Group | N | % | Ever Quit (N) | Ever Quit % | Mean Yrs Quit | Short Term (n) | Short Term % |
|---|---|---|---|---|---|---|---|---|---|
| 10 | 18-19 | Never Any | 0 | . | | | | | |
| 10 | 18-19 | CC | 0 | . | 0 | . | | | |
| 10 | 18-19 | Former | 0 | . | | | . | 0 | . |

Year of Follow-up: 2000, Age Group: 20-24

| Period | Age Group | Smoking Group | N | % | Ever Quit (N) | Ever Quit % | Mean Yrs Quit | Short Term (n) | Short Term % |
|---|---|---|---|---|---|---|---|---|---|
| 10 | 20-24 | Never Any | 4845 | 54.78 | | | | | |
| 10 | 20-24 | CC | 3091 | 34.95 | 0 | 0.00 | | | |
| 10 | 20-24 | Former | 909 | 10.28 | | | 3.29 | 273 | 30.03 |

Year of Follow-up: 2000, Age Group: 25-29

| Period | Age Group | Smoking Group | N | % | Ever Quit (N) | Ever Quit % | Mean Yrs Quit | Short Term (n) | Short Term % |
|---|---|---|---|---|---|---|---|---|---|
| 10 | 25-29 | Never Any | 5316 | 58.81 | | | | | |
| 10 | 25-29 | CC | 2773 | 30.67 | 0 | 0.00 | | | |
| 10 | 25-29 | Former | 951 | 10.52 | | | 5.07 | 174 | 18.30 |

Year of Follow-up: 2000, Age Group: 30-34

| Period | Age Group | Smoking Group | N | % | Ever Quit (N) | Ever Quit % | Mean Yrs Quit | Short Term (n) | Short Term % |
|---|---|---|---|---|---|---|---|---|---|
| 10 | 30-34 | Never Any | 5256 | 53.55 | | | | | |
| 10 | 30-34 | CC | 3060 | 31.17 | 0 | 0.00 | | | |
| 10 | 30-34 | Former | 1500 | 15.28 | | | 8.63 | 125 | 8.33 |

Year of Follow-up: 2000, Age Group: 35-39

| Period | Age Group | Smoking Group | N | % | Ever Quit (N) | Ever Quit % | Mean Yrs Quit | Short Term (n) | Short Term % |
|---|---|---|---|---|---|---|---|---|---|
| 10 | 35-39 | Never Any | 4989 | 46.78 | | | | | |
| 10 | 35-39 | CC | 3629 | 34.03 | 0 | 0.00 | | | |
| 10 | 35-39 | Former | 2046 | 19.19 | | | 10.71 | 110 | 5.38 |

Year of Follow-up: 2000, Age Group: 40-44

| Period | Age Group | Smoking Group | N | % | Ever Quit (N) | Ever Quit % | Mean Yrs Quit | Short Term (n) | Short Term % |
|---|---|---|---|---|---|---|---|---|---|
| 10 | 40-44 | Never Any | 4711 | 43.16 | | | | | |
| 10 | 40-44 | CC | 3416 | 31.29 | 0 | 0.00 | | | |
| 10 | 40-44 | Former | 2789 | 25.55 | | | 11.76 | 131 | 4.70 |

Year of Follow-up: 2000, Age Group: 45-49

| Period | Age Group | Smoking Group | N | % | Ever Quit (N) | Ever Quit % | Mean Yrs Quit | Short Term (n) | Short Term % |
|---|---|---|---|---|---|---|---|---|---|
| 10 | 45-49 | Never Any | 3941 | 39.85 | | | | | |
| 10 | 45-49 | CC | 2816 | 28.48 | 0 | 0.00 | | | |
| 10 | 45-49 | Former | 3132 | 31.67 | | | 15.29 | 160 | 5.11 |

Year of Follow-up: 2000, Age Group: 50-54

| Period | Age Group | Smoking Group | N | % | Ever Quit (N) | Ever Quit % | Mean Yrs Quit | Short Term (n) | Short Term % |
|---|---|---|---|---|---|---|---|---|---|
| 10 | 50-54 | Never Any | 2912 | 33.65 | | | | | |
| 10 | 50-54 | CC | 2237 | 25.85 | 0 | 0.00 | | | |
| 10 | 50-54 | Former | 3506 | 40.51 | | | 16.59 | 184 | 5.25 |

Year of Follow-up: 2000, Age Group: 55-59

| Period | Age Group | Smoking Group | N | % | Ever Quit (N) | Ever Quit % | Mean Yrs Quit | Short Term (n) | Short Term % |
|---|---|---|---|---|---|---|---|---|---|

# Results for PHIM for Run 1 (Main_Analysis.RTF)

| | | | | | | | | | |
|---|---|---|---|---|---|---|---|---|---|
| 10 | 55-59 | Never Any | 1919 | 29.46 | | | | | |
| 10 | 55-59 | CC | 1538 | 23.61 | 0 | 0.00 | | | |
| 10 | 55-59 | Former | 3058 | 46.94 | | | 18.41 | 158 | 5.17 |

Year of Follow-up: 2000, Age Group: 60-64

| Period | Age Group | Smoking Group | N | % | Ever Quit (N) | Ever Quit % | Mean Yrs Quit | Short Term (n) | Short Term % |
|---|---|---|---|---|---|---|---|---|---|
| 10 | 60-64 | Never Any | 1611 | 29.41 | | | | | |
| 10 | 60-64 | CC | 1140 | 20.81 | 0 | 0.00 | | | |
| 10 | 60-64 | Former | 2726 | 49.77 | | | 19.59 | 122 | 4.48 |

Year of Follow-up: 2000, Age Group: 65-69

| Period | Age Group | Smoking Group | N | % | Ever Quit (N) | Ever Quit % | Mean Yrs Quit | Short Term (n) | Short Term % |
|---|---|---|---|---|---|---|---|---|---|
| 10 | 65-69 | Never Any | 1337 | 26.83 | | | | | |
| 10 | 65-69 | CC | 809 | 16.24 | 0 | 0.00 | | | |
| 10 | 65-69 | Former | 2837 | 56.93 | | | 19.73 | 124 | 4.37 |

Year of Follow-up: 2000, Age Group: 70-74

| Period | Age Group | Smoking Group | N | % | Ever Quit (N) | Ever Quit % | Mean Yrs Quit | Short Term (n) | Short Term % |
|---|---|---|---|---|---|---|---|---|---|
| 10 | 70-74 | Never Any | 1169 | 23.83 | | | | | |
| 10 | 70-74 | CC | 570 | 11.62 | 0 | 0.00 | | | |
| 10 | 70-74 | Former | 3166 | 64.55 | | | 23.50 | 80 | 2.53 |

Year of Follow-up: 2000, Age Group: 75-79

| Period | Age Group | Smoking Group | N | % | Ever Quit (N) | Ever Quit % | Mean Yrs Quit | Short Term (n) | Short Term % |
|---|---|---|---|---|---|---|---|---|---|
| 10 | 75-79 | Never Any | 1023 | 23.32 | | | | | |
| 10 | 75-79 | CC | 426 | 9.71 | 0 | 0.00 | | | |
| 10 | 75-79 | Former | 2938 | 66.97 | | | 25.29 | 65 | 2.21 |

Year of Follow-up: 2005, Age Group: All Ages

| Period | Age Group | Smoking Group | N | % | Ever Quit (N) | Ever Quit % | Mean Yrs Quit | Short Term (n) | Short Term % |
|---|---|---|---|---|---|---|---|---|---|
| 15 | All Ages | Never Any | 37254 | 41.53 | | | | | |
| 15 | All Ages | CC | 21889 | 24.40 | 0 | 0.00 | | | |
| 15 | All Ages | Former | 30562 | 34.07 | | | 18.41 | 1493 | 4.89 |

Year of Follow-up: 2005, Age Group: 18-19

| Period | Age Group | Smoking Group | N | % | Ever Quit (N) | Ever Quit % | Mean Yrs Quit | Short Term (n) | Short Term % |
|---|---|---|---|---|---|---|---|---|---|
| 15 | 18-19 | Never Any | 0 | . | | | | | |
| 15 | 18-19 | CC | 0 | . | 0 | . | | | |
| 15 | 18-19 | Former | 0 | . | | | . | 0 | . |

Year of Follow-up: 2005, Age Group: 20-24

| Period | Age Group | Smoking Group | N | % | Ever Quit (N) | Ever Quit % | Mean Yrs Quit | Short Term (n) | Short Term % |
|---|---|---|---|---|---|---|---|---|---|
| 15 | 20-24 | Never Any | 0 | . | | | | | |
| 15 | 20-24 | CC | 0 | . | 0 | . | | | |
| 15 | 20-24 | Former | 0 | . | | | . | 0 | . |

Year of Follow-up: 2005, Age Group: 25-29

| Period | Age Group | Smoking Group | N | % | Ever Quit (N) | Ever Quit % | Mean Yrs Quit | Short Term (n) | Short Term % |
|---|---|---|---|---|---|---|---|---|---|
| 15 | 25-29 | Never Any | 4521 | 51.11 | | | | | |
| 15 | 25-29 | CC | 2890 | 32.67 | 0 | 0.00 | | | |
| 15 | 25-29 | Former | 1434 | 16.21 | | | 6.06 | 189 | 13.18 |

Year of Follow-up: 2005, Age Group: 30-34

| Period | Age Group | Smoking Group | N | % | Ever Quit (N) | Ever Quit % | Mean Yrs Quit | Short Term (n) | Short Term % |
|---|---|---|---|---|---|---|---|---|---|
| 15 | 30-34 | Never Any | 4999 | 55.30 | | | | | |
| 15 | 30-34 | CC | 2700 | 29.87 | 0 | 0.00 | | | |
| 15 | 30-34 | Former | 1341 | 14.83 | | | 7.70 | 161 | 12.01 |

Year of Follow-up: 2005, Age Group: 35-39

| Period | Age Group | Smoking Group | N | % | Ever Quit (N) | Ever Quit % | Mean Yrs Quit | Short Term (n) | Short Term % |
|---|---|---|---|---|---|---|---|---|---|
| 15 | 35-39 | Never Any | 5145 | 52.41 | | | | | |
| 15 | 35-39 | CC | 2789 | 28.41 | 0 | 0.00 | | | |
| 15 | 35-39 | Former | 1882 | 19.17 | | | 11.26 | 146 | 7.76 |

Year of Follow-up: 2005, Age Group: 40-44

| Period | Age Group | Smoking Group | N | % | Ever Quit (N) | Ever Quit % | Mean Yrs Quit | Short Term (n) | Short Term % |
|---|---|---|---|---|---|---|---|---|---|
| 15 | 40-44 | Never Any | 4989 | 46.78 | | | | | |
| 15 | 40-44 | CC | 3202 | 30.03 | 0 | 0.00 | | | |
| 15 | 40-44 | Former | 2473 | 23.19 | | | 13.31 | 182 | 7.36 |

Year of Follow-up: 2005, Age Group: 45-49

| Period | Age Group | Smoking Group | N | % | Ever Quit (N) | Ever Quit % | Mean Yrs Quit | Short Term (n) | Short Term % |
|---|---|---|---|---|---|---|---|---|---|
| 15 | 45-49 | Never Any | 4711 | 43.16 | | | | | |
| 15 | 45-49 | CC | 3012 | 27.59 | 0 | 0.00 | | | |
| 15 | 45-49 | Former | 3193 | 29.25 | | | 14.88 | 167 | 5.23 |

Year of Follow-up: 2005, Age Group: 50-54

| Period | Age Group | Smoking Group | N | % | Ever Quit (N) | Ever Quit % | Mean Yrs Quit | Short Term (n) | Short Term % |
|---|---|---|---|---|---|---|---|---|---|

# Results for PHIM for Run 1 (Main_Analysis.RTF)

| | | | | | | | | | |
|---|---|---|---|---|---|---|---|---|---|
| 15 | 50-54 | Never Any | 3941 | 39.85 | | | | | |
| 15 | 50-54 | CC | 2432 | 24.59 | 0 | 0.00 | | | |
| 15 | 50-54 | Former | 3516 | 35.55 | | | 18.30 | 155 | 4.41 |

Year of Follow-up: 2005, Age Group: 55-59

| Period | Age Group | Smoking Group | N | % | Ever Quit (N) | Ever Quit % | Mean Yrs Quit | Short Term (n) | Short Term % |
|---|---|---|---|---|---|---|---|---|---|
| 15 | 55-59 | Never Any | 2912 | 33.65 | | | | | |
| 15 | 55-59 | CC | 1928 | 22.28 | 0 | 0.00 | | | |
| 15 | 55-59 | Former | 3815 | 44.08 | | | 20.03 | 78 | 2.04 |

Year of Follow-up: 2005, Age Group: 60-64

| Period | Age Group | Smoking Group | N | % | Ever Quit (N) | Ever Quit % | Mean Yrs Quit | Short Term (n) | Short Term % |
|---|---|---|---|---|---|---|---|---|---|
| 15 | 60-64 | Never Any | 1919 | 29.46 | | | | | |
| 15 | 60-64 | CC | 1202 | 18.45 | 0 | 0.00 | | | |
| 15 | 60-64 | Former | 3394 | 52.10 | | | 21.28 | 142 | 4.18 |

Year of Follow-up: 2005, Age Group: 65-69

| Period | Age Group | Smoking Group | N | % | Ever Quit (N) | Ever Quit % | Mean Yrs Quit | Short Term (n) | Short Term % |
|---|---|---|---|---|---|---|---|---|---|
| 15 | 65-69 | Never Any | 1611 | 29.41 | | | | | |
| 15 | 65-69 | CC | 813 | 14.84 | 0 | 0.00 | | | |
| 15 | 65-69 | Former | 3053 | 55.74 | | | 22.18 | 121 | 3.96 |

Year of Follow-up: 2005, Age Group: 70-74

| Period | Age Group | Smoking Group | N | % | Ever Quit (N) | Ever Quit % | Mean Yrs Quit | Short Term (n) | Short Term % |
|---|---|---|---|---|---|---|---|---|---|
| 15 | 70-74 | Never Any | 1337 | 26.83 | | | | | |
| 15 | 70-74 | CC | 543 | 10.90 | 0 | 0.00 | | | |
| 15 | 70-74 | Former | 3103 | 62.27 | | | 22.80 | 96 | 3.09 |

Year of Follow-up: 2005, Age Group: 75-79

| Period | Age Group | Smoking Group | N | % | Ever Quit (N) | Ever Quit % | Mean Yrs Quit | Short Term (n) | Short Term % |
|---|---|---|---|---|---|---|---|---|---|
| 15 | 75-79 | Never Any | 1169 | 23.83 | | | | | |
| 15 | 75-79 | CC | 378 | 7.71 | 0 | 0.00 | | | |
| 15 | 75-79 | Former | 3358 | 68.46 | | | 27.00 | 56 | 1.67 |

Year of Follow-up: 2010, Age Group: All Ages

| Period | Age Group | Smoking Group | N | % | Ever Quit (N) | Ever Quit % | Mean Yrs Quit | Short Term (n) | Short Term % |
|---|---|---|---|---|---|---|---|---|---|
| 20 | All Ages | Never Any | 35903 | 42.34 | | | | | |
| 20 | All Ages | CC | 17853 | 21.05 | 0 | 0.00 | | | |
| 20 | All Ages | Former | 31044 | 36.61 | | | 19.83 | 1550 | 4.99 |

Year of Follow-up: 2010, Age Group: 18-19

| Period | Age Group | Smoking Group | N | % | Ever Quit (N) | Ever Quit % | Mean Yrs Quit | Short Term (n) | Short Term % |
|---|---|---|---|---|---|---|---|---|---|
| 20 | 18-19 | Never Any | 0 | . | | | | | |
| 20 | 18-19 | CC | 0 | . | 0 | . | | | |
| 20 | 18-19 | Former | 0 | . | | | . | 0 | . |

Year of Follow-up: 2010, Age Group: 20-24

| Period | Age Group | Smoking Group | N | % | Ever Quit (N) | Ever Quit % | Mean Yrs Quit | Short Term (n) | Short Term % |
|---|---|---|---|---|---|---|---|---|---|
| 20 | 20-24 | Never Any | 0 | . | | | | | |
| 20 | 20-24 | CC | 0 | . | 0 | . | | | |
| 20 | 20-24 | Former | 0 | . | | | . | 0 | . |

Year of Follow-up: 2010, Age Group: 25-29

| Period | Age Group | Smoking Group | N | % | Ever Quit (N) | Ever Quit % | Mean Yrs Quit | Short Term (n) | Short Term % |
|---|---|---|---|---|---|---|---|---|---|
| 20 | 25-29 | Never Any | 0 | . | | | | | |
| 20 | 25-29 | CC | 0 | . | 0 | . | | | |
| 20 | 25-29 | Former | 0 | . | | | . | 0 | . |

Year of Follow-up: 2010, Age Group: 30-34

| Period | Age Group | Smoking Group | N | % | Ever Quit (N) | Ever Quit % | Mean Yrs Quit | Short Term (n) | Short Term % |
|---|---|---|---|---|---|---|---|---|---|
| 20 | 30-34 | Never Any | 4390 | 49.63 | | | | | |
| 20 | 30-34 | CC | 2501 | 28.28 | 0 | 0.00 | | | |
| 20 | 30-34 | Former | 1954 | 22.09 | | | 8.61 | 240 | 12.28 |

Year of Follow-up: 2010, Age Group: 35-39

| Period | Age Group | Smoking Group | N | % | Ever Quit (N) | Ever Quit % | Mean Yrs Quit | Short Term (n) | Short Term % |
|---|---|---|---|---|---|---|---|---|---|
| 20 | 35-39 | Never Any | 4948 | 54.73 | | | | | |
| 20 | 35-39 | CC | 2261 | 25.01 | 0 | 0.00 | | | |
| 20 | 35-39 | Former | 1831 | 20.25 | | | 9.87 | 174 | 9.50 |

Year of Follow-up: 2010, Age Group: 40-44

| Period | Age Group | Smoking Group | N | % | Ever Quit (N) | Ever Quit % | Mean Yrs Quit | Short Term (n) | Short Term % |
|---|---|---|---|---|---|---|---|---|---|
| 20 | 40-44 | Never Any | 5145 | 52.41 | | | | | |
| 20 | 40-44 | CC | 2437 | 24.83 | 0 | 0.00 | | | |
| 20 | 40-44 | Former | 2234 | 22.76 | | | 13.99 | 167 | 7.48 |

Year of Follow-up: 2010, Age Group: 45-49

| Period | Age Group | Smoking Group | N | % | Ever Quit (N) | Ever Quit % | Mean Yrs Quit | Short Term (n) | Short Term % |
|---|---|---|---|---|---|---|---|---|---|

# Results for PHIM for Run 1 (Main_Analysis.RTF)

| | | | | | | | | | |
|---|---|---|---|---|---|---|---|---|---|
| 20 | 45-49 | Never Any | 4989 | 46.78 | | | | | |
| 20 | 45-49 | CC | 2794 | 26.20 | 0 | 0.00 | | | |
| 20 | 45-49 | Former | 2881 | 27.02 | | | 16.01 | 148 | 5.14 |

Year of Follow-up: 2010, Age Group: 50-54

| Period | Age Group | Smoking Group | N | % | Ever Quit (N) | Ever Quit % | Mean Yrs Quit | Short Term (n) | Short Term % |
|---|---|---|---|---|---|---|---|---|---|
| 20 | 50-54 | Never Any | 4711 | 43.16 | | | | | |
| 20 | 50-54 | CC | 2568 | 23.53 | 0 | 0.00 | | | |
| 20 | 50-54 | Former | 3637 | 33.32 | | | 17.69 | 169 | 4.65 |

Year of Follow-up: 2010, Age Group: 55-59

| Period | Age Group | Smoking Group | N | % | Ever Quit (N) | Ever Quit % | Mean Yrs Quit | Short Term (n) | Short Term % |
|---|---|---|---|---|---|---|---|---|---|
| 20 | 55-59 | Never Any | 3941 | 39.85 | | | | | |
| 20 | 55-59 | CC | 2005 | 20.28 | 0 | 0.00 | | | |
| 20 | 55-59 | Former | 3943 | 39.87 | | | 20.98 | 188 | 4.77 |

Year of Follow-up: 2010, Age Group: 60-64

| Period | Age Group | Smoking Group | N | % | Ever Quit (N) | Ever Quit % | Mean Yrs Quit | Short Term (n) | Short Term % |
|---|---|---|---|---|---|---|---|---|---|
| 20 | 60-64 | Never Any | 2912 | 33.65 | | | | | |
| 20 | 60-64 | CC | 1438 | 16.61 | 0 | 0.00 | | | |
| 20 | 60-64 | Former | 4305 | 49.74 | | | 22.38 | 221 | 5.13 |

Year of Follow-up: 2010, Age Group: 65-69

| Period | Age Group | Smoking Group | N | % | Ever Quit (N) | Ever Quit % | Mean Yrs Quit | Short Term (n) | Short Term % |
|---|---|---|---|---|---|---|---|---|---|
| 20 | 65-69 | Never Any | 1919 | 29.46 | | | | | |
| 20 | 65-69 | CC | 844 | 12.95 | 0 | 0.00 | | | |
| 20 | 65-69 | Former | 3752 | 57.59 | | | 23.99 | 116 | 3.09 |

Year of Follow-up: 2010, Age Group: 70-74

| Period | Age Group | Smoking Group | N | % | Ever Quit (N) | Ever Quit % | Mean Yrs Quit | Short Term (n) | Short Term % |
|---|---|---|---|---|---|---|---|---|---|
| 20 | 70-74 | Never Any | 1611 | 29.41 | | | | | |
| 20 | 70-74 | CC | 602 | 10.99 | 0 | 0.00 | | | |
| 20 | 70-74 | Former | 3264 | 59.59 | | | 25.56 | 74 | 2.27 |

Year of Follow-up: 2010, Age Group: 75-79

| Period | Age Group | Smoking Group | N | % | Ever Quit (N) | Ever Quit % | Mean Yrs Quit | Short Term (n) | Short Term % |
|---|---|---|---|---|---|---|---|---|---|
| 20 | 75-79 | Never Any | 1337 | 26.83 | | | | | |
| 20 | 75-79 | CC | 403 | 8.09 | 0 | 0.00 | | | |
| 20 | 75-79 | Former | 3243 | 65.08 | | | 26.69 | 53 | 1.63 |

Year of Follow-up: 2015, Age Group: All Ages

| Period | Age Group | Smoking Group | N | % | Ever Quit (N) | Ever Quit % | Mean Yrs Quit | Short Term (n) | Short Term % |
|---|---|---|---|---|---|---|---|---|---|
| 25 | All Ages | Never Any | 34539 | 43.27 | | | | | |
| 25 | All Ages | CC | 14583 | 18.27 | 0 | 0.00 | | | |
| 25 | All Ages | Former | 30695 | 38.46 | | | 21.97 | 1073 | 3.50 |

Year of Follow-up: 2015, Age Group: 18-19

| Period | Age Group | Smoking Group | N | % | Ever Quit (N) | Ever Quit % | Mean Yrs Quit | Short Term (n) | Short Term % |
|---|---|---|---|---|---|---|---|---|---|
| 25 | 18-19 | Never Any | 0 | . | | | | | |
| 25 | 18-19 | CC | 0 | . | 0 | . | | | |
| 25 | 18-19 | Former | 0 | . | | | . | 0 | . |

Year of Follow-up: 2015, Age Group: 20-24

| Period | Age Group | Smoking Group | N | % | Ever Quit (N) | Ever Quit % | Mean Yrs Quit | Short Term (n) | Short Term % |
|---|---|---|---|---|---|---|---|---|---|
| 25 | 20-24 | Never Any | 0 | . | | | | | |
| 25 | 20-24 | CC | 0 | . | 0 | . | | | |
| 25 | 20-24 | Former | 0 | . | | | . | 0 | . |

Year of Follow-up: 2015, Age Group: 25-29

| Period | Age Group | Smoking Group | N | % | Ever Quit (N) | Ever Quit % | Mean Yrs Quit | Short Term (n) | Short Term % |
|---|---|---|---|---|---|---|---|---|---|
| 25 | 25-29 | Never Any | 0 | . | | | | | |
| 25 | 25-29 | CC | 0 | . | 0 | . | | | |
| 25 | 25-29 | Former | 0 | . | | | . | 0 | . |

Year of Follow-up: 2015, Age Group: 30-34

| Period | Age Group | Smoking Group | N | % | Ever Quit (N) | Ever Quit % | Mean Yrs Quit | Short Term (n) | Short Term % |
|---|---|---|---|---|---|---|---|---|---|
| 25 | 30-34 | Never Any | 0 | . | | | | | |
| 25 | 30-34 | CC | 0 | . | 0 | . | | | |
| 25 | 30-34 | Former | 0 | . | | | . | 0 | . |

Year of Follow-up: 2015, Age Group: 35-39

| Period | Age Group | Smoking Group | N | % | Ever Quit (N) | Ever Quit % | Mean Yrs Quit | Short Term (n) | Short Term % |
|---|---|---|---|---|---|---|---|---|---|
| 25 | 35-39 | Never Any | 4363 | 49.33 | | | | | |
| 25 | 35-39 | CC | 2079 | 23.50 | 0 | 0.00 | | | |
| 25 | 35-39 | Former | 2403 | 27.17 | | | 11.50 | 140 | 5.83 |

Year of Follow-up: 2015, Age Group: 40-44

| Period | Age Group | Smoking Group | N | % | Ever Quit (N) | Ever Quit % | Mean Yrs Quit | Short Term (n) | Short Term % |
|---|---|---|---|---|---|---|---|---|---|

# Results for PHIM for Run 1 (Main_Analysis.RTF)

| | | | | | | | | | |
|---|---|---|---|---|---|---|---|---|---|
| 25 | 40-44 | Never Any | 4948 | 54.73 | | | | | |
| 25 | 40-44 | CC | 1988 | 21.99 | 0 | 0.00 | | | |
| 25 | 40-44 | Former | 2104 | 23.27 | | | 13.19 | 101 | 4.80 |

Year of Follow-up: 2015, Age Group: 45-49

| Period | Age Group | Smoking Group | N | % | Ever Quit (N) | Ever Quit % | Mean Yrs Quit | Short Term (n) | Short Term % |
|---|---|---|---|---|---|---|---|---|---|
| 25 | 45-49 | Never Any | 5145 | 52.41 | | | | | |
| 25 | 45-49 | CC | 2186 | 22.27 | 0 | 0.00 | | | |
| 25 | 45-49 | Former | 2485 | 25.32 | | | 17.30 | 81 | 3.26 |

Year of Follow-up: 2015, Age Group: 50-54

| Period | Age Group | Smoking Group | N | % | Ever Quit (N) | Ever Quit % | Mean Yrs Quit | Short Term (n) | Short Term % |
|---|---|---|---|---|---|---|---|---|---|
| 25 | 50-54 | Never Any | 4989 | 46.78 | | | | | |
| 25 | 50-54 | CC | 2518 | 23.61 | 0 | 0.00 | | | |
| 25 | 50-54 | Former | 3157 | 29.60 | | | 19.36 | 117 | 3.71 |

Year of Follow-up: 2015, Age Group: 55-59

| Period | Age Group | Smoking Group | N | % | Ever Quit (N) | Ever Quit % | Mean Yrs Quit | Short Term (n) | Short Term % |
|---|---|---|---|---|---|---|---|---|---|
| 25 | 55-59 | Never Any | 4711 | 43.16 | | | | | |
| 25 | 55-59 | CC | 2129 | 19.50 | 0 | 0.00 | | | |
| 25 | 55-59 | Former | 4076 | 37.34 | | | 20.47 | 180 | 4.42 |

Year of Follow-up: 2015, Age Group: 60-64

| Period | Age Group | Smoking Group | N | % | Ever Quit (N) | Ever Quit % | Mean Yrs Quit | Short Term (n) | Short Term % |
|---|---|---|---|---|---|---|---|---|---|
| 25 | 60-64 | Never Any | 3941 | 39.85 | | | | | |
| 25 | 60-64 | CC | 1565 | 15.83 | 0 | 0.00 | | | |
| 25 | 60-64 | Former | 4383 | 44.32 | | | 23.56 | 197 | 4.49 |

Year of Follow-up: 2015, Age Group: 65-69

| Period | Age Group | Smoking Group | N | % | Ever Quit (N) | Ever Quit % | Mean Yrs Quit | Short Term (n) | Short Term % |
|---|---|---|---|---|---|---|---|---|---|
| 25 | 65-69 | Never Any | 2912 | 33.65 | | | | | |
| 25 | 65-69 | CC | 1017 | 11.75 | 0 | 0.00 | | | |
| 25 | 65-69 | Former | 4726 | 54.60 | | | 25.14 | 136 | 2.88 |

Year of Follow-up: 2015, Age Group: 70-74

| Period | Age Group | Smoking Group | N | % | Ever Quit (N) | Ever Quit % | Mean Yrs Quit | Short Term (n) | Short Term % |
|---|---|---|---|---|---|---|---|---|---|
| 25 | 70-74 | Never Any | 1919 | 29.46 | | | | | |
| 25 | 70-74 | CC | 638 | 9.79 | 0 | 0.00 | | | |
| 25 | 70-74 | Former | 3958 | 60.75 | | | 27.59 | 75 | 1.89 |

Year of Follow-up: 2015, Age Group: 75-79

| Period | Age Group | Smoking Group | N | % | Ever Quit (N) | Ever Quit % | Mean Yrs Quit | Short Term (n) | Short Term % |
|---|---|---|---|---|---|---|---|---|---|
| 25 | 75-79 | Never Any | 1611 | 29.41 | | | | | |
| 25 | 75-79 | CC | 463 | 8.45 | 0 | 0.00 | | | |
| 25 | 75-79 | Former | 3403 | 62.13 | | | 29.40 | 46 | 1.35 |

Year of Follow-up: 2020, Age Group: All Ages

| Period | Age Group | Smoking Group | N | % | Ever Quit (N) | Ever Quit % | Mean Yrs Quit | Short Term (n) | Short Term % |
|---|---|---|---|---|---|---|---|---|---|
| 30 | All Ages | Never Any | 32928 | 44.29 | | | | | |
| 30 | All Ages | CC | 11664 | 15.69 | 0 | 0.00 | | | |
| 30 | All Ages | Former | 29748 | 40.02 | | | 24.06 | 964 | 3.24 |

Year of Follow-up: 2020, Age Group: 18-19

| Period | Age Group | Smoking Group | N | % | Ever Quit (N) | Ever Quit % | Mean Yrs Quit | Short Term (n) | Short Term % |
|---|---|---|---|---|---|---|---|---|---|
| 30 | 18-19 | Never Any | 0 | . | | | | | |
| 30 | 18-19 | CC | 0 | . | 0 | . | | | |
| 30 | 18-19 | Former | 0 | . | | | . | 0 | . |

Year of Follow-up: 2020, Age Group: 20-24

| Period | Age Group | Smoking Group | N | % | Ever Quit (N) | Ever Quit % | Mean Yrs Quit | Short Term (n) | Short Term % |
|---|---|---|---|---|---|---|---|---|---|
| 30 | 20-24 | Never Any | 0 | . | | | | | |
| 30 | 20-24 | CC | 0 | . | 0 | . | | | |
| 30 | 20-24 | Former | 0 | . | | | . | 0 | . |

Year of Follow-up: 2020, Age Group: 25-29

| Period | Age Group | Smoking Group | N | % | Ever Quit (N) | Ever Quit % | Mean Yrs Quit | Short Term (n) | Short Term % |
|---|---|---|---|---|---|---|---|---|---|
| 30 | 25-29 | Never Any | 0 | . | | | | | |
| 30 | 25-29 | CC | 0 | . | 0 | . | | | |
| 30 | 25-29 | Former | 0 | . | | | . | 0 | . |

Year of Follow-up: 2020, Age Group: 30-34

| Period | Age Group | Smoking Group | N | % | Ever Quit (N) | Ever Quit % | Mean Yrs Quit | Short Term (n) | Short Term % |
|---|---|---|---|---|---|---|---|---|---|
| 30 | 30-34 | Never Any | 0 | . | | | | | |
| 30 | 30-34 | CC | 0 | . | 0 | . | | | |
| 30 | 30-34 | Former | 0 | . | | | . | 0 | . |

Year of Follow-up: 2020, Age Group: 35-39

| Period | Age Group | Smoking Group | N | % | Ever Quit (N) | Ever Quit % | Mean Yrs Quit | Short Term (n) | Short Term % |
|---|---|---|---|---|---|---|---|---|---|

# Results for PHIM for Run 1 (Main_Analysis.RTF)

| | | | | | | | | | |
|---|---|---|---|---|---|---|---|---|---|
| 30 | 35-39 | Never Any | 0 | . | | | | | |
| 30 | 35-39 | CC | 0 | . | 0 | . | | | |
| 30 | 35-39 | Former | 0 | . | | | . | 0 | . |

Year of Follow-up: 2020, Age Group: 40-44

| Period | Age Group | Smoking Group | N | % | Ever Quit (N) | Ever Quit % | Mean Yrs Quit | Short Term (n) | Short Term % |
|---|---|---|---|---|---|---|---|---|---|
| 30 | 40-44 | Never Any | 4363 | 49.33 | | | | | |
| 30 | 40-44 | CC | 1810 | 20.46 | 0 | 0.00 | | | |
| 30 | 40-44 | Former | 2672 | 30.21 | | | 15.03 | 113 | 4.23 |

Year of Follow-up: 2020, Age Group: 45-49

| Period | Age Group | Smoking Group | N | % | Ever Quit (N) | Ever Quit % | Mean Yrs Quit | Short Term (n) | Short Term % |
|---|---|---|---|---|---|---|---|---|---|
| 30 | 45-49 | Never Any | 4948 | 54.73 | | | | | |
| 30 | 45-49 | CC | 1753 | 19.39 | 0 | 0.00 | | | |
| 30 | 45-49 | Former | 2339 | 25.87 | | | 16.60 | 68 | 2.91 |

Year of Follow-up: 2020, Age Group: 50-54

| Period | Age Group | Smoking Group | N | % | Ever Quit (N) | Ever Quit % | Mean Yrs Quit | Short Term (n) | Short Term % |
|---|---|---|---|---|---|---|---|---|---|
| 30 | 50-54 | Never Any | 5145 | 52.41 | | | | | |
| 30 | 50-54 | CC | 1976 | 20.13 | 0 | 0.00 | | | |
| 30 | 50-54 | Former | 2695 | 27.46 | | | 20.69 | 108 | 4.01 |

Year of Follow-up: 2020, Age Group: 55-59

| Period | Age Group | Smoking Group | N | % | Ever Quit (N) | Ever Quit % | Mean Yrs Quit | Short Term (n) | Short Term % |
|---|---|---|---|---|---|---|---|---|---|
| 30 | 55-59 | Never Any | 4989 | 46.78 | | | | | |
| 30 | 55-59 | CC | 2119 | 19.87 | 0 | 0.00 | | | |
| 30 | 55-59 | Former | 3556 | 33.35 | | | 21.84 | 166 | 4.67 |

Year of Follow-up: 2020, Age Group: 60-64

| Period | Age Group | Smoking Group | N | % | Ever Quit (N) | Ever Quit % | Mean Yrs Quit | Short Term (n) | Short Term % |
|---|---|---|---|---|---|---|---|---|---|
| 30 | 60-64 | Never Any | 4711 | 43.16 | | | | | |
| 30 | 60-64 | CC | 1618 | 14.82 | 0 | 0.00 | | | |
| 30 | 60-64 | Former | 4587 | 42.02 | | | 22.85 | 203 | 4.43 |

Year of Follow-up: 2020, Age Group: 65-69

| Period | Age Group | Smoking Group | N | % | Ever Quit (N) | Ever Quit % | Mean Yrs Quit | Short Term (n) | Short Term % |
|---|---|---|---|---|---|---|---|---|---|
| 30 | 65-69 | Never Any | 3941 | 39.85 | | | | | |
| 30 | 65-69 | CC | 1133 | 11.46 | 0 | 0.00 | | | |
| 30 | 65-69 | Former | 4815 | 48.69 | | | 26.19 | 150 | 3.12 |

Year of Follow-up: 2020, Age Group: 70-74

| Period | Age Group | Smoking Group | N | % | Ever Quit (N) | Ever Quit % | Mean Yrs Quit | Short Term (n) | Short Term % |
|---|---|---|---|---|---|---|---|---|---|
| 30 | 70-74 | Never Any | 2912 | 33.65 | | | | | |
| 30 | 70-74 | CC | 769 | 8.89 | 0 | 0.00 | | | |
| 30 | 70-74 | Former | 4974 | 57.47 | | | 28.74 | 95 | 1.91 |

Year of Follow-up: 2020, Age Group: 75-79

| Period | Age Group | Smoking Group | N | % | Ever Quit (N) | Ever Quit % | Mean Yrs Quit | Short Term (n) | Short Term % |
|---|---|---|---|---|---|---|---|---|---|
| 30 | 75-79 | Never Any | 1919 | 29.46 | | | | | |
| 30 | 75-79 | CC | 486 | 7.46 | 0 | 0.00 | | | |
| 30 | 75-79 | Former | 4110 | 63.09 | | | 31.46 | 61 | 1.48 |

Year of Follow-up: 2025, Age Group: All Ages

| Period | Age Group | Smoking Group | N | % | Ever Quit (N) | Ever Quit % | Mean Yrs Quit | Short Term (n) | Short Term % |
|---|---|---|---|---|---|---|---|---|---|
| 35 | All Ages | Never Any | 31009 | 45.72 | | | | | |
| 35 | All Ages | CC | 9033 | 13.32 | 0 | 0.00 | | | |
| 35 | All Ages | Former | 27783 | 40.96 | | | 25.88 | 811 | 2.92 |

Year of Follow-up: 2025, Age Group: 18-19

| Period | Age Group | Smoking Group | N | % | Ever Quit (N) | Ever Quit % | Mean Yrs Quit | Short Term (n) | Short Term % |
|---|---|---|---|---|---|---|---|---|---|
| 35 | 18-19 | Never Any | 0 | . | | | | | |
| 35 | 18-19 | CC | 0 | . | 0 | . | | | |
| 35 | 18-19 | Former | 0 | . | | | . | 0 | . |

Year of Follow-up: 2025, Age Group: 20-24

| Period | Age Group | Smoking Group | N | % | Ever Quit (N) | Ever Quit % | Mean Yrs Quit | Short Term (n) | Short Term % |
|---|---|---|---|---|---|---|---|---|---|
| 35 | 20-24 | Never Any | 0 | . | | | | | |
| 35 | 20-24 | CC | 0 | . | 0 | . | | | |
| 35 | 20-24 | Former | 0 | . | | | . | 0 | . |

Year of Follow-up: 2025, Age Group: 25-29

| Period | Age Group | Smoking Group | N | % | Ever Quit (N) | Ever Quit % | Mean Yrs Quit | Short Term (n) | Short Term % |
|---|---|---|---|---|---|---|---|---|---|
| 35 | 25-29 | Never Any | 0 | . | | | | | |
| 35 | 25-29 | CC | 0 | . | 0 | . | | | |
| 35 | 25-29 | Former | 0 | . | | | . | 0 | . |

Year of Follow-up: 2025, Age Group: 30-34

| Period | Age Group | Smoking Group | N | % | Ever Quit (N) | Ever Quit % | Mean Yrs Quit | Short Term (n) | Short Term % |
|---|---|---|---|---|---|---|---|---|---|

# Results for PHIM for Run 1 (Main_Analysis.RTF)

| | | | | | | | | | |
|---|---|---|---|---|---|---|---|---|---|
| 35 | 30-34 | Never Any | 0 | . | | | | | |
| 35 | 30-34 | CC | 0 | . | 0 | . | | | |
| 35 | 30-34 | Former | 0 | . | | | . | 0 | . |

Year of Follow-up: 2025, Age Group: 35-39

| Period | Age Group | Smoking Group | N | % | Ever Quit (N) | Ever Quit % | Mean Yrs Quit | Short Term (n) | Short Term % |
|---|---|---|---|---|---|---|---|---|---|
| 35 | 35-39 | Never Any | 0 | . | | | | | |
| 35 | 35-39 | CC | 0 | . | 0 | . | | | |
| 35 | 35-39 | Former | 0 | . | | | . | 0 | . |

Year of Follow-up: 2025, Age Group: 40-44

| Period | Age Group | Smoking Group | N | % | Ever Quit (N) | Ever Quit % | Mean Yrs Quit | Short Term (n) | Short Term % |
|---|---|---|---|---|---|---|---|---|---|
| 35 | 40-44 | Never Any | 0 | . | | | | | |
| 35 | 40-44 | CC | 0 | . | 0 | . | | | |
| 35 | 40-44 | Former | 0 | . | | | . | 0 | . |

Year of Follow-up: 2025, Age Group: 45-49

| Period | Age Group | Smoking Group | N | % | Ever Quit (N) | Ever Quit % | Mean Yrs Quit | Short Term (n) | Short Term % |
|---|---|---|---|---|---|---|---|---|---|
| 35 | 45-49 | Never Any | 4363 | 49.33 | | | | | |
| 35 | 45-49 | CC | 1611 | 18.21 | 0 | 0.00 | | | |
| 35 | 45-49 | Former | 2871 | 32.46 | | | 18.79 | 66 | 2.30 |

Year of Follow-up: 2025, Age Group: 50-54

| Period | Age Group | Smoking Group | N | % | Ever Quit (N) | Ever Quit % | Mean Yrs Quit | Short Term (n) | Short Term % |
|---|---|---|---|---|---|---|---|---|---|
| 35 | 50-54 | Never Any | 4948 | 54.73 | | | | | |
| 35 | 50-54 | CC | 1590 | 17.59 | 0 | 0.00 | | | |
| 35 | 50-54 | Former | 2502 | 27.68 | | | 20.31 | 78 | 3.12 |

Year of Follow-up: 2025, Age Group: 55-59

| Period | Age Group | Smoking Group | N | % | Ever Quit (N) | Ever Quit % | Mean Yrs Quit | Short Term (n) | Short Term % |
|---|---|---|---|---|---|---|---|---|---|
| 35 | 55-59 | Never Any | 5145 | 52.41 | | | | | |
| 35 | 55-59 | CC | 1654 | 16.85 | 0 | 0.00 | | | |
| 35 | 55-59 | Former | 3017 | 30.74 | | | 23.14 | 143 | 4.74 |

Year of Follow-up: 2025, Age Group: 60-64

| Period | Age Group | Smoking Group | N | % | Ever Quit (N) | Ever Quit % | Mean Yrs Quit | Short Term (n) | Short Term % |
|---|---|---|---|---|---|---|---|---|---|
| 35 | 60-64 | Never Any | 4989 | 46.78 | | | | | |
| 35 | 60-64 | CC | 1665 | 15.61 | 0 | 0.00 | | | |
| 35 | 60-64 | Former | 4010 | 37.60 | | | 24.03 | 181 | 4.51 |

Year of Follow-up: 2025, Age Group: 65-69

| Period | Age Group | Smoking Group | N | % | Ever Quit (N) | Ever Quit % | Mean Yrs Quit | Short Term (n) | Short Term % |
|---|---|---|---|---|---|---|---|---|---|
| 35 | 65-69 | Never Any | 4711 | 43.16 | | | | | |
| 35 | 65-69 | CC | 1116 | 10.22 | 0 | 0.00 | | | |
| 35 | 65-69 | Former | 5089 | 46.62 | | | 25.33 | 163 | 3.20 |

Year of Follow-up: 2025, Age Group: 70-74

| Period | Age Group | Smoking Group | N | % | Ever Quit (N) | Ever Quit % | Mean Yrs Quit | Short Term (n) | Short Term % |
|---|---|---|---|---|---|---|---|---|---|
| 35 | 70-74 | Never Any | 3941 | 39.85 | | | | | |
| 35 | 70-74 | CC | 838 | 8.47 | 0 | 0.00 | | | |
| 35 | 70-74 | Former | 5110 | 51.67 | | | 29.52 | 105 | 2.05 |

Year of Follow-up: 2025, Age Group: 75-79

| Period | Age Group | Smoking Group | N | % | Ever Quit (N) | Ever Quit % | Mean Yrs Quit | Short Term (n) | Short Term % |
|---|---|---|---|---|---|---|---|---|---|
| 35 | 75-79 | Never Any | 2912 | 33.65 | | | | | |
| 35 | 75-79 | CC | 559 | 6.46 | 0 | 0.00 | | | |
| 35 | 75-79 | Former | 5184 | 59.90 | | | 32.46 | 75 | 1.45 |

Year of Follow-up: 2030, Age Group: All Ages

| Period | Age Group | Smoking Group | N | % | Ever Quit (N) | Ever Quit % | Mean Yrs Quit | Short Term (n) | Short Term % |
|---|---|---|---|---|---|---|---|---|---|
| 40 | All Ages | Never Any | 28097 | 47.49 | | | | | |
| 40 | All Ages | CC | 6677 | 11.28 | 0 | 0.00 | | | |
| 40 | All Ages | Former | 24396 | 41.23 | | | 27.35 | 754 | 3.09 |

Year of Follow-up: 2030, Age Group: 18-19

| Period | Age Group | Smoking Group | N | % | Ever Quit (N) | Ever Quit % | Mean Yrs Quit | Short Term (n) | Short Term % |
|---|---|---|---|---|---|---|---|---|---|
| 40 | 18-19 | Never Any | 0 | . | | | | | |
| 40 | 18-19 | CC | 0 | . | 0 | . | | | |
| 40 | 18-19 | Former | 0 | . | | | . | 0 | . |

Year of Follow-up: 2030, Age Group: 20-24

| Period | Age Group | Smoking Group | N | % | Ever Quit (N) | Ever Quit % | Mean Yrs Quit | Short Term (n) | Short Term % |
|---|---|---|---|---|---|---|---|---|---|
| 40 | 20-24 | Never Any | 0 | . | | | | | |
| 40 | 20-24 | CC | 0 | . | 0 | . | | | |
| 40 | 20-24 | Former | 0 | . | | | . | 0 | . |

Year of Follow-up: 2030, Age Group: 25-29

| Period | Age Group | Smoking Group | N | % | Ever Quit (N) | Ever Quit % | Mean Yrs Quit | Short Term (n) | Short Term % |
|---|---|---|---|---|---|---|---|---|---|

# Results for PHIM for Run 1 (Main_Analysis.RTF)

| | | | | | | | | | |
|---|---|---|---|---|---|---|---|---|---|
| 40 | 25-29 | Never Any | 0 | . | | | | | |
| 40 | 25-29 | CC | 0 | . | 0 | . | | | |
| 40 | 25-29 | Former | 0 | . | | | . | 0 | . |

Year of Follow-up: 2030, Age Group: 30-34

| Period | Age Group | Smoking Group | N | % | Ever Quit (N) | Ever Quit % | Mean Yrs Quit | Short Term (n) | Short Term % |
|---|---|---|---|---|---|---|---|---|---|
| 40 | 30-34 | Never Any | 0 | . | | | | | |
| 40 | 30-34 | CC | 0 | . | 0 | . | | | |
| 40 | 30-34 | Former | 0 | . | | | . | 0 | . |

Year of Follow-up: 2030, Age Group: 35-39

| Period | Age Group | Smoking Group | N | % | Ever Quit (N) | Ever Quit % | Mean Yrs Quit | Short Term (n) | Short Term % |
|---|---|---|---|---|---|---|---|---|---|
| 40 | 35-39 | Never Any | 0 | . | | | | | |
| 40 | 35-39 | CC | 0 | . | 0 | . | | | |
| 40 | 35-39 | Former | 0 | . | | | . | 0 | . |

Year of Follow-up: 2030, Age Group: 40-44

| Period | Age Group | Smoking Group | N | % | Ever Quit (N) | Ever Quit % | Mean Yrs Quit | Short Term (n) | Short Term % |
|---|---|---|---|---|---|---|---|---|---|
| 40 | 40-44 | Never Any | 0 | . | | | | | |
| 40 | 40-44 | CC | 0 | . | 0 | . | | | |
| 40 | 40-44 | Former | 0 | . | | | . | 0 | . |

Year of Follow-up: 2030, Age Group: 45-49

| Period | Age Group | Smoking Group | N | % | Ever Quit (N) | Ever Quit % | Mean Yrs Quit | Short Term (n) | Short Term % |
|---|---|---|---|---|---|---|---|---|---|
| 40 | 45-49 | Never Any | 0 | . | | | | | |
| 40 | 45-49 | CC | 0 | . | 0 | . | | | |
| 40 | 45-49 | Former | 0 | . | | | . | 0 | . |

Year of Follow-up: 2030, Age Group: 50-54

| Period | Age Group | Smoking Group | N | % | Ever Quit (N) | Ever Quit % | Mean Yrs Quit | Short Term (n) | Short Term % |
|---|---|---|---|---|---|---|---|---|---|
| 40 | 50-54 | Never Any | 4363 | 49.33 | | | | | |
| 40 | 50-54 | CC | 1474 | 16.66 | 0 | 0.00 | | | |
| 40 | 50-54 | Former | 3008 | 34.01 | | | 22.78 | 73 | 2.43 |

Year of Follow-up: 2030, Age Group: 55-59

| Period | Age Group | Smoking Group | N | % | Ever Quit (N) | Ever Quit % | Mean Yrs Quit | Short Term (n) | Short Term % |
|---|---|---|---|---|---|---|---|---|---|
| 40 | 55-59 | Never Any | 4948 | 54.73 | | | | | |
| 40 | 55-59 | CC | 1338 | 14.80 | 0 | 0.00 | | | |
| 40 | 55-59 | Former | 2754 | 30.46 | | | 23.16 | 111 | 4.03 |

Year of Follow-up: 2030, Age Group: 60-64

| Period | Age Group | Smoking Group | N | % | Ever Quit (N) | Ever Quit % | Mean Yrs Quit | Short Term (n) | Short Term % |
|---|---|---|---|---|---|---|---|---|---|
| 40 | 60-64 | Never Any | 5145 | 52.41 | | | | | |
| 40 | 60-64 | CC | 1258 | 12.82 | 0 | 0.00 | | | |
| 40 | 60-64 | Former | 3413 | 34.77 | | | 25.09 | 185 | 5.42 |

Year of Follow-up: 2030, Age Group: 65-69

| Period | Age Group | Smoking Group | N | % | Ever Quit (N) | Ever Quit % | Mean Yrs Quit | Short Term (n) | Short Term % |
|---|---|---|---|---|---|---|---|---|---|
| 40 | 65-69 | Never Any | 4989 | 46.78 | | | | | |
| 40 | 65-69 | CC | 1177 | 11.04 | 0 | 0.00 | | | |
| 40 | 65-69 | Former | 4498 | 42.18 | | | 26.11 | 178 | 3.96 |

Year of Follow-up: 2030, Age Group: 70-74

| Period | Age Group | Smoking Group | N | % | Ever Quit (N) | Ever Quit % | Mean Yrs Quit | Short Term (n) | Short Term % |
|---|---|---|---|---|---|---|---|---|---|
| 40 | 70-74 | Never Any | 4711 | 43.16 | | | | | |
| 40 | 70-74 | CC | 835 | 7.65 | 0 | 0.00 | | | |
| 40 | 70-74 | Former | 5370 | 49.19 | | | 28.84 | 113 | 2.10 |

Year of Follow-up: 2030, Age Group: 75-79

| Period | Age Group | Smoking Group | N | % | Ever Quit (N) | Ever Quit % | Mean Yrs Quit | Short Term (n) | Short Term % |
|---|---|---|---|---|---|---|---|---|---|
| 40 | 75-79 | Never Any | 3941 | 39.85 | | | | | |
| 40 | 75-79 | CC | 595 | 6.02 | 0 | 0.00 | | | |
| 40 | 75-79 | Former | 5353 | 54.13 | | | 33.05 | 94 | 1.76 |

Year of Follow-up: 2035, Age Group: All Ages

| Period | Age Group | Smoking Group | N | % | Ever Quit (N) | Ever Quit % | Mean Yrs Quit | Short Term (n) | Short Term % |
|---|---|---|---|---|---|---|---|---|---|
| 45 | All Ages | Never Any | 24156 | 49.02 | | | | | |
| 45 | All Ages | CC | 4673 | 9.48 | 0 | 0.00 | | | |
| 45 | All Ages | Former | 20452 | 41.50 | | | 28.77 | 524 | 2.56 |

Year of Follow-up: 2035, Age Group: 18-19

| Period | Age Group | Smoking Group | N | % | Ever Quit (N) | Ever Quit % | Mean Yrs Quit | Short Term (n) | Short Term % |
|---|---|---|---|---|---|---|---|---|---|
| 45 | 18-19 | Never Any | 0 | . | | | | | |
| 45 | 18-19 | CC | 0 | . | 0 | . | | | |
| 45 | 18-19 | Former | 0 | . | | | . | 0 | . |

Year of Follow-up: 2035, Age Group: 20-24

| Period | Age Group | Smoking Group | N | % | Ever Quit (N) | Ever Quit % | Mean Yrs Quit | Short Term (n) | Short Term % |
|---|---|---|---|---|---|---|---|---|---|

# Results for PHIM for Run 1 (Main_Analysis.RTF)

| | | | | | | | | | |
|---|---|---|---|---|---|---|---|---|---|
| 45 | 20-24 | Never Any | 0 | . | | | | | |
| 45 | 20-24 | CC | 0 | . | 0 | . | | | |
| 45 | 20-24 | Former | 0 | . | | | . | 0 | . |

Year of Follow-up: 2035, Age Group: 25-29

| Period | Age Group | Smoking Group | N | % | Ever Quit (N) | Ever Quit % | Mean Yrs Quit | Short Term (n) | Short Term % |
|---|---|---|---|---|---|---|---|---|---|
| 45 | 25-29 | Never Any | 0 | . | | | | | |
| 45 | 25-29 | CC | 0 | . | 0 | . | | | |
| 45 | 25-29 | Former | 0 | . | | | . | 0 | . |

Year of Follow-up: 2035, Age Group: 30-34

| Period | Age Group | Smoking Group | N | % | Ever Quit (N) | Ever Quit % | Mean Yrs Quit | Short Term (n) | Short Term % |
|---|---|---|---|---|---|---|---|---|---|
| 45 | 30-34 | Never Any | 0 | . | | | | | |
| 45 | 30-34 | CC | 0 | . | 0 | . | | | |
| 45 | 30-34 | Former | 0 | . | | | . | 0 | . |

Year of Follow-up: 2035, Age Group: 35-39

| Period | Age Group | Smoking Group | N | % | Ever Quit (N) | Ever Quit % | Mean Yrs Quit | Short Term (n) | Short Term % |
|---|---|---|---|---|---|---|---|---|---|
| 45 | 35-39 | Never Any | 0 | . | | | | | |
| 45 | 35-39 | CC | 0 | . | 0 | . | | | |
| 45 | 35-39 | Former | 0 | . | | | . | 0 | . |

Year of Follow-up: 2035, Age Group: 40-44

| Period | Age Group | Smoking Group | N | % | Ever Quit (N) | Ever Quit % | Mean Yrs Quit | Short Term (n) | Short Term % |
|---|---|---|---|---|---|---|---|---|---|
| 45 | 40-44 | Never Any | 0 | . | | | | | |
| 45 | 40-44 | CC | 0 | . | 0 | . | | | |
| 45 | 40-44 | Former | 0 | . | | | . | 0 | . |

Year of Follow-up: 2035, Age Group: 45-49

| Period | Age Group | Smoking Group | N | % | Ever Quit (N) | Ever Quit % | Mean Yrs Quit | Short Term (n) | Short Term % |
|---|---|---|---|---|---|---|---|---|---|
| 45 | 45-49 | Never Any | 0 | . | | | | | |
| 45 | 45-49 | CC | 0 | . | 0 | . | | | |
| 45 | 45-49 | Former | 0 | . | | | . | 0 | . |

Year of Follow-up: 2035, Age Group: 50-54

| Period | Age Group | Smoking Group | N | % | Ever Quit (N) | Ever Quit % | Mean Yrs Quit | Short Term (n) | Short Term % |
|---|---|---|---|---|---|---|---|---|---|
| 45 | 50-54 | Never Any | 0 | . | | | | | |
| 45 | 50-54 | CC | 0 | . | 0 | . | | | |
| 45 | 50-54 | Former | 0 | . | | | . | 0 | . |

Year of Follow-up: 2035, Age Group: 55-59

| Period | Age Group | Smoking Group | N | % | Ever Quit (N) | Ever Quit % | Mean Yrs Quit | Short Term (n) | Short Term % |
|---|---|---|---|---|---|---|---|---|---|
| 45 | 55-59 | Never Any | 4363 | 49.33 | | | | | |
| 45 | 55-59 | CC | 1233 | 13.94 | 0 | 0.00 | | | |
| 45 | 55-59 | Former | 3249 | 36.73 | | | 25.87 | 94 | 2.89 |

Year of Follow-up: 2035, Age Group: 60-64

| Period | Age Group | Smoking Group | N | % | Ever Quit (N) | Ever Quit % | Mean Yrs Quit | Short Term (n) | Short Term % |
|---|---|---|---|---|---|---|---|---|---|
| 45 | 60-64 | Never Any | 4948 | 54.73 | | | | | |
| 45 | 60-64 | CC | 1020 | 11.28 | 0 | 0.00 | | | |
| 45 | 60-64 | Former | 3072 | 33.98 | | | 25.45 | 128 | 4.17 |

Year of Follow-up: 2035, Age Group: 65-69

| Period | Age Group | Smoking Group | N | % | Ever Quit (N) | Ever Quit % | Mean Yrs Quit | Short Term (n) | Short Term % |
|---|---|---|---|---|---|---|---|---|---|
| 45 | 65-69 | Never Any | 5145 | 52.41 | | | | | |
| 45 | 65-69 | CC | 923 | 9.40 | 0 | 0.00 | | | |
| 45 | 65-69 | Former | 3748 | 38.18 | | | 27.59 | 116 | 3.09 |

Year of Follow-up: 2035, Age Group: 70-74

| Period | Age Group | Smoking Group | N | % | Ever Quit (N) | Ever Quit % | Mean Yrs Quit | Short Term (n) | Short Term % |
|---|---|---|---|---|---|---|---|---|---|
| 45 | 70-74 | Never Any | 4989 | 46.78 | | | | | |
| 45 | 70-74 | CC | 865 | 8.11 | 0 | 0.00 | | | |
| 45 | 70-74 | Former | 4810 | 45.11 | | | 29.23 | 111 | 2.31 |

Year of Follow-up: 2035, Age Group: 75-79

| Period | Age Group | Smoking Group | N | % | Ever Quit (N) | Ever Quit % | Mean Yrs Quit | Short Term (n) | Short Term % |
|---|---|---|---|---|---|---|---|---|---|
| 45 | 75-79 | Never Any | 4711 | 43.16 | | | | | |
| 45 | 75-79 | CC | 632 | 5.79 | 0 | 0.00 | | | |
| 45 | 75-79 | Former | 5573 | 51.05 | | | 32.69 | 75 | 1.35 |

Year of Follow-up: 2040, Age Group: All Ages

| Period | Age Group | Smoking Group | N | % | Ever Quit (N) | Ever Quit % | Mean Yrs Quit | Short Term (n) | Short Term % |
|---|---|---|---|---|---|---|---|---|---|
| 50 | All Ages | Never Any | 19445 | 50.68 | | | | | |
| 50 | All Ages | CC | 3024 | 7.88 | 0 | 0.00 | | | |
| 50 | All Ages | Former | 15896 | 41.43 | | | 30.37 | 356 | 2.24 |

Year of Follow-up: 2040, Age Group: 18-19

| Period | Age Group | Smoking Group | N | % | Ever Quit (N) | Ever Quit % | Mean Yrs Quit | Short Term (n) | Short Term % |
|---|---|---|---|---|---|---|---|---|---|

# Results for PHIM for Run 1 (Main_Analysis.RTF)

| | | | | | | | | | |
|---|---|---|---|---|---|---|---|---|---|
| 50 | 18-19 | Never Any | 0 | . | | | | | |
| 50 | 18-19 | CC | 0 | . | 0 | . | | | |
| 50 | 18-19 | Former | 0 | . | | | . | 0 | . |

Year of Follow-up: 2040, Age Group: 20-24

| Period | Age Group | Smoking Group | N | % | Ever Quit (N) | Ever Quit % | Mean Yrs Quit | Short Term (n) | Short Term % |
|---|---|---|---|---|---|---|---|---|---|
| 50 | 20-24 | Never Any | 0 | . | | | | | |
| 50 | 20-24 | CC | 0 | . | 0 | . | | | |
| 50 | 20-24 | Former | 0 | . | | | . | 0 | . |

Year of Follow-up: 2040, Age Group: 25-29

| Period | Age Group | Smoking Group | N | % | Ever Quit (N) | Ever Quit % | Mean Yrs Quit | Short Term (n) | Short Term % |
|---|---|---|---|---|---|---|---|---|---|
| 50 | 25-29 | Never Any | 0 | . | | | | | |
| 50 | 25-29 | CC | 0 | . | 0 | . | | | |
| 50 | 25-29 | Former | 0 | . | | | . | 0 | . |

Year of Follow-up: 2040, Age Group: 30-34

| Period | Age Group | Smoking Group | N | % | Ever Quit (N) | Ever Quit % | Mean Yrs Quit | Short Term (n) | Short Term % |
|---|---|---|---|---|---|---|---|---|---|
| 50 | 30-34 | Never Any | 0 | . | | | | | |
| 50 | 30-34 | CC | 0 | . | 0 | . | | | |
| 50 | 30-34 | Former | 0 | . | | | . | 0 | . |

Year of Follow-up: 2040, Age Group: 35-39

| Period | Age Group | Smoking Group | N | % | Ever Quit (N) | Ever Quit % | Mean Yrs Quit | Short Term (n) | Short Term % |
|---|---|---|---|---|---|---|---|---|---|
| 50 | 35-39 | Never Any | 0 | . | | | | | |
| 50 | 35-39 | CC | 0 | . | 0 | . | | | |
| 50 | 35-39 | Former | 0 | . | | | . | 0 | . |

Year of Follow-up: 2040, Age Group: 40-44

| Period | Age Group | Smoking Group | N | % | Ever Quit (N) | Ever Quit % | Mean Yrs Quit | Short Term (n) | Short Term % |
|---|---|---|---|---|---|---|---|---|---|
| 50 | 40-44 | Never Any | 0 | . | | | | | |
| 50 | 40-44 | CC | 0 | . | 0 | . | | | |
| 50 | 40-44 | Former | 0 | . | | | . | 0 | . |

Year of Follow-up: 2040, Age Group: 45-49

| Period | Age Group | Smoking Group | N | % | Ever Quit (N) | Ever Quit % | Mean Yrs Quit | Short Term (n) | Short Term % |
|---|---|---|---|---|---|---|---|---|---|
| 50 | 45-49 | Never Any | 0 | . | | | | | |
| 50 | 45-49 | CC | 0 | . | 0 | . | | | |
| 50 | 45-49 | Former | 0 | . | | | . | 0 | . |

Year of Follow-up: 2040, Age Group: 50-54

| Period | Age Group | Smoking Group | N | % | Ever Quit (N) | Ever Quit % | Mean Yrs Quit | Short Term (n) | Short Term % |
|---|---|---|---|---|---|---|---|---|---|
| 50 | 50-54 | Never Any | 0 | . | | | | | |
| 50 | 50-54 | CC | 0 | . | 0 | . | | | |
| 50 | 50-54 | Former | 0 | . | | | . | 0 | . |

Year of Follow-up: 2040, Age Group: 55-59

| Period | Age Group | Smoking Group | N | % | Ever Quit (N) | Ever Quit % | Mean Yrs Quit | Short Term (n) | Short Term % |
|---|---|---|---|---|---|---|---|---|---|
| 50 | 55-59 | Never Any | 0 | . | | | | | |
| 50 | 55-59 | CC | 0 | . | 0 | . | | | |
| 50 | 55-59 | Former | 0 | . | | | . | 0 | . |

Year of Follow-up: 2040, Age Group: 60-64

| Period | Age Group | Smoking Group | N | % | Ever Quit (N) | Ever Quit % | Mean Yrs Quit | Short Term (n) | Short Term % |
|---|---|---|---|---|---|---|---|---|---|
| 50 | 60-64 | Never Any | 4363 | 49.33 | | | | | |
| 50 | 60-64 | CC | 934 | 10.56 | 0 | 0.00 | | | |
| 50 | 60-64 | Former | 3548 | 40.11 | | | 28.44 | 109 | 3.07 |

Year of Follow-up: 2040, Age Group: 65-69

| Period | Age Group | Smoking Group | N | % | Ever Quit (N) | Ever Quit % | Mean Yrs Quit | Short Term (n) | Short Term % |
|---|---|---|---|---|---|---|---|---|---|
| 50 | 65-69 | Never Any | 4948 | 54.73 | | | | | |
| 50 | 65-69 | CC | 734 | 8.12 | 0 | 0.00 | | | |
| 50 | 65-69 | Former | 3358 | 37.15 | | | 28.04 | 94 | 2.80 |

Year of Follow-up: 2040, Age Group: 70-74

| Period | Age Group | Smoking Group | N | % | Ever Quit (N) | Ever Quit % | Mean Yrs Quit | Short Term (n) | Short Term % |
|---|---|---|---|---|---|---|---|---|---|
| 50 | 70-74 | Never Any | 5145 | 52.41 | | | | | |
| 50 | 70-74 | CC | 688 | 7.01 | 0 | 0.00 | | | |
| 50 | 70-74 | Former | 3983 | 40.58 | | | 30.80 | 81 | 2.03 |

Year of Follow-up: 2040, Age Group: 75-79

| Period | Age Group | Smoking Group | N | % | Ever Quit (N) | Ever Quit % | Mean Yrs Quit | Short Term (n) | Short Term % |
|---|---|---|---|---|---|---|---|---|---|
| 50 | 75-79 | Never Any | 4989 | 46.78 | | | | | |
| 50 | 75-79 | CC | 668 | 6.26 | 0 | 0.00 | | | |
| 50 | 75-79 | Former | 5007 | 46.95 | | | 32.97 | 72 | 1.44 |

# Results for PHIM for Run 1 (Main_Analysis.RTF)

************************************************************************************

**Main Output for the Tobacco History for Run: 1, Simulation: 1, Scenario: Ecig**

| **Variable Parameter** | **Value** |
|---|---|
| 1. Country | US (United States) |
| 2. Sex | M (Males) |
| 3. Year of start of process | 1990 |

Year of Follow-up: 1990, Age Group: All Ages

| Period | Age Group | Smoking Group | N | % | Ever Quit (N) | Ever Quit % | Mean Yrs Quit | Short Term (n) | Short Term % |
|---|---|---|---|---|---|---|---|---|---|
| 0 | All Ages | Never Any | 34075 | 39.78 | | | | | |
| 0 | All Ages | CC | 27323 | 31.90 | 0 | 0.00 | | | |
| 0 | All Ages | Ecig | 0 | 0.00 | | | | | |
| 0 | All Ages | Dual | 0 | 0.00 | | | | | |
| 0 | All Ages | Former | 24266 | 28.33 | | | 13.08 | 3623 | 14.93 |

Year of Follow-up: 1990, Age Group: 18-19

| Period | Age Group | Smoking Group | N | % | Ever Quit (N) | Ever Quit % | Mean Yrs Quit | Short Term (n) | Short Term % |
|---|---|---|---|---|---|---|---|---|---|
| 0 | 18-19 | Never Any | 2783 | 78.42 | | | | | |
| 0 | 18-19 | CC | 634 | 17.86 | 0 | 0.00 | | | |
| 0 | 18-19 | Ecig | 0 | 0.00 | | | | | |
| 0 | 18-19 | Dual | 0 | 0.00 | | | | | |
| 0 | 18-19 | Former | 132 | 3.72 | | | 0.11 | 132 | 100.00 |

Year of Follow-up: 1990, Age Group: 20-24

| Period | Age Group | Smoking Group | N | % | Ever Quit (N) | Ever Quit % | Mean Yrs Quit | Short Term (n) | Short Term % |
|---|---|---|---|---|---|---|---|---|---|
| 0 | 20-24 | Never Any | 5722 | 58.29 | | | | | |
| 0 | 20-24 | CC | 3199 | 32.59 | 0 | 0.00 | | | |
| 0 | 20-24 | Ecig | 0 | 0.00 | | | | | |
| 0 | 20-24 | Dual | 0 | 0.00 | | | | | |
| 0 | 20-24 | Former | 895 | 9.12 | | | 1.46 | 506 | 56.54 |

Year of Follow-up: 1990, Age Group: 25-29

| Period | Age Group | Smoking Group | N | % | Ever Quit (N) | Ever Quit % | Mean Yrs Quit | Short Term (n) | Short Term % |
|---|---|---|---|---|---|---|---|---|---|
| 0 | 25-29 | Never Any | 5232 | 49.06 | | | | | |
| 0 | 25-29 | CC | 3914 | 36.70 | 0 | 0.00 | | | |
| 0 | 25-29 | Ecig | 0 | 0.00 | | | | | |
| 0 | 25-29 | Dual | 0 | 0.00 | | | | | |
| 0 | 25-29 | Former | 1518 | 14.23 | | | 2.87 | 643 | 42.36 |

Year of Follow-up: 1990, Age Group: 30-34

| Period | Age Group | Smoking Group | N | % | Ever Quit (N) | Ever Quit % | Mean Yrs Quit | Short Term (n) | Short Term % |
|---|---|---|---|---|---|---|---|---|---|
| 0 | 30-34 | Never Any | 4765 | 43.65 | | | | | |
| 0 | 30-34 | CC | 4023 | 36.85 | 0 | 0.00 | | | |
| 0 | 30-34 | Ecig | 0 | 0.00 | | | | | |
| 0 | 30-34 | Dual | 0 | 0.00 | | | | | |
| 0 | 30-34 | Former | 2128 | 19.49 | | | 4.06 | 592 | 27.82 |

Year of Follow-up: 1990, Age Group: 35-39

| Period | Age Group | Smoking Group | N | % | Ever Quit (N) | Ever Quit % | Mean Yrs Quit | Short Term (n) | Short Term % |
|---|---|---|---|---|---|---|---|---|---|
| 0 | 35-39 | Never Any | 3941 | 39.85 | | | | | |
| 0 | 35-39 | CC | 3474 | 35.13 | 0 | 0.00 | | | |
| 0 | 35-39 | Ecig | 0 | 0.00 | | | | | |
| 0 | 35-39 | Dual | 0 | 0.00 | | | | | |
| 0 | 35-39 | Former | 2474 | 25.02 | | | 8.31 | 425 | 17.18 |

Year of Follow-up: 1990, Age Group: 40-44

| Period | Age Group | Smoking Group | N | % | Ever Quit (N) | Ever Quit % | Mean Yrs Quit | Short Term (n) | Short Term % |
|---|---|---|---|---|---|---|---|---|---|
| 0 | 40-44 | Never Any | 2912 | 33.65 | | | | | |
| 0 | 40-44 | CC | 3113 | 35.97 | 0 | 0.00 | | | |
| 0 | 40-44 | Ecig | 0 | 0.00 | | | | | |
| 0 | 40-44 | Dual | 0 | 0.00 | | | | | |
| 0 | 40-44 | Former | 2630 | 30.39 | | | 10.69 | 317 | 12.05 |

Year of Follow-up: 1990, Age Group: 45-49

| Period | Age Group | Smoking Group | N | % | Ever Quit (N) | Ever Quit % | Mean Yrs Quit | Short Term (n) | Short Term % |
|---|---|---|---|---|---|---|---|---|---|
| 0 | 45-49 | Never Any | 1919 | 29.46 | | | | | |
| 0 | 45-49 | CC | 2318 | 35.58 | 0 | 0.00 | | | |
| 0 | 45-49 | Ecig | 0 | 0.00 | | | | | |
| 0 | 45-49 | Dual | 0 | 0.00 | | | | | |
| 0 | 45-49 | Former | 2278 | 34.97 | | | 13.26 | 221 | 9.70 |

Year of Follow-up: 1990, Age Group: 50-54

| Period | Age Group | Smoking Group | N | % | Ever Quit (N) | Ever Quit % | Mean Yrs Quit | Short Term (n) | Short Term % |
|---|---|---|---|---|---|---|---|---|---|
| 0 | 50-54 | Never Any | 1611 | 29.41 | | | | | |
| 0 | 50-54 | CC | 1816 | 33.16 | 0 | 0.00 | | | |

# Results for PHIM for Run 1 (Main_Analysis.RTF)

| | | | | | | | | | |
|---|---|---|---|---|---|---|---|---|---|
| 0 | 50-54 | Ecig | 0 | 0.00 | | | | | |
| 0 | 50-54 | Dual | 0 | 0.00 | | | | | |
| 0 | 50-54 | Former | 2050 | 37.43 | | | 14.55 | 261 | 12.73 |

Year of Follow-up: 1990, Age Group: 55-59

| Period | Age Group | Smoking Group | N | % | Ever Quit (N) | Ever Quit % | Mean Yrs Quit | Short Term (n) | Short Term % |
|---|---|---|---|---|---|---|---|---|---|
| 0 | 55-59 | Never Any | 1337 | 26.83 | | | | | |
| 0 | 55-59 | CC | 1520 | 30.50 | 0 | 0.00 | | | |
| 0 | 55-59 | Ecig | 0 | 0.00 | | | | | |
| 0 | 55-59 | Dual | 0 | 0.00 | | | | | |
| 0 | 55-59 | Former | 2126 | 42.67 | | | 14.80 | 216 | 10.16 |

Year of Follow-up: 1990, Age Group: 60-64

| Period | Age Group | Smoking Group | N | % | Ever Quit (N) | Ever Quit % | Mean Yrs Quit | Short Term (n) | Short Term % |
|---|---|---|---|---|---|---|---|---|---|
| 0 | 60-64 | Never Any | 1169 | 23.83 | | | | | |
| 0 | 60-64 | CC | 1287 | 26.24 | 0 | 0.00 | | | |
| 0 | 60-64 | Ecig | 0 | 0.00 | | | | | |
| 0 | 60-64 | Dual | 0 | 0.00 | | | | | |
| 0 | 60-64 | Former | 2449 | 49.93 | | | 18.87 | 106 | 4.33 |

Year of Follow-up: 1990, Age Group: 65-69

| Period | Age Group | Smoking Group | N | % | Ever Quit (N) | Ever Quit % | Mean Yrs Quit | Short Term (n) | Short Term % |
|---|---|---|---|---|---|---|---|---|---|
| 0 | 65-69 | Never Any | 1023 | 23.32 | | | | | |
| 0 | 65-69 | CC | 979 | 22.32 | 0 | 0.00 | | | |
| 0 | 65-69 | Ecig | 0 | 0.00 | | | | | |
| 0 | 65-69 | Dual | 0 | 0.00 | | | | | |
| 0 | 65-69 | Former | 2385 | 54.37 | | | 19.91 | 113 | 4.74 |

Year of Follow-up: 1990, Age Group: 70-74

| Period | Age Group | Smoking Group | N | % | Ever Quit (N) | Ever Quit % | Mean Yrs Quit | Short Term (n) | Short Term % |
|---|---|---|---|---|---|---|---|---|---|
| 0 | 70-74 | Never Any | 961 | 27.46 | | | | | |
| 0 | 70-74 | CC | 675 | 19.29 | 0 | 0.00 | | | |
| 0 | 70-74 | Ecig | 0 | 0.00 | | | | | |
| 0 | 70-74 | Dual | 0 | 0.00 | | | | | |
| 0 | 70-74 | Former | 1863 | 53.24 | | | 20.69 | 23 | 1.23 |

Year of Follow-up: 1990, Age Group: 75-79

| Period | Age Group | Smoking Group | N | % | Ever Quit (N) | Ever Quit % | Mean Yrs Quit | Short Term (n) | Short Term % |
|---|---|---|---|---|---|---|---|---|---|
| 0 | 75-79 | Never Any | 700 | 29.06 | | | | | |
| 0 | 75-79 | CC | 371 | 15.40 | 0 | 0.00 | | | |
| 0 | 75-79 | Ecig | 0 | 0.00 | | | | | |
| 0 | 75-79 | Dual | 0 | 0.00 | | | | | |
| 0 | 75-79 | Former | 1338 | 55.54 | | | 22.88 | 68 | 5.08 |

Year of Follow-up: 1995, Age Group: All Ages

| Period | Age Group | Smoking Group | N | % | Ever Quit (N) | Ever Quit % | Mean Yrs Quit | Short Term (n) | Short Term % |
|---|---|---|---|---|---|---|---|---|---|
| 5 | All Ages | Never Any | 38368 | 41.57 | | | | | |
| 5 | All Ages | CC | 11508 | 12.47 | 0 | 0.00 | | | |
| 5 | All Ages | Ecig | 15517 | 16.81 | | | | | |
| 5 | All Ages | Dual | 0 | 0.00 | | | | | |
| 5 | All Ages | Former | 26897 | 29.14 | | | 15.19 | 1705 | 6.34 |

Year of Follow-up: 1995, Age Group: 18-19

| Period | Age Group | Smoking Group | N | % | Ever Quit (N) | Ever Quit % | Mean Yrs Quit | Short Term (n) | Short Term % |
|---|---|---|---|---|---|---|---|---|---|
| 5 | 18-19 | Never Any | 2448 | 69.07 | | | | | |
| 5 | 18-19 | CC | 320 | 9.03 | 0 | 0.00 | | | |
| 5 | 18-19 | Ecig | 659 | 18.59 | | | | | |
| 5 | 18-19 | Dual | 0 | 0.00 | | | | | |
| 5 | 18-19 | Former | 117 | 3.30 | | | 1.11 | 80 | 68.38 |

Year of Follow-up: 1995, Age Group: 20-24

| Period | Age Group | Smoking Group | N | % | Ever Quit (N) | Ever Quit % | Mean Yrs Quit | Short Term (n) | Short Term % |
|---|---|---|---|---|---|---|---|---|---|
| 5 | 20-24 | Never Any | 5787 | 64.02 | | | | | |
| 5 | 20-24 | CC | 946 | 10.46 | 0 | 0.00 | | | |
| 5 | 20-24 | Ecig | 1826 | 20.20 | | | | | |
| 5 | 20-24 | Dual | 0 | 0.00 | | | | | |
| 5 | 20-24 | Former | 481 | 5.32 | | | 2.81 | 142 | 29.52 |

Year of Follow-up: 1995, Age Group: 25-29

| Period | Age Group | Smoking Group | N | % | Ever Quit (N) | Ever Quit % | Mean Yrs Quit | Short Term (n) | Short Term % |
|---|---|---|---|---|---|---|---|---|---|
| 5 | 25-29 | Never Any | 5471 | 55.74 | | | | | |
| 5 | 25-29 | CC | 1255 | 12.79 | 0 | 0.00 | | | |
| 5 | 25-29 | Ecig | 1942 | 19.78 | | | | | |
| 5 | 25-29 | Dual | 0 | 0.00 | | | | | |
| 5 | 25-29 | Former | 1148 | 11.70 | | | 5.43 | 115 | 10.02 |

Year of Follow-up: 1995, Age Group: 30-34

| Period | Age Group | Smoking Group | N | % | Ever Quit (N) | Ever Quit % | Mean Yrs Quit | Short Term (n) | Short Term % |
|---|---|---|---|---|---|---|---|---|---|

# Results for PHIM for Run 1 (Main_Analysis.RTF)

| | | | | | | | | | |
|---|---|---|---|---|---|---|---|---|---|
| 5 | 30-34 | Never Any | 5065 | 47.50 | | | | | |
| 5 | 30-34 | CC | 1480 | 13.88 | 0 | 0.00 | | | |
| 5 | 30-34 | Ecig | 2319 | 21.75 | | | | | |
| 5 | 30-34 | Dual | 0 | 0.00 | | | | | |
| 5 | 30-34 | Former | 1800 | 16.88 | | | 6.94 | 106 | 5.89 |

Year of Follow-up: 1995, Age Group: 35-39

| Period | Age Group | Smoking Group | N | % | Ever Quit (N) | Ever Quit % | Mean Yrs Quit | Short Term (n) | Short Term % |
|---|---|---|---|---|---|---|---|---|---|
| 5 | 35-39 | Never Any | 4724 | 43.28 | | | | | |
| 5 | 35-39 | CC | 1496 | 13.70 | 0 | 0.00 | | | |
| 5 | 35-39 | Ecig | 2275 | 20.84 | | | | | |
| 5 | 35-39 | Dual | 0 | 0.00 | | | | | |
| 5 | 35-39 | Former | 2421 | 22.18 | | | 8.20 | 114 | 4.71 |

Year of Follow-up: 1995, Age Group: 40-44

| Period | Age Group | Smoking Group | N | % | Ever Quit (N) | Ever Quit % | Mean Yrs Quit | Short Term (n) | Short Term % |
|---|---|---|---|---|---|---|---|---|---|
| 5 | 40-44 | Never Any | 3941 | 39.85 | | | | | |
| 5 | 40-44 | CC | 1280 | 12.94 | 0 | 0.00 | | | |
| 5 | 40-44 | Ecig | 1894 | 19.15 | | | | | |
| 5 | 40-44 | Dual | 0 | 0.00 | | | | | |
| 5 | 40-44 | Former | 2774 | 28.05 | | | 12.06 | 130 | 4.69 |

Year of Follow-up: 1995, Age Group: 45-49

| Period | Age Group | Smoking Group | N | % | Ever Quit (N) | Ever Quit % | Mean Yrs Quit | Short Term (n) | Short Term % |
|---|---|---|---|---|---|---|---|---|---|
| 5 | 45-49 | Never Any | 2912 | 33.65 | | | | | |
| 5 | 45-49 | CC | 1236 | 14.28 | 0 | 0.00 | | | |
| 5 | 45-49 | Ecig | 1562 | 18.05 | | | | | |
| 5 | 45-49 | Dual | 0 | 0.00 | | | | | |
| 5 | 45-49 | Former | 2945 | 34.03 | | | 14.20 | 148 | 5.03 |

Year of Follow-up: 1995, Age Group: 50-54

| Period | Age Group | Smoking Group | N | % | Ever Quit (N) | Ever Quit % | Mean Yrs Quit | Short Term (n) | Short Term % |
|---|---|---|---|---|---|---|---|---|---|
| 5 | 50-54 | Never Any | 1919 | 29.46 | | | | | |
| 5 | 50-54 | CC | 940 | 14.43 | 0 | 0.00 | | | |
| 5 | 50-54 | Ecig | 1015 | 15.58 | | | | | |
| 5 | 50-54 | Dual | 0 | 0.00 | | | | | |
| 5 | 50-54 | Former | 2641 | 40.54 | | | 15.98 | 175 | 6.63 |

Year of Follow-up: 1995, Age Group: 55-59

| Period | Age Group | Smoking Group | N | % | Ever Quit (N) | Ever Quit % | Mean Yrs Quit | Short Term (n) | Short Term % |
|---|---|---|---|---|---|---|---|---|---|
| 5 | 55-59 | Never Any | 1611 | 29.41 | | | | | |
| 5 | 55-59 | CC | 740 | 13.51 | 0 | 0.00 | | | |
| 5 | 55-59 | Ecig | 762 | 13.91 | | | | | |
| 5 | 55-59 | Dual | 0 | 0.00 | | | | | |
| 5 | 55-59 | Former | 2364 | 43.16 | | | 17.21 | 122 | 5.16 |

Year of Follow-up: 1995, Age Group: 60-64

| Period | Age Group | Smoking Group | N | % | Ever Quit (N) | Ever Quit % | Mean Yrs Quit | Short Term (n) | Short Term % |
|---|---|---|---|---|---|---|---|---|---|
| 5 | 60-64 | Never Any | 1337 | 26.83 | | | | | |
| 5 | 60-64 | CC | 597 | 11.98 | 0 | 0.00 | | | |
| 5 | 60-64 | Ecig | 572 | 11.48 | | | | | |
| 5 | 60-64 | Dual | 0 | 0.00 | | | | | |
| 5 | 60-64 | Former | 2477 | 49.71 | | | 17.27 | 143 | 5.77 |

Year of Follow-up: 1995, Age Group: 65-69

| Period | Age Group | Smoking Group | N | % | Ever Quit (N) | Ever Quit % | Mean Yrs Quit | Short Term (n) | Short Term % |
|---|---|---|---|---|---|---|---|---|---|
| 5 | 65-69 | Never Any | 1169 | 23.83 | | | | | |
| 5 | 65-69 | CC | 477 | 9.72 | 0 | 0.00 | | | |
| 5 | 65-69 | Ecig | 360 | 7.34 | | | | | |
| 5 | 65-69 | Dual | 0 | 0.00 | | | | | |
| 5 | 65-69 | Former | 2899 | 59.10 | | | 20.44 | 196 | 6.76 |

Year of Follow-up: 1995, Age Group: 70-74

| Period | Age Group | Smoking Group | N | % | Ever Quit (N) | Ever Quit % | Mean Yrs Quit | Short Term (n) | Short Term % |
|---|---|---|---|---|---|---|---|---|---|
| 5 | 70-74 | Never Any | 1023 | 23.32 | | | | | |
| 5 | 70-74 | CC | 438 | 9.98 | 0 | 0.00 | | | |
| 5 | 70-74 | Ecig | 196 | 4.47 | | | | | |
| 5 | 70-74 | Dual | 0 | 0.00 | | | | | |
| 5 | 70-74 | Former | 2730 | 62.23 | | | 22.00 | 137 | 5.02 |

Year of Follow-up: 1995, Age Group: 75-79

| Period | Age Group | Smoking Group | N | % | Ever Quit (N) | Ever Quit % | Mean Yrs Quit | Short Term (n) | Short Term % |
|---|---|---|---|---|---|---|---|---|---|
| 5 | 75-79 | Never Any | 961 | 27.46 | | | | | |
| 5 | 75-79 | CC | 303 | 8.66 | 0 | 0.00 | | | |
| 5 | 75-79 | Ecig | 135 | 3.86 | | | | | |
| 5 | 75-79 | Dual | 0 | 0.00 | | | | | |
| 5 | 75-79 | Former | 2100 | 60.02 | | | 23.02 | 97 | 4.62 |

Year of Follow-up: 2000, Age Group: All Ages

## Results for PHIM for Run 1 (Main_Analysis.RTF)

| Period | Age Group | Smoking Group | N | % | Ever Quit (N) | Ever Quit % | Mean Yrs Quit | Short Term (n) | Short Term % |
|---|---|---|---|---|---|---|---|---|---|
| 10 | All Ages | Never Any | 39177 | 41.64 | | | | | |
| 10 | All Ages | CC | 4843 | 5.15 | 0 | 0.00 | | | |
| 10 | All Ages | Ecig | 20724 | 22.03 | | | | | |
| 10 | All Ages | Dual | 0 | 0.00 | | | | | |
| 10 | All Ages | Former | 29348 | 31.19 | | | 16.84 | 1724 | 5.87 |

Year of Follow-up: 2000, Age Group: 18-19

| Period | Age Group | Smoking Group | N | % | Ever Quit (N) | Ever Quit % | Mean Yrs Quit | Short Term (n) | Short Term % |
|---|---|---|---|---|---|---|---|---|---|
| 10 | 18-19 | Never Any | 0 | . | | | | | |
| 10 | 18-19 | CC | 0 | . | 0 | . | | | |
| 10 | 18-19 | Ecig | 0 | . | | | | | |
| 10 | 18-19 | Dual | 0 | . | | | | | |
| 10 | 18-19 | Former | 0 | . | | | . | 0 | . |

Year of Follow-up: 2000, Age Group: 20-24

| Period | Age Group | Smoking Group | N | % | Ever Quit (N) | Ever Quit % | Mean Yrs Quit | Short Term (n) | Short Term % |
|---|---|---|---|---|---|---|---|---|---|
| 10 | 20-24 | Never Any | 4927 | 55.70 | | | | | |
| 10 | 20-24 | CC | 462 | 5.22 | 0 | 0.00 | | | |
| 10 | 20-24 | Ecig | 2544 | 28.76 | | | | | |
| 10 | 20-24 | Dual | 0 | 0.00 | | | | | |
| 10 | 20-24 | Former | 912 | 10.31 | | | 3.41 | 258 | 28.29 |

Year of Follow-up: 2000, Age Group: 25-29

| Period | Age Group | Smoking Group | N | % | Ever Quit (N) | Ever Quit % | Mean Yrs Quit | Short Term (n) | Short Term % |
|---|---|---|---|---|---|---|---|---|---|
| 10 | 25-29 | Never Any | 5341 | 59.08 | | | | | |
| 10 | 25-29 | CC | 404 | 4.47 | 0 | 0.00 | | | |
| 10 | 25-29 | Ecig | 2359 | 26.10 | | | | | |
| 10 | 25-29 | Dual | 0 | 0.00 | | | | | |
| 10 | 25-29 | Former | 936 | 10.35 | | | 4.91 | 195 | 20.83 |

Year of Follow-up: 2000, Age Group: 30-34

| Period | Age Group | Smoking Group | N | % | Ever Quit (N) | Ever Quit % | Mean Yrs Quit | Short Term (n) | Short Term % |
|---|---|---|---|---|---|---|---|---|---|
| 10 | 30-34 | Never Any | 5276 | 53.75 | | | | | |
| 10 | 30-34 | CC | 481 | 4.90 | 0 | 0.00 | | | |
| 10 | 30-34 | Ecig | 2608 | 26.57 | | | | | |
| 10 | 30-34 | Dual | 0 | 0.00 | | | | | |
| 10 | 30-34 | Former | 1451 | 14.78 | | | 8.66 | 118 | 8.13 |

Year of Follow-up: 2000, Age Group: 35-39

| Period | Age Group | Smoking Group | N | % | Ever Quit (N) | Ever Quit % | Mean Yrs Quit | Short Term (n) | Short Term % |
|---|---|---|---|---|---|---|---|---|---|
| 10 | 35-39 | Never Any | 4997 | 46.86 | | | | | |
| 10 | 35-39 | CC | 609 | 5.71 | 0 | 0.00 | | | |
| 10 | 35-39 | Ecig | 2980 | 27.94 | | | | | |
| 10 | 35-39 | Dual | 0 | 0.00 | | | | | |
| 10 | 35-39 | Former | 2078 | 19.49 | | | 10.61 | 109 | 5.25 |

Year of Follow-up: 2000, Age Group: 40-44

| Period | Age Group | Smoking Group | N | % | Ever Quit (N) | Ever Quit % | Mean Yrs Quit | Short Term (n) | Short Term % |
|---|---|---|---|---|---|---|---|---|---|
| 10 | 40-44 | Never Any | 4724 | 43.28 | | | | | |
| 10 | 40-44 | CC | 567 | 5.19 | 0 | 0.00 | | | |
| 10 | 40-44 | Ecig | 2857 | 26.17 | | | | | |
| 10 | 40-44 | Dual | 0 | 0.00 | | | | | |
| 10 | 40-44 | Former | 2768 | 25.36 | | | 11.82 | 124 | 4.48 |

Year of Follow-up: 2000, Age Group: 45-49

| Period | Age Group | Smoking Group | N | % | Ever Quit (N) | Ever Quit % | Mean Yrs Quit | Short Term (n) | Short Term % |
|---|---|---|---|---|---|---|---|---|---|
| 10 | 45-49 | Never Any | 3941 | 39.85 | | | | | |
| 10 | 45-49 | CC | 484 | 4.89 | 0 | 0.00 | | | |
| 10 | 45-49 | Ecig | 2312 | 23.38 | | | | | |
| 10 | 45-49 | Dual | 0 | 0.00 | | | | | |
| 10 | 45-49 | Former | 3152 | 31.87 | | | 15.25 | 163 | 5.17 |

Year of Follow-up: 2000, Age Group: 50-54

| Period | Age Group | Smoking Group | N | % | Ever Quit (N) | Ever Quit % | Mean Yrs Quit | Short Term (n) | Short Term % |
|---|---|---|---|---|---|---|---|---|---|
| 10 | 50-54 | Never Any | 2912 | 33.65 | | | | | |
| 10 | 50-54 | CC | 473 | 5.47 | 0 | 0.00 | | | |
| 10 | 50-54 | Ecig | 1859 | 21.48 | | | | | |
| 10 | 50-54 | Dual | 0 | 0.00 | | | | | |
| 10 | 50-54 | Former | 3411 | 39.41 | | | 16.85 | 186 | 5.45 |

Year of Follow-up: 2000, Age Group: 55-59

| Period | Age Group | Smoking Group | N | % | Ever Quit (N) | Ever Quit % | Mean Yrs Quit | Short Term (n) | Short Term % |
|---|---|---|---|---|---|---|---|---|---|
| 10 | 55-59 | Never Any | 1919 | 29.46 | | | | | |
| 10 | 55-59 | CC | 363 | 5.57 | 0 | 0.00 | | | |
| 10 | 55-59 | Ecig | 1194 | 18.33 | | | | | |
| 10 | 55-59 | Dual | 0 | 0.00 | | | | | |

| | | | | | | | | | |
|---|---|---|---|---|---|---|---|---|---|
| 10 | 55-59 | Former | 3039 | 46.65 | | | 18.50 | 146 | 4.80 |

Year of Follow-up: 2000, Age Group: 60-64

| Period | Age Group | Smoking Group | N | % | Ever Quit (N) | Ever Quit % | Mean Yrs Quit | Short Term (n) | Short Term % |
|---|---|---|---|---|---|---|---|---|---|
| 10 | 60-64 | Never Any | 1611 | 29.41 | | | | | |
| 10 | 60-64 | CC | 306 | 5.59 | 0 | 0.00 | | | |
| 10 | 60-64 | Ecig | 852 | 15.56 | | | | | |
| 10 | 60-64 | Dual | 0 | 0.00 | | | | | |
| 10 | 60-64 | Former | 2708 | 49.44 | | | 19.63 | 144 | 5.32 |

Year of Follow-up: 2000, Age Group: 65-69

| Period | Age Group | Smoking Group | N | % | Ever Quit (N) | Ever Quit % | Mean Yrs Quit | Short Term (n) | Short Term % |
|---|---|---|---|---|---|---|---|---|---|
| 10 | 65-69 | Never Any | 1337 | 26.83 | | | | | |
| 10 | 65-69 | CC | 244 | 4.90 | 0 | 0.00 | | | |
| 10 | 65-69 | Ecig | 603 | 12.10 | | | | | |
| 10 | 65-69 | Dual | 0 | 0.00 | | | | | |
| 10 | 65-69 | Former | 2799 | 56.17 | | | 19.95 | 120 | 4.29 |

Year of Follow-up: 2000, Age Group: 70-74

| Period | Age Group | Smoking Group | N | % | Ever Quit (N) | Ever Quit % | Mean Yrs Quit | Short Term (n) | Short Term % |
|---|---|---|---|---|---|---|---|---|---|
| 10 | 70-74 | Never Any | 1169 | 23.83 | | | | | |
| 10 | 70-74 | CC | 236 | 4.81 | 0 | 0.00 | | | |
| 10 | 70-74 | Ecig | 327 | 6.67 | | | | | |
| 10 | 70-74 | Dual | 0 | 0.00 | | | | | |
| 10 | 70-74 | Former | 3173 | 64.69 | | | 23.44 | 94 | 2.96 |

Year of Follow-up: 2000, Age Group: 75-79

| Period | Age Group | Smoking Group | N | % | Ever Quit (N) | Ever Quit % | Mean Yrs Quit | Short Term (n) | Short Term % |
|---|---|---|---|---|---|---|---|---|---|
| 10 | 75-79 | Never Any | 1023 | 23.32 | | | | | |
| 10 | 75-79 | CC | 214 | 4.88 | 0 | 0.00 | | | |
| 10 | 75-79 | Ecig | 229 | 5.22 | | | | | |
| 10 | 75-79 | Dual | 0 | 0.00 | | | | | |
| 10 | 75-79 | Former | 2921 | 66.58 | | | 25.38 | 67 | 2.29 |

Year of Follow-up: 2005, Age Group: All Ages

| Period | Age Group | Smoking Group | N | % | Ever Quit (N) | Ever Quit % | Mean Yrs Quit | Short Term (n) | Short Term % |
|---|---|---|---|---|---|---|---|---|---|
| 15 | All Ages | Never Any | 37449 | 41.75 | | | | | |
| 15 | All Ages | CC | 1911 | 2.13 | 0 | 0.00 | | | |
| 15 | All Ages | Ecig | 20003 | 22.30 | | | | | |
| 15 | All Ages | Dual | 0 | 0.00 | | | | | |
| 15 | All Ages | Former | 30342 | 33.82 | | | 18.46 | 1574 | 5.19 |

Year of Follow-up: 2005, Age Group: 18-19

| Period | Age Group | Smoking Group | N | % | Ever Quit (N) | Ever Quit % | Mean Yrs Quit | Short Term (n) | Short Term % |
|---|---|---|---|---|---|---|---|---|---|
| 15 | 18-19 | Never Any | 0 | . | | | | | |
| 15 | 18-19 | CC | 0 | . | 0 | . | | | |
| 15 | 18-19 | Ecig | 0 | . | | | | | |
| 15 | 18-19 | Dual | 0 | . | | | | | |
| 15 | 18-19 | Former | 0 | . | | | . | 0 | . |

Year of Follow-up: 2005, Age Group: 20-24

| Period | Age Group | Smoking Group | N | % | Ever Quit (N) | Ever Quit % | Mean Yrs Quit | Short Term (n) | Short Term % |
|---|---|---|---|---|---|---|---|---|---|
| 15 | 20-24 | Never Any | 0 | . | | | | | |
| 15 | 20-24 | CC | 0 | . | 0 | . | | | |
| 15 | 20-24 | Ecig | 0 | . | | | | | |
| 15 | 20-24 | Dual | 0 | . | | | | | |
| 15 | 20-24 | Former | 0 | . | | | . | 0 | . |

Year of Follow-up: 2005, Age Group: 25-29

| Period | Age Group | Smoking Group | N | % | Ever Quit (N) | Ever Quit % | Mean Yrs Quit | Short Term (n) | Short Term % |
|---|---|---|---|---|---|---|---|---|---|
| 15 | 25-29 | Never Any | 4634 | 52.39 | | | | | |
| 15 | 25-29 | CC | 179 | 2.02 | 0 | 0.00 | | | |
| 15 | 25-29 | Ecig | 2661 | 30.08 | | | | | |
| 15 | 25-29 | Dual | 0 | 0.00 | | | | | |
| 15 | 25-29 | Former | 1371 | 15.50 | | | 6.32 | 174 | 12.69 |

Year of Follow-up: 2005, Age Group: 30-34

| Period | Age Group | Smoking Group | N | % | Ever Quit (N) | Ever Quit % | Mean Yrs Quit | Short Term (n) | Short Term % |
|---|---|---|---|---|---|---|---|---|---|
| 15 | 30-34 | Never Any | 5042 | 55.77 | | | | | |
| 15 | 30-34 | CC | 153 | 1.69 | 0 | 0.00 | | | |
| 15 | 30-34 | Ecig | 2479 | 27.42 | | | | | |
| 15 | 30-34 | Dual | 0 | 0.00 | | | | | |
| 15 | 30-34 | Former | 1366 | 15.11 | | | 7.38 | 171 | 12.52 |

Year of Follow-up: 2005, Age Group: 35-39

| Period | Age Group | Smoking Group | N | % | Ever Quit (N) | Ever Quit % | Mean Yrs Quit | Short Term (n) | Short Term % |
|---|---|---|---|---|---|---|---|---|---|
| 15 | 35-39 | Never Any | 5163 | 52.60 | | | | | |
| 15 | 35-39 | CC | 188 | 1.92 | 0 | 0.00 | | | |

## Results for PHIM for Run 1 (Main_Analysis.RTF)

| | | | | | | | | | |
|---|---|---|---|---|---|---|---|---|---|
| 15 | 35-39 | Ecig | 2654 | 27.04 | | | | | |
| 15 | 35-39 | Dual | 0 | 0.00 | | | | | |
| 15 | 35-39 | Former | 1811 | 18.45 | | | 11.33 | 147 | 8.12 |

Year of Follow-up: 2005, Age Group: 40-44

| Period | Age Group | Smoking Group | N | % | Ever Quit (N) | Ever Quit % | Mean Yrs Quit | Short Term (n) | Short Term % |
|---|---|---|---|---|---|---|---|---|---|
| 15 | 40-44 | Never Any | 4997 | 46.86 | | | | | |
| 15 | 40-44 | CC | 231 | 2.17 | 0 | 0.00 | | | |
| 15 | 40-44 | Ecig | 2948 | 27.64 | | | | | |
| 15 | 40-44 | Dual | 0 | 0.00 | | | | | |
| 15 | 40-44 | Former | 2488 | 23.33 | | | 13.32 | 199 | 8.00 |

Year of Follow-up: 2005, Age Group: 45-49

| Period | Age Group | Smoking Group | N | % | Ever Quit (N) | Ever Quit % | Mean Yrs Quit | Short Term (n) | Short Term % |
|---|---|---|---|---|---|---|---|---|---|
| 15 | 45-49 | Never Any | 4724 | 43.28 | | | | | |
| 15 | 45-49 | CC | 216 | 1.98 | 0 | 0.00 | | | |
| 15 | 45-49 | Ecig | 2793 | 25.59 | | | | | |
| 15 | 45-49 | Dual | 0 | 0.00 | | | | | |
| 15 | 45-49 | Former | 3183 | 29.16 | | | 14.88 | 160 | 5.03 |

Year of Follow-up: 2005, Age Group: 50-54

| Period | Age Group | Smoking Group | N | % | Ever Quit (N) | Ever Quit % | Mean Yrs Quit | Short Term (n) | Short Term % |
|---|---|---|---|---|---|---|---|---|---|
| 15 | 50-54 | Never Any | 3941 | 39.85 | | | | | |
| 15 | 50-54 | CC | 213 | 2.15 | 0 | 0.00 | | | |
| 15 | 50-54 | Ecig | 2180 | 22.04 | | | | | |
| 15 | 50-54 | Dual | 0 | 0.00 | | | | | |
| 15 | 50-54 | Former | 3555 | 35.95 | | | 18.19 | 154 | 4.33 |

Year of Follow-up: 2005, Age Group: 55-59

| Period | Age Group | Smoking Group | N | % | Ever Quit (N) | Ever Quit % | Mean Yrs Quit | Short Term (n) | Short Term % |
|---|---|---|---|---|---|---|---|---|---|
| 15 | 55-59 | Never Any | 2912 | 33.65 | | | | | |
| 15 | 55-59 | CC | 215 | 2.48 | 0 | 0.00 | | | |
| 15 | 55-59 | Ecig | 1781 | 20.58 | | | | | |
| 15 | 55-59 | Dual | 0 | 0.00 | | | | | |
| 15 | 55-59 | Former | 3747 | 43.29 | | | 20.09 | 117 | 3.12 |

Year of Follow-up: 2005, Age Group: 60-64

| Period | Age Group | Smoking Group | N | % | Ever Quit (N) | Ever Quit % | Mean Yrs Quit | Short Term (n) | Short Term % |
|---|---|---|---|---|---|---|---|---|---|
| 15 | 60-64 | Never Any | 1919 | 29.46 | | | | | |
| 15 | 60-64 | CC | 143 | 2.19 | 0 | 0.00 | | | |
| 15 | 60-64 | Ecig | 1100 | 16.88 | | | | | |
| 15 | 60-64 | Dual | 0 | 0.00 | | | | | |
| 15 | 60-64 | Former | 3353 | 51.47 | | | 21.47 | 156 | 4.65 |

Year of Follow-up: 2005, Age Group: 65-69

| Period | Age Group | Smoking Group | N | % | Ever Quit (N) | Ever Quit % | Mean Yrs Quit | Short Term (n) | Short Term % |
|---|---|---|---|---|---|---|---|---|---|
| 15 | 65-69 | Never Any | 1611 | 29.41 | | | | | |
| 15 | 65-69 | CC | 124 | 2.26 | 0 | 0.00 | | | |
| 15 | 65-69 | Ecig | 705 | 12.87 | | | | | |
| 15 | 65-69 | Dual | 0 | 0.00 | | | | | |
| 15 | 65-69 | Former | 3037 | 55.45 | | | 22.19 | 118 | 3.89 |

Year of Follow-up: 2005, Age Group: 70-74

| Period | Age Group | Smoking Group | N | % | Ever Quit (N) | Ever Quit % | Mean Yrs Quit | Short Term (n) | Short Term % |
|---|---|---|---|---|---|---|---|---|---|
| 15 | 70-74 | Never Any | 1337 | 26.83 | | | | | |
| 15 | 70-74 | CC | 129 | 2.59 | 0 | 0.00 | | | |
| 15 | 70-74 | Ecig | 441 | 8.85 | | | | | |
| 15 | 70-74 | Dual | 0 | 0.00 | | | | | |
| 15 | 70-74 | Former | 3076 | 61.73 | | | 22.89 | 112 | 3.64 |

Year of Follow-up: 2005, Age Group: 75-79

| Period | Age Group | Smoking Group | N | % | Ever Quit (N) | Ever Quit % | Mean Yrs Quit | Short Term (n) | Short Term % |
|---|---|---|---|---|---|---|---|---|---|
| 15 | 75-79 | Never Any | 1169 | 23.83 | | | | | |
| 15 | 75-79 | CC | 120 | 2.45 | 0 | 0.00 | | | |
| 15 | 75-79 | Ecig | 261 | 5.32 | | | | | |
| 15 | 75-79 | Dual | 0 | 0.00 | | | | | |
| 15 | 75-79 | Former | 3355 | 68.40 | | | 27.01 | 66 | 1.97 |

Year of Follow-up: 2010, Age Group: All Ages

| Period | Age Group | Smoking Group | N | % | Ever Quit (N) | Ever Quit % | Mean Yrs Quit | Short Term (n) | Short Term % |
|---|---|---|---|---|---|---|---|---|---|
| 20 | All Ages | Never Any | 36103 | 42.57 | | | | | |
| 20 | All Ages | CC | 738 | 0.87 | 0 | 0.00 | | | |
| 20 | All Ages | Ecig | 17076 | 20.14 | | | | | |
| 20 | All Ages | Dual | 0 | 0.00 | | | | | |
| 20 | All Ages | Former | 30883 | 36.42 | | | 19.82 | 1518 | 4.92 |

Year of Follow-up: 2010, Age Group: 18-19

| Period | Age Group | Smoking Group | N | % | Ever Quit (N) | Ever Quit % | Mean Yrs Quit | Short Term (n) | Short Term % |
|---|---|---|---|---|---|---|---|---|---|

# Results for PHIM for Run 1 (Main_Analysis.RTF)

| | | | | | | | | | |
|---|---|---|---|---|---|---|---|---|---|
| 20 | 18-19 | Never Any | 0 | . | | | | | |
| 20 | 18-19 | CC | 0 | . | 0 | . | | | |
| 20 | 18-19 | Ecig | 0 | . | | | | | |
| 20 | 18-19 | Dual | 0 | . | | | | | |
| 20 | 18-19 | Former | 0 | . | | | . | 0 | . |

Year of Follow-up: 2010, Age Group: 20-24

| Period | Age Group | Smoking Group | N | % | Ever Quit (N) | Ever Quit % | Mean Yrs Quit | Short Term (n) | Short Term % |
|---|---|---|---|---|---|---|---|---|---|
| 20 | 20-24 | Never Any | 0 | . | | | | | |
| 20 | 20-24 | CC | 0 | . | 0 | . | | | |
| 20 | 20-24 | Ecig | 0 | . | | | | | |
| 20 | 20-24 | Dual | 0 | . | | | | | |
| 20 | 20-24 | Former | 0 | . | | | . | 0 | . |

Year of Follow-up: 2010, Age Group: 25-29

| Period | Age Group | Smoking Group | N | % | Ever Quit (N) | Ever Quit % | Mean Yrs Quit | Short Term (n) | Short Term % |
|---|---|---|---|---|---|---|---|---|---|
| 20 | 25-29 | Never Any | 0 | . | | | | | |
| 20 | 25-29 | CC | 0 | . | 0 | . | | | |
| 20 | 25-29 | Ecig | 0 | . | | | | | |
| 20 | 25-29 | Dual | 0 | . | | | | | |
| 20 | 25-29 | Former | 0 | . | | | . | 0 | . |

Year of Follow-up: 2010, Age Group: 30-34

| Period | Age Group | Smoking Group | N | % | Ever Quit (N) | Ever Quit % | Mean Yrs Quit | Short Term (n) | Short Term % |
|---|---|---|---|---|---|---|---|---|---|
| 20 | 30-34 | Never Any | 4508 | 50.97 | | | | | |
| 20 | 30-34 | CC | 71 | 0.80 | 0 | 0.00 | | | |
| 20 | 30-34 | Ecig | 2376 | 26.86 | | | | | |
| 20 | 30-34 | Dual | 0 | 0.00 | | | | | |
| 20 | 30-34 | Former | 1890 | 21.37 | | | 8.72 | 230 | 12.17 |

Year of Follow-up: 2010, Age Group: 35-39

| Period | Age Group | Smoking Group | N | % | Ever Quit (N) | Ever Quit % | Mean Yrs Quit | Short Term (n) | Short Term % |
|---|---|---|---|---|---|---|---|---|---|
| 20 | 35-39 | Never Any | 4991 | 55.21 | | | | | |
| 20 | 35-39 | CC | 59 | 0.65 | 0 | 0.00 | | | |
| 20 | 35-39 | Ecig | 2154 | 23.83 | | | | | |
| 20 | 35-39 | Dual | 0 | 0.00 | | | | | |
| 20 | 35-39 | Former | 1836 | 20.31 | | | 9.77 | 141 | 7.68 |

Year of Follow-up: 2010, Age Group: 40-44

| Period | Age Group | Smoking Group | N | % | Ever Quit (N) | Ever Quit % | Mean Yrs Quit | Short Term (n) | Short Term % |
|---|---|---|---|---|---|---|---|---|---|
| 20 | 40-44 | Never Any | 5163 | 52.60 | | | | | |
| 20 | 40-44 | CC | 68 | 0.69 | 0 | 0.00 | | | |
| 20 | 40-44 | Ecig | 2416 | 24.61 | | | | | |
| 20 | 40-44 | Dual | 0 | 0.00 | | | | | |
| 20 | 40-44 | Former | 2169 | 22.10 | | | 13.96 | 143 | 6.59 |

Year of Follow-up: 2010, Age Group: 45-49

| Period | Age Group | Smoking Group | N | % | Ever Quit (N) | Ever Quit % | Mean Yrs Quit | Short Term (n) | Short Term % |
|---|---|---|---|---|---|---|---|---|---|
| 20 | 45-49 | Never Any | 4997 | 46.86 | | | | | |
| 20 | 45-49 | CC | 89 | 0.83 | 0 | 0.00 | | | |
| 20 | 45-49 | Ecig | 2619 | 24.56 | | | | | |
| 20 | 45-49 | Dual | 0 | 0.00 | | | | | |
| 20 | 45-49 | Former | 2959 | 27.75 | | | 15.74 | 171 | 5.78 |

Year of Follow-up: 2010, Age Group: 50-54

| Period | Age Group | Smoking Group | N | % | Ever Quit (N) | Ever Quit % | Mean Yrs Quit | Short Term (n) | Short Term % |
|---|---|---|---|---|---|---|---|---|---|
| 20 | 50-54 | Never Any | 4724 | 43.28 | | | | | |
| 20 | 50-54 | CC | 87 | 0.80 | 0 | 0.00 | | | |
| 20 | 50-54 | Ecig | 2490 | 22.81 | | | | | |
| 20 | 50-54 | Dual | 0 | 0.00 | | | | | |
| 20 | 50-54 | Former | 3615 | 33.12 | | | 17.75 | 174 | 4.81 |

Year of Follow-up: 2010, Age Group: 55-59

| Period | Age Group | Smoking Group | N | % | Ever Quit (N) | Ever Quit % | Mean Yrs Quit | Short Term (n) | Short Term % |
|---|---|---|---|---|---|---|---|---|---|
| 20 | 55-59 | Never Any | 3941 | 39.85 | | | | | |
| 20 | 55-59 | CC | 85 | 0.86 | 0 | 0.00 | | | |
| 20 | 55-59 | Ecig | 1859 | 18.80 | | | | | |
| 20 | 55-59 | Dual | 0 | 0.00 | | | | | |
| 20 | 55-59 | Former | 4004 | 40.49 | | | 20.81 | 181 | 4.52 |

Year of Follow-up: 2010, Age Group: 60-64

| Period | Age Group | Smoking Group | N | % | Ever Quit (N) | Ever Quit % | Mean Yrs Quit | Short Term (n) | Short Term % |
|---|---|---|---|---|---|---|---|---|---|
| 20 | 60-64 | Never Any | 2912 | 33.65 | | | | | |
| 20 | 60-64 | CC | 87 | 1.01 | 0 | 0.00 | | | |
| 20 | 60-64 | Ecig | 1428 | 16.50 | | | | | |
| 20 | 60-64 | Dual | 0 | 0.00 | | | | | |
| 20 | 60-64 | Former | 4228 | 48.85 | | | 22.41 | 241 | 5.70 |

Year of Follow-up: 2010, Age Group: 65-69

# Results for PHIM for Run 1 (Main_Analysis.RTF)

| Period | Age Group | Smoking Group | N | % | Ever Quit (N) | Ever Quit % | Mean Yrs Quit | Short Term (n) | Short Term % |
|---|---|---|---|---|---|---|---|---|---|
| 20 | 65-69 | Never Any | 1919 | 29.46 | | | | | |
| 20 | 65-69 | CC | 60 | 0.92 | 0 | 0.00 | | | |
| 20 | 65-69 | Ecig | 809 | 12.42 | | | | | |
| 20 | 65-69 | Dual | 0 | 0.00 | | | | | |
| 20 | 65-69 | Former | 3727 | 57.21 | | | 24.03 | 133 | 3.57 |

Year of Follow-up: 2010, Age Group: 70-74

| Period | Age Group | Smoking Group | N | % | Ever Quit (N) | Ever Quit % | Mean Yrs Quit | Short Term (n) | Short Term % |
|---|---|---|---|---|---|---|---|---|---|
| 20 | 70-74 | Never Any | 1611 | 29.41 | | | | | |
| 20 | 70-74 | CC | 69 | 1.26 | 0 | 0.00 | | | |
| 20 | 70-74 | Ecig | 574 | 10.48 | | | | | |
| 20 | 70-74 | Dual | 0 | 0.00 | | | | | |
| 20 | 70-74 | Former | 3223 | 58.85 | | | 25.75 | 61 | 1.89 |

Year of Follow-up: 2010, Age Group: 75-79

| Period | Age Group | Smoking Group | N | % | Ever Quit (N) | Ever Quit % | Mean Yrs Quit | Short Term (n) | Short Term % |
|---|---|---|---|---|---|---|---|---|---|
| 20 | 75-79 | Never Any | 1337 | 26.83 | | | | | |
| 20 | 75-79 | CC | 63 | 1.26 | 0 | 0.00 | | | |
| 20 | 75-79 | Ecig | 351 | 7.04 | | | | | |
| 20 | 75-79 | Dual | 0 | 0.00 | | | | | |
| 20 | 75-79 | Former | 3232 | 64.86 | | | 26.66 | 43 | 1.33 |

Year of Follow-up: 2015, Age Group: All Ages

| Period | Age Group | Smoking Group | N | % | Ever Quit (N) | Ever Quit % | Mean Yrs Quit | Short Term (n) | Short Term % |
|---|---|---|---|---|---|---|---|---|---|
| 25 | All Ages | Never Any | 34747 | 43.53 | | | | | |
| 25 | All Ages | CC | 289 | 0.36 | 0 | 0.00 | | | |
| 25 | All Ages | Ecig | 14107 | 17.67 | | | | | |
| 25 | All Ages | Dual | 0 | 0.00 | | | | | |
| 25 | All Ages | Former | 30674 | 38.43 | | | 21.87 | 1088 | 3.55 |

Year of Follow-up: 2015, Age Group: 18-19

| Period | Age Group | Smoking Group | N | % | Ever Quit (N) | Ever Quit % | Mean Yrs Quit | Short Term (n) | Short Term % |
|---|---|---|---|---|---|---|---|---|---|
| 25 | 18-19 | Never Any | 0 | . | | | | | |
| 25 | 18-19 | CC | 0 | . | 0 | . | | | |
| 25 | 18-19 | Ecig | 0 | . | | | | | |
| 25 | 18-19 | Dual | 0 | . | | | | | |
| 25 | 18-19 | Former | 0 | . | | | . | 0 | . |

Year of Follow-up: 2015, Age Group: 20-24

| Period | Age Group | Smoking Group | N | % | Ever Quit (N) | Ever Quit % | Mean Yrs Quit | Short Term (n) | Short Term % |
|---|---|---|---|---|---|---|---|---|---|
| 25 | 20-24 | Never Any | 0 | . | | | | | |
| 25 | 20-24 | CC | 0 | . | 0 | . | | | |
| 25 | 20-24 | Ecig | 0 | . | | | | | |
| 25 | 20-24 | Dual | 0 | . | | | | | |
| 25 | 20-24 | Former | 0 | . | | | . | 0 | . |

Year of Follow-up: 2015, Age Group: 25-29

| Period | Age Group | Smoking Group | N | % | Ever Quit (N) | Ever Quit % | Mean Yrs Quit | Short Term (n) | Short Term % |
|---|---|---|---|---|---|---|---|---|---|
| 25 | 25-29 | Never Any | 0 | . | | | | | |
| 25 | 25-29 | CC | 0 | . | 0 | . | | | |
| 25 | 25-29 | Ecig | 0 | . | | | | | |
| 25 | 25-29 | Dual | 0 | . | | | | | |
| 25 | 25-29 | Former | 0 | . | | | . | 0 | . |

Year of Follow-up: 2015, Age Group: 30-34

| Period | Age Group | Smoking Group | N | % | Ever Quit (N) | Ever Quit % | Mean Yrs Quit | Short Term (n) | Short Term % |
|---|---|---|---|---|---|---|---|---|---|
| 25 | 30-34 | Never Any | 0 | . | | | | | |
| 25 | 30-34 | CC | 0 | . | 0 | . | | | |
| 25 | 30-34 | Ecig | 0 | . | | | | | |
| 25 | 30-34 | Dual | 0 | . | | | | | |
| 25 | 30-34 | Former | 0 | . | | | . | 0 | . |

Year of Follow-up: 2015, Age Group: 35-39

| Period | Age Group | Smoking Group | N | % | Ever Quit (N) | Ever Quit % | Mean Yrs Quit | Short Term (n) | Short Term % |
|---|---|---|---|---|---|---|---|---|---|
| 25 | 35-39 | Never Any | 4489 | 50.75 | | | | | |
| 25 | 35-39 | CC | 28 | 0.32 | 0 | 0.00 | | | |
| 25 | 35-39 | Ecig | 1990 | 22.50 | | | | | |
| 25 | 35-39 | Dual | 0 | 0.00 | | | | | |
| 25 | 35-39 | Former | 2338 | 26.43 | | | 11.53 | 142 | 6.07 |

Year of Follow-up: 2015, Age Group: 40-44

| Period | Age Group | Smoking Group | N | % | Ever Quit (N) | Ever Quit % | Mean Yrs Quit | Short Term (n) | Short Term % |
|---|---|---|---|---|---|---|---|---|---|
| 25 | 40-44 | Never Any | 4991 | 55.21 | | | | | |
| 25 | 40-44 | CC | 20 | 0.22 | 0 | 0.00 | | | |
| 25 | 40-44 | Ecig | 1897 | 20.98 | | | | | |
| 25 | 40-44 | Dual | 0 | 0.00 | | | | | |

## Results for PHIM for Run 1 (Main_Analysis.RTF)

| | | | | | | | | | |
|---|---|---|---|---|---|---|---|---|---|
| 25 | 40-44 | Former | 2132 | 23.58 | | | 13.01 | 112 | 5.25 |

Year of Follow-up: 2015, Age Group: 45-49

| Period | Age Group | Smoking Group | N | % | Ever Quit (N) | Ever Quit % | Mean Yrs Quit | Short Term (n) | Short Term % |
|---|---|---|---|---|---|---|---|---|---|
| 25 | 45-49 | Never Any | 5163 | 52.60 | | | | | |
| 25 | 45-49 | CC | 29 | 0.30 | 0 | 0.00 | | | |
| 25 | 45-49 | Ecig | 2195 | 22.36 | | | | | |
| 25 | 45-49 | Dual | 0 | 0.00 | | | | | |
| 25 | 45-49 | Former | 2429 | 24.75 | | | 17.18 | 81 | 3.33 |

Year of Follow-up: 2015, Age Group: 50-54

| Period | Age Group | Smoking Group | N | % | Ever Quit (N) | Ever Quit % | Mean Yrs Quit | Short Term (n) | Short Term % |
|---|---|---|---|---|---|---|---|---|---|
| 25 | 50-54 | Never Any | 4997 | 46.86 | | | | | |
| 25 | 50-54 | CC | 37 | 0.35 | 0 | 0.00 | | | |
| 25 | 50-54 | Ecig | 2387 | 22.38 | | | | | |
| 25 | 50-54 | Dual | 0 | 0.00 | | | | | |
| 25 | 50-54 | Former | 3243 | 30.41 | | | 19.10 | 130 | 4.01 |

Year of Follow-up: 2015, Age Group: 55-59

| Period | Age Group | Smoking Group | N | % | Ever Quit (N) | Ever Quit % | Mean Yrs Quit | Short Term (n) | Short Term % |
|---|---|---|---|---|---|---|---|---|---|
| 25 | 55-59 | Never Any | 4724 | 43.28 | | | | | |
| 25 | 55-59 | CC | 33 | 0.30 | 0 | 0.00 | | | |
| 25 | 55-59 | Ecig | 2103 | 19.27 | | | | | |
| 25 | 55-59 | Dual | 0 | 0.00 | | | | | |
| 25 | 55-59 | Former | 4056 | 37.16 | | | 20.50 | 162 | 3.99 |

Year of Follow-up: 2015, Age Group: 60-64

| Period | Age Group | Smoking Group | N | % | Ever Quit (N) | Ever Quit % | Mean Yrs Quit | Short Term (n) | Short Term % |
|---|---|---|---|---|---|---|---|---|---|
| 25 | 60-64 | Never Any | 3941 | 39.85 | | | | | |
| 25 | 60-64 | CC | 28 | 0.28 | 0 | 0.00 | | | |
| 25 | 60-64 | Ecig | 1443 | 14.59 | | | | | |
| 25 | 60-64 | Dual | 0 | 0.00 | | | | | |
| 25 | 60-64 | Former | 4477 | 45.27 | | | 23.28 | 205 | 4.58 |

Year of Follow-up: 2015, Age Group: 65-69

| Period | Age Group | Smoking Group | N | % | Ever Quit (N) | Ever Quit % | Mean Yrs Quit | Short Term (n) | Short Term % |
|---|---|---|---|---|---|---|---|---|---|
| 25 | 65-69 | Never Any | 2912 | 33.65 | | | | | |
| 25 | 65-69 | CC | 43 | 0.50 | 0 | 0.00 | | | |
| 25 | 65-69 | Ecig | 1019 | 11.77 | | | | | |
| 25 | 65-69 | Dual | 0 | 0.00 | | | | | |
| 25 | 65-69 | Former | 4681 | 54.08 | | | 24.99 | 134 | 2.86 |

Year of Follow-up: 2015, Age Group: 70-74

| Period | Age Group | Smoking Group | N | % | Ever Quit (N) | Ever Quit % | Mean Yrs Quit | Short Term (n) | Short Term % |
|---|---|---|---|---|---|---|---|---|---|
| 25 | 70-74 | Never Any | 1919 | 29.46 | | | | | |
| 25 | 70-74 | CC | 35 | 0.54 | 0 | 0.00 | | | |
| 25 | 70-74 | Ecig | 616 | 9.46 | | | | | |
| 25 | 70-74 | Dual | 0 | 0.00 | | | | | |
| 25 | 70-74 | Former | 3945 | 60.55 | | | 27.55 | 70 | 1.77 |

Year of Follow-up: 2015, Age Group: 75-79

| Period | Age Group | Smoking Group | N | % | Ever Quit (N) | Ever Quit % | Mean Yrs Quit | Short Term (n) | Short Term % |
|---|---|---|---|---|---|---|---|---|---|
| 25 | 75-79 | Never Any | 1611 | 29.41 | | | | | |
| 25 | 75-79 | CC | 36 | 0.66 | 0 | 0.00 | | | |
| 25 | 75-79 | Ecig | 457 | 8.34 | | | | | |
| 25 | 75-79 | Dual | 0 | 0.00 | | | | | |
| 25 | 75-79 | Former | 3373 | 61.58 | | | 29.48 | 52 | 1.54 |

Year of Follow-up: 2020, Age Group: All Ages

| Period | Age Group | Smoking Group | N | % | Ever Quit (N) | Ever Quit % | Mean Yrs Quit | Short Term (n) | Short Term % |
|---|---|---|---|---|---|---|---|---|---|
| 30 | All Ages | Never Any | 33136 | 44.57 | | | | | |
| 30 | All Ages | CC | 100 | 0.13 | 0 | 0.00 | | | |
| 30 | All Ages | Ecig | 11358 | 15.28 | | | | | |
| 30 | All Ages | Dual | 0 | 0.00 | | | | | |
| 30 | All Ages | Former | 29746 | 40.01 | | | 23.97 | 918 | 3.09 |

Year of Follow-up: 2020, Age Group: 18-19

| Period | Age Group | Smoking Group | N | % | Ever Quit (N) | Ever Quit % | Mean Yrs Quit | Short Term (n) | Short Term % |
|---|---|---|---|---|---|---|---|---|---|
| 30 | 18-19 | Never Any | 0 | . | | | | | |
| 30 | 18-19 | CC | 0 | . | 0 | . | | | |
| 30 | 18-19 | Ecig | 0 | . | | | | | |
| 30 | 18-19 | Dual | 0 | . | | | | | |
| 30 | 18-19 | Former | 0 | . | | | . | 0 | . |

Year of Follow-up: 2020, Age Group: 20-24

| Period | Age Group | Smoking Group | N | % | Ever Quit (N) | Ever Quit % | Mean Yrs Quit | Short Term (n) | Short Term % |
|---|---|---|---|---|---|---|---|---|---|
| 30 | 20-24 | Never Any | 0 | . | | | | | |
| 30 | 20-24 | CC | 0 | . | 0 | . | | | |

# Results for PHIM for Run 1 (Main_Analysis.RTF)

| | | | | | | | | | |
|---|---|---|---|---|---|---|---|---|---|
| 30 | 20-24 | Ecig | 0 | . | | | | | |
| 30 | 20-24 | Dual | 0 | . | | | | | |
| 30 | 20-24 | Former | 0 | . | | | . | 0 | . |

Year of Follow-up: 2020, Age Group: 25-29

| Period | Age Group | Smoking Group | N | % | Ever Quit (N) | Ever Quit % | Mean Yrs Quit | Short Term (n) | Short Term % |
|---|---|---|---|---|---|---|---|---|---|
| 30 | 25-29 | Never Any | 0 | . | | | | | |
| 30 | 25-29 | CC | 0 | . | 0 | . | | | |
| 30 | 25-29 | Ecig | 0 | . | | | | | |
| 30 | 25-29 | Dual | 0 | . | | | | | |
| 30 | 25-29 | Former | 0 | . | | | . | 0 | . |

Year of Follow-up: 2020, Age Group: 30-34

| Period | Age Group | Smoking Group | N | % | Ever Quit (N) | Ever Quit % | Mean Yrs Quit | Short Term (n) | Short Term % |
|---|---|---|---|---|---|---|---|---|---|
| 30 | 30-34 | Never Any | 0 | . | | | | | |
| 30 | 30-34 | CC | 0 | . | 0 | . | | | |
| 30 | 30-34 | Ecig | 0 | . | | | | | |
| 30 | 30-34 | Dual | 0 | . | | | | | |
| 30 | 30-34 | Former | 0 | . | | | . | 0 | . |

Year of Follow-up: 2020, Age Group: 35-39

| Period | Age Group | Smoking Group | N | % | Ever Quit (N) | Ever Quit % | Mean Yrs Quit | Short Term (n) | Short Term % |
|---|---|---|---|---|---|---|---|---|---|
| 30 | 35-39 | Never Any | 0 | . | | | | | |
| 30 | 35-39 | CC | 0 | . | 0 | . | | | |
| 30 | 35-39 | Ecig | 0 | . | | | | | |
| 30 | 35-39 | Dual | 0 | . | | | | | |
| 30 | 35-39 | Former | 0 | . | | | . | 0 | . |

Year of Follow-up: 2020, Age Group: 40-44

| Period | Age Group | Smoking Group | N | % | Ever Quit (N) | Ever Quit % | Mean Yrs Quit | Short Term (n) | Short Term % |
|---|---|---|---|---|---|---|---|---|---|
| 30 | 40-44 | Never Any | 4489 | 50.75 | | | | | |
| 30 | 40-44 | CC | 12 | 0.14 | 0 | 0.00 | | | |
| 30 | 40-44 | Ecig | 1743 | 19.71 | | | | | |
| 30 | 40-44 | Dual | 0 | 0.00 | | | | | |
| 30 | 40-44 | Former | 2601 | 29.41 | | | 15.05 | 107 | 4.11 |

Year of Follow-up: 2020, Age Group: 45-49

| Period | Age Group | Smoking Group | N | % | Ever Quit (N) | Ever Quit % | Mean Yrs Quit | Short Term (n) | Short Term % |
|---|---|---|---|---|---|---|---|---|---|
| 30 | 45-49 | Never Any | 4991 | 55.21 | | | | | |
| 30 | 45-49 | CC | 5 | 0.06 | 0 | 0.00 | | | |
| 30 | 45-49 | Ecig | 1690 | 18.69 | | | | | |
| 30 | 45-49 | Dual | 0 | 0.00 | | | | | |
| 30 | 45-49 | Former | 2354 | 26.04 | | | 16.54 | 63 | 2.68 |

Year of Follow-up: 2020, Age Group: 50-54

| Period | Age Group | Smoking Group | N | % | Ever Quit (N) | Ever Quit % | Mean Yrs Quit | Short Term (n) | Short Term % |
|---|---|---|---|---|---|---|---|---|---|
| 30 | 50-54 | Never Any | 5163 | 52.60 | | | | | |
| 30 | 50-54 | CC | 9 | 0.09 | 0 | 0.00 | | | |
| 30 | 50-54 | Ecig | 2021 | 20.59 | | | | | |
| 30 | 50-54 | Dual | 0 | 0.00 | | | | | |
| 30 | 50-54 | Former | 2623 | 26.72 | | | 20.66 | 99 | 3.77 |

Year of Follow-up: 2020, Age Group: 55-59

| Period | Age Group | Smoking Group | N | % | Ever Quit (N) | Ever Quit % | Mean Yrs Quit | Short Term (n) | Short Term % |
|---|---|---|---|---|---|---|---|---|---|
| 30 | 55-59 | Never Any | 4997 | 46.86 | | | | | |
| 30 | 55-59 | CC | 18 | 0.17 | 0 | 0.00 | | | |
| 30 | 55-59 | Ecig | 2002 | 18.77 | | | | | |
| 30 | 55-59 | Dual | 0 | 0.00 | | | | | |
| 30 | 55-59 | Former | 3647 | 34.20 | | | 21.65 | 157 | 4.30 |

Year of Follow-up: 2020, Age Group: 60-64

| Period | Age Group | Smoking Group | N | % | Ever Quit (N) | Ever Quit % | Mean Yrs Quit | Short Term (n) | Short Term % |
|---|---|---|---|---|---|---|---|---|---|
| 30 | 60-64 | Never Any | 4724 | 43.28 | | | | | |
| 30 | 60-64 | CC | 10 | 0.09 | 0 | 0.00 | | | |
| 30 | 60-64 | Ecig | 1614 | 14.79 | | | | | |
| 30 | 60-64 | Dual | 0 | 0.00 | | | | | |
| 30 | 60-64 | Former | 4568 | 41.85 | | | 22.87 | 203 | 4.44 |

Year of Follow-up: 2020, Age Group: 65-69

| Period | Age Group | Smoking Group | N | % | Ever Quit (N) | Ever Quit % | Mean Yrs Quit | Short Term (n) | Short Term % |
|---|---|---|---|---|---|---|---|---|---|
| 30 | 65-69 | Never Any | 3941 | 39.85 | | | | | |
| 30 | 65-69 | CC | 13 | 0.13 | 0 | 0.00 | | | |
| 30 | 65-69 | Ecig | 1039 | 10.51 | | | | | |
| 30 | 65-69 | Dual | 0 | 0.00 | | | | | |
| 30 | 65-69 | Former | 4896 | 49.51 | | | 26.05 | 145 | 2.96 |

Year of Follow-up: 2020, Age Group: 70-74

| Period | Age Group | Smoking Group | N | % | Ever Quit (N) | Ever Quit % | Mean Yrs Quit | Short Term (n) | Short Term % |
|---|---|---|---|---|---|---|---|---|---|

| | | | | | | | | | |
|---|---|---|---|---|---|---|---|---|---|
| 30 | 70-74 | Never Any | 2912 | 33.65 | | | | | |
| 30 | 70-74 | CC | 18 | 0.21 | 0 | 0.00 | | | |
| 30 | 70-74 | Ecig | 770 | 8.90 | | | | | |
| 30 | 70-74 | Dual | 0 | 0.00 | | | | | |
| 30 | 70-74 | Former | 4955 | 57.25 | | | 28.45 | 93 | 1.88 |

Year of Follow-up: 2020, Age Group: 75-79

| Period | Age Group | Smoking Group | N | % | Ever Quit (N) | Ever Quit % | Mean Yrs Quit | Short Term (n) | Short Term % |
|---|---|---|---|---|---|---|---|---|---|
| 30 | 75-79 | Never Any | 1919 | 29.46 | | | | | |
| 30 | 75-79 | CC | 15 | 0.23 | 0 | 0.00 | | | |
| 30 | 75-79 | Ecig | 479 | 7.35 | | | | | |
| 30 | 75-79 | Dual | 0 | 0.00 | | | | | |
| 30 | 75-79 | Former | 4102 | 62.96 | | | 31.39 | 51 | 1.24 |

Year of Follow-up: 2025, Age Group: All Ages

| Period | Age Group | Smoking Group | N | % | Ever Quit (N) | Ever Quit % | Mean Yrs Quit | Short Term (n) | Short Term % |
|---|---|---|---|---|---|---|---|---|---|
| 35 | All Ages | Never Any | 31217 | 46.03 | | | | | |
| 35 | All Ages | CC | 38 | 0.06 | 0 | 0.00 | | | |
| 35 | All Ages | Ecig | 8864 | 13.07 | | | | | |
| 35 | All Ages | Dual | 0 | 0.00 | | | | | |
| 35 | All Ages | Former | 27706 | 40.85 | | | 25.87 | 785 | 2.83 |

Year of Follow-up: 2025, Age Group: 18-19

| Period | Age Group | Smoking Group | N | % | Ever Quit (N) | Ever Quit % | Mean Yrs Quit | Short Term (n) | Short Term % |
|---|---|---|---|---|---|---|---|---|---|
| 35 | 18-19 | Never Any | 0 | . | | | | | |
| 35 | 18-19 | CC | 0 | . | 0 | . | | | |
| 35 | 18-19 | Ecig | 0 | . | | | | | |
| 35 | 18-19 | Dual | 0 | . | | | | | |
| 35 | 18-19 | Former | 0 | . | | | . | 0 | . |

Year of Follow-up: 2025, Age Group: 20-24

| Period | Age Group | Smoking Group | N | % | Ever Quit (N) | Ever Quit % | Mean Yrs Quit | Short Term (n) | Short Term % |
|---|---|---|---|---|---|---|---|---|---|
| 35 | 20-24 | Never Any | 0 | . | | | | | |
| 35 | 20-24 | CC | 0 | . | 0 | . | | | |
| 35 | 20-24 | Ecig | 0 | . | | | | | |
| 35 | 20-24 | Dual | 0 | . | | | | | |
| 35 | 20-24 | Former | 0 | . | | | . | 0 | . |

Year of Follow-up: 2025, Age Group: 25-29

| Period | Age Group | Smoking Group | N | % | Ever Quit (N) | Ever Quit % | Mean Yrs Quit | Short Term (n) | Short Term % |
|---|---|---|---|---|---|---|---|---|---|
| 35 | 25-29 | Never Any | 0 | . | | | | | |
| 35 | 25-29 | CC | 0 | . | 0 | . | | | |
| 35 | 25-29 | Ecig | 0 | . | | | | | |
| 35 | 25-29 | Dual | 0 | . | | | | | |
| 35 | 25-29 | Former | 0 | . | | | . | 0 | . |

Year of Follow-up: 2025, Age Group: 30-34

| Period | Age Group | Smoking Group | N | % | Ever Quit (N) | Ever Quit % | Mean Yrs Quit | Short Term (n) | Short Term % |
|---|---|---|---|---|---|---|---|---|---|
| 35 | 30-34 | Never Any | 0 | . | | | | | |
| 35 | 30-34 | CC | 0 | . | 0 | . | | | |
| 35 | 30-34 | Ecig | 0 | . | | | | | |
| 35 | 30-34 | Dual | 0 | . | | | | | |
| 35 | 30-34 | Former | 0 | . | | | . | 0 | . |

Year of Follow-up: 2025, Age Group: 35-39

| Period | Age Group | Smoking Group | N | % | Ever Quit (N) | Ever Quit % | Mean Yrs Quit | Short Term (n) | Short Term % |
|---|---|---|---|---|---|---|---|---|---|
| 35 | 35-39 | Never Any | 0 | . | | | | | |
| 35 | 35-39 | CC | 0 | . | 0 | . | | | |
| 35 | 35-39 | Ecig | 0 | . | | | | | |
| 35 | 35-39 | Dual | 0 | . | | | | | |
| 35 | 35-39 | Former | 0 | . | | | . | 0 | . |

Year of Follow-up: 2025, Age Group: 40-44

| Period | Age Group | Smoking Group | N | % | Ever Quit (N) | Ever Quit % | Mean Yrs Quit | Short Term (n) | Short Term % |
|---|---|---|---|---|---|---|---|---|---|
| 35 | 40-44 | Never Any | 0 | . | | | | | |
| 35 | 40-44 | CC | 0 | . | 0 | . | | | |
| 35 | 40-44 | Ecig | 0 | . | | | | | |
| 35 | 40-44 | Dual | 0 | . | | | | | |
| 35 | 40-44 | Former | 0 | . | | | . | 0 | . |

Year of Follow-up: 2025, Age Group: 45-49

| Period | Age Group | Smoking Group | N | % | Ever Quit (N) | Ever Quit % | Mean Yrs Quit | Short Term (n) | Short Term % |
|---|---|---|---|---|---|---|---|---|---|
| 35 | 45-49 | Never Any | 4489 | 50.75 | | | | | |
| 35 | 45-49 | CC | 7 | 0.08 | 0 | 0.00 | | | |
| 35 | 45-49 | Ecig | 1541 | 17.42 | | | | | |
| 35 | 45-49 | Dual | 0 | 0.00 | | | | | |
| 35 | 45-49 | Former | 2808 | 31.75 | | | 18.76 | 60 | 2.14 |

Year of Follow-up: 2025, Age Group: 50-54

# Results for PHIM for Run 1 (Main_Analysis.RTF)

| Period | Age Group | Smoking Group | N | % | Ever Quit (N) | Ever Quit % | Mean Yrs Quit | Short Term (n) | Short Term % |
|---|---|---|---|---|---|---|---|---|---|
| 35 | 50-54 | Never Any | 4991 | 55.21 | | | | | |
| 35 | 50-54 | CC | 3 | 0.03 | 0 | 0.00 | | | |
| 35 | 50-54 | Ecig | 1554 | 17.19 | | | | | |
| 35 | 50-54 | Dual | 0 | 0.00 | | | | | |
| 35 | 50-54 | Former | 2492 | 27.57 | | | 20.44 | 75 | 3.01 |

Year of Follow-up: 2025, Age Group: 55-59

| Period | Age Group | Smoking Group | N | % | Ever Quit (N) | Ever Quit % | Mean Yrs Quit | Short Term (n) | Short Term % |
|---|---|---|---|---|---|---|---|---|---|
| 35 | 55-59 | Never Any | 5163 | 52.60 | | | | | |
| 35 | 55-59 | CC | 0 | 0.00 | 0 | 0.00 | | | |
| 35 | 55-59 | Ecig | 1664 | 16.95 | | | | | |
| 35 | 55-59 | Dual | 0 | 0.00 | | | | | |
| 35 | 55-59 | Former | 2989 | 30.45 | | | 22.76 | 155 | 5.19 |

Year of Follow-up: 2025, Age Group: 60-64

| Period | Age Group | Smoking Group | N | % | Ever Quit (N) | Ever Quit % | Mean Yrs Quit | Short Term (n) | Short Term % |
|---|---|---|---|---|---|---|---|---|---|
| 35 | 60-64 | Never Any | 4997 | 46.86 | | | | | |
| 35 | 60-64 | CC | 9 | 0.08 | 0 | 0.00 | | | |
| 35 | 60-64 | Ecig | 1532 | 14.37 | | | | | |
| 35 | 60-64 | Dual | 0 | 0.00 | | | | | |
| 35 | 60-64 | Former | 4126 | 38.69 | | | 23.79 | 186 | 4.51 |

Year of Follow-up: 2025, Age Group: 65-69

| Period | Age Group | Smoking Group | N | % | Ever Quit (N) | Ever Quit % | Mean Yrs Quit | Short Term (n) | Short Term % |
|---|---|---|---|---|---|---|---|---|---|
| 35 | 65-69 | Never Any | 4724 | 43.28 | | | | | |
| 35 | 65-69 | CC | 5 | 0.05 | 0 | 0.00 | | | |
| 35 | 65-69 | Ecig | 1169 | 10.71 | | | | | |
| 35 | 65-69 | Dual | 0 | 0.00 | | | | | |
| 35 | 65-69 | Former | 5018 | 45.97 | | | 25.57 | 158 | 3.15 |

Year of Follow-up: 2025, Age Group: 70-74

| Period | Age Group | Smoking Group | N | % | Ever Quit (N) | Ever Quit % | Mean Yrs Quit | Short Term (n) | Short Term % |
|---|---|---|---|---|---|---|---|---|---|
| 35 | 70-74 | Never Any | 3941 | 39.85 | | | | | |
| 35 | 70-74 | CC | 5 | 0.05 | 0 | 0.00 | | | |
| 35 | 70-74 | Ecig | 811 | 8.20 | | | | | |
| 35 | 70-74 | Dual | 0 | 0.00 | | | | | |
| 35 | 70-74 | Former | 5132 | 51.90 | | | 29.73 | 79 | 1.54 |

Year of Follow-up: 2025, Age Group: 75-79

| Period | Age Group | Smoking Group | N | % | Ever Quit (N) | Ever Quit % | Mean Yrs Quit | Short Term (n) | Short Term % |
|---|---|---|---|---|---|---|---|---|---|
| 35 | 75-79 | Never Any | 2912 | 33.65 | | | | | |
| 35 | 75-79 | CC | 9 | 0.10 | 0 | 0.00 | | | |
| 35 | 75-79 | Ecig | 593 | 6.85 | | | | | |
| 35 | 75-79 | Dual | 0 | 0.00 | | | | | |
| 35 | 75-79 | Former | 5141 | 59.40 | | | 32.31 | 72 | 1.40 |

Year of Follow-up: 2030, Age Group: All Ages

| Period | Age Group | Smoking Group | N | % | Ever Quit (N) | Ever Quit % | Mean Yrs Quit | Short Term (n) | Short Term % |
|---|---|---|---|---|---|---|---|---|---|
| 40 | All Ages | Never Any | 28305 | 47.84 | | | | | |
| 40 | All Ages | CC | 15 | 0.03 | 0 | 0.00 | | | |
| 40 | All Ages | Ecig | 6535 | 11.04 | | | | | |
| 40 | All Ages | Dual | 0 | 0.00 | | | | | |
| 40 | All Ages | Former | 24315 | 41.09 | | | 27.43 | 691 | 2.84 |

Year of Follow-up: 2030, Age Group: 18-19

| Period | Age Group | Smoking Group | N | % | Ever Quit (N) | Ever Quit % | Mean Yrs Quit | Short Term (n) | Short Term % |
|---|---|---|---|---|---|---|---|---|---|
| 40 | 18-19 | Never Any | 0 | . | | | | | |
| 40 | 18-19 | CC | 0 | . | 0 | . | | | |
| 40 | 18-19 | Ecig | 0 | . | | | | | |
| 40 | 18-19 | Dual | 0 | . | | | | | |
| 40 | 18-19 | Former | 0 | . | | | . | 0 | . |

Year of Follow-up: 2030, Age Group: 20-24

| Period | Age Group | Smoking Group | N | % | Ever Quit (N) | Ever Quit % | Mean Yrs Quit | Short Term (n) | Short Term % |
|---|---|---|---|---|---|---|---|---|---|
| 40 | 20-24 | Never Any | 0 | . | | | | | |
| 40 | 20-24 | CC | 0 | . | 0 | . | | | |
| 40 | 20-24 | Ecig | 0 | . | | | | | |
| 40 | 20-24 | Dual | 0 | . | | | | | |
| 40 | 20-24 | Former | 0 | . | | | . | 0 | . |

Year of Follow-up: 2030, Age Group: 25-29

| Period | Age Group | Smoking Group | N | % | Ever Quit (N) | Ever Quit % | Mean Yrs Quit | Short Term (n) | Short Term % |
|---|---|---|---|---|---|---|---|---|---|
| 40 | 25-29 | Never Any | 0 | . | | | | | |
| 40 | 25-29 | CC | 0 | . | 0 | . | | | |
| 40 | 25-29 | Ecig | 0 | . | | | | | |
| 40 | 25-29 | Dual | 0 | . | | | | | |

# Results for PHIM for Run 1 (Main_Analysis.RTF)

| | | | | | | | | | |
|---|---|---|---|---|---|---|---|---|---|
| 40 | 25-29 | Former | 0 | . | | | . | 0 | . |

Year of Follow-up: 2030, Age Group: 30-34

| Period | Age Group | Smoking Group | N | % | Ever Quit (N) | Ever Quit % | Mean Yrs Quit | Short Term (n) | Short Term % |
|---|---|---|---|---|---|---|---|---|---|
| 40 | 30-34 | Never Any | 0 | . | | | | | |
| 40 | 30-34 | CC | 0 | . | 0 | . | | | |
| 40 | 30-34 | Ecig | 0 | . | | | | | |
| 40 | 30-34 | Dual | 0 | . | | | | | |
| 40 | 30-34 | Former | 0 | . | | | . | 0 | . |

Year of Follow-up: 2030, Age Group: 35-39

| Period | Age Group | Smoking Group | N | % | Ever Quit (N) | Ever Quit % | Mean Yrs Quit | Short Term (n) | Short Term % |
|---|---|---|---|---|---|---|---|---|---|
| 40 | 35-39 | Never Any | 0 | . | | | | | |
| 40 | 35-39 | CC | 0 | . | 0 | . | | | |
| 40 | 35-39 | Ecig | 0 | . | | | | | |
| 40 | 35-39 | Dual | 0 | . | | | | | |
| 40 | 35-39 | Former | 0 | . | | | . | 0 | . |

Year of Follow-up: 2030, Age Group: 40-44

| Period | Age Group | Smoking Group | N | % | Ever Quit (N) | Ever Quit % | Mean Yrs Quit | Short Term (n) | Short Term % |
|---|---|---|---|---|---|---|---|---|---|
| 40 | 40-44 | Never Any | 0 | . | | | | | |
| 40 | 40-44 | CC | 0 | . | 0 | . | | | |
| 40 | 40-44 | Ecig | 0 | . | | | | | |
| 40 | 40-44 | Dual | 0 | . | | | | | |
| 40 | 40-44 | Former | 0 | . | | | . | 0 | . |

Year of Follow-up: 2030, Age Group: 45-49

| Period | Age Group | Smoking Group | N | % | Ever Quit (N) | Ever Quit % | Mean Yrs Quit | Short Term (n) | Short Term % |
|---|---|---|---|---|---|---|---|---|---|
| 40 | 45-49 | Never Any | 0 | . | | | | | |
| 40 | 45-49 | CC | 0 | . | 0 | . | | | |
| 40 | 45-49 | Ecig | 0 | . | | | | | |
| 40 | 45-49 | Dual | 0 | . | | | | | |
| 40 | 45-49 | Former | 0 | . | | | . | 0 | . |

Year of Follow-up: 2030, Age Group: 50-54

| Period | Age Group | Smoking Group | N | % | Ever Quit (N) | Ever Quit % | Mean Yrs Quit | Short Term (n) | Short Term % |
|---|---|---|---|---|---|---|---|---|---|
| 40 | 50-54 | Never Any | 4489 | 50.75 | | | | | |
| 40 | 50-54 | CC | 3 | 0.03 | 0 | 0.00 | | | |
| 40 | 50-54 | Ecig | 1392 | 15.74 | | | | | |
| 40 | 50-54 | Dual | 0 | 0.00 | | | | | |
| 40 | 50-54 | Former | 2961 | 33.48 | | | 22.61 | 80 | 2.70 |

Year of Follow-up: 2030, Age Group: 55-59

| Period | Age Group | Smoking Group | N | % | Ever Quit (N) | Ever Quit % | Mean Yrs Quit | Short Term (n) | Short Term % |
|---|---|---|---|---|---|---|---|---|---|
| 40 | 55-59 | Never Any | 4991 | 55.21 | | | | | |
| 40 | 55-59 | CC | 2 | 0.02 | 0 | 0.00 | | | |
| 40 | 55-59 | Ecig | 1291 | 14.28 | | | | | |
| 40 | 55-59 | Dual | 0 | 0.00 | | | | | |
| 40 | 55-59 | Former | 2756 | 30.49 | | | 23.19 | 114 | 4.14 |

Year of Follow-up: 2030, Age Group: 60-64

| Period | Age Group | Smoking Group | N | % | Ever Quit (N) | Ever Quit % | Mean Yrs Quit | Short Term (n) | Short Term % |
|---|---|---|---|---|---|---|---|---|---|
| 40 | 60-64 | Never Any | 5163 | 52.60 | | | | | |
| 40 | 60-64 | CC | 0 | 0.00 | 0 | 0.00 | | | |
| 40 | 60-64 | Ecig | 1238 | 12.61 | | | | | |
| 40 | 60-64 | Dual | 0 | 0.00 | | | | | |
| 40 | 60-64 | Former | 3415 | 34.79 | | | 24.53 | 183 | 5.36 |

Year of Follow-up: 2030, Age Group: 65-69

| Period | Age Group | Smoking Group | N | % | Ever Quit (N) | Ever Quit % | Mean Yrs Quit | Short Term (n) | Short Term % |
|---|---|---|---|---|---|---|---|---|---|
| 40 | 65-69 | Never Any | 4997 | 46.86 | | | | | |
| 40 | 65-69 | CC | 4 | 0.04 | 0 | 0.00 | | | |
| 40 | 65-69 | Ecig | 1119 | 10.49 | | | | | |
| 40 | 65-69 | Dual | 0 | 0.00 | | | | | |
| 40 | 65-69 | Former | 4544 | 42.61 | | | 26.35 | 136 | 2.99 |

Year of Follow-up: 2030, Age Group: 70-74

| Period | Age Group | Smoking Group | N | % | Ever Quit (N) | Ever Quit % | Mean Yrs Quit | Short Term (n) | Short Term % |
|---|---|---|---|---|---|---|---|---|---|
| 40 | 70-74 | Never Any | 4724 | 43.28 | | | | | |
| 40 | 70-74 | CC | 3 | 0.03 | 0 | 0.00 | | | |
| 40 | 70-74 | Ecig | 880 | 8.06 | | | | | |
| 40 | 70-74 | Dual | 0 | 0.00 | | | | | |
| 40 | 70-74 | Former | 5309 | 48.64 | | | 29.01 | 95 | 1.79 |

Year of Follow-up: 2030, Age Group: 75-79

| Period | Age Group | Smoking Group | N | % | Ever Quit (N) | Ever Quit % | Mean Yrs Quit | Short Term (n) | Short Term % |
|---|---|---|---|---|---|---|---|---|---|
| 40 | 75-79 | Never Any | 3941 | 39.85 | | | | | |
| 40 | 75-79 | CC | 3 | 0.03 | 0 | 0.00 | | | |

| | | | | | | | | | |
|---|---|---|---|---|---|---|---|---|---|
| 40 | 75-79 | Ecig | 615 | 6.22 | | | | | |
| 40 | 75-79 | Dual | 0 | 0.00 | | | | | |
| 40 | 75-79 | Former | 5330 | 53.90 | | | 33.51 | 83 | 1.56 |

Year of Follow-up: 2035, Age Group: All Ages

| Period | Age Group | Smoking Group | N | % | Ever Quit (N) | Ever Quit % | Mean Yrs Quit | Short Term (n) | Short Term % |
|---|---|---|---|---|---|---|---|---|---|
| 45 | All Ages | Never Any | 24364 | 49.44 | | | | | |
| 45 | All Ages | CC | 6 | 0.01 | 0 | 0.00 | | | |
| 45 | All Ages | Ecig | 4578 | 9.29 | | | | | |
| 45 | All Ages | Dual | 0 | 0.00 | | | | | |
| 45 | All Ages | Former | 20333 | 41.26 | | | 28.82 | 538 | 2.65 |

Year of Follow-up: 2035, Age Group: 18-19

| Period | Age Group | Smoking Group | N | % | Ever Quit (N) | Ever Quit % | Mean Yrs Quit | Short Term (n) | Short Term % |
|---|---|---|---|---|---|---|---|---|---|
| 45 | 18-19 | Never Any | 0 | . | | | | | |
| 45 | 18-19 | CC | 0 | . | 0 | . | | | |
| 45 | 18-19 | Ecig | 0 | . | | | | | |
| 45 | 18-19 | Dual | 0 | . | | | | | |
| 45 | 18-19 | Former | 0 | . | | | . | 0 | . |

Year of Follow-up: 2035, Age Group: 20-24

| Period | Age Group | Smoking Group | N | % | Ever Quit (N) | Ever Quit % | Mean Yrs Quit | Short Term (n) | Short Term % |
|---|---|---|---|---|---|---|---|---|---|
| 45 | 20-24 | Never Any | 0 | . | | | | | |
| 45 | 20-24 | CC | 0 | . | 0 | . | | | |
| 45 | 20-24 | Ecig | 0 | . | | | | | |
| 45 | 20-24 | Dual | 0 | . | | | | | |
| 45 | 20-24 | Former | 0 | . | | | . | 0 | . |

Year of Follow-up: 2035, Age Group: 25-29

| Period | Age Group | Smoking Group | N | % | Ever Quit (N) | Ever Quit % | Mean Yrs Quit | Short Term (n) | Short Term % |
|---|---|---|---|---|---|---|---|---|---|
| 45 | 25-29 | Never Any | 0 | . | | | | | |
| 45 | 25-29 | CC | 0 | . | 0 | . | | | |
| 45 | 25-29 | Ecig | 0 | . | | | | | |
| 45 | 25-29 | Dual | 0 | . | | | | | |
| 45 | 25-29 | Former | 0 | . | | | . | 0 | . |

Year of Follow-up: 2035, Age Group: 30-34

| Period | Age Group | Smoking Group | N | % | Ever Quit (N) | Ever Quit % | Mean Yrs Quit | Short Term (n) | Short Term % |
|---|---|---|---|---|---|---|---|---|---|
| 45 | 30-34 | Never Any | 0 | . | | | | | |
| 45 | 30-34 | CC | 0 | . | 0 | . | | | |
| 45 | 30-34 | Ecig | 0 | . | | | | | |
| 45 | 30-34 | Dual | 0 | . | | | | | |
| 45 | 30-34 | Former | 0 | . | | | . | 0 | . |

Year of Follow-up: 2035, Age Group: 35-39

| Period | Age Group | Smoking Group | N | % | Ever Quit (N) | Ever Quit % | Mean Yrs Quit | Short Term (n) | Short Term % |
|---|---|---|---|---|---|---|---|---|---|
| 45 | 35-39 | Never Any | 0 | . | | | | | |
| 45 | 35-39 | CC | 0 | . | 0 | . | | | |
| 45 | 35-39 | Ecig | 0 | . | | | | | |
| 45 | 35-39 | Dual | 0 | . | | | | | |
| 45 | 35-39 | Former | 0 | . | | | . | 0 | . |

Year of Follow-up: 2035, Age Group: 40-44

| Period | Age Group | Smoking Group | N | % | Ever Quit (N) | Ever Quit % | Mean Yrs Quit | Short Term (n) | Short Term % |
|---|---|---|---|---|---|---|---|---|---|
| 45 | 40-44 | Never Any | 0 | . | | | | | |
| 45 | 40-44 | CC | 0 | . | 0 | . | | | |
| 45 | 40-44 | Ecig | 0 | . | | | | | |
| 45 | 40-44 | Dual | 0 | . | | | | | |
| 45 | 40-44 | Former | 0 | . | | | . | 0 | . |

Year of Follow-up: 2035, Age Group: 45-49

| Period | Age Group | Smoking Group | N | % | Ever Quit (N) | Ever Quit % | Mean Yrs Quit | Short Term (n) | Short Term % |
|---|---|---|---|---|---|---|---|---|---|
| 45 | 45-49 | Never Any | 0 | . | | | | | |
| 45 | 45-49 | CC | 0 | . | 0 | . | | | |
| 45 | 45-49 | Ecig | 0 | . | | | | | |
| 45 | 45-49 | Dual | 0 | . | | | | | |
| 45 | 45-49 | Former | 0 | . | | | . | 0 | . |

Year of Follow-up: 2035, Age Group: 50-54

| Period | Age Group | Smoking Group | N | % | Ever Quit (N) | Ever Quit % | Mean Yrs Quit | Short Term (n) | Short Term % |
|---|---|---|---|---|---|---|---|---|---|
| 45 | 50-54 | Never Any | 0 | . | | | | | |
| 45 | 50-54 | CC | 0 | . | 0 | . | | | |
| 45 | 50-54 | Ecig | 0 | . | | | | | |
| 45 | 50-54 | Dual | 0 | . | | | | | |
| 45 | 50-54 | Former | 0 | . | | | . | 0 | . |

Year of Follow-up: 2035, Age Group: 55-59

| Period | Age Group | Smoking Group | N | % | Ever Quit (N) | Ever Quit % | Mean Yrs Quit | Short Term (n) | Short Term % |
|---|---|---|---|---|---|---|---|---|---|

## Results for PHIM for Run 1 (Main_Analysis.RTF)

| | | | | | | | | | |
|---|---|---|---|---|---|---|---|---|---|
| 45 | 55-59 | Never Any | 4489 | 50.75 | | | | | |
| 45 | 55-59 | CC | 2 | 0.02 | 0 | 0.00 | | | |
| 45 | 55-59 | Ecig | 1171 | 13.24 | | | | | |
| 45 | 55-59 | Dual | 0 | 0.00 | | | | | |
| 45 | 55-59 | Former | 3183 | 35.99 | | | 25.82 | 100 | 3.14 |

Year of Follow-up: 2035, Age Group: 60-64

| Period | Age Group | Smoking Group | N | % | Ever Quit (N) | Ever Quit % | Mean Yrs Quit | Short Term (n) | Short Term % |
|---|---|---|---|---|---|---|---|---|---|
| 45 | 60-64 | Never Any | 4991 | 55.21 | | | | | |
| 45 | 60-64 | CC | 0 | 0.00 | 0 | 0.00 | | | |
| 45 | 60-64 | Ecig | 1020 | 11.28 | | | | | |
| 45 | 60-64 | Dual | 0 | 0.00 | | | | | |
| 45 | 60-64 | Former | 3029 | 33.51 | | | 25.81 | 129 | 4.26 |

Year of Follow-up: 2035, Age Group: 65-69

| Period | Age Group | Smoking Group | N | % | Ever Quit (N) | Ever Quit % | Mean Yrs Quit | Short Term (n) | Short Term % |
|---|---|---|---|---|---|---|---|---|---|
| 45 | 65-69 | Never Any | 5163 | 52.60 | | | | | |
| 45 | 65-69 | CC | 0 | 0.00 | 0 | 0.00 | | | |
| 45 | 65-69 | Ecig | 902 | 9.19 | | | | | |
| 45 | 65-69 | Dual | 0 | 0.00 | | | | | |
| 45 | 65-69 | Former | 3751 | 38.21 | | | 27.09 | 106 | 2.83 |

Year of Follow-up: 2035, Age Group: 70-74

| Period | Age Group | Smoking Group | N | % | Ever Quit (N) | Ever Quit % | Mean Yrs Quit | Short Term (n) | Short Term % |
|---|---|---|---|---|---|---|---|---|---|
| 45 | 70-74 | Never Any | 4997 | 46.86 | | | | | |
| 45 | 70-74 | CC | 1 | 0.01 | 0 | 0.00 | | | |
| 45 | 70-74 | Ecig | 850 | 7.97 | | | | | |
| 45 | 70-74 | Dual | 0 | 0.00 | | | | | |
| 45 | 70-74 | Former | 4816 | 45.16 | | | 29.70 | 104 | 2.16 |

Year of Follow-up: 2035, Age Group: 75-79

| Period | Age Group | Smoking Group | N | % | Ever Quit (N) | Ever Quit % | Mean Yrs Quit | Short Term (n) | Short Term % |
|---|---|---|---|---|---|---|---|---|---|
| 45 | 75-79 | Never Any | 4724 | 43.28 | | | | | |
| 45 | 75-79 | CC | 3 | 0.03 | 0 | 0.00 | | | |
| 45 | 75-79 | Ecig | 635 | 5.82 | | | | | |
| 45 | 75-79 | Dual | 0 | 0.00 | | | | | |
| 45 | 75-79 | Former | 5554 | 50.88 | | | 32.60 | 99 | 1.78 |

Year of Follow-up: 2040, Age Group: All Ages

| Period | Age Group | Smoking Group | N | % | Ever Quit (N) | Ever Quit % | Mean Yrs Quit | Short Term (n) | Short Term % |
|---|---|---|---|---|---|---|---|---|---|
| 50 | All Ages | Never Any | 19640 | 51.19 | | | | | |
| 50 | All Ages | CC | 1 | 0.00 | 0 | 0.00 | | | |
| 50 | All Ages | Ecig | 2868 | 7.48 | | | | | |
| 50 | All Ages | Dual | 0 | 0.00 | | | | | |
| 50 | All Ages | Former | 15856 | 41.33 | | | 30.34 | 408 | 2.57 |

Year of Follow-up: 2040, Age Group: 18-19

| Period | Age Group | Smoking Group | N | % | Ever Quit (N) | Ever Quit % | Mean Yrs Quit | Short Term (n) | Short Term % |
|---|---|---|---|---|---|---|---|---|---|
| 50 | 18-19 | Never Any | 0 | . | | | | | |
| 50 | 18-19 | CC | 0 | . | 0 | . | | | |
| 50 | 18-19 | Ecig | 0 | . | | | | | |
| 50 | 18-19 | Dual | 0 | . | | | | | |
| 50 | 18-19 | Former | 0 | . | | | . | 0 | . |

Year of Follow-up: 2040, Age Group: 20-24

| Period | Age Group | Smoking Group | N | % | Ever Quit (N) | Ever Quit % | Mean Yrs Quit | Short Term (n) | Short Term % |
|---|---|---|---|---|---|---|---|---|---|
| 50 | 20-24 | Never Any | 0 | . | | | | | |
| 50 | 20-24 | CC | 0 | . | 0 | . | | | |
| 50 | 20-24 | Ecig | 0 | . | | | | | |
| 50 | 20-24 | Dual | 0 | . | | | | | |
| 50 | 20-24 | Former | 0 | . | | | . | 0 | . |

Year of Follow-up: 2040, Age Group: 25-29

| Period | Age Group | Smoking Group | N | % | Ever Quit (N) | Ever Quit % | Mean Yrs Quit | Short Term (n) | Short Term % |
|---|---|---|---|---|---|---|---|---|---|
| 50 | 25-29 | Never Any | 0 | . | | | | | |
| 50 | 25-29 | CC | 0 | . | 0 | . | | | |
| 50 | 25-29 | Ecig | 0 | . | | | | | |
| 50 | 25-29 | Dual | 0 | . | | | | | |
| 50 | 25-29 | Former | 0 | . | | | . | 0 | . |

Year of Follow-up: 2040, Age Group: 30-34

| Period | Age Group | Smoking Group | N | % | Ever Quit (N) | Ever Quit % | Mean Yrs Quit | Short Term (n) | Short Term % |
|---|---|---|---|---|---|---|---|---|---|
| 50 | 30-34 | Never Any | 0 | . | | | | | |
| 50 | 30-34 | CC | 0 | . | 0 | . | | | |
| 50 | 30-34 | Ecig | 0 | . | | | | | |
| 50 | 30-34 | Dual | 0 | . | | | | | |
| 50 | 30-34 | Former | 0 | . | | | . | 0 | . |

Year of Follow-up: 2040, Age Group: 35-39

# Results for PHIM for Run 1 (Main_Analysis.RTF)

| Period | Age Group | Smoking Group | N | % | Ever Quit (N) | Ever Quit % | Mean Yrs Quit | Short Term (n) | Short Term % |
|---|---|---|---|---|---|---|---|---|---|
| 50 | 35-39 | Never Any | 0 | . | | | | | |
| 50 | 35-39 | CC | 0 | . | 0 | . | | | |
| 50 | 35-39 | Ecig | 0 | . | | | | | |
| 50 | 35-39 | Dual | 0 | . | | | | | |
| 50 | 35-39 | Former | 0 | . | | | . | 0 | . |

Year of Follow-up: 2040, Age Group: 40-44

| Period | Age Group | Smoking Group | N | % | Ever Quit (N) | Ever Quit % | Mean Yrs Quit | Short Term (n) | Short Term % |
|---|---|---|---|---|---|---|---|---|---|
| 50 | 40-44 | Never Any | 0 | . | | | | | |
| 50 | 40-44 | CC | 0 | . | 0 | . | | | |
| 50 | 40-44 | Ecig | 0 | . | | | | | |
| 50 | 40-44 | Dual | 0 | . | | | | | |
| 50 | 40-44 | Former | 0 | . | | | . | 0 | . |

Year of Follow-up: 2040, Age Group: 45-49

| Period | Age Group | Smoking Group | N | % | Ever Quit (N) | Ever Quit % | Mean Yrs Quit | Short Term (n) | Short Term % |
|---|---|---|---|---|---|---|---|---|---|
| 50 | 45-49 | Never Any | 0 | . | | | | | |
| 50 | 45-49 | CC | 0 | . | 0 | . | | | |
| 50 | 45-49 | Ecig | 0 | . | | | | | |
| 50 | 45-49 | Dual | 0 | . | | | | | |
| 50 | 45-49 | Former | 0 | . | | | . | 0 | . |

Year of Follow-up: 2040, Age Group: 50-54

| Period | Age Group | Smoking Group | N | % | Ever Quit (N) | Ever Quit % | Mean Yrs Quit | Short Term (n) | Short Term % |
|---|---|---|---|---|---|---|---|---|---|
| 50 | 50-54 | Never Any | 0 | . | | | | | |
| 50 | 50-54 | CC | 0 | . | 0 | . | | | |
| 50 | 50-54 | Ecig | 0 | . | | | | | |
| 50 | 50-54 | Dual | 0 | . | | | | | |
| 50 | 50-54 | Former | 0 | . | | | . | 0 | . |

Year of Follow-up: 2040, Age Group: 55-59

| Period | Age Group | Smoking Group | N | % | Ever Quit (N) | Ever Quit % | Mean Yrs Quit | Short Term (n) | Short Term % |
|---|---|---|---|---|---|---|---|---|---|
| 50 | 55-59 | Never Any | 0 | . | | | | | |
| 50 | 55-59 | CC | 0 | . | 0 | . | | | |
| 50 | 55-59 | Ecig | 0 | . | | | | | |
| 50 | 55-59 | Dual | 0 | . | | | | | |
| 50 | 55-59 | Former | 0 | . | | | . | 0 | . |

Year of Follow-up: 2040, Age Group: 60-64

| Period | Age Group | Smoking Group | N | % | Ever Quit (N) | Ever Quit % | Mean Yrs Quit | Short Term (n) | Short Term % |
|---|---|---|---|---|---|---|---|---|---|
| 50 | 60-64 | Never Any | 4489 | 50.75 | | | | | |
| 50 | 60-64 | CC | 0 | 0.00 | 0 | 0.00 | | | |
| 50 | 60-64 | Ecig | 865 | 9.78 | | | | | |
| 50 | 60-64 | Dual | 0 | 0.00 | | | | | |
| 50 | 60-64 | Former | 3491 | 39.47 | | | 28.27 | 129 | 3.70 |

Year of Follow-up: 2040, Age Group: 65-69

| Period | Age Group | Smoking Group | N | % | Ever Quit (N) | Ever Quit % | Mean Yrs Quit | Short Term (n) | Short Term % |
|---|---|---|---|---|---|---|---|---|---|
| 50 | 65-69 | Never Any | 4991 | 55.21 | | | | | |
| 50 | 65-69 | CC | 0 | 0.00 | 0 | 0.00 | | | |
| 50 | 65-69 | Ecig | 705 | 7.80 | | | | | |
| 50 | 65-69 | Dual | 0 | 0.00 | | | | | |
| 50 | 65-69 | Former | 3344 | 36.99 | | | 28.11 | 120 | 3.59 |

Year of Follow-up: 2040, Age Group: 70-74

| Period | Age Group | Smoking Group | N | % | Ever Quit (N) | Ever Quit % | Mean Yrs Quit | Short Term (n) | Short Term % |
|---|---|---|---|---|---|---|---|---|---|
| 50 | 70-74 | Never Any | 5163 | 52.60 | | | | | |
| 50 | 70-74 | CC | 0 | 0.00 | 0 | 0.00 | | | |
| 50 | 70-74 | Ecig | 665 | 6.77 | | | | | |
| 50 | 70-74 | Dual | 0 | 0.00 | | | | | |
| 50 | 70-74 | Former | 3988 | 40.63 | | | 30.31 | 80 | 2.01 |

Year of Follow-up: 2040, Age Group: 75-79

| Period | Age Group | Smoking Group | N | % | Ever Quit (N) | Ever Quit % | Mean Yrs Quit | Short Term (n) | Short Term % |
|---|---|---|---|---|---|---|---|---|---|
| 50 | 75-79 | Never Any | 4997 | 46.86 | | | | | |
| 50 | 75-79 | CC | 1 | 0.01 | 0 | 0.00 | | | |
| 50 | 75-79 | Ecig | 633 | 5.94 | | | | | |
| 50 | 75-79 | Dual | 0 | 0.00 | | | | | |
| 50 | 75-79 | Former | 5033 | 47.20 | | | 33.29 | 79 | 1.57 |

# Results for PHIM for Run 1 (Main_Analysis.RTF)

************************************************************************************

**Main Results for E-Component for Run: 1, Simulation: 1, COD: Four Diseases Combined**

| Variable Parameter | Value |
|---|---|
| 1. Country | US (United States) |
| 2. Sex | M (Males) |
| 3. Year of start of process | 1990 |
| 4. Cause of Death | Four Diseases Combined |
| 5. Effective Dose NULL | CC:1 |
| 6. Effective Dose Ecig | CC:1 Ecig:0.05 Dual:0.525 |
| Note: | Death rates are per million population |

------------------------------------------------------------------------------------

Results for Age Groups Combined and Years combined, Population unadjusted for deaths in Ecig scenario,

| Age Group:All | | NULL | Ecig | | | %Drop | | NULL | Ecig | |
|---|---|---|---|---|---|---|---|---|---|---|
| Year | Number | Attrib | Attrib | Drop | %Drop | Attrib | Death | Attrib | Attrib | Drop |
| | Deaths | Deaths | Deaths | Deaths | Deaths | Deaths | Rate | Rates | Rates | Rates |
| All Comb | 13148871 | 4509390.583 | 2984679.112 | 1524711 | 11.596 | 33.812 | 3806.31 | 1305.37 | 864.000 | 441.371 |

------------------------------------------------------------------------------------

Results for Age Groups Combined, Population unadjusted for deaths in Ecig scenario,

| Age Group:All | | NULL | Ecig | | | %Drop | | NULL | Ecig | |
|---|---|---|---|---|---|---|---|---|---|---|
| Year | Number | Attrib | Attrib | Drop | %Drop | Attrib | Death | Attrib | Attrib | Drop |
| | Deaths | Deaths | Deaths | Deaths | Deaths | Deaths | Rate | Rates | Rates | Rates |
| 1990 | 317161 | 125773.065 | 125773.065 | 0.000 | 0.000 | 0.000 | 5027.71 | 1993.78 | 1993.78 | 0.000 |
| 1991 | 313217 | 123999.442 | 123575.252 | 424.190 | 0.135 | 0.342 | 4883.81 | 1933.45 | 1926.84 | 6.614 |
| 1992 | 308577 | 122014.015 | 120877.258 | 1136.757 | 0.368 | 0.932 | 4729.73 | 1870.18 | 1852.75 | 17.424 |
| 1993 | 311713 | 122810.618 | 120675.272 | 2135.346 | 0.685 | 1.739 | 4692.62 | 1848.83 | 1816.68 | 32.146 |
| 1994 | 305700 | 119925.592 | 116642.213 | 3283.379 | 1.074 | 2.738 | 4516.19 | 1771.69 | 1723.19 | 48.506 |
| 1995 | 303225 | 117896.496 | 113270.222 | 4626.275 | 1.526 | 3.924 | 4393.80 | 1708.35 | 1641.31 | 67.036 |
| 1996 | 299559 | 115698.645 | 109592.838 | 6105.807 | 2.038 | 5.277 | 4273.16 | 1650.42 | 1563.32 | 87.098 |
| 1997 | 294226 | 113359.847 | 105764.237 | 7595.609 | 2.582 | 6.700 | 4128.67 | 1590.70 | 1484.12 | 106.584 |
| 1998 | 289258 | 111444.212 | 102354.614 | 9089.598 | 3.142 | 8.156 | 3992.19 | 1538.10 | 1412.65 | 125.450 |
| 1999 | 311002 | 114877.722 | 102717.729 | 12159.99 | 3.910 | 10.585 | 4224.60 | 1560.48 | 1395.30 | 165.179 |
| 2000 | 303786 | 112379.704 | 98503.172 | 13876.53 | 4.568 | 12.348 | 4066.21 | 1504.22 | 1318.48 | 185.739 |
| 2001 | 296294 | 110024.943 | 94313.120 | 15711.82 | 5.303 | 14.280 | 3914.28 | 1453.52 | 1245.95 | 207.566 |
| 2002 | 291898 | 108289.173 | 90517.550 | 17771.62 | 6.088 | 16.411 | 3813.00 | 1414.56 | 1182.41 | 232.147 |
| 2003 | 285865 | 106539.023 | 86946.352 | 19592.67 | 6.854 | 18.390 | 3695.36 | 1377.22 | 1123.95 | 253.273 |
| 2004 | 274177 | 103037.251 | 82110.840 | 20926.41 | 7.632 | 20.310 | 3506.10 | 1317.61 | 1050.01 | 267.601 |
| 2005 | 272379 | 103044.562 | 80197.351 | 22847.21 | 8.388 | 22.172 | 3442.69 | 1302.41 | 1013.64 | 288.773 |
| 2006 | 264118 | 99672.555 | 75189.882 | 24482.67 | 9.270 | 24.563 | 3307.76 | 1248.28 | 941.663 | 306.616 |
| 2007 | 258419 | 97839.152 | 71981.795 | 25857.36 | 10.006 | 26.428 | 3203.20 | 1212.75 | 892.240 | 320.511 |
| 2008 | 259461 | 98075.852 | 70477.901 | 27597.95 | 10.637 | 28.139 | 3180.09 | 1202.07 | 863.815 | 338.255 |
| 2009 | 253907 | 95617.338 | 66805.356 | 28811.98 | 11.347 | 30.133 | 3075.31 | 1158.11 | 809.144 | 348.970 |
| 2010 | 251878 | 94607.988 | 64431.426 | 30176.56 | 11.981 | 31.896 | 3013.72 | 1131.98 | 770.922 | 361.062 |
| 2011 | 251779 | 93633.119 | 61788.680 | 31844.44 | 12.648 | 34.010 | 2982.76 | 1109.25 | 731.996 | 377.253 |
| 2012 | 252643 | 92775.175 | 59296.371 | 33478.80 | 13.251 | 36.086 | 2960.61 | 1087.19 | 694.867 | 392.323 |
| 2013 | 256175 | 92345.780 | 57192.739 | 35153.04 | 13.722 | 38.067 | 2967.76 | 1069.82 | 662.572 | 407.244 |
| 2014 | 257393 | 91379.215 | 54826.416 | 36552.80 | 14.201 | 40.001 | 2947.05 | 1046.26 | 627.741 | 418.515 |
| 2015 | 259782 | 90750.242 | 52936.177 | 37814.07 | 14.556 | 41.668 | 3346.10 | 1168.90 | 681.840 | 487.061 |
| 2016 | 261453 | 88811.996 | 49699.461 | 39112.53 | 14.960 | 44.040 | 3336.15 | 1133.24 | 634.167 | 499.078 |
| 2017 | 263685 | 87325.126 | 47066.158 | 40258.97 | 15.268 | 46.102 | 3333.85 | 1104.08 | 595.072 | 509.007 |
| 2018 | 258714 | 87612.144 | 46315.614 | 41296.53 | 15.962 | 47.136 | 3240.73 | 1097.46 | 580.163 | 517.293 |
| 2019 | 261438 | 87797.760 | 44988.643 | 42809.12 | 16.374 | 48.759 | 3242.99 | 1089.08 | 558.059 | 531.023 |
| 2020 | 262288 | 87221.042 | 43536.345 | 43684.70 | 16.655 | 50.085 | 3715.29 | 1235.48 | 616.689 | 618.790 |
| 2021 | 255079 | 82001.292 | 38768.306 | 43232.99 | 16.949 | 52.722 | 3587.82 | 1153.39 | 545.296 | 608.094 |
| 2022 | 257097 | 81915.977 | 37292.841 | 44623.14 | 17.357 | 54.474 | 3588.53 | 1143.37 | 520.530 | 622.845 |
| 2023 | 259080 | 81810.810 | 35853.657 | 45957.15 | 17.739 | 56.175 | 3586.88 | 1132.64 | 496.383 | 636.262 |
| 2024 | 260719 | 81528.303 | 34430.663 | 47097.64 | 18.065 | 57.768 | 3579.65 | 1119.38 | 472.731 | 646.647 |
| 2025 | 257308 | 79599.213 | 32763.035 | 46836.18 | 18.202 | 58.840 | 4112.08 | 1272.09 | 523.591 | 748.496 |
| 2026 | 251528 | 75082.462 | 29110.480 | 45971.98 | 18.277 | 61.229 | 4003.24 | 1194.99 | 463.313 | 731.675 |
| 2027 | 250988 | 74444.425 | 27620.624 | 46823.80 | 18.656 | 62.898 | 3976.53 | 1179.46 | 437.608 | 741.853 |
| 2028 | 250285 | 73699.585 | 26060.257 | 47639.33 | 19.034 | 64.640 | 3945.35 | 1161.76 | 410.800 | 750.960 |
| 2029 | 249525 | 72819.703 | 24567.072 | 48252.63 | 19.338 | 66.263 | 3910.99 | 1141.36 | 385.058 | 756.299 |
| 2030 | 240528 | 69309.277 | 22886.854 | 46422.42 | 19.300 | 66.979 | 4504.36 | 1297.95 | 428.600 | 869.349 |
| 2031 | 235442 | 65433.368 | 20269.324 | 45164.04 | 19.183 | 69.023 | 4397.65 | 1222.18 | 378.596 | 843.586 |
| 2032 | 234017 | 64643.552 | 19038.140 | 45605.41 | 19.488 | 70.549 | 4356.12 | 1203.31 | 354.386 | 848.923 |
| 2033 | 232559 | 63793.514 | 17876.751 | 45916.76 | 19.744 | 71.977 | 4310.93 | 1182.54 | 331.380 | 851.156 |
| 2034 | 230963 | 63032.151 | 16784.296 | 46247.86 | 20.024 | 73.372 | 4260.75 | 1162.80 | 309.632 | 853.168 |
| 2035 | 215822 | 57653.301 | 15283.522 | 42369.78 | 19.632 | 73.491 | 4924.38 | 1315.47 | 348.723 | 966.748 |
| 2036 | 208183 | 52988.733 | 12794.436 | 40194.30 | 19.307 | 75.854 | 4748.94 | 1208.75 | 291.859 | 916.887 |
| 2037 | 205491 | 52026.921 | 11850.994 | 40175.93 | 19.551 | 77.221 | 4682.40 | 1185.50 | 270.041 | 915.462 |
| 2038 | 202834 | 50943.211 | 10923.876 | 40019.34 | 19.730 | 78.557 | 4612.18 | 1158.38 | 248.395 | 909.988 |
| 2039 | 200251 | 50085.991 | 10139.935 | 39946.06 | 19.948 | 79.755 | 4538.65 | 1135.19 | 229.820 | 905.371 |

# Results for PHIM for Run 1 (Main_Analysis.RTF)

*************************************************************************************
**Main Results for E-Component for Run: 1, Simulation: 1, COD: LUNG CANCER**

| **Variable Parameter** | **Value** |
|---|---|
| 1. Country | US (United States) |
| 2. Sex | M (Males) |
| 3. Year of start of process | 1990 |
| 4. Cause of Death | LUNG CANCER |
| 5. Effective Dose NULL | CC:1 |
| 6. Effective Dose Ecig | CC:1 Ecig:0.05 Dual:0.525 |
| Note: | Death rates are per million population |

-----------------------------------------------------------------------------------------
Results for Age Groups Combined and Years combined, Population unadjusted for deaths in Ecig scenario,

| Age Group:All | | NULL | Ecig | | | %Drop | | NULL | Ecig | |
|---|---|---|---|---|---|---|---|---|---|---|
| Year | Number | Attrib | Attrib | Drop | %Drop | Attrib | Death | Attrib | Attrib | Drop |
| | Deaths | Deaths | Deaths | Deaths | Deaths | Deaths | Rate | Rates | Rates | Rates |
| All Comb | 3065988 | 2244988.113 | 1722967.276 | 522020.8 | 17.026 | 23.253 | 887.537 | 649.875 | 498.762 | 151.114 |

-----------------------------------------------------------------------------------------
Results for Age Groups Combined, Population unadjusted for deaths in Ecig scenario,

| Age Group:All | | NULL | Ecig | | | %Drop | | NULL | Ecig | |
|---|---|---|---|---|---|---|---|---|---|---|
| Year | Number | Attrib | Attrib | Drop | %Drop | Attrib | Death | Attrib | Attrib | Drop |
| | Deaths | Deaths | Deaths | Deaths | Deaths | Deaths | Rate | Rates | Rates | Rates |
| 1990 | 78822 | 63385.736 | 63385.736 | 0.000 | 0.000 | 0.000 | 1249.50 | 1004.81 | 1004.81 | 0.000 |
| 1991 | 78462 | 62918.402 | 62870.284 | 48.118 | 0.061 | 0.076 | 1223.41 | 981.049 | 980.299 | 0.750 |
| 1992 | 77987 | 62366.766 | 62237.572 | 129.194 | 0.166 | 0.207 | 1195.35 | 955.930 | 953.949 | 1.980 |
| 1993 | 78542 | 62652.888 | 62398.514 | 254.374 | 0.324 | 0.406 | 1182.39 | 943.195 | 939.365 | 3.829 |
| 1994 | 77437 | 61565.227 | 61163.821 | 401.406 | 0.518 | 0.652 | 1144.00 | 909.520 | 903.590 | 5.930 |
| 1995 | 76904 | 60851.961 | 60267.833 | 584.128 | 0.760 | 0.960 | 1114.36 | 881.759 | 873.295 | 8.464 |
| 1996 | 76232 | 60122.922 | 59308.518 | 814.404 | 1.068 | 1.355 | 1087.44 | 857.643 | 846.026 | 11.617 |
| 1997 | 75473 | 59237.155 | 58175.080 | 1062.074 | 1.407 | 1.793 | 1059.06 | 831.234 | 816.331 | 14.903 |
| 1998 | 75381 | 58906.696 | 57537.293 | 1369.403 | 1.817 | 2.325 | 1040.37 | 813.001 | 794.101 | 18.900 |
| 1999 | 73159 | 56930.073 | 55258.820 | 1671.253 | 2.284 | 2.936 | 993.781 | 773.329 | 750.627 | 22.702 |
| 2000 | 73784 | 57137.121 | 55072.463 | 2064.658 | 2.798 | 3.614 | 987.608 | 764.787 | 737.152 | 27.636 |
| 2001 | 73294 | 56481.726 | 53973.185 | 2508.541 | 3.423 | 4.441 | 968.273 | 746.169 | 713.030 | 33.140 |
| 2002 | 72442 | 55588.365 | 52613.222 | 2975.143 | 4.107 | 5.352 | 946.293 | 726.138 | 687.274 | 38.864 |
| 2003 | 71942 | 54945.856 | 51454.115 | 3491.742 | 4.854 | 6.355 | 929.989 | 710.281 | 665.143 | 45.137 |
| 2004 | 71301 | 54203.126 | 50142.148 | 4060.977 | 5.696 | 7.492 | 911.778 | 693.135 | 641.204 | 51.931 |
| 2005 | 70964 | 53744.603 | 49094.615 | 4649.988 | 6.553 | 8.652 | 896.937 | 679.296 | 620.523 | 58.773 |
| 2006 | 69658 | 52535.572 | 47267.564 | 5268.008 | 7.563 | 10.028 | 872.383 | 657.945 | 591.969 | 65.975 |
| 2007 | 68565 | 51492.124 | 45613.004 | 5879.120 | 8.575 | 11.418 | 849.888 | 638.264 | 565.390 | 72.874 |
| 2008 | 68226 | 51030.736 | 44410.892 | 6619.843 | 9.703 | 12.972 | 836.215 | 625.460 | 544.324 | 81.136 |
| 2009 | 67509 | 50276.199 | 42915.597 | 7360.602 | 10.903 | 14.640 | 817.667 | 608.943 | 519.792 | 89.151 |
| 2010 | 67196 | 49840.933 | 41737.184 | 8103.749 | 12.060 | 16.259 | 804.000 | 596.347 | 499.385 | 96.961 |
| 2011 | 66506 | 49072.841 | 40150.989 | 8921.852 | 13.415 | 18.181 | 787.880 | 581.354 | 475.659 | 105.695 |
| 2012 | 66155 | 48539.512 | 38739.555 | 9799.957 | 14.814 | 20.190 | 775.240 | 568.812 | 453.971 | 114.841 |
| 2013 | 65333 | 47675.741 | 37094.574 | 10581.17 | 16.196 | 22.194 | 756.876 | 552.318 | 429.737 | 122.582 |
| 2014 | 65033 | 47194.534 | 35798.099 | 11396.44 | 17.524 | 24.148 | 744.602 | 540.359 | 409.874 | 130.485 |
| 2015 | 64145 | 46305.842 | 34200.531 | 12105.31 | 18.872 | 26.142 | 826.215 | 596.439 | 440.517 | 155.922 |
| 2016 | 61618 | 44208.867 | 31690.376 | 12518.49 | 20.316 | 28.317 | 786.248 | 564.107 | 404.371 | 159.736 |
| 2017 | 59897 | 42702.624 | 29702.634 | 12999.99 | 21.704 | 30.443 | 757.297 | 539.903 | 375.540 | 164.363 |
| 2018 | 62468 | 44321.202 | 29714.738 | 14606.46 | 23.382 | 32.956 | 782.490 | 555.181 | 372.216 | 182.965 |
| 2019 | 63045 | 44493.088 | 28815.278 | 15677.81 | 24.868 | 35.237 | 782.035 | 551.912 | 357.437 | 194.474 |
| 2020 | 63466 | 44529.551 | 27852.542 | 16677.01 | 26.277 | 37.452 | 898.991 | 630.757 | 394.529 | 236.228 |
| 2021 | 57113 | 39852.556 | 23958.784 | 15893.77 | 27.829 | 39.881 | 803.323 | 560.546 | 336.992 | 223.554 |
| 2022 | 57440 | 39853.685 | 22965.786 | 16887.90 | 29.401 | 42.375 | 801.745 | 556.274 | 320.554 | 235.720 |
| 2023 | 57738 | 39847.050 | 21980.631 | 17866.42 | 30.944 | 44.837 | 799.366 | 551.670 | 304.315 | 247.355 |
| 2024 | 57942 | 39726.010 | 20989.883 | 18736.13 | 32.336 | 47.163 | 795.539 | 545.435 | 288.190 | 257.246 |
| 2025 | 57521 | 39207.348 | 19886.366 | 19320.98 | 33.589 | 49.279 | 919.257 | 626.579 | 317.807 | 308.772 |
| 2026 | 51428 | 34803.960 | 16841.985 | 17961.97 | 34.927 | 51.609 | 818.510 | 553.929 | 268.052 | 285.877 |
| 2027 | 51172 | 34484.595 | 15859.227 | 18625.37 | 36.398 | 54.011 | 810.740 | 546.357 | 251.266 | 295.091 |
| 2028 | 50870 | 34100.638 | 14849.719 | 19250.92 | 37.843 | 56.453 | 801.884 | 537.544 | 234.083 | 303.461 |
| 2029 | 50540 | 33669.838 | 13893.199 | 19776.64 | 39.131 | 58.737 | 792.146 | 527.732 | 217.758 | 309.974 |
| 2030 | 49109 | 32557.688 | 12861.081 | 19696.61 | 40.108 | 60.498 | 919.662 | 609.705 | 240.848 | 368.857 |
| 2031 | 43585 | 28646.393 | 10735.554 | 17910.84 | 41.094 | 62.524 | 814.096 | 535.065 | 200.521 | 334.543 |
| 2032 | 43163 | 28227.160 | 9993.007 | 18234.15 | 42.245 | 64.598 | 803.454 | 525.435 | 186.015 | 339.420 |
| 2033 | 42725 | 27786.623 | 9298.038 | 18488.58 | 43.273 | 66.538 | 791.996 | 515.079 | 172.357 | 342.722 |
| 2034 | 42256 | 27348.299 | 8653.437 | 18694.86 | 44.242 | 68.358 | 779.523 | 504.514 | 159.636 | 344.878 |
| 2035 | 39748 | 25570.077 | 7820.113 | 17749.96 | 44.656 | 69.417 | 906.933 | 583.430 | 178.431 | 404.999 |
| 2036 | 34030 | 21717.319 | 6135.387 | 15581.93 | 45.789 | 71.749 | 776.269 | 495.402 | 139.957 | 355.445 |
| 2037 | 33454 | 21256.024 | 5642.911 | 15613.11 | 46.671 | 73.453 | 762.289 | 484.347 | 128.581 | 355.766 |
| 2038 | 32883 | 20747.175 | 5168.957 | 15578.22 | 47.374 | 75.086 | 747.720 | 471.764 | 117.535 | 354.229 |
| 2039 | 32325 | 20327.688 | 4776.433 | 15551.25 | 48.109 | 76.503 | 732.644 | 460.724 | 108.257 | 352.467 |

# Results for PHIM for Run 1 (Main_Analysis.RTF)

********************************************************************************

**Main Results for E-Component for Run: 1, Simulation: 1, COD: IHD**

| **Variable Parameter** | **Value** |
|---|---|
| 1. Country | US (United States) |
| 2. Sex | M (Males) |
| 3. Year of start of process | 1990 |
| 4. Cause of Death | IHD |
| 5. Effective Dose NULL | CC:1 |
| 6. Effective Dose Ecig | CC:1 Ecig:0.05 Dual:0.525 |
| Note: | Death rates are per million population |

-------------------------------------------------------------------------------------

Results for Age Groups Combined and Years combined, Population unadjusted for deaths in Ecig scenario,

| Age Group:All | | NULL | Ecig | | | %Drop | | NULL | Ecig | |
|---|---|---|---|---|---|---|---|---|---|---|
| Year | Number | Attrib | Attrib | Drop | %Drop | Attrib | Death | Attrib | Attrib | Drop |
| | Deaths | Deaths | Deaths | Deaths | Deaths | Deaths | Rate | Rates | Rates | Rates |
| All Comb | 6360041 | 1098877.421 | 515692.894 | 583184.5 | 9.170 | 53.071 | 1841.09 | 318.101 | 149.282 | 168.819 |

-------------------------------------------------------------------------------------

Results for Age Groups Combined, Population unadjusted for deaths in Ecig scenario,

| Age Group:All | | NULL | Ecig | | | %Drop | | NULL | Ecig | |
|---|---|---|---|---|---|---|---|---|---|---|
| Year | Number | Attrib | Attrib | Drop | %Drop | Attrib | Death | Attrib | Attrib | Drop |
| | Deaths | Deaths | Deaths | Deaths | Deaths | Deaths | Rate | Rates | Rates | Rates |
| 1990 | 171484 | 38511.725 | 38511.725 | 0.000 | 0.000 | 0.000 | 2718.40 | 610.497 | 610.497 | 0.000 |
| 1991 | 168044 | 37309.838 | 36996.011 | 313.827 | 0.187 | 0.841 | 2620.21 | 581.750 | 576.857 | 4.893 |
| 1992 | 164548 | 36137.488 | 35292.956 | 844.532 | 0.513 | 2.337 | 2522.12 | 553.899 | 540.955 | 12.945 |
| 1993 | 164362 | 35599.588 | 34035.919 | 1563.668 | 0.951 | 4.392 | 2474.35 | 535.926 | 512.387 | 23.540 |
| 1994 | 159866 | 34336.757 | 31950.205 | 2386.552 | 1.493 | 6.950 | 2361.75 | 507.267 | 472.009 | 35.257 |
| 1995 | 158083 | 33455.759 | 30119.059 | 3336.700 | 2.111 | 9.973 | 2290.66 | 484.782 | 436.432 | 48.350 |
| 1996 | 154875 | 32193.824 | 27848.795 | 4345.029 | 2.806 | 13.496 | 2209.26 | 459.239 | 397.258 | 61.981 |
| 1997 | 149970 | 30665.509 | 25343.155 | 5322.354 | 3.549 | 17.356 | 2104.43 | 430.308 | 355.623 | 74.685 |
| 1998 | 145407 | 29188.430 | 22951.010 | 6237.421 | 4.290 | 21.369 | 2006.83 | 402.844 | 316.758 | 86.086 |
| 1999 | 165952 | 33375.386 | 24696.433 | 8678.953 | 5.230 | 26.004 | 2254.27 | 453.366 | 335.473 | 117.894 |
| 2000 | 160260 | 32024.754 | 22305.964 | 9718.790 | 6.064 | 30.348 | 2145.10 | 428.655 | 298.568 | 130.087 |
| 2001 | 154444 | 30682.393 | 19884.180 | 10798.21 | 6.992 | 35.194 | 2040.33 | 405.339 | 262.686 | 142.653 |
| 2002 | 151887 | 30218.370 | 18154.596 | 12063.77 | 7.943 | 39.922 | 1984.06 | 394.736 | 237.149 | 157.586 |
| 2003 | 146733 | 29175.202 | 16178.320 | 12996.88 | 8.858 | 44.548 | 1896.81 | 377.146 | 209.136 | 168.010 |
| 2004 | 138381 | 27488.687 | 14008.217 | 13480.47 | 9.742 | 49.040 | 1769.58 | 351.518 | 179.133 | 172.385 |
| 2005 | 135881 | 27017.439 | 12666.767 | 14350.67 | 10.561 | 53.116 | 1717.44 | 341.482 | 160.099 | 181.383 |
| 2006 | 131922 | 26231.435 | 11140.901 | 15090.53 | 11.439 | 57.528 | 1652.16 | 328.517 | 139.526 | 188.991 |
| 2007 | 126502 | 25103.049 | 9701.108 | 15401.94 | 12.175 | 61.355 | 1568.04 | 311.161 | 120.249 | 190.913 |
| 2008 | 125859 | 24621.740 | 8736.544 | 15885.20 | 12.621 | 64.517 | 1542.60 | 301.777 | 107.080 | 194.698 |
| 2009 | 122428 | 23741.177 | 7701.208 | 16039.97 | 13.102 | 67.562 | 1482.84 | 287.552 | 93.277 | 194.276 |
| 2010 | 120537 | 23189.160 | 6895.061 | 16294.10 | 13.518 | 70.266 | 1442.22 | 277.458 | 82.499 | 194.959 |
| 2011 | 120221 | 22786.927 | 6145.166 | 16641.76 | 13.843 | 73.032 | 1424.23 | 269.951 | 72.800 | 197.151 |
| 2012 | 120927 | 22485.295 | 5535.829 | 16949.47 | 14.016 | 75.380 | 1417.09 | 263.495 | 64.872 | 198.623 |
| 2013 | 122648 | 22251.884 | 5023.153 | 17228.73 | 14.047 | 77.426 | 1420.86 | 257.786 | 58.193 | 199.593 |
| 2014 | 123229 | 21894.593 | 4537.468 | 17357.12 | 14.085 | 79.276 | 1410.92 | 250.685 | 51.952 | 198.732 |
| 2015 | 123774 | 21471.109 | 4115.852 | 17355.26 | 14.022 | 80.831 | 1594.26 | 276.557 | 53.014 | 223.543 |
| 2016 | 126218 | 21411.568 | 3741.021 | 17670.55 | 14.000 | 82.528 | 1610.55 | 273.213 | 47.736 | 225.477 |
| 2017 | 128265 | 21169.399 | 3404.678 | 17764.72 | 13.850 | 83.917 | 1621.69 | 267.651 | 43.046 | 224.605 |
| 2018 | 121429 | 19830.576 | 2905.328 | 16925.25 | 13.938 | 85.349 | 1521.05 | 248.404 | 36.393 | 212.011 |
| 2019 | 121832 | 19431.014 | 2647.024 | 16783.99 | 13.776 | 86.377 | 1511.26 | 241.031 | 32.835 | 208.196 |
| 2020 | 120811 | 18532.612 | 2364.275 | 16168.34 | 13.383 | 87.243 | 1711.27 | 262.513 | 33.490 | 229.023 |
| 2021 | 119407 | 18234.128 | 2129.607 | 16104.52 | 13.487 | 88.321 | 1679.51 | 256.472 | 29.954 | 226.518 |
| 2022 | 119438 | 17812.559 | 1932.519 | 15880.04 | 13.296 | 89.151 | 1667.10 | 248.626 | 26.974 | 221.652 |
| 2023 | 119445 | 17361.668 | 1753.701 | 15607.97 | 13.067 | 89.899 | 1653.68 | 240.367 | 24.279 | 216.087 |
| 2024 | 119305 | 16929.676 | 1612.037 | 15317.64 | 12.839 | 90.478 | 1638.05 | 232.443 | 22.133 | 210.310 |
| 2025 | 115685 | 15501.666 | 1409.242 | 14092.42 | 12.182 | 90.909 | 1848.78 | 247.735 | 22.521 | 225.213 |
| 2026 | 115073 | 15523.814 | 1323.705 | 14200.11 | 12.340 | 91.473 | 1831.46 | 247.072 | 21.068 | 226.004 |
| 2027 | 113989 | 15073.070 | 1226.503 | 13846.57 | 12.147 | 91.863 | 1805.98 | 238.810 | 19.432 | 219.378 |
| 2028 | 112855 | 14636.710 | 1129.785 | 13506.92 | 11.968 | 92.281 | 1778.98 | 230.725 | 17.809 | 212.916 |
| 2029 | 111718 | 14180.166 | 1049.525 | 13130.64 | 11.753 | 92.599 | 1751.04 | 222.256 | 16.450 | 205.806 |
| 2030 | 104851 | 12111.772 | 874.312 | 11237.46 | 10.718 | 92.781 | 1963.54 | 226.816 | 16.373 | 210.443 |
| 2031 | 105289 | 12417.367 | 868.428 | 11548.94 | 10.969 | 93.006 | 1966.62 | 231.935 | 16.221 | 215.714 |
| 2032 | 103879 | 12054.064 | 816.882 | 11237.18 | 10.818 | 93.223 | 1933.67 | 224.381 | 15.206 | 209.175 |
| 2033 | 102485 | 11672.389 | 761.751 | 10910.64 | 10.646 | 93.474 | 1899.76 | 216.370 | 14.121 | 202.250 |
| 2034 | 101070 | 11365.762 | 724.458 | 10641.30 | 10.529 | 93.626 | 1864.51 | 209.673 | 13.365 | 196.308 |
| 2035 | 90820 | 8580.360 | 535.436 | 8044.924 | 8.858 | 93.760 | 2072.23 | 195.777 | 12.217 | 183.560 |
| 2036 | 92117 | 8948.841 | 542.755 | 8406.086 | 9.125 | 93.935 | 2101.31 | 204.135 | 12.381 | 191.754 |
| 2037 | 90327 | 8624.570 | 517.892 | 8106.678 | 8.975 | 93.995 | 2058.21 | 196.522 | 11.801 | 184.721 |
| 2038 | 88590 | 8315.017 | 486.985 | 7828.032 | 8.836 | 94.143 | 2014.42 | 189.073 | 11.073 | 177.999 |
| 2039 | 86921 | 8001.132 | 459.442 | 7541.690 | 8.676 | 94.258 | 1970.05 | 181.344 | 10.413 | 170.931 |

# Results for PHIM for Run 1 (Main_Analysis.RTF)

********************************************************************************

**Main Results for E-Component for Run: 1, Simulation: 1, COD: STROKE**

| Variable Parameter | Value |
|---|---|
| 1. Country | US (United States) |
| 2. Sex | M (Males) |
| 3. Year of start of process | 1990 |
| 4. Cause of Death | STROKE |
| 5. Effective Dose NULL | CC:1 |
| 6. Effective Dose Ecig | CC:1 Ecig:0.05 Dual:0.525 |
| Note: | Death rates are per million population |

-------------------------------------------------------------------------------------

Results for Age Groups Combined and Years combined, Population unadjusted for deaths in Ecig scenario,

| Age Group:All Year | Number Deaths | NULL Attrib Deaths | Ecig Attrib Deaths | Drop Deaths | %Drop Deaths | %Drop Attrib Deaths | Death Rate | NULL Attrib Rates | Ecig Attrib Rates | Drop Rates |
|---|---|---|---|---|---|---|---|---|---|---|
| All Comb | 1489699 | 149918.462 | 69563.177 | 80355.29 | 5.394 | 53.599 | 431.236 | 43.398 | 20.137 | 23.261 |

-------------------------------------------------------------------------------------

Results for Age Groups Combined, Population unadjusted for deaths in Ecig scenario,

| Age Group:All Year | Number Deaths | NULL Attrib Deaths | Ecig Attrib Deaths | Drop Deaths | %Drop Deaths | %Drop Attrib Deaths | Death Rate | NULL Attrib Rates | Ecig Attrib Rates | Drop Rates |
|---|---|---|---|---|---|---|---|---|---|---|
| 1990 | 32862 | 4613.166 | 4613.166 | 0.000 | 0.000 | 0.000 | 520.936 | 73.129 | 73.129 | 0.000 |
| 1991 | 32619 | 4555.566 | 4515.509 | 40.057 | 0.123 | 0.879 | 508.609 | 71.032 | 70.408 | 0.625 |
| 1992 | 32100 | 4453.572 | 4348.504 | 105.068 | 0.327 | 2.359 | 492.014 | 68.262 | 66.652 | 1.610 |
| 1993 | 32896 | 4462.473 | 4263.946 | 198.527 | 0.603 | 4.449 | 495.226 | 67.179 | 64.191 | 2.989 |
| 1994 | 33323 | 4499.513 | 4186.127 | 313.387 | 0.940 | 6.965 | 492.290 | 66.473 | 61.843 | 4.630 |
| 1995 | 33569 | 4472.774 | 4028.406 | 444.368 | 1.324 | 9.935 | 486.422 | 64.812 | 58.373 | 6.439 |
| 1996 | 33870 | 4406.878 | 3817.675 | 589.204 | 1.740 | 13.370 | 483.150 | 62.863 | 54.458 | 8.405 |
| 1997 | 33516 | 4288.158 | 3551.996 | 736.162 | 2.196 | 17.167 | 470.307 | 60.173 | 49.843 | 10.330 |
| 1998 | 32795 | 4120.148 | 3249.742 | 870.406 | 2.654 | 21.126 | 452.620 | 56.864 | 44.851 | 12.013 |
| 1999 | 33375 | 3982.443 | 2978.317 | 1004.125 | 3.009 | 25.214 | 453.361 | 54.097 | 40.457 | 13.640 |
| 2000 | 33459 | 4021.411 | 2841.047 | 1180.365 | 3.528 | 29.352 | 447.853 | 53.827 | 38.028 | 15.799 |
| 2001 | 32275 | 3848.092 | 2549.160 | 1298.932 | 4.025 | 33.755 | 426.379 | 50.836 | 33.676 | 17.160 |
| 2002 | 31586 | 3783.081 | 2345.597 | 1437.484 | 4.551 | 37.998 | 412.601 | 49.418 | 30.640 | 18.778 |
| 2003 | 30985 | 3754.415 | 2163.500 | 1590.915 | 5.134 | 42.375 | 400.541 | 48.533 | 27.967 | 20.566 |
| 2004 | 29884 | 3666.274 | 1959.474 | 1706.801 | 5.711 | 46.554 | 382.149 | 46.883 | 25.057 | 21.826 |
| 2005 | 28708 | 3589.688 | 1772.555 | 1817.134 | 6.330 | 50.621 | 362.850 | 45.371 | 22.404 | 22.967 |
| 2006 | 28188 | 3596.985 | 1628.628 | 1968.356 | 6.983 | 54.722 | 353.021 | 45.048 | 20.397 | 24.651 |
| 2007 | 28026 | 3560.798 | 1480.452 | 2080.346 | 7.423 | 58.424 | 347.392 | 44.137 | 18.351 | 25.787 |
| 2008 | 27128 | 3417.001 | 1304.470 | 2112.532 | 7.787 | 61.824 | 332.495 | 41.881 | 15.988 | 25.892 |
| 2009 | 27068 | 3394.390 | 1189.173 | 2205.216 | 8.147 | 64.967 | 327.847 | 41.113 | 14.403 | 26.709 |
| 2010 | 26911 | 3340.137 | 1078.162 | 2261.975 | 8.405 | 67.721 | 321.990 | 39.965 | 12.900 | 27.065 |
| 2011 | 26959 | 3285.435 | 973.719 | 2311.716 | 8.575 | 70.363 | 319.377 | 38.922 | 11.535 | 27.386 |
| 2012 | 27057 | 3259.209 | 880.447 | 2378.763 | 8.792 | 72.986 | 317.068 | 38.193 | 10.318 | 27.876 |
| 2013 | 27904 | 3274.703 | 810.288 | 2464.414 | 8.832 | 75.256 | 323.265 | 37.937 | 9.387 | 28.550 |
| 2014 | 28792 | 3298.083 | 745.491 | 2552.592 | 8.866 | 77.396 | 329.657 | 37.762 | 8.536 | 29.226 |
| 2015 | 29560 | 3244.406 | 677.792 | 2566.614 | 8.683 | 79.109 | 380.745 | 41.789 | 8.730 | 33.059 |
| 2016 | 30513 | 3314.836 | 634.348 | 2680.489 | 8.785 | 80.863 | 389.347 | 42.297 | 8.094 | 34.203 |
| 2017 | 31416 | 3333.314 | 585.609 | 2747.705 | 8.746 | 82.432 | 397.202 | 42.144 | 7.404 | 34.740 |
| 2018 | 29348 | 2925.242 | 476.835 | 2448.407 | 8.343 | 83.699 | 367.626 | 36.642 | 5.973 | 30.669 |
| 2019 | 29598 | 2864.628 | 433.082 | 2431.546 | 8.215 | 84.882 | 367.149 | 35.534 | 5.372 | 30.162 |
| 2020 | 29592 | 2730.366 | 384.634 | 2345.732 | 7.927 | 85.913 | 419.174 | 38.675 | 5.448 | 33.227 |
| 2021 | 30097 | 2647.911 | 346.581 | 2301.329 | 7.646 | 86.911 | 423.328 | 37.244 | 4.875 | 32.369 |
| 2022 | 30304 | 2586.165 | 313.384 | 2272.780 | 7.500 | 87.882 | 422.974 | 36.097 | 4.374 | 31.723 |
| 2023 | 30505 | 2517.960 | 283.847 | 2234.113 | 7.324 | 88.727 | 422.337 | 34.860 | 3.930 | 30.931 |
| 2024 | 30661 | 2454.161 | 259.791 | 2194.370 | 7.157 | 89.414 | 420.968 | 33.695 | 3.567 | 30.129 |
| 2025 | 30254 | 2275.364 | 227.321 | 2048.043 | 6.770 | 90.009 | 483.488 | 36.363 | 3.633 | 32.730 |
| 2026 | 30685 | 2183.166 | 205.664 | 1977.502 | 6.444 | 90.580 | 488.380 | 34.747 | 3.273 | 31.473 |
| 2027 | 30561 | 2112.345 | 188.826 | 1923.519 | 6.294 | 91.061 | 484.196 | 33.467 | 2.992 | 30.475 |
| 2028 | 30412 | 2046.677 | 173.460 | 1873.217 | 6.160 | 91.525 | 479.392 | 32.263 | 2.734 | 29.528 |
| 2029 | 30254 | 1977.671 | 159.798 | 1817.874 | 6.009 | 91.920 | 474.195 | 30.997 | 2.505 | 28.493 |
| 2030 | 29330 | 1744.457 | 135.628 | 1608.829 | 5.485 | 92.225 | 549.260 | 32.668 | 2.540 | 30.128 |
| 2031 | 28976 | 1653.738 | 123.967 | 1529.770 | 5.279 | 92.504 | 541.223 | 30.889 | 2.315 | 28.573 |
| 2032 | 28724 | 1603.120 | 114.929 | 1488.191 | 5.181 | 92.831 | 534.690 | 29.841 | 2.139 | 27.702 |
| 2033 | 28464 | 1548.245 | 106.814 | 1441.431 | 5.064 | 93.101 | 527.627 | 28.700 | 1.980 | 26.720 |
| 2034 | 28178 | 1511.985 | 101.001 | 1410.983 | 5.007 | 93.320 | 519.826 | 27.893 | 1.863 | 26.029 |
| 2035 | 26737 | 1239.866 | 80.697 | 1159.168 | 4.335 | 93.491 | 610.065 | 28.290 | 1.841 | 26.449 |
| 2036 | 25591 | 1182.522 | 74.571 | 1107.951 | 4.329 | 93.694 | 583.762 | 26.975 | 1.701 | 25.274 |
| 2037 | 25143 | 1134.483 | 70.737 | 1063.746 | 4.231 | 93.765 | 572.916 | 25.851 | 1.612 | 24.239 |
| 2038 | 24693 | 1093.537 | 66.302 | 1027.235 | 4.160 | 93.937 | 561.491 | 24.866 | 1.508 | 23.358 |
| 2039 | 24247 | 1047.905 | 62.007 | 985.898 | 4.066 | 94.083 | 549.553 | 23.751 | 1.405 | 22.345 |

# Results for PHIM for Run 1 (Main_Analysis.RTF)

*******************************************************************************
**Main Results for E-Component for Run: 1, Simulation: 1, COD: COPD**

| Variable Parameter | Value |
|---|---|
| 1. Country | US (United States) |
| 2. Sex | M (Males) |
| 3. Year of start of process | 1990 |
| 4. Cause of Death | COPD |
| 5. Effective Dose NULL | CC:1 |
| 6. Effective Dose Ecig | CC:1 Ecig:0.05 Dual:0.525 |
| Note: | Death rates are per million population |

--------------------------------------------------------------------------------------
Results for Age Groups Combined and Years combined, Population unadjusted for deaths in Ecig scenario,

| Age Group:All | | NULL | Ecig | | | %Drop | | NULL | Ecig | |
|---|---|---|---|---|---|---|---|---|---|---|
| Year | Number Deaths | Attrib Deaths | Attrib Deaths | Drop Deaths | %Drop Deaths | Attrib Deaths | Death Rate | Attrib Rates | Attrib Rates | Drop Rates |
| All Comb | 2233143 | 1015606.587 | 676455.765 | 339150.8 | 15.187 | 33.394 | 646.447 | 293.996 | 195.819 | 98.177 |

--------------------------------------------------------------------------------------
Results for Age Groups Combined, Population unadjusted for deaths in Ecig scenario,

| Age Group:All | | NULL | Ecig | | | %Drop | | NULL | Ecig | |
|---|---|---|---|---|---|---|---|---|---|---|
| Year | Number Deaths | Attrib Deaths | Attrib Deaths | Drop Deaths | %Drop Deaths | Attrib Deaths | Death Rate | Attrib Rates | Attrib Rates | Drop Rates |
| 1990 | 33993 | 19262.437 | 19262.437 | 0.000 | 0.000 | 0.000 | 538.865 | 305.353 | 305.353 | 0.000 |
| 1991 | 34092 | 19215.636 | 19193.448 | 22.188 | 0.065 | 0.115 | 531.576 | 299.618 | 299.272 | 0.346 |
| 1992 | 33942 | 19056.189 | 18998.226 | 57.963 | 0.171 | 0.304 | 520.248 | 292.085 | 291.196 | 0.888 |
| 1993 | 35913 | 20095.670 | 19976.893 | 118.777 | 0.331 | 0.591 | 540.645 | 302.526 | 300.738 | 1.788 |
| 1994 | 35074 | 19524.095 | 19342.060 | 182.034 | 0.519 | 0.932 | 518.158 | 288.435 | 285.746 | 2.689 |
| 1995 | 34669 | 19116.002 | 18854.923 | 261.079 | 0.753 | 1.366 | 502.362 | 276.995 | 273.212 | 3.783 |
| 1996 | 34582 | 18975.021 | 18617.851 | 357.170 | 1.033 | 1.882 | 493.306 | 270.675 | 265.580 | 5.095 |
| 1997 | 35267 | 19169.025 | 18694.006 | 475.019 | 1.347 | 2.478 | 494.878 | 268.986 | 262.320 | 6.666 |
| 1998 | 35675 | 19228.937 | 18616.569 | 612.368 | 1.717 | 3.185 | 492.368 | 265.388 | 256.937 | 8.452 |
| 1999 | 38516 | 20589.820 | 19784.158 | 805.662 | 2.092 | 3.913 | 523.195 | 279.689 | 268.745 | 10.944 |
| 2000 | 36283 | 19196.417 | 18283.698 | 912.719 | 2.516 | 4.755 | 485.652 | 256.946 | 244.730 | 12.217 |
| 2001 | 36281 | 19012.731 | 17906.595 | 1106.137 | 3.049 | 5.818 | 479.301 | 251.174 | 236.561 | 14.613 |
| 2002 | 35983 | 18699.357 | 17404.135 | 1295.222 | 3.600 | 6.927 | 470.038 | 244.265 | 227.346 | 16.919 |
| 2003 | 36205 | 18663.550 | 17150.417 | 1513.133 | 4.179 | 8.107 | 468.019 | 241.262 | 221.702 | 19.560 |
| 2004 | 34611 | 17679.164 | 16001.001 | 1678.162 | 4.849 | 9.492 | 442.596 | 226.076 | 204.616 | 21.460 |
| 2005 | 36826 | 18692.832 | 16663.414 | 2029.418 | 5.511 | 10.857 | 465.456 | 236.265 | 210.614 | 25.650 |
| 2006 | 34350 | 17308.564 | 15152.789 | 2155.774 | 6.276 | 12.455 | 430.192 | 216.769 | 189.770 | 26.998 |
| 2007 | 35326 | 17683.181 | 15187.232 | 2495.949 | 7.065 | 14.115 | 437.879 | 219.189 | 188.251 | 30.938 |
| 2008 | 38248 | 19006.374 | 16025.995 | 2980.379 | 7.792 | 15.681 | 468.788 | 232.952 | 196.423 | 36.529 |
| 2009 | 36902 | 18205.573 | 14999.378 | 3206.194 | 8.688 | 17.611 | 446.956 | 220.505 | 181.672 | 38.833 |
| 2010 | 37234 | 18237.758 | 14721.018 | 3516.740 | 9.445 | 19.283 | 445.505 | 218.215 | 176.137 | 42.078 |
| 2011 | 38093 | 18487.916 | 14518.806 | 3969.111 | 10.420 | 21.469 | 451.279 | 219.022 | 172.001 | 47.021 |
| 2012 | 38504 | 18491.158 | 14140.540 | 4350.618 | 11.299 | 23.528 | 451.211 | 216.689 | 165.706 | 50.983 |
| 2013 | 40290 | 19143.452 | 14264.724 | 4878.728 | 12.109 | 25.485 | 466.755 | 221.775 | 165.255 | 56.520 |
| 2014 | 40339 | 18992.005 | 13745.357 | 5246.647 | 13.006 | 27.626 | 461.866 | 217.451 | 157.379 | 60.072 |
| 2015 | 42303 | 19728.885 | 13942.001 | 5786.884 | 13.680 | 29.332 | 544.880 | 254.116 | 179.579 | 74.538 |
| 2016 | 43104 | 19876.724 | 13633.716 | 6243.008 | 14.484 | 31.409 | 550.009 | 253.628 | 173.967 | 79.661 |
| 2017 | 44107 | 20119.790 | 13373.237 | 6746.553 | 15.296 | 33.532 | 557.659 | 254.381 | 169.082 | 85.299 |
| 2018 | 45469 | 20535.123 | 13218.713 | 7316.410 | 16.091 | 35.629 | 569.564 | 257.229 | 165.582 | 91.648 |
| 2019 | 46963 | 21009.029 | 13093.260 | 7915.769 | 16.855 | 37.678 | 582.552 | 260.605 | 162.415 | 98.191 |
| 2020 | 48419 | 21428.512 | 12934.893 | 8493.619 | 17.542 | 39.637 | 685.853 | 303.533 | 183.222 | 120.311 |
| 2021 | 48463 | 21266.697 | 12333.333 | 8933.364 | 18.433 | 42.006 | 681.652 | 299.127 | 173.474 | 125.652 |
| 2022 | 49915 | 21663.568 | 12081.152 | 9582.416 | 19.197 | 44.233 | 696.712 | 302.378 | 168.628 | 133.750 |
| 2023 | 51391 | 22084.131 | 11835.478 | 10248.65 | 19.943 | 46.407 | 711.491 | 305.748 | 163.858 | 141.889 |
| 2024 | 52811 | 22418.456 | 11568.951 | 10849.50 | 20.544 | 48.395 | 725.093 | 307.804 | 158.841 | 148.963 |
| 2025 | 53848 | 22614.834 | 11240.107 | 11374.73 | 21.124 | 50.298 | 860.553 | 361.411 | 179.630 | 181.781 |
| 2026 | 54342 | 22571.522 | 10739.126 | 11832.40 | 21.774 | 52.422 | 864.886 | 359.241 | 170.921 | 188.321 |
| 2027 | 55267 | 22774.416 | 10346.068 | 12428.35 | 22.488 | 54.572 | 875.619 | 360.827 | 163.918 | 196.909 |
| 2028 | 56149 | 22915.561 | 9907.294 | 13008.27 | 23.167 | 56.766 | 885.102 | 361.228 | 156.173 | 205.055 |
| 2029 | 57014 | 22992.027 | 9464.550 | 13527.48 | 23.727 | 58.836 | 893.616 | 360.371 | 148.345 | 212.026 |
| 2030 | 57238 | 22895.360 | 9015.833 | 13879.53 | 24.249 | 60.622 | 1071.90 | 428.760 | 168.839 | 259.921 |
| 2031 | 57592 | 22715.871 | 8541.375 | 14174.50 | 24.612 | 62.399 | 1075.71 | 424.293 | 159.538 | 264.755 |
| 2032 | 58251 | 22759.207 | 8113.322 | 14645.89 | 25.143 | 64.351 | 1084.31 | 423.652 | 151.026 | 272.626 |
| 2033 | 58885 | 22786.258 | 7710.147 | 15076.11 | 25.603 | 66.163 | 1091.54 | 422.387 | 142.923 | 279.465 |
| 2034 | 59460 | 22806.106 | 7305.399 | 15500.71 | 26.069 | 67.967 | 1096.89 | 420.721 | 134.768 | 285.953 |
| 2035 | 58516 | 22262.998 | 6847.275 | 15415.72 | 26.344 | 69.244 | 1335.15 | 507.973 | 156.234 | 351.739 |
| 2036 | 56446 | 21140.051 | 6041.723 | 15098.33 | 26.748 | 71.420 | 1287.60 | 482.234 | 137.820 | 344.414 |
| 2037 | 56568 | 21011.845 | 5619.454 | 15392.39 | 27.210 | 73.256 | 1288.98 | 478.783 | 128.047 | 350.736 |
| 2038 | 56668 | 20787.483 | 5201.632 | 15585.85 | 27.504 | 74.977 | 1288.55 | 472.681 | 118.278 | 354.402 |
| 2039 | 56758 | 20709.267 | 4842.053 | 15867.21 | 27.956 | 76.619 | 1286.40 | 469.372 | 109.744 | 359.628 |

## Results for PHIM for Run 1 (Main_Analysis.RTF)

*************************************************************************************

**Main Results for E-Component for Run: 1, Simulation: 1, COD: Four Diseases Combined, Adjusted**

| Variable Parameter | Value |
|---|---|
| 1. Country | US (United States) |
| 2. Sex | M (Males) |
| 3. Year of start of process | 1990 |
| 4. Cause of Death | Four Diseases Combined |
| 5. Effective Dose NULL | CC:1 |
| 6. Effective Dose Ecig | CC:1 Ecig:0.05 Dual:0.525 |
| Note: | Death rates are per million population |

-------------------------------------------------------------------------------------

Results for Age Groups Combined and Years combined, Population and Number deaths adjusted for Ecig scenario,

| Age Group:All Year | Number Deaths | NULL Attrib Deaths | Ecig Attrib Deaths | Drop Deaths | %Drop Deaths | %Drop Attrib Deaths | Death Rate | NULL Attrib Rates | Ecig Attrib Rates | Drop Rates |
|---|---|---|---|---|---|---|---|---|---|---|
| All Comb | 13291564 | 4509390.583 | 3002576.182 | 1506814 | 11.337 | 33.415 | 3815.59 | 1305.37 | 861.944 | 443.427 |

-------------------------------------------------------------------------------------

Results for Age Groups Combined, Population and Number deaths adjusted for Ecig scenario,

| Age Group:All Year | Number Deaths | NULL Attrib Deaths | Ecig Attrib Deaths | Drop Deaths | %Drop Deaths | %Drop Attrib Deaths | Death Rate | NULL Attrib Rates | Ecig Attrib Rates | Drop Rates |
|---|---|---|---|---|---|---|---|---|---|---|
| 1990 | 317161 | 125773.065 | 125773.065 | 0.000 | 0.000 | 0.000 | 5027.71 | 1993.78 | 1993.78 | 0.000 |
| 1991 | 313217 | 123999.442 | 123575.252 | 424.190 | 0.135 | 0.342 | 4883.81 | 1933.45 | 1926.84 | 6.614 |
| 1992 | 308580 | 122014.015 | 120878.376 | 1135.639 | 0.368 | 0.931 | 4729.74 | 1870.18 | 1852.76 | 17.419 |
| 1993 | 311723 | 122810.618 | 120679.258 | 2131.360 | 0.684 | 1.735 | 4692.65 | 1848.83 | 1816.70 | 32.131 |
| 1994 | 305723 | 119925.592 | 116651.381 | 3274.211 | 1.071 | 2.730 | 4516.28 | 1771.69 | 1723.23 | 48.469 |
| 1995 | 303268 | 117896.496 | 113286.850 | 4609.647 | 1.520 | 3.910 | 4393.95 | 1708.35 | 1641.38 | 66.970 |
| 1996 | 299629 | 115698.645 | 109619.386 | 6079.259 | 2.029 | 5.254 | 4273.40 | 1650.42 | 1563.43 | 86.994 |
| 1997 | 294330 | 113359.847 | 105803.241 | 7556.606 | 2.567 | 6.666 | 4129.04 | 1590.70 | 1484.27 | 106.431 |
| 1998 | 289405 | 111444.212 | 102408.577 | 9035.635 | 3.122 | 8.108 | 3992.71 | 1538.10 | 1412.86 | 125.237 |
| 1999 | 311218 | 114877.722 | 102791.891 | 12085.83 | 3.883 | 10.521 | 4225.40 | 1560.48 | 1395.60 | 164.880 |
| 2000 | 304071 | 112379.704 | 98599.233 | 13780.47 | 4.532 | 12.262 | 4067.26 | 1504.22 | 1318.86 | 185.351 |
| 2001 | 296654 | 110024.943 | 94431.956 | 15592.99 | 5.256 | 14.172 | 3915.60 | 1453.52 | 1246.43 | 207.089 |
| 2002 | 292345 | 108289.173 | 90661.379 | 17627.79 | 6.030 | 16.278 | 3814.65 | 1414.56 | 1182.99 | 231.566 |
| 2003 | 286406 | 106539.023 | 87116.574 | 19422.45 | 6.781 | 18.230 | 3697.37 | 1377.22 | 1124.64 | 252.586 |
| 2004 | 274805 | 103037.251 | 82305.225 | 20732.03 | 7.544 | 20.121 | 3508.45 | 1317.61 | 1050.80 | 266.817 |
| 2005 | 273120 | 103044.562 | 80422.573 | 22621.99 | 8.283 | 21.954 | 3445.47 | 1302.41 | 1014.55 | 287.865 |
| 2006 | 264961 | 99672.555 | 75437.151 | 24235.40 | 9.147 | 24.315 | 3310.95 | 1248.28 | 942.662 | 305.617 |
| 2007 | 259376 | 97839.152 | 72255.840 | 25583.31 | 9.863 | 26.148 | 3206.84 | 1212.75 | 893.348 | 319.403 |
| 2008 | 260562 | 98075.852 | 70785.159 | 27290.69 | 10.474 | 27.826 | 3184.31 | 1202.07 | 865.058 | 337.012 |
| 2009 | 255134 | 95617.338 | 67136.888 | 28480.45 | 11.163 | 29.786 | 3080.04 | 1158.11 | 810.492 | 347.621 |
| 2010 | 253248 | 94607.988 | 64791.819 | 29816.17 | 11.774 | 31.515 | 3019.01 | 1131.98 | 772.394 | 359.591 |
| 2011 | 253310 | 93633.119 | 62175.287 | 31457.83 | 12.419 | 33.597 | 2988.73 | 1109.25 | 733.589 | 375.660 |
| 2012 | 254350 | 92775.175 | 59708.597 | 33066.58 | 13.000 | 35.642 | 2967.32 | 1087.19 | 696.576 | 390.613 |
| 2013 | 258091 | 92345.780 | 57632.987 | 34712.79 | 13.450 | 37.590 | 2975.36 | 1069.82 | 664.412 | 405.404 |
| 2014 | 259513 | 91379.215 | 55292.072 | 36087.14 | 13.906 | 39.492 | 2955.52 | 1046.26 | 629.706 | 416.551 |
| 2015 | 262135 | 90750.242 | 53429.343 | 37320.90 | 14.237 | 41.125 | 3354.48 | 1168.90 | 683.724 | 485.178 |
| 2016 | 264033 | 88811.996 | 50204.850 | 38607.15 | 14.622 | 43.471 | 3345.44 | 1133.24 | 636.123 | 497.121 |
| 2017 | 266514 | 87325.126 | 47586.473 | 39738.65 | 14.911 | 45.507 | 3344.20 | 1104.08 | 597.112 | 506.967 |
| 2018 | 261706 | 87612.144 | 46869.666 | 40742.48 | 15.568 | 46.503 | 3251.71 | 1097.46 | 582.358 | 515.098 |
| 2019 | 264699 | 87797.760 | 45569.732 | 42228.03 | 15.953 | 48.097 | 3255.11 | 1089.08 | 560.390 | 528.692 |
| 2020 | 265832 | 87221.042 | 44143.179 | 43077.86 | 16.205 | 49.389 | 3726.42 | 1235.48 | 618.798 | 616.681 |
| 2021 | 258761 | 82001.292 | 39348.726 | 42652.57 | 16.483 | 52.015 | 3599.51 | 1153.39 | 547.362 | 606.027 |
| 2022 | 261054 | 81915.977 | 37888.836 | 44027.14 | 16.865 | 53.747 | 3601.33 | 1143.37 | 522.691 | 620.684 |
| 2023 | 263324 | 81810.810 | 36464.063 | 45346.75 | 17.221 | 55.429 | 3600.87 | 1132.64 | 498.634 | 634.011 |
| 2024 | 265258 | 81528.303 | 35054.186 | 46474.12 | 17.520 | 57.004 | 3594.91 | 1119.38 | 475.071 | 644.307 |
| 2025 | 262127 | 79599.213 | 33395.942 | 46203.27 | 17.626 | 58.045 | 4125.11 | 1272.09 | 525.553 | 746.534 |
| 2026 | 256490 | 75082.462 | 29707.887 | 45374.57 | 17.691 | 60.433 | 4016.80 | 1194.99 | 465.244 | 729.745 |
| 2027 | 256211 | 74444.425 | 28219.439 | 46224.99 | 18.042 | 62.093 | 3991.28 | 1179.46 | 439.605 | 739.856 |
| 2028 | 255771 | 73699.585 | 26656.824 | 47042.76 | 18.393 | 63.830 | 3961.35 | 1161.76 | 412.858 | 748.902 |
| 2029 | 255278 | 72819.703 | 25159.430 | 47660.27 | 18.670 | 65.450 | 3928.29 | 1141.36 | 387.161 | 754.196 |
| 2030 | 246482 | 69309.277 | 23472.738 | 45836.54 | 18.596 | 66.133 | 4518.49 | 1297.95 | 430.300 | 867.649 |
| 2031 | 241507 | 65433.368 | 20812.640 | 44620.73 | 18.476 | 68.193 | 4411.95 | 1222.18 | 380.215 | 841.967 |
| 2032 | 240318 | 64643.552 | 19572.221 | 45071.33 | 18.755 | 69.723 | 4371.61 | 1203.31 | 356.037 | 847.271 |
| 2033 | 239095 | 63793.514 | 18401.504 | 45392.01 | 18.985 | 71.155 | 4327.66 | 1182.54 | 333.070 | 849.466 |
| 2034 | 237730 | 63032.151 | 17299.174 | 45732.98 | 19.237 | 72.555 | 4278.74 | 1162.80 | 311.356 | 851.445 |
| 2035 | 222602 | 57653.301 | 15780.529 | 41872.77 | 18.811 | 72.629 | 4939.41 | 1315.47 | 350.161 | 965.309 |
| 2036 | 214890 | 52988.733 | 13223.027 | 39765.71 | 18.505 | 75.046 | 4762.71 | 1208.75 | 293.068 | 915.677 |
| 2037 | 212343 | 52026.921 | 12262.379 | 39764.54 | 18.727 | 76.431 | 4697.04 | 1185.50 | 271.245 | 914.258 |
| 2038 | 209826 | 50943.211 | 11316.701 | 39626.51 | 18.885 | 77.786 | 4627.73 | 1158.38 | 249.591 | 908.792 |
| 2039 | 207379 | 50085.991 | 10516.675 | 39569.32 | 19.081 | 79.003 | 4555.10 | 1135.19 | 231.000 | 904.191 |

# Results for PHIM for Run 1 (Main_Analysis.RTF)

************************************************************************************

**Main Results for E-Component for Run: 1, Simulation: 1, COD: LUNG CANCER, Adjusted**

| **Variable Parameter** | **Value** |
|---|---|
| 1. Country | US (United States) |
| 2. Sex | M (Males) |
| 3. Year of start of process | 1990 |
| 4. Cause of Death | LUNG CANCER |
| 5. Effective Dose NULL | CC:1 |
| 6. Effective Dose Ecig | CC:1 Ecig:0.05 Dual:0.525 |
| Note: | Death rates are per million population |

------------------------------------------------------------------------------------------

Results for Age Groups Combined and Years combined, Population and Number deaths adjusted for Ecig scenario,

| Age Group:All | | NULL | Ecig | | | %Drop | | NULL | Ecig | |
|---|---|---|---|---|---|---|---|---|---|---|
| Year | Number | Attrib | Attrib | Drop | %Drop | Attrib | Death | Attrib | Attrib | Drop |
| | Deaths | Deaths | Deaths | Deaths | Deaths | Deaths | Rate | Rates | Rates | Rates |
| All Comb | 3095731 | 2244988.113 | 1733526.139 | 511462.0 | 16.522 | 22.782 | 888.686 | 649.875 | 497.640 | 152.235 |

------------------------------------------------------------------------------------------

Results for Age Groups Combined, Population and Number deaths adjusted for Ecig scenario,

| Age Group:All | | NULL | Ecig | | | %Drop | | NULL | Ecig | |
|---|---|---|---|---|---|---|---|---|---|---|
| Year | Number | Attrib | Attrib | Drop | %Drop | Attrib | Death | Attrib | Attrib | Drop |
| | Deaths | Deaths | Deaths | Deaths | Deaths | Deaths | Rate | Rates | Rates | Rates |
| 1990 | 78822 | 63385.736 | 63385.736 | 0.000 | 0.000 | 0.000 | 1249.50 | 1004.81 | 1004.81 | 0.000 |
| 1991 | 78462 | 62918.402 | 62870.284 | 48.118 | 0.061 | 0.076 | 1223.41 | 981.049 | 980.299 | 0.750 |
| 1992 | 77988 | 62366.766 | 62238.154 | 128.612 | 0.165 | 0.206 | 1195.35 | 955.930 | 953.952 | 1.978 |
| 1993 | 78545 | 62652.888 | 62400.588 | 252.300 | 0.321 | 0.403 | 1182.40 | 943.195 | 939.373 | 3.821 |
| 1994 | 77443 | 61565.227 | 61168.659 | 396.567 | 0.512 | 0.644 | 1144.02 | 909.520 | 903.610 | 5.910 |
| 1995 | 76915 | 60851.961 | 60276.730 | 575.231 | 0.748 | 0.945 | 1114.40 | 881.759 | 873.330 | 8.428 |
| 1996 | 76251 | 60122.922 | 59322.964 | 799.958 | 1.049 | 1.331 | 1087.51 | 857.643 | 846.083 | 11.560 |
| 1997 | 75501 | 59237.155 | 58196.647 | 1040.508 | 1.378 | 1.757 | 1059.17 | 831.234 | 816.417 | 14.817 |
| 1998 | 75421 | 58906.696 | 57567.804 | 1338.892 | 1.775 | 2.273 | 1040.53 | 813.001 | 794.223 | 18.778 |
| 1999 | 73212 | 56930.073 | 55298.971 | 1631.102 | 2.228 | 2.865 | 993.996 | 773.329 | 750.792 | 22.538 |
| 2000 | 73856 | 57137.121 | 55126.429 | 2010.692 | 2.722 | 3.519 | 987.900 | 764.787 | 737.372 | 27.415 |
| 2001 | 73387 | 56481.726 | 54041.555 | 2440.172 | 3.325 | 4.320 | 968.647 | 746.169 | 713.307 | 32.862 |
| 2002 | 72557 | 55588.365 | 52697.356 | 2891.010 | 3.984 | 5.201 | 946.763 | 726.138 | 687.619 | 38.519 |
| 2003 | 72083 | 54945.856 | 51555.378 | 3390.478 | 4.704 | 6.171 | 930.560 | 710.281 | 665.557 | 44.724 |
| 2004 | 71470 | 54203.126 | 50261.518 | 3941.607 | 5.515 | 7.272 | 912.463 | 693.135 | 641.692 | 51.443 |
| 2005 | 71164 | 53744.603 | 49233.335 | 4511.269 | 6.339 | 8.394 | 897.747 | 679.296 | 621.090 | 58.206 |
| 2006 | 69888 | 52535.572 | 47424.012 | 5111.560 | 7.314 | 9.730 | 873.316 | 657.945 | 592.610 | 65.335 |
| 2007 | 68827 | 51492.124 | 45787.823 | 5704.301 | 8.288 | 11.078 | 850.955 | 638.264 | 566.106 | 72.157 |
| 2008 | 68524 | 51030.736 | 44605.800 | 6424.936 | 9.376 | 12.590 | 837.430 | 625.460 | 545.123 | 80.337 |
| 2009 | 67845 | 50276.199 | 43129.834 | 7146.365 | 10.533 | 14.214 | 819.037 | 608.943 | 520.673 | 88.270 |
| 2010 | 67572 | 49840.933 | 41972.027 | 7868.906 | 11.645 | 15.788 | 805.542 | 596.347 | 500.355 | 95.991 |
| 2011 | 66923 | 49072.841 | 40403.735 | 8669.106 | 12.954 | 17.666 | 789.601 | 581.354 | 476.712 | 104.642 |
| 2012 | 66614 | 48539.512 | 39010.320 | 9529.191 | 14.305 | 19.632 | 777.141 | 568.812 | 455.104 | 113.708 |
| 2013 | 65835 | 47675.741 | 37381.462 | 10294.28 | 15.637 | 21.592 | 758.963 | 552.318 | 430.946 | 121.373 |
| 2014 | 65583 | 47194.534 | 36103.670 | 11090.86 | 16.911 | 23.500 | 746.911 | 540.359 | 411.174 | 129.185 |
| 2015 | 64740 | 46305.842 | 34520.386 | 11785.46 | 18.204 | 25.451 | 828.462 | 596.439 | 441.750 | 154.689 |
| 2016 | 62241 | 44208.867 | 32013.927 | 12194.94 | 19.593 | 27.585 | 788.628 | 564.107 | 405.634 | 158.473 |
| 2017 | 60555 | 42702.624 | 30032.201 | 12670.42 | 20.924 | 29.671 | 759.838 | 539.903 | 376.842 | 163.061 |
| 2018 | 63208 | 44321.202 | 30071.572 | 14249.63 | 22.544 | 32.151 | 785.367 | 555.181 | 373.641 | 181.540 |
| 2019 | 63850 | 44493.088 | 29188.786 | 15304.30 | 23.969 | 34.397 | 785.194 | 551.912 | 358.947 | 192.965 |
| 2020 | 64340 | 44529.551 | 28241.670 | 16287.88 | 25.315 | 36.578 | 901.914 | 630.757 | 395.891 | 234.867 |
| 2021 | 57957 | 39852.556 | 24318.688 | 15533.87 | 26.803 | 38.978 | 806.210 | 560.546 | 338.286 | 222.260 |
| 2022 | 58345 | 39853.685 | 23334.004 | 16519.68 | 28.314 | 41.451 | 804.886 | 556.274 | 321.901 | 234.372 |
| 2023 | 58705 | 39847.050 | 22355.999 | 17491.05 | 29.795 | 43.895 | 802.776 | 551.670 | 305.711 | 245.959 |
| 2024 | 58973 | 39726.010 | 21371.112 | 18354.90 | 31.124 | 46.204 | 799.232 | 545.435 | 289.632 | 255.804 |
| 2025 | 58614 | 39207.348 | 20271.094 | 18936.25 | 32.307 | 48.298 | 922.414 | 626.579 | 319.007 | 307.572 |
| 2026 | 52463 | 34803.960 | 17188.478 | 17615.48 | 33.577 | 50.613 | 821.595 | 553.929 | 269.182 | 284.747 |
| 2027 | 52257 | 34484.595 | 16203.865 | 18280.73 | 34.982 | 53.011 | 814.070 | 546.357 | 252.425 | 293.932 |
| 2028 | 52006 | 34100.638 | 15190.338 | 18910.30 | 36.361 | 55.454 | 805.470 | 537.544 | 235.266 | 302.278 |
| 2029 | 51727 | 33669.838 | 14228.762 | 19441.08 | 37.584 | 57.740 | 795.994 | 527.732 | 218.956 | 308.776 |
| 2030 | 50338 | 32557.688 | 13190.340 | 19367.35 | 38.474 | 59.486 | 922.797 | 609.705 | 241.804 | 367.901 |
| 2031 | 44723 | 28646.393 | 11023.526 | 17622.87 | 39.404 | 61.519 | 817.022 | 535.065 | 201.383 | 333.682 |
| 2032 | 44340 | 28227.160 | 10273.450 | 17953.71 | 40.491 | 63.604 | 806.596 | 525.435 | 186.884 | 338.551 |
| 2033 | 43942 | 27786.623 | 9570.950 | 18215.67 | 41.454 | 65.556 | 795.361 | 515.079 | 173.236 | 341.843 |
| 2034 | 43510 | 27348.299 | 8918.752 | 18429.55 | 42.357 | 67.388 | 783.111 | 504.514 | 160.522 | 343.992 |
| 2035 | 41007 | 25570.077 | 8073.938 | 17496.14 | 42.666 | 68.424 | 909.922 | 583.430 | 179.156 | 404.274 |
| 2036 | 35129 | 21717.319 | 6339.777 | 15377.54 | 43.774 | 70.808 | 778.589 | 495.402 | 140.512 | 354.890 |
| 2037 | 34572 | 21256.024 | 5837.586 | 15418.44 | 44.598 | 72.537 | 764.741 | 484.347 | 129.128 | 355.219 |
| 2038 | 34020 | 20747.175 | 5353.592 | 15393.58 | 45.249 | 74.196 | 750.307 | 471.764 | 118.074 | 353.690 |
| 2039 | 33479 | 20327.688 | 4952.557 | 15375.13 | 45.925 | 75.636 | 735.362 | 460.724 | 108.783 | 351.940 |

# Results for PHIM for Run 1 (Main_Analysis.RTF)

************************************************************************************

**Main Results for E-Component for Run: 1, Simulation: 1, COD: IHD, Adjusted**

| Variable Parameter | Value |
|---|---|
| 1. Country | US (United States) |
| 2. Sex | M (Males) |
| 3. Year of start of process | 1990 |
| 4. Cause of Death | IHD |
| 5. Effective Dose NULL | CC:1 |
| 6. Effective Dose Ecig | CC:1 Ecig:0.05 Dual:0.525 |
| Note: | Death rates are per million population |

-----------------------------------------------------------------------------------------

Results for Age Groups Combined and Years combined, Population and Number deaths adjusted for Ecig scenario,

| Age Group:All | | NULL | Ecig | | | %Drop | | NULL | Ecig | |
|---|---|---|---|---|---|---|---|---|---|---|
| Year | Number | Attrib | Attrib | Drop | %Drop | Attrib | Death | Attrib | Attrib | Drop |
| | Deaths | Deaths | Deaths | Deaths | Deaths | Deaths | Rate | Rates | Rates | Rates |
| All Comb | 6423083 | 1098877.421 | 516869.102 | 582008.3 | 9.061 | 52.964 | 1843.86 | 318.101 | 148.377 | 169.725 |

-----------------------------------------------------------------------------------------

Results for Age Groups Combined, Population and Number deaths adjusted for Ecig scenario,

| Age Group:All | | NULL | Ecig | | | %Drop | | NULL | Ecig | |
|---|---|---|---|---|---|---|---|---|---|---|
| Year | Number | Attrib | Attrib | Drop | %Drop | Attrib | Death | Attrib | Attrib | Drop |
| | Deaths | Deaths | Deaths | Deaths | Deaths | Deaths | Rate | Rates | Rates | Rates |
| 1990 | 171484 | 38511.725 | 38511.725 | 0.000 | 0.000 | 0.000 | 2718.40 | 610.497 | 610.497 | 0.000 |
| 1991 | 168044 | 37309.838 | 36996.011 | 313.827 | 0.187 | 0.841 | 2620.21 | 581.750 | 576.857 | 4.893 |
| 1992 | 164549 | 36137.488 | 35293.287 | 844.201 | 0.513 | 2.336 | 2522.12 | 553.899 | 540.956 | 12.943 |
| 1993 | 164367 | 35599.588 | 34037.071 | 1562.516 | 0.951 | 4.389 | 2474.37 | 535.926 | 512.391 | 23.535 |
| 1994 | 159878 | 34336.757 | 31952.773 | 2383.985 | 1.491 | 6.943 | 2361.79 | 507.267 | 472.020 | 35.246 |
| 1995 | 158105 | 33455.759 | 30123.602 | 3332.157 | 2.108 | 9.960 | 2290.74 | 484.782 | 436.451 | 48.330 |
| 1996 | 154911 | 32193.824 | 27855.739 | 4338.085 | 2.800 | 13.475 | 2209.39 | 459.239 | 397.287 | 61.952 |
| 1997 | 150023 | 30665.509 | 25352.796 | 5312.713 | 3.541 | 17.325 | 2104.61 | 430.308 | 355.664 | 74.644 |
| 1998 | 145480 | 29188.430 | 22963.494 | 6224.936 | 4.279 | 21.327 | 2007.09 | 402.844 | 316.811 | 86.033 |
| 1999 | 166067 | 33375.386 | 24714.776 | 8660.610 | 5.215 | 25.949 | 2254.68 | 453.366 | 335.551 | 117.815 |
| 2000 | 160410 | 32024.754 | 22328.379 | 9696.376 | 6.045 | 30.278 | 2145.65 | 428.655 | 298.665 | 129.991 |
| 2001 | 154631 | 30682.393 | 19910.004 | 10772.39 | 6.967 | 35.109 | 2041.01 | 405.339 | 262.797 | 142.542 |
| 2002 | 152119 | 30218.370 | 18184.244 | 12034.13 | 7.911 | 39.824 | 1984.91 | 394.736 | 237.276 | 157.459 |
| 2003 | 147009 | 29175.202 | 16210.799 | 12964.40 | 8.819 | 44.436 | 1897.82 | 377.146 | 209.274 | 167.872 |
| 2004 | 138696 | 27488.687 | 14042.122 | 13446.57 | 9.695 | 48.917 | 1770.73 | 351.518 | 179.277 | 172.241 |
| 2005 | 136248 | 27017.439 | 12703.002 | 14314.44 | 10.506 | 52.982 | 1718.80 | 341.482 | 160.251 | 181.231 |
| 2006 | 132339 | 26231.435 | 11177.999 | 15053.44 | 11.375 | 57.387 | 1653.71 | 328.517 | 139.680 | 188.837 |
| 2007 | 126966 | 25103.049 | 9738.335 | 15364.71 | 12.101 | 61.207 | 1569.76 | 311.161 | 120.402 | 190.760 |
| 2008 | 126388 | 24621.740 | 8774.835 | 15846.91 | 12.538 | 64.361 | 1544.57 | 301.777 | 107.236 | 194.541 |
| 2009 | 123013 | 23741.177 | 7739.451 | 16001.73 | 13.008 | 67.401 | 1485.04 | 287.552 | 93.432 | 194.120 |
| 2010 | 121184 | 23189.160 | 6933.395 | 16255.77 | 13.414 | 70.101 | 1444.65 | 277.458 | 82.654 | 194.804 |
| 2011 | 120941 | 22786.927 | 6183.129 | 16603.80 | 13.729 | 72.865 | 1426.95 | 269.951 | 72.953 | 196.998 |
| 2012 | 121732 | 22485.295 | 5573.671 | 16911.62 | 13.892 | 75.212 | 1420.16 | 263.495 | 65.024 | 198.471 |
| 2013 | 123551 | 22251.884 | 5060.925 | 17190.96 | 13.914 | 77.256 | 1424.34 | 257.786 | 58.344 | 199.442 |
| 2014 | 124228 | 21894.593 | 4574.847 | 17319.75 | 13.942 | 79.105 | 1414.79 | 250.685 | 52.102 | 198.583 |
| 2015 | 124877 | 21471.109 | 4152.948 | 17318.16 | 13.868 | 80.658 | 1598.02 | 276.557 | 53.144 | 223.413 |
| 2016 | 127443 | 21411.568 | 3777.592 | 17633.98 | 13.837 | 82.357 | 1614.78 | 273.213 | 47.864 | 225.348 |
| 2017 | 129619 | 21169.399 | 3440.644 | 17728.76 | 13.678 | 83.747 | 1626.45 | 267.651 | 43.173 | 224.478 |
| 2018 | 122801 | 19830.576 | 2937.931 | 16892.64 | 13.756 | 85.185 | 1525.81 | 248.404 | 36.504 | 211.900 |
| 2019 | 123315 | 19431.014 | 2678.804 | 16752.21 | 13.585 | 86.214 | 1516.46 | 241.031 | 32.942 | 208.088 |
| 2020 | 122409 | 18532.612 | 2395.078 | 16137.53 | 13.183 | 87.076 | 1715.92 | 262.513 | 33.574 | 228.939 |
| 2021 | 121084 | 18234.128 | 2158.705 | 16075.42 | 13.276 | 88.161 | 1684.35 | 256.472 | 30.029 | 226.443 |
| 2022 | 121226 | 17812.559 | 1960.341 | 15852.22 | 13.077 | 88.995 | 1672.35 | 248.626 | 27.044 | 221.582 |
| 2023 | 121347 | 17361.668 | 1780.294 | 15581.37 | 12.840 | 89.746 | 1659.38 | 240.367 | 24.345 | 216.022 |
| 2024 | 121322 | 16929.676 | 1637.739 | 15291.94 | 12.604 | 90.326 | 1644.21 | 232.443 | 22.195 | 210.248 |
| 2025 | 117804 | 15501.666 | 1433.770 | 14067.90 | 11.942 | 90.751 | 1853.89 | 247.735 | 22.563 | 225.171 |
| 2026 | 117276 | 15523.814 | 1347.314 | 14176.50 | 12.088 | 91.321 | 1836.61 | 247.072 | 21.100 | 225.972 |
| 2027 | 116287 | 15073.070 | 1249.289 | 13823.78 | 11.888 | 91.712 | 1811.53 | 238.810 | 19.462 | 219.349 |
| 2028 | 115248 | 14636.710 | 1151.707 | 13485.00 | 11.701 | 92.131 | 1784.95 | 230.725 | 17.837 | 212.888 |
| 2029 | 114207 | 14180.166 | 1070.677 | 13109.49 | 11.479 | 92.449 | 1757.45 | 222.256 | 16.476 | 205.780 |
| 2030 | 107387 | 12111.772 | 893.945 | 11217.83 | 10.446 | 92.619 | 1968.62 | 226.816 | 16.388 | 210.428 |
| 2031 | 107921 | 12417.367 | 888.138 | 11529.23 | 10.683 | 92.848 | 1971.56 | 231.935 | 16.225 | 215.710 |
| 2032 | 106590 | 12054.064 | 835.991 | 11218.07 | 10.525 | 93.065 | 1938.98 | 224.381 | 15.207 | 209.173 |
| 2033 | 105273 | 11672.389 | 780.250 | 10892.14 | 10.347 | 93.315 | 1905.45 | 216.370 | 14.123 | 202.248 |
| 2034 | 103931 | 11365.762 | 742.678 | 10623.08 | 10.221 | 93.466 | 1870.57 | 209.673 | 13.367 | 196.306 |
| 2035 | 93612 | 8580.360 | 550.937 | 8029.423 | 8.577 | 93.579 | 2077.21 | 195.777 | 12.225 | 183.552 |
| 2036 | 95007 | 8948.841 | 558.540 | 8390.301 | 8.831 | 93.759 | 2105.69 | 204.135 | 12.379 | 191.756 |
| 2037 | 93256 | 8624.570 | 533.388 | 8091.181 | 8.676 | 93.815 | 2062.83 | 196.522 | 11.799 | 184.724 |
| 2038 | 91556 | 8315.017 | 501.997 | 7813.019 | 8.534 | 93.963 | 2019.28 | 189.073 | 11.072 | 178.001 |
| 2039 | 89922 | 8001.132 | 473.991 | 7527.141 | 8.371 | 94.076 | 1975.16 | 181.344 | 10.411 | 170.933 |

# Results for PHIM for Run 1 (Main_Analysis.RTF)

************************************************************************************

**Main Results for E-Component for Run: 1, Simulation: 1, COD: STROKE, Adjusted**

| Variable Parameter | Value |
|---|---|
| 1. Country | US (United States) |
| 2. Sex | M (Males) |
| 3. Year of start of process | 1990 |
| 4. Cause of Death | STROKE |
| 5. Effective Dose NULL | CC:1 |
| 6. Effective Dose Ecig | CC:1 Ecig:0.05 Dual:0.525 |
| Note: | Death rates are per million population |

-----------------------------------------------------------------------------------------

Results for Age Groups Combined and Years combined, Population and Number deaths adjusted for Ecig scenario,

| Age Group:All | | NULL | Ecig | | | %Drop | | NULL | Ecig | |
|---|---|---|---|---|---|---|---|---|---|---|
| Year | Number | Attrib | Attrib | Drop | %Drop | Attrib | Death | Attrib | Attrib | Drop |
| | Deaths | Deaths | Deaths | Deaths | Deaths | Deaths | Rate | Rates | Rates | Rates |
| All Comb | 1506815 | 149918.462 | 69737.075 | 80181.39 | 5.321 | 53.483 | 432.559 | 43.398 | 20.019 | 23.379 |

-----------------------------------------------------------------------------------------

Results for Age Groups Combined, Population and Number deaths adjusted for Ecig scenario,

| Age Group:All | | NULL | Ecig | | | %Drop | | NULL | Ecig | |
|---|---|---|---|---|---|---|---|---|---|---|
| Year | Number | Attrib | Attrib | Drop | %Drop | Attrib | Death | Attrib | Attrib | Drop |
| | Deaths | Deaths | Deaths | Deaths | Deaths | Deaths | Rate | Rates | Rates | Rates |
| 1990 | 32862 | 4613.166 | 4613.166 | 0.000 | 0.000 | 0.000 | 520.936 | 73.129 | 73.129 | 0.000 |
| 1991 | 32619 | 4555.566 | 4515.509 | 40.057 | 0.123 | 0.879 | 508.609 | 71.032 | 70.408 | 0.625 |
| 1992 | 32100 | 4453.572 | 4348.544 | 105.028 | 0.327 | 2.358 | 492.015 | 68.262 | 66.652 | 1.610 |
| 1993 | 32897 | 4462.473 | 4264.090 | 198.384 | 0.603 | 4.446 | 495.229 | 67.179 | 64.191 | 2.988 |
| 1994 | 33325 | 4499.513 | 4186.460 | 313.053 | 0.939 | 6.957 | 492.297 | 66.473 | 61.844 | 4.628 |
| 1995 | 33574 | 4472.774 | 4029.008 | 443.766 | 1.322 | 9.921 | 486.436 | 64.812 | 58.375 | 6.436 |
| 1996 | 33877 | 4406.878 | 3818.620 | 588.258 | 1.736 | 13.349 | 483.172 | 62.863 | 54.462 | 8.401 |
| 1997 | 33527 | 4288.158 | 3553.336 | 734.822 | 2.192 | 17.136 | 470.341 | 60.173 | 49.848 | 10.324 |
| 1998 | 32811 | 4120.148 | 3251.495 | 868.653 | 2.647 | 21.083 | 452.668 | 56.864 | 44.859 | 12.006 |
| 1999 | 33397 | 3982.443 | 2980.498 | 1001.944 | 3.000 | 25.159 | 453.430 | 54.097 | 40.466 | 13.631 |
| 2000 | 33489 | 4021.411 | 2843.864 | 1177.548 | 3.516 | 29.282 | 447.949 | 53.827 | 38.040 | 15.788 |
| 2001 | 32312 | 3848.092 | 2552.413 | 1295.680 | 4.010 | 33.671 | 426.499 | 50.836 | 33.690 | 17.147 |
| 2002 | 31632 | 3783.081 | 2349.366 | 1433.715 | 4.532 | 37.898 | 412.754 | 49.418 | 30.656 | 18.762 |
| 2003 | 31041 | 3754.415 | 2167.726 | 1586.689 | 5.112 | 42.262 | 400.725 | 48.533 | 27.984 | 20.549 |
| 2004 | 29950 | 3666.274 | 1964.083 | 1702.192 | 5.683 | 46.428 | 382.370 | 46.883 | 25.076 | 21.808 |
| 2005 | 28783 | 3589.688 | 1777.469 | 1812.219 | 6.296 | 50.484 | 363.108 | 45.371 | 22.423 | 22.948 |
| 2006 | 28275 | 3596.985 | 1633.871 | 1963.113 | 6.943 | 54.577 | 353.323 | 45.048 | 20.417 | 24.631 |
| 2007 | 28126 | 3560.798 | 1485.921 | 2074.876 | 7.377 | 58.270 | 347.744 | 44.137 | 18.371 | 25.766 |
| 2008 | 27239 | 3417.001 | 1309.951 | 2107.051 | 7.735 | 61.664 | 332.889 | 41.881 | 16.009 | 25.872 |
| 2009 | 27195 | 3394.390 | 1194.810 | 2199.580 | 8.088 | 64.800 | 328.299 | 41.113 | 14.424 | 26.689 |
| 2010 | 27053 | 3340.137 | 1083.882 | 2256.255 | 8.340 | 67.550 | 322.502 | 39.965 | 12.921 | 27.044 |
| 2011 | 27118 | 3285.435 | 979.483 | 2305.952 | 8.503 | 70.187 | 319.963 | 38.922 | 11.557 | 27.365 |
| 2012 | 27235 | 3259.209 | 886.159 | 2373.051 | 8.713 | 72.811 | 317.728 | 38.193 | 10.338 | 27.855 |
| 2013 | 28108 | 3274.703 | 816.076 | 2458.627 | 8.747 | 75.079 | 324.034 | 37.937 | 9.408 | 28.529 |
| 2014 | 29023 | 3298.083 | 751.279 | 2546.804 | 8.775 | 77.221 | 330.534 | 37.762 | 8.556 | 29.206 |
| 2015 | 29822 | 3244.406 | 683.589 | 2560.817 | 8.587 | 78.930 | 381.630 | 41.789 | 8.748 | 33.042 |
| 2016 | 30808 | 3314.836 | 640.200 | 2674.637 | 8.682 | 80.687 | 390.350 | 42.297 | 8.112 | 34.186 |
| 2017 | 31746 | 3333.314 | 591.436 | 2741.878 | 8.637 | 82.257 | 398.346 | 42.144 | 7.421 | 34.723 |
| 2018 | 29685 | 2925.242 | 482.021 | 2443.221 | 8.230 | 83.522 | 368.842 | 36.642 | 5.989 | 30.653 |
| 2019 | 29965 | 2864.628 | 438.117 | 2426.511 | 8.098 | 84.706 | 368.493 | 35.534 | 5.388 | 30.146 |
| 2020 | 29991 | 2730.366 | 389.496 | 2340.870 | 7.805 | 85.735 | 420.408 | 38.675 | 5.460 | 33.215 |
| 2021 | 30533 | 2647.911 | 351.250 | 2296.661 | 7.522 | 86.735 | 424.730 | 37.244 | 4.886 | 32.358 |
| 2022 | 30772 | 2586.165 | 317.840 | 2268.325 | 7.371 | 87.710 | 424.511 | 36.097 | 4.385 | 31.713 |
| 2023 | 31008 | 2517.960 | 288.107 | 2229.854 | 7.191 | 88.558 | 424.018 | 34.860 | 3.940 | 30.921 |
| 2024 | 31197 | 2454.161 | 263.895 | 2190.266 | 7.021 | 89.247 | 422.802 | 33.695 | 3.576 | 30.119 |
| 2025 | 30823 | 2275.364 | 231.230 | 2044.134 | 6.632 | 89.838 | 485.065 | 36.363 | 3.639 | 32.724 |
| 2026 | 31298 | 2183.166 | 209.373 | 1973.793 | 6.306 | 90.410 | 490.151 | 34.747 | 3.279 | 31.468 |
| 2027 | 31205 | 2112.345 | 192.381 | 1919.964 | 6.153 | 90.893 | 486.121 | 33.467 | 2.997 | 30.470 |
| 2028 | 31087 | 2046.677 | 176.876 | 1869.801 | 6.015 | 91.358 | 481.478 | 32.263 | 2.739 | 29.523 |
| 2029 | 30962 | 1977.671 | 163.065 | 1814.607 | 5.861 | 91.755 | 476.450 | 30.997 | 2.509 | 28.488 |
| 2030 | 30063 | 1744.457 | 138.669 | 1605.788 | 5.341 | 92.051 | 551.120 | 32.668 | 2.542 | 30.126 |
| 2031 | 29736 | 1653.738 | 126.840 | 1526.898 | 5.135 | 92.330 | 543.231 | 30.889 | 2.317 | 28.572 |
| 2032 | 29513 | 1603.120 | 117.676 | 1485.445 | 5.033 | 92.660 | 536.862 | 29.841 | 2.141 | 27.701 |
| 2033 | 29280 | 1548.245 | 109.458 | 1438.786 | 4.914 | 92.930 | 529.967 | 28.700 | 1.981 | 26.719 |
| 2034 | 29021 | 1511.985 | 103.589 | 1408.396 | 4.853 | 93.149 | 522.336 | 27.893 | 1.864 | 26.028 |
| 2035 | 27588 | 1239.866 | 82.992 | 1156.874 | 4.193 | 93.306 | 612.170 | 28.290 | 1.842 | 26.448 |
| 2036 | 26436 | 1182.522 | 76.741 | 1105.781 | 4.183 | 93.510 | 585.916 | 26.975 | 1.701 | 25.274 |
| 2037 | 26003 | 1134.483 | 72.854 | 1061.629 | 4.083 | 93.578 | 575.193 | 25.851 | 1.612 | 24.239 |
| 2038 | 25568 | 1093.537 | 68.342 | 1025.195 | 4.010 | 93.750 | 563.894 | 24.866 | 1.507 | 23.358 |
| 2039 | 25134 | 1047.905 | 63.961 | 983.944 | 3.915 | 93.896 | 552.079 | 23.751 | 1.405 | 22.346 |

# Results for PHIM for Run 1 (Main_Analysis.RTF)

**************************************************************************************

**Main Results for E-Component for Run: 1, Simulation: 1, COD: COPD, Adjusted**

| Variable Parameter | Value |
|---|---|
| 1. Country | US (United States) |
| 2. Sex | M (Males) |
| 3. Year of start of process | 1990 |
| 4. Cause of Death | COPD |
| 5. Effective Dose NULL | CC:1 |
| 6. Effective Dose Ecig | CC:1 Ecig:0.05 Dual:0.525 |
| Note: | Death rates are per million population |

-----------------------------------------------------------------------------------------

Results for Age Groups Combined and Years combined, Population and Number deaths adjusted for Ecig scenario,

| Age Group:All | | NULL | Ecig | | | %Drop | | NULL | Ecig | |
|---|---|---|---|---|---|---|---|---|---|---|
| Year | Number | Attrib | Attrib | Drop | %Drop | Attrib | Death | Attrib | Attrib | Drop |
| | Deaths | Deaths | Deaths | Deaths | Deaths | Deaths | Rate | Rates | Rates | Rates |
| All Comb | 2265935 | 1015606.587 | 682443.867 | 333162.7 | 14.703 | 32.804 | 650.478 | 293.996 | 195.908 | 98.088 |

-----------------------------------------------------------------------------------------

Results for Age Groups Combined, Population and Number deaths adjusted for Ecig scenario,

| Age Group:All | | NULL | Ecig | | | %Drop | | NULL | Ecig | |
|---|---|---|---|---|---|---|---|---|---|---|
| Year | Number | Attrib | Attrib | Drop | %Drop | Attrib | Death | Attrib | Attrib | Drop |
| | Deaths | Deaths | Deaths | Deaths | Deaths | Deaths | Rate | Rates | Rates | Rates |
| 1990 | 33993 | 19262.437 | 19262.437 | 0.000 | 0.000 | 0.000 | 538.865 | 305.353 | 305.353 | 0.000 |
| 1991 | 34092 | 19215.636 | 19193.448 | 22.188 | 0.065 | 0.115 | 531.576 | 299.618 | 299.272 | 0.346 |
| 1992 | 33942 | 19056.189 | 18998.391 | 57.798 | 0.170 | 0.303 | 520.249 | 292.085 | 291.197 | 0.888 |
| 1993 | 35914 | 20095.670 | 19977.509 | 118.160 | 0.329 | 0.588 | 540.648 | 302.526 | 300.740 | 1.786 |
| 1994 | 35077 | 19524.095 | 19343.489 | 180.605 | 0.515 | 0.925 | 518.166 | 288.435 | 285.750 | 2.684 |
| 1995 | 34674 | 19116.002 | 18857.509 | 258.493 | 0.746 | 1.352 | 502.376 | 276.995 | 273.220 | 3.775 |
| 1996 | 34590 | 18975.021 | 18622.063 | 352.959 | 1.020 | 1.860 | 493.330 | 270.675 | 265.594 | 5.082 |
| 1997 | 35279 | 19169.025 | 18700.462 | 468.563 | 1.328 | 2.444 | 494.916 | 268.986 | 262.341 | 6.645 |
| 1998 | 35693 | 19228.937 | 18625.784 | 603.154 | 1.690 | 3.137 | 492.425 | 265.388 | 256.967 | 8.421 |
| 1999 | 38542 | 20589.820 | 19797.645 | 792.175 | 2.055 | 3.847 | 523.284 | 279.689 | 268.792 | 10.897 |
| 2000 | 36316 | 19196.417 | 18300.562 | 895.856 | 2.467 | 4.667 | 485.767 | 256.946 | 244.789 | 12.158 |
| 2001 | 36324 | 19012.731 | 17927.985 | 1084.746 | 2.986 | 5.705 | 479.449 | 251.174 | 236.636 | 14.538 |
| 2002 | 36037 | 18699.357 | 17430.413 | 1268.944 | 3.521 | 6.786 | 470.226 | 244.265 | 227.440 | 16.826 |
| 2003 | 36273 | 18663.550 | 17182.671 | 1480.879 | 4.083 | 7.935 | 468.262 | 241.262 | 221.821 | 19.442 |
| 2004 | 34689 | 17679.164 | 16037.502 | 1641.662 | 4.732 | 9.286 | 442.880 | 226.076 | 204.752 | 21.325 |
| 2005 | 36925 | 18692.832 | 16708.767 | 1984.065 | 5.373 | 10.614 | 465.823 | 236.265 | 210.785 | 25.480 |
| 2006 | 34459 | 17308.564 | 15201.269 | 2107.294 | 6.115 | 12.175 | 430.602 | 216.769 | 189.955 | 26.814 |
| 2007 | 35457 | 17683.181 | 15243.761 | 2439.421 | 6.880 | 13.795 | 438.377 | 219.189 | 188.469 | 30.720 |
| 2008 | 38411 | 19006.374 | 16094.573 | 2911.801 | 7.581 | 15.320 | 469.416 | 232.952 | 196.690 | 36.262 |
| 2009 | 37082 | 18205.573 | 15072.793 | 3132.780 | 8.448 | 17.208 | 447.660 | 220.505 | 181.962 | 38.543 |
| 2010 | 37439 | 18237.758 | 14802.515 | 3435.243 | 9.176 | 18.836 | 446.317 | 218.215 | 176.463 | 41.752 |
| 2011 | 38328 | 18487.916 | 14608.940 | 3878.976 | 10.120 | 20.981 | 452.222 | 219.022 | 172.367 | 46.655 |
| 2012 | 38769 | 18491.158 | 14238.447 | 4252.711 | 10.969 | 22.999 | 452.285 | 216.689 | 166.109 | 50.580 |
| 2013 | 40598 | 19143.452 | 14374.525 | 4768.928 | 11.747 | 24.912 | 468.022 | 221.775 | 165.714 | 56.061 |
| 2014 | 40679 | 18992.005 | 13862.276 | 5129.729 | 12.610 | 27.010 | 463.279 | 217.451 | 157.874 | 59.578 |
| 2015 | 42696 | 19728.885 | 14072.420 | 5656.465 | 13.248 | 28.671 | 546.368 | 254.116 | 180.082 | 74.035 |
| 2016 | 43541 | 19876.724 | 13773.132 | 6103.592 | 14.018 | 30.707 | 551.685 | 253.628 | 174.513 | 79.115 |
| 2017 | 44594 | 20119.790 | 13522.192 | 6597.598 | 14.795 | 32.792 | 559.561 | 254.381 | 169.676 | 84.705 |
| 2018 | 46012 | 20535.123 | 13378.141 | 7156.982 | 15.555 | 34.852 | 571.699 | 257.229 | 166.224 | 91.005 |
| 2019 | 47568 | 21009.029 | 13264.025 | 7745.004 | 16.282 | 36.865 | 584.967 | 260.605 | 163.113 | 97.492 |
| 2020 | 49093 | 21428.512 | 13116.934 | 8311.578 | 16.930 | 38.787 | 688.181 | 303.533 | 183.873 | 119.660 |
| 2021 | 49187 | 21266.697 | 12520.083 | 8746.614 | 17.782 | 41.128 | 684.220 | 299.127 | 174.161 | 124.966 |
| 2022 | 50712 | 21663.568 | 12276.650 | 9386.918 | 18.510 | 43.330 | 699.584 | 302.378 | 169.361 | 133.017 |
| 2023 | 52264 | 22084.131 | 12039.664 | 10044.47 | 19.219 | 45.483 | 714.697 | 305.748 | 164.638 | 141.109 |
| 2024 | 53766 | 22418.456 | 11781.441 | 10637.01 | 19.784 | 47.448 | 728.661 | 307.804 | 159.668 | 148.136 |
| 2025 | 54886 | 22614.834 | 11459.849 | 11154.99 | 20.324 | 49.326 | 863.742 | 361.411 | 180.344 | 181.067 |
| 2026 | 55454 | 22571.522 | 10962.722 | 11608.80 | 20.934 | 51.431 | 868.444 | 359.241 | 171.683 | 187.558 |
| 2027 | 56461 | 22774.416 | 10573.905 | 12200.51 | 21.609 | 53.571 | 879.556 | 360.827 | 164.721 | 196.106 |
| 2028 | 57429 | 22915.561 | 10137.904 | 12777.66 | 22.250 | 55.760 | 889.447 | 361.228 | 157.015 | 204.214 |
| 2029 | 58382 | 22992.027 | 9696.925 | 13295.10 | 22.773 | 57.825 | 898.397 | 360.371 | 149.219 | 211.152 |
| 2030 | 58693 | 22895.360 | 9249.784 | 13645.58 | 23.249 | 59.600 | 1075.96 | 428.760 | 169.566 | 259.193 |
| 2031 | 59126 | 22715.871 | 8774.137 | 13941.73 | 23.580 | 61.374 | 1080.14 | 424.293 | 160.290 | 264.003 |
| 2032 | 59875 | 22759.207 | 8345.104 | 14414.10 | 24.074 | 63.333 | 1089.18 | 423.652 | 151.805 | 271.846 |
| 2033 | 60600 | 22786.258 | 7940.846 | 14845.41 | 24.497 | 65.151 | 1096.88 | 422.387 | 143.730 | 278.657 |
| 2034 | 61268 | 22806.106 | 7534.156 | 15271.95 | 24.926 | 66.964 | 1102.72 | 420.721 | 135.602 | 285.119 |
| 2035 | 60394 | 22262.998 | 7072.662 | 15190.34 | 25.152 | 68.231 | 1340.11 | 507.973 | 156.938 | 351.035 |
| 2036 | 58318 | 21140.051 | 6247.969 | 14892.08 | 25.536 | 70.445 | 1292.52 | 482.234 | 138.477 | 343.757 |
| 2037 | 58511 | 21011.845 | 5818.550 | 15193.29 | 25.966 | 72.308 | 1294.28 | 478.783 | 128.707 | 350.076 |
| 2038 | 58682 | 20787.483 | 5392.771 | 15394.71 | 26.234 | 74.058 | 1294.25 | 472.681 | 118.938 | 353.743 |
| 2039 | 58844 | 20709.267 | 5026.167 | 15683.10 | 26.652 | 75.730 | 1292.50 | 469.372 | 110.400 | 358.972 |

# Results for PHIM for Run 2 (Main_Analysis.RTF)

---

-----------------------------------------------------------------------------------

**Control File Details for Run: 2**

| Variable Parameter | Value |
|---|---|
| 1. Name for Reference Scenario | NULL |
| 2. Name for Test Scenario | Ecig |
| 3. Name for Product 1 | CC |
| 4. Name for Product 2 | Ecig |
| 5. Name for Product 3 | - |
| 6. Name for Product 4 | Dual |
| 7. Country | US (United States) |
| 8. Sex | F (Females) |
| 9. Year of start of process | 1990 |
| 10. Number of months of follow-up | 600 |
| 11. Follow-up interval length (in months) | 12 |
| 12. Lower age for risk estimation | 10 |
| 13. Upper age for risk estimation | 79 |
| 14. The effective dose for Product 1 (F1) - Ref | 1 |
| 15. The effective dose for Product 2 (F2) - Ref | - |
| 16. The effective dose for Product 3 (F3) - Ref | - |
| 17. The effective dose for Product 4 (F4) - Ref | - |
| 18. The effective dose for Product 1 (F1) - Test | 1 |
| 19. The effective dose for Product 2 (F2) - Test | 0.05 |
| 20. The effective dose for Product 3 (F3) - Test | - |
| 21. The effective dose for Product 4 (F4) - Test | FROMF2 |
| 22. Number in population to be simulated | 100000 |
| 23. Number of MC simulations | 1 |
| 24. The random number seed for the first simulation | 15975263 |
| 25. Source for the population file (POP) | UN4 |
| 26. Source for the socioeconomic prevalence file (SEP) | - |
| 27. Source for the current smoking prevalence file (CSP) | ISS3 |
| 28. Source for the former smoking prevalence file (FSP) | ISS3 |
| 29. Source for the quit-time distribution file (QTD) | NHIS2006 |
| 30. Source for the death file (MORT) | APR-20 |
| 31. Source for the relative risk file (RR) | PNLEST |
| 32. Source for the half-life file (H) | PNLEST |
| 33. Assumption Set for TTP Factor Reference | F1 |
| 34. Assumption Set for TTP Factor Test | F1 |
| 35. Assumption Set for TTP Reference | ISS3 |
| 36. Assumption Set for TTP Test | ISS3 |
| 37. Assumption Set for TP for socioeconomic group | - |
| 38. Source for Output Choice file (OUTC) | BASIC |
| 39. Output for DTP P1 | N |
| 40. Output for DTP P2 | N |
| 41. Output for Main P Component | Y |
| 42. Output for DTP E1 | N |
| 43. Output for DTP E2 | N |
| 44. Output for DTP E3 | N |
| 45. Output for Main E Component | Y |
| 46. Output file name | Main_Analysis |
| 47. Run number for P-Component | - |
| 48. Results file for E-Component | - |
| 49. Components required | PE |
| 50. Results folder for P to be used in E-Component | - |
| 51. Fixed age of starting product use | 16 |
| 52. Output absolute risk file? | N |

# Results for PHIM for Run 2 (Main_Analysis.RTF)

*************************************************************************************
**TTP values for Run: 2, Scenario: NULL**

| **Variable Parameter** | **Value** |
|---|---|
| 1. Country | US (United States) |
| 2. Sex | F (Females) |
| 3. Assumption Set | ISS3 |
| Note: | Period is in Months |

| | | **Initiation** | **Cessation** | **Re-initiation** |
|---|---|---|---|---|
| Age | Period | Never CC | CC Former | Former CC |
| 10-14 | 1-12 | 0.003758 | 0.008742 | 0.000000 |
| 15-19 | 1-12 | 0.003469 | 0.002515 | 0.000000 |
| 20-24 | 1-12 | 0.000276 | 0.000761 | 0.000000 |
| 25-29 | 1-12 | 0.000137 | 0.001375 | 0.000000 |
| 30-34 | 1-12 | 0.000069 | 0.001149 | 0.000000 |
| 35-39 | 1-12 | 0.000000 | 0.000991 | 0.000000 |
| 40-44 | 1-12 | 0.000000 | 0.000833 | 0.000000 |
| 45-49 | 1-12 | 0.000000 | 0.001723 | 0.000000 |
| 50-54 | 1-12 | 0.000000 | 0.002336 | 0.000000 |
| 55-59 | 1-12 | 0.000000 | 0.002233 | 0.000000 |
| 60-64 | 1-12 | 0.000000 | 0.004634 | 0.000000 |
| 65-69 | 1-12 | 0.000000 | 0.003505 | 0.000000 |
| 70-74 | 1-12 | 0.000000 | 0.004329 | 0.000000 |
| 75-79 | 1-12 | 0.000000 | 0.004329 | 0.000000 |
| 10-14 | 13-72 | 0.004794 | 0.008742 | 0.000000 |
| 15-19 | 13-72 | 0.004527 | 0.004082 | 0.000000 |
| 20-24 | 13-72 | 0.000105 | 0.001261 | 0.000000 |
| 25-29 | 13-72 | 0.000053 | 0.000952 | 0.000000 |
| 30-34 | 13-72 | 0.000026 | 0.000733 | 0.000000 |
| 35-39 | 13-72 | 0.000000 | 0.000753 | 0.000000 |
| 40-44 | 13-72 | 0.000000 | 0.001535 | 0.000000 |
| 45-49 | 13-72 | 0.000000 | 0.002998 | 0.000000 |
| 50-54 | 13-72 | 0.000000 | 0.003159 | 0.000000 |
| 55-59 | 13-72 | 0.000000 | 0.002965 | 0.000000 |
| 60-64 | 13-72 | 0.000000 | 0.003633 | 0.000000 |
| 65-69 | 13-72 | 0.000000 | 0.004522 | 0.000000 |
| 70-74 | 13-72 | 0.000000 | 0.005208 | 0.000000 |
| 75-79 | 13-72 | 0.000000 | 0.005208 | 0.000000 |
| 10-14 | 73-132 | 0.003969 | 0.008742 | 0.000000 |
| 15-19 | 73-132 | 0.003056 | 0.003373 | 0.000000 |
| 20-24 | 73-132 | 0.001459 | 0.003881 | 0.000000 |
| 25-29 | 73-132 | 0.000714 | 0.001953 | 0.000000 |
| 30-34 | 73-132 | 0.000111 | 0.001338 | 0.000000 |
| 35-39 | 73-132 | 0.000000 | 0.001507 | 0.000000 |
| 40-44 | 73-132 | 0.000000 | 0.002080 | 0.000000 |
| 45-49 | 73-132 | 0.000000 | 0.003809 | 0.000000 |
| 50-54 | 73-132 | 0.000000 | 0.004871 | 0.000000 |
| 55-59 | 73-132 | 0.000000 | 0.004560 | 0.000000 |
| 60-64 | 73-132 | 0.000000 | 0.007192 | 0.000000 |
| 65-69 | 73-132 | 0.000000 | 0.007033 | 0.000000 |
| 70-74 | 73-132 | 0.000000 | 0.008206 | 0.000000 |
| 75-79 | 73-132 | 0.000000 | 0.008206 | 0.000000 |
| 10-14 | 133-192 | 0.002861 | 0.008742 | 0.000000 |
| 15-19 | 133-192 | 0.002594 | 0.003996 | 0.000000 |
| 20-24 | 133-192 | 0.001247 | 0.002805 | 0.000000 |
| 25-29 | 133-192 | 0.000289 | 0.001574 | 0.000000 |
| 30-34 | 133-192 | 0.000132 | 0.001293 | 0.000000 |
| 35-39 | 133-192 | 0.000000 | 0.001219 | 0.000000 |
| 40-44 | 133-192 | 0.000000 | 0.001657 | 0.000000 |
| 45-49 | 133-192 | 0.000000 | 0.001995 | 0.000000 |
| 50-54 | 133-192 | 0.000000 | 0.002154 | 0.000000 |
| 55-59 | 133-192 | 0.000000 | 0.002072 | 0.000000 |
| 60-64 | 133-192 | 0.000000 | 0.005049 | 0.000000 |
| 65-69 | 133-192 | 0.000000 | 0.003680 | 0.000000 |
| 70-74 | 133-192 | 0.000000 | 0.006144 | 0.000000 |
| 75-79 | 133-192 | 0.000000 | 0.006144 | 0.000000 |
| 10-14 | 193-252 | 0.001859 | 0.008742 | 0.000000 |
| 15-19 | 193-252 | 0.002232 | 0.005770 | 0.000000 |
| 20-24 | 193-252 | 0.001079 | 0.003262 | 0.000000 |
| 25-29 | 193-252 | 0.000531 | 0.002002 | 0.000000 |
| 30-34 | 193-252 | 0.000263 | 0.003240 | 0.000000 |
| 35-39 | 193-252 | 0.000000 | 0.001771 | 0.000000 |
| 40-44 | 193-252 | 0.000000 | 0.002173 | 0.000000 |
| 45-49 | 193-252 | 0.000000 | 0.002075 | 0.000000 |
| 50-54 | 193-252 | 0.000000 | 0.003431 | 0.000000 |

## Results for PHIM for Run 2 (Main_Analysis.RTF)

| | | | | |
|---|---|---|---|---|
| 55-59 | 193-252 | 0.000000 | 0.002906 | 0.000000 |
| 60-64 | 193-252 | 0.000000 | 0.007139 | 0.000000 |
| 65-69 | 193-252 | 0.000000 | 0.004963 | 0.000000 |
| 70-74 | 193-252 | 0.000000 | 0.006269 | 0.000000 |
| 75-79 | 193-252 | 0.000000 | 0.006269 | 0.000000 |
| 10-14 | 253+ | 0.000931 | 0.008742 | 0.000000 |
| 15-19 | 253+ | 0.001798 | 0.009440 | 0.000000 |
| 20-24 | 253+ | 0.000023 | 0.002831 | 0.000000 |
| 25-29 | 253+ | 0.000073 | 0.002655 | 0.000000 |
| 30-34 | 253+ | 0.000037 | 0.004614 | 0.000000 |
| 35-39 | 253+ | 0.000000 | 0.002607 | 0.000000 |
| 40-44 | 253+ | 0.000000 | 0.003115 | 0.000000 |
| 45-49 | 253+ | 0.000000 | 0.002132 | 0.000000 |
| 50-54 | 253+ | 0.000000 | 0.003497 | 0.000000 |
| 55-59 | 253+ | 0.000000 | 0.002942 | 0.000000 |
| 60-64 | 253+ | 0.000000 | 0.005376 | 0.000000 |
| 65-69 | 253+ | 0.000000 | 0.001789 | 0.000000 |
| 70-74 | 253+ | 0.000000 | 0.004077 | 0.000000 |
| 75-79 | 253+ | 0.000000 | 0.004077 | 0.000000 |

# Results for PHIM for Run 2 (Main_Analysis.RTF)

**************************************************************************************

**TTP Factors**

| Factor | TTP Affected | Relates to | Value | < or > | Years |
|---|---|---|---|---|---|
| 1a | Current PR2 to Current PR1 | Previous PR1 use | 2 | - | - |
| 1b | Current PR3 to Current PR1 | Previous PR1 use | 2 | - | - |
| 1c | Current PR4 to Current PR1 | Previous PR1 use | 2 | - | - |
| 2a | Current PR1 to Former | Previously quit | 2 | - | - |
| 2b | Current PR2 to Former | Previously quit | 2 | - | - |
| 2c | Current PR3 to Former | Previously quit | 2 | - | - |
| 2d | Current PR4 to Former | Previously quit | 2 | - | - |
| 3a | Former to Current PR1 | Time quit | 2 | < | 2 |
| 3b | Former to Current PR2 | Time quit | 2 | < | 2 |
| 3c | Former to Current PR3 | Time quit | 2 | < | 2 |
| 3d | Former to Current PR4 | Time quit | 2 | < | 2 |
| 4a | Current PR2 to Current PR1 | Time PR2 use | 0 | > | 2 |
| 4b | Current PR2 to Current PR3 | Time PR2 use | 0 | > | 2 |
| 4c | Current PR2 to Current PR4 | Time PR2 use | 0 | > | 2 |
| 5a | Current PR3 to Current PR1 | Time PR3 use | 0 | > | 2 |
| 5b | Current PR3 to Current PR2 | Time PR3 use | 0 | > | 2 |
| 5c | Current PR3 to Current PR4 | Time PR3 use | 0 | > | 2 |
| 6a | Current PR4 to Current PR1 | Time PR4 use | - | - | - |
| 6b | Current PR4 to Current PR2 | Time PR4 use | - | - | - |
| 6c | Current PR4 to Current PR3 | Time PR4 use | - | - | - |
| 7a | Current PR1 to Current PR2 | Time PR1 use | - | - | - |
| 7b | Current PR1 to Current PR3 | Time PR1 use | - | - | - |
| 7c | Current PR1 to Current PR4 | Time PR1 use | - | - | - |

# Results for PHIM for Run 2 (Main_Analysis.RTF)

************************************************************************************

**TTP values for Run: 2, Scenario: Ecig**

| **Variable Parameter** | **Value** |
|---|---|
| 1. Country | US (United States) |
| 2. Sex | F (Females) |
| 3. Assumption Set | ISS3 |
| Note: | Period is in Months |

| Age | Period | **Initiation** Never CC | Never Ecig | Never Dual | **Cessation** CC Former | Ecig Former | Dual Former | **Re-initiation** Former CC | Former Ecig | Former Dual |
|---|---|---|---|---|---|---|---|---|---|---|
| 10-14 | 1-12 | 0.001879 | 0.001879 | 0.000000 | 0.008742 | 0.008742 | 0.000000 | 0.000000 | 0.000000 | 0.000000 |
| 15-19 | 1-12 | 0.001735 | 0.001735 | 0.000000 | 0.002515 | 0.002515 | 0.000000 | 0.000000 | 0.000000 | 0.000000 |
| 20-24 | 1-12 | 0.000138 | 0.000138 | 0.000000 | 0.000761 | 0.000761 | 0.000000 | 0.000000 | 0.000000 | 0.000000 |
| 25-29 | 1-12 | 0.000069 | 0.000069 | 0.000000 | 0.001376 | 0.001376 | 0.000000 | 0.000000 | 0.000000 | 0.000000 |
| 30-34 | 1-12 | 0.000034 | 0.000034 | 0.000000 | 0.001149 | 0.001149 | 0.000000 | 0.000000 | 0.000000 | 0.000000 |
| 35-39 | 1-12 | 0.000000 | 0.000000 | 0.000000 | 0.000991 | 0.000991 | 0.000000 | 0.000000 | 0.000000 | 0.000000 |
| 40-44 | 1-12 | 0.000000 | 0.000000 | 0.000000 | 0.000833 | 0.000833 | 0.000000 | 0.000000 | 0.000000 | 0.000000 |
| 45-49 | 1-12 | 0.000000 | 0.000000 | 0.000000 | 0.001723 | 0.001723 | 0.000000 | 0.000000 | 0.000000 | 0.000000 |
| 50-54 | 1-12 | 0.000000 | 0.000000 | 0.000000 | 0.002336 | 0.002336 | 0.000000 | 0.000000 | 0.000000 | 0.000000 |
| 55-59 | 1-12 | 0.000000 | 0.000000 | 0.000000 | 0.002233 | 0.002233 | 0.000000 | 0.000000 | 0.000000 | 0.000000 |
| 60-64 | 1-12 | 0.000000 | 0.000000 | 0.000000 | 0.004635 | 0.004635 | 0.000000 | 0.000000 | 0.000000 | 0.000000 |
| 65-69 | 1-12 | 0.000000 | 0.000000 | 0.000000 | 0.003505 | 0.003505 | 0.000000 | 0.000000 | 0.000000 | 0.000000 |
| 70-74 | 1-12 | 0.000000 | 0.000000 | 0.000000 | 0.004329 | 0.004329 | 0.000000 | 0.000000 | 0.000000 | 0.000000 |
| 75-79 | 1-12 | 0.000000 | 0.000000 | 0.000000 | 0.004329 | 0.004329 | 0.000000 | 0.000000 | 0.000000 | 0.000000 |
| 10-14 | 13-72 | 0.001918 | 0.002877 | 0.000000 | 0.008742 | 0.008742 | 0.000000 | 0.000000 | 0.000000 | 0.000000 |
| 15-19 | 13-72 | 0.001811 | 0.002716 | 0.000000 | 0.004083 | 0.004083 | 0.000000 | 0.000000 | 0.000000 | 0.000000 |
| 20-24 | 13-72 | 0.000042 | 0.000063 | 0.000000 | 0.001261 | 0.001261 | 0.000000 | 0.000000 | 0.000000 | 0.000000 |
| 25-29 | 13-72 | 0.000021 | 0.000032 | 0.000000 | 0.000952 | 0.000952 | 0.000000 | 0.000000 | 0.000000 | 0.000000 |
| 30-34 | 13-72 | 0.000011 | 0.000016 | 0.000000 | 0.000733 | 0.000733 | 0.000000 | 0.000000 | 0.000000 | 0.000000 |
| 35-39 | 13-72 | 0.000000 | 0.000000 | 0.000000 | 0.000754 | 0.000754 | 0.000000 | 0.000000 | 0.000000 | 0.000000 |
| 40-44 | 13-72 | 0.000000 | 0.000000 | 0.000000 | 0.001535 | 0.001535 | 0.000000 | 0.000000 | 0.000000 | 0.000000 |
| 45-49 | 13-72 | 0.000000 | 0.000000 | 0.000000 | 0.002998 | 0.002998 | 0.000000 | 0.000000 | 0.000000 | 0.000000 |
| 50-54 | 13-72 | 0.000000 | 0.000000 | 0.000000 | 0.003160 | 0.003160 | 0.000000 | 0.000000 | 0.000000 | 0.000000 |
| 55-59 | 13-72 | 0.000000 | 0.000000 | 0.000000 | 0.002965 | 0.002965 | 0.000000 | 0.000000 | 0.000000 | 0.000000 |
| 60-64 | 13-72 | 0.000000 | 0.000000 | 0.000000 | 0.003633 | 0.003633 | 0.000000 | 0.000000 | 0.000000 | 0.000000 |
| 65-69 | 13-72 | 0.000000 | 0.000000 | 0.000000 | 0.004522 | 0.004522 | 0.000000 | 0.000000 | 0.000000 | 0.000000 |
| 70-74 | 13-72 | 0.000000 | 0.000000 | 0.000000 | 0.005208 | 0.005208 | 0.000000 | 0.000000 | 0.000000 | 0.000000 |
| 75-79 | 13-72 | 0.000000 | 0.000000 | 0.000000 | 0.005208 | 0.005208 | 0.000000 | 0.000000 | 0.000000 | 0.000000 |
| 10-14 | 73-132 | 0.000794 | 0.003175 | 0.000000 | 0.008742 | 0.008742 | 0.000000 | 0.000000 | 0.000000 | 0.000000 |
| 15-19 | 73-132 | 0.000611 | 0.002445 | 0.000000 | 0.003373 | 0.003373 | 0.000000 | 0.000000 | 0.000000 | 0.000000 |
| 20-24 | 73-132 | 0.000292 | 0.001167 | 0.000000 | 0.003881 | 0.003881 | 0.000000 | 0.000000 | 0.000000 | 0.000000 |
| 25-29 | 73-132 | 0.000143 | 0.000571 | 0.000000 | 0.001954 | 0.001954 | 0.000000 | 0.000000 | 0.000000 | 0.000000 |
| 30-34 | 73-132 | 0.000022 | 0.000089 | 0.000000 | 0.001338 | 0.001338 | 0.000000 | 0.000000 | 0.000000 | 0.000000 |
| 35-39 | 73-132 | 0.000000 | 0.000000 | 0.000000 | 0.001507 | 0.001507 | 0.000000 | 0.000000 | 0.000000 | 0.000000 |
| 40-44 | 73-132 | 0.000000 | 0.000000 | 0.000000 | 0.002080 | 0.002080 | 0.000000 | 0.000000 | 0.000000 | 0.000000 |
| 45-49 | 73-132 | 0.000000 | 0.000000 | 0.000000 | 0.003809 | 0.003809 | 0.000000 | 0.000000 | 0.000000 | 0.000000 |
| 50-54 | 73-132 | 0.000000 | 0.000000 | 0.000000 | 0.004871 | 0.004871 | 0.000000 | 0.000000 | 0.000000 | 0.000000 |
| 55-59 | 73-132 | 0.000000 | 0.000000 | 0.000000 | 0.004560 | 0.004560 | 0.000000 | 0.000000 | 0.000000 | 0.000000 |
| 60-64 | 73-132 | 0.000000 | 0.000000 | 0.000000 | 0.007192 | 0.007192 | 0.000000 | 0.000000 | 0.000000 | 0.000000 |
| 65-69 | 73-132 | 0.000000 | 0.000000 | 0.000000 | 0.007033 | 0.007033 | 0.000000 | 0.000000 | 0.000000 | 0.000000 |
| 70-74 | 73-132 | 0.000000 | 0.000000 | 0.000000 | 0.008206 | 0.008206 | 0.000000 | 0.000000 | 0.000000 | 0.000000 |
| 75-79 | 73-132 | 0.000000 | 0.000000 | 0.000000 | 0.008206 | 0.008206 | 0.000000 | 0.000000 | 0.000000 | 0.000000 |
| 10-14 | 133-192 | 0.000143 | 0.002718 | 0.000000 | 0.008742 | 0.008742 | 0.000000 | 0.000000 | 0.000000 | 0.000000 |
| 15-19 | 133-192 | 0.000130 | 0.002465 | 0.000000 | 0.003996 | 0.003996 | 0.000000 | 0.000000 | 0.000000 | 0.000000 |
| 20-24 | 133-192 | 0.000062 | 0.001185 | 0.000000 | 0.002805 | 0.002805 | 0.000000 | 0.000000 | 0.000000 | 0.000000 |
| 25-29 | 133-192 | 0.000015 | 0.000275 | 0.000000 | 0.001574 | 0.001574 | 0.000000 | 0.000000 | 0.000000 | 0.000000 |
| 30-34 | 133-192 | 0.000007 | 0.000125 | 0.000000 | 0.001293 | 0.001293 | 0.000000 | 0.000000 | 0.000000 | 0.000000 |
| 35-39 | 133-192 | 0.000000 | 0.000000 | 0.000000 | 0.001219 | 0.001219 | 0.000000 | 0.000000 | 0.000000 | 0.000000 |
| 40-44 | 133-192 | 0.000000 | 0.000000 | 0.000000 | 0.001657 | 0.001657 | 0.000000 | 0.000000 | 0.000000 | 0.000000 |
| 45-49 | 133-192 | 0.000000 | 0.000000 | 0.000000 | 0.001995 | 0.001995 | 0.000000 | 0.000000 | 0.000000 | 0.000000 |
| 50-54 | 133-192 | 0.000000 | 0.000000 | 0.000000 | 0.002154 | 0.002154 | 0.000000 | 0.000000 | 0.000000 | 0.000000 |
| 55-59 | 133-192 | 0.000000 | 0.000000 | 0.000000 | 0.002072 | 0.002072 | 0.000000 | 0.000000 | 0.000000 | 0.000000 |
| 60-64 | 133-192 | 0.000000 | 0.000000 | 0.000000 | 0.005049 | 0.005049 | 0.000000 | 0.000000 | 0.000000 | 0.000000 |
| 65-69 | 133-192 | 0.000000 | 0.000000 | 0.000000 | 0.003681 | 0.003681 | 0.000000 | 0.000000 | 0.000000 | 0.000000 |
| 70-74 | 133-192 | 0.000000 | 0.000000 | 0.000000 | 0.006144 | 0.006144 | 0.000000 | 0.000000 | 0.000000 | 0.000000 |
| 75-79 | 133-192 | 0.000000 | 0.000000 | 0.000000 | 0.006144 | 0.006144 | 0.000000 | 0.000000 | 0.000000 | 0.000000 |
| 10-14 | 193-252 | 0.000093 | 0.001766 | 0.000000 | 0.008742 | 0.008742 | 0.000000 | 0.000000 | 0.000000 | 0.000000 |
| 15-19 | 193-252 | 0.000112 | 0.002120 | 0.000000 | 0.005770 | 0.005770 | 0.000000 | 0.000000 | 0.000000 | 0.000000 |
| 20-24 | 193-252 | 0.000054 | 0.001025 | 0.000000 | 0.003262 | 0.003262 | 0.000000 | 0.000000 | 0.000000 | 0.000000 |
| 25-29 | 193-252 | 0.000027 | 0.000504 | 0.000000 | 0.002002 | 0.002002 | 0.000000 | 0.000000 | 0.000000 | 0.000000 |
| 30-34 | 193-252 | 0.000013 | 0.000250 | 0.000000 | 0.003241 | 0.003241 | 0.000000 | 0.000000 | 0.000000 | 0.000000 |
| 35-39 | 193-252 | 0.000000 | 0.000000 | 0.000000 | 0.001771 | 0.001771 | 0.000000 | 0.000000 | 0.000000 | 0.000000 |
| 40-44 | 193-252 | 0.000000 | 0.000000 | 0.000000 | 0.002173 | 0.002173 | 0.000000 | 0.000000 | 0.000000 | 0.000000 |
| 45-49 | 193-252 | 0.000000 | 0.000000 | 0.000000 | 0.002075 | 0.002075 | 0.000000 | 0.000000 | 0.000000 | 0.000000 |
| 50-54 | 193-252 | 0.000000 | 0.000000 | 0.000000 | 0.003432 | 0.003432 | 0.000000 | 0.000000 | 0.000000 | 0.000000 |

## Results for PHIM for Run 2 (Main_Analysis.RTF)

| | | | | | | | | | | |
|---|---|---|---|---|---|---|---|---|---|---|
| 55-59 | 193-252 | 0.000000 | 0.000000 | 0.000000 | 0.002906 | 0.002906 | 0.000000 | 0.000000 | 0.000000 | 0.000000 |
| 60-64 | 193-252 | 0.000000 | 0.000000 | 0.000000 | 0.007139 | 0.007139 | 0.000000 | 0.000000 | 0.000000 | 0.000000 |
| 65-69 | 193-252 | 0.000000 | 0.000000 | 0.000000 | 0.004963 | 0.004963 | 0.000000 | 0.000000 | 0.000000 | 0.000000 |
| 70-74 | 193-252 | 0.000000 | 0.000000 | 0.000000 | 0.006269 | 0.006269 | 0.000000 | 0.000000 | 0.000000 | 0.000000 |
| 75-79 | 193-252 | 0.000000 | 0.000000 | 0.000000 | 0.006269 | 0.006269 | 0.000000 | 0.000000 | 0.000000 | 0.000000 |
| 10-14 | 253+ | 0.000047 | 0.000884 | 0.000000 | 0.008742 | 0.008742 | 0.000000 | 0.000000 | 0.000000 | 0.000000 |
| 15-19 | 253+ | 0.000090 | 0.001708 | 0.000000 | 0.009440 | 0.009440 | 0.000000 | 0.000000 | 0.000000 | 0.000000 |
| 20-24 | 253+ | 0.000001 | 0.000022 | 0.000000 | 0.002831 | 0.002831 | 0.000000 | 0.000000 | 0.000000 | 0.000000 |
| 25-29 | 253+ | 0.000004 | 0.000070 | 0.000000 | 0.002656 | 0.002656 | 0.000000 | 0.000000 | 0.000000 | 0.000000 |
| 30-34 | 253+ | 0.000002 | 0.000035 | 0.000000 | 0.004614 | 0.004614 | 0.000000 | 0.000000 | 0.000000 | 0.000000 |
| 35-39 | 253+ | 0.000000 | 0.000000 | 0.000000 | 0.002608 | 0.002608 | 0.000000 | 0.000000 | 0.000000 | 0.000000 |
| 40-44 | 253+ | 0.000000 | 0.000000 | 0.000000 | 0.003115 | 0.003115 | 0.000000 | 0.000000 | 0.000000 | 0.000000 |
| 45-49 | 253+ | 0.000000 | 0.000000 | 0.000000 | 0.002132 | 0.002132 | 0.000000 | 0.000000 | 0.000000 | 0.000000 |
| 50-54 | 253+ | 0.000000 | 0.000000 | 0.000000 | 0.003497 | 0.003497 | 0.000000 | 0.000000 | 0.000000 | 0.000000 |
| 55-59 | 253+ | 0.000000 | 0.000000 | 0.000000 | 0.002942 | 0.002942 | 0.000000 | 0.000000 | 0.000000 | 0.000000 |
| 60-64 | 253+ | 0.000000 | 0.000000 | 0.000000 | 0.005376 | 0.005376 | 0.000000 | 0.000000 | 0.000000 | 0.000000 |
| 65-69 | 253+ | 0.000000 | 0.000000 | 0.000000 | 0.001789 | 0.001789 | 0.000000 | 0.000000 | 0.000000 | 0.000000 |
| 70-74 | 253+ | 0.000000 | 0.000000 | 0.000000 | 0.004077 | 0.004077 | 0.000000 | 0.000000 | 0.000000 | 0.000000 |
| 75-79 | 253+ | 0.000000 | 0.000000 | 0.000000 | 0.004077 | 0.004077 | 0.000000 | 0.000000 | 0.000000 | 0.000000 |

# Results for PHIM for Run 2 (Main_Analysis.RTF)

************************************************************************************

| | | Switch from CC | | Switch from Ecig | | Switch from Dual | |
|---|---|---|---|---|---|---|---|
| | | **CC** | **CC** | **Ecig** | **Ecig** | **Dual** | **Dual** |
| Age | Period | Ecig | Dual | CC | Dual | CC | Ecig |
| 10-14 | 1-12 | 0.009055 | 0.000000 | 0.000000 | 0.000000 | 0.000000 | 0.000000 |
| 15-19 | 1-12 | 0.011817 | 0.000000 | 0.000000 | 0.000000 | 0.000000 | 0.000000 |
| 20-24 | 1-12 | 0.013405 | 0.000000 | 0.000000 | 0.000000 | 0.000000 | 0.000000 |
| 25-29 | 1-12 | 0.014127 | 0.000000 | 0.000000 | 0.000000 | 0.000000 | 0.000000 |
| 30-34 | 1-12 | 0.013889 | 0.000000 | 0.000000 | 0.000000 | 0.000000 | 0.000000 |
| 35-39 | 1-12 | 0.012929 | 0.000000 | 0.000000 | 0.000000 | 0.000000 | 0.000000 |
| 40-44 | 1-12 | 0.012785 | 0.000000 | 0.000000 | 0.000000 | 0.000000 | 0.000000 |
| 45-49 | 1-12 | 0.012295 | 0.000000 | 0.000000 | 0.000000 | 0.000000 | 0.000000 |
| 50-54 | 1-12 | 0.011536 | 0.000000 | 0.000000 | 0.000000 | 0.000000 | 0.000000 |
| 55-59 | 1-12 | 0.008988 | 0.000000 | 0.000000 | 0.000000 | 0.000000 | 0.000000 |
| 60-64 | 1-12 | 0.008098 | 0.000000 | 0.000000 | 0.000000 | 0.000000 | 0.000000 |
| 65-69 | 1-12 | 0.006274 | 0.000000 | 0.000000 | 0.000000 | 0.000000 | 0.000000 |
| 70-74 | 1-12 | 0.004141 | 0.000000 | 0.000000 | 0.000000 | 0.000000 | 0.000000 |
| 75-79 | 1-12 | 0.004141 | 0.000000 | 0.000000 | 0.000000 | 0.000000 | 0.000000 |
| 10-14 | 13-72 | 0.009055 | 0.000000 | 0.000000 | 0.000000 | 0.000000 | 0.000000 |
| 15-19 | 13-72 | 0.011817 | 0.000000 | 0.000000 | 0.000000 | 0.000000 | 0.000000 |
| 20-24 | 13-72 | 0.013405 | 0.000000 | 0.000000 | 0.000000 | 0.000000 | 0.000000 |
| 25-29 | 13-72 | 0.014127 | 0.000000 | 0.000000 | 0.000000 | 0.000000 | 0.000000 |
| 30-34 | 13-72 | 0.013889 | 0.000000 | 0.000000 | 0.000000 | 0.000000 | 0.000000 |
| 35-39 | 13-72 | 0.012929 | 0.000000 | 0.000000 | 0.000000 | 0.000000 | 0.000000 |
| 40-44 | 13-72 | 0.012785 | 0.000000 | 0.000000 | 0.000000 | 0.000000 | 0.000000 |
| 45-49 | 13-72 | 0.012295 | 0.000000 | 0.000000 | 0.000000 | 0.000000 | 0.000000 |
| 50-54 | 13-72 | 0.011536 | 0.000000 | 0.000000 | 0.000000 | 0.000000 | 0.000000 |
| 55-59 | 13-72 | 0.008988 | 0.000000 | 0.000000 | 0.000000 | 0.000000 | 0.000000 |
| 60-64 | 13-72 | 0.008098 | 0.000000 | 0.000000 | 0.000000 | 0.000000 | 0.000000 |
| 65-69 | 13-72 | 0.006274 | 0.000000 | 0.000000 | 0.000000 | 0.000000 | 0.000000 |
| 70-74 | 13-72 | 0.004141 | 0.000000 | 0.000000 | 0.000000 | 0.000000 | 0.000000 |
| 75-79 | 13-72 | 0.004141 | 0.000000 | 0.000000 | 0.000000 | 0.000000 | 0.000000 |
| 10-14 | 73-132 | 0.009055 | 0.000000 | 0.000000 | 0.000000 | 0.000000 | 0.000000 |
| 15-19 | 73-132 | 0.011817 | 0.000000 | 0.000000 | 0.000000 | 0.000000 | 0.000000 |
| 20-24 | 73-132 | 0.013405 | 0.000000 | 0.000000 | 0.000000 | 0.000000 | 0.000000 |
| 25-29 | 73-132 | 0.014127 | 0.000000 | 0.000000 | 0.000000 | 0.000000 | 0.000000 |
| 30-34 | 73-132 | 0.013889 | 0.000000 | 0.000000 | 0.000000 | 0.000000 | 0.000000 |
| 35-39 | 73-132 | 0.012929 | 0.000000 | 0.000000 | 0.000000 | 0.000000 | 0.000000 |
| 40-44 | 73-132 | 0.012785 | 0.000000 | 0.000000 | 0.000000 | 0.000000 | 0.000000 |
| 45-49 | 73-132 | 0.012295 | 0.000000 | 0.000000 | 0.000000 | 0.000000 | 0.000000 |
| 50-54 | 73-132 | 0.011536 | 0.000000 | 0.000000 | 0.000000 | 0.000000 | 0.000000 |
| 55-59 | 73-132 | 0.008988 | 0.000000 | 0.000000 | 0.000000 | 0.000000 | 0.000000 |
| 60-64 | 73-132 | 0.008098 | 0.000000 | 0.000000 | 0.000000 | 0.000000 | 0.000000 |
| 65-69 | 73-132 | 0.006274 | 0.000000 | 0.000000 | 0.000000 | 0.000000 | 0.000000 |
| 70-74 | 73-132 | 0.004141 | 0.000000 | 0.000000 | 0.000000 | 0.000000 | 0.000000 |
| 75-79 | 73-132 | 0.004141 | 0.000000 | 0.000000 | 0.000000 | 0.000000 | 0.000000 |
| 10-14 | 133-192 | 0.009055 | 0.000000 | 0.000000 | 0.000000 | 0.000000 | 0.000000 |
| 15-19 | 133-192 | 0.011817 | 0.000000 | 0.000000 | 0.000000 | 0.000000 | 0.000000 |
| 20-24 | 133-192 | 0.013405 | 0.000000 | 0.000000 | 0.000000 | 0.000000 | 0.000000 |
| 25-29 | 133-192 | 0.014127 | 0.000000 | 0.000000 | 0.000000 | 0.000000 | 0.000000 |
| 30-34 | 133-192 | 0.013889 | 0.000000 | 0.000000 | 0.000000 | 0.000000 | 0.000000 |
| 35-39 | 133-192 | 0.012929 | 0.000000 | 0.000000 | 0.000000 | 0.000000 | 0.000000 |
| 40-44 | 133-192 | 0.012785 | 0.000000 | 0.000000 | 0.000000 | 0.000000 | 0.000000 |
| 45-49 | 133-192 | 0.012295 | 0.000000 | 0.000000 | 0.000000 | 0.000000 | 0.000000 |
| 50-54 | 133-192 | 0.011536 | 0.000000 | 0.000000 | 0.000000 | 0.000000 | 0.000000 |
| 55-59 | 133-192 | 0.008988 | 0.000000 | 0.000000 | 0.000000 | 0.000000 | 0.000000 |
| 60-64 | 133-192 | 0.008098 | 0.000000 | 0.000000 | 0.000000 | 0.000000 | 0.000000 |
| 65-69 | 133-192 | 0.006274 | 0.000000 | 0.000000 | 0.000000 | 0.000000 | 0.000000 |
| 70-74 | 133-192 | 0.004141 | 0.000000 | 0.000000 | 0.000000 | 0.000000 | 0.000000 |
| 75-79 | 133-192 | 0.004141 | 0.000000 | 0.000000 | 0.000000 | 0.000000 | 0.000000 |
| 10-14 | 193-252 | 0.009055 | 0.000000 | 0.000000 | 0.000000 | 0.000000 | 0.000000 |
| 15-19 | 193-252 | 0.011817 | 0.000000 | 0.000000 | 0.000000 | 0.000000 | 0.000000 |
| 20-24 | 193-252 | 0.013405 | 0.000000 | 0.000000 | 0.000000 | 0.000000 | 0.000000 |
| 25-29 | 193-252 | 0.014127 | 0.000000 | 0.000000 | 0.000000 | 0.000000 | 0.000000 |
| 30-34 | 193-252 | 0.013889 | 0.000000 | 0.000000 | 0.000000 | 0.000000 | 0.000000 |
| 35-39 | 193-252 | 0.012929 | 0.000000 | 0.000000 | 0.000000 | 0.000000 | 0.000000 |
| 40-44 | 193-252 | 0.012785 | 0.000000 | 0.000000 | 0.000000 | 0.000000 | 0.000000 |
| 45-49 | 193-252 | 0.012295 | 0.000000 | 0.000000 | 0.000000 | 0.000000 | 0.000000 |
| 50-54 | 193-252 | 0.011536 | 0.000000 | 0.000000 | 0.000000 | 0.000000 | 0.000000 |
| 55-59 | 193-252 | 0.008988 | 0.000000 | 0.000000 | 0.000000 | 0.000000 | 0.000000 |
| 60-64 | 193-252 | 0.008098 | 0.000000 | 0.000000 | 0.000000 | 0.000000 | 0.000000 |
| 65-69 | 193-252 | 0.006274 | 0.000000 | 0.000000 | 0.000000 | 0.000000 | 0.000000 |
| 70-74 | 193-252 | 0.004141 | 0.000000 | 0.000000 | 0.000000 | 0.000000 | 0.000000 |
| 75-79 | 193-252 | 0.004141 | 0.000000 | 0.000000 | 0.000000 | 0.000000 | 0.000000 |
| 10-14 | 253+ | 0.009055 | 0.000000 | 0.000000 | 0.000000 | 0.000000 | 0.000000 |
| 15-19 | 253+ | 0.011817 | 0.000000 | 0.000000 | 0.000000 | 0.000000 | 0.000000 |
| 20-24 | 253+ | 0.013405 | 0.000000 | 0.000000 | 0.000000 | 0.000000 | 0.000000 |

## Results for PHIM for Run 2 (Main_Analysis.RTF)

| | | | | | | | |
|---|---|---|---|---|---|---|---|
| 25-29 | 253+ | 0.014127 | 0.000000 | 0.000000 | 0.000000 | 0.000000 | 0.000000 |
| 30-34 | 253+ | 0.013889 | 0.000000 | 0.000000 | 0.000000 | 0.000000 | 0.000000 |
| 35-39 | 253+ | 0.012929 | 0.000000 | 0.000000 | 0.000000 | 0.000000 | 0.000000 |
| 40-44 | 253+ | 0.012785 | 0.000000 | 0.000000 | 0.000000 | 0.000000 | 0.000000 |
| 45-49 | 253+ | 0.012295 | 0.000000 | 0.000000 | 0.000000 | 0.000000 | 0.000000 |
| 50-54 | 253+ | 0.011536 | 0.000000 | 0.000000 | 0.000000 | 0.000000 | 0.000000 |
| 55-59 | 253+ | 0.008988 | 0.000000 | 0.000000 | 0.000000 | 0.000000 | 0.000000 |
| 60-64 | 253+ | 0.008098 | 0.000000 | 0.000000 | 0.000000 | 0.000000 | 0.000000 |
| 65-69 | 253+ | 0.006274 | 0.000000 | 0.000000 | 0.000000 | 0.000000 | 0.000000 |
| 70-74 | 253+ | 0.004141 | 0.000000 | 0.000000 | 0.000000 | 0.000000 | 0.000000 |
| 75-79 | 253+ | 0.004141 | 0.000000 | 0.000000 | 0.000000 | 0.000000 | 0.000000 |

# Results for PHIM for Run 2 (Main_Analysis.RTF)

*************************************************************************************

**TTP Factors**

| Factor | TTP Affected | Relates to | Value | < or > | Years |
|---|---|---|---|---|---|
| 1a | Current PR2 to Current PR1 | Previous PR1 use | 2 | - | - |
| 1b | Current PR3 to Current PR1 | Previous PR1 use | 2 | - | - |
| 1c | Current PR4 to Current PR1 | Previous PR1 use | 2 | - | - |
| 2a | Current PR1 to Former | Previously quit | 2 | - | - |
| 2b | Current PR2 to Former | Previously quit | 2 | - | - |
| 2c | Current PR3 to Former | Previously quit | 2 | - | - |
| 2d | Current PR4 to Former | Previously quit | 2 | - | - |
| 3a | Former to Current PR1 | Time quit | 2 | < | 2 |
| 3b | Former to Current PR2 | Time quit | 2 | < | 2 |
| 3c | Former to Current PR3 | Time quit | 2 | < | 2 |
| 3d | Former to Current PR4 | Time quit | 2 | < | 2 |
| 4a | Current PR2 to Current PR1 | Time PR2 use | 0 | > | 2 |
| 4b | Current PR2 to Current PR3 | Time PR2 use | 0 | > | 2 |
| 4c | Current PR2 to Current PR4 | Time PR2 use | 0 | > | 2 |
| 5a | Current PR3 to Current PR1 | Time PR3 use | 0 | > | 2 |
| 5b | Current PR3 to Current PR2 | Time PR3 use | 0 | > | 2 |
| 5c | Current PR3 to Current PR4 | Time PR3 use | 0 | > | 2 |
| 6a | Current PR4 to Current PR1 | Time PR4 use | - | - | - |
| 6b | Current PR4 to Current PR2 | Time PR4 use | - | - | - |
| 6c | Current PR4 to Current PR3 | Time PR4 use | - | - | - |
| 7a | Current PR1 to Current PR2 | Time PR1 use | - | - | - |
| 7b | Current PR1 to Current PR3 | Time PR1 use | - | - | - |
| 7c | Current PR1 to Current PR4 | Time PR1 use | - | - | - |

# Results for PHIM for Run 2 (Main_Analysis.RTF)

************************************************************************************
**Main Output for the Tobacco History for Run: 2, Simulation: 1, Scenario: NULL**

| **Variable Parameter** | **Value** |
|---|---|
| 1. Country | US (United States) |
| 2. Sex | F (Females) |
| 3. Year of start of process | 1990 |

Year of Follow-up: 1990, Age Group: All Ages

| Period | Age Group | Smoking Group | N | % | Ever Quit (N) | Ever Quit % | Mean Yrs Quit | Short Term (n) | Short Term % |
|---|---|---|---|---|---|---|---|---|---|
| 0 | All Ages | Never Any | 47776 | 54.92 | | | | | |
| 0 | All Ages | CC | 22737 | 26.14 | 0 | 0.00 | | | |
| 0 | All Ages | Former | 16484 | 18.95 | | | 11.90 | 2815 | 17.08 |

Year of Follow-up: 1990, Age Group: 18-19

| Period | Age Group | Smoking Group | N | % | Ever Quit (N) | Ever Quit % | Mean Yrs Quit | Short Term (n) | Short Term % |
|---|---|---|---|---|---|---|---|---|---|
| 0 | 18-19 | Never Any | 2570 | 78.43 | | | | | |
| 0 | 18-19 | CC | 535 | 16.33 | 0 | 0.00 | | | |
| 0 | 18-19 | Former | 172 | 5.25 | | | 0.20 | 172 | 100.00 |

Year of Follow-up: 1990, Age Group: 20-24

| Period | Age Group | Smoking Group | N | % | Ever Quit (N) | Ever Quit % | Mean Yrs Quit | Short Term (n) | Short Term % |
|---|---|---|---|---|---|---|---|---|---|
| 0 | 20-24 | Never Any | 5354 | 60.89 | | | | | |
| 0 | 20-24 | CC | 2560 | 29.11 | 0 | 0.00 | | | |
| 0 | 20-24 | Former | 879 | 10.00 | | | 1.58 | 420 | 47.78 |

Year of Follow-up: 1990, Age Group: 25-29

| Period | Age Group | Smoking Group | N | % | Ever Quit (N) | Ever Quit % | Mean Yrs Quit | Short Term (n) | Short Term % |
|---|---|---|---|---|---|---|---|---|---|
| 0 | 25-29 | Never Any | 5318 | 52.77 | | | | | |
| 0 | 25-29 | CC | 3335 | 33.09 | 0 | 0.00 | | | |
| 0 | 25-29 | Former | 1425 | 14.14 | | | 3.91 | 404 | 28.35 |

Year of Follow-up: 1990, Age Group: 30-34

| Period | Age Group | Smoking Group | N | % | Ever Quit (N) | Ever Quit % | Mean Yrs Quit | Short Term (n) | Short Term % |
|---|---|---|---|---|---|---|---|---|---|
| 0 | 30-34 | Never Any | 5287 | 50.36 | | | | | |
| 0 | 30-34 | CC | 3326 | 31.68 | 0 | 0.00 | | | |
| 0 | 30-34 | Former | 1886 | 17.96 | | | 4.36 | 566 | 30.01 |

Year of Follow-up: 1990, Age Group: 35-39

| Period | Age Group | Smoking Group | N | % | Ever Quit (N) | Ever Quit % | Mean Yrs Quit | Short Term (n) | Short Term % |
|---|---|---|---|---|---|---|---|---|---|
| 0 | 35-39 | Never Any | 5018 | 52.27 | | | | | |
| 0 | 35-39 | CC | 2653 | 27.64 | 0 | 0.00 | | | |
| 0 | 35-39 | Former | 1929 | 20.09 | | | 7.96 | 324 | 16.80 |

Year of Follow-up: 1990, Age Group: 40-44

| Period | Age Group | Smoking Group | N | % | Ever Quit (N) | Ever Quit % | Mean Yrs Quit | Short Term (n) | Short Term % |
|---|---|---|---|---|---|---|---|---|---|
| 0 | 40-44 | Never Any | 4356 | 51.27 | | | | | |
| 0 | 40-44 | CC | 2397 | 28.21 | 0 | 0.00 | | | |
| 0 | 40-44 | Former | 1744 | 20.52 | | | 7.33 | 339 | 19.44 |

Year of Follow-up: 1990, Age Group: 45-49

| Period | Age Group | Smoking Group | N | % | Ever Quit (N) | Ever Quit % | Mean Yrs Quit | Short Term (n) | Short Term % |
|---|---|---|---|---|---|---|---|---|---|
| 0 | 45-49 | Never Any | 3116 | 47.67 | | | | | |
| 0 | 45-49 | CC | 1875 | 28.69 | 0 | 0.00 | | | |
| 0 | 45-49 | Former | 1545 | 23.64 | | | 12.55 | 194 | 12.56 |

Year of Follow-up: 1990, Age Group: 50-54

| Period | Age Group | Smoking Group | N | % | Ever Quit (N) | Ever Quit % | Mean Yrs Quit | Short Term (n) | Short Term % |
|---|---|---|---|---|---|---|---|---|---|
| 0 | 50-54 | Never Any | 2684 | 48.50 | | | | | |
| 0 | 50-54 | CC | 1572 | 28.41 | 0 | 0.00 | | | |
| 0 | 50-54 | Former | 1278 | 23.09 | | | 13.28 | 126 | 9.86 |

Year of Follow-up: 1990, Age Group: 55-59

| Period | Age Group | Smoking Group | N | % | Ever Quit (N) | Ever Quit % | Mean Yrs Quit | Short Term (n) | Short Term % |
|---|---|---|---|---|---|---|---|---|---|
| 0 | 55-59 | Never Any | 2732 | 51.90 | | | | | |
| 0 | 55-59 | CC | 1295 | 24.60 | 0 | 0.00 | | | |
| 0 | 55-59 | Former | 1237 | 23.50 | | | 17.26 | 90 | 7.28 |

Year of Follow-up: 1990, Age Group: 60-64

| Period | Age Group | Smoking Group | N | % | Ever Quit (N) | Ever Quit % | Mean Yrs Quit | Short Term (n) | Short Term % |
|---|---|---|---|---|---|---|---|---|---|
| 0 | 60-64 | Never Any | 3078 | 55.72 | | | | | |
| 0 | 60-64 | CC | 1249 | 22.61 | 0 | 0.00 | | | |
| 0 | 60-64 | Former | 1197 | 21.67 | | | 18.28 | 61 | 5.10 |

Year of Follow-up: 1990, Age Group: 65-69

| Period | Age Group | Smoking Group | N | % | Ever Quit (N) | Ever Quit % | Mean Yrs Quit | Short Term (n) | Short Term % |
|---|---|---|---|---|---|---|---|---|---|

## Results for PHIM for Run 2 (Main_Analysis.RTF)

| | | | | | | | | | |
|---|---|---|---|---|---|---|---|---|---|
| 0 | 65-69 | Never Any | 3042 | 57.36 | | | | | |
| 0 | 65-69 | CC | 976 | 18.40 | 0 | 0.00 | | | |
| 0 | 65-69 | Former | 1285 | 24.23 | | | 20.65 | 68 | 5.29 |

Year of Follow-up: 1990, Age Group: 70-74

| Period | Age Group | Smoking Group | N | % | Ever Quit (N) | Ever Quit % | Mean Yrs Quit | Short Term (n) | Short Term % |
|---|---|---|---|---|---|---|---|---|---|
| 0 | 70-74 | Never Any | 2738 | 61.05 | | | | | |
| 0 | 70-74 | CC | 610 | 13.60 | 0 | 0.00 | | | |
| 0 | 70-74 | Former | 1137 | 25.35 | | | 24.48 | 27 | 2.37 |

Year of Follow-up: 1990, Age Group: 75-79

| Period | Age Group | Smoking Group | N | % | Ever Quit (N) | Ever Quit % | Mean Yrs Quit | Short Term (n) | Short Term % |
|---|---|---|---|---|---|---|---|---|---|
| 0 | 75-79 | Never Any | 2483 | 68.84 | | | | | |
| 0 | 75-79 | CC | 354 | 9.81 | 0 | 0.00 | | | |
| 0 | 75-79 | Former | 770 | 21.35 | | | 24.48 | 24 | 3.12 |

Year of Follow-up: 1995, Age Group: All Ages

| Period | Age Group | Smoking Group | N | % | Ever Quit (N) | Ever Quit % | Mean Yrs Quit | Short Term (n) | Short Term % |
|---|---|---|---|---|---|---|---|---|---|
| 5 | All Ages | Never Any | 50981 | 55.63 | | | | | |
| 5 | All Ages | CC | 22244 | 24.27 | 0 | 0.00 | | | |
| 5 | All Ages | Former | 18414 | 20.09 | | | 14.18 | 1112 | 6.04 |

Year of Follow-up: 1995, Age Group: 18-19

| Period | Age Group | Smoking Group | N | % | Ever Quit (N) | Ever Quit % | Mean Yrs Quit | Short Term (n) | Short Term % |
|---|---|---|---|---|---|---|---|---|---|
| 5 | 18-19 | Never Any | 2456 | 77.33 | | | | | |
| 5 | 18-19 | CC | 655 | 20.62 | 0 | 0.00 | | | |
| 5 | 18-19 | Former | 65 | 2.05 | | | 1.03 | 44 | 67.69 |

Year of Follow-up: 1995, Age Group: 20-24

| Period | Age Group | Smoking Group | N | % | Ever Quit (N) | Ever Quit % | Mean Yrs Quit | Short Term (n) | Short Term % |
|---|---|---|---|---|---|---|---|---|---|
| 5 | 20-24 | Never Any | 5863 | 70.22 | | | | | |
| 5 | 20-24 | CC | 2044 | 24.48 | 0 | 0.00 | | | |
| 5 | 20-24 | Former | 443 | 5.31 | | | 3.18 | 111 | 25.06 |

Year of Follow-up: 1995, Age Group: 25-29

| Period | Age Group | Smoking Group | N | % | Ever Quit (N) | Ever Quit % | Mean Yrs Quit | Short Term (n) | Short Term % |
|---|---|---|---|---|---|---|---|---|---|
| 5 | 25-29 | Never Any | 5317 | 60.47 | | | | | |
| 5 | 25-29 | CC | 2445 | 27.81 | 0 | 0.00 | | | |
| 5 | 25-29 | Former | 1031 | 11.73 | | | 5.90 | 56 | 5.43 |

Year of Follow-up: 1995, Age Group: 30-34

| Period | Age Group | Smoking Group | N | % | Ever Quit (N) | Ever Quit % | Mean Yrs Quit | Short Term (n) | Short Term % |
|---|---|---|---|---|---|---|---|---|---|
| 5 | 30-34 | Never Any | 5299 | 52.58 | | | | | |
| 5 | 30-34 | CC | 3133 | 31.09 | 0 | 0.00 | | | |
| 5 | 30-34 | Former | 1646 | 16.33 | | | 8.04 | 60 | 3.65 |

Year of Follow-up: 1995, Age Group: 35-39

| Period | Age Group | Smoking Group | N | % | Ever Quit (N) | Ever Quit % | Mean Yrs Quit | Short Term (n) | Short Term % |
|---|---|---|---|---|---|---|---|---|---|
| 5 | 35-39 | Never Any | 5282 | 50.31 | | | | | |
| 5 | 35-39 | CC | 3194 | 30.42 | 0 | 0.00 | | | |
| 5 | 35-39 | Former | 2023 | 19.27 | | | 8.86 | 56 | 2.77 |

Year of Follow-up: 1995, Age Group: 40-44

| Period | Age Group | Smoking Group | N | % | Ever Quit (N) | Ever Quit % | Mean Yrs Quit | Short Term (n) | Short Term % |
|---|---|---|---|---|---|---|---|---|---|
| 5 | 40-44 | Never Any | 5018 | 52.27 | | | | | |
| 5 | 40-44 | CC | 2469 | 25.72 | 0 | 0.00 | | | |
| 5 | 40-44 | Former | 2113 | 22.01 | | | 11.98 | 88 | 4.16 |

Year of Follow-up: 1995, Age Group: 45-49

| Period | Age Group | Smoking Group | N | % | Ever Quit (N) | Ever Quit % | Mean Yrs Quit | Short Term (n) | Short Term % |
|---|---|---|---|---|---|---|---|---|---|
| 5 | 45-49 | Never Any | 4356 | 51.27 | | | | | |
| 5 | 45-49 | CC | 2119 | 24.94 | 0 | 0.00 | | | |
| 5 | 45-49 | Former | 2022 | 23.80 | | | 10.86 | 131 | 6.48 |

Year of Follow-up: 1995, Age Group: 50-54

| Period | Age Group | Smoking Group | N | % | Ever Quit (N) | Ever Quit % | Mean Yrs Quit | Short Term (n) | Short Term % |
|---|---|---|---|---|---|---|---|---|---|
| 5 | 50-54 | Never Any | 3116 | 47.67 | | | | | |
| 5 | 50-54 | CC | 1609 | 24.62 | 0 | 0.00 | | | |
| 5 | 50-54 | Former | 1811 | 27.71 | | | 15.25 | 103 | 5.69 |

Year of Follow-up: 1995, Age Group: 55-59

| Period | Age Group | Smoking Group | N | % | Ever Quit (N) | Ever Quit % | Mean Yrs Quit | Short Term (n) | Short Term % |
|---|---|---|---|---|---|---|---|---|---|
| 5 | 55-59 | Never Any | 2684 | 48.50 | | | | | |
| 5 | 55-59 | CC | 1332 | 24.07 | 0 | 0.00 | | | |
| 5 | 55-59 | Former | 1518 | 27.43 | | | 15.70 | 98 | 6.46 |

Year of Follow-up: 1995, Age Group: 60-64

| Period | Age Group | Smoking Group | N | % | Ever Quit (N) | Ever Quit % | Mean Yrs Quit | Short Term (n) | Short Term % |
|---|---|---|---|---|---|---|---|---|---|

# Results for PHIM for Run 2 (Main_Analysis.RTF)

| | | | | | | | | | |
|---|---|---|---|---|---|---|---|---|---|
| 5 | 60-64 | Never Any | 2732 | 51.90 | | | | | |
| 5 | 60-64 | CC | 1075 | 20.42 | 0 | 0.00 | | | |
| 5 | 60-64 | Former | 1457 | 27.68 | | | 19.19 | 91 | 6.25 |

Year of Follow-up: 1995, Age Group: 65-69

| Period | Age Group | Smoking Group | N | % | Ever Quit (N) | Ever Quit % | Mean Yrs Quit | Short Term (n) | Short Term % |
|---|---|---|---|---|---|---|---|---|---|
| 5 | 65-69 | Never Any | 3078 | 55.72 | | | | | |
| 5 | 65-69 | CC | 973 | 17.61 | 0 | 0.00 | | | |
| 5 | 65-69 | Former | 1473 | 26.67 | | | 19.32 | 103 | 6.99 |

Year of Follow-up: 1995, Age Group: 70-74

| Period | Age Group | Smoking Group | N | % | Ever Quit (N) | Ever Quit % | Mean Yrs Quit | Short Term (n) | Short Term % |
|---|---|---|---|---|---|---|---|---|---|
| 5 | 70-74 | Never Any | 3042 | 57.36 | | | | | |
| 5 | 70-74 | CC | 743 | 14.01 | 0 | 0.00 | | | |
| 5 | 70-74 | Former | 1518 | 28.63 | | | 22.00 | 101 | 6.65 |

Year of Follow-up: 1995, Age Group: 75-79

| Period | Age Group | Smoking Group | N | % | Ever Quit (N) | Ever Quit % | Mean Yrs Quit | Short Term (n) | Short Term % |
|---|---|---|---|---|---|---|---|---|---|
| 5 | 75-79 | Never Any | 2738 | 61.05 | | | | | |
| 5 | 75-79 | CC | 453 | 10.10 | 0 | 0.00 | | | |
| 5 | 75-79 | Former | 1294 | 28.85 | | | 26.12 | 70 | 5.41 |

Year of Follow-up: 2000, Age Group: All Ages

| Period | Age Group | Smoking Group | N | % | Ever Quit (N) | Ever Quit % | Mean Yrs Quit | Short Term (n) | Short Term % |
|---|---|---|---|---|---|---|---|---|---|
| 10 | All Ages | Never Any | 50547 | 55.00 | | | | | |
| 10 | All Ages | CC | 20170 | 21.95 | 0 | 0.00 | | | |
| 10 | All Ages | Former | 21191 | 23.06 | | | 15.16 | 1629 | 7.69 |

Year of Follow-up: 2000, Age Group: 18-19

| Period | Age Group | Smoking Group | N | % | Ever Quit (N) | Ever Quit % | Mean Yrs Quit | Short Term (n) | Short Term % |
|---|---|---|---|---|---|---|---|---|---|
| 10 | 18-19 | Never Any | 0 | . | | | | | |
| 10 | 18-19 | CC | 0 | . | 0 | . | | | |
| 10 | 18-19 | Former | 0 | . | | | . | 0 | . |

Year of Follow-up: 2000, Age Group: 20-24

| Period | Age Group | Smoking Group | N | % | Ever Quit (N) | Ever Quit % | Mean Yrs Quit | Short Term (n) | Short Term % |
|---|---|---|---|---|---|---|---|---|---|
| 10 | 20-24 | Never Any | 5202 | 65.60 | | | | | |
| 10 | 20-24 | CC | 2090 | 26.36 | 0 | 0.00 | | | |
| 10 | 20-24 | Former | 638 | 8.05 | | | 3.29 | 203 | 31.82 |

Year of Follow-up: 2000, Age Group: 25-29

| Period | Age Group | Smoking Group | N | % | Ever Quit (N) | Ever Quit % | Mean Yrs Quit | Short Term (n) | Short Term % |
|---|---|---|---|---|---|---|---|---|---|
| 10 | 25-29 | Never Any | 5551 | 66.48 | | | | | |
| 10 | 25-29 | CC | 2028 | 24.29 | 0 | 0.00 | | | |
| 10 | 25-29 | Former | 771 | 9.23 | | | 5.52 | 121 | 15.69 |

Year of Follow-up: 2000, Age Group: 30-34

| Period | Age Group | Smoking Group | N | % | Ever Quit (N) | Ever Quit % | Mean Yrs Quit | Short Term (n) | Short Term % |
|---|---|---|---|---|---|---|---|---|---|
| 10 | 30-34 | Never Any | 5207 | 59.22 | | | | | |
| 10 | 30-34 | CC | 2340 | 26.61 | 0 | 0.00 | | | |
| 10 | 30-34 | Former | 1246 | 14.17 | | | 9.37 | 75 | 6.02 |

Year of Follow-up: 2000, Age Group: 35-39

| Period | Age Group | Smoking Group | N | % | Ever Quit (N) | Ever Quit % | Mean Yrs Quit | Short Term (n) | Short Term % |
|---|---|---|---|---|---|---|---|---|---|
| 10 | 35-39 | Never Any | 5279 | 52.38 | | | | | |
| 10 | 35-39 | CC | 2932 | 29.09 | 0 | 0.00 | | | |
| 10 | 35-39 | Former | 1867 | 18.53 | | | 11.71 | 99 | 5.30 |

Year of Follow-up: 2000, Age Group: 40-44

| Period | Age Group | Smoking Group | N | % | Ever Quit (N) | Ever Quit % | Mean Yrs Quit | Short Term (n) | Short Term % |
|---|---|---|---|---|---|---|---|---|---|
| 10 | 40-44 | Never Any | 5282 | 50.31 | | | | | |
| 10 | 40-44 | CC | 2909 | 27.71 | 0 | 0.00 | | | |
| 10 | 40-44 | Former | 2308 | 21.98 | | | 12.36 | 121 | 5.24 |

Year of Follow-up: 2000, Age Group: 45-49

| Period | Age Group | Smoking Group | N | % | Ever Quit (N) | Ever Quit % | Mean Yrs Quit | Short Term (n) | Short Term % |
|---|---|---|---|---|---|---|---|---|---|
| 10 | 45-49 | Never Any | 5018 | 52.27 | | | | | |
| 10 | 45-49 | CC | 2119 | 22.07 | 0 | 0.00 | | | |
| 10 | 45-49 | Former | 2463 | 25.66 | | | 14.83 | 164 | 6.66 |

Year of Follow-up: 2000, Age Group: 50-54

| Period | Age Group | Smoking Group | N | % | Ever Quit (N) | Ever Quit % | Mean Yrs Quit | Short Term (n) | Short Term % |
|---|---|---|---|---|---|---|---|---|---|
| 10 | 50-54 | Never Any | 4356 | 51.27 | | | | | |
| 10 | 50-54 | CC | 1637 | 19.27 | 0 | 0.00 | | | |
| 10 | 50-54 | Former | 2504 | 29.47 | | | 13.17 | 199 | 7.95 |

Year of Follow-up: 2000, Age Group: 55-59

| Period | Age Group | Smoking Group | N | % | Ever Quit (N) | Ever Quit % | Mean Yrs Quit | Short Term (n) | Short Term % |
|---|---|---|---|---|---|---|---|---|---|

# Results for PHIM for Run 2 (Main_Analysis.RTF)

| | | | | | | | | | |
|---|---|---|---|---|---|---|---|---|---|
| 10 | 55-59 | Never Any | 3116 | 47.67 | | | | | |
| 10 | 55-59 | CC | 1238 | 18.94 | 0 | 0.00 | | | |
| 10 | 55-59 | Former | 2182 | 33.38 | | | 17.12 | 160 | 7.33 |

Year of Follow-up: 2000, Age Group: 60-64

| Period | Age Group | Smoking Group | N | % | Ever Quit (N) | Ever Quit % | Mean Yrs Quit | Short Term (n) | Short Term % |
|---|---|---|---|---|---|---|---|---|---|
| 10 | 60-64 | Never Any | 2684 | 48.50 | | | | | |
| 10 | 60-64 | CC | 986 | 17.82 | 0 | 0.00 | | | |
| 10 | 60-64 | Former | 1864 | 33.68 | | | 17.20 | 136 | 7.30 |

Year of Follow-up: 2000, Age Group: 65-69

| Period | Age Group | Smoking Group | N | % | Ever Quit (N) | Ever Quit % | Mean Yrs Quit | Short Term (n) | Short Term % |
|---|---|---|---|---|---|---|---|---|---|
| 10 | 65-69 | Never Any | 2732 | 51.90 | | | | | |
| 10 | 65-69 | CC | 745 | 14.15 | 0 | 0.00 | | | |
| 10 | 65-69 | Former | 1787 | 33.95 | | | 20.07 | 142 | 7.95 |

Year of Follow-up: 2000, Age Group: 70-74

| Period | Age Group | Smoking Group | N | % | Ever Quit (N) | Ever Quit % | Mean Yrs Quit | Short Term (n) | Short Term % |
|---|---|---|---|---|---|---|---|---|---|
| 10 | 70-74 | Never Any | 3078 | 55.72 | | | | | |
| 10 | 70-74 | CC | 669 | 12.11 | 0 | 0.00 | | | |
| 10 | 70-74 | Former | 1777 | 32.17 | | | 20.49 | 122 | 6.87 |

Year of Follow-up: 2000, Age Group: 75-79

| Period | Age Group | Smoking Group | N | % | Ever Quit (N) | Ever Quit % | Mean Yrs Quit | Short Term (n) | Short Term % |
|---|---|---|---|---|---|---|---|---|---|
| 10 | 75-79 | Never Any | 3042 | 57.36 | | | | | |
| 10 | 75-79 | CC | 477 | 8.99 | 0 | 0.00 | | | |
| 10 | 75-79 | Former | 1784 | 33.64 | | | 23.30 | 87 | 4.88 |

Year of Follow-up: 2005, Age Group: All Ages

| Period | Age Group | Smoking Group | N | % | Ever Quit (N) | Ever Quit % | Mean Yrs Quit | Short Term (n) | Short Term % |
|---|---|---|---|---|---|---|---|---|---|
| 15 | All Ages | Never Any | 47103 | 54.39 | | | | | |
| 15 | All Ages | CC | 17388 | 20.08 | 0 | 0.00 | | | |
| 15 | All Ages | Former | 22114 | 25.53 | | | 17.31 | 946 | 4.28 |

Year of Follow-up: 2005, Age Group: 18-19

| Period | Age Group | Smoking Group | N | % | Ever Quit (N) | Ever Quit % | Mean Yrs Quit | Short Term (n) | Short Term % |
|---|---|---|---|---|---|---|---|---|---|
| 15 | 18-19 | Never Any | 0 | . | | | | | |
| 15 | 18-19 | CC | 0 | . | 0 | . | | | |
| 15 | 18-19 | Former | 0 | . | | | . | 0 | . |

Year of Follow-up: 2005, Age Group: 20-24

| Period | Age Group | Smoking Group | N | % | Ever Quit (N) | Ever Quit % | Mean Yrs Quit | Short Term (n) | Short Term % |
|---|---|---|---|---|---|---|---|---|---|
| 15 | 20-24 | Never Any | 0 | . | | | | | |
| 15 | 20-24 | CC | 0 | . | 0 | . | | | |
| 15 | 20-24 | Former | 0 | . | | | . | 0 | . |

Year of Follow-up: 2005, Age Group: 25-29

| Period | Age Group | Smoking Group | N | % | Ever Quit (N) | Ever Quit % | Mean Yrs Quit | Short Term (n) | Short Term % |
|---|---|---|---|---|---|---|---|---|---|
| 15 | 25-29 | Never Any | 4932 | 62.19 | | | | | |
| 15 | 25-29 | CC | 2046 | 25.80 | 0 | 0.00 | | | |
| 15 | 25-29 | Former | 952 | 12.01 | | | 6.37 | 91 | 9.56 |

Year of Follow-up: 2005, Age Group: 30-34

| Period | Age Group | Smoking Group | N | % | Ever Quit (N) | Ever Quit % | Mean Yrs Quit | Short Term (n) | Short Term % |
|---|---|---|---|---|---|---|---|---|---|
| 15 | 30-34 | Never Any | 5445 | 65.21 | | | | | |
| 15 | 30-34 | CC | 1941 | 23.25 | 0 | 0.00 | | | |
| 15 | 30-34 | Former | 964 | 11.54 | | | 8.81 | 80 | 8.30 |

Year of Follow-up: 2005, Age Group: 35-39

| Period | Age Group | Smoking Group | N | % | Ever Quit (N) | Ever Quit % | Mean Yrs Quit | Short Term (n) | Short Term % |
|---|---|---|---|---|---|---|---|---|---|
| 15 | 35-39 | Never Any | 5181 | 58.92 | | | | | |
| 15 | 35-39 | CC | 2182 | 24.82 | 0 | 0.00 | | | |
| 15 | 35-39 | Former | 1430 | 16.26 | | | 12.82 | 53 | 3.71 |

Year of Follow-up: 2005, Age Group: 40-44

| Period | Age Group | Smoking Group | N | % | Ever Quit (N) | Ever Quit % | Mean Yrs Quit | Short Term (n) | Short Term % |
|---|---|---|---|---|---|---|---|---|---|
| 15 | 40-44 | Never Any | 5279 | 52.38 | | | | | |
| 15 | 40-44 | CC | 2692 | 26.71 | 0 | 0.00 | | | |
| 15 | 40-44 | Former | 2107 | 20.91 | | | 15.03 | 104 | 4.94 |

Year of Follow-up: 2005, Age Group: 45-49

| Period | Age Group | Smoking Group | N | % | Ever Quit (N) | Ever Quit % | Mean Yrs Quit | Short Term (n) | Short Term % |
|---|---|---|---|---|---|---|---|---|---|
| 15 | 45-49 | Never Any | 5282 | 50.31 | | | | | |
| 15 | 45-49 | CC | 2582 | 24.59 | 0 | 0.00 | | | |
| 15 | 45-49 | Former | 2635 | 25.10 | | | 15.46 | 132 | 5.01 |

Year of Follow-up: 2005, Age Group: 50-54

| Period | Age Group | Smoking Group | N | % | Ever Quit (N) | Ever Quit % | Mean Yrs Quit | Short Term (n) | Short Term % |
|---|---|---|---|---|---|---|---|---|---|

# Results for PHIM for Run 2 (Main_Analysis.RTF)

| | | | | | | | | | |
|---|---|---|---|---|---|---|---|---|---|
| 15 | 50-54 | Never Any | 5018 | 52.27 | | | | | |
| 15 | 50-54 | CC | 1831 | 19.07 | 0 | 0.00 | | | |
| 15 | 50-54 | Former | 2751 | 28.66 | | | 18.00 | 96 | 3.49 |

Year of Follow-up: 2005, Age Group: 55-59

| Period | Age Group | Smoking Group | N | % | Ever Quit (N) | Ever Quit % | Mean Yrs Quit | Short Term (n) | Short Term % |
|---|---|---|---|---|---|---|---|---|---|
| 15 | 55-59 | Never Any | 4356 | 51.27 | | | | | |
| 15 | 55-59 | CC | 1398 | 16.45 | 0 | 0.00 | | | |
| 15 | 55-59 | Former | 2743 | 32.28 | | | 16.81 | 63 | 2.30 |

Year of Follow-up: 2005, Age Group: 60-64

| Period | Age Group | Smoking Group | N | % | Ever Quit (N) | Ever Quit % | Mean Yrs Quit | Short Term (n) | Short Term % |
|---|---|---|---|---|---|---|---|---|---|
| 15 | 60-64 | Never Any | 3116 | 47.67 | | | | | |
| 15 | 60-64 | CC | 972 | 14.87 | 0 | 0.00 | | | |
| 15 | 60-64 | Former | 2448 | 37.45 | | | 19.94 | 116 | 4.74 |

Year of Follow-up: 2005, Age Group: 65-69

| Period | Age Group | Smoking Group | N | % | Ever Quit (N) | Ever Quit % | Mean Yrs Quit | Short Term (n) | Short Term % |
|---|---|---|---|---|---|---|---|---|---|
| 15 | 65-69 | Never Any | 2684 | 48.50 | | | | | |
| 15 | 65-69 | CC | 725 | 13.10 | 0 | 0.00 | | | |
| 15 | 65-69 | Former | 2125 | 38.40 | | | 19.76 | 83 | 3.91 |

Year of Follow-up: 2005, Age Group: 70-74

| Period | Age Group | Smoking Group | N | % | Ever Quit (N) | Ever Quit % | Mean Yrs Quit | Short Term (n) | Short Term % |
|---|---|---|---|---|---|---|---|---|---|
| 15 | 70-74 | Never Any | 2732 | 51.90 | | | | | |
| 15 | 70-74 | CC | 555 | 10.54 | 0 | 0.00 | | | |
| 15 | 70-74 | Former | 1977 | 37.56 | | | 22.86 | 71 | 3.59 |

Year of Follow-up: 2005, Age Group: 75-79

| Period | Age Group | Smoking Group | N | % | Ever Quit (N) | Ever Quit % | Mean Yrs Quit | Short Term (n) | Short Term % |
|---|---|---|---|---|---|---|---|---|---|
| 15 | 75-79 | Never Any | 3078 | 55.72 | | | | | |
| 15 | 75-79 | CC | 464 | 8.40 | 0 | 0.00 | | | |
| 15 | 75-79 | Former | 1982 | 35.88 | | | 23.10 | 57 | 2.88 |

Year of Follow-up: 2010, Age Group: All Ages

| Period | Age Group | Smoking Group | N | % | Ever Quit (N) | Ever Quit % | Mean Yrs Quit | Short Term (n) | Short Term % |
|---|---|---|---|---|---|---|---|---|---|
| 20 | All Ages | Never Any | 43878 | 54.12 | | | | | |
| 20 | All Ages | CC | 14309 | 17.65 | 0 | 0.00 | | | |
| 20 | All Ages | Former | 22894 | 28.24 | | | 19.34 | 1178 | 5.15 |

Year of Follow-up: 2010, Age Group: 18-19

| Period | Age Group | Smoking Group | N | % | Ever Quit (N) | Ever Quit % | Mean Yrs Quit | Short Term (n) | Short Term % |
|---|---|---|---|---|---|---|---|---|---|
| 20 | 18-19 | Never Any | 0 | . | | | | | |
| 20 | 18-19 | CC | 0 | . | 0 | . | | | |
| 20 | 18-19 | Former | 0 | . | | | . | 0 | . |

Year of Follow-up: 2010, Age Group: 20-24

| Period | Age Group | Smoking Group | N | % | Ever Quit (N) | Ever Quit % | Mean Yrs Quit | Short Term (n) | Short Term % |
|---|---|---|---|---|---|---|---|---|---|
| 20 | 20-24 | Never Any | 0 | . | | | | | |
| 20 | 20-24 | CC | 0 | . | 0 | . | | | |
| 20 | 20-24 | Former | 0 | . | | | . | 0 | . |

Year of Follow-up: 2010, Age Group: 25-29

| Period | Age Group | Smoking Group | N | % | Ever Quit (N) | Ever Quit % | Mean Yrs Quit | Short Term (n) | Short Term % |
|---|---|---|---|---|---|---|---|---|---|
| 20 | 25-29 | Never Any | 0 | . | | | | | |
| 20 | 25-29 | CC | 0 | . | 0 | . | | | |
| 20 | 25-29 | Former | 0 | . | | | . | 0 | . |

Year of Follow-up: 2010, Age Group: 30-34

| Period | Age Group | Smoking Group | N | % | Ever Quit (N) | Ever Quit % | Mean Yrs Quit | Short Term (n) | Short Term % |
|---|---|---|---|---|---|---|---|---|---|
| 20 | 30-34 | Never Any | 4823 | 60.82 | | | | | |
| 20 | 30-34 | CC | 1886 | 23.78 | 0 | 0.00 | | | |
| 20 | 30-34 | Former | 1221 | 15.40 | | | 9.25 | 131 | 10.73 |

Year of Follow-up: 2010, Age Group: 35-39

| Period | Age Group | Smoking Group | N | % | Ever Quit (N) | Ever Quit % | Mean Yrs Quit | Short Term (n) | Short Term % |
|---|---|---|---|---|---|---|---|---|---|
| 20 | 35-39 | Never Any | 5407 | 64.75 | | | | | |
| 20 | 35-39 | CC | 1717 | 20.56 | 0 | 0.00 | | | |
| 20 | 35-39 | Former | 1226 | 14.68 | | | 11.27 | 97 | 7.91 |

Year of Follow-up: 2010, Age Group: 40-44

| Period | Age Group | Smoking Group | N | % | Ever Quit (N) | Ever Quit % | Mean Yrs Quit | Short Term (n) | Short Term % |
|---|---|---|---|---|---|---|---|---|---|
| 20 | 40-44 | Never Any | 5181 | 58.92 | | | | | |
| 20 | 40-44 | CC | 1927 | 21.92 | 0 | 0.00 | | | |
| 20 | 40-44 | Former | 1685 | 19.16 | | | 15.41 | 106 | 6.29 |

Year of Follow-up: 2010, Age Group: 45-49

| Period | Age Group | Smoking Group | N | % | Ever Quit (N) | Ever Quit % | Mean Yrs Quit | Short Term (n) | Short Term % |
|---|---|---|---|---|---|---|---|---|---|

# Results for PHIM for Run 2 (Main_Analysis.RTF)

| | | | | | | | | | |
|---|---|---|---|---|---|---|---|---|---|
| 20 | 45-49 | Never Any | 5279 | 52.38 | | | | | |
| 20 | 45-49 | CC | 2378 | 23.60 | 0 | 0.00 | | | |
| 20 | 45-49 | Former | 2421 | 24.02 | | | 17.69 | 128 | 5.29 |

Year of Follow-up: 2010, Age Group: 50-54

| Period | Age Group | Smoking Group | N | % | Ever Quit (N) | Ever Quit % | Mean Yrs Quit | Short Term (n) | Short Term % |
|---|---|---|---|---|---|---|---|---|---|
| 20 | 50-54 | Never Any | 5282 | 50.31 | | | | | |
| 20 | 50-54 | CC | 2192 | 20.88 | 0 | 0.00 | | | |
| 20 | 50-54 | Former | 3025 | 28.81 | | | 18.05 | 181 | 5.98 |

Year of Follow-up: 2010, Age Group: 55-59

| Period | Age Group | Smoking Group | N | % | Ever Quit (N) | Ever Quit % | Mean Yrs Quit | Short Term (n) | Short Term % |
|---|---|---|---|---|---|---|---|---|---|
| 20 | 55-59 | Never Any | 5018 | 52.27 | | | | | |
| 20 | 55-59 | CC | 1529 | 15.93 | 0 | 0.00 | | | |
| 20 | 55-59 | Former | 3053 | 31.80 | | | 20.92 | 129 | 4.23 |

Year of Follow-up: 2010, Age Group: 60-64

| Period | Age Group | Smoking Group | N | % | Ever Quit (N) | Ever Quit % | Mean Yrs Quit | Short Term (n) | Short Term % |
|---|---|---|---|---|---|---|---|---|---|
| 20 | 60-64 | Never Any | 4356 | 51.27 | | | | | |
| 20 | 60-64 | CC | 1087 | 12.79 | 0 | 0.00 | | | |
| 20 | 60-64 | Former | 3054 | 35.94 | | | 19.75 | 163 | 5.34 |

Year of Follow-up: 2010, Age Group: 65-69

| Period | Age Group | Smoking Group | N | % | Ever Quit (N) | Ever Quit % | Mean Yrs Quit | Short Term (n) | Short Term % |
|---|---|---|---|---|---|---|---|---|---|
| 20 | 65-69 | Never Any | 3116 | 47.67 | | | | | |
| 20 | 65-69 | CC | 677 | 10.36 | 0 | 0.00 | | | |
| 20 | 65-69 | Former | 2743 | 41.97 | | | 22.49 | 97 | 3.54 |

Year of Follow-up: 2010, Age Group: 70-74

| Period | Age Group | Smoking Group | N | % | Ever Quit (N) | Ever Quit % | Mean Yrs Quit | Short Term (n) | Short Term % |
|---|---|---|---|---|---|---|---|---|---|
| 20 | 70-74 | Never Any | 2684 | 48.50 | | | | | |
| 20 | 70-74 | CC | 527 | 9.52 | 0 | 0.00 | | | |
| 20 | 70-74 | Former | 2323 | 41.98 | | | 22.81 | 85 | 3.66 |

Year of Follow-up: 2010, Age Group: 75-79

| Period | Age Group | Smoking Group | N | % | Ever Quit (N) | Ever Quit % | Mean Yrs Quit | Short Term (n) | Short Term % |
|---|---|---|---|---|---|---|---|---|---|
| 20 | 75-79 | Never Any | 2732 | 51.90 | | | | | |
| 20 | 75-79 | CC | 389 | 7.39 | 0 | 0.00 | | | |
| 20 | 75-79 | Former | 2143 | 40.71 | | | 25.87 | 61 | 2.85 |

Year of Follow-up: 2015, Age Group: All Ages

| Period | Age Group | Smoking Group | N | % | Ever Quit (N) | Ever Quit % | Mean Yrs Quit | Short Term (n) | Short Term % |
|---|---|---|---|---|---|---|---|---|---|
| 25 | All Ages | Never Any | 41126 | 54.24 | | | | | |
| 25 | All Ages | CC | 11487 | 15.15 | 0 | 0.00 | | | |
| 25 | All Ages | Former | 23204 | 30.61 | | | 21.38 | 942 | 4.06 |

Year of Follow-up: 2015, Age Group: 18-19

| Period | Age Group | Smoking Group | N | % | Ever Quit (N) | Ever Quit % | Mean Yrs Quit | Short Term (n) | Short Term % |
|---|---|---|---|---|---|---|---|---|---|
| 25 | 18-19 | Never Any | 0 | . | | | | | |
| 25 | 18-19 | CC | 0 | . | 0 | . | | | |
| 25 | 18-19 | Former | 0 | . | | | . | 0 | . |

Year of Follow-up: 2015, Age Group: 20-24

| Period | Age Group | Smoking Group | N | % | Ever Quit (N) | Ever Quit % | Mean Yrs Quit | Short Term (n) | Short Term % |
|---|---|---|---|---|---|---|---|---|---|
| 25 | 20-24 | Never Any | 0 | . | | | | | |
| 25 | 20-24 | CC | 0 | . | 0 | . | | | |
| 25 | 20-24 | Former | 0 | . | | | . | 0 | . |

Year of Follow-up: 2015, Age Group: 25-29

| Period | Age Group | Smoking Group | N | % | Ever Quit (N) | Ever Quit % | Mean Yrs Quit | Short Term (n) | Short Term % |
|---|---|---|---|---|---|---|---|---|---|
| 25 | 25-29 | Never Any | 0 | . | | | | | |
| 25 | 25-29 | CC | 0 | . | 0 | . | | | |
| 25 | 25-29 | Former | 0 | . | | | . | 0 | . |

Year of Follow-up: 2015, Age Group: 30-34

| Period | Age Group | Smoking Group | N | % | Ever Quit (N) | Ever Quit % | Mean Yrs Quit | Short Term (n) | Short Term % |
|---|---|---|---|---|---|---|---|---|---|
| 25 | 30-34 | Never Any | 0 | . | | | | | |
| 25 | 30-34 | CC | 0 | . | 0 | . | | | |
| 25 | 30-34 | Former | 0 | . | | | . | 0 | . |

Year of Follow-up: 2015, Age Group: 35-39

| Period | Age Group | Smoking Group | N | % | Ever Quit (N) | Ever Quit % | Mean Yrs Quit | Short Term (n) | Short Term % |
|---|---|---|---|---|---|---|---|---|---|
| 25 | 35-39 | Never Any | 4803 | 60.57 | | | | | |
| 25 | 35-39 | CC | 1520 | 19.17 | 0 | 0.00 | | | |
| 25 | 35-39 | Former | 1607 | 20.26 | | | 11.32 | 140 | 8.71 |

Year of Follow-up: 2015, Age Group: 40-44

| Period | Age Group | Smoking Group | N | % | Ever Quit (N) | Ever Quit % | Mean Yrs Quit | Short Term (n) | Short Term % |
|---|---|---|---|---|---|---|---|---|---|

# Results for PHIM for Run 2 (Main_Analysis.RTF)

| | | | | | | | | | |
|---|---|---|---|---|---|---|---|---|---|
| 25 | 40-44 | Never Any | 5407 | 64.75 | | | | | |
| 25 | 40-44 | CC | 1456 | 17.44 | 0 | 0.00 | | | |
| 25 | 40-44 | Former | 1487 | 17.81 | | | 13.77 | 95 | 6.39 |

Year of Follow-up: 2015, Age Group: 45-49

| Period | Age Group | Smoking Group | N | % | Ever Quit (N) | Ever Quit % | Mean Yrs Quit | Short Term (n) | Short Term % |
|---|---|---|---|---|---|---|---|---|---|
| 25 | 45-49 | Never Any | 5181 | 58.92 | | | | | |
| 25 | 45-49 | CC | 1616 | 18.38 | 0 | 0.00 | | | |
| 25 | 45-49 | Former | 1996 | 22.70 | | | 17.55 | 115 | 5.76 |

Year of Follow-up: 2015, Age Group: 50-54

| Period | Age Group | Smoking Group | N | % | Ever Quit (N) | Ever Quit % | Mean Yrs Quit | Short Term (n) | Short Term % |
|---|---|---|---|---|---|---|---|---|---|
| 25 | 50-54 | Never Any | 5279 | 52.38 | | | | | |
| 25 | 50-54 | CC | 2034 | 20.18 | 0 | 0.00 | | | |
| 25 | 50-54 | Former | 2765 | 27.44 | | | 20.11 | 143 | 5.17 |

Year of Follow-up: 2015, Age Group: 55-59

| Period | Age Group | Smoking Group | N | % | Ever Quit (N) | Ever Quit % | Mean Yrs Quit | Short Term (n) | Short Term % |
|---|---|---|---|---|---|---|---|---|---|
| 25 | 55-59 | Never Any | 5282 | 50.31 | | | | | |
| 25 | 55-59 | CC | 1817 | 17.31 | 0 | 0.00 | | | |
| 25 | 55-59 | Former | 3400 | 32.38 | | | 20.73 | 148 | 4.35 |

Year of Follow-up: 2015, Age Group: 60-64

| Period | Age Group | Smoking Group | N | % | Ever Quit (N) | Ever Quit % | Mean Yrs Quit | Short Term (n) | Short Term % |
|---|---|---|---|---|---|---|---|---|---|
| 25 | 60-64 | Never Any | 5018 | 52.27 | | | | | |
| 25 | 60-64 | CC | 1220 | 12.71 | 0 | 0.00 | | | |
| 25 | 60-64 | Former | 3362 | 35.02 | | | 23.69 | 151 | 4.49 |

Year of Follow-up: 2015, Age Group: 65-69

| Period | Age Group | Smoking Group | N | % | Ever Quit (N) | Ever Quit % | Mean Yrs Quit | Short Term (n) | Short Term % |
|---|---|---|---|---|---|---|---|---|---|
| 25 | 65-69 | Never Any | 4356 | 51.27 | | | | | |
| 25 | 65-69 | CC | 840 | 9.89 | 0 | 0.00 | | | |
| 25 | 65-69 | Former | 3301 | 38.85 | | | 23.09 | 63 | 1.91 |

Year of Follow-up: 2015, Age Group: 70-74

| Period | Age Group | Smoking Group | N | % | Ever Quit (N) | Ever Quit % | Mean Yrs Quit | Short Term (n) | Short Term % |
|---|---|---|---|---|---|---|---|---|---|
| 25 | 70-74 | Never Any | 3116 | 47.67 | | | | | |
| 25 | 70-74 | CC | 565 | 8.64 | 0 | 0.00 | | | |
| 25 | 70-74 | Former | 2855 | 43.68 | | | 26.49 | 43 | 1.51 |

Year of Follow-up: 2015, Age Group: 75-79

| Period | Age Group | Smoking Group | N | % | Ever Quit (N) | Ever Quit % | Mean Yrs Quit | Short Term (n) | Short Term % |
|---|---|---|---|---|---|---|---|---|---|
| 25 | 75-79 | Never Any | 2684 | 48.50 | | | | | |
| 25 | 75-79 | CC | 419 | 7.57 | 0 | 0.00 | | | |
| 25 | 75-79 | Former | 2431 | 43.93 | | | 26.67 | 44 | 1.81 |

Year of Follow-up: 2020, Age Group: All Ages

| Period | Age Group | Smoking Group | N | % | Ever Quit (N) | Ever Quit % | Mean Yrs Quit | Short Term (n) | Short Term % |
|---|---|---|---|---|---|---|---|---|---|
| 30 | All Ages | Never Any | 38442 | 54.70 | | | | | |
| 30 | All Ages | CC | 9164 | 13.04 | 0 | 0.00 | | | |
| 30 | All Ages | Former | 22677 | 32.27 | | | 23.77 | 745 | 3.29 |

Year of Follow-up: 2020, Age Group: 18-19

| Period | Age Group | Smoking Group | N | % | Ever Quit (N) | Ever Quit % | Mean Yrs Quit | Short Term (n) | Short Term % |
|---|---|---|---|---|---|---|---|---|---|
| 30 | 18-19 | Never Any | 0 | . | | | | | |
| 30 | 18-19 | CC | 0 | . | 0 | . | | | |
| 30 | 18-19 | Former | 0 | . | | | . | 0 | . |

Year of Follow-up: 2020, Age Group: 20-24

| Period | Age Group | Smoking Group | N | % | Ever Quit (N) | Ever Quit % | Mean Yrs Quit | Short Term (n) | Short Term % |
|---|---|---|---|---|---|---|---|---|---|
| 30 | 20-24 | Never Any | 0 | . | | | | | |
| 30 | 20-24 | CC | 0 | . | 0 | . | | | |
| 30 | 20-24 | Former | 0 | . | | | . | 0 | . |

Year of Follow-up: 2020, Age Group: 25-29

| Period | Age Group | Smoking Group | N | % | Ever Quit (N) | Ever Quit % | Mean Yrs Quit | Short Term (n) | Short Term % |
|---|---|---|---|---|---|---|---|---|---|
| 30 | 25-29 | Never Any | 0 | . | | | | | |
| 30 | 25-29 | CC | 0 | . | 0 | . | | | |
| 30 | 25-29 | Former | 0 | . | | | . | 0 | . |

Year of Follow-up: 2020, Age Group: 30-34

| Period | Age Group | Smoking Group | N | % | Ever Quit (N) | Ever Quit % | Mean Yrs Quit | Short Term (n) | Short Term % |
|---|---|---|---|---|---|---|---|---|---|
| 30 | 30-34 | Never Any | 0 | . | | | | | |
| 30 | 30-34 | CC | 0 | . | 0 | . | | | |
| 30 | 30-34 | Former | 0 | . | | | . | 0 | . |

Year of Follow-up: 2020, Age Group: 35-39

| Period | Age Group | Smoking Group | N | % | Ever Quit (N) | Ever Quit % | Mean Yrs Quit | Short Term (n) | Short Term % |
|---|---|---|---|---|---|---|---|---|---|

# Results for PHIM for Run 2 (Main_Analysis.RTF)

| | | | | | | | | | |
|---|---|---|---|---|---|---|---|---|---|
| 30 | 35-39 | Never Any | 0 | . | | | | | |
| 30 | 35-39 | CC | 0 | . | 0 | . | | | |
| 30 | 35-39 | Former | 0 | . | | | . | 0 | . |

Year of Follow-up: 2020, Age Group: 40-44

| Period | Age Group | Smoking Group | N | % | Ever Quit (N) | Ever Quit % | Mean Yrs Quit | Short Term (n) | Short Term % |
|---|---|---|---|---|---|---|---|---|---|
| 30 | 40-44 | Never Any | 4803 | 60.57 | | | | | |
| 30 | 40-44 | CC | 1279 | 16.13 | 0 | 0.00 | | | |
| 30 | 40-44 | Former | 1848 | 23.30 | | | 14.45 | 108 | 5.84 |

Year of Follow-up: 2020, Age Group: 45-49

| Period | Age Group | Smoking Group | N | % | Ever Quit (N) | Ever Quit % | Mean Yrs Quit | Short Term (n) | Short Term % |
|---|---|---|---|---|---|---|---|---|---|
| 30 | 45-49 | Never Any | 5407 | 64.75 | | | | | |
| 30 | 45-49 | CC | 1247 | 14.93 | 0 | 0.00 | | | |
| 30 | 45-49 | Former | 1696 | 20.31 | | | 16.75 | 65 | 3.83 |

Year of Follow-up: 2020, Age Group: 50-54

| Period | Age Group | Smoking Group | N | % | Ever Quit (N) | Ever Quit % | Mean Yrs Quit | Short Term (n) | Short Term % |
|---|---|---|---|---|---|---|---|---|---|
| 30 | 50-54 | Never Any | 5181 | 58.92 | | | | | |
| 30 | 50-54 | CC | 1363 | 15.50 | 0 | 0.00 | | | |
| 30 | 50-54 | Former | 2249 | 25.58 | | | 20.22 | 115 | 5.11 |

Year of Follow-up: 2020, Age Group: 55-59

| Period | Age Group | Smoking Group | N | % | Ever Quit (N) | Ever Quit % | Mean Yrs Quit | Short Term (n) | Short Term % |
|---|---|---|---|---|---|---|---|---|---|
| 30 | 55-59 | Never Any | 5279 | 52.38 | | | | | |
| 30 | 55-59 | CC | 1698 | 16.85 | 0 | 0.00 | | | |
| 30 | 55-59 | Former | 3101 | 30.77 | | | 22.63 | 108 | 3.48 |

Year of Follow-up: 2020, Age Group: 60-64

| Period | Age Group | Smoking Group | N | % | Ever Quit (N) | Ever Quit % | Mean Yrs Quit | Short Term (n) | Short Term % |
|---|---|---|---|---|---|---|---|---|---|
| 30 | 60-64 | Never Any | 5282 | 50.31 | | | | | |
| 30 | 60-64 | CC | 1451 | 13.82 | 0 | 0.00 | | | |
| 30 | 60-64 | Former | 3766 | 35.87 | | | 23.40 | 173 | 4.59 |

Year of Follow-up: 2020, Age Group: 65-69

| Period | Age Group | Smoking Group | N | % | Ever Quit (N) | Ever Quit % | Mean Yrs Quit | Short Term (n) | Short Term % |
|---|---|---|---|---|---|---|---|---|---|
| 30 | 65-69 | Never Any | 5018 | 52.27 | | | | | |
| 30 | 65-69 | CC | 961 | 10.01 | 0 | 0.00 | | | |
| 30 | 65-69 | Former | 3621 | 37.72 | | | 26.81 | 78 | 2.15 |

Year of Follow-up: 2020, Age Group: 70-74

| Period | Age Group | Smoking Group | N | % | Ever Quit (N) | Ever Quit % | Mean Yrs Quit | Short Term (n) | Short Term % |
|---|---|---|---|---|---|---|---|---|---|
| 30 | 70-74 | Never Any | 4356 | 51.27 | | | | | |
| 30 | 70-74 | CC | 720 | 8.47 | 0 | 0.00 | | | |
| 30 | 70-74 | Former | 3421 | 40.26 | | | 27.18 | 45 | 1.32 |

Year of Follow-up: 2020, Age Group: 75-79

| Period | Age Group | Smoking Group | N | % | Ever Quit (N) | Ever Quit % | Mean Yrs Quit | Short Term (n) | Short Term % |
|---|---|---|---|---|---|---|---|---|---|
| 30 | 75-79 | Never Any | 3116 | 47.67 | | | | | |
| 30 | 75-79 | CC | 445 | 6.81 | 0 | 0.00 | | | |
| 30 | 75-79 | Former | 2975 | 45.52 | | | 30.30 | 53 | 1.78 |

Year of Follow-up: 2025, Age Group: All Ages

| Period | Age Group | Smoking Group | N | % | Ever Quit (N) | Ever Quit % | Mean Yrs Quit | Short Term (n) | Short Term % |
|---|---|---|---|---|---|---|---|---|---|
| 35 | All Ages | Never Any | 35326 | 55.42 | | | | | |
| 35 | All Ages | CC | 7117 | 11.16 | 0 | 0.00 | | | |
| 35 | All Ages | Former | 21304 | 33.42 | | | 25.85 | 631 | 2.96 |

Year of Follow-up: 2025, Age Group: 18-19

| Period | Age Group | Smoking Group | N | % | Ever Quit (N) | Ever Quit % | Mean Yrs Quit | Short Term (n) | Short Term % |
|---|---|---|---|---|---|---|---|---|---|
| 35 | 18-19 | Never Any | 0 | . | | | | | |
| 35 | 18-19 | CC | 0 | . | 0 | . | | | |
| 35 | 18-19 | Former | 0 | . | | | . | 0 | . |

Year of Follow-up: 2025, Age Group: 20-24

| Period | Age Group | Smoking Group | N | % | Ever Quit (N) | Ever Quit % | Mean Yrs Quit | Short Term (n) | Short Term % |
|---|---|---|---|---|---|---|---|---|---|
| 35 | 20-24 | Never Any | 0 | . | | | | | |
| 35 | 20-24 | CC | 0 | . | 0 | . | | | |
| 35 | 20-24 | Former | 0 | . | | | . | 0 | . |

Year of Follow-up: 2025, Age Group: 25-29

| Period | Age Group | Smoking Group | N | % | Ever Quit (N) | Ever Quit % | Mean Yrs Quit | Short Term (n) | Short Term % |
|---|---|---|---|---|---|---|---|---|---|
| 35 | 25-29 | Never Any | 0 | . | | | | | |
| 35 | 25-29 | CC | 0 | . | 0 | . | | | |
| 35 | 25-29 | Former | 0 | . | | | . | 0 | . |

Year of Follow-up: 2025, Age Group: 30-34

| Period | Age Group | Smoking Group | N | % | Ever Quit (N) | Ever Quit % | Mean Yrs Quit | Short Term (n) | Short Term % |
|---|---|---|---|---|---|---|---|---|---|

# Results for PHIM for Run 2 (Main_Analysis.RTF)

| | | | | | | | | | |
|---|---|---|---|---|---|---|---|---|---|
| 35 | 30-34 | Never Any | 0 | . | | | | | |
| 35 | 30-34 | CC | 0 | . | 0 | . | | | |
| 35 | 30-34 | Former | 0 | . | | | . | 0 | . |

Year of Follow-up: 2025, Age Group: 35-39

| Period | Age Group | Smoking Group | N | % | Ever Quit (N) | Ever Quit % | Mean Yrs Quit | Short Term (n) | Short Term % |
|---|---|---|---|---|---|---|---|---|---|
| 35 | 35-39 | Never Any | 0 | . | | | | | |
| 35 | 35-39 | CC | 0 | . | 0 | . | | | |
| 35 | 35-39 | Former | 0 | . | | | . | 0 | . |

Year of Follow-up: 2025, Age Group: 40-44

| Period | Age Group | Smoking Group | N | % | Ever Quit (N) | Ever Quit % | Mean Yrs Quit | Short Term (n) | Short Term % |
|---|---|---|---|---|---|---|---|---|---|
| 35 | 40-44 | Never Any | 0 | . | | | | | |
| 35 | 40-44 | CC | 0 | . | 0 | . | | | |
| 35 | 40-44 | Former | 0 | . | | | . | 0 | . |

Year of Follow-up: 2025, Age Group: 45-49

| Period | Age Group | Smoking Group | N | % | Ever Quit (N) | Ever Quit % | Mean Yrs Quit | Short Term (n) | Short Term % |
|---|---|---|---|---|---|---|---|---|---|
| 35 | 45-49 | Never Any | 4803 | 60.57 | | | | | |
| 35 | 45-49 | CC | 1077 | 13.58 | 0 | 0.00 | | | |
| 35 | 45-49 | Former | 2050 | 25.85 | | | 17.75 | 79 | 3.85 |

Year of Follow-up: 2025, Age Group: 50-54

| Period | Age Group | Smoking Group | N | % | Ever Quit (N) | Ever Quit % | Mean Yrs Quit | Short Term (n) | Short Term % |
|---|---|---|---|---|---|---|---|---|---|
| 35 | 50-54 | Never Any | 5407 | 64.75 | | | | | |
| 35 | 50-54 | CC | 1071 | 12.83 | 0 | 0.00 | | | |
| 35 | 50-54 | Former | 1872 | 22.42 | | | 19.88 | 75 | 4.01 |

Year of Follow-up: 2025, Age Group: 55-59

| Period | Age Group | Smoking Group | N | % | Ever Quit (N) | Ever Quit % | Mean Yrs Quit | Short Term (n) | Short Term % |
|---|---|---|---|---|---|---|---|---|---|
| 35 | 55-59 | Never Any | 5181 | 58.92 | | | | | |
| 35 | 55-59 | CC | 1098 | 12.49 | 0 | 0.00 | | | |
| 35 | 55-59 | Former | 2514 | 28.59 | | | 22.77 | 110 | 4.38 |

Year of Follow-up: 2025, Age Group: 60-64

| Period | Age Group | Smoking Group | N | % | Ever Quit (N) | Ever Quit % | Mean Yrs Quit | Short Term (n) | Short Term % |
|---|---|---|---|---|---|---|---|---|---|
| 35 | 60-64 | Never Any | 5279 | 52.38 | | | | | |
| 35 | 60-64 | CC | 1327 | 13.17 | 0 | 0.00 | | | |
| 35 | 60-64 | Former | 3472 | 34.45 | | | 24.88 | 166 | 4.78 |

Year of Follow-up: 2025, Age Group: 65-69

| Period | Age Group | Smoking Group | N | % | Ever Quit (N) | Ever Quit % | Mean Yrs Quit | Short Term (n) | Short Term % |
|---|---|---|---|---|---|---|---|---|---|
| 35 | 65-69 | Never Any | 5282 | 50.31 | | | | | |
| 35 | 65-69 | CC | 1147 | 10.92 | 0 | 0.00 | | | |
| 35 | 65-69 | Former | 4070 | 38.77 | | | 26.47 | 82 | 2.01 |

Year of Follow-up: 2025, Age Group: 70-74

| Period | Age Group | Smoking Group | N | % | Ever Quit (N) | Ever Quit % | Mean Yrs Quit | Short Term (n) | Short Term % |
|---|---|---|---|---|---|---|---|---|---|
| 35 | 70-74 | Never Any | 5018 | 52.27 | | | | | |
| 35 | 70-74 | CC | 817 | 8.51 | 0 | 0.00 | | | |
| 35 | 70-74 | Former | 3765 | 39.22 | | | 30.66 | 59 | 1.57 |

Year of Follow-up: 2025, Age Group: 75-79

| Period | Age Group | Smoking Group | N | % | Ever Quit (N) | Ever Quit % | Mean Yrs Quit | Short Term (n) | Short Term % |
|---|---|---|---|---|---|---|---|---|---|
| 35 | 75-79 | Never Any | 4356 | 51.27 | | | | | |
| 35 | 75-79 | CC | 580 | 6.83 | 0 | 0.00 | | | |
| 35 | 75-79 | Former | 3561 | 41.91 | | | 30.99 | 60 | 1.68 |

Year of Follow-up: 2030, Age Group: All Ages

| Period | Age Group | Smoking Group | N | % | Ever Quit (N) | Ever Quit % | Mean Yrs Quit | Short Term (n) | Short Term % |
|---|---|---|---|---|---|---|---|---|---|
| 40 | All Ages | Never Any | 30970 | 56.05 | | | | | |
| 40 | All Ages | CC | 5318 | 9.63 | 0 | 0.00 | | | |
| 40 | All Ages | Former | 18962 | 34.32 | | | 28.04 | 458 | 2.42 |

Year of Follow-up: 2030, Age Group: 18-19

| Period | Age Group | Smoking Group | N | % | Ever Quit (N) | Ever Quit % | Mean Yrs Quit | Short Term (n) | Short Term % |
|---|---|---|---|---|---|---|---|---|---|
| 40 | 18-19 | Never Any | 0 | . | | | | | |
| 40 | 18-19 | CC | 0 | . | 0 | . | | | |
| 40 | 18-19 | Former | 0 | . | | | . | 0 | . |

Year of Follow-up: 2030, Age Group: 20-24

| Period | Age Group | Smoking Group | N | % | Ever Quit (N) | Ever Quit % | Mean Yrs Quit | Short Term (n) | Short Term % |
|---|---|---|---|---|---|---|---|---|---|
| 40 | 20-24 | Never Any | 0 | . | | | | | |
| 40 | 20-24 | CC | 0 | . | 0 | . | | | |
| 40 | 20-24 | Former | 0 | . | | | . | 0 | . |

Year of Follow-up: 2030, Age Group: 25-29

| Period | Age Group | Smoking Group | N | % | Ever Quit (N) | Ever Quit % | Mean Yrs Quit | Short Term (n) | Short Term % |
|---|---|---|---|---|---|---|---|---|---|

# Results for PHIM for Run 2 (Main_Analysis.RTF)

| | | | | | | | | | |
|---|---|---|---|---|---|---|---|---|---|
| 40 | 25-29 | Never Any | 0 | . | | | | | |
| 40 | 25-29 | CC | 0 | . | 0 | . | | | |
| 40 | 25-29 | Former | 0 | . | | | . | 0 | . |

Year of Follow-up: 2030, Age Group: 30-34

| Period | Age Group | Smoking Group | N | % | Ever Quit (N) | Ever Quit % | Mean Yrs Quit | Short Term (n) | Short Term % |
|---|---|---|---|---|---|---|---|---|---|
| 40 | 30-34 | Never Any | 0 | . | | | | | |
| 40 | 30-34 | CC | 0 | . | 0 | . | | | |
| 40 | 30-34 | Former | 0 | . | | | . | 0 | . |

Year of Follow-up: 2030, Age Group: 35-39

| Period | Age Group | Smoking Group | N | % | Ever Quit (N) | Ever Quit % | Mean Yrs Quit | Short Term (n) | Short Term % |
|---|---|---|---|---|---|---|---|---|---|
| 40 | 35-39 | Never Any | 0 | . | | | | | |
| 40 | 35-39 | CC | 0 | . | 0 | . | | | |
| 40 | 35-39 | Former | 0 | . | | | . | 0 | . |

Year of Follow-up: 2030, Age Group: 40-44

| Period | Age Group | Smoking Group | N | % | Ever Quit (N) | Ever Quit % | Mean Yrs Quit | Short Term (n) | Short Term % |
|---|---|---|---|---|---|---|---|---|---|
| 40 | 40-44 | Never Any | 0 | . | | | | | |
| 40 | 40-44 | CC | 0 | . | 0 | . | | | |
| 40 | 40-44 | Former | 0 | . | | | . | 0 | . |

Year of Follow-up: 2030, Age Group: 45-49

| Period | Age Group | Smoking Group | N | % | Ever Quit (N) | Ever Quit % | Mean Yrs Quit | Short Term (n) | Short Term % |
|---|---|---|---|---|---|---|---|---|---|
| 40 | 45-49 | Never Any | 0 | . | | | | | |
| 40 | 45-49 | CC | 0 | . | 0 | . | | | |
| 40 | 45-49 | Former | 0 | . | | | . | 0 | . |

Year of Follow-up: 2030, Age Group: 50-54

| Period | Age Group | Smoking Group | N | % | Ever Quit (N) | Ever Quit % | Mean Yrs Quit | Short Term (n) | Short Term % |
|---|---|---|---|---|---|---|---|---|---|
| 40 | 50-54 | Never Any | 4803 | 60.57 | | | | | |
| 40 | 50-54 | CC | 918 | 11.58 | 0 | 0.00 | | | |
| 40 | 50-54 | Former | 2209 | 27.86 | | | 21.26 | 61 | 2.76 |

Year of Follow-up: 2030, Age Group: 55-59

| Period | Age Group | Smoking Group | N | % | Ever Quit (N) | Ever Quit % | Mean Yrs Quit | Short Term (n) | Short Term % |
|---|---|---|---|---|---|---|---|---|---|
| 40 | 55-59 | Never Any | 5407 | 64.75 | | | | | |
| 40 | 55-59 | CC | 881 | 10.55 | 0 | 0.00 | | | |
| 40 | 55-59 | Former | 2062 | 24.69 | | | 22.78 | 70 | 3.39 |

Year of Follow-up: 2030, Age Group: 60-64

| Period | Age Group | Smoking Group | N | % | Ever Quit (N) | Ever Quit % | Mean Yrs Quit | Short Term (n) | Short Term % |
|---|---|---|---|---|---|---|---|---|---|
| 40 | 60-64 | Never Any | 5181 | 58.92 | | | | | |
| 40 | 60-64 | CC | 870 | 9.89 | 0 | 0.00 | | | |
| 40 | 60-64 | Former | 2742 | 31.18 | | | 25.61 | 111 | 4.05 |

Year of Follow-up: 2030, Age Group: 65-69

| Period | Age Group | Smoking Group | N | % | Ever Quit (N) | Ever Quit % | Mean Yrs Quit | Short Term (n) | Short Term % |
|---|---|---|---|---|---|---|---|---|---|
| 40 | 65-69 | Never Any | 5279 | 52.38 | | | | | |
| 40 | 65-69 | CC | 1041 | 10.33 | 0 | 0.00 | | | |
| 40 | 65-69 | Former | 3758 | 37.29 | | | 27.80 | 71 | 1.89 |

Year of Follow-up: 2030, Age Group: 70-74

| Period | Age Group | Smoking Group | N | % | Ever Quit (N) | Ever Quit % | Mean Yrs Quit | Short Term (n) | Short Term % |
|---|---|---|---|---|---|---|---|---|---|
| 40 | 70-74 | Never Any | 5282 | 50.31 | | | | | |
| 40 | 70-74 | CC | 965 | 9.19 | 0 | 0.00 | | | |
| 40 | 70-74 | Former | 4252 | 40.50 | | | 30.20 | 80 | 1.88 |

Year of Follow-up: 2030, Age Group: 75-79

| Period | Age Group | Smoking Group | N | % | Ever Quit (N) | Ever Quit % | Mean Yrs Quit | Short Term (n) | Short Term % |
|---|---|---|---|---|---|---|---|---|---|
| 40 | 75-79 | Never Any | 5018 | 52.27 | | | | | |
| 40 | 75-79 | CC | 643 | 6.70 | 0 | 0.00 | | | |
| 40 | 75-79 | Former | 3939 | 41.03 | | | 34.18 | 65 | 1.65 |

Year of Follow-up: 2035, Age Group: All Ages

| Period | Age Group | Smoking Group | N | % | Ever Quit (N) | Ever Quit % | Mean Yrs Quit | Short Term (n) | Short Term % |
|---|---|---|---|---|---|---|---|---|---|
| 45 | All Ages | Never Any | 25952 | 56.85 | | | | | |
| 45 | All Ages | CC | 3767 | 8.25 | 0 | 0.00 | | | |
| 45 | All Ages | Former | 15931 | 34.90 | | | 29.76 | 350 | 2.20 |

Year of Follow-up: 2035, Age Group: 18-19

| Period | Age Group | Smoking Group | N | % | Ever Quit (N) | Ever Quit % | Mean Yrs Quit | Short Term (n) | Short Term % |
|---|---|---|---|---|---|---|---|---|---|
| 45 | 18-19 | Never Any | 0 | . | | | | | |
| 45 | 18-19 | CC | 0 | . | 0 | . | | | |
| 45 | 18-19 | Former | 0 | . | | | . | 0 | . |

Year of Follow-up: 2035, Age Group: 20-24

| Period | Age Group | Smoking Group | N | % | Ever Quit (N) | Ever Quit % | Mean Yrs Quit | Short Term (n) | Short Term % |
|---|---|---|---|---|---|---|---|---|---|

| | | | | | | | | | |
|---|---|---|---|---|---|---|---|---|---|
| 45 | 20-24 | Never Any | 0 | . | | | | | |
| 45 | 20-24 | CC | 0 | . | 0 | . | | | |
| 45 | 20-24 | Former | 0 | . | | | . | 0 | . |

Year of Follow-up: 2035, Age Group: 25-29

| Period | Age Group | Smoking Group | N | % | Ever Quit (N) | Ever Quit % | Mean Yrs Quit | Short Term (n) | Short Term % |
|---|---|---|---|---|---|---|---|---|---|
| 45 | 25-29 | Never Any | 0 | . | | | | | |
| 45 | 25-29 | CC | 0 | . | 0 | . | | | |
| 45 | 25-29 | Former | 0 | . | | | . | 0 | . |

Year of Follow-up: 2035, Age Group: 30-34

| Period | Age Group | Smoking Group | N | % | Ever Quit (N) | Ever Quit % | Mean Yrs Quit | Short Term (n) | Short Term % |
|---|---|---|---|---|---|---|---|---|---|
| 45 | 30-34 | Never Any | 0 | . | | | | | |
| 45 | 30-34 | CC | 0 | . | 0 | . | | | |
| 45 | 30-34 | Former | 0 | . | | | . | 0 | . |

Year of Follow-up: 2035, Age Group: 35-39

| Period | Age Group | Smoking Group | N | % | Ever Quit (N) | Ever Quit % | Mean Yrs Quit | Short Term (n) | Short Term % |
|---|---|---|---|---|---|---|---|---|---|
| 45 | 35-39 | Never Any | 0 | . | | | | | |
| 45 | 35-39 | CC | 0 | . | 0 | . | | | |
| 45 | 35-39 | Former | 0 | . | | | . | 0 | . |

Year of Follow-up: 2035, Age Group: 40-44

| Period | Age Group | Smoking Group | N | % | Ever Quit (N) | Ever Quit % | Mean Yrs Quit | Short Term (n) | Short Term % |
|---|---|---|---|---|---|---|---|---|---|
| 45 | 40-44 | Never Any | 0 | . | | | | | |
| 45 | 40-44 | CC | 0 | . | 0 | . | | | |
| 45 | 40-44 | Former | 0 | . | | | . | 0 | . |

Year of Follow-up: 2035, Age Group: 45-49

| Period | Age Group | Smoking Group | N | % | Ever Quit (N) | Ever Quit % | Mean Yrs Quit | Short Term (n) | Short Term % |
|---|---|---|---|---|---|---|---|---|---|
| 45 | 45-49 | Never Any | 0 | . | | | | | |
| 45 | 45-49 | CC | 0 | . | 0 | . | | | |
| 45 | 45-49 | Former | 0 | . | | | . | 0 | . |

Year of Follow-up: 2035, Age Group: 50-54

| Period | Age Group | Smoking Group | N | % | Ever Quit (N) | Ever Quit % | Mean Yrs Quit | Short Term (n) | Short Term % |
|---|---|---|---|---|---|---|---|---|---|
| 45 | 50-54 | Never Any | 0 | . | | | | | |
| 45 | 50-54 | CC | 0 | . | 0 | . | | | |
| 45 | 50-54 | Former | 0 | . | | | . | 0 | . |

Year of Follow-up: 2035, Age Group: 55-59

| Period | Age Group | Smoking Group | N | % | Ever Quit (N) | Ever Quit % | Mean Yrs Quit | Short Term (n) | Short Term % |
|---|---|---|---|---|---|---|---|---|---|
| 45 | 55-59 | Never Any | 4803 | 60.57 | | | | | |
| 45 | 55-59 | CC | 763 | 9.62 | 0 | 0.00 | | | |
| 45 | 55-59 | Former | 2364 | 29.81 | | | 24.66 | 71 | 3.00 |

Year of Follow-up: 2035, Age Group: 60-64

| Period | Age Group | Smoking Group | N | % | Ever Quit (N) | Ever Quit % | Mean Yrs Quit | Short Term (n) | Short Term % |
|---|---|---|---|---|---|---|---|---|---|
| 45 | 60-64 | Never Any | 5407 | 64.75 | | | | | |
| 45 | 60-64 | CC | 691 | 8.28 | 0 | 0.00 | | | |
| 45 | 60-64 | Former | 2252 | 26.97 | | | 25.59 | 91 | 4.04 |

Year of Follow-up: 2035, Age Group: 65-69

| Period | Age Group | Smoking Group | N | % | Ever Quit (N) | Ever Quit % | Mean Yrs Quit | Short Term (n) | Short Term % |
|---|---|---|---|---|---|---|---|---|---|
| 45 | 65-69 | Never Any | 5181 | 58.92 | | | | | |
| 45 | 65-69 | CC | 702 | 7.98 | 0 | 0.00 | | | |
| 45 | 65-69 | Former | 2910 | 33.09 | | | 28.98 | 48 | 1.65 |

Year of Follow-up: 2035, Age Group: 70-74

| Period | Age Group | Smoking Group | N | % | Ever Quit (N) | Ever Quit % | Mean Yrs Quit | Short Term (n) | Short Term % |
|---|---|---|---|---|---|---|---|---|---|
| 45 | 70-74 | Never Any | 5279 | 52.38 | | | | | |
| 45 | 70-74 | CC | 880 | 8.73 | 0 | 0.00 | | | |
| 45 | 70-74 | Former | 3919 | 38.89 | | | 31.53 | 68 | 1.74 |

Year of Follow-up: 2035, Age Group: 75-79

| Period | Age Group | Smoking Group | N | % | Ever Quit (N) | Ever Quit % | Mean Yrs Quit | Short Term (n) | Short Term % |
|---|---|---|---|---|---|---|---|---|---|
| 45 | 75-79 | Never Any | 5282 | 50.31 | | | | | |
| 45 | 75-79 | CC | 731 | 6.96 | 0 | 0.00 | | | |
| 45 | 75-79 | Former | 4486 | 42.73 | | | 33.48 | 72 | 1.60 |

Year of Follow-up: 2040, Age Group: All Ages

| Period | Age Group | Smoking Group | N | % | Ever Quit (N) | Ever Quit % | Mean Yrs Quit | Short Term (n) | Short Term % |
|---|---|---|---|---|---|---|---|---|---|
| 50 | All Ages | Never Any | 20670 | 58.80 | | | | | |
| 50 | All Ages | CC | 2462 | 7.00 | 0 | 0.00 | | | |
| 50 | All Ages | Former | 12019 | 34.19 | | | 31.80 | 230 | 1.91 |

Year of Follow-up: 2040, Age Group: 18-19

| Period | Age Group | Smoking Group | N | % | Ever Quit (N) | Ever Quit % | Mean Yrs Quit | Short Term (n) | Short Term % |
|---|---|---|---|---|---|---|---|---|---|

# Results for PHIM for Run 2 (Main_Analysis.RTF)

| | | | | | | | | | |
|---|---|---|---|---|---|---|---|---|---|
| 50 | 18-19 | Never Any | 0 | . | | | | | |
| 50 | 18-19 | CC | 0 | . | 0 | . | | | |
| 50 | 18-19 | Former | 0 | . | | | . | 0 | . |

Year of Follow-up: 2040, Age Group: 20-24

| Period | Age Group | Smoking Group | N | % | Ever Quit (N) | Ever Quit % | Mean Yrs Quit | Short Term (n) | Short Term % |
|---|---|---|---|---|---|---|---|---|---|
| 50 | 20-24 | Never Any | 0 | . | | | | | |
| 50 | 20-24 | CC | 0 | . | 0 | . | | | |
| 50 | 20-24 | Former | 0 | . | | | . | 0 | . |

Year of Follow-up: 2040, Age Group: 25-29

| Period | Age Group | Smoking Group | N | % | Ever Quit (N) | Ever Quit % | Mean Yrs Quit | Short Term (n) | Short Term % |
|---|---|---|---|---|---|---|---|---|---|
| 50 | 25-29 | Never Any | 0 | . | | | | | |
| 50 | 25-29 | CC | 0 | . | 0 | . | | | |
| 50 | 25-29 | Former | 0 | . | | | . | 0 | . |

Year of Follow-up: 2040, Age Group: 30-34

| Period | Age Group | Smoking Group | N | % | Ever Quit (N) | Ever Quit % | Mean Yrs Quit | Short Term (n) | Short Term % |
|---|---|---|---|---|---|---|---|---|---|
| 50 | 30-34 | Never Any | 0 | . | | | | | |
| 50 | 30-34 | CC | 0 | . | 0 | . | | | |
| 50 | 30-34 | Former | 0 | . | | | . | 0 | . |

Year of Follow-up: 2040, Age Group: 35-39

| Period | Age Group | Smoking Group | N | % | Ever Quit (N) | Ever Quit % | Mean Yrs Quit | Short Term (n) | Short Term % |
|---|---|---|---|---|---|---|---|---|---|
| 50 | 35-39 | Never Any | 0 | . | | | | | |
| 50 | 35-39 | CC | 0 | . | 0 | . | | | |
| 50 | 35-39 | Former | 0 | . | | | . | 0 | . |

Year of Follow-up: 2040, Age Group: 40-44

| Period | Age Group | Smoking Group | N | % | Ever Quit (N) | Ever Quit % | Mean Yrs Quit | Short Term (n) | Short Term % |
|---|---|---|---|---|---|---|---|---|---|
| 50 | 40-44 | Never Any | 0 | . | | | | | |
| 50 | 40-44 | CC | 0 | . | 0 | . | | | |
| 50 | 40-44 | Former | 0 | . | | | . | 0 | . |

Year of Follow-up: 2040, Age Group: 45-49

| Period | Age Group | Smoking Group | N | % | Ever Quit (N) | Ever Quit % | Mean Yrs Quit | Short Term (n) | Short Term % |
|---|---|---|---|---|---|---|---|---|---|
| 50 | 45-49 | Never Any | 0 | . | | | | | |
| 50 | 45-49 | CC | 0 | . | 0 | . | | | |
| 50 | 45-49 | Former | 0 | . | | | . | 0 | . |

Year of Follow-up: 2040, Age Group: 50-54

| Period | Age Group | Smoking Group | N | % | Ever Quit (N) | Ever Quit % | Mean Yrs Quit | Short Term (n) | Short Term % |
|---|---|---|---|---|---|---|---|---|---|
| 50 | 50-54 | Never Any | 0 | . | | | | | |
| 50 | 50-54 | CC | 0 | . | 0 | . | | | |
| 50 | 50-54 | Former | 0 | . | | | . | 0 | . |

Year of Follow-up: 2040, Age Group: 55-59

| Period | Age Group | Smoking Group | N | % | Ever Quit (N) | Ever Quit % | Mean Yrs Quit | Short Term (n) | Short Term % |
|---|---|---|---|---|---|---|---|---|---|
| 50 | 55-59 | Never Any | 0 | . | | | | | |
| 50 | 55-59 | CC | 0 | . | 0 | . | | | |
| 50 | 55-59 | Former | 0 | . | | | . | 0 | . |

Year of Follow-up: 2040, Age Group: 60-64

| Period | Age Group | Smoking Group | N | % | Ever Quit (N) | Ever Quit % | Mean Yrs Quit | Short Term (n) | Short Term % |
|---|---|---|---|---|---|---|---|---|---|
| 50 | 60-64 | Never Any | 4803 | 60.57 | | | | | |
| 50 | 60-64 | CC | 621 | 7.83 | 0 | 0.00 | | | |
| 50 | 60-64 | Former | 2506 | 31.60 | | | 28.08 | 64 | 2.55 |

Year of Follow-up: 2040, Age Group: 65-69

| Period | Age Group | Smoking Group | N | % | Ever Quit (N) | Ever Quit % | Mean Yrs Quit | Short Term (n) | Short Term % |
|---|---|---|---|---|---|---|---|---|---|
| 50 | 65-69 | Never Any | 5407 | 64.75 | | | | | |
| 50 | 65-69 | CC | 538 | 6.44 | 0 | 0.00 | | | |
| 50 | 65-69 | Former | 2405 | 28.80 | | | 28.80 | 40 | 1.66 |

Year of Follow-up: 2040, Age Group: 70-74

| Period | Age Group | Smoking Group | N | % | Ever Quit (N) | Ever Quit % | Mean Yrs Quit | Short Term (n) | Short Term % |
|---|---|---|---|---|---|---|---|---|---|
| 50 | 70-74 | Never Any | 5181 | 58.92 | | | | | |
| 50 | 70-74 | CC | 605 | 6.88 | 0 | 0.00 | | | |
| 50 | 70-74 | Former | 3007 | 34.20 | | | 32.94 | 48 | 1.60 |

Year of Follow-up: 2040, Age Group: 75-79

| Period | Age Group | Smoking Group | N | % | Ever Quit (N) | Ever Quit % | Mean Yrs Quit | Short Term (n) | Short Term % |
|---|---|---|---|---|---|---|---|---|---|
| 50 | 75-79 | Never Any | 5279 | 52.38 | | | | | |
| 50 | 75-79 | CC | 698 | 6.93 | 0 | 0.00 | | | |
| 50 | 75-79 | Former | 4101 | 40.69 | | | 34.99 | 78 | 1.90 |

# Results for PHIM for Run 2 (Main_Analysis.RTF)

************************************************************************************
**Main Output for the Tobacco History for Run: 2, Simulation: 1, Scenario: Ecig**

| Variable Parameter | Value |
|---|---|
| 1. Country | US (United States) |
| 2. Sex | F (Females) |
| 3. Year of start of process | 1990 |

Year of Follow-up: 1990, Age Group: All Ages

| Period | Age Group | Smoking Group | N | % | Ever Quit (N) | Ever Quit % | Mean Yrs Quit | Short Term (n) | Short Term % |
|---|---|---|---|---|---|---|---|---|---|
| 0 | All Ages | Never Any | 47776 | 54.92 | | | | | |
| 0 | All Ages | CC | 22737 | 26.14 | 0 | 0.00 | | | |
| 0 | All Ages | Ecig | 0 | 0.00 | | | | | |
| 0 | All Ages | Dual | 0 | 0.00 | | | | | |
| 0 | All Ages | Former | 16484 | 18.95 | | | 11.90 | 2815 | 17.08 |

Year of Follow-up: 1990, Age Group: 18-19

| Period | Age Group | Smoking Group | N | % | Ever Quit (N) | Ever Quit % | Mean Yrs Quit | Short Term (n) | Short Term % |
|---|---|---|---|---|---|---|---|---|---|
| 0 | 18-19 | Never Any | 2570 | 78.43 | | | | | |
| 0 | 18-19 | CC | 535 | 16.33 | 0 | 0.00 | | | |
| 0 | 18-19 | Ecig | 0 | 0.00 | | | | | |
| 0 | 18-19 | Dual | 0 | 0.00 | | | | | |
| 0 | 18-19 | Former | 172 | 5.25 | | | 0.20 | 172 | 100.00 |

Year of Follow-up: 1990, Age Group: 20-24

| Period | Age Group | Smoking Group | N | % | Ever Quit (N) | Ever Quit % | Mean Yrs Quit | Short Term (n) | Short Term % |
|---|---|---|---|---|---|---|---|---|---|
| 0 | 20-24 | Never Any | 5354 | 60.89 | | | | | |
| 0 | 20-24 | CC | 2560 | 29.11 | 0 | 0.00 | | | |
| 0 | 20-24 | Ecig | 0 | 0.00 | | | | | |
| 0 | 20-24 | Dual | 0 | 0.00 | | | | | |
| 0 | 20-24 | Former | 879 | 10.00 | | | 1.58 | 420 | 47.78 |

Year of Follow-up: 1990, Age Group: 25-29

| Period | Age Group | Smoking Group | N | % | Ever Quit (N) | Ever Quit % | Mean Yrs Quit | Short Term (n) | Short Term % |
|---|---|---|---|---|---|---|---|---|---|
| 0 | 25-29 | Never Any | 5318 | 52.77 | | | | | |
| 0 | 25-29 | CC | 3335 | 33.09 | 0 | 0.00 | | | |
| 0 | 25-29 | Ecig | 0 | 0.00 | | | | | |
| 0 | 25-29 | Dual | 0 | 0.00 | | | | | |
| 0 | 25-29 | Former | 1425 | 14.14 | | | 3.91 | 404 | 28.35 |

Year of Follow-up: 1990, Age Group: 30-34

| Period | Age Group | Smoking Group | N | % | Ever Quit (N) | Ever Quit % | Mean Yrs Quit | Short Term (n) | Short Term % |
|---|---|---|---|---|---|---|---|---|---|
| 0 | 30-34 | Never Any | 5287 | 50.36 | | | | | |
| 0 | 30-34 | CC | 3326 | 31.68 | 0 | 0.00 | | | |
| 0 | 30-34 | Ecig | 0 | 0.00 | | | | | |
| 0 | 30-34 | Dual | 0 | 0.00 | | | | | |
| 0 | 30-34 | Former | 1886 | 17.96 | | | 4.36 | 566 | 30.01 |

Year of Follow-up: 1990, Age Group: 35-39

| Period | Age Group | Smoking Group | N | % | Ever Quit (N) | Ever Quit % | Mean Yrs Quit | Short Term (n) | Short Term % |
|---|---|---|---|---|---|---|---|---|---|
| 0 | 35-39 | Never Any | 5018 | 52.27 | | | | | |
| 0 | 35-39 | CC | 2653 | 27.64 | 0 | 0.00 | | | |
| 0 | 35-39 | Ecig | 0 | 0.00 | | | | | |
| 0 | 35-39 | Dual | 0 | 0.00 | | | | | |
| 0 | 35-39 | Former | 1929 | 20.09 | | | 7.96 | 324 | 16.80 |

Year of Follow-up: 1990, Age Group: 40-44

| Period | Age Group | Smoking Group | N | % | Ever Quit (N) | Ever Quit % | Mean Yrs Quit | Short Term (n) | Short Term % |
|---|---|---|---|---|---|---|---|---|---|
| 0 | 40-44 | Never Any | 4356 | 51.27 | | | | | |
| 0 | 40-44 | CC | 2397 | 28.21 | 0 | 0.00 | | | |
| 0 | 40-44 | Ecig | 0 | 0.00 | | | | | |
| 0 | 40-44 | Dual | 0 | 0.00 | | | | | |
| 0 | 40-44 | Former | 1744 | 20.52 | | | 7.33 | 339 | 19.44 |

Year of Follow-up: 1990, Age Group: 45-49

| Period | Age Group | Smoking Group | N | % | Ever Quit (N) | Ever Quit % | Mean Yrs Quit | Short Term (n) | Short Term % |
|---|---|---|---|---|---|---|---|---|---|
| 0 | 45-49 | Never Any | 3116 | 47.67 | | | | | |
| 0 | 45-49 | CC | 1875 | 28.69 | 0 | 0.00 | | | |
| 0 | 45-49 | Ecig | 0 | 0.00 | | | | | |
| 0 | 45-49 | Dual | 0 | 0.00 | | | | | |
| 0 | 45-49 | Former | 1545 | 23.64 | | | 12.55 | 194 | 12.56 |

Year of Follow-up: 1990, Age Group: 50-54

| Period | Age Group | Smoking Group | N | % | Ever Quit (N) | Ever Quit % | Mean Yrs Quit | Short Term (n) | Short Term % |
|---|---|---|---|---|---|---|---|---|---|
| 0 | 50-54 | Never Any | 2684 | 48.50 | | | | | |
| 0 | 50-54 | CC | 1572 | 28.41 | 0 | 0.00 | | | |

# Results for PHIM for Run 2 (Main_Analysis.RTF)

| | | | | | | | | | |
|---|---|---|---|---|---|---|---|---|---|
| 0 | 50-54 | Ecig | 0 | 0.00 | | | | | |
| 0 | 50-54 | Dual | 0 | 0.00 | | | | | |
| 0 | 50-54 | Former | 1278 | 23.09 | | | 13.28 | 126 | 9.86 |

Year of Follow-up: 1990, Age Group: 55-59

| Period | Age Group | Smoking Group | N | % | Ever Quit (N) | Ever Quit % | Mean Yrs Quit | Short Term (n) | Short Term % |
|---|---|---|---|---|---|---|---|---|---|
| 0 | 55-59 | Never Any | 2732 | 51.90 | | | | | |
| 0 | 55-59 | CC | 1295 | 24.60 | 0 | 0.00 | | | |
| 0 | 55-59 | Ecig | 0 | 0.00 | | | | | |
| 0 | 55-59 | Dual | 0 | 0.00 | | | | | |
| 0 | 55-59 | Former | 1237 | 23.50 | | | 17.26 | 90 | 7.28 |

Year of Follow-up: 1990, Age Group: 60-64

| Period | Age Group | Smoking Group | N | % | Ever Quit (N) | Ever Quit % | Mean Yrs Quit | Short Term (n) | Short Term % |
|---|---|---|---|---|---|---|---|---|---|
| 0 | 60-64 | Never Any | 3078 | 55.72 | | | | | |
| 0 | 60-64 | CC | 1249 | 22.61 | 0 | 0.00 | | | |
| 0 | 60-64 | Ecig | 0 | 0.00 | | | | | |
| 0 | 60-64 | Dual | 0 | 0.00 | | | | | |
| 0 | 60-64 | Former | 1197 | 21.67 | | | 18.28 | 61 | 5.10 |

Year of Follow-up: 1990, Age Group: 65-69

| Period | Age Group | Smoking Group | N | % | Ever Quit (N) | Ever Quit % | Mean Yrs Quit | Short Term (n) | Short Term % |
|---|---|---|---|---|---|---|---|---|---|
| 0 | 65-69 | Never Any | 3042 | 57.36 | | | | | |
| 0 | 65-69 | CC | 976 | 18.40 | 0 | 0.00 | | | |
| 0 | 65-69 | Ecig | 0 | 0.00 | | | | | |
| 0 | 65-69 | Dual | 0 | 0.00 | | | | | |
| 0 | 65-69 | Former | 1285 | 24.23 | | | 20.65 | 68 | 5.29 |

Year of Follow-up: 1990, Age Group: 70-74

| Period | Age Group | Smoking Group | N | % | Ever Quit (N) | Ever Quit % | Mean Yrs Quit | Short Term (n) | Short Term % |
|---|---|---|---|---|---|---|---|---|---|
| 0 | 70-74 | Never Any | 2738 | 61.05 | | | | | |
| 0 | 70-74 | CC | 610 | 13.60 | 0 | 0.00 | | | |
| 0 | 70-74 | Ecig | 0 | 0.00 | | | | | |
| 0 | 70-74 | Dual | 0 | 0.00 | | | | | |
| 0 | 70-74 | Former | 1137 | 25.35 | | | 24.48 | 27 | 2.37 |

Year of Follow-up: 1990, Age Group: 75-79

| Period | Age Group | Smoking Group | N | % | Ever Quit (N) | Ever Quit % | Mean Yrs Quit | Short Term (n) | Short Term % |
|---|---|---|---|---|---|---|---|---|---|
| 0 | 75-79 | Never Any | 2483 | 68.84 | | | | | |
| 0 | 75-79 | CC | 354 | 9.81 | 0 | 0.00 | | | |
| 0 | 75-79 | Ecig | 0 | 0.00 | | | | | |
| 0 | 75-79 | Dual | 0 | 0.00 | | | | | |
| 0 | 75-79 | Former | 770 | 21.35 | | | 24.48 | 24 | 3.12 |

Year of Follow-up: 1995, Age Group: All Ages

| Period | Age Group | Smoking Group | N | % | Ever Quit (N) | Ever Quit % | Mean Yrs Quit | Short Term (n) | Short Term % |
|---|---|---|---|---|---|---|---|---|---|
| 5 | All Ages | Never Any | 50887 | 55.53 | | | | | |
| 5 | All Ages | CC | 10659 | 11.63 | 0 | 0.00 | | | |
| 5 | All Ages | Ecig | 11697 | 12.76 | | | | | |
| 5 | All Ages | Dual | 0 | 0.00 | | | | | |
| 5 | All Ages | Former | 18396 | 20.07 | | | 14.19 | 1129 | 6.14 |

Year of Follow-up: 1995, Age Group: 18-19

| Period | Age Group | Smoking Group | N | % | Ever Quit (N) | Ever Quit % | Mean Yrs Quit | Short Term (n) | Short Term % |
|---|---|---|---|---|---|---|---|---|---|
| 5 | 18-19 | Never Any | 2434 | 76.64 | | | | | |
| 5 | 18-19 | CC | 233 | 7.34 | 0 | 0.00 | | | |
| 5 | 18-19 | Ecig | 427 | 13.44 | | | | | |
| 5 | 18-19 | Dual | 0 | 0.00 | | | | | |
| 5 | 18-19 | Former | 82 | 2.58 | | | 1.15 | 52 | 63.41 |

Year of Follow-up: 1995, Age Group: 20-24

| Period | Age Group | Smoking Group | N | % | Ever Quit (N) | Ever Quit % | Mean Yrs Quit | Short Term (n) | Short Term % |
|---|---|---|---|---|---|---|---|---|---|
| 5 | 20-24 | Never Any | 5794 | 69.39 | | | | | |
| 5 | 20-24 | CC | 862 | 10.32 | 0 | 0.00 | | | |
| 5 | 20-24 | Ecig | 1250 | 14.97 | | | | | |
| 5 | 20-24 | Dual | 0 | 0.00 | | | | | |
| 5 | 20-24 | Former | 444 | 5.32 | | | 3.23 | 106 | 23.87 |

Year of Follow-up: 1995, Age Group: 25-29

| Period | Age Group | Smoking Group | N | % | Ever Quit (N) | Ever Quit % | Mean Yrs Quit | Short Term (n) | Short Term % |
|---|---|---|---|---|---|---|---|---|---|
| 5 | 25-29 | Never Any | 5313 | 60.42 | | | | | |
| 5 | 25-29 | CC | 1062 | 12.08 | 0 | 0.00 | | | |
| 5 | 25-29 | Ecig | 1395 | 15.86 | | | | | |
| 5 | 25-29 | Dual | 0 | 0.00 | | | | | |
| 5 | 25-29 | Former | 1023 | 11.63 | | | 5.94 | 54 | 5.28 |

Year of Follow-up: 1995, Age Group: 30-34

| Period | Age Group | Smoking Group | N | % | Ever Quit (N) | Ever Quit % | Mean Yrs Quit | Short Term (n) | Short Term % |
|---|---|---|---|---|---|---|---|---|---|

# Results for PHIM for Run 2 (Main_Analysis.RTF)

| | | | | | | | | | |
|---|---|---|---|---|---|---|---|---|---|
| 5 | 30-34 | Never Any | 5304 | 52.63 | | | | | |
| 5 | 30-34 | CC | 1373 | 13.62 | 0 | 0.00 | | | |
| 5 | 30-34 | Ecig | 1802 | 17.88 | | | | | |
| 5 | 30-34 | Dual | 0 | 0.00 | | | | | |
| 5 | 30-34 | Former | 1599 | 15.87 | | | 8.16 | 67 | 4.19 |

Year of Follow-up: 1995, Age Group: 35-39

| Period | Age Group | Smoking Group | N | % | Ever Quit (N) | Ever Quit % | Mean Yrs Quit | Short Term (n) | Short Term % |
|---|---|---|---|---|---|---|---|---|---|
| 5 | 35-39 | Never Any | 5278 | 50.27 | | | | | |
| 5 | 35-39 | CC | 1410 | 13.43 | 0 | 0.00 | | | |
| 5 | 35-39 | Ecig | 1746 | 16.63 | | | | | |
| 5 | 35-39 | Dual | 0 | 0.00 | | | | | |
| 5 | 35-39 | Former | 2065 | 19.67 | | | 8.75 | 62 | 3.00 |

Year of Follow-up: 1995, Age Group: 40-44

| Period | Age Group | Smoking Group | N | % | Ever Quit (N) | Ever Quit % | Mean Yrs Quit | Short Term (n) | Short Term % |
|---|---|---|---|---|---|---|---|---|---|
| 5 | 40-44 | Never Any | 5018 | 52.27 | | | | | |
| 5 | 40-44 | CC | 1135 | 11.82 | 0 | 0.00 | | | |
| 5 | 40-44 | Ecig | 1356 | 14.13 | | | | | |
| 5 | 40-44 | Dual | 0 | 0.00 | | | | | |
| 5 | 40-44 | Former | 2091 | 21.78 | | | 12.09 | 81 | 3.87 |

Year of Follow-up: 1995, Age Group: 45-49

| Period | Age Group | Smoking Group | N | % | Ever Quit (N) | Ever Quit % | Mean Yrs Quit | Short Term (n) | Short Term % |
|---|---|---|---|---|---|---|---|---|---|
| 5 | 45-49 | Never Any | 4356 | 51.27 | | | | | |
| 5 | 45-49 | CC | 974 | 11.46 | 0 | 0.00 | | | |
| 5 | 45-49 | Ecig | 1161 | 13.66 | | | | | |
| 5 | 45-49 | Dual | 0 | 0.00 | | | | | |
| 5 | 45-49 | Former | 2006 | 23.61 | | | 10.93 | 133 | 6.63 |

Year of Follow-up: 1995, Age Group: 50-54

| Period | Age Group | Smoking Group | N | % | Ever Quit (N) | Ever Quit % | Mean Yrs Quit | Short Term (n) | Short Term % |
|---|---|---|---|---|---|---|---|---|---|
| 5 | 50-54 | Never Any | 3116 | 47.67 | | | | | |
| 5 | 50-54 | CC | 739 | 11.31 | 0 | 0.00 | | | |
| 5 | 50-54 | Ecig | 826 | 12.64 | | | | | |
| 5 | 50-54 | Dual | 0 | 0.00 | | | | | |
| 5 | 50-54 | Former | 1855 | 28.38 | | | 14.92 | 140 | 7.55 |

Year of Follow-up: 1995, Age Group: 55-59

| Period | Age Group | Smoking Group | N | % | Ever Quit (N) | Ever Quit % | Mean Yrs Quit | Short Term (n) | Short Term % |
|---|---|---|---|---|---|---|---|---|---|
| 5 | 55-59 | Never Any | 2684 | 48.50 | | | | | |
| 5 | 55-59 | CC | 722 | 13.05 | 0 | 0.00 | | | |
| 5 | 55-59 | Ecig | 627 | 11.33 | | | | | |
| 5 | 55-59 | Dual | 0 | 0.00 | | | | | |
| 5 | 55-59 | Former | 1501 | 27.12 | | | 15.84 | 93 | 6.20 |

Year of Follow-up: 1995, Age Group: 60-64

| Period | Age Group | Smoking Group | N | % | Ever Quit (N) | Ever Quit % | Mean Yrs Quit | Short Term (n) | Short Term % |
|---|---|---|---|---|---|---|---|---|---|
| 5 | 60-64 | Never Any | 2732 | 51.90 | | | | | |
| 5 | 60-64 | CC | 633 | 12.03 | 0 | 0.00 | | | |
| 5 | 60-64 | Ecig | 444 | 8.43 | | | | | |
| 5 | 60-64 | Dual | 0 | 0.00 | | | | | |
| 5 | 60-64 | Former | 1455 | 27.64 | | | 19.22 | 87 | 5.98 |

Year of Follow-up: 1995, Age Group: 65-69

| Period | Age Group | Smoking Group | N | % | Ever Quit (N) | Ever Quit % | Mean Yrs Quit | Short Term (n) | Short Term % |
|---|---|---|---|---|---|---|---|---|---|
| 5 | 65-69 | Never Any | 3078 | 55.72 | | | | | |
| 5 | 65-69 | CC | 602 | 10.90 | 0 | 0.00 | | | |
| 5 | 65-69 | Ecig | 374 | 6.77 | | | | | |
| 5 | 65-69 | Dual | 0 | 0.00 | | | | | |
| 5 | 65-69 | Former | 1470 | 26.61 | | | 19.34 | 107 | 7.28 |

Year of Follow-up: 1995, Age Group: 70-74

| Period | Age Group | Smoking Group | N | % | Ever Quit (N) | Ever Quit % | Mean Yrs Quit | Short Term (n) | Short Term % |
|---|---|---|---|---|---|---|---|---|---|
| 5 | 70-74 | Never Any | 3042 | 57.36 | | | | | |
| 5 | 70-74 | CC | 577 | 10.88 | 0 | 0.00 | | | |
| 5 | 70-74 | Ecig | 188 | 3.55 | | | | | |
| 5 | 70-74 | Dual | 0 | 0.00 | | | | | |
| 5 | 70-74 | Former | 1496 | 28.21 | | | 22.32 | 77 | 5.15 |

Year of Follow-up: 1995, Age Group: 75-79

| Period | Age Group | Smoking Group | N | % | Ever Quit (N) | Ever Quit % | Mean Yrs Quit | Short Term (n) | Short Term % |
|---|---|---|---|---|---|---|---|---|---|
| 5 | 75-79 | Never Any | 2738 | 61.05 | | | | | |
| 5 | 75-79 | CC | 337 | 7.51 | 0 | 0.00 | | | |
| 5 | 75-79 | Ecig | 101 | 2.25 | | | | | |
| 5 | 75-79 | Dual | 0 | 0.00 | | | | | |
| 5 | 75-79 | Former | 1309 | 29.19 | | | 25.87 | 70 | 5.35 |

Year of Follow-up: 2000, Age Group: All Ages

# Results for PHIM for Run 2 (Main_Analysis.RTF)

| Period | Age Group | Smoking Group | N | % | Ever Quit (N) | Ever Quit % | Mean Yrs Quit | Short Term (n) | Short Term % |
|---|---|---|---|---|---|---|---|---|---|
| 10 | All Ages | Never Any | 50423 | 54.86 | | | | | |
| 10 | All Ages | CC | 4595 | 5.00 | 0 | 0.00 | | | |
| 10 | All Ages | Ecig | 15644 | 17.02 | | | | | |
| 10 | All Ages | Dual | 0 | 0.00 | | | | | |
| 10 | All Ages | Former | 21246 | 23.12 | | | 15.11 | 1717 | 8.08 |

Year of Follow-up: 2000, Age Group: 18-19

| Period | Age Group | Smoking Group | N | % | Ever Quit (N) | Ever Quit % | Mean Yrs Quit | Short Term (n) | Short Term % |
|---|---|---|---|---|---|---|---|---|---|
| 10 | 18-19 | Never Any | 0 | . | | | | | |
| 10 | 18-19 | CC | 0 | . | 0 | . | | | |
| 10 | 18-19 | Ecig | 0 | . | | | | | |
| 10 | 18-19 | Dual | 0 | . | | | | | |
| 10 | 18-19 | Former | 0 | . | | | . | 0 | . |

Year of Follow-up: 2000, Age Group: 20-24

| Period | Age Group | Smoking Group | N | % | Ever Quit (N) | Ever Quit % | Mean Yrs Quit | Short Term (n) | Short Term % |
|---|---|---|---|---|---|---|---|---|---|
| 10 | 20-24 | Never Any | 5128 | 64.67 | | | | | |
| 10 | 20-24 | CC | 370 | 4.67 | 0 | 0.00 | | | |
| 10 | 20-24 | Ecig | 1780 | 22.45 | | | | | |
| 10 | 20-24 | Dual | 0 | 0.00 | | | | | |
| 10 | 20-24 | Former | 652 | 8.22 | | | 3.55 | 169 | 25.92 |

Year of Follow-up: 2000, Age Group: 25-29

| Period | Age Group | Smoking Group | N | % | Ever Quit (N) | Ever Quit % | Mean Yrs Quit | Short Term (n) | Short Term % |
|---|---|---|---|---|---|---|---|---|---|
| 10 | 25-29 | Never Any | 5493 | 65.78 | | | | | |
| 10 | 25-29 | CC | 400 | 4.79 | 0 | 0.00 | | | |
| 10 | 25-29 | Ecig | 1690 | 20.24 | | | | | |
| 10 | 25-29 | Dual | 0 | 0.00 | | | | | |
| 10 | 25-29 | Former | 767 | 9.19 | | | 5.59 | 126 | 16.43 |

Year of Follow-up: 2000, Age Group: 30-34

| Period | Age Group | Smoking Group | N | % | Ever Quit (N) | Ever Quit % | Mean Yrs Quit | Short Term (n) | Short Term % |
|---|---|---|---|---|---|---|---|---|---|
| 10 | 30-34 | Never Any | 5214 | 59.30 | | | | | |
| 10 | 30-34 | CC | 417 | 4.74 | 0 | 0.00 | | | |
| 10 | 30-34 | Ecig | 1908 | 21.70 | | | | | |
| 10 | 30-34 | Dual | 0 | 0.00 | | | | | |
| 10 | 30-34 | Former | 1254 | 14.26 | | | 9.28 | 91 | 7.26 |

Year of Follow-up: 2000, Age Group: 35-39

| Period | Age Group | Smoking Group | N | % | Ever Quit (N) | Ever Quit % | Mean Yrs Quit | Short Term (n) | Short Term % |
|---|---|---|---|---|---|---|---|---|---|
| 10 | 35-39 | Never Any | 5284 | 52.43 | | | | | |
| 10 | 35-39 | CC | 561 | 5.57 | 0 | 0.00 | | | |
| 10 | 35-39 | Ecig | 2413 | 23.94 | | | | | |
| 10 | 35-39 | Dual | 0 | 0.00 | | | | | |
| 10 | 35-39 | Former | 1820 | 18.06 | | | 11.77 | 105 | 5.77 |

Year of Follow-up: 2000, Age Group: 40-44

| Period | Age Group | Smoking Group | N | % | Ever Quit (N) | Ever Quit % | Mean Yrs Quit | Short Term (n) | Short Term % |
|---|---|---|---|---|---|---|---|---|---|
| 10 | 40-44 | Never Any | 5278 | 50.27 | | | | | |
| 10 | 40-44 | CC | 584 | 5.56 | 0 | 0.00 | | | |
| 10 | 40-44 | Ecig | 2292 | 21.83 | | | | | |
| 10 | 40-44 | Dual | 0 | 0.00 | | | | | |
| 10 | 40-44 | Former | 2345 | 22.34 | | | 12.30 | 143 | 6.10 |

Year of Follow-up: 2000, Age Group: 45-49

| Period | Age Group | Smoking Group | N | % | Ever Quit (N) | Ever Quit % | Mean Yrs Quit | Short Term (n) | Short Term % |
|---|---|---|---|---|---|---|---|---|---|
| 10 | 45-49 | Never Any | 5018 | 52.27 | | | | | |
| 10 | 45-49 | CC | 446 | 4.65 | 0 | 0.00 | | | |
| 10 | 45-49 | Ecig | 1673 | 17.43 | | | | | |
| 10 | 45-49 | Dual | 0 | 0.00 | | | | | |
| 10 | 45-49 | Former | 2463 | 25.66 | | | 14.76 | 181 | 7.35 |

Year of Follow-up: 2000, Age Group: 50-54

| Period | Age Group | Smoking Group | N | % | Ever Quit (N) | Ever Quit % | Mean Yrs Quit | Short Term (n) | Short Term % |
|---|---|---|---|---|---|---|---|---|---|
| 10 | 50-54 | Never Any | 4356 | 51.27 | | | | | |
| 10 | 50-54 | CC | 378 | 4.45 | 0 | 0.00 | | | |
| 10 | 50-54 | Ecig | 1299 | 15.29 | | | | | |
| 10 | 50-54 | Dual | 0 | 0.00 | | | | | |
| 10 | 50-54 | Former | 2464 | 29.00 | | | 13.32 | 188 | 7.63 |

Year of Follow-up: 2000, Age Group: 55-59

| Period | Age Group | Smoking Group | N | % | Ever Quit (N) | Ever Quit % | Mean Yrs Quit | Short Term (n) | Short Term % |
|---|---|---|---|---|---|---|---|---|---|
| 10 | 55-59 | Never Any | 3116 | 47.67 | | | | | |
| 10 | 55-59 | CC | 283 | 4.33 | 0 | 0.00 | | | |
| 10 | 55-59 | Ecig | 912 | 13.95 | | | | | |
| 10 | 55-59 | Dual | 0 | 0.00 | | | | | |

# Results for PHIM for Run 2 (Main_Analysis.RTF)

| | | | | | | | | | |
|---|---|---|---|---|---|---|---|---|---|
| 10 | 55-59 | Former | 2225 | 34.04 | | | 16.92 | 157 | 7.06 |

Year of Follow-up: 2000, Age Group: 60-64

| Period | Age Group | Smoking Group | N | % | Ever Quit (N) | Ever Quit % | Mean Yrs Quit | Short Term (n) | Short Term % |
|---|---|---|---|---|---|---|---|---|---|
| 10 | 60-64 | Never Any | 2684 | 48.50 | | | | | |
| 10 | 60-64 | CC | 334 | 6.04 | 0 | 0.00 | | | |
| 10 | 60-64 | Ecig | 663 | 11.98 | | | | | |
| 10 | 60-64 | Dual | 0 | 0.00 | | | | | |
| 10 | 60-64 | Former | 1853 | 33.48 | | | 17.23 | 149 | 8.04 |

Year of Follow-up: 2000, Age Group: 65-69

| Period | Age Group | Smoking Group | N | % | Ever Quit (N) | Ever Quit % | Mean Yrs Quit | Short Term (n) | Short Term % |
|---|---|---|---|---|---|---|---|---|---|
| 10 | 65-69 | Never Any | 2732 | 51.90 | | | | | |
| 10 | 65-69 | CC | 280 | 5.32 | 0 | 0.00 | | | |
| 10 | 65-69 | Ecig | 443 | 8.42 | | | | | |
| 10 | 65-69 | Dual | 0 | 0.00 | | | | | |
| 10 | 65-69 | Former | 1809 | 34.37 | | | 19.85 | 146 | 8.07 |

Year of Follow-up: 2000, Age Group: 70-74

| Period | Age Group | Smoking Group | N | % | Ever Quit (N) | Ever Quit % | Mean Yrs Quit | Short Term (n) | Short Term % |
|---|---|---|---|---|---|---|---|---|---|
| 10 | 70-74 | Never Any | 3078 | 55.72 | | | | | |
| 10 | 70-74 | CC | 274 | 4.96 | 0 | 0.00 | | | |
| 10 | 70-74 | Ecig | 368 | 6.66 | | | | | |
| 10 | 70-74 | Dual | 0 | 0.00 | | | | | |
| 10 | 70-74 | Former | 1804 | 32.66 | | | 20.18 | 154 | 8.54 |

Year of Follow-up: 2000, Age Group: 75-79

| Period | Age Group | Smoking Group | N | % | Ever Quit (N) | Ever Quit % | Mean Yrs Quit | Short Term (n) | Short Term % |
|---|---|---|---|---|---|---|---|---|---|
| 10 | 75-79 | Never Any | 3042 | 57.36 | | | | | |
| 10 | 75-79 | CC | 268 | 5.05 | 0 | 0.00 | | | |
| 10 | 75-79 | Ecig | 203 | 3.83 | | | | | |
| 10 | 75-79 | Dual | 0 | 0.00 | | | | | |
| 10 | 75-79 | Former | 1790 | 33.75 | | | 23.17 | 108 | 6.03 |

Year of Follow-up: 2005, Age Group: All Ages

| Period | Age Group | Smoking Group | N | % | Ever Quit (N) | Ever Quit % | Mean Yrs Quit | Short Term (n) | Short Term % |
|---|---|---|---|---|---|---|---|---|---|
| 15 | All Ages | Never Any | 47002 | 54.27 | | | | | |
| 15 | All Ages | CC | 1890 | 2.18 | 0 | 0.00 | | | |
| 15 | All Ages | Ecig | 15617 | 18.03 | | | | | |
| 15 | All Ages | Dual | 0 | 0.00 | | | | | |
| 15 | All Ages | Former | 22096 | 25.51 | | | 17.32 | 941 | 4.26 |

Year of Follow-up: 2005, Age Group: 18-19

| Period | Age Group | Smoking Group | N | % | Ever Quit (N) | Ever Quit % | Mean Yrs Quit | Short Term (n) | Short Term % |
|---|---|---|---|---|---|---|---|---|---|
| 15 | 18-19 | Never Any | 0 | . | | | | | |
| 15 | 18-19 | CC | 0 | . | 0 | . | | | |
| 15 | 18-19 | Ecig | 0 | . | | | | | |
| 15 | 18-19 | Dual | 0 | . | | | | | |
| 15 | 18-19 | Former | 0 | . | | | . | 0 | . |

Year of Follow-up: 2005, Age Group: 20-24

| Period | Age Group | Smoking Group | N | % | Ever Quit (N) | Ever Quit % | Mean Yrs Quit | Short Term (n) | Short Term % |
|---|---|---|---|---|---|---|---|---|---|
| 15 | 20-24 | Never Any | 0 | . | | | | | |
| 15 | 20-24 | CC | 0 | . | 0 | . | | | |
| 15 | 20-24 | Ecig | 0 | . | | | | | |
| 15 | 20-24 | Dual | 0 | . | | | | | |
| 15 | 20-24 | Former | 0 | . | | | . | 0 | . |

Year of Follow-up: 2005, Age Group: 25-29

| Period | Age Group | Smoking Group | N | % | Ever Quit (N) | Ever Quit % | Mean Yrs Quit | Short Term (n) | Short Term % |
|---|---|---|---|---|---|---|---|---|---|
| 15 | 25-29 | Never Any | 4860 | 61.29 | | | | | |
| 15 | 25-29 | CC | 166 | 2.09 | 0 | 0.00 | | | |
| 15 | 25-29 | Ecig | 1941 | 24.48 | | | | | |
| 15 | 25-29 | Dual | 0 | 0.00 | | | | | |
| 15 | 25-29 | Former | 963 | 12.14 | | | 6.57 | 96 | 9.97 |

Year of Follow-up: 2005, Age Group: 30-34

| Period | Age Group | Smoking Group | N | % | Ever Quit (N) | Ever Quit % | Mean Yrs Quit | Short Term (n) | Short Term % |
|---|---|---|---|---|---|---|---|---|---|
| 15 | 30-34 | Never Any | 5407 | 64.75 | | | | | |
| 15 | 30-34 | CC | 149 | 1.78 | 0 | 0.00 | | | |
| 15 | 30-34 | Ecig | 1822 | 21.82 | | | | | |
| 15 | 30-34 | Dual | 0 | 0.00 | | | | | |
| 15 | 30-34 | Former | 972 | 11.64 | | | 8.77 | 80 | 8.23 |

Year of Follow-up: 2005, Age Group: 35-39

| Period | Age Group | Smoking Group | N | % | Ever Quit (N) | Ever Quit % | Mean Yrs Quit | Short Term (n) | Short Term % |
|---|---|---|---|---|---|---|---|---|---|
| 15 | 35-39 | Never Any | 5189 | 59.01 | | | | | |
| 15 | 35-39 | CC | 167 | 1.90 | 0 | 0.00 | | | |

# Results for PHIM for Run 2 (Main_Analysis.RTF)

| | | | | | | | | | |
|---|---|---|---|---|---|---|---|---|---|
| 15 | 35-39 | Ecig | 2002 | 22.77 | | | | | |
| 15 | 35-39 | Dual | 0 | 0.00 | | | | | |
| 15 | 35-39 | Former | 1435 | 16.32 | | | 12.75 | 66 | 4.60 |

Year of Follow-up: 2005, Age Group: 40-44

| Period | Age Group | Smoking Group | N | % | Ever Quit (N) | Ever Quit % | Mean Yrs Quit | Short Term (n) | Short Term % |
|---|---|---|---|---|---|---|---|---|---|
| 15 | 40-44 | Never Any | 5284 | 52.43 | | | | | |
| 15 | 40-44 | CC | 238 | 2.36 | 0 | 0.00 | | | |
| 15 | 40-44 | Ecig | 2500 | 24.81 | | | | | |
| 15 | 40-44 | Dual | 0 | 0.00 | | | | | |
| 15 | 40-44 | Former | 2056 | 20.40 | | | 15.08 | 101 | 4.91 |

Year of Follow-up: 2005, Age Group: 45-49

| Period | Age Group | Smoking Group | N | % | Ever Quit (N) | Ever Quit % | Mean Yrs Quit | Short Term (n) | Short Term % |
|---|---|---|---|---|---|---|---|---|---|
| 15 | 45-49 | Never Any | 5278 | 50.27 | | | | | |
| 15 | 45-49 | CC | 237 | 2.26 | 0 | 0.00 | | | |
| 15 | 45-49 | Ecig | 2318 | 22.08 | | | | | |
| 15 | 45-49 | Dual | 0 | 0.00 | | | | | |
| 15 | 45-49 | Former | 2666 | 25.39 | | | 15.45 | 135 | 5.06 |

Year of Follow-up: 2005, Age Group: 50-54

| Period | Age Group | Smoking Group | N | % | Ever Quit (N) | Ever Quit % | Mean Yrs Quit | Short Term (n) | Short Term % |
|---|---|---|---|---|---|---|---|---|---|
| 15 | 50-54 | Never Any | 5018 | 52.27 | | | | | |
| 15 | 50-54 | CC | 191 | 1.99 | 0 | 0.00 | | | |
| 15 | 50-54 | Ecig | 1622 | 16.90 | | | | | |
| 15 | 50-54 | Dual | 0 | 0.00 | | | | | |
| 15 | 50-54 | Former | 2769 | 28.84 | | | 17.84 | 99 | 3.58 |

Year of Follow-up: 2005, Age Group: 55-59

| Period | Age Group | Smoking Group | N | % | Ever Quit (N) | Ever Quit % | Mean Yrs Quit | Short Term (n) | Short Term % |
|---|---|---|---|---|---|---|---|---|---|
| 15 | 55-59 | Never Any | 4356 | 51.27 | | | | | |
| 15 | 55-59 | CC | 166 | 1.95 | 0 | 0.00 | | | |
| 15 | 55-59 | Ecig | 1281 | 15.08 | | | | | |
| 15 | 55-59 | Dual | 0 | 0.00 | | | | | |
| 15 | 55-59 | Former | 2694 | 31.71 | | | 16.95 | 78 | 2.90 |

Year of Follow-up: 2005, Age Group: 60-64

| Period | Age Group | Smoking Group | N | % | Ever Quit (N) | Ever Quit % | Mean Yrs Quit | Short Term (n) | Short Term % |
|---|---|---|---|---|---|---|---|---|---|
| 15 | 60-64 | Never Any | 3116 | 47.67 | | | | | |
| 15 | 60-64 | CC | 131 | 2.00 | 0 | 0.00 | | | |
| 15 | 60-64 | Ecig | 852 | 13.04 | | | | | |
| 15 | 60-64 | Dual | 0 | 0.00 | | | | | |
| 15 | 60-64 | Former | 2437 | 37.29 | | | 20.18 | 93 | 3.82 |

Year of Follow-up: 2005, Age Group: 65-69

| Period | Age Group | Smoking Group | N | % | Ever Quit (N) | Ever Quit % | Mean Yrs Quit | Short Term (n) | Short Term % |
|---|---|---|---|---|---|---|---|---|---|
| 15 | 65-69 | Never Any | 2684 | 48.50 | | | | | |
| 15 | 65-69 | CC | 160 | 2.89 | 0 | 0.00 | | | |
| 15 | 65-69 | Ecig | 591 | 10.68 | | | | | |
| 15 | 65-69 | Dual | 0 | 0.00 | | | | | |
| 15 | 65-69 | Former | 2099 | 37.93 | | | 19.92 | 68 | 3.24 |

Year of Follow-up: 2005, Age Group: 70-74

| Period | Age Group | Smoking Group | N | % | Ever Quit (N) | Ever Quit % | Mean Yrs Quit | Short Term (n) | Short Term % |
|---|---|---|---|---|---|---|---|---|---|
| 15 | 70-74 | Never Any | 2732 | 51.90 | | | | | |
| 15 | 70-74 | CC | 139 | 2.64 | 0 | 0.00 | | | |
| 15 | 70-74 | Ecig | 391 | 7.43 | | | | | |
| 15 | 70-74 | Dual | 0 | 0.00 | | | | | |
| 15 | 70-74 | Former | 2002 | 38.03 | | | 22.67 | 69 | 3.45 |

Year of Follow-up: 2005, Age Group: 75-79

| Period | Age Group | Smoking Group | N | % | Ever Quit (N) | Ever Quit % | Mean Yrs Quit | Short Term (n) | Short Term % |
|---|---|---|---|---|---|---|---|---|---|
| 15 | 75-79 | Never Any | 3078 | 55.72 | | | | | |
| 15 | 75-79 | CC | 146 | 2.64 | 0 | 0.00 | | | |
| 15 | 75-79 | Ecig | 297 | 5.38 | | | | | |
| 15 | 75-79 | Dual | 0 | 0.00 | | | | | |
| 15 | 75-79 | Former | 2003 | 36.26 | | | 22.91 | 56 | 2.80 |

Year of Follow-up: 2010, Age Group: All Ages

| Period | Age Group | Smoking Group | N | % | Ever Quit (N) | Ever Quit % | Mean Yrs Quit | Short Term (n) | Short Term % |
|---|---|---|---|---|---|---|---|---|---|
| 20 | All Ages | Never Any | 43759 | 53.97 | | | | | |
| 20 | All Ages | CC | 765 | 0.94 | 0 | 0.00 | | | |
| 20 | All Ages | Ecig | 13785 | 17.00 | | | | | |
| 20 | All Ages | Dual | 0 | 0.00 | | | | | |
| 20 | All Ages | Former | 22772 | 28.09 | | | 19.42 | 1117 | 4.91 |

Year of Follow-up: 2010, Age Group: 18-19

| Period | Age Group | Smoking Group | N | % | Ever Quit (N) | Ever Quit % | Mean Yrs Quit | Short Term (n) | Short Term % |
|---|---|---|---|---|---|---|---|---|---|

# Results for PHIM for Run 2 (Main_Analysis.RTF)

| | | | | | | | | | |
|---|---|---|---|---|---|---|---|---|---|
| 20 | 18-19 | Never Any | 0 | . | | | | | |
| 20 | 18-19 | CC | 0 | . | 0 | . | | | |
| 20 | 18-19 | Ecig | 0 | . | | | | | |
| 20 | 18-19 | Dual | 0 | . | | | | | |
| 20 | 18-19 | Former | 0 | . | | | . | 0 | . |

Year of Follow-up: 2010, Age Group: 20-24

| Period | Age Group | Smoking Group | N | % | Ever Quit (N) | Ever Quit % | Mean Yrs Quit | Short Term (n) | Short Term % |
|---|---|---|---|---|---|---|---|---|---|
| 20 | 20-24 | Never Any | 0 | . | | | | | |
| 20 | 20-24 | CC | 0 | . | 0 | . | | | |
| 20 | 20-24 | Ecig | 0 | . | | | | | |
| 20 | 20-24 | Dual | 0 | . | | | | | |
| 20 | 20-24 | Former | 0 | . | | | . | 0 | . |

Year of Follow-up: 2010, Age Group: 25-29

| Period | Age Group | Smoking Group | N | % | Ever Quit (N) | Ever Quit % | Mean Yrs Quit | Short Term (n) | Short Term % |
|---|---|---|---|---|---|---|---|---|---|
| 20 | 25-29 | Never Any | 0 | . | | | | | |
| 20 | 25-29 | CC | 0 | . | 0 | . | | | |
| 20 | 25-29 | Ecig | 0 | . | | | | | |
| 20 | 25-29 | Dual | 0 | . | | | | | |
| 20 | 25-29 | Former | 0 | . | | | . | 0 | . |

Year of Follow-up: 2010, Age Group: 30-34

| Period | Age Group | Smoking Group | N | % | Ever Quit (N) | Ever Quit % | Mean Yrs Quit | Short Term (n) | Short Term % |
|---|---|---|---|---|---|---|---|---|---|
| 20 | 30-34 | Never Any | 4743 | 59.81 | | | | | |
| 20 | 30-34 | CC | 65 | 0.82 | 0 | 0.00 | | | |
| 20 | 30-34 | Ecig | 1881 | 23.72 | | | | | |
| 20 | 30-34 | Dual | 0 | 0.00 | | | | | |
| 20 | 30-34 | Former | 1241 | 15.65 | | | 9.36 | 130 | 10.48 |

Year of Follow-up: 2010, Age Group: 35-39

| Period | Age Group | Smoking Group | N | % | Ever Quit (N) | Ever Quit % | Mean Yrs Quit | Short Term (n) | Short Term % |
|---|---|---|---|---|---|---|---|---|---|
| 20 | 35-39 | Never Any | 5359 | 64.18 | | | | | |
| 20 | 35-39 | CC | 58 | 0.69 | 0 | 0.00 | | | |
| 20 | 35-39 | Ecig | 1717 | 20.56 | | | | | |
| 20 | 35-39 | Dual | 0 | 0.00 | | | | | |
| 20 | 35-39 | Former | 1216 | 14.56 | | | 11.38 | 98 | 8.06 |

Year of Follow-up: 2010, Age Group: 40-44

| Period | Age Group | Smoking Group | N | % | Ever Quit (N) | Ever Quit % | Mean Yrs Quit | Short Term (n) | Short Term % |
|---|---|---|---|---|---|---|---|---|---|
| 20 | 40-44 | Never Any | 5189 | 59.01 | | | | | |
| 20 | 40-44 | CC | 64 | 0.73 | 0 | 0.00 | | | |
| 20 | 40-44 | Ecig | 1849 | 21.03 | | | | | |
| 20 | 40-44 | Dual | 0 | 0.00 | | | | | |
| 20 | 40-44 | Former | 1691 | 19.23 | | | 15.34 | 112 | 6.62 |

Year of Follow-up: 2010, Age Group: 45-49

| Period | Age Group | Smoking Group | N | % | Ever Quit (N) | Ever Quit % | Mean Yrs Quit | Short Term (n) | Short Term % |
|---|---|---|---|---|---|---|---|---|---|
| 20 | 45-49 | Never Any | 5284 | 52.43 | | | | | |
| 20 | 45-49 | CC | 105 | 1.04 | 0 | 0.00 | | | |
| 20 | 45-49 | Ecig | 2327 | 23.09 | | | | | |
| 20 | 45-49 | Dual | 0 | 0.00 | | | | | |
| 20 | 45-49 | Former | 2362 | 23.44 | | | 17.71 | 131 | 5.55 |

Year of Follow-up: 2010, Age Group: 50-54

| Period | Age Group | Smoking Group | N | % | Ever Quit (N) | Ever Quit % | Mean Yrs Quit | Short Term (n) | Short Term % |
|---|---|---|---|---|---|---|---|---|---|
| 20 | 50-54 | Never Any | 5278 | 50.27 | | | | | |
| 20 | 50-54 | CC | 101 | 0.96 | 0 | 0.00 | | | |
| 20 | 50-54 | Ecig | 2101 | 20.01 | | | | | |
| 20 | 50-54 | Dual | 0 | 0.00 | | | | | |
| 20 | 50-54 | Former | 3019 | 28.76 | | | 18.27 | 163 | 5.40 |

Year of Follow-up: 2010, Age Group: 55-59

| Period | Age Group | Smoking Group | N | % | Ever Quit (N) | Ever Quit % | Mean Yrs Quit | Short Term (n) | Short Term % |
|---|---|---|---|---|---|---|---|---|---|
| 20 | 55-59 | Never Any | 5018 | 52.27 | | | | | |
| 20 | 55-59 | CC | 91 | 0.95 | 0 | 0.00 | | | |
| 20 | 55-59 | Ecig | 1433 | 14.93 | | | | | |
| 20 | 55-59 | Dual | 0 | 0.00 | | | | | |
| 20 | 55-59 | Former | 3058 | 31.85 | | | 20.88 | 103 | 3.37 |

Year of Follow-up: 2010, Age Group: 60-64

| Period | Age Group | Smoking Group | N | % | Ever Quit (N) | Ever Quit % | Mean Yrs Quit | Short Term (n) | Short Term % |
|---|---|---|---|---|---|---|---|---|---|
| 20 | 60-64 | Never Any | 4356 | 51.27 | | | | | |
| 20 | 60-64 | CC | 71 | 0.84 | 0 | 0.00 | | | |
| 20 | 60-64 | Ecig | 1079 | 12.70 | | | | | |
| 20 | 60-64 | Dual | 0 | 0.00 | | | | | |
| 20 | 60-64 | Former | 2991 | 35.20 | | | 19.94 | 144 | 4.81 |

Year of Follow-up: 2010, Age Group: 65-69

# Results for PHIM for Run 2 (Main_Analysis.RTF)

| Period | Age Group | Smoking Group | N | % | Ever Quit (N) | Ever Quit % | Mean Yrs Quit | Short Term (n) | Short Term % |
|---|---|---|---|---|---|---|---|---|---|
| 20 | 65-69 | Never Any | 3116 | 47.67 | | | | | |
| 20 | 65-69 | CC | 47 | 0.72 | 0 | 0.00 | | | |
| 20 | 65-69 | Ecig | 645 | 9.87 | | | | | |
| 20 | 65-69 | Dual | 0 | 0.00 | | | | | |
| 20 | 65-69 | Former | 2728 | 41.74 | | | 22.72 | 95 | 3.48 |

Year of Follow-up: 2010, Age Group: 70-74

| Period | Age Group | Smoking Group | N | % | Ever Quit (N) | Ever Quit % | Mean Yrs Quit | Short Term (n) | Short Term % |
|---|---|---|---|---|---|---|---|---|---|
| 20 | 70-74 | Never Any | 2684 | 48.50 | | | | | |
| 20 | 70-74 | CC | 88 | 1.59 | 0 | 0.00 | | | |
| 20 | 70-74 | Ecig | 477 | 8.62 | | | | | |
| 20 | 70-74 | Dual | 0 | 0.00 | | | | | |
| 20 | 70-74 | Former | 2285 | 41.29 | | | 23.05 | 69 | 3.02 |

Year of Follow-up: 2010, Age Group: 75-79

| Period | Age Group | Smoking Group | N | % | Ever Quit (N) | Ever Quit % | Mean Yrs Quit | Short Term (n) | Short Term % |
|---|---|---|---|---|---|---|---|---|---|
| 20 | 75-79 | Never Any | 2732 | 51.90 | | | | | |
| 20 | 75-79 | CC | 75 | 1.42 | 0 | 0.00 | | | |
| 20 | 75-79 | Ecig | 276 | 5.24 | | | | | |
| 20 | 75-79 | Dual | 0 | 0.00 | | | | | |
| 20 | 75-79 | Former | 2181 | 41.43 | | | 25.56 | 72 | 3.30 |

Year of Follow-up: 2015, Age Group: All Ages

| Period | Age Group | Smoking Group | N | % | Ever Quit (N) | Ever Quit % | Mean Yrs Quit | Short Term (n) | Short Term % |
|---|---|---|---|---|---|---|---|---|---|
| 25 | All Ages | Never Any | 41010 | 54.09 | | | | | |
| 25 | All Ages | CC | 340 | 0.45 | 0 | 0.00 | | | |
| 25 | All Ages | Ecig | 11438 | 15.09 | | | | | |
| 25 | All Ages | Dual | 0 | 0.00 | | | | | |
| 25 | All Ages | Former | 23029 | 30.37 | | | 21.48 | 914 | 3.97 |

Year of Follow-up: 2015, Age Group: 18-19

| Period | Age Group | Smoking Group | N | % | Ever Quit (N) | Ever Quit % | Mean Yrs Quit | Short Term (n) | Short Term % |
|---|---|---|---|---|---|---|---|---|---|
| 25 | 18-19 | Never Any | 0 | . | | | | | |
| 25 | 18-19 | CC | 0 | . | 0 | . | | | |
| 25 | 18-19 | Ecig | 0 | . | | | | | |
| 25 | 18-19 | Dual | 0 | . | | | | | |
| 25 | 18-19 | Former | 0 | . | | | . | 0 | . |

Year of Follow-up: 2015, Age Group: 20-24

| Period | Age Group | Smoking Group | N | % | Ever Quit (N) | Ever Quit % | Mean Yrs Quit | Short Term (n) | Short Term % |
|---|---|---|---|---|---|---|---|---|---|
| 25 | 20-24 | Never Any | 0 | . | | | | | |
| 25 | 20-24 | CC | 0 | . | 0 | . | | | |
| 25 | 20-24 | Ecig | 0 | . | | | | | |
| 25 | 20-24 | Dual | 0 | . | | | | | |
| 25 | 20-24 | Former | 0 | . | | | . | 0 | . |

Year of Follow-up: 2015, Age Group: 25-29

| Period | Age Group | Smoking Group | N | % | Ever Quit (N) | Ever Quit % | Mean Yrs Quit | Short Term (n) | Short Term % |
|---|---|---|---|---|---|---|---|---|---|
| 25 | 25-29 | Never Any | 0 | . | | | | | |
| 25 | 25-29 | CC | 0 | . | 0 | . | | | |
| 25 | 25-29 | Ecig | 0 | . | | | | | |
| 25 | 25-29 | Dual | 0 | . | | | | | |
| 25 | 25-29 | Former | 0 | . | | | . | 0 | . |

Year of Follow-up: 2015, Age Group: 30-34

| Period | Age Group | Smoking Group | N | % | Ever Quit (N) | Ever Quit % | Mean Yrs Quit | Short Term (n) | Short Term % |
|---|---|---|---|---|---|---|---|---|---|
| 25 | 30-34 | Never Any | 0 | . | | | | | |
| 25 | 30-34 | CC | 0 | . | 0 | . | | | |
| 25 | 30-34 | Ecig | 0 | . | | | | | |
| 25 | 30-34 | Dual | 0 | . | | | | | |
| 25 | 30-34 | Former | 0 | . | | | . | 0 | . |

Year of Follow-up: 2015, Age Group: 35-39

| Period | Age Group | Smoking Group | N | % | Ever Quit (N) | Ever Quit % | Mean Yrs Quit | Short Term (n) | Short Term % |
|---|---|---|---|---|---|---|---|---|---|
| 25 | 35-39 | Never Any | 4726 | 59.60 | | | | | |
| 25 | 35-39 | CC | 20 | 0.25 | 0 | 0.00 | | | |
| 25 | 35-39 | Ecig | 1567 | 19.76 | | | | | |
| 25 | 35-39 | Dual | 0 | 0.00 | | | | | |
| 25 | 35-39 | Former | 1617 | 20.39 | | | 11.52 | 129 | 7.98 |

Year of Follow-up: 2015, Age Group: 40-44

| Period | Age Group | Smoking Group | N | % | Ever Quit (N) | Ever Quit % | Mean Yrs Quit | Short Term (n) | Short Term % |
|---|---|---|---|---|---|---|---|---|---|
| 25 | 40-44 | Never Any | 5359 | 64.18 | | | | | |
| 25 | 40-44 | CC | 33 | 0.40 | 0 | 0.00 | | | |
| 25 | 40-44 | Ecig | 1502 | 17.99 | | | | | |
| 25 | 40-44 | Dual | 0 | 0.00 | | | | | |

# Results for PHIM for Run 2 (Main_Analysis.RTF)

| | | | | | | | | | |
|---|---|---|---|---|---|---|---|---|---|
| 25 | 40-44 | Former | 1456 | 17.44 | | | 14.00 | 103 | 7.07 |

Year of Follow-up: 2015, Age Group: 45-49

| Period | Age Group | Smoking Group | N | % | Ever Quit (N) | Ever Quit % | Mean Yrs Quit | Short Term (n) | Short Term % |
|---|---|---|---|---|---|---|---|---|---|
| 25 | 45-49 | Never Any | 5189 | 59.01 | | | | | |
| 25 | 45-49 | CC | 32 | 0.36 | 0 | 0.00 | | | |
| 25 | 45-49 | Ecig | 1627 | 18.50 | | | | | |
| 25 | 45-49 | Dual | 0 | 0.00 | | | | | |
| 25 | 45-49 | Former | 1945 | 22.12 | | | 17.95 | 96 | 4.94 |

Year of Follow-up: 2015, Age Group: 50-54

| Period | Age Group | Smoking Group | N | % | Ever Quit (N) | Ever Quit % | Mean Yrs Quit | Short Term (n) | Short Term % |
|---|---|---|---|---|---|---|---|---|---|
| 25 | 50-54 | Never Any | 5284 | 52.43 | | | | | |
| 25 | 50-54 | CC | 45 | 0.45 | 0 | 0.00 | | | |
| 25 | 50-54 | Ecig | 2059 | 20.43 | | | | | |
| 25 | 50-54 | Dual | 0 | 0.00 | | | | | |
| 25 | 50-54 | Former | 2690 | 26.69 | | | 20.17 | 150 | 5.58 |

Year of Follow-up: 2015, Age Group: 55-59

| Period | Age Group | Smoking Group | N | % | Ever Quit (N) | Ever Quit % | Mean Yrs Quit | Short Term (n) | Short Term % |
|---|---|---|---|---|---|---|---|---|---|
| 25 | 55-59 | Never Any | 5278 | 50.27 | | | | | |
| 25 | 55-59 | CC | 37 | 0.35 | 0 | 0.00 | | | |
| 25 | 55-59 | Ecig | 1773 | 16.89 | | | | | |
| 25 | 55-59 | Dual | 0 | 0.00 | | | | | |
| 25 | 55-59 | Former | 3411 | 32.49 | | | 20.84 | 140 | 4.10 |

Year of Follow-up: 2015, Age Group: 60-64

| Period | Age Group | Smoking Group | N | % | Ever Quit (N) | Ever Quit % | Mean Yrs Quit | Short Term (n) | Short Term % |
|---|---|---|---|---|---|---|---|---|---|
| 25 | 60-64 | Never Any | 5018 | 52.27 | | | | | |
| 25 | 60-64 | CC | 42 | 0.44 | 0 | 0.00 | | | |
| 25 | 60-64 | Ecig | 1154 | 12.02 | | | | | |
| 25 | 60-64 | Dual | 0 | 0.00 | | | | | |
| 25 | 60-64 | Former | 3386 | 35.27 | | | 23.56 | 144 | 4.25 |

Year of Follow-up: 2015, Age Group: 65-69

| Period | Age Group | Smoking Group | N | % | Ever Quit (N) | Ever Quit % | Mean Yrs Quit | Short Term (n) | Short Term % |
|---|---|---|---|---|---|---|---|---|---|
| 25 | 65-69 | Never Any | 4356 | 51.27 | | | | | |
| 25 | 65-69 | CC | 41 | 0.48 | 0 | 0.00 | | | |
| 25 | 65-69 | Ecig | 844 | 9.93 | | | | | |
| 25 | 65-69 | Dual | 0 | 0.00 | | | | | |
| 25 | 65-69 | Former | 3256 | 38.32 | | | 23.12 | 70 | 2.15 |

Year of Follow-up: 2015, Age Group: 70-74

| Period | Age Group | Smoking Group | N | % | Ever Quit (N) | Ever Quit % | Mean Yrs Quit | Short Term (n) | Short Term % |
|---|---|---|---|---|---|---|---|---|---|
| 25 | 70-74 | Never Any | 3116 | 47.67 | | | | | |
| 25 | 70-74 | CC | 31 | 0.47 | 0 | 0.00 | | | |
| 25 | 70-74 | Ecig | 527 | 8.06 | | | | | |
| 25 | 70-74 | Dual | 0 | 0.00 | | | | | |
| 25 | 70-74 | Former | 2862 | 43.79 | | | 26.54 | 42 | 1.47 |

Year of Follow-up: 2015, Age Group: 75-79

| Period | Age Group | Smoking Group | N | % | Ever Quit (N) | Ever Quit % | Mean Yrs Quit | Short Term (n) | Short Term % |
|---|---|---|---|---|---|---|---|---|---|
| 25 | 75-79 | Never Any | 2684 | 48.50 | | | | | |
| 25 | 75-79 | CC | 59 | 1.07 | 0 | 0.00 | | | |
| 25 | 75-79 | Ecig | 385 | 6.96 | | | | | |
| 25 | 75-79 | Dual | 0 | 0.00 | | | | | |
| 25 | 75-79 | Former | 2406 | 43.48 | | | 26.76 | 40 | 1.66 |

Year of Follow-up: 2020, Age Group: All Ages

| Period | Age Group | Smoking Group | N | % | Ever Quit (N) | Ever Quit % | Mean Yrs Quit | Short Term (n) | Short Term % |
|---|---|---|---|---|---|---|---|---|---|
| 30 | All Ages | Never Any | 38326 | 54.53 | | | | | |
| 30 | All Ages | CC | 115 | 0.16 | 0 | 0.00 | | | |
| 30 | All Ages | Ecig | 9272 | 13.19 | | | | | |
| 30 | All Ages | Dual | 0 | 0.00 | | | | | |
| 30 | All Ages | Former | 22570 | 32.11 | | | 23.81 | 756 | 3.35 |

Year of Follow-up: 2020, Age Group: 18-19

| Period | Age Group | Smoking Group | N | % | Ever Quit (N) | Ever Quit % | Mean Yrs Quit | Short Term (n) | Short Term % |
|---|---|---|---|---|---|---|---|---|---|
| 30 | 18-19 | Never Any | 0 | . | | | | | |
| 30 | 18-19 | CC | 0 | . | 0 | . | | | |
| 30 | 18-19 | Ecig | 0 | . | | | | | |
| 30 | 18-19 | Dual | 0 | . | | | | | |
| 30 | 18-19 | Former | 0 | . | | | . | 0 | . |

Year of Follow-up: 2020, Age Group: 20-24

| Period | Age Group | Smoking Group | N | % | Ever Quit (N) | Ever Quit % | Mean Yrs Quit | Short Term (n) | Short Term % |
|---|---|---|---|---|---|---|---|---|---|
| 30 | 20-24 | Never Any | 0 | . | | | | | |
| 30 | 20-24 | CC | 0 | . | 0 | . | | | |

# Results for PHIM for Run 2 (Main_Analysis.RTF)

| | | | | | | | | | |
|---|---|---|---|---|---|---|---|---|---|
| 30 | 20-24 | Ecig | 0 | . | | | | | |
| 30 | 20-24 | Dual | 0 | . | | | | | |
| 30 | 20-24 | Former | 0 | . | | | . | 0 | . |

Year of Follow-up: 2020, Age Group: 25-29

| Period | Age Group | Smoking Group | N | % | Ever Quit (N) | Ever Quit % | Mean Yrs Quit | Short Term (n) | Short Term % |
|---|---|---|---|---|---|---|---|---|---|
| 30 | 25-29 | Never Any | 0 | . | | | | | |
| 30 | 25-29 | CC | 0 | . | 0 | . | | | |
| 30 | 25-29 | Ecig | 0 | . | | | | | |
| 30 | 25-29 | Dual | 0 | . | | | | | |
| 30 | 25-29 | Former | 0 | . | | | . | 0 | . |

Year of Follow-up: 2020, Age Group: 30-34

| Period | Age Group | Smoking Group | N | % | Ever Quit (N) | Ever Quit % | Mean Yrs Quit | Short Term (n) | Short Term % |
|---|---|---|---|---|---|---|---|---|---|
| 30 | 30-34 | Never Any | 0 | . | | | | | |
| 30 | 30-34 | CC | 0 | . | 0 | . | | | |
| 30 | 30-34 | Ecig | 0 | . | | | | | |
| 30 | 30-34 | Dual | 0 | . | | | | | |
| 30 | 30-34 | Former | 0 | . | | | . | 0 | . |

Year of Follow-up: 2020, Age Group: 35-39

| Period | Age Group | Smoking Group | N | % | Ever Quit (N) | Ever Quit % | Mean Yrs Quit | Short Term (n) | Short Term % |
|---|---|---|---|---|---|---|---|---|---|
| 30 | 35-39 | Never Any | 0 | . | | | | | |
| 30 | 35-39 | CC | 0 | . | 0 | . | | | |
| 30 | 35-39 | Ecig | 0 | . | | | | | |
| 30 | 35-39 | Dual | 0 | . | | | | | |
| 30 | 35-39 | Former | 0 | . | | | . | 0 | . |

Year of Follow-up: 2020, Age Group: 40-44

| Period | Age Group | Smoking Group | N | % | Ever Quit (N) | Ever Quit % | Mean Yrs Quit | Short Term (n) | Short Term % |
|---|---|---|---|---|---|---|---|---|---|
| 30 | 40-44 | Never Any | 4726 | 59.60 | | | | | |
| 30 | 40-44 | CC | 6 | 0.08 | 0 | 0.00 | | | |
| 30 | 40-44 | Ecig | 1345 | 16.96 | | | | | |
| 30 | 40-44 | Dual | 0 | 0.00 | | | | | |
| 30 | 40-44 | Former | 1853 | 23.37 | | | 14.68 | 82 | 4.43 |

Year of Follow-up: 2020, Age Group: 45-49

| Period | Age Group | Smoking Group | N | % | Ever Quit (N) | Ever Quit % | Mean Yrs Quit | Short Term (n) | Short Term % |
|---|---|---|---|---|---|---|---|---|---|
| 30 | 45-49 | Never Any | 5359 | 64.18 | | | | | |
| 30 | 45-49 | CC | 13 | 0.16 | 0 | 0.00 | | | |
| 30 | 45-49 | Ecig | 1317 | 15.77 | | | | | |
| 30 | 45-49 | Dual | 0 | 0.00 | | | | | |
| 30 | 45-49 | Former | 1661 | 19.89 | | | 16.93 | 71 | 4.27 |

Year of Follow-up: 2020, Age Group: 50-54

| Period | Age Group | Smoking Group | N | % | Ever Quit (N) | Ever Quit % | Mean Yrs Quit | Short Term (n) | Short Term % |
|---|---|---|---|---|---|---|---|---|---|
| 30 | 50-54 | Never Any | 5189 | 59.01 | | | | | |
| 30 | 50-54 | CC | 9 | 0.10 | 0 | 0.00 | | | |
| 30 | 50-54 | Ecig | 1395 | 15.86 | | | | | |
| 30 | 50-54 | Dual | 0 | 0.00 | | | | | |
| 30 | 50-54 | Former | 2200 | 25.02 | | | 20.50 | 121 | 5.50 |

Year of Follow-up: 2020, Age Group: 55-59

| Period | Age Group | Smoking Group | N | % | Ever Quit (N) | Ever Quit % | Mean Yrs Quit | Short Term (n) | Short Term % |
|---|---|---|---|---|---|---|---|---|---|
| 30 | 55-59 | Never Any | 5284 | 52.43 | | | | | |
| 30 | 55-59 | CC | 14 | 0.14 | 0 | 0.00 | | | |
| 30 | 55-59 | Ecig | 1708 | 16.95 | | | | | |
| 30 | 55-59 | Dual | 0 | 0.00 | | | | | |
| 30 | 55-59 | Former | 3072 | 30.48 | | | 22.30 | 140 | 4.56 |

Year of Follow-up: 2020, Age Group: 60-64

| Period | Age Group | Smoking Group | N | % | Ever Quit (N) | Ever Quit % | Mean Yrs Quit | Short Term (n) | Short Term % |
|---|---|---|---|---|---|---|---|---|---|
| 30 | 60-64 | Never Any | 5278 | 50.27 | | | | | |
| 30 | 60-64 | CC | 15 | 0.14 | 0 | 0.00 | | | |
| 30 | 60-64 | Ecig | 1428 | 13.60 | | | | | |
| 30 | 60-64 | Dual | 0 | 0.00 | | | | | |
| 30 | 60-64 | Former | 3778 | 35.98 | | | 23.52 | 153 | 4.05 |

Year of Follow-up: 2020, Age Group: 65-69

| Period | Age Group | Smoking Group | N | % | Ever Quit (N) | Ever Quit % | Mean Yrs Quit | Short Term (n) | Short Term % |
|---|---|---|---|---|---|---|---|---|---|
| 30 | 65-69 | Never Any | 5018 | 52.27 | | | | | |
| 30 | 65-69 | CC | 18 | 0.19 | 0 | 0.00 | | | |
| 30 | 65-69 | Ecig | 917 | 9.55 | | | | | |
| 30 | 65-69 | Dual | 0 | 0.00 | | | | | |
| 30 | 65-69 | Former | 3647 | 37.99 | | | 26.68 | 75 | 2.06 |

Year of Follow-up: 2020, Age Group: 70-74

| Period | Age Group | Smoking Group | N | % | Ever Quit (N) | Ever Quit % | Mean Yrs Quit | Short Term (n) | Short Term % |
|---|---|---|---|---|---|---|---|---|---|

| | | | | | | | | | |
|---|---|---|---|---|---|---|---|---|---|
| 30 | 70-74 | Never Any | 4356 | 51.27 | | | | | |
| 30 | 70-74 | CC | 22 | 0.26 | 0 | 0.00 | | | |
| 30 | 70-74 | Ecig | 727 | 8.56 | | | | | |
| 30 | 70-74 | Dual | 0 | 0.00 | | | | | |
| 30 | 70-74 | Former | 3392 | 39.92 | | | 27.06 | 66 | 1.95 |

Year of Follow-up: 2020, Age Group: 75-79

| Period | Age Group | Smoking Group | N | % | Ever Quit (N) | Ever Quit % | Mean Yrs Quit | Short Term (n) | Short Term % |
|---|---|---|---|---|---|---|---|---|---|
| 30 | 75-79 | Never Any | 3116 | 47.67 | | | | | |
| 30 | 75-79 | CC | 18 | 0.28 | 0 | 0.00 | | | |
| 30 | 75-79 | Ecig | 435 | 6.66 | | | | | |
| 30 | 75-79 | Dual | 0 | 0.00 | | | | | |
| 30 | 75-79 | Former | 2967 | 45.39 | | | 30.49 | 48 | 1.62 |

Year of Follow-up: 2025, Age Group: All Ages

| Period | Age Group | Smoking Group | N | % | Ever Quit (N) | Ever Quit % | Mean Yrs Quit | Short Term (n) | Short Term % |
|---|---|---|---|---|---|---|---|---|---|
| 35 | All Ages | Never Any | 35210 | 55.23 | | | | | |
| 35 | All Ages | CC | 47 | 0.07 | 0 | 0.00 | | | |
| 35 | All Ages | Ecig | 7305 | 11.46 | | | | | |
| 35 | All Ages | Dual | 0 | 0.00 | | | | | |
| 35 | All Ages | Former | 21185 | 33.23 | | | 25.87 | 616 | 2.91 |

Year of Follow-up: 2025, Age Group: 18-19

| Period | Age Group | Smoking Group | N | % | Ever Quit (N) | Ever Quit % | Mean Yrs Quit | Short Term (n) | Short Term % |
|---|---|---|---|---|---|---|---|---|---|
| 35 | 18-19 | Never Any | 0 | . | | | | | |
| 35 | 18-19 | CC | 0 | . | 0 | . | | | |
| 35 | 18-19 | Ecig | 0 | . | | | | | |
| 35 | 18-19 | Dual | 0 | . | | | | | |
| 35 | 18-19 | Former | 0 | . | | | . | 0 | . |

Year of Follow-up: 2025, Age Group: 20-24

| Period | Age Group | Smoking Group | N | % | Ever Quit (N) | Ever Quit % | Mean Yrs Quit | Short Term (n) | Short Term % |
|---|---|---|---|---|---|---|---|---|---|
| 35 | 20-24 | Never Any | 0 | . | | | | | |
| 35 | 20-24 | CC | 0 | . | 0 | . | | | |
| 35 | 20-24 | Ecig | 0 | . | | | | | |
| 35 | 20-24 | Dual | 0 | . | | | | | |
| 35 | 20-24 | Former | 0 | . | | | . | 0 | . |

Year of Follow-up: 2025, Age Group: 25-29

| Period | Age Group | Smoking Group | N | % | Ever Quit (N) | Ever Quit % | Mean Yrs Quit | Short Term (n) | Short Term % |
|---|---|---|---|---|---|---|---|---|---|
| 35 | 25-29 | Never Any | 0 | . | | | | | |
| 35 | 25-29 | CC | 0 | . | 0 | . | | | |
| 35 | 25-29 | Ecig | 0 | . | | | | | |
| 35 | 25-29 | Dual | 0 | . | | | | | |
| 35 | 25-29 | Former | 0 | . | | | . | 0 | . |

Year of Follow-up: 2025, Age Group: 30-34

| Period | Age Group | Smoking Group | N | % | Ever Quit (N) | Ever Quit % | Mean Yrs Quit | Short Term (n) | Short Term % |
|---|---|---|---|---|---|---|---|---|---|
| 35 | 30-34 | Never Any | 0 | . | | | | | |
| 35 | 30-34 | CC | 0 | . | 0 | . | | | |
| 35 | 30-34 | Ecig | 0 | . | | | | | |
| 35 | 30-34 | Dual | 0 | . | | | | | |
| 35 | 30-34 | Former | 0 | . | | | . | 0 | . |

Year of Follow-up: 2025, Age Group: 35-39

| Period | Age Group | Smoking Group | N | % | Ever Quit (N) | Ever Quit % | Mean Yrs Quit | Short Term (n) | Short Term % |
|---|---|---|---|---|---|---|---|---|---|
| 35 | 35-39 | Never Any | 0 | . | | | | | |
| 35 | 35-39 | CC | 0 | . | 0 | . | | | |
| 35 | 35-39 | Ecig | 0 | . | | | | | |
| 35 | 35-39 | Dual | 0 | . | | | | | |
| 35 | 35-39 | Former | 0 | . | | | . | 0 | . |

Year of Follow-up: 2025, Age Group: 40-44

| Period | Age Group | Smoking Group | N | % | Ever Quit (N) | Ever Quit % | Mean Yrs Quit | Short Term (n) | Short Term % |
|---|---|---|---|---|---|---|---|---|---|
| 35 | 40-44 | Never Any | 0 | . | | | | | |
| 35 | 40-44 | CC | 0 | . | 0 | . | | | |
| 35 | 40-44 | Ecig | 0 | . | | | | | |
| 35 | 40-44 | Dual | 0 | . | | | | | |
| 35 | 40-44 | Former | 0 | . | | | . | 0 | . |

Year of Follow-up: 2025, Age Group: 45-49

| Period | Age Group | Smoking Group | N | % | Ever Quit (N) | Ever Quit % | Mean Yrs Quit | Short Term (n) | Short Term % |
|---|---|---|---|---|---|---|---|---|---|
| 35 | 45-49 | Never Any | 4726 | 59.60 | | | | | |
| 35 | 45-49 | CC | 1 | 0.01 | 0 | 0.00 | | | |
| 35 | 45-49 | Ecig | 1146 | 14.45 | | | | | |
| 35 | 45-49 | Dual | 0 | 0.00 | | | | | |
| 35 | 45-49 | Former | 2057 | 25.94 | | | 17.95 | 74 | 3.60 |

Year of Follow-up: 2025, Age Group: 50-54

# Results for PHIM for Run 2 (Main_Analysis.RTF)

| Period | Age Group | Smoking Group | N | % | Ever Quit (N) | Ever Quit % | Mean Yrs Quit | Short Term (n) | Short Term % |
|---|---|---|---|---|---|---|---|---|---|
| 35 | 50-54 | Never Any | 5359 | 64.18 | | | | | |
| 35 | 50-54 | CC | 3 | 0.04 | 0 | 0.00 | | | |
| 35 | 50-54 | Ecig | 1112 | 13.32 | | | | | |
| 35 | 50-54 | Dual | 0 | 0.00 | | | | | |
| 35 | 50-54 | Former | 1876 | 22.47 | | | 19.62 | 104 | 5.54 |

Year of Follow-up: 2025, Age Group: 55-59

| Period | Age Group | Smoking Group | N | % | Ever Quit (N) | Ever Quit % | Mean Yrs Quit | Short Term (n) | Short Term % |
|---|---|---|---|---|---|---|---|---|---|
| 35 | 55-59 | Never Any | 5189 | 59.01 | | | | | |
| 35 | 55-59 | CC | 4 | 0.05 | 0 | 0.00 | | | |
| 35 | 55-59 | Ecig | 1153 | 13.11 | | | | | |
| 35 | 55-59 | Dual | 0 | 0.00 | | | | | |
| 35 | 55-59 | Former | 2447 | 27.83 | | | 23.13 | 98 | 4.00 |

Year of Follow-up: 2025, Age Group: 60-64

| Period | Age Group | Smoking Group | N | % | Ever Quit (N) | Ever Quit % | Mean Yrs Quit | Short Term (n) | Short Term % |
|---|---|---|---|---|---|---|---|---|---|
| 35 | 60-64 | Never Any | 5284 | 52.43 | | | | | |
| 35 | 60-64 | CC | 7 | 0.07 | 0 | 0.00 | | | |
| 35 | 60-64 | Ecig | 1378 | 13.67 | | | | | |
| 35 | 60-64 | Dual | 0 | 0.00 | | | | | |
| 35 | 60-64 | Former | 3409 | 33.83 | | | 24.79 | 139 | 4.08 |

Year of Follow-up: 2025, Age Group: 65-69

| Period | Age Group | Smoking Group | N | % | Ever Quit (N) | Ever Quit % | Mean Yrs Quit | Short Term (n) | Short Term % |
|---|---|---|---|---|---|---|---|---|---|
| 35 | 65-69 | Never Any | 5278 | 50.27 | | | | | |
| 35 | 65-69 | CC | 7 | 0.07 | 0 | 0.00 | | | |
| 35 | 65-69 | Ecig | 1139 | 10.85 | | | | | |
| 35 | 65-69 | Dual | 0 | 0.00 | | | | | |
| 35 | 65-69 | Former | 4075 | 38.81 | | | 26.61 | 83 | 2.04 |

Year of Follow-up: 2025, Age Group: 70-74

| Period | Age Group | Smoking Group | N | % | Ever Quit (N) | Ever Quit % | Mean Yrs Quit | Short Term (n) | Short Term % |
|---|---|---|---|---|---|---|---|---|---|
| 35 | 70-74 | Never Any | 5018 | 52.27 | | | | | |
| 35 | 70-74 | CC | 11 | 0.11 | 0 | 0.00 | | | |
| 35 | 70-74 | Ecig | 789 | 8.22 | | | | | |
| 35 | 70-74 | Dual | 0 | 0.00 | | | | | |
| 35 | 70-74 | Former | 3782 | 39.40 | | | 30.61 | 65 | 1.72 |

Year of Follow-up: 2025, Age Group: 75-79

| Period | Age Group | Smoking Group | N | % | Ever Quit (N) | Ever Quit % | Mean Yrs Quit | Short Term (n) | Short Term % |
|---|---|---|---|---|---|---|---|---|---|
| 35 | 75-79 | Never Any | 4356 | 51.27 | | | | | |
| 35 | 75-79 | CC | 14 | 0.16 | 0 | 0.00 | | | |
| 35 | 75-79 | Ecig | 588 | 6.92 | | | | | |
| 35 | 75-79 | Dual | 0 | 0.00 | | | | | |
| 35 | 75-79 | Former | 3539 | 41.65 | | | 30.81 | 53 | 1.50 |

Year of Follow-up: 2030, Age Group: All Ages

| Period | Age Group | Smoking Group | N | % | Ever Quit (N) | Ever Quit % | Mean Yrs Quit | Short Term (n) | Short Term % |
|---|---|---|---|---|---|---|---|---|---|
| 40 | All Ages | Never Any | 30854 | 55.84 | | | | | |
| 40 | All Ages | CC | 14 | 0.03 | 0 | 0.00 | | | |
| 40 | All Ages | Ecig | 5391 | 9.76 | | | | | |
| 40 | All Ages | Dual | 0 | 0.00 | | | | | |
| 40 | All Ages | Former | 18991 | 34.37 | | | 27.90 | 543 | 2.86 |

Year of Follow-up: 2030, Age Group: 18-19

| Period | Age Group | Smoking Group | N | % | Ever Quit (N) | Ever Quit % | Mean Yrs Quit | Short Term (n) | Short Term % |
|---|---|---|---|---|---|---|---|---|---|
| 40 | 18-19 | Never Any | 0 | . | | | | | |
| 40 | 18-19 | CC | 0 | . | 0 | . | | | |
| 40 | 18-19 | Ecig | 0 | . | | | | | |
| 40 | 18-19 | Dual | 0 | . | | | | | |
| 40 | 18-19 | Former | 0 | . | | | . | 0 | . |

Year of Follow-up: 2030, Age Group: 20-24

| Period | Age Group | Smoking Group | N | % | Ever Quit (N) | Ever Quit % | Mean Yrs Quit | Short Term (n) | Short Term % |
|---|---|---|---|---|---|---|---|---|---|
| 40 | 20-24 | Never Any | 0 | . | | | | | |
| 40 | 20-24 | CC | 0 | . | 0 | . | | | |
| 40 | 20-24 | Ecig | 0 | . | | | | | |
| 40 | 20-24 | Dual | 0 | . | | | | | |
| 40 | 20-24 | Former | 0 | . | | | . | 0 | . |

Year of Follow-up: 2030, Age Group: 25-29

| Period | Age Group | Smoking Group | N | % | Ever Quit (N) | Ever Quit % | Mean Yrs Quit | Short Term (n) | Short Term % |
|---|---|---|---|---|---|---|---|---|---|
| 40 | 25-29 | Never Any | 0 | . | | | | | |
| 40 | 25-29 | CC | 0 | . | 0 | . | | | |
| 40 | 25-29 | Ecig | 0 | . | | | | | |
| 40 | 25-29 | Dual | 0 | . | | | | | |

# Results for PHIM for Run 2 (Main_Analysis.RTF)

| | | | | | | | | | |
|---|---|---|---|---|---|---|---|---|---|
| 40 | 25-29 | Former | 0 | . | | | . | 0 | . |
| Year of Follow-up: 2030, Age Group: 30-34 | | | | | | | | | |
| Period | Age Group | Smoking Group | N | % | Ever Quit (N) | Ever Quit % | Mean Yrs Quit | Short Term (n) | Short Term % |
| 40 | 30-34 | Never Any | 0 | . | | | | | |
| 40 | 30-34 | CC | 0 | . | 0 | . | | | |
| 40 | 30-34 | Ecig | 0 | . | | | | | |
| 40 | 30-34 | Dual | 0 | . | | | | | |
| 40 | 30-34 | Former | 0 | . | | | . | 0 | . |
| Year of Follow-up: 2030, Age Group: 35-39 | | | | | | | | | |
| Period | Age Group | Smoking Group | N | % | Ever Quit (N) | Ever Quit % | Mean Yrs Quit | Short Term (n) | Short Term % |
| 40 | 35-39 | Never Any | 0 | . | | | | | |
| 40 | 35-39 | CC | 0 | . | 0 | . | | | |
| 40 | 35-39 | Ecig | 0 | . | | | | | |
| 40 | 35-39 | Dual | 0 | . | | | | | |
| 40 | 35-39 | Former | 0 | . | | | . | 0 | . |
| Year of Follow-up: 2030, Age Group: 40-44 | | | | | | | | | |
| Period | Age Group | Smoking Group | N | % | Ever Quit (N) | Ever Quit % | Mean Yrs Quit | Short Term (n) | Short Term % |
| 40 | 40-44 | Never Any | 0 | . | | | | | |
| 40 | 40-44 | CC | 0 | . | 0 | . | | | |
| 40 | 40-44 | Ecig | 0 | . | | | | | |
| 40 | 40-44 | Dual | 0 | . | | | | | |
| 40 | 40-44 | Former | 0 | . | | | . | 0 | . |
| Year of Follow-up: 2030, Age Group: 45-49 | | | | | | | | | |
| Period | Age Group | Smoking Group | N | % | Ever Quit (N) | Ever Quit % | Mean Yrs Quit | Short Term (n) | Short Term % |
| 40 | 45-49 | Never Any | 0 | . | | | | | |
| 40 | 45-49 | CC | 0 | . | 0 | . | | | |
| 40 | 45-49 | Ecig | 0 | . | | | | | |
| 40 | 45-49 | Dual | 0 | . | | | | | |
| 40 | 45-49 | Former | 0 | . | | | . | 0 | . |
| Year of Follow-up: 2030, Age Group: 50-54 | | | | | | | | | |
| Period | Age Group | Smoking Group | N | % | Ever Quit (N) | Ever Quit % | Mean Yrs Quit | Short Term (n) | Short Term % |
| 40 | 50-54 | Never Any | 4726 | 59.60 | | | | | |
| 40 | 50-54 | CC | 0 | 0.00 | 0 | 0.00 | | | |
| 40 | 50-54 | Ecig | 959 | 12.09 | | | | | |
| 40 | 50-54 | Dual | 0 | 0.00 | | | | | |
| 40 | 50-54 | Former | 2245 | 28.31 | | | 21.16 | 100 | 4.45 |
| Year of Follow-up: 2030, Age Group: 55-59 | | | | | | | | | |
| Period | Age Group | Smoking Group | N | % | Ever Quit (N) | Ever Quit % | Mean Yrs Quit | Short Term (n) | Short Term % |
| 40 | 55-59 | Never Any | 5359 | 64.18 | | | | | |
| 40 | 55-59 | CC | 0 | 0.00 | 0 | 0.00 | | | |
| 40 | 55-59 | Ecig | 897 | 10.74 | | | | | |
| 40 | 55-59 | Dual | 0 | 0.00 | | | | | |
| 40 | 55-59 | Former | 2094 | 25.08 | | | 22.27 | 80 | 3.82 |
| Year of Follow-up: 2030, Age Group: 60-64 | | | | | | | | | |
| Period | Age Group | Smoking Group | N | % | Ever Quit (N) | Ever Quit % | Mean Yrs Quit | Short Term (n) | Short Term % |
| 40 | 60-64 | Never Any | 5189 | 59.01 | | | | | |
| 40 | 60-64 | CC | 3 | 0.03 | 0 | 0.00 | | | |
| 40 | 60-64 | Ecig | 886 | 10.08 | | | | | |
| 40 | 60-64 | Dual | 0 | 0.00 | | | | | |
| 40 | 60-64 | Former | 2715 | 30.88 | | | 25.52 | 128 | 4.71 |
| Year of Follow-up: 2030, Age Group: 65-69 | | | | | | | | | |
| Period | Age Group | Smoking Group | N | % | Ever Quit (N) | Ever Quit % | Mean Yrs Quit | Short Term (n) | Short Term % |
| 40 | 65-69 | Never Any | 5284 | 52.43 | | | | | |
| 40 | 65-69 | CC | 3 | 0.03 | 0 | 0.00 | | | |
| 40 | 65-69 | Ecig | 1080 | 10.72 | | | | | |
| 40 | 65-69 | Dual | 0 | 0.00 | | | | | |
| 40 | 65-69 | Former | 3711 | 36.82 | | | 27.57 | 73 | 1.97 |
| Year of Follow-up: 2030, Age Group: 70-74 | | | | | | | | | |
| Period | Age Group | Smoking Group | N | % | Ever Quit (N) | Ever Quit % | Mean Yrs Quit | Short Term (n) | Short Term % |
| 40 | 70-74 | Never Any | 5278 | 50.27 | | | | | |
| 40 | 70-74 | CC | 4 | 0.04 | 0 | 0.00 | | | |
| 40 | 70-74 | Ecig | 957 | 9.12 | | | | | |
| 40 | 70-74 | Dual | 0 | 0.00 | | | | | |
| 40 | 70-74 | Former | 4260 | 40.58 | | | 30.32 | 86 | 2.02 |
| Year of Follow-up: 2030, Age Group: 75-79 | | | | | | | | | |
| Period | Age Group | Smoking Group | N | % | Ever Quit (N) | Ever Quit % | Mean Yrs Quit | Short Term (n) | Short Term % |
| 40 | 75-79 | Never Any | 5018 | 52.27 | | | | | |
| 40 | 75-79 | CC | 4 | 0.04 | 0 | 0.00 | | | |

# Results for PHIM for Run 2 (Main_Analysis.RTF)

| | | | | | | | | | |
|---|---|---|---|---|---|---|---|---|---|
| 40 | 75-79 | Ecig | 612 | 6.38 | | | | | |
| 40 | 75-79 | Dual | 0 | 0.00 | | | | | |
| 40 | 75-79 | Former | 3966 | 41.31 | | | 34.05 | 76 | 1.92 |

Year of Follow-up: 2035, Age Group: All Ages

| Period | Age Group | Smoking Group | N | % | Ever Quit (N) | Ever Quit % | Mean Yrs Quit | Short Term (n) | Short Term % |
|---|---|---|---|---|---|---|---|---|---|
| 45 | All Ages | Never Any | 25836 | 56.60 | | | | | |
| 45 | All Ages | CC | 5 | 0.01 | 0 | 0.00 | | | |
| 45 | All Ages | Ecig | 3886 | 8.51 | | | | | |
| 45 | All Ages | Dual | 0 | 0.00 | | | | | |
| 45 | All Ages | Former | 15923 | 34.88 | | | 29.63 | 351 | 2.20 |

Year of Follow-up: 2035, Age Group: 18-19

| Period | Age Group | Smoking Group | N | % | Ever Quit (N) | Ever Quit % | Mean Yrs Quit | Short Term (n) | Short Term % |
|---|---|---|---|---|---|---|---|---|---|
| 45 | 18-19 | Never Any | 0 | . | | | | | |
| 45 | 18-19 | CC | 0 | . | 0 | . | | | |
| 45 | 18-19 | Ecig | 0 | . | | | | | |
| 45 | 18-19 | Dual | 0 | . | | | | | |
| 45 | 18-19 | Former | 0 | . | | | . | 0 | . |

Year of Follow-up: 2035, Age Group: 20-24

| Period | Age Group | Smoking Group | N | % | Ever Quit (N) | Ever Quit % | Mean Yrs Quit | Short Term (n) | Short Term % |
|---|---|---|---|---|---|---|---|---|---|
| 45 | 20-24 | Never Any | 0 | . | | | | | |
| 45 | 20-24 | CC | 0 | . | 0 | . | | | |
| 45 | 20-24 | Ecig | 0 | . | | | | | |
| 45 | 20-24 | Dual | 0 | . | | | | | |
| 45 | 20-24 | Former | 0 | . | | | . | 0 | . |

Year of Follow-up: 2035, Age Group: 25-29

| Period | Age Group | Smoking Group | N | % | Ever Quit (N) | Ever Quit % | Mean Yrs Quit | Short Term (n) | Short Term % |
|---|---|---|---|---|---|---|---|---|---|
| 45 | 25-29 | Never Any | 0 | . | | | | | |
| 45 | 25-29 | CC | 0 | . | 0 | . | | | |
| 45 | 25-29 | Ecig | 0 | . | | | | | |
| 45 | 25-29 | Dual | 0 | . | | | | | |
| 45 | 25-29 | Former | 0 | . | | | . | 0 | . |

Year of Follow-up: 2035, Age Group: 30-34

| Period | Age Group | Smoking Group | N | % | Ever Quit (N) | Ever Quit % | Mean Yrs Quit | Short Term (n) | Short Term % |
|---|---|---|---|---|---|---|---|---|---|
| 45 | 30-34 | Never Any | 0 | . | | | | | |
| 45 | 30-34 | CC | 0 | . | 0 | . | | | |
| 45 | 30-34 | Ecig | 0 | . | | | | | |
| 45 | 30-34 | Dual | 0 | . | | | | | |
| 45 | 30-34 | Former | 0 | . | | | . | 0 | . |

Year of Follow-up: 2035, Age Group: 35-39

| Period | Age Group | Smoking Group | N | % | Ever Quit (N) | Ever Quit % | Mean Yrs Quit | Short Term (n) | Short Term % |
|---|---|---|---|---|---|---|---|---|---|
| 45 | 35-39 | Never Any | 0 | . | | | | | |
| 45 | 35-39 | CC | 0 | . | 0 | . | | | |
| 45 | 35-39 | Ecig | 0 | . | | | | | |
| 45 | 35-39 | Dual | 0 | . | | | | | |
| 45 | 35-39 | Former | 0 | . | | | . | 0 | . |

Year of Follow-up: 2035, Age Group: 40-44

| Period | Age Group | Smoking Group | N | % | Ever Quit (N) | Ever Quit % | Mean Yrs Quit | Short Term (n) | Short Term % |
|---|---|---|---|---|---|---|---|---|---|
| 45 | 40-44 | Never Any | 0 | . | | | | | |
| 45 | 40-44 | CC | 0 | . | 0 | . | | | |
| 45 | 40-44 | Ecig | 0 | . | | | | | |
| 45 | 40-44 | Dual | 0 | . | | | | | |
| 45 | 40-44 | Former | 0 | . | | | . | 0 | . |

Year of Follow-up: 2035, Age Group: 45-49

| Period | Age Group | Smoking Group | N | % | Ever Quit (N) | Ever Quit % | Mean Yrs Quit | Short Term (n) | Short Term % |
|---|---|---|---|---|---|---|---|---|---|
| 45 | 45-49 | Never Any | 0 | . | | | | | |
| 45 | 45-49 | CC | 0 | . | 0 | . | | | |
| 45 | 45-49 | Ecig | 0 | . | | | | | |
| 45 | 45-49 | Dual | 0 | . | | | | | |
| 45 | 45-49 | Former | 0 | . | | | . | 0 | . |

Year of Follow-up: 2035, Age Group: 50-54

| Period | Age Group | Smoking Group | N | % | Ever Quit (N) | Ever Quit % | Mean Yrs Quit | Short Term (n) | Short Term % |
|---|---|---|---|---|---|---|---|---|---|
| 45 | 50-54 | Never Any | 0 | . | | | | | |
| 45 | 50-54 | CC | 0 | . | 0 | . | | | |
| 45 | 50-54 | Ecig | 0 | . | | | | | |
| 45 | 50-54 | Dual | 0 | . | | | | | |
| 45 | 50-54 | Former | 0 | . | | | . | 0 | . |

Year of Follow-up: 2035, Age Group: 55-59

| Period | Age Group | Smoking Group | N | % | Ever Quit (N) | Ever Quit % | Mean Yrs Quit | Short Term (n) | Short Term % |
|---|---|---|---|---|---|---|---|---|---|

# Results for PHIM for Run 2 (Main_Analysis.RTF)

| | | | | | | | | | |
|---|---|---|---|---|---|---|---|---|---|
| 45 | 55-59 | Never Any | 4726 | 59.60 | | | | | |
| 45 | 55-59 | CC | 0 | 0.00 | 0 | 0.00 | | | |
| 45 | 55-59 | Ecig | 792 | 9.99 | | | | | |
| 45 | 55-59 | Dual | 0 | 0.00 | | | | | |
| 45 | 55-59 | Former | 2412 | 30.42 | | | 24.49 | 64 | 2.65 |

Year of Follow-up: 2035, Age Group: 60-64

| Period | Age Group | Smoking Group | N | % | Ever Quit (N) | Ever Quit % | Mean Yrs Quit | Short Term (n) | Short Term % |
|---|---|---|---|---|---|---|---|---|---|
| 45 | 60-64 | Never Any | 5359 | 64.18 | | | | | |
| 45 | 60-64 | CC | 0 | 0.00 | 0 | 0.00 | | | |
| 45 | 60-64 | Ecig | 719 | 8.61 | | | | | |
| 45 | 60-64 | Dual | 0 | 0.00 | | | | | |
| 45 | 60-64 | Former | 2272 | 27.21 | | | 25.28 | 77 | 3.39 |

Year of Follow-up: 2035, Age Group: 65-69

| Period | Age Group | Smoking Group | N | % | Ever Quit (N) | Ever Quit % | Mean Yrs Quit | Short Term (n) | Short Term % |
|---|---|---|---|---|---|---|---|---|---|
| 45 | 65-69 | Never Any | 5189 | 59.01 | | | | | |
| 45 | 65-69 | CC | 3 | 0.03 | 0 | 0.00 | | | |
| 45 | 65-69 | Ecig | 694 | 7.89 | | | | | |
| 45 | 65-69 | Dual | 0 | 0.00 | | | | | |
| 45 | 65-69 | Former | 2907 | 33.06 | | | 28.66 | 55 | 1.89 |

Year of Follow-up: 2035, Age Group: 70-74

| Period | Age Group | Smoking Group | N | % | Ever Quit (N) | Ever Quit % | Mean Yrs Quit | Short Term (n) | Short Term % |
|---|---|---|---|---|---|---|---|---|---|
| 45 | 70-74 | Never Any | 5284 | 52.43 | | | | | |
| 45 | 70-74 | CC | 1 | 0.01 | 0 | 0.00 | | | |
| 45 | 70-74 | Ecig | 934 | 9.27 | | | | | |
| 45 | 70-74 | Dual | 0 | 0.00 | | | | | |
| 45 | 70-74 | Former | 3859 | 38.29 | | | 31.38 | 76 | 1.97 |

Year of Follow-up: 2035, Age Group: 75-79

| Period | Age Group | Smoking Group | N | % | Ever Quit (N) | Ever Quit % | Mean Yrs Quit | Short Term (n) | Short Term % |
|---|---|---|---|---|---|---|---|---|---|
| 45 | 75-79 | Never Any | 5278 | 50.27 | | | | | |
| 45 | 75-79 | CC | 1 | 0.01 | 0 | 0.00 | | | |
| 45 | 75-79 | Ecig | 747 | 7.11 | | | | | |
| 45 | 75-79 | Dual | 0 | 0.00 | | | | | |
| 45 | 75-79 | Former | 4473 | 42.60 | | | 33.73 | 79 | 1.77 |

Year of Follow-up: 2040, Age Group: All Ages

| Period | Age Group | Smoking Group | N | % | Ever Quit (N) | Ever Quit % | Mean Yrs Quit | Short Term (n) | Short Term % |
|---|---|---|---|---|---|---|---|---|---|
| 50 | All Ages | Never Any | 20558 | 58.48 | | | | | |
| 50 | All Ages | CC | 4 | 0.01 | 0 | 0.00 | | | |
| 50 | All Ages | Ecig | 2497 | 7.10 | | | | | |
| 50 | All Ages | Dual | 0 | 0.00 | | | | | |
| 50 | All Ages | Former | 12092 | 34.40 | | | 31.39 | 238 | 1.97 |

Year of Follow-up: 2040, Age Group: 18-19

| Period | Age Group | Smoking Group | N | % | Ever Quit (N) | Ever Quit % | Mean Yrs Quit | Short Term (n) | Short Term % |
|---|---|---|---|---|---|---|---|---|---|
| 50 | 18-19 | Never Any | 0 | . | | | | | |
| 50 | 18-19 | CC | 0 | . | 0 | . | | | |
| 50 | 18-19 | Ecig | 0 | . | | | | | |
| 50 | 18-19 | Dual | 0 | . | | | | | |
| 50 | 18-19 | Former | 0 | . | | | . | 0 | . |

Year of Follow-up: 2040, Age Group: 20-24

| Period | Age Group | Smoking Group | N | % | Ever Quit (N) | Ever Quit % | Mean Yrs Quit | Short Term (n) | Short Term % |
|---|---|---|---|---|---|---|---|---|---|
| 50 | 20-24 | Never Any | 0 | . | | | | | |
| 50 | 20-24 | CC | 0 | . | 0 | . | | | |
| 50 | 20-24 | Ecig | 0 | . | | | | | |
| 50 | 20-24 | Dual | 0 | . | | | | | |
| 50 | 20-24 | Former | 0 | . | | | . | 0 | . |

Year of Follow-up: 2040, Age Group: 25-29

| Period | Age Group | Smoking Group | N | % | Ever Quit (N) | Ever Quit % | Mean Yrs Quit | Short Term (n) | Short Term % |
|---|---|---|---|---|---|---|---|---|---|
| 50 | 25-29 | Never Any | 0 | . | | | | | |
| 50 | 25-29 | CC | 0 | . | 0 | . | | | |
| 50 | 25-29 | Ecig | 0 | . | | | | | |
| 50 | 25-29 | Dual | 0 | . | | | | | |
| 50 | 25-29 | Former | 0 | . | | | . | 0 | . |

Year of Follow-up: 2040, Age Group: 30-34

| Period | Age Group | Smoking Group | N | % | Ever Quit (N) | Ever Quit % | Mean Yrs Quit | Short Term (n) | Short Term % |
|---|---|---|---|---|---|---|---|---|---|
| 50 | 30-34 | Never Any | 0 | . | | | | | |
| 50 | 30-34 | CC | 0 | . | 0 | . | | | |
| 50 | 30-34 | Ecig | 0 | . | | | | | |
| 50 | 30-34 | Dual | 0 | . | | | | | |
| 50 | 30-34 | Former | 0 | . | | | . | 0 | . |

Year of Follow-up: 2040, Age Group: 35-39

# Results for PHIM for Run 2 (Main_Analysis.RTF)

| Period | Age Group | Smoking Group | N | % | Ever Quit (N) | Ever Quit % | Mean Yrs Quit | Short Term (n) | Short Term % |
|---|---|---|---|---|---|---|---|---|---|
| 50 | 35-39 | Never Any | 0 | . | | | | | |
| 50 | 35-39 | CC | 0 | . | 0 | . | | | |
| 50 | 35-39 | Ecig | 0 | . | | | | | |
| 50 | 35-39 | Dual | 0 | . | | | | | |
| 50 | 35-39 | Former | 0 | . | | | . | 0 | . |

Year of Follow-up: 2040, Age Group: 40-44

| Period | Age Group | Smoking Group | N | % | Ever Quit (N) | Ever Quit % | Mean Yrs Quit | Short Term (n) | Short Term % |
|---|---|---|---|---|---|---|---|---|---|
| 50 | 40-44 | Never Any | 0 | . | | | | | |
| 50 | 40-44 | CC | 0 | . | 0 | . | | | |
| 50 | 40-44 | Ecig | 0 | . | | | | | |
| 50 | 40-44 | Dual | 0 | . | | | | | |
| 50 | 40-44 | Former | 0 | . | | | . | 0 | . |

Year of Follow-up: 2040, Age Group: 45-49

| Period | Age Group | Smoking Group | N | % | Ever Quit (N) | Ever Quit % | Mean Yrs Quit | Short Term (n) | Short Term % |
|---|---|---|---|---|---|---|---|---|---|
| 50 | 45-49 | Never Any | 0 | . | | | | | |
| 50 | 45-49 | CC | 0 | . | 0 | . | | | |
| 50 | 45-49 | Ecig | 0 | . | | | | | |
| 50 | 45-49 | Dual | 0 | . | | | | | |
| 50 | 45-49 | Former | 0 | . | | | . | 0 | . |

Year of Follow-up: 2040, Age Group: 50-54

| Period | Age Group | Smoking Group | N | % | Ever Quit (N) | Ever Quit % | Mean Yrs Quit | Short Term (n) | Short Term % |
|---|---|---|---|---|---|---|---|---|---|
| 50 | 50-54 | Never Any | 0 | . | | | | | |
| 50 | 50-54 | CC | 0 | . | 0 | . | | | |
| 50 | 50-54 | Ecig | 0 | . | | | | | |
| 50 | 50-54 | Dual | 0 | . | | | | | |
| 50 | 50-54 | Former | 0 | . | | | . | 0 | . |

Year of Follow-up: 2040, Age Group: 55-59

| Period | Age Group | Smoking Group | N | % | Ever Quit (N) | Ever Quit % | Mean Yrs Quit | Short Term (n) | Short Term % |
|---|---|---|---|---|---|---|---|---|---|
| 50 | 55-59 | Never Any | 0 | . | | | | | |
| 50 | 55-59 | CC | 0 | . | 0 | . | | | |
| 50 | 55-59 | Ecig | 0 | . | | | | | |
| 50 | 55-59 | Dual | 0 | . | | | | | |
| 50 | 55-59 | Former | 0 | . | | | . | 0 | . |

Year of Follow-up: 2040, Age Group: 60-64

| Period | Age Group | Smoking Group | N | % | Ever Quit (N) | Ever Quit % | Mean Yrs Quit | Short Term (n) | Short Term % |
|---|---|---|---|---|---|---|---|---|---|
| 50 | 60-64 | Never Any | 4726 | 59.60 | | | | | |
| 50 | 60-64 | CC | 0 | 0.00 | 0 | 0.00 | | | |
| 50 | 60-64 | Ecig | 613 | 7.73 | | | | | |
| 50 | 60-64 | Dual | 0 | 0.00 | | | | | |
| 50 | 60-64 | Former | 2591 | 32.67 | | | 27.58 | 77 | 2.97 |

Year of Follow-up: 2040, Age Group: 65-69

| Period | Age Group | Smoking Group | N | % | Ever Quit (N) | Ever Quit % | Mean Yrs Quit | Short Term (n) | Short Term % |
|---|---|---|---|---|---|---|---|---|---|
| 50 | 65-69 | Never Any | 5359 | 64.18 | | | | | |
| 50 | 65-69 | CC | 0 | 0.00 | 0 | 0.00 | | | |
| 50 | 65-69 | Ecig | 578 | 6.92 | | | | | |
| 50 | 65-69 | Dual | 0 | 0.00 | | | | | |
| 50 | 65-69 | Former | 2413 | 28.90 | | | 28.67 | 34 | 1.41 |

Year of Follow-up: 2040, Age Group: 70-74

| Period | Age Group | Smoking Group | N | % | Ever Quit (N) | Ever Quit % | Mean Yrs Quit | Short Term (n) | Short Term % |
|---|---|---|---|---|---|---|---|---|---|
| 50 | 70-74 | Never Any | 5189 | 59.01 | | | | | |
| 50 | 70-74 | CC | 3 | 0.03 | 0 | 0.00 | | | |
| 50 | 70-74 | Ecig | 583 | 6.63 | | | | | |
| 50 | 70-74 | Dual | 0 | 0.00 | | | | | |
| 50 | 70-74 | Former | 3018 | 34.32 | | | 32.48 | 56 | 1.86 |

Year of Follow-up: 2040, Age Group: 75-79

| Period | Age Group | Smoking Group | N | % | Ever Quit (N) | Ever Quit % | Mean Yrs Quit | Short Term (n) | Short Term % |
|---|---|---|---|---|---|---|---|---|---|
| 50 | 75-79 | Never Any | 5284 | 52.43 | | | | | |
| 50 | 75-79 | CC | 1 | 0.01 | 0 | 0.00 | | | |
| 50 | 75-79 | Ecig | 723 | 7.17 | | | | | |
| 50 | 75-79 | Dual | 0 | 0.00 | | | | | |
| 50 | 75-79 | Former | 4070 | 40.38 | | | 34.61 | 71 | 1.74 |

# Results for PHIM for Run 2 (Main_Analysis.RTF)

************************************************************************************

**Main Results for E-Component for Run: 2, Simulation: 1, COD: Four Diseases Combined**

| Variable Parameter | Value |
|---|---|
| 1. Country | US (United States) |
| 2. Sex | F (Females) |
| 3. Year of start of process | 1990 |
| 4. Cause of Death | Four Diseases Combined |
| 5. Effective Dose NULL | CC:1 |
| 6. Effective Dose Ecig | CC:1 Ecig:0.05 Dual:0.525 |
| Note: | Death rates are per million population |

-----------------------------------------------------------------------------------------

Results for Age Groups Combined and Years combined, Population unadjusted for deaths in Ecig scenario,

| Age Group:All | | NULL | Ecig | | | %Drop | | NULL | Ecig | |
|---|---|---|---|---|---|---|---|---|---|---|
| Year | Number Deaths | Attrib Deaths | Attrib Deaths | Drop Deaths | %Drop Deaths | Attrib Deaths | Death Rate | Attrib Rates | Attrib Rates | Drop Rates |
| All Comb | 9082078 | 2946250.006 | 1945986.460 | 1000264 | 11.014 | 33.950 | 2493.45 | 808.881 | 534.263 | 274.618 |

-----------------------------------------------------------------------------------------

Results for Age Groups Combined, Population unadjusted for deaths in Ecig scenario,

| Age Group:All | | NULL | Ecig | | | %Drop | | NULL | Ecig | |
|---|---|---|---|---|---|---|---|---|---|---|
| Year | Number Deaths | Attrib Deaths | Attrib Deaths | Drop Deaths | %Drop Deaths | Attrib Deaths | Death Rate | Attrib Rates | Attrib Rates | Drop Rates |
| 1990 | 199848 | 57524.822 | 57524.822 | 0.000 | 0.000 | 0.000 | 2926.84 | 842.471 | 842.471 | 0.000 |
| 1991 | 199162 | 59078.486 | 58910.022 | 168.464 | 0.085 | 0.285 | 2877.87 | 853.678 | 851.244 | 2.434 |
| 1992 | 198532 | 60494.150 | 60022.454 | 471.695 | 0.238 | 0.780 | 2829.84 | 862.271 | 855.548 | 6.723 |
| 1993 | 202512 | 62694.848 | 61776.738 | 918.109 | 0.453 | 1.464 | 2845.85 | 881.034 | 868.132 | 12.902 |
| 1994 | 200658 | 63202.598 | 61751.785 | 1450.813 | 0.723 | 2.295 | 2777.87 | 874.966 | 854.881 | 20.085 |
| 1995 | 201023 | 63766.862 | 61670.717 | 2096.145 | 1.043 | 3.287 | 2739.80 | 869.097 | 840.528 | 28.569 |
| 1996 | 201180 | 64591.163 | 61718.469 | 2872.693 | 1.428 | 4.448 | 2706.36 | 868.908 | 830.263 | 38.645 |
| 1997 | 198639 | 64686.912 | 61088.629 | 3598.283 | 1.811 | 5.563 | 2635.18 | 858.147 | 810.412 | 47.735 |
| 1998 | 197573 | 65295.961 | 60857.310 | 4438.651 | 2.247 | 6.798 | 2584.18 | 854.046 | 795.990 | 58.056 |
| 1999 | 211329 | 67327.521 | 61593.161 | 5734.360 | 2.713 | 8.517 | 2726.91 | 868.771 | 794.776 | 73.994 |
| 2000 | 208496 | 67596.897 | 60917.212 | 6679.684 | 3.204 | 9.882 | 2656.96 | 861.419 | 776.297 | 85.122 |
| 2001 | 203606 | 67008.811 | 59328.640 | 7680.170 | 3.772 | 11.461 | 2565.88 | 844.458 | 747.671 | 96.787 |
| 2002 | 200828 | 67067.744 | 58272.836 | 8794.908 | 4.379 | 13.113 | 2506.09 | 836.925 | 727.175 | 109.750 |
| 2003 | 196352 | 66756.217 | 56953.992 | 9802.225 | 4.992 | 14.684 | 2427.31 | 825.244 | 704.068 | 121.176 |
| 2004 | 188160 | 65058.725 | 54404.731 | 10653.99 | 5.662 | 16.376 | 2302.83 | 796.234 | 665.843 | 130.391 |
| 2005 | 186416 | 65214.724 | 53412.806 | 11801.92 | 6.331 | 18.097 | 2256.27 | 789.321 | 646.477 | 142.843 |
| 2006 | 180088 | 63771.146 | 51098.360 | 12672.79 | 7.037 | 19.872 | 2160.67 | 765.117 | 613.071 | 152.046 |
| 2007 | 175644 | 63110.718 | 49543.854 | 13566.86 | 7.724 | 21.497 | 2086.36 | 749.650 | 588.498 | 161.152 |
| 2008 | 177570 | 63718.624 | 48858.922 | 14859.70 | 8.368 | 23.321 | 2086.39 | 748.674 | 574.077 | 174.597 |
| 2009 | 172806 | 62794.698 | 47101.532 | 15693.17 | 9.081 | 24.991 | 2007.57 | 729.515 | 547.200 | 182.315 |
| 2010 | 170489 | 61917.074 | 45348.407 | 16568.67 | 9.718 | 26.759 | 1957.97 | 711.083 | 520.801 | 190.282 |
| 2011 | 170305 | 61680.114 | 43937.404 | 17742.71 | 10.418 | 28.766 | 1937.46 | 701.700 | 499.851 | 201.849 |
| 2012 | 171421 | 62023.127 | 42978.209 | 19044.92 | 11.110 | 30.706 | 1930.22 | 698.386 | 483.939 | 214.448 |
| 2013 | 172699 | 62037.765 | 41820.587 | 20217.18 | 11.707 | 32.589 | 1923.75 | 691.059 | 465.853 | 225.206 |
| 2014 | 174169 | 61921.017 | 40417.468 | 21503.55 | 12.346 | 34.727 | 1918.85 | 682.195 | 445.286 | 236.908 |
| 2015 | 177354 | 61856.204 | 39257.952 | 22598.25 | 12.742 | 36.534 | 2184.51 | 761.899 | 483.550 | 278.348 |
| 2016 | 176853 | 60464.119 | 36987.444 | 23476.67 | 13.275 | 38.827 | 2157.59 | 737.655 | 451.243 | 286.413 |
| 2017 | 179309 | 60243.839 | 35585.731 | 24658.11 | 13.752 | 40.931 | 2166.86 | 728.017 | 430.036 | 297.981 |
| 2018 | 177157 | 60713.327 | 34986.207 | 25727.12 | 14.522 | 42.375 | 2120.51 | 726.721 | 418.775 | 307.946 |
| 2019 | 179227 | 61148.172 | 34050.786 | 27097.39 | 15.119 | 44.314 | 2124.53 | 724.841 | 403.633 | 321.208 |
| 2020 | 180467 | 61149.317 | 33100.796 | 28048.52 | 15.542 | 45.869 | 2424.25 | 821.432 | 444.650 | 376.782 |
| 2021 | 178116 | 58727.786 | 30280.704 | 28447.08 | 15.971 | 48.439 | 2375.42 | 783.216 | 403.835 | 379.381 |
| 2022 | 179408 | 58848.802 | 29245.268 | 29603.53 | 16.501 | 50.304 | 2374.52 | 778.881 | 387.069 | 391.811 |
| 2023 | 180640 | 58816.149 | 28199.145 | 30617.00 | 16.949 | 52.055 | 2372.26 | 772.407 | 370.327 | 402.080 |
| 2024 | 181610 | 58955.232 | 27162.218 | 31793.01 | 17.506 | 53.927 | 2366.77 | 768.316 | 353.983 | 414.333 |
| 2025 | 180418 | 58297.744 | 26007.992 | 32289.75 | 17.897 | 55.388 | 2713.69 | 876.861 | 391.188 | 485.673 |
| 2026 | 180313 | 56469.971 | 23968.749 | 32501.22 | 18.025 | 57.555 | 2701.83 | 846.150 | 359.149 | 487.000 |
| 2027 | 179619 | 56209.427 | 22822.059 | 33387.37 | 18.588 | 59.398 | 2680.68 | 838.883 | 340.602 | 498.281 |
| 2028 | 178762 | 55733.105 | 21620.876 | 34112.23 | 19.082 | 61.206 | 2656.74 | 828.298 | 321.327 | 506.971 |
| 2029 | 177808 | 55145.838 | 20403.767 | 34742.07 | 19.539 | 63.000 | 2630.89 | 815.951 | 301.899 | 514.052 |
| 2030 | 173587 | 53615.097 | 19159.325 | 34455.77 | 19.849 | 64.265 | 3040.33 | 939.056 | 335.571 | 603.485 |
| 2031 | 174985 | 52046.561 | 17935.340 | 34111.22 | 19.494 | 65.540 | 3060.73 | 910.364 | 313.713 | 596.651 |
| 2032 | 173400 | 51582.636 | 16946.423 | 34636.21 | 19.975 | 67.147 | 3027.10 | 900.496 | 295.839 | 604.656 |
| 2033 | 171756 | 50839.758 | 15943.578 | 34896.18 | 20.317 | 68.640 | 2991.14 | 885.375 | 277.657 | 607.717 |
| 2034 | 169973 | 50215.451 | 14970.006 | 35245.45 | 20.736 | 70.188 | 2951.96 | 872.104 | 259.988 | 612.116 |
| 2035 | 162644 | 47773.075 | 13853.280 | 33919.79 | 20.855 | 71.002 | 3459.14 | 1016.05 | 294.634 | 721.412 |
| 2036 | 156254 | 43298.709 | 11869.439 | 31429.27 | 20.114 | 72.587 | 3328.25 | 922.274 | 252.822 | 669.452 |
| 2037 | 153593 | 42241.563 | 10908.835 | 31332.73 | 20.400 | 74.175 | 3274.10 | 900.450 | 232.540 | 667.910 |
| 2038 | 150947 | 41356.353 | 10137.398 | 31218.95 | 20.682 | 75.488 | 3217.50 | 881.531 | 216.084 | 665.447 |
| 2039 | 148373 | 40161.050 | 9274.074 | 30886.98 | 20.817 | 76.908 | 3159.42 | 855.178 | 197.479 | 657.698 |

## Results for PHIM for Run 2 (Main_Analysis.RTF)

************************************************************************************

**Main Results for E-Component for Run: 2, Simulation: 1, COD: LUNG CANCER**

| Variable Parameter | Value |
|---|---|
| 1. Country | US (United States) |
| 2. Sex | F (Females) |
| 3. Year of start of process | 1990 |
| 4. Cause of Death | LUNG CANCER |
| 5. Effective Dose NULL | CC:1 |
| 6. Effective Dose Ecig | CC:1 Ecig:0.05 Dual:0.525 |
| Note: | Death rates are per million population |

----------------------------------------------------------------------------------------

Results for Age Groups Combined and Years combined, Population unadjusted for deaths in Ecig scenario,

| Age Group:All<br>Year | Number<br>Deaths | NULL<br>Attrib<br>Deaths | Ecig<br>Attrib<br>Deaths | Drop<br>Deaths | %Drop<br>Deaths | %Drop<br>Attrib<br>Deaths | Death<br>Rate | NULL<br>Attrib<br>Rates | Ecig<br>Attrib<br>Rates | Drop<br>Rates |
|---|---|---|---|---|---|---|---|---|---|---|
| All Comb | 2327764 | 1580465.217 | 1143412.495 | 437052.7 | 18.776 | 27.653 | 639.078 | 433.910 | 313.919 | 119.991 |

----------------------------------------------------------------------------------------

Results for Age Groups Combined, Population unadjusted for deaths in Ecig scenario,

| Age Group:All<br>Year | Number<br>Deaths | NULL<br>Attrib<br>Deaths | Ecig<br>Attrib<br>Deaths | Drop<br>Deaths | %Drop<br>Deaths | %Drop<br>Attrib<br>Deaths | Death<br>Rate | NULL<br>Attrib<br>Rates | Ecig<br>Attrib<br>Rates | Drop<br>Rates |
|---|---|---|---|---|---|---|---|---|---|---|
| 1990 | 42690 | 30614.201 | 30614.201 | 0.000 | 0.000 | 0.000 | 625.210 | 448.355 | 448.355 | 0.000 |
| 1991 | 43692 | 31412.010 | 31379.684 | 32.326 | 0.074 | 0.103 | 631.345 | 453.901 | 453.433 | 0.467 |
| 1992 | 45423 | 32782.407 | 32688.427 | 93.980 | 0.207 | 0.287 | 647.450 | 467.274 | 465.934 | 1.340 |
| 1993 | 46291 | 33491.183 | 33305.336 | 185.847 | 0.401 | 0.555 | 650.515 | 470.643 | 468.031 | 2.612 |
| 1994 | 47017 | 34074.347 | 33772.924 | 301.424 | 0.641 | 0.885 | 650.895 | 471.719 | 467.546 | 4.173 |
| 1995 | 47840 | 34694.454 | 34239.732 | 454.722 | 0.951 | 1.311 | 652.025 | 472.861 | 466.663 | 6.198 |
| 1996 | 48244 | 35027.269 | 34397.689 | 629.580 | 1.305 | 1.797 | 648.999 | 471.202 | 462.732 | 8.469 |
| 1997 | 49035 | 35590.763 | 34752.669 | 838.093 | 1.709 | 2.355 | 650.506 | 472.153 | 461.035 | 11.118 |
| 1998 | 49686 | 36100.785 | 35016.164 | 1084.620 | 2.183 | 3.004 | 649.874 | 472.184 | 457.998 | 14.186 |
| 1999 | 48883 | 35451.030 | 34143.611 | 1307.418 | 2.675 | 3.688 | 630.769 | 457.448 | 440.577 | 16.870 |
| 2000 | 50331 | 36447.986 | 34810.942 | 1637.044 | 3.253 | 4.491 | 641.392 | 464.474 | 443.612 | 20.862 |
| 2001 | 50389 | 36411.326 | 34439.844 | 1971.482 | 3.913 | 5.414 | 635.012 | 458.863 | 434.018 | 24.845 |
| 2002 | 51445 | 37078.545 | 34691.561 | 2386.984 | 4.640 | 6.438 | 641.972 | 462.696 | 432.909 | 29.787 |
| 2003 | 51408 | 36999.876 | 34215.919 | 2783.956 | 5.415 | 7.524 | 635.509 | 457.395 | 422.979 | 34.415 |
| 2004 | 51399 | 36861.555 | 33637.298 | 3224.257 | 6.273 | 8.747 | 629.057 | 451.138 | 411.677 | 39.461 |
| 2005 | 51421 | 36689.529 | 32999.409 | 3690.119 | 7.176 | 10.058 | 622.370 | 444.069 | 399.406 | 44.663 |
| 2006 | 51355 | 36504.774 | 32325.093 | 4179.681 | 8.139 | 11.450 | 616.150 | 437.979 | 387.832 | 50.147 |
| 2007 | 51839 | 36663.202 | 31900.792 | 4762.410 | 9.187 | 12.990 | 615.761 | 435.498 | 378.928 | 56.570 |
| 2008 | 50976 | 35898.803 | 30697.995 | 5200.808 | 10.202 | 14.487 | 598.952 | 421.800 | 360.692 | 61.108 |
| 2009 | 51193 | 35934.525 | 30107.924 | 5826.602 | 11.382 | 16.214 | 594.733 | 417.468 | 349.778 | 67.690 |
| 2010 | 50853 | 35502.829 | 29162.994 | 6339.836 | 12.467 | 17.857 | 584.018 | 407.730 | 334.921 | 72.809 |
| 2011 | 50509 | 35136.342 | 28199.604 | 6936.738 | 13.734 | 19.742 | 574.613 | 399.726 | 320.811 | 78.915 |
| 2012 | 51245 | 35450.201 | 27770.845 | 7679.356 | 14.986 | 21.662 | 577.023 | 399.173 | 312.702 | 86.470 |
| 2013 | 51069 | 35123.224 | 26792.547 | 8330.677 | 16.313 | 23.718 | 568.874 | 391.249 | 298.451 | 92.798 |
| 2014 | 51251 | 35079.370 | 26051.341 | 9028.029 | 17.615 | 25.736 | 564.641 | 386.476 | 287.012 | 99.463 |
| 2015 | 50443 | 34324.171 | 24840.755 | 9483.416 | 18.800 | 27.629 | 621.319 | 422.780 | 305.970 | 116.810 |
| 2016 | 48854 | 33054.753 | 23174.732 | 9880.021 | 20.224 | 29.890 | 596.013 | 403.264 | 282.729 | 120.535 |
| 2017 | 48325 | 32471.234 | 22019.651 | 10451.58 | 21.628 | 32.187 | 583.983 | 392.399 | 266.096 | 126.302 |
| 2018 | 50465 | 33657.595 | 21983.043 | 11674.55 | 23.134 | 34.686 | 604.052 | 402.871 | 263.131 | 139.741 |
| 2019 | 51036 | 33863.424 | 21320.199 | 12543.23 | 24.577 | 37.041 | 604.975 | 401.412 | 252.726 | 148.685 |
| 2020 | 51444 | 33910.830 | 20644.971 | 13265.86 | 25.787 | 39.120 | 691.053 | 455.532 | 277.329 | 178.203 |
| 2021 | 48037 | 31531.880 | 18418.652 | 13113.23 | 27.298 | 41.587 | 640.639 | 420.521 | 245.638 | 174.883 |
| 2022 | 48335 | 31560.212 | 17690.096 | 13870.12 | 28.696 | 43.948 | 639.727 | 417.708 | 234.133 | 183.575 |
| 2023 | 48599 | 31508.781 | 16951.480 | 14557.30 | 29.954 | 46.201 | 638.227 | 413.791 | 222.616 | 191.175 |
| 2024 | 48784 | 31517.293 | 16210.852 | 15306.44 | 31.376 | 48.565 | 635.757 | 410.740 | 211.263 | 199.477 |
| 2025 | 48477 | 31175.389 | 15408.304 | 15767.08 | 32.525 | 50.575 | 729.141 | 468.912 | 231.758 | 237.154 |
| 2026 | 45324 | 29044.598 | 13710.768 | 15333.83 | 33.832 | 52.794 | 679.138 | 435.206 | 205.443 | 229.763 |
| 2027 | 45107 | 28825.765 | 12948.464 | 15877.30 | 35.199 | 55.080 | 673.191 | 430.203 | 193.246 | 236.957 |
| 2028 | 44842 | 28513.846 | 12170.255 | 16343.59 | 36.447 | 57.318 | 666.430 | 423.769 | 180.873 | 242.896 |
| 2029 | 44539 | 28148.109 | 11387.707 | 16760.40 | 37.631 | 59.544 | 659.013 | 416.486 | 168.495 | 247.991 |
| 2030 | 43393 | 27314.475 | 10589.226 | 16725.25 | 38.544 | 61.232 | 760.018 | 478.407 | 185.468 | 292.939 |
| 2031 | 40748 | 25423.102 | 9508.069 | 15915.03 | 39.058 | 62.601 | 712.731 | 444.684 | 166.309 | 278.375 |
| 2032 | 40325 | 25085.902 | 8892.058 | 16193.84 | 40.158 | 64.554 | 703.969 | 437.933 | 155.232 | 282.701 |
| 2033 | 39882 | 24645.067 | 8289.126 | 16355.94 | 41.011 | 66.366 | 694.543 | 429.194 | 144.355 | 284.839 |
| 2034 | 39404 | 24239.126 | 7709.207 | 16529.92 | 41.949 | 68.195 | 684.347 | 420.967 | 133.888 | 287.079 |
| 2035 | 37454 | 22944.147 | 7036.732 | 15907.41 | 42.471 | 69.331 | 796.588 | 487.980 | 149.658 | 338.322 |
| 2036 | 32988 | 19950.581 | 5790.589 | 14159.99 | 42.924 | 70.975 | 702.663 | 424.953 | 123.341 | 301.612 |
| 2037 | 32403 | 19415.334 | 5282.513 | 14132.82 | 43.615 | 72.792 | 690.731 | 413.871 | 112.606 | 301.265 |
| 2038 | 31820 | 18939.317 | 4879.891 | 14059.43 | 44.184 | 74.234 | 678.257 | 403.701 | 104.017 | 299.684 |
| 2039 | 31251 | 18379.752 | 4440.611 | 13939.14 | 44.603 | 75.840 | 665.457 | 391.373 | 94.557 | 296.816 |

# Results for PHIM for Run 2 (Main_Analysis.RTF)

*************************************************************************************

**Main Results for E-Component for Run: 2, Simulation: 1, COD: IHD**

| Variable Parameter | Value |
|---|---|
| 1. Country | US (United States) |
| 2. Sex | F (Females) |
| 3. Year of start of process | 1990 |
| 4. Cause of Death | IHD |
| 5. Effective Dose NULL | CC:1 |
| 6. Effective Dose Ecig | CC:1 Ecig:0.05 Dual:0.525 |
| Note: | Death rates are per million population |

-----------------------------------------------------------------------------------------

Results for Age Groups Combined and Years combined, Population unadjusted for deaths in Ecig scenario,

| Age Group:All | | NULL | Ecig | | | %Drop | | NULL | Ecig | |
|---|---|---|---|---|---|---|---|---|---|---|
| Year | Number | Attrib | Attrib | Drop | %Drop | Attrib | Death | Attrib | Attrib | Drop |
| | Deaths | Deaths | Deaths | Deaths | Deaths | Deaths | Rate | Rates | Rates | Rates |
| All Comb | 3242167 | 398833.796 | 199495.584 | 199338.2 | 6.148 | 49.980 | 890.124 | 109.498 | 54.771 | 54.728 |

-----------------------------------------------------------------------------------------

Results for Age Groups Combined, Population unadjusted for deaths in Ecig scenario,

| Age Group:All | | NULL | Ecig | | | %Drop | | NULL | Ecig | |
|---|---|---|---|---|---|---|---|---|---|---|
| Year | Number | Attrib | Attrib | Drop | %Drop | Attrib | Death | Attrib | Attrib | Drop |
| | Deaths | Deaths | Deaths | Deaths | Deaths | Deaths | Rate | Rates | Rates | Rates |
| 1990 | 98916 | 12922.892 | 12922.892 | 0.000 | 0.000 | 0.000 | 1448.66 | 189.260 | 189.260 | 0.000 |
| 1991 | 96403 | 12856.479 | 12766.075 | 90.404 | 0.094 | 0.703 | 1393.01 | 185.775 | 184.469 | 1.306 |
| 1992 | 93721 | 12662.244 | 12411.645 | 250.598 | 0.267 | 1.979 | 1335.88 | 180.485 | 176.913 | 3.572 |
| 1993 | 93679 | 12730.613 | 12252.269 | 478.344 | 0.511 | 3.757 | 1316.45 | 178.900 | 172.178 | 6.722 |
| 1994 | 90832 | 12495.323 | 11750.260 | 745.063 | 0.820 | 5.963 | 1257.46 | 172.983 | 162.669 | 10.315 |
| 1995 | 89698 | 12365.590 | 11312.560 | 1053.030 | 1.174 | 8.516 | 1222.52 | 168.534 | 154.182 | 14.352 |
| 1996 | 88286 | 12290.551 | 10854.600 | 1435.951 | 1.626 | 11.683 | 1187.66 | 165.338 | 146.021 | 19.317 |
| 1997 | 84777 | 11719.531 | 9989.885 | 1729.646 | 2.040 | 14.759 | 1124.67 | 155.473 | 132.527 | 22.946 |
| 1998 | 82517 | 11416.794 | 9346.031 | 2070.763 | 2.509 | 18.138 | 1079.29 | 149.327 | 122.243 | 27.085 |
| 1999 | 93939 | 13028.038 | 10177.102 | 2850.936 | 3.035 | 21.883 | 1212.16 | 168.109 | 131.322 | 36.787 |
| 2000 | 90039 | 12426.299 | 9243.118 | 3183.181 | 3.535 | 25.616 | 1147.41 | 158.354 | 117.789 | 40.565 |
| 2001 | 86119 | 11939.281 | 8351.446 | 3587.835 | 4.166 | 30.051 | 1085.29 | 150.461 | 105.247 | 45.215 |
| 2002 | 83014 | 11528.794 | 7547.387 | 3981.407 | 4.796 | 34.534 | 1035.91 | 143.865 | 94.182 | 49.683 |
| 2003 | 79302 | 11048.407 | 6769.312 | 4279.095 | 5.396 | 38.730 | 980.336 | 136.581 | 83.683 | 52.898 |
| 2004 | 73861 | 10375.132 | 5895.069 | 4480.063 | 6.066 | 43.181 | 903.963 | 126.978 | 72.148 | 54.830 |
| 2005 | 71649 | 10051.029 | 5297.510 | 4753.520 | 6.634 | 47.294 | 867.197 | 121.652 | 64.118 | 57.534 |
| 2006 | 67551 | 9542.523 | 4614.220 | 4928.303 | 7.296 | 51.646 | 810.467 | 114.490 | 55.361 | 59.129 |
| 2007 | 63503 | 8936.533 | 3972.675 | 4963.858 | 7.817 | 55.546 | 754.310 | 106.151 | 47.189 | 58.962 |
| 2008 | 63171 | 8944.180 | 3646.455 | 5297.726 | 8.386 | 59.231 | 742.239 | 105.091 | 42.845 | 62.247 |
| 2009 | 59747 | 8390.033 | 3157.331 | 5232.702 | 8.758 | 62.368 | 694.108 | 97.471 | 36.680 | 60.791 |
| 2010 | 58008 | 8157.570 | 2817.796 | 5339.773 | 9.205 | 65.458 | 666.189 | 93.685 | 32.361 | 61.324 |
| 2011 | 57368 | 8035.356 | 2526.732 | 5508.624 | 9.602 | 68.555 | 652.644 | 91.414 | 28.745 | 62.669 |
| 2012 | 57411 | 7970.766 | 2271.713 | 5699.053 | 9.927 | 71.499 | 646.453 | 89.752 | 25.580 | 64.172 |
| 2013 | 57067 | 7743.216 | 2027.909 | 5715.307 | 10.015 | 73.811 | 635.688 | 86.254 | 22.590 | 63.665 |
| 2014 | 57538 | 7774.935 | 1850.157 | 5924.778 | 10.297 | 76.204 | 633.906 | 85.658 | 20.384 | 65.274 |
| 2015 | 58395 | 7722.535 | 1705.608 | 6016.927 | 10.304 | 77.914 | 719.266 | 95.120 | 21.008 | 74.112 |
| 2016 | 58658 | 7600.353 | 1527.004 | 6073.349 | 10.354 | 79.909 | 715.621 | 92.723 | 18.629 | 74.094 |
| 2017 | 59004 | 7470.266 | 1373.755 | 6096.512 | 10.332 | 81.610 | 713.034 | 90.274 | 16.601 | 73.673 |
| 2018 | 56077 | 6907.050 | 1161.948 | 5745.102 | 10.245 | 83.177 | 671.224 | 82.675 | 13.908 | 68.767 |
| 2019 | 55886 | 6732.046 | 1043.761 | 5688.285 | 10.178 | 84.496 | 662.462 | 79.801 | 12.373 | 67.428 |
| 2020 | 55313 | 6429.836 | 931.733 | 5498.102 | 9.940 | 85.509 | 743.037 | 86.373 | 12.516 | 73.857 |
| 2021 | 56292 | 6562.016 | 859.600 | 5702.415 | 10.130 | 86.900 | 750.730 | 87.513 | 11.464 | 76.050 |
| 2022 | 55827 | 6352.366 | 772.433 | 5579.933 | 9.995 | 87.840 | 738.885 | 84.075 | 10.223 | 73.852 |
| 2023 | 55338 | 6134.123 | 698.988 | 5435.136 | 9.822 | 88.605 | 726.728 | 80.557 | 9.179 | 71.377 |
| 2024 | 54770 | 5922.193 | 630.685 | 5291.508 | 9.661 | 89.350 | 713.771 | 77.179 | 8.219 | 68.960 |
| 2025 | 53295 | 5504.188 | 558.358 | 4945.830 | 9.280 | 89.856 | 801.612 | 82.789 | 8.398 | 74.391 |
| 2026 | 55278 | 5641.075 | 536.622 | 5104.454 | 9.234 | 90.487 | 828.283 | 84.526 | 8.041 | 76.485 |
| 2027 | 54199 | 5426.216 | 489.706 | 4936.510 | 9.108 | 90.975 | 808.875 | 80.982 | 7.308 | 73.674 |
| 2028 | 53090 | 5197.830 | 445.214 | 4752.616 | 8.952 | 91.435 | 789.020 | 77.249 | 6.617 | 70.633 |
| 2029 | 51974 | 4973.547 | 406.751 | 4566.796 | 8.787 | 91.822 | 769.023 | 73.590 | 6.018 | 67.571 |
| 2030 | 49479 | 4450.618 | 350.673 | 4099.945 | 8.286 | 92.121 | 866.605 | 77.952 | 6.142 | 71.810 |
| 2031 | 51987 | 4555.052 | 347.120 | 4207.932 | 8.094 | 92.379 | 909.317 | 79.674 | 6.072 | 73.602 |
| 2032 | 50677 | 4349.999 | 318.996 | 4031.003 | 7.954 | 92.667 | 884.689 | 75.939 | 5.569 | 70.371 |
| 2033 | 49380 | 4147.948 | 296.451 | 3851.497 | 7.800 | 92.853 | 859.955 | 72.237 | 5.163 | 67.074 |
| 2034 | 48076 | 3966.133 | 275.159 | 3690.974 | 7.677 | 93.062 | 834.943 | 68.881 | 4.779 | 64.102 |
| 2035 | 44518 | 3289.064 | 222.276 | 3066.788 | 6.889 | 93.242 | 946.825 | 69.952 | 4.727 | 65.225 |
| 2036 | 46347 | 3313.192 | 216.323 | 3096.869 | 6.682 | 93.471 | 987.211 | 70.572 | 4.608 | 65.964 |
| 2037 | 44841 | 3117.951 | 198.215 | 2919.736 | 6.511 | 93.643 | 955.859 | 66.464 | 4.225 | 62.239 |
| 2038 | 43378 | 2949.775 | 184.628 | 2765.147 | 6.375 | 93.741 | 924.621 | 62.876 | 3.935 | 58.940 |
| 2039 | 41973 | 2766.312 | 171.425 | 2594.887 | 6.182 | 93.803 | 893.753 | 58.905 | 3.650 | 55.255 |

# Results for PHIM for Run 2 (Main_Analysis.RTF)

********************************************************************************
**Main Results for E-Component for Run: 2, Simulation: 1, COD: STROKE**

| Variable Parameter | Value |
|---|---|
| 1. Country | US (United States) |
| 2. Sex | F (Females) |
| 3. Year of start of process | 1990 |
| 4. Cause of Death | STROKE |
| 5. Effective Dose NULL | CC:1 |
| 6. Effective Dose Ecig | CC:1 Ecig:0.05 Dual:0.525 |
| Note: | Death rates are per million population |

-----------------------------------------------------------------------------------------
Results for Age Groups Combined and Years combined, Population unadjusted for deaths in Ecig scenario,

| Age Group:All Year | Number Deaths | NULL Attrib Deaths | Ecig Attrib Deaths | Drop Deaths | %Drop Deaths | %Drop Attrib Deaths | Death Rate | NULL Attrib Rates | Ecig Attrib Rates | Drop Rates |
|---|---|---|---|---|---|---|---|---|---|---|
| All Comb | 1352207 | 104899.750 | 51393.873 | 53505.88 | 3.957 | 51.007 | 371.243 | 28.800 | 14.110 | 14.690 |

-----------------------------------------------------------------------------------------
Results for Age Groups Combined, Population unadjusted for deaths in Ecig scenario,

| Age Group:All Year | Number Deaths | NULL Attrib Deaths | Ecig Attrib Deaths | Drop Deaths | %Drop Deaths | %Drop Attrib Deaths | Death Rate | NULL Attrib Rates | Ecig Attrib Rates | Drop Rates |
|---|---|---|---|---|---|---|---|---|---|---|
| 1990 | 33504 | 3293.017 | 3293.017 | 0.000 | 0.000 | 0.000 | 490.678 | 48.227 | 48.227 | 0.000 |
| 1991 | 32505 | 3207.063 | 3179.556 | 27.507 | 0.085 | 0.858 | 469.694 | 46.342 | 45.944 | 0.397 |
| 1992 | 32560 | 3218.125 | 3141.225 | 76.899 | 0.236 | 2.390 | 464.104 | 45.871 | 44.774 | 1.096 |
| 1993 | 32768 | 3226.680 | 3081.071 | 145.609 | 0.444 | 4.513 | 460.480 | 45.344 | 43.297 | 2.046 |
| 1994 | 33005 | 3277.266 | 3045.067 | 232.199 | 0.704 | 7.085 | 456.915 | 45.370 | 42.155 | 3.215 |
| 1995 | 33730 | 3309.378 | 2976.639 | 332.739 | 0.986 | 10.054 | 459.716 | 45.104 | 40.569 | 4.535 |
| 1996 | 33750 | 3312.987 | 2863.115 | 449.871 | 1.333 | 13.579 | 454.019 | 44.568 | 38.516 | 6.052 |
| 1997 | 33699 | 3267.066 | 2703.734 | 563.332 | 1.672 | 17.243 | 447.056 | 43.341 | 35.868 | 7.473 |
| 1998 | 33274 | 3178.725 | 2507.134 | 671.591 | 2.018 | 21.128 | 435.211 | 41.577 | 32.792 | 8.784 |
| 1999 | 33891 | 3120.351 | 2338.538 | 781.813 | 2.307 | 25.055 | 437.317 | 40.264 | 30.176 | 10.088 |
| 2000 | 33827 | 3142.808 | 2228.404 | 914.404 | 2.703 | 29.095 | 431.074 | 40.050 | 28.398 | 11.653 |
| 2001 | 32506 | 2961.588 | 1979.726 | 981.862 | 3.021 | 33.153 | 409.647 | 37.323 | 24.949 | 12.374 |
| 2002 | 32078 | 2940.627 | 1836.883 | 1103.744 | 3.441 | 37.534 | 400.295 | 36.695 | 22.922 | 13.773 |
| 2003 | 30568 | 2819.545 | 1644.379 | 1175.166 | 3.844 | 41.679 | 377.883 | 34.855 | 20.328 | 14.527 |
| 2004 | 29388 | 2730.466 | 1479.270 | 1251.196 | 4.258 | 45.824 | 359.671 | 33.417 | 18.104 | 15.313 |
| 2005 | 27954 | 2670.510 | 1342.312 | 1328.198 | 4.751 | 49.736 | 338.339 | 32.322 | 16.247 | 16.076 |
| 2006 | 27201 | 2623.285 | 1220.899 | 1402.387 | 5.156 | 53.459 | 326.354 | 31.474 | 14.648 | 16.826 |
| 2007 | 26302 | 2536.599 | 1089.111 | 1447.488 | 5.503 | 57.064 | 312.424 | 30.131 | 12.937 | 17.194 |
| 2008 | 25998 | 2469.443 | 980.367 | 1489.077 | 5.728 | 60.300 | 305.468 | 29.015 | 11.519 | 17.496 |
| 2009 | 25040 | 2386.945 | 873.650 | 1513.295 | 6.044 | 63.399 | 290.901 | 27.730 | 10.150 | 17.581 |
| 2010 | 24840 | 2329.274 | 786.008 | 1543.267 | 6.213 | 66.255 | 285.273 | 26.750 | 9.027 | 17.724 |
| 2011 | 24792 | 2286.581 | 706.526 | 1580.055 | 6.373 | 69.101 | 282.045 | 26.013 | 8.038 | 17.975 |
| 2012 | 24500 | 2243.311 | 634.417 | 1608.894 | 6.567 | 71.720 | 275.872 | 25.260 | 7.144 | 18.116 |
| 2013 | 24451 | 2176.503 | 563.638 | 1612.866 | 6.596 | 74.104 | 272.368 | 24.245 | 6.279 | 17.966 |
| 2014 | 25296 | 2211.619 | 525.463 | 1686.156 | 6.666 | 76.241 | 278.691 | 24.366 | 5.789 | 18.577 |
| 2015 | 26316 | 2237.016 | 493.301 | 1743.715 | 6.626 | 77.948 | 324.141 | 27.554 | 6.076 | 21.478 |
| 2016 | 26735 | 2229.057 | 452.970 | 1776.087 | 6.643 | 79.679 | 326.164 | 27.194 | 5.526 | 21.668 |
| 2017 | 27616 | 2221.649 | 417.118 | 1804.531 | 6.534 | 81.225 | 333.726 | 26.848 | 5.041 | 21.807 |
| 2018 | 25256 | 1939.420 | 338.305 | 1601.115 | 6.340 | 82.556 | 302.304 | 23.214 | 4.049 | 19.165 |
| 2019 | 25429 | 1904.400 | 306.072 | 1598.328 | 6.285 | 83.928 | 301.434 | 22.574 | 3.628 | 18.946 |
| 2020 | 25379 | 1806.054 | 271.196 | 1534.859 | 6.048 | 84.984 | 340.922 | 24.261 | 3.643 | 20.618 |
| 2021 | 25448 | 1748.172 | 242.963 | 1505.209 | 5.915 | 86.102 | 339.386 | 23.314 | 3.240 | 20.074 |
| 2022 | 25539 | 1701.148 | 219.987 | 1481.161 | 5.800 | 87.068 | 338.015 | 22.515 | 2.912 | 19.604 |
| 2023 | 25620 | 1652.087 | 200.308 | 1451.779 | 5.667 | 87.875 | 336.461 | 21.696 | 2.631 | 19.066 |
| 2024 | 25659 | 1597.127 | 180.903 | 1416.224 | 5.519 | 88.673 | 334.391 | 20.814 | 2.358 | 18.457 |
| 2025 | 25260 | 1467.622 | 157.696 | 1309.926 | 5.186 | 89.255 | 379.931 | 22.075 | 2.372 | 19.703 |
| 2026 | 25480 | 1390.505 | 141.337 | 1249.169 | 4.903 | 89.836 | 381.790 | 20.835 | 2.118 | 18.718 |
| 2027 | 25264 | 1342.095 | 128.856 | 1213.239 | 4.802 | 90.399 | 377.042 | 20.030 | 1.923 | 18.107 |
| 2028 | 25022 | 1289.536 | 117.622 | 1171.914 | 4.684 | 90.879 | 371.870 | 19.165 | 1.748 | 17.417 |
| 2029 | 24767 | 1242.216 | 107.730 | 1134.486 | 4.581 | 91.328 | 366.459 | 18.380 | 1.594 | 16.786 |
| 2030 | 23959 | 1096.803 | 90.911 | 1005.892 | 4.198 | 91.711 | 419.641 | 19.210 | 1.592 | 17.618 |
| 2031 | 23963 | 1019.991 | 81.416 | 938.575 | 3.917 | 92.018 | 419.142 | 17.841 | 1.424 | 16.417 |
| 2032 | 23615 | 977.406 | 74.813 | 902.593 | 3.822 | 92.346 | 412.259 | 17.063 | 1.306 | 15.757 |
| 2033 | 23259 | 937.450 | 69.681 | 867.768 | 3.731 | 92.567 | 405.051 | 16.326 | 1.213 | 15.112 |
| 2034 | 22879 | 900.763 | 64.722 | 836.041 | 3.654 | 92.815 | 397.339 | 15.644 | 1.124 | 14.520 |
| 2035 | 21733 | 745.023 | 51.968 | 693.055 | 3.189 | 93.025 | 462.224 | 15.845 | 1.105 | 14.740 |
| 2036 | 20904 | 680.360 | 45.611 | 634.749 | 3.036 | 93.296 | 445.263 | 14.492 | 0.972 | 13.520 |
| 2037 | 20397 | 644.109 | 42.149 | 601.960 | 2.951 | 93.456 | 434.791 | 13.730 | 0.898 | 12.832 |
| 2038 | 19890 | 612.448 | 39.375 | 573.073 | 2.881 | 93.571 | 423.966 | 13.055 | 0.839 | 12.215 |
| 2039 | 19392 | 577.529 | 36.715 | 540.815 | 2.789 | 93.643 | 412.930 | 12.298 | 0.782 | 11.516 |

# Results for PHIM for Run 2 (Main_Analysis.RTF)

************************************************************************************
**Main Results for E-Component for Run: 2, Simulation: 1, COD: COPD**

| Variable Parameter | Value |
|---|---|
| 1. Country | US (United States) |
| 2. Sex | F (Females) |
| 3. Year of start of process | 1990 |
| 4. Cause of Death | COPD |
| 5. Effective Dose NULL | CC:1 |
| 6. Effective Dose Ecig | CC:1 Ecig:0.05 Dual:0.525 |
| Note: | Death rates are per million population |

------------------------------------------------------------------------------------------
Results for Age Groups Combined and Years combined, Population unadjusted for deaths in Ecig scenario,

| Age Group:All Year | Number Deaths | NULL Attrib Deaths | Ecig Attrib Deaths | Drop Deaths | %Drop Deaths | %Drop Attrib Deaths | Death Rate | NULL Attrib Rates | Ecig Attrib Rates | Drop Rates |
|---|---|---|---|---|---|---|---|---|---|---|
| All Comb | 2159941 | 862051.244 | 551684.508 | 310366.7 | 14.369 | 36.003 | 593.003 | 236.673 | 151.463 | 85.210 |

------------------------------------------------------------------------------------------
Results for Age Groups Combined, Population unadjusted for deaths in Ecig scenario,

| Age Group:All Year | Number Deaths | NULL Attrib Deaths | Ecig Attrib Deaths | Drop Deaths | %Drop Deaths | %Drop Attrib Deaths | Death Rate | NULL Attrib Rates | Ecig Attrib Rates | Drop Rates |
|---|---|---|---|---|---|---|---|---|---|---|
| 1990 | 24738 | 10694.712 | 10694.712 | 0.000 | 0.000 | 0.000 | 362.296 | 156.628 | 156.628 | 0.000 |
| 1991 | 26562 | 11602.933 | 11584.706 | 18.227 | 0.069 | 0.157 | 383.818 | 167.661 | 167.398 | 0.263 |
| 1992 | 26828 | 11831.375 | 11781.157 | 50.218 | 0.187 | 0.424 | 382.401 | 168.642 | 167.926 | 0.716 |
| 1993 | 29774 | 13246.371 | 13138.062 | 108.309 | 0.364 | 0.818 | 418.406 | 186.148 | 184.626 | 1.522 |
| 1994 | 29804 | 13355.662 | 13183.534 | 172.127 | 0.578 | 1.289 | 412.601 | 184.893 | 182.511 | 2.383 |
| 1995 | 29755 | 13397.439 | 13141.785 | 255.654 | 0.859 | 1.908 | 405.540 | 182.598 | 179.113 | 3.484 |
| 1996 | 30900 | 13960.356 | 13603.065 | 357.291 | 1.156 | 2.559 | 415.680 | 187.801 | 182.994 | 4.806 |
| 1997 | 31128 | 14109.552 | 13642.341 | 467.211 | 1.501 | 3.311 | 412.949 | 187.180 | 180.981 | 6.198 |
| 1998 | 32096 | 14599.657 | 13987.980 | 611.677 | 1.906 | 4.190 | 419.803 | 190.958 | 182.957 | 8.000 |
| 1999 | 34616 | 15728.102 | 14933.909 | 794.193 | 2.294 | 5.050 | 446.673 | 202.950 | 192.702 | 10.248 |
| 2000 | 34299 | 15579.803 | 14634.748 | 945.056 | 2.755 | 6.066 | 437.088 | 198.541 | 186.498 | 12.043 |
| 2001 | 34592 | 15696.614 | 14557.623 | 1138.991 | 3.293 | 7.256 | 435.935 | 197.812 | 183.458 | 14.354 |
| 2002 | 34291 | 15519.777 | 14197.005 | 1322.773 | 3.857 | 8.523 | 427.910 | 193.668 | 177.162 | 16.507 |
| 2003 | 35074 | 15888.389 | 14324.381 | 1564.008 | 4.459 | 9.844 | 433.587 | 196.413 | 177.079 | 19.334 |
| 2004 | 33512 | 15091.573 | 13393.094 | 1698.478 | 5.068 | 11.254 | 410.143 | 184.701 | 163.914 | 20.787 |
| 2005 | 35392 | 15803.656 | 13773.575 | 2030.081 | 5.736 | 12.846 | 428.364 | 191.278 | 166.707 | 24.571 |
| 2006 | 33981 | 15100.563 | 12938.149 | 2162.415 | 6.364 | 14.320 | 407.699 | 181.174 | 155.230 | 25.944 |
| 2007 | 34000 | 14974.384 | 12581.276 | 2393.108 | 7.039 | 15.981 | 403.863 | 177.871 | 149.445 | 28.426 |
| 2008 | 37425 | 16406.197 | 13534.105 | 2872.092 | 7.674 | 17.506 | 439.732 | 192.768 | 159.021 | 33.746 |
| 2009 | 36826 | 16083.194 | 12962.627 | 3120.567 | 8.474 | 19.403 | 427.825 | 186.846 | 150.593 | 36.253 |
| 2010 | 36788 | 15927.400 | 12581.609 | 3345.791 | 9.095 | 21.007 | 422.489 | 182.917 | 144.493 | 38.425 |
| 2011 | 37636 | 16221.836 | 12504.543 | 3717.293 | 9.877 | 22.915 | 428.164 | 184.547 | 142.257 | 42.290 |
| 2012 | 38265 | 16358.849 | 12301.235 | 4057.615 | 10.604 | 24.804 | 430.867 | 184.202 | 138.513 | 45.689 |
| 2013 | 40112 | 16994.822 | 12436.493 | 4558.329 | 11.364 | 26.822 | 446.821 | 189.311 | 138.534 | 50.777 |
| 2014 | 40084 | 16855.093 | 11990.508 | 4864.585 | 12.136 | 28.861 | 441.613 | 185.696 | 132.102 | 53.594 |
| 2015 | 42200 | 17572.483 | 12218.289 | 5354.194 | 12.688 | 30.469 | 519.788 | 216.445 | 150.496 | 65.949 |
| 2016 | 42606 | 17579.955 | 11832.738 | 5747.218 | 13.489 | 32.692 | 519.788 | 214.473 | 144.358 | 70.115 |
| 2017 | 44364 | 18080.688 | 11775.207 | 6305.481 | 14.213 | 34.874 | 536.117 | 218.496 | 142.297 | 76.199 |
| 2018 | 45359 | 18209.262 | 11502.911 | 6706.351 | 14.785 | 36.829 | 542.932 | 217.960 | 137.686 | 80.273 |
| 2019 | 46876 | 18648.302 | 11380.754 | 7267.548 | 15.504 | 38.972 | 555.657 | 221.054 | 134.906 | 86.148 |
| 2020 | 48331 | 19002.597 | 11252.896 | 7749.701 | 16.035 | 40.782 | 649.239 | 255.266 | 151.163 | 104.103 |
| 2021 | 48338 | 18885.717 | 10759.489 | 8126.229 | 16.811 | 43.028 | 644.660 | 251.867 | 143.493 | 108.374 |
| 2022 | 49708 | 19235.076 | 10562.751 | 8672.325 | 17.447 | 45.086 | 657.895 | 254.582 | 139.801 | 114.781 |
| 2023 | 51083 | 19521.158 | 10348.370 | 9172.788 | 17.957 | 46.989 | 670.849 | 256.363 | 135.901 | 120.462 |
| 2024 | 52397 | 19918.620 | 10139.779 | 9778.841 | 18.663 | 49.094 | 682.854 | 259.583 | 132.144 | 127.440 |
| 2025 | 53387 | 20150.545 | 9883.634 | 10266.91 | 19.231 | 50.951 | 803.004 | 303.086 | 148.661 | 154.425 |
| 2026 | 54232 | 20393.792 | 9580.022 | 10813.77 | 19.940 | 53.025 | 812.615 | 305.582 | 143.548 | 162.034 |
| 2027 | 55049 | 20615.351 | 9255.033 | 11360.32 | 20.637 | 55.106 | 821.568 | 307.668 | 138.124 | 169.544 |
| 2028 | 55809 | 20731.894 | 8887.786 | 11844.11 | 21.223 | 57.130 | 829.419 | 308.115 | 132.089 | 176.026 |
| 2029 | 56527 | 20781.967 | 8501.578 | 12280.39 | 21.725 | 59.092 | 836.392 | 307.495 | 125.791 | 181.704 |
| 2030 | 56756 | 20753.201 | 8128.515 | 12624.69 | 22.244 | 60.832 | 994.067 | 363.487 | 142.369 | 221.118 |
| 2031 | 58288 | 21048.417 | 7998.735 | 13049.68 | 22.388 | 61.998 | 1019.54 | 368.165 | 139.909 | 228.256 |
| 2032 | 58782 | 21169.329 | 7660.556 | 13508.77 | 22.981 | 63.813 | 1026.18 | 369.560 | 133.733 | 235.827 |
| 2033 | 59236 | 21109.293 | 7288.320 | 13820.97 | 23.332 | 65.473 | 1031.59 | 367.618 | 126.926 | 240.692 |
| 2034 | 59614 | 21109.429 | 6920.918 | 14188.51 | 23.801 | 67.214 | 1035.33 | 366.613 | 120.197 | 246.415 |
| 2035 | 58938 | 20794.840 | 6542.303 | 14252.54 | 24.182 | 68.539 | 1253.51 | 442.268 | 139.143 | 303.125 |
| 2036 | 56014 | 19354.577 | 5816.916 | 13537.66 | 24.168 | 69.946 | 1193.11 | 412.258 | 123.902 | 288.356 |
| 2037 | 55953 | 19064.169 | 5385.959 | 13678.21 | 24.446 | 71.748 | 1192.72 | 406.385 | 114.811 | 291.574 |
| 2038 | 55859 | 18854.812 | 5033.504 | 13821.31 | 24.743 | 73.304 | 1190.66 | 401.900 | 107.292 | 294.608 |
| 2039 | 55757 | 18437.457 | 4625.323 | 13812.13 | 24.772 | 74.913 | 1187.28 | 392.602 | 98.490 | 294.111 |

# Results for PHIM for Run 2 (Main_Analysis.RTF)

************************************************************************************

**Main Results for E-Component for Run: 2, Simulation: 1, COD: Four Diseases Combined, Adjusted**

| Variable Parameter | Value |
|---|---|
| 1. Country | US (United States) |
| 2. Sex | F (Females) |
| 3. Year of start of process | 1990 |
| 4. Cause of Death | Four Diseases Combined |
| 5. Effective Dose NULL | CC:1 |
| 6. Effective Dose Ecig | CC:1 Ecig:0.05 Dual:0.525 |
| Note: | Death rates are per million population |

------------------------------------------------------------------------------------------

Results for Age Groups Combined and Years combined, Population and Number deaths adjusted for Ecig scenario,

| Age Group:All Year | Number Deaths | NULL Attrib Deaths | Ecig Attrib Deaths | Drop Deaths | %Drop Deaths | %Drop Attrib Deaths | Death Rate | NULL Attrib Rates | Ecig Attrib Rates | Drop Rates |
|---|---|---|---|---|---|---|---|---|---|---|
| All Comb | 9148407 | 2946250.006 | 1954823.387 | 991426.6 | 10.837 | 33.650 | 2499.62 | 808.881 | 534.116 | 274.766 |

------------------------------------------------------------------------------------------

Results for Age Groups Combined, Population and Number deaths adjusted for Ecig scenario,

| Age Group:All Year | Number Deaths | NULL Attrib Deaths | Ecig Attrib Deaths | Drop Deaths | %Drop Deaths | %Drop Attrib Deaths | Death Rate | NULL Attrib Rates | Ecig Attrib Rates | Drop Rates |
|---|---|---|---|---|---|---|---|---|---|---|
| 1990 | 199848 | 57524.822 | 57524.822 | 0.000 | 0.000 | 0.000 | 2926.84 | 842.471 | 842.471 | 0.000 |
| 1991 | 199162 | 59078.486 | 58910.022 | 168.464 | 0.085 | 0.285 | 2877.87 | 853.678 | 851.244 | 2.434 |
| 1992 | 198533 | 60494.150 | 60022.679 | 471.471 | 0.237 | 0.779 | 2829.84 | 862.271 | 855.549 | 6.722 |
| 1993 | 202515 | 62694.848 | 61777.616 | 917.231 | 0.453 | 1.463 | 2845.86 | 881.034 | 868.136 | 12.898 |
| 1994 | 200665 | 63202.598 | 61753.940 | 1448.657 | 0.722 | 2.292 | 2777.91 | 874.966 | 854.892 | 20.074 |
| 1995 | 201036 | 63766.862 | 61674.857 | 2092.005 | 1.041 | 3.281 | 2739.86 | 869.097 | 840.549 | 28.548 |
| 1996 | 201202 | 64591.163 | 61725.514 | 2865.648 | 1.424 | 4.437 | 2706.47 | 868.908 | 830.299 | 38.609 |
| 1997 | 198673 | 64686.912 | 61099.550 | 3587.362 | 1.806 | 5.546 | 2635.34 | 858.147 | 810.467 | 47.680 |
| 1998 | 197623 | 65295.961 | 60873.258 | 4422.704 | 2.238 | 6.773 | 2584.42 | 854.046 | 796.072 | 57.975 |
| 1999 | 211404 | 67327.521 | 61615.666 | 5711.855 | 2.702 | 8.484 | 2727.28 | 868.771 | 794.892 | 73.878 |
| 2000 | 208596 | 67596.897 | 60947.459 | 6649.437 | 3.188 | 9.837 | 2657.45 | 861.419 | 776.453 | 84.967 |
| 2001 | 203735 | 67008.811 | 59367.234 | 7641.577 | 3.751 | 11.404 | 2566.52 | 844.458 | 747.870 | 96.588 |
| 2002 | 200990 | 67067.744 | 58321.004 | 8746.739 | 4.352 | 13.042 | 2506.90 | 836.925 | 727.423 | 109.501 |
| 2003 | 196550 | 66756.217 | 57012.856 | 9743.361 | 4.957 | 14.595 | 2428.31 | 825.244 | 704.374 | 120.870 |
| 2004 | 188394 | 65058.725 | 54473.689 | 10585.04 | 5.619 | 16.270 | 2304.02 | 796.234 | 666.202 | 130.032 |
| 2005 | 186694 | 65214.724 | 53493.802 | 11720.92 | 6.278 | 17.973 | 2257.67 | 789.321 | 646.897 | 142.424 |
| 2006 | 180406 | 63771.146 | 51190.155 | 12580.99 | 6.974 | 19.728 | 2162.28 | 765.117 | 613.547 | 151.571 |
| 2007 | 176006 | 63110.718 | 49647.768 | 13462.95 | 7.649 | 21.332 | 2088.19 | 749.650 | 589.036 | 160.615 |
| 2008 | 177993 | 63718.624 | 48977.494 | 14741.13 | 8.282 | 23.135 | 2088.52 | 748.674 | 574.689 | 173.984 |
| 2009 | 173277 | 62794.698 | 47232.424 | 15562.27 | 8.981 | 24.783 | 2009.93 | 729.515 | 547.872 | 181.643 |
| 2010 | 171016 | 61917.074 | 45491.241 | 16425.83 | 9.605 | 26.529 | 1960.59 | 711.083 | 521.529 | 189.554 |
| 2011 | 170895 | 61680.114 | 44092.757 | 17587.36 | 10.291 | 28.514 | 1940.38 | 701.700 | 500.638 | 201.062 |
| 2012 | 172084 | 62023.127 | 43148.010 | 18875.12 | 10.969 | 30.432 | 1933.47 | 698.386 | 484.794 | 213.593 |
| 2013 | 173443 | 62037.765 | 42004.473 | 20033.29 | 11.550 | 32.292 | 1927.38 | 691.059 | 466.773 | 224.285 |
| 2014 | 174998 | 61921.017 | 40614.209 | 21306.81 | 12.175 | 34.410 | 1922.86 | 682.195 | 446.264 | 235.931 |
| 2015 | 178288 | 61856.204 | 39469.169 | 22387.04 | 12.557 | 36.192 | 2188.82 | 761.899 | 484.560 | 277.338 |
| 2016 | 177872 | 60464.119 | 37205.628 | 23258.49 | 13.076 | 38.467 | 2162.26 | 737.655 | 452.279 | 285.376 |
| 2017 | 180440 | 60243.839 | 35815.740 | 24428.10 | 13.538 | 40.549 | 2172.04 | 728.017 | 431.129 | 296.887 |
| 2018 | 178377 | 60713.327 | 35233.084 | 25480.24 | 14.284 | 41.968 | 2126.10 | 726.721 | 419.948 | 306.772 |
| 2019 | 180571 | 61148.172 | 34312.773 | 26835.40 | 14.861 | 43.886 | 2130.69 | 724.841 | 404.883 | 319.959 |
| 2020 | 181943 | 61149.317 | 33378.065 | 27771.25 | 15.264 | 45.415 | 2430.54 | 821.432 | 445.892 | 375.541 |
| 2021 | 179686 | 58727.786 | 30554.645 | 28173.14 | 15.679 | 47.972 | 2382.11 | 783.216 | 405.065 | 378.151 |
| 2022 | 181110 | 58848.802 | 29530.122 | 29318.68 | 16.188 | 49.820 | 2381.81 | 778.881 | 388.355 | 390.525 |
| 2023 | 182478 | 58816.149 | 28494.241 | 30321.91 | 16.617 | 51.554 | 2380.17 | 772.407 | 371.667 | 400.740 |
| 2024 | 183588 | 58955.232 | 27466.918 | 31488.31 | 17.152 | 53.411 | 2375.32 | 768.316 | 355.376 | 412.940 |
| 2025 | 182536 | 58297.744 | 26321.127 | 31976.62 | 17.518 | 54.851 | 2721.85 | 876.861 | 392.483 | 484.378 |
| 2026 | 182579 | 56469.971 | 24279.267 | 32190.70 | 17.631 | 57.005 | 2710.80 | 846.150 | 360.480 | 485.669 |
| 2027 | 182020 | 56209.427 | 23137.418 | 33072.01 | 18.169 | 58.837 | 2690.34 | 838.883 | 341.983 | 496.900 |
| 2028 | 181299 | 55733.105 | 21938.573 | 33794.53 | 18.640 | 60.636 | 2667.13 | 828.298 | 322.744 | 505.555 |
| 2029 | 180482 | 55145.838 | 20722.296 | 34423.54 | 19.073 | 62.423 | 2642.03 | 815.951 | 303.348 | 512.602 |
| 2030 | 176388 | 53615.097 | 19478.584 | 34136.51 | 19.353 | 63.670 | 3050.43 | 939.056 | 336.860 | 602.196 |
| 2031 | 177997 | 52046.561 | 18256.129 | 33790.43 | 18.984 | 64.923 | 3072.27 | 910.364 | 315.105 | 595.258 |
| 2032 | 176549 | 51582.636 | 17267.357 | 34315.28 | 19.437 | 66.525 | 3039.56 | 900.496 | 297.283 | 603.213 |
| 2033 | 175044 | 50839.758 | 16262.688 | 34577.07 | 19.753 | 68.012 | 3004.54 | 885.375 | 279.141 | 606.234 |
| 2034 | 173393 | 50215.451 | 15286.085 | 34929.37 | 20.145 | 69.559 | 2966.30 | 872.104 | 261.504 | 610.600 |
| 2035 | 166157 | 47773.075 | 14165.206 | 33607.87 | 20.227 | 70.349 | 3471.86 | 1016.05 | 295.983 | 720.062 |
| 2036 | 159795 | 43298.709 | 12150.966 | 31147.74 | 19.492 | 71.937 | 3341.66 | 922.274 | 254.104 | 668.170 |
| 2037 | 157227 | 42241.563 | 11179.493 | 31062.07 | 19.756 | 73.534 | 3288.45 | 900.450 | 233.822 | 666.628 |
| 2038 | 154669 | 41356.353 | 10400.445 | 30955.91 | 20.014 | 74.852 | 3232.78 | 881.531 | 217.383 | 664.148 |
| 2039 | 152181 | 40161.050 | 9524.869 | 30636.18 | 20.131 | 76.283 | 3175.61 | 855.178 | 198.759 | 656.419 |

# Results for PHIM for Run 2 (Main_Analysis.RTF)

***********************************************************************************

**Main Results for E-Component for Run: 2, Simulation: 1, COD: LUNG CANCER, Adjusted**

| Variable Parameter | Value |
|---|---|
| 1. Country | US (United States) |
| 2. Sex | F (Females) |
| 3. Year of start of process | 1990 |
| 4. Cause of Death | LUNG CANCER |
| 5. Effective Dose NULL | CC:1 |
| 6. Effective Dose Ecig | CC:1 Ecig:0.05 Dual:0.525 |
| Note: | Death rates are per million population |

-----------------------------------------------------------------------------------------

Results for Age Groups Combined and Years combined, Population and Number deaths adjusted for Ecig scenario,

| Age Group:All | | NULL | Ecig | | | %Drop | | NULL | Ecig | |
|---|---|---|---|---|---|---|---|---|---|---|
| Year | Number | Attrib | Attrib | Drop | %Drop | Attrib | Death | Attrib | Attrib | Drop |
| | Deaths | Deaths | Deaths | Deaths | Deaths | Deaths | Rate | Rates | Rates | Rates |
| All Comb | 2343934 | 1580465.217 | 1148450.411 | 432014.8 | 18.431 | 27.335 | 640.432 | 433.910 | 313.791 | 120.120 |

-----------------------------------------------------------------------------------------

Results for Age Groups Combined, Population and Number deaths adjusted for Ecig scenario,

| Age Group:All | | NULL | Ecig | | | %Drop | | NULL | Ecig | |
|---|---|---|---|---|---|---|---|---|---|---|
| Year | Number | Attrib | Attrib | Drop | %Drop | Attrib | Death | Attrib | Attrib | Drop |
| | Deaths | Deaths | Deaths | Deaths | Deaths | Deaths | Rate | Rates | Rates | Rates |
| 1990 | 42690 | 30614.201 | 30614.201 | 0.000 | 0.000 | 0.000 | 625.210 | 448.355 | 448.355 | 0.000 |
| 1991 | 43692 | 31412.010 | 31379.684 | 32.326 | 0.074 | 0.103 | 631.345 | 453.901 | 453.433 | 0.467 |
| 1992 | 45423 | 32782.407 | 32688.550 | 93.857 | 0.207 | 0.286 | 647.451 | 467.274 | 465.935 | 1.339 |
| 1993 | 46292 | 33491.183 | 33305.813 | 185.370 | 0.400 | 0.553 | 650.518 | 470.643 | 468.033 | 2.609 |
| 1994 | 47019 | 34074.347 | 33774.114 | 300.233 | 0.639 | 0.881 | 650.904 | 471.719 | 467.553 | 4.167 |
| 1995 | 47843 | 34694.454 | 34242.053 | 452.401 | 0.946 | 1.304 | 652.042 | 472.861 | 466.675 | 6.186 |
| 1996 | 48250 | 35027.269 | 34401.657 | 625.612 | 1.297 | 1.786 | 649.027 | 471.202 | 462.753 | 8.449 |
| 1997 | 49044 | 35590.763 | 34758.927 | 831.836 | 1.696 | 2.337 | 650.551 | 472.153 | 461.066 | 11.086 |
| 1998 | 49699 | 36100.785 | 35025.397 | 1075.388 | 2.164 | 2.979 | 649.940 | 472.184 | 458.046 | 14.139 |
| 1999 | 48901 | 35451.030 | 34156.135 | 1294.894 | 2.648 | 3.653 | 630.861 | 457.448 | 440.642 | 16.806 |
| 2000 | 50356 | 36447.986 | 34828.291 | 1619.695 | 3.216 | 4.444 | 641.520 | 464.474 | 443.702 | 20.772 |
| 2001 | 50422 | 36411.326 | 34462.282 | 1949.045 | 3.865 | 5.353 | 635.180 | 458.863 | 434.134 | 24.729 |
| 2002 | 51487 | 37078.545 | 34720.291 | 2358.254 | 4.580 | 6.360 | 642.190 | 462.696 | 433.058 | 29.638 |
| 2003 | 51461 | 36999.876 | 34251.317 | 2748.559 | 5.341 | 7.429 | 635.782 | 457.395 | 423.163 | 34.232 |
| 2004 | 51464 | 36861.555 | 33679.922 | 3181.633 | 6.182 | 8.631 | 629.391 | 451.138 | 411.899 | 39.239 |
| 2005 | 51498 | 36689.529 | 33049.362 | 3640.167 | 7.069 | 9.922 | 622.765 | 444.069 | 399.664 | 44.405 |
| 2006 | 51446 | 36504.774 | 32382.996 | 4121.778 | 8.012 | 11.291 | 616.615 | 437.979 | 388.131 | 49.848 |
| 2007 | 51946 | 36663.202 | 31967.477 | 4695.725 | 9.040 | 12.808 | 616.306 | 435.498 | 379.272 | 56.226 |
| 2008 | 51098 | 35898.803 | 30772.110 | 5126.693 | 10.033 | 14.281 | 599.566 | 421.800 | 361.072 | 60.728 |
| 2009 | 51333 | 35934.525 | 30191.194 | 5743.331 | 11.188 | 15.983 | 595.432 | 417.468 | 350.202 | 67.266 |
| 2010 | 51010 | 35502.829 | 29254.342 | 6248.488 | 12.250 | 17.600 | 584.795 | 407.730 | 335.383 | 72.347 |
| 2011 | 50684 | 35136.342 | 28298.729 | 6837.613 | 13.491 | 19.460 | 575.471 | 399.726 | 321.309 | 78.417 |
| 2012 | 51443 | 35450.201 | 27879.940 | 7570.261 | 14.716 | 21.355 | 577.989 | 399.173 | 313.248 | 85.925 |
| 2013 | 51288 | 35123.224 | 26909.619 | 8213.605 | 16.015 | 23.385 | 569.935 | 391.249 | 299.032 | 92.217 |
| 2014 | 51494 | 35079.370 | 26177.440 | 8901.930 | 17.287 | 25.377 | 565.810 | 386.476 | 287.635 | 98.841 |
| 2015 | 50707 | 34324.171 | 24973.591 | 9350.579 | 18.440 | 27.242 | 622.527 | 422.780 | 306.599 | 116.180 |
| 2016 | 49134 | 33054.753 | 23310.695 | 9744.057 | 19.832 | 29.479 | 597.284 | 403.264 | 283.370 | 119.894 |
| 2017 | 48628 | 32471.234 | 22161.218 | 10310.02 | 21.202 | 31.751 | 585.360 | 392.399 | 266.764 | 125.635 |
| 2018 | 50809 | 33657.595 | 22136.895 | 11520.70 | 22.674 | 34.229 | 605.601 | 402.871 | 263.853 | 139.019 |
| 2019 | 51414 | 33863.424 | 21482.826 | 12380.60 | 24.080 | 36.560 | 606.676 | 401.412 | 253.492 | 147.920 |
| 2020 | 51858 | 33910.830 | 20816.314 | 13094.52 | 25.251 | 38.615 | 692.762 | 455.532 | 278.081 | 177.450 |
| 2021 | 48455 | 31531.880 | 18583.832 | 12948.05 | 26.722 | 41.063 | 642.374 | 420.521 | 246.367 | 174.154 |
| 2022 | 48787 | 31560.212 | 17860.891 | 13699.32 | 28.080 | 43.407 | 641.609 | 417.708 | 234.891 | 182.817 |
| 2023 | 49087 | 31508.781 | 17127.298 | 14381.48 | 29.298 | 45.643 | 640.264 | 413.791 | 223.401 | 190.390 |
| 2024 | 49307 | 31517.293 | 16391.065 | 15126.23 | 30.678 | 47.993 | 637.953 | 410.740 | 212.073 | 198.667 |
| 2025 | 49036 | 31175.389 | 15592.140 | 15583.25 | 31.779 | 49.986 | 731.193 | 468.912 | 232.499 | 236.412 |
| 2026 | 45887 | 29044.598 | 13886.873 | 15157.72 | 33.033 | 52.188 | 681.289 | 435.206 | 206.182 | 229.024 |
| 2027 | 45702 | 28825.765 | 13125.800 | 15699.96 | 34.353 | 54.465 | 675.504 | 430.203 | 194.006 | 236.197 |
| 2028 | 45469 | 28513.846 | 12347.422 | 16166.42 | 35.554 | 56.697 | 668.912 | 423.769 | 181.646 | 242.123 |
| 2029 | 45200 | 28148.109 | 11563.755 | 16584.35 | 36.691 | 58.918 | 661.667 | 416.486 | 169.279 | 247.207 |
| 2030 | 44083 | 27314.475 | 10764.030 | 16550.45 | 37.544 | 60.592 | 762.366 | 478.407 | 186.152 | 292.255 |
| 2031 | 41442 | 25423.102 | 9676.647 | 15746.45 | 37.996 | 61.938 | 715.304 | 444.684 | 167.021 | 277.662 |
| 2032 | 41050 | 25085.902 | 9058.840 | 16027.06 | 39.043 | 63.889 | 706.733 | 437.933 | 155.961 | 281.972 |
| 2033 | 40636 | 24645.067 | 8453.303 | 16191.76 | 39.845 | 65.700 | 697.504 | 429.194 | 145.097 | 284.097 |
| 2034 | 40188 | 24239.126 | 7870.133 | 16368.99 | 40.731 | 67.531 | 687.502 | 420.967 | 134.637 | 286.330 |
| 2035 | 38255 | 22944.147 | 7193.640 | 15750.51 | 41.172 | 68.647 | 799.343 | 487.980 | 150.312 | 337.668 |
| 2036 | 33726 | 19950.581 | 5926.294 | 14024.29 | 41.583 | 70.295 | 705.291 | 424.953 | 123.932 | 301.021 |
| 2037 | 33159 | 19415.334 | 5411.787 | 14003.55 | 42.232 | 72.126 | 693.530 | 413.871 | 113.189 | 300.682 |
| 2038 | 32593 | 18939.317 | 5004.607 | 13934.71 | 42.754 | 73.576 | 681.227 | 403.701 | 104.603 | 299.098 |
| 2039 | 32040 | 18379.752 | 4558.672 | 13821.08 | 43.137 | 75.197 | 668.593 | 391.373 | 95.127 | 296.246 |

# Results for PHIM for Run 2 (Main_Analysis.RTF)

************************************************************************************

**Main Results for E-Component for Run: 2, Simulation: 1, COD: IHD, Adjusted**

| Variable Parameter | Value |
|---|---|
| 1. Country | US (United States) |
| 2. Sex | F (Females) |
| 3. Year of start of process | 1990 |
| 4. Cause of Death | IHD |
| 5. Effective Dose NULL | CC:1 |
| 6. Effective Dose Ecig | CC:1 Ecig:0.05 Dual:0.525 |
| Note: | Death rates are per million population |

-----------------------------------------------------------------------------------------

Results for Age Groups Combined and Years combined, Population and Number deaths adjusted for Ecig scenario,

| Age Group:All<br>Year | Number<br>Deaths | NULL<br>Attrib<br>Deaths | Ecig<br>Attrib<br>Deaths | Drop<br>Deaths | %Drop<br>Deaths | %Drop<br>Attrib<br>Deaths | Death<br>Rate | NULL<br>Attrib<br>Rates | Ecig<br>Attrib<br>Rates | Drop<br>Rates |
|---|---|---|---|---|---|---|---|---|---|---|
| All Comb | 3261640 | 398833.796 | 199770.684 | 199063.1 | 6.103 | 49.911 | 891.176 | 109.498 | 54.583 | 54.915 |

-----------------------------------------------------------------------------------------

Results for Age Groups Combined, Population and Number deaths adjusted for Ecig scenario,

| Age Group:All<br>Year | Number<br>Deaths | NULL<br>Attrib<br>Deaths | Ecig<br>Attrib<br>Deaths | Drop<br>Deaths | %Drop<br>Deaths | %Drop<br>Attrib<br>Deaths | Death<br>Rate | NULL<br>Attrib<br>Rates | Ecig<br>Attrib<br>Rates | Drop<br>Rates |
|---|---|---|---|---|---|---|---|---|---|---|
| 1990 | 98916 | 12922.892 | 12922.892 | 0.000 | 0.000 | 0.000 | 1448.66 | 189.260 | 189.260 | 0.000 |
| 1991 | 96403 | 12856.479 | 12766.075 | 90.404 | 0.094 | 0.703 | 1393.01 | 185.775 | 184.469 | 1.306 |
| 1992 | 93721 | 12662.244 | 12411.692 | 250.552 | 0.267 | 1.979 | 1335.88 | 180.485 | 176.913 | 3.572 |
| 1993 | 93680 | 12730.613 | 12252.443 | 478.170 | 0.510 | 3.756 | 1316.45 | 178.900 | 172.179 | 6.721 |
| 1994 | 90835 | 12495.323 | 11750.669 | 744.654 | 0.820 | 5.959 | 1257.48 | 172.983 | 162.671 | 10.312 |
| 1995 | 89704 | 12365.590 | 11313.321 | 1052.269 | 1.173 | 8.510 | 1222.55 | 168.534 | 154.186 | 14.348 |
| 1996 | 88296 | 12290.551 | 10855.838 | 1434.714 | 1.625 | 11.673 | 1187.71 | 165.338 | 146.027 | 19.311 |
| 1997 | 84792 | 11719.531 | 9991.683 | 1727.848 | 2.038 | 14.743 | 1124.73 | 155.473 | 132.537 | 22.937 |
| 1998 | 82538 | 11416.794 | 9348.504 | 2068.291 | 2.506 | 18.116 | 1079.39 | 149.327 | 122.255 | 27.072 |
| 1999 | 93972 | 13028.038 | 10180.868 | 2847.170 | 3.030 | 21.854 | 1212.32 | 168.109 | 131.341 | 36.768 |
| 2000 | 90082 | 12426.299 | 9247.769 | 3178.530 | 3.528 | 25.579 | 1147.62 | 158.354 | 117.814 | 40.540 |
| 2001 | 86173 | 11939.281 | 8356.945 | 3582.336 | 4.157 | 30.005 | 1085.55 | 150.461 | 105.275 | 45.186 |
| 2002 | 83080 | 11528.794 | 7553.679 | 3975.115 | 4.785 | 34.480 | 1036.24 | 143.865 | 94.215 | 49.650 |
| 2003 | 79382 | 11048.407 | 6776.349 | 4272.058 | 5.382 | 38.667 | 980.731 | 136.581 | 83.719 | 52.862 |
| 2004 | 73952 | 10375.132 | 5902.554 | 4472.577 | 6.048 | 43.109 | 904.420 | 126.978 | 72.187 | 54.791 |
| 2005 | 71755 | 10051.029 | 5305.549 | 4745.481 | 6.613 | 47.214 | 867.730 | 121.652 | 64.160 | 57.492 |
| 2006 | 67670 | 9542.523 | 4622.475 | 4920.048 | 7.271 | 51.559 | 811.063 | 114.490 | 55.403 | 59.087 |
| 2007 | 63633 | 8936.533 | 3980.938 | 4955.595 | 7.788 | 55.453 | 754.960 | 106.151 | 47.231 | 58.920 |
| 2008 | 63320 | 8944.180 | 3655.167 | 5289.013 | 8.353 | 59.134 | 742.981 | 105.091 | 42.889 | 62.203 |
| 2009 | 59909 | 8390.033 | 3165.961 | 5224.073 | 8.720 | 62.265 | 694.908 | 97.471 | 36.724 | 60.747 |
| 2010 | 58185 | 8157.570 | 2826.488 | 5331.081 | 9.162 | 65.351 | 667.060 | 93.685 | 32.404 | 61.281 |
| 2011 | 57565 | 8035.356 | 2535.443 | 5499.912 | 9.554 | 68.446 | 653.604 | 91.414 | 28.788 | 62.626 |
| 2012 | 57630 | 7970.766 | 2280.366 | 5690.399 | 9.874 | 71.391 | 647.510 | 89.752 | 25.621 | 64.130 |
| 2013 | 57310 | 7743.216 | 2036.461 | 5706.755 | 9.958 | 73.700 | 636.855 | 86.254 | 22.630 | 63.624 |
| 2014 | 57808 | 7774.935 | 1858.730 | 5916.205 | 10.234 | 76.093 | 635.189 | 85.658 | 20.424 | 65.234 |
| 2015 | 58698 | 7722.535 | 1714.316 | 6008.219 | 10.236 | 77.801 | 720.628 | 95.120 | 21.047 | 74.074 |
| 2016 | 58991 | 7600.353 | 1535.501 | 6064.852 | 10.281 | 79.797 | 717.111 | 92.723 | 18.666 | 74.058 |
| 2017 | 59370 | 7470.266 | 1382.058 | 6088.208 | 10.255 | 81.499 | 714.666 | 90.274 | 16.636 | 73.638 |
| 2018 | 56456 | 6907.050 | 1169.537 | 5737.513 | 10.163 | 83.067 | 672.908 | 82.675 | 13.940 | 68.735 |
| 2019 | 56297 | 6732.046 | 1051.133 | 5680.914 | 10.091 | 84.386 | 664.292 | 79.801 | 12.403 | 67.398 |
| 2020 | 55758 | 6429.836 | 938.891 | 5490.944 | 9.848 | 85.398 | 744.863 | 86.373 | 12.542 | 73.831 |
| 2021 | 56777 | 6562.016 | 866.607 | 5695.408 | 10.031 | 86.794 | 752.699 | 87.513 | 11.489 | 76.025 |
| 2022 | 56344 | 6352.366 | 779.157 | 5573.209 | 9.891 | 87.734 | 740.994 | 84.075 | 10.247 | 73.829 |
| 2023 | 55888 | 6134.123 | 705.465 | 5428.658 | 9.713 | 88.499 | 728.982 | 80.557 | 9.202 | 71.355 |
| 2024 | 55353 | 5922.193 | 636.884 | 5285.309 | 9.548 | 89.246 | 716.170 | 77.179 | 8.240 | 68.939 |
| 2025 | 53908 | 5504.188 | 564.314 | 4939.873 | 9.163 | 89.748 | 803.846 | 82.789 | 8.415 | 74.374 |
| 2026 | 55953 | 5641.075 | 542.669 | 5098.406 | 9.112 | 90.380 | 830.754 | 84.526 | 8.057 | 76.469 |
| 2027 | 54903 | 5426.216 | 495.543 | 4930.673 | 8.981 | 90.868 | 811.493 | 80.982 | 7.324 | 73.658 |
| 2028 | 53822 | 5197.830 | 450.788 | 4747.042 | 8.820 | 91.327 | 791.789 | 77.249 | 6.632 | 70.618 |
| 2029 | 52733 | 4973.547 | 412.095 | 4561.452 | 8.650 | 91.714 | 771.943 | 73.590 | 6.033 | 67.557 |
| 2030 | 50258 | 4450.618 | 355.747 | 4094.871 | 8.148 | 92.007 | 869.160 | 77.952 | 6.152 | 71.799 |
| 2031 | 52852 | 4555.052 | 352.379 | 4202.674 | 7.952 | 92.264 | 912.245 | 79.674 | 6.082 | 73.592 |
| 2032 | 51566 | 4349.999 | 324.041 | 4025.959 | 7.807 | 92.551 | 887.792 | 75.939 | 5.579 | 70.361 |
| 2033 | 50292 | 4147.948 | 301.349 | 3846.599 | 7.649 | 92.735 | 863.234 | 72.237 | 5.172 | 67.064 |
| 2034 | 49008 | 3966.133 | 279.883 | 3686.250 | 7.522 | 92.943 | 838.389 | 68.881 | 4.788 | 64.093 |
| 2035 | 45453 | 3289.064 | 226.646 | 3062.418 | 6.738 | 93.109 | 949.745 | 69.952 | 4.736 | 65.217 |
| 2036 | 47364 | 3313.192 | 220.698 | 3092.494 | 6.529 | 93.339 | 990.481 | 70.572 | 4.615 | 65.957 |
| 2037 | 45866 | 3117.951 | 202.340 | 2915.612 | 6.357 | 93.510 | 959.290 | 66.464 | 4.232 | 62.232 |
| 2038 | 44409 | 2949.775 | 188.587 | 2761.187 | 6.218 | 93.607 | 928.206 | 62.876 | 3.942 | 58.934 |
| 2039 | 43009 | 2766.312 | 175.195 | 2591.118 | 6.025 | 93.667 | 897.481 | 58.905 | 3.656 | 55.249 |

# Results for PHIM for Run 2 (Main_Analysis.RTF)

************************************************************************************

**Main Results for E-Component for Run: 2, Simulation: 1, COD: STROKE, Adjusted**

| Variable Parameter | Value |
|---|---|
| 1. Country | US (United States) |
| 2. Sex | F (Females) |
| 3. Year of start of process | 1990 |
| 4. Cause of Death | STROKE |
| 5. Effective Dose NULL | CC:1 |
| 6. Effective Dose Ecig | CC:1 Ecig:0.05 Dual:0.525 |
| Note: | Death rates are per million population |

-----------------------------------------------------------------------------------------

Results for Age Groups Combined and Years combined, Population and Number deaths adjusted for Ecig scenario,

| Age Group:All | | NULL | Ecig | | | %Drop | | NULL | Ecig | |
|---|---|---|---|---|---|---|---|---|---|---|
| Year | Number Deaths | Attrib Deaths | Attrib Deaths | Drop Deaths | %Drop Deaths | Attrib Deaths | Death Rate | Attrib Rates | Attrib Rates | Drop Rates |
| All Comb | 1361482 | 104899.750 | 51457.219 | 53442.53 | 3.925 | 50.946 | 371.997 | 28.800 | 14.060 | 14.740 |

-----------------------------------------------------------------------------------------

Results for Age Groups Combined, Population and Number deaths adjusted for Ecig scenario,

| Age Group:All | | NULL | Ecig | | | %Drop | | NULL | Ecig | |
|---|---|---|---|---|---|---|---|---|---|---|
| Year | Number Deaths | Attrib Deaths | Attrib Deaths | Drop Deaths | %Drop Deaths | Attrib Deaths | Death Rate | Attrib Rates | Attrib Rates | Drop Rates |
| 1990 | 33504 | 3293.017 | 3293.017 | 0.000 | 0.000 | 0.000 | 490.678 | 48.227 | 48.227 | 0.000 |
| 1991 | 32505 | 3207.063 | 3179.556 | 27.507 | 0.085 | 0.858 | 469.694 | 46.342 | 45.944 | 0.397 |
| 1992 | 32560 | 3218.125 | 3141.236 | 76.889 | 0.236 | 2.389 | 464.104 | 45.871 | 44.774 | 1.096 |
| 1993 | 32768 | 3226.680 | 3081.112 | 145.569 | 0.444 | 4.511 | 460.482 | 45.344 | 43.298 | 2.046 |
| 1994 | 33006 | 3277.266 | 3045.164 | 232.102 | 0.703 | 7.082 | 456.920 | 45.370 | 42.156 | 3.214 |
| 1995 | 33732 | 3309.378 | 2976.822 | 332.557 | 0.986 | 10.049 | 459.725 | 45.104 | 40.570 | 4.534 |
| 1996 | 33754 | 3312.987 | 2863.411 | 449.575 | 1.332 | 13.570 | 454.035 | 44.568 | 38.517 | 6.051 |
| 1997 | 33705 | 3267.066 | 2704.171 | 562.895 | 1.670 | 17.229 | 447.080 | 43.341 | 35.870 | 7.471 |
| 1998 | 33282 | 3178.725 | 2507.729 | 670.996 | 2.016 | 21.109 | 435.246 | 41.577 | 32.795 | 8.782 |
| 1999 | 33902 | 3120.351 | 2339.308 | 781.043 | 2.304 | 25.031 | 437.368 | 40.264 | 30.179 | 10.085 |
| 2000 | 33842 | 3142.808 | 2229.393 | 913.415 | 2.699 | 29.064 | 431.143 | 40.050 | 28.402 | 11.648 |
| 2001 | 32525 | 2961.588 | 1980.871 | 980.718 | 3.015 | 33.115 | 409.735 | 37.323 | 24.954 | 12.369 |
| 2002 | 32103 | 2940.627 | 1838.210 | 1102.418 | 3.434 | 37.489 | 400.409 | 36.695 | 22.928 | 13.768 |
| 2003 | 30598 | 2819.545 | 1645.842 | 1173.703 | 3.836 | 41.627 | 378.021 | 34.855 | 20.334 | 14.522 |
| 2004 | 29423 | 2730.466 | 1480.857 | 1249.609 | 4.247 | 45.765 | 359.838 | 33.417 | 18.111 | 15.307 |
| 2005 | 27994 | 2670.510 | 1344.000 | 1326.510 | 4.739 | 49.673 | 338.529 | 32.322 | 16.253 | 16.069 |
| 2006 | 27247 | 2623.285 | 1222.709 | 1400.576 | 5.140 | 53.390 | 326.577 | 31.474 | 14.655 | 16.819 |
| 2007 | 26354 | 2536.599 | 1090.972 | 1445.628 | 5.485 | 56.991 | 312.675 | 30.131 | 12.944 | 17.187 |
| 2008 | 26058 | 2469.443 | 982.288 | 1487.156 | 5.707 | 60.222 | 305.757 | 29.015 | 11.526 | 17.489 |
| 2009 | 25106 | 2386.945 | 875.597 | 1511.348 | 6.020 | 63.317 | 291.220 | 27.730 | 10.156 | 17.574 |
| 2010 | 24915 | 2329.274 | 787.985 | 1541.289 | 6.186 | 66.170 | 285.632 | 26.750 | 9.034 | 17.717 |
| 2011 | 24876 | 2286.581 | 708.512 | 1578.069 | 6.344 | 69.014 | 282.447 | 26.013 | 8.045 | 17.969 |
| 2012 | 24593 | 2243.311 | 636.382 | 1606.929 | 6.534 | 71.632 | 276.315 | 25.260 | 7.150 | 18.110 |
| 2013 | 24554 | 2176.503 | 565.569 | 1610.934 | 6.561 | 74.015 | 272.859 | 24.245 | 6.285 | 17.960 |
| 2014 | 25415 | 2211.619 | 527.451 | 1684.168 | 6.627 | 76.151 | 279.253 | 24.366 | 5.796 | 18.570 |
| 2015 | 26453 | 2237.016 | 495.368 | 1741.648 | 6.584 | 77.856 | 324.759 | 27.554 | 6.082 | 21.472 |
| 2016 | 26887 | 2229.057 | 455.049 | 1774.008 | 6.598 | 79.586 | 326.848 | 27.194 | 5.532 | 21.663 |
| 2017 | 27789 | 2221.649 | 419.226 | 1802.423 | 6.486 | 81.130 | 334.505 | 26.848 | 5.046 | 21.801 |
| 2018 | 25429 | 1939.420 | 340.180 | 1599.240 | 6.289 | 82.460 | 303.090 | 23.214 | 4.055 | 19.160 |
| 2019 | 25619 | 1904.400 | 307.909 | 1596.490 | 6.232 | 83.832 | 302.300 | 22.574 | 3.633 | 18.941 |
| 2020 | 25587 | 1806.054 | 272.988 | 1533.066 | 5.992 | 84.885 | 341.810 | 24.261 | 3.647 | 20.614 |
| 2021 | 25674 | 1748.172 | 244.707 | 1503.466 | 5.856 | 86.002 | 340.366 | 23.314 | 3.244 | 20.070 |
| 2022 | 25783 | 1701.148 | 221.681 | 1479.467 | 5.738 | 86.969 | 339.077 | 22.515 | 2.915 | 19.600 |
| 2023 | 25883 | 1652.087 | 201.955 | 1450.131 | 5.603 | 87.776 | 337.609 | 21.696 | 2.634 | 19.062 |
| 2024 | 25941 | 1597.127 | 182.484 | 1414.642 | 5.453 | 88.574 | 335.627 | 20.814 | 2.361 | 18.453 |
| 2025 | 25560 | 1467.622 | 159.217 | 1308.405 | 5.119 | 89.151 | 381.129 | 22.075 | 2.374 | 19.700 |
| 2026 | 25804 | 1390.505 | 142.800 | 1247.705 | 4.835 | 89.730 | 383.124 | 20.835 | 2.120 | 18.715 |
| 2027 | 25606 | 1342.095 | 130.268 | 1211.827 | 4.733 | 90.294 | 378.470 | 20.030 | 1.925 | 18.104 |
| 2028 | 25382 | 1289.536 | 118.979 | 1170.557 | 4.612 | 90.774 | 373.396 | 19.165 | 1.750 | 17.415 |
| 2029 | 25145 | 1242.216 | 109.037 | 1133.179 | 4.507 | 91.222 | 368.085 | 18.380 | 1.596 | 16.784 |
| 2030 | 24353 | 1096.803 | 92.145 | 1004.658 | 4.125 | 91.599 | 421.149 | 19.210 | 1.594 | 17.617 |
| 2031 | 24381 | 1019.991 | 82.579 | 937.411 | 3.845 | 91.904 | 420.823 | 17.841 | 1.425 | 16.416 |
| 2032 | 24050 | 977.406 | 75.928 | 901.478 | 3.748 | 92.232 | 414.064 | 17.063 | 1.307 | 15.756 |
| 2033 | 23711 | 937.450 | 70.767 | 866.682 | 3.655 | 92.451 | 406.982 | 16.326 | 1.215 | 15.111 |
| 2034 | 23346 | 900.763 | 65.771 | 834.992 | 3.577 | 92.698 | 399.394 | 15.644 | 1.125 | 14.519 |
| 2035 | 22211 | 745.023 | 52.933 | 692.090 | 3.116 | 92.895 | 464.092 | 15.845 | 1.106 | 14.739 |
| 2036 | 21387 | 680.360 | 46.480 | 633.880 | 2.964 | 93.168 | 447.244 | 14.492 | 0.972 | 13.520 |
| 2037 | 20889 | 644.109 | 42.974 | 601.136 | 2.878 | 93.328 | 436.896 | 13.730 | 0.899 | 12.831 |
| 2038 | 20391 | 612.448 | 40.165 | 572.283 | 2.807 | 93.442 | 426.194 | 13.055 | 0.840 | 12.215 |
| 2039 | 19901 | 577.529 | 37.467 | 540.062 | 2.714 | 93.512 | 415.275 | 12.298 | 0.782 | 11.516 |

# Results for PHIM for Run 2 (Main_Analysis.RTF)

************************************************************************************
**Main Results for E-Component for Run: 2, Simulation: 1, COD: COPD, Adjusted**

| Variable Parameter | Value |
|---|---|
| 1. Country | US (United States) |
| 2. Sex | F (Females) |
| 3. Year of start of process | 1990 |
| 4. Cause of Death | COPD |
| 5. Effective Dose NULL | CC:1 |
| 6. Effective Dose Ecig | CC:1 Ecig:0.05 Dual:0.525 |
| Note: | Death rates are per million population |

-----------------------------------------------------------------------------------------
Results for Age Groups Combined and Years combined, Population and Number deaths adjusted for Ecig scenario,

| Age Group:All | | NULL | Ecig | | | %Drop | | NULL | Ecig | |
|---|---|---|---|---|---|---|---|---|---|---|
| Year | Number Deaths | Attrib Deaths | Attrib Deaths | Drop Deaths | %Drop Deaths | Attrib Deaths | Death Rate | Attrib Rates | Attrib Rates | Drop Rates |
| All Comb | 2181351 | 862051.244 | 555145.073 | 306906.2 | 14.070 | 35.602 | 596.010 | 236.673 | 151.682 | 84.991 |

-----------------------------------------------------------------------------------------
Results for Age Groups Combined, Population and Number deaths adjusted for Ecig scenario,

| Age Group:All | | NULL | Ecig | | | %Drop | | NULL | Ecig | |
|---|---|---|---|---|---|---|---|---|---|---|
| Year | Number Deaths | Attrib Deaths | Attrib Deaths | Drop Deaths | %Drop Deaths | Attrib Deaths | Death Rate | Attrib Rates | Attrib Rates | Drop Rates |
| 1990 | 24738 | 10694.712 | 10694.712 | 0.000 | 0.000 | 0.000 | 362.296 | 156.628 | 156.628 | 0.000 |
| 1991 | 26562 | 11602.933 | 11584.706 | 18.227 | 0.069 | 0.157 | 383.818 | 167.661 | 167.398 | 0.263 |
| 1992 | 26828 | 11831.375 | 11781.201 | 50.173 | 0.187 | 0.424 | 382.401 | 168.642 | 167.926 | 0.716 |
| 1993 | 29774 | 13246.371 | 13138.248 | 108.123 | 0.363 | 0.816 | 418.408 | 186.148 | 184.627 | 1.521 |
| 1994 | 29805 | 13355.662 | 13183.994 | 171.668 | 0.576 | 1.285 | 412.606 | 184.893 | 182.513 | 2.381 |
| 1995 | 29757 | 13397.439 | 13142.661 | 254.778 | 0.856 | 1.902 | 405.549 | 182.598 | 179.118 | 3.480 |
| 1996 | 30903 | 13960.356 | 13604.608 | 355.748 | 1.151 | 2.548 | 415.697 | 187.801 | 183.002 | 4.799 |
| 1997 | 31133 | 14109.552 | 13644.769 | 464.783 | 1.493 | 3.294 | 412.976 | 187.180 | 180.994 | 6.186 |
| 1998 | 32104 | 14599.657 | 13991.628 | 608.029 | 1.894 | 4.165 | 419.844 | 190.958 | 182.976 | 7.982 |
| 1999 | 34628 | 15728.102 | 14939.356 | 788.747 | 2.278 | 5.015 | 446.736 | 202.950 | 192.730 | 10.220 |
| 2000 | 34316 | 15579.803 | 14642.006 | 937.797 | 2.733 | 6.019 | 437.174 | 198.541 | 186.535 | 12.006 |
| 2001 | 34614 | 15696.614 | 14567.136 | 1129.478 | 3.263 | 7.196 | 436.050 | 197.812 | 183.507 | 14.304 |
| 2002 | 34319 | 15519.777 | 14208.824 | 1310.953 | 3.820 | 8.447 | 428.057 | 193.668 | 177.223 | 16.445 |
| 2003 | 35110 | 15888.389 | 14339.348 | 1549.041 | 4.412 | 9.750 | 433.777 | 196.413 | 177.158 | 19.256 |
| 2004 | 33555 | 15091.573 | 13410.356 | 1681.217 | 5.010 | 11.140 | 410.370 | 184.701 | 164.006 | 20.695 |
| 2005 | 35446 | 15803.656 | 13794.891 | 2008.765 | 5.667 | 12.711 | 428.650 | 191.278 | 166.821 | 24.458 |
| 2006 | 34043 | 15100.563 | 12961.975 | 2138.589 | 6.282 | 14.162 | 408.027 | 181.174 | 155.358 | 25.817 |
| 2007 | 34073 | 14974.384 | 12608.381 | 2366.003 | 6.944 | 15.800 | 404.246 | 177.871 | 149.590 | 28.281 |
| 2008 | 37518 | 16406.197 | 13567.928 | 2838.269 | 7.565 | 17.300 | 440.221 | 192.768 | 159.203 | 33.565 |
| 2009 | 36930 | 16083.194 | 12999.672 | 3083.522 | 8.350 | 19.172 | 428.368 | 186.846 | 150.790 | 36.056 |
| 2010 | 36906 | 15927.400 | 12622.425 | 3304.975 | 8.955 | 20.750 | 423.101 | 182.917 | 144.708 | 38.209 |
| 2011 | 37771 | 16221.836 | 12550.073 | 3671.762 | 9.721 | 22.635 | 428.860 | 184.547 | 142.496 | 42.051 |
| 2012 | 38419 | 16358.849 | 12351.321 | 4007.528 | 10.431 | 24.498 | 431.655 | 184.202 | 138.774 | 45.428 |
| 2013 | 40291 | 16994.822 | 12492.823 | 4501.999 | 11.174 | 26.490 | 447.732 | 189.311 | 138.826 | 50.485 |
| 2014 | 40282 | 16855.093 | 12050.588 | 4804.505 | 11.927 | 28.505 | 442.609 | 185.696 | 132.410 | 53.285 |
| 2015 | 42430 | 17572.483 | 12285.894 | 5286.589 | 12.460 | 30.084 | 520.910 | 216.445 | 150.833 | 65.612 |
| 2016 | 42860 | 17579.955 | 11904.383 | 5675.573 | 13.242 | 32.284 | 521.011 | 214.473 | 144.712 | 69.761 |
| 2017 | 44653 | 18080.688 | 11853.238 | 6227.451 | 13.946 | 34.443 | 537.504 | 218.496 | 142.682 | 75.814 |
| 2018 | 45683 | 18209.262 | 11586.472 | 6622.790 | 14.497 | 36.370 | 544.501 | 217.960 | 138.101 | 79.859 |
| 2019 | 47240 | 18648.302 | 11470.906 | 7177.396 | 15.193 | 38.488 | 557.422 | 221.054 | 135.354 | 85.700 |
| 2020 | 48740 | 19002.597 | 11349.872 | 7652.725 | 15.701 | 40.272 | 651.104 | 255.266 | 151.621 | 103.645 |
| 2021 | 48780 | 18885.717 | 10859.498 | 8026.219 | 16.454 | 42.499 | 646.673 | 251.867 | 143.965 | 107.902 |
| 2022 | 50195 | 19235.076 | 10668.394 | 8566.682 | 17.067 | 44.537 | 660.126 | 254.582 | 140.302 | 114.280 |
| 2023 | 51620 | 19521.158 | 10459.522 | 9061.636 | 17.554 | 46.420 | 673.315 | 256.363 | 136.430 | 119.933 |
| 2024 | 52987 | 19918.620 | 10256.485 | 9662.135 | 18.235 | 48.508 | 685.568 | 259.583 | 132.702 | 126.882 |
| 2025 | 54032 | 20150.545 | 10005.456 | 10145.09 | 18.776 | 50.346 | 805.685 | 303.086 | 149.195 | 153.891 |
| 2026 | 54935 | 20393.792 | 9706.924 | 10686.87 | 19.454 | 52.403 | 815.636 | 305.582 | 144.121 | 161.461 |
| 2027 | 55808 | 20615.351 | 9385.807 | 11229.54 | 20.122 | 54.472 | 824.877 | 307.668 | 138.727 | 168.941 |
| 2028 | 56626 | 20731.894 | 9021.384 | 11710.51 | 20.681 | 56.485 | 833.037 | 308.115 | 132.716 | 175.399 |
| 2029 | 57405 | 20781.967 | 8637.409 | 12144.56 | 21.156 | 58.438 | 840.335 | 307.495 | 126.441 | 181.054 |
| 2030 | 57694 | 20753.201 | 8266.662 | 12486.54 | 21.643 | 60.167 | 997.750 | 363.487 | 142.963 | 220.525 |
| 2031 | 59321 | 21048.417 | 8144.524 | 12903.89 | 21.753 | 61.306 | 1023.90 | 368.165 | 140.577 | 227.588 |
| 2032 | 59883 | 21169.329 | 7808.549 | 13360.78 | 22.312 | 63.114 | 1030.97 | 369.560 | 134.436 | 235.125 |
| 2033 | 60405 | 21109.293 | 7437.269 | 13672.02 | 22.634 | 64.768 | 1036.82 | 367.618 | 127.657 | 239.961 |
| 2034 | 60852 | 21109.429 | 7070.298 | 14039.13 | 23.071 | 66.506 | 1041.01 | 366.613 | 120.954 | 245.659 |
| 2035 | 60238 | 20794.840 | 6691.987 | 14102.85 | 23.412 | 67.819 | 1258.68 | 442.268 | 139.830 | 302.438 |
| 2036 | 57318 | 19354.577 | 5957.494 | 13397.08 | 23.373 | 69.219 | 1198.65 | 412.258 | 124.585 | 287.673 |
| 2037 | 57314 | 19064.169 | 5522.393 | 13541.78 | 23.627 | 71.033 | 1198.73 | 406.385 | 115.502 | 290.883 |
| 2038 | 57277 | 18854.812 | 5167.085 | 13687.73 | 23.898 | 72.595 | 1197.15 | 401.900 | 107.999 | 293.901 |
| 2039 | 57231 | 18437.457 | 4753.536 | 13683.92 | 23.910 | 74.218 | 1194.27 | 392.602 | 99.194 | 293.408 |

# Results for PHIM for Run 2 (ELL Summary Main_Analysis.RTF)

**********************************************************************************

**Estimated Loss of Life from the E Component for Run: 2, Simulation: 1**

Country: US (United States)

Follow-up Period: 1990 to 2039

**Table 1: Estimated Loss of Life, at the end of follow-up, all age groups combined, Population unadjusted for deaths in Ecig scenario**

| Disease | Sex | Year | Years of life lost NULL | Years of life lost Ecig | Years of life Saved |
|---|---|---|---|---|---|
| All Diseases | Males | 2039 | 42093547.949 | 24844842.920 | 17248705.0 |
| | Female | 2039 | 23817891.273 | 14841321.981 | 8976569.29 |
| Lung cancer | Males | 2039 | 18222549.860 | 13673132.793 | 4549417.07 |
| | Female | 2039 | 12512468.845 | 8943697.068 | 3568771.78 |
| IHD | Males | 2039 | 15635992.616 | 6388971.725 | 9247020.89 |
| | Female | 2039 | 4798769.065 | 2031004.418 | 2767764.65 |
| Stroke | Males | 2039 | 2389546.238 | 1130931.546 | 1258614.69 |
| | Female | 2039 | 1665361.569 | 838551.181 | 826810.388 |
| COPD | Males | 2039 | 5845459.234 | 3651806.856 | 2193652.38 |
| | Female | 2039 | 4841291.793 | 3028069.314 | 1813222.48 |

# Results for PHIM for Run 2 (ELL Summary Main_Analysis.RTF)

************************************************************************************

**Table 2: Estimated Loss of Life, at the end of follow-up, all age groups combined, Population and Number deaths adjusted for Ecig scenario**

| Disease | Sex | Year | Years of life lost NULL | Years of life lost Ecig | Years of life Saved |
|---|---|---|---|---|---|
| All Diseases | Males | 2039 | 42093547.949 | 24944049.345 | 17149498.6 |
| | Female | 2039 | 23817891.273 | 14878472.973 | 8939418.30 |
| Lung cancer | Males | 2039 | 18222549.860 | 13734071.508 | 4488478.35 |
| | Female | 2039 | 12512468.845 | 8966835.101 | 3545633.74 |
| IHD | Males | 2039 | 15635992.616 | 6400411.543 | 9235581.07 |
| | Female | 2039 | 4798769.065 | 2032897.444 | 2765871.62 |
| Stroke | Males | 2039 | 2389546.238 | 1133120.630 | 1256425.61 |
| | Female | 2039 | 1665361.569 | 839227.001 | 826134.569 |
| COPD | Males | 2039 | 5845459.234 | 3676445.664 | 2169013.57 |
| | Female | 2039 | 4841291.793 | 3039513.428 | 1801778.37 |

# Results for PHIM for Run 2 (ELL Summary Main_Analysis.RTF)

*************************************************************************************

**Table 3: Estimated Loss of Life, All age groups combined, Population unadjusted for deaths in Ecig scenario, cumulative over years**

| Disease | Sex | Year | Years of life lost NULL | Years of life lost Ecig | Years of life Saved |
|---|---|---|---|---|---|
| All Diseases | Males | 1990 | 1304839.645 | 1304839.645 | 0.000 |
| | | 1991 | 2579865.358 | 2571191.563 | 8673.794 |
| | | 1992 | 3830683.831 | 3798434.278 | 32249.553 |
| | | 1993 | 5076567.803 | 5000945.334 | 75622.469 |
| | | 1994 | 6292145.526 | 6149972.450 | 142173.076 |
| | | 1995 | 7485537.010 | 7250147.137 | 235389.872 |
| | | 1996 | 8655955.028 | 8299455.316 | 356499.712 |
| | | 1997 | 9791729.713 | 9287416.800 | 504312.913 |
| | | 1998 | 10899134.186 | 10221705.069 | 677429.116 |
| | | 1999 | 12070409.674 | 11161557.359 | 908852.316 |
| | | 2000 | 13231384.595 | 12060869.628 | 1170514.97 |
| | | 2001 | 14371761.018 | 12909860.896 | 1461900.12 |
| | | 2002 | 15508906.386 | 13721118.233 | 1787788.15 |
| | | 2003 | 16638100.422 | 14494953.295 | 2143147.13 |
| | | 2004 | 17736465.088 | 15221016.767 | 2515448.32 |
| | | 2005 | 18834658.606 | 15920770.426 | 2913888.18 |
| | | 2006 | 19909100.501 | 16574333.431 | 3334767.07 |
| | | 2007 | 20958321.463 | 17189500.048 | 3768821.42 |
| | | 2008 | 22000068.148 | 17780542.528 | 4219525.62 |
| | | 2009 | 23018816.903 | 18338833.779 | 4679983.12 |
| | | 2010 | 24018480.537 | 18865893.612 | 5152586.92 |
| | | 2011 | 25003034.574 | 19365460.142 | 5637574.43 |
| | | 2012 | 25969871.429 | 19836253.379 | 6133618.05 |
| | | 2013 | 26920275.180 | 20280410.566 | 6639864.61 |
| | | 2014 | 27849005.227 | 20696280.124 | 7152725.10 |
| | | 2015 | 28749620.400 | 21087450.541 | 7662169.86 |
| | | 2016 | 29622417.562 | 21445334.867 | 8177082.69 |
| | | 2017 | 30462559.724 | 21772633.062 | 8689926.66 |
| | | 2018 | 31293299.299 | 22086805.483 | 9206493.82 |
| | | 2019 | 32109993.427 | 22383308.149 | 9726685.28 |
| | | 2020 | 32883323.977 | 22658766.923 | 10224557.1 |
| | | 2021 | 33610102.554 | 22897203.212 | 10712899.3 |
| | | 2022 | 34322010.448 | 23120423.621 | 11201586.8 |
| | | 2023 | 35018383.550 | 23328545.924 | 11689837.6 |
| | | 2024 | 35700177.955 | 23522841.800 | 12177336.2 |
| | | 2025 | 36315500.346 | 23697896.667 | 12617603.7 |
| | | 2026 | 36896159.343 | 23846299.741 | 13049859.6 |
| | | 2027 | 37462570.116 | 23983904.847 | 13478665.3 |
| | | 2028 | 38015337.780 | 24110842.845 | 13904494.9 |
| | | 2029 | 38554814.764 | 24228461.159 | 14326353.6 |
| | | 2030 | 39003805.400 | 24328914.052 | 14674891.3 |
| | | 2031 | 39436509.703 | 24414150.996 | 15022358.7 |
| | | 2032 | 39858470.458 | 24492813.925 | 15365656.5 |
| | | 2033 | 40270147.082 | 24564731.518 | 15705415.6 |
| | | 2034 | 40672665.743 | 24631241.939 | 16041423.8 |
| | | 2035 | 40962732.664 | 24681878.285 | 16280854.4 |
| | | 2036 | 41255021.653 | 24727475.101 | 16527546.6 |
| | | 2037 | 41540573.023 | 24769779.637 | 16770793.4 |
| | | 2038 | 41820158.066 | 24808653.227 | 17011504.8 |
| | | 2039 | 42093547.949 | 24844842.920 | 17248705.0 |
| | Female | 1990 | 588979.406 | 588979.406 | 0.000 |
| | | 1991 | 1182571.252 | 1179606.280 | 2964.972 |
| | | 1992 | 1777346.679 | 1766096.264 | 11250.415 |
| | | 1993 | 2380261.899 | 2353262.976 | 26998.923 |
| | | 1994 | 2983130.039 | 2931319.535 | 51810.504 |
| | | 1995 | 3588982.584 | 3501953.135 | 87029.448 |
| | | 1996 | 4195430.235 | 4060559.156 | 134871.078 |
| | | 1997 | 4793866.694 | 4600669.542 | 193197.152 |
| | | 1998 | 5389747.381 | 5126522.729 | 263224.652 |
| | | 1999 | 6006722.467 | 5652709.202 | 354013.265 |
| | | 2000 | 6625758.871 | 6168008.445 | 457750.425 |
| | | 2001 | 7245135.411 | 6668469.982 | 576665.429 |
| | | 2002 | 7867492.021 | 7156230.900 | 711261.121 |
| | | 2003 | 8485051.793 | 7626509.909 | 858541.884 |
| | | 2004 | 9092994.774 | 8075889.851 | 1017104.92 |
| | | 2005 | 9706032.803 | 8515624.851 | 1190407.95 |
| | | 2006 | 10311907.548 | 8936036.634 | 1375870.91 |
| | | 2007 | 10909437.502 | 9339199.145 | 1570238.36 |
| | | 2008 | 11506462.491 | 9726998.701 | 1779463.79 |
| | | 2009 | 12098393.287 | 10101269.888 | 1997123.40 |
| | | 2010 | 12681188.981 | 10457302.966 | 2223886.02 |

## Results for PHIM for Run 2 (ELL Summary Main_Analysis.RTF)

| | | | | | |
|---|---|---|---|---|---|
| | | 2011 | 13260062.991 | 10798106.476 | 2461956.52 |
| | | 2012 | 13839417.441 | 11126309.813 | 2713107.63 |
| | | 2013 | 14409614.758 | 11438724.543 | 2970890.22 |
| | | 2014 | 14976658.746 | 11735791.599 | 3240867.15 |
| | | 2015 | 15529977.821 | 12016036.561 | 3513941.26 |
| | | 2016 | 16066640.764 | 12275711.741 | 3790929.02 |
| | | 2017 | 16590035.078 | 12518427.703 | 4071607.38 |
| | | 2018 | 17098716.137 | 12747664.327 | 4351051.81 |
| | | 2019 | 17599868.850 | 12964026.696 | 4635842.15 |
| | | 2020 | 18081346.241 | 13166276.826 | 4915069.42 |
| | | 2021 | 18537594.460 | 13348102.615 | 5189491.84 |
| | | 2022 | 18985300.234 | 13518565.788 | 5466734.45 |
| | | 2023 | 19423958.747 | 13678091.569 | 5745867.18 |
| | | 2024 | 19853347.772 | 13826545.792 | 6026801.98 |
| | | 2025 | 20252822.245 | 13961902.146 | 6290920.10 |
| | | 2026 | 20619307.063 | 14078701.719 | 6540605.34 |
| | | 2027 | 20976063.406 | 14186622.182 | 6789441.22 |
| | | 2028 | 21323641.947 | 14286616.429 | 7037025.52 |
| | | 2029 | 21662117.106 | 14378588.022 | 7283529.08 |
| | | 2030 | 21960971.694 | 14459104.607 | 7501867.09 |
| | | 2031 | 22224951.983 | 14524390.922 | 7700561.06 |
| | | 2032 | 22480397.853 | 14583926.600 | 7896471.25 |
| | | 2033 | 22728639.455 | 14638591.108 | 8090048.35 |
| | | 2034 | 22970448.888 | 14688423.591 | 8282025.30 |
| | | 2035 | 23164154.188 | 14728542.093 | 8435612.10 |
| | | 2036 | 23334538.616 | 14760471.560 | 8574067.06 |
| | | 2037 | 23499760.723 | 14789694.462 | 8710066.26 |
| | | 2038 | 23660308.401 | 14816547.837 | 8843760.56 |
| | | 2039 | 23817891.273 | 14841321.981 | 8976569.29 |
| Lung cancer | Males | 1990 | 595621.791 | 595621.791 | 0.000 |
| | | 1991 | 1177744.113 | 1177096.215 | 647.898 |
| | | 1992 | 1750034.892 | 1747588.524 | 2446.368 |
| | | 1993 | 2319752.308 | 2313793.474 | 5958.833 |
| | | 1994 | 2872597.339 | 2861044.943 | 11552.396 |
| | | 1995 | 3411494.825 | 3391837.345 | 19657.480 |
| | | 1996 | 3945569.726 | 3914495.006 | 31074.720 |
| | | 1997 | 4463316.362 | 4417529.473 | 45786.889 |
| | | 1998 | 4975301.180 | 4910809.033 | 64492.147 |
| | | 1999 | 5473691.125 | 5386270.379 | 87420.746 |
| | | 2000 | 5977500.570 | 5861408.635 | 116091.935 |
| | | 2001 | 6474795.516 | 6324339.217 | 150456.299 |
| | | 2002 | 6968769.705 | 6777551.543 | 191218.162 |
| | | 2003 | 7460282.140 | 7221078.886 | 239203.254 |
| | | 2004 | 7947048.275 | 7652813.485 | 294234.791 |
| | | 2005 | 8430571.669 | 8073726.972 | 356844.697 |
| | | 2006 | 8903945.390 | 8477112.119 | 426833.271 |
| | | 2007 | 9364177.982 | 8860731.444 | 503446.538 |
| | | 2008 | 9822525.458 | 9233176.188 | 589349.271 |
| | | 2009 | 10275228.886 | 9591550.883 | 683678.003 |
| | | 2010 | 10720275.500 | 9934556.553 | 785718.947 |
| | | 2011 | 11155356.373 | 10260302.038 | 895054.335 |
| | | 2012 | 11582611.617 | 10569495.530 | 1013116.09 |
| | | 2013 | 11998803.791 | 10860600.203 | 1138203.59 |
| | | 2014 | 12402087.090 | 11133009.920 | 1269077.17 |
| | | 2015 | 12793451.706 | 11388523.018 | 1404928.69 |
| | | 2016 | 13160101.653 | 11618462.876 | 1541638.78 |
| | | 2017 | 13503583.414 | 11825261.361 | 1678322.05 |
| | | 2018 | 13859755.737 | 12028890.032 | 1830865.71 |
| | | 2019 | 14211625.176 | 12220929.355 | 1990695.82 |
| | | 2020 | 14553935.288 | 12400132.143 | 2153803.14 |
| | | 2021 | 14851747.495 | 12548213.981 | 2303533.51 |
| | | 2022 | 15144531.004 | 12686347.277 | 2458183.73 |
| | | 2023 | 15431932.496 | 12814666.588 | 2617265.91 |
| | | 2024 | 15713920.740 | 12933847.277 | 2780073.46 |
| | | 2025 | 15979112.816 | 13041642.796 | 2937470.02 |
| | | 2026 | 16206550.153 | 13127605.870 | 3078944.28 |
| | | 2027 | 16428826.050 | 13206742.693 | 3222083.36 |
| | | 2028 | 16645902.186 | 13279304.459 | 3366597.73 |
| | | 2029 | 16858126.517 | 13346132.562 | 3511993.95 |
| | | 2030 | 17045220.687 | 13403560.703 | 3641659.98 |
| | | 2031 | 17208482.610 | 13449035.864 | 3759446.75 |
| | | 2032 | 17367586.074 | 13490760.199 | 3876825.88 |
| | | 2033 | 17522716.280 | 13528751.798 | 3993964.48 |
| | | 2034 | 17674226.555 | 13563727.351 | 4110499.20 |
| | | 2035 | 17792201.734 | 13590647.600 | 4201554.13 |
| | | 2036 | 17903508.236 | 13613766.039 | 4289742.20 |
| | | 2037 | 18012162.345 | 13635190.297 | 4376972.05 |
| | | 2038 | 18118464.470 | 13654828.273 | 4463636.20 |

# Results for PHIM for Run 2 (ELL Summary Main_Analysis.RTF)

| | | | | | |
|---|---|---|---|---|---|
| | | 2039 | 18222549.860 | 13673132.793 | 4549417.07 |
| | Female | 1990 | 307897.357 | 307897.357 | 0.000 |
| | | 1991 | 616547.418 | 616130.924 | 416.494 |
| | | 1992 | 931086.773 | 929437.087 | 1649.686 |
| | | 1993 | 1246155.671 | 1242093.622 | 4062.049 |
| | | 1994 | 1561212.934 | 1553213.610 | 7999.324 |
| | | 1995 | 1879967.742 | 1866031.995 | 13935.747 |
| | | 1996 | 2197488.109 | 2175330.941 | 22157.168 |
| | | 1997 | 2515698.453 | 2482608.212 | 33090.240 |
| | | 1998 | 2836249.556 | 2789166.728 | 47082.828 |
| | | 1999 | 3150201.390 | 3086075.629 | 64125.761 |
| | | 2000 | 3472722.342 | 3387451.783 | 85270.560 |
| | | 2001 | 3796994.889 | 3686142.879 | 110852.010 |
| | | 2002 | 4126619.935 | 3984994.427 | 141625.508 |
| | | 2003 | 4454982.533 | 4277792.995 | 177189.538 |
| | | 2004 | 4784343.030 | 4565968.922 | 218374.109 |
| | | 2005 | 5113443.121 | 4848172.807 | 265270.314 |
| | | 2006 | 5442543.963 | 5123799.513 | 318744.449 |
| | | 2007 | 5772441.497 | 5393546.115 | 378895.382 |
| | | 2008 | 6091911.943 | 5648112.112 | 443799.831 |
| | | 2009 | 6413671.330 | 5897096.830 | 516574.500 |
| | | 2010 | 6730763.530 | 6135774.383 | 594989.146 |
| | | 2011 | 7043614.101 | 6363893.211 | 679720.890 |
| | | 2012 | 7356270.780 | 6584383.784 | 771886.996 |
| | | 2013 | 7662551.964 | 6793036.181 | 869515.782 |
| | | 2014 | 7964551.465 | 6991439.356 | 973112.109 |
| | | 2015 | 8254895.403 | 7176068.627 | 1078826.78 |
| | | 2016 | 8530661.848 | 7344333.377 | 1186328.47 |
| | | 2017 | 8795826.122 | 7499811.619 | 1296014.50 |
| | | 2018 | 9067233.707 | 7651200.460 | 1416033.25 |
| | | 2019 | 9335343.270 | 7793747.034 | 1541596.24 |
| | | 2020 | 9596110.883 | 7926871.666 | 1669239.22 |
| | | 2021 | 9833186.859 | 8043051.101 | 1790135.76 |
| | | 2022 | 10066452.037 | 8151529.510 | 1914922.53 |
| | | 2023 | 10295503.609 | 8252603.152 | 2042900.46 |
| | | 2024 | 10520125.318 | 8346209.147 | 2173916.17 |
| | | 2025 | 10731915.863 | 8431162.800 | 2300753.06 |
| | | 2026 | 10917495.549 | 8501919.930 | 2415575.62 |
| | | 2027 | 11098620.885 | 8566938.175 | 2531682.71 |
| | | 2028 | 11275441.234 | 8626869.944 | 2648571.29 |
| | | 2029 | 11447962.710 | 8681691.318 | 2766271.39 |
| | | 2030 | 11601867.623 | 8729270.165 | 2872597.46 |
| | | 2031 | 11731666.516 | 8766524.676 | 2965141.84 |
| | | 2032 | 11857585.969 | 8800389.551 | 3057196.42 |
| | | 2033 | 11980128.625 | 8831382.206 | 3148746.42 |
| | | 2034 | 12099562.464 | 8859569.651 | 3239992.81 |
| | | 2035 | 12195919.946 | 8881900.533 | 3314019.41 |
| | | 2036 | 12278201.969 | 8899335.753 | 3378866.22 |
| | | 2037 | 12358146.336 | 8915337.369 | 3442808.97 |
| | | 2038 | 12435978.633 | 8930069.880 | 3505908.75 |
| | | 2039 | 12512468.845 | 8943697.068 | 3568771.78 |
| IHD | Males | 1990 | 517209.093 | 517209.093 | 0.000 |
| | | 1991 | 1019219.395 | 1012116.414 | 7102.981 |
| | | 1992 | 1509598.614 | 1483223.780 | 26374.834 |
| | | 1993 | 1992735.299 | 1931293.560 | 61441.739 |
| | | 1994 | 2462877.575 | 2348236.427 | 114641.147 |
| | | 1995 | 2928249.390 | 2739587.604 | 188661.786 |
| | | 1996 | 3377377.730 | 3093769.594 | 283608.136 |
| | | 1997 | 3809062.472 | 3410983.614 | 398078.858 |
| | | 1998 | 4222213.867 | 3691999.343 | 530214.525 |
| | | 1999 | 4708317.743 | 3995842.935 | 712474.808 |
| | | 2000 | 5182084.396 | 4267611.500 | 914472.896 |
| | | 2001 | 5644097.705 | 4507654.531 | 1136443.17 |
| | | 2002 | 6107232.129 | 4725171.439 | 1382060.69 |
| | | 2003 | 6561785.704 | 4917367.588 | 1644418.12 |
| | | 2004 | 6996156.348 | 5083038.074 | 1913118.27 |
| | | 2005 | 7426261.361 | 5231275.656 | 2194985.71 |
| | | 2006 | 7849012.694 | 5361233.683 | 2487779.01 |
| | | 2007 | 8255300.530 | 5473563.830 | 2781736.70 |
| | | 2008 | 8650662.040 | 5572263.316 | 3078398.72 |
| | | 2009 | 9030818.513 | 5658314.697 | 3372503.82 |
| | | 2010 | 9403026.327 | 5734767.765 | 3668258.56 |
| | | 2011 | 9767535.506 | 5802661.524 | 3964873.98 |
| | | 2012 | 10123044.281 | 5863226.883 | 4259817.40 |
| | | 2013 | 10470104.786 | 5917213.307 | 4552891.48 |
| | | 2014 | 10807756.112 | 5965604.967 | 4842151.15 |
| | | 2015 | 11130681.425 | 6008570.192 | 5122111.23 |
| | | 2016 | 11448966.506 | 6047704.281 | 5401262.22 |

# Results for PHIM for Run 2 (ELL Summary Main_Analysis.RTF)

| | | | | | |
|---|---|---|---|---|---|
| | | 2017 | 11758164.882 | 6083249.110 | 5674915.77 |
| | | 2018 | 12054448.241 | 6114587.734 | 5939860.51 |
| | | 2019 | 12341606.261 | 6143268.022 | 6198338.24 |
| | | 2020 | 12600728.939 | 6167887.690 | 6432841.25 |
| | | 2021 | 12861767.105 | 6191301.895 | 6670465.21 |
| | | 2022 | 13114168.616 | 6212951.237 | 6901217.38 |
| | | 2023 | 13357894.797 | 6232859.489 | 7125035.31 |
| | | 2024 | 13593848.521 | 6251462.470 | 7342386.05 |
| | | 2025 | 13789177.359 | 6266464.753 | 7522712.61 |
| | | 2026 | 13993401.656 | 6281594.134 | 7711807.52 |
| | | 2027 | 14190519.224 | 6295912.057 | 7894607.17 |
| | | 2028 | 14381193.356 | 6309234.044 | 8071959.31 |
| | | 2029 | 14565171.927 | 6321780.927 | 8243391.00 |
| | | 2030 | 14697381.605 | 6330713.522 | 8366668.08 |
| | | 2031 | 14842203.512 | 6340380.062 | 8501823.45 |
| | | 2032 | 14982485.826 | 6349625.811 | 8632860.01 |
| | | 2033 | 15118410.647 | 6358208.273 | 8760202.37 |
| | | 2034 | 15250284.398 | 6366399.959 | 8883884.44 |
| | | 2035 | 15322911.166 | 6370776.265 | 8952134.90 |
| | | 2036 | 15405115.031 | 6375654.323 | 9029460.71 |
| | | 2037 | 15484709.137 | 6380358.809 | 9104350.33 |
| | | 2038 | 15561812.193 | 6384788.800 | 9177023.39 |
| | | 2039 | 15635992.616 | 6388971.725 | 9247020.89 |
| | Female | 1990 | 147299.110 | 147299.110 | 0.000 |
| | | 1991 | 295122.462 | 293257.343 | 1865.120 |
| | | 1992 | 439962.920 | 432980.418 | 6982.502 |
| | | 1993 | 584735.615 | 568176.357 | 16559.258 |
| | | 1994 | 727640.783 | 696325.065 | 31315.717 |
| | | 1995 | 868486.937 | 816723.530 | 51763.407 |
| | | 1996 | 1010321.847 | 930874.715 | 79447.132 |
| | | 1997 | 1144230.930 | 1032428.216 | 111802.713 |
| | | 1998 | 1274513.622 | 1124660.899 | 149852.722 |
| | | 1999 | 1426725.938 | 1224827.253 | 201898.685 |
| | | 2000 | 1573462.680 | 1314391.947 | 259070.733 |
| | | 2001 | 1719132.015 | 1395197.136 | 323934.879 |
| | | 2002 | 1863006.732 | 1467654.366 | 395352.366 |
| | | 2003 | 2002943.227 | 1531544.847 | 471398.381 |
| | | 2004 | 2137798.705 | 1587240.173 | 550558.532 |
| | | 2005 | 2271071.245 | 1637284.865 | 633786.381 |
| | | 2006 | 2400707.731 | 1680843.235 | 719864.496 |
| | | 2007 | 2523634.512 | 1718179.358 | 805455.154 |
| | | 2008 | 2648752.232 | 1752373.351 | 896378.881 |
| | | 2009 | 2765973.961 | 1781570.120 | 984403.841 |
| | | 2010 | 2881329.235 | 1807703.688 | 1073625.55 |
| | | 2011 | 2994972.694 | 1831017.606 | 1163955.09 |
| | | 2012 | 3108958.829 | 1852205.036 | 1256753.79 |
| | | 2013 | 3218140.416 | 1870994.327 | 1347146.09 |
| | | 2014 | 3328387.914 | 1888351.743 | 1440036.17 |
| | | 2015 | 3435342.796 | 1904126.035 | 1531216.76 |
| | | 2016 | 3539189.408 | 1918341.043 | 1620848.37 |
| | | 2017 | 3640041.363 | 1931171.774 | 1708869.59 |
| | | 2018 | 3732645.362 | 1942128.745 | 1790516.62 |
| | | 2019 | 3821421.978 | 1951959.221 | 1869462.76 |
| | | 2020 | 3902115.950 | 1960442.965 | 1941672.99 |
| | | 2021 | 3984301.053 | 1968588.745 | 2015712.31 |
| | | 2022 | 4062629.299 | 1975923.341 | 2086705.96 |
| | | 2023 | 4137348.729 | 1982600.137 | 2154748.59 |
| | | 2024 | 4208611.493 | 1988661.061 | 2219950.43 |
| | | 2025 | 4269419.492 | 1993701.540 | 2275717.95 |
| | | 2026 | 4330969.998 | 1998593.325 | 2332376.67 |
| | | 2027 | 4389134.306 | 2003055.260 | 2386079.05 |
| | | 2028 | 4444222.970 | 2007162.144 | 2437060.83 |
| | | 2029 | 4496447.877 | 2010961.378 | 2485486.50 |
| | | 2030 | 4536975.698 | 2013836.656 | 2523139.04 |
| | | 2031 | 4578338.586 | 2016712.196 | 2561626.39 |
| | | 2032 | 4617397.784 | 2019384.771 | 2598013.01 |
| | | 2033 | 4654407.697 | 2021889.930 | 2632517.77 |
| | | 2034 | 4689913.454 | 2024250.640 | 2665662.81 |
| | | 2035 | 4712518.190 | 2025681.025 | 2686837.16 |
| | | 2036 | 4735845.827 | 2027129.806 | 2708716.02 |
| | | 2037 | 4757958.806 | 2028491.318 | 2729467.49 |
| | | 2038 | 4778813.806 | 2029778.356 | 2749035.45 |
| | | 2039 | 4798769.065 | 2031004.418 | 2767764.65 |
| Stroke | Males | 1990 | 75005.854 | 75005.854 | 0.000 |
| | | 1991 | 149463.017 | 148743.133 | 719.885 |
| | | 1992 | 222966.278 | 220289.469 | 2676.810 |
| | | 1993 | 296801.879 | 290425.407 | 6376.472 |
| | | 1994 | 372453.706 | 360103.582 | 12350.124 |

## Results for PHIM for Run 2 (ELL Summary Main_Analysis.RTF)

| | | | | |
|---|---|---|---|---|
| | 1995 | 448401.338 | 427518.754 | 20882.584 |
| | 1996 | 523536.301 | 491400.873 | 32135.428 |
| | 1997 | 596763.211 | 550666.507 | 46096.704 |
| | 1998 | 667546.681 | 604952.842 | 62593.839 |
| | 1999 | 734925.407 | 653716.943 | 81208.464 |
| | 2000 | 803749.361 | 700561.059 | 103188.302 |
| | 2001 | 870081.588 | 742674.246 | 127407.343 |
| | 2002 | 935347.140 | 781277.487 | 154069.653 |
| | 2003 | 1001627.349 | 817465.778 | 184161.572 |
| | 2004 | 1066587.976 | 850306.480 | 216281.496 |
| | 2005 | 1130548.052 | 880061.563 | 250486.489 |
| | 2006 | 1195132.454 | 907588.589 | 287543.865 |
| | 2007 | 1258974.043 | 932518.398 | 326455.645 |
| | 2008 | 1320124.867 | 954280.138 | 365844.729 |
| | 2009 | 1380776.123 | 974032.396 | 406743.727 |
| | 2010 | 1440048.533 | 991731.884 | 448316.649 |
| | 2011 | 1497484.847 | 1007458.785 | 490026.062 |
| | 2012 | 1554496.526 | 1021639.478 | 532857.048 |
| | 2013 | 1610819.598 | 1034456.307 | 576363.291 |
| | 2014 | 1667484.890 | 1046195.419 | 621289.471 |
| | 2015 | 1720948.287 | 1056475.234 | 664473.053 |
| | 2016 | 1775699.228 | 1066095.981 | 709603.247 |
| | 2017 | 1830250.175 | 1074919.927 | 755330.248 |
| | 2018 | 1875958.950 | 1081771.422 | 794187.528 |
| | 2019 | 1920359.232 | 1087941.738 | 832417.493 |
| | 2020 | 1960605.297 | 1093193.599 | 867411.698 |
| | 2021 | 1998912.887 | 1097839.741 | 901073.147 |
| | 2022 | 2036003.696 | 1102030.521 | 933973.175 |
| | 2023 | 2071857.536 | 1105812.477 | 966045.060 |
| | 2024 | 2106636.468 | 1109277.008 | 997359.460 |
| | 2025 | 2136450.873 | 1112108.644 | 1024342.23 |
| | 2026 | 2164796.470 | 1114649.808 | 1050146.66 |
| | 2027 | 2192079.608 | 1116990.155 | 1075089.45 |
| | 2028 | 2218404.367 | 1119127.749 | 1099276.62 |
| | 2029 | 2243769.140 | 1121105.350 | 1122663.79 |
| | 2030 | 2263570.213 | 1122597.818 | 1140972.39 |
| | 2031 | 2282521.590 | 1123980.619 | 1158540.97 |
| | 2032 | 2300804.945 | 1125272.581 | 1175532.36 |
| | 2033 | 2318464.018 | 1126467.716 | 1191996.30 |
| | 2034 | 2335611.973 | 1127598.460 | 1208013.51 |
| | 2035 | 2347097.290 | 1128337.696 | 1218759.59 |
| | 2036 | 2358248.747 | 1129038.631 | 1229210.12 |
| | 2037 | 2369033.655 | 1129708.358 | 1239325.30 |
| | 2038 | 2379490.114 | 1130338.450 | 1249151.66 |
| | 2039 | 2389546.238 | 1130931.546 | 1258614.69 |
| Female | 1990 | 56348.092 | 56348.092 | 0.000 |
| | 1991 | 110871.862 | 110356.693 | 515.169 |
| | 1992 | 165654.454 | 163676.511 | 1977.943 |
| | 1993 | 220008.519 | 215283.502 | 4725.018 |
| | 1994 | 275706.807 | 266523.785 | 9183.022 |
| | 1995 | 331417.547 | 315917.897 | 15499.650 |
| | 1996 | 387580.161 | 363579.386 | 24000.774 |
| | 1997 | 442778.242 | 408222.520 | 34555.722 |
| | 1998 | 496117.305 | 449177.869 | 46939.435 |
| | 1999 | 548268.214 | 487022.220 | 61245.995 |
| | 2000 | 601693.202 | 523576.314 | 78116.888 |
| | 2001 | 652221.103 | 555966.299 | 96254.804 |
| | 2002 | 703022.600 | 586296.080 | 116726.520 |
| | 2003 | 752023.351 | 613473.084 | 138550.267 |
| | 2004 | 799826.733 | 637985.805 | 161840.927 |
| | 2005 | 847563.928 | 660622.448 | 186941.481 |
| | 2006 | 894205.027 | 680984.758 | 213220.269 |
| | 2007 | 939571.282 | 699160.730 | 240410.552 |
| | 2008 | 983437.578 | 715316.542 | 268121.036 |
| | 2009 | 1025803.786 | 729647.054 | 296156.732 |
| | 2010 | 1066964.934 | 742381.777 | 324583.157 |
| | 2011 | 1106954.044 | 753699.091 | 353254.953 |
| | 2012 | 1146108.461 | 763870.354 | 382238.107 |
| | 2013 | 1183544.689 | 772820.741 | 410723.948 |
| | 2014 | 1221220.945 | 781069.226 | 440151.720 |
| | 2015 | 1258289.311 | 788624.395 | 469664.916 |
| | 2016 | 1294830.207 | 795481.871 | 499348.336 |
| | 2017 | 1330332.626 | 801635.078 | 528697.548 |
| | 2018 | 1360571.987 | 806504.624 | 554067.363 |
| | 2019 | 1389848.723 | 810852.754 | 578995.968 |
| | 2020 | 1416046.192 | 814520.721 | 601525.472 |
| | 2021 | 1440588.850 | 817717.425 | 622871.425 |
| | 2022 | 1464157.934 | 820577.251 | 643580.683 |

## Results for PHIM for Run 2 (ELL Summary Main_Analysis.RTF)

| | | | | | |
|---|---|---|---|---|---|
| | | 2023 | 1486810.237 | 823161.957 | 663648.280 |
| | | 2024 | 1508591.729 | 825488.519 | 683103.211 |
| | | 2025 | 1526875.621 | 827363.468 | 699512.153 |
| | | 2026 | 1543659.810 | 828987.557 | 714672.252 |
| | | 2027 | 1559623.774 | 830455.771 | 729168.003 |
| | | 2028 | 1574852.850 | 831795.191 | 743057.659 |
| | | 2029 | 1589437.452 | 833020.917 | 756416.535 |
| | | 2030 | 1600568.199 | 833923.008 | 766645.191 |
| | | 2031 | 1610776.297 | 834722.434 | 776053.862 |
| | | 2032 | 1620483.563 | 835460.615 | 785022.948 |
| | | 2033 | 1629745.442 | 836146.781 | 793598.661 |
| | | 2034 | 1638721.425 | 836788.579 | 801932.846 |
| | | 2035 | 1644649.404 | 837194.641 | 807454.763 |
| | | 2036 | 1650181.590 | 837562.894 | 812618.695 |
| | | 2037 | 1655476.066 | 837909.646 | 817566.420 |
| | | 2038 | 1660507.914 | 838238.210 | 822269.704 |
| | | 2039 | 1665361.569 | 838551.181 | 826810.388 |
| COPD | Males | 1990 | 117002.906 | 117002.906 | 0.000 |
| | | 1991 | 233438.833 | 233235.802 | 203.031 |
| | | 1992 | 348084.047 | 347332.506 | 751.541 |
| | | 1993 | 467278.318 | 465432.893 | 1845.425 |
| | | 1994 | 584216.907 | 580587.498 | 3629.409 |
| | | 1995 | 697391.457 | 691203.434 | 6188.022 |
| | | 1996 | 809471.271 | 799789.842 | 9681.428 |
| | | 1997 | 922587.668 | 908237.206 | 14350.462 |
| | | 1998 | 1034072.457 | 1013943.852 | 20128.605 |
| | | 1999 | 1153475.399 | 1125727.102 | 27748.298 |
| | | 2000 | 1268050.268 | 1231288.434 | 36761.834 |
| | | 2001 | 1382786.209 | 1335192.903 | 47593.307 |
| | | 2002 | 1497557.412 | 1437117.764 | 60439.648 |
| | | 2003 | 1614405.229 | 1539041.044 | 75364.185 |
| | | 2004 | 1726672.489 | 1634858.729 | 91813.760 |
| | | 2005 | 1847277.524 | 1735706.236 | 111571.289 |
| | | 2006 | 1961009.963 | 1828399.040 | 132610.923 |
| | | 2007 | 2079868.908 | 1922686.375 | 157182.533 |
| | | 2008 | 2206755.783 | 2020822.886 | 185932.896 |
| | | 2009 | 2331993.380 | 2114935.803 | 217057.578 |
| | | 2010 | 2455130.176 | 2204837.410 | 250292.766 |
| | | 2011 | 2582657.849 | 2295037.795 | 287620.053 |
| | | 2012 | 2709719.005 | 2381891.487 | 327827.518 |
| | | 2013 | 2840547.005 | 2468140.749 | 372406.256 |
| | | 2014 | 2971677.135 | 2551469.818 | 420207.317 |
| | | 2015 | 3104538.983 | 2633882.097 | 470656.886 |
| | | 2016 | 3237650.175 | 2713071.730 | 524578.446 |
| | | 2017 | 3370561.253 | 2789202.664 | 581358.589 |
| | | 2018 | 3503136.371 | 2861556.296 | 641580.076 |
| | | 2019 | 3636402.758 | 2931169.034 | 705233.724 |
| | | 2020 | 3768054.453 | 2997553.491 | 770500.962 |
| | | 2021 | 3897675.067 | 3059847.595 | 837827.472 |
| | | 2022 | 4027307.132 | 3119094.585 | 908212.547 |
| | | 2023 | 4156698.720 | 3175207.371 | 981491.350 |
| | | 2024 | 4285772.226 | 3228255.044 | 1057517.18 |
| | | 2025 | 4410759.298 | 3277680.474 | 1133078.82 |
| | | 2026 | 4531411.064 | 3322449.929 | 1208961.14 |
| | | 2027 | 4651145.233 | 3364259.942 | 1286885.29 |
| | | 2028 | 4769837.872 | 3403176.594 | 1366661.28 |
| | | 2029 | 4887747.179 | 3439442.319 | 1448304.86 |
| | | 2030 | 4997632.895 | 3472042.009 | 1525590.89 |
| | | 2031 | 5103301.991 | 3500754.451 | 1602547.54 |
| | | 2032 | 5207593.613 | 3527155.334 | 1680438.28 |
| | | 2033 | 5310556.138 | 3551303.731 | 1759252.41 |
| | | 2034 | 5412542.816 | 3573516.169 | 1839026.65 |
| | | 2035 | 5500522.473 | 3592116.723 | 1908405.75 |
| | | 2036 | 5588149.640 | 3609016.108 | 1979133.53 |
| | | 2037 | 5674667.886 | 3624522.174 | 2050145.71 |
| | | 2038 | 5760391.289 | 3638697.704 | 2121693.58 |
| | | 2039 | 5845459.234 | 3651806.856 | 2193652.38 |
| | Female | 1990 | 77434.846 | 77434.846 | 0.000 |
| | | 1991 | 160029.510 | 159861.321 | 168.190 |
| | | 1992 | 240642.531 | 240002.248 | 640.284 |
| | | 1993 | 329362.093 | 327709.495 | 1652.598 |
| | | 1994 | 418569.515 | 415257.075 | 3312.440 |
| | | 1995 | 509110.357 | 503279.713 | 5830.644 |
| | | 1996 | 600040.117 | 590774.114 | 9266.004 |
| | | 1997 | 691159.071 | 677410.594 | 13748.477 |
| | | 1998 | 782866.899 | 763517.233 | 19349.666 |
| | | 1999 | 881526.925 | 854784.101 | 26742.824 |
| | | 2000 | 977880.647 | 942588.402 | 35292.245 |

## Results for PHIM for Run 2 (ELL Summary Main_Analysis.RTF)

| | | | |
|---|---|---|---|
| 2001 | 1076787.404 | 1031163.667 | 45623.737 |
| 2002 | 1174842.754 | 1117286.026 | 57556.727 |
| 2003 | 1275102.681 | 1203698.984 | 71403.697 |
| 2004 | 1371026.306 | 1284694.950 | 86331.355 |
| 2005 | 1473954.509 | 1369544.732 | 104409.777 |
| 2006 | 1574450.828 | 1450409.128 | 124041.700 |
| 2007 | 1673790.211 | 1528312.941 | 145477.270 |
| 2008 | 1782360.738 | 1611196.696 | 171164.042 |
| 2009 | 1892944.209 | 1692955.884 | 199988.325 |
| 2010 | 2002131.282 | 1771443.116 | 230688.166 |
| 2011 | 2114522.152 | 1849496.567 | 265025.585 |
| 2012 | 2228079.372 | 1925850.640 | 302228.732 |
| 2013 | 2345377.689 | 2001873.293 | 343504.396 |
| 2014 | 2462498.422 | 2074931.275 | 387567.147 |
| 2015 | 2581450.312 | 2147217.505 | 434232.807 |
| 2016 | 2701959.301 | 2217555.449 | 484403.852 |
| 2017 | 2823834.967 | 2285809.232 | 538025.736 |
| 2018 | 2938265.081 | 2347830.498 | 590434.582 |
| 2019 | 3053254.880 | 2407467.687 | 645787.193 |
| 2020 | 3167073.216 | 2464441.474 | 702631.742 |
| 2021 | 3279517.699 | 2518745.344 | 760772.356 |
| 2022 | 3392060.964 | 2570535.685 | 821525.278 |
| 2023 | 3504296.171 | 2619726.322 | 884569.850 |
| 2024 | 3616019.231 | 2666187.066 | 949832.165 |
| 2025 | 3724611.269 | 2709674.338 | 1014936.93 |
| 2026 | 3827181.707 | 2749200.906 | 1077980.80 |
| 2027 | 3928684.441 | 2786172.976 | 1142511.46 |
| 2028 | 4029124.892 | 2820789.149 | 1208335.74 |
| 2029 | 4128269.068 | 2852914.409 | 1275354.66 |
| 2030 | 4221560.174 | 2882074.778 | 1339485.40 |
| 2031 | 4304170.584 | 2906431.616 | 1397738.97 |
| 2032 | 4384930.537 | 2928691.664 | 1456238.87 |
| 2033 | 4464357.691 | 2949172.191 | 1515185.50 |
| 2034 | 4542251.545 | 2967814.721 | 1574436.82 |
| 2035 | 4611066.648 | 2983765.893 | 1627300.75 |
| 2036 | 4670309.230 | 2996443.107 | 1673866.12 |
| 2037 | 4728179.514 | 3007956.130 | 1720223.38 |
| 2038 | 4785008.048 | 3018461.391 | 1766546.66 |
| 2039 | 4841291.793 | 3028069.314 | 1813222.48 |

# Results for PHIM for Run 2 (ELL Summary Main_Analysis.RTF)

***************************************************************************************

**Table 4: Estimated Loss of Life, All age groups combined, Population and Number deaths adjusted for Ecig scenario, cumulative over years**

| Disease | Sex | Year | Years of life lost NULL | Years of life lost Ecig | Years of life Saved |
|---|---|---|---|---|---|
| All Diseases | Males | 1990 | 1304839.645 | 1304839.645 | 0.000 |
| | | 1991 | 2579865.358 | 2571191.563 | 8673.794 |
| | | 1992 | 3830683.831 | 3798446.216 | 32237.615 |
| | | 1993 | 5076567.803 | 5000999.590 | 75568.213 |
| | | 1994 | 6292145.526 | 6150122.035 | 142023.492 |
| | | 1995 | 7485537.010 | 7250468.764 | 235068.245 |
| | | 1996 | 8655955.028 | 8300049.303 | 355905.725 |
| | | 1997 | 9791729.713 | 9288401.804 | 503327.909 |
| | | 1998 | 10899134.186 | 10223216.561 | 675917.625 |
| | | 1999 | 12070409.674 | 11163784.337 | 906625.338 |
| | | 2000 | 13231384.595 | 12064014.243 | 1167370.35 |
| | | 2001 | 14371761.018 | 12914122.231 | 1457638.79 |
| | | 2002 | 15508906.386 | 13726715.633 | 1782190.75 |
| | | 2003 | 16638100.422 | 14502109.591 | 2135990.83 |
| | | 2004 | 17736465.088 | 15229931.517 | 2506533.57 |
| | | 2005 | 18834658.606 | 15931683.532 | 2902975.07 |
| | | 2006 | 19909100.501 | 16587416.172 | 3321684.33 |
| | | 2007 | 20958321.463 | 17204934.202 | 3753387.26 |
| | | 2008 | 22000068.148 | 17798549.966 | 4201518.18 |
| | | 2009 | 23018816.903 | 18359583.298 | 4659233.61 |
| | | 2010 | 24018480.537 | 18889541.562 | 5128938.97 |
| | | 2011 | 25003034.574 | 19392159.479 | 5610875.10 |
| | | 2012 | 25969871.429 | 19866117.792 | 6103753.64 |
| | | 2013 | 26920275.180 | 20313553.922 | 6606721.26 |
| | | 2014 | 27849005.227 | 20732780.841 | 7116224.39 |
| | | 2015 | 28749620.400 | 21127411.399 | 7622209.00 |
| | | 2016 | 29622417.562 | 21488726.936 | 8133690.63 |
| | | 2017 | 30462559.724 | 21819422.499 | 8643137.23 |
| | | 2018 | 31293299.299 | 22137089.451 | 9156209.85 |
| | | 2019 | 32109993.427 | 22437123.004 | 9672870.42 |
| | | 2020 | 32883323.977 | 22716131.636 | 10167192.3 |
| | | 2021 | 33610102.554 | 22957831.316 | 10652271.2 |
| | | 2022 | 34322010.448 | 23184288.281 | 11137722.2 |
| | | 2023 | 35018383.550 | 23395605.268 | 11622778.3 |
| | | 2024 | 35700177.955 | 23593049.343 | 12107128.6 |
| | | 2025 | 36315500.346 | 23771173.995 | 12544326.4 |
| | | 2026 | 36896159.343 | 23922277.482 | 12973881.9 |
| | | 2027 | 37462570.116 | 24062501.748 | 13400068.4 |
| | | 2028 | 38015337.780 | 24191968.767 | 13823369.0 |
| | | 2029 | 38554814.764 | 24312027.250 | 14242787.5 |
| | | 2030 | 39003805.400 | 24414757.530 | 14589047.9 |
| | | 2031 | 39436509.703 | 24501954.120 | 14934555.6 |
| | | 2032 | 39858470.458 | 24582481.566 | 15275988.9 |
| | | 2033 | 40270147.082 | 24656159.192 | 15613987.9 |
| | | 2034 | 40672665.743 | 24724344.026 | 15948321.7 |
| | | 2035 | 40962732.664 | 24776397.561 | 16186335.1 |
| | | 2036 | 41255021.653 | 24823265.382 | 16431756.3 |
| | | 2037 | 41540573.023 | 24866775.507 | 16673797.5 |
| | | 2038 | 41820158.066 | 24906784.462 | 16913373.6 |
| | | 2039 | 42093547.949 | 24944049.345 | 17149498.6 |
| | Female | 1990 | 588979.406 | 588979.406 | 0.000 |
| | | 1991 | 1182571.252 | 1179606.280 | 2964.972 |
| | | 1992 | 1777346.679 | 1766098.293 | 11248.386 |
| | | 1993 | 2380261.899 | 2353272.844 | 26989.055 |
| | | 1994 | 2983130.039 | 2931348.284 | 51781.755 |
| | | 1995 | 3588982.584 | 3502017.856 | 86964.728 |
| | | 1996 | 4195430.235 | 4060683.539 | 134746.695 |
| | | 1997 | 4793866.694 | 4600883.769 | 192982.926 |
| | | 1998 | 5389747.381 | 5126864.206 | 262883.175 |
| | | 1999 | 6006722.467 | 5653225.387 | 353497.080 |
| | | 2000 | 6625758.871 | 6168755.643 | 457003.228 |
| | | 2001 | 7245135.411 | 6669506.678 | 575628.733 |
| | | 2002 | 7867492.021 | 7157621.042 | 709870.979 |
| | | 2003 | 8485051.793 | 7628322.364 | 856729.429 |
| | | 2004 | 9092994.774 | 8078188.922 | 1014805.85 |
| | | 2005 | 9706032.803 | 8518486.010 | 1187546.79 |
| | | 2006 | 10311907.548 | 8939525.790 | 1372381.76 |
| | | 2007 | 10909437.502 | 9343386.471 | 1566051.03 |
| | | 2008 | 11506462.491 | 9731957.443 | 1774505.05 |
| | | 2009 | 12098393.287 | 10107071.964 | 1991321.32 |
| | | 2010 | 12681188.981 | 10464009.317 | 2217179.66 |

# Results for PHIM for Run 2 (ELL Summary Main_Analysis.RTF)

| | | | | | |
|---|---|---|---|---|---|
| | | 2011 | 13260062.991 | 10805779.853 | 2454283.14 |
| | | 2012 | 13839417.441 | 11135018.447 | 2704398.99 |
| | | 2013 | 14409614.758 | 11448525.204 | 2961089.55 |
| | | 2014 | 14976658.746 | 11746736.502 | 3229922.24 |
| | | 2015 | 15529977.821 | 12028176.575 | 3501801.25 |
| | | 2016 | 16066640.764 | 12289066.766 | 3777574.00 |
| | | 2017 | 16590035.078 | 12533029.998 | 4057005.08 |
| | | 2018 | 17098716.137 | 12763548.255 | 4335167.88 |
| | | 2019 | 17599868.850 | 12981221.513 | 4618647.34 |
| | | 2020 | 18081346.241 | 13184807.328 | 4896538.91 |
| | | 2021 | 18537594.460 | 13367946.050 | 5169648.41 |
| | | 2022 | 18985300.234 | 13539731.862 | 5445568.37 |
| | | 2023 | 19423958.747 | 13700583.577 | 5723375.17 |
| | | 2024 | 19853347.772 | 13850356.373 | 6002991.40 |
| | | 2025 | 20252822.245 | 13987019.698 | 6265802.55 |
| | | 2026 | 20619307.063 | 14105036.806 | 6514270.26 |
| | | 2027 | 20976063.406 | 14214150.226 | 6761913.18 |
| | | 2028 | 21323641.947 | 14315313.849 | 7008328.10 |
| | | 2029 | 21662117.106 | 14408420.330 | 7253696.78 |
| | | 2030 | 21960971.694 | 14490025.520 | 7470946.17 |
| | | 2031 | 22224951.983 | 14556239.544 | 7668712.44 |
| | | 2032 | 22480397.853 | 14616655.459 | 7863742.39 |
| | | 2033 | 22728639.455 | 14672161.209 | 8056478.25 |
| | | 2034 | 22970448.888 | 14722787.650 | 8247661.24 |
| | | 2035 | 23164154.188 | 14763621.031 | 8400533.16 |
| | | 2036 | 23334538.616 | 14796122.902 | 8538415.71 |
| | | 2037 | 23499760.723 | 14825879.278 | 8673881.44 |
| | | 2038 | 23660308.401 | 14853231.741 | 8807076.66 |
| | | 2039 | 23817891.273 | 14878472.973 | 8939418.30 |
| Lung cancer | Males | 1990 | 595621.791 | 595621.791 | 0.000 |
| | | 1991 | 1177744.113 | 1177096.215 | 647.898 |
| | | 1992 | 1750034.892 | 1747594.305 | 2440.587 |
| | | 1993 | 2319752.308 | 2313819.857 | 5932.450 |
| | | 1994 | 2872597.339 | 2861118.286 | 11479.052 |
| | | 1995 | 3411494.825 | 3391996.555 | 19498.271 |
| | | 1996 | 3945569.726 | 3914793.909 | 30775.816 |
| | | 1997 | 4463316.362 | 4418033.301 | 45283.061 |
| | | 1998 | 4975301.180 | 4911598.470 | 63702.710 |
| | | 1999 | 5473691.125 | 5387431.814 | 86259.311 |
| | | 2000 | 5977500.570 | 5863066.309 | 114434.261 |
| | | 2001 | 6474795.516 | 6326620.248 | 148175.268 |
| | | 2002 | 6968769.705 | 6780596.847 | 188172.858 |
| | | 2003 | 7460282.140 | 7225037.116 | 235245.024 |
| | | 2004 | 7947048.275 | 7657839.350 | 289208.925 |
| | | 2005 | 8430571.669 | 8079977.552 | 350594.117 |
| | | 2006 | 8903945.390 | 8484728.563 | 419216.827 |
| | | 2007 | 9364177.982 | 8869843.501 | 494334.480 |
| | | 2008 | 9822525.458 | 9243937.015 | 578588.443 |
| | | 2009 | 10275228.886 | 9604100.581 | 671128.305 |
| | | 2010 | 10720275.500 | 9949023.521 | 771251.979 |
| | | 2011 | 11155356.373 | 10276792.516 | 878563.857 |
| | | 2012 | 11582611.617 | 10588098.479 | 994513.138 |
| | | 2013 | 11998803.791 | 10881386.232 | 1117417.56 |
| | | 2014 | 12402087.090 | 11156032.938 | 1246054.15 |
| | | 2015 | 12793451.706 | 11413837.357 | 1379614.35 |
| | | 2016 | 13160101.653 | 11646016.408 | 1514085.24 |
| | | 2017 | 13503583.414 | 11854996.911 | 1648586.50 |
| | | 2018 | 13859755.737 | 12060928.797 | 1798826.94 |
| | | 2019 | 14211625.176 | 12255294.199 | 1956330.98 |
| | | 2020 | 14553935.288 | 12436833.593 | 2117101.70 |
| | | 2021 | 14851747.495 | 12586976.727 | 2264770.77 |
| | | 2022 | 15144531.004 | 12727148.519 | 2417382.49 |
| | | 2023 | 15431932.496 | 12857472.730 | 2574459.77 |
| | | 2024 | 15713920.740 | 12978619.787 | 2735300.95 |
| | | 2025 | 15979112.816 | 13088326.525 | 2890786.29 |
| | | 2026 | 16206550.153 | 13175876.174 | 3030673.98 |
| | | 2027 | 16428826.050 | 13256541.653 | 3172284.40 |
| | | 2028 | 16645902.186 | 13330570.262 | 3315331.92 |
| | | 2029 | 16858126.517 | 13398805.322 | 3459321.20 |
| | | 2030 | 17045220.687 | 13457544.816 | 3587675.87 |
| | | 2031 | 17208482.610 | 13504070.926 | 3704411.68 |
| | | 2032 | 17367586.074 | 13546789.518 | 3820796.56 |
| | | 2033 | 17522716.280 | 13585715.369 | 3937000.91 |
| | | 2034 | 17674226.555 | 13621575.338 | 4052651.22 |
| | | 2035 | 17792201.734 | 13649247.315 | 4142954.42 |
| | | 2036 | 17903508.236 | 13673001.354 | 4230506.88 |
| | | 2037 | 18012162.345 | 13695027.545 | 4317134.80 |
| | | 2038 | 18118464.470 | 13715231.081 | 4403233.39 |

## Results for PHIM for Run 2 (ELL Summary Main_Analysis.RTF)

| | | | | | |
|---|---|---|---|---|---|
| | | 2039 | 18222549.860 | 13734071.508 | 4488478.35 |
| | Female | 1990 | 307897.357 | 307897.357 | 0.000 |
| | | 1991 | 616547.418 | 616130.924 | 416.494 |
| | | 1992 | 931086.773 | 929438.203 | 1648.571 |
| | | 1993 | 1246155.671 | 1242099.033 | 4056.638 |
| | | 1994 | 1561212.934 | 1553229.491 | 7983.444 |
| | | 1995 | 1879967.742 | 1866068.175 | 13899.567 |
| | | 1996 | 2197488.109 | 2175401.079 | 22087.031 |
| | | 1997 | 2515698.453 | 2482730.562 | 32967.891 |
| | | 1998 | 2836249.556 | 2789364.617 | 46884.940 |
| | | 1999 | 3150201.390 | 3086373.458 | 63827.932 |
| | | 2000 | 3472722.342 | 3387886.446 | 84835.897 |
| | | 2001 | 3796994.889 | 3686751.835 | 110243.054 |
| | | 2002 | 4126619.935 | 3985821.974 | 140797.962 |
| | | 2003 | 4454982.533 | 4278885.687 | 176096.847 |
| | | 2004 | 4784343.030 | 4567375.615 | 216967.415 |
| | | 2005 | 5113443.121 | 4849941.924 | 263501.197 |
| | | 2006 | 5442543.963 | 5125982.138 | 316561.825 |
| | | 2007 | 5772441.497 | 5396197.542 | 376243.954 |
| | | 2008 | 6091911.943 | 5651271.110 | 440640.833 |
| | | 2009 | 6413671.330 | 5900817.659 | 512853.671 |
| | | 2010 | 6730763.530 | 6140102.704 | 590660.826 |
| | | 2011 | 7043614.101 | 6368870.335 | 674743.766 |
| | | 2012 | 7356270.780 | 6590058.476 | 766212.304 |
| | | 2013 | 7662551.964 | 6799441.966 | 863109.998 |
| | | 2014 | 7964551.465 | 6998612.901 | 965938.564 |
| | | 2015 | 8254895.403 | 7184031.876 | 1070863.53 |
| | | 2016 | 8530661.848 | 7353086.955 | 1177574.89 |
| | | 2017 | 8795826.122 | 7509367.467 | 1286458.66 |
| | | 2018 | 9067233.707 | 7661600.426 | 1405633.28 |
| | | 2019 | 9335343.270 | 7805007.849 | 1530335.42 |
| | | 2020 | 9596110.883 | 7939006.659 | 1657104.22 |
| | | 2021 | 9833186.859 | 8056021.601 | 1777165.26 |
| | | 2022 | 10066452.037 | 8165337.769 | 1901114.27 |
| | | 2023 | 10295503.609 | 8267247.085 | 2028256.52 |
| | | 2024 | 10520125.318 | 8361679.641 | 2158445.68 |
| | | 2025 | 10731915.863 | 8447447.676 | 2284468.19 |
| | | 2026 | 10917495.549 | 8518936.556 | 2398558.99 |
| | | 2027 | 11098620.885 | 8584666.857 | 2513954.03 |
| | | 2028 | 11275441.234 | 8645292.217 | 2630149.02 |
| | | 2029 | 11447962.710 | 8700782.146 | 2747180.56 |
| | | 2030 | 11601867.623 | 8748997.324 | 2852870.30 |
| | | 2031 | 11731666.516 | 8786772.888 | 2944893.63 |
| | | 2032 | 11857585.969 | 8821129.507 | 3036456.46 |
| | | 2033 | 11980128.625 | 8852589.710 | 3127538.91 |
| | | 2034 | 12099562.464 | 8881216.574 | 3218345.89 |
| | | 2035 | 12195919.946 | 8903939.129 | 3291980.82 |
| | | 2036 | 12278201.969 | 8921677.802 | 3356524.17 |
| | | 2037 | 12358146.336 | 8937962.388 | 3420183.95 |
| | | 2038 | 12435978.633 | 8952959.672 | 3483018.96 |
| | | 2039 | 12512468.845 | 8966835.101 | 3545633.74 |
| IHD | Males | 1990 | 517209.093 | 517209.093 | 0.000 |
| | | 1991 | 1019219.395 | 1012116.414 | 7102.981 |
| | | 1992 | 1509598.614 | 1483228.170 | 26370.445 |
| | | 1993 | 1992735.299 | 1931313.195 | 61422.104 |
| | | 1994 | 2462877.575 | 2348289.509 | 114588.066 |
| | | 1995 | 2928249.390 | 2739699.863 | 188549.527 |
| | | 1996 | 3377377.730 | 3093970.875 | 283406.855 |
| | | 1997 | 3809062.472 | 3411306.451 | 397756.021 |
| | | 1998 | 4222213.867 | 3692475.151 | 529738.716 |
| | | 1999 | 4708317.743 | 3996541.764 | 711775.979 |
| | | 2000 | 5182084.396 | 4268578.824 | 913505.572 |
| | | 2001 | 5644097.705 | 4508926.177 | 1135171.53 |
| | | 2002 | 6107232.129 | 4726786.586 | 1380445.54 |
| | | 2003 | 6561785.704 | 4919352.611 | 1642433.09 |
| | | 2004 | 6996156.348 | 5085404.558 | 1910751.79 |
| | | 2005 | 7426261.361 | 5234043.552 | 2192217.81 |
| | | 2006 | 7849012.694 | 5364407.456 | 2484605.24 |
| | | 2007 | 8255300.530 | 5477139.204 | 2778161.33 |
| | | 2008 | 8650662.040 | 5576239.715 | 3074422.33 |
| | | 2009 | 9030818.513 | 5662683.579 | 3368134.93 |
| | | 2010 | 9403026.327 | 5739522.784 | 3663503.54 |
| | | 2011 | 9767535.506 | 5807793.488 | 3959742.02 |
| | | 2012 | 10123044.281 | 5868727.078 | 4254317.20 |
| | | 2013 | 10470104.786 | 5923070.665 | 4547034.12 |
| | | 2014 | 10807756.112 | 5971808.731 | 4835947.38 |
| | | 2015 | 11130681.425 | 6015110.406 | 5115571.02 |
| | | 2016 | 11448966.506 | 6054572.859 | 5394393.65 |

## Results for PHIM for Run 2 (ELL Summary Main_Analysis.RTF)

| | | | | | |
|---|---|---|---|---|---|
| | | 2017 | 11758164.882 | 6090436.370 | 5667728.51 |
| | | 2018 | 12054448.241 | 6122062.377 | 5932385.86 |
| | | 2019 | 12341606.261 | 6151019.415 | 6190586.85 |
| | | 2020 | 12600728.939 | 6175903.695 | 6424825.24 |
| | | 2021 | 12861767.105 | 6199573.496 | 6662193.61 |
| | | 2022 | 13114168.616 | 6221468.010 | 6892700.61 |
| | | 2023 | 13357894.797 | 6241611.778 | 7116283.02 |
| | | 2024 | 13593848.521 | 6260443.886 | 7333404.64 |
| | | 2025 | 13789177.359 | 6275659.749 | 7513517.61 |
| | | 2026 | 13993401.656 | 6291003.321 | 7702398.33 |
| | | 2027 | 14190519.224 | 6305530.500 | 7884988.72 |
| | | 2028 | 14381193.356 | 6319054.862 | 8062138.49 |
| | | 2029 | 14565171.927 | 6331797.936 | 8233373.99 |
| | | 2030 | 14697381.605 | 6340896.520 | 8356485.09 |
| | | 2031 | 14842203.512 | 6350741.964 | 8491461.55 |
| | | 2032 | 14982485.826 | 6360162.936 | 8622322.89 |
| | | 2033 | 15118410.647 | 6368913.243 | 8749497.40 |
| | | 2034 | 15250284.398 | 6377269.451 | 8873014.95 |
| | | 2035 | 15322911.166 | 6381752.773 | 8941158.39 |
| | | 2036 | 15405115.031 | 6386750.292 | 9018364.74 |
| | | 2037 | 15484709.137 | 6391572.984 | 9093136.15 |
| | | 2038 | 15561812.193 | 6396117.691 | 9165694.50 |
| | | 2039 | 15635992.616 | 6400411.543 | 9235581.07 |
| | Female | 1990 | 147299.110 | 147299.110 | 0.000 |
| | | 1991 | 295122.462 | 293257.343 | 1865.120 |
| | | 1992 | 439962.920 | 432980.887 | 6982.033 |
| | | 1993 | 584735.615 | 568178.568 | 16557.047 |
| | | 1994 | 727640.783 | 696331.324 | 31309.459 |
| | | 1995 | 868486.937 | 816737.160 | 51749.777 |
| | | 1996 | 1010321.847 | 930900.151 | 79421.697 |
| | | 1997 | 1144230.930 | 1032470.161 | 111760.769 |
| | | 1998 | 1274513.622 | 1124724.572 | 149789.050 |
| | | 1999 | 1426725.938 | 1224923.489 | 201802.449 |
| | | 2000 | 1573462.680 | 1314527.573 | 258935.107 |
| | | 2001 | 1719132.015 | 1395378.307 | 323753.708 |
| | | 2002 | 1863006.732 | 1467886.511 | 395120.221 |
| | | 2003 | 2002943.227 | 1531832.121 | 471111.106 |
| | | 2004 | 2137798.705 | 1587585.252 | 550213.453 |
| | | 2005 | 2271071.245 | 1637691.437 | 633379.809 |
| | | 2006 | 2400707.731 | 1681311.723 | 719396.008 |
| | | 2007 | 2523634.512 | 1718708.931 | 804925.582 |
| | | 2008 | 2648752.232 | 1752966.104 | 895786.128 |
| | | 2009 | 2765973.961 | 1782224.032 | 983749.929 |
| | | 2010 | 2881329.235 | 1808418.686 | 1072910.55 |
| | | 2011 | 2994972.694 | 1831792.976 | 1163179.72 |
| | | 2012 | 3108958.829 | 1853040.208 | 1255918.62 |
| | | 2013 | 3218140.416 | 1871887.835 | 1346252.58 |
| | | 2014 | 3328387.914 | 1889303.660 | 1439084.25 |
| | | 2015 | 3435342.796 | 1905136.772 | 1530206.02 |
| | | 2016 | 3539189.408 | 1919409.556 | 1619779.85 |
| | | 2017 | 3640041.363 | 1932296.736 | 1707744.63 |
| | | 2018 | 3732645.362 | 1943305.134 | 1789340.23 |
| | | 2019 | 3821421.978 | 1953184.899 | 1868237.08 |
| | | 2020 | 3902115.950 | 1961715.823 | 1940400.13 |
| | | 2021 | 3984301.053 | 1969909.629 | 2014391.42 |
| | | 2022 | 4062629.299 | 1977290.000 | 2085339.30 |
| | | 2023 | 4137348.729 | 1984010.869 | 2153337.86 |
| | | 2024 | 4208611.493 | 1990113.798 | 2218497.70 |
| | | 2025 | 4269419.492 | 1995194.021 | 2274225.47 |
| | | 2026 | 4330969.998 | 2000125.893 | 2330844.11 |
| | | 2027 | 4389134.306 | 2004625.783 | 2384508.52 |
| | | 2028 | 4444222.970 | 2008768.890 | 2435454.08 |
| | | 2029 | 4496447.877 | 2012602.747 | 2483845.13 |
| | | 2030 | 4536975.698 | 2015508.997 | 2521466.70 |
| | | 2031 | 4578338.586 | 2018416.109 | 2559922.48 |
| | | 2032 | 4617397.784 | 2021118.606 | 2596279.18 |
| | | 2033 | 4654407.697 | 2023652.510 | 2630755.19 |
| | | 2034 | 4689913.454 | 2026040.842 | 2663872.61 |
| | | 2035 | 4712518.190 | 2027492.516 | 2685025.67 |
| | | 2036 | 4735845.827 | 2028963.048 | 2706882.78 |
| | | 2037 | 4757958.806 | 2030345.289 | 2727613.52 |
| | | 2038 | 4778813.806 | 2031652.192 | 2747161.61 |
| | | 2039 | 4798769.065 | 2032897.444 | 2765871.62 |
| Stroke | Males | 1990 | 75005.854 | 75005.854 | 0.000 |
| | | 1991 | 149463.017 | 148743.133 | 719.885 |
| | | 1992 | 222966.278 | 220290.087 | 2676.192 |
| | | 1993 | 296801.879 | 290428.236 | 6373.643 |
| | | 1994 | 372453.706 | 360111.565 | 12342.141 |

## Results for PHIM for Run 2 (ELL Summary Main_Analysis.RTF)

| | | | | |
|---|---|---|---|---|
| | 1995 | 448401.338 | 427536.079 | 20865.259 |
| | 1996 | 523536.301 | 491432.928 | 32103.373 |
| | 1997 | 596763.211 | 550719.256 | 46043.955 |
| | 1998 | 667546.681 | 605032.443 | 62514.238 |
| | 1999 | 734925.407 | 653829.062 | 81096.345 |
| | 2000 | 803749.361 | 700715.235 | 103034.126 |
| | 2001 | 870081.588 | 742876.682 | 127204.906 |
| | 2002 | 935347.140 | 781535.649 | 153811.491 |
| | 2003 | 1001627.349 | 817786.716 | 183840.633 |
| | 2004 | 1066587.976 | 850696.034 | 215891.942 |
| | 2005 | 1130548.052 | 880524.399 | 250023.653 |
| | 2006 | 1195132.454 | 908129.950 | 287002.504 |
| | 2007 | 1258974.043 | 933141.147 | 325832.896 |
| | 2008 | 1320124.867 | 954983.147 | 365141.719 |
| | 2009 | 1380776.123 | 974817.314 | 405958.809 |
| | 2010 | 1440048.533 | 992598.724 | 447449.809 |
| | 2011 | 1497484.847 | 1008406.908 | 489077.939 |
| | 2012 | 1554496.526 | 1022667.142 | 531829.384 |
| | 2013 | 1610819.598 | 1035563.084 | 575256.514 |
| | 2014 | 1667484.890 | 1047379.990 | 620104.899 |
| | 2015 | 1720948.287 | 1057735.870 | 663212.416 |
| | 2016 | 1775699.228 | 1067432.465 | 708266.763 |
| | 2017 | 1830250.175 | 1076330.968 | 753919.207 |
| | 2018 | 1875958.950 | 1083245.687 | 792713.262 |
| | 2019 | 1920359.232 | 1089476.244 | 830882.988 |
| | 2020 | 1960605.297 | 1094785.174 | 865820.124 |
| | 2021 | 1998912.887 | 1099484.348 | 899428.540 |
| | 2022 | 2036003.696 | 1103725.121 | 932278.575 |
| | 2023 | 2071857.536 | 1107554.383 | 964303.154 |
| | 2024 | 2106636.468 | 1111064.113 | 995572.356 |
| | 2025 | 2136450.873 | 1113937.599 | 1022513.27 |
| | 2026 | 2164796.470 | 1116517.465 | 1048279.01 |
| | 2027 | 2192079.608 | 1118894.676 | 1073184.93 |
| | 2028 | 2218404.367 | 1121067.193 | 1097337.17 |
| | 2029 | 2243769.140 | 1123077.959 | 1120691.18 |
| | 2030 | 2263570.213 | 1124599.156 | 1138971.06 |
| | 2031 | 2282521.590 | 1126009.188 | 1156512.40 |
| | 2032 | 2300804.945 | 1127327.194 | 1173477.75 |
| | 2033 | 2318464.018 | 1128547.130 | 1189916.89 |
| | 2034 | 2335611.973 | 1129701.942 | 1205910.03 |
| | 2035 | 2347097.290 | 1130459.343 | 1216637.95 |
| | 2036 | 2358248.747 | 1131177.824 | 1227070.92 |
| | 2037 | 2369033.655 | 1131864.746 | 1237168.91 |
| | 2038 | 2379490.114 | 1132511.498 | 1246978.62 |
| | 2039 | 2389546.238 | 1133120.630 | 1256425.61 |
| Female | 1990 | 56348.092 | 56348.092 | 0.000 |
| | 1991 | 110871.862 | 110356.693 | 515.169 |
| | 1992 | 165654.454 | 163676.662 | 1977.792 |
| | 1993 | 220008.519 | 215284.214 | 4724.305 |
| | 1994 | 275706.807 | 266525.818 | 9180.988 |
| | 1995 | 331417.547 | 315922.396 | 15495.151 |
| | 1996 | 387580.161 | 363587.877 | 23992.284 |
| | 1997 | 442778.242 | 408236.762 | 34541.479 |
| | 1998 | 496117.305 | 449199.774 | 46917.531 |
| | 1999 | 548268.214 | 487053.793 | 61214.421 |
| | 2000 | 601693.202 | 523620.373 | 78072.829 |
| | 2001 | 652221.103 | 556024.633 | 96196.470 |
| | 2002 | 703022.600 | 586370.987 | 116651.613 |
| | 2003 | 752023.351 | 613566.293 | 138457.058 |
| | 2004 | 799826.733 | 638098.830 | 161727.903 |
| | 2005 | 847563.928 | 660756.943 | 186806.985 |
| | 2006 | 894205.027 | 681142.109 | 213062.918 |
| | 2007 | 939571.282 | 699341.610 | 240229.672 |
| | 2008 | 983437.578 | 715521.359 | 267916.219 |
| | 2009 | 1025803.786 | 729876.054 | 295927.732 |
| | 2010 | 1066964.934 | 742634.945 | 324329.989 |
| | 2011 | 1106954.044 | 753976.396 | 352977.648 |
| | 2012 | 1146108.461 | 764171.646 | 381936.815 |
| | 2013 | 1183544.689 | 773145.525 | 410399.164 |
| | 2014 | 1221220.945 | 781417.845 | 439803.101 |
| | 2015 | 1258289.311 | 788997.466 | 469291.845 |
| | 2016 | 1294830.207 | 795879.146 | 498951.061 |
| | 2017 | 1330332.626 | 802056.235 | 528276.390 |
| | 2018 | 1360571.987 | 806946.462 | 553625.526 |
| | 2019 | 1389848.723 | 811314.418 | 578534.305 |
| | 2020 | 1416046.192 | 815001.306 | 601044.887 |
| | 2021 | 1440588.850 | 818215.773 | 622373.077 |
| | 2022 | 1464157.934 | 821092.444 | 643065.491 |

## Results for PHIM for Run 2 (ELL Summary Main_Analysis.RTF)

| | | | | | |
|---|---|---|---|---|---|
| | | 2023 | 1486810.237 | 823693.287 | 663116.950 |
| | | 2024 | 1508591.729 | 826035.138 | 682556.592 |
| | | 2025 | 1526875.621 | 827924.344 | 698951.277 |
| | | 2026 | 1543659.810 | 829561.338 | 714098.471 |
| | | 2027 | 1559623.774 | 831041.647 | 728582.127 |
| | | 2028 | 1574852.850 | 832392.517 | 742460.333 |
| | | 2029 | 1589437.452 | 833629.081 | 755808.372 |
| | | 2030 | 1600568.199 | 834540.697 | 766027.502 |
| | | 2031 | 1610776.297 | 835348.778 | 775427.518 |
| | | 2032 | 1620483.563 | 836095.128 | 784388.435 |
| | | 2033 | 1629745.442 | 836789.087 | 792956.355 |
| | | 2034 | 1638721.425 | 837438.342 | 801283.083 |
| | | 2035 | 1644649.404 | 837850.253 | 806799.151 |
| | | 2036 | 1650181.590 | 838223.873 | 811957.717 |
| | | 2037 | 1655476.066 | 838575.753 | 816900.313 |
| | | 2038 | 1660507.914 | 838909.255 | 821598.659 |
| | | 2039 | 1665361.569 | 839227.001 | 826134.569 |
| COPD | Males | 1990 | 117002.906 | 117002.906 | 0.000 |
| | | 1991 | 233438.833 | 233235.802 | 203.031 |
| | | 1992 | 348084.047 | 347333.655 | 750.392 |
| | | 1993 | 467278.318 | 465438.303 | 1840.015 |
| | | 1994 | 584216.907 | 580602.674 | 3614.233 |
| | | 1995 | 697391.457 | 691236.268 | 6155.189 |
| | | 1996 | 809471.271 | 799851.591 | 9619.680 |
| | | 1997 | 922587.668 | 908342.795 | 14244.873 |
| | | 1998 | 1034072.457 | 1014110.497 | 19961.960 |
| | | 1999 | 1153475.399 | 1125981.696 | 27493.703 |
| | | 2000 | 1268050.268 | 1231653.876 | 36396.392 |
| | | 2001 | 1382786.209 | 1335699.125 | 47087.084 |
| | | 2002 | 1497557.412 | 1437796.551 | 59760.861 |
| | | 2003 | 1614405.229 | 1539933.149 | 74472.081 |
| | | 2004 | 1726672.489 | 1635991.575 | 90680.914 |
| | | 2005 | 1847277.524 | 1737138.030 | 110139.495 |
| | | 2006 | 1961009.963 | 1830150.203 | 130859.760 |
| | | 2007 | 2079868.908 | 1924810.350 | 155058.558 |
| | | 2008 | 2206755.783 | 2023390.089 | 183365.694 |
| | | 2009 | 2331993.380 | 2117981.823 | 214011.557 |
| | | 2010 | 2455130.176 | 2208396.533 | 246733.644 |
| | | 2011 | 2582657.849 | 2299166.567 | 283491.282 |
| | | 2012 | 2709719.005 | 2386625.093 | 323093.912 |
| | | 2013 | 2840547.005 | 2473533.941 | 367013.065 |
| | | 2014 | 2971677.135 | 2557559.181 | 414117.954 |
| | | 2015 | 3104538.983 | 2640727.766 | 463811.217 |
| | | 2016 | 3237650.175 | 2720705.205 | 516944.971 |
| | | 2017 | 3370561.253 | 2797658.250 | 572903.003 |
| | | 2018 | 3503136.371 | 2870852.589 | 632283.782 |
| | | 2019 | 3636402.758 | 2941333.146 | 695069.612 |
| | | 2020 | 3768054.453 | 3008609.175 | 759445.278 |
| | | 2021 | 3897675.067 | 3071796.745 | 825878.322 |
| | | 2022 | 4027307.132 | 3131946.632 | 895360.500 |
| | | 2023 | 4156698.720 | 3188966.378 | 967732.343 |
| | | 2024 | 4285772.226 | 3242921.557 | 1042850.67 |
| | | 2025 | 4410759.298 | 3293250.121 | 1117509.18 |
| | | 2026 | 4531411.064 | 3338880.521 | 1192530.54 |
| | | 2027 | 4651145.233 | 3381534.919 | 1269610.31 |
| | | 2028 | 4769837.872 | 3421276.450 | 1348561.42 |
| | | 2029 | 4887747.179 | 3458346.033 | 1429401.15 |
| | | 2030 | 4997632.895 | 3491717.038 | 1505915.86 |
| | | 2031 | 5103301.991 | 3521132.043 | 1582169.95 |
| | | 2032 | 5207593.613 | 3548201.919 | 1659391.69 |
| | | 2033 | 5310556.138 | 3572983.450 | 1737572.69 |
| | | 2034 | 5412542.816 | 3595797.295 | 1816745.52 |
| | | 2035 | 5500522.473 | 3614938.130 | 1885584.34 |
| | | 2036 | 5588149.640 | 3632335.913 | 1955813.73 |
| | | 2037 | 5674667.886 | 3648310.231 | 2026357.65 |
| | | 2038 | 5760391.289 | 3662924.192 | 2097467.10 |
| | | 2039 | 5845459.234 | 3676445.664 | 2169013.57 |
| | Female | 1990 | 77434.846 | 77434.846 | 0.000 |
| | | 1991 | 160029.510 | 159861.321 | 168.190 |
| | | 1992 | 240642.531 | 240002.541 | 639.990 |
| | | 1993 | 329362.093 | 327711.028 | 1651.065 |
| | | 1994 | 418569.515 | 415261.651 | 3307.864 |
| | | 1995 | 509110.357 | 503290.124 | 5820.233 |
| | | 1996 | 600040.117 | 590794.433 | 9245.684 |
| | | 1997 | 691159.071 | 677446.284 | 13712.786 |
| | | 1998 | 782866.899 | 763575.243 | 19291.655 |
| | | 1999 | 881526.925 | 854874.647 | 26652.278 |
| | | 2000 | 977880.647 | 942721.251 | 35159.396 |

## Results for PHIM for Run 2 (ELL Summary Main_Analysis.RTF)

| | | | |
|---|---|---|---|
| 2001 | 1076787.404 | 1031351.903 | 45435.501 |
| 2002 | 1174842.754 | 1117541.570 | 57301.184 |
| 2003 | 1275102.681 | 1204038.263 | 71064.418 |
| 2004 | 1371026.306 | 1285129.225 | 85897.080 |
| 2005 | 1473954.509 | 1370095.706 | 103858.802 |
| 2006 | 1574450.828 | 1451089.820 | 123361.008 |
| 2007 | 1673790.211 | 1529138.388 | 144651.823 |
| 2008 | 1782360.738 | 1612198.870 | 170161.867 |
| 2009 | 1892944.209 | 1694154.218 | 198789.992 |
| 2010 | 2002131.282 | 1772852.982 | 229278.300 |
| 2011 | 2114522.152 | 1851140.146 | 263382.006 |
| 2012 | 2228079.372 | 1927748.117 | 300331.255 |
| 2013 | 2345377.689 | 2004049.878 | 341327.811 |
| 2014 | 2462498.422 | 2077402.096 | 385096.326 |
| 2015 | 2581450.312 | 2150010.460 | 431439.852 |
| 2016 | 2701959.301 | 2220691.109 | 481268.192 |
| 2017 | 2823834.967 | 2289309.559 | 534525.408 |
| 2018 | 2938265.081 | 2351696.233 | 586568.847 |
| 2019 | 3053254.880 | 2411714.348 | 641540.532 |
| 2020 | 3167073.216 | 2469083.541 | 697989.675 |
| 2021 | 3279517.699 | 2523799.047 | 755718.652 |
| 2022 | 3392060.964 | 2576011.649 | 816049.314 |
| 2023 | 3504296.171 | 2625632.336 | 878663.835 |
| 2024 | 3616019.231 | 2672527.796 | 943491.435 |
| 2025 | 3724611.269 | 2716453.658 | 1008157.61 |
| 2026 | 3827181.707 | 2756413.018 | 1070768.69 |
| 2027 | 3928684.441 | 2793815.938 | 1134868.50 |
| 2028 | 4029124.892 | 2828860.225 | 1200264.67 |
| 2029 | 4128269.068 | 2861406.356 | 1266862.71 |
| 2030 | 4221560.174 | 2890978.501 | 1330581.67 |
| 2031 | 4304170.584 | 2915701.768 | 1388468.82 |
| 2032 | 4384930.537 | 2938312.218 | 1446618.32 |
| 2033 | 4464357.691 | 2959129.901 | 1505227.79 |
| 2034 | 4542251.545 | 2978091.893 | 1564159.65 |
| 2035 | 4611066.648 | 2994339.134 | 1616727.51 |
| 2036 | 4670309.230 | 3007258.180 | 1663051.05 |
| 2037 | 4728179.514 | 3018995.848 | 1709183.67 |
| 2038 | 4785008.048 | 3029710.622 | 1755297.43 |
| 2039 | 4841291.793 | 3039513.428 | 1801778.37 |

## Results for PHIM for Run 2 (ELL Summary Main_Analysis.RTF)

************************************************************************************

**Table 5: Estimated Loss of Life, at the end of follow-up, Population unadjusted for deaths in Ecig scenario**

| Disease | Sex | Age Group | Years of life lost NULL | Years of life lost Ecig | Years of life Saved |
|---|---|---|---|---|---|
| All Diseases | Males | 30-34 | 443911.034 | 238683.977 | 205227.057 |
| | | 35-39 | 1146798.555 | 610243.444 | 536555.111 |
| | | 40-44 | 2609591.408 | 1349167.978 | 1260423.43 |
| | | 45-49 | 4775841.753 | 2428118.032 | 2347723.72 |
| | | 50-54 | 7446969.205 | 3925842.223 | 3521126.98 |
| | | 55-59 | 8240205.925 | 4771295.184 | 3468910.74 |
| | | 60-64 | 8772829.118 | 5499674.699 | 3273154.42 |
| | | 65-69 | 6161494.678 | 4243210.109 | 1918284.57 |
| | | 70-74 | 2495906.274 | 1778607.274 | 717298.999 |
| | | 75-79 | 0.000 | 0.000 | 0.000 |
| | Female | 30-34 | 229140.561 | 142114.245 | 87026.316 |
| | | 35-39 | 570217.942 | 347146.463 | 223071.478 |
| | | 40-44 | 1267538.938 | 748645.249 | 518893.689 |
| | | 45-49 | 2340070.698 | 1359164.050 | 980906.648 |
| | | 50-54 | 3743803.613 | 2189604.741 | 1554198.87 |
| | | 55-59 | 4591478.168 | 2807126.419 | 1784351.75 |
| | | 60-64 | 5207367.788 | 3305062.784 | 1902305.00 |
| | | 65-69 | 4092181.394 | 2736939.809 | 1355241.59 |
| | | 70-74 | 1776092.171 | 1205518.221 | 570573.949 |
| | | 75-79 | 0.000 | 0.000 | 0.000 |
| Lung cancer | Males | 30-34 | 66065.017 | 51712.541 | 14352.476 |
| | | 35-39 | 232509.414 | 183484.631 | 49024.784 |
| | | 40-44 | 688628.041 | 531489.729 | 157138.312 |
| | | 45-49 | 1528300.367 | 1142312.132 | 385988.235 |
| | | 50-54 | 2730187.229 | 2007521.021 | 722666.209 |
| | | 55-59 | 3909101.673 | 2855854.172 | 1053247.50 |
| | | 60-64 | 4287716.660 | 3223303.002 | 1064413.66 |
| | | 65-69 | 3467897.115 | 2656175.004 | 811722.111 |
| | | 70-74 | 1312144.343 | 1021280.562 | 290863.782 |
| | | 75-79 | 0.000 | 0.000 | 0.000 |
| | Female | 30-34 | 51913.113 | 40645.069 | 11268.044 |
| | | 35-39 | 189792.904 | 148856.758 | 40936.146 |
| | | 40-44 | 527596.573 | 396619.330 | 130977.243 |
| | | 45-49 | 1122524.269 | 812310.482 | 310213.786 |
| | | 50-54 | 1882214.322 | 1331788.765 | 550425.557 |
| | | 55-59 | 2595581.943 | 1811859.595 | 783722.348 |
| | | 60-64 | 2846273.090 | 2024084.478 | 822188.612 |
| | | 65-69 | 2358758.394 | 1695538.427 | 663219.967 |
| | | 70-74 | 937814.238 | 681994.164 | 255820.073 |
| | | 75-79 | 0.000 | 0.000 | 0.000 |
| IHD | Males | 30-34 | 263834.566 | 117992.888 | 145841.678 |
| | | 35-39 | 692140.150 | 295160.109 | 396980.041 |
| | | 40-44 | 1519708.432 | 588864.931 | 930843.501 |
| | | 45-49 | 2568546.697 | 911115.964 | 1657430.73 |
| | | 50-54 | 3632929.903 | 1340974.189 | 2291955.71 |
| | | 55-59 | 2807892.356 | 1098170.591 | 1709721.77 |
| | | 60-64 | 2599825.934 | 1191664.301 | 1408161.63 |
| | | 65-69 | 1113578.307 | 583540.251 | 530038.056 |
| | | 70-74 | 437536.271 | 261488.501 | 176047.769 |
| | | 75-79 | 0.000 | 0.000 | 0.000 |
| | Female | 30-34 | 79649.097 | 37852.193 | 41796.904 |
| | | 35-39 | 193965.908 | 81447.686 | 112518.221 |
| | | 40-44 | 403380.974 | 151123.428 | 252257.546 |
| | | 45-49 | 671385.408 | 232831.376 | 438554.032 |
| | | 50-54 | 1011875.989 | 376040.561 | 635835.429 |
| | | 55-59 | 849875.715 | 343991.407 | 505884.309 |
| | | 60-64 | 925490.876 | 436192.169 | 489298.708 |
| | | 65-69 | 455463.881 | 246010.438 | 209453.443 |
| | | 70-74 | 207681.216 | 125515.161 | 82166.056 |
| | | 75-79 | 0.000 | 0.000 | 0.000 |
| Stroke | Males | 30-34 | 73463.650 | 43068.115 | 30395.535 |
| | | 35-39 | 151934.057 | 86388.815 | 65545.242 |
| | | 40-44 | 262845.974 | 141430.433 | 121415.541 |
| | | 45-49 | 383421.246 | 192597.008 | 190824.238 |
| | | 50-54 | 479872.634 | 218809.696 | 261062.938 |
| | | 55-59 | 437124.903 | 184459.622 | 252665.282 |
| | | 60-64 | 419457.235 | 180370.148 | 239087.087 |
| | | 65-69 | 128180.574 | 57699.210 | 70481.364 |
| | | 70-74 | 53245.965 | 26108.500 | 27137.465 |
| | | 75-79 | 0.000 | 0.000 | 0.000 |
| | Female | 30-34 | 62919.090 | 40065.099 | 22853.991 |

# Results for PHIM for Run 2 (ELL Summary Main_Analysis.RTF)

| | | | | | |
|---|---|---|---|---|---|
| | | 35-39 | 120806.205 | 72695.725 | 48110.480 |
| | | 40-44 | 203391.885 | 113506.261 | 89885.624 |
| | | 45-49 | 278172.097 | 143983.320 | 134188.777 |
| | | 50-54 | 325979.181 | 157261.008 | 168718.173 |
| | | 55-59 | 275161.340 | 124712.430 | 150448.910 |
| | | 60-64 | 268920.493 | 123470.229 | 145450.264 |
| | | 65-69 | 87956.054 | 41969.550 | 45986.504 |
| | | 70-74 | 42055.224 | 20887.558 | 21167.665 |
| | | 75-79 | 0.000 | 0.000 | 0.000 |
| COPD | Males | 30-34 | 40547.801 | 25910.433 | 14637.368 |
| | | 35-39 | 70214.933 | 45209.890 | 25005.044 |
| | | 40-44 | 138408.960 | 87382.885 | 51026.075 |
| | | 45-49 | 295573.443 | 182092.928 | 113480.515 |
| | | 50-54 | 603979.438 | 358537.317 | 245442.121 |
| | | 55-59 | 1086086.992 | 632810.800 | 453276.192 |
| | | 60-64 | 1465829.290 | 904337.248 | 561492.042 |
| | | 65-69 | 1451838.682 | 945795.644 | 506043.038 |
| | | 70-74 | 692979.695 | 469729.712 | 223249.983 |
| | | 75-79 | 0.000 | 0.000 | 0.000 |
| | Female | 30-34 | 34659.261 | 23551.884 | 11107.377 |
| | | 35-39 | 65652.924 | 44146.294 | 21506.631 |
| | | 40-44 | 133169.505 | 87396.230 | 45773.275 |
| | | 45-49 | 267988.924 | 170038.872 | 97950.053 |
| | | 50-54 | 523734.121 | 324514.407 | 199219.714 |
| | | 55-59 | 870859.170 | 526562.987 | 344296.182 |
| | | 60-64 | 1166683.329 | 721315.908 | 445367.421 |
| | | 65-69 | 1190003.066 | 753421.394 | 436581.672 |
| | | 70-74 | 588541.493 | 377121.338 | 211420.155 |
| | | 75-79 | 0.000 | 0.000 | 0.000 |

## Results for PHIM for Run 2 (ELL Summary Main_Analysis.RTF)

*************************************************************************************

**Table 6: Estimated Loss of Life, at the end of follow-up, Population and Number deaths adjusted for Ecig scenario**

| Disease | Sex | Age Group | Years of life lost NULL | Years of life lost Ecig | Years of life Saved |
|---|---|---|---|---|---|
| All Diseases | Males | 30-34 | 443911.034 | 238699.292 | 205211.742 |
| | | 35-39 | 1146798.555 | 610359.649 | 536438.906 |
| | | 40-44 | 2609591.408 | 1349924.882 | 1259666.53 |
| | | 45-49 | 4775841.753 | 2431509.736 | 2344332.02 |
| | | 50-54 | 7446969.205 | 3936197.522 | 3510771.68 |
| | | 55-59 | 8240205.925 | 4788241.179 | 3451964.75 |
| | | 60-64 | 8772829.118 | 5528603.499 | 3244225.62 |
| | | 65-69 | 6161494.678 | 4268174.993 | 1893319.69 |
| | | 70-74 | 2495906.274 | 1792338.592 | 703567.681 |
| | | 75-79 | 0.000 | 0.000 | 0.000 |
| | Female | 30-34 | 229140.561 | 142118.173 | 87022.387 |
| | | 35-39 | 570217.942 | 347175.885 | 223042.057 |
| | | 40-44 | 1267538.938 | 748834.778 | 518704.160 |
| | | 45-49 | 2340070.698 | 1360010.092 | 980060.607 |
| | | 50-54 | 3743803.613 | 2192291.881 | 1551511.73 |
| | | 55-59 | 4591478.168 | 2812347.624 | 1779130.54 |
| | | 60-64 | 5207367.788 | 3315180.334 | 1892187.45 |
| | | 65-69 | 4092181.394 | 2747910.770 | 1344270.62 |
| | | 70-74 | 1776092.171 | 1212603.436 | 563488.734 |
| | | 75-79 | 0.000 | 0.000 | 0.000 |
| Lung cancer | Males | 30-34 | 66065.017 | 51716.159 | 14348.858 |
| | | 35-39 | 232509.414 | 183524.180 | 48985.234 |
| | | 40-44 | 688628.041 | 531833.027 | 156795.014 |
| | | 45-49 | 1528300.367 | 1144140.708 | 384159.659 |
| | | 50-54 | 2730187.229 | 2013471.176 | 716716.053 |
| | | 55-59 | 3909101.673 | 2866796.630 | 1042305.04 |
| | | 60-64 | 4287716.660 | 3241425.968 | 1046290.69 |
| | | 65-69 | 3467897.115 | 2671917.726 | 795979.389 |
| | | 70-74 | 1312144.343 | 1029245.934 | 282898.409 |
| | | 75-79 | 0.000 | 0.000 | 0.000 |
| | Female | 30-34 | 51913.113 | 40646.356 | 11266.756 |
| | | 35-39 | 189792.904 | 148870.579 | 40922.325 |
| | | 40-44 | 527596.573 | 396729.948 | 130866.625 |
| | | 45-49 | 1122524.269 | 812853.665 | 309670.604 |
| | | 50-54 | 1882214.322 | 1333534.926 | 548679.396 |
| | | 55-59 | 2595581.943 | 1815374.024 | 780207.919 |
| | | 60-64 | 2846273.090 | 2030547.851 | 815725.239 |
| | | 65-69 | 2358758.394 | 1702293.438 | 656464.956 |
| | | 70-74 | 937814.238 | 685984.313 | 251829.924 |
| | | 75-79 | 0.000 | 0.000 | 0.000 |
| IHD | Males | 30-34 | 263834.566 | 118000.103 | 145834.462 |
| | | 35-39 | 692140.150 | 295209.655 | 396930.495 |
| | | 40-44 | 1519708.432 | 589135.039 | 930573.393 |
| | | 45-49 | 2568546.697 | 912069.594 | 1656477.10 |
| | | 50-54 | 3632929.903 | 1343452.082 | 2289477.82 |
| | | 55-59 | 2807892.356 | 1100407.237 | 1707485.12 |
| | | 60-64 | 2599825.934 | 1194919.554 | 1404906.38 |
| | | 65-69 | 1113578.307 | 584963.142 | 528615.166 |
| | | 70-74 | 437536.271 | 262255.136 | 175281.134 |
| | | 75-79 | 0.000 | 0.000 | 0.000 |
| | Female | 30-34 | 79649.097 | 37853.176 | 41795.920 |
| | | 35-39 | 193965.908 | 81453.727 | 112512.180 |
| | | 40-44 | 403380.974 | 151153.112 | 252227.862 |
| | | 45-49 | 671385.408 | 232932.262 | 438453.146 |
| | | 50-54 | 1011875.989 | 376327.213 | 635548.776 |
| | | 55-59 | 849875.715 | 344304.860 | 505570.855 |
| | | 60-64 | 925490.876 | 436774.710 | 488716.166 |
| | | 65-69 | 455463.881 | 246347.224 | 209116.657 |
| | | 70-74 | 207681.216 | 125751.158 | 81930.058 |
| | | 75-79 | 0.000 | 0.000 | 0.000 |
| Stroke | Males | 30-34 | 73463.650 | 43070.555 | 30393.095 |
| | | 35-39 | 151934.057 | 86404.109 | 65529.948 |
| | | 40-44 | 262845.974 | 141502.760 | 121343.214 |
| | | 45-49 | 383421.246 | 192832.541 | 190588.705 |
| | | 50-54 | 479872.634 | 219294.720 | 260577.914 |
| | | 55-59 | 437124.903 | 184941.796 | 252183.108 |
| | | 60-64 | 419457.235 | 181008.217 | 238449.018 |
| | | 65-69 | 128180.574 | 57871.667 | 70308.907 |
| | | 70-74 | 53245.965 | 26194.265 | 27051.700 |
| | | 75-79 | 0.000 | 0.000 | 0.000 |
| | Female | 30-34 | 62919.090 | 40066.003 | 22853.087 |

## Results for PHIM for Run 2 (ELL Summary Main_Analysis.RTF)

| | | | | | |
|---|---|---|---|---|---|
| | | 35-39 | 120806.205 | 72700.709 | 48105.496 |
| | | 40-44 | 203391.885 | 113528.310 | 89863.576 |
| | | 45-49 | 278172.097 | 144048.534 | 134123.563 |
| | | 50-54 | 325979.181 | 157392.218 | 168586.963 |
| | | 55-59 | 275161.340 | 124849.429 | 150311.911 |
| | | 60-64 | 268920.493 | 123672.215 | 145248.278 |
| | | 65-69 | 87956.054 | 42037.992 | 45918.061 |
| | | 70-74 | 42055.224 | 20931.590 | 21123.634 |
| | | 75-79 | 0.000 | 0.000 | 0.000 |
| COPD | Males | 30-34 | 40547.801 | 25912.474 | 14635.327 |
| | | 35-39 | 70214.933 | 45221.705 | 24993.229 |
| | | 40-44 | 138408.960 | 87454.055 | 50954.905 |
| | | 45-49 | 295573.443 | 182466.892 | 113106.551 |
| | | 50-54 | 603979.438 | 359979.544 | 243999.894 |
| | | 55-59 | 1086086.992 | 636095.518 | 449991.474 |
| | | 60-64 | 1465829.290 | 911249.761 | 554579.529 |
| | | 65-69 | 1451838.682 | 953422.458 | 498416.224 |
| | | 70-74 | 692979.695 | 474643.257 | 218336.438 |
| | | 75-79 | 0.000 | 0.000 | 0.000 |
| | Female | 30-34 | 34659.261 | 23552.637 | 11106.624 |
| | | 35-39 | 65652.924 | 44150.869 | 21502.055 |
| | | 40-44 | 133169.505 | 87423.408 | 45746.097 |
| | | 45-49 | 267988.924 | 170175.630 | 97813.294 |
| | | 50-54 | 523734.121 | 325037.523 | 198696.597 |
| | | 55-59 | 870859.170 | 527819.311 | 343039.859 |
| | | 60-64 | 1166683.329 | 724185.558 | 442497.771 |
| | | 65-69 | 1190003.066 | 757232.116 | 432770.950 |
| | | 70-74 | 588541.493 | 379936.375 | 208605.118 |
| | | 75-79 | 0.000 | 0.000 | 0.000 |

## Results for PHIM for Run 1 (Pessimistic_Analysis.RTF)

## )

---

System Date and Time: 20210220T083445
User: JohnS
SAS Version: 9.4
Project: Population Health Impact Model 7.3

The System is running in Test Mode

Maximum Upper Age for P Component = 79
Upper Age for ELL Calculations = 75
Report in E-component: Social Classes A and B combined
Description: Pessimistic Analysis for VeneBio on US
Assumption Files:
Options Control File: C:\VeneBio\OG_Test\\US_Control_V_PA.CSV
TTP Test File: C:\VeneBio\OG_Test\\TTP_Test_PA.csv
TTP Reference File: C:\VeneBio\OG_Test\\TTP_Reference_V.csv
TTP Factor Test File: C:\VeneBio\OG_Test\\TTP_FACTOR_Test.csv
TTP Factor Reference File: C:\VeneBio\OG_Test\\TTP_FACTOR_Test.csv
TP Socioeconomic Groups: Not Used
Output Choices:C:\VeneBio\OG_Test\\OUTC_PA.csv

Main Results will be sent to the folder C:\VeneBio\Main_Results\
Plots will be sent to the folder C:\VeneBio\Plot_Results\Plots_20210220T083445
Saved P, E and/or A Component results will be sent to the folder C:\VeneBio\SAVED\Saved_20210220T083445\

# Table of Contents


Control File Details for Run: 1 ........ 124
TTP values for Run: 1, Scenario: NULL ........ 125
TTP values for Run: 1, Scenario: Ecig ........ 127
Main Output for the Tobacco History for Run: 1, Simulation: 1, Scenario: NULL ........ 131
Main Output for the Tobacco History for Run: 1, Simulation: 1, Scenario: Ecig ........ 133
Main Results for E-Component for Run: 1, Simulation: 1, COD: Four Diseases Combined ........ 149
Main Results for E-Component for Run: 1, Simulation: 1, COD: LUNG CANCER ........ 150
Main Results for E-Component for Run: 1, Simulation: 1, COD: IHD ........ 151
Main Results for E-Component for Run: 1, Simulation: 1, COD: STROKE ........ 152
Main Results for E-Component for Run: 1, Simulation: 1, COD: COPD ........ 153
Main Results for E-Component for Run: 1, Simulation: 1, COD: Four Diseases Combined, Adjusted ........ 154
Main Results for E-Component for Run: 1, Simulation: 1, COD: LUNG CANCER, Adjusted ........ 155
Main Results for E-Component for Run: 1, Simulation: 1, COD: IHD, Adjusted ........ 156
Main Results for E-Component for Run: 1, Simulation: 1, COD: STROKE, Adjusted ........ 157
Main Results for E-Component for Run: 1, Simulation: 1, COD: COPD, Adjusted ........ 158
Control File Details for Run: 2 ........ 159
TTP values for Run: 2, Scenario: NULL ........ 160
TTP values for Run: 2, Scenario: Ecig ........ 162
Main Output for the Tobacco History for Run: 2, Simulation: 1, Scenario: NULL ........ 166
Main Output for the Tobacco History for Run: 2, Simulation: 1, Scenario: Ecig ........ 168
Main Results for E-Component for Run: 2, Simulation: 1, COD: Four Diseases Combined ........ 184
Main Results for E-Component for Run: 2, Simulation: 1, COD: LUNG CANCER ........ 185
Main Results for E-Component for Run: 2, Simulation: 1, COD: IHD ........ 186
Main Results for E-Component for Run: 2, Simulation: 1, COD: STROKE ........ 187
Main Results for E-Component for Run: 2, Simulation: 1, COD: COPD ........ 188
Main Results for E-Component for Run: 2, Simulation: 1, COD: Four Diseases Combined, Adjusted ........ 189
Main Results for E-Component for Run: 2, Simulation: 1, COD: LUNG CANCER, Adjusted ........ 190
Main Results for E-Component for Run: 2, Simulation: 1, COD: IHD, Adjusted ........ 191
Main Results for E-Component for Run: 2, Simulation: 1, COD: STROKE, Adjusted ........ 192
Main Results for E-Component for Run: 2, Simulation: 1, COD: COPD, Adjusted ........ 193
Estimated Loss of Life from the E Component for Run: 2, Simulation: 1 ........ 194
Table 1: Estimated Loss of Life, at the end of follow-up, all age groups combined, Population unadjusted for deaths in Ecig scenario ........ 194
Table 2: Estimated Loss of Life, at the end of follow-up, all age groups combined, Population and Number deaths adjusted for Ecig scenario ........ 195
Table 3: Estimated Loss of Life, All age groups combined, Population unadjusted for deaths in Ecig scenario, cumulative over years ........ 196
Table 4: Estimated Loss of Life, All age groups combined, Population and Number deaths adjusted for Ecig scenario, cumulative over years ........ 203
Table 5: Estimated Loss of Life, at the end of follow-up, Population unadjusted for deaths in Ecig scenari ........ 210
Table 6: Estimated Loss of Life, at the end of follow-up, Population and Number deaths adjusted for Ecig scenario ........ 212

## Results for PHIM for Run 1 (Pessimistic_Analysis.RTF)

---
--------------------------------------------------------------------------------------

**Control File Details for Run: 1**

| Variable Parameter | Value |
|---|---|
| 1. Name for Reference Scenario | NULL |
| 2. Name for Test Scenario | Ecig |
| 3. Name for Product 1 | CC |
| 4. Name for Product 2 | Ecig |
| 5. Name for Product 3 | - |
| 6. Name for Product 4 | Dual |
| 7. Country | US (United States) |
| 8. Sex | M (Males) |
| 9. Year of start of process | 1990 |
| 10. Number of months of follow-up | 600 |
| 11. Follow-up interval length (in months) | 12 |
| 12. Lower age for risk estimation | 10 |
| 13. Upper age for risk estimation | 79 |
| 14. The effective dose for Product 1 (F1) - Ref | 1 |
| 15. The effective dose for Product 2 (F2) - Ref | - |
| 16. The effective dose for Product 3 (F3) - Ref | - |
| 17. The effective dose for Product 4 (F4) - Ref | - |
| 18. The effective dose for Product 1 (F1) - Test | 1 |
| 19. The effective dose for Product 2 (F2) - Test | 0.4 |
| 20. The effective dose for Product 3 (F3) - Test | - |
| 21. The effective dose for Product 4 (F4) - Test | FROMF2 |
| 22. Number in population to be simulated | 100000 |
| 23. Number of MC simulations | 1 |
| 24. The random number seed for the first simulation | 15975263 |
| 25. Source for the population file (POP) | UN4 |
| 26. Source for the socioeconomic prevalence file (SEP) | - |
| 27. Source for the current smoking prevalence file (CSP) | ISS3 |
| 28. Source for the former smoking prevalence file (FSP) | ISS3 |
| 29. Source for the quit-time distribution file (QTD) | NHIS2006 |
| 30. Source for the death file (MORT) | APR-20 |
| 31. Source for the relative risk file (RR) | PNLEST |
| 32. Source for the half-life file (H) | PNLEST |
| 33. Assumption Set for TTP Factor Reference | F1 |
| 34. Assumption Set for TTP Factor Test | F1 |
| 35. Assumption Set for TTP Reference | ISS3 |
| 36. Assumption Set for TTP Test | ISS3 |
| 37. Assumption Set for TP for socioeconomic group | - |
| 38. Source for Output Choice file (OUTC) | BASIC |
| 39. Output for DTP P1 | N |
| 40. Output for DTP P2 | N |
| 41. Output for Main P Component | Y |
| 42. Output for DTP E1 | N |
| 43. Output for DTP E2 | N |
| 44. Output for DTP E3 | N |
| 45. Output for Main E Component | Y |
| 46. Output file name | Pessimistic_Analysis |
| 47. Run number for P-Component | - |
| 48. Results file for E-Component | - |
| 49. Components required | PE |
| 50. Results folder for P to be used in E-Component | - |
| 51. Fixed age of starting product use | 16 |
| 52. Output absolute risk file? | N |

# Results for PHIM for Run 1 (Pessimistic_Analysis.RTF)

************************************************************************************

**TTP values for Run: 1, Scenario: NULL**

| **Variable Parameter** | **Value** |
|---|---|
| 1. Country | US (United States) |
| 2. Sex | M (Males) |
| 3. Assumption Set | ISS3 |
| Note: | Period is in Months |

| Age | Period | **Initiation** Never CC | **Cessation** CC Former | **Re-initiation** Former CC |
|---|---|---|---|---|
| 10-14 | 1-12 | 0.003842 | 0.008742 | 0.000000 |
| 15-19 | 1-12 | 0.004720 | 0.003614 | 0.000000 |
| 20-24 | 1-12 | 0.000376 | 0.000943 | 0.000000 |
| 25-29 | 1-12 | 0.000187 | 0.001143 | 0.000000 |
| 30-34 | 1-12 | 0.000093 | 0.000871 | 0.000000 |
| 35-39 | 1-12 | 0.000000 | 0.000867 | 0.000000 |
| 40-44 | 1-12 | 0.000000 | 0.001399 | 0.000000 |
| 45-49 | 1-12 | 0.000000 | 0.002033 | 0.000000 |
| 50-54 | 1-12 | 0.000000 | 0.003209 | 0.000000 |
| 55-59 | 1-12 | 0.000000 | 0.003574 | 0.000000 |
| 60-64 | 1-12 | 0.000000 | 0.003460 | 0.000000 |
| 65-69 | 1-12 | 0.000000 | 0.004136 | 0.000000 |
| 70-74 | 1-12 | 0.000000 | 0.005592 | 0.000000 |
| 75-79 | 1-12 | 0.000000 | 0.005592 | 0.000000 |
| 10-14 | 13-72 | 0.004906 | 0.008742 | 0.000000 |
| 15-19 | 13-72 | 0.006989 | 0.004426 | 0.000000 |
| 20-24 | 13-72 | 0.000687 | 0.001527 | 0.000000 |
| 25-29 | 13-72 | 0.000962 | 0.001515 | 0.000000 |
| 30-34 | 13-72 | 0.000364 | 0.001227 | 0.000000 |
| 35-39 | 13-72 | 0.000000 | 0.001630 | 0.000000 |
| 40-44 | 13-72 | 0.000000 | 0.001852 | 0.000000 |
| 45-49 | 13-72 | 0.000000 | 0.002667 | 0.000000 |
| 50-54 | 13-72 | 0.000000 | 0.003208 | 0.000000 |
| 55-59 | 13-72 | 0.000000 | 0.003978 | 0.000000 |
| 60-64 | 13-72 | 0.000000 | 0.005771 | 0.000000 |
| 65-69 | 13-72 | 0.000000 | 0.010432 | 0.000000 |
| 70-74 | 13-72 | 0.000000 | 0.007079 | 0.000000 |
| 75-79 | 13-72 | 0.000000 | 0.007079 | 0.000000 |
| 10-14 | 73-132 | 0.004312 | 0.008742 | 0.000000 |
| 15-19 | 73-132 | 0.005330 | 0.003289 | 0.000000 |
| 20-24 | 73-132 | 0.002456 | 0.003844 | 0.000000 |
| 25-29 | 73-132 | 0.000839 | 0.002006 | 0.000000 |
| 30-34 | 73-132 | 0.000414 | 0.001329 | 0.000000 |
| 35-39 | 73-132 | 0.000000 | 0.001278 | 0.000000 |
| 40-44 | 73-132 | 0.000000 | 0.001691 | 0.000000 |
| 45-49 | 73-132 | 0.000000 | 0.002904 | 0.000000 |
| 50-54 | 73-132 | 0.000000 | 0.003319 | 0.000000 |
| 55-59 | 73-132 | 0.000000 | 0.003976 | 0.000000 |
| 60-64 | 73-132 | 0.000000 | 0.005235 | 0.000000 |
| 65-69 | 73-132 | 0.000000 | 0.005901 | 0.000000 |
| 70-74 | 73-132 | 0.000000 | 0.005615 | 0.000000 |
| 75-79 | 73-132 | 0.000000 | 0.005615 | 0.000000 |
| 10-14 | 133-192 | 0.003468 | 0.008742 | 0.000000 |
| 15-19 | 133-192 | 0.004675 | 0.003732 | 0.000000 |
| 20-24 | 133-192 | 0.000062 | 0.002260 | 0.000000 |
| 25-29 | 133-192 | 0.001422 | 0.002822 | 0.000000 |
| 30-34 | 133-192 | 0.000696 | 0.002610 | 0.000000 |
| 35-39 | 133-192 | 0.000000 | 0.002058 | 0.000000 |
| 40-44 | 133-192 | 0.000000 | 0.002310 | 0.000000 |
| 45-49 | 133-192 | 0.000000 | 0.002256 | 0.000000 |
| 50-54 | 133-192 | 0.000000 | 0.002663 | 0.000000 |
| 55-59 | 133-192 | 0.000000 | 0.001746 | 0.000000 |
| 60-64 | 133-192 | 0.000000 | 0.005809 | 0.000000 |
| 65-69 | 133-192 | 0.000000 | 0.005896 | 0.000000 |
| 70-74 | 133-192 | 0.000000 | 0.007323 | 0.000000 |
| 75-79 | 133-192 | 0.000000 | 0.007323 | 0.000000 |
| 10-14 | 193-252 | 0.002314 | 0.008742 | 0.000000 |
| 15-19 | 193-252 | 0.003987 | 0.005036 | 0.000000 |
| 20-24 | 193-252 | 0.000320 | 0.002857 | 0.000000 |
| 25-29 | 193-252 | 0.000251 | 0.002314 | 0.000000 |
| 30-34 | 193-252 | 0.000125 | 0.004343 | 0.000000 |
| 35-39 | 193-252 | 0.000000 | 0.002092 | 0.000000 |
| 40-44 | 193-252 | 0.000000 | 0.002566 | 0.000000 |
| 45-49 | 193-252 | 0.000000 | 0.002311 | 0.000000 |
| 50-54 | 193-252 | 0.000000 | 0.003209 | 0.000000 |

## Results for PHIM for Run 1 (Pessimistic_Analysis.RTF)

| | | | | |
|---|---|---|---|---|
| 55-59 | 193-252 | 0.000000 | 0.004057 | 0.000000 |
| 60-64 | 193-252 | 0.000000 | 0.007166 | 0.000000 |
| 65-69 | 193-252 | 0.000000 | 0.004281 | 0.000000 |
| 70-74 | 193-252 | 0.000000 | 0.004580 | 0.000000 |
| 75-79 | 193-252 | 0.000000 | 0.004580 | 0.000000 |
| 10-14 | 253+ | 0.001325 | 0.008742 | 0.000000 |
| 15-19 | 253+ | 0.002984 | 0.010602 | 0.000000 |
| 20-24 | 253+ | 0.000452 | 0.004314 | 0.000000 |
| 25-29 | 253+ | 0.000225 | 0.003645 | 0.000000 |
| 30-34 | 253+ | 0.000112 | 0.004512 | 0.000000 |
| 35-39 | 253+ | 0.000000 | 0.002147 | 0.000000 |
| 40-44 | 253+ | 0.000000 | 0.002636 | 0.000000 |
| 45-49 | 253+ | 0.000000 | 0.001051 | 0.000000 |
| 50-54 | 253+ | 0.000000 | 0.002519 | 0.000000 |
| 55-59 | 253+ | 0.000000 | 0.003693 | 0.000000 |
| 60-64 | 253+ | 0.000000 | 0.005888 | 0.000000 |
| 65-69 | 253+ | 0.000000 | 0.004823 | 0.000000 |
| 70-74 | 253+ | 0.000000 | 0.004733 | 0.000000 |
| 75-79 | 253+ | 0.000000 | 0.004733 | 0.000000 |

# Results for PHIM for Run 1 (Pessimistic_Analysis.RTF)

************************************************************************************

**TTP values for Run: 1, Scenario: Ecig**

| **Variable Parameter** | **Value** |
|---|---|
| 1. Country | US (United States) |
| 2. Sex | M (Males) |
| 3. Assumption Set | ISS3 |
| Note: | Period is in Months |

| | | **Initiation** | | | **Cessation** | | | **Re-initiation** | | |
|---|---|---|---|---|---|---|---|---|---|---|
| Age | Period | Never CC | Never Ecig | Never Dual | CC Former | Ecig Former | Dual Former | Former CC | Former Ecig | Former Dual |
| 10-14 | 1-12 | 0.001921 | 0.002882 | 0.000000 | 0.008742 | 0.004371 | 0.000000 | 0.000000 | 0.000000 | 0.000000 |
| 15-19 | 1-12 | 0.002360 | 0.003540 | 0.000000 | 0.003614 | 0.001807 | 0.000000 | 0.000000 | 0.000000 | 0.000000 |
| 20-24 | 1-12 | 0.000188 | 0.000282 | 0.000000 | 0.000943 | 0.000472 | 0.000000 | 0.000000 | 0.000000 | 0.000000 |
| 25-29 | 1-12 | 0.000093 | 0.000140 | 0.000000 | 0.001143 | 0.000571 | 0.000000 | 0.000000 | 0.000000 | 0.000000 |
| 30-34 | 1-12 | 0.000047 | 0.000070 | 0.000000 | 0.000871 | 0.000435 | 0.000000 | 0.000000 | 0.000000 | 0.000000 |
| 35-39 | 1-12 | 0.000000 | 0.000000 | 0.000000 | 0.000867 | 0.000433 | 0.000000 | 0.000000 | 0.000000 | 0.000000 |
| 40-44 | 1-12 | 0.000000 | 0.000000 | 0.000000 | 0.001399 | 0.000699 | 0.000000 | 0.000000 | 0.000000 | 0.000000 |
| 45-49 | 1-12 | 0.000000 | 0.000000 | 0.000000 | 0.002033 | 0.001016 | 0.000000 | 0.000000 | 0.000000 | 0.000000 |
| 50-54 | 1-12 | 0.000000 | 0.000000 | 0.000000 | 0.003209 | 0.001604 | 0.000000 | 0.000000 | 0.000000 | 0.000000 |
| 55-59 | 1-12 | 0.000000 | 0.000000 | 0.000000 | 0.003574 | 0.001787 | 0.000000 | 0.000000 | 0.000000 | 0.000000 |
| 60-64 | 1-12 | 0.000000 | 0.000000 | 0.000000 | 0.003460 | 0.001730 | 0.000000 | 0.000000 | 0.000000 | 0.000000 |
| 65-69 | 1-12 | 0.000000 | 0.000000 | 0.000000 | 0.004136 | 0.002068 | 0.000000 | 0.000000 | 0.000000 | 0.000000 |
| 70-74 | 1-12 | 0.000000 | 0.000000 | 0.000000 | 0.005592 | 0.002796 | 0.000000 | 0.000000 | 0.000000 | 0.000000 |
| 75-79 | 1-12 | 0.000000 | 0.000000 | 0.000000 | 0.005592 | 0.002796 | 0.000000 | 0.000000 | 0.000000 | 0.000000 |
| 10-14 | 13-72 | 0.001962 | 0.004415 | 0.000000 | 0.008742 | 0.004371 | 0.000000 | 0.000000 | 0.000000 | 0.000000 |
| 15-19 | 13-72 | 0.002796 | 0.006290 | 0.000000 | 0.004426 | 0.002213 | 0.000000 | 0.000000 | 0.000000 | 0.000000 |
| 20-24 | 13-72 | 0.000275 | 0.000618 | 0.000000 | 0.001527 | 0.000764 | 0.000000 | 0.000000 | 0.000000 | 0.000000 |
| 25-29 | 13-72 | 0.000385 | 0.000866 | 0.000000 | 0.001515 | 0.000758 | 0.000000 | 0.000000 | 0.000000 | 0.000000 |
| 30-34 | 13-72 | 0.000146 | 0.000328 | 0.000000 | 0.001227 | 0.000613 | 0.000000 | 0.000000 | 0.000000 | 0.000000 |
| 35-39 | 13-72 | 0.000000 | 0.000000 | 0.000000 | 0.001630 | 0.000815 | 0.000000 | 0.000000 | 0.000000 | 0.000000 |
| 40-44 | 13-72 | 0.000000 | 0.000000 | 0.000000 | 0.001852 | 0.000926 | 0.000000 | 0.000000 | 0.000000 | 0.000000 |
| 45-49 | 13-72 | 0.000000 | 0.000000 | 0.000000 | 0.002667 | 0.001333 | 0.000000 | 0.000000 | 0.000000 | 0.000000 |
| 50-54 | 13-72 | 0.000000 | 0.000000 | 0.000000 | 0.003208 | 0.001604 | 0.000000 | 0.000000 | 0.000000 | 0.000000 |
| 55-59 | 13-72 | 0.000000 | 0.000000 | 0.000000 | 0.003978 | 0.001989 | 0.000000 | 0.000000 | 0.000000 | 0.000000 |
| 60-64 | 13-72 | 0.000000 | 0.000000 | 0.000000 | 0.005771 | 0.002886 | 0.000000 | 0.000000 | 0.000000 | 0.000000 |
| 65-69 | 13-72 | 0.000000 | 0.000000 | 0.000000 | 0.010432 | 0.005216 | 0.000000 | 0.000000 | 0.000000 | 0.000000 |
| 70-74 | 13-72 | 0.000000 | 0.000000 | 0.000000 | 0.007079 | 0.003540 | 0.000000 | 0.000000 | 0.000000 | 0.000000 |
| 75-79 | 13-72 | 0.000000 | 0.000000 | 0.000000 | 0.007079 | 0.003540 | 0.000000 | 0.000000 | 0.000000 | 0.000000 |
| 10-14 | 73-132 | 0.000862 | 0.005174 | 0.000000 | 0.008742 | 0.004371 | 0.000000 | 0.000000 | 0.000000 | 0.000000 |
| 15-19 | 73-132 | 0.001066 | 0.006396 | 0.000000 | 0.003289 | 0.001645 | 0.000000 | 0.000000 | 0.000000 | 0.000000 |
| 20-24 | 73-132 | 0.000491 | 0.002947 | 0.000000 | 0.003844 | 0.001922 | 0.000000 | 0.000000 | 0.000000 | 0.000000 |
| 25-29 | 73-132 | 0.000168 | 0.001007 | 0.000000 | 0.002006 | 0.001003 | 0.000000 | 0.000000 | 0.000000 | 0.000000 |
| 30-34 | 73-132 | 0.000083 | 0.000497 | 0.000000 | 0.001329 | 0.000664 | 0.000000 | 0.000000 | 0.000000 | 0.000000 |
| 35-39 | 73-132 | 0.000000 | 0.000000 | 0.000000 | 0.001278 | 0.000639 | 0.000000 | 0.000000 | 0.000000 | 0.000000 |
| 40-44 | 73-132 | 0.000000 | 0.000000 | 0.000000 | 0.001691 | 0.000846 | 0.000000 | 0.000000 | 0.000000 | 0.000000 |
| 45-49 | 73-132 | 0.000000 | 0.000000 | 0.000000 | 0.002904 | 0.001452 | 0.000000 | 0.000000 | 0.000000 | 0.000000 |
| 50-54 | 73-132 | 0.000000 | 0.000000 | 0.000000 | 0.003319 | 0.001660 | 0.000000 | 0.000000 | 0.000000 | 0.000000 |
| 55-59 | 73-132 | 0.000000 | 0.000000 | 0.000000 | 0.003976 | 0.001988 | 0.000000 | 0.000000 | 0.000000 | 0.000000 |
| 60-64 | 73-132 | 0.000000 | 0.000000 | 0.000000 | 0.005235 | 0.002617 | 0.000000 | 0.000000 | 0.000000 | 0.000000 |
| 65-69 | 73-132 | 0.000000 | 0.000000 | 0.000000 | 0.005901 | 0.002951 | 0.000000 | 0.000000 | 0.000000 | 0.000000 |
| 70-74 | 73-132 | 0.000000 | 0.000000 | 0.000000 | 0.005615 | 0.002808 | 0.000000 | 0.000000 | 0.000000 | 0.000000 |
| 75-79 | 73-132 | 0.000000 | 0.000000 | 0.000000 | 0.005615 | 0.002808 | 0.000000 | 0.000000 | 0.000000 | 0.000000 |
| 10-14 | 133-192 | 0.000173 | 0.004941 | 0.000000 | 0.008742 | 0.004371 | 0.000000 | 0.000000 | 0.000000 | 0.000000 |
| 15-19 | 133-192 | 0.000234 | 0.006663 | 0.000000 | 0.003732 | 0.001866 | 0.000000 | 0.000000 | 0.000000 | 0.000000 |
| 20-24 | 133-192 | 0.000003 | 0.000089 | 0.000000 | 0.002260 | 0.001130 | 0.000000 | 0.000000 | 0.000000 | 0.000000 |
| 25-29 | 133-192 | 0.000071 | 0.002027 | 0.000000 | 0.002822 | 0.001411 | 0.000000 | 0.000000 | 0.000000 | 0.000000 |
| 30-34 | 133-192 | 0.000035 | 0.000992 | 0.000000 | 0.002610 | 0.001305 | 0.000000 | 0.000000 | 0.000000 | 0.000000 |
| 35-39 | 133-192 | 0.000000 | 0.000000 | 0.000000 | 0.002058 | 0.001029 | 0.000000 | 0.000000 | 0.000000 | 0.000000 |
| 40-44 | 133-192 | 0.000000 | 0.000000 | 0.000000 | 0.002310 | 0.001155 | 0.000000 | 0.000000 | 0.000000 | 0.000000 |
| 45-49 | 133-192 | 0.000000 | 0.000000 | 0.000000 | 0.002256 | 0.001128 | 0.000000 | 0.000000 | 0.000000 | 0.000000 |
| 50-54 | 133-192 | 0.000000 | 0.000000 | 0.000000 | 0.002663 | 0.001332 | 0.000000 | 0.000000 | 0.000000 | 0.000000 |
| 55-59 | 133-192 | 0.000000 | 0.000000 | 0.000000 | 0.001747 | 0.000873 | 0.000000 | 0.000000 | 0.000000 | 0.000000 |
| 60-64 | 133-192 | 0.000000 | 0.000000 | 0.000000 | 0.005809 | 0.002905 | 0.000000 | 0.000000 | 0.000000 | 0.000000 |
| 65-69 | 133-192 | 0.000000 | 0.000000 | 0.000000 | 0.005896 | 0.002948 | 0.000000 | 0.000000 | 0.000000 | 0.000000 |
| 70-74 | 133-192 | 0.000000 | 0.000000 | 0.000000 | 0.007323 | 0.003661 | 0.000000 | 0.000000 | 0.000000 | 0.000000 |
| 75-79 | 133-192 | 0.000000 | 0.000000 | 0.000000 | 0.007323 | 0.003661 | 0.000000 | 0.000000 | 0.000000 | 0.000000 |
| 10-14 | 193-252 | 0.000116 | 0.003298 | 0.000000 | 0.008742 | 0.004371 | 0.000000 | 0.000000 | 0.000000 | 0.000000 |
| 15-19 | 193-252 | 0.000199 | 0.005682 | 0.000000 | 0.005036 | 0.002518 | 0.000000 | 0.000000 | 0.000000 | 0.000000 |
| 20-24 | 193-252 | 0.000016 | 0.000456 | 0.000000 | 0.002857 | 0.001429 | 0.000000 | 0.000000 | 0.000000 | 0.000000 |
| 25-29 | 193-252 | 0.000013 | 0.000358 | 0.000000 | 0.002314 | 0.001157 | 0.000000 | 0.000000 | 0.000000 | 0.000000 |
| 30-34 | 193-252 | 0.000006 | 0.000178 | 0.000000 | 0.004343 | 0.002171 | 0.000000 | 0.000000 | 0.000000 | 0.000000 |
| 35-39 | 193-252 | 0.000000 | 0.000000 | 0.000000 | 0.002092 | 0.001046 | 0.000000 | 0.000000 | 0.000000 | 0.000000 |
| 40-44 | 193-252 | 0.000000 | 0.000000 | 0.000000 | 0.002566 | 0.001283 | 0.000000 | 0.000000 | 0.000000 | 0.000000 |
| 45-49 | 193-252 | 0.000000 | 0.000000 | 0.000000 | 0.002311 | 0.001156 | 0.000000 | 0.000000 | 0.000000 | 0.000000 |
| 50-54 | 193-252 | 0.000000 | 0.000000 | 0.000000 | 0.003209 | 0.001604 | 0.000000 | 0.000000 | 0.000000 | 0.000000 |

## Results for PHIM for Run 1 (Pessimistic_Analysis.RTF)

| | | | | | | | | | | |
|---|---|---|---|---|---|---|---|---|---|---|
| 55-59 | 193-252 | 0.000000 | 0.000000 | 0.000000 | 0.004057 | 0.002029 | 0.000000 | 0.000000 | 0.000000 | 0.000000 |
| 60-64 | 193-252 | 0.000000 | 0.000000 | 0.000000 | 0.007166 | 0.003583 | 0.000000 | 0.000000 | 0.000000 | 0.000000 |
| 65-69 | 193-252 | 0.000000 | 0.000000 | 0.000000 | 0.004281 | 0.002141 | 0.000000 | 0.000000 | 0.000000 | 0.000000 |
| 70-74 | 193-252 | 0.000000 | 0.000000 | 0.000000 | 0.004580 | 0.002290 | 0.000000 | 0.000000 | 0.000000 | 0.000000 |
| 75-79 | 193-252 | 0.000000 | 0.000000 | 0.000000 | 0.004580 | 0.002290 | 0.000000 | 0.000000 | 0.000000 | 0.000000 |
| 10-14 | 253+ | 0.000066 | 0.001888 | 0.000000 | 0.008742 | 0.004371 | 0.000000 | 0.000000 | 0.000000 | 0.000000 |
| 15-19 | 253+ | 0.000149 | 0.004253 | 0.000000 | 0.010602 | 0.005301 | 0.000000 | 0.000000 | 0.000000 | 0.000000 |
| 20-24 | 253+ | 0.000023 | 0.000644 | 0.000000 | 0.004314 | 0.002157 | 0.000000 | 0.000000 | 0.000000 | 0.000000 |
| 25-29 | 253+ | 0.000011 | 0.000320 | 0.000000 | 0.003645 | 0.001823 | 0.000000 | 0.000000 | 0.000000 | 0.000000 |
| 30-34 | 253+ | 0.000006 | 0.000160 | 0.000000 | 0.004512 | 0.002256 | 0.000000 | 0.000000 | 0.000000 | 0.000000 |
| 35-39 | 253+ | 0.000000 | 0.000000 | 0.000000 | 0.002147 | 0.001073 | 0.000000 | 0.000000 | 0.000000 | 0.000000 |
| 40-44 | 253+ | 0.000000 | 0.000000 | 0.000000 | 0.002636 | 0.001318 | 0.000000 | 0.000000 | 0.000000 | 0.000000 |
| 45-49 | 253+ | 0.000000 | 0.000000 | 0.000000 | 0.001051 | 0.000525 | 0.000000 | 0.000000 | 0.000000 | 0.000000 |
| 50-54 | 253+ | 0.000000 | 0.000000 | 0.000000 | 0.002519 | 0.001259 | 0.000000 | 0.000000 | 0.000000 | 0.000000 |
| 55-59 | 253+ | 0.000000 | 0.000000 | 0.000000 | 0.003693 | 0.001847 | 0.000000 | 0.000000 | 0.000000 | 0.000000 |
| 60-64 | 253+ | 0.000000 | 0.000000 | 0.000000 | 0.005888 | 0.002944 | 0.000000 | 0.000000 | 0.000000 | 0.000000 |
| 65-69 | 253+ | 0.000000 | 0.000000 | 0.000000 | 0.004823 | 0.002412 | 0.000000 | 0.000000 | 0.000000 | 0.000000 |
| 70-74 | 253+ | 0.000000 | 0.000000 | 0.000000 | 0.004733 | 0.002366 | 0.000000 | 0.000000 | 0.000000 | 0.000000 |
| 75-79 | 253+ | 0.000000 | 0.000000 | 0.000000 | 0.004733 | 0.002366 | 0.000000 | 0.000000 | 0.000000 | 0.000000 |

# Results for PHIM for Run 1 (Pessimistic_Analysis.RTF)

*******************************************************************************************

| | | Switch from CC | | Switch from Ecig | | Switch from Dual | |
|---|---|---|---|---|---|---|---|
| | | **CC** | **CC** | **Ecig** | **Ecig** | **Dual** | **Dual** |
| Age | Period | Ecig | Dual | CC | Dual | CC | Ecig |
| 10-14 | 1-12 | 0.000000 | 0.000000 | 0.000000 | 0.000000 | 0.000000 | 0.000000 |
| 15-19 | 1-12 | 0.004422 | 0.000000 | 0.000000 | 0.000000 | 0.000000 | 0.000000 |
| 20-24 | 1-12 | 0.008449 | 0.000000 | 0.000000 | 0.000000 | 0.000000 | 0.000000 |
| 25-29 | 1-12 | 0.009232 | 0.000000 | 0.000000 | 0.000000 | 0.000000 | 0.000000 |
| 30-34 | 1-12 | 0.009517 | 0.000000 | 0.000000 | 0.000000 | 0.000000 | 0.000000 |
| 35-39 | 1-12 | 0.008769 | 0.000000 | 0.000000 | 0.000000 | 0.000000 | 0.000000 |
| 40-44 | 1-12 | 0.008295 | 0.000000 | 0.000000 | 0.000000 | 0.000000 | 0.000000 |
| 45-49 | 1-12 | 0.007061 | 0.000000 | 0.000000 | 0.000000 | 0.000000 | 0.000000 |
| 50-54 | 1-12 | 0.005807 | 0.000000 | 0.000000 | 0.000000 | 0.000000 | 0.000000 |
| 55-59 | 1-12 | 0.005067 | 0.000000 | 0.000000 | 0.000000 | 0.000000 | 0.000000 |
| 60-64 | 1-12 | 0.003551 | 0.000000 | 0.000000 | 0.000000 | 0.000000 | 0.000000 |
| 65-69 | 1-12 | 0.000907 | 0.000000 | 0.000000 | 0.000000 | 0.000000 | 0.000000 |
| 70-74 | 1-12 | 0.000000 | 0.000000 | 0.000000 | 0.000000 | 0.000000 | 0.000000 |
| 75-79 | 1-12 | 0.000000 | 0.000000 | 0.000000 | 0.000000 | 0.000000 | 0.000000 |
| 10-14 | 13-72 | 0.000000 | 0.000000 | 0.000000 | 0.000000 | 0.000000 | 0.000000 |
| 15-19 | 13-72 | 0.004422 | 0.000000 | 0.000000 | 0.000000 | 0.000000 | 0.000000 |
| 20-24 | 13-72 | 0.008449 | 0.000000 | 0.000000 | 0.000000 | 0.000000 | 0.000000 |
| 25-29 | 13-72 | 0.009232 | 0.000000 | 0.000000 | 0.000000 | 0.000000 | 0.000000 |
| 30-34 | 13-72 | 0.009517 | 0.000000 | 0.000000 | 0.000000 | 0.000000 | 0.000000 |
| 35-39 | 13-72 | 0.008769 | 0.000000 | 0.000000 | 0.000000 | 0.000000 | 0.000000 |
| 40-44 | 13-72 | 0.008295 | 0.000000 | 0.000000 | 0.000000 | 0.000000 | 0.000000 |
| 45-49 | 13-72 | 0.007061 | 0.000000 | 0.000000 | 0.000000 | 0.000000 | 0.000000 |
| 50-54 | 13-72 | 0.005807 | 0.000000 | 0.000000 | 0.000000 | 0.000000 | 0.000000 |
| 55-59 | 13-72 | 0.005067 | 0.000000 | 0.000000 | 0.000000 | 0.000000 | 0.000000 |
| 60-64 | 13-72 | 0.003551 | 0.000000 | 0.000000 | 0.000000 | 0.000000 | 0.000000 |
| 65-69 | 13-72 | 0.000907 | 0.000000 | 0.000000 | 0.000000 | 0.000000 | 0.000000 |
| 70-74 | 13-72 | 0.000000 | 0.000000 | 0.000000 | 0.000000 | 0.000000 | 0.000000 |
| 75-79 | 13-72 | 0.000000 | 0.000000 | 0.000000 | 0.000000 | 0.000000 | 0.000000 |
| 10-14 | 73-132 | 0.000000 | 0.000000 | 0.000000 | 0.000000 | 0.000000 | 0.000000 |
| 15-19 | 73-132 | 0.004422 | 0.000000 | 0.000000 | 0.000000 | 0.000000 | 0.000000 |
| 20-24 | 73-132 | 0.008449 | 0.000000 | 0.000000 | 0.000000 | 0.000000 | 0.000000 |
| 25-29 | 73-132 | 0.009232 | 0.000000 | 0.000000 | 0.000000 | 0.000000 | 0.000000 |
| 30-34 | 73-132 | 0.009517 | 0.000000 | 0.000000 | 0.000000 | 0.000000 | 0.000000 |
| 35-39 | 73-132 | 0.008769 | 0.000000 | 0.000000 | 0.000000 | 0.000000 | 0.000000 |
| 40-44 | 73-132 | 0.008295 | 0.000000 | 0.000000 | 0.000000 | 0.000000 | 0.000000 |
| 45-49 | 73-132 | 0.007061 | 0.000000 | 0.000000 | 0.000000 | 0.000000 | 0.000000 |
| 50-54 | 73-132 | 0.005807 | 0.000000 | 0.000000 | 0.000000 | 0.000000 | 0.000000 |
| 55-59 | 73-132 | 0.005067 | 0.000000 | 0.000000 | 0.000000 | 0.000000 | 0.000000 |
| 60-64 | 73-132 | 0.003551 | 0.000000 | 0.000000 | 0.000000 | 0.000000 | 0.000000 |
| 65-69 | 73-132 | 0.000907 | 0.000000 | 0.000000 | 0.000000 | 0.000000 | 0.000000 |
| 70-74 | 73-132 | 0.000000 | 0.000000 | 0.000000 | 0.000000 | 0.000000 | 0.000000 |
| 75-79 | 73-132 | 0.000000 | 0.000000 | 0.000000 | 0.000000 | 0.000000 | 0.000000 |
| 10-14 | 133-192 | 0.000000 | 0.000000 | 0.000000 | 0.000000 | 0.000000 | 0.000000 |
| 15-19 | 133-192 | 0.004422 | 0.000000 | 0.000000 | 0.000000 | 0.000000 | 0.000000 |
| 20-24 | 133-192 | 0.008449 | 0.000000 | 0.000000 | 0.000000 | 0.000000 | 0.000000 |
| 25-29 | 133-192 | 0.009232 | 0.000000 | 0.000000 | 0.000000 | 0.000000 | 0.000000 |
| 30-34 | 133-192 | 0.009517 | 0.000000 | 0.000000 | 0.000000 | 0.000000 | 0.000000 |
| 35-39 | 133-192 | 0.008769 | 0.000000 | 0.000000 | 0.000000 | 0.000000 | 0.000000 |
| 40-44 | 133-192 | 0.008295 | 0.000000 | 0.000000 | 0.000000 | 0.000000 | 0.000000 |
| 45-49 | 133-192 | 0.007061 | 0.000000 | 0.000000 | 0.000000 | 0.000000 | 0.000000 |
| 50-54 | 133-192 | 0.005807 | 0.000000 | 0.000000 | 0.000000 | 0.000000 | 0.000000 |
| 55-59 | 133-192 | 0.005067 | 0.000000 | 0.000000 | 0.000000 | 0.000000 | 0.000000 |
| 60-64 | 133-192 | 0.003551 | 0.000000 | 0.000000 | 0.000000 | 0.000000 | 0.000000 |
| 65-69 | 133-192 | 0.000907 | 0.000000 | 0.000000 | 0.000000 | 0.000000 | 0.000000 |
| 70-74 | 133-192 | 0.000000 | 0.000000 | 0.000000 | 0.000000 | 0.000000 | 0.000000 |
| 75-79 | 133-192 | 0.000000 | 0.000000 | 0.000000 | 0.000000 | 0.000000 | 0.000000 |
| 10-14 | 193-252 | 0.000000 | 0.000000 | 0.000000 | 0.000000 | 0.000000 | 0.000000 |
| 15-19 | 193-252 | 0.004422 | 0.000000 | 0.000000 | 0.000000 | 0.000000 | 0.000000 |
| 20-24 | 193-252 | 0.008449 | 0.000000 | 0.000000 | 0.000000 | 0.000000 | 0.000000 |
| 25-29 | 193-252 | 0.009232 | 0.000000 | 0.000000 | 0.000000 | 0.000000 | 0.000000 |
| 30-34 | 193-252 | 0.009517 | 0.000000 | 0.000000 | 0.000000 | 0.000000 | 0.000000 |
| 35-39 | 193-252 | 0.008769 | 0.000000 | 0.000000 | 0.000000 | 0.000000 | 0.000000 |
| 40-44 | 193-252 | 0.008295 | 0.000000 | 0.000000 | 0.000000 | 0.000000 | 0.000000 |
| 45-49 | 193-252 | 0.007061 | 0.000000 | 0.000000 | 0.000000 | 0.000000 | 0.000000 |
| 50-54 | 193-252 | 0.005807 | 0.000000 | 0.000000 | 0.000000 | 0.000000 | 0.000000 |
| 55-59 | 193-252 | 0.005067 | 0.000000 | 0.000000 | 0.000000 | 0.000000 | 0.000000 |
| 60-64 | 193-252 | 0.003551 | 0.000000 | 0.000000 | 0.000000 | 0.000000 | 0.000000 |
| 65-69 | 193-252 | 0.000907 | 0.000000 | 0.000000 | 0.000000 | 0.000000 | 0.000000 |
| 70-74 | 193-252 | 0.000000 | 0.000000 | 0.000000 | 0.000000 | 0.000000 | 0.000000 |
| 75-79 | 193-252 | 0.000000 | 0.000000 | 0.000000 | 0.000000 | 0.000000 | 0.000000 |
| 10-14 | 253+ | 0.000000 | 0.000000 | 0.000000 | 0.000000 | 0.000000 | 0.000000 |
| 15-19 | 253+ | 0.004422 | 0.000000 | 0.000000 | 0.000000 | 0.000000 | 0.000000 |
| 20-24 | 253+ | 0.008449 | 0.000000 | 0.000000 | 0.000000 | 0.000000 | 0.000000 |

## Results for PHIM for Run 1 (Pessimistic_Analysis.RTF)

| | | | | | | | |
|---|---|---|---|---|---|---|---|
| 25-29 | 253+ | 0.009232 | 0.000000 | 0.000000 | 0.000000 | 0.000000 | 0.000000 |
| 30-34 | 253+ | 0.009517 | 0.000000 | 0.000000 | 0.000000 | 0.000000 | 0.000000 |
| 35-39 | 253+ | 0.008769 | 0.000000 | 0.000000 | 0.000000 | 0.000000 | 0.000000 |
| 40-44 | 253+ | 0.008295 | 0.000000 | 0.000000 | 0.000000 | 0.000000 | 0.000000 |
| 45-49 | 253+ | 0.007061 | 0.000000 | 0.000000 | 0.000000 | 0.000000 | 0.000000 |
| 50-54 | 253+ | 0.005807 | 0.000000 | 0.000000 | 0.000000 | 0.000000 | 0.000000 |
| 55-59 | 253+ | 0.005067 | 0.000000 | 0.000000 | 0.000000 | 0.000000 | 0.000000 |
| 60-64 | 253+ | 0.003551 | 0.000000 | 0.000000 | 0.000000 | 0.000000 | 0.000000 |
| 65-69 | 253+ | 0.000907 | 0.000000 | 0.000000 | 0.000000 | 0.000000 | 0.000000 |
| 70-74 | 253+ | 0.000000 | 0.000000 | 0.000000 | 0.000000 | 0.000000 | 0.000000 |
| 75-79 | 253+ | 0.000000 | 0.000000 | 0.000000 | 0.000000 | 0.000000 | 0.000000 |

# Results for PHIM for Run 1 (Pessimistic_Analysis.RTF)

*************************************************************************************

**Main Output for the Tobacco History for Run: 1, Simulation: 1, Scenario: NULL**

| Variable Parameter | Value |
|---|---|
| 1. Country | US (United States) |
| 2. Sex | M (Males) |
| 3. Year of start of process | 1990 |

Year of Follow-up: 2040, Age Group: All Ages

| Period | Age Group | Smoking Group | N | % |
|---|---|---|---|---|
| 50 | All Ages | Never Any | 19445 | 50.68 |
| 50 | All Ages | CC | 3024 | 7.88 |
| 50 | All Ages | Former | 15896 | 41.43 |

Year of Follow-up: 2040, Age Group: 18-19

| Period | Age Group | Smoking Group | N | % |
|---|---|---|---|---|
| 50 | 18-19 | Never Any | 0 | . |
| 50 | 18-19 | CC | 0 | . |
| 50 | 18-19 | Former | 0 | . |

Year of Follow-up: 2040, Age Group: 20-24

| Period | Age Group | Smoking Group | N | % |
|---|---|---|---|---|
| 50 | 20-24 | Never Any | 0 | . |
| 50 | 20-24 | CC | 0 | . |
| 50 | 20-24 | Former | 0 | . |

Year of Follow-up: 2040, Age Group: 25-29

| Period | Age Group | Smoking Group | N | % |
|---|---|---|---|---|
| 50 | 25-29 | Never Any | 0 | . |
| 50 | 25-29 | CC | 0 | . |
| 50 | 25-29 | Former | 0 | . |

Year of Follow-up: 2040, Age Group: 30-34

| Period | Age Group | Smoking Group | N | % |
|---|---|---|---|---|
| 50 | 30-34 | Never Any | 0 | . |
| 50 | 30-34 | CC | 0 | . |
| 50 | 30-34 | Former | 0 | . |

Year of Follow-up: 2040, Age Group: 35-39

| Period | Age Group | Smoking Group | N | % |
|---|---|---|---|---|
| 50 | 35-39 | Never Any | 0 | . |
| 50 | 35-39 | CC | 0 | . |
| 50 | 35-39 | Former | 0 | . |

Year of Follow-up: 2040, Age Group: 40-44

| Period | Age Group | Smoking Group | N | % |
|---|---|---|---|---|
| 50 | 40-44 | Never Any | 0 | . |
| 50 | 40-44 | CC | 0 | . |
| 50 | 40-44 | Former | 0 | . |

Year of Follow-up: 2040, Age Group: 45-49

| Period | Age Group | Smoking Group | N | % |
|---|---|---|---|---|
| 50 | 45-49 | Never Any | 0 | . |
| 50 | 45-49 | CC | 0 | . |
| 50 | 45-49 | Former | 0 | . |

Year of Follow-up: 2040, Age Group: 50-54

| Period | Age Group | Smoking Group | N | % |
|---|---|---|---|---|
| 50 | 50-54 | Never Any | 0 | . |
| 50 | 50-54 | CC | 0 | . |
| 50 | 50-54 | Former | 0 | . |

Year of Follow-up: 2040, Age Group: 55-59

| Period | Age Group | Smoking Group | N | % |
|---|---|---|---|---|
| 50 | 55-59 | Never Any | 0 | . |
| 50 | 55-59 | CC | 0 | . |
| 50 | 55-59 | Former | 0 | . |

Year of Follow-up: 2040, Age Group: 60-64

| Period | Age Group | Smoking Group | N | % |
|---|---|---|---|---|

## Results for PHIM for Run 1 (Pessimistic_Analysis.RTF)

| | | | | |
|---|---|---|---|---|
| 50 | 60-64 | Never Any | 4363 | 49.33 |
| 50 | 60-64 | CC | 934 | 10.56 |
| 50 | 60-64 | Former | 3548 | 40.11 |

Year of Follow-up: 2040, Age Group: 65-69

| Period | Age Group | Smoking Group | N | % |
|---|---|---|---|---|
| 50 | 65-69 | Never Any | 4948 | 54.73 |
| 50 | 65-69 | CC | 734 | 8.12 |
| 50 | 65-69 | Former | 3358 | 37.15 |

Year of Follow-up: 2040, Age Group: 70-74

| Period | Age Group | Smoking Group | N | % |
|---|---|---|---|---|
| 50 | 70-74 | Never Any | 5145 | 52.41 |
| 50 | 70-74 | CC | 688 | 7.01 |
| 50 | 70-74 | Former | 3983 | 40.58 |

Year of Follow-up: 2040, Age Group: 75-79

| Period | Age Group | Smoking Group | N | % |
|---|---|---|---|---|
| 50 | 75-79 | Never Any | 4989 | 46.78 |
| 50 | 75-79 | CC | 668 | 6.26 |
| 50 | 75-79 | Former | 5007 | 46.95 |

# Results for PHIM for Run 1 (Pessimistic_Analysis.RTF)

************************************************************************************

**Main Output for the Tobacco History for Run: 1, Simulation: 1, Scenario: Ecig**

| **Variable Parameter** | **Value** |
|---|---|
| 1. Country | US (United States) |
| 2. Sex | M (Males) |
| 3. Year of start of process | 1990 |

Year of Follow-up: 1990, Age Group: All Ages

| Period | Age Group | Smoking Group | N | % | Ever Quit (N) | Ever Quit % | Mean Yrs Quit | Short Term (n) | Short Term % |
|---|---|---|---|---|---|---|---|---|---|
| 0 | All Ages | Never Any | 34075 | 39.78 | | | | | |
| 0 | All Ages | CC | 27323 | 31.90 | 0 | 0.00 | | | |
| 0 | All Ages | Ecig | 0 | 0.00 | | | | | |
| 0 | All Ages | Dual | 0 | 0.00 | | | | | |
| 0 | All Ages | Former | 24266 | 28.33 | | | 13.08 | 3623 | 14.93 |

Year of Follow-up: 1990, Age Group: 18-19

| Period | Age Group | Smoking Group | N | % | Ever Quit (N) | Ever Quit % | Mean Yrs Quit | Short Term (n) | Short Term % |
|---|---|---|---|---|---|---|---|---|---|
| 0 | 18-19 | Never Any | 2783 | 78.42 | | | | | |
| 0 | 18-19 | CC | 634 | 17.86 | 0 | 0.00 | | | |
| 0 | 18-19 | Ecig | 0 | 0.00 | | | | | |
| 0 | 18-19 | Dual | 0 | 0.00 | | | | | |
| 0 | 18-19 | Former | 132 | 3.72 | | | 0.11 | 132 | 100.00 |

Year of Follow-up: 1990, Age Group: 20-24

| Period | Age Group | Smoking Group | N | % | Ever Quit (N) | Ever Quit % | Mean Yrs Quit | Short Term (n) | Short Term % |
|---|---|---|---|---|---|---|---|---|---|
| 0 | 20-24 | Never Any | 5722 | 58.29 | | | | | |
| 0 | 20-24 | CC | 3199 | 32.59 | 0 | 0.00 | | | |
| 0 | 20-24 | Ecig | 0 | 0.00 | | | | | |
| 0 | 20-24 | Dual | 0 | 0.00 | | | | | |
| 0 | 20-24 | Former | 895 | 9.12 | | | 1.46 | 506 | 56.54 |

Year of Follow-up: 1990, Age Group: 25-29

| Period | Age Group | Smoking Group | N | % | Ever Quit (N) | Ever Quit % | Mean Yrs Quit | Short Term (n) | Short Term % |
|---|---|---|---|---|---|---|---|---|---|
| 0 | 25-29 | Never Any | 5232 | 49.06 | | | | | |
| 0 | 25-29 | CC | 3914 | 36.70 | 0 | 0.00 | | | |
| 0 | 25-29 | Ecig | 0 | 0.00 | | | | | |
| 0 | 25-29 | Dual | 0 | 0.00 | | | | | |
| 0 | 25-29 | Former | 1518 | 14.23 | | | 2.87 | 643 | 42.36 |

Year of Follow-up: 1990, Age Group: 30-34

| Period | Age Group | Smoking Group | N | % | Ever Quit (N) | Ever Quit % | Mean Yrs Quit | Short Term (n) | Short Term % |
|---|---|---|---|---|---|---|---|---|---|
| 0 | 30-34 | Never Any | 4765 | 43.65 | | | | | |
| 0 | 30-34 | CC | 4023 | 36.85 | 0 | 0.00 | | | |
| 0 | 30-34 | Ecig | 0 | 0.00 | | | | | |
| 0 | 30-34 | Dual | 0 | 0.00 | | | | | |
| 0 | 30-34 | Former | 2128 | 19.49 | | | 4.06 | 592 | 27.82 |

Year of Follow-up: 1990, Age Group: 35-39

| Period | Age Group | Smoking Group | N | % | Ever Quit (N) | Ever Quit % | Mean Yrs Quit | Short Term (n) | Short Term % |
|---|---|---|---|---|---|---|---|---|---|
| 0 | 35-39 | Never Any | 3941 | 39.85 | | | | | |
| 0 | 35-39 | CC | 3474 | 35.13 | 0 | 0.00 | | | |
| 0 | 35-39 | Ecig | 0 | 0.00 | | | | | |
| 0 | 35-39 | Dual | 0 | 0.00 | | | | | |
| 0 | 35-39 | Former | 2474 | 25.02 | | | 8.31 | 425 | 17.18 |

Year of Follow-up: 1990, Age Group: 40-44

| Period | Age Group | Smoking Group | N | % | Ever Quit (N) | Ever Quit % | Mean Yrs Quit | Short Term (n) | Short Term % |
|---|---|---|---|---|---|---|---|---|---|
| 0 | 40-44 | Never Any | 2912 | 33.65 | | | | | |
| 0 | 40-44 | CC | 3113 | 35.97 | 0 | 0.00 | | | |
| 0 | 40-44 | Ecig | 0 | 0.00 | | | | | |
| 0 | 40-44 | Dual | 0 | 0.00 | | | | | |
| 0 | 40-44 | Former | 2630 | 30.39 | | | 10.69 | 317 | 12.05 |

Year of Follow-up: 1990, Age Group: 45-49

| Period | Age Group | Smoking Group | N | % | Ever Quit (N) | Ever Quit % | Mean Yrs Quit | Short Term (n) | Short Term % |
|---|---|---|---|---|---|---|---|---|---|
| 0 | 45-49 | Never Any | 1919 | 29.46 | | | | | |
| 0 | 45-49 | CC | 2318 | 35.58 | 0 | 0.00 | | | |
| 0 | 45-49 | Ecig | 0 | 0.00 | | | | | |
| 0 | 45-49 | Dual | 0 | 0.00 | | | | | |
| 0 | 45-49 | Former | 2278 | 34.97 | | | 13.26 | 221 | 9.70 |

Year of Follow-up: 1990, Age Group: 50-54

| Period | Age Group | Smoking Group | N | % | Ever Quit (N) | Ever Quit % | Mean Yrs Quit | Short Term (n) | Short Term % |
|---|---|---|---|---|---|---|---|---|---|
| 0 | 50-54 | Never Any | 1611 | 29.41 | | | | | |
| 0 | 50-54 | CC | 1816 | 33.16 | 0 | 0.00 | | | |

# Results for PHIM for Run 1 (Pessimistic_Analysis.RTF)

| | | | | | | | | | |
|---|---|---|---|---|---|---|---|---|---|
| 0 | 50-54 | Ecig | 0 | 0.00 | | | | | |
| 0 | 50-54 | Dual | 0 | 0.00 | | | | | |
| 0 | 50-54 | Former | 2050 | 37.43 | | | 14.55 | 261 | 12.73 |

Year of Follow-up: 1990, Age Group: 55-59

| Period | Age Group | Smoking Group | N | % | Ever Quit (N) | Ever Quit % | Mean Yrs Quit | Short Term (n) | Short Term % |
|---|---|---|---|---|---|---|---|---|---|
| 0 | 55-59 | Never Any | 1337 | 26.83 | | | | | |
| 0 | 55-59 | CC | 1520 | 30.50 | 0 | 0.00 | | | |
| 0 | 55-59 | Ecig | 0 | 0.00 | | | | | |
| 0 | 55-59 | Dual | 0 | 0.00 | | | | | |
| 0 | 55-59 | Former | 2126 | 42.67 | | | 14.80 | 216 | 10.16 |

Year of Follow-up: 1990, Age Group: 60-64

| Period | Age Group | Smoking Group | N | % | Ever Quit (N) | Ever Quit % | Mean Yrs Quit | Short Term (n) | Short Term % |
|---|---|---|---|---|---|---|---|---|---|
| 0 | 60-64 | Never Any | 1169 | 23.83 | | | | | |
| 0 | 60-64 | CC | 1287 | 26.24 | 0 | 0.00 | | | |
| 0 | 60-64 | Ecig | 0 | 0.00 | | | | | |
| 0 | 60-64 | Dual | 0 | 0.00 | | | | | |
| 0 | 60-64 | Former | 2449 | 49.93 | | | 18.87 | 106 | 4.33 |

Year of Follow-up: 1990, Age Group: 65-69

| Period | Age Group | Smoking Group | N | % | Ever Quit (N) | Ever Quit % | Mean Yrs Quit | Short Term (n) | Short Term % |
|---|---|---|---|---|---|---|---|---|---|
| 0 | 65-69 | Never Any | 1023 | 23.32 | | | | | |
| 0 | 65-69 | CC | 979 | 22.32 | 0 | 0.00 | | | |
| 0 | 65-69 | Ecig | 0 | 0.00 | | | | | |
| 0 | 65-69 | Dual | 0 | 0.00 | | | | | |
| 0 | 65-69 | Former | 2385 | 54.37 | | | 19.91 | 113 | 4.74 |

Year of Follow-up: 1990, Age Group: 70-74

| Period | Age Group | Smoking Group | N | % | Ever Quit (N) | Ever Quit % | Mean Yrs Quit | Short Term (n) | Short Term % |
|---|---|---|---|---|---|---|---|---|---|
| 0 | 70-74 | Never Any | 961 | 27.46 | | | | | |
| 0 | 70-74 | CC | 675 | 19.29 | 0 | 0.00 | | | |
| 0 | 70-74 | Ecig | 0 | 0.00 | | | | | |
| 0 | 70-74 | Dual | 0 | 0.00 | | | | | |
| 0 | 70-74 | Former | 1863 | 53.24 | | | 20.69 | 23 | 1.23 |

Year of Follow-up: 1990, Age Group: 75-79

| Period | Age Group | Smoking Group | N | % | Ever Quit (N) | Ever Quit % | Mean Yrs Quit | Short Term (n) | Short Term % |
|---|---|---|---|---|---|---|---|---|---|
| 0 | 75-79 | Never Any | 700 | 29.06 | | | | | |
| 0 | 75-79 | CC | 371 | 15.40 | 0 | 0.00 | | | |
| 0 | 75-79 | Ecig | 0 | 0.00 | | | | | |
| 0 | 75-79 | Dual | 0 | 0.00 | | | | | |
| 0 | 75-79 | Former | 1338 | 55.54 | | | 22.88 | 68 | 5.08 |

Year of Follow-up: 1995, Age Group: All Ages

| Period | Age Group | Smoking Group | N | % | Ever Quit (N) | Ever Quit % | Mean Yrs Quit | Short Term (n) | Short Term % |
|---|---|---|---|---|---|---|---|---|---|
| 5 | All Ages | Never Any | 37668 | 40.81 | | | | | |
| 5 | All Ages | CC | 16332 | 17.70 | 0 | 0.00 | | | |
| 5 | All Ages | Ecig | 11519 | 12.48 | | | | | |
| 5 | All Ages | Dual | 0 | 0.00 | | | | | |
| 5 | All Ages | Former | 26771 | 29.01 | | | 15.26 | 1588 | 5.93 |

Year of Follow-up: 1995, Age Group: 18-19

| Period | Age Group | Smoking Group | N | % | Ever Quit (N) | Ever Quit % | Mean Yrs Quit | Short Term (n) | Short Term % |
|---|---|---|---|---|---|---|---|---|---|
| 5 | 18-19 | Never Any | 2215 | 62.50 | | | | | |
| 5 | 18-19 | CC | 361 | 10.19 | 0 | 0.00 | | | |
| 5 | 18-19 | Ecig | 884 | 24.94 | | | | | |
| 5 | 18-19 | Dual | 0 | 0.00 | | | | | |
| 5 | 18-19 | Former | 84 | 2.37 | | | 0.95 | 63 | 75.00 |

Year of Follow-up: 1995, Age Group: 20-24

| Period | Age Group | Smoking Group | N | % | Ever Quit (N) | Ever Quit % | Mean Yrs Quit | Short Term (n) | Short Term % |
|---|---|---|---|---|---|---|---|---|---|
| 5 | 20-24 | Never Any | 5440 | 60.18 | | | | | |
| 5 | 20-24 | CC | 1327 | 14.68 | 0 | 0.00 | | | |
| 5 | 20-24 | Ecig | 1734 | 19.18 | | | | | |
| 5 | 20-24 | Dual | 0 | 0.00 | | | | | |
| 5 | 20-24 | Former | 539 | 5.96 | | | 2.77 | 160 | 29.68 |

Year of Follow-up: 1995, Age Group: 25-29

| Period | Age Group | Smoking Group | N | % | Ever Quit (N) | Ever Quit % | Mean Yrs Quit | Short Term (n) | Short Term % |
|---|---|---|---|---|---|---|---|---|---|
| 5 | 25-29 | Never Any | 5406 | 55.07 | | | | | |
| 5 | 25-29 | CC | 1806 | 18.40 | 0 | 0.00 | | | |
| 5 | 25-29 | Ecig | 1496 | 15.24 | | | | | |
| 5 | 25-29 | Dual | 0 | 0.00 | | | | | |
| 5 | 25-29 | Former | 1108 | 11.29 | | | 5.57 | 90 | 8.12 |

Year of Follow-up: 1995, Age Group: 30-34

| Period | Age Group | Smoking Group | N | % | Ever Quit (N) | Ever Quit % | Mean Yrs Quit | Short Term (n) | Short Term % |
|---|---|---|---|---|---|---|---|---|---|

# Results for PHIM for Run 1 (Pessimistic_Analysis.RTF)

| | | | | | | | | | |
|---|---|---|---|---|---|---|---|---|---|
| 5 | 30-34 | Never Any | 5022 | 47.09 | | | | | |
| 5 | 30-34 | CC | 2076 | 19.47 | 0 | 0.00 | | | |
| 5 | 30-34 | Ecig | 1797 | 16.85 | | | | | |
| 5 | 30-34 | Dual | 0 | 0.00 | | | | | |
| 5 | 30-34 | Former | 1769 | 16.59 | | | 7.05 | 87 | 4.92 |

Year of Follow-up: 1995, Age Group: 35-39

| Period | Age Group | Smoking Group | N | % | Ever Quit (N) | Ever Quit % | Mean Yrs Quit | Short Term (n) | Short Term % |
|---|---|---|---|---|---|---|---|---|---|
| 5 | 35-39 | Never Any | 4712 | 43.17 | | | | | |
| 5 | 35-39 | CC | 2143 | 19.63 | 0 | 0.00 | | | |
| 5 | 35-39 | Ecig | 1653 | 15.14 | | | | | |
| 5 | 35-39 | Dual | 0 | 0.00 | | | | | |
| 5 | 35-39 | Former | 2408 | 22.06 | | | 8.24 | 112 | 4.65 |

Year of Follow-up: 1995, Age Group: 40-44

| Period | Age Group | Smoking Group | N | % | Ever Quit (N) | Ever Quit % | Mean Yrs Quit | Short Term (n) | Short Term % |
|---|---|---|---|---|---|---|---|---|---|
| 5 | 40-44 | Never Any | 3941 | 39.85 | | | | | |
| 5 | 40-44 | CC | 1852 | 18.73 | 0 | 0.00 | | | |
| 5 | 40-44 | Ecig | 1336 | 13.51 | | | | | |
| 5 | 40-44 | Dual | 0 | 0.00 | | | | | |
| 5 | 40-44 | Former | 2760 | 27.91 | | | 12.10 | 137 | 4.96 |

Year of Follow-up: 1995, Age Group: 45-49

| Period | Age Group | Smoking Group | N | % | Ever Quit (N) | Ever Quit % | Mean Yrs Quit | Short Term (n) | Short Term % |
|---|---|---|---|---|---|---|---|---|---|
| 5 | 45-49 | Never Any | 2912 | 33.65 | | | | | |
| 5 | 45-49 | CC | 1733 | 20.02 | 0 | 0.00 | | | |
| 5 | 45-49 | Ecig | 1056 | 12.20 | | | | | |
| 5 | 45-49 | Dual | 0 | 0.00 | | | | | |
| 5 | 45-49 | Former | 2954 | 34.13 | | | 14.16 | 141 | 4.77 |

Year of Follow-up: 1995, Age Group: 50-54

| Period | Age Group | Smoking Group | N | % | Ever Quit (N) | Ever Quit % | Mean Yrs Quit | Short Term (n) | Short Term % |
|---|---|---|---|---|---|---|---|---|---|
| 5 | 50-54 | Never Any | 1919 | 29.46 | | | | | |
| 5 | 50-54 | CC | 1375 | 21.11 | 0 | 0.00 | | | |
| 5 | 50-54 | Ecig | 649 | 9.96 | | | | | |
| 5 | 50-54 | Dual | 0 | 0.00 | | | | | |
| 5 | 50-54 | Former | 2572 | 39.48 | | | 16.39 | 111 | 4.32 |

Year of Follow-up: 1995, Age Group: 55-59

| Period | Age Group | Smoking Group | N | % | Ever Quit (N) | Ever Quit % | Mean Yrs Quit | Short Term (n) | Short Term % |
|---|---|---|---|---|---|---|---|---|---|
| 5 | 55-59 | Never Any | 1611 | 29.41 | | | | | |
| 5 | 55-59 | CC | 1051 | 19.19 | 0 | 0.00 | | | |
| 5 | 55-59 | Ecig | 434 | 7.92 | | | | | |
| 5 | 55-59 | Dual | 0 | 0.00 | | | | | |
| 5 | 55-59 | Former | 2381 | 43.47 | | | 17.12 | 121 | 5.08 |

Year of Follow-up: 1995, Age Group: 60-64

| Period | Age Group | Smoking Group | N | % | Ever Quit (N) | Ever Quit % | Mean Yrs Quit | Short Term (n) | Short Term % |
|---|---|---|---|---|---|---|---|---|---|
| 5 | 60-64 | Never Any | 1337 | 26.83 | | | | | |
| 5 | 60-64 | CC | 869 | 17.44 | 0 | 0.00 | | | |
| 5 | 60-64 | Ecig | 306 | 6.14 | | | | | |
| 5 | 60-64 | Dual | 0 | 0.00 | | | | | |
| 5 | 60-64 | Former | 2471 | 49.59 | | | 17.31 | 133 | 5.38 |

Year of Follow-up: 1995, Age Group: 65-69

| Period | Age Group | Smoking Group | N | % | Ever Quit (N) | Ever Quit % | Mean Yrs Quit | Short Term (n) | Short Term % |
|---|---|---|---|---|---|---|---|---|---|
| 5 | 65-69 | Never Any | 1169 | 23.83 | | | | | |
| 5 | 65-69 | CC | 708 | 14.43 | 0 | 0.00 | | | |
| 5 | 65-69 | Ecig | 156 | 3.18 | | | | | |
| 5 | 65-69 | Dual | 0 | 0.00 | | | | | |
| 5 | 65-69 | Former | 2872 | 58.55 | | | 20.61 | 202 | 7.03 |

Year of Follow-up: 1995, Age Group: 70-74

| Period | Age Group | Smoking Group | N | % | Ever Quit (N) | Ever Quit % | Mean Yrs Quit | Short Term (n) | Short Term % |
|---|---|---|---|---|---|---|---|---|---|
| 5 | 70-74 | Never Any | 1023 | 23.32 | | | | | |
| 5 | 70-74 | CC | 595 | 13.56 | 0 | 0.00 | | | |
| 5 | 70-74 | Ecig | 18 | 0.41 | | | | | |
| 5 | 70-74 | Dual | 0 | 0.00 | | | | | |
| 5 | 70-74 | Former | 2751 | 62.71 | | | 21.85 | 137 | 4.98 |

Year of Follow-up: 1995, Age Group: 75-79

| Period | Age Group | Smoking Group | N | % | Ever Quit (N) | Ever Quit % | Mean Yrs Quit | Short Term (n) | Short Term % |
|---|---|---|---|---|---|---|---|---|---|
| 5 | 75-79 | Never Any | 961 | 27.46 | | | | | |
| 5 | 75-79 | CC | 436 | 12.46 | 0 | 0.00 | | | |
| 5 | 75-79 | Ecig | 0 | 0.00 | | | | | |
| 5 | 75-79 | Dual | 0 | 0.00 | | | | | |
| 5 | 75-79 | Former | 2102 | 60.07 | | | 23.00 | 94 | 4.47 |

Year of Follow-up: 2000, Age Group: All Ages

# Results for PHIM for Run 1 (Pessimistic_Analysis.RTF)

| Period | Age Group | Smoking Group | N | % | Ever Quit (N) | Ever Quit % | Mean Yrs Quit | Short Term (n) | Short Term % |
|---|---|---|---|---|---|---|---|---|---|
| 10 | All Ages | Never Any | 37583 | 39.94 | | | | | |
| 10 | All Ages | CC | 9539 | 10.14 | 0 | 0.00 | | | |
| 10 | All Ages | Ecig | 18758 | 19.94 | | | | | |
| 10 | All Ages | Dual | 0 | 0.00 | | | | | |
| 10 | All Ages | Former | 28212 | 29.98 | | | 17.42 | 1320 | 4.68 |

Year of Follow-up: 2000, Age Group: 18-19

| Period | Age Group | Smoking Group | N | % | Ever Quit (N) | Ever Quit % | Mean Yrs Quit | Short Term (n) | Short Term % |
|---|---|---|---|---|---|---|---|---|---|
| 10 | 18-19 | Never Any | 0 | . | | | | | |
| 10 | 18-19 | CC | 0 | . | 0 | . | | | |
| 10 | 18-19 | Ecig | 0 | . | | | | | |
| 10 | 18-19 | Dual | 0 | . | | | | | |
| 10 | 18-19 | Former | 0 | . | | | . | 0 | . |

Year of Follow-up: 2000, Age Group: 20-24

| Period | Age Group | Smoking Group | N | % | Ever Quit (N) | Ever Quit % | Mean Yrs Quit | Short Term (n) | Short Term % |
|---|---|---|---|---|---|---|---|---|---|
| 10 | 20-24 | Never Any | 4038 | 45.65 | | | | | |
| 10 | 20-24 | CC | 664 | 7.51 | 0 | 0.00 | | | |
| 10 | 20-24 | Ecig | 3423 | 38.70 | | | | | |
| 10 | 20-24 | Dual | 0 | 0.00 | | | | | |
| 10 | 20-24 | Former | 720 | 8.14 | | | 3.45 | 196 | 27.22 |

Year of Follow-up: 2000, Age Group: 25-29

| Period | Age Group | Smoking Group | N | % | Ever Quit (N) | Ever Quit % | Mean Yrs Quit | Short Term (n) | Short Term % |
|---|---|---|---|---|---|---|---|---|---|
| 10 | 25-29 | Never Any | 4870 | 53.87 | | | | | |
| 10 | 25-29 | CC | 750 | 8.30 | 0 | 0.00 | | | |
| 10 | 25-29 | Ecig | 2557 | 28.29 | | | | | |
| 10 | 25-29 | Dual | 0 | 0.00 | | | | | |
| 10 | 25-29 | Former | 863 | 9.55 | | | 5.57 | 131 | 15.18 |

Year of Follow-up: 2000, Age Group: 30-34

| Period | Age Group | Smoking Group | N | % | Ever Quit (N) | Ever Quit % | Mean Yrs Quit | Short Term (n) | Short Term % |
|---|---|---|---|---|---|---|---|---|---|
| 10 | 30-34 | Never Any | 5125 | 52.21 | | | | | |
| 10 | 30-34 | CC | 979 | 9.97 | 0 | 0.00 | | | |
| 10 | 30-34 | Ecig | 2359 | 24.03 | | | | | |
| 10 | 30-34 | Dual | 0 | 0.00 | | | | | |
| 10 | 30-34 | Former | 1353 | 13.78 | | | 9.04 | 88 | 6.50 |

Year of Follow-up: 2000, Age Group: 35-39

| Period | Age Group | Smoking Group | N | % | Ever Quit (N) | Ever Quit % | Mean Yrs Quit | Short Term (n) | Short Term % |
|---|---|---|---|---|---|---|---|---|---|
| 10 | 35-39 | Never Any | 4926 | 46.19 | | | | | |
| 10 | 35-39 | CC | 1168 | 10.95 | 0 | 0.00 | | | |
| 10 | 35-39 | Ecig | 2621 | 24.58 | | | | | |
| 10 | 35-39 | Dual | 0 | 0.00 | | | | | |
| 10 | 35-39 | Former | 1949 | 18.28 | | | 11.12 | 64 | 3.28 |

Year of Follow-up: 2000, Age Group: 40-44

| Period | Age Group | Smoking Group | N | % | Ever Quit (N) | Ever Quit % | Mean Yrs Quit | Short Term (n) | Short Term % |
|---|---|---|---|---|---|---|---|---|---|
| 10 | 40-44 | Never Any | 4712 | 43.17 | | | | | |
| 10 | 40-44 | CC | 1178 | 10.79 | 0 | 0.00 | | | |
| 10 | 40-44 | Ecig | 2393 | 21.92 | | | | | |
| 10 | 40-44 | Dual | 0 | 0.00 | | | | | |
| 10 | 40-44 | Former | 2633 | 24.12 | | | 12.29 | 81 | 3.08 |

Year of Follow-up: 2000, Age Group: 45-49

| Period | Age Group | Smoking Group | N | % | Ever Quit (N) | Ever Quit % | Mean Yrs Quit | Short Term (n) | Short Term % |
|---|---|---|---|---|---|---|---|---|---|
| 10 | 45-49 | Never Any | 3941 | 39.85 | | | | | |
| 10 | 45-49 | CC | 1039 | 10.51 | 0 | 0.00 | | | |
| 10 | 45-49 | Ecig | 1880 | 19.01 | | | | | |
| 10 | 45-49 | Dual | 0 | 0.00 | | | | | |
| 10 | 45-49 | Former | 3029 | 30.63 | | | 15.75 | 116 | 3.83 |

Year of Follow-up: 2000, Age Group: 50-54

| Period | Age Group | Smoking Group | N | % | Ever Quit (N) | Ever Quit % | Mean Yrs Quit | Short Term (n) | Short Term % |
|---|---|---|---|---|---|---|---|---|---|
| 10 | 50-54 | Never Any | 2912 | 33.65 | | | | | |
| 10 | 50-54 | CC | 953 | 11.01 | 0 | 0.00 | | | |
| 10 | 50-54 | Ecig | 1495 | 17.27 | | | | | |
| 10 | 50-54 | Dual | 0 | 0.00 | | | | | |
| 10 | 50-54 | Former | 3295 | 38.07 | | | 17.39 | 132 | 4.01 |

Year of Follow-up: 2000, Age Group: 55-59

| Period | Age Group | Smoking Group | N | % | Ever Quit (N) | Ever Quit % | Mean Yrs Quit | Short Term (n) | Short Term % |
|---|---|---|---|---|---|---|---|---|---|
| 10 | 55-59 | Never Any | 1919 | 29.46 | | | | | |
| 10 | 55-59 | CC | 787 | 12.08 | 0 | 0.00 | | | |
| 10 | 55-59 | Ecig | 911 | 13.98 | | | | | |
| 10 | 55-59 | Dual | 0 | 0.00 | | | | | |

# Results for PHIM for Run 1 (Pessimistic_Analysis.RTF)

| | | | | | | | | | |
|---|---|---|---|---|---|---|---|---|---|
| 10 | 55-59 | Former | 2898 | 44.48 | | | 19.22 | 124 | 4.28 |

Year of Follow-up: 2000, Age Group: 60-64

| Period | Age Group | Smoking Group | N | % | Ever Quit (N) | Ever Quit % | Mean Yrs Quit | Short Term (n) | Short Term % |
|---|---|---|---|---|---|---|---|---|---|
| 10 | 60-64 | Never Any | 1611 | 29.41 | | | | | |
| 10 | 60-64 | CC | 626 | 11.43 | 0 | 0.00 | | | |
| 10 | 60-64 | Ecig | 597 | 10.90 | | | | | |
| 10 | 60-64 | Dual | 0 | 0.00 | | | | | |
| 10 | 60-64 | Former | 2643 | 48.26 | | | 20.11 | 119 | 4.50 |

Year of Follow-up: 2000, Age Group: 65-69

| Period | Age Group | Smoking Group | N | % | Ever Quit (N) | Ever Quit % | Mean Yrs Quit | Short Term (n) | Short Term % |
|---|---|---|---|---|---|---|---|---|---|
| 10 | 65-69 | Never Any | 1337 | 26.83 | | | | | |
| 10 | 65-69 | CC | 519 | 10.42 | 0 | 0.00 | | | |
| 10 | 65-69 | Ecig | 364 | 7.30 | | | | | |
| 10 | 65-69 | Dual | 0 | 0.00 | | | | | |
| 10 | 65-69 | Former | 2763 | 55.45 | | | 20.16 | 127 | 4.60 |

Year of Follow-up: 2000, Age Group: 70-74

| Period | Age Group | Smoking Group | N | % | Ever Quit (N) | Ever Quit % | Mean Yrs Quit | Short Term (n) | Short Term % |
|---|---|---|---|---|---|---|---|---|---|
| 10 | 70-74 | Never Any | 1169 | 23.83 | | | | | |
| 10 | 70-74 | CC | 474 | 9.66 | 0 | 0.00 | | | |
| 10 | 70-74 | Ecig | 145 | 2.96 | | | | | |
| 10 | 70-74 | Dual | 0 | 0.00 | | | | | |
| 10 | 70-74 | Former | 3117 | 63.55 | | | 23.79 | 69 | 2.21 |

Year of Follow-up: 2000, Age Group: 75-79

| Period | Age Group | Smoking Group | N | % | Ever Quit (N) | Ever Quit % | Mean Yrs Quit | Short Term (n) | Short Term % |
|---|---|---|---|---|---|---|---|---|---|
| 10 | 75-79 | Never Any | 1023 | 23.32 | | | | | |
| 10 | 75-79 | CC | 402 | 9.16 | 0 | 0.00 | | | |
| 10 | 75-79 | Ecig | 13 | 0.30 | | | | | |
| 10 | 75-79 | Dual | 0 | 0.00 | | | | | |
| 10 | 75-79 | Former | 2949 | 67.22 | | | 25.19 | 73 | 2.48 |

Year of Follow-up: 2005, Age Group: All Ages

| Period | Age Group | Smoking Group | N | % | Ever Quit (N) | Ever Quit % | Mean Yrs Quit | Short Term (n) | Short Term % |
|---|---|---|---|---|---|---|---|---|---|
| 15 | All Ages | Never Any | 35646 | 39.74 | | | | | |
| 15 | All Ages | CC | 5286 | 5.89 | 0 | 0.00 | | | |
| 15 | All Ages | Ecig | 20616 | 22.98 | | | | | |
| 15 | All Ages | Dual | 0 | 0.00 | | | | | |
| 15 | All Ages | Former | 28157 | 31.39 | | | 19.52 | 1141 | 4.05 |

Year of Follow-up: 2005, Age Group: 18-19

| Period | Age Group | Smoking Group | N | % | Ever Quit (N) | Ever Quit % | Mean Yrs Quit | Short Term (n) | Short Term % |
|---|---|---|---|---|---|---|---|---|---|
| 15 | 18-19 | Never Any | 0 | . | | | | | |
| 15 | 18-19 | CC | 0 | . | 0 | . | | | |
| 15 | 18-19 | Ecig | 0 | . | | | | | |
| 15 | 18-19 | Dual | 0 | . | | | | | |
| 15 | 18-19 | Former | 0 | . | | | . | 0 | . |

Year of Follow-up: 2005, Age Group: 20-24

| Period | Age Group | Smoking Group | N | % | Ever Quit (N) | Ever Quit % | Mean Yrs Quit | Short Term (n) | Short Term % |
|---|---|---|---|---|---|---|---|---|---|
| 15 | 20-24 | Never Any | 0 | . | | | | | |
| 15 | 20-24 | CC | 0 | . | 0 | . | | | |
| 15 | 20-24 | Ecig | 0 | . | | | | | |
| 15 | 20-24 | Dual | 0 | . | | | | | |
| 15 | 20-24 | Former | 0 | . | | | . | 0 | . |

Year of Follow-up: 2005, Age Group: 25-29

| Period | Age Group | Smoking Group | N | % | Ever Quit (N) | Ever Quit % | Mean Yrs Quit | Short Term (n) | Short Term % |
|---|---|---|---|---|---|---|---|---|---|
| 15 | 25-29 | Never Any | 3679 | 41.59 | | | | | |
| 15 | 25-29 | CC | 350 | 3.96 | 0 | 0.00 | | | |
| 15 | 25-29 | Ecig | 3734 | 42.22 | | | | | |
| 15 | 25-29 | Dual | 0 | 0.00 | | | | | |
| 15 | 25-29 | Former | 1082 | 12.23 | | | 6.34 | 139 | 12.85 |

Year of Follow-up: 2005, Age Group: 30-34

| Period | Age Group | Smoking Group | N | % | Ever Quit (N) | Ever Quit % | Mean Yrs Quit | Short Term (n) | Short Term % |
|---|---|---|---|---|---|---|---|---|---|
| 15 | 30-34 | Never Any | 4479 | 49.55 | | | | | |
| 15 | 30-34 | CC | 373 | 4.13 | 0 | 0.00 | | | |
| 15 | 30-34 | Ecig | 2990 | 33.08 | | | | | |
| 15 | 30-34 | Dual | 0 | 0.00 | | | | | |
| 15 | 30-34 | Former | 1198 | 13.25 | | | 8.14 | 140 | 11.69 |

Year of Follow-up: 2005, Age Group: 35-39

| Period | Age Group | Smoking Group | N | % | Ever Quit (N) | Ever Quit % | Mean Yrs Quit | Short Term (n) | Short Term % |
|---|---|---|---|---|---|---|---|---|---|
| 15 | 35-39 | Never Any | 4961 | 50.54 | | | | | |
| 15 | 35-39 | CC | 532 | 5.42 | 0 | 0.00 | | | |

# Results for PHIM for Run 1 (Pessimistic_Analysis.RTF)

| | | | | | | | | | |
|---|---|---|---|---|---|---|---|---|---|
| 15 | 35-39 | Ecig | 2742 | 27.93 | | | | | |
| 15 | 35-39 | Dual | 0 | 0.00 | | | | | |
| 15 | 35-39 | Former | 1581 | 16.11 | | | 12.30 | 93 | 5.88 |

Year of Follow-up: 2005, Age Group: 40-44

| Period | Age Group | Smoking Group | N | % | Ever Quit (N) | Ever Quit % | Mean Yrs Quit | Short Term (n) | Short Term % |
|---|---|---|---|---|---|---|---|---|---|
| 15 | 40-44 | Never Any | 4926 | 46.19 | | | | | |
| 15 | 40-44 | CC | 622 | 5.83 | 0 | 0.00 | | | |
| 15 | 40-44 | Ecig | 2883 | 27.03 | | | | | |
| 15 | 40-44 | Dual | 0 | 0.00 | | | | | |
| 15 | 40-44 | Former | 2233 | 20.94 | | | 14.31 | 124 | 5.55 |

Year of Follow-up: 2005, Age Group: 45-49

| Period | Age Group | Smoking Group | N | % | Ever Quit (N) | Ever Quit % | Mean Yrs Quit | Short Term (n) | Short Term % |
|---|---|---|---|---|---|---|---|---|---|
| 15 | 45-49 | Never Any | 4712 | 43.17 | | | | | |
| 15 | 45-49 | CC | 657 | 6.02 | 0 | 0.00 | | | |
| 15 | 45-49 | Ecig | 2637 | 24.16 | | | | | |
| 15 | 45-49 | Dual | 0 | 0.00 | | | | | |
| 15 | 45-49 | Former | 2910 | 26.66 | | | 15.83 | 109 | 3.75 |

Year of Follow-up: 2005, Age Group: 50-54

| Period | Age Group | Smoking Group | N | % | Ever Quit (N) | Ever Quit % | Mean Yrs Quit | Short Term (n) | Short Term % |
|---|---|---|---|---|---|---|---|---|---|
| 15 | 50-54 | Never Any | 3941 | 39.85 | | | | | |
| 15 | 50-54 | CC | 637 | 6.44 | 0 | 0.00 | | | |
| 15 | 50-54 | Ecig | 2030 | 20.53 | | | | | |
| 15 | 50-54 | Dual | 0 | 0.00 | | | | | |
| 15 | 50-54 | Former | 3281 | 33.18 | | | 19.31 | 100 | 3.05 |

Year of Follow-up: 2005, Age Group: 55-59

| Period | Age Group | Smoking Group | N | % | Ever Quit (N) | Ever Quit % | Mean Yrs Quit | Short Term (n) | Short Term % |
|---|---|---|---|---|---|---|---|---|---|
| 15 | 55-59 | Never Any | 2912 | 33.65 | | | | | |
| 15 | 55-59 | CC | 596 | 6.89 | 0 | 0.00 | | | |
| 15 | 55-59 | Ecig | 1633 | 18.87 | | | | | |
| 15 | 55-59 | Dual | 0 | 0.00 | | | | | |
| 15 | 55-59 | Former | 3514 | 40.60 | | | 21.14 | 72 | 2.05 |

Year of Follow-up: 2005, Age Group: 60-64

| Period | Age Group | Smoking Group | N | % | Ever Quit (N) | Ever Quit % | Mean Yrs Quit | Short Term (n) | Short Term % |
|---|---|---|---|---|---|---|---|---|---|
| 15 | 60-64 | Never Any | 1919 | 29.46 | | | | | |
| 15 | 60-64 | CC | 469 | 7.20 | 0 | 0.00 | | | |
| 15 | 60-64 | Ecig | 973 | 14.93 | | | | | |
| 15 | 60-64 | Dual | 0 | 0.00 | | | | | |
| 15 | 60-64 | Former | 3154 | 48.41 | | | 22.41 | 118 | 3.74 |

Year of Follow-up: 2005, Age Group: 65-69

| Period | Age Group | Smoking Group | N | % | Ever Quit (N) | Ever Quit % | Mean Yrs Quit | Short Term (n) | Short Term % |
|---|---|---|---|---|---|---|---|---|---|
| 15 | 65-69 | Never Any | 1611 | 29.41 | | | | | |
| 15 | 65-69 | CC | 374 | 6.83 | 0 | 0.00 | | | |
| 15 | 65-69 | Ecig | 564 | 10.30 | | | | | |
| 15 | 65-69 | Dual | 0 | 0.00 | | | | | |
| 15 | 65-69 | Former | 2928 | 53.46 | | | 22.88 | 103 | 3.52 |

Year of Follow-up: 2005, Age Group: 70-74

| Period | Age Group | Smoking Group | N | % | Ever Quit (N) | Ever Quit % | Mean Yrs Quit | Short Term (n) | Short Term % |
|---|---|---|---|---|---|---|---|---|---|
| 15 | 70-74 | Never Any | 1337 | 26.83 | | | | | |
| 15 | 70-74 | CC | 358 | 7.18 | 0 | 0.00 | | | |
| 15 | 70-74 | Ecig | 315 | 6.32 | | | | | |
| 15 | 70-74 | Dual | 0 | 0.00 | | | | | |
| 15 | 70-74 | Former | 2973 | 59.66 | | | 23.54 | 69 | 2.32 |

Year of Follow-up: 2005, Age Group: 75-79

| Period | Age Group | Smoking Group | N | % | Ever Quit (N) | Ever Quit % | Mean Yrs Quit | Short Term (n) | Short Term % |
|---|---|---|---|---|---|---|---|---|---|
| 15 | 75-79 | Never Any | 1169 | 23.83 | | | | | |
| 15 | 75-79 | CC | 318 | 6.48 | 0 | 0.00 | | | |
| 15 | 75-79 | Ecig | 115 | 2.34 | | | | | |
| 15 | 75-79 | Dual | 0 | 0.00 | | | | | |
| 15 | 75-79 | Former | 3303 | 67.34 | | | 27.28 | 74 | 2.24 |

Year of Follow-up: 2010, Age Group: All Ages

| Period | Age Group | Smoking Group | N | % | Ever Quit (N) | Ever Quit % | Mean Yrs Quit | Short Term (n) | Short Term % |
|---|---|---|---|---|---|---|---|---|---|
| 20 | All Ages | Never Any | 34261 | 40.40 | | | | | |
| 20 | All Ages | CC | 2878 | 3.39 | 0 | 0.00 | | | |
| 20 | All Ages | Ecig | 19984 | 23.57 | | | | | |
| 20 | All Ages | Dual | 0 | 0.00 | | | | | |
| 20 | All Ages | Former | 27677 | 32.64 | | | 21.29 | 1139 | 4.12 |

Year of Follow-up: 2010, Age Group: 18-19

| Period | Age Group | Smoking Group | N | % | Ever Quit (N) | Ever Quit % | Mean Yrs Quit | Short Term (n) | Short Term % |
|---|---|---|---|---|---|---|---|---|---|

# Results for PHIM for Run 1 (Pessimistic_Analysis.RTF)

| | | | | | | | | | |
|---|---|---|---|---|---|---|---|---|---|
| 20 | 18-19 | Never Any | 0 | . | | | | | |
| 20 | 18-19 | CC | 0 | . | 0 | . | | | |
| 20 | 18-19 | Ecig | 0 | . | | | | | |
| 20 | 18-19 | Dual | 0 | . | | | | | |
| 20 | 18-19 | Former | 0 | . | | | . | 0 | . |

Year of Follow-up: 2010, Age Group: 20-24

| Period | Age Group | Smoking Group | N | % | Ever Quit (N) | Ever Quit % | Mean Yrs Quit | Short Term (n) | Short Term % |
|---|---|---|---|---|---|---|---|---|---|
| 20 | 20-24 | Never Any | 0 | . | | | | | |
| 20 | 20-24 | CC | 0 | . | 0 | . | | | |
| 20 | 20-24 | Ecig | 0 | . | | | | | |
| 20 | 20-24 | Dual | 0 | . | | | | | |
| 20 | 20-24 | Former | 0 | . | | | . | 0 | . |

Year of Follow-up: 2010, Age Group: 25-29

| Period | Age Group | Smoking Group | N | % | Ever Quit (N) | Ever Quit % | Mean Yrs Quit | Short Term (n) | Short Term % |
|---|---|---|---|---|---|---|---|---|---|
| 20 | 25-29 | Never Any | 0 | . | | | | | |
| 20 | 25-29 | CC | 0 | . | 0 | . | | | |
| 20 | 25-29 | Ecig | 0 | . | | | | | |
| 20 | 25-29 | Dual | 0 | . | | | | | |
| 20 | 25-29 | Former | 0 | . | | | . | 0 | . |

Year of Follow-up: 2010, Age Group: 30-34

| Period | Age Group | Smoking Group | N | % | Ever Quit (N) | Ever Quit % | Mean Yrs Quit | Short Term (n) | Short Term % |
|---|---|---|---|---|---|---|---|---|---|
| 20 | 30-34 | Never Any | 3537 | 39.99 | | | | | |
| 20 | 30-34 | CC | 155 | 1.75 | 0 | 0.00 | | | |
| 20 | 30-34 | Ecig | 3656 | 41.33 | | | | | |
| 20 | 30-34 | Dual | 0 | 0.00 | | | | | |
| 20 | 30-34 | Former | 1497 | 16.92 | | | 8.72 | 190 | 12.69 |

Year of Follow-up: 2010, Age Group: 35-39

| Period | Age Group | Smoking Group | N | % | Ever Quit (N) | Ever Quit % | Mean Yrs Quit | Short Term (n) | Short Term % |
|---|---|---|---|---|---|---|---|---|---|
| 20 | 35-39 | Never Any | 4405 | 48.73 | | | | | |
| 20 | 35-39 | CC | 188 | 2.08 | 0 | 0.00 | | | |
| 20 | 35-39 | Ecig | 2887 | 31.94 | | | | | |
| 20 | 35-39 | Dual | 0 | 0.00 | | | | | |
| 20 | 35-39 | Former | 1560 | 17.26 | | | 10.58 | 123 | 7.88 |

Year of Follow-up: 2010, Age Group: 40-44

| Period | Age Group | Smoking Group | N | % | Ever Quit (N) | Ever Quit % | Mean Yrs Quit | Short Term (n) | Short Term % |
|---|---|---|---|---|---|---|---|---|---|
| 20 | 40-44 | Never Any | 4961 | 50.54 | | | | | |
| 20 | 40-44 | CC | 295 | 3.01 | 0 | 0.00 | | | |
| 20 | 40-44 | Ecig | 2735 | 27.86 | | | | | |
| 20 | 40-44 | Dual | 0 | 0.00 | | | | | |
| 20 | 40-44 | Former | 1825 | 18.59 | | | 15.26 | 97 | 5.32 |

Year of Follow-up: 2010, Age Group: 45-49

| Period | Age Group | Smoking Group | N | % | Ever Quit (N) | Ever Quit % | Mean Yrs Quit | Short Term (n) | Short Term % |
|---|---|---|---|---|---|---|---|---|---|
| 20 | 45-49 | Never Any | 4926 | 46.19 | | | | | |
| 20 | 45-49 | CC | 342 | 3.21 | 0 | 0.00 | | | |
| 20 | 45-49 | Ecig | 2877 | 26.98 | | | | | |
| 20 | 45-49 | Dual | 0 | 0.00 | | | | | |
| 20 | 45-49 | Former | 2519 | 23.62 | | | 17.35 | 110 | 4.37 |

Year of Follow-up: 2010, Age Group: 50-54

| Period | Age Group | Smoking Group | N | % | Ever Quit (N) | Ever Quit % | Mean Yrs Quit | Short Term (n) | Short Term % |
|---|---|---|---|---|---|---|---|---|---|
| 20 | 50-54 | Never Any | 4712 | 43.17 | | | | | |
| 20 | 50-54 | CC | 377 | 3.45 | 0 | 0.00 | | | |
| 20 | 50-54 | Ecig | 2629 | 24.08 | | | | | |
| 20 | 50-54 | Dual | 0 | 0.00 | | | | | |
| 20 | 50-54 | Former | 3198 | 29.30 | | | 19.14 | 117 | 3.66 |

Year of Follow-up: 2010, Age Group: 55-59

| Period | Age Group | Smoking Group | N | % | Ever Quit (N) | Ever Quit % | Mean Yrs Quit | Short Term (n) | Short Term % |
|---|---|---|---|---|---|---|---|---|---|
| 20 | 55-59 | Never Any | 3941 | 39.85 | | | | | |
| 20 | 55-59 | CC | 372 | 3.76 | 0 | 0.00 | | | |
| 20 | 55-59 | Ecig | 1976 | 19.98 | | | | | |
| 20 | 55-59 | Dual | 0 | 0.00 | | | | | |
| 20 | 55-59 | Former | 3600 | 36.40 | | | 22.33 | 123 | 3.42 |

Year of Follow-up: 2010, Age Group: 60-64

| Period | Age Group | Smoking Group | N | % | Ever Quit (N) | Ever Quit % | Mean Yrs Quit | Short Term (n) | Short Term % |
|---|---|---|---|---|---|---|---|---|---|
| 20 | 60-64 | Never Any | 2912 | 33.65 | | | | | |
| 20 | 60-64 | CC | 332 | 3.84 | 0 | 0.00 | | | |
| 20 | 60-64 | Ecig | 1547 | 17.87 | | | | | |
| 20 | 60-64 | Dual | 0 | 0.00 | | | | | |
| 20 | 60-64 | Former | 3864 | 44.64 | | | 23.92 | 186 | 4.81 |

Year of Follow-up: 2010, Age Group: 65-69

# Results for PHIM for Run 1 (Pessimistic_Analysis.RTF)

| Period | Age Group | Smoking Group | N | % | Ever Quit (N) | Ever Quit % | Mean Yrs Quit | Short Term (n) | Short Term % |
|---|---|---|---|---|---|---|---|---|---|
| 20 | 65-69 | Never Any | 1919 | 29.46 | | | | | |
| 20 | 65-69 | CC | 283 | 4.34 | 0 | 0.00 | | | |
| 20 | 65-69 | Ecig | 890 | 13.66 | | | | | |
| 20 | 65-69 | Dual | 0 | 0.00 | | | | | |
| 20 | 65-69 | Former | 3423 | 52.54 | | | 25.43 | 89 | 2.60 |

Year of Follow-up: 2010, Age Group: 70-74

| Period | Age Group | Smoking Group | N | % | Ever Quit (N) | Ever Quit % | Mean Yrs Quit | Short Term (n) | Short Term % |
|---|---|---|---|---|---|---|---|---|---|
| 20 | 70-74 | Never Any | 1611 | 29.41 | | | | | |
| 20 | 70-74 | CC | 270 | 4.93 | 0 | 0.00 | | | |
| 20 | 70-74 | Ecig | 510 | 9.31 | | | | | |
| 20 | 70-74 | Dual | 0 | 0.00 | | | | | |
| 20 | 70-74 | Former | 3086 | 56.34 | | | 26.56 | 60 | 1.94 |

Year of Follow-up: 2010, Age Group: 75-79

| Period | Age Group | Smoking Group | N | % | Ever Quit (N) | Ever Quit % | Mean Yrs Quit | Short Term (n) | Short Term % |
|---|---|---|---|---|---|---|---|---|---|
| 20 | 75-79 | Never Any | 1337 | 26.83 | | | | | |
| 20 | 75-79 | CC | 264 | 5.30 | 0 | 0.00 | | | |
| 20 | 75-79 | Ecig | 277 | 5.56 | | | | | |
| 20 | 75-79 | Dual | 0 | 0.00 | | | | | |
| 20 | 75-79 | Former | 3105 | 62.31 | | | 27.42 | 44 | 1.42 |

Year of Follow-up: 2015, Age Group: All Ages

| Period | Age Group | Smoking Group | N | % | Ever Quit (N) | Ever Quit % | Mean Yrs Quit | Short Term (n) | Short Term % |
|---|---|---|---|---|---|---|---|---|---|
| 25 | All Ages | Never Any | 32900 | 41.22 | | | | | |
| 25 | All Ages | CC | 1555 | 1.95 | 0 | 0.00 | | | |
| 25 | All Ages | Ecig | 18489 | 23.16 | | | | | |
| 25 | All Ages | Dual | 0 | 0.00 | | | | | |
| 25 | All Ages | Former | 26873 | 33.67 | | | 23.51 | 830 | 3.09 |

Year of Follow-up: 2015, Age Group: 18-19

| Period | Age Group | Smoking Group | N | % | Ever Quit (N) | Ever Quit % | Mean Yrs Quit | Short Term (n) | Short Term % |
|---|---|---|---|---|---|---|---|---|---|
| 25 | 18-19 | Never Any | 0 | . | | | | | |
| 25 | 18-19 | CC | 0 | . | 0 | . | | | |
| 25 | 18-19 | Ecig | 0 | . | | | | | |
| 25 | 18-19 | Dual | 0 | . | | | | | |
| 25 | 18-19 | Former | 0 | . | | | . | 0 | . |

Year of Follow-up: 2015, Age Group: 20-24

| Period | Age Group | Smoking Group | N | % | Ever Quit (N) | Ever Quit % | Mean Yrs Quit | Short Term (n) | Short Term % |
|---|---|---|---|---|---|---|---|---|---|
| 25 | 20-24 | Never Any | 0 | . | | | | | |
| 25 | 20-24 | CC | 0 | . | 0 | . | | | |
| 25 | 20-24 | Ecig | 0 | . | | | | | |
| 25 | 20-24 | Dual | 0 | . | | | | | |
| 25 | 20-24 | Former | 0 | . | | | . | 0 | . |

Year of Follow-up: 2015, Age Group: 25-29

| Period | Age Group | Smoking Group | N | % | Ever Quit (N) | Ever Quit % | Mean Yrs Quit | Short Term (n) | Short Term % |
|---|---|---|---|---|---|---|---|---|---|
| 25 | 25-29 | Never Any | 0 | . | | | | | |
| 25 | 25-29 | CC | 0 | . | 0 | . | | | |
| 25 | 25-29 | Ecig | 0 | . | | | | | |
| 25 | 25-29 | Dual | 0 | . | | | | | |
| 25 | 25-29 | Former | 0 | . | | | . | 0 | . |

Year of Follow-up: 2015, Age Group: 30-34

| Period | Age Group | Smoking Group | N | % | Ever Quit (N) | Ever Quit % | Mean Yrs Quit | Short Term (n) | Short Term % |
|---|---|---|---|---|---|---|---|---|---|
| 25 | 30-34 | Never Any | 0 | . | | | | | |
| 25 | 30-34 | CC | 0 | . | 0 | . | | | |
| 25 | 30-34 | Ecig | 0 | . | | | | | |
| 25 | 30-34 | Dual | 0 | . | | | | | |
| 25 | 30-34 | Former | 0 | . | | | . | 0 | . |

Year of Follow-up: 2015, Age Group: 35-39

| Period | Age Group | Smoking Group | N | % | Ever Quit (N) | Ever Quit % | Mean Yrs Quit | Short Term (n) | Short Term % |
|---|---|---|---|---|---|---|---|---|---|
| 25 | 35-39 | Never Any | 3513 | 39.72 | | | | | |
| 25 | 35-39 | CC | 70 | 0.79 | 0 | 0.00 | | | |
| 25 | 35-39 | Ecig | 3376 | 38.17 | | | | | |
| 25 | 35-39 | Dual | 0 | 0.00 | | | | | |
| 25 | 35-39 | Former | 1886 | 21.32 | | | 11.35 | 131 | 6.95 |

Year of Follow-up: 2015, Age Group: 40-44

| Period | Age Group | Smoking Group | N | % | Ever Quit (N) | Ever Quit % | Mean Yrs Quit | Short Term (n) | Short Term % |
|---|---|---|---|---|---|---|---|---|---|
| 25 | 40-44 | Never Any | 4405 | 48.73 | | | | | |
| 25 | 40-44 | CC | 87 | 0.96 | 0 | 0.00 | | | |
| 25 | 40-44 | Ecig | 2774 | 30.69 | | | | | |
| 25 | 40-44 | Dual | 0 | 0.00 | | | | | |

# Results for PHIM for Run 1 (Pessimistic_Analysis.RTF)

| | | | | | | | | | |
|---|---|---|---|---|---|---|---|---|---|
| 25 | 40-44 | Former | 1774 | 19.62 | | | 13.94 | 89 | 5.02 |

Year of Follow-up: 2015, Age Group: 45-49

| Period | Age Group | Smoking Group | N | % | Ever Quit (N) | Ever Quit % | Mean Yrs Quit | Short Term (n) | Short Term % |
|---|---|---|---|---|---|---|---|---|---|
| 25 | 45-49 | Never Any | 4961 | 50.54 | | | | | |
| 25 | 45-49 | CC | 166 | 1.69 | 0 | 0.00 | | | |
| 25 | 45-49 | Ecig | 2676 | 27.26 | | | | | |
| 25 | 45-49 | Dual | 0 | 0.00 | | | | | |
| 25 | 45-49 | Former | 2013 | 20.51 | | | 18.58 | 56 | 2.78 |

Year of Follow-up: 2015, Age Group: 50-54

| Period | Age Group | Smoking Group | N | % | Ever Quit (N) | Ever Quit % | Mean Yrs Quit | Short Term (n) | Short Term % |
|---|---|---|---|---|---|---|---|---|---|
| 25 | 50-54 | Never Any | 4926 | 46.19 | | | | | |
| 25 | 50-54 | CC | 194 | 1.82 | 0 | 0.00 | | | |
| 25 | 50-54 | Ecig | 2831 | 26.55 | | | | | |
| 25 | 50-54 | Dual | 0 | 0.00 | | | | | |
| 25 | 50-54 | Former | 2713 | 25.44 | | | 20.88 | 94 | 3.46 |

Year of Follow-up: 2015, Age Group: 55-59

| Period | Age Group | Smoking Group | N | % | Ever Quit (N) | Ever Quit % | Mean Yrs Quit | Short Term (n) | Short Term % |
|---|---|---|---|---|---|---|---|---|---|
| 25 | 55-59 | Never Any | 4712 | 43.17 | | | | | |
| 25 | 55-59 | CC | 202 | 1.85 | 0 | 0.00 | | | |
| 25 | 55-59 | Ecig | 2493 | 22.84 | | | | | |
| 25 | 55-59 | Dual | 0 | 0.00 | | | | | |
| 25 | 55-59 | Former | 3509 | 32.15 | | | 22.19 | 110 | 3.13 |

Year of Follow-up: 2015, Age Group: 60-64

| Period | Age Group | Smoking Group | N | % | Ever Quit (N) | Ever Quit % | Mean Yrs Quit | Short Term (n) | Short Term % |
|---|---|---|---|---|---|---|---|---|---|
| 25 | 60-64 | Never Any | 3941 | 39.85 | | | | | |
| 25 | 60-64 | CC | 202 | 2.04 | 0 | 0.00 | | | |
| 25 | 60-64 | Ecig | 1785 | 18.05 | | | | | |
| 25 | 60-64 | Dual | 0 | 0.00 | | | | | |
| 25 | 60-64 | Former | 3961 | 40.05 | | | 25.03 | 135 | 3.41 |

Year of Follow-up: 2015, Age Group: 65-69

| Period | Age Group | Smoking Group | N | % | Ever Quit (N) | Ever Quit % | Mean Yrs Quit | Short Term (n) | Short Term % |
|---|---|---|---|---|---|---|---|---|---|
| 25 | 65-69 | Never Any | 2912 | 33.65 | | | | | |
| 25 | 65-69 | CC | 219 | 2.53 | 0 | 0.00 | | | |
| 25 | 65-69 | Ecig | 1325 | 15.31 | | | | | |
| 25 | 65-69 | Dual | 0 | 0.00 | | | | | |
| 25 | 65-69 | Former | 4199 | 48.52 | | | 26.79 | 109 | 2.60 |

Year of Follow-up: 2015, Age Group: 70-74

| Period | Age Group | Smoking Group | N | % | Ever Quit (N) | Ever Quit % | Mean Yrs Quit | Short Term (n) | Short Term % |
|---|---|---|---|---|---|---|---|---|---|
| 25 | 70-74 | Never Any | 1919 | 29.46 | | | | | |
| 25 | 70-74 | CC | 216 | 3.32 | 0 | 0.00 | | | |
| 25 | 70-74 | Ecig | 779 | 11.96 | | | | | |
| 25 | 70-74 | Dual | 0 | 0.00 | | | | | |
| 25 | 70-74 | Former | 3601 | 55.27 | | | 29.03 | 61 | 1.69 |

Year of Follow-up: 2015, Age Group: 75-79

| Period | Age Group | Smoking Group | N | % | Ever Quit (N) | Ever Quit % | Mean Yrs Quit | Short Term (n) | Short Term % |
|---|---|---|---|---|---|---|---|---|---|
| 25 | 75-79 | Never Any | 1611 | 29.41 | | | | | |
| 25 | 75-79 | CC | 199 | 3.63 | 0 | 0.00 | | | |
| 25 | 75-79 | Ecig | 450 | 8.22 | | | | | |
| 25 | 75-79 | Dual | 0 | 0.00 | | | | | |
| 25 | 75-79 | Former | 3217 | 58.74 | | | 30.36 | 45 | 1.40 |

Year of Follow-up: 2020, Age Group: All Ages

| Period | Age Group | Smoking Group | N | % | Ever Quit (N) | Ever Quit % | Mean Yrs Quit | Short Term (n) | Short Term % |
|---|---|---|---|---|---|---|---|---|---|
| 30 | All Ages | Never Any | 31289 | 42.09 | | | | | |
| 30 | All Ages | CC | 861 | 1.16 | 0 | 0.00 | | | |
| 30 | All Ages | Ecig | 16609 | 22.34 | | | | | |
| 30 | All Ages | Dual | 0 | 0.00 | | | | | |
| 30 | All Ages | Former | 25581 | 34.41 | | | 25.66 | 740 | 2.89 |

Year of Follow-up: 2020, Age Group: 18-19

| Period | Age Group | Smoking Group | N | % | Ever Quit (N) | Ever Quit % | Mean Yrs Quit | Short Term (n) | Short Term % |
|---|---|---|---|---|---|---|---|---|---|
| 30 | 18-19 | Never Any | 0 | . | | | | | |
| 30 | 18-19 | CC | 0 | . | 0 | . | | | |
| 30 | 18-19 | Ecig | 0 | . | | | | | |
| 30 | 18-19 | Dual | 0 | . | | | | | |
| 30 | 18-19 | Former | 0 | . | | | . | 0 | . |

Year of Follow-up: 2020, Age Group: 20-24

| Period | Age Group | Smoking Group | N | % | Ever Quit (N) | Ever Quit % | Mean Yrs Quit | Short Term (n) | Short Term % |
|---|---|---|---|---|---|---|---|---|---|
| 30 | 20-24 | Never Any | 0 | . | | | | | |
| 30 | 20-24 | CC | 0 | . | 0 | . | | | |

# Results for PHIM for Run 1 (Pessimistic_Analysis.RTF)

| | | | | | | | | | |
|---|---|---|---|---|---|---|---|---|---|
| 30 | 20-24 | Ecig | 0 | . | | | | | |
| 30 | 20-24 | Dual | 0 | . | | | | | |
| 30 | 20-24 | Former | 0 | . | | | . | 0 | . |

Year of Follow-up: 2020, Age Group: 25-29

| Period | Age Group | Smoking Group | N | % | Ever Quit (N) | Ever Quit % | Mean Yrs Quit | Short Term (n) | Short Term % |
|---|---|---|---|---|---|---|---|---|---|
| 30 | 25-29 | Never Any | 0 | . | | | | | |
| 30 | 25-29 | CC | 0 | . | 0 | . | | | |
| 30 | 25-29 | Ecig | 0 | . | | | | | |
| 30 | 25-29 | Dual | 0 | . | | | | | |
| 30 | 25-29 | Former | 0 | . | | | . | 0 | . |

Year of Follow-up: 2020, Age Group: 30-34

| Period | Age Group | Smoking Group | N | % | Ever Quit (N) | Ever Quit % | Mean Yrs Quit | Short Term (n) | Short Term % |
|---|---|---|---|---|---|---|---|---|---|
| 30 | 30-34 | Never Any | 0 | . | | | | | |
| 30 | 30-34 | CC | 0 | . | 0 | . | | | |
| 30 | 30-34 | Ecig | 0 | . | | | | | |
| 30 | 30-34 | Dual | 0 | . | | | | | |
| 30 | 30-34 | Former | 0 | . | | | . | 0 | . |

Year of Follow-up: 2020, Age Group: 35-39

| Period | Age Group | Smoking Group | N | % | Ever Quit (N) | Ever Quit % | Mean Yrs Quit | Short Term (n) | Short Term % |
|---|---|---|---|---|---|---|---|---|---|
| 30 | 35-39 | Never Any | 0 | . | | | | | |
| 30 | 35-39 | CC | 0 | . | 0 | . | | | |
| 30 | 35-39 | Ecig | 0 | . | | | | | |
| 30 | 35-39 | Dual | 0 | . | | | | | |
| 30 | 35-39 | Former | 0 | . | | | . | 0 | . |

Year of Follow-up: 2020, Age Group: 40-44

| Period | Age Group | Smoking Group | N | % | Ever Quit (N) | Ever Quit % | Mean Yrs Quit | Short Term (n) | Short Term % |
|---|---|---|---|---|---|---|---|---|---|
| 30 | 40-44 | Never Any | 3513 | 39.72 | | | | | |
| 30 | 40-44 | CC | 34 | 0.38 | 0 | 0.00 | | | |
| 30 | 40-44 | Ecig | 3160 | 35.73 | | | | | |
| 30 | 40-44 | Dual | 0 | 0.00 | | | | | |
| 30 | 40-44 | Former | 2138 | 24.17 | | | 14.67 | 103 | 4.82 |

Year of Follow-up: 2020, Age Group: 45-49

| Period | Age Group | Smoking Group | N | % | Ever Quit (N) | Ever Quit % | Mean Yrs Quit | Short Term (n) | Short Term % |
|---|---|---|---|---|---|---|---|---|---|
| 30 | 45-49 | Never Any | 4405 | 48.73 | | | | | |
| 30 | 45-49 | CC | 52 | 0.58 | 0 | 0.00 | | | |
| 30 | 45-49 | Ecig | 2637 | 29.17 | | | | | |
| 30 | 45-49 | Dual | 0 | 0.00 | | | | | |
| 30 | 45-49 | Former | 1946 | 21.53 | | | 17.47 | 53 | 2.72 |

Year of Follow-up: 2020, Age Group: 50-54

| Period | Age Group | Smoking Group | N | % | Ever Quit (N) | Ever Quit % | Mean Yrs Quit | Short Term (n) | Short Term % |
|---|---|---|---|---|---|---|---|---|---|
| 30 | 50-54 | Never Any | 4961 | 50.54 | | | | | |
| 30 | 50-54 | CC | 99 | 1.01 | 0 | 0.00 | | | |
| 30 | 50-54 | Ecig | 2606 | 26.55 | | | | | |
| 30 | 50-54 | Dual | 0 | 0.00 | | | | | |
| 30 | 50-54 | Former | 2150 | 21.90 | | | 22.19 | 68 | 3.16 |

Year of Follow-up: 2020, Age Group: 55-59

| Period | Age Group | Smoking Group | N | % | Ever Quit (N) | Ever Quit % | Mean Yrs Quit | Short Term (n) | Short Term % |
|---|---|---|---|---|---|---|---|---|---|
| 30 | 55-59 | Never Any | 4926 | 46.19 | | | | | |
| 30 | 55-59 | CC | 114 | 1.07 | 0 | 0.00 | | | |
| 30 | 55-59 | Ecig | 2629 | 24.65 | | | | | |
| 30 | 55-59 | Dual | 0 | 0.00 | | | | | |
| 30 | 55-59 | Former | 2995 | 28.09 | | | 23.64 | 100 | 3.34 |

Year of Follow-up: 2020, Age Group: 60-64

| Period | Age Group | Smoking Group | N | % | Ever Quit (N) | Ever Quit % | Mean Yrs Quit | Short Term (n) | Short Term % |
|---|---|---|---|---|---|---|---|---|---|
| 30 | 60-64 | Never Any | 4712 | 43.17 | | | | | |
| 30 | 60-64 | CC | 115 | 1.05 | 0 | 0.00 | | | |
| 30 | 60-64 | Ecig | 2199 | 20.14 | | | | | |
| 30 | 60-64 | Dual | 0 | 0.00 | | | | | |
| 30 | 60-64 | Former | 3890 | 35.64 | | | 24.71 | 165 | 4.24 |

Year of Follow-up: 2020, Age Group: 65-69

| Period | Age Group | Smoking Group | N | % | Ever Quit (N) | Ever Quit % | Mean Yrs Quit | Short Term (n) | Short Term % |
|---|---|---|---|---|---|---|---|---|---|
| 30 | 65-69 | Never Any | 3941 | 39.85 | | | | | |
| 30 | 65-69 | CC | 133 | 1.34 | 0 | 0.00 | | | |
| 30 | 65-69 | Ecig | 1543 | 15.60 | | | | | |
| 30 | 65-69 | Dual | 0 | 0.00 | | | | | |
| 30 | 65-69 | Former | 4272 | 43.20 | | | 28.00 | 119 | 2.79 |

Year of Follow-up: 2020, Age Group: 70-74

| Period | Age Group | Smoking Group | N | % | Ever Quit (N) | Ever Quit % | Mean Yrs Quit | Short Term (n) | Short Term % |
|---|---|---|---|---|---|---|---|---|---|

# Results for PHIM for Run 1 (Pessimistic_Analysis.RTF)

| | | | | | | | | | |
|---|---|---|---|---|---|---|---|---|---|
| 30 | 70-74 | Never Any | 2912 | 33.65 | | | | | |
| 30 | 70-74 | CC | 154 | 1.78 | 0 | 0.00 | | | |
| 30 | 70-74 | Ecig | 1144 | 13.22 | | | | | |
| 30 | 70-74 | Dual | 0 | 0.00 | | | | | |
| 30 | 70-74 | Former | 4445 | 51.36 | | | 30.15 | 80 | 1.80 |

Year of Follow-up: 2020, Age Group: 75-79

| Period | Age Group | Smoking Group | N | % | Ever Quit (N) | Ever Quit % | Mean Yrs Quit | Short Term (n) | Short Term % |
|---|---|---|---|---|---|---|---|---|---|
| 30 | 75-79 | Never Any | 1919 | 29.46 | | | | | |
| 30 | 75-79 | CC | 160 | 2.46 | 0 | 0.00 | | | |
| 30 | 75-79 | Ecig | 691 | 10.61 | | | | | |
| 30 | 75-79 | Dual | 0 | 0.00 | | | | | |
| 30 | 75-79 | Former | 3745 | 57.48 | | | 32.81 | 52 | 1.39 |

Year of Follow-up: 2025, Age Group: All Ages

| Period | Age Group | Smoking Group | N | % | Ever Quit (N) | Ever Quit % | Mean Yrs Quit | Short Term (n) | Short Term % |
|---|---|---|---|---|---|---|---|---|---|
| 35 | All Ages | Never Any | 29370 | 43.30 | | | | | |
| 35 | All Ages | CC | 473 | 0.70 | 0 | 0.00 | | | |
| 35 | All Ages | Ecig | 14438 | 21.29 | | | | | |
| 35 | All Ages | Dual | 0 | 0.00 | | | | | |
| 35 | All Ages | Former | 23544 | 34.71 | | | 27.45 | 662 | 2.81 |

Year of Follow-up: 2025, Age Group: 18-19

| Period | Age Group | Smoking Group | N | % | Ever Quit (N) | Ever Quit % | Mean Yrs Quit | Short Term (n) | Short Term % |
|---|---|---|---|---|---|---|---|---|---|
| 35 | 18-19 | Never Any | 0 | . | | | | | |
| 35 | 18-19 | CC | 0 | . | 0 | . | | | |
| 35 | 18-19 | Ecig | 0 | . | | | | | |
| 35 | 18-19 | Dual | 0 | . | | | | | |
| 35 | 18-19 | Former | 0 | . | | | . | 0 | . |

Year of Follow-up: 2025, Age Group: 20-24

| Period | Age Group | Smoking Group | N | % | Ever Quit (N) | Ever Quit % | Mean Yrs Quit | Short Term (n) | Short Term % |
|---|---|---|---|---|---|---|---|---|---|
| 35 | 20-24 | Never Any | 0 | . | | | | | |
| 35 | 20-24 | CC | 0 | . | 0 | . | | | |
| 35 | 20-24 | Ecig | 0 | . | | | | | |
| 35 | 20-24 | Dual | 0 | . | | | | | |
| 35 | 20-24 | Former | 0 | . | | | . | 0 | . |

Year of Follow-up: 2025, Age Group: 25-29

| Period | Age Group | Smoking Group | N | % | Ever Quit (N) | Ever Quit % | Mean Yrs Quit | Short Term (n) | Short Term % |
|---|---|---|---|---|---|---|---|---|---|
| 35 | 25-29 | Never Any | 0 | . | | | | | |
| 35 | 25-29 | CC | 0 | . | 0 | . | | | |
| 35 | 25-29 | Ecig | 0 | . | | | | | |
| 35 | 25-29 | Dual | 0 | . | | | | | |
| 35 | 25-29 | Former | 0 | . | | | . | 0 | . |

Year of Follow-up: 2025, Age Group: 30-34

| Period | Age Group | Smoking Group | N | % | Ever Quit (N) | Ever Quit % | Mean Yrs Quit | Short Term (n) | Short Term % |
|---|---|---|---|---|---|---|---|---|---|
| 35 | 30-34 | Never Any | 0 | . | | | | | |
| 35 | 30-34 | CC | 0 | . | 0 | . | | | |
| 35 | 30-34 | Ecig | 0 | . | | | | | |
| 35 | 30-34 | Dual | 0 | . | | | | | |
| 35 | 30-34 | Former | 0 | . | | | . | 0 | . |

Year of Follow-up: 2025, Age Group: 35-39

| Period | Age Group | Smoking Group | N | % | Ever Quit (N) | Ever Quit % | Mean Yrs Quit | Short Term (n) | Short Term % |
|---|---|---|---|---|---|---|---|---|---|
| 35 | 35-39 | Never Any | 0 | . | | | | | |
| 35 | 35-39 | CC | 0 | . | 0 | . | | | |
| 35 | 35-39 | Ecig | 0 | . | | | | | |
| 35 | 35-39 | Dual | 0 | . | | | | | |
| 35 | 35-39 | Former | 0 | . | | | . | 0 | . |

Year of Follow-up: 2025, Age Group: 40-44

| Period | Age Group | Smoking Group | N | % | Ever Quit (N) | Ever Quit % | Mean Yrs Quit | Short Term (n) | Short Term % |
|---|---|---|---|---|---|---|---|---|---|
| 35 | 40-44 | Never Any | 0 | . | | | | | |
| 35 | 40-44 | CC | 0 | . | 0 | . | | | |
| 35 | 40-44 | Ecig | 0 | . | | | | | |
| 35 | 40-44 | Dual | 0 | . | | | | | |
| 35 | 40-44 | Former | 0 | . | | | . | 0 | . |

Year of Follow-up: 2025, Age Group: 45-49

| Period | Age Group | Smoking Group | N | % | Ever Quit (N) | Ever Quit % | Mean Yrs Quit | Short Term (n) | Short Term % |
|---|---|---|---|---|---|---|---|---|---|
| 35 | 45-49 | Never Any | 3513 | 39.72 | | | | | |
| 35 | 45-49 | CC | 20 | 0.23 | 0 | 0.00 | | | |
| 35 | 45-49 | Ecig | 2991 | 33.82 | | | | | |
| 35 | 45-49 | Dual | 0 | 0.00 | | | | | |
| 35 | 45-49 | Former | 2321 | 26.24 | | | 18.31 | 49 | 2.11 |

Year of Follow-up: 2025, Age Group: 50-54

# Results for PHIM for Run 1 (Pessimistic_Analysis.RTF)

| Period | Age Group | Smoking Group | N | % | Ever Quit (N) | Ever Quit % | Mean Yrs Quit | Short Term (n) | Short Term % |
|---|---|---|---|---|---|---|---|---|---|
| 35 | 50-54 | Never Any | 4405 | 48.73 | | | | | |
| 35 | 50-54 | CC | 33 | 0.37 | 0 | 0.00 | | | |
| 35 | 50-54 | Ecig | 2537 | 28.06 | | | | | |
| 35 | 50-54 | Dual | 0 | 0.00 | | | | | |
| 35 | 50-54 | Former | 2065 | 22.84 | | | 21.27 | 57 | 2.76 |

Year of Follow-up: 2025, Age Group: 55-59

| Period | Age Group | Smoking Group | N | % | Ever Quit (N) | Ever Quit % | Mean Yrs Quit | Short Term (n) | Short Term % |
|---|---|---|---|---|---|---|---|---|---|
| 35 | 55-59 | Never Any | 4961 | 50.54 | | | | | |
| 35 | 55-59 | CC | 58 | 0.59 | 0 | 0.00 | | | |
| 35 | 55-59 | Ecig | 2368 | 24.12 | | | | | |
| 35 | 55-59 | Dual | 0 | 0.00 | | | | | |
| 35 | 55-59 | Former | 2429 | 24.75 | | | 24.29 | 123 | 5.06 |

Year of Follow-up: 2025, Age Group: 60-64

| Period | Age Group | Smoking Group | N | % | Ever Quit (N) | Ever Quit % | Mean Yrs Quit | Short Term (n) | Short Term % |
|---|---|---|---|---|---|---|---|---|---|
| 35 | 60-64 | Never Any | 4926 | 46.19 | | | | | |
| 35 | 60-64 | CC | 65 | 0.61 | 0 | 0.00 | | | |
| 35 | 60-64 | Ecig | 2309 | 21.65 | | | | | |
| 35 | 60-64 | Dual | 0 | 0.00 | | | | | |
| 35 | 60-64 | Former | 3364 | 31.55 | | | 25.72 | 148 | 4.40 |

Year of Follow-up: 2025, Age Group: 65-69

| Period | Age Group | Smoking Group | N | % | Ever Quit (N) | Ever Quit % | Mean Yrs Quit | Short Term (n) | Short Term % |
|---|---|---|---|---|---|---|---|---|---|
| 35 | 65-69 | Never Any | 4712 | 43.17 | | | | | |
| 35 | 65-69 | CC | 73 | 0.67 | 0 | 0.00 | | | |
| 35 | 65-69 | Ecig | 1870 | 17.13 | | | | | |
| 35 | 65-69 | Dual | 0 | 0.00 | | | | | |
| 35 | 65-69 | Former | 4261 | 39.03 | | | 27.31 | 134 | 3.14 |

Year of Follow-up: 2025, Age Group: 70-74

| Period | Age Group | Smoking Group | N | % | Ever Quit (N) | Ever Quit % | Mean Yrs Quit | Short Term (n) | Short Term % |
|---|---|---|---|---|---|---|---|---|---|
| 35 | 70-74 | Never Any | 3941 | 39.85 | | | | | |
| 35 | 70-74 | CC | 104 | 1.05 | 0 | 0.00 | | | |
| 35 | 70-74 | Ecig | 1362 | 13.77 | | | | | |
| 35 | 70-74 | Dual | 0 | 0.00 | | | | | |
| 35 | 70-74 | Former | 4482 | 45.32 | | | 31.55 | 75 | 1.67 |

Year of Follow-up: 2025, Age Group: 75-79

| Period | Age Group | Smoking Group | N | % | Ever Quit (N) | Ever Quit % | Mean Yrs Quit | Short Term (n) | Short Term % |
|---|---|---|---|---|---|---|---|---|---|
| 35 | 75-79 | Never Any | 2912 | 33.65 | | | | | |
| 35 | 75-79 | CC | 120 | 1.39 | 0 | 0.00 | | | |
| 35 | 75-79 | Ecig | 1001 | 11.57 | | | | | |
| 35 | 75-79 | Dual | 0 | 0.00 | | | | | |
| 35 | 75-79 | Former | 4622 | 53.40 | | | 33.88 | 76 | 1.64 |

Year of Follow-up: 2030, Age Group: All Ages

| Period | Age Group | Smoking Group | N | % | Ever Quit (N) | Ever Quit % | Mean Yrs Quit | Short Term (n) | Short Term % |
|---|---|---|---|---|---|---|---|---|---|
| 40 | All Ages | Never Any | 26458 | 44.72 | | | | | |
| 40 | All Ages | CC | 228 | 0.39 | 0 | 0.00 | | | |
| 40 | All Ages | Ecig | 12012 | 20.30 | | | | | |
| 40 | All Ages | Dual | 0 | 0.00 | | | | | |
| 40 | All Ages | Former | 20472 | 34.60 | | | 28.69 | 609 | 2.97 |

Year of Follow-up: 2030, Age Group: 18-19

| Period | Age Group | Smoking Group | N | % | Ever Quit (N) | Ever Quit % | Mean Yrs Quit | Short Term (n) | Short Term % |
|---|---|---|---|---|---|---|---|---|---|
| 40 | 18-19 | Never Any | 0 | . | | | | | |
| 40 | 18-19 | CC | 0 | . | 0 | . | | | |
| 40 | 18-19 | Ecig | 0 | . | | | | | |
| 40 | 18-19 | Dual | 0 | . | | | | | |
| 40 | 18-19 | Former | 0 | . | | | . | 0 | . |

Year of Follow-up: 2030, Age Group: 20-24

| Period | Age Group | Smoking Group | N | % | Ever Quit (N) | Ever Quit % | Mean Yrs Quit | Short Term (n) | Short Term % |
|---|---|---|---|---|---|---|---|---|---|
| 40 | 20-24 | Never Any | 0 | . | | | | | |
| 40 | 20-24 | CC | 0 | . | 0 | . | | | |
| 40 | 20-24 | Ecig | 0 | . | | | | | |
| 40 | 20-24 | Dual | 0 | . | | | | | |
| 40 | 20-24 | Former | 0 | . | | | . | 0 | . |

Year of Follow-up: 2030, Age Group: 25-29

| Period | Age Group | Smoking Group | N | % | Ever Quit (N) | Ever Quit % | Mean Yrs Quit | Short Term (n) | Short Term % |
|---|---|---|---|---|---|---|---|---|---|
| 40 | 25-29 | Never Any | 0 | . | | | | | |
| 40 | 25-29 | CC | 0 | . | 0 | . | | | |
| 40 | 25-29 | Ecig | 0 | . | | | | | |
| 40 | 25-29 | Dual | 0 | . | | | | | |

# Results for PHIM for Run 1 (Pessimistic_Analysis.RTF)

| | | | | | | | | | |
|---|---|---|---|---|---|---|---|---|---|
| 40 | 25-29 | Former | 0 | . | | | . | 0 | . |

Year of Follow-up: 2030, Age Group: 30-34

| Period | Age Group | Smoking Group | N | % | Ever Quit (N) | Ever Quit % | Mean Yrs Quit | Short Term (n) | Short Term % |
|---|---|---|---|---|---|---|---|---|---|
| 40 | 30-34 | Never Any | 0 | . | | | | | |
| 40 | 30-34 | CC | 0 | . | 0 | . | | | |
| 40 | 30-34 | Ecig | 0 | . | | | | | |
| 40 | 30-34 | Dual | 0 | . | | | | | |
| 40 | 30-34 | Former | 0 | . | | | . | 0 | . |

Year of Follow-up: 2030, Age Group: 35-39

| Period | Age Group | Smoking Group | N | % | Ever Quit (N) | Ever Quit % | Mean Yrs Quit | Short Term (n) | Short Term % |
|---|---|---|---|---|---|---|---|---|---|
| 40 | 35-39 | Never Any | 0 | . | | | | | |
| 40 | 35-39 | CC | 0 | . | 0 | . | | | |
| 40 | 35-39 | Ecig | 0 | . | | | | | |
| 40 | 35-39 | Dual | 0 | . | | | | | |
| 40 | 35-39 | Former | 0 | . | | | . | 0 | . |

Year of Follow-up: 2030, Age Group: 40-44

| Period | Age Group | Smoking Group | N | % | Ever Quit (N) | Ever Quit % | Mean Yrs Quit | Short Term (n) | Short Term % |
|---|---|---|---|---|---|---|---|---|---|
| 40 | 40-44 | Never Any | 0 | . | | | | | |
| 40 | 40-44 | CC | 0 | . | 0 | . | | | |
| 40 | 40-44 | Ecig | 0 | . | | | | | |
| 40 | 40-44 | Dual | 0 | . | | | | | |
| 40 | 40-44 | Former | 0 | . | | | . | 0 | . |

Year of Follow-up: 2030, Age Group: 45-49

| Period | Age Group | Smoking Group | N | % | Ever Quit (N) | Ever Quit % | Mean Yrs Quit | Short Term (n) | Short Term % |
|---|---|---|---|---|---|---|---|---|---|
| 40 | 45-49 | Never Any | 0 | . | | | | | |
| 40 | 45-49 | CC | 0 | . | 0 | . | | | |
| 40 | 45-49 | Ecig | 0 | . | | | | | |
| 40 | 45-49 | Dual | 0 | . | | | | | |
| 40 | 45-49 | Former | 0 | . | | | . | 0 | . |

Year of Follow-up: 2030, Age Group: 50-54

| Period | Age Group | Smoking Group | N | % | Ever Quit (N) | Ever Quit % | Mean Yrs Quit | Short Term (n) | Short Term % |
|---|---|---|---|---|---|---|---|---|---|
| 40 | 50-54 | Never Any | 3513 | 39.72 | | | | | |
| 40 | 50-54 | CC | 14 | 0.16 | 0 | 0.00 | | | |
| 40 | 50-54 | Ecig | 2852 | 32.24 | | | | | |
| 40 | 50-54 | Dual | 0 | 0.00 | | | | | |
| 40 | 50-54 | Former | 2466 | 27.88 | | | 22.05 | 69 | 2.80 |

Year of Follow-up: 2030, Age Group: 55-59

| Period | Age Group | Smoking Group | N | % | Ever Quit (N) | Ever Quit % | Mean Yrs Quit | Short Term (n) | Short Term % |
|---|---|---|---|---|---|---|---|---|---|
| 40 | 55-59 | Never Any | 4405 | 48.73 | | | | | |
| 40 | 55-59 | CC | 20 | 0.22 | 0 | 0.00 | | | |
| 40 | 55-59 | Ecig | 2308 | 25.53 | | | | | |
| 40 | 55-59 | Dual | 0 | 0.00 | | | | | |
| 40 | 55-59 | Former | 2307 | 25.52 | | | 23.73 | 89 | 3.86 |

Year of Follow-up: 2030, Age Group: 60-64

| Period | Age Group | Smoking Group | N | % | Ever Quit (N) | Ever Quit % | Mean Yrs Quit | Short Term (n) | Short Term % |
|---|---|---|---|---|---|---|---|---|---|
| 40 | 60-64 | Never Any | 4961 | 50.54 | | | | | |
| 40 | 60-64 | CC | 30 | 0.31 | 0 | 0.00 | | | |
| 40 | 60-64 | Ecig | 2059 | 20.98 | | | | | |
| 40 | 60-64 | Dual | 0 | 0.00 | | | | | |
| 40 | 60-64 | Former | 2766 | 28.18 | | | 25.94 | 155 | 5.60 |

Year of Follow-up: 2030, Age Group: 65-69

| Period | Age Group | Smoking Group | N | % | Ever Quit (N) | Ever Quit % | Mean Yrs Quit | Short Term (n) | Short Term % |
|---|---|---|---|---|---|---|---|---|---|
| 40 | 65-69 | Never Any | 4926 | 46.19 | | | | | |
| 40 | 65-69 | CC | 33 | 0.31 | 0 | 0.00 | | | |
| 40 | 65-69 | Ecig | 1969 | 18.46 | | | | | |
| 40 | 65-69 | Dual | 0 | 0.00 | | | | | |
| 40 | 65-69 | Former | 3736 | 35.03 | | | 27.87 | 127 | 3.40 |

Year of Follow-up: 2030, Age Group: 70-74

| Period | Age Group | Smoking Group | N | % | Ever Quit (N) | Ever Quit % | Mean Yrs Quit | Short Term (n) | Short Term % |
|---|---|---|---|---|---|---|---|---|---|
| 40 | 70-74 | Never Any | 4712 | 43.17 | | | | | |
| 40 | 70-74 | CC | 50 | 0.46 | 0 | 0.00 | | | |
| 40 | 70-74 | Ecig | 1631 | 14.94 | | | | | |
| 40 | 70-74 | Dual | 0 | 0.00 | | | | | |
| 40 | 70-74 | Former | 4523 | 41.43 | | | 30.55 | 96 | 2.12 |

Year of Follow-up: 2030, Age Group: 75-79

| Period | Age Group | Smoking Group | N | % | Ever Quit (N) | Ever Quit % | Mean Yrs Quit | Short Term (n) | Short Term % |
|---|---|---|---|---|---|---|---|---|---|
| 40 | 75-79 | Never Any | 3941 | 39.85 | | | | | |
| 40 | 75-79 | CC | 81 | 0.82 | 0 | 0.00 | | | |

# Results for PHIM for Run 1 (Pessimistic_Analysis.RTF)

| | | | | | | | | | |
|---|---|---|---|---|---|---|---|---|---|
| 40 | 75-79 | Ecig | 1193 | 12.06 | | | | | |
| 40 | 75-79 | Dual | 0 | 0.00 | | | | | |
| 40 | 75-79 | Former | 4674 | 47.26 | | | 35.13 | 73 | 1.56 |

Year of Follow-up: 2035, Age Group: All Ages

| Period | Age Group | Smoking Group | N | % | Ever Quit (N) | Ever Quit % | Mean Yrs Quit | Short Term (n) | Short Term % |
|---|---|---|---|---|---|---|---|---|---|
| 45 | All Ages | Never Any | 22517 | 45.69 | | | | | |
| 45 | All Ages | CC | 102 | 0.21 | 0 | 0.00 | | | |
| 45 | All Ages | Ecig | 9520 | 19.32 | | | | | |
| 45 | All Ages | Dual | 0 | 0.00 | | | | | |
| 45 | All Ages | Former | 17142 | 34.78 | | | 29.45 | 563 | 3.28 |

Year of Follow-up: 2035, Age Group: 18-19

| Period | Age Group | Smoking Group | N | % | Ever Quit (N) | Ever Quit % | Mean Yrs Quit | Short Term (n) | Short Term % |
|---|---|---|---|---|---|---|---|---|---|
| 45 | 18-19 | Never Any | 0 | . | | | | | |
| 45 | 18-19 | CC | 0 | . | 0 | . | | | |
| 45 | 18-19 | Ecig | 0 | . | | | | | |
| 45 | 18-19 | Dual | 0 | . | | | | | |
| 45 | 18-19 | Former | 0 | . | | | . | 0 | . |

Year of Follow-up: 2035, Age Group: 20-24

| Period | Age Group | Smoking Group | N | % | Ever Quit (N) | Ever Quit % | Mean Yrs Quit | Short Term (n) | Short Term % |
|---|---|---|---|---|---|---|---|---|---|
| 45 | 20-24 | Never Any | 0 | . | | | | | |
| 45 | 20-24 | CC | 0 | . | 0 | . | | | |
| 45 | 20-24 | Ecig | 0 | . | | | | | |
| 45 | 20-24 | Dual | 0 | . | | | | | |
| 45 | 20-24 | Former | 0 | . | | | . | 0 | . |

Year of Follow-up: 2035, Age Group: 25-29

| Period | Age Group | Smoking Group | N | % | Ever Quit (N) | Ever Quit % | Mean Yrs Quit | Short Term (n) | Short Term % |
|---|---|---|---|---|---|---|---|---|---|
| 45 | 25-29 | Never Any | 0 | . | | | | | |
| 45 | 25-29 | CC | 0 | . | 0 | . | | | |
| 45 | 25-29 | Ecig | 0 | . | | | | | |
| 45 | 25-29 | Dual | 0 | . | | | | | |
| 45 | 25-29 | Former | 0 | . | | | . | 0 | . |

Year of Follow-up: 2035, Age Group: 30-34

| Period | Age Group | Smoking Group | N | % | Ever Quit (N) | Ever Quit % | Mean Yrs Quit | Short Term (n) | Short Term % |
|---|---|---|---|---|---|---|---|---|---|
| 45 | 30-34 | Never Any | 0 | . | | | | | |
| 45 | 30-34 | CC | 0 | . | 0 | . | | | |
| 45 | 30-34 | Ecig | 0 | . | | | | | |
| 45 | 30-34 | Dual | 0 | . | | | | | |
| 45 | 30-34 | Former | 0 | . | | | . | 0 | . |

Year of Follow-up: 2035, Age Group: 35-39

| Period | Age Group | Smoking Group | N | % | Ever Quit (N) | Ever Quit % | Mean Yrs Quit | Short Term (n) | Short Term % |
|---|---|---|---|---|---|---|---|---|---|
| 45 | 35-39 | Never Any | 0 | . | | | | | |
| 45 | 35-39 | CC | 0 | . | 0 | . | | | |
| 45 | 35-39 | Ecig | 0 | . | | | | | |
| 45 | 35-39 | Dual | 0 | . | | | | | |
| 45 | 35-39 | Former | 0 | . | | | . | 0 | . |

Year of Follow-up: 2035, Age Group: 40-44

| Period | Age Group | Smoking Group | N | % | Ever Quit (N) | Ever Quit % | Mean Yrs Quit | Short Term (n) | Short Term % |
|---|---|---|---|---|---|---|---|---|---|
| 45 | 40-44 | Never Any | 0 | . | | | | | |
| 45 | 40-44 | CC | 0 | . | 0 | . | | | |
| 45 | 40-44 | Ecig | 0 | . | | | | | |
| 45 | 40-44 | Dual | 0 | . | | | | | |
| 45 | 40-44 | Former | 0 | . | | | . | 0 | . |

Year of Follow-up: 2035, Age Group: 45-49

| Period | Age Group | Smoking Group | N | % | Ever Quit (N) | Ever Quit % | Mean Yrs Quit | Short Term (n) | Short Term % |
|---|---|---|---|---|---|---|---|---|---|
| 45 | 45-49 | Never Any | 0 | . | | | | | |
| 45 | 45-49 | CC | 0 | . | 0 | . | | | |
| 45 | 45-49 | Ecig | 0 | . | | | | | |
| 45 | 45-49 | Dual | 0 | . | | | | | |
| 45 | 45-49 | Former | 0 | . | | | . | 0 | . |

Year of Follow-up: 2035, Age Group: 50-54

| Period | Age Group | Smoking Group | N | % | Ever Quit (N) | Ever Quit % | Mean Yrs Quit | Short Term (n) | Short Term % |
|---|---|---|---|---|---|---|---|---|---|
| 45 | 50-54 | Never Any | 0 | . | | | | | |
| 45 | 50-54 | CC | 0 | . | 0 | . | | | |
| 45 | 50-54 | Ecig | 0 | . | | | | | |
| 45 | 50-54 | Dual | 0 | . | | | | | |
| 45 | 50-54 | Former | 0 | . | | | . | 0 | . |

Year of Follow-up: 2035, Age Group: 55-59

| Period | Age Group | Smoking Group | N | % | Ever Quit (N) | Ever Quit % | Mean Yrs Quit | Short Term (n) | Short Term % |
|---|---|---|---|---|---|---|---|---|---|

# Results for PHIM for Run 1 (Pessimistic_Analysis.RTF)

| | | | | | | | | | |
|---|---|---|---|---|---|---|---|---|---|
| 45 | 55-59 | Never Any | 3513 | 39.72 | | | | | |
| 45 | 55-59 | CC | 9 | 0.10 | 0 | 0.00 | | | |
| 45 | 55-59 | Ecig | 2636 | 29.80 | | | | | |
| 45 | 55-59 | Dual | 0 | 0.00 | | | | | |
| 45 | 55-59 | Former | 2687 | 30.38 | | | 24.97 | 103 | 3.83 |

Year of Follow-up: 2035, Age Group: 60-64

| Period | Age Group | Smoking Group | N | % | Ever Quit (N) | Ever Quit % | Mean Yrs Quit | Short Term (n) | Short Term % |
|---|---|---|---|---|---|---|---|---|---|
| 45 | 60-64 | Never Any | 4405 | 48.73 | | | | | |
| 45 | 60-64 | CC | 14 | 0.15 | 0 | 0.00 | | | |
| 45 | 60-64 | Ecig | 2027 | 22.42 | | | | | |
| 45 | 60-64 | Dual | 0 | 0.00 | | | | | |
| 45 | 60-64 | Former | 2594 | 28.69 | | | 25.75 | 125 | 4.82 |

Year of Follow-up: 2035, Age Group: 65-69

| Period | Age Group | Smoking Group | N | % | Ever Quit (N) | Ever Quit % | Mean Yrs Quit | Short Term (n) | Short Term % |
|---|---|---|---|---|---|---|---|---|---|
| 45 | 65-69 | Never Any | 4961 | 50.54 | | | | | |
| 45 | 65-69 | CC | 18 | 0.18 | 0 | 0.00 | | | |
| 45 | 65-69 | Ecig | 1756 | 17.89 | | | | | |
| 45 | 65-69 | Dual | 0 | 0.00 | | | | | |
| 45 | 65-69 | Former | 3081 | 31.39 | | | 27.99 | 117 | 3.80 |

Year of Follow-up: 2035, Age Group: 70-74

| Period | Age Group | Smoking Group | N | % | Ever Quit (N) | Ever Quit % | Mean Yrs Quit | Short Term (n) | Short Term % |
|---|---|---|---|---|---|---|---|---|---|
| 45 | 70-74 | Never Any | 4926 | 46.19 | | | | | |
| 45 | 70-74 | CC | 26 | 0.24 | 0 | 0.00 | | | |
| 45 | 70-74 | Ecig | 1709 | 16.03 | | | | | |
| 45 | 70-74 | Dual | 0 | 0.00 | | | | | |
| 45 | 70-74 | Former | 4003 | 37.54 | | | 30.81 | 115 | 2.87 |

Year of Follow-up: 2035, Age Group: 75-79

| Period | Age Group | Smoking Group | N | % | Ever Quit (N) | Ever Quit % | Mean Yrs Quit | Short Term (n) | Short Term % |
|---|---|---|---|---|---|---|---|---|---|
| 45 | 75-79 | Never Any | 4712 | 43.17 | | | | | |
| 45 | 75-79 | CC | 35 | 0.32 | 0 | 0.00 | | | |
| 45 | 75-79 | Ecig | 1392 | 12.75 | | | | | |
| 45 | 75-79 | Dual | 0 | 0.00 | | | | | |
| 45 | 75-79 | Former | 4777 | 43.76 | | | 33.77 | 103 | 2.16 |

Year of Follow-up: 2040, Age Group: All Ages

| Period | Age Group | Smoking Group | N | % | Ever Quit (N) | Ever Quit % | Mean Yrs Quit | Short Term (n) | Short Term % |
|---|---|---|---|---|---|---|---|---|---|
| 50 | All Ages | Never Any | 17805 | 46.41 | | | | | |
| 50 | All Ages | CC | 46 | 0.12 | 0 | 0.00 | | | |
| 50 | All Ages | Ecig | 7017 | 18.29 | | | | | |
| 50 | All Ages | Dual | 0 | 0.00 | | | | | |
| 50 | All Ages | Former | 13497 | 35.18 | | | 30.19 | 465 | 3.45 |

Year of Follow-up: 2040, Age Group: 18-19

| Period | Age Group | Smoking Group | N | % | Ever Quit (N) | Ever Quit % | Mean Yrs Quit | Short Term (n) | Short Term % |
|---|---|---|---|---|---|---|---|---|---|
| 50 | 18-19 | Never Any | 0 | . | | | | | |
| 50 | 18-19 | CC | 0 | . | 0 | . | | | |
| 50 | 18-19 | Ecig | 0 | . | | | | | |
| 50 | 18-19 | Dual | 0 | . | | | | | |
| 50 | 18-19 | Former | 0 | . | | | . | 0 | . |

Year of Follow-up: 2040, Age Group: 20-24

| Period | Age Group | Smoking Group | N | % | Ever Quit (N) | Ever Quit % | Mean Yrs Quit | Short Term (n) | Short Term % |
|---|---|---|---|---|---|---|---|---|---|
| 50 | 20-24 | Never Any | 0 | . | | | | | |
| 50 | 20-24 | CC | 0 | . | 0 | . | | | |
| 50 | 20-24 | Ecig | 0 | . | | | | | |
| 50 | 20-24 | Dual | 0 | . | | | | | |
| 50 | 20-24 | Former | 0 | . | | | . | 0 | . |

Year of Follow-up: 2040, Age Group: 25-29

| Period | Age Group | Smoking Group | N | % | Ever Quit (N) | Ever Quit % | Mean Yrs Quit | Short Term (n) | Short Term % |
|---|---|---|---|---|---|---|---|---|---|
| 50 | 25-29 | Never Any | 0 | . | | | | | |
| 50 | 25-29 | CC | 0 | . | 0 | . | | | |
| 50 | 25-29 | Ecig | 0 | . | | | | | |
| 50 | 25-29 | Dual | 0 | . | | | | | |
| 50 | 25-29 | Former | 0 | . | | | . | 0 | . |

Year of Follow-up: 2040, Age Group: 30-34

| Period | Age Group | Smoking Group | N | % | Ever Quit (N) | Ever Quit % | Mean Yrs Quit | Short Term (n) | Short Term % |
|---|---|---|---|---|---|---|---|---|---|
| 50 | 30-34 | Never Any | 0 | . | | | | | |
| 50 | 30-34 | CC | 0 | . | 0 | . | | | |
| 50 | 30-34 | Ecig | 0 | . | | | | | |
| 50 | 30-34 | Dual | 0 | . | | | | | |
| 50 | 30-34 | Former | 0 | . | | | . | 0 | . |

Year of Follow-up: 2040, Age Group: 35-39

## Results for PHIM for Run 1 (Pessimistic_Analysis.RTF)

| Period | Age Group | Smoking Group | N | % | Ever Quit (N) | Ever Quit % | Mean Yrs Quit | Short Term (n) | Short Term % |
|---|---|---|---|---|---|---|---|---|---|
| 50 | 35-39 | Never Any | 0 | . | | | | | |
| 50 | 35-39 | CC | 0 | . | 0 | . | | | |
| 50 | 35-39 | Ecig | 0 | . | | | | | |
| 50 | 35-39 | Dual | 0 | . | | | | | |
| 50 | 35-39 | Former | 0 | . | | | . | 0 | . |

Year of Follow-up: 2040, Age Group: 40-44

| Period | Age Group | Smoking Group | N | % | Ever Quit (N) | Ever Quit % | Mean Yrs Quit | Short Term (n) | Short Term % |
|---|---|---|---|---|---|---|---|---|---|
| 50 | 40-44 | Never Any | 0 | . | | | | | |
| 50 | 40-44 | CC | 0 | . | 0 | . | | | |
| 50 | 40-44 | Ecig | 0 | . | | | | | |
| 50 | 40-44 | Dual | 0 | . | | | | | |
| 50 | 40-44 | Former | 0 | . | | | . | 0 | . |

Year of Follow-up: 2040, Age Group: 45-49

| Period | Age Group | Smoking Group | N | % | Ever Quit (N) | Ever Quit % | Mean Yrs Quit | Short Term (n) | Short Term % |
|---|---|---|---|---|---|---|---|---|---|
| 50 | 45-49 | Never Any | 0 | . | | | | | |
| 50 | 45-49 | CC | 0 | . | 0 | . | | | |
| 50 | 45-49 | Ecig | 0 | . | | | | | |
| 50 | 45-49 | Dual | 0 | . | | | | | |
| 50 | 45-49 | Former | 0 | . | | | . | 0 | . |

Year of Follow-up: 2040, Age Group: 50-54

| Period | Age Group | Smoking Group | N | % | Ever Quit (N) | Ever Quit % | Mean Yrs Quit | Short Term (n) | Short Term % |
|---|---|---|---|---|---|---|---|---|---|
| 50 | 50-54 | Never Any | 0 | . | | | | | |
| 50 | 50-54 | CC | 0 | . | 0 | . | | | |
| 50 | 50-54 | Ecig | 0 | . | | | | | |
| 50 | 50-54 | Dual | 0 | . | | | | | |
| 50 | 50-54 | Former | 0 | . | | | . | 0 | . |

Year of Follow-up: 2040, Age Group: 55-59

| Period | Age Group | Smoking Group | N | % | Ever Quit (N) | Ever Quit % | Mean Yrs Quit | Short Term (n) | Short Term % |
|---|---|---|---|---|---|---|---|---|---|
| 50 | 55-59 | Never Any | 0 | . | | | | | |
| 50 | 55-59 | CC | 0 | . | 0 | . | | | |
| 50 | 55-59 | Ecig | 0 | . | | | | | |
| 50 | 55-59 | Dual | 0 | . | | | | | |
| 50 | 55-59 | Former | 0 | . | | | . | 0 | . |

Year of Follow-up: 2040, Age Group: 60-64

| Period | Age Group | Smoking Group | N | % | Ever Quit (N) | Ever Quit % | Mean Yrs Quit | Short Term (n) | Short Term % |
|---|---|---|---|---|---|---|---|---|---|
| 50 | 60-64 | Never Any | 3513 | 39.72 | | | | | |
| 50 | 60-64 | CC | 7 | 0.08 | 0 | 0.00 | | | |
| 50 | 60-64 | Ecig | 2285 | 25.83 | | | | | |
| 50 | 60-64 | Dual | 0 | 0.00 | | | | | |
| 50 | 60-64 | Former | 3040 | 34.37 | | | 26.70 | 163 | 5.36 |

Year of Follow-up: 2040, Age Group: 65-69

| Period | Age Group | Smoking Group | N | % | Ever Quit (N) | Ever Quit % | Mean Yrs Quit | Short Term (n) | Short Term % |
|---|---|---|---|---|---|---|---|---|---|
| 50 | 65-69 | Never Any | 4405 | 48.73 | | | | | |
| 50 | 65-69 | CC | 6 | 0.07 | 0 | 0.00 | | | |
| 50 | 65-69 | Ecig | 1729 | 19.13 | | | | | |
| 50 | 65-69 | Dual | 0 | 0.00 | | | | | |
| 50 | 65-69 | Former | 2900 | 32.08 | | | 27.73 | 118 | 4.07 |

Year of Follow-up: 2040, Age Group: 70-74

| Period | Age Group | Smoking Group | N | % | Ever Quit (N) | Ever Quit % | Mean Yrs Quit | Short Term (n) | Short Term % |
|---|---|---|---|---|---|---|---|---|---|
| 50 | 70-74 | Never Any | 4961 | 50.54 | | | | | |
| 50 | 70-74 | CC | 13 | 0.13 | 0 | 0.00 | | | |
| 50 | 70-74 | Ecig | 1530 | 15.59 | | | | | |
| 50 | 70-74 | Dual | 0 | 0.00 | | | | | |
| 50 | 70-74 | Former | 3312 | 33.74 | | | 30.84 | 83 | 2.51 |

Year of Follow-up: 2040, Age Group: 75-79

| Period | Age Group | Smoking Group | N | % | Ever Quit (N) | Ever Quit % | Mean Yrs Quit | Short Term (n) | Short Term % |
|---|---|---|---|---|---|---|---|---|---|
| 50 | 75-79 | Never Any | 4926 | 46.19 | | | | | |
| 50 | 75-79 | CC | 20 | 0.19 | 0 | 0.00 | | | |
| 50 | 75-79 | Ecig | 1473 | 13.81 | | | | | |
| 50 | 75-79 | Dual | 0 | 0.00 | | | | | |
| 50 | 75-79 | Former | 4245 | 39.81 | | | 33.87 | 101 | 2.38 |

# Results for PHIM for Run 1 (Pessimistic_Analysis.RTF)

**************************************************************************************
**Main Results for E-Component for Run: 1, Simulation: 1, COD: Four Diseases Combined**

| Variable Parameter | Value |
|---|---|
| 1. Country | US (United States) |
| 2. Sex | M (Males) |
| 3. Year of start of process | 1990 |
| 4. Cause of Death | Four Diseases Combined |
| 5. Effective Dose NULL | CC:1 |
| 6. Effective Dose Ecig | CC:1 Ecig:0.4 Dual:0.7 |
| Note: | Death rates are per million population |

------------------------------------------------------------------------------------------
Results for Age Groups Combined and Years combined, Population unadjusted for deaths in Ecig scenario,

| Age Group:All | | NULL | Ecig | | | %Drop | | NULL | Ecig | |
|---|---|---|---|---|---|---|---|---|---|---|
| Year | Number Deaths | Attrib Deaths | Attrib Deaths | Drop Deaths | %Drop Deaths | Attrib Deaths | Death Rate | Attrib Rates | Attrib Rates | Drop Rates |
| All Comb | 13148871 | 4509390.583 | 4037084.197 | 472306.4 | 3.592 | 10.474 | 3806.31 | 1305.37 | 1168.65 | 136.722 |

------------------------------------------------------------------------------------------
Results for Age Groups Combined, Population unadjusted for deaths in Ecig scenario,

| Age Group:All | | NULL | Ecig | | | %Drop | | NULL | Ecig | |
|---|---|---|---|---|---|---|---|---|---|---|
| Year | Number Deaths | Attrib Deaths | Attrib Deaths | Drop Deaths | %Drop Deaths | Attrib Deaths | Death Rate | Attrib Rates | Attrib Rates | Drop Rates |
| 1990 | 317161 | 125773.065 | 125773.065 | 0.000 | 0.000 | 0.000 | 5027.71 | 1993.78 | 1993.78 | 0.000 |
| 1991 | 313217 | 123999.442 | 123873.164 | 126.278 | 0.040 | 0.102 | 4883.81 | 1933.45 | 1931.48 | 1.969 |
| 1992 | 308577 | 122014.015 | 121689.663 | 324.352 | 0.105 | 0.266 | 4729.73 | 1870.18 | 1865.20 | 4.972 |
| 1993 | 311713 | 122810.618 | 122195.247 | 615.371 | 0.197 | 0.501 | 4692.62 | 1848.83 | 1839.56 | 9.264 |
| 1994 | 305700 | 119925.592 | 118971.566 | 954.026 | 0.312 | 0.796 | 4516.19 | 1771.69 | 1757.60 | 14.094 |
| 1995 | 303225 | 117896.496 | 116520.681 | 1375.815 | 0.454 | 1.167 | 4393.80 | 1708.35 | 1688.41 | 19.936 |
| 1996 | 299559 | 115698.645 | 113866.225 | 1832.420 | 0.612 | 1.584 | 4273.16 | 1650.42 | 1624.28 | 26.139 |
| 1997 | 294226 | 113359.847 | 111063.249 | 2296.598 | 0.781 | 2.026 | 4128.67 | 1590.70 | 1558.47 | 32.227 |
| 1998 | 289258 | 111444.212 | 108678.734 | 2765.478 | 0.956 | 2.481 | 3992.19 | 1538.10 | 1499.93 | 38.168 |
| 1999 | 311002 | 114877.722 | 111117.570 | 3760.151 | 1.209 | 3.273 | 4224.60 | 1560.48 | 1509.40 | 51.077 |
| 2000 | 303786 | 112379.704 | 108017.543 | 4362.161 | 1.436 | 3.882 | 4066.21 | 1504.22 | 1445.83 | 58.388 |
| 2001 | 296294 | 110024.943 | 105019.397 | 5005.546 | 1.689 | 4.549 | 3914.28 | 1453.52 | 1387.39 | 66.127 |
| 2002 | 291898 | 108289.173 | 102554.454 | 5734.719 | 1.965 | 5.296 | 3813.00 | 1414.56 | 1339.65 | 74.911 |
| 2003 | 285865 | 106539.023 | 100187.979 | 6351.044 | 2.222 | 5.961 | 3695.36 | 1377.22 | 1295.12 | 82.099 |
| 2004 | 274177 | 103037.251 | 96162.823 | 6874.428 | 2.507 | 6.672 | 3506.10 | 1317.61 | 1229.70 | 87.908 |
| 2005 | 272379 | 103044.562 | 95502.265 | 7542.297 | 2.769 | 7.319 | 3442.69 | 1302.41 | 1207.08 | 95.330 |
| 2006 | 264118 | 99672.555 | 91436.308 | 8236.247 | 3.118 | 8.263 | 3307.76 | 1248.28 | 1145.13 | 103.149 |
| 2007 | 258419 | 97839.152 | 89078.066 | 8761.086 | 3.390 | 8.955 | 3203.20 | 1212.75 | 1104.15 | 108.597 |
| 2008 | 259461 | 98075.852 | 88714.450 | 9361.402 | 3.608 | 9.545 | 3180.09 | 1202.07 | 1087.33 | 114.738 |
| 2009 | 253907 | 95617.338 | 85794.454 | 9822.884 | 3.869 | 10.273 | 3075.31 | 1158.11 | 1039.14 | 118.974 |
| 2010 | 251878 | 94607.988 | 84367.449 | 10240.54 | 4.066 | 10.824 | 3013.72 | 1131.98 | 1009.46 | 122.528 |
| 2011 | 251779 | 93633.119 | 82762.832 | 10870.29 | 4.317 | 11.609 | 2982.76 | 1109.25 | 980.471 | 128.778 |
| 2012 | 252643 | 92775.175 | 81346.552 | 11428.62 | 4.524 | 12.319 | 2960.61 | 1087.19 | 953.263 | 133.927 |
| 2013 | 256175 | 92345.780 | 80305.701 | 12040.08 | 4.700 | 13.038 | 2967.76 | 1069.82 | 930.333 | 139.483 |
| 2014 | 257393 | 91379.215 | 78924.444 | 12454.77 | 4.839 | 13.630 | 2947.05 | 1046.26 | 903.654 | 142.602 |
| 2015 | 259782 | 90750.242 | 77919.415 | 12830.83 | 4.939 | 14.139 | 3346.10 | 1168.90 | 1003.63 | 165.266 |
| 2016 | 261453 | 88811.996 | 75587.022 | 13224.97 | 5.058 | 14.891 | 3336.15 | 1133.24 | 964.494 | 168.751 |
| 2017 | 263685 | 87325.126 | 73749.469 | 13575.66 | 5.148 | 15.546 | 3333.85 | 1104.08 | 932.438 | 171.641 |
| 2018 | 258714 | 87612.144 | 73899.715 | 13712.43 | 5.300 | 15.651 | 3240.73 | 1097.46 | 925.690 | 171.766 |
| 2019 | 261438 | 87797.760 | 73726.752 | 14071.01 | 5.382 | 16.027 | 3242.99 | 1089.08 | 914.539 | 174.543 |
| 2020 | 262288 | 87221.042 | 72992.945 | 14228.10 | 5.425 | 16.313 | 3715.29 | 1235.48 | 1033.94 | 201.540 |
| 2021 | 255079 | 82001.292 | 67941.638 | 14059.65 | 5.512 | 17.146 | 3587.82 | 1153.39 | 955.633 | 197.756 |
| 2022 | 257097 | 81915.977 | 67474.072 | 14441.91 | 5.617 | 17.630 | 3588.53 | 1143.37 | 941.796 | 201.579 |
| 2023 | 259080 | 81810.810 | 67060.731 | 14750.08 | 5.693 | 18.029 | 3586.88 | 1132.64 | 928.435 | 204.210 |
| 2024 | 260719 | 81528.303 | 66657.718 | 14870.58 | 5.704 | 18.240 | 3579.65 | 1119.38 | 915.206 | 204.172 |
| 2025 | 257308 | 79599.213 | 64881.940 | 14717.27 | 5.720 | 18.489 | 4112.08 | 1272.09 | 1036.89 | 235.199 |
| 2026 | 251528 | 75082.462 | 60855.609 | 14226.85 | 5.656 | 18.948 | 4003.24 | 1194.99 | 968.558 | 226.430 |
| 2027 | 250988 | 74444.425 | 60137.888 | 14306.54 | 5.700 | 19.218 | 3976.53 | 1179.46 | 952.795 | 226.666 |
| 2028 | 250285 | 73699.585 | 59300.454 | 14399.13 | 5.753 | 19.538 | 3945.35 | 1161.76 | 934.780 | 226.980 |
| 2029 | 249525 | 72819.703 | 58532.836 | 14286.87 | 5.726 | 19.620 | 3910.99 | 1141.36 | 917.428 | 223.929 |
| 2030 | 240528 | 69309.277 | 55515.726 | 13793.55 | 5.735 | 19.901 | 4504.36 | 1297.95 | 1039.64 | 258.311 |
| 2031 | 235442 | 65433.368 | 52338.296 | 13095.07 | 5.562 | 20.013 | 4397.65 | 1222.18 | 977.588 | 244.593 |
| 2032 | 234017 | 64643.552 | 51618.159 | 13025.39 | 5.566 | 20.150 | 4356.12 | 1203.31 | 960.847 | 242.461 |
| 2033 | 232559 | 63793.514 | 51021.495 | 12772.02 | 5.492 | 20.021 | 4310.93 | 1182.54 | 945.782 | 236.754 |
| 2034 | 230963 | 63032.151 | 50484.587 | 12547.56 | 5.433 | 19.907 | 4260.75 | 1162.80 | 931.326 | 231.474 |
| 2035 | 215822 | 57653.301 | 46033.427 | 11619.87 | 5.384 | 20.155 | 4924.38 | 1315.47 | 1050.34 | 265.130 |
| 2036 | 208183 | 52988.733 | 42241.847 | 10746.89 | 5.162 | 20.281 | 4748.94 | 1208.75 | 963.595 | 245.151 |
| 2037 | 205491 | 52026.921 | 41651.392 | 10375.53 | 5.049 | 19.943 | 4682.40 | 1185.50 | 949.083 | 236.420 |
| 2038 | 202834 | 50943.211 | 40969.014 | 9974.198 | 4.917 | 19.579 | 4612.18 | 1158.38 | 931.583 | 226.800 |
| 2039 | 200251 | 50085.991 | 40568.170 | 9517.820 | 4.753 | 19.003 | 4538.65 | 1135.19 | 919.471 | 215.720 |

# Results for PHIM for Run 1 (Pessimistic_Analysis.RTF)

************************************************************************************

**Main Results for E-Component for Run: 1, Simulation: 1, COD: LUNG CANCER**

| Variable Parameter | Value |
|---|---|
| 1. Country | US (United States) |
| 2. Sex | M (Males) |
| 3. Year of start of process | 1990 |
| 4. Cause of Death | LUNG CANCER |
| 5. Effective Dose NULL | CC:1 |
| 6. Effective Dose Ecig | CC:1 Ecig:0.4 Dual:0.7 |
| Note: | Death rates are per million population |

-----------------------------------------------------------------------------------------

Results for Age Groups Combined and Years combined, Population unadjusted for deaths in Ecig scenario,

| Age Group:All | | NULL | Ecig | | | %Drop | | NULL | Ecig | |
|---|---|---|---|---|---|---|---|---|---|---|
| Year | Number Deaths | Attrib Deaths | Attrib Deaths | Drop Deaths | %Drop Deaths | Attrib Deaths | Death Rate | Attrib Rates | Attrib Rates | Drop Rates |
| All Comb | 3065988 | 2244988.113 | 2114227.357 | 130760.8 | 4.265 | 5.825 | 887.537 | 649.875 | 612.023 | 37.852 |

-----------------------------------------------------------------------------------------

Results for Age Groups Combined, Population unadjusted for deaths in Ecig scenario,

| Age Group:All | | NULL | Ecig | | | %Drop | | NULL | Ecig | |
|---|---|---|---|---|---|---|---|---|---|---|
| Year | Number Deaths | Attrib Deaths | Attrib Deaths | Drop Deaths | %Drop Deaths | Attrib Deaths | Death Rate | Attrib Rates | Attrib Rates | Drop Rates |
| 1990 | 78822 | 63385.736 | 63385.736 | 0.000 | 0.000 | 0.000 | 1249.50 | 1004.81 | 1004.81 | 0.000 |
| 1991 | 78462 | 62918.402 | 62905.561 | 12.841 | 0.016 | 0.020 | 1223.41 | 981.049 | 980.849 | 0.200 |
| 1992 | 77987 | 62366.766 | 62336.739 | 30.027 | 0.039 | 0.048 | 1195.35 | 955.930 | 955.469 | 0.460 |
| 1993 | 78542 | 62652.888 | 62592.993 | 59.895 | 0.076 | 0.096 | 1182.39 | 943.195 | 942.293 | 0.902 |
| 1994 | 77437 | 61565.227 | 61470.526 | 94.701 | 0.122 | 0.154 | 1144.00 | 909.520 | 908.121 | 1.399 |
| 1995 | 76904 | 60851.961 | 60707.978 | 143.983 | 0.187 | 0.237 | 1114.36 | 881.759 | 879.672 | 2.086 |
| 1996 | 76232 | 60122.922 | 59917.035 | 205.886 | 0.270 | 0.342 | 1087.44 | 857.643 | 854.706 | 2.937 |
| 1997 | 75473 | 59237.155 | 58964.385 | 272.770 | 0.361 | 0.460 | 1059.06 | 831.234 | 827.407 | 3.828 |
| 1998 | 75381 | 58906.696 | 58547.480 | 359.216 | 0.477 | 0.610 | 1040.37 | 813.001 | 808.043 | 4.958 |
| 1999 | 73159 | 56930.073 | 56486.341 | 443.732 | 0.607 | 0.779 | 993.781 | 773.329 | 767.302 | 6.028 |
| 2000 | 73784 | 57137.121 | 56573.793 | 563.328 | 0.763 | 0.986 | 987.608 | 764.787 | 757.247 | 7.540 |
| 2001 | 73294 | 56481.726 | 55785.480 | 696.247 | 0.950 | 1.233 | 968.273 | 746.169 | 736.971 | 9.198 |
| 2002 | 72442 | 55588.365 | 54754.397 | 833.968 | 1.151 | 1.500 | 946.293 | 726.138 | 715.244 | 10.894 |
| 2003 | 71942 | 54945.856 | 53973.182 | 972.674 | 1.352 | 1.770 | 929.989 | 710.281 | 697.707 | 12.574 |
| 2004 | 71301 | 54203.126 | 53053.422 | 1149.703 | 1.612 | 2.121 | 911.778 | 693.135 | 678.433 | 14.702 |
| 2005 | 70964 | 53744.603 | 52425.113 | 1319.490 | 1.859 | 2.455 | 896.937 | 679.296 | 662.618 | 16.677 |
| 2006 | 69658 | 52535.572 | 51019.725 | 1515.847 | 2.176 | 2.885 | 872.383 | 657.945 | 638.961 | 18.984 |
| 2007 | 68565 | 51492.124 | 49796.954 | 1695.170 | 2.472 | 3.292 | 849.888 | 638.264 | 617.251 | 21.012 |
| 2008 | 68226 | 51030.736 | 49125.174 | 1905.562 | 2.793 | 3.734 | 836.215 | 625.460 | 602.105 | 23.356 |
| 2009 | 67509 | 50276.199 | 48152.138 | 2124.061 | 3.146 | 4.225 | 817.667 | 608.943 | 583.217 | 25.727 |
| 2010 | 67196 | 49840.933 | 47530.604 | 2310.329 | 3.438 | 4.635 | 804.000 | 596.347 | 568.704 | 27.643 |
| 2011 | 66506 | 49072.841 | 46514.379 | 2558.462 | 3.847 | 5.214 | 787.880 | 581.354 | 551.045 | 30.309 |
| 2012 | 66155 | 48539.512 | 45747.649 | 2791.863 | 4.220 | 5.752 | 775.240 | 568.812 | 536.096 | 32.717 |
| 2013 | 65333 | 47675.741 | 44665.145 | 3010.596 | 4.608 | 6.315 | 756.876 | 552.318 | 517.441 | 34.877 |
| 2014 | 65033 | 47194.534 | 43995.722 | 3198.812 | 4.919 | 6.778 | 744.602 | 540.359 | 503.734 | 36.625 |
| 2015 | 64145 | 46305.842 | 42940.126 | 3365.716 | 5.247 | 7.268 | 826.215 | 596.439 | 553.087 | 43.352 |
| 2016 | 61618 | 44208.867 | 40773.496 | 3435.371 | 5.575 | 7.771 | 786.248 | 564.107 | 520.272 | 43.835 |
| 2017 | 59897 | 42702.624 | 39168.310 | 3534.313 | 5.901 | 8.277 | 757.297 | 539.903 | 495.217 | 44.685 |
| 2018 | 62468 | 44321.202 | 40366.295 | 3954.908 | 6.331 | 8.923 | 782.490 | 555.181 | 505.640 | 49.540 |
| 2019 | 63045 | 44493.088 | 40288.344 | 4204.744 | 6.669 | 9.450 | 782.035 | 551.912 | 499.754 | 52.157 |
| 2020 | 63466 | 44529.551 | 40112.455 | 4417.096 | 6.960 | 9.919 | 898.991 | 630.757 | 568.190 | 62.568 |
| 2021 | 57113 | 39852.556 | 35667.294 | 4185.262 | 7.328 | 10.502 | 803.323 | 560.546 | 501.678 | 58.868 |
| 2022 | 57440 | 39853.685 | 35414.822 | 4438.863 | 7.728 | 11.138 | 801.745 | 556.274 | 494.317 | 61.957 |
| 2023 | 57738 | 39847.050 | 35181.275 | 4665.775 | 8.081 | 11.709 | 799.366 | 551.670 | 487.074 | 64.596 |
| 2024 | 57942 | 39726.010 | 34902.789 | 4823.221 | 8.324 | 12.141 | 795.539 | 545.435 | 479.213 | 66.223 |
| 2025 | 57521 | 39207.348 | 34279.922 | 4927.426 | 8.566 | 12.568 | 919.257 | 626.579 | 547.833 | 78.746 |
| 2026 | 51428 | 34803.960 | 30313.564 | 4490.397 | 8.731 | 12.902 | 818.510 | 553.929 | 482.461 | 71.468 |
| 2027 | 51172 | 34484.595 | 29862.506 | 4622.088 | 9.032 | 13.403 | 810.740 | 546.357 | 473.127 | 73.230 |
| 2028 | 50870 | 34100.638 | 29359.620 | 4741.018 | 9.320 | 13.903 | 801.884 | 537.544 | 462.809 | 74.735 |
| 2029 | 50540 | 33669.838 | 28876.633 | 4793.205 | 9.484 | 14.236 | 792.146 | 527.732 | 452.605 | 75.127 |
| 2030 | 49109 | 32557.688 | 27800.697 | 4756.991 | 9.687 | 14.611 | 919.662 | 609.705 | 520.621 | 89.084 |
| 2031 | 43585 | 28646.393 | 24460.806 | 4185.587 | 9.603 | 14.611 | 814.096 | 535.065 | 456.885 | 78.179 |
| 2032 | 43163 | 28227.160 | 24021.736 | 4205.424 | 9.743 | 14.899 | 803.454 | 525.435 | 447.153 | 78.282 |
| 2033 | 42725 | 27786.623 | 23631.889 | 4154.734 | 9.724 | 14.952 | 791.996 | 515.079 | 438.063 | 77.016 |
| 2034 | 42256 | 27348.299 | 23228.643 | 4119.655 | 9.749 | 15.064 | 779.523 | 504.514 | 428.516 | 75.998 |
| 2035 | 39748 | 25570.077 | 21661.529 | 3908.549 | 9.833 | 15.286 | 906.933 | 583.430 | 494.249 | 89.181 |
| 2036 | 34030 | 21717.319 | 18420.584 | 3296.735 | 9.688 | 15.180 | 776.269 | 495.402 | 420.199 | 75.203 |
| 2037 | 33454 | 21256.024 | 18048.008 | 3208.016 | 9.589 | 15.092 | 762.289 | 484.347 | 411.248 | 73.099 |
| 2038 | 32883 | 20747.175 | 17653.749 | 3093.426 | 9.407 | 14.910 | 747.720 | 471.764 | 401.424 | 70.341 |
| 2039 | 32325 | 20327.688 | 17364.617 | 2963.070 | 9.166 | 14.577 | 732.644 | 460.724 | 393.566 | 67.158 |

# Results for PHIM for Run 1 (Pessimistic_Analysis.RTF)

**********************************************************************************

**Main Results for E-Component for Run: 1, Simulation: 1, COD: IHD**

| Variable Parameter | Value |
|---|---|
| 1. Country | US (United States) |
| 2. Sex | M (Males) |
| 3. Year of start of process | 1990 |
| 4. Cause of Death | IHD |
| 5. Effective Dose NULL | CC:1 |
| 6. Effective Dose Ecig | CC:1 Ecig:0.4 Dual:0.7 |
| Note: | Death rates are per million population |

-----------------------------------------------------------------------------------------

Results for Age Groups Combined and Years combined, Population unadjusted for deaths in Ecig scenario,

| Age Group:All | | NULL | Ecig | | | %Drop | | NULL | Ecig | |
|---|---|---|---|---|---|---|---|---|---|---|
| Year | Number | Attrib | Attrib | Drop | %Drop | Attrib | Death | Attrib | Attrib | Drop |
| | Deaths | Deaths | Deaths | Deaths | Deaths | Deaths | Rate | Rates | Rates | Rates |
| All Comb | 6360041 | 1098877.421 | 894508.089 | 204369.3 | 3.213 | 18.598 | 1841.09 | 318.101 | 258.941 | 59.160 |

-----------------------------------------------------------------------------------------

Results for Age Groups Combined, Population unadjusted for deaths in Ecig scenario,

| Age Group:All | | NULL | Ecig | | | %Drop | | NULL | Ecig | |
|---|---|---|---|---|---|---|---|---|---|---|
| Year | Number | Attrib | Attrib | Drop | %Drop | Attrib | Death | Attrib | Attrib | Drop |
| | Deaths | Deaths | Deaths | Deaths | Deaths | Deaths | Rate | Rates | Rates | Rates |
| 1990 | 171484 | 38511.725 | 38511.725 | 0.000 | 0.000 | 0.000 | 2718.40 | 610.497 | 610.497 | 0.000 |
| 1991 | 168044 | 37309.838 | 37213.274 | 96.564 | 0.057 | 0.259 | 2620.21 | 581.750 | 580.245 | 1.506 |
| 1992 | 164548 | 36137.488 | 35882.632 | 254.856 | 0.155 | 0.705 | 2522.12 | 553.899 | 549.993 | 3.906 |
| 1993 | 164362 | 35599.588 | 35122.407 | 477.181 | 0.290 | 1.340 | 2474.35 | 535.926 | 528.743 | 7.184 |
| 1994 | 159866 | 34336.757 | 33600.934 | 735.823 | 0.460 | 2.143 | 2361.75 | 507.267 | 496.396 | 10.871 |
| 1995 | 158083 | 33455.759 | 32409.461 | 1046.298 | 0.662 | 3.127 | 2290.66 | 484.782 | 469.620 | 15.161 |
| 1996 | 154875 | 32193.824 | 30819.797 | 1374.027 | 0.887 | 4.268 | 2209.26 | 459.239 | 439.639 | 19.600 |
| 1997 | 149970 | 30665.509 | 28972.696 | 1692.813 | 1.129 | 5.520 | 2104.43 | 430.308 | 406.554 | 23.754 |
| 1998 | 145407 | 29188.430 | 27196.817 | 1991.613 | 1.370 | 6.823 | 2006.83 | 402.844 | 375.357 | 27.487 |
| 1999 | 165952 | 33375.386 | 30563.488 | 2811.898 | 1.694 | 8.425 | 2254.27 | 453.366 | 415.170 | 38.196 |
| 2000 | 160260 | 32024.754 | 28836.473 | 3188.281 | 1.989 | 9.956 | 2145.10 | 428.655 | 385.980 | 42.676 |
| 2001 | 154444 | 30682.393 | 27089.949 | 3592.444 | 2.326 | 11.708 | 2040.33 | 405.339 | 357.880 | 47.459 |
| 2002 | 151887 | 30218.370 | 26147.623 | 4070.747 | 2.680 | 13.471 | 1984.06 | 394.736 | 341.560 | 53.175 |
| 2003 | 146733 | 29175.202 | 24744.808 | 4430.394 | 3.019 | 15.185 | 1896.81 | 377.146 | 319.874 | 57.271 |
| 2004 | 138381 | 27488.687 | 22829.241 | 4659.446 | 3.367 | 16.950 | 1769.58 | 351.518 | 291.934 | 59.584 |
| 2005 | 135881 | 27017.439 | 22017.260 | 5000.179 | 3.680 | 18.507 | 1717.44 | 341.482 | 278.283 | 63.199 |
| 2006 | 131922 | 26231.435 | 20869.756 | 5361.679 | 4.064 | 20.440 | 1652.16 | 328.517 | 261.369 | 67.149 |
| 2007 | 126502 | 25103.049 | 19567.055 | 5535.994 | 4.376 | 22.053 | 1568.04 | 311.161 | 242.541 | 68.621 |
| 2008 | 125859 | 24621.740 | 18864.858 | 5756.882 | 4.574 | 23.381 | 1542.60 | 301.777 | 231.218 | 70.559 |
| 2009 | 122428 | 23741.177 | 17882.082 | 5859.095 | 4.786 | 24.679 | 1482.84 | 287.552 | 216.587 | 70.965 |
| 2010 | 120537 | 23189.160 | 17213.506 | 5975.654 | 4.958 | 25.769 | 1442.22 | 277.458 | 205.960 | 71.499 |
| 2011 | 120221 | 22786.927 | 16634.659 | 6152.268 | 5.117 | 26.999 | 1424.23 | 269.951 | 197.067 | 72.884 |
| 2012 | 120927 | 22485.295 | 16172.104 | 6313.191 | 5.221 | 28.077 | 1417.09 | 263.495 | 189.513 | 73.981 |
| 2013 | 122648 | 22251.884 | 15776.420 | 6475.465 | 5.280 | 29.101 | 1420.86 | 257.786 | 182.768 | 75.018 |
| 2014 | 123229 | 21894.593 | 15347.945 | 6546.648 | 5.313 | 29.901 | 1410.92 | 250.685 | 175.728 | 74.957 |
| 2015 | 123774 | 21471.109 | 14903.180 | 6567.929 | 5.306 | 30.590 | 1594.26 | 276.557 | 191.959 | 84.598 |
| 2016 | 126218 | 21411.568 | 14707.434 | 6704.134 | 5.312 | 31.311 | 1610.55 | 273.213 | 187.667 | 85.545 |
| 2017 | 128265 | 21169.399 | 14415.109 | 6754.290 | 5.266 | 31.906 | 1621.69 | 267.651 | 182.255 | 85.397 |
| 2018 | 121429 | 19830.576 | 13444.914 | 6385.662 | 5.259 | 32.201 | 1521.05 | 248.404 | 168.415 | 79.989 |
| 2019 | 121832 | 19431.014 | 13126.288 | 6304.726 | 5.175 | 32.447 | 1511.26 | 241.031 | 162.824 | 78.207 |
| 2020 | 120811 | 18532.612 | 12431.147 | 6101.466 | 5.050 | 32.923 | 1711.27 | 262.513 | 176.086 | 86.427 |
| 2021 | 119407 | 18234.128 | 12230.942 | 6003.186 | 5.028 | 32.923 | 1679.51 | 256.472 | 172.034 | 84.438 |
| 2022 | 119438 | 17812.559 | 11915.385 | 5897.174 | 4.937 | 33.107 | 1667.10 | 248.626 | 166.314 | 82.312 |
| 2023 | 119445 | 17361.668 | 11605.861 | 5755.807 | 4.819 | 33.152 | 1653.68 | 240.367 | 160.679 | 79.687 |
| 2024 | 119305 | 16929.676 | 11365.986 | 5563.690 | 4.663 | 32.864 | 1638.05 | 232.443 | 156.054 | 76.389 |
| 2025 | 115685 | 15501.666 | 10310.285 | 5191.381 | 4.488 | 33.489 | 1848.78 | 247.735 | 164.770 | 82.964 |
| 2026 | 115073 | 15523.814 | 10471.173 | 5052.641 | 4.391 | 32.548 | 1831.46 | 247.072 | 166.656 | 80.416 |
| 2027 | 113989 | 15073.070 | 10243.633 | 4829.437 | 4.237 | 32.040 | 1805.98 | 238.810 | 162.295 | 76.515 |
| 2028 | 112855 | 14636.710 | 10009.251 | 4627.458 | 4.100 | 31.615 | 1778.98 | 230.725 | 157.780 | 72.945 |
| 2029 | 111718 | 14180.166 | 9818.836 | 4361.330 | 3.904 | 30.757 | 1751.04 | 222.256 | 153.898 | 68.358 |
| 2030 | 104851 | 12111.772 | 8242.260 | 3869.512 | 3.690 | 31.948 | 1963.54 | 226.816 | 154.352 | 72.464 |
| 2031 | 105289 | 12417.367 | 8640.278 | 3777.088 | 3.587 | 30.418 | 1966.62 | 231.935 | 161.385 | 70.549 |
| 2032 | 103879 | 12054.064 | 8469.321 | 3584.743 | 3.451 | 29.739 | 1933.67 | 224.381 | 157.652 | 66.728 |
| 2033 | 102485 | 11672.389 | 8304.301 | 3368.088 | 3.286 | 28.855 | 1899.76 | 216.370 | 153.936 | 62.434 |
| 2034 | 101070 | 11365.762 | 8245.972 | 3119.790 | 3.087 | 27.449 | 1864.51 | 209.673 | 152.119 | 57.553 |
| 2035 | 90820 | 8580.360 | 6035.279 | 2545.081 | 2.802 | 29.662 | 2072.23 | 195.777 | 137.706 | 58.071 |
| 2036 | 92117 | 8948.841 | 6463.404 | 2485.436 | 2.698 | 27.774 | 2101.31 | 204.135 | 147.439 | 56.696 |
| 2037 | 90327 | 8624.570 | 6373.458 | 2251.112 | 2.492 | 26.101 | 2058.21 | 196.522 | 145.228 | 51.295 |
| 2038 | 88590 | 8315.017 | 6255.073 | 2059.944 | 2.325 | 24.774 | 2014.42 | 189.073 | 142.232 | 46.840 |
| 2039 | 86921 | 8001.132 | 6192.347 | 1808.784 | 2.081 | 22.607 | 1970.05 | 181.344 | 140.349 | 40.996 |

# Results for PHIM for Run 1 (Pessimistic_Analysis.RTF)

************************************************************************************
**Main Results for E-Component for Run: 1, Simulation: 1, COD: STROKE**

| Variable Parameter | Value |
|---|---|
| 1. Country | US (United States) |
| 2. Sex | M (Males) |
| 3. Year of start of process | 1990 |
| 4. Cause of Death | STROKE |
| 5. Effective Dose NULL | CC:1 |
| 6. Effective Dose Ecig | CC:1 Ecig:0.4 Dual:0.7 |
| Note: | Death rates are per million population |

-----------------------------------------------------------------------------------------
Results for Age Groups Combined and Years combined, Population unadjusted for deaths in Ecig scenario,

| Age Group:All | | NULL | Ecig | | | %Drop | | NULL | Ecig | |
|---|---|---|---|---|---|---|---|---|---|---|
| Year | Number Deaths | Attrib Deaths | Attrib Deaths | Drop Deaths | %Drop Deaths | Attrib Deaths | Death Rate | Attrib Rates | Attrib Rates | Drop Rates |
| All Comb | 1489699 | 149918.462 | 120982.566 | 28935.90 | 1.942 | 19.301 | 431.236 | 43.398 | 35.022 | 8.376 |

-----------------------------------------------------------------------------------------
Results for Age Groups Combined, Population unadjusted for deaths in Ecig scenario,

| Age Group:All | | NULL | Ecig | | | %Drop | | NULL | Ecig | |
|---|---|---|---|---|---|---|---|---|---|---|
| Year | Number Deaths | Attrib Deaths | Attrib Deaths | Drop Deaths | %Drop Deaths | Attrib Deaths | Death Rate | Attrib Rates | Attrib Rates | Drop Rates |
| 1990 | 32862 | 4613.166 | 4613.166 | 0.000 | 0.000 | 0.000 | 520.936 | 73.129 | 73.129 | 0.000 |
| 1991 | 32619 | 4555.566 | 4544.170 | 11.396 | 0.035 | 0.250 | 508.609 | 71.032 | 70.855 | 0.178 |
| 1992 | 32100 | 4453.572 | 4424.763 | 28.809 | 0.090 | 0.647 | 492.014 | 68.262 | 67.821 | 0.442 |
| 1993 | 32896 | 4462.473 | 4406.237 | 56.237 | 0.171 | 1.260 | 495.226 | 67.179 | 66.333 | 0.847 |
| 1994 | 33323 | 4499.513 | 4409.321 | 90.192 | 0.271 | 2.004 | 492.290 | 66.473 | 65.140 | 1.332 |
| 1995 | 33569 | 4472.774 | 4339.160 | 133.615 | 0.398 | 2.987 | 486.422 | 64.812 | 62.875 | 1.936 |
| 1996 | 33870 | 4406.878 | 4225.271 | 181.607 | 0.536 | 4.121 | 483.150 | 62.863 | 60.273 | 2.591 |
| 1997 | 33516 | 4288.158 | 4056.440 | 231.718 | 0.691 | 5.404 | 470.307 | 60.173 | 56.921 | 3.252 |
| 1998 | 32795 | 4120.148 | 3842.479 | 277.669 | 0.847 | 6.739 | 452.620 | 56.864 | 53.032 | 3.832 |
| 1999 | 33375 | 3982.443 | 3657.649 | 324.793 | 0.973 | 8.156 | 453.361 | 54.097 | 49.685 | 4.412 |
| 2000 | 33459 | 4021.411 | 3630.953 | 390.458 | 1.167 | 9.709 | 447.853 | 53.827 | 48.601 | 5.226 |
| 2001 | 32275 | 3848.092 | 3409.250 | 438.843 | 1.360 | 11.404 | 426.379 | 50.836 | 45.039 | 5.797 |
| 2002 | 31586 | 3783.081 | 3288.031 | 495.050 | 1.567 | 13.086 | 412.601 | 49.418 | 42.951 | 6.467 |
| 2003 | 30985 | 3754.415 | 3196.398 | 558.017 | 1.801 | 14.863 | 400.541 | 48.533 | 41.320 | 7.213 |
| 2004 | 29884 | 3666.274 | 3055.630 | 610.645 | 2.043 | 16.656 | 382.149 | 46.883 | 39.075 | 7.809 |
| 2005 | 28708 | 3589.688 | 2933.114 | 656.574 | 2.287 | 18.291 | 362.850 | 45.371 | 37.073 | 8.299 |
| 2006 | 28188 | 3596.985 | 2865.974 | 731.011 | 2.593 | 20.323 | 353.021 | 45.048 | 35.893 | 9.155 |
| 2007 | 28026 | 3560.798 | 2778.148 | 782.650 | 2.793 | 21.980 | 347.392 | 44.137 | 34.436 | 9.701 |
| 2008 | 27128 | 3417.001 | 2615.667 | 801.334 | 2.954 | 23.451 | 332.495 | 41.881 | 32.059 | 9.822 |
| 2009 | 27068 | 3394.390 | 2552.424 | 841.965 | 3.111 | 24.805 | 327.847 | 41.113 | 30.915 | 10.198 |
| 2010 | 26911 | 3340.137 | 2473.219 | 866.918 | 3.221 | 25.955 | 321.990 | 39.965 | 29.592 | 10.373 |
| 2011 | 26959 | 3285.435 | 2396.669 | 888.766 | 3.297 | 27.052 | 319.377 | 38.922 | 28.393 | 10.529 |
| 2012 | 27057 | 3259.209 | 2338.944 | 920.266 | 3.401 | 28.236 | 317.068 | 38.193 | 27.409 | 10.784 |
| 2013 | 27904 | 3274.703 | 2311.747 | 962.955 | 3.451 | 29.406 | 323.265 | 37.937 | 26.781 | 11.156 |
| 2014 | 28792 | 3298.083 | 2300.359 | 997.724 | 3.465 | 30.252 | 329.657 | 37.762 | 26.338 | 11.424 |
| 2015 | 29560 | 3244.406 | 2238.171 | 1006.235 | 3.404 | 31.014 | 380.745 | 41.789 | 28.829 | 12.961 |
| 2016 | 30513 | 3314.836 | 2262.762 | 1052.074 | 3.448 | 31.738 | 389.347 | 42.297 | 28.873 | 13.425 |
| 2017 | 31416 | 3333.314 | 2251.377 | 1081.936 | 3.444 | 32.458 | 397.202 | 42.144 | 28.465 | 13.679 |
| 2018 | 29348 | 2925.242 | 1962.889 | 962.352 | 3.279 | 32.898 | 367.626 | 36.642 | 24.588 | 12.055 |
| 2019 | 29598 | 2864.628 | 1914.447 | 950.181 | 3.210 | 33.169 | 367.149 | 35.534 | 23.748 | 11.786 |
| 2020 | 29592 | 2730.366 | 1813.877 | 916.489 | 3.097 | 33.567 | 419.174 | 38.675 | 25.693 | 12.982 |
| 2021 | 30097 | 2647.911 | 1754.873 | 893.037 | 2.967 | 33.726 | 423.328 | 37.244 | 24.683 | 12.561 |
| 2022 | 30304 | 2586.165 | 1709.787 | 876.377 | 2.892 | 33.887 | 422.974 | 36.097 | 23.865 | 12.232 |
| 2023 | 30505 | 2517.960 | 1662.318 | 855.642 | 2.805 | 33.982 | 422.337 | 34.860 | 23.014 | 11.846 |
| 2024 | 30661 | 2454.161 | 1626.349 | 827.812 | 2.700 | 33.731 | 420.968 | 33.695 | 22.330 | 11.366 |
| 2025 | 30254 | 2275.364 | 1500.328 | 775.036 | 2.562 | 34.062 | 483.488 | 36.363 | 23.977 | 12.386 |
| 2026 | 30685 | 2183.166 | 1452.374 | 730.792 | 2.382 | 33.474 | 488.380 | 34.747 | 23.116 | 11.631 |
| 2027 | 30561 | 2112.345 | 1418.703 | 693.642 | 2.270 | 32.838 | 484.196 | 33.467 | 22.477 | 10.990 |
| 2028 | 30412 | 2046.677 | 1387.748 | 658.928 | 2.167 | 32.195 | 479.392 | 32.263 | 21.876 | 10.387 |
| 2029 | 30254 | 1977.671 | 1357.151 | 620.521 | 2.051 | 31.376 | 474.195 | 30.997 | 21.272 | 9.726 |
| 2030 | 29330 | 1744.457 | 1195.940 | 548.517 | 1.870 | 31.443 | 549.260 | 32.668 | 22.396 | 10.272 |
| 2031 | 28976 | 1653.738 | 1149.489 | 504.249 | 1.740 | 30.491 | 541.223 | 30.889 | 21.470 | 9.418 |
| 2032 | 28724 | 1603.120 | 1126.212 | 476.908 | 1.660 | 29.749 | 534.690 | 29.841 | 20.964 | 8.877 |
| 2033 | 28464 | 1548.245 | 1105.974 | 442.271 | 1.554 | 28.566 | 527.627 | 28.700 | 20.501 | 8.198 |
| 2034 | 28178 | 1511.985 | 1099.399 | 412.585 | 1.464 | 27.288 | 519.826 | 27.893 | 20.281 | 7.611 |
| 2035 | 26737 | 1239.866 | 898.724 | 341.142 | 1.276 | 27.514 | 610.065 | 28.290 | 20.506 | 7.784 |
| 2036 | 25591 | 1182.522 | 873.829 | 308.693 | 1.206 | 26.105 | 583.762 | 26.975 | 19.933 | 7.042 |
| 2037 | 25143 | 1134.483 | 864.069 | 270.414 | 1.076 | 23.836 | 572.916 | 25.851 | 19.689 | 6.162 |
| 2038 | 24693 | 1093.537 | 850.679 | 242.857 | 0.984 | 22.208 | 561.491 | 24.866 | 19.343 | 5.522 |
| 2039 | 24247 | 1047.905 | 839.914 | 207.991 | 0.858 | 19.848 | 549.553 | 23.751 | 19.037 | 4.714 |

# Results for PHIM for Run 1 (Pessimistic_Analysis.RTF)

********************************************************************************
**Main Results for E-Component for Run: 1, Simulation: 1, COD: COPD**

| Variable Parameter | Value |
|---|---|
| 1. Country | US (United States) |
| 2. Sex | M (Males) |
| 3. Year of start of process | 1990 |
| 4. Cause of Death | COPD |
| 5. Effective Dose NULL | CC:1 |
| 6. Effective Dose Ecig | CC:1 Ecig:0.4 Dual:0.7 |
| Note: | Death rates are per million population |

-----------------------------------------------------------------------------------------
Results for Age Groups Combined and Years combined, Population unadjusted for deaths in Ecig scenario,

| Age Group:All Year | Number Deaths | NULL Attrib Deaths | Ecig Attrib Deaths | Drop Deaths | %Drop Deaths | %Drop Attrib Deaths | Death Rate | NULL Attrib Rates | Ecig Attrib Rates | Drop Rates |
|---|---|---|---|---|---|---|---|---|---|---|
| All Comb | 2233143 | 1015606.587 | 907366.185 | 108240.4 | 4.847 | 10.658 | 646.447 | 293.996 | 262.663 | 31.333 |

-----------------------------------------------------------------------------------------
Results for Age Groups Combined, Population unadjusted for deaths in Ecig scenario,

| Age Group:All Year | Number Deaths | NULL Attrib Deaths | Ecig Attrib Deaths | Drop Deaths | %Drop Deaths | %Drop Attrib Deaths | Death Rate | NULL Attrib Rates | Ecig Attrib Rates | Drop Rates |
|---|---|---|---|---|---|---|---|---|---|---|
| 1990 | 33993 | 19262.437 | 19262.437 | 0.000 | 0.000 | 0.000 | 538.865 | 305.353 | 305.353 | 0.000 |
| 1991 | 34092 | 19215.636 | 19210.159 | 5.477 | 0.016 | 0.029 | 531.576 | 299.618 | 299.533 | 0.085 |
| 1992 | 33942 | 19056.189 | 19045.530 | 10.660 | 0.031 | 0.056 | 520.248 | 292.085 | 291.921 | 0.163 |
| 1993 | 35913 | 20095.670 | 20073.611 | 22.059 | 0.061 | 0.110 | 540.645 | 302.526 | 302.194 | 0.332 |
| 1994 | 35074 | 19524.095 | 19490.785 | 33.309 | 0.095 | 0.171 | 518.158 | 288.435 | 287.943 | 0.492 |
| 1995 | 34669 | 19116.002 | 19064.083 | 51.919 | 0.150 | 0.272 | 502.362 | 276.995 | 276.243 | 0.752 |
| 1996 | 34582 | 18975.021 | 18904.122 | 70.899 | 0.205 | 0.374 | 493.306 | 270.675 | 269.664 | 1.011 |
| 1997 | 35267 | 19169.025 | 19069.727 | 99.298 | 0.282 | 0.518 | 494.878 | 268.986 | 267.592 | 1.393 |
| 1998 | 35675 | 19228.937 | 19091.958 | 136.979 | 0.384 | 0.712 | 492.368 | 265.388 | 263.498 | 1.891 |
| 1999 | 38516 | 20589.820 | 20410.092 | 179.729 | 0.467 | 0.873 | 523.195 | 279.689 | 277.248 | 2.441 |
| 2000 | 36283 | 19196.417 | 18976.324 | 220.094 | 0.607 | 1.147 | 485.652 | 256.946 | 254.000 | 2.946 |
| 2001 | 36281 | 19012.731 | 18734.719 | 278.012 | 0.766 | 1.462 | 479.301 | 251.174 | 247.501 | 3.673 |
| 2002 | 35983 | 18699.357 | 18364.403 | 334.954 | 0.931 | 1.791 | 470.038 | 244.265 | 239.890 | 4.375 |
| 2003 | 36205 | 18663.550 | 18273.590 | 389.959 | 1.077 | 2.089 | 468.019 | 241.262 | 236.221 | 5.041 |
| 2004 | 34611 | 17679.164 | 17224.531 | 454.633 | 1.314 | 2.572 | 442.596 | 226.076 | 220.263 | 5.814 |
| 2005 | 36826 | 18692.832 | 18126.778 | 566.054 | 1.537 | 3.028 | 465.456 | 236.265 | 229.110 | 7.155 |
| 2006 | 34350 | 17308.564 | 16680.854 | 627.710 | 1.827 | 3.627 | 430.192 | 216.769 | 208.908 | 7.861 |
| 2007 | 35326 | 17683.181 | 16935.909 | 747.272 | 2.115 | 4.226 | 437.879 | 219.189 | 209.927 | 9.263 |
| 2008 | 38248 | 19006.374 | 18108.751 | 897.624 | 2.347 | 4.723 | 468.788 | 232.952 | 221.951 | 11.002 |
| 2009 | 36902 | 18205.573 | 17207.810 | 997.763 | 2.704 | 5.481 | 446.956 | 220.505 | 208.420 | 12.085 |
| 2010 | 37234 | 18237.758 | 17150.120 | 1087.638 | 2.921 | 5.964 | 445.505 | 218.215 | 205.201 | 13.014 |
| 2011 | 38093 | 18487.916 | 17217.126 | 1270.790 | 3.336 | 6.874 | 451.279 | 219.022 | 203.967 | 15.055 |
| 2012 | 38504 | 18491.158 | 17087.855 | 1403.303 | 3.645 | 7.589 | 451.211 | 216.689 | 200.245 | 16.445 |
| 2013 | 40290 | 19143.452 | 17552.389 | 1591.063 | 3.949 | 8.311 | 466.755 | 221.775 | 203.343 | 18.432 |
| 2014 | 40339 | 18992.005 | 17280.418 | 1711.587 | 4.243 | 9.012 | 461.866 | 217.451 | 197.854 | 19.597 |
| 2015 | 42303 | 19728.885 | 17837.937 | 1890.947 | 4.470 | 9.585 | 544.880 | 254.116 | 229.760 | 24.356 |
| 2016 | 43104 | 19876.724 | 17843.329 | 2033.395 | 4.717 | 10.230 | 550.009 | 253.628 | 227.682 | 25.946 |
| 2017 | 44107 | 20119.790 | 17914.672 | 2205.118 | 4.999 | 10.960 | 557.659 | 254.381 | 226.501 | 27.880 |
| 2018 | 45469 | 20535.123 | 18125.617 | 2409.506 | 5.299 | 11.734 | 569.564 | 257.229 | 227.047 | 30.182 |
| 2019 | 46963 | 21009.029 | 18397.672 | 2611.357 | 5.560 | 12.430 | 582.552 | 260.605 | 228.213 | 32.392 |
| 2020 | 48419 | 21428.512 | 18635.465 | 2793.047 | 5.768 | 13.034 | 685.853 | 303.533 | 263.970 | 39.563 |
| 2021 | 48463 | 21266.697 | 18288.529 | 2978.168 | 6.145 | 14.004 | 681.652 | 299.127 | 257.237 | 41.889 |
| 2022 | 49915 | 21663.568 | 18434.077 | 3229.491 | 6.470 | 14.907 | 696.712 | 302.378 | 257.301 | 45.077 |
| 2023 | 51391 | 22084.131 | 18611.277 | 3472.854 | 6.758 | 15.726 | 711.491 | 305.748 | 257.667 | 48.081 |
| 2024 | 52811 | 22418.456 | 18762.595 | 3655.861 | 6.923 | 16.307 | 725.093 | 307.804 | 257.609 | 50.195 |
| 2025 | 53848 | 22614.834 | 18791.405 | 3823.429 | 7.100 | 16.907 | 860.553 | 361.411 | 300.308 | 61.103 |
| 2026 | 54342 | 22571.522 | 18618.498 | 3953.023 | 7.274 | 17.513 | 864.886 | 359.241 | 296.326 | 62.915 |
| 2027 | 55267 | 22774.416 | 18613.045 | 4161.371 | 7.530 | 18.272 | 875.619 | 360.827 | 294.896 | 65.931 |
| 2028 | 56149 | 22915.561 | 18543.835 | 4371.726 | 7.786 | 19.078 | 885.102 | 361.228 | 292.315 | 68.913 |
| 2029 | 57014 | 22992.027 | 18480.217 | 4511.811 | 7.914 | 19.623 | 893.616 | 360.371 | 289.654 | 70.717 |
| 2030 | 57238 | 22895.360 | 18276.829 | 4618.531 | 8.069 | 20.172 | 1071.90 | 428.760 | 342.269 | 86.491 |
| 2031 | 57592 | 22715.871 | 18087.723 | 4628.148 | 8.036 | 20.374 | 1075.71 | 424.293 | 337.847 | 86.446 |
| 2032 | 58251 | 22759.207 | 18000.889 | 4758.318 | 8.169 | 20.907 | 1084.31 | 423.652 | 335.078 | 88.574 |
| 2033 | 58885 | 22786.258 | 17979.332 | 4806.925 | 8.163 | 21.096 | 1091.54 | 422.387 | 333.282 | 89.106 |
| 2034 | 59460 | 22806.106 | 17910.572 | 4895.534 | 8.233 | 21.466 | 1096.89 | 420.721 | 330.409 | 90.311 |
| 2035 | 58516 | 22262.998 | 17437.896 | 4825.102 | 8.246 | 21.673 | 1335.15 | 507.973 | 397.879 | 110.094 |
| 2036 | 56446 | 21140.051 | 16484.031 | 4656.020 | 8.249 | 22.025 | 1287.60 | 482.234 | 376.023 | 106.210 |
| 2037 | 56568 | 21011.845 | 16365.857 | 4645.988 | 8.213 | 22.111 | 1288.98 | 478.783 | 372.918 | 105.865 |
| 2038 | 56668 | 20787.483 | 16209.512 | 4577.971 | 8.079 | 22.023 | 1288.55 | 472.681 | 368.583 | 104.097 |
| 2039 | 56758 | 20709.267 | 16171.292 | 4537.975 | 7.995 | 21.913 | 1286.40 | 469.372 | 366.520 | 102.852 |

# Results for PHIM for Run 1 (Pessimistic_Analysis.RTF)

************************************************************************************

**Main Results for E-Component for Run: 1, Simulation: 1, COD: Four Diseases Combined, Adjusted**

| Variable Parameter | Value |
|---|---|
| 1. Country | US (United States) |
| 2. Sex | M (Males) |
| 3. Year of start of process | 1990 |
| 4. Cause of Death | Four Diseases Combined |
| 5. Effective Dose NULL | CC:1 |
| 6. Effective Dose Ecig | CC:1 Ecig:0.4 Dual:0.7 |
| Note: | Death rates are per million population |

-------------------------------------------------------------------------------------

Results for Age Groups Combined and Years combined, Population and Number deaths adjusted for Ecig scenario,

| Age Group:All | | NULL | Ecig | | | %Drop | | NULL | Ecig | |
|---|---|---|---|---|---|---|---|---|---|---|
| Year | Number | Attrib | Attrib | Drop | %Drop | Attrib | Death | Attrib | Attrib | Drop |
| | Deaths | Deaths | Deaths | Deaths | Deaths | Deaths | Rate | Rates | Rates | Rates |
| All Comb | 13188829 | 4509390.583 | 4046974.432 | 462416.2 | 3.506 | 10.255 | 3807.87 | 1305.37 | 1168.44 | 136.933 |

-------------------------------------------------------------------------------------

Results for Age Groups Combined, Population and Number deaths adjusted for Ecig scenario,

| Age Group:All | | NULL | Ecig | | | %Drop | | NULL | Ecig | |
|---|---|---|---|---|---|---|---|---|---|---|
| Year | Number | Attrib | Attrib | Drop | %Drop | Attrib | Death | Attrib | Attrib | Drop |
| | Deaths | Deaths | Deaths | Deaths | Deaths | Deaths | Rate | Rates | Rates | Rates |
| 1990 | 317161 | 125773.065 | 125773.065 | 0.000 | 0.000 | 0.000 | 5027.71 | 1993.78 | 1993.78 | 0.000 |
| 1991 | 313217 | 123999.442 | 123873.164 | 126.278 | 0.040 | 0.102 | 4883.81 | 1933.45 | 1931.48 | 1.969 |
| 1992 | 308578 | 122014.015 | 121689.944 | 324.071 | 0.105 | 0.266 | 4729.73 | 1870.18 | 1865.20 | 4.971 |
| 1993 | 311715 | 122810.618 | 122196.117 | 614.501 | 0.197 | 0.500 | 4692.61 | 1848.83 | 1839.56 | 9.264 |
| 1994 | 305704 | 119925.592 | 118973.511 | 952.081 | 0.311 | 0.794 | 4516.18 | 1771.69 | 1757.60 | 14.094 |
| 1995 | 303233 | 117896.496 | 116524.149 | 1372.347 | 0.453 | 1.164 | 4393.78 | 1708.35 | 1688.41 | 19.938 |
| 1996 | 299572 | 115698.645 | 113871.915 | 1826.730 | 0.610 | 1.579 | 4273.12 | 1650.42 | 1624.28 | 26.142 |
| 1997 | 294246 | 113359.847 | 111071.742 | 2288.104 | 0.778 | 2.018 | 4128.62 | 1590.70 | 1558.47 | 32.230 |
| 1998 | 289286 | 111444.212 | 108690.733 | 2753.479 | 0.952 | 2.471 | 3992.13 | 1538.10 | 1499.93 | 38.170 |
| 1999 | 311045 | 114877.722 | 111134.871 | 3742.850 | 1.203 | 3.258 | 4224.54 | 1560.48 | 1509.41 | 51.072 |
| 2000 | 303844 | 112379.704 | 108040.828 | 4338.876 | 1.428 | 3.861 | 4066.15 | 1504.22 | 1445.84 | 58.374 |
| 2001 | 296370 | 110024.943 | 105049.391 | 4975.551 | 1.679 | 4.522 | 3914.23 | 1453.52 | 1387.42 | 66.103 |
| 2002 | 291996 | 108289.173 | 102592.439 | 5696.734 | 1.951 | 5.261 | 3812.98 | 1414.56 | 1339.69 | 74.869 |
| 2003 | 285987 | 106539.023 | 100234.729 | 6304.294 | 2.204 | 5.917 | 3695.38 | 1377.22 | 1295.18 | 82.038 |
| 2004 | 274322 | 103037.251 | 96217.977 | 6819.275 | 2.486 | 6.618 | 3506.17 | 1317.61 | 1229.79 | 87.827 |
| 2005 | 272554 | 103044.562 | 95568.317 | 7476.246 | 2.743 | 7.255 | 3442.83 | 1302.41 | 1207.19 | 95.222 |
| 2006 | 264323 | 99672.555 | 91511.785 | 8160.770 | 3.087 | 8.188 | 3307.99 | 1248.28 | 1145.27 | 103.013 |
| 2007 | 258658 | 97839.152 | 89164.799 | 8674.353 | 3.354 | 8.866 | 3203.53 | 1212.75 | 1104.33 | 108.426 |
| 2008 | 259742 | 98075.852 | 88814.850 | 9261.002 | 3.565 | 9.443 | 3180.55 | 1202.07 | 1087.54 | 114.527 |
| 2009 | 254226 | 95617.338 | 85906.785 | 9710.553 | 3.820 | 10.156 | 3075.91 | 1158.11 | 1039.40 | 118.718 |
| 2010 | 252241 | 94607.988 | 84493.196 | 10114.79 | 4.010 | 10.691 | 3014.46 | 1131.98 | 1009.76 | 122.227 |
| 2011 | 252190 | 93633.119 | 82902.289 | 10730.83 | 4.255 | 11.461 | 2983.68 | 1109.25 | 980.826 | 128.423 |
| 2012 | 253108 | 92775.175 | 81500.156 | 11275.02 | 4.455 | 12.153 | 2961.73 | 1087.19 | 953.671 | 133.518 |
| 2013 | 256702 | 92345.780 | 80474.763 | 11871.02 | 4.624 | 12.855 | 2969.11 | 1069.82 | 930.802 | 139.014 |
| 2014 | 257981 | 91379.215 | 79108.808 | 12270.41 | 4.756 | 13.428 | 2948.64 | 1046.26 | 904.187 | 142.070 |
| 2015 | 260440 | 90750.242 | 78120.607 | 12629.63 | 4.849 | 13.917 | 3347.44 | 1168.90 | 1004.08 | 164.818 |
| 2016 | 262179 | 88811.996 | 75800.576 | 13011.42 | 4.963 | 14.651 | 3337.73 | 1133.24 | 964.994 | 168.251 |
| 2017 | 264484 | 87325.126 | 73976.048 | 13349.08 | 5.047 | 15.287 | 3335.68 | 1104.08 | 932.989 | 171.090 |
| 2018 | 259560 | 87612.144 | 74144.541 | 13467.60 | 5.189 | 15.372 | 3242.71 | 1097.46 | 926.297 | 171.160 |
| 2019 | 262361 | 87797.760 | 73990.170 | 13807.59 | 5.263 | 15.727 | 3245.26 | 1089.08 | 915.216 | 173.866 |
| 2020 | 263292 | 87221.042 | 73275.511 | 13945.53 | 5.297 | 15.989 | 3716.88 | 1235.48 | 1034.43 | 201.052 |
| 2021 | 256124 | 82001.292 | 68222.606 | 13778.69 | 5.380 | 16.803 | 3589.59 | 1153.39 | 956.143 | 197.246 |
| 2022 | 258220 | 81915.977 | 67771.642 | 14144.34 | 5.478 | 17.267 | 3590.59 | 1143.37 | 942.373 | 201.002 |
| 2023 | 260286 | 81810.810 | 67375.435 | 14435.38 | 5.546 | 17.645 | 3589.25 | 1132.64 | 929.082 | 203.563 |
| 2024 | 262010 | 81528.303 | 66990.158 | 14538.14 | 5.549 | 17.832 | 3582.37 | 1119.38 | 915.932 | 203.447 |
| 2025 | 258680 | 79599.213 | 65230.620 | 14368.59 | 5.555 | 18.051 | 4113.68 | 1272.09 | 1037.34 | 234.751 |
| 2026 | 252939 | 75082.462 | 61198.821 | 13883.64 | 5.489 | 18.491 | 4004.99 | 1194.99 | 969.011 | 225.977 |
| 2027 | 252474 | 74444.425 | 60495.436 | 13948.99 | 5.525 | 18.737 | 3978.63 | 1179.46 | 953.322 | 226.139 |
| 2028 | 251847 | 73699.585 | 59671.954 | 14027.63 | 5.570 | 19.034 | 3947.84 | 1161.76 | 935.391 | 226.368 |
| 2029 | 251165 | 72819.703 | 58918.002 | 13901.70 | 5.535 | 19.091 | 3913.90 | 1141.36 | 918.119 | 223.237 |
| 2030 | 242223 | 69309.277 | 55909.036 | 13400.24 | 5.532 | 19.334 | 4505.84 | 1297.95 | 1040.02 | 257.925 |
| 2031 | 237165 | 65433.368 | 52720.322 | 12713.05 | 5.360 | 19.429 | 4399.21 | 1222.18 | 977.917 | 244.264 |
| 2032 | 235805 | 64643.552 | 52011.118 | 12632.43 | 5.357 | 19.542 | 4358.06 | 1203.31 | 961.247 | 242.061 |
| 2033 | 234412 | 63793.514 | 51426.052 | 12367.46 | 5.276 | 19.387 | 4313.27 | 1182.54 | 946.258 | 236.278 |
| 2034 | 232879 | 63032.151 | 50900.829 | 12131.32 | 5.209 | 19.246 | 4263.51 | 1162.80 | 931.882 | 230.918 |
| 2035 | 217731 | 57653.301 | 46441.622 | 11211.68 | 5.149 | 19.447 | 4925.81 | 1315.47 | 1050.67 | 264.803 |
| 2036 | 210077 | 52988.733 | 42625.303 | 10363.43 | 4.933 | 19.558 | 4750.33 | 1208.75 | 963.855 | 244.891 |
| 2037 | 207424 | 52026.921 | 42041.855 | 9985.066 | 4.814 | 19.192 | 4684.11 | 1185.50 | 949.403 | 236.100 |
| 2038 | 204802 | 50943.211 | 41364.952 | 9578.260 | 4.677 | 18.802 | 4614.24 | 1158.38 | 931.964 | 226.419 |
| 2039 | 202252 | 50085.991 | 40970.894 | 9115.097 | 4.507 | 18.199 | 4541.07 | 1135.19 | 919.898 | 215.293 |

# Results for PHIM for Run 1 (Pessimistic_Analysis.RTF)

***************************************************************************************

**Main Results for E-Component for Run: 1, Simulation: 1, COD: LUNG CANCER, Adjusted**

| Variable Parameter | Value |
|---|---|
| 1. Country | US (United States) |
| 2. Sex | M (Males) |
| 3. Year of start of process | 1990 |
| 4. Cause of Death | LUNG CANCER |
| 5. Effective Dose NULL | CC:1 |
| 6. Effective Dose Ecig | CC:1 Ecig:0.4 Dual:0.7 |
| Note: | Death rates are per million population |

-----------------------------------------------------------------------------------------

Results for Age Groups Combined and Years combined, Population and Number deaths adjusted for Ecig scenario,

| Age Group:All Year | Number Deaths | NULL Attrib Deaths | Ecig Attrib Deaths | Drop Deaths | %Drop Deaths | %Drop Attrib Deaths | Death Rate | NULL Attrib Rates | Ecig Attrib Rates | Drop Rates |
|---|---|---|---|---|---|---|---|---|---|---|
| All Comb | 3074319 | 2244988.113 | 2119222.685 | 125765.4 | 4.091 | 5.602 | 887.614 | 649.875 | 611.860 | 38.016 |

-----------------------------------------------------------------------------------------

Results for Age Groups Combined, Population and Number deaths adjusted for Ecig scenario,

| Age Group:All Year | Number Deaths | NULL Attrib Deaths | Ecig Attrib Deaths | Drop Deaths | %Drop Deaths | %Drop Attrib Deaths | Death Rate | NULL Attrib Rates | Ecig Attrib Rates | Drop Rates |
|---|---|---|---|---|---|---|---|---|---|---|
| 1990 | 78822 | 63385.736 | 63385.736 | 0.000 | 0.000 | 0.000 | 1249.50 | 1004.81 | 1004.81 | 0.000 |
| 1991 | 78462 | 62918.402 | 62905.561 | 12.841 | 0.016 | 0.020 | 1223.41 | 981.049 | 980.849 | 0.200 |
| 1992 | 77987 | 62366.766 | 62336.882 | 29.884 | 0.038 | 0.048 | 1195.35 | 955.930 | 955.470 | 0.460 |
| 1993 | 78543 | 62652.888 | 62593.429 | 59.458 | 0.076 | 0.095 | 1182.39 | 943.195 | 942.293 | 0.902 |
| 1994 | 77438 | 61565.227 | 61471.500 | 93.727 | 0.121 | 0.152 | 1144.00 | 909.520 | 908.121 | 1.400 |
| 1995 | 76906 | 60851.961 | 60709.716 | 142.245 | 0.185 | 0.234 | 1114.35 | 881.759 | 879.670 | 2.088 |
| 1996 | 76236 | 60122.922 | 59919.930 | 202.991 | 0.266 | 0.338 | 1087.43 | 857.643 | 854.703 | 2.940 |
| 1997 | 75479 | 59237.155 | 58968.755 | 268.400 | 0.356 | 0.453 | 1059.06 | 831.234 | 827.403 | 3.831 |
| 1998 | 75389 | 58906.696 | 58553.781 | 352.915 | 0.468 | 0.599 | 1040.36 | 813.001 | 808.039 | 4.961 |
| 1999 | 73170 | 56930.073 | 56494.913 | 435.160 | 0.595 | 0.764 | 993.778 | 773.329 | 767.301 | 6.028 |
| 2000 | 73799 | 57137.121 | 56585.647 | 551.474 | 0.747 | 0.965 | 987.609 | 764.787 | 757.250 | 7.538 |
| 2001 | 73314 | 56481.726 | 55801.033 | 680.694 | 0.928 | 1.205 | 968.281 | 746.169 | 736.979 | 9.190 |
| 2002 | 72468 | 55588.365 | 54774.305 | 814.060 | 1.123 | 1.464 | 946.314 | 726.138 | 715.262 | 10.876 |
| 2003 | 71975 | 54945.856 | 53997.936 | 947.921 | 1.317 | 1.725 | 930.023 | 710.281 | 697.735 | 12.546 |
| 2004 | 71341 | 54203.126 | 53083.398 | 1119.727 | 1.570 | 2.066 | 911.827 | 693.135 | 678.472 | 14.663 |
| 2005 | 71012 | 53744.603 | 52460.957 | 1283.646 | 1.808 | 2.388 | 897.006 | 679.296 | 662.672 | 16.624 |
| 2006 | 69715 | 52535.572 | 51061.423 | 1474.149 | 2.115 | 2.806 | 872.476 | 657.945 | 639.032 | 18.913 |
| 2007 | 68631 | 51492.124 | 49845.055 | 1647.069 | 2.400 | 3.199 | 850.010 | 638.264 | 617.342 | 20.921 |
| 2008 | 68303 | 51030.736 | 49180.632 | 1850.104 | 2.709 | 3.625 | 836.373 | 625.460 | 602.220 | 23.240 |
| 2009 | 67597 | 50276.199 | 48215.065 | 2061.134 | 3.049 | 4.100 | 817.865 | 608.943 | 583.359 | 25.584 |
| 2010 | 67296 | 49840.933 | 47601.576 | 2239.357 | 3.328 | 4.493 | 804.242 | 596.347 | 568.875 | 27.472 |
| 2011 | 66618 | 49072.841 | 46593.032 | 2479.809 | 3.722 | 5.053 | 788.170 | 581.354 | 551.247 | 30.107 |
| 2012 | 66280 | 48539.512 | 45834.424 | 2705.088 | 4.081 | 5.573 | 775.579 | 568.812 | 536.330 | 32.482 |
| 2013 | 65471 | 47675.741 | 44759.830 | 2915.911 | 4.454 | 6.116 | 757.269 | 552.318 | 517.710 | 34.609 |
| 2014 | 65186 | 47194.534 | 44099.279 | 3095.255 | 4.748 | 6.559 | 745.055 | 540.359 | 504.040 | 36.319 |
| 2015 | 64312 | 46305.842 | 43051.843 | 3253.999 | 5.060 | 7.027 | 826.602 | 596.439 | 553.345 | 43.094 |
| 2016 | 61794 | 44208.867 | 40889.714 | 3319.153 | 5.371 | 7.508 | 786.676 | 564.107 | 520.554 | 43.553 |
| 2017 | 60083 | 42702.624 | 39289.619 | 3413.005 | 5.681 | 7.992 | 757.765 | 539.903 | 495.522 | 44.381 |
| 2018 | 62678 | 44321.202 | 40501.960 | 3819.243 | 6.093 | 8.617 | 783.043 | 555.181 | 505.996 | 49.185 |
| 2019 | 63274 | 44493.088 | 40434.605 | 4058.483 | 6.414 | 9.122 | 782.662 | 551.912 | 500.153 | 51.759 |
| 2020 | 63715 | 44529.551 | 40269.612 | 4259.940 | 6.686 | 9.567 | 899.461 | 630.757 | 568.484 | 62.273 |
| 2021 | 57353 | 39852.556 | 35817.225 | 4035.331 | 7.036 | 10.126 | 803.810 | 560.546 | 501.980 | 58.566 |
| 2022 | 57698 | 39853.685 | 35573.770 | 4279.916 | 7.418 | 10.739 | 802.301 | 556.274 | 494.658 | 61.616 |
| 2023 | 58014 | 39847.050 | 35349.510 | 4497.540 | 7.752 | 11.287 | 799.998 | 551.670 | 487.457 | 64.213 |
| 2024 | 58237 | 39726.010 | 35080.403 | 4645.607 | 7.977 | 11.694 | 796.254 | 545.435 | 479.641 | 65.794 |
| 2025 | 57834 | 39207.348 | 34466.479 | 4740.869 | 8.197 | 12.092 | 919.718 | 626.579 | 548.106 | 78.472 |
| 2026 | 51723 | 34803.960 | 30487.271 | 4316.689 | 8.346 | 12.403 | 818.969 | 553.929 | 482.730 | 71.199 |
| 2027 | 51482 | 34484.595 | 30043.155 | 4441.439 | 8.627 | 12.879 | 811.277 | 546.357 | 473.437 | 72.919 |
| 2028 | 51195 | 34100.638 | 29547.000 | 4553.638 | 8.895 | 13.354 | 802.505 | 537.544 | 463.166 | 74.378 |
| 2029 | 50879 | 33669.838 | 29070.595 | 4599.242 | 9.039 | 13.660 | 792.855 | 527.732 | 453.007 | 74.725 |
| 2030 | 49461 | 32557.688 | 27999.738 | 4557.950 | 9.215 | 14.000 | 920.070 | 609.705 | 520.853 | 88.852 |
| 2031 | 43908 | 28646.393 | 24641.474 | 4004.919 | 9.121 | 13.981 | 814.452 | 535.065 | 457.078 | 77.986 |
| 2032 | 43496 | 28227.160 | 24207.048 | 4020.112 | 9.242 | 14.242 | 803.883 | 525.435 | 447.384 | 78.051 |
| 2033 | 43070 | 27786.623 | 23821.997 | 3964.626 | 9.205 | 14.268 | 792.503 | 515.079 | 438.334 | 76.745 |
| 2034 | 42611 | 27348.299 | 23423.217 | 3925.082 | 9.211 | 14.352 | 780.108 | 504.514 | 428.828 | 75.686 |
| 2035 | 40103 | 25570.077 | 21854.582 | 3715.495 | 9.265 | 14.531 | 907.264 | 583.430 | 494.425 | 89.005 |
| 2036 | 34340 | 21717.319 | 18588.058 | 3129.262 | 9.113 | 14.409 | 776.503 | 495.402 | 420.318 | 75.084 |
| 2037 | 33769 | 21256.024 | 18217.549 | 3038.475 | 8.998 | 14.295 | 762.577 | 484.347 | 411.395 | 72.952 |
| 2038 | 33203 | 20747.175 | 17824.809 | 2922.365 | 8.802 | 14.086 | 748.064 | 471.764 | 401.598 | 70.166 |
| 2039 | 32649 | 20327.688 | 17537.657 | 2790.030 | 8.546 | 13.725 | 733.043 | 460.724 | 393.764 | 66.960 |

# Results for PHIM for Run 1 (Pessimistic_Analysis.RTF)

*********************************************************************************************

**Main Results for E-Component for Run: 1, Simulation: 1, COD: IHD, Adjusted**

| Variable Parameter | Value |
|---|---|
| 1. Country | US (United States) |
| 2. Sex | M (Males) |
| 3. Year of start of process | 1990 |
| 4. Cause of Death | IHD |
| 5. Effective Dose NULL | CC:1 |
| 6. Effective Dose Ecig | CC:1 Ecig:0.4 Dual:0.7 |
| Note: | Death rates are per million population |

-----------------------------------------------------------------------------------------

Results for Age Groups Combined and Years combined, Population and Number deaths adjusted for Ecig scenario,

| Age Group:All | | NULL | Ecig | | | %Drop | | NULL | Ecig | |
|---|---|---|---|---|---|---|---|---|---|---|
| Year | Number Deaths | Attrib Deaths | Attrib Deaths | Drop Deaths | %Drop Deaths | Attrib Deaths | Death Rate | Attrib Rates | Attrib Rates | Drop Rates |
| All Comb | 6377985 | 1098877.421 | 896176.760 | 202700.7 | 3.178 | 18.446 | 1841.45 | 318.101 | 258.743 | 59.358 |

-----------------------------------------------------------------------------------------

Results for Age Groups Combined, Population and Number deaths adjusted for Ecig scenario,

| Age Group:All | | NULL | Ecig | | | %Drop | | NULL | Ecig | |
|---|---|---|---|---|---|---|---|---|---|---|
| Year | Number Deaths | Attrib Deaths | Attrib Deaths | Drop Deaths | %Drop Deaths | Attrib Deaths | Death Rate | Attrib Rates | Attrib Rates | Drop Rates |
| 1990 | 171484 | 38511.725 | 38511.725 | 0.000 | 0.000 | 0.000 | 2718.40 | 610.497 | 610.497 | 0.000 |
| 1991 | 168044 | 37309.838 | 37213.274 | 96.564 | 0.057 | 0.259 | 2620.21 | 581.750 | 580.245 | 1.506 |
| 1992 | 164548 | 36137.488 | 35882.720 | 254.768 | 0.155 | 0.705 | 2522.12 | 553.899 | 549.993 | 3.906 |
| 1993 | 164363 | 35599.588 | 35122.691 | 476.897 | 0.290 | 1.340 | 2474.35 | 535.926 | 528.743 | 7.183 |
| 1994 | 159868 | 34336.757 | 33601.572 | 735.185 | 0.460 | 2.141 | 2361.74 | 507.267 | 496.397 | 10.869 |
| 1995 | 158087 | 33455.759 | 32410.609 | 1045.150 | 0.661 | 3.124 | 2290.65 | 484.782 | 469.623 | 15.159 |
| 1996 | 154882 | 32193.824 | 30821.627 | 1372.197 | 0.886 | 4.262 | 2209.25 | 459.239 | 439.642 | 19.597 |
| 1997 | 149980 | 30665.509 | 28975.361 | 1690.149 | 1.127 | 5.512 | 2104.40 | 430.308 | 406.559 | 23.749 |
| 1998 | 145421 | 29188.430 | 27200.428 | 1988.003 | 1.367 | 6.811 | 2006.80 | 402.844 | 375.364 | 27.480 |
| 1999 | 165975 | 33375.386 | 30569.146 | 2806.240 | 1.691 | 8.408 | 2254.24 | 453.366 | 415.183 | 38.183 |
| 2000 | 160291 | 32024.754 | 28843.842 | 3180.912 | 1.984 | 9.933 | 2145.07 | 428.655 | 385.999 | 42.656 |
| 2001 | 154484 | 30682.393 | 27099.062 | 3583.331 | 2.320 | 11.679 | 2040.32 | 405.339 | 357.905 | 47.435 |
| 2002 | 151939 | 30218.370 | 26158.884 | 4059.485 | 2.672 | 13.434 | 1984.07 | 394.736 | 341.592 | 53.144 |
| 2003 | 146797 | 29175.202 | 24758.115 | 4417.087 | 3.009 | 15.140 | 1896.84 | 377.146 | 319.912 | 57.233 |
| 2004 | 138455 | 27488.687 | 22844.251 | 4644.437 | 3.354 | 16.896 | 1769.63 | 351.518 | 291.978 | 59.540 |
| 2005 | 135970 | 27017.439 | 22034.598 | 4982.841 | 3.665 | 18.443 | 1717.53 | 341.482 | 278.335 | 63.148 |
| 2006 | 132026 | 26231.435 | 20889.111 | 5342.325 | 4.046 | 20.366 | 1652.30 | 328.517 | 261.426 | 67.091 |
| 2007 | 126621 | 25103.049 | 19588.284 | 5514.765 | 4.355 | 21.969 | 1568.23 | 311.161 | 242.605 | 68.556 |
| 2008 | 125997 | 24621.740 | 18888.433 | 5733.307 | 4.550 | 23.286 | 1542.85 | 301.777 | 231.290 | 70.487 |
| 2009 | 122584 | 23741.177 | 17907.581 | 5833.596 | 4.759 | 24.572 | 1483.16 | 287.552 | 216.666 | 70.887 |
| 2010 | 120713 | 23189.160 | 17241.094 | 5948.066 | 4.927 | 25.650 | 1442.61 | 277.458 | 206.044 | 71.414 |
| 2011 | 120419 | 22786.927 | 16664.430 | 6122.497 | 5.084 | 26.868 | 1424.69 | 269.951 | 197.159 | 72.793 |
| 2012 | 121152 | 22485.295 | 16204.249 | 6281.045 | 5.184 | 27.934 | 1417.65 | 263.495 | 189.613 | 73.881 |
| 2013 | 122902 | 22251.884 | 15810.944 | 6440.940 | 5.241 | 28.946 | 1421.53 | 257.786 | 182.876 | 74.910 |
| 2014 | 123512 | 21894.593 | 15384.808 | 6509.786 | 5.271 | 29.732 | 1411.70 | 250.685 | 175.843 | 74.841 |
| 2015 | 124089 | 21471.109 | 14942.568 | 6528.542 | 5.261 | 30.406 | 1594.92 | 276.557 | 192.057 | 84.500 |
| 2016 | 126570 | 21411.568 | 14749.671 | 6661.898 | 5.263 | 31.114 | 1611.33 | 273.213 | 187.773 | 85.439 |
| 2017 | 128655 | 21169.399 | 14459.924 | 6709.475 | 5.215 | 31.694 | 1622.60 | 267.651 | 182.369 | 85.282 |
| 2018 | 121824 | 19830.576 | 13488.875 | 6341.702 | 5.206 | 31.979 | 1521.96 | 248.404 | 168.518 | 79.886 |
| 2019 | 122260 | 19431.014 | 13172.137 | 6258.877 | 5.119 | 32.211 | 1512.28 | 241.031 | 162.932 | 78.099 |
| 2020 | 121272 | 18532.612 | 12478.795 | 6053.817 | 4.992 | 32.666 | 1711.98 | 262.513 | 176.163 | 86.350 |
| 2021 | 119892 | 18234.128 | 12279.768 | 5954.360 | 4.966 | 32.655 | 1680.29 | 256.472 | 172.102 | 84.371 |
| 2022 | 119954 | 17812.559 | 11965.650 | 5846.909 | 4.874 | 32.825 | 1667.98 | 248.626 | 166.384 | 82.242 |
| 2023 | 119995 | 17361.668 | 11657.474 | 5704.195 | 4.754 | 32.855 | 1654.68 | 240.367 | 160.752 | 79.615 |
| 2024 | 119888 | 16929.676 | 11419.152 | 5510.525 | 4.596 | 32.549 | 1639.19 | 232.443 | 156.130 | 76.313 |
| 2025 | 116297 | 15501.666 | 10364.014 | 5137.652 | 4.418 | 33.143 | 1849.43 | 247.735 | 164.815 | 82.920 |
| 2026 | 115711 | 15523.814 | 10526.942 | 4996.872 | 4.318 | 32.188 | 1832.14 | 247.072 | 166.682 | 80.390 |
| 2027 | 114654 | 15073.070 | 10300.545 | 4772.526 | 4.163 | 31.663 | 1806.79 | 238.810 | 162.322 | 76.488 |
| 2028 | 113547 | 14636.710 | 10067.169 | 4569.540 | 4.024 | 31.220 | 1779.92 | 230.725 | 157.809 | 72.917 |
| 2029 | 112438 | 14180.166 | 9877.630 | 4302.537 | 3.827 | 30.342 | 1752.13 | 222.256 | 153.923 | 68.333 |
| 2030 | 105583 | 12111.772 | 8298.262 | 3813.510 | 3.612 | 31.486 | 1964.06 | 226.816 | 154.365 | 72.451 |
| 2031 | 106051 | 12417.367 | 8699.569 | 3717.798 | 3.506 | 29.940 | 1967.16 | 231.935 | 161.370 | 70.565 |
| 2032 | 104663 | 12054.064 | 8529.230 | 3524.835 | 3.368 | 29.242 | 1934.34 | 224.381 | 157.634 | 66.747 |
| 2033 | 103290 | 11672.389 | 8364.838 | 3307.552 | 3.202 | 28.337 | 1900.57 | 216.370 | 153.916 | 62.454 |
| 2034 | 101893 | 11365.762 | 8307.734 | 3058.028 | 3.001 | 26.906 | 1865.44 | 209.673 | 152.096 | 57.576 |
| 2035 | 91618 | 8580.360 | 6087.386 | 2492.974 | 2.721 | 29.054 | 2072.70 | 195.777 | 137.717 | 58.060 |
| 2036 | 92947 | 8948.841 | 6519.793 | 2429.048 | 2.613 | 27.144 | 2101.73 | 204.135 | 147.427 | 56.708 |
| 2037 | 91166 | 8624.570 | 6430.475 | 2194.095 | 2.407 | 25.440 | 2058.74 | 196.522 | 145.215 | 51.307 |
| 2038 | 89437 | 8315.017 | 6312.333 | 2002.684 | 2.239 | 24.085 | 2015.05 | 189.073 | 142.219 | 46.854 |
| 2039 | 87776 | 8001.132 | 6249.991 | 1751.141 | 1.995 | 21.886 | 1970.79 | 181.344 | 140.328 | 41.017 |

# Results for PHIM for Run 1 (Pessimistic_Analysis.RTF)

************************************************************************************

**Main Results for E-Component for Run: 1, Simulation: 1, COD: STROKE, Adjusted**

| Variable Parameter | Value |
|---|---|
| 1. Country | US (United States) |
| 2. Sex | M (Males) |
| 3. Year of start of process | 1990 |
| 4. Cause of Death | STROKE |
| 5. Effective Dose NULL | CC:1 |
| 6. Effective Dose Ecig | CC:1 Ecig:0.4 Dual:0.7 |
| Note: | Death rates are per million population |

-----------------------------------------------------------------------------------------

Results for Age Groups Combined and Years combined, Population and Number deaths adjusted for Ecig scenario,

| Age Group:All | | NULL | Ecig | | | %Drop | | NULL | Ecig | |
|---|---|---|---|---|---|---|---|---|---|---|
| Year | Number | Attrib | Attrib | Drop | %Drop | Attrib | Death | Attrib | Attrib | Drop |
| | Deaths | Deaths | Deaths | Deaths | Deaths | Deaths | Rate | Rates | Rates | Rates |
| All Comb | 1494409 | 149918.462 | 121226.790 | 28691.67 | 1.920 | 19.138 | 431.464 | 43.398 | 35.000 | 8.398 |

-----------------------------------------------------------------------------------------

Results for Age Groups Combined, Population and Number deaths adjusted for Ecig scenario,

| Age Group:All | | NULL | Ecig | | | %Drop | | NULL | Ecig | |
|---|---|---|---|---|---|---|---|---|---|---|
| Year | Number | Attrib | Attrib | Drop | %Drop | Attrib | Death | Attrib | Attrib | Drop |
| | Deaths | Deaths | Deaths | Deaths | Deaths | Deaths | Rate | Rates | Rates | Rates |
| 1990 | 32862 | 4613.166 | 4613.166 | 0.000 | 0.000 | 0.000 | 520.936 | 73.129 | 73.129 | 0.000 |
| 1991 | 32619 | 4555.566 | 4544.170 | 11.396 | 0.035 | 0.250 | 508.609 | 71.032 | 70.855 | 0.178 |
| 1992 | 32100 | 4453.572 | 4424.774 | 28.798 | 0.090 | 0.647 | 492.014 | 68.262 | 67.821 | 0.442 |
| 1993 | 32896 | 4462.473 | 4406.275 | 56.199 | 0.171 | 1.259 | 495.225 | 67.179 | 66.333 | 0.846 |
| 1994 | 33323 | 4499.513 | 4409.412 | 90.102 | 0.270 | 2.002 | 492.288 | 66.473 | 65.140 | 1.332 |
| 1995 | 33570 | 4472.774 | 4339.328 | 133.446 | 0.398 | 2.984 | 486.419 | 64.812 | 62.876 | 1.936 |
| 1996 | 33871 | 4406.878 | 4225.547 | 181.331 | 0.535 | 4.115 | 483.144 | 62.863 | 60.274 | 2.590 |
| 1997 | 33518 | 4288.158 | 4056.851 | 231.307 | 0.690 | 5.394 | 470.298 | 60.173 | 56.923 | 3.250 |
| 1998 | 32798 | 4120.148 | 3843.043 | 277.105 | 0.845 | 6.726 | 452.608 | 56.864 | 53.034 | 3.830 |
| 1999 | 33379 | 3982.443 | 3658.378 | 324.064 | 0.971 | 8.137 | 453.346 | 54.097 | 49.687 | 4.410 |
| 2000 | 33465 | 4021.411 | 3631.955 | 389.456 | 1.164 | 9.685 | 447.836 | 53.827 | 48.604 | 5.223 |
| 2001 | 32282 | 3848.092 | 3410.473 | 437.619 | 1.356 | 11.372 | 426.361 | 50.836 | 45.043 | 5.793 |
| 2002 | 31595 | 3783.081 | 3289.545 | 493.536 | 1.562 | 13.046 | 412.585 | 49.418 | 42.956 | 6.461 |
| 2003 | 30997 | 3754.415 | 3198.213 | 556.202 | 1.794 | 14.815 | 400.526 | 48.533 | 41.326 | 7.207 |
| 2004 | 29898 | 3666.274 | 3057.753 | 608.521 | 2.035 | 16.598 | 382.138 | 46.883 | 39.082 | 7.801 |
| 2005 | 28725 | 3589.688 | 2935.548 | 654.140 | 2.277 | 18.223 | 362.847 | 45.371 | 37.081 | 8.290 |
| 2006 | 28208 | 3596.985 | 2868.768 | 728.217 | 2.582 | 20.245 | 353.027 | 45.048 | 35.903 | 9.145 |
| 2007 | 28050 | 3560.798 | 2781.287 | 779.511 | 2.779 | 21.891 | 347.408 | 44.137 | 34.447 | 9.691 |
| 2008 | 27156 | 3417.001 | 2619.045 | 797.956 | 2.938 | 23.353 | 332.522 | 41.881 | 32.070 | 9.810 |
| 2009 | 27100 | 3394.390 | 2556.166 | 838.224 | 3.093 | 24.694 | 327.886 | 41.113 | 30.927 | 10.185 |
| 2010 | 26948 | 3340.137 | 2477.293 | 862.845 | 3.202 | 25.833 | 322.044 | 39.965 | 29.606 | 10.359 |
| 2011 | 27001 | 3285.435 | 2401.092 | 884.343 | 3.275 | 26.917 | 319.448 | 38.922 | 28.408 | 10.514 |
| 2012 | 27104 | 3259.209 | 2343.673 | 915.536 | 3.378 | 28.091 | 317.161 | 38.193 | 27.424 | 10.769 |
| 2013 | 27959 | 3274.703 | 2316.903 | 957.800 | 3.426 | 29.248 | 323.383 | 37.937 | 26.798 | 11.139 |
| 2014 | 28855 | 3298.083 | 2305.926 | 992.157 | 3.438 | 30.083 | 329.801 | 37.762 | 26.356 | 11.406 |
| 2015 | 29632 | 3244.406 | 2244.183 | 1000.223 | 3.375 | 30.829 | 380.859 | 41.789 | 28.844 | 12.945 |
| 2016 | 30595 | 3314.836 | 2269.311 | 1045.525 | 3.417 | 31.541 | 389.490 | 42.297 | 28.890 | 13.408 |
| 2017 | 31508 | 3333.314 | 2258.412 | 1074.901 | 3.412 | 32.247 | 397.376 | 42.144 | 28.483 | 13.661 |
| 2018 | 29442 | 2925.242 | 1969.577 | 955.665 | 3.246 | 32.670 | 367.822 | 36.642 | 24.606 | 12.036 |
| 2019 | 29700 | 2864.628 | 1921.406 | 943.222 | 3.176 | 32.927 | 367.376 | 35.534 | 23.767 | 11.767 |
| 2020 | 29703 | 2730.366 | 1821.105 | 909.261 | 3.061 | 33.302 | 419.321 | 38.675 | 25.708 | 12.967 |
| 2021 | 30218 | 2647.911 | 1762.267 | 885.643 | 2.931 | 33.447 | 423.509 | 37.244 | 24.698 | 12.546 |
| 2022 | 30434 | 2586.165 | 1717.396 | 868.768 | 2.855 | 33.593 | 423.187 | 36.097 | 23.881 | 12.217 |
| 2023 | 30645 | 2517.960 | 1670.113 | 847.847 | 2.767 | 33.672 | 422.588 | 34.860 | 23.030 | 11.830 |
| 2024 | 30811 | 2454.161 | 1634.369 | 819.791 | 2.661 | 33.404 | 421.261 | 33.695 | 22.346 | 11.349 |
| 2025 | 30413 | 2275.364 | 1508.464 | 766.900 | 2.522 | 33.704 | 483.643 | 36.363 | 23.988 | 12.374 |
| 2026 | 30856 | 2183.166 | 1460.589 | 722.577 | 2.342 | 33.098 | 488.567 | 34.747 | 23.127 | 11.620 |
| 2027 | 30741 | 2112.345 | 1427.075 | 685.269 | 2.229 | 32.441 | 484.428 | 33.467 | 22.489 | 10.978 |
| 2028 | 30600 | 2046.677 | 1396.273 | 650.404 | 2.125 | 31.779 | 479.674 | 32.263 | 21.887 | 10.375 |
| 2029 | 30452 | 1977.671 | 1365.777 | 611.895 | 2.009 | 30.940 | 474.533 | 30.997 | 21.283 | 9.715 |
| 2030 | 29535 | 1744.457 | 1204.328 | 540.129 | 1.829 | 30.963 | 549.410 | 32.668 | 22.403 | 10.265 |
| 2031 | 29188 | 1653.738 | 1157.777 | 495.961 | 1.699 | 29.990 | 541.411 | 30.889 | 21.476 | 9.413 |
| 2032 | 28944 | 1603.120 | 1134.578 | 468.543 | 1.619 | 29.227 | 534.931 | 29.841 | 20.969 | 8.873 |
| 2033 | 28691 | 1548.245 | 1114.436 | 433.808 | 1.512 | 28.019 | 527.925 | 28.700 | 20.506 | 8.194 |
| 2034 | 28413 | 1511.985 | 1108.052 | 403.933 | 1.422 | 26.715 | 520.183 | 27.893 | 20.286 | 7.607 |
| 2035 | 26974 | 1239.866 | 906.574 | 333.291 | 1.236 | 26.881 | 610.233 | 28.290 | 20.510 | 7.780 |
| 2036 | 25825 | 1182.522 | 881.634 | 300.888 | 1.165 | 25.445 | 583.972 | 26.975 | 19.936 | 7.039 |
| 2037 | 25382 | 1134.483 | 871.978 | 262.505 | 1.034 | 23.139 | 573.177 | 25.851 | 19.691 | 6.159 |
| 2038 | 24936 | 1093.537 | 858.637 | 234.900 | 0.942 | 21.481 | 561.804 | 24.866 | 19.345 | 5.520 |
| 2039 | 24493 | 1047.905 | 847.896 | 200.009 | 0.817 | 19.087 | 549.919 | 23.751 | 19.037 | 4.713 |

# Results for PHIM for Run 1 (Pessimistic_Analysis.RTF)

************************************************************************************

**Main Results for E-Component for Run: 1, Simulation: 1, COD: COPD, Adjusted**

| **Variable Parameter** | **Value** |
|---|---|
| 1. Country | US (United States) |
| 2. Sex | M (Males) |
| 3. Year of start of process | 1990 |
| 4. Cause of Death | COPD |
| 5. Effective Dose NULL | CC:1 |
| 6. Effective Dose Ecig | CC:1 Ecig:0.4 Dual:0.7 |
| Note: | Death rates are per million population |

------------------------------------------------------------------------------------------

Results for Age Groups Combined and Years combined, Population and Number deaths adjusted for Ecig scenario,

| Age Group:All | | NULL | Ecig | | | %Drop | | NULL | Ecig | |
|---|---|---|---|---|---|---|---|---|---|---|
| Year | Number Deaths | Attrib Deaths | Attrib Deaths | Drop Deaths | %Drop Deaths | Attrib Deaths | Death Rate | Attrib Rates | Attrib Rates | Drop Rates |
| All Comb | 2242116 | 1015606.587 | 910348.198 | 105258.4 | 4.695 | 10.364 | 647.342 | 293.996 | 262.835 | 31.161 |

------------------------------------------------------------------------------------------

Results for Age Groups Combined, Population and Number deaths adjusted for Ecig scenario,

| Age Group:All | | NULL | Ecig | | | %Drop | | NULL | Ecig | |
|---|---|---|---|---|---|---|---|---|---|---|
| Year | Number Deaths | Attrib Deaths | Attrib Deaths | Drop Deaths | %Drop Deaths | Attrib Deaths | Death Rate | Attrib Rates | Attrib Rates | Drop Rates |
| 1990 | 33993 | 19262.437 | 19262.437 | 0.000 | 0.000 | 0.000 | 538.865 | 305.353 | 305.353 | 0.000 |
| 1991 | 34092 | 19215.636 | 19210.159 | 5.477 | 0.016 | 0.029 | 531.576 | 299.618 | 299.533 | 0.085 |
| 1992 | 33942 | 19056.189 | 19045.568 | 10.621 | 0.031 | 0.056 | 520.248 | 292.085 | 291.921 | 0.163 |
| 1993 | 35913 | 20095.670 | 20073.722 | 21.947 | 0.061 | 0.109 | 540.644 | 302.526 | 302.193 | 0.333 |
| 1994 | 35074 | 19524.095 | 19491.027 | 33.067 | 0.094 | 0.169 | 518.156 | 288.435 | 287.942 | 0.493 |
| 1995 | 34670 | 19116.002 | 19064.496 | 51.506 | 0.149 | 0.269 | 502.357 | 276.995 | 276.240 | 0.755 |
| 1996 | 34583 | 18975.021 | 18904.811 | 70.211 | 0.203 | 0.370 | 493.298 | 270.675 | 269.660 | 1.015 |
| 1997 | 35269 | 19169.025 | 19070.776 | 98.249 | 0.279 | 0.513 | 494.865 | 268.986 | 267.586 | 1.400 |
| 1998 | 35678 | 19228.937 | 19093.482 | 135.456 | 0.380 | 0.704 | 492.351 | 265.388 | 263.489 | 1.899 |
| 1999 | 38520 | 20589.820 | 20412.434 | 177.387 | 0.461 | 0.862 | 523.174 | 279.689 | 277.237 | 2.452 |
| 2000 | 36289 | 19196.417 | 18979.383 | 217.034 | 0.598 | 1.131 | 485.629 | 256.946 | 253.989 | 2.957 |
| 2001 | 36289 | 19012.731 | 18738.824 | 273.907 | 0.755 | 1.441 | 479.276 | 251.174 | 247.489 | 3.685 |
| 2002 | 35993 | 18699.357 | 18369.705 | 329.653 | 0.916 | 1.763 | 470.012 | 244.265 | 239.878 | 4.387 |
| 2003 | 36218 | 18663.550 | 18280.465 | 383.084 | 1.058 | 2.053 | 467.996 | 241.262 | 236.211 | 5.051 |
| 2004 | 34627 | 17679.164 | 17232.575 | 446.589 | 1.290 | 2.526 | 442.574 | 226.076 | 220.254 | 5.823 |
| 2005 | 36847 | 18692.832 | 18137.213 | 555.619 | 1.508 | 2.972 | 465.439 | 236.265 | 229.104 | 7.161 |
| 2006 | 34374 | 17308.564 | 16692.483 | 616.080 | 1.792 | 3.559 | 430.185 | 216.769 | 208.906 | 7.863 |
| 2007 | 35355 | 17683.181 | 16950.173 | 733.008 | 2.073 | 4.145 | 437.885 | 219.189 | 209.932 | 9.258 |
| 2008 | 38286 | 19006.374 | 18126.739 | 879.635 | 2.298 | 4.628 | 468.811 | 232.952 | 221.963 | 10.989 |
| 2009 | 36945 | 18205.573 | 17227.973 | 977.599 | 2.646 | 5.370 | 447.002 | 220.505 | 208.443 | 12.062 |
| 2010 | 37284 | 18237.758 | 17173.234 | 1064.524 | 2.855 | 5.837 | 445.572 | 218.215 | 205.233 | 12.982 |
| 2011 | 38152 | 18487.916 | 17243.735 | 1244.181 | 3.261 | 6.730 | 451.377 | 219.022 | 204.013 | 15.009 |
| 2012 | 38571 | 18491.158 | 17117.810 | 1373.348 | 3.561 | 7.427 | 451.340 | 216.689 | 200.303 | 16.386 |
| 2013 | 40369 | 19143.452 | 17587.086 | 1556.366 | 3.855 | 8.130 | 466.928 | 221.775 | 203.419 | 18.356 |
| 2014 | 40428 | 18992.005 | 17318.795 | 1673.210 | 4.139 | 8.810 | 462.082 | 217.451 | 197.948 | 19.503 |
| 2015 | 42407 | 19728.885 | 17882.014 | 1846.871 | 4.355 | 9.361 | 545.062 | 254.116 | 229.837 | 24.279 |
| 2016 | 43221 | 19876.724 | 17891.880 | 1984.844 | 4.592 | 9.986 | 550.234 | 253.628 | 227.776 | 25.852 |
| 2017 | 44238 | 20119.790 | 17968.094 | 2151.697 | 4.864 | 10.694 | 557.937 | 254.381 | 226.614 | 27.767 |
| 2018 | 45616 | 20535.123 | 18184.129 | 2350.994 | 5.154 | 11.449 | 569.887 | 257.229 | 227.176 | 30.053 |
| 2019 | 47127 | 21009.029 | 18462.022 | 2547.007 | 5.405 | 12.123 | 582.939 | 260.605 | 228.365 | 32.241 |
| 2020 | 48602 | 21428.512 | 18705.999 | 2722.513 | 5.602 | 12.705 | 686.118 | 303.533 | 264.072 | 39.461 |
| 2021 | 48661 | 21266.697 | 18363.346 | 2903.351 | 5.966 | 13.652 | 681.987 | 299.127 | 257.363 | 41.764 |
| 2022 | 50134 | 21663.568 | 18514.825 | 3148.743 | 6.281 | 14.535 | 697.116 | 302.378 | 257.451 | 44.927 |
| 2023 | 51631 | 22084.131 | 18698.337 | 3385.793 | 6.558 | 15.331 | 711.975 | 305.748 | 257.843 | 47.905 |
| 2024 | 53075 | 22418.456 | 18856.235 | 3562.221 | 6.712 | 15.890 | 725.670 | 307.804 | 257.814 | 49.990 |
| 2025 | 54135 | 22614.834 | 18891.662 | 3723.172 | 6.878 | 16.463 | 860.889 | 361.411 | 300.426 | 60.985 |
| 2026 | 54649 | 22571.522 | 18724.019 | 3847.503 | 7.040 | 17.046 | 865.310 | 359.241 | 296.473 | 62.768 |
| 2027 | 55598 | 22774.416 | 18724.661 | 4049.754 | 7.284 | 17.782 | 876.145 | 360.827 | 295.074 | 65.753 |
| 2028 | 56505 | 22915.561 | 18661.513 | 4254.048 | 7.529 | 18.564 | 885.742 | 361.228 | 292.530 | 68.699 |
| 2029 | 57395 | 22992.027 | 18604.001 | 4388.027 | 7.645 | 19.085 | 894.385 | 360.371 | 289.906 | 70.465 |
| 2030 | 57644 | 22895.360 | 18406.709 | 4488.651 | 7.787 | 19.605 | 1072.30 | 428.760 | 342.403 | 86.357 |
| 2031 | 58018 | 22715.871 | 18221.502 | 4494.369 | 7.747 | 19.785 | 1076.18 | 424.293 | 337.993 | 86.299 |
| 2032 | 58702 | 22759.207 | 18140.262 | 4618.945 | 7.868 | 20.295 | 1084.91 | 423.652 | 335.261 | 88.391 |
| 2033 | 59361 | 22786.258 | 18124.781 | 4661.476 | 7.853 | 20.457 | 1092.27 | 422.387 | 333.503 | 88.885 |
| 2034 | 59962 | 22806.106 | 18061.826 | 4744.280 | 7.912 | 20.803 | 1097.77 | 420.721 | 330.672 | 90.049 |
| 2035 | 59037 | 22262.998 | 17593.080 | 4669.918 | 7.910 | 20.976 | 1335.61 | 507.973 | 398.015 | 109.958 |
| 2036 | 56965 | 21140.051 | 16635.819 | 4504.233 | 7.907 | 21.307 | 1288.12 | 482.234 | 376.174 | 106.060 |
| 2037 | 57107 | 21011.845 | 16521.852 | 4489.992 | 7.862 | 21.369 | 1289.62 | 478.783 | 373.102 | 105.681 |
| 2038 | 57226 | 20787.483 | 16369.173 | 4418.311 | 7.721 | 21.255 | 1289.33 | 472.681 | 368.802 | 103.879 |
| 2039 | 57335 | 20709.267 | 16335.350 | 4373.917 | 7.629 | 21.121 | 1287.32 | 469.372 | 366.769 | 102.603 |

# Results for PHIM for Run 2 (Pessimistic_Analysis.RTF)

---

-------------------------------------------------------------------------------------

**Control File Details for Run: 2**

| **Variable Parameter** | **Value** |
|---|---|
| 1. Name for Reference Scenario | NULL |
| 2. Name for Test Scenario | Ecig |
| 3. Name for Product 1 | CC |
| 4. Name for Product 2 | Ecig |
| 5. Name for Product 3 | - |
| 6. Name for Product 4 | Dual |
| 7. Country | US (United States) |
| 8. Sex | F (Females) |
| 9. Year of start of process | 1990 |
| 10. Number of months of follow-up | 600 |
| 11. Follow-up interval length (in months) | 12 |
| 12. Lower age for risk estimation | 10 |
| 13. Upper age for risk estimation | 79 |
| 14. The effective dose for Product 1 (F1) - Ref | 1 |
| 15. The effective dose for Product 2 (F2) - Ref | - |
| 16. The effective dose for Product 3 (F3) - Ref | - |
| 17. The effective dose for Product 4 (F4) - Ref | - |
| 18. The effective dose for Product 1 (F1) - Test | 1 |
| 19. The effective dose for Product 2 (F2) - Test | 0.4 |
| 20. The effective dose for Product 3 (F3) - Test | - |
| 21. The effective dose for Product 4 (F4) - Test | FROMF2 |
| 22. Number in population to be simulated | 100000 |
| 23. Number of MC simulations | 1 |
| 24. The random number seed for the first simulation | 15975263 |
| 25. Source for the population file (POP) | UN4 |
| 26. Source for the socioeconomic prevalence file (SEP) | - |
| 27. Source for the current smoking prevalence file (CSP) | ISS3 |
| 28. Source for the former smoking prevalence file (FSP) | ISS3 |
| 29. Source for the quit-time distribution file (QTD) | NHIS2006 |
| 30. Source for the death file (MORT) | APR-20 |
| 31. Source for the relative risk file (RR) | PNLEST |
| 32. Source for the half-life file (H) | PNLEST |
| 33. Assumption Set for TTP Factor Reference | F1 |
| 34. Assumption Set for TTP Factor Test | F1 |
| 35. Assumption Set for TTP Reference | ISS3 |
| 36. Assumption Set for TTP Test | ISS3 |
| 37. Assumption Set for TP for socioeconomic group | - |
| 38. Source for Output Choice file (OUTC) | BASIC |
| 39. Output for DTP P1 | N |
| 40. Output for DTP P2 | N |
| 41. Output for Main P Component | Y |
| 42. Output for DTP E1 | N |
| 43. Output for DTP E2 | N |
| 44. Output for DTP E3 | N |
| 45. Output for Main E Component | Y |
| 46. Output file name | Pessimistic_Analysis |
| 47. Run number for P-Component | - |
| 48. Results file for E-Component | - |
| 49. Components required | PE |
| 50. Results folder for P to be used in E-Component | - |
| 51. Fixed age of starting product use | 16 |
| 52. Output absolute risk file? | N |

# Results for PHIM for Run 2 (Pessimistic_Analysis.RTF)

************************************************************************************

**TTP values for Run: 2, Scenario: NULL**

| **Variable Parameter** | **Value** |
|---|---|
| 1. Country | US (United States) |
| 2. Sex | F (Females) |
| 3. Assumption Set | ISS3 |
| Note: | Period is in Months |

| Age | Period | **Initiation** Never CC | **Cessation** CC Former | **Re-initiation** Former CC |
|---|---|---|---|---|
| 10-14 | 1-12 | 0.003758 | 0.008742 | 0.000000 |
| 15-19 | 1-12 | 0.003469 | 0.002515 | 0.000000 |
| 20-24 | 1-12 | 0.000276 | 0.000761 | 0.000000 |
| 25-29 | 1-12 | 0.000137 | 0.001375 | 0.000000 |
| 30-34 | 1-12 | 0.000069 | 0.001149 | 0.000000 |
| 35-39 | 1-12 | 0.000000 | 0.000991 | 0.000000 |
| 40-44 | 1-12 | 0.000000 | 0.000833 | 0.000000 |
| 45-49 | 1-12 | 0.000000 | 0.001723 | 0.000000 |
| 50-54 | 1-12 | 0.000000 | 0.002336 | 0.000000 |
| 55-59 | 1-12 | 0.000000 | 0.002233 | 0.000000 |
| 60-64 | 1-12 | 0.000000 | 0.004634 | 0.000000 |
| 65-69 | 1-12 | 0.000000 | 0.003505 | 0.000000 |
| 70-74 | 1-12 | 0.000000 | 0.004329 | 0.000000 |
| 75-79 | 1-12 | 0.000000 | 0.004329 | 0.000000 |
| 10-14 | 13-72 | 0.004794 | 0.008742 | 0.000000 |
| 15-19 | 13-72 | 0.004527 | 0.004082 | 0.000000 |
| 20-24 | 13-72 | 0.000105 | 0.001261 | 0.000000 |
| 25-29 | 13-72 | 0.000053 | 0.000952 | 0.000000 |
| 30-34 | 13-72 | 0.000026 | 0.000733 | 0.000000 |
| 35-39 | 13-72 | 0.000000 | 0.000753 | 0.000000 |
| 40-44 | 13-72 | 0.000000 | 0.001535 | 0.000000 |
| 45-49 | 13-72 | 0.000000 | 0.002998 | 0.000000 |
| 50-54 | 13-72 | 0.000000 | 0.003159 | 0.000000 |
| 55-59 | 13-72 | 0.000000 | 0.002965 | 0.000000 |
| 60-64 | 13-72 | 0.000000 | 0.003633 | 0.000000 |
| 65-69 | 13-72 | 0.000000 | 0.004522 | 0.000000 |
| 70-74 | 13-72 | 0.000000 | 0.005208 | 0.000000 |
| 75-79 | 13-72 | 0.000000 | 0.005208 | 0.000000 |
| 10-14 | 73-132 | 0.003969 | 0.008742 | 0.000000 |
| 15-19 | 73-132 | 0.003056 | 0.003373 | 0.000000 |
| 20-24 | 73-132 | 0.001459 | 0.003881 | 0.000000 |
| 25-29 | 73-132 | 0.000714 | 0.001953 | 0.000000 |
| 30-34 | 73-132 | 0.000111 | 0.001338 | 0.000000 |
| 35-39 | 73-132 | 0.000000 | 0.001507 | 0.000000 |
| 40-44 | 73-132 | 0.000000 | 0.002080 | 0.000000 |
| 45-49 | 73-132 | 0.000000 | 0.003809 | 0.000000 |
| 50-54 | 73-132 | 0.000000 | 0.004871 | 0.000000 |
| 55-59 | 73-132 | 0.000000 | 0.004560 | 0.000000 |
| 60-64 | 73-132 | 0.000000 | 0.007192 | 0.000000 |
| 65-69 | 73-132 | 0.000000 | 0.007033 | 0.000000 |
| 70-74 | 73-132 | 0.000000 | 0.008206 | 0.000000 |
| 75-79 | 73-132 | 0.000000 | 0.008206 | 0.000000 |
| 10-14 | 133-192 | 0.002861 | 0.008742 | 0.000000 |
| 15-19 | 133-192 | 0.002594 | 0.003996 | 0.000000 |
| 20-24 | 133-192 | 0.001247 | 0.002805 | 0.000000 |
| 25-29 | 133-192 | 0.000289 | 0.001574 | 0.000000 |
| 30-34 | 133-192 | 0.000132 | 0.001293 | 0.000000 |
| 35-39 | 133-192 | 0.000000 | 0.001219 | 0.000000 |
| 40-44 | 133-192 | 0.000000 | 0.001657 | 0.000000 |
| 45-49 | 133-192 | 0.000000 | 0.001995 | 0.000000 |
| 50-54 | 133-192 | 0.000000 | 0.002154 | 0.000000 |
| 55-59 | 133-192 | 0.000000 | 0.002072 | 0.000000 |
| 60-64 | 133-192 | 0.000000 | 0.005049 | 0.000000 |
| 65-69 | 133-192 | 0.000000 | 0.003680 | 0.000000 |
| 70-74 | 133-192 | 0.000000 | 0.006144 | 0.000000 |
| 75-79 | 133-192 | 0.000000 | 0.006144 | 0.000000 |
| 10-14 | 193-252 | 0.001859 | 0.008742 | 0.000000 |
| 15-19 | 193-252 | 0.002232 | 0.005770 | 0.000000 |
| 20-24 | 193-252 | 0.001079 | 0.003262 | 0.000000 |
| 25-29 | 193-252 | 0.000531 | 0.002002 | 0.000000 |
| 30-34 | 193-252 | 0.000263 | 0.003240 | 0.000000 |
| 35-39 | 193-252 | 0.000000 | 0.001771 | 0.000000 |
| 40-44 | 193-252 | 0.000000 | 0.002173 | 0.000000 |
| 45-49 | 193-252 | 0.000000 | 0.002075 | 0.000000 |
| 50-54 | 193-252 | 0.000000 | 0.003431 | 0.000000 |

## Results for PHIM for Run 2 (Pessimistic_Analysis.RTF)

| | | | | |
|---|---|---|---|---|
| 55-59 | 193-252 | 0.000000 | 0.002906 | 0.000000 |
| 60-64 | 193-252 | 0.000000 | 0.007139 | 0.000000 |
| 65-69 | 193-252 | 0.000000 | 0.004963 | 0.000000 |
| 70-74 | 193-252 | 0.000000 | 0.006269 | 0.000000 |
| 75-79 | 193-252 | 0.000000 | 0.006269 | 0.000000 |
| 10-14 | 253+ | 0.000931 | 0.008742 | 0.000000 |
| 15-19 | 253+ | 0.001798 | 0.009440 | 0.000000 |
| 20-24 | 253+ | 0.000023 | 0.002831 | 0.000000 |
| 25-29 | 253+ | 0.000073 | 0.002655 | 0.000000 |
| 30-34 | 253+ | 0.000037 | 0.004614 | 0.000000 |
| 35-39 | 253+ | 0.000000 | 0.002607 | 0.000000 |
| 40-44 | 253+ | 0.000000 | 0.003115 | 0.000000 |
| 45-49 | 253+ | 0.000000 | 0.002132 | 0.000000 |
| 50-54 | 253+ | 0.000000 | 0.003497 | 0.000000 |
| 55-59 | 253+ | 0.000000 | 0.002942 | 0.000000 |
| 60-64 | 253+ | 0.000000 | 0.005376 | 0.000000 |
| 65-69 | 253+ | 0.000000 | 0.001789 | 0.000000 |
| 70-74 | 253+ | 0.000000 | 0.004077 | 0.000000 |
| 75-79 | 253+ | 0.000000 | 0.004077 | 0.000000 |

# Results for PHIM for Run 2 (Pessimistic_Analysis.RTF)

*********************************************************************************
**TTP values for Run: 2, Scenario: Ecig**

| **Variable Parameter** | **Value** |
|---|---|
| 1. Country | US (United States) |
| 2. Sex | F (Females) |
| 3. Assumption Set | ISS3 |
| Note: | Period is in Months |

| | | **Initiation** | | | **Cessation** | | | **Re-initiation** | | |
|---|---|---|---|---|---|---|---|---|---|---|
| Age | Period | Never CC | Never Ecig | Never Dual | CC Former | Ecig Former | Dual Former | Former CC | Former Ecig | Former Dual |
| 10-14 | 1-12 | 0.001879 | 0.002819 | 0.000000 | 0.008742 | 0.004371 | 0.000000 | 0.000000 | 0.000000 | 0.000000 |
| 15-19 | 1-12 | 0.001735 | 0.002602 | 0.000000 | 0.002515 | 0.001258 | 0.000000 | 0.000000 | 0.000000 | 0.000000 |
| 20-24 | 1-12 | 0.000138 | 0.000207 | 0.000000 | 0.000761 | 0.000381 | 0.000000 | 0.000000 | 0.000000 | 0.000000 |
| 25-29 | 1-12 | 0.000069 | 0.000103 | 0.000000 | 0.001376 | 0.000688 | 0.000000 | 0.000000 | 0.000000 | 0.000000 |
| 30-34 | 1-12 | 0.000034 | 0.000051 | 0.000000 | 0.001149 | 0.000575 | 0.000000 | 0.000000 | 0.000000 | 0.000000 |
| 35-39 | 1-12 | 0.000000 | 0.000000 | 0.000000 | 0.000991 | 0.000496 | 0.000000 | 0.000000 | 0.000000 | 0.000000 |
| 40-44 | 1-12 | 0.000000 | 0.000000 | 0.000000 | 0.000833 | 0.000417 | 0.000000 | 0.000000 | 0.000000 | 0.000000 |
| 45-49 | 1-12 | 0.000000 | 0.000000 | 0.000000 | 0.001723 | 0.000862 | 0.000000 | 0.000000 | 0.000000 | 0.000000 |
| 50-54 | 1-12 | 0.000000 | 0.000000 | 0.000000 | 0.002336 | 0.001168 | 0.000000 | 0.000000 | 0.000000 | 0.000000 |
| 55-59 | 1-12 | 0.000000 | 0.000000 | 0.000000 | 0.002233 | 0.001116 | 0.000000 | 0.000000 | 0.000000 | 0.000000 |
| 60-64 | 1-12 | 0.000000 | 0.000000 | 0.000000 | 0.004635 | 0.002317 | 0.000000 | 0.000000 | 0.000000 | 0.000000 |
| 65-69 | 1-12 | 0.000000 | 0.000000 | 0.000000 | 0.003505 | 0.001752 | 0.000000 | 0.000000 | 0.000000 | 0.000000 |
| 70-74 | 1-12 | 0.000000 | 0.000000 | 0.000000 | 0.004329 | 0.002164 | 0.000000 | 0.000000 | 0.000000 | 0.000000 |
| 75-79 | 1-12 | 0.000000 | 0.000000 | 0.000000 | 0.004329 | 0.002164 | 0.000000 | 0.000000 | 0.000000 | 0.000000 |
| 10-14 | 13-72 | 0.001918 | 0.004315 | 0.000000 | 0.008742 | 0.004371 | 0.000000 | 0.000000 | 0.000000 | 0.000000 |
| 15-19 | 13-72 | 0.001811 | 0.004075 | 0.000000 | 0.004083 | 0.002041 | 0.000000 | 0.000000 | 0.000000 | 0.000000 |
| 20-24 | 13-72 | 0.000042 | 0.000095 | 0.000000 | 0.001261 | 0.000631 | 0.000000 | 0.000000 | 0.000000 | 0.000000 |
| 25-29 | 13-72 | 0.000021 | 0.000047 | 0.000000 | 0.000952 | 0.000476 | 0.000000 | 0.000000 | 0.000000 | 0.000000 |
| 30-34 | 13-72 | 0.000011 | 0.000024 | 0.000000 | 0.000733 | 0.000367 | 0.000000 | 0.000000 | 0.000000 | 0.000000 |
| 35-39 | 13-72 | 0.000000 | 0.000000 | 0.000000 | 0.000754 | 0.000377 | 0.000000 | 0.000000 | 0.000000 | 0.000000 |
| 40-44 | 13-72 | 0.000000 | 0.000000 | 0.000000 | 0.001535 | 0.000767 | 0.000000 | 0.000000 | 0.000000 | 0.000000 |
| 45-49 | 13-72 | 0.000000 | 0.000000 | 0.000000 | 0.002998 | 0.001499 | 0.000000 | 0.000000 | 0.000000 | 0.000000 |
| 50-54 | 13-72 | 0.000000 | 0.000000 | 0.000000 | 0.003160 | 0.001580 | 0.000000 | 0.000000 | 0.000000 | 0.000000 |
| 55-59 | 13-72 | 0.000000 | 0.000000 | 0.000000 | 0.002965 | 0.001482 | 0.000000 | 0.000000 | 0.000000 | 0.000000 |
| 60-64 | 13-72 | 0.000000 | 0.000000 | 0.000000 | 0.003633 | 0.001816 | 0.000000 | 0.000000 | 0.000000 | 0.000000 |
| 65-69 | 13-72 | 0.000000 | 0.000000 | 0.000000 | 0.004522 | 0.002261 | 0.000000 | 0.000000 | 0.000000 | 0.000000 |
| 70-74 | 13-72 | 0.000000 | 0.000000 | 0.000000 | 0.005208 | 0.002604 | 0.000000 | 0.000000 | 0.000000 | 0.000000 |
| 75-79 | 13-72 | 0.000000 | 0.000000 | 0.000000 | 0.005208 | 0.002604 | 0.000000 | 0.000000 | 0.000000 | 0.000000 |
| 10-14 | 73-132 | 0.000794 | 0.004763 | 0.000000 | 0.008742 | 0.004371 | 0.000000 | 0.000000 | 0.000000 | 0.000000 |
| 15-19 | 73-132 | 0.000611 | 0.003668 | 0.000000 | 0.003373 | 0.001686 | 0.000000 | 0.000000 | 0.000000 | 0.000000 |
| 20-24 | 73-132 | 0.000292 | 0.001751 | 0.000000 | 0.003881 | 0.001940 | 0.000000 | 0.000000 | 0.000000 | 0.000000 |
| 25-29 | 73-132 | 0.000143 | 0.000857 | 0.000000 | 0.001954 | 0.000977 | 0.000000 | 0.000000 | 0.000000 | 0.000000 |
| 30-34 | 73-132 | 0.000022 | 0.000134 | 0.000000 | 0.001338 | 0.000669 | 0.000000 | 0.000000 | 0.000000 | 0.000000 |
| 35-39 | 73-132 | 0.000000 | 0.000000 | 0.000000 | 0.001507 | 0.000753 | 0.000000 | 0.000000 | 0.000000 | 0.000000 |
| 40-44 | 73-132 | 0.000000 | 0.000000 | 0.000000 | 0.002080 | 0.001040 | 0.000000 | 0.000000 | 0.000000 | 0.000000 |
| 45-49 | 73-132 | 0.000000 | 0.000000 | 0.000000 | 0.003809 | 0.001904 | 0.000000 | 0.000000 | 0.000000 | 0.000000 |
| 50-54 | 73-132 | 0.000000 | 0.000000 | 0.000000 | 0.004871 | 0.002436 | 0.000000 | 0.000000 | 0.000000 | 0.000000 |
| 55-59 | 73-132 | 0.000000 | 0.000000 | 0.000000 | 0.004560 | 0.002280 | 0.000000 | 0.000000 | 0.000000 | 0.000000 |
| 60-64 | 73-132 | 0.000000 | 0.000000 | 0.000000 | 0.007192 | 0.003596 | 0.000000 | 0.000000 | 0.000000 | 0.000000 |
| 65-69 | 73-132 | 0.000000 | 0.000000 | 0.000000 | 0.007033 | 0.003517 | 0.000000 | 0.000000 | 0.000000 | 0.000000 |
| 70-74 | 73-132 | 0.000000 | 0.000000 | 0.000000 | 0.008206 | 0.004103 | 0.000000 | 0.000000 | 0.000000 | 0.000000 |
| 75-79 | 73-132 | 0.000000 | 0.000000 | 0.000000 | 0.008206 | 0.004103 | 0.000000 | 0.000000 | 0.000000 | 0.000000 |
| 10-14 | 133-192 | 0.000143 | 0.004077 | 0.000000 | 0.008742 | 0.004371 | 0.000000 | 0.000000 | 0.000000 | 0.000000 |
| 15-19 | 133-192 | 0.000130 | 0.003697 | 0.000000 | 0.003996 | 0.001998 | 0.000000 | 0.000000 | 0.000000 | 0.000000 |
| 20-24 | 133-192 | 0.000062 | 0.001778 | 0.000000 | 0.002805 | 0.001403 | 0.000000 | 0.000000 | 0.000000 | 0.000000 |
| 25-29 | 133-192 | 0.000015 | 0.000412 | 0.000000 | 0.001574 | 0.000787 | 0.000000 | 0.000000 | 0.000000 | 0.000000 |
| 30-34 | 133-192 | 0.000007 | 0.000188 | 0.000000 | 0.001293 | 0.000646 | 0.000000 | 0.000000 | 0.000000 | 0.000000 |
| 35-39 | 133-192 | 0.000000 | 0.000000 | 0.000000 | 0.001219 | 0.000610 | 0.000000 | 0.000000 | 0.000000 | 0.000000 |
| 40-44 | 133-192 | 0.000000 | 0.000000 | 0.000000 | 0.001657 | 0.000828 | 0.000000 | 0.000000 | 0.000000 | 0.000000 |
| 45-49 | 133-192 | 0.000000 | 0.000000 | 0.000000 | 0.001995 | 0.000997 | 0.000000 | 0.000000 | 0.000000 | 0.000000 |
| 50-54 | 133-192 | 0.000000 | 0.000000 | 0.000000 | 0.002154 | 0.001077 | 0.000000 | 0.000000 | 0.000000 | 0.000000 |
| 55-59 | 133-192 | 0.000000 | 0.000000 | 0.000000 | 0.002072 | 0.001036 | 0.000000 | 0.000000 | 0.000000 | 0.000000 |
| 60-64 | 133-192 | 0.000000 | 0.000000 | 0.000000 | 0.005049 | 0.002524 | 0.000000 | 0.000000 | 0.000000 | 0.000000 |
| 65-69 | 133-192 | 0.000000 | 0.000000 | 0.000000 | 0.003681 | 0.001840 | 0.000000 | 0.000000 | 0.000000 | 0.000000 |
| 70-74 | 133-192 | 0.000000 | 0.000000 | 0.000000 | 0.006144 | 0.003072 | 0.000000 | 0.000000 | 0.000000 | 0.000000 |
| 75-79 | 133-192 | 0.000000 | 0.000000 | 0.000000 | 0.006144 | 0.003072 | 0.000000 | 0.000000 | 0.000000 | 0.000000 |
| 10-14 | 193-252 | 0.000093 | 0.002649 | 0.000000 | 0.008742 | 0.004371 | 0.000000 | 0.000000 | 0.000000 | 0.000000 |
| 15-19 | 193-252 | 0.000112 | 0.003180 | 0.000000 | 0.005770 | 0.002885 | 0.000000 | 0.000000 | 0.000000 | 0.000000 |
| 20-24 | 193-252 | 0.000054 | 0.001538 | 0.000000 | 0.003262 | 0.001631 | 0.000000 | 0.000000 | 0.000000 | 0.000000 |
| 25-29 | 193-252 | 0.000027 | 0.000757 | 0.000000 | 0.002002 | 0.001001 | 0.000000 | 0.000000 | 0.000000 | 0.000000 |
| 30-34 | 193-252 | 0.000013 | 0.000375 | 0.000000 | 0.003241 | 0.001620 | 0.000000 | 0.000000 | 0.000000 | 0.000000 |
| 35-39 | 193-252 | 0.000000 | 0.000000 | 0.000000 | 0.001771 | 0.000885 | 0.000000 | 0.000000 | 0.000000 | 0.000000 |
| 40-44 | 193-252 | 0.000000 | 0.000000 | 0.000000 | 0.002173 | 0.001087 | 0.000000 | 0.000000 | 0.000000 | 0.000000 |
| 45-49 | 193-252 | 0.000000 | 0.000000 | 0.000000 | 0.002075 | 0.001037 | 0.000000 | 0.000000 | 0.000000 | 0.000000 |
| 50-54 | 193-252 | 0.000000 | 0.000000 | 0.000000 | 0.003432 | 0.001716 | 0.000000 | 0.000000 | 0.000000 | 0.000000 |

## Results for PHIM for Run 2 (Pessimistic_Analysis.RTF)

| | | | | | | | | | | |
|---|---|---|---|---|---|---|---|---|---|---|
| 55-59 | 193-252 | 0.000000 | 0.000000 | 0.000000 | 0.002906 | 0.001453 | 0.000000 | 0.000000 | 0.000000 | 0.000000 |
| 60-64 | 193-252 | 0.000000 | 0.000000 | 0.000000 | 0.007139 | 0.003569 | 0.000000 | 0.000000 | 0.000000 | 0.000000 |
| 65-69 | 193-252 | 0.000000 | 0.000000 | 0.000000 | 0.004963 | 0.002481 | 0.000000 | 0.000000 | 0.000000 | 0.000000 |
| 70-74 | 193-252 | 0.000000 | 0.000000 | 0.000000 | 0.006269 | 0.003134 | 0.000000 | 0.000000 | 0.000000 | 0.000000 |
| 75-79 | 193-252 | 0.000000 | 0.000000 | 0.000000 | 0.006269 | 0.003134 | 0.000000 | 0.000000 | 0.000000 | 0.000000 |
| 10-14 | 253+ | 0.000047 | 0.001326 | 0.000000 | 0.008742 | 0.004371 | 0.000000 | 0.000000 | 0.000000 | 0.000000 |
| 15-19 | 253+ | 0.000090 | 0.002562 | 0.000000 | 0.009440 | 0.004720 | 0.000000 | 0.000000 | 0.000000 | 0.000000 |
| 20-24 | 253+ | 0.000001 | 0.000033 | 0.000000 | 0.002831 | 0.001415 | 0.000000 | 0.000000 | 0.000000 | 0.000000 |
| 25-29 | 253+ | 0.000004 | 0.000105 | 0.000000 | 0.002656 | 0.001328 | 0.000000 | 0.000000 | 0.000000 | 0.000000 |
| 30-34 | 253+ | 0.000002 | 0.000052 | 0.000000 | 0.004614 | 0.002307 | 0.000000 | 0.000000 | 0.000000 | 0.000000 |
| 35-39 | 253+ | 0.000000 | 0.000000 | 0.000000 | 0.002608 | 0.001304 | 0.000000 | 0.000000 | 0.000000 | 0.000000 |
| 40-44 | 253+ | 0.000000 | 0.000000 | 0.000000 | 0.003115 | 0.001558 | 0.000000 | 0.000000 | 0.000000 | 0.000000 |
| 45-49 | 253+ | 0.000000 | 0.000000 | 0.000000 | 0.002132 | 0.001066 | 0.000000 | 0.000000 | 0.000000 | 0.000000 |
| 50-54 | 253+ | 0.000000 | 0.000000 | 0.000000 | 0.003497 | 0.001749 | 0.000000 | 0.000000 | 0.000000 | 0.000000 |
| 55-59 | 253+ | 0.000000 | 0.000000 | 0.000000 | 0.002942 | 0.001471 | 0.000000 | 0.000000 | 0.000000 | 0.000000 |
| 60-64 | 253+ | 0.000000 | 0.000000 | 0.000000 | 0.005376 | 0.002688 | 0.000000 | 0.000000 | 0.000000 | 0.000000 |
| 65-69 | 253+ | 0.000000 | 0.000000 | 0.000000 | 0.001789 | 0.000894 | 0.000000 | 0.000000 | 0.000000 | 0.000000 |
| 70-74 | 253+ | 0.000000 | 0.000000 | 0.000000 | 0.004077 | 0.002039 | 0.000000 | 0.000000 | 0.000000 | 0.000000 |
| 75-79 | 253+ | 0.000000 | 0.000000 | 0.000000 | 0.004077 | 0.002039 | 0.000000 | 0.000000 | 0.000000 | 0.000000 |

# Results for PHIM for Run 2 (Pessimistic_Analysis.RTF)

**********************************************************************************

| | | Switch from CC | | Switch from Ecig | | Switch from Dual | |
|---|---|---|---|---|---|---|---|
| | | **CC** | **CC** | **Ecig** | **Ecig** | **Dual** | **Dual** |
| Age | Period | Ecig | Dual | CC | Dual | CC | Ecig |
| 10-14 | 1-12 | 0.000000 | 0.000000 | 0.000000 | 0.000000 | 0.000000 | 0.000000 |
| 15-19 | 1-12 | 0.003771 | 0.000000 | 0.000000 | 0.000000 | 0.000000 | 0.000000 |
| 20-24 | 1-12 | 0.007376 | 0.000000 | 0.000000 | 0.000000 | 0.000000 | 0.000000 |
| 25-29 | 1-12 | 0.008145 | 0.000000 | 0.000000 | 0.000000 | 0.000000 | 0.000000 |
| 30-34 | 1-12 | 0.007936 | 0.000000 | 0.000000 | 0.000000 | 0.000000 | 0.000000 |
| 35-39 | 1-12 | 0.006975 | 0.000000 | 0.000000 | 0.000000 | 0.000000 | 0.000000 |
| 40-44 | 1-12 | 0.006830 | 0.000000 | 0.000000 | 0.000000 | 0.000000 | 0.000000 |
| 45-49 | 1-12 | 0.006338 | 0.000000 | 0.000000 | 0.000000 | 0.000000 | 0.000000 |
| 50-54 | 1-12 | 0.005574 | 0.000000 | 0.000000 | 0.000000 | 0.000000 | 0.000000 |
| 55-59 | 1-12 | 0.003011 | 0.000000 | 0.000000 | 0.000000 | 0.000000 | 0.000000 |
| 60-64 | 1-12 | 0.002115 | 0.000000 | 0.000000 | 0.000000 | 0.000000 | 0.000000 |
| 65-69 | 1-12 | 0.000281 | 0.000000 | 0.000000 | 0.000000 | 0.000000 | 0.000000 |
| 70-74 | 1-12 | 0.000000 | 0.000000 | 0.000000 | 0.000000 | 0.000000 | 0.000000 |
| 75-79 | 1-12 | 0.000000 | 0.000000 | 0.000000 | 0.000000 | 0.000000 | 0.000000 |
| 10-14 | 13-72 | 0.000000 | 0.000000 | 0.000000 | 0.000000 | 0.000000 | 0.000000 |
| 15-19 | 13-72 | 0.003771 | 0.000000 | 0.000000 | 0.000000 | 0.000000 | 0.000000 |
| 20-24 | 13-72 | 0.007376 | 0.000000 | 0.000000 | 0.000000 | 0.000000 | 0.000000 |
| 25-29 | 13-72 | 0.008145 | 0.000000 | 0.000000 | 0.000000 | 0.000000 | 0.000000 |
| 30-34 | 13-72 | 0.007936 | 0.000000 | 0.000000 | 0.000000 | 0.000000 | 0.000000 |
| 35-39 | 13-72 | 0.006975 | 0.000000 | 0.000000 | 0.000000 | 0.000000 | 0.000000 |
| 40-44 | 13-72 | 0.006830 | 0.000000 | 0.000000 | 0.000000 | 0.000000 | 0.000000 |
| 45-49 | 13-72 | 0.006338 | 0.000000 | 0.000000 | 0.000000 | 0.000000 | 0.000000 |
| 50-54 | 13-72 | 0.005574 | 0.000000 | 0.000000 | 0.000000 | 0.000000 | 0.000000 |
| 55-59 | 13-72 | 0.003011 | 0.000000 | 0.000000 | 0.000000 | 0.000000 | 0.000000 |
| 60-64 | 13-72 | 0.002115 | 0.000000 | 0.000000 | 0.000000 | 0.000000 | 0.000000 |
| 65-69 | 13-72 | 0.000281 | 0.000000 | 0.000000 | 0.000000 | 0.000000 | 0.000000 |
| 70-74 | 13-72 | 0.000000 | 0.000000 | 0.000000 | 0.000000 | 0.000000 | 0.000000 |
| 75-79 | 13-72 | 0.000000 | 0.000000 | 0.000000 | 0.000000 | 0.000000 | 0.000000 |
| 10-14 | 73-132 | 0.000000 | 0.000000 | 0.000000 | 0.000000 | 0.000000 | 0.000000 |
| 15-19 | 73-132 | 0.003771 | 0.000000 | 0.000000 | 0.000000 | 0.000000 | 0.000000 |
| 20-24 | 73-132 | 0.007376 | 0.000000 | 0.000000 | 0.000000 | 0.000000 | 0.000000 |
| 25-29 | 73-132 | 0.008145 | 0.000000 | 0.000000 | 0.000000 | 0.000000 | 0.000000 |
| 30-34 | 73-132 | 0.007936 | 0.000000 | 0.000000 | 0.000000 | 0.000000 | 0.000000 |
| 35-39 | 73-132 | 0.006975 | 0.000000 | 0.000000 | 0.000000 | 0.000000 | 0.000000 |
| 40-44 | 73-132 | 0.006830 | 0.000000 | 0.000000 | 0.000000 | 0.000000 | 0.000000 |
| 45-49 | 73-132 | 0.006338 | 0.000000 | 0.000000 | 0.000000 | 0.000000 | 0.000000 |
| 50-54 | 73-132 | 0.005574 | 0.000000 | 0.000000 | 0.000000 | 0.000000 | 0.000000 |
| 55-59 | 73-132 | 0.003011 | 0.000000 | 0.000000 | 0.000000 | 0.000000 | 0.000000 |
| 60-64 | 73-132 | 0.002115 | 0.000000 | 0.000000 | 0.000000 | 0.000000 | 0.000000 |
| 65-69 | 73-132 | 0.000281 | 0.000000 | 0.000000 | 0.000000 | 0.000000 | 0.000000 |
| 70-74 | 73-132 | 0.000000 | 0.000000 | 0.000000 | 0.000000 | 0.000000 | 0.000000 |
| 75-79 | 73-132 | 0.000000 | 0.000000 | 0.000000 | 0.000000 | 0.000000 | 0.000000 |
| 10-14 | 133-192 | 0.000000 | 0.000000 | 0.000000 | 0.000000 | 0.000000 | 0.000000 |
| 15-19 | 133-192 | 0.003771 | 0.000000 | 0.000000 | 0.000000 | 0.000000 | 0.000000 |
| 20-24 | 133-192 | 0.007376 | 0.000000 | 0.000000 | 0.000000 | 0.000000 | 0.000000 |
| 25-29 | 133-192 | 0.008145 | 0.000000 | 0.000000 | 0.000000 | 0.000000 | 0.000000 |
| 30-34 | 133-192 | 0.007936 | 0.000000 | 0.000000 | 0.000000 | 0.000000 | 0.000000 |
| 35-39 | 133-192 | 0.006975 | 0.000000 | 0.000000 | 0.000000 | 0.000000 | 0.000000 |
| 40-44 | 133-192 | 0.006830 | 0.000000 | 0.000000 | 0.000000 | 0.000000 | 0.000000 |
| 45-49 | 133-192 | 0.006338 | 0.000000 | 0.000000 | 0.000000 | 0.000000 | 0.000000 |
| 50-54 | 133-192 | 0.005574 | 0.000000 | 0.000000 | 0.000000 | 0.000000 | 0.000000 |
| 55-59 | 133-192 | 0.003011 | 0.000000 | 0.000000 | 0.000000 | 0.000000 | 0.000000 |
| 60-64 | 133-192 | 0.002115 | 0.000000 | 0.000000 | 0.000000 | 0.000000 | 0.000000 |
| 65-69 | 133-192 | 0.000281 | 0.000000 | 0.000000 | 0.000000 | 0.000000 | 0.000000 |
| 70-74 | 133-192 | 0.000000 | 0.000000 | 0.000000 | 0.000000 | 0.000000 | 0.000000 |
| 75-79 | 133-192 | 0.000000 | 0.000000 | 0.000000 | 0.000000 | 0.000000 | 0.000000 |
| 10-14 | 193-252 | 0.000000 | 0.000000 | 0.000000 | 0.000000 | 0.000000 | 0.000000 |
| 15-19 | 193-252 | 0.003771 | 0.000000 | 0.000000 | 0.000000 | 0.000000 | 0.000000 |
| 20-24 | 193-252 | 0.007376 | 0.000000 | 0.000000 | 0.000000 | 0.000000 | 0.000000 |
| 25-29 | 193-252 | 0.008145 | 0.000000 | 0.000000 | 0.000000 | 0.000000 | 0.000000 |
| 30-34 | 193-252 | 0.007936 | 0.000000 | 0.000000 | 0.000000 | 0.000000 | 0.000000 |
| 35-39 | 193-252 | 0.006975 | 0.000000 | 0.000000 | 0.000000 | 0.000000 | 0.000000 |
| 40-44 | 193-252 | 0.006830 | 0.000000 | 0.000000 | 0.000000 | 0.000000 | 0.000000 |
| 45-49 | 193-252 | 0.006338 | 0.000000 | 0.000000 | 0.000000 | 0.000000 | 0.000000 |
| 50-54 | 193-252 | 0.005574 | 0.000000 | 0.000000 | 0.000000 | 0.000000 | 0.000000 |
| 55-59 | 193-252 | 0.003011 | 0.000000 | 0.000000 | 0.000000 | 0.000000 | 0.000000 |
| 60-64 | 193-252 | 0.002115 | 0.000000 | 0.000000 | 0.000000 | 0.000000 | 0.000000 |
| 65-69 | 193-252 | 0.000281 | 0.000000 | 0.000000 | 0.000000 | 0.000000 | 0.000000 |
| 70-74 | 193-252 | 0.000000 | 0.000000 | 0.000000 | 0.000000 | 0.000000 | 0.000000 |
| 75-79 | 193-252 | 0.000000 | 0.000000 | 0.000000 | 0.000000 | 0.000000 | 0.000000 |
| 10-14 | 253+ | 0.000000 | 0.000000 | 0.000000 | 0.000000 | 0.000000 | 0.000000 |
| 15-19 | 253+ | 0.003771 | 0.000000 | 0.000000 | 0.000000 | 0.000000 | 0.000000 |
| 20-24 | 253+ | 0.007376 | 0.000000 | 0.000000 | 0.000000 | 0.000000 | 0.000000 |

## Results for PHIM for Run 2 (Pessimistic_Analysis.RTF)

| | | | | | | | |
|---|---|---|---|---|---|---|---|
| 25-29 | 253+ | 0.008145 | 0.000000 | 0.000000 | 0.000000 | 0.000000 | 0.000000 |
| 30-34 | 253+ | 0.007936 | 0.000000 | 0.000000 | 0.000000 | 0.000000 | 0.000000 |
| 35-39 | 253+ | 0.006975 | 0.000000 | 0.000000 | 0.000000 | 0.000000 | 0.000000 |
| 40-44 | 253+ | 0.006830 | 0.000000 | 0.000000 | 0.000000 | 0.000000 | 0.000000 |
| 45-49 | 253+ | 0.006338 | 0.000000 | 0.000000 | 0.000000 | 0.000000 | 0.000000 |
| 50-54 | 253+ | 0.005574 | 0.000000 | 0.000000 | 0.000000 | 0.000000 | 0.000000 |
| 55-59 | 253+ | 0.003011 | 0.000000 | 0.000000 | 0.000000 | 0.000000 | 0.000000 |
| 60-64 | 253+ | 0.002115 | 0.000000 | 0.000000 | 0.000000 | 0.000000 | 0.000000 |
| 65-69 | 253+ | 0.000281 | 0.000000 | 0.000000 | 0.000000 | 0.000000 | 0.000000 |
| 70-74 | 253+ | 0.000000 | 0.000000 | 0.000000 | 0.000000 | 0.000000 | 0.000000 |
| 75-79 | 253+ | 0.000000 | 0.000000 | 0.000000 | 0.000000 | 0.000000 | 0.000000 |

# Results for PHIM for Run 2 (Pessimistic_Analysis.RTF)

************************************************************************************

**Main Output for the Tobacco History for Run: 2, Simulation: 1, Scenario: NULL**

| Variable Parameter | Value |
|---|---|
| 1. Country | US (United States) |
| 2. Sex | F (Females) |
| 3. Year of start of process | 1990 |

Year of Follow-up: 2040, Age Group: All Ages

| Period | Age Group | Smoking Group | N | % |
|---|---|---|---|---|
| 50 | All Ages | Never Any | 20670 | 58.80 |
| 50 | All Ages | CC | 2462 | 7.00 |
| 50 | All Ages | Former | 12019 | 34.19 |

Year of Follow-up: 2040, Age Group: 18-19

| Period | Age Group | Smoking Group | N | % |
|---|---|---|---|---|
| 50 | 18-19 | Never Any | 0 | . |
| 50 | 18-19 | CC | 0 | . |
| 50 | 18-19 | Former | 0 | . |

Year of Follow-up: 2040, Age Group: 20-24

| Period | Age Group | Smoking Group | N | % |
|---|---|---|---|---|
| 50 | 20-24 | Never Any | 0 | . |
| 50 | 20-24 | CC | 0 | . |
| 50 | 20-24 | Former | 0 | . |

Year of Follow-up: 2040, Age Group: 25-29

| Period | Age Group | Smoking Group | N | % |
|---|---|---|---|---|
| 50 | 25-29 | Never Any | 0 | . |
| 50 | 25-29 | CC | 0 | . |
| 50 | 25-29 | Former | 0 | . |

Year of Follow-up: 2040, Age Group: 30-34

| Period | Age Group | Smoking Group | N | % |
|---|---|---|---|---|
| 50 | 30-34 | Never Any | 0 | . |
| 50 | 30-34 | CC | 0 | . |
| 50 | 30-34 | Former | 0 | . |

Year of Follow-up: 2040, Age Group: 35-39

| Period | Age Group | Smoking Group | N | % |
|---|---|---|---|---|
| 50 | 35-39 | Never Any | 0 | . |
| 50 | 35-39 | CC | 0 | . |
| 50 | 35-39 | Former | 0 | . |

Year of Follow-up: 2040, Age Group: 40-44

| Period | Age Group | Smoking Group | N | % |
|---|---|---|---|---|
| 50 | 40-44 | Never Any | 0 | . |
| 50 | 40-44 | CC | 0 | . |
| 50 | 40-44 | Former | 0 | . |

Year of Follow-up: 2040, Age Group: 45-49

| Period | Age Group | Smoking Group | N | % |
|---|---|---|---|---|
| 50 | 45-49 | Never Any | 0 | . |
| 50 | 45-49 | CC | 0 | . |
| 50 | 45-49 | Former | 0 | . |

Year of Follow-up: 2040, Age Group: 50-54

| Period | Age Group | Smoking Group | N | % |
|---|---|---|---|---|
| 50 | 50-54 | Never Any | 0 | . |
| 50 | 50-54 | CC | 0 | . |
| 50 | 50-54 | Former | 0 | . |

Year of Follow-up: 2040, Age Group: 55-59

| Period | Age Group | Smoking Group | N | % |
|---|---|---|---|---|
| 50 | 55-59 | Never Any | 0 | . |
| 50 | 55-59 | CC | 0 | . |
| 50 | 55-59 | Former | 0 | . |

Year of Follow-up: 2040, Age Group: 60-64

| Period | Age Group | Smoking Group | N | % |
|---|---|---|---|---|

## Results for PHIM for Run 2 (Pessimistic_Analysis.RTF)

| | | | | |
|---|---|---|---|---|
| 50 | 60-64 | Never Any | 4803 | 60.57 |
| 50 | 60-64 | CC | 621 | 7.83 |
| 50 | 60-64 | Former | 2506 | 31.60 |

Year of Follow-up: 2040, Age Group: 65-69

| Period | Age Group | Smoking Group | N | % |
|---|---|---|---|---|
| 50 | 65-69 | Never Any | 5407 | 64.75 |
| 50 | 65-69 | CC | 538 | 6.44 |
| 50 | 65-69 | Former | 2405 | 28.80 |

Year of Follow-up: 2040, Age Group: 70-74

| Period | Age Group | Smoking Group | N | % |
|---|---|---|---|---|
| 50 | 70-74 | Never Any | 5181 | 58.92 |
| 50 | 70-74 | CC | 605 | 6.88 |
| 50 | 70-74 | Former | 3007 | 34.20 |

Year of Follow-up: 2040, Age Group: 75-79

| Period | Age Group | Smoking Group | N | % |
|---|---|---|---|---|
| 50 | 75-79 | Never Any | 5279 | 52.38 |
| 50 | 75-79 | CC | 698 | 6.93 |
| 50 | 75-79 | Former | 4101 | 40.69 |

# Results for PHIM for Run 2 (Pessimistic_Analysis.RTF)

***************************************************************************************

**Main Output for the Tobacco History for Run: 2, Simulation: 1, Scenario: Ecig**

| **Variable Parameter** | **Value** |
|---|---|
| 1. Country | US (United States) |
| 2. Sex | F (Females) |
| 3. Year of start of process | 1990 |

Year of Follow-up: 1990, Age Group: All Ages

| Period | Age Group | Smoking Group | N | % | Ever Quit (N) | Ever Quit % | Mean Yrs Quit | Short Term (n) | Short Term % |
|---|---|---|---|---|---|---|---|---|---|
| 0 | All Ages | Never Any | 47776 | 54.92 | | | | | |
| 0 | All Ages | CC | 22737 | 26.14 | 0 | 0.00 | | | |
| 0 | All Ages | Ecig | 0 | 0.00 | | | | | |
| 0 | All Ages | Dual | 0 | 0.00 | | | | | |
| 0 | All Ages | Former | 16484 | 18.95 | | | 11.90 | 2815 | 17.08 |

Year of Follow-up: 1990, Age Group: 18-19

| Period | Age Group | Smoking Group | N | % | Ever Quit (N) | Ever Quit % | Mean Yrs Quit | Short Term (n) | Short Term % |
|---|---|---|---|---|---|---|---|---|---|
| 0 | 18-19 | Never Any | 2570 | 78.43 | | | | | |
| 0 | 18-19 | CC | 535 | 16.33 | 0 | 0.00 | | | |
| 0 | 18-19 | Ecig | 0 | 0.00 | | | | | |
| 0 | 18-19 | Dual | 0 | 0.00 | | | | | |
| 0 | 18-19 | Former | 172 | 5.25 | | | 0.20 | 172 | 100.00 |

Year of Follow-up: 1990, Age Group: 20-24

| Period | Age Group | Smoking Group | N | % | Ever Quit (N) | Ever Quit % | Mean Yrs Quit | Short Term (n) | Short Term % |
|---|---|---|---|---|---|---|---|---|---|
| 0 | 20-24 | Never Any | 5354 | 60.89 | | | | | |
| 0 | 20-24 | CC | 2560 | 29.11 | 0 | 0.00 | | | |
| 0 | 20-24 | Ecig | 0 | 0.00 | | | | | |
| 0 | 20-24 | Dual | 0 | 0.00 | | | | | |
| 0 | 20-24 | Former | 879 | 10.00 | | | 1.58 | 420 | 47.78 |

Year of Follow-up: 1990, Age Group: 25-29

| Period | Age Group | Smoking Group | N | % | Ever Quit (N) | Ever Quit % | Mean Yrs Quit | Short Term (n) | Short Term % |
|---|---|---|---|---|---|---|---|---|---|
| 0 | 25-29 | Never Any | 5318 | 52.77 | | | | | |
| 0 | 25-29 | CC | 3335 | 33.09 | 0 | 0.00 | | | |
| 0 | 25-29 | Ecig | 0 | 0.00 | | | | | |
| 0 | 25-29 | Dual | 0 | 0.00 | | | | | |
| 0 | 25-29 | Former | 1425 | 14.14 | | | 3.91 | 404 | 28.35 |

Year of Follow-up: 1990, Age Group: 30-34

| Period | Age Group | Smoking Group | N | % | Ever Quit (N) | Ever Quit % | Mean Yrs Quit | Short Term (n) | Short Term % |
|---|---|---|---|---|---|---|---|---|---|
| 0 | 30-34 | Never Any | 5287 | 50.36 | | | | | |
| 0 | 30-34 | CC | 3326 | 31.68 | 0 | 0.00 | | | |
| 0 | 30-34 | Ecig | 0 | 0.00 | | | | | |
| 0 | 30-34 | Dual | 0 | 0.00 | | | | | |
| 0 | 30-34 | Former | 1886 | 17.96 | | | 4.36 | 566 | 30.01 |

Year of Follow-up: 1990, Age Group: 35-39

| Period | Age Group | Smoking Group | N | % | Ever Quit (N) | Ever Quit % | Mean Yrs Quit | Short Term (n) | Short Term % |
|---|---|---|---|---|---|---|---|---|---|
| 0 | 35-39 | Never Any | 5018 | 52.27 | | | | | |
| 0 | 35-39 | CC | 2653 | 27.64 | 0 | 0.00 | | | |
| 0 | 35-39 | Ecig | 0 | 0.00 | | | | | |
| 0 | 35-39 | Dual | 0 | 0.00 | | | | | |
| 0 | 35-39 | Former | 1929 | 20.09 | | | 7.96 | 324 | 16.80 |

Year of Follow-up: 1990, Age Group: 40-44

| Period | Age Group | Smoking Group | N | % | Ever Quit (N) | Ever Quit % | Mean Yrs Quit | Short Term (n) | Short Term % |
|---|---|---|---|---|---|---|---|---|---|
| 0 | 40-44 | Never Any | 4356 | 51.27 | | | | | |
| 0 | 40-44 | CC | 2397 | 28.21 | 0 | 0.00 | | | |
| 0 | 40-44 | Ecig | 0 | 0.00 | | | | | |
| 0 | 40-44 | Dual | 0 | 0.00 | | | | | |
| 0 | 40-44 | Former | 1744 | 20.52 | | | 7.33 | 339 | 19.44 |

Year of Follow-up: 1990, Age Group: 45-49

| Period | Age Group | Smoking Group | N | % | Ever Quit (N) | Ever Quit % | Mean Yrs Quit | Short Term (n) | Short Term % |
|---|---|---|---|---|---|---|---|---|---|
| 0 | 45-49 | Never Any | 3116 | 47.67 | | | | | |
| 0 | 45-49 | CC | 1875 | 28.69 | 0 | 0.00 | | | |
| 0 | 45-49 | Ecig | 0 | 0.00 | | | | | |
| 0 | 45-49 | Dual | 0 | 0.00 | | | | | |
| 0 | 45-49 | Former | 1545 | 23.64 | | | 12.55 | 194 | 12.56 |

Year of Follow-up: 1990, Age Group: 50-54

| Period | Age Group | Smoking Group | N | % | Ever Quit (N) | Ever Quit % | Mean Yrs Quit | Short Term (n) | Short Term % |
|---|---|---|---|---|---|---|---|---|---|
| 0 | 50-54 | Never Any | 2684 | 48.50 | | | | | |
| 0 | 50-54 | CC | 1572 | 28.41 | 0 | 0.00 | | | |

# Results for PHIM for Run 2 (Pessimistic_Analysis.RTF)

| | | | | | | | | | |
|---|---|---|---|---|---|---|---|---|---|
| 0 | 50-54 | Ecig | 0 | 0.00 | | | | | |
| 0 | 50-54 | Dual | 0 | 0.00 | | | | | |
| 0 | 50-54 | Former | 1278 | 23.09 | | | 13.28 | 126 | 9.86 |

Year of Follow-up: 1990, Age Group: 55-59

| Period | Age Group | Smoking Group | N | % | Ever Quit (N) | Ever Quit % | Mean Yrs Quit | Short Term (n) | Short Term % |
|---|---|---|---|---|---|---|---|---|---|
| 0 | 55-59 | Never Any | 2732 | 51.90 | | | | | |
| 0 | 55-59 | CC | 1295 | 24.60 | 0 | 0.00 | | | |
| 0 | 55-59 | Ecig | 0 | 0.00 | | | | | |
| 0 | 55-59 | Dual | 0 | 0.00 | | | | | |
| 0 | 55-59 | Former | 1237 | 23.50 | | | 17.26 | 90 | 7.28 |

Year of Follow-up: 1990, Age Group: 60-64

| Period | Age Group | Smoking Group | N | % | Ever Quit (N) | Ever Quit % | Mean Yrs Quit | Short Term (n) | Short Term % |
|---|---|---|---|---|---|---|---|---|---|
| 0 | 60-64 | Never Any | 3078 | 55.72 | | | | | |
| 0 | 60-64 | CC | 1249 | 22.61 | 0 | 0.00 | | | |
| 0 | 60-64 | Ecig | 0 | 0.00 | | | | | |
| 0 | 60-64 | Dual | 0 | 0.00 | | | | | |
| 0 | 60-64 | Former | 1197 | 21.67 | | | 18.28 | 61 | 5.10 |

Year of Follow-up: 1990, Age Group: 65-69

| Period | Age Group | Smoking Group | N | % | Ever Quit (N) | Ever Quit % | Mean Yrs Quit | Short Term (n) | Short Term % |
|---|---|---|---|---|---|---|---|---|---|
| 0 | 65-69 | Never Any | 3042 | 57.36 | | | | | |
| 0 | 65-69 | CC | 976 | 18.40 | 0 | 0.00 | | | |
| 0 | 65-69 | Ecig | 0 | 0.00 | | | | | |
| 0 | 65-69 | Dual | 0 | 0.00 | | | | | |
| 0 | 65-69 | Former | 1285 | 24.23 | | | 20.65 | 68 | 5.29 |

Year of Follow-up: 1990, Age Group: 70-74

| Period | Age Group | Smoking Group | N | % | Ever Quit (N) | Ever Quit % | Mean Yrs Quit | Short Term (n) | Short Term % |
|---|---|---|---|---|---|---|---|---|---|
| 0 | 70-74 | Never Any | 2738 | 61.05 | | | | | |
| 0 | 70-74 | CC | 610 | 13.60 | 0 | 0.00 | | | |
| 0 | 70-74 | Ecig | 0 | 0.00 | | | | | |
| 0 | 70-74 | Dual | 0 | 0.00 | | | | | |
| 0 | 70-74 | Former | 1137 | 25.35 | | | 24.48 | 27 | 2.37 |

Year of Follow-up: 1990, Age Group: 75-79

| Period | Age Group | Smoking Group | N | % | Ever Quit (N) | Ever Quit % | Mean Yrs Quit | Short Term (n) | Short Term % |
|---|---|---|---|---|---|---|---|---|---|
| 0 | 75-79 | Never Any | 2483 | 68.84 | | | | | |
| 0 | 75-79 | CC | 354 | 9.81 | 0 | 0.00 | | | |
| 0 | 75-79 | Ecig | 0 | 0.00 | | | | | |
| 0 | 75-79 | Dual | 0 | 0.00 | | | | | |
| 0 | 75-79 | Former | 770 | 21.35 | | | 24.48 | 24 | 3.12 |

Year of Follow-up: 1995, Age Group: All Ages

| Period | Age Group | Smoking Group | N | % | Ever Quit (N) | Ever Quit % | Mean Yrs Quit | Short Term (n) | Short Term % |
|---|---|---|---|---|---|---|---|---|---|
| 5 | All Ages | Never Any | 50436 | 55.04 | | | | | |
| 5 | All Ages | CC | 15052 | 16.43 | 0 | 0.00 | | | |
| 5 | All Ages | Ecig | 7906 | 8.63 | | | | | |
| 5 | All Ages | Dual | 0 | 0.00 | | | | | |
| 5 | All Ages | Former | 18245 | 19.91 | | | 14.29 | 1028 | 5.63 |

Year of Follow-up: 1995, Age Group: 18-19

| Period | Age Group | Smoking Group | N | % | Ever Quit (N) | Ever Quit % | Mean Yrs Quit | Short Term (n) | Short Term % |
|---|---|---|---|---|---|---|---|---|---|
| 5 | 18-19 | Never Any | 2249 | 70.81 | | | | | |
| 5 | 18-19 | CC | 276 | 8.69 | 0 | 0.00 | | | |
| 5 | 18-19 | Ecig | 594 | 18.70 | | | | | |
| 5 | 18-19 | Dual | 0 | 0.00 | | | | | |
| 5 | 18-19 | Former | 57 | 1.79 | | | 1.04 | 37 | 64.91 |

Year of Follow-up: 1995, Age Group: 20-24

| Period | Age Group | Smoking Group | N | % | Ever Quit (N) | Ever Quit % | Mean Yrs Quit | Short Term (n) | Short Term % |
|---|---|---|---|---|---|---|---|---|---|
| 5 | 20-24 | Never Any | 5544 | 66.40 | | | | | |
| 5 | 20-24 | CC | 1213 | 14.53 | 0 | 0.00 | | | |
| 5 | 20-24 | Ecig | 1173 | 14.05 | | | | | |
| 5 | 20-24 | Dual | 0 | 0.00 | | | | | |
| 5 | 20-24 | Former | 420 | 5.03 | | | 3.32 | 91 | 21.67 |

Year of Follow-up: 1995, Age Group: 25-29

| Period | Age Group | Smoking Group | N | % | Ever Quit (N) | Ever Quit % | Mean Yrs Quit | Short Term (n) | Short Term % |
|---|---|---|---|---|---|---|---|---|---|
| 5 | 25-29 | Never Any | 5302 | 60.30 | | | | | |
| 5 | 25-29 | CC | 1528 | 17.38 | 0 | 0.00 | | | |
| 5 | 25-29 | Ecig | 964 | 10.96 | | | | | |
| 5 | 25-29 | Dual | 0 | 0.00 | | | | | |
| 5 | 25-29 | Former | 999 | 11.36 | | | 6.02 | 49 | 4.90 |

Year of Follow-up: 1995, Age Group: 30-34

| Period | Age Group | Smoking Group | N | % | Ever Quit (N) | Ever Quit % | Mean Yrs Quit | Short Term (n) | Short Term % |
|---|---|---|---|---|---|---|---|---|---|

## Results for PHIM for Run 2 (Pessimistic_Analysis.RTF)

| | | | | | | | | | |
|---|---|---|---|---|---|---|---|---|---|
| 5 | 30-34 | Never Any | 5301 | 52.60 | | | | | |
| 5 | 30-34 | CC | 1922 | 19.07 | 0 | 0.00 | | | |
| 5 | 30-34 | Ecig | 1253 | 12.43 | | | | | |
| 5 | 30-34 | Dual | 0 | 0.00 | | | | | |
| 5 | 30-34 | Former | 1602 | 15.90 | | | 8.17 | 64 | 4.00 |

Year of Follow-up: 1995, Age Group: 35-39

| Period | Age Group | Smoking Group | N | % | Ever Quit (N) | Ever Quit % | Mean Yrs Quit | Short Term (n) | Short Term % |
|---|---|---|---|---|---|---|---|---|---|
| 5 | 35-39 | Never Any | 5276 | 50.25 | | | | | |
| 5 | 35-39 | CC | 2029 | 19.33 | 0 | 0.00 | | | |
| 5 | 35-39 | Ecig | 1184 | 11.28 | | | | | |
| 5 | 35-39 | Dual | 0 | 0.00 | | | | | |
| 5 | 35-39 | Former | 2010 | 19.14 | | | 8.93 | 40 | 1.99 |

Year of Follow-up: 1995, Age Group: 40-44

| Period | Age Group | Smoking Group | N | % | Ever Quit (N) | Ever Quit % | Mean Yrs Quit | Short Term (n) | Short Term % |
|---|---|---|---|---|---|---|---|---|---|
| 5 | 40-44 | Never Any | 5018 | 52.27 | | | | | |
| 5 | 40-44 | CC | 1610 | 16.77 | 0 | 0.00 | | | |
| 5 | 40-44 | Ecig | 876 | 9.13 | | | | | |
| 5 | 40-44 | Dual | 0 | 0.00 | | | | | |
| 5 | 40-44 | Former | 2096 | 21.83 | | | 12.07 | 73 | 3.48 |

Year of Follow-up: 1995, Age Group: 45-49

| Period | Age Group | Smoking Group | N | % | Ever Quit (N) | Ever Quit % | Mean Yrs Quit | Short Term (n) | Short Term % |
|---|---|---|---|---|---|---|---|---|---|
| 5 | 45-49 | Never Any | 4356 | 51.27 | | | | | |
| 5 | 45-49 | CC | 1404 | 16.52 | 0 | 0.00 | | | |
| 5 | 45-49 | Ecig | 734 | 8.64 | | | | | |
| 5 | 45-49 | Dual | 0 | 0.00 | | | | | |
| 5 | 45-49 | Former | 2003 | 23.57 | | | 10.95 | 124 | 6.19 |

Year of Follow-up: 1995, Age Group: 50-54

| Period | Age Group | Smoking Group | N | % | Ever Quit (N) | Ever Quit % | Mean Yrs Quit | Short Term (n) | Short Term % |
|---|---|---|---|---|---|---|---|---|---|
| 5 | 50-54 | Never Any | 3116 | 47.67 | | | | | |
| 5 | 50-54 | CC | 1067 | 16.32 | 0 | 0.00 | | | |
| 5 | 50-54 | Ecig | 536 | 8.20 | | | | | |
| 5 | 50-54 | Dual | 0 | 0.00 | | | | | |
| 5 | 50-54 | Former | 1817 | 27.80 | | | 15.21 | 119 | 6.55 |

Year of Follow-up: 1995, Age Group: 55-59

| Period | Age Group | Smoking Group | N | % | Ever Quit (N) | Ever Quit % | Mean Yrs Quit | Short Term (n) | Short Term % |
|---|---|---|---|---|---|---|---|---|---|
| 5 | 55-59 | Never Any | 2684 | 48.50 | | | | | |
| 5 | 55-59 | CC | 1039 | 18.77 | 0 | 0.00 | | | |
| 5 | 55-59 | Ecig | 305 | 5.51 | | | | | |
| 5 | 55-59 | Dual | 0 | 0.00 | | | | | |
| 5 | 55-59 | Former | 1506 | 27.21 | | | 15.78 | 100 | 6.64 |

Year of Follow-up: 1995, Age Group: 60-64

| Period | Age Group | Smoking Group | N | % | Ever Quit (N) | Ever Quit % | Mean Yrs Quit | Short Term (n) | Short Term % |
|---|---|---|---|---|---|---|---|---|---|
| 5 | 60-64 | Never Any | 2732 | 51.90 | | | | | |
| 5 | 60-64 | CC | 900 | 17.10 | 0 | 0.00 | | | |
| 5 | 60-64 | Ecig | 182 | 3.46 | | | | | |
| 5 | 60-64 | Dual | 0 | 0.00 | | | | | |
| 5 | 60-64 | Former | 1450 | 27.55 | | | 19.27 | 84 | 5.79 |

Year of Follow-up: 1995, Age Group: 65-69

| Period | Age Group | Smoking Group | N | % | Ever Quit (N) | Ever Quit % | Mean Yrs Quit | Short Term (n) | Short Term % |
|---|---|---|---|---|---|---|---|---|---|
| 5 | 65-69 | Never Any | 3078 | 55.72 | | | | | |
| 5 | 65-69 | CC | 866 | 15.68 | 0 | 0.00 | | | |
| 5 | 65-69 | Ecig | 95 | 1.72 | | | | | |
| 5 | 65-69 | Dual | 0 | 0.00 | | | | | |
| 5 | 65-69 | Former | 1485 | 26.88 | | | 19.19 | 97 | 6.53 |

Year of Follow-up: 1995, Age Group: 70-74

| Period | Age Group | Smoking Group | N | % | Ever Quit (N) | Ever Quit % | Mean Yrs Quit | Short Term (n) | Short Term % |
|---|---|---|---|---|---|---|---|---|---|
| 5 | 70-74 | Never Any | 3042 | 57.36 | | | | | |
| 5 | 70-74 | CC | 743 | 14.01 | 0 | 0.00 | | | |
| 5 | 70-74 | Ecig | 10 | 0.19 | | | | | |
| 5 | 70-74 | Dual | 0 | 0.00 | | | | | |
| 5 | 70-74 | Former | 1508 | 28.44 | | | 22.14 | 87 | 5.77 |

Year of Follow-up: 1995, Age Group: 75-79

| Period | Age Group | Smoking Group | N | % | Ever Quit (N) | Ever Quit % | Mean Yrs Quit | Short Term (n) | Short Term % |
|---|---|---|---|---|---|---|---|---|---|
| 5 | 75-79 | Never Any | 2738 | 61.05 | | | | | |
| 5 | 75-79 | CC | 455 | 10.14 | 0 | 0.00 | | | |
| 5 | 75-79 | Ecig | 0 | 0.00 | | | | | |
| 5 | 75-79 | Dual | 0 | 0.00 | | | | | |
| 5 | 75-79 | Former | 1292 | 28.81 | | | 26.18 | 63 | 4.88 |

Year of Follow-up: 2000, Age Group: All Ages

# Results for PHIM for Run 2 (Pessimistic_Analysis.RTF)

| Period | Age Group | Smoking Group | N | % | Ever Quit (N) | Ever Quit % | Mean Yrs Quit | Short Term (n) | Short Term % |
|---|---|---|---|---|---|---|---|---|---|
| 10 | All Ages | Never Any | 49386 | 53.73 | | | | | |
| 10 | All Ages | CC | 9072 | 9.87 | 0 | 0.00 | | | |
| 10 | All Ages | Ecig | 13000 | 14.14 | | | | | |
| 10 | All Ages | Dual | 0 | 0.00 | | | | | |
| 10 | All Ages | Former | 20450 | 22.25 | | | 15.60 | 1372 | 6.71 |

Year of Follow-up: 2000, Age Group: 18-19

| Period | Age Group | Smoking Group | N | % | Ever Quit (N) | Ever Quit % | Mean Yrs Quit | Short Term (n) | Short Term % |
|---|---|---|---|---|---|---|---|---|---|
| 10 | 18-19 | Never Any | 0 | . | | | | | |
| 10 | 18-19 | CC | 0 | . | 0 | . | | | |
| 10 | 18-19 | Ecig | 0 | . | | | | | |
| 10 | 18-19 | Dual | 0 | . | | | | | |
| 10 | 18-19 | Former | 0 | . | | | . | 0 | . |

Year of Follow-up: 2000, Age Group: 20-24

| Period | Age Group | Smoking Group | N | % | Ever Quit (N) | Ever Quit % | Mean Yrs Quit | Short Term (n) | Short Term % |
|---|---|---|---|---|---|---|---|---|---|
| 10 | 20-24 | Never Any | 4505 | 56.81 | | | | | |
| 10 | 20-24 | CC | 580 | 7.31 | 0 | 0.00 | | | |
| 10 | 20-24 | Ecig | 2296 | 28.95 | | | | | |
| 10 | 20-24 | Dual | 0 | 0.00 | | | | | |
| 10 | 20-24 | Former | 549 | 6.92 | | | 3.53 | 150 | 27.32 |

Year of Follow-up: 2000, Age Group: 25-29

| Period | Age Group | Smoking Group | N | % | Ever Quit (N) | Ever Quit % | Mean Yrs Quit | Short Term (n) | Short Term % |
|---|---|---|---|---|---|---|---|---|---|
| 10 | 25-29 | Never Any | 5140 | 61.56 | | | | | |
| 10 | 25-29 | CC | 711 | 8.51 | 0 | 0.00 | | | |
| 10 | 25-29 | Ecig | 1817 | 21.76 | | | | | |
| 10 | 25-29 | Dual | 0 | 0.00 | | | | | |
| 10 | 25-29 | Former | 682 | 8.17 | | | 5.88 | 94 | 13.78 |

Year of Follow-up: 2000, Age Group: 30-34

| Period | Age Group | Smoking Group | N | % | Ever Quit (N) | Ever Quit % | Mean Yrs Quit | Short Term (n) | Short Term % |
|---|---|---|---|---|---|---|---|---|---|
| 10 | 30-34 | Never Any | 5169 | 58.79 | | | | | |
| 10 | 30-34 | CC | 856 | 9.74 | 0 | 0.00 | | | |
| 10 | 30-34 | Ecig | 1579 | 17.96 | | | | | |
| 10 | 30-34 | Dual | 0 | 0.00 | | | | | |
| 10 | 30-34 | Former | 1189 | 13.52 | | | 9.57 | 70 | 5.89 |

Year of Follow-up: 2000, Age Group: 35-39

| Period | Age Group | Smoking Group | N | % | Ever Quit (N) | Ever Quit % | Mean Yrs Quit | Short Term (n) | Short Term % |
|---|---|---|---|---|---|---|---|---|---|
| 10 | 35-39 | Never Any | 5270 | 52.29 | | | | | |
| 10 | 35-39 | CC | 1115 | 11.06 | 0 | 0.00 | | | |
| 10 | 35-39 | Ecig | 1904 | 18.89 | | | | | |
| 10 | 35-39 | Dual | 0 | 0.00 | | | | | |
| 10 | 35-39 | Former | 1789 | 17.75 | | | 11.98 | 87 | 4.86 |

Year of Follow-up: 2000, Age Group: 40-44

| Period | Age Group | Smoking Group | N | % | Ever Quit (N) | Ever Quit % | Mean Yrs Quit | Short Term (n) | Short Term % |
|---|---|---|---|---|---|---|---|---|---|
| 10 | 40-44 | Never Any | 5276 | 50.25 | | | | | |
| 10 | 40-44 | CC | 1227 | 11.69 | 0 | 0.00 | | | |
| 10 | 40-44 | Ecig | 1749 | 16.66 | | | | | |
| 10 | 40-44 | Dual | 0 | 0.00 | | | | | |
| 10 | 40-44 | Former | 2247 | 21.40 | | | 12.63 | 115 | 5.12 |

Year of Follow-up: 2000, Age Group: 45-49

| Period | Age Group | Smoking Group | N | % | Ever Quit (N) | Ever Quit % | Mean Yrs Quit | Short Term (n) | Short Term % |
|---|---|---|---|---|---|---|---|---|---|
| 10 | 45-49 | Never Any | 5018 | 52.27 | | | | | |
| 10 | 45-49 | CC | 949 | 9.89 | 0 | 0.00 | | | |
| 10 | 45-49 | Ecig | 1275 | 13.28 | | | | | |
| 10 | 45-49 | Dual | 0 | 0.00 | | | | | |
| 10 | 45-49 | Former | 2358 | 24.56 | | | 15.37 | 118 | 5.00 |

Year of Follow-up: 2000, Age Group: 50-54

| Period | Age Group | Smoking Group | N | % | Ever Quit (N) | Ever Quit % | Mean Yrs Quit | Short Term (n) | Short Term % |
|---|---|---|---|---|---|---|---|---|---|
| 10 | 50-54 | Never Any | 4356 | 51.27 | | | | | |
| 10 | 50-54 | CC | 772 | 9.09 | 0 | 0.00 | | | |
| 10 | 50-54 | Ecig | 1029 | 12.11 | | | | | |
| 10 | 50-54 | Dual | 0 | 0.00 | | | | | |
| 10 | 50-54 | Former | 2340 | 27.54 | | | 13.93 | 130 | 5.56 |

Year of Follow-up: 2000, Age Group: 55-59

| Period | Age Group | Smoking Group | N | % | Ever Quit (N) | Ever Quit % | Mean Yrs Quit | Short Term (n) | Short Term % |
|---|---|---|---|---|---|---|---|---|---|
| 10 | 55-59 | Never Any | 3116 | 47.67 | | | | | |
| 10 | 55-59 | CC | 614 | 9.39 | 0 | 0.00 | | | |
| 10 | 55-59 | Ecig | 678 | 10.37 | | | | | |
| 10 | 55-59 | Dual | 0 | 0.00 | | | | | |

| | | | | | | | | | |
|---|---|---|---|---|---|---|---|---|---|
| 10 | 55-59 | Former | 2128 | 32.56 | | | 17.55 | 123 | 5.78 |

Year of Follow-up: 2000, Age Group: 60-64

| Period | Age Group | Smoking Group | N | % | Ever Quit (N) | Ever Quit % | Mean Yrs Quit | Short Term (n) | Short Term % |
|---|---|---|---|---|---|---|---|---|---|
| 10 | 60-64 | Never Any | 2684 | 48.50 | | | | | |
| 10 | 60-64 | CC | 661 | 11.94 | 0 | 0.00 | | | |
| 10 | 60-64 | Ecig | 377 | 6.81 | | | | | |
| 10 | 60-64 | Dual | 0 | 0.00 | | | | | |
| 10 | 60-64 | Former | 1812 | 32.74 | | | 17.58 | 137 | 7.56 |

Year of Follow-up: 2000, Age Group: 65-69

| Period | Age Group | Smoking Group | N | % | Ever Quit (N) | Ever Quit % | Mean Yrs Quit | Short Term (n) | Short Term % |
|---|---|---|---|---|---|---|---|---|---|
| 10 | 65-69 | Never Any | 2732 | 51.90 | | | | | |
| 10 | 65-69 | CC | 581 | 11.04 | 0 | 0.00 | | | |
| 10 | 65-69 | Ecig | 201 | 3.82 | | | | | |
| 10 | 65-69 | Dual | 0 | 0.00 | | | | | |
| 10 | 65-69 | Former | 1750 | 33.24 | | | 20.45 | 112 | 6.40 |

Year of Follow-up: 2000, Age Group: 70-74

| Period | Age Group | Smoking Group | N | % | Ever Quit (N) | Ever Quit % | Mean Yrs Quit | Short Term (n) | Short Term % |
|---|---|---|---|---|---|---|---|---|---|
| 10 | 70-74 | Never Any | 3078 | 55.72 | | | | | |
| 10 | 70-74 | CC | 558 | 10.10 | 0 | 0.00 | | | |
| 10 | 70-74 | Ecig | 86 | 1.56 | | | | | |
| 10 | 70-74 | Dual | 0 | 0.00 | | | | | |
| 10 | 70-74 | Former | 1802 | 32.62 | | | 20.28 | 132 | 7.33 |

Year of Follow-up: 2000, Age Group: 75-79

| Period | Age Group | Smoking Group | N | % | Ever Quit (N) | Ever Quit % | Mean Yrs Quit | Short Term (n) | Short Term % |
|---|---|---|---|---|---|---|---|---|---|
| 10 | 75-79 | Never Any | 3042 | 57.36 | | | | | |
| 10 | 75-79 | CC | 448 | 8.45 | 0 | 0.00 | | | |
| 10 | 75-79 | Ecig | 9 | 0.17 | | | | | |
| 10 | 75-79 | Dual | 0 | 0.00 | | | | | |
| 10 | 75-79 | Former | 1804 | 34.02 | | | 23.03 | 104 | 5.76 |

Year of Follow-up: 2005, Age Group: All Ages

| Period | Age Group | Smoking Group | N | % | Ever Quit (N) | Ever Quit % | Mean Yrs Quit | Short Term (n) | Short Term % |
|---|---|---|---|---|---|---|---|---|---|
| 15 | All Ages | Never Any | 45867 | 52.96 | | | | | |
| 15 | All Ages | CC | 5423 | 6.26 | 0 | 0.00 | | | |
| 15 | All Ages | Ecig | 14616 | 16.88 | | | | | |
| 15 | All Ages | Dual | 0 | 0.00 | | | | | |
| 15 | All Ages | Former | 20699 | 23.90 | | | 18.13 | 737 | 3.56 |

Year of Follow-up: 2005, Age Group: 18-19

| Period | Age Group | Smoking Group | N | % | Ever Quit (N) | Ever Quit % | Mean Yrs Quit | Short Term (n) | Short Term % |
|---|---|---|---|---|---|---|---|---|---|
| 15 | 18-19 | Never Any | 0 | . | | | | | |
| 15 | 18-19 | CC | 0 | . | 0 | . | | | |
| 15 | 18-19 | Ecig | 0 | . | | | | | |
| 15 | 18-19 | Dual | 0 | . | | | | | |
| 15 | 18-19 | Former | 0 | . | | | . | 0 | . |

Year of Follow-up: 2005, Age Group: 20-24

| Period | Age Group | Smoking Group | N | % | Ever Quit (N) | Ever Quit % | Mean Yrs Quit | Short Term (n) | Short Term % |
|---|---|---|---|---|---|---|---|---|---|
| 15 | 20-24 | Never Any | 0 | . | | | | | |
| 15 | 20-24 | CC | 0 | . | 0 | . | | | |
| 15 | 20-24 | Ecig | 0 | . | | | | | |
| 15 | 20-24 | Dual | 0 | . | | | | | |
| 15 | 20-24 | Former | 0 | . | | | . | 0 | . |

Year of Follow-up: 2005, Age Group: 25-29

| Period | Age Group | Smoking Group | N | % | Ever Quit (N) | Ever Quit % | Mean Yrs Quit | Short Term (n) | Short Term % |
|---|---|---|---|---|---|---|---|---|---|
| 15 | 25-29 | Never Any | 4174 | 52.64 | | | | | |
| 15 | 25-29 | CC | 341 | 4.30 | 0 | 0.00 | | | |
| 15 | 25-29 | Ecig | 2625 | 33.10 | | | | | |
| 15 | 25-29 | Dual | 0 | 0.00 | | | | | |
| 15 | 25-29 | Former | 790 | 9.96 | | | 6.60 | 90 | 11.39 |

Year of Follow-up: 2005, Age Group: 30-34

| Period | Age Group | Smoking Group | N | % | Ever Quit (N) | Ever Quit % | Mean Yrs Quit | Short Term (n) | Short Term % |
|---|---|---|---|---|---|---|---|---|---|
| 15 | 30-34 | Never Any | 5026 | 60.19 | | | | | |
| 15 | 30-34 | CC | 410 | 4.91 | 0 | 0.00 | | | |
| 15 | 30-34 | Ecig | 2088 | 25.01 | | | | | |
| 15 | 30-34 | Dual | 0 | 0.00 | | | | | |
| 15 | 30-34 | Former | 826 | 9.89 | | | 9.38 | 42 | 5.08 |

Year of Follow-up: 2005, Age Group: 35-39

| Period | Age Group | Smoking Group | N | % | Ever Quit (N) | Ever Quit % | Mean Yrs Quit | Short Term (n) | Short Term % |
|---|---|---|---|---|---|---|---|---|---|
| 15 | 35-39 | Never Any | 5137 | 58.42 | | | | | |
| 15 | 35-39 | CC | 503 | 5.72 | 0 | 0.00 | | | |

# Results for PHIM for Run 2 (Pessimistic_Analysis.RTF)

| | | | | | | | | | |
|---|---|---|---|---|---|---|---|---|---|
| 15 | 35-39 | Ecig | 1854 | 21.08 | | | | | |
| 15 | 35-39 | Dual | 0 | 0.00 | | | | | |
| 15 | 35-39 | Former | 1299 | 14.77 | | | 13.52 | 43 | 3.31 |
| Year of Follow-up: 2005, Age Group: 40-44 | | | | | | | | | |
| Period | Age Group | Smoking Group | N | % | Ever Quit (N) | Ever Quit % | Mean Yrs Quit | Short Term (n) | Short Term % |
| 15 | 40-44 | Never Any | 5270 | 52.29 | | | | | |
| 15 | 40-44 | CC | 674 | 6.69 | 0 | 0.00 | | | |
| 15 | 40-44 | Ecig | 2168 | 21.51 | | | | | |
| 15 | 40-44 | Dual | 0 | 0.00 | | | | | |
| 15 | 40-44 | Former | 1966 | 19.51 | | | 15.64 | 74 | 3.76 |
| Year of Follow-up: 2005, Age Group: 45-49 | | | | | | | | | |
| Period | Age Group | Smoking Group | N | % | Ever Quit (N) | Ever Quit % | Mean Yrs Quit | Short Term (n) | Short Term % |
| 15 | 45-49 | Never Any | 5276 | 50.25 | | | | | |
| 15 | 45-49 | CC | 757 | 7.21 | 0 | 0.00 | | | |
| 15 | 45-49 | Ecig | 2017 | 19.21 | | | | | |
| 15 | 45-49 | Dual | 0 | 0.00 | | | | | |
| 15 | 45-49 | Former | 2449 | 23.33 | | | 16.34 | 91 | 3.72 |
| Year of Follow-up: 2005, Age Group: 50-54 | | | | | | | | | |
| Period | Age Group | Smoking Group | N | % | Ever Quit (N) | Ever Quit % | Mean Yrs Quit | Short Term (n) | Short Term % |
| 15 | 50-54 | Never Any | 5018 | 52.27 | | | | | |
| 15 | 50-54 | CC | 583 | 6.07 | 0 | 0.00 | | | |
| 15 | 50-54 | Ecig | 1428 | 14.88 | | | | | |
| 15 | 50-54 | Dual | 0 | 0.00 | | | | | |
| 15 | 50-54 | Former | 2571 | 26.78 | | | 18.87 | 69 | 2.68 |
| Year of Follow-up: 2005, Age Group: 55-59 | | | | | | | | | |
| Period | Age Group | Smoking Group | N | % | Ever Quit (N) | Ever Quit % | Mean Yrs Quit | Short Term (n) | Short Term % |
| 15 | 55-59 | Never Any | 4356 | 51.27 | | | | | |
| 15 | 55-59 | CC | 510 | 6.00 | 0 | 0.00 | | | |
| 15 | 55-59 | Ecig | 1124 | 13.23 | | | | | |
| 15 | 55-59 | Dual | 0 | 0.00 | | | | | |
| 15 | 55-59 | Former | 2507 | 29.50 | | | 17.82 | 55 | 2.19 |
| Year of Follow-up: 2005, Age Group: 60-64 | | | | | | | | | |
| Period | Age Group | Smoking Group | N | % | Ever Quit (N) | Ever Quit % | Mean Yrs Quit | Short Term (n) | Short Term % |
| 15 | 60-64 | Never Any | 3116 | 47.67 | | | | | |
| 15 | 60-64 | CC | 423 | 6.47 | 0 | 0.00 | | | |
| 15 | 60-64 | Ecig | 686 | 10.50 | | | | | |
| 15 | 60-64 | Dual | 0 | 0.00 | | | | | |
| 15 | 60-64 | Former | 2311 | 35.36 | | | 20.91 | 81 | 3.50 |
| Year of Follow-up: 2005, Age Group: 65-69 | | | | | | | | | |
| Period | Age Group | Smoking Group | N | % | Ever Quit (N) | Ever Quit % | Mean Yrs Quit | Short Term (n) | Short Term % |
| 15 | 65-69 | Never Any | 2684 | 48.50 | | | | | |
| 15 | 65-69 | CC | 457 | 8.26 | 0 | 0.00 | | | |
| 15 | 65-69 | Ecig | 374 | 6.76 | | | | | |
| 15 | 65-69 | Dual | 0 | 0.00 | | | | | |
| 15 | 65-69 | Former | 2019 | 36.48 | | | 20.52 | 55 | 2.72 |
| Year of Follow-up: 2005, Age Group: 70-74 | | | | | | | | | |
| Period | Age Group | Smoking Group | N | % | Ever Quit (N) | Ever Quit % | Mean Yrs Quit | Short Term (n) | Short Term % |
| 15 | 70-74 | Never Any | 2732 | 51.90 | | | | | |
| 15 | 70-74 | CC | 387 | 7.35 | 0 | 0.00 | | | |
| 15 | 70-74 | Ecig | 177 | 3.36 | | | | | |
| 15 | 70-74 | Dual | 0 | 0.00 | | | | | |
| 15 | 70-74 | Former | 1968 | 37.39 | | | 22.87 | 77 | 3.91 |
| Year of Follow-up: 2005, Age Group: 75-79 | | | | | | | | | |
| Period | Age Group | Smoking Group | N | % | Ever Quit (N) | Ever Quit % | Mean Yrs Quit | Short Term (n) | Short Term % |
| 15 | 75-79 | Never Any | 3078 | 55.72 | | | | | |
| 15 | 75-79 | CC | 378 | 6.84 | 0 | 0.00 | | | |
| 15 | 75-79 | Ecig | 75 | 1.36 | | | | | |
| 15 | 75-79 | Dual | 0 | 0.00 | | | | | |
| 15 | 75-79 | Former | 1993 | 36.08 | | | 23.08 | 60 | 3.01 |
| Year of Follow-up: 2010, Age Group: All Ages | | | | | | | | | |
| Period | Age Group | Smoking Group | N | % | Ever Quit (N) | Ever Quit % | Mean Yrs Quit | Short Term (n) | Short Term % |
| 20 | All Ages | Never Any | 42596 | 52.54 | | | | | |
| 20 | All Ages | CC | 3145 | 3.88 | 0 | 0.00 | | | |
| 20 | All Ages | Ecig | 14615 | 18.03 | | | | | |
| 20 | All Ages | Dual | 0 | 0.00 | | | | | |
| 20 | All Ages | Former | 20725 | 25.56 | | | 20.58 | 862 | 4.16 |
| Year of Follow-up: 2010, Age Group: 18-19 | | | | | | | | | |
| Period | Age Group | Smoking Group | N | % | Ever Quit (N) | Ever Quit % | Mean Yrs Quit | Short Term (n) | Short Term % |

# Results for PHIM for Run 2 (Pessimistic_Analysis.RTF)

| | | | | | | | | | |
|---|---|---|---|---|---|---|---|---|---|
| 20 | 18-19 | Never Any | 0 | . | | | | | |
| 20 | 18-19 | CC | 0 | . | 0 | . | | | |
| 20 | 18-19 | Ecig | 0 | . | | | | | |
| 20 | 18-19 | Dual | 0 | . | | | | | |
| 20 | 18-19 | Former | 0 | . | | | . | 0 | . |

Year of Follow-up: 2010, Age Group: 20-24

| Period | Age Group | Smoking Group | N | % | Ever Quit (N) | Ever Quit % | Mean Yrs Quit | Short Term (n) | Short Term % |
|---|---|---|---|---|---|---|---|---|---|
| 20 | 20-24 | Never Any | 0 | . | | | | | |
| 20 | 20-24 | CC | 0 | . | 0 | . | | | |
| 20 | 20-24 | Ecig | 0 | . | | | | | |
| 20 | 20-24 | Dual | 0 | . | | | | | |
| 20 | 20-24 | Former | 0 | . | | | . | 0 | . |

Year of Follow-up: 2010, Age Group: 25-29

| Period | Age Group | Smoking Group | N | % | Ever Quit (N) | Ever Quit % | Mean Yrs Quit | Short Term (n) | Short Term % |
|---|---|---|---|---|---|---|---|---|---|
| 20 | 25-29 | Never Any | 0 | . | | | | | |
| 20 | 25-29 | CC | 0 | . | 0 | . | | | |
| 20 | 25-29 | Ecig | 0 | . | | | | | |
| 20 | 25-29 | Dual | 0 | . | | | | | |
| 20 | 25-29 | Former | 0 | . | | | . | 0 | . |

Year of Follow-up: 2010, Age Group: 30-34

| Period | Age Group | Smoking Group | N | % | Ever Quit (N) | Ever Quit % | Mean Yrs Quit | Short Term (n) | Short Term % |
|---|---|---|---|---|---|---|---|---|---|
| 20 | 30-34 | Never Any | 4039 | 50.93 | | | | | |
| 20 | 30-34 | CC | 177 | 2.23 | 0 | 0.00 | | | |
| 20 | 30-34 | Ecig | 2697 | 34.01 | | | | | |
| 20 | 30-34 | Dual | 0 | 0.00 | | | | | |
| 20 | 30-34 | Former | 1017 | 12.82 | | | 9.38 | 105 | 10.32 |

Year of Follow-up: 2010, Age Group: 35-39

| Period | Age Group | Smoking Group | N | % | Ever Quit (N) | Ever Quit % | Mean Yrs Quit | Short Term (n) | Short Term % |
|---|---|---|---|---|---|---|---|---|---|
| 20 | 35-39 | Never Any | 4968 | 59.50 | | | | | |
| 20 | 35-39 | CC | 230 | 2.75 | 0 | 0.00 | | | |
| 20 | 35-39 | Ecig | 2138 | 25.60 | | | | | |
| 20 | 35-39 | Dual | 0 | 0.00 | | | | | |
| 20 | 35-39 | Former | 1014 | 12.14 | | | 12.07 | 72 | 7.10 |

Year of Follow-up: 2010, Age Group: 40-44

| Period | Age Group | Smoking Group | N | % | Ever Quit (N) | Ever Quit % | Mean Yrs Quit | Short Term (n) | Short Term % |
|---|---|---|---|---|---|---|---|---|---|
| 20 | 40-44 | Never Any | 5137 | 58.42 | | | | | |
| 20 | 40-44 | CC | 274 | 3.12 | 0 | 0.00 | | | |
| 20 | 40-44 | Ecig | 1909 | 21.71 | | | | | |
| 20 | 40-44 | Dual | 0 | 0.00 | | | | | |
| 20 | 40-44 | Former | 1473 | 16.75 | | | 16.55 | 77 | 5.23 |

Year of Follow-up: 2010, Age Group: 45-49

| Period | Age Group | Smoking Group | N | % | Ever Quit (N) | Ever Quit % | Mean Yrs Quit | Short Term (n) | Short Term % |
|---|---|---|---|---|---|---|---|---|---|
| 20 | 45-49 | Never Any | 5270 | 52.29 | | | | | |
| 20 | 45-49 | CC | 407 | 4.04 | 0 | 0.00 | | | |
| 20 | 45-49 | Ecig | 2262 | 22.44 | | | | | |
| 20 | 45-49 | Dual | 0 | 0.00 | | | | | |
| 20 | 45-49 | Former | 2139 | 21.22 | | | 19.12 | 74 | 3.46 |

Year of Follow-up: 2010, Age Group: 50-54

| Period | Age Group | Smoking Group | N | % | Ever Quit (N) | Ever Quit % | Mean Yrs Quit | Short Term (n) | Short Term % |
|---|---|---|---|---|---|---|---|---|---|
| 20 | 50-54 | Never Any | 5276 | 50.25 | | | | | |
| 20 | 50-54 | CC | 458 | 4.36 | 0 | 0.00 | | | |
| 20 | 50-54 | Ecig | 2072 | 19.74 | | | | | |
| 20 | 50-54 | Dual | 0 | 0.00 | | | | | |
| 20 | 50-54 | Former | 2693 | 25.65 | | | 19.56 | 123 | 4.57 |

Year of Follow-up: 2010, Age Group: 55-59

| Period | Age Group | Smoking Group | N | % | Ever Quit (N) | Ever Quit % | Mean Yrs Quit | Short Term (n) | Short Term % |
|---|---|---|---|---|---|---|---|---|---|
| 20 | 55-59 | Never Any | 5018 | 52.27 | | | | | |
| 20 | 55-59 | CC | 386 | 4.02 | 0 | 0.00 | | | |
| 20 | 55-59 | Ecig | 1434 | 14.94 | | | | | |
| 20 | 55-59 | Dual | 0 | 0.00 | | | | | |
| 20 | 55-59 | Former | 2762 | 28.77 | | | 22.37 | 68 | 2.46 |

Year of Follow-up: 2010, Age Group: 60-64

| Period | Age Group | Smoking Group | N | % | Ever Quit (N) | Ever Quit % | Mean Yrs Quit | Short Term (n) | Short Term % |
|---|---|---|---|---|---|---|---|---|---|
| 20 | 60-64 | Never Any | 4356 | 51.27 | | | | | |
| 20 | 60-64 | CC | 334 | 3.93 | 0 | 0.00 | | | |
| 20 | 60-64 | Ecig | 1049 | 12.35 | | | | | |
| 20 | 60-64 | Dual | 0 | 0.00 | | | | | |
| 20 | 60-64 | Former | 2758 | 32.46 | | | 20.89 | 129 | 4.68 |

Year of Follow-up: 2010, Age Group: 65-69

# Results for PHIM for Run 2 (Pessimistic_Analysis.RTF)

| Period | Age Group | Smoking Group | N | % | Ever Quit (N) | Ever Quit % | Mean Yrs Quit | Short Term (n) | Short Term % |
|---|---|---|---|---|---|---|---|---|---|
| 20 | 65-69 | Never Any | 3116 | 47.67 | | | | | |
| 20 | 65-69 | CC | 276 | 4.22 | 0 | 0.00 | | | |
| 20 | 65-69 | Ecig | 583 | 8.92 | | | | | |
| 20 | 65-69 | Dual | 0 | 0.00 | | | | | |
| 20 | 65-69 | Former | 2561 | 39.18 | | | 23.59 | 90 | 3.51 |

Year of Follow-up: 2010, Age Group: 70-74

| Period | Age Group | Smoking Group | N | % | Ever Quit (N) | Ever Quit % | Mean Yrs Quit | Short Term (n) | Short Term % |
|---|---|---|---|---|---|---|---|---|---|
| 20 | 70-74 | Never Any | 2684 | 48.50 | | | | | |
| 20 | 70-74 | CC | 327 | 5.91 | 0 | 0.00 | | | |
| 20 | 70-74 | Ecig | 333 | 6.02 | | | | | |
| 20 | 70-74 | Dual | 0 | 0.00 | | | | | |
| 20 | 70-74 | Former | 2190 | 39.57 | | | 23.68 | 64 | 2.92 |

Year of Follow-up: 2010, Age Group: 75-79

| Period | Age Group | Smoking Group | N | % | Ever Quit (N) | Ever Quit % | Mean Yrs Quit | Short Term (n) | Short Term % |
|---|---|---|---|---|---|---|---|---|---|
| 20 | 75-79 | Never Any | 2732 | 51.90 | | | | | |
| 20 | 75-79 | CC | 276 | 5.24 | 0 | 0.00 | | | |
| 20 | 75-79 | Ecig | 138 | 2.62 | | | | | |
| 20 | 75-79 | Dual | 0 | 0.00 | | | | | |
| 20 | 75-79 | Former | 2118 | 40.24 | | | 26.04 | 60 | 2.83 |

Year of Follow-up: 2015, Age Group: All Ages

| Period | Age Group | Smoking Group | N | % | Ever Quit (N) | Ever Quit % | Mean Yrs Quit | Short Term (n) | Short Term % |
|---|---|---|---|---|---|---|---|---|---|
| 25 | All Ages | Never Any | 39842 | 52.55 | | | | | |
| 25 | All Ages | CC | 1922 | 2.54 | 0 | 0.00 | | | |
| 25 | All Ages | Ecig | 13689 | 18.06 | | | | | |
| 25 | All Ages | Dual | 0 | 0.00 | | | | | |
| 25 | All Ages | Former | 20364 | 26.86 | | | 22.98 | 668 | 3.28 |

Year of Follow-up: 2015, Age Group: 18-19

| Period | Age Group | Smoking Group | N | % | Ever Quit (N) | Ever Quit % | Mean Yrs Quit | Short Term (n) | Short Term % |
|---|---|---|---|---|---|---|---|---|---|
| 25 | 18-19 | Never Any | 0 | . | | | | | |
| 25 | 18-19 | CC | 0 | . | 0 | . | | | |
| 25 | 18-19 | Ecig | 0 | . | | | | | |
| 25 | 18-19 | Dual | 0 | . | | | | | |
| 25 | 18-19 | Former | 0 | . | | | . | 0 | . |

Year of Follow-up: 2015, Age Group: 20-24

| Period | Age Group | Smoking Group | N | % | Ever Quit (N) | Ever Quit % | Mean Yrs Quit | Short Term (n) | Short Term % |
|---|---|---|---|---|---|---|---|---|---|
| 25 | 20-24 | Never Any | 0 | . | | | | | |
| 25 | 20-24 | CC | 0 | . | 0 | . | | | |
| 25 | 20-24 | Ecig | 0 | . | | | | | |
| 25 | 20-24 | Dual | 0 | . | | | | | |
| 25 | 20-24 | Former | 0 | . | | | . | 0 | . |

Year of Follow-up: 2015, Age Group: 25-29

| Period | Age Group | Smoking Group | N | % | Ever Quit (N) | Ever Quit % | Mean Yrs Quit | Short Term (n) | Short Term % |
|---|---|---|---|---|---|---|---|---|---|
| 25 | 25-29 | Never Any | 0 | . | | | | | |
| 25 | 25-29 | CC | 0 | . | 0 | . | | | |
| 25 | 25-29 | Ecig | 0 | . | | | | | |
| 25 | 25-29 | Dual | 0 | . | | | | | |
| 25 | 25-29 | Former | 0 | . | | | . | 0 | . |

Year of Follow-up: 2015, Age Group: 30-34

| Period | Age Group | Smoking Group | N | % | Ever Quit (N) | Ever Quit % | Mean Yrs Quit | Short Term (n) | Short Term % |
|---|---|---|---|---|---|---|---|---|---|
| 25 | 30-34 | Never Any | 0 | . | | | | | |
| 25 | 30-34 | CC | 0 | . | 0 | . | | | |
| 25 | 30-34 | Ecig | 0 | . | | | | | |
| 25 | 30-34 | Dual | 0 | . | | | | | |
| 25 | 30-34 | Former | 0 | . | | | . | 0 | . |

Year of Follow-up: 2015, Age Group: 35-39

| Period | Age Group | Smoking Group | N | % | Ever Quit (N) | Ever Quit % | Mean Yrs Quit | Short Term (n) | Short Term % |
|---|---|---|---|---|---|---|---|---|---|
| 25 | 35-39 | Never Any | 4017 | 50.66 | | | | | |
| 25 | 35-39 | CC | 85 | 1.07 | 0 | 0.00 | | | |
| 25 | 35-39 | Ecig | 2516 | 31.73 | | | | | |
| 25 | 35-39 | Dual | 0 | 0.00 | | | | | |
| 25 | 35-39 | Former | 1312 | 16.54 | | | 11.63 | 101 | 7.70 |

Year of Follow-up: 2015, Age Group: 40-44

| Period | Age Group | Smoking Group | N | % | Ever Quit (N) | Ever Quit % | Mean Yrs Quit | Short Term (n) | Short Term % |
|---|---|---|---|---|---|---|---|---|---|
| 25 | 40-44 | Never Any | 4968 | 59.50 | | | | | |
| 25 | 40-44 | CC | 145 | 1.74 | 0 | 0.00 | | | |
| 25 | 40-44 | Ecig | 2040 | 24.43 | | | | | |
| 25 | 40-44 | Dual | 0 | 0.00 | | | | | |

# Results for PHIM for Run 2 (Pessimistic_Analysis.RTF)

| | | | | | | | | | |
|---|---|---|---|---|---|---|---|---|---|
| 25 | 40-44 | Former | 1197 | 14.34 | | | 14.76 | 75 | 6.27 |

Year of Follow-up: 2015, Age Group: 45-49

| Period | Age Group | Smoking Group | N | % | Ever Quit (N) | Ever Quit % | Mean Yrs Quit | Short Term (n) | Short Term % |
|---|---|---|---|---|---|---|---|---|---|
| 25 | 45-49 | Never Any | 5137 | 58.42 | | | | | |
| 25 | 45-49 | CC | 168 | 1.91 | 0 | 0.00 | | | |
| 25 | 45-49 | Ecig | 1860 | 21.15 | | | | | |
| 25 | 45-49 | Dual | 0 | 0.00 | | | | | |
| 25 | 45-49 | Former | 1628 | 18.51 | | | 19.68 | 59 | 3.62 |

Year of Follow-up: 2015, Age Group: 50-54

| Period | Age Group | Smoking Group | N | % | Ever Quit (N) | Ever Quit % | Mean Yrs Quit | Short Term (n) | Short Term % |
|---|---|---|---|---|---|---|---|---|---|
| 25 | 50-54 | Never Any | 5270 | 52.29 | | | | | |
| 25 | 50-54 | CC | 234 | 2.32 | 0 | 0.00 | | | |
| 25 | 50-54 | Ecig | 2220 | 22.03 | | | | | |
| 25 | 50-54 | Dual | 0 | 0.00 | | | | | |
| 25 | 50-54 | Former | 2354 | 23.36 | | | 22.09 | 95 | 4.04 |

Year of Follow-up: 2015, Age Group: 55-59

| Period | Age Group | Smoking Group | N | % | Ever Quit (N) | Ever Quit % | Mean Yrs Quit | Short Term (n) | Short Term % |
|---|---|---|---|---|---|---|---|---|---|
| 25 | 55-59 | Never Any | 5276 | 50.25 | | | | | |
| 25 | 55-59 | CC | 295 | 2.81 | 0 | 0.00 | | | |
| 25 | 55-59 | Ecig | 1984 | 18.90 | | | | | |
| 25 | 55-59 | Dual | 0 | 0.00 | | | | | |
| 25 | 55-59 | Former | 2944 | 28.04 | | | 22.63 | 104 | 3.53 |

Year of Follow-up: 2015, Age Group: 60-64

| Period | Age Group | Smoking Group | N | % | Ever Quit (N) | Ever Quit % | Mean Yrs Quit | Short Term (n) | Short Term % |
|---|---|---|---|---|---|---|---|---|---|
| 25 | 60-64 | Never Any | 5018 | 52.27 | | | | | |
| 25 | 60-64 | CC | 261 | 2.72 | 0 | 0.00 | | | |
| 25 | 60-64 | Ecig | 1312 | 13.67 | | | | | |
| 25 | 60-64 | Dual | 0 | 0.00 | | | | | |
| 25 | 60-64 | Former | 3009 | 31.34 | | | 25.27 | 118 | 3.92 |

Year of Follow-up: 2015, Age Group: 65-69

| Period | Age Group | Smoking Group | N | % | Ever Quit (N) | Ever Quit % | Mean Yrs Quit | Short Term (n) | Short Term % |
|---|---|---|---|---|---|---|---|---|---|
| 25 | 65-69 | Never Any | 4356 | 51.27 | | | | | |
| 25 | 65-69 | CC | 242 | 2.85 | 0 | 0.00 | | | |
| 25 | 65-69 | Ecig | 948 | 11.16 | | | | | |
| 25 | 65-69 | Dual | 0 | 0.00 | | | | | |
| 25 | 65-69 | Former | 2951 | 34.73 | | | 24.37 | 44 | 1.49 |

Year of Follow-up: 2015, Age Group: 70-74

| Period | Age Group | Smoking Group | N | % | Ever Quit (N) | Ever Quit % | Mean Yrs Quit | Short Term (n) | Short Term % |
|---|---|---|---|---|---|---|---|---|---|
| 25 | 70-74 | Never Any | 3116 | 47.67 | | | | | |
| 25 | 70-74 | CC | 227 | 3.47 | 0 | 0.00 | | | |
| 25 | 70-74 | Ecig | 521 | 7.97 | | | | | |
| 25 | 70-74 | Dual | 0 | 0.00 | | | | | |
| 25 | 70-74 | Former | 2672 | 40.88 | | | 27.49 | 42 | 1.57 |

Year of Follow-up: 2015, Age Group: 75-79

| Period | Age Group | Smoking Group | N | % | Ever Quit (N) | Ever Quit % | Mean Yrs Quit | Short Term (n) | Short Term % |
|---|---|---|---|---|---|---|---|---|---|
| 25 | 75-79 | Never Any | 2684 | 48.50 | | | | | |
| 25 | 75-79 | CC | 265 | 4.79 | 0 | 0.00 | | | |
| 25 | 75-79 | Ecig | 288 | 5.20 | | | | | |
| 25 | 75-79 | Dual | 0 | 0.00 | | | | | |
| 25 | 75-79 | Former | 2297 | 41.51 | | | 27.46 | 30 | 1.31 |

Year of Follow-up: 2020, Age Group: All Ages

| Period | Age Group | Smoking Group | N | % | Ever Quit (N) | Ever Quit % | Mean Yrs Quit | Short Term (n) | Short Term % |
|---|---|---|---|---|---|---|---|---|---|
| 30 | All Ages | Never Any | 37158 | 52.87 | | | | | |
| 30 | All Ages | CC | 1139 | 1.62 | 0 | 0.00 | | | |
| 30 | All Ages | Ecig | 12414 | 17.66 | | | | | |
| 30 | All Ages | Dual | 0 | 0.00 | | | | | |
| 30 | All Ages | Former | 19572 | 27.85 | | | 25.46 | 595 | 3.04 |

Year of Follow-up: 2020, Age Group: 18-19

| Period | Age Group | Smoking Group | N | % | Ever Quit (N) | Ever Quit % | Mean Yrs Quit | Short Term (n) | Short Term % |
|---|---|---|---|---|---|---|---|---|---|
| 30 | 18-19 | Never Any | 0 | . | | | | | |
| 30 | 18-19 | CC | 0 | . | 0 | . | | | |
| 30 | 18-19 | Ecig | 0 | . | | | | | |
| 30 | 18-19 | Dual | 0 | . | | | | | |
| 30 | 18-19 | Former | 0 | . | | | . | 0 | . |

Year of Follow-up: 2020, Age Group: 20-24

| Period | Age Group | Smoking Group | N | % | Ever Quit (N) | Ever Quit % | Mean Yrs Quit | Short Term (n) | Short Term % |
|---|---|---|---|---|---|---|---|---|---|
| 30 | 20-24 | Never Any | 0 | . | | | | | |
| 30 | 20-24 | CC | 0 | . | 0 | . | | | |

# Results for PHIM for Run 2 (Pessimistic_Analysis.RTF)

| | | | | | | | | | |
|---|---|---|---|---|---|---|---|---|---|
| 30 | 20-24 | Ecig | 0 | . | | | | | |
| 30 | 20-24 | Dual | 0 | . | | | | | |
| 30 | 20-24 | Former | 0 | . | | | . | 0 | . |

Year of Follow-up: 2020, Age Group: 25-29

| Period | Age Group | Smoking Group | N | % | Ever Quit (N) | Ever Quit % | Mean Yrs Quit | Short Term (n) | Short Term % |
|---|---|---|---|---|---|---|---|---|---|
| 30 | 25-29 | Never Any | 0 | . | | | | | |
| 30 | 25-29 | CC | 0 | . | 0 | . | | | |
| 30 | 25-29 | Ecig | 0 | . | | | | | |
| 30 | 25-29 | Dual | 0 | . | | | | | |
| 30 | 25-29 | Former | 0 | . | | | . | 0 | . |

Year of Follow-up: 2020, Age Group: 30-34

| Period | Age Group | Smoking Group | N | % | Ever Quit (N) | Ever Quit % | Mean Yrs Quit | Short Term (n) | Short Term % |
|---|---|---|---|---|---|---|---|---|---|
| 30 | 30-34 | Never Any | 0 | . | | | | | |
| 30 | 30-34 | CC | 0 | . | 0 | . | | | |
| 30 | 30-34 | Ecig | 0 | . | | | | | |
| 30 | 30-34 | Dual | 0 | . | | | | | |
| 30 | 30-34 | Former | 0 | . | | | . | 0 | . |

Year of Follow-up: 2020, Age Group: 35-39

| Period | Age Group | Smoking Group | N | % | Ever Quit (N) | Ever Quit % | Mean Yrs Quit | Short Term (n) | Short Term % |
|---|---|---|---|---|---|---|---|---|---|
| 30 | 35-39 | Never Any | 0 | . | | | | | |
| 30 | 35-39 | CC | 0 | . | 0 | . | | | |
| 30 | 35-39 | Ecig | 0 | . | | | | | |
| 30 | 35-39 | Dual | 0 | . | | | | | |
| 30 | 35-39 | Former | 0 | . | | | . | 0 | . |

Year of Follow-up: 2020, Age Group: 40-44

| Period | Age Group | Smoking Group | N | % | Ever Quit (N) | Ever Quit % | Mean Yrs Quit | Short Term (n) | Short Term % |
|---|---|---|---|---|---|---|---|---|---|
| 30 | 40-44 | Never Any | 4017 | 50.66 | | | | | |
| 30 | 40-44 | CC | 48 | 0.61 | 0 | 0.00 | | | |
| 30 | 40-44 | Ecig | 2328 | 29.36 | | | | | |
| 30 | 40-44 | Dual | 0 | 0.00 | | | | | |
| 30 | 40-44 | Former | 1537 | 19.38 | | | 14.49 | 90 | 5.86 |

Year of Follow-up: 2020, Age Group: 45-49

| Period | Age Group | Smoking Group | N | % | Ever Quit (N) | Ever Quit % | Mean Yrs Quit | Short Term (n) | Short Term % |
|---|---|---|---|---|---|---|---|---|---|
| 30 | 45-49 | Never Any | 4968 | 59.50 | | | | | |
| 30 | 45-49 | CC | 84 | 1.01 | 0 | 0.00 | | | |
| 30 | 45-49 | Ecig | 1935 | 23.17 | | | | | |
| 30 | 45-49 | Dual | 0 | 0.00 | | | | | |
| 30 | 45-49 | Former | 1363 | 16.32 | | | 17.61 | 64 | 4.70 |

Year of Follow-up: 2020, Age Group: 50-54

| Period | Age Group | Smoking Group | N | % | Ever Quit (N) | Ever Quit % | Mean Yrs Quit | Short Term (n) | Short Term % |
|---|---|---|---|---|---|---|---|---|---|
| 30 | 50-54 | Never Any | 5137 | 58.42 | | | | | |
| 30 | 50-54 | CC | 92 | 1.05 | 0 | 0.00 | | | |
| 30 | 50-54 | Ecig | 1766 | 20.08 | | | | | |
| 30 | 50-54 | Dual | 0 | 0.00 | | | | | |
| 30 | 50-54 | Former | 1798 | 20.45 | | | 22.52 | 79 | 4.39 |

Year of Follow-up: 2020, Age Group: 55-59

| Period | Age Group | Smoking Group | N | % | Ever Quit (N) | Ever Quit % | Mean Yrs Quit | Short Term (n) | Short Term % |
|---|---|---|---|---|---|---|---|---|---|
| 30 | 55-59 | Never Any | 5270 | 52.29 | | | | | |
| 30 | 55-59 | CC | 141 | 1.40 | 0 | 0.00 | | | |
| 30 | 55-59 | Ecig | 2078 | 20.62 | | | | | |
| 30 | 55-59 | Dual | 0 | 0.00 | | | | | |
| 30 | 55-59 | Former | 2589 | 25.69 | | | 24.82 | 88 | 3.40 |

Year of Follow-up: 2020, Age Group: 60-64

| Period | Age Group | Smoking Group | N | % | Ever Quit (N) | Ever Quit % | Mean Yrs Quit | Short Term (n) | Short Term % |
|---|---|---|---|---|---|---|---|---|---|
| 30 | 60-64 | Never Any | 5276 | 50.25 | | | | | |
| 30 | 60-64 | CC | 213 | 2.03 | 0 | 0.00 | | | |
| 30 | 60-64 | Ecig | 1790 | 17.05 | | | | | |
| 30 | 60-64 | Dual | 0 | 0.00 | | | | | |
| 30 | 60-64 | Former | 3220 | 30.67 | | | 25.43 | 113 | 3.51 |

Year of Follow-up: 2020, Age Group: 65-69

| Period | Age Group | Smoking Group | N | % | Ever Quit (N) | Ever Quit % | Mean Yrs Quit | Short Term (n) | Short Term % |
|---|---|---|---|---|---|---|---|---|---|
| 30 | 65-69 | Never Any | 5018 | 52.27 | | | | | |
| 30 | 65-69 | CC | 183 | 1.91 | 0 | 0.00 | | | |
| 30 | 65-69 | Ecig | 1185 | 12.34 | | | | | |
| 30 | 65-69 | Dual | 0 | 0.00 | | | | | |
| 30 | 65-69 | Former | 3214 | 33.48 | | | 28.49 | 61 | 1.90 |

Year of Follow-up: 2020, Age Group: 70-74

| Period | Age Group | Smoking Group | N | % | Ever Quit (N) | Ever Quit % | Mean Yrs Quit | Short Term (n) | Short Term % |
|---|---|---|---|---|---|---|---|---|---|

# Results for PHIM for Run 2 (Pessimistic_Analysis.RTF)

| | | | | | | | | | |
|---|---|---|---|---|---|---|---|---|---|
| 30 | 70-74 | Never Any | 4356 | 51.27 | | | | | |
| 30 | 70-74 | CC | 200 | 2.35 | 0 | 0.00 | | | |
| 30 | 70-74 | Ecig | 884 | 10.40 | | | | | |
| 30 | 70-74 | Dual | 0 | 0.00 | | | | | |
| 30 | 70-74 | Former | 3057 | 35.98 | | | 28.40 | 54 | 1.77 |

Year of Follow-up: 2020, Age Group: 75-79

| Period | Age Group | Smoking Group | N | % | Ever Quit (N) | Ever Quit % | Mean Yrs Quit | Short Term (n) | Short Term % |
|---|---|---|---|---|---|---|---|---|---|
| 30 | 75-79 | Never Any | 3116 | 47.67 | | | | | |
| 30 | 75-79 | CC | 178 | 2.72 | 0 | 0.00 | | | |
| 30 | 75-79 | Ecig | 448 | 6.85 | | | | | |
| 30 | 75-79 | Dual | 0 | 0.00 | | | | | |
| 30 | 75-79 | Former | 2794 | 42.75 | | | 31.16 | 46 | 1.65 |

Year of Follow-up: 2025, Age Group: All Ages

| Period | Age Group | Smoking Group | N | % | Ever Quit (N) | Ever Quit % | Mean Yrs Quit | Short Term (n) | Short Term % |
|---|---|---|---|---|---|---|---|---|---|
| 35 | All Ages | Never Any | 34042 | 53.40 | | | | | |
| 35 | All Ages | CC | 707 | 1.11 | 0 | 0.00 | | | |
| 35 | All Ages | Ecig | 10971 | 17.21 | | | | | |
| 35 | All Ages | Dual | 0 | 0.00 | | | | | |
| 35 | All Ages | Former | 18027 | 28.28 | | | 27.61 | 476 | 2.64 |

Year of Follow-up: 2025, Age Group: 18-19

| Period | Age Group | Smoking Group | N | % | Ever Quit (N) | Ever Quit % | Mean Yrs Quit | Short Term (n) | Short Term % |
|---|---|---|---|---|---|---|---|---|---|
| 35 | 18-19 | Never Any | 0 | . | | | | | |
| 35 | 18-19 | CC | 0 | . | 0 | . | | | |
| 35 | 18-19 | Ecig | 0 | . | | | | | |
| 35 | 18-19 | Dual | 0 | . | | | | | |
| 35 | 18-19 | Former | 0 | . | | | . | 0 | . |

Year of Follow-up: 2025, Age Group: 20-24

| Period | Age Group | Smoking Group | N | % | Ever Quit (N) | Ever Quit % | Mean Yrs Quit | Short Term (n) | Short Term % |
|---|---|---|---|---|---|---|---|---|---|
| 35 | 20-24 | Never Any | 0 | . | | | | | |
| 35 | 20-24 | CC | 0 | . | 0 | . | | | |
| 35 | 20-24 | Ecig | 0 | . | | | | | |
| 35 | 20-24 | Dual | 0 | . | | | | | |
| 35 | 20-24 | Former | 0 | . | | | . | 0 | . |

Year of Follow-up: 2025, Age Group: 25-29

| Period | Age Group | Smoking Group | N | % | Ever Quit (N) | Ever Quit % | Mean Yrs Quit | Short Term (n) | Short Term % |
|---|---|---|---|---|---|---|---|---|---|
| 35 | 25-29 | Never Any | 0 | . | | | | | |
| 35 | 25-29 | CC | 0 | . | 0 | . | | | |
| 35 | 25-29 | Ecig | 0 | . | | | | | |
| 35 | 25-29 | Dual | 0 | . | | | | | |
| 35 | 25-29 | Former | 0 | . | | | . | 0 | . |

Year of Follow-up: 2025, Age Group: 30-34

| Period | Age Group | Smoking Group | N | % | Ever Quit (N) | Ever Quit % | Mean Yrs Quit | Short Term (n) | Short Term % |
|---|---|---|---|---|---|---|---|---|---|
| 35 | 30-34 | Never Any | 0 | . | | | | | |
| 35 | 30-34 | CC | 0 | . | 0 | . | | | |
| 35 | 30-34 | Ecig | 0 | . | | | | | |
| 35 | 30-34 | Dual | 0 | . | | | | | |
| 35 | 30-34 | Former | 0 | . | | | . | 0 | . |

Year of Follow-up: 2025, Age Group: 35-39

| Period | Age Group | Smoking Group | N | % | Ever Quit (N) | Ever Quit % | Mean Yrs Quit | Short Term (n) | Short Term % |
|---|---|---|---|---|---|---|---|---|---|
| 35 | 35-39 | Never Any | 0 | . | | | | | |
| 35 | 35-39 | CC | 0 | . | 0 | . | | | |
| 35 | 35-39 | Ecig | 0 | . | | | | | |
| 35 | 35-39 | Dual | 0 | . | | | | | |
| 35 | 35-39 | Former | 0 | . | | | . | 0 | . |

Year of Follow-up: 2025, Age Group: 40-44

| Period | Age Group | Smoking Group | N | % | Ever Quit (N) | Ever Quit % | Mean Yrs Quit | Short Term (n) | Short Term % |
|---|---|---|---|---|---|---|---|---|---|
| 35 | 40-44 | Never Any | 0 | . | | | | | |
| 35 | 40-44 | CC | 0 | . | 0 | . | | | |
| 35 | 40-44 | Ecig | 0 | . | | | | | |
| 35 | 40-44 | Dual | 0 | . | | | | | |
| 35 | 40-44 | Former | 0 | . | | | . | 0 | . |

Year of Follow-up: 2025, Age Group: 45-49

| Period | Age Group | Smoking Group | N | % | Ever Quit (N) | Ever Quit % | Mean Yrs Quit | Short Term (n) | Short Term % |
|---|---|---|---|---|---|---|---|---|---|
| 35 | 45-49 | Never Any | 4017 | 50.66 | | | | | |
| 35 | 45-49 | CC | 24 | 0.30 | 0 | 0.00 | | | |
| 35 | 45-49 | Ecig | 2179 | 27.48 | | | | | |
| 35 | 45-49 | Dual | 0 | 0.00 | | | | | |
| 35 | 45-49 | Former | 1710 | 21.56 | | | 17.75 | 54 | 3.16 |

Year of Follow-up: 2025, Age Group: 50-54

# Results for PHIM for Run 2 (Pessimistic_Analysis.RTF)

| Period | Age Group | Smoking Group | N | % | Ever Quit (N) | Ever Quit % | Mean Yrs Quit | Short Term (n) | Short Term % |
|---|---|---|---|---|---|---|---|---|---|
| 35 | 50-54 | Never Any | 4968 | 59.50 | | | | | |
| 35 | 50-54 | CC | 51 | 0.61 | 0 | 0.00 | | | |
| 35 | 50-54 | Ecig | 1808 | 21.65 | | | | | |
| 35 | 50-54 | Dual | 0 | 0.00 | | | | | |
| 35 | 50-54 | Former | 1523 | 18.24 | | | 20.43 | 71 | 4.66 |

Year of Follow-up: 2025, Age Group: 55-59

| Period | Age Group | Smoking Group | N | % | Ever Quit (N) | Ever Quit % | Mean Yrs Quit | Short Term (n) | Short Term % |
|---|---|---|---|---|---|---|---|---|---|
| 35 | 55-59 | Never Any | 5137 | 58.42 | | | | | |
| 35 | 55-59 | CC | 57 | 0.65 | 0 | 0.00 | | | |
| 35 | 55-59 | Ecig | 1626 | 18.49 | | | | | |
| 35 | 55-59 | Dual | 0 | 0.00 | | | | | |
| 35 | 55-59 | Former | 1973 | 22.44 | | | 25.25 | 68 | 3.45 |

Year of Follow-up: 2025, Age Group: 60-64

| Period | Age Group | Smoking Group | N | % | Ever Quit (N) | Ever Quit % | Mean Yrs Quit | Short Term (n) | Short Term % |
|---|---|---|---|---|---|---|---|---|---|
| 35 | 60-64 | Never Any | 5270 | 52.29 | | | | | |
| 35 | 60-64 | CC | 99 | 0.98 | 0 | 0.00 | | | |
| 35 | 60-64 | Ecig | 1856 | 18.42 | | | | | |
| 35 | 60-64 | Dual | 0 | 0.00 | | | | | |
| 35 | 60-64 | Former | 2853 | 28.31 | | | 27.22 | 123 | 4.31 |

Year of Follow-up: 2025, Age Group: 65-69

| Period | Age Group | Smoking Group | N | % | Ever Quit (N) | Ever Quit % | Mean Yrs Quit | Short Term (n) | Short Term % |
|---|---|---|---|---|---|---|---|---|---|
| 35 | 65-69 | Never Any | 5276 | 50.25 | | | | | |
| 35 | 65-69 | CC | 162 | 1.54 | 0 | 0.00 | | | |
| 35 | 65-69 | Ecig | 1615 | 15.38 | | | | | |
| 35 | 65-69 | Dual | 0 | 0.00 | | | | | |
| 35 | 65-69 | Former | 3446 | 32.82 | | | 28.59 | 62 | 1.80 |

Year of Follow-up: 2025, Age Group: 70-74

| Period | Age Group | Smoking Group | N | % | Ever Quit (N) | Ever Quit % | Mean Yrs Quit | Short Term (n) | Short Term % |
|---|---|---|---|---|---|---|---|---|---|
| 35 | 70-74 | Never Any | 5018 | 52.27 | | | | | |
| 35 | 70-74 | CC | 153 | 1.59 | 0 | 0.00 | | | |
| 35 | 70-74 | Ecig | 1094 | 11.40 | | | | | |
| 35 | 70-74 | Dual | 0 | 0.00 | | | | | |
| 35 | 70-74 | Former | 3335 | 34.74 | | | 32.34 | 53 | 1.59 |

Year of Follow-up: 2025, Age Group: 75-79

| Period | Age Group | Smoking Group | N | % | Ever Quit (N) | Ever Quit % | Mean Yrs Quit | Short Term (n) | Short Term % |
|---|---|---|---|---|---|---|---|---|---|
| 35 | 75-79 | Never Any | 4356 | 51.27 | | | | | |
| 35 | 75-79 | CC | 161 | 1.89 | 0 | 0.00 | | | |
| 35 | 75-79 | Ecig | 793 | 9.33 | | | | | |
| 35 | 75-79 | Dual | 0 | 0.00 | | | | | |
| 35 | 75-79 | Former | 3187 | 37.51 | | | 32.13 | 45 | 1.41 |

Year of Follow-up: 2030, Age Group: All Ages

| Period | Age Group | Smoking Group | N | % | Ever Quit (N) | Ever Quit % | Mean Yrs Quit | Short Term (n) | Short Term % |
|---|---|---|---|---|---|---|---|---|---|
| 40 | All Ages | Never Any | 29686 | 53.73 | | | | | |
| 40 | All Ages | CC | 399 | 0.72 | 0 | 0.00 | | | |
| 40 | All Ages | Ecig | 9240 | 16.72 | | | | | |
| 40 | All Ages | Dual | 0 | 0.00 | | | | | |
| 40 | All Ages | Former | 15925 | 28.82 | | | 29.62 | 431 | 2.71 |

Year of Follow-up: 2030, Age Group: 18-19

| Period | Age Group | Smoking Group | N | % | Ever Quit (N) | Ever Quit % | Mean Yrs Quit | Short Term (n) | Short Term % |
|---|---|---|---|---|---|---|---|---|---|
| 40 | 18-19 | Never Any | 0 | . | | | | | |
| 40 | 18-19 | CC | 0 | . | 0 | . | | | |
| 40 | 18-19 | Ecig | 0 | . | | | | | |
| 40 | 18-19 | Dual | 0 | . | | | | | |
| 40 | 18-19 | Former | 0 | . | | | . | 0 | . |

Year of Follow-up: 2030, Age Group: 20-24

| Period | Age Group | Smoking Group | N | % | Ever Quit (N) | Ever Quit % | Mean Yrs Quit | Short Term (n) | Short Term % |
|---|---|---|---|---|---|---|---|---|---|
| 40 | 20-24 | Never Any | 0 | . | | | | | |
| 40 | 20-24 | CC | 0 | . | 0 | . | | | |
| 40 | 20-24 | Ecig | 0 | . | | | | | |
| 40 | 20-24 | Dual | 0 | . | | | | | |
| 40 | 20-24 | Former | 0 | . | | | . | 0 | . |

Year of Follow-up: 2030, Age Group: 25-29

| Period | Age Group | Smoking Group | N | % | Ever Quit (N) | Ever Quit % | Mean Yrs Quit | Short Term (n) | Short Term % |
|---|---|---|---|---|---|---|---|---|---|
| 40 | 25-29 | Never Any | 0 | . | | | | | |
| 40 | 25-29 | CC | 0 | . | 0 | . | | | |
| 40 | 25-29 | Ecig | 0 | . | | | | | |
| 40 | 25-29 | Dual | 0 | . | | | | | |

# Results for PHIM for Run 2 (Pessimistic_Analysis.RTF)

| | | | | | | | | | |
|---|---|---|---|---|---|---|---|---|---|
| 40 | 25-29 | Former | 0 | . | | | . | 0 | . |

Year of Follow-up: 2030, Age Group: 30-34

| Period | Age Group | Smoking Group | N | % | Ever Quit (N) | Ever Quit % | Mean Yrs Quit | Short Term (n) | Short Term % |
|---|---|---|---|---|---|---|---|---|---|
| 40 | 30-34 | Never Any | 0 | . | | | | | |
| 40 | 30-34 | CC | 0 | . | 0 | . | | | |
| 40 | 30-34 | Ecig | 0 | . | | | | | |
| 40 | 30-34 | Dual | 0 | . | | | | | |
| 40 | 30-34 | Former | 0 | . | | | . | 0 | . |

Year of Follow-up: 2030, Age Group: 35-39

| Period | Age Group | Smoking Group | N | % | Ever Quit (N) | Ever Quit % | Mean Yrs Quit | Short Term (n) | Short Term % |
|---|---|---|---|---|---|---|---|---|---|
| 40 | 35-39 | Never Any | 0 | . | | | | | |
| 40 | 35-39 | CC | 0 | . | 0 | . | | | |
| 40 | 35-39 | Ecig | 0 | . | | | | | |
| 40 | 35-39 | Dual | 0 | . | | | | | |
| 40 | 35-39 | Former | 0 | . | | | . | 0 | . |

Year of Follow-up: 2030, Age Group: 40-44

| Period | Age Group | Smoking Group | N | % | Ever Quit (N) | Ever Quit % | Mean Yrs Quit | Short Term (n) | Short Term % |
|---|---|---|---|---|---|---|---|---|---|
| 40 | 40-44 | Never Any | 0 | . | | | | | |
| 40 | 40-44 | CC | 0 | . | 0 | . | | | |
| 40 | 40-44 | Ecig | 0 | . | | | | | |
| 40 | 40-44 | Dual | 0 | . | | | | | |
| 40 | 40-44 | Former | 0 | . | | | . | 0 | . |

Year of Follow-up: 2030, Age Group: 45-49

| Period | Age Group | Smoking Group | N | % | Ever Quit (N) | Ever Quit % | Mean Yrs Quit | Short Term (n) | Short Term % |
|---|---|---|---|---|---|---|---|---|---|
| 40 | 45-49 | Never Any | 0 | . | | | | | |
| 40 | 45-49 | CC | 0 | . | 0 | . | | | |
| 40 | 45-49 | Ecig | 0 | . | | | | | |
| 40 | 45-49 | Dual | 0 | . | | | | | |
| 40 | 45-49 | Former | 0 | . | | | . | 0 | . |

Year of Follow-up: 2030, Age Group: 50-54

| Period | Age Group | Smoking Group | N | % | Ever Quit (N) | Ever Quit % | Mean Yrs Quit | Short Term (n) | Short Term % |
|---|---|---|---|---|---|---|---|---|---|
| 40 | 50-54 | Never Any | 4017 | 50.66 | | | | | |
| 40 | 50-54 | CC | 11 | 0.14 | 0 | 0.00 | | | |
| 40 | 50-54 | Ecig | 2007 | 25.31 | | | | | |
| 40 | 50-54 | Dual | 0 | 0.00 | | | | | |
| 40 | 50-54 | Former | 1895 | 23.90 | | | 20.69 | 99 | 5.22 |

Year of Follow-up: 2030, Age Group: 55-59

| Period | Age Group | Smoking Group | N | % | Ever Quit (N) | Ever Quit % | Mean Yrs Quit | Short Term (n) | Short Term % |
|---|---|---|---|---|---|---|---|---|---|
| 40 | 55-59 | Never Any | 4968 | 59.50 | | | | | |
| 40 | 55-59 | CC | 25 | 0.30 | 0 | 0.00 | | | |
| 40 | 55-59 | Ecig | 1658 | 19.86 | | | | | |
| 40 | 55-59 | Dual | 0 | 0.00 | | | | | |
| 40 | 55-59 | Former | 1699 | 20.35 | | | 23.03 | 55 | 3.24 |

Year of Follow-up: 2030, Age Group: 60-64

| Period | Age Group | Smoking Group | N | % | Ever Quit (N) | Ever Quit % | Mean Yrs Quit | Short Term (n) | Short Term % |
|---|---|---|---|---|---|---|---|---|---|
| 40 | 60-64 | Never Any | 5137 | 58.42 | | | | | |
| 40 | 60-64 | CC | 35 | 0.40 | 0 | 0.00 | | | |
| 40 | 60-64 | Ecig | 1453 | 16.52 | | | | | |
| 40 | 60-64 | Dual | 0 | 0.00 | | | | | |
| 40 | 60-64 | Former | 2168 | 24.66 | | | 27.71 | 79 | 3.64 |

Year of Follow-up: 2030, Age Group: 65-69

| Period | Age Group | Smoking Group | N | % | Ever Quit (N) | Ever Quit % | Mean Yrs Quit | Short Term (n) | Short Term % |
|---|---|---|---|---|---|---|---|---|---|
| 40 | 65-69 | Never Any | 5270 | 52.29 | | | | | |
| 40 | 65-69 | CC | 77 | 0.76 | 0 | 0.00 | | | |
| 40 | 65-69 | Ecig | 1646 | 16.33 | | | | | |
| 40 | 65-69 | Dual | 0 | 0.00 | | | | | |
| 40 | 65-69 | Former | 3085 | 30.61 | | | 29.98 | 70 | 2.27 |

Year of Follow-up: 2030, Age Group: 70-74

| Period | Age Group | Smoking Group | N | % | Ever Quit (N) | Ever Quit % | Mean Yrs Quit | Short Term (n) | Short Term % |
|---|---|---|---|---|---|---|---|---|---|
| 40 | 70-74 | Never Any | 5276 | 50.25 | | | | | |
| 40 | 70-74 | CC | 132 | 1.26 | 0 | 0.00 | | | |
| 40 | 70-74 | Ecig | 1491 | 14.20 | | | | | |
| 40 | 70-74 | Dual | 0 | 0.00 | | | | | |
| 40 | 70-74 | Former | 3600 | 34.29 | | | 32.23 | 74 | 2.06 |

Year of Follow-up: 2030, Age Group: 75-79

| Period | Age Group | Smoking Group | N | % | Ever Quit (N) | Ever Quit % | Mean Yrs Quit | Short Term (n) | Short Term % |
|---|---|---|---|---|---|---|---|---|---|
| 40 | 75-79 | Never Any | 5018 | 52.27 | | | | | |
| 40 | 75-79 | CC | 119 | 1.24 | 0 | 0.00 | | | |

# Results for PHIM for Run 2 (Pessimistic_Analysis.RTF)

| | | | | | | | | | |
|---|---|---|---|---|---|---|---|---|---|
| 40 | 75-79 | Ecig | 985 | 10.26 | | | | | |
| 40 | 75-79 | Dual | 0 | 0.00 | | | | | |
| 40 | 75-79 | Former | 3478 | 36.23 | | | 35.88 | 54 | 1.55 |

Year of Follow-up: 2035, Age Group: All Ages

| Period | Age Group | Smoking Group | N | % | Ever Quit (N) | Ever Quit % | Mean Yrs Quit | Short Term (n) | Short Term % |
|---|---|---|---|---|---|---|---|---|---|
| 45 | All Ages | Never Any | 24668 | 54.04 | | | | | |
| 45 | All Ages | CC | 203 | 0.44 | 0 | 0.00 | | | |
| 45 | All Ages | Ecig | 7433 | 16.28 | | | | | |
| 45 | All Ages | Dual | 0 | 0.00 | | | | | |
| 45 | All Ages | Former | 13346 | 29.24 | | | 30.79 | 366 | 2.74 |

Year of Follow-up: 2035, Age Group: 18-19

| Period | Age Group | Smoking Group | N | % | Ever Quit (N) | Ever Quit % | Mean Yrs Quit | Short Term (n) | Short Term % |
|---|---|---|---|---|---|---|---|---|---|
| 45 | 18-19 | Never Any | 0 | . | | | | | |
| 45 | 18-19 | CC | 0 | . | 0 | . | | | |
| 45 | 18-19 | Ecig | 0 | . | | | | | |
| 45 | 18-19 | Dual | 0 | . | | | | | |
| 45 | 18-19 | Former | 0 | . | | | . | 0 | . |

Year of Follow-up: 2035, Age Group: 20-24

| Period | Age Group | Smoking Group | N | % | Ever Quit (N) | Ever Quit % | Mean Yrs Quit | Short Term (n) | Short Term % |
|---|---|---|---|---|---|---|---|---|---|
| 45 | 20-24 | Never Any | 0 | . | | | | | |
| 45 | 20-24 | CC | 0 | . | 0 | . | | | |
| 45 | 20-24 | Ecig | 0 | . | | | | | |
| 45 | 20-24 | Dual | 0 | . | | | | | |
| 45 | 20-24 | Former | 0 | . | | | . | 0 | . |

Year of Follow-up: 2035, Age Group: 25-29

| Period | Age Group | Smoking Group | N | % | Ever Quit (N) | Ever Quit % | Mean Yrs Quit | Short Term (n) | Short Term % |
|---|---|---|---|---|---|---|---|---|---|
| 45 | 25-29 | Never Any | 0 | . | | | | | |
| 45 | 25-29 | CC | 0 | . | 0 | . | | | |
| 45 | 25-29 | Ecig | 0 | . | | | | | |
| 45 | 25-29 | Dual | 0 | . | | | | | |
| 45 | 25-29 | Former | 0 | . | | | . | 0 | . |

Year of Follow-up: 2035, Age Group: 30-34

| Period | Age Group | Smoking Group | N | % | Ever Quit (N) | Ever Quit % | Mean Yrs Quit | Short Term (n) | Short Term % |
|---|---|---|---|---|---|---|---|---|---|
| 45 | 30-34 | Never Any | 0 | . | | | | | |
| 45 | 30-34 | CC | 0 | . | 0 | . | | | |
| 45 | 30-34 | Ecig | 0 | . | | | | | |
| 45 | 30-34 | Dual | 0 | . | | | | | |
| 45 | 30-34 | Former | 0 | . | | | . | 0 | . |

Year of Follow-up: 2035, Age Group: 35-39

| Period | Age Group | Smoking Group | N | % | Ever Quit (N) | Ever Quit % | Mean Yrs Quit | Short Term (n) | Short Term % |
|---|---|---|---|---|---|---|---|---|---|
| 45 | 35-39 | Never Any | 0 | . | | | | | |
| 45 | 35-39 | CC | 0 | . | 0 | . | | | |
| 45 | 35-39 | Ecig | 0 | . | | | | | |
| 45 | 35-39 | Dual | 0 | . | | | | | |
| 45 | 35-39 | Former | 0 | . | | | . | 0 | . |

Year of Follow-up: 2035, Age Group: 40-44

| Period | Age Group | Smoking Group | N | % | Ever Quit (N) | Ever Quit % | Mean Yrs Quit | Short Term (n) | Short Term % |
|---|---|---|---|---|---|---|---|---|---|
| 45 | 40-44 | Never Any | 0 | . | | | | | |
| 45 | 40-44 | CC | 0 | . | 0 | . | | | |
| 45 | 40-44 | Ecig | 0 | . | | | | | |
| 45 | 40-44 | Dual | 0 | . | | | | | |
| 45 | 40-44 | Former | 0 | . | | | . | 0 | . |

Year of Follow-up: 2035, Age Group: 45-49

| Period | Age Group | Smoking Group | N | % | Ever Quit (N) | Ever Quit % | Mean Yrs Quit | Short Term (n) | Short Term % |
|---|---|---|---|---|---|---|---|---|---|
| 45 | 45-49 | Never Any | 0 | . | | | | | |
| 45 | 45-49 | CC | 0 | . | 0 | . | | | |
| 45 | 45-49 | Ecig | 0 | . | | | | | |
| 45 | 45-49 | Dual | 0 | . | | | | | |
| 45 | 45-49 | Former | 0 | . | | | . | 0 | . |

Year of Follow-up: 2035, Age Group: 50-54

| Period | Age Group | Smoking Group | N | % | Ever Quit (N) | Ever Quit % | Mean Yrs Quit | Short Term (n) | Short Term % |
|---|---|---|---|---|---|---|---|---|---|
| 45 | 50-54 | Never Any | 0 | . | | | | | |
| 45 | 50-54 | CC | 0 | . | 0 | . | | | |
| 45 | 50-54 | Ecig | 0 | . | | | | | |
| 45 | 50-54 | Dual | 0 | . | | | | | |
| 45 | 50-54 | Former | 0 | . | | | . | 0 | . |

Year of Follow-up: 2035, Age Group: 55-59

| Period | Age Group | Smoking Group | N | % | Ever Quit (N) | Ever Quit % | Mean Yrs Quit | Short Term (n) | Short Term % |
|---|---|---|---|---|---|---|---|---|---|

# Results for PHIM for Run 2 (Pessimistic_Analysis.RTF)

| | | | | | | | | | |
|---|---|---|---|---|---|---|---|---|---|
| 45 | 55-59 | Never Any | 4017 | 50.66 | | | | | |
| 45 | 55-59 | CC | 8 | 0.10 | 0 | 0.00 | | | |
| 45 | 55-59 | Ecig | 1806 | 22.77 | | | | | |
| 45 | 55-59 | Dual | 0 | 0.00 | | | | | |
| 45 | 55-59 | Former | 2099 | 26.47 | | | 23.38 | 86 | 4.10 |

Year of Follow-up: 2035, Age Group: 60-64

| Period | Age Group | Smoking Group | N | % | Ever Quit (N) | Ever Quit % | Mean Yrs Quit | Short Term (n) | Short Term % |
|---|---|---|---|---|---|---|---|---|---|
| 45 | 60-64 | Never Any | 4968 | 59.50 | | | | | |
| 45 | 60-64 | CC | 15 | 0.18 | 0 | 0.00 | | | |
| 45 | 60-64 | Ecig | 1482 | 17.75 | | | | | |
| 45 | 60-64 | Dual | 0 | 0.00 | | | | | |
| 45 | 60-64 | Former | 1885 | 22.57 | | | 25.43 | 90 | 4.77 |

Year of Follow-up: 2035, Age Group: 65-69

| Period | Age Group | Smoking Group | N | % | Ever Quit (N) | Ever Quit % | Mean Yrs Quit | Short Term (n) | Short Term % |
|---|---|---|---|---|---|---|---|---|---|
| 45 | 65-69 | Never Any | 5137 | 58.42 | | | | | |
| 45 | 65-69 | CC | 26 | 0.30 | 0 | 0.00 | | | |
| 45 | 65-69 | Ecig | 1299 | 14.77 | | | | | |
| 45 | 65-69 | Dual | 0 | 0.00 | | | | | |
| 45 | 65-69 | Former | 2331 | 26.51 | | | 30.59 | 47 | 2.02 |

Year of Follow-up: 2035, Age Group: 70-74

| Period | Age Group | Smoking Group | N | % | Ever Quit (N) | Ever Quit % | Mean Yrs Quit | Short Term (n) | Short Term % |
|---|---|---|---|---|---|---|---|---|---|
| 45 | 70-74 | Never Any | 5270 | 52.29 | | | | | |
| 45 | 70-74 | CC | 59 | 0.59 | 0 | 0.00 | | | |
| 45 | 70-74 | Ecig | 1525 | 15.13 | | | | | |
| 45 | 70-74 | Dual | 0 | 0.00 | | | | | |
| 45 | 70-74 | Former | 3224 | 31.99 | | | 33.54 | 69 | 2.14 |

Year of Follow-up: 2035, Age Group: 75-79

| Period | Age Group | Smoking Group | N | % | Ever Quit (N) | Ever Quit % | Mean Yrs Quit | Short Term (n) | Short Term % |
|---|---|---|---|---|---|---|---|---|---|
| 45 | 75-79 | Never Any | 5276 | 50.25 | | | | | |
| 45 | 75-79 | CC | 95 | 0.90 | 0 | 0.00 | | | |
| 45 | 75-79 | Ecig | 1321 | 12.58 | | | | | |
| 45 | 75-79 | Dual | 0 | 0.00 | | | | | |
| 45 | 75-79 | Former | 3807 | 36.26 | | | 35.32 | 74 | 1.94 |

Year of Follow-up: 2040, Age Group: All Ages

| Period | Age Group | Smoking Group | N | % | Ever Quit (N) | Ever Quit % | Mean Yrs Quit | Short Term (n) | Short Term % |
|---|---|---|---|---|---|---|---|---|---|
| 50 | All Ages | Never Any | 19392 | 55.17 | | | | | |
| 50 | All Ages | CC | 83 | 0.24 | 0 | 0.00 | | | |
| 50 | All Ages | Ecig | 5518 | 15.70 | | | | | |
| 50 | All Ages | Dual | 0 | 0.00 | | | | | |
| 50 | All Ages | Former | 10158 | 28.90 | | | 32.03 | 263 | 2.59 |

Year of Follow-up: 2040, Age Group: 18-19

| Period | Age Group | Smoking Group | N | % | Ever Quit (N) | Ever Quit % | Mean Yrs Quit | Short Term (n) | Short Term % |
|---|---|---|---|---|---|---|---|---|---|
| 50 | 18-19 | Never Any | 0 | . | | | | | |
| 50 | 18-19 | CC | 0 | . | 0 | . | | | |
| 50 | 18-19 | Ecig | 0 | . | | | | | |
| 50 | 18-19 | Dual | 0 | . | | | | | |
| 50 | 18-19 | Former | 0 | . | | | . | 0 | . |

Year of Follow-up: 2040, Age Group: 20-24

| Period | Age Group | Smoking Group | N | % | Ever Quit (N) | Ever Quit % | Mean Yrs Quit | Short Term (n) | Short Term % |
|---|---|---|---|---|---|---|---|---|---|
| 50 | 20-24 | Never Any | 0 | . | | | | | |
| 50 | 20-24 | CC | 0 | . | 0 | . | | | |
| 50 | 20-24 | Ecig | 0 | . | | | | | |
| 50 | 20-24 | Dual | 0 | . | | | | | |
| 50 | 20-24 | Former | 0 | . | | | . | 0 | . |

Year of Follow-up: 2040, Age Group: 25-29

| Period | Age Group | Smoking Group | N | % | Ever Quit (N) | Ever Quit % | Mean Yrs Quit | Short Term (n) | Short Term % |
|---|---|---|---|---|---|---|---|---|---|
| 50 | 25-29 | Never Any | 0 | . | | | | | |
| 50 | 25-29 | CC | 0 | . | 0 | . | | | |
| 50 | 25-29 | Ecig | 0 | . | | | | | |
| 50 | 25-29 | Dual | 0 | . | | | | | |
| 50 | 25-29 | Former | 0 | . | | | . | 0 | . |

Year of Follow-up: 2040, Age Group: 30-34

| Period | Age Group | Smoking Group | N | % | Ever Quit (N) | Ever Quit % | Mean Yrs Quit | Short Term (n) | Short Term % |
|---|---|---|---|---|---|---|---|---|---|
| 50 | 30-34 | Never Any | 0 | . | | | | | |
| 50 | 30-34 | CC | 0 | . | 0 | . | | | |
| 50 | 30-34 | Ecig | 0 | . | | | | | |
| 50 | 30-34 | Dual | 0 | . | | | | | |
| 50 | 30-34 | Former | 0 | . | | | . | 0 | . |

Year of Follow-up: 2040, Age Group: 35-39

# Results for PHIM for Run 2 (Pessimistic_Analysis.RTF)

| Period | Age Group | Smoking Group | N | % | Ever Quit (N) | Ever Quit % | Mean Yrs Quit | Short Term (n) | Short Term % |
|---|---|---|---|---|---|---|---|---|---|
| 50 | 35-39 | Never Any | 0 | . | | | | | |
| 50 | 35-39 | CC | 0 | . | 0 | . | | | |
| 50 | 35-39 | Ecig | 0 | . | | | | | |
| 50 | 35-39 | Dual | 0 | . | | | | | |
| 50 | 35-39 | Former | 0 | . | | | . | 0 | . |

Year of Follow-up: 2040, Age Group: 40-44

| Period | Age Group | Smoking Group | N | % | Ever Quit (N) | Ever Quit % | Mean Yrs Quit | Short Term (n) | Short Term % |
|---|---|---|---|---|---|---|---|---|---|
| 50 | 40-44 | Never Any | 0 | . | | | | | |
| 50 | 40-44 | CC | 0 | . | 0 | . | | | |
| 50 | 40-44 | Ecig | 0 | . | | | | | |
| 50 | 40-44 | Dual | 0 | . | | | | | |
| 50 | 40-44 | Former | 0 | . | | | . | 0 | . |

Year of Follow-up: 2040, Age Group: 45-49

| Period | Age Group | Smoking Group | N | % | Ever Quit (N) | Ever Quit % | Mean Yrs Quit | Short Term (n) | Short Term % |
|---|---|---|---|---|---|---|---|---|---|
| 50 | 45-49 | Never Any | 0 | . | | | | | |
| 50 | 45-49 | CC | 0 | . | 0 | . | | | |
| 50 | 45-49 | Ecig | 0 | . | | | | | |
| 50 | 45-49 | Dual | 0 | . | | | | | |
| 50 | 45-49 | Former | 0 | . | | | . | 0 | . |

Year of Follow-up: 2040, Age Group: 50-54

| Period | Age Group | Smoking Group | N | % | Ever Quit (N) | Ever Quit % | Mean Yrs Quit | Short Term (n) | Short Term % |
|---|---|---|---|---|---|---|---|---|---|
| 50 | 50-54 | Never Any | 0 | . | | | | | |
| 50 | 50-54 | CC | 0 | . | 0 | . | | | |
| 50 | 50-54 | Ecig | 0 | . | | | | | |
| 50 | 50-54 | Dual | 0 | . | | | | | |
| 50 | 50-54 | Former | 0 | . | | | . | 0 | . |

Year of Follow-up: 2040, Age Group: 55-59

| Period | Age Group | Smoking Group | N | % | Ever Quit (N) | Ever Quit % | Mean Yrs Quit | Short Term (n) | Short Term % |
|---|---|---|---|---|---|---|---|---|---|
| 50 | 55-59 | Never Any | 0 | . | | | | | |
| 50 | 55-59 | CC | 0 | . | 0 | . | | | |
| 50 | 55-59 | Ecig | 0 | . | | | | | |
| 50 | 55-59 | Dual | 0 | . | | | | | |
| 50 | 55-59 | Former | 0 | . | | | . | 0 | . |

Year of Follow-up: 2040, Age Group: 60-64

| Period | Age Group | Smoking Group | N | % | Ever Quit (N) | Ever Quit % | Mean Yrs Quit | Short Term (n) | Short Term % |
|---|---|---|---|---|---|---|---|---|---|
| 50 | 60-64 | Never Any | 4017 | 50.66 | | | | | |
| 50 | 60-64 | CC | 6 | 0.08 | 0 | 0.00 | | | |
| 50 | 60-64 | Ecig | 1617 | 20.39 | | | | | |
| 50 | 60-64 | Dual | 0 | 0.00 | | | | | |
| 50 | 60-64 | Former | 2290 | 28.88 | | | 26.16 | 95 | 4.15 |

Year of Follow-up: 2040, Age Group: 65-69

| Period | Age Group | Smoking Group | N | % | Ever Quit (N) | Ever Quit % | Mean Yrs Quit | Short Term (n) | Short Term % |
|---|---|---|---|---|---|---|---|---|---|
| 50 | 65-69 | Never Any | 4968 | 59.50 | | | | | |
| 50 | 65-69 | CC | 7 | 0.08 | 0 | 0.00 | | | |
| 50 | 65-69 | Ecig | 1343 | 16.08 | | | | | |
| 50 | 65-69 | Dual | 0 | 0.00 | | | | | |
| 50 | 65-69 | Former | 2032 | 24.34 | | | 28.40 | 53 | 2.61 |

Year of Follow-up: 2040, Age Group: 70-74

| Period | Age Group | Smoking Group | N | % | Ever Quit (N) | Ever Quit % | Mean Yrs Quit | Short Term (n) | Short Term % |
|---|---|---|---|---|---|---|---|---|---|
| 50 | 70-74 | Never Any | 5137 | 58.42 | | | | | |
| 50 | 70-74 | CC | 22 | 0.25 | 0 | 0.00 | | | |
| 50 | 70-74 | Ecig | 1200 | 13.65 | | | | | |
| 50 | 70-74 | Dual | 0 | 0.00 | | | | | |
| 50 | 70-74 | Former | 2434 | 27.68 | | | 34.15 | 50 | 2.05 |

Year of Follow-up: 2040, Age Group: 75-79

| Period | Age Group | Smoking Group | N | % | Ever Quit (N) | Ever Quit % | Mean Yrs Quit | Short Term (n) | Short Term % |
|---|---|---|---|---|---|---|---|---|---|
| 50 | 75-79 | Never Any | 5270 | 52.29 | | | | | |
| 50 | 75-79 | CC | 48 | 0.48 | 0 | 0.00 | | | |
| 50 | 75-79 | Ecig | 1358 | 13.47 | | | | | |
| 50 | 75-79 | Dual | 0 | 0.00 | | | | | |
| 50 | 75-79 | Former | 3402 | 33.76 | | | 36.63 | 65 | 1.91 |

# Results for PHIM for Run 2 (Pessimistic_Analysis.RTF)

************************************************************************************

**Main Results for E-Component for Run: 2, Simulation: 1, COD: Four Diseases Combined**

| Variable Parameter | Value |
|---|---|
| 1. Country | US (United States) |
| 2. Sex | F (Females) |
| 3. Year of start of process | 1990 |
| 4. Cause of Death | Four Diseases Combined |
| 5. Effective Dose NULL | CC:1 |
| 6. Effective Dose Ecig | CC:1 Ecig:0.4 Dual:0.7 |
| Note: | Death rates are per million population |

------------------------------------------------------------------------------------------

Results for Age Groups Combined and Years combined, Population unadjusted for deaths in Ecig scenario,

| Age Group:All | | NULL | Ecig | | | %Drop | | NULL | Ecig | |
|---|---|---|---|---|---|---|---|---|---|---|
| Year | Number | Attrib | Attrib | Drop | %Drop | Attrib | Death | Attrib | Attrib | Drop |
| | Deaths | Deaths | Deaths | Deaths | Deaths | Deaths | Rate | Rates | Rates | Rates |
| All Comb | 9082078 | 2946250.006 | 2659500.065 | 286749.9 | 3.157 | 9.733 | 2493.45 | 808.881 | 730.155 | 78.726 |

------------------------------------------------------------------------------------------

Results for Age Groups Combined, Population unadjusted for deaths in Ecig scenario,

| Age Group:All | | NULL | Ecig | | | %Drop | | NULL | Ecig | |
|---|---|---|---|---|---|---|---|---|---|---|
| Year | Number | Attrib | Attrib | Drop | %Drop | Attrib | Death | Attrib | Attrib | Drop |
| | Deaths | Deaths | Deaths | Deaths | Deaths | Deaths | Rate | Rates | Rates | Rates |
| 1990 | 199848 | 57524.822 | 57524.822 | 0.000 | 0.000 | 0.000 | 2926.84 | 842.471 | 842.471 | 0.000 |
| 1991 | 199162 | 59078.486 | 59034.753 | 43.732 | 0.022 | 0.074 | 2877.87 | 853.678 | 853.047 | 0.632 |
| 1992 | 198532 | 60494.150 | 60380.223 | 113.927 | 0.057 | 0.188 | 2829.84 | 862.271 | 860.648 | 1.624 |
| 1993 | 202512 | 62694.848 | 62470.729 | 224.119 | 0.111 | 0.357 | 2845.85 | 881.034 | 877.884 | 3.149 |
| 1994 | 200658 | 63202.598 | 62848.417 | 354.181 | 0.177 | 0.560 | 2777.87 | 874.966 | 870.063 | 4.903 |
| 1995 | 201023 | 63766.862 | 63246.921 | 519.941 | 0.259 | 0.815 | 2739.80 | 869.097 | 862.011 | 7.086 |
| 1996 | 201180 | 64591.163 | 63880.417 | 710.745 | 0.353 | 1.100 | 2706.36 | 868.908 | 859.347 | 9.561 |
| 1997 | 198639 | 64686.912 | 63775.903 | 911.009 | 0.459 | 1.408 | 2635.18 | 858.147 | 846.061 | 12.086 |
| 1998 | 197573 | 65295.961 | 64135.880 | 1160.081 | 0.587 | 1.777 | 2584.18 | 854.046 | 838.873 | 15.173 |
| 1999 | 211329 | 67327.521 | 65782.998 | 1544.523 | 0.731 | 2.294 | 2726.91 | 868.771 | 848.841 | 19.930 |
| 2000 | 208496 | 67596.897 | 65748.523 | 1848.373 | 0.887 | 2.734 | 2656.96 | 861.419 | 837.865 | 23.555 |
| 2001 | 203606 | 67008.811 | 64868.504 | 2140.307 | 1.051 | 3.194 | 2565.88 | 844.458 | 817.486 | 26.973 |
| 2002 | 200828 | 67067.744 | 64545.210 | 2522.534 | 1.256 | 3.761 | 2506.09 | 836.925 | 805.447 | 31.478 |
| 2003 | 196352 | 66756.217 | 63904.094 | 2852.123 | 1.453 | 4.272 | 2427.31 | 825.244 | 789.986 | 35.258 |
| 2004 | 188160 | 65058.725 | 61903.935 | 3154.790 | 1.677 | 4.849 | 2302.83 | 796.234 | 757.624 | 38.611 |
| 2005 | 186416 | 65214.724 | 61686.062 | 3528.661 | 1.893 | 5.411 | 2256.27 | 789.321 | 746.612 | 42.709 |
| 2006 | 180088 | 63771.146 | 59923.078 | 3848.068 | 2.137 | 6.034 | 2160.67 | 765.117 | 718.949 | 46.169 |
| 2007 | 175644 | 63110.718 | 58952.387 | 4158.331 | 2.367 | 6.589 | 2086.36 | 749.650 | 700.256 | 49.394 |
| 2008 | 177570 | 63718.624 | 59166.725 | 4551.899 | 2.563 | 7.144 | 2086.39 | 748.674 | 695.190 | 53.483 |
| 2009 | 172806 | 62794.698 | 58005.753 | 4788.945 | 2.771 | 7.626 | 2007.57 | 729.515 | 673.880 | 55.635 |
| 2010 | 170489 | 61917.074 | 56845.832 | 5071.241 | 2.975 | 8.190 | 1957.97 | 711.083 | 652.842 | 58.240 |
| 2011 | 170305 | 61680.114 | 56188.063 | 5492.052 | 3.225 | 8.904 | 1937.46 | 701.700 | 639.220 | 62.480 |
| 2012 | 171421 | 62023.127 | 56131.698 | 5891.429 | 3.437 | 9.499 | 1930.22 | 698.386 | 632.048 | 66.338 |
| 2013 | 172699 | 62037.765 | 55791.971 | 6245.794 | 3.617 | 10.068 | 1923.75 | 691.059 | 621.485 | 69.574 |
| 2014 | 174169 | 61921.017 | 55276.316 | 6644.701 | 3.815 | 10.731 | 1918.85 | 682.195 | 608.989 | 73.206 |
| 2015 | 177354 | 61856.204 | 54883.160 | 6973.044 | 3.932 | 11.273 | 2184.51 | 761.899 | 676.010 | 85.889 |
| 2016 | 176853 | 60464.119 | 53216.690 | 7247.429 | 4.098 | 11.986 | 2157.59 | 737.655 | 649.237 | 88.418 |
| 2017 | 179309 | 60243.839 | 52640.748 | 7603.091 | 4.240 | 12.621 | 2166.86 | 728.017 | 636.137 | 91.880 |
| 2018 | 177157 | 60713.327 | 52823.825 | 7889.502 | 4.453 | 12.995 | 2120.51 | 726.721 | 632.286 | 94.435 |
| 2019 | 179227 | 61148.172 | 52886.830 | 8261.342 | 4.609 | 13.510 | 2124.53 | 724.841 | 626.912 | 97.929 |
| 2020 | 180467 | 61149.317 | 52643.017 | 8506.299 | 4.713 | 13.911 | 2424.25 | 821.432 | 707.165 | 114.267 |
| 2021 | 178116 | 58727.786 | 50072.452 | 8655.334 | 4.859 | 14.738 | 2375.42 | 783.216 | 667.785 | 115.431 |
| 2022 | 179408 | 58848.802 | 49884.885 | 8963.917 | 4.996 | 15.232 | 2374.52 | 778.881 | 660.241 | 118.640 |
| 2023 | 180640 | 58816.149 | 49669.571 | 9146.579 | 5.063 | 15.551 | 2372.26 | 772.407 | 652.289 | 120.118 |
| 2024 | 181610 | 58955.232 | 49551.429 | 9403.803 | 5.178 | 15.951 | 2366.77 | 768.316 | 645.764 | 122.552 |
| 2025 | 180418 | 58297.744 | 48837.823 | 9459.921 | 5.243 | 16.227 | 2713.69 | 876.861 | 734.574 | 142.287 |
| 2026 | 180313 | 56469.971 | 46941.391 | 9528.580 | 5.284 | 16.874 | 2701.83 | 846.150 | 703.373 | 142.777 |
| 2027 | 179619 | 56209.427 | 46487.295 | 9722.132 | 5.413 | 17.296 | 2680.68 | 838.883 | 693.788 | 145.095 |
| 2028 | 178762 | 55733.105 | 45952.390 | 9780.715 | 5.471 | 17.549 | 2656.74 | 828.298 | 682.938 | 145.360 |
| 2029 | 177808 | 55145.838 | 45338.881 | 9806.958 | 5.515 | 17.784 | 2630.89 | 815.951 | 670.845 | 145.106 |
| 2030 | 173587 | 53615.097 | 43862.851 | 9752.246 | 5.618 | 18.189 | 3040.33 | 939.056 | 768.248 | 170.808 |
| 2031 | 174985 | 52046.561 | 42557.131 | 9489.430 | 5.423 | 18.233 | 3060.73 | 910.364 | 744.381 | 165.983 |
| 2032 | 173400 | 51582.636 | 42076.328 | 9506.308 | 5.482 | 18.429 | 3027.10 | 900.496 | 734.541 | 165.955 |
| 2033 | 171756 | 50839.758 | 41535.129 | 9304.629 | 5.417 | 18.302 | 2991.14 | 885.375 | 723.334 | 162.040 |
| 2034 | 169973 | 50215.451 | 41022.491 | 9192.961 | 5.408 | 18.307 | 2951.96 | 872.104 | 712.447 | 159.656 |
| 2035 | 162644 | 47773.075 | 38791.884 | 8981.191 | 5.522 | 18.800 | 3459.14 | 1016.05 | 825.032 | 191.013 |
| 2036 | 156254 | 43298.709 | 35129.189 | 8169.520 | 5.228 | 18.868 | 3328.25 | 922.274 | 748.261 | 174.013 |
| 2037 | 153593 | 42241.563 | 34260.564 | 7980.999 | 5.196 | 18.894 | 3274.10 | 900.450 | 730.322 | 170.129 |
| 2038 | 150947 | 41356.353 | 33631.251 | 7725.101 | 5.118 | 18.679 | 3217.50 | 881.531 | 716.867 | 164.664 |
| 2039 | 148373 | 40161.050 | 32783.644 | 7377.406 | 4.972 | 18.370 | 3159.42 | 855.178 | 698.085 | 157.092 |

# Results for PHIM for Run 2 (Pessimistic_Analysis.RTF)

*********************************************************************************

**Main Results for E-Component for Run: 2, Simulation: 1, COD: LUNG CANCER**

| Variable Parameter | Value |
|---|---|
| 1. Country | US (United States) |
| 2. Sex | F (Females) |
| 3. Year of start of process | 1990 |
| 4. Cause of Death | LUNG CANCER |
| 5. Effective Dose NULL | CC:1 |
| 6. Effective Dose Ecig | CC:1 Ecig:0.4 Dual:0.7 |
| Note: | Death rates are per million population |

-----------------------------------------------------------------------------------------

Results for Age Groups Combined and Years combined, Population unadjusted for deaths in Ecig scenario,

| Age Group:All Year | Number Deaths | NULL Attrib Deaths | Ecig Attrib Deaths | Drop Deaths | %Drop Deaths | %Drop Attrib Deaths | Death Rate | NULL Attrib Rates | Ecig Attrib Rates | Drop Rates |
|---|---|---|---|---|---|---|---|---|---|---|
| All Comb | 2327764 | 1580465.217 | 1473995.308 | 106469.9 | 4.574 | 6.737 | 639.078 | 433.910 | 404.680 | 29.231 |

-----------------------------------------------------------------------------------------

Results for Age Groups Combined, Population unadjusted for deaths in Ecig scenario,

| Age Group:All Year | Number Deaths | NULL Attrib Deaths | Ecig Attrib Deaths | Drop Deaths | %Drop Deaths | %Drop Attrib Deaths | Death Rate | NULL Attrib Rates | Ecig Attrib Rates | Drop Rates |
|---|---|---|---|---|---|---|---|---|---|---|
| 1990 | 42690 | 30614.201 | 30614.201 | 0.000 | 0.000 | 0.000 | 625.210 | 448.355 | 448.355 | 0.000 |
| 1991 | 43692 | 31412.010 | 31404.310 | 7.700 | 0.018 | 0.025 | 631.345 | 453.901 | 453.789 | 0.111 |
| 1992 | 45423 | 32782.407 | 32763.024 | 19.383 | 0.043 | 0.059 | 647.450 | 467.274 | 466.998 | 0.276 |
| 1993 | 46291 | 33491.183 | 33451.795 | 39.388 | 0.085 | 0.118 | 650.515 | 470.643 | 470.089 | 0.554 |
| 1994 | 47017 | 34074.347 | 34011.713 | 62.634 | 0.133 | 0.184 | 650.895 | 471.719 | 470.852 | 0.867 |
| 1995 | 47840 | 34694.454 | 34598.207 | 96.247 | 0.201 | 0.277 | 652.025 | 472.861 | 471.549 | 1.312 |
| 1996 | 48244 | 35027.269 | 34896.426 | 130.842 | 0.271 | 0.374 | 648.999 | 471.202 | 469.442 | 1.760 |
| 1997 | 49035 | 35590.763 | 35410.199 | 180.564 | 0.368 | 0.507 | 650.506 | 472.153 | 469.757 | 2.395 |
| 1998 | 49686 | 36100.785 | 35850.972 | 249.813 | 0.503 | 0.692 | 649.874 | 472.184 | 468.917 | 3.267 |
| 1999 | 48883 | 35451.030 | 35139.426 | 311.604 | 0.637 | 0.879 | 630.769 | 457.448 | 453.427 | 4.021 |
| 2000 | 50331 | 36447.986 | 36042.617 | 405.369 | 0.805 | 1.112 | 641.392 | 464.474 | 459.308 | 5.166 |
| 2001 | 50389 | 36411.326 | 35920.159 | 491.167 | 0.975 | 1.349 | 635.012 | 458.863 | 452.673 | 6.190 |
| 2002 | 51445 | 37078.545 | 36464.657 | 613.888 | 1.193 | 1.656 | 641.972 | 462.696 | 455.035 | 7.661 |
| 2003 | 51408 | 36999.876 | 36273.227 | 726.649 | 1.413 | 1.964 | 635.509 | 457.395 | 448.412 | 8.983 |
| 2004 | 51399 | 36861.555 | 36005.637 | 855.919 | 1.665 | 2.322 | 629.057 | 451.138 | 440.662 | 10.475 |
| 2005 | 51421 | 36689.529 | 35702.884 | 986.645 | 1.919 | 2.689 | 622.370 | 444.069 | 432.127 | 11.942 |
| 2006 | 51355 | 36504.774 | 35375.712 | 1129.062 | 2.199 | 3.093 | 616.150 | 437.979 | 424.433 | 13.546 |
| 2007 | 51839 | 36663.202 | 35364.339 | 1298.863 | 2.506 | 3.543 | 615.761 | 435.498 | 420.069 | 15.428 |
| 2008 | 50976 | 35898.803 | 34488.508 | 1410.296 | 2.767 | 3.929 | 598.952 | 421.800 | 405.229 | 16.571 |
| 2009 | 51193 | 35934.525 | 34364.837 | 1569.689 | 3.066 | 4.368 | 594.733 | 417.468 | 399.232 | 18.236 |
| 2010 | 50853 | 35502.829 | 33794.796 | 1708.033 | 3.359 | 4.811 | 584.018 | 407.730 | 388.114 | 19.616 |
| 2011 | 50509 | 35136.342 | 33251.050 | 1885.292 | 3.733 | 5.366 | 574.613 | 399.726 | 378.279 | 21.448 |
| 2012 | 51245 | 35450.201 | 33379.079 | 2071.122 | 4.042 | 5.842 | 577.023 | 399.173 | 375.852 | 23.321 |
| 2013 | 51069 | 35123.224 | 32885.333 | 2237.891 | 4.382 | 6.372 | 568.874 | 391.249 | 366.320 | 24.929 |
| 2014 | 51251 | 35079.370 | 32666.461 | 2412.909 | 4.708 | 6.878 | 564.641 | 386.476 | 359.892 | 26.583 |
| 2015 | 50443 | 34324.171 | 31806.955 | 2517.215 | 4.990 | 7.334 | 621.319 | 422.780 | 391.774 | 31.005 |
| 2016 | 48854 | 33054.753 | 30445.622 | 2609.130 | 5.341 | 7.893 | 596.013 | 403.264 | 371.433 | 31.831 |
| 2017 | 48325 | 32471.234 | 29719.224 | 2752.011 | 5.695 | 8.475 | 583.983 | 392.399 | 359.142 | 33.257 |
| 2018 | 50465 | 33657.595 | 30575.385 | 3082.210 | 6.108 | 9.158 | 604.052 | 402.871 | 365.978 | 36.893 |
| 2019 | 51036 | 33863.424 | 30571.369 | 3292.055 | 6.450 | 9.722 | 604.975 | 401.412 | 362.388 | 39.024 |
| 2020 | 51444 | 33910.830 | 30453.613 | 3457.217 | 6.720 | 10.195 | 691.053 | 455.532 | 409.090 | 46.442 |
| 2021 | 48037 | 31531.880 | 28130.980 | 3400.900 | 7.080 | 10.786 | 640.639 | 420.521 | 375.165 | 45.356 |
| 2022 | 48335 | 31560.212 | 27981.263 | 3578.950 | 7.404 | 11.340 | 639.727 | 417.708 | 370.340 | 47.368 |
| 2023 | 48599 | 31508.781 | 27804.284 | 3704.497 | 7.623 | 11.757 | 638.227 | 413.791 | 365.141 | 48.650 |
| 2024 | 48784 | 31517.293 | 27659.458 | 3857.835 | 7.908 | 12.240 | 635.757 | 410.740 | 360.463 | 50.276 |
| 2025 | 48477 | 31175.389 | 27246.422 | 3928.967 | 8.105 | 12.603 | 729.141 | 468.912 | 409.816 | 59.096 |
| 2026 | 45324 | 29044.598 | 25244.664 | 3799.933 | 8.384 | 13.083 | 679.138 | 435.206 | 378.268 | 56.938 |
| 2027 | 45107 | 28825.765 | 24916.044 | 3909.721 | 8.668 | 13.563 | 673.191 | 430.203 | 371.853 | 58.350 |
| 2028 | 44842 | 28513.846 | 24551.552 | 3962.293 | 8.836 | 13.896 | 666.430 | 423.769 | 364.882 | 58.887 |
| 2029 | 44539 | 28148.109 | 24144.045 | 4004.064 | 8.990 | 14.225 | 659.013 | 416.486 | 357.241 | 59.245 |
| 2030 | 43393 | 27314.475 | 23323.576 | 3990.899 | 9.197 | 14.611 | 760.018 | 478.407 | 408.507 | 69.900 |
| 2031 | 40748 | 25423.102 | 21715.993 | 3707.108 | 9.098 | 14.582 | 712.731 | 444.684 | 379.842 | 64.842 |
| 2032 | 40325 | 25085.902 | 21367.191 | 3718.711 | 9.222 | 14.824 | 703.969 | 437.933 | 373.014 | 64.919 |
| 2033 | 39882 | 24645.067 | 20997.669 | 3647.398 | 9.145 | 14.800 | 694.543 | 429.194 | 365.674 | 63.519 |
| 2034 | 39404 | 24239.126 | 20637.699 | 3601.427 | 9.140 | 14.858 | 684.347 | 420.967 | 358.420 | 62.547 |
| 2035 | 37454 | 22944.147 | 19444.639 | 3499.507 | 9.343 | 15.252 | 796.588 | 487.980 | 413.552 | 74.428 |
| 2036 | 32988 | 19950.581 | 16929.831 | 3020.750 | 9.157 | 15.141 | 702.663 | 424.953 | 360.610 | 64.343 |
| 2037 | 32403 | 19415.334 | 16460.477 | 2954.857 | 9.119 | 15.219 | 690.731 | 413.871 | 350.883 | 62.988 |
| 2038 | 31820 | 18939.317 | 16094.400 | 2844.918 | 8.941 | 15.021 | 678.257 | 403.701 | 343.060 | 60.641 |
| 2039 | 31251 | 18379.752 | 15653.384 | 2726.368 | 8.724 | 14.834 | 665.457 | 391.373 | 333.319 | 58.054 |

# Results for PHIM for Run 2 (Pessimistic_Analysis.RTF)

********************************************************************************
**Main Results for E-Component for Run: 2, Simulation: 1, COD: IHD**

| Variable Parameter | Value |
|---|---|
| 1. Country | US (United States) |
| 2. Sex | F (Females) |
| 3. Year of start of process | 1990 |
| 4. Cause of Death | IHD |
| 5. Effective Dose NULL | CC:1 |
| 6. Effective Dose Ecig | CC:1 Ecig:0.4 Dual:0.7 |
| Note: | Death rates are per million population |

------------------------------------------------------------------------------------------
Results for Age Groups Combined and Years combined, Population unadjusted for deaths in Ecig scenario,

| Age Group:All Year | Number Deaths | NULL Attrib Deaths | Ecig Attrib Deaths | Drop Deaths | %Drop Deaths | %Drop Attrib Deaths | Death Rate | NULL Attrib Rates | Ecig Attrib Rates | Drop Rates |
|---|---|---|---|---|---|---|---|---|---|---|
| All Comb | 3242167 | 398833.796 | 332460.338 | 66373.46 | 2.047 | 16.642 | 890.124 | 109.498 | 91.276 | 18.223 |

------------------------------------------------------------------------------------------
Results for Age Groups Combined, Population unadjusted for deaths in Ecig scenario,

| Age Group:All Year | Number Deaths | NULL Attrib Deaths | Ecig Attrib Deaths | Drop Deaths | %Drop Deaths | %Drop Attrib Deaths | Death Rate | NULL Attrib Rates | Ecig Attrib Rates | Drop Rates |
|---|---|---|---|---|---|---|---|---|---|---|
| 1990 | 98916 | 12922.892 | 12922.892 | 0.000 | 0.000 | 0.000 | 1448.66 | 189.260 | 189.260 | 0.000 |
| 1991 | 96403 | 12856.479 | 12831.863 | 24.616 | 0.026 | 0.191 | 1393.01 | 185.775 | 185.419 | 0.356 |
| 1992 | 93721 | 12662.244 | 12595.504 | 66.740 | 0.071 | 0.527 | 1335.88 | 180.485 | 179.534 | 0.951 |
| 1993 | 93679 | 12730.613 | 12602.358 | 128.255 | 0.137 | 1.007 | 1316.45 | 178.900 | 177.098 | 1.802 |
| 1994 | 90832 | 12495.323 | 12293.736 | 201.586 | 0.222 | 1.613 | 1257.46 | 172.983 | 170.192 | 2.791 |
| 1995 | 89698 | 12365.590 | 12075.407 | 290.184 | 0.324 | 2.347 | 1222.52 | 168.534 | 164.579 | 3.955 |
| 1996 | 88286 | 12290.551 | 11893.052 | 397.500 | 0.450 | 3.234 | 1187.66 | 165.338 | 159.990 | 5.347 |
| 1997 | 84777 | 11719.531 | 11231.564 | 487.967 | 0.576 | 4.164 | 1124.67 | 155.473 | 149.000 | 6.473 |
| 1998 | 82517 | 11416.794 | 10822.861 | 593.933 | 0.720 | 5.202 | 1079.29 | 149.327 | 141.559 | 7.768 |
| 1999 | 93939 | 13028.038 | 12194.062 | 833.976 | 0.888 | 6.401 | 1212.16 | 168.109 | 157.348 | 10.761 |
| 2000 | 90039 | 12426.299 | 11474.877 | 951.422 | 1.057 | 7.657 | 1147.41 | 158.354 | 146.230 | 12.124 |
| 2001 | 86119 | 11939.281 | 10853.946 | 1085.336 | 1.260 | 9.090 | 1085.29 | 150.461 | 136.784 | 13.678 |
| 2002 | 83014 | 11528.794 | 10295.143 | 1233.651 | 1.486 | 10.701 | 1035.91 | 143.865 | 128.471 | 15.394 |
| 2003 | 79302 | 11048.407 | 9700.532 | 1347.875 | 1.700 | 12.200 | 980.336 | 136.581 | 119.919 | 16.663 |
| 2004 | 73861 | 10375.132 | 8937.805 | 1437.327 | 1.946 | 13.854 | 903.963 | 126.978 | 109.387 | 17.591 |
| 2005 | 71649 | 10051.029 | 8505.117 | 1545.913 | 2.158 | 15.381 | 867.197 | 121.652 | 102.941 | 18.711 |
| 2006 | 67551 | 9542.523 | 7906.784 | 1635.740 | 2.421 | 17.142 | 810.467 | 114.490 | 94.864 | 19.625 |
| 2007 | 63503 | 8936.533 | 7266.125 | 1670.408 | 2.630 | 18.692 | 754.310 | 106.151 | 86.309 | 19.842 |
| 2008 | 63171 | 8944.180 | 7147.303 | 1796.878 | 2.844 | 20.090 | 742.239 | 105.091 | 83.979 | 21.113 |
| 2009 | 59747 | 8390.033 | 6606.912 | 1783.121 | 2.984 | 21.253 | 694.108 | 97.471 | 76.756 | 20.715 |
| 2010 | 58008 | 8157.570 | 6320.312 | 1837.257 | 3.167 | 22.522 | 666.189 | 93.685 | 72.585 | 21.100 |
| 2011 | 57368 | 8035.356 | 6113.220 | 1922.136 | 3.351 | 23.921 | 652.644 | 91.414 | 69.547 | 21.867 |
| 2012 | 57411 | 7970.766 | 5958.935 | 2011.831 | 3.504 | 25.240 | 646.453 | 89.752 | 67.098 | 22.653 |
| 2013 | 57067 | 7743.216 | 5710.217 | 2032.998 | 3.562 | 26.255 | 635.688 | 86.254 | 63.608 | 22.646 |
| 2014 | 57538 | 7774.935 | 5655.763 | 2119.172 | 3.683 | 27.256 | 633.906 | 85.658 | 62.311 | 23.347 |
| 2015 | 58395 | 7722.535 | 5557.884 | 2164.651 | 3.707 | 28.030 | 719.266 | 95.120 | 68.458 | 26.663 |
| 2016 | 58658 | 7600.353 | 5405.046 | 2195.307 | 3.743 | 28.884 | 715.621 | 92.723 | 65.941 | 26.782 |
| 2017 | 59004 | 7470.266 | 5259.435 | 2210.832 | 3.747 | 29.595 | 713.034 | 90.274 | 63.558 | 26.717 |
| 2018 | 56077 | 6907.050 | 4822.813 | 2084.237 | 3.717 | 30.175 | 671.224 | 82.675 | 57.728 | 24.948 |
| 2019 | 55886 | 6732.046 | 4677.009 | 2055.037 | 3.677 | 30.526 | 662.462 | 79.801 | 55.441 | 24.360 |
| 2020 | 55313 | 6429.836 | 4435.363 | 1994.473 | 3.606 | 31.019 | 743.037 | 86.373 | 59.581 | 26.792 |
| 2021 | 56292 | 6562.016 | 4493.310 | 2068.706 | 3.675 | 31.525 | 750.730 | 87.513 | 59.924 | 27.589 |
| 2022 | 55827 | 6352.366 | 4332.974 | 2019.391 | 3.617 | 31.790 | 738.885 | 84.075 | 57.348 | 26.727 |
| 2023 | 55338 | 6134.123 | 4189.897 | 1944.226 | 3.513 | 31.695 | 726.728 | 80.557 | 55.024 | 25.533 |
| 2024 | 54770 | 5922.193 | 4053.302 | 1868.891 | 3.412 | 31.557 | 713.771 | 77.179 | 52.823 | 24.356 |
| 2025 | 53295 | 5504.188 | 3748.484 | 1755.704 | 3.294 | 31.898 | 801.612 | 82.789 | 56.381 | 26.408 |
| 2026 | 55278 | 5641.075 | 3860.288 | 1780.788 | 3.222 | 31.568 | 828.283 | 84.526 | 57.843 | 26.683 |
| 2027 | 54199 | 5426.216 | 3732.326 | 1693.890 | 3.125 | 31.217 | 808.875 | 80.982 | 55.702 | 25.280 |
| 2028 | 53090 | 5197.830 | 3609.218 | 1588.612 | 2.992 | 30.563 | 789.020 | 77.249 | 53.640 | 23.610 |
| 2029 | 51974 | 4973.547 | 3493.924 | 1479.623 | 2.847 | 29.750 | 769.023 | 73.590 | 51.697 | 21.893 |
| 2030 | 49479 | 4450.618 | 3082.609 | 1368.009 | 2.765 | 30.737 | 866.605 | 77.952 | 53.991 | 23.960 |
| 2031 | 51987 | 4555.052 | 3205.952 | 1349.100 | 2.595 | 29.618 | 909.317 | 79.674 | 56.076 | 23.598 |
| 2032 | 50677 | 4349.999 | 3096.683 | 1253.316 | 2.473 | 28.812 | 884.689 | 75.939 | 54.060 | 21.880 |
| 2033 | 49380 | 4147.948 | 3009.564 | 1138.384 | 2.305 | 27.445 | 859.955 | 72.237 | 52.412 | 19.825 |
| 2034 | 48076 | 3966.133 | 2923.265 | 1042.868 | 2.169 | 26.294 | 834.943 | 68.881 | 50.769 | 18.112 |
| 2035 | 44518 | 3289.064 | 2359.424 | 929.640 | 2.088 | 28.265 | 946.825 | 69.952 | 50.181 | 19.772 |
| 2036 | 46347 | 3313.192 | 2424.657 | 888.535 | 1.917 | 26.818 | 987.211 | 70.572 | 51.646 | 18.926 |
| 2037 | 44841 | 3117.951 | 2330.141 | 787.810 | 1.757 | 25.267 | 955.859 | 66.464 | 49.671 | 16.794 |
| 2038 | 43378 | 2949.775 | 2257.882 | 691.893 | 1.595 | 23.456 | 924.621 | 62.876 | 48.128 | 14.748 |
| 2039 | 41973 | 2766.312 | 2182.601 | 583.711 | 1.391 | 21.101 | 893.753 | 58.905 | 46.476 | 12.429 |

# Results for PHIM for Run 2 (Pessimistic_Analysis.RTF)

************************************************************************************
**Main Results for E-Component for Run: 2, Simulation: 1, COD: STROKE**

| Variable Parameter | Value |
|---|---|
| 1. Country | US (United States) |
| 2. Sex | F (Females) |
| 3. Year of start of process | 1990 |
| 4. Cause of Death | STROKE |
| 5. Effective Dose NULL | CC:1 |
| 6. Effective Dose Ecig | CC:1 Ecig:0.4 Dual:0.7 |
| Note: | Death rates are per million population |

---------------------------------------------------------------------------------------
Results for Age Groups Combined and Years combined, Population unadjusted for deaths in Ecig scenario,

| Age Group:All | | NULL | Ecig | | | %Drop | | NULL | Ecig | |
|---|---|---|---|---|---|---|---|---|---|---|
| Year | Number | Attrib | Attrib | Drop | %Drop | Attrib | Death | Attrib | Attrib | Drop |
| | Deaths | Deaths | Deaths | Deaths | Deaths | Deaths | Rate | Rates | Rates | Rates |
| All Comb | 1352207 | 104899.750 | 86668.100 | 18231.65 | 1.348 | 17.380 | 371.243 | 28.800 | 23.794 | 5.005 |

---------------------------------------------------------------------------------------
Results for Age Groups Combined, Population unadjusted for deaths in Ecig scenario,

| Age Group:All | | NULL | Ecig | | | %Drop | | NULL | Ecig | |
|---|---|---|---|---|---|---|---|---|---|---|
| Year | Number | Attrib | Attrib | Drop | %Drop | Attrib | Death | Attrib | Attrib | Drop |
| | Deaths | Deaths | Deaths | Deaths | Deaths | Deaths | Rate | Rates | Rates | Rates |
| 1990 | 33504 | 3293.017 | 3293.017 | 0.000 | 0.000 | 0.000 | 490.678 | 48.227 | 48.227 | 0.000 |
| 1991 | 32505 | 3207.063 | 3200.117 | 6.946 | 0.021 | 0.217 | 469.694 | 46.342 | 46.241 | 0.100 |
| 1992 | 32560 | 3218.125 | 3199.463 | 18.662 | 0.057 | 0.580 | 464.104 | 45.871 | 45.604 | 0.266 |
| 1993 | 32768 | 3226.680 | 3189.786 | 36.894 | 0.113 | 1.143 | 460.480 | 45.344 | 44.825 | 0.518 |
| 1994 | 33005 | 3277.266 | 3216.480 | 60.786 | 0.184 | 1.855 | 456.915 | 45.370 | 44.528 | 0.842 |
| 1995 | 33730 | 3309.378 | 3219.287 | 90.092 | 0.267 | 2.722 | 459.716 | 45.104 | 43.877 | 1.228 |
| 1996 | 33750 | 3312.987 | 3186.961 | 126.025 | 0.373 | 3.804 | 454.019 | 44.568 | 42.872 | 1.695 |
| 1997 | 33699 | 3267.066 | 3102.281 | 164.785 | 0.489 | 5.044 | 447.056 | 43.341 | 41.155 | 2.186 |
| 1998 | 33274 | 3178.725 | 2977.942 | 200.783 | 0.603 | 6.316 | 435.211 | 41.577 | 38.950 | 2.626 |
| 1999 | 33891 | 3120.351 | 2881.900 | 238.451 | 0.704 | 7.642 | 437.317 | 40.264 | 37.187 | 3.077 |
| 2000 | 33827 | 3142.808 | 2856.575 | 286.233 | 0.846 | 9.108 | 431.074 | 40.050 | 36.403 | 3.648 |
| 2001 | 32506 | 2961.588 | 2649.251 | 312.337 | 0.961 | 10.546 | 409.647 | 37.323 | 33.386 | 3.936 |
| 2002 | 32078 | 2940.627 | 2579.506 | 361.121 | 1.126 | 12.280 | 400.295 | 36.695 | 32.189 | 4.506 |
| 2003 | 30568 | 2819.545 | 2426.835 | 392.710 | 1.285 | 13.928 | 377.883 | 34.855 | 30.001 | 4.855 |
| 2004 | 29388 | 2730.466 | 2306.957 | 423.509 | 1.441 | 15.510 | 359.671 | 33.417 | 28.234 | 5.183 |
| 2005 | 27954 | 2670.510 | 2211.948 | 458.562 | 1.640 | 17.171 | 338.339 | 32.322 | 26.772 | 5.550 |
| 2006 | 27201 | 2623.285 | 2129.687 | 493.598 | 1.815 | 18.816 | 326.354 | 31.474 | 25.552 | 5.922 |
| 2007 | 26302 | 2536.599 | 2021.476 | 515.123 | 1.958 | 20.308 | 312.424 | 30.131 | 24.012 | 6.119 |
| 2008 | 25998 | 2469.443 | 1936.676 | 532.767 | 2.049 | 21.574 | 305.468 | 29.015 | 22.755 | 6.260 |
| 2009 | 25040 | 2386.945 | 1842.544 | 544.401 | 2.174 | 22.807 | 290.901 | 27.730 | 21.406 | 6.325 |
| 2010 | 24840 | 2329.274 | 1773.409 | 555.865 | 2.238 | 23.864 | 285.273 | 26.750 | 20.367 | 6.384 |
| 2011 | 24792 | 2286.581 | 1712.204 | 574.376 | 2.317 | 25.119 | 282.045 | 26.013 | 19.479 | 6.534 |
| 2012 | 24500 | 2243.311 | 1651.225 | 592.086 | 2.417 | 26.393 | 275.872 | 25.260 | 18.593 | 6.667 |
| 2013 | 24451 | 2176.503 | 1577.365 | 599.138 | 2.450 | 27.528 | 272.368 | 24.245 | 17.571 | 6.674 |
| 2014 | 25296 | 2211.619 | 1583.657 | 627.962 | 2.482 | 28.394 | 278.691 | 24.366 | 17.447 | 6.918 |
| 2015 | 26316 | 2237.016 | 1584.981 | 652.035 | 2.478 | 29.148 | 324.141 | 27.554 | 19.523 | 8.031 |
| 2016 | 26735 | 2229.057 | 1564.464 | 664.593 | 2.486 | 29.815 | 326.164 | 27.194 | 19.086 | 8.108 |
| 2017 | 27616 | 2221.649 | 1546.221 | 675.429 | 2.446 | 30.402 | 333.726 | 26.848 | 18.685 | 8.162 |
| 2018 | 25256 | 1939.420 | 1343.006 | 596.414 | 2.362 | 30.752 | 302.304 | 23.214 | 16.075 | 7.139 |
| 2019 | 25429 | 1904.400 | 1312.092 | 592.307 | 2.329 | 31.102 | 301.434 | 22.574 | 15.553 | 7.021 |
| 2020 | 25379 | 1806.054 | 1235.962 | 570.092 | 2.246 | 31.566 | 340.922 | 24.261 | 16.603 | 7.658 |
| 2021 | 25448 | 1748.172 | 1190.540 | 557.632 | 2.191 | 31.898 | 339.386 | 23.314 | 15.877 | 7.437 |
| 2022 | 25539 | 1701.148 | 1155.373 | 545.775 | 2.137 | 32.083 | 338.015 | 22.515 | 15.292 | 7.223 |
| 2023 | 25620 | 1652.087 | 1124.113 | 527.973 | 2.061 | 31.958 | 336.461 | 21.696 | 14.762 | 6.934 |
| 2024 | 25659 | 1597.127 | 1089.786 | 507.341 | 1.977 | 31.766 | 334.391 | 20.814 | 14.202 | 6.612 |
| 2025 | 25260 | 1467.622 | 996.876 | 470.746 | 1.864 | 32.075 | 379.931 | 22.075 | 14.994 | 7.081 |
| 2026 | 25480 | 1390.505 | 953.000 | 437.505 | 1.717 | 31.464 | 381.790 | 20.835 | 14.280 | 6.556 |
| 2027 | 25264 | 1342.095 | 926.500 | 415.596 | 1.645 | 30.966 | 377.042 | 20.030 | 13.827 | 6.202 |
| 2028 | 25022 | 1289.536 | 901.952 | 387.584 | 1.549 | 30.056 | 371.870 | 19.165 | 13.405 | 5.760 |
| 2029 | 24767 | 1242.216 | 880.882 | 361.334 | 1.459 | 29.088 | 366.459 | 18.380 | 13.034 | 5.346 |
| 2030 | 23959 | 1096.803 | 771.704 | 325.099 | 1.357 | 29.641 | 419.641 | 19.210 | 13.516 | 5.694 |
| 2031 | 23963 | 1019.991 | 731.189 | 288.802 | 1.205 | 28.314 | 419.142 | 17.841 | 12.789 | 5.052 |
| 2032 | 23615 | 977.406 | 710.267 | 267.139 | 1.131 | 27.331 | 412.259 | 17.063 | 12.399 | 4.664 |
| 2033 | 23259 | 937.450 | 696.071 | 241.378 | 1.038 | 25.748 | 405.051 | 16.326 | 12.122 | 4.204 |
| 2034 | 22879 | 900.763 | 680.583 | 220.180 | 0.962 | 24.444 | 397.339 | 15.644 | 11.820 | 3.824 |
| 2035 | 21733 | 745.023 | 556.299 | 188.724 | 0.868 | 25.331 | 462.224 | 15.845 | 11.831 | 4.014 |
| 2036 | 20904 | 680.360 | 517.977 | 162.382 | 0.777 | 23.867 | 445.263 | 14.492 | 11.033 | 3.459 |
| 2037 | 20397 | 644.109 | 502.424 | 141.685 | 0.695 | 21.997 | 434.791 | 13.730 | 10.710 | 3.020 |
| 2038 | 19890 | 612.448 | 490.377 | 122.071 | 0.614 | 19.932 | 423.966 | 13.055 | 10.453 | 2.602 |
| 2039 | 19392 | 577.529 | 478.907 | 98.622 | 0.509 | 17.077 | 412.930 | 12.298 | 10.198 | 2.100 |

# Results for PHIM for Run 2 (Pessimistic_Analysis.RTF)

********************************************************************************
**Main Results for E-Component for Run: 2, Simulation: 1, COD: COPD**

| Variable Parameter | Value |
|---|---|
| 1. Country | US (United States) |
| 2. Sex | F (Females) |
| 3. Year of start of process | 1990 |
| 4. Cause of Death | COPD |
| 5. Effective Dose NULL | CC:1 |
| 6. Effective Dose Ecig | CC:1 Ecig:0.4 Dual:0.7 |
| Note: | Death rates are per million population |

--------------------------------------------------------------------------------------
Results for Age Groups Combined and Years combined, Population unadjusted for deaths in Ecig scenario,

| Age Group:All Year | Number Deaths | NULL Attrib Deaths | Ecig Attrib Deaths | Drop Deaths | %Drop Deaths | %Drop Attrib Deaths | Death Rate | NULL Attrib Rates | Ecig Attrib Rates | Drop Rates |
|---|---|---|---|---|---|---|---|---|---|---|
| All Comb | 2159941 | 862051.244 | 766376.319 | 95674.92 | 4.430 | 11.099 | 593.003 | 236.673 | 210.406 | 26.267 |

--------------------------------------------------------------------------------------
Results for Age Groups Combined, Population unadjusted for deaths in Ecig scenario,

| Age Group:All Year | Number Deaths | NULL Attrib Deaths | Ecig Attrib Deaths | Drop Deaths | %Drop Deaths | %Drop Attrib Deaths | Death Rate | NULL Attrib Rates | Ecig Attrib Rates | Drop Rates |
|---|---|---|---|---|---|---|---|---|---|---|
| 1990 | 24738 | 10694.712 | 10694.712 | 0.000 | 0.000 | 0.000 | 362.296 | 156.628 | 156.628 | 0.000 |
| 1991 | 26562 | 11602.933 | 11598.462 | 4.471 | 0.017 | 0.039 | 383.818 | 167.661 | 167.597 | 0.065 |
| 1992 | 26828 | 11831.375 | 11822.232 | 9.142 | 0.034 | 0.077 | 382.401 | 168.642 | 168.512 | 0.130 |
| 1993 | 29774 | 13246.371 | 13226.791 | 19.581 | 0.066 | 0.148 | 418.406 | 186.148 | 185.873 | 0.275 |
| 1994 | 29804 | 13355.662 | 13326.487 | 29.175 | 0.098 | 0.218 | 412.601 | 184.893 | 184.490 | 0.404 |
| 1995 | 29755 | 13397.439 | 13354.021 | 43.419 | 0.146 | 0.324 | 405.540 | 182.598 | 182.006 | 0.592 |
| 1996 | 30900 | 13960.356 | 13903.978 | 56.378 | 0.182 | 0.404 | 415.680 | 187.801 | 187.042 | 0.758 |
| 1997 | 31128 | 14109.552 | 14031.859 | 77.693 | 0.250 | 0.551 | 412.949 | 187.180 | 186.149 | 1.031 |
| 1998 | 32096 | 14599.657 | 14484.105 | 115.552 | 0.360 | 0.791 | 419.803 | 190.958 | 189.447 | 1.511 |
| 1999 | 34616 | 15728.102 | 15567.611 | 160.492 | 0.464 | 1.020 | 446.673 | 202.950 | 200.879 | 2.071 |
| 2000 | 34299 | 15579.803 | 15374.454 | 205.349 | 0.599 | 1.318 | 437.088 | 198.541 | 195.924 | 2.617 |
| 2001 | 34592 | 15696.614 | 15445.148 | 251.466 | 0.727 | 1.602 | 435.935 | 197.812 | 194.643 | 3.169 |
| 2002 | 34291 | 15519.777 | 15205.904 | 313.873 | 0.915 | 2.022 | 427.910 | 193.668 | 189.751 | 3.917 |
| 2003 | 35074 | 15888.389 | 15503.500 | 384.889 | 1.097 | 2.422 | 433.587 | 196.413 | 191.655 | 4.758 |
| 2004 | 33512 | 15091.573 | 14653.537 | 438.036 | 1.307 | 2.903 | 410.143 | 184.701 | 179.340 | 5.361 |
| 2005 | 35392 | 15803.656 | 15266.114 | 537.542 | 1.519 | 3.401 | 428.364 | 191.278 | 184.772 | 6.506 |
| 2006 | 33981 | 15100.563 | 14510.895 | 589.669 | 1.735 | 3.905 | 407.699 | 181.174 | 174.100 | 7.075 |
| 2007 | 34000 | 14974.384 | 14300.446 | 673.937 | 1.982 | 4.501 | 403.863 | 177.871 | 169.865 | 8.005 |
| 2008 | 37425 | 16406.197 | 15594.239 | 811.958 | 2.170 | 4.949 | 439.732 | 192.768 | 183.227 | 9.540 |
| 2009 | 36826 | 16083.194 | 15191.460 | 891.734 | 2.421 | 5.545 | 427.825 | 186.846 | 176.486 | 10.360 |
| 2010 | 36788 | 15927.400 | 14957.315 | 970.085 | 2.637 | 6.091 | 422.489 | 182.917 | 171.776 | 11.141 |
| 2011 | 37636 | 16221.836 | 15111.588 | 1110.248 | 2.950 | 6.844 | 428.164 | 184.547 | 171.916 | 12.631 |
| 2012 | 38265 | 16358.849 | 15142.459 | 1216.390 | 3.179 | 7.436 | 430.867 | 184.202 | 170.506 | 13.697 |
| 2013 | 40112 | 16994.822 | 15619.056 | 1375.767 | 3.430 | 8.095 | 446.821 | 189.311 | 173.986 | 15.325 |
| 2014 | 40084 | 16855.093 | 15370.435 | 1484.658 | 3.704 | 8.808 | 441.613 | 185.696 | 169.339 | 16.357 |
| 2015 | 42200 | 17572.483 | 15933.340 | 1639.143 | 3.884 | 9.328 | 519.788 | 216.445 | 196.255 | 20.190 |
| 2016 | 42606 | 17579.955 | 15801.557 | 1778.398 | 4.174 | 10.116 | 519.788 | 214.473 | 192.777 | 21.696 |
| 2017 | 44364 | 18080.688 | 16115.869 | 1964.820 | 4.429 | 10.867 | 536.117 | 218.496 | 194.752 | 23.744 |
| 2018 | 45359 | 18209.262 | 16082.621 | 2126.641 | 4.688 | 11.679 | 542.932 | 217.960 | 192.504 | 25.455 |
| 2019 | 46876 | 18648.302 | 16326.360 | 2321.942 | 4.953 | 12.451 | 555.657 | 221.054 | 193.530 | 27.524 |
| 2020 | 48331 | 19002.597 | 16518.080 | 2484.517 | 5.141 | 13.075 | 649.239 | 255.266 | 221.891 | 33.375 |
| 2021 | 48338 | 18885.717 | 16257.622 | 2628.095 | 5.437 | 13.916 | 644.660 | 251.867 | 216.818 | 35.049 |
| 2022 | 49708 | 19235.076 | 16415.275 | 2819.801 | 5.673 | 14.660 | 657.895 | 254.582 | 217.261 | 37.321 |
| 2023 | 51083 | 19521.158 | 16551.276 | 2969.882 | 5.814 | 15.214 | 670.849 | 256.363 | 217.361 | 39.002 |
| 2024 | 52397 | 19918.620 | 16748.882 | 3169.737 | 6.049 | 15.913 | 682.854 | 259.583 | 218.275 | 41.309 |
| 2025 | 53387 | 20150.545 | 16846.041 | 3304.504 | 6.190 | 16.399 | 803.004 | 303.086 | 253.383 | 49.703 |
| 2026 | 54232 | 20393.792 | 16883.439 | 3510.354 | 6.473 | 17.213 | 812.615 | 305.582 | 252.983 | 52.599 |
| 2027 | 55049 | 20615.351 | 16912.426 | 3702.925 | 6.727 | 17.962 | 821.568 | 307.668 | 252.405 | 55.263 |
| 2028 | 55809 | 20731.894 | 16889.667 | 3842.227 | 6.885 | 18.533 | 829.419 | 308.115 | 251.012 | 57.103 |
| 2029 | 56527 | 20781.967 | 16820.029 | 3961.938 | 7.009 | 19.064 | 836.392 | 307.495 | 248.873 | 58.622 |
| 2030 | 56756 | 20753.201 | 16684.962 | 4068.239 | 7.168 | 19.603 | 994.067 | 363.487 | 292.233 | 71.254 |
| 2031 | 58288 | 21048.417 | 16903.997 | 4144.420 | 7.110 | 19.690 | 1019.54 | 368.165 | 295.673 | 72.491 |
| 2032 | 58782 | 21169.329 | 16902.187 | 4267.142 | 7.259 | 20.157 | 1026.18 | 369.560 | 295.067 | 74.493 |
| 2033 | 59236 | 21109.293 | 16831.825 | 4277.468 | 7.221 | 20.263 | 1031.59 | 367.618 | 293.126 | 74.492 |
| 2034 | 59614 | 21109.429 | 16780.942 | 4328.486 | 7.261 | 20.505 | 1035.33 | 366.613 | 291.439 | 75.174 |
| 2035 | 58938 | 20794.840 | 16431.521 | 4363.319 | 7.403 | 20.983 | 1253.51 | 442.268 | 349.468 | 92.800 |
| 2036 | 56014 | 19354.577 | 15256.724 | 4097.853 | 7.316 | 21.173 | 1193.11 | 412.258 | 324.972 | 87.285 |
| 2037 | 55953 | 19064.169 | 14967.521 | 4096.648 | 7.322 | 21.489 | 1192.72 | 406.385 | 319.058 | 87.327 |
| 2038 | 55859 | 18854.812 | 14788.593 | 4066.219 | 7.279 | 21.566 | 1190.66 | 401.900 | 315.226 | 86.673 |
| 2039 | 55757 | 18437.457 | 14468.753 | 3968.704 | 7.118 | 21.525 | 1187.28 | 392.602 | 308.093 | 84.508 |

# Results for PHIM for Run 2 (Pessimistic_Analysis.RTF)

************************************************************************************

**Main Results for E-Component for Run: 2, Simulation: 1, COD: Four Diseases Combined, Adjusted**

| Variable Parameter | Value |
|---|---|
| 1. Country | US (United States) |
| 2. Sex | F (Females) |
| 3. Year of start of process | 1990 |
| 4. Cause of Death | Four Diseases Combined |
| 5. Effective Dose NULL | CC:1 |
| 6. Effective Dose Ecig | CC:1 Ecig:0.4 Dual:0.7 |
| Note: | Death rates are per million population |

------------------------------------------------------------------------------------------

Results for Age Groups Combined and Years combined, Population and Number deaths adjusted for Ecig scenario,

| Age Group:All | | NULL | Ecig | | | %Drop | | NULL | Ecig | |
|---|---|---|---|---|---|---|---|---|---|---|
| Year | Number Deaths | Attrib Deaths | Attrib Deaths | Drop Deaths | %Drop Deaths | Attrib Deaths | Death Rate | Attrib Rates | Attrib Rates | Drop Rates |
| All Comb | 9098863 | 2946250.006 | 2663836.376 | 282413.6 | 3.104 | 9.586 | 2494.62 | 808.881 | 730.341 | 78.540 |

------------------------------------------------------------------------------------------

Results for Age Groups Combined, Population and Number deaths adjusted for Ecig scenario,

| Age Group:All | | NULL | Ecig | | | %Drop | | NULL | Ecig | |
|---|---|---|---|---|---|---|---|---|---|---|
| Year | Number Deaths | Attrib Deaths | Attrib Deaths | Drop Deaths | %Drop Deaths | Attrib Deaths | Death Rate | Attrib Rates | Attrib Rates | Drop Rates |
| 1990 | 199848 | 57524.822 | 57524.822 | 0.000 | 0.000 | 0.000 | 2926.84 | 842.471 | 842.471 | 0.000 |
| 1991 | 199162 | 59078.486 | 59034.753 | 43.732 | 0.022 | 0.074 | 2877.87 | 853.678 | 853.047 | 0.632 |
| 1992 | 198532 | 60494.150 | 60380.268 | 113.881 | 0.057 | 0.188 | 2829.84 | 862.271 | 860.648 | 1.624 |
| 1993 | 202513 | 62694.848 | 62470.874 | 223.973 | 0.111 | 0.357 | 2845.85 | 881.034 | 877.884 | 3.149 |
| 1994 | 200659 | 63202.598 | 62848.772 | 353.825 | 0.176 | 0.560 | 2777.87 | 874.966 | 870.063 | 4.903 |
| 1995 | 201025 | 63766.862 | 63247.599 | 519.263 | 0.258 | 0.814 | 2739.80 | 869.097 | 862.011 | 7.086 |
| 1996 | 201183 | 64591.163 | 63881.571 | 709.592 | 0.353 | 1.099 | 2706.35 | 868.908 | 859.347 | 9.561 |
| 1997 | 198644 | 64686.912 | 63777.657 | 909.255 | 0.458 | 1.406 | 2635.16 | 858.147 | 846.061 | 12.086 |
| 1998 | 197579 | 65295.961 | 64138.468 | 1157.493 | 0.586 | 1.773 | 2584.16 | 854.046 | 838.873 | 15.173 |
| 1999 | 211339 | 67327.521 | 65786.860 | 1540.661 | 0.729 | 2.288 | 2726.89 | 868.771 | 848.843 | 19.928 |
| 2000 | 208510 | 67596.897 | 65754.023 | 1842.874 | 0.884 | 2.726 | 2656.94 | 861.419 | 837.870 | 23.549 |
| 2001 | 203626 | 67008.811 | 64875.978 | 2132.832 | 1.047 | 3.183 | 2565.87 | 844.458 | 817.497 | 26.962 |
| 2002 | 200854 | 67067.744 | 64554.936 | 2512.807 | 1.251 | 3.747 | 2506.09 | 836.925 | 805.463 | 31.462 |
| 2003 | 196386 | 66756.217 | 63916.588 | 2839.628 | 1.446 | 4.254 | 2427.34 | 825.244 | 790.011 | 35.233 |
| 2004 | 188202 | 65058.725 | 61919.351 | 3139.374 | 1.668 | 4.825 | 2302.89 | 796.234 | 757.660 | 38.575 |
| 2005 | 186469 | 65214.724 | 61705.025 | 3509.699 | 1.882 | 5.382 | 2256.36 | 789.321 | 746.660 | 42.661 |
| 2006 | 180151 | 63771.146 | 59945.585 | 3825.560 | 2.124 | 5.999 | 2160.80 | 765.117 | 719.011 | 46.106 |
| 2007 | 175719 | 63110.718 | 58978.874 | 4131.844 | 2.351 | 6.547 | 2086.54 | 749.650 | 700.333 | 49.317 |
| 2008 | 177660 | 63718.624 | 59198.056 | 4520.568 | 2.545 | 7.095 | 2086.63 | 748.674 | 695.285 | 53.388 |
| 2009 | 172909 | 62794.698 | 58041.613 | 4753.085 | 2.749 | 7.569 | 2007.86 | 729.515 | 673.993 | 55.522 |
| 2010 | 170606 | 61917.074 | 56886.172 | 5030.901 | 2.949 | 8.125 | 1958.32 | 711.083 | 652.972 | 58.111 |
| 2011 | 170439 | 61680.114 | 56233.386 | 5446.728 | 3.196 | 8.831 | 1937.88 | 701.700 | 639.369 | 62.331 |
| 2012 | 171574 | 62023.127 | 56182.893 | 5840.234 | 3.404 | 9.416 | 1930.71 | 698.386 | 632.220 | 66.166 |
| 2013 | 172873 | 62037.765 | 55849.059 | 6188.706 | 3.580 | 9.976 | 1924.32 | 691.059 | 621.679 | 69.380 |
| 2014 | 174366 | 61921.017 | 55339.472 | 6581.546 | 3.775 | 10.629 | 1919.51 | 682.195 | 609.207 | 72.988 |
| 2015 | 177578 | 61856.204 | 54953.073 | 6903.131 | 3.887 | 11.160 | 2185.16 | 761.899 | 676.217 | 85.681 |
| 2016 | 177100 | 60464.119 | 53291.635 | 7172.483 | 4.050 | 11.862 | 2158.31 | 737.655 | 649.465 | 88.190 |
| 2017 | 179584 | 60243.839 | 52722.402 | 7521.437 | 4.188 | 12.485 | 2167.69 | 728.017 | 636.392 | 91.625 |
| 2018 | 177454 | 60713.327 | 52913.294 | 7800.033 | 4.396 | 12.847 | 2121.43 | 726.721 | 632.568 | 94.153 |
| 2019 | 179557 | 61148.172 | 52984.961 | 8163.211 | 4.546 | 13.350 | 2125.59 | 724.841 | 627.232 | 97.609 |
| 2020 | 180832 | 61149.317 | 52750.196 | 8399.120 | 4.645 | 13.735 | 2425.21 | 821.432 | 707.452 | 113.980 |
| 2021 | 178510 | 58727.786 | 50183.784 | 8544.002 | 4.786 | 14.548 | 2376.52 | 783.216 | 668.102 | 115.114 |
| 2022 | 179838 | 58848.802 | 50004.781 | 8844.022 | 4.918 | 15.028 | 2375.78 | 778.881 | 660.594 | 118.286 |
| 2023 | 181107 | 58816.149 | 49798.215 | 9017.935 | 4.979 | 15.332 | 2373.68 | 772.407 | 652.681 | 119.726 |
| 2024 | 182114 | 58955.232 | 49689.114 | 9266.118 | 5.088 | 15.717 | 2368.35 | 768.316 | 646.195 | 122.121 |
| 2025 | 180961 | 58297.744 | 48984.638 | 9313.106 | 5.146 | 15.975 | 2715.00 | 876.861 | 734.931 | 141.930 |
| 2026 | 180895 | 56469.971 | 47093.011 | 9376.959 | 5.184 | 16.605 | 2703.35 | 846.150 | 703.772 | 142.378 |
| 2027 | 180238 | 56209.427 | 46647.689 | 9561.739 | 5.305 | 17.011 | 2682.40 | 838.883 | 694.235 | 144.648 |
| 2028 | 179420 | 55733.105 | 46121.334 | 9611.771 | 5.357 | 17.246 | 2658.68 | 828.298 | 683.435 | 144.863 |
| 2029 | 178504 | 55145.838 | 45516.071 | 9629.767 | 5.395 | 17.462 | 2633.05 | 815.951 | 671.392 | 144.559 |
| 2030 | 174318 | 53615.097 | 44047.718 | 9567.378 | 5.488 | 17.845 | 3042.05 | 939.056 | 768.684 | 170.372 |
| 2031 | 175768 | 52046.561 | 42747.036 | 9299.525 | 5.291 | 17.868 | 3062.73 | 910.364 | 744.862 | 165.501 |
| 2032 | 174220 | 51582.636 | 42274.754 | 9307.882 | 5.343 | 18.045 | 3029.37 | 900.496 | 735.083 | 165.413 |
| 2033 | 172614 | 50839.758 | 41741.794 | 9097.964 | 5.271 | 17.895 | 2993.70 | 885.375 | 723.940 | 161.435 |
| 2034 | 170866 | 50215.451 | 41236.926 | 8978.526 | 5.255 | 17.880 | 2954.80 | 872.104 | 713.112 | 158.991 |
| 2035 | 163561 | 47773.075 | 39010.468 | 8762.607 | 5.357 | 18.342 | 3461.37 | 1016.05 | 825.562 | 190.483 |
| 2036 | 157174 | 43298.709 | 35335.090 | 7963.619 | 5.067 | 18.392 | 3330.62 | 922.274 | 748.775 | 173.499 |
| 2037 | 154539 | 42241.563 | 34470.033 | 7771.530 | 5.029 | 18.398 | 3276.78 | 900.450 | 730.889 | 169.562 |
| 2038 | 151916 | 41356.353 | 33845.392 | 7510.960 | 4.944 | 18.162 | 3220.49 | 881.531 | 717.492 | 164.039 |
| 2039 | 149366 | 40161.050 | 33000.309 | 7160.741 | 4.794 | 17.830 | 3162.71 | 855.178 | 698.759 | 156.419 |

# Results for PHIM for Run 2 (Pessimistic_Analysis.RTF)

*************************************************************************************
**Main Results for E-Component for Run: 2, Simulation: 1, COD: LUNG CANCER, Adjusted**

| Variable Parameter | Value |
|---|---|
| 1. Country | US (United States) |
| 2. Sex | F (Females) |
| 3. Year of start of process | 1990 |
| 4. Cause of Death | LUNG CANCER |
| 5. Effective Dose NULL | CC:1 |
| 6. Effective Dose Ecig | CC:1 Ecig:0.4 Dual:0.7 |
| Note: | Death rates are per million population |

-----------------------------------------------------------------------------------------
Results for Age Groups Combined and Years combined, Population and Number deaths adjusted for Ecig scenario,

| Age Group:All | | NULL | Ecig | | | %Drop | | NULL | Ecig | |
|---|---|---|---|---|---|---|---|---|---|---|
| Year | Number Deaths | Attrib Deaths | Attrib Deaths | Drop Deaths | %Drop Deaths | Attrib Deaths | Death Rate | Attrib Rates | Attrib Rates | Drop Rates |
| All Comb | 2331914 | 1580465.217 | 1476306.165 | 104159.1 | 4.467 | 6.590 | 639.338 | 433.910 | 404.757 | 29.153 |

-----------------------------------------------------------------------------------------
Results for Age Groups Combined, Population and Number deaths adjusted for Ecig scenario,

| Age Group:All | | NULL | Ecig | | | %Drop | | NULL | Ecig | |
|---|---|---|---|---|---|---|---|---|---|---|
| Year | Number Deaths | Attrib Deaths | Attrib Deaths | Drop Deaths | %Drop Deaths | Attrib Deaths | Death Rate | Attrib Rates | Attrib Rates | Drop Rates |
| 1990 | 42690 | 30614.201 | 30614.201 | 0.000 | 0.000 | 0.000 | 625.210 | 448.355 | 448.355 | 0.000 |
| 1991 | 43692 | 31412.010 | 31404.310 | 7.700 | 0.018 | 0.025 | 631.345 | 453.901 | 453.789 | 0.111 |
| 1992 | 45423 | 32782.407 | 32763.048 | 19.358 | 0.043 | 0.059 | 647.450 | 467.274 | 466.998 | 0.276 |
| 1993 | 46291 | 33491.183 | 33451.874 | 39.309 | 0.085 | 0.117 | 650.515 | 470.643 | 470.089 | 0.553 |
| 1994 | 47017 | 34074.347 | 34011.909 | 62.438 | 0.133 | 0.183 | 650.895 | 471.719 | 470.852 | 0.867 |
| 1995 | 47841 | 34694.454 | 34598.585 | 95.869 | 0.200 | 0.276 | 652.026 | 472.861 | 471.549 | 1.312 |
| 1996 | 48245 | 35027.269 | 34897.071 | 130.197 | 0.270 | 0.372 | 648.999 | 471.202 | 469.442 | 1.760 |
| 1997 | 49036 | 35590.763 | 35411.191 | 179.571 | 0.366 | 0.505 | 650.506 | 472.153 | 469.758 | 2.395 |
| 1998 | 49688 | 36100.785 | 35852.454 | 248.331 | 0.500 | 0.688 | 649.874 | 472.184 | 468.917 | 3.267 |
| 1999 | 48886 | 35451.030 | 35141.527 | 309.502 | 0.633 | 0.873 | 630.770 | 457.448 | 453.428 | 4.019 |
| 2000 | 50335 | 36447.986 | 36045.693 | 402.293 | 0.799 | 1.104 | 641.396 | 464.474 | 459.312 | 5.162 |
| 2001 | 50395 | 36411.326 | 35924.369 | 486.957 | 0.966 | 1.337 | 635.021 | 458.863 | 452.680 | 6.183 |
| 2002 | 51453 | 37078.545 | 36470.249 | 608.296 | 1.182 | 1.641 | 641.985 | 462.696 | 455.045 | 7.650 |
| 2003 | 51418 | 36999.876 | 36280.460 | 719.416 | 1.399 | 1.944 | 635.530 | 457.395 | 448.428 | 8.967 |
| 2004 | 51412 | 36861.555 | 36014.758 | 846.797 | 1.647 | 2.297 | 629.088 | 451.138 | 440.685 | 10.453 |
| 2005 | 51437 | 36689.529 | 35714.027 | 975.502 | 1.897 | 2.659 | 622.411 | 444.069 | 432.157 | 11.912 |
| 2006 | 51374 | 36504.774 | 35389.170 | 1115.604 | 2.172 | 3.056 | 616.205 | 437.979 | 424.471 | 13.508 |
| 2007 | 51862 | 36663.202 | 35380.436 | 1282.766 | 2.473 | 3.499 | 615.831 | 435.498 | 420.118 | 15.380 |
| 2008 | 51003 | 35898.803 | 34507.001 | 1391.802 | 2.729 | 3.877 | 599.037 | 421.800 | 405.287 | 16.512 |
| 2009 | 51225 | 35934.525 | 34386.328 | 1548.198 | 3.022 | 4.308 | 594.836 | 417.468 | 399.302 | 18.166 |
| 2010 | 50889 | 35502.829 | 33819.070 | 1683.759 | 3.309 | 4.743 | 584.138 | 407.730 | 388.195 | 19.535 |
| 2011 | 50550 | 35136.342 | 33278.198 | 1858.144 | 3.676 | 5.288 | 574.751 | 399.726 | 378.370 | 21.356 |
| 2012 | 51292 | 35450.201 | 33409.887 | 2040.314 | 3.978 | 5.755 | 577.186 | 399.173 | 375.958 | 23.215 |
| 2013 | 51122 | 35123.224 | 32919.385 | 2203.839 | 4.311 | 6.275 | 569.059 | 391.249 | 366.439 | 24.810 |
| 2014 | 51310 | 35079.370 | 32704.233 | 2375.137 | 4.629 | 6.771 | 564.852 | 386.476 | 360.026 | 26.450 |
| 2015 | 50508 | 34324.171 | 31847.947 | 2476.224 | 4.903 | 7.214 | 621.520 | 422.780 | 391.901 | 30.879 |
| 2016 | 48924 | 33054.753 | 30488.959 | 2565.793 | 5.244 | 7.762 | 596.232 | 403.264 | 371.569 | 31.695 |
| 2017 | 48401 | 32471.234 | 29765.855 | 2705.379 | 5.590 | 8.332 | 584.228 | 392.399 | 359.292 | 33.107 |
| 2018 | 50551 | 33657.595 | 30627.658 | 3029.937 | 5.994 | 9.002 | 604.332 | 402.871 | 366.147 | 36.724 |
| 2019 | 51132 | 33863.424 | 30628.587 | 3234.837 | 6.326 | 9.553 | 605.292 | 401.412 | 362.579 | 38.833 |
| 2020 | 51549 | 33910.830 | 30515.991 | 3394.839 | 6.586 | 10.011 | 691.340 | 455.532 | 409.261 | 46.271 |
| 2021 | 48144 | 31531.880 | 28193.957 | 3337.923 | 6.933 | 10.586 | 640.952 | 420.521 | 375.349 | 45.172 |
| 2022 | 48452 | 31560.212 | 28048.962 | 3511.251 | 7.247 | 11.126 | 640.078 | 417.708 | 370.544 | 47.164 |
| 2023 | 48725 | 31508.781 | 27876.764 | 3632.018 | 7.454 | 11.527 | 638.620 | 413.791 | 365.367 | 48.424 |
| 2024 | 48920 | 31517.293 | 27736.814 | 3780.480 | 7.728 | 11.995 | 636.193 | 410.740 | 360.711 | 50.029 |
| 2025 | 48623 | 31175.389 | 27328.649 | 3846.740 | 7.911 | 12.339 | 729.503 | 468.912 | 410.020 | 58.892 |
| 2026 | 45471 | 29044.598 | 25326.583 | 3718.015 | 8.177 | 12.801 | 679.533 | 435.206 | 378.488 | 56.718 |
| 2027 | 45263 | 28825.765 | 25002.387 | 3823.378 | 8.447 | 13.264 | 673.634 | 430.203 | 372.098 | 58.104 |
| 2028 | 45007 | 28513.846 | 24642.193 | 3871.653 | 8.602 | 13.578 | 666.925 | 423.769 | 365.153 | 58.616 |
| 2029 | 44714 | 28148.109 | 24238.762 | 3909.346 | 8.743 | 13.888 | 659.561 | 416.486 | 357.537 | 58.949 |
| 2030 | 43576 | 27314.475 | 23422.018 | 3892.457 | 8.933 | 14.251 | 760.450 | 478.407 | 408.741 | 69.665 |
| 2031 | 40930 | 25423.102 | 21813.035 | 3610.066 | 8.820 | 14.200 | 713.198 | 444.684 | 380.090 | 64.594 |
| 2032 | 40516 | 25085.902 | 21468.063 | 3617.839 | 8.929 | 14.422 | 704.494 | 437.933 | 373.291 | 64.642 |
| 2033 | 40081 | 24645.067 | 21102.209 | 3542.858 | 8.839 | 14.376 | 695.131 | 429.194 | 365.981 | 63.212 |
| 2034 | 39611 | 24239.126 | 20745.610 | 3493.516 | 8.820 | 14.413 | 684.995 | 420.967 | 358.755 | 62.212 |
| 2035 | 37665 | 22944.147 | 19554.069 | 3390.077 | 9.001 | 14.775 | 797.095 | 487.980 | 413.815 | 74.165 |
| 2036 | 33181 | 19950.581 | 17028.592 | 2921.988 | 8.806 | 14.646 | 703.134 | 424.953 | 360.848 | 64.105 |
| 2037 | 32601 | 19415.334 | 16560.594 | 2854.740 | 8.757 | 14.704 | 691.259 | 413.871 | 351.144 | 62.726 |
| 2038 | 32022 | 18939.317 | 16196.283 | 2743.034 | 8.566 | 14.483 | 678.843 | 403.701 | 343.347 | 60.354 |
| 2039 | 31458 | 18379.752 | 15756.188 | 2623.564 | 8.340 | 14.274 | 666.102 | 391.373 | 333.626 | 57.747 |

# Results for PHIM for Run 2 (Pessimistic_Analysis.RTF)

************************************************************************************

**Main Results for E-Component for Run: 2, Simulation: 1, COD: IHD, Adjusted**

| Variable Parameter | Value |
|---|---|
| 1. Country | US (United States) |
| 2. Sex | F (Females) |
| 3. Year of start of process | 1990 |
| 4. Cause of Death | IHD |
| 5. Effective Dose NULL | CC:1 |
| 6. Effective Dose Ecig | CC:1 Ecig:0.4 Dual:0.7 |
| Note: | Death rates are per million population |

-----------------------------------------------------------------------------------------

Results for Age Groups Combined and Years combined, Population and Number deaths adjusted for Ecig scenario,

| Age Group:All | | NULL | Ecig | | | %Drop | | NULL | Ecig | |
|---|---|---|---|---|---|---|---|---|---|---|
| Year | Number | Attrib | Attrib | Drop | %Drop | Attrib | Death | Attrib | Attrib | Drop |
| | Deaths | Deaths | Deaths | Deaths | Deaths | Deaths | Rate | Rates | Rates | Rates |
| All Comb | 3247109 | 398833.796 | 332784.329 | 66049.47 | 2.034 | 16.561 | 890.256 | 109.498 | 91.239 | 18.259 |

-----------------------------------------------------------------------------------------

Results for Age Groups Combined, Population and Number deaths adjusted for Ecig scenario,

| Age Group:All | | NULL | Ecig | | | %Drop | | NULL | Ecig | |
|---|---|---|---|---|---|---|---|---|---|---|
| Year | Number | Attrib | Attrib | Drop | %Drop | Attrib | Death | Attrib | Attrib | Drop |
| | Deaths | Deaths | Deaths | Deaths | Deaths | Deaths | Rate | Rates | Rates | Rates |
| 1990 | 98916 | 12922.892 | 12922.892 | 0.000 | 0.000 | 0.000 | 1448.66 | 189.260 | 189.260 | 0.000 |
| 1991 | 96403 | 12856.479 | 12831.863 | 24.616 | 0.026 | 0.191 | 1393.01 | 185.775 | 185.419 | 0.356 |
| 1992 | 93721 | 12662.244 | 12595.513 | 66.731 | 0.071 | 0.527 | 1335.88 | 180.485 | 179.534 | 0.951 |
| 1993 | 93679 | 12730.613 | 12602.386 | 128.227 | 0.137 | 1.007 | 1316.45 | 178.900 | 177.098 | 1.802 |
| 1994 | 90833 | 12495.323 | 12293.806 | 201.517 | 0.222 | 1.613 | 1257.46 | 172.983 | 170.192 | 2.791 |
| 1995 | 89699 | 12365.590 | 12075.539 | 290.051 | 0.323 | 2.346 | 1222.52 | 168.534 | 164.579 | 3.955 |
| 1996 | 88287 | 12290.551 | 11893.277 | 397.274 | 0.450 | 3.232 | 1187.66 | 165.338 | 159.991 | 5.347 |
| 1997 | 84779 | 11719.531 | 11231.899 | 487.632 | 0.575 | 4.161 | 1124.66 | 155.473 | 149.000 | 6.473 |
| 1998 | 82519 | 11416.794 | 10823.344 | 593.451 | 0.719 | 5.198 | 1079.28 | 149.327 | 141.559 | 7.768 |
| 1999 | 93943 | 13028.038 | 12194.865 | 833.174 | 0.887 | 6.395 | 1212.14 | 168.109 | 157.349 | 10.760 |
| 2000 | 90045 | 12426.299 | 11475.948 | 950.351 | 1.055 | 7.648 | 1147.40 | 158.354 | 146.232 | 12.122 |
| 2001 | 86127 | 11939.281 | 10855.325 | 1083.957 | 1.259 | 9.079 | 1085.28 | 150.461 | 136.787 | 13.674 |
| 2002 | 83024 | 11528.794 | 10296.841 | 1231.953 | 1.484 | 10.686 | 1035.91 | 143.865 | 128.475 | 15.390 |
| 2003 | 79315 | 11048.407 | 9702.575 | 1345.832 | 1.697 | 12.181 | 980.337 | 136.581 | 119.924 | 16.657 |
| 2004 | 73877 | 10375.132 | 8940.175 | 1434.957 | 1.942 | 13.831 | 903.976 | 126.978 | 109.394 | 17.584 |
| 2005 | 71669 | 10051.029 | 8507.880 | 1543.149 | 2.153 | 15.353 | 867.225 | 121.652 | 102.949 | 18.702 |
| 2006 | 67574 | 9542.523 | 7909.870 | 1632.653 | 2.416 | 17.109 | 810.511 | 114.490 | 94.874 | 19.616 |
| 2007 | 63529 | 8936.533 | 7269.486 | 1667.047 | 2.624 | 18.654 | 754.367 | 106.151 | 86.320 | 19.831 |
| 2008 | 63202 | 8944.180 | 7151.150 | 1793.030 | 2.837 | 20.047 | 742.318 | 105.091 | 83.991 | 21.101 |
| 2009 | 59782 | 8390.033 | 6611.016 | 1779.018 | 2.976 | 21.204 | 694.203 | 97.471 | 76.769 | 20.702 |
| 2010 | 58047 | 8157.570 | 6324.782 | 1832.787 | 3.157 | 22.467 | 666.302 | 93.685 | 72.599 | 21.086 |
| 2011 | 57413 | 8035.356 | 6118.097 | 1917.259 | 3.339 | 23.860 | 652.777 | 91.414 | 69.562 | 21.851 |
| 2012 | 57462 | 7970.766 | 5964.233 | 2006.533 | 3.492 | 25.174 | 646.611 | 89.752 | 67.115 | 22.637 |
| 2013 | 57124 | 7743.216 | 5715.882 | 2027.334 | 3.549 | 26.182 | 635.870 | 86.254 | 63.626 | 22.628 |
| 2014 | 57602 | 7774.935 | 5661.974 | 2112.962 | 3.668 | 27.177 | 634.117 | 85.658 | 62.330 | 23.328 |
| 2015 | 58468 | 7722.535 | 5564.696 | 2157.839 | 3.691 | 27.942 | 719.469 | 95.120 | 68.476 | 26.645 |
| 2016 | 58739 | 7600.353 | 5412.359 | 2187.994 | 3.725 | 28.788 | 715.854 | 92.723 | 65.960 | 26.763 |
| 2017 | 59094 | 7470.266 | 5267.224 | 2203.042 | 3.728 | 29.491 | 713.298 | 90.274 | 63.579 | 26.696 |
| 2018 | 56170 | 6907.050 | 4830.564 | 2076.486 | 3.697 | 30.063 | 671.503 | 82.675 | 57.748 | 24.927 |
| 2019 | 55988 | 6732.046 | 4685.169 | 2046.878 | 3.656 | 30.405 | 662.778 | 79.801 | 55.463 | 24.338 |
| 2020 | 55424 | 6429.836 | 4443.901 | 1985.935 | 3.583 | 30.886 | 743.315 | 86.373 | 59.599 | 26.775 |
| 2021 | 56415 | 6562.016 | 4502.605 | 2059.410 | 3.650 | 31.384 | 751.057 | 87.513 | 59.944 | 27.570 |
| 2022 | 55959 | 6352.366 | 4342.586 | 2009.780 | 3.592 | 31.638 | 739.250 | 84.075 | 57.368 | 26.707 |
| 2023 | 55479 | 6134.123 | 4199.831 | 1934.293 | 3.487 | 31.533 | 727.133 | 80.557 | 55.045 | 25.512 |
| 2024 | 54920 | 5922.193 | 4063.515 | 1858.677 | 3.384 | 31.385 | 714.216 | 77.179 | 52.845 | 24.334 |
| 2025 | 53453 | 5504.188 | 3758.930 | 1745.257 | 3.265 | 31.708 | 801.972 | 82.789 | 56.396 | 26.393 |
| 2026 | 55453 | 5641.075 | 3871.642 | 1769.434 | 3.191 | 31.367 | 828.705 | 84.526 | 57.859 | 26.667 |
| 2027 | 54382 | 5426.216 | 3743.922 | 1682.294 | 3.093 | 31.003 | 809.344 | 80.982 | 55.719 | 25.263 |
| 2028 | 53282 | 5197.830 | 3621.025 | 1576.805 | 2.959 | 30.336 | 789.539 | 77.249 | 53.657 | 23.592 |
| 2029 | 52173 | 4973.547 | 3505.927 | 1467.620 | 2.813 | 29.509 | 769.592 | 73.590 | 51.715 | 21.875 |
| 2030 | 49684 | 4450.618 | 3094.490 | 1356.128 | 2.730 | 30.471 | 867.036 | 77.952 | 54.002 | 23.949 |
| 2031 | 52215 | 4555.052 | 3218.798 | 1336.255 | 2.559 | 29.336 | 909.834 | 79.674 | 56.087 | 23.587 |
| 2032 | 50912 | 4349.999 | 3109.611 | 1240.388 | 2.436 | 28.515 | 885.265 | 75.939 | 54.071 | 21.869 |
| 2033 | 49621 | 4147.948 | 3022.643 | 1125.305 | 2.268 | 27.129 | 860.591 | 72.237 | 52.423 | 19.814 |
| 2034 | 48322 | 3966.133 | 2936.423 | 1029.711 | 2.131 | 25.963 | 835.635 | 68.881 | 50.780 | 18.101 |
| 2035 | 44765 | 3289.064 | 2371.692 | 917.372 | 2.049 | 27.892 | 947.339 | 69.952 | 50.191 | 19.761 |
| 2036 | 46615 | 3313.192 | 2437.583 | 875.610 | 1.878 | 26.428 | 987.801 | 70.572 | 51.654 | 18.918 |
| 2037 | 45111 | 3117.951 | 2342.899 | 775.052 | 1.718 | 24.858 | 956.511 | 66.464 | 49.678 | 16.787 |
| 2038 | 43650 | 2949.775 | 2270.557 | 679.217 | 1.556 | 23.026 | 925.332 | 62.876 | 48.134 | 14.742 |
| 2039 | 42246 | 2766.312 | 2195.120 | 571.192 | 1.352 | 20.648 | 894.523 | 58.905 | 46.480 | 12.425 |

# Results for PHIM for Run 2 (Pessimistic_Analysis.RTF)

************************************************************************************
**Main Results for E-Component for Run: 2, Simulation: 1, COD: STROKE, Adjusted**

| Variable Parameter | Value |
|---|---|
| 1. Country | US (United States) |
| 2. Sex | F (Females) |
| 3. Year of start of process | 1990 |
| 4. Cause of Death | STROKE |
| 5. Effective Dose NULL | CC:1 |
| 6. Effective Dose Ecig | CC:1 Ecig:0.4 Dual:0.7 |
| Note: | Death rates are per million population |

-----------------------------------------------------------------------------------------
Results for Age Groups Combined and Years combined, Population and Number deaths adjusted for Ecig scenario,

| Age Group:All | | NULL | Ecig | | | %Drop | | NULL | Ecig | |
|---|---|---|---|---|---|---|---|---|---|---|
| Year | Number Deaths | Attrib Deaths | Attrib Deaths | Drop Deaths | %Drop Deaths | Attrib Deaths | Death Rate | Attrib Rates | Attrib Rates | Drop Rates |
| All Comb | 1354509 | 104899.750 | 86748.638 | 18151.11 | 1.340 | 17.303 | 371.364 | 28.800 | 23.784 | 5.016 |

-----------------------------------------------------------------------------------------
Results for Age Groups Combined, Population and Number deaths adjusted for Ecig scenario,

| Age Group:All | | NULL | Ecig | | | %Drop | | NULL | Ecig | |
|---|---|---|---|---|---|---|---|---|---|---|
| Year | Number Deaths | Attrib Deaths | Attrib Deaths | Drop Deaths | %Drop Deaths | Attrib Deaths | Death Rate | Attrib Rates | Attrib Rates | Drop Rates |
| 1990 | 33504 | 3293.017 | 3293.017 | 0.000 | 0.000 | 0.000 | 490.678 | 48.227 | 48.227 | 0.000 |
| 1991 | 32505 | 3207.063 | 3200.117 | 6.946 | 0.021 | 0.217 | 469.694 | 46.342 | 46.241 | 0.100 |
| 1992 | 32560 | 3218.125 | 3199.465 | 18.660 | 0.057 | 0.580 | 464.104 | 45.871 | 45.605 | 0.266 |
| 1993 | 32768 | 3226.680 | 3189.794 | 36.886 | 0.113 | 1.143 | 460.480 | 45.344 | 44.825 | 0.518 |
| 1994 | 33005 | 3277.266 | 3216.500 | 60.766 | 0.184 | 1.854 | 456.916 | 45.370 | 44.528 | 0.841 |
| 1995 | 33730 | 3309.378 | 3219.326 | 90.052 | 0.267 | 2.721 | 459.716 | 45.104 | 43.877 | 1.228 |
| 1996 | 33751 | 3312.987 | 3187.030 | 125.957 | 0.373 | 3.802 | 454.018 | 44.568 | 42.873 | 1.695 |
| 1997 | 33700 | 3267.066 | 3102.386 | 164.680 | 0.489 | 5.041 | 447.053 | 43.341 | 41.156 | 2.186 |
| 1998 | 33275 | 3178.725 | 2978.096 | 200.629 | 0.603 | 6.312 | 435.206 | 41.577 | 38.951 | 2.626 |
| 1999 | 33892 | 3120.351 | 2882.112 | 238.239 | 0.703 | 7.635 | 437.311 | 40.264 | 37.188 | 3.076 |
| 2000 | 33829 | 3142.808 | 2856.870 | 285.938 | 0.845 | 9.098 | 431.066 | 40.050 | 36.404 | 3.647 |
| 2001 | 32509 | 2961.588 | 2649.617 | 311.971 | 0.960 | 10.534 | 409.640 | 37.323 | 33.388 | 3.935 |
| 2002 | 32082 | 2940.627 | 2579.963 | 360.665 | 1.124 | 12.265 | 400.289 | 36.695 | 32.191 | 4.505 |
| 2003 | 30573 | 2819.545 | 2427.379 | 392.166 | 1.283 | 13.909 | 377.880 | 34.855 | 30.002 | 4.853 |
| 2004 | 29394 | 2730.466 | 2307.594 | 422.872 | 1.439 | 15.487 | 359.672 | 33.417 | 28.236 | 5.181 |
| 2005 | 27961 | 2670.510 | 2212.681 | 457.829 | 1.637 | 17.144 | 338.345 | 32.322 | 26.774 | 5.548 |
| 2006 | 27210 | 2623.285 | 2130.531 | 492.754 | 1.811 | 18.784 | 326.367 | 31.474 | 25.554 | 5.919 |
| 2007 | 26312 | 2536.599 | 2022.412 | 514.187 | 1.954 | 20.271 | 312.442 | 30.131 | 24.015 | 6.116 |
| 2008 | 26010 | 2469.443 | 1937.711 | 531.733 | 2.044 | 21.532 | 305.494 | 29.015 | 22.759 | 6.257 |
| 2009 | 25054 | 2386.945 | 1843.673 | 543.272 | 2.168 | 22.760 | 290.934 | 27.730 | 21.409 | 6.321 |
| 2010 | 24856 | 2329.274 | 1774.637 | 554.637 | 2.231 | 23.812 | 285.314 | 26.750 | 20.370 | 6.380 |
| 2011 | 24811 | 2286.581 | 1713.539 | 573.042 | 2.310 | 25.061 | 282.094 | 26.013 | 19.483 | 6.530 |
| 2012 | 24521 | 2243.311 | 1652.654 | 590.657 | 2.409 | 26.330 | 275.931 | 25.260 | 18.597 | 6.663 |
| 2013 | 24475 | 2176.503 | 1578.885 | 597.618 | 2.442 | 27.458 | 272.437 | 24.245 | 17.575 | 6.670 |
| 2014 | 25323 | 2211.619 | 1585.347 | 626.272 | 2.473 | 28.317 | 278.774 | 24.366 | 17.452 | 6.913 |
| 2015 | 26348 | 2237.016 | 1586.868 | 650.148 | 2.468 | 29.063 | 324.221 | 27.554 | 19.527 | 8.027 |
| 2016 | 26771 | 2229.057 | 1566.506 | 662.552 | 2.475 | 29.723 | 326.258 | 27.194 | 19.091 | 8.103 |
| 2017 | 27657 | 2221.649 | 1548.442 | 673.207 | 2.434 | 30.302 | 333.838 | 26.848 | 18.691 | 8.157 |
| 2018 | 25297 | 1939.420 | 1345.120 | 594.300 | 2.349 | 30.643 | 302.421 | 23.214 | 16.081 | 7.134 |
| 2019 | 25475 | 1904.400 | 1314.325 | 590.075 | 2.316 | 30.985 | 301.570 | 22.574 | 15.559 | 7.016 |
| 2020 | 25429 | 1806.054 | 1238.300 | 567.754 | 2.233 | 31.436 | 341.043 | 24.261 | 16.607 | 7.654 |
| 2021 | 25504 | 1748.172 | 1192.972 | 555.201 | 2.177 | 31.759 | 339.532 | 23.314 | 15.882 | 7.432 |
| 2022 | 25599 | 1701.148 | 1157.897 | 543.251 | 2.122 | 31.934 | 338.181 | 22.515 | 15.297 | 7.219 |
| 2023 | 25686 | 1652.087 | 1126.731 | 525.355 | 2.045 | 31.800 | 336.648 | 21.696 | 14.768 | 6.929 |
| 2024 | 25729 | 1597.127 | 1092.476 | 504.651 | 1.961 | 31.597 | 334.601 | 20.814 | 14.207 | 6.607 |
| 2025 | 25335 | 1467.622 | 999.622 | 468.000 | 1.847 | 31.888 | 380.105 | 22.075 | 14.998 | 7.077 |
| 2026 | 25561 | 1390.505 | 955.782 | 434.723 | 1.701 | 31.264 | 381.994 | 20.835 | 14.283 | 6.552 |
| 2027 | 25350 | 1342.095 | 929.350 | 412.746 | 1.628 | 30.754 | 377.273 | 20.030 | 13.831 | 6.199 |
| 2028 | 25113 | 1289.536 | 904.866 | 384.669 | 1.532 | 29.830 | 372.131 | 19.165 | 13.408 | 5.756 |
| 2029 | 24863 | 1242.216 | 883.863 | 358.353 | 1.441 | 28.848 | 366.751 | 18.380 | 13.038 | 5.343 |
| 2030 | 24060 | 1096.803 | 774.653 | 322.150 | 1.339 | 29.372 | 419.875 | 19.210 | 13.519 | 5.692 |
| 2031 | 24070 | 1019.991 | 734.101 | 285.889 | 1.188 | 28.029 | 419.413 | 17.841 | 12.792 | 5.049 |
| 2032 | 23727 | 977.406 | 713.208 | 264.198 | 1.113 | 27.031 | 412.569 | 17.063 | 12.401 | 4.661 |
| 2033 | 23375 | 937.450 | 699.066 | 238.384 | 1.020 | 25.429 | 405.400 | 16.326 | 12.124 | 4.202 |
| 2034 | 22999 | 900.763 | 683.608 | 217.155 | 0.944 | 24.108 | 397.727 | 15.644 | 11.822 | 3.822 |
| 2035 | 21856 | 745.023 | 559.139 | 185.885 | 0.850 | 24.950 | 462.531 | 15.845 | 11.833 | 4.012 |
| 2036 | 21028 | 680.360 | 520.682 | 159.677 | 0.759 | 23.470 | 445.600 | 14.492 | 11.034 | 3.458 |
| 2037 | 20523 | 644.109 | 505.108 | 139.001 | 0.677 | 21.580 | 435.171 | 13.730 | 10.710 | 3.020 |
| 2038 | 20019 | 612.448 | 493.051 | 119.397 | 0.596 | 19.495 | 424.389 | 13.055 | 10.452 | 2.602 |
| 2039 | 19523 | 577.529 | 481.566 | 95.963 | 0.492 | 16.616 | 413.396 | 12.298 | 10.197 | 2.101 |

# Results for PHIM for Run 2 (Pessimistic_Analysis.RTF)

********************************************************************************
**Main Results for E-Component for Run: 2, Simulation: 1, COD: COPD, Adjusted**

| Variable Parameter | Value |
|---|---|
| 1. Country | US (United States) |
| 2. Sex | F (Females) |
| 3. Year of start of process | 1990 |
| 4. Cause of Death | COPD |
| 5. Effective Dose NULL | CC:1 |
| 6. Effective Dose Ecig | CC:1 Ecig:0.4 Dual:0.7 |
| Note: | Death rates are per million population |

------------------------------------------------------------------------------------
Results for Age Groups Combined and Years combined, Population and Number deaths adjusted for Ecig scenario,

| Age Group:All Year | Number Deaths | NULL Attrib Deaths | Ecig Attrib Deaths | Drop Deaths | %Drop Deaths | %Drop Attrib Deaths | Death Rate | NULL Attrib Rates | Ecig Attrib Rates | Drop Rates |
|---|---|---|---|---|---|---|---|---|---|---|
| All Comb | 2165331 | 862051.244 | 767997.244 | 94054.00 | 4.344 | 10.910 | 593.666 | 236.673 | 210.561 | 26.112 |

------------------------------------------------------------------------------------
Results for Age Groups Combined, Population and Number deaths adjusted for Ecig scenario,

| Age Group:All Year | Number Deaths | NULL Attrib Deaths | Ecig Attrib Deaths | Drop Deaths | %Drop Deaths | %Drop Attrib Deaths | Death Rate | NULL Attrib Rates | Ecig Attrib Rates | Drop Rates |
|---|---|---|---|---|---|---|---|---|---|---|
| 1990 | 24738 | 10694.712 | 10694.712 | 0.000 | 0.000 | 0.000 | 362.296 | 156.628 | 156.628 | 0.000 |
| 1991 | 26562 | 11602.933 | 11598.462 | 4.471 | 0.017 | 0.039 | 383.818 | 167.661 | 167.597 | 0.065 |
| 1992 | 26828 | 11831.375 | 11822.242 | 9.133 | 0.034 | 0.077 | 382.401 | 168.642 | 168.512 | 0.130 |
| 1993 | 29774 | 13246.371 | 13226.820 | 19.551 | 0.066 | 0.148 | 418.406 | 186.148 | 185.873 | 0.275 |
| 1994 | 29804 | 13355.662 | 13326.558 | 29.104 | 0.098 | 0.218 | 412.601 | 184.893 | 184.490 | 0.404 |
| 1995 | 29755 | 13397.439 | 13354.149 | 43.291 | 0.145 | 0.323 | 405.539 | 182.598 | 182.006 | 0.592 |
| 1996 | 30900 | 13960.356 | 13904.192 | 56.163 | 0.182 | 0.402 | 415.679 | 187.801 | 187.042 | 0.759 |
| 1997 | 31129 | 14109.552 | 14032.180 | 77.372 | 0.249 | 0.548 | 412.947 | 187.180 | 186.148 | 1.032 |
| 1998 | 32097 | 14599.657 | 14484.575 | 115.082 | 0.359 | 0.788 | 419.799 | 190.958 | 189.445 | 1.513 |
| 1999 | 34618 | 15728.102 | 15568.356 | 159.746 | 0.461 | 1.016 | 446.668 | 202.950 | 200.877 | 2.073 |
| 2000 | 34301 | 15579.803 | 15375.512 | 204.292 | 0.596 | 1.311 | 437.083 | 198.541 | 195.922 | 2.618 |
| 2001 | 34595 | 15696.614 | 15446.667 | 249.948 | 0.722 | 1.592 | 435.932 | 197.812 | 194.642 | 3.170 |
| 2002 | 34295 | 15519.777 | 15207.884 | 311.894 | 0.909 | 2.010 | 427.909 | 193.668 | 189.751 | 3.917 |
| 2003 | 35080 | 15888.389 | 15506.174 | 382.215 | 1.090 | 2.406 | 433.589 | 196.413 | 191.657 | 4.756 |
| 2004 | 33519 | 15091.573 | 14656.824 | 434.749 | 1.297 | 2.881 | 410.151 | 184.701 | 179.344 | 5.357 |
| 2005 | 35402 | 15803.656 | 15270.437 | 533.219 | 1.506 | 3.374 | 428.379 | 191.278 | 184.779 | 6.499 |
| 2006 | 33993 | 15100.563 | 14516.014 | 584.549 | 1.720 | 3.871 | 407.723 | 181.174 | 174.111 | 7.064 |
| 2007 | 34014 | 14974.384 | 14306.540 | 667.844 | 1.963 | 4.460 | 403.896 | 177.871 | 169.880 | 7.990 |
| 2008 | 37444 | 16406.197 | 15602.195 | 804.003 | 2.147 | 4.901 | 439.781 | 192.768 | 183.249 | 9.519 |
| 2009 | 36848 | 16083.194 | 15200.597 | 882.597 | 2.395 | 5.488 | 427.887 | 186.846 | 176.513 | 10.333 |
| 2010 | 36813 | 15927.400 | 14967.683 | 959.717 | 2.607 | 6.026 | 422.564 | 182.917 | 171.808 | 11.110 |
| 2011 | 37666 | 16221.836 | 15123.552 | 1098.284 | 2.916 | 6.770 | 428.255 | 184.547 | 171.954 | 12.593 |
| 2012 | 38299 | 16358.849 | 15156.118 | 1202.731 | 3.140 | 7.352 | 430.979 | 184.202 | 170.550 | 13.652 |
| 2013 | 40153 | 16994.822 | 15634.907 | 1359.915 | 3.387 | 8.002 | 446.955 | 189.311 | 174.039 | 15.272 |
| 2014 | 40130 | 16855.093 | 15387.918 | 1467.175 | 3.656 | 8.705 | 441.768 | 185.696 | 169.399 | 16.297 |
| 2015 | 42254 | 17572.483 | 15953.562 | 1618.921 | 3.831 | 9.213 | 519.945 | 216.445 | 196.314 | 20.130 |
| 2016 | 42666 | 17579.955 | 15823.811 | 1756.144 | 4.116 | 9.989 | 519.970 | 214.473 | 192.845 | 21.629 |
| 2017 | 44433 | 18080.688 | 16140.880 | 1939.808 | 4.366 | 10.729 | 536.331 | 218.496 | 194.830 | 23.666 |
| 2018 | 45436 | 18209.262 | 16109.952 | 2099.310 | 4.620 | 11.529 | 543.176 | 217.960 | 192.591 | 25.368 |
| 2019 | 46963 | 18648.302 | 16356.881 | 2291.421 | 4.879 | 12.288 | 555.945 | 221.054 | 193.631 | 27.423 |
| 2020 | 48430 | 19002.597 | 16552.005 | 2450.592 | 5.060 | 12.896 | 649.509 | 255.266 | 221.985 | 33.281 |
| 2021 | 48447 | 18885.717 | 16294.250 | 2591.468 | 5.349 | 13.722 | 644.980 | 251.867 | 216.927 | 34.940 |
| 2022 | 49829 | 19235.076 | 16455.336 | 2779.740 | 5.579 | 14.451 | 658.266 | 254.582 | 217.385 | 37.196 |
| 2023 | 51217 | 19521.158 | 16594.889 | 2926.269 | 5.713 | 14.990 | 671.276 | 256.363 | 217.501 | 38.862 |
| 2024 | 52545 | 19918.620 | 16796.310 | 3122.310 | 5.942 | 15.675 | 683.341 | 259.583 | 218.432 | 41.151 |
| 2025 | 53550 | 20150.545 | 16897.436 | 3253.109 | 6.075 | 16.144 | 803.425 | 303.086 | 253.517 | 49.569 |
| 2026 | 54410 | 20393.792 | 16939.005 | 3454.788 | 6.350 | 16.940 | 813.119 | 305.582 | 253.141 | 52.441 |
| 2027 | 55243 | 20615.351 | 16972.030 | 3643.321 | 6.595 | 17.673 | 822.149 | 307.668 | 252.586 | 55.082 |
| 2028 | 56018 | 20731.894 | 16953.250 | 3778.644 | 6.745 | 18.226 | 830.085 | 308.115 | 251.216 | 56.898 |
| 2029 | 56753 | 20781.967 | 16887.519 | 3894.448 | 6.862 | 18.740 | 837.149 | 307.495 | 249.102 | 58.393 |
| 2030 | 56998 | 20753.201 | 16756.556 | 3996.644 | 7.012 | 19.258 | 994.688 | 363.487 | 292.421 | 71.066 |
| 2031 | 58553 | 21048.417 | 16981.102 | 4067.314 | 6.946 | 19.324 | 1020.28 | 368.165 | 295.894 | 72.271 |
| 2032 | 59066 | 21169.329 | 16983.871 | 4185.458 | 7.086 | 19.771 | 1027.05 | 369.560 | 295.319 | 74.241 |
| 2033 | 59538 | 21109.293 | 16917.876 | 4191.417 | 7.040 | 19.856 | 1032.58 | 367.618 | 293.411 | 74.207 |
| 2034 | 59934 | 21109.429 | 16871.285 | 4238.144 | 7.071 | 20.077 | 1036.45 | 366.613 | 291.756 | 74.857 |
| 2035 | 59275 | 20794.840 | 16525.567 | 4269.273 | 7.203 | 20.530 | 1254.40 | 442.268 | 349.724 | 92.544 |
| 2036 | 56350 | 19354.577 | 15348.234 | 4006.343 | 7.110 | 20.700 | 1194.09 | 412.258 | 325.240 | 87.018 |
| 2037 | 56303 | 19064.169 | 15061.432 | 4002.737 | 7.109 | 20.996 | 1193.84 | 406.385 | 319.357 | 87.028 |
| 2038 | 56225 | 18854.812 | 14885.500 | 3969.312 | 7.060 | 21.052 | 1191.92 | 401.900 | 315.559 | 86.340 |
| 2039 | 56138 | 18437.457 | 14567.435 | 3870.022 | 6.894 | 20.990 | 1188.69 | 392.602 | 308.455 | 84.146 |

# Results for PHIM for Run 2 (ELL Summary Pessimistic_Analysis.RTF)

**********************************************************************************

**Estimated Loss of Life from the E Component for Run: 2, Simulation: 1**

Country: US (United States)

Follow-up Period: 1990 to 2039

**Table 1: Estimated Loss of Life, at the end of follow-up, all age groups combined, Population unadjusted for deaths in Ecig scenario**

| Disease | Sex | Year | Years of life lost NULL | Years of life lost Ecig | Years of life Saved |
|---|---|---|---|---|---|
| All Diseases | Males | 2039 | 42093547.949 | 36501440.925 | 5592107.02 |
| | Female | 2039 | 23817891.273 | 21104833.012 | 2713058.26 |
| Lung cancer | Males | 2039 | 18222549.860 | 17057566.947 | 1164982.91 |
| | Female | 2039 | 12512468.845 | 11616087.724 | 896381.122 |
| IHD | Males | 2039 | 15635992.616 | 12406132.581 | 3229860.04 |
| | Female | 2039 | 4798769.065 | 3865706.661 | 933062.405 |
| Stroke | Males | 2039 | 2389546.238 | 1920183.109 | 469363.128 |
| | Female | 2039 | 1665361.569 | 1370650.422 | 294711.147 |
| COPD | Males | 2039 | 5845459.234 | 5117558.287 | 727900.947 |
| | Female | 2039 | 4841291.793 | 4252388.206 | 588903.587 |

# Results for PHIM for Run 2 (ELL Summary Pessimistic_Analysis.RTF)

************************************************************************************

**Table 2: Estimated Loss of Life, at the end of follow-up, all age groups combined, Population and Number deaths adjusted for Ecig scenario**

| Disease | Sex | Year | Years of life lost NULL | Years of life lost Ecig | Years of life Saved |
|---|---|---|---|---|---|
| All Diseases | Males | 2039 | 42093547.949 | 36575271.380 | 5518276.57 |
| | Female | 2039 | 23817891.273 | 21128264.569 | 2689626.70 |
| Lung cancer | Males | 2039 | 18222549.860 | 17092593.671 | 1129956.19 |
| | Female | 2039 | 12512468.845 | 11629065.776 | 883403.070 |
| IHD | Males | 2039 | 15635992.616 | 12426196.035 | 3209796.58 |
| | Female | 2039 | 4798769.065 | 3868680.028 | 930089.038 |
| Stroke | Males | 2039 | 2389546.238 | 1923208.547 | 466337.691 |
| | Female | 2039 | 1665361.569 | 1371516.889 | 293844.681 |
| COPD | Males | 2039 | 5845459.234 | 5133273.128 | 712186.106 |
| | Female | 2039 | 4841291.793 | 4259001.877 | 582289.915 |

## Results for PHIM for Run 2 (ELL Summary Pessimistic_Analysis.RTF)

**************************************************************************************

**Table 3: Estimated Loss of Life, All age groups combined, Population unadjusted for deaths in Ecig scenario, cumulative over years**

| Disease | Sex | Year | Years of life lost NULL | Years of life lost Ecig | Years of life Saved |
|---|---|---|---|---|---|
| All Diseases | Males | 1990 | 1304839.645 | 1304839.645 | 0.000 |
| | | 1991 | 2579865.358 | 2577032.488 | 2832.870 |
| | | 1992 | 3830683.831 | 3820089.698 | 10594.133 |
| | | 1993 | 5076567.803 | 5051440.774 | 25127.028 |
| | | 1994 | 6292145.526 | 6244458.839 | 47686.687 |
| | | 1995 | 7485537.010 | 7405922.781 | 79614.229 |
| | | 1996 | 8655955.028 | 8534518.085 | 121436.943 |
| | | 1997 | 9791729.713 | 9619102.296 | 172627.417 |
| | | 1998 | 10899134.186 | 10666365.492 | 232768.693 |
| | | 1999 | 12070409.674 | 11756805.077 | 313604.597 |
| | | 2000 | 13231384.595 | 12825994.379 | 405390.216 |
| | | 2001 | 14371761.018 | 13863259.939 | 508501.079 |
| | | 2002 | 15508906.386 | 14884081.064 | 624825.322 |
| | | 2003 | 16638100.422 | 15885469.649 | 752630.773 |
| | | 2004 | 17736465.088 | 16849455.775 | 887009.314 |
| | | 2005 | 18834658.606 | 17803650.819 | 1031007.79 |
| | | 2006 | 19909100.501 | 18724838.275 | 1184262.23 |
| | | 2007 | 20958321.463 | 19615555.718 | 1342765.75 |
| | | 2008 | 22000068.148 | 20492514.340 | 1507553.81 |
| | | 2009 | 23018816.903 | 21343268.561 | 1675548.34 |
| | | 2010 | 24018480.537 | 22171224.791 | 1847255.75 |
| | | 2011 | 25003034.574 | 22979746.393 | 2023288.18 |
| | | 2012 | 25969871.429 | 23766971.921 | 2202899.51 |
| | | 2013 | 26920275.180 | 24534260.331 | 2386014.85 |
| | | 2014 | 27849005.227 | 25278562.237 | 2570442.99 |
| | | 2015 | 28749620.400 | 25996196.864 | 2753423.54 |
| | | 2016 | 29622417.562 | 26685049.029 | 2937368.53 |
| | | 2017 | 30462559.724 | 27343130.343 | 3119429.38 |
| | | 2018 | 31293299.299 | 27995624.491 | 3297674.81 |
| | | 2019 | 32109993.427 | 28635806.506 | 3474186.92 |
| | | 2020 | 32883323.977 | 29240469.430 | 3642854.55 |
| | | 2021 | 33610102.554 | 29805244.016 | 3804858.54 |
| | | 2022 | 34322010.448 | 30358169.415 | 3963841.03 |
| | | 2023 | 35018383.550 | 30899260.069 | 4119123.48 |
| | | 2024 | 35700177.955 | 31430317.139 | 4269860.82 |
| | | 2025 | 36315500.346 | 31908347.670 | 4407152.68 |
| | | 2026 | 36896159.343 | 32358891.750 | 4537267.59 |
| | | 2027 | 37462570.116 | 32800347.882 | 4662222.23 |
| | | 2028 | 38015337.780 | 33232534.617 | 4782803.16 |
| | | 2029 | 38554814.764 | 33657876.512 | 4896938.25 |
| | | 2030 | 39003805.400 | 34009486.170 | 4994319.23 |
| | | 2031 | 39436509.703 | 34349897.472 | 5086612.23 |
| | | 2032 | 39858470.458 | 34684145.884 | 5174324.57 |
| | | 2033 | 40270147.082 | 35012184.737 | 5257962.34 |
| | | 2034 | 40672665.743 | 35337740.990 | 5334924.75 |
| | | 2035 | 40962732.664 | 35569642.610 | 5393090.05 |
| | | 2036 | 41255021.653 | 35806146.957 | 5448874.70 |
| | | 2037 | 41540573.023 | 36039900.187 | 5500672.84 |
| | | 2038 | 41820158.066 | 36270909.142 | 5549248.92 |
| | | 2039 | 42093547.949 | 36501440.925 | 5592107.02 |
| | Female | 1990 | 588979.406 | 588979.406 | 0.000 |
| | | 1991 | 1182571.252 | 1181666.191 | 905.061 |
| | | 1992 | 1777346.679 | 1773859.750 | 3486.929 |
| | | 1993 | 2380261.899 | 2371859.441 | 8402.458 |
| | | 1994 | 2983130.039 | 2966951.849 | 16178.190 |
| | | 1995 | 3588982.584 | 3561502.537 | 27480.047 |
| | | 1996 | 4195430.235 | 4152341.381 | 43088.853 |
| | | 1997 | 4793866.694 | 4731536.380 | 62330.314 |
| | | 1998 | 5389747.381 | 5304192.195 | 85555.186 |
| | | 1999 | 6006722.467 | 5890967.387 | 115755.080 |
| | | 2000 | 6625758.871 | 6475249.479 | 150509.392 |
| | | 2001 | 7245135.411 | 7054390.300 | 190745.111 |
| | | 2002 | 7867492.021 | 7630879.877 | 236612.144 |
| | | 2003 | 8485051.793 | 8198013.219 | 287038.574 |
| | | 2004 | 9092994.774 | 8751487.060 | 341507.714 |
| | | 2005 | 9706032.803 | 9304859.859 | 401172.945 |
| | | 2006 | 10311907.548 | 9846553.240 | 465354.308 |
| | | 2007 | 10909437.502 | 10376770.648 | 532666.854 |
| | | 2008 | 11506462.491 | 10901220.059 | 605242.432 |
| | | 2009 | 12098393.287 | 11418151.358 | 680241.928 |
| | | 2010 | 12681188.981 | 11923143.179 | 758045.801 |

## Results for PHIM for Run 2 (ELL Summary Pessimistic_Analysis.RTF)

| | | | | | |
|---|---|---|---|---|---|
| | | 2011 | 13260062.991 | 12420356.784 | 839706.207 |
| | | 2012 | 13839417.441 | 12913688.477 | 925728.964 |
| | | 2013 | 14409614.758 | 13396214.144 | 1013400.61 |
| | | 2014 | 14976658.746 | 13872121.557 | 1104537.19 |
| | | 2015 | 15529977.821 | 14333462.623 | 1196515.20 |
| | | 2016 | 16066640.764 | 14777191.163 | 1289449.60 |
| | | 2017 | 16590035.078 | 15207041.958 | 1382993.12 |
| | | 2018 | 17098716.137 | 15625075.669 | 1473640.47 |
| | | 2019 | 17599868.850 | 16035494.314 | 1564374.54 |
| | | 2020 | 18081346.241 | 16428362.700 | 1652983.54 |
| | | 2021 | 18537594.460 | 16798482.956 | 1739111.50 |
| | | 2022 | 18985300.234 | 17160595.412 | 1824704.82 |
| | | 2023 | 19423958.747 | 17515195.326 | 1908763.42 |
| | | 2024 | 19853347.772 | 17862078.654 | 1991269.12 |
| | | 2025 | 20252822.245 | 18183685.191 | 2069137.05 |
| | | 2026 | 20619307.063 | 18478517.894 | 2140789.17 |
| | | 2027 | 20976063.406 | 18765911.074 | 2210152.33 |
| | | 2028 | 21323641.947 | 19047026.696 | 2276615.25 |
| | | 2029 | 21662117.106 | 19321777.713 | 2340339.39 |
| | | 2030 | 21960971.694 | 19562649.048 | 2398322.65 |
| | | 2031 | 22224951.983 | 19776625.142 | 2448326.84 |
| | | 2032 | 22480397.853 | 19984905.162 | 2495492.69 |
| | | 2033 | 22728639.455 | 20189259.493 | 2539379.96 |
| | | 2034 | 22970448.888 | 20389914.807 | 2580534.08 |
| | | 2035 | 23164154.188 | 20548660.320 | 2615493.87 |
| | | 2036 | 23334538.616 | 20690431.092 | 2644107.52 |
| | | 2037 | 23499760.723 | 20829689.134 | 2670071.59 |
| | | 2038 | 23660308.401 | 20967566.185 | 2692742.22 |
| | | 2039 | 23817891.273 | 21104833.012 | 2713058.26 |
| Lung cancer | Males | 1990 | 595621.791 | 595621.791 | 0.000 |
| | | 1991 | 1177744.113 | 1177548.728 | 195.384 |
| | | 1992 | 1750034.892 | 1749299.108 | 735.784 |
| | | 1993 | 2319752.308 | 2317935.178 | 1817.130 |
| | | 1994 | 2872597.339 | 2869024.623 | 3572.716 |
| | | 1995 | 3411494.825 | 3405330.604 | 6164.221 |
| | | 1996 | 3945569.726 | 3935657.135 | 9912.590 |
| | | 1997 | 4463316.362 | 4448551.009 | 14765.354 |
| | | 1998 | 4975301.180 | 4954365.937 | 20935.243 |
| | | 1999 | 5473691.125 | 5445164.742 | 28526.383 |
| | | 2000 | 5977500.570 | 5939499.207 | 38001.363 |
| | | 2001 | 6474795.516 | 6425394.117 | 49401.398 |
| | | 2002 | 6968769.705 | 6905890.132 | 62879.573 |
| | | 2003 | 7460282.140 | 7381572.267 | 78709.873 |
| | | 2004 | 7947048.275 | 7850331.518 | 96716.757 |
| | | 2005 | 8430571.669 | 8313619.487 | 116952.182 |
| | | 2006 | 8903945.390 | 8764520.286 | 139425.104 |
| | | 2007 | 9364177.982 | 9200395.608 | 163782.374 |
| | | 2008 | 9822525.458 | 9631632.176 | 190893.283 |
| | | 2009 | 10275228.886 | 10055008.513 | 220220.373 |
| | | 2010 | 10720275.500 | 10468779.791 | 251495.709 |
| | | 2011 | 11155356.373 | 10870655.507 | 284700.866 |
| | | 2012 | 11582611.617 | 11262468.106 | 320143.510 |
| | | 2013 | 11998803.791 | 11641393.284 | 357410.507 |
| | | 2014 | 12402087.090 | 12006270.915 | 395816.175 |
| | | 2015 | 12793451.706 | 12358196.548 | 435255.158 |
| | | 2016 | 13160101.653 | 12685664.139 | 474437.514 |
| | | 2017 | 13503583.414 | 12990405.829 | 513177.585 |
| | | 2018 | 13859755.737 | 13304208.649 | 555547.088 |
| | | 2019 | 14211625.176 | 13612390.245 | 599234.931 |
| | | 2020 | 14553935.288 | 13910532.492 | 643402.796 |
| | | 2021 | 14851747.495 | 14168880.596 | 682866.899 |
| | | 2022 | 15144531.004 | 14421604.946 | 722926.058 |
| | | 2023 | 15431932.496 | 14668672.069 | 763260.427 |
| | | 2024 | 15713920.740 | 14910262.456 | 803658.284 |
| | | 2025 | 15979112.816 | 15136464.962 | 842647.854 |
| | | 2026 | 16206550.153 | 15330315.655 | 876234.498 |
| | | 2027 | 16428826.050 | 15519475.996 | 909350.054 |
| | | 2028 | 16645902.186 | 15704047.422 | 941854.764 |
| | | 2029 | 16858126.517 | 15884626.227 | 973500.291 |
| | | 2030 | 17045220.687 | 16043012.207 | 1002208.48 |
| | | 2031 | 17208482.610 | 16181882.297 | 1026600.31 |
| | | 2032 | 17367586.074 | 16317625.865 | 1049960.21 |
| | | 2033 | 17522716.280 | 16450307.853 | 1072408.43 |
| | | 2034 | 17674226.555 | 16580751.671 | 1093474.88 |
| | | 2035 | 17792201.734 | 16681583.203 | 1110618.53 |
| | | 2036 | 17903508.236 | 16777846.881 | 1125661.35 |
| | | 2037 | 18012162.345 | 16872408.053 | 1139754.29 |
| | | 2038 | 18118464.470 | 16965400.116 | 1153064.35 |

# Results for PHIM for Run 2 (ELL Summary Pessimistic_Analysis.RTF)

| | | | | | |
|---|---|---|---|---|---|
| | | 2039 | 18222549.860 | 17057566.947 | 1164982.91 |
| | Female | 1990 | 307897.357 | 307897.357 | 0.000 |
| | | 1991 | 616547.418 | 616436.677 | 110.741 |
| | | 1992 | 931086.773 | 930632.576 | 454.197 |
| | | 1993 | 1246155.671 | 1245012.803 | 1142.868 |
| | | 1994 | 1561212.934 | 1558930.543 | 2282.392 |
| | | 1995 | 1879967.742 | 1875903.733 | 4064.009 |
| | | 1996 | 2197488.109 | 2190911.519 | 6576.590 |
| | | 1997 | 2515698.453 | 2505718.043 | 9980.410 |
| | | 1998 | 2836249.556 | 2821881.469 | 14368.087 |
| | | 1999 | 3150201.390 | 3130476.235 | 19725.154 |
| | | 2000 | 3472722.342 | 3446337.323 | 26385.019 |
| | | 2001 | 3796994.889 | 3762497.580 | 34497.310 |
| | | 2002 | 4126619.935 | 4082391.452 | 44228.483 |
| | | 2003 | 4454982.533 | 4399574.498 | 55408.035 |
| | | 2004 | 4784343.030 | 4716075.733 | 68267.297 |
| | | 2005 | 5113443.121 | 5030610.299 | 82832.822 |
| | | 2006 | 5442543.963 | 5343180.073 | 99363.890 |
| | | 2007 | 5772441.497 | 5654614.070 | 117827.426 |
| | | 2008 | 6091911.943 | 5954327.204 | 137584.739 |
| | | 2009 | 6413671.330 | 6254189.837 | 159481.493 |
| | | 2010 | 6730763.530 | 6547986.264 | 182777.266 |
| | | 2011 | 7043614.101 | 6835796.374 | 207817.727 |
| | | 2012 | 7356270.780 | 7121467.557 | 234803.223 |
| | | 2013 | 7662551.964 | 7399448.045 | 263103.919 |
| | | 2014 | 7964551.465 | 7671793.223 | 292758.242 |
| | | 2015 | 8254895.403 | 7932241.248 | 322654.154 |
| | | 2016 | 8530661.848 | 8177819.635 | 352842.212 |
| | | 2017 | 8795826.122 | 8412414.746 | 383411.376 |
| | | 2018 | 9067233.707 | 8651105.411 | 416128.296 |
| | | 2019 | 9335343.270 | 8885572.097 | 449771.173 |
| | | 2020 | 9596110.883 | 9112401.734 | 483709.149 |
| | | 2021 | 9833186.859 | 9317897.016 | 515289.843 |
| | | 2022 | 10066452.037 | 9519006.650 | 547445.387 |
| | | 2023 | 10295503.609 | 9715816.974 | 579686.636 |
| | | 2024 | 10520125.318 | 9908176.191 | 611949.127 |
| | | 2025 | 10731915.863 | 10088678.052 | 643237.811 |
| | | 2026 | 10917495.549 | 10246895.823 | 670599.725 |
| | | 2027 | 11098620.885 | 10400975.660 | 697645.225 |
| | | 2028 | 11275441.234 | 10551467.597 | 723973.637 |
| | | 2029 | 11447962.710 | 10698304.710 | 749657.999 |
| | | 2030 | 11601867.623 | 10828543.357 | 773324.266 |
| | | 2031 | 11731666.516 | 10939029.764 | 792636.752 |
| | | 2032 | 11857585.969 | 11046456.250 | 811129.719 |
| | | 2033 | 11980128.625 | 11151580.568 | 828548.057 |
| | | 2034 | 12099562.464 | 11254532.955 | 845029.510 |
| | | 2035 | 12195919.946 | 11336911.291 | 859008.655 |
| | | 2036 | 12278201.969 | 11408349.568 | 869852.402 |
| | | 2037 | 12358146.336 | 11478366.300 | 879780.036 |
| | | 2038 | 12435978.633 | 11547497.459 | 888481.174 |
| | | 2039 | 12512468.845 | 11616087.724 | 896381.122 |
| IHD | Males | 1990 | 517209.093 | 517209.093 | 0.000 |
| | | 1991 | 1019219.395 | 1016872.731 | 2346.664 |
| | | 1992 | 1509598.614 | 1500828.384 | 8770.230 |
| | | 1993 | 1992735.299 | 1972080.183 | 20655.116 |
| | | 1994 | 2462877.575 | 2424019.152 | 38858.422 |
| | | 1995 | 2928249.390 | 2863870.443 | 64378.946 |
| | | 1996 | 3377377.730 | 3280101.348 | 97276.382 |
| | | 1997 | 3809062.472 | 3672069.475 | 136992.997 |
| | | 1998 | 4222213.867 | 4039229.287 | 182984.580 |
| | | 1999 | 4708317.743 | 4461535.068 | 246782.675 |
| | | 2000 | 5182084.396 | 4864257.604 | 317826.792 |
| | | 2001 | 5644097.705 | 5247487.715 | 396609.990 |
| | | 2002 | 6107232.129 | 5622487.116 | 484745.013 |
| | | 2003 | 6561785.704 | 5981991.070 | 579794.634 |
| | | 2004 | 6996156.348 | 6318370.555 | 677785.793 |
| | | 2005 | 7426261.361 | 6645153.832 | 781107.529 |
| | | 2006 | 7849012.694 | 6959389.073 | 889623.621 |
| | | 2007 | 8255300.530 | 7255959.728 | 999340.802 |
| | | 2008 | 8650662.040 | 7539870.435 | 1110791.60 |
| | | 2009 | 9030818.513 | 7809109.508 | 1221709.00 |
| | | 2010 | 9403026.327 | 8069611.628 | 1333414.70 |
| | | 2011 | 9767535.506 | 8321660.179 | 1445875.33 |
| | | 2012 | 10123044.281 | 8564930.397 | 1558113.88 |
| | | 2013 | 10470104.786 | 8800062.445 | 1670042.34 |
| | | 2014 | 10807756.112 | 9027190.028 | 1780566.08 |
| | | 2015 | 11130681.425 | 9242603.053 | 1888078.37 |
| | | 2016 | 11448966.506 | 9454045.950 | 1994920.56 |

## Results for PHIM for Run 2 (ELL Summary Pessimistic_Analysis.RTF)

| | | | | | |
|---|---|---|---|---|---|
| | | 2017 | 11758164.882 | 9659109.318 | 2099055.56 |
| | | 2018 | 12054448.241 | 9857047.024 | 2197401.22 |
| | | 2019 | 12341606.261 | 10049602.668 | 2292003.59 |
| | | 2020 | 12600728.939 | 10221905.481 | 2378823.46 |
| | | 2021 | 12861767.105 | 10397849.194 | 2463917.91 |
| | | 2022 | 13114168.616 | 10569202.539 | 2544966.08 |
| | | 2023 | 13357894.797 | 10736071.474 | 2621823.32 |
| | | 2024 | 13593848.521 | 10899871.892 | 2693976.63 |
| | | 2025 | 13789177.359 | 11033459.378 | 2755717.98 |
| | | 2026 | 13993401.656 | 11176632.265 | 2816769.39 |
| | | 2027 | 14190519.224 | 11317008.877 | 2873510.35 |
| | | 2028 | 14381193.356 | 11454278.679 | 2926914.68 |
| | | 2029 | 14565171.927 | 11589870.180 | 2975301.75 |
| | | 2030 | 14697381.605 | 11684626.285 | 3012755.32 |
| | | 2031 | 14842203.512 | 11791416.886 | 3050786.63 |
| | | 2032 | 14982485.826 | 11896319.931 | 3086165.89 |
| | | 2033 | 15118410.647 | 11999166.786 | 3119243.86 |
| | | 2034 | 15250284.398 | 12102120.743 | 3148163.65 |
| | | 2035 | 15322911.166 | 12156574.690 | 3166336.48 |
| | | 2036 | 15405115.031 | 12220011.608 | 3185103.42 |
| | | 2037 | 15484709.137 | 12282739.924 | 3201969.21 |
| | | 2038 | 15561812.193 | 12344480.150 | 3217332.04 |
| | | 2039 | 15635992.616 | 12406132.581 | 3229860.04 |
| | Female | 1990 | 147299.110 | 147299.110 | 0.000 |
| | | 1991 | 295122.462 | 294526.629 | 595.834 |
| | | 1992 | 439962.920 | 437705.670 | 2257.250 |
| | | 1993 | 584735.615 | 579392.968 | 5342.647 |
| | | 1994 | 727640.783 | 717561.270 | 10079.513 |
| | | 1995 | 868486.937 | 851721.743 | 16765.194 |
| | | 1996 | 1010321.847 | 984391.531 | 25930.316 |
| | | 1997 | 1144230.930 | 1107519.344 | 36711.585 |
| | | 1998 | 1274513.622 | 1225072.427 | 49441.194 |
| | | 1999 | 1426725.938 | 1359861.049 | 66864.889 |
| | | 2000 | 1573462.680 | 1487299.399 | 86163.281 |
| | | 2001 | 1719132.015 | 1610812.639 | 108319.376 |
| | | 2002 | 1863006.732 | 1730041.269 | 132965.463 |
| | | 2003 | 2002943.227 | 1843464.644 | 159478.584 |
| | | 2004 | 2137798.705 | 1950446.851 | 187351.855 |
| | | 2005 | 2271071.245 | 2054242.255 | 216828.991 |
| | | 2006 | 2400707.731 | 2152997.132 | 247710.599 |
| | | 2007 | 2523634.512 | 2244928.728 | 278705.784 |
| | | 2008 | 2648752.232 | 2336881.832 | 311870.400 |
| | | 2009 | 2765973.961 | 2421925.001 | 344048.960 |
| | | 2010 | 2881329.235 | 2504503.916 | 376825.318 |
| | | 2011 | 2994972.694 | 2584730.514 | 410242.179 |
| | | 2012 | 3108958.829 | 2664150.652 | 444808.177 |
| | | 2013 | 3218140.416 | 2739655.984 | 478484.432 |
| | | 2014 | 3328387.914 | 2815433.409 | 512954.505 |
| | | 2015 | 3435342.796 | 2888380.878 | 546961.918 |
| | | 2016 | 3539189.408 | 2958891.076 | 580298.332 |
| | | 2017 | 3640041.363 | 3027161.717 | 612879.645 |
| | | 2018 | 3732645.362 | 3089991.299 | 642654.063 |
| | | 2019 | 3821421.978 | 3150451.286 | 670970.692 |
| | | 2020 | 3902115.950 | 3204989.537 | 697126.413 |
| | | 2021 | 3984301.053 | 3260914.454 | 723386.599 |
| | | 2022 | 4062629.299 | 3314552.154 | 748077.144 |
| | | 2023 | 4137348.729 | 3366311.493 | 771037.237 |
| | | 2024 | 4208611.493 | 3416282.660 | 792328.833 |
| | | 2025 | 4269419.492 | 3458507.498 | 810911.994 |
| | | 2026 | 4330969.998 | 3502086.124 | 828883.874 |
| | | 2027 | 4389134.306 | 3544044.664 | 845089.642 |
| | | 2028 | 4444222.970 | 3584663.137 | 859559.832 |
| | | 2029 | 4496447.877 | 3624053.540 | 872394.337 |
| | | 2030 | 4536975.698 | 3653806.567 | 883169.130 |
| | | 2031 | 4578338.586 | 3685153.832 | 893184.754 |
| | | 2032 | 4617397.784 | 3715512.648 | 901885.136 |
| | | 2033 | 4654407.697 | 3745220.254 | 909187.442 |
| | | 2034 | 4689913.454 | 3774390.191 | 915523.263 |
| | | 2035 | 4712518.190 | 3792086.983 | 920431.207 |
| | | 2036 | 4735845.827 | 3810985.579 | 924860.248 |
| | | 2037 | 4757958.806 | 3829501.471 | 928457.335 |
| | | 2038 | 4778813.806 | 3847720.855 | 931092.950 |
| | | 2039 | 4798769.065 | 3865706.661 | 933062.405 |
| Stroke | Males | 1990 | 75005.854 | 75005.854 | 0.000 |
| | | 1991 | 149463.017 | 149231.128 | 231.889 |
| | | 1992 | 222966.278 | 222092.327 | 873.951 |
| | | 1993 | 296801.879 | 294674.600 | 2127.278 |
| | | 1994 | 372453.706 | 368258.010 | 4195.696 |

## Results for PHIM for Run 2 (ELL Summary Pessimistic_Analysis.RTF)

| | | | | |
|---|---|---|---|---|
| | 1995 | 448401.338 | 441186.641 | 7214.696 |
| | 1996 | 523536.301 | 512273.354 | 11262.948 |
| | 1997 | 596763.211 | 580415.495 | 16347.716 |
| | 1998 | 667546.681 | 645126.974 | 22419.707 |
| | 1999 | 734925.407 | 705614.108 | 29311.299 |
| | 2000 | 803749.361 | 766243.806 | 37505.555 |
| | 2001 | 870081.588 | 823425.257 | 46656.331 |
| | 2002 | 935347.140 | 878530.045 | 56817.095 |
| | 2003 | 1001627.349 | 933190.124 | 68437.226 |
| | 2004 | 1066587.976 | 985665.633 | 80922.343 |
| | 2005 | 1130548.052 | 1036267.158 | 94280.894 |
| | 2006 | 1195132.454 | 1086217.871 | 108914.583 |
| | 2007 | 1258974.043 | 1134578.653 | 124395.390 |
| | 2008 | 1320124.867 | 1180010.199 | 140114.668 |
| | 2009 | 1380776.123 | 1224303.881 | 156472.243 |
| | 2010 | 1440048.533 | 1266939.650 | 173108.883 |
| | 2011 | 1497484.847 | 1307677.513 | 189807.334 |
| | 2012 | 1554496.526 | 1347522.569 | 206973.957 |
| | 2013 | 1610819.598 | 1386348.454 | 224471.144 |
| | 2014 | 1667484.890 | 1425053.897 | 242430.993 |
| | 2015 | 1720948.287 | 1461105.867 | 259842.420 |
| | 2016 | 1775699.228 | 1497804.165 | 277895.063 |
| | 2017 | 1830250.175 | 1534165.590 | 296084.585 |
| | 2018 | 1875958.950 | 1564501.890 | 311457.060 |
| | 2019 | 1920359.232 | 1593963.131 | 326396.101 |
| | 2020 | 1960605.297 | 1620442.087 | 340163.211 |
| | 2021 | 1998912.887 | 1645702.338 | 353210.550 |
| | 2022 | 2036003.696 | 1670254.675 | 365749.020 |
| | 2023 | 2071857.536 | 1694118.950 | 377738.586 |
| | 2024 | 2106636.468 | 1717485.354 | 389151.114 |
| | 2025 | 2136450.873 | 1737328.771 | 399122.103 |
| | 2026 | 2164796.470 | 1756414.491 | 408381.979 |
| | 2027 | 2192079.608 | 1775070.136 | 417009.472 |
| | 2028 | 2218404.367 | 1793277.588 | 425126.779 |
| | 2029 | 2243769.140 | 1811175.169 | 432593.972 |
| | 2030 | 2263570.213 | 1825002.572 | 438567.641 |
| | 2031 | 2282521.590 | 1838497.828 | 444023.762 |
| | 2032 | 2300804.945 | 1851733.663 | 449071.282 |
| | 2033 | 2318464.018 | 1864737.794 | 453726.224 |
| | 2034 | 2335611.973 | 1877723.460 | 457888.512 |
| | 2035 | 2347097.290 | 1886339.967 | 460757.324 |
| | 2036 | 2358248.747 | 1894906.485 | 463342.262 |
| | 2037 | 2369033.655 | 1903407.231 | 465626.425 |
| | 2038 | 2379490.114 | 1911810.495 | 467679.618 |
| | 2039 | 2389546.238 | 1920183.109 | 469363.128 |
| Female | 1990 | 56348.092 | 56348.092 | 0.000 |
| | 1991 | 110871.862 | 110712.449 | 159.413 |
| | 1992 | 165654.454 | 165033.661 | 620.794 |
| | 1993 | 220008.519 | 218507.923 | 1500.597 |
| | 1994 | 275706.807 | 272755.205 | 2951.602 |
| | 1995 | 331417.547 | 326357.573 | 5059.974 |
| | 1996 | 387580.161 | 379598.488 | 7981.672 |
| | 1997 | 442778.242 | 431103.063 | 11675.179 |
| | 1998 | 496117.305 | 480066.913 | 16050.391 |
| | 1999 | 548268.214 | 527134.114 | 21134.101 |
| | 2000 | 601693.202 | 574504.842 | 27188.359 |
| | 2001 | 652221.103 | 618448.141 | 33772.962 |
| | 2002 | 703022.600 | 661734.096 | 41288.504 |
| | 2003 | 752023.351 | 702644.219 | 49379.132 |
| | 2004 | 799826.733 | 741745.481 | 58081.251 |
| | 2005 | 847563.928 | 780020.571 | 67543.357 |
| | 2006 | 894205.027 | 816670.423 | 77534.604 |
| | 2007 | 939571.282 | 851623.648 | 87947.634 |
| | 2008 | 983437.578 | 884806.674 | 98630.904 |
| | 2009 | 1025803.786 | 916350.060 | 109453.726 |
| | 2010 | 1066964.934 | 946561.469 | 120403.465 |
| | 2011 | 1106954.044 | 975485.278 | 131468.767 |
| | 2012 | 1146108.461 | 1003366.351 | 142742.110 |
| | 2013 | 1183544.689 | 1029764.559 | 153780.131 |
| | 2014 | 1221220.945 | 1056074.445 | 165146.500 |
| | 2015 | 1258289.311 | 1081686.575 | 176602.737 |
| | 2016 | 1294830.207 | 1106770.988 | 188059.219 |
| | 2017 | 1330332.626 | 1131025.749 | 199306.877 |
| | 2018 | 1360571.987 | 1151667.872 | 208904.115 |
| | 2019 | 1389848.723 | 1171658.011 | 218190.712 |
| | 2020 | 1416046.192 | 1189366.997 | 226679.195 |
| | 2021 | 1440588.850 | 1206014.962 | 234573.888 |
| | 2022 | 1464157.934 | 1222076.537 | 242081.398 |

## Results for PHIM for Run 2 (ELL Summary Pessimistic_Analysis.RTF)

| | | | | | |
|---|---|---|---|---|---|
| | | 2023 | 1486810.237 | 1237665.983 | 249144.254 |
| | | 2024 | 1508591.729 | 1252789.715 | 255802.014 |
| | | 2025 | 1526875.621 | 1265360.831 | 261514.790 |
| | | 2026 | 1543659.810 | 1277091.611 | 266568.199 |
| | | 2027 | 1559623.774 | 1288436.024 | 271187.751 |
| | | 2028 | 1574852.850 | 1299481.460 | 275371.390 |
| | | 2029 | 1589437.452 | 1310272.461 | 279164.991 |
| | | 2030 | 1600568.199 | 1318378.102 | 282190.097 |
| | | 2031 | 1610776.297 | 1326034.101 | 284742.196 |
| | | 2032 | 1620483.563 | 1333496.047 | 286987.516 |
| | | 2033 | 1629745.442 | 1340849.468 | 288895.974 |
| | | 2034 | 1638721.425 | 1348132.858 | 290588.567 |
| | | 2035 | 1644649.404 | 1352829.266 | 291820.138 |
| | | 2036 | 1650181.590 | 1357346.560 | 292835.030 |
| | | 2037 | 1655476.066 | 1361815.602 | 293660.464 |
| | | 2038 | 1660507.914 | 1366245.141 | 294262.773 |
| | | 2039 | 1665361.569 | 1370650.422 | 294711.147 |
| COPD | Males | 1990 | 117002.906 | 117002.906 | 0.000 |
| | | 1991 | 233438.833 | 233379.900 | 58.932 |
| | | 1992 | 348084.047 | 347869.879 | 214.168 |
| | | 1993 | 467278.318 | 466750.813 | 527.504 |
| | | 1994 | 584216.907 | 583157.054 | 1059.853 |
| | | 1995 | 697391.457 | 695535.091 | 1856.365 |
| | | 1996 | 809471.271 | 806486.248 | 2985.023 |
| | | 1997 | 922587.668 | 918066.318 | 4521.350 |
| | | 1998 | 1034072.457 | 1027643.293 | 6429.163 |
| | | 1999 | 1153475.399 | 1144491.160 | 8984.239 |
| | | 2000 | 1268050.268 | 1255993.762 | 12056.506 |
| | | 2001 | 1382786.209 | 1366952.849 | 15833.360 |
| | | 2002 | 1497557.412 | 1477173.771 | 20383.641 |
| | | 2003 | 1614405.229 | 1588716.189 | 25689.040 |
| | | 2004 | 1726672.489 | 1695088.069 | 31584.420 |
| | | 2005 | 1847277.524 | 1808610.342 | 38667.182 |
| | | 2006 | 1961009.963 | 1914711.044 | 46298.919 |
| | | 2007 | 2079868.908 | 2024621.729 | 55247.179 |
| | | 2008 | 2206755.783 | 2141001.530 | 65754.252 |
| | | 2009 | 2331993.380 | 2254846.659 | 77146.721 |
| | | 2010 | 2455130.176 | 2365893.723 | 89236.454 |
| | | 2011 | 2582657.849 | 2479753.194 | 102904.655 |
| | | 2012 | 2709719.005 | 2592050.849 | 117668.156 |
| | | 2013 | 2840547.005 | 2706456.149 | 134090.857 |
| | | 2014 | 2971677.135 | 2820047.397 | 151629.739 |
| | | 2015 | 3104538.983 | 2934291.395 | 170247.588 |
| | | 2016 | 3237650.175 | 3047534.775 | 190115.400 |
| | | 2017 | 3370561.253 | 3159449.607 | 211111.646 |
| | | 2018 | 3503136.371 | 3269866.929 | 233269.442 |
| | | 2019 | 3636402.758 | 3379850.462 | 256552.296 |
| | | 2020 | 3768054.453 | 3487589.371 | 280465.082 |
| | | 2021 | 3897675.067 | 3592811.888 | 304863.178 |
| | | 2022 | 4027307.132 | 3697107.255 | 330199.877 |
| | | 2023 | 4156698.720 | 3800397.574 | 356301.146 |
| | | 2024 | 4285772.226 | 3902697.437 | 383074.789 |
| | | 2025 | 4410759.298 | 4001094.559 | 409664.738 |
| | | 2026 | 4531411.064 | 4095529.339 | 435881.726 |
| | | 2027 | 4651145.233 | 4188792.873 | 462352.360 |
| | | 2028 | 4769837.872 | 4280930.928 | 488906.944 |
| | | 2029 | 4887747.179 | 4372204.937 | 515542.242 |
| | | 2030 | 4997632.895 | 4456845.106 | 540787.790 |
| | | 2031 | 5103301.991 | 4538100.461 | 565201.531 |
| | | 2032 | 5207593.613 | 4618466.425 | 589127.188 |
| | | 2033 | 5310556.138 | 4697972.304 | 612583.833 |
| | | 2034 | 5412542.816 | 4777145.115 | 635397.701 |
| | | 2035 | 5500522.473 | 4845144.751 | 655377.722 |
| | | 2036 | 5588149.640 | 4913381.983 | 674767.657 |
| | | 2037 | 5674667.886 | 4981344.980 | 693322.907 |
| | | 2038 | 5760391.289 | 5049218.380 | 711172.909 |
| | | 2039 | 5845459.234 | 5117558.287 | 727900.947 |
| | Female | 1990 | 77434.846 | 77434.846 | 0.000 |
| | | 1991 | 160029.510 | 159990.437 | 39.074 |
| | | 1992 | 240642.531 | 240487.844 | 154.687 |
| | | 1993 | 329362.093 | 328945.748 | 416.346 |
| | | 1994 | 418569.515 | 417704.831 | 864.685 |
| | | 1995 | 509110.357 | 507519.488 | 1590.869 |
| | | 1996 | 600040.117 | 597439.842 | 2600.275 |
| | | 1997 | 691159.071 | 687195.930 | 3963.140 |
| | | 1998 | 782866.899 | 777171.385 | 5695.514 |
| | | 1999 | 881526.925 | 873495.989 | 8030.937 |
| | | 2000 | 977880.647 | 967107.914 | 10772.733 |

## Results for PHIM for Run 2 (ELL Summary Pessimistic_Analysis.RTF)

| | | | |
|---|---|---|---|
| 2001 | 1076787.404 | 1062631.941 | 14155.463 |
| 2002 | 1174842.754 | 1156713.059 | 18129.694 |
| 2003 | 1275102.681 | 1252329.858 | 22772.823 |
| 2004 | 1371026.306 | 1343218.995 | 27807.310 |
| 2005 | 1473954.509 | 1439986.735 | 33967.774 |
| 2006 | 1574450.828 | 1533705.613 | 40745.215 |
| 2007 | 1673790.211 | 1625604.202 | 48186.009 |
| 2008 | 1782360.738 | 1725204.349 | 57156.388 |
| 2009 | 1892944.209 | 1825686.460 | 67257.749 |
| 2010 | 2002131.282 | 1924091.530 | 78039.752 |
| 2011 | 2114522.152 | 2024344.619 | 90177.533 |
| 2012 | 2228079.372 | 2124703.917 | 103375.455 |
| 2013 | 2345377.689 | 2227345.556 | 118032.133 |
| 2014 | 2462498.422 | 2328820.479 | 133677.943 |
| 2015 | 2581450.312 | 2431153.922 | 150296.390 |
| 2016 | 2701959.301 | 2533709.463 | 168249.838 |
| 2017 | 2823834.967 | 2636439.746 | 187395.221 |
| 2018 | 2938265.081 | 2732311.087 | 205953.994 |
| 2019 | 3053254.880 | 2827812.920 | 225441.959 |
| 2020 | 3167073.216 | 2921604.431 | 245468.785 |
| 2021 | 3279517.699 | 3013656.525 | 265861.174 |
| 2022 | 3392060.964 | 3104960.072 | 287100.892 |
| 2023 | 3504296.171 | 3195400.877 | 308895.295 |
| 2024 | 3616019.231 | 3284830.087 | 331189.144 |
| 2025 | 3724611.269 | 3371138.810 | 353472.459 |
| 2026 | 3827181.707 | 3452444.336 | 374737.371 |
| 2027 | 3928684.441 | 3532454.726 | 396229.715 |
| 2028 | 4029124.892 | 3611414.501 | 417710.391 |
| 2029 | 4128269.068 | 3689147.001 | 439122.066 |
| 2030 | 4221560.174 | 3761921.022 | 459639.152 |
| 2031 | 4304170.584 | 3826407.446 | 477763.139 |
| 2032 | 4384930.537 | 3889440.218 | 495490.320 |
| 2033 | 4464357.691 | 3951609.203 | 512748.489 |
| 2034 | 4542251.545 | 4012858.803 | 529392.741 |
| 2035 | 4611066.648 | 4066832.780 | 544233.868 |
| 2036 | 4670309.230 | 4113749.385 | 556559.845 |
| 2037 | 4728179.514 | 4160005.760 | 568173.754 |
| 2038 | 4785008.048 | 4206102.730 | 578905.318 |
| 2039 | 4841291.793 | 4252388.206 | 588903.587 |

## Results for PHIM for Run 2 (ELL Summary Pessimistic_Analysis.RTF)

*************************************************************************************

**Table 4: Estimated Loss of Life, All age groups combined, Population and Number deaths adjusted for Ecig scenario, cumulative over years**

| Disease | Sex | Year | Years of life lost NULL | Years of life lost Ecig | Years of life Saved |
|---|---|---|---|---|---|
| All Diseases | Males | 1990 | 1304839.645 | 1304839.645 | 0.000 |
| | | 1991 | 2579865.358 | 2577032.488 | 2832.870 |
| | | 1992 | 3830683.831 | 3820093.127 | 10590.704 |
| | | 1993 | 5076567.803 | 5051456.133 | 25111.670 |
| | | 1994 | 6292145.526 | 6244501.481 | 47644.045 |
| | | 1995 | 7485537.010 | 7406016.033 | 79520.976 |
| | | 1996 | 8655955.028 | 8534694.242 | 121260.786 |
| | | 1997 | 9791729.713 | 9619402.087 | 172327.626 |
| | | 1998 | 10899134.186 | 10666837.108 | 232297.078 |
| | | 1999 | 12070409.674 | 11757520.530 | 312889.144 |
| | | 2000 | 13231384.595 | 12827033.650 | 404350.945 |
| | | 2001 | 14371761.018 | 13864706.220 | 507054.798 |
| | | 2002 | 15508906.386 | 14886033.072 | 622873.313 |
| | | 2003 | 16638100.422 | 15888032.373 | 750068.049 |
| | | 2004 | 17736465.088 | 16852729.545 | 883735.543 |
| | | 2005 | 18834658.606 | 17807758.568 | 1026900.04 |
| | | 2006 | 19909100.501 | 18729884.485 | 1179216.02 |
| | | 2007 | 20958321.463 | 19621650.695 | 1336670.77 |
| | | 2008 | 22000068.148 | 20499788.700 | 1500279.45 |
| | | 2009 | 23018816.903 | 21351836.413 | 1666980.49 |
| | | 2010 | 24018480.537 | 22181199.835 | 1837280.70 |
| | | 2011 | 25003034.574 | 22991248.935 | 2011785.64 |
| | | 2012 | 25969871.429 | 23780110.204 | 2189761.23 |
| | | 2013 | 26920275.180 | 24549149.316 | 2371125.86 |
| | | 2014 | 27849005.227 | 25295302.755 | 2553702.47 |
| | | 2015 | 28749620.400 | 26014908.417 | 2734711.98 |
| | | 2016 | 29622417.562 | 26705801.665 | 2916615.90 |
| | | 2017 | 30462559.724 | 27365987.076 | 3096572.65 |
| | | 2018 | 31293299.299 | 28020687.418 | 3272611.88 |
| | | 2019 | 32109993.427 | 28663176.610 | 3446816.82 |
| | | 2020 | 32883323.977 | 29270238.412 | 3613085.56 |
| | | 2021 | 33610102.554 | 29837335.934 | 3772766.62 |
| | | 2022 | 34322010.448 | 30392658.452 | 3929352.00 |
| | | 2023 | 35018383.550 | 30936216.641 | 4082166.91 |
| | | 2024 | 35700177.955 | 31469812.014 | 4230365.94 |
| | | 2025 | 36315500.346 | 31950397.026 | 4365103.32 |
| | | 2026 | 36896159.343 | 32403373.037 | 4492786.31 |
| | | 2027 | 37462570.116 | 32847304.946 | 4615265.17 |
| | | 2028 | 38015337.780 | 33282003.853 | 4733333.93 |
| | | 2029 | 38554814.764 | 33709894.463 | 4844920.30 |
| | | 2030 | 39003805.400 | 34063905.418 | 4939899.98 |
| | | 2031 | 39436509.703 | 34406638.239 | 5029871.46 |
| | | 2032 | 39858470.458 | 34743224.330 | 5115246.13 |
| | | 2033 | 40270147.082 | 35073614.547 | 5196532.54 |
| | | 2034 | 40672665.743 | 35401554.365 | 5271111.38 |
| | | 2035 | 40962732.664 | 35635410.330 | 5327322.33 |
| | | 2036 | 41255021.653 | 35873907.047 | 5381114.61 |
| | | 2037 | 41540573.023 | 36109668.024 | 5430905.00 |
| | | 2038 | 41820158.066 | 36342697.408 | 5477460.66 |
| | | 2039 | 42093547.949 | 36575271.380 | 5518276.57 |
| | Female | 1990 | 588979.406 | 588979.406 | 0.000 |
| | | 1991 | 1182571.252 | 1181666.191 | 905.061 |
| | | 1992 | 1777346.679 | 1773860.176 | 3486.503 |
| | | 1993 | 2380261.899 | 2371861.572 | 8400.327 |
| | | 1994 | 2983130.039 | 2966958.320 | 16171.719 |
| | | 1995 | 3588982.584 | 3561517.691 | 27464.893 |
| | | 1996 | 4195430.235 | 4152371.653 | 43058.581 |
| | | 1997 | 4793866.694 | 4731590.219 | 62276.475 |
| | | 1998 | 5389747.381 | 5304280.620 | 85466.761 |
| | | 1999 | 6006722.467 | 5891105.533 | 115616.934 |
| | | 2000 | 6625758.871 | 6475455.707 | 150303.164 |
| | | 2001 | 7245135.411 | 7054684.972 | 190450.439 |
| | | 2002 | 7867492.021 | 7631286.384 | 236205.637 |
| | | 2003 | 8485051.793 | 8198557.411 | 286494.382 |
| | | 2004 | 9092994.774 | 8752195.139 | 340799.635 |
| | | 2005 | 9706032.803 | 9305763.398 | 400269.406 |
| | | 2006 | 10311907.548 | 9847682.112 | 464225.436 |
| | | 2007 | 10909437.502 | 10378156.942 | 531280.560 |
| | | 2008 | 11506462.491 | 10902898.892 | 603563.598 |
| | | 2009 | 12098393.287 | 11420158.986 | 678234.301 |
| | | 2010 | 12681188.981 | 11925514.140 | 755674.840 |

# Results for PHIM for Run 2 (ELL Summary Pessimistic_Analysis.RTF)

| | | | | | |
|---|---|---|---|---|---|
| | | 2011 | 13260062.991 | 12423129.096 | 836933.895 |
| | | 2012 | 13839417.441 | 12916903.566 | 922513.875 |
| | | 2013 | 14409614.758 | 13399910.015 | 1009704.74 |
| | | 2014 | 14976658.746 | 13876340.436 | 1100318.31 |
| | | 2015 | 15529977.821 | 14338246.267 | 1191731.55 |
| | | 2016 | 16066640.764 | 14782571.551 | 1284069.21 |
| | | 2017 | 16590035.078 | 15213054.972 | 1376980.11 |
| | | 2018 | 17098716.137 | 15631756.082 | 1466960.05 |
| | | 2019 | 17599868.850 | 16042881.430 | 1556987.42 |
| | | 2020 | 18081346.241 | 16436494.086 | 1644852.15 |
| | | 2021 | 18537594.460 | 16807371.067 | 1730223.39 |
| | | 2022 | 18985300.234 | 17170274.811 | 1815025.42 |
| | | 2023 | 19423958.747 | 17525700.293 | 1898258.45 |
| | | 2024 | 19853347.772 | 17873440.014 | 1979907.76 |
| | | 2025 | 20252822.245 | 18195925.172 | 2056897.07 |
| | | 2026 | 20619307.063 | 18491604.448 | 2127702.61 |
| | | 2027 | 20976063.406 | 18779864.783 | 2196198.62 |
| | | 2028 | 21323641.947 | 19061869.318 | 2261772.63 |
| | | 2029 | 21662117.106 | 19337526.924 | 2324590.18 |
| | | 2030 | 21960971.694 | 19579293.028 | 2381678.67 |
| | | 2031 | 22224951.983 | 19794083.236 | 2430868.75 |
| | | 2032 | 22480397.853 | 20003181.028 | 2477216.83 |
| | | 2033 | 22728639.455 | 20208362.794 | 2520276.66 |
| | | 2034 | 22970448.888 | 20409852.756 | 2560596.13 |
| | | 2035 | 23164154.188 | 20569362.276 | 2594791.91 |
| | | 2036 | 23334538.616 | 20711813.094 | 2622725.52 |
| | | 2037 | 23499760.723 | 20851750.027 | 2648010.70 |
| | | 2038 | 23660308.401 | 20990309.373 | 2669999.03 |
| | | 2039 | 23817891.273 | 21128264.569 | 2689626.70 |
| Lung cancer | Males | 1990 | 595621.791 | 595621.791 | 0.000 |
| | | 1991 | 1177744.113 | 1177548.728 | 195.384 |
| | | 1992 | 1750034.892 | 1749300.720 | 734.171 |
| | | 1993 | 2319752.308 | 2317942.342 | 1809.966 |
| | | 1994 | 2872597.339 | 2869044.430 | 3552.909 |
| | | 1995 | 3411494.825 | 3405373.902 | 6120.923 |
| | | 1996 | 3945569.726 | 3935739.681 | 9830.045 |
| | | 1997 | 4463316.362 | 4448692.826 | 14623.536 |
| | | 1998 | 4975301.180 | 4954592.300 | 20708.880 |
| | | 1999 | 5473691.125 | 5445503.357 | 28187.768 |
| | | 2000 | 5977500.570 | 5939990.803 | 37509.768 |
| | | 2001 | 6474795.516 | 6426081.575 | 48713.941 |
| | | 2002 | 6968769.705 | 6906823.363 | 61946.342 |
| | | 2003 | 7460282.140 | 7382805.850 | 77476.290 |
| | | 2004 | 7947048.275 | 7851923.879 | 95124.396 |
| | | 2005 | 8430571.669 | 8315632.054 | 114939.615 |
| | | 2006 | 8903945.390 | 8767011.598 | 136933.792 |
| | | 2007 | 9364177.982 | 9203422.735 | 160755.247 |
| | | 2008 | 9822525.458 | 9635264.719 | 187260.739 |
| | | 2009 | 10275228.886 | 10059313.979 | 215914.908 |
| | | 2010 | 10720275.500 | 10473823.582 | 246451.918 |
| | | 2011 | 11155356.373 | 10876498.841 | 278857.532 |
| | | 2012 | 11582611.617 | 11269169.337 | 313442.279 |
| | | 2013 | 11998803.791 | 11649007.820 | 349795.971 |
| | | 2014 | 12402087.090 | 12014847.446 | 387239.644 |
| | | 2015 | 12793451.706 | 12367790.556 | 425661.150 |
| | | 2016 | 13160101.653 | 12696285.649 | 463816.004 |
| | | 2017 | 13503583.414 | 13002062.042 | 501521.372 |
| | | 2018 | 13859755.737 | 13317002.056 | 542753.681 |
| | | 2019 | 14211625.176 | 13626377.726 | 585247.451 |
| | | 2020 | 14553935.288 | 13925768.365 | 628166.923 |
| | | 2021 | 14851747.495 | 14185255.569 | 666491.926 |
| | | 2022 | 15144531.004 | 14439157.998 | 705373.006 |
| | | 2023 | 15431932.496 | 14687439.862 | 744492.635 |
| | | 2024 | 15713920.740 | 14930279.449 | 783641.291 |
| | | 2025 | 15979112.816 | 15157752.156 | 821360.660 |
| | | 2026 | 16206550.153 | 15352712.418 | 853837.735 |
| | | 2027 | 16428826.050 | 15543000.474 | 885825.576 |
| | | 2028 | 16645902.186 | 15728715.048 | 917187.138 |
| | | 2029 | 16858126.517 | 15910451.521 | 947674.996 |
| | | 2030 | 17045220.687 | 16069958.678 | 975262.009 |
| | | 2031 | 17208482.610 | 16209810.761 | 998671.849 |
| | | 2032 | 17367586.074 | 16346540.488 | 1021045.59 |
| | | 2033 | 17522716.280 | 16480211.310 | 1042504.97 |
| | | 2034 | 17674226.555 | 16611650.032 | 1062576.52 |
| | | 2035 | 17792201.734 | 16713343.224 | 1078858.51 |
| | | 2036 | 17903508.236 | 16810421.806 | 1093086.43 |
| | | 2037 | 18012162.345 | 16905798.727 | 1106363.62 |
| | | 2038 | 18118464.470 | 16999606.974 | 1118857.50 |

## Results for PHIM for Run 2 (ELL Summary Pessimistic_Analysis.RTF)

| | | | | | |
|---|---|---|---|---|---|
| | | 2039 | 18222549.860 | 17092593.671 | 1129956.19 |
| | Female | 1990 | 307897.357 | 307897.357 | 0.000 |
| | | 1991 | 616547.418 | 616436.677 | 110.741 |
| | | 1992 | 931086.773 | 930632.803 | 453.970 |
| | | 1993 | 1246155.671 | 1245013.947 | 1141.724 |
| | | 1994 | 1561212.934 | 1558934.029 | 2278.905 |
| | | 1995 | 1879967.742 | 1875911.971 | 4055.771 |
| | | 1996 | 2197488.109 | 2190928.038 | 6560.072 |
| | | 1997 | 2515698.453 | 2505747.688 | 9950.764 |
| | | 1998 | 2836249.556 | 2821930.716 | 14318.840 |
| | | 1999 | 3150201.390 | 3130552.290 | 19649.100 |
| | | 2000 | 3472722.342 | 3446451.171 | 26271.171 |
| | | 2001 | 3796994.889 | 3762661.031 | 34333.859 |
| | | 2002 | 4126619.935 | 4082618.752 | 44001.184 |
| | | 2003 | 4454982.533 | 4399881.206 | 55101.327 |
| | | 2004 | 4784343.030 | 4716478.917 | 67864.114 |
| | | 2005 | 5113443.121 | 5031127.832 | 82315.289 |
| | | 2006 | 5442543.963 | 5343831.435 | 98712.527 |
| | | 2007 | 5772441.497 | 5655420.956 | 117020.540 |
| | | 2008 | 6091911.943 | 5955306.335 | 136605.608 |
| | | 2009 | 6413671.330 | 6255365.064 | 158306.266 |
| | | 2010 | 6730763.530 | 6549379.189 | 181384.341 |
| | | 2011 | 7043614.101 | 6837428.902 | 206185.199 |
| | | 2012 | 7356270.780 | 7123364.973 | 232905.807 |
| | | 2013 | 7662551.964 | 7401631.531 | 260920.432 |
| | | 2014 | 7964551.465 | 7674287.184 | 290264.281 |
| | | 2015 | 8254895.403 | 7935064.128 | 319831.275 |
| | | 2016 | 8530661.848 | 8180983.831 | 349678.017 |
| | | 2017 | 8795826.122 | 8415936.625 | 379889.498 |
| | | 2018 | 9067233.707 | 8655019.398 | 412214.308 |
| | | 2019 | 9335343.270 | 8889901.635 | 445441.635 |
| | | 2020 | 9596110.883 | 9117169.470 | 478941.412 |
| | | 2021 | 9833186.859 | 9323095.292 | 510091.567 |
| | | 2022 | 10066452.037 | 9524655.517 | 541796.520 |
| | | 2023 | 10295503.609 | 9721935.902 | 573567.707 |
| | | 2024 | 10520125.318 | 9914782.755 | 605342.563 |
| | | 2025 | 10731915.863 | 10095785.686 | 636130.177 |
| | | 2026 | 10917495.549 | 10254465.836 | 663029.712 |
| | | 2027 | 11098620.885 | 10409019.291 | 689601.594 |
| | | 2028 | 11275441.234 | 10559996.379 | 715444.855 |
| | | 2029 | 11447962.710 | 10707327.891 | 740634.819 |
| | | 2030 | 11601867.623 | 10838055.955 | 763811.668 |
| | | 2031 | 11731666.516 | 10948966.268 | 782700.248 |
| | | 2032 | 11857585.969 | 11056818.430 | 800767.539 |
| | | 2033 | 11980128.625 | 11162372.448 | 817756.177 |
| | | 2034 | 12099562.464 | 11265757.362 | 833805.102 |
| | | 2035 | 12195919.946 | 11348533.289 | 847386.657 |
| | | 2036 | 12278201.969 | 11420310.677 | 857891.292 |
| | | 2037 | 12358146.336 | 11490665.243 | 867481.093 |
| | | 2038 | 12435978.633 | 11560135.032 | 875843.601 |
| | | 2039 | 12512468.845 | 11629065.776 | 883403.070 |
| IHD | Males | 1990 | 517209.093 | 517209.093 | 0.000 |
| | | 1991 | 1019219.395 | 1016872.731 | 2346.664 |
| | | 1992 | 1509598.614 | 1500829.708 | 8768.906 |
| | | 1993 | 1992735.299 | 1972086.148 | 20649.151 |
| | | 1994 | 2462877.575 | 2424035.705 | 38841.870 |
| | | 1995 | 2928249.390 | 2863906.606 | 64342.783 |
| | | 1996 | 3377377.730 | 3280168.571 | 97209.159 |
| | | 1997 | 3809062.472 | 3672181.806 | 136880.666 |
| | | 1998 | 4222213.867 | 4039401.868 | 182811.999 |
| | | 1999 | 4708317.743 | 4461801.448 | 246516.295 |
| | | 2000 | 5182084.396 | 4864644.837 | 317439.560 |
| | | 2001 | 5644097.705 | 5248022.545 | 396075.160 |
| | | 2002 | 6107232.129 | 5623202.414 | 484029.715 |
| | | 2003 | 6561785.704 | 5982917.596 | 578868.108 |
| | | 2004 | 6996156.348 | 6319534.320 | 676622.028 |
| | | 2005 | 7426261.361 | 6646589.564 | 779671.797 |
| | | 2006 | 7849012.694 | 6961126.995 | 887885.699 |
| | | 2007 | 8255300.530 | 7258025.672 | 997274.857 |
| | | 2008 | 8650662.040 | 7542293.783 | 1108368.26 |
| | | 2009 | 9030818.513 | 7811913.668 | 1218904.84 |
| | | 2010 | 9403026.327 | 8072823.903 | 1330202.42 |
| | | 2011 | 9767535.506 | 8325307.382 | 1442228.12 |
| | | 2012 | 10123044.281 | 8569038.637 | 1554005.64 |
| | | 2013 | 10470104.786 | 8804656.859 | 1665447.93 |
| | | 2014 | 10807756.112 | 9032294.446 | 1775461.67 |
| | | 2015 | 11130681.425 | 9248242.431 | 1882438.99 |
| | | 2016 | 11448966.506 | 9460249.240 | 1988717.27 |

## Results for PHIM for Run 2 (ELL Summary Pessimistic_Analysis.RTF)

| | | | | | |
|---|---|---|---|---|---|
| | | 2017 | 11758164.882 | 9665899.659 | 2092265.22 |
| | | 2018 | 12054448.241 | 9864411.658 | 2190036.58 |
| | | 2019 | 12341606.261 | 10057556.422 | 2284049.84 |
| | | 2020 | 12600728.939 | 10230457.090 | 2370271.85 |
| | | 2021 | 12861767.105 | 10407016.338 | 2454750.77 |
| | | 2022 | 13114168.616 | 10578995.297 | 2535173.32 |
| | | 2023 | 13357894.797 | 10746499.118 | 2611395.68 |
| | | 2024 | 13593848.521 | 10910946.195 | 2682902.33 |
| | | 2025 | 13789177.359 | 11045157.865 | 2744019.49 |
| | | 2026 | 13993401.656 | 11188995.866 | 2804405.79 |
| | | 2027 | 14190519.224 | 11330045.142 | 2860474.08 |
| | | 2028 | 14381193.356 | 11467991.721 | 2913201.63 |
| | | 2029 | 14565171.927 | 11604265.377 | 2960906.55 |
| | | 2030 | 14697381.605 | 11699602.320 | 2997779.28 |
| | | 2031 | 14842203.512 | 11807043.597 | 3035159.92 |
| | | 2032 | 14982485.826 | 11912598.844 | 3069886.98 |
| | | 2033 | 15118410.647 | 12016098.716 | 3102311.93 |
| | | 2034 | 15250284.398 | 12119717.400 | 3130567.00 |
| | | 2035 | 15322911.166 | 12174603.180 | 3148307.99 |
| | | 2036 | 15405115.031 | 12238544.060 | 3166570.97 |
| | | 2037 | 15484709.137 | 12301780.282 | 3182928.85 |
| | | 2038 | 15561812.193 | 12364029.729 | 3197782.46 |
| | | 2039 | 15635992.616 | 12426196.035 | 3209796.58 |
| | Female | 1990 | 147299.110 | 147299.110 | 0.000 |
| | | 1991 | 295122.462 | 294526.629 | 595.834 |
| | | 1992 | 439962.920 | 437705.779 | 2257.142 |
| | | 1993 | 584735.615 | 579393.492 | 5342.124 |
| | | 1994 | 727640.783 | 717562.822 | 10077.961 |
| | | 1995 | 868486.937 | 851725.267 | 16761.671 |
| | | 1996 | 1010321.847 | 984398.453 | 25923.394 |
| | | 1997 | 1144230.930 | 1107531.290 | 36699.640 |
| | | 1998 | 1274513.622 | 1225091.387 | 49422.235 |
| | | 1999 | 1426725.938 | 1359891.293 | 66834.644 |
| | | 2000 | 1573462.680 | 1487344.204 | 86118.475 |
| | | 2001 | 1719132.015 | 1610875.635 | 108256.380 |
| | | 2002 | 1863006.732 | 1730126.351 | 132880.381 |
| | | 2003 | 2002943.227 | 1843575.551 | 159367.677 |
| | | 2004 | 2137798.705 | 1950587.282 | 187211.424 |
| | | 2005 | 2271071.245 | 2054416.783 | 216654.462 |
| | | 2006 | 2400707.731 | 2153209.510 | 247498.221 |
| | | 2007 | 2523634.512 | 2245181.980 | 278452.532 |
| | | 2008 | 2648752.232 | 2337181.484 | 311570.748 |
| | | 2009 | 2765973.961 | 2422273.404 | 343700.557 |
| | | 2010 | 2881329.235 | 2504905.548 | 376423.686 |
| | | 2011 | 2994972.694 | 2585189.851 | 409782.843 |
| | | 2012 | 3108958.829 | 2664672.651 | 444286.178 |
| | | 2013 | 3218140.416 | 2740244.005 | 477896.411 |
| | | 2014 | 3328387.914 | 2816093.535 | 512294.379 |
| | | 2015 | 3435342.796 | 2889119.176 | 546223.620 |
| | | 2016 | 3539189.408 | 2959712.162 | 579477.246 |
| | | 2017 | 3640041.363 | 3028069.604 | 611971.758 |
| | | 2018 | 3732645.362 | 3090984.405 | 641660.958 |
| | | 2019 | 3821421.978 | 3151532.024 | 669889.954 |
| | | 2020 | 3902115.950 | 3206159.526 | 695956.424 |
| | | 2021 | 3984301.053 | 3262181.049 | 722120.004 |
| | | 2022 | 4062629.299 | 3315916.594 | 746712.705 |
| | | 2023 | 4137348.729 | 3367775.385 | 769573.345 |
| | | 2024 | 4208611.493 | 3417846.989 | 790764.504 |
| | | 2025 | 4269419.492 | 3460170.467 | 809249.025 |
| | | 2026 | 4330969.998 | 3503853.848 | 827116.150 |
| | | 2027 | 4389134.306 | 3545916.640 | 843217.666 |
| | | 2028 | 4444222.970 | 3586639.245 | 857583.725 |
| | | 2029 | 4496447.877 | 3626133.396 | 870314.480 |
| | | 2030 | 4536975.698 | 3655980.926 | 880994.772 |
| | | 2031 | 4578338.586 | 3687428.836 | 890909.750 |
| | | 2032 | 4617397.784 | 3717886.839 | 899510.945 |
| | | 2033 | 4654407.697 | 3747693.402 | 906714.295 |
| | | 2034 | 4689913.454 | 3776962.196 | 912951.257 |
| | | 2035 | 4712518.190 | 3794735.505 | 917782.685 |
| | | 2036 | 4735845.827 | 3813716.152 | 922129.674 |
| | | 2037 | 4757958.806 | 3832313.411 | 925645.396 |
| | | 2038 | 4778813.806 | 3850613.631 | 928200.175 |
| | | 2039 | 4798769.065 | 3868680.028 | 930089.038 |
| Stroke | Males | 1990 | 75005.854 | 75005.854 | 0.000 |
| | | 1991 | 149463.017 | 149231.128 | 231.889 |
| | | 1992 | 222966.278 | 222092.516 | 873.762 |
| | | 1993 | 296801.879 | 294675.475 | 2126.403 |
| | | 1994 | 372453.706 | 368260.542 | 4193.164 |

## Results for PHIM for Run 2 (ELL Summary Pessimistic_Analysis.RTF)

| | | | | |
|---|---|---|---|---|
| | 1995 | 448401.338 | 441192.296 | 7209.042 |
| | 1996 | 523536.301 | 512284.127 | 11252.174 |
| | 1997 | 596763.211 | 580433.781 | 16329.430 |
| | 1998 | 667546.681 | 645155.453 | 22391.228 |
| | 1999 | 734925.407 | 705655.461 | 29269.946 |
| | 2000 | 803749.361 | 766302.652 | 37446.709 |
| | 2001 | 870081.588 | 823505.218 | 46576.370 |
| | 2002 | 935347.140 | 878635.760 | 56711.379 |
| | 2003 | 1001627.349 | 933326.659 | 68300.690 |
| | 2004 | 1066587.976 | 985838.009 | 80749.967 |
| | 2005 | 1130548.052 | 1036480.343 | 94067.708 |
| | 2006 | 1195132.454 | 1086477.777 | 108654.676 |
| | 2007 | 1258974.043 | 1134890.501 | 124083.542 |
| | 2008 | 1320124.867 | 1180376.990 | 139747.877 |
| | 2009 | 1380776.123 | 1224730.866 | 156045.257 |
| | 2010 | 1440048.533 | 1267431.275 | 172617.258 |
| | 2011 | 1497484.847 | 1308238.020 | 189246.826 |
| | 2012 | 1554496.526 | 1348155.837 | 206340.689 |
| | 2013 | 1610819.598 | 1387059.748 | 223759.849 |
| | 2014 | 1667484.890 | 1425848.168 | 241636.722 |
| | 2015 | 1720948.287 | 1461987.782 | 258960.504 |
| | 2016 | 1775699.228 | 1498780.634 | 276918.594 |
| | 2017 | 1830250.175 | 1535242.485 | 295007.690 |
| | 2018 | 1875958.950 | 1565670.173 | 310288.777 |
| | 2019 | 1920359.232 | 1595225.090 | 325134.142 |
| | 2020 | 1960605.297 | 1621799.318 | 338805.980 |
| | 2021 | 1998912.887 | 1647154.338 | 351758.550 |
| | 2022 | 2036003.696 | 1671802.976 | 364200.719 |
| | 2023 | 2071857.536 | 1695764.914 | 376092.623 |
| | 2024 | 2106636.468 | 1719230.743 | 387405.725 |
| | 2025 | 2136450.873 | 1739171.466 | 397279.407 |
| | 2026 | 2164796.470 | 1758353.368 | 406443.103 |
| | 2027 | 2192079.608 | 1777106.129 | 414973.479 |
| | 2028 | 2218404.367 | 1795411.166 | 422993.201 |
| | 2029 | 2243769.140 | 1813406.840 | 430362.301 |
| | 2030 | 2263570.213 | 1827322.473 | 436247.740 |
| | 2031 | 2282521.590 | 1840905.341 | 441616.249 |
| | 2032 | 2300804.945 | 1854228.889 | 446576.056 |
| | 2033 | 2318464.018 | 1867321.097 | 451142.921 |
| | 2034 | 2335611.973 | 1880396.393 | 455215.580 |
| | 2035 | 2347097.290 | 1889082.120 | 458015.170 |
| | 2036 | 2358248.747 | 1897718.451 | 460530.296 |
| | 2037 | 2369033.655 | 1906289.842 | 462743.813 |
| | 2038 | 2379490.114 | 1914764.258 | 464725.855 |
| | 2039 | 2389546.238 | 1923208.547 | 466337.691 |
| Female | 1990 | 56348.092 | 56348.092 | 0.000 |
| | 1991 | 110871.862 | 110712.449 | 159.413 |
| | 1992 | 165654.454 | 165033.702 | 620.752 |
| | 1993 | 220008.519 | 218508.122 | 1500.397 |
| | 1994 | 275706.807 | 272755.789 | 2951.018 |
| | 1995 | 331417.547 | 326358.898 | 5058.649 |
| | 1996 | 387580.161 | 379601.078 | 7979.083 |
| | 1997 | 442778.242 | 431107.562 | 11670.680 |
| | 1998 | 496117.305 | 480074.092 | 16043.212 |
| | 1999 | 548268.214 | 527144.842 | 21123.373 |
| | 2000 | 601693.202 | 574520.395 | 27172.807 |
| | 2001 | 652221.103 | 618469.521 | 33751.582 |
| | 2002 | 703022.600 | 661762.670 | 41259.930 |
| | 2003 | 752023.351 | 702681.261 | 49342.090 |
| | 2004 | 799826.733 | 741792.298 | 58034.434 |
| | 2005 | 847563.928 | 780078.719 | 67485.209 |
| | 2006 | 894205.027 | 816741.431 | 77463.596 |
| | 2007 | 939571.282 | 851708.825 | 87862.457 |
| | 2008 | 983437.578 | 884907.264 | 98530.314 |
| | 2009 | 1025803.786 | 916467.348 | 109336.438 |
| | 2010 | 1066964.934 | 946696.625 | 120268.309 |
| | 2011 | 1106954.044 | 975639.602 | 131314.442 |
| | 2012 | 1146108.461 | 1003541.171 | 142567.290 |
| | 2013 | 1183544.689 | 1029960.880 | 153583.809 |
| | 2014 | 1221220.945 | 1056294.235 | 164926.711 |
| | 2015 | 1258289.311 | 1081932.243 | 176357.069 |
| | 2016 | 1294830.207 | 1107044.132 | 187786.076 |
| | 2017 | 1330332.626 | 1131327.913 | 199004.713 |
| | 2018 | 1360571.987 | 1151996.946 | 208575.041 |
| | 2019 | 1389848.723 | 1172014.884 | 217833.838 |
| | 2020 | 1416046.192 | 1189752.231 | 226293.961 |
| | 2021 | 1440588.850 | 1206428.538 | 234160.311 |
| | 2022 | 1464157.934 | 1222518.946 | 241638.988 |

## Results for PHIM for Run 2 (ELL Summary Pessimistic_Analysis.RTF)

| | | | | | |
|---|---|---|---|---|---|
| | | 2023 | 1486810.237 | 1238137.837 | 248672.400 |
| | | 2024 | 1508591.729 | 1253291.393 | 255300.336 |
| | | 2025 | 1526875.621 | 1265891.754 | 260983.867 |
| | | 2026 | 1543659.810 | 1277650.728 | 266009.082 |
| | | 2027 | 1559623.774 | 1289023.281 | 270600.494 |
| | | 2028 | 1574852.850 | 1300096.959 | 274755.891 |
| | | 2029 | 1589437.452 | 1310916.273 | 278521.179 |
| | | 2030 | 1600568.199 | 1319047.741 | 281520.458 |
| | | 2031 | 1610776.297 | 1326728.518 | 284047.779 |
| | | 2032 | 1620483.563 | 1334215.040 | 286268.523 |
| | | 2033 | 1629745.442 | 1341593.177 | 288152.265 |
| | | 2034 | 1638721.425 | 1348901.494 | 289819.931 |
| | | 2035 | 1644649.404 | 1353617.930 | 291031.475 |
| | | 2036 | 1650181.590 | 1358154.628 | 292026.962 |
| | | 2037 | 1655476.066 | 1362643.104 | 292832.962 |
| | | 2038 | 1660507.914 | 1367092.086 | 293415.828 |
| | | 2039 | 1665361.569 | 1371516.889 | 293844.681 |
| COPD | Males | 1990 | 117002.906 | 117002.906 | 0.000 |
| | | 1991 | 233438.833 | 233379.900 | 58.932 |
| | | 1992 | 348084.047 | 347870.183 | 213.864 |
| | | 1993 | 467278.318 | 466752.168 | 526.150 |
| | | 1994 | 584216.907 | 583160.805 | 1056.102 |
| | | 1995 | 697391.457 | 695543.229 | 1848.227 |
| | | 1996 | 809471.271 | 806501.863 | 2969.408 |
| | | 1997 | 922587.668 | 918093.674 | 4493.993 |
| | | 1998 | 1034072.457 | 1027687.487 | 6384.970 |
| | | 1999 | 1153475.399 | 1144560.264 | 8915.135 |
| | | 2000 | 1268050.268 | 1256095.359 | 11954.909 |
| | | 2001 | 1382786.209 | 1367096.882 | 15689.328 |
| | | 2002 | 1497557.412 | 1477371.536 | 20185.876 |
| | | 2003 | 1614405.229 | 1588982.269 | 25422.961 |
| | | 2004 | 1726672.489 | 1695433.337 | 31239.152 |
| | | 2005 | 1847277.524 | 1809056.606 | 38220.918 |
| | | 2006 | 1961009.963 | 1915268.115 | 45741.848 |
| | | 2007 | 2079868.908 | 2025311.787 | 54557.122 |
| | | 2008 | 2206755.783 | 2141853.208 | 64902.575 |
| | | 2009 | 2331993.380 | 2255877.900 | 76115.480 |
| | | 2010 | 2455130.176 | 2367121.075 | 88009.101 |
| | | 2011 | 2582657.849 | 2481204.691 | 101453.158 |
| | | 2012 | 2709719.005 | 2593746.393 | 115972.612 |
| | | 2013 | 2840547.005 | 2708424.889 | 132122.117 |
| | | 2014 | 2971677.135 | 2822312.696 | 149364.440 |
| | | 2015 | 3104538.983 | 2936887.648 | 167651.335 |
| | | 2016 | 3237650.175 | 3050486.142 | 187164.033 |
| | | 2017 | 3370561.253 | 3162782.890 | 207778.363 |
| | | 2018 | 3503136.371 | 3273603.531 | 229532.841 |
| | | 2019 | 3636402.758 | 3384017.372 | 252385.386 |
| | | 2020 | 3768054.453 | 3492213.639 | 275840.813 |
| | | 2021 | 3897675.067 | 3597909.689 | 299765.378 |
| | | 2022 | 4027307.132 | 3702702.180 | 324604.952 |
| | | 2023 | 4156698.720 | 3806512.747 | 350185.973 |
| | | 2024 | 4285772.226 | 3909355.627 | 376416.598 |
| | | 2025 | 4410759.298 | 4008315.539 | 402443.759 |
| | | 2026 | 4531411.064 | 4103311.386 | 428099.678 |
| | | 2027 | 4651145.233 | 4197153.200 | 453992.033 |
| | | 2028 | 4769837.872 | 4289885.918 | 479951.954 |
| | | 2029 | 4887747.179 | 4381770.725 | 505976.454 |
| | | 2030 | 4997632.895 | 4467021.946 | 530610.949 |
| | | 2031 | 5103301.991 | 4548878.541 | 554423.450 |
| | | 2032 | 5207593.613 | 4629856.110 | 577737.503 |
| | | 2033 | 5310556.138 | 4709983.423 | 600572.714 |
| | | 2034 | 5412542.816 | 4789790.540 | 622752.276 |
| | | 2035 | 5500522.473 | 4858381.805 | 642140.668 |
| | | 2036 | 5588149.640 | 4927222.730 | 660926.910 |
| | | 2037 | 5674667.886 | 4995799.173 | 678868.713 |
| | | 2038 | 5760391.289 | 5064296.447 | 696094.841 |
| | | 2039 | 5845459.234 | 5133273.128 | 712186.106 |
| | Female | 1990 | 77434.846 | 77434.846 | 0.000 |
| | | 1991 | 160029.510 | 159990.437 | 39.074 |
| | | 1992 | 240642.531 | 240487.892 | 154.639 |
| | | 1993 | 329362.093 | 328946.011 | 416.083 |
| | | 1994 | 418569.515 | 417705.680 | 863.835 |
| | | 1995 | 509110.357 | 507521.555 | 1588.802 |
| | | 1996 | 600040.117 | 597444.084 | 2596.033 |
| | | 1997 | 691159.071 | 687203.680 | 3955.391 |
| | | 1998 | 782866.899 | 777184.425 | 5682.474 |
| | | 1999 | 881526.925 | 873517.108 | 8009.817 |
| | | 2000 | 977880.647 | 967139.937 | 10740.711 |

## Results for PHIM for Run 2 (ELL Summary Pessimistic_Analysis.RTF)

| | | | |
|---|---|---|---|
| 2001 | 1076787.404 | 1062678.786 | 14108.618 |
| 2002 | 1174842.754 | 1156778.611 | 18064.143 |
| 2003 | 1275102.681 | 1252419.393 | 22683.288 |
| 2004 | 1371026.306 | 1343336.642 | 27689.664 |
| 2005 | 1473954.509 | 1440140.064 | 33814.445 |
| 2006 | 1574450.828 | 1533899.736 | 40551.092 |
| 2007 | 1673790.211 | 1625845.181 | 47945.030 |
| 2008 | 1782360.738 | 1725503.809 | 56856.928 |
| 2009 | 1892944.209 | 1826053.171 | 66891.039 |
| 2010 | 2002131.282 | 1924532.778 | 77598.504 |
| 2011 | 2114522.152 | 2024870.741 | 89651.412 |
| 2012 | 2228079.372 | 2125324.771 | 102754.601 |
| 2013 | 2345377.689 | 2228073.598 | 117304.091 |
| 2014 | 2462498.422 | 2329665.483 | 132832.939 |
| 2015 | 2581450.312 | 2432130.721 | 149319.591 |
| 2016 | 2701959.301 | 2534831.427 | 167127.874 |
| 2017 | 2823834.967 | 2637720.830 | 186114.137 |
| 2018 | 2938265.081 | 2733755.333 | 204509.748 |
| 2019 | 3053254.880 | 2829432.886 | 223821.994 |
| 2020 | 3167073.216 | 2923412.858 | 243660.357 |
| 2021 | 3279517.699 | 3015666.188 | 263851.511 |
| 2022 | 3392060.964 | 3107183.754 | 284877.210 |
| 2023 | 3504296.171 | 3197851.169 | 306445.002 |
| 2024 | 3616019.231 | 3287518.876 | 328500.355 |
| 2025 | 3724611.269 | 3374077.265 | 350534.004 |
| 2026 | 3827181.707 | 3455634.036 | 371547.671 |
| 2027 | 3928684.441 | 3535905.572 | 392778.869 |
| 2028 | 4029124.892 | 3615136.735 | 413988.158 |
| 2029 | 4128269.068 | 3693149.364 | 435119.704 |
| 2030 | 4221560.174 | 3766208.406 | 455351.768 |
| 2031 | 4304170.584 | 3830959.615 | 473210.970 |
| 2032 | 4384930.537 | 3894260.719 | 490669.818 |
| 2033 | 4464357.691 | 3956703.767 | 507653.924 |
| 2034 | 4542251.545 | 4018231.703 | 524019.841 |
| 2035 | 4611066.648 | 4072475.553 | 538591.095 |
| 2036 | 4670309.230 | 4119631.637 | 550677.593 |
| 2037 | 4728179.514 | 4166128.269 | 562051.245 |
| 2038 | 4785008.048 | 4212468.624 | 572539.424 |
| 2039 | 4841291.793 | 4259001.877 | 582289.915 |

# Results for PHIM for Run 2 (ELL Summary Pessimistic_Analysis.RTF)

*************************************************************************************

**Table 5: Estimated Loss of Life, at the end of follow-up, Population unadjusted for deaths in Ecig scenario**

| Disease | Sex | Age Group | Years of life lost NULL | Years of life lost Ecig | Years of life Saved |
|---|---|---|---|---|---|
| All Diseases | Males | 30-34 | 443911.034 | 375714.313 | 68196.720 |
| | | 35-39 | 1146798.555 | 966951.329 | 179847.226 |
| | | 40-44 | 2609591.408 | 2190451.513 | 419139.895 |
| | | 45-49 | 4775841.753 | 4003538.532 | 772303.221 |
| | | 50-54 | 7446969.205 | 6309468.925 | 1137500.28 |
| | | 55-59 | 8240205.925 | 7117767.847 | 1122438.08 |
| | | 60-64 | 8772829.118 | 7699950.326 | 1072878.79 |
| | | 65-69 | 6161494.678 | 5554521.986 | 606972.692 |
| | | 70-74 | 2495906.274 | 2283076.153 | 212830.120 |
| | | 75-79 | 0.000 | 0.000 | 0.000 |
| | Female | 30-34 | 229140.561 | 201838.358 | 27302.203 |
| | | 35-39 | 570217.942 | 498986.428 | 71231.514 |
| | | 40-44 | 1267538.938 | 1102790.874 | 164748.064 |
| | | 45-49 | 2340070.698 | 2035727.180 | 304343.518 |
| | | 50-54 | 3743803.613 | 3270929.125 | 472874.488 |
| | | 55-59 | 4591478.168 | 4060430.894 | 531047.274 |
| | | 60-64 | 5207367.788 | 4625493.137 | 581874.650 |
| | | 65-69 | 4092181.394 | 3692652.702 | 399528.692 |
| | | 70-74 | 1776092.171 | 1615984.313 | 160107.858 |
| | | 75-79 | 0.000 | 0.000 | 0.000 |
| Lung cancer | Males | 30-34 | 66065.017 | 62447.790 | 3617.228 |
| | | 35-39 | 232509.414 | 220041.730 | 12467.684 |
| | | 40-44 | 688628.041 | 648789.215 | 39838.826 |
| | | 45-49 | 1528300.367 | 1430409.886 | 97890.482 |
| | | 50-54 | 2730187.229 | 2547439.495 | 182747.734 |
| | | 55-59 | 3909101.673 | 3645942.383 | 263159.290 |
| | | 60-64 | 4287716.660 | 4008286.522 | 279430.138 |
| | | 65-69 | 3467897.115 | 3254635.855 | 213261.260 |
| | | 70-74 | 1312144.343 | 1239574.071 | 72570.272 |
| | | 75-79 | 0.000 | 0.000 | 0.000 |
| | Female | 30-34 | 51913.113 | 49204.937 | 2708.176 |
| | | 35-39 | 189792.904 | 179504.453 | 10288.451 |
| | | 40-44 | 527596.573 | 494565.039 | 33031.534 |
| | | 45-49 | 1122524.269 | 1044666.406 | 77857.863 |
| | | 50-54 | 1882214.322 | 1744847.660 | 137366.662 |
| | | 55-59 | 2595581.943 | 2403314.583 | 192267.360 |
| | | 60-64 | 2846273.090 | 2634005.717 | 212267.372 |
| | | 65-69 | 2358758.394 | 2190048.276 | 168710.118 |
| | | 70-74 | 937814.238 | 875930.652 | 61883.585 |
| | | 75-79 | 0.000 | 0.000 | 0.000 |
| IHD | Males | 30-34 | 263834.566 | 215372.781 | 48461.785 |
| | | 35-39 | 692140.150 | 557555.898 | 134584.253 |
| | | 40-44 | 1519708.432 | 1203399.181 | 316309.251 |
| | | 45-49 | 2568546.697 | 2004301.730 | 564244.967 |
| | | 50-54 | 3632929.903 | 2857036.822 | 775893.082 |
| | | 55-59 | 2807892.356 | 2189053.194 | 618839.162 |
| | | 60-64 | 2599825.934 | 2083694.874 | 516131.059 |
| | | 65-69 | 1113578.307 | 917959.439 | 195618.868 |
| | | 70-74 | 437536.271 | 377758.660 | 59777.610 |
| | | 75-79 | 0.000 | 0.000 | 0.000 |
| | Female | 30-34 | 79649.097 | 66374.850 | 13274.247 |
| | | 35-39 | 193965.908 | 157033.521 | 36932.387 |
| | | 40-44 | 403380.974 | 319497.935 | 83883.039 |
| | | 45-49 | 671385.408 | 526342.880 | 145042.528 |
| | | 50-54 | 1011875.989 | 802613.315 | 209262.674 |
| | | 55-59 | 849875.715 | 674373.378 | 175502.338 |
| | | 60-64 | 925490.876 | 754260.361 | 171230.515 |
| | | 65-69 | 455463.881 | 383607.404 | 71856.478 |
| | | 70-74 | 207681.216 | 181603.016 | 26078.200 |
| | | 75-79 | 0.000 | 0.000 | 0.000 |
| Stroke | Males | 30-34 | 73463.650 | 62326.949 | 11136.701 |
| | | 35-39 | 151934.057 | 127582.332 | 24351.725 |
| | | 40-44 | 262845.974 | 216954.578 | 45891.396 |
| | | 45-49 | 383421.246 | 310908.685 | 72512.561 |
| | | 50-54 | 479872.634 | 381700.024 | 98172.610 |
| | | 55-59 | 437124.903 | 342138.390 | 94986.514 |
| | | 60-64 | 419457.235 | 330911.881 | 88545.354 |
| | | 65-69 | 128180.574 | 102641.171 | 25539.403 |
| | | 70-74 | 53245.965 | 45019.100 | 8226.865 |
| | | 75-79 | 0.000 | 0.000 | 0.000 |
| | Female | 30-34 | 62919.090 | 55104.294 | 7814.796 |

## Results for PHIM for Run 2 (ELL Summary Pessimistic_Analysis.RTF)

| | | | | | |
|---|---|---|---|---|---|
| | | 35-39 | 120806.205 | 103732.523 | 17073.682 |
| | | 40-44 | 203391.885 | 170514.335 | 32877.550 |
| | | 45-49 | 278172.097 | 228948.433 | 49223.664 |
| | | 50-54 | 325979.181 | 264813.689 | 61165.492 |
| | | 55-59 | 275161.340 | 221115.046 | 54046.294 |
| | | 60-64 | 268920.493 | 217833.927 | 51086.566 |
| | | 65-69 | 87956.054 | 72664.757 | 15291.296 |
| | | 70-74 | 42055.224 | 35923.418 | 6131.806 |
| | | 75-79 | 0.000 | 0.000 | 0.000 |
| COPD | Males | 30-34 | 40547.801 | 35566.793 | 4981.008 |
| | | 35-39 | 70214.933 | 61771.369 | 8443.565 |
| | | 40-44 | 138408.960 | 121308.539 | 17100.421 |
| | | 45-49 | 295573.443 | 257918.231 | 37655.212 |
| | | 50-54 | 603979.438 | 523292.584 | 80686.854 |
| | | 55-59 | 1086086.992 | 940633.880 | 145453.112 |
| | | 60-64 | 1465829.290 | 1277057.049 | 188772.241 |
| | | 65-69 | 1451838.682 | 1279285.521 | 172553.161 |
| | | 70-74 | 692979.695 | 620724.321 | 72255.374 |
| | | 75-79 | 0.000 | 0.000 | 0.000 |
| | Female | 30-34 | 34659.261 | 31154.277 | 3504.984 |
| | | 35-39 | 65652.924 | 58715.931 | 6936.993 |
| | | 40-44 | 133169.505 | 118213.565 | 14955.940 |
| | | 45-49 | 267988.924 | 235769.461 | 32219.463 |
| | | 50-54 | 523734.121 | 458654.460 | 65079.660 |
| | | 55-59 | 870859.170 | 761627.887 | 109231.282 |
| | | 60-64 | 1166683.329 | 1019393.132 | 147290.197 |
| | | 65-69 | 1190003.066 | 1046332.265 | 143670.800 |
| | | 70-74 | 588541.493 | 522527.227 | 66014.267 |
| | | 75-79 | 0.000 | 0.000 | 0.000 |

## Results for PHIM for Run 2 (ELL Summary Pessimistic_Analysis.RTF)

*************************************************************************************

**Table 6: Estimated Loss of Life, at the end of follow-up, Population and Number deaths adjusted for Ecig scenario**

| Disease | Sex | Age Group | Years of life lost NULL | Years of life lost Ecig | Years of life Saved |
|---|---|---|---|---|---|
| All Diseases | Males | 30-34 | 443911.034 | 375731.167 | 68179.867 |
| | | 35-39 | 1146798.555 | 967081.754 | 179716.801 |
| | | 40-44 | 2609591.408 | 2191293.860 | 418297.548 |
| | | 45-49 | 4775841.753 | 4007165.135 | 768676.618 |
| | | 50-54 | 7446969.205 | 6319900.081 | 1127069.12 |
| | | 55-59 | 8240205.925 | 7133021.341 | 1107184.58 |
| | | 60-64 | 8772829.118 | 7721783.482 | 1051045.64 |
| | | 65-69 | 6161494.678 | 5569414.801 | 592079.877 |
| | | 70-74 | 2495906.274 | 2289879.759 | 206026.514 |
| | | 75-79 | 0.000 | 0.000 | 0.000 |
| | Female | 30-34 | 229140.561 | 201841.798 | 27298.763 |
| | | 35-39 | 570217.942 | 499012.531 | 71205.411 |
| | | 40-44 | 1267538.938 | 1102955.049 | 164583.889 |
| | | 45-49 | 2340070.698 | 2036437.365 | 303633.333 |
| | | 50-54 | 3743803.613 | 3273154.781 | 470648.832 |
| | | 55-59 | 4591478.168 | 4064511.539 | 526966.629 |
| | | 60-64 | 5207367.788 | 4632328.621 | 575039.167 |
| | | 65-69 | 4092181.394 | 3698692.902 | 393488.492 |
| | | 70-74 | 1776092.171 | 1619329.983 | 156762.187 |
| | | 75-79 | 0.000 | 0.000 | 0.000 |
| Lung cancer | Males | 30-34 | 66065.017 | 62450.117 | 3614.900 |
| | | 35-39 | 232509.414 | 220067.106 | 12442.308 |
| | | 40-44 | 688628.041 | 649009.921 | 39618.120 |
| | | 45-49 | 1528300.367 | 1431590.356 | 96710.011 |
| | | 50-54 | 2730187.229 | 2551283.518 | 178903.711 |
| | | 55-59 | 3909101.673 | 3653217.503 | 255884.170 |
| | | 60-64 | 4287716.660 | 4019004.571 | 268712.089 |
| | | 65-69 | 3467897.115 | 3262881.237 | 205015.877 |
| | | 70-74 | 1312144.343 | 1243089.342 | 69055.001 |
| | | 75-79 | 0.000 | 0.000 | 0.000 |
| | Female | 30-34 | 51913.113 | 49205.721 | 2707.391 |
| | | 35-39 | 189792.904 | 179512.991 | 10279.914 |
| | | 40-44 | 527596.573 | 494635.193 | 32961.380 |
| | | 45-49 | 1122524.269 | 1045017.792 | 77506.477 |
| | | 50-54 | 1882214.322 | 1745999.380 | 136214.942 |
| | | 55-59 | 2595581.943 | 2405671.928 | 189910.015 |
| | | 60-64 | 2846273.090 | 2637823.586 | 208449.504 |
| | | 65-69 | 2358758.394 | 2193513.376 | 165245.018 |
| | | 70-74 | 937814.238 | 877685.810 | 60128.428 |
| | | 75-79 | 0.000 | 0.000 | 0.000 |
| IHD | Males | 30-34 | 263834.566 | 215383.101 | 48451.465 |
| | | 35-39 | 692140.150 | 557635.204 | 134504.947 |
| | | 40-44 | 1519708.432 | 1203887.699 | 315820.733 |
| | | 45-49 | 2568546.697 | 2006199.949 | 562346.748 |
| | | 50-54 | 3632929.903 | 2861912.000 | 771017.903 |
| | | 55-59 | 2807892.356 | 2193661.285 | 614231.072 |
| | | 60-64 | 2599825.934 | 2089073.161 | 510752.773 |
| | | 65-69 | 1113578.307 | 919894.997 | 193683.311 |
| | | 70-74 | 437536.271 | 378548.640 | 58987.631 |
| | | 75-79 | 0.000 | 0.000 | 0.000 |
| | Female | 30-34 | 79649.097 | 66376.117 | 13272.979 |
| | | 35-39 | 193965.908 | 157042.801 | 36923.107 |
| | | 40-44 | 403380.974 | 319549.823 | 83831.151 |
| | | 45-49 | 671385.408 | 526535.393 | 144850.015 |
| | | 50-54 | 1011875.989 | 803160.585 | 208715.404 |
| | | 55-59 | 849875.715 | 674990.298 | 174885.417 |
| | | 60-64 | 925490.876 | 755171.297 | 170319.580 |
| | | 65-69 | 455463.881 | 384028.625 | 71435.257 |
| | | 70-74 | 207681.216 | 181825.088 | 25856.128 |
| | | 75-79 | 0.000 | 0.000 | 0.000 |
| Stroke | Males | 30-34 | 73463.650 | 62329.484 | 11134.165 |
| | | 35-39 | 151934.057 | 127598.676 | 24335.380 |
| | | 40-44 | 262845.974 | 217032.801 | 45813.174 |
| | | 45-49 | 383421.246 | 311173.630 | 72247.616 |
| | | 50-54 | 479872.634 | 382314.149 | 97558.485 |
| | | 55-59 | 437124.903 | 342867.851 | 94257.052 |
| | | 60-64 | 419457.235 | 331859.368 | 87597.866 |
| | | 65-69 | 128180.574 | 102896.570 | 25284.004 |
| | | 70-74 | 53245.965 | 45136.017 | 8109.948 |
| | | 75-79 | 0.000 | 0.000 | 0.000 |
| | Female | 30-34 | 62919.090 | 55105.123 | 7813.967 |

## Results for PHIM for Run 2 (ELL Summary Pessimistic_Analysis.RTF)

| | | | | | |
|---|---|---|---|---|---|
| | | 35-39 | 120806.205 | 103737.427 | 17068.778 |
| | | 40-44 | 203391.885 | 170536.781 | 32855.105 |
| | | 45-49 | 278172.097 | 229018.954 | 49153.143 |
| | | 50-54 | 325979.181 | 264972.934 | 61006.247 |
| | | 55-59 | 275161.340 | 221307.765 | 53853.575 |
| | | 60-64 | 268920.493 | 218107.258 | 50813.235 |
| | | 65-69 | 87956.054 | 72754.078 | 15201.976 |
| | | 70-74 | 42055.224 | 35976.568 | 6078.655 |
| | | 75-79 | 0.000 | 0.000 | 0.000 |
| COPD | Males | 30-34 | 40547.801 | 35568.464 | 4979.337 |
| | | 35-39 | 70214.933 | 61780.768 | 8434.165 |
| | | 40-44 | 138408.960 | 121363.439 | 17045.521 |
| | | 45-49 | 295573.443 | 258201.200 | 37372.243 |
| | | 50-54 | 603979.438 | 524390.413 | 79589.025 |
| | | 55-59 | 1086086.992 | 943274.703 | 142812.289 |
| | | 60-64 | 1465829.290 | 1281846.382 | 183982.908 |
| | | 65-69 | 1451838.682 | 1283741.997 | 168096.685 |
| | | 70-74 | 692979.695 | 623105.760 | 69873.934 |
| | | 75-79 | 0.000 | 0.000 | 0.000 |
| | Female | 30-34 | 34659.261 | 31154.836 | 3504.425 |
| | | 35-39 | 65652.924 | 58719.312 | 6933.612 |
| | | 40-44 | 133169.505 | 118233.252 | 14936.253 |
| | | 45-49 | 267988.924 | 235865.226 | 32123.698 |
| | | 50-54 | 523734.121 | 459021.882 | 64712.239 |
| | | 55-59 | 870859.170 | 762541.548 | 108317.622 |
| | | 60-64 | 1166683.329 | 1021226.480 | 145456.849 |
| | | 65-69 | 1190003.066 | 1048396.824 | 141606.241 |
| | | 70-74 | 588541.493 | 523842.517 | 64698.976 |
| | | 75-79 | 0.000 | 0.000 | 0.000 |

## Results for PHIM for Run 1 (Sensitivity_Analysis_1.RTF)

---

System Date and Time: 20210220T085818
User: JohnS
SAS Version: 9.4
Project: Population Health Impact Model 7.3

The System is running in Test Mode

Maximum Upper Age for P Component = 79
Upper Age for ELL Calculations = 75
Report in E-component: Social Classes A and B combined
Description: Sensitivity Analysis 1 for VeneBio, Change F value
Assumption Files:
Options Control File: C:\VeneBio\OG_Test\\US_Control_V_S1.CSV
TTP Test File: C:\VeneBio\OG_Test\\TTP_Test_MA.csv
TTP Reference File: C:\VeneBio\OG_Test\\TTP_Reference_V.csv
TTP Factor Test File: C:\VeneBio\OG_Test\\TTP_FACTOR_Test.csv
TTP Factor Reference File: C:\VeneBio\OG_Test\\TTP_FACTOR_Test.csv
TP Socioeconomic Groups: Not Used
Output Choices:C:\VeneBio\OG_Test\\OUTC_PA.csv

Main Results will be sent to the folder C:\VeneBio\Main_Results\
Plots will be sent to the folder C:\VeneBio\Plot_Results\Plots_20210220T085818
Saved P, E and/or A Component results will be sent to the folder C:\VeneBio\SAVED\Saved_20210220T085818\

# Table of Contents


Control File Details for Run: 1 ....217
TTP values for Run: 1, Scenario: NULL ....218
TTP values for Run: 1, Scenario: Ecig ....220
Main Output for the Tobacco History for Run: 1, Simulation: 1, Scenario: NULL ....224
Main Output for the Tobacco History for Run: 1, Simulation: 1, Scenario: Ecig ....226
Main Results for E-Component for Run: 1, Simulation: 1, COD: Four Diseases Combined ....242
Main Results for E-Component for Run: 1, Simulation: 1, COD: LUNG CANCER ....243
Main Results for E-Component for Run: 1, Simulation: 1, COD: IHD ....244
Main Results for E-Component for Run: 1, Simulation: 1, COD: STROKE ....245
Main Results for E-Component for Run: 1, Simulation: 1, COD: COPD ....246
Main Results for E-Component for Run: 1, Simulation: 1, COD: Four Diseases Combined, Adjusted ....247
Main Results for E-Component for Run: 1, Simulation: 1, COD: LUNG CANCER, Adjusted ....248
Main Results for E-Component for Run: 1, Simulation: 1, COD: IHD, Adjusted ....249
Main Results for E-Component for Run: 1, Simulation: 1, COD: STROKE, Adjusted ....250
Main Results for E-Component for Run: 1, Simulation: 1, COD: COPD, Adjusted ....251
Control File Details for Run: 2 ....252
TTP values for Run: 2, Scenario: NULL ....253
TTP values for Run: 2, Scenario: Ecig ....255
Main Output for the Tobacco History for Run: 2, Simulation: 1, Scenario: NULL ....259
Main Output for the Tobacco History for Run: 2, Simulation: 1, Scenario: Ecig ....261
Main Results for E-Component for Run: 2, Simulation: 1, COD: Four Diseases Combined ....277
Main Results for E-Component for Run: 2, Simulation: 1, COD: LUNG CANCER ....278
Main Results for E-Component for Run: 2, Simulation: 1, COD: IHD ....279
Main Results for E-Component for Run: 2, Simulation: 1, COD: STROKE ....280
Main Results for E-Component for Run: 2, Simulation: 1, COD: COPD ....281
Main Results for E-Component for Run: 2, Simulation: 1, COD: Four Diseases Combined, Adjusted ....282
Main Results for E-Component for Run: 2, Simulation: 1, COD: LUNG CANCER, Adjusted ....283
Main Results for E-Component for Run: 2, Simulation: 1, COD: IHD, Adjusted ....284
Main Results for E-Component for Run: 2, Simulation: 1, COD: STROKE, Adjusted ....285
Main Results for E-Component for Run: 2, Simulation: 1, COD: COPD, Adjusted ....286
Control File Details for Run: 3 ....287
TTP values for Run: 3, Scenario: NULL ....288
TTP values for Run: 3, Scenario: Ecig ....290
Main Output for the Tobacco History for Run: 3, Simulation: 1, Scenario: NULL ....294
Main Output for the Tobacco History for Run: 3, Simulation: 1, Scenario: Ecig ....296
Main Results for E-Component for Run: 3, Simulation: 1, COD: Four Diseases Combined ....312
Main Results for E-Component for Run: 3, Simulation: 1, COD: LUNG CANCER ....313
Main Results for E-Component for Run: 3, Simulation: 1, COD: IHD ....314
Main Results for E-Component for Run: 3, Simulation: 1, COD: STROKE ....315
Main Results for E-Component for Run: 3, Simulation: 1, COD: COPD ....316
Main Results for E-Component for Run: 3, Simulation: 1, COD: Four Diseases Combined, Adjusted ....317
Main Results for E-Component for Run: 3, Simulation: 1, COD: LUNG CANCER, Adjusted ....318
Main Results for E-Component for Run: 3, Simulation: 1, COD: IHD, Adjusted ....319
Main Results for E-Component for Run: 3, Simulation: 1, COD: STROKE, Adjusted ....320
Main Results for E-Component for Run: 3, Simulation: 1, COD: COPD, Adjusted ....321
Control File Details for Run: 4 ....322
TTP values for Run: 4, Scenario: NULL ....323

## Results for PHIM for Run 1 (Sensitivity_Analysis_1.RTF)


TTP values for Run: 4, Scenario: Ecig....325
Main Output for the Tobacco History for Run: 4, Simulation: 1, Scenario: NULL....329
Main Output for the Tobacco History for Run: 4, Simulation: 1, Scenario: Ecig....331
Main Results for E-Component for Run: 4, Simulation: 1, COD: Four Diseases Combined....347
Main Results for E-Component for Run: 4, Simulation: 1, COD: LUNG CANCER....348
Main Results for E-Component for Run: 4, Simulation: 1, COD: IHD....349
Main Results for E-Component for Run: 4, Simulation: 1, COD: STROKE....350
Main Results for E-Component for Run: 4, Simulation: 1, COD: COPD....351
Main Results for E-Component for Run: 4, Simulation: 1, COD: Four Diseases Combined, Adjusted....352
Main Results for E-Component for Run: 4, Simulation: 1, COD: LUNG CANCER, Adjusted....353
Main Results for E-Component for Run: 4, Simulation: 1, COD: IHD, Adjusted....354
Main Results for E-Component for Run: 4, Simulation: 1, COD: STROKE, Adjusted....355
Main Results for E-Component for Run: 4, Simulation: 1, COD: COPD, Adjusted....356
Control File Details for Run: 5....357
TTP values for Run: 5, Scenario: NULL....358
TTP values for Run: 5, Scenario: Ecig....360
Main Output for the Tobacco History for Run: 5, Simulation: 1, Scenario: NULL....364
Main Output for the Tobacco History for Run: 5, Simulation: 1, Scenario: Ecig....366
Main Results for E-Component for Run: 5, Simulation: 1, COD: Four Diseases Combined....382
Main Results for E-Component for Run: 5, Simulation: 1, COD: LUNG CANCER....383
Main Results for E-Component for Run: 5, Simulation: 1, COD: IHD....384
Main Results for E-Component for Run: 5, Simulation: 1, COD: STROKE....385
Main Results for E-Component for Run: 5, Simulation: 1, COD: COPD....386
Main Results for E-Component for Run: 5, Simulation: 1, COD: Four Diseases Combined, Adjusted....387
Main Results for E-Component for Run: 5, Simulation: 1, COD: LUNG CANCER, Adjusted....388
Main Results for E-Component for Run: 5, Simulation: 1, COD: IHD, Adjusted....389
Main Results for E-Component for Run: 5, Simulation: 1, COD: STROKE, Adjusted....390
Main Results for E-Component for Run: 5, Simulation: 1, COD: COPD, Adjusted....391
Control File Details for Run: 6....392
TTP values for Run: 6, Scenario: NULL....393
TTP values for Run: 6, Scenario: Ecig....395
Main Output for the Tobacco History for Run: 6, Simulation: 1, Scenario: NULL....399
Main Output for the Tobacco History for Run: 6, Simulation: 1, Scenario: Ecig....401
Main Results for E-Component for Run: 6, Simulation: 1, COD: Four Diseases Combined....417
Main Results for E-Component for Run: 6, Simulation: 1, COD: LUNG CANCER....418
Main Results for E-Component for Run: 6, Simulation: 1, COD: IHD....419
Main Results for E-Component for Run: 6, Simulation: 1, COD: STROKE....420
Main Results for E-Component for Run: 6, Simulation: 1, COD: COPD....421
Main Results for E-Component for Run: 6, Simulation: 1, COD: Four Diseases Combined, Adjusted....422
Main Results for E-Component for Run: 6, Simulation: 1, COD: LUNG CANCER, Adjusted....423
Main Results for E-Component for Run: 6, Simulation: 1, COD: IHD, Adjusted....424
Main Results for E-Component for Run: 6, Simulation: 1, COD: STROKE, Adjusted....425
Main Results for E-Component for Run: 6, Simulation: 1, COD: COPD, Adjusted....426
Control File Details for Run: 7....427
TTP values for Run: 7, Scenario: NULL....428
TTP values for Run: 7, Scenario: Ecig....430
Main Output for the Tobacco History for Run: 7, Simulation: 1, Scenario: NULL....434
Main Output for the Tobacco History for Run: 7, Simulation: 1, Scenario: Ecig....436
Main Results for E-Component for Run: 7, Simulation: 1, COD: Four Diseases Combined....452
Main Results for E-Component for Run: 7, Simulation: 1, COD: LUNG CANCER....453
Main Results for E-Component for Run: 7, Simulation: 1, COD: IHD....454
Main Results for E-Component for Run: 7, Simulation: 1, COD: STROKE....455
Main Results for E-Component for Run: 7, Simulation: 1, COD: COPD....456
Main Results for E-Component for Run: 7, Simulation: 1, COD: Four Diseases Combined, Adjusted....457
Main Results for E-Component for Run: 7, Simulation: 1, COD: LUNG CANCER, Adjusted....458
Main Results for E-Component for Run: 7, Simulation: 1, COD: IHD, Adjusted....459
Main Results for E-Component for Run: 7, Simulation: 1, COD: STROKE, Adjusted....460
Main Results for E-Component for Run: 7, Simulation: 1, COD: COPD, Adjusted....461
Control File Details for Run: 8....462
TTP values for Run: 8, Scenario: NULL....463
TTP values for Run: 8, Scenario: Ecig....465
Main Output for the Tobacco History for Run: 8, Simulation: 1, Scenario: NULL....469
Main Output for the Tobacco History for Run: 8, Simulation: 1, Scenario: Ecig....471
Main Results for E-Component for Run: 8, Simulation: 1, COD: Four Diseases Combined....487
Main Results for E-Component for Run: 8, Simulation: 1, COD: LUNG CANCER....488
Main Results for E-Component for Run: 8, Simulation: 1, COD: IHD....489
Main Results for E-Component for Run: 8, Simulation: 1, COD: STROKE....490
Main Results for E-Component for Run: 8, Simulation: 1, COD: COPD....491
Main Results for E-Component for Run: 8, Simulation: 1, COD: Four Diseases Combined, Adjusted....492
Main Results for E-Component for Run: 8, Simulation: 1, COD: LUNG CANCER, Adjusted....493
Main Results for E-Component for Run: 8, Simulation: 1, COD: IHD, Adjusted....494
Main Results for E-Component for Run: 8, Simulation: 1, COD: STROKE, Adjusted....495
Main Results for E-Component for Run: 8, Simulation: 1, COD: COPD, Adjusted....496

# Results for PHIM for Run 1 (Sensitivity_Analysis_1.RTF)


Control File Details for Run: 9.....497
TTP values for Run: 9, Scenario: NULL.....498
TTP values for Run: 9, Scenario: Ecig.....500
Main Output for the Tobacco History for Run: 9, Simulation: 1, Scenario: NULL.....504
Main Output for the Tobacco History for Run: 9, Simulation: 1, Scenario: Ecig.....506
Main Results for E-Component for Run: 9, Simulation: 1, COD: Four Diseases Combined.....522
Main Results for E-Component for Run: 9, Simulation: 1, COD: LUNG CANCER.....523
Main Results for E-Component for Run: 9, Simulation: 1, COD: IHD.....524
Main Results for E-Component for Run: 9, Simulation: 1, COD: STROKE.....525
Main Results for E-Component for Run: 9, Simulation: 1, COD: COPD.....526
Main Results for E-Component for Run: 9, Simulation: 1, COD: Four Diseases Combined, Adjusted.....527
Main Results for E-Component for Run: 9, Simulation: 1, COD: LUNG CANCER, Adjusted.....528
Main Results for E-Component for Run: 9, Simulation: 1, COD: IHD, Adjusted.....529
Main Results for E-Component for Run: 9, Simulation: 1, COD: STROKE, Adjusted.....530
Main Results for E-Component for Run: 9, Simulation: 1, COD: COPD, Adjusted.....531
Control File Details for Run: 10.....532
TTP values for Run: 10, Scenario: NULL.....533
TTP values for Run: 10, Scenario: Ecig.....535
Main Output for the Tobacco History for Run: 10, Simulation: 1, Scenario: NULL.....539
Main Output for the Tobacco History for Run: 10, Simulation: 1, Scenario: Ecig.....541
Main Results for E-Component for Run: 10, Simulation: 1, COD: Four Diseases Combined.....557
Main Results for E-Component for Run: 10, Simulation: 1, COD: LUNG CANCER.....558
Main Results for E-Component for Run: 10, Simulation: 1, COD: IHD.....559
Main Results for E-Component for Run: 10, Simulation: 1, COD: STROKE.....560
Main Results for E-Component for Run: 10, Simulation: 1, COD: COPD.....561
Main Results for E-Component for Run: 10, Simulation: 1, COD: Four Diseases Combined, Adjusted.....562
Main Results for E-Component for Run: 10, Simulation: 1, COD: LUNG CANCER, Adjusted.....563
Main Results for E-Component for Run: 10, Simulation: 1, COD: IHD, Adjusted.....564
Main Results for E-Component for Run: 10, Simulation: 1, COD: STROKE, Adjusted.....565
Main Results for E-Component for Run: 10, Simulation: 1, COD: COPD, Adjusted.....566
Estimated Loss of Life from the E Component for Run: 2, Simulation: 1.....567
Table 1: Estimated Loss of Life, at the end of follow-up, all age groups combined, Population unadjusted for deaths in Ecig scenario.....567
Table 2: Estimated Loss of Life, at the end of follow-up, all age groups combined, Population and Number deaths adjusted for Ecig scenario.....568
Table 3: Estimated Loss of Life, All age groups combined, Population unadjusted for deaths in Ecig scenario, cumulative over years.....569
Table 4: Estimated Loss of Life, All age groups combined, Population and Number deaths adjusted for Ecig scenario, cumulative over years.....576
Table 5: Estimated Loss of Life, at the end of follow-up, Population unadjusted for deaths in Ecig scenario.....583
Table 6: Estimated Loss of Life, at the end of follow-up, Population and Number deaths adjusted for Ecig scenario.....585
Estimated Loss of Life from the E Component for Run: 4, Simulation: 1.....587
Table 1: Estimated Loss of Life, at the end of follow-up, all age groups combined, Population unadjusted for deaths in Ecig scenario.....587
Table 2: Estimated Loss of Life, at the end of follow-up, all age groups combined, Population and Number deaths adjusted for Ecig scenario.....588
Table 3: Estimated Loss of Life, All age groups combined, Population unadjusted for deaths in Ecig scenario, cumulative over years.....589
Table 4: Estimated Loss of Life, All age groups combined, Population and Number deaths adjusted for Ecig scenario, cumulative over years.....596
Table 5: Estimated Loss of Life, at the end of follow-up, Population unadjusted for deaths in Ecig scenario.....603
Table 6: Estimated Loss of Life, at the end of follow-up, Population and Number deaths adjusted for Ecig scenario.....605
Estimated Loss of Life from the E Component for Run: 6, Simulation: 1.....607
Table 1: Estimated Loss of Life, at the end of follow-up, all age groups combined, Population unadjusted for deaths in Ecig scenario.....607
Table 2: Estimated Loss of Life, at the end of follow-up, all age groups combined, Population and Number deaths adjusted for Ecig scenario.....608
Table 3: Estimated Loss of Life, All age groups combined, Population unadjusted for deaths in Ecig scenario, cumulative over years.....609
Table 4: Estimated Loss of Life, All age groups combined, Population and Number deaths adjusted for Ecig scenario, cumulative over years.....616
Table 5: Estimated Loss of Life, at the end of follow-up, Population unadjusted for deaths in Ecig scenario.....623
Table 6: Estimated Loss of Life, at the end of follow-up, Population and Number deaths adjusted for Ecig scenario.....625
Estimated Loss of Life from the E Component for Run: 8, Simulation: 1.....627
Table 1: Estimated Loss of Life, at the end of follow-up, all age groups combined, Population unadjusted for deaths in Ecig scenario.....627
Table 2: Estimated Loss of Life, at the end of follow-up, all age groups combined, Population and Number deaths adjusted for Ecig scenario.....628
Table 3: Estimated Loss of Life, All age groups combined, Population unadjusted for deaths in Ecig scenario, cumulative over years.....629
Table 4: Estimated Loss of Life, All age groups combined, Population and Number deaths adjusted for Ecig scenario, cumulative over years.....636
Table 5: Estimated Loss of Life, at the end of follow-up, Population unadjusted for deaths in Ecig scenario.....643
Table 6: Estimated Loss of Life, at the end of follow-up, Population and Number deaths adjusted for Ecig scenario.....645
Estimated Loss of Life from the E Component for Run: 10, Simulation: 1.....647
Table 1: Estimated Loss of Life, at the end of follow-up, all age groups combined, Population unadjusted for deaths in Ecig scenario.....647
Table 2: Estimated Loss of Life, at the end of follow-up, all age groups combined, Population and Number deaths adjusted for Ecig scenario.....648
Table 3: Estimated Loss of Life, All age groups combined, Population unadjusted for deaths in Ecig scenario, cumulative over years.....649
Table 4: Estimated Loss of Life, All age groups combined, Population and Number deaths adjusted for Ecig scenario, cumulative over years.....656
Table 5: Estimated Loss of Life, at the end of follow-up, Population unadjusted for deaths in Ecig scenario.....663
Table 6: Estimated Loss of Life, at the end of follow-up, Population and Number deaths adjusted for Ecig scenario.....665

## Results for PHIM for Run 1 (Sensitivity_Analysis_1.RTF)

---

-------------------------------------------------------------------------------------

**Control File Details for Run: 1**

| Variable Parameter | Value |
|---|---|
| 1. Name for Reference Scenario | NULL |
| 2. Name for Test Scenario | Ecig |
| 3. Name for Product 1 | CC |
| 4. Name for Product 2 | Ecig |
| 5. Name for Product 3 | - |
| 6. Name for Product 4 | Dual |
| 7. Country | US (United States) |
| 8. Sex | M (Males) |
| 9. Year of start of process | 1990 |
| 10. Number of months of follow-up | 600 |
| 11. Follow-up interval length (in months) | 12 |
| 12. Lower age for risk estimation | 10 |
| 13. Upper age for risk estimation | 79 |
| 14. The effective dose for Product 1 (F1) - Ref | 1 |
| 15. The effective dose for Product 2 (F2) - Ref | - |
| 16. The effective dose for Product 3 (F3) - Ref | - |
| 17. The effective dose for Product 4 (F4) - Ref | - |
| 18. The effective dose for Product 1 (F1) - Test | 1 |
| 19. The effective dose for Product 2 (F2) - Test | 0.4 |
| 20. The effective dose for Product 3 (F3) - Test | - |
| 21. The effective dose for Product 4 (F4) - Test | FROMF2 |
| 22. Number in population to be simulated | 100000 |
| 23. Number of MC simulations | 1 |
| 24. The random number seed for the first simulation | 15975263 |
| 25. Source for the population file (POP) | UN4 |
| 26. Source for the socioeconomic prevalence file (SEP) | - |
| 27. Source for the current smoking prevalence file (CSP) | ISS3 |
| 28. Source for the former smoking prevalence file (FSP) | ISS3 |
| 29. Source for the quit-time distribution file (QTD) | NHIS2006 |
| 30. Source for the death file (MORT) | APR-20 |
| 31. Source for the relative risk file (RR) | PNLEST |
| 32. Source for the half-life file (H) | PNLEST |
| 33. Assumption Set for TTP Factor Reference | F1 |
| 34. Assumption Set for TTP Factor Test | F1 |
| 35. Assumption Set for TTP Reference | ISS3 |
| 36. Assumption Set for TTP Test | ISS3 |
| 37. Assumption Set for TP for socioeconomic group | - |
| 38. Source for Output Choice file (OUTC) | BASIC |
| 39. Output for DTP P1 | N |
| 40. Output for DTP P2 | N |
| 41. Output for Main P Component | Y |
| 42. Output for DTP E1 | N |
| 43. Output for DTP E2 | N |
| 44. Output for DTP E3 | N |
| 45. Output for Main E Component | Y |
| 46. Output file name | Sensitivity_Analysis_1 |
| 47. Run number for P-Component | - |
| 48. Results file for E-Component | - |
| 49. Components required | PE |
| 50. Results folder for P to be used in E-Component | - |
| 51. Fixed age of starting product use | 16 |
| 52. Output absolute risk file? | N |

# Results for PHIM for Run 1 (Sensitivity_Analysis_1.RTF)

************************************************************************************
**TTP values for Run: 1, Scenario: NULL**

| **Variable Parameter** | **Value** |
|---|---|
| 1. Country | US (United States) |
| 2. Sex | M (Males) |
| 3. Assumption Set | ISS3 |
| Note: | Period is in Months |

| Age | Period | **Initiation** Never CC | **Cessation** CC Former | **Re-initiation** Former CC |
|---|---|---|---|---|
| 10-14 | 1-12 | 0.003842 | 0.008742 | 0.000000 |
| 15-19 | 1-12 | 0.004720 | 0.003614 | 0.000000 |
| 20-24 | 1-12 | 0.000376 | 0.000943 | 0.000000 |
| 25-29 | 1-12 | 0.000187 | 0.001143 | 0.000000 |
| 30-34 | 1-12 | 0.000093 | 0.000871 | 0.000000 |
| 35-39 | 1-12 | 0.000000 | 0.000867 | 0.000000 |
| 40-44 | 1-12 | 0.000000 | 0.001399 | 0.000000 |
| 45-49 | 1-12 | 0.000000 | 0.002033 | 0.000000 |
| 50-54 | 1-12 | 0.000000 | 0.003209 | 0.000000 |
| 55-59 | 1-12 | 0.000000 | 0.003574 | 0.000000 |
| 60-64 | 1-12 | 0.000000 | 0.003460 | 0.000000 |
| 65-69 | 1-12 | 0.000000 | 0.004136 | 0.000000 |
| 70-74 | 1-12 | 0.000000 | 0.005592 | 0.000000 |
| 75-79 | 1-12 | 0.000000 | 0.005592 | 0.000000 |
| 10-14 | 13-72 | 0.004906 | 0.008742 | 0.000000 |
| 15-19 | 13-72 | 0.006989 | 0.004426 | 0.000000 |
| 20-24 | 13-72 | 0.000687 | 0.001527 | 0.000000 |
| 25-29 | 13-72 | 0.000962 | 0.001515 | 0.000000 |
| 30-34 | 13-72 | 0.000364 | 0.001227 | 0.000000 |
| 35-39 | 13-72 | 0.000000 | 0.001630 | 0.000000 |
| 40-44 | 13-72 | 0.000000 | 0.001852 | 0.000000 |
| 45-49 | 13-72 | 0.000000 | 0.002667 | 0.000000 |
| 50-54 | 13-72 | 0.000000 | 0.003208 | 0.000000 |
| 55-59 | 13-72 | 0.000000 | 0.003978 | 0.000000 |
| 60-64 | 13-72 | 0.000000 | 0.005771 | 0.000000 |
| 65-69 | 13-72 | 0.000000 | 0.010432 | 0.000000 |
| 70-74 | 13-72 | 0.000000 | 0.007079 | 0.000000 |
| 75-79 | 13-72 | 0.000000 | 0.007079 | 0.000000 |
| 10-14 | 73-132 | 0.004312 | 0.008742 | 0.000000 |
| 15-19 | 73-132 | 0.005330 | 0.003289 | 0.000000 |
| 20-24 | 73-132 | 0.002456 | 0.003844 | 0.000000 |
| 25-29 | 73-132 | 0.000839 | 0.002006 | 0.000000 |
| 30-34 | 73-132 | 0.000414 | 0.001329 | 0.000000 |
| 35-39 | 73-132 | 0.000000 | 0.001278 | 0.000000 |
| 40-44 | 73-132 | 0.000000 | 0.001691 | 0.000000 |
| 45-49 | 73-132 | 0.000000 | 0.002904 | 0.000000 |
| 50-54 | 73-132 | 0.000000 | 0.003319 | 0.000000 |
| 55-59 | 73-132 | 0.000000 | 0.003976 | 0.000000 |
| 60-64 | 73-132 | 0.000000 | 0.005235 | 0.000000 |
| 65-69 | 73-132 | 0.000000 | 0.005901 | 0.000000 |
| 70-74 | 73-132 | 0.000000 | 0.005615 | 0.000000 |
| 75-79 | 73-132 | 0.000000 | 0.005615 | 0.000000 |
| 10-14 | 133-192 | 0.003468 | 0.008742 | 0.000000 |
| 15-19 | 133-192 | 0.004675 | 0.003732 | 0.000000 |
| 20-24 | 133-192 | 0.000062 | 0.002260 | 0.000000 |
| 25-29 | 133-192 | 0.001422 | 0.002822 | 0.000000 |
| 30-34 | 133-192 | 0.000696 | 0.002610 | 0.000000 |
| 35-39 | 133-192 | 0.000000 | 0.002058 | 0.000000 |
| 40-44 | 133-192 | 0.000000 | 0.002310 | 0.000000 |
| 45-49 | 133-192 | 0.000000 | 0.002256 | 0.000000 |
| 50-54 | 133-192 | 0.000000 | 0.002663 | 0.000000 |
| 55-59 | 133-192 | 0.000000 | 0.001746 | 0.000000 |
| 60-64 | 133-192 | 0.000000 | 0.005809 | 0.000000 |
| 65-69 | 133-192 | 0.000000 | 0.005896 | 0.000000 |
| 70-74 | 133-192 | 0.000000 | 0.007323 | 0.000000 |
| 75-79 | 133-192 | 0.000000 | 0.007323 | 0.000000 |
| 10-14 | 193-252 | 0.002314 | 0.008742 | 0.000000 |
| 15-19 | 193-252 | 0.003987 | 0.005036 | 0.000000 |
| 20-24 | 193-252 | 0.000320 | 0.002857 | 0.000000 |
| 25-29 | 193-252 | 0.000251 | 0.002314 | 0.000000 |
| 30-34 | 193-252 | 0.000125 | 0.004343 | 0.000000 |
| 35-39 | 193-252 | 0.000000 | 0.002092 | 0.000000 |
| 40-44 | 193-252 | 0.000000 | 0.002566 | 0.000000 |
| 45-49 | 193-252 | 0.000000 | 0.002311 | 0.000000 |
| 50-54 | 193-252 | 0.000000 | 0.003209 | 0.000000 |

## Results for PHIM for Run 1 (Sensitivity_Analysis_1.RTF)

| | | | | |
|---|---|---|---|---|
| 55-59 | 193-252 | 0.000000 | 0.004057 | 0.000000 |
| 60-64 | 193-252 | 0.000000 | 0.007166 | 0.000000 |
| 65-69 | 193-252 | 0.000000 | 0.004281 | 0.000000 |
| 70-74 | 193-252 | 0.000000 | 0.004580 | 0.000000 |
| 75-79 | 193-252 | 0.000000 | 0.004580 | 0.000000 |
| 10-14 | 253+ | 0.001325 | 0.008742 | 0.000000 |
| 15-19 | 253+ | 0.002984 | 0.010602 | 0.000000 |
| 20-24 | 253+ | 0.000452 | 0.004314 | 0.000000 |
| 25-29 | 253+ | 0.000225 | 0.003645 | 0.000000 |
| 30-34 | 253+ | 0.000112 | 0.004512 | 0.000000 |
| 35-39 | 253+ | 0.000000 | 0.002147 | 0.000000 |
| 40-44 | 253+ | 0.000000 | 0.002636 | 0.000000 |
| 45-49 | 253+ | 0.000000 | 0.001051 | 0.000000 |
| 50-54 | 253+ | 0.000000 | 0.002519 | 0.000000 |
| 55-59 | 253+ | 0.000000 | 0.003693 | 0.000000 |
| 60-64 | 253+ | 0.000000 | 0.005888 | 0.000000 |
| 65-69 | 253+ | 0.000000 | 0.004823 | 0.000000 |
| 70-74 | 253+ | 0.000000 | 0.004733 | 0.000000 |
| 75-79 | 253+ | 0.000000 | 0.004733 | 0.000000 |

## Results for PHIM for Run 1 (Sensitivity_Analysis_1.RTF)

************************************************************************************
**TTP values for Run: 1, Scenario: Ecig**

| **Variable Parameter** | **Value** |
|---|---|
| 1. Country | US (United States) |
| 2. Sex | M (Males) |
| 3. Assumption Set | ISS3 |
| Note: | Period is in Months |

| | | **Initiation** | | | **Cessation** | | | **Re-initiation** | | |
|---|---|---|---|---|---|---|---|---|---|---|
| Age | Period | Never CC | Never Ecig | Never Dual | CC Former | Ecig Former | Dual Former | Former CC | Former Ecig | Former Dual |
| 10-14 | 1-12 | 0.001921 | 0.001921 | 0.000000 | 0.008742 | 0.008742 | 0.000000 | 0.000000 | 0.000000 | 0.000000 |
| 15-19 | 1-12 | 0.002360 | 0.002360 | 0.000000 | 0.003614 | 0.003614 | 0.000000 | 0.000000 | 0.000000 | 0.000000 |
| 20-24 | 1-12 | 0.000188 | 0.000188 | 0.000000 | 0.000943 | 0.000943 | 0.000000 | 0.000000 | 0.000000 | 0.000000 |
| 25-29 | 1-12 | 0.000093 | 0.000093 | 0.000000 | 0.001143 | 0.001143 | 0.000000 | 0.000000 | 0.000000 | 0.000000 |
| 30-34 | 1-12 | 0.000047 | 0.000047 | 0.000000 | 0.000871 | 0.000871 | 0.000000 | 0.000000 | 0.000000 | 0.000000 |
| 35-39 | 1-12 | 0.000000 | 0.000000 | 0.000000 | 0.000867 | 0.000867 | 0.000000 | 0.000000 | 0.000000 | 0.000000 |
| 40-44 | 1-12 | 0.000000 | 0.000000 | 0.000000 | 0.001399 | 0.001399 | 0.000000 | 0.000000 | 0.000000 | 0.000000 |
| 45-49 | 1-12 | 0.000000 | 0.000000 | 0.000000 | 0.002033 | 0.002033 | 0.000000 | 0.000000 | 0.000000 | 0.000000 |
| 50-54 | 1-12 | 0.000000 | 0.000000 | 0.000000 | 0.003209 | 0.003209 | 0.000000 | 0.000000 | 0.000000 | 0.000000 |
| 55-59 | 1-12 | 0.000000 | 0.000000 | 0.000000 | 0.003574 | 0.003574 | 0.000000 | 0.000000 | 0.000000 | 0.000000 |
| 60-64 | 1-12 | 0.000000 | 0.000000 | 0.000000 | 0.003460 | 0.003460 | 0.000000 | 0.000000 | 0.000000 | 0.000000 |
| 65-69 | 1-12 | 0.000000 | 0.000000 | 0.000000 | 0.004136 | 0.004136 | 0.000000 | 0.000000 | 0.000000 | 0.000000 |
| 70-74 | 1-12 | 0.000000 | 0.000000 | 0.000000 | 0.005592 | 0.005592 | 0.000000 | 0.000000 | 0.000000 | 0.000000 |
| 75-79 | 1-12 | 0.000000 | 0.000000 | 0.000000 | 0.005592 | 0.005592 | 0.000000 | 0.000000 | 0.000000 | 0.000000 |
| 10-14 | 13-72 | 0.001962 | 0.002944 | 0.000000 | 0.008742 | 0.008742 | 0.000000 | 0.000000 | 0.000000 | 0.000000 |
| 15-19 | 13-72 | 0.002796 | 0.004193 | 0.000000 | 0.004426 | 0.004426 | 0.000000 | 0.000000 | 0.000000 | 0.000000 |
| 20-24 | 13-72 | 0.000275 | 0.000412 | 0.000000 | 0.001527 | 0.001527 | 0.000000 | 0.000000 | 0.000000 | 0.000000 |
| 25-29 | 13-72 | 0.000385 | 0.000578 | 0.000000 | 0.001515 | 0.001515 | 0.000000 | 0.000000 | 0.000000 | 0.000000 |
| 30-34 | 13-72 | 0.000146 | 0.000218 | 0.000000 | 0.001227 | 0.001227 | 0.000000 | 0.000000 | 0.000000 | 0.000000 |
| 35-39 | 13-72 | 0.000000 | 0.000000 | 0.000000 | 0.001630 | 0.001630 | 0.000000 | 0.000000 | 0.000000 | 0.000000 |
| 40-44 | 13-72 | 0.000000 | 0.000000 | 0.000000 | 0.001852 | 0.001852 | 0.000000 | 0.000000 | 0.000000 | 0.000000 |
| 45-49 | 13-72 | 0.000000 | 0.000000 | 0.000000 | 0.002667 | 0.002667 | 0.000000 | 0.000000 | 0.000000 | 0.000000 |
| 50-54 | 13-72 | 0.000000 | 0.000000 | 0.000000 | 0.003208 | 0.003208 | 0.000000 | 0.000000 | 0.000000 | 0.000000 |
| 55-59 | 13-72 | 0.000000 | 0.000000 | 0.000000 | 0.003978 | 0.003978 | 0.000000 | 0.000000 | 0.000000 | 0.000000 |
| 60-64 | 13-72 | 0.000000 | 0.000000 | 0.000000 | 0.005771 | 0.005771 | 0.000000 | 0.000000 | 0.000000 | 0.000000 |
| 65-69 | 13-72 | 0.000000 | 0.000000 | 0.000000 | 0.010432 | 0.010432 | 0.000000 | 0.000000 | 0.000000 | 0.000000 |
| 70-74 | 13-72 | 0.000000 | 0.000000 | 0.000000 | 0.007079 | 0.007079 | 0.000000 | 0.000000 | 0.000000 | 0.000000 |
| 75-79 | 13-72 | 0.000000 | 0.000000 | 0.000000 | 0.007079 | 0.007079 | 0.000000 | 0.000000 | 0.000000 | 0.000000 |
| 10-14 | 73-132 | 0.000862 | 0.003449 | 0.000000 | 0.008742 | 0.008742 | 0.000000 | 0.000000 | 0.000000 | 0.000000 |
| 15-19 | 73-132 | 0.001066 | 0.004264 | 0.000000 | 0.003289 | 0.003289 | 0.000000 | 0.000000 | 0.000000 | 0.000000 |
| 20-24 | 73-132 | 0.000491 | 0.001965 | 0.000000 | 0.003844 | 0.003844 | 0.000000 | 0.000000 | 0.000000 | 0.000000 |
| 25-29 | 73-132 | 0.000168 | 0.000671 | 0.000000 | 0.002006 | 0.002006 | 0.000000 | 0.000000 | 0.000000 | 0.000000 |
| 30-34 | 73-132 | 0.000083 | 0.000332 | 0.000000 | 0.001329 | 0.001329 | 0.000000 | 0.000000 | 0.000000 | 0.000000 |
| 35-39 | 73-132 | 0.000000 | 0.000000 | 0.000000 | 0.001278 | 0.001278 | 0.000000 | 0.000000 | 0.000000 | 0.000000 |
| 40-44 | 73-132 | 0.000000 | 0.000000 | 0.000000 | 0.001691 | 0.001691 | 0.000000 | 0.000000 | 0.000000 | 0.000000 |
| 45-49 | 73-132 | 0.000000 | 0.000000 | 0.000000 | 0.002904 | 0.002904 | 0.000000 | 0.000000 | 0.000000 | 0.000000 |
| 50-54 | 73-132 | 0.000000 | 0.000000 | 0.000000 | 0.003319 | 0.003319 | 0.000000 | 0.000000 | 0.000000 | 0.000000 |
| 55-59 | 73-132 | 0.000000 | 0.000000 | 0.000000 | 0.003976 | 0.003976 | 0.000000 | 0.000000 | 0.000000 | 0.000000 |
| 60-64 | 73-132 | 0.000000 | 0.000000 | 0.000000 | 0.005235 | 0.005235 | 0.000000 | 0.000000 | 0.000000 | 0.000000 |
| 65-69 | 73-132 | 0.000000 | 0.000000 | 0.000000 | 0.005901 | 0.005901 | 0.000000 | 0.000000 | 0.000000 | 0.000000 |
| 70-74 | 73-132 | 0.000000 | 0.000000 | 0.000000 | 0.005615 | 0.005615 | 0.000000 | 0.000000 | 0.000000 | 0.000000 |
| 75-79 | 73-132 | 0.000000 | 0.000000 | 0.000000 | 0.005615 | 0.005615 | 0.000000 | 0.000000 | 0.000000 | 0.000000 |
| 10-14 | 133-192 | 0.000173 | 0.003294 | 0.000000 | 0.008742 | 0.008742 | 0.000000 | 0.000000 | 0.000000 | 0.000000 |
| 15-19 | 133-192 | 0.000234 | 0.004442 | 0.000000 | 0.003732 | 0.003732 | 0.000000 | 0.000000 | 0.000000 | 0.000000 |
| 20-24 | 133-192 | 0.000003 | 0.000059 | 0.000000 | 0.002260 | 0.002260 | 0.000000 | 0.000000 | 0.000000 | 0.000000 |
| 25-29 | 133-192 | 0.000071 | 0.001351 | 0.000000 | 0.002822 | 0.002822 | 0.000000 | 0.000000 | 0.000000 | 0.000000 |
| 30-34 | 133-192 | 0.000035 | 0.000661 | 0.000000 | 0.002610 | 0.002610 | 0.000000 | 0.000000 | 0.000000 | 0.000000 |
| 35-39 | 133-192 | 0.000000 | 0.000000 | 0.000000 | 0.002058 | 0.002058 | 0.000000 | 0.000000 | 0.000000 | 0.000000 |
| 40-44 | 133-192 | 0.000000 | 0.000000 | 0.000000 | 0.002310 | 0.002310 | 0.000000 | 0.000000 | 0.000000 | 0.000000 |
| 45-49 | 133-192 | 0.000000 | 0.000000 | 0.000000 | 0.002256 | 0.002256 | 0.000000 | 0.000000 | 0.000000 | 0.000000 |
| 50-54 | 133-192 | 0.000000 | 0.000000 | 0.000000 | 0.002663 | 0.002663 | 0.000000 | 0.000000 | 0.000000 | 0.000000 |
| 55-59 | 133-192 | 0.000000 | 0.000000 | 0.000000 | 0.001747 | 0.001747 | 0.000000 | 0.000000 | 0.000000 | 0.000000 |
| 60-64 | 133-192 | 0.000000 | 0.000000 | 0.000000 | 0.005809 | 0.005809 | 0.000000 | 0.000000 | 0.000000 | 0.000000 |
| 65-69 | 133-192 | 0.000000 | 0.000000 | 0.000000 | 0.005896 | 0.005896 | 0.000000 | 0.000000 | 0.000000 | 0.000000 |
| 70-74 | 133-192 | 0.000000 | 0.000000 | 0.000000 | 0.007323 | 0.007323 | 0.000000 | 0.000000 | 0.000000 | 0.000000 |
| 75-79 | 133-192 | 0.000000 | 0.000000 | 0.000000 | 0.007323 | 0.007323 | 0.000000 | 0.000000 | 0.000000 | 0.000000 |
| 10-14 | 193-252 | 0.000116 | 0.002198 | 0.000000 | 0.008742 | 0.008742 | 0.000000 | 0.000000 | 0.000000 | 0.000000 |
| 15-19 | 193-252 | 0.000199 | 0.003788 | 0.000000 | 0.005036 | 0.005036 | 0.000000 | 0.000000 | 0.000000 | 0.000000 |
| 20-24 | 193-252 | 0.000016 | 0.000304 | 0.000000 | 0.002857 | 0.002857 | 0.000000 | 0.000000 | 0.000000 | 0.000000 |
| 25-29 | 193-252 | 0.000013 | 0.000239 | 0.000000 | 0.002314 | 0.002314 | 0.000000 | 0.000000 | 0.000000 | 0.000000 |
| 30-34 | 193-252 | 0.000006 | 0.000119 | 0.000000 | 0.004343 | 0.004343 | 0.000000 | 0.000000 | 0.000000 | 0.000000 |
| 35-39 | 193-252 | 0.000000 | 0.000000 | 0.000000 | 0.002092 | 0.002092 | 0.000000 | 0.000000 | 0.000000 | 0.000000 |
| 40-44 | 193-252 | 0.000000 | 0.000000 | 0.000000 | 0.002566 | 0.002566 | 0.000000 | 0.000000 | 0.000000 | 0.000000 |
| 45-49 | 193-252 | 0.000000 | 0.000000 | 0.000000 | 0.002311 | 0.002311 | 0.000000 | 0.000000 | 0.000000 | 0.000000 |
| 50-54 | 193-252 | 0.000000 | 0.000000 | 0.000000 | 0.003209 | 0.003209 | 0.000000 | 0.000000 | 0.000000 | 0.000000 |

## Results for PHIM for Run 1 (Sensitivity_Analysis_1.RTF)

| | | | | | | | | | | |
|---|---|---|---|---|---|---|---|---|---|---|
| 55-59 | 193-252 | 0.000000 | 0.000000 | 0.000000 | 0.004057 | 0.004057 | 0.000000 | 0.000000 | 0.000000 | 0.000000 |
| 60-64 | 193-252 | 0.000000 | 0.000000 | 0.000000 | 0.007166 | 0.007166 | 0.000000 | 0.000000 | 0.000000 | 0.000000 |
| 65-69 | 193-252 | 0.000000 | 0.000000 | 0.000000 | 0.004281 | 0.004281 | 0.000000 | 0.000000 | 0.000000 | 0.000000 |
| 70-74 | 193-252 | 0.000000 | 0.000000 | 0.000000 | 0.004580 | 0.004580 | 0.000000 | 0.000000 | 0.000000 | 0.000000 |
| 75-79 | 193-252 | 0.000000 | 0.000000 | 0.000000 | 0.004580 | 0.004580 | 0.000000 | 0.000000 | 0.000000 | 0.000000 |
| 10-14 | 253+ | 0.000066 | 0.001259 | 0.000000 | 0.008742 | 0.008742 | 0.000000 | 0.000000 | 0.000000 | 0.000000 |
| 15-19 | 253+ | 0.000149 | 0.002835 | 0.000000 | 0.010602 | 0.010602 | 0.000000 | 0.000000 | 0.000000 | 0.000000 |
| 20-24 | 253+ | 0.000023 | 0.000430 | 0.000000 | 0.004314 | 0.004314 | 0.000000 | 0.000000 | 0.000000 | 0.000000 |
| 25-29 | 253+ | 0.000011 | 0.000213 | 0.000000 | 0.003645 | 0.003645 | 0.000000 | 0.000000 | 0.000000 | 0.000000 |
| 30-34 | 253+ | 0.000006 | 0.000106 | 0.000000 | 0.004512 | 0.004512 | 0.000000 | 0.000000 | 0.000000 | 0.000000 |
| 35-39 | 253+ | 0.000000 | 0.000000 | 0.000000 | 0.002147 | 0.002147 | 0.000000 | 0.000000 | 0.000000 | 0.000000 |
| 40-44 | 253+ | 0.000000 | 0.000000 | 0.000000 | 0.002636 | 0.002636 | 0.000000 | 0.000000 | 0.000000 | 0.000000 |
| 45-49 | 253+ | 0.000000 | 0.000000 | 0.000000 | 0.001051 | 0.001051 | 0.000000 | 0.000000 | 0.000000 | 0.000000 |
| 50-54 | 253+ | 0.000000 | 0.000000 | 0.000000 | 0.002519 | 0.002519 | 0.000000 | 0.000000 | 0.000000 | 0.000000 |
| 55-59 | 253+ | 0.000000 | 0.000000 | 0.000000 | 0.003693 | 0.003693 | 0.000000 | 0.000000 | 0.000000 | 0.000000 |
| 60-64 | 253+ | 0.000000 | 0.000000 | 0.000000 | 0.005888 | 0.005888 | 0.000000 | 0.000000 | 0.000000 | 0.000000 |
| 65-69 | 253+ | 0.000000 | 0.000000 | 0.000000 | 0.004823 | 0.004823 | 0.000000 | 0.000000 | 0.000000 | 0.000000 |
| 70-74 | 253+ | 0.000000 | 0.000000 | 0.000000 | 0.004733 | 0.004733 | 0.000000 | 0.000000 | 0.000000 | 0.000000 |
| 75-79 | 253+ | 0.000000 | 0.000000 | 0.000000 | 0.004733 | 0.004733 | 0.000000 | 0.000000 | 0.000000 | 0.000000 |

# Results for PHIM for Run 1 (Sensitivity_Analysis_1.RTF)

*************************************************************************************

| | | Switch from CC | | Switch from Ecig | | Switch from Dual | |
|---|---|---|---|---|---|---|---|
| | | **CC** | **CC** | **Ecig** | **Ecig** | **Dual** | **Dual** |
| Age | Period | Ecig | Dual | CC | Dual | CC | Ecig |
| 10-14 | 1-12 | 0.009549 | 0.000000 | 0.000000 | 0.000000 | 0.000000 | 0.000000 |
| 15-19 | 1-12 | 0.012695 | 0.000000 | 0.000000 | 0.000000 | 0.000000 | 0.000000 |
| 20-24 | 1-12 | 0.014490 | 0.000000 | 0.000000 | 0.000000 | 0.000000 | 0.000000 |
| 25-29 | 1-12 | 0.015212 | 0.000000 | 0.000000 | 0.000000 | 0.000000 | 0.000000 |
| 30-34 | 1-12 | 0.015460 | 0.000000 | 0.000000 | 0.000000 | 0.000000 | 0.000000 |
| 35-39 | 1-12 | 0.014711 | 0.000000 | 0.000000 | 0.000000 | 0.000000 | 0.000000 |
| 40-44 | 1-12 | 0.014241 | 0.000000 | 0.000000 | 0.000000 | 0.000000 | 0.000000 |
| 45-49 | 1-12 | 0.013014 | 0.000000 | 0.000000 | 0.000000 | 0.000000 | 0.000000 |
| 50-54 | 1-12 | 0.011768 | 0.000000 | 0.000000 | 0.000000 | 0.000000 | 0.000000 |
| 55-59 | 1-12 | 0.011032 | 0.000000 | 0.000000 | 0.000000 | 0.000000 | 0.000000 |
| 60-64 | 1-12 | 0.009525 | 0.000000 | 0.000000 | 0.000000 | 0.000000 | 0.000000 |
| 65-69 | 1-12 | 0.006897 | 0.000000 | 0.000000 | 0.000000 | 0.000000 | 0.000000 |
| 70-74 | 1-12 | 0.005435 | 0.000000 | 0.000000 | 0.000000 | 0.000000 | 0.000000 |
| 75-79 | 1-12 | 0.005435 | 0.000000 | 0.000000 | 0.000000 | 0.000000 | 0.000000 |
| 10-14 | 13-72 | 0.009549 | 0.000000 | 0.000000 | 0.000000 | 0.000000 | 0.000000 |
| 15-19 | 13-72 | 0.012695 | 0.000000 | 0.000000 | 0.000000 | 0.000000 | 0.000000 |
| 20-24 | 13-72 | 0.014490 | 0.000000 | 0.000000 | 0.000000 | 0.000000 | 0.000000 |
| 25-29 | 13-72 | 0.015212 | 0.000000 | 0.000000 | 0.000000 | 0.000000 | 0.000000 |
| 30-34 | 13-72 | 0.015460 | 0.000000 | 0.000000 | 0.000000 | 0.000000 | 0.000000 |
| 35-39 | 13-72 | 0.014711 | 0.000000 | 0.000000 | 0.000000 | 0.000000 | 0.000000 |
| 40-44 | 13-72 | 0.014241 | 0.000000 | 0.000000 | 0.000000 | 0.000000 | 0.000000 |
| 45-49 | 13-72 | 0.013014 | 0.000000 | 0.000000 | 0.000000 | 0.000000 | 0.000000 |
| 50-54 | 13-72 | 0.011768 | 0.000000 | 0.000000 | 0.000000 | 0.000000 | 0.000000 |
| 55-59 | 13-72 | 0.011032 | 0.000000 | 0.000000 | 0.000000 | 0.000000 | 0.000000 |
| 60-64 | 13-72 | 0.009525 | 0.000000 | 0.000000 | 0.000000 | 0.000000 | 0.000000 |
| 65-69 | 13-72 | 0.006897 | 0.000000 | 0.000000 | 0.000000 | 0.000000 | 0.000000 |
| 70-74 | 13-72 | 0.005435 | 0.000000 | 0.000000 | 0.000000 | 0.000000 | 0.000000 |
| 75-79 | 13-72 | 0.005435 | 0.000000 | 0.000000 | 0.000000 | 0.000000 | 0.000000 |
| 10-14 | 73-132 | 0.009549 | 0.000000 | 0.000000 | 0.000000 | 0.000000 | 0.000000 |
| 15-19 | 73-132 | 0.012695 | 0.000000 | 0.000000 | 0.000000 | 0.000000 | 0.000000 |
| 20-24 | 73-132 | 0.014490 | 0.000000 | 0.000000 | 0.000000 | 0.000000 | 0.000000 |
| 25-29 | 73-132 | 0.015212 | 0.000000 | 0.000000 | 0.000000 | 0.000000 | 0.000000 |
| 30-34 | 73-132 | 0.015460 | 0.000000 | 0.000000 | 0.000000 | 0.000000 | 0.000000 |
| 35-39 | 73-132 | 0.014711 | 0.000000 | 0.000000 | 0.000000 | 0.000000 | 0.000000 |
| 40-44 | 73-132 | 0.014241 | 0.000000 | 0.000000 | 0.000000 | 0.000000 | 0.000000 |
| 45-49 | 73-132 | 0.013014 | 0.000000 | 0.000000 | 0.000000 | 0.000000 | 0.000000 |
| 50-54 | 73-132 | 0.011768 | 0.000000 | 0.000000 | 0.000000 | 0.000000 | 0.000000 |
| 55-59 | 73-132 | 0.011032 | 0.000000 | 0.000000 | 0.000000 | 0.000000 | 0.000000 |
| 60-64 | 73-132 | 0.009525 | 0.000000 | 0.000000 | 0.000000 | 0.000000 | 0.000000 |
| 65-69 | 73-132 | 0.006897 | 0.000000 | 0.000000 | 0.000000 | 0.000000 | 0.000000 |
| 70-74 | 73-132 | 0.005435 | 0.000000 | 0.000000 | 0.000000 | 0.000000 | 0.000000 |
| 75-79 | 73-132 | 0.005435 | 0.000000 | 0.000000 | 0.000000 | 0.000000 | 0.000000 |
| 10-14 | 133-192 | 0.009549 | 0.000000 | 0.000000 | 0.000000 | 0.000000 | 0.000000 |
| 15-19 | 133-192 | 0.012695 | 0.000000 | 0.000000 | 0.000000 | 0.000000 | 0.000000 |
| 20-24 | 133-192 | 0.014490 | 0.000000 | 0.000000 | 0.000000 | 0.000000 | 0.000000 |
| 25-29 | 133-192 | 0.015212 | 0.000000 | 0.000000 | 0.000000 | 0.000000 | 0.000000 |
| 30-34 | 133-192 | 0.015460 | 0.000000 | 0.000000 | 0.000000 | 0.000000 | 0.000000 |
| 35-39 | 133-192 | 0.014711 | 0.000000 | 0.000000 | 0.000000 | 0.000000 | 0.000000 |
| 40-44 | 133-192 | 0.014241 | 0.000000 | 0.000000 | 0.000000 | 0.000000 | 0.000000 |
| 45-49 | 133-192 | 0.013014 | 0.000000 | 0.000000 | 0.000000 | 0.000000 | 0.000000 |
| 50-54 | 133-192 | 0.011768 | 0.000000 | 0.000000 | 0.000000 | 0.000000 | 0.000000 |
| 55-59 | 133-192 | 0.011032 | 0.000000 | 0.000000 | 0.000000 | 0.000000 | 0.000000 |
| 60-64 | 133-192 | 0.009525 | 0.000000 | 0.000000 | 0.000000 | 0.000000 | 0.000000 |
| 65-69 | 133-192 | 0.006897 | 0.000000 | 0.000000 | 0.000000 | 0.000000 | 0.000000 |
| 70-74 | 133-192 | 0.005435 | 0.000000 | 0.000000 | 0.000000 | 0.000000 | 0.000000 |
| 75-79 | 133-192 | 0.005435 | 0.000000 | 0.000000 | 0.000000 | 0.000000 | 0.000000 |
| 10-14 | 193-252 | 0.009549 | 0.000000 | 0.000000 | 0.000000 | 0.000000 | 0.000000 |
| 15-19 | 193-252 | 0.012695 | 0.000000 | 0.000000 | 0.000000 | 0.000000 | 0.000000 |
| 20-24 | 193-252 | 0.014490 | 0.000000 | 0.000000 | 0.000000 | 0.000000 | 0.000000 |
| 25-29 | 193-252 | 0.015212 | 0.000000 | 0.000000 | 0.000000 | 0.000000 | 0.000000 |
| 30-34 | 193-252 | 0.015460 | 0.000000 | 0.000000 | 0.000000 | 0.000000 | 0.000000 |
| 35-39 | 193-252 | 0.014711 | 0.000000 | 0.000000 | 0.000000 | 0.000000 | 0.000000 |
| 40-44 | 193-252 | 0.014241 | 0.000000 | 0.000000 | 0.000000 | 0.000000 | 0.000000 |
| 45-49 | 193-252 | 0.013014 | 0.000000 | 0.000000 | 0.000000 | 0.000000 | 0.000000 |
| 50-54 | 193-252 | 0.011768 | 0.000000 | 0.000000 | 0.000000 | 0.000000 | 0.000000 |
| 55-59 | 193-252 | 0.011032 | 0.000000 | 0.000000 | 0.000000 | 0.000000 | 0.000000 |
| 60-64 | 193-252 | 0.009525 | 0.000000 | 0.000000 | 0.000000 | 0.000000 | 0.000000 |
| 65-69 | 193-252 | 0.006897 | 0.000000 | 0.000000 | 0.000000 | 0.000000 | 0.000000 |
| 70-74 | 193-252 | 0.005435 | 0.000000 | 0.000000 | 0.000000 | 0.000000 | 0.000000 |
| 75-79 | 193-252 | 0.005435 | 0.000000 | 0.000000 | 0.000000 | 0.000000 | 0.000000 |
| 10-14 | 253+ | 0.009549 | 0.000000 | 0.000000 | 0.000000 | 0.000000 | 0.000000 |
| 15-19 | 253+ | 0.012695 | 0.000000 | 0.000000 | 0.000000 | 0.000000 | 0.000000 |
| 20-24 | 253+ | 0.014490 | 0.000000 | 0.000000 | 0.000000 | 0.000000 | 0.000000 |

## Results for PHIM for Run 1 (Sensitivity_Analysis_1.RTF)

| | | | | | | | |
|---|---|---|---|---|---|---|---|
| 25-29 | 253+ | 0.015212 | 0.000000 | 0.000000 | 0.000000 | 0.000000 | 0.000000 |
| 30-34 | 253+ | 0.015460 | 0.000000 | 0.000000 | 0.000000 | 0.000000 | 0.000000 |
| 35-39 | 253+ | 0.014711 | 0.000000 | 0.000000 | 0.000000 | 0.000000 | 0.000000 |
| 40-44 | 253+ | 0.014241 | 0.000000 | 0.000000 | 0.000000 | 0.000000 | 0.000000 |
| 45-49 | 253+ | 0.013014 | 0.000000 | 0.000000 | 0.000000 | 0.000000 | 0.000000 |
| 50-54 | 253+ | 0.011768 | 0.000000 | 0.000000 | 0.000000 | 0.000000 | 0.000000 |
| 55-59 | 253+ | 0.011032 | 0.000000 | 0.000000 | 0.000000 | 0.000000 | 0.000000 |
| 60-64 | 253+ | 0.009525 | 0.000000 | 0.000000 | 0.000000 | 0.000000 | 0.000000 |
| 65-69 | 253+ | 0.006897 | 0.000000 | 0.000000 | 0.000000 | 0.000000 | 0.000000 |
| 70-74 | 253+ | 0.005435 | 0.000000 | 0.000000 | 0.000000 | 0.000000 | 0.000000 |
| 75-79 | 253+ | 0.005435 | 0.000000 | 0.000000 | 0.000000 | 0.000000 | 0.000000 |

# Results for PHIM for Run 1 (Sensitivity_Analysis_1.RTF)

************************************************************************************
**Main Output for the Tobacco History for Run: 1, Simulation: 1, Scenario: NULL**

| Variable Parameter | Value |
|---|---|
| 1. Country | US (United States) |
| 2. Sex | M (Males) |
| 3. Year of start of process | 1990 |

Year of Follow-up: 2040, Age Group: All Ages

| Period | Age Group | Smoking Group | N | % |
|---|---|---|---|---|
| 50 | All Ages | Never Any | 19445 | 50.68 |
| 50 | All Ages | CC | 3024 | 7.88 |
| 50 | All Ages | Former | 15896 | 41.43 |

Year of Follow-up: 2040, Age Group: 18-19

| Period | Age Group | Smoking Group | N | % |
|---|---|---|---|---|
| 50 | 18-19 | Never Any | 0 | . |
| 50 | 18-19 | CC | 0 | . |
| 50 | 18-19 | Former | 0 | . |

Year of Follow-up: 2040, Age Group: 20-24

| Period | Age Group | Smoking Group | N | % |
|---|---|---|---|---|
| 50 | 20-24 | Never Any | 0 | . |
| 50 | 20-24 | CC | 0 | . |
| 50 | 20-24 | Former | 0 | . |

Year of Follow-up: 2040, Age Group: 25-29

| Period | Age Group | Smoking Group | N | % |
|---|---|---|---|---|
| 50 | 25-29 | Never Any | 0 | . |
| 50 | 25-29 | CC | 0 | . |
| 50 | 25-29 | Former | 0 | . |

Year of Follow-up: 2040, Age Group: 30-34

| Period | Age Group | Smoking Group | N | % |
|---|---|---|---|---|
| 50 | 30-34 | Never Any | 0 | . |
| 50 | 30-34 | CC | 0 | . |
| 50 | 30-34 | Former | 0 | . |

Year of Follow-up: 2040, Age Group: 35-39

| Period | Age Group | Smoking Group | N | % |
|---|---|---|---|---|
| 50 | 35-39 | Never Any | 0 | . |
| 50 | 35-39 | CC | 0 | . |
| 50 | 35-39 | Former | 0 | . |

Year of Follow-up: 2040, Age Group: 40-44

| Period | Age Group | Smoking Group | N | % |
|---|---|---|---|---|
| 50 | 40-44 | Never Any | 0 | . |
| 50 | 40-44 | CC | 0 | . |
| 50 | 40-44 | Former | 0 | . |

Year of Follow-up: 2040, Age Group: 45-49

| Period | Age Group | Smoking Group | N | % |
|---|---|---|---|---|
| 50 | 45-49 | Never Any | 0 | . |
| 50 | 45-49 | CC | 0 | . |
| 50 | 45-49 | Former | 0 | . |

Year of Follow-up: 2040, Age Group: 50-54

| Period | Age Group | Smoking Group | N | % |
|---|---|---|---|---|
| 50 | 50-54 | Never Any | 0 | . |
| 50 | 50-54 | CC | 0 | . |
| 50 | 50-54 | Former | 0 | . |

Year of Follow-up: 2040, Age Group: 55-59

| Period | Age Group | Smoking Group | N | % |
|---|---|---|---|---|
| 50 | 55-59 | Never Any | 0 | . |
| 50 | 55-59 | CC | 0 | . |
| 50 | 55-59 | Former | 0 | . |

Year of Follow-up: 2040, Age Group: 60-64

| Period | Age Group | Smoking Group | N | % |
|---|---|---|---|---|

## Results for PHIM for Run 1 (Sensitivity_Analysis_1.RTF)

| | | | | |
|---|---|---|---|---|
| 50 | 60-64 | Never Any | 4363 | 49.33 |
| 50 | 60-64 | CC | 934 | 10.56 |
| 50 | 60-64 | Former | 3548 | 40.11 |

Year of Follow-up: 2040, Age Group: 65-69

| Period | Age Group | Smoking Group | N | % |
|---|---|---|---|---|
| 50 | 65-69 | Never Any | 4948 | 54.73 |
| 50 | 65-69 | CC | 734 | 8.12 |
| 50 | 65-69 | Former | 3358 | 37.15 |

Year of Follow-up: 2040, Age Group: 70-74

| Period | Age Group | Smoking Group | N | % |
|---|---|---|---|---|
| 50 | 70-74 | Never Any | 5145 | 52.41 |
| 50 | 70-74 | CC | 688 | 7.01 |
| 50 | 70-74 | Former | 3983 | 40.58 |

Year of Follow-up: 2040, Age Group: 75-79

| Period | Age Group | Smoking Group | N | % |
|---|---|---|---|---|
| 50 | 75-79 | Never Any | 4989 | 46.78 |
| 50 | 75-79 | CC | 668 | 6.26 |
| 50 | 75-79 | Former | 5007 | 46.95 |

# Results for PHIM for Run 1 (Sensitivity_Analysis_1.RTF)

************************************************************************************
**Main Output for the Tobacco History for Run: 1, Simulation: 1, Scenario: Ecig**

| Variable Parameter | Value |
|---|---|
| 1. Country | US (United States) |
| 2. Sex | M (Males) |
| 3. Year of start of process | 1990 |

Year of Follow-up: 1990, Age Group: All Ages

| Period | Age Group | Smoking Group | N | % | Ever Quit (N) | Ever Quit % | Mean Yrs Quit | Short Term (n) | Short Term % |
|---|---|---|---|---|---|---|---|---|---|
| 0 | All Ages | Never Any | 34075 | 39.78 | | | | | |
| 0 | All Ages | CC | 27323 | 31.90 | 0 | 0.00 | | | |
| 0 | All Ages | Ecig | 0 | 0.00 | | | | | |
| 0 | All Ages | Dual | 0 | 0.00 | | | | | |
| 0 | All Ages | Former | 24266 | 28.33 | | | 13.08 | 3623 | 14.93 |

Year of Follow-up: 1990, Age Group: 18-19

| Period | Age Group | Smoking Group | N | % | Ever Quit (N) | Ever Quit % | Mean Yrs Quit | Short Term (n) | Short Term % |
|---|---|---|---|---|---|---|---|---|---|
| 0 | 18-19 | Never Any | 2783 | 78.42 | | | | | |
| 0 | 18-19 | CC | 634 | 17.86 | 0 | 0.00 | | | |
| 0 | 18-19 | Ecig | 0 | 0.00 | | | | | |
| 0 | 18-19 | Dual | 0 | 0.00 | | | | | |
| 0 | 18-19 | Former | 132 | 3.72 | | | 0.11 | 132 | 100.00 |

Year of Follow-up: 1990, Age Group: 20-24

| Period | Age Group | Smoking Group | N | % | Ever Quit (N) | Ever Quit % | Mean Yrs Quit | Short Term (n) | Short Term % |
|---|---|---|---|---|---|---|---|---|---|
| 0 | 20-24 | Never Any | 5722 | 58.29 | | | | | |
| 0 | 20-24 | CC | 3199 | 32.59 | 0 | 0.00 | | | |
| 0 | 20-24 | Ecig | 0 | 0.00 | | | | | |
| 0 | 20-24 | Dual | 0 | 0.00 | | | | | |
| 0 | 20-24 | Former | 895 | 9.12 | | | 1.46 | 506 | 56.54 |

Year of Follow-up: 1990, Age Group: 25-29

| Period | Age Group | Smoking Group | N | % | Ever Quit (N) | Ever Quit % | Mean Yrs Quit | Short Term (n) | Short Term % |
|---|---|---|---|---|---|---|---|---|---|
| 0 | 25-29 | Never Any | 5232 | 49.06 | | | | | |
| 0 | 25-29 | CC | 3914 | 36.70 | 0 | 0.00 | | | |
| 0 | 25-29 | Ecig | 0 | 0.00 | | | | | |
| 0 | 25-29 | Dual | 0 | 0.00 | | | | | |
| 0 | 25-29 | Former | 1518 | 14.23 | | | 2.87 | 643 | 42.36 |

Year of Follow-up: 1990, Age Group: 30-34

| Period | Age Group | Smoking Group | N | % | Ever Quit (N) | Ever Quit % | Mean Yrs Quit | Short Term (n) | Short Term % |
|---|---|---|---|---|---|---|---|---|---|
| 0 | 30-34 | Never Any | 4765 | 43.65 | | | | | |
| 0 | 30-34 | CC | 4023 | 36.85 | 0 | 0.00 | | | |
| 0 | 30-34 | Ecig | 0 | 0.00 | | | | | |
| 0 | 30-34 | Dual | 0 | 0.00 | | | | | |
| 0 | 30-34 | Former | 2128 | 19.49 | | | 4.06 | 592 | 27.82 |

Year of Follow-up: 1990, Age Group: 35-39

| Period | Age Group | Smoking Group | N | % | Ever Quit (N) | Ever Quit % | Mean Yrs Quit | Short Term (n) | Short Term % |
|---|---|---|---|---|---|---|---|---|---|
| 0 | 35-39 | Never Any | 3941 | 39.85 | | | | | |
| 0 | 35-39 | CC | 3474 | 35.13 | 0 | 0.00 | | | |
| 0 | 35-39 | Ecig | 0 | 0.00 | | | | | |
| 0 | 35-39 | Dual | 0 | 0.00 | | | | | |
| 0 | 35-39 | Former | 2474 | 25.02 | | | 8.31 | 425 | 17.18 |

Year of Follow-up: 1990, Age Group: 40-44

| Period | Age Group | Smoking Group | N | % | Ever Quit (N) | Ever Quit % | Mean Yrs Quit | Short Term (n) | Short Term % |
|---|---|---|---|---|---|---|---|---|---|
| 0 | 40-44 | Never Any | 2912 | 33.65 | | | | | |
| 0 | 40-44 | CC | 3113 | 35.97 | 0 | 0.00 | | | |
| 0 | 40-44 | Ecig | 0 | 0.00 | | | | | |
| 0 | 40-44 | Dual | 0 | 0.00 | | | | | |
| 0 | 40-44 | Former | 2630 | 30.39 | | | 10.69 | 317 | 12.05 |

Year of Follow-up: 1990, Age Group: 45-49

| Period | Age Group | Smoking Group | N | % | Ever Quit (N) | Ever Quit % | Mean Yrs Quit | Short Term (n) | Short Term % |
|---|---|---|---|---|---|---|---|---|---|
| 0 | 45-49 | Never Any | 1919 | 29.46 | | | | | |
| 0 | 45-49 | CC | 2318 | 35.58 | 0 | 0.00 | | | |
| 0 | 45-49 | Ecig | 0 | 0.00 | | | | | |
| 0 | 45-49 | Dual | 0 | 0.00 | | | | | |
| 0 | 45-49 | Former | 2278 | 34.97 | | | 13.26 | 221 | 9.70 |

Year of Follow-up: 1990, Age Group: 50-54

| Period | Age Group | Smoking Group | N | % | Ever Quit (N) | Ever Quit % | Mean Yrs Quit | Short Term (n) | Short Term % |
|---|---|---|---|---|---|---|---|---|---|
| 0 | 50-54 | Never Any | 1611 | 29.41 | | | | | |
| 0 | 50-54 | CC | 1816 | 33.16 | 0 | 0.00 | | | |

# Results for PHIM for Run 1 (Sensitivity_Analysis_1.RTF)

| | | | | | | | | | |
|---|---|---|---|---|---|---|---|---|---|
| 0 | 50-54 | Ecig | 0 | 0.00 | | | | | |
| 0 | 50-54 | Dual | 0 | 0.00 | | | | | |
| 0 | 50-54 | Former | 2050 | 37.43 | | | 14.55 | 261 | 12.73 |

Year of Follow-up: 1990, Age Group: 55-59

| Period | Age Group | Smoking Group | N | % | Ever Quit (N) | Ever Quit % | Mean Yrs Quit | Short Term (n) | Short Term % |
|---|---|---|---|---|---|---|---|---|---|
| 0 | 55-59 | Never Any | 1337 | 26.83 | | | | | |
| 0 | 55-59 | CC | 1520 | 30.50 | 0 | 0.00 | | | |
| 0 | 55-59 | Ecig | 0 | 0.00 | | | | | |
| 0 | 55-59 | Dual | 0 | 0.00 | | | | | |
| 0 | 55-59 | Former | 2126 | 42.67 | | | 14.80 | 216 | 10.16 |

Year of Follow-up: 1990, Age Group: 60-64

| Period | Age Group | Smoking Group | N | % | Ever Quit (N) | Ever Quit % | Mean Yrs Quit | Short Term (n) | Short Term % |
|---|---|---|---|---|---|---|---|---|---|
| 0 | 60-64 | Never Any | 1169 | 23.83 | | | | | |
| 0 | 60-64 | CC | 1287 | 26.24 | 0 | 0.00 | | | |
| 0 | 60-64 | Ecig | 0 | 0.00 | | | | | |
| 0 | 60-64 | Dual | 0 | 0.00 | | | | | |
| 0 | 60-64 | Former | 2449 | 49.93 | | | 18.87 | 106 | 4.33 |

Year of Follow-up: 1990, Age Group: 65-69

| Period | Age Group | Smoking Group | N | % | Ever Quit (N) | Ever Quit % | Mean Yrs Quit | Short Term (n) | Short Term % |
|---|---|---|---|---|---|---|---|---|---|
| 0 | 65-69 | Never Any | 1023 | 23.32 | | | | | |
| 0 | 65-69 | CC | 979 | 22.32 | 0 | 0.00 | | | |
| 0 | 65-69 | Ecig | 0 | 0.00 | | | | | |
| 0 | 65-69 | Dual | 0 | 0.00 | | | | | |
| 0 | 65-69 | Former | 2385 | 54.37 | | | 19.91 | 113 | 4.74 |

Year of Follow-up: 1990, Age Group: 70-74

| Period | Age Group | Smoking Group | N | % | Ever Quit (N) | Ever Quit % | Mean Yrs Quit | Short Term (n) | Short Term % |
|---|---|---|---|---|---|---|---|---|---|
| 0 | 70-74 | Never Any | 961 | 27.46 | | | | | |
| 0 | 70-74 | CC | 675 | 19.29 | 0 | 0.00 | | | |
| 0 | 70-74 | Ecig | 0 | 0.00 | | | | | |
| 0 | 70-74 | Dual | 0 | 0.00 | | | | | |
| 0 | 70-74 | Former | 1863 | 53.24 | | | 20.69 | 23 | 1.23 |

Year of Follow-up: 1990, Age Group: 75-79

| Period | Age Group | Smoking Group | N | % | Ever Quit (N) | Ever Quit % | Mean Yrs Quit | Short Term (n) | Short Term % |
|---|---|---|---|---|---|---|---|---|---|
| 0 | 75-79 | Never Any | 700 | 29.06 | | | | | |
| 0 | 75-79 | CC | 371 | 15.40 | 0 | 0.00 | | | |
| 0 | 75-79 | Ecig | 0 | 0.00 | | | | | |
| 0 | 75-79 | Dual | 0 | 0.00 | | | | | |
| 0 | 75-79 | Former | 1338 | 55.54 | | | 22.88 | 68 | 5.08 |

Year of Follow-up: 1995, Age Group: All Ages

| Period | Age Group | Smoking Group | N | % | Ever Quit (N) | Ever Quit % | Mean Yrs Quit | Short Term (n) | Short Term % |
|---|---|---|---|---|---|---|---|---|---|
| 5 | All Ages | Never Any | 38368 | 41.57 | | | | | |
| 5 | All Ages | CC | 11508 | 12.47 | 0 | 0.00 | | | |
| 5 | All Ages | Ecig | 15517 | 16.81 | | | | | |
| 5 | All Ages | Dual | 0 | 0.00 | | | | | |
| 5 | All Ages | Former | 26897 | 29.14 | | | 15.19 | 1705 | 6.34 |

Year of Follow-up: 1995, Age Group: 18-19

| Period | Age Group | Smoking Group | N | % | Ever Quit (N) | Ever Quit % | Mean Yrs Quit | Short Term (n) | Short Term % |
|---|---|---|---|---|---|---|---|---|---|
| 5 | 18-19 | Never Any | 2448 | 69.07 | | | | | |
| 5 | 18-19 | CC | 320 | 9.03 | 0 | 0.00 | | | |
| 5 | 18-19 | Ecig | 659 | 18.59 | | | | | |
| 5 | 18-19 | Dual | 0 | 0.00 | | | | | |
| 5 | 18-19 | Former | 117 | 3.30 | | | 1.11 | 80 | 68.38 |

Year of Follow-up: 1995, Age Group: 20-24

| Period | Age Group | Smoking Group | N | % | Ever Quit (N) | Ever Quit % | Mean Yrs Quit | Short Term (n) | Short Term % |
|---|---|---|---|---|---|---|---|---|---|
| 5 | 20-24 | Never Any | 5787 | 64.02 | | | | | |
| 5 | 20-24 | CC | 946 | 10.46 | 0 | 0.00 | | | |
| 5 | 20-24 | Ecig | 1826 | 20.20 | | | | | |
| 5 | 20-24 | Dual | 0 | 0.00 | | | | | |
| 5 | 20-24 | Former | 481 | 5.32 | | | 2.81 | 142 | 29.52 |

Year of Follow-up: 1995, Age Group: 25-29

| Period | Age Group | Smoking Group | N | % | Ever Quit (N) | Ever Quit % | Mean Yrs Quit | Short Term (n) | Short Term % |
|---|---|---|---|---|---|---|---|---|---|
| 5 | 25-29 | Never Any | 5471 | 55.74 | | | | | |
| 5 | 25-29 | CC | 1255 | 12.79 | 0 | 0.00 | | | |
| 5 | 25-29 | Ecig | 1942 | 19.78 | | | | | |
| 5 | 25-29 | Dual | 0 | 0.00 | | | | | |
| 5 | 25-29 | Former | 1148 | 11.70 | | | 5.43 | 115 | 10.02 |

Year of Follow-up: 1995, Age Group: 30-34

| Period | Age Group | Smoking Group | N | % | Ever Quit (N) | Ever Quit % | Mean Yrs Quit | Short Term (n) | Short Term % |
|---|---|---|---|---|---|---|---|---|---|

# Results for PHIM for Run 1 (Sensitivity_Analysis_1.RTF)

| | | | | | | | | | |
|---|---|---|---|---|---|---|---|---|---|
| 5 | 30-34 | Never Any | 5065 | 47.50 | | | | | |
| 5 | 30-34 | CC | 1480 | 13.88 | 0 | 0.00 | | | |
| 5 | 30-34 | Ecig | 2319 | 21.75 | | | | | |
| 5 | 30-34 | Dual | 0 | 0.00 | | | | | |
| 5 | 30-34 | Former | 1800 | 16.88 | | | 6.94 | 106 | 5.89 |

Year of Follow-up: 1995, Age Group: 35-39

| Period | Age Group | Smoking Group | N | % | Ever Quit (N) | Ever Quit % | Mean Yrs Quit | Short Term (n) | Short Term % |
|---|---|---|---|---|---|---|---|---|---|
| 5 | 35-39 | Never Any | 4724 | 43.28 | | | | | |
| 5 | 35-39 | CC | 1496 | 13.70 | 0 | 0.00 | | | |
| 5 | 35-39 | Ecig | 2275 | 20.84 | | | | | |
| 5 | 35-39 | Dual | 0 | 0.00 | | | | | |
| 5 | 35-39 | Former | 2421 | 22.18 | | | 8.20 | 114 | 4.71 |

Year of Follow-up: 1995, Age Group: 40-44

| Period | Age Group | Smoking Group | N | % | Ever Quit (N) | Ever Quit % | Mean Yrs Quit | Short Term (n) | Short Term % |
|---|---|---|---|---|---|---|---|---|---|
| 5 | 40-44 | Never Any | 3941 | 39.85 | | | | | |
| 5 | 40-44 | CC | 1280 | 12.94 | 0 | 0.00 | | | |
| 5 | 40-44 | Ecig | 1894 | 19.15 | | | | | |
| 5 | 40-44 | Dual | 0 | 0.00 | | | | | |
| 5 | 40-44 | Former | 2774 | 28.05 | | | 12.06 | 130 | 4.69 |

Year of Follow-up: 1995, Age Group: 45-49

| Period | Age Group | Smoking Group | N | % | Ever Quit (N) | Ever Quit % | Mean Yrs Quit | Short Term (n) | Short Term % |
|---|---|---|---|---|---|---|---|---|---|
| 5 | 45-49 | Never Any | 2912 | 33.65 | | | | | |
| 5 | 45-49 | CC | 1236 | 14.28 | 0 | 0.00 | | | |
| 5 | 45-49 | Ecig | 1562 | 18.05 | | | | | |
| 5 | 45-49 | Dual | 0 | 0.00 | | | | | |
| 5 | 45-49 | Former | 2945 | 34.03 | | | 14.20 | 148 | 5.03 |

Year of Follow-up: 1995, Age Group: 50-54

| Period | Age Group | Smoking Group | N | % | Ever Quit (N) | Ever Quit % | Mean Yrs Quit | Short Term (n) | Short Term % |
|---|---|---|---|---|---|---|---|---|---|
| 5 | 50-54 | Never Any | 1919 | 29.46 | | | | | |
| 5 | 50-54 | CC | 940 | 14.43 | 0 | 0.00 | | | |
| 5 | 50-54 | Ecig | 1015 | 15.58 | | | | | |
| 5 | 50-54 | Dual | 0 | 0.00 | | | | | |
| 5 | 50-54 | Former | 2641 | 40.54 | | | 15.98 | 175 | 6.63 |

Year of Follow-up: 1995, Age Group: 55-59

| Period | Age Group | Smoking Group | N | % | Ever Quit (N) | Ever Quit % | Mean Yrs Quit | Short Term (n) | Short Term % |
|---|---|---|---|---|---|---|---|---|---|
| 5 | 55-59 | Never Any | 1611 | 29.41 | | | | | |
| 5 | 55-59 | CC | 740 | 13.51 | 0 | 0.00 | | | |
| 5 | 55-59 | Ecig | 762 | 13.91 | | | | | |
| 5 | 55-59 | Dual | 0 | 0.00 | | | | | |
| 5 | 55-59 | Former | 2364 | 43.16 | | | 17.21 | 122 | 5.16 |

Year of Follow-up: 1995, Age Group: 60-64

| Period | Age Group | Smoking Group | N | % | Ever Quit (N) | Ever Quit % | Mean Yrs Quit | Short Term (n) | Short Term % |
|---|---|---|---|---|---|---|---|---|---|
| 5 | 60-64 | Never Any | 1337 | 26.83 | | | | | |
| 5 | 60-64 | CC | 597 | 11.98 | 0 | 0.00 | | | |
| 5 | 60-64 | Ecig | 572 | 11.48 | | | | | |
| 5 | 60-64 | Dual | 0 | 0.00 | | | | | |
| 5 | 60-64 | Former | 2477 | 49.71 | | | 17.27 | 143 | 5.77 |

Year of Follow-up: 1995, Age Group: 65-69

| Period | Age Group | Smoking Group | N | % | Ever Quit (N) | Ever Quit % | Mean Yrs Quit | Short Term (n) | Short Term % |
|---|---|---|---|---|---|---|---|---|---|
| 5 | 65-69 | Never Any | 1169 | 23.83 | | | | | |
| 5 | 65-69 | CC | 477 | 9.72 | 0 | 0.00 | | | |
| 5 | 65-69 | Ecig | 360 | 7.34 | | | | | |
| 5 | 65-69 | Dual | 0 | 0.00 | | | | | |
| 5 | 65-69 | Former | 2899 | 59.10 | | | 20.44 | 196 | 6.76 |

Year of Follow-up: 1995, Age Group: 70-74

| Period | Age Group | Smoking Group | N | % | Ever Quit (N) | Ever Quit % | Mean Yrs Quit | Short Term (n) | Short Term % |
|---|---|---|---|---|---|---|---|---|---|
| 5 | 70-74 | Never Any | 1023 | 23.32 | | | | | |
| 5 | 70-74 | CC | 438 | 9.98 | 0 | 0.00 | | | |
| 5 | 70-74 | Ecig | 196 | 4.47 | | | | | |
| 5 | 70-74 | Dual | 0 | 0.00 | | | | | |
| 5 | 70-74 | Former | 2730 | 62.23 | | | 22.00 | 137 | 5.02 |

Year of Follow-up: 1995, Age Group: 75-79

| Period | Age Group | Smoking Group | N | % | Ever Quit (N) | Ever Quit % | Mean Yrs Quit | Short Term (n) | Short Term % |
|---|---|---|---|---|---|---|---|---|---|
| 5 | 75-79 | Never Any | 961 | 27.46 | | | | | |
| 5 | 75-79 | CC | 303 | 8.66 | 0 | 0.00 | | | |
| 5 | 75-79 | Ecig | 135 | 3.86 | | | | | |
| 5 | 75-79 | Dual | 0 | 0.00 | | | | | |
| 5 | 75-79 | Former | 2100 | 60.02 | | | 23.02 | 97 | 4.62 |

Year of Follow-up: 2000, Age Group: All Ages

# Results for PHIM for Run 1 (Sensitivity_Analysis_1.RTF)

| Period | Age Group | Smoking Group | N | % | Ever Quit (N) | Ever Quit % | Mean Yrs Quit | Short Term (n) | Short Term % |
|---|---|---|---|---|---|---|---|---|---|
| 10 | All Ages | Never Any | 39177 | 41.64 | | | | | |
| 10 | All Ages | CC | 4843 | 5.15 | 0 | 0.00 | | | |
| 10 | All Ages | Ecig | 20724 | 22.03 | | | | | |
| 10 | All Ages | Dual | 0 | 0.00 | | | | | |
| 10 | All Ages | Former | 29348 | 31.19 | | | 16.84 | 1724 | 5.87 |

Year of Follow-up: 2000, Age Group: 18-19

| Period | Age Group | Smoking Group | N | % | Ever Quit (N) | Ever Quit % | Mean Yrs Quit | Short Term (n) | Short Term % |
|---|---|---|---|---|---|---|---|---|---|
| 10 | 18-19 | Never Any | 0 | . | | | | | |
| 10 | 18-19 | CC | 0 | . | 0 | . | | | |
| 10 | 18-19 | Ecig | 0 | . | | | | | |
| 10 | 18-19 | Dual | 0 | . | | | | | |
| 10 | 18-19 | Former | 0 | . | | | . | 0 | . |

Year of Follow-up: 2000, Age Group: 20-24

| Period | Age Group | Smoking Group | N | % | Ever Quit (N) | Ever Quit % | Mean Yrs Quit | Short Term (n) | Short Term % |
|---|---|---|---|---|---|---|---|---|---|
| 10 | 20-24 | Never Any | 4927 | 55.70 | | | | | |
| 10 | 20-24 | CC | 462 | 5.22 | 0 | 0.00 | | | |
| 10 | 20-24 | Ecig | 2544 | 28.76 | | | | | |
| 10 | 20-24 | Dual | 0 | 0.00 | | | | | |
| 10 | 20-24 | Former | 912 | 10.31 | | | 3.41 | 258 | 28.29 |

Year of Follow-up: 2000, Age Group: 25-29

| Period | Age Group | Smoking Group | N | % | Ever Quit (N) | Ever Quit % | Mean Yrs Quit | Short Term (n) | Short Term % |
|---|---|---|---|---|---|---|---|---|---|
| 10 | 25-29 | Never Any | 5341 | 59.08 | | | | | |
| 10 | 25-29 | CC | 404 | 4.47 | 0 | 0.00 | | | |
| 10 | 25-29 | Ecig | 2359 | 26.10 | | | | | |
| 10 | 25-29 | Dual | 0 | 0.00 | | | | | |
| 10 | 25-29 | Former | 936 | 10.35 | | | 4.91 | 195 | 20.83 |

Year of Follow-up: 2000, Age Group: 30-34

| Period | Age Group | Smoking Group | N | % | Ever Quit (N) | Ever Quit % | Mean Yrs Quit | Short Term (n) | Short Term % |
|---|---|---|---|---|---|---|---|---|---|
| 10 | 30-34 | Never Any | 5276 | 53.75 | | | | | |
| 10 | 30-34 | CC | 481 | 4.90 | 0 | 0.00 | | | |
| 10 | 30-34 | Ecig | 2608 | 26.57 | | | | | |
| 10 | 30-34 | Dual | 0 | 0.00 | | | | | |
| 10 | 30-34 | Former | 1451 | 14.78 | | | 8.66 | 118 | 8.13 |

Year of Follow-up: 2000, Age Group: 35-39

| Period | Age Group | Smoking Group | N | % | Ever Quit (N) | Ever Quit % | Mean Yrs Quit | Short Term (n) | Short Term % |
|---|---|---|---|---|---|---|---|---|---|
| 10 | 35-39 | Never Any | 4997 | 46.86 | | | | | |
| 10 | 35-39 | CC | 609 | 5.71 | 0 | 0.00 | | | |
| 10 | 35-39 | Ecig | 2980 | 27.94 | | | | | |
| 10 | 35-39 | Dual | 0 | 0.00 | | | | | |
| 10 | 35-39 | Former | 2078 | 19.49 | | | 10.61 | 109 | 5.25 |

Year of Follow-up: 2000, Age Group: 40-44

| Period | Age Group | Smoking Group | N | % | Ever Quit (N) | Ever Quit % | Mean Yrs Quit | Short Term (n) | Short Term % |
|---|---|---|---|---|---|---|---|---|---|
| 10 | 40-44 | Never Any | 4724 | 43.28 | | | | | |
| 10 | 40-44 | CC | 567 | 5.19 | 0 | 0.00 | | | |
| 10 | 40-44 | Ecig | 2857 | 26.17 | | | | | |
| 10 | 40-44 | Dual | 0 | 0.00 | | | | | |
| 10 | 40-44 | Former | 2768 | 25.36 | | | 11.82 | 124 | 4.48 |

Year of Follow-up: 2000, Age Group: 45-49

| Period | Age Group | Smoking Group | N | % | Ever Quit (N) | Ever Quit % | Mean Yrs Quit | Short Term (n) | Short Term % |
|---|---|---|---|---|---|---|---|---|---|
| 10 | 45-49 | Never Any | 3941 | 39.85 | | | | | |
| 10 | 45-49 | CC | 484 | 4.89 | 0 | 0.00 | | | |
| 10 | 45-49 | Ecig | 2312 | 23.38 | | | | | |
| 10 | 45-49 | Dual | 0 | 0.00 | | | | | |
| 10 | 45-49 | Former | 3152 | 31.87 | | | 15.25 | 163 | 5.17 |

Year of Follow-up: 2000, Age Group: 50-54

| Period | Age Group | Smoking Group | N | % | Ever Quit (N) | Ever Quit % | Mean Yrs Quit | Short Term (n) | Short Term % |
|---|---|---|---|---|---|---|---|---|---|
| 10 | 50-54 | Never Any | 2912 | 33.65 | | | | | |
| 10 | 50-54 | CC | 473 | 5.47 | 0 | 0.00 | | | |
| 10 | 50-54 | Ecig | 1859 | 21.48 | | | | | |
| 10 | 50-54 | Dual | 0 | 0.00 | | | | | |
| 10 | 50-54 | Former | 3411 | 39.41 | | | 16.85 | 186 | 5.45 |

Year of Follow-up: 2000, Age Group: 55-59

| Period | Age Group | Smoking Group | N | % | Ever Quit (N) | Ever Quit % | Mean Yrs Quit | Short Term (n) | Short Term % |
|---|---|---|---|---|---|---|---|---|---|
| 10 | 55-59 | Never Any | 1919 | 29.46 | | | | | |
| 10 | 55-59 | CC | 363 | 5.57 | 0 | 0.00 | | | |
| 10 | 55-59 | Ecig | 1194 | 18.33 | | | | | |
| 10 | 55-59 | Dual | 0 | 0.00 | | | | | |

# Results for PHIM for Run 1 (Sensitivity_Analysis_1.RTF)

| | | | | | | | | | |
|---|---|---|---|---|---|---|---|---|---|
| 10 | 55-59 | Former | 3039 | 46.65 | | | 18.50 | 146 | 4.80 |

Year of Follow-up: 2000, Age Group: 60-64

| Period | Age Group | Smoking Group | N | % | Ever Quit (N) | Ever Quit % | Mean Yrs Quit | Short Term (n) | Short Term % |
|---|---|---|---|---|---|---|---|---|---|
| 10 | 60-64 | Never Any | 1611 | 29.41 | | | | | |
| 10 | 60-64 | CC | 306 | 5.59 | 0 | 0.00 | | | |
| 10 | 60-64 | Ecig | 852 | 15.56 | | | | | |
| 10 | 60-64 | Dual | 0 | 0.00 | | | | | |
| 10 | 60-64 | Former | 2708 | 49.44 | | | 19.63 | 144 | 5.32 |

Year of Follow-up: 2000, Age Group: 65-69

| Period | Age Group | Smoking Group | N | % | Ever Quit (N) | Ever Quit % | Mean Yrs Quit | Short Term (n) | Short Term % |
|---|---|---|---|---|---|---|---|---|---|
| 10 | 65-69 | Never Any | 1337 | 26.83 | | | | | |
| 10 | 65-69 | CC | 244 | 4.90 | 0 | 0.00 | | | |
| 10 | 65-69 | Ecig | 603 | 12.10 | | | | | |
| 10 | 65-69 | Dual | 0 | 0.00 | | | | | |
| 10 | 65-69 | Former | 2799 | 56.17 | | | 19.95 | 120 | 4.29 |

Year of Follow-up: 2000, Age Group: 70-74

| Period | Age Group | Smoking Group | N | % | Ever Quit (N) | Ever Quit % | Mean Yrs Quit | Short Term (n) | Short Term % |
|---|---|---|---|---|---|---|---|---|---|
| 10 | 70-74 | Never Any | 1169 | 23.83 | | | | | |
| 10 | 70-74 | CC | 236 | 4.81 | 0 | 0.00 | | | |
| 10 | 70-74 | Ecig | 327 | 6.67 | | | | | |
| 10 | 70-74 | Dual | 0 | 0.00 | | | | | |
| 10 | 70-74 | Former | 3173 | 64.69 | | | 23.44 | 94 | 2.96 |

Year of Follow-up: 2000, Age Group: 75-79

| Period | Age Group | Smoking Group | N | % | Ever Quit (N) | Ever Quit % | Mean Yrs Quit | Short Term (n) | Short Term % |
|---|---|---|---|---|---|---|---|---|---|
| 10 | 75-79 | Never Any | 1023 | 23.32 | | | | | |
| 10 | 75-79 | CC | 214 | 4.88 | 0 | 0.00 | | | |
| 10 | 75-79 | Ecig | 229 | 5.22 | | | | | |
| 10 | 75-79 | Dual | 0 | 0.00 | | | | | |
| 10 | 75-79 | Former | 2921 | 66.58 | | | 25.38 | 67 | 2.29 |

Year of Follow-up: 2005, Age Group: All Ages

| Period | Age Group | Smoking Group | N | % | Ever Quit (N) | Ever Quit % | Mean Yrs Quit | Short Term (n) | Short Term % |
|---|---|---|---|---|---|---|---|---|---|
| 15 | All Ages | Never Any | 37449 | 41.75 | | | | | |
| 15 | All Ages | CC | 1911 | 2.13 | 0 | 0.00 | | | |
| 15 | All Ages | Ecig | 20003 | 22.30 | | | | | |
| 15 | All Ages | Dual | 0 | 0.00 | | | | | |
| 15 | All Ages | Former | 30342 | 33.82 | | | 18.46 | 1574 | 5.19 |

Year of Follow-up: 2005, Age Group: 18-19

| Period | Age Group | Smoking Group | N | % | Ever Quit (N) | Ever Quit % | Mean Yrs Quit | Short Term (n) | Short Term % |
|---|---|---|---|---|---|---|---|---|---|
| 15 | 18-19 | Never Any | 0 | . | | | | | |
| 15 | 18-19 | CC | 0 | . | 0 | . | | | |
| 15 | 18-19 | Ecig | 0 | . | | | | | |
| 15 | 18-19 | Dual | 0 | . | | | | | |
| 15 | 18-19 | Former | 0 | . | | | . | 0 | . |

Year of Follow-up: 2005, Age Group: 20-24

| Period | Age Group | Smoking Group | N | % | Ever Quit (N) | Ever Quit % | Mean Yrs Quit | Short Term (n) | Short Term % |
|---|---|---|---|---|---|---|---|---|---|
| 15 | 20-24 | Never Any | 0 | . | | | | | |
| 15 | 20-24 | CC | 0 | . | 0 | . | | | |
| 15 | 20-24 | Ecig | 0 | . | | | | | |
| 15 | 20-24 | Dual | 0 | . | | | | | |
| 15 | 20-24 | Former | 0 | . | | | . | 0 | . |

Year of Follow-up: 2005, Age Group: 25-29

| Period | Age Group | Smoking Group | N | % | Ever Quit (N) | Ever Quit % | Mean Yrs Quit | Short Term (n) | Short Term % |
|---|---|---|---|---|---|---|---|---|---|
| 15 | 25-29 | Never Any | 4634 | 52.39 | | | | | |
| 15 | 25-29 | CC | 179 | 2.02 | 0 | 0.00 | | | |
| 15 | 25-29 | Ecig | 2661 | 30.08 | | | | | |
| 15 | 25-29 | Dual | 0 | 0.00 | | | | | |
| 15 | 25-29 | Former | 1371 | 15.50 | | | 6.32 | 174 | 12.69 |

Year of Follow-up: 2005, Age Group: 30-34

| Period | Age Group | Smoking Group | N | % | Ever Quit (N) | Ever Quit % | Mean Yrs Quit | Short Term (n) | Short Term % |
|---|---|---|---|---|---|---|---|---|---|
| 15 | 30-34 | Never Any | 5042 | 55.77 | | | | | |
| 15 | 30-34 | CC | 153 | 1.69 | 0 | 0.00 | | | |
| 15 | 30-34 | Ecig | 2479 | 27.42 | | | | | |
| 15 | 30-34 | Dual | 0 | 0.00 | | | | | |
| 15 | 30-34 | Former | 1366 | 15.11 | | | 7.38 | 171 | 12.52 |

Year of Follow-up: 2005, Age Group: 35-39

| Period | Age Group | Smoking Group | N | % | Ever Quit (N) | Ever Quit % | Mean Yrs Quit | Short Term (n) | Short Term % |
|---|---|---|---|---|---|---|---|---|---|
| 15 | 35-39 | Never Any | 5163 | 52.60 | | | | | |
| 15 | 35-39 | CC | 188 | 1.92 | 0 | 0.00 | | | |

# Results for PHIM for Run 1 (Sensitivity_Analysis_1.RTF)

| | | | | | | | | | |
|---|---|---|---|---|---|---|---|---|---|
| 15 | 35-39 | Ecig | 2654 | 27.04 | | | | | |
| 15 | 35-39 | Dual | 0 | 0.00 | | | | | |
| 15 | 35-39 | Former | 1811 | 18.45 | | | 11.33 | 147 | 8.12 |

Year of Follow-up: 2005, Age Group: 40-44

| Period | Age Group | Smoking Group | N | % | Ever Quit (N) | Ever Quit % | Mean Yrs Quit | Short Term (n) | Short Term % |
|---|---|---|---|---|---|---|---|---|---|
| 15 | 40-44 | Never Any | 4997 | 46.86 | | | | | |
| 15 | 40-44 | CC | 231 | 2.17 | 0 | 0.00 | | | |
| 15 | 40-44 | Ecig | 2948 | 27.64 | | | | | |
| 15 | 40-44 | Dual | 0 | 0.00 | | | | | |
| 15 | 40-44 | Former | 2488 | 23.33 | | | 13.32 | 199 | 8.00 |

Year of Follow-up: 2005, Age Group: 45-49

| Period | Age Group | Smoking Group | N | % | Ever Quit (N) | Ever Quit % | Mean Yrs Quit | Short Term (n) | Short Term % |
|---|---|---|---|---|---|---|---|---|---|
| 15 | 45-49 | Never Any | 4724 | 43.28 | | | | | |
| 15 | 45-49 | CC | 216 | 1.98 | 0 | 0.00 | | | |
| 15 | 45-49 | Ecig | 2793 | 25.59 | | | | | |
| 15 | 45-49 | Dual | 0 | 0.00 | | | | | |
| 15 | 45-49 | Former | 3183 | 29.16 | | | 14.88 | 160 | 5.03 |

Year of Follow-up: 2005, Age Group: 50-54

| Period | Age Group | Smoking Group | N | % | Ever Quit (N) | Ever Quit % | Mean Yrs Quit | Short Term (n) | Short Term % |
|---|---|---|---|---|---|---|---|---|---|
| 15 | 50-54 | Never Any | 3941 | 39.85 | | | | | |
| 15 | 50-54 | CC | 213 | 2.15 | 0 | 0.00 | | | |
| 15 | 50-54 | Ecig | 2180 | 22.04 | | | | | |
| 15 | 50-54 | Dual | 0 | 0.00 | | | | | |
| 15 | 50-54 | Former | 3555 | 35.95 | | | 18.19 | 154 | 4.33 |

Year of Follow-up: 2005, Age Group: 55-59

| Period | Age Group | Smoking Group | N | % | Ever Quit (N) | Ever Quit % | Mean Yrs Quit | Short Term (n) | Short Term % |
|---|---|---|---|---|---|---|---|---|---|
| 15 | 55-59 | Never Any | 2912 | 33.65 | | | | | |
| 15 | 55-59 | CC | 215 | 2.48 | 0 | 0.00 | | | |
| 15 | 55-59 | Ecig | 1781 | 20.58 | | | | | |
| 15 | 55-59 | Dual | 0 | 0.00 | | | | | |
| 15 | 55-59 | Former | 3747 | 43.29 | | | 20.09 | 117 | 3.12 |

Year of Follow-up: 2005, Age Group: 60-64

| Period | Age Group | Smoking Group | N | % | Ever Quit (N) | Ever Quit % | Mean Yrs Quit | Short Term (n) | Short Term % |
|---|---|---|---|---|---|---|---|---|---|
| 15 | 60-64 | Never Any | 1919 | 29.46 | | | | | |
| 15 | 60-64 | CC | 143 | 2.19 | 0 | 0.00 | | | |
| 15 | 60-64 | Ecig | 1100 | 16.88 | | | | | |
| 15 | 60-64 | Dual | 0 | 0.00 | | | | | |
| 15 | 60-64 | Former | 3353 | 51.47 | | | 21.47 | 156 | 4.65 |

Year of Follow-up: 2005, Age Group: 65-69

| Period | Age Group | Smoking Group | N | % | Ever Quit (N) | Ever Quit % | Mean Yrs Quit | Short Term (n) | Short Term % |
|---|---|---|---|---|---|---|---|---|---|
| 15 | 65-69 | Never Any | 1611 | 29.41 | | | | | |
| 15 | 65-69 | CC | 124 | 2.26 | 0 | 0.00 | | | |
| 15 | 65-69 | Ecig | 705 | 12.87 | | | | | |
| 15 | 65-69 | Dual | 0 | 0.00 | | | | | |
| 15 | 65-69 | Former | 3037 | 55.45 | | | 22.19 | 118 | 3.89 |

Year of Follow-up: 2005, Age Group: 70-74

| Period | Age Group | Smoking Group | N | % | Ever Quit (N) | Ever Quit % | Mean Yrs Quit | Short Term (n) | Short Term % |
|---|---|---|---|---|---|---|---|---|---|
| 15 | 70-74 | Never Any | 1337 | 26.83 | | | | | |
| 15 | 70-74 | CC | 129 | 2.59 | 0 | 0.00 | | | |
| 15 | 70-74 | Ecig | 441 | 8.85 | | | | | |
| 15 | 70-74 | Dual | 0 | 0.00 | | | | | |
| 15 | 70-74 | Former | 3076 | 61.73 | | | 22.89 | 112 | 3.64 |

Year of Follow-up: 2005, Age Group: 75-79

| Period | Age Group | Smoking Group | N | % | Ever Quit (N) | Ever Quit % | Mean Yrs Quit | Short Term (n) | Short Term % |
|---|---|---|---|---|---|---|---|---|---|
| 15 | 75-79 | Never Any | 1169 | 23.83 | | | | | |
| 15 | 75-79 | CC | 120 | 2.45 | 0 | 0.00 | | | |
| 15 | 75-79 | Ecig | 261 | 5.32 | | | | | |
| 15 | 75-79 | Dual | 0 | 0.00 | | | | | |
| 15 | 75-79 | Former | 3355 | 68.40 | | | 27.01 | 66 | 1.97 |

Year of Follow-up: 2010, Age Group: All Ages

| Period | Age Group | Smoking Group | N | % | Ever Quit (N) | Ever Quit % | Mean Yrs Quit | Short Term (n) | Short Term % |
|---|---|---|---|---|---|---|---|---|---|
| 20 | All Ages | Never Any | 36103 | 42.57 | | | | | |
| 20 | All Ages | CC | 738 | 0.87 | 0 | 0.00 | | | |
| 20 | All Ages | Ecig | 17076 | 20.14 | | | | | |
| 20 | All Ages | Dual | 0 | 0.00 | | | | | |
| 20 | All Ages | Former | 30883 | 36.42 | | | 19.82 | 1518 | 4.92 |

Year of Follow-up: 2010, Age Group: 18-19

| Period | Age Group | Smoking Group | N | % | Ever Quit (N) | Ever Quit % | Mean Yrs Quit | Short Term (n) | Short Term % |
|---|---|---|---|---|---|---|---|---|---|

# Results for PHIM for Run 1 (Sensitivity_Analysis_1.RTF)

| | | | | | | | | | |
|---|---|---|---|---|---|---|---|---|---|
| 20 | 18-19 | Never Any | 0 | . | | | | | |
| 20 | 18-19 | CC | 0 | . | 0 | . | | | |
| 20 | 18-19 | Ecig | 0 | . | | | | | |
| 20 | 18-19 | Dual | 0 | . | | | | | |
| 20 | 18-19 | Former | 0 | . | | | . | 0 | . |

Year of Follow-up: 2010, Age Group: 20-24

| Period | Age Group | Smoking Group | N | % | Ever Quit (N) | Ever Quit % | Mean Yrs Quit | Short Term (n) | Short Term % |
|---|---|---|---|---|---|---|---|---|---|
| 20 | 20-24 | Never Any | 0 | . | | | | | |
| 20 | 20-24 | CC | 0 | . | 0 | . | | | |
| 20 | 20-24 | Ecig | 0 | . | | | | | |
| 20 | 20-24 | Dual | 0 | . | | | | | |
| 20 | 20-24 | Former | 0 | . | | | . | 0 | . |

Year of Follow-up: 2010, Age Group: 25-29

| Period | Age Group | Smoking Group | N | % | Ever Quit (N) | Ever Quit % | Mean Yrs Quit | Short Term (n) | Short Term % |
|---|---|---|---|---|---|---|---|---|---|
| 20 | 25-29 | Never Any | 0 | . | | | | | |
| 20 | 25-29 | CC | 0 | . | 0 | . | | | |
| 20 | 25-29 | Ecig | 0 | . | | | | | |
| 20 | 25-29 | Dual | 0 | . | | | | | |
| 20 | 25-29 | Former | 0 | . | | | . | 0 | . |

Year of Follow-up: 2010, Age Group: 30-34

| Period | Age Group | Smoking Group | N | % | Ever Quit (N) | Ever Quit % | Mean Yrs Quit | Short Term (n) | Short Term % |
|---|---|---|---|---|---|---|---|---|---|
| 20 | 30-34 | Never Any | 4508 | 50.97 | | | | | |
| 20 | 30-34 | CC | 71 | 0.80 | 0 | 0.00 | | | |
| 20 | 30-34 | Ecig | 2376 | 26.86 | | | | | |
| 20 | 30-34 | Dual | 0 | 0.00 | | | | | |
| 20 | 30-34 | Former | 1890 | 21.37 | | | 8.72 | 230 | 12.17 |

Year of Follow-up: 2010, Age Group: 35-39

| Period | Age Group | Smoking Group | N | % | Ever Quit (N) | Ever Quit % | Mean Yrs Quit | Short Term (n) | Short Term % |
|---|---|---|---|---|---|---|---|---|---|
| 20 | 35-39 | Never Any | 4991 | 55.21 | | | | | |
| 20 | 35-39 | CC | 59 | 0.65 | 0 | 0.00 | | | |
| 20 | 35-39 | Ecig | 2154 | 23.83 | | | | | |
| 20 | 35-39 | Dual | 0 | 0.00 | | | | | |
| 20 | 35-39 | Former | 1836 | 20.31 | | | 9.77 | 141 | 7.68 |

Year of Follow-up: 2010, Age Group: 40-44

| Period | Age Group | Smoking Group | N | % | Ever Quit (N) | Ever Quit % | Mean Yrs Quit | Short Term (n) | Short Term % |
|---|---|---|---|---|---|---|---|---|---|
| 20 | 40-44 | Never Any | 5163 | 52.60 | | | | | |
| 20 | 40-44 | CC | 68 | 0.69 | 0 | 0.00 | | | |
| 20 | 40-44 | Ecig | 2416 | 24.61 | | | | | |
| 20 | 40-44 | Dual | 0 | 0.00 | | | | | |
| 20 | 40-44 | Former | 2169 | 22.10 | | | 13.96 | 143 | 6.59 |

Year of Follow-up: 2010, Age Group: 45-49

| Period | Age Group | Smoking Group | N | % | Ever Quit (N) | Ever Quit % | Mean Yrs Quit | Short Term (n) | Short Term % |
|---|---|---|---|---|---|---|---|---|---|
| 20 | 45-49 | Never Any | 4997 | 46.86 | | | | | |
| 20 | 45-49 | CC | 89 | 0.83 | 0 | 0.00 | | | |
| 20 | 45-49 | Ecig | 2619 | 24.56 | | | | | |
| 20 | 45-49 | Dual | 0 | 0.00 | | | | | |
| 20 | 45-49 | Former | 2959 | 27.75 | | | 15.74 | 171 | 5.78 |

Year of Follow-up: 2010, Age Group: 50-54

| Period | Age Group | Smoking Group | N | % | Ever Quit (N) | Ever Quit % | Mean Yrs Quit | Short Term (n) | Short Term % |
|---|---|---|---|---|---|---|---|---|---|
| 20 | 50-54 | Never Any | 4724 | 43.28 | | | | | |
| 20 | 50-54 | CC | 87 | 0.80 | 0 | 0.00 | | | |
| 20 | 50-54 | Ecig | 2490 | 22.81 | | | | | |
| 20 | 50-54 | Dual | 0 | 0.00 | | | | | |
| 20 | 50-54 | Former | 3615 | 33.12 | | | 17.75 | 174 | 4.81 |

Year of Follow-up: 2010, Age Group: 55-59

| Period | Age Group | Smoking Group | N | % | Ever Quit (N) | Ever Quit % | Mean Yrs Quit | Short Term (n) | Short Term % |
|---|---|---|---|---|---|---|---|---|---|
| 20 | 55-59 | Never Any | 3941 | 39.85 | | | | | |
| 20 | 55-59 | CC | 85 | 0.86 | 0 | 0.00 | | | |
| 20 | 55-59 | Ecig | 1859 | 18.80 | | | | | |
| 20 | 55-59 | Dual | 0 | 0.00 | | | | | |
| 20 | 55-59 | Former | 4004 | 40.49 | | | 20.81 | 181 | 4.52 |

Year of Follow-up: 2010, Age Group: 60-64

| Period | Age Group | Smoking Group | N | % | Ever Quit (N) | Ever Quit % | Mean Yrs Quit | Short Term (n) | Short Term % |
|---|---|---|---|---|---|---|---|---|---|
| 20 | 60-64 | Never Any | 2912 | 33.65 | | | | | |
| 20 | 60-64 | CC | 87 | 1.01 | 0 | 0.00 | | | |
| 20 | 60-64 | Ecig | 1428 | 16.50 | | | | | |
| 20 | 60-64 | Dual | 0 | 0.00 | | | | | |
| 20 | 60-64 | Former | 4228 | 48.85 | | | 22.41 | 241 | 5.70 |

Year of Follow-up: 2010, Age Group: 65-69

# Results for PHIM for Run 1 (Sensitivity_Analysis_1.RTF)

| Period | Age Group | Smoking Group | N | % | Ever Quit (N) | Ever Quit % | Mean Yrs Quit | Short Term (n) | Short Term % |
|---|---|---|---|---|---|---|---|---|---|
| 20 | 65-69 | Never Any | 1919 | 29.46 | | | | | |
| 20 | 65-69 | CC | 60 | 0.92 | 0 | 0.00 | | | |
| 20 | 65-69 | Ecig | 809 | 12.42 | | | | | |
| 20 | 65-69 | Dual | 0 | 0.00 | | | | | |
| 20 | 65-69 | Former | 3727 | 57.21 | | | 24.03 | 133 | 3.57 |

Year of Follow-up: 2010, Age Group: 70-74

| Period | Age Group | Smoking Group | N | % | Ever Quit (N) | Ever Quit % | Mean Yrs Quit | Short Term (n) | Short Term % |
|---|---|---|---|---|---|---|---|---|---|
| 20 | 70-74 | Never Any | 1611 | 29.41 | | | | | |
| 20 | 70-74 | CC | 69 | 1.26 | 0 | 0.00 | | | |
| 20 | 70-74 | Ecig | 574 | 10.48 | | | | | |
| 20 | 70-74 | Dual | 0 | 0.00 | | | | | |
| 20 | 70-74 | Former | 3223 | 58.85 | | | 25.75 | 61 | 1.89 |

Year of Follow-up: 2010, Age Group: 75-79

| Period | Age Group | Smoking Group | N | % | Ever Quit (N) | Ever Quit % | Mean Yrs Quit | Short Term (n) | Short Term % |
|---|---|---|---|---|---|---|---|---|---|
| 20 | 75-79 | Never Any | 1337 | 26.83 | | | | | |
| 20 | 75-79 | CC | 63 | 1.26 | 0 | 0.00 | | | |
| 20 | 75-79 | Ecig | 351 | 7.04 | | | | | |
| 20 | 75-79 | Dual | 0 | 0.00 | | | | | |
| 20 | 75-79 | Former | 3232 | 64.86 | | | 26.66 | 43 | 1.33 |

Year of Follow-up: 2015, Age Group: All Ages

| Period | Age Group | Smoking Group | N | % | Ever Quit (N) | Ever Quit % | Mean Yrs Quit | Short Term (n) | Short Term % |
|---|---|---|---|---|---|---|---|---|---|
| 25 | All Ages | Never Any | 34747 | 43.53 | | | | | |
| 25 | All Ages | CC | 289 | 0.36 | 0 | 0.00 | | | |
| 25 | All Ages | Ecig | 14107 | 17.67 | | | | | |
| 25 | All Ages | Dual | 0 | 0.00 | | | | | |
| 25 | All Ages | Former | 30674 | 38.43 | | | 21.87 | 1088 | 3.55 |

Year of Follow-up: 2015, Age Group: 18-19

| Period | Age Group | Smoking Group | N | % | Ever Quit (N) | Ever Quit % | Mean Yrs Quit | Short Term (n) | Short Term % |
|---|---|---|---|---|---|---|---|---|---|
| 25 | 18-19 | Never Any | 0 | . | | | | | |
| 25 | 18-19 | CC | 0 | . | 0 | . | | | |
| 25 | 18-19 | Ecig | 0 | . | | | | | |
| 25 | 18-19 | Dual | 0 | . | | | | | |
| 25 | 18-19 | Former | 0 | . | | | . | 0 | . |

Year of Follow-up: 2015, Age Group: 20-24

| Period | Age Group | Smoking Group | N | % | Ever Quit (N) | Ever Quit % | Mean Yrs Quit | Short Term (n) | Short Term % |
|---|---|---|---|---|---|---|---|---|---|
| 25 | 20-24 | Never Any | 0 | . | | | | | |
| 25 | 20-24 | CC | 0 | . | 0 | . | | | |
| 25 | 20-24 | Ecig | 0 | . | | | | | |
| 25 | 20-24 | Dual | 0 | . | | | | | |
| 25 | 20-24 | Former | 0 | . | | | . | 0 | . |

Year of Follow-up: 2015, Age Group: 25-29

| Period | Age Group | Smoking Group | N | % | Ever Quit (N) | Ever Quit % | Mean Yrs Quit | Short Term (n) | Short Term % |
|---|---|---|---|---|---|---|---|---|---|
| 25 | 25-29 | Never Any | 0 | . | | | | | |
| 25 | 25-29 | CC | 0 | . | 0 | . | | | |
| 25 | 25-29 | Ecig | 0 | . | | | | | |
| 25 | 25-29 | Dual | 0 | . | | | | | |
| 25 | 25-29 | Former | 0 | . | | | . | 0 | . |

Year of Follow-up: 2015, Age Group: 30-34

| Period | Age Group | Smoking Group | N | % | Ever Quit (N) | Ever Quit % | Mean Yrs Quit | Short Term (n) | Short Term % |
|---|---|---|---|---|---|---|---|---|---|
| 25 | 30-34 | Never Any | 0 | . | | | | | |
| 25 | 30-34 | CC | 0 | . | 0 | . | | | |
| 25 | 30-34 | Ecig | 0 | . | | | | | |
| 25 | 30-34 | Dual | 0 | . | | | | | |
| 25 | 30-34 | Former | 0 | . | | | . | 0 | . |

Year of Follow-up: 2015, Age Group: 35-39

| Period | Age Group | Smoking Group | N | % | Ever Quit (N) | Ever Quit % | Mean Yrs Quit | Short Term (n) | Short Term % |
|---|---|---|---|---|---|---|---|---|---|
| 25 | 35-39 | Never Any | 4489 | 50.75 | | | | | |
| 25 | 35-39 | CC | 28 | 0.32 | 0 | 0.00 | | | |
| 25 | 35-39 | Ecig | 1990 | 22.50 | | | | | |
| 25 | 35-39 | Dual | 0 | 0.00 | | | | | |
| 25 | 35-39 | Former | 2338 | 26.43 | | | 11.53 | 142 | 6.07 |

Year of Follow-up: 2015, Age Group: 40-44

| Period | Age Group | Smoking Group | N | % | Ever Quit (N) | Ever Quit % | Mean Yrs Quit | Short Term (n) | Short Term % |
|---|---|---|---|---|---|---|---|---|---|
| 25 | 40-44 | Never Any | 4991 | 55.21 | | | | | |
| 25 | 40-44 | CC | 20 | 0.22 | 0 | 0.00 | | | |
| 25 | 40-44 | Ecig | 1897 | 20.98 | | | | | |
| 25 | 40-44 | Dual | 0 | 0.00 | | | | | |

# Results for PHIM for Run 1 (Sensitivity_Analysis_1.RTF)

| | | | | | | | | | |
|---|---|---|---|---|---|---|---|---|---|
| 25 | 40-44 | Former | 2132 | 23.58 | | | 13.01 | 112 | 5.25 |

Year of Follow-up: 2015, Age Group: 45-49

| Period | Age Group | Smoking Group | N | % | Ever Quit (N) | Ever Quit % | Mean Yrs Quit | Short Term (n) | Short Term % |
|---|---|---|---|---|---|---|---|---|---|
| 25 | 45-49 | Never Any | 5163 | 52.60 | | | | | |
| 25 | 45-49 | CC | 29 | 0.30 | 0 | 0.00 | | | |
| 25 | 45-49 | Ecig | 2195 | 22.36 | | | | | |
| 25 | 45-49 | Dual | 0 | 0.00 | | | | | |
| 25 | 45-49 | Former | 2429 | 24.75 | | | 17.18 | 81 | 3.33 |

Year of Follow-up: 2015, Age Group: 50-54

| Period | Age Group | Smoking Group | N | % | Ever Quit (N) | Ever Quit % | Mean Yrs Quit | Short Term (n) | Short Term % |
|---|---|---|---|---|---|---|---|---|---|
| 25 | 50-54 | Never Any | 4997 | 46.86 | | | | | |
| 25 | 50-54 | CC | 37 | 0.35 | 0 | 0.00 | | | |
| 25 | 50-54 | Ecig | 2387 | 22.38 | | | | | |
| 25 | 50-54 | Dual | 0 | 0.00 | | | | | |
| 25 | 50-54 | Former | 3243 | 30.41 | | | 19.10 | 130 | 4.01 |

Year of Follow-up: 2015, Age Group: 55-59

| Period | Age Group | Smoking Group | N | % | Ever Quit (N) | Ever Quit % | Mean Yrs Quit | Short Term (n) | Short Term % |
|---|---|---|---|---|---|---|---|---|---|
| 25 | 55-59 | Never Any | 4724 | 43.28 | | | | | |
| 25 | 55-59 | CC | 33 | 0.30 | 0 | 0.00 | | | |
| 25 | 55-59 | Ecig | 2103 | 19.27 | | | | | |
| 25 | 55-59 | Dual | 0 | 0.00 | | | | | |
| 25 | 55-59 | Former | 4056 | 37.16 | | | 20.50 | 162 | 3.99 |

Year of Follow-up: 2015, Age Group: 60-64

| Period | Age Group | Smoking Group | N | % | Ever Quit (N) | Ever Quit % | Mean Yrs Quit | Short Term (n) | Short Term % |
|---|---|---|---|---|---|---|---|---|---|
| 25 | 60-64 | Never Any | 3941 | 39.85 | | | | | |
| 25 | 60-64 | CC | 28 | 0.28 | 0 | 0.00 | | | |
| 25 | 60-64 | Ecig | 1443 | 14.59 | | | | | |
| 25 | 60-64 | Dual | 0 | 0.00 | | | | | |
| 25 | 60-64 | Former | 4477 | 45.27 | | | 23.28 | 205 | 4.58 |

Year of Follow-up: 2015, Age Group: 65-69

| Period | Age Group | Smoking Group | N | % | Ever Quit (N) | Ever Quit % | Mean Yrs Quit | Short Term (n) | Short Term % |
|---|---|---|---|---|---|---|---|---|---|
| 25 | 65-69 | Never Any | 2912 | 33.65 | | | | | |
| 25 | 65-69 | CC | 43 | 0.50 | 0 | 0.00 | | | |
| 25 | 65-69 | Ecig | 1019 | 11.77 | | | | | |
| 25 | 65-69 | Dual | 0 | 0.00 | | | | | |
| 25 | 65-69 | Former | 4681 | 54.08 | | | 24.99 | 134 | 2.86 |

Year of Follow-up: 2015, Age Group: 70-74

| Period | Age Group | Smoking Group | N | % | Ever Quit (N) | Ever Quit % | Mean Yrs Quit | Short Term (n) | Short Term % |
|---|---|---|---|---|---|---|---|---|---|
| 25 | 70-74 | Never Any | 1919 | 29.46 | | | | | |
| 25 | 70-74 | CC | 35 | 0.54 | 0 | 0.00 | | | |
| 25 | 70-74 | Ecig | 616 | 9.46 | | | | | |
| 25 | 70-74 | Dual | 0 | 0.00 | | | | | |
| 25 | 70-74 | Former | 3945 | 60.55 | | | 27.55 | 70 | 1.77 |

Year of Follow-up: 2015, Age Group: 75-79

| Period | Age Group | Smoking Group | N | % | Ever Quit (N) | Ever Quit % | Mean Yrs Quit | Short Term (n) | Short Term % |
|---|---|---|---|---|---|---|---|---|---|
| 25 | 75-79 | Never Any | 1611 | 29.41 | | | | | |
| 25 | 75-79 | CC | 36 | 0.66 | 0 | 0.00 | | | |
| 25 | 75-79 | Ecig | 457 | 8.34 | | | | | |
| 25 | 75-79 | Dual | 0 | 0.00 | | | | | |
| 25 | 75-79 | Former | 3373 | 61.58 | | | 29.48 | 52 | 1.54 |

Year of Follow-up: 2020, Age Group: All Ages

| Period | Age Group | Smoking Group | N | % | Ever Quit (N) | Ever Quit % | Mean Yrs Quit | Short Term (n) | Short Term % |
|---|---|---|---|---|---|---|---|---|---|
| 30 | All Ages | Never Any | 33136 | 44.57 | | | | | |
| 30 | All Ages | CC | 100 | 0.13 | 0 | 0.00 | | | |
| 30 | All Ages | Ecig | 11358 | 15.28 | | | | | |
| 30 | All Ages | Dual | 0 | 0.00 | | | | | |
| 30 | All Ages | Former | 29746 | 40.01 | | | 23.97 | 918 | 3.09 |

Year of Follow-up: 2020, Age Group: 18-19

| Period | Age Group | Smoking Group | N | % | Ever Quit (N) | Ever Quit % | Mean Yrs Quit | Short Term (n) | Short Term % |
|---|---|---|---|---|---|---|---|---|---|
| 30 | 18-19 | Never Any | 0 | . | | | | | |
| 30 | 18-19 | CC | 0 | . | 0 | . | | | |
| 30 | 18-19 | Ecig | 0 | . | | | | | |
| 30 | 18-19 | Dual | 0 | . | | | | | |
| 30 | 18-19 | Former | 0 | . | | | . | 0 | . |

Year of Follow-up: 2020, Age Group: 20-24

| Period | Age Group | Smoking Group | N | % | Ever Quit (N) | Ever Quit % | Mean Yrs Quit | Short Term (n) | Short Term % |
|---|---|---|---|---|---|---|---|---|---|
| 30 | 20-24 | Never Any | 0 | . | | | | | |
| 30 | 20-24 | CC | 0 | . | 0 | . | | | |

# Results for PHIM for Run 1 (Sensitivity_Analysis_1.RTF)

| | | | | | | | | | |
|---|---|---|---|---|---|---|---|---|---|
| 30 | 20-24 | Ecig | 0 | . | | | | | |
| 30 | 20-24 | Dual | 0 | . | | | | | |
| 30 | 20-24 | Former | 0 | . | | | . | 0 | . |

Year of Follow-up: 2020, Age Group: 25-29

| Period | Age Group | Smoking Group | N | % | Ever Quit (N) | Ever Quit % | Mean Yrs Quit | Short Term (n) | Short Term % |
|---|---|---|---|---|---|---|---|---|---|
| 30 | 25-29 | Never Any | 0 | . | | | | | |
| 30 | 25-29 | CC | 0 | . | 0 | . | | | |
| 30 | 25-29 | Ecig | 0 | . | | | | | |
| 30 | 25-29 | Dual | 0 | . | | | | | |
| 30 | 25-29 | Former | 0 | . | | | . | 0 | . |

Year of Follow-up: 2020, Age Group: 30-34

| Period | Age Group | Smoking Group | N | % | Ever Quit (N) | Ever Quit % | Mean Yrs Quit | Short Term (n) | Short Term % |
|---|---|---|---|---|---|---|---|---|---|
| 30 | 30-34 | Never Any | 0 | . | | | | | |
| 30 | 30-34 | CC | 0 | . | 0 | . | | | |
| 30 | 30-34 | Ecig | 0 | . | | | | | |
| 30 | 30-34 | Dual | 0 | . | | | | | |
| 30 | 30-34 | Former | 0 | . | | | . | 0 | . |

Year of Follow-up: 2020, Age Group: 35-39

| Period | Age Group | Smoking Group | N | % | Ever Quit (N) | Ever Quit % | Mean Yrs Quit | Short Term (n) | Short Term % |
|---|---|---|---|---|---|---|---|---|---|
| 30 | 35-39 | Never Any | 0 | . | | | | | |
| 30 | 35-39 | CC | 0 | . | 0 | . | | | |
| 30 | 35-39 | Ecig | 0 | . | | | | | |
| 30 | 35-39 | Dual | 0 | . | | | | | |
| 30 | 35-39 | Former | 0 | . | | | . | 0 | . |

Year of Follow-up: 2020, Age Group: 40-44

| Period | Age Group | Smoking Group | N | % | Ever Quit (N) | Ever Quit % | Mean Yrs Quit | Short Term (n) | Short Term % |
|---|---|---|---|---|---|---|---|---|---|
| 30 | 40-44 | Never Any | 4489 | 50.75 | | | | | |
| 30 | 40-44 | CC | 12 | 0.14 | 0 | 0.00 | | | |
| 30 | 40-44 | Ecig | 1743 | 19.71 | | | | | |
| 30 | 40-44 | Dual | 0 | 0.00 | | | | | |
| 30 | 40-44 | Former | 2601 | 29.41 | | | 15.05 | 107 | 4.11 |

Year of Follow-up: 2020, Age Group: 45-49

| Period | Age Group | Smoking Group | N | % | Ever Quit (N) | Ever Quit % | Mean Yrs Quit | Short Term (n) | Short Term % |
|---|---|---|---|---|---|---|---|---|---|
| 30 | 45-49 | Never Any | 4991 | 55.21 | | | | | |
| 30 | 45-49 | CC | 5 | 0.06 | 0 | 0.00 | | | |
| 30 | 45-49 | Ecig | 1690 | 18.69 | | | | | |
| 30 | 45-49 | Dual | 0 | 0.00 | | | | | |
| 30 | 45-49 | Former | 2354 | 26.04 | | | 16.54 | 63 | 2.68 |

Year of Follow-up: 2020, Age Group: 50-54

| Period | Age Group | Smoking Group | N | % | Ever Quit (N) | Ever Quit % | Mean Yrs Quit | Short Term (n) | Short Term % |
|---|---|---|---|---|---|---|---|---|---|
| 30 | 50-54 | Never Any | 5163 | 52.60 | | | | | |
| 30 | 50-54 | CC | 9 | 0.09 | 0 | 0.00 | | | |
| 30 | 50-54 | Ecig | 2021 | 20.59 | | | | | |
| 30 | 50-54 | Dual | 0 | 0.00 | | | | | |
| 30 | 50-54 | Former | 2623 | 26.72 | | | 20.66 | 99 | 3.77 |

Year of Follow-up: 2020, Age Group: 55-59

| Period | Age Group | Smoking Group | N | % | Ever Quit (N) | Ever Quit % | Mean Yrs Quit | Short Term (n) | Short Term % |
|---|---|---|---|---|---|---|---|---|---|
| 30 | 55-59 | Never Any | 4997 | 46.86 | | | | | |
| 30 | 55-59 | CC | 18 | 0.17 | 0 | 0.00 | | | |
| 30 | 55-59 | Ecig | 2002 | 18.77 | | | | | |
| 30 | 55-59 | Dual | 0 | 0.00 | | | | | |
| 30 | 55-59 | Former | 3647 | 34.20 | | | 21.65 | 157 | 4.30 |

Year of Follow-up: 2020, Age Group: 60-64

| Period | Age Group | Smoking Group | N | % | Ever Quit (N) | Ever Quit % | Mean Yrs Quit | Short Term (n) | Short Term % |
|---|---|---|---|---|---|---|---|---|---|
| 30 | 60-64 | Never Any | 4724 | 43.28 | | | | | |
| 30 | 60-64 | CC | 10 | 0.09 | 0 | 0.00 | | | |
| 30 | 60-64 | Ecig | 1614 | 14.79 | | | | | |
| 30 | 60-64 | Dual | 0 | 0.00 | | | | | |
| 30 | 60-64 | Former | 4568 | 41.85 | | | 22.87 | 203 | 4.44 |

Year of Follow-up: 2020, Age Group: 65-69

| Period | Age Group | Smoking Group | N | % | Ever Quit (N) | Ever Quit % | Mean Yrs Quit | Short Term (n) | Short Term % |
|---|---|---|---|---|---|---|---|---|---|
| 30 | 65-69 | Never Any | 3941 | 39.85 | | | | | |
| 30 | 65-69 | CC | 13 | 0.13 | 0 | 0.00 | | | |
| 30 | 65-69 | Ecig | 1039 | 10.51 | | | | | |
| 30 | 65-69 | Dual | 0 | 0.00 | | | | | |
| 30 | 65-69 | Former | 4896 | 49.51 | | | 26.05 | 145 | 2.96 |

Year of Follow-up: 2020, Age Group: 70-74

| Period | Age Group | Smoking Group | N | % | Ever Quit (N) | Ever Quit % | Mean Yrs Quit | Short Term (n) | Short Term % |
|---|---|---|---|---|---|---|---|---|---|

# Results for PHIM for Run 1 (Sensitivity_Analysis_1.RTF)

| | | | | | | | | | |
|---|---|---|---|---|---|---|---|---|---|
| 30 | 70-74 | Never Any | 2912 | 33.65 | | | | | |
| 30 | 70-74 | CC | 18 | 0.21 | 0 | 0.00 | | | |
| 30 | 70-74 | Ecig | 770 | 8.90 | | | | | |
| 30 | 70-74 | Dual | 0 | 0.00 | | | | | |
| 30 | 70-74 | Former | 4955 | 57.25 | | | 28.45 | 93 | 1.88 |

Year of Follow-up: 2020, Age Group: 75-79

| Period | Age Group | Smoking Group | N | % | Ever Quit (N) | Ever Quit % | Mean Yrs Quit | Short Term (n) | Short Term % |
|---|---|---|---|---|---|---|---|---|---|
| 30 | 75-79 | Never Any | 1919 | 29.46 | | | | | |
| 30 | 75-79 | CC | 15 | 0.23 | 0 | 0.00 | | | |
| 30 | 75-79 | Ecig | 479 | 7.35 | | | | | |
| 30 | 75-79 | Dual | 0 | 0.00 | | | | | |
| 30 | 75-79 | Former | 4102 | 62.96 | | | 31.39 | 51 | 1.24 |

Year of Follow-up: 2025, Age Group: All Ages

| Period | Age Group | Smoking Group | N | % | Ever Quit (N) | Ever Quit % | Mean Yrs Quit | Short Term (n) | Short Term % |
|---|---|---|---|---|---|---|---|---|---|
| 35 | All Ages | Never Any | 31217 | 46.03 | | | | | |
| 35 | All Ages | CC | 38 | 0.06 | 0 | 0.00 | | | |
| 35 | All Ages | Ecig | 8864 | 13.07 | | | | | |
| 35 | All Ages | Dual | 0 | 0.00 | | | | | |
| 35 | All Ages | Former | 27706 | 40.85 | | | 25.87 | 785 | 2.83 |

Year of Follow-up: 2025, Age Group: 18-19

| Period | Age Group | Smoking Group | N | % | Ever Quit (N) | Ever Quit % | Mean Yrs Quit | Short Term (n) | Short Term % |
|---|---|---|---|---|---|---|---|---|---|
| 35 | 18-19 | Never Any | 0 | . | | | | | |
| 35 | 18-19 | CC | 0 | . | 0 | . | | | |
| 35 | 18-19 | Ecig | 0 | . | | | | | |
| 35 | 18-19 | Dual | 0 | . | | | | | |
| 35 | 18-19 | Former | 0 | . | | | . | 0 | . |

Year of Follow-up: 2025, Age Group: 20-24

| Period | Age Group | Smoking Group | N | % | Ever Quit (N) | Ever Quit % | Mean Yrs Quit | Short Term (n) | Short Term % |
|---|---|---|---|---|---|---|---|---|---|
| 35 | 20-24 | Never Any | 0 | . | | | | | |
| 35 | 20-24 | CC | 0 | . | 0 | . | | | |
| 35 | 20-24 | Ecig | 0 | . | | | | | |
| 35 | 20-24 | Dual | 0 | . | | | | | |
| 35 | 20-24 | Former | 0 | . | | | . | 0 | . |

Year of Follow-up: 2025, Age Group: 25-29

| Period | Age Group | Smoking Group | N | % | Ever Quit (N) | Ever Quit % | Mean Yrs Quit | Short Term (n) | Short Term % |
|---|---|---|---|---|---|---|---|---|---|
| 35 | 25-29 | Never Any | 0 | . | | | | | |
| 35 | 25-29 | CC | 0 | . | 0 | . | | | |
| 35 | 25-29 | Ecig | 0 | . | | | | | |
| 35 | 25-29 | Dual | 0 | . | | | | | |
| 35 | 25-29 | Former | 0 | . | | | . | 0 | . |

Year of Follow-up: 2025, Age Group: 30-34

| Period | Age Group | Smoking Group | N | % | Ever Quit (N) | Ever Quit % | Mean Yrs Quit | Short Term (n) | Short Term % |
|---|---|---|---|---|---|---|---|---|---|
| 35 | 30-34 | Never Any | 0 | . | | | | | |
| 35 | 30-34 | CC | 0 | . | 0 | . | | | |
| 35 | 30-34 | Ecig | 0 | . | | | | | |
| 35 | 30-34 | Dual | 0 | . | | | | | |
| 35 | 30-34 | Former | 0 | . | | | . | 0 | . |

Year of Follow-up: 2025, Age Group: 35-39

| Period | Age Group | Smoking Group | N | % | Ever Quit (N) | Ever Quit % | Mean Yrs Quit | Short Term (n) | Short Term % |
|---|---|---|---|---|---|---|---|---|---|
| 35 | 35-39 | Never Any | 0 | . | | | | | |
| 35 | 35-39 | CC | 0 | . | 0 | . | | | |
| 35 | 35-39 | Ecig | 0 | . | | | | | |
| 35 | 35-39 | Dual | 0 | . | | | | | |
| 35 | 35-39 | Former | 0 | . | | | . | 0 | . |

Year of Follow-up: 2025, Age Group: 40-44

| Period | Age Group | Smoking Group | N | % | Ever Quit (N) | Ever Quit % | Mean Yrs Quit | Short Term (n) | Short Term % |
|---|---|---|---|---|---|---|---|---|---|
| 35 | 40-44 | Never Any | 0 | . | | | | | |
| 35 | 40-44 | CC | 0 | . | 0 | . | | | |
| 35 | 40-44 | Ecig | 0 | . | | | | | |
| 35 | 40-44 | Dual | 0 | . | | | | | |
| 35 | 40-44 | Former | 0 | . | | | . | 0 | . |

Year of Follow-up: 2025, Age Group: 45-49

| Period | Age Group | Smoking Group | N | % | Ever Quit (N) | Ever Quit % | Mean Yrs Quit | Short Term (n) | Short Term % |
|---|---|---|---|---|---|---|---|---|---|
| 35 | 45-49 | Never Any | 4489 | 50.75 | | | | | |
| 35 | 45-49 | CC | 7 | 0.08 | 0 | 0.00 | | | |
| 35 | 45-49 | Ecig | 1541 | 17.42 | | | | | |
| 35 | 45-49 | Dual | 0 | 0.00 | | | | | |
| 35 | 45-49 | Former | 2808 | 31.75 | | | 18.76 | 60 | 2.14 |

Year of Follow-up: 2025, Age Group: 50-54

## Results for PHIM for Run 1 (Sensitivity_Analysis_1.RTF)

| Period | Age Group | Smoking Group | N | % | Ever Quit (N) | Ever Quit % | Mean Yrs Quit | Short Term (n) | Short Term % |
|---|---|---|---|---|---|---|---|---|---|
| 35 | 50-54 | Never Any | 4991 | 55.21 | | | | | |
| 35 | 50-54 | CC | 3 | 0.03 | 0 | 0.00 | | | |
| 35 | 50-54 | Ecig | 1554 | 17.19 | | | | | |
| 35 | 50-54 | Dual | 0 | 0.00 | | | | | |
| 35 | 50-54 | Former | 2492 | 27.57 | | | 20.44 | 75 | 3.01 |

Year of Follow-up: 2025, Age Group: 55-59

| Period | Age Group | Smoking Group | N | % | Ever Quit (N) | Ever Quit % | Mean Yrs Quit | Short Term (n) | Short Term % |
|---|---|---|---|---|---|---|---|---|---|
| 35 | 55-59 | Never Any | 5163 | 52.60 | | | | | |
| 35 | 55-59 | CC | 0 | 0.00 | 0 | 0.00 | | | |
| 35 | 55-59 | Ecig | 1664 | 16.95 | | | | | |
| 35 | 55-59 | Dual | 0 | 0.00 | | | | | |
| 35 | 55-59 | Former | 2989 | 30.45 | | | 22.76 | 155 | 5.19 |

Year of Follow-up: 2025, Age Group: 60-64

| Period | Age Group | Smoking Group | N | % | Ever Quit (N) | Ever Quit % | Mean Yrs Quit | Short Term (n) | Short Term % |
|---|---|---|---|---|---|---|---|---|---|
| 35 | 60-64 | Never Any | 4997 | 46.86 | | | | | |
| 35 | 60-64 | CC | 9 | 0.08 | 0 | 0.00 | | | |
| 35 | 60-64 | Ecig | 1532 | 14.37 | | | | | |
| 35 | 60-64 | Dual | 0 | 0.00 | | | | | |
| 35 | 60-64 | Former | 4126 | 38.69 | | | 23.79 | 186 | 4.51 |

Year of Follow-up: 2025, Age Group: 65-69

| Period | Age Group | Smoking Group | N | % | Ever Quit (N) | Ever Quit % | Mean Yrs Quit | Short Term (n) | Short Term % |
|---|---|---|---|---|---|---|---|---|---|
| 35 | 65-69 | Never Any | 4724 | 43.28 | | | | | |
| 35 | 65-69 | CC | 5 | 0.05 | 0 | 0.00 | | | |
| 35 | 65-69 | Ecig | 1169 | 10.71 | | | | | |
| 35 | 65-69 | Dual | 0 | 0.00 | | | | | |
| 35 | 65-69 | Former | 5018 | 45.97 | | | 25.57 | 158 | 3.15 |

Year of Follow-up: 2025, Age Group: 70-74

| Period | Age Group | Smoking Group | N | % | Ever Quit (N) | Ever Quit % | Mean Yrs Quit | Short Term (n) | Short Term % |
|---|---|---|---|---|---|---|---|---|---|
| 35 | 70-74 | Never Any | 3941 | 39.85 | | | | | |
| 35 | 70-74 | CC | 5 | 0.05 | 0 | 0.00 | | | |
| 35 | 70-74 | Ecig | 811 | 8.20 | | | | | |
| 35 | 70-74 | Dual | 0 | 0.00 | | | | | |
| 35 | 70-74 | Former | 5132 | 51.90 | | | 29.73 | 79 | 1.54 |

Year of Follow-up: 2025, Age Group: 75-79

| Period | Age Group | Smoking Group | N | % | Ever Quit (N) | Ever Quit % | Mean Yrs Quit | Short Term (n) | Short Term % |
|---|---|---|---|---|---|---|---|---|---|
| 35 | 75-79 | Never Any | 2912 | 33.65 | | | | | |
| 35 | 75-79 | CC | 9 | 0.10 | 0 | 0.00 | | | |
| 35 | 75-79 | Ecig | 593 | 6.85 | | | | | |
| 35 | 75-79 | Dual | 0 | 0.00 | | | | | |
| 35 | 75-79 | Former | 5141 | 59.40 | | | 32.31 | 72 | 1.40 |

Year of Follow-up: 2030, Age Group: All Ages

| Period | Age Group | Smoking Group | N | % | Ever Quit (N) | Ever Quit % | Mean Yrs Quit | Short Term (n) | Short Term % |
|---|---|---|---|---|---|---|---|---|---|
| 40 | All Ages | Never Any | 28305 | 47.84 | | | | | |
| 40 | All Ages | CC | 15 | 0.03 | 0 | 0.00 | | | |
| 40 | All Ages | Ecig | 6535 | 11.04 | | | | | |
| 40 | All Ages | Dual | 0 | 0.00 | | | | | |
| 40 | All Ages | Former | 24315 | 41.09 | | | 27.43 | 691 | 2.84 |

Year of Follow-up: 2030, Age Group: 18-19

| Period | Age Group | Smoking Group | N | % | Ever Quit (N) | Ever Quit % | Mean Yrs Quit | Short Term (n) | Short Term % |
|---|---|---|---|---|---|---|---|---|---|
| 40 | 18-19 | Never Any | 0 | . | | | | | |
| 40 | 18-19 | CC | 0 | . | 0 | . | | | |
| 40 | 18-19 | Ecig | 0 | . | | | | | |
| 40 | 18-19 | Dual | 0 | . | | | | | |
| 40 | 18-19 | Former | 0 | . | | | . | 0 | . |

Year of Follow-up: 2030, Age Group: 20-24

| Period | Age Group | Smoking Group | N | % | Ever Quit (N) | Ever Quit % | Mean Yrs Quit | Short Term (n) | Short Term % |
|---|---|---|---|---|---|---|---|---|---|
| 40 | 20-24 | Never Any | 0 | . | | | | | |
| 40 | 20-24 | CC | 0 | . | 0 | . | | | |
| 40 | 20-24 | Ecig | 0 | . | | | | | |
| 40 | 20-24 | Dual | 0 | . | | | | | |
| 40 | 20-24 | Former | 0 | . | | | . | 0 | . |

Year of Follow-up: 2030, Age Group: 25-29

| Period | Age Group | Smoking Group | N | % | Ever Quit (N) | Ever Quit % | Mean Yrs Quit | Short Term (n) | Short Term % |
|---|---|---|---|---|---|---|---|---|---|
| 40 | 25-29 | Never Any | 0 | . | | | | | |
| 40 | 25-29 | CC | 0 | . | 0 | . | | | |
| 40 | 25-29 | Ecig | 0 | . | | | | | |
| 40 | 25-29 | Dual | 0 | . | | | | | |

# Results for PHIM for Run 1 (Sensitivity_Analysis_1.RTF)

| | | | | | | | | | |
|---|---|---|---|---|---|---|---|---|---|
| 40 | 25-29 | Former | 0 | . | | | . | 0 | . |

Year of Follow-up: 2030, Age Group: 30-34

| Period | Age Group | Smoking Group | N | % | Ever Quit (N) | Ever Quit % | Mean Yrs Quit | Short Term (n) | Short Term % |
|---|---|---|---|---|---|---|---|---|---|
| 40 | 30-34 | Never Any | 0 | . | | | | | |
| 40 | 30-34 | CC | 0 | . | 0 | . | | | |
| 40 | 30-34 | Ecig | 0 | . | | | | | |
| 40 | 30-34 | Dual | 0 | . | | | | | |
| 40 | 30-34 | Former | 0 | . | | | . | 0 | . |

Year of Follow-up: 2030, Age Group: 35-39

| Period | Age Group | Smoking Group | N | % | Ever Quit (N) | Ever Quit % | Mean Yrs Quit | Short Term (n) | Short Term % |
|---|---|---|---|---|---|---|---|---|---|
| 40 | 35-39 | Never Any | 0 | . | | | | | |
| 40 | 35-39 | CC | 0 | . | 0 | . | | | |
| 40 | 35-39 | Ecig | 0 | . | | | | | |
| 40 | 35-39 | Dual | 0 | . | | | | | |
| 40 | 35-39 | Former | 0 | . | | | . | 0 | . |

Year of Follow-up: 2030, Age Group: 40-44

| Period | Age Group | Smoking Group | N | % | Ever Quit (N) | Ever Quit % | Mean Yrs Quit | Short Term (n) | Short Term % |
|---|---|---|---|---|---|---|---|---|---|
| 40 | 40-44 | Never Any | 0 | . | | | | | |
| 40 | 40-44 | CC | 0 | . | 0 | . | | | |
| 40 | 40-44 | Ecig | 0 | . | | | | | |
| 40 | 40-44 | Dual | 0 | . | | | | | |
| 40 | 40-44 | Former | 0 | . | | | . | 0 | . |

Year of Follow-up: 2030, Age Group: 45-49

| Period | Age Group | Smoking Group | N | % | Ever Quit (N) | Ever Quit % | Mean Yrs Quit | Short Term (n) | Short Term % |
|---|---|---|---|---|---|---|---|---|---|
| 40 | 45-49 | Never Any | 0 | . | | | | | |
| 40 | 45-49 | CC | 0 | . | 0 | . | | | |
| 40 | 45-49 | Ecig | 0 | . | | | | | |
| 40 | 45-49 | Dual | 0 | . | | | | | |
| 40 | 45-49 | Former | 0 | . | | | . | 0 | . |

Year of Follow-up: 2030, Age Group: 50-54

| Period | Age Group | Smoking Group | N | % | Ever Quit (N) | Ever Quit % | Mean Yrs Quit | Short Term (n) | Short Term % |
|---|---|---|---|---|---|---|---|---|---|
| 40 | 50-54 | Never Any | 4489 | 50.75 | | | | | |
| 40 | 50-54 | CC | 3 | 0.03 | 0 | 0.00 | | | |
| 40 | 50-54 | Ecig | 1392 | 15.74 | | | | | |
| 40 | 50-54 | Dual | 0 | 0.00 | | | | | |
| 40 | 50-54 | Former | 2961 | 33.48 | | | 22.61 | 80 | 2.70 |

Year of Follow-up: 2030, Age Group: 55-59

| Period | Age Group | Smoking Group | N | % | Ever Quit (N) | Ever Quit % | Mean Yrs Quit | Short Term (n) | Short Term % |
|---|---|---|---|---|---|---|---|---|---|
| 40 | 55-59 | Never Any | 4991 | 55.21 | | | | | |
| 40 | 55-59 | CC | 2 | 0.02 | 0 | 0.00 | | | |
| 40 | 55-59 | Ecig | 1291 | 14.28 | | | | | |
| 40 | 55-59 | Dual | 0 | 0.00 | | | | | |
| 40 | 55-59 | Former | 2756 | 30.49 | | | 23.19 | 114 | 4.14 |

Year of Follow-up: 2030, Age Group: 60-64

| Period | Age Group | Smoking Group | N | % | Ever Quit (N) | Ever Quit % | Mean Yrs Quit | Short Term (n) | Short Term % |
|---|---|---|---|---|---|---|---|---|---|
| 40 | 60-64 | Never Any | 5163 | 52.60 | | | | | |
| 40 | 60-64 | CC | 0 | 0.00 | 0 | 0.00 | | | |
| 40 | 60-64 | Ecig | 1238 | 12.61 | | | | | |
| 40 | 60-64 | Dual | 0 | 0.00 | | | | | |
| 40 | 60-64 | Former | 3415 | 34.79 | | | 24.53 | 183 | 5.36 |

Year of Follow-up: 2030, Age Group: 65-69

| Period | Age Group | Smoking Group | N | % | Ever Quit (N) | Ever Quit % | Mean Yrs Quit | Short Term (n) | Short Term % |
|---|---|---|---|---|---|---|---|---|---|
| 40 | 65-69 | Never Any | 4997 | 46.86 | | | | | |
| 40 | 65-69 | CC | 4 | 0.04 | 0 | 0.00 | | | |
| 40 | 65-69 | Ecig | 1119 | 10.49 | | | | | |
| 40 | 65-69 | Dual | 0 | 0.00 | | | | | |
| 40 | 65-69 | Former | 4544 | 42.61 | | | 26.35 | 136 | 2.99 |

Year of Follow-up: 2030, Age Group: 70-74

| Period | Age Group | Smoking Group | N | % | Ever Quit (N) | Ever Quit % | Mean Yrs Quit | Short Term (n) | Short Term % |
|---|---|---|---|---|---|---|---|---|---|
| 40 | 70-74 | Never Any | 4724 | 43.28 | | | | | |
| 40 | 70-74 | CC | 3 | 0.03 | 0 | 0.00 | | | |
| 40 | 70-74 | Ecig | 880 | 8.06 | | | | | |
| 40 | 70-74 | Dual | 0 | 0.00 | | | | | |
| 40 | 70-74 | Former | 5309 | 48.64 | | | 29.01 | 95 | 1.79 |

Year of Follow-up: 2030, Age Group: 75-79

| Period | Age Group | Smoking Group | N | % | Ever Quit (N) | Ever Quit % | Mean Yrs Quit | Short Term (n) | Short Term % |
|---|---|---|---|---|---|---|---|---|---|
| 40 | 75-79 | Never Any | 3941 | 39.85 | | | | | |
| 40 | 75-79 | CC | 3 | 0.03 | 0 | 0.00 | | | |

# Results for PHIM for Run 1 (Sensitivity_Analysis_1.RTF)

| | | | | | | | | | |
|---|---|---|---|---|---|---|---|---|---|
| 40 | 75-79 | Ecig | 615 | 6.22 | | | | | |
| 40 | 75-79 | Dual | 0 | 0.00 | | | | | |
| 40 | 75-79 | Former | 5330 | 53.90 | | | 33.51 | 83 | 1.56 |

Year of Follow-up: 2035, Age Group: All Ages

| Period | Age Group | Smoking Group | N | % | Ever Quit (N) | Ever Quit % | Mean Yrs Quit | Short Term (n) | Short Term % |
|---|---|---|---|---|---|---|---|---|---|
| 45 | All Ages | Never Any | 24364 | 49.44 | | | | | |
| 45 | All Ages | CC | 6 | 0.01 | 0 | 0.00 | | | |
| 45 | All Ages | Ecig | 4578 | 9.29 | | | | | |
| 45 | All Ages | Dual | 0 | 0.00 | | | | | |
| 45 | All Ages | Former | 20333 | 41.26 | | | 28.82 | 538 | 2.65 |

Year of Follow-up: 2035, Age Group: 18-19

| Period | Age Group | Smoking Group | N | % | Ever Quit (N) | Ever Quit % | Mean Yrs Quit | Short Term (n) | Short Term % |
|---|---|---|---|---|---|---|---|---|---|
| 45 | 18-19 | Never Any | 0 | . | | | | | |
| 45 | 18-19 | CC | 0 | . | 0 | . | | | |
| 45 | 18-19 | Ecig | 0 | . | | | | | |
| 45 | 18-19 | Dual | 0 | . | | | | | |
| 45 | 18-19 | Former | 0 | . | | | . | 0 | . |

Year of Follow-up: 2035, Age Group: 20-24

| Period | Age Group | Smoking Group | N | % | Ever Quit (N) | Ever Quit % | Mean Yrs Quit | Short Term (n) | Short Term % |
|---|---|---|---|---|---|---|---|---|---|
| 45 | 20-24 | Never Any | 0 | . | | | | | |
| 45 | 20-24 | CC | 0 | . | 0 | . | | | |
| 45 | 20-24 | Ecig | 0 | . | | | | | |
| 45 | 20-24 | Dual | 0 | . | | | | | |
| 45 | 20-24 | Former | 0 | . | | | . | 0 | . |

Year of Follow-up: 2035, Age Group: 25-29

| Period | Age Group | Smoking Group | N | % | Ever Quit (N) | Ever Quit % | Mean Yrs Quit | Short Term (n) | Short Term % |
|---|---|---|---|---|---|---|---|---|---|
| 45 | 25-29 | Never Any | 0 | . | | | | | |
| 45 | 25-29 | CC | 0 | . | 0 | . | | | |
| 45 | 25-29 | Ecig | 0 | . | | | | | |
| 45 | 25-29 | Dual | 0 | . | | | | | |
| 45 | 25-29 | Former | 0 | . | | | . | 0 | . |

Year of Follow-up: 2035, Age Group: 30-34

| Period | Age Group | Smoking Group | N | % | Ever Quit (N) | Ever Quit % | Mean Yrs Quit | Short Term (n) | Short Term % |
|---|---|---|---|---|---|---|---|---|---|
| 45 | 30-34 | Never Any | 0 | . | | | | | |
| 45 | 30-34 | CC | 0 | . | 0 | . | | | |
| 45 | 30-34 | Ecig | 0 | . | | | | | |
| 45 | 30-34 | Dual | 0 | . | | | | | |
| 45 | 30-34 | Former | 0 | . | | | . | 0 | . |

Year of Follow-up: 2035, Age Group: 35-39

| Period | Age Group | Smoking Group | N | % | Ever Quit (N) | Ever Quit % | Mean Yrs Quit | Short Term (n) | Short Term % |
|---|---|---|---|---|---|---|---|---|---|
| 45 | 35-39 | Never Any | 0 | . | | | | | |
| 45 | 35-39 | CC | 0 | . | 0 | . | | | |
| 45 | 35-39 | Ecig | 0 | . | | | | | |
| 45 | 35-39 | Dual | 0 | . | | | | | |
| 45 | 35-39 | Former | 0 | . | | | . | 0 | . |

Year of Follow-up: 2035, Age Group: 40-44

| Period | Age Group | Smoking Group | N | % | Ever Quit (N) | Ever Quit % | Mean Yrs Quit | Short Term (n) | Short Term % |
|---|---|---|---|---|---|---|---|---|---|
| 45 | 40-44 | Never Any | 0 | . | | | | | |
| 45 | 40-44 | CC | 0 | . | 0 | . | | | |
| 45 | 40-44 | Ecig | 0 | . | | | | | |
| 45 | 40-44 | Dual | 0 | . | | | | | |
| 45 | 40-44 | Former | 0 | . | | | . | 0 | . |

Year of Follow-up: 2035, Age Group: 45-49

| Period | Age Group | Smoking Group | N | % | Ever Quit (N) | Ever Quit % | Mean Yrs Quit | Short Term (n) | Short Term % |
|---|---|---|---|---|---|---|---|---|---|
| 45 | 45-49 | Never Any | 0 | . | | | | | |
| 45 | 45-49 | CC | 0 | . | 0 | . | | | |
| 45 | 45-49 | Ecig | 0 | . | | | | | |
| 45 | 45-49 | Dual | 0 | . | | | | | |
| 45 | 45-49 | Former | 0 | . | | | . | 0 | . |

Year of Follow-up: 2035, Age Group: 50-54

| Period | Age Group | Smoking Group | N | % | Ever Quit (N) | Ever Quit % | Mean Yrs Quit | Short Term (n) | Short Term % |
|---|---|---|---|---|---|---|---|---|---|
| 45 | 50-54 | Never Any | 0 | . | | | | | |
| 45 | 50-54 | CC | 0 | . | 0 | . | | | |
| 45 | 50-54 | Ecig | 0 | . | | | | | |
| 45 | 50-54 | Dual | 0 | . | | | | | |
| 45 | 50-54 | Former | 0 | . | | | . | 0 | . |

Year of Follow-up: 2035, Age Group: 55-59

| Period | Age Group | Smoking Group | N | % | Ever Quit (N) | Ever Quit % | Mean Yrs Quit | Short Term (n) | Short Term % |
|---|---|---|---|---|---|---|---|---|---|

# Results for PHIM for Run 1 (Sensitivity_Analysis_1.RTF)

| | | | | | | | | | |
|---|---|---|---|---|---|---|---|---|---|
| 45 | 55-59 | Never Any | 4489 | 50.75 | | | | | |
| 45 | 55-59 | CC | 2 | 0.02 | 0 | 0.00 | | | |
| 45 | 55-59 | Ecig | 1171 | 13.24 | | | | | |
| 45 | 55-59 | Dual | 0 | 0.00 | | | | | |
| 45 | 55-59 | Former | 3183 | 35.99 | | | 25.82 | 100 | 3.14 |

Year of Follow-up: 2035, Age Group: 60-64

| Period | Age Group | Smoking Group | N | % | Ever Quit (N) | Ever Quit % | Mean Yrs Quit | Short Term (n) | Short Term % |
|---|---|---|---|---|---|---|---|---|---|
| 45 | 60-64 | Never Any | 4991 | 55.21 | | | | | |
| 45 | 60-64 | CC | 0 | 0.00 | 0 | 0.00 | | | |
| 45 | 60-64 | Ecig | 1020 | 11.28 | | | | | |
| 45 | 60-64 | Dual | 0 | 0.00 | | | | | |
| 45 | 60-64 | Former | 3029 | 33.51 | | | 25.81 | 129 | 4.26 |

Year of Follow-up: 2035, Age Group: 65-69

| Period | Age Group | Smoking Group | N | % | Ever Quit (N) | Ever Quit % | Mean Yrs Quit | Short Term (n) | Short Term % |
|---|---|---|---|---|---|---|---|---|---|
| 45 | 65-69 | Never Any | 5163 | 52.60 | | | | | |
| 45 | 65-69 | CC | 0 | 0.00 | 0 | 0.00 | | | |
| 45 | 65-69 | Ecig | 902 | 9.19 | | | | | |
| 45 | 65-69 | Dual | 0 | 0.00 | | | | | |
| 45 | 65-69 | Former | 3751 | 38.21 | | | 27.09 | 106 | 2.83 |

Year of Follow-up: 2035, Age Group: 70-74

| Period | Age Group | Smoking Group | N | % | Ever Quit (N) | Ever Quit % | Mean Yrs Quit | Short Term (n) | Short Term % |
|---|---|---|---|---|---|---|---|---|---|
| 45 | 70-74 | Never Any | 4997 | 46.86 | | | | | |
| 45 | 70-74 | CC | 1 | 0.01 | 0 | 0.00 | | | |
| 45 | 70-74 | Ecig | 850 | 7.97 | | | | | |
| 45 | 70-74 | Dual | 0 | 0.00 | | | | | |
| 45 | 70-74 | Former | 4816 | 45.16 | | | 29.70 | 104 | 2.16 |

Year of Follow-up: 2035, Age Group: 75-79

| Period | Age Group | Smoking Group | N | % | Ever Quit (N) | Ever Quit % | Mean Yrs Quit | Short Term (n) | Short Term % |
|---|---|---|---|---|---|---|---|---|---|
| 45 | 75-79 | Never Any | 4724 | 43.28 | | | | | |
| 45 | 75-79 | CC | 3 | 0.03 | 0 | 0.00 | | | |
| 45 | 75-79 | Ecig | 635 | 5.82 | | | | | |
| 45 | 75-79 | Dual | 0 | 0.00 | | | | | |
| 45 | 75-79 | Former | 5554 | 50.88 | | | 32.60 | 99 | 1.78 |

Year of Follow-up: 2040, Age Group: All Ages

| Period | Age Group | Smoking Group | N | % | Ever Quit (N) | Ever Quit % | Mean Yrs Quit | Short Term (n) | Short Term % |
|---|---|---|---|---|---|---|---|---|---|
| 50 | All Ages | Never Any | 19640 | 51.19 | | | | | |
| 50 | All Ages | CC | 1 | 0.00 | 0 | 0.00 | | | |
| 50 | All Ages | Ecig | 2868 | 7.48 | | | | | |
| 50 | All Ages | Dual | 0 | 0.00 | | | | | |
| 50 | All Ages | Former | 15856 | 41.33 | | | 30.34 | 408 | 2.57 |

Year of Follow-up: 2040, Age Group: 18-19

| Period | Age Group | Smoking Group | N | % | Ever Quit (N) | Ever Quit % | Mean Yrs Quit | Short Term (n) | Short Term % |
|---|---|---|---|---|---|---|---|---|---|
| 50 | 18-19 | Never Any | 0 | . | | | | | |
| 50 | 18-19 | CC | 0 | . | 0 | . | | | |
| 50 | 18-19 | Ecig | 0 | . | | | | | |
| 50 | 18-19 | Dual | 0 | . | | | | | |
| 50 | 18-19 | Former | 0 | . | | | . | 0 | . |

Year of Follow-up: 2040, Age Group: 20-24

| Period | Age Group | Smoking Group | N | % | Ever Quit (N) | Ever Quit % | Mean Yrs Quit | Short Term (n) | Short Term % |
|---|---|---|---|---|---|---|---|---|---|
| 50 | 20-24 | Never Any | 0 | . | | | | | |
| 50 | 20-24 | CC | 0 | . | 0 | . | | | |
| 50 | 20-24 | Ecig | 0 | . | | | | | |
| 50 | 20-24 | Dual | 0 | . | | | | | |
| 50 | 20-24 | Former | 0 | . | | | . | 0 | . |

Year of Follow-up: 2040, Age Group: 25-29

| Period | Age Group | Smoking Group | N | % | Ever Quit (N) | Ever Quit % | Mean Yrs Quit | Short Term (n) | Short Term % |
|---|---|---|---|---|---|---|---|---|---|
| 50 | 25-29 | Never Any | 0 | . | | | | | |
| 50 | 25-29 | CC | 0 | . | 0 | . | | | |
| 50 | 25-29 | Ecig | 0 | . | | | | | |
| 50 | 25-29 | Dual | 0 | . | | | | | |
| 50 | 25-29 | Former | 0 | . | | | . | 0 | . |

Year of Follow-up: 2040, Age Group: 30-34

| Period | Age Group | Smoking Group | N | % | Ever Quit (N) | Ever Quit % | Mean Yrs Quit | Short Term (n) | Short Term % |
|---|---|---|---|---|---|---|---|---|---|
| 50 | 30-34 | Never Any | 0 | . | | | | | |
| 50 | 30-34 | CC | 0 | . | 0 | . | | | |
| 50 | 30-34 | Ecig | 0 | . | | | | | |
| 50 | 30-34 | Dual | 0 | . | | | | | |
| 50 | 30-34 | Former | 0 | . | | | . | 0 | . |

Year of Follow-up: 2040, Age Group: 35-39

## Results for PHIM for Run 1 (Sensitivity_Analysis_1.RTF)

| Period | Age Group | Smoking Group | N | % | Ever Quit (N) | Ever Quit % | Mean Yrs Quit | Short Term (n) | Short Term % |
|---|---|---|---|---|---|---|---|---|---|
| 50 | 35-39 | Never Any | 0 | . | | | | | |
| 50 | 35-39 | CC | 0 | . | 0 | . | | | |
| 50 | 35-39 | Ecig | 0 | . | | | | | |
| 50 | 35-39 | Dual | 0 | . | | | | | |
| 50 | 35-39 | Former | 0 | . | | | . | 0 | . |

Year of Follow-up: 2040, Age Group: 40-44

| Period | Age Group | Smoking Group | N | % | Ever Quit (N) | Ever Quit % | Mean Yrs Quit | Short Term (n) | Short Term % |
|---|---|---|---|---|---|---|---|---|---|
| 50 | 40-44 | Never Any | 0 | . | | | | | |
| 50 | 40-44 | CC | 0 | . | 0 | . | | | |
| 50 | 40-44 | Ecig | 0 | . | | | | | |
| 50 | 40-44 | Dual | 0 | . | | | | | |
| 50 | 40-44 | Former | 0 | . | | | . | 0 | . |

Year of Follow-up: 2040, Age Group: 45-49

| Period | Age Group | Smoking Group | N | % | Ever Quit (N) | Ever Quit % | Mean Yrs Quit | Short Term (n) | Short Term % |
|---|---|---|---|---|---|---|---|---|---|
| 50 | 45-49 | Never Any | 0 | . | | | | | |
| 50 | 45-49 | CC | 0 | . | 0 | . | | | |
| 50 | 45-49 | Ecig | 0 | . | | | | | |
| 50 | 45-49 | Dual | 0 | . | | | | | |
| 50 | 45-49 | Former | 0 | . | | | . | 0 | . |

Year of Follow-up: 2040, Age Group: 50-54

| Period | Age Group | Smoking Group | N | % | Ever Quit (N) | Ever Quit % | Mean Yrs Quit | Short Term (n) | Short Term % |
|---|---|---|---|---|---|---|---|---|---|
| 50 | 50-54 | Never Any | 0 | . | | | | | |
| 50 | 50-54 | CC | 0 | . | 0 | . | | | |
| 50 | 50-54 | Ecig | 0 | . | | | | | |
| 50 | 50-54 | Dual | 0 | . | | | | | |
| 50 | 50-54 | Former | 0 | . | | | . | 0 | . |

Year of Follow-up: 2040, Age Group: 55-59

| Period | Age Group | Smoking Group | N | % | Ever Quit (N) | Ever Quit % | Mean Yrs Quit | Short Term (n) | Short Term % |
|---|---|---|---|---|---|---|---|---|---|
| 50 | 55-59 | Never Any | 0 | . | | | | | |
| 50 | 55-59 | CC | 0 | . | 0 | . | | | |
| 50 | 55-59 | Ecig | 0 | . | | | | | |
| 50 | 55-59 | Dual | 0 | . | | | | | |
| 50 | 55-59 | Former | 0 | . | | | . | 0 | . |

Year of Follow-up: 2040, Age Group: 60-64

| Period | Age Group | Smoking Group | N | % | Ever Quit (N) | Ever Quit % | Mean Yrs Quit | Short Term (n) | Short Term % |
|---|---|---|---|---|---|---|---|---|---|
| 50 | 60-64 | Never Any | 4489 | 50.75 | | | | | |
| 50 | 60-64 | CC | 0 | 0.00 | 0 | 0.00 | | | |
| 50 | 60-64 | Ecig | 865 | 9.78 | | | | | |
| 50 | 60-64 | Dual | 0 | 0.00 | | | | | |
| 50 | 60-64 | Former | 3491 | 39.47 | | | 28.27 | 129 | 3.70 |

Year of Follow-up: 2040, Age Group: 65-69

| Period | Age Group | Smoking Group | N | % | Ever Quit (N) | Ever Quit % | Mean Yrs Quit | Short Term (n) | Short Term % |
|---|---|---|---|---|---|---|---|---|---|
| 50 | 65-69 | Never Any | 4991 | 55.21 | | | | | |
| 50 | 65-69 | CC | 0 | 0.00 | 0 | 0.00 | | | |
| 50 | 65-69 | Ecig | 705 | 7.80 | | | | | |
| 50 | 65-69 | Dual | 0 | 0.00 | | | | | |
| 50 | 65-69 | Former | 3344 | 36.99 | | | 28.11 | 120 | 3.59 |

Year of Follow-up: 2040, Age Group: 70-74

| Period | Age Group | Smoking Group | N | % | Ever Quit (N) | Ever Quit % | Mean Yrs Quit | Short Term (n) | Short Term % |
|---|---|---|---|---|---|---|---|---|---|
| 50 | 70-74 | Never Any | 5163 | 52.60 | | | | | |
| 50 | 70-74 | CC | 0 | 0.00 | 0 | 0.00 | | | |
| 50 | 70-74 | Ecig | 665 | 6.77 | | | | | |
| 50 | 70-74 | Dual | 0 | 0.00 | | | | | |
| 50 | 70-74 | Former | 3988 | 40.63 | | | 30.31 | 80 | 2.01 |

Year of Follow-up: 2040, Age Group: 75-79

| Period | Age Group | Smoking Group | N | % | Ever Quit (N) | Ever Quit % | Mean Yrs Quit | Short Term (n) | Short Term % |
|---|---|---|---|---|---|---|---|---|---|
| 50 | 75-79 | Never Any | 4997 | 46.86 | | | | | |
| 50 | 75-79 | CC | 1 | 0.01 | 0 | 0.00 | | | |
| 50 | 75-79 | Ecig | 633 | 5.94 | | | | | |
| 50 | 75-79 | Dual | 0 | 0.00 | | | | | |
| 50 | 75-79 | Former | 5033 | 47.20 | | | 33.29 | 79 | 1.57 |

# Results for PHIM for Run 1 (Sensitivity_Analysis_1.RTF)

************************************************************************************

**Main Results for E-Component for Run: 1, Simulation: 1, COD: Four Diseases Combined**

| Variable Parameter | Value |
|---|---|
| 1. Country | US (United States) |
| 2. Sex | M (Males) |
| 3. Year of start of process | 1990 |
| 4. Cause of Death | Four Diseases Combined |
| 5. Effective Dose NULL | CC:1 |
| 6. Effective Dose Ecig | CC:1 Ecig:0.4 Dual:0.7 |
| Note: | Death rates are per million population |

-----------------------------------------------------------------------------------------

Results for Age Groups Combined and Years combined, Population unadjusted for deaths in Ecig scenario,

| Age Group:All Year | Number Deaths | NULL Attrib Deaths | Ecig Attrib Deaths | Drop Deaths | %Drop Deaths | %Drop Attrib Deaths | Death Rate | NULL Attrib Rates | Ecig Attrib Rates | Drop Rates |
|---|---|---|---|---|---|---|---|---|---|---|
| All Comb | 13148871 | 4509390.583 | 3692121.713 | 817268.9 | 6.216 | 18.124 | 3806.31 | 1305.37 | 1068.79 | 236.582 |

-----------------------------------------------------------------------------------------

Results for Age Groups Combined, Population unadjusted for deaths in Ecig scenario,

| Age Group:All Year | Number Deaths | NULL Attrib Deaths | Ecig Attrib Deaths | Drop Deaths | %Drop Deaths | %Drop Attrib Deaths | Death Rate | NULL Attrib Rates | Ecig Attrib Rates | Drop Rates |
|---|---|---|---|---|---|---|---|---|---|---|
| 1990 | 317161 | 125773.065 | 125773.065 | 0.000 | 0.000 | 0.000 | 5027.71 | 1993.78 | 1993.78 | 0.000 |
| 1991 | 313217 | 123999.442 | 123730.577 | 268.866 | 0.086 | 0.217 | 4883.81 | 1933.45 | 1929.26 | 4.192 |
| 1992 | 308577 | 122014.015 | 121308.909 | 705.107 | 0.229 | 0.578 | 4729.73 | 1870.18 | 1859.37 | 10.808 |
| 1993 | 311713 | 122810.618 | 121500.897 | 1309.721 | 0.420 | 1.066 | 4692.62 | 1848.83 | 1829.11 | 19.717 |
| 1994 | 305700 | 119925.592 | 117938.639 | 1986.953 | 0.650 | 1.657 | 4516.19 | 1771.69 | 1742.34 | 29.354 |
| 1995 | 303225 | 117896.496 | 115123.838 | 2772.658 | 0.914 | 2.352 | 4393.80 | 1708.35 | 1668.17 | 40.176 |
| 1996 | 299559 | 115698.645 | 112070.850 | 3627.795 | 1.211 | 3.136 | 4273.16 | 1650.42 | 1598.67 | 51.750 |
| 1997 | 294226 | 113359.847 | 108883.214 | 4476.632 | 1.521 | 3.949 | 4128.67 | 1590.70 | 1527.88 | 62.818 |
| 1998 | 289258 | 111444.212 | 106111.638 | 5332.574 | 1.844 | 4.785 | 3992.19 | 1538.10 | 1464.50 | 73.598 |
| 1999 | 311002 | 114877.722 | 107802.858 | 7074.864 | 2.275 | 6.159 | 4224.60 | 1560.48 | 1464.38 | 96.104 |
| 2000 | 303786 | 112379.704 | 104387.314 | 7992.390 | 2.631 | 7.112 | 4066.21 | 1504.22 | 1397.24 | 106.979 |
| 2001 | 296294 | 110024.943 | 101015.392 | 9009.551 | 3.041 | 8.189 | 3914.28 | 1453.52 | 1334.50 | 119.023 |
| 2002 | 291898 | 108289.173 | 98169.699 | 10119.47 | 3.467 | 9.345 | 3813.00 | 1414.56 | 1282.37 | 132.188 |
| 2003 | 285865 | 106539.023 | 95456.769 | 11082.25 | 3.877 | 10.402 | 3695.36 | 1377.22 | 1233.96 | 143.259 |
| 2004 | 274177 | 103037.251 | 91223.556 | 11813.69 | 4.309 | 11.465 | 3506.10 | 1317.61 | 1166.54 | 151.070 |
| 2005 | 272379 | 103044.562 | 90200.271 | 12844.29 | 4.716 | 12.465 | 3442.69 | 1302.41 | 1140.07 | 162.343 |
| 2006 | 264118 | 99672.555 | 85966.619 | 13705.94 | 5.189 | 13.751 | 3307.76 | 1248.28 | 1076.63 | 171.650 |
| 2007 | 258419 | 97839.152 | 83410.595 | 14428.56 | 5.583 | 14.747 | 3203.20 | 1212.75 | 1033.90 | 178.847 |
| 2008 | 259461 | 98075.852 | 82747.547 | 15328.31 | 5.908 | 15.629 | 3180.09 | 1202.07 | 1014.20 | 187.872 |
| 2009 | 253907 | 95617.338 | 79671.919 | 15945.42 | 6.280 | 16.676 | 3075.31 | 1158.11 | 964.983 | 193.130 |
| 2010 | 251878 | 94607.988 | 77981.406 | 16626.58 | 6.601 | 17.574 | 3013.72 | 1131.98 | 933.047 | 198.937 |
| 2011 | 251779 | 93633.119 | 76140.482 | 17492.64 | 6.948 | 18.682 | 2982.76 | 1109.25 | 902.018 | 207.231 |
| 2012 | 252643 | 92775.175 | 74430.140 | 18345.03 | 7.261 | 19.774 | 2960.61 | 1087.19 | 872.212 | 214.977 |
| 2013 | 256175 | 92345.780 | 73121.645 | 19224.14 | 7.504 | 20.818 | 2967.76 | 1069.82 | 847.106 | 222.710 |
| 2014 | 257393 | 91379.215 | 71430.861 | 19948.35 | 7.750 | 21.830 | 2947.05 | 1046.26 | 817.856 | 228.401 |
| 2015 | 259782 | 90750.242 | 70193.402 | 20556.84 | 7.913 | 22.652 | 3346.10 | 1168.90 | 904.121 | 264.781 |
| 2016 | 261453 | 88811.996 | 67646.515 | 21165.48 | 8.095 | 23.832 | 3336.15 | 1133.24 | 863.172 | 270.072 |
| 2017 | 263685 | 87325.126 | 65532.963 | 21792.16 | 8.264 | 24.955 | 3333.85 | 1104.08 | 828.554 | 275.525 |
| 2018 | 258714 | 87612.144 | 65450.748 | 22161.40 | 8.566 | 25.295 | 3240.73 | 1097.46 | 819.856 | 277.600 |
| 2019 | 261438 | 87797.760 | 64926.578 | 22871.18 | 8.748 | 26.050 | 3242.99 | 1089.08 | 805.378 | 283.704 |
| 2020 | 262288 | 87221.042 | 63972.925 | 23248.12 | 8.864 | 26.654 | 3715.29 | 1235.48 | 906.171 | 329.308 |
| 2021 | 255079 | 82001.292 | 58982.820 | 23018.47 | 9.024 | 28.071 | 3587.82 | 1153.39 | 829.623 | 323.766 |
| 2022 | 257097 | 81915.977 | 58207.001 | 23708.98 | 9.222 | 28.943 | 3588.53 | 1143.37 | 812.447 | 330.927 |
| 2023 | 259080 | 81810.810 | 57481.824 | 24328.99 | 9.391 | 29.738 | 3586.88 | 1132.64 | 795.818 | 336.827 |
| 2024 | 260719 | 81528.303 | 56712.019 | 24816.28 | 9.518 | 30.439 | 3579.65 | 1119.38 | 778.652 | 340.726 |
| 2025 | 257308 | 79599.213 | 55066.570 | 24532.64 | 9.534 | 30.820 | 4112.08 | 1272.09 | 880.027 | 392.060 |
| 2026 | 251528 | 75082.462 | 50936.261 | 24146.20 | 9.600 | 32.160 | 4003.24 | 1194.99 | 810.685 | 384.303 |
| 2027 | 250988 | 74444.425 | 49915.584 | 24528.84 | 9.773 | 32.949 | 3976.53 | 1179.46 | 790.838 | 388.623 |
| 2028 | 250285 | 73699.585 | 48813.675 | 24885.91 | 9.943 | 33.767 | 3945.35 | 1161.76 | 769.472 | 392.288 |
| 2029 | 249525 | 72819.703 | 47675.401 | 25144.30 | 10.077 | 34.530 | 3910.99 | 1141.36 | 747.252 | 394.105 |
| 2030 | 240528 | 69309.277 | 45228.072 | 24081.21 | 10.012 | 34.745 | 4504.36 | 1297.95 | 846.983 | 450.967 |
| 2031 | 235442 | 65433.368 | 41925.116 | 23508.25 | 9.985 | 35.927 | 4397.65 | 1222.18 | 783.088 | 439.093 |
| 2032 | 234017 | 64643.552 | 40856.714 | 23786.84 | 10.165 | 36.797 | 4356.12 | 1203.31 | 760.528 | 442.781 |
| 2033 | 232559 | 63793.514 | 39927.416 | 23866.10 | 10.262 | 37.411 | 4310.93 | 1182.54 | 740.132 | 442.404 |
| 2034 | 230963 | 63032.151 | 39010.729 | 24021.42 | 10.401 | 38.110 | 4260.75 | 1162.80 | 719.659 | 443.141 |
| 2035 | 215822 | 57653.301 | 35832.722 | 21820.58 | 10.110 | 37.848 | 4924.38 | 1315.47 | 817.592 | 497.878 |
| 2036 | 208183 | 52988.733 | 32031.376 | 20957.36 | 10.067 | 39.551 | 4748.94 | 1208.75 | 730.680 | 478.066 |
| 2037 | 205491 | 52026.921 | 31024.288 | 21002.63 | 10.221 | 40.369 | 4682.40 | 1185.50 | 706.930 | 478.573 |
| 2038 | 202834 | 50943.211 | 30005.859 | 20937.35 | 10.322 | 41.099 | 4612.18 | 1158.38 | 682.295 | 476.088 |
| 2039 | 200251 | 50085.991 | 29166.437 | 20919.55 | 10.447 | 41.767 | 4538.65 | 1135.19 | 661.053 | 474.138 |

# Results for PHIM for Run 1 (Sensitivity_Analysis_1.RTF)

************************************************************************************
**Main Results for E-Component for Run: 1, Simulation: 1, COD: LUNG CANCER**

| Variable Parameter | Value |
|---|---|
| 1. Country | US (United States) |
| 2. Sex | M (Males) |
| 3. Year of start of process | 1990 |
| 4. Cause of Death | LUNG CANCER |
| 5. Effective Dose NULL | CC:1 |
| 6. Effective Dose Ecig | CC:1 Ecig:0.4 Dual:0.7 |
| Note: | Death rates are per million population |

-----------------------------------------------------------------------------------------
Results for Age Groups Combined and Years combined, Population unadjusted for deaths in Ecig scenario,

| Age Group:All | | NULL | Ecig | | | %Drop | | NULL | Ecig | |
|---|---|---|---|---|---|---|---|---|---|---|
| Year | Number | Attrib | Attrib | Drop | %Drop | Attrib | Death | Attrib | Attrib | Drop |
| | Deaths | Deaths | Deaths | Deaths | Deaths | Deaths | Rate | Rates | Rates | Rates |
| All Comb | 3065988 | 2244988.113 | 2002713.165 | 242274.9 | 7.902 | 10.792 | 887.537 | 649.875 | 579.742 | 70.133 |

-----------------------------------------------------------------------------------------
Results for Age Groups Combined, Population unadjusted for deaths in Ecig scenario,

| Age Group:All | | NULL | Ecig | | | %Drop | | NULL | Ecig | |
|---|---|---|---|---|---|---|---|---|---|---|
| Year | Number | Attrib | Attrib | Drop | %Drop | Attrib | Death | Attrib | Attrib | Drop |
| | Deaths | Deaths | Deaths | Deaths | Deaths | Deaths | Rate | Rates | Rates | Rates |
| 1990 | 78822 | 63385.736 | 63385.736 | 0.000 | 0.000 | 0.000 | 1249.50 | 1004.81 | 1004.81 | 0.000 |
| 1991 | 78462 | 62918.402 | 62887.194 | 31.208 | 0.040 | 0.050 | 1223.41 | 981.049 | 980.563 | 0.487 |
| 1992 | 77987 | 62366.766 | 62285.514 | 81.252 | 0.104 | 0.130 | 1195.35 | 955.930 | 954.684 | 1.245 |
| 1993 | 78542 | 62652.888 | 62494.447 | 158.440 | 0.202 | 0.253 | 1182.39 | 943.195 | 940.809 | 2.385 |
| 1994 | 77437 | 61565.227 | 61319.172 | 246.054 | 0.318 | 0.400 | 1144.00 | 909.520 | 905.885 | 3.635 |
| 1995 | 76904 | 60851.961 | 60496.037 | 355.924 | 0.463 | 0.585 | 1114.36 | 881.759 | 876.601 | 5.157 |
| 1996 | 76232 | 60122.922 | 59629.916 | 493.006 | 0.647 | 0.820 | 1087.44 | 857.643 | 850.610 | 7.033 |
| 1997 | 75473 | 59237.155 | 58600.818 | 636.337 | 0.843 | 1.074 | 1059.06 | 831.234 | 822.305 | 8.929 |
| 1998 | 75381 | 58906.696 | 58088.409 | 818.287 | 1.086 | 1.389 | 1040.37 | 813.001 | 801.707 | 11.294 |
| 1999 | 73159 | 56930.073 | 55942.669 | 987.404 | 1.350 | 1.734 | 993.781 | 773.329 | 759.917 | 13.413 |
| 2000 | 73784 | 57137.121 | 55937.740 | 1199.381 | 1.626 | 2.099 | 987.608 | 764.787 | 748.734 | 16.054 |
| 2001 | 73294 | 56481.726 | 55035.979 | 1445.747 | 1.973 | 2.560 | 968.273 | 746.169 | 727.070 | 19.099 |
| 2002 | 72442 | 55588.365 | 53896.774 | 1691.592 | 2.335 | 3.043 | 946.293 | 726.138 | 704.041 | 22.097 |
| 2003 | 71942 | 54945.856 | 52988.486 | 1957.370 | 2.721 | 3.562 | 929.989 | 710.281 | 684.978 | 25.303 |
| 2004 | 71301 | 54203.126 | 51944.757 | 2258.368 | 3.167 | 4.166 | 911.778 | 693.135 | 664.256 | 28.879 |
| 2005 | 70964 | 53744.603 | 51195.381 | 2549.222 | 3.592 | 4.743 | 896.937 | 679.296 | 647.075 | 32.220 |
| 2006 | 69658 | 52535.572 | 49682.260 | 2853.312 | 4.096 | 5.431 | 872.383 | 657.945 | 622.211 | 35.734 |
| 2007 | 68565 | 51492.124 | 48348.050 | 3144.074 | 4.586 | 6.106 | 849.888 | 638.264 | 599.292 | 38.972 |
| 2008 | 68226 | 51030.736 | 47547.164 | 3483.571 | 5.106 | 6.826 | 836.215 | 625.460 | 582.764 | 42.697 |
| 2009 | 67509 | 50276.199 | 46451.450 | 3824.749 | 5.666 | 7.607 | 817.667 | 608.943 | 562.618 | 46.325 |
| 2010 | 67196 | 49840.933 | 45682.776 | 4158.157 | 6.188 | 8.343 | 804.000 | 596.347 | 546.594 | 49.752 |
| 2011 | 66506 | 49072.841 | 44550.465 | 4522.376 | 6.800 | 9.216 | 787.880 | 581.354 | 527.779 | 53.575 |
| 2012 | 66155 | 48539.512 | 43629.568 | 4909.944 | 7.422 | 10.115 | 775.240 | 568.812 | 511.275 | 57.537 |
| 2013 | 65333 | 47675.741 | 42433.098 | 5242.643 | 8.024 | 10.996 | 756.876 | 552.318 | 491.583 | 60.735 |
| 2014 | 65033 | 47194.534 | 41601.098 | 5593.436 | 8.601 | 11.852 | 744.602 | 540.359 | 476.316 | 64.043 |
| 2015 | 64145 | 46305.842 | 40435.374 | 5870.468 | 9.152 | 12.678 | 826.215 | 596.439 | 520.825 | 75.614 |
| 2016 | 61618 | 44208.867 | 38219.951 | 5988.916 | 9.719 | 13.547 | 786.248 | 564.107 | 487.688 | 76.419 |
| 2017 | 59897 | 42702.624 | 36521.861 | 6180.763 | 10.319 | 14.474 | 757.297 | 539.903 | 461.757 | 78.145 |
| 2018 | 62468 | 44321.202 | 37447.158 | 6874.044 | 11.004 | 15.510 | 782.490 | 555.181 | 469.074 | 86.106 |
| 2019 | 63045 | 44493.088 | 37172.187 | 7320.901 | 11.612 | 16.454 | 782.035 | 551.912 | 461.100 | 90.812 |
| 2020 | 63466 | 44529.551 | 36790.751 | 7738.800 | 12.194 | 17.379 | 898.991 | 630.757 | 521.138 | 109.619 |
| 2021 | 57113 | 39852.556 | 32538.159 | 7314.397 | 12.807 | 18.354 | 803.323 | 560.546 | 457.665 | 102.881 |
| 2022 | 57440 | 39853.685 | 32122.244 | 7731.441 | 13.460 | 19.400 | 801.745 | 556.274 | 448.359 | 107.915 |
| 2023 | 57738 | 39847.050 | 31723.071 | 8123.979 | 14.070 | 20.388 | 799.366 | 551.670 | 439.196 | 112.474 |
| 2024 | 57942 | 39726.010 | 31268.335 | 8457.675 | 14.597 | 21.290 | 795.539 | 545.435 | 429.312 | 116.123 |
| 2025 | 57521 | 39207.348 | 30543.210 | 8664.138 | 15.062 | 22.098 | 919.257 | 626.579 | 488.116 | 138.463 |
| 2026 | 51428 | 34803.960 | 26784.585 | 8019.375 | 15.593 | 23.042 | 818.510 | 553.929 | 426.295 | 127.634 |
| 2027 | 51172 | 34484.595 | 26194.146 | 8290.449 | 16.201 | 24.041 | 810.740 | 546.357 | 415.007 | 131.350 |
| 2028 | 50870 | 34100.638 | 25575.923 | 8524.715 | 16.758 | 24.999 | 801.884 | 537.544 | 403.165 | 134.379 |
| 2029 | 50540 | 33669.838 | 24935.429 | 8734.409 | 17.282 | 25.941 | 792.146 | 527.732 | 390.831 | 136.901 |
| 2030 | 49109 | 32557.688 | 23872.656 | 8685.032 | 17.685 | 26.676 | 919.662 | 609.705 | 447.061 | 162.644 |
| 2031 | 43585 | 28646.393 | 20793.271 | 7853.122 | 18.018 | 27.414 | 814.096 | 535.065 | 388.382 | 146.683 |
| 2032 | 43163 | 28227.160 | 20218.625 | 8008.535 | 18.554 | 28.372 | 803.454 | 525.435 | 376.360 | 149.075 |
| 2033 | 42725 | 27786.623 | 19713.232 | 8073.390 | 18.896 | 29.055 | 791.996 | 515.079 | 365.423 | 149.656 |
| 2034 | 42256 | 27348.299 | 19192.356 | 8155.943 | 19.301 | 29.822 | 779.523 | 504.514 | 354.055 | 150.459 |
| 2035 | 39748 | 25570.077 | 17850.716 | 7719.361 | 19.421 | 30.189 | 906.933 | 583.430 | 407.298 | 176.132 |
| 2036 | 34030 | 21717.319 | 14926.996 | 6790.323 | 19.954 | 31.267 | 776.269 | 495.402 | 340.505 | 154.897 |
| 2037 | 33454 | 21256.024 | 14412.915 | 6843.109 | 20.455 | 32.194 | 762.289 | 484.347 | 328.417 | 155.929 |
| 2038 | 32883 | 20747.175 | 13912.997 | 6834.178 | 20.783 | 32.940 | 747.720 | 471.764 | 316.364 | 155.400 |
| 2039 | 32325 | 20327.688 | 13492.060 | 6835.627 | 21.146 | 33.627 | 732.644 | 460.724 | 305.795 | 154.928 |

# Results for PHIM for Run 1 (Sensitivity_Analysis_1.RTF)

**********************************************************************************
**Main Results for E-Component for Run: 1, Simulation: 1, COD: IHD**

| **Variable Parameter** | **Value** |
|---|---|
| 1. Country | US (United States) |
| 2. Sex | M (Males) |
| 3. Year of start of process | 1990 |
| 4. Cause of Death | IHD |
| 5. Effective Dose NULL | CC:1 |
| 6. Effective Dose Ecig | CC:1 Ecig:0.4 Dual:0.7 |
| Note: | Death rates are per million population |

-----------------------------------------------------------------------------------------
Results for Age Groups Combined and Years combined, Population unadjusted for deaths in Ecig scenario,

| Age Group:All | | NULL | Ecig | | | %Drop | | NULL | Ecig | |
|---|---|---|---|---|---|---|---|---|---|---|
| Year | Number | Attrib | Attrib | Drop | %Drop | Attrib | Death | Attrib | Attrib | Drop |
| | Deaths | Deaths | Deaths | Deaths | Deaths | Deaths | Rate | Rates | Rates | Rates |
| All Comb | 6360041 | 1098877.421 | 760965.061 | 337912.4 | 5.313 | 30.751 | 1841.09 | 318.101 | 220.283 | 97.818 |

-----------------------------------------------------------------------------------------
Results for Age Groups Combined, Population unadjusted for deaths in Ecig scenario,

| Age Group:All | | NULL | Ecig | | | %Drop | | NULL | Ecig | |
|---|---|---|---|---|---|---|---|---|---|---|
| Year | Number | Attrib | Attrib | Drop | %Drop | Attrib | Death | Attrib | Attrib | Drop |
| | Deaths | Deaths | Deaths | Deaths | Deaths | Deaths | Rate | Rates | Rates | Rates |
| 1990 | 171484 | 38511.725 | 38511.725 | 0.000 | 0.000 | 0.000 | 2718.40 | 610.497 | 610.497 | 0.000 |
| 1991 | 168044 | 37309.838 | 37112.529 | 197.309 | 0.117 | 0.529 | 2620.21 | 581.750 | 578.674 | 3.077 |
| 1992 | 164548 | 36137.488 | 35616.322 | 521.166 | 0.317 | 1.442 | 2522.12 | 553.899 | 545.911 | 7.988 |
| 1993 | 164362 | 35599.588 | 34646.209 | 953.379 | 0.580 | 2.678 | 2474.35 | 535.926 | 521.574 | 14.352 |
| 1994 | 159866 | 34336.757 | 32900.110 | 1436.647 | 0.899 | 4.184 | 2361.75 | 507.267 | 486.043 | 21.224 |
| 1995 | 158083 | 33455.759 | 31471.215 | 1984.544 | 1.255 | 5.932 | 2290.66 | 484.782 | 456.025 | 28.756 |
| 1996 | 154875 | 32193.824 | 29636.547 | 2557.277 | 1.651 | 7.943 | 2209.26 | 459.239 | 422.760 | 36.479 |
| 1997 | 149970 | 30665.509 | 27561.126 | 3104.383 | 2.070 | 10.123 | 2104.43 | 430.308 | 386.746 | 43.562 |
| 1998 | 145407 | 29188.430 | 25577.819 | 3610.611 | 2.483 | 12.370 | 2006.83 | 402.844 | 353.012 | 49.832 |
| 1999 | 165952 | 33375.386 | 28385.999 | 4989.386 | 3.007 | 14.949 | 2254.27 | 453.366 | 385.591 | 67.775 |
| 2000 | 160260 | 32024.754 | 26486.651 | 5538.103 | 3.456 | 17.293 | 2145.10 | 428.655 | 354.527 | 74.128 |
| 2001 | 154444 | 30682.393 | 24558.805 | 6123.588 | 3.965 | 19.958 | 2040.33 | 405.339 | 324.442 | 80.898 |
| 2002 | 151887 | 30218.370 | 23417.213 | 6801.157 | 4.478 | 22.507 | 1984.06 | 394.736 | 305.894 | 88.842 |
| 2003 | 146733 | 29175.202 | 21885.941 | 7289.261 | 4.968 | 24.984 | 1896.81 | 377.146 | 282.918 | 94.228 |
| 2004 | 138381 | 27488.687 | 19935.129 | 7553.559 | 5.459 | 27.479 | 1769.58 | 351.518 | 254.925 | 96.593 |
| 2005 | 135881 | 27017.439 | 18986.715 | 8030.724 | 5.910 | 29.724 | 1717.44 | 341.482 | 239.979 | 101.503 |
| 2006 | 131922 | 26231.435 | 17801.060 | 8430.375 | 6.390 | 32.138 | 1652.16 | 328.517 | 222.937 | 105.580 |
| 2007 | 126502 | 25103.049 | 16497.301 | 8605.749 | 6.803 | 34.282 | 1568.04 | 311.161 | 204.490 | 106.671 |
| 2008 | 125859 | 24621.740 | 15741.758 | 8879.982 | 7.056 | 36.066 | 1542.60 | 301.777 | 192.939 | 108.838 |
| 2009 | 122428 | 23741.177 | 14759.830 | 8981.347 | 7.336 | 37.830 | 1482.84 | 287.552 | 178.771 | 108.782 |
| 2010 | 120537 | 23189.160 | 14060.510 | 9128.650 | 7.573 | 39.366 | 1442.22 | 277.458 | 168.234 | 109.224 |
| 2011 | 120221 | 22786.927 | 13439.007 | 9347.920 | 7.776 | 41.023 | 1424.23 | 269.951 | 159.209 | 110.743 |
| 2012 | 120927 | 22485.295 | 12923.671 | 9561.624 | 7.907 | 42.524 | 1417.09 | 263.495 | 151.447 | 112.048 |
| 2013 | 122648 | 22251.884 | 12491.789 | 9760.095 | 7.958 | 43.862 | 1420.86 | 257.786 | 144.716 | 113.070 |
| 2014 | 123229 | 21894.593 | 12015.922 | 9878.672 | 8.017 | 45.119 | 1410.92 | 250.685 | 137.578 | 113.107 |
| 2015 | 123774 | 21471.109 | 11563.169 | 9907.940 | 8.005 | 46.145 | 1594.26 | 276.557 | 148.939 | 127.618 |
| 2016 | 126218 | 21411.568 | 11310.527 | 10101.04 | 8.003 | 47.176 | 1610.55 | 273.213 | 144.323 | 128.890 |
| 2017 | 128265 | 21169.399 | 10961.897 | 10207.50 | 7.958 | 48.218 | 1621.69 | 267.651 | 138.595 | 129.057 |
| 2018 | 121429 | 19830.576 | 10090.355 | 9740.222 | 8.021 | 49.117 | 1521.05 | 248.404 | 126.395 | 122.009 |
| 2019 | 121832 | 19431.014 | 9749.286 | 9681.729 | 7.947 | 49.826 | 1511.26 | 241.031 | 120.934 | 120.096 |
| 2020 | 120811 | 18532.612 | 9166.962 | 9365.650 | 7.752 | 50.536 | 1711.27 | 262.513 | 129.849 | 132.664 |
| 2021 | 119407 | 18234.128 | 8881.545 | 9352.582 | 7.833 | 51.292 | 1679.51 | 256.472 | 124.923 | 131.549 |
| 2022 | 119438 | 17812.559 | 8539.299 | 9273.260 | 7.764 | 52.060 | 1667.10 | 248.626 | 119.191 | 129.435 |
| 2023 | 119445 | 17361.668 | 8209.130 | 9152.538 | 7.663 | 52.717 | 1653.68 | 240.367 | 113.653 | 126.714 |
| 2024 | 119305 | 16929.676 | 7918.262 | 9011.414 | 7.553 | 53.229 | 1638.05 | 232.443 | 108.717 | 123.726 |
| 2025 | 115685 | 15501.666 | 7170.064 | 8331.602 | 7.202 | 53.746 | 1848.78 | 247.735 | 114.586 | 133.149 |
| 2026 | 115073 | 15523.814 | 7136.085 | 8387.729 | 7.289 | 54.031 | 1831.46 | 247.072 | 113.576 | 133.496 |
| 2027 | 113989 | 15073.070 | 6874.301 | 8198.769 | 7.193 | 54.393 | 1805.98 | 238.810 | 108.913 | 129.897 |
| 2028 | 112855 | 14636.710 | 6602.188 | 8034.522 | 7.119 | 54.893 | 1778.98 | 230.725 | 104.073 | 126.652 |
| 2029 | 111718 | 14180.166 | 6353.864 | 7826.302 | 7.005 | 55.192 | 1751.04 | 222.256 | 99.589 | 122.667 |
| 2030 | 104851 | 12111.772 | 5376.550 | 6735.222 | 6.424 | 55.609 | 1963.54 | 226.816 | 100.686 | 126.130 |
| 2031 | 105289 | 12417.367 | 5502.150 | 6915.217 | 6.568 | 55.690 | 1966.62 | 231.935 | 102.771 | 129.164 |
| 2032 | 103879 | 12054.064 | 5281.733 | 6772.332 | 6.519 | 56.183 | 1933.67 | 224.381 | 98.317 | 126.064 |
| 2033 | 102485 | 11672.389 | 5058.525 | 6613.864 | 6.453 | 56.662 | 1899.76 | 216.370 | 93.770 | 122.601 |
| 2034 | 101070 | 11365.762 | 4905.408 | 6460.354 | 6.392 | 56.840 | 1864.51 | 209.673 | 90.494 | 119.179 |
| 2035 | 90820 | 8580.360 | 3655.601 | 4924.759 | 5.423 | 57.396 | 2072.23 | 195.777 | 83.410 | 112.368 |
| 2036 | 92117 | 8948.841 | 3786.827 | 5162.013 | 5.604 | 57.684 | 2101.31 | 204.135 | 86.383 | 117.753 |
| 2037 | 90327 | 8624.570 | 3646.441 | 4978.129 | 5.511 | 57.720 | 2058.21 | 196.522 | 83.089 | 113.433 |
| 2038 | 88590 | 8315.017 | 3472.434 | 4842.582 | 5.466 | 58.239 | 2014.42 | 189.073 | 78.959 | 110.114 |
| 2039 | 86921 | 8001.132 | 3331.555 | 4669.577 | 5.372 | 58.361 | 1970.05 | 181.344 | 75.509 | 105.835 |

# Results for PHIM for Run 1 (Sensitivity_Analysis_1.RTF)

************************************************************************************

**Main Results for E-Component for Run: 1, Simulation: 1, COD: STROKE**

| Variable Parameter | Value |
|---|---|
| 1. Country | US (United States) |
| 2. Sex | M (Males) |
| 3. Year of start of process | 1990 |
| 4. Cause of Death | STROKE |
| 5. Effective Dose NULL | CC:1 |
| 6. Effective Dose Ecig | CC:1 Ecig:0.4 Dual:0.7 |
| Note: | Death rates are per million population |

-----------------------------------------------------------------------------------------

Results for Age Groups Combined and Years combined, Population unadjusted for deaths in Ecig scenario,

| Age Group:All Year | Number Deaths | NULL Attrib Deaths | Ecig Attrib Deaths | Drop Deaths | %Drop Deaths | %Drop Attrib Deaths | Death Rate | NULL Attrib Rates | Ecig Attrib Rates | Drop Rates |
|---|---|---|---|---|---|---|---|---|---|---|
| All Comb | 1489699 | 149918.462 | 101962.098 | 47956.36 | 3.219 | 31.988 | 431.236 | 43.398 | 29.516 | 13.882 |

-----------------------------------------------------------------------------------------

Results for Age Groups Combined, Population unadjusted for deaths in Ecig scenario,

| Age Group:All Year | Number Deaths | NULL Attrib Deaths | Ecig Attrib Deaths | Drop Deaths | %Drop Deaths | %Drop Attrib Deaths | Death Rate | NULL Attrib Rates | Ecig Attrib Rates | Drop Rates |
|---|---|---|---|---|---|---|---|---|---|---|
| 1990 | 32862 | 4613.166 | 4613.166 | 0.000 | 0.000 | 0.000 | 520.936 | 73.129 | 73.129 | 0.000 |
| 1991 | 32619 | 4555.566 | 4529.884 | 25.682 | 0.079 | 0.564 | 508.609 | 71.032 | 70.632 | 0.400 |
| 1992 | 32100 | 4453.572 | 4387.821 | 65.752 | 0.205 | 1.476 | 492.014 | 68.262 | 67.255 | 1.008 |
| 1993 | 32896 | 4462.473 | 4339.168 | 123.305 | 0.375 | 2.763 | 495.226 | 67.179 | 65.323 | 1.856 |
| 1994 | 33323 | 4499.513 | 4307.041 | 192.473 | 0.578 | 4.278 | 492.290 | 66.473 | 63.629 | 2.843 |
| 1995 | 33569 | 4472.774 | 4201.557 | 271.217 | 0.808 | 6.064 | 486.422 | 64.812 | 60.882 | 3.930 |
| 1996 | 33870 | 4406.878 | 4048.913 | 357.965 | 1.057 | 8.123 | 483.150 | 62.863 | 57.757 | 5.106 |
| 1997 | 33516 | 4288.158 | 3842.071 | 446.087 | 1.331 | 10.403 | 470.307 | 60.173 | 53.913 | 6.260 |
| 1998 | 32795 | 4120.148 | 3594.339 | 525.809 | 1.603 | 12.762 | 452.620 | 56.864 | 49.607 | 7.257 |
| 1999 | 33375 | 3982.443 | 3376.645 | 605.797 | 1.815 | 15.212 | 453.361 | 54.097 | 45.868 | 8.229 |
| 2000 | 33459 | 4021.411 | 3315.232 | 706.179 | 2.111 | 17.560 | 447.853 | 53.827 | 44.375 | 9.452 |
| 2001 | 32275 | 3848.092 | 3073.636 | 774.456 | 2.400 | 20.126 | 426.379 | 50.836 | 40.605 | 10.231 |
| 2002 | 31586 | 3783.081 | 2930.452 | 852.629 | 2.699 | 22.538 | 412.601 | 49.418 | 38.280 | 11.138 |
| 2003 | 30985 | 3754.415 | 2817.327 | 937.088 | 3.024 | 24.960 | 400.541 | 48.533 | 36.419 | 12.114 |
| 2004 | 29884 | 3666.274 | 2663.120 | 1003.154 | 3.357 | 27.362 | 382.149 | 46.883 | 34.055 | 12.828 |
| 2005 | 28708 | 3589.688 | 2524.071 | 1065.617 | 3.712 | 29.685 | 362.850 | 45.371 | 31.903 | 13.469 |
| 2006 | 28188 | 3596.985 | 2445.278 | 1151.707 | 4.086 | 32.019 | 353.021 | 45.048 | 30.624 | 14.424 |
| 2007 | 28026 | 3560.798 | 2344.412 | 1216.385 | 4.340 | 34.160 | 347.392 | 44.137 | 29.060 | 15.078 |
| 2008 | 27128 | 3417.001 | 2183.430 | 1233.571 | 4.547 | 36.101 | 332.495 | 41.881 | 26.761 | 15.119 |
| 2009 | 27068 | 3394.390 | 2108.499 | 1285.891 | 4.751 | 37.883 | 327.847 | 41.113 | 25.538 | 15.575 |
| 2010 | 26911 | 3340.137 | 2023.051 | 1317.086 | 4.894 | 39.432 | 321.990 | 39.965 | 24.206 | 15.759 |
| 2011 | 26959 | 3285.435 | 1939.351 | 1346.084 | 4.993 | 40.971 | 319.377 | 38.922 | 22.975 | 15.947 |
| 2012 | 27057 | 3259.209 | 1871.276 | 1387.934 | 5.130 | 42.585 | 317.068 | 38.193 | 21.929 | 16.265 |
| 2013 | 27904 | 3274.703 | 1832.091 | 1442.612 | 5.170 | 44.053 | 323.265 | 37.937 | 21.225 | 16.713 |
| 2014 | 28792 | 3298.083 | 1799.531 | 1498.552 | 5.205 | 45.437 | 329.657 | 37.762 | 20.604 | 17.158 |
| 2015 | 29560 | 3244.406 | 1733.182 | 1511.224 | 5.112 | 46.579 | 380.745 | 41.789 | 22.324 | 19.465 |
| 2016 | 30513 | 3314.836 | 1738.419 | 1576.417 | 5.166 | 47.556 | 389.347 | 42.297 | 22.182 | 20.115 |
| 2017 | 31416 | 3333.314 | 1710.930 | 1622.384 | 5.164 | 48.672 | 397.202 | 42.144 | 21.632 | 20.512 |
| 2018 | 29348 | 2925.242 | 1474.302 | 1450.940 | 4.944 | 49.601 | 367.626 | 36.642 | 18.468 | 18.175 |
| 2019 | 29598 | 2864.628 | 1422.295 | 1442.333 | 4.873 | 50.350 | 367.149 | 35.534 | 17.643 | 17.891 |
| 2020 | 29592 | 2730.366 | 1337.306 | 1393.061 | 4.707 | 51.021 | 419.174 | 38.675 | 18.943 | 19.733 |
| 2021 | 30097 | 2647.911 | 1275.298 | 1372.613 | 4.561 | 51.838 | 423.328 | 37.244 | 17.938 | 19.306 |
| 2022 | 30304 | 2586.165 | 1224.588 | 1361.576 | 4.493 | 52.648 | 422.974 | 36.097 | 17.093 | 19.005 |
| 2023 | 30505 | 2517.960 | 1176.880 | 1341.080 | 4.396 | 53.261 | 422.337 | 34.860 | 16.294 | 18.567 |
| 2024 | 30661 | 2454.161 | 1133.658 | 1320.502 | 4.307 | 53.807 | 420.968 | 33.695 | 15.565 | 18.130 |
| 2025 | 30254 | 2275.364 | 1038.562 | 1236.803 | 4.088 | 54.356 | 483.488 | 36.363 | 16.597 | 19.766 |
| 2026 | 30685 | 2183.166 | 988.769 | 1194.397 | 3.892 | 54.709 | 488.380 | 34.747 | 15.737 | 19.010 |
| 2027 | 30561 | 2112.345 | 949.695 | 1162.650 | 3.804 | 55.041 | 484.196 | 33.467 | 15.046 | 18.420 |
| 2028 | 30412 | 2046.677 | 912.520 | 1134.156 | 3.729 | 55.415 | 479.392 | 32.263 | 14.384 | 17.878 |
| 2029 | 30254 | 1977.671 | 876.128 | 1101.543 | 3.641 | 55.699 | 474.195 | 30.997 | 13.732 | 17.265 |
| 2030 | 29330 | 1744.457 | 766.828 | 977.629 | 3.333 | 56.042 | 549.260 | 32.668 | 14.360 | 18.308 |
| 2031 | 28976 | 1653.738 | 721.678 | 932.059 | 3.217 | 56.361 | 541.223 | 30.889 | 13.480 | 17.409 |
| 2032 | 28724 | 1603.120 | 689.317 | 913.803 | 3.181 | 57.002 | 534.690 | 29.841 | 12.831 | 17.010 |
| 2033 | 28464 | 1548.245 | 661.256 | 886.988 | 3.116 | 57.290 | 527.627 | 28.700 | 12.258 | 16.442 |
| 2034 | 28178 | 1511.985 | 639.995 | 871.990 | 3.095 | 57.672 | 519.826 | 27.893 | 11.806 | 16.086 |
| 2035 | 26737 | 1239.866 | 522.330 | 717.536 | 2.684 | 57.872 | 610.065 | 28.290 | 11.918 | 16.372 |
| 2036 | 25591 | 1182.522 | 493.513 | 689.009 | 2.692 | 58.266 | 583.762 | 26.975 | 11.258 | 15.717 |
| 2037 | 25143 | 1134.483 | 476.451 | 658.032 | 2.617 | 58.003 | 572.916 | 25.851 | 10.857 | 14.994 |
| 2038 | 24693 | 1093.537 | 453.601 | 639.935 | 2.592 | 58.520 | 561.491 | 24.866 | 10.314 | 14.551 |
| 2039 | 24247 | 1047.905 | 433.232 | 614.673 | 2.535 | 58.657 | 549.553 | 23.751 | 9.819 | 13.931 |

# Results for PHIM for Run 1 (Sensitivity_Analysis_1.RTF)

************************************************************************************

**Main Results for E-Component for Run: 1, Simulation: 1, COD: COPD**

| **Variable Parameter** | **Value** |
|---|---|
| 1. Country | US (United States) |
| 2. Sex | M (Males) |
| 3. Year of start of process | 1990 |
| 4. Cause of Death | COPD |
| 5. Effective Dose NULL | CC:1 |
| 6. Effective Dose Ecig | CC:1 Ecig:0.4 Dual:0.7 |
| Note: | Death rates are per million population |

-----------------------------------------------------------------------------------------

Results for Age Groups Combined and Years combined, Population unadjusted for deaths in Ecig scenario,

| Age Group:All | | NULL | Ecig | | | %Drop | | NULL | Ecig | |
|---|---|---|---|---|---|---|---|---|---|---|
| Year | Number | Attrib | Attrib | Drop | %Drop | Attrib | Death | Attrib | Attrib | Drop |
| | Deaths | Deaths | Deaths | Deaths | Deaths | Deaths | Rate | Rates | Rates | Rates |
| All Comb | 2233143 | 1015606.587 | 826481.389 | 189125.2 | 8.469 | 18.622 | 646.447 | 293.996 | 239.248 | 54.748 |

-----------------------------------------------------------------------------------------

Results for Age Groups Combined, Population unadjusted for deaths in Ecig scenario,

| Age Group:All | | NULL | Ecig | | | %Drop | | NULL | Ecig | |
|---|---|---|---|---|---|---|---|---|---|---|
| Year | Number | Attrib | Attrib | Drop | %Drop | Attrib | Death | Attrib | Attrib | Drop |
| | Deaths | Deaths | Deaths | Deaths | Deaths | Deaths | Rate | Rates | Rates | Rates |
| 1990 | 33993 | 19262.437 | 19262.437 | 0.000 | 0.000 | 0.000 | 538.865 | 305.353 | 305.353 | 0.000 |
| 1991 | 34092 | 19215.636 | 19200.970 | 14.666 | 0.043 | 0.076 | 531.576 | 299.618 | 299.389 | 0.229 |
| 1992 | 33942 | 19056.189 | 19019.252 | 36.937 | 0.109 | 0.194 | 520.248 | 292.085 | 291.519 | 0.566 |
| 1993 | 35913 | 20095.670 | 20021.073 | 74.597 | 0.208 | 0.371 | 540.645 | 302.526 | 301.403 | 1.123 |
| 1994 | 35074 | 19524.095 | 19412.316 | 111.778 | 0.319 | 0.573 | 518.158 | 288.435 | 286.784 | 1.651 |
| 1995 | 34669 | 19116.002 | 18955.029 | 160.973 | 0.464 | 0.842 | 502.362 | 276.995 | 274.663 | 2.333 |
| 1996 | 34582 | 18975.021 | 18755.475 | 219.547 | 0.635 | 1.157 | 493.306 | 270.675 | 267.544 | 3.132 |
| 1997 | 35267 | 19169.025 | 18879.200 | 289.825 | 0.822 | 1.512 | 494.878 | 268.986 | 264.919 | 4.067 |
| 1998 | 35675 | 19228.937 | 18851.070 | 377.867 | 1.059 | 1.965 | 492.368 | 265.388 | 260.173 | 5.215 |
| 1999 | 38516 | 20589.820 | 20097.544 | 492.276 | 1.278 | 2.391 | 523.195 | 279.689 | 273.002 | 6.687 |
| 2000 | 36283 | 19196.417 | 18647.691 | 548.726 | 1.512 | 2.858 | 485.652 | 256.946 | 249.602 | 7.345 |
| 2001 | 36281 | 19012.731 | 18346.971 | 665.760 | 1.835 | 3.502 | 479.301 | 251.174 | 242.378 | 8.795 |
| 2002 | 35983 | 18699.357 | 17925.261 | 774.097 | 2.151 | 4.140 | 470.038 | 244.265 | 234.154 | 10.112 |
| 2003 | 36205 | 18663.550 | 17765.014 | 898.535 | 2.482 | 4.814 | 468.019 | 241.262 | 229.647 | 11.615 |
| 2004 | 34611 | 17679.164 | 16680.550 | 998.614 | 2.885 | 5.649 | 442.596 | 226.076 | 213.306 | 12.770 |
| 2005 | 36826 | 18692.832 | 17494.104 | 1198.728 | 3.255 | 6.413 | 465.456 | 236.265 | 221.114 | 15.151 |
| 2006 | 34350 | 17308.564 | 16038.021 | 1270.543 | 3.699 | 7.341 | 430.192 | 216.769 | 200.857 | 15.912 |
| 2007 | 35326 | 17683.181 | 16220.833 | 1462.348 | 4.140 | 8.270 | 437.879 | 219.189 | 201.063 | 18.126 |
| 2008 | 38248 | 19006.374 | 17275.194 | 1731.180 | 4.526 | 9.108 | 468.788 | 232.952 | 211.734 | 21.218 |
| 2009 | 36902 | 18205.573 | 16352.140 | 1853.432 | 5.023 | 10.181 | 446.956 | 220.505 | 198.057 | 22.449 |
| 2010 | 37234 | 18237.758 | 16215.069 | 2022.690 | 5.432 | 11.091 | 445.505 | 218.215 | 194.013 | 24.201 |
| 2011 | 38093 | 18487.916 | 16211.660 | 2276.256 | 5.976 | 12.312 | 451.279 | 219.022 | 192.056 | 26.966 |
| 2012 | 38504 | 18491.158 | 16005.626 | 2485.533 | 6.455 | 13.442 | 451.211 | 216.689 | 187.563 | 29.127 |
| 2013 | 40290 | 19143.452 | 16364.666 | 2778.786 | 6.897 | 14.516 | 466.755 | 221.775 | 189.583 | 32.192 |
| 2014 | 40339 | 18992.005 | 16014.309 | 2977.695 | 7.382 | 15.679 | 461.866 | 217.451 | 183.358 | 34.093 |
| 2015 | 42303 | 19728.885 | 16461.676 | 3267.208 | 7.723 | 16.561 | 544.880 | 254.116 | 212.033 | 42.083 |
| 2016 | 43104 | 19876.724 | 16377.618 | 3499.106 | 8.118 | 17.604 | 550.009 | 253.628 | 208.979 | 44.649 |
| 2017 | 44107 | 20119.790 | 16338.276 | 3781.515 | 8.574 | 18.795 | 557.659 | 254.381 | 206.570 | 47.811 |
| 2018 | 45469 | 20535.123 | 16438.933 | 4096.190 | 9.009 | 19.947 | 569.564 | 257.229 | 205.919 | 51.310 |
| 2019 | 46963 | 21009.029 | 16582.809 | 4426.220 | 9.425 | 21.068 | 582.552 | 260.605 | 205.700 | 54.905 |
| 2020 | 48419 | 21428.512 | 16677.906 | 4750.606 | 9.811 | 22.170 | 685.853 | 303.533 | 236.241 | 67.292 |
| 2021 | 48463 | 21266.697 | 16287.818 | 4978.879 | 10.274 | 23.412 | 681.652 | 299.127 | 229.096 | 70.030 |
| 2022 | 49915 | 21663.568 | 16320.870 | 5342.698 | 10.704 | 24.662 | 696.712 | 302.378 | 227.805 | 74.573 |
| 2023 | 51391 | 22084.131 | 16372.742 | 5711.389 | 11.114 | 25.862 | 711.491 | 305.748 | 226.675 | 79.072 |
| 2024 | 52811 | 22418.456 | 16391.763 | 6026.692 | 11.412 | 26.883 | 725.093 | 307.804 | 225.058 | 82.746 |
| 2025 | 53848 | 22614.834 | 16314.735 | 6300.099 | 11.700 | 27.858 | 860.553 | 361.411 | 260.728 | 100.683 |
| 2026 | 54342 | 22571.522 | 16026.821 | 6544.701 | 12.044 | 28.995 | 864.886 | 359.241 | 255.078 | 104.163 |
| 2027 | 55267 | 22774.416 | 15897.442 | 6876.974 | 12.443 | 30.196 | 875.619 | 360.827 | 251.871 | 108.955 |
| 2028 | 56149 | 22915.561 | 15723.044 | 7192.517 | 12.810 | 31.387 | 885.102 | 361.228 | 247.849 | 113.379 |
| 2029 | 57014 | 22992.027 | 15509.981 | 7482.047 | 13.123 | 32.542 | 893.616 | 360.371 | 243.099 | 117.272 |
| 2030 | 57238 | 22895.360 | 15212.038 | 7683.321 | 13.423 | 33.558 | 1071.90 | 428.760 | 284.875 | 143.885 |
| 2031 | 57592 | 22715.871 | 14908.017 | 7807.854 | 13.557 | 34.372 | 1075.71 | 424.293 | 278.456 | 145.837 |
| 2032 | 58251 | 22759.207 | 14667.039 | 8092.168 | 13.892 | 35.556 | 1084.31 | 423.652 | 273.020 | 150.632 |
| 2033 | 58885 | 22786.258 | 14494.402 | 8291.855 | 14.082 | 36.390 | 1091.54 | 422.387 | 268.682 | 153.706 |
| 2034 | 59460 | 22806.106 | 14272.971 | 8533.135 | 14.351 | 37.416 | 1096.89 | 420.721 | 263.304 | 157.417 |
| 2035 | 58516 | 22262.998 | 13804.076 | 8458.922 | 14.456 | 37.995 | 1335.15 | 507.973 | 314.966 | 193.007 |
| 2036 | 56446 | 21140.051 | 12824.039 | 8316.012 | 14.733 | 39.338 | 1287.60 | 482.234 | 292.534 | 189.700 |
| 2037 | 56568 | 21011.845 | 12488.481 | 8523.363 | 15.067 | 40.565 | 1288.98 | 478.783 | 284.567 | 194.216 |
| 2038 | 56668 | 20787.483 | 12166.827 | 8620.656 | 15.213 | 41.470 | 1288.55 | 472.681 | 276.658 | 196.023 |
| 2039 | 56758 | 20709.267 | 11909.589 | 8799.678 | 15.504 | 42.491 | 1286.40 | 469.372 | 269.929 | 199.443 |

# Results for PHIM for Run 1 (Sensitivity_Analysis_1.RTF)

************************************************************************************

**Main Results for E-Component for Run: 1, Simulation: 1, COD: Four Diseases Combined, Adjusted**

| Variable Parameter | Value |
|---|---|
| 1. Country | US (United States) |
| 2. Sex | M (Males) |
| 3. Year of start of process | 1990 |
| 4. Cause of Death | Four Diseases Combined |
| 5. Effective Dose NULL | CC:1 |
| 6. Effective Dose Ecig | CC:1 Ecig:0.4 Dual:0.7 |
| Note: | Death rates are per million population |

-----------------------------------------------------------------------------------------

Results for Age Groups Combined and Years combined, Population and Number deaths adjusted for Ecig scenario,

| Age Group:All Year | Number Deaths | NULL Attrib Deaths | Ecig Attrib Deaths | Drop Deaths | %Drop Deaths | %Drop Attrib Deaths | Death Rate | NULL Attrib Rates | Ecig Attrib Rates | Drop Rates |
|---|---|---|---|---|---|---|---|---|---|---|
| All Comb | 13228515 | 4509390.583 | 3708665.052 | 800725.5 | 6.053 | 17.757 | 3811.74 | 1305.37 | 1068.63 | 236.736 |

-----------------------------------------------------------------------------------------

Results for Age Groups Combined, Population and Number deaths adjusted for Ecig scenario,

| Age Group:All Year | Number Deaths | NULL Attrib Deaths | Ecig Attrib Deaths | Drop Deaths | %Drop Deaths | %Drop Attrib Deaths | Death Rate | NULL Attrib Rates | Ecig Attrib Rates | Drop Rates |
|---|---|---|---|---|---|---|---|---|---|---|
| 1990 | 317161 | 125773.065 | 125773.065 | 0.000 | 0.000 | 0.000 | 5027.71 | 1993.78 | 1993.78 | 0.000 |
| 1991 | 313217 | 123999.442 | 123730.577 | 268.866 | 0.086 | 0.217 | 4883.81 | 1933.45 | 1929.26 | 4.192 |
| 1992 | 308579 | 122014.015 | 121309.643 | 704.372 | 0.228 | 0.577 | 4729.74 | 1870.18 | 1859.37 | 10.804 |
| 1993 | 311719 | 122810.618 | 121503.461 | 1307.157 | 0.419 | 1.064 | 4692.64 | 1848.83 | 1829.12 | 19.706 |
| 1994 | 305715 | 119925.592 | 117944.483 | 1981.108 | 0.648 | 1.652 | 4516.25 | 1771.69 | 1742.37 | 29.329 |
| 1995 | 303252 | 117896.496 | 115134.347 | 2762.149 | 0.911 | 2.343 | 4393.90 | 1708.35 | 1668.21 | 40.133 |
| 1996 | 299602 | 115698.645 | 112087.607 | 3611.038 | 1.205 | 3.121 | 4273.32 | 1650.42 | 1598.74 | 51.681 |
| 1997 | 294290 | 113359.847 | 108907.872 | 4451.975 | 1.513 | 3.927 | 4128.91 | 1590.70 | 1527.99 | 62.716 |
| 1998 | 289348 | 111444.212 | 106145.812 | 5298.400 | 1.831 | 4.754 | 3992.53 | 1538.10 | 1464.64 | 73.456 |
| 1999 | 311134 | 114877.722 | 107850.303 | 7027.419 | 2.259 | 6.117 | 4225.12 | 1560.48 | 1464.58 | 95.902 |
| 2000 | 303960 | 112379.704 | 104449.162 | 7930.543 | 2.609 | 7.057 | 4066.90 | 1504.22 | 1397.50 | 106.714 |
| 2001 | 296512 | 110024.943 | 101092.295 | 8932.648 | 3.013 | 8.119 | 3915.15 | 1453.52 | 1334.82 | 118.696 |
| 2002 | 292169 | 108289.173 | 98263.533 | 10025.64 | 3.431 | 9.258 | 3814.08 | 1414.56 | 1282.77 | 131.787 |
| 2003 | 286190 | 106539.023 | 95568.612 | 10970.41 | 3.833 | 10.297 | 3696.66 | 1377.22 | 1234.44 | 142.782 |
| 2004 | 274553 | 103037.251 | 91352.096 | 11685.16 | 4.256 | 11.341 | 3507.62 | 1317.61 | 1167.09 | 150.523 |
| 2005 | 272822 | 103044.562 | 90350.413 | 12694.15 | 4.653 | 12.319 | 3444.48 | 1302.41 | 1140.71 | 161.705 |
| 2006 | 264620 | 99672.555 | 86133.388 | 13539.17 | 5.116 | 13.584 | 3309.80 | 1248.28 | 1077.34 | 170.942 |
| 2007 | 258987 | 97839.152 | 83597.266 | 14241.89 | 5.499 | 14.556 | 3205.52 | 1212.75 | 1034.70 | 178.055 |
| 2008 | 260113 | 98075.852 | 82958.810 | 15117.04 | 5.812 | 15.414 | 3182.77 | 1202.07 | 1015.09 | 186.977 |
| 2009 | 254630 | 95617.338 | 79902.230 | 15715.11 | 6.172 | 16.435 | 3078.29 | 1158.11 | 965.958 | 192.155 |
| 2010 | 252683 | 94607.988 | 78234.173 | 16373.82 | 6.480 | 17.307 | 3017.03 | 1131.98 | 934.116 | 197.868 |
| 2011 | 252675 | 93633.119 | 76415.111 | 17218.01 | 6.814 | 18.389 | 2986.47 | 1109.25 | 903.183 | 206.066 |
| 2012 | 253639 | 92775.175 | 74726.962 | 18048.21 | 7.116 | 19.454 | 2964.75 | 1087.19 | 873.473 | 213.716 |
| 2013 | 257289 | 92345.780 | 73442.945 | 18902.84 | 7.347 | 20.470 | 2972.42 | 1069.82 | 848.476 | 221.340 |
| 2014 | 258622 | 91379.215 | 71775.338 | 19603.88 | 7.580 | 21.453 | 2952.21 | 1046.26 | 819.329 | 226.928 |
| 2015 | 261141 | 90750.242 | 70563.584 | 20186.66 | 7.730 | 22.244 | 3351.28 | 1168.90 | 905.556 | 263.346 |
| 2016 | 262939 | 88811.996 | 68033.947 | 20778.05 | 7.902 | 23.396 | 3341.84 | 1133.24 | 864.684 | 268.561 |
| 2017 | 265309 | 87325.126 | 65939.171 | 21385.95 | 8.061 | 24.490 | 3340.14 | 1104.08 | 830.151 | 273.928 |
| 2018 | 260426 | 87612.144 | 65887.088 | 21725.06 | 8.342 | 24.797 | 3247.37 | 1097.46 | 821.574 | 275.882 |
| 2019 | 263298 | 87797.760 | 65391.570 | 22406.19 | 8.510 | 25.520 | 3250.26 | 1089.08 | 807.223 | 281.859 |
| 2020 | 264302 | 87221.042 | 64466.774 | 22754.27 | 8.609 | 26.088 | 3722.07 | 1235.48 | 907.861 | 327.618 |
| 2021 | 257165 | 82001.292 | 59467.679 | 22533.61 | 8.762 | 27.480 | 3594.89 | 1153.39 | 831.293 | 322.096 |
| 2022 | 259332 | 81915.977 | 58715.525 | 23200.45 | 8.946 | 28.322 | 3596.22 | 1143.37 | 814.223 | 329.152 |
| 2023 | 261470 | 81810.810 | 58014.569 | 23796.24 | 9.101 | 29.087 | 3595.22 | 1132.64 | 797.702 | 334.943 |
| 2024 | 263268 | 81528.303 | 57268.542 | 24259.76 | 9.215 | 29.756 | 3588.69 | 1119.38 | 780.646 | 338.732 |
| 2025 | 260006 | 79599.213 | 55644.855 | 23954.36 | 9.213 | 30.094 | 4119.93 | 1272.09 | 881.720 | 390.367 |
| 2026 | 254297 | 75082.462 | 51499.119 | 23583.34 | 9.274 | 31.410 | 4011.35 | 1194.99 | 812.360 | 382.629 |
| 2027 | 253895 | 74444.425 | 50495.550 | 23948.88 | 9.433 | 32.170 | 3985.29 | 1179.46 | 792.609 | 386.852 |
| 2028 | 253329 | 73699.585 | 49409.711 | 24289.87 | 9.588 | 32.958 | 3954.77 | 1161.76 | 771.346 | 390.413 |
| 2029 | 252708 | 72819.703 | 48285.391 | 24534.31 | 9.709 | 33.692 | 3921.11 | 1141.36 | 749.213 | 392.143 |
| 2030 | 243814 | 69309.277 | 45847.185 | 23462.09 | 9.623 | 33.851 | 4512.73 | 1297.95 | 848.580 | 449.369 |
| 2031 | 238780 | 65433.368 | 42519.934 | 22913.43 | 9.596 | 35.018 | 4406.08 | 1222.18 | 784.599 | 437.582 |
| 2032 | 237476 | 64643.552 | 41460.504 | 23183.05 | 9.762 | 35.863 | 4365.18 | 1203.31 | 762.109 | 441.199 |
| 2033 | 236138 | 63793.514 | 40542.528 | 23250.99 | 9.846 | 36.447 | 4320.63 | 1182.54 | 741.810 | 440.726 |
| 2034 | 234659 | 63032.151 | 39635.655 | 23396.50 | 9.970 | 37.118 | 4271.11 | 1162.80 | 721.421 | 441.379 |
| 2035 | 219519 | 57653.301 | 36449.511 | 21203.79 | 9.659 | 36.778 | 4932.99 | 1315.47 | 819.087 | 496.384 |
| 2036 | 211829 | 52988.733 | 32593.375 | 20395.36 | 9.628 | 38.490 | 4756.77 | 1208.75 | 731.907 | 476.839 |
| 2037 | 209209 | 52026.921 | 31586.741 | 20440.18 | 9.770 | 39.288 | 4690.68 | 1185.50 | 708.207 | 477.296 |
| 2038 | 206622 | 50943.211 | 30567.764 | 20375.45 | 9.861 | 39.996 | 4620.95 | 1158.38 | 683.626 | 474.757 |
| 2039 | 204106 | 50085.991 | 29729.279 | 20356.71 | 9.974 | 40.644 | 4547.88 | 1135.19 | 662.427 | 472.764 |

# Results for PHIM for Run 1 (Sensitivity_Analysis_1.RTF)

**************************************************************************************
**Main Results for E-Component for Run: 1, Simulation: 1, COD: LUNG CANCER, Adjusted**

| Variable Parameter | Value |
|---|---|
| 1. Country | US (United States) |
| 2. Sex | M (Males) |
| 3. Year of start of process | 1990 |
| 4. Cause of Death | LUNG CANCER |
| 5. Effective Dose NULL | CC:1 |
| 6. Effective Dose Ecig | CC:1 Ecig:0.4 Dual:0.7 |
| Note: | Death rates are per million population |

---------------------------------------------------------------------------------------
Results for Age Groups Combined and Years combined, Population and Number deaths adjusted for Ecig scenario,

| Age Group:All | | NULL | Ecig | | | %Drop | | NULL | Ecig | |
|---|---|---|---|---|---|---|---|---|---|---|
| Year | Number Deaths | Attrib Deaths | Attrib Deaths | Drop Deaths | %Drop Deaths | Attrib Deaths | Death Rate | Attrib Rates | Attrib Rates | Drop Rates |
| All Comb | 3082667 | 2244988.113 | 2011642.868 | 233345.2 | 7.570 | 10.394 | 888.256 | 649.875 | 579.646 | 70.230 |

---------------------------------------------------------------------------------------
Results for Age Groups Combined, Population and Number deaths adjusted for Ecig scenario,

| Age Group:All | | NULL | Ecig | | | %Drop | | NULL | Ecig | |
|---|---|---|---|---|---|---|---|---|---|---|
| Year | Number Deaths | Attrib Deaths | Attrib Deaths | Drop Deaths | %Drop Deaths | Attrib Deaths | Death Rate | Attrib Rates | Attrib Rates | Drop Rates |
| 1990 | 78822 | 63385.736 | 63385.736 | 0.000 | 0.000 | 0.000 | 1249.50 | 1004.81 | 1004.81 | 0.000 |
| 1991 | 78462 | 62918.402 | 62887.194 | 31.208 | 0.040 | 0.050 | 1223.41 | 981.049 | 980.563 | 0.487 |
| 1992 | 77987 | 62366.766 | 62285.896 | 80.870 | 0.104 | 0.130 | 1195.35 | 955.930 | 954.686 | 1.244 |
| 1993 | 78544 | 62652.888 | 62495.777 | 157.111 | 0.200 | 0.251 | 1182.40 | 943.195 | 940.815 | 2.380 |
| 1994 | 77441 | 61565.227 | 61322.238 | 242.989 | 0.314 | 0.395 | 1144.02 | 909.520 | 905.899 | 3.622 |
| 1995 | 76911 | 60851.961 | 60501.604 | 350.357 | 0.456 | 0.576 | 1114.39 | 881.759 | 876.625 | 5.134 |
| 1996 | 76243 | 60122.922 | 59638.904 | 484.018 | 0.635 | 0.805 | 1087.48 | 857.643 | 850.648 | 6.995 |
| 1997 | 75490 | 59237.155 | 58614.197 | 622.958 | 0.825 | 1.052 | 1059.13 | 831.234 | 822.361 | 8.873 |
| 1998 | 75405 | 58906.696 | 58107.281 | 799.415 | 1.060 | 1.357 | 1040.47 | 813.001 | 801.787 | 11.214 |
| 1999 | 73191 | 56930.073 | 55967.547 | 962.526 | 1.315 | 1.691 | 993.921 | 773.329 | 760.025 | 13.305 |
| 2000 | 73828 | 57137.121 | 55971.193 | 1165.928 | 1.579 | 2.041 | 987.799 | 764.787 | 748.879 | 15.908 |
| 2001 | 73350 | 56481.726 | 55078.321 | 1403.405 | 1.913 | 2.485 | 968.516 | 746.169 | 727.254 | 18.915 |
| 2002 | 72512 | 55588.365 | 53948.937 | 1639.429 | 2.261 | 2.949 | 946.598 | 726.138 | 704.270 | 21.868 |
| 2003 | 72027 | 54945.856 | 53051.330 | 1894.527 | 2.630 | 3.448 | 930.358 | 710.281 | 685.253 | 25.028 |
| 2004 | 71402 | 54203.126 | 52018.937 | 2184.189 | 3.059 | 4.030 | 912.218 | 693.135 | 664.580 | 28.555 |
| 2005 | 71083 | 53744.603 | 51281.849 | 2462.755 | 3.465 | 4.582 | 897.456 | 679.296 | 647.453 | 31.842 |
| 2006 | 69795 | 52535.572 | 49780.209 | 2755.363 | 3.948 | 5.245 | 872.978 | 657.945 | 622.639 | 35.306 |
| 2007 | 68721 | 51492.124 | 48458.108 | 3034.015 | 4.415 | 5.892 | 850.567 | 638.264 | 599.774 | 38.490 |
| 2008 | 68403 | 51030.736 | 47670.628 | 3360.108 | 4.912 | 6.584 | 836.984 | 625.460 | 583.303 | 42.157 |
| 2009 | 67707 | 50276.199 | 46588.051 | 3688.148 | 5.447 | 7.336 | 818.528 | 608.943 | 563.215 | 45.729 |
| 2010 | 67417 | 49840.933 | 45833.575 | 4007.357 | 5.944 | 8.040 | 804.963 | 596.347 | 547.253 | 49.094 |
| 2011 | 66750 | 49072.841 | 44714.301 | 4358.540 | 6.530 | 8.882 | 788.947 | 581.354 | 528.498 | 52.856 |
| 2012 | 66423 | 48539.512 | 43806.924 | 4732.588 | 7.125 | 9.750 | 776.412 | 568.812 | 512.053 | 56.759 |
| 2013 | 65625 | 47675.741 | 42623.150 | 5052.591 | 7.699 | 10.598 | 758.154 | 552.318 | 492.419 | 59.899 |
| 2014 | 65352 | 47194.534 | 41805.886 | 5388.648 | 8.246 | 11.418 | 746.009 | 540.359 | 477.222 | 63.137 |
| 2015 | 64489 | 46305.842 | 40652.643 | 5653.199 | 8.766 | 12.208 | 827.599 | 596.439 | 521.703 | 74.736 |
| 2016 | 61977 | 44208.867 | 38443.249 | 5765.617 | 9.303 | 13.042 | 787.703 | 564.107 | 488.598 | 75.509 |
| 2017 | 60275 | 42702.624 | 36752.845 | 5949.779 | 9.871 | 13.933 | 758.841 | 539.903 | 462.706 | 77.197 |
| 2018 | 62892 | 44321.202 | 37702.044 | 6619.159 | 10.525 | 14.935 | 784.225 | 555.181 | 470.123 | 85.058 |
| 2019 | 63504 | 44493.088 | 37443.960 | 7049.128 | 11.100 | 15.843 | 783.927 | 551.912 | 462.225 | 89.687 |
| 2020 | 63963 | 44529.551 | 37079.549 | 7450.002 | 11.647 | 16.730 | 900.765 | 630.757 | 522.177 | 108.580 |
| 2021 | 57591 | 39852.556 | 32811.398 | 7041.158 | 12.226 | 17.668 | 805.064 | 560.546 | 458.667 | 101.879 |
| 2022 | 57951 | 39853.685 | 32408.877 | 7444.808 | 12.847 | 18.680 | 803.626 | 556.274 | 449.422 | 106.852 |
| 2023 | 58283 | 39847.050 | 32023.254 | 7823.797 | 13.424 | 19.635 | 801.395 | 551.670 | 440.321 | 111.349 |
| 2024 | 58521 | 39726.010 | 31581.577 | 8144.433 | 13.917 | 20.502 | 797.722 | 545.435 | 430.499 | 114.937 |
| 2025 | 58133 | 39207.348 | 30868.643 | 8338.705 | 14.344 | 21.268 | 921.152 | 626.579 | 489.129 | 137.450 |
| 2026 | 52006 | 34803.960 | 27086.184 | 7717.776 | 14.840 | 22.175 | 820.351 | 553.929 | 427.264 | 126.665 |
| 2027 | 51776 | 34484.595 | 26504.247 | 7980.348 | 15.413 | 23.142 | 812.712 | 546.357 | 416.027 | 130.330 |
| 2028 | 51501 | 34100.638 | 25893.977 | 8206.661 | 15.935 | 24.066 | 803.991 | 537.544 | 404.237 | 133.307 |
| 2029 | 51197 | 33669.838 | 25260.371 | 8409.467 | 16.426 | 24.976 | 794.390 | 527.732 | 391.949 | 135.783 |
| 2030 | 49788 | 32557.688 | 24202.940 | 8354.748 | 16.781 | 25.661 | 921.512 | 609.705 | 447.969 | 161.736 |
| 2031 | 44212 | 28646.393 | 21092.400 | 7553.993 | 17.086 | 26.370 | 815.817 | 535.065 | 389.207 | 145.857 |
| 2032 | 43809 | 28227.160 | 20521.617 | 7705.543 | 17.589 | 27.298 | 805.287 | 525.435 | 377.220 | 148.215 |
| 2033 | 43392 | 27786.623 | 20021.027 | 7765.596 | 17.896 | 27.947 | 793.946 | 515.079 | 366.326 | 148.752 |
| 2034 | 42941 | 27348.299 | 19503.943 | 7844.356 | 18.268 | 28.683 | 781.586 | 504.514 | 354.997 | 149.517 |
| 2035 | 40435 | 25570.077 | 18159.480 | 7410.597 | 18.327 | 28.982 | 908.644 | 583.430 | 408.076 | 175.354 |
| 2036 | 34628 | 21717.319 | 15189.439 | 6527.880 | 18.852 | 30.058 | 777.589 | 495.402 | 341.089 | 154.313 |
| 2037 | 34061 | 21256.024 | 14674.695 | 6581.328 | 19.322 | 30.962 | 763.677 | 484.347 | 329.022 | 155.325 |
| 2038 | 33499 | 20747.175 | 14173.946 | 6573.229 | 19.622 | 31.683 | 749.179 | 471.764 | 316.990 | 154.774 |
| 2039 | 32949 | 20327.688 | 13752.792 | 6574.895 | 19.955 | 32.345 | 734.170 | 460.724 | 306.439 | 154.284 |

# Results for PHIM for Run 1 (Sensitivity_Analysis_1.RTF)

************************************************************************************

**Main Results for E-Component for Run: 1, Simulation: 1, COD: IHD, Adjusted**

| Variable Parameter | Value |
|---|---|
| 1. Country | US (United States) |
| 2. Sex | M (Males) |
| 3. Year of start of process | 1990 |
| 4. Cause of Death | IHD |
| 5. Effective Dose NULL | CC:1 |
| 6. Effective Dose Ecig | CC:1 Ecig:0.4 Dual:0.7 |
| Note: | Death rates are per million population |

-----------------------------------------------------------------------------------------

Results for Age Groups Combined and Years combined, Population and Number deaths adjusted for Ecig scenario,

| Age Group:All Year | Number Deaths | NULL Attrib Deaths | Ecig Attrib Deaths | Drop Deaths | %Drop Deaths | %Drop Attrib Deaths | Death Rate | NULL Attrib Rates | Ecig Attrib Rates | Drop Rates |
|---|---|---|---|---|---|---|---|---|---|---|
| All Comb | 6395210 | 1098877.421 | 763102.519 | 335774.9 | 5.250 | 30.556 | 1842.75 | 318.101 | 219.884 | 98.217 |

-----------------------------------------------------------------------------------------

Results for Age Groups Combined, Population and Number deaths adjusted for Ecig scenario,

| Age Group:All Year | Number Deaths | NULL Attrib Deaths | Ecig Attrib Deaths | Drop Deaths | %Drop Deaths | %Drop Attrib Deaths | Death Rate | NULL Attrib Rates | Ecig Attrib Rates | Drop Rates |
|---|---|---|---|---|---|---|---|---|---|---|
| 1990 | 171484 | 38511.725 | 38511.725 | 0.000 | 0.000 | 0.000 | 2718.40 | 610.497 | 610.497 | 0.000 |
| 1991 | 168044 | 37309.838 | 37112.529 | 197.309 | 0.117 | 0.529 | 2620.21 | 581.750 | 578.674 | 3.077 |
| 1992 | 164549 | 36137.488 | 35616.539 | 520.949 | 0.317 | 1.442 | 2522.12 | 553.899 | 545.912 | 7.987 |
| 1993 | 164365 | 35599.588 | 34646.952 | 952.636 | 0.580 | 2.676 | 2474.37 | 535.926 | 521.577 | 14.349 |
| 1994 | 159874 | 34336.757 | 32901.763 | 1434.994 | 0.898 | 4.179 | 2361.77 | 507.267 | 486.050 | 21.217 |
| 1995 | 158097 | 33455.759 | 31474.144 | 1981.615 | 1.253 | 5.923 | 2290.71 | 484.782 | 456.038 | 28.744 |
| 1996 | 154897 | 32193.824 | 29641.070 | 2552.755 | 1.648 | 7.929 | 2209.35 | 459.239 | 422.780 | 36.460 |
| 1997 | 150003 | 30665.509 | 27567.498 | 3098.011 | 2.065 | 10.103 | 2104.55 | 430.308 | 386.774 | 43.534 |
| 1998 | 145452 | 29188.430 | 25586.217 | 3602.214 | 2.477 | 12.341 | 2007.00 | 402.844 | 353.049 | 49.795 |
| 1999 | 166022 | 33375.386 | 28398.647 | 4976.739 | 2.998 | 14.911 | 2254.54 | 453.366 | 385.646 | 67.720 |
| 2000 | 160351 | 32024.754 | 26502.512 | 5522.242 | 3.444 | 17.244 | 2145.46 | 428.655 | 354.596 | 74.059 |
| 2001 | 154557 | 30682.393 | 24577.643 | 6104.750 | 3.950 | 19.897 | 2040.77 | 405.339 | 324.523 | 80.816 |
| 2002 | 152027 | 30218.370 | 23439.614 | 6778.756 | 4.459 | 22.433 | 1984.61 | 394.736 | 305.990 | 88.746 |
| 2003 | 146899 | 29175.202 | 21911.444 | 7263.757 | 4.945 | 24.897 | 1897.46 | 377.146 | 283.026 | 94.120 |
| 2004 | 138569 | 27488.687 | 19962.859 | 7525.829 | 5.431 | 27.378 | 1770.32 | 351.518 | 255.040 | 96.478 |
| 2005 | 136099 | 27017.439 | 19017.671 | 7999.769 | 5.878 | 29.610 | 1718.31 | 341.482 | 240.105 | 101.377 |
| 2006 | 132169 | 26231.435 | 17834.563 | 8396.872 | 6.353 | 32.011 | 1653.14 | 328.517 | 223.071 | 105.447 |
| 2007 | 126776 | 25103.049 | 16532.834 | 8570.215 | 6.760 | 34.140 | 1569.13 | 311.161 | 204.629 | 106.532 |
| 2008 | 126171 | 24621.740 | 15780.247 | 8841.494 | 7.008 | 35.909 | 1543.84 | 301.777 | 193.089 | 108.688 |
| 2009 | 122772 | 23741.177 | 14800.386 | 8940.791 | 7.282 | 37.659 | 1484.22 | 287.552 | 178.926 | 108.627 |
| 2010 | 120916 | 23189.160 | 14103.369 | 9085.791 | 7.514 | 39.181 | 1443.73 | 277.458 | 168.394 | 109.064 |
| 2011 | 120641 | 22786.927 | 13484.160 | 9302.767 | 7.711 | 40.825 | 1425.91 | 269.951 | 159.375 | 110.576 |
| 2012 | 121395 | 22485.295 | 12971.465 | 9513.829 | 7.837 | 42.311 | 1418.97 | 263.495 | 151.622 | 111.873 |
| 2013 | 123172 | 22251.884 | 12542.315 | 9709.569 | 7.883 | 43.635 | 1422.98 | 257.786 | 144.900 | 112.886 |
| 2014 | 123806 | 21894.593 | 12068.850 | 9825.744 | 7.936 | 44.877 | 1413.27 | 250.685 | 137.768 | 112.916 |
| 2015 | 124410 | 21471.109 | 11618.782 | 9852.327 | 7.919 | 45.886 | 1596.57 | 276.557 | 149.106 | 127.451 |
| 2016 | 126922 | 21411.568 | 11369.349 | 10042.22 | 7.912 | 46.901 | 1613.13 | 273.213 | 144.500 | 128.713 |
| 2017 | 129041 | 21169.399 | 11023.394 | 10146.01 | 7.863 | 47.928 | 1624.58 | 267.651 | 138.781 | 128.871 |
| 2018 | 122212 | 19830.576 | 10149.777 | 9680.799 | 7.921 | 48.818 | 1523.91 | 248.404 | 126.562 | 121.842 |
| 2019 | 122676 | 19431.014 | 9810.510 | 9620.504 | 7.842 | 49.511 | 1514.37 | 241.031 | 121.105 | 119.925 |
| 2020 | 121717 | 18532.612 | 9229.774 | 9302.838 | 7.643 | 50.197 | 1714.09 | 262.513 | 129.979 | 132.534 |
| 2021 | 120355 | 18234.128 | 8944.769 | 9289.359 | 7.718 | 50.945 | 1682.43 | 256.472 | 125.038 | 131.434 |
| 2022 | 120445 | 17812.559 | 8603.437 | 9209.122 | 7.646 | 51.700 | 1670.25 | 248.626 | 119.306 | 129.320 |
| 2023 | 120514 | 17361.668 | 8274.162 | 9087.507 | 7.541 | 52.342 | 1657.07 | 240.367 | 113.770 | 126.597 |
| 2024 | 120435 | 16929.676 | 7984.201 | 8945.475 | 7.428 | 52.839 | 1641.69 | 232.443 | 108.835 | 123.608 |
| 2025 | 116870 | 15501.666 | 7236.061 | 8265.606 | 7.073 | 53.321 | 1851.86 | 247.735 | 114.659 | 133.076 |
| 2026 | 116300 | 15523.814 | 7203.189 | 8320.625 | 7.154 | 53.599 | 1834.54 | 247.072 | 113.625 | 133.447 |
| 2027 | 115265 | 15073.070 | 6941.801 | 8131.270 | 7.054 | 53.946 | 1809.27 | 238.811 | 108.963 | 129.848 |
| 2028 | 114180 | 14636.710 | 6669.971 | 7966.739 | 6.977 | 54.430 | 1782.49 | 230.725 | 104.126 | 126.599 |
| 2029 | 113092 | 14180.166 | 6421.666 | 7758.500 | 6.860 | 54.714 | 1754.78 | 222.256 | 99.641 | 122.615 |
| 2030 | 106249 | 12111.772 | 5441.216 | 6670.556 | 6.278 | 55.075 | 1966.55 | 226.816 | 100.711 | 126.105 |
| 2031 | 106735 | 12417.367 | 5568.772 | 6848.595 | 6.416 | 55.153 | 1969.53 | 231.935 | 102.758 | 129.177 |
| 2032 | 105365 | 12054.064 | 5347.794 | 6706.271 | 6.365 | 55.635 | 1936.77 | 224.381 | 98.301 | 126.080 |
| 2033 | 104009 | 11672.389 | 5124.197 | 6548.192 | 6.296 | 56.100 | 1903.06 | 216.370 | 93.758 | 122.612 |
| 2034 | 102630 | 11365.762 | 4971.177 | 6394.585 | 6.231 | 56.262 | 1868.00 | 209.673 | 90.482 | 119.191 |
| 2035 | 92342 | 8580.360 | 3712.449 | 4867.911 | 5.272 | 56.733 | 2075.08 | 195.777 | 83.425 | 112.352 |
| 2036 | 93687 | 8948.841 | 3845.939 | 5102.902 | 5.447 | 57.023 | 2103.80 | 204.135 | 86.363 | 117.772 |
| 2037 | 91915 | 8624.570 | 3705.064 | 4919.506 | 5.352 | 57.041 | 2060.83 | 196.522 | 83.071 | 113.451 |
| 2038 | 90196 | 8315.017 | 3529.927 | 4785.090 | 5.305 | 57.548 | 2017.16 | 189.073 | 78.944 | 110.128 |
| 2039 | 88543 | 8001.132 | 3388.126 | 4613.006 | 5.210 | 57.654 | 1972.92 | 181.344 | 75.494 | 105.850 |

# Results for PHIM for Run 1 (Sensitivity_Analysis_1.RTF)

************************************************************************************
**Main Results for E-Component for Run: 1, Simulation: 1, COD: STROKE, Adjusted**

| Variable Parameter | Value |
|---|---|
| 1. Country | US (United States) |
| 2. Sex | M (Males) |
| 3. Year of start of process | 1990 |
| 4. Cause of Death | STROKE |
| 5. Effective Dose NULL | CC:1 |
| 6. Effective Dose Ecig | CC:1 Ecig:0.4 Dual:0.7 |
| Note: | Death rates are per million population |

-----------------------------------------------------------------------------------------
Results for Age Groups Combined and Years combined, Population and Number deaths adjusted for Ecig scenario,

| Age Group:All | | NULL | Ecig | | | %Drop | | NULL | Ecig | |
|---|---|---|---|---|---|---|---|---|---|---|
| Year | Number | Attrib | Attrib | Drop | %Drop | Attrib | Death | Attrib | Attrib | Drop |
| | Deaths | Deaths | Deaths | Deaths | Deaths | Deaths | Rate | Rates | Rates | Rates |
| All Comb | 1499248 | 149918.462 | 102260.563 | 47657.90 | 3.179 | 31.789 | 432.001 | 43.398 | 29.466 | 13.932 |

-----------------------------------------------------------------------------------------
Results for Age Groups Combined, Population and Number deaths adjusted for Ecig scenario,

| Age Group:All | | NULL | Ecig | | | %Drop | | NULL | Ecig | |
|---|---|---|---|---|---|---|---|---|---|---|
| Year | Number | Attrib | Attrib | Drop | %Drop | Attrib | Death | Attrib | Attrib | Drop |
| | Deaths | Deaths | Deaths | Deaths | Deaths | Deaths | Rate | Rates | Rates | Rates |
| 1990 | 32862 | 4613.166 | 4613.166 | 0.000 | 0.000 | 0.000 | 520.936 | 73.129 | 73.129 | 0.000 |
| 1991 | 32619 | 4555.566 | 4529.884 | 25.682 | 0.079 | 0.564 | 508.609 | 71.032 | 70.632 | 0.400 |
| 1992 | 32100 | 4453.572 | 4387.847 | 65.725 | 0.205 | 1.476 | 492.015 | 68.262 | 67.255 | 1.008 |
| 1993 | 32897 | 4462.473 | 4339.260 | 123.213 | 0.375 | 2.761 | 495.228 | 67.179 | 65.323 | 1.856 |
| 1994 | 33325 | 4499.513 | 4307.254 | 192.259 | 0.577 | 4.273 | 492.295 | 66.473 | 63.630 | 2.843 |
| 1995 | 33572 | 4472.774 | 4201.944 | 270.830 | 0.807 | 6.055 | 486.431 | 64.812 | 60.883 | 3.928 |
| 1996 | 33875 | 4406.878 | 4049.525 | 357.353 | 1.055 | 8.109 | 483.164 | 62.863 | 57.760 | 5.104 |
| 1997 | 33523 | 4288.158 | 3842.950 | 445.208 | 1.328 | 10.382 | 470.330 | 60.173 | 53.917 | 6.256 |
| 1998 | 32805 | 4120.148 | 3595.508 | 524.640 | 1.599 | 12.734 | 452.652 | 56.864 | 49.612 | 7.252 |
| 1999 | 33389 | 3982.443 | 3378.130 | 604.313 | 1.810 | 15.174 | 453.408 | 54.097 | 45.874 | 8.223 |
| 2000 | 33477 | 4021.411 | 3317.195 | 704.216 | 2.104 | 17.512 | 447.918 | 53.827 | 44.383 | 9.444 |
| 2001 | 32298 | 3848.092 | 3075.961 | 772.132 | 2.391 | 20.065 | 426.459 | 50.836 | 40.615 | 10.221 |
| 2002 | 31614 | 3783.081 | 2933.228 | 849.853 | 2.688 | 22.465 | 412.702 | 49.418 | 38.291 | 11.126 |
| 2003 | 31019 | 3754.415 | 2820.545 | 933.870 | 3.011 | 24.874 | 400.663 | 48.533 | 36.432 | 12.101 |
| 2004 | 29923 | 3666.274 | 2666.757 | 999.517 | 3.340 | 27.262 | 382.293 | 46.883 | 34.070 | 12.814 |
| 2005 | 28753 | 3589.688 | 2528.105 | 1061.583 | 3.692 | 29.573 | 363.018 | 45.371 | 31.918 | 13.453 |
| 2006 | 28240 | 3596.985 | 2449.786 | 1147.198 | 4.062 | 31.893 | 353.216 | 45.048 | 30.641 | 14.407 |
| 2007 | 28086 | 3560.798 | 2349.344 | 1211.454 | 4.313 | 34.022 | 347.619 | 44.137 | 29.078 | 15.059 |
| 2008 | 27194 | 3417.001 | 2188.616 | 1228.385 | 4.517 | 35.949 | 332.748 | 41.881 | 26.780 | 15.100 |
| 2009 | 27143 | 3394.390 | 2114.111 | 1280.278 | 4.717 | 37.717 | 328.134 | 41.113 | 25.558 | 15.555 |
| 2010 | 26994 | 3340.137 | 2029.043 | 1311.095 | 4.857 | 39.253 | 322.313 | 39.965 | 24.227 | 15.738 |
| 2011 | 27052 | 3285.435 | 1945.729 | 1339.706 | 4.952 | 40.777 | 319.743 | 38.922 | 22.997 | 15.924 |
| 2012 | 27161 | 3259.209 | 1877.975 | 1381.235 | 5.085 | 42.379 | 317.479 | 38.193 | 21.951 | 16.242 |
| 2013 | 28022 | 3274.703 | 1839.284 | 1435.419 | 5.122 | 43.834 | 323.739 | 37.937 | 21.249 | 16.688 |
| 2014 | 28926 | 3298.083 | 1807.159 | 1490.924 | 5.154 | 45.206 | 330.195 | 37.762 | 20.629 | 17.133 |
| 2015 | 29712 | 3244.406 | 1741.285 | 1503.121 | 5.059 | 46.330 | 381.296 | 41.789 | 22.346 | 19.443 |
| 2016 | 30683 | 3314.836 | 1747.143 | 1567.693 | 5.109 | 47.293 | 389.966 | 42.297 | 22.205 | 20.092 |
| 2017 | 31605 | 3333.314 | 1720.157 | 1613.157 | 5.104 | 48.395 | 397.902 | 42.144 | 21.656 | 20.488 |
| 2018 | 29541 | 2925.242 | 1482.996 | 1442.246 | 4.882 | 49.303 | 368.364 | 36.642 | 18.492 | 18.150 |
| 2019 | 29808 | 2864.628 | 1431.226 | 1433.402 | 4.809 | 50.038 | 367.959 | 35.534 | 17.668 | 17.866 |
| 2020 | 29819 | 2730.366 | 1346.459 | 1383.907 | 4.641 | 50.686 | 419.930 | 38.675 | 18.962 | 19.714 |
| 2021 | 30344 | 2647.911 | 1284.556 | 1363.355 | 4.493 | 51.488 | 424.179 | 37.244 | 17.957 | 19.288 |
| 2022 | 30568 | 2586.165 | 1233.959 | 1352.206 | 4.424 | 52.286 | 423.900 | 36.097 | 17.112 | 18.986 |
| 2023 | 30789 | 2517.960 | 1186.367 | 1331.593 | 4.325 | 52.884 | 423.343 | 34.860 | 16.313 | 18.548 |
| 2024 | 30962 | 2454.161 | 1143.253 | 1310.908 | 4.234 | 53.416 | 422.059 | 33.695 | 15.584 | 18.111 |
| 2025 | 30573 | 2275.364 | 1048.175 | 1227.189 | 4.014 | 53.934 | 484.443 | 36.363 | 16.609 | 19.754 |
| 2026 | 31028 | 2183.166 | 998.364 | 1184.802 | 3.818 | 54.270 | 489.444 | 34.747 | 15.748 | 18.998 |
| 2027 | 30920 | 2112.345 | 959.313 | 1153.032 | 3.729 | 54.585 | 485.342 | 33.467 | 15.058 | 18.409 |
| 2028 | 30787 | 2046.677 | 922.170 | 1124.506 | 3.653 | 54.943 | 480.624 | 32.263 | 14.396 | 17.866 |
| 2029 | 30646 | 1977.671 | 885.743 | 1091.929 | 3.563 | 55.213 | 475.517 | 30.997 | 13.744 | 17.254 |
| 2030 | 29735 | 1744.457 | 776.090 | 968.367 | 3.257 | 55.511 | 550.365 | 32.668 | 14.365 | 18.304 |
| 2031 | 29395 | 1653.738 | 730.655 | 923.082 | 3.140 | 55.818 | 542.409 | 30.889 | 13.482 | 17.407 |
| 2032 | 29158 | 1603.120 | 698.160 | 904.961 | 3.104 | 56.450 | 535.962 | 29.841 | 12.833 | 17.008 |
| 2033 | 28911 | 1548.245 | 670.043 | 878.202 | 3.038 | 56.722 | 528.987 | 28.700 | 12.260 | 16.440 |
| 2034 | 28639 | 1511.985 | 648.775 | 863.210 | 3.014 | 57.091 | 521.273 | 27.893 | 11.809 | 16.084 |
| 2035 | 27202 | 1239.866 | 530.323 | 709.542 | 2.608 | 57.227 | 611.273 | 28.290 | 11.917 | 16.373 |
| 2036 | 26051 | 1182.522 | 501.237 | 681.285 | 2.615 | 57.613 | 584.987 | 26.975 | 11.256 | 15.719 |
| 2037 | 25610 | 1134.483 | 484.128 | 650.355 | 2.539 | 57.326 | 574.204 | 25.851 | 10.855 | 14.996 |
| 2038 | 25167 | 1093.537 | 461.110 | 632.427 | 2.513 | 57.833 | 562.846 | 24.866 | 10.312 | 14.553 |
| 2039 | 24727 | 1047.905 | 440.575 | 607.330 | 2.456 | 57.957 | 550.971 | 23.751 | 9.817 | 13.934 |

# Results for PHIM for Run 1 (Sensitivity_Analysis_1.RTF)

************************************************************************************

**Main Results for E-Component for Run: 1, Simulation: 1, COD: COPD, Adjusted**

| Variable Parameter | Value |
|---|---|
| 1. Country | US (United States) |
| 2. Sex | M (Males) |
| 3. Year of start of process | 1990 |
| 4. Cause of Death | COPD |
| 5. Effective Dose NULL | CC:1 |
| 6. Effective Dose Ecig | CC:1 Ecig:0.4 Dual:0.7 |
| Note: | Death rates are per million population |

-----------------------------------------------------------------------------------------

Results for Age Groups Combined and Years combined, Population and Number deaths adjusted for Ecig scenario,

| Age Group:All | | NULL | Ecig | | | %Drop | | NULL | Ecig | |
|---|---|---|---|---|---|---|---|---|---|---|
| Year | Number | Attrib | Attrib | Drop | %Drop | Attrib | Death | Attrib | Attrib | Drop |
| | Deaths | Deaths | Deaths | Deaths | Deaths | Deaths | Rate | Rates | Rates | Rates |
| All Comb | 2251391 | 1015606.587 | 831659.102 | 183947.5 | 8.170 | 18.112 | 648.728 | 293.996 | 239.639 | 54.357 |

-----------------------------------------------------------------------------------------

Results for Age Groups Combined, Population and Number deaths adjusted for Ecig scenario,

| Age Group:All | | NULL | Ecig | | | %Drop | | NULL | Ecig | |
|---|---|---|---|---|---|---|---|---|---|---|
| Year | Number | Attrib | Attrib | Drop | %Drop | Attrib | Death | Attrib | Attrib | Drop |
| | Deaths | Deaths | Deaths | Deaths | Deaths | Deaths | Rate | Rates | Rates | Rates |
| 1990 | 33993 | 19262.437 | 19262.437 | 0.000 | 0.000 | 0.000 | 538.865 | 305.353 | 305.353 | 0.000 |
| 1991 | 34092 | 19215.636 | 19200.970 | 14.666 | 0.043 | 0.076 | 531.576 | 299.618 | 299.389 | 0.229 |
| 1992 | 33942 | 19056.189 | 19019.362 | 36.828 | 0.109 | 0.193 | 520.248 | 292.085 | 291.519 | 0.566 |
| 1993 | 35914 | 20095.670 | 20021.472 | 74.197 | 0.207 | 0.369 | 540.647 | 302.526 | 301.404 | 1.122 |
| 1994 | 35076 | 19524.095 | 19413.229 | 110.866 | 0.316 | 0.568 | 518.164 | 288.435 | 286.787 | 1.648 |
| 1995 | 34672 | 19116.002 | 18956.654 | 159.348 | 0.460 | 0.834 | 502.372 | 276.995 | 274.668 | 2.327 |
| 1996 | 34587 | 18975.021 | 18758.109 | 216.913 | 0.627 | 1.143 | 493.322 | 270.675 | 267.553 | 3.123 |
| 1997 | 35274 | 19169.025 | 18883.227 | 285.798 | 0.810 | 1.491 | 494.903 | 268.986 | 264.933 | 4.053 |
| 1998 | 35686 | 19228.937 | 18856.807 | 372.130 | 1.043 | 1.935 | 492.406 | 265.388 | 260.194 | 5.195 |
| 1999 | 38532 | 20589.820 | 20105.980 | 483.841 | 1.256 | 2.350 | 523.255 | 279.689 | 273.034 | 6.655 |
| 2000 | 36303 | 19196.417 | 18658.261 | 538.157 | 1.482 | 2.803 | 485.730 | 256.946 | 249.642 | 7.304 |
| 2001 | 36307 | 19012.731 | 18360.371 | 652.361 | 1.797 | 3.431 | 479.401 | 251.174 | 242.430 | 8.743 |
| 2002 | 36016 | 18699.357 | 17941.754 | 757.603 | 2.104 | 4.051 | 470.164 | 244.265 | 234.218 | 10.047 |
| 2003 | 36246 | 18663.550 | 17785.293 | 878.257 | 2.423 | 4.706 | 468.182 | 241.262 | 229.729 | 11.533 |
| 2004 | 34658 | 17679.164 | 16703.543 | 975.621 | 2.815 | 5.518 | 442.785 | 226.076 | 213.400 | 12.676 |
| 2005 | 36886 | 18692.832 | 17522.789 | 1170.043 | 3.172 | 6.259 | 465.698 | 236.265 | 221.232 | 15.033 |
| 2006 | 34415 | 17308.564 | 16068.829 | 1239.734 | 3.602 | 7.163 | 430.461 | 216.769 | 200.985 | 15.784 |
| 2007 | 35404 | 17683.181 | 16256.980 | 1426.201 | 4.028 | 8.065 | 438.204 | 219.189 | 201.215 | 17.974 |
| 2008 | 38345 | 19006.374 | 17319.319 | 1687.055 | 4.400 | 8.876 | 469.196 | 232.952 | 211.921 | 21.031 |
| 2009 | 37009 | 18205.573 | 16399.682 | 1805.891 | 4.880 | 9.919 | 447.408 | 220.505 | 198.260 | 22.245 |
| 2010 | 37355 | 18237.758 | 16268.186 | 1969.573 | 5.273 | 10.799 | 446.023 | 218.215 | 194.242 | 23.973 |
| 2011 | 38232 | 18487.916 | 16270.920 | 2216.996 | 5.799 | 11.992 | 451.875 | 219.022 | 192.313 | 26.709 |
| 2012 | 38659 | 18491.158 | 16070.599 | 2420.560 | 6.261 | 13.090 | 451.885 | 216.689 | 187.847 | 28.842 |
| 2013 | 40470 | 19143.452 | 16438.196 | 2705.256 | 6.685 | 14.131 | 467.544 | 221.775 | 189.908 | 31.867 |
| 2014 | 40537 | 18992.005 | 16093.444 | 2898.561 | 7.150 | 15.262 | 462.738 | 217.451 | 183.710 | 33.741 |
| 2015 | 42531 | 19728.885 | 16550.873 | 3178.011 | 7.472 | 16.108 | 545.811 | 254.116 | 212.400 | 41.716 |
| 2016 | 43357 | 19876.724 | 16474.206 | 3402.518 | 7.848 | 17.118 | 551.048 | 253.628 | 209.381 | 44.247 |
| 2017 | 44388 | 20119.790 | 16442.776 | 3677.014 | 8.284 | 18.276 | 558.826 | 254.381 | 207.009 | 47.372 |
| 2018 | 45781 | 20535.123 | 16552.272 | 3982.851 | 8.700 | 19.395 | 570.865 | 257.229 | 206.397 | 50.832 |
| 2019 | 47310 | 21009.029 | 16705.873 | 4303.156 | 9.096 | 20.482 | 584.012 | 260.605 | 206.225 | 54.380 |
| 2020 | 48803 | 21428.512 | 16810.992 | 4617.520 | 9.461 | 21.548 | 687.281 | 303.533 | 236.743 | 66.790 |
| 2021 | 48875 | 21266.697 | 16426.956 | 4839.741 | 9.902 | 22.757 | 683.213 | 299.127 | 229.631 | 69.496 |
| 2022 | 50367 | 21663.568 | 16469.252 | 5194.316 | 10.313 | 23.977 | 698.445 | 302.378 | 228.383 | 73.995 |
| 2023 | 51884 | 22084.131 | 16530.787 | 5553.344 | 10.703 | 25.146 | 713.411 | 305.748 | 227.299 | 78.449 |
| 2024 | 53349 | 22418.456 | 16559.510 | 5858.946 | 10.982 | 26.134 | 727.215 | 307.804 | 225.728 | 82.076 |
| 2025 | 54431 | 22614.834 | 16491.977 | 6122.858 | 11.249 | 27.075 | 862.482 | 361.411 | 261.324 | 100.088 |
| 2026 | 54964 | 22571.522 | 16211.382 | 6360.140 | 11.571 | 28.178 | 867.020 | 359.241 | 255.722 | 103.519 |
| 2027 | 55933 | 22774.416 | 16090.189 | 6684.226 | 11.950 | 29.350 | 877.960 | 360.827 | 252.561 | 108.265 |
| 2028 | 56861 | 22915.561 | 15923.593 | 6991.967 | 12.297 | 30.512 | 887.664 | 361.228 | 248.587 | 112.641 |
| 2029 | 57772 | 22992.027 | 15717.611 | 7274.416 | 12.592 | 31.639 | 896.415 | 360.371 | 243.880 | 116.491 |
| 2030 | 58043 | 22895.360 | 15426.939 | 7468.420 | 12.867 | 32.620 | 1074.30 | 428.760 | 285.535 | 143.224 |
| 2031 | 58438 | 22715.871 | 15128.107 | 7587.764 | 12.984 | 33.403 | 1078.32 | 424.293 | 279.151 | 145.142 |
| 2032 | 59144 | 22759.207 | 14892.933 | 7866.274 | 13.300 | 34.563 | 1087.16 | 423.652 | 273.756 | 149.896 |
| 2033 | 59826 | 22786.258 | 14727.261 | 8058.996 | 13.471 | 35.368 | 1094.64 | 422.387 | 269.466 | 152.922 |
| 2034 | 60449 | 22806.106 | 14511.760 | 8294.346 | 13.721 | 36.369 | 1100.25 | 420.721 | 264.133 | 156.588 |
| 2035 | 59541 | 22262.998 | 14047.258 | 8215.739 | 13.798 | 36.903 | 1337.99 | 507.973 | 315.667 | 192.306 |
| 2036 | 57464 | 21140.051 | 13056.760 | 8083.291 | 14.067 | 38.237 | 1290.40 | 482.234 | 293.199 | 189.035 |
| 2037 | 57623 | 21011.845 | 12722.854 | 8288.991 | 14.385 | 39.449 | 1291.97 | 478.783 | 285.259 | 193.524 |
| 2038 | 57760 | 20787.483 | 12402.781 | 8384.703 | 14.516 | 40.335 | 1291.76 | 472.681 | 277.379 | 195.301 |
| 2039 | 57886 | 20709.267 | 12147.787 | 8561.480 | 14.790 | 41.341 | 1289.83 | 469.372 | 270.677 | 198.696 |

# Results for PHIM for Run 2 (Sensitivity_Analysis_1.RTF)

---
--------------------------------------------------------------------------------------

**Control File Details for Run: 2**

| Variable Parameter | Value |
|---|---|
| 1. Name for Reference Scenario | NULL |
| 2. Name for Test Scenario | Ecig |
| 3. Name for Product 1 | CC |
| 4. Name for Product 2 | Ecig |
| 5. Name for Product 3 | - |
| 6. Name for Product 4 | Dual |
| 7. Country | US (United States) |
| 8. Sex | F (Females) |
| 9. Year of start of process | 1990 |
| 10. Number of months of follow-up | 600 |
| 11. Follow-up interval length (in months) | 12 |
| 12. Lower age for risk estimation | 10 |
| 13. Upper age for risk estimation | 79 |
| 14. The effective dose for Product 1 (F1) - Ref | 1 |
| 15. The effective dose for Product 2 (F2) - Ref | - |
| 16. The effective dose for Product 3 (F3) - Ref | - |
| 17. The effective dose for Product 4 (F4) - Ref | - |
| 18. The effective dose for Product 1 (F1) - Test | 1 |
| 19. The effective dose for Product 2 (F2) - Test | 0.4 |
| 20. The effective dose for Product 3 (F3) - Test | - |
| 21. The effective dose for Product 4 (F4) - Test | FROMF2 |
| 22. Number in population to be simulated | 100000 |
| 23. Number of MC simulations | 1 |
| 24. The random number seed for the first simulation | 15975263 |
| 25. Source for the population file (POP) | UN4 |
| 26. Source for the socioeconomic prevalence file (SEP) | - |
| 27. Source for the current smoking prevalence file (CSP) | ISS3 |
| 28. Source for the former smoking prevalence file (FSP) | ISS3 |
| 29. Source for the quit-time distribution file (QTD) | NHIS2006 |
| 30. Source for the death file (MORT) | APR-20 |
| 31. Source for the relative risk file (RR) | PNLEST |
| 32. Source for the half-life file (H) | PNLEST |
| 33. Assumption Set for TTP Factor Reference | F1 |
| 34. Assumption Set for TTP Factor Test | F1 |
| 35. Assumption Set for TTP Reference | ISS3 |
| 36. Assumption Set for TTP Test | ISS3 |
| 37. Assumption Set for TP for socioeconomic group | - |
| 38. Source for Output Choice file (OUTC) | BASIC |
| 39. Output for DTP P1 | N |
| 40. Output for DTP P2 | N |
| 41. Output for Main P Component | Y |
| 42. Output for DTP E1 | N |
| 43. Output for DTP E2 | N |
| 44. Output for DTP E3 | N |
| 45. Output for Main E Component | Y |
| 46. Output file name | Sensitivity_Analysis_1 |
| 47. Run number for P-Component | - |
| 48. Results file for E-Component | - |
| 49. Components required | PE |
| 50. Results folder for P to be used in E-Component | - |
| 51. Fixed age of starting product use | 16 |
| 52. Output absolute risk file? | N |

# Results for PHIM for Run 2 (Sensitivity_Analysis_1.RTF)

************************************************************************************

**TTP values for Run: 2, Scenario: NULL**

| **Variable Parameter** | **Value** |
|---|---|
| 1. Country | US (United States) |
| 2. Sex | F (Females) |
| 3. Assumption Set | ISS3 |
| Note: | Period is in Months |

| Age | Period | **Initiation** Never CC | **Cessation** CC Former | **Re-initiation** Former CC |
|---|---|---|---|---|
| 10-14 | 1-12 | 0.003758 | 0.008742 | 0.000000 |
| 15-19 | 1-12 | 0.003469 | 0.002515 | 0.000000 |
| 20-24 | 1-12 | 0.000276 | 0.000761 | 0.000000 |
| 25-29 | 1-12 | 0.000137 | 0.001375 | 0.000000 |
| 30-34 | 1-12 | 0.000069 | 0.001149 | 0.000000 |
| 35-39 | 1-12 | 0.000000 | 0.000991 | 0.000000 |
| 40-44 | 1-12 | 0.000000 | 0.000833 | 0.000000 |
| 45-49 | 1-12 | 0.000000 | 0.001723 | 0.000000 |
| 50-54 | 1-12 | 0.000000 | 0.002336 | 0.000000 |
| 55-59 | 1-12 | 0.000000 | 0.002233 | 0.000000 |
| 60-64 | 1-12 | 0.000000 | 0.004634 | 0.000000 |
| 65-69 | 1-12 | 0.000000 | 0.003505 | 0.000000 |
| 70-74 | 1-12 | 0.000000 | 0.004329 | 0.000000 |
| 75-79 | 1-12 | 0.000000 | 0.004329 | 0.000000 |
| 10-14 | 13-72 | 0.004794 | 0.008742 | 0.000000 |
| 15-19 | 13-72 | 0.004527 | 0.004082 | 0.000000 |
| 20-24 | 13-72 | 0.000105 | 0.001261 | 0.000000 |
| 25-29 | 13-72 | 0.000053 | 0.000952 | 0.000000 |
| 30-34 | 13-72 | 0.000026 | 0.000733 | 0.000000 |
| 35-39 | 13-72 | 0.000000 | 0.000753 | 0.000000 |
| 40-44 | 13-72 | 0.000000 | 0.001535 | 0.000000 |
| 45-49 | 13-72 | 0.000000 | 0.002998 | 0.000000 |
| 50-54 | 13-72 | 0.000000 | 0.003159 | 0.000000 |
| 55-59 | 13-72 | 0.000000 | 0.002965 | 0.000000 |
| 60-64 | 13-72 | 0.000000 | 0.003633 | 0.000000 |
| 65-69 | 13-72 | 0.000000 | 0.004522 | 0.000000 |
| 70-74 | 13-72 | 0.000000 | 0.005208 | 0.000000 |
| 75-79 | 13-72 | 0.000000 | 0.005208 | 0.000000 |
| 10-14 | 73-132 | 0.003969 | 0.008742 | 0.000000 |
| 15-19 | 73-132 | 0.003056 | 0.003373 | 0.000000 |
| 20-24 | 73-132 | 0.001459 | 0.003881 | 0.000000 |
| 25-29 | 73-132 | 0.000714 | 0.001953 | 0.000000 |
| 30-34 | 73-132 | 0.000111 | 0.001338 | 0.000000 |
| 35-39 | 73-132 | 0.000000 | 0.001507 | 0.000000 |
| 40-44 | 73-132 | 0.000000 | 0.002080 | 0.000000 |
| 45-49 | 73-132 | 0.000000 | 0.003809 | 0.000000 |
| 50-54 | 73-132 | 0.000000 | 0.004871 | 0.000000 |
| 55-59 | 73-132 | 0.000000 | 0.004560 | 0.000000 |
| 60-64 | 73-132 | 0.000000 | 0.007192 | 0.000000 |
| 65-69 | 73-132 | 0.000000 | 0.007033 | 0.000000 |
| 70-74 | 73-132 | 0.000000 | 0.008206 | 0.000000 |
| 75-79 | 73-132 | 0.000000 | 0.008206 | 0.000000 |
| 10-14 | 133-192 | 0.002861 | 0.008742 | 0.000000 |
| 15-19 | 133-192 | 0.002594 | 0.003996 | 0.000000 |
| 20-24 | 133-192 | 0.001247 | 0.002805 | 0.000000 |
| 25-29 | 133-192 | 0.000289 | 0.001574 | 0.000000 |
| 30-34 | 133-192 | 0.000132 | 0.001293 | 0.000000 |
| 35-39 | 133-192 | 0.000000 | 0.001219 | 0.000000 |
| 40-44 | 133-192 | 0.000000 | 0.001657 | 0.000000 |
| 45-49 | 133-192 | 0.000000 | 0.001995 | 0.000000 |
| 50-54 | 133-192 | 0.000000 | 0.002154 | 0.000000 |
| 55-59 | 133-192 | 0.000000 | 0.002072 | 0.000000 |
| 60-64 | 133-192 | 0.000000 | 0.005049 | 0.000000 |
| 65-69 | 133-192 | 0.000000 | 0.003680 | 0.000000 |
| 70-74 | 133-192 | 0.000000 | 0.006144 | 0.000000 |
| 75-79 | 133-192 | 0.000000 | 0.006144 | 0.000000 |
| 10-14 | 193-252 | 0.001859 | 0.008742 | 0.000000 |
| 15-19 | 193-252 | 0.002232 | 0.005770 | 0.000000 |
| 20-24 | 193-252 | 0.001079 | 0.003262 | 0.000000 |
| 25-29 | 193-252 | 0.000531 | 0.002002 | 0.000000 |
| 30-34 | 193-252 | 0.000263 | 0.003240 | 0.000000 |
| 35-39 | 193-252 | 0.000000 | 0.001771 | 0.000000 |
| 40-44 | 193-252 | 0.000000 | 0.002173 | 0.000000 |
| 45-49 | 193-252 | 0.000000 | 0.002075 | 0.000000 |
| 50-54 | 193-252 | 0.000000 | 0.003431 | 0.000000 |

# Results for PHIM for Run 2 (Sensitivity_Analysis_1.RTF)

| | | | | |
|---|---|---|---|---|
| 55-59 | 193-252 | 0.000000 | 0.002906 | 0.000000 |
| 60-64 | 193-252 | 0.000000 | 0.007139 | 0.000000 |
| 65-69 | 193-252 | 0.000000 | 0.004963 | 0.000000 |
| 70-74 | 193-252 | 0.000000 | 0.006269 | 0.000000 |
| 75-79 | 193-252 | 0.000000 | 0.006269 | 0.000000 |
| 10-14 | 253+ | 0.000931 | 0.008742 | 0.000000 |
| 15-19 | 253+ | 0.001798 | 0.009440 | 0.000000 |
| 20-24 | 253+ | 0.000023 | 0.002831 | 0.000000 |
| 25-29 | 253+ | 0.000073 | 0.002655 | 0.000000 |
| 30-34 | 253+ | 0.000037 | 0.004614 | 0.000000 |
| 35-39 | 253+ | 0.000000 | 0.002607 | 0.000000 |
| 40-44 | 253+ | 0.000000 | 0.003115 | 0.000000 |
| 45-49 | 253+ | 0.000000 | 0.002132 | 0.000000 |
| 50-54 | 253+ | 0.000000 | 0.003497 | 0.000000 |
| 55-59 | 253+ | 0.000000 | 0.002942 | 0.000000 |
| 60-64 | 253+ | 0.000000 | 0.005376 | 0.000000 |
| 65-69 | 253+ | 0.000000 | 0.001789 | 0.000000 |
| 70-74 | 253+ | 0.000000 | 0.004077 | 0.000000 |
| 75-79 | 253+ | 0.000000 | 0.004077 | 0.000000 |

# Results for PHIM for Run 2 (Sensitivity_Analysis_1.RTF)

************************************************************************************

**TTP values for Run: 2, Scenario: Ecig**

| **Variable Parameter** | **Value** |
|---|---|
| 1. Country | US (United States) |
| 2. Sex | F (Females) |
| 3. Assumption Set | ISS3 |
| Note: | Period is in Months |

| | | **Initiation** | | | **Cessation** | | | **Re-initiation** | | |
|---|---|---|---|---|---|---|---|---|---|---|
| Age | Period | Never CC | Never Ecig | Never Dual | CC Former | Ecig Former | Dual Former | Former CC | Former Ecig | Former Dual |
| 10-14 | 1-12 | 0.001879 | 0.001879 | 0.000000 | 0.008742 | 0.008742 | 0.000000 | 0.000000 | 0.000000 | 0.000000 |
| 15-19 | 1-12 | 0.001735 | 0.001735 | 0.000000 | 0.002515 | 0.002515 | 0.000000 | 0.000000 | 0.000000 | 0.000000 |
| 20-24 | 1-12 | 0.000138 | 0.000138 | 0.000000 | 0.000761 | 0.000761 | 0.000000 | 0.000000 | 0.000000 | 0.000000 |
| 25-29 | 1-12 | 0.000069 | 0.000069 | 0.000000 | 0.001376 | 0.001376 | 0.000000 | 0.000000 | 0.000000 | 0.000000 |
| 30-34 | 1-12 | 0.000034 | 0.000034 | 0.000000 | 0.001149 | 0.001149 | 0.000000 | 0.000000 | 0.000000 | 0.000000 |
| 35-39 | 1-12 | 0.000000 | 0.000000 | 0.000000 | 0.000991 | 0.000991 | 0.000000 | 0.000000 | 0.000000 | 0.000000 |
| 40-44 | 1-12 | 0.000000 | 0.000000 | 0.000000 | 0.000833 | 0.000833 | 0.000000 | 0.000000 | 0.000000 | 0.000000 |
| 45-49 | 1-12 | 0.000000 | 0.000000 | 0.000000 | 0.001723 | 0.001723 | 0.000000 | 0.000000 | 0.000000 | 0.000000 |
| 50-54 | 1-12 | 0.000000 | 0.000000 | 0.000000 | 0.002336 | 0.002336 | 0.000000 | 0.000000 | 0.000000 | 0.000000 |
| 55-59 | 1-12 | 0.000000 | 0.000000 | 0.000000 | 0.002233 | 0.002233 | 0.000000 | 0.000000 | 0.000000 | 0.000000 |
| 60-64 | 1-12 | 0.000000 | 0.000000 | 0.000000 | 0.004635 | 0.004635 | 0.000000 | 0.000000 | 0.000000 | 0.000000 |
| 65-69 | 1-12 | 0.000000 | 0.000000 | 0.000000 | 0.003505 | 0.003505 | 0.000000 | 0.000000 | 0.000000 | 0.000000 |
| 70-74 | 1-12 | 0.000000 | 0.000000 | 0.000000 | 0.004329 | 0.004329 | 0.000000 | 0.000000 | 0.000000 | 0.000000 |
| 75-79 | 1-12 | 0.000000 | 0.000000 | 0.000000 | 0.004329 | 0.004329 | 0.000000 | 0.000000 | 0.000000 | 0.000000 |
| 10-14 | 13-72 | 0.001918 | 0.002877 | 0.000000 | 0.008742 | 0.008742 | 0.000000 | 0.000000 | 0.000000 | 0.000000 |
| 15-19 | 13-72 | 0.001811 | 0.002716 | 0.000000 | 0.004083 | 0.004083 | 0.000000 | 0.000000 | 0.000000 | 0.000000 |
| 20-24 | 13-72 | 0.000042 | 0.000063 | 0.000000 | 0.001261 | 0.001261 | 0.000000 | 0.000000 | 0.000000 | 0.000000 |
| 25-29 | 13-72 | 0.000021 | 0.000032 | 0.000000 | 0.000952 | 0.000952 | 0.000000 | 0.000000 | 0.000000 | 0.000000 |
| 30-34 | 13-72 | 0.000011 | 0.000016 | 0.000000 | 0.000733 | 0.000733 | 0.000000 | 0.000000 | 0.000000 | 0.000000 |
| 35-39 | 13-72 | 0.000000 | 0.000000 | 0.000000 | 0.000754 | 0.000754 | 0.000000 | 0.000000 | 0.000000 | 0.000000 |
| 40-44 | 13-72 | 0.000000 | 0.000000 | 0.000000 | 0.001535 | 0.001535 | 0.000000 | 0.000000 | 0.000000 | 0.000000 |
| 45-49 | 13-72 | 0.000000 | 0.000000 | 0.000000 | 0.002998 | 0.002998 | 0.000000 | 0.000000 | 0.000000 | 0.000000 |
| 50-54 | 13-72 | 0.000000 | 0.000000 | 0.000000 | 0.003160 | 0.003160 | 0.000000 | 0.000000 | 0.000000 | 0.000000 |
| 55-59 | 13-72 | 0.000000 | 0.000000 | 0.000000 | 0.002965 | 0.002965 | 0.000000 | 0.000000 | 0.000000 | 0.000000 |
| 60-64 | 13-72 | 0.000000 | 0.000000 | 0.000000 | 0.003633 | 0.003633 | 0.000000 | 0.000000 | 0.000000 | 0.000000 |
| 65-69 | 13-72 | 0.000000 | 0.000000 | 0.000000 | 0.004522 | 0.004522 | 0.000000 | 0.000000 | 0.000000 | 0.000000 |
| 70-74 | 13-72 | 0.000000 | 0.000000 | 0.000000 | 0.005208 | 0.005208 | 0.000000 | 0.000000 | 0.000000 | 0.000000 |
| 75-79 | 13-72 | 0.000000 | 0.000000 | 0.000000 | 0.005208 | 0.005208 | 0.000000 | 0.000000 | 0.000000 | 0.000000 |
| 10-14 | 73-132 | 0.000794 | 0.003175 | 0.000000 | 0.008742 | 0.008742 | 0.000000 | 0.000000 | 0.000000 | 0.000000 |
| 15-19 | 73-132 | 0.000611 | 0.002445 | 0.000000 | 0.003373 | 0.003373 | 0.000000 | 0.000000 | 0.000000 | 0.000000 |
| 20-24 | 73-132 | 0.000292 | 0.001167 | 0.000000 | 0.003881 | 0.003881 | 0.000000 | 0.000000 | 0.000000 | 0.000000 |
| 25-29 | 73-132 | 0.000143 | 0.000571 | 0.000000 | 0.001954 | 0.001954 | 0.000000 | 0.000000 | 0.000000 | 0.000000 |
| 30-34 | 73-132 | 0.000022 | 0.000089 | 0.000000 | 0.001338 | 0.001338 | 0.000000 | 0.000000 | 0.000000 | 0.000000 |
| 35-39 | 73-132 | 0.000000 | 0.000000 | 0.000000 | 0.001507 | 0.001507 | 0.000000 | 0.000000 | 0.000000 | 0.000000 |
| 40-44 | 73-132 | 0.000000 | 0.000000 | 0.000000 | 0.002080 | 0.002080 | 0.000000 | 0.000000 | 0.000000 | 0.000000 |
| 45-49 | 73-132 | 0.000000 | 0.000000 | 0.000000 | 0.003809 | 0.003809 | 0.000000 | 0.000000 | 0.000000 | 0.000000 |
| 50-54 | 73-132 | 0.000000 | 0.000000 | 0.000000 | 0.004871 | 0.004871 | 0.000000 | 0.000000 | 0.000000 | 0.000000 |
| 55-59 | 73-132 | 0.000000 | 0.000000 | 0.000000 | 0.004560 | 0.004560 | 0.000000 | 0.000000 | 0.000000 | 0.000000 |
| 60-64 | 73-132 | 0.000000 | 0.000000 | 0.000000 | 0.007192 | 0.007192 | 0.000000 | 0.000000 | 0.000000 | 0.000000 |
| 65-69 | 73-132 | 0.000000 | 0.000000 | 0.000000 | 0.007033 | 0.007033 | 0.000000 | 0.000000 | 0.000000 | 0.000000 |
| 70-74 | 73-132 | 0.000000 | 0.000000 | 0.000000 | 0.008206 | 0.008206 | 0.000000 | 0.000000 | 0.000000 | 0.000000 |
| 75-79 | 73-132 | 0.000000 | 0.000000 | 0.000000 | 0.008206 | 0.008206 | 0.000000 | 0.000000 | 0.000000 | 0.000000 |
| 10-14 | 133-192 | 0.000143 | 0.002718 | 0.000000 | 0.008742 | 0.008742 | 0.000000 | 0.000000 | 0.000000 | 0.000000 |
| 15-19 | 133-192 | 0.000130 | 0.002465 | 0.000000 | 0.003996 | 0.003996 | 0.000000 | 0.000000 | 0.000000 | 0.000000 |
| 20-24 | 133-192 | 0.000062 | 0.001185 | 0.000000 | 0.002805 | 0.002805 | 0.000000 | 0.000000 | 0.000000 | 0.000000 |
| 25-29 | 133-192 | 0.000015 | 0.000275 | 0.000000 | 0.001574 | 0.001574 | 0.000000 | 0.000000 | 0.000000 | 0.000000 |
| 30-34 | 133-192 | 0.000007 | 0.000125 | 0.000000 | 0.001293 | 0.001293 | 0.000000 | 0.000000 | 0.000000 | 0.000000 |
| 35-39 | 133-192 | 0.000000 | 0.000000 | 0.000000 | 0.001219 | 0.001219 | 0.000000 | 0.000000 | 0.000000 | 0.000000 |
| 40-44 | 133-192 | 0.000000 | 0.000000 | 0.000000 | 0.001657 | 0.001657 | 0.000000 | 0.000000 | 0.000000 | 0.000000 |
| 45-49 | 133-192 | 0.000000 | 0.000000 | 0.000000 | 0.001995 | 0.001995 | 0.000000 | 0.000000 | 0.000000 | 0.000000 |
| 50-54 | 133-192 | 0.000000 | 0.000000 | 0.000000 | 0.002154 | 0.002154 | 0.000000 | 0.000000 | 0.000000 | 0.000000 |
| 55-59 | 133-192 | 0.000000 | 0.000000 | 0.000000 | 0.002072 | 0.002072 | 0.000000 | 0.000000 | 0.000000 | 0.000000 |
| 60-64 | 133-192 | 0.000000 | 0.000000 | 0.000000 | 0.005049 | 0.005049 | 0.000000 | 0.000000 | 0.000000 | 0.000000 |
| 65-69 | 133-192 | 0.000000 | 0.000000 | 0.000000 | 0.003681 | 0.003681 | 0.000000 | 0.000000 | 0.000000 | 0.000000 |
| 70-74 | 133-192 | 0.000000 | 0.000000 | 0.000000 | 0.006144 | 0.006144 | 0.000000 | 0.000000 | 0.000000 | 0.000000 |
| 75-79 | 133-192 | 0.000000 | 0.000000 | 0.000000 | 0.006144 | 0.006144 | 0.000000 | 0.000000 | 0.000000 | 0.000000 |
| 10-14 | 193-252 | 0.000093 | 0.001766 | 0.000000 | 0.008742 | 0.008742 | 0.000000 | 0.000000 | 0.000000 | 0.000000 |
| 15-19 | 193-252 | 0.000112 | 0.002120 | 0.000000 | 0.005770 | 0.005770 | 0.000000 | 0.000000 | 0.000000 | 0.000000 |
| 20-24 | 193-252 | 0.000054 | 0.001025 | 0.000000 | 0.003262 | 0.003262 | 0.000000 | 0.000000 | 0.000000 | 0.000000 |
| 25-29 | 193-252 | 0.000027 | 0.000504 | 0.000000 | 0.002002 | 0.002002 | 0.000000 | 0.000000 | 0.000000 | 0.000000 |
| 30-34 | 193-252 | 0.000013 | 0.000250 | 0.000000 | 0.003241 | 0.003241 | 0.000000 | 0.000000 | 0.000000 | 0.000000 |
| 35-39 | 193-252 | 0.000000 | 0.000000 | 0.000000 | 0.001771 | 0.001771 | 0.000000 | 0.000000 | 0.000000 | 0.000000 |
| 40-44 | 193-252 | 0.000000 | 0.000000 | 0.000000 | 0.002173 | 0.002173 | 0.000000 | 0.000000 | 0.000000 | 0.000000 |
| 45-49 | 193-252 | 0.000000 | 0.000000 | 0.000000 | 0.002075 | 0.002075 | 0.000000 | 0.000000 | 0.000000 | 0.000000 |
| 50-54 | 193-252 | 0.000000 | 0.000000 | 0.000000 | 0.003432 | 0.003432 | 0.000000 | 0.000000 | 0.000000 | 0.000000 |

## Results for PHIM for Run 2 (Sensitivity_Analysis_1.RTF)

| | | | | | | | | | | |
|---|---|---|---|---|---|---|---|---|---|---|
| 55-59 | 193-252 | 0.000000 | 0.000000 | 0.000000 | 0.002906 | 0.002906 | 0.000000 | 0.000000 | 0.000000 | 0.000000 |
| 60-64 | 193-252 | 0.000000 | 0.000000 | 0.000000 | 0.007139 | 0.007139 | 0.000000 | 0.000000 | 0.000000 | 0.000000 |
| 65-69 | 193-252 | 0.000000 | 0.000000 | 0.000000 | 0.004963 | 0.004963 | 0.000000 | 0.000000 | 0.000000 | 0.000000 |
| 70-74 | 193-252 | 0.000000 | 0.000000 | 0.000000 | 0.006269 | 0.006269 | 0.000000 | 0.000000 | 0.000000 | 0.000000 |
| 75-79 | 193-252 | 0.000000 | 0.000000 | 0.000000 | 0.006269 | 0.006269 | 0.000000 | 0.000000 | 0.000000 | 0.000000 |
| 10-14 | 253+ | 0.000047 | 0.000884 | 0.000000 | 0.008742 | 0.008742 | 0.000000 | 0.000000 | 0.000000 | 0.000000 |
| 15-19 | 253+ | 0.000090 | 0.001708 | 0.000000 | 0.009440 | 0.009440 | 0.000000 | 0.000000 | 0.000000 | 0.000000 |
| 20-24 | 253+ | 0.000001 | 0.000022 | 0.000000 | 0.002831 | 0.002831 | 0.000000 | 0.000000 | 0.000000 | 0.000000 |
| 25-29 | 253+ | 0.000004 | 0.000070 | 0.000000 | 0.002656 | 0.002656 | 0.000000 | 0.000000 | 0.000000 | 0.000000 |
| 30-34 | 253+ | 0.000002 | 0.000035 | 0.000000 | 0.004614 | 0.004614 | 0.000000 | 0.000000 | 0.000000 | 0.000000 |
| 35-39 | 253+ | 0.000000 | 0.000000 | 0.000000 | 0.002608 | 0.002608 | 0.000000 | 0.000000 | 0.000000 | 0.000000 |
| 40-44 | 253+ | 0.000000 | 0.000000 | 0.000000 | 0.003115 | 0.003115 | 0.000000 | 0.000000 | 0.000000 | 0.000000 |
| 45-49 | 253+ | 0.000000 | 0.000000 | 0.000000 | 0.002132 | 0.002132 | 0.000000 | 0.000000 | 0.000000 | 0.000000 |
| 50-54 | 253+ | 0.000000 | 0.000000 | 0.000000 | 0.003497 | 0.003497 | 0.000000 | 0.000000 | 0.000000 | 0.000000 |
| 55-59 | 253+ | 0.000000 | 0.000000 | 0.000000 | 0.002942 | 0.002942 | 0.000000 | 0.000000 | 0.000000 | 0.000000 |
| 60-64 | 253+ | 0.000000 | 0.000000 | 0.000000 | 0.005376 | 0.005376 | 0.000000 | 0.000000 | 0.000000 | 0.000000 |
| 65-69 | 253+ | 0.000000 | 0.000000 | 0.000000 | 0.001789 | 0.001789 | 0.000000 | 0.000000 | 0.000000 | 0.000000 |
| 70-74 | 253+ | 0.000000 | 0.000000 | 0.000000 | 0.004077 | 0.004077 | 0.000000 | 0.000000 | 0.000000 | 0.000000 |
| 75-79 | 253+ | 0.000000 | 0.000000 | 0.000000 | 0.004077 | 0.004077 | 0.000000 | 0.000000 | 0.000000 | 0.000000 |

# Results for PHIM for Run 2 (Sensitivity_Analysis_1.RTF)

************************************************************************************

| | | Switch from CC | | Switch from Ecig | | Switch from Dual | |
|---|---|---|---|---|---|---|---|
| | | **CC** | **CC** | **Ecig** | **Ecig** | **Dual** | **Dual** |
| Age | Period | Ecig | Dual | CC | Dual | CC | Ecig |
| 10-14 | 1-12 | 0.009055 | 0.000000 | 0.000000 | 0.000000 | 0.000000 | 0.000000 |
| 15-19 | 1-12 | 0.011817 | 0.000000 | 0.000000 | 0.000000 | 0.000000 | 0.000000 |
| 20-24 | 1-12 | 0.013405 | 0.000000 | 0.000000 | 0.000000 | 0.000000 | 0.000000 |
| 25-29 | 1-12 | 0.014127 | 0.000000 | 0.000000 | 0.000000 | 0.000000 | 0.000000 |
| 30-34 | 1-12 | 0.013889 | 0.000000 | 0.000000 | 0.000000 | 0.000000 | 0.000000 |
| 35-39 | 1-12 | 0.012929 | 0.000000 | 0.000000 | 0.000000 | 0.000000 | 0.000000 |
| 40-44 | 1-12 | 0.012785 | 0.000000 | 0.000000 | 0.000000 | 0.000000 | 0.000000 |
| 45-49 | 1-12 | 0.012295 | 0.000000 | 0.000000 | 0.000000 | 0.000000 | 0.000000 |
| 50-54 | 1-12 | 0.011536 | 0.000000 | 0.000000 | 0.000000 | 0.000000 | 0.000000 |
| 55-59 | 1-12 | 0.008988 | 0.000000 | 0.000000 | 0.000000 | 0.000000 | 0.000000 |
| 60-64 | 1-12 | 0.008098 | 0.000000 | 0.000000 | 0.000000 | 0.000000 | 0.000000 |
| 65-69 | 1-12 | 0.006274 | 0.000000 | 0.000000 | 0.000000 | 0.000000 | 0.000000 |
| 70-74 | 1-12 | 0.004141 | 0.000000 | 0.000000 | 0.000000 | 0.000000 | 0.000000 |
| 75-79 | 1-12 | 0.004141 | 0.000000 | 0.000000 | 0.000000 | 0.000000 | 0.000000 |
| 10-14 | 13-72 | 0.009055 | 0.000000 | 0.000000 | 0.000000 | 0.000000 | 0.000000 |
| 15-19 | 13-72 | 0.011817 | 0.000000 | 0.000000 | 0.000000 | 0.000000 | 0.000000 |
| 20-24 | 13-72 | 0.013405 | 0.000000 | 0.000000 | 0.000000 | 0.000000 | 0.000000 |
| 25-29 | 13-72 | 0.014127 | 0.000000 | 0.000000 | 0.000000 | 0.000000 | 0.000000 |
| 30-34 | 13-72 | 0.013889 | 0.000000 | 0.000000 | 0.000000 | 0.000000 | 0.000000 |
| 35-39 | 13-72 | 0.012929 | 0.000000 | 0.000000 | 0.000000 | 0.000000 | 0.000000 |
| 40-44 | 13-72 | 0.012785 | 0.000000 | 0.000000 | 0.000000 | 0.000000 | 0.000000 |
| 45-49 | 13-72 | 0.012295 | 0.000000 | 0.000000 | 0.000000 | 0.000000 | 0.000000 |
| 50-54 | 13-72 | 0.011536 | 0.000000 | 0.000000 | 0.000000 | 0.000000 | 0.000000 |
| 55-59 | 13-72 | 0.008988 | 0.000000 | 0.000000 | 0.000000 | 0.000000 | 0.000000 |
| 60-64 | 13-72 | 0.008098 | 0.000000 | 0.000000 | 0.000000 | 0.000000 | 0.000000 |
| 65-69 | 13-72 | 0.006274 | 0.000000 | 0.000000 | 0.000000 | 0.000000 | 0.000000 |
| 70-74 | 13-72 | 0.004141 | 0.000000 | 0.000000 | 0.000000 | 0.000000 | 0.000000 |
| 75-79 | 13-72 | 0.004141 | 0.000000 | 0.000000 | 0.000000 | 0.000000 | 0.000000 |
| 10-14 | 73-132 | 0.009055 | 0.000000 | 0.000000 | 0.000000 | 0.000000 | 0.000000 |
| 15-19 | 73-132 | 0.011817 | 0.000000 | 0.000000 | 0.000000 | 0.000000 | 0.000000 |
| 20-24 | 73-132 | 0.013405 | 0.000000 | 0.000000 | 0.000000 | 0.000000 | 0.000000 |
| 25-29 | 73-132 | 0.014127 | 0.000000 | 0.000000 | 0.000000 | 0.000000 | 0.000000 |
| 30-34 | 73-132 | 0.013889 | 0.000000 | 0.000000 | 0.000000 | 0.000000 | 0.000000 |
| 35-39 | 73-132 | 0.012929 | 0.000000 | 0.000000 | 0.000000 | 0.000000 | 0.000000 |
| 40-44 | 73-132 | 0.012785 | 0.000000 | 0.000000 | 0.000000 | 0.000000 | 0.000000 |
| 45-49 | 73-132 | 0.012295 | 0.000000 | 0.000000 | 0.000000 | 0.000000 | 0.000000 |
| 50-54 | 73-132 | 0.011536 | 0.000000 | 0.000000 | 0.000000 | 0.000000 | 0.000000 |
| 55-59 | 73-132 | 0.008988 | 0.000000 | 0.000000 | 0.000000 | 0.000000 | 0.000000 |
| 60-64 | 73-132 | 0.008098 | 0.000000 | 0.000000 | 0.000000 | 0.000000 | 0.000000 |
| 65-69 | 73-132 | 0.006274 | 0.000000 | 0.000000 | 0.000000 | 0.000000 | 0.000000 |
| 70-74 | 73-132 | 0.004141 | 0.000000 | 0.000000 | 0.000000 | 0.000000 | 0.000000 |
| 75-79 | 73-132 | 0.004141 | 0.000000 | 0.000000 | 0.000000 | 0.000000 | 0.000000 |
| 10-14 | 133-192 | 0.009055 | 0.000000 | 0.000000 | 0.000000 | 0.000000 | 0.000000 |
| 15-19 | 133-192 | 0.011817 | 0.000000 | 0.000000 | 0.000000 | 0.000000 | 0.000000 |
| 20-24 | 133-192 | 0.013405 | 0.000000 | 0.000000 | 0.000000 | 0.000000 | 0.000000 |
| 25-29 | 133-192 | 0.014127 | 0.000000 | 0.000000 | 0.000000 | 0.000000 | 0.000000 |
| 30-34 | 133-192 | 0.013889 | 0.000000 | 0.000000 | 0.000000 | 0.000000 | 0.000000 |
| 35-39 | 133-192 | 0.012929 | 0.000000 | 0.000000 | 0.000000 | 0.000000 | 0.000000 |
| 40-44 | 133-192 | 0.012785 | 0.000000 | 0.000000 | 0.000000 | 0.000000 | 0.000000 |
| 45-49 | 133-192 | 0.012295 | 0.000000 | 0.000000 | 0.000000 | 0.000000 | 0.000000 |
| 50-54 | 133-192 | 0.011536 | 0.000000 | 0.000000 | 0.000000 | 0.000000 | 0.000000 |
| 55-59 | 133-192 | 0.008988 | 0.000000 | 0.000000 | 0.000000 | 0.000000 | 0.000000 |
| 60-64 | 133-192 | 0.008098 | 0.000000 | 0.000000 | 0.000000 | 0.000000 | 0.000000 |
| 65-69 | 133-192 | 0.006274 | 0.000000 | 0.000000 | 0.000000 | 0.000000 | 0.000000 |
| 70-74 | 133-192 | 0.004141 | 0.000000 | 0.000000 | 0.000000 | 0.000000 | 0.000000 |
| 75-79 | 133-192 | 0.004141 | 0.000000 | 0.000000 | 0.000000 | 0.000000 | 0.000000 |
| 10-14 | 193-252 | 0.009055 | 0.000000 | 0.000000 | 0.000000 | 0.000000 | 0.000000 |
| 15-19 | 193-252 | 0.011817 | 0.000000 | 0.000000 | 0.000000 | 0.000000 | 0.000000 |
| 20-24 | 193-252 | 0.013405 | 0.000000 | 0.000000 | 0.000000 | 0.000000 | 0.000000 |
| 25-29 | 193-252 | 0.014127 | 0.000000 | 0.000000 | 0.000000 | 0.000000 | 0.000000 |
| 30-34 | 193-252 | 0.013889 | 0.000000 | 0.000000 | 0.000000 | 0.000000 | 0.000000 |
| 35-39 | 193-252 | 0.012929 | 0.000000 | 0.000000 | 0.000000 | 0.000000 | 0.000000 |
| 40-44 | 193-252 | 0.012785 | 0.000000 | 0.000000 | 0.000000 | 0.000000 | 0.000000 |
| 45-49 | 193-252 | 0.012295 | 0.000000 | 0.000000 | 0.000000 | 0.000000 | 0.000000 |
| 50-54 | 193-252 | 0.011536 | 0.000000 | 0.000000 | 0.000000 | 0.000000 | 0.000000 |
| 55-59 | 193-252 | 0.008988 | 0.000000 | 0.000000 | 0.000000 | 0.000000 | 0.000000 |
| 60-64 | 193-252 | 0.008098 | 0.000000 | 0.000000 | 0.000000 | 0.000000 | 0.000000 |
| 65-69 | 193-252 | 0.006274 | 0.000000 | 0.000000 | 0.000000 | 0.000000 | 0.000000 |
| 70-74 | 193-252 | 0.004141 | 0.000000 | 0.000000 | 0.000000 | 0.000000 | 0.000000 |
| 75-79 | 193-252 | 0.004141 | 0.000000 | 0.000000 | 0.000000 | 0.000000 | 0.000000 |
| 10-14 | 253+ | 0.009055 | 0.000000 | 0.000000 | 0.000000 | 0.000000 | 0.000000 |
| 15-19 | 253+ | 0.011817 | 0.000000 | 0.000000 | 0.000000 | 0.000000 | 0.000000 |
| 20-24 | 253+ | 0.013405 | 0.000000 | 0.000000 | 0.000000 | 0.000000 | 0.000000 |

## Results for PHIM for Run 2 (Sensitivity_Analysis_1.RTF)

| | | | | | | | |
|---|---|---|---|---|---|---|---|
| 25-29 | 253+ | 0.014127 | 0.000000 | 0.000000 | 0.000000 | 0.000000 | 0.000000 |
| 30-34 | 253+ | 0.013889 | 0.000000 | 0.000000 | 0.000000 | 0.000000 | 0.000000 |
| 35-39 | 253+ | 0.012929 | 0.000000 | 0.000000 | 0.000000 | 0.000000 | 0.000000 |
| 40-44 | 253+ | 0.012785 | 0.000000 | 0.000000 | 0.000000 | 0.000000 | 0.000000 |
| 45-49 | 253+ | 0.012295 | 0.000000 | 0.000000 | 0.000000 | 0.000000 | 0.000000 |
| 50-54 | 253+ | 0.011536 | 0.000000 | 0.000000 | 0.000000 | 0.000000 | 0.000000 |
| 55-59 | 253+ | 0.008988 | 0.000000 | 0.000000 | 0.000000 | 0.000000 | 0.000000 |
| 60-64 | 253+ | 0.008098 | 0.000000 | 0.000000 | 0.000000 | 0.000000 | 0.000000 |
| 65-69 | 253+ | 0.006274 | 0.000000 | 0.000000 | 0.000000 | 0.000000 | 0.000000 |
| 70-74 | 253+ | 0.004141 | 0.000000 | 0.000000 | 0.000000 | 0.000000 | 0.000000 |
| 75-79 | 253+ | 0.004141 | 0.000000 | 0.000000 | 0.000000 | 0.000000 | 0.000000 |

# Results for PHIM for Run 2 (Sensitivity_Analysis_1.RTF)

************************************************************************************

**Main Output for the Tobacco History for Run: 2, Simulation: 1, Scenario: NULL**

| Variable Parameter | Value |
|---|---|
| 1. Country | US (United States) |
| 2. Sex | F (Females) |
| 3. Year of start of process | 1990 |

Year of Follow-up: 2040, Age Group: All Ages

| Period | Age Group | Smoking Group | N | % |
|---|---|---|---|---|
| 50 | All Ages | Never Any | 20670 | 58.80 |
| 50 | All Ages | CC | 2462 | 7.00 |
| 50 | All Ages | Former | 12019 | 34.19 |

Year of Follow-up: 2040, Age Group: 18-19

| Period | Age Group | Smoking Group | N | % |
|---|---|---|---|---|
| 50 | 18-19 | Never Any | 0 | . |
| 50 | 18-19 | CC | 0 | . |
| 50 | 18-19 | Former | 0 | . |

Year of Follow-up: 2040, Age Group: 20-24

| Period | Age Group | Smoking Group | N | % |
|---|---|---|---|---|
| 50 | 20-24 | Never Any | 0 | . |
| 50 | 20-24 | CC | 0 | . |
| 50 | 20-24 | Former | 0 | . |

Year of Follow-up: 2040, Age Group: 25-29

| Period | Age Group | Smoking Group | N | % |
|---|---|---|---|---|
| 50 | 25-29 | Never Any | 0 | . |
| 50 | 25-29 | CC | 0 | . |
| 50 | 25-29 | Former | 0 | . |

Year of Follow-up: 2040, Age Group: 30-34

| Period | Age Group | Smoking Group | N | % |
|---|---|---|---|---|
| 50 | 30-34 | Never Any | 0 | . |
| 50 | 30-34 | CC | 0 | . |
| 50 | 30-34 | Former | 0 | . |

Year of Follow-up: 2040, Age Group: 35-39

| Period | Age Group | Smoking Group | N | % |
|---|---|---|---|---|
| 50 | 35-39 | Never Any | 0 | . |
| 50 | 35-39 | CC | 0 | . |
| 50 | 35-39 | Former | 0 | . |

Year of Follow-up: 2040, Age Group: 40-44

| Period | Age Group | Smoking Group | N | % |
|---|---|---|---|---|
| 50 | 40-44 | Never Any | 0 | . |
| 50 | 40-44 | CC | 0 | . |
| 50 | 40-44 | Former | 0 | . |

Year of Follow-up: 2040, Age Group: 45-49

| Period | Age Group | Smoking Group | N | % |
|---|---|---|---|---|
| 50 | 45-49 | Never Any | 0 | . |
| 50 | 45-49 | CC | 0 | . |
| 50 | 45-49 | Former | 0 | . |

Year of Follow-up: 2040, Age Group: 50-54

| Period | Age Group | Smoking Group | N | % |
|---|---|---|---|---|
| 50 | 50-54 | Never Any | 0 | . |
| 50 | 50-54 | CC | 0 | . |
| 50 | 50-54 | Former | 0 | . |

Year of Follow-up: 2040, Age Group: 55-59

| Period | Age Group | Smoking Group | N | % |
|---|---|---|---|---|
| 50 | 55-59 | Never Any | 0 | . |
| 50 | 55-59 | CC | 0 | . |
| 50 | 55-59 | Former | 0 | . |

Year of Follow-up: 2040, Age Group: 60-64

| Period | Age Group | Smoking Group | N | % |
|---|---|---|---|---|

# Results for PHIM for Run 2 (Sensitivity_Analysis_1.RTF)

| | | | | |
|---|---|---|---|---|
| 50 | 60-64 | Never Any | 4803 | 60.57 |
| 50 | 60-64 | CC | 621 | 7.83 |
| 50 | 60-64 | Former | 2506 | 31.60 |

Year of Follow-up: 2040, Age Group: 65-69

| Period | Age Group | Smoking Group | N | % |
|---|---|---|---|---|
| 50 | 65-69 | Never Any | 5407 | 64.75 |
| 50 | 65-69 | CC | 538 | 6.44 |
| 50 | 65-69 | Former | 2405 | 28.80 |

Year of Follow-up: 2040, Age Group: 70-74

| Period | Age Group | Smoking Group | N | % |
|---|---|---|---|---|
| 50 | 70-74 | Never Any | 5181 | 58.92 |
| 50 | 70-74 | CC | 605 | 6.88 |
| 50 | 70-74 | Former | 3007 | 34.20 |

Year of Follow-up: 2040, Age Group: 75-79

| Period | Age Group | Smoking Group | N | % |
|---|---|---|---|---|
| 50 | 75-79 | Never Any | 5279 | 52.38 |
| 50 | 75-79 | CC | 698 | 6.93 |
| 50 | 75-79 | Former | 4101 | 40.69 |

# Results for PHIM for Run 2 (Sensitivity_Analysis_1.RTF)

**************************************************************************************
**Main Output for the Tobacco History for Run: 2, Simulation: 1, Scenario: Ecig**

| Variable Parameter | Value |
|---|---|
| 1. Country | US (United States) |
| 2. Sex | F (Females) |
| 3. Year of start of process | 1990 |

Year of Follow-up: 1990, Age Group: All Ages

| Period | Age Group | Smoking Group | N | % | Ever Quit (N) | Ever Quit % | Mean Yrs Quit | Short Term (n) | Short Term % |
|---|---|---|---|---|---|---|---|---|---|
| 0 | All Ages | Never Any | 47776 | 54.92 | | | | | |
| 0 | All Ages | CC | 22737 | 26.14 | 0 | 0.00 | | | |
| 0 | All Ages | Ecig | 0 | 0.00 | | | | | |
| 0 | All Ages | Dual | 0 | 0.00 | | | | | |
| 0 | All Ages | Former | 16484 | 18.95 | | | 11.90 | 2815 | 17.08 |

Year of Follow-up: 1990, Age Group: 18-19

| Period | Age Group | Smoking Group | N | % | Ever Quit (N) | Ever Quit % | Mean Yrs Quit | Short Term (n) | Short Term % |
|---|---|---|---|---|---|---|---|---|---|
| 0 | 18-19 | Never Any | 2570 | 78.43 | | | | | |
| 0 | 18-19 | CC | 535 | 16.33 | 0 | 0.00 | | | |
| 0 | 18-19 | Ecig | 0 | 0.00 | | | | | |
| 0 | 18-19 | Dual | 0 | 0.00 | | | | | |
| 0 | 18-19 | Former | 172 | 5.25 | | | 0.20 | 172 | 100.00 |

Year of Follow-up: 1990, Age Group: 20-24

| Period | Age Group | Smoking Group | N | % | Ever Quit (N) | Ever Quit % | Mean Yrs Quit | Short Term (n) | Short Term % |
|---|---|---|---|---|---|---|---|---|---|
| 0 | 20-24 | Never Any | 5354 | 60.89 | | | | | |
| 0 | 20-24 | CC | 2560 | 29.11 | 0 | 0.00 | | | |
| 0 | 20-24 | Ecig | 0 | 0.00 | | | | | |
| 0 | 20-24 | Dual | 0 | 0.00 | | | | | |
| 0 | 20-24 | Former | 879 | 10.00 | | | 1.58 | 420 | 47.78 |

Year of Follow-up: 1990, Age Group: 25-29

| Period | Age Group | Smoking Group | N | % | Ever Quit (N) | Ever Quit % | Mean Yrs Quit | Short Term (n) | Short Term % |
|---|---|---|---|---|---|---|---|---|---|
| 0 | 25-29 | Never Any | 5318 | 52.77 | | | | | |
| 0 | 25-29 | CC | 3335 | 33.09 | 0 | 0.00 | | | |
| 0 | 25-29 | Ecig | 0 | 0.00 | | | | | |
| 0 | 25-29 | Dual | 0 | 0.00 | | | | | |
| 0 | 25-29 | Former | 1425 | 14.14 | | | 3.91 | 404 | 28.35 |

Year of Follow-up: 1990, Age Group: 30-34

| Period | Age Group | Smoking Group | N | % | Ever Quit (N) | Ever Quit % | Mean Yrs Quit | Short Term (n) | Short Term % |
|---|---|---|---|---|---|---|---|---|---|
| 0 | 30-34 | Never Any | 5287 | 50.36 | | | | | |
| 0 | 30-34 | CC | 3326 | 31.68 | 0 | 0.00 | | | |
| 0 | 30-34 | Ecig | 0 | 0.00 | | | | | |
| 0 | 30-34 | Dual | 0 | 0.00 | | | | | |
| 0 | 30-34 | Former | 1886 | 17.96 | | | 4.36 | 566 | 30.01 |

Year of Follow-up: 1990, Age Group: 35-39

| Period | Age Group | Smoking Group | N | % | Ever Quit (N) | Ever Quit % | Mean Yrs Quit | Short Term (n) | Short Term % |
|---|---|---|---|---|---|---|---|---|---|
| 0 | 35-39 | Never Any | 5018 | 52.27 | | | | | |
| 0 | 35-39 | CC | 2653 | 27.64 | 0 | 0.00 | | | |
| 0 | 35-39 | Ecig | 0 | 0.00 | | | | | |
| 0 | 35-39 | Dual | 0 | 0.00 | | | | | |
| 0 | 35-39 | Former | 1929 | 20.09 | | | 7.96 | 324 | 16.80 |

Year of Follow-up: 1990, Age Group: 40-44

| Period | Age Group | Smoking Group | N | % | Ever Quit (N) | Ever Quit % | Mean Yrs Quit | Short Term (n) | Short Term % |
|---|---|---|---|---|---|---|---|---|---|
| 0 | 40-44 | Never Any | 4356 | 51.27 | | | | | |
| 0 | 40-44 | CC | 2397 | 28.21 | 0 | 0.00 | | | |
| 0 | 40-44 | Ecig | 0 | 0.00 | | | | | |
| 0 | 40-44 | Dual | 0 | 0.00 | | | | | |
| 0 | 40-44 | Former | 1744 | 20.52 | | | 7.33 | 339 | 19.44 |

Year of Follow-up: 1990, Age Group: 45-49

| Period | Age Group | Smoking Group | N | % | Ever Quit (N) | Ever Quit % | Mean Yrs Quit | Short Term (n) | Short Term % |
|---|---|---|---|---|---|---|---|---|---|
| 0 | 45-49 | Never Any | 3116 | 47.67 | | | | | |
| 0 | 45-49 | CC | 1875 | 28.69 | 0 | 0.00 | | | |
| 0 | 45-49 | Ecig | 0 | 0.00 | | | | | |
| 0 | 45-49 | Dual | 0 | 0.00 | | | | | |
| 0 | 45-49 | Former | 1545 | 23.64 | | | 12.55 | 194 | 12.56 |

Year of Follow-up: 1990, Age Group: 50-54

| Period | Age Group | Smoking Group | N | % | Ever Quit (N) | Ever Quit % | Mean Yrs Quit | Short Term (n) | Short Term % |
|---|---|---|---|---|---|---|---|---|---|
| 0 | 50-54 | Never Any | 2684 | 48.50 | | | | | |
| 0 | 50-54 | CC | 1572 | 28.41 | 0 | 0.00 | | | |

# Results for PHIM for Run 2 (Sensitivity_Analysis_1.RTF)

| | | | | | | | | | |
|---|---|---|---|---|---|---|---|---|---|
| 0 | 50-54 | Ecig | 0 | 0.00 | | | | | |
| 0 | 50-54 | Dual | 0 | 0.00 | | | | | |
| 0 | 50-54 | Former | 1278 | 23.09 | | | 13.28 | 126 | 9.86 |

Year of Follow-up: 1990, Age Group: 55-59

| Period | Age Group | Smoking Group | N | % | Ever Quit (N) | Ever Quit % | Mean Yrs Quit | Short Term (n) | Short Term % |
|---|---|---|---|---|---|---|---|---|---|
| 0 | 55-59 | Never Any | 2732 | 51.90 | | | | | |
| 0 | 55-59 | CC | 1295 | 24.60 | 0 | 0.00 | | | |
| 0 | 55-59 | Ecig | 0 | 0.00 | | | | | |
| 0 | 55-59 | Dual | 0 | 0.00 | | | | | |
| 0 | 55-59 | Former | 1237 | 23.50 | | | 17.26 | 90 | 7.28 |

Year of Follow-up: 1990, Age Group: 60-64

| Period | Age Group | Smoking Group | N | % | Ever Quit (N) | Ever Quit % | Mean Yrs Quit | Short Term (n) | Short Term % |
|---|---|---|---|---|---|---|---|---|---|
| 0 | 60-64 | Never Any | 3078 | 55.72 | | | | | |
| 0 | 60-64 | CC | 1249 | 22.61 | 0 | 0.00 | | | |
| 0 | 60-64 | Ecig | 0 | 0.00 | | | | | |
| 0 | 60-64 | Dual | 0 | 0.00 | | | | | |
| 0 | 60-64 | Former | 1197 | 21.67 | | | 18.28 | 61 | 5.10 |

Year of Follow-up: 1990, Age Group: 65-69

| Period | Age Group | Smoking Group | N | % | Ever Quit (N) | Ever Quit % | Mean Yrs Quit | Short Term (n) | Short Term % |
|---|---|---|---|---|---|---|---|---|---|
| 0 | 65-69 | Never Any | 3042 | 57.36 | | | | | |
| 0 | 65-69 | CC | 976 | 18.40 | 0 | 0.00 | | | |
| 0 | 65-69 | Ecig | 0 | 0.00 | | | | | |
| 0 | 65-69 | Dual | 0 | 0.00 | | | | | |
| 0 | 65-69 | Former | 1285 | 24.23 | | | 20.65 | 68 | 5.29 |

Year of Follow-up: 1990, Age Group: 70-74

| Period | Age Group | Smoking Group | N | % | Ever Quit (N) | Ever Quit % | Mean Yrs Quit | Short Term (n) | Short Term % |
|---|---|---|---|---|---|---|---|---|---|
| 0 | 70-74 | Never Any | 2738 | 61.05 | | | | | |
| 0 | 70-74 | CC | 610 | 13.60 | 0 | 0.00 | | | |
| 0 | 70-74 | Ecig | 0 | 0.00 | | | | | |
| 0 | 70-74 | Dual | 0 | 0.00 | | | | | |
| 0 | 70-74 | Former | 1137 | 25.35 | | | 24.48 | 27 | 2.37 |

Year of Follow-up: 1990, Age Group: 75-79

| Period | Age Group | Smoking Group | N | % | Ever Quit (N) | Ever Quit % | Mean Yrs Quit | Short Term (n) | Short Term % |
|---|---|---|---|---|---|---|---|---|---|
| 0 | 75-79 | Never Any | 2483 | 68.84 | | | | | |
| 0 | 75-79 | CC | 354 | 9.81 | 0 | 0.00 | | | |
| 0 | 75-79 | Ecig | 0 | 0.00 | | | | | |
| 0 | 75-79 | Dual | 0 | 0.00 | | | | | |
| 0 | 75-79 | Former | 770 | 21.35 | | | 24.48 | 24 | 3.12 |

Year of Follow-up: 1995, Age Group: All Ages

| Period | Age Group | Smoking Group | N | % | Ever Quit (N) | Ever Quit % | Mean Yrs Quit | Short Term (n) | Short Term % |
|---|---|---|---|---|---|---|---|---|---|
| 5 | All Ages | Never Any | 50887 | 55.53 | | | | | |
| 5 | All Ages | CC | 10659 | 11.63 | 0 | 0.00 | | | |
| 5 | All Ages | Ecig | 11697 | 12.76 | | | | | |
| 5 | All Ages | Dual | 0 | 0.00 | | | | | |
| 5 | All Ages | Former | 18396 | 20.07 | | | 14.19 | 1129 | 6.14 |

Year of Follow-up: 1995, Age Group: 18-19

| Period | Age Group | Smoking Group | N | % | Ever Quit (N) | Ever Quit % | Mean Yrs Quit | Short Term (n) | Short Term % |
|---|---|---|---|---|---|---|---|---|---|
| 5 | 18-19 | Never Any | 2434 | 76.64 | | | | | |
| 5 | 18-19 | CC | 233 | 7.34 | 0 | 0.00 | | | |
| 5 | 18-19 | Ecig | 427 | 13.44 | | | | | |
| 5 | 18-19 | Dual | 0 | 0.00 | | | | | |
| 5 | 18-19 | Former | 82 | 2.58 | | | 1.15 | 52 | 63.41 |

Year of Follow-up: 1995, Age Group: 20-24

| Period | Age Group | Smoking Group | N | % | Ever Quit (N) | Ever Quit % | Mean Yrs Quit | Short Term (n) | Short Term % |
|---|---|---|---|---|---|---|---|---|---|
| 5 | 20-24 | Never Any | 5794 | 69.39 | | | | | |
| 5 | 20-24 | CC | 862 | 10.32 | 0 | 0.00 | | | |
| 5 | 20-24 | Ecig | 1250 | 14.97 | | | | | |
| 5 | 20-24 | Dual | 0 | 0.00 | | | | | |
| 5 | 20-24 | Former | 444 | 5.32 | | | 3.23 | 106 | 23.87 |

Year of Follow-up: 1995, Age Group: 25-29

| Period | Age Group | Smoking Group | N | % | Ever Quit (N) | Ever Quit % | Mean Yrs Quit | Short Term (n) | Short Term % |
|---|---|---|---|---|---|---|---|---|---|
| 5 | 25-29 | Never Any | 5313 | 60.42 | | | | | |
| 5 | 25-29 | CC | 1062 | 12.08 | 0 | 0.00 | | | |
| 5 | 25-29 | Ecig | 1395 | 15.86 | | | | | |
| 5 | 25-29 | Dual | 0 | 0.00 | | | | | |
| 5 | 25-29 | Former | 1023 | 11.63 | | | 5.94 | 54 | 5.28 |

Year of Follow-up: 1995, Age Group: 30-34

| Period | Age Group | Smoking Group | N | % | Ever Quit (N) | Ever Quit % | Mean Yrs Quit | Short Term (n) | Short Term % |
|---|---|---|---|---|---|---|---|---|---|

# Results for PHIM for Run 2 (Sensitivity_Analysis_1.RTF)

| | | | | | | | | | |
|---|---|---|---|---|---|---|---|---|---|
| 5 | 30-34 | Never Any | 5304 | 52.63 | | | | | |
| 5 | 30-34 | CC | 1373 | 13.62 | 0 | 0.00 | | | |
| 5 | 30-34 | Ecig | 1802 | 17.88 | | | | | |
| 5 | 30-34 | Dual | 0 | 0.00 | | | | | |
| 5 | 30-34 | Former | 1599 | 15.87 | | | 8.16 | 67 | 4.19 |

Year of Follow-up: 1995, Age Group: 35-39

| Period | Age Group | Smoking Group | N | % | Ever Quit (N) | Ever Quit % | Mean Yrs Quit | Short Term (n) | Short Term % |
|---|---|---|---|---|---|---|---|---|---|
| 5 | 35-39 | Never Any | 5278 | 50.27 | | | | | |
| 5 | 35-39 | CC | 1410 | 13.43 | 0 | 0.00 | | | |
| 5 | 35-39 | Ecig | 1746 | 16.63 | | | | | |
| 5 | 35-39 | Dual | 0 | 0.00 | | | | | |
| 5 | 35-39 | Former | 2065 | 19.67 | | | 8.75 | 62 | 3.00 |

Year of Follow-up: 1995, Age Group: 40-44

| Period | Age Group | Smoking Group | N | % | Ever Quit (N) | Ever Quit % | Mean Yrs Quit | Short Term (n) | Short Term % |
|---|---|---|---|---|---|---|---|---|---|
| 5 | 40-44 | Never Any | 5018 | 52.27 | | | | | |
| 5 | 40-44 | CC | 1135 | 11.82 | 0 | 0.00 | | | |
| 5 | 40-44 | Ecig | 1356 | 14.13 | | | | | |
| 5 | 40-44 | Dual | 0 | 0.00 | | | | | |
| 5 | 40-44 | Former | 2091 | 21.78 | | | 12.09 | 81 | 3.87 |

Year of Follow-up: 1995, Age Group: 45-49

| Period | Age Group | Smoking Group | N | % | Ever Quit (N) | Ever Quit % | Mean Yrs Quit | Short Term (n) | Short Term % |
|---|---|---|---|---|---|---|---|---|---|
| 5 | 45-49 | Never Any | 4356 | 51.27 | | | | | |
| 5 | 45-49 | CC | 974 | 11.46 | 0 | 0.00 | | | |
| 5 | 45-49 | Ecig | 1161 | 13.66 | | | | | |
| 5 | 45-49 | Dual | 0 | 0.00 | | | | | |
| 5 | 45-49 | Former | 2006 | 23.61 | | | 10.93 | 133 | 6.63 |

Year of Follow-up: 1995, Age Group: 50-54

| Period | Age Group | Smoking Group | N | % | Ever Quit (N) | Ever Quit % | Mean Yrs Quit | Short Term (n) | Short Term % |
|---|---|---|---|---|---|---|---|---|---|
| 5 | 50-54 | Never Any | 3116 | 47.67 | | | | | |
| 5 | 50-54 | CC | 739 | 11.31 | 0 | 0.00 | | | |
| 5 | 50-54 | Ecig | 826 | 12.64 | | | | | |
| 5 | 50-54 | Dual | 0 | 0.00 | | | | | |
| 5 | 50-54 | Former | 1855 | 28.38 | | | 14.92 | 140 | 7.55 |

Year of Follow-up: 1995, Age Group: 55-59

| Period | Age Group | Smoking Group | N | % | Ever Quit (N) | Ever Quit % | Mean Yrs Quit | Short Term (n) | Short Term % |
|---|---|---|---|---|---|---|---|---|---|
| 5 | 55-59 | Never Any | 2684 | 48.50 | | | | | |
| 5 | 55-59 | CC | 722 | 13.05 | 0 | 0.00 | | | |
| 5 | 55-59 | Ecig | 627 | 11.33 | | | | | |
| 5 | 55-59 | Dual | 0 | 0.00 | | | | | |
| 5 | 55-59 | Former | 1501 | 27.12 | | | 15.84 | 93 | 6.20 |

Year of Follow-up: 1995, Age Group: 60-64

| Period | Age Group | Smoking Group | N | % | Ever Quit (N) | Ever Quit % | Mean Yrs Quit | Short Term (n) | Short Term % |
|---|---|---|---|---|---|---|---|---|---|
| 5 | 60-64 | Never Any | 2732 | 51.90 | | | | | |
| 5 | 60-64 | CC | 633 | 12.03 | 0 | 0.00 | | | |
| 5 | 60-64 | Ecig | 444 | 8.43 | | | | | |
| 5 | 60-64 | Dual | 0 | 0.00 | | | | | |
| 5 | 60-64 | Former | 1455 | 27.64 | | | 19.22 | 87 | 5.98 |

Year of Follow-up: 1995, Age Group: 65-69

| Period | Age Group | Smoking Group | N | % | Ever Quit (N) | Ever Quit % | Mean Yrs Quit | Short Term (n) | Short Term % |
|---|---|---|---|---|---|---|---|---|---|
| 5 | 65-69 | Never Any | 3078 | 55.72 | | | | | |
| 5 | 65-69 | CC | 602 | 10.90 | 0 | 0.00 | | | |
| 5 | 65-69 | Ecig | 374 | 6.77 | | | | | |
| 5 | 65-69 | Dual | 0 | 0.00 | | | | | |
| 5 | 65-69 | Former | 1470 | 26.61 | | | 19.34 | 107 | 7.28 |

Year of Follow-up: 1995, Age Group: 70-74

| Period | Age Group | Smoking Group | N | % | Ever Quit (N) | Ever Quit % | Mean Yrs Quit | Short Term (n) | Short Term % |
|---|---|---|---|---|---|---|---|---|---|
| 5 | 70-74 | Never Any | 3042 | 57.36 | | | | | |
| 5 | 70-74 | CC | 577 | 10.88 | 0 | 0.00 | | | |
| 5 | 70-74 | Ecig | 188 | 3.55 | | | | | |
| 5 | 70-74 | Dual | 0 | 0.00 | | | | | |
| 5 | 70-74 | Former | 1496 | 28.21 | | | 22.32 | 77 | 5.15 |

Year of Follow-up: 1995, Age Group: 75-79

| Period | Age Group | Smoking Group | N | % | Ever Quit (N) | Ever Quit % | Mean Yrs Quit | Short Term (n) | Short Term % |
|---|---|---|---|---|---|---|---|---|---|
| 5 | 75-79 | Never Any | 2738 | 61.05 | | | | | |
| 5 | 75-79 | CC | 337 | 7.51 | 0 | 0.00 | | | |
| 5 | 75-79 | Ecig | 101 | 2.25 | | | | | |
| 5 | 75-79 | Dual | 0 | 0.00 | | | | | |
| 5 | 75-79 | Former | 1309 | 29.19 | | | 25.87 | 70 | 5.35 |

Year of Follow-up: 2000, Age Group: All Ages

# Results for PHIM for Run 2 (Sensitivity_Analysis_1.RTF)

| Period | Age Group | Smoking Group | N | % | Ever Quit (N) | Ever Quit % | Mean Yrs Quit | Short Term (n) | Short Term % |
|---|---|---|---|---|---|---|---|---|---|
| 10 | All Ages | Never Any | 50423 | 54.86 | | | | | |
| 10 | All Ages | CC | 4595 | 5.00 | 0 | 0.00 | | | |
| 10 | All Ages | Ecig | 15644 | 17.02 | | | | | |
| 10 | All Ages | Dual | 0 | 0.00 | | | | | |
| 10 | All Ages | Former | 21246 | 23.12 | | | 15.11 | 1717 | 8.08 |

Year of Follow-up: 2000, Age Group: 18-19

| Period | Age Group | Smoking Group | N | % | Ever Quit (N) | Ever Quit % | Mean Yrs Quit | Short Term (n) | Short Term % |
|---|---|---|---|---|---|---|---|---|---|
| 10 | 18-19 | Never Any | 0 | . | | | | | |
| 10 | 18-19 | CC | 0 | . | 0 | . | | | |
| 10 | 18-19 | Ecig | 0 | . | | | | | |
| 10 | 18-19 | Dual | 0 | . | | | | | |
| 10 | 18-19 | Former | 0 | . | | | . | 0 | . |

Year of Follow-up: 2000, Age Group: 20-24

| Period | Age Group | Smoking Group | N | % | Ever Quit (N) | Ever Quit % | Mean Yrs Quit | Short Term (n) | Short Term % |
|---|---|---|---|---|---|---|---|---|---|
| 10 | 20-24 | Never Any | 5128 | 64.67 | | | | | |
| 10 | 20-24 | CC | 370 | 4.67 | 0 | 0.00 | | | |
| 10 | 20-24 | Ecig | 1780 | 22.45 | | | | | |
| 10 | 20-24 | Dual | 0 | 0.00 | | | | | |
| 10 | 20-24 | Former | 652 | 8.22 | | | 3.55 | 169 | 25.92 |

Year of Follow-up: 2000, Age Group: 25-29

| Period | Age Group | Smoking Group | N | % | Ever Quit (N) | Ever Quit % | Mean Yrs Quit | Short Term (n) | Short Term % |
|---|---|---|---|---|---|---|---|---|---|
| 10 | 25-29 | Never Any | 5493 | 65.78 | | | | | |
| 10 | 25-29 | CC | 400 | 4.79 | 0 | 0.00 | | | |
| 10 | 25-29 | Ecig | 1690 | 20.24 | | | | | |
| 10 | 25-29 | Dual | 0 | 0.00 | | | | | |
| 10 | 25-29 | Former | 767 | 9.19 | | | 5.59 | 126 | 16.43 |

Year of Follow-up: 2000, Age Group: 30-34

| Period | Age Group | Smoking Group | N | % | Ever Quit (N) | Ever Quit % | Mean Yrs Quit | Short Term (n) | Short Term % |
|---|---|---|---|---|---|---|---|---|---|
| 10 | 30-34 | Never Any | 5214 | 59.30 | | | | | |
| 10 | 30-34 | CC | 417 | 4.74 | 0 | 0.00 | | | |
| 10 | 30-34 | Ecig | 1908 | 21.70 | | | | | |
| 10 | 30-34 | Dual | 0 | 0.00 | | | | | |
| 10 | 30-34 | Former | 1254 | 14.26 | | | 9.28 | 91 | 7.26 |

Year of Follow-up: 2000, Age Group: 35-39

| Period | Age Group | Smoking Group | N | % | Ever Quit (N) | Ever Quit % | Mean Yrs Quit | Short Term (n) | Short Term % |
|---|---|---|---|---|---|---|---|---|---|
| 10 | 35-39 | Never Any | 5284 | 52.43 | | | | | |
| 10 | 35-39 | CC | 561 | 5.57 | 0 | 0.00 | | | |
| 10 | 35-39 | Ecig | 2413 | 23.94 | | | | | |
| 10 | 35-39 | Dual | 0 | 0.00 | | | | | |
| 10 | 35-39 | Former | 1820 | 18.06 | | | 11.77 | 105 | 5.77 |

Year of Follow-up: 2000, Age Group: 40-44

| Period | Age Group | Smoking Group | N | % | Ever Quit (N) | Ever Quit % | Mean Yrs Quit | Short Term (n) | Short Term % |
|---|---|---|---|---|---|---|---|---|---|
| 10 | 40-44 | Never Any | 5278 | 50.27 | | | | | |
| 10 | 40-44 | CC | 584 | 5.56 | 0 | 0.00 | | | |
| 10 | 40-44 | Ecig | 2292 | 21.83 | | | | | |
| 10 | 40-44 | Dual | 0 | 0.00 | | | | | |
| 10 | 40-44 | Former | 2345 | 22.34 | | | 12.30 | 143 | 6.10 |

Year of Follow-up: 2000, Age Group: 45-49

| Period | Age Group | Smoking Group | N | % | Ever Quit (N) | Ever Quit % | Mean Yrs Quit | Short Term (n) | Short Term % |
|---|---|---|---|---|---|---|---|---|---|
| 10 | 45-49 | Never Any | 5018 | 52.27 | | | | | |
| 10 | 45-49 | CC | 446 | 4.65 | 0 | 0.00 | | | |
| 10 | 45-49 | Ecig | 1673 | 17.43 | | | | | |
| 10 | 45-49 | Dual | 0 | 0.00 | | | | | |
| 10 | 45-49 | Former | 2463 | 25.66 | | | 14.76 | 181 | 7.35 |

Year of Follow-up: 2000, Age Group: 50-54

| Period | Age Group | Smoking Group | N | % | Ever Quit (N) | Ever Quit % | Mean Yrs Quit | Short Term (n) | Short Term % |
|---|---|---|---|---|---|---|---|---|---|
| 10 | 50-54 | Never Any | 4356 | 51.27 | | | | | |
| 10 | 50-54 | CC | 378 | 4.45 | 0 | 0.00 | | | |
| 10 | 50-54 | Ecig | 1299 | 15.29 | | | | | |
| 10 | 50-54 | Dual | 0 | 0.00 | | | | | |
| 10 | 50-54 | Former | 2464 | 29.00 | | | 13.32 | 188 | 7.63 |

Year of Follow-up: 2000, Age Group: 55-59

| Period | Age Group | Smoking Group | N | % | Ever Quit (N) | Ever Quit % | Mean Yrs Quit | Short Term (n) | Short Term % |
|---|---|---|---|---|---|---|---|---|---|
| 10 | 55-59 | Never Any | 3116 | 47.67 | | | | | |
| 10 | 55-59 | CC | 283 | 4.33 | 0 | 0.00 | | | |
| 10 | 55-59 | Ecig | 912 | 13.95 | | | | | |
| 10 | 55-59 | Dual | 0 | 0.00 | | | | | |

# Results for PHIM for Run 2 (Sensitivity_Analysis_1.RTF)

| | | | | | | | | | |
|---|---|---|---|---|---|---|---|---|---|
| 10 | 55-59 | Former | 2225 | 34.04 | | | 16.92 | 157 | 7.06 |

Year of Follow-up: 2000, Age Group: 60-64

| Period | Age Group | Smoking Group | N | % | Ever Quit (N) | Ever Quit % | Mean Yrs Quit | Short Term (n) | Short Term % |
|---|---|---|---|---|---|---|---|---|---|
| 10 | 60-64 | Never Any | 2684 | 48.50 | | | | | |
| 10 | 60-64 | CC | 334 | 6.04 | 0 | 0.00 | | | |
| 10 | 60-64 | Ecig | 663 | 11.98 | | | | | |
| 10 | 60-64 | Dual | 0 | 0.00 | | | | | |
| 10 | 60-64 | Former | 1853 | 33.48 | | | 17.23 | 149 | 8.04 |

Year of Follow-up: 2000, Age Group: 65-69

| Period | Age Group | Smoking Group | N | % | Ever Quit (N) | Ever Quit % | Mean Yrs Quit | Short Term (n) | Short Term % |
|---|---|---|---|---|---|---|---|---|---|
| 10 | 65-69 | Never Any | 2732 | 51.90 | | | | | |
| 10 | 65-69 | CC | 280 | 5.32 | 0 | 0.00 | | | |
| 10 | 65-69 | Ecig | 443 | 8.42 | | | | | |
| 10 | 65-69 | Dual | 0 | 0.00 | | | | | |
| 10 | 65-69 | Former | 1809 | 34.37 | | | 19.85 | 146 | 8.07 |

Year of Follow-up: 2000, Age Group: 70-74

| Period | Age Group | Smoking Group | N | % | Ever Quit (N) | Ever Quit % | Mean Yrs Quit | Short Term (n) | Short Term % |
|---|---|---|---|---|---|---|---|---|---|
| 10 | 70-74 | Never Any | 3078 | 55.72 | | | | | |
| 10 | 70-74 | CC | 274 | 4.96 | 0 | 0.00 | | | |
| 10 | 70-74 | Ecig | 368 | 6.66 | | | | | |
| 10 | 70-74 | Dual | 0 | 0.00 | | | | | |
| 10 | 70-74 | Former | 1804 | 32.66 | | | 20.18 | 154 | 8.54 |

Year of Follow-up: 2000, Age Group: 75-79

| Period | Age Group | Smoking Group | N | % | Ever Quit (N) | Ever Quit % | Mean Yrs Quit | Short Term (n) | Short Term % |
|---|---|---|---|---|---|---|---|---|---|
| 10 | 75-79 | Never Any | 3042 | 57.36 | | | | | |
| 10 | 75-79 | CC | 268 | 5.05 | 0 | 0.00 | | | |
| 10 | 75-79 | Ecig | 203 | 3.83 | | | | | |
| 10 | 75-79 | Dual | 0 | 0.00 | | | | | |
| 10 | 75-79 | Former | 1790 | 33.75 | | | 23.17 | 108 | 6.03 |

Year of Follow-up: 2005, Age Group: All Ages

| Period | Age Group | Smoking Group | N | % | Ever Quit (N) | Ever Quit % | Mean Yrs Quit | Short Term (n) | Short Term % |
|---|---|---|---|---|---|---|---|---|---|
| 15 | All Ages | Never Any | 47002 | 54.27 | | | | | |
| 15 | All Ages | CC | 1890 | 2.18 | 0 | 0.00 | | | |
| 15 | All Ages | Ecig | 15617 | 18.03 | | | | | |
| 15 | All Ages | Dual | 0 | 0.00 | | | | | |
| 15 | All Ages | Former | 22096 | 25.51 | | | 17.32 | 941 | 4.26 |

Year of Follow-up: 2005, Age Group: 18-19

| Period | Age Group | Smoking Group | N | % | Ever Quit (N) | Ever Quit % | Mean Yrs Quit | Short Term (n) | Short Term % |
|---|---|---|---|---|---|---|---|---|---|
| 15 | 18-19 | Never Any | 0 | . | | | | | |
| 15 | 18-19 | CC | 0 | . | 0 | . | | | |
| 15 | 18-19 | Ecig | 0 | . | | | | | |
| 15 | 18-19 | Dual | 0 | . | | | | | |
| 15 | 18-19 | Former | 0 | . | | | . | 0 | . |

Year of Follow-up: 2005, Age Group: 20-24

| Period | Age Group | Smoking Group | N | % | Ever Quit (N) | Ever Quit % | Mean Yrs Quit | Short Term (n) | Short Term % |
|---|---|---|---|---|---|---|---|---|---|
| 15 | 20-24 | Never Any | 0 | . | | | | | |
| 15 | 20-24 | CC | 0 | . | 0 | . | | | |
| 15 | 20-24 | Ecig | 0 | . | | | | | |
| 15 | 20-24 | Dual | 0 | . | | | | | |
| 15 | 20-24 | Former | 0 | . | | | . | 0 | . |

Year of Follow-up: 2005, Age Group: 25-29

| Period | Age Group | Smoking Group | N | % | Ever Quit (N) | Ever Quit % | Mean Yrs Quit | Short Term (n) | Short Term % |
|---|---|---|---|---|---|---|---|---|---|
| 15 | 25-29 | Never Any | 4860 | 61.29 | | | | | |
| 15 | 25-29 | CC | 166 | 2.09 | 0 | 0.00 | | | |
| 15 | 25-29 | Ecig | 1941 | 24.48 | | | | | |
| 15 | 25-29 | Dual | 0 | 0.00 | | | | | |
| 15 | 25-29 | Former | 963 | 12.14 | | | 6.57 | 96 | 9.97 |

Year of Follow-up: 2005, Age Group: 30-34

| Period | Age Group | Smoking Group | N | % | Ever Quit (N) | Ever Quit % | Mean Yrs Quit | Short Term (n) | Short Term % |
|---|---|---|---|---|---|---|---|---|---|
| 15 | 30-34 | Never Any | 5407 | 64.75 | | | | | |
| 15 | 30-34 | CC | 149 | 1.78 | 0 | 0.00 | | | |
| 15 | 30-34 | Ecig | 1822 | 21.82 | | | | | |
| 15 | 30-34 | Dual | 0 | 0.00 | | | | | |
| 15 | 30-34 | Former | 972 | 11.64 | | | 8.77 | 80 | 8.23 |

Year of Follow-up: 2005, Age Group: 35-39

| Period | Age Group | Smoking Group | N | % | Ever Quit (N) | Ever Quit % | Mean Yrs Quit | Short Term (n) | Short Term % |
|---|---|---|---|---|---|---|---|---|---|
| 15 | 35-39 | Never Any | 5189 | 59.01 | | | | | |
| 15 | 35-39 | CC | 167 | 1.90 | 0 | 0.00 | | | |

# Results for PHIM for Run 2 (Sensitivity_Analysis_1.RTF)

| | | | | | | | | | |
|---|---|---|---|---|---|---|---|---|---|
| 15 | 35-39 | Ecig | 2002 | 22.77 | | | | | |
| 15 | 35-39 | Dual | 0 | 0.00 | | | | | |
| 15 | 35-39 | Former | 1435 | 16.32 | | | 12.75 | 66 | 4.60 |

Year of Follow-up: 2005, Age Group: 40-44

| Period | Age Group | Smoking Group | N | % | Ever Quit (N) | Ever Quit % | Mean Yrs Quit | Short Term (n) | Short Term % |
|---|---|---|---|---|---|---|---|---|---|
| 15 | 40-44 | Never Any | 5284 | 52.43 | | | | | |
| 15 | 40-44 | CC | 238 | 2.36 | 0 | 0.00 | | | |
| 15 | 40-44 | Ecig | 2500 | 24.81 | | | | | |
| 15 | 40-44 | Dual | 0 | 0.00 | | | | | |
| 15 | 40-44 | Former | 2056 | 20.40 | | | 15.08 | 101 | 4.91 |

Year of Follow-up: 2005, Age Group: 45-49

| Period | Age Group | Smoking Group | N | % | Ever Quit (N) | Ever Quit % | Mean Yrs Quit | Short Term (n) | Short Term % |
|---|---|---|---|---|---|---|---|---|---|
| 15 | 45-49 | Never Any | 5278 | 50.27 | | | | | |
| 15 | 45-49 | CC | 237 | 2.26 | 0 | 0.00 | | | |
| 15 | 45-49 | Ecig | 2318 | 22.08 | | | | | |
| 15 | 45-49 | Dual | 0 | 0.00 | | | | | |
| 15 | 45-49 | Former | 2666 | 25.39 | | | 15.45 | 135 | 5.06 |

Year of Follow-up: 2005, Age Group: 50-54

| Period | Age Group | Smoking Group | N | % | Ever Quit (N) | Ever Quit % | Mean Yrs Quit | Short Term (n) | Short Term % |
|---|---|---|---|---|---|---|---|---|---|
| 15 | 50-54 | Never Any | 5018 | 52.27 | | | | | |
| 15 | 50-54 | CC | 191 | 1.99 | 0 | 0.00 | | | |
| 15 | 50-54 | Ecig | 1622 | 16.90 | | | | | |
| 15 | 50-54 | Dual | 0 | 0.00 | | | | | |
| 15 | 50-54 | Former | 2769 | 28.84 | | | 17.84 | 99 | 3.58 |

Year of Follow-up: 2005, Age Group: 55-59

| Period | Age Group | Smoking Group | N | % | Ever Quit (N) | Ever Quit % | Mean Yrs Quit | Short Term (n) | Short Term % |
|---|---|---|---|---|---|---|---|---|---|
| 15 | 55-59 | Never Any | 4356 | 51.27 | | | | | |
| 15 | 55-59 | CC | 166 | 1.95 | 0 | 0.00 | | | |
| 15 | 55-59 | Ecig | 1281 | 15.08 | | | | | |
| 15 | 55-59 | Dual | 0 | 0.00 | | | | | |
| 15 | 55-59 | Former | 2694 | 31.71 | | | 16.95 | 78 | 2.90 |

Year of Follow-up: 2005, Age Group: 60-64

| Period | Age Group | Smoking Group | N | % | Ever Quit (N) | Ever Quit % | Mean Yrs Quit | Short Term (n) | Short Term % |
|---|---|---|---|---|---|---|---|---|---|
| 15 | 60-64 | Never Any | 3116 | 47.67 | | | | | |
| 15 | 60-64 | CC | 131 | 2.00 | 0 | 0.00 | | | |
| 15 | 60-64 | Ecig | 852 | 13.04 | | | | | |
| 15 | 60-64 | Dual | 0 | 0.00 | | | | | |
| 15 | 60-64 | Former | 2437 | 37.29 | | | 20.18 | 93 | 3.82 |

Year of Follow-up: 2005, Age Group: 65-69

| Period | Age Group | Smoking Group | N | % | Ever Quit (N) | Ever Quit % | Mean Yrs Quit | Short Term (n) | Short Term % |
|---|---|---|---|---|---|---|---|---|---|
| 15 | 65-69 | Never Any | 2684 | 48.50 | | | | | |
| 15 | 65-69 | CC | 160 | 2.89 | 0 | 0.00 | | | |
| 15 | 65-69 | Ecig | 591 | 10.68 | | | | | |
| 15 | 65-69 | Dual | 0 | 0.00 | | | | | |
| 15 | 65-69 | Former | 2099 | 37.93 | | | 19.92 | 68 | 3.24 |

Year of Follow-up: 2005, Age Group: 70-74

| Period | Age Group | Smoking Group | N | % | Ever Quit (N) | Ever Quit % | Mean Yrs Quit | Short Term (n) | Short Term % |
|---|---|---|---|---|---|---|---|---|---|
| 15 | 70-74 | Never Any | 2732 | 51.90 | | | | | |
| 15 | 70-74 | CC | 139 | 2.64 | 0 | 0.00 | | | |
| 15 | 70-74 | Ecig | 391 | 7.43 | | | | | |
| 15 | 70-74 | Dual | 0 | 0.00 | | | | | |
| 15 | 70-74 | Former | 2002 | 38.03 | | | 22.67 | 69 | 3.45 |

Year of Follow-up: 2005, Age Group: 75-79

| Period | Age Group | Smoking Group | N | % | Ever Quit (N) | Ever Quit % | Mean Yrs Quit | Short Term (n) | Short Term % |
|---|---|---|---|---|---|---|---|---|---|
| 15 | 75-79 | Never Any | 3078 | 55.72 | | | | | |
| 15 | 75-79 | CC | 146 | 2.64 | 0 | 0.00 | | | |
| 15 | 75-79 | Ecig | 297 | 5.38 | | | | | |
| 15 | 75-79 | Dual | 0 | 0.00 | | | | | |
| 15 | 75-79 | Former | 2003 | 36.26 | | | 22.91 | 56 | 2.80 |

Year of Follow-up: 2010, Age Group: All Ages

| Period | Age Group | Smoking Group | N | % | Ever Quit (N) | Ever Quit % | Mean Yrs Quit | Short Term (n) | Short Term % |
|---|---|---|---|---|---|---|---|---|---|
| 20 | All Ages | Never Any | 43759 | 53.97 | | | | | |
| 20 | All Ages | CC | 765 | 0.94 | 0 | 0.00 | | | |
| 20 | All Ages | Ecig | 13785 | 17.00 | | | | | |
| 20 | All Ages | Dual | 0 | 0.00 | | | | | |
| 20 | All Ages | Former | 22772 | 28.09 | | | 19.42 | 1117 | 4.91 |

Year of Follow-up: 2010, Age Group: 18-19

| Period | Age Group | Smoking Group | N | % | Ever Quit (N) | Ever Quit % | Mean Yrs Quit | Short Term (n) | Short Term % |
|---|---|---|---|---|---|---|---|---|---|

# Results for PHIM for Run 2 (Sensitivity_Analysis_1.RTF)

| | | | | | | | | | |
|---|---|---|---|---|---|---|---|---|---|
| 20 | 18-19 | Never Any | 0 | . | | | | | |
| 20 | 18-19 | CC | 0 | . | 0 | . | | | |
| 20 | 18-19 | Ecig | 0 | . | | | | | |
| 20 | 18-19 | Dual | 0 | . | | | | | |
| 20 | 18-19 | Former | 0 | . | | | . | 0 | . |

Year of Follow-up: 2010, Age Group: 20-24

| Period | Age Group | Smoking Group | N | % | Ever Quit (N) | Ever Quit % | Mean Yrs Quit | Short Term (n) | Short Term % |
|---|---|---|---|---|---|---|---|---|---|
| 20 | 20-24 | Never Any | 0 | . | | | | | |
| 20 | 20-24 | CC | 0 | . | 0 | . | | | |
| 20 | 20-24 | Ecig | 0 | . | | | | | |
| 20 | 20-24 | Dual | 0 | . | | | | | |
| 20 | 20-24 | Former | 0 | . | | | . | 0 | . |

Year of Follow-up: 2010, Age Group: 25-29

| Period | Age Group | Smoking Group | N | % | Ever Quit (N) | Ever Quit % | Mean Yrs Quit | Short Term (n) | Short Term % |
|---|---|---|---|---|---|---|---|---|---|
| 20 | 25-29 | Never Any | 0 | . | | | | | |
| 20 | 25-29 | CC | 0 | . | 0 | . | | | |
| 20 | 25-29 | Ecig | 0 | . | | | | | |
| 20 | 25-29 | Dual | 0 | . | | | | | |
| 20 | 25-29 | Former | 0 | . | | | . | 0 | . |

Year of Follow-up: 2010, Age Group: 30-34

| Period | Age Group | Smoking Group | N | % | Ever Quit (N) | Ever Quit % | Mean Yrs Quit | Short Term (n) | Short Term % |
|---|---|---|---|---|---|---|---|---|---|
| 20 | 30-34 | Never Any | 4743 | 59.81 | | | | | |
| 20 | 30-34 | CC | 65 | 0.82 | 0 | 0.00 | | | |
| 20 | 30-34 | Ecig | 1881 | 23.72 | | | | | |
| 20 | 30-34 | Dual | 0 | 0.00 | | | | | |
| 20 | 30-34 | Former | 1241 | 15.65 | | | 9.36 | 130 | 10.48 |

Year of Follow-up: 2010, Age Group: 35-39

| Period | Age Group | Smoking Group | N | % | Ever Quit (N) | Ever Quit % | Mean Yrs Quit | Short Term (n) | Short Term % |
|---|---|---|---|---|---|---|---|---|---|
| 20 | 35-39 | Never Any | 5359 | 64.18 | | | | | |
| 20 | 35-39 | CC | 58 | 0.69 | 0 | 0.00 | | | |
| 20 | 35-39 | Ecig | 1717 | 20.56 | | | | | |
| 20 | 35-39 | Dual | 0 | 0.00 | | | | | |
| 20 | 35-39 | Former | 1216 | 14.56 | | | 11.38 | 98 | 8.06 |

Year of Follow-up: 2010, Age Group: 40-44

| Period | Age Group | Smoking Group | N | % | Ever Quit (N) | Ever Quit % | Mean Yrs Quit | Short Term (n) | Short Term % |
|---|---|---|---|---|---|---|---|---|---|
| 20 | 40-44 | Never Any | 5189 | 59.01 | | | | | |
| 20 | 40-44 | CC | 64 | 0.73 | 0 | 0.00 | | | |
| 20 | 40-44 | Ecig | 1849 | 21.03 | | | | | |
| 20 | 40-44 | Dual | 0 | 0.00 | | | | | |
| 20 | 40-44 | Former | 1691 | 19.23 | | | 15.34 | 112 | 6.62 |

Year of Follow-up: 2010, Age Group: 45-49

| Period | Age Group | Smoking Group | N | % | Ever Quit (N) | Ever Quit % | Mean Yrs Quit | Short Term (n) | Short Term % |
|---|---|---|---|---|---|---|---|---|---|
| 20 | 45-49 | Never Any | 5284 | 52.43 | | | | | |
| 20 | 45-49 | CC | 105 | 1.04 | 0 | 0.00 | | | |
| 20 | 45-49 | Ecig | 2327 | 23.09 | | | | | |
| 20 | 45-49 | Dual | 0 | 0.00 | | | | | |
| 20 | 45-49 | Former | 2362 | 23.44 | | | 17.71 | 131 | 5.55 |

Year of Follow-up: 2010, Age Group: 50-54

| Period | Age Group | Smoking Group | N | % | Ever Quit (N) | Ever Quit % | Mean Yrs Quit | Short Term (n) | Short Term % |
|---|---|---|---|---|---|---|---|---|---|
| 20 | 50-54 | Never Any | 5278 | 50.27 | | | | | |
| 20 | 50-54 | CC | 101 | 0.96 | 0 | 0.00 | | | |
| 20 | 50-54 | Ecig | 2101 | 20.01 | | | | | |
| 20 | 50-54 | Dual | 0 | 0.00 | | | | | |
| 20 | 50-54 | Former | 3019 | 28.76 | | | 18.27 | 163 | 5.40 |

Year of Follow-up: 2010, Age Group: 55-59

| Period | Age Group | Smoking Group | N | % | Ever Quit (N) | Ever Quit % | Mean Yrs Quit | Short Term (n) | Short Term % |
|---|---|---|---|---|---|---|---|---|---|
| 20 | 55-59 | Never Any | 5018 | 52.27 | | | | | |
| 20 | 55-59 | CC | 91 | 0.95 | 0 | 0.00 | | | |
| 20 | 55-59 | Ecig | 1433 | 14.93 | | | | | |
| 20 | 55-59 | Dual | 0 | 0.00 | | | | | |
| 20 | 55-59 | Former | 3058 | 31.85 | | | 20.88 | 103 | 3.37 |

Year of Follow-up: 2010, Age Group: 60-64

| Period | Age Group | Smoking Group | N | % | Ever Quit (N) | Ever Quit % | Mean Yrs Quit | Short Term (n) | Short Term % |
|---|---|---|---|---|---|---|---|---|---|
| 20 | 60-64 | Never Any | 4356 | 51.27 | | | | | |
| 20 | 60-64 | CC | 71 | 0.84 | 0 | 0.00 | | | |
| 20 | 60-64 | Ecig | 1079 | 12.70 | | | | | |
| 20 | 60-64 | Dual | 0 | 0.00 | | | | | |
| 20 | 60-64 | Former | 2991 | 35.20 | | | 19.94 | 144 | 4.81 |

Year of Follow-up: 2010, Age Group: 65-69

## Results for PHIM for Run 2 (Sensitivity_Analysis_1.RTF)

| Period | Age Group | Smoking Group | N | % | Ever Quit (N) | Ever Quit % | Mean Yrs Quit | Short Term (n) | Short Term % |
|---|---|---|---|---|---|---|---|---|---|
| 20 | 65-69 | Never Any | 3116 | 47.67 | | | | | |
| 20 | 65-69 | CC | 47 | 0.72 | 0 | 0.00 | | | |
| 20 | 65-69 | Ecig | 645 | 9.87 | | | | | |
| 20 | 65-69 | Dual | 0 | 0.00 | | | | | |
| 20 | 65-69 | Former | 2728 | 41.74 | | | 22.72 | 95 | 3.48 |

Year of Follow-up: 2010, Age Group: 70-74

| Period | Age Group | Smoking Group | N | % | Ever Quit (N) | Ever Quit % | Mean Yrs Quit | Short Term (n) | Short Term % |
|---|---|---|---|---|---|---|---|---|---|
| 20 | 70-74 | Never Any | 2684 | 48.50 | | | | | |
| 20 | 70-74 | CC | 88 | 1.59 | 0 | 0.00 | | | |
| 20 | 70-74 | Ecig | 477 | 8.62 | | | | | |
| 20 | 70-74 | Dual | 0 | 0.00 | | | | | |
| 20 | 70-74 | Former | 2285 | 41.29 | | | 23.05 | 69 | 3.02 |

Year of Follow-up: 2010, Age Group: 75-79

| Period | Age Group | Smoking Group | N | % | Ever Quit (N) | Ever Quit % | Mean Yrs Quit | Short Term (n) | Short Term % |
|---|---|---|---|---|---|---|---|---|---|
| 20 | 75-79 | Never Any | 2732 | 51.90 | | | | | |
| 20 | 75-79 | CC | 75 | 1.42 | 0 | 0.00 | | | |
| 20 | 75-79 | Ecig | 276 | 5.24 | | | | | |
| 20 | 75-79 | Dual | 0 | 0.00 | | | | | |
| 20 | 75-79 | Former | 2181 | 41.43 | | | 25.56 | 72 | 3.30 |

Year of Follow-up: 2015, Age Group: All Ages

| Period | Age Group | Smoking Group | N | % | Ever Quit (N) | Ever Quit % | Mean Yrs Quit | Short Term (n) | Short Term % |
|---|---|---|---|---|---|---|---|---|---|
| 25 | All Ages | Never Any | 41010 | 54.09 | | | | | |
| 25 | All Ages | CC | 340 | 0.45 | 0 | 0.00 | | | |
| 25 | All Ages | Ecig | 11438 | 15.09 | | | | | |
| 25 | All Ages | Dual | 0 | 0.00 | | | | | |
| 25 | All Ages | Former | 23029 | 30.37 | | | 21.48 | 914 | 3.97 |

Year of Follow-up: 2015, Age Group: 18-19

| Period | Age Group | Smoking Group | N | % | Ever Quit (N) | Ever Quit % | Mean Yrs Quit | Short Term (n) | Short Term % |
|---|---|---|---|---|---|---|---|---|---|
| 25 | 18-19 | Never Any | 0 | . | | | | | |
| 25 | 18-19 | CC | 0 | . | 0 | . | | | |
| 25 | 18-19 | Ecig | 0 | . | | | | | |
| 25 | 18-19 | Dual | 0 | . | | | | | |
| 25 | 18-19 | Former | 0 | . | | | . | 0 | . |

Year of Follow-up: 2015, Age Group: 20-24

| Period | Age Group | Smoking Group | N | % | Ever Quit (N) | Ever Quit % | Mean Yrs Quit | Short Term (n) | Short Term % |
|---|---|---|---|---|---|---|---|---|---|
| 25 | 20-24 | Never Any | 0 | . | | | | | |
| 25 | 20-24 | CC | 0 | . | 0 | . | | | |
| 25 | 20-24 | Ecig | 0 | . | | | | | |
| 25 | 20-24 | Dual | 0 | . | | | | | |
| 25 | 20-24 | Former | 0 | . | | | . | 0 | . |

Year of Follow-up: 2015, Age Group: 25-29

| Period | Age Group | Smoking Group | N | % | Ever Quit (N) | Ever Quit % | Mean Yrs Quit | Short Term (n) | Short Term % |
|---|---|---|---|---|---|---|---|---|---|
| 25 | 25-29 | Never Any | 0 | . | | | | | |
| 25 | 25-29 | CC | 0 | . | 0 | . | | | |
| 25 | 25-29 | Ecig | 0 | . | | | | | |
| 25 | 25-29 | Dual | 0 | . | | | | | |
| 25 | 25-29 | Former | 0 | . | | | . | 0 | . |

Year of Follow-up: 2015, Age Group: 30-34

| Period | Age Group | Smoking Group | N | % | Ever Quit (N) | Ever Quit % | Mean Yrs Quit | Short Term (n) | Short Term % |
|---|---|---|---|---|---|---|---|---|---|
| 25 | 30-34 | Never Any | 0 | . | | | | | |
| 25 | 30-34 | CC | 0 | . | 0 | . | | | |
| 25 | 30-34 | Ecig | 0 | . | | | | | |
| 25 | 30-34 | Dual | 0 | . | | | | | |
| 25 | 30-34 | Former | 0 | . | | | . | 0 | . |

Year of Follow-up: 2015, Age Group: 35-39

| Period | Age Group | Smoking Group | N | % | Ever Quit (N) | Ever Quit % | Mean Yrs Quit | Short Term (n) | Short Term % |
|---|---|---|---|---|---|---|---|---|---|
| 25 | 35-39 | Never Any | 4726 | 59.60 | | | | | |
| 25 | 35-39 | CC | 20 | 0.25 | 0 | 0.00 | | | |
| 25 | 35-39 | Ecig | 1567 | 19.76 | | | | | |
| 25 | 35-39 | Dual | 0 | 0.00 | | | | | |
| 25 | 35-39 | Former | 1617 | 20.39 | | | 11.52 | 129 | 7.98 |

Year of Follow-up: 2015, Age Group: 40-44

| Period | Age Group | Smoking Group | N | % | Ever Quit (N) | Ever Quit % | Mean Yrs Quit | Short Term (n) | Short Term % |
|---|---|---|---|---|---|---|---|---|---|
| 25 | 40-44 | Never Any | 5359 | 64.18 | | | | | |
| 25 | 40-44 | CC | 33 | 0.40 | 0 | 0.00 | | | |
| 25 | 40-44 | Ecig | 1502 | 17.99 | | | | | |
| 25 | 40-44 | Dual | 0 | 0.00 | | | | | |

# Results for PHIM for Run 2 (Sensitivity_Analysis_1.RTF)

| | | | | | | | | | |
|---|---|---|---|---|---|---|---|---|---|
| 25 | 40-44 | Former | 1456 | 17.44 | | | 14.00 | 103 | 7.07 |

Year of Follow-up: 2015, Age Group: 45-49

| Period | Age Group | Smoking Group | N | % | Ever Quit (N) | Ever Quit % | Mean Yrs Quit | Short Term (n) | Short Term % |
|---|---|---|---|---|---|---|---|---|---|
| 25 | 45-49 | Never Any | 5189 | 59.01 | | | | | |
| 25 | 45-49 | CC | 32 | 0.36 | 0 | 0.00 | | | |
| 25 | 45-49 | Ecig | 1627 | 18.50 | | | | | |
| 25 | 45-49 | Dual | 0 | 0.00 | | | | | |
| 25 | 45-49 | Former | 1945 | 22.12 | | | 17.95 | 96 | 4.94 |

Year of Follow-up: 2015, Age Group: 50-54

| Period | Age Group | Smoking Group | N | % | Ever Quit (N) | Ever Quit % | Mean Yrs Quit | Short Term (n) | Short Term % |
|---|---|---|---|---|---|---|---|---|---|
| 25 | 50-54 | Never Any | 5284 | 52.43 | | | | | |
| 25 | 50-54 | CC | 45 | 0.45 | 0 | 0.00 | | | |
| 25 | 50-54 | Ecig | 2059 | 20.43 | | | | | |
| 25 | 50-54 | Dual | 0 | 0.00 | | | | | |
| 25 | 50-54 | Former | 2690 | 26.69 | | | 20.17 | 150 | 5.58 |

Year of Follow-up: 2015, Age Group: 55-59

| Period | Age Group | Smoking Group | N | % | Ever Quit (N) | Ever Quit % | Mean Yrs Quit | Short Term (n) | Short Term % |
|---|---|---|---|---|---|---|---|---|---|
| 25 | 55-59 | Never Any | 5278 | 50.27 | | | | | |
| 25 | 55-59 | CC | 37 | 0.35 | 0 | 0.00 | | | |
| 25 | 55-59 | Ecig | 1773 | 16.89 | | | | | |
| 25 | 55-59 | Dual | 0 | 0.00 | | | | | |
| 25 | 55-59 | Former | 3411 | 32.49 | | | 20.84 | 140 | 4.10 |

Year of Follow-up: 2015, Age Group: 60-64

| Period | Age Group | Smoking Group | N | % | Ever Quit (N) | Ever Quit % | Mean Yrs Quit | Short Term (n) | Short Term % |
|---|---|---|---|---|---|---|---|---|---|
| 25 | 60-64 | Never Any | 5018 | 52.27 | | | | | |
| 25 | 60-64 | CC | 42 | 0.44 | 0 | 0.00 | | | |
| 25 | 60-64 | Ecig | 1154 | 12.02 | | | | | |
| 25 | 60-64 | Dual | 0 | 0.00 | | | | | |
| 25 | 60-64 | Former | 3386 | 35.27 | | | 23.56 | 144 | 4.25 |

Year of Follow-up: 2015, Age Group: 65-69

| Period | Age Group | Smoking Group | N | % | Ever Quit (N) | Ever Quit % | Mean Yrs Quit | Short Term (n) | Short Term % |
|---|---|---|---|---|---|---|---|---|---|
| 25 | 65-69 | Never Any | 4356 | 51.27 | | | | | |
| 25 | 65-69 | CC | 41 | 0.48 | 0 | 0.00 | | | |
| 25 | 65-69 | Ecig | 844 | 9.93 | | | | | |
| 25 | 65-69 | Dual | 0 | 0.00 | | | | | |
| 25 | 65-69 | Former | 3256 | 38.32 | | | 23.12 | 70 | 2.15 |

Year of Follow-up: 2015, Age Group: 70-74

| Period | Age Group | Smoking Group | N | % | Ever Quit (N) | Ever Quit % | Mean Yrs Quit | Short Term (n) | Short Term % |
|---|---|---|---|---|---|---|---|---|---|
| 25 | 70-74 | Never Any | 3116 | 47.67 | | | | | |
| 25 | 70-74 | CC | 31 | 0.47 | 0 | 0.00 | | | |
| 25 | 70-74 | Ecig | 527 | 8.06 | | | | | |
| 25 | 70-74 | Dual | 0 | 0.00 | | | | | |
| 25 | 70-74 | Former | 2862 | 43.79 | | | 26.54 | 42 | 1.47 |

Year of Follow-up: 2015, Age Group: 75-79

| Period | Age Group | Smoking Group | N | % | Ever Quit (N) | Ever Quit % | Mean Yrs Quit | Short Term (n) | Short Term % |
|---|---|---|---|---|---|---|---|---|---|
| 25 | 75-79 | Never Any | 2684 | 48.50 | | | | | |
| 25 | 75-79 | CC | 59 | 1.07 | 0 | 0.00 | | | |
| 25 | 75-79 | Ecig | 385 | 6.96 | | | | | |
| 25 | 75-79 | Dual | 0 | 0.00 | | | | | |
| 25 | 75-79 | Former | 2406 | 43.48 | | | 26.76 | 40 | 1.66 |

Year of Follow-up: 2020, Age Group: All Ages

| Period | Age Group | Smoking Group | N | % | Ever Quit (N) | Ever Quit % | Mean Yrs Quit | Short Term (n) | Short Term % |
|---|---|---|---|---|---|---|---|---|---|
| 30 | All Ages | Never Any | 38326 | 54.53 | | | | | |
| 30 | All Ages | CC | 115 | 0.16 | 0 | 0.00 | | | |
| 30 | All Ages | Ecig | 9272 | 13.19 | | | | | |
| 30 | All Ages | Dual | 0 | 0.00 | | | | | |
| 30 | All Ages | Former | 22570 | 32.11 | | | 23.81 | 756 | 3.35 |

Year of Follow-up: 2020, Age Group: 18-19

| Period | Age Group | Smoking Group | N | % | Ever Quit (N) | Ever Quit % | Mean Yrs Quit | Short Term (n) | Short Term % |
|---|---|---|---|---|---|---|---|---|---|
| 30 | 18-19 | Never Any | 0 | . | | | | | |
| 30 | 18-19 | CC | 0 | . | 0 | . | | | |
| 30 | 18-19 | Ecig | 0 | . | | | | | |
| 30 | 18-19 | Dual | 0 | . | | | | | |
| 30 | 18-19 | Former | 0 | . | | | . | 0 | . |

Year of Follow-up: 2020, Age Group: 20-24

| Period | Age Group | Smoking Group | N | % | Ever Quit (N) | Ever Quit % | Mean Yrs Quit | Short Term (n) | Short Term % |
|---|---|---|---|---|---|---|---|---|---|
| 30 | 20-24 | Never Any | 0 | . | | | | | |
| 30 | 20-24 | CC | 0 | . | 0 | . | | | |

# Results for PHIM for Run 2 (Sensitivity_Analysis_1.RTF)

| | | | | | | | | | |
|---|---|---|---|---|---|---|---|---|---|
| 30 | 20-24 | Ecig | 0 | . | | | | | |
| 30 | 20-24 | Dual | 0 | . | | | | | |
| 30 | 20-24 | Former | 0 | . | | | . | 0 | . |

Year of Follow-up: 2020, Age Group: 25-29

| Period | Age Group | Smoking Group | N | % | Ever Quit (N) | Ever Quit % | Mean Yrs Quit | Short Term (n) | Short Term % |
|---|---|---|---|---|---|---|---|---|---|
| 30 | 25-29 | Never Any | 0 | . | | | | | |
| 30 | 25-29 | CC | 0 | . | 0 | . | | | |
| 30 | 25-29 | Ecig | 0 | . | | | | | |
| 30 | 25-29 | Dual | 0 | . | | | | | |
| 30 | 25-29 | Former | 0 | . | | | . | 0 | . |

Year of Follow-up: 2020, Age Group: 30-34

| Period | Age Group | Smoking Group | N | % | Ever Quit (N) | Ever Quit % | Mean Yrs Quit | Short Term (n) | Short Term % |
|---|---|---|---|---|---|---|---|---|---|
| 30 | 30-34 | Never Any | 0 | . | | | | | |
| 30 | 30-34 | CC | 0 | . | 0 | . | | | |
| 30 | 30-34 | Ecig | 0 | . | | | | | |
| 30 | 30-34 | Dual | 0 | . | | | | | |
| 30 | 30-34 | Former | 0 | . | | | . | 0 | . |

Year of Follow-up: 2020, Age Group: 35-39

| Period | Age Group | Smoking Group | N | % | Ever Quit (N) | Ever Quit % | Mean Yrs Quit | Short Term (n) | Short Term % |
|---|---|---|---|---|---|---|---|---|---|
| 30 | 35-39 | Never Any | 0 | . | | | | | |
| 30 | 35-39 | CC | 0 | . | 0 | . | | | |
| 30 | 35-39 | Ecig | 0 | . | | | | | |
| 30 | 35-39 | Dual | 0 | . | | | | | |
| 30 | 35-39 | Former | 0 | . | | | . | 0 | . |

Year of Follow-up: 2020, Age Group: 40-44

| Period | Age Group | Smoking Group | N | % | Ever Quit (N) | Ever Quit % | Mean Yrs Quit | Short Term (n) | Short Term % |
|---|---|---|---|---|---|---|---|---|---|
| 30 | 40-44 | Never Any | 4726 | 59.60 | | | | | |
| 30 | 40-44 | CC | 6 | 0.08 | 0 | 0.00 | | | |
| 30 | 40-44 | Ecig | 1345 | 16.96 | | | | | |
| 30 | 40-44 | Dual | 0 | 0.00 | | | | | |
| 30 | 40-44 | Former | 1853 | 23.37 | | | 14.68 | 82 | 4.43 |

Year of Follow-up: 2020, Age Group: 45-49

| Period | Age Group | Smoking Group | N | % | Ever Quit (N) | Ever Quit % | Mean Yrs Quit | Short Term (n) | Short Term % |
|---|---|---|---|---|---|---|---|---|---|
| 30 | 45-49 | Never Any | 5359 | 64.18 | | | | | |
| 30 | 45-49 | CC | 13 | 0.16 | 0 | 0.00 | | | |
| 30 | 45-49 | Ecig | 1317 | 15.77 | | | | | |
| 30 | 45-49 | Dual | 0 | 0.00 | | | | | |
| 30 | 45-49 | Former | 1661 | 19.89 | | | 16.93 | 71 | 4.27 |

Year of Follow-up: 2020, Age Group: 50-54

| Period | Age Group | Smoking Group | N | % | Ever Quit (N) | Ever Quit % | Mean Yrs Quit | Short Term (n) | Short Term % |
|---|---|---|---|---|---|---|---|---|---|
| 30 | 50-54 | Never Any | 5189 | 59.01 | | | | | |
| 30 | 50-54 | CC | 9 | 0.10 | 0 | 0.00 | | | |
| 30 | 50-54 | Ecig | 1395 | 15.86 | | | | | |
| 30 | 50-54 | Dual | 0 | 0.00 | | | | | |
| 30 | 50-54 | Former | 2200 | 25.02 | | | 20.50 | 121 | 5.50 |

Year of Follow-up: 2020, Age Group: 55-59

| Period | Age Group | Smoking Group | N | % | Ever Quit (N) | Ever Quit % | Mean Yrs Quit | Short Term (n) | Short Term % |
|---|---|---|---|---|---|---|---|---|---|
| 30 | 55-59 | Never Any | 5284 | 52.43 | | | | | |
| 30 | 55-59 | CC | 14 | 0.14 | 0 | 0.00 | | | |
| 30 | 55-59 | Ecig | 1708 | 16.95 | | | | | |
| 30 | 55-59 | Dual | 0 | 0.00 | | | | | |
| 30 | 55-59 | Former | 3072 | 30.48 | | | 22.30 | 140 | 4.56 |

Year of Follow-up: 2020, Age Group: 60-64

| Period | Age Group | Smoking Group | N | % | Ever Quit (N) | Ever Quit % | Mean Yrs Quit | Short Term (n) | Short Term % |
|---|---|---|---|---|---|---|---|---|---|
| 30 | 60-64 | Never Any | 5278 | 50.27 | | | | | |
| 30 | 60-64 | CC | 15 | 0.14 | 0 | 0.00 | | | |
| 30 | 60-64 | Ecig | 1428 | 13.60 | | | | | |
| 30 | 60-64 | Dual | 0 | 0.00 | | | | | |
| 30 | 60-64 | Former | 3778 | 35.98 | | | 23.52 | 153 | 4.05 |

Year of Follow-up: 2020, Age Group: 65-69

| Period | Age Group | Smoking Group | N | % | Ever Quit (N) | Ever Quit % | Mean Yrs Quit | Short Term (n) | Short Term % |
|---|---|---|---|---|---|---|---|---|---|
| 30 | 65-69 | Never Any | 5018 | 52.27 | | | | | |
| 30 | 65-69 | CC | 18 | 0.19 | 0 | 0.00 | | | |
| 30 | 65-69 | Ecig | 917 | 9.55 | | | | | |
| 30 | 65-69 | Dual | 0 | 0.00 | | | | | |
| 30 | 65-69 | Former | 3647 | 37.99 | | | 26.68 | 75 | 2.06 |

Year of Follow-up: 2020, Age Group: 70-74

| Period | Age Group | Smoking Group | N | % | Ever Quit (N) | Ever Quit % | Mean Yrs Quit | Short Term (n) | Short Term % |
|---|---|---|---|---|---|---|---|---|---|

# Results for PHIM for Run 2 (Sensitivity_Analysis_1.RTF)

| | | | | | | | | | |
|---|---|---|---|---|---|---|---|---|---|
| 30 | 70-74 | Never Any | 4356 | 51.27 | | | | | |
| 30 | 70-74 | CC | 22 | 0.26 | 0 | 0.00 | | | |
| 30 | 70-74 | Ecig | 727 | 8.56 | | | | | |
| 30 | 70-74 | Dual | 0 | 0.00 | | | | | |
| 30 | 70-74 | Former | 3392 | 39.92 | | | 27.06 | 66 | 1.95 |

Year of Follow-up: 2020, Age Group: 75-79

| Period | Age Group | Smoking Group | N | % | Ever Quit (N) | Ever Quit % | Mean Yrs Quit | Short Term (n) | Short Term % |
|---|---|---|---|---|---|---|---|---|---|
| 30 | 75-79 | Never Any | 3116 | 47.67 | | | | | |
| 30 | 75-79 | CC | 18 | 0.28 | 0 | 0.00 | | | |
| 30 | 75-79 | Ecig | 435 | 6.66 | | | | | |
| 30 | 75-79 | Dual | 0 | 0.00 | | | | | |
| 30 | 75-79 | Former | 2967 | 45.39 | | | 30.49 | 48 | 1.62 |

Year of Follow-up: 2025, Age Group: All Ages

| Period | Age Group | Smoking Group | N | % | Ever Quit (N) | Ever Quit % | Mean Yrs Quit | Short Term (n) | Short Term % |
|---|---|---|---|---|---|---|---|---|---|
| 35 | All Ages | Never Any | 35210 | 55.23 | | | | | |
| 35 | All Ages | CC | 47 | 0.07 | 0 | 0.00 | | | |
| 35 | All Ages | Ecig | 7305 | 11.46 | | | | | |
| 35 | All Ages | Dual | 0 | 0.00 | | | | | |
| 35 | All Ages | Former | 21185 | 33.23 | | | 25.87 | 616 | 2.91 |

Year of Follow-up: 2025, Age Group: 18-19

| Period | Age Group | Smoking Group | N | % | Ever Quit (N) | Ever Quit % | Mean Yrs Quit | Short Term (n) | Short Term % |
|---|---|---|---|---|---|---|---|---|---|
| 35 | 18-19 | Never Any | 0 | . | | | | | |
| 35 | 18-19 | CC | 0 | . | 0 | . | | | |
| 35 | 18-19 | Ecig | 0 | . | | | | | |
| 35 | 18-19 | Dual | 0 | . | | | | | |
| 35 | 18-19 | Former | 0 | . | | | . | 0 | . |

Year of Follow-up: 2025, Age Group: 20-24

| Period | Age Group | Smoking Group | N | % | Ever Quit (N) | Ever Quit % | Mean Yrs Quit | Short Term (n) | Short Term % |
|---|---|---|---|---|---|---|---|---|---|
| 35 | 20-24 | Never Any | 0 | . | | | | | |
| 35 | 20-24 | CC | 0 | . | 0 | . | | | |
| 35 | 20-24 | Ecig | 0 | . | | | | | |
| 35 | 20-24 | Dual | 0 | . | | | | | |
| 35 | 20-24 | Former | 0 | . | | | . | 0 | . |

Year of Follow-up: 2025, Age Group: 25-29

| Period | Age Group | Smoking Group | N | % | Ever Quit (N) | Ever Quit % | Mean Yrs Quit | Short Term (n) | Short Term % |
|---|---|---|---|---|---|---|---|---|---|
| 35 | 25-29 | Never Any | 0 | . | | | | | |
| 35 | 25-29 | CC | 0 | . | 0 | . | | | |
| 35 | 25-29 | Ecig | 0 | . | | | | | |
| 35 | 25-29 | Dual | 0 | . | | | | | |
| 35 | 25-29 | Former | 0 | . | | | . | 0 | . |

Year of Follow-up: 2025, Age Group: 30-34

| Period | Age Group | Smoking Group | N | % | Ever Quit (N) | Ever Quit % | Mean Yrs Quit | Short Term (n) | Short Term % |
|---|---|---|---|---|---|---|---|---|---|
| 35 | 30-34 | Never Any | 0 | . | | | | | |
| 35 | 30-34 | CC | 0 | . | 0 | . | | | |
| 35 | 30-34 | Ecig | 0 | . | | | | | |
| 35 | 30-34 | Dual | 0 | . | | | | | |
| 35 | 30-34 | Former | 0 | . | | | . | 0 | . |

Year of Follow-up: 2025, Age Group: 35-39

| Period | Age Group | Smoking Group | N | % | Ever Quit (N) | Ever Quit % | Mean Yrs Quit | Short Term (n) | Short Term % |
|---|---|---|---|---|---|---|---|---|---|
| 35 | 35-39 | Never Any | 0 | . | | | | | |
| 35 | 35-39 | CC | 0 | . | 0 | . | | | |
| 35 | 35-39 | Ecig | 0 | . | | | | | |
| 35 | 35-39 | Dual | 0 | . | | | | | |
| 35 | 35-39 | Former | 0 | . | | | . | 0 | . |

Year of Follow-up: 2025, Age Group: 40-44

| Period | Age Group | Smoking Group | N | % | Ever Quit (N) | Ever Quit % | Mean Yrs Quit | Short Term (n) | Short Term % |
|---|---|---|---|---|---|---|---|---|---|
| 35 | 40-44 | Never Any | 0 | . | | | | | |
| 35 | 40-44 | CC | 0 | . | 0 | . | | | |
| 35 | 40-44 | Ecig | 0 | . | | | | | |
| 35 | 40-44 | Dual | 0 | . | | | | | |
| 35 | 40-44 | Former | 0 | . | | | . | 0 | . |

Year of Follow-up: 2025, Age Group: 45-49

| Period | Age Group | Smoking Group | N | % | Ever Quit (N) | Ever Quit % | Mean Yrs Quit | Short Term (n) | Short Term % |
|---|---|---|---|---|---|---|---|---|---|
| 35 | 45-49 | Never Any | 4726 | 59.60 | | | | | |
| 35 | 45-49 | CC | 1 | 0.01 | 0 | 0.00 | | | |
| 35 | 45-49 | Ecig | 1146 | 14.45 | | | | | |
| 35 | 45-49 | Dual | 0 | 0.00 | | | | | |
| 35 | 45-49 | Former | 2057 | 25.94 | | | 17.95 | 74 | 3.60 |

Year of Follow-up: 2025, Age Group: 50-54

# Results for PHIM for Run 2 (Sensitivity_Analysis_1.RTF)

| Period | Age Group | Smoking Group | N | % | Ever Quit (N) | Ever Quit % | Mean Yrs Quit | Short Term (n) | Short Term % |
|---|---|---|---|---|---|---|---|---|---|
| 35 | 50-54 | Never Any | 5359 | 64.18 | | | | | |
| 35 | 50-54 | CC | 3 | 0.04 | 0 | 0.00 | | | |
| 35 | 50-54 | Ecig | 1112 | 13.32 | | | | | |
| 35 | 50-54 | Dual | 0 | 0.00 | | | | | |
| 35 | 50-54 | Former | 1876 | 22.47 | | | 19.62 | 104 | 5.54 |

Year of Follow-up: 2025, Age Group: 55-59

| Period | Age Group | Smoking Group | N | % | Ever Quit (N) | Ever Quit % | Mean Yrs Quit | Short Term (n) | Short Term % |
|---|---|---|---|---|---|---|---|---|---|
| 35 | 55-59 | Never Any | 5189 | 59.01 | | | | | |
| 35 | 55-59 | CC | 4 | 0.05 | 0 | 0.00 | | | |
| 35 | 55-59 | Ecig | 1153 | 13.11 | | | | | |
| 35 | 55-59 | Dual | 0 | 0.00 | | | | | |
| 35 | 55-59 | Former | 2447 | 27.83 | | | 23.13 | 98 | 4.00 |

Year of Follow-up: 2025, Age Group: 60-64

| Period | Age Group | Smoking Group | N | % | Ever Quit (N) | Ever Quit % | Mean Yrs Quit | Short Term (n) | Short Term % |
|---|---|---|---|---|---|---|---|---|---|
| 35 | 60-64 | Never Any | 5284 | 52.43 | | | | | |
| 35 | 60-64 | CC | 7 | 0.07 | 0 | 0.00 | | | |
| 35 | 60-64 | Ecig | 1378 | 13.67 | | | | | |
| 35 | 60-64 | Dual | 0 | 0.00 | | | | | |
| 35 | 60-64 | Former | 3409 | 33.83 | | | 24.79 | 139 | 4.08 |

Year of Follow-up: 2025, Age Group: 65-69

| Period | Age Group | Smoking Group | N | % | Ever Quit (N) | Ever Quit % | Mean Yrs Quit | Short Term (n) | Short Term % |
|---|---|---|---|---|---|---|---|---|---|
| 35 | 65-69 | Never Any | 5278 | 50.27 | | | | | |
| 35 | 65-69 | CC | 7 | 0.07 | 0 | 0.00 | | | |
| 35 | 65-69 | Ecig | 1139 | 10.85 | | | | | |
| 35 | 65-69 | Dual | 0 | 0.00 | | | | | |
| 35 | 65-69 | Former | 4075 | 38.81 | | | 26.61 | 83 | 2.04 |

Year of Follow-up: 2025, Age Group: 70-74

| Period | Age Group | Smoking Group | N | % | Ever Quit (N) | Ever Quit % | Mean Yrs Quit | Short Term (n) | Short Term % |
|---|---|---|---|---|---|---|---|---|---|
| 35 | 70-74 | Never Any | 5018 | 52.27 | | | | | |
| 35 | 70-74 | CC | 11 | 0.11 | 0 | 0.00 | | | |
| 35 | 70-74 | Ecig | 789 | 8.22 | | | | | |
| 35 | 70-74 | Dual | 0 | 0.00 | | | | | |
| 35 | 70-74 | Former | 3782 | 39.40 | | | 30.61 | 65 | 1.72 |

Year of Follow-up: 2025, Age Group: 75-79

| Period | Age Group | Smoking Group | N | % | Ever Quit (N) | Ever Quit % | Mean Yrs Quit | Short Term (n) | Short Term % |
|---|---|---|---|---|---|---|---|---|---|
| 35 | 75-79 | Never Any | 4356 | 51.27 | | | | | |
| 35 | 75-79 | CC | 14 | 0.16 | 0 | 0.00 | | | |
| 35 | 75-79 | Ecig | 588 | 6.92 | | | | | |
| 35 | 75-79 | Dual | 0 | 0.00 | | | | | |
| 35 | 75-79 | Former | 3539 | 41.65 | | | 30.81 | 53 | 1.50 |

Year of Follow-up: 2030, Age Group: All Ages

| Period | Age Group | Smoking Group | N | % | Ever Quit (N) | Ever Quit % | Mean Yrs Quit | Short Term (n) | Short Term % |
|---|---|---|---|---|---|---|---|---|---|
| 40 | All Ages | Never Any | 30854 | 55.84 | | | | | |
| 40 | All Ages | CC | 14 | 0.03 | 0 | 0.00 | | | |
| 40 | All Ages | Ecig | 5391 | 9.76 | | | | | |
| 40 | All Ages | Dual | 0 | 0.00 | | | | | |
| 40 | All Ages | Former | 18991 | 34.37 | | | 27.90 | 543 | 2.86 |

Year of Follow-up: 2030, Age Group: 18-19

| Period | Age Group | Smoking Group | N | % | Ever Quit (N) | Ever Quit % | Mean Yrs Quit | Short Term (n) | Short Term % |
|---|---|---|---|---|---|---|---|---|---|
| 40 | 18-19 | Never Any | 0 | . | | | | | |
| 40 | 18-19 | CC | 0 | . | 0 | . | | | |
| 40 | 18-19 | Ecig | 0 | . | | | | | |
| 40 | 18-19 | Dual | 0 | . | | | | | |
| 40 | 18-19 | Former | 0 | . | | | . | 0 | . |

Year of Follow-up: 2030, Age Group: 20-24

| Period | Age Group | Smoking Group | N | % | Ever Quit (N) | Ever Quit % | Mean Yrs Quit | Short Term (n) | Short Term % |
|---|---|---|---|---|---|---|---|---|---|
| 40 | 20-24 | Never Any | 0 | . | | | | | |
| 40 | 20-24 | CC | 0 | . | 0 | . | | | |
| 40 | 20-24 | Ecig | 0 | . | | | | | |
| 40 | 20-24 | Dual | 0 | . | | | | | |
| 40 | 20-24 | Former | 0 | . | | | . | 0 | . |

Year of Follow-up: 2030, Age Group: 25-29

| Period | Age Group | Smoking Group | N | % | Ever Quit (N) | Ever Quit % | Mean Yrs Quit | Short Term (n) | Short Term % |
|---|---|---|---|---|---|---|---|---|---|
| 40 | 25-29 | Never Any | 0 | . | | | | | |
| 40 | 25-29 | CC | 0 | . | 0 | . | | | |
| 40 | 25-29 | Ecig | 0 | . | | | | | |
| 40 | 25-29 | Dual | 0 | . | | | | | |

# Results for PHIM for Run 2 (Sensitivity_Analysis_1.RTF)

| | | | | | | | | | |
|---|---|---|---|---|---|---|---|---|---|
| 40 | 25-29 | Former | 0 | . | | | . | 0 | . |

Year of Follow-up: 2030, Age Group: 30-34

| Period | Age Group | Smoking Group | N | % | Ever Quit (N) | Ever Quit % | Mean Yrs Quit | Short Term (n) | Short Term % |
|---|---|---|---|---|---|---|---|---|---|
| 40 | 30-34 | Never Any | 0 | . | | | | | |
| 40 | 30-34 | CC | 0 | . | 0 | . | | | |
| 40 | 30-34 | Ecig | 0 | . | | | | | |
| 40 | 30-34 | Dual | 0 | . | | | | | |
| 40 | 30-34 | Former | 0 | . | | | . | 0 | . |

Year of Follow-up: 2030, Age Group: 35-39

| Period | Age Group | Smoking Group | N | % | Ever Quit (N) | Ever Quit % | Mean Yrs Quit | Short Term (n) | Short Term % |
|---|---|---|---|---|---|---|---|---|---|
| 40 | 35-39 | Never Any | 0 | . | | | | | |
| 40 | 35-39 | CC | 0 | . | 0 | . | | | |
| 40 | 35-39 | Ecig | 0 | . | | | | | |
| 40 | 35-39 | Dual | 0 | . | | | | | |
| 40 | 35-39 | Former | 0 | . | | | . | 0 | . |

Year of Follow-up: 2030, Age Group: 40-44

| Period | Age Group | Smoking Group | N | % | Ever Quit (N) | Ever Quit % | Mean Yrs Quit | Short Term (n) | Short Term % |
|---|---|---|---|---|---|---|---|---|---|
| 40 | 40-44 | Never Any | 0 | . | | | | | |
| 40 | 40-44 | CC | 0 | . | 0 | . | | | |
| 40 | 40-44 | Ecig | 0 | . | | | | | |
| 40 | 40-44 | Dual | 0 | . | | | | | |
| 40 | 40-44 | Former | 0 | . | | | . | 0 | . |

Year of Follow-up: 2030, Age Group: 45-49

| Period | Age Group | Smoking Group | N | % | Ever Quit (N) | Ever Quit % | Mean Yrs Quit | Short Term (n) | Short Term % |
|---|---|---|---|---|---|---|---|---|---|
| 40 | 45-49 | Never Any | 0 | . | | | | | |
| 40 | 45-49 | CC | 0 | . | 0 | . | | | |
| 40 | 45-49 | Ecig | 0 | . | | | | | |
| 40 | 45-49 | Dual | 0 | . | | | | | |
| 40 | 45-49 | Former | 0 | . | | | . | 0 | . |

Year of Follow-up: 2030, Age Group: 50-54

| Period | Age Group | Smoking Group | N | % | Ever Quit (N) | Ever Quit % | Mean Yrs Quit | Short Term (n) | Short Term % |
|---|---|---|---|---|---|---|---|---|---|
| 40 | 50-54 | Never Any | 4726 | 59.60 | | | | | |
| 40 | 50-54 | CC | 0 | 0.00 | 0 | 0.00 | | | |
| 40 | 50-54 | Ecig | 959 | 12.09 | | | | | |
| 40 | 50-54 | Dual | 0 | 0.00 | | | | | |
| 40 | 50-54 | Former | 2245 | 28.31 | | | 21.16 | 100 | 4.45 |

Year of Follow-up: 2030, Age Group: 55-59

| Period | Age Group | Smoking Group | N | % | Ever Quit (N) | Ever Quit % | Mean Yrs Quit | Short Term (n) | Short Term % |
|---|---|---|---|---|---|---|---|---|---|
| 40 | 55-59 | Never Any | 5359 | 64.18 | | | | | |
| 40 | 55-59 | CC | 0 | 0.00 | 0 | 0.00 | | | |
| 40 | 55-59 | Ecig | 897 | 10.74 | | | | | |
| 40 | 55-59 | Dual | 0 | 0.00 | | | | | |
| 40 | 55-59 | Former | 2094 | 25.08 | | | 22.27 | 80 | 3.82 |

Year of Follow-up: 2030, Age Group: 60-64

| Period | Age Group | Smoking Group | N | % | Ever Quit (N) | Ever Quit % | Mean Yrs Quit | Short Term (n) | Short Term % |
|---|---|---|---|---|---|---|---|---|---|
| 40 | 60-64 | Never Any | 5189 | 59.01 | | | | | |
| 40 | 60-64 | CC | 3 | 0.03 | 0 | 0.00 | | | |
| 40 | 60-64 | Ecig | 886 | 10.08 | | | | | |
| 40 | 60-64 | Dual | 0 | 0.00 | | | | | |
| 40 | 60-64 | Former | 2715 | 30.88 | | | 25.52 | 128 | 4.71 |

Year of Follow-up: 2030, Age Group: 65-69

| Period | Age Group | Smoking Group | N | % | Ever Quit (N) | Ever Quit % | Mean Yrs Quit | Short Term (n) | Short Term % |
|---|---|---|---|---|---|---|---|---|---|
| 40 | 65-69 | Never Any | 5284 | 52.43 | | | | | |
| 40 | 65-69 | CC | 3 | 0.03 | 0 | 0.00 | | | |
| 40 | 65-69 | Ecig | 1080 | 10.72 | | | | | |
| 40 | 65-69 | Dual | 0 | 0.00 | | | | | |
| 40 | 65-69 | Former | 3711 | 36.82 | | | 27.57 | 73 | 1.97 |

Year of Follow-up: 2030, Age Group: 70-74

| Period | Age Group | Smoking Group | N | % | Ever Quit (N) | Ever Quit % | Mean Yrs Quit | Short Term (n) | Short Term % |
|---|---|---|---|---|---|---|---|---|---|
| 40 | 70-74 | Never Any | 5278 | 50.27 | | | | | |
| 40 | 70-74 | CC | 4 | 0.04 | 0 | 0.00 | | | |
| 40 | 70-74 | Ecig | 957 | 9.12 | | | | | |
| 40 | 70-74 | Dual | 0 | 0.00 | | | | | |
| 40 | 70-74 | Former | 4260 | 40.58 | | | 30.32 | 86 | 2.02 |

Year of Follow-up: 2030, Age Group: 75-79

| Period | Age Group | Smoking Group | N | % | Ever Quit (N) | Ever Quit % | Mean Yrs Quit | Short Term (n) | Short Term % |
|---|---|---|---|---|---|---|---|---|---|
| 40 | 75-79 | Never Any | 5018 | 52.27 | | | | | |
| 40 | 75-79 | CC | 4 | 0.04 | 0 | 0.00 | | | |

# Results for PHIM for Run 2 (Sensitivity_Analysis_1.RTF)

| | | | | | | | | | |
|---|---|---|---|---|---|---|---|---|---|
| 40 | 75-79 | Ecig | 612 | 6.38 | | | | | |
| 40 | 75-79 | Dual | 0 | 0.00 | | | | | |
| 40 | 75-79 | Former | 3966 | 41.31 | | | 34.05 | 76 | 1.92 |

Year of Follow-up: 2035, Age Group: All Ages

| Period | Age Group | Smoking Group | N | % | Ever Quit (N) | Ever Quit % | Mean Yrs Quit | Short Term (n) | Short Term % |
|---|---|---|---|---|---|---|---|---|---|
| 45 | All Ages | Never Any | 25836 | 56.60 | | | | | |
| 45 | All Ages | CC | 5 | 0.01 | 0 | 0.00 | | | |
| 45 | All Ages | Ecig | 3886 | 8.51 | | | | | |
| 45 | All Ages | Dual | 0 | 0.00 | | | | | |
| 45 | All Ages | Former | 15923 | 34.88 | | | 29.63 | 351 | 2.20 |

Year of Follow-up: 2035, Age Group: 18-19

| Period | Age Group | Smoking Group | N | % | Ever Quit (N) | Ever Quit % | Mean Yrs Quit | Short Term (n) | Short Term % |
|---|---|---|---|---|---|---|---|---|---|
| 45 | 18-19 | Never Any | 0 | . | | | | | |
| 45 | 18-19 | CC | 0 | . | 0 | . | | | |
| 45 | 18-19 | Ecig | 0 | . | | | | | |
| 45 | 18-19 | Dual | 0 | . | | | | | |
| 45 | 18-19 | Former | 0 | . | | | . | 0 | . |

Year of Follow-up: 2035, Age Group: 20-24

| Period | Age Group | Smoking Group | N | % | Ever Quit (N) | Ever Quit % | Mean Yrs Quit | Short Term (n) | Short Term % |
|---|---|---|---|---|---|---|---|---|---|
| 45 | 20-24 | Never Any | 0 | . | | | | | |
| 45 | 20-24 | CC | 0 | . | 0 | . | | | |
| 45 | 20-24 | Ecig | 0 | . | | | | | |
| 45 | 20-24 | Dual | 0 | . | | | | | |
| 45 | 20-24 | Former | 0 | . | | | . | 0 | . |

Year of Follow-up: 2035, Age Group: 25-29

| Period | Age Group | Smoking Group | N | % | Ever Quit (N) | Ever Quit % | Mean Yrs Quit | Short Term (n) | Short Term % |
|---|---|---|---|---|---|---|---|---|---|
| 45 | 25-29 | Never Any | 0 | . | | | | | |
| 45 | 25-29 | CC | 0 | . | 0 | . | | | |
| 45 | 25-29 | Ecig | 0 | . | | | | | |
| 45 | 25-29 | Dual | 0 | . | | | | | |
| 45 | 25-29 | Former | 0 | . | | | . | 0 | . |

Year of Follow-up: 2035, Age Group: 30-34

| Period | Age Group | Smoking Group | N | % | Ever Quit (N) | Ever Quit % | Mean Yrs Quit | Short Term (n) | Short Term % |
|---|---|---|---|---|---|---|---|---|---|
| 45 | 30-34 | Never Any | 0 | . | | | | | |
| 45 | 30-34 | CC | 0 | . | 0 | . | | | |
| 45 | 30-34 | Ecig | 0 | . | | | | | |
| 45 | 30-34 | Dual | 0 | . | | | | | |
| 45 | 30-34 | Former | 0 | . | | | . | 0 | . |

Year of Follow-up: 2035, Age Group: 35-39

| Period | Age Group | Smoking Group | N | % | Ever Quit (N) | Ever Quit % | Mean Yrs Quit | Short Term (n) | Short Term % |
|---|---|---|---|---|---|---|---|---|---|
| 45 | 35-39 | Never Any | 0 | . | | | | | |
| 45 | 35-39 | CC | 0 | . | 0 | . | | | |
| 45 | 35-39 | Ecig | 0 | . | | | | | |
| 45 | 35-39 | Dual | 0 | . | | | | | |
| 45 | 35-39 | Former | 0 | . | | | . | 0 | . |

Year of Follow-up: 2035, Age Group: 40-44

| Period | Age Group | Smoking Group | N | % | Ever Quit (N) | Ever Quit % | Mean Yrs Quit | Short Term (n) | Short Term % |
|---|---|---|---|---|---|---|---|---|---|
| 45 | 40-44 | Never Any | 0 | . | | | | | |
| 45 | 40-44 | CC | 0 | . | 0 | . | | | |
| 45 | 40-44 | Ecig | 0 | . | | | | | |
| 45 | 40-44 | Dual | 0 | . | | | | | |
| 45 | 40-44 | Former | 0 | . | | | . | 0 | . |

Year of Follow-up: 2035, Age Group: 45-49

| Period | Age Group | Smoking Group | N | % | Ever Quit (N) | Ever Quit % | Mean Yrs Quit | Short Term (n) | Short Term % |
|---|---|---|---|---|---|---|---|---|---|
| 45 | 45-49 | Never Any | 0 | . | | | | | |
| 45 | 45-49 | CC | 0 | . | 0 | . | | | |
| 45 | 45-49 | Ecig | 0 | . | | | | | |
| 45 | 45-49 | Dual | 0 | . | | | | | |
| 45 | 45-49 | Former | 0 | . | | | . | 0 | . |

Year of Follow-up: 2035, Age Group: 50-54

| Period | Age Group | Smoking Group | N | % | Ever Quit (N) | Ever Quit % | Mean Yrs Quit | Short Term (n) | Short Term % |
|---|---|---|---|---|---|---|---|---|---|
| 45 | 50-54 | Never Any | 0 | . | | | | | |
| 45 | 50-54 | CC | 0 | . | 0 | . | | | |
| 45 | 50-54 | Ecig | 0 | . | | | | | |
| 45 | 50-54 | Dual | 0 | . | | | | | |
| 45 | 50-54 | Former | 0 | . | | | . | 0 | . |

Year of Follow-up: 2035, Age Group: 55-59

| Period | Age Group | Smoking Group | N | % | Ever Quit (N) | Ever Quit % | Mean Yrs Quit | Short Term (n) | Short Term % |
|---|---|---|---|---|---|---|---|---|---|

# Results for PHIM for Run 2 (Sensitivity_Analysis_1.RTF)

| | | | | | | | | | |
|---|---|---|---|---|---|---|---|---|---|
| 45 | 55-59 | Never Any | 4726 | 59.60 | | | | | |
| 45 | 55-59 | CC | 0 | 0.00 | 0 | 0.00 | | | |
| 45 | 55-59 | Ecig | 792 | 9.99 | | | | | |
| 45 | 55-59 | Dual | 0 | 0.00 | | | | | |
| 45 | 55-59 | Former | 2412 | 30.42 | | | 24.49 | 64 | 2.65 |

Year of Follow-up: 2035, Age Group: 60-64

| Period | Age Group | Smoking Group | N | % | Ever Quit (N) | Ever Quit % | Mean Yrs Quit | Short Term (n) | Short Term % |
|---|---|---|---|---|---|---|---|---|---|
| 45 | 60-64 | Never Any | 5359 | 64.18 | | | | | |
| 45 | 60-64 | CC | 0 | 0.00 | 0 | 0.00 | | | |
| 45 | 60-64 | Ecig | 719 | 8.61 | | | | | |
| 45 | 60-64 | Dual | 0 | 0.00 | | | | | |
| 45 | 60-64 | Former | 2272 | 27.21 | | | 25.28 | 77 | 3.39 |

Year of Follow-up: 2035, Age Group: 65-69

| Period | Age Group | Smoking Group | N | % | Ever Quit (N) | Ever Quit % | Mean Yrs Quit | Short Term (n) | Short Term % |
|---|---|---|---|---|---|---|---|---|---|
| 45 | 65-69 | Never Any | 5189 | 59.01 | | | | | |
| 45 | 65-69 | CC | 3 | 0.03 | 0 | 0.00 | | | |
| 45 | 65-69 | Ecig | 694 | 7.89 | | | | | |
| 45 | 65-69 | Dual | 0 | 0.00 | | | | | |
| 45 | 65-69 | Former | 2907 | 33.06 | | | 28.66 | 55 | 1.89 |

Year of Follow-up: 2035, Age Group: 70-74

| Period | Age Group | Smoking Group | N | % | Ever Quit (N) | Ever Quit % | Mean Yrs Quit | Short Term (n) | Short Term % |
|---|---|---|---|---|---|---|---|---|---|
| 45 | 70-74 | Never Any | 5284 | 52.43 | | | | | |
| 45 | 70-74 | CC | 1 | 0.01 | 0 | 0.00 | | | |
| 45 | 70-74 | Ecig | 934 | 9.27 | | | | | |
| 45 | 70-74 | Dual | 0 | 0.00 | | | | | |
| 45 | 70-74 | Former | 3859 | 38.29 | | | 31.38 | 76 | 1.97 |

Year of Follow-up: 2035, Age Group: 75-79

| Period | Age Group | Smoking Group | N | % | Ever Quit (N) | Ever Quit % | Mean Yrs Quit | Short Term (n) | Short Term % |
|---|---|---|---|---|---|---|---|---|---|
| 45 | 75-79 | Never Any | 5278 | 50.27 | | | | | |
| 45 | 75-79 | CC | 1 | 0.01 | 0 | 0.00 | | | |
| 45 | 75-79 | Ecig | 747 | 7.11 | | | | | |
| 45 | 75-79 | Dual | 0 | 0.00 | | | | | |
| 45 | 75-79 | Former | 4473 | 42.60 | | | 33.73 | 79 | 1.77 |

Year of Follow-up: 2040, Age Group: All Ages

| Period | Age Group | Smoking Group | N | % | Ever Quit (N) | Ever Quit % | Mean Yrs Quit | Short Term (n) | Short Term % |
|---|---|---|---|---|---|---|---|---|---|
| 50 | All Ages | Never Any | 20558 | 58.48 | | | | | |
| 50 | All Ages | CC | 4 | 0.01 | 0 | 0.00 | | | |
| 50 | All Ages | Ecig | 2497 | 7.10 | | | | | |
| 50 | All Ages | Dual | 0 | 0.00 | | | | | |
| 50 | All Ages | Former | 12092 | 34.40 | | | 31.39 | 238 | 1.97 |

Year of Follow-up: 2040, Age Group: 18-19

| Period | Age Group | Smoking Group | N | % | Ever Quit (N) | Ever Quit % | Mean Yrs Quit | Short Term (n) | Short Term % |
|---|---|---|---|---|---|---|---|---|---|
| 50 | 18-19 | Never Any | 0 | . | | | | | |
| 50 | 18-19 | CC | 0 | . | 0 | . | | | |
| 50 | 18-19 | Ecig | 0 | . | | | | | |
| 50 | 18-19 | Dual | 0 | . | | | | | |
| 50 | 18-19 | Former | 0 | . | | | . | 0 | . |

Year of Follow-up: 2040, Age Group: 20-24

| Period | Age Group | Smoking Group | N | % | Ever Quit (N) | Ever Quit % | Mean Yrs Quit | Short Term (n) | Short Term % |
|---|---|---|---|---|---|---|---|---|---|
| 50 | 20-24 | Never Any | 0 | . | | | | | |
| 50 | 20-24 | CC | 0 | . | 0 | . | | | |
| 50 | 20-24 | Ecig | 0 | . | | | | | |
| 50 | 20-24 | Dual | 0 | . | | | | | |
| 50 | 20-24 | Former | 0 | . | | | . | 0 | . |

Year of Follow-up: 2040, Age Group: 25-29

| Period | Age Group | Smoking Group | N | % | Ever Quit (N) | Ever Quit % | Mean Yrs Quit | Short Term (n) | Short Term % |
|---|---|---|---|---|---|---|---|---|---|
| 50 | 25-29 | Never Any | 0 | . | | | | | |
| 50 | 25-29 | CC | 0 | . | 0 | . | | | |
| 50 | 25-29 | Ecig | 0 | . | | | | | |
| 50 | 25-29 | Dual | 0 | . | | | | | |
| 50 | 25-29 | Former | 0 | . | | | . | 0 | . |

Year of Follow-up: 2040, Age Group: 30-34

| Period | Age Group | Smoking Group | N | % | Ever Quit (N) | Ever Quit % | Mean Yrs Quit | Short Term (n) | Short Term % |
|---|---|---|---|---|---|---|---|---|---|
| 50 | 30-34 | Never Any | 0 | . | | | | | |
| 50 | 30-34 | CC | 0 | . | 0 | . | | | |
| 50 | 30-34 | Ecig | 0 | . | | | | | |
| 50 | 30-34 | Dual | 0 | . | | | | | |
| 50 | 30-34 | Former | 0 | . | | | . | 0 | . |

Year of Follow-up: 2040, Age Group: 35-39

# Results for PHIM for Run 2 (Sensitivity_Analysis_1.RTF)

| Period | Age Group | Smoking Group | N | % | Ever Quit (N) | Ever Quit % | Mean Yrs Quit | Short Term (n) | Short Term % |
|---|---|---|---|---|---|---|---|---|---|
| 50 | 35-39 | Never Any | 0 | . | | | | | |
| 50 | 35-39 | CC | 0 | . | 0 | . | | | |
| 50 | 35-39 | Ecig | 0 | . | | | | | |
| 50 | 35-39 | Dual | 0 | . | | | | | |
| 50 | 35-39 | Former | 0 | . | | | . | 0 | . |

Year of Follow-up: 2040, Age Group: 40-44

| Period | Age Group | Smoking Group | N | % | Ever Quit (N) | Ever Quit % | Mean Yrs Quit | Short Term (n) | Short Term % |
|---|---|---|---|---|---|---|---|---|---|
| 50 | 40-44 | Never Any | 0 | . | | | | | |
| 50 | 40-44 | CC | 0 | . | 0 | . | | | |
| 50 | 40-44 | Ecig | 0 | . | | | | | |
| 50 | 40-44 | Dual | 0 | . | | | | | |
| 50 | 40-44 | Former | 0 | . | | | . | 0 | . |

Year of Follow-up: 2040, Age Group: 45-49

| Period | Age Group | Smoking Group | N | % | Ever Quit (N) | Ever Quit % | Mean Yrs Quit | Short Term (n) | Short Term % |
|---|---|---|---|---|---|---|---|---|---|
| 50 | 45-49 | Never Any | 0 | . | | | | | |
| 50 | 45-49 | CC | 0 | . | 0 | . | | | |
| 50 | 45-49 | Ecig | 0 | . | | | | | |
| 50 | 45-49 | Dual | 0 | . | | | | | |
| 50 | 45-49 | Former | 0 | . | | | . | 0 | . |

Year of Follow-up: 2040, Age Group: 50-54

| Period | Age Group | Smoking Group | N | % | Ever Quit (N) | Ever Quit % | Mean Yrs Quit | Short Term (n) | Short Term % |
|---|---|---|---|---|---|---|---|---|---|
| 50 | 50-54 | Never Any | 0 | . | | | | | |
| 50 | 50-54 | CC | 0 | . | 0 | . | | | |
| 50 | 50-54 | Ecig | 0 | . | | | | | |
| 50 | 50-54 | Dual | 0 | . | | | | | |
| 50 | 50-54 | Former | 0 | . | | | . | 0 | . |

Year of Follow-up: 2040, Age Group: 55-59

| Period | Age Group | Smoking Group | N | % | Ever Quit (N) | Ever Quit % | Mean Yrs Quit | Short Term (n) | Short Term % |
|---|---|---|---|---|---|---|---|---|---|
| 50 | 55-59 | Never Any | 0 | . | | | | | |
| 50 | 55-59 | CC | 0 | . | 0 | . | | | |
| 50 | 55-59 | Ecig | 0 | . | | | | | |
| 50 | 55-59 | Dual | 0 | . | | | | | |
| 50 | 55-59 | Former | 0 | . | | | . | 0 | . |

Year of Follow-up: 2040, Age Group: 60-64

| Period | Age Group | Smoking Group | N | % | Ever Quit (N) | Ever Quit % | Mean Yrs Quit | Short Term (n) | Short Term % |
|---|---|---|---|---|---|---|---|---|---|
| 50 | 60-64 | Never Any | 4726 | 59.60 | | | | | |
| 50 | 60-64 | CC | 0 | 0.00 | 0 | 0.00 | | | |
| 50 | 60-64 | Ecig | 613 | 7.73 | | | | | |
| 50 | 60-64 | Dual | 0 | 0.00 | | | | | |
| 50 | 60-64 | Former | 2591 | 32.67 | | | 27.58 | 77 | 2.97 |

Year of Follow-up: 2040, Age Group: 65-69

| Period | Age Group | Smoking Group | N | % | Ever Quit (N) | Ever Quit % | Mean Yrs Quit | Short Term (n) | Short Term % |
|---|---|---|---|---|---|---|---|---|---|
| 50 | 65-69 | Never Any | 5359 | 64.18 | | | | | |
| 50 | 65-69 | CC | 0 | 0.00 | 0 | 0.00 | | | |
| 50 | 65-69 | Ecig | 578 | 6.92 | | | | | |
| 50 | 65-69 | Dual | 0 | 0.00 | | | | | |
| 50 | 65-69 | Former | 2413 | 28.90 | | | 28.67 | 34 | 1.41 |

Year of Follow-up: 2040, Age Group: 70-74

| Period | Age Group | Smoking Group | N | % | Ever Quit (N) | Ever Quit % | Mean Yrs Quit | Short Term (n) | Short Term % |
|---|---|---|---|---|---|---|---|---|---|
| 50 | 70-74 | Never Any | 5189 | 59.01 | | | | | |
| 50 | 70-74 | CC | 3 | 0.03 | 0 | 0.00 | | | |
| 50 | 70-74 | Ecig | 583 | 6.63 | | | | | |
| 50 | 70-74 | Dual | 0 | 0.00 | | | | | |
| 50 | 70-74 | Former | 3018 | 34.32 | | | 32.48 | 56 | 1.86 |

Year of Follow-up: 2040, Age Group: 75-79

| Period | Age Group | Smoking Group | N | % | Ever Quit (N) | Ever Quit % | Mean Yrs Quit | Short Term (n) | Short Term % |
|---|---|---|---|---|---|---|---|---|---|
| 50 | 75-79 | Never Any | 5284 | 52.43 | | | | | |
| 50 | 75-79 | CC | 1 | 0.01 | 0 | 0.00 | | | |
| 50 | 75-79 | Ecig | 723 | 7.17 | | | | | |
| 50 | 75-79 | Dual | 0 | 0.00 | | | | | |
| 50 | 75-79 | Former | 4070 | 40.38 | | | 34.61 | 71 | 1.74 |

# Results for PHIM for Run 2 (Sensitivity_Analysis_1.RTF)

************************************************************************************
**Main Results for E-Component for Run: 2, Simulation: 1, COD: Four Diseases Combined**

| **Variable Parameter** | **Value** |
|---|---|
| 1. Country | US (United States) |
| 2. Sex | F (Females) |
| 3. Year of start of process | 1990 |
| 4. Cause of Death | Four Diseases Combined |
| 5. Effective Dose NULL | CC:1 |
| 6. Effective Dose Ecig | CC:1 Ecig:0.4 Dual:0.7 |
| Note: | Death rates are per million population |

-----------------------------------------------------------------------------------------
Results for Age Groups Combined and Years combined, Population unadjusted for deaths in Ecig scenario,

| Age Group:All | | NULL | Ecig | | | %Drop | | NULL | Ecig | |
|---|---|---|---|---|---|---|---|---|---|---|
| Year | Number | Attrib | Attrib | Drop | %Drop | Attrib | Death | Attrib | Attrib | Drop |
| | Deaths | Deaths | Deaths | Deaths | Deaths | Deaths | Rate | Rates | Rates | Rates |
| All Comb | 9082078 | 2946250.006 | 2416528.159 | 529721.8 | 5.833 | 17.980 | 2493.45 | 808.881 | 663.448 | 145.433 |

-----------------------------------------------------------------------------------------
Results for Age Groups Combined, Population unadjusted for deaths in Ecig scenario,

| Age Group:All | | NULL | Ecig | | | %Drop | | NULL | Ecig | |
|---|---|---|---|---|---|---|---|---|---|---|
| Year | Number | Attrib | Attrib | Drop | %Drop | Attrib | Death | Attrib | Attrib | Drop |
| | Deaths | Deaths | Deaths | Deaths | Deaths | Deaths | Rate | Rates | Rates | Rates |
| 1990 | 199848 | 57524.822 | 57524.822 | 0.000 | 0.000 | 0.000 | 2926.84 | 842.471 | 842.471 | 0.000 |
| 1991 | 199162 | 59078.486 | 58970.930 | 107.556 | 0.054 | 0.182 | 2877.87 | 853.678 | 852.124 | 1.554 |
| 1992 | 198532 | 60494.150 | 60197.996 | 296.153 | 0.149 | 0.490 | 2829.84 | 862.271 | 858.050 | 4.221 |
| 1993 | 202512 | 62694.848 | 62121.218 | 573.629 | 0.283 | 0.915 | 2845.85 | 881.034 | 872.973 | 8.061 |
| 1994 | 200658 | 63202.598 | 62303.100 | 899.498 | 0.448 | 1.423 | 2777.87 | 874.966 | 862.513 | 12.452 |
| 1995 | 201023 | 63766.862 | 62470.595 | 1296.267 | 0.645 | 2.033 | 2739.80 | 869.097 | 851.430 | 17.667 |
| 1996 | 201180 | 64591.163 | 62826.784 | 1764.378 | 0.877 | 2.732 | 2706.36 | 868.908 | 845.173 | 23.735 |
| 1997 | 198639 | 64686.912 | 62494.562 | 2192.350 | 1.104 | 3.389 | 2635.18 | 858.147 | 829.063 | 29.084 |
| 1998 | 197573 | 65295.961 | 62604.665 | 2691.296 | 1.362 | 4.122 | 2584.18 | 854.046 | 818.845 | 35.201 |
| 1999 | 211329 | 67327.521 | 63882.307 | 3445.214 | 1.630 | 5.117 | 2726.91 | 868.771 | 824.315 | 44.456 |
| 2000 | 208496 | 67596.897 | 63587.436 | 4009.460 | 1.923 | 5.931 | 2656.96 | 861.419 | 810.325 | 51.094 |
| 2001 | 203606 | 67008.811 | 62422.143 | 4586.668 | 2.253 | 6.845 | 2565.88 | 844.458 | 786.656 | 57.802 |
| 2002 | 200828 | 67067.744 | 61829.255 | 5238.488 | 2.608 | 7.811 | 2506.09 | 836.925 | 771.555 | 65.370 |
| 2003 | 196352 | 66756.217 | 60940.712 | 5815.505 | 2.962 | 8.712 | 2427.31 | 825.244 | 753.353 | 71.892 |
| 2004 | 188160 | 65058.725 | 58775.962 | 6282.763 | 3.339 | 9.657 | 2302.83 | 796.234 | 719.342 | 76.893 |
| 2005 | 186416 | 65214.724 | 58303.979 | 6910.745 | 3.707 | 10.597 | 2256.27 | 789.321 | 705.677 | 83.644 |
| 2006 | 180088 | 63771.146 | 56405.526 | 7365.620 | 4.090 | 11.550 | 2160.67 | 765.117 | 676.745 | 88.372 |
| 2007 | 175644 | 63110.718 | 55287.415 | 7823.303 | 4.454 | 12.396 | 2086.36 | 749.650 | 656.722 | 92.928 |
| 2008 | 177570 | 63718.624 | 55209.170 | 8509.455 | 4.792 | 13.355 | 2086.39 | 748.674 | 648.690 | 99.983 |
| 2009 | 172806 | 62794.698 | 53868.398 | 8926.300 | 5.166 | 14.215 | 2007.57 | 729.515 | 625.814 | 103.701 |
| 2010 | 170489 | 61917.074 | 52583.313 | 9333.761 | 5.475 | 15.075 | 1957.97 | 711.083 | 603.890 | 107.193 |
| 2011 | 170305 | 61680.114 | 51747.558 | 9932.556 | 5.832 | 16.103 | 1937.46 | 701.700 | 588.703 | 112.997 |
| 2012 | 171421 | 62023.127 | 51461.914 | 10561.21 | 6.161 | 17.028 | 1930.22 | 698.386 | 579.466 | 118.920 |
| 2013 | 172699 | 62037.765 | 50878.198 | 11159.57 | 6.462 | 17.988 | 1923.75 | 691.059 | 566.749 | 124.310 |
| 2014 | 174169 | 61921.017 | 50122.614 | 11798.40 | 6.774 | 19.054 | 1918.85 | 682.195 | 552.210 | 129.985 |
| 2015 | 177354 | 61856.204 | 49543.255 | 12312.95 | 6.943 | 19.906 | 2184.51 | 761.899 | 610.237 | 151.662 |
| 2016 | 176853 | 60464.119 | 47732.844 | 12731.27 | 7.199 | 21.056 | 2157.59 | 737.655 | 582.335 | 155.320 |
| 2017 | 179309 | 60243.839 | 46928.227 | 13315.61 | 7.426 | 22.103 | 2166.86 | 728.017 | 567.104 | 160.912 |
| 2018 | 177157 | 60713.327 | 46904.288 | 13809.04 | 7.795 | 22.745 | 2120.51 | 726.721 | 561.431 | 165.290 |
| 2019 | 179227 | 61148.172 | 46669.922 | 14478.25 | 8.078 | 23.677 | 2124.53 | 724.841 | 553.218 | 171.623 |
| 2020 | 180467 | 61149.317 | 46307.966 | 14841.35 | 8.224 | 24.271 | 2424.25 | 821.432 | 622.065 | 199.367 |
| 2021 | 178116 | 58727.786 | 43663.462 | 15064.32 | 8.458 | 25.651 | 2375.42 | 783.216 | 582.312 | 200.903 |
| 2022 | 179408 | 58848.802 | 43295.615 | 15553.19 | 8.669 | 26.429 | 2374.52 | 778.881 | 573.030 | 205.851 |
| 2023 | 180640 | 58816.149 | 42838.573 | 15977.58 | 8.845 | 27.165 | 2372.26 | 772.407 | 562.580 | 209.827 |
| 2024 | 181610 | 58955.232 | 42474.245 | 16480.99 | 9.075 | 27.955 | 2366.77 | 768.316 | 553.533 | 214.783 |
| 2025 | 180418 | 58297.744 | 41656.125 | 16641.62 | 9.224 | 28.546 | 2713.69 | 876.861 | 626.553 | 250.308 |
| 2026 | 180313 | 56469.971 | 39686.346 | 16783.62 | 9.308 | 29.721 | 2701.83 | 846.150 | 594.663 | 251.487 |
| 2027 | 179619 | 56209.427 | 39032.859 | 17176.57 | 9.563 | 30.558 | 2680.68 | 838.883 | 582.536 | 256.347 |
| 2028 | 178762 | 55733.105 | 38292.762 | 17440.34 | 9.756 | 31.293 | 2656.74 | 828.298 | 569.102 | 259.196 |
| 2029 | 177808 | 55145.838 | 37458.905 | 17686.93 | 9.947 | 32.073 | 2630.89 | 815.951 | 554.251 | 261.700 |
| 2030 | 173587 | 53615.097 | 36066.003 | 17549.09 | 10.110 | 32.732 | 3040.33 | 939.056 | 631.688 | 307.368 |
| 2031 | 174985 | 52046.561 | 34661.629 | 17384.93 | 9.935 | 33.403 | 3060.73 | 910.364 | 606.278 | 304.086 |
| 2032 | 173400 | 51582.636 | 33997.205 | 17585.43 | 10.142 | 34.092 | 3027.10 | 900.496 | 593.501 | 306.995 |
| 2033 | 171756 | 50839.758 | 33238.439 | 17601.32 | 10.248 | 34.621 | 2991.14 | 885.375 | 578.847 | 306.527 |
| 2034 | 169973 | 50215.451 | 32531.236 | 17684.22 | 10.404 | 35.217 | 2951.96 | 872.104 | 564.978 | 307.126 |
| 2035 | 162644 | 47773.075 | 30726.459 | 17046.62 | 10.481 | 35.682 | 3459.14 | 1016.05 | 653.495 | 362.550 |
| 2036 | 156254 | 43298.709 | 27381.748 | 15916.96 | 10.187 | 36.761 | 3328.25 | 922.274 | 583.239 | 339.036 |
| 2037 | 153593 | 42241.563 | 26387.681 | 15853.88 | 10.322 | 37.531 | 3274.10 | 900.450 | 562.498 | 337.952 |
| 2038 | 150947 | 41356.353 | 25611.427 | 15744.93 | 10.431 | 38.071 | 3217.50 | 881.531 | 545.920 | 335.611 |
| 2039 | 148373 | 40161.050 | 24620.365 | 15540.68 | 10.474 | 38.696 | 3159.42 | 855.178 | 524.259 | 330.919 |

# Results for PHIM for Run 2 (Sensitivity_Analysis_1.RTF)

************************************************************************************

**Main Results for E-Component for Run: 2, Simulation: 1, COD: LUNG CANCER**

| Variable Parameter | Value |
|---|---|
| 1. Country | US (United States) |
| 2. Sex | F (Females) |
| 3. Year of start of process | 1990 |
| 4. Cause of Death | LUNG CANCER |
| 5. Effective Dose NULL | CC:1 |
| 6. Effective Dose Ecig | CC:1 Ecig:0.4 Dual:0.7 |
| Note: | Death rates are per million population |

-----------------------------------------------------------------------------------------

Results for Age Groups Combined and Years combined, Population unadjusted for deaths in Ecig scenario,

| Age Group:All | | NULL | Ecig | | | %Drop | | NULL | Ecig | |
|---|---|---|---|---|---|---|---|---|---|---|
| Year | Number | Attrib | Attrib | Drop | %Drop | Attrib | Death | Attrib | Attrib | Drop |
| | Deaths | Deaths | Deaths | Deaths | Deaths | Deaths | Rate | Rates | Rates | Rates |
| All Comb | 2327764 | 1580465.217 | 1373633.475 | 206831.7 | 8.885 | 13.087 | 639.078 | 433.910 | 377.126 | 56.785 |

-----------------------------------------------------------------------------------------

Results for Age Groups Combined, Population unadjusted for deaths in Ecig scenario,

| Age Group:All | | NULL | Ecig | | | %Drop | | NULL | Ecig | |
|---|---|---|---|---|---|---|---|---|---|---|
| Year | Number | Attrib | Attrib | Drop | %Drop | Attrib | Death | Attrib | Attrib | Drop |
| | Deaths | Deaths | Deaths | Deaths | Deaths | Deaths | Rate | Rates | Rates | Rates |
| 1990 | 42690 | 30614.201 | 30614.201 | 0.000 | 0.000 | 0.000 | 625.210 | 448.355 | 448.355 | 0.000 |
| 1991 | 43692 | 31412.010 | 31391.193 | 20.818 | 0.048 | 0.066 | 631.345 | 453.901 | 453.600 | 0.301 |
| 1992 | 45423 | 32782.407 | 32723.146 | 59.261 | 0.130 | 0.181 | 647.450 | 467.274 | 466.429 | 0.845 |
| 1993 | 46291 | 33491.183 | 33374.294 | 116.889 | 0.253 | 0.349 | 650.515 | 470.643 | 469.000 | 1.643 |
| 1994 | 47017 | 34074.347 | 33886.411 | 187.936 | 0.400 | 0.552 | 650.895 | 471.719 | 469.118 | 2.602 |
| 1995 | 47840 | 34694.454 | 34411.198 | 283.256 | 0.592 | 0.816 | 652.025 | 472.861 | 469.000 | 3.861 |
| 1996 | 48244 | 35027.269 | 34638.666 | 388.603 | 0.805 | 1.109 | 648.999 | 471.202 | 465.974 | 5.228 |
| 1997 | 49035 | 35590.763 | 35079.055 | 511.708 | 1.044 | 1.438 | 650.506 | 472.153 | 465.364 | 6.788 |
| 1998 | 49686 | 36100.785 | 35443.870 | 656.915 | 1.322 | 1.820 | 649.874 | 472.184 | 463.592 | 8.592 |
| 1999 | 48883 | 35451.030 | 34669.445 | 781.584 | 1.599 | 2.205 | 630.769 | 457.448 | 447.362 | 10.085 |
| 2000 | 50331 | 36447.986 | 35472.086 | 975.900 | 1.939 | 2.678 | 641.392 | 464.474 | 452.038 | 12.436 |
| 2001 | 50389 | 36411.326 | 35245.043 | 1166.283 | 2.315 | 3.203 | 635.012 | 458.863 | 444.165 | 14.698 |
| 2002 | 51445 | 37078.545 | 35676.866 | 1401.679 | 2.725 | 3.780 | 641.972 | 462.696 | 445.204 | 17.491 |
| 2003 | 51408 | 36999.876 | 35379.010 | 1620.866 | 3.153 | 4.381 | 635.509 | 457.395 | 437.357 | 20.037 |
| 2004 | 51399 | 36861.555 | 35005.896 | 1855.659 | 3.610 | 5.034 | 629.057 | 451.138 | 428.427 | 22.711 |
| 2005 | 51421 | 36689.529 | 34591.770 | 2097.759 | 4.080 | 5.718 | 622.370 | 444.069 | 418.679 | 25.390 |
| 2006 | 51355 | 36504.774 | 34160.217 | 2344.557 | 4.565 | 6.423 | 616.150 | 437.979 | 409.849 | 28.130 |
| 2007 | 51839 | 36663.202 | 34024.884 | 2638.318 | 5.089 | 7.196 | 615.761 | 435.498 | 404.159 | 31.339 |
| 2008 | 50976 | 35898.803 | 33056.642 | 2842.161 | 5.575 | 7.917 | 598.952 | 421.800 | 388.405 | 33.394 |
| 2009 | 51193 | 35934.525 | 32788.223 | 3146.302 | 6.146 | 8.756 | 594.733 | 417.468 | 380.916 | 36.552 |
| 2010 | 50853 | 35502.829 | 32130.836 | 3371.993 | 6.631 | 9.498 | 584.018 | 407.730 | 369.005 | 38.725 |
| 2011 | 50509 | 35136.342 | 31493.468 | 3642.873 | 7.212 | 10.368 | 574.613 | 399.726 | 358.284 | 41.443 |
| 2012 | 51245 | 35450.201 | 31480.586 | 3969.615 | 7.746 | 11.198 | 577.023 | 399.173 | 354.474 | 44.698 |
| 2013 | 51069 | 35123.224 | 30858.074 | 4265.149 | 8.352 | 12.143 | 568.874 | 391.249 | 343.738 | 47.511 |
| 2014 | 51251 | 35079.370 | 30508.580 | 4570.790 | 8.918 | 13.030 | 564.641 | 386.476 | 336.118 | 50.357 |
| 2015 | 50443 | 34324.171 | 29585.243 | 4738.928 | 9.395 | 13.806 | 621.319 | 422.780 | 364.409 | 58.371 |
| 2016 | 48854 | 33054.753 | 28168.638 | 4886.115 | 10.001 | 14.782 | 596.013 | 403.264 | 343.654 | 59.610 |
| 2017 | 48325 | 32471.234 | 27347.059 | 5124.175 | 10.604 | 15.781 | 583.983 | 392.399 | 330.475 | 61.923 |
| 2018 | 50465 | 33657.595 | 27967.874 | 5689.720 | 11.275 | 16.905 | 604.052 | 402.871 | 334.767 | 68.104 |
| 2019 | 51036 | 33863.424 | 27795.561 | 6067.863 | 11.889 | 17.919 | 604.975 | 401.412 | 329.484 | 71.928 |
| 2020 | 51444 | 33910.830 | 27574.470 | 6336.359 | 12.317 | 18.685 | 691.053 | 455.532 | 370.414 | 85.118 |
| 2021 | 48037 | 31531.880 | 25300.130 | 6231.750 | 12.973 | 19.763 | 640.639 | 420.521 | 337.412 | 83.109 |
| 2022 | 48335 | 31560.212 | 25040.680 | 6519.532 | 13.488 | 20.657 | 639.727 | 417.708 | 331.420 | 86.288 |
| 2023 | 48599 | 31508.781 | 24724.777 | 6784.005 | 13.959 | 21.531 | 638.227 | 413.791 | 324.700 | 89.091 |
| 2024 | 48784 | 31517.293 | 24447.513 | 7069.780 | 14.492 | 22.431 | 635.757 | 410.740 | 318.605 | 92.135 |
| 2025 | 48477 | 31175.389 | 23943.092 | 7232.297 | 14.919 | 23.199 | 729.141 | 468.912 | 360.130 | 108.782 |
| 2026 | 45324 | 29044.598 | 22033.598 | 7010.999 | 15.469 | 24.139 | 679.138 | 435.206 | 330.153 | 105.053 |
| 2027 | 45107 | 28825.765 | 21607.611 | 7218.153 | 16.002 | 25.041 | 673.191 | 430.203 | 322.477 | 107.725 |
| 2028 | 44842 | 28513.846 | 21142.352 | 7371.493 | 16.439 | 25.852 | 666.430 | 423.769 | 314.215 | 109.554 |
| 2029 | 44539 | 28148.109 | 20627.887 | 7520.221 | 16.884 | 26.717 | 659.013 | 416.486 | 305.215 | 111.271 |
| 2030 | 43393 | 27314.475 | 19809.966 | 7504.509 | 17.294 | 27.474 | 760.018 | 478.407 | 346.967 | 131.440 |
| 2031 | 40748 | 25423.102 | 18303.813 | 7119.289 | 17.472 | 28.003 | 712.731 | 444.684 | 320.158 | 124.526 |
| 2032 | 40325 | 25085.902 | 17878.842 | 7207.060 | 17.872 | 28.730 | 703.969 | 437.933 | 312.117 | 125.816 |
| 2033 | 39882 | 24645.067 | 17418.485 | 7226.581 | 18.120 | 29.323 | 694.543 | 429.194 | 303.343 | 125.851 |
| 2034 | 39404 | 24239.126 | 16981.108 | 7258.018 | 18.419 | 29.943 | 684.347 | 420.967 | 294.915 | 126.052 |
| 2035 | 37454 | 22944.147 | 15942.846 | 7001.300 | 18.693 | 30.515 | 796.588 | 487.980 | 339.075 | 148.905 |
| 2036 | 32988 | 19950.581 | 13698.778 | 6251.802 | 18.951 | 31.336 | 702.663 | 424.953 | 291.788 | 133.165 |
| 2037 | 32403 | 19415.334 | 13179.607 | 6235.727 | 19.244 | 32.118 | 690.731 | 413.871 | 280.946 | 132.925 |
| 2038 | 31820 | 18939.317 | 12757.790 | 6181.527 | 19.427 | 32.639 | 678.257 | 403.701 | 271.939 | 131.762 |
| 2039 | 31251 | 18379.752 | 12252.089 | 6127.663 | 19.608 | 33.339 | 665.457 | 391.373 | 260.892 | 130.481 |

# Results for PHIM for Run 2 (Sensitivity_Analysis_1.RTF)

************************************************************************************

**Main Results for E-Component for Run: 2, Simulation: 1, COD: IHD**

| **Variable Parameter** | **Value** |
|---|---|
| 1. Country | US (United States) |
| 2. Sex | F (Females) |
| 3. Year of start of process | 1990 |
| 4. Cause of Death | IHD |
| 5. Effective Dose NULL | CC:1 |
| 6. Effective Dose Ecig | CC:1 Ecig:0.4 Dual:0.7 |
| Note: | Death rates are per million population |

-----------------------------------------------------------------------------------------

Results for Age Groups Combined and Years combined, Population unadjusted for deaths in Ecig scenario,

| Age Group:All | | NULL | Ecig | | | %Drop | | NULL | Ecig | |
|---|---|---|---|---|---|---|---|---|---|---|
| Year | Number | Attrib | Attrib | Drop | %Drop | Attrib | Death | Attrib | Attrib | Drop |
| | Deaths | Deaths | Deaths | Deaths | Deaths | Deaths | Rate | Rates | Rates | Rates |
| All Comb | 3242167 | 398833.796 | 282177.202 | 116656.6 | 3.598 | 29.249 | 890.124 | 109.498 | 77.471 | 32.028 |

-----------------------------------------------------------------------------------------

Results for Age Groups Combined, Population unadjusted for deaths in Ecig scenario,

| Age Group:All | | NULL | Ecig | | | %Drop | | NULL | Ecig | |
|---|---|---|---|---|---|---|---|---|---|---|
| Year | Number | Attrib | Attrib | Drop | %Drop | Attrib | Death | Attrib | Attrib | Drop |
| | Deaths | Deaths | Deaths | Deaths | Deaths | Deaths | Rate | Rates | Rates | Rates |
| 1990 | 98916 | 12922.892 | 12922.892 | 0.000 | 0.000 | 0.000 | 1448.66 | 189.260 | 189.260 | 0.000 |
| 1991 | 96403 | 12856.479 | 12799.224 | 57.256 | 0.059 | 0.445 | 1393.01 | 185.775 | 184.948 | 0.827 |
| 1992 | 93721 | 12662.244 | 12505.888 | 156.355 | 0.167 | 1.235 | 1335.88 | 180.485 | 178.256 | 2.229 |
| 1993 | 93679 | 12730.613 | 12434.248 | 296.365 | 0.316 | 2.328 | 1316.45 | 178.900 | 174.735 | 4.165 |
| 1994 | 90832 | 12495.323 | 12037.119 | 458.204 | 0.504 | 3.667 | 1257.46 | 172.983 | 166.640 | 6.343 |
| 1995 | 89698 | 12365.590 | 11721.075 | 644.515 | 0.719 | 5.212 | 1222.52 | 168.534 | 159.750 | 8.784 |
| 1996 | 88286 | 12290.551 | 11418.114 | 872.437 | 0.988 | 7.098 | 1187.66 | 165.338 | 153.601 | 11.736 |
| 1997 | 84777 | 11719.531 | 10676.301 | 1043.230 | 1.231 | 8.902 | 1124.67 | 155.473 | 141.634 | 13.840 |
| 1998 | 82517 | 11416.794 | 10174.253 | 1242.541 | 1.506 | 10.883 | 1079.29 | 149.327 | 133.075 | 16.252 |
| 1999 | 93939 | 13028.038 | 11329.161 | 1698.877 | 1.808 | 13.040 | 1212.16 | 168.109 | 146.187 | 21.922 |
| 2000 | 90039 | 12426.299 | 10533.572 | 1892.727 | 2.102 | 15.232 | 1147.41 | 158.354 | 134.234 | 24.120 |
| 2001 | 86119 | 11939.281 | 9817.779 | 2121.502 | 2.463 | 17.769 | 1085.29 | 150.461 | 123.726 | 26.736 |
| 2002 | 83014 | 11528.794 | 9178.591 | 2350.203 | 2.831 | 20.386 | 1035.91 | 143.865 | 114.538 | 29.328 |
| 2003 | 79302 | 11048.407 | 8529.482 | 2518.924 | 3.176 | 22.799 | 980.336 | 136.581 | 105.442 | 31.139 |
| 2004 | 73861 | 10375.132 | 7746.374 | 2628.757 | 3.559 | 25.337 | 903.963 | 126.978 | 94.806 | 32.173 |
| 2005 | 71649 | 10051.029 | 7275.788 | 2775.241 | 3.873 | 27.612 | 867.197 | 121.652 | 88.062 | 33.590 |
| 2006 | 67551 | 9542.523 | 6675.027 | 2867.496 | 4.245 | 30.050 | 810.467 | 114.490 | 80.086 | 34.404 |
| 2007 | 63503 | 8936.533 | 6061.523 | 2875.010 | 4.527 | 32.171 | 754.310 | 106.151 | 72.001 | 34.150 |
| 2008 | 63171 | 8944.180 | 5883.614 | 3060.566 | 4.845 | 34.219 | 742.239 | 105.091 | 69.131 | 35.961 |
| 2009 | 59747 | 8390.033 | 5371.045 | 3018.988 | 5.053 | 35.983 | 694.108 | 97.471 | 62.398 | 35.073 |
| 2010 | 58008 | 8157.570 | 5089.817 | 3067.752 | 5.288 | 37.606 | 666.189 | 93.685 | 58.454 | 35.231 |
| 2011 | 57368 | 8035.356 | 4868.251 | 3167.104 | 5.521 | 39.415 | 652.644 | 91.414 | 55.383 | 36.030 |
| 2012 | 57411 | 7970.766 | 4694.893 | 3275.872 | 5.706 | 41.099 | 646.453 | 89.752 | 52.865 | 36.887 |
| 2013 | 57067 | 7743.216 | 4450.745 | 3292.471 | 5.769 | 42.521 | 635.688 | 86.254 | 49.578 | 36.676 |
| 2014 | 57538 | 7774.935 | 4359.371 | 3415.564 | 5.936 | 43.930 | 633.906 | 85.658 | 48.028 | 37.630 |
| 2015 | 58395 | 7722.535 | 4254.205 | 3468.329 | 5.939 | 44.912 | 719.266 | 95.120 | 52.400 | 42.720 |
| 2016 | 58658 | 7600.353 | 4091.397 | 3508.956 | 5.982 | 46.168 | 715.621 | 92.723 | 49.915 | 42.809 |
| 2017 | 59004 | 7470.266 | 3943.068 | 3527.198 | 5.978 | 47.216 | 713.034 | 90.274 | 47.650 | 42.624 |
| 2018 | 56077 | 6907.050 | 3576.073 | 3330.977 | 5.940 | 48.226 | 671.224 | 82.675 | 42.805 | 39.871 |
| 2019 | 55886 | 6732.046 | 3429.648 | 3302.399 | 5.909 | 49.055 | 662.462 | 79.801 | 40.655 | 39.146 |
| 2020 | 55313 | 6429.836 | 3231.714 | 3198.121 | 5.782 | 49.739 | 743.037 | 86.373 | 43.412 | 42.961 |
| 2021 | 56292 | 6562.016 | 3236.875 | 3325.141 | 5.907 | 50.673 | 750.730 | 87.513 | 43.168 | 44.345 |
| 2022 | 55827 | 6352.366 | 3093.251 | 3259.115 | 5.838 | 51.306 | 738.885 | 84.075 | 40.940 | 43.135 |
| 2023 | 55338 | 6134.123 | 2960.352 | 3173.772 | 5.735 | 51.740 | 726.728 | 80.557 | 38.877 | 41.680 |
| 2024 | 54770 | 5922.193 | 2829.562 | 3092.630 | 5.647 | 52.221 | 713.771 | 77.179 | 36.875 | 40.304 |
| 2025 | 53295 | 5504.188 | 2601.482 | 2902.706 | 5.447 | 52.736 | 801.612 | 82.789 | 39.129 | 43.660 |
| 2026 | 55278 | 5641.075 | 2643.898 | 2997.178 | 5.422 | 53.131 | 828.283 | 84.526 | 39.616 | 44.910 |
| 2027 | 54199 | 5426.216 | 2523.436 | 2902.780 | 5.356 | 53.495 | 808.875 | 80.982 | 37.660 | 43.322 |
| 2028 | 53090 | 5197.830 | 2405.304 | 2792.526 | 5.260 | 53.725 | 789.020 | 77.249 | 35.747 | 41.502 |
| 2029 | 51974 | 4973.547 | 2291.453 | 2682.093 | 5.160 | 53.927 | 769.023 | 73.590 | 33.905 | 39.685 |
| 2030 | 49479 | 4450.618 | 2017.318 | 2433.300 | 4.918 | 54.673 | 866.605 | 77.952 | 35.333 | 42.619 |
| 2031 | 51987 | 4555.052 | 2064.934 | 2490.119 | 4.790 | 54.667 | 909.317 | 79.674 | 36.118 | 43.555 |
| 2032 | 50677 | 4349.999 | 1965.830 | 2384.169 | 4.705 | 54.809 | 884.689 | 75.939 | 34.318 | 41.621 |
| 2033 | 49380 | 4147.948 | 1877.256 | 2270.692 | 4.598 | 54.743 | 859.955 | 72.237 | 32.692 | 39.544 |
| 2034 | 48076 | 3966.133 | 1790.237 | 2175.896 | 4.526 | 54.862 | 834.943 | 68.881 | 31.091 | 37.789 |
| 2035 | 44518 | 3289.064 | 1455.751 | 1833.314 | 4.118 | 55.740 | 946.825 | 69.952 | 30.961 | 38.991 |
| 2036 | 46347 | 3313.192 | 1456.764 | 1856.428 | 4.005 | 56.031 | 987.211 | 70.572 | 31.029 | 39.542 |
| 2037 | 44841 | 3117.951 | 1368.495 | 1749.456 | 3.901 | 56.109 | 955.859 | 66.464 | 29.172 | 37.293 |
| 2038 | 43378 | 2949.775 | 1295.726 | 1654.049 | 3.813 | 56.074 | 924.621 | 62.876 | 27.619 | 35.257 |
| 2039 | 41973 | 2766.312 | 1219.025 | 1547.287 | 3.686 | 55.933 | 893.753 | 58.905 | 25.958 | 32.947 |

# Results for PHIM for Run 2 (Sensitivity_Analysis_1.RTF)

*********************************************************************************
**Main Results for E-Component for Run: 2, Simulation: 1, COD: STROKE**

| Variable Parameter | Value |
|---|---|
| 1. Country | US (United States) |
| 2. Sex | F (Females) |
| 3. Year of start of process | 1990 |
| 4. Cause of Death | STROKE |
| 5. Effective Dose NULL | CC:1 |
| 6. Effective Dose Ecig | CC:1 Ecig:0.4 Dual:0.7 |
| Note: | Death rates are per million population |

-----------------------------------------------------------------------------------------
Results for Age Groups Combined and Years combined, Population unadjusted for deaths in Ecig scenario,

| Age Group:All | | NULL | Ecig | | | %Drop | | NULL | Ecig | |
|---|---|---|---|---|---|---|---|---|---|---|
| Year | Number | Attrib | Attrib | Drop | %Drop | Attrib | Death | Attrib | Attrib | Drop |
| | Deaths | Deaths | Deaths | Deaths | Deaths | Deaths | Rate | Rates | Rates | Rates |
| All Comb | 1352207 | 104899.750 | 72985.607 | 31914.14 | 2.360 | 30.423 | 371.243 | 28.800 | 20.038 | 8.762 |

-----------------------------------------------------------------------------------------
Results for Age Groups Combined, Population unadjusted for deaths in Ecig scenario,

| Age Group:All | | NULL | Ecig | | | %Drop | | NULL | Ecig | |
|---|---|---|---|---|---|---|---|---|---|---|
| Year | Number | Attrib | Attrib | Drop | %Drop | Attrib | Death | Attrib | Attrib | Drop |
| | Deaths | Deaths | Deaths | Deaths | Deaths | Deaths | Rate | Rates | Rates | Rates |
| 1990 | 33504 | 3293.017 | 3293.017 | 0.000 | 0.000 | 0.000 | 490.678 | 48.227 | 48.227 | 0.000 |
| 1991 | 32505 | 3207.063 | 3189.553 | 17.510 | 0.054 | 0.546 | 469.694 | 46.342 | 46.089 | 0.253 |
| 1992 | 32560 | 3218.125 | 3169.595 | 48.529 | 0.149 | 1.508 | 464.104 | 45.871 | 45.179 | 0.692 |
| 1993 | 32768 | 3226.680 | 3135.233 | 91.447 | 0.279 | 2.834 | 460.480 | 45.344 | 44.059 | 1.285 |
| 1994 | 33005 | 3277.266 | 3132.503 | 144.763 | 0.439 | 4.417 | 456.915 | 45.370 | 43.366 | 2.004 |
| 1995 | 33730 | 3309.378 | 3102.802 | 206.576 | 0.612 | 6.242 | 459.716 | 45.104 | 42.289 | 2.815 |
| 1996 | 33750 | 3312.987 | 3034.184 | 278.802 | 0.826 | 8.415 | 454.019 | 44.568 | 40.817 | 3.751 |
| 1997 | 33699 | 3267.066 | 2919.678 | 347.388 | 1.031 | 10.633 | 447.056 | 43.341 | 38.733 | 4.609 |
| 1998 | 33274 | 3178.725 | 2765.267 | 413.458 | 1.243 | 13.007 | 435.211 | 41.577 | 36.169 | 5.408 |
| 1999 | 33891 | 3120.351 | 2640.822 | 479.529 | 1.415 | 15.368 | 437.317 | 40.264 | 34.076 | 6.188 |
| 2000 | 33827 | 3142.808 | 2583.369 | 559.439 | 1.654 | 17.801 | 431.074 | 40.050 | 32.921 | 7.129 |
| 2001 | 32506 | 2961.588 | 2362.778 | 598.810 | 1.842 | 20.219 | 409.647 | 37.323 | 29.776 | 7.546 |
| 2002 | 32078 | 2940.627 | 2267.671 | 672.957 | 2.098 | 22.885 | 400.295 | 36.695 | 28.298 | 8.398 |
| 2003 | 30568 | 2819.545 | 2105.910 | 713.635 | 2.335 | 25.310 | 377.883 | 34.855 | 26.033 | 8.822 |
| 2004 | 29388 | 2730.466 | 1972.530 | 757.936 | 2.579 | 27.758 | 359.671 | 33.417 | 24.141 | 9.276 |
| 2005 | 27954 | 2670.510 | 1871.750 | 798.760 | 2.857 | 29.910 | 338.339 | 32.322 | 22.655 | 9.668 |
| 2006 | 27201 | 2623.285 | 1782.189 | 841.096 | 3.092 | 32.063 | 326.354 | 31.474 | 21.382 | 10.091 |
| 2007 | 26302 | 2536.599 | 1674.241 | 862.358 | 3.279 | 33.997 | 312.424 | 30.131 | 19.887 | 10.243 |
| 2008 | 25998 | 2469.443 | 1584.294 | 885.150 | 3.405 | 35.844 | 305.468 | 29.015 | 18.615 | 10.400 |
| 2009 | 25040 | 2386.945 | 1491.204 | 895.741 | 3.577 | 37.527 | 290.901 | 27.730 | 17.324 | 10.406 |
| 2010 | 24840 | 2329.274 | 1419.583 | 909.691 | 3.662 | 39.055 | 285.273 | 26.750 | 16.303 | 10.447 |
| 2011 | 24792 | 2286.581 | 1356.572 | 930.009 | 3.751 | 40.672 | 282.045 | 26.013 | 15.433 | 10.580 |
| 2012 | 24500 | 2243.311 | 1296.194 | 947.117 | 3.866 | 42.220 | 275.872 | 25.260 | 14.595 | 10.665 |
| 2013 | 24451 | 2176.503 | 1225.545 | 950.958 | 3.889 | 43.692 | 272.368 | 24.245 | 13.652 | 10.593 |
| 2014 | 25296 | 2211.619 | 1215.884 | 995.735 | 3.936 | 45.023 | 278.691 | 24.366 | 13.396 | 10.970 |
| 2015 | 26316 | 2237.016 | 1209.202 | 1027.814 | 3.906 | 45.946 | 324.141 | 27.554 | 14.894 | 12.660 |
| 2016 | 26735 | 2229.057 | 1182.466 | 1046.592 | 3.915 | 46.952 | 326.164 | 27.194 | 14.426 | 12.768 |
| 2017 | 27616 | 2221.649 | 1158.439 | 1063.210 | 3.850 | 47.857 | 333.726 | 26.848 | 13.999 | 12.848 |
| 2018 | 25256 | 1939.420 | 995.973 | 943.447 | 3.736 | 48.646 | 302.304 | 23.214 | 11.921 | 11.293 |
| 2019 | 25429 | 1904.400 | 962.171 | 942.229 | 3.705 | 49.476 | 301.434 | 22.574 | 11.405 | 11.169 |
| 2020 | 25379 | 1806.054 | 900.119 | 905.936 | 3.570 | 50.161 | 340.922 | 24.261 | 12.091 | 12.170 |
| 2021 | 25448 | 1748.172 | 855.863 | 892.309 | 3.506 | 51.042 | 339.386 | 23.314 | 11.414 | 11.900 |
| 2022 | 25539 | 1701.148 | 822.310 | 878.838 | 3.441 | 51.661 | 338.015 | 22.515 | 10.883 | 11.632 |
| 2023 | 25620 | 1652.087 | 792.260 | 859.827 | 3.356 | 52.045 | 336.461 | 21.696 | 10.404 | 11.292 |
| 2024 | 25659 | 1597.127 | 758.076 | 839.051 | 3.270 | 52.535 | 334.391 | 20.814 | 9.879 | 10.935 |
| 2025 | 25260 | 1467.622 | 688.871 | 778.751 | 3.083 | 53.062 | 379.931 | 22.075 | 10.361 | 11.713 |
| 2026 | 25480 | 1390.505 | 647.754 | 742.752 | 2.915 | 53.416 | 381.790 | 20.835 | 9.706 | 11.129 |
| 2027 | 25264 | 1342.095 | 620.167 | 721.928 | 2.858 | 53.791 | 377.042 | 20.030 | 9.256 | 10.774 |
| 2028 | 25022 | 1289.536 | 592.945 | 696.591 | 2.784 | 54.019 | 371.870 | 19.165 | 8.812 | 10.353 |
| 2029 | 24767 | 1242.216 | 568.354 | 673.862 | 2.721 | 54.247 | 366.459 | 18.380 | 8.410 | 9.971 |
| 2030 | 23959 | 1096.803 | 493.566 | 603.237 | 2.518 | 55.000 | 419.641 | 19.210 | 8.645 | 10.566 |
| 2031 | 23963 | 1019.991 | 458.796 | 561.195 | 2.342 | 55.020 | 419.142 | 17.841 | 8.025 | 9.816 |
| 2032 | 23615 | 977.406 | 438.192 | 539.214 | 2.283 | 55.168 | 412.259 | 17.063 | 7.650 | 9.413 |
| 2033 | 23259 | 937.450 | 420.538 | 516.912 | 2.222 | 55.140 | 405.051 | 16.326 | 7.324 | 9.002 |
| 2034 | 22879 | 900.763 | 402.616 | 498.147 | 2.177 | 55.303 | 397.339 | 15.644 | 6.992 | 8.651 |
| 2035 | 21733 | 745.023 | 328.547 | 416.476 | 1.916 | 55.901 | 462.224 | 15.845 | 6.988 | 8.858 |
| 2036 | 20904 | 680.360 | 296.763 | 383.597 | 1.835 | 56.381 | 445.263 | 14.492 | 6.321 | 8.171 |
| 2037 | 20397 | 644.109 | 280.383 | 363.726 | 1.783 | 56.470 | 434.791 | 13.730 | 5.977 | 7.753 |
| 2038 | 19890 | 612.448 | 266.542 | 345.906 | 1.739 | 56.479 | 423.966 | 13.055 | 5.681 | 7.373 |
| 2039 | 19392 | 577.529 | 252.327 | 325.202 | 1.677 | 56.309 | 412.930 | 12.298 | 5.373 | 6.925 |

# Results for PHIM for Run 2 (Sensitivity_Analysis_1.RTF)

********************************************************************************

**Main Results for E-Component for Run: 2, Simulation: 1, COD: COPD**

| Variable Parameter | Value |
|---|---|
| 1. Country | US (United States) |
| 2. Sex | F (Females) |
| 3. Year of start of process | 1990 |
| 4. Cause of Death | COPD |
| 5. Effective Dose NULL | CC:1 |
| 6. Effective Dose Ecig | CC:1 Ecig:0.4 Dual:0.7 |
| Note: | Death rates are per million population |

-------------------------------------------------------------------------------------

Results for Age Groups Combined and Years combined, Population unadjusted for deaths in Ecig scenario,

| Age Group:All | | NULL | Ecig | | | %Drop | | NULL | Ecig | |
|---|---|---|---|---|---|---|---|---|---|---|
| Year | Number Deaths | Attrib Deaths | Attrib Deaths | Drop Deaths | %Drop Deaths | Attrib Deaths | Death Rate | Attrib Rates | Attrib Rates | Drop Rates |
| All Comb | 2159941 | 862051.244 | 687731.875 | 174319.4 | 8.071 | 20.221 | 593.003 | 236.673 | 188.814 | 47.859 |

-------------------------------------------------------------------------------------

Results for Age Groups Combined, Population unadjusted for deaths in Ecig scenario,

| Age Group:All | | NULL | Ecig | | | %Drop | | NULL | Ecig | |
|---|---|---|---|---|---|---|---|---|---|---|
| Year | Number Deaths | Attrib Deaths | Attrib Deaths | Drop Deaths | %Drop Deaths | Attrib Deaths | Death Rate | Attrib Rates | Attrib Rates | Drop Rates |
| 1990 | 24738 | 10694.712 | 10694.712 | 0.000 | 0.000 | 0.000 | 362.296 | 156.628 | 156.628 | 0.000 |
| 1991 | 26562 | 11602.933 | 11590.960 | 11.972 | 0.045 | 0.103 | 383.818 | 167.661 | 167.488 | 0.173 |
| 1992 | 26828 | 11831.375 | 11799.367 | 32.008 | 0.119 | 0.271 | 382.401 | 168.642 | 168.186 | 0.456 |
| 1993 | 29774 | 13246.371 | 13177.443 | 68.928 | 0.232 | 0.520 | 418.406 | 186.148 | 185.179 | 0.969 |
| 1994 | 29804 | 13355.662 | 13247.068 | 108.594 | 0.364 | 0.813 | 412.601 | 184.893 | 183.390 | 1.503 |
| 1995 | 29755 | 13397.439 | 13235.521 | 161.919 | 0.544 | 1.209 | 405.540 | 182.598 | 180.391 | 2.207 |
| 1996 | 30900 | 13960.356 | 13735.820 | 224.536 | 0.727 | 1.608 | 415.680 | 187.801 | 184.780 | 3.021 |
| 1997 | 31128 | 14109.552 | 13819.528 | 290.024 | 0.932 | 2.056 | 412.949 | 187.180 | 183.332 | 3.848 |
| 1998 | 32096 | 14599.657 | 14221.275 | 378.383 | 1.179 | 2.592 | 419.803 | 190.958 | 186.009 | 4.949 |
| 1999 | 34616 | 15728.102 | 15242.878 | 485.224 | 1.402 | 3.085 | 446.673 | 202.950 | 196.689 | 6.261 |
| 2000 | 34299 | 15579.803 | 14998.410 | 581.393 | 1.695 | 3.732 | 437.088 | 198.541 | 191.132 | 7.409 |
| 2001 | 34592 | 15696.614 | 14996.542 | 700.072 | 2.024 | 4.460 | 435.935 | 197.812 | 188.989 | 8.822 |
| 2002 | 34291 | 15519.777 | 14706.128 | 813.649 | 2.373 | 5.243 | 427.910 | 193.668 | 183.515 | 10.153 |
| 2003 | 35074 | 15888.389 | 14926.310 | 962.080 | 2.743 | 6.055 | 433.587 | 196.413 | 184.520 | 11.893 |
| 2004 | 33512 | 15091.573 | 14051.162 | 1040.411 | 3.105 | 6.894 | 410.143 | 184.701 | 171.968 | 12.733 |
| 2005 | 35392 | 15803.656 | 14564.672 | 1238.984 | 3.501 | 7.840 | 428.364 | 191.278 | 176.282 | 14.996 |
| 2006 | 33981 | 15100.563 | 13788.093 | 1312.471 | 3.862 | 8.692 | 407.699 | 181.174 | 165.428 | 15.747 |
| 2007 | 34000 | 14974.384 | 13526.766 | 1447.618 | 4.258 | 9.667 | 403.863 | 177.871 | 160.675 | 17.195 |
| 2008 | 37425 | 16406.197 | 14684.619 | 1721.578 | 4.600 | 10.493 | 439.732 | 192.768 | 172.540 | 20.228 |
| 2009 | 36826 | 16083.194 | 14217.926 | 1865.268 | 5.065 | 11.598 | 427.825 | 186.846 | 165.176 | 21.670 |
| 2010 | 36788 | 15927.400 | 13943.077 | 1984.324 | 5.394 | 12.459 | 422.489 | 182.917 | 160.128 | 22.789 |
| 2011 | 37636 | 16221.836 | 14029.266 | 2192.570 | 5.826 | 13.516 | 428.164 | 184.547 | 159.603 | 24.944 |
| 2012 | 38265 | 16358.849 | 13990.241 | 2368.609 | 6.190 | 14.479 | 430.867 | 184.202 | 157.531 | 26.671 |
| 2013 | 40112 | 16994.822 | 14343.834 | 2650.988 | 6.609 | 15.599 | 446.821 | 189.311 | 159.781 | 29.530 |
| 2014 | 40084 | 16855.093 | 14038.779 | 2816.314 | 7.026 | 16.709 | 441.613 | 185.696 | 154.668 | 31.028 |
| 2015 | 42200 | 17572.483 | 14494.605 | 3077.878 | 7.294 | 17.515 | 519.788 | 216.445 | 178.534 | 37.911 |
| 2016 | 42606 | 17579.955 | 14290.343 | 3289.612 | 7.721 | 18.712 | 519.788 | 214.473 | 174.341 | 40.133 |
| 2017 | 44364 | 18080.688 | 14479.661 | 3601.028 | 8.117 | 19.916 | 536.117 | 218.496 | 174.979 | 43.517 |
| 2018 | 45359 | 18209.262 | 14364.368 | 3844.894 | 8.477 | 21.115 | 542.932 | 217.960 | 171.937 | 46.022 |
| 2019 | 46876 | 18648.302 | 14482.542 | 4165.760 | 8.887 | 22.339 | 555.657 | 221.054 | 171.674 | 49.380 |
| 2020 | 48331 | 19002.597 | 14601.663 | 4400.934 | 9.106 | 23.160 | 649.239 | 255.266 | 196.147 | 59.119 |
| 2021 | 48338 | 18885.717 | 14270.594 | 4615.123 | 9.548 | 24.437 | 644.660 | 251.867 | 190.318 | 61.549 |
| 2022 | 49708 | 19235.076 | 14339.375 | 4895.702 | 9.849 | 25.452 | 657.895 | 254.582 | 189.786 | 64.796 |
| 2023 | 51083 | 19521.158 | 14361.185 | 5159.973 | 10.101 | 26.433 | 670.849 | 256.363 | 188.599 | 67.764 |
| 2024 | 52397 | 19918.620 | 14439.094 | 5479.526 | 10.458 | 27.510 | 682.854 | 259.583 | 188.173 | 71.410 |
| 2025 | 53387 | 20150.545 | 14422.680 | 5727.865 | 10.729 | 28.425 | 803.004 | 303.086 | 216.933 | 86.153 |
| 2026 | 54232 | 20393.792 | 14361.097 | 6032.696 | 11.124 | 29.581 | 812.615 | 305.582 | 215.188 | 90.394 |
| 2027 | 55049 | 20615.351 | 14281.644 | 6333.707 | 11.506 | 30.723 | 821.568 | 307.668 | 213.143 | 94.526 |
| 2028 | 55809 | 20731.894 | 14152.161 | 6579.733 | 11.790 | 31.737 | 829.419 | 308.115 | 210.328 | 97.787 |
| 2029 | 56527 | 20781.967 | 13971.209 | 6810.758 | 12.049 | 32.772 | 836.392 | 307.495 | 206.721 | 100.774 |
| 2030 | 56756 | 20753.201 | 13745.153 | 7008.048 | 12.348 | 33.769 | 994.067 | 363.487 | 240.743 | 122.744 |
| 2031 | 58288 | 21048.417 | 13834.087 | 7214.330 | 12.377 | 34.275 | 1019.54 | 368.165 | 241.977 | 126.188 |
| 2032 | 58782 | 21169.329 | 13714.341 | 7454.988 | 12.682 | 35.216 | 1026.18 | 369.560 | 239.416 | 130.144 |
| 2033 | 59236 | 21109.293 | 13522.160 | 7587.134 | 12.808 | 35.942 | 1031.59 | 367.618 | 235.488 | 132.130 |
| 2034 | 59614 | 21109.429 | 13357.274 | 7752.155 | 13.004 | 36.724 | 1035.33 | 366.613 | 231.979 | 134.634 |
| 2035 | 58938 | 20794.840 | 12999.315 | 7795.525 | 13.227 | 37.488 | 1253.51 | 442.268 | 276.472 | 165.797 |
| 2036 | 56014 | 19354.577 | 11929.443 | 7425.134 | 13.256 | 38.364 | 1193.11 | 412.258 | 254.100 | 158.157 |
| 2037 | 55953 | 19064.169 | 11559.196 | 7504.974 | 13.413 | 39.367 | 1192.72 | 406.385 | 246.404 | 159.981 |
| 2038 | 55859 | 18854.812 | 11291.369 | 7563.443 | 13.540 | 40.114 | 1190.66 | 401.900 | 240.681 | 161.218 |
| 2039 | 55757 | 18437.457 | 10896.923 | 7540.533 | 13.524 | 40.898 | 1187.28 | 392.602 | 232.036 | 160.566 |

# Results for PHIM for Run 2 (Sensitivity_Analysis_1.RTF)

************************************************************************************

**Main Results for E-Component for Run: 2, Simulation: 1, COD: Four Diseases Combined, Adjusted**

| Variable Parameter | Value |
|---|---|
| 1. Country | US (United States) |
| 2. Sex | F (Females) |
| 3. Year of start of process | 1990 |
| 4. Cause of Death | Four Diseases Combined |
| 5. Effective Dose NULL | CC:1 |
| 6. Effective Dose Ecig | CC:1 Ecig:0.4 Dual:0.7 |
| Note: | Death rates are per million population |

------------------------------------------------------------------------------------------

Results for Age Groups Combined and Years combined, Population and Number deaths adjusted for Ecig scenario,

| Age Group:All Year | Number Deaths | NULL Attrib Deaths | Ecig Attrib Deaths | Drop Deaths | %Drop Deaths | %Drop Attrib Deaths | Death Rate | NULL Attrib Rates | Ecig Attrib Rates | Drop Rates |
|---|---|---|---|---|---|---|---|---|---|---|
| All Comb | 9119610 | 2946250.006 | 2424661.893 | 521588.1 | 5.719 | 17.703 | 2497.07 | 808.881 | 663.905 | 144.977 |

------------------------------------------------------------------------------------------

Results for Age Groups Combined, Population and Number deaths adjusted for Ecig scenario,

| Age Group:All Year | Number Deaths | NULL Attrib Deaths | Ecig Attrib Deaths | Drop Deaths | %Drop Deaths | %Drop Attrib Deaths | Death Rate | NULL Attrib Rates | Ecig Attrib Rates | Drop Rates |
|---|---|---|---|---|---|---|---|---|---|---|
| 1990 | 199848 | 57524.822 | 57524.822 | 0.000 | 0.000 | 0.000 | 2926.84 | 842.471 | 842.471 | 0.000 |
| 1991 | 199162 | 59078.486 | 58970.930 | 107.556 | 0.054 | 0.182 | 2877.87 | 853.678 | 852.124 | 1.554 |
| 1992 | 198532 | 60494.150 | 60198.144 | 296.006 | 0.149 | 0.489 | 2829.84 | 862.271 | 858.051 | 4.221 |
| 1993 | 202514 | 62694.848 | 62121.784 | 573.063 | 0.283 | 0.914 | 2845.86 | 881.034 | 872.976 | 8.058 |
| 1994 | 200662 | 63202.598 | 62304.484 | 898.114 | 0.448 | 1.421 | 2777.90 | 874.966 | 862.520 | 12.445 |
| 1995 | 201031 | 63766.862 | 62473.241 | 1293.621 | 0.643 | 2.029 | 2739.84 | 869.097 | 851.444 | 17.654 |
| 1996 | 201194 | 64591.163 | 62831.300 | 1759.863 | 0.875 | 2.725 | 2706.43 | 868.908 | 845.196 | 23.712 |
| 1997 | 198661 | 64686.912 | 62501.575 | 2185.337 | 1.100 | 3.378 | 2635.29 | 858.147 | 829.099 | 29.048 |
| 1998 | 197604 | 65295.961 | 62614.899 | 2681.062 | 1.357 | 4.106 | 2584.34 | 854.046 | 818.898 | 35.148 |
| 1999 | 211376 | 67327.521 | 63896.793 | 3430.729 | 1.623 | 5.096 | 2727.15 | 868.771 | 824.391 | 44.380 |
| 2000 | 208558 | 67596.897 | 63606.898 | 3989.999 | 1.913 | 5.903 | 2657.28 | 861.419 | 810.427 | 50.993 |
| 2001 | 203686 | 67008.811 | 62447.078 | 4561.732 | 2.240 | 6.808 | 2566.29 | 844.458 | 786.787 | 57.672 |
| 2002 | 200928 | 67067.744 | 61860.533 | 5207.211 | 2.592 | 7.764 | 2506.60 | 836.925 | 771.718 | 65.206 |
| 2003 | 196474 | 66756.217 | 60979.171 | 5777.046 | 2.940 | 8.654 | 2427.95 | 825.244 | 753.555 | 71.689 |
| 2004 | 188304 | 65058.725 | 58821.319 | 6237.406 | 3.312 | 9.587 | 2303.59 | 796.234 | 719.581 | 76.653 |
| 2005 | 186587 | 65214.724 | 58357.622 | 6857.102 | 3.675 | 10.515 | 2257.17 | 789.321 | 705.959 | 83.362 |
| 2006 | 180284 | 63771.146 | 56466.750 | 7304.396 | 4.052 | 11.454 | 2161.70 | 765.117 | 677.067 | 88.050 |
| 2007 | 175866 | 63110.718 | 55357.222 | 7753.496 | 4.409 | 12.286 | 2087.52 | 749.650 | 657.088 | 92.562 |
| 2008 | 177829 | 63718.624 | 55289.456 | 8429.168 | 4.740 | 13.229 | 2087.75 | 748.674 | 649.110 | 99.564 |
| 2009 | 173093 | 62794.698 | 53957.594 | 8837.104 | 5.105 | 14.073 | 2009.06 | 729.515 | 626.276 | 103.239 |
| 2010 | 170809 | 61917.074 | 52681.449 | 9235.624 | 5.407 | 14.916 | 1959.63 | 711.083 | 604.393 | 106.690 |
| 2011 | 170663 | 61680.114 | 51855.377 | 9824.737 | 5.757 | 15.929 | 1939.30 | 701.700 | 589.252 | 112.448 |
| 2012 | 171821 | 62023.127 | 51580.999 | 10442.13 | 6.077 | 16.836 | 1932.26 | 698.386 | 580.067 | 118.319 |
| 2013 | 173146 | 62037.765 | 51008.482 | 11029.28 | 6.370 | 17.778 | 1926.02 | 691.059 | 567.401 | 123.657 |
| 2014 | 174665 | 61921.017 | 50263.695 | 11657.32 | 6.674 | 18.826 | 1921.34 | 682.195 | 552.909 | 129.286 |
| 2015 | 177910 | 61856.204 | 49696.620 | 12159.58 | 6.835 | 19.658 | 2187.20 | 761.899 | 610.966 | 150.933 |
| 2016 | 177457 | 60464.119 | 47893.765 | 12570.35 | 7.084 | 20.790 | 2160.48 | 737.655 | 583.092 | 154.563 |
| 2017 | 179976 | 60243.839 | 47100.633 | 13143.21 | 7.303 | 21.817 | 2170.05 | 728.017 | 567.913 | 160.103 |
| 2018 | 177873 | 60713.327 | 47091.502 | 13621.82 | 7.658 | 22.436 | 2123.94 | 726.721 | 562.308 | 164.413 |
| 2019 | 180013 | 61148.172 | 46871.897 | 14276.27 | 7.931 | 23.347 | 2128.29 | 724.841 | 554.166 | 170.675 |
| 2020 | 181327 | 61149.317 | 46525.527 | 14623.79 | 8.065 | 23.915 | 2428.13 | 821.432 | 623.017 | 198.416 |
| 2021 | 179027 | 58727.786 | 43883.716 | 14844.07 | 8.292 | 25.276 | 2379.52 | 783.216 | 583.276 | 199.940 |
| 2022 | 180392 | 58848.802 | 43529.568 | 15319.23 | 8.492 | 26.032 | 2378.96 | 778.881 | 574.056 | 204.825 |
| 2023 | 181699 | 58816.149 | 43085.853 | 15730.30 | 8.657 | 26.745 | 2377.06 | 772.407 | 563.667 | 208.739 |
| 2024 | 182744 | 58955.232 | 42735.631 | 16219.60 | 8.876 | 27.512 | 2371.93 | 768.316 | 554.687 | 213.629 |
| 2025 | 181628 | 58297.744 | 41931.085 | 16366.66 | 9.011 | 28.074 | 2718.67 | 876.861 | 627.638 | 249.223 |
| 2026 | 181602 | 56469.971 | 39967.101 | 16502.87 | 9.087 | 29.224 | 2707.26 | 846.150 | 595.815 | 250.334 |
| 2027 | 180979 | 56209.427 | 39325.681 | 16883.75 | 9.329 | 30.037 | 2686.49 | 838.883 | 583.760 | 255.124 |
| 2028 | 180193 | 55733.105 | 38596.534 | 17136.57 | 9.510 | 30.748 | 2662.95 | 828.298 | 570.391 | 257.907 |
| 2029 | 179310 | 55145.838 | 37772.730 | 17373.11 | 9.689 | 31.504 | 2637.50 | 815.951 | 555.604 | 260.346 |
| 2030 | 175155 | 53615.097 | 36389.340 | 17225.76 | 9.835 | 32.129 | 3046.39 | 939.056 | 632.904 | 306.152 |
| 2031 | 176667 | 52046.561 | 34994.357 | 17052.20 | 9.652 | 32.763 | 3067.64 | 910.364 | 607.639 | 302.725 |
| 2032 | 175152 | 51582.636 | 34340.899 | 17241.74 | 9.844 | 33.425 | 3034.49 | 900.496 | 594.953 | 305.543 |
| 2033 | 173578 | 50839.758 | 33591.495 | 17248.26 | 9.937 | 33.927 | 2999.03 | 885.375 | 580.383 | 304.992 |
| 2034 | 171861 | 50215.451 | 32893.679 | 17321.77 | 10.079 | 34.495 | 2960.34 | 872.104 | 566.600 | 305.504 |
| 2035 | 164576 | 47773.075 | 31093.939 | 16679.14 | 10.135 | 34.913 | 3466.61 | 1016.05 | 654.957 | 361.088 |
| 2036 | 158196 | 43298.709 | 27724.203 | 15574.51 | 9.845 | 35.970 | 3336.09 | 922.274 | 584.659 | 337.616 |
| 2037 | 155580 | 42241.563 | 26731.029 | 15510.53 | 9.970 | 36.719 | 3282.42 | 900.450 | 563.972 | 336.478 |
| 2038 | 152975 | 41356.353 | 25957.958 | 15398.39 | 10.066 | 37.233 | 3226.30 | 881.531 | 547.463 | 334.067 |
| 2039 | 150441 | 40161.050 | 24965.535 | 15195.52 | 10.101 | 37.836 | 3168.68 | 855.178 | 525.837 | 329.340 |

# Results for PHIM for Run 2 (Sensitivity_Analysis_1.RTF)

************************************************************************************

**Main Results for E-Component for Run: 2, Simulation: 1, COD: LUNG CANCER, Adjusted**

| Variable Parameter | Value |
|---|---|
| 1. Country | US (United States) |
| 2. Sex | F (Females) |
| 3. Year of start of process | 1990 |
| 4. Cause of Death | LUNG CANCER |
| 5. Effective Dose NULL | CC:1 |
| 6. Effective Dose Ecig | CC:1 Ecig:0.4 Dual:0.7 |
| Note: | Death rates are per million population |

------------------------------------------------------------------------------------

Results for Age Groups Combined and Years combined, Population and Number deaths adjusted for Ecig scenario,

| Age Group:All Year | Number Deaths | NULL Attrib Deaths | Ecig Attrib Deaths | Drop Deaths | %Drop Deaths | %Drop Attrib Deaths | Death Rate | NULL Attrib Rates | Ecig Attrib Rates | Drop Rates |
|---|---|---|---|---|---|---|---|---|---|---|
| All Comb | 2336928 | 1580465.217 | 1378116.696 | 202348.5 | 8.659 | 12.803 | 639.882 | 433.910 | 377.347 | 56.564 |

------------------------------------------------------------------------------------

Results for Age Groups Combined, Population and Number deaths adjusted for Ecig scenario,

| Age Group:All Year | Number Deaths | NULL Attrib Deaths | Ecig Attrib Deaths | Drop Deaths | %Drop Deaths | %Drop Attrib Deaths | Death Rate | NULL Attrib Rates | Ecig Attrib Rates | Drop Rates |
|---|---|---|---|---|---|---|---|---|---|---|
| 1990 | 42690 | 30614.201 | 30614.201 | 0.000 | 0.000 | 0.000 | 625.210 | 448.355 | 448.355 | 0.000 |
| 1991 | 43692 | 31412.010 | 31391.193 | 20.818 | 0.048 | 0.066 | 631.345 | 453.901 | 453.600 | 0.301 |
| 1992 | 45423 | 32782.407 | 32723.227 | 59.180 | 0.130 | 0.181 | 647.451 | 467.274 | 466.430 | 0.844 |
| 1993 | 46291 | 33491.183 | 33374.600 | 116.583 | 0.252 | 0.348 | 650.517 | 470.643 | 469.001 | 1.641 |
| 1994 | 47018 | 34074.347 | 33887.170 | 187.177 | 0.398 | 0.549 | 650.901 | 471.719 | 469.122 | 2.598 |
| 1995 | 47842 | 34694.454 | 34412.670 | 281.784 | 0.589 | 0.812 | 652.036 | 472.861 | 469.008 | 3.853 |
| 1996 | 48247 | 35027.269 | 34641.184 | 386.085 | 0.800 | 1.102 | 649.017 | 471.202 | 465.987 | 5.215 |
| 1997 | 49041 | 35590.763 | 35083.025 | 507.737 | 1.035 | 1.427 | 650.535 | 472.153 | 465.385 | 6.768 |
| 1998 | 49694 | 36100.785 | 35449.713 | 651.072 | 1.310 | 1.803 | 649.917 | 472.184 | 463.623 | 8.561 |
| 1999 | 48894 | 35451.030 | 34677.363 | 773.666 | 1.582 | 2.182 | 630.828 | 457.448 | 447.404 | 10.043 |
| 2000 | 50346 | 36447.986 | 35483.027 | 964.959 | 1.917 | 2.647 | 641.473 | 464.474 | 452.095 | 12.379 |
| 2001 | 50409 | 36411.326 | 35259.216 | 1152.111 | 2.286 | 3.164 | 635.118 | 458.863 | 444.240 | 14.623 |
| 2002 | 51471 | 37078.545 | 35695.057 | 1383.487 | 2.688 | 3.731 | 642.110 | 462.696 | 445.301 | 17.395 |
| 2003 | 51441 | 36999.876 | 35401.511 | 1598.365 | 3.107 | 4.320 | 635.682 | 457.395 | 437.477 | 19.918 |
| 2004 | 51439 | 36861.555 | 35033.111 | 1828.444 | 3.555 | 4.960 | 629.269 | 451.138 | 428.572 | 22.566 |
| 2005 | 51469 | 36689.529 | 34623.807 | 2065.721 | 4.014 | 5.630 | 622.621 | 444.069 | 418.848 | 25.220 |
| 2006 | 51411 | 36504.774 | 34197.534 | 2307.240 | 4.488 | 6.320 | 616.445 | 437.979 | 410.047 | 27.932 |
| 2007 | 51905 | 36663.202 | 34068.110 | 2595.092 | 5.000 | 7.078 | 616.106 | 435.498 | 404.387 | 31.111 |
| 2008 | 51050 | 35898.803 | 33104.956 | 2793.847 | 5.473 | 7.783 | 599.341 | 421.800 | 388.659 | 33.140 |
| 2009 | 51278 | 35934.525 | 32842.815 | 3091.710 | 6.029 | 8.604 | 595.174 | 417.468 | 381.201 | 36.267 |
| 2010 | 50948 | 35502.829 | 32191.123 | 3311.707 | 6.500 | 9.328 | 584.507 | 407.730 | 369.316 | 38.414 |
| 2011 | 50614 | 35136.342 | 31559.429 | 3576.913 | 7.067 | 10.180 | 575.150 | 399.726 | 358.622 | 41.105 |
| 2012 | 51364 | 35450.201 | 31553.872 | 3896.329 | 7.586 | 10.991 | 577.626 | 399.173 | 354.847 | 44.326 |
| 2013 | 51200 | 35123.224 | 30937.490 | 4185.733 | 8.175 | 11.917 | 569.533 | 391.249 | 344.138 | 47.111 |
| 2014 | 51396 | 35079.370 | 30595.026 | 4484.344 | 8.725 | 12.783 | 565.363 | 386.476 | 336.550 | 49.925 |
| 2015 | 50600 | 34324.171 | 29677.342 | 4646.829 | 9.184 | 13.538 | 622.069 | 422.780 | 364.851 | 57.929 |
| 2016 | 49020 | 33054.753 | 28264.232 | 4790.521 | 9.773 | 14.493 | 596.798 | 403.264 | 344.108 | 59.156 |
| 2017 | 48504 | 32471.234 | 27448.190 | 5023.044 | 10.356 | 15.469 | 584.829 | 392.399 | 330.955 | 61.443 |
| 2018 | 50667 | 33657.595 | 28079.658 | 5577.937 | 11.009 | 16.573 | 604.999 | 402.871 | 335.292 | 67.579 |
| 2019 | 51257 | 33863.424 | 27915.888 | 5947.536 | 11.603 | 17.563 | 606.009 | 401.412 | 330.049 | 71.363 |
| 2020 | 51685 | 33910.830 | 27703.752 | 6207.078 | 12.010 | 18.304 | 692.101 | 455.532 | 370.977 | 84.555 |
| 2021 | 48279 | 31531.880 | 25427.693 | 6104.187 | 12.644 | 19.359 | 641.697 | 420.521 | 337.969 | 82.552 |
| 2022 | 48596 | 31560.212 | 25175.831 | 6384.382 | 13.138 | 20.229 | 640.869 | 417.708 | 332.012 | 85.696 |
| 2023 | 48879 | 31508.781 | 24867.235 | 6641.547 | 13.588 | 21.078 | 639.456 | 413.791 | 325.324 | 88.467 |
| 2024 | 49083 | 31517.293 | 24597.575 | 6919.718 | 14.098 | 21.955 | 637.074 | 410.740 | 319.264 | 91.475 |
| 2025 | 48795 | 31175.389 | 24100.398 | 7074.991 | 14.499 | 22.694 | 730.384 | 468.912 | 360.743 | 108.169 |
| 2026 | 45643 | 29044.598 | 22188.932 | 6855.666 | 15.020 | 23.604 | 680.434 | 435.206 | 330.785 | 104.421 |
| 2027 | 45444 | 28825.765 | 21769.013 | 7056.752 | 15.529 | 24.481 | 674.575 | 430.203 | 323.144 | 107.058 |
| 2028 | 45195 | 28513.846 | 21309.235 | 7204.611 | 15.941 | 25.267 | 667.905 | 423.769 | 314.914 | 108.855 |
| 2029 | 44909 | 28148.109 | 20799.700 | 7348.409 | 16.363 | 26.106 | 660.579 | 416.486 | 305.946 | 110.540 |
| 2030 | 43778 | 27314.475 | 19986.296 | 7328.179 | 16.739 | 26.829 | 761.417 | 478.407 | 347.613 | 130.794 |
| 2031 | 41135 | 25423.102 | 18478.419 | 6944.683 | 16.883 | 27.316 | 714.264 | 444.684 | 320.858 | 123.826 |
| 2032 | 40728 | 25085.902 | 18058.266 | 7027.636 | 17.255 | 28.014 | 705.603 | 437.933 | 312.858 | 125.075 |
| 2033 | 40299 | 24645.067 | 17601.978 | 7043.089 | 17.477 | 28.578 | 696.280 | 429.194 | 304.121 | 125.073 |
| 2034 | 39836 | 24239.126 | 17168.557 | 7070.570 | 17.749 | 29.170 | 686.184 | 420.967 | 295.732 | 125.235 |
| 2035 | 37894 | 22944.147 | 16131.756 | 6812.391 | 17.977 | 29.691 | 798.197 | 487.980 | 339.797 | 148.183 |
| 2036 | 33393 | 19950.581 | 13868.080 | 6082.500 | 18.215 | 30.488 | 704.197 | 424.953 | 292.455 | 132.497 |
| 2037 | 32816 | 19415.334 | 13348.898 | 6066.436 | 18.486 | 31.246 | 692.352 | 413.871 | 281.635 | 132.235 |
| 2038 | 32241 | 18939.317 | 12928.018 | 6011.299 | 18.645 | 31.740 | 679.965 | 403.701 | 272.657 | 131.044 |
| 2039 | 31679 | 18379.752 | 12421.327 | 5958.425 | 18.809 | 32.418 | 667.249 | 391.373 | 261.625 | 129.748 |

## Results for PHIM for Run 2 (Sensitivity_Analysis_1.RTF)

**********************************************************************************
**Main Results for E-Component for Run: 2, Simulation: 1, COD: IHD, Adjusted**

| Variable Parameter | Value |
|---|---|
| 1. Country | US (United States) |
| 2. Sex | F (Females) |
| 3. Year of start of process | 1990 |
| 4. Cause of Death | IHD |
| 5. Effective Dose NULL | CC:1 |
| 6. Effective Dose Ecig | CC:1 Ecig:0.4 Dual:0.7 |
| Note: | Death rates are per million population |

---------------------------------------------------------------------------------------

Results for Age Groups Combined and Years combined, Population and Number deaths adjusted for Ecig scenario,

| Age Group:All Year | Number Deaths | NULL Attrib Deaths | Ecig Attrib Deaths | Drop Deaths | %Drop Deaths | %Drop Attrib Deaths | Death Rate | NULL Attrib Rates | Ecig Attrib Rates | Drop Rates |
|---|---|---|---|---|---|---|---|---|---|---|
| All Comb | 3253198 | 398833.796 | 282649.619 | 116184.2 | 3.571 | 29.131 | 890.769 | 109.498 | 77.393 | 32.105 |

---------------------------------------------------------------------------------------

Results for Age Groups Combined, Population and Number deaths adjusted for Ecig scenario,

| Age Group:All Year | Number Deaths | NULL Attrib Deaths | Ecig Attrib Deaths | Drop Deaths | %Drop Deaths | %Drop Attrib Deaths | Death Rate | NULL Attrib Rates | Ecig Attrib Rates | Drop Rates |
|---|---|---|---|---|---|---|---|---|---|---|
| 1990 | 98916 | 12922.892 | 12922.892 | 0.000 | 0.000 | 0.000 | 1448.66 | 189.260 | 189.260 | 0.000 |
| 1991 | 96403 | 12856.479 | 12799.224 | 57.256 | 0.059 | 0.445 | 1393.01 | 185.775 | 184.948 | 0.827 |
| 1992 | 93721 | 12662.244 | 12505.919 | 156.325 | 0.167 | 1.235 | 1335.88 | 180.485 | 178.257 | 2.229 |
| 1993 | 93680 | 12730.613 | 12434.361 | 296.252 | 0.316 | 2.327 | 1316.45 | 178.900 | 174.736 | 4.164 |
| 1994 | 90834 | 12495.323 | 12037.384 | 457.939 | 0.504 | 3.665 | 1257.47 | 172.983 | 166.641 | 6.342 |
| 1995 | 89702 | 12365.590 | 11721.569 | 644.021 | 0.718 | 5.208 | 1222.54 | 168.534 | 159.752 | 8.782 |
| 1996 | 88292 | 12290.551 | 11418.926 | 871.626 | 0.987 | 7.092 | 1187.69 | 165.338 | 153.605 | 11.732 |
| 1997 | 84786 | 11719.531 | 10677.493 | 1042.038 | 1.229 | 8.891 | 1124.71 | 155.473 | 141.640 | 13.834 |
| 1998 | 82530 | 11416.794 | 10175.908 | 1240.887 | 1.504 | 10.869 | 1079.36 | 149.327 | 133.084 | 16.243 |
| 1999 | 93960 | 13028.038 | 11331.718 | 1696.321 | 1.805 | 13.021 | 1212.26 | 168.109 | 146.201 | 21.908 |
| 2000 | 90066 | 12426.299 | 10536.774 | 1889.525 | 2.098 | 15.206 | 1147.54 | 158.354 | 134.251 | 24.103 |
| 2001 | 86152 | 11939.281 | 9821.646 | 2117.636 | 2.458 | 17.737 | 1085.46 | 150.461 | 123.745 | 26.716 |
| 2002 | 83055 | 11528.794 | 9183.124 | 2345.670 | 2.824 | 20.346 | 1036.12 | 143.865 | 114.561 | 29.305 |
| 2003 | 79351 | 11048.407 | 8534.690 | 2513.717 | 3.168 | 22.752 | 980.588 | 136.581 | 105.468 | 31.113 |
| 2004 | 73917 | 10375.132 | 7752.089 | 2623.042 | 3.549 | 25.282 | 904.255 | 126.978 | 94.834 | 32.144 |
| 2005 | 71714 | 10051.029 | 7282.134 | 2768.895 | 3.861 | 27.548 | 867.537 | 121.652 | 88.093 | 33.559 |
| 2006 | 67624 | 9542.523 | 6681.796 | 2860.727 | 4.230 | 29.979 | 810.848 | 114.490 | 80.118 | 34.371 |
| 2007 | 63583 | 8936.533 | 6068.590 | 2867.943 | 4.511 | 32.092 | 754.725 | 106.151 | 72.034 | 34.117 |
| 2008 | 63262 | 8944.180 | 5891.380 | 3052.800 | 4.826 | 34.132 | 742.712 | 105.091 | 69.166 | 35.925 |
| 2009 | 59846 | 8390.033 | 5379.061 | 3010.972 | 5.031 | 35.887 | 694.617 | 97.471 | 62.434 | 35.037 |
| 2010 | 58116 | 8157.570 | 5098.272 | 3059.298 | 5.264 | 37.503 | 666.740 | 93.685 | 58.490 | 35.195 |
| 2011 | 57487 | 8035.356 | 4877.198 | 3158.158 | 5.494 | 39.303 | 653.250 | 91.414 | 55.421 | 35.992 |
| 2012 | 57543 | 7970.766 | 4704.307 | 3266.459 | 5.677 | 40.980 | 647.117 | 89.752 | 52.903 | 36.848 |
| 2013 | 57213 | 7743.216 | 4460.557 | 3282.659 | 5.738 | 42.394 | 636.417 | 86.254 | 49.618 | 36.636 |
| 2014 | 57699 | 7774.935 | 4369.808 | 3405.127 | 5.901 | 43.796 | 634.703 | 85.658 | 48.069 | 37.589 |
| 2015 | 58575 | 7722.535 | 4265.398 | 3457.137 | 5.902 | 44.767 | 720.116 | 95.120 | 52.438 | 42.682 |
| 2016 | 58855 | 7600.353 | 4103.093 | 3497.261 | 5.942 | 46.014 | 716.545 | 92.723 | 49.954 | 42.770 |
| 2017 | 59220 | 7470.266 | 3955.278 | 3514.989 | 5.935 | 47.053 | 714.040 | 90.274 | 47.691 | 42.584 |
| 2018 | 56299 | 6907.050 | 3587.968 | 3319.082 | 5.895 | 48.054 | 672.256 | 82.675 | 42.843 | 39.832 |
| 2019 | 56126 | 6732.046 | 3441.955 | 3290.091 | 5.862 | 48.872 | 663.579 | 79.801 | 40.694 | 39.106 |
| 2020 | 55572 | 6429.836 | 3244.410 | 3185.426 | 5.732 | 49.541 | 744.163 | 86.373 | 43.445 | 42.928 |
| 2021 | 56573 | 6562.016 | 3250.369 | 3311.647 | 5.854 | 50.467 | 751.935 | 87.513 | 43.202 | 44.312 |
| 2022 | 56126 | 6352.366 | 3107.014 | 3245.352 | 5.782 | 51.089 | 740.170 | 84.075 | 40.974 | 43.101 |
| 2023 | 55654 | 6134.123 | 2974.368 | 3159.755 | 5.677 | 51.511 | 728.094 | 80.557 | 38.912 | 41.645 |
| 2024 | 55104 | 5922.193 | 2843.779 | 3078.414 | 5.587 | 51.981 | 715.217 | 77.179 | 36.911 | 40.268 |
| 2025 | 53645 | 5504.188 | 2615.850 | 2888.338 | 5.384 | 52.475 | 802.974 | 82.789 | 39.155 | 43.634 |
| 2026 | 55661 | 5641.075 | 2659.337 | 2981.738 | 5.357 | 52.858 | 829.778 | 84.526 | 39.644 | 44.882 |
| 2027 | 54597 | 5426.216 | 2539.010 | 2887.206 | 5.288 | 53.208 | 810.448 | 80.982 | 37.690 | 43.292 |
| 2028 | 53502 | 5197.830 | 2420.927 | 2776.903 | 5.190 | 53.424 | 790.673 | 77.249 | 35.777 | 41.472 |
| 2029 | 52400 | 4973.547 | 2307.109 | 2666.438 | 5.089 | 53.612 | 770.755 | 73.590 | 33.936 | 39.654 |
| 2030 | 49914 | 4450.618 | 2032.702 | 2417.916 | 4.844 | 54.328 | 868.139 | 77.952 | 35.354 | 42.598 |
| 2031 | 52469 | 4555.052 | 2081.396 | 2473.657 | 4.715 | 54.306 | 911.067 | 79.674 | 36.141 | 43.533 |
| 2032 | 51171 | 4349.999 | 1982.206 | 2367.793 | 4.627 | 54.432 | 886.529 | 75.939 | 34.342 | 41.598 |
| 2033 | 49884 | 4147.948 | 1893.583 | 2254.365 | 4.519 | 54.349 | 861.884 | 72.237 | 32.717 | 39.520 |
| 2034 | 48589 | 3966.133 | 1806.437 | 2159.696 | 4.445 | 54.453 | 836.955 | 68.881 | 31.116 | 37.765 |
| 2035 | 45032 | 3289.064 | 1470.970 | 1818.094 | 4.037 | 55.277 | 948.538 | 69.952 | 30.984 | 38.968 |
| 2036 | 46903 | 3313.192 | 1472.395 | 1840.798 | 3.925 | 55.560 | 989.118 | 70.572 | 31.050 | 39.521 |
| 2037 | 45400 | 3117.951 | 1383.602 | 1734.349 | 3.820 | 55.625 | 957.845 | 66.464 | 29.191 | 37.273 |
| 2038 | 43939 | 2949.775 | 1310.443 | 1639.332 | 3.731 | 55.575 | 926.682 | 62.876 | 27.638 | 35.238 |
| 2039 | 42534 | 2766.312 | 1233.201 | 1533.111 | 3.604 | 55.421 | 895.882 | 58.905 | 25.974 | 32.931 |

# Results for PHIM for Run 2 (Sensitivity_Analysis_1.RTF)

************************************************************************************
**Main Results for E-Component for Run: 2, Simulation: 1, COD: STROKE, Adjusted**

| Variable Parameter | Value |
|---|---|
| 1. Country | US (United States) |
| 2. Sex | F (Females) |
| 3. Year of start of process | 1990 |
| 4. Cause of Death | STROKE |
| 5. Effective Dose NULL | CC:1 |
| 6. Effective Dose Ecig | CC:1 Ecig:0.4 Dual:0.7 |
| Note: | Death rates are per million population |

------------------------------------------------------------------------------------------
Results for Age Groups Combined and Years combined, Population and Number deaths adjusted for Ecig scenario,

| Age Group:All Year | Number Deaths | NULL Attrib Deaths | Ecig Attrib Deaths | Drop Deaths | %Drop Deaths | %Drop Attrib Deaths | Death Rate | NULL Attrib Rates | Ecig Attrib Rates | Drop Rates |
|---|---|---|---|---|---|---|---|---|---|---|
| All Comb | 1357470 | 104899.750 | 73094.278 | 31805.47 | 2.343 | 30.320 | 371.693 | 28.800 | 20.014 | 8.786 |

------------------------------------------------------------------------------------------
Results for Age Groups Combined, Population and Number deaths adjusted for Ecig scenario,

| Age Group:All Year | Number Deaths | NULL Attrib Deaths | Ecig Attrib Deaths | Drop Deaths | %Drop Deaths | %Drop Attrib Deaths | Death Rate | NULL Attrib Rates | Ecig Attrib Rates | Drop Rates |
|---|---|---|---|---|---|---|---|---|---|---|
| 1990 | 33504 | 3293.017 | 3293.017 | 0.000 | 0.000 | 0.000 | 490.678 | 48.227 | 48.227 | 0.000 |
| 1991 | 32505 | 3207.063 | 3189.553 | 17.510 | 0.054 | 0.546 | 469.694 | 46.342 | 46.089 | 0.253 |
| 1992 | 32560 | 3218.125 | 3169.602 | 48.522 | 0.149 | 1.508 | 464.104 | 45.871 | 45.179 | 0.692 |
| 1993 | 32768 | 3226.680 | 3135.259 | 91.421 | 0.279 | 2.833 | 460.481 | 45.344 | 44.059 | 1.285 |
| 1994 | 33006 | 3277.266 | 3132.565 | 144.700 | 0.438 | 4.415 | 456.919 | 45.370 | 43.366 | 2.004 |
| 1995 | 33731 | 3309.378 | 3102.921 | 206.458 | 0.612 | 6.239 | 459.722 | 45.104 | 42.289 | 2.815 |
| 1996 | 33752 | 3312.987 | 3034.379 | 278.608 | 0.825 | 8.410 | 454.030 | 44.568 | 40.818 | 3.750 |
| 1997 | 33703 | 3267.066 | 2919.970 | 347.096 | 1.030 | 10.624 | 447.073 | 43.341 | 38.734 | 4.607 |
| 1998 | 33279 | 3178.725 | 2765.671 | 413.054 | 1.241 | 12.994 | 435.234 | 41.577 | 36.170 | 5.406 |
| 1999 | 33898 | 3120.351 | 2641.354 | 478.997 | 1.413 | 15.351 | 437.351 | 40.264 | 34.079 | 6.185 |
| 2000 | 33837 | 3142.808 | 2584.067 | 558.742 | 1.651 | 17.778 | 431.118 | 40.050 | 32.924 | 7.126 |
| 2001 | 32518 | 2961.588 | 2363.604 | 597.985 | 1.839 | 20.191 | 409.704 | 37.323 | 29.780 | 7.543 |
| 2002 | 32093 | 2940.627 | 2268.655 | 671.972 | 2.094 | 22.851 | 400.368 | 36.695 | 28.302 | 8.394 |
| 2003 | 30586 | 2819.545 | 2107.031 | 712.514 | 2.330 | 25.271 | 377.971 | 34.855 | 26.038 | 8.818 |
| 2004 | 29410 | 2730.466 | 1973.787 | 756.678 | 2.573 | 27.712 | 359.777 | 33.417 | 24.146 | 9.271 |
| 2005 | 27979 | 2670.510 | 1873.138 | 797.372 | 2.850 | 29.858 | 338.460 | 32.322 | 22.660 | 9.663 |
| 2006 | 27230 | 2623.285 | 1783.737 | 839.549 | 3.083 | 32.004 | 326.497 | 31.474 | 21.388 | 10.086 |
| 2007 | 26334 | 2536.599 | 1675.904 | 860.696 | 3.268 | 33.931 | 312.585 | 30.131 | 19.893 | 10.238 |
| 2008 | 26035 | 2469.443 | 1586.082 | 883.362 | 3.393 | 35.772 | 305.653 | 29.015 | 18.621 | 10.394 |
| 2009 | 25080 | 2386.945 | 1493.101 | 893.844 | 3.564 | 37.447 | 291.104 | 27.730 | 17.330 | 10.400 |
| 2010 | 24885 | 2329.274 | 1421.604 | 907.670 | 3.647 | 38.968 | 285.501 | 26.750 | 16.309 | 10.441 |
| 2011 | 24843 | 2286.581 | 1358.717 | 927.864 | 3.735 | 40.579 | 282.300 | 26.013 | 15.440 | 10.574 |
| 2012 | 24556 | 2243.311 | 1298.437 | 944.874 | 3.848 | 42.120 | 276.152 | 25.260 | 14.602 | 10.658 |
| 2013 | 24513 | 2176.503 | 1227.879 | 948.625 | 3.870 | 43.585 | 272.676 | 24.245 | 13.659 | 10.586 |
| 2014 | 25367 | 2211.619 | 1218.421 | 993.198 | 3.915 | 44.908 | 279.042 | 24.366 | 13.403 | 10.963 |
| 2015 | 26398 | 2237.016 | 1211.989 | 1025.026 | 3.883 | 45.821 | 324.529 | 27.554 | 14.900 | 12.654 |
| 2016 | 26825 | 2229.057 | 1185.432 | 1043.625 | 3.890 | 46.819 | 326.590 | 27.194 | 14.432 | 12.762 |
| 2017 | 27718 | 2221.649 | 1161.630 | 1060.019 | 3.824 | 47.713 | 334.208 | 26.848 | 14.006 | 12.841 |
| 2018 | 25358 | 1939.420 | 998.967 | 940.453 | 3.709 | 48.491 | 302.788 | 23.214 | 11.928 | 11.286 |
| 2019 | 25540 | 1904.400 | 965.292 | 939.107 | 3.677 | 49.313 | 301.965 | 22.574 | 11.413 | 11.162 |
| 2020 | 25500 | 1806.054 | 903.347 | 902.707 | 3.540 | 49.982 | 341.472 | 24.261 | 12.097 | 12.165 |
| 2021 | 25580 | 1748.172 | 859.189 | 888.984 | 3.475 | 50.852 | 339.989 | 23.314 | 11.420 | 11.894 |
| 2022 | 25680 | 1701.148 | 825.720 | 875.428 | 3.409 | 51.461 | 338.666 | 22.515 | 10.889 | 11.626 |
| 2023 | 25772 | 1652.087 | 795.756 | 856.330 | 3.323 | 51.833 | 337.161 | 21.696 | 10.410 | 11.286 |
| 2024 | 25821 | 1597.127 | 761.617 | 835.510 | 3.236 | 52.313 | 335.140 | 20.814 | 9.885 | 10.929 |
| 2025 | 25431 | 1467.622 | 692.445 | 775.178 | 3.048 | 52.819 | 380.666 | 22.075 | 10.365 | 11.710 |
| 2026 | 25665 | 1390.505 | 651.352 | 739.153 | 2.880 | 53.157 | 382.603 | 20.835 | 9.710 | 11.125 |
| 2027 | 25458 | 1342.095 | 623.803 | 718.293 | 2.821 | 53.520 | 377.906 | 20.030 | 9.260 | 10.770 |
| 2028 | 25225 | 1289.536 | 596.596 | 692.940 | 2.747 | 53.736 | 372.787 | 19.165 | 8.817 | 10.348 |
| 2029 | 24980 | 1242.216 | 572.031 | 670.185 | 2.683 | 53.951 | 367.430 | 18.380 | 8.414 | 9.966 |
| 2030 | 24180 | 1096.803 | 497.164 | 599.640 | 2.480 | 54.672 | 420.553 | 19.210 | 8.647 | 10.563 |
| 2031 | 24197 | 1019.991 | 462.309 | 557.681 | 2.305 | 54.675 | 420.155 | 17.841 | 8.027 | 9.813 |
| 2032 | 23858 | 977.406 | 441.692 | 535.714 | 2.245 | 54.810 | 413.337 | 17.063 | 7.652 | 9.411 |
| 2033 | 23510 | 937.450 | 424.039 | 513.410 | 2.184 | 54.767 | 406.195 | 16.326 | 7.326 | 8.999 |
| 2034 | 23137 | 900.763 | 406.093 | 494.670 | 2.138 | 54.917 | 398.547 | 15.644 | 6.995 | 8.649 |
| 2035 | 21996 | 745.023 | 331.817 | 413.207 | 1.879 | 55.462 | 463.327 | 15.845 | 6.989 | 8.856 |
| 2036 | 21169 | 680.360 | 299.783 | 380.576 | 1.798 | 55.938 | 446.425 | 14.492 | 6.322 | 8.170 |
| 2037 | 20666 | 644.109 | 283.306 | 360.803 | 1.746 | 56.016 | 436.016 | 13.730 | 5.977 | 7.753 |
| 2038 | 20163 | 612.448 | 269.385 | 343.063 | 1.701 | 56.015 | 425.253 | 13.055 | 5.681 | 7.373 |
| 2039 | 19669 | 577.529 | 255.069 | 322.461 | 1.639 | 55.835 | 414.274 | 12.298 | 5.372 | 6.925 |

## Results for PHIM for Run 2 (Sensitivity_Analysis_1.RTF)

************************************************************************************
**Main Results for E-Component for Run: 2, Simulation: 1, COD: COPD, Adjusted**

| Variable Parameter | Value |
|---|---|
| 1. Country | US (United States) |
| 2. Sex | F (Females) |
| 3. Year of start of process | 1990 |
| 4. Cause of Death | COPD |
| 5. Effective Dose NULL | CC:1 |
| 6. Effective Dose Ecig | CC:1 Ecig:0.4 Dual:0.7 |
| Note: | Death rates are per million population |

------------------------------------------------------------------------------------------

Results for Age Groups Combined and Years combined, Population and Number deaths adjusted for Ecig scenario,

| Age Group:All | | NULL | Ecig | | | %Drop | | NULL | Ecig | |
|---|---|---|---|---|---|---|---|---|---|---|
| Year | Number | Attrib | Attrib | Drop | %Drop | Attrib | Death | Attrib | Attrib | Drop |
| | Deaths | Deaths | Deaths | Deaths | Deaths | Deaths | Rate | Rates | Rates | Rates |
| All Comb | 2172014 | 862051.244 | 690801.300 | 171249.9 | 7.884 | 19.865 | 594.726 | 236.673 | 189.151 | 47.522 |

------------------------------------------------------------------------------------------

Results for Age Groups Combined, Population and Number deaths adjusted for Ecig scenario,

| Age Group:All | | NULL | Ecig | | | %Drop | | NULL | Ecig | |
|---|---|---|---|---|---|---|---|---|---|---|
| Year | Number | Attrib | Attrib | Drop | %Drop | Attrib | Death | Attrib | Attrib | Drop |
| | Deaths | Deaths | Deaths | Deaths | Deaths | Deaths | Rate | Rates | Rates | Rates |
| 1990 | 24738 | 10694.712 | 10694.712 | 0.000 | 0.000 | 0.000 | 362.296 | 156.628 | 156.628 | 0.000 |
| 1991 | 26562 | 11602.933 | 11590.960 | 11.972 | 0.045 | 0.103 | 383.818 | 167.661 | 167.488 | 0.173 |
| 1992 | 26828 | 11831.375 | 11799.396 | 31.979 | 0.119 | 0.270 | 382.401 | 168.642 | 168.186 | 0.456 |
| 1993 | 29774 | 13246.371 | 13177.564 | 68.807 | 0.231 | 0.519 | 418.407 | 186.148 | 185.180 | 0.968 |
| 1994 | 29805 | 13355.662 | 13247.364 | 108.298 | 0.363 | 0.811 | 412.605 | 184.893 | 183.392 | 1.502 |
| 1995 | 29756 | 13397.439 | 13236.082 | 161.357 | 0.542 | 1.204 | 405.546 | 182.598 | 180.394 | 2.204 |
| 1996 | 30902 | 13960.356 | 13736.810 | 223.545 | 0.723 | 1.601 | 415.691 | 187.801 | 184.785 | 3.015 |
| 1997 | 31131 | 14109.552 | 13821.087 | 288.466 | 0.927 | 2.044 | 412.967 | 187.180 | 183.340 | 3.839 |
| 1998 | 32101 | 14599.657 | 14223.608 | 376.049 | 1.171 | 2.576 | 419.830 | 190.958 | 186.021 | 4.937 |
| 1999 | 34624 | 15728.102 | 15246.358 | 481.745 | 1.391 | 3.063 | 446.713 | 202.950 | 196.707 | 6.243 |
| 2000 | 34309 | 15579.803 | 15003.031 | 576.772 | 1.681 | 3.702 | 437.143 | 198.541 | 191.156 | 7.385 |
| 2001 | 34606 | 15696.614 | 15002.613 | 694.001 | 2.005 | 4.421 | 436.008 | 197.812 | 189.022 | 8.790 |
| 2002 | 34309 | 15519.777 | 14713.696 | 806.081 | 2.350 | 5.194 | 428.004 | 193.668 | 183.555 | 10.113 |
| 2003 | 35097 | 15888.389 | 14935.939 | 952.450 | 2.714 | 5.995 | 433.708 | 196.413 | 184.572 | 11.841 |
| 2004 | 33539 | 15091.573 | 14062.332 | 1029.241 | 3.069 | 6.820 | 410.288 | 184.701 | 172.029 | 12.672 |
| 2005 | 35426 | 15803.656 | 14578.543 | 1225.113 | 3.458 | 7.752 | 428.547 | 191.278 | 176.358 | 14.920 |
| 2006 | 34019 | 15100.563 | 13803.684 | 1296.879 | 3.812 | 8.588 | 407.909 | 181.174 | 165.514 | 15.661 |
| 2007 | 34045 | 14974.384 | 13544.618 | 1429.766 | 4.200 | 9.548 | 404.108 | 177.871 | 160.774 | 17.097 |
| 2008 | 37482 | 16406.197 | 14707.038 | 1699.160 | 4.533 | 10.357 | 440.044 | 192.768 | 172.664 | 20.104 |
| 2009 | 36890 | 16083.194 | 14242.616 | 1840.578 | 4.989 | 11.444 | 428.170 | 186.846 | 165.311 | 21.534 |
| 2010 | 36860 | 15927.400 | 13970.451 | 1956.950 | 5.309 | 12.287 | 422.877 | 182.917 | 160.277 | 22.640 |
| 2011 | 37718 | 16221.836 | 14060.033 | 2161.803 | 5.731 | 13.327 | 428.604 | 184.547 | 159.769 | 24.777 |
| 2012 | 38358 | 16358.849 | 14024.384 | 2334.465 | 6.086 | 14.270 | 431.363 | 184.202 | 157.715 | 26.487 |
| 2013 | 40220 | 16994.822 | 14382.556 | 2612.266 | 6.495 | 15.371 | 447.391 | 189.311 | 159.987 | 29.324 |
| 2014 | 40202 | 16855.093 | 14080.440 | 2774.654 | 6.902 | 16.462 | 442.233 | 185.696 | 154.887 | 30.808 |
| 2015 | 42337 | 17572.483 | 14541.891 | 3030.592 | 7.158 | 17.246 | 520.490 | 216.445 | 178.777 | 37.668 |
| 2016 | 42757 | 17579.955 | 14341.009 | 3238.946 | 7.575 | 18.424 | 520.548 | 214.473 | 174.597 | 39.876 |
| 2017 | 44535 | 18080.688 | 14535.534 | 3545.154 | 7.960 | 19.607 | 536.974 | 218.496 | 175.261 | 43.235 |
| 2018 | 45550 | 18209.262 | 14424.909 | 3784.353 | 8.308 | 20.783 | 543.898 | 217.960 | 172.244 | 45.715 |
| 2019 | 47089 | 18648.302 | 14548.761 | 4099.541 | 8.706 | 21.983 | 556.738 | 221.054 | 172.010 | 49.044 |
| 2020 | 48570 | 19002.597 | 14674.018 | 4328.579 | 8.912 | 22.779 | 650.392 | 255.266 | 196.498 | 58.768 |
| 2021 | 48595 | 18885.717 | 14346.465 | 4539.252 | 9.341 | 24.035 | 645.896 | 251.867 | 190.684 | 61.183 |
| 2022 | 49990 | 19235.076 | 14421.004 | 4814.072 | 9.630 | 25.028 | 659.258 | 254.582 | 190.180 | 64.401 |
| 2023 | 51393 | 19521.158 | 14448.494 | 5072.664 | 9.870 | 25.985 | 672.347 | 256.363 | 189.021 | 67.341 |
| 2024 | 52737 | 19918.620 | 14532.660 | 5385.960 | 10.213 | 27.040 | 684.495 | 259.583 | 188.627 | 70.957 |
| 2025 | 53756 | 20150.545 | 14522.392 | 5628.153 | 10.470 | 27.931 | 804.641 | 303.086 | 217.376 | 85.710 |
| 2026 | 54633 | 20393.792 | 14467.480 | 5926.312 | 10.848 | 29.059 | 814.447 | 305.582 | 215.676 | 89.906 |
| 2027 | 55480 | 20615.351 | 14393.855 | 6221.497 | 11.214 | 30.179 | 823.561 | 307.668 | 213.666 | 94.003 |
| 2028 | 56271 | 20731.894 | 14269.777 | 6462.117 | 11.484 | 31.170 | 831.583 | 308.115 | 210.883 | 97.232 |
| 2029 | 57021 | 20781.967 | 14093.890 | 6688.077 | 11.729 | 32.182 | 838.735 | 307.495 | 207.309 | 100.186 |
| 2030 | 57282 | 20753.201 | 13873.178 | 6880.023 | 12.011 | 33.152 | 996.279 | 363.487 | 241.290 | 122.197 |
| 2031 | 58867 | 21048.417 | 13972.234 | 7076.183 | 12.021 | 33.619 | 1022.15 | 368.165 | 242.613 | 125.552 |
| 2032 | 59396 | 21169.329 | 13858.735 | 7310.594 | 12.308 | 34.534 | 1029.03 | 369.560 | 240.101 | 129.459 |
| 2033 | 59885 | 21109.293 | 13671.895 | 7437.399 | 12.420 | 35.233 | 1034.67 | 367.618 | 236.219 | 131.400 |
| 2034 | 60298 | 21109.429 | 13512.592 | 7596.836 | 12.599 | 35.988 | 1038.65 | 366.613 | 232.757 | 133.856 |
| 2035 | 59654 | 20794.840 | 13159.396 | 7635.444 | 12.800 | 36.718 | 1256.54 | 442.268 | 277.187 | 165.081 |
| 2036 | 56730 | 19354.577 | 12083.945 | 7270.632 | 12.816 | 37.565 | 1196.35 | 412.258 | 254.831 | 157.427 |
| 2037 | 56698 | 19064.169 | 11715.223 | 7348.946 | 12.962 | 38.548 | 1196.21 | 406.385 | 247.168 | 159.217 |
| 2038 | 56632 | 18854.812 | 11450.111 | 7404.701 | 13.075 | 39.272 | 1194.40 | 401.900 | 241.487 | 160.412 |
| 2039 | 56559 | 18437.457 | 11055.938 | 7381.519 | 13.051 | 40.035 | 1191.27 | 392.602 | 232.866 | 159.736 |

## Results for PHIM for Run 3 (Sensitivity_Analysis_1.RTF)

---
------------------------------------------------------------------------------------------

**Control File Details for Run: 3**

| Variable Parameter | Value |
|---|---|
| 1. Name for Reference Scenario | NULL |
| 2. Name for Test Scenario | Ecig |
| 3. Name for Product 1 | CC |
| 4. Name for Product 2 | Ecig |
| 5. Name for Product 3 | - |
| 6. Name for Product 4 | Dual |
| 7. Country | US (United States) |
| 8. Sex | M (Males) |
| 9. Year of start of process | 1990 |
| 10. Number of months of follow-up | 600 |
| 11. Follow-up interval length (in months) | 12 |
| 12. Lower age for risk estimation | 10 |
| 13. Upper age for risk estimation | 79 |
| 14. The effective dose for Product 1 (F1) - Ref | 1 |
| 15. The effective dose for Product 2 (F2) - Ref | - |
| 16. The effective dose for Product 3 (F3) - Ref | - |
| 17. The effective dose for Product 4 (F4) - Ref | - |
| 18. The effective dose for Product 1 (F1) - Test | 1 |
| 19. The effective dose for Product 2 (F2) - Test | 0 |
| 20. The effective dose for Product 3 (F3) - Test | - |
| 21. The effective dose for Product 4 (F4) - Test | FROMF2 |
| 22. Number in population to be simulated | 100000 |
| 23. Number of MC simulations | 1 |
| 24. The random number seed for the first simulation | 15975263 |
| 25. Source for the population file (POP) | UN4 |
| 26. Source for the socioeconomic prevalence file (SEP) | - |
| 27. Source for the current smoking prevalence file (CSP) | ISS3 |
| 28. Source for the former smoking prevalence file (FSP) | ISS3 |
| 29. Source for the quit-time distribution file (QTD) | NHIS2006 |
| 30. Source for the death file (MORT) | APR-20 |
| 31. Source for the relative risk file (RR) | PNLEST |
| 32. Source for the half-life file (H) | PNLEST |
| 33. Assumption Set for TTP Factor Reference | F1 |
| 34. Assumption Set for TTP Factor Test | F1 |
| 35. Assumption Set for TTP Reference | ISS3 |
| 36. Assumption Set for TTP Test | ISS3 |
| 37. Assumption Set for TP for socioeconomic group | - |
| 38. Source for Output Choice file (OUTC) | BASIC |
| 39. Output for DTP P1 | N |
| 40. Output for DTP P2 | N |
| 41. Output for Main P Component | Y |
| 42. Output for DTP E1 | N |
| 43. Output for DTP E2 | N |
| 44. Output for DTP E3 | N |
| 45. Output for Main E Component | Y |
| 46. Output file name | Sensitivity_Analysis_1 |
| 47. Run number for P-Component | - |
| 48. Results file for E-Component | - |
| 49. Components required | PE |
| 50. Results folder for P to be used in E-Component | - |
| 51. Fixed age of starting product use | 16 |
| 52. Output absolute risk file? | N |

## Results for PHIM for Run 3 (Sensitivity_Analysis_1.RTF)

**************************************************************************************
**TTP values for Run: 3, Scenario: NULL**

| **Variable Parameter** | **Value** |
|---|---|
| 1. Country | US (United States) |
| 2. Sex | M (Males) |
| 3. Assumption Set | ISS3 |
| Note: | Period is in Months |

| Age | Period | **Initiation** Never CC | **Cessation** CC Former | **Re-initiation** Former CC |
|---|---|---|---|---|
| 10-14 | 1-12 | 0.003842 | 0.008742 | 0.000000 |
| 15-19 | 1-12 | 0.004720 | 0.003614 | 0.000000 |
| 20-24 | 1-12 | 0.000376 | 0.000943 | 0.000000 |
| 25-29 | 1-12 | 0.000187 | 0.001143 | 0.000000 |
| 30-34 | 1-12 | 0.000093 | 0.000871 | 0.000000 |
| 35-39 | 1-12 | 0.000000 | 0.000867 | 0.000000 |
| 40-44 | 1-12 | 0.000000 | 0.001399 | 0.000000 |
| 45-49 | 1-12 | 0.000000 | 0.002033 | 0.000000 |
| 50-54 | 1-12 | 0.000000 | 0.003209 | 0.000000 |
| 55-59 | 1-12 | 0.000000 | 0.003574 | 0.000000 |
| 60-64 | 1-12 | 0.000000 | 0.003460 | 0.000000 |
| 65-69 | 1-12 | 0.000000 | 0.004136 | 0.000000 |
| 70-74 | 1-12 | 0.000000 | 0.005592 | 0.000000 |
| 75-79 | 1-12 | 0.000000 | 0.005592 | 0.000000 |
| 10-14 | 13-72 | 0.004906 | 0.008742 | 0.000000 |
| 15-19 | 13-72 | 0.006989 | 0.004426 | 0.000000 |
| 20-24 | 13-72 | 0.000687 | 0.001527 | 0.000000 |
| 25-29 | 13-72 | 0.000962 | 0.001515 | 0.000000 |
| 30-34 | 13-72 | 0.000364 | 0.001227 | 0.000000 |
| 35-39 | 13-72 | 0.000000 | 0.001630 | 0.000000 |
| 40-44 | 13-72 | 0.000000 | 0.001852 | 0.000000 |
| 45-49 | 13-72 | 0.000000 | 0.002667 | 0.000000 |
| 50-54 | 13-72 | 0.000000 | 0.003208 | 0.000000 |
| 55-59 | 13-72 | 0.000000 | 0.003978 | 0.000000 |
| 60-64 | 13-72 | 0.000000 | 0.005771 | 0.000000 |
| 65-69 | 13-72 | 0.000000 | 0.010432 | 0.000000 |
| 70-74 | 13-72 | 0.000000 | 0.007079 | 0.000000 |
| 75-79 | 13-72 | 0.000000 | 0.007079 | 0.000000 |
| 10-14 | 73-132 | 0.004312 | 0.008742 | 0.000000 |
| 15-19 | 73-132 | 0.005330 | 0.003289 | 0.000000 |
| 20-24 | 73-132 | 0.002456 | 0.003844 | 0.000000 |
| 25-29 | 73-132 | 0.000839 | 0.002006 | 0.000000 |
| 30-34 | 73-132 | 0.000414 | 0.001329 | 0.000000 |
| 35-39 | 73-132 | 0.000000 | 0.001278 | 0.000000 |
| 40-44 | 73-132 | 0.000000 | 0.001691 | 0.000000 |
| 45-49 | 73-132 | 0.000000 | 0.002904 | 0.000000 |
| 50-54 | 73-132 | 0.000000 | 0.003319 | 0.000000 |
| 55-59 | 73-132 | 0.000000 | 0.003976 | 0.000000 |
| 60-64 | 73-132 | 0.000000 | 0.005235 | 0.000000 |
| 65-69 | 73-132 | 0.000000 | 0.005901 | 0.000000 |
| 70-74 | 73-132 | 0.000000 | 0.005615 | 0.000000 |
| 75-79 | 73-132 | 0.000000 | 0.005615 | 0.000000 |
| 10-14 | 133-192 | 0.003468 | 0.008742 | 0.000000 |
| 15-19 | 133-192 | 0.004675 | 0.003732 | 0.000000 |
| 20-24 | 133-192 | 0.000062 | 0.002260 | 0.000000 |
| 25-29 | 133-192 | 0.001422 | 0.002822 | 0.000000 |
| 30-34 | 133-192 | 0.000696 | 0.002610 | 0.000000 |
| 35-39 | 133-192 | 0.000000 | 0.002058 | 0.000000 |
| 40-44 | 133-192 | 0.000000 | 0.002310 | 0.000000 |
| 45-49 | 133-192 | 0.000000 | 0.002256 | 0.000000 |
| 50-54 | 133-192 | 0.000000 | 0.002663 | 0.000000 |
| 55-59 | 133-192 | 0.000000 | 0.001746 | 0.000000 |
| 60-64 | 133-192 | 0.000000 | 0.005809 | 0.000000 |
| 65-69 | 133-192 | 0.000000 | 0.005896 | 0.000000 |
| 70-74 | 133-192 | 0.000000 | 0.007323 | 0.000000 |
| 75-79 | 133-192 | 0.000000 | 0.007323 | 0.000000 |
| 10-14 | 193-252 | 0.002314 | 0.008742 | 0.000000 |
| 15-19 | 193-252 | 0.003987 | 0.005036 | 0.000000 |
| 20-24 | 193-252 | 0.000320 | 0.002857 | 0.000000 |
| 25-29 | 193-252 | 0.000251 | 0.002314 | 0.000000 |
| 30-34 | 193-252 | 0.000125 | 0.004343 | 0.000000 |
| 35-39 | 193-252 | 0.000000 | 0.002092 | 0.000000 |
| 40-44 | 193-252 | 0.000000 | 0.002566 | 0.000000 |
| 45-49 | 193-252 | 0.000000 | 0.002311 | 0.000000 |
| 50-54 | 193-252 | 0.000000 | 0.003209 | 0.000000 |

## Results for PHIM for Run 3 (Sensitivity_Analysis_1.RTF)

| | | | | |
|---|---|---|---|---|
| 55-59 | 193-252 | 0.000000 | 0.004057 | 0.000000 |
| 60-64 | 193-252 | 0.000000 | 0.007166 | 0.000000 |
| 65-69 | 193-252 | 0.000000 | 0.004281 | 0.000000 |
| 70-74 | 193-252 | 0.000000 | 0.004580 | 0.000000 |
| 75-79 | 193-252 | 0.000000 | 0.004580 | 0.000000 |
| 10-14 | 253+ | 0.001325 | 0.008742 | 0.000000 |
| 15-19 | 253+ | 0.002984 | 0.010602 | 0.000000 |
| 20-24 | 253+ | 0.000452 | 0.004314 | 0.000000 |
| 25-29 | 253+ | 0.000225 | 0.003645 | 0.000000 |
| 30-34 | 253+ | 0.000112 | 0.004512 | 0.000000 |
| 35-39 | 253+ | 0.000000 | 0.002147 | 0.000000 |
| 40-44 | 253+ | 0.000000 | 0.002636 | 0.000000 |
| 45-49 | 253+ | 0.000000 | 0.001051 | 0.000000 |
| 50-54 | 253+ | 0.000000 | 0.002519 | 0.000000 |
| 55-59 | 253+ | 0.000000 | 0.003693 | 0.000000 |
| 60-64 | 253+ | 0.000000 | 0.005888 | 0.000000 |
| 65-69 | 253+ | 0.000000 | 0.004823 | 0.000000 |
| 70-74 | 253+ | 0.000000 | 0.004733 | 0.000000 |
| 75-79 | 253+ | 0.000000 | 0.004733 | 0.000000 |

# Results for PHIM for Run 3 (Sensitivity_Analysis_1.RTF)

************************************************************************************

**TTP values for Run: 3, Scenario: Ecig**

| **Variable Parameter** | **Value** |
|---|---|
| 1. Country | US (United States) |
| 2. Sex | M (Males) |
| 3. Assumption Set | ISS3 |
| Note: | Period is in Months |

| | | **Initiation** | | | **Cessation** | | | **Re-initiation** | | |
|---|---|---|---|---|---|---|---|---|---|---|
| Age | Period | Never CC | Never Ecig | Never Dual | CC Former | Ecig Former | Dual Former | Former CC | Former Ecig | Former Dual |
| 10-14 | 1-12 | 0.001921 | 0.001921 | 0.000000 | 0.008742 | 0.008742 | 0.000000 | 0.000000 | 0.000000 | 0.000000 |
| 15-19 | 1-12 | 0.002360 | 0.002360 | 0.000000 | 0.003614 | 0.003614 | 0.000000 | 0.000000 | 0.000000 | 0.000000 |
| 20-24 | 1-12 | 0.000188 | 0.000188 | 0.000000 | 0.000943 | 0.000943 | 0.000000 | 0.000000 | 0.000000 | 0.000000 |
| 25-29 | 1-12 | 0.000093 | 0.000093 | 0.000000 | 0.001143 | 0.001143 | 0.000000 | 0.000000 | 0.000000 | 0.000000 |
| 30-34 | 1-12 | 0.000047 | 0.000047 | 0.000000 | 0.000871 | 0.000871 | 0.000000 | 0.000000 | 0.000000 | 0.000000 |
| 35-39 | 1-12 | 0.000000 | 0.000000 | 0.000000 | 0.000867 | 0.000867 | 0.000000 | 0.000000 | 0.000000 | 0.000000 |
| 40-44 | 1-12 | 0.000000 | 0.000000 | 0.000000 | 0.001399 | 0.001399 | 0.000000 | 0.000000 | 0.000000 | 0.000000 |
| 45-49 | 1-12 | 0.000000 | 0.000000 | 0.000000 | 0.002033 | 0.002033 | 0.000000 | 0.000000 | 0.000000 | 0.000000 |
| 50-54 | 1-12 | 0.000000 | 0.000000 | 0.000000 | 0.003209 | 0.003209 | 0.000000 | 0.000000 | 0.000000 | 0.000000 |
| 55-59 | 1-12 | 0.000000 | 0.000000 | 0.000000 | 0.003574 | 0.003574 | 0.000000 | 0.000000 | 0.000000 | 0.000000 |
| 60-64 | 1-12 | 0.000000 | 0.000000 | 0.000000 | 0.003460 | 0.003460 | 0.000000 | 0.000000 | 0.000000 | 0.000000 |
| 65-69 | 1-12 | 0.000000 | 0.000000 | 0.000000 | 0.004136 | 0.004136 | 0.000000 | 0.000000 | 0.000000 | 0.000000 |
| 70-74 | 1-12 | 0.000000 | 0.000000 | 0.000000 | 0.005592 | 0.005592 | 0.000000 | 0.000000 | 0.000000 | 0.000000 |
| 75-79 | 1-12 | 0.000000 | 0.000000 | 0.000000 | 0.005592 | 0.005592 | 0.000000 | 0.000000 | 0.000000 | 0.000000 |
| 10-14 | 13-72 | 0.001962 | 0.002944 | 0.000000 | 0.008742 | 0.008742 | 0.000000 | 0.000000 | 0.000000 | 0.000000 |
| 15-19 | 13-72 | 0.002796 | 0.004193 | 0.000000 | 0.004426 | 0.004426 | 0.000000 | 0.000000 | 0.000000 | 0.000000 |
| 20-24 | 13-72 | 0.000275 | 0.000412 | 0.000000 | 0.001527 | 0.001527 | 0.000000 | 0.000000 | 0.000000 | 0.000000 |
| 25-29 | 13-72 | 0.000385 | 0.000578 | 0.000000 | 0.001515 | 0.001515 | 0.000000 | 0.000000 | 0.000000 | 0.000000 |
| 30-34 | 13-72 | 0.000146 | 0.000218 | 0.000000 | 0.001227 | 0.001227 | 0.000000 | 0.000000 | 0.000000 | 0.000000 |
| 35-39 | 13-72 | 0.000000 | 0.000000 | 0.000000 | 0.001630 | 0.001630 | 0.000000 | 0.000000 | 0.000000 | 0.000000 |
| 40-44 | 13-72 | 0.000000 | 0.000000 | 0.000000 | 0.001852 | 0.001852 | 0.000000 | 0.000000 | 0.000000 | 0.000000 |
| 45-49 | 13-72 | 0.000000 | 0.000000 | 0.000000 | 0.002667 | 0.002667 | 0.000000 | 0.000000 | 0.000000 | 0.000000 |
| 50-54 | 13-72 | 0.000000 | 0.000000 | 0.000000 | 0.003208 | 0.003208 | 0.000000 | 0.000000 | 0.000000 | 0.000000 |
| 55-59 | 13-72 | 0.000000 | 0.000000 | 0.000000 | 0.003978 | 0.003978 | 0.000000 | 0.000000 | 0.000000 | 0.000000 |
| 60-64 | 13-72 | 0.000000 | 0.000000 | 0.000000 | 0.005771 | 0.005771 | 0.000000 | 0.000000 | 0.000000 | 0.000000 |
| 65-69 | 13-72 | 0.000000 | 0.000000 | 0.000000 | 0.010432 | 0.010432 | 0.000000 | 0.000000 | 0.000000 | 0.000000 |
| 70-74 | 13-72 | 0.000000 | 0.000000 | 0.000000 | 0.007079 | 0.007079 | 0.000000 | 0.000000 | 0.000000 | 0.000000 |
| 75-79 | 13-72 | 0.000000 | 0.000000 | 0.000000 | 0.007079 | 0.007079 | 0.000000 | 0.000000 | 0.000000 | 0.000000 |
| 10-14 | 73-132 | 0.000862 | 0.003449 | 0.000000 | 0.008742 | 0.008742 | 0.000000 | 0.000000 | 0.000000 | 0.000000 |
| 15-19 | 73-132 | 0.001066 | 0.004264 | 0.000000 | 0.003289 | 0.003289 | 0.000000 | 0.000000 | 0.000000 | 0.000000 |
| 20-24 | 73-132 | 0.000491 | 0.001965 | 0.000000 | 0.003844 | 0.003844 | 0.000000 | 0.000000 | 0.000000 | 0.000000 |
| 25-29 | 73-132 | 0.000168 | 0.000671 | 0.000000 | 0.002006 | 0.002006 | 0.000000 | 0.000000 | 0.000000 | 0.000000 |
| 30-34 | 73-132 | 0.000083 | 0.000332 | 0.000000 | 0.001329 | 0.001329 | 0.000000 | 0.000000 | 0.000000 | 0.000000 |
| 35-39 | 73-132 | 0.000000 | 0.000000 | 0.000000 | 0.001278 | 0.001278 | 0.000000 | 0.000000 | 0.000000 | 0.000000 |
| 40-44 | 73-132 | 0.000000 | 0.000000 | 0.000000 | 0.001691 | 0.001691 | 0.000000 | 0.000000 | 0.000000 | 0.000000 |
| 45-49 | 73-132 | 0.000000 | 0.000000 | 0.000000 | 0.002904 | 0.002904 | 0.000000 | 0.000000 | 0.000000 | 0.000000 |
| 50-54 | 73-132 | 0.000000 | 0.000000 | 0.000000 | 0.003319 | 0.003319 | 0.000000 | 0.000000 | 0.000000 | 0.000000 |
| 55-59 | 73-132 | 0.000000 | 0.000000 | 0.000000 | 0.003976 | 0.003976 | 0.000000 | 0.000000 | 0.000000 | 0.000000 |
| 60-64 | 73-132 | 0.000000 | 0.000000 | 0.000000 | 0.005235 | 0.005235 | 0.000000 | 0.000000 | 0.000000 | 0.000000 |
| 65-69 | 73-132 | 0.000000 | 0.000000 | 0.000000 | 0.005901 | 0.005901 | 0.000000 | 0.000000 | 0.000000 | 0.000000 |
| 70-74 | 73-132 | 0.000000 | 0.000000 | 0.000000 | 0.005615 | 0.005615 | 0.000000 | 0.000000 | 0.000000 | 0.000000 |
| 75-79 | 73-132 | 0.000000 | 0.000000 | 0.000000 | 0.005615 | 0.005615 | 0.000000 | 0.000000 | 0.000000 | 0.000000 |
| 10-14 | 133-192 | 0.000173 | 0.003294 | 0.000000 | 0.008742 | 0.008742 | 0.000000 | 0.000000 | 0.000000 | 0.000000 |
| 15-19 | 133-192 | 0.000234 | 0.004442 | 0.000000 | 0.003732 | 0.003732 | 0.000000 | 0.000000 | 0.000000 | 0.000000 |
| 20-24 | 133-192 | 0.000003 | 0.000059 | 0.000000 | 0.002260 | 0.002260 | 0.000000 | 0.000000 | 0.000000 | 0.000000 |
| 25-29 | 133-192 | 0.000071 | 0.001351 | 0.000000 | 0.002822 | 0.002822 | 0.000000 | 0.000000 | 0.000000 | 0.000000 |
| 30-34 | 133-192 | 0.000035 | 0.000661 | 0.000000 | 0.002610 | 0.002610 | 0.000000 | 0.000000 | 0.000000 | 0.000000 |
| 35-39 | 133-192 | 0.000000 | 0.000000 | 0.000000 | 0.002058 | 0.002058 | 0.000000 | 0.000000 | 0.000000 | 0.000000 |
| 40-44 | 133-192 | 0.000000 | 0.000000 | 0.000000 | 0.002310 | 0.002310 | 0.000000 | 0.000000 | 0.000000 | 0.000000 |
| 45-49 | 133-192 | 0.000000 | 0.000000 | 0.000000 | 0.002256 | 0.002256 | 0.000000 | 0.000000 | 0.000000 | 0.000000 |
| 50-54 | 133-192 | 0.000000 | 0.000000 | 0.000000 | 0.002663 | 0.002663 | 0.000000 | 0.000000 | 0.000000 | 0.000000 |
| 55-59 | 133-192 | 0.000000 | 0.000000 | 0.000000 | 0.001747 | 0.001747 | 0.000000 | 0.000000 | 0.000000 | 0.000000 |
| 60-64 | 133-192 | 0.000000 | 0.000000 | 0.000000 | 0.005809 | 0.005809 | 0.000000 | 0.000000 | 0.000000 | 0.000000 |
| 65-69 | 133-192 | 0.000000 | 0.000000 | 0.000000 | 0.005896 | 0.005896 | 0.000000 | 0.000000 | 0.000000 | 0.000000 |
| 70-74 | 133-192 | 0.000000 | 0.000000 | 0.000000 | 0.007323 | 0.007323 | 0.000000 | 0.000000 | 0.000000 | 0.000000 |
| 75-79 | 133-192 | 0.000000 | 0.000000 | 0.000000 | 0.007323 | 0.007323 | 0.000000 | 0.000000 | 0.000000 | 0.000000 |
| 10-14 | 193-252 | 0.000116 | 0.002198 | 0.000000 | 0.008742 | 0.008742 | 0.000000 | 0.000000 | 0.000000 | 0.000000 |
| 15-19 | 193-252 | 0.000199 | 0.003788 | 0.000000 | 0.005036 | 0.005036 | 0.000000 | 0.000000 | 0.000000 | 0.000000 |
| 20-24 | 193-252 | 0.000016 | 0.000304 | 0.000000 | 0.002857 | 0.002857 | 0.000000 | 0.000000 | 0.000000 | 0.000000 |
| 25-29 | 193-252 | 0.000013 | 0.000239 | 0.000000 | 0.002314 | 0.002314 | 0.000000 | 0.000000 | 0.000000 | 0.000000 |
| 30-34 | 193-252 | 0.000006 | 0.000119 | 0.000000 | 0.004343 | 0.004343 | 0.000000 | 0.000000 | 0.000000 | 0.000000 |
| 35-39 | 193-252 | 0.000000 | 0.000000 | 0.000000 | 0.002092 | 0.002092 | 0.000000 | 0.000000 | 0.000000 | 0.000000 |
| 40-44 | 193-252 | 0.000000 | 0.000000 | 0.000000 | 0.002566 | 0.002566 | 0.000000 | 0.000000 | 0.000000 | 0.000000 |
| 45-49 | 193-252 | 0.000000 | 0.000000 | 0.000000 | 0.002311 | 0.002311 | 0.000000 | 0.000000 | 0.000000 | 0.000000 |
| 50-54 | 193-252 | 0.000000 | 0.000000 | 0.000000 | 0.003209 | 0.003209 | 0.000000 | 0.000000 | 0.000000 | 0.000000 |

## Results for PHIM for Run 3 (Sensitivity_Analysis_1.RTF)

| | | | | | | | | | | |
|---|---|---|---|---|---|---|---|---|---|---|
| 55-59 | 193-252 | 0.000000 | 0.000000 | 0.000000 | 0.004057 | 0.004057 | 0.000000 | 0.000000 | 0.000000 | 0.000000 |
| 60-64 | 193-252 | 0.000000 | 0.000000 | 0.000000 | 0.007166 | 0.007166 | 0.000000 | 0.000000 | 0.000000 | 0.000000 |
| 65-69 | 193-252 | 0.000000 | 0.000000 | 0.000000 | 0.004281 | 0.004281 | 0.000000 | 0.000000 | 0.000000 | 0.000000 |
| 70-74 | 193-252 | 0.000000 | 0.000000 | 0.000000 | 0.004580 | 0.004580 | 0.000000 | 0.000000 | 0.000000 | 0.000000 |
| 75-79 | 193-252 | 0.000000 | 0.000000 | 0.000000 | 0.004580 | 0.004580 | 0.000000 | 0.000000 | 0.000000 | 0.000000 |
| 10-14 | 253+ | 0.000066 | 0.001259 | 0.000000 | 0.008742 | 0.008742 | 0.000000 | 0.000000 | 0.000000 | 0.000000 |
| 15-19 | 253+ | 0.000149 | 0.002835 | 0.000000 | 0.010602 | 0.010602 | 0.000000 | 0.000000 | 0.000000 | 0.000000 |
| 20-24 | 253+ | 0.000023 | 0.000430 | 0.000000 | 0.004314 | 0.004314 | 0.000000 | 0.000000 | 0.000000 | 0.000000 |
| 25-29 | 253+ | 0.000011 | 0.000213 | 0.000000 | 0.003645 | 0.003645 | 0.000000 | 0.000000 | 0.000000 | 0.000000 |
| 30-34 | 253+ | 0.000006 | 0.000106 | 0.000000 | 0.004512 | 0.004512 | 0.000000 | 0.000000 | 0.000000 | 0.000000 |
| 35-39 | 253+ | 0.000000 | 0.000000 | 0.000000 | 0.002147 | 0.002147 | 0.000000 | 0.000000 | 0.000000 | 0.000000 |
| 40-44 | 253+ | 0.000000 | 0.000000 | 0.000000 | 0.002636 | 0.002636 | 0.000000 | 0.000000 | 0.000000 | 0.000000 |
| 45-49 | 253+ | 0.000000 | 0.000000 | 0.000000 | 0.001051 | 0.001051 | 0.000000 | 0.000000 | 0.000000 | 0.000000 |
| 50-54 | 253+ | 0.000000 | 0.000000 | 0.000000 | 0.002519 | 0.002519 | 0.000000 | 0.000000 | 0.000000 | 0.000000 |
| 55-59 | 253+ | 0.000000 | 0.000000 | 0.000000 | 0.003693 | 0.003693 | 0.000000 | 0.000000 | 0.000000 | 0.000000 |
| 60-64 | 253+ | 0.000000 | 0.000000 | 0.000000 | 0.005888 | 0.005888 | 0.000000 | 0.000000 | 0.000000 | 0.000000 |
| 65-69 | 253+ | 0.000000 | 0.000000 | 0.000000 | 0.004823 | 0.004823 | 0.000000 | 0.000000 | 0.000000 | 0.000000 |
| 70-74 | 253+ | 0.000000 | 0.000000 | 0.000000 | 0.004733 | 0.004733 | 0.000000 | 0.000000 | 0.000000 | 0.000000 |
| 75-79 | 253+ | 0.000000 | 0.000000 | 0.000000 | 0.004733 | 0.004733 | 0.000000 | 0.000000 | 0.000000 | 0.000000 |

# Results for PHIM for Run 3 (Sensitivity_Analysis_1.RTF)

*************************************************************************************

| | | **Switch from CC** | | **Switch from Ecig** | | **Switch from Dual** | |
|---|---|---|---|---|---|---|---|
| | | **CC** | **CC** | **Ecig** | **Ecig** | **Dual** | **Dual** |
| Age | Period | Ecig | Dual | CC | Dual | CC | Ecig |
| 10-14 | 1-12 | 0.009549 | 0.000000 | 0.000000 | 0.000000 | 0.000000 | 0.000000 |
| 15-19 | 1-12 | 0.012695 | 0.000000 | 0.000000 | 0.000000 | 0.000000 | 0.000000 |
| 20-24 | 1-12 | 0.014490 | 0.000000 | 0.000000 | 0.000000 | 0.000000 | 0.000000 |
| 25-29 | 1-12 | 0.015212 | 0.000000 | 0.000000 | 0.000000 | 0.000000 | 0.000000 |
| 30-34 | 1-12 | 0.015460 | 0.000000 | 0.000000 | 0.000000 | 0.000000 | 0.000000 |
| 35-39 | 1-12 | 0.014711 | 0.000000 | 0.000000 | 0.000000 | 0.000000 | 0.000000 |
| 40-44 | 1-12 | 0.014241 | 0.000000 | 0.000000 | 0.000000 | 0.000000 | 0.000000 |
| 45-49 | 1-12 | 0.013014 | 0.000000 | 0.000000 | 0.000000 | 0.000000 | 0.000000 |
| 50-54 | 1-12 | 0.011768 | 0.000000 | 0.000000 | 0.000000 | 0.000000 | 0.000000 |
| 55-59 | 1-12 | 0.011032 | 0.000000 | 0.000000 | 0.000000 | 0.000000 | 0.000000 |
| 60-64 | 1-12 | 0.009525 | 0.000000 | 0.000000 | 0.000000 | 0.000000 | 0.000000 |
| 65-69 | 1-12 | 0.006897 | 0.000000 | 0.000000 | 0.000000 | 0.000000 | 0.000000 |
| 70-74 | 1-12 | 0.005435 | 0.000000 | 0.000000 | 0.000000 | 0.000000 | 0.000000 |
| 75-79 | 1-12 | 0.005435 | 0.000000 | 0.000000 | 0.000000 | 0.000000 | 0.000000 |
| 10-14 | 13-72 | 0.009549 | 0.000000 | 0.000000 | 0.000000 | 0.000000 | 0.000000 |
| 15-19 | 13-72 | 0.012695 | 0.000000 | 0.000000 | 0.000000 | 0.000000 | 0.000000 |
| 20-24 | 13-72 | 0.014490 | 0.000000 | 0.000000 | 0.000000 | 0.000000 | 0.000000 |
| 25-29 | 13-72 | 0.015212 | 0.000000 | 0.000000 | 0.000000 | 0.000000 | 0.000000 |
| 30-34 | 13-72 | 0.015460 | 0.000000 | 0.000000 | 0.000000 | 0.000000 | 0.000000 |
| 35-39 | 13-72 | 0.014711 | 0.000000 | 0.000000 | 0.000000 | 0.000000 | 0.000000 |
| 40-44 | 13-72 | 0.014241 | 0.000000 | 0.000000 | 0.000000 | 0.000000 | 0.000000 |
| 45-49 | 13-72 | 0.013014 | 0.000000 | 0.000000 | 0.000000 | 0.000000 | 0.000000 |
| 50-54 | 13-72 | 0.011768 | 0.000000 | 0.000000 | 0.000000 | 0.000000 | 0.000000 |
| 55-59 | 13-72 | 0.011032 | 0.000000 | 0.000000 | 0.000000 | 0.000000 | 0.000000 |
| 60-64 | 13-72 | 0.009525 | 0.000000 | 0.000000 | 0.000000 | 0.000000 | 0.000000 |
| 65-69 | 13-72 | 0.006897 | 0.000000 | 0.000000 | 0.000000 | 0.000000 | 0.000000 |
| 70-74 | 13-72 | 0.005435 | 0.000000 | 0.000000 | 0.000000 | 0.000000 | 0.000000 |
| 75-79 | 13-72 | 0.005435 | 0.000000 | 0.000000 | 0.000000 | 0.000000 | 0.000000 |
| 10-14 | 73-132 | 0.009549 | 0.000000 | 0.000000 | 0.000000 | 0.000000 | 0.000000 |
| 15-19 | 73-132 | 0.012695 | 0.000000 | 0.000000 | 0.000000 | 0.000000 | 0.000000 |
| 20-24 | 73-132 | 0.014490 | 0.000000 | 0.000000 | 0.000000 | 0.000000 | 0.000000 |
| 25-29 | 73-132 | 0.015212 | 0.000000 | 0.000000 | 0.000000 | 0.000000 | 0.000000 |
| 30-34 | 73-132 | 0.015460 | 0.000000 | 0.000000 | 0.000000 | 0.000000 | 0.000000 |
| 35-39 | 73-132 | 0.014711 | 0.000000 | 0.000000 | 0.000000 | 0.000000 | 0.000000 |
| 40-44 | 73-132 | 0.014241 | 0.000000 | 0.000000 | 0.000000 | 0.000000 | 0.000000 |
| 45-49 | 73-132 | 0.013014 | 0.000000 | 0.000000 | 0.000000 | 0.000000 | 0.000000 |
| 50-54 | 73-132 | 0.011768 | 0.000000 | 0.000000 | 0.000000 | 0.000000 | 0.000000 |
| 55-59 | 73-132 | 0.011032 | 0.000000 | 0.000000 | 0.000000 | 0.000000 | 0.000000 |
| 60-64 | 73-132 | 0.009525 | 0.000000 | 0.000000 | 0.000000 | 0.000000 | 0.000000 |
| 65-69 | 73-132 | 0.006897 | 0.000000 | 0.000000 | 0.000000 | 0.000000 | 0.000000 |
| 70-74 | 73-132 | 0.005435 | 0.000000 | 0.000000 | 0.000000 | 0.000000 | 0.000000 |
| 75-79 | 73-132 | 0.005435 | 0.000000 | 0.000000 | 0.000000 | 0.000000 | 0.000000 |
| 10-14 | 133-192 | 0.009549 | 0.000000 | 0.000000 | 0.000000 | 0.000000 | 0.000000 |
| 15-19 | 133-192 | 0.012695 | 0.000000 | 0.000000 | 0.000000 | 0.000000 | 0.000000 |
| 20-24 | 133-192 | 0.014490 | 0.000000 | 0.000000 | 0.000000 | 0.000000 | 0.000000 |
| 25-29 | 133-192 | 0.015212 | 0.000000 | 0.000000 | 0.000000 | 0.000000 | 0.000000 |
| 30-34 | 133-192 | 0.015460 | 0.000000 | 0.000000 | 0.000000 | 0.000000 | 0.000000 |
| 35-39 | 133-192 | 0.014711 | 0.000000 | 0.000000 | 0.000000 | 0.000000 | 0.000000 |
| 40-44 | 133-192 | 0.014241 | 0.000000 | 0.000000 | 0.000000 | 0.000000 | 0.000000 |
| 45-49 | 133-192 | 0.013014 | 0.000000 | 0.000000 | 0.000000 | 0.000000 | 0.000000 |
| 50-54 | 133-192 | 0.011768 | 0.000000 | 0.000000 | 0.000000 | 0.000000 | 0.000000 |
| 55-59 | 133-192 | 0.011032 | 0.000000 | 0.000000 | 0.000000 | 0.000000 | 0.000000 |
| 60-64 | 133-192 | 0.009525 | 0.000000 | 0.000000 | 0.000000 | 0.000000 | 0.000000 |
| 65-69 | 133-192 | 0.006897 | 0.000000 | 0.000000 | 0.000000 | 0.000000 | 0.000000 |
| 70-74 | 133-192 | 0.005435 | 0.000000 | 0.000000 | 0.000000 | 0.000000 | 0.000000 |
| 75-79 | 133-192 | 0.005435 | 0.000000 | 0.000000 | 0.000000 | 0.000000 | 0.000000 |
| 10-14 | 193-252 | 0.009549 | 0.000000 | 0.000000 | 0.000000 | 0.000000 | 0.000000 |
| 15-19 | 193-252 | 0.012695 | 0.000000 | 0.000000 | 0.000000 | 0.000000 | 0.000000 |
| 20-24 | 193-252 | 0.014490 | 0.000000 | 0.000000 | 0.000000 | 0.000000 | 0.000000 |
| 25-29 | 193-252 | 0.015212 | 0.000000 | 0.000000 | 0.000000 | 0.000000 | 0.000000 |
| 30-34 | 193-252 | 0.015460 | 0.000000 | 0.000000 | 0.000000 | 0.000000 | 0.000000 |
| 35-39 | 193-252 | 0.014711 | 0.000000 | 0.000000 | 0.000000 | 0.000000 | 0.000000 |
| 40-44 | 193-252 | 0.014241 | 0.000000 | 0.000000 | 0.000000 | 0.000000 | 0.000000 |
| 45-49 | 193-252 | 0.013014 | 0.000000 | 0.000000 | 0.000000 | 0.000000 | 0.000000 |
| 50-54 | 193-252 | 0.011768 | 0.000000 | 0.000000 | 0.000000 | 0.000000 | 0.000000 |
| 55-59 | 193-252 | 0.011032 | 0.000000 | 0.000000 | 0.000000 | 0.000000 | 0.000000 |
| 60-64 | 193-252 | 0.009525 | 0.000000 | 0.000000 | 0.000000 | 0.000000 | 0.000000 |
| 65-69 | 193-252 | 0.006897 | 0.000000 | 0.000000 | 0.000000 | 0.000000 | 0.000000 |
| 70-74 | 193-252 | 0.005435 | 0.000000 | 0.000000 | 0.000000 | 0.000000 | 0.000000 |
| 75-79 | 193-252 | 0.005435 | 0.000000 | 0.000000 | 0.000000 | 0.000000 | 0.000000 |
| 10-14 | 253+ | 0.009549 | 0.000000 | 0.000000 | 0.000000 | 0.000000 | 0.000000 |
| 15-19 | 253+ | 0.012695 | 0.000000 | 0.000000 | 0.000000 | 0.000000 | 0.000000 |
| 20-24 | 253+ | 0.014490 | 0.000000 | 0.000000 | 0.000000 | 0.000000 | 0.000000 |

## Results for PHIM for Run 3 (Sensitivity_Analysis_1.RTF)

| | | | | | | | |
|---|---|---|---|---|---|---|---|
| 25-29 | 253+ | 0.015212 | 0.000000 | 0.000000 | 0.000000 | 0.000000 | 0.000000 |
| 30-34 | 253+ | 0.015460 | 0.000000 | 0.000000 | 0.000000 | 0.000000 | 0.000000 |
| 35-39 | 253+ | 0.014711 | 0.000000 | 0.000000 | 0.000000 | 0.000000 | 0.000000 |
| 40-44 | 253+ | 0.014241 | 0.000000 | 0.000000 | 0.000000 | 0.000000 | 0.000000 |
| 45-49 | 253+ | 0.013014 | 0.000000 | 0.000000 | 0.000000 | 0.000000 | 0.000000 |
| 50-54 | 253+ | 0.011768 | 0.000000 | 0.000000 | 0.000000 | 0.000000 | 0.000000 |
| 55-59 | 253+ | 0.011032 | 0.000000 | 0.000000 | 0.000000 | 0.000000 | 0.000000 |
| 60-64 | 253+ | 0.009525 | 0.000000 | 0.000000 | 0.000000 | 0.000000 | 0.000000 |
| 65-69 | 253+ | 0.006897 | 0.000000 | 0.000000 | 0.000000 | 0.000000 | 0.000000 |
| 70-74 | 253+ | 0.005435 | 0.000000 | 0.000000 | 0.000000 | 0.000000 | 0.000000 |
| 75-79 | 253+ | 0.005435 | 0.000000 | 0.000000 | 0.000000 | 0.000000 | 0.000000 |

# Results for PHIM for Run 3 (Sensitivity_Analysis_1.RTF)

*************************************************************************************

**Main Output for the Tobacco History for Run: 3, Simulation: 1, Scenario: NULL**

| Variable Parameter | Value |
|---|---|
| 1. Country | US (United States) |
| 2. Sex | M (Males) |
| 3. Year of start of process | 1990 |

Year of Follow-up: 2040, Age Group: All Ages

| Period | Age Group | Smoking Group | N | % |
|---|---|---|---|---|
| 50 | All Ages | Never Any | 19445 | 50.68 |
| 50 | All Ages | CC | 3024 | 7.88 |
| 50 | All Ages | Former | 15896 | 41.43 |

Year of Follow-up: 2040, Age Group: 18-19

| Period | Age Group | Smoking Group | N | % |
|---|---|---|---|---|
| 50 | 18-19 | Never Any | 0 | . |
| 50 | 18-19 | CC | 0 | . |
| 50 | 18-19 | Former | 0 | . |

Year of Follow-up: 2040, Age Group: 20-24

| Period | Age Group | Smoking Group | N | % |
|---|---|---|---|---|
| 50 | 20-24 | Never Any | 0 | . |
| 50 | 20-24 | CC | 0 | . |
| 50 | 20-24 | Former | 0 | . |

Year of Follow-up: 2040, Age Group: 25-29

| Period | Age Group | Smoking Group | N | % |
|---|---|---|---|---|
| 50 | 25-29 | Never Any | 0 | . |
| 50 | 25-29 | CC | 0 | . |
| 50 | 25-29 | Former | 0 | . |

Year of Follow-up: 2040, Age Group: 30-34

| Period | Age Group | Smoking Group | N | % |
|---|---|---|---|---|
| 50 | 30-34 | Never Any | 0 | . |
| 50 | 30-34 | CC | 0 | . |
| 50 | 30-34 | Former | 0 | . |

Year of Follow-up: 2040, Age Group: 35-39

| Period | Age Group | Smoking Group | N | % |
|---|---|---|---|---|
| 50 | 35-39 | Never Any | 0 | . |
| 50 | 35-39 | CC | 0 | . |
| 50 | 35-39 | Former | 0 | . |

Year of Follow-up: 2040, Age Group: 40-44

| Period | Age Group | Smoking Group | N | % |
|---|---|---|---|---|
| 50 | 40-44 | Never Any | 0 | . |
| 50 | 40-44 | CC | 0 | . |
| 50 | 40-44 | Former | 0 | . |

Year of Follow-up: 2040, Age Group: 45-49

| Period | Age Group | Smoking Group | N | % |
|---|---|---|---|---|
| 50 | 45-49 | Never Any | 0 | . |
| 50 | 45-49 | CC | 0 | . |
| 50 | 45-49 | Former | 0 | . |

Year of Follow-up: 2040, Age Group: 50-54

| Period | Age Group | Smoking Group | N | % |
|---|---|---|---|---|
| 50 | 50-54 | Never Any | 0 | . |
| 50 | 50-54 | CC | 0 | . |
| 50 | 50-54 | Former | 0 | . |

Year of Follow-up: 2040, Age Group: 55-59

| Period | Age Group | Smoking Group | N | % |
|---|---|---|---|---|
| 50 | 55-59 | Never Any | 0 | . |
| 50 | 55-59 | CC | 0 | . |
| 50 | 55-59 | Former | 0 | . |

Year of Follow-up: 2040, Age Group: 60-64

| Period | Age Group | Smoking Group | N | % |
|---|---|---|---|---|

# Results for PHIM for Run 3 (Sensitivity_Analysis_1.RTF)

| | | | | |
|---|---|---|---|---|
| 50 | 60-64 | Never Any | 4363 | 49.33 |
| 50 | 60-64 | CC | 934 | 10.56 |
| 50 | 60-64 | Former | 3548 | 40.11 |

Year of Follow-up: 2040, Age Group: 65-69

| Period | Age Group | Smoking Group | N | % |
|---|---|---|---|---|
| 50 | 65-69 | Never Any | 4948 | 54.73 |
| 50 | 65-69 | CC | 734 | 8.12 |
| 50 | 65-69 | Former | 3358 | 37.15 |

Year of Follow-up: 2040, Age Group: 70-74

| Period | Age Group | Smoking Group | N | % |
|---|---|---|---|---|
| 50 | 70-74 | Never Any | 5145 | 52.41 |
| 50 | 70-74 | CC | 688 | 7.01 |
| 50 | 70-74 | Former | 3983 | 40.58 |

Year of Follow-up: 2040, Age Group: 75-79

| Period | Age Group | Smoking Group | N | % |
|---|---|---|---|---|
| 50 | 75-79 | Never Any | 4989 | 46.78 |
| 50 | 75-79 | CC | 668 | 6.26 |
| 50 | 75-79 | Former | 5007 | 46.95 |

# Results for PHIM for Run 3 (Sensitivity_Analysis_1.RTF)

*************************************************************************************
**Main Output for the Tobacco History for Run: 3, Simulation: 1, Scenario: Ecig**

| Variable Parameter | Value |
|---|---|
| 1. Country | US (United States) |
| 2. Sex | M (Males) |
| 3. Year of start of process | 1990 |

Year of Follow-up: 1990, Age Group: All Ages

| Period | Age Group | Smoking Group | N | % | Ever Quit (N) | Ever Quit % | Mean Yrs Quit | Short Term (n) | Short Term % |
|---|---|---|---|---|---|---|---|---|---|
| 0 | All Ages | Never Any | 34075 | 39.78 | | | | | |
| 0 | All Ages | CC | 27323 | 31.90 | 0 | 0.00 | | | |
| 0 | All Ages | Ecig | 0 | 0.00 | | | | | |
| 0 | All Ages | Dual | 0 | 0.00 | | | | | |
| 0 | All Ages | Former | 24266 | 28.33 | | | 13.08 | 3623 | 14.93 |

Year of Follow-up: 1990, Age Group: 18-19

| Period | Age Group | Smoking Group | N | % | Ever Quit (N) | Ever Quit % | Mean Yrs Quit | Short Term (n) | Short Term % |
|---|---|---|---|---|---|---|---|---|---|
| 0 | 18-19 | Never Any | 2783 | 78.42 | | | | | |
| 0 | 18-19 | CC | 634 | 17.86 | 0 | 0.00 | | | |
| 0 | 18-19 | Ecig | 0 | 0.00 | | | | | |
| 0 | 18-19 | Dual | 0 | 0.00 | | | | | |
| 0 | 18-19 | Former | 132 | 3.72 | | | 0.11 | 132 | 100.00 |

Year of Follow-up: 1990, Age Group: 20-24

| Period | Age Group | Smoking Group | N | % | Ever Quit (N) | Ever Quit % | Mean Yrs Quit | Short Term (n) | Short Term % |
|---|---|---|---|---|---|---|---|---|---|
| 0 | 20-24 | Never Any | 5722 | 58.29 | | | | | |
| 0 | 20-24 | CC | 3199 | 32.59 | 0 | 0.00 | | | |
| 0 | 20-24 | Ecig | 0 | 0.00 | | | | | |
| 0 | 20-24 | Dual | 0 | 0.00 | | | | | |
| 0 | 20-24 | Former | 895 | 9.12 | | | 1.46 | 506 | 56.54 |

Year of Follow-up: 1990, Age Group: 25-29

| Period | Age Group | Smoking Group | N | % | Ever Quit (N) | Ever Quit % | Mean Yrs Quit | Short Term (n) | Short Term % |
|---|---|---|---|---|---|---|---|---|---|
| 0 | 25-29 | Never Any | 5232 | 49.06 | | | | | |
| 0 | 25-29 | CC | 3914 | 36.70 | 0 | 0.00 | | | |
| 0 | 25-29 | Ecig | 0 | 0.00 | | | | | |
| 0 | 25-29 | Dual | 0 | 0.00 | | | | | |
| 0 | 25-29 | Former | 1518 | 14.23 | | | 2.87 | 643 | 42.36 |

Year of Follow-up: 1990, Age Group: 30-34

| Period | Age Group | Smoking Group | N | % | Ever Quit (N) | Ever Quit % | Mean Yrs Quit | Short Term (n) | Short Term % |
|---|---|---|---|---|---|---|---|---|---|
| 0 | 30-34 | Never Any | 4765 | 43.65 | | | | | |
| 0 | 30-34 | CC | 4023 | 36.85 | 0 | 0.00 | | | |
| 0 | 30-34 | Ecig | 0 | 0.00 | | | | | |
| 0 | 30-34 | Dual | 0 | 0.00 | | | | | |
| 0 | 30-34 | Former | 2128 | 19.49 | | | 4.06 | 592 | 27.82 |

Year of Follow-up: 1990, Age Group: 35-39

| Period | Age Group | Smoking Group | N | % | Ever Quit (N) | Ever Quit % | Mean Yrs Quit | Short Term (n) | Short Term % |
|---|---|---|---|---|---|---|---|---|---|
| 0 | 35-39 | Never Any | 3941 | 39.85 | | | | | |
| 0 | 35-39 | CC | 3474 | 35.13 | 0 | 0.00 | | | |
| 0 | 35-39 | Ecig | 0 | 0.00 | | | | | |
| 0 | 35-39 | Dual | 0 | 0.00 | | | | | |
| 0 | 35-39 | Former | 2474 | 25.02 | | | 8.31 | 425 | 17.18 |

Year of Follow-up: 1990, Age Group: 40-44

| Period | Age Group | Smoking Group | N | % | Ever Quit (N) | Ever Quit % | Mean Yrs Quit | Short Term (n) | Short Term % |
|---|---|---|---|---|---|---|---|---|---|
| 0 | 40-44 | Never Any | 2912 | 33.65 | | | | | |
| 0 | 40-44 | CC | 3113 | 35.97 | 0 | 0.00 | | | |
| 0 | 40-44 | Ecig | 0 | 0.00 | | | | | |
| 0 | 40-44 | Dual | 0 | 0.00 | | | | | |
| 0 | 40-44 | Former | 2630 | 30.39 | | | 10.69 | 317 | 12.05 |

Year of Follow-up: 1990, Age Group: 45-49

| Period | Age Group | Smoking Group | N | % | Ever Quit (N) | Ever Quit % | Mean Yrs Quit | Short Term (n) | Short Term % |
|---|---|---|---|---|---|---|---|---|---|
| 0 | 45-49 | Never Any | 1919 | 29.46 | | | | | |
| 0 | 45-49 | CC | 2318 | 35.58 | 0 | 0.00 | | | |
| 0 | 45-49 | Ecig | 0 | 0.00 | | | | | |
| 0 | 45-49 | Dual | 0 | 0.00 | | | | | |
| 0 | 45-49 | Former | 2278 | 34.97 | | | 13.26 | 221 | 9.70 |

Year of Follow-up: 1990, Age Group: 50-54

| Period | Age Group | Smoking Group | N | % | Ever Quit (N) | Ever Quit % | Mean Yrs Quit | Short Term (n) | Short Term % |
|---|---|---|---|---|---|---|---|---|---|
| 0 | 50-54 | Never Any | 1611 | 29.41 | | | | | |
| 0 | 50-54 | CC | 1816 | 33.16 | 0 | 0.00 | | | |

# Results for PHIM for Run 3 (Sensitivity_Analysis_1.RTF)

| | | | | | | | | | |
|---|---|---|---|---|---|---|---|---|---|
| 0 | 50-54 | Ecig | 0 | 0.00 | | | | | |
| 0 | 50-54 | Dual | 0 | 0.00 | | | | | |
| 0 | 50-54 | Former | 2050 | 37.43 | | | 14.55 | 261 | 12.73 |

Year of Follow-up: 1990, Age Group: 55-59

| Period | Age Group | Smoking Group | N | % | Ever Quit (N) | Ever Quit % | Mean Yrs Quit | Short Term (n) | Short Term % |
|---|---|---|---|---|---|---|---|---|---|
| 0 | 55-59 | Never Any | 1337 | 26.83 | | | | | |
| 0 | 55-59 | CC | 1520 | 30.50 | 0 | 0.00 | | | |
| 0 | 55-59 | Ecig | 0 | 0.00 | | | | | |
| 0 | 55-59 | Dual | 0 | 0.00 | | | | | |
| 0 | 55-59 | Former | 2126 | 42.67 | | | 14.80 | 216 | 10.16 |

Year of Follow-up: 1990, Age Group: 60-64

| Period | Age Group | Smoking Group | N | % | Ever Quit (N) | Ever Quit % | Mean Yrs Quit | Short Term (n) | Short Term % |
|---|---|---|---|---|---|---|---|---|---|
| 0 | 60-64 | Never Any | 1169 | 23.83 | | | | | |
| 0 | 60-64 | CC | 1287 | 26.24 | 0 | 0.00 | | | |
| 0 | 60-64 | Ecig | 0 | 0.00 | | | | | |
| 0 | 60-64 | Dual | 0 | 0.00 | | | | | |
| 0 | 60-64 | Former | 2449 | 49.93 | | | 18.87 | 106 | 4.33 |

Year of Follow-up: 1990, Age Group: 65-69

| Period | Age Group | Smoking Group | N | % | Ever Quit (N) | Ever Quit % | Mean Yrs Quit | Short Term (n) | Short Term % |
|---|---|---|---|---|---|---|---|---|---|
| 0 | 65-69 | Never Any | 1023 | 23.32 | | | | | |
| 0 | 65-69 | CC | 979 | 22.32 | 0 | 0.00 | | | |
| 0 | 65-69 | Ecig | 0 | 0.00 | | | | | |
| 0 | 65-69 | Dual | 0 | 0.00 | | | | | |
| 0 | 65-69 | Former | 2385 | 54.37 | | | 19.91 | 113 | 4.74 |

Year of Follow-up: 1990, Age Group: 70-74

| Period | Age Group | Smoking Group | N | % | Ever Quit (N) | Ever Quit % | Mean Yrs Quit | Short Term (n) | Short Term % |
|---|---|---|---|---|---|---|---|---|---|
| 0 | 70-74 | Never Any | 961 | 27.46 | | | | | |
| 0 | 70-74 | CC | 675 | 19.29 | 0 | 0.00 | | | |
| 0 | 70-74 | Ecig | 0 | 0.00 | | | | | |
| 0 | 70-74 | Dual | 0 | 0.00 | | | | | |
| 0 | 70-74 | Former | 1863 | 53.24 | | | 20.69 | 23 | 1.23 |

Year of Follow-up: 1990, Age Group: 75-79

| Period | Age Group | Smoking Group | N | % | Ever Quit (N) | Ever Quit % | Mean Yrs Quit | Short Term (n) | Short Term % |
|---|---|---|---|---|---|---|---|---|---|
| 0 | 75-79 | Never Any | 700 | 29.06 | | | | | |
| 0 | 75-79 | CC | 371 | 15.40 | 0 | 0.00 | | | |
| 0 | 75-79 | Ecig | 0 | 0.00 | | | | | |
| 0 | 75-79 | Dual | 0 | 0.00 | | | | | |
| 0 | 75-79 | Former | 1338 | 55.54 | | | 22.88 | 68 | 5.08 |

Year of Follow-up: 1995, Age Group: All Ages

| Period | Age Group | Smoking Group | N | % | Ever Quit (N) | Ever Quit % | Mean Yrs Quit | Short Term (n) | Short Term % |
|---|---|---|---|---|---|---|---|---|---|
| 5 | All Ages | Never Any | 38368 | 41.57 | | | | | |
| 5 | All Ages | CC | 11508 | 12.47 | 0 | 0.00 | | | |
| 5 | All Ages | Ecig | 15517 | 16.81 | | | | | |
| 5 | All Ages | Dual | 0 | 0.00 | | | | | |
| 5 | All Ages | Former | 26897 | 29.14 | | | 15.19 | 1705 | 6.34 |

Year of Follow-up: 1995, Age Group: 18-19

| Period | Age Group | Smoking Group | N | % | Ever Quit (N) | Ever Quit % | Mean Yrs Quit | Short Term (n) | Short Term % |
|---|---|---|---|---|---|---|---|---|---|
| 5 | 18-19 | Never Any | 2448 | 69.07 | | | | | |
| 5 | 18-19 | CC | 320 | 9.03 | 0 | 0.00 | | | |
| 5 | 18-19 | Ecig | 659 | 18.59 | | | | | |
| 5 | 18-19 | Dual | 0 | 0.00 | | | | | |
| 5 | 18-19 | Former | 117 | 3.30 | | | 1.11 | 80 | 68.38 |

Year of Follow-up: 1995, Age Group: 20-24

| Period | Age Group | Smoking Group | N | % | Ever Quit (N) | Ever Quit % | Mean Yrs Quit | Short Term (n) | Short Term % |
|---|---|---|---|---|---|---|---|---|---|
| 5 | 20-24 | Never Any | 5787 | 64.02 | | | | | |
| 5 | 20-24 | CC | 946 | 10.46 | 0 | 0.00 | | | |
| 5 | 20-24 | Ecig | 1826 | 20.20 | | | | | |
| 5 | 20-24 | Dual | 0 | 0.00 | | | | | |
| 5 | 20-24 | Former | 481 | 5.32 | | | 2.81 | 142 | 29.52 |

Year of Follow-up: 1995, Age Group: 25-29

| Period | Age Group | Smoking Group | N | % | Ever Quit (N) | Ever Quit % | Mean Yrs Quit | Short Term (n) | Short Term % |
|---|---|---|---|---|---|---|---|---|---|
| 5 | 25-29 | Never Any | 5471 | 55.74 | | | | | |
| 5 | 25-29 | CC | 1255 | 12.79 | 0 | 0.00 | | | |
| 5 | 25-29 | Ecig | 1942 | 19.78 | | | | | |
| 5 | 25-29 | Dual | 0 | 0.00 | | | | | |
| 5 | 25-29 | Former | 1148 | 11.70 | | | 5.43 | 115 | 10.02 |

Year of Follow-up: 1995, Age Group: 30-34

| Period | Age Group | Smoking Group | N | % | Ever Quit (N) | Ever Quit % | Mean Yrs Quit | Short Term (n) | Short Term % |
|---|---|---|---|---|---|---|---|---|---|

## Results for PHIM for Run 3 (Sensitivity_Analysis_1.RTF)

| | | | | | | | | | |
|---|---|---|---|---|---|---|---|---|---|
| 5 | 30-34 | Never Any | 5065 | 47.50 | | | | | |
| 5 | 30-34 | CC | 1480 | 13.88 | 0 | 0.00 | | | |
| 5 | 30-34 | Ecig | 2319 | 21.75 | | | | | |
| 5 | 30-34 | Dual | 0 | 0.00 | | | | | |
| 5 | 30-34 | Former | 1800 | 16.88 | | | 6.94 | 106 | 5.89 |

Year of Follow-up: 1995, Age Group: 35-39

| Period | Age Group | Smoking Group | N | % | Ever Quit (N) | Ever Quit % | Mean Yrs Quit | Short Term (n) | Short Term % |
|---|---|---|---|---|---|---|---|---|---|
| 5 | 35-39 | Never Any | 4724 | 43.28 | | | | | |
| 5 | 35-39 | CC | 1496 | 13.70 | 0 | 0.00 | | | |
| 5 | 35-39 | Ecig | 2275 | 20.84 | | | | | |
| 5 | 35-39 | Dual | 0 | 0.00 | | | | | |
| 5 | 35-39 | Former | 2421 | 22.18 | | | 8.20 | 114 | 4.71 |

Year of Follow-up: 1995, Age Group: 40-44

| Period | Age Group | Smoking Group | N | % | Ever Quit (N) | Ever Quit % | Mean Yrs Quit | Short Term (n) | Short Term % |
|---|---|---|---|---|---|---|---|---|---|
| 5 | 40-44 | Never Any | 3941 | 39.85 | | | | | |
| 5 | 40-44 | CC | 1280 | 12.94 | 0 | 0.00 | | | |
| 5 | 40-44 | Ecig | 1894 | 19.15 | | | | | |
| 5 | 40-44 | Dual | 0 | 0.00 | | | | | |
| 5 | 40-44 | Former | 2774 | 28.05 | | | 12.06 | 130 | 4.69 |

Year of Follow-up: 1995, Age Group: 45-49

| Period | Age Group | Smoking Group | N | % | Ever Quit (N) | Ever Quit % | Mean Yrs Quit | Short Term (n) | Short Term % |
|---|---|---|---|---|---|---|---|---|---|
| 5 | 45-49 | Never Any | 2912 | 33.65 | | | | | |
| 5 | 45-49 | CC | 1236 | 14.28 | 0 | 0.00 | | | |
| 5 | 45-49 | Ecig | 1562 | 18.05 | | | | | |
| 5 | 45-49 | Dual | 0 | 0.00 | | | | | |
| 5 | 45-49 | Former | 2945 | 34.03 | | | 14.20 | 148 | 5.03 |

Year of Follow-up: 1995, Age Group: 50-54

| Period | Age Group | Smoking Group | N | % | Ever Quit (N) | Ever Quit % | Mean Yrs Quit | Short Term (n) | Short Term % |
|---|---|---|---|---|---|---|---|---|---|
| 5 | 50-54 | Never Any | 1919 | 29.46 | | | | | |
| 5 | 50-54 | CC | 940 | 14.43 | 0 | 0.00 | | | |
| 5 | 50-54 | Ecig | 1015 | 15.58 | | | | | |
| 5 | 50-54 | Dual | 0 | 0.00 | | | | | |
| 5 | 50-54 | Former | 2641 | 40.54 | | | 15.98 | 175 | 6.63 |

Year of Follow-up: 1995, Age Group: 55-59

| Period | Age Group | Smoking Group | N | % | Ever Quit (N) | Ever Quit % | Mean Yrs Quit | Short Term (n) | Short Term % |
|---|---|---|---|---|---|---|---|---|---|
| 5 | 55-59 | Never Any | 1611 | 29.41 | | | | | |
| 5 | 55-59 | CC | 740 | 13.51 | 0 | 0.00 | | | |
| 5 | 55-59 | Ecig | 762 | 13.91 | | | | | |
| 5 | 55-59 | Dual | 0 | 0.00 | | | | | |
| 5 | 55-59 | Former | 2364 | 43.16 | | | 17.21 | 122 | 5.16 |

Year of Follow-up: 1995, Age Group: 60-64

| Period | Age Group | Smoking Group | N | % | Ever Quit (N) | Ever Quit % | Mean Yrs Quit | Short Term (n) | Short Term % |
|---|---|---|---|---|---|---|---|---|---|
| 5 | 60-64 | Never Any | 1337 | 26.83 | | | | | |
| 5 | 60-64 | CC | 597 | 11.98 | 0 | 0.00 | | | |
| 5 | 60-64 | Ecig | 572 | 11.48 | | | | | |
| 5 | 60-64 | Dual | 0 | 0.00 | | | | | |
| 5 | 60-64 | Former | 2477 | 49.71 | | | 17.27 | 143 | 5.77 |

Year of Follow-up: 1995, Age Group: 65-69

| Period | Age Group | Smoking Group | N | % | Ever Quit (N) | Ever Quit % | Mean Yrs Quit | Short Term (n) | Short Term % |
|---|---|---|---|---|---|---|---|---|---|
| 5 | 65-69 | Never Any | 1169 | 23.83 | | | | | |
| 5 | 65-69 | CC | 477 | 9.72 | 0 | 0.00 | | | |
| 5 | 65-69 | Ecig | 360 | 7.34 | | | | | |
| 5 | 65-69 | Dual | 0 | 0.00 | | | | | |
| 5 | 65-69 | Former | 2899 | 59.10 | | | 20.44 | 196 | 6.76 |

Year of Follow-up: 1995, Age Group: 70-74

| Period | Age Group | Smoking Group | N | % | Ever Quit (N) | Ever Quit % | Mean Yrs Quit | Short Term (n) | Short Term % |
|---|---|---|---|---|---|---|---|---|---|
| 5 | 70-74 | Never Any | 1023 | 23.32 | | | | | |
| 5 | 70-74 | CC | 438 | 9.98 | 0 | 0.00 | | | |
| 5 | 70-74 | Ecig | 196 | 4.47 | | | | | |
| 5 | 70-74 | Dual | 0 | 0.00 | | | | | |
| 5 | 70-74 | Former | 2730 | 62.23 | | | 22.00 | 137 | 5.02 |

Year of Follow-up: 1995, Age Group: 75-79

| Period | Age Group | Smoking Group | N | % | Ever Quit (N) | Ever Quit % | Mean Yrs Quit | Short Term (n) | Short Term % |
|---|---|---|---|---|---|---|---|---|---|
| 5 | 75-79 | Never Any | 961 | 27.46 | | | | | |
| 5 | 75-79 | CC | 303 | 8.66 | 0 | 0.00 | | | |
| 5 | 75-79 | Ecig | 135 | 3.86 | | | | | |
| 5 | 75-79 | Dual | 0 | 0.00 | | | | | |
| 5 | 75-79 | Former | 2100 | 60.02 | | | 23.02 | 97 | 4.62 |

Year of Follow-up: 2000, Age Group: All Ages

# Results for PHIM for Run 3 (Sensitivity_Analysis_1.RTF)

| Period | Age Group | Smoking Group | N | % | Ever Quit (N) | Ever Quit % | Mean Yrs Quit | Short Term (n) | Short Term % |
|---|---|---|---|---|---|---|---|---|---|
| 10 | All Ages | Never Any | 39177 | 41.64 | | | | | |
| 10 | All Ages | CC | 4843 | 5.15 | 0 | 0.00 | | | |
| 10 | All Ages | Ecig | 20724 | 22.03 | | | | | |
| 10 | All Ages | Dual | 0 | 0.00 | | | | | |
| 10 | All Ages | Former | 29348 | 31.19 | | | 16.84 | 1724 | 5.87 |

Year of Follow-up: 2000, Age Group: 18-19

| Period | Age Group | Smoking Group | N | % | Ever Quit (N) | Ever Quit % | Mean Yrs Quit | Short Term (n) | Short Term % |
|---|---|---|---|---|---|---|---|---|---|
| 10 | 18-19 | Never Any | 0 | . | | | | | |
| 10 | 18-19 | CC | 0 | . | 0 | . | | | |
| 10 | 18-19 | Ecig | 0 | . | | | | | |
| 10 | 18-19 | Dual | 0 | . | | | | | |
| 10 | 18-19 | Former | 0 | . | | | . | 0 | . |

Year of Follow-up: 2000, Age Group: 20-24

| Period | Age Group | Smoking Group | N | % | Ever Quit (N) | Ever Quit % | Mean Yrs Quit | Short Term (n) | Short Term % |
|---|---|---|---|---|---|---|---|---|---|
| 10 | 20-24 | Never Any | 4927 | 55.70 | | | | | |
| 10 | 20-24 | CC | 462 | 5.22 | 0 | 0.00 | | | |
| 10 | 20-24 | Ecig | 2544 | 28.76 | | | | | |
| 10 | 20-24 | Dual | 0 | 0.00 | | | | | |
| 10 | 20-24 | Former | 912 | 10.31 | | | 3.41 | 258 | 28.29 |

Year of Follow-up: 2000, Age Group: 25-29

| Period | Age Group | Smoking Group | N | % | Ever Quit (N) | Ever Quit % | Mean Yrs Quit | Short Term (n) | Short Term % |
|---|---|---|---|---|---|---|---|---|---|
| 10 | 25-29 | Never Any | 5341 | 59.08 | | | | | |
| 10 | 25-29 | CC | 404 | 4.47 | 0 | 0.00 | | | |
| 10 | 25-29 | Ecig | 2359 | 26.10 | | | | | |
| 10 | 25-29 | Dual | 0 | 0.00 | | | | | |
| 10 | 25-29 | Former | 936 | 10.35 | | | 4.91 | 195 | 20.83 |

Year of Follow-up: 2000, Age Group: 30-34

| Period | Age Group | Smoking Group | N | % | Ever Quit (N) | Ever Quit % | Mean Yrs Quit | Short Term (n) | Short Term % |
|---|---|---|---|---|---|---|---|---|---|
| 10 | 30-34 | Never Any | 5276 | 53.75 | | | | | |
| 10 | 30-34 | CC | 481 | 4.90 | 0 | 0.00 | | | |
| 10 | 30-34 | Ecig | 2608 | 26.57 | | | | | |
| 10 | 30-34 | Dual | 0 | 0.00 | | | | | |
| 10 | 30-34 | Former | 1451 | 14.78 | | | 8.66 | 118 | 8.13 |

Year of Follow-up: 2000, Age Group: 35-39

| Period | Age Group | Smoking Group | N | % | Ever Quit (N) | Ever Quit % | Mean Yrs Quit | Short Term (n) | Short Term % |
|---|---|---|---|---|---|---|---|---|---|
| 10 | 35-39 | Never Any | 4997 | 46.86 | | | | | |
| 10 | 35-39 | CC | 609 | 5.71 | 0 | 0.00 | | | |
| 10 | 35-39 | Ecig | 2980 | 27.94 | | | | | |
| 10 | 35-39 | Dual | 0 | 0.00 | | | | | |
| 10 | 35-39 | Former | 2078 | 19.49 | | | 10.61 | 109 | 5.25 |

Year of Follow-up: 2000, Age Group: 40-44

| Period | Age Group | Smoking Group | N | % | Ever Quit (N) | Ever Quit % | Mean Yrs Quit | Short Term (n) | Short Term % |
|---|---|---|---|---|---|---|---|---|---|
| 10 | 40-44 | Never Any | 4724 | 43.28 | | | | | |
| 10 | 40-44 | CC | 567 | 5.19 | 0 | 0.00 | | | |
| 10 | 40-44 | Ecig | 2857 | 26.17 | | | | | |
| 10 | 40-44 | Dual | 0 | 0.00 | | | | | |
| 10 | 40-44 | Former | 2768 | 25.36 | | | 11.82 | 124 | 4.48 |

Year of Follow-up: 2000, Age Group: 45-49

| Period | Age Group | Smoking Group | N | % | Ever Quit (N) | Ever Quit % | Mean Yrs Quit | Short Term (n) | Short Term % |
|---|---|---|---|---|---|---|---|---|---|
| 10 | 45-49 | Never Any | 3941 | 39.85 | | | | | |
| 10 | 45-49 | CC | 484 | 4.89 | 0 | 0.00 | | | |
| 10 | 45-49 | Ecig | 2312 | 23.38 | | | | | |
| 10 | 45-49 | Dual | 0 | 0.00 | | | | | |
| 10 | 45-49 | Former | 3152 | 31.87 | | | 15.25 | 163 | 5.17 |

Year of Follow-up: 2000, Age Group: 50-54

| Period | Age Group | Smoking Group | N | % | Ever Quit (N) | Ever Quit % | Mean Yrs Quit | Short Term (n) | Short Term % |
|---|---|---|---|---|---|---|---|---|---|
| 10 | 50-54 | Never Any | 2912 | 33.65 | | | | | |
| 10 | 50-54 | CC | 473 | 5.47 | 0 | 0.00 | | | |
| 10 | 50-54 | Ecig | 1859 | 21.48 | | | | | |
| 10 | 50-54 | Dual | 0 | 0.00 | | | | | |
| 10 | 50-54 | Former | 3411 | 39.41 | | | 16.85 | 186 | 5.45 |

Year of Follow-up: 2000, Age Group: 55-59

| Period | Age Group | Smoking Group | N | % | Ever Quit (N) | Ever Quit % | Mean Yrs Quit | Short Term (n) | Short Term % |
|---|---|---|---|---|---|---|---|---|---|
| 10 | 55-59 | Never Any | 1919 | 29.46 | | | | | |
| 10 | 55-59 | CC | 363 | 5.57 | 0 | 0.00 | | | |
| 10 | 55-59 | Ecig | 1194 | 18.33 | | | | | |
| 10 | 55-59 | Dual | 0 | 0.00 | | | | | |

# Results for PHIM for Run 3 (Sensitivity_Analysis_1.RTF)

| | | | | | | | | | |
|---|---|---|---|---|---|---|---|---|---|
| 10 | 55-59 | Former | 3039 | 46.65 | | | 18.50 | 146 | 4.80 |

Year of Follow-up: 2000, Age Group: 60-64

| Period | Age Group | Smoking Group | N | % | Ever Quit (N) | Ever Quit % | Mean Yrs Quit | Short Term (n) | Short Term % |
|---|---|---|---|---|---|---|---|---|---|
| 10 | 60-64 | Never Any | 1611 | 29.41 | | | | | |
| 10 | 60-64 | CC | 306 | 5.59 | 0 | 0.00 | | | |
| 10 | 60-64 | Ecig | 852 | 15.56 | | | | | |
| 10 | 60-64 | Dual | 0 | 0.00 | | | | | |
| 10 | 60-64 | Former | 2708 | 49.44 | | | 19.63 | 144 | 5.32 |

Year of Follow-up: 2000, Age Group: 65-69

| Period | Age Group | Smoking Group | N | % | Ever Quit (N) | Ever Quit % | Mean Yrs Quit | Short Term (n) | Short Term % |
|---|---|---|---|---|---|---|---|---|---|
| 10 | 65-69 | Never Any | 1337 | 26.83 | | | | | |
| 10 | 65-69 | CC | 244 | 4.90 | 0 | 0.00 | | | |
| 10 | 65-69 | Ecig | 603 | 12.10 | | | | | |
| 10 | 65-69 | Dual | 0 | 0.00 | | | | | |
| 10 | 65-69 | Former | 2799 | 56.17 | | | 19.95 | 120 | 4.29 |

Year of Follow-up: 2000, Age Group: 70-74

| Period | Age Group | Smoking Group | N | % | Ever Quit (N) | Ever Quit % | Mean Yrs Quit | Short Term (n) | Short Term % |
|---|---|---|---|---|---|---|---|---|---|
| 10 | 70-74 | Never Any | 1169 | 23.83 | | | | | |
| 10 | 70-74 | CC | 236 | 4.81 | 0 | 0.00 | | | |
| 10 | 70-74 | Ecig | 327 | 6.67 | | | | | |
| 10 | 70-74 | Dual | 0 | 0.00 | | | | | |
| 10 | 70-74 | Former | 3173 | 64.69 | | | 23.44 | 94 | 2.96 |

Year of Follow-up: 2000, Age Group: 75-79

| Period | Age Group | Smoking Group | N | % | Ever Quit (N) | Ever Quit % | Mean Yrs Quit | Short Term (n) | Short Term % |
|---|---|---|---|---|---|---|---|---|---|
| 10 | 75-79 | Never Any | 1023 | 23.32 | | | | | |
| 10 | 75-79 | CC | 214 | 4.88 | 0 | 0.00 | | | |
| 10 | 75-79 | Ecig | 229 | 5.22 | | | | | |
| 10 | 75-79 | Dual | 0 | 0.00 | | | | | |
| 10 | 75-79 | Former | 2921 | 66.58 | | | 25.38 | 67 | 2.29 |

Year of Follow-up: 2005, Age Group: All Ages

| Period | Age Group | Smoking Group | N | % | Ever Quit (N) | Ever Quit % | Mean Yrs Quit | Short Term (n) | Short Term % |
|---|---|---|---|---|---|---|---|---|---|
| 15 | All Ages | Never Any | 37449 | 41.75 | | | | | |
| 15 | All Ages | CC | 1911 | 2.13 | 0 | 0.00 | | | |
| 15 | All Ages | Ecig | 20003 | 22.30 | | | | | |
| 15 | All Ages | Dual | 0 | 0.00 | | | | | |
| 15 | All Ages | Former | 30342 | 33.82 | | | 18.46 | 1574 | 5.19 |

Year of Follow-up: 2005, Age Group: 18-19

| Period | Age Group | Smoking Group | N | % | Ever Quit (N) | Ever Quit % | Mean Yrs Quit | Short Term (n) | Short Term % |
|---|---|---|---|---|---|---|---|---|---|
| 15 | 18-19 | Never Any | 0 | . | | | | | |
| 15 | 18-19 | CC | 0 | . | 0 | . | | | |
| 15 | 18-19 | Ecig | 0 | . | | | | | |
| 15 | 18-19 | Dual | 0 | . | | | | | |
| 15 | 18-19 | Former | 0 | . | | | . | 0 | . |

Year of Follow-up: 2005, Age Group: 20-24

| Period | Age Group | Smoking Group | N | % | Ever Quit (N) | Ever Quit % | Mean Yrs Quit | Short Term (n) | Short Term % |
|---|---|---|---|---|---|---|---|---|---|
| 15 | 20-24 | Never Any | 0 | . | | | | | |
| 15 | 20-24 | CC | 0 | . | 0 | . | | | |
| 15 | 20-24 | Ecig | 0 | . | | | | | |
| 15 | 20-24 | Dual | 0 | . | | | | | |
| 15 | 20-24 | Former | 0 | . | | | . | 0 | . |

Year of Follow-up: 2005, Age Group: 25-29

| Period | Age Group | Smoking Group | N | % | Ever Quit (N) | Ever Quit % | Mean Yrs Quit | Short Term (n) | Short Term % |
|---|---|---|---|---|---|---|---|---|---|
| 15 | 25-29 | Never Any | 4634 | 52.39 | | | | | |
| 15 | 25-29 | CC | 179 | 2.02 | 0 | 0.00 | | | |
| 15 | 25-29 | Ecig | 2661 | 30.08 | | | | | |
| 15 | 25-29 | Dual | 0 | 0.00 | | | | | |
| 15 | 25-29 | Former | 1371 | 15.50 | | | 6.32 | 174 | 12.69 |

Year of Follow-up: 2005, Age Group: 30-34

| Period | Age Group | Smoking Group | N | % | Ever Quit (N) | Ever Quit % | Mean Yrs Quit | Short Term (n) | Short Term % |
|---|---|---|---|---|---|---|---|---|---|
| 15 | 30-34 | Never Any | 5042 | 55.77 | | | | | |
| 15 | 30-34 | CC | 153 | 1.69 | 0 | 0.00 | | | |
| 15 | 30-34 | Ecig | 2479 | 27.42 | | | | | |
| 15 | 30-34 | Dual | 0 | 0.00 | | | | | |
| 15 | 30-34 | Former | 1366 | 15.11 | | | 7.38 | 171 | 12.52 |

Year of Follow-up: 2005, Age Group: 35-39

| Period | Age Group | Smoking Group | N | % | Ever Quit (N) | Ever Quit % | Mean Yrs Quit | Short Term (n) | Short Term % |
|---|---|---|---|---|---|---|---|---|---|
| 15 | 35-39 | Never Any | 5163 | 52.60 | | | | | |
| 15 | 35-39 | CC | 188 | 1.92 | 0 | 0.00 | | | |

## Results for PHIM for Run 3 (Sensitivity_Analysis_1.RTF)

| | | | | | | | | | |
|---|---|---|---|---|---|---|---|---|---|
| 15 | 35-39 | Ecig | 2654 | 27.04 | | | | | |
| 15 | 35-39 | Dual | 0 | 0.00 | | | | | |
| 15 | 35-39 | Former | 1811 | 18.45 | | | 11.33 | 147 | 8.12 |

Year of Follow-up: 2005, Age Group: 40-44

| Period | Age Group | Smoking Group | N | % | Ever Quit (N) | Ever Quit % | Mean Yrs Quit | Short Term (n) | Short Term % |
|---|---|---|---|---|---|---|---|---|---|
| 15 | 40-44 | Never Any | 4997 | 46.86 | | | | | |
| 15 | 40-44 | CC | 231 | 2.17 | 0 | 0.00 | | | |
| 15 | 40-44 | Ecig | 2948 | 27.64 | | | | | |
| 15 | 40-44 | Dual | 0 | 0.00 | | | | | |
| 15 | 40-44 | Former | 2488 | 23.33 | | | 13.32 | 199 | 8.00 |

Year of Follow-up: 2005, Age Group: 45-49

| Period | Age Group | Smoking Group | N | % | Ever Quit (N) | Ever Quit % | Mean Yrs Quit | Short Term (n) | Short Term % |
|---|---|---|---|---|---|---|---|---|---|
| 15 | 45-49 | Never Any | 4724 | 43.28 | | | | | |
| 15 | 45-49 | CC | 216 | 1.98 | 0 | 0.00 | | | |
| 15 | 45-49 | Ecig | 2793 | 25.59 | | | | | |
| 15 | 45-49 | Dual | 0 | 0.00 | | | | | |
| 15 | 45-49 | Former | 3183 | 29.16 | | | 14.88 | 160 | 5.03 |

Year of Follow-up: 2005, Age Group: 50-54

| Period | Age Group | Smoking Group | N | % | Ever Quit (N) | Ever Quit % | Mean Yrs Quit | Short Term (n) | Short Term % |
|---|---|---|---|---|---|---|---|---|---|
| 15 | 50-54 | Never Any | 3941 | 39.85 | | | | | |
| 15 | 50-54 | CC | 213 | 2.15 | 0 | 0.00 | | | |
| 15 | 50-54 | Ecig | 2180 | 22.04 | | | | | |
| 15 | 50-54 | Dual | 0 | 0.00 | | | | | |
| 15 | 50-54 | Former | 3555 | 35.95 | | | 18.19 | 154 | 4.33 |

Year of Follow-up: 2005, Age Group: 55-59

| Period | Age Group | Smoking Group | N | % | Ever Quit (N) | Ever Quit % | Mean Yrs Quit | Short Term (n) | Short Term % |
|---|---|---|---|---|---|---|---|---|---|
| 15 | 55-59 | Never Any | 2912 | 33.65 | | | | | |
| 15 | 55-59 | CC | 215 | 2.48 | 0 | 0.00 | | | |
| 15 | 55-59 | Ecig | 1781 | 20.58 | | | | | |
| 15 | 55-59 | Dual | 0 | 0.00 | | | | | |
| 15 | 55-59 | Former | 3747 | 43.29 | | | 20.09 | 117 | 3.12 |

Year of Follow-up: 2005, Age Group: 60-64

| Period | Age Group | Smoking Group | N | % | Ever Quit (N) | Ever Quit % | Mean Yrs Quit | Short Term (n) | Short Term % |
|---|---|---|---|---|---|---|---|---|---|
| 15 | 60-64 | Never Any | 1919 | 29.46 | | | | | |
| 15 | 60-64 | CC | 143 | 2.19 | 0 | 0.00 | | | |
| 15 | 60-64 | Ecig | 1100 | 16.88 | | | | | |
| 15 | 60-64 | Dual | 0 | 0.00 | | | | | |
| 15 | 60-64 | Former | 3353 | 51.47 | | | 21.47 | 156 | 4.65 |

Year of Follow-up: 2005, Age Group: 65-69

| Period | Age Group | Smoking Group | N | % | Ever Quit (N) | Ever Quit % | Mean Yrs Quit | Short Term (n) | Short Term % |
|---|---|---|---|---|---|---|---|---|---|
| 15 | 65-69 | Never Any | 1611 | 29.41 | | | | | |
| 15 | 65-69 | CC | 124 | 2.26 | 0 | 0.00 | | | |
| 15 | 65-69 | Ecig | 705 | 12.87 | | | | | |
| 15 | 65-69 | Dual | 0 | 0.00 | | | | | |
| 15 | 65-69 | Former | 3037 | 55.45 | | | 22.19 | 118 | 3.89 |

Year of Follow-up: 2005, Age Group: 70-74

| Period | Age Group | Smoking Group | N | % | Ever Quit (N) | Ever Quit % | Mean Yrs Quit | Short Term (n) | Short Term % |
|---|---|---|---|---|---|---|---|---|---|
| 15 | 70-74 | Never Any | 1337 | 26.83 | | | | | |
| 15 | 70-74 | CC | 129 | 2.59 | 0 | 0.00 | | | |
| 15 | 70-74 | Ecig | 441 | 8.85 | | | | | |
| 15 | 70-74 | Dual | 0 | 0.00 | | | | | |
| 15 | 70-74 | Former | 3076 | 61.73 | | | 22.89 | 112 | 3.64 |

Year of Follow-up: 2005, Age Group: 75-79

| Period | Age Group | Smoking Group | N | % | Ever Quit (N) | Ever Quit % | Mean Yrs Quit | Short Term (n) | Short Term % |
|---|---|---|---|---|---|---|---|---|---|
| 15 | 75-79 | Never Any | 1169 | 23.83 | | | | | |
| 15 | 75-79 | CC | 120 | 2.45 | 0 | 0.00 | | | |
| 15 | 75-79 | Ecig | 261 | 5.32 | | | | | |
| 15 | 75-79 | Dual | 0 | 0.00 | | | | | |
| 15 | 75-79 | Former | 3355 | 68.40 | | | 27.01 | 66 | 1.97 |

Year of Follow-up: 2010, Age Group: All Ages

| Period | Age Group | Smoking Group | N | % | Ever Quit (N) | Ever Quit % | Mean Yrs Quit | Short Term (n) | Short Term % |
|---|---|---|---|---|---|---|---|---|---|
| 20 | All Ages | Never Any | 36103 | 42.57 | | | | | |
| 20 | All Ages | CC | 738 | 0.87 | 0 | 0.00 | | | |
| 20 | All Ages | Ecig | 17076 | 20.14 | | | | | |
| 20 | All Ages | Dual | 0 | 0.00 | | | | | |
| 20 | All Ages | Former | 30883 | 36.42 | | | 19.82 | 1518 | 4.92 |

Year of Follow-up: 2010, Age Group: 18-19

| Period | Age Group | Smoking Group | N | % | Ever Quit (N) | Ever Quit % | Mean Yrs Quit | Short Term (n) | Short Term % |
|---|---|---|---|---|---|---|---|---|---|

# Results for PHIM for Run 3 (Sensitivity_Analysis_1.RTF)

| Period | Age Group | Smoking Group | N | % | Ever Quit (N) | Ever Quit % | Mean Yrs Quit | Short Term (n) | Short Term % |
|---|---|---|---|---|---|---|---|---|---|
| 20 | 18-19 | Never Any | 0 | . | | | | | |
| 20 | 18-19 | CC | 0 | . | 0 | . | | | |
| 20 | 18-19 | Ecig | 0 | . | | | | | |
| 20 | 18-19 | Dual | 0 | . | | | | | |
| 20 | 18-19 | Former | 0 | . | | | . | 0 | . |

Year of Follow-up: 2010, Age Group: 20-24

| Period | Age Group | Smoking Group | N | % | Ever Quit (N) | Ever Quit % | Mean Yrs Quit | Short Term (n) | Short Term % |
|---|---|---|---|---|---|---|---|---|---|
| 20 | 20-24 | Never Any | 0 | . | | | | | |
| 20 | 20-24 | CC | 0 | . | 0 | . | | | |
| 20 | 20-24 | Ecig | 0 | . | | | | | |
| 20 | 20-24 | Dual | 0 | . | | | | | |
| 20 | 20-24 | Former | 0 | . | | | . | 0 | . |

Year of Follow-up: 2010, Age Group: 25-29

| Period | Age Group | Smoking Group | N | % | Ever Quit (N) | Ever Quit % | Mean Yrs Quit | Short Term (n) | Short Term % |
|---|---|---|---|---|---|---|---|---|---|
| 20 | 25-29 | Never Any | 0 | . | | | | | |
| 20 | 25-29 | CC | 0 | . | 0 | . | | | |
| 20 | 25-29 | Ecig | 0 | . | | | | | |
| 20 | 25-29 | Dual | 0 | . | | | | | |
| 20 | 25-29 | Former | 0 | . | | | . | 0 | . |

Year of Follow-up: 2010, Age Group: 30-34

| Period | Age Group | Smoking Group | N | % | Ever Quit (N) | Ever Quit % | Mean Yrs Quit | Short Term (n) | Short Term % |
|---|---|---|---|---|---|---|---|---|---|
| 20 | 30-34 | Never Any | 4508 | 50.97 | | | | | |
| 20 | 30-34 | CC | 71 | 0.80 | 0 | 0.00 | | | |
| 20 | 30-34 | Ecig | 2376 | 26.86 | | | | | |
| 20 | 30-34 | Dual | 0 | 0.00 | | | | | |
| 20 | 30-34 | Former | 1890 | 21.37 | | | 8.72 | 230 | 12.17 |

Year of Follow-up: 2010, Age Group: 35-39

| Period | Age Group | Smoking Group | N | % | Ever Quit (N) | Ever Quit % | Mean Yrs Quit | Short Term (n) | Short Term % |
|---|---|---|---|---|---|---|---|---|---|
| 20 | 35-39 | Never Any | 4991 | 55.21 | | | | | |
| 20 | 35-39 | CC | 59 | 0.65 | 0 | 0.00 | | | |
| 20 | 35-39 | Ecig | 2154 | 23.83 | | | | | |
| 20 | 35-39 | Dual | 0 | 0.00 | | | | | |
| 20 | 35-39 | Former | 1836 | 20.31 | | | 9.77 | 141 | 7.68 |

Year of Follow-up: 2010, Age Group: 40-44

| Period | Age Group | Smoking Group | N | % | Ever Quit (N) | Ever Quit % | Mean Yrs Quit | Short Term (n) | Short Term % |
|---|---|---|---|---|---|---|---|---|---|
| 20 | 40-44 | Never Any | 5163 | 52.60 | | | | | |
| 20 | 40-44 | CC | 68 | 0.69 | 0 | 0.00 | | | |
| 20 | 40-44 | Ecig | 2416 | 24.61 | | | | | |
| 20 | 40-44 | Dual | 0 | 0.00 | | | | | |
| 20 | 40-44 | Former | 2169 | 22.10 | | | 13.96 | 143 | 6.59 |

Year of Follow-up: 2010, Age Group: 45-49

| Period | Age Group | Smoking Group | N | % | Ever Quit (N) | Ever Quit % | Mean Yrs Quit | Short Term (n) | Short Term % |
|---|---|---|---|---|---|---|---|---|---|
| 20 | 45-49 | Never Any | 4997 | 46.86 | | | | | |
| 20 | 45-49 | CC | 89 | 0.83 | 0 | 0.00 | | | |
| 20 | 45-49 | Ecig | 2619 | 24.56 | | | | | |
| 20 | 45-49 | Dual | 0 | 0.00 | | | | | |
| 20 | 45-49 | Former | 2959 | 27.75 | | | 15.74 | 171 | 5.78 |

Year of Follow-up: 2010, Age Group: 50-54

| Period | Age Group | Smoking Group | N | % | Ever Quit (N) | Ever Quit % | Mean Yrs Quit | Short Term (n) | Short Term % |
|---|---|---|---|---|---|---|---|---|---|
| 20 | 50-54 | Never Any | 4724 | 43.28 | | | | | |
| 20 | 50-54 | CC | 87 | 0.80 | 0 | 0.00 | | | |
| 20 | 50-54 | Ecig | 2490 | 22.81 | | | | | |
| 20 | 50-54 | Dual | 0 | 0.00 | | | | | |
| 20 | 50-54 | Former | 3615 | 33.12 | | | 17.75 | 174 | 4.81 |

Year of Follow-up: 2010, Age Group: 55-59

| Period | Age Group | Smoking Group | N | % | Ever Quit (N) | Ever Quit % | Mean Yrs Quit | Short Term (n) | Short Term % |
|---|---|---|---|---|---|---|---|---|---|
| 20 | 55-59 | Never Any | 3941 | 39.85 | | | | | |
| 20 | 55-59 | CC | 85 | 0.86 | 0 | 0.00 | | | |
| 20 | 55-59 | Ecig | 1859 | 18.80 | | | | | |
| 20 | 55-59 | Dual | 0 | 0.00 | | | | | |
| 20 | 55-59 | Former | 4004 | 40.49 | | | 20.81 | 181 | 4.52 |

Year of Follow-up: 2010, Age Group: 60-64

| Period | Age Group | Smoking Group | N | % | Ever Quit (N) | Ever Quit % | Mean Yrs Quit | Short Term (n) | Short Term % |
|---|---|---|---|---|---|---|---|---|---|
| 20 | 60-64 | Never Any | 2912 | 33.65 | | | | | |
| 20 | 60-64 | CC | 87 | 1.01 | 0 | 0.00 | | | |
| 20 | 60-64 | Ecig | 1428 | 16.50 | | | | | |
| 20 | 60-64 | Dual | 0 | 0.00 | | | | | |
| 20 | 60-64 | Former | 4228 | 48.85 | | | 22.41 | 241 | 5.70 |

Year of Follow-up: 2010, Age Group: 65-69

# Results for PHIM for Run 3 (Sensitivity_Analysis_1.RTF)

| Period | Age Group | Smoking Group | N | % | Ever Quit (N) | Ever Quit % | Mean Yrs Quit | Short Term (n) | Short Term % |
|---|---|---|---|---|---|---|---|---|---|
| 20 | 65-69 | Never Any | 1919 | 29.46 | | | | | |
| 20 | 65-69 | CC | 60 | 0.92 | 0 | 0.00 | | | |
| 20 | 65-69 | Ecig | 809 | 12.42 | | | | | |
| 20 | 65-69 | Dual | 0 | 0.00 | | | | | |
| 20 | 65-69 | Former | 3727 | 57.21 | | | 24.03 | 133 | 3.57 |

Year of Follow-up: 2010, Age Group: 70-74

| Period | Age Group | Smoking Group | N | % | Ever Quit (N) | Ever Quit % | Mean Yrs Quit | Short Term (n) | Short Term % |
|---|---|---|---|---|---|---|---|---|---|
| 20 | 70-74 | Never Any | 1611 | 29.41 | | | | | |
| 20 | 70-74 | CC | 69 | 1.26 | 0 | 0.00 | | | |
| 20 | 70-74 | Ecig | 574 | 10.48 | | | | | |
| 20 | 70-74 | Dual | 0 | 0.00 | | | | | |
| 20 | 70-74 | Former | 3223 | 58.85 | | | 25.75 | 61 | 1.89 |

Year of Follow-up: 2010, Age Group: 75-79

| Period | Age Group | Smoking Group | N | % | Ever Quit (N) | Ever Quit % | Mean Yrs Quit | Short Term (n) | Short Term % |
|---|---|---|---|---|---|---|---|---|---|
| 20 | 75-79 | Never Any | 1337 | 26.83 | | | | | |
| 20 | 75-79 | CC | 63 | 1.26 | 0 | 0.00 | | | |
| 20 | 75-79 | Ecig | 351 | 7.04 | | | | | |
| 20 | 75-79 | Dual | 0 | 0.00 | | | | | |
| 20 | 75-79 | Former | 3232 | 64.86 | | | 26.66 | 43 | 1.33 |

Year of Follow-up: 2015, Age Group: All Ages

| Period | Age Group | Smoking Group | N | % | Ever Quit (N) | Ever Quit % | Mean Yrs Quit | Short Term (n) | Short Term % |
|---|---|---|---|---|---|---|---|---|---|
| 25 | All Ages | Never Any | 34747 | 43.53 | | | | | |
| 25 | All Ages | CC | 289 | 0.36 | 0 | 0.00 | | | |
| 25 | All Ages | Ecig | 14107 | 17.67 | | | | | |
| 25 | All Ages | Dual | 0 | 0.00 | | | | | |
| 25 | All Ages | Former | 30674 | 38.43 | | | 21.87 | 1088 | 3.55 |

Year of Follow-up: 2015, Age Group: 18-19

| Period | Age Group | Smoking Group | N | % | Ever Quit (N) | Ever Quit % | Mean Yrs Quit | Short Term (n) | Short Term % |
|---|---|---|---|---|---|---|---|---|---|
| 25 | 18-19 | Never Any | 0 | . | | | | | |
| 25 | 18-19 | CC | 0 | . | 0 | . | | | |
| 25 | 18-19 | Ecig | 0 | . | | | | | |
| 25 | 18-19 | Dual | 0 | . | | | | | |
| 25 | 18-19 | Former | 0 | . | | | . | 0 | . |

Year of Follow-up: 2015, Age Group: 20-24

| Period | Age Group | Smoking Group | N | % | Ever Quit (N) | Ever Quit % | Mean Yrs Quit | Short Term (n) | Short Term % |
|---|---|---|---|---|---|---|---|---|---|
| 25 | 20-24 | Never Any | 0 | . | | | | | |
| 25 | 20-24 | CC | 0 | . | 0 | . | | | |
| 25 | 20-24 | Ecig | 0 | . | | | | | |
| 25 | 20-24 | Dual | 0 | . | | | | | |
| 25 | 20-24 | Former | 0 | . | | | . | 0 | . |

Year of Follow-up: 2015, Age Group: 25-29

| Period | Age Group | Smoking Group | N | % | Ever Quit (N) | Ever Quit % | Mean Yrs Quit | Short Term (n) | Short Term % |
|---|---|---|---|---|---|---|---|---|---|
| 25 | 25-29 | Never Any | 0 | . | | | | | |
| 25 | 25-29 | CC | 0 | . | 0 | . | | | |
| 25 | 25-29 | Ecig | 0 | . | | | | | |
| 25 | 25-29 | Dual | 0 | . | | | | | |
| 25 | 25-29 | Former | 0 | . | | | . | 0 | . |

Year of Follow-up: 2015, Age Group: 30-34

| Period | Age Group | Smoking Group | N | % | Ever Quit (N) | Ever Quit % | Mean Yrs Quit | Short Term (n) | Short Term % |
|---|---|---|---|---|---|---|---|---|---|
| 25 | 30-34 | Never Any | 0 | . | | | | | |
| 25 | 30-34 | CC | 0 | . | 0 | . | | | |
| 25 | 30-34 | Ecig | 0 | . | | | | | |
| 25 | 30-34 | Dual | 0 | . | | | | | |
| 25 | 30-34 | Former | 0 | . | | | . | 0 | . |

Year of Follow-up: 2015, Age Group: 35-39

| Period | Age Group | Smoking Group | N | % | Ever Quit (N) | Ever Quit % | Mean Yrs Quit | Short Term (n) | Short Term % |
|---|---|---|---|---|---|---|---|---|---|
| 25 | 35-39 | Never Any | 4489 | 50.75 | | | | | |
| 25 | 35-39 | CC | 28 | 0.32 | 0 | 0.00 | | | |
| 25 | 35-39 | Ecig | 1990 | 22.50 | | | | | |
| 25 | 35-39 | Dual | 0 | 0.00 | | | | | |
| 25 | 35-39 | Former | 2338 | 26.43 | | | 11.53 | 142 | 6.07 |

Year of Follow-up: 2015, Age Group: 40-44

| Period | Age Group | Smoking Group | N | % | Ever Quit (N) | Ever Quit % | Mean Yrs Quit | Short Term (n) | Short Term % |
|---|---|---|---|---|---|---|---|---|---|
| 25 | 40-44 | Never Any | 4991 | 55.21 | | | | | |
| 25 | 40-44 | CC | 20 | 0.22 | 0 | 0.00 | | | |
| 25 | 40-44 | Ecig | 1897 | 20.98 | | | | | |
| 25 | 40-44 | Dual | 0 | 0.00 | | | | | |

# Results for PHIM for Run 3 (Sensitivity_Analysis_1.RTF)

| | | | | | | | | | |
|---|---|---|---|---|---|---|---|---|---|
| 25 | 40-44 | Former | 2132 | 23.58 | | | 13.01 | 112 | 5.25 |

Year of Follow-up: 2015, Age Group: 45-49

| Period | Age Group | Smoking Group | N | % | Ever Quit (N) | Ever Quit % | Mean Yrs Quit | Short Term (n) | Short Term % |
|---|---|---|---|---|---|---|---|---|---|
| 25 | 45-49 | Never Any | 5163 | 52.60 | | | | | |
| 25 | 45-49 | CC | 29 | 0.30 | 0 | 0.00 | | | |
| 25 | 45-49 | Ecig | 2195 | 22.36 | | | | | |
| 25 | 45-49 | Dual | 0 | 0.00 | | | | | |
| 25 | 45-49 | Former | 2429 | 24.75 | | | 17.18 | 81 | 3.33 |

Year of Follow-up: 2015, Age Group: 50-54

| Period | Age Group | Smoking Group | N | % | Ever Quit (N) | Ever Quit % | Mean Yrs Quit | Short Term (n) | Short Term % |
|---|---|---|---|---|---|---|---|---|---|
| 25 | 50-54 | Never Any | 4997 | 46.86 | | | | | |
| 25 | 50-54 | CC | 37 | 0.35 | 0 | 0.00 | | | |
| 25 | 50-54 | Ecig | 2387 | 22.38 | | | | | |
| 25 | 50-54 | Dual | 0 | 0.00 | | | | | |
| 25 | 50-54 | Former | 3243 | 30.41 | | | 19.10 | 130 | 4.01 |

Year of Follow-up: 2015, Age Group: 55-59

| Period | Age Group | Smoking Group | N | % | Ever Quit (N) | Ever Quit % | Mean Yrs Quit | Short Term (n) | Short Term % |
|---|---|---|---|---|---|---|---|---|---|
| 25 | 55-59 | Never Any | 4724 | 43.28 | | | | | |
| 25 | 55-59 | CC | 33 | 0.30 | 0 | 0.00 | | | |
| 25 | 55-59 | Ecig | 2103 | 19.27 | | | | | |
| 25 | 55-59 | Dual | 0 | 0.00 | | | | | |
| 25 | 55-59 | Former | 4056 | 37.16 | | | 20.50 | 162 | 3.99 |

Year of Follow-up: 2015, Age Group: 60-64

| Period | Age Group | Smoking Group | N | % | Ever Quit (N) | Ever Quit % | Mean Yrs Quit | Short Term (n) | Short Term % |
|---|---|---|---|---|---|---|---|---|---|
| 25 | 60-64 | Never Any | 3941 | 39.85 | | | | | |
| 25 | 60-64 | CC | 28 | 0.28 | 0 | 0.00 | | | |
| 25 | 60-64 | Ecig | 1443 | 14.59 | | | | | |
| 25 | 60-64 | Dual | 0 | 0.00 | | | | | |
| 25 | 60-64 | Former | 4477 | 45.27 | | | 23.28 | 205 | 4.58 |

Year of Follow-up: 2015, Age Group: 65-69

| Period | Age Group | Smoking Group | N | % | Ever Quit (N) | Ever Quit % | Mean Yrs Quit | Short Term (n) | Short Term % |
|---|---|---|---|---|---|---|---|---|---|
| 25 | 65-69 | Never Any | 2912 | 33.65 | | | | | |
| 25 | 65-69 | CC | 43 | 0.50 | 0 | 0.00 | | | |
| 25 | 65-69 | Ecig | 1019 | 11.77 | | | | | |
| 25 | 65-69 | Dual | 0 | 0.00 | | | | | |
| 25 | 65-69 | Former | 4681 | 54.08 | | | 24.99 | 134 | 2.86 |

Year of Follow-up: 2015, Age Group: 70-74

| Period | Age Group | Smoking Group | N | % | Ever Quit (N) | Ever Quit % | Mean Yrs Quit | Short Term (n) | Short Term % |
|---|---|---|---|---|---|---|---|---|---|
| 25 | 70-74 | Never Any | 1919 | 29.46 | | | | | |
| 25 | 70-74 | CC | 35 | 0.54 | 0 | 0.00 | | | |
| 25 | 70-74 | Ecig | 616 | 9.46 | | | | | |
| 25 | 70-74 | Dual | 0 | 0.00 | | | | | |
| 25 | 70-74 | Former | 3945 | 60.55 | | | 27.55 | 70 | 1.77 |

Year of Follow-up: 2015, Age Group: 75-79

| Period | Age Group | Smoking Group | N | % | Ever Quit (N) | Ever Quit % | Mean Yrs Quit | Short Term (n) | Short Term % |
|---|---|---|---|---|---|---|---|---|---|
| 25 | 75-79 | Never Any | 1611 | 29.41 | | | | | |
| 25 | 75-79 | CC | 36 | 0.66 | 0 | 0.00 | | | |
| 25 | 75-79 | Ecig | 457 | 8.34 | | | | | |
| 25 | 75-79 | Dual | 0 | 0.00 | | | | | |
| 25 | 75-79 | Former | 3373 | 61.58 | | | 29.48 | 52 | 1.54 |

Year of Follow-up: 2020, Age Group: All Ages

| Period | Age Group | Smoking Group | N | % | Ever Quit (N) | Ever Quit % | Mean Yrs Quit | Short Term (n) | Short Term % |
|---|---|---|---|---|---|---|---|---|---|
| 30 | All Ages | Never Any | 33136 | 44.57 | | | | | |
| 30 | All Ages | CC | 100 | 0.13 | 0 | 0.00 | | | |
| 30 | All Ages | Ecig | 11358 | 15.28 | | | | | |
| 30 | All Ages | Dual | 0 | 0.00 | | | | | |
| 30 | All Ages | Former | 29746 | 40.01 | | | 23.97 | 918 | 3.09 |

Year of Follow-up: 2020, Age Group: 18-19

| Period | Age Group | Smoking Group | N | % | Ever Quit (N) | Ever Quit % | Mean Yrs Quit | Short Term (n) | Short Term % |
|---|---|---|---|---|---|---|---|---|---|
| 30 | 18-19 | Never Any | 0 | . | | | | | |
| 30 | 18-19 | CC | 0 | . | 0 | . | | | |
| 30 | 18-19 | Ecig | 0 | . | | | | | |
| 30 | 18-19 | Dual | 0 | . | | | | | |
| 30 | 18-19 | Former | 0 | . | | | . | 0 | . |

Year of Follow-up: 2020, Age Group: 20-24

| Period | Age Group | Smoking Group | N | % | Ever Quit (N) | Ever Quit % | Mean Yrs Quit | Short Term (n) | Short Term % |
|---|---|---|---|---|---|---|---|---|---|
| 30 | 20-24 | Never Any | 0 | . | | | | | |
| 30 | 20-24 | CC | 0 | . | 0 | . | | | |

# Results for PHIM for Run 3 (Sensitivity_Analysis_1.RTF)

| | | | | | | | | | |
|---|---|---|---|---|---|---|---|---|---|
| 30 | 20-24 | Ecig | 0 | . | | | | | |
| 30 | 20-24 | Dual | 0 | . | | | | | |
| 30 | 20-24 | Former | 0 | . | | | . | 0 | . |

Year of Follow-up: 2020, Age Group: 25-29

| Period | Age Group | Smoking Group | N | % | Ever Quit (N) | Ever Quit % | Mean Yrs Quit | Short Term (n) | Short Term % |
|---|---|---|---|---|---|---|---|---|---|
| 30 | 25-29 | Never Any | 0 | . | | | | | |
| 30 | 25-29 | CC | 0 | . | 0 | . | | | |
| 30 | 25-29 | Ecig | 0 | . | | | | | |
| 30 | 25-29 | Dual | 0 | . | | | | | |
| 30 | 25-29 | Former | 0 | . | | | . | 0 | . |

Year of Follow-up: 2020, Age Group: 30-34

| Period | Age Group | Smoking Group | N | % | Ever Quit (N) | Ever Quit % | Mean Yrs Quit | Short Term (n) | Short Term % |
|---|---|---|---|---|---|---|---|---|---|
| 30 | 30-34 | Never Any | 0 | . | | | | | |
| 30 | 30-34 | CC | 0 | . | 0 | . | | | |
| 30 | 30-34 | Ecig | 0 | . | | | | | |
| 30 | 30-34 | Dual | 0 | . | | | | | |
| 30 | 30-34 | Former | 0 | . | | | . | 0 | . |

Year of Follow-up: 2020, Age Group: 35-39

| Period | Age Group | Smoking Group | N | % | Ever Quit (N) | Ever Quit % | Mean Yrs Quit | Short Term (n) | Short Term % |
|---|---|---|---|---|---|---|---|---|---|
| 30 | 35-39 | Never Any | 0 | . | | | | | |
| 30 | 35-39 | CC | 0 | . | 0 | . | | | |
| 30 | 35-39 | Ecig | 0 | . | | | | | |
| 30 | 35-39 | Dual | 0 | . | | | | | |
| 30 | 35-39 | Former | 0 | . | | | . | 0 | . |

Year of Follow-up: 2020, Age Group: 40-44

| Period | Age Group | Smoking Group | N | % | Ever Quit (N) | Ever Quit % | Mean Yrs Quit | Short Term (n) | Short Term % |
|---|---|---|---|---|---|---|---|---|---|
| 30 | 40-44 | Never Any | 4489 | 50.75 | | | | | |
| 30 | 40-44 | CC | 12 | 0.14 | 0 | 0.00 | | | |
| 30 | 40-44 | Ecig | 1743 | 19.71 | | | | | |
| 30 | 40-44 | Dual | 0 | 0.00 | | | | | |
| 30 | 40-44 | Former | 2601 | 29.41 | | | 15.05 | 107 | 4.11 |

Year of Follow-up: 2020, Age Group: 45-49

| Period | Age Group | Smoking Group | N | % | Ever Quit (N) | Ever Quit % | Mean Yrs Quit | Short Term (n) | Short Term % |
|---|---|---|---|---|---|---|---|---|---|
| 30 | 45-49 | Never Any | 4991 | 55.21 | | | | | |
| 30 | 45-49 | CC | 5 | 0.06 | 0 | 0.00 | | | |
| 30 | 45-49 | Ecig | 1690 | 18.69 | | | | | |
| 30 | 45-49 | Dual | 0 | 0.00 | | | | | |
| 30 | 45-49 | Former | 2354 | 26.04 | | | 16.54 | 63 | 2.68 |

Year of Follow-up: 2020, Age Group: 50-54

| Period | Age Group | Smoking Group | N | % | Ever Quit (N) | Ever Quit % | Mean Yrs Quit | Short Term (n) | Short Term % |
|---|---|---|---|---|---|---|---|---|---|
| 30 | 50-54 | Never Any | 5163 | 52.60 | | | | | |
| 30 | 50-54 | CC | 9 | 0.09 | 0 | 0.00 | | | |
| 30 | 50-54 | Ecig | 2021 | 20.59 | | | | | |
| 30 | 50-54 | Dual | 0 | 0.00 | | | | | |
| 30 | 50-54 | Former | 2623 | 26.72 | | | 20.66 | 99 | 3.77 |

Year of Follow-up: 2020, Age Group: 55-59

| Period | Age Group | Smoking Group | N | % | Ever Quit (N) | Ever Quit % | Mean Yrs Quit | Short Term (n) | Short Term % |
|---|---|---|---|---|---|---|---|---|---|
| 30 | 55-59 | Never Any | 4997 | 46.86 | | | | | |
| 30 | 55-59 | CC | 18 | 0.17 | 0 | 0.00 | | | |
| 30 | 55-59 | Ecig | 2002 | 18.77 | | | | | |
| 30 | 55-59 | Dual | 0 | 0.00 | | | | | |
| 30 | 55-59 | Former | 3647 | 34.20 | | | 21.65 | 157 | 4.30 |

Year of Follow-up: 2020, Age Group: 60-64

| Period | Age Group | Smoking Group | N | % | Ever Quit (N) | Ever Quit % | Mean Yrs Quit | Short Term (n) | Short Term % |
|---|---|---|---|---|---|---|---|---|---|
| 30 | 60-64 | Never Any | 4724 | 43.28 | | | | | |
| 30 | 60-64 | CC | 10 | 0.09 | 0 | 0.00 | | | |
| 30 | 60-64 | Ecig | 1614 | 14.79 | | | | | |
| 30 | 60-64 | Dual | 0 | 0.00 | | | | | |
| 30 | 60-64 | Former | 4568 | 41.85 | | | 22.87 | 203 | 4.44 |

Year of Follow-up: 2020, Age Group: 65-69

| Period | Age Group | Smoking Group | N | % | Ever Quit (N) | Ever Quit % | Mean Yrs Quit | Short Term (n) | Short Term % |
|---|---|---|---|---|---|---|---|---|---|
| 30 | 65-69 | Never Any | 3941 | 39.85 | | | | | |
| 30 | 65-69 | CC | 13 | 0.13 | 0 | 0.00 | | | |
| 30 | 65-69 | Ecig | 1039 | 10.51 | | | | | |
| 30 | 65-69 | Dual | 0 | 0.00 | | | | | |
| 30 | 65-69 | Former | 4896 | 49.51 | | | 26.05 | 145 | 2.96 |

Year of Follow-up: 2020, Age Group: 70-74

| Period | Age Group | Smoking Group | N | % | Ever Quit (N) | Ever Quit % | Mean Yrs Quit | Short Term (n) | Short Term % |
|---|---|---|---|---|---|---|---|---|---|

# Results for PHIM for Run 3 (Sensitivity_Analysis_1.RTF)

| | | | | | | | | | |
|---|---|---|---|---|---|---|---|---|---|
| 30 | 70-74 | Never Any | 2912 | 33.65 | | | | | |
| 30 | 70-74 | CC | 18 | 0.21 | 0 | 0.00 | | | |
| 30 | 70-74 | Ecig | 770 | 8.90 | | | | | |
| 30 | 70-74 | Dual | 0 | 0.00 | | | | | |
| 30 | 70-74 | Former | 4955 | 57.25 | | | 28.45 | 93 | 1.88 |

Year of Follow-up: 2020, Age Group: 75-79

| Period | Age Group | Smoking Group | N | % | Ever Quit (N) | Ever Quit % | Mean Yrs Quit | Short Term (n) | Short Term % |
|---|---|---|---|---|---|---|---|---|---|
| 30 | 75-79 | Never Any | 1919 | 29.46 | | | | | |
| 30 | 75-79 | CC | 15 | 0.23 | 0 | 0.00 | | | |
| 30 | 75-79 | Ecig | 479 | 7.35 | | | | | |
| 30 | 75-79 | Dual | 0 | 0.00 | | | | | |
| 30 | 75-79 | Former | 4102 | 62.96 | | | 31.39 | 51 | 1.24 |

Year of Follow-up: 2025, Age Group: All Ages

| Period | Age Group | Smoking Group | N | % | Ever Quit (N) | Ever Quit % | Mean Yrs Quit | Short Term (n) | Short Term % |
|---|---|---|---|---|---|---|---|---|---|
| 35 | All Ages | Never Any | 31217 | 46.03 | | | | | |
| 35 | All Ages | CC | 38 | 0.06 | 0 | 0.00 | | | |
| 35 | All Ages | Ecig | 8864 | 13.07 | | | | | |
| 35 | All Ages | Dual | 0 | 0.00 | | | | | |
| 35 | All Ages | Former | 27706 | 40.85 | | | 25.87 | 785 | 2.83 |

Year of Follow-up: 2025, Age Group: 18-19

| Period | Age Group | Smoking Group | N | % | Ever Quit (N) | Ever Quit % | Mean Yrs Quit | Short Term (n) | Short Term % |
|---|---|---|---|---|---|---|---|---|---|
| 35 | 18-19 | Never Any | 0 | . | | | | | |
| 35 | 18-19 | CC | 0 | . | 0 | . | | | |
| 35 | 18-19 | Ecig | 0 | . | | | | | |
| 35 | 18-19 | Dual | 0 | . | | | | | |
| 35 | 18-19 | Former | 0 | . | | | . | 0 | . |

Year of Follow-up: 2025, Age Group: 20-24

| Period | Age Group | Smoking Group | N | % | Ever Quit (N) | Ever Quit % | Mean Yrs Quit | Short Term (n) | Short Term % |
|---|---|---|---|---|---|---|---|---|---|
| 35 | 20-24 | Never Any | 0 | . | | | | | |
| 35 | 20-24 | CC | 0 | . | 0 | . | | | |
| 35 | 20-24 | Ecig | 0 | . | | | | | |
| 35 | 20-24 | Dual | 0 | . | | | | | |
| 35 | 20-24 | Former | 0 | . | | | . | 0 | . |

Year of Follow-up: 2025, Age Group: 25-29

| Period | Age Group | Smoking Group | N | % | Ever Quit (N) | Ever Quit % | Mean Yrs Quit | Short Term (n) | Short Term % |
|---|---|---|---|---|---|---|---|---|---|
| 35 | 25-29 | Never Any | 0 | . | | | | | |
| 35 | 25-29 | CC | 0 | . | 0 | . | | | |
| 35 | 25-29 | Ecig | 0 | . | | | | | |
| 35 | 25-29 | Dual | 0 | . | | | | | |
| 35 | 25-29 | Former | 0 | . | | | . | 0 | . |

Year of Follow-up: 2025, Age Group: 30-34

| Period | Age Group | Smoking Group | N | % | Ever Quit (N) | Ever Quit % | Mean Yrs Quit | Short Term (n) | Short Term % |
|---|---|---|---|---|---|---|---|---|---|
| 35 | 30-34 | Never Any | 0 | . | | | | | |
| 35 | 30-34 | CC | 0 | . | 0 | . | | | |
| 35 | 30-34 | Ecig | 0 | . | | | | | |
| 35 | 30-34 | Dual | 0 | . | | | | | |
| 35 | 30-34 | Former | 0 | . | | | . | 0 | . |

Year of Follow-up: 2025, Age Group: 35-39

| Period | Age Group | Smoking Group | N | % | Ever Quit (N) | Ever Quit % | Mean Yrs Quit | Short Term (n) | Short Term % |
|---|---|---|---|---|---|---|---|---|---|
| 35 | 35-39 | Never Any | 0 | . | | | | | |
| 35 | 35-39 | CC | 0 | . | 0 | . | | | |
| 35 | 35-39 | Ecig | 0 | . | | | | | |
| 35 | 35-39 | Dual | 0 | . | | | | | |
| 35 | 35-39 | Former | 0 | . | | | . | 0 | . |

Year of Follow-up: 2025, Age Group: 40-44

| Period | Age Group | Smoking Group | N | % | Ever Quit (N) | Ever Quit % | Mean Yrs Quit | Short Term (n) | Short Term % |
|---|---|---|---|---|---|---|---|---|---|
| 35 | 40-44 | Never Any | 0 | . | | | | | |
| 35 | 40-44 | CC | 0 | . | 0 | . | | | |
| 35 | 40-44 | Ecig | 0 | . | | | | | |
| 35 | 40-44 | Dual | 0 | . | | | | | |
| 35 | 40-44 | Former | 0 | . | | | . | 0 | . |

Year of Follow-up: 2025, Age Group: 45-49

| Period | Age Group | Smoking Group | N | % | Ever Quit (N) | Ever Quit % | Mean Yrs Quit | Short Term (n) | Short Term % |
|---|---|---|---|---|---|---|---|---|---|
| 35 | 45-49 | Never Any | 4489 | 50.75 | | | | | |
| 35 | 45-49 | CC | 7 | 0.08 | 0 | 0.00 | | | |
| 35 | 45-49 | Ecig | 1541 | 17.42 | | | | | |
| 35 | 45-49 | Dual | 0 | 0.00 | | | | | |
| 35 | 45-49 | Former | 2808 | 31.75 | | | 18.76 | 60 | 2.14 |

Year of Follow-up: 2025, Age Group: 50-54

# Results for PHIM for Run 3 (Sensitivity_Analysis_1.RTF)

| Period | Age Group | Smoking Group | N | % | Ever Quit (N) | Ever Quit % | Mean Yrs Quit | Short Term (n) | Short Term % |
|---|---|---|---|---|---|---|---|---|---|
| 35 | 50-54 | Never Any | 4991 | 55.21 | | | | | |
| 35 | 50-54 | CC | 3 | 0.03 | 0 | 0.00 | | | |
| 35 | 50-54 | Ecig | 1554 | 17.19 | | | | | |
| 35 | 50-54 | Dual | 0 | 0.00 | | | | | |
| 35 | 50-54 | Former | 2492 | 27.57 | | | 20.44 | 75 | 3.01 |

Year of Follow-up: 2025, Age Group: 55-59

| Period | Age Group | Smoking Group | N | % | Ever Quit (N) | Ever Quit % | Mean Yrs Quit | Short Term (n) | Short Term % |
|---|---|---|---|---|---|---|---|---|---|
| 35 | 55-59 | Never Any | 5163 | 52.60 | | | | | |
| 35 | 55-59 | CC | 0 | 0.00 | 0 | 0.00 | | | |
| 35 | 55-59 | Ecig | 1664 | 16.95 | | | | | |
| 35 | 55-59 | Dual | 0 | 0.00 | | | | | |
| 35 | 55-59 | Former | 2989 | 30.45 | | | 22.76 | 155 | 5.19 |

Year of Follow-up: 2025, Age Group: 60-64

| Period | Age Group | Smoking Group | N | % | Ever Quit (N) | Ever Quit % | Mean Yrs Quit | Short Term (n) | Short Term % |
|---|---|---|---|---|---|---|---|---|---|
| 35 | 60-64 | Never Any | 4997 | 46.86 | | | | | |
| 35 | 60-64 | CC | 9 | 0.08 | 0 | 0.00 | | | |
| 35 | 60-64 | Ecig | 1532 | 14.37 | | | | | |
| 35 | 60-64 | Dual | 0 | 0.00 | | | | | |
| 35 | 60-64 | Former | 4126 | 38.69 | | | 23.79 | 186 | 4.51 |

Year of Follow-up: 2025, Age Group: 65-69

| Period | Age Group | Smoking Group | N | % | Ever Quit (N) | Ever Quit % | Mean Yrs Quit | Short Term (n) | Short Term % |
|---|---|---|---|---|---|---|---|---|---|
| 35 | 65-69 | Never Any | 4724 | 43.28 | | | | | |
| 35 | 65-69 | CC | 5 | 0.05 | 0 | 0.00 | | | |
| 35 | 65-69 | Ecig | 1169 | 10.71 | | | | | |
| 35 | 65-69 | Dual | 0 | 0.00 | | | | | |
| 35 | 65-69 | Former | 5018 | 45.97 | | | 25.57 | 158 | 3.15 |

Year of Follow-up: 2025, Age Group: 70-74

| Period | Age Group | Smoking Group | N | % | Ever Quit (N) | Ever Quit % | Mean Yrs Quit | Short Term (n) | Short Term % |
|---|---|---|---|---|---|---|---|---|---|
| 35 | 70-74 | Never Any | 3941 | 39.85 | | | | | |
| 35 | 70-74 | CC | 5 | 0.05 | 0 | 0.00 | | | |
| 35 | 70-74 | Ecig | 811 | 8.20 | | | | | |
| 35 | 70-74 | Dual | 0 | 0.00 | | | | | |
| 35 | 70-74 | Former | 5132 | 51.90 | | | 29.73 | 79 | 1.54 |

Year of Follow-up: 2025, Age Group: 75-79

| Period | Age Group | Smoking Group | N | % | Ever Quit (N) | Ever Quit % | Mean Yrs Quit | Short Term (n) | Short Term % |
|---|---|---|---|---|---|---|---|---|---|
| 35 | 75-79 | Never Any | 2912 | 33.65 | | | | | |
| 35 | 75-79 | CC | 9 | 0.10 | 0 | 0.00 | | | |
| 35 | 75-79 | Ecig | 593 | 6.85 | | | | | |
| 35 | 75-79 | Dual | 0 | 0.00 | | | | | |
| 35 | 75-79 | Former | 5141 | 59.40 | | | 32.31 | 72 | 1.40 |

Year of Follow-up: 2030, Age Group: All Ages

| Period | Age Group | Smoking Group | N | % | Ever Quit (N) | Ever Quit % | Mean Yrs Quit | Short Term (n) | Short Term % |
|---|---|---|---|---|---|---|---|---|---|
| 40 | All Ages | Never Any | 28305 | 47.84 | | | | | |
| 40 | All Ages | CC | 15 | 0.03 | 0 | 0.00 | | | |
| 40 | All Ages | Ecig | 6535 | 11.04 | | | | | |
| 40 | All Ages | Dual | 0 | 0.00 | | | | | |
| 40 | All Ages | Former | 24315 | 41.09 | | | 27.43 | 691 | 2.84 |

Year of Follow-up: 2030, Age Group: 18-19

| Period | Age Group | Smoking Group | N | % | Ever Quit (N) | Ever Quit % | Mean Yrs Quit | Short Term (n) | Short Term % |
|---|---|---|---|---|---|---|---|---|---|
| 40 | 18-19 | Never Any | 0 | . | | | | | |
| 40 | 18-19 | CC | 0 | . | 0 | . | | | |
| 40 | 18-19 | Ecig | 0 | . | | | | | |
| 40 | 18-19 | Dual | 0 | . | | | | | |
| 40 | 18-19 | Former | 0 | . | | | . | 0 | . |

Year of Follow-up: 2030, Age Group: 20-24

| Period | Age Group | Smoking Group | N | % | Ever Quit (N) | Ever Quit % | Mean Yrs Quit | Short Term (n) | Short Term % |
|---|---|---|---|---|---|---|---|---|---|
| 40 | 20-24 | Never Any | 0 | . | | | | | |
| 40 | 20-24 | CC | 0 | . | 0 | . | | | |
| 40 | 20-24 | Ecig | 0 | . | | | | | |
| 40 | 20-24 | Dual | 0 | . | | | | | |
| 40 | 20-24 | Former | 0 | . | | | . | 0 | . |

Year of Follow-up: 2030, Age Group: 25-29

| Period | Age Group | Smoking Group | N | % | Ever Quit (N) | Ever Quit % | Mean Yrs Quit | Short Term (n) | Short Term % |
|---|---|---|---|---|---|---|---|---|---|
| 40 | 25-29 | Never Any | 0 | . | | | | | |
| 40 | 25-29 | CC | 0 | . | 0 | . | | | |
| 40 | 25-29 | Ecig | 0 | . | | | | | |
| 40 | 25-29 | Dual | 0 | . | | | | | |

# Results for PHIM for Run 3 (Sensitivity_Analysis_1.RTF)

| | | | | | | | | | |
|---|---|---|---|---|---|---|---|---|---|
| 40 | 25-29 | Former | 0 | . | | | . | 0 | . |

Year of Follow-up: 2030, Age Group: 30-34

| Period | Age Group | Smoking Group | N | % | Ever Quit (N) | Ever Quit % | Mean Yrs Quit | Short Term (n) | Short Term % |
|---|---|---|---|---|---|---|---|---|---|
| 40 | 30-34 | Never Any | 0 | . | | | | | |
| 40 | 30-34 | CC | 0 | . | 0 | . | | | |
| 40 | 30-34 | Ecig | 0 | . | | | | | |
| 40 | 30-34 | Dual | 0 | . | | | | | |
| 40 | 30-34 | Former | 0 | . | | | . | 0 | . |

Year of Follow-up: 2030, Age Group: 35-39

| Period | Age Group | Smoking Group | N | % | Ever Quit (N) | Ever Quit % | Mean Yrs Quit | Short Term (n) | Short Term % |
|---|---|---|---|---|---|---|---|---|---|
| 40 | 35-39 | Never Any | 0 | . | | | | | |
| 40 | 35-39 | CC | 0 | . | 0 | . | | | |
| 40 | 35-39 | Ecig | 0 | . | | | | | |
| 40 | 35-39 | Dual | 0 | . | | | | | |
| 40 | 35-39 | Former | 0 | . | | | . | 0 | . |

Year of Follow-up: 2030, Age Group: 40-44

| Period | Age Group | Smoking Group | N | % | Ever Quit (N) | Ever Quit % | Mean Yrs Quit | Short Term (n) | Short Term % |
|---|---|---|---|---|---|---|---|---|---|
| 40 | 40-44 | Never Any | 0 | . | | | | | |
| 40 | 40-44 | CC | 0 | . | 0 | . | | | |
| 40 | 40-44 | Ecig | 0 | . | | | | | |
| 40 | 40-44 | Dual | 0 | . | | | | | |
| 40 | 40-44 | Former | 0 | . | | | . | 0 | . |

Year of Follow-up: 2030, Age Group: 45-49

| Period | Age Group | Smoking Group | N | % | Ever Quit (N) | Ever Quit % | Mean Yrs Quit | Short Term (n) | Short Term % |
|---|---|---|---|---|---|---|---|---|---|
| 40 | 45-49 | Never Any | 0 | . | | | | | |
| 40 | 45-49 | CC | 0 | . | 0 | . | | | |
| 40 | 45-49 | Ecig | 0 | . | | | | | |
| 40 | 45-49 | Dual | 0 | . | | | | | |
| 40 | 45-49 | Former | 0 | . | | | . | 0 | . |

Year of Follow-up: 2030, Age Group: 50-54

| Period | Age Group | Smoking Group | N | % | Ever Quit (N) | Ever Quit % | Mean Yrs Quit | Short Term (n) | Short Term % |
|---|---|---|---|---|---|---|---|---|---|
| 40 | 50-54 | Never Any | 4489 | 50.75 | | | | | |
| 40 | 50-54 | CC | 3 | 0.03 | 0 | 0.00 | | | |
| 40 | 50-54 | Ecig | 1392 | 15.74 | | | | | |
| 40 | 50-54 | Dual | 0 | 0.00 | | | | | |
| 40 | 50-54 | Former | 2961 | 33.48 | | | 22.61 | 80 | 2.70 |

Year of Follow-up: 2030, Age Group: 55-59

| Period | Age Group | Smoking Group | N | % | Ever Quit (N) | Ever Quit % | Mean Yrs Quit | Short Term (n) | Short Term % |
|---|---|---|---|---|---|---|---|---|---|
| 40 | 55-59 | Never Any | 4991 | 55.21 | | | | | |
| 40 | 55-59 | CC | 2 | 0.02 | 0 | 0.00 | | | |
| 40 | 55-59 | Ecig | 1291 | 14.28 | | | | | |
| 40 | 55-59 | Dual | 0 | 0.00 | | | | | |
| 40 | 55-59 | Former | 2756 | 30.49 | | | 23.19 | 114 | 4.14 |

Year of Follow-up: 2030, Age Group: 60-64

| Period | Age Group | Smoking Group | N | % | Ever Quit (N) | Ever Quit % | Mean Yrs Quit | Short Term (n) | Short Term % |
|---|---|---|---|---|---|---|---|---|---|
| 40 | 60-64 | Never Any | 5163 | 52.60 | | | | | |
| 40 | 60-64 | CC | 0 | 0.00 | 0 | 0.00 | | | |
| 40 | 60-64 | Ecig | 1238 | 12.61 | | | | | |
| 40 | 60-64 | Dual | 0 | 0.00 | | | | | |
| 40 | 60-64 | Former | 3415 | 34.79 | | | 24.53 | 183 | 5.36 |

Year of Follow-up: 2030, Age Group: 65-69

| Period | Age Group | Smoking Group | N | % | Ever Quit (N) | Ever Quit % | Mean Yrs Quit | Short Term (n) | Short Term % |
|---|---|---|---|---|---|---|---|---|---|
| 40 | 65-69 | Never Any | 4997 | 46.86 | | | | | |
| 40 | 65-69 | CC | 4 | 0.04 | 0 | 0.00 | | | |
| 40 | 65-69 | Ecig | 1119 | 10.49 | | | | | |
| 40 | 65-69 | Dual | 0 | 0.00 | | | | | |
| 40 | 65-69 | Former | 4544 | 42.61 | | | 26.35 | 136 | 2.99 |

Year of Follow-up: 2030, Age Group: 70-74

| Period | Age Group | Smoking Group | N | % | Ever Quit (N) | Ever Quit % | Mean Yrs Quit | Short Term (n) | Short Term % |
|---|---|---|---|---|---|---|---|---|---|
| 40 | 70-74 | Never Any | 4724 | 43.28 | | | | | |
| 40 | 70-74 | CC | 3 | 0.03 | 0 | 0.00 | | | |
| 40 | 70-74 | Ecig | 880 | 8.06 | | | | | |
| 40 | 70-74 | Dual | 0 | 0.00 | | | | | |
| 40 | 70-74 | Former | 5309 | 48.64 | | | 29.01 | 95 | 1.79 |

Year of Follow-up: 2030, Age Group: 75-79

| Period | Age Group | Smoking Group | N | % | Ever Quit (N) | Ever Quit % | Mean Yrs Quit | Short Term (n) | Short Term % |
|---|---|---|---|---|---|---|---|---|---|
| 40 | 75-79 | Never Any | 3941 | 39.85 | | | | | |
| 40 | 75-79 | CC | 3 | 0.03 | 0 | 0.00 | | | |

## Results for PHIM for Run 3 (Sensitivity_Analysis_1.RTF)

| | | | | | | | | | |
|---|---|---|---|---|---|---|---|---|---|
| 40 | 75-79 | Ecig | 615 | 6.22 | | | | | |
| 40 | 75-79 | Dual | 0 | 0.00 | | | | | |
| 40 | 75-79 | Former | 5330 | 53.90 | | | 33.51 | 83 | 1.56 |

Year of Follow-up: 2035, Age Group: All Ages

| Period | Age Group | Smoking Group | N | % | Ever Quit (N) | Ever Quit % | Mean Yrs Quit | Short Term (n) | Short Term % |
|---|---|---|---|---|---|---|---|---|---|
| 45 | All Ages | Never Any | 24364 | 49.44 | | | | | |
| 45 | All Ages | CC | 6 | 0.01 | 0 | 0.00 | | | |
| 45 | All Ages | Ecig | 4578 | 9.29 | | | | | |
| 45 | All Ages | Dual | 0 | 0.00 | | | | | |
| 45 | All Ages | Former | 20333 | 41.26 | | | 28.82 | 538 | 2.65 |

Year of Follow-up: 2035, Age Group: 18-19

| Period | Age Group | Smoking Group | N | % | Ever Quit (N) | Ever Quit % | Mean Yrs Quit | Short Term (n) | Short Term % |
|---|---|---|---|---|---|---|---|---|---|
| 45 | 18-19 | Never Any | 0 | . | | | | | |
| 45 | 18-19 | CC | 0 | . | 0 | . | | | |
| 45 | 18-19 | Ecig | 0 | . | | | | | |
| 45 | 18-19 | Dual | 0 | . | | | | | |
| 45 | 18-19 | Former | 0 | . | | | . | 0 | . |

Year of Follow-up: 2035, Age Group: 20-24

| Period | Age Group | Smoking Group | N | % | Ever Quit (N) | Ever Quit % | Mean Yrs Quit | Short Term (n) | Short Term % |
|---|---|---|---|---|---|---|---|---|---|
| 45 | 20-24 | Never Any | 0 | . | | | | | |
| 45 | 20-24 | CC | 0 | . | 0 | . | | | |
| 45 | 20-24 | Ecig | 0 | . | | | | | |
| 45 | 20-24 | Dual | 0 | . | | | | | |
| 45 | 20-24 | Former | 0 | . | | | . | 0 | . |

Year of Follow-up: 2035, Age Group: 25-29

| Period | Age Group | Smoking Group | N | % | Ever Quit (N) | Ever Quit % | Mean Yrs Quit | Short Term (n) | Short Term % |
|---|---|---|---|---|---|---|---|---|---|
| 45 | 25-29 | Never Any | 0 | . | | | | | |
| 45 | 25-29 | CC | 0 | . | 0 | . | | | |
| 45 | 25-29 | Ecig | 0 | . | | | | | |
| 45 | 25-29 | Dual | 0 | . | | | | | |
| 45 | 25-29 | Former | 0 | . | | | . | 0 | . |

Year of Follow-up: 2035, Age Group: 30-34

| Period | Age Group | Smoking Group | N | % | Ever Quit (N) | Ever Quit % | Mean Yrs Quit | Short Term (n) | Short Term % |
|---|---|---|---|---|---|---|---|---|---|
| 45 | 30-34 | Never Any | 0 | . | | | | | |
| 45 | 30-34 | CC | 0 | . | 0 | . | | | |
| 45 | 30-34 | Ecig | 0 | . | | | | | |
| 45 | 30-34 | Dual | 0 | . | | | | | |
| 45 | 30-34 | Former | 0 | . | | | . | 0 | . |

Year of Follow-up: 2035, Age Group: 35-39

| Period | Age Group | Smoking Group | N | % | Ever Quit (N) | Ever Quit % | Mean Yrs Quit | Short Term (n) | Short Term % |
|---|---|---|---|---|---|---|---|---|---|
| 45 | 35-39 | Never Any | 0 | . | | | | | |
| 45 | 35-39 | CC | 0 | . | 0 | . | | | |
| 45 | 35-39 | Ecig | 0 | . | | | | | |
| 45 | 35-39 | Dual | 0 | . | | | | | |
| 45 | 35-39 | Former | 0 | . | | | . | 0 | . |

Year of Follow-up: 2035, Age Group: 40-44

| Period | Age Group | Smoking Group | N | % | Ever Quit (N) | Ever Quit % | Mean Yrs Quit | Short Term (n) | Short Term % |
|---|---|---|---|---|---|---|---|---|---|
| 45 | 40-44 | Never Any | 0 | . | | | | | |
| 45 | 40-44 | CC | 0 | . | 0 | . | | | |
| 45 | 40-44 | Ecig | 0 | . | | | | | |
| 45 | 40-44 | Dual | 0 | . | | | | | |
| 45 | 40-44 | Former | 0 | . | | | . | 0 | . |

Year of Follow-up: 2035, Age Group: 45-49

| Period | Age Group | Smoking Group | N | % | Ever Quit (N) | Ever Quit % | Mean Yrs Quit | Short Term (n) | Short Term % |
|---|---|---|---|---|---|---|---|---|---|
| 45 | 45-49 | Never Any | 0 | . | | | | | |
| 45 | 45-49 | CC | 0 | . | 0 | . | | | |
| 45 | 45-49 | Ecig | 0 | . | | | | | |
| 45 | 45-49 | Dual | 0 | . | | | | | |
| 45 | 45-49 | Former | 0 | . | | | . | 0 | . |

Year of Follow-up: 2035, Age Group: 50-54

| Period | Age Group | Smoking Group | N | % | Ever Quit (N) | Ever Quit % | Mean Yrs Quit | Short Term (n) | Short Term % |
|---|---|---|---|---|---|---|---|---|---|
| 45 | 50-54 | Never Any | 0 | . | | | | | |
| 45 | 50-54 | CC | 0 | . | 0 | . | | | |
| 45 | 50-54 | Ecig | 0 | . | | | | | |
| 45 | 50-54 | Dual | 0 | . | | | | | |
| 45 | 50-54 | Former | 0 | . | | | . | 0 | . |

Year of Follow-up: 2035, Age Group: 55-59

| Period | Age Group | Smoking Group | N | % | Ever Quit (N) | Ever Quit % | Mean Yrs Quit | Short Term (n) | Short Term % |
|---|---|---|---|---|---|---|---|---|---|

# Results for PHIM for Run 3 (Sensitivity_Analysis_1.RTF)

| | | | | | | | | | |
|---|---|---|---|---|---|---|---|---|---|
| 45 | 55-59 | Never Any | 4489 | 50.75 | | | | | |
| 45 | 55-59 | CC | 2 | 0.02 | 0 | 0.00 | | | |
| 45 | 55-59 | Ecig | 1171 | 13.24 | | | | | |
| 45 | 55-59 | Dual | 0 | 0.00 | | | | | |
| 45 | 55-59 | Former | 3183 | 35.99 | | | 25.82 | 100 | 3.14 |

Year of Follow-up: 2035, Age Group: 60-64

| Period | Age Group | Smoking Group | N | % | Ever Quit (N) | Ever Quit % | Mean Yrs Quit | Short Term (n) | Short Term % |
|---|---|---|---|---|---|---|---|---|---|
| 45 | 60-64 | Never Any | 4991 | 55.21 | | | | | |
| 45 | 60-64 | CC | 0 | 0.00 | 0 | 0.00 | | | |
| 45 | 60-64 | Ecig | 1020 | 11.28 | | | | | |
| 45 | 60-64 | Dual | 0 | 0.00 | | | | | |
| 45 | 60-64 | Former | 3029 | 33.51 | | | 25.81 | 129 | 4.26 |

Year of Follow-up: 2035, Age Group: 65-69

| Period | Age Group | Smoking Group | N | % | Ever Quit (N) | Ever Quit % | Mean Yrs Quit | Short Term (n) | Short Term % |
|---|---|---|---|---|---|---|---|---|---|
| 45 | 65-69 | Never Any | 5163 | 52.60 | | | | | |
| 45 | 65-69 | CC | 0 | 0.00 | 0 | 0.00 | | | |
| 45 | 65-69 | Ecig | 902 | 9.19 | | | | | |
| 45 | 65-69 | Dual | 0 | 0.00 | | | | | |
| 45 | 65-69 | Former | 3751 | 38.21 | | | 27.09 | 106 | 2.83 |

Year of Follow-up: 2035, Age Group: 70-74

| Period | Age Group | Smoking Group | N | % | Ever Quit (N) | Ever Quit % | Mean Yrs Quit | Short Term (n) | Short Term % |
|---|---|---|---|---|---|---|---|---|---|
| 45 | 70-74 | Never Any | 4997 | 46.86 | | | | | |
| 45 | 70-74 | CC | 1 | 0.01 | 0 | 0.00 | | | |
| 45 | 70-74 | Ecig | 850 | 7.97 | | | | | |
| 45 | 70-74 | Dual | 0 | 0.00 | | | | | |
| 45 | 70-74 | Former | 4816 | 45.16 | | | 29.70 | 104 | 2.16 |

Year of Follow-up: 2035, Age Group: 75-79

| Period | Age Group | Smoking Group | N | % | Ever Quit (N) | Ever Quit % | Mean Yrs Quit | Short Term (n) | Short Term % |
|---|---|---|---|---|---|---|---|---|---|
| 45 | 75-79 | Never Any | 4724 | 43.28 | | | | | |
| 45 | 75-79 | CC | 3 | 0.03 | 0 | 0.00 | | | |
| 45 | 75-79 | Ecig | 635 | 5.82 | | | | | |
| 45 | 75-79 | Dual | 0 | 0.00 | | | | | |
| 45 | 75-79 | Former | 5554 | 50.88 | | | 32.60 | 99 | 1.78 |

Year of Follow-up: 2040, Age Group: All Ages

| Period | Age Group | Smoking Group | N | % | Ever Quit (N) | Ever Quit % | Mean Yrs Quit | Short Term (n) | Short Term % |
|---|---|---|---|---|---|---|---|---|---|
| 50 | All Ages | Never Any | 19640 | 51.19 | | | | | |
| 50 | All Ages | CC | 1 | 0.00 | 0 | 0.00 | | | |
| 50 | All Ages | Ecig | 2868 | 7.48 | | | | | |
| 50 | All Ages | Dual | 0 | 0.00 | | | | | |
| 50 | All Ages | Former | 15856 | 41.33 | | | 30.34 | 408 | 2.57 |

Year of Follow-up: 2040, Age Group: 18-19

| Period | Age Group | Smoking Group | N | % | Ever Quit (N) | Ever Quit % | Mean Yrs Quit | Short Term (n) | Short Term % |
|---|---|---|---|---|---|---|---|---|---|
| 50 | 18-19 | Never Any | 0 | . | | | | | |
| 50 | 18-19 | CC | 0 | . | 0 | . | | | |
| 50 | 18-19 | Ecig | 0 | . | | | | | |
| 50 | 18-19 | Dual | 0 | . | | | | | |
| 50 | 18-19 | Former | 0 | . | | | . | 0 | . |

Year of Follow-up: 2040, Age Group: 20-24

| Period | Age Group | Smoking Group | N | % | Ever Quit (N) | Ever Quit % | Mean Yrs Quit | Short Term (n) | Short Term % |
|---|---|---|---|---|---|---|---|---|---|
| 50 | 20-24 | Never Any | 0 | . | | | | | |
| 50 | 20-24 | CC | 0 | . | 0 | . | | | |
| 50 | 20-24 | Ecig | 0 | . | | | | | |
| 50 | 20-24 | Dual | 0 | . | | | | | |
| 50 | 20-24 | Former | 0 | . | | | . | 0 | . |

Year of Follow-up: 2040, Age Group: 25-29

| Period | Age Group | Smoking Group | N | % | Ever Quit (N) | Ever Quit % | Mean Yrs Quit | Short Term (n) | Short Term % |
|---|---|---|---|---|---|---|---|---|---|
| 50 | 25-29 | Never Any | 0 | . | | | | | |
| 50 | 25-29 | CC | 0 | . | 0 | . | | | |
| 50 | 25-29 | Ecig | 0 | . | | | | | |
| 50 | 25-29 | Dual | 0 | . | | | | | |
| 50 | 25-29 | Former | 0 | . | | | . | 0 | . |

Year of Follow-up: 2040, Age Group: 30-34

| Period | Age Group | Smoking Group | N | % | Ever Quit (N) | Ever Quit % | Mean Yrs Quit | Short Term (n) | Short Term % |
|---|---|---|---|---|---|---|---|---|---|
| 50 | 30-34 | Never Any | 0 | . | | | | | |
| 50 | 30-34 | CC | 0 | . | 0 | . | | | |
| 50 | 30-34 | Ecig | 0 | . | | | | | |
| 50 | 30-34 | Dual | 0 | . | | | | | |
| 50 | 30-34 | Former | 0 | . | | | . | 0 | . |

Year of Follow-up: 2040, Age Group: 35-39

# Results for PHIM for Run 3 (Sensitivity_Analysis_1.RTF)

| Period | Age Group | Smoking Group | N | % | Ever Quit (N) | Ever Quit % | Mean Yrs Quit | Short Term (n) | Short Term % |
|---|---|---|---|---|---|---|---|---|---|
| 50 | 35-39 | Never Any | 0 | . | | | | | |
| 50 | 35-39 | CC | 0 | . | 0 | . | | | |
| 50 | 35-39 | Ecig | 0 | . | | | | | |
| 50 | 35-39 | Dual | 0 | . | | | | | |
| 50 | 35-39 | Former | 0 | . | | | . | 0 | . |

Year of Follow-up: 2040, Age Group: 40-44

| Period | Age Group | Smoking Group | N | % | Ever Quit (N) | Ever Quit % | Mean Yrs Quit | Short Term (n) | Short Term % |
|---|---|---|---|---|---|---|---|---|---|
| 50 | 40-44 | Never Any | 0 | . | | | | | |
| 50 | 40-44 | CC | 0 | . | 0 | . | | | |
| 50 | 40-44 | Ecig | 0 | . | | | | | |
| 50 | 40-44 | Dual | 0 | . | | | | | |
| 50 | 40-44 | Former | 0 | . | | | . | 0 | . |

Year of Follow-up: 2040, Age Group: 45-49

| Period | Age Group | Smoking Group | N | % | Ever Quit (N) | Ever Quit % | Mean Yrs Quit | Short Term (n) | Short Term % |
|---|---|---|---|---|---|---|---|---|---|
| 50 | 45-49 | Never Any | 0 | . | | | | | |
| 50 | 45-49 | CC | 0 | . | 0 | . | | | |
| 50 | 45-49 | Ecig | 0 | . | | | | | |
| 50 | 45-49 | Dual | 0 | . | | | | | |
| 50 | 45-49 | Former | 0 | . | | | . | 0 | . |

Year of Follow-up: 2040, Age Group: 50-54

| Period | Age Group | Smoking Group | N | % | Ever Quit (N) | Ever Quit % | Mean Yrs Quit | Short Term (n) | Short Term % |
|---|---|---|---|---|---|---|---|---|---|
| 50 | 50-54 | Never Any | 0 | . | | | | | |
| 50 | 50-54 | CC | 0 | . | 0 | . | | | |
| 50 | 50-54 | Ecig | 0 | . | | | | | |
| 50 | 50-54 | Dual | 0 | . | | | | | |
| 50 | 50-54 | Former | 0 | . | | | . | 0 | . |

Year of Follow-up: 2040, Age Group: 55-59

| Period | Age Group | Smoking Group | N | % | Ever Quit (N) | Ever Quit % | Mean Yrs Quit | Short Term (n) | Short Term % |
|---|---|---|---|---|---|---|---|---|---|
| 50 | 55-59 | Never Any | 0 | . | | | | | |
| 50 | 55-59 | CC | 0 | . | 0 | . | | | |
| 50 | 55-59 | Ecig | 0 | . | | | | | |
| 50 | 55-59 | Dual | 0 | . | | | | | |
| 50 | 55-59 | Former | 0 | . | | | . | 0 | . |

Year of Follow-up: 2040, Age Group: 60-64

| Period | Age Group | Smoking Group | N | % | Ever Quit (N) | Ever Quit % | Mean Yrs Quit | Short Term (n) | Short Term % |
|---|---|---|---|---|---|---|---|---|---|
| 50 | 60-64 | Never Any | 4489 | 50.75 | | | | | |
| 50 | 60-64 | CC | 0 | 0.00 | 0 | 0.00 | | | |
| 50 | 60-64 | Ecig | 865 | 9.78 | | | | | |
| 50 | 60-64 | Dual | 0 | 0.00 | | | | | |
| 50 | 60-64 | Former | 3491 | 39.47 | | | 28.27 | 129 | 3.70 |

Year of Follow-up: 2040, Age Group: 65-69

| Period | Age Group | Smoking Group | N | % | Ever Quit (N) | Ever Quit % | Mean Yrs Quit | Short Term (n) | Short Term % |
|---|---|---|---|---|---|---|---|---|---|
| 50 | 65-69 | Never Any | 4991 | 55.21 | | | | | |
| 50 | 65-69 | CC | 0 | 0.00 | 0 | 0.00 | | | |
| 50 | 65-69 | Ecig | 705 | 7.80 | | | | | |
| 50 | 65-69 | Dual | 0 | 0.00 | | | | | |
| 50 | 65-69 | Former | 3344 | 36.99 | | | 28.11 | 120 | 3.59 |

Year of Follow-up: 2040, Age Group: 70-74

| Period | Age Group | Smoking Group | N | % | Ever Quit (N) | Ever Quit % | Mean Yrs Quit | Short Term (n) | Short Term % |
|---|---|---|---|---|---|---|---|---|---|
| 50 | 70-74 | Never Any | 5163 | 52.60 | | | | | |
| 50 | 70-74 | CC | 0 | 0.00 | 0 | 0.00 | | | |
| 50 | 70-74 | Ecig | 665 | 6.77 | | | | | |
| 50 | 70-74 | Dual | 0 | 0.00 | | | | | |
| 50 | 70-74 | Former | 3988 | 40.63 | | | 30.31 | 80 | 2.01 |

Year of Follow-up: 2040, Age Group: 75-79

| Period | Age Group | Smoking Group | N | % | Ever Quit (N) | Ever Quit % | Mean Yrs Quit | Short Term (n) | Short Term % |
|---|---|---|---|---|---|---|---|---|---|
| 50 | 75-79 | Never Any | 4997 | 46.86 | | | | | |
| 50 | 75-79 | CC | 1 | 0.01 | 0 | 0.00 | | | |
| 50 | 75-79 | Ecig | 633 | 5.94 | | | | | |
| 50 | 75-79 | Dual | 0 | 0.00 | | | | | |
| 50 | 75-79 | Former | 5033 | 47.20 | | | 33.29 | 79 | 1.57 |

# Results for PHIM for Run 3 (Sensitivity_Analysis_1.RTF)

************************************************************************************

**Main Results for E-Component for Run: 3, Simulation: 1, COD: Four Diseases Combined**

| **Variable Parameter** | **Value** |
|---|---|
| 1. Country | US (United States) |
| 2. Sex | M (Males) |
| 3. Year of start of process | 1990 |
| 4. Cause of Death | Four Diseases Combined |
| 5. Effective Dose NULL | CC:1 |
| 6. Effective Dose Ecig | CC:1 Ecig:0 Dual:0.5 |
| Note: | Death rates are per million population |

-----------------------------------------------------------------------------------------

Results for Age Groups Combined and Years combined, Population unadjusted for deaths in Ecig scenario,

| Age Group:All | | NULL | Ecig | | | %Drop | | NULL | Ecig | |
|---|---|---|---|---|---|---|---|---|---|---|
| Year | Number Deaths | Attrib Deaths | Attrib Deaths | Drop Deaths | %Drop Deaths | Attrib Deaths | Death Rate | Attrib Rates | Attrib Rates | Drop Rates |
| All Comb | 13148871 | 4509390.583 | 2856614.918 | 1652776 | 12.570 | 36.652 | 3806.31 | 1305.37 | 826.928 | 478.443 |

-----------------------------------------------------------------------------------------

Results for Age Groups Combined, Population unadjusted for deaths in Ecig scenario,

| Age Group:All | | NULL | Ecig | | | %Drop | | NULL | Ecig | |
|---|---|---|---|---|---|---|---|---|---|---|
| Year | Number Deaths | Attrib Deaths | Attrib Deaths | Drop Deaths | %Drop Deaths | Attrib Deaths | Death Rate | Attrib Rates | Attrib Rates | Drop Rates |
| 1990 | 317161 | 125773.065 | 125773.065 | 0.000 | 0.000 | 0.000 | 5027.71 | 1993.78 | 1993.78 | 0.000 |
| 1991 | 313217 | 123999.442 | 123552.947 | 446.495 | 0.143 | 0.360 | 4883.81 | 1933.45 | 1926.49 | 6.962 |
| 1992 | 308577 | 122014.015 | 120814.764 | 1199.251 | 0.389 | 0.983 | 4729.73 | 1870.18 | 1851.79 | 18.382 |
| 1993 | 311713 | 122810.618 | 120554.604 | 2256.014 | 0.724 | 1.837 | 4692.62 | 1848.83 | 1814.86 | 33.963 |
| 1994 | 305700 | 119925.592 | 116450.703 | 3474.889 | 1.137 | 2.898 | 4516.19 | 1771.69 | 1720.36 | 51.336 |
| 1995 | 303225 | 117896.496 | 112993.274 | 4903.222 | 1.617 | 4.159 | 4393.80 | 1708.35 | 1637.30 | 71.049 |
| 1996 | 299559 | 115698.645 | 109218.297 | 6480.349 | 2.163 | 5.601 | 4273.16 | 1650.42 | 1557.98 | 92.441 |
| 1997 | 294226 | 113359.847 | 105287.828 | 8072.019 | 2.743 | 7.121 | 4128.67 | 1590.70 | 1477.43 | 113.269 |
| 1998 | 289258 | 111444.212 | 101774.972 | 9669.240 | 3.343 | 8.676 | 3992.19 | 1538.10 | 1404.65 | 133.450 |
| 1999 | 311002 | 114877.722 | 101924.420 | 12953.30 | 4.165 | 11.276 | 4224.60 | 1560.48 | 1384.53 | 175.956 |
| 2000 | 303786 | 112379.704 | 97577.455 | 14802.25 | 4.873 | 13.172 | 4066.21 | 1504.22 | 1306.09 | 198.130 |
| 2001 | 296294 | 110024.943 | 93250.230 | 16774.71 | 5.662 | 15.246 | 3914.28 | 1453.52 | 1231.91 | 221.608 |
| 2002 | 291898 | 108289.173 | 89294.516 | 18994.66 | 6.507 | 17.541 | 3813.00 | 1414.56 | 1166.43 | 248.123 |
| 2003 | 285865 | 106539.023 | 85576.545 | 20962.48 | 7.333 | 19.676 | 3695.36 | 1377.22 | 1106.24 | 270.980 |
| 2004 | 274177 | 103037.251 | 80635.435 | 22401.82 | 8.171 | 21.741 | 3506.10 | 1317.61 | 1031.14 | 286.468 |
| 2005 | 272379 | 103044.562 | 78568.549 | 24476.01 | 8.986 | 23.753 | 3442.69 | 1302.41 | 993.054 | 309.360 |
| 2006 | 264118 | 99672.555 | 73422.734 | 26249.82 | 9.939 | 26.336 | 3307.76 | 1248.28 | 919.531 | 328.747 |
| 2007 | 258419 | 97839.152 | 70098.169 | 27740.98 | 10.735 | 28.354 | 3203.20 | 1212.75 | 868.892 | 343.860 |
| 2008 | 259461 | 98075.852 | 68443.911 | 29631.94 | 11.421 | 30.213 | 3180.09 | 1202.07 | 838.886 | 363.185 |
| 2009 | 253907 | 95617.338 | 64658.682 | 30958.66 | 12.193 | 32.378 | 3075.31 | 1158.11 | 783.144 | 374.970 |
| 2010 | 251878 | 94607.988 | 62155.127 | 32452.86 | 12.884 | 34.302 | 3013.72 | 1131.98 | 743.686 | 388.298 |
| 2011 | 251779 | 93633.119 | 59363.942 | 34269.18 | 13.611 | 36.599 | 2982.76 | 1109.25 | 703.270 | 405.979 |
| 2012 | 252643 | 92775.175 | 56721.337 | 36053.84 | 14.271 | 38.862 | 2960.61 | 1087.19 | 664.691 | 422.498 |
| 2013 | 256175 | 92345.780 | 54463.985 | 37881.79 | 14.787 | 41.022 | 2967.76 | 1069.82 | 630.959 | 438.857 |
| 2014 | 257393 | 91379.215 | 51961.698 | 39417.52 | 15.314 | 43.136 | 2947.05 | 1046.26 | 594.941 | 451.315 |
| 2015 | 259782 | 90750.242 | 49937.651 | 40812.59 | 15.710 | 44.972 | 3346.10 | 1168.90 | 643.218 | 525.683 |
| 2016 | 261453 | 88811.996 | 46563.415 | 42248.58 | 16.159 | 47.571 | 3336.15 | 1133.24 | 594.151 | 539.094 |
| 2017 | 263685 | 87325.126 | 43822.789 | 43502.34 | 16.498 | 49.817 | 3333.85 | 1104.08 | 554.065 | 550.014 |
| 2018 | 258714 | 87612.144 | 42900.765 | 44711.38 | 17.282 | 51.033 | 3240.73 | 1097.46 | 537.388 | 560.068 |
| 2019 | 261438 | 87797.760 | 41397.987 | 46399.77 | 17.748 | 52.848 | 3242.99 | 1089.08 | 513.519 | 575.563 |
| 2020 | 262288 | 87221.042 | 39822.614 | 47398.43 | 18.071 | 54.343 | 3715.29 | 1235.48 | 564.084 | 671.395 |
| 2021 | 255079 | 82001.292 | 35090.341 | 46910.95 | 18.391 | 57.208 | 3587.82 | 1153.39 | 493.563 | 659.826 |
| 2022 | 257097 | 81915.977 | 33455.320 | 48460.66 | 18.849 | 59.159 | 3588.53 | 1143.37 | 466.966 | 676.409 |
| 2023 | 259080 | 81810.810 | 31849.675 | 49961.14 | 19.284 | 61.069 | 3586.88 | 1132.64 | 440.949 | 691.696 |
| 2024 | 260719 | 81528.303 | 30272.735 | 51255.57 | 19.659 | 62.868 | 3579.65 | 1119.38 | 415.643 | 703.736 |
| 2025 | 257308 | 79599.213 | 28566.385 | 51032.83 | 19.833 | 64.112 | 4112.08 | 1272.09 | 456.524 | 815.564 |
| 2026 | 251528 | 75082.462 | 25016.974 | 50065.49 | 19.905 | 66.681 | 4003.24 | 1194.99 | 398.162 | 796.826 |
| 2027 | 250988 | 74444.425 | 23411.364 | 51033.06 | 20.333 | 68.552 | 3976.53 | 1179.46 | 370.918 | 808.543 |
| 2028 | 250285 | 73699.585 | 21736.178 | 51963.41 | 20.762 | 70.507 | 3945.35 | 1161.76 | 342.637 | 819.122 |
| 2029 | 249525 | 72819.703 | 20151.964 | 52667.74 | 21.107 | 72.326 | 3910.99 | 1141.36 | 315.857 | 825.500 |
| 2030 | 240528 | 69309.277 | 18592.541 | 50716.74 | 21.086 | 73.175 | 4504.36 | 1297.95 | 348.181 | 949.768 |
| 2031 | 235442 | 65433.368 | 16136.330 | 49297.04 | 20.938 | 75.339 | 4397.65 | 1222.18 | 301.399 | 920.783 |
| 2032 | 234017 | 64643.552 | 14860.237 | 49783.32 | 21.273 | 77.012 | 4356.12 | 1203.31 | 276.616 | 926.692 |
| 2033 | 232559 | 63793.514 | 13640.344 | 50153.17 | 21.566 | 78.618 | 4310.93 | 1182.54 | 252.850 | 929.686 |
| 2034 | 230963 | 63032.151 | 12502.378 | 50529.77 | 21.878 | 80.165 | 4260.75 | 1162.80 | 230.641 | 932.160 |
| 2035 | 215822 | 57653.301 | 11310.760 | 46342.54 | 21.473 | 80.381 | 4924.38 | 1315.47 | 258.077 | 1057.39 |
| 2036 | 208183 | 52988.733 | 9107.963 | 43880.77 | 21.078 | 82.812 | 4748.94 | 1208.75 | 207.765 | 1000.98 |
| 2037 | 205491 | 52026.921 | 8174.189 | 43852.73 | 21.340 | 84.289 | 4682.40 | 1185.50 | 186.260 | 999.243 |
| 2038 | 202834 | 50943.211 | 7265.698 | 43677.51 | 21.534 | 85.738 | 4612.18 | 1158.38 | 165.213 | 993.171 |
| 2039 | 200251 | 50085.991 | 6491.129 | 43594.86 | 21.770 | 87.040 | 4538.65 | 1135.19 | 147.120 | 988.071 |

# Results for PHIM for Run 3 (Sensitivity_Analysis_1.RTF)

************************************************************************************

**Main Results for E-Component for Run: 3, Simulation: 1, COD: LUNG CANCER**

| Variable Parameter | Value |
|---|---|
| 1. Country | US (United States) |
| 2. Sex | M (Males) |
| 3. Year of start of process | 1990 |
| 4. Cause of Death | LUNG CANCER |
| 5. Effective Dose NULL | CC:1 |
| 6. Effective Dose Ecig | CC:1 Ecig:0 Dual:0.5 |
| Note: | Death rates are per million population |

---------------------------------------------------------------------------------------

Results for Age Groups Combined and Years combined, Population unadjusted for deaths in Ecig scenario,

| Age Group:All | | NULL | Ecig | | | %Drop | | NULL | Ecig | |
|---|---|---|---|---|---|---|---|---|---|---|
| Year | Number | Attrib | Attrib | Drop | %Drop | Attrib | Death | Attrib | Attrib | Drop |
| | Deaths | Deaths | Deaths | Deaths | Deaths | Deaths | Rate | Rates | Rates | Rates |
| All Comb | 3065988 | 2244988.113 | 1663881.390 | 581106.7 | 18.953 | 25.885 | 887.537 | 649.875 | 481.658 | 168.218 |

---------------------------------------------------------------------------------------

Results for Age Groups Combined, Population unadjusted for deaths in Ecig scenario,

| Age Group:All | | NULL | Ecig | | | %Drop | | NULL | Ecig | |
|---|---|---|---|---|---|---|---|---|---|---|
| Year | Number | Attrib | Attrib | Drop | %Drop | Attrib | Death | Attrib | Attrib | Drop |
| | Deaths | Deaths | Deaths | Deaths | Deaths | Deaths | Rate | Rates | Rates | Rates |
| 1990 | 78822 | 63385.736 | 63385.736 | 0.000 | 0.000 | 0.000 | 1249.50 | 1004.81 | 1004.81 | 0.000 |
| 1991 | 78462 | 62918.402 | 62867.864 | 50.538 | 0.064 | 0.080 | 1223.41 | 981.049 | 980.261 | 0.788 |
| 1992 | 77987 | 62366.766 | 62230.688 | 136.078 | 0.174 | 0.218 | 1195.35 | 955.930 | 953.844 | 2.086 |
| 1993 | 78542 | 62652.888 | 62384.672 | 268.216 | 0.341 | 0.428 | 1182.39 | 943.195 | 939.157 | 4.038 |
| 1994 | 77437 | 61565.227 | 61141.270 | 423.957 | 0.547 | 0.689 | 1144.00 | 909.520 | 903.257 | 6.263 |
| 1995 | 76904 | 60851.961 | 60234.465 | 617.496 | 0.803 | 1.015 | 1114.36 | 881.759 | 872.811 | 8.948 |
| 1996 | 76232 | 60122.922 | 59261.097 | 861.824 | 1.131 | 1.433 | 1087.44 | 857.643 | 845.349 | 12.294 |
| 1997 | 75473 | 59237.155 | 58111.669 | 1125.486 | 1.491 | 1.900 | 1059.06 | 831.234 | 815.441 | 15.793 |
| 1998 | 75381 | 58906.696 | 57454.311 | 1452.385 | 1.927 | 2.466 | 1040.37 | 813.001 | 792.955 | 20.045 |
| 1999 | 73159 | 56930.073 | 55154.606 | 1775.467 | 2.427 | 3.119 | 993.781 | 773.329 | 749.212 | 24.118 |
| 2000 | 73784 | 57137.121 | 54938.811 | 2198.310 | 2.979 | 3.847 | 987.608 | 764.787 | 735.363 | 29.425 |
| 2001 | 73294 | 56481.726 | 53806.700 | 2675.027 | 3.650 | 4.736 | 968.273 | 746.169 | 710.830 | 35.339 |
| 2002 | 72442 | 55588.365 | 52409.108 | 3179.258 | 4.389 | 5.719 | 946.293 | 726.138 | 684.608 | 41.530 |
| 2003 | 71942 | 54945.856 | 51206.020 | 3739.837 | 5.198 | 6.806 | 929.989 | 710.281 | 661.936 | 48.345 |
| 2004 | 71301 | 54203.126 | 49846.005 | 4357.121 | 6.111 | 8.039 | 911.778 | 693.135 | 637.417 | 55.718 |
| 2005 | 70964 | 53744.603 | 48743.701 | 5000.902 | 7.047 | 9.305 | 896.937 | 679.296 | 616.088 | 63.208 |
| 2006 | 69658 | 52535.572 | 46856.966 | 5678.605 | 8.152 | 10.809 | 872.383 | 657.945 | 586.827 | 71.118 |
| 2007 | 68565 | 51492.124 | 45140.298 | 6351.826 | 9.264 | 12.336 | 849.888 | 638.264 | 559.530 | 78.733 |
| 2008 | 68226 | 51030.736 | 43858.818 | 7171.918 | 10.512 | 14.054 | 836.215 | 625.460 | 537.557 | 87.903 |
| 2009 | 67509 | 50276.199 | 42282.418 | 7993.781 | 11.841 | 15.900 | 817.667 | 608.943 | 512.123 | 96.820 |
| 2010 | 67196 | 49840.933 | 41019.119 | 8821.814 | 13.128 | 17.700 | 804.000 | 596.347 | 490.794 | 105.553 |
| 2011 | 66506 | 49072.841 | 39337.603 | 9735.238 | 14.638 | 19.838 | 787.880 | 581.354 | 466.023 | 115.331 |
| 2012 | 66155 | 48539.512 | 37820.384 | 10719.13 | 16.203 | 22.083 | 775.240 | 568.812 | 443.200 | 125.613 |
| 2013 | 65333 | 47675.741 | 36075.045 | 11600.70 | 17.756 | 24.332 | 756.876 | 552.318 | 417.926 | 134.393 |
| 2014 | 65033 | 47194.534 | 34674.797 | 12519.74 | 19.251 | 26.528 | 744.602 | 540.359 | 397.013 | 143.346 |
| 2015 | 64145 | 46305.842 | 32976.627 | 13329.21 | 20.780 | 28.785 | 826.215 | 596.439 | 424.753 | 171.686 |
| 2016 | 61618 | 44208.867 | 30390.901 | 13817.97 | 22.425 | 31.256 | 786.248 | 564.107 | 387.789 | 176.318 |
| 2017 | 59897 | 42702.624 | 28330.400 | 14372.22 | 23.995 | 33.657 | 757.297 | 539.903 | 358.190 | 181.713 |
| 2018 | 62468 | 44321.202 | 28133.909 | 16187.29 | 25.913 | 36.523 | 782.490 | 555.181 | 352.414 | 202.767 |
| 2019 | 63045 | 44493.088 | 27086.766 | 17406.32 | 27.609 | 39.121 | 782.035 | 551.912 | 335.996 | 215.916 |
| 2020 | 63466 | 44529.551 | 25984.551 | 18545.00 | 29.220 | 41.647 | 898.991 | 630.757 | 368.069 | 262.688 |
| 2021 | 57113 | 39852.556 | 22150.268 | 17702.29 | 30.995 | 44.419 | 803.323 | 560.546 | 311.555 | 248.992 |
| 2022 | 57440 | 39853.685 | 21017.447 | 18836.24 | 32.793 | 47.263 | 801.745 | 556.274 | 293.359 | 262.914 |
| 2023 | 57738 | 39847.050 | 19887.343 | 19959.71 | 34.569 | 50.091 | 799.366 | 551.670 | 275.334 | 276.336 |
| 2024 | 57942 | 39726.010 | 18763.494 | 20962.52 | 36.178 | 52.768 | 795.539 | 545.435 | 257.622 | 287.814 |
| 2025 | 57521 | 39207.348 | 17563.186 | 21644.16 | 37.628 | 55.204 | 919.257 | 626.579 | 280.680 | 345.899 |
| 2026 | 51428 | 34803.960 | 14664.793 | 20139.17 | 39.160 | 57.865 | 818.510 | 553.929 | 233.400 | 320.529 |
| 2027 | 51172 | 34484.595 | 13582.059 | 20902.54 | 40.848 | 60.614 | 810.740 | 546.357 | 215.187 | 331.169 |
| 2028 | 50870 | 34100.638 | 12471.121 | 21629.52 | 42.519 | 63.428 | 801.884 | 537.544 | 196.588 | 340.956 |
| 2029 | 50540 | 33669.838 | 11432.114 | 22237.72 | 44.000 | 66.046 | 792.146 | 527.732 | 179.184 | 348.548 |
| 2030 | 49109 | 32557.688 | 10401.638 | 22156.05 | 45.116 | 68.052 | 919.662 | 609.705 | 194.791 | 414.915 |
| 2031 | 43585 | 28646.393 | 8484.152 | 20162.24 | 46.259 | 70.383 | 814.096 | 535.065 | 158.469 | 376.595 |
| 2032 | 43163 | 28227.160 | 7698.172 | 20528.99 | 47.562 | 72.728 | 803.454 | 525.435 | 143.298 | 382.137 |
| 2033 | 42725 | 27786.623 | 6954.106 | 20832.52 | 48.759 | 74.973 | 791.996 | 515.079 | 128.908 | 386.171 |
| 2034 | 42256 | 27348.299 | 6275.922 | 21072.38 | 49.869 | 77.052 | 779.523 | 504.514 | 115.777 | 388.738 |
| 2035 | 39748 | 25570.077 | 5562.507 | 20007.57 | 50.336 | 78.246 | 906.933 | 583.430 | 126.919 | 456.511 |
| 2036 | 34030 | 21717.319 | 4150.983 | 17566.34 | 51.620 | 80.886 | 776.269 | 495.402 | 94.690 | 400.712 |
| 2037 | 33454 | 21256.024 | 3663.632 | 17592.39 | 52.587 | 82.764 | 762.289 | 484.347 | 83.481 | 400.866 |
| 2038 | 32883 | 20747.175 | 3199.358 | 17547.82 | 53.364 | 84.579 | 747.720 | 471.764 | 72.749 | 399.015 |
| 2039 | 32325 | 20327.688 | 2813.771 | 17513.92 | 54.181 | 86.158 | 732.644 | 460.724 | 63.774 | 396.950 |

# Results for PHIM for Run 3 (Sensitivity_Analysis_1.RTF)

********************************************************************************

**Main Results for E-Component for Run: 3, Simulation: 1, COD: IHD**

| Variable Parameter | Value |
|---|---|
| 1. Country | US (United States) |
| 2. Sex | M (Males) |
| 3. Year of start of process | 1990 |
| 4. Cause of Death | IHD |
| 5. Effective Dose NULL | CC:1 |
| 6. Effective Dose Ecig | CC:1 Ecig:0 Dual:0.5 |
| Note: | Death rates are per million population |

---

Results for Age Groups Combined and Years combined, Population unadjusted for deaths in Ecig scenario,

| Age Group:All Year | Number Deaths | NULL Attrib Deaths | Ecig Attrib Deaths | Drop Deaths | %Drop Deaths | %Drop Attrib Deaths | Death Rate | NULL Attrib Rates | Ecig Attrib Rates | Drop Rates |
|---|---|---|---|---|---|---|---|---|---|---|
| All Comb | 6360041 | 1098877.421 | 476566.196 | 622311.2 | 9.785 | 56.632 | 1841.09 | 318.101 | 137.956 | 180.146 |

---

Results for Age Groups Combined, Population unadjusted for deaths in Ecig scenario,

| Age Group:All Year | Number Deaths | NULL Attrib Deaths | Ecig Attrib Deaths | Drop Deaths | %Drop Deaths | %Drop Attrib Deaths | Death Rate | NULL Attrib Rates | Ecig Attrib Rates | Drop Rates |
|---|---|---|---|---|---|---|---|---|---|---|
| 1990 | 171484 | 38511.725 | 38511.725 | 0.000 | 0.000 | 0.000 | 2718.40 | 610.497 | 610.497 | 0.000 |
| 1991 | 168044 | 37309.838 | 36979.259 | 330.579 | 0.197 | 0.886 | 2620.21 | 581.750 | 576.596 | 5.155 |
| 1992 | 164548 | 36137.488 | 35246.000 | 891.488 | 0.542 | 2.467 | 2522.12 | 553.899 | 540.235 | 13.664 |
| 1993 | 164362 | 35599.588 | 33946.271 | 1653.317 | 1.006 | 4.644 | 2474.35 | 535.926 | 511.037 | 24.890 |
| 1994 | 159866 | 34336.757 | 31808.860 | 2527.897 | 1.581 | 7.362 | 2361.75 | 507.267 | 469.921 | 37.345 |
| 1995 | 158083 | 33455.759 | 29915.129 | 3540.630 | 2.240 | 10.583 | 2290.66 | 484.782 | 433.477 | 51.305 |
| 1996 | 154875 | 32193.824 | 27575.456 | 4618.369 | 2.982 | 14.346 | 2209.26 | 459.239 | 393.359 | 65.880 |
| 1997 | 149970 | 30665.509 | 24999.792 | 5665.718 | 3.778 | 18.476 | 2104.43 | 430.308 | 350.805 | 79.503 |
| 1998 | 145407 | 29188.430 | 22539.620 | 6648.810 | 4.573 | 22.779 | 2006.83 | 402.844 | 311.080 | 91.764 |
| 1999 | 165952 | 33375.386 | 24112.583 | 9262.803 | 5.582 | 27.753 | 2254.27 | 453.366 | 327.542 | 125.824 |
| 2000 | 160260 | 32024.754 | 21638.548 | 10386.21 | 6.481 | 32.432 | 2145.10 | 428.655 | 289.635 | 139.021 |
| 2001 | 154444 | 30682.393 | 19131.991 | 11550.40 | 7.479 | 37.645 | 2040.33 | 405.339 | 252.749 | 152.590 |
| 2002 | 151887 | 30218.370 | 17301.832 | 12916.54 | 8.504 | 42.744 | 1984.06 | 394.736 | 226.010 | 168.726 |
| 2003 | 146733 | 29175.202 | 15248.232 | 13926.97 | 9.491 | 47.736 | 1896.81 | 377.146 | 197.113 | 180.033 |
| 2004 | 138381 | 27488.687 | 13038.979 | 14449.71 | 10.442 | 52.566 | 1769.58 | 351.518 | 166.739 | 184.779 |
| 2005 | 135881 | 27017.439 | 11630.228 | 15387.21 | 11.324 | 56.953 | 1717.44 | 341.482 | 146.998 | 194.484 |
| 2006 | 131922 | 26231.435 | 10045.441 | 16185.99 | 12.269 | 61.705 | 1652.16 | 328.517 | 125.807 | 202.710 |
| 2007 | 126502 | 25103.049 | 8582.641 | 16520.41 | 13.059 | 65.810 | 1568.04 | 311.161 | 106.385 | 204.776 |
| 2008 | 125859 | 24621.740 | 7583.940 | 17037.80 | 13.537 | 69.198 | 1542.60 | 301.777 | 92.953 | 208.824 |
| 2009 | 122428 | 23741.177 | 6540.958 | 17200.22 | 14.049 | 72.449 | 1482.84 | 287.552 | 79.224 | 208.328 |
| 2010 | 120537 | 23189.160 | 5717.460 | 17471.70 | 14.495 | 75.344 | 1442.22 | 277.458 | 68.409 | 209.049 |
| 2011 | 120221 | 22786.927 | 4950.478 | 17836.45 | 14.836 | 78.275 | 1424.23 | 269.951 | 58.647 | 211.304 |
| 2012 | 120927 | 22485.295 | 4329.406 | 18155.89 | 15.014 | 80.746 | 1417.09 | 263.495 | 50.734 | 212.760 |
| 2013 | 122648 | 22251.884 | 3807.095 | 18444.79 | 15.039 | 82.891 | 1420.86 | 257.786 | 44.105 | 213.681 |
| 2014 | 123229 | 21894.593 | 3323.136 | 18571.46 | 15.071 | 84.822 | 1410.92 | 250.685 | 38.049 | 212.636 |
| 2015 | 123774 | 21471.109 | 2909.966 | 18561.14 | 14.996 | 86.447 | 1594.26 | 276.557 | 37.482 | 239.075 |
| 2016 | 126218 | 21411.568 | 2519.178 | 18892.39 | 14.968 | 88.235 | 1610.55 | 273.213 | 32.145 | 241.068 |
| 2017 | 128265 | 21169.399 | 2189.175 | 18980.22 | 14.798 | 89.659 | 1621.69 | 267.651 | 27.678 | 239.973 |
| 2018 | 121429 | 19830.576 | 1750.180 | 18080.40 | 14.890 | 91.174 | 1521.05 | 248.404 | 21.923 | 226.480 |
| 2019 | 121832 | 19431.014 | 1508.386 | 17922.63 | 14.711 | 92.237 | 1511.26 | 241.031 | 18.711 | 222.320 |
| 2020 | 120811 | 18532.612 | 1279.520 | 17253.09 | 14.281 | 93.096 | 1711.27 | 262.513 | 18.124 | 244.389 |
| 2021 | 119407 | 18234.128 | 1054.484 | 17179.64 | 14.388 | 94.217 | 1679.51 | 256.472 | 14.832 | 241.640 |
| 2022 | 119438 | 17812.559 | 884.051 | 16928.51 | 14.173 | 95.037 | 1667.10 | 248.626 | 12.339 | 236.286 |
| 2023 | 119445 | 17361.668 | 732.450 | 16629.22 | 13.922 | 95.781 | 1653.68 | 240.367 | 10.141 | 230.226 |
| 2024 | 119305 | 16929.676 | 616.463 | 16313.21 | 13.674 | 96.359 | 1638.05 | 232.443 | 8.464 | 223.979 |
| 2025 | 115685 | 15501.666 | 505.852 | 14995.81 | 12.963 | 96.737 | 1848.78 | 247.735 | 8.084 | 239.650 |
| 2026 | 115073 | 15523.814 | 410.429 | 15113.39 | 13.134 | 97.356 | 1831.46 | 247.072 | 6.532 | 240.540 |
| 2027 | 113989 | 15073.070 | 340.359 | 14732.71 | 12.925 | 97.742 | 1805.98 | 238.810 | 5.392 | 233.418 |
| 2028 | 112855 | 14636.710 | 273.564 | 14363.15 | 12.727 | 98.131 | 1778.98 | 230.725 | 4.312 | 226.413 |
| 2029 | 111718 | 14180.166 | 221.619 | 13958.55 | 12.494 | 98.437 | 1751.04 | 222.256 | 3.474 | 218.783 |
| 2030 | 104851 | 12111.772 | 179.027 | 11932.74 | 11.381 | 98.522 | 1963.54 | 226.816 | 3.353 | 223.464 |
| 2031 | 105289 | 12417.367 | 150.914 | 12266.45 | 11.650 | 98.785 | 1966.62 | 231.935 | 2.819 | 229.116 |
| 2032 | 103879 | 12054.064 | 127.074 | 11926.99 | 11.482 | 98.946 | 1933.67 | 224.381 | 2.365 | 222.015 |
| 2033 | 102485 | 11672.389 | 100.261 | 11572.13 | 11.291 | 99.141 | 1899.76 | 216.370 | 1.859 | 214.512 |
| 2034 | 101070 | 11365.762 | 81.063 | 11284.70 | 11.165 | 99.287 | 1864.51 | 209.673 | 1.495 | 208.177 |
| 2035 | 90820 | 8580.360 | 64.810 | 8515.550 | 9.376 | 99.245 | 2072.23 | 195.777 | 1.479 | 194.299 |
| 2036 | 92117 | 8948.841 | 52.548 | 8896.293 | 9.658 | 99.413 | 2101.31 | 204.135 | 1.199 | 202.937 |
| 2037 | 90327 | 8624.570 | 45.460 | 8579.110 | 9.498 | 99.473 | 2058.21 | 196.522 | 1.036 | 195.486 |
| 2038 | 88590 | 8315.017 | 37.008 | 8278.009 | 9.344 | 99.555 | 2014.42 | 189.073 | 0.842 | 188.231 |
| 2039 | 86921 | 8001.132 | 27.296 | 7973.835 | 9.174 | 99.659 | 1970.05 | 181.344 | 0.619 | 180.726 |

# Results for PHIM for Run 3 (Sensitivity_Analysis_1.RTF)

********************************************************************************
**Main Results for E-Component for Run: 3, Simulation: 1, COD: STROKE**

| Variable Parameter | Value |
|---|---|
| 1. Country | US (United States) |
| 2. Sex | M (Males) |
| 3. Year of start of process | 1990 |
| 4. Cause of Death | STROKE |
| 5. Effective Dose NULL | CC:1 |
| 6. Effective Dose Ecig | CC:1 Ecig:0 Dual:0.5 |
| Note: | Death rates are per million population |

-----------------------------------------------------------------------------------------
Results for Age Groups Combined and Years combined, Population unadjusted for deaths in Ecig scenario,

| Age Group:All | | NULL | Ecig | | | %Drop | | NULL | Ecig | |
|---|---|---|---|---|---|---|---|---|---|---|
| Year | Number | Attrib | Attrib | Drop | %Drop | Attrib | Death | Attrib | Attrib | Drop |
| | Deaths | Deaths | Deaths | Deaths | Deaths | Deaths | Rate | Rates | Rates | Rates |
| All Comb | 1489699 | 149918.462 | 64595.015 | 85323.45 | 5.728 | 56.913 | 431.236 | 43.398 | 18.699 | 24.699 |

-----------------------------------------------------------------------------------------
Results for Age Groups Combined, Population unadjusted for deaths in Ecig scenario,

| Age Group:All | | NULL | Ecig | | | %Drop | | NULL | Ecig | |
|---|---|---|---|---|---|---|---|---|---|---|
| Year | Number | Attrib | Attrib | Drop | %Drop | Attrib | Death | Attrib | Attrib | Drop |
| | Deaths | Deaths | Deaths | Deaths | Deaths | Deaths | Rate | Rates | Rates | Rates |
| 1990 | 32862 | 4613.166 | 4613.166 | 0.000 | 0.000 | 0.000 | 520.936 | 73.129 | 73.129 | 0.000 |
| 1991 | 32619 | 4555.566 | 4513.452 | 42.114 | 0.129 | 0.924 | 508.609 | 71.032 | 70.376 | 0.657 |
| 1992 | 32100 | 4453.572 | 4342.861 | 110.712 | 0.345 | 2.486 | 492.014 | 68.262 | 66.565 | 1.697 |
| 1993 | 32896 | 4462.473 | 4253.106 | 209.367 | 0.636 | 4.692 | 495.226 | 67.179 | 64.027 | 3.152 |
| 1994 | 33323 | 4499.513 | 4168.617 | 330.897 | 0.993 | 7.354 | 492.290 | 66.473 | 61.584 | 4.888 |
| 1995 | 33569 | 4472.774 | 4003.197 | 469.577 | 1.399 | 10.499 | 486.422 | 64.812 | 58.007 | 6.804 |
| 1996 | 33870 | 4406.878 | 3783.811 | 623.067 | 1.840 | 14.139 | 483.150 | 62.863 | 53.975 | 8.888 |
| 1997 | 33516 | 4288.158 | 3509.267 | 778.890 | 2.324 | 18.164 | 470.307 | 60.173 | 49.243 | 10.930 |
| 1998 | 32795 | 4120.148 | 3198.676 | 921.472 | 2.810 | 22.365 | 452.620 | 56.864 | 44.147 | 12.718 |
| 1999 | 33375 | 3982.443 | 2918.982 | 1063.460 | 3.186 | 26.704 | 453.361 | 54.097 | 39.651 | 14.446 |
| 2000 | 33459 | 4021.411 | 2770.001 | 1251.410 | 3.740 | 31.119 | 447.853 | 53.827 | 37.077 | 16.750 |
| 2001 | 32275 | 3848.092 | 2470.139 | 1377.953 | 4.269 | 35.809 | 426.379 | 50.836 | 32.633 | 18.204 |
| 2002 | 31586 | 3783.081 | 2257.026 | 1526.055 | 4.831 | 40.339 | 412.601 | 49.418 | 29.483 | 19.935 |
| 2003 | 30985 | 3754.415 | 2063.890 | 1690.525 | 5.456 | 45.028 | 400.541 | 48.533 | 26.680 | 21.853 |
| 2004 | 29884 | 3666.274 | 1851.790 | 1814.484 | 6.072 | 49.491 | 382.149 | 46.883 | 23.680 | 23.203 |
| 2005 | 28708 | 3589.688 | 1657.038 | 1932.650 | 6.732 | 53.839 | 362.850 | 45.371 | 20.944 | 24.427 |
| 2006 | 28188 | 3596.985 | 1502.526 | 2094.459 | 7.430 | 58.228 | 353.021 | 45.048 | 18.817 | 26.231 |
| 2007 | 28026 | 3560.798 | 1346.654 | 2214.144 | 7.900 | 62.181 | 347.392 | 44.137 | 16.692 | 27.445 |
| 2008 | 27128 | 3417.001 | 1167.979 | 2249.023 | 8.290 | 65.819 | 332.495 | 41.881 | 14.315 | 27.565 |
| 2009 | 27068 | 3394.390 | 1046.116 | 2348.274 | 8.675 | 69.181 | 327.847 | 41.113 | 12.671 | 28.442 |
| 2010 | 26911 | 3340.137 | 930.903 | 2409.234 | 8.953 | 72.130 | 321.990 | 39.965 | 11.138 | 28.826 |
| 2011 | 26959 | 3285.435 | 823.221 | 2462.214 | 9.133 | 74.943 | 319.377 | 38.922 | 9.752 | 29.169 |
| 2012 | 27057 | 3259.209 | 725.882 | 2533.328 | 9.363 | 77.728 | 317.068 | 38.193 | 8.506 | 29.687 |
| 2013 | 27904 | 3274.703 | 650.941 | 2623.762 | 9.403 | 80.122 | 323.265 | 37.937 | 7.541 | 30.396 |
| 2014 | 28792 | 3298.083 | 581.111 | 2716.972 | 9.437 | 82.380 | 329.657 | 37.762 | 6.653 | 31.108 |
| 2015 | 29560 | 3244.406 | 513.437 | 2730.969 | 9.239 | 84.175 | 380.745 | 41.789 | 6.613 | 35.176 |
| 2016 | 30513 | 3314.836 | 462.366 | 2852.470 | 9.348 | 86.052 | 389.347 | 42.297 | 5.900 | 36.398 |
| 2017 | 31416 | 3333.314 | 410.439 | 2922.874 | 9.304 | 87.687 | 397.202 | 42.144 | 5.189 | 36.955 |
| 2018 | 29348 | 2925.242 | 322.055 | 2603.187 | 8.870 | 88.990 | 367.626 | 36.642 | 4.034 | 32.608 |
| 2019 | 29598 | 2864.628 | 279.727 | 2584.901 | 8.733 | 90.235 | 367.149 | 35.534 | 3.470 | 32.064 |
| 2020 | 29592 | 2730.366 | 237.257 | 2493.109 | 8.425 | 91.310 | 419.174 | 38.675 | 3.361 | 35.315 |
| 2021 | 30097 | 2647.911 | 203.328 | 2444.582 | 8.122 | 92.321 | 423.328 | 37.244 | 2.860 | 34.384 |
| 2022 | 30304 | 2586.165 | 173.048 | 2413.117 | 7.963 | 93.309 | 422.974 | 36.097 | 2.415 | 33.682 |
| 2023 | 30505 | 2517.960 | 146.504 | 2371.456 | 7.774 | 94.182 | 422.337 | 34.860 | 2.028 | 32.832 |
| 2024 | 30661 | 2454.161 | 125.508 | 2328.652 | 7.595 | 94.886 | 420.968 | 33.695 | 1.723 | 31.972 |
| 2025 | 30254 | 2275.364 | 103.084 | 2172.280 | 7.180 | 95.470 | 483.488 | 36.363 | 1.647 | 34.716 |
| 2026 | 30685 | 2183.166 | 85.941 | 2097.225 | 6.835 | 96.063 | 488.380 | 34.747 | 1.368 | 33.379 |
| 2027 | 30561 | 2112.345 | 72.612 | 2039.733 | 6.674 | 96.563 | 484.196 | 33.467 | 1.150 | 32.317 |
| 2028 | 30412 | 2046.677 | 60.804 | 1985.873 | 6.530 | 97.029 | 479.392 | 32.263 | 0.958 | 31.304 |
| 2029 | 30254 | 1977.671 | 50.769 | 1926.903 | 6.369 | 97.433 | 474.195 | 30.997 | 0.796 | 30.202 |
| 2030 | 29330 | 1744.457 | 40.161 | 1704.296 | 5.811 | 97.698 | 549.260 | 32.668 | 0.752 | 31.916 |
| 2031 | 28976 | 1653.738 | 33.663 | 1620.074 | 5.591 | 97.964 | 541.223 | 30.889 | 0.629 | 30.260 |
| 2032 | 28724 | 1603.120 | 28.290 | 1574.831 | 5.483 | 98.235 | 534.690 | 29.841 | 0.527 | 29.315 |
| 2033 | 28464 | 1548.245 | 23.337 | 1524.908 | 5.357 | 98.493 | 527.627 | 28.700 | 0.433 | 28.267 |
| 2034 | 28178 | 1511.985 | 19.902 | 1492.083 | 5.295 | 98.684 | 519.826 | 27.893 | 0.367 | 27.526 |
| 2035 | 26737 | 1239.866 | 14.806 | 1225.059 | 4.582 | 98.806 | 610.065 | 28.290 | 0.338 | 27.952 |
| 2036 | 25591 | 1182.522 | 12.042 | 1170.480 | 4.574 | 98.982 | 583.762 | 26.975 | 0.275 | 26.700 |
| 2037 | 25143 | 1134.483 | 10.208 | 1124.275 | 4.472 | 99.100 | 572.916 | 25.851 | 0.233 | 25.618 |
| 2038 | 24693 | 1093.537 | 8.594 | 1084.943 | 4.394 | 99.214 | 561.491 | 24.866 | 0.195 | 24.670 |
| 2039 | 24247 | 1047.905 | 6.783 | 1041.122 | 4.294 | 99.353 | 549.553 | 23.751 | 0.154 | 23.597 |

# Results for PHIM for Run 3 (Sensitivity_Analysis_1.RTF)

************************************************************************************

**Main Results for E-Component for Run: 3, Simulation: 1, COD: COPD**

| Variable Parameter | Value |
|---|---|
| 1. Country | US (United States) |
| 2. Sex | M (Males) |
| 3. Year of start of process | 1990 |
| 4. Cause of Death | COPD |
| 5. Effective Dose NULL | CC:1 |
| 6. Effective Dose Ecig | CC:1 Ecig:0 Dual:0.5 |
| Note: | Death rates are per million population |

------------------------------------------------------------------------------------------

Results for Age Groups Combined and Years combined, Population unadjusted for deaths in Ecig scenario,

| Age Group:All<br>Year | Number<br>Deaths | NULL<br>Attrib<br>Deaths | Ecig<br>Attrib<br>Deaths | Drop<br>Deaths | %Drop<br>Deaths | %Drop<br>Attrib<br>Deaths | Death<br>Rate | NULL<br>Attrib<br>Rates | Ecig<br>Attrib<br>Rates | Drop<br>Rates |
|---|---|---|---|---|---|---|---|---|---|---|
| All Comb | 2233143 | 1015606.587 | 651572.317 | 364034.3 | 16.301 | 35.844 | 646.447 | 293.996 | 188.616 | 105.380 |

------------------------------------------------------------------------------------------

Results for Age Groups Combined, Population unadjusted for deaths in Ecig scenario,

| Age Group:All<br>Year | Number<br>Deaths | NULL<br>Attrib<br>Deaths | Ecig<br>Attrib<br>Deaths | Drop<br>Deaths | %Drop<br>Deaths | %Drop<br>Attrib<br>Deaths | Death<br>Rate | NULL<br>Attrib<br>Rates | Ecig<br>Attrib<br>Rates | Drop<br>Rates |
|---|---|---|---|---|---|---|---|---|---|---|
| 1990 | 33993 | 19262.437 | 19262.437 | 0.000 | 0.000 | 0.000 | 538.865 | 305.353 | 305.353 | 0.000 |
| 1991 | 34092 | 19215.636 | 19192.373 | 23.264 | 0.068 | 0.121 | 531.576 | 299.618 | 299.255 | 0.363 |
| 1992 | 33942 | 19056.189 | 18995.216 | 60.973 | 0.180 | 0.320 | 520.248 | 292.085 | 291.150 | 0.935 |
| 1993 | 35913 | 20095.670 | 19970.556 | 125.114 | 0.348 | 0.623 | 540.645 | 302.526 | 300.643 | 1.884 |
| 1994 | 35074 | 19524.095 | 19331.957 | 192.138 | 0.548 | 0.984 | 518.158 | 288.435 | 285.596 | 2.839 |
| 1995 | 34669 | 19116.002 | 18840.483 | 275.519 | 0.795 | 1.441 | 502.362 | 276.995 | 273.003 | 3.992 |
| 1996 | 34582 | 18975.021 | 18597.933 | 377.089 | 1.090 | 1.987 | 493.306 | 270.675 | 265.296 | 5.379 |
| 1997 | 35267 | 19169.025 | 18667.100 | 501.925 | 1.423 | 2.618 | 494.878 | 268.986 | 261.943 | 7.043 |
| 1998 | 35675 | 19228.937 | 18582.365 | 646.573 | 1.812 | 3.362 | 492.368 | 265.388 | 256.464 | 8.924 |
| 1999 | 38516 | 20589.820 | 19738.248 | 851.572 | 2.211 | 4.136 | 523.195 | 279.689 | 268.121 | 11.568 |
| 2000 | 36283 | 19196.417 | 18230.095 | 966.322 | 2.663 | 5.034 | 485.652 | 256.946 | 244.012 | 12.934 |
| 2001 | 36281 | 19012.731 | 17841.401 | 1171.331 | 3.228 | 6.161 | 479.301 | 251.174 | 235.699 | 15.474 |
| 2002 | 35983 | 18699.357 | 17326.551 | 1372.806 | 3.815 | 7.341 | 470.038 | 244.265 | 226.333 | 17.933 |
| 2003 | 36205 | 18663.550 | 17058.404 | 1605.146 | 4.433 | 8.600 | 468.019 | 241.262 | 220.513 | 20.750 |
| 2004 | 34611 | 17679.164 | 15898.661 | 1780.503 | 5.144 | 10.071 | 442.596 | 226.076 | 203.308 | 22.769 |
| 2005 | 36826 | 18692.832 | 16537.581 | 2155.251 | 5.853 | 11.530 | 465.456 | 236.265 | 209.024 | 27.241 |
| 2006 | 34350 | 17308.564 | 15017.801 | 2290.762 | 6.669 | 13.235 | 430.192 | 216.769 | 188.080 | 28.689 |
| 2007 | 35326 | 17683.181 | 15028.577 | 2654.605 | 7.515 | 15.012 | 437.879 | 219.189 | 186.285 | 32.905 |
| 2008 | 38248 | 19006.374 | 15833.174 | 3173.200 | 8.296 | 16.695 | 468.788 | 232.952 | 194.060 | 38.892 |
| 2009 | 36902 | 18205.573 | 14789.190 | 3416.382 | 9.258 | 18.766 | 446.956 | 220.505 | 179.126 | 41.379 |
| 2010 | 37234 | 18237.758 | 14487.644 | 3750.114 | 10.072 | 20.562 | 445.505 | 218.215 | 173.345 | 44.870 |
| 2011 | 38093 | 18487.916 | 14252.640 | 4235.276 | 11.118 | 22.908 | 451.279 | 219.022 | 168.848 | 50.174 |
| 2012 | 38504 | 18491.158 | 13845.665 | 4645.493 | 12.065 | 25.123 | 451.211 | 216.689 | 162.251 | 54.438 |
| 2013 | 40290 | 19143.452 | 13930.904 | 5212.548 | 12.938 | 27.229 | 466.755 | 221.775 | 161.388 | 60.387 |
| 2014 | 40339 | 18992.005 | 13382.655 | 5609.350 | 13.906 | 29.535 | 461.866 | 217.451 | 153.226 | 64.225 |
| 2015 | 42303 | 19728.885 | 13537.621 | 6191.264 | 14.636 | 31.382 | 544.880 | 254.116 | 174.370 | 79.746 |
| 2016 | 43104 | 19876.724 | 13190.969 | 6685.755 | 15.511 | 33.636 | 550.009 | 253.628 | 168.317 | 85.311 |
| 2017 | 44107 | 20119.790 | 12892.775 | 7227.016 | 16.385 | 35.920 | 557.659 | 254.381 | 163.007 | 91.373 |
| 2018 | 45469 | 20535.123 | 12694.622 | 7840.501 | 17.243 | 38.181 | 569.564 | 257.229 | 159.017 | 98.212 |
| 2019 | 46963 | 21009.029 | 12523.109 | 8485.920 | 18.069 | 40.392 | 582.552 | 260.605 | 155.342 | 105.263 |
| 2020 | 48419 | 21428.512 | 12321.286 | 9107.226 | 18.809 | 42.501 | 685.853 | 303.533 | 174.530 | 129.003 |
| 2021 | 48463 | 21266.697 | 11682.261 | 9584.436 | 19.777 | 45.068 | 681.652 | 299.127 | 164.317 | 134.810 |
| 2022 | 49915 | 21663.568 | 11380.774 | 10282.79 | 20.600 | 47.466 | 696.712 | 302.378 | 158.852 | 143.526 |
| 2023 | 51391 | 22084.131 | 11083.377 | 11000.75 | 21.406 | 49.813 | 711.491 | 305.748 | 153.446 | 152.302 |
| 2024 | 52811 | 22418.456 | 10767.270 | 11651.19 | 22.062 | 51.971 | 725.093 | 307.804 | 147.834 | 159.970 |
| 2025 | 53848 | 22614.834 | 10394.263 | 12220.57 | 22.695 | 54.038 | 860.553 | 361.411 | 166.112 | 195.299 |
| 2026 | 54342 | 22571.522 | 9855.812 | 12715.71 | 23.400 | 56.335 | 864.886 | 359.241 | 156.862 | 202.379 |
| 2027 | 55267 | 22774.416 | 9416.334 | 13358.08 | 24.170 | 58.654 | 875.619 | 360.827 | 149.188 | 211.639 |
| 2028 | 56149 | 22915.561 | 8930.690 | 13984.87 | 24.907 | 61.028 | 885.102 | 361.228 | 140.778 | 220.450 |
| 2029 | 57014 | 22992.027 | 8447.463 | 14544.56 | 25.511 | 63.259 | 893.616 | 360.371 | 132.403 | 227.968 |
| 2030 | 57238 | 22895.360 | 7971.714 | 14923.65 | 26.073 | 65.182 | 1071.90 | 428.760 | 149.286 | 279.474 |
| 2031 | 57592 | 22715.871 | 7467.601 | 15248.27 | 26.476 | 67.126 | 1075.71 | 424.293 | 139.482 | 284.811 |
| 2032 | 58251 | 22759.207 | 7006.701 | 15752.51 | 27.043 | 69.214 | 1084.31 | 423.652 | 130.426 | 293.225 |
| 2033 | 58885 | 22786.258 | 6562.641 | 16223.62 | 27.552 | 71.199 | 1091.54 | 422.387 | 121.651 | 300.736 |
| 2034 | 59460 | 22806.106 | 6125.492 | 16680.61 | 28.054 | 73.141 | 1096.89 | 420.721 | 113.001 | 307.719 |
| 2035 | 58516 | 22262.998 | 5668.636 | 16594.36 | 28.359 | 74.538 | 1335.15 | 507.973 | 129.341 | 378.632 |
| 2036 | 56446 | 21140.051 | 4892.390 | 16247.66 | 28.785 | 76.857 | 1287.60 | 482.234 | 111.602 | 370.631 |
| 2037 | 56568 | 21011.845 | 4454.890 | 16556.96 | 29.269 | 78.798 | 1288.98 | 478.783 | 101.511 | 377.272 |
| 2038 | 56668 | 20787.483 | 4020.739 | 16766.74 | 29.588 | 80.658 | 1288.55 | 472.681 | 91.426 | 381.254 |
| 2039 | 56758 | 20709.267 | 3643.279 | 17065.99 | 30.068 | 82.407 | 1286.40 | 469.372 | 82.574 | 386.798 |

# Results for PHIM for Run 3 (Sensitivity_Analysis_1.RTF)

************************************************************************************
**Main Results for E-Component for Run: 3, Simulation: 1, COD: Four Diseases Combined, Adjusted**

| **Variable Parameter** | **Value** |
|---|---|
| 1. Country | US (United States) |
| 2. Sex | M (Males) |
| 3. Year of start of process | 1990 |
| 4. Cause of Death | Four Diseases Combined |
| 5. Effective Dose NULL | CC:1 |
| 6. Effective Dose Ecig | CC:1 Ecig:0 Dual:0.5 |
| Note: | Death rates are per million population |

-------------------------------------------------------------------------------------------

Results for Age Groups Combined and Years combined, Population and Number deaths adjusted for Ecig scenario,

| Age Group:All | | NULL | Ecig | | | %Drop | | NULL | Ecig | |
|---|---|---|---|---|---|---|---|---|---|---|
| Year | Number | Attrib | Attrib | Drop | %Drop | Attrib | Death | Attrib | Attrib | Drop |
| | Deaths | Deaths | Deaths | Deaths | Deaths | Deaths | Rate | Rates | Rates | Rates |
| All Comb | 13302112 | 4509390.583 | 2873449.699 | 1635941 | 12.298 | 36.279 | 3816.17 | 1305.37 | 824.348 | 481.023 |

-------------------------------------------------------------------------------------------

Results for Age Groups Combined, Population and Number deaths adjusted for Ecig scenario,

| Age Group:All | | NULL | Ecig | | | %Drop | | NULL | Ecig | |
|---|---|---|---|---|---|---|---|---|---|---|
| Year | Number | Attrib | Attrib | Drop | %Drop | Attrib | Death | Attrib | Attrib | Drop |
| | Deaths | Deaths | Deaths | Deaths | Deaths | Deaths | Rate | Rates | Rates | Rates |
| 1990 | 317161 | 125773.065 | 125773.065 | 0.000 | 0.000 | 0.000 | 5027.71 | 1993.78 | 1993.78 | 0.000 |
| 1991 | 313217 | 123999.442 | 123552.947 | 446.495 | 0.143 | 0.360 | 4883.81 | 1933.45 | 1926.49 | 6.962 |
| 1992 | 308580 | 122014.015 | 120815.937 | 1198.078 | 0.388 | 0.982 | 4729.74 | 1870.18 | 1851.80 | 18.377 |
| 1993 | 311723 | 122810.618 | 120558.793 | 2251.825 | 0.722 | 1.834 | 4692.65 | 1848.83 | 1814.88 | 33.946 |
| 1994 | 305724 | 119925.592 | 116460.343 | 3465.249 | 1.133 | 2.889 | 4516.28 | 1771.69 | 1720.40 | 51.297 |
| 1995 | 303270 | 117896.496 | 113010.766 | 4885.730 | 1.611 | 4.144 | 4393.96 | 1708.35 | 1637.37 | 70.980 |
| 1996 | 299633 | 115698.645 | 109246.218 | 6452.427 | 2.153 | 5.577 | 4273.41 | 1650.42 | 1558.09 | 92.332 |
| 1997 | 294336 | 113359.847 | 105328.830 | 8031.017 | 2.729 | 7.085 | 4129.06 | 1590.70 | 1477.59 | 113.109 |
| 1998 | 289413 | 111444.212 | 101831.670 | 9612.542 | 3.321 | 8.625 | 3992.74 | 1538.10 | 1404.87 | 133.228 |
| 1999 | 311230 | 114877.722 | 102002.212 | 12875.51 | 4.137 | 11.208 | 4225.43 | 1560.48 | 1384.84 | 175.645 |
| 2000 | 304088 | 112379.704 | 97678.104 | 14701.60 | 4.835 | 13.082 | 4067.31 | 1504.22 | 1306.49 | 197.727 |
| 2001 | 296674 | 110024.943 | 93374.596 | 16650.35 | 5.612 | 15.133 | 3915.66 | 1453.52 | 1232.41 | 221.113 |
| 2002 | 292371 | 108289.173 | 89444.796 | 18844.38 | 6.445 | 17.402 | 3814.73 | 1414.56 | 1167.03 | 247.523 |
| 2003 | 286437 | 106539.023 | 85754.130 | 20784.89 | 7.256 | 19.509 | 3697.46 | 1377.22 | 1106.95 | 270.271 |
| 2004 | 274842 | 103037.251 | 80837.932 | 22199.32 | 8.077 | 21.545 | 3508.55 | 1317.61 | 1031.95 | 285.659 |
| 2005 | 273165 | 103044.562 | 78802.766 | 24241.80 | 8.874 | 23.526 | 3445.60 | 1302.41 | 993.989 | 308.425 |
| 2006 | 265012 | 99672.555 | 73679.242 | 25993.31 | 9.808 | 26.079 | 3311.10 | 1248.28 | 920.558 | 327.720 |
| 2007 | 259434 | 97839.152 | 70381.852 | 27457.30 | 10.584 | 28.064 | 3207.01 | 1212.75 | 870.029 | 342.723 |
| 2008 | 260631 | 98075.852 | 68761.307 | 29314.55 | 11.248 | 29.890 | 3184.51 | 1202.07 | 840.159 | 361.912 |
| 2009 | 255211 | 95617.338 | 65000.327 | 30617.01 | 11.997 | 32.020 | 3080.27 | 1158.11 | 784.523 | 373.591 |
| 2010 | 253335 | 94607.988 | 62525.645 | 32082.34 | 12.664 | 33.911 | 3019.28 | 1131.98 | 745.190 | 386.795 |
| 2011 | 253408 | 93633.119 | 59760.194 | 33872.93 | 13.367 | 36.176 | 2989.04 | 1109.25 | 704.895 | 404.354 |
| 2012 | 254460 | 92775.175 | 57142.433 | 35632.74 | 14.003 | 38.408 | 2967.67 | 1087.19 | 666.430 | 420.759 |
| 2013 | 258216 | 92345.780 | 54912.160 | 37433.62 | 14.497 | 40.536 | 2975.77 | 1069.82 | 632.828 | 436.988 |
| 2014 | 259652 | 91379.215 | 52434.051 | 38945.16 | 14.999 | 42.619 | 2955.99 | 1046.26 | 596.931 | 449.325 |
| 2015 | 262291 | 90750.242 | 50435.932 | 40314.31 | 15.370 | 44.423 | 3354.92 | 1168.90 | 645.119 | 523.783 |
| 2016 | 264205 | 88811.996 | 47071.171 | 41740.82 | 15.799 | 46.999 | 3345.94 | 1133.24 | 596.118 | 537.127 |
| 2017 | 266705 | 87325.126 | 44342.863 | 42982.26 | 16.116 | 49.221 | 3344.77 | 1104.08 | 556.108 | 547.971 |
| 2018 | 261910 | 87612.144 | 43452.622 | 44159.52 | 16.861 | 50.403 | 3252.33 | 1097.46 | 539.583 | 557.873 |
| 2019 | 264923 | 87797.760 | 41973.787 | 45823.97 | 17.297 | 52.193 | 3255.81 | 1089.08 | 515.843 | 573.239 |
| 2020 | 266078 | 87221.042 | 40420.512 | 46800.53 | 17.589 | 53.657 | 3727.02 | 1235.48 | 566.180 | 669.298 |
| 2021 | 259019 | 82001.292 | 35657.714 | 46343.58 | 17.892 | 56.516 | 3600.15 | 1153.39 | 495.612 | 657.777 |
| 2022 | 261334 | 81915.977 | 34033.599 | 47882.38 | 18.322 | 58.453 | 3602.06 | 1143.37 | 469.097 | 674.278 |
| 2023 | 263627 | 81810.810 | 32437.019 | 49373.79 | 18.729 | 60.351 | 3601.68 | 1132.64 | 443.155 | 689.490 |
| 2024 | 265585 | 81528.303 | 30867.589 | 50660.71 | 19.075 | 62.139 | 3595.81 | 1119.38 | 417.922 | 701.456 |
| 2025 | 262478 | 79599.213 | 29164.509 | 50434.70 | 19.215 | 63.361 | 4125.82 | 1272.09 | 458.429 | 813.659 |
| 2026 | 256855 | 75082.462 | 25575.404 | 49507.06 | 19.274 | 65.937 | 4017.56 | 1194.99 | 400.033 | 794.955 |
| 2027 | 256599 | 74444.425 | 23964.564 | 50479.86 | 19.673 | 67.809 | 3992.13 | 1179.46 | 372.838 | 806.623 |
| 2028 | 256182 | 73699.585 | 22279.713 | 51419.87 | 20.072 | 69.770 | 3962.30 | 1161.76 | 344.595 | 817.164 |
| 2029 | 255713 | 72819.703 | 20684.120 | 52135.58 | 20.388 | 71.595 | 3929.36 | 1141.36 | 317.838 | 823.519 |
| 2030 | 246936 | 69309.277 | 19112.112 | 50197.16 | 20.328 | 72.425 | 4519.30 | 1297.95 | 349.781 | 948.169 |
| 2031 | 241973 | 65433.368 | 16610.897 | 48822.47 | 20.177 | 74.614 | 4412.78 | 1222.18 | 302.928 | 919.253 |
| 2032 | 240806 | 64643.552 | 15318.934 | 49324.62 | 20.483 | 76.302 | 4372.55 | 1203.31 | 278.161 | 925.148 |
| 2033 | 239605 | 63793.514 | 14082.066 | 49711.45 | 20.747 | 77.926 | 4328.69 | 1182.54 | 254.406 | 928.130 |
| 2034 | 238262 | 63032.151 | 12926.954 | 50105.20 | 21.029 | 79.491 | 4279.89 | 1162.80 | 232.206 | 930.594 |
| 2035 | 223137 | 57653.301 | 11714.816 | 45938.48 | 20.588 | 79.681 | 4940.35 | 1315.47 | 259.371 | 1056.10 |
| 2036 | 215425 | 52988.733 | 9445.675 | 43543.06 | 20.213 | 82.174 | 4763.60 | 1208.75 | 208.868 | 999.878 |
| 2037 | 212893 | 52026.921 | 8489.199 | 43537.72 | 20.451 | 83.683 | 4698.00 | 1185.50 | 187.335 | 998.168 |
| 2038 | 210390 | 50943.211 | 7556.781 | 43386.43 | 20.622 | 85.166 | 4628.77 | 1158.38 | 166.256 | 992.127 |
| 2039 | 207957 | 50085.991 | 6760.832 | 43325.16 | 20.834 | 86.502 | 4556.22 | 1135.19 | 148.126 | 987.065 |

# Results for PHIM for Run 3 (Sensitivity_Analysis_1.RTF)

**********************************************************************************
**Main Results for E-Component for Run: 3, Simulation: 1, COD: LUNG CANCER, Adjusted**

| Variable Parameter | Value |
|---|---|
| 1. Country | US (United States) |
| 2. Sex | M (Males) |
| 3. Year of start of process | 1990 |
| 4. Cause of Death | LUNG CANCER |
| 5. Effective Dose NULL | CC:1 |
| 6. Effective Dose Ecig | CC:1 Ecig:0 Dual:0.5 |
| Note: | Death rates are per million population |

-----------------------------------------------------------------------------------------
Results for Age Groups Combined and Years combined, Population and Number deaths adjusted for Ecig scenario,

| Age Group:All | | NULL | Ecig | | | %Drop | | NULL | Ecig | |
|---|---|---|---|---|---|---|---|---|---|---|
| Year | Number | Attrib | Attrib | Drop | %Drop | Attrib | Death | Attrib | Attrib | Drop |
| | Deaths | Deaths | Deaths | Deaths | Deaths | Deaths | Rate | Rates | Rates | Rates |
| All Comb | 3097900 | 2244988.113 | 1673945.285 | 571042.8 | 18.433 | 25.436 | 888.739 | 649.875 | 480.229 | 169.647 |

-----------------------------------------------------------------------------------------
Results for Age Groups Combined, Population and Number deaths adjusted for Ecig scenario,

| Age Group:All | | NULL | Ecig | | | %Drop | | NULL | Ecig | |
|---|---|---|---|---|---|---|---|---|---|---|
| Year | Number | Attrib | Attrib | Drop | %Drop | Attrib | Death | Attrib | Attrib | Drop |
| | Deaths | Deaths | Deaths | Deaths | Deaths | Deaths | Rate | Rates | Rates | Rates |
| 1990 | 78822 | 63385.736 | 63385.736 | 0.000 | 0.000 | 0.000 | 1249.50 | 1004.81 | 1004.81 | 0.000 |
| 1991 | 78462 | 62918.402 | 62867.864 | 50.538 | 0.064 | 0.080 | 1223.41 | 981.049 | 980.261 | 0.788 |
| 1992 | 77988 | 62366.766 | 62231.297 | 135.468 | 0.174 | 0.217 | 1195.35 | 955.930 | 953.846 | 2.083 |
| 1993 | 78545 | 62652.888 | 62386.853 | 266.035 | 0.339 | 0.425 | 1182.40 | 943.195 | 939.165 | 4.029 |
| 1994 | 77443 | 61565.227 | 61146.363 | 418.864 | 0.541 | 0.680 | 1144.02 | 909.520 | 903.278 | 6.243 |
| 1995 | 76916 | 60851.961 | 60243.839 | 608.122 | 0.791 | 0.999 | 1114.40 | 881.759 | 872.848 | 8.910 |
| 1996 | 76252 | 60122.922 | 59276.326 | 846.595 | 1.110 | 1.408 | 1087.51 | 857.643 | 845.409 | 12.234 |
| 1997 | 75502 | 59237.155 | 58134.410 | 1102.744 | 1.461 | 1.862 | 1059.18 | 831.234 | 815.531 | 15.703 |
| 1998 | 75423 | 58906.696 | 57486.494 | 1420.202 | 1.883 | 2.411 | 1040.54 | 813.001 | 793.083 | 19.917 |
| 1999 | 73215 | 56930.073 | 55196.949 | 1733.124 | 2.367 | 3.044 | 994.006 | 773.329 | 749.383 | 23.946 |
| 2000 | 73860 | 57137.121 | 54995.718 | 2141.403 | 2.899 | 3.748 | 987.914 | 764.787 | 735.593 | 29.195 |
| 2001 | 73392 | 56481.726 | 53878.792 | 2602.935 | 3.547 | 4.608 | 968.664 | 746.169 | 711.120 | 35.050 |
| 2002 | 72564 | 55588.365 | 52497.797 | 3090.568 | 4.259 | 5.560 | 946.784 | 726.138 | 684.967 | 41.171 |
| 2003 | 72091 | 54945.856 | 51312.730 | 3633.126 | 5.040 | 6.612 | 930.587 | 710.281 | 662.366 | 47.914 |
| 2004 | 71480 | 54203.126 | 49971.739 | 4231.387 | 5.920 | 7.807 | 912.495 | 693.135 | 637.924 | 55.210 |
| 2005 | 71176 | 53744.603 | 48889.714 | 4854.889 | 6.821 | 9.033 | 897.785 | 679.296 | 616.677 | 62.619 |
| 2006 | 69902 | 52535.572 | 47021.474 | 5514.098 | 7.888 | 10.496 | 873.361 | 657.945 | 587.493 | 70.452 |
| 2007 | 68843 | 51492.124 | 45323.902 | 6168.222 | 8.960 | 11.979 | 851.006 | 638.264 | 560.274 | 77.990 |
| 2008 | 68543 | 51030.736 | 44063.225 | 6967.511 | 10.165 | 13.654 | 837.490 | 625.460 | 538.386 | 87.075 |
| 2009 | 67866 | 50276.199 | 42506.732 | 7769.467 | 11.448 | 15.454 | 819.106 | 608.943 | 513.036 | 95.908 |
| 2010 | 67596 | 49840.933 | 41264.572 | 8576.361 | 12.688 | 17.207 | 805.621 | 596.347 | 491.797 | 104.550 |
| 2011 | 66949 | 49072.841 | 39601.137 | 9471.704 | 14.148 | 19.301 | 789.690 | 581.354 | 467.111 | 114.243 |
| 2012 | 66644 | 48539.512 | 38101.952 | 10437.56 | 15.662 | 21.503 | 777.243 | 568.812 | 444.368 | 124.444 |
| 2013 | 65867 | 47675.741 | 36372.481 | 11303.26 | 17.161 | 23.709 | 759.077 | 552.318 | 419.170 | 133.149 |
| 2014 | 65619 | 47194.534 | 34990.618 | 12203.92 | 18.598 | 25.859 | 747.039 | 540.359 | 398.348 | 142.011 |
| 2015 | 64779 | 46305.842 | 33305.967 | 12999.88 | 20.068 | 28.074 | 828.581 | 596.439 | 426.012 | 170.427 |
| 2016 | 62283 | 44208.867 | 30722.535 | 13486.33 | 21.653 | 30.506 | 788.757 | 564.107 | 389.076 | 175.031 |
| 2017 | 60599 | 42702.624 | 28666.663 | 14035.96 | 23.162 | 32.869 | 759.979 | 539.903 | 359.511 | 180.391 |
| 2018 | 63259 | 44321.202 | 28495.857 | 15825.35 | 25.017 | 35.706 | 785.530 | 555.181 | 353.854 | 201.327 |
| 2019 | 63906 | 44493.088 | 27463.396 | 17029.69 | 26.648 | 38.275 | 785.377 | 551.912 | 337.515 | 214.396 |
| 2020 | 64400 | 44529.551 | 26374.343 | 18155.21 | 28.191 | 40.771 | 902.074 | 630.757 | 369.432 | 261.325 |
| 2021 | 58016 | 39852.556 | 22508.109 | 17344.45 | 29.896 | 43.522 | 806.372 | 560.546 | 312.844 | 247.702 |
| 2022 | 58409 | 39853.685 | 21380.360 | 18473.33 | 31.628 | 46.353 | 805.067 | 556.274 | 294.693 | 261.580 |
| 2023 | 58774 | 39847.050 | 20253.639 | 19593.41 | 33.337 | 49.172 | 802.977 | 551.670 | 276.706 | 274.964 |
| 2024 | 59047 | 39726.010 | 19131.664 | 20594.35 | 34.878 | 51.841 | 799.454 | 545.435 | 259.027 | 286.408 |
| 2025 | 58694 | 39207.348 | 17930.353 | 21277.00 | 36.251 | 54.268 | 922.591 | 626.579 | 281.842 | 344.736 |
| 2026 | 52538 | 34803.960 | 14991.643 | 19812.32 | 37.710 | 56.925 | 821.772 | 553.929 | 234.489 | 319.440 |
| 2027 | 52338 | 34484.595 | 13902.447 | 20582.15 | 39.325 | 59.685 | 814.267 | 546.357 | 216.293 | 330.064 |
| 2028 | 52092 | 34100.638 | 12782.333 | 21318.31 | 40.925 | 62.516 | 805.688 | 537.544 | 197.701 | 339.842 |
| 2029 | 51817 | 33669.838 | 11733.265 | 21936.57 | 42.335 | 65.152 | 796.235 | 527.732 | 180.297 | 347.435 |
| 2030 | 50432 | 32557.688 | 10691.719 | 21865.97 | 43.357 | 67.161 | 922.981 | 609.705 | 195.675 | 414.031 |
| 2031 | 44810 | 28646.393 | 8733.220 | 19913.17 | 44.439 | 69.514 | 817.195 | 535.065 | 159.265 | 375.800 |
| 2032 | 44432 | 28227.160 | 7935.309 | 20291.85 | 45.670 | 71.888 | 806.788 | 525.435 | 144.089 | 381.346 |
| 2033 | 44037 | 27786.623 | 7178.792 | 20607.83 | 46.797 | 74.165 | 795.572 | 515.079 | 129.692 | 385.387 |
| 2034 | 43609 | 27348.299 | 6488.486 | 20859.81 | 47.834 | 76.275 | 783.342 | 504.514 | 116.552 | 387.962 |
| 2035 | 41106 | 25570.077 | 5760.935 | 19809.14 | 48.190 | 77.470 | 910.112 | 583.430 | 127.549 | 455.881 |
| 2036 | 35217 | 21717.319 | 4304.298 | 17413.02 | 49.445 | 80.180 | 778.738 | 495.402 | 95.179 | 400.223 |
| 2037 | 34662 | 21256.024 | 3804.203 | 17451.82 | 50.349 | 82.103 | 764.902 | 484.347 | 83.949 | 400.398 |
| 2038 | 34111 | 20747.175 | 3326.963 | 17420.21 | 51.069 | 83.964 | 750.480 | 471.764 | 73.196 | 398.568 |
| 2039 | 33572 | 20327.688 | 2930.074 | 17397.61 | 51.822 | 85.586 | 735.546 | 460.724 | 64.196 | 396.527 |

# Results for PHIM for Run 3 (Sensitivity_Analysis_1.RTF)

*********************************************************************************

**Main Results for E-Component for Run: 3, Simulation: 1, COD: IHD, Adjusted**

| Variable Parameter | Value |
|---|---|
| 1. Country | US (United States) |
| 2. Sex | M (Males) |
| 3. Year of start of process | 1990 |
| 4. Cause of Death | IHD |
| 5. Effective Dose NULL | CC:1 |
| 6. Effective Dose Ecig | CC:1 Ecig:0 Dual:0.5 |
| Note: | Death rates are per million population |

-------------------------------------------------------------------------------------

Results for Age Groups Combined and Years combined, Population and Number deaths adjusted for Ecig scenario,

| Age Group:All | | NULL | Ecig | | | %Drop | | NULL | Ecig | |
|---|---|---|---|---|---|---|---|---|---|---|
| Year | Number | Attrib | Attrib | Drop | %Drop | Attrib | Death | Attrib | Attrib | Drop |
| | Deaths | Deaths | Deaths | Deaths | Deaths | Deaths | Rate | Rates | Rates | Rates |
| All Comb | 6427754 | 1098877.421 | 477380.920 | 621496.5 | 9.669 | 56.557 | 1844.02 | 318.101 | 136.953 | 181.148 |

-------------------------------------------------------------------------------------

Results for Age Groups Combined, Population and Number deaths adjusted for Ecig scenario,

| Age Group:All | | NULL | Ecig | | | %Drop | | NULL | Ecig | |
|---|---|---|---|---|---|---|---|---|---|---|
| Year | Number | Attrib | Attrib | Drop | %Drop | Attrib | Death | Attrib | Attrib | Drop |
| | Deaths | Deaths | Deaths | Deaths | Deaths | Deaths | Rate | Rates | Rates | Rates |
| 1990 | 171484 | 38511.725 | 38511.725 | 0.000 | 0.000 | 0.000 | 2718.40 | 610.497 | 610.497 | 0.000 |
| 1991 | 168044 | 37309.838 | 36979.259 | 330.579 | 0.197 | 0.886 | 2620.21 | 581.750 | 576.596 | 5.155 |
| 1992 | 164550 | 36137.488 | 35246.348 | 891.140 | 0.542 | 2.466 | 2522.12 | 553.899 | 540.236 | 13.663 |
| 1993 | 164367 | 35599.588 | 33947.480 | 1652.107 | 1.005 | 4.641 | 2474.37 | 535.926 | 511.042 | 24.884 |
| 1994 | 159879 | 34336.757 | 31811.554 | 2525.203 | 1.579 | 7.354 | 2361.79 | 507.267 | 469.933 | 37.334 |
| 1995 | 158106 | 33455.759 | 29919.891 | 3535.868 | 2.236 | 10.569 | 2290.74 | 484.782 | 433.497 | 51.284 |
| 1996 | 154913 | 32193.824 | 27582.721 | 4611.103 | 2.977 | 14.323 | 2209.39 | 459.239 | 393.390 | 65.850 |
| 1997 | 150026 | 30665.509 | 25009.850 | 5655.660 | 3.770 | 18.443 | 2104.62 | 430.308 | 350.847 | 79.461 |
| 1998 | 145484 | 29188.430 | 22552.600 | 6635.831 | 4.561 | 22.734 | 2007.10 | 402.844 | 311.135 | 91.709 |
| 1999 | 166074 | 33375.386 | 24131.564 | 9243.822 | 5.566 | 27.697 | 2254.70 | 453.366 | 327.623 | 125.743 |
| 2000 | 160419 | 32024.754 | 21661.621 | 10363.13 | 6.460 | 32.360 | 2145.67 | 428.655 | 289.734 | 138.921 |
| 2001 | 154641 | 30682.393 | 19158.398 | 11523.99 | 7.452 | 37.559 | 2041.04 | 405.339 | 252.862 | 152.477 |
| 2002 | 152132 | 30218.370 | 17331.912 | 12886.46 | 8.471 | 42.644 | 1984.95 | 394.736 | 226.139 | 168.597 |
| 2003 | 147025 | 29175.202 | 15280.884 | 13894.32 | 9.450 | 47.624 | 1897.86 | 377.146 | 197.252 | 179.894 |
| 2004 | 138714 | 27488.687 | 13072.718 | 14415.97 | 10.393 | 52.443 | 1770.79 | 351.518 | 166.882 | 184.635 |
| 2005 | 136270 | 27017.439 | 11665.872 | 15351.57 | 11.266 | 56.821 | 1718.86 | 341.482 | 147.149 | 194.333 |
| 2006 | 132365 | 26231.435 | 10081.372 | 16150.06 | 12.201 | 61.568 | 1653.78 | 328.517 | 125.958 | 202.559 |
| 2007 | 126994 | 25103.049 | 8618.107 | 16484.94 | 12.981 | 65.669 | 1569.85 | 311.161 | 106.533 | 204.628 |
| 2008 | 126421 | 24621.740 | 7619.822 | 17001.92 | 13.449 | 69.052 | 1544.67 | 301.777 | 93.103 | 208.675 |
| 2009 | 123050 | 23741.177 | 6576.146 | 17165.03 | 13.950 | 72.301 | 1485.16 | 287.552 | 79.371 | 208.181 |
| 2010 | 121225 | 23189.160 | 5752.044 | 17437.12 | 14.384 | 75.195 | 1444.78 | 277.458 | 68.554 | 208.905 |
| 2011 | 120987 | 22786.927 | 4983.913 | 17803.01 | 14.715 | 78.128 | 1427.09 | 269.951 | 58.787 | 211.164 |
| 2012 | 121785 | 22485.295 | 4361.893 | 18123.40 | 14.881 | 80.601 | 1420.33 | 263.495 | 50.871 | 212.624 |
| 2013 | 123610 | 22251.884 | 3838.674 | 18413.21 | 14.896 | 82.749 | 1424.53 | 257.786 | 44.238 | 213.547 |
| 2014 | 124294 | 21894.593 | 3353.505 | 18541.09 | 14.917 | 84.683 | 1415.01 | 250.685 | 38.178 | 212.507 |
| 2015 | 124951 | 21471.109 | 2939.171 | 18531.94 | 14.831 | 86.311 | 1598.22 | 276.557 | 37.595 | 238.962 |
| 2016 | 127526 | 21411.568 | 2546.775 | 18864.79 | 14.793 | 88.106 | 1615.01 | 273.213 | 32.253 | 240.960 |
| 2017 | 129711 | 21169.399 | 2215.231 | 18954.17 | 14.613 | 89.536 | 1626.72 | 267.651 | 27.781 | 239.870 |
| 2018 | 122895 | 19830.576 | 1772.681 | 18057.90 | 14.694 | 91.061 | 1526.08 | 248.404 | 22.013 | 226.391 |
| 2019 | 123418 | 19431.014 | 1529.328 | 17901.69 | 14.505 | 92.129 | 1516.76 | 241.031 | 18.795 | 222.236 |
| 2020 | 122520 | 18532.612 | 1298.783 | 17233.83 | 14.066 | 92.992 | 1716.18 | 262.513 | 18.192 | 244.321 |
| 2021 | 121203 | 18234.128 | 1071.500 | 17162.63 | 14.160 | 94.124 | 1684.62 | 256.472 | 14.893 | 241.579 |
| 2022 | 121353 | 17812.559 | 899.226 | 16913.33 | 13.937 | 94.952 | 1672.65 | 248.626 | 12.394 | 236.232 |
| 2023 | 121484 | 17361.668 | 745.837 | 16615.83 | 13.677 | 95.704 | 1659.71 | 240.367 | 10.190 | 230.177 |
| 2024 | 121468 | 16929.676 | 628.459 | 16301.22 | 13.420 | 96.288 | 1644.58 | 232.443 | 8.509 | 223.934 |
| 2025 | 117959 | 15501.666 | 516.398 | 14985.27 | 12.704 | 96.669 | 1854.17 | 247.735 | 8.117 | 239.617 |
| 2026 | 117439 | 15523.814 | 419.431 | 15104.38 | 12.861 | 97.298 | 1836.90 | 247.072 | 6.560 | 240.512 |
| 2027 | 116459 | 15073.070 | 348.203 | 14724.87 | 12.644 | 97.690 | 1811.85 | 238.810 | 5.417 | 233.393 |
| 2028 | 115429 | 14636.710 | 280.251 | 14356.46 | 12.438 | 98.085 | 1785.31 | 230.725 | 4.335 | 226.390 |
| 2029 | 114396 | 14180.166 | 227.344 | 13952.82 | 12.197 | 98.397 | 1757.85 | 222.256 | 3.493 | 218.763 |
| 2030 | 107582 | 12111.772 | 183.912 | 11927.86 | 11.087 | 98.482 | 1968.91 | 226.816 | 3.366 | 223.450 |
| 2031 | 108125 | 12417.367 | 155.209 | 12262.16 | 11.341 | 98.750 | 1971.84 | 231.935 | 2.831 | 229.104 |
| 2032 | 106801 | 12054.064 | 130.794 | 11923.27 | 11.164 | 98.915 | 1939.30 | 224.381 | 2.375 | 222.006 |
| 2033 | 105492 | 11672.389 | 103.362 | 11569.03 | 10.967 | 99.114 | 1905.80 | 216.370 | 1.867 | 214.503 |
| 2034 | 104157 | 11365.762 | 83.763 | 11282.00 | 10.832 | 99.263 | 1870.96 | 209.673 | 1.505 | 208.168 |
| 2035 | 93834 | 8580.360 | 67.080 | 8513.280 | 9.073 | 99.218 | 2077.52 | 195.777 | 1.485 | 194.292 |
| 2036 | 95238 | 8948.841 | 54.424 | 8894.416 | 9.339 | 99.392 | 2105.97 | 204.135 | 1.203 | 202.932 |
| 2037 | 93492 | 8624.570 | 47.108 | 8577.461 | 9.175 | 99.454 | 2063.13 | 196.522 | 1.040 | 195.483 |
| 2038 | 91796 | 8315.017 | 38.392 | 8276.625 | 9.016 | 99.538 | 2019.61 | 189.073 | 0.845 | 188.228 |
| 2039 | 90167 | 8001.132 | 28.384 | 7972.747 | 8.842 | 99.645 | 1975.50 | 181.344 | 0.622 | 180.722 |

# Results for PHIM for Run 3 (Sensitivity_Analysis_1.RTF)

************************************************************************************

**Main Results for E-Component for Run: 3, Simulation: 1, COD: STROKE, Adjusted**

| Variable Parameter | Value |
|---|---|
| 1. Country | US (United States) |
| 2. Sex | M (Males) |
| 3. Year of start of process | 1990 |
| 4. Cause of Death | STROKE |
| 5. Effective Dose NULL | CC:1 |
| 6. Effective Dose Ecig | CC:1 Ecig:0 Dual:0.5 |
| Note: | Death rates are per million population |

-----------------------------------------------------------------------------------------

Results for Age Groups Combined and Years combined, Population and Number deaths adjusted for Ecig scenario,

| Age Group:All Year | Number Deaths | NULL Attrib Deaths | Ecig Attrib Deaths | Drop Deaths | %Drop Deaths | %Drop Attrib Deaths | Death Rate | NULL Attrib Rates | Ecig Attrib Rates | Drop Rates |
|---|---|---|---|---|---|---|---|---|---|---|
| All Comb | 1508081 | 149918.462 | 64722.288 | 85196.17 | 5.649 | 56.828 | 432.645 | 43.398 | 18.568 | 24.830 |

-----------------------------------------------------------------------------------------

Results for Age Groups Combined, Population and Number deaths adjusted for Ecig scenario,

| Age Group:All Year | Number Deaths | NULL Attrib Deaths | Ecig Attrib Deaths | Drop Deaths | %Drop Deaths | %Drop Attrib Deaths | Death Rate | NULL Attrib Rates | Ecig Attrib Rates | Drop Rates |
|---|---|---|---|---|---|---|---|---|---|---|
| 1990 | 32862 | 4613.166 | 4613.166 | 0.000 | 0.000 | 0.000 | 520.936 | 73.129 | 73.129 | 0.000 |
| 1991 | 32619 | 4555.566 | 4513.452 | 42.114 | 0.129 | 0.924 | 508.609 | 71.032 | 70.376 | 0.657 |
| 1992 | 32100 | 4453.572 | 4342.903 | 110.669 | 0.345 | 2.485 | 492.015 | 68.262 | 66.566 | 1.697 |
| 1993 | 32897 | 4462.473 | 4253.257 | 209.216 | 0.636 | 4.688 | 495.229 | 67.179 | 64.028 | 3.151 |
| 1994 | 33326 | 4499.513 | 4168.967 | 330.547 | 0.992 | 7.346 | 492.298 | 66.473 | 61.586 | 4.887 |
| 1995 | 33574 | 4472.774 | 4003.829 | 468.945 | 1.397 | 10.484 | 486.436 | 64.812 | 58.010 | 6.802 |
| 1996 | 33878 | 4406.878 | 3784.802 | 622.076 | 1.836 | 14.116 | 483.173 | 62.863 | 53.980 | 8.884 |
| 1997 | 33528 | 4288.158 | 3510.668 | 777.489 | 2.319 | 18.131 | 470.342 | 60.173 | 49.249 | 10.924 |
| 1998 | 32812 | 4120.148 | 3200.503 | 919.645 | 2.803 | 22.321 | 452.670 | 56.864 | 44.154 | 12.710 |
| 1999 | 33398 | 3982.443 | 2921.247 | 1061.195 | 3.177 | 26.647 | 453.433 | 54.097 | 39.660 | 14.436 |
| 2000 | 33491 | 4021.411 | 2772.914 | 1248.497 | 3.728 | 31.046 | 447.953 | 53.827 | 37.089 | 16.738 |
| 2001 | 32315 | 3848.092 | 2473.486 | 1374.606 | 4.254 | 35.722 | 426.504 | 50.836 | 32.646 | 18.190 |
| 2002 | 31635 | 3783.081 | 2260.882 | 1522.199 | 4.812 | 40.237 | 412.760 | 49.418 | 29.499 | 19.919 |
| 2003 | 31044 | 3754.415 | 2068.181 | 1686.234 | 5.432 | 44.913 | 400.733 | 48.533 | 26.697 | 21.836 |
| 2004 | 29954 | 3666.274 | 1856.434 | 1809.841 | 6.042 | 49.365 | 382.379 | 46.883 | 23.699 | 23.185 |
| 2005 | 28788 | 3589.688 | 1661.944 | 1927.745 | 6.696 | 53.702 | 363.119 | 45.371 | 20.963 | 24.408 |
| 2006 | 28280 | 3596.985 | 1507.698 | 2089.286 | 7.388 | 58.084 | 353.337 | 45.048 | 18.837 | 26.211 |
| 2007 | 28132 | 3560.798 | 1351.982 | 2208.815 | 7.851 | 62.031 | 347.761 | 44.137 | 16.713 | 27.425 |
| 2008 | 27246 | 3417.001 | 1173.246 | 2243.756 | 8.235 | 65.664 | 332.908 | 41.881 | 14.335 | 27.545 |
| 2009 | 27202 | 3394.390 | 1051.449 | 2342.940 | 8.613 | 69.024 | 328.320 | 41.113 | 12.690 | 28.422 |
| 2010 | 27062 | 3340.137 | 936.227 | 2403.910 | 8.883 | 71.970 | 322.527 | 39.965 | 11.158 | 28.807 |
| 2011 | 27129 | 3285.435 | 828.485 | 2456.950 | 9.057 | 74.783 | 319.993 | 38.922 | 9.772 | 29.149 |
| 2012 | 27246 | 3259.209 | 730.985 | 2528.224 | 9.279 | 77.572 | 317.763 | 38.193 | 8.525 | 29.668 |
| 2013 | 28121 | 3274.703 | 655.992 | 2618.711 | 9.312 | 79.968 | 324.075 | 37.937 | 7.560 | 30.377 |
| 2014 | 29038 | 3298.083 | 586.033 | 2712.050 | 9.340 | 82.231 | 330.582 | 37.762 | 6.672 | 31.090 |
| 2015 | 29840 | 3244.406 | 518.230 | 2726.176 | 9.136 | 84.027 | 381.675 | 41.789 | 6.629 | 35.161 |
| 2016 | 30827 | 3314.836 | 467.042 | 2847.794 | 9.238 | 85.911 | 390.402 | 42.297 | 5.915 | 36.383 |
| 2017 | 31768 | 3333.314 | 414.933 | 2918.380 | 9.186 | 87.552 | 398.407 | 42.144 | 5.204 | 36.940 |
| 2018 | 29708 | 2925.242 | 325.916 | 2599.326 | 8.750 | 88.858 | 368.909 | 36.642 | 4.047 | 32.595 |
| 2019 | 29990 | 2864.628 | 283.329 | 2581.299 | 8.607 | 90.109 | 368.569 | 35.534 | 3.482 | 32.052 |
| 2020 | 30018 | 2730.366 | 240.577 | 2489.789 | 8.294 | 91.189 | 420.473 | 38.675 | 3.370 | 35.306 |
| 2021 | 30563 | 2647.911 | 206.380 | 2441.531 | 7.988 | 92.206 | 424.805 | 37.244 | 2.869 | 34.376 |
| 2022 | 30805 | 2586.165 | 175.802 | 2410.363 | 7.825 | 93.202 | 424.596 | 36.097 | 2.423 | 33.674 |
| 2023 | 31043 | 2517.960 | 148.978 | 2368.982 | 7.631 | 94.083 | 424.114 | 34.860 | 2.035 | 32.825 |
| 2024 | 31236 | 2454.161 | 127.754 | 2326.407 | 7.448 | 94.794 | 422.909 | 33.695 | 1.730 | 31.966 |
| 2025 | 30864 | 2275.364 | 105.081 | 2170.283 | 7.032 | 95.382 | 485.150 | 36.363 | 1.652 | 34.711 |
| 2026 | 31343 | 2183.166 | 87.695 | 2095.471 | 6.686 | 95.983 | 490.249 | 34.747 | 1.372 | 33.375 |
| 2027 | 31253 | 2112.345 | 74.166 | 2038.179 | 6.522 | 96.489 | 486.230 | 33.467 | 1.154 | 32.313 |
| 2028 | 31138 | 2046.677 | 62.175 | 1984.502 | 6.373 | 96.962 | 481.601 | 32.263 | 0.962 | 31.301 |
| 2029 | 31015 | 1977.671 | 51.962 | 1925.709 | 6.209 | 97.373 | 476.586 | 30.997 | 0.798 | 30.199 |
| 2030 | 30119 | 1744.457 | 41.186 | 1703.271 | 5.655 | 97.639 | 551.223 | 32.668 | 0.754 | 31.915 |
| 2031 | 29794 | 1653.738 | 34.560 | 1619.177 | 5.435 | 97.910 | 543.346 | 30.889 | 0.630 | 30.259 |
| 2032 | 29573 | 1603.120 | 29.069 | 1574.051 | 5.323 | 98.187 | 536.991 | 29.841 | 0.528 | 29.313 |
| 2033 | 29343 | 1548.245 | 24.006 | 1524.239 | 5.195 | 98.449 | 530.110 | 28.700 | 0.434 | 28.266 |
| 2034 | 29087 | 1511.985 | 20.499 | 1491.485 | 5.128 | 98.644 | 522.495 | 27.893 | 0.368 | 27.524 |
| 2035 | 27655 | 1239.866 | 15.290 | 1224.576 | 4.428 | 98.767 | 612.300 | 28.290 | 0.339 | 27.951 |
| 2036 | 26503 | 1182.522 | 12.445 | 1170.077 | 4.415 | 98.948 | 586.054 | 26.975 | 0.275 | 26.700 |
| 2037 | 26072 | 1134.483 | 10.556 | 1123.927 | 4.311 | 99.070 | 575.342 | 25.851 | 0.233 | 25.618 |
| 2038 | 25638 | 1093.537 | 8.895 | 1084.641 | 4.231 | 99.187 | 564.054 | 24.866 | 0.196 | 24.670 |
| 2039 | 25206 | 1047.905 | 7.027 | 1040.878 | 4.129 | 99.329 | 552.251 | 23.751 | 0.154 | 23.597 |

# Results for PHIM for Run 3 (Sensitivity_Analysis_1.RTF)

************************************************************************************

**Main Results for E-Component for Run: 3, Simulation: 1, COD: COPD, Adjusted**

| Variable Parameter | Value |
|---|---|
| 1. Country | US (United States) |
| 2. Sex | M (Males) |
| 3. Year of start of process | 1990 |
| 4. Cause of Death | COPD |
| 5. Effective Dose NULL | CC:1 |
| 6. Effective Dose Ecig | CC:1 Ecig:0 Dual:0.5 |
| Note: | Death rates are per million population |

-----------------------------------------------------------------------------------------

Results for Age Groups Combined and Years combined, Population and Number deaths adjusted for Ecig scenario,

| Age Group:All | | NULL | Ecig | | | %Drop | | NULL | Ecig | |
|---|---|---|---|---|---|---|---|---|---|---|
| Year | Number | Attrib | Attrib | Drop | %Drop | Attrib | Death | Attrib | Attrib | Drop |
| | Deaths | Deaths | Deaths | Deaths | Deaths | Deaths | Rate | Rates | Rates | Rates |
| All Comb | 2268378 | 1015606.587 | 657401.205 | 358205.4 | 15.791 | 35.270 | 650.762 | 293.996 | 188.598 | 105.398 |

-----------------------------------------------------------------------------------------

Results for Age Groups Combined, Population and Number deaths adjusted for Ecig scenario,

| Age Group:All | | NULL | Ecig | | | %Drop | | NULL | Ecig | |
|---|---|---|---|---|---|---|---|---|---|---|
| Year | Number | Attrib | Attrib | Drop | %Drop | Attrib | Death | Attrib | Attrib | Drop |
| | Deaths | Deaths | Deaths | Deaths | Deaths | Deaths | Rate | Rates | Rates | Rates |
| 1990 | 33993 | 19262.437 | 19262.437 | 0.000 | 0.000 | 0.000 | 538.865 | 305.353 | 305.353 | 0.000 |
| 1991 | 34092 | 19215.636 | 19192.373 | 23.264 | 0.068 | 0.121 | 531.576 | 299.618 | 299.255 | 0.363 |
| 1992 | 33942 | 19056.189 | 18995.389 | 60.801 | 0.179 | 0.319 | 520.249 | 292.085 | 291.151 | 0.934 |
| 1993 | 35914 | 20095.670 | 19971.203 | 124.467 | 0.347 | 0.619 | 540.648 | 302.526 | 300.645 | 1.882 |
| 1994 | 35077 | 19524.095 | 19333.459 | 190.635 | 0.543 | 0.976 | 518.167 | 288.435 | 285.601 | 2.834 |
| 1995 | 34674 | 19116.002 | 18843.207 | 272.795 | 0.787 | 1.427 | 502.377 | 276.995 | 273.012 | 3.984 |
| 1996 | 34590 | 18975.021 | 18602.369 | 372.652 | 1.077 | 1.964 | 493.331 | 270.675 | 265.310 | 5.365 |
| 1997 | 35280 | 19169.025 | 18673.901 | 495.123 | 1.403 | 2.583 | 494.918 | 268.986 | 261.964 | 7.021 |
| 1998 | 35694 | 19228.937 | 18592.074 | 636.863 | 1.784 | 3.312 | 492.427 | 265.388 | 256.496 | 8.892 |
| 1999 | 38544 | 20589.820 | 19752.453 | 837.368 | 2.173 | 4.067 | 523.288 | 279.689 | 268.170 | 11.519 |
| 2000 | 36318 | 19196.417 | 18247.851 | 948.567 | 2.612 | 4.941 | 485.771 | 256.946 | 244.073 | 12.873 |
| 2001 | 36326 | 19012.731 | 17863.920 | 1148.811 | 3.162 | 6.042 | 479.455 | 251.174 | 235.777 | 15.397 |
| 2002 | 36040 | 18699.357 | 17354.206 | 1345.151 | 3.732 | 7.194 | 470.233 | 244.265 | 226.430 | 17.836 |
| 2003 | 36276 | 18663.550 | 17092.334 | 1571.215 | 4.331 | 8.419 | 468.272 | 241.262 | 220.635 | 20.627 |
| 2004 | 34694 | 17679.164 | 15937.041 | 1742.122 | 5.021 | 9.854 | 442.892 | 226.076 | 203.448 | 22.629 |
| 2005 | 36931 | 18692.832 | 16585.237 | 2107.595 | 5.707 | 11.275 | 465.838 | 236.265 | 209.200 | 27.065 |
| 2006 | 34466 | 17308.564 | 15068.698 | 2239.866 | 6.499 | 12.941 | 430.619 | 216.769 | 188.270 | 28.499 |
| 2007 | 35465 | 17683.181 | 15087.861 | 2595.320 | 7.318 | 14.677 | 438.398 | 219.189 | 186.509 | 32.680 |
| 2008 | 38421 | 19006.374 | 15905.014 | 3101.360 | 8.072 | 16.317 | 469.444 | 232.952 | 194.335 | 38.617 |
| 2009 | 37093 | 18205.573 | 14865.999 | 3339.573 | 9.003 | 18.344 | 447.692 | 220.505 | 179.425 | 41.080 |
| 2010 | 37452 | 18237.758 | 14572.802 | 3664.957 | 9.786 | 20.095 | 446.355 | 218.215 | 173.681 | 44.534 |
| 2011 | 38343 | 18487.916 | 14346.659 | 4141.257 | 10.801 | 22.400 | 452.268 | 219.022 | 169.225 | 49.797 |
| 2012 | 38785 | 18491.158 | 13947.603 | 4543.556 | 11.715 | 24.572 | 452.339 | 216.689 | 162.665 | 54.024 |
| 2013 | 40617 | 19143.452 | 14045.013 | 5098.440 | 12.552 | 26.633 | 468.087 | 221.775 | 161.860 | 59.915 |
| 2014 | 40701 | 18992.005 | 13503.894 | 5488.110 | 13.484 | 28.897 | 463.354 | 217.451 | 153.734 | 63.717 |
| 2015 | 42721 | 19728.885 | 13672.564 | 6056.320 | 14.176 | 30.698 | 546.442 | 254.116 | 174.884 | 79.233 |
| 2016 | 43569 | 19876.724 | 13334.819 | 6541.905 | 15.015 | 32.912 | 551.771 | 253.628 | 168.874 | 84.753 |
| 2017 | 44626 | 20119.790 | 13046.036 | 7073.755 | 15.851 | 35.158 | 559.662 | 254.381 | 163.612 | 90.769 |
| 2018 | 46048 | 20535.123 | 12858.168 | 7676.955 | 16.672 | 37.385 | 571.815 | 257.229 | 159.669 | 97.560 |
| 2019 | 47609 | 21009.029 | 12697.734 | 8311.296 | 17.457 | 39.561 | 585.102 | 260.605 | 156.051 | 104.555 |
| 2020 | 49139 | 21428.512 | 12506.810 | 8921.702 | 18.156 | 41.635 | 688.302 | 303.533 | 175.186 | 128.347 |
| 2021 | 49237 | 21266.697 | 11871.725 | 9394.972 | 19.081 | 44.177 | 684.358 | 299.127 | 165.007 | 134.120 |
| 2022 | 50767 | 21663.568 | 11578.211 | 10085.36 | 19.866 | 46.554 | 699.743 | 302.378 | 159.587 | 142.791 |
| 2023 | 52326 | 22084.131 | 11288.564 | 10795.57 | 20.631 | 48.884 | 714.879 | 305.748 | 154.225 | 151.523 |
| 2024 | 53834 | 22418.456 | 10979.712 | 11438.74 | 21.248 | 51.024 | 728.869 | 307.804 | 148.656 | 159.147 |
| 2025 | 54961 | 22614.834 | 10612.676 | 12002.16 | 21.838 | 53.072 | 863.914 | 361.411 | 166.818 | 194.593 |
| 2026 | 55535 | 22571.522 | 10076.635 | 12494.89 | 22.499 | 55.357 | 868.642 | 359.241 | 157.612 | 201.629 |
| 2027 | 56549 | 22774.416 | 9639.749 | 13134.67 | 23.227 | 57.673 | 879.782 | 360.827 | 149.974 | 210.853 |
| 2028 | 57524 | 22915.561 | 9154.955 | 13760.61 | 23.922 | 60.049 | 889.704 | 361.228 | 141.598 | 219.631 |
| 2029 | 58484 | 22992.027 | 8671.548 | 14320.48 | 24.486 | 62.285 | 898.689 | 360.371 | 133.249 | 227.121 |
| 2030 | 58803 | 22895.360 | 8195.295 | 14700.06 | 24.999 | 64.205 | 1076.19 | 428.760 | 149.986 | 278.773 |
| 2031 | 59243 | 22715.871 | 7687.907 | 15027.96 | 25.367 | 66.156 | 1080.40 | 424.293 | 140.202 | 284.091 |
| 2032 | 60000 | 22759.207 | 7223.761 | 15535.45 | 25.893 | 68.260 | 1089.47 | 423.652 | 131.169 | 292.483 |
| 2033 | 60733 | 22786.258 | 6775.906 | 16010.35 | 26.362 | 70.263 | 1097.21 | 422.387 | 122.413 | 299.974 |
| 2034 | 61409 | 22806.106 | 6334.204 | 16471.90 | 26.823 | 72.226 | 1103.09 | 420.721 | 113.781 | 306.940 |
| 2035 | 60542 | 22262.998 | 5871.512 | 16391.49 | 27.075 | 73.627 | 1340.42 | 507.973 | 129.998 | 377.975 |
| 2036 | 58466 | 21140.051 | 5074.508 | 16065.54 | 27.478 | 75.996 | 1292.84 | 482.234 | 112.210 | 370.023 |
| 2037 | 58667 | 21011.845 | 4627.331 | 16384.51 | 27.928 | 77.978 | 1294.62 | 478.783 | 102.114 | 376.669 |
| 2038 | 58844 | 20787.483 | 4182.531 | 16604.95 | 28.218 | 79.880 | 1294.63 | 472.681 | 92.020 | 380.661 |
| 2039 | 59012 | 20709.267 | 3795.347 | 16913.92 | 28.662 | 81.673 | 1292.92 | 469.372 | 83.154 | 386.218 |

# Results for PHIM for Run 4 (Sensitivity_Analysis_1.RTF)

---

-------------------------------------------------------------------------------------

**Control File Details for Run: 4**

| Variable Parameter | Value |
|---|---|
| 1. Name for Reference Scenario | NULL |
| 2. Name for Test Scenario | Ecig |
| 3. Name for Product 1 | CC |
| 4. Name for Product 2 | Ecig |
| 5. Name for Product 3 | - |
| 6. Name for Product 4 | Dual |
| 7. Country | US (United States) |
| 8. Sex | F (Females) |
| 9. Year of start of process | 1990 |
| 10. Number of months of follow-up | 600 |
| 11. Follow-up interval length (in months) | 12 |
| 12. Lower age for risk estimation | 10 |
| 13. Upper age for risk estimation | 79 |
| 14. The effective dose for Product 1 (F1) - Ref | 1 |
| 15. The effective dose for Product 2 (F2) - Ref | - |
| 16. The effective dose for Product 3 (F3) - Ref | - |
| 17. The effective dose for Product 4 (F4) - Ref | - |
| 18. The effective dose for Product 1 (F1) - Test | 1 |
| 19. The effective dose for Product 2 (F2) - Test | 0 |
| 20. The effective dose for Product 3 (F3) - Test | - |
| 21. The effective dose for Product 4 (F4) - Test | FROMF2 |
| 22. Number in population to be simulated | 100000 |
| 23. Number of MC simulations | 1 |
| 24. The random number seed for the first simulation | 15975263 |
| 25. Source for the population file (POP) | UN4 |
| 26. Source for the socioeconomic prevalence file (SEP) | - |
| 27. Source for the current smoking prevalence file (CSP) | ISS3 |
| 28. Source for the former smoking prevalence file (FSP) | ISS3 |
| 29. Source for the quit-time distribution file (QTD) | NHIS2006 |
| 30. Source for the death file (MORT) | APR-20 |
| 31. Source for the relative risk file (RR) | PNLEST |
| 32. Source for the half-life file (H) | PNLEST |
| 33. Assumption Set for TTP Factor Reference | F1 |
| 34. Assumption Set for TTP Factor Test | F1 |
| 35. Assumption Set for TTP Reference | ISS3 |
| 36. Assumption Set for TTP Test | ISS3 |
| 37. Assumption Set for TP for socioeconomic group | - |
| 38. Source for Output Choice file (OUTC) | BASIC |
| 39. Output for DTP P1 | N |
| 40. Output for DTP P2 | N |
| 41. Output for Main P Component | Y |
| 42. Output for DTP E1 | N |
| 43. Output for DTP E2 | N |
| 44. Output for DTP E3 | N |
| 45. Output for Main E Component | Y |
| 46. Output file name | Sensitivity_Analysis_1 |
| 47. Run number for P-Component | - |
| 48. Results file for E-Component | - |
| 49. Components required | PE |
| 50. Results folder for P to be used in E-Component | - |
| 51. Fixed age of starting product use | 16 |
| 52. Output absolute risk file? | N |

# Results for PHIM for Run 4 (Sensitivity_Analysis_1.RTF)

************************************************************************************

**TTP values for Run: 4, Scenario: NULL**

| **Variable Parameter** | **Value** |
|---|---|
| 1. Country | US (United States) |
| 2. Sex | F (Females) |
| 3. Assumption Set | ISS3 |
| Note: | Period is in Months |

| Age | Period | **Initiation** Never CC | **Cessation** CC Former | **Re-initiation** Former CC |
|---|---|---|---|---|
| 10-14 | 1-12 | 0.003758 | 0.008742 | 0.000000 |
| 15-19 | 1-12 | 0.003469 | 0.002515 | 0.000000 |
| 20-24 | 1-12 | 0.000276 | 0.000761 | 0.000000 |
| 25-29 | 1-12 | 0.000137 | 0.001375 | 0.000000 |
| 30-34 | 1-12 | 0.000069 | 0.001149 | 0.000000 |
| 35-39 | 1-12 | 0.000000 | 0.000991 | 0.000000 |
| 40-44 | 1-12 | 0.000000 | 0.000833 | 0.000000 |
| 45-49 | 1-12 | 0.000000 | 0.001723 | 0.000000 |
| 50-54 | 1-12 | 0.000000 | 0.002336 | 0.000000 |
| 55-59 | 1-12 | 0.000000 | 0.002233 | 0.000000 |
| 60-64 | 1-12 | 0.000000 | 0.004634 | 0.000000 |
| 65-69 | 1-12 | 0.000000 | 0.003505 | 0.000000 |
| 70-74 | 1-12 | 0.000000 | 0.004329 | 0.000000 |
| 75-79 | 1-12 | 0.000000 | 0.004329 | 0.000000 |
| 10-14 | 13-72 | 0.004794 | 0.008742 | 0.000000 |
| 15-19 | 13-72 | 0.004527 | 0.004082 | 0.000000 |
| 20-24 | 13-72 | 0.000105 | 0.001261 | 0.000000 |
| 25-29 | 13-72 | 0.000053 | 0.000952 | 0.000000 |
| 30-34 | 13-72 | 0.000026 | 0.000733 | 0.000000 |
| 35-39 | 13-72 | 0.000000 | 0.000753 | 0.000000 |
| 40-44 | 13-72 | 0.000000 | 0.001535 | 0.000000 |
| 45-49 | 13-72 | 0.000000 | 0.002998 | 0.000000 |
| 50-54 | 13-72 | 0.000000 | 0.003159 | 0.000000 |
| 55-59 | 13-72 | 0.000000 | 0.002965 | 0.000000 |
| 60-64 | 13-72 | 0.000000 | 0.003633 | 0.000000 |
| 65-69 | 13-72 | 0.000000 | 0.004522 | 0.000000 |
| 70-74 | 13-72 | 0.000000 | 0.005208 | 0.000000 |
| 75-79 | 13-72 | 0.000000 | 0.005208 | 0.000000 |
| 10-14 | 73-132 | 0.003969 | 0.008742 | 0.000000 |
| 15-19 | 73-132 | 0.003056 | 0.003373 | 0.000000 |
| 20-24 | 73-132 | 0.001459 | 0.003881 | 0.000000 |
| 25-29 | 73-132 | 0.000714 | 0.001953 | 0.000000 |
| 30-34 | 73-132 | 0.000111 | 0.001338 | 0.000000 |
| 35-39 | 73-132 | 0.000000 | 0.001507 | 0.000000 |
| 40-44 | 73-132 | 0.000000 | 0.002080 | 0.000000 |
| 45-49 | 73-132 | 0.000000 | 0.003809 | 0.000000 |
| 50-54 | 73-132 | 0.000000 | 0.004871 | 0.000000 |
| 55-59 | 73-132 | 0.000000 | 0.004560 | 0.000000 |
| 60-64 | 73-132 | 0.000000 | 0.007192 | 0.000000 |
| 65-69 | 73-132 | 0.000000 | 0.007033 | 0.000000 |
| 70-74 | 73-132 | 0.000000 | 0.008206 | 0.000000 |
| 75-79 | 73-132 | 0.000000 | 0.008206 | 0.000000 |
| 10-14 | 133-192 | 0.002861 | 0.008742 | 0.000000 |
| 15-19 | 133-192 | 0.002594 | 0.003996 | 0.000000 |
| 20-24 | 133-192 | 0.001247 | 0.002805 | 0.000000 |
| 25-29 | 133-192 | 0.000289 | 0.001574 | 0.000000 |
| 30-34 | 133-192 | 0.000132 | 0.001293 | 0.000000 |
| 35-39 | 133-192 | 0.000000 | 0.001219 | 0.000000 |
| 40-44 | 133-192 | 0.000000 | 0.001657 | 0.000000 |
| 45-49 | 133-192 | 0.000000 | 0.001995 | 0.000000 |
| 50-54 | 133-192 | 0.000000 | 0.002154 | 0.000000 |
| 55-59 | 133-192 | 0.000000 | 0.002072 | 0.000000 |
| 60-64 | 133-192 | 0.000000 | 0.005049 | 0.000000 |
| 65-69 | 133-192 | 0.000000 | 0.003680 | 0.000000 |
| 70-74 | 133-192 | 0.000000 | 0.006144 | 0.000000 |
| 75-79 | 133-192 | 0.000000 | 0.006144 | 0.000000 |
| 10-14 | 193-252 | 0.001859 | 0.008742 | 0.000000 |
| 15-19 | 193-252 | 0.002232 | 0.005770 | 0.000000 |
| 20-24 | 193-252 | 0.001079 | 0.003262 | 0.000000 |
| 25-29 | 193-252 | 0.000531 | 0.002002 | 0.000000 |
| 30-34 | 193-252 | 0.000263 | 0.003240 | 0.000000 |
| 35-39 | 193-252 | 0.000000 | 0.001771 | 0.000000 |
| 40-44 | 193-252 | 0.000000 | 0.002173 | 0.000000 |
| 45-49 | 193-252 | 0.000000 | 0.002075 | 0.000000 |
| 50-54 | 193-252 | 0.000000 | 0.003431 | 0.000000 |

## Results for PHIM for Run 4 (Sensitivity_Analysis_1.RTF)

| | | | | |
|---|---|---|---|---|
| 55-59 | 193-252 | 0.000000 | 0.002906 | 0.000000 |
| 60-64 | 193-252 | 0.000000 | 0.007139 | 0.000000 |
| 65-69 | 193-252 | 0.000000 | 0.004963 | 0.000000 |
| 70-74 | 193-252 | 0.000000 | 0.006269 | 0.000000 |
| 75-79 | 193-252 | 0.000000 | 0.006269 | 0.000000 |
| 10-14 | 253+ | 0.000931 | 0.008742 | 0.000000 |
| 15-19 | 253+ | 0.001798 | 0.009440 | 0.000000 |
| 20-24 | 253+ | 0.000023 | 0.002831 | 0.000000 |
| 25-29 | 253+ | 0.000073 | 0.002655 | 0.000000 |
| 30-34 | 253+ | 0.000037 | 0.004614 | 0.000000 |
| 35-39 | 253+ | 0.000000 | 0.002607 | 0.000000 |
| 40-44 | 253+ | 0.000000 | 0.003115 | 0.000000 |
| 45-49 | 253+ | 0.000000 | 0.002132 | 0.000000 |
| 50-54 | 253+ | 0.000000 | 0.003497 | 0.000000 |
| 55-59 | 253+ | 0.000000 | 0.002942 | 0.000000 |
| 60-64 | 253+ | 0.000000 | 0.005376 | 0.000000 |
| 65-69 | 253+ | 0.000000 | 0.001789 | 0.000000 |
| 70-74 | 253+ | 0.000000 | 0.004077 | 0.000000 |
| 75-79 | 253+ | 0.000000 | 0.004077 | 0.000000 |

# Results for PHIM for Run 4 (Sensitivity_Analysis_1.RTF)

************************************************************************************

**TTP values for Run: 4, Scenario: Ecig**

| **Variable Parameter** | **Value** |
|---|---|
| 1. Country | US (United States) |
| 2. Sex | F (Females) |
| 3. Assumption Set | ISS3 |
| Note: | Period is in Months |

| | | **Initiation** | | | **Cessation** | | | **Re-initiation** | | |
|---|---|---|---|---|---|---|---|---|---|---|
| Age | Period | Never CC | Never Ecig | Never Dual | CC Former | Ecig Former | Dual Former | Former CC | Former Ecig | Former Dual |
| 10-14 | 1-12 | 0.001879 | 0.001879 | 0.000000 | 0.008742 | 0.008742 | 0.000000 | 0.000000 | 0.000000 | 0.000000 |
| 15-19 | 1-12 | 0.001735 | 0.001735 | 0.000000 | 0.002515 | 0.002515 | 0.000000 | 0.000000 | 0.000000 | 0.000000 |
| 20-24 | 1-12 | 0.000138 | 0.000138 | 0.000000 | 0.000761 | 0.000761 | 0.000000 | 0.000000 | 0.000000 | 0.000000 |
| 25-29 | 1-12 | 0.000069 | 0.000069 | 0.000000 | 0.001376 | 0.001376 | 0.000000 | 0.000000 | 0.000000 | 0.000000 |
| 30-34 | 1-12 | 0.000034 | 0.000034 | 0.000000 | 0.001149 | 0.001149 | 0.000000 | 0.000000 | 0.000000 | 0.000000 |
| 35-39 | 1-12 | 0.000000 | 0.000000 | 0.000000 | 0.000991 | 0.000991 | 0.000000 | 0.000000 | 0.000000 | 0.000000 |
| 40-44 | 1-12 | 0.000000 | 0.000000 | 0.000000 | 0.000833 | 0.000833 | 0.000000 | 0.000000 | 0.000000 | 0.000000 |
| 45-49 | 1-12 | 0.000000 | 0.000000 | 0.000000 | 0.001723 | 0.001723 | 0.000000 | 0.000000 | 0.000000 | 0.000000 |
| 50-54 | 1-12 | 0.000000 | 0.000000 | 0.000000 | 0.002336 | 0.002336 | 0.000000 | 0.000000 | 0.000000 | 0.000000 |
| 55-59 | 1-12 | 0.000000 | 0.000000 | 0.000000 | 0.002233 | 0.002233 | 0.000000 | 0.000000 | 0.000000 | 0.000000 |
| 60-64 | 1-12 | 0.000000 | 0.000000 | 0.000000 | 0.004635 | 0.004635 | 0.000000 | 0.000000 | 0.000000 | 0.000000 |
| 65-69 | 1-12 | 0.000000 | 0.000000 | 0.000000 | 0.003505 | 0.003505 | 0.000000 | 0.000000 | 0.000000 | 0.000000 |
| 70-74 | 1-12 | 0.000000 | 0.000000 | 0.000000 | 0.004329 | 0.004329 | 0.000000 | 0.000000 | 0.000000 | 0.000000 |
| 75-79 | 1-12 | 0.000000 | 0.000000 | 0.000000 | 0.004329 | 0.004329 | 0.000000 | 0.000000 | 0.000000 | 0.000000 |
| 10-14 | 13-72 | 0.001918 | 0.002877 | 0.000000 | 0.008742 | 0.008742 | 0.000000 | 0.000000 | 0.000000 | 0.000000 |
| 15-19 | 13-72 | 0.001811 | 0.002716 | 0.000000 | 0.004083 | 0.004083 | 0.000000 | 0.000000 | 0.000000 | 0.000000 |
| 20-24 | 13-72 | 0.000042 | 0.000063 | 0.000000 | 0.001261 | 0.001261 | 0.000000 | 0.000000 | 0.000000 | 0.000000 |
| 25-29 | 13-72 | 0.000021 | 0.000032 | 0.000000 | 0.000952 | 0.000952 | 0.000000 | 0.000000 | 0.000000 | 0.000000 |
| 30-34 | 13-72 | 0.000011 | 0.000016 | 0.000000 | 0.000733 | 0.000733 | 0.000000 | 0.000000 | 0.000000 | 0.000000 |
| 35-39 | 13-72 | 0.000000 | 0.000000 | 0.000000 | 0.000754 | 0.000754 | 0.000000 | 0.000000 | 0.000000 | 0.000000 |
| 40-44 | 13-72 | 0.000000 | 0.000000 | 0.000000 | 0.001535 | 0.001535 | 0.000000 | 0.000000 | 0.000000 | 0.000000 |
| 45-49 | 13-72 | 0.000000 | 0.000000 | 0.000000 | 0.002998 | 0.002998 | 0.000000 | 0.000000 | 0.000000 | 0.000000 |
| 50-54 | 13-72 | 0.000000 | 0.000000 | 0.000000 | 0.003160 | 0.003160 | 0.000000 | 0.000000 | 0.000000 | 0.000000 |
| 55-59 | 13-72 | 0.000000 | 0.000000 | 0.000000 | 0.002965 | 0.002965 | 0.000000 | 0.000000 | 0.000000 | 0.000000 |
| 60-64 | 13-72 | 0.000000 | 0.000000 | 0.000000 | 0.003633 | 0.003633 | 0.000000 | 0.000000 | 0.000000 | 0.000000 |
| 65-69 | 13-72 | 0.000000 | 0.000000 | 0.000000 | 0.004522 | 0.004522 | 0.000000 | 0.000000 | 0.000000 | 0.000000 |
| 70-74 | 13-72 | 0.000000 | 0.000000 | 0.000000 | 0.005208 | 0.005208 | 0.000000 | 0.000000 | 0.000000 | 0.000000 |
| 75-79 | 13-72 | 0.000000 | 0.000000 | 0.000000 | 0.005208 | 0.005208 | 0.000000 | 0.000000 | 0.000000 | 0.000000 |
| 10-14 | 73-132 | 0.000794 | 0.003175 | 0.000000 | 0.008742 | 0.008742 | 0.000000 | 0.000000 | 0.000000 | 0.000000 |
| 15-19 | 73-132 | 0.000611 | 0.002445 | 0.000000 | 0.003373 | 0.003373 | 0.000000 | 0.000000 | 0.000000 | 0.000000 |
| 20-24 | 73-132 | 0.000292 | 0.001167 | 0.000000 | 0.003881 | 0.003881 | 0.000000 | 0.000000 | 0.000000 | 0.000000 |
| 25-29 | 73-132 | 0.000143 | 0.000571 | 0.000000 | 0.001954 | 0.001954 | 0.000000 | 0.000000 | 0.000000 | 0.000000 |
| 30-34 | 73-132 | 0.000022 | 0.000089 | 0.000000 | 0.001338 | 0.001338 | 0.000000 | 0.000000 | 0.000000 | 0.000000 |
| 35-39 | 73-132 | 0.000000 | 0.000000 | 0.000000 | 0.001507 | 0.001507 | 0.000000 | 0.000000 | 0.000000 | 0.000000 |
| 40-44 | 73-132 | 0.000000 | 0.000000 | 0.000000 | 0.002080 | 0.002080 | 0.000000 | 0.000000 | 0.000000 | 0.000000 |
| 45-49 | 73-132 | 0.000000 | 0.000000 | 0.000000 | 0.003809 | 0.003809 | 0.000000 | 0.000000 | 0.000000 | 0.000000 |
| 50-54 | 73-132 | 0.000000 | 0.000000 | 0.000000 | 0.004871 | 0.004871 | 0.000000 | 0.000000 | 0.000000 | 0.000000 |
| 55-59 | 73-132 | 0.000000 | 0.000000 | 0.000000 | 0.004560 | 0.004560 | 0.000000 | 0.000000 | 0.000000 | 0.000000 |
| 60-64 | 73-132 | 0.000000 | 0.000000 | 0.000000 | 0.007192 | 0.007192 | 0.000000 | 0.000000 | 0.000000 | 0.000000 |
| 65-69 | 73-132 | 0.000000 | 0.000000 | 0.000000 | 0.007033 | 0.007033 | 0.000000 | 0.000000 | 0.000000 | 0.000000 |
| 70-74 | 73-132 | 0.000000 | 0.000000 | 0.000000 | 0.008206 | 0.008206 | 0.000000 | 0.000000 | 0.000000 | 0.000000 |
| 75-79 | 73-132 | 0.000000 | 0.000000 | 0.000000 | 0.008206 | 0.008206 | 0.000000 | 0.000000 | 0.000000 | 0.000000 |
| 10-14 | 133-192 | 0.000143 | 0.002718 | 0.000000 | 0.008742 | 0.008742 | 0.000000 | 0.000000 | 0.000000 | 0.000000 |
| 15-19 | 133-192 | 0.000130 | 0.002465 | 0.000000 | 0.003996 | 0.003996 | 0.000000 | 0.000000 | 0.000000 | 0.000000 |
| 20-24 | 133-192 | 0.000062 | 0.001185 | 0.000000 | 0.002805 | 0.002805 | 0.000000 | 0.000000 | 0.000000 | 0.000000 |
| 25-29 | 133-192 | 0.000015 | 0.000275 | 0.000000 | 0.001574 | 0.001574 | 0.000000 | 0.000000 | 0.000000 | 0.000000 |
| 30-34 | 133-192 | 0.000007 | 0.000125 | 0.000000 | 0.001293 | 0.001293 | 0.000000 | 0.000000 | 0.000000 | 0.000000 |
| 35-39 | 133-192 | 0.000000 | 0.000000 | 0.000000 | 0.001219 | 0.001219 | 0.000000 | 0.000000 | 0.000000 | 0.000000 |
| 40-44 | 133-192 | 0.000000 | 0.000000 | 0.000000 | 0.001657 | 0.001657 | 0.000000 | 0.000000 | 0.000000 | 0.000000 |
| 45-49 | 133-192 | 0.000000 | 0.000000 | 0.000000 | 0.001995 | 0.001995 | 0.000000 | 0.000000 | 0.000000 | 0.000000 |
| 50-54 | 133-192 | 0.000000 | 0.000000 | 0.000000 | 0.002154 | 0.002154 | 0.000000 | 0.000000 | 0.000000 | 0.000000 |
| 55-59 | 133-192 | 0.000000 | 0.000000 | 0.000000 | 0.002072 | 0.002072 | 0.000000 | 0.000000 | 0.000000 | 0.000000 |
| 60-64 | 133-192 | 0.000000 | 0.000000 | 0.000000 | 0.005049 | 0.005049 | 0.000000 | 0.000000 | 0.000000 | 0.000000 |
| 65-69 | 133-192 | 0.000000 | 0.000000 | 0.000000 | 0.003681 | 0.003681 | 0.000000 | 0.000000 | 0.000000 | 0.000000 |
| 70-74 | 133-192 | 0.000000 | 0.000000 | 0.000000 | 0.006144 | 0.006144 | 0.000000 | 0.000000 | 0.000000 | 0.000000 |
| 75-79 | 133-192 | 0.000000 | 0.000000 | 0.000000 | 0.006144 | 0.006144 | 0.000000 | 0.000000 | 0.000000 | 0.000000 |
| 10-14 | 193-252 | 0.000093 | 0.001766 | 0.000000 | 0.008742 | 0.008742 | 0.000000 | 0.000000 | 0.000000 | 0.000000 |
| 15-19 | 193-252 | 0.000112 | 0.002120 | 0.000000 | 0.005770 | 0.005770 | 0.000000 | 0.000000 | 0.000000 | 0.000000 |
| 20-24 | 193-252 | 0.000054 | 0.001025 | 0.000000 | 0.003262 | 0.003262 | 0.000000 | 0.000000 | 0.000000 | 0.000000 |
| 25-29 | 193-252 | 0.000027 | 0.000504 | 0.000000 | 0.002002 | 0.002002 | 0.000000 | 0.000000 | 0.000000 | 0.000000 |
| 30-34 | 193-252 | 0.000013 | 0.000250 | 0.000000 | 0.003241 | 0.003241 | 0.000000 | 0.000000 | 0.000000 | 0.000000 |
| 35-39 | 193-252 | 0.000000 | 0.000000 | 0.000000 | 0.001771 | 0.001771 | 0.000000 | 0.000000 | 0.000000 | 0.000000 |
| 40-44 | 193-252 | 0.000000 | 0.000000 | 0.000000 | 0.002173 | 0.002173 | 0.000000 | 0.000000 | 0.000000 | 0.000000 |
| 45-49 | 193-252 | 0.000000 | 0.000000 | 0.000000 | 0.002075 | 0.002075 | 0.000000 | 0.000000 | 0.000000 | 0.000000 |
| 50-54 | 193-252 | 0.000000 | 0.000000 | 0.000000 | 0.003432 | 0.003432 | 0.000000 | 0.000000 | 0.000000 | 0.000000 |

## Results for PHIM for Run 4 (Sensitivity_Analysis_1.RTF)

| | | | | | | | | | | |
|---|---|---|---|---|---|---|---|---|---|---|
| 55-59 | 193-252 | 0.000000 | 0.000000 | 0.000000 | 0.002906 | 0.002906 | 0.000000 | 0.000000 | 0.000000 | 0.000000 |
| 60-64 | 193-252 | 0.000000 | 0.000000 | 0.000000 | 0.007139 | 0.007139 | 0.000000 | 0.000000 | 0.000000 | 0.000000 |
| 65-69 | 193-252 | 0.000000 | 0.000000 | 0.000000 | 0.004963 | 0.004963 | 0.000000 | 0.000000 | 0.000000 | 0.000000 |
| 70-74 | 193-252 | 0.000000 | 0.000000 | 0.000000 | 0.006269 | 0.006269 | 0.000000 | 0.000000 | 0.000000 | 0.000000 |
| 75-79 | 193-252 | 0.000000 | 0.000000 | 0.000000 | 0.006269 | 0.006269 | 0.000000 | 0.000000 | 0.000000 | 0.000000 |
| 10-14 | 253+ | 0.000047 | 0.000884 | 0.000000 | 0.008742 | 0.008742 | 0.000000 | 0.000000 | 0.000000 | 0.000000 |
| 15-19 | 253+ | 0.000090 | 0.001708 | 0.000000 | 0.009440 | 0.009440 | 0.000000 | 0.000000 | 0.000000 | 0.000000 |
| 20-24 | 253+ | 0.000001 | 0.000022 | 0.000000 | 0.002831 | 0.002831 | 0.000000 | 0.000000 | 0.000000 | 0.000000 |
| 25-29 | 253+ | 0.000004 | 0.000070 | 0.000000 | 0.002656 | 0.002656 | 0.000000 | 0.000000 | 0.000000 | 0.000000 |
| 30-34 | 253+ | 0.000002 | 0.000035 | 0.000000 | 0.004614 | 0.004614 | 0.000000 | 0.000000 | 0.000000 | 0.000000 |
| 35-39 | 253+ | 0.000000 | 0.000000 | 0.000000 | 0.002608 | 0.002608 | 0.000000 | 0.000000 | 0.000000 | 0.000000 |
| 40-44 | 253+ | 0.000000 | 0.000000 | 0.000000 | 0.003115 | 0.003115 | 0.000000 | 0.000000 | 0.000000 | 0.000000 |
| 45-49 | 253+ | 0.000000 | 0.000000 | 0.000000 | 0.002132 | 0.002132 | 0.000000 | 0.000000 | 0.000000 | 0.000000 |
| 50-54 | 253+ | 0.000000 | 0.000000 | 0.000000 | 0.003497 | 0.003497 | 0.000000 | 0.000000 | 0.000000 | 0.000000 |
| 55-59 | 253+ | 0.000000 | 0.000000 | 0.000000 | 0.002942 | 0.002942 | 0.000000 | 0.000000 | 0.000000 | 0.000000 |
| 60-64 | 253+ | 0.000000 | 0.000000 | 0.000000 | 0.005376 | 0.005376 | 0.000000 | 0.000000 | 0.000000 | 0.000000 |
| 65-69 | 253+ | 0.000000 | 0.000000 | 0.000000 | 0.001789 | 0.001789 | 0.000000 | 0.000000 | 0.000000 | 0.000000 |
| 70-74 | 253+ | 0.000000 | 0.000000 | 0.000000 | 0.004077 | 0.004077 | 0.000000 | 0.000000 | 0.000000 | 0.000000 |
| 75-79 | 253+ | 0.000000 | 0.000000 | 0.000000 | 0.004077 | 0.004077 | 0.000000 | 0.000000 | 0.000000 | 0.000000 |

# Results for PHIM for Run 4 (Sensitivity_Analysis_1.RTF)

************************************************************************************

| | | Switch from CC | | Switch from Ecig | | Switch from Dual | |
|---|---|---|---|---|---|---|---|
| | | **CC** | **CC** | **Ecig** | **Ecig** | **Dual** | **Dual** |
| Age | Period | Ecig | Dual | CC | Dual | CC | Ecig |
| 10-14 | 1-12 | 0.009055 | 0.000000 | 0.000000 | 0.000000 | 0.000000 | 0.000000 |
| 15-19 | 1-12 | 0.011817 | 0.000000 | 0.000000 | 0.000000 | 0.000000 | 0.000000 |
| 20-24 | 1-12 | 0.013405 | 0.000000 | 0.000000 | 0.000000 | 0.000000 | 0.000000 |
| 25-29 | 1-12 | 0.014127 | 0.000000 | 0.000000 | 0.000000 | 0.000000 | 0.000000 |
| 30-34 | 1-12 | 0.013889 | 0.000000 | 0.000000 | 0.000000 | 0.000000 | 0.000000 |
| 35-39 | 1-12 | 0.012929 | 0.000000 | 0.000000 | 0.000000 | 0.000000 | 0.000000 |
| 40-44 | 1-12 | 0.012785 | 0.000000 | 0.000000 | 0.000000 | 0.000000 | 0.000000 |
| 45-49 | 1-12 | 0.012295 | 0.000000 | 0.000000 | 0.000000 | 0.000000 | 0.000000 |
| 50-54 | 1-12 | 0.011536 | 0.000000 | 0.000000 | 0.000000 | 0.000000 | 0.000000 |
| 55-59 | 1-12 | 0.008988 | 0.000000 | 0.000000 | 0.000000 | 0.000000 | 0.000000 |
| 60-64 | 1-12 | 0.008098 | 0.000000 | 0.000000 | 0.000000 | 0.000000 | 0.000000 |
| 65-69 | 1-12 | 0.006274 | 0.000000 | 0.000000 | 0.000000 | 0.000000 | 0.000000 |
| 70-74 | 1-12 | 0.004141 | 0.000000 | 0.000000 | 0.000000 | 0.000000 | 0.000000 |
| 75-79 | 1-12 | 0.004141 | 0.000000 | 0.000000 | 0.000000 | 0.000000 | 0.000000 |
| 10-14 | 13-72 | 0.009055 | 0.000000 | 0.000000 | 0.000000 | 0.000000 | 0.000000 |
| 15-19 | 13-72 | 0.011817 | 0.000000 | 0.000000 | 0.000000 | 0.000000 | 0.000000 |
| 20-24 | 13-72 | 0.013405 | 0.000000 | 0.000000 | 0.000000 | 0.000000 | 0.000000 |
| 25-29 | 13-72 | 0.014127 | 0.000000 | 0.000000 | 0.000000 | 0.000000 | 0.000000 |
| 30-34 | 13-72 | 0.013889 | 0.000000 | 0.000000 | 0.000000 | 0.000000 | 0.000000 |
| 35-39 | 13-72 | 0.012929 | 0.000000 | 0.000000 | 0.000000 | 0.000000 | 0.000000 |
| 40-44 | 13-72 | 0.012785 | 0.000000 | 0.000000 | 0.000000 | 0.000000 | 0.000000 |
| 45-49 | 13-72 | 0.012295 | 0.000000 | 0.000000 | 0.000000 | 0.000000 | 0.000000 |
| 50-54 | 13-72 | 0.011536 | 0.000000 | 0.000000 | 0.000000 | 0.000000 | 0.000000 |
| 55-59 | 13-72 | 0.008988 | 0.000000 | 0.000000 | 0.000000 | 0.000000 | 0.000000 |
| 60-64 | 13-72 | 0.008098 | 0.000000 | 0.000000 | 0.000000 | 0.000000 | 0.000000 |
| 65-69 | 13-72 | 0.006274 | 0.000000 | 0.000000 | 0.000000 | 0.000000 | 0.000000 |
| 70-74 | 13-72 | 0.004141 | 0.000000 | 0.000000 | 0.000000 | 0.000000 | 0.000000 |
| 75-79 | 13-72 | 0.004141 | 0.000000 | 0.000000 | 0.000000 | 0.000000 | 0.000000 |
| 10-14 | 73-132 | 0.009055 | 0.000000 | 0.000000 | 0.000000 | 0.000000 | 0.000000 |
| 15-19 | 73-132 | 0.011817 | 0.000000 | 0.000000 | 0.000000 | 0.000000 | 0.000000 |
| 20-24 | 73-132 | 0.013405 | 0.000000 | 0.000000 | 0.000000 | 0.000000 | 0.000000 |
| 25-29 | 73-132 | 0.014127 | 0.000000 | 0.000000 | 0.000000 | 0.000000 | 0.000000 |
| 30-34 | 73-132 | 0.013889 | 0.000000 | 0.000000 | 0.000000 | 0.000000 | 0.000000 |
| 35-39 | 73-132 | 0.012929 | 0.000000 | 0.000000 | 0.000000 | 0.000000 | 0.000000 |
| 40-44 | 73-132 | 0.012785 | 0.000000 | 0.000000 | 0.000000 | 0.000000 | 0.000000 |
| 45-49 | 73-132 | 0.012295 | 0.000000 | 0.000000 | 0.000000 | 0.000000 | 0.000000 |
| 50-54 | 73-132 | 0.011536 | 0.000000 | 0.000000 | 0.000000 | 0.000000 | 0.000000 |
| 55-59 | 73-132 | 0.008988 | 0.000000 | 0.000000 | 0.000000 | 0.000000 | 0.000000 |
| 60-64 | 73-132 | 0.008098 | 0.000000 | 0.000000 | 0.000000 | 0.000000 | 0.000000 |
| 65-69 | 73-132 | 0.006274 | 0.000000 | 0.000000 | 0.000000 | 0.000000 | 0.000000 |
| 70-74 | 73-132 | 0.004141 | 0.000000 | 0.000000 | 0.000000 | 0.000000 | 0.000000 |
| 75-79 | 73-132 | 0.004141 | 0.000000 | 0.000000 | 0.000000 | 0.000000 | 0.000000 |
| 10-14 | 133-192 | 0.009055 | 0.000000 | 0.000000 | 0.000000 | 0.000000 | 0.000000 |
| 15-19 | 133-192 | 0.011817 | 0.000000 | 0.000000 | 0.000000 | 0.000000 | 0.000000 |
| 20-24 | 133-192 | 0.013405 | 0.000000 | 0.000000 | 0.000000 | 0.000000 | 0.000000 |
| 25-29 | 133-192 | 0.014127 | 0.000000 | 0.000000 | 0.000000 | 0.000000 | 0.000000 |
| 30-34 | 133-192 | 0.013889 | 0.000000 | 0.000000 | 0.000000 | 0.000000 | 0.000000 |
| 35-39 | 133-192 | 0.012929 | 0.000000 | 0.000000 | 0.000000 | 0.000000 | 0.000000 |
| 40-44 | 133-192 | 0.012785 | 0.000000 | 0.000000 | 0.000000 | 0.000000 | 0.000000 |
| 45-49 | 133-192 | 0.012295 | 0.000000 | 0.000000 | 0.000000 | 0.000000 | 0.000000 |
| 50-54 | 133-192 | 0.011536 | 0.000000 | 0.000000 | 0.000000 | 0.000000 | 0.000000 |
| 55-59 | 133-192 | 0.008988 | 0.000000 | 0.000000 | 0.000000 | 0.000000 | 0.000000 |
| 60-64 | 133-192 | 0.008098 | 0.000000 | 0.000000 | 0.000000 | 0.000000 | 0.000000 |
| 65-69 | 133-192 | 0.006274 | 0.000000 | 0.000000 | 0.000000 | 0.000000 | 0.000000 |
| 70-74 | 133-192 | 0.004141 | 0.000000 | 0.000000 | 0.000000 | 0.000000 | 0.000000 |
| 75-79 | 133-192 | 0.004141 | 0.000000 | 0.000000 | 0.000000 | 0.000000 | 0.000000 |
| 10-14 | 193-252 | 0.009055 | 0.000000 | 0.000000 | 0.000000 | 0.000000 | 0.000000 |
| 15-19 | 193-252 | 0.011817 | 0.000000 | 0.000000 | 0.000000 | 0.000000 | 0.000000 |
| 20-24 | 193-252 | 0.013405 | 0.000000 | 0.000000 | 0.000000 | 0.000000 | 0.000000 |
| 25-29 | 193-252 | 0.014127 | 0.000000 | 0.000000 | 0.000000 | 0.000000 | 0.000000 |
| 30-34 | 193-252 | 0.013889 | 0.000000 | 0.000000 | 0.000000 | 0.000000 | 0.000000 |
| 35-39 | 193-252 | 0.012929 | 0.000000 | 0.000000 | 0.000000 | 0.000000 | 0.000000 |
| 40-44 | 193-252 | 0.012785 | 0.000000 | 0.000000 | 0.000000 | 0.000000 | 0.000000 |
| 45-49 | 193-252 | 0.012295 | 0.000000 | 0.000000 | 0.000000 | 0.000000 | 0.000000 |
| 50-54 | 193-252 | 0.011536 | 0.000000 | 0.000000 | 0.000000 | 0.000000 | 0.000000 |
| 55-59 | 193-252 | 0.008988 | 0.000000 | 0.000000 | 0.000000 | 0.000000 | 0.000000 |
| 60-64 | 193-252 | 0.008098 | 0.000000 | 0.000000 | 0.000000 | 0.000000 | 0.000000 |
| 65-69 | 193-252 | 0.006274 | 0.000000 | 0.000000 | 0.000000 | 0.000000 | 0.000000 |
| 70-74 | 193-252 | 0.004141 | 0.000000 | 0.000000 | 0.000000 | 0.000000 | 0.000000 |
| 75-79 | 193-252 | 0.004141 | 0.000000 | 0.000000 | 0.000000 | 0.000000 | 0.000000 |
| 10-14 | 253+ | 0.009055 | 0.000000 | 0.000000 | 0.000000 | 0.000000 | 0.000000 |
| 15-19 | 253+ | 0.011817 | 0.000000 | 0.000000 | 0.000000 | 0.000000 | 0.000000 |
| 20-24 | 253+ | 0.013405 | 0.000000 | 0.000000 | 0.000000 | 0.000000 | 0.000000 |

## Results for PHIM for Run 4 (Sensitivity_Analysis_1.RTF)

| | | | | | | | |
|---|---|---|---|---|---|---|---|
| 25-29 | 253+ | 0.014127 | 0.000000 | 0.000000 | 0.000000 | 0.000000 | 0.000000 |
| 30-34 | 253+ | 0.013889 | 0.000000 | 0.000000 | 0.000000 | 0.000000 | 0.000000 |
| 35-39 | 253+ | 0.012929 | 0.000000 | 0.000000 | 0.000000 | 0.000000 | 0.000000 |
| 40-44 | 253+ | 0.012785 | 0.000000 | 0.000000 | 0.000000 | 0.000000 | 0.000000 |
| 45-49 | 253+ | 0.012295 | 0.000000 | 0.000000 | 0.000000 | 0.000000 | 0.000000 |
| 50-54 | 253+ | 0.011536 | 0.000000 | 0.000000 | 0.000000 | 0.000000 | 0.000000 |
| 55-59 | 253+ | 0.008988 | 0.000000 | 0.000000 | 0.000000 | 0.000000 | 0.000000 |
| 60-64 | 253+ | 0.008098 | 0.000000 | 0.000000 | 0.000000 | 0.000000 | 0.000000 |
| 65-69 | 253+ | 0.006274 | 0.000000 | 0.000000 | 0.000000 | 0.000000 | 0.000000 |
| 70-74 | 253+ | 0.004141 | 0.000000 | 0.000000 | 0.000000 | 0.000000 | 0.000000 |
| 75-79 | 253+ | 0.004141 | 0.000000 | 0.000000 | 0.000000 | 0.000000 | 0.000000 |

# Results for PHIM for Run 4 (Sensitivity_Analysis_1.RTF)

************************************************************************************

**Main Output for the Tobacco History for Run: 4, Simulation: 1, Scenario: NULL**

| Variable Parameter | Value |
|---|---|
| 1. Country | US (United States) |
| 2. Sex | F (Females) |
| 3. Year of start of process | 1990 |

Year of Follow-up: 2040, Age Group: All Ages

| Period | Age Group | Smoking Group | N | % |
|---|---|---|---|---|
| 50 | All Ages | Never Any | 20670 | 58.80 |
| 50 | All Ages | CC | 2462 | 7.00 |
| 50 | All Ages | Former | 12019 | 34.19 |

Year of Follow-up: 2040, Age Group: 18-19

| Period | Age Group | Smoking Group | N | % |
|---|---|---|---|---|
| 50 | 18-19 | Never Any | 0 | . |
| 50 | 18-19 | CC | 0 | . |
| 50 | 18-19 | Former | 0 | . |

Year of Follow-up: 2040, Age Group: 20-24

| Period | Age Group | Smoking Group | N | % |
|---|---|---|---|---|
| 50 | 20-24 | Never Any | 0 | . |
| 50 | 20-24 | CC | 0 | . |
| 50 | 20-24 | Former | 0 | . |

Year of Follow-up: 2040, Age Group: 25-29

| Period | Age Group | Smoking Group | N | % |
|---|---|---|---|---|
| 50 | 25-29 | Never Any | 0 | . |
| 50 | 25-29 | CC | 0 | . |
| 50 | 25-29 | Former | 0 | . |

Year of Follow-up: 2040, Age Group: 30-34

| Period | Age Group | Smoking Group | N | % |
|---|---|---|---|---|
| 50 | 30-34 | Never Any | 0 | . |
| 50 | 30-34 | CC | 0 | . |
| 50 | 30-34 | Former | 0 | . |

Year of Follow-up: 2040, Age Group: 35-39

| Period | Age Group | Smoking Group | N | % |
|---|---|---|---|---|
| 50 | 35-39 | Never Any | 0 | . |
| 50 | 35-39 | CC | 0 | . |
| 50 | 35-39 | Former | 0 | . |

Year of Follow-up: 2040, Age Group: 40-44

| Period | Age Group | Smoking Group | N | % |
|---|---|---|---|---|
| 50 | 40-44 | Never Any | 0 | . |
| 50 | 40-44 | CC | 0 | . |
| 50 | 40-44 | Former | 0 | . |

Year of Follow-up: 2040, Age Group: 45-49

| Period | Age Group | Smoking Group | N | % |
|---|---|---|---|---|
| 50 | 45-49 | Never Any | 0 | . |
| 50 | 45-49 | CC | 0 | . |
| 50 | 45-49 | Former | 0 | . |

Year of Follow-up: 2040, Age Group: 50-54

| Period | Age Group | Smoking Group | N | % |
|---|---|---|---|---|
| 50 | 50-54 | Never Any | 0 | . |
| 50 | 50-54 | CC | 0 | . |
| 50 | 50-54 | Former | 0 | . |

Year of Follow-up: 2040, Age Group: 55-59

| Period | Age Group | Smoking Group | N | % |
|---|---|---|---|---|
| 50 | 55-59 | Never Any | 0 | . |
| 50 | 55-59 | CC | 0 | . |
| 50 | 55-59 | Former | 0 | . |

Year of Follow-up: 2040, Age Group: 60-64

| Period | Age Group | Smoking Group | N | % |
|---|---|---|---|---|

## Results for PHIM for Run 4 (Sensitivity_Analysis_1.RTF)

| | | | | |
|---|---|---|---|---|
| 50 | 60-64 | Never Any | 4803 | 60.57 |
| 50 | 60-64 | CC | 621 | 7.83 |
| 50 | 60-64 | Former | 2506 | 31.60 |

Year of Follow-up: 2040, Age Group: 65-69

| Period | Age Group | Smoking Group | N | % |
|---|---|---|---|---|
| 50 | 65-69 | Never Any | 5407 | 64.75 |
| 50 | 65-69 | CC | 538 | 6.44 |
| 50 | 65-69 | Former | 2405 | 28.80 |

Year of Follow-up: 2040, Age Group: 70-74

| Period | Age Group | Smoking Group | N | % |
|---|---|---|---|---|
| 50 | 70-74 | Never Any | 5181 | 58.92 |
| 50 | 70-74 | CC | 605 | 6.88 |
| 50 | 70-74 | Former | 3007 | 34.20 |

Year of Follow-up: 2040, Age Group: 75-79

| Period | Age Group | Smoking Group | N | % |
|---|---|---|---|---|
| 50 | 75-79 | Never Any | 5279 | 52.38 |
| 50 | 75-79 | CC | 698 | 6.93 |
| 50 | 75-79 | Former | 4101 | 40.69 |

# Results for PHIM for Run 4 (Sensitivity_Analysis_1.RTF)

*************************************************************************************
**Main Output for the Tobacco History for Run: 4, Simulation: 1, Scenario: Ecig**

| Variable Parameter | Value |
|---|---|
| 1. Country | US (United States) |
| 2. Sex | F (Females) |
| 3. Year of start of process | 1990 |

Year of Follow-up: 1990, Age Group: All Ages

| Period | Age Group | Smoking Group | N | % | Ever Quit (N) | Ever Quit % | Mean Yrs Quit | Short Term (n) | Short Term % |
|---|---|---|---|---|---|---|---|---|---|
| 0 | All Ages | Never Any | 47776 | 54.92 | | | | | |
| 0 | All Ages | CC | 22737 | 26.14 | 0 | 0.00 | | | |
| 0 | All Ages | Ecig | 0 | 0.00 | | | | | |
| 0 | All Ages | Dual | 0 | 0.00 | | | | | |
| 0 | All Ages | Former | 16484 | 18.95 | | | 11.90 | 2815 | 17.08 |

Year of Follow-up: 1990, Age Group: 18-19

| Period | Age Group | Smoking Group | N | % | Ever Quit (N) | Ever Quit % | Mean Yrs Quit | Short Term (n) | Short Term % |
|---|---|---|---|---|---|---|---|---|---|
| 0 | 18-19 | Never Any | 2570 | 78.43 | | | | | |
| 0 | 18-19 | CC | 535 | 16.33 | 0 | 0.00 | | | |
| 0 | 18-19 | Ecig | 0 | 0.00 | | | | | |
| 0 | 18-19 | Dual | 0 | 0.00 | | | | | |
| 0 | 18-19 | Former | 172 | 5.25 | | | 0.20 | 172 | 100.00 |

Year of Follow-up: 1990, Age Group: 20-24

| Period | Age Group | Smoking Group | N | % | Ever Quit (N) | Ever Quit % | Mean Yrs Quit | Short Term (n) | Short Term % |
|---|---|---|---|---|---|---|---|---|---|
| 0 | 20-24 | Never Any | 5354 | 60.89 | | | | | |
| 0 | 20-24 | CC | 2560 | 29.11 | 0 | 0.00 | | | |
| 0 | 20-24 | Ecig | 0 | 0.00 | | | | | |
| 0 | 20-24 | Dual | 0 | 0.00 | | | | | |
| 0 | 20-24 | Former | 879 | 10.00 | | | 1.58 | 420 | 47.78 |

Year of Follow-up: 1990, Age Group: 25-29

| Period | Age Group | Smoking Group | N | % | Ever Quit (N) | Ever Quit % | Mean Yrs Quit | Short Term (n) | Short Term % |
|---|---|---|---|---|---|---|---|---|---|
| 0 | 25-29 | Never Any | 5318 | 52.77 | | | | | |
| 0 | 25-29 | CC | 3335 | 33.09 | 0 | 0.00 | | | |
| 0 | 25-29 | Ecig | 0 | 0.00 | | | | | |
| 0 | 25-29 | Dual | 0 | 0.00 | | | | | |
| 0 | 25-29 | Former | 1425 | 14.14 | | | 3.91 | 404 | 28.35 |

Year of Follow-up: 1990, Age Group: 30-34

| Period | Age Group | Smoking Group | N | % | Ever Quit (N) | Ever Quit % | Mean Yrs Quit | Short Term (n) | Short Term % |
|---|---|---|---|---|---|---|---|---|---|
| 0 | 30-34 | Never Any | 5287 | 50.36 | | | | | |
| 0 | 30-34 | CC | 3326 | 31.68 | 0 | 0.00 | | | |
| 0 | 30-34 | Ecig | 0 | 0.00 | | | | | |
| 0 | 30-34 | Dual | 0 | 0.00 | | | | | |
| 0 | 30-34 | Former | 1886 | 17.96 | | | 4.36 | 566 | 30.01 |

Year of Follow-up: 1990, Age Group: 35-39

| Period | Age Group | Smoking Group | N | % | Ever Quit (N) | Ever Quit % | Mean Yrs Quit | Short Term (n) | Short Term % |
|---|---|---|---|---|---|---|---|---|---|
| 0 | 35-39 | Never Any | 5018 | 52.27 | | | | | |
| 0 | 35-39 | CC | 2653 | 27.64 | 0 | 0.00 | | | |
| 0 | 35-39 | Ecig | 0 | 0.00 | | | | | |
| 0 | 35-39 | Dual | 0 | 0.00 | | | | | |
| 0 | 35-39 | Former | 1929 | 20.09 | | | 7.96 | 324 | 16.80 |

Year of Follow-up: 1990, Age Group: 40-44

| Period | Age Group | Smoking Group | N | % | Ever Quit (N) | Ever Quit % | Mean Yrs Quit | Short Term (n) | Short Term % |
|---|---|---|---|---|---|---|---|---|---|
| 0 | 40-44 | Never Any | 4356 | 51.27 | | | | | |
| 0 | 40-44 | CC | 2397 | 28.21 | 0 | 0.00 | | | |
| 0 | 40-44 | Ecig | 0 | 0.00 | | | | | |
| 0 | 40-44 | Dual | 0 | 0.00 | | | | | |
| 0 | 40-44 | Former | 1744 | 20.52 | | | 7.33 | 339 | 19.44 |

Year of Follow-up: 1990, Age Group: 45-49

| Period | Age Group | Smoking Group | N | % | Ever Quit (N) | Ever Quit % | Mean Yrs Quit | Short Term (n) | Short Term % |
|---|---|---|---|---|---|---|---|---|---|
| 0 | 45-49 | Never Any | 3116 | 47.67 | | | | | |
| 0 | 45-49 | CC | 1875 | 28.69 | 0 | 0.00 | | | |
| 0 | 45-49 | Ecig | 0 | 0.00 | | | | | |
| 0 | 45-49 | Dual | 0 | 0.00 | | | | | |
| 0 | 45-49 | Former | 1545 | 23.64 | | | 12.55 | 194 | 12.56 |

Year of Follow-up: 1990, Age Group: 50-54

| Period | Age Group | Smoking Group | N | % | Ever Quit (N) | Ever Quit % | Mean Yrs Quit | Short Term (n) | Short Term % |
|---|---|---|---|---|---|---|---|---|---|
| 0 | 50-54 | Never Any | 2684 | 48.50 | | | | | |
| 0 | 50-54 | CC | 1572 | 28.41 | 0 | 0.00 | | | |

# Results for PHIM for Run 4 (Sensitivity_Analysis_1.RTF)

| | | | | | | | | | |
|---|---|---|---|---|---|---|---|---|---|
| 0 | 50-54 | Ecig | 0 | 0.00 | | | | | |
| 0 | 50-54 | Dual | 0 | 0.00 | | | | | |
| 0 | 50-54 | Former | 1278 | 23.09 | | | 13.28 | 126 | 9.86 |

Year of Follow-up: 1990, Age Group: 55-59

| Period | Age Group | Smoking Group | N | % | Ever Quit (N) | Ever Quit % | Mean Yrs Quit | Short Term (n) | Short Term % |
|---|---|---|---|---|---|---|---|---|---|
| 0 | 55-59 | Never Any | 2732 | 51.90 | | | | | |
| 0 | 55-59 | CC | 1295 | 24.60 | 0 | 0.00 | | | |
| 0 | 55-59 | Ecig | 0 | 0.00 | | | | | |
| 0 | 55-59 | Dual | 0 | 0.00 | | | | | |
| 0 | 55-59 | Former | 1237 | 23.50 | | | 17.26 | 90 | 7.28 |

Year of Follow-up: 1990, Age Group: 60-64

| Period | Age Group | Smoking Group | N | % | Ever Quit (N) | Ever Quit % | Mean Yrs Quit | Short Term (n) | Short Term % |
|---|---|---|---|---|---|---|---|---|---|
| 0 | 60-64 | Never Any | 3078 | 55.72 | | | | | |
| 0 | 60-64 | CC | 1249 | 22.61 | 0 | 0.00 | | | |
| 0 | 60-64 | Ecig | 0 | 0.00 | | | | | |
| 0 | 60-64 | Dual | 0 | 0.00 | | | | | |
| 0 | 60-64 | Former | 1197 | 21.67 | | | 18.28 | 61 | 5.10 |

Year of Follow-up: 1990, Age Group: 65-69

| Period | Age Group | Smoking Group | N | % | Ever Quit (N) | Ever Quit % | Mean Yrs Quit | Short Term (n) | Short Term % |
|---|---|---|---|---|---|---|---|---|---|
| 0 | 65-69 | Never Any | 3042 | 57.36 | | | | | |
| 0 | 65-69 | CC | 976 | 18.40 | 0 | 0.00 | | | |
| 0 | 65-69 | Ecig | 0 | 0.00 | | | | | |
| 0 | 65-69 | Dual | 0 | 0.00 | | | | | |
| 0 | 65-69 | Former | 1285 | 24.23 | | | 20.65 | 68 | 5.29 |

Year of Follow-up: 1990, Age Group: 70-74

| Period | Age Group | Smoking Group | N | % | Ever Quit (N) | Ever Quit % | Mean Yrs Quit | Short Term (n) | Short Term % |
|---|---|---|---|---|---|---|---|---|---|
| 0 | 70-74 | Never Any | 2738 | 61.05 | | | | | |
| 0 | 70-74 | CC | 610 | 13.60 | 0 | 0.00 | | | |
| 0 | 70-74 | Ecig | 0 | 0.00 | | | | | |
| 0 | 70-74 | Dual | 0 | 0.00 | | | | | |
| 0 | 70-74 | Former | 1137 | 25.35 | | | 24.48 | 27 | 2.37 |

Year of Follow-up: 1990, Age Group: 75-79

| Period | Age Group | Smoking Group | N | % | Ever Quit (N) | Ever Quit % | Mean Yrs Quit | Short Term (n) | Short Term % |
|---|---|---|---|---|---|---|---|---|---|
| 0 | 75-79 | Never Any | 2483 | 68.84 | | | | | |
| 0 | 75-79 | CC | 354 | 9.81 | 0 | 0.00 | | | |
| 0 | 75-79 | Ecig | 0 | 0.00 | | | | | |
| 0 | 75-79 | Dual | 0 | 0.00 | | | | | |
| 0 | 75-79 | Former | 770 | 21.35 | | | 24.48 | 24 | 3.12 |

Year of Follow-up: 1995, Age Group: All Ages

| Period | Age Group | Smoking Group | N | % | Ever Quit (N) | Ever Quit % | Mean Yrs Quit | Short Term (n) | Short Term % |
|---|---|---|---|---|---|---|---|---|---|
| 5 | All Ages | Never Any | 50887 | 55.53 | | | | | |
| 5 | All Ages | CC | 10659 | 11.63 | 0 | 0.00 | | | |
| 5 | All Ages | Ecig | 11697 | 12.76 | | | | | |
| 5 | All Ages | Dual | 0 | 0.00 | | | | | |
| 5 | All Ages | Former | 18396 | 20.07 | | | 14.19 | 1129 | 6.14 |

Year of Follow-up: 1995, Age Group: 18-19

| Period | Age Group | Smoking Group | N | % | Ever Quit (N) | Ever Quit % | Mean Yrs Quit | Short Term (n) | Short Term % |
|---|---|---|---|---|---|---|---|---|---|
| 5 | 18-19 | Never Any | 2434 | 76.64 | | | | | |
| 5 | 18-19 | CC | 233 | 7.34 | 0 | 0.00 | | | |
| 5 | 18-19 | Ecig | 427 | 13.44 | | | | | |
| 5 | 18-19 | Dual | 0 | 0.00 | | | | | |
| 5 | 18-19 | Former | 82 | 2.58 | | | 1.15 | 52 | 63.41 |

Year of Follow-up: 1995, Age Group: 20-24

| Period | Age Group | Smoking Group | N | % | Ever Quit (N) | Ever Quit % | Mean Yrs Quit | Short Term (n) | Short Term % |
|---|---|---|---|---|---|---|---|---|---|
| 5 | 20-24 | Never Any | 5794 | 69.39 | | | | | |
| 5 | 20-24 | CC | 862 | 10.32 | 0 | 0.00 | | | |
| 5 | 20-24 | Ecig | 1250 | 14.97 | | | | | |
| 5 | 20-24 | Dual | 0 | 0.00 | | | | | |
| 5 | 20-24 | Former | 444 | 5.32 | | | 3.23 | 106 | 23.87 |

Year of Follow-up: 1995, Age Group: 25-29

| Period | Age Group | Smoking Group | N | % | Ever Quit (N) | Ever Quit % | Mean Yrs Quit | Short Term (n) | Short Term % |
|---|---|---|---|---|---|---|---|---|---|
| 5 | 25-29 | Never Any | 5313 | 60.42 | | | | | |
| 5 | 25-29 | CC | 1062 | 12.08 | 0 | 0.00 | | | |
| 5 | 25-29 | Ecig | 1395 | 15.86 | | | | | |
| 5 | 25-29 | Dual | 0 | 0.00 | | | | | |
| 5 | 25-29 | Former | 1023 | 11.63 | | | 5.94 | 54 | 5.28 |

Year of Follow-up: 1995, Age Group: 30-34

| Period | Age Group | Smoking Group | N | % | Ever Quit (N) | Ever Quit % | Mean Yrs Quit | Short Term (n) | Short Term % |
|---|---|---|---|---|---|---|---|---|---|

# Results for PHIM for Run 4 (Sensitivity_Analysis_1.RTF)

| | | | | | | | | | |
|---|---|---|---|---|---|---|---|---|---|
| 5 | 30-34 | Never Any | 5304 | 52.63 | | | | | |
| 5 | 30-34 | CC | 1373 | 13.62 | 0 | 0.00 | | | |
| 5 | 30-34 | Ecig | 1802 | 17.88 | | | | | |
| 5 | 30-34 | Dual | 0 | 0.00 | | | | | |
| 5 | 30-34 | Former | 1599 | 15.87 | | | 8.16 | 67 | 4.19 |
| Year of Follow-up: 1995, Age Group: 35-39 | | | | | | | | | |
| Period | Age Group | Smoking Group | N | % | Ever Quit (N) | Ever Quit % | Mean Yrs Quit | Short Term (n) | Short Term % |
| 5 | 35-39 | Never Any | 5278 | 50.27 | | | | | |
| 5 | 35-39 | CC | 1410 | 13.43 | 0 | 0.00 | | | |
| 5 | 35-39 | Ecig | 1746 | 16.63 | | | | | |
| 5 | 35-39 | Dual | 0 | 0.00 | | | | | |
| 5 | 35-39 | Former | 2065 | 19.67 | | | 8.75 | 62 | 3.00 |
| Year of Follow-up: 1995, Age Group: 40-44 | | | | | | | | | |
| Period | Age Group | Smoking Group | N | % | Ever Quit (N) | Ever Quit % | Mean Yrs Quit | Short Term (n) | Short Term % |
| 5 | 40-44 | Never Any | 5018 | 52.27 | | | | | |
| 5 | 40-44 | CC | 1135 | 11.82 | 0 | 0.00 | | | |
| 5 | 40-44 | Ecig | 1356 | 14.13 | | | | | |
| 5 | 40-44 | Dual | 0 | 0.00 | | | | | |
| 5 | 40-44 | Former | 2091 | 21.78 | | | 12.09 | 81 | 3.87 |
| Year of Follow-up: 1995, Age Group: 45-49 | | | | | | | | | |
| Period | Age Group | Smoking Group | N | % | Ever Quit (N) | Ever Quit % | Mean Yrs Quit | Short Term (n) | Short Term % |
| 5 | 45-49 | Never Any | 4356 | 51.27 | | | | | |
| 5 | 45-49 | CC | 974 | 11.46 | 0 | 0.00 | | | |
| 5 | 45-49 | Ecig | 1161 | 13.66 | | | | | |
| 5 | 45-49 | Dual | 0 | 0.00 | | | | | |
| 5 | 45-49 | Former | 2006 | 23.61 | | | 10.93 | 133 | 6.63 |
| Year of Follow-up: 1995, Age Group: 50-54 | | | | | | | | | |
| Period | Age Group | Smoking Group | N | % | Ever Quit (N) | Ever Quit % | Mean Yrs Quit | Short Term (n) | Short Term % |
| 5 | 50-54 | Never Any | 3116 | 47.67 | | | | | |
| 5 | 50-54 | CC | 739 | 11.31 | 0 | 0.00 | | | |
| 5 | 50-54 | Ecig | 826 | 12.64 | | | | | |
| 5 | 50-54 | Dual | 0 | 0.00 | | | | | |
| 5 | 50-54 | Former | 1855 | 28.38 | | | 14.92 | 140 | 7.55 |
| Year of Follow-up: 1995, Age Group: 55-59 | | | | | | | | | |
| Period | Age Group | Smoking Group | N | % | Ever Quit (N) | Ever Quit % | Mean Yrs Quit | Short Term (n) | Short Term % |
| 5 | 55-59 | Never Any | 2684 | 48.50 | | | | | |
| 5 | 55-59 | CC | 722 | 13.05 | 0 | 0.00 | | | |
| 5 | 55-59 | Ecig | 627 | 11.33 | | | | | |
| 5 | 55-59 | Dual | 0 | 0.00 | | | | | |
| 5 | 55-59 | Former | 1501 | 27.12 | | | 15.84 | 93 | 6.20 |
| Year of Follow-up: 1995, Age Group: 60-64 | | | | | | | | | |
| Period | Age Group | Smoking Group | N | % | Ever Quit (N) | Ever Quit % | Mean Yrs Quit | Short Term (n) | Short Term % |
| 5 | 60-64 | Never Any | 2732 | 51.90 | | | | | |
| 5 | 60-64 | CC | 633 | 12.03 | 0 | 0.00 | | | |
| 5 | 60-64 | Ecig | 444 | 8.43 | | | | | |
| 5 | 60-64 | Dual | 0 | 0.00 | | | | | |
| 5 | 60-64 | Former | 1455 | 27.64 | | | 19.22 | 87 | 5.98 |
| Year of Follow-up: 1995, Age Group: 65-69 | | | | | | | | | |
| Period | Age Group | Smoking Group | N | % | Ever Quit (N) | Ever Quit % | Mean Yrs Quit | Short Term (n) | Short Term % |
| 5 | 65-69 | Never Any | 3078 | 55.72 | | | | | |
| 5 | 65-69 | CC | 602 | 10.90 | 0 | 0.00 | | | |
| 5 | 65-69 | Ecig | 374 | 6.77 | | | | | |
| 5 | 65-69 | Dual | 0 | 0.00 | | | | | |
| 5 | 65-69 | Former | 1470 | 26.61 | | | 19.34 | 107 | 7.28 |
| Year of Follow-up: 1995, Age Group: 70-74 | | | | | | | | | |
| Period | Age Group | Smoking Group | N | % | Ever Quit (N) | Ever Quit % | Mean Yrs Quit | Short Term (n) | Short Term % |
| 5 | 70-74 | Never Any | 3042 | 57.36 | | | | | |
| 5 | 70-74 | CC | 577 | 10.88 | 0 | 0.00 | | | |
| 5 | 70-74 | Ecig | 188 | 3.55 | | | | | |
| 5 | 70-74 | Dual | 0 | 0.00 | | | | | |
| 5 | 70-74 | Former | 1496 | 28.21 | | | 22.32 | 77 | 5.15 |
| Year of Follow-up: 1995, Age Group: 75-79 | | | | | | | | | |
| Period | Age Group | Smoking Group | N | % | Ever Quit (N) | Ever Quit % | Mean Yrs Quit | Short Term (n) | Short Term % |
| 5 | 75-79 | Never Any | 2738 | 61.05 | | | | | |
| 5 | 75-79 | CC | 337 | 7.51 | 0 | 0.00 | | | |
| 5 | 75-79 | Ecig | 101 | 2.25 | | | | | |
| 5 | 75-79 | Dual | 0 | 0.00 | | | | | |
| 5 | 75-79 | Former | 1309 | 29.19 | | | 25.87 | 70 | 5.35 |

Year of Follow-up: 2000, Age Group: All Ages

# Results for PHIM for Run 4 (Sensitivity_Analysis_1.RTF)

| Period | Age Group | Smoking Group | N | % | Ever Quit (N) | Ever Quit % | Mean Yrs Quit | Short Term (n) | Short Term % |
|---|---|---|---|---|---|---|---|---|---|
| 10 | All Ages | Never Any | 50423 | 54.86 | | | | | |
| 10 | All Ages | CC | 4595 | 5.00 | 0 | 0.00 | | | |
| 10 | All Ages | Ecig | 15644 | 17.02 | | | | | |
| 10 | All Ages | Dual | 0 | 0.00 | | | | | |
| 10 | All Ages | Former | 21246 | 23.12 | | | 15.11 | 1717 | 8.08 |

Year of Follow-up: 2000, Age Group: 18-19

| Period | Age Group | Smoking Group | N | % | Ever Quit (N) | Ever Quit % | Mean Yrs Quit | Short Term (n) | Short Term % |
|---|---|---|---|---|---|---|---|---|---|
| 10 | 18-19 | Never Any | 0 | . | | | | | |
| 10 | 18-19 | CC | 0 | . | 0 | . | | | |
| 10 | 18-19 | Ecig | 0 | . | | | | | |
| 10 | 18-19 | Dual | 0 | . | | | | | |
| 10 | 18-19 | Former | 0 | . | | | . | 0 | . |

Year of Follow-up: 2000, Age Group: 20-24

| Period | Age Group | Smoking Group | N | % | Ever Quit (N) | Ever Quit % | Mean Yrs Quit | Short Term (n) | Short Term % |
|---|---|---|---|---|---|---|---|---|---|
| 10 | 20-24 | Never Any | 5128 | 64.67 | | | | | |
| 10 | 20-24 | CC | 370 | 4.67 | 0 | 0.00 | | | |
| 10 | 20-24 | Ecig | 1780 | 22.45 | | | | | |
| 10 | 20-24 | Dual | 0 | 0.00 | | | | | |
| 10 | 20-24 | Former | 652 | 8.22 | | | 3.55 | 169 | 25.92 |

Year of Follow-up: 2000, Age Group: 25-29

| Period | Age Group | Smoking Group | N | % | Ever Quit (N) | Ever Quit % | Mean Yrs Quit | Short Term (n) | Short Term % |
|---|---|---|---|---|---|---|---|---|---|
| 10 | 25-29 | Never Any | 5493 | 65.78 | | | | | |
| 10 | 25-29 | CC | 400 | 4.79 | 0 | 0.00 | | | |
| 10 | 25-29 | Ecig | 1690 | 20.24 | | | | | |
| 10 | 25-29 | Dual | 0 | 0.00 | | | | | |
| 10 | 25-29 | Former | 767 | 9.19 | | | 5.59 | 126 | 16.43 |

Year of Follow-up: 2000, Age Group: 30-34

| Period | Age Group | Smoking Group | N | % | Ever Quit (N) | Ever Quit % | Mean Yrs Quit | Short Term (n) | Short Term % |
|---|---|---|---|---|---|---|---|---|---|
| 10 | 30-34 | Never Any | 5214 | 59.30 | | | | | |
| 10 | 30-34 | CC | 417 | 4.74 | 0 | 0.00 | | | |
| 10 | 30-34 | Ecig | 1908 | 21.70 | | | | | |
| 10 | 30-34 | Dual | 0 | 0.00 | | | | | |
| 10 | 30-34 | Former | 1254 | 14.26 | | | 9.28 | 91 | 7.26 |

Year of Follow-up: 2000, Age Group: 35-39

| Period | Age Group | Smoking Group | N | % | Ever Quit (N) | Ever Quit % | Mean Yrs Quit | Short Term (n) | Short Term % |
|---|---|---|---|---|---|---|---|---|---|
| 10 | 35-39 | Never Any | 5284 | 52.43 | | | | | |
| 10 | 35-39 | CC | 561 | 5.57 | 0 | 0.00 | | | |
| 10 | 35-39 | Ecig | 2413 | 23.94 | | | | | |
| 10 | 35-39 | Dual | 0 | 0.00 | | | | | |
| 10 | 35-39 | Former | 1820 | 18.06 | | | 11.77 | 105 | 5.77 |

Year of Follow-up: 2000, Age Group: 40-44

| Period | Age Group | Smoking Group | N | % | Ever Quit (N) | Ever Quit % | Mean Yrs Quit | Short Term (n) | Short Term % |
|---|---|---|---|---|---|---|---|---|---|
| 10 | 40-44 | Never Any | 5278 | 50.27 | | | | | |
| 10 | 40-44 | CC | 584 | 5.56 | 0 | 0.00 | | | |
| 10 | 40-44 | Ecig | 2292 | 21.83 | | | | | |
| 10 | 40-44 | Dual | 0 | 0.00 | | | | | |
| 10 | 40-44 | Former | 2345 | 22.34 | | | 12.30 | 143 | 6.10 |

Year of Follow-up: 2000, Age Group: 45-49

| Period | Age Group | Smoking Group | N | % | Ever Quit (N) | Ever Quit % | Mean Yrs Quit | Short Term (n) | Short Term % |
|---|---|---|---|---|---|---|---|---|---|
| 10 | 45-49 | Never Any | 5018 | 52.27 | | | | | |
| 10 | 45-49 | CC | 446 | 4.65 | 0 | 0.00 | | | |
| 10 | 45-49 | Ecig | 1673 | 17.43 | | | | | |
| 10 | 45-49 | Dual | 0 | 0.00 | | | | | |
| 10 | 45-49 | Former | 2463 | 25.66 | | | 14.76 | 181 | 7.35 |

Year of Follow-up: 2000, Age Group: 50-54

| Period | Age Group | Smoking Group | N | % | Ever Quit (N) | Ever Quit % | Mean Yrs Quit | Short Term (n) | Short Term % |
|---|---|---|---|---|---|---|---|---|---|
| 10 | 50-54 | Never Any | 4356 | 51.27 | | | | | |
| 10 | 50-54 | CC | 378 | 4.45 | 0 | 0.00 | | | |
| 10 | 50-54 | Ecig | 1299 | 15.29 | | | | | |
| 10 | 50-54 | Dual | 0 | 0.00 | | | | | |
| 10 | 50-54 | Former | 2464 | 29.00 | | | 13.32 | 188 | 7.63 |

Year of Follow-up: 2000, Age Group: 55-59

| Period | Age Group | Smoking Group | N | % | Ever Quit (N) | Ever Quit % | Mean Yrs Quit | Short Term (n) | Short Term % |
|---|---|---|---|---|---|---|---|---|---|
| 10 | 55-59 | Never Any | 3116 | 47.67 | | | | | |
| 10 | 55-59 | CC | 283 | 4.33 | 0 | 0.00 | | | |
| 10 | 55-59 | Ecig | 912 | 13.95 | | | | | |
| 10 | 55-59 | Dual | 0 | 0.00 | | | | | |

# Results for PHIM for Run 4 (Sensitivity_Analysis_1.RTF)

| | | | | | | | | | |
|---|---|---|---|---|---|---|---|---|---|
| 10 | 55-59 | Former | 2225 | 34.04 | | | 16.92 | 157 | 7.06 |

Year of Follow-up: 2000, Age Group: 60-64

| Period | Age Group | Smoking Group | N | % | Ever Quit (N) | Ever Quit % | Mean Yrs Quit | Short Term (n) | Short Term % |
|---|---|---|---|---|---|---|---|---|---|
| 10 | 60-64 | Never Any | 2684 | 48.50 | | | | | |
| 10 | 60-64 | CC | 334 | 6.04 | 0 | 0.00 | | | |
| 10 | 60-64 | Ecig | 663 | 11.98 | | | | | |
| 10 | 60-64 | Dual | 0 | 0.00 | | | | | |
| 10 | 60-64 | Former | 1853 | 33.48 | | | 17.23 | 149 | 8.04 |

Year of Follow-up: 2000, Age Group: 65-69

| Period | Age Group | Smoking Group | N | % | Ever Quit (N) | Ever Quit % | Mean Yrs Quit | Short Term (n) | Short Term % |
|---|---|---|---|---|---|---|---|---|---|
| 10 | 65-69 | Never Any | 2732 | 51.90 | | | | | |
| 10 | 65-69 | CC | 280 | 5.32 | 0 | 0.00 | | | |
| 10 | 65-69 | Ecig | 443 | 8.42 | | | | | |
| 10 | 65-69 | Dual | 0 | 0.00 | | | | | |
| 10 | 65-69 | Former | 1809 | 34.37 | | | 19.85 | 146 | 8.07 |

Year of Follow-up: 2000, Age Group: 70-74

| Period | Age Group | Smoking Group | N | % | Ever Quit (N) | Ever Quit % | Mean Yrs Quit | Short Term (n) | Short Term % |
|---|---|---|---|---|---|---|---|---|---|
| 10 | 70-74 | Never Any | 3078 | 55.72 | | | | | |
| 10 | 70-74 | CC | 274 | 4.96 | 0 | 0.00 | | | |
| 10 | 70-74 | Ecig | 368 | 6.66 | | | | | |
| 10 | 70-74 | Dual | 0 | 0.00 | | | | | |
| 10 | 70-74 | Former | 1804 | 32.66 | | | 20.18 | 154 | 8.54 |

Year of Follow-up: 2000, Age Group: 75-79

| Period | Age Group | Smoking Group | N | % | Ever Quit (N) | Ever Quit % | Mean Yrs Quit | Short Term (n) | Short Term % |
|---|---|---|---|---|---|---|---|---|---|
| 10 | 75-79 | Never Any | 3042 | 57.36 | | | | | |
| 10 | 75-79 | CC | 268 | 5.05 | 0 | 0.00 | | | |
| 10 | 75-79 | Ecig | 203 | 3.83 | | | | | |
| 10 | 75-79 | Dual | 0 | 0.00 | | | | | |
| 10 | 75-79 | Former | 1790 | 33.75 | | | 23.17 | 108 | 6.03 |

Year of Follow-up: 2005, Age Group: All Ages

| Period | Age Group | Smoking Group | N | % | Ever Quit (N) | Ever Quit % | Mean Yrs Quit | Short Term (n) | Short Term % |
|---|---|---|---|---|---|---|---|---|---|
| 15 | All Ages | Never Any | 47002 | 54.27 | | | | | |
| 15 | All Ages | CC | 1890 | 2.18 | 0 | 0.00 | | | |
| 15 | All Ages | Ecig | 15617 | 18.03 | | | | | |
| 15 | All Ages | Dual | 0 | 0.00 | | | | | |
| 15 | All Ages | Former | 22096 | 25.51 | | | 17.32 | 941 | 4.26 |

Year of Follow-up: 2005, Age Group: 18-19

| Period | Age Group | Smoking Group | N | % | Ever Quit (N) | Ever Quit % | Mean Yrs Quit | Short Term (n) | Short Term % |
|---|---|---|---|---|---|---|---|---|---|
| 15 | 18-19 | Never Any | 0 | . | | | | | |
| 15 | 18-19 | CC | 0 | . | 0 | . | | | |
| 15 | 18-19 | Ecig | 0 | . | | | | | |
| 15 | 18-19 | Dual | 0 | . | | | | | |
| 15 | 18-19 | Former | 0 | . | | | . | 0 | . |

Year of Follow-up: 2005, Age Group: 20-24

| Period | Age Group | Smoking Group | N | % | Ever Quit (N) | Ever Quit % | Mean Yrs Quit | Short Term (n) | Short Term % |
|---|---|---|---|---|---|---|---|---|---|
| 15 | 20-24 | Never Any | 0 | . | | | | | |
| 15 | 20-24 | CC | 0 | . | 0 | . | | | |
| 15 | 20-24 | Ecig | 0 | . | | | | | |
| 15 | 20-24 | Dual | 0 | . | | | | | |
| 15 | 20-24 | Former | 0 | . | | | . | 0 | . |

Year of Follow-up: 2005, Age Group: 25-29

| Period | Age Group | Smoking Group | N | % | Ever Quit (N) | Ever Quit % | Mean Yrs Quit | Short Term (n) | Short Term % |
|---|---|---|---|---|---|---|---|---|---|
| 15 | 25-29 | Never Any | 4860 | 61.29 | | | | | |
| 15 | 25-29 | CC | 166 | 2.09 | 0 | 0.00 | | | |
| 15 | 25-29 | Ecig | 1941 | 24.48 | | | | | |
| 15 | 25-29 | Dual | 0 | 0.00 | | | | | |
| 15 | 25-29 | Former | 963 | 12.14 | | | 6.57 | 96 | 9.97 |

Year of Follow-up: 2005, Age Group: 30-34

| Period | Age Group | Smoking Group | N | % | Ever Quit (N) | Ever Quit % | Mean Yrs Quit | Short Term (n) | Short Term % |
|---|---|---|---|---|---|---|---|---|---|
| 15 | 30-34 | Never Any | 5407 | 64.75 | | | | | |
| 15 | 30-34 | CC | 149 | 1.78 | 0 | 0.00 | | | |
| 15 | 30-34 | Ecig | 1822 | 21.82 | | | | | |
| 15 | 30-34 | Dual | 0 | 0.00 | | | | | |
| 15 | 30-34 | Former | 972 | 11.64 | | | 8.77 | 80 | 8.23 |

Year of Follow-up: 2005, Age Group: 35-39

| Period | Age Group | Smoking Group | N | % | Ever Quit (N) | Ever Quit % | Mean Yrs Quit | Short Term (n) | Short Term % |
|---|---|---|---|---|---|---|---|---|---|
| 15 | 35-39 | Never Any | 5189 | 59.01 | | | | | |
| 15 | 35-39 | CC | 167 | 1.90 | 0 | 0.00 | | | |

# Results for PHIM for Run 4 (Sensitivity_Analysis_1.RTF)

| | | | | | | | | | |
|---|---|---|---|---|---|---|---|---|---|
| 15 | 35-39 | Ecig | 2002 | 22.77 | | | | | |
| 15 | 35-39 | Dual | 0 | 0.00 | | | | | |
| 15 | 35-39 | Former | 1435 | 16.32 | | | 12.75 | 66 | 4.60 |

Year of Follow-up: 2005, Age Group: 40-44

| Period | Age Group | Smoking Group | N | % | Ever Quit (N) | Ever Quit % | Mean Yrs Quit | Short Term (n) | Short Term % |
|---|---|---|---|---|---|---|---|---|---|
| 15 | 40-44 | Never Any | 5284 | 52.43 | | | | | |
| 15 | 40-44 | CC | 238 | 2.36 | 0 | 0.00 | | | |
| 15 | 40-44 | Ecig | 2500 | 24.81 | | | | | |
| 15 | 40-44 | Dual | 0 | 0.00 | | | | | |
| 15 | 40-44 | Former | 2056 | 20.40 | | | 15.08 | 101 | 4.91 |

Year of Follow-up: 2005, Age Group: 45-49

| Period | Age Group | Smoking Group | N | % | Ever Quit (N) | Ever Quit % | Mean Yrs Quit | Short Term (n) | Short Term % |
|---|---|---|---|---|---|---|---|---|---|
| 15 | 45-49 | Never Any | 5278 | 50.27 | | | | | |
| 15 | 45-49 | CC | 237 | 2.26 | 0 | 0.00 | | | |
| 15 | 45-49 | Ecig | 2318 | 22.08 | | | | | |
| 15 | 45-49 | Dual | 0 | 0.00 | | | | | |
| 15 | 45-49 | Former | 2666 | 25.39 | | | 15.45 | 135 | 5.06 |

Year of Follow-up: 2005, Age Group: 50-54

| Period | Age Group | Smoking Group | N | % | Ever Quit (N) | Ever Quit % | Mean Yrs Quit | Short Term (n) | Short Term % |
|---|---|---|---|---|---|---|---|---|---|
| 15 | 50-54 | Never Any | 5018 | 52.27 | | | | | |
| 15 | 50-54 | CC | 191 | 1.99 | 0 | 0.00 | | | |
| 15 | 50-54 | Ecig | 1622 | 16.90 | | | | | |
| 15 | 50-54 | Dual | 0 | 0.00 | | | | | |
| 15 | 50-54 | Former | 2769 | 28.84 | | | 17.84 | 99 | 3.58 |

Year of Follow-up: 2005, Age Group: 55-59

| Period | Age Group | Smoking Group | N | % | Ever Quit (N) | Ever Quit % | Mean Yrs Quit | Short Term (n) | Short Term % |
|---|---|---|---|---|---|---|---|---|---|
| 15 | 55-59 | Never Any | 4356 | 51.27 | | | | | |
| 15 | 55-59 | CC | 166 | 1.95 | 0 | 0.00 | | | |
| 15 | 55-59 | Ecig | 1281 | 15.08 | | | | | |
| 15 | 55-59 | Dual | 0 | 0.00 | | | | | |
| 15 | 55-59 | Former | 2694 | 31.71 | | | 16.95 | 78 | 2.90 |

Year of Follow-up: 2005, Age Group: 60-64

| Period | Age Group | Smoking Group | N | % | Ever Quit (N) | Ever Quit % | Mean Yrs Quit | Short Term (n) | Short Term % |
|---|---|---|---|---|---|---|---|---|---|
| 15 | 60-64 | Never Any | 3116 | 47.67 | | | | | |
| 15 | 60-64 | CC | 131 | 2.00 | 0 | 0.00 | | | |
| 15 | 60-64 | Ecig | 852 | 13.04 | | | | | |
| 15 | 60-64 | Dual | 0 | 0.00 | | | | | |
| 15 | 60-64 | Former | 2437 | 37.29 | | | 20.18 | 93 | 3.82 |

Year of Follow-up: 2005, Age Group: 65-69

| Period | Age Group | Smoking Group | N | % | Ever Quit (N) | Ever Quit % | Mean Yrs Quit | Short Term (n) | Short Term % |
|---|---|---|---|---|---|---|---|---|---|
| 15 | 65-69 | Never Any | 2684 | 48.50 | | | | | |
| 15 | 65-69 | CC | 160 | 2.89 | 0 | 0.00 | | | |
| 15 | 65-69 | Ecig | 591 | 10.68 | | | | | |
| 15 | 65-69 | Dual | 0 | 0.00 | | | | | |
| 15 | 65-69 | Former | 2099 | 37.93 | | | 19.92 | 68 | 3.24 |

Year of Follow-up: 2005, Age Group: 70-74

| Period | Age Group | Smoking Group | N | % | Ever Quit (N) | Ever Quit % | Mean Yrs Quit | Short Term (n) | Short Term % |
|---|---|---|---|---|---|---|---|---|---|
| 15 | 70-74 | Never Any | 2732 | 51.90 | | | | | |
| 15 | 70-74 | CC | 139 | 2.64 | 0 | 0.00 | | | |
| 15 | 70-74 | Ecig | 391 | 7.43 | | | | | |
| 15 | 70-74 | Dual | 0 | 0.00 | | | | | |
| 15 | 70-74 | Former | 2002 | 38.03 | | | 22.67 | 69 | 3.45 |

Year of Follow-up: 2005, Age Group: 75-79

| Period | Age Group | Smoking Group | N | % | Ever Quit (N) | Ever Quit % | Mean Yrs Quit | Short Term (n) | Short Term % |
|---|---|---|---|---|---|---|---|---|---|
| 15 | 75-79 | Never Any | 3078 | 55.72 | | | | | |
| 15 | 75-79 | CC | 146 | 2.64 | 0 | 0.00 | | | |
| 15 | 75-79 | Ecig | 297 | 5.38 | | | | | |
| 15 | 75-79 | Dual | 0 | 0.00 | | | | | |
| 15 | 75-79 | Former | 2003 | 36.26 | | | 22.91 | 56 | 2.80 |

Year of Follow-up: 2010, Age Group: All Ages

| Period | Age Group | Smoking Group | N | % | Ever Quit (N) | Ever Quit % | Mean Yrs Quit | Short Term (n) | Short Term % |
|---|---|---|---|---|---|---|---|---|---|
| 20 | All Ages | Never Any | 43759 | 53.97 | | | | | |
| 20 | All Ages | CC | 765 | 0.94 | 0 | 0.00 | | | |
| 20 | All Ages | Ecig | 13785 | 17.00 | | | | | |
| 20 | All Ages | Dual | 0 | 0.00 | | | | | |
| 20 | All Ages | Former | 22772 | 28.09 | | | 19.42 | 1117 | 4.91 |

Year of Follow-up: 2010, Age Group: 18-19

| Period | Age Group | Smoking Group | N | % | Ever Quit (N) | Ever Quit % | Mean Yrs Quit | Short Term (n) | Short Term % |
|---|---|---|---|---|---|---|---|---|---|

# Results for PHIM for Run 4 (Sensitivity_Analysis_1.RTF)

| | | | | | | | | | |
|---|---|---|---|---|---|---|---|---|---|
| 20 | 18-19 | Never Any | 0 | . | | | | | |
| 20 | 18-19 | CC | 0 | . | 0 | . | | | |
| 20 | 18-19 | Ecig | 0 | . | | | | | |
| 20 | 18-19 | Dual | 0 | . | | | | | |
| 20 | 18-19 | Former | 0 | . | | | . | 0 | . |

Year of Follow-up: 2010, Age Group: 20-24

| Period | Age Group | Smoking Group | N | % | Ever Quit (N) | Ever Quit % | Mean Yrs Quit | Short Term (n) | Short Term % |
|---|---|---|---|---|---|---|---|---|---|
| 20 | 20-24 | Never Any | 0 | . | | | | | |
| 20 | 20-24 | CC | 0 | . | 0 | . | | | |
| 20 | 20-24 | Ecig | 0 | . | | | | | |
| 20 | 20-24 | Dual | 0 | . | | | | | |
| 20 | 20-24 | Former | 0 | . | | | . | 0 | . |

Year of Follow-up: 2010, Age Group: 25-29

| Period | Age Group | Smoking Group | N | % | Ever Quit (N) | Ever Quit % | Mean Yrs Quit | Short Term (n) | Short Term % |
|---|---|---|---|---|---|---|---|---|---|
| 20 | 25-29 | Never Any | 0 | . | | | | | |
| 20 | 25-29 | CC | 0 | . | 0 | . | | | |
| 20 | 25-29 | Ecig | 0 | . | | | | | |
| 20 | 25-29 | Dual | 0 | . | | | | | |
| 20 | 25-29 | Former | 0 | . | | | . | 0 | . |

Year of Follow-up: 2010, Age Group: 30-34

| Period | Age Group | Smoking Group | N | % | Ever Quit (N) | Ever Quit % | Mean Yrs Quit | Short Term (n) | Short Term % |
|---|---|---|---|---|---|---|---|---|---|
| 20 | 30-34 | Never Any | 4743 | 59.81 | | | | | |
| 20 | 30-34 | CC | 65 | 0.82 | 0 | 0.00 | | | |
| 20 | 30-34 | Ecig | 1881 | 23.72 | | | | | |
| 20 | 30-34 | Dual | 0 | 0.00 | | | | | |
| 20 | 30-34 | Former | 1241 | 15.65 | | | 9.36 | 130 | 10.48 |

Year of Follow-up: 2010, Age Group: 35-39

| Period | Age Group | Smoking Group | N | % | Ever Quit (N) | Ever Quit % | Mean Yrs Quit | Short Term (n) | Short Term % |
|---|---|---|---|---|---|---|---|---|---|
| 20 | 35-39 | Never Any | 5359 | 64.18 | | | | | |
| 20 | 35-39 | CC | 58 | 0.69 | 0 | 0.00 | | | |
| 20 | 35-39 | Ecig | 1717 | 20.56 | | | | | |
| 20 | 35-39 | Dual | 0 | 0.00 | | | | | |
| 20 | 35-39 | Former | 1216 | 14.56 | | | 11.38 | 98 | 8.06 |

Year of Follow-up: 2010, Age Group: 40-44

| Period | Age Group | Smoking Group | N | % | Ever Quit (N) | Ever Quit % | Mean Yrs Quit | Short Term (n) | Short Term % |
|---|---|---|---|---|---|---|---|---|---|
| 20 | 40-44 | Never Any | 5189 | 59.01 | | | | | |
| 20 | 40-44 | CC | 64 | 0.73 | 0 | 0.00 | | | |
| 20 | 40-44 | Ecig | 1849 | 21.03 | | | | | |
| 20 | 40-44 | Dual | 0 | 0.00 | | | | | |
| 20 | 40-44 | Former | 1691 | 19.23 | | | 15.34 | 112 | 6.62 |

Year of Follow-up: 2010, Age Group: 45-49

| Period | Age Group | Smoking Group | N | % | Ever Quit (N) | Ever Quit % | Mean Yrs Quit | Short Term (n) | Short Term % |
|---|---|---|---|---|---|---|---|---|---|
| 20 | 45-49 | Never Any | 5284 | 52.43 | | | | | |
| 20 | 45-49 | CC | 105 | 1.04 | 0 | 0.00 | | | |
| 20 | 45-49 | Ecig | 2327 | 23.09 | | | | | |
| 20 | 45-49 | Dual | 0 | 0.00 | | | | | |
| 20 | 45-49 | Former | 2362 | 23.44 | | | 17.71 | 131 | 5.55 |

Year of Follow-up: 2010, Age Group: 50-54

| Period | Age Group | Smoking Group | N | % | Ever Quit (N) | Ever Quit % | Mean Yrs Quit | Short Term (n) | Short Term % |
|---|---|---|---|---|---|---|---|---|---|
| 20 | 50-54 | Never Any | 5278 | 50.27 | | | | | |
| 20 | 50-54 | CC | 101 | 0.96 | 0 | 0.00 | | | |
| 20 | 50-54 | Ecig | 2101 | 20.01 | | | | | |
| 20 | 50-54 | Dual | 0 | 0.00 | | | | | |
| 20 | 50-54 | Former | 3019 | 28.76 | | | 18.27 | 163 | 5.40 |

Year of Follow-up: 2010, Age Group: 55-59

| Period | Age Group | Smoking Group | N | % | Ever Quit (N) | Ever Quit % | Mean Yrs Quit | Short Term (n) | Short Term % |
|---|---|---|---|---|---|---|---|---|---|
| 20 | 55-59 | Never Any | 5018 | 52.27 | | | | | |
| 20 | 55-59 | CC | 91 | 0.95 | 0 | 0.00 | | | |
| 20 | 55-59 | Ecig | 1433 | 14.93 | | | | | |
| 20 | 55-59 | Dual | 0 | 0.00 | | | | | |
| 20 | 55-59 | Former | 3058 | 31.85 | | | 20.88 | 103 | 3.37 |

Year of Follow-up: 2010, Age Group: 60-64

| Period | Age Group | Smoking Group | N | % | Ever Quit (N) | Ever Quit % | Mean Yrs Quit | Short Term (n) | Short Term % |
|---|---|---|---|---|---|---|---|---|---|
| 20 | 60-64 | Never Any | 4356 | 51.27 | | | | | |
| 20 | 60-64 | CC | 71 | 0.84 | 0 | 0.00 | | | |
| 20 | 60-64 | Ecig | 1079 | 12.70 | | | | | |
| 20 | 60-64 | Dual | 0 | 0.00 | | | | | |
| 20 | 60-64 | Former | 2991 | 35.20 | | | 19.94 | 144 | 4.81 |

Year of Follow-up: 2010, Age Group: 65-69

# Results for PHIM for Run 4 (Sensitivity_Analysis_1.RTF)

| Period | Age Group | Smoking Group | N | % | Ever Quit (N) | Ever Quit % | Mean Yrs Quit | Short Term (n) | Short Term % |
|---|---|---|---|---|---|---|---|---|---|
| 20 | 65-69 | Never Any | 3116 | 47.67 | | | | | |
| 20 | 65-69 | CC | 47 | 0.72 | 0 | 0.00 | | | |
| 20 | 65-69 | Ecig | 645 | 9.87 | | | | | |
| 20 | 65-69 | Dual | 0 | 0.00 | | | | | |
| 20 | 65-69 | Former | 2728 | 41.74 | | | 22.72 | 95 | 3.48 |

Year of Follow-up: 2010, Age Group: 70-74

| Period | Age Group | Smoking Group | N | % | Ever Quit (N) | Ever Quit % | Mean Yrs Quit | Short Term (n) | Short Term % |
|---|---|---|---|---|---|---|---|---|---|
| 20 | 70-74 | Never Any | 2684 | 48.50 | | | | | |
| 20 | 70-74 | CC | 88 | 1.59 | 0 | 0.00 | | | |
| 20 | 70-74 | Ecig | 477 | 8.62 | | | | | |
| 20 | 70-74 | Dual | 0 | 0.00 | | | | | |
| 20 | 70-74 | Former | 2285 | 41.29 | | | 23.05 | 69 | 3.02 |

Year of Follow-up: 2010, Age Group: 75-79

| Period | Age Group | Smoking Group | N | % | Ever Quit (N) | Ever Quit % | Mean Yrs Quit | Short Term (n) | Short Term % |
|---|---|---|---|---|---|---|---|---|---|
| 20 | 75-79 | Never Any | 2732 | 51.90 | | | | | |
| 20 | 75-79 | CC | 75 | 1.42 | 0 | 0.00 | | | |
| 20 | 75-79 | Ecig | 276 | 5.24 | | | | | |
| 20 | 75-79 | Dual | 0 | 0.00 | | | | | |
| 20 | 75-79 | Former | 2181 | 41.43 | | | 25.56 | 72 | 3.30 |

Year of Follow-up: 2015, Age Group: All Ages

| Period | Age Group | Smoking Group | N | % | Ever Quit (N) | Ever Quit % | Mean Yrs Quit | Short Term (n) | Short Term % |
|---|---|---|---|---|---|---|---|---|---|
| 25 | All Ages | Never Any | 41010 | 54.09 | | | | | |
| 25 | All Ages | CC | 340 | 0.45 | 0 | 0.00 | | | |
| 25 | All Ages | Ecig | 11438 | 15.09 | | | | | |
| 25 | All Ages | Dual | 0 | 0.00 | | | | | |
| 25 | All Ages | Former | 23029 | 30.37 | | | 21.48 | 914 | 3.97 |

Year of Follow-up: 2015, Age Group: 18-19

| Period | Age Group | Smoking Group | N | % | Ever Quit (N) | Ever Quit % | Mean Yrs Quit | Short Term (n) | Short Term % |
|---|---|---|---|---|---|---|---|---|---|
| 25 | 18-19 | Never Any | 0 | . | | | | | |
| 25 | 18-19 | CC | 0 | . | 0 | . | | | |
| 25 | 18-19 | Ecig | 0 | . | | | | | |
| 25 | 18-19 | Dual | 0 | . | | | | | |
| 25 | 18-19 | Former | 0 | . | | | . | 0 | . |

Year of Follow-up: 2015, Age Group: 20-24

| Period | Age Group | Smoking Group | N | % | Ever Quit (N) | Ever Quit % | Mean Yrs Quit | Short Term (n) | Short Term % |
|---|---|---|---|---|---|---|---|---|---|
| 25 | 20-24 | Never Any | 0 | . | | | | | |
| 25 | 20-24 | CC | 0 | . | 0 | . | | | |
| 25 | 20-24 | Ecig | 0 | . | | | | | |
| 25 | 20-24 | Dual | 0 | . | | | | | |
| 25 | 20-24 | Former | 0 | . | | | . | 0 | . |

Year of Follow-up: 2015, Age Group: 25-29

| Period | Age Group | Smoking Group | N | % | Ever Quit (N) | Ever Quit % | Mean Yrs Quit | Short Term (n) | Short Term % |
|---|---|---|---|---|---|---|---|---|---|
| 25 | 25-29 | Never Any | 0 | . | | | | | |
| 25 | 25-29 | CC | 0 | . | 0 | . | | | |
| 25 | 25-29 | Ecig | 0 | . | | | | | |
| 25 | 25-29 | Dual | 0 | . | | | | | |
| 25 | 25-29 | Former | 0 | . | | | . | 0 | . |

Year of Follow-up: 2015, Age Group: 30-34

| Period | Age Group | Smoking Group | N | % | Ever Quit (N) | Ever Quit % | Mean Yrs Quit | Short Term (n) | Short Term % |
|---|---|---|---|---|---|---|---|---|---|
| 25 | 30-34 | Never Any | 0 | . | | | | | |
| 25 | 30-34 | CC | 0 | . | 0 | . | | | |
| 25 | 30-34 | Ecig | 0 | . | | | | | |
| 25 | 30-34 | Dual | 0 | . | | | | | |
| 25 | 30-34 | Former | 0 | . | | | . | 0 | . |

Year of Follow-up: 2015, Age Group: 35-39

| Period | Age Group | Smoking Group | N | % | Ever Quit (N) | Ever Quit % | Mean Yrs Quit | Short Term (n) | Short Term % |
|---|---|---|---|---|---|---|---|---|---|
| 25 | 35-39 | Never Any | 4726 | 59.60 | | | | | |
| 25 | 35-39 | CC | 20 | 0.25 | 0 | 0.00 | | | |
| 25 | 35-39 | Ecig | 1567 | 19.76 | | | | | |
| 25 | 35-39 | Dual | 0 | 0.00 | | | | | |
| 25 | 35-39 | Former | 1617 | 20.39 | | | 11.52 | 129 | 7.98 |

Year of Follow-up: 2015, Age Group: 40-44

| Period | Age Group | Smoking Group | N | % | Ever Quit (N) | Ever Quit % | Mean Yrs Quit | Short Term (n) | Short Term % |
|---|---|---|---|---|---|---|---|---|---|
| 25 | 40-44 | Never Any | 5359 | 64.18 | | | | | |
| 25 | 40-44 | CC | 33 | 0.40 | 0 | 0.00 | | | |
| 25 | 40-44 | Ecig | 1502 | 17.99 | | | | | |
| 25 | 40-44 | Dual | 0 | 0.00 | | | | | |

# Results for PHIM for Run 4 (Sensitivity_Analysis_1.RTF)

| | | | | | | | | | | |
|---|---|---|---|---|---|---|---|---|---|---|
| 25 | 40-44 | Former | 1456 | 17.44 | | | 14.00 | 103 | 7.07 |

Year of Follow-up: 2015, Age Group: 45-49

| Period | Age Group | Smoking Group | N | % | Ever Quit (N) | Ever Quit % | Mean Yrs Quit | Short Term (n) | Short Term % |
|---|---|---|---|---|---|---|---|---|---|
| 25 | 45-49 | Never Any | 5189 | 59.01 | | | | | |
| 25 | 45-49 | CC | 32 | 0.36 | 0 | 0.00 | | | |
| 25 | 45-49 | Ecig | 1627 | 18.50 | | | | | |
| 25 | 45-49 | Dual | 0 | 0.00 | | | | | |
| 25 | 45-49 | Former | 1945 | 22.12 | | | 17.95 | 96 | 4.94 |

Year of Follow-up: 2015, Age Group: 50-54

| Period | Age Group | Smoking Group | N | % | Ever Quit (N) | Ever Quit % | Mean Yrs Quit | Short Term (n) | Short Term % |
|---|---|---|---|---|---|---|---|---|---|
| 25 | 50-54 | Never Any | 5284 | 52.43 | | | | | |
| 25 | 50-54 | CC | 45 | 0.45 | 0 | 0.00 | | | |
| 25 | 50-54 | Ecig | 2059 | 20.43 | | | | | |
| 25 | 50-54 | Dual | 0 | 0.00 | | | | | |
| 25 | 50-54 | Former | 2690 | 26.69 | | | 20.17 | 150 | 5.58 |

Year of Follow-up: 2015, Age Group: 55-59

| Period | Age Group | Smoking Group | N | % | Ever Quit (N) | Ever Quit % | Mean Yrs Quit | Short Term (n) | Short Term % |
|---|---|---|---|---|---|---|---|---|---|
| 25 | 55-59 | Never Any | 5278 | 50.27 | | | | | |
| 25 | 55-59 | CC | 37 | 0.35 | 0 | 0.00 | | | |
| 25 | 55-59 | Ecig | 1773 | 16.89 | | | | | |
| 25 | 55-59 | Dual | 0 | 0.00 | | | | | |
| 25 | 55-59 | Former | 3411 | 32.49 | | | 20.84 | 140 | 4.10 |

Year of Follow-up: 2015, Age Group: 60-64

| Period | Age Group | Smoking Group | N | % | Ever Quit (N) | Ever Quit % | Mean Yrs Quit | Short Term (n) | Short Term % |
|---|---|---|---|---|---|---|---|---|---|
| 25 | 60-64 | Never Any | 5018 | 52.27 | | | | | |
| 25 | 60-64 | CC | 42 | 0.44 | 0 | 0.00 | | | |
| 25 | 60-64 | Ecig | 1154 | 12.02 | | | | | |
| 25 | 60-64 | Dual | 0 | 0.00 | | | | | |
| 25 | 60-64 | Former | 3386 | 35.27 | | | 23.56 | 144 | 4.25 |

Year of Follow-up: 2015, Age Group: 65-69

| Period | Age Group | Smoking Group | N | % | Ever Quit (N) | Ever Quit % | Mean Yrs Quit | Short Term (n) | Short Term % |
|---|---|---|---|---|---|---|---|---|---|
| 25 | 65-69 | Never Any | 4356 | 51.27 | | | | | |
| 25 | 65-69 | CC | 41 | 0.48 | 0 | 0.00 | | | |
| 25 | 65-69 | Ecig | 844 | 9.93 | | | | | |
| 25 | 65-69 | Dual | 0 | 0.00 | | | | | |
| 25 | 65-69 | Former | 3256 | 38.32 | | | 23.12 | 70 | 2.15 |

Year of Follow-up: 2015, Age Group: 70-74

| Period | Age Group | Smoking Group | N | % | Ever Quit (N) | Ever Quit % | Mean Yrs Quit | Short Term (n) | Short Term % |
|---|---|---|---|---|---|---|---|---|---|
| 25 | 70-74 | Never Any | 3116 | 47.67 | | | | | |
| 25 | 70-74 | CC | 31 | 0.47 | 0 | 0.00 | | | |
| 25 | 70-74 | Ecig | 527 | 8.06 | | | | | |
| 25 | 70-74 | Dual | 0 | 0.00 | | | | | |
| 25 | 70-74 | Former | 2862 | 43.79 | | | 26.54 | 42 | 1.47 |

Year of Follow-up: 2015, Age Group: 75-79

| Period | Age Group | Smoking Group | N | % | Ever Quit (N) | Ever Quit % | Mean Yrs Quit | Short Term (n) | Short Term % |
|---|---|---|---|---|---|---|---|---|---|
| 25 | 75-79 | Never Any | 2684 | 48.50 | | | | | |
| 25 | 75-79 | CC | 59 | 1.07 | 0 | 0.00 | | | |
| 25 | 75-79 | Ecig | 385 | 6.96 | | | | | |
| 25 | 75-79 | Dual | 0 | 0.00 | | | | | |
| 25 | 75-79 | Former | 2406 | 43.48 | | | 26.76 | 40 | 1.66 |

Year of Follow-up: 2020, Age Group: All Ages

| Period | Age Group | Smoking Group | N | % | Ever Quit (N) | Ever Quit % | Mean Yrs Quit | Short Term (n) | Short Term % |
|---|---|---|---|---|---|---|---|---|---|
| 30 | All Ages | Never Any | 38326 | 54.53 | | | | | |
| 30 | All Ages | CC | 115 | 0.16 | 0 | 0.00 | | | |
| 30 | All Ages | Ecig | 9272 | 13.19 | | | | | |
| 30 | All Ages | Dual | 0 | 0.00 | | | | | |
| 30 | All Ages | Former | 22570 | 32.11 | | | 23.81 | 756 | 3.35 |

Year of Follow-up: 2020, Age Group: 18-19

| Period | Age Group | Smoking Group | N | % | Ever Quit (N) | Ever Quit % | Mean Yrs Quit | Short Term (n) | Short Term % |
|---|---|---|---|---|---|---|---|---|---|
| 30 | 18-19 | Never Any | 0 | . | | | | | |
| 30 | 18-19 | CC | 0 | . | 0 | . | | | |
| 30 | 18-19 | Ecig | 0 | . | | | | | |
| 30 | 18-19 | Dual | 0 | . | | | | | |
| 30 | 18-19 | Former | 0 | . | | | . | 0 | . |

Year of Follow-up: 2020, Age Group: 20-24

| Period | Age Group | Smoking Group | N | % | Ever Quit (N) | Ever Quit % | Mean Yrs Quit | Short Term (n) | Short Term % |
|---|---|---|---|---|---|---|---|---|---|
| 30 | 20-24 | Never Any | 0 | . | | | | | |
| 30 | 20-24 | CC | 0 | . | 0 | . | | | |

# Results for PHIM for Run 4 (Sensitivity_Analysis_1.RTF)

| | | | | | | | | | |
|---|---|---|---|---|---|---|---|---|---|
| 30 | 20-24 | Ecig | 0 | . | | | | | |
| 30 | 20-24 | Dual | 0 | . | | | | | |
| 30 | 20-24 | Former | 0 | . | | | . | 0 | . |

Year of Follow-up: 2020, Age Group: 25-29

| Period | Age Group | Smoking Group | N | % | Ever Quit (N) | Ever Quit % | Mean Yrs Quit | Short Term (n) | Short Term % |
|---|---|---|---|---|---|---|---|---|---|
| 30 | 25-29 | Never Any | 0 | . | | | | | |
| 30 | 25-29 | CC | 0 | . | 0 | . | | | |
| 30 | 25-29 | Ecig | 0 | . | | | | | |
| 30 | 25-29 | Dual | 0 | . | | | | | |
| 30 | 25-29 | Former | 0 | . | | | . | 0 | . |

Year of Follow-up: 2020, Age Group: 30-34

| Period | Age Group | Smoking Group | N | % | Ever Quit (N) | Ever Quit % | Mean Yrs Quit | Short Term (n) | Short Term % |
|---|---|---|---|---|---|---|---|---|---|
| 30 | 30-34 | Never Any | 0 | . | | | | | |
| 30 | 30-34 | CC | 0 | . | 0 | . | | | |
| 30 | 30-34 | Ecig | 0 | . | | | | | |
| 30 | 30-34 | Dual | 0 | . | | | | | |
| 30 | 30-34 | Former | 0 | . | | | . | 0 | . |

Year of Follow-up: 2020, Age Group: 35-39

| Period | Age Group | Smoking Group | N | % | Ever Quit (N) | Ever Quit % | Mean Yrs Quit | Short Term (n) | Short Term % |
|---|---|---|---|---|---|---|---|---|---|
| 30 | 35-39 | Never Any | 0 | . | | | | | |
| 30 | 35-39 | CC | 0 | . | 0 | . | | | |
| 30 | 35-39 | Ecig | 0 | . | | | | | |
| 30 | 35-39 | Dual | 0 | . | | | | | |
| 30 | 35-39 | Former | 0 | . | | | . | 0 | . |

Year of Follow-up: 2020, Age Group: 40-44

| Period | Age Group | Smoking Group | N | % | Ever Quit (N) | Ever Quit % | Mean Yrs Quit | Short Term (n) | Short Term % |
|---|---|---|---|---|---|---|---|---|---|
| 30 | 40-44 | Never Any | 4726 | 59.60 | | | | | |
| 30 | 40-44 | CC | 6 | 0.08 | 0 | 0.00 | | | |
| 30 | 40-44 | Ecig | 1345 | 16.96 | | | | | |
| 30 | 40-44 | Dual | 0 | 0.00 | | | | | |
| 30 | 40-44 | Former | 1853 | 23.37 | | | 14.68 | 82 | 4.43 |

Year of Follow-up: 2020, Age Group: 45-49

| Period | Age Group | Smoking Group | N | % | Ever Quit (N) | Ever Quit % | Mean Yrs Quit | Short Term (n) | Short Term % |
|---|---|---|---|---|---|---|---|---|---|
| 30 | 45-49 | Never Any | 5359 | 64.18 | | | | | |
| 30 | 45-49 | CC | 13 | 0.16 | 0 | 0.00 | | | |
| 30 | 45-49 | Ecig | 1317 | 15.77 | | | | | |
| 30 | 45-49 | Dual | 0 | 0.00 | | | | | |
| 30 | 45-49 | Former | 1661 | 19.89 | | | 16.93 | 71 | 4.27 |

Year of Follow-up: 2020, Age Group: 50-54

| Period | Age Group | Smoking Group | N | % | Ever Quit (N) | Ever Quit % | Mean Yrs Quit | Short Term (n) | Short Term % |
|---|---|---|---|---|---|---|---|---|---|
| 30 | 50-54 | Never Any | 5189 | 59.01 | | | | | |
| 30 | 50-54 | CC | 9 | 0.10 | 0 | 0.00 | | | |
| 30 | 50-54 | Ecig | 1395 | 15.86 | | | | | |
| 30 | 50-54 | Dual | 0 | 0.00 | | | | | |
| 30 | 50-54 | Former | 2200 | 25.02 | | | 20.50 | 121 | 5.50 |

Year of Follow-up: 2020, Age Group: 55-59

| Period | Age Group | Smoking Group | N | % | Ever Quit (N) | Ever Quit % | Mean Yrs Quit | Short Term (n) | Short Term % |
|---|---|---|---|---|---|---|---|---|---|
| 30 | 55-59 | Never Any | 5284 | 52.43 | | | | | |
| 30 | 55-59 | CC | 14 | 0.14 | 0 | 0.00 | | | |
| 30 | 55-59 | Ecig | 1708 | 16.95 | | | | | |
| 30 | 55-59 | Dual | 0 | 0.00 | | | | | |
| 30 | 55-59 | Former | 3072 | 30.48 | | | 22.30 | 140 | 4.56 |

Year of Follow-up: 2020, Age Group: 60-64

| Period | Age Group | Smoking Group | N | % | Ever Quit (N) | Ever Quit % | Mean Yrs Quit | Short Term (n) | Short Term % |
|---|---|---|---|---|---|---|---|---|---|
| 30 | 60-64 | Never Any | 5278 | 50.27 | | | | | |
| 30 | 60-64 | CC | 15 | 0.14 | 0 | 0.00 | | | |
| 30 | 60-64 | Ecig | 1428 | 13.60 | | | | | |
| 30 | 60-64 | Dual | 0 | 0.00 | | | | | |
| 30 | 60-64 | Former | 3778 | 35.98 | | | 23.52 | 153 | 4.05 |

Year of Follow-up: 2020, Age Group: 65-69

| Period | Age Group | Smoking Group | N | % | Ever Quit (N) | Ever Quit % | Mean Yrs Quit | Short Term (n) | Short Term % |
|---|---|---|---|---|---|---|---|---|---|
| 30 | 65-69 | Never Any | 5018 | 52.27 | | | | | |
| 30 | 65-69 | CC | 18 | 0.19 | 0 | 0.00 | | | |
| 30 | 65-69 | Ecig | 917 | 9.55 | | | | | |
| 30 | 65-69 | Dual | 0 | 0.00 | | | | | |
| 30 | 65-69 | Former | 3647 | 37.99 | | | 26.68 | 75 | 2.06 |

Year of Follow-up: 2020, Age Group: 70-74

| Period | Age Group | Smoking Group | N | % | Ever Quit (N) | Ever Quit % | Mean Yrs Quit | Short Term (n) | Short Term % |
|---|---|---|---|---|---|---|---|---|---|

## Results for PHIM for Run 4 (Sensitivity_Analysis_1.RTF)

| | | | | | | | | | |
|---|---|---|---|---|---|---|---|---|---|
| 30 | 70-74 | Never Any | 4356 | 51.27 | | | | | |
| 30 | 70-74 | CC | 22 | 0.26 | 0 | 0.00 | | | |
| 30 | 70-74 | Ecig | 727 | 8.56 | | | | | |
| 30 | 70-74 | Dual | 0 | 0.00 | | | | | |
| 30 | 70-74 | Former | 3392 | 39.92 | | | 27.06 | 66 | 1.95 |

Year of Follow-up: 2020, Age Group: 75-79

| Period | Age Group | Smoking Group | N | % | Ever Quit (N) | Ever Quit % | Mean Yrs Quit | Short Term (n) | Short Term % |
|---|---|---|---|---|---|---|---|---|---|
| 30 | 75-79 | Never Any | 3116 | 47.67 | | | | | |
| 30 | 75-79 | CC | 18 | 0.28 | 0 | 0.00 | | | |
| 30 | 75-79 | Ecig | 435 | 6.66 | | | | | |
| 30 | 75-79 | Dual | 0 | 0.00 | | | | | |
| 30 | 75-79 | Former | 2967 | 45.39 | | | 30.49 | 48 | 1.62 |

Year of Follow-up: 2025, Age Group: All Ages

| Period | Age Group | Smoking Group | N | % | Ever Quit (N) | Ever Quit % | Mean Yrs Quit | Short Term (n) | Short Term % |
|---|---|---|---|---|---|---|---|---|---|
| 35 | All Ages | Never Any | 35210 | 55.23 | | | | | |
| 35 | All Ages | CC | 47 | 0.07 | 0 | 0.00 | | | |
| 35 | All Ages | Ecig | 7305 | 11.46 | | | | | |
| 35 | All Ages | Dual | 0 | 0.00 | | | | | |
| 35 | All Ages | Former | 21185 | 33.23 | | | 25.87 | 616 | 2.91 |

Year of Follow-up: 2025, Age Group: 18-19

| Period | Age Group | Smoking Group | N | % | Ever Quit (N) | Ever Quit % | Mean Yrs Quit | Short Term (n) | Short Term % |
|---|---|---|---|---|---|---|---|---|---|
| 35 | 18-19 | Never Any | 0 | . | | | | | |
| 35 | 18-19 | CC | 0 | . | 0 | . | | | |
| 35 | 18-19 | Ecig | 0 | . | | | | | |
| 35 | 18-19 | Dual | 0 | . | | | | | |
| 35 | 18-19 | Former | 0 | . | | | . | 0 | . |

Year of Follow-up: 2025, Age Group: 20-24

| Period | Age Group | Smoking Group | N | % | Ever Quit (N) | Ever Quit % | Mean Yrs Quit | Short Term (n) | Short Term % |
|---|---|---|---|---|---|---|---|---|---|
| 35 | 20-24 | Never Any | 0 | . | | | | | |
| 35 | 20-24 | CC | 0 | . | 0 | . | | | |
| 35 | 20-24 | Ecig | 0 | . | | | | | |
| 35 | 20-24 | Dual | 0 | . | | | | | |
| 35 | 20-24 | Former | 0 | . | | | . | 0 | . |

Year of Follow-up: 2025, Age Group: 25-29

| Period | Age Group | Smoking Group | N | % | Ever Quit (N) | Ever Quit % | Mean Yrs Quit | Short Term (n) | Short Term % |
|---|---|---|---|---|---|---|---|---|---|
| 35 | 25-29 | Never Any | 0 | . | | | | | |
| 35 | 25-29 | CC | 0 | . | 0 | . | | | |
| 35 | 25-29 | Ecig | 0 | . | | | | | |
| 35 | 25-29 | Dual | 0 | . | | | | | |
| 35 | 25-29 | Former | 0 | . | | | . | 0 | . |

Year of Follow-up: 2025, Age Group: 30-34

| Period | Age Group | Smoking Group | N | % | Ever Quit (N) | Ever Quit % | Mean Yrs Quit | Short Term (n) | Short Term % |
|---|---|---|---|---|---|---|---|---|---|
| 35 | 30-34 | Never Any | 0 | . | | | | | |
| 35 | 30-34 | CC | 0 | . | 0 | . | | | |
| 35 | 30-34 | Ecig | 0 | . | | | | | |
| 35 | 30-34 | Dual | 0 | . | | | | | |
| 35 | 30-34 | Former | 0 | . | | | . | 0 | . |

Year of Follow-up: 2025, Age Group: 35-39

| Period | Age Group | Smoking Group | N | % | Ever Quit (N) | Ever Quit % | Mean Yrs Quit | Short Term (n) | Short Term % |
|---|---|---|---|---|---|---|---|---|---|
| 35 | 35-39 | Never Any | 0 | . | | | | | |
| 35 | 35-39 | CC | 0 | . | 0 | . | | | |
| 35 | 35-39 | Ecig | 0 | . | | | | | |
| 35 | 35-39 | Dual | 0 | . | | | | | |
| 35 | 35-39 | Former | 0 | . | | | . | 0 | . |

Year of Follow-up: 2025, Age Group: 40-44

| Period | Age Group | Smoking Group | N | % | Ever Quit (N) | Ever Quit % | Mean Yrs Quit | Short Term (n) | Short Term % |
|---|---|---|---|---|---|---|---|---|---|
| 35 | 40-44 | Never Any | 0 | . | | | | | |
| 35 | 40-44 | CC | 0 | . | 0 | . | | | |
| 35 | 40-44 | Ecig | 0 | . | | | | | |
| 35 | 40-44 | Dual | 0 | . | | | | | |
| 35 | 40-44 | Former | 0 | . | | | . | 0 | . |

Year of Follow-up: 2025, Age Group: 45-49

| Period | Age Group | Smoking Group | N | % | Ever Quit (N) | Ever Quit % | Mean Yrs Quit | Short Term (n) | Short Term % |
|---|---|---|---|---|---|---|---|---|---|
| 35 | 45-49 | Never Any | 4726 | 59.60 | | | | | |
| 35 | 45-49 | CC | 1 | 0.01 | 0 | 0.00 | | | |
| 35 | 45-49 | Ecig | 1146 | 14.45 | | | | | |
| 35 | 45-49 | Dual | 0 | 0.00 | | | | | |
| 35 | 45-49 | Former | 2057 | 25.94 | | | 17.95 | 74 | 3.60 |

Year of Follow-up: 2025, Age Group: 50-54

# Results for PHIM for Run 4 (Sensitivity_Analysis_1.RTF)

| Period | Age Group | Smoking Group | N | % | Ever Quit (N) | Ever Quit % | Mean Yrs Quit | Short Term (n) | Short Term % |
|---|---|---|---|---|---|---|---|---|---|
| 35 | 50-54 | Never Any | 5359 | 64.18 | | | | | |
| 35 | 50-54 | CC | 3 | 0.04 | 0 | 0.00 | | | |
| 35 | 50-54 | Ecig | 1112 | 13.32 | | | | | |
| 35 | 50-54 | Dual | 0 | 0.00 | | | | | |
| 35 | 50-54 | Former | 1876 | 22.47 | | | 19.62 | 104 | 5.54 |

Year of Follow-up: 2025, Age Group: 55-59

| Period | Age Group | Smoking Group | N | % | Ever Quit (N) | Ever Quit % | Mean Yrs Quit | Short Term (n) | Short Term % |
|---|---|---|---|---|---|---|---|---|---|
| 35 | 55-59 | Never Any | 5189 | 59.01 | | | | | |
| 35 | 55-59 | CC | 4 | 0.05 | 0 | 0.00 | | | |
| 35 | 55-59 | Ecig | 1153 | 13.11 | | | | | |
| 35 | 55-59 | Dual | 0 | 0.00 | | | | | |
| 35 | 55-59 | Former | 2447 | 27.83 | | | 23.13 | 98 | 4.00 |

Year of Follow-up: 2025, Age Group: 60-64

| Period | Age Group | Smoking Group | N | % | Ever Quit (N) | Ever Quit % | Mean Yrs Quit | Short Term (n) | Short Term % |
|---|---|---|---|---|---|---|---|---|---|
| 35 | 60-64 | Never Any | 5284 | 52.43 | | | | | |
| 35 | 60-64 | CC | 7 | 0.07 | 0 | 0.00 | | | |
| 35 | 60-64 | Ecig | 1378 | 13.67 | | | | | |
| 35 | 60-64 | Dual | 0 | 0.00 | | | | | |
| 35 | 60-64 | Former | 3409 | 33.83 | | | 24.79 | 139 | 4.08 |

Year of Follow-up: 2025, Age Group: 65-69

| Period | Age Group | Smoking Group | N | % | Ever Quit (N) | Ever Quit % | Mean Yrs Quit | Short Term (n) | Short Term % |
|---|---|---|---|---|---|---|---|---|---|
| 35 | 65-69 | Never Any | 5278 | 50.27 | | | | | |
| 35 | 65-69 | CC | 7 | 0.07 | 0 | 0.00 | | | |
| 35 | 65-69 | Ecig | 1139 | 10.85 | | | | | |
| 35 | 65-69 | Dual | 0 | 0.00 | | | | | |
| 35 | 65-69 | Former | 4075 | 38.81 | | | 26.61 | 83 | 2.04 |

Year of Follow-up: 2025, Age Group: 70-74

| Period | Age Group | Smoking Group | N | % | Ever Quit (N) | Ever Quit % | Mean Yrs Quit | Short Term (n) | Short Term % |
|---|---|---|---|---|---|---|---|---|---|
| 35 | 70-74 | Never Any | 5018 | 52.27 | | | | | |
| 35 | 70-74 | CC | 11 | 0.11 | 0 | 0.00 | | | |
| 35 | 70-74 | Ecig | 789 | 8.22 | | | | | |
| 35 | 70-74 | Dual | 0 | 0.00 | | | | | |
| 35 | 70-74 | Former | 3782 | 39.40 | | | 30.61 | 65 | 1.72 |

Year of Follow-up: 2025, Age Group: 75-79

| Period | Age Group | Smoking Group | N | % | Ever Quit (N) | Ever Quit % | Mean Yrs Quit | Short Term (n) | Short Term % |
|---|---|---|---|---|---|---|---|---|---|
| 35 | 75-79 | Never Any | 4356 | 51.27 | | | | | |
| 35 | 75-79 | CC | 14 | 0.16 | 0 | 0.00 | | | |
| 35 | 75-79 | Ecig | 588 | 6.92 | | | | | |
| 35 | 75-79 | Dual | 0 | 0.00 | | | | | |
| 35 | 75-79 | Former | 3539 | 41.65 | | | 30.81 | 53 | 1.50 |

Year of Follow-up: 2030, Age Group: All Ages

| Period | Age Group | Smoking Group | N | % | Ever Quit (N) | Ever Quit % | Mean Yrs Quit | Short Term (n) | Short Term % |
|---|---|---|---|---|---|---|---|---|---|
| 40 | All Ages | Never Any | 30854 | 55.84 | | | | | |
| 40 | All Ages | CC | 14 | 0.03 | 0 | 0.00 | | | |
| 40 | All Ages | Ecig | 5391 | 9.76 | | | | | |
| 40 | All Ages | Dual | 0 | 0.00 | | | | | |
| 40 | All Ages | Former | 18991 | 34.37 | | | 27.90 | 543 | 2.86 |

Year of Follow-up: 2030, Age Group: 18-19

| Period | Age Group | Smoking Group | N | % | Ever Quit (N) | Ever Quit % | Mean Yrs Quit | Short Term (n) | Short Term % |
|---|---|---|---|---|---|---|---|---|---|
| 40 | 18-19 | Never Any | 0 | . | | | | | |
| 40 | 18-19 | CC | 0 | . | 0 | . | | | |
| 40 | 18-19 | Ecig | 0 | . | | | | | |
| 40 | 18-19 | Dual | 0 | . | | | | | |
| 40 | 18-19 | Former | 0 | . | | | . | 0 | . |

Year of Follow-up: 2030, Age Group: 20-24

| Period | Age Group | Smoking Group | N | % | Ever Quit (N) | Ever Quit % | Mean Yrs Quit | Short Term (n) | Short Term % |
|---|---|---|---|---|---|---|---|---|---|
| 40 | 20-24 | Never Any | 0 | . | | | | | |
| 40 | 20-24 | CC | 0 | . | 0 | . | | | |
| 40 | 20-24 | Ecig | 0 | . | | | | | |
| 40 | 20-24 | Dual | 0 | . | | | | | |
| 40 | 20-24 | Former | 0 | . | | | . | 0 | . |

Year of Follow-up: 2030, Age Group: 25-29

| Period | Age Group | Smoking Group | N | % | Ever Quit (N) | Ever Quit % | Mean Yrs Quit | Short Term (n) | Short Term % |
|---|---|---|---|---|---|---|---|---|---|
| 40 | 25-29 | Never Any | 0 | . | | | | | |
| 40 | 25-29 | CC | 0 | . | 0 | . | | | |
| 40 | 25-29 | Ecig | 0 | . | | | | | |
| 40 | 25-29 | Dual | 0 | . | | | | | |

# Results for PHIM for Run 4 (Sensitivity_Analysis_1.RTF)

| | | | | | | | | | |
|---|---|---|---|---|---|---|---|---|---|
| 40 | 25-29 | Former | 0 | . | | | . | 0 | . |

Year of Follow-up: 2030, Age Group: 30-34

| Period | Age Group | Smoking Group | N | % | Ever Quit (N) | Ever Quit % | Mean Yrs Quit | Short Term (n) | Short Term % |
|---|---|---|---|---|---|---|---|---|---|
| 40 | 30-34 | Never Any | 0 | . | | | | | |
| 40 | 30-34 | CC | 0 | . | 0 | . | | | |
| 40 | 30-34 | Ecig | 0 | . | | | | | |
| 40 | 30-34 | Dual | 0 | . | | | | | |
| 40 | 30-34 | Former | 0 | . | | | . | 0 | . |

Year of Follow-up: 2030, Age Group: 35-39

| Period | Age Group | Smoking Group | N | % | Ever Quit (N) | Ever Quit % | Mean Yrs Quit | Short Term (n) | Short Term % |
|---|---|---|---|---|---|---|---|---|---|
| 40 | 35-39 | Never Any | 0 | . | | | | | |
| 40 | 35-39 | CC | 0 | . | 0 | . | | | |
| 40 | 35-39 | Ecig | 0 | . | | | | | |
| 40 | 35-39 | Dual | 0 | . | | | | | |
| 40 | 35-39 | Former | 0 | . | | | . | 0 | . |

Year of Follow-up: 2030, Age Group: 40-44

| Period | Age Group | Smoking Group | N | % | Ever Quit (N) | Ever Quit % | Mean Yrs Quit | Short Term (n) | Short Term % |
|---|---|---|---|---|---|---|---|---|---|
| 40 | 40-44 | Never Any | 0 | . | | | | | |
| 40 | 40-44 | CC | 0 | . | 0 | . | | | |
| 40 | 40-44 | Ecig | 0 | . | | | | | |
| 40 | 40-44 | Dual | 0 | . | | | | | |
| 40 | 40-44 | Former | 0 | . | | | . | 0 | . |

Year of Follow-up: 2030, Age Group: 45-49

| Period | Age Group | Smoking Group | N | % | Ever Quit (N) | Ever Quit % | Mean Yrs Quit | Short Term (n) | Short Term % |
|---|---|---|---|---|---|---|---|---|---|
| 40 | 45-49 | Never Any | 0 | . | | | | | |
| 40 | 45-49 | CC | 0 | . | 0 | . | | | |
| 40 | 45-49 | Ecig | 0 | . | | | | | |
| 40 | 45-49 | Dual | 0 | . | | | | | |
| 40 | 45-49 | Former | 0 | . | | | . | 0 | . |

Year of Follow-up: 2030, Age Group: 50-54

| Period | Age Group | Smoking Group | N | % | Ever Quit (N) | Ever Quit % | Mean Yrs Quit | Short Term (n) | Short Term % |
|---|---|---|---|---|---|---|---|---|---|
| 40 | 50-54 | Never Any | 4726 | 59.60 | | | | | |
| 40 | 50-54 | CC | 0 | 0.00 | 0 | 0.00 | | | |
| 40 | 50-54 | Ecig | 959 | 12.09 | | | | | |
| 40 | 50-54 | Dual | 0 | 0.00 | | | | | |
| 40 | 50-54 | Former | 2245 | 28.31 | | | 21.16 | 100 | 4.45 |

Year of Follow-up: 2030, Age Group: 55-59

| Period | Age Group | Smoking Group | N | % | Ever Quit (N) | Ever Quit % | Mean Yrs Quit | Short Term (n) | Short Term % |
|---|---|---|---|---|---|---|---|---|---|
| 40 | 55-59 | Never Any | 5359 | 64.18 | | | | | |
| 40 | 55-59 | CC | 0 | 0.00 | 0 | 0.00 | | | |
| 40 | 55-59 | Ecig | 897 | 10.74 | | | | | |
| 40 | 55-59 | Dual | 0 | 0.00 | | | | | |
| 40 | 55-59 | Former | 2094 | 25.08 | | | 22.27 | 80 | 3.82 |

Year of Follow-up: 2030, Age Group: 60-64

| Period | Age Group | Smoking Group | N | % | Ever Quit (N) | Ever Quit % | Mean Yrs Quit | Short Term (n) | Short Term % |
|---|---|---|---|---|---|---|---|---|---|
| 40 | 60-64 | Never Any | 5189 | 59.01 | | | | | |
| 40 | 60-64 | CC | 3 | 0.03 | 0 | 0.00 | | | |
| 40 | 60-64 | Ecig | 886 | 10.08 | | | | | |
| 40 | 60-64 | Dual | 0 | 0.00 | | | | | |
| 40 | 60-64 | Former | 2715 | 30.88 | | | 25.52 | 128 | 4.71 |

Year of Follow-up: 2030, Age Group: 65-69

| Period | Age Group | Smoking Group | N | % | Ever Quit (N) | Ever Quit % | Mean Yrs Quit | Short Term (n) | Short Term % |
|---|---|---|---|---|---|---|---|---|---|
| 40 | 65-69 | Never Any | 5284 | 52.43 | | | | | |
| 40 | 65-69 | CC | 3 | 0.03 | 0 | 0.00 | | | |
| 40 | 65-69 | Ecig | 1080 | 10.72 | | | | | |
| 40 | 65-69 | Dual | 0 | 0.00 | | | | | |
| 40 | 65-69 | Former | 3711 | 36.82 | | | 27.57 | 73 | 1.97 |

Year of Follow-up: 2030, Age Group: 70-74

| Period | Age Group | Smoking Group | N | % | Ever Quit (N) | Ever Quit % | Mean Yrs Quit | Short Term (n) | Short Term % |
|---|---|---|---|---|---|---|---|---|---|
| 40 | 70-74 | Never Any | 5278 | 50.27 | | | | | |
| 40 | 70-74 | CC | 4 | 0.04 | 0 | 0.00 | | | |
| 40 | 70-74 | Ecig | 957 | 9.12 | | | | | |
| 40 | 70-74 | Dual | 0 | 0.00 | | | | | |
| 40 | 70-74 | Former | 4260 | 40.58 | | | 30.32 | 86 | 2.02 |

Year of Follow-up: 2030, Age Group: 75-79

| Period | Age Group | Smoking Group | N | % | Ever Quit (N) | Ever Quit % | Mean Yrs Quit | Short Term (n) | Short Term % |
|---|---|---|---|---|---|---|---|---|---|
| 40 | 75-79 | Never Any | 5018 | 52.27 | | | | | |
| 40 | 75-79 | CC | 4 | 0.04 | 0 | 0.00 | | | |

# Results for PHIM for Run 4 (Sensitivity_Analysis_1.RTF)

| | | | | | | | | | |
|---|---|---|---|---|---|---|---|---|---|
| 40 | 75-79 | Ecig | 612 | 6.38 | | | | | |
| 40 | 75-79 | Dual | 0 | 0.00 | | | | | |
| 40 | 75-79 | Former | 3966 | 41.31 | | | 34.05 | 76 | 1.92 |

Year of Follow-up: 2035, Age Group: All Ages

| Period | Age Group | Smoking Group | N | % | Ever Quit (N) | Ever Quit % | Mean Yrs Quit | Short Term (n) | Short Term % |
|---|---|---|---|---|---|---|---|---|---|
| 45 | All Ages | Never Any | 25836 | 56.60 | | | | | |
| 45 | All Ages | CC | 5 | 0.01 | 0 | 0.00 | | | |
| 45 | All Ages | Ecig | 3886 | 8.51 | | | | | |
| 45 | All Ages | Dual | 0 | 0.00 | | | | | |
| 45 | All Ages | Former | 15923 | 34.88 | | | 29.63 | 351 | 2.20 |

Year of Follow-up: 2035, Age Group: 18-19

| Period | Age Group | Smoking Group | N | % | Ever Quit (N) | Ever Quit % | Mean Yrs Quit | Short Term (n) | Short Term % |
|---|---|---|---|---|---|---|---|---|---|
| 45 | 18-19 | Never Any | 0 | . | | | | | |
| 45 | 18-19 | CC | 0 | . | 0 | . | | | |
| 45 | 18-19 | Ecig | 0 | . | | | | | |
| 45 | 18-19 | Dual | 0 | . | | | | | |
| 45 | 18-19 | Former | 0 | . | | | . | 0 | . |

Year of Follow-up: 2035, Age Group: 20-24

| Period | Age Group | Smoking Group | N | % | Ever Quit (N) | Ever Quit % | Mean Yrs Quit | Short Term (n) | Short Term % |
|---|---|---|---|---|---|---|---|---|---|
| 45 | 20-24 | Never Any | 0 | . | | | | | |
| 45 | 20-24 | CC | 0 | . | 0 | . | | | |
| 45 | 20-24 | Ecig | 0 | . | | | | | |
| 45 | 20-24 | Dual | 0 | . | | | | | |
| 45 | 20-24 | Former | 0 | . | | | . | 0 | . |

Year of Follow-up: 2035, Age Group: 25-29

| Period | Age Group | Smoking Group | N | % | Ever Quit (N) | Ever Quit % | Mean Yrs Quit | Short Term (n) | Short Term % |
|---|---|---|---|---|---|---|---|---|---|
| 45 | 25-29 | Never Any | 0 | . | | | | | |
| 45 | 25-29 | CC | 0 | . | 0 | . | | | |
| 45 | 25-29 | Ecig | 0 | . | | | | | |
| 45 | 25-29 | Dual | 0 | . | | | | | |
| 45 | 25-29 | Former | 0 | . | | | . | 0 | . |

Year of Follow-up: 2035, Age Group: 30-34

| Period | Age Group | Smoking Group | N | % | Ever Quit (N) | Ever Quit % | Mean Yrs Quit | Short Term (n) | Short Term % |
|---|---|---|---|---|---|---|---|---|---|
| 45 | 30-34 | Never Any | 0 | . | | | | | |
| 45 | 30-34 | CC | 0 | . | 0 | . | | | |
| 45 | 30-34 | Ecig | 0 | . | | | | | |
| 45 | 30-34 | Dual | 0 | . | | | | | |
| 45 | 30-34 | Former | 0 | . | | | . | 0 | . |

Year of Follow-up: 2035, Age Group: 35-39

| Period | Age Group | Smoking Group | N | % | Ever Quit (N) | Ever Quit % | Mean Yrs Quit | Short Term (n) | Short Term % |
|---|---|---|---|---|---|---|---|---|---|
| 45 | 35-39 | Never Any | 0 | . | | | | | |
| 45 | 35-39 | CC | 0 | . | 0 | . | | | |
| 45 | 35-39 | Ecig | 0 | . | | | | | |
| 45 | 35-39 | Dual | 0 | . | | | | | |
| 45 | 35-39 | Former | 0 | . | | | . | 0 | . |

Year of Follow-up: 2035, Age Group: 40-44

| Period | Age Group | Smoking Group | N | % | Ever Quit (N) | Ever Quit % | Mean Yrs Quit | Short Term (n) | Short Term % |
|---|---|---|---|---|---|---|---|---|---|
| 45 | 40-44 | Never Any | 0 | . | | | | | |
| 45 | 40-44 | CC | 0 | . | 0 | . | | | |
| 45 | 40-44 | Ecig | 0 | . | | | | | |
| 45 | 40-44 | Dual | 0 | . | | | | | |
| 45 | 40-44 | Former | 0 | . | | | . | 0 | . |

Year of Follow-up: 2035, Age Group: 45-49

| Period | Age Group | Smoking Group | N | % | Ever Quit (N) | Ever Quit % | Mean Yrs Quit | Short Term (n) | Short Term % |
|---|---|---|---|---|---|---|---|---|---|
| 45 | 45-49 | Never Any | 0 | . | | | | | |
| 45 | 45-49 | CC | 0 | . | 0 | . | | | |
| 45 | 45-49 | Ecig | 0 | . | | | | | |
| 45 | 45-49 | Dual | 0 | . | | | | | |
| 45 | 45-49 | Former | 0 | . | | | . | 0 | . |

Year of Follow-up: 2035, Age Group: 50-54

| Period | Age Group | Smoking Group | N | % | Ever Quit (N) | Ever Quit % | Mean Yrs Quit | Short Term (n) | Short Term % |
|---|---|---|---|---|---|---|---|---|---|
| 45 | 50-54 | Never Any | 0 | . | | | | | |
| 45 | 50-54 | CC | 0 | . | 0 | . | | | |
| 45 | 50-54 | Ecig | 0 | . | | | | | |
| 45 | 50-54 | Dual | 0 | . | | | | | |
| 45 | 50-54 | Former | 0 | . | | | . | 0 | . |

Year of Follow-up: 2035, Age Group: 55-59

| Period | Age Group | Smoking Group | N | % | Ever Quit (N) | Ever Quit % | Mean Yrs Quit | Short Term (n) | Short Term % |
|---|---|---|---|---|---|---|---|---|---|

## Results for PHIM for Run 4 (Sensitivity_Analysis_1.RTF)

| | | | | | | | | | |
|---|---|---|---|---|---|---|---|---|---|
| 45 | 55-59 | Never Any | 4726 | 59.60 | | | | | |
| 45 | 55-59 | CC | 0 | 0.00 | 0 | 0.00 | | | |
| 45 | 55-59 | Ecig | 792 | 9.99 | | | | | |
| 45 | 55-59 | Dual | 0 | 0.00 | | | | | |
| 45 | 55-59 | Former | 2412 | 30.42 | | | 24.49 | 64 | 2.65 |

Year of Follow-up: 2035, Age Group: 60-64

| Period | Age Group | Smoking Group | N | % | Ever Quit (N) | Ever Quit % | Mean Yrs Quit | Short Term (n) | Short Term % |
|---|---|---|---|---|---|---|---|---|---|
| 45 | 60-64 | Never Any | 5359 | 64.18 | | | | | |
| 45 | 60-64 | CC | 0 | 0.00 | 0 | 0.00 | | | |
| 45 | 60-64 | Ecig | 719 | 8.61 | | | | | |
| 45 | 60-64 | Dual | 0 | 0.00 | | | | | |
| 45 | 60-64 | Former | 2272 | 27.21 | | | 25.28 | 77 | 3.39 |

Year of Follow-up: 2035, Age Group: 65-69

| Period | Age Group | Smoking Group | N | % | Ever Quit (N) | Ever Quit % | Mean Yrs Quit | Short Term (n) | Short Term % |
|---|---|---|---|---|---|---|---|---|---|
| 45 | 65-69 | Never Any | 5189 | 59.01 | | | | | |
| 45 | 65-69 | CC | 3 | 0.03 | 0 | 0.00 | | | |
| 45 | 65-69 | Ecig | 694 | 7.89 | | | | | |
| 45 | 65-69 | Dual | 0 | 0.00 | | | | | |
| 45 | 65-69 | Former | 2907 | 33.06 | | | 28.66 | 55 | 1.89 |

Year of Follow-up: 2035, Age Group: 70-74

| Period | Age Group | Smoking Group | N | % | Ever Quit (N) | Ever Quit % | Mean Yrs Quit | Short Term (n) | Short Term % |
|---|---|---|---|---|---|---|---|---|---|
| 45 | 70-74 | Never Any | 5284 | 52.43 | | | | | |
| 45 | 70-74 | CC | 1 | 0.01 | 0 | 0.00 | | | |
| 45 | 70-74 | Ecig | 934 | 9.27 | | | | | |
| 45 | 70-74 | Dual | 0 | 0.00 | | | | | |
| 45 | 70-74 | Former | 3859 | 38.29 | | | 31.38 | 76 | 1.97 |

Year of Follow-up: 2035, Age Group: 75-79

| Period | Age Group | Smoking Group | N | % | Ever Quit (N) | Ever Quit % | Mean Yrs Quit | Short Term (n) | Short Term % |
|---|---|---|---|---|---|---|---|---|---|
| 45 | 75-79 | Never Any | 5278 | 50.27 | | | | | |
| 45 | 75-79 | CC | 1 | 0.01 | 0 | 0.00 | | | |
| 45 | 75-79 | Ecig | 747 | 7.11 | | | | | |
| 45 | 75-79 | Dual | 0 | 0.00 | | | | | |
| 45 | 75-79 | Former | 4473 | 42.60 | | | 33.73 | 79 | 1.77 |

Year of Follow-up: 2040, Age Group: All Ages

| Period | Age Group | Smoking Group | N | % | Ever Quit (N) | Ever Quit % | Mean Yrs Quit | Short Term (n) | Short Term % |
|---|---|---|---|---|---|---|---|---|---|
| 50 | All Ages | Never Any | 20558 | 58.48 | | | | | |
| 50 | All Ages | CC | 4 | 0.01 | 0 | 0.00 | | | |
| 50 | All Ages | Ecig | 2497 | 7.10 | | | | | |
| 50 | All Ages | Dual | 0 | 0.00 | | | | | |
| 50 | All Ages | Former | 12092 | 34.40 | | | 31.39 | 238 | 1.97 |

Year of Follow-up: 2040, Age Group: 18-19

| Period | Age Group | Smoking Group | N | % | Ever Quit (N) | Ever Quit % | Mean Yrs Quit | Short Term (n) | Short Term % |
|---|---|---|---|---|---|---|---|---|---|
| 50 | 18-19 | Never Any | 0 | . | | | | | |
| 50 | 18-19 | CC | 0 | . | 0 | . | | | |
| 50 | 18-19 | Ecig | 0 | . | | | | | |
| 50 | 18-19 | Dual | 0 | . | | | | | |
| 50 | 18-19 | Former | 0 | . | | | . | 0 | . |

Year of Follow-up: 2040, Age Group: 20-24

| Period | Age Group | Smoking Group | N | % | Ever Quit (N) | Ever Quit % | Mean Yrs Quit | Short Term (n) | Short Term % |
|---|---|---|---|---|---|---|---|---|---|
| 50 | 20-24 | Never Any | 0 | . | | | | | |
| 50 | 20-24 | CC | 0 | . | 0 | . | | | |
| 50 | 20-24 | Ecig | 0 | . | | | | | |
| 50 | 20-24 | Dual | 0 | . | | | | | |
| 50 | 20-24 | Former | 0 | . | | | . | 0 | . |

Year of Follow-up: 2040, Age Group: 25-29

| Period | Age Group | Smoking Group | N | % | Ever Quit (N) | Ever Quit % | Mean Yrs Quit | Short Term (n) | Short Term % |
|---|---|---|---|---|---|---|---|---|---|
| 50 | 25-29 | Never Any | 0 | . | | | | | |
| 50 | 25-29 | CC | 0 | . | 0 | . | | | |
| 50 | 25-29 | Ecig | 0 | . | | | | | |
| 50 | 25-29 | Dual | 0 | . | | | | | |
| 50 | 25-29 | Former | 0 | . | | | . | 0 | . |

Year of Follow-up: 2040, Age Group: 30-34

| Period | Age Group | Smoking Group | N | % | Ever Quit (N) | Ever Quit % | Mean Yrs Quit | Short Term (n) | Short Term % |
|---|---|---|---|---|---|---|---|---|---|
| 50 | 30-34 | Never Any | 0 | . | | | | | |
| 50 | 30-34 | CC | 0 | . | 0 | . | | | |
| 50 | 30-34 | Ecig | 0 | . | | | | | |
| 50 | 30-34 | Dual | 0 | . | | | | | |
| 50 | 30-34 | Former | 0 | . | | | . | 0 | . |

Year of Follow-up: 2040, Age Group: 35-39

# Results for PHIM for Run 4 (Sensitivity_Analysis_1.RTF)

| Period | Age Group | Smoking Group | N | % | Ever Quit (N) | Ever Quit % | Mean Yrs Quit | Short Term (n) | Short Term % |
|---|---|---|---|---|---|---|---|---|---|
| 50 | 35-39 | Never Any | 0 | . | | | | | |
| 50 | 35-39 | CC | 0 | . | 0 | . | | | |
| 50 | 35-39 | Ecig | 0 | . | | | | | |
| 50 | 35-39 | Dual | 0 | . | | | | | |
| 50 | 35-39 | Former | 0 | . | | | . | 0 | . |

Year of Follow-up: 2040, Age Group: 40-44

| Period | Age Group | Smoking Group | N | % | Ever Quit (N) | Ever Quit % | Mean Yrs Quit | Short Term (n) | Short Term % |
|---|---|---|---|---|---|---|---|---|---|
| 50 | 40-44 | Never Any | 0 | . | | | | | |
| 50 | 40-44 | CC | 0 | . | 0 | . | | | |
| 50 | 40-44 | Ecig | 0 | . | | | | | |
| 50 | 40-44 | Dual | 0 | . | | | | | |
| 50 | 40-44 | Former | 0 | . | | | . | 0 | . |

Year of Follow-up: 2040, Age Group: 45-49

| Period | Age Group | Smoking Group | N | % | Ever Quit (N) | Ever Quit % | Mean Yrs Quit | Short Term (n) | Short Term % |
|---|---|---|---|---|---|---|---|---|---|
| 50 | 45-49 | Never Any | 0 | . | | | | | |
| 50 | 45-49 | CC | 0 | . | 0 | . | | | |
| 50 | 45-49 | Ecig | 0 | . | | | | | |
| 50 | 45-49 | Dual | 0 | . | | | | | |
| 50 | 45-49 | Former | 0 | . | | | . | 0 | . |

Year of Follow-up: 2040, Age Group: 50-54

| Period | Age Group | Smoking Group | N | % | Ever Quit (N) | Ever Quit % | Mean Yrs Quit | Short Term (n) | Short Term % |
|---|---|---|---|---|---|---|---|---|---|
| 50 | 50-54 | Never Any | 0 | . | | | | | |
| 50 | 50-54 | CC | 0 | . | 0 | . | | | |
| 50 | 50-54 | Ecig | 0 | . | | | | | |
| 50 | 50-54 | Dual | 0 | . | | | | | |
| 50 | 50-54 | Former | 0 | . | | | . | 0 | . |

Year of Follow-up: 2040, Age Group: 55-59

| Period | Age Group | Smoking Group | N | % | Ever Quit (N) | Ever Quit % | Mean Yrs Quit | Short Term (n) | Short Term % |
|---|---|---|---|---|---|---|---|---|---|
| 50 | 55-59 | Never Any | 0 | . | | | | | |
| 50 | 55-59 | CC | 0 | . | 0 | . | | | |
| 50 | 55-59 | Ecig | 0 | . | | | | | |
| 50 | 55-59 | Dual | 0 | . | | | | | |
| 50 | 55-59 | Former | 0 | . | | | . | 0 | . |

Year of Follow-up: 2040, Age Group: 60-64

| Period | Age Group | Smoking Group | N | % | Ever Quit (N) | Ever Quit % | Mean Yrs Quit | Short Term (n) | Short Term % |
|---|---|---|---|---|---|---|---|---|---|
| 50 | 60-64 | Never Any | 4726 | 59.60 | | | | | |
| 50 | 60-64 | CC | 0 | 0.00 | 0 | 0.00 | | | |
| 50 | 60-64 | Ecig | 613 | 7.73 | | | | | |
| 50 | 60-64 | Dual | 0 | 0.00 | | | | | |
| 50 | 60-64 | Former | 2591 | 32.67 | | | 27.58 | 77 | 2.97 |

Year of Follow-up: 2040, Age Group: 65-69

| Period | Age Group | Smoking Group | N | % | Ever Quit (N) | Ever Quit % | Mean Yrs Quit | Short Term (n) | Short Term % |
|---|---|---|---|---|---|---|---|---|---|
| 50 | 65-69 | Never Any | 5359 | 64.18 | | | | | |
| 50 | 65-69 | CC | 0 | 0.00 | 0 | 0.00 | | | |
| 50 | 65-69 | Ecig | 578 | 6.92 | | | | | |
| 50 | 65-69 | Dual | 0 | 0.00 | | | | | |
| 50 | 65-69 | Former | 2413 | 28.90 | | | 28.67 | 34 | 1.41 |

Year of Follow-up: 2040, Age Group: 70-74

| Period | Age Group | Smoking Group | N | % | Ever Quit (N) | Ever Quit % | Mean Yrs Quit | Short Term (n) | Short Term % |
|---|---|---|---|---|---|---|---|---|---|
| 50 | 70-74 | Never Any | 5189 | 59.01 | | | | | |
| 50 | 70-74 | CC | 3 | 0.03 | 0 | 0.00 | | | |
| 50 | 70-74 | Ecig | 583 | 6.63 | | | | | |
| 50 | 70-74 | Dual | 0 | 0.00 | | | | | |
| 50 | 70-74 | Former | 3018 | 34.32 | | | 32.48 | 56 | 1.86 |

Year of Follow-up: 2040, Age Group: 75-79

| Period | Age Group | Smoking Group | N | % | Ever Quit (N) | Ever Quit % | Mean Yrs Quit | Short Term (n) | Short Term % |
|---|---|---|---|---|---|---|---|---|---|
| 50 | 75-79 | Never Any | 5284 | 52.43 | | | | | |
| 50 | 75-79 | CC | 1 | 0.01 | 0 | 0.00 | | | |
| 50 | 75-79 | Ecig | 723 | 7.17 | | | | | |
| 50 | 75-79 | Dual | 0 | 0.00 | | | | | |
| 50 | 75-79 | Former | 4070 | 40.38 | | | 34.61 | 71 | 1.74 |

# Results for PHIM for Run 4 (Sensitivity_Analysis_1.RTF)

************************************************************************************
**Main Results for E-Component for Run: 4, Simulation: 1, COD: Four Diseases Combined**

| Variable Parameter | Value |
|---|---|
| 1. Country | US (United States) |
| 2. Sex | F (Females) |
| 3. Year of start of process | 1990 |
| 4. Cause of Death | Four Diseases Combined |
| 5. Effective Dose NULL | CC:1 |
| 6. Effective Dose Ecig | CC:1 Ecig:0 Dual:0.5 |
| Note: | Death rates are per million population |

------------------------------------------------------------------------------------------
Results for Age Groups Combined and Years combined, Population unadjusted for deaths in Ecig scenario,

| Age Group:All Year | Number Deaths | NULL Attrib Deaths | Ecig Attrib Deaths | Drop Deaths | %Drop Deaths | %Drop Attrib Deaths | Death Rate | NULL Attrib Rates | Ecig Attrib Rates | Drop Rates |
|---|---|---|---|---|---|---|---|---|---|---|
| All Comb | 9082078 | 2946250.006 | 1860886.698 | 1085363 | 11.951 | 36.839 | 2493.45 | 808.881 | 510.899 | 297.982 |

------------------------------------------------------------------------------------------
Results for Age Groups Combined, Population unadjusted for deaths in Ecig scenario,

| Age Group:All Year | Number Deaths | NULL Attrib Deaths | Ecig Attrib Deaths | Drop Deaths | %Drop Deaths | %Drop Attrib Deaths | Death Rate | NULL Attrib Rates | Ecig Attrib Rates | Drop Rates |
|---|---|---|---|---|---|---|---|---|---|---|
| 1990 | 199848 | 57524.822 | 57524.822 | 0.000 | 0.000 | 0.000 | 2926.84 | 842.471 | 842.471 | 0.000 |
| 1991 | 199162 | 59078.486 | 58901.294 | 177.192 | 0.089 | 0.300 | 2877.87 | 853.678 | 851.118 | 2.560 |
| 1992 | 198532 | 60494.150 | 59997.182 | 496.968 | 0.250 | 0.822 | 2829.84 | 862.271 | 855.188 | 7.084 |
| 1993 | 202512 | 62694.848 | 61726.865 | 967.983 | 0.478 | 1.544 | 2845.85 | 881.034 | 867.431 | 13.603 |
| 1994 | 200658 | 63202.598 | 61671.454 | 1531.144 | 0.763 | 2.423 | 2777.87 | 874.966 | 853.769 | 21.197 |
| 1995 | 201023 | 63766.862 | 61553.356 | 2213.506 | 1.101 | 3.471 | 2739.80 | 869.097 | 838.929 | 30.169 |
| 1996 | 201180 | 64591.163 | 61554.552 | 3036.611 | 1.509 | 4.701 | 2706.36 | 868.908 | 828.058 | 40.850 |
| 1997 | 198639 | 64686.912 | 60879.386 | 3807.526 | 1.917 | 5.886 | 2635.18 | 858.147 | 807.636 | 50.511 |
| 1998 | 197573 | 65295.961 | 60595.574 | 4700.387 | 2.379 | 7.199 | 2584.18 | 854.046 | 792.567 | 61.479 |
| 1999 | 211329 | 67327.521 | 61247.607 | 6079.915 | 2.877 | 9.030 | 2726.91 | 868.771 | 790.317 | 78.453 |
| 2000 | 208496 | 67596.897 | 60511.435 | 7085.462 | 3.398 | 10.482 | 2656.96 | 861.419 | 771.126 | 90.293 |
| 2001 | 203606 | 67008.811 | 58854.674 | 8154.137 | 4.005 | 12.169 | 2565.88 | 844.458 | 741.698 | 102.760 |
| 2002 | 200828 | 67067.744 | 57723.791 | 9343.952 | 4.653 | 13.932 | 2506.09 | 836.925 | 720.324 | 116.601 |
| 2003 | 196352 | 66756.217 | 56334.234 | 10421.98 | 5.308 | 15.612 | 2427.31 | 825.244 | 696.407 | 128.837 |
| 2004 | 188160 | 65058.725 | 53719.770 | 11338.95 | 6.026 | 17.429 | 2302.83 | 796.234 | 657.460 | 138.774 |
| 2005 | 186416 | 65214.724 | 52640.432 | 12574.29 | 6.745 | 19.281 | 2256.27 | 789.321 | 637.129 | 152.192 |
| 2006 | 180088 | 63771.146 | 50252.345 | 13518.80 | 7.507 | 21.199 | 2160.67 | 765.117 | 602.920 | 162.197 |
| 2007 | 175644 | 63110.718 | 48619.911 | 14490.81 | 8.250 | 22.961 | 2086.36 | 749.650 | 577.524 | 172.127 |
| 2008 | 177570 | 63718.624 | 47829.926 | 15888.70 | 8.948 | 24.936 | 2086.39 | 748.674 | 561.986 | 186.687 |
| 2009 | 172806 | 62794.698 | 45993.592 | 16801.11 | 9.723 | 26.756 | 2007.57 | 729.515 | 534.329 | 195.186 |
| 2010 | 170489 | 61917.074 | 44152.580 | 17764.49 | 10.420 | 28.691 | 1957.97 | 711.083 | 507.068 | 204.015 |
| 2011 | 170305 | 61680.114 | 42635.339 | 19044.77 | 11.183 | 30.877 | 1937.46 | 701.700 | 485.038 | 216.662 |
| 2012 | 171421 | 62023.127 | 41550.340 | 20472.79 | 11.943 | 33.008 | 1930.22 | 698.386 | 467.861 | 230.525 |
| 2013 | 172699 | 62037.765 | 40282.898 | 21754.87 | 12.597 | 35.067 | 1923.75 | 691.059 | 448.724 | 242.334 |
| 2014 | 174169 | 61921.017 | 38754.656 | 23166.36 | 13.301 | 37.413 | 1918.85 | 682.195 | 426.967 | 255.228 |
| 2015 | 177354 | 61856.204 | 37484.859 | 24371.35 | 13.742 | 39.400 | 2184.51 | 761.899 | 461.711 | 300.188 |
| 2016 | 176853 | 60464.119 | 35121.569 | 25342.55 | 14.330 | 41.913 | 2157.59 | 737.655 | 428.479 | 309.176 |
| 2017 | 179309 | 60243.839 | 33602.989 | 26640.85 | 14.858 | 44.222 | 2166.86 | 728.017 | 406.075 | 321.941 |
| 2018 | 177157 | 60713.327 | 32873.591 | 27839.74 | 15.715 | 45.854 | 2120.51 | 726.721 | 393.487 | 333.233 |
| 2019 | 179227 | 61148.172 | 31792.895 | 29355.28 | 16.379 | 48.007 | 2124.53 | 724.841 | 376.868 | 347.973 |
| 2020 | 180467 | 61149.317 | 30718.704 | 30430.61 | 16.862 | 49.764 | 2424.25 | 821.432 | 412.651 | 408.781 |
| 2021 | 178116 | 58727.786 | 27861.551 | 30866.24 | 17.329 | 52.558 | 2375.42 | 783.216 | 371.572 | 411.644 |
| 2022 | 179408 | 58848.802 | 26682.894 | 32165.91 | 17.929 | 54.659 | 2374.52 | 778.881 | 353.156 | 425.725 |
| 2023 | 180640 | 58816.149 | 25507.010 | 33309.14 | 18.440 | 56.633 | 2372.26 | 772.407 | 334.972 | 437.434 |
| 2024 | 181610 | 58955.232 | 24323.636 | 34631.60 | 19.069 | 58.742 | 2366.77 | 768.316 | 316.990 | 451.326 |
| 2025 | 180418 | 58297.744 | 23090.125 | 35207.62 | 19.514 | 60.393 | 2713.69 | 876.861 | 347.300 | 529.561 |
| 2026 | 180313 | 56469.971 | 21044.921 | 35425.05 | 19.646 | 62.733 | 2701.83 | 846.150 | 315.338 | 530.811 |
| 2027 | 179619 | 56209.427 | 19786.036 | 36423.39 | 20.278 | 64.799 | 2680.68 | 838.883 | 295.292 | 543.591 |
| 2028 | 178762 | 55733.105 | 18478.197 | 37254.91 | 20.840 | 66.845 | 2656.74 | 828.298 | 274.621 | 553.678 |
| 2029 | 177808 | 55145.838 | 17171.394 | 37974.44 | 21.357 | 68.862 | 2630.89 | 815.951 | 254.072 | 561.879 |
| 2030 | 173587 | 53615.097 | 15946.546 | 37668.55 | 21.700 | 70.257 | 3040.33 | 939.056 | 279.300 | 659.756 |
| 2031 | 174985 | 52046.561 | 14779.582 | 37266.98 | 21.297 | 71.603 | 3060.73 | 910.364 | 258.515 | 651.849 |
| 2032 | 173400 | 51582.636 | 13716.137 | 37866.50 | 21.838 | 73.409 | 3027.10 | 900.496 | 239.447 | 661.048 |
| 2033 | 171756 | 50839.758 | 12654.899 | 38184.86 | 22.232 | 75.108 | 2991.14 | 885.375 | 220.385 | 664.989 |
| 2034 | 169973 | 50215.451 | 11619.530 | 38595.92 | 22.707 | 76.861 | 2951.96 | 872.104 | 201.799 | 670.305 |
| 2035 | 162644 | 47773.075 | 10634.793 | 37138.28 | 22.834 | 77.739 | 3459.14 | 1016.05 | 226.183 | 789.863 |
| 2036 | 156254 | 43298.709 | 8934.456 | 34364.25 | 21.993 | 79.366 | 3328.25 | 922.274 | 190.306 | 731.968 |
| 2037 | 153593 | 42241.563 | 7979.108 | 34262.45 | 22.307 | 81.111 | 3274.10 | 900.450 | 170.088 | 730.362 |
| 2038 | 150947 | 41356.353 | 7204.660 | 34151.69 | 22.625 | 82.579 | 3217.50 | 881.531 | 153.571 | 727.960 |
| 2039 | 148373 | 40161.050 | 6368.601 | 33792.45 | 22.775 | 84.142 | 3159.42 | 855.178 | 135.611 | 719.566 |

# Results for PHIM for Run 4 (Sensitivity_Analysis_1.RTF)

************************************************************************************
**Main Results for E-Component for Run: 4, Simulation: 1, COD: LUNG CANCER**

| **Variable Parameter** | **Value** |
|---|---|
| 1. Country | US (United States) |
| 2. Sex | F (Females) |
| 3. Year of start of process | 1990 |
| 4. Cause of Death | LUNG CANCER |
| 5. Effective Dose NULL | CC:1 |
| 6. Effective Dose Ecig | CC:1 Ecig:0 Dual:0.5 |
| Note: | Death rates are per million population |

-----------------------------------------------------------------------------------------
Results for Age Groups Combined and Years combined, Population unadjusted for deaths in Ecig scenario,

| Age Group:All | | NULL | Ecig | | | %Drop | | NULL | Ecig | |
|---|---|---|---|---|---|---|---|---|---|---|
| Year | Number Deaths | Attrib Deaths | Attrib Deaths | Drop Deaths | %Drop Deaths | Attrib Deaths | Death Rate | Attrib Rates | Attrib Rates | Drop Rates |
| All Comb | 2327764 | 1580465.217 | 1096584.929 | 483880.3 | 20.787 | 30.616 | 639.078 | 433.910 | 301.063 | 132.847 |

-----------------------------------------------------------------------------------------
Results for Age Groups Combined, Population unadjusted for deaths in Ecig scenario,

| Age Group:All | | NULL | Ecig | | | %Drop | | NULL | Ecig | |
|---|---|---|---|---|---|---|---|---|---|---|
| Year | Number Deaths | Attrib Deaths | Attrib Deaths | Drop Deaths | %Drop Deaths | Attrib Deaths | Death Rate | Attrib Rates | Attrib Rates | Drop Rates |
| 1990 | 42690 | 30614.201 | 30614.201 | 0.000 | 0.000 | 0.000 | 625.210 | 448.355 | 448.355 | 0.000 |
| 1991 | 43692 | 31412.010 | 31378.038 | 33.973 | 0.078 | 0.108 | 631.345 | 453.901 | 453.410 | 0.491 |
| 1992 | 45423 | 32782.407 | 32683.444 | 98.963 | 0.218 | 0.302 | 647.450 | 467.274 | 465.863 | 1.411 |
| 1993 | 46291 | 33491.183 | 33295.398 | 195.785 | 0.423 | 0.585 | 650.515 | 470.643 | 467.891 | 2.751 |
| 1994 | 47017 | 34074.347 | 33756.478 | 317.869 | 0.676 | 0.933 | 650.895 | 471.719 | 467.319 | 4.401 |
| 1995 | 47840 | 34694.454 | 34214.717 | 479.737 | 1.003 | 1.383 | 652.025 | 472.861 | 466.322 | 6.538 |
| 1996 | 48244 | 35027.269 | 34362.259 | 665.009 | 1.378 | 1.899 | 648.999 | 471.202 | 462.256 | 8.946 |
| 1997 | 49035 | 35590.763 | 34704.273 | 886.490 | 1.808 | 2.491 | 650.506 | 472.153 | 460.392 | 11.760 |
| 1998 | 49686 | 36100.785 | 34952.155 | 1148.630 | 2.312 | 3.182 | 649.874 | 472.184 | 457.161 | 15.024 |
| 1999 | 48883 | 35451.030 | 34064.096 | 1386.933 | 2.837 | 3.912 | 630.769 | 457.448 | 439.551 | 17.896 |
| 2000 | 50331 | 36447.986 | 34709.865 | 1738.121 | 3.453 | 4.769 | 641.392 | 464.474 | 442.324 | 22.150 |
| 2001 | 50389 | 36411.326 | 34315.212 | 2096.114 | 4.160 | 5.757 | 635.012 | 458.863 | 432.447 | 26.416 |
| 2002 | 51445 | 37078.545 | 34537.058 | 2541.487 | 4.940 | 6.854 | 641.972 | 462.696 | 430.981 | 31.715 |
| 2003 | 51408 | 36999.876 | 34031.159 | 2968.716 | 5.775 | 8.024 | 635.509 | 457.395 | 420.695 | 36.699 |
| 2004 | 51399 | 36861.555 | 33416.729 | 3444.826 | 6.702 | 9.345 | 629.057 | 451.138 | 408.977 | 42.160 |
| 2005 | 51421 | 36689.529 | 32738.907 | 3950.622 | 7.683 | 10.768 | 622.370 | 444.069 | 396.253 | 47.816 |
| 2006 | 51355 | 36504.774 | 32019.889 | 4484.885 | 8.733 | 12.286 | 616.150 | 437.979 | 384.170 | 53.809 |
| 2007 | 51839 | 36663.202 | 31542.011 | 5121.191 | 9.879 | 13.968 | 615.761 | 435.498 | 374.667 | 60.831 |
| 2008 | 50976 | 35898.803 | 30293.726 | 5605.077 | 10.996 | 15.614 | 598.952 | 421.800 | 355.942 | 65.858 |
| 2009 | 51193 | 35934.525 | 29640.586 | 6293.939 | 12.295 | 17.515 | 594.733 | 417.468 | 344.348 | 73.120 |
| 2010 | 50853 | 35502.829 | 28637.682 | 6865.147 | 13.500 | 19.337 | 584.018 | 407.730 | 328.888 | 78.842 |
| 2011 | 50509 | 35136.342 | 27607.592 | 7528.750 | 14.906 | 21.427 | 574.613 | 399.726 | 314.076 | 85.650 |
| 2012 | 51245 | 35450.201 | 27094.465 | 8355.736 | 16.305 | 23.570 | 577.023 | 399.173 | 305.086 | 94.086 |
| 2013 | 51069 | 35123.224 | 26041.249 | 9081.975 | 17.784 | 25.857 | 568.874 | 391.249 | 290.082 | 101.167 |
| 2014 | 51251 | 35079.370 | 25216.314 | 9863.056 | 19.245 | 28.116 | 564.641 | 386.476 | 277.813 | 108.663 |
| 2015 | 50443 | 34324.171 | 23941.487 | 10382.68 | 20.583 | 30.249 | 621.319 | 422.780 | 294.893 | 127.886 |
| 2016 | 48854 | 33054.753 | 22216.289 | 10838.46 | 22.185 | 32.789 | 596.013 | 403.264 | 271.036 | 132.228 |
| 2017 | 48325 | 32471.234 | 20986.230 | 11485.00 | 23.766 | 35.370 | 583.983 | 392.399 | 253.608 | 138.791 |
| 2018 | 50465 | 33657.595 | 20807.356 | 12850.24 | 25.464 | 38.179 | 604.052 | 402.871 | 249.058 | 153.814 |
| 2019 | 51036 | 33863.424 | 20033.678 | 13829.75 | 27.098 | 40.840 | 604.975 | 401.412 | 237.476 | 163.936 |
| 2020 | 51444 | 33910.830 | 19256.059 | 14654.77 | 28.487 | 43.216 | 691.053 | 455.532 | 258.671 | 196.861 |
| 2021 | 48037 | 31531.880 | 17029.248 | 14502.63 | 30.191 | 45.994 | 640.639 | 420.521 | 227.108 | 193.412 |
| 2022 | 48335 | 31560.212 | 16191.188 | 15369.02 | 31.797 | 48.697 | 639.727 | 417.708 | 214.295 | 203.413 |
| 2023 | 48599 | 31508.781 | 15351.652 | 16157.13 | 33.246 | 51.278 | 638.227 | 413.791 | 201.607 | 212.185 |
| 2024 | 48784 | 31517.293 | 14500.754 | 17016.54 | 34.882 | 53.991 | 635.757 | 410.740 | 188.977 | 221.763 |
| 2025 | 48477 | 31175.389 | 13627.039 | 17548.35 | 36.200 | 56.289 | 729.141 | 468.912 | 204.965 | 263.946 |
| 2026 | 45324 | 29044.598 | 11969.656 | 17074.94 | 37.673 | 58.789 | 679.138 | 435.206 | 179.354 | 255.852 |
| 2027 | 45107 | 28825.765 | 11123.591 | 17702.17 | 39.245 | 61.411 | 673.191 | 430.203 | 166.011 | 264.192 |
| 2028 | 44842 | 28513.846 | 10266.020 | 18247.83 | 40.694 | 63.996 | 666.430 | 423.769 | 152.572 | 271.197 |
| 2029 | 44539 | 28148.109 | 9415.305 | 18732.80 | 42.059 | 66.551 | 659.013 | 416.486 | 139.311 | 277.175 |
| 2030 | 43393 | 27314.475 | 8617.999 | 18696.48 | 43.086 | 68.449 | 760.018 | 478.407 | 150.942 | 327.464 |
| 2031 | 40748 | 25423.102 | 7632.832 | 17790.27 | 43.660 | 69.977 | 712.731 | 444.684 | 133.508 | 311.176 |
| 2032 | 40325 | 25085.902 | 6967.521 | 18118.38 | 44.931 | 72.225 | 703.969 | 437.933 | 121.634 | 316.299 |
| 2033 | 39882 | 24645.067 | 6326.005 | 18319.06 | 45.933 | 74.332 | 694.543 | 429.194 | 110.167 | 319.027 |
| 2034 | 39404 | 24239.126 | 5708.042 | 18531.08 | 47.028 | 76.451 | 684.347 | 420.967 | 99.133 | 321.834 |
| 2035 | 37454 | 22944.147 | 5117.691 | 17826.46 | 47.595 | 77.695 | 796.588 | 487.980 | 108.844 | 379.136 |
| 2036 | 32988 | 19950.581 | 4092.741 | 15857.84 | 48.071 | 79.486 | 702.663 | 424.953 | 87.176 | 337.776 |
| 2037 | 32403 | 19415.334 | 3587.541 | 15827.79 | 48.846 | 81.522 | 690.731 | 413.871 | 76.475 | 337.396 |
| 2038 | 31820 | 18939.317 | 3186.596 | 15752.72 | 49.506 | 83.175 | 678.257 | 403.701 | 67.924 | 335.777 |
| 2039 | 31251 | 18379.752 | 2764.908 | 15614.84 | 49.965 | 84.957 | 665.457 | 391.373 | 58.875 | 332.498 |

# Results for PHIM for Run 4 (Sensitivity_Analysis_1.RTF)

*******************************************************************************

**Main Results for E-Component for Run: 4, Simulation: 1, COD: IHD**

| Variable Parameter | Value |
|---|---|
| 1. Country | US (United States) |
| 2. Sex | F (Females) |
| 3. Year of start of process | 1990 |
| 4. Cause of Death | IHD |
| 5. Effective Dose NULL | CC:1 |
| 6. Effective Dose Ecig | CC:1 Ecig:0 Dual:0.5 |
| Note: | Death rates are per million population |

-----------------------------------------------------------------------------------------

Results for Age Groups Combined and Years combined, Population unadjusted for deaths in Ecig scenario,

| Age Group:All |  | NULL | Ecig |  |  | %Drop |  | NULL | Ecig |  |
|---|---|---|---|---|---|---|---|---|---|---|
| Year | Number | Attrib | Attrib | Drop | %Drop | Attrib | Death | Attrib | Attrib | Drop |
|  | Deaths | Deaths | Deaths | Deaths | Deaths | Deaths | Rate | Rates | Rates | Rates |
| All Comb | 3242167 | 398833.796 | 186644.106 | 212189.7 | 6.545 | 53.203 | 890.124 | 109.498 | 51.242 | 58.256 |

-----------------------------------------------------------------------------------------

Results for Age Groups Combined, Population unadjusted for deaths in Ecig scenario,

| Age Group:All |  | NULL | Ecig |  |  | %Drop |  | NULL | Ecig |  |
|---|---|---|---|---|---|---|---|---|---|---|
| Year | Number | Attrib | Attrib | Drop | %Drop | Attrib | Death | Attrib | Attrib | Drop |
|  | Deaths | Deaths | Deaths | Deaths | Deaths | Deaths | Rate | Rates | Rates | Rates |
| 1990 | 98916 | 12922.892 | 12922.892 | 0.000 | 0.000 | 0.000 | 1448.66 | 189.260 | 189.260 | 0.000 |
| 1991 | 96403 | 12856.479 | 12761.318 | 95.161 | 0.099 | 0.740 | 1393.01 | 185.775 | 184.400 | 1.375 |
| 1992 | 93721 | 12662.244 | 12398.030 | 264.213 | 0.282 | 2.087 | 1335.88 | 180.485 | 176.719 | 3.766 |
| 1993 | 93679 | 12730.613 | 12225.772 | 504.841 | 0.539 | 3.966 | 1316.45 | 178.900 | 171.805 | 7.094 |
| 1994 | 90832 | 12495.323 | 11708.132 | 787.191 | 0.867 | 6.300 | 1257.46 | 172.983 | 162.085 | 10.898 |
| 1995 | 89698 | 12365.590 | 11252.022 | 1113.569 | 1.241 | 9.005 | 1222.52 | 168.534 | 153.357 | 15.177 |
| 1996 | 88286 | 12290.551 | 10770.229 | 1520.322 | 1.722 | 12.370 | 1187.66 | 165.338 | 144.886 | 20.452 |
| 1997 | 84777 | 11719.531 | 9886.351 | 1833.181 | 2.162 | 15.642 | 1124.67 | 155.473 | 131.154 | 24.319 |
| 1998 | 82517 | 11416.794 | 9220.211 | 2196.584 | 2.662 | 19.240 | 1079.29 | 149.327 | 120.597 | 28.730 |
| 1999 | 93939 | 13028.038 | 10000.842 | 3027.196 | 3.223 | 23.236 | 1212.16 | 168.109 | 129.047 | 39.062 |
| 2000 | 90039 | 12426.299 | 9044.426 | 3381.873 | 3.756 | 27.215 | 1147.41 | 158.354 | 115.257 | 43.097 |
| 2001 | 86119 | 11939.281 | 8123.854 | 3815.427 | 4.430 | 31.957 | 1085.29 | 150.461 | 102.378 | 48.083 |
| 2002 | 83014 | 11528.794 | 7292.673 | 4236.121 | 5.103 | 36.744 | 1035.91 | 143.865 | 91.004 | 52.862 |
| 2003 | 79302 | 11048.407 | 6493.168 | 4555.239 | 5.744 | 41.230 | 980.336 | 136.581 | 80.269 | 56.312 |
| 2004 | 73861 | 10375.132 | 5603.394 | 4771.738 | 6.460 | 45.992 | 903.963 | 126.978 | 68.578 | 58.400 |
| 2005 | 71649 | 10051.029 | 4984.676 | 5066.354 | 7.071 | 50.406 | 867.197 | 121.652 | 60.332 | 61.320 |
| 2006 | 67551 | 9542.523 | 4287.043 | 5255.481 | 7.780 | 55.074 | 810.467 | 114.490 | 51.435 | 63.055 |
| 2007 | 63503 | 8936.533 | 3640.194 | 5296.339 | 8.340 | 59.266 | 754.310 | 106.151 | 43.239 | 62.912 |
| 2008 | 63171 | 8944.180 | 3289.433 | 5654.747 | 8.951 | 63.223 | 742.239 | 105.091 | 38.650 | 66.441 |
| 2009 | 59747 | 8390.033 | 2803.936 | 5586.098 | 9.350 | 66.580 | 694.108 | 97.471 | 32.575 | 64.896 |
| 2010 | 58008 | 8157.570 | 2454.421 | 5703.149 | 9.832 | 69.912 | 666.189 | 93.685 | 28.188 | 65.497 |
| 2011 | 57368 | 8035.356 | 2152.897 | 5882.459 | 10.254 | 73.207 | 652.644 | 91.414 | 24.492 | 66.921 |
| 2012 | 57411 | 7970.766 | 1885.147 | 6085.619 | 10.600 | 76.349 | 646.453 | 89.752 | 21.227 | 68.525 |
| 2013 | 57067 | 7743.216 | 1641.938 | 6101.278 | 10.691 | 78.795 | 635.688 | 86.254 | 18.290 | 67.964 |
| 2014 | 57538 | 7774.935 | 1450.900 | 6324.035 | 10.991 | 81.339 | 633.906 | 85.658 | 15.985 | 69.673 |
| 2015 | 58395 | 7722.535 | 1301.374 | 6421.160 | 10.996 | 83.148 | 719.266 | 95.120 | 16.029 | 79.091 |
| 2016 | 58658 | 7600.353 | 1121.766 | 6478.587 | 11.045 | 85.241 | 715.621 | 92.723 | 13.685 | 79.038 |
| 2017 | 59004 | 7470.266 | 969.164 | 6501.102 | 11.018 | 87.026 | 713.034 | 90.274 | 11.712 | 78.563 |
| 2018 | 56077 | 6907.050 | 782.604 | 6124.447 | 10.922 | 88.669 | 671.224 | 82.675 | 9.368 | 73.308 |
| 2019 | 55886 | 6732.046 | 669.912 | 6062.135 | 10.847 | 90.049 | 662.462 | 79.801 | 7.941 | 71.860 |
| 2020 | 55313 | 6429.836 | 573.076 | 5856.760 | 10.588 | 91.087 | 743.037 | 86.373 | 7.698 | 78.675 |
| 2021 | 56292 | 6562.016 | 489.761 | 6072.254 | 10.787 | 92.536 | 750.730 | 87.513 | 6.532 | 80.982 |
| 2022 | 55827 | 6352.366 | 412.271 | 5940.094 | 10.640 | 93.510 | 738.885 | 84.075 | 5.457 | 78.619 |
| 2023 | 55338 | 6134.123 | 348.647 | 5785.476 | 10.455 | 94.316 | 726.728 | 80.557 | 4.579 | 75.978 |
| 2024 | 54770 | 5922.193 | 290.761 | 5631.432 | 10.282 | 95.090 | 713.771 | 77.179 | 3.789 | 73.390 |
| 2025 | 53295 | 5504.188 | 244.186 | 5260.002 | 9.870 | 95.564 | 801.612 | 82.789 | 3.673 | 79.116 |
| 2026 | 55278 | 5641.075 | 213.293 | 5427.782 | 9.819 | 96.219 | 828.283 | 84.526 | 3.196 | 81.330 |
| 2027 | 54199 | 5426.216 | 178.282 | 5247.934 | 9.683 | 96.714 | 808.875 | 80.982 | 2.661 | 78.321 |
| 2028 | 53090 | 5197.830 | 145.595 | 5052.235 | 9.516 | 97.199 | 789.020 | 77.249 | 2.164 | 75.086 |
| 2029 | 51974 | 4973.547 | 119.191 | 4854.355 | 9.340 | 97.603 | 769.023 | 73.590 | 1.764 | 71.826 |
| 2030 | 49479 | 4450.618 | 98.376 | 4352.242 | 8.796 | 97.790 | 866.605 | 77.952 | 1.723 | 76.228 |
| 2031 | 51987 | 4555.052 | 87.350 | 4467.702 | 8.594 | 98.082 | 909.317 | 79.674 | 1.528 | 78.146 |
| 2032 | 50677 | 4349.999 | 70.196 | 4279.803 | 8.445 | 98.386 | 884.689 | 75.939 | 1.225 | 74.714 |
| 2033 | 49380 | 4147.948 | 57.797 | 4090.151 | 8.283 | 98.607 | 859.955 | 72.237 | 1.007 | 71.230 |
| 2034 | 48076 | 3966.133 | 46.592 | 3919.542 | 8.153 | 98.825 | 834.943 | 68.881 | 0.809 | 68.072 |
| 2035 | 44518 | 3289.064 | 38.597 | 3250.468 | 7.301 | 98.827 | 946.825 | 69.952 | 0.821 | 69.131 |
| 2036 | 46347 | 3313.192 | 31.734 | 3281.459 | 7.080 | 99.042 | 987.211 | 70.572 | 0.676 | 69.896 |
| 2037 | 44841 | 3117.951 | 24.219 | 3093.732 | 6.899 | 99.223 | 955.859 | 66.464 | 0.516 | 65.948 |
| 2038 | 43378 | 2949.775 | 19.554 | 2930.221 | 6.755 | 99.337 | 924.621 | 62.876 | 0.417 | 62.459 |
| 2039 | 41973 | 2766.312 | 15.910 | 2750.402 | 6.553 | 99.425 | 893.753 | 58.905 | 0.339 | 58.566 |

# Results for PHIM for Run 4 (Sensitivity_Analysis_1.RTF)

************************************************************************************
**Main Results for E-Component for Run: 4, Simulation: 1, COD: STROKE**

| Variable Parameter | Value |
|---|---|
| 1. Country | US (United States) |
| 2. Sex | F (Females) |
| 3. Year of start of process | 1990 |
| 4. Cause of Death | STROKE |
| 5. Effective Dose NULL | CC:1 |
| 6. Effective Dose Ecig | CC:1 Ecig:0 Dual:0.5 |
| Note: | Death rates are per million population |

--------------------------------------------------------------------------------------------

Results for Age Groups Combined and Years combined, Population unadjusted for deaths in Ecig scenario,

| Age Group:All | | NULL | Ecig | | | %Drop | | NULL | Ecig | |
|---|---|---|---|---|---|---|---|---|---|---|
| Year | Number | Attrib | Attrib | Drop | %Drop | Attrib | Death | Attrib | Attrib | Drop |
| | Deaths | Deaths | Deaths | Deaths | Deaths | Deaths | Rate | Rates | Rates | Rates |
| All Comb | 1352207 | 104899.750 | 48122.626 | 56777.12 | 4.199 | 54.125 | 371.243 | 28.800 | 13.212 | 15.588 |

--------------------------------------------------------------------------------------------

Results for Age Groups Combined, Population unadjusted for deaths in Ecig scenario,

| Age Group:All | | NULL | Ecig | | | %Drop | | NULL | Ecig | |
|---|---|---|---|---|---|---|---|---|---|---|
| Year | Number | Attrib | Attrib | Drop | %Drop | Attrib | Death | Attrib | Attrib | Drop |
| | Deaths | Deaths | Deaths | Deaths | Deaths | Deaths | Rate | Rates | Rates | Rates |
| 1990 | 33504 | 3293.017 | 3293.017 | 0.000 | 0.000 | 0.000 | 490.678 | 48.227 | 48.227 | 0.000 |
| 1991 | 32505 | 3207.063 | 3178.126 | 28.937 | 0.089 | 0.902 | 469.694 | 46.342 | 45.924 | 0.418 |
| 1992 | 32560 | 3218.125 | 3137.157 | 80.968 | 0.249 | 2.516 | 464.104 | 45.871 | 44.716 | 1.154 |
| 1993 | 32768 | 3226.680 | 3073.280 | 153.401 | 0.468 | 4.754 | 460.480 | 45.344 | 43.188 | 2.156 |
| 1994 | 33005 | 3277.266 | 3032.440 | 244.826 | 0.742 | 7.470 | 456.915 | 45.370 | 41.981 | 3.389 |
| 1995 | 33730 | 3309.378 | 2958.341 | 351.037 | 1.041 | 10.607 | 459.716 | 45.104 | 40.320 | 4.784 |
| 1996 | 33750 | 3312.987 | 2838.187 | 474.800 | 1.407 | 14.331 | 454.019 | 44.568 | 38.181 | 6.387 |
| 1997 | 33699 | 3267.066 | 2672.119 | 594.947 | 1.765 | 18.210 | 447.056 | 43.341 | 35.449 | 7.893 |
| 1998 | 33274 | 3178.725 | 2469.179 | 709.546 | 2.132 | 22.322 | 435.211 | 41.577 | 32.296 | 9.281 |
| 1999 | 33891 | 3120.351 | 2293.897 | 826.455 | 2.439 | 26.486 | 437.317 | 40.264 | 29.600 | 10.664 |
| 2000 | 33827 | 3142.808 | 2175.729 | 967.079 | 2.859 | 30.771 | 431.074 | 40.050 | 27.726 | 12.324 |
| 2001 | 32506 | 2961.588 | 1922.624 | 1038.965 | 3.196 | 35.081 | 409.647 | 37.323 | 24.229 | 13.093 |
| 2002 | 32078 | 2940.627 | 1772.370 | 1168.257 | 3.642 | 39.728 | 400.295 | 36.695 | 22.117 | 14.578 |
| 2003 | 30568 | 2819.545 | 1574.976 | 1244.569 | 4.071 | 44.141 | 377.883 | 34.855 | 19.470 | 15.385 |
| 2004 | 29388 | 2730.466 | 1404.804 | 1325.662 | 4.511 | 48.551 | 359.671 | 33.417 | 17.193 | 16.224 |
| 2005 | 27954 | 2670.510 | 1262.005 | 1408.505 | 5.039 | 52.743 | 338.339 | 32.322 | 15.275 | 17.048 |
| 2006 | 27201 | 2623.285 | 1135.506 | 1487.780 | 5.470 | 56.714 | 326.354 | 31.474 | 13.624 | 17.850 |
| 2007 | 26302 | 2536.599 | 999.800 | 1536.799 | 5.843 | 60.585 | 312.424 | 30.131 | 11.876 | 18.255 |
| 2008 | 25998 | 2469.443 | 887.989 | 1581.454 | 6.083 | 64.041 | 305.468 | 29.015 | 10.434 | 18.582 |
| 2009 | 25040 | 2386.945 | 778.906 | 1608.039 | 6.422 | 67.368 | 290.901 | 27.730 | 9.049 | 18.681 |
| 2010 | 24840 | 2329.274 | 688.626 | 1640.649 | 6.605 | 70.436 | 285.273 | 26.750 | 7.908 | 18.842 |
| 2011 | 24792 | 2286.581 | 606.524 | 1680.056 | 6.777 | 73.475 | 282.045 | 26.013 | 6.900 | 19.113 |
| 2012 | 24500 | 2243.311 | 532.483 | 1710.828 | 6.983 | 76.264 | 275.872 | 25.260 | 5.996 | 19.264 |
| 2013 | 24451 | 2176.503 | 461.642 | 1714.861 | 7.013 | 78.790 | 272.368 | 24.245 | 5.142 | 19.102 |
| 2014 | 25296 | 2211.619 | 419.067 | 1792.552 | 7.086 | 81.052 | 278.691 | 24.366 | 4.617 | 19.749 |
| 2015 | 26316 | 2237.016 | 383.040 | 1853.976 | 7.045 | 82.877 | 324.141 | 27.554 | 4.718 | 22.836 |
| 2016 | 26735 | 2229.057 | 340.697 | 1888.360 | 7.063 | 84.716 | 326.164 | 27.194 | 4.156 | 23.038 |
| 2017 | 27616 | 2221.649 | 303.202 | 1918.447 | 6.947 | 86.352 | 333.726 | 26.848 | 3.664 | 23.183 |
| 2018 | 25256 | 1939.420 | 237.360 | 1702.060 | 6.739 | 87.761 | 302.304 | 23.214 | 2.841 | 20.373 |
| 2019 | 25429 | 1904.400 | 205.443 | 1698.956 | 6.681 | 89.212 | 301.434 | 22.574 | 2.435 | 20.139 |
| 2020 | 25379 | 1806.054 | 174.995 | 1631.059 | 6.427 | 90.311 | 340.922 | 24.261 | 2.351 | 21.910 |
| 2021 | 25448 | 1748.172 | 149.485 | 1598.688 | 6.282 | 91.449 | 339.386 | 23.314 | 1.994 | 21.321 |
| 2022 | 25539 | 1701.148 | 128.233 | 1572.915 | 6.159 | 92.462 | 338.015 | 22.515 | 1.697 | 20.818 |
| 2023 | 25620 | 1652.087 | 110.192 | 1541.895 | 6.018 | 93.330 | 336.461 | 21.696 | 1.447 | 20.249 |
| 2024 | 25659 | 1597.127 | 93.153 | 1503.973 | 5.861 | 94.167 | 334.391 | 20.814 | 1.214 | 19.600 |
| 2025 | 25260 | 1467.622 | 77.261 | 1390.361 | 5.504 | 94.736 | 379.931 | 22.075 | 1.162 | 20.913 |
| 2026 | 25480 | 1390.505 | 64.903 | 1325.602 | 5.203 | 95.332 | 381.790 | 20.835 | 0.973 | 19.863 |
| 2027 | 25264 | 1342.095 | 54.838 | 1287.257 | 5.095 | 95.914 | 377.042 | 20.030 | 0.818 | 19.211 |
| 2028 | 25022 | 1289.536 | 46.120 | 1243.416 | 4.969 | 96.424 | 371.870 | 19.165 | 0.685 | 18.479 |
| 2029 | 24767 | 1242.216 | 38.550 | 1203.666 | 4.860 | 96.897 | 366.459 | 18.380 | 0.570 | 17.810 |
| 2030 | 23959 | 1096.803 | 30.832 | 1065.971 | 4.449 | 97.189 | 419.641 | 19.210 | 0.540 | 18.670 |
| 2031 | 23963 | 1019.991 | 25.190 | 994.801 | 4.151 | 97.530 | 419.142 | 17.841 | 0.441 | 17.400 |
| 2032 | 23615 | 977.406 | 20.724 | 956.682 | 4.051 | 97.880 | 412.259 | 17.063 | 0.362 | 16.701 |
| 2033 | 23259 | 937.450 | 17.494 | 919.955 | 3.955 | 98.134 | 405.051 | 16.326 | 0.305 | 16.021 |
| 2034 | 22879 | 900.763 | 14.486 | 886.277 | 3.874 | 98.392 | 397.339 | 15.644 | 0.252 | 15.392 |
| 2035 | 21733 | 745.023 | 11.188 | 733.835 | 3.377 | 98.498 | 462.224 | 15.845 | 0.238 | 15.607 |
| 2036 | 20904 | 680.360 | 8.577 | 671.783 | 3.214 | 98.739 | 445.263 | 14.492 | 0.183 | 14.309 |
| 2037 | 20397 | 644.109 | 7.030 | 637.079 | 3.123 | 98.909 | 434.791 | 13.730 | 0.150 | 13.580 |
| 2038 | 19890 | 612.448 | 5.895 | 606.553 | 3.050 | 99.037 | 423.966 | 13.055 | 0.126 | 12.929 |
| 2039 | 19392 | 577.529 | 4.944 | 572.585 | 2.953 | 99.144 | 412.930 | 12.298 | 0.105 | 12.192 |

# Results for PHIM for Run 4 (Sensitivity_Analysis_1.RTF)

*******************************************************************************

**Main Results for E-Component for Run: 4, Simulation: 1, COD: COPD**

| Variable Parameter | Value |
|---|---|
| 1. Country | US (United States) |
| 2. Sex | F (Females) |
| 3. Year of start of process | 1990 |
| 4. Cause of Death | COPD |
| 5. Effective Dose NULL | CC:1 |
| 6. Effective Dose Ecig | CC:1 Ecig:0 Dual:0.5 |
| Note: | Death rates are per million population |

-----------------------------------------------------------------------------------------

Results for Age Groups Combined and Years combined, Population unadjusted for deaths in Ecig scenario,

| Age Group:All<br>Year | Number<br>Deaths | NULL<br>Attrib<br>Deaths | Ecig<br>Attrib<br>Deaths | Drop<br>Deaths | %Drop<br>Deaths | %Drop<br>Attrib<br>Deaths | Death<br>Rate | NULL<br>Attrib<br>Rates | Ecig<br>Attrib<br>Rates | Drop<br>Rates |
|---|---|---|---|---|---|---|---|---|---|---|
| All Comb | 2159941 | 862051.244 | 529535.037 | 332516.2 | 15.395 | 38.573 | 593.003 | 236.673 | 145.382 | 91.291 |

-----------------------------------------------------------------------------------------

Results for Age Groups Combined, Population unadjusted for deaths in Ecig scenario,

| Age Group:All<br>Year | Number<br>Deaths | NULL<br>Attrib<br>Deaths | Ecig<br>Attrib<br>Deaths | Drop<br>Deaths | %Drop<br>Deaths | %Drop<br>Attrib<br>Deaths | Death<br>Rate | NULL<br>Attrib<br>Rates | Ecig<br>Attrib<br>Rates | Drop<br>Rates |
|---|---|---|---|---|---|---|---|---|---|---|
| 1990 | 24738 | 10694.712 | 10694.712 | 0.000 | 0.000 | 0.000 | 362.296 | 156.628 | 156.628 | 0.000 |
| 1991 | 26562 | 11602.933 | 11583.812 | 19.121 | 0.072 | 0.165 | 383.818 | 167.661 | 167.385 | 0.276 |
| 1992 | 26828 | 11831.375 | 11778.551 | 52.824 | 0.197 | 0.446 | 382.401 | 168.642 | 167.889 | 0.753 |
| 1993 | 29774 | 13246.371 | 13132.416 | 113.955 | 0.383 | 0.860 | 418.406 | 186.148 | 184.546 | 1.601 |
| 1994 | 29804 | 13355.662 | 13174.404 | 181.258 | 0.608 | 1.357 | 412.601 | 184.893 | 182.384 | 2.509 |
| 1995 | 29755 | 13397.439 | 13128.276 | 269.163 | 0.905 | 2.009 | 405.540 | 182.598 | 178.929 | 3.669 |
| 1996 | 30900 | 13960.356 | 13583.876 | 376.480 | 1.218 | 2.697 | 415.680 | 187.801 | 182.736 | 5.065 |
| 1997 | 31128 | 14109.552 | 13616.644 | 492.908 | 1.583 | 3.493 | 412.949 | 187.180 | 180.641 | 6.539 |
| 1998 | 32096 | 14599.657 | 13954.030 | 645.627 | 2.012 | 4.422 | 419.803 | 190.958 | 182.513 | 8.445 |
| 1999 | 34616 | 15728.102 | 14888.772 | 839.330 | 2.425 | 5.337 | 446.673 | 202.950 | 192.119 | 10.830 |
| 2000 | 34299 | 15579.803 | 14581.415 | 998.388 | 2.911 | 6.408 | 437.088 | 198.541 | 185.818 | 12.723 |
| 2001 | 34592 | 15696.614 | 14492.984 | 1203.630 | 3.480 | 7.668 | 435.935 | 197.812 | 182.643 | 15.168 |
| 2002 | 34291 | 15519.777 | 14121.691 | 1398.086 | 4.077 | 9.008 | 427.910 | 193.668 | 176.222 | 17.446 |
| 2003 | 35074 | 15888.389 | 14234.931 | 1653.458 | 4.714 | 10.407 | 433.587 | 196.413 | 175.973 | 20.440 |
| 2004 | 33512 | 15091.573 | 13294.844 | 1796.729 | 5.361 | 11.906 | 410.143 | 184.701 | 162.712 | 21.990 |
| 2005 | 35392 | 15803.656 | 13654.845 | 2148.811 | 6.071 | 13.597 | 428.364 | 191.278 | 165.270 | 26.008 |
| 2006 | 33981 | 15100.563 | 12809.908 | 2290.655 | 6.741 | 15.169 | 407.699 | 181.174 | 153.691 | 27.483 |
| 2007 | 34000 | 14974.384 | 12437.906 | 2536.478 | 7.460 | 16.939 | 403.863 | 177.871 | 147.742 | 30.129 |
| 2008 | 37425 | 16406.197 | 13358.778 | 3047.420 | 8.143 | 18.575 | 439.732 | 192.768 | 156.961 | 35.806 |
| 2009 | 36826 | 16083.194 | 12770.164 | 3313.030 | 8.996 | 20.599 | 427.825 | 186.846 | 148.357 | 38.489 |
| 2010 | 36788 | 15927.400 | 12371.851 | 3555.550 | 9.665 | 22.323 | 422.489 | 182.917 | 142.084 | 40.833 |
| 2011 | 37636 | 16221.836 | 12268.327 | 3953.509 | 10.505 | 24.372 | 428.164 | 184.547 | 139.570 | 44.977 |
| 2012 | 38265 | 16358.849 | 12038.245 | 4320.605 | 11.291 | 26.411 | 430.867 | 184.202 | 135.552 | 48.650 |
| 2013 | 40112 | 16994.822 | 12138.069 | 4856.753 | 12.108 | 28.578 | 446.821 | 189.311 | 135.210 | 54.101 |
| 2014 | 40084 | 16855.093 | 11668.375 | 5186.719 | 12.940 | 30.772 | 441.613 | 185.696 | 128.553 | 57.143 |
| 2015 | 42200 | 17572.483 | 11858.958 | 5713.525 | 13.539 | 32.514 | 519.788 | 216.445 | 146.070 | 70.375 |
| 2016 | 42606 | 17579.955 | 11442.817 | 6137.138 | 14.404 | 34.910 | 519.788 | 214.473 | 139.601 | 74.872 |
| 2017 | 44364 | 18080.688 | 11344.393 | 6736.296 | 15.184 | 37.257 | 536.117 | 218.496 | 137.091 | 81.405 |
| 2018 | 45359 | 18209.262 | 11046.272 | 7162.991 | 15.792 | 39.337 | 542.932 | 217.960 | 132.221 | 85.739 |
| 2019 | 46876 | 18648.302 | 10883.862 | 7764.440 | 16.564 | 41.636 | 555.657 | 221.054 | 129.016 | 92.038 |
| 2020 | 48331 | 19002.597 | 10714.573 | 8288.024 | 17.149 | 43.615 | 649.239 | 255.266 | 143.931 | 111.335 |
| 2021 | 48338 | 18885.717 | 10193.057 | 8692.661 | 17.983 | 46.028 | 644.660 | 251.867 | 135.938 | 115.929 |
| 2022 | 49708 | 19235.076 | 9951.201 | 9283.875 | 18.677 | 48.265 | 657.895 | 254.582 | 131.707 | 122.875 |
| 2023 | 51083 | 19521.158 | 9696.519 | 9824.639 | 19.233 | 50.328 | 670.849 | 256.363 | 127.340 | 129.023 |
| 2024 | 52397 | 19918.620 | 9438.968 | 10479.65 | 20.000 | 52.612 | 682.854 | 259.583 | 123.010 | 136.573 |
| 2025 | 53387 | 20150.545 | 9141.639 | 11008.91 | 20.621 | 54.633 | 803.004 | 303.086 | 137.500 | 165.586 |
| 2026 | 54232 | 20393.792 | 8797.068 | 11596.72 | 21.384 | 56.864 | 812.615 | 305.582 | 131.816 | 173.766 |
| 2027 | 55049 | 20615.351 | 8429.324 | 12186.03 | 22.137 | 59.111 | 821.568 | 307.668 | 125.801 | 181.867 |
| 2028 | 55809 | 20731.894 | 8020.462 | 12711.43 | 22.777 | 61.313 | 829.419 | 308.115 | 119.199 | 188.916 |
| 2029 | 56527 | 20781.967 | 7598.347 | 13183.62 | 23.323 | 63.438 | 836.392 | 307.495 | 112.427 | 195.068 |
| 2030 | 56756 | 20753.201 | 7199.339 | 13553.86 | 23.881 | 65.310 | 994.067 | 363.487 | 126.095 | 237.393 |
| 2031 | 58288 | 21048.417 | 7034.211 | 14014.21 | 24.043 | 66.581 | 1019.54 | 368.165 | 123.038 | 245.127 |
| 2032 | 58782 | 21169.329 | 6657.696 | 14511.63 | 24.687 | 68.550 | 1026.18 | 369.560 | 116.226 | 253.335 |
| 2033 | 59236 | 21109.293 | 6253.603 | 14855.69 | 25.079 | 70.375 | 1031.59 | 367.618 | 108.907 | 258.712 |
| 2034 | 59614 | 21109.429 | 5850.411 | 15259.02 | 25.596 | 72.285 | 1035.33 | 366.613 | 101.605 | 265.007 |
| 2035 | 58938 | 20794.840 | 5467.317 | 15327.52 | 26.006 | 73.708 | 1253.51 | 442.268 | 116.280 | 325.988 |
| 2036 | 56014 | 19354.577 | 4801.405 | 14553.17 | 25.981 | 75.192 | 1193.11 | 412.258 | 102.271 | 309.986 |
| 2037 | 55953 | 19064.169 | 4360.319 | 14703.85 | 26.279 | 77.128 | 1192.72 | 406.385 | 92.948 | 313.437 |
| 2038 | 55859 | 18854.812 | 3992.614 | 14862.20 | 26.607 | 78.824 | 1190.66 | 401.900 | 85.105 | 316.795 |
| 2039 | 55757 | 18437.457 | 3582.838 | 14854.62 | 26.642 | 80.568 | 1187.28 | 392.602 | 76.292 | 316.310 |

# Results for PHIM for Run 4 (Sensitivity_Analysis_1.RTF)

************************************************************************************
**Main Results for E-Component for Run: 4, Simulation: 1, COD: Four Diseases Combined, Adjusted**

| Variable Parameter | Value |
|---|---|
| 1. Country | US (United States) |
| 2. Sex | F (Females) |
| 3. Year of start of process | 1990 |
| 4. Cause of Death | Four Diseases Combined |
| 5. Effective Dose NULL | CC:1 |
| 6. Effective Dose Ecig | CC:1 Ecig:0 Dual:0.5 |
| Note: | Death rates are per million population |

------------------------------------------------------------------------------------------
Results for Age Groups Combined and Years combined, Population and Number deaths adjusted for Ecig scenario,

| Age Group:All Year | Number Deaths | NULL Attrib Deaths | Ecig Attrib Deaths | Drop Deaths | %Drop Deaths | %Drop Attrib Deaths | Death Rate | NULL Attrib Rates | Ecig Attrib Rates | Drop Rates |
|---|---|---|---|---|---|---|---|---|---|---|
| All Comb | 9153195 | 2946250.006 | 1869245.975 | 1077004 | 11.766 | 36.555 | 2500.01 | 808.881 | 510.547 | 298.334 |

------------------------------------------------------------------------------------------
Results for Age Groups Combined, Population and Number deaths adjusted for Ecig scenario,

| Age Group:All Year | Number Deaths | NULL Attrib Deaths | Ecig Attrib Deaths | Drop Deaths | %Drop Deaths | %Drop Attrib Deaths | Death Rate | NULL Attrib Rates | Ecig Attrib Rates | Drop Rates |
|---|---|---|---|---|---|---|---|---|---|---|
| 1990 | 199848 | 57524.822 | 57524.822 | 0.000 | 0.000 | 0.000 | 2926.84 | 842.471 | 842.471 | 0.000 |
| 1991 | 199162 | 59078.486 | 58901.294 | 177.192 | 0.089 | 0.300 | 2877.87 | 853.678 | 851.118 | 2.560 |
| 1992 | 198533 | 60494.150 | 59997.417 | 496.732 | 0.250 | 0.821 | 2829.84 | 862.271 | 855.189 | 7.083 |
| 1993 | 202515 | 62694.848 | 61727.787 | 967.060 | 0.478 | 1.542 | 2845.86 | 881.034 | 867.435 | 13.598 |
| 1994 | 200665 | 63202.598 | 61673.719 | 1528.878 | 0.762 | 2.419 | 2777.91 | 874.966 | 853.780 | 21.186 |
| 1995 | 201037 | 63766.862 | 61557.707 | 2209.155 | 1.099 | 3.464 | 2739.87 | 869.097 | 838.950 | 30.147 |
| 1996 | 201203 | 64591.163 | 61561.954 | 3029.209 | 1.506 | 4.690 | 2706.47 | 868.908 | 828.095 | 40.813 |
| 1997 | 198675 | 64686.912 | 60890.856 | 3796.056 | 1.911 | 5.868 | 2635.35 | 858.147 | 807.693 | 50.454 |
| 1998 | 197626 | 65295.961 | 60612.321 | 4683.640 | 2.370 | 7.173 | 2584.43 | 854.046 | 792.652 | 61.394 |
| 1999 | 211408 | 67327.521 | 61271.226 | 6056.296 | 2.865 | 8.995 | 2727.30 | 868.771 | 790.439 | 78.332 |
| 2000 | 208602 | 67596.897 | 60543.171 | 7053.725 | 3.381 | 10.435 | 2657.48 | 861.419 | 771.289 | 90.130 |
| 2001 | 203742 | 67008.811 | 58895.138 | 8113.672 | 3.982 | 12.108 | 2566.55 | 844.458 | 741.906 | 102.552 |
| 2002 | 200999 | 67067.744 | 57774.253 | 9293.490 | 4.624 | 13.857 | 2506.94 | 836.925 | 720.583 | 116.342 |
| 2003 | 196562 | 66756.217 | 56395.842 | 10360.38 | 5.271 | 15.520 | 2428.36 | 825.244 | 696.726 | 128.518 |
| 2004 | 188407 | 65058.725 | 53791.863 | 11266.86 | 5.980 | 17.318 | 2304.08 | 796.234 | 657.835 | 138.400 |
| 2005 | 186710 | 65214.724 | 52725.010 | 12489.71 | 6.689 | 19.152 | 2257.75 | 789.321 | 637.566 | 151.755 |
| 2006 | 180424 | 63771.146 | 50348.076 | 13423.07 | 7.440 | 21.049 | 2162.37 | 765.117 | 603.416 | 161.701 |
| 2007 | 176027 | 63110.718 | 48728.130 | 14382.59 | 8.171 | 22.789 | 2088.28 | 749.650 | 578.082 | 171.568 |
| 2008 | 178018 | 63718.624 | 47953.217 | 15765.41 | 8.856 | 24.742 | 2088.63 | 748.674 | 562.623 | 186.051 |
| 2009 | 173305 | 62794.698 | 46129.495 | 16665.20 | 9.616 | 26.539 | 2010.05 | 729.515 | 535.026 | 194.489 |
| 2010 | 171047 | 61917.074 | 44300.611 | 17616.46 | 10.299 | 28.452 | 1960.73 | 711.083 | 507.822 | 203.261 |
| 2011 | 170931 | 61680.114 | 42795.979 | 18884.14 | 11.048 | 30.616 | 1940.54 | 701.700 | 485.852 | 215.848 |
| 2012 | 172124 | 62023.127 | 41725.486 | 20297.64 | 11.792 | 32.726 | 1933.64 | 698.386 | 468.744 | 229.643 |
| 2013 | 173489 | 62037.765 | 40472.092 | 21565.67 | 12.431 | 34.762 | 1927.58 | 691.059 | 449.673 | 241.386 |
| 2014 | 175050 | 61921.017 | 38956.459 | 22964.56 | 13.119 | 37.087 | 1923.08 | 682.195 | 427.973 | 254.222 |
| 2015 | 178346 | 61856.204 | 37700.825 | 24155.38 | 13.544 | 39.051 | 2189.06 | 761.899 | 462.748 | 299.151 |
| 2016 | 177937 | 60464.119 | 35343.749 | 25120.37 | 14.118 | 41.546 | 2162.51 | 737.655 | 429.541 | 308.115 |
| 2017 | 180513 | 60243.839 | 33836.204 | 26407.63 | 14.629 | 43.835 | 2172.32 | 728.017 | 407.191 | 320.826 |
| 2018 | 178456 | 60713.327 | 33123.003 | 27590.32 | 15.461 | 45.444 | 2126.41 | 726.721 | 394.681 | 332.039 |
| 2019 | 180659 | 61148.172 | 32056.310 | 29091.86 | 16.103 | 47.576 | 2131.04 | 724.841 | 378.135 | 346.706 |
| 2020 | 182040 | 61149.317 | 30996.028 | 30153.29 | 16.564 | 49.311 | 2430.89 | 821.432 | 413.907 | 407.525 |
| 2021 | 179791 | 58727.786 | 28133.607 | 30594.18 | 17.016 | 52.095 | 2382.49 | 783.216 | 372.810 | 410.406 |
| 2022 | 181225 | 58848.802 | 26963.856 | 31884.95 | 17.594 | 54.181 | 2382.22 | 778.881 | 354.442 | 424.438 |
| 2023 | 182604 | 58816.149 | 25796.082 | 33020.07 | 18.083 | 56.141 | 2380.63 | 772.407 | 336.306 | 436.101 |
| 2024 | 183725 | 58955.232 | 24619.675 | 34335.56 | 18.689 | 58.240 | 2375.82 | 768.316 | 318.367 | 449.949 |
| 2025 | 182684 | 58297.744 | 23391.767 | 34905.98 | 19.107 | 59.875 | 2722.31 | 876.861 | 348.578 | 528.283 |
| 2026 | 182740 | 56469.971 | 21341.104 | 35128.87 | 19.223 | 62.208 | 2711.32 | 846.150 | 316.639 | 529.511 |
| 2027 | 182192 | 56209.427 | 20083.674 | 36125.75 | 19.828 | 64.270 | 2690.91 | 838.883 | 296.630 | 542.254 |
| 2028 | 181483 | 55733.105 | 18774.441 | 36958.66 | 20.365 | 66.314 | 2667.76 | 828.298 | 275.980 | 552.318 |
| 2029 | 180678 | 55145.838 | 17464.597 | 37681.24 | 20.855 | 68.330 | 2642.72 | 815.951 | 255.449 | 560.502 |
| 2030 | 176595 | 53615.097 | 16236.778 | 37378.32 | 21.166 | 69.716 | 3051.02 | 939.056 | 280.522 | 658.534 |
| 2031 | 178221 | 52046.561 | 15068.634 | 36977.93 | 20.748 | 71.048 | 3072.96 | 910.364 | 259.819 | 650.545 |
| 2032 | 176786 | 51582.636 | 14000.875 | 37581.76 | 21.258 | 72.857 | 3040.32 | 900.496 | 240.783 | 659.713 |
| 2033 | 175294 | 50839.758 | 12933.341 | 37906.42 | 21.625 | 74.561 | 3005.38 | 885.375 | 221.740 | 663.635 |
| 2034 | 173656 | 50215.451 | 11890.059 | 38325.39 | 22.070 | 76.322 | 2967.22 | 872.104 | 203.163 | 668.941 |
| 2035 | 166429 | 47773.075 | 10897.692 | 36875.38 | 22.157 | 77.189 | 3472.66 | 1016.05 | 227.388 | 788.657 |
| 2036 | 160071 | 43298.709 | 9168.101 | 34130.61 | 21.322 | 78.826 | 3342.51 | 922.274 | 191.443 | 730.831 |
| 2037 | 157513 | 42241.563 | 8198.213 | 34043.35 | 21.613 | 80.592 | 3289.39 | 900.450 | 171.205 | 729.245 |
| 2038 | 154964 | 41356.353 | 7412.443 | 33943.91 | 21.904 | 82.077 | 3233.80 | 881.531 | 154.683 | 726.847 |
| 2039 | 152485 | 40161.050 | 6560.993 | 33600.06 | 22.035 | 83.663 | 3176.72 | 855.178 | 136.685 | 718.492 |

# Results for PHIM for Run 4 (Sensitivity_Analysis_1.RTF)

************************************************************************************
**Main Results for E-Component for Run: 4, Simulation: 1, COD: LUNG CANCER, Adjusted**

| Variable Parameter | Value |
|---|---|
| 1. Country | US (United States) |
| 2. Sex | F (Females) |
| 3. Year of start of process | 1990 |
| 4. Cause of Death | LUNG CANCER |
| 5. Effective Dose NULL | CC:1 |
| 6. Effective Dose Ecig | CC:1 Ecig:0 Dual:0.5 |
| Note: | Death rates are per million population |

------------------------------------------------------------------------------------------
Results for Age Groups Combined and Years combined, Population and Number deaths adjusted for Ecig scenario,

| Age Group:All | | NULL | Ecig | | | %Drop | | NULL | Ecig | |
|---|---|---|---|---|---|---|---|---|---|---|
| Year | Number Deaths | Attrib Deaths | Attrib Deaths | Drop Deaths | %Drop Deaths | Attrib Deaths | Death Rate | Attrib Rates | Attrib Rates | Drop Rates |
| All Comb | 2345097 | 1580465.217 | 1101336.308 | 479128.9 | 20.431 | 30.316 | 640.517 | 433.910 | 300.808 | 133.102 |

------------------------------------------------------------------------------------------
Results for Age Groups Combined, Population and Number deaths adjusted for Ecig scenario,

| Age Group:All | | NULL | Ecig | | | %Drop | | NULL | Ecig | |
|---|---|---|---|---|---|---|---|---|---|---|
| Year | Number Deaths | Attrib Deaths | Attrib Deaths | Drop Deaths | %Drop Deaths | Attrib Deaths | Death Rate | Attrib Rates | Attrib Rates | Drop Rates |
| 1990 | 42690 | 30614.201 | 30614.201 | 0.000 | 0.000 | 0.000 | 625.210 | 448.355 | 448.355 | 0.000 |
| 1991 | 43692 | 31412.010 | 31378.038 | 33.973 | 0.078 | 0.108 | 631.345 | 453.901 | 453.410 | 0.491 |
| 1992 | 45423 | 32782.407 | 32683.573 | 98.833 | 0.218 | 0.301 | 647.451 | 467.274 | 465.864 | 1.410 |
| 1993 | 46292 | 33491.183 | 33295.899 | 195.284 | 0.422 | 0.583 | 650.518 | 470.643 | 467.894 | 2.749 |
| 1994 | 47019 | 34074.347 | 33757.730 | 316.617 | 0.673 | 0.929 | 650.904 | 471.719 | 467.325 | 4.394 |
| 1995 | 47843 | 34694.454 | 34217.159 | 477.295 | 0.998 | 1.376 | 652.042 | 472.861 | 466.335 | 6.526 |
| 1996 | 48250 | 35027.269 | 34366.434 | 660.834 | 1.370 | 1.887 | 649.028 | 471.202 | 462.277 | 8.925 |
| 1997 | 49044 | 35590.763 | 34710.856 | 879.906 | 1.794 | 2.472 | 650.553 | 472.153 | 460.426 | 11.727 |
| 1998 | 49700 | 36100.785 | 34961.871 | 1138.914 | 2.292 | 3.155 | 649.944 | 472.184 | 457.211 | 14.974 |
| 1999 | 48902 | 35451.030 | 34077.276 | 1373.753 | 2.809 | 3.875 | 630.865 | 457.448 | 439.619 | 17.828 |
| 2000 | 50357 | 36447.986 | 34728.124 | 1719.862 | 3.415 | 4.719 | 641.527 | 464.474 | 442.418 | 22.056 |
| 2001 | 50424 | 36411.326 | 34338.822 | 2072.505 | 4.110 | 5.692 | 635.189 | 458.863 | 432.569 | 26.294 |
| 2002 | 51490 | 37078.545 | 34567.276 | 2511.269 | 4.877 | 6.773 | 642.201 | 462.696 | 431.137 | 31.559 |
| 2003 | 51464 | 36999.876 | 34068.370 | 2931.506 | 5.696 | 7.923 | 635.796 | 457.395 | 420.888 | 36.507 |
| 2004 | 51467 | 36861.555 | 33461.504 | 3400.051 | 6.606 | 9.224 | 629.409 | 451.138 | 409.210 | 41.928 |
| 2005 | 51503 | 36689.529 | 32791.340 | 3898.189 | 7.569 | 10.625 | 622.786 | 444.069 | 396.522 | 47.546 |
| 2006 | 51451 | 36504.774 | 32080.613 | 4424.161 | 8.599 | 12.119 | 616.639 | 437.979 | 384.482 | 53.497 |
| 2007 | 51952 | 36663.202 | 31611.866 | 5051.336 | 9.723 | 13.778 | 616.334 | 435.498 | 375.025 | 60.473 |
| 2008 | 51105 | 35898.803 | 30371.271 | 5527.532 | 10.816 | 15.398 | 599.598 | 421.800 | 356.338 | 65.461 |
| 2009 | 51341 | 35934.525 | 29727.593 | 6206.933 | 12.090 | 17.273 | 595.469 | 417.468 | 344.791 | 72.677 |
| 2010 | 51019 | 35502.829 | 28732.981 | 6769.848 | 13.269 | 19.068 | 584.837 | 407.730 | 329.369 | 78.361 |
| 2011 | 50694 | 35136.342 | 27710.796 | 7425.546 | 14.648 | 21.134 | 575.517 | 399.726 | 314.594 | 85.132 |
| 2012 | 51455 | 35450.201 | 27207.786 | 8242.414 | 16.019 | 23.251 | 578.041 | 399.173 | 305.652 | 93.521 |
| 2013 | 51301 | 35123.224 | 26162.546 | 8960.678 | 17.467 | 25.512 | 569.993 | 391.249 | 290.684 | 100.565 |
| 2014 | 51509 | 35079.370 | 25346.593 | 9732.777 | 18.895 | 27.745 | 565.875 | 386.476 | 278.456 | 108.020 |
| 2015 | 50724 | 34324.171 | 24078.299 | 10245.87 | 20.199 | 29.850 | 622.593 | 422.780 | 295.542 | 127.238 |
| 2016 | 49152 | 33054.753 | 22355.767 | 10698.99 | 21.767 | 32.367 | 597.355 | 403.264 | 271.695 | 131.569 |
| 2017 | 48648 | 32471.234 | 21130.800 | 11340.43 | 23.311 | 34.925 | 585.438 | 392.399 | 254.292 | 138.107 |
| 2018 | 50832 | 33657.595 | 20963.690 | 12693.91 | 24.972 | 37.715 | 605.690 | 402.871 | 249.795 | 153.076 |
| 2019 | 51439 | 33863.424 | 20198.020 | 13665.40 | 26.566 | 40.354 | 606.775 | 401.412 | 238.255 | 163.157 |
| 2020 | 51886 | 33910.830 | 19428.156 | 14482.67 | 27.913 | 42.708 | 692.857 | 455.532 | 259.435 | 196.097 |
| 2021 | 48483 | 31531.880 | 17193.912 | 14337.97 | 29.573 | 45.471 | 642.473 | 420.521 | 227.843 | 192.678 |
| 2022 | 48818 | 31560.212 | 16360.046 | 15200.17 | 31.136 | 48.162 | 641.718 | 417.708 | 215.054 | 202.654 |
| 2023 | 49120 | 31508.781 | 15524.000 | 15984.78 | 32.542 | 50.731 | 640.385 | 413.791 | 202.388 | 211.403 |
| 2024 | 49344 | 31517.293 | 14675.619 | 16841.67 | 34.131 | 53.436 | 638.085 | 410.740 | 189.777 | 220.963 |
| 2025 | 49076 | 31175.389 | 13803.512 | 17371.88 | 35.398 | 55.723 | 731.311 | 468.912 | 205.696 | 263.215 |
| 2026 | 45927 | 29044.598 | 12136.733 | 16907.86 | 36.815 | 58.213 | 681.416 | 435.206 | 180.073 | 255.133 |
| 2027 | 45745 | 28825.765 | 11289.559 | 17536.21 | 38.334 | 60.835 | 675.643 | 430.203 | 166.743 | 263.459 |
| 2028 | 45515 | 28513.846 | 10429.258 | 18084.59 | 39.733 | 63.424 | 669.064 | 423.769 | 153.308 | 270.461 |
| 2029 | 45248 | 28148.109 | 9574.765 | 18573.34 | 41.047 | 65.984 | 661.834 | 416.486 | 140.047 | 276.439 |
| 2030 | 44134 | 27314.475 | 8773.690 | 18540.79 | 42.010 | 67.879 | 762.509 | 478.407 | 151.582 | 326.824 |
| 2031 | 41494 | 25423.102 | 7781.158 | 17641.94 | 42.517 | 69.393 | 715.460 | 444.684 | 134.166 | 310.518 |
| 2032 | 41105 | 25085.902 | 7111.186 | 17974.72 | 43.729 | 71.653 | 706.905 | 437.933 | 122.296 | 315.637 |
| 2033 | 40694 | 24645.067 | 6464.226 | 18180.84 | 44.677 | 73.771 | 697.693 | 429.194 | 110.828 | 318.366 |
| 2034 | 40248 | 24239.126 | 5839.983 | 18399.14 | 45.714 | 75.907 | 687.709 | 420.967 | 99.786 | 321.180 |
| 2035 | 38317 | 22944.147 | 5243.476 | 17700.67 | 46.195 | 77.147 | 799.519 | 487.980 | 109.409 | 378.571 |
| 2036 | 33784 | 19950.581 | 4199.091 | 15751.49 | 46.624 | 78.953 | 705.459 | 424.953 | 87.683 | 337.270 |
| 2037 | 33219 | 19415.334 | 3685.333 | 15730.00 | 47.353 | 81.018 | 693.714 | 413.871 | 76.962 | 336.909 |
| 2038 | 32654 | 18939.317 | 3277.784 | 15661.53 | 47.962 | 82.693 | 681.427 | 403.701 | 68.401 | 335.300 |
| 2039 | 32103 | 18379.752 | 2847.727 | 15532.02 | 48.381 | 84.506 | 668.809 | 391.373 | 59.327 | 332.046 |

# Results for PHIM for Run 4 (Sensitivity_Analysis_1.RTF)

************************************************************************************
**Main Results for E-Component for Run: 4, Simulation: 1, COD: IHD, Adjusted**

| Variable Parameter | Value |
|---|---|
| 1. Country | US (United States) |
| 2. Sex | F (Females) |
| 3. Year of start of process | 1990 |
| 4. Cause of Death | IHD |
| 5. Effective Dose NULL | CC:1 |
| 6. Effective Dose Ecig | CC:1 Ecig:0 Dual:0.5 |
| Note: | Death rates are per million population |

-----------------------------------------------------------------------------------------
Results for Age Groups Combined and Years combined, Population and Number deaths adjusted for Ecig scenario,

| Age Group:All | | NULL | Ecig | | | %Drop | | NULL | Ecig | |
|---|---|---|---|---|---|---|---|---|---|---|
| Year | Number | Attrib | Attrib | Drop | %Drop | Attrib | Death | Attrib | Attrib | Drop |
| | Deaths | Deaths | Deaths | Deaths | Deaths | Deaths | Rate | Rates | Rates | Rates |
| All Comb | 3263043 | 398833.796 | 186846.829 | 211987.0 | 6.497 | 53.152 | 891.235 | 109.498 | 51.033 | 58.465 |

-----------------------------------------------------------------------------------------
Results for Age Groups Combined, Population and Number deaths adjusted for Ecig scenario,

| Age Group:All | | NULL | Ecig | | | %Drop | | NULL | Ecig | |
|---|---|---|---|---|---|---|---|---|---|---|
| Year | Number | Attrib | Attrib | Drop | %Drop | Attrib | Death | Attrib | Attrib | Drop |
| | Deaths | Deaths | Deaths | Deaths | Deaths | Deaths | Rate | Rates | Rates | Rates |
| 1990 | 98916 | 12922.892 | 12922.892 | 0.000 | 0.000 | 0.000 | 1448.66 | 189.260 | 189.260 | 0.000 |
| 1991 | 96403 | 12856.479 | 12761.318 | 95.161 | 0.099 | 0.740 | 1393.01 | 185.775 | 184.400 | 1.375 |
| 1992 | 93721 | 12662.244 | 12398.079 | 264.165 | 0.282 | 2.086 | 1335.88 | 180.485 | 176.719 | 3.766 |
| 1993 | 93680 | 12730.613 | 12225.954 | 504.659 | 0.539 | 3.964 | 1316.45 | 178.900 | 171.806 | 7.094 |
| 1994 | 90835 | 12495.323 | 11708.561 | 786.761 | 0.866 | 6.296 | 1257.48 | 172.983 | 162.087 | 10.896 |
| 1995 | 89704 | 12365.590 | 11252.819 | 1112.771 | 1.240 | 8.999 | 1222.55 | 168.534 | 153.361 | 15.173 |
| 1996 | 88296 | 12290.551 | 10771.525 | 1519.026 | 1.720 | 12.359 | 1187.71 | 165.338 | 144.892 | 20.446 |
| 1997 | 84792 | 11719.531 | 9888.229 | 1831.302 | 2.160 | 15.626 | 1124.74 | 155.473 | 131.163 | 24.310 |
| 1998 | 82539 | 11416.794 | 9222.789 | 2194.005 | 2.658 | 19.217 | 1079.39 | 149.327 | 120.610 | 28.717 |
| 1999 | 93974 | 13028.038 | 10004.759 | 3023.279 | 3.217 | 23.206 | 1212.32 | 168.109 | 129.068 | 39.041 |
| 2000 | 90084 | 12426.299 | 9049.251 | 3377.048 | 3.749 | 27.177 | 1147.63 | 158.354 | 115.283 | 43.071 |
| 2001 | 86176 | 11939.281 | 8129.536 | 3809.745 | 4.421 | 31.909 | 1085.56 | 150.461 | 102.408 | 48.053 |
| 2002 | 83084 | 11528.794 | 7299.145 | 4229.649 | 5.091 | 36.688 | 1036.26 | 143.865 | 91.038 | 52.828 |
| 2003 | 79386 | 11048.407 | 6500.370 | 4548.037 | 5.729 | 41.165 | 980.751 | 136.581 | 80.307 | 56.274 |
| 2004 | 73957 | 10375.132 | 5611.007 | 4764.125 | 6.442 | 45.919 | 904.443 | 126.978 | 68.618 | 58.360 |
| 2005 | 71761 | 10051.029 | 4992.794 | 5058.235 | 7.049 | 50.326 | 867.757 | 121.652 | 60.374 | 61.277 |
| 2006 | 67676 | 9542.523 | 4295.308 | 5247.215 | 7.753 | 54.988 | 811.094 | 114.490 | 51.479 | 63.011 |
| 2007 | 63640 | 8936.533 | 3648.384 | 5288.149 | 8.309 | 59.175 | 754.993 | 106.151 | 43.282 | 62.869 |
| 2008 | 63329 | 8944.180 | 3297.978 | 5646.202 | 8.916 | 63.127 | 743.019 | 105.091 | 38.694 | 66.397 |
| 2009 | 59918 | 8390.033 | 2812.306 | 5577.728 | 9.309 | 66.480 | 694.950 | 97.471 | 32.618 | 64.853 |
| 2010 | 58196 | 8157.570 | 2462.738 | 5694.831 | 9.786 | 69.810 | 667.105 | 93.685 | 28.231 | 65.455 |
| 2011 | 57577 | 8035.356 | 2161.096 | 5874.260 | 10.202 | 73.105 | 653.655 | 91.414 | 24.534 | 66.879 |
| 2012 | 57643 | 7970.766 | 1893.139 | 6077.627 | 10.543 | 76.249 | 647.566 | 89.752 | 21.268 | 68.484 |
| 2013 | 57325 | 7743.216 | 1649.687 | 6093.529 | 10.630 | 78.695 | 636.918 | 86.254 | 18.329 | 67.925 |
| 2014 | 57825 | 7774.935 | 1458.491 | 6316.444 | 10.923 | 81.241 | 635.260 | 85.658 | 16.023 | 69.635 |
| 2015 | 58717 | 7722.535 | 1308.912 | 6413.623 | 10.923 | 83.051 | 720.702 | 95.120 | 16.066 | 79.055 |
| 2016 | 59013 | 7600.353 | 1128.897 | 6471.456 | 10.966 | 85.147 | 717.193 | 92.723 | 13.720 | 79.004 |
| 2017 | 59394 | 7470.266 | 975.908 | 6494.359 | 10.934 | 86.936 | 714.757 | 90.274 | 11.744 | 78.530 |
| 2018 | 56481 | 6907.050 | 788.551 | 6118.499 | 10.833 | 88.583 | 673.003 | 82.675 | 9.396 | 73.279 |
| 2019 | 56324 | 6732.046 | 675.469 | 6056.577 | 10.753 | 89.966 | 664.397 | 79.801 | 7.968 | 71.833 |
| 2020 | 55788 | 6429.836 | 578.257 | 5851.579 | 10.489 | 91.007 | 744.964 | 86.373 | 7.722 | 78.652 |
| 2021 | 56810 | 6562.016 | 494.517 | 6067.499 | 10.680 | 92.464 | 752.810 | 87.513 | 6.553 | 80.960 |
| 2022 | 56380 | 6352.366 | 416.595 | 5935.771 | 10.528 | 93.442 | 741.115 | 84.075 | 5.476 | 78.599 |
| 2023 | 55926 | 6134.123 | 352.585 | 5781.538 | 10.338 | 94.252 | 729.114 | 80.557 | 4.597 | 75.960 |
| 2024 | 55393 | 5922.193 | 294.290 | 5627.903 | 10.160 | 95.031 | 716.312 | 77.179 | 3.806 | 73.374 |
| 2025 | 53952 | 5504.188 | 247.367 | 5256.821 | 9.744 | 95.506 | 803.972 | 82.789 | 3.686 | 79.103 |
| 2026 | 56002 | 5641.075 | 216.270 | 5424.805 | 9.687 | 96.166 | 830.897 | 84.526 | 3.209 | 81.317 |
| 2027 | 54954 | 5426.216 | 180.954 | 5245.262 | 9.545 | 96.665 | 811.648 | 80.982 | 2.673 | 78.310 |
| 2028 | 53875 | 5197.830 | 147.920 | 5049.910 | 9.373 | 97.154 | 791.956 | 77.249 | 2.174 | 75.075 |
| 2029 | 52789 | 4973.547 | 121.214 | 4852.332 | 9.192 | 97.563 | 772.123 | 73.590 | 1.773 | 71.817 |
| 2030 | 50316 | 4450.618 | 100.150 | 4350.468 | 8.646 | 97.750 | 869.310 | 77.952 | 1.730 | 76.221 |
| 2031 | 52917 | 4555.052 | 89.030 | 4466.023 | 8.440 | 98.045 | 912.420 | 79.674 | 1.535 | 78.139 |
| 2032 | 51634 | 4349.999 | 71.628 | 4278.371 | 8.286 | 98.353 | 887.983 | 75.939 | 1.232 | 74.708 |
| 2033 | 50362 | 4147.948 | 59.052 | 4088.896 | 8.119 | 98.576 | 863.441 | 72.237 | 1.012 | 71.224 |
| 2034 | 49080 | 3966.133 | 47.659 | 3918.474 | 7.984 | 98.798 | 838.612 | 68.881 | 0.814 | 68.066 |
| 2035 | 45526 | 3289.064 | 39.537 | 3249.527 | 7.138 | 98.798 | 949.929 | 69.952 | 0.825 | 69.127 |
| 2036 | 47444 | 3313.192 | 32.547 | 3280.645 | 6.915 | 99.018 | 990.691 | 70.572 | 0.680 | 69.892 |
| 2037 | 45947 | 3117.951 | 24.870 | 3093.081 | 6.732 | 99.202 | 959.516 | 66.464 | 0.519 | 65.945 |
| 2038 | 44491 | 2949.775 | 20.109 | 2929.666 | 6.585 | 99.318 | 928.447 | 62.876 | 0.420 | 62.456 |
| 2039 | 43092 | 2766.312 | 16.382 | 2749.930 | 6.382 | 99.408 | 897.738 | 58.905 | 0.341 | 58.564 |

# Results for PHIM for Run 4 (Sensitivity_Analysis_1.RTF)

************************************************************************************
**Main Results for E-Component for Run: 4, Simulation: 1, COD: STROKE, Adjusted**

| Variable Parameter | Value |
|---|---|
| 1. Country | US (United States) |
| 2. Sex | F (Females) |
| 3. Year of start of process | 1990 |
| 4. Cause of Death | STROKE |
| 5. Effective Dose NULL | CC:1 |
| 6. Effective Dose Ecig | CC:1 Ecig:0 Dual:0.5 |
| Note: | Death rates are per million population |

-----------------------------------------------------------------------------------------
Results for Age Groups Combined and Years combined, Population and Number deaths adjusted for Ecig scenario,

| Age Group:All | | NULL | Ecig | | | %Drop | | NULL | Ecig | |
|---|---|---|---|---|---|---|---|---|---|---|
| Year | Number | Attrib | Attrib | Drop | %Drop | Attrib | Death | Attrib | Attrib | Drop |
| | Deaths | Deaths | Deaths | Deaths | Deaths | Deaths | Rate | Rates | Rates | Rates |
| All Comb | 1362146 | 104899.750 | 48169.601 | 56730.15 | 4.165 | 54.080 | 372.043 | 28.800 | 13.157 | 15.643 |

-----------------------------------------------------------------------------------------
Results for Age Groups Combined, Population and Number deaths adjusted for Ecig scenario,

| Age Group:All | | NULL | Ecig | | | %Drop | | NULL | Ecig | |
|---|---|---|---|---|---|---|---|---|---|---|
| Year | Number | Attrib | Attrib | Drop | %Drop | Attrib | Death | Attrib | Attrib | Drop |
| | Deaths | Deaths | Deaths | Deaths | Deaths | Deaths | Rate | Rates | Rates | Rates |
| 1990 | 33504 | 3293.017 | 3293.017 | 0.000 | 0.000 | 0.000 | 490.678 | 48.227 | 48.227 | 0.000 |
| 1991 | 32505 | 3207.063 | 3178.126 | 28.937 | 0.089 | 0.902 | 469.694 | 46.342 | 45.924 | 0.418 |
| 1992 | 32560 | 3218.125 | 3137.168 | 80.956 | 0.249 | 2.516 | 464.104 | 45.871 | 44.716 | 1.154 |
| 1993 | 32768 | 3226.680 | 3073.322 | 153.358 | 0.468 | 4.753 | 460.482 | 45.344 | 43.188 | 2.156 |
| 1994 | 33006 | 3277.266 | 3032.541 | 244.725 | 0.741 | 7.467 | 456.920 | 45.370 | 41.981 | 3.389 |
| 1995 | 33732 | 3309.378 | 2958.532 | 350.846 | 1.040 | 10.602 | 459.725 | 45.104 | 40.321 | 4.784 |
| 1996 | 33754 | 3312.987 | 2838.497 | 474.490 | 1.406 | 14.322 | 454.036 | 44.568 | 38.182 | 6.386 |
| 1997 | 33705 | 3267.066 | 2672.575 | 594.491 | 1.764 | 18.196 | 447.081 | 43.341 | 35.451 | 7.891 |
| 1998 | 33282 | 3178.725 | 2469.798 | 708.927 | 2.130 | 22.302 | 435.248 | 41.577 | 32.299 | 9.278 |
| 1999 | 33903 | 3120.351 | 2294.695 | 825.656 | 2.435 | 26.460 | 437.371 | 40.264 | 29.603 | 10.661 |
| 2000 | 33843 | 3142.808 | 2176.752 | 966.056 | 2.854 | 30.739 | 431.146 | 40.050 | 27.731 | 12.320 |
| 2001 | 32527 | 2961.588 | 1923.801 | 1037.787 | 3.191 | 35.042 | 409.740 | 37.323 | 24.234 | 13.088 |
| 2002 | 32104 | 2940.627 | 1773.729 | 1166.898 | 3.635 | 39.682 | 400.415 | 36.695 | 22.123 | 14.573 |
| 2003 | 30599 | 2819.545 | 1576.464 | 1243.080 | 4.062 | 44.088 | 378.028 | 34.855 | 19.476 | 15.379 |
| 2004 | 29425 | 2730.466 | 1406.407 | 1324.059 | 4.500 | 48.492 | 359.846 | 33.417 | 17.199 | 16.218 |
| 2005 | 27996 | 2670.510 | 1263.697 | 1406.813 | 5.025 | 52.680 | 338.539 | 32.322 | 15.281 | 17.041 |
| 2006 | 27250 | 2623.285 | 1137.304 | 1485.981 | 5.453 | 56.646 | 326.588 | 31.474 | 13.630 | 17.843 |
| 2007 | 26357 | 2536.599 | 1001.629 | 1534.970 | 5.824 | 60.513 | 312.688 | 30.131 | 11.883 | 18.248 |
| 2008 | 26061 | 2469.443 | 889.857 | 1579.586 | 6.061 | 63.965 | 305.771 | 29.015 | 10.440 | 18.575 |
| 2009 | 25110 | 2386.945 | 780.775 | 1606.170 | 6.396 | 67.290 | 291.236 | 27.730 | 9.056 | 18.675 |
| 2010 | 24919 | 2329.274 | 690.497 | 1638.777 | 6.576 | 70.356 | 285.651 | 26.750 | 7.915 | 18.835 |
| 2011 | 24881 | 2286.581 | 608.371 | 1678.210 | 6.745 | 73.394 | 282.468 | 26.013 | 6.907 | 19.106 |
| 2012 | 24598 | 2243.311 | 534.275 | 1709.036 | 6.948 | 76.184 | 276.339 | 25.260 | 6.002 | 19.258 |
| 2013 | 24561 | 2176.503 | 463.365 | 1713.138 | 6.975 | 78.711 | 272.885 | 24.245 | 5.148 | 19.096 |
| 2014 | 25422 | 2211.619 | 420.802 | 1790.817 | 7.044 | 80.973 | 279.284 | 24.366 | 4.623 | 19.743 |
| 2015 | 26461 | 2237.016 | 384.799 | 1852.217 | 7.000 | 82.799 | 324.792 | 27.554 | 4.723 | 22.831 |
| 2016 | 26897 | 2229.057 | 342.420 | 1886.638 | 7.014 | 84.638 | 326.885 | 27.194 | 4.162 | 23.033 |
| 2017 | 27800 | 2221.649 | 304.896 | 1916.753 | 6.895 | 86.276 | 334.548 | 26.848 | 3.669 | 23.178 |
| 2018 | 25440 | 1939.420 | 238.821 | 1700.599 | 6.685 | 87.686 | 303.134 | 23.214 | 2.846 | 20.369 |
| 2019 | 25632 | 1904.400 | 206.820 | 1697.579 | 6.623 | 89.140 | 302.349 | 22.574 | 2.440 | 20.135 |
| 2020 | 25601 | 1806.054 | 176.284 | 1629.771 | 6.366 | 90.239 | 341.858 | 24.261 | 2.354 | 21.907 |
| 2021 | 25689 | 1748.172 | 150.685 | 1597.488 | 6.218 | 91.380 | 340.420 | 23.314 | 1.997 | 21.317 |
| 2022 | 25800 | 1701.148 | 129.346 | 1571.802 | 6.092 | 92.397 | 339.137 | 22.515 | 1.700 | 20.815 |
| 2023 | 25901 | 1652.087 | 111.220 | 1540.867 | 5.949 | 93.268 | 337.674 | 21.696 | 1.450 | 20.246 |
| 2024 | 25960 | 1597.127 | 94.085 | 1503.042 | 5.790 | 94.109 | 335.698 | 20.814 | 1.217 | 19.597 |
| 2025 | 25581 | 1467.622 | 78.107 | 1389.515 | 5.432 | 94.678 | 381.195 | 22.075 | 1.164 | 20.911 |
| 2026 | 25827 | 1390.505 | 65.672 | 1324.833 | 5.130 | 95.277 | 383.199 | 20.835 | 0.974 | 19.861 |
| 2027 | 25630 | 1342.095 | 55.532 | 1286.564 | 5.020 | 95.862 | 378.552 | 20.030 | 0.820 | 19.210 |
| 2028 | 25408 | 1289.536 | 46.739 | 1242.797 | 4.891 | 96.376 | 373.485 | 19.165 | 0.687 | 18.478 |
| 2029 | 25172 | 1242.216 | 39.098 | 1203.118 | 4.780 | 96.853 | 368.182 | 18.380 | 0.572 | 17.808 |
| 2030 | 24381 | 1096.803 | 31.313 | 1065.490 | 4.370 | 97.145 | 421.235 | 19.210 | 0.541 | 18.669 |
| 2031 | 24412 | 1019.991 | 25.608 | 994.383 | 4.073 | 97.489 | 420.920 | 17.841 | 0.442 | 17.399 |
| 2032 | 24083 | 977.406 | 21.084 | 956.322 | 3.971 | 97.843 | 414.171 | 17.063 | 0.363 | 16.700 |
| 2033 | 23745 | 937.450 | 17.816 | 919.633 | 3.873 | 98.100 | 407.101 | 16.326 | 0.305 | 16.020 |
| 2034 | 23382 | 900.763 | 14.765 | 885.998 | 3.789 | 98.361 | 399.524 | 15.644 | 0.252 | 15.391 |
| 2035 | 22247 | 745.023 | 11.429 | 733.595 | 3.297 | 98.466 | 464.207 | 15.845 | 0.238 | 15.607 |
| 2036 | 21424 | 680.360 | 8.768 | 671.592 | 3.135 | 98.711 | 447.368 | 14.492 | 0.183 | 14.309 |
| 2037 | 20927 | 644.109 | 7.192 | 636.917 | 3.043 | 98.883 | 437.032 | 13.730 | 0.150 | 13.580 |
| 2038 | 20430 | 612.448 | 6.037 | 606.411 | 2.968 | 99.014 | 426.342 | 13.055 | 0.126 | 12.929 |
| 2039 | 19941 | 577.529 | 5.068 | 572.461 | 2.871 | 99.122 | 415.434 | 12.298 | 0.106 | 12.192 |

# Results for PHIM for Run 4 (Sensitivity_Analysis_1.RTF)

************************************************************************************
**Main Results for E-Component for Run: 4, Simulation: 1, COD: COPD, Adjusted**

| Variable Parameter | Value |
|---|---|
| 1. Country | US (United States) |
| 2. Sex | F (Females) |
| 3. Year of start of process | 1990 |
| 4. Cause of Death | COPD |
| 5. Effective Dose NULL | CC:1 |
| 6. Effective Dose Ecig | CC:1 Ecig:0 Dual:0.5 |
| Note: | Death rates are per million population |

-----------------------------------------------------------------------------------------
Results for Age Groups Combined and Years combined, Population and Number deaths adjusted for Ecig scenario,

| Age Group:All | | NULL | Ecig | | | %Drop | | NULL | Ecig | |
|---|---|---|---|---|---|---|---|---|---|---|
| Year | Number | Attrib | Attrib | Drop | %Drop | Attrib | Death | Attrib | Attrib | Drop |
| | Deaths | Deaths | Deaths | Deaths | Deaths | Deaths | Rate | Rates | Rates | Rates |
| All Comb | 2182910 | 862051.244 | 532893.238 | 329158.0 | 15.079 | 38.183 | 596.218 | 236.673 | 145.549 | 91.124 |

-----------------------------------------------------------------------------------------
Results for Age Groups Combined, Population and Number deaths adjusted for Ecig scenario,

| Age Group:All | | NULL | Ecig | | | %Drop | | NULL | Ecig | |
|---|---|---|---|---|---|---|---|---|---|---|
| Year | Number | Attrib | Attrib | Drop | %Drop | Attrib | Death | Attrib | Attrib | Drop |
| | Deaths | Deaths | Deaths | Deaths | Deaths | Deaths | Rate | Rates | Rates | Rates |
| 1990 | 24738 | 10694.712 | 10694.712 | 0.000 | 0.000 | 0.000 | 362.296 | 156.628 | 156.628 | 0.000 |
| 1991 | 26562 | 11602.933 | 11583.812 | 19.121 | 0.072 | 0.165 | 383.818 | 167.661 | 167.385 | 0.276 |
| 1992 | 26828 | 11831.375 | 11778.597 | 52.778 | 0.197 | 0.446 | 382.401 | 168.642 | 167.889 | 0.753 |
| 1993 | 29774 | 13246.371 | 13132.611 | 113.760 | 0.382 | 0.859 | 418.408 | 186.148 | 184.547 | 1.600 |
| 1994 | 29805 | 13355.662 | 13174.887 | 180.775 | 0.607 | 1.354 | 412.607 | 184.893 | 182.387 | 2.507 |
| 1995 | 29757 | 13397.439 | 13129.197 | 268.242 | 0.901 | 2.002 | 405.549 | 182.598 | 178.934 | 3.664 |
| 1996 | 30904 | 13960.356 | 13585.497 | 374.858 | 1.213 | 2.685 | 415.698 | 187.801 | 182.744 | 5.057 |
| 1997 | 31134 | 14109.552 | 13619.195 | 490.357 | 1.575 | 3.475 | 412.977 | 187.180 | 180.653 | 6.526 |
| 1998 | 32105 | 14599.657 | 13957.864 | 641.793 | 1.999 | 4.396 | 419.846 | 190.958 | 182.533 | 8.425 |
| 1999 | 34629 | 15728.102 | 14894.495 | 833.607 | 2.407 | 5.300 | 446.739 | 202.950 | 192.149 | 10.801 |
| 2000 | 34317 | 15579.803 | 14589.044 | 990.759 | 2.887 | 6.359 | 437.178 | 198.541 | 185.857 | 12.684 |
| 2001 | 34616 | 15696.614 | 14502.979 | 1193.635 | 3.448 | 7.604 | 436.056 | 197.812 | 182.695 | 15.117 |
| 2002 | 34321 | 15519.777 | 14134.104 | 1385.674 | 4.037 | 8.928 | 428.064 | 193.668 | 176.286 | 17.382 |
| 2003 | 35112 | 15888.389 | 14250.638 | 1637.751 | 4.664 | 10.308 | 433.786 | 196.413 | 176.055 | 20.358 |
| 2004 | 33557 | 15091.573 | 13312.945 | 1778.628 | 5.300 | 11.786 | 410.381 | 184.701 | 162.808 | 21.894 |
| 2005 | 35449 | 15803.656 | 13677.179 | 2126.477 | 5.999 | 13.456 | 428.664 | 191.278 | 165.388 | 25.890 |
| 2006 | 34047 | 15100.563 | 12834.851 | 2265.713 | 6.655 | 15.004 | 408.044 | 181.174 | 153.824 | 27.350 |
| 2007 | 34077 | 14974.384 | 12466.251 | 2508.133 | 7.360 | 16.749 | 404.266 | 177.871 | 147.892 | 29.978 |
| 2008 | 37523 | 16406.197 | 13394.111 | 3012.086 | 8.027 | 18.359 | 440.246 | 192.768 | 157.150 | 35.618 |
| 2009 | 36936 | 16083.194 | 12808.822 | 3274.372 | 8.865 | 20.359 | 428.396 | 186.846 | 148.561 | 38.285 |
| 2010 | 36913 | 15927.400 | 12414.395 | 3513.005 | 9.517 | 22.056 | 423.133 | 182.917 | 142.307 | 40.610 |
| 2011 | 37779 | 16221.836 | 12315.716 | 3906.120 | 10.339 | 24.079 | 428.896 | 184.547 | 139.817 | 44.729 |
| 2012 | 38428 | 16358.849 | 12090.286 | 4268.563 | 11.108 | 26.093 | 431.697 | 184.202 | 135.822 | 48.380 |
| 2013 | 40302 | 16994.822 | 12196.494 | 4798.328 | 11.906 | 28.234 | 447.781 | 189.311 | 135.511 | 53.799 |
| 2014 | 40294 | 16855.093 | 11730.573 | 5124.520 | 12.718 | 30.403 | 442.664 | 185.696 | 128.871 | 56.824 |
| 2015 | 42444 | 17572.483 | 11928.815 | 5643.668 | 13.297 | 32.117 | 520.970 | 216.445 | 146.417 | 70.028 |
| 2016 | 42876 | 17579.955 | 11516.665 | 6063.291 | 14.142 | 34.490 | 521.078 | 214.473 | 139.965 | 74.509 |
| 2017 | 44671 | 18080.688 | 11424.600 | 6656.089 | 14.900 | 36.813 | 537.581 | 218.496 | 137.486 | 81.010 |
| 2018 | 45704 | 18209.262 | 11131.941 | 7077.322 | 15.485 | 38.867 | 544.588 | 217.960 | 132.644 | 85.315 |
| 2019 | 47264 | 18648.302 | 10976.000 | 7672.301 | 16.233 | 41.142 | 557.521 | 221.054 | 129.472 | 91.582 |
| 2020 | 48766 | 19002.597 | 10813.332 | 8189.265 | 16.793 | 43.096 | 651.206 | 255.266 | 144.396 | 110.870 |
| 2021 | 48809 | 18885.717 | 10294.492 | 8591.225 | 17.602 | 45.491 | 646.786 | 251.867 | 136.416 | 115.451 |
| 2022 | 50228 | 19235.076 | 10057.869 | 9177.207 | 18.271 | 47.711 | 660.252 | 254.582 | 132.212 | 122.370 |
| 2023 | 51657 | 19521.158 | 9808.277 | 9712.881 | 18.803 | 49.756 | 673.457 | 256.363 | 127.871 | 128.491 |
| 2024 | 53028 | 19918.620 | 9555.681 | 10362.94 | 19.542 | 52.026 | 685.727 | 259.583 | 123.569 | 136.015 |
| 2025 | 54077 | 20150.545 | 9262.781 | 10887.76 | 20.134 | 54.032 | 805.835 | 303.086 | 138.031 | 165.055 |
| 2026 | 54985 | 20393.792 | 8922.428 | 11471.36 | 20.863 | 56.249 | 815.808 | 305.582 | 132.382 | 173.200 |
| 2027 | 55862 | 20615.351 | 8557.629 | 12057.72 | 21.585 | 58.489 | 825.071 | 307.668 | 126.393 | 181.275 |
| 2028 | 56685 | 20731.894 | 8150.523 | 12581.37 | 22.195 | 60.686 | 833.253 | 308.115 | 119.811 | 188.304 |
| 2029 | 57469 | 20781.967 | 7729.519 | 13052.45 | 22.712 | 62.807 | 840.576 | 307.495 | 113.057 | 194.438 |
| 2030 | 57763 | 20753.201 | 7331.624 | 13421.58 | 23.236 | 64.672 | 997.966 | 363.487 | 126.668 | 236.819 |
| 2031 | 59398 | 21048.417 | 7172.838 | 13875.58 | 23.360 | 65.922 | 1024.16 | 368.165 | 123.677 | 244.488 |
| 2032 | 59965 | 21169.329 | 6796.977 | 14372.35 | 23.968 | 67.892 | 1031.26 | 369.560 | 116.892 | 252.668 |
| 2033 | 60493 | 21109.293 | 6392.246 | 14717.05 | 24.328 | 69.718 | 1037.14 | 367.618 | 109.594 | 258.025 |
| 2034 | 60946 | 21109.429 | 5987.652 | 15121.78 | 24.812 | 71.635 | 1041.38 | 366.613 | 102.310 | 264.303 |
| 2035 | 60338 | 20794.840 | 5603.250 | 15191.59 | 25.177 | 73.055 | 1259.00 | 442.268 | 116.916 | 325.352 |
| 2036 | 57419 | 19354.577 | 4927.695 | 14426.88 | 25.126 | 74.540 | 1199.00 | 412.258 | 102.897 | 309.360 |
| 2037 | 57420 | 19064.169 | 4480.817 | 14583.35 | 25.398 | 76.496 | 1199.12 | 406.385 | 93.574 | 312.811 |
| 2038 | 57389 | 18854.812 | 4108.513 | 14746.30 | 25.696 | 78.210 | 1197.59 | 401.900 | 85.737 | 316.163 |
| 2039 | 57349 | 18437.457 | 3691.816 | 14745.64 | 25.712 | 79.977 | 1194.74 | 392.602 | 76.912 | 315.690 |

# Results for PHIM for Run 5 (Sensitivity_Analysis_1.RTF)

---

-----------------------------------------------------------------------------------------

**Control File Details for Run: 5**

| Variable Parameter | Value |
|---|---|
| 1. Name for Reference Scenario | NULL |
| 2. Name for Test Scenario | Ecig |
| 3. Name for Product 1 | CC |
| 4. Name for Product 2 | Ecig |
| 5. Name for Product 3 | - |
| 6. Name for Product 4 | Dual |
| 7. Country | US (United States) |
| 8. Sex | M (Males) |
| 9. Year of start of process | 1990 |
| 10. Number of months of follow-up | 600 |
| 11. Follow-up interval length (in months) | 12 |
| 12. Lower age for risk estimation | 10 |
| 13. Upper age for risk estimation | 79 |
| 14. The effective dose for Product 1 (F1) - Ref | 1 |
| 15. The effective dose for Product 2 (F2) - Ref | - |
| 16. The effective dose for Product 3 (F3) - Ref | - |
| 17. The effective dose for Product 4 (F4) - Ref | - |
| 18. The effective dose for Product 1 (F1) - Test | 1 |
| 19. The effective dose for Product 2 (F2) - Test | 0.1 |
| 20. The effective dose for Product 3 (F3) - Test | - |
| 21. The effective dose for Product 4 (F4) - Test | FROMF2 |
| 22. Number in population to be simulated | 100000 |
| 23. Number of MC simulations | 1 |
| 24. The random number seed for the first simulation | 15975263 |
| 25. Source for the population file (POP) | UN4 |
| 26. Source for the socioeconomic prevalence file (SEP) | - |
| 27. Source for the current smoking prevalence file (CSP) | ISS3 |
| 28. Source for the former smoking prevalence file (FSP) | ISS3 |
| 29. Source for the quit-time distribution file (QTD) | NHIS2006 |
| 30. Source for the death file (MORT) | APR-20 |
| 31. Source for the relative risk file (RR) | PNLEST |
| 32. Source for the half-life file (H) | PNLEST |
| 33. Assumption Set for TTP Factor Reference | F1 |
| 34. Assumption Set for TTP Factor Test | F1 |
| 35. Assumption Set for TTP Reference | ISS3 |
| 36. Assumption Set for TTP Test | ISS3 |
| 37. Assumption Set for TP for socioeconomic group | - |
| 38. Source for Output Choice file (OUTC) | BASIC |
| 39. Output for DTP P1 | N |
| 40. Output for DTP P2 | N |
| 41. Output for Main P Component | Y |
| 42. Output for DTP E1 | N |
| 43. Output for DTP E2 | N |
| 44. Output for DTP E3 | N |
| 45. Output for Main E Component | Y |
| 46. Output file name | Sensitivity_Analysis_1 |
| 47. Run number for P-Component | - |
| 48. Results file for E-Component | - |
| 49. Components required | PE |
| 50. Results folder for P to be used in E-Component | - |
| 51. Fixed age of starting product use | 16 |
| 52. Output absolute risk file? | N |

# Results for PHIM for Run 5 (Sensitivity_Analysis_1.RTF)

************************************************************************************
**TTP values for Run: 5, Scenario: NULL**

| **Variable Parameter** | **Value** |
|---|---|
| 1. Country | US (United States) |
| 2. Sex | M (Males) |
| 3. Assumption Set | ISS3 |
| Note: | Period is in Months |

| Age | Period | **Initiation** Never CC | **Cessation** CC Former | **Re-initiation** Former CC |
|---|---|---|---|---|
| 10-14 | 1-12 | 0.003842 | 0.008742 | 0.000000 |
| 15-19 | 1-12 | 0.004720 | 0.003614 | 0.000000 |
| 20-24 | 1-12 | 0.000376 | 0.000943 | 0.000000 |
| 25-29 | 1-12 | 0.000187 | 0.001143 | 0.000000 |
| 30-34 | 1-12 | 0.000093 | 0.000871 | 0.000000 |
| 35-39 | 1-12 | 0.000000 | 0.000867 | 0.000000 |
| 40-44 | 1-12 | 0.000000 | 0.001399 | 0.000000 |
| 45-49 | 1-12 | 0.000000 | 0.002033 | 0.000000 |
| 50-54 | 1-12 | 0.000000 | 0.003209 | 0.000000 |
| 55-59 | 1-12 | 0.000000 | 0.003574 | 0.000000 |
| 60-64 | 1-12 | 0.000000 | 0.003460 | 0.000000 |
| 65-69 | 1-12 | 0.000000 | 0.004136 | 0.000000 |
| 70-74 | 1-12 | 0.000000 | 0.005592 | 0.000000 |
| 75-79 | 1-12 | 0.000000 | 0.005592 | 0.000000 |
| 10-14 | 13-72 | 0.004906 | 0.008742 | 0.000000 |
| 15-19 | 13-72 | 0.006989 | 0.004426 | 0.000000 |
| 20-24 | 13-72 | 0.000687 | 0.001527 | 0.000000 |
| 25-29 | 13-72 | 0.000962 | 0.001515 | 0.000000 |
| 30-34 | 13-72 | 0.000364 | 0.001227 | 0.000000 |
| 35-39 | 13-72 | 0.000000 | 0.001630 | 0.000000 |
| 40-44 | 13-72 | 0.000000 | 0.001852 | 0.000000 |
| 45-49 | 13-72 | 0.000000 | 0.002667 | 0.000000 |
| 50-54 | 13-72 | 0.000000 | 0.003208 | 0.000000 |
| 55-59 | 13-72 | 0.000000 | 0.003978 | 0.000000 |
| 60-64 | 13-72 | 0.000000 | 0.005771 | 0.000000 |
| 65-69 | 13-72 | 0.000000 | 0.010432 | 0.000000 |
| 70-74 | 13-72 | 0.000000 | 0.007079 | 0.000000 |
| 75-79 | 13-72 | 0.000000 | 0.007079 | 0.000000 |
| 10-14 | 73-132 | 0.004312 | 0.008742 | 0.000000 |
| 15-19 | 73-132 | 0.005330 | 0.003289 | 0.000000 |
| 20-24 | 73-132 | 0.002456 | 0.003844 | 0.000000 |
| 25-29 | 73-132 | 0.000839 | 0.002006 | 0.000000 |
| 30-34 | 73-132 | 0.000414 | 0.001329 | 0.000000 |
| 35-39 | 73-132 | 0.000000 | 0.001278 | 0.000000 |
| 40-44 | 73-132 | 0.000000 | 0.001691 | 0.000000 |
| 45-49 | 73-132 | 0.000000 | 0.002904 | 0.000000 |
| 50-54 | 73-132 | 0.000000 | 0.003319 | 0.000000 |
| 55-59 | 73-132 | 0.000000 | 0.003976 | 0.000000 |
| 60-64 | 73-132 | 0.000000 | 0.005235 | 0.000000 |
| 65-69 | 73-132 | 0.000000 | 0.005901 | 0.000000 |
| 70-74 | 73-132 | 0.000000 | 0.005615 | 0.000000 |
| 75-79 | 73-132 | 0.000000 | 0.005615 | 0.000000 |
| 10-14 | 133-192 | 0.003468 | 0.008742 | 0.000000 |
| 15-19 | 133-192 | 0.004675 | 0.003732 | 0.000000 |
| 20-24 | 133-192 | 0.000062 | 0.002260 | 0.000000 |
| 25-29 | 133-192 | 0.001422 | 0.002822 | 0.000000 |
| 30-34 | 133-192 | 0.000696 | 0.002610 | 0.000000 |
| 35-39 | 133-192 | 0.000000 | 0.002058 | 0.000000 |
| 40-44 | 133-192 | 0.000000 | 0.002310 | 0.000000 |
| 45-49 | 133-192 | 0.000000 | 0.002256 | 0.000000 |
| 50-54 | 133-192 | 0.000000 | 0.002663 | 0.000000 |
| 55-59 | 133-192 | 0.000000 | 0.001746 | 0.000000 |
| 60-64 | 133-192 | 0.000000 | 0.005809 | 0.000000 |
| 65-69 | 133-192 | 0.000000 | 0.005896 | 0.000000 |
| 70-74 | 133-192 | 0.000000 | 0.007323 | 0.000000 |
| 75-79 | 133-192 | 0.000000 | 0.007323 | 0.000000 |
| 10-14 | 193-252 | 0.002314 | 0.008742 | 0.000000 |
| 15-19 | 193-252 | 0.003987 | 0.005036 | 0.000000 |
| 20-24 | 193-252 | 0.000320 | 0.002857 | 0.000000 |
| 25-29 | 193-252 | 0.000251 | 0.002314 | 0.000000 |
| 30-34 | 193-252 | 0.000125 | 0.004343 | 0.000000 |
| 35-39 | 193-252 | 0.000000 | 0.002092 | 0.000000 |
| 40-44 | 193-252 | 0.000000 | 0.002566 | 0.000000 |
| 45-49 | 193-252 | 0.000000 | 0.002311 | 0.000000 |
| 50-54 | 193-252 | 0.000000 | 0.003209 | 0.000000 |

## Results for PHIM for Run 5 (Sensitivity_Analysis_1.RTF)

| | | | | |
|---|---|---|---|---|
| 55-59 | 193-252 | 0.000000 | 0.004057 | 0.000000 |
| 60-64 | 193-252 | 0.000000 | 0.007166 | 0.000000 |
| 65-69 | 193-252 | 0.000000 | 0.004281 | 0.000000 |
| 70-74 | 193-252 | 0.000000 | 0.004580 | 0.000000 |
| 75-79 | 193-252 | 0.000000 | 0.004580 | 0.000000 |
| 10-14 | 253+ | 0.001325 | 0.008742 | 0.000000 |
| 15-19 | 253+ | 0.002984 | 0.010602 | 0.000000 |
| 20-24 | 253+ | 0.000452 | 0.004314 | 0.000000 |
| 25-29 | 253+ | 0.000225 | 0.003645 | 0.000000 |
| 30-34 | 253+ | 0.000112 | 0.004512 | 0.000000 |
| 35-39 | 253+ | 0.000000 | 0.002147 | 0.000000 |
| 40-44 | 253+ | 0.000000 | 0.002636 | 0.000000 |
| 45-49 | 253+ | 0.000000 | 0.001051 | 0.000000 |
| 50-54 | 253+ | 0.000000 | 0.002519 | 0.000000 |
| 55-59 | 253+ | 0.000000 | 0.003693 | 0.000000 |
| 60-64 | 253+ | 0.000000 | 0.005888 | 0.000000 |
| 65-69 | 253+ | 0.000000 | 0.004823 | 0.000000 |
| 70-74 | 253+ | 0.000000 | 0.004733 | 0.000000 |
| 75-79 | 253+ | 0.000000 | 0.004733 | 0.000000 |

# Results for PHIM for Run 5 (Sensitivity_Analysis_1.RTF)

************************************************************************************

**TTP values for Run: 5, Scenario: Ecig**

| **Variable Parameter** | **Value** |
|---|---|
| 1. Country | US (United States) |
| 2. Sex | M (Males) |
| 3. Assumption Set | ISS3 |
| Note: | Period is in Months |

| | | **Initiation** | | | **Cessation** | | | **Re-initiation** | | |
|---|---|---|---|---|---|---|---|---|---|---|
| Age | Period | Never CC | Never Ecig | Never Dual | CC Former | Ecig Former | Dual Former | Former CC | Former Ecig | Former Dual |
| 10-14 | 1-12 | 0.001921 | 0.001921 | 0.000000 | 0.008742 | 0.008742 | 0.000000 | 0.000000 | 0.000000 | 0.000000 |
| 15-19 | 1-12 | 0.002360 | 0.002360 | 0.000000 | 0.003614 | 0.003614 | 0.000000 | 0.000000 | 0.000000 | 0.000000 |
| 20-24 | 1-12 | 0.000188 | 0.000188 | 0.000000 | 0.000943 | 0.000943 | 0.000000 | 0.000000 | 0.000000 | 0.000000 |
| 25-29 | 1-12 | 0.000093 | 0.000093 | 0.000000 | 0.001143 | 0.001143 | 0.000000 | 0.000000 | 0.000000 | 0.000000 |
| 30-34 | 1-12 | 0.000047 | 0.000047 | 0.000000 | 0.000871 | 0.000871 | 0.000000 | 0.000000 | 0.000000 | 0.000000 |
| 35-39 | 1-12 | 0.000000 | 0.000000 | 0.000000 | 0.000867 | 0.000867 | 0.000000 | 0.000000 | 0.000000 | 0.000000 |
| 40-44 | 1-12 | 0.000000 | 0.000000 | 0.000000 | 0.001399 | 0.001399 | 0.000000 | 0.000000 | 0.000000 | 0.000000 |
| 45-49 | 1-12 | 0.000000 | 0.000000 | 0.000000 | 0.002033 | 0.002033 | 0.000000 | 0.000000 | 0.000000 | 0.000000 |
| 50-54 | 1-12 | 0.000000 | 0.000000 | 0.000000 | 0.003209 | 0.003209 | 0.000000 | 0.000000 | 0.000000 | 0.000000 |
| 55-59 | 1-12 | 0.000000 | 0.000000 | 0.000000 | 0.003574 | 0.003574 | 0.000000 | 0.000000 | 0.000000 | 0.000000 |
| 60-64 | 1-12 | 0.000000 | 0.000000 | 0.000000 | 0.003460 | 0.003460 | 0.000000 | 0.000000 | 0.000000 | 0.000000 |
| 65-69 | 1-12 | 0.000000 | 0.000000 | 0.000000 | 0.004136 | 0.004136 | 0.000000 | 0.000000 | 0.000000 | 0.000000 |
| 70-74 | 1-12 | 0.000000 | 0.000000 | 0.000000 | 0.005592 | 0.005592 | 0.000000 | 0.000000 | 0.000000 | 0.000000 |
| 75-79 | 1-12 | 0.000000 | 0.000000 | 0.000000 | 0.005592 | 0.005592 | 0.000000 | 0.000000 | 0.000000 | 0.000000 |
| 10-14 | 13-72 | 0.001962 | 0.002944 | 0.000000 | 0.008742 | 0.008742 | 0.000000 | 0.000000 | 0.000000 | 0.000000 |
| 15-19 | 13-72 | 0.002796 | 0.004193 | 0.000000 | 0.004426 | 0.004426 | 0.000000 | 0.000000 | 0.000000 | 0.000000 |
| 20-24 | 13-72 | 0.000275 | 0.000412 | 0.000000 | 0.001527 | 0.001527 | 0.000000 | 0.000000 | 0.000000 | 0.000000 |
| 25-29 | 13-72 | 0.000385 | 0.000578 | 0.000000 | 0.001515 | 0.001515 | 0.000000 | 0.000000 | 0.000000 | 0.000000 |
| 30-34 | 13-72 | 0.000146 | 0.000218 | 0.000000 | 0.001227 | 0.001227 | 0.000000 | 0.000000 | 0.000000 | 0.000000 |
| 35-39 | 13-72 | 0.000000 | 0.000000 | 0.000000 | 0.001630 | 0.001630 | 0.000000 | 0.000000 | 0.000000 | 0.000000 |
| 40-44 | 13-72 | 0.000000 | 0.000000 | 0.000000 | 0.001852 | 0.001852 | 0.000000 | 0.000000 | 0.000000 | 0.000000 |
| 45-49 | 13-72 | 0.000000 | 0.000000 | 0.000000 | 0.002667 | 0.002667 | 0.000000 | 0.000000 | 0.000000 | 0.000000 |
| 50-54 | 13-72 | 0.000000 | 0.000000 | 0.000000 | 0.003208 | 0.003208 | 0.000000 | 0.000000 | 0.000000 | 0.000000 |
| 55-59 | 13-72 | 0.000000 | 0.000000 | 0.000000 | 0.003978 | 0.003978 | 0.000000 | 0.000000 | 0.000000 | 0.000000 |
| 60-64 | 13-72 | 0.000000 | 0.000000 | 0.000000 | 0.005771 | 0.005771 | 0.000000 | 0.000000 | 0.000000 | 0.000000 |
| 65-69 | 13-72 | 0.000000 | 0.000000 | 0.000000 | 0.010432 | 0.010432 | 0.000000 | 0.000000 | 0.000000 | 0.000000 |
| 70-74 | 13-72 | 0.000000 | 0.000000 | 0.000000 | 0.007079 | 0.007079 | 0.000000 | 0.000000 | 0.000000 | 0.000000 |
| 75-79 | 13-72 | 0.000000 | 0.000000 | 0.000000 | 0.007079 | 0.007079 | 0.000000 | 0.000000 | 0.000000 | 0.000000 |
| 10-14 | 73-132 | 0.000862 | 0.003449 | 0.000000 | 0.008742 | 0.008742 | 0.000000 | 0.000000 | 0.000000 | 0.000000 |
| 15-19 | 73-132 | 0.001066 | 0.004264 | 0.000000 | 0.003289 | 0.003289 | 0.000000 | 0.000000 | 0.000000 | 0.000000 |
| 20-24 | 73-132 | 0.000491 | 0.001965 | 0.000000 | 0.003844 | 0.003844 | 0.000000 | 0.000000 | 0.000000 | 0.000000 |
| 25-29 | 73-132 | 0.000168 | 0.000671 | 0.000000 | 0.002006 | 0.002006 | 0.000000 | 0.000000 | 0.000000 | 0.000000 |
| 30-34 | 73-132 | 0.000083 | 0.000332 | 0.000000 | 0.001329 | 0.001329 | 0.000000 | 0.000000 | 0.000000 | 0.000000 |
| 35-39 | 73-132 | 0.000000 | 0.000000 | 0.000000 | 0.001278 | 0.001278 | 0.000000 | 0.000000 | 0.000000 | 0.000000 |
| 40-44 | 73-132 | 0.000000 | 0.000000 | 0.000000 | 0.001691 | 0.001691 | 0.000000 | 0.000000 | 0.000000 | 0.000000 |
| 45-49 | 73-132 | 0.000000 | 0.000000 | 0.000000 | 0.002904 | 0.002904 | 0.000000 | 0.000000 | 0.000000 | 0.000000 |
| 50-54 | 73-132 | 0.000000 | 0.000000 | 0.000000 | 0.003319 | 0.003319 | 0.000000 | 0.000000 | 0.000000 | 0.000000 |
| 55-59 | 73-132 | 0.000000 | 0.000000 | 0.000000 | 0.003976 | 0.003976 | 0.000000 | 0.000000 | 0.000000 | 0.000000 |
| 60-64 | 73-132 | 0.000000 | 0.000000 | 0.000000 | 0.005235 | 0.005235 | 0.000000 | 0.000000 | 0.000000 | 0.000000 |
| 65-69 | 73-132 | 0.000000 | 0.000000 | 0.000000 | 0.005901 | 0.005901 | 0.000000 | 0.000000 | 0.000000 | 0.000000 |
| 70-74 | 73-132 | 0.000000 | 0.000000 | 0.000000 | 0.005615 | 0.005615 | 0.000000 | 0.000000 | 0.000000 | 0.000000 |
| 75-79 | 73-132 | 0.000000 | 0.000000 | 0.000000 | 0.005615 | 0.005615 | 0.000000 | 0.000000 | 0.000000 | 0.000000 |
| 10-14 | 133-192 | 0.000173 | 0.003294 | 0.000000 | 0.008742 | 0.008742 | 0.000000 | 0.000000 | 0.000000 | 0.000000 |
| 15-19 | 133-192 | 0.000234 | 0.004442 | 0.000000 | 0.003732 | 0.003732 | 0.000000 | 0.000000 | 0.000000 | 0.000000 |
| 20-24 | 133-192 | 0.000003 | 0.000059 | 0.000000 | 0.002260 | 0.002260 | 0.000000 | 0.000000 | 0.000000 | 0.000000 |
| 25-29 | 133-192 | 0.000071 | 0.001351 | 0.000000 | 0.002822 | 0.002822 | 0.000000 | 0.000000 | 0.000000 | 0.000000 |
| 30-34 | 133-192 | 0.000035 | 0.000661 | 0.000000 | 0.002610 | 0.002610 | 0.000000 | 0.000000 | 0.000000 | 0.000000 |
| 35-39 | 133-192 | 0.000000 | 0.000000 | 0.000000 | 0.002058 | 0.002058 | 0.000000 | 0.000000 | 0.000000 | 0.000000 |
| 40-44 | 133-192 | 0.000000 | 0.000000 | 0.000000 | 0.002310 | 0.002310 | 0.000000 | 0.000000 | 0.000000 | 0.000000 |
| 45-49 | 133-192 | 0.000000 | 0.000000 | 0.000000 | 0.002256 | 0.002256 | 0.000000 | 0.000000 | 0.000000 | 0.000000 |
| 50-54 | 133-192 | 0.000000 | 0.000000 | 0.000000 | 0.002663 | 0.002663 | 0.000000 | 0.000000 | 0.000000 | 0.000000 |
| 55-59 | 133-192 | 0.000000 | 0.000000 | 0.000000 | 0.001747 | 0.001747 | 0.000000 | 0.000000 | 0.000000 | 0.000000 |
| 60-64 | 133-192 | 0.000000 | 0.000000 | 0.000000 | 0.005809 | 0.005809 | 0.000000 | 0.000000 | 0.000000 | 0.000000 |
| 65-69 | 133-192 | 0.000000 | 0.000000 | 0.000000 | 0.005896 | 0.005896 | 0.000000 | 0.000000 | 0.000000 | 0.000000 |
| 70-74 | 133-192 | 0.000000 | 0.000000 | 0.000000 | 0.007323 | 0.007323 | 0.000000 | 0.000000 | 0.000000 | 0.000000 |
| 75-79 | 133-192 | 0.000000 | 0.000000 | 0.000000 | 0.007323 | 0.007323 | 0.000000 | 0.000000 | 0.000000 | 0.000000 |
| 10-14 | 193-252 | 0.000116 | 0.002198 | 0.000000 | 0.008742 | 0.008742 | 0.000000 | 0.000000 | 0.000000 | 0.000000 |
| 15-19 | 193-252 | 0.000199 | 0.003788 | 0.000000 | 0.005036 | 0.005036 | 0.000000 | 0.000000 | 0.000000 | 0.000000 |
| 20-24 | 193-252 | 0.000016 | 0.000304 | 0.000000 | 0.002857 | 0.002857 | 0.000000 | 0.000000 | 0.000000 | 0.000000 |
| 25-29 | 193-252 | 0.000013 | 0.000239 | 0.000000 | 0.002314 | 0.002314 | 0.000000 | 0.000000 | 0.000000 | 0.000000 |
| 30-34 | 193-252 | 0.000006 | 0.000119 | 0.000000 | 0.004343 | 0.004343 | 0.000000 | 0.000000 | 0.000000 | 0.000000 |
| 35-39 | 193-252 | 0.000000 | 0.000000 | 0.000000 | 0.002092 | 0.002092 | 0.000000 | 0.000000 | 0.000000 | 0.000000 |
| 40-44 | 193-252 | 0.000000 | 0.000000 | 0.000000 | 0.002566 | 0.002566 | 0.000000 | 0.000000 | 0.000000 | 0.000000 |
| 45-49 | 193-252 | 0.000000 | 0.000000 | 0.000000 | 0.002311 | 0.002311 | 0.000000 | 0.000000 | 0.000000 | 0.000000 |
| 50-54 | 193-252 | 0.000000 | 0.000000 | 0.000000 | 0.003209 | 0.003209 | 0.000000 | 0.000000 | 0.000000 | 0.000000 |

## Results for PHIM for Run 5 (Sensitivity_Analysis_1.RTF)

| | | | | | | | | | | |
|---|---|---|---|---|---|---|---|---|---|---|
| 55-59 | 193-252 | 0.000000 | 0.000000 | 0.000000 | 0.004057 | 0.004057 | 0.000000 | 0.000000 | 0.000000 | 0.000000 |
| 60-64 | 193-252 | 0.000000 | 0.000000 | 0.000000 | 0.007166 | 0.007166 | 0.000000 | 0.000000 | 0.000000 | 0.000000 |
| 65-69 | 193-252 | 0.000000 | 0.000000 | 0.000000 | 0.004281 | 0.004281 | 0.000000 | 0.000000 | 0.000000 | 0.000000 |
| 70-74 | 193-252 | 0.000000 | 0.000000 | 0.000000 | 0.004580 | 0.004580 | 0.000000 | 0.000000 | 0.000000 | 0.000000 |
| 75-79 | 193-252 | 0.000000 | 0.000000 | 0.000000 | 0.004580 | 0.004580 | 0.000000 | 0.000000 | 0.000000 | 0.000000 |
| 10-14 | 253+ | 0.000066 | 0.001259 | 0.000000 | 0.008742 | 0.008742 | 0.000000 | 0.000000 | 0.000000 | 0.000000 |
| 15-19 | 253+ | 0.000149 | 0.002835 | 0.000000 | 0.010602 | 0.010602 | 0.000000 | 0.000000 | 0.000000 | 0.000000 |
| 20-24 | 253+ | 0.000023 | 0.000430 | 0.000000 | 0.004314 | 0.004314 | 0.000000 | 0.000000 | 0.000000 | 0.000000 |
| 25-29 | 253+ | 0.000011 | 0.000213 | 0.000000 | 0.003645 | 0.003645 | 0.000000 | 0.000000 | 0.000000 | 0.000000 |
| 30-34 | 253+ | 0.000006 | 0.000106 | 0.000000 | 0.004512 | 0.004512 | 0.000000 | 0.000000 | 0.000000 | 0.000000 |
| 35-39 | 253+ | 0.000000 | 0.000000 | 0.000000 | 0.002147 | 0.002147 | 0.000000 | 0.000000 | 0.000000 | 0.000000 |
| 40-44 | 253+ | 0.000000 | 0.000000 | 0.000000 | 0.002636 | 0.002636 | 0.000000 | 0.000000 | 0.000000 | 0.000000 |
| 45-49 | 253+ | 0.000000 | 0.000000 | 0.000000 | 0.001051 | 0.001051 | 0.000000 | 0.000000 | 0.000000 | 0.000000 |
| 50-54 | 253+ | 0.000000 | 0.000000 | 0.000000 | 0.002519 | 0.002519 | 0.000000 | 0.000000 | 0.000000 | 0.000000 |
| 55-59 | 253+ | 0.000000 | 0.000000 | 0.000000 | 0.003693 | 0.003693 | 0.000000 | 0.000000 | 0.000000 | 0.000000 |
| 60-64 | 253+ | 0.000000 | 0.000000 | 0.000000 | 0.005888 | 0.005888 | 0.000000 | 0.000000 | 0.000000 | 0.000000 |
| 65-69 | 253+ | 0.000000 | 0.000000 | 0.000000 | 0.004823 | 0.004823 | 0.000000 | 0.000000 | 0.000000 | 0.000000 |
| 70-74 | 253+ | 0.000000 | 0.000000 | 0.000000 | 0.004733 | 0.004733 | 0.000000 | 0.000000 | 0.000000 | 0.000000 |
| 75-79 | 253+ | 0.000000 | 0.000000 | 0.000000 | 0.004733 | 0.004733 | 0.000000 | 0.000000 | 0.000000 | 0.000000 |

# Results for PHIM for Run 5 (Sensitivity_Analysis_1.RTF)

************************************************************************************

| | | Switch from CC | | Switch from Ecig | | Switch from Dual | |
|---|---|---|---|---|---|---|---|
| | | **CC** | **CC** | **Ecig** | **Ecig** | **Dual** | **Dual** |
| Age | Period | Ecig | Dual | CC | Dual | CC | Ecig |
| 10-14 | 1-12 | 0.009549 | 0.000000 | 0.000000 | 0.000000 | 0.000000 | 0.000000 |
| 15-19 | 1-12 | 0.012695 | 0.000000 | 0.000000 | 0.000000 | 0.000000 | 0.000000 |
| 20-24 | 1-12 | 0.014490 | 0.000000 | 0.000000 | 0.000000 | 0.000000 | 0.000000 |
| 25-29 | 1-12 | 0.015212 | 0.000000 | 0.000000 | 0.000000 | 0.000000 | 0.000000 |
| 30-34 | 1-12 | 0.015460 | 0.000000 | 0.000000 | 0.000000 | 0.000000 | 0.000000 |
| 35-39 | 1-12 | 0.014711 | 0.000000 | 0.000000 | 0.000000 | 0.000000 | 0.000000 |
| 40-44 | 1-12 | 0.014241 | 0.000000 | 0.000000 | 0.000000 | 0.000000 | 0.000000 |
| 45-49 | 1-12 | 0.013014 | 0.000000 | 0.000000 | 0.000000 | 0.000000 | 0.000000 |
| 50-54 | 1-12 | 0.011768 | 0.000000 | 0.000000 | 0.000000 | 0.000000 | 0.000000 |
| 55-59 | 1-12 | 0.011032 | 0.000000 | 0.000000 | 0.000000 | 0.000000 | 0.000000 |
| 60-64 | 1-12 | 0.009525 | 0.000000 | 0.000000 | 0.000000 | 0.000000 | 0.000000 |
| 65-69 | 1-12 | 0.006897 | 0.000000 | 0.000000 | 0.000000 | 0.000000 | 0.000000 |
| 70-74 | 1-12 | 0.005435 | 0.000000 | 0.000000 | 0.000000 | 0.000000 | 0.000000 |
| 75-79 | 1-12 | 0.005435 | 0.000000 | 0.000000 | 0.000000 | 0.000000 | 0.000000 |
| 10-14 | 13-72 | 0.009549 | 0.000000 | 0.000000 | 0.000000 | 0.000000 | 0.000000 |
| 15-19 | 13-72 | 0.012695 | 0.000000 | 0.000000 | 0.000000 | 0.000000 | 0.000000 |
| 20-24 | 13-72 | 0.014490 | 0.000000 | 0.000000 | 0.000000 | 0.000000 | 0.000000 |
| 25-29 | 13-72 | 0.015212 | 0.000000 | 0.000000 | 0.000000 | 0.000000 | 0.000000 |
| 30-34 | 13-72 | 0.015460 | 0.000000 | 0.000000 | 0.000000 | 0.000000 | 0.000000 |
| 35-39 | 13-72 | 0.014711 | 0.000000 | 0.000000 | 0.000000 | 0.000000 | 0.000000 |
| 40-44 | 13-72 | 0.014241 | 0.000000 | 0.000000 | 0.000000 | 0.000000 | 0.000000 |
| 45-49 | 13-72 | 0.013014 | 0.000000 | 0.000000 | 0.000000 | 0.000000 | 0.000000 |
| 50-54 | 13-72 | 0.011768 | 0.000000 | 0.000000 | 0.000000 | 0.000000 | 0.000000 |
| 55-59 | 13-72 | 0.011032 | 0.000000 | 0.000000 | 0.000000 | 0.000000 | 0.000000 |
| 60-64 | 13-72 | 0.009525 | 0.000000 | 0.000000 | 0.000000 | 0.000000 | 0.000000 |
| 65-69 | 13-72 | 0.006897 | 0.000000 | 0.000000 | 0.000000 | 0.000000 | 0.000000 |
| 70-74 | 13-72 | 0.005435 | 0.000000 | 0.000000 | 0.000000 | 0.000000 | 0.000000 |
| 75-79 | 13-72 | 0.005435 | 0.000000 | 0.000000 | 0.000000 | 0.000000 | 0.000000 |
| 10-14 | 73-132 | 0.009549 | 0.000000 | 0.000000 | 0.000000 | 0.000000 | 0.000000 |
| 15-19 | 73-132 | 0.012695 | 0.000000 | 0.000000 | 0.000000 | 0.000000 | 0.000000 |
| 20-24 | 73-132 | 0.014490 | 0.000000 | 0.000000 | 0.000000 | 0.000000 | 0.000000 |
| 25-29 | 73-132 | 0.015212 | 0.000000 | 0.000000 | 0.000000 | 0.000000 | 0.000000 |
| 30-34 | 73-132 | 0.015460 | 0.000000 | 0.000000 | 0.000000 | 0.000000 | 0.000000 |
| 35-39 | 73-132 | 0.014711 | 0.000000 | 0.000000 | 0.000000 | 0.000000 | 0.000000 |
| 40-44 | 73-132 | 0.014241 | 0.000000 | 0.000000 | 0.000000 | 0.000000 | 0.000000 |
| 45-49 | 73-132 | 0.013014 | 0.000000 | 0.000000 | 0.000000 | 0.000000 | 0.000000 |
| 50-54 | 73-132 | 0.011768 | 0.000000 | 0.000000 | 0.000000 | 0.000000 | 0.000000 |
| 55-59 | 73-132 | 0.011032 | 0.000000 | 0.000000 | 0.000000 | 0.000000 | 0.000000 |
| 60-64 | 73-132 | 0.009525 | 0.000000 | 0.000000 | 0.000000 | 0.000000 | 0.000000 |
| 65-69 | 73-132 | 0.006897 | 0.000000 | 0.000000 | 0.000000 | 0.000000 | 0.000000 |
| 70-74 | 73-132 | 0.005435 | 0.000000 | 0.000000 | 0.000000 | 0.000000 | 0.000000 |
| 75-79 | 73-132 | 0.005435 | 0.000000 | 0.000000 | 0.000000 | 0.000000 | 0.000000 |
| 10-14 | 133-192 | 0.009549 | 0.000000 | 0.000000 | 0.000000 | 0.000000 | 0.000000 |
| 15-19 | 133-192 | 0.012695 | 0.000000 | 0.000000 | 0.000000 | 0.000000 | 0.000000 |
| 20-24 | 133-192 | 0.014490 | 0.000000 | 0.000000 | 0.000000 | 0.000000 | 0.000000 |
| 25-29 | 133-192 | 0.015212 | 0.000000 | 0.000000 | 0.000000 | 0.000000 | 0.000000 |
| 30-34 | 133-192 | 0.015460 | 0.000000 | 0.000000 | 0.000000 | 0.000000 | 0.000000 |
| 35-39 | 133-192 | 0.014711 | 0.000000 | 0.000000 | 0.000000 | 0.000000 | 0.000000 |
| 40-44 | 133-192 | 0.014241 | 0.000000 | 0.000000 | 0.000000 | 0.000000 | 0.000000 |
| 45-49 | 133-192 | 0.013014 | 0.000000 | 0.000000 | 0.000000 | 0.000000 | 0.000000 |
| 50-54 | 133-192 | 0.011768 | 0.000000 | 0.000000 | 0.000000 | 0.000000 | 0.000000 |
| 55-59 | 133-192 | 0.011032 | 0.000000 | 0.000000 | 0.000000 | 0.000000 | 0.000000 |
| 60-64 | 133-192 | 0.009525 | 0.000000 | 0.000000 | 0.000000 | 0.000000 | 0.000000 |
| 65-69 | 133-192 | 0.006897 | 0.000000 | 0.000000 | 0.000000 | 0.000000 | 0.000000 |
| 70-74 | 133-192 | 0.005435 | 0.000000 | 0.000000 | 0.000000 | 0.000000 | 0.000000 |
| 75-79 | 133-192 | 0.005435 | 0.000000 | 0.000000 | 0.000000 | 0.000000 | 0.000000 |
| 10-14 | 193-252 | 0.009549 | 0.000000 | 0.000000 | 0.000000 | 0.000000 | 0.000000 |
| 15-19 | 193-252 | 0.012695 | 0.000000 | 0.000000 | 0.000000 | 0.000000 | 0.000000 |
| 20-24 | 193-252 | 0.014490 | 0.000000 | 0.000000 | 0.000000 | 0.000000 | 0.000000 |
| 25-29 | 193-252 | 0.015212 | 0.000000 | 0.000000 | 0.000000 | 0.000000 | 0.000000 |
| 30-34 | 193-252 | 0.015460 | 0.000000 | 0.000000 | 0.000000 | 0.000000 | 0.000000 |
| 35-39 | 193-252 | 0.014711 | 0.000000 | 0.000000 | 0.000000 | 0.000000 | 0.000000 |
| 40-44 | 193-252 | 0.014241 | 0.000000 | 0.000000 | 0.000000 | 0.000000 | 0.000000 |
| 45-49 | 193-252 | 0.013014 | 0.000000 | 0.000000 | 0.000000 | 0.000000 | 0.000000 |
| 50-54 | 193-252 | 0.011768 | 0.000000 | 0.000000 | 0.000000 | 0.000000 | 0.000000 |
| 55-59 | 193-252 | 0.011032 | 0.000000 | 0.000000 | 0.000000 | 0.000000 | 0.000000 |
| 60-64 | 193-252 | 0.009525 | 0.000000 | 0.000000 | 0.000000 | 0.000000 | 0.000000 |
| 65-69 | 193-252 | 0.006897 | 0.000000 | 0.000000 | 0.000000 | 0.000000 | 0.000000 |
| 70-74 | 193-252 | 0.005435 | 0.000000 | 0.000000 | 0.000000 | 0.000000 | 0.000000 |
| 75-79 | 193-252 | 0.005435 | 0.000000 | 0.000000 | 0.000000 | 0.000000 | 0.000000 |
| 10-14 | 253+ | 0.009549 | 0.000000 | 0.000000 | 0.000000 | 0.000000 | 0.000000 |
| 15-19 | 253+ | 0.012695 | 0.000000 | 0.000000 | 0.000000 | 0.000000 | 0.000000 |
| 20-24 | 253+ | 0.014490 | 0.000000 | 0.000000 | 0.000000 | 0.000000 | 0.000000 |

## Results for PHIM for Run 5 (Sensitivity_Analysis_1.RTF)

| | | | | | | | |
|---|---|---|---|---|---|---|---|
| 25-29 | 253+ | 0.015212 | 0.000000 | 0.000000 | 0.000000 | 0.000000 | 0.000000 |
| 30-34 | 253+ | 0.015460 | 0.000000 | 0.000000 | 0.000000 | 0.000000 | 0.000000 |
| 35-39 | 253+ | 0.014711 | 0.000000 | 0.000000 | 0.000000 | 0.000000 | 0.000000 |
| 40-44 | 253+ | 0.014241 | 0.000000 | 0.000000 | 0.000000 | 0.000000 | 0.000000 |
| 45-49 | 253+ | 0.013014 | 0.000000 | 0.000000 | 0.000000 | 0.000000 | 0.000000 |
| 50-54 | 253+ | 0.011768 | 0.000000 | 0.000000 | 0.000000 | 0.000000 | 0.000000 |
| 55-59 | 253+ | 0.011032 | 0.000000 | 0.000000 | 0.000000 | 0.000000 | 0.000000 |
| 60-64 | 253+ | 0.009525 | 0.000000 | 0.000000 | 0.000000 | 0.000000 | 0.000000 |
| 65-69 | 253+ | 0.006897 | 0.000000 | 0.000000 | 0.000000 | 0.000000 | 0.000000 |
| 70-74 | 253+ | 0.005435 | 0.000000 | 0.000000 | 0.000000 | 0.000000 | 0.000000 |
| 75-79 | 253+ | 0.005435 | 0.000000 | 0.000000 | 0.000000 | 0.000000 | 0.000000 |

# Results for PHIM for Run 5 (Sensitivity_Analysis_1.RTF)

**************************************************************************************

**Main Output for the Tobacco History for Run: 5, Simulation: 1, Scenario: NULL**

| Variable Parameter | Value |
|---|---|
| 1. Country | US (United States) |
| 2. Sex | M (Males) |
| 3. Year of start of process | 1990 |

Year of Follow-up: 2040, Age Group: All Ages

| Period | Age Group | Smoking Group | N | % |
|---|---|---|---|---|
| 50 | All Ages | Never Any | 19445 | 50.68 |
| 50 | All Ages | CC | 3024 | 7.88 |
| 50 | All Ages | Former | 15896 | 41.43 |

Year of Follow-up: 2040, Age Group: 18-19

| Period | Age Group | Smoking Group | N | % |
|---|---|---|---|---|
| 50 | 18-19 | Never Any | 0 | . |
| 50 | 18-19 | CC | 0 | . |
| 50 | 18-19 | Former | 0 | . |

Year of Follow-up: 2040, Age Group: 20-24

| Period | Age Group | Smoking Group | N | % |
|---|---|---|---|---|
| 50 | 20-24 | Never Any | 0 | . |
| 50 | 20-24 | CC | 0 | . |
| 50 | 20-24 | Former | 0 | . |

Year of Follow-up: 2040, Age Group: 25-29

| Period | Age Group | Smoking Group | N | % |
|---|---|---|---|---|
| 50 | 25-29 | Never Any | 0 | . |
| 50 | 25-29 | CC | 0 | . |
| 50 | 25-29 | Former | 0 | . |

Year of Follow-up: 2040, Age Group: 30-34

| Period | Age Group | Smoking Group | N | % |
|---|---|---|---|---|
| 50 | 30-34 | Never Any | 0 | . |
| 50 | 30-34 | CC | 0 | . |
| 50 | 30-34 | Former | 0 | . |

Year of Follow-up: 2040, Age Group: 35-39

| Period | Age Group | Smoking Group | N | % |
|---|---|---|---|---|
| 50 | 35-39 | Never Any | 0 | . |
| 50 | 35-39 | CC | 0 | . |
| 50 | 35-39 | Former | 0 | . |

Year of Follow-up: 2040, Age Group: 40-44

| Period | Age Group | Smoking Group | N | % |
|---|---|---|---|---|
| 50 | 40-44 | Never Any | 0 | . |
| 50 | 40-44 | CC | 0 | . |
| 50 | 40-44 | Former | 0 | . |

Year of Follow-up: 2040, Age Group: 45-49

| Period | Age Group | Smoking Group | N | % |
|---|---|---|---|---|
| 50 | 45-49 | Never Any | 0 | . |
| 50 | 45-49 | CC | 0 | . |
| 50 | 45-49 | Former | 0 | . |

Year of Follow-up: 2040, Age Group: 50-54

| Period | Age Group | Smoking Group | N | % |
|---|---|---|---|---|
| 50 | 50-54 | Never Any | 0 | . |
| 50 | 50-54 | CC | 0 | . |
| 50 | 50-54 | Former | 0 | . |

Year of Follow-up: 2040, Age Group: 55-59

| Period | Age Group | Smoking Group | N | % |
|---|---|---|---|---|
| 50 | 55-59 | Never Any | 0 | . |
| 50 | 55-59 | CC | 0 | . |
| 50 | 55-59 | Former | 0 | . |

Year of Follow-up: 2040, Age Group: 60-64

| Period | Age Group | Smoking Group | N | % |
|---|---|---|---|---|

# Results for PHIM for Run 5 (Sensitivity_Analysis_1.RTF)

| | | | | |
|---|---|---|---|---|
| 50 | 60-64 | Never Any | 4363 | 49.33 |
| 50 | 60-64 | CC | 934 | 10.56 |
| 50 | 60-64 | Former | 3548 | 40.11 |

Year of Follow-up: 2040, Age Group: 65-69

| Period | Age Group | Smoking Group | N | % |
|---|---|---|---|---|
| 50 | 65-69 | Never Any | 4948 | 54.73 |
| 50 | 65-69 | CC | 734 | 8.12 |
| 50 | 65-69 | Former | 3358 | 37.15 |

Year of Follow-up: 2040, Age Group: 70-74

| Period | Age Group | Smoking Group | N | % |
|---|---|---|---|---|
| 50 | 70-74 | Never Any | 5145 | 52.41 |
| 50 | 70-74 | CC | 688 | 7.01 |
| 50 | 70-74 | Former | 3983 | 40.58 |

Year of Follow-up: 2040, Age Group: 75-79

| Period | Age Group | Smoking Group | N | % |
|---|---|---|---|---|
| 50 | 75-79 | Never Any | 4989 | 46.78 |
| 50 | 75-79 | CC | 668 | 6.26 |
| 50 | 75-79 | Former | 5007 | 46.95 |

# Results for PHIM for Run 5 (Sensitivity_Analysis_1.RTF)

************************************************************************************
**Main Output for the Tobacco History for Run: 5, Simulation: 1, Scenario: Ecig**

| Variable Parameter | Value |
|---|---|
| 1. Country | US (United States) |
| 2. Sex | M (Males) |
| 3. Year of start of process | 1990 |

Year of Follow-up: 1990, Age Group: All Ages

| Period | Age Group | Smoking Group | N | % | Ever Quit (N) | Ever Quit % | Mean Yrs Quit | Short Term (n) | Short Term % |
|---|---|---|---|---|---|---|---|---|---|
| 0 | All Ages | Never Any | 34075 | 39.78 | | | | | |
| 0 | All Ages | CC | 27323 | 31.90 | 0 | 0.00 | | | |
| 0 | All Ages | Ecig | 0 | 0.00 | | | | | |
| 0 | All Ages | Dual | 0 | 0.00 | | | | | |
| 0 | All Ages | Former | 24266 | 28.33 | | | 13.08 | 3623 | 14.93 |

Year of Follow-up: 1990, Age Group: 18-19

| Period | Age Group | Smoking Group | N | % | Ever Quit (N) | Ever Quit % | Mean Yrs Quit | Short Term (n) | Short Term % |
|---|---|---|---|---|---|---|---|---|---|
| 0 | 18-19 | Never Any | 2783 | 78.42 | | | | | |
| 0 | 18-19 | CC | 634 | 17.86 | 0 | 0.00 | | | |
| 0 | 18-19 | Ecig | 0 | 0.00 | | | | | |
| 0 | 18-19 | Dual | 0 | 0.00 | | | | | |
| 0 | 18-19 | Former | 132 | 3.72 | | | 0.11 | 132 | 100.00 |

Year of Follow-up: 1990, Age Group: 20-24

| Period | Age Group | Smoking Group | N | % | Ever Quit (N) | Ever Quit % | Mean Yrs Quit | Short Term (n) | Short Term % |
|---|---|---|---|---|---|---|---|---|---|
| 0 | 20-24 | Never Any | 5722 | 58.29 | | | | | |
| 0 | 20-24 | CC | 3199 | 32.59 | 0 | 0.00 | | | |
| 0 | 20-24 | Ecig | 0 | 0.00 | | | | | |
| 0 | 20-24 | Dual | 0 | 0.00 | | | | | |
| 0 | 20-24 | Former | 895 | 9.12 | | | 1.46 | 506 | 56.54 |

Year of Follow-up: 1990, Age Group: 25-29

| Period | Age Group | Smoking Group | N | % | Ever Quit (N) | Ever Quit % | Mean Yrs Quit | Short Term (n) | Short Term % |
|---|---|---|---|---|---|---|---|---|---|
| 0 | 25-29 | Never Any | 5232 | 49.06 | | | | | |
| 0 | 25-29 | CC | 3914 | 36.70 | 0 | 0.00 | | | |
| 0 | 25-29 | Ecig | 0 | 0.00 | | | | | |
| 0 | 25-29 | Dual | 0 | 0.00 | | | | | |
| 0 | 25-29 | Former | 1518 | 14.23 | | | 2.87 | 643 | 42.36 |

Year of Follow-up: 1990, Age Group: 30-34

| Period | Age Group | Smoking Group | N | % | Ever Quit (N) | Ever Quit % | Mean Yrs Quit | Short Term (n) | Short Term % |
|---|---|---|---|---|---|---|---|---|---|
| 0 | 30-34 | Never Any | 4765 | 43.65 | | | | | |
| 0 | 30-34 | CC | 4023 | 36.85 | 0 | 0.00 | | | |
| 0 | 30-34 | Ecig | 0 | 0.00 | | | | | |
| 0 | 30-34 | Dual | 0 | 0.00 | | | | | |
| 0 | 30-34 | Former | 2128 | 19.49 | | | 4.06 | 592 | 27.82 |

Year of Follow-up: 1990, Age Group: 35-39

| Period | Age Group | Smoking Group | N | % | Ever Quit (N) | Ever Quit % | Mean Yrs Quit | Short Term (n) | Short Term % |
|---|---|---|---|---|---|---|---|---|---|
| 0 | 35-39 | Never Any | 3941 | 39.85 | | | | | |
| 0 | 35-39 | CC | 3474 | 35.13 | 0 | 0.00 | | | |
| 0 | 35-39 | Ecig | 0 | 0.00 | | | | | |
| 0 | 35-39 | Dual | 0 | 0.00 | | | | | |
| 0 | 35-39 | Former | 2474 | 25.02 | | | 8.31 | 425 | 17.18 |

Year of Follow-up: 1990, Age Group: 40-44

| Period | Age Group | Smoking Group | N | % | Ever Quit (N) | Ever Quit % | Mean Yrs Quit | Short Term (n) | Short Term % |
|---|---|---|---|---|---|---|---|---|---|
| 0 | 40-44 | Never Any | 2912 | 33.65 | | | | | |
| 0 | 40-44 | CC | 3113 | 35.97 | 0 | 0.00 | | | |
| 0 | 40-44 | Ecig | 0 | 0.00 | | | | | |
| 0 | 40-44 | Dual | 0 | 0.00 | | | | | |
| 0 | 40-44 | Former | 2630 | 30.39 | | | 10.69 | 317 | 12.05 |

Year of Follow-up: 1990, Age Group: 45-49

| Period | Age Group | Smoking Group | N | % | Ever Quit (N) | Ever Quit % | Mean Yrs Quit | Short Term (n) | Short Term % |
|---|---|---|---|---|---|---|---|---|---|
| 0 | 45-49 | Never Any | 1919 | 29.46 | | | | | |
| 0 | 45-49 | CC | 2318 | 35.58 | 0 | 0.00 | | | |
| 0 | 45-49 | Ecig | 0 | 0.00 | | | | | |
| 0 | 45-49 | Dual | 0 | 0.00 | | | | | |
| 0 | 45-49 | Former | 2278 | 34.97 | | | 13.26 | 221 | 9.70 |

Year of Follow-up: 1990, Age Group: 50-54

| Period | Age Group | Smoking Group | N | % | Ever Quit (N) | Ever Quit % | Mean Yrs Quit | Short Term (n) | Short Term % |
|---|---|---|---|---|---|---|---|---|---|
| 0 | 50-54 | Never Any | 1611 | 29.41 | | | | | |
| 0 | 50-54 | CC | 1816 | 33.16 | 0 | 0.00 | | | |

# Results for PHIM for Run 5 (Sensitivity_Analysis_1.RTF)

| | | | | | | | | | |
|---|---|---|---|---|---|---|---|---|---|
| 0 | 50-54 | Ecig | 0 | 0.00 | | | | | |
| 0 | 50-54 | Dual | 0 | 0.00 | | | | | |
| 0 | 50-54 | Former | 2050 | 37.43 | | | 14.55 | 261 | 12.73 |

Year of Follow-up: 1990, Age Group: 55-59

| Period | Age Group | Smoking Group | N | % | Ever Quit (N) | Ever Quit % | Mean Yrs Quit | Short Term (n) | Short Term % |
|---|---|---|---|---|---|---|---|---|---|
| 0 | 55-59 | Never Any | 1337 | 26.83 | | | | | |
| 0 | 55-59 | CC | 1520 | 30.50 | 0 | 0.00 | | | |
| 0 | 55-59 | Ecig | 0 | 0.00 | | | | | |
| 0 | 55-59 | Dual | 0 | 0.00 | | | | | |
| 0 | 55-59 | Former | 2126 | 42.67 | | | 14.80 | 216 | 10.16 |

Year of Follow-up: 1990, Age Group: 60-64

| Period | Age Group | Smoking Group | N | % | Ever Quit (N) | Ever Quit % | Mean Yrs Quit | Short Term (n) | Short Term % |
|---|---|---|---|---|---|---|---|---|---|
| 0 | 60-64 | Never Any | 1169 | 23.83 | | | | | |
| 0 | 60-64 | CC | 1287 | 26.24 | 0 | 0.00 | | | |
| 0 | 60-64 | Ecig | 0 | 0.00 | | | | | |
| 0 | 60-64 | Dual | 0 | 0.00 | | | | | |
| 0 | 60-64 | Former | 2449 | 49.93 | | | 18.87 | 106 | 4.33 |

Year of Follow-up: 1990, Age Group: 65-69

| Period | Age Group | Smoking Group | N | % | Ever Quit (N) | Ever Quit % | Mean Yrs Quit | Short Term (n) | Short Term % |
|---|---|---|---|---|---|---|---|---|---|
| 0 | 65-69 | Never Any | 1023 | 23.32 | | | | | |
| 0 | 65-69 | CC | 979 | 22.32 | 0 | 0.00 | | | |
| 0 | 65-69 | Ecig | 0 | 0.00 | | | | | |
| 0 | 65-69 | Dual | 0 | 0.00 | | | | | |
| 0 | 65-69 | Former | 2385 | 54.37 | | | 19.91 | 113 | 4.74 |

Year of Follow-up: 1990, Age Group: 70-74

| Period | Age Group | Smoking Group | N | % | Ever Quit (N) | Ever Quit % | Mean Yrs Quit | Short Term (n) | Short Term % |
|---|---|---|---|---|---|---|---|---|---|
| 0 | 70-74 | Never Any | 961 | 27.46 | | | | | |
| 0 | 70-74 | CC | 675 | 19.29 | 0 | 0.00 | | | |
| 0 | 70-74 | Ecig | 0 | 0.00 | | | | | |
| 0 | 70-74 | Dual | 0 | 0.00 | | | | | |
| 0 | 70-74 | Former | 1863 | 53.24 | | | 20.69 | 23 | 1.23 |

Year of Follow-up: 1990, Age Group: 75-79

| Period | Age Group | Smoking Group | N | % | Ever Quit (N) | Ever Quit % | Mean Yrs Quit | Short Term (n) | Short Term % |
|---|---|---|---|---|---|---|---|---|---|
| 0 | 75-79 | Never Any | 700 | 29.06 | | | | | |
| 0 | 75-79 | CC | 371 | 15.40 | 0 | 0.00 | | | |
| 0 | 75-79 | Ecig | 0 | 0.00 | | | | | |
| 0 | 75-79 | Dual | 0 | 0.00 | | | | | |
| 0 | 75-79 | Former | 1338 | 55.54 | | | 22.88 | 68 | 5.08 |

Year of Follow-up: 1995, Age Group: All Ages

| Period | Age Group | Smoking Group | N | % | Ever Quit (N) | Ever Quit % | Mean Yrs Quit | Short Term (n) | Short Term % |
|---|---|---|---|---|---|---|---|---|---|
| 5 | All Ages | Never Any | 38368 | 41.57 | | | | | |
| 5 | All Ages | CC | 11508 | 12.47 | 0 | 0.00 | | | |
| 5 | All Ages | Ecig | 15517 | 16.81 | | | | | |
| 5 | All Ages | Dual | 0 | 0.00 | | | | | |
| 5 | All Ages | Former | 26897 | 29.14 | | | 15.19 | 1705 | 6.34 |

Year of Follow-up: 1995, Age Group: 18-19

| Period | Age Group | Smoking Group | N | % | Ever Quit (N) | Ever Quit % | Mean Yrs Quit | Short Term (n) | Short Term % |
|---|---|---|---|---|---|---|---|---|---|
| 5 | 18-19 | Never Any | 2448 | 69.07 | | | | | |
| 5 | 18-19 | CC | 320 | 9.03 | 0 | 0.00 | | | |
| 5 | 18-19 | Ecig | 659 | 18.59 | | | | | |
| 5 | 18-19 | Dual | 0 | 0.00 | | | | | |
| 5 | 18-19 | Former | 117 | 3.30 | | | 1.11 | 80 | 68.38 |

Year of Follow-up: 1995, Age Group: 20-24

| Period | Age Group | Smoking Group | N | % | Ever Quit (N) | Ever Quit % | Mean Yrs Quit | Short Term (n) | Short Term % |
|---|---|---|---|---|---|---|---|---|---|
| 5 | 20-24 | Never Any | 5787 | 64.02 | | | | | |
| 5 | 20-24 | CC | 946 | 10.46 | 0 | 0.00 | | | |
| 5 | 20-24 | Ecig | 1826 | 20.20 | | | | | |
| 5 | 20-24 | Dual | 0 | 0.00 | | | | | |
| 5 | 20-24 | Former | 481 | 5.32 | | | 2.81 | 142 | 29.52 |

Year of Follow-up: 1995, Age Group: 25-29

| Period | Age Group | Smoking Group | N | % | Ever Quit (N) | Ever Quit % | Mean Yrs Quit | Short Term (n) | Short Term % |
|---|---|---|---|---|---|---|---|---|---|
| 5 | 25-29 | Never Any | 5471 | 55.74 | | | | | |
| 5 | 25-29 | CC | 1255 | 12.79 | 0 | 0.00 | | | |
| 5 | 25-29 | Ecig | 1942 | 19.78 | | | | | |
| 5 | 25-29 | Dual | 0 | 0.00 | | | | | |
| 5 | 25-29 | Former | 1148 | 11.70 | | | 5.43 | 115 | 10.02 |

Year of Follow-up: 1995, Age Group: 30-34

| Period | Age Group | Smoking Group | N | % | Ever Quit (N) | Ever Quit % | Mean Yrs Quit | Short Term (n) | Short Term % |
|---|---|---|---|---|---|---|---|---|---|

# Results for PHIM for Run 5 (Sensitivity_Analysis_1.RTF)

| | | | | | | | | | |
|---|---|---|---|---|---|---|---|---|---|
| 5 | 30-34 | Never Any | 5065 | 47.50 | | | | | |
| 5 | 30-34 | CC | 1480 | 13.88 | 0 | 0.00 | | | |
| 5 | 30-34 | Ecig | 2319 | 21.75 | | | | | |
| 5 | 30-34 | Dual | 0 | 0.00 | | | | | |
| 5 | 30-34 | Former | 1800 | 16.88 | | | 6.94 | 106 | 5.89 |

Year of Follow-up: 1995, Age Group: 35-39

| Period | Age Group | Smoking Group | N | % | Ever Quit (N) | Ever Quit % | Mean Yrs Quit | Short Term (n) | Short Term % |
|---|---|---|---|---|---|---|---|---|---|
| 5 | 35-39 | Never Any | 4724 | 43.28 | | | | | |
| 5 | 35-39 | CC | 1496 | 13.70 | 0 | 0.00 | | | |
| 5 | 35-39 | Ecig | 2275 | 20.84 | | | | | |
| 5 | 35-39 | Dual | 0 | 0.00 | | | | | |
| 5 | 35-39 | Former | 2421 | 22.18 | | | 8.20 | 114 | 4.71 |

Year of Follow-up: 1995, Age Group: 40-44

| Period | Age Group | Smoking Group | N | % | Ever Quit (N) | Ever Quit % | Mean Yrs Quit | Short Term (n) | Short Term % |
|---|---|---|---|---|---|---|---|---|---|
| 5 | 40-44 | Never Any | 3941 | 39.85 | | | | | |
| 5 | 40-44 | CC | 1280 | 12.94 | 0 | 0.00 | | | |
| 5 | 40-44 | Ecig | 1894 | 19.15 | | | | | |
| 5 | 40-44 | Dual | 0 | 0.00 | | | | | |
| 5 | 40-44 | Former | 2774 | 28.05 | | | 12.06 | 130 | 4.69 |

Year of Follow-up: 1995, Age Group: 45-49

| Period | Age Group | Smoking Group | N | % | Ever Quit (N) | Ever Quit % | Mean Yrs Quit | Short Term (n) | Short Term % |
|---|---|---|---|---|---|---|---|---|---|
| 5 | 45-49 | Never Any | 2912 | 33.65 | | | | | |
| 5 | 45-49 | CC | 1236 | 14.28 | 0 | 0.00 | | | |
| 5 | 45-49 | Ecig | 1562 | 18.05 | | | | | |
| 5 | 45-49 | Dual | 0 | 0.00 | | | | | |
| 5 | 45-49 | Former | 2945 | 34.03 | | | 14.20 | 148 | 5.03 |

Year of Follow-up: 1995, Age Group: 50-54

| Period | Age Group | Smoking Group | N | % | Ever Quit (N) | Ever Quit % | Mean Yrs Quit | Short Term (n) | Short Term % |
|---|---|---|---|---|---|---|---|---|---|
| 5 | 50-54 | Never Any | 1919 | 29.46 | | | | | |
| 5 | 50-54 | CC | 940 | 14.43 | 0 | 0.00 | | | |
| 5 | 50-54 | Ecig | 1015 | 15.58 | | | | | |
| 5 | 50-54 | Dual | 0 | 0.00 | | | | | |
| 5 | 50-54 | Former | 2641 | 40.54 | | | 15.98 | 175 | 6.63 |

Year of Follow-up: 1995, Age Group: 55-59

| Period | Age Group | Smoking Group | N | % | Ever Quit (N) | Ever Quit % | Mean Yrs Quit | Short Term (n) | Short Term % |
|---|---|---|---|---|---|---|---|---|---|
| 5 | 55-59 | Never Any | 1611 | 29.41 | | | | | |
| 5 | 55-59 | CC | 740 | 13.51 | 0 | 0.00 | | | |
| 5 | 55-59 | Ecig | 762 | 13.91 | | | | | |
| 5 | 55-59 | Dual | 0 | 0.00 | | | | | |
| 5 | 55-59 | Former | 2364 | 43.16 | | | 17.21 | 122 | 5.16 |

Year of Follow-up: 1995, Age Group: 60-64

| Period | Age Group | Smoking Group | N | % | Ever Quit (N) | Ever Quit % | Mean Yrs Quit | Short Term (n) | Short Term % |
|---|---|---|---|---|---|---|---|---|---|
| 5 | 60-64 | Never Any | 1337 | 26.83 | | | | | |
| 5 | 60-64 | CC | 597 | 11.98 | 0 | 0.00 | | | |
| 5 | 60-64 | Ecig | 572 | 11.48 | | | | | |
| 5 | 60-64 | Dual | 0 | 0.00 | | | | | |
| 5 | 60-64 | Former | 2477 | 49.71 | | | 17.27 | 143 | 5.77 |

Year of Follow-up: 1995, Age Group: 65-69

| Period | Age Group | Smoking Group | N | % | Ever Quit (N) | Ever Quit % | Mean Yrs Quit | Short Term (n) | Short Term % |
|---|---|---|---|---|---|---|---|---|---|
| 5 | 65-69 | Never Any | 1169 | 23.83 | | | | | |
| 5 | 65-69 | CC | 477 | 9.72 | 0 | 0.00 | | | |
| 5 | 65-69 | Ecig | 360 | 7.34 | | | | | |
| 5 | 65-69 | Dual | 0 | 0.00 | | | | | |
| 5 | 65-69 | Former | 2899 | 59.10 | | | 20.44 | 196 | 6.76 |

Year of Follow-up: 1995, Age Group: 70-74

| Period | Age Group | Smoking Group | N | % | Ever Quit (N) | Ever Quit % | Mean Yrs Quit | Short Term (n) | Short Term % |
|---|---|---|---|---|---|---|---|---|---|
| 5 | 70-74 | Never Any | 1023 | 23.32 | | | | | |
| 5 | 70-74 | CC | 438 | 9.98 | 0 | 0.00 | | | |
| 5 | 70-74 | Ecig | 196 | 4.47 | | | | | |
| 5 | 70-74 | Dual | 0 | 0.00 | | | | | |
| 5 | 70-74 | Former | 2730 | 62.23 | | | 22.00 | 137 | 5.02 |

Year of Follow-up: 1995, Age Group: 75-79

| Period | Age Group | Smoking Group | N | % | Ever Quit (N) | Ever Quit % | Mean Yrs Quit | Short Term (n) | Short Term % |
|---|---|---|---|---|---|---|---|---|---|
| 5 | 75-79 | Never Any | 961 | 27.46 | | | | | |
| 5 | 75-79 | CC | 303 | 8.66 | 0 | 0.00 | | | |
| 5 | 75-79 | Ecig | 135 | 3.86 | | | | | |
| 5 | 75-79 | Dual | 0 | 0.00 | | | | | |
| 5 | 75-79 | Former | 2100 | 60.02 | | | 23.02 | 97 | 4.62 |

Year of Follow-up: 2000, Age Group: All Ages

## Results for PHIM for Run 5 (Sensitivity_Analysis_1.RTF)

| Period | Age Group | Smoking Group | N | % | Ever Quit (N) | Ever Quit % | Mean Yrs Quit | Short Term (n) | Short Term % |
|---|---|---|---|---|---|---|---|---|---|
| 10 | All Ages | Never Any | 39177 | 41.64 | | | | | |
| 10 | All Ages | CC | 4843 | 5.15 | 0 | 0.00 | | | |
| 10 | All Ages | Ecig | 20724 | 22.03 | | | | | |
| 10 | All Ages | Dual | 0 | 0.00 | | | | | |
| 10 | All Ages | Former | 29348 | 31.19 | | | 16.84 | 1724 | 5.87 |

Year of Follow-up: 2000, Age Group: 18-19

| Period | Age Group | Smoking Group | N | % | Ever Quit (N) | Ever Quit % | Mean Yrs Quit | Short Term (n) | Short Term % |
|---|---|---|---|---|---|---|---|---|---|
| 10 | 18-19 | Never Any | 0 | . | | | | | |
| 10 | 18-19 | CC | 0 | . | 0 | . | | | |
| 10 | 18-19 | Ecig | 0 | . | | | | | |
| 10 | 18-19 | Dual | 0 | . | | | | | |
| 10 | 18-19 | Former | 0 | . | | | . | 0 | . |

Year of Follow-up: 2000, Age Group: 20-24

| Period | Age Group | Smoking Group | N | % | Ever Quit (N) | Ever Quit % | Mean Yrs Quit | Short Term (n) | Short Term % |
|---|---|---|---|---|---|---|---|---|---|
| 10 | 20-24 | Never Any | 4927 | 55.70 | | | | | |
| 10 | 20-24 | CC | 462 | 5.22 | 0 | 0.00 | | | |
| 10 | 20-24 | Ecig | 2544 | 28.76 | | | | | |
| 10 | 20-24 | Dual | 0 | 0.00 | | | | | |
| 10 | 20-24 | Former | 912 | 10.31 | | | 3.41 | 258 | 28.29 |

Year of Follow-up: 2000, Age Group: 25-29

| Period | Age Group | Smoking Group | N | % | Ever Quit (N) | Ever Quit % | Mean Yrs Quit | Short Term (n) | Short Term % |
|---|---|---|---|---|---|---|---|---|---|
| 10 | 25-29 | Never Any | 5341 | 59.08 | | | | | |
| 10 | 25-29 | CC | 404 | 4.47 | 0 | 0.00 | | | |
| 10 | 25-29 | Ecig | 2359 | 26.10 | | | | | |
| 10 | 25-29 | Dual | 0 | 0.00 | | | | | |
| 10 | 25-29 | Former | 936 | 10.35 | | | 4.91 | 195 | 20.83 |

Year of Follow-up: 2000, Age Group: 30-34

| Period | Age Group | Smoking Group | N | % | Ever Quit (N) | Ever Quit % | Mean Yrs Quit | Short Term (n) | Short Term % |
|---|---|---|---|---|---|---|---|---|---|
| 10 | 30-34 | Never Any | 5276 | 53.75 | | | | | |
| 10 | 30-34 | CC | 481 | 4.90 | 0 | 0.00 | | | |
| 10 | 30-34 | Ecig | 2608 | 26.57 | | | | | |
| 10 | 30-34 | Dual | 0 | 0.00 | | | | | |
| 10 | 30-34 | Former | 1451 | 14.78 | | | 8.66 | 118 | 8.13 |

Year of Follow-up: 2000, Age Group: 35-39

| Period | Age Group | Smoking Group | N | % | Ever Quit (N) | Ever Quit % | Mean Yrs Quit | Short Term (n) | Short Term % |
|---|---|---|---|---|---|---|---|---|---|
| 10 | 35-39 | Never Any | 4997 | 46.86 | | | | | |
| 10 | 35-39 | CC | 609 | 5.71 | 0 | 0.00 | | | |
| 10 | 35-39 | Ecig | 2980 | 27.94 | | | | | |
| 10 | 35-39 | Dual | 0 | 0.00 | | | | | |
| 10 | 35-39 | Former | 2078 | 19.49 | | | 10.61 | 109 | 5.25 |

Year of Follow-up: 2000, Age Group: 40-44

| Period | Age Group | Smoking Group | N | % | Ever Quit (N) | Ever Quit % | Mean Yrs Quit | Short Term (n) | Short Term % |
|---|---|---|---|---|---|---|---|---|---|
| 10 | 40-44 | Never Any | 4724 | 43.28 | | | | | |
| 10 | 40-44 | CC | 567 | 5.19 | 0 | 0.00 | | | |
| 10 | 40-44 | Ecig | 2857 | 26.17 | | | | | |
| 10 | 40-44 | Dual | 0 | 0.00 | | | | | |
| 10 | 40-44 | Former | 2768 | 25.36 | | | 11.82 | 124 | 4.48 |

Year of Follow-up: 2000, Age Group: 45-49

| Period | Age Group | Smoking Group | N | % | Ever Quit (N) | Ever Quit % | Mean Yrs Quit | Short Term (n) | Short Term % |
|---|---|---|---|---|---|---|---|---|---|
| 10 | 45-49 | Never Any | 3941 | 39.85 | | | | | |
| 10 | 45-49 | CC | 484 | 4.89 | 0 | 0.00 | | | |
| 10 | 45-49 | Ecig | 2312 | 23.38 | | | | | |
| 10 | 45-49 | Dual | 0 | 0.00 | | | | | |
| 10 | 45-49 | Former | 3152 | 31.87 | | | 15.25 | 163 | 5.17 |

Year of Follow-up: 2000, Age Group: 50-54

| Period | Age Group | Smoking Group | N | % | Ever Quit (N) | Ever Quit % | Mean Yrs Quit | Short Term (n) | Short Term % |
|---|---|---|---|---|---|---|---|---|---|
| 10 | 50-54 | Never Any | 2912 | 33.65 | | | | | |
| 10 | 50-54 | CC | 473 | 5.47 | 0 | 0.00 | | | |
| 10 | 50-54 | Ecig | 1859 | 21.48 | | | | | |
| 10 | 50-54 | Dual | 0 | 0.00 | | | | | |
| 10 | 50-54 | Former | 3411 | 39.41 | | | 16.85 | 186 | 5.45 |

Year of Follow-up: 2000, Age Group: 55-59

| Period | Age Group | Smoking Group | N | % | Ever Quit (N) | Ever Quit % | Mean Yrs Quit | Short Term (n) | Short Term % |
|---|---|---|---|---|---|---|---|---|---|
| 10 | 55-59 | Never Any | 1919 | 29.46 | | | | | |
| 10 | 55-59 | CC | 363 | 5.57 | 0 | 0.00 | | | |
| 10 | 55-59 | Ecig | 1194 | 18.33 | | | | | |
| 10 | 55-59 | Dual | 0 | 0.00 | | | | | |

# Results for PHIM for Run 5 (Sensitivity_Analysis_1.RTF)

| | | | | | | | | | |
|---|---|---|---|---|---|---|---|---|---|
| 10 | 55-59 | Former | 3039 | 46.65 | | | 18.50 | 146 | 4.80 |

Year of Follow-up: 2000, Age Group: 60-64

| Period | Age Group | Smoking Group | N | % | Ever Quit (N) | Ever Quit % | Mean Yrs Quit | Short Term (n) | Short Term % |
|---|---|---|---|---|---|---|---|---|---|
| 10 | 60-64 | Never Any | 1611 | 29.41 | | | | | |
| 10 | 60-64 | CC | 306 | 5.59 | 0 | 0.00 | | | |
| 10 | 60-64 | Ecig | 852 | 15.56 | | | | | |
| 10 | 60-64 | Dual | 0 | 0.00 | | | | | |
| 10 | 60-64 | Former | 2708 | 49.44 | | | 19.63 | 144 | 5.32 |

Year of Follow-up: 2000, Age Group: 65-69

| Period | Age Group | Smoking Group | N | % | Ever Quit (N) | Ever Quit % | Mean Yrs Quit | Short Term (n) | Short Term % |
|---|---|---|---|---|---|---|---|---|---|
| 10 | 65-69 | Never Any | 1337 | 26.83 | | | | | |
| 10 | 65-69 | CC | 244 | 4.90 | 0 | 0.00 | | | |
| 10 | 65-69 | Ecig | 603 | 12.10 | | | | | |
| 10 | 65-69 | Dual | 0 | 0.00 | | | | | |
| 10 | 65-69 | Former | 2799 | 56.17 | | | 19.95 | 120 | 4.29 |

Year of Follow-up: 2000, Age Group: 70-74

| Period | Age Group | Smoking Group | N | % | Ever Quit (N) | Ever Quit % | Mean Yrs Quit | Short Term (n) | Short Term % |
|---|---|---|---|---|---|---|---|---|---|
| 10 | 70-74 | Never Any | 1169 | 23.83 | | | | | |
| 10 | 70-74 | CC | 236 | 4.81 | 0 | 0.00 | | | |
| 10 | 70-74 | Ecig | 327 | 6.67 | | | | | |
| 10 | 70-74 | Dual | 0 | 0.00 | | | | | |
| 10 | 70-74 | Former | 3173 | 64.69 | | | 23.44 | 94 | 2.96 |

Year of Follow-up: 2000, Age Group: 75-79

| Period | Age Group | Smoking Group | N | % | Ever Quit (N) | Ever Quit % | Mean Yrs Quit | Short Term (n) | Short Term % |
|---|---|---|---|---|---|---|---|---|---|
| 10 | 75-79 | Never Any | 1023 | 23.32 | | | | | |
| 10 | 75-79 | CC | 214 | 4.88 | 0 | 0.00 | | | |
| 10 | 75-79 | Ecig | 229 | 5.22 | | | | | |
| 10 | 75-79 | Dual | 0 | 0.00 | | | | | |
| 10 | 75-79 | Former | 2921 | 66.58 | | | 25.38 | 67 | 2.29 |

Year of Follow-up: 2005, Age Group: All Ages

| Period | Age Group | Smoking Group | N | % | Ever Quit (N) | Ever Quit % | Mean Yrs Quit | Short Term (n) | Short Term % |
|---|---|---|---|---|---|---|---|---|---|
| 15 | All Ages | Never Any | 37449 | 41.75 | | | | | |
| 15 | All Ages | CC | 1911 | 2.13 | 0 | 0.00 | | | |
| 15 | All Ages | Ecig | 20003 | 22.30 | | | | | |
| 15 | All Ages | Dual | 0 | 0.00 | | | | | |
| 15 | All Ages | Former | 30342 | 33.82 | | | 18.46 | 1574 | 5.19 |

Year of Follow-up: 2005, Age Group: 18-19

| Period | Age Group | Smoking Group | N | % | Ever Quit (N) | Ever Quit % | Mean Yrs Quit | Short Term (n) | Short Term % |
|---|---|---|---|---|---|---|---|---|---|
| 15 | 18-19 | Never Any | 0 | . | | | | | |
| 15 | 18-19 | CC | 0 | . | 0 | . | | | |
| 15 | 18-19 | Ecig | 0 | . | | | | | |
| 15 | 18-19 | Dual | 0 | . | | | | | |
| 15 | 18-19 | Former | 0 | . | | | . | 0 | . |

Year of Follow-up: 2005, Age Group: 20-24

| Period | Age Group | Smoking Group | N | % | Ever Quit (N) | Ever Quit % | Mean Yrs Quit | Short Term (n) | Short Term % |
|---|---|---|---|---|---|---|---|---|---|
| 15 | 20-24 | Never Any | 0 | . | | | | | |
| 15 | 20-24 | CC | 0 | . | 0 | . | | | |
| 15 | 20-24 | Ecig | 0 | . | | | | | |
| 15 | 20-24 | Dual | 0 | . | | | | | |
| 15 | 20-24 | Former | 0 | . | | | . | 0 | . |

Year of Follow-up: 2005, Age Group: 25-29

| Period | Age Group | Smoking Group | N | % | Ever Quit (N) | Ever Quit % | Mean Yrs Quit | Short Term (n) | Short Term % |
|---|---|---|---|---|---|---|---|---|---|
| 15 | 25-29 | Never Any | 4634 | 52.39 | | | | | |
| 15 | 25-29 | CC | 179 | 2.02 | 0 | 0.00 | | | |
| 15 | 25-29 | Ecig | 2661 | 30.08 | | | | | |
| 15 | 25-29 | Dual | 0 | 0.00 | | | | | |
| 15 | 25-29 | Former | 1371 | 15.50 | | | 6.32 | 174 | 12.69 |

Year of Follow-up: 2005, Age Group: 30-34

| Period | Age Group | Smoking Group | N | % | Ever Quit (N) | Ever Quit % | Mean Yrs Quit | Short Term (n) | Short Term % |
|---|---|---|---|---|---|---|---|---|---|
| 15 | 30-34 | Never Any | 5042 | 55.77 | | | | | |
| 15 | 30-34 | CC | 153 | 1.69 | 0 | 0.00 | | | |
| 15 | 30-34 | Ecig | 2479 | 27.42 | | | | | |
| 15 | 30-34 | Dual | 0 | 0.00 | | | | | |
| 15 | 30-34 | Former | 1366 | 15.11 | | | 7.38 | 171 | 12.52 |

Year of Follow-up: 2005, Age Group: 35-39

| Period | Age Group | Smoking Group | N | % | Ever Quit (N) | Ever Quit % | Mean Yrs Quit | Short Term (n) | Short Term % |
|---|---|---|---|---|---|---|---|---|---|
| 15 | 35-39 | Never Any | 5163 | 52.60 | | | | | |
| 15 | 35-39 | CC | 188 | 1.92 | 0 | 0.00 | | | |

# Results for PHIM for Run 5 (Sensitivity_Analysis_1.RTF)

| | | | | | | | | | |
|---|---|---|---|---|---|---|---|---|---|
| 15 | 35-39 | Ecig | 2654 | 27.04 | | | | | |
| 15 | 35-39 | Dual | 0 | 0.00 | | | | | |
| 15 | 35-39 | Former | 1811 | 18.45 | | | 11.33 | 147 | 8.12 |

Year of Follow-up: 2005, Age Group: 40-44

| Period | Age Group | Smoking Group | N | % | Ever Quit (N) | Ever Quit % | Mean Yrs Quit | Short Term (n) | Short Term % |
|---|---|---|---|---|---|---|---|---|---|
| 15 | 40-44 | Never Any | 4997 | 46.86 | | | | | |
| 15 | 40-44 | CC | 231 | 2.17 | 0 | 0.00 | | | |
| 15 | 40-44 | Ecig | 2948 | 27.64 | | | | | |
| 15 | 40-44 | Dual | 0 | 0.00 | | | | | |
| 15 | 40-44 | Former | 2488 | 23.33 | | | 13.32 | 199 | 8.00 |

Year of Follow-up: 2005, Age Group: 45-49

| Period | Age Group | Smoking Group | N | % | Ever Quit (N) | Ever Quit % | Mean Yrs Quit | Short Term (n) | Short Term % |
|---|---|---|---|---|---|---|---|---|---|
| 15 | 45-49 | Never Any | 4724 | 43.28 | | | | | |
| 15 | 45-49 | CC | 216 | 1.98 | 0 | 0.00 | | | |
| 15 | 45-49 | Ecig | 2793 | 25.59 | | | | | |
| 15 | 45-49 | Dual | 0 | 0.00 | | | | | |
| 15 | 45-49 | Former | 3183 | 29.16 | | | 14.88 | 160 | 5.03 |

Year of Follow-up: 2005, Age Group: 50-54

| Period | Age Group | Smoking Group | N | % | Ever Quit (N) | Ever Quit % | Mean Yrs Quit | Short Term (n) | Short Term % |
|---|---|---|---|---|---|---|---|---|---|
| 15 | 50-54 | Never Any | 3941 | 39.85 | | | | | |
| 15 | 50-54 | CC | 213 | 2.15 | 0 | 0.00 | | | |
| 15 | 50-54 | Ecig | 2180 | 22.04 | | | | | |
| 15 | 50-54 | Dual | 0 | 0.00 | | | | | |
| 15 | 50-54 | Former | 3555 | 35.95 | | | 18.19 | 154 | 4.33 |

Year of Follow-up: 2005, Age Group: 55-59

| Period | Age Group | Smoking Group | N | % | Ever Quit (N) | Ever Quit % | Mean Yrs Quit | Short Term (n) | Short Term % |
|---|---|---|---|---|---|---|---|---|---|
| 15 | 55-59 | Never Any | 2912 | 33.65 | | | | | |
| 15 | 55-59 | CC | 215 | 2.48 | 0 | 0.00 | | | |
| 15 | 55-59 | Ecig | 1781 | 20.58 | | | | | |
| 15 | 55-59 | Dual | 0 | 0.00 | | | | | |
| 15 | 55-59 | Former | 3747 | 43.29 | | | 20.09 | 117 | 3.12 |

Year of Follow-up: 2005, Age Group: 60-64

| Period | Age Group | Smoking Group | N | % | Ever Quit (N) | Ever Quit % | Mean Yrs Quit | Short Term (n) | Short Term % |
|---|---|---|---|---|---|---|---|---|---|
| 15 | 60-64 | Never Any | 1919 | 29.46 | | | | | |
| 15 | 60-64 | CC | 143 | 2.19 | 0 | 0.00 | | | |
| 15 | 60-64 | Ecig | 1100 | 16.88 | | | | | |
| 15 | 60-64 | Dual | 0 | 0.00 | | | | | |
| 15 | 60-64 | Former | 3353 | 51.47 | | | 21.47 | 156 | 4.65 |

Year of Follow-up: 2005, Age Group: 65-69

| Period | Age Group | Smoking Group | N | % | Ever Quit (N) | Ever Quit % | Mean Yrs Quit | Short Term (n) | Short Term % |
|---|---|---|---|---|---|---|---|---|---|
| 15 | 65-69 | Never Any | 1611 | 29.41 | | | | | |
| 15 | 65-69 | CC | 124 | 2.26 | 0 | 0.00 | | | |
| 15 | 65-69 | Ecig | 705 | 12.87 | | | | | |
| 15 | 65-69 | Dual | 0 | 0.00 | | | | | |
| 15 | 65-69 | Former | 3037 | 55.45 | | | 22.19 | 118 | 3.89 |

Year of Follow-up: 2005, Age Group: 70-74

| Period | Age Group | Smoking Group | N | % | Ever Quit (N) | Ever Quit % | Mean Yrs Quit | Short Term (n) | Short Term % |
|---|---|---|---|---|---|---|---|---|---|
| 15 | 70-74 | Never Any | 1337 | 26.83 | | | | | |
| 15 | 70-74 | CC | 129 | 2.59 | 0 | 0.00 | | | |
| 15 | 70-74 | Ecig | 441 | 8.85 | | | | | |
| 15 | 70-74 | Dual | 0 | 0.00 | | | | | |
| 15 | 70-74 | Former | 3076 | 61.73 | | | 22.89 | 112 | 3.64 |

Year of Follow-up: 2005, Age Group: 75-79

| Period | Age Group | Smoking Group | N | % | Ever Quit (N) | Ever Quit % | Mean Yrs Quit | Short Term (n) | Short Term % |
|---|---|---|---|---|---|---|---|---|---|
| 15 | 75-79 | Never Any | 1169 | 23.83 | | | | | |
| 15 | 75-79 | CC | 120 | 2.45 | 0 | 0.00 | | | |
| 15 | 75-79 | Ecig | 261 | 5.32 | | | | | |
| 15 | 75-79 | Dual | 0 | 0.00 | | | | | |
| 15 | 75-79 | Former | 3355 | 68.40 | | | 27.01 | 66 | 1.97 |

Year of Follow-up: 2010, Age Group: All Ages

| Period | Age Group | Smoking Group | N | % | Ever Quit (N) | Ever Quit % | Mean Yrs Quit | Short Term (n) | Short Term % |
|---|---|---|---|---|---|---|---|---|---|
| 20 | All Ages | Never Any | 36103 | 42.57 | | | | | |
| 20 | All Ages | CC | 738 | 0.87 | 0 | 0.00 | | | |
| 20 | All Ages | Ecig | 17076 | 20.14 | | | | | |
| 20 | All Ages | Dual | 0 | 0.00 | | | | | |
| 20 | All Ages | Former | 30883 | 36.42 | | | 19.82 | 1518 | 4.92 |

Year of Follow-up: 2010, Age Group: 18-19

| Period | Age Group | Smoking Group | N | % | Ever Quit (N) | Ever Quit % | Mean Yrs Quit | Short Term (n) | Short Term % |
|---|---|---|---|---|---|---|---|---|---|

# Results for PHIM for Run 5 (Sensitivity_Analysis_1.RTF)

| | | | | | | | | | |
|---|---|---|---|---|---|---|---|---|---|
| 20 | 18-19 | Never Any | 0 | . | | | | | |
| 20 | 18-19 | CC | 0 | . | 0 | . | | | |
| 20 | 18-19 | Ecig | 0 | . | | | | | |
| 20 | 18-19 | Dual | 0 | . | | | | | |
| 20 | 18-19 | Former | 0 | . | | | . | 0 | . |

Year of Follow-up: 2010, Age Group: 20-24

| Period | Age Group | Smoking Group | N | % | Ever Quit (N) | Ever Quit % | Mean Yrs Quit | Short Term (n) | Short Term % |
|---|---|---|---|---|---|---|---|---|---|
| 20 | 20-24 | Never Any | 0 | . | | | | | |
| 20 | 20-24 | CC | 0 | . | 0 | . | | | |
| 20 | 20-24 | Ecig | 0 | . | | | | | |
| 20 | 20-24 | Dual | 0 | . | | | | | |
| 20 | 20-24 | Former | 0 | . | | | . | 0 | . |

Year of Follow-up: 2010, Age Group: 25-29

| Period | Age Group | Smoking Group | N | % | Ever Quit (N) | Ever Quit % | Mean Yrs Quit | Short Term (n) | Short Term % |
|---|---|---|---|---|---|---|---|---|---|
| 20 | 25-29 | Never Any | 0 | . | | | | | |
| 20 | 25-29 | CC | 0 | . | 0 | . | | | |
| 20 | 25-29 | Ecig | 0 | . | | | | | |
| 20 | 25-29 | Dual | 0 | . | | | | | |
| 20 | 25-29 | Former | 0 | . | | | . | 0 | . |

Year of Follow-up: 2010, Age Group: 30-34

| Period | Age Group | Smoking Group | N | % | Ever Quit (N) | Ever Quit % | Mean Yrs Quit | Short Term (n) | Short Term % |
|---|---|---|---|---|---|---|---|---|---|
| 20 | 30-34 | Never Any | 4508 | 50.97 | | | | | |
| 20 | 30-34 | CC | 71 | 0.80 | 0 | 0.00 | | | |
| 20 | 30-34 | Ecig | 2376 | 26.86 | | | | | |
| 20 | 30-34 | Dual | 0 | 0.00 | | | | | |
| 20 | 30-34 | Former | 1890 | 21.37 | | | 8.72 | 230 | 12.17 |

Year of Follow-up: 2010, Age Group: 35-39

| Period | Age Group | Smoking Group | N | % | Ever Quit (N) | Ever Quit % | Mean Yrs Quit | Short Term (n) | Short Term % |
|---|---|---|---|---|---|---|---|---|---|
| 20 | 35-39 | Never Any | 4991 | 55.21 | | | | | |
| 20 | 35-39 | CC | 59 | 0.65 | 0 | 0.00 | | | |
| 20 | 35-39 | Ecig | 2154 | 23.83 | | | | | |
| 20 | 35-39 | Dual | 0 | 0.00 | | | | | |
| 20 | 35-39 | Former | 1836 | 20.31 | | | 9.77 | 141 | 7.68 |

Year of Follow-up: 2010, Age Group: 40-44

| Period | Age Group | Smoking Group | N | % | Ever Quit (N) | Ever Quit % | Mean Yrs Quit | Short Term (n) | Short Term % |
|---|---|---|---|---|---|---|---|---|---|
| 20 | 40-44 | Never Any | 5163 | 52.60 | | | | | |
| 20 | 40-44 | CC | 68 | 0.69 | 0 | 0.00 | | | |
| 20 | 40-44 | Ecig | 2416 | 24.61 | | | | | |
| 20 | 40-44 | Dual | 0 | 0.00 | | | | | |
| 20 | 40-44 | Former | 2169 | 22.10 | | | 13.96 | 143 | 6.59 |

Year of Follow-up: 2010, Age Group: 45-49

| Period | Age Group | Smoking Group | N | % | Ever Quit (N) | Ever Quit % | Mean Yrs Quit | Short Term (n) | Short Term % |
|---|---|---|---|---|---|---|---|---|---|
| 20 | 45-49 | Never Any | 4997 | 46.86 | | | | | |
| 20 | 45-49 | CC | 89 | 0.83 | 0 | 0.00 | | | |
| 20 | 45-49 | Ecig | 2619 | 24.56 | | | | | |
| 20 | 45-49 | Dual | 0 | 0.00 | | | | | |
| 20 | 45-49 | Former | 2959 | 27.75 | | | 15.74 | 171 | 5.78 |

Year of Follow-up: 2010, Age Group: 50-54

| Period | Age Group | Smoking Group | N | % | Ever Quit (N) | Ever Quit % | Mean Yrs Quit | Short Term (n) | Short Term % |
|---|---|---|---|---|---|---|---|---|---|
| 20 | 50-54 | Never Any | 4724 | 43.28 | | | | | |
| 20 | 50-54 | CC | 87 | 0.80 | 0 | 0.00 | | | |
| 20 | 50-54 | Ecig | 2490 | 22.81 | | | | | |
| 20 | 50-54 | Dual | 0 | 0.00 | | | | | |
| 20 | 50-54 | Former | 3615 | 33.12 | | | 17.75 | 174 | 4.81 |

Year of Follow-up: 2010, Age Group: 55-59

| Period | Age Group | Smoking Group | N | % | Ever Quit (N) | Ever Quit % | Mean Yrs Quit | Short Term (n) | Short Term % |
|---|---|---|---|---|---|---|---|---|---|
| 20 | 55-59 | Never Any | 3941 | 39.85 | | | | | |
| 20 | 55-59 | CC | 85 | 0.86 | 0 | 0.00 | | | |
| 20 | 55-59 | Ecig | 1859 | 18.80 | | | | | |
| 20 | 55-59 | Dual | 0 | 0.00 | | | | | |
| 20 | 55-59 | Former | 4004 | 40.49 | | | 20.81 | 181 | 4.52 |

Year of Follow-up: 2010, Age Group: 60-64

| Period | Age Group | Smoking Group | N | % | Ever Quit (N) | Ever Quit % | Mean Yrs Quit | Short Term (n) | Short Term % |
|---|---|---|---|---|---|---|---|---|---|
| 20 | 60-64 | Never Any | 2912 | 33.65 | | | | | |
| 20 | 60-64 | CC | 87 | 1.01 | 0 | 0.00 | | | |
| 20 | 60-64 | Ecig | 1428 | 16.50 | | | | | |
| 20 | 60-64 | Dual | 0 | 0.00 | | | | | |
| 20 | 60-64 | Former | 4228 | 48.85 | | | 22.41 | 241 | 5.70 |

Year of Follow-up: 2010, Age Group: 65-69

# Results for PHIM for Run 5 (Sensitivity_Analysis_1.RTF)

| Period | Age Group | Smoking Group | N | % | Ever Quit (N) | Ever Quit % | Mean Yrs Quit | Short Term (n) | Short Term % |
|---|---|---|---|---|---|---|---|---|---|
| 20 | 65-69 | Never Any | 1919 | 29.46 | | | | | |
| 20 | 65-69 | CC | 60 | 0.92 | 0 | 0.00 | | | |
| 20 | 65-69 | Ecig | 809 | 12.42 | | | | | |
| 20 | 65-69 | Dual | 0 | 0.00 | | | | | |
| 20 | 65-69 | Former | 3727 | 57.21 | | | 24.03 | 133 | 3.57 |

Year of Follow-up: 2010, Age Group: 70-74

| Period | Age Group | Smoking Group | N | % | Ever Quit (N) | Ever Quit % | Mean Yrs Quit | Short Term (n) | Short Term % |
|---|---|---|---|---|---|---|---|---|---|
| 20 | 70-74 | Never Any | 1611 | 29.41 | | | | | |
| 20 | 70-74 | CC | 69 | 1.26 | 0 | 0.00 | | | |
| 20 | 70-74 | Ecig | 574 | 10.48 | | | | | |
| 20 | 70-74 | Dual | 0 | 0.00 | | | | | |
| 20 | 70-74 | Former | 3223 | 58.85 | | | 25.75 | 61 | 1.89 |

Year of Follow-up: 2010, Age Group: 75-79

| Period | Age Group | Smoking Group | N | % | Ever Quit (N) | Ever Quit % | Mean Yrs Quit | Short Term (n) | Short Term % |
|---|---|---|---|---|---|---|---|---|---|
| 20 | 75-79 | Never Any | 1337 | 26.83 | | | | | |
| 20 | 75-79 | CC | 63 | 1.26 | 0 | 0.00 | | | |
| 20 | 75-79 | Ecig | 351 | 7.04 | | | | | |
| 20 | 75-79 | Dual | 0 | 0.00 | | | | | |
| 20 | 75-79 | Former | 3232 | 64.86 | | | 26.66 | 43 | 1.33 |

Year of Follow-up: 2015, Age Group: All Ages

| Period | Age Group | Smoking Group | N | % | Ever Quit (N) | Ever Quit % | Mean Yrs Quit | Short Term (n) | Short Term % |
|---|---|---|---|---|---|---|---|---|---|
| 25 | All Ages | Never Any | 34747 | 43.53 | | | | | |
| 25 | All Ages | CC | 289 | 0.36 | 0 | 0.00 | | | |
| 25 | All Ages | Ecig | 14107 | 17.67 | | | | | |
| 25 | All Ages | Dual | 0 | 0.00 | | | | | |
| 25 | All Ages | Former | 30674 | 38.43 | | | 21.87 | 1088 | 3.55 |

Year of Follow-up: 2015, Age Group: 18-19

| Period | Age Group | Smoking Group | N | % | Ever Quit (N) | Ever Quit % | Mean Yrs Quit | Short Term (n) | Short Term % |
|---|---|---|---|---|---|---|---|---|---|
| 25 | 18-19 | Never Any | 0 | . | | | | | |
| 25 | 18-19 | CC | 0 | . | 0 | . | | | |
| 25 | 18-19 | Ecig | 0 | . | | | | | |
| 25 | 18-19 | Dual | 0 | . | | | | | |
| 25 | 18-19 | Former | 0 | . | | | . | 0 | . |

Year of Follow-up: 2015, Age Group: 20-24

| Period | Age Group | Smoking Group | N | % | Ever Quit (N) | Ever Quit % | Mean Yrs Quit | Short Term (n) | Short Term % |
|---|---|---|---|---|---|---|---|---|---|
| 25 | 20-24 | Never Any | 0 | . | | | | | |
| 25 | 20-24 | CC | 0 | . | 0 | . | | | |
| 25 | 20-24 | Ecig | 0 | . | | | | | |
| 25 | 20-24 | Dual | 0 | . | | | | | |
| 25 | 20-24 | Former | 0 | . | | | . | 0 | . |

Year of Follow-up: 2015, Age Group: 25-29

| Period | Age Group | Smoking Group | N | % | Ever Quit (N) | Ever Quit % | Mean Yrs Quit | Short Term (n) | Short Term % |
|---|---|---|---|---|---|---|---|---|---|
| 25 | 25-29 | Never Any | 0 | . | | | | | |
| 25 | 25-29 | CC | 0 | . | 0 | . | | | |
| 25 | 25-29 | Ecig | 0 | . | | | | | |
| 25 | 25-29 | Dual | 0 | . | | | | | |
| 25 | 25-29 | Former | 0 | . | | | . | 0 | . |

Year of Follow-up: 2015, Age Group: 30-34

| Period | Age Group | Smoking Group | N | % | Ever Quit (N) | Ever Quit % | Mean Yrs Quit | Short Term (n) | Short Term % |
|---|---|---|---|---|---|---|---|---|---|
| 25 | 30-34 | Never Any | 0 | . | | | | | |
| 25 | 30-34 | CC | 0 | . | 0 | . | | | |
| 25 | 30-34 | Ecig | 0 | . | | | | | |
| 25 | 30-34 | Dual | 0 | . | | | | | |
| 25 | 30-34 | Former | 0 | . | | | . | 0 | . |

Year of Follow-up: 2015, Age Group: 35-39

| Period | Age Group | Smoking Group | N | % | Ever Quit (N) | Ever Quit % | Mean Yrs Quit | Short Term (n) | Short Term % |
|---|---|---|---|---|---|---|---|---|---|
| 25 | 35-39 | Never Any | 4489 | 50.75 | | | | | |
| 25 | 35-39 | CC | 28 | 0.32 | 0 | 0.00 | | | |
| 25 | 35-39 | Ecig | 1990 | 22.50 | | | | | |
| 25 | 35-39 | Dual | 0 | 0.00 | | | | | |
| 25 | 35-39 | Former | 2338 | 26.43 | | | 11.53 | 142 | 6.07 |

Year of Follow-up: 2015, Age Group: 40-44

| Period | Age Group | Smoking Group | N | % | Ever Quit (N) | Ever Quit % | Mean Yrs Quit | Short Term (n) | Short Term % |
|---|---|---|---|---|---|---|---|---|---|
| 25 | 40-44 | Never Any | 4991 | 55.21 | | | | | |
| 25 | 40-44 | CC | 20 | 0.22 | 0 | 0.00 | | | |
| 25 | 40-44 | Ecig | 1897 | 20.98 | | | | | |
| 25 | 40-44 | Dual | 0 | 0.00 | | | | | |

# Results for PHIM for Run 5 (Sensitivity_Analysis_1.RTF)

| | | | | | | | | | |
|---|---|---|---|---|---|---|---|---|---|
| 25 | 40-44 | Former | 2132 | 23.58 | | | 13.01 | 112 | 5.25 |

Year of Follow-up: 2015, Age Group: 45-49

| Period | Age Group | Smoking Group | N | % | Ever Quit (N) | Ever Quit % | Mean Yrs Quit | Short Term (n) | Short Term % |
|---|---|---|---|---|---|---|---|---|---|
| 25 | 45-49 | Never Any | 5163 | 52.60 | | | | | |
| 25 | 45-49 | CC | 29 | 0.30 | 0 | 0.00 | | | |
| 25 | 45-49 | Ecig | 2195 | 22.36 | | | | | |
| 25 | 45-49 | Dual | 0 | 0.00 | | | | | |
| 25 | 45-49 | Former | 2429 | 24.75 | | | 17.18 | 81 | 3.33 |

Year of Follow-up: 2015, Age Group: 50-54

| Period | Age Group | Smoking Group | N | % | Ever Quit (N) | Ever Quit % | Mean Yrs Quit | Short Term (n) | Short Term % |
|---|---|---|---|---|---|---|---|---|---|
| 25 | 50-54 | Never Any | 4997 | 46.86 | | | | | |
| 25 | 50-54 | CC | 37 | 0.35 | 0 | 0.00 | | | |
| 25 | 50-54 | Ecig | 2387 | 22.38 | | | | | |
| 25 | 50-54 | Dual | 0 | 0.00 | | | | | |
| 25 | 50-54 | Former | 3243 | 30.41 | | | 19.10 | 130 | 4.01 |

Year of Follow-up: 2015, Age Group: 55-59

| Period | Age Group | Smoking Group | N | % | Ever Quit (N) | Ever Quit % | Mean Yrs Quit | Short Term (n) | Short Term % |
|---|---|---|---|---|---|---|---|---|---|
| 25 | 55-59 | Never Any | 4724 | 43.28 | | | | | |
| 25 | 55-59 | CC | 33 | 0.30 | 0 | 0.00 | | | |
| 25 | 55-59 | Ecig | 2103 | 19.27 | | | | | |
| 25 | 55-59 | Dual | 0 | 0.00 | | | | | |
| 25 | 55-59 | Former | 4056 | 37.16 | | | 20.50 | 162 | 3.99 |

Year of Follow-up: 2015, Age Group: 60-64

| Period | Age Group | Smoking Group | N | % | Ever Quit (N) | Ever Quit % | Mean Yrs Quit | Short Term (n) | Short Term % |
|---|---|---|---|---|---|---|---|---|---|
| 25 | 60-64 | Never Any | 3941 | 39.85 | | | | | |
| 25 | 60-64 | CC | 28 | 0.28 | 0 | 0.00 | | | |
| 25 | 60-64 | Ecig | 1443 | 14.59 | | | | | |
| 25 | 60-64 | Dual | 0 | 0.00 | | | | | |
| 25 | 60-64 | Former | 4477 | 45.27 | | | 23.28 | 205 | 4.58 |

Year of Follow-up: 2015, Age Group: 65-69

| Period | Age Group | Smoking Group | N | % | Ever Quit (N) | Ever Quit % | Mean Yrs Quit | Short Term (n) | Short Term % |
|---|---|---|---|---|---|---|---|---|---|
| 25 | 65-69 | Never Any | 2912 | 33.65 | | | | | |
| 25 | 65-69 | CC | 43 | 0.50 | 0 | 0.00 | | | |
| 25 | 65-69 | Ecig | 1019 | 11.77 | | | | | |
| 25 | 65-69 | Dual | 0 | 0.00 | | | | | |
| 25 | 65-69 | Former | 4681 | 54.08 | | | 24.99 | 134 | 2.86 |

Year of Follow-up: 2015, Age Group: 70-74

| Period | Age Group | Smoking Group | N | % | Ever Quit (N) | Ever Quit % | Mean Yrs Quit | Short Term (n) | Short Term % |
|---|---|---|---|---|---|---|---|---|---|
| 25 | 70-74 | Never Any | 1919 | 29.46 | | | | | |
| 25 | 70-74 | CC | 35 | 0.54 | 0 | 0.00 | | | |
| 25 | 70-74 | Ecig | 616 | 9.46 | | | | | |
| 25 | 70-74 | Dual | 0 | 0.00 | | | | | |
| 25 | 70-74 | Former | 3945 | 60.55 | | | 27.55 | 70 | 1.77 |

Year of Follow-up: 2015, Age Group: 75-79

| Period | Age Group | Smoking Group | N | % | Ever Quit (N) | Ever Quit % | Mean Yrs Quit | Short Term (n) | Short Term % |
|---|---|---|---|---|---|---|---|---|---|
| 25 | 75-79 | Never Any | 1611 | 29.41 | | | | | |
| 25 | 75-79 | CC | 36 | 0.66 | 0 | 0.00 | | | |
| 25 | 75-79 | Ecig | 457 | 8.34 | | | | | |
| 25 | 75-79 | Dual | 0 | 0.00 | | | | | |
| 25 | 75-79 | Former | 3373 | 61.58 | | | 29.48 | 52 | 1.54 |

Year of Follow-up: 2020, Age Group: All Ages

| Period | Age Group | Smoking Group | N | % | Ever Quit (N) | Ever Quit % | Mean Yrs Quit | Short Term (n) | Short Term % |
|---|---|---|---|---|---|---|---|---|---|
| 30 | All Ages | Never Any | 33136 | 44.57 | | | | | |
| 30 | All Ages | CC | 100 | 0.13 | 0 | 0.00 | | | |
| 30 | All Ages | Ecig | 11358 | 15.28 | | | | | |
| 30 | All Ages | Dual | 0 | 0.00 | | | | | |
| 30 | All Ages | Former | 29746 | 40.01 | | | 23.97 | 918 | 3.09 |

Year of Follow-up: 2020, Age Group: 18-19

| Period | Age Group | Smoking Group | N | % | Ever Quit (N) | Ever Quit % | Mean Yrs Quit | Short Term (n) | Short Term % |
|---|---|---|---|---|---|---|---|---|---|
| 30 | 18-19 | Never Any | 0 | . | | | | | |
| 30 | 18-19 | CC | 0 | . | 0 | . | | | |
| 30 | 18-19 | Ecig | 0 | . | | | | | |
| 30 | 18-19 | Dual | 0 | . | | | | | |
| 30 | 18-19 | Former | 0 | . | | | . | 0 | . |

Year of Follow-up: 2020, Age Group: 20-24

| Period | Age Group | Smoking Group | N | % | Ever Quit (N) | Ever Quit % | Mean Yrs Quit | Short Term (n) | Short Term % |
|---|---|---|---|---|---|---|---|---|---|
| 30 | 20-24 | Never Any | 0 | . | | | | | |
| 30 | 20-24 | CC | 0 | . | 0 | . | | | |

# Results for PHIM for Run 5 (Sensitivity_Analysis_1.RTF)

| | | | | | | | | | |
|---|---|---|---|---|---|---|---|---|---|
| 30 | 20-24 | Ecig | 0 | . | | | | | |
| 30 | 20-24 | Dual | 0 | . | | | | | |
| 30 | 20-24 | Former | 0 | . | | | . | 0 | . |

Year of Follow-up: 2020, Age Group: 25-29

| Period | Age Group | Smoking Group | N | % | Ever Quit (N) | Ever Quit % | Mean Yrs Quit | Short Term (n) | Short Term % |
|---|---|---|---|---|---|---|---|---|---|
| 30 | 25-29 | Never Any | 0 | . | | | | | |
| 30 | 25-29 | CC | 0 | . | 0 | . | | | |
| 30 | 25-29 | Ecig | 0 | . | | | | | |
| 30 | 25-29 | Dual | 0 | . | | | | | |
| 30 | 25-29 | Former | 0 | . | | | . | 0 | . |

Year of Follow-up: 2020, Age Group: 30-34

| Period | Age Group | Smoking Group | N | % | Ever Quit (N) | Ever Quit % | Mean Yrs Quit | Short Term (n) | Short Term % |
|---|---|---|---|---|---|---|---|---|---|
| 30 | 30-34 | Never Any | 0 | . | | | | | |
| 30 | 30-34 | CC | 0 | . | 0 | . | | | |
| 30 | 30-34 | Ecig | 0 | . | | | | | |
| 30 | 30-34 | Dual | 0 | . | | | | | |
| 30 | 30-34 | Former | 0 | . | | | . | 0 | . |

Year of Follow-up: 2020, Age Group: 35-39

| Period | Age Group | Smoking Group | N | % | Ever Quit (N) | Ever Quit % | Mean Yrs Quit | Short Term (n) | Short Term % |
|---|---|---|---|---|---|---|---|---|---|
| 30 | 35-39 | Never Any | 0 | . | | | | | |
| 30 | 35-39 | CC | 0 | . | 0 | . | | | |
| 30 | 35-39 | Ecig | 0 | . | | | | | |
| 30 | 35-39 | Dual | 0 | . | | | | | |
| 30 | 35-39 | Former | 0 | . | | | . | 0 | . |

Year of Follow-up: 2020, Age Group: 40-44

| Period | Age Group | Smoking Group | N | % | Ever Quit (N) | Ever Quit % | Mean Yrs Quit | Short Term (n) | Short Term % |
|---|---|---|---|---|---|---|---|---|---|
| 30 | 40-44 | Never Any | 4489 | 50.75 | | | | | |
| 30 | 40-44 | CC | 12 | 0.14 | 0 | 0.00 | | | |
| 30 | 40-44 | Ecig | 1743 | 19.71 | | | | | |
| 30 | 40-44 | Dual | 0 | 0.00 | | | | | |
| 30 | 40-44 | Former | 2601 | 29.41 | | | 15.05 | 107 | 4.11 |

Year of Follow-up: 2020, Age Group: 45-49

| Period | Age Group | Smoking Group | N | % | Ever Quit (N) | Ever Quit % | Mean Yrs Quit | Short Term (n) | Short Term % |
|---|---|---|---|---|---|---|---|---|---|
| 30 | 45-49 | Never Any | 4991 | 55.21 | | | | | |
| 30 | 45-49 | CC | 5 | 0.06 | 0 | 0.00 | | | |
| 30 | 45-49 | Ecig | 1690 | 18.69 | | | | | |
| 30 | 45-49 | Dual | 0 | 0.00 | | | | | |
| 30 | 45-49 | Former | 2354 | 26.04 | | | 16.54 | 63 | 2.68 |

Year of Follow-up: 2020, Age Group: 50-54

| Period | Age Group | Smoking Group | N | % | Ever Quit (N) | Ever Quit % | Mean Yrs Quit | Short Term (n) | Short Term % |
|---|---|---|---|---|---|---|---|---|---|
| 30 | 50-54 | Never Any | 5163 | 52.60 | | | | | |
| 30 | 50-54 | CC | 9 | 0.09 | 0 | 0.00 | | | |
| 30 | 50-54 | Ecig | 2021 | 20.59 | | | | | |
| 30 | 50-54 | Dual | 0 | 0.00 | | | | | |
| 30 | 50-54 | Former | 2623 | 26.72 | | | 20.66 | 99 | 3.77 |

Year of Follow-up: 2020, Age Group: 55-59

| Period | Age Group | Smoking Group | N | % | Ever Quit (N) | Ever Quit % | Mean Yrs Quit | Short Term (n) | Short Term % |
|---|---|---|---|---|---|---|---|---|---|
| 30 | 55-59 | Never Any | 4997 | 46.86 | | | | | |
| 30 | 55-59 | CC | 18 | 0.17 | 0 | 0.00 | | | |
| 30 | 55-59 | Ecig | 2002 | 18.77 | | | | | |
| 30 | 55-59 | Dual | 0 | 0.00 | | | | | |
| 30 | 55-59 | Former | 3647 | 34.20 | | | 21.65 | 157 | 4.30 |

Year of Follow-up: 2020, Age Group: 60-64

| Period | Age Group | Smoking Group | N | % | Ever Quit (N) | Ever Quit % | Mean Yrs Quit | Short Term (n) | Short Term % |
|---|---|---|---|---|---|---|---|---|---|
| 30 | 60-64 | Never Any | 4724 | 43.28 | | | | | |
| 30 | 60-64 | CC | 10 | 0.09 | 0 | 0.00 | | | |
| 30 | 60-64 | Ecig | 1614 | 14.79 | | | | | |
| 30 | 60-64 | Dual | 0 | 0.00 | | | | | |
| 30 | 60-64 | Former | 4568 | 41.85 | | | 22.87 | 203 | 4.44 |

Year of Follow-up: 2020, Age Group: 65-69

| Period | Age Group | Smoking Group | N | % | Ever Quit (N) | Ever Quit % | Mean Yrs Quit | Short Term (n) | Short Term % |
|---|---|---|---|---|---|---|---|---|---|
| 30 | 65-69 | Never Any | 3941 | 39.85 | | | | | |
| 30 | 65-69 | CC | 13 | 0.13 | 0 | 0.00 | | | |
| 30 | 65-69 | Ecig | 1039 | 10.51 | | | | | |
| 30 | 65-69 | Dual | 0 | 0.00 | | | | | |
| 30 | 65-69 | Former | 4896 | 49.51 | | | 26.05 | 145 | 2.96 |

Year of Follow-up: 2020, Age Group: 70-74

| Period | Age Group | Smoking Group | N | % | Ever Quit (N) | Ever Quit % | Mean Yrs Quit | Short Term (n) | Short Term % |
|---|---|---|---|---|---|---|---|---|---|

# Results for PHIM for Run 5 (Sensitivity_Analysis_1.RTF)

| | | | | | | | | | |
|---|---|---|---|---|---|---|---|---|---|
| 30 | 70-74 | Never Any | 2912 | 33.65 | | | | | |
| 30 | 70-74 | CC | 18 | 0.21 | 0 | 0.00 | | | |
| 30 | 70-74 | Ecig | 770 | 8.90 | | | | | |
| 30 | 70-74 | Dual | 0 | 0.00 | | | | | |
| 30 | 70-74 | Former | 4955 | 57.25 | | | 28.45 | 93 | 1.88 |

Year of Follow-up: 2020, Age Group: 75-79

| Period | Age Group | Smoking Group | N | % | Ever Quit (N) | Ever Quit % | Mean Yrs Quit | Short Term (n) | Short Term % |
|---|---|---|---|---|---|---|---|---|---|
| 30 | 75-79 | Never Any | 1919 | 29.46 | | | | | |
| 30 | 75-79 | CC | 15 | 0.23 | 0 | 0.00 | | | |
| 30 | 75-79 | Ecig | 479 | 7.35 | | | | | |
| 30 | 75-79 | Dual | 0 | 0.00 | | | | | |
| 30 | 75-79 | Former | 4102 | 62.96 | | | 31.39 | 51 | 1.24 |

Year of Follow-up: 2025, Age Group: All Ages

| Period | Age Group | Smoking Group | N | % | Ever Quit (N) | Ever Quit % | Mean Yrs Quit | Short Term (n) | Short Term % |
|---|---|---|---|---|---|---|---|---|---|
| 35 | All Ages | Never Any | 31217 | 46.03 | | | | | |
| 35 | All Ages | CC | 38 | 0.06 | 0 | 0.00 | | | |
| 35 | All Ages | Ecig | 8864 | 13.07 | | | | | |
| 35 | All Ages | Dual | 0 | 0.00 | | | | | |
| 35 | All Ages | Former | 27706 | 40.85 | | | 25.87 | 785 | 2.83 |

Year of Follow-up: 2025, Age Group: 18-19

| Period | Age Group | Smoking Group | N | % | Ever Quit (N) | Ever Quit % | Mean Yrs Quit | Short Term (n) | Short Term % |
|---|---|---|---|---|---|---|---|---|---|
| 35 | 18-19 | Never Any | 0 | . | | | | | |
| 35 | 18-19 | CC | 0 | . | 0 | . | | | |
| 35 | 18-19 | Ecig | 0 | . | | | | | |
| 35 | 18-19 | Dual | 0 | . | | | | | |
| 35 | 18-19 | Former | 0 | . | | | . | 0 | . |

Year of Follow-up: 2025, Age Group: 20-24

| Period | Age Group | Smoking Group | N | % | Ever Quit (N) | Ever Quit % | Mean Yrs Quit | Short Term (n) | Short Term % |
|---|---|---|---|---|---|---|---|---|---|
| 35 | 20-24 | Never Any | 0 | . | | | | | |
| 35 | 20-24 | CC | 0 | . | 0 | . | | | |
| 35 | 20-24 | Ecig | 0 | . | | | | | |
| 35 | 20-24 | Dual | 0 | . | | | | | |
| 35 | 20-24 | Former | 0 | . | | | . | 0 | . |

Year of Follow-up: 2025, Age Group: 25-29

| Period | Age Group | Smoking Group | N | % | Ever Quit (N) | Ever Quit % | Mean Yrs Quit | Short Term (n) | Short Term % |
|---|---|---|---|---|---|---|---|---|---|
| 35 | 25-29 | Never Any | 0 | . | | | | | |
| 35 | 25-29 | CC | 0 | . | 0 | . | | | |
| 35 | 25-29 | Ecig | 0 | . | | | | | |
| 35 | 25-29 | Dual | 0 | . | | | | | |
| 35 | 25-29 | Former | 0 | . | | | . | 0 | . |

Year of Follow-up: 2025, Age Group: 30-34

| Period | Age Group | Smoking Group | N | % | Ever Quit (N) | Ever Quit % | Mean Yrs Quit | Short Term (n) | Short Term % |
|---|---|---|---|---|---|---|---|---|---|
| 35 | 30-34 | Never Any | 0 | . | | | | | |
| 35 | 30-34 | CC | 0 | . | 0 | . | | | |
| 35 | 30-34 | Ecig | 0 | . | | | | | |
| 35 | 30-34 | Dual | 0 | . | | | | | |
| 35 | 30-34 | Former | 0 | . | | | . | 0 | . |

Year of Follow-up: 2025, Age Group: 35-39

| Period | Age Group | Smoking Group | N | % | Ever Quit (N) | Ever Quit % | Mean Yrs Quit | Short Term (n) | Short Term % |
|---|---|---|---|---|---|---|---|---|---|
| 35 | 35-39 | Never Any | 0 | . | | | | | |
| 35 | 35-39 | CC | 0 | . | 0 | . | | | |
| 35 | 35-39 | Ecig | 0 | . | | | | | |
| 35 | 35-39 | Dual | 0 | . | | | | | |
| 35 | 35-39 | Former | 0 | . | | | . | 0 | . |

Year of Follow-up: 2025, Age Group: 40-44

| Period | Age Group | Smoking Group | N | % | Ever Quit (N) | Ever Quit % | Mean Yrs Quit | Short Term (n) | Short Term % |
|---|---|---|---|---|---|---|---|---|---|
| 35 | 40-44 | Never Any | 0 | . | | | | | |
| 35 | 40-44 | CC | 0 | . | 0 | . | | | |
| 35 | 40-44 | Ecig | 0 | . | | | | | |
| 35 | 40-44 | Dual | 0 | . | | | | | |
| 35 | 40-44 | Former | 0 | . | | | . | 0 | . |

Year of Follow-up: 2025, Age Group: 45-49

| Period | Age Group | Smoking Group | N | % | Ever Quit (N) | Ever Quit % | Mean Yrs Quit | Short Term (n) | Short Term % |
|---|---|---|---|---|---|---|---|---|---|
| 35 | 45-49 | Never Any | 4489 | 50.75 | | | | | |
| 35 | 45-49 | CC | 7 | 0.08 | 0 | 0.00 | | | |
| 35 | 45-49 | Ecig | 1541 | 17.42 | | | | | |
| 35 | 45-49 | Dual | 0 | 0.00 | | | | | |
| 35 | 45-49 | Former | 2808 | 31.75 | | | 18.76 | 60 | 2.14 |

Year of Follow-up: 2025, Age Group: 50-54

# Results for PHIM for Run 5 (Sensitivity_Analysis_1.RTF)

| Period | Age Group | Smoking Group | N | % | Ever Quit (N) | Ever Quit % | Mean Yrs Quit | Short Term (n) | Short Term % |
|---|---|---|---|---|---|---|---|---|---|
| 35 | 50-54 | Never Any | 4991 | 55.21 | | | | | |
| 35 | 50-54 | CC | 3 | 0.03 | 0 | 0.00 | | | |
| 35 | 50-54 | Ecig | 1554 | 17.19 | | | | | |
| 35 | 50-54 | Dual | 0 | 0.00 | | | | | |
| 35 | 50-54 | Former | 2492 | 27.57 | | | 20.44 | 75 | 3.01 |

Year of Follow-up: 2025, Age Group: 55-59

| Period | Age Group | Smoking Group | N | % | Ever Quit (N) | Ever Quit % | Mean Yrs Quit | Short Term (n) | Short Term % |
|---|---|---|---|---|---|---|---|---|---|
| 35 | 55-59 | Never Any | 5163 | 52.60 | | | | | |
| 35 | 55-59 | CC | 0 | 0.00 | 0 | 0.00 | | | |
| 35 | 55-59 | Ecig | 1664 | 16.95 | | | | | |
| 35 | 55-59 | Dual | 0 | 0.00 | | | | | |
| 35 | 55-59 | Former | 2989 | 30.45 | | | 22.76 | 155 | 5.19 |

Year of Follow-up: 2025, Age Group: 60-64

| Period | Age Group | Smoking Group | N | % | Ever Quit (N) | Ever Quit % | Mean Yrs Quit | Short Term (n) | Short Term % |
|---|---|---|---|---|---|---|---|---|---|
| 35 | 60-64 | Never Any | 4997 | 46.86 | | | | | |
| 35 | 60-64 | CC | 9 | 0.08 | 0 | 0.00 | | | |
| 35 | 60-64 | Ecig | 1532 | 14.37 | | | | | |
| 35 | 60-64 | Dual | 0 | 0.00 | | | | | |
| 35 | 60-64 | Former | 4126 | 38.69 | | | 23.79 | 186 | 4.51 |

Year of Follow-up: 2025, Age Group: 65-69

| Period | Age Group | Smoking Group | N | % | Ever Quit (N) | Ever Quit % | Mean Yrs Quit | Short Term (n) | Short Term % |
|---|---|---|---|---|---|---|---|---|---|
| 35 | 65-69 | Never Any | 4724 | 43.28 | | | | | |
| 35 | 65-69 | CC | 5 | 0.05 | 0 | 0.00 | | | |
| 35 | 65-69 | Ecig | 1169 | 10.71 | | | | | |
| 35 | 65-69 | Dual | 0 | 0.00 | | | | | |
| 35 | 65-69 | Former | 5018 | 45.97 | | | 25.57 | 158 | 3.15 |

Year of Follow-up: 2025, Age Group: 70-74

| Period | Age Group | Smoking Group | N | % | Ever Quit (N) | Ever Quit % | Mean Yrs Quit | Short Term (n) | Short Term % |
|---|---|---|---|---|---|---|---|---|---|
| 35 | 70-74 | Never Any | 3941 | 39.85 | | | | | |
| 35 | 70-74 | CC | 5 | 0.05 | 0 | 0.00 | | | |
| 35 | 70-74 | Ecig | 811 | 8.20 | | | | | |
| 35 | 70-74 | Dual | 0 | 0.00 | | | | | |
| 35 | 70-74 | Former | 5132 | 51.90 | | | 29.73 | 79 | 1.54 |

Year of Follow-up: 2025, Age Group: 75-79

| Period | Age Group | Smoking Group | N | % | Ever Quit (N) | Ever Quit % | Mean Yrs Quit | Short Term (n) | Short Term % |
|---|---|---|---|---|---|---|---|---|---|
| 35 | 75-79 | Never Any | 2912 | 33.65 | | | | | |
| 35 | 75-79 | CC | 9 | 0.10 | 0 | 0.00 | | | |
| 35 | 75-79 | Ecig | 593 | 6.85 | | | | | |
| 35 | 75-79 | Dual | 0 | 0.00 | | | | | |
| 35 | 75-79 | Former | 5141 | 59.40 | | | 32.31 | 72 | 1.40 |

Year of Follow-up: 2030, Age Group: All Ages

| Period | Age Group | Smoking Group | N | % | Ever Quit (N) | Ever Quit % | Mean Yrs Quit | Short Term (n) | Short Term % |
|---|---|---|---|---|---|---|---|---|---|
| 40 | All Ages | Never Any | 28305 | 47.84 | | | | | |
| 40 | All Ages | CC | 15 | 0.03 | 0 | 0.00 | | | |
| 40 | All Ages | Ecig | 6535 | 11.04 | | | | | |
| 40 | All Ages | Dual | 0 | 0.00 | | | | | |
| 40 | All Ages | Former | 24315 | 41.09 | | | 27.43 | 691 | 2.84 |

Year of Follow-up: 2030, Age Group: 18-19

| Period | Age Group | Smoking Group | N | % | Ever Quit (N) | Ever Quit % | Mean Yrs Quit | Short Term (n) | Short Term % |
|---|---|---|---|---|---|---|---|---|---|
| 40 | 18-19 | Never Any | 0 | . | | | | | |
| 40 | 18-19 | CC | 0 | . | 0 | . | | | |
| 40 | 18-19 | Ecig | 0 | . | | | | | |
| 40 | 18-19 | Dual | 0 | . | | | | | |
| 40 | 18-19 | Former | 0 | . | | | . | 0 | . |

Year of Follow-up: 2030, Age Group: 20-24

| Period | Age Group | Smoking Group | N | % | Ever Quit (N) | Ever Quit % | Mean Yrs Quit | Short Term (n) | Short Term % |
|---|---|---|---|---|---|---|---|---|---|
| 40 | 20-24 | Never Any | 0 | . | | | | | |
| 40 | 20-24 | CC | 0 | . | 0 | . | | | |
| 40 | 20-24 | Ecig | 0 | . | | | | | |
| 40 | 20-24 | Dual | 0 | . | | | | | |
| 40 | 20-24 | Former | 0 | . | | | . | 0 | . |

Year of Follow-up: 2030, Age Group: 25-29

| Period | Age Group | Smoking Group | N | % | Ever Quit (N) | Ever Quit % | Mean Yrs Quit | Short Term (n) | Short Term % |
|---|---|---|---|---|---|---|---|---|---|
| 40 | 25-29 | Never Any | 0 | . | | | | | |
| 40 | 25-29 | CC | 0 | . | 0 | . | | | |
| 40 | 25-29 | Ecig | 0 | . | | | | | |
| 40 | 25-29 | Dual | 0 | . | | | | | |

# Results for PHIM for Run 5 (Sensitivity_Analysis_1.RTF)

| | | | | | | | | | |
|---|---|---|---|---|---|---|---|---|---|
| 40 | 25-29 | Former | 0 | . | | | . | 0 | . |

Year of Follow-up: 2030, Age Group: 30-34

| Period | Age Group | Smoking Group | N | % | Ever Quit (N) | Ever Quit % | Mean Yrs Quit | Short Term (n) | Short Term % |
|---|---|---|---|---|---|---|---|---|---|
| 40 | 30-34 | Never Any | 0 | . | | | | | |
| 40 | 30-34 | CC | 0 | . | 0 | . | | | |
| 40 | 30-34 | Ecig | 0 | . | | | | | |
| 40 | 30-34 | Dual | 0 | . | | | | | |
| 40 | 30-34 | Former | 0 | . | | | . | 0 | . |

Year of Follow-up: 2030, Age Group: 35-39

| Period | Age Group | Smoking Group | N | % | Ever Quit (N) | Ever Quit % | Mean Yrs Quit | Short Term (n) | Short Term % |
|---|---|---|---|---|---|---|---|---|---|
| 40 | 35-39 | Never Any | 0 | . | | | | | |
| 40 | 35-39 | CC | 0 | . | 0 | . | | | |
| 40 | 35-39 | Ecig | 0 | . | | | | | |
| 40 | 35-39 | Dual | 0 | . | | | | | |
| 40 | 35-39 | Former | 0 | . | | | . | 0 | . |

Year of Follow-up: 2030, Age Group: 40-44

| Period | Age Group | Smoking Group | N | % | Ever Quit (N) | Ever Quit % | Mean Yrs Quit | Short Term (n) | Short Term % |
|---|---|---|---|---|---|---|---|---|---|
| 40 | 40-44 | Never Any | 0 | . | | | | | |
| 40 | 40-44 | CC | 0 | . | 0 | . | | | |
| 40 | 40-44 | Ecig | 0 | . | | | | | |
| 40 | 40-44 | Dual | 0 | . | | | | | |
| 40 | 40-44 | Former | 0 | . | | | . | 0 | . |

Year of Follow-up: 2030, Age Group: 45-49

| Period | Age Group | Smoking Group | N | % | Ever Quit (N) | Ever Quit % | Mean Yrs Quit | Short Term (n) | Short Term % |
|---|---|---|---|---|---|---|---|---|---|
| 40 | 45-49 | Never Any | 0 | . | | | | | |
| 40 | 45-49 | CC | 0 | . | 0 | . | | | |
| 40 | 45-49 | Ecig | 0 | . | | | | | |
| 40 | 45-49 | Dual | 0 | . | | | | | |
| 40 | 45-49 | Former | 0 | . | | | . | 0 | . |

Year of Follow-up: 2030, Age Group: 50-54

| Period | Age Group | Smoking Group | N | % | Ever Quit (N) | Ever Quit % | Mean Yrs Quit | Short Term (n) | Short Term % |
|---|---|---|---|---|---|---|---|---|---|
| 40 | 50-54 | Never Any | 4489 | 50.75 | | | | | |
| 40 | 50-54 | CC | 3 | 0.03 | 0 | 0.00 | | | |
| 40 | 50-54 | Ecig | 1392 | 15.74 | | | | | |
| 40 | 50-54 | Dual | 0 | 0.00 | | | | | |
| 40 | 50-54 | Former | 2961 | 33.48 | | | 22.61 | 80 | 2.70 |

Year of Follow-up: 2030, Age Group: 55-59

| Period | Age Group | Smoking Group | N | % | Ever Quit (N) | Ever Quit % | Mean Yrs Quit | Short Term (n) | Short Term % |
|---|---|---|---|---|---|---|---|---|---|
| 40 | 55-59 | Never Any | 4991 | 55.21 | | | | | |
| 40 | 55-59 | CC | 2 | 0.02 | 0 | 0.00 | | | |
| 40 | 55-59 | Ecig | 1291 | 14.28 | | | | | |
| 40 | 55-59 | Dual | 0 | 0.00 | | | | | |
| 40 | 55-59 | Former | 2756 | 30.49 | | | 23.19 | 114 | 4.14 |

Year of Follow-up: 2030, Age Group: 60-64

| Period | Age Group | Smoking Group | N | % | Ever Quit (N) | Ever Quit % | Mean Yrs Quit | Short Term (n) | Short Term % |
|---|---|---|---|---|---|---|---|---|---|
| 40 | 60-64 | Never Any | 5163 | 52.60 | | | | | |
| 40 | 60-64 | CC | 0 | 0.00 | 0 | 0.00 | | | |
| 40 | 60-64 | Ecig | 1238 | 12.61 | | | | | |
| 40 | 60-64 | Dual | 0 | 0.00 | | | | | |
| 40 | 60-64 | Former | 3415 | 34.79 | | | 24.53 | 183 | 5.36 |

Year of Follow-up: 2030, Age Group: 65-69

| Period | Age Group | Smoking Group | N | % | Ever Quit (N) | Ever Quit % | Mean Yrs Quit | Short Term (n) | Short Term % |
|---|---|---|---|---|---|---|---|---|---|
| 40 | 65-69 | Never Any | 4997 | 46.86 | | | | | |
| 40 | 65-69 | CC | 4 | 0.04 | 0 | 0.00 | | | |
| 40 | 65-69 | Ecig | 1119 | 10.49 | | | | | |
| 40 | 65-69 | Dual | 0 | 0.00 | | | | | |
| 40 | 65-69 | Former | 4544 | 42.61 | | | 26.35 | 136 | 2.99 |

Year of Follow-up: 2030, Age Group: 70-74

| Period | Age Group | Smoking Group | N | % | Ever Quit (N) | Ever Quit % | Mean Yrs Quit | Short Term (n) | Short Term % |
|---|---|---|---|---|---|---|---|---|---|
| 40 | 70-74 | Never Any | 4724 | 43.28 | | | | | |
| 40 | 70-74 | CC | 3 | 0.03 | 0 | 0.00 | | | |
| 40 | 70-74 | Ecig | 880 | 8.06 | | | | | |
| 40 | 70-74 | Dual | 0 | 0.00 | | | | | |
| 40 | 70-74 | Former | 5309 | 48.64 | | | 29.01 | 95 | 1.79 |

Year of Follow-up: 2030, Age Group: 75-79

| Period | Age Group | Smoking Group | N | % | Ever Quit (N) | Ever Quit % | Mean Yrs Quit | Short Term (n) | Short Term % |
|---|---|---|---|---|---|---|---|---|---|
| 40 | 75-79 | Never Any | 3941 | 39.85 | | | | | |
| 40 | 75-79 | CC | 3 | 0.03 | 0 | 0.00 | | | |

# Results for PHIM for Run 5 (Sensitivity_Analysis_1.RTF)

| | | | | | | | | | |
|---|---|---|---|---|---|---|---|---|---|
| 40 | 75-79 | Ecig | 615 | 6.22 | | | | | |
| 40 | 75-79 | Dual | 0 | 0.00 | | | | | |
| 40 | 75-79 | Former | 5330 | 53.90 | | | 33.51 | 83 | 1.56 |

Year of Follow-up: 2035, Age Group: All Ages

| Period | Age Group | Smoking Group | N | % | Ever Quit (N) | Ever Quit % | Mean Yrs Quit | Short Term (n) | Short Term % |
|---|---|---|---|---|---|---|---|---|---|
| 45 | All Ages | Never Any | 24364 | 49.44 | | | | | |
| 45 | All Ages | CC | 6 | 0.01 | 0 | 0.00 | | | |
| 45 | All Ages | Ecig | 4578 | 9.29 | | | | | |
| 45 | All Ages | Dual | 0 | 0.00 | | | | | |
| 45 | All Ages | Former | 20333 | 41.26 | | | 28.82 | 538 | 2.65 |

Year of Follow-up: 2035, Age Group: 18-19

| Period | Age Group | Smoking Group | N | % | Ever Quit (N) | Ever Quit % | Mean Yrs Quit | Short Term (n) | Short Term % |
|---|---|---|---|---|---|---|---|---|---|
| 45 | 18-19 | Never Any | 0 | . | | | | | |
| 45 | 18-19 | CC | 0 | . | 0 | . | | | |
| 45 | 18-19 | Ecig | 0 | . | | | | | |
| 45 | 18-19 | Dual | 0 | . | | | | | |
| 45 | 18-19 | Former | 0 | . | | | . | 0 | . |

Year of Follow-up: 2035, Age Group: 20-24

| Period | Age Group | Smoking Group | N | % | Ever Quit (N) | Ever Quit % | Mean Yrs Quit | Short Term (n) | Short Term % |
|---|---|---|---|---|---|---|---|---|---|
| 45 | 20-24 | Never Any | 0 | . | | | | | |
| 45 | 20-24 | CC | 0 | . | 0 | . | | | |
| 45 | 20-24 | Ecig | 0 | . | | | | | |
| 45 | 20-24 | Dual | 0 | . | | | | | |
| 45 | 20-24 | Former | 0 | . | | | . | 0 | . |

Year of Follow-up: 2035, Age Group: 25-29

| Period | Age Group | Smoking Group | N | % | Ever Quit (N) | Ever Quit % | Mean Yrs Quit | Short Term (n) | Short Term % |
|---|---|---|---|---|---|---|---|---|---|
| 45 | 25-29 | Never Any | 0 | . | | | | | |
| 45 | 25-29 | CC | 0 | . | 0 | . | | | |
| 45 | 25-29 | Ecig | 0 | . | | | | | |
| 45 | 25-29 | Dual | 0 | . | | | | | |
| 45 | 25-29 | Former | 0 | . | | | . | 0 | . |

Year of Follow-up: 2035, Age Group: 30-34

| Period | Age Group | Smoking Group | N | % | Ever Quit (N) | Ever Quit % | Mean Yrs Quit | Short Term (n) | Short Term % |
|---|---|---|---|---|---|---|---|---|---|
| 45 | 30-34 | Never Any | 0 | . | | | | | |
| 45 | 30-34 | CC | 0 | . | 0 | . | | | |
| 45 | 30-34 | Ecig | 0 | . | | | | | |
| 45 | 30-34 | Dual | 0 | . | | | | | |
| 45 | 30-34 | Former | 0 | . | | | . | 0 | . |

Year of Follow-up: 2035, Age Group: 35-39

| Period | Age Group | Smoking Group | N | % | Ever Quit (N) | Ever Quit % | Mean Yrs Quit | Short Term (n) | Short Term % |
|---|---|---|---|---|---|---|---|---|---|
| 45 | 35-39 | Never Any | 0 | . | | | | | |
| 45 | 35-39 | CC | 0 | . | 0 | . | | | |
| 45 | 35-39 | Ecig | 0 | . | | | | | |
| 45 | 35-39 | Dual | 0 | . | | | | | |
| 45 | 35-39 | Former | 0 | . | | | . | 0 | . |

Year of Follow-up: 2035, Age Group: 40-44

| Period | Age Group | Smoking Group | N | % | Ever Quit (N) | Ever Quit % | Mean Yrs Quit | Short Term (n) | Short Term % |
|---|---|---|---|---|---|---|---|---|---|
| 45 | 40-44 | Never Any | 0 | . | | | | | |
| 45 | 40-44 | CC | 0 | . | 0 | . | | | |
| 45 | 40-44 | Ecig | 0 | . | | | | | |
| 45 | 40-44 | Dual | 0 | . | | | | | |
| 45 | 40-44 | Former | 0 | . | | | . | 0 | . |

Year of Follow-up: 2035, Age Group: 45-49

| Period | Age Group | Smoking Group | N | % | Ever Quit (N) | Ever Quit % | Mean Yrs Quit | Short Term (n) | Short Term % |
|---|---|---|---|---|---|---|---|---|---|
| 45 | 45-49 | Never Any | 0 | . | | | | | |
| 45 | 45-49 | CC | 0 | . | 0 | . | | | |
| 45 | 45-49 | Ecig | 0 | . | | | | | |
| 45 | 45-49 | Dual | 0 | . | | | | | |
| 45 | 45-49 | Former | 0 | . | | | . | 0 | . |

Year of Follow-up: 2035, Age Group: 50-54

| Period | Age Group | Smoking Group | N | % | Ever Quit (N) | Ever Quit % | Mean Yrs Quit | Short Term (n) | Short Term % |
|---|---|---|---|---|---|---|---|---|---|
| 45 | 50-54 | Never Any | 0 | . | | | | | |
| 45 | 50-54 | CC | 0 | . | 0 | . | | | |
| 45 | 50-54 | Ecig | 0 | . | | | | | |
| 45 | 50-54 | Dual | 0 | . | | | | | |
| 45 | 50-54 | Former | 0 | . | | | . | 0 | . |

Year of Follow-up: 2035, Age Group: 55-59

| Period | Age Group | Smoking Group | N | % | Ever Quit (N) | Ever Quit % | Mean Yrs Quit | Short Term (n) | Short Term % |
|---|---|---|---|---|---|---|---|---|---|

# Results for PHIM for Run 5 (Sensitivity_Analysis_1.RTF)

| | | | | | | | | | |
|---|---|---|---|---|---|---|---|---|---|
| 45 | 55-59 | Never Any | 4489 | 50.75 | | | | | |
| 45 | 55-59 | CC | 2 | 0.02 | 0 | 0.00 | | | |
| 45 | 55-59 | Ecig | 1171 | 13.24 | | | | | |
| 45 | 55-59 | Dual | 0 | 0.00 | | | | | |
| 45 | 55-59 | Former | 3183 | 35.99 | | | 25.82 | 100 | 3.14 |

Year of Follow-up: 2035, Age Group: 60-64

| Period | Age Group | Smoking Group | N | % | Ever Quit (N) | Ever Quit % | Mean Yrs Quit | Short Term (n) | Short Term % |
|---|---|---|---|---|---|---|---|---|---|
| 45 | 60-64 | Never Any | 4991 | 55.21 | | | | | |
| 45 | 60-64 | CC | 0 | 0.00 | 0 | 0.00 | | | |
| 45 | 60-64 | Ecig | 1020 | 11.28 | | | | | |
| 45 | 60-64 | Dual | 0 | 0.00 | | | | | |
| 45 | 60-64 | Former | 3029 | 33.51 | | | 25.81 | 129 | 4.26 |

Year of Follow-up: 2035, Age Group: 65-69

| Period | Age Group | Smoking Group | N | % | Ever Quit (N) | Ever Quit % | Mean Yrs Quit | Short Term (n) | Short Term % |
|---|---|---|---|---|---|---|---|---|---|
| 45 | 65-69 | Never Any | 5163 | 52.60 | | | | | |
| 45 | 65-69 | CC | 0 | 0.00 | 0 | 0.00 | | | |
| 45 | 65-69 | Ecig | 902 | 9.19 | | | | | |
| 45 | 65-69 | Dual | 0 | 0.00 | | | | | |
| 45 | 65-69 | Former | 3751 | 38.21 | | | 27.09 | 106 | 2.83 |

Year of Follow-up: 2035, Age Group: 70-74

| Period | Age Group | Smoking Group | N | % | Ever Quit (N) | Ever Quit % | Mean Yrs Quit | Short Term (n) | Short Term % |
|---|---|---|---|---|---|---|---|---|---|
| 45 | 70-74 | Never Any | 4997 | 46.86 | | | | | |
| 45 | 70-74 | CC | 1 | 0.01 | 0 | 0.00 | | | |
| 45 | 70-74 | Ecig | 850 | 7.97 | | | | | |
| 45 | 70-74 | Dual | 0 | 0.00 | | | | | |
| 45 | 70-74 | Former | 4816 | 45.16 | | | 29.70 | 104 | 2.16 |

Year of Follow-up: 2035, Age Group: 75-79

| Period | Age Group | Smoking Group | N | % | Ever Quit (N) | Ever Quit % | Mean Yrs Quit | Short Term (n) | Short Term % |
|---|---|---|---|---|---|---|---|---|---|
| 45 | 75-79 | Never Any | 4724 | 43.28 | | | | | |
| 45 | 75-79 | CC | 3 | 0.03 | 0 | 0.00 | | | |
| 45 | 75-79 | Ecig | 635 | 5.82 | | | | | |
| 45 | 75-79 | Dual | 0 | 0.00 | | | | | |
| 45 | 75-79 | Former | 5554 | 50.88 | | | 32.60 | 99 | 1.78 |

Year of Follow-up: 2040, Age Group: All Ages

| Period | Age Group | Smoking Group | N | % | Ever Quit (N) | Ever Quit % | Mean Yrs Quit | Short Term (n) | Short Term % |
|---|---|---|---|---|---|---|---|---|---|
| 50 | All Ages | Never Any | 19640 | 51.19 | | | | | |
| 50 | All Ages | CC | 1 | 0.00 | 0 | 0.00 | | | |
| 50 | All Ages | Ecig | 2868 | 7.48 | | | | | |
| 50 | All Ages | Dual | 0 | 0.00 | | | | | |
| 50 | All Ages | Former | 15856 | 41.33 | | | 30.34 | 408 | 2.57 |

Year of Follow-up: 2040, Age Group: 18-19

| Period | Age Group | Smoking Group | N | % | Ever Quit (N) | Ever Quit % | Mean Yrs Quit | Short Term (n) | Short Term % |
|---|---|---|---|---|---|---|---|---|---|
| 50 | 18-19 | Never Any | 0 | . | | | | | |
| 50 | 18-19 | CC | 0 | . | 0 | . | | | |
| 50 | 18-19 | Ecig | 0 | . | | | | | |
| 50 | 18-19 | Dual | 0 | . | | | | | |
| 50 | 18-19 | Former | 0 | . | | | . | 0 | . |

Year of Follow-up: 2040, Age Group: 20-24

| Period | Age Group | Smoking Group | N | % | Ever Quit (N) | Ever Quit % | Mean Yrs Quit | Short Term (n) | Short Term % |
|---|---|---|---|---|---|---|---|---|---|
| 50 | 20-24 | Never Any | 0 | . | | | | | |
| 50 | 20-24 | CC | 0 | . | 0 | . | | | |
| 50 | 20-24 | Ecig | 0 | . | | | | | |
| 50 | 20-24 | Dual | 0 | . | | | | | |
| 50 | 20-24 | Former | 0 | . | | | . | 0 | . |

Year of Follow-up: 2040, Age Group: 25-29

| Period | Age Group | Smoking Group | N | % | Ever Quit (N) | Ever Quit % | Mean Yrs Quit | Short Term (n) | Short Term % |
|---|---|---|---|---|---|---|---|---|---|
| 50 | 25-29 | Never Any | 0 | . | | | | | |
| 50 | 25-29 | CC | 0 | . | 0 | . | | | |
| 50 | 25-29 | Ecig | 0 | . | | | | | |
| 50 | 25-29 | Dual | 0 | . | | | | | |
| 50 | 25-29 | Former | 0 | . | | | . | 0 | . |

Year of Follow-up: 2040, Age Group: 30-34

| Period | Age Group | Smoking Group | N | % | Ever Quit (N) | Ever Quit % | Mean Yrs Quit | Short Term (n) | Short Term % |
|---|---|---|---|---|---|---|---|---|---|
| 50 | 30-34 | Never Any | 0 | . | | | | | |
| 50 | 30-34 | CC | 0 | . | 0 | . | | | |
| 50 | 30-34 | Ecig | 0 | . | | | | | |
| 50 | 30-34 | Dual | 0 | . | | | | | |
| 50 | 30-34 | Former | 0 | . | | | . | 0 | . |

Year of Follow-up: 2040, Age Group: 35-39

# Results for PHIM for Run 5 (Sensitivity_Analysis_1.RTF)

| Period | Age Group | Smoking Group | N | % | Ever Quit (N) | Ever Quit % | Mean Yrs Quit | Short Term (n) | Short Term % |
|---|---|---|---|---|---|---|---|---|---|
| 50 | 35-39 | Never Any | 0 | . | | | | | |
| 50 | 35-39 | CC | 0 | . | 0 | . | | | |
| 50 | 35-39 | Ecig | 0 | . | | | | | |
| 50 | 35-39 | Dual | 0 | . | | | | | |
| 50 | 35-39 | Former | 0 | . | | | . | 0 | . |

Year of Follow-up: 2040, Age Group: 40-44

| Period | Age Group | Smoking Group | N | % | Ever Quit (N) | Ever Quit % | Mean Yrs Quit | Short Term (n) | Short Term % |
|---|---|---|---|---|---|---|---|---|---|
| 50 | 40-44 | Never Any | 0 | . | | | | | |
| 50 | 40-44 | CC | 0 | . | 0 | . | | | |
| 50 | 40-44 | Ecig | 0 | . | | | | | |
| 50 | 40-44 | Dual | 0 | . | | | | | |
| 50 | 40-44 | Former | 0 | . | | | . | 0 | . |

Year of Follow-up: 2040, Age Group: 45-49

| Period | Age Group | Smoking Group | N | % | Ever Quit (N) | Ever Quit % | Mean Yrs Quit | Short Term (n) | Short Term % |
|---|---|---|---|---|---|---|---|---|---|
| 50 | 45-49 | Never Any | 0 | . | | | | | |
| 50 | 45-49 | CC | 0 | . | 0 | . | | | |
| 50 | 45-49 | Ecig | 0 | . | | | | | |
| 50 | 45-49 | Dual | 0 | . | | | | | |
| 50 | 45-49 | Former | 0 | . | | | . | 0 | . |

Year of Follow-up: 2040, Age Group: 50-54

| Period | Age Group | Smoking Group | N | % | Ever Quit (N) | Ever Quit % | Mean Yrs Quit | Short Term (n) | Short Term % |
|---|---|---|---|---|---|---|---|---|---|
| 50 | 50-54 | Never Any | 0 | . | | | | | |
| 50 | 50-54 | CC | 0 | . | 0 | . | | | |
| 50 | 50-54 | Ecig | 0 | . | | | | | |
| 50 | 50-54 | Dual | 0 | . | | | | | |
| 50 | 50-54 | Former | 0 | . | | | . | 0 | . |

Year of Follow-up: 2040, Age Group: 55-59

| Period | Age Group | Smoking Group | N | % | Ever Quit (N) | Ever Quit % | Mean Yrs Quit | Short Term (n) | Short Term % |
|---|---|---|---|---|---|---|---|---|---|
| 50 | 55-59 | Never Any | 0 | . | | | | | |
| 50 | 55-59 | CC | 0 | . | 0 | . | | | |
| 50 | 55-59 | Ecig | 0 | . | | | | | |
| 50 | 55-59 | Dual | 0 | . | | | | | |
| 50 | 55-59 | Former | 0 | . | | | . | 0 | . |

Year of Follow-up: 2040, Age Group: 60-64

| Period | Age Group | Smoking Group | N | % | Ever Quit (N) | Ever Quit % | Mean Yrs Quit | Short Term (n) | Short Term % |
|---|---|---|---|---|---|---|---|---|---|
| 50 | 60-64 | Never Any | 4489 | 50.75 | | | | | |
| 50 | 60-64 | CC | 0 | 0.00 | 0 | 0.00 | | | |
| 50 | 60-64 | Ecig | 865 | 9.78 | | | | | |
| 50 | 60-64 | Dual | 0 | 0.00 | | | | | |
| 50 | 60-64 | Former | 3491 | 39.47 | | | 28.27 | 129 | 3.70 |

Year of Follow-up: 2040, Age Group: 65-69

| Period | Age Group | Smoking Group | N | % | Ever Quit (N) | Ever Quit % | Mean Yrs Quit | Short Term (n) | Short Term % |
|---|---|---|---|---|---|---|---|---|---|
| 50 | 65-69 | Never Any | 4991 | 55.21 | | | | | |
| 50 | 65-69 | CC | 0 | 0.00 | 0 | 0.00 | | | |
| 50 | 65-69 | Ecig | 705 | 7.80 | | | | | |
| 50 | 65-69 | Dual | 0 | 0.00 | | | | | |
| 50 | 65-69 | Former | 3344 | 36.99 | | | 28.11 | 120 | 3.59 |

Year of Follow-up: 2040, Age Group: 70-74

| Period | Age Group | Smoking Group | N | % | Ever Quit (N) | Ever Quit % | Mean Yrs Quit | Short Term (n) | Short Term % |
|---|---|---|---|---|---|---|---|---|---|
| 50 | 70-74 | Never Any | 5163 | 52.60 | | | | | |
| 50 | 70-74 | CC | 0 | 0.00 | 0 | 0.00 | | | |
| 50 | 70-74 | Ecig | 665 | 6.77 | | | | | |
| 50 | 70-74 | Dual | 0 | 0.00 | | | | | |
| 50 | 70-74 | Former | 3988 | 40.63 | | | 30.31 | 80 | 2.01 |

Year of Follow-up: 2040, Age Group: 75-79

| Period | Age Group | Smoking Group | N | % | Ever Quit (N) | Ever Quit % | Mean Yrs Quit | Short Term (n) | Short Term % |
|---|---|---|---|---|---|---|---|---|---|
| 50 | 75-79 | Never Any | 4997 | 46.86 | | | | | |
| 50 | 75-79 | CC | 1 | 0.01 | 0 | 0.00 | | | |
| 50 | 75-79 | Ecig | 633 | 5.94 | | | | | |
| 50 | 75-79 | Dual | 0 | 0.00 | | | | | |
| 50 | 75-79 | Former | 5033 | 47.20 | | | 33.29 | 79 | 1.57 |

# Results for PHIM for Run 5 (Sensitivity_Analysis_1.RTF)

************************************************************************************

**Main Results for E-Component for Run: 5, Simulation: 1, COD: Four Diseases Combined**

| Variable Parameter | Value |
|---|---|
| 1. Country | US (United States) |
| 2. Sex | M (Males) |
| 3. Year of start of process | 1990 |
| 4. Cause of Death | Four Diseases Combined |
| 5. Effective Dose NULL | CC:1 |
| 6. Effective Dose Ecig | CC:1 Ecig:0.1 Dual:0.55 |
| Note: | Death rates are per million population |

-----------------------------------------------------------------------------------------

Results for Age Groups Combined and Years combined, Population unadjusted for deaths in Ecig scenario,

| Age Group:All | | NULL | Ecig | | | %Drop | | NULL | Ecig | |
|---|---|---|---|---|---|---|---|---|---|---|
| Year | Number | Attrib | Attrib | Drop | %Drop | Attrib | Death | Attrib | Attrib | Drop |
| | Deaths | Deaths | Deaths | Deaths | Deaths | Deaths | Rate | Rates | Rates | Rates |
| All Comb | 13148871 | 4509390.583 | 3104046.546 | 1405344 | 10.688 | 31.165 | 3806.31 | 1305.37 | 898.554 | 406.817 |

-----------------------------------------------------------------------------------------

Results for Age Groups Combined, Population unadjusted for deaths in Ecig scenario,

| Age Group:All | | NULL | Ecig | | | %Drop | | NULL | Ecig | |
|---|---|---|---|---|---|---|---|---|---|---|
| Year | Number | Attrib | Attrib | Drop | %Drop | Attrib | Death | Attrib | Attrib | Drop |
| | Deaths | Deaths | Deaths | Deaths | Deaths | Deaths | Rate | Rates | Rates | Rates |
| 1990 | 317161 | 125773.065 | 125773.065 | 0.000 | 0.000 | 0.000 | 5027.71 | 1993.78 | 1993.78 | 0.000 |
| 1991 | 313217 | 123999.442 | 123597.528 | 401.915 | 0.128 | 0.324 | 4883.81 | 1933.45 | 1927.18 | 6.267 |
| 1992 | 308577 | 122014.015 | 120939.541 | 1074.474 | 0.348 | 0.881 | 4729.73 | 1870.18 | 1853.71 | 16.469 |
| 1993 | 311713 | 122810.618 | 120795.239 | 2015.379 | 0.647 | 1.641 | 4692.62 | 1848.83 | 1818.49 | 30.340 |
| 1994 | 305700 | 119925.592 | 116832.080 | 3093.512 | 1.012 | 2.580 | 4516.19 | 1771.69 | 1725.99 | 45.701 |
| 1995 | 303225 | 117896.496 | 113543.966 | 4352.530 | 1.435 | 3.692 | 4393.80 | 1708.35 | 1645.28 | 63.069 |
| 1996 | 299559 | 115698.645 | 109961.901 | 5736.744 | 1.915 | 4.958 | 4273.16 | 1650.42 | 1568.59 | 81.834 |
| 1997 | 294226 | 113359.847 | 106232.334 | 7127.513 | 2.422 | 6.288 | 4128.67 | 1590.70 | 1490.69 | 100.016 |
| 1998 | 289258 | 111444.212 | 102922.575 | 8521.637 | 2.946 | 7.647 | 3992.19 | 1538.10 | 1420.49 | 117.611 |
| 1999 | 311002 | 114877.722 | 103492.683 | 11385.04 | 3.661 | 9.911 | 4224.60 | 1560.48 | 1405.83 | 154.653 |
| 2000 | 303786 | 112379.704 | 99405.356 | 12974.35 | 4.271 | 11.545 | 4066.21 | 1504.22 | 1330.55 | 173.663 |
| 2001 | 296294 | 110024.943 | 95346.688 | 14678.25 | 4.954 | 13.341 | 3914.28 | 1453.52 | 1259.61 | 193.912 |
| 2002 | 291898 | 108289.173 | 91704.255 | 16584.92 | 5.682 | 15.315 | 3813.00 | 1414.56 | 1197.91 | 216.645 |
| 2003 | 285865 | 106539.023 | 88272.843 | 18266.18 | 6.390 | 17.145 | 3695.36 | 1377.22 | 1141.10 | 236.126 |
| 2004 | 274177 | 103037.251 | 83537.219 | 19500.03 | 7.112 | 18.925 | 3506.10 | 1317.61 | 1068.25 | 249.361 |
| 2005 | 272379 | 103044.562 | 81769.435 | 21275.13 | 7.811 | 20.647 | 3442.69 | 1302.41 | 1033.51 | 268.903 |
| 2006 | 264118 | 99672.555 | 76892.062 | 22780.49 | 8.625 | 22.855 | 3307.76 | 1248.28 | 962.981 | 285.298 |
| 2007 | 258419 | 97839.152 | 73793.443 | 24045.71 | 9.305 | 24.577 | 3203.20 | 1212.75 | 914.696 | 298.055 |
| 2008 | 259461 | 98075.852 | 72430.681 | 25645.17 | 9.884 | 26.148 | 3180.09 | 1202.07 | 887.750 | 314.321 |
| 2009 | 253907 | 95617.338 | 68862.223 | 26755.11 | 10.537 | 27.981 | 3075.31 | 1158.11 | 834.057 | 324.057 |
| 2010 | 251878 | 94607.988 | 66607.772 | 28000.22 | 11.117 | 29.596 | 3013.72 | 1131.98 | 796.962 | 335.022 |
| 2011 | 251779 | 93633.119 | 64102.773 | 29530.35 | 11.729 | 31.538 | 2982.76 | 1109.25 | 759.410 | 349.839 |
| 2012 | 252643 | 92775.175 | 61748.187 | 31026.99 | 12.281 | 33.443 | 2960.61 | 1087.19 | 723.598 | 363.591 |
| 2013 | 256175 | 92345.780 | 59785.077 | 32560.70 | 12.710 | 35.260 | 2967.76 | 1069.82 | 692.604 | 377.212 |
| 2014 | 257393 | 91379.215 | 57541.473 | 33837.74 | 13.146 | 37.030 | 2947.05 | 1046.26 | 658.827 | 387.429 |
| 2015 | 259782 | 90750.242 | 55771.258 | 34978.98 | 13.465 | 38.544 | 3346.10 | 1168.90 | 718.357 | 450.544 |
| 2016 | 261453 | 88811.996 | 52658.840 | 36153.16 | 13.828 | 40.708 | 3336.15 | 1133.24 | 671.929 | 461.316 |
| 2017 | 263685 | 87325.126 | 50121.358 | 37203.77 | 14.109 | 42.604 | 3333.85 | 1104.08 | 633.700 | 470.379 |
| 2018 | 258714 | 87612.144 | 49515.640 | 38096.50 | 14.725 | 43.483 | 3240.73 | 1097.46 | 620.248 | 477.208 |
| 2019 | 261438 | 87797.760 | 48343.140 | 39454.62 | 15.091 | 44.938 | 3242.99 | 1089.08 | 599.669 | 489.412 |
| 2020 | 262288 | 87221.042 | 46995.269 | 40225.77 | 15.336 | 46.119 | 3715.29 | 1235.48 | 665.684 | 569.795 |
| 2021 | 255079 | 82001.292 | 42192.265 | 39809.03 | 15.607 | 48.547 | 3587.82 | 1153.39 | 593.455 | 559.934 |
| 2022 | 257097 | 81915.977 | 40855.313 | 41060.66 | 15.971 | 50.125 | 3588.53 | 1143.37 | 570.254 | 573.121 |
| 2023 | 259080 | 81810.810 | 39559.704 | 42251.11 | 16.308 | 51.645 | 3586.88 | 1132.64 | 547.692 | 584.953 |
| 2024 | 260719 | 81528.303 | 38269.029 | 43259.27 | 16.592 | 53.060 | 3579.65 | 1119.38 | 525.431 | 593.947 |
| 2025 | 257308 | 79599.213 | 36626.715 | 42972.50 | 16.701 | 53.986 | 4112.08 | 1272.09 | 585.337 | 686.750 |
| 2026 | 251528 | 75082.462 | 32882.997 | 42199.47 | 16.777 | 56.204 | 4003.24 | 1194.99 | 523.355 | 671.633 |
| 2027 | 250988 | 74444.425 | 31491.593 | 42952.83 | 17.113 | 57.698 | 3976.53 | 1179.46 | 498.937 | 680.523 |
| 2028 | 250285 | 73699.585 | 30028.465 | 43671.12 | 17.449 | 59.256 | 3945.35 | 1161.76 | 473.352 | 688.407 |
| 2029 | 249525 | 72819.703 | 28611.739 | 44207.96 | 17.717 | 60.709 | 3910.99 | 1141.36 | 448.453 | 692.904 |
| 2030 | 240528 | 69309.277 | 26813.646 | 42495.63 | 17.668 | 61.313 | 4504.36 | 1297.95 | 502.137 | 795.812 |
| 2031 | 235442 | 65433.368 | 24057.012 | 41376.36 | 17.574 | 63.234 | 4397.65 | 1222.18 | 449.343 | 772.838 |
| 2032 | 234017 | 64643.552 | 22863.000 | 41780.55 | 17.854 | 64.632 | 4356.12 | 1203.31 | 425.584 | 777.725 |
| 2033 | 232559 | 63793.514 | 21751.443 | 42042.07 | 18.078 | 65.903 | 4310.93 | 1182.54 | 403.205 | 779.331 |
| 2034 | 230963 | 63032.151 | 20697.423 | 42334.73 | 18.330 | 67.164 | 4260.75 | 1162.80 | 381.821 | 780.980 |
| 2035 | 215822 | 57653.301 | 18911.031 | 38742.27 | 17.951 | 67.199 | 4924.38 | 1315.47 | 431.491 | 883.979 |
| 2036 | 208183 | 52988.733 | 16169.110 | 36819.62 | 17.686 | 69.486 | 4748.94 | 1208.75 | 368.840 | 839.906 |
| 2037 | 205491 | 52026.921 | 15216.330 | 36810.59 | 17.913 | 70.753 | 4682.40 | 1185.50 | 346.724 | 838.778 |
| 2038 | 202834 | 50943.211 | 14273.132 | 36670.08 | 18.079 | 71.982 | 4612.18 | 1158.38 | 324.553 | 833.831 |
| 2039 | 200251 | 50085.991 | 13480.696 | 36605.30 | 18.280 | 73.085 | 4538.65 | 1135.19 | 305.538 | 829.653 |

# Results for PHIM for Run 5 (Sensitivity_Analysis_1.RTF)

************************************************************************************
**Main Results for E-Component for Run: 5, Simulation: 1, COD: LUNG CANCER**

| Variable Parameter | Value |
|---|---|
| 1. Country | US (United States) |
| 2. Sex | M (Males) |
| 3. Year of start of process | 1990 |
| 4. Cause of Death | LUNG CANCER |
| 5. Effective Dose NULL | CC:1 |
| 6. Effective Dose Ecig | CC:1 Ecig:0.1 Dual:0.55 |
| Note: | Death rates are per million population |

--------------------------------------------------------------------------------------
Results for Age Groups Combined and Years combined, Population unadjusted for deaths in Ecig scenario,

| Age Group:All | | NULL | Ecig | | | %Drop | | NULL | Ecig | |
|---|---|---|---|---|---|---|---|---|---|---|
| Year | Number Deaths | Attrib Deaths | Attrib Deaths | Drop Deaths | %Drop Deaths | Attrib Deaths | Death Rate | Attrib Rates | Attrib Rates | Drop Rates |
| All Comb | 3065988 | 2244988.113 | 1775558.458 | 469429.7 | 15.311 | 20.910 | 887.537 | 649.875 | 513.986 | 135.890 |

--------------------------------------------------------------------------------------
Results for Age Groups Combined, Population unadjusted for deaths in Ecig scenario,

| Age Group:All | | NULL | Ecig | | | %Drop | | NULL | Ecig | |
|---|---|---|---|---|---|---|---|---|---|---|
| Year | Number Deaths | Attrib Deaths | Attrib Deaths | Drop Deaths | %Drop Deaths | Attrib Deaths | Death Rate | Attrib Rates | Attrib Rates | Drop Rates |
| 1990 | 78822 | 63385.736 | 63385.736 | 0.000 | 0.000 | 0.000 | 1249.50 | 1004.81 | 1004.81 | 0.000 |
| 1991 | 78462 | 62918.402 | 62872.703 | 45.699 | 0.058 | 0.073 | 1223.41 | 981.049 | 980.337 | 0.713 |
| 1992 | 77987 | 62366.766 | 62244.448 | 122.318 | 0.157 | 0.196 | 1195.35 | 955.930 | 954.055 | 1.875 |
| 1993 | 78542 | 62652.888 | 62412.321 | 240.567 | 0.306 | 0.384 | 1182.39 | 943.195 | 939.573 | 3.622 |
| 1994 | 77437 | 61565.227 | 61186.281 | 378.946 | 0.489 | 0.616 | 1144.00 | 909.520 | 903.922 | 5.598 |
| 1995 | 76904 | 60851.961 | 60301.005 | 550.956 | 0.716 | 0.905 | 1114.36 | 881.759 | 873.775 | 7.983 |
| 1996 | 76232 | 60122.922 | 59355.549 | 767.372 | 1.007 | 1.276 | 1087.44 | 857.643 | 846.696 | 10.946 |
| 1997 | 75473 | 59237.155 | 58237.816 | 999.338 | 1.324 | 1.687 | 1059.06 | 831.234 | 817.211 | 14.023 |
| 1998 | 75381 | 58906.696 | 57619.155 | 1287.541 | 1.708 | 2.186 | 1040.37 | 813.001 | 795.231 | 17.770 |
| 1999 | 73159 | 56930.073 | 55361.297 | 1568.776 | 2.144 | 2.756 | 993.781 | 773.329 | 752.019 | 21.310 |
| 2000 | 73784 | 57137.121 | 55203.407 | 1933.714 | 2.621 | 3.384 | 987.608 | 764.787 | 738.904 | 25.883 |
| 2001 | 73294 | 56481.726 | 54135.668 | 2346.058 | 3.201 | 4.154 | 968.273 | 746.169 | 715.176 | 30.993 |
| 2002 | 72442 | 55588.365 | 52811.598 | 2776.768 | 3.833 | 4.995 | 946.293 | 726.138 | 689.866 | 36.272 |
| 2003 | 71942 | 54945.856 | 51694.100 | 3251.756 | 4.520 | 5.918 | 929.989 | 710.281 | 668.246 | 42.035 |
| 2004 | 71301 | 54203.126 | 50427.308 | 3775.818 | 5.296 | 6.966 | 911.778 | 693.135 | 644.851 | 48.284 |
| 2005 | 70964 | 53744.603 | 49430.878 | 4313.725 | 6.079 | 8.026 | 896.937 | 679.296 | 624.773 | 54.523 |
| 2006 | 69658 | 52535.572 | 47658.972 | 4876.600 | 7.001 | 9.282 | 872.383 | 657.945 | 596.871 | 61.074 |
| 2007 | 68565 | 51492.124 | 46061.448 | 5430.676 | 7.920 | 10.547 | 849.888 | 638.264 | 570.948 | 67.315 |
| 2008 | 68226 | 51030.736 | 44931.755 | 6098.980 | 8.939 | 11.952 | 836.215 | 625.460 | 550.708 | 74.752 |
| 2009 | 67509 | 50276.199 | 43509.866 | 6766.333 | 10.023 | 13.458 | 817.667 | 608.943 | 526.990 | 81.954 |
| 2010 | 67196 | 49840.933 | 42407.763 | 7433.170 | 11.062 | 14.914 | 804.000 | 596.347 | 507.409 | 88.938 |
| 2011 | 66506 | 49072.841 | 40906.971 | 8165.870 | 12.278 | 16.640 | 787.880 | 581.354 | 484.615 | 96.739 |
| 2012 | 66155 | 48539.512 | 39589.429 | 8950.083 | 13.529 | 18.439 | 775.240 | 568.812 | 463.930 | 104.882 |
| 2013 | 65333 | 47675.741 | 38032.573 | 9643.168 | 14.760 | 20.227 | 756.876 | 552.318 | 440.603 | 111.715 |
| 2014 | 65033 | 47194.534 | 36827.121 | 10367.41 | 15.942 | 21.967 | 744.602 | 540.359 | 421.656 | 118.703 |
| 2015 | 64145 | 46305.842 | 35316.747 | 10989.09 | 17.132 | 23.732 | 826.215 | 596.439 | 454.895 | 141.544 |
| 2016 | 61618 | 44208.867 | 32870.384 | 11338.48 | 18.401 | 25.648 | 786.248 | 564.107 | 419.427 | 144.680 |
| 2017 | 59897 | 42702.624 | 30944.286 | 11758.34 | 19.631 | 27.535 | 757.297 | 539.903 | 391.238 | 148.664 |
| 2018 | 62468 | 44321.202 | 31137.742 | 13183.46 | 21.104 | 29.745 | 782.490 | 555.181 | 390.041 | 165.140 |
| 2019 | 63045 | 44493.088 | 30365.323 | 14127.77 | 22.409 | 31.753 | 782.035 | 551.912 | 376.665 | 175.247 |
| 2020 | 63466 | 44529.551 | 29522.026 | 15007.53 | 23.647 | 33.702 | 898.991 | 630.757 | 418.177 | 212.580 |
| 2021 | 57113 | 39852.556 | 25570.761 | 14281.80 | 25.006 | 35.837 | 803.323 | 560.546 | 359.666 | 200.881 |
| 2022 | 57440 | 39853.685 | 24697.146 | 15156.54 | 26.387 | 38.030 | 801.745 | 556.274 | 344.720 | 211.553 |
| 2023 | 57738 | 39847.050 | 23834.970 | 16012.08 | 27.732 | 40.184 | 799.366 | 551.670 | 329.988 | 221.682 |
| 2024 | 57942 | 39726.010 | 22956.895 | 16769.11 | 28.941 | 42.212 | 795.539 | 545.435 | 315.197 | 230.239 |
| 2025 | 57521 | 39207.348 | 21934.786 | 17272.56 | 30.028 | 44.054 | 919.257 | 626.579 | 350.543 | 276.036 |
| 2026 | 51428 | 34803.960 | 18759.021 | 16044.94 | 31.199 | 46.101 | 818.510 | 553.929 | 298.563 | 255.366 |
| 2027 | 51172 | 34484.595 | 17860.372 | 16624.22 | 32.487 | 48.208 | 810.740 | 546.357 | 282.971 | 263.386 |
| 2028 | 50870 | 34100.638 | 16935.849 | 17164.79 | 33.743 | 50.336 | 801.884 | 537.544 | 266.967 | 270.576 |
| 2029 | 50540 | 33669.838 | 16048.144 | 17621.69 | 34.867 | 52.337 | 792.146 | 527.732 | 251.534 | 276.198 |
| 2030 | 49109 | 32557.688 | 15013.496 | 17544.19 | 35.725 | 53.886 | 919.662 | 609.705 | 281.157 | 328.549 |
| 2031 | 43585 | 28646.393 | 12704.522 | 15941.87 | 36.576 | 55.651 | 814.096 | 535.065 | 237.298 | 297.766 |
| 2032 | 43163 | 28227.160 | 11998.356 | 16228.80 | 37.599 | 57.494 | 803.454 | 525.435 | 223.344 | 302.091 |
| 2033 | 42725 | 27786.623 | 11344.801 | 16441.82 | 38.483 | 59.172 | 791.996 | 515.079 | 210.298 | 304.781 |
| 2034 | 42256 | 27348.299 | 10728.044 | 16620.25 | 39.333 | 60.773 | 779.523 | 504.514 | 197.908 | 306.606 |
| 2035 | 39748 | 25570.077 | 9792.207 | 15777.87 | 39.694 | 61.704 | 906.933 | 583.430 | 223.428 | 360.002 |
| 2036 | 34030 | 21717.319 | 7866.979 | 13850.34 | 40.700 | 63.776 | 776.269 | 495.402 | 179.457 | 315.945 |
| 2037 | 33454 | 21256.024 | 7370.170 | 13885.85 | 41.508 | 65.327 | 762.289 | 484.347 | 167.939 | 316.408 |
| 2038 | 32883 | 20747.175 | 6889.209 | 13857.97 | 42.143 | 66.794 | 747.720 | 471.764 | 156.652 | 315.112 |
| 2039 | 32325 | 20327.688 | 6491.055 | 13836.63 | 42.805 | 68.068 | 732.644 | 460.724 | 147.119 | 313.605 |

# Results for PHIM for Run 5 (Sensitivity_Analysis_1.RTF)

*********************************************************************************************
**Main Results for E-Component for Run: 5, Simulation: 1, COD: IHD**

| Variable Parameter | Value |
|---|---|
| 1. Country | US (United States) |
| 2. Sex | M (Males) |
| 3. Year of start of process | 1990 |
| 4. Cause of Death | IHD |
| 5. Effective Dose NULL | CC:1 |
| 6. Effective Dose Ecig | CC:1 Ecig:0.1 Dual:0.55 |
| Note: | Death rates are per million population |

-----------------------------------------------------------------------------------------
Results for Age Groups Combined and Years combined, Population unadjusted for deaths in Ecig scenario,

| Age Group:All | | NULL | Ecig | | | %Drop | | NULL | Ecig | |
|---|---|---|---|---|---|---|---|---|---|---|
| Year | Number Deaths | Attrib Deaths | Attrib Deaths | Drop Deaths | %Drop Deaths | Attrib Deaths | Death Rate | Attrib Rates | Attrib Rates | Drop Rates |
| All Comb | 6360041 | 1098877.421 | 553680.078 | 545197.3 | 8.572 | 49.614 | 1841.09 | 318.101 | 160.278 | 157.823 |

-----------------------------------------------------------------------------------------
Results for Age Groups Combined, Population unadjusted for deaths in Ecig scenario,

| Age Group:All | | NULL | Ecig | | | %Drop | | NULL | Ecig | |
|---|---|---|---|---|---|---|---|---|---|---|
| Year | Number Deaths | Attrib Deaths | Attrib Deaths | Drop Deaths | %Drop Deaths | Attrib Deaths | Death Rate | Attrib Rates | Attrib Rates | Drop Rates |
| 1990 | 171484 | 38511.725 | 38511.725 | 0.000 | 0.000 | 0.000 | 2718.40 | 610.497 | 610.497 | 0.000 |
| 1991 | 168044 | 37309.838 | 37012.736 | 297.102 | 0.177 | 0.796 | 2620.21 | 581.750 | 577.118 | 4.633 |
| 1992 | 164548 | 36137.488 | 35339.718 | 797.770 | 0.485 | 2.208 | 2522.12 | 553.899 | 541.671 | 12.228 |
| 1993 | 164362 | 35599.588 | 34124.933 | 1474.655 | 0.897 | 4.142 | 2474.35 | 535.926 | 513.727 | 22.200 |
| 1994 | 159866 | 34336.757 | 32090.076 | 2246.682 | 1.405 | 6.543 | 2361.75 | 507.267 | 474.076 | 33.191 |
| 1995 | 158083 | 33455.759 | 30320.139 | 3135.620 | 1.984 | 9.372 | 2290.66 | 484.782 | 439.346 | 45.436 |
| 1996 | 154875 | 32193.824 | 28117.322 | 4076.503 | 2.632 | 12.662 | 2209.26 | 459.239 | 401.089 | 58.151 |
| 1997 | 149970 | 30665.509 | 25679.327 | 4986.182 | 3.325 | 16.260 | 2104.43 | 430.308 | 360.340 | 69.968 |
| 1998 | 145407 | 29188.430 | 23352.493 | 5835.937 | 4.014 | 19.994 | 2006.83 | 402.844 | 322.299 | 80.545 |
| 1999 | 165952 | 33375.386 | 25264.589 | 8110.797 | 4.887 | 24.302 | 2254.27 | 453.366 | 343.190 | 110.176 |
| 2000 | 160260 | 32024.754 | 22953.831 | 9070.923 | 5.660 | 28.325 | 2145.10 | 428.655 | 307.240 | 121.415 |
| 2001 | 154444 | 30682.393 | 20612.716 | 10069.68 | 6.520 | 32.819 | 2040.33 | 405.339 | 272.311 | 133.029 |
| 2002 | 151887 | 30218.370 | 18978.910 | 11239.46 | 7.400 | 37.194 | 1984.06 | 394.736 | 247.917 | 146.818 |
| 2003 | 146733 | 29175.202 | 17075.945 | 12099.26 | 8.246 | 41.471 | 1896.81 | 377.146 | 220.739 | 156.406 |
| 2004 | 138381 | 27488.687 | 14942.696 | 12545.99 | 9.066 | 45.641 | 1769.58 | 351.518 | 191.083 | 160.435 |
| 2005 | 135881 | 27017.439 | 13665.301 | 13352.14 | 9.826 | 49.420 | 1717.44 | 341.482 | 172.720 | 168.762 |
| 2006 | 131922 | 26231.435 | 12195.366 | 14036.07 | 10.640 | 53.509 | 1652.16 | 328.517 | 152.732 | 175.785 |
| 2007 | 126502 | 25103.049 | 10777.568 | 14325.48 | 11.324 | 57.067 | 1568.04 | 311.161 | 133.592 | 177.570 |
| 2008 | 125859 | 24621.740 | 9845.924 | 14775.82 | 11.740 | 60.011 | 1542.60 | 301.777 | 120.677 | 181.100 |
| 2009 | 122428 | 23741.177 | 8818.264 | 14922.91 | 12.189 | 62.857 | 1482.84 | 287.552 | 106.806 | 180.746 |
| 2010 | 120537 | 23189.160 | 8028.880 | 15160.28 | 12.577 | 65.377 | 1442.22 | 277.458 | 96.066 | 181.393 |
| 2011 | 120221 | 22786.927 | 7296.583 | 15490.34 | 12.885 | 67.979 | 1424.23 | 269.951 | 86.441 | 183.510 |
| 2012 | 120927 | 22485.295 | 6699.570 | 15785.73 | 13.054 | 70.205 | 1417.09 | 263.495 | 78.509 | 184.986 |
| 2013 | 122648 | 22251.884 | 6197.161 | 16054.72 | 13.090 | 72.150 | 1420.86 | 257.786 | 71.793 | 185.992 |
| 2014 | 123229 | 21894.593 | 5710.708 | 16183.89 | 13.133 | 73.917 | 1410.92 | 250.685 | 65.385 | 185.299 |
| 2015 | 123774 | 21471.109 | 5281.842 | 16189.27 | 13.080 | 75.400 | 1594.26 | 276.557 | 68.032 | 208.525 |
| 2016 | 126218 | 21411.568 | 4923.496 | 16488.07 | 13.063 | 77.005 | 1610.55 | 273.213 | 62.824 | 210.388 |
| 2017 | 128265 | 21169.399 | 4582.200 | 16587.20 | 12.932 | 78.355 | 1621.69 | 267.651 | 57.934 | 209.717 |
| 2018 | 121429 | 19830.576 | 4024.543 | 15806.03 | 13.017 | 79.705 | 1521.05 | 248.404 | 50.413 | 197.991 |
| 2019 | 121832 | 19431.014 | 3751.122 | 15679.89 | 12.870 | 80.695 | 1511.26 | 241.031 | 46.531 | 194.500 |
| 2020 | 120811 | 18532.612 | 3417.742 | 15114.87 | 12.511 | 81.558 | 1711.27 | 262.513 | 48.412 | 214.101 |
| 2021 | 119407 | 18234.128 | 3174.162 | 15059.97 | 12.612 | 82.592 | 1679.51 | 256.472 | 44.646 | 211.826 |
| 2022 | 119438 | 17812.559 | 2952.151 | 14860.41 | 12.442 | 83.427 | 1667.10 | 248.626 | 41.206 | 207.420 |
| 2023 | 119445 | 17361.668 | 2747.736 | 14613.93 | 12.235 | 84.174 | 1653.68 | 240.367 | 38.042 | 202.325 |
| 2024 | 119305 | 16929.676 | 2581.639 | 14348.04 | 12.026 | 84.751 | 1638.05 | 232.443 | 35.446 | 196.998 |
| 2025 | 115685 | 15501.666 | 2290.695 | 13210.97 | 11.420 | 85.223 | 1848.78 | 247.735 | 36.608 | 211.127 |
| 2026 | 115073 | 15523.814 | 2214.311 | 13309.50 | 11.566 | 85.736 | 1831.46 | 247.072 | 35.242 | 211.830 |
| 2027 | 113989 | 15073.070 | 2090.980 | 12982.09 | 11.389 | 86.128 | 1805.98 | 238.810 | 33.128 | 205.682 |
| 2028 | 112855 | 14636.710 | 1965.717 | 12670.99 | 11.228 | 86.570 | 1778.98 | 230.725 | 30.986 | 199.739 |
| 2029 | 111718 | 14180.166 | 1858.369 | 12321.80 | 11.029 | 86.895 | 1751.04 | 222.256 | 29.128 | 193.129 |
| 2030 | 104851 | 12111.772 | 1555.555 | 10556.22 | 10.068 | 87.157 | 1963.54 | 226.816 | 29.131 | 197.685 |
| 2031 | 105289 | 12417.367 | 1570.934 | 10846.43 | 10.302 | 87.349 | 1966.62 | 231.935 | 29.342 | 202.592 |
| 2032 | 103879 | 12054.064 | 1492.682 | 10561.38 | 10.167 | 87.617 | 1933.67 | 224.381 | 27.786 | 196.595 |
| 2033 | 102485 | 11672.389 | 1410.452 | 10261.94 | 10.013 | 87.916 | 1899.76 | 216.370 | 26.145 | 190.225 |
| 2034 | 101070 | 11365.762 | 1355.478 | 10010.28 | 9.904 | 88.074 | 1864.51 | 209.673 | 25.005 | 184.667 |
| 2035 | 90820 | 8580.360 | 999.537 | 7580.823 | 8.347 | 88.351 | 2072.23 | 195.777 | 22.806 | 172.971 |
| 2036 | 92117 | 8948.841 | 1025.935 | 7922.906 | 8.601 | 88.536 | 2101.31 | 204.135 | 23.403 | 180.732 |
| 2037 | 90327 | 8624.570 | 983.634 | 7640.935 | 8.459 | 88.595 | 2058.21 | 196.522 | 22.413 | 174.109 |
| 2038 | 88590 | 8315.017 | 930.810 | 7384.206 | 8.335 | 88.806 | 2014.42 | 189.073 | 21.165 | 167.907 |
| 2039 | 86921 | 8001.132 | 885.876 | 7115.256 | 8.186 | 88.928 | 1970.05 | 181.344 | 20.078 | 161.266 |

# Results for PHIM for Run 5 (Sensitivity_Analysis_1.RTF)

************************************************************************************

**Main Results for E-Component for Run: 5, Simulation: 1, COD: STROKE**

| **Variable Parameter** | **Value** |
|---|---|
| 1. Country | US (United States) |
| 2. Sex | M (Males) |
| 3. Year of start of process | 1990 |
| 4. Cause of Death | STROKE |
| 5. Effective Dose NULL | CC:1 |
| 6. Effective Dose Ecig | CC:1 Ecig:0.1 Dual:0.55 |
| Note: | Death rates are per million population |

------------------------------------------------------------------------------------------

Results for Age Groups Combined and Years combined, Population unadjusted for deaths in Ecig scenario,

| Age Group:All | | NULL | Ecig | | | %Drop | | NULL | Ecig | |
|---|---|---|---|---|---|---|---|---|---|---|
| Year | Number Deaths | Attrib Deaths | Attrib Deaths | Drop Deaths | %Drop Deaths | Attrib Deaths | Death Rate | Attrib Rates | Attrib Rates | Drop Rates |
| All Comb | 1489699 | 149918.462 | 74440.445 | 75478.02 | 5.067 | 50.346 | 431.236 | 43.398 | 21.549 | 21.849 |

------------------------------------------------------------------------------------------

Results for Age Groups Combined, Population unadjusted for deaths in Ecig scenario,

| Age Group:All | | NULL | Ecig | | | %Drop | | NULL | Ecig | |
|---|---|---|---|---|---|---|---|---|---|---|
| Year | Number Deaths | Attrib Deaths | Attrib Deaths | Drop Deaths | %Drop Deaths | Attrib Deaths | Death Rate | Attrib Rates | Attrib Rates | Drop Rates |
| 1990 | 32862 | 4613.166 | 4613.166 | 0.000 | 0.000 | 0.000 | 520.936 | 73.129 | 73.129 | 0.000 |
| 1991 | 32619 | 4555.566 | 4517.565 | 38.001 | 0.116 | 0.834 | 508.609 | 71.032 | 70.440 | 0.593 |
| 1992 | 32100 | 4453.572 | 4354.141 | 99.432 | 0.310 | 2.233 | 492.014 | 68.262 | 66.738 | 1.524 |
| 1993 | 32896 | 4462.473 | 4274.762 | 187.711 | 0.571 | 4.206 | 495.226 | 67.179 | 64.354 | 2.826 |
| 1994 | 33323 | 4499.513 | 4203.577 | 295.937 | 0.888 | 6.577 | 492.290 | 66.473 | 62.101 | 4.372 |
| 1995 | 33569 | 4472.774 | 4053.495 | 419.279 | 1.249 | 9.374 | 486.422 | 64.812 | 58.736 | 6.075 |
| 1996 | 33870 | 4406.878 | 3851.326 | 555.553 | 1.640 | 12.606 | 483.150 | 62.863 | 54.938 | 7.925 |
| 1997 | 33516 | 4288.158 | 3594.393 | 693.765 | 2.070 | 16.179 | 470.307 | 60.173 | 50.438 | 9.735 |
| 1998 | 32795 | 4120.148 | 3300.333 | 819.815 | 2.500 | 19.898 | 452.620 | 56.864 | 45.550 | 11.315 |
| 1999 | 33375 | 3982.443 | 3037.020 | 945.422 | 2.833 | 23.740 | 453.361 | 54.097 | 41.254 | 12.842 |
| 2000 | 33459 | 4021.411 | 2911.229 | 1110.183 | 3.318 | 27.607 | 447.853 | 53.827 | 38.967 | 14.860 |
| 2001 | 32275 | 3848.092 | 2627.106 | 1220.986 | 3.783 | 31.730 | 426.379 | 50.836 | 34.706 | 16.130 |
| 2002 | 31586 | 3783.081 | 2432.844 | 1350.237 | 4.275 | 35.691 | 412.601 | 49.418 | 31.780 | 17.638 |
| 2003 | 30985 | 3754.415 | 2261.466 | 1492.949 | 4.818 | 39.765 | 400.541 | 48.533 | 29.234 | 19.299 |
| 2004 | 29884 | 3666.274 | 2065.252 | 1601.022 | 5.357 | 43.669 | 382.149 | 46.883 | 26.410 | 20.473 |
| 2005 | 28708 | 3589.688 | 1885.894 | 1703.794 | 5.935 | 47.464 | 362.850 | 45.371 | 23.836 | 21.535 |
| 2006 | 28188 | 3596.985 | 1752.204 | 1844.781 | 6.545 | 51.287 | 353.021 | 45.048 | 21.944 | 23.104 |
| 2007 | 28026 | 3560.798 | 1611.465 | 1949.332 | 6.955 | 54.744 | 347.392 | 44.137 | 19.975 | 24.163 |
| 2008 | 27128 | 3417.001 | 1438.022 | 1978.980 | 7.295 | 57.916 | 332.495 | 41.881 | 17.625 | 24.255 |
| 2009 | 27068 | 3394.390 | 1329.071 | 2065.319 | 7.630 | 60.845 | 327.847 | 41.113 | 16.098 | 25.015 |
| 2010 | 26911 | 3340.137 | 1222.108 | 2118.029 | 7.870 | 63.411 | 321.990 | 39.965 | 14.623 | 25.342 |
| 2011 | 26959 | 3285.435 | 1120.830 | 2164.604 | 8.029 | 65.885 | 319.377 | 38.922 | 13.278 | 25.644 |
| 2012 | 27057 | 3259.209 | 1031.496 | 2227.713 | 8.233 | 68.351 | 317.068 | 38.193 | 12.088 | 26.106 |
| 2013 | 27904 | 3274.703 | 966.024 | 2308.679 | 8.274 | 70.500 | 323.265 | 37.937 | 11.191 | 26.746 |
| 2014 | 28792 | 3298.083 | 906.142 | 2391.941 | 8.308 | 72.525 | 329.657 | 37.762 | 10.375 | 27.387 |
| 2015 | 29560 | 3244.406 | 838.479 | 2405.927 | 8.139 | 74.156 | 380.745 | 41.789 | 10.800 | 30.989 |
| 2016 | 30513 | 3314.836 | 802.479 | 2512.357 | 8.234 | 75.791 | 389.347 | 42.297 | 10.240 | 32.058 |
| 2017 | 31416 | 3333.314 | 756.889 | 2576.425 | 8.201 | 77.293 | 397.202 | 42.144 | 9.570 | 32.575 |
| 2018 | 29348 | 2925.242 | 628.306 | 2296.936 | 7.826 | 78.521 | 367.626 | 36.642 | 7.870 | 28.772 |
| 2019 | 29598 | 2864.628 | 583.196 | 2281.432 | 7.708 | 79.641 | 367.149 | 35.534 | 7.234 | 28.300 |
| 2020 | 29592 | 2730.366 | 528.980 | 2201.387 | 7.439 | 80.626 | 419.174 | 38.675 | 7.493 | 31.182 |
| 2021 | 30097 | 2647.911 | 486.996 | 2160.914 | 7.180 | 81.608 | 423.328 | 37.244 | 6.850 | 30.394 |
| 2022 | 30304 | 2586.165 | 450.997 | 2135.168 | 7.046 | 82.561 | 422.974 | 36.097 | 6.295 | 29.802 |
| 2023 | 30505 | 2517.960 | 418.576 | 2099.384 | 6.882 | 83.376 | 422.337 | 34.860 | 5.795 | 29.065 |
| 2024 | 30661 | 2454.161 | 391.548 | 2062.613 | 6.727 | 84.046 | 420.968 | 33.695 | 5.376 | 28.320 |
| 2025 | 30254 | 2275.364 | 349.332 | 1926.032 | 6.366 | 84.647 | 483.488 | 36.363 | 5.583 | 30.780 |
| 2026 | 30685 | 2183.166 | 323.295 | 1859.871 | 6.061 | 85.191 | 488.380 | 34.747 | 5.145 | 29.601 |
| 2027 | 30561 | 2112.345 | 303.036 | 1809.309 | 5.920 | 85.654 | 484.196 | 33.467 | 4.801 | 28.666 |
| 2028 | 30412 | 2046.677 | 284.234 | 1762.443 | 5.795 | 86.112 | 479.392 | 32.263 | 4.481 | 27.782 |
| 2029 | 30254 | 1977.671 | 267.049 | 1710.623 | 5.654 | 86.497 | 474.195 | 30.997 | 4.186 | 26.812 |
| 2030 | 29330 | 1744.457 | 229.696 | 1514.761 | 5.165 | 86.833 | 549.260 | 32.668 | 4.301 | 28.367 |
| 2031 | 28976 | 1653.738 | 212.973 | 1440.765 | 4.972 | 87.122 | 541.223 | 30.889 | 3.978 | 26.911 |
| 2032 | 28724 | 1603.120 | 200.360 | 1402.760 | 4.884 | 87.502 | 534.690 | 29.841 | 3.730 | 26.112 |
| 2033 | 28464 | 1548.245 | 189.168 | 1359.077 | 4.775 | 87.782 | 527.627 | 28.700 | 3.507 | 25.193 |
| 2034 | 28178 | 1511.985 | 181.022 | 1330.963 | 4.723 | 88.028 | 519.826 | 27.893 | 3.339 | 24.553 |
| 2035 | 26737 | 1239.866 | 145.857 | 1094.008 | 4.092 | 88.236 | 610.065 | 28.290 | 3.328 | 24.962 |
| 2036 | 25591 | 1182.522 | 136.400 | 1046.122 | 4.088 | 88.465 | 583.762 | 26.975 | 3.111 | 23.863 |
| 2037 | 25143 | 1134.483 | 130.596 | 1003.887 | 3.993 | 88.488 | 572.916 | 25.851 | 2.976 | 22.875 |
| 2038 | 24693 | 1093.537 | 123.390 | 970.147 | 3.929 | 88.716 | 561.491 | 24.866 | 2.806 | 22.060 |
| 2039 | 24247 | 1047.905 | 116.661 | 931.244 | 3.841 | 88.867 | 549.553 | 23.751 | 2.644 | 21.106 |

# Results for PHIM for Run 5 (Sensitivity_Analysis_1.RTF)

************************************************************************************
**Main Results for E-Component for Run: 5, Simulation: 1, COD: COPD**

| Variable Parameter | Value |
|---|---|
| 1. Country | US (United States) |
| 2. Sex | M (Males) |
| 3. Year of start of process | 1990 |
| 4. Cause of Death | COPD |
| 5. Effective Dose NULL | CC:1 |
| 6. Effective Dose Ecig | CC:1 Ecig:0.1 Dual:0.55 |
| Note: | Death rates are per million population |

------------------------------------------------------------------------------------------
Results for Age Groups Combined and Years combined, Population unadjusted for deaths in Ecig scenario,

| Age Group:All Year | Number Deaths | NULL Attrib Deaths | Ecig Attrib Deaths | Drop Deaths | %Drop Deaths | %Drop Attrib Deaths | Death Rate | NULL Attrib Rates | Ecig Attrib Rates | Drop Rates |
|---|---|---|---|---|---|---|---|---|---|---|
| All Comb | 2233143 | 1015606.587 | 700367.564 | 315239.0 | 14.116 | 31.039 | 646.447 | 293.996 | 202.741 | 91.255 |

------------------------------------------------------------------------------------------
Results for Age Groups Combined, Population unadjusted for deaths in Ecig scenario,

| Age Group:All Year | Number Deaths | NULL Attrib Deaths | Ecig Attrib Deaths | Drop Deaths | %Drop Deaths | %Drop Attrib Deaths | Death Rate | NULL Attrib Rates | Ecig Attrib Rates | Drop Rates |
|---|---|---|---|---|---|---|---|---|---|---|
| 1990 | 33993 | 19262.437 | 19262.437 | 0.000 | 0.000 | 0.000 | 538.865 | 305.353 | 305.353 | 0.000 |
| 1991 | 34092 | 19215.636 | 19194.523 | 21.113 | 0.062 | 0.110 | 531.576 | 299.618 | 299.289 | 0.329 |
| 1992 | 33942 | 19056.189 | 19001.235 | 54.955 | 0.162 | 0.288 | 520.248 | 292.085 | 291.242 | 0.842 |
| 1993 | 35913 | 20095.670 | 19983.224 | 112.446 | 0.313 | 0.560 | 540.645 | 302.526 | 300.833 | 1.693 |
| 1994 | 35074 | 19524.095 | 19352.147 | 171.948 | 0.490 | 0.881 | 518.158 | 288.435 | 285.895 | 2.540 |
| 1995 | 34669 | 19116.002 | 18869.328 | 246.674 | 0.712 | 1.290 | 502.362 | 276.995 | 273.421 | 3.574 |
| 1996 | 34582 | 18975.021 | 18637.705 | 337.317 | 0.975 | 1.778 | 493.306 | 270.675 | 265.864 | 4.812 |
| 1997 | 35267 | 19169.025 | 18720.798 | 448.227 | 1.271 | 2.338 | 494.878 | 268.986 | 262.696 | 6.290 |
| 1998 | 35675 | 19228.937 | 18650.594 | 578.344 | 1.621 | 3.008 | 492.368 | 265.388 | 257.406 | 7.982 |
| 1999 | 38516 | 20589.820 | 19829.776 | 760.044 | 1.973 | 3.691 | 523.195 | 279.689 | 269.365 | 10.324 |
| 2000 | 36283 | 19196.417 | 18336.888 | 859.529 | 2.369 | 4.478 | 485.652 | 256.946 | 245.442 | 11.505 |
| 2001 | 36281 | 19012.731 | 17971.198 | 1041.533 | 2.871 | 5.478 | 479.301 | 251.174 | 237.414 | 13.759 |
| 2002 | 35983 | 18699.357 | 17480.904 | 1218.453 | 3.386 | 6.516 | 470.038 | 244.265 | 228.349 | 15.916 |
| 2003 | 36205 | 18663.550 | 17241.331 | 1422.219 | 3.928 | 7.620 | 468.019 | 241.262 | 222.877 | 18.385 |
| 2004 | 34611 | 17679.164 | 16101.963 | 1577.201 | 4.557 | 8.921 | 442.596 | 226.076 | 205.908 | 20.169 |
| 2005 | 36826 | 18692.832 | 16787.361 | 1905.470 | 5.174 | 10.194 | 465.456 | 236.265 | 212.181 | 24.084 |
| 2006 | 34350 | 17308.564 | 15285.521 | 2023.043 | 5.889 | 11.688 | 430.192 | 216.769 | 191.433 | 25.336 |
| 2007 | 35326 | 17683.181 | 15342.961 | 2340.220 | 6.625 | 13.234 | 437.879 | 219.189 | 190.182 | 29.008 |
| 2008 | 38248 | 19006.374 | 16214.980 | 2791.394 | 7.298 | 14.687 | 468.788 | 232.952 | 198.740 | 34.213 |
| 2009 | 36902 | 18205.573 | 15205.022 | 3000.550 | 8.131 | 16.481 | 446.956 | 220.505 | 184.163 | 36.343 |
| 2010 | 37234 | 18237.758 | 14949.021 | 3288.737 | 8.833 | 18.033 | 445.505 | 218.215 | 178.865 | 39.350 |
| 2011 | 38093 | 18487.916 | 14778.389 | 3709.528 | 9.738 | 20.065 | 451.279 | 219.022 | 175.076 | 43.946 |
| 2012 | 38504 | 18491.158 | 14427.692 | 4063.466 | 10.553 | 21.975 | 451.211 | 216.689 | 169.071 | 47.618 |
| 2013 | 40290 | 19143.452 | 14589.319 | 4554.134 | 11.303 | 23.790 | 466.755 | 221.775 | 169.016 | 52.759 |
| 2014 | 40339 | 18992.005 | 14097.502 | 4894.503 | 12.133 | 25.771 | 461.866 | 217.451 | 161.411 | 56.040 |
| 2015 | 42303 | 19728.885 | 14334.189 | 5394.696 | 12.753 | 27.344 | 544.880 | 254.116 | 184.630 | 69.486 |
| 2016 | 43104 | 19876.724 | 14062.480 | 5814.244 | 13.489 | 29.252 | 550.009 | 253.628 | 179.438 | 74.190 |
| 2017 | 44107 | 20119.790 | 13837.984 | 6281.807 | 14.242 | 31.222 | 557.659 | 254.381 | 174.958 | 79.423 |
| 2018 | 45469 | 20535.123 | 13725.049 | 6810.074 | 14.977 | 33.163 | 569.564 | 257.229 | 171.924 | 85.305 |
| 2019 | 46963 | 21009.029 | 13643.500 | 7365.529 | 15.684 | 35.059 | 582.552 | 260.605 | 169.240 | 91.365 |
| 2020 | 48419 | 21428.512 | 13526.522 | 7901.990 | 16.320 | 36.876 | 685.853 | 303.533 | 191.602 | 111.931 |
| 2021 | 48463 | 21266.697 | 12960.346 | 8306.351 | 17.140 | 39.058 | 681.652 | 299.127 | 182.294 | 116.833 |
| 2022 | 49915 | 21663.568 | 12755.019 | 8908.549 | 17.847 | 41.122 | 696.712 | 302.378 | 178.033 | 124.345 |
| 2023 | 51391 | 22084.131 | 12558.421 | 9525.710 | 18.536 | 43.134 | 711.491 | 305.748 | 173.867 | 131.880 |
| 2024 | 52811 | 22418.456 | 12338.947 | 10079.51 | 19.086 | 44.961 | 725.093 | 307.804 | 169.413 | 138.391 |
| 2025 | 53848 | 22614.834 | 12051.902 | 10562.93 | 19.616 | 46.708 | 860.553 | 361.411 | 192.603 | 168.808 |
| 2026 | 54342 | 22571.522 | 11586.370 | 10985.15 | 20.215 | 48.668 | 864.886 | 359.241 | 184.405 | 174.836 |
| 2027 | 55267 | 22774.416 | 11237.205 | 11537.21 | 20.876 | 50.659 | 875.619 | 360.827 | 178.037 | 182.790 |
| 2028 | 56149 | 22915.561 | 10842.665 | 12072.90 | 21.502 | 52.684 | 885.102 | 361.228 | 170.918 | 190.310 |
| 2029 | 57014 | 22992.027 | 10438.178 | 12553.85 | 22.019 | 54.601 | 893.616 | 360.371 | 163.605 | 196.766 |
| 2030 | 57238 | 22895.360 | 10014.900 | 12880.46 | 22.503 | 56.258 | 1071.90 | 428.760 | 187.548 | 241.211 |
| 2031 | 57592 | 22715.871 | 9568.583 | 13147.29 | 22.828 | 57.877 | 1075.71 | 424.293 | 178.724 | 245.568 |
| 2032 | 58251 | 22759.207 | 9171.602 | 13587.61 | 23.326 | 59.702 | 1084.31 | 423.652 | 170.725 | 252.927 |
| 2033 | 58885 | 22786.258 | 8807.023 | 13979.23 | 23.740 | 61.349 | 1091.54 | 422.387 | 163.255 | 259.132 |
| 2034 | 59460 | 22806.106 | 8432.879 | 14373.23 | 24.173 | 63.024 | 1096.89 | 420.721 | 155.567 | 265.153 |
| 2035 | 58516 | 22262.998 | 7973.430 | 14289.57 | 24.420 | 64.185 | 1335.15 | 507.973 | 181.929 | 326.044 |
| 2036 | 56446 | 21140.051 | 7139.796 | 14000.26 | 24.803 | 66.226 | 1287.60 | 482.234 | 162.869 | 319.365 |
| 2037 | 56568 | 21011.845 | 6731.929 | 14279.92 | 25.244 | 67.961 | 1288.98 | 478.783 | 153.396 | 325.387 |
| 2038 | 56668 | 20787.483 | 6329.722 | 14457.76 | 25.513 | 69.550 | 1288.55 | 472.681 | 143.930 | 328.751 |
| 2039 | 56758 | 20709.267 | 5987.105 | 14722.16 | 25.939 | 71.090 | 1286.40 | 469.372 | 135.697 | 333.675 |

# Results for PHIM for Run 5 (Sensitivity_Analysis_1.RTF)

************************************************************************************

**Main Results for E-Component for Run: 5, Simulation: 1, COD: Four Diseases Combined, Adjusted**

| Variable Parameter | Value |
|---|---|
| 1. Country | US (United States) |
| 2. Sex | M (Males) |
| 3. Year of start of process | 1990 |
| 4. Cause of Death | Four Diseases Combined |
| 5. Effective Dose NULL | CC:1 |
| 6. Effective Dose Ecig | CC:1 Ecig:0.1 Dual:0.55 |
| Note: | Death rates are per million population |

--------------------------------------------------------------------------------------

Results for Age Groups Combined and Years combined, Population and Number deaths adjusted for Ecig scenario,

| Age Group:All | | NULL | Ecig | | | %Drop | | NULL | Ecig | |
|---|---|---|---|---|---|---|---|---|---|---|
| Year | Number Deaths | Attrib Deaths | Attrib Deaths | Drop Deaths | %Drop Deaths | Attrib Deaths | Death Rate | Attrib Rates | Attrib Rates | Drop Rates |
| All Comb | 13281485 | 4509390.583 | 3122577.274 | 1386813 | 10.442 | 30.754 | 3815.01 | 1305.37 | 896.937 | 408.434 |

--------------------------------------------------------------------------------------

Results for Age Groups Combined, Population and Number deaths adjusted for Ecig scenario,

| Age Group:All | | NULL | Ecig | | | %Drop | | NULL | Ecig | |
|---|---|---|---|---|---|---|---|---|---|---|
| Year | Number Deaths | Attrib Deaths | Attrib Deaths | Drop Deaths | %Drop Deaths | Attrib Deaths | Death Rate | Attrib Rates | Attrib Rates | Drop Rates |
| 1990 | 317161 | 125773.065 | 125773.065 | 0.000 | 0.000 | 0.000 | 5027.71 | 1993.78 | 1993.78 | 0.000 |
| 1991 | 313217 | 123999.442 | 123597.528 | 401.915 | 0.128 | 0.324 | 4883.81 | 1933.45 | 1927.18 | 6.267 |
| 1992 | 308580 | 122014.015 | 120940.605 | 1073.411 | 0.348 | 0.880 | 4729.74 | 1870.18 | 1853.71 | 16.465 |
| 1993 | 311722 | 122810.618 | 120799.023 | 2011.595 | 0.645 | 1.638 | 4692.65 | 1848.83 | 1818.50 | 30.325 |
| 1994 | 305722 | 119925.592 | 116840.776 | 3084.816 | 1.009 | 2.572 | 4516.27 | 1771.69 | 1726.03 | 45.666 |
| 1995 | 303265 | 117896.496 | 113559.728 | 4336.768 | 1.430 | 3.678 | 4393.94 | 1708.35 | 1645.34 | 63.006 |
| 1996 | 299625 | 115698.645 | 109987.068 | 5711.578 | 1.906 | 4.937 | 4273.39 | 1650.42 | 1568.69 | 81.734 |
| 1997 | 294324 | 113359.847 | 106269.325 | 7090.522 | 2.409 | 6.255 | 4129.02 | 1590.70 | 1490.83 | 99.869 |
| 1998 | 289396 | 111444.212 | 102973.777 | 8470.435 | 2.927 | 7.601 | 3992.69 | 1538.10 | 1420.69 | 117.407 |
| 1999 | 311206 | 114877.722 | 103563.162 | 11314.56 | 3.636 | 9.849 | 4225.36 | 1560.48 | 1406.12 | 154.366 |
| 2000 | 304055 | 112379.704 | 99496.744 | 12882.96 | 4.237 | 11.464 | 4067.22 | 1504.22 | 1330.93 | 173.291 |
| 2001 | 296633 | 110024.943 | 95459.861 | 14565.08 | 4.910 | 13.238 | 3915.54 | 1453.52 | 1260.07 | 193.453 |
| 2002 | 292319 | 108289.173 | 91841.432 | 16447.74 | 5.627 | 15.189 | 3814.58 | 1414.56 | 1198.47 | 216.086 |
| 2003 | 286374 | 106539.023 | 88435.416 | 18103.61 | 6.322 | 16.992 | 3697.27 | 1377.22 | 1141.76 | 235.463 |
| 2004 | 274768 | 103037.251 | 83723.112 | 19314.14 | 7.029 | 18.745 | 3508.34 | 1317.61 | 1069.01 | 248.604 |
| 2005 | 273076 | 103044.562 | 81985.148 | 21059.41 | 7.712 | 20.437 | 3445.34 | 1302.41 | 1034.39 | 268.025 |
| 2006 | 264910 | 99672.555 | 77129.419 | 22543.14 | 8.510 | 22.617 | 3310.79 | 1248.28 | 963.948 | 284.331 |
| 2007 | 259318 | 97839.152 | 74056.999 | 23782.15 | 9.171 | 24.307 | 3206.66 | 1212.75 | 915.771 | 296.981 |
| 2008 | 260495 | 98075.852 | 72726.724 | 25349.13 | 9.731 | 25.846 | 3184.10 | 1202.07 | 888.957 | 313.114 |
| 2009 | 255059 | 95617.338 | 69182.325 | 26435.01 | 10.364 | 27.647 | 3079.80 | 1158.11 | 835.368 | 322.746 |
| 2010 | 253163 | 94607.988 | 66956.437 | 27651.55 | 10.922 | 29.228 | 3018.74 | 1131.98 | 798.394 | 333.590 |
| 2011 | 253214 | 93633.119 | 64477.786 | 29155.33 | 11.514 | 31.138 | 2988.42 | 1109.25 | 760.963 | 348.286 |
| 2012 | 254243 | 92775.175 | 62149.190 | 30625.98 | 12.046 | 33.011 | 2966.96 | 1087.19 | 725.268 | 361.922 |
| 2013 | 257969 | 92345.780 | 60214.578 | 32131.20 | 12.455 | 34.794 | 2974.94 | 1069.82 | 694.404 | 375.411 |
| 2014 | 259377 | 91379.215 | 57997.107 | 33382.11 | 12.870 | 36.531 | 2955.05 | 1046.26 | 660.753 | 385.503 |
| 2015 | 261983 | 90750.242 | 56255.377 | 34494.86 | 13.167 | 38.011 | 3354.04 | 1168.90 | 720.210 | 448.692 |
| 2016 | 263865 | 88811.996 | 53157.263 | 35654.73 | 13.512 | 40.146 | 3344.94 | 1133.24 | 673.859 | 459.386 |
| 2017 | 266328 | 87325.126 | 50636.634 | 36688.49 | 13.776 | 42.014 | 3343.62 | 1104.08 | 635.718 | 468.361 |
| 2018 | 261509 | 87612.144 | 50065.737 | 37546.41 | 14.358 | 42.855 | 3251.09 | 1097.46 | 622.421 | 475.036 |
| 2019 | 264482 | 87797.760 | 48922.382 | 38875.38 | 14.699 | 44.278 | 3254.41 | 1089.08 | 601.984 | 487.098 |
| 2020 | 265594 | 87221.042 | 47602.784 | 39618.26 | 14.917 | 45.423 | 3725.81 | 1235.48 | 667.783 | 567.696 |
| 2021 | 258512 | 82001.292 | 42776.927 | 39224.36 | 15.173 | 47.834 | 3598.85 | 1153.39 | 595.517 | 557.873 |
| 2022 | 260784 | 81915.977 | 41458.985 | 40456.99 | 15.514 | 49.388 | 3600.60 | 1143.37 | 572.419 | 570.956 |
| 2023 | 263032 | 81810.810 | 40181.731 | 41629.08 | 15.827 | 50.885 | 3600.05 | 1132.64 | 549.957 | 582.688 |
| 2024 | 264943 | 81528.303 | 38908.319 | 42619.98 | 16.086 | 52.276 | 3594.00 | 1119.38 | 527.798 | 591.580 |
| 2025 | 261791 | 79599.213 | 37279.925 | 42319.29 | 16.165 | 53.165 | 4124.38 | 1272.09 | 587.326 | 684.761 |
| 2026 | 256141 | 75082.462 | 33504.465 | 41578.00 | 16.232 | 55.376 | 4016.02 | 1194.99 | 525.315 | 669.674 |
| 2027 | 255841 | 74444.425 | 32119.522 | 42324.90 | 16.543 | 56.854 | 3990.41 | 1179.46 | 500.977 | 678.484 |
| 2028 | 255379 | 73699.585 | 30659.821 | 43039.76 | 16.853 | 58.399 | 3960.39 | 1161.76 | 475.469 | 686.290 |
| 2029 | 254864 | 72819.703 | 29244.386 | 43575.32 | 17.097 | 59.840 | 3927.23 | 1141.36 | 450.631 | 690.726 |
| 2030 | 246051 | 69309.277 | 27444.491 | 41864.79 | 17.015 | 60.403 | 4517.67 | 1297.95 | 503.900 | 794.049 |
| 2031 | 241064 | 65433.368 | 24647.859 | 40785.51 | 16.919 | 62.331 | 4411.11 | 1222.18 | 451.018 | 771.163 |
| 2032 | 239855 | 64643.552 | 23449.785 | 41193.77 | 17.174 | 63.724 | 4370.68 | 1203.31 | 427.305 | 776.003 |
| 2033 | 238612 | 63793.514 | 22334.848 | 41458.67 | 17.375 | 64.989 | 4326.62 | 1182.54 | 404.985 | 777.551 |
| 2034 | 237227 | 63032.151 | 21276.599 | 41755.55 | 17.602 | 66.245 | 4277.60 | 1162.80 | 383.652 | 779.148 |
| 2035 | 222096 | 57653.301 | 19474.494 | 38178.81 | 17.190 | 66.221 | 4938.46 | 1315.47 | 433.029 | 882.441 |
| 2036 | 214386 | 52988.733 | 16663.570 | 36325.16 | 16.944 | 68.553 | 4761.83 | 1208.75 | 370.123 | 838.623 |
| 2037 | 211825 | 52026.921 | 15698.010 | 36328.91 | 17.150 | 69.827 | 4696.09 | 1185.50 | 348.019 | 837.484 |
| 2038 | 209295 | 50943.211 | 14740.636 | 36202.58 | 17.297 | 71.065 | 4626.70 | 1158.38 | 325.858 | 832.525 |
| 2039 | 206836 | 50085.991 | 13936.392 | 36149.60 | 17.477 | 72.175 | 4554.00 | 1135.19 | 306.844 | 828.347 |

# Results for PHIM for Run 5 (Sensitivity_Analysis_1.RTF)

************************************************************************************

**Main Results for E-Component for Run: 5, Simulation: 1, COD: LUNG CANCER, Adjusted**

| **Variable Parameter** | **Value** |
|---|---|
| 1. Country | US (United States) |
| 2. Sex | M (Males) |
| 3. Year of start of process | 1990 |
| 4. Cause of Death | LUNG CANCER |
| 5. Effective Dose NULL | CC:1 |
| 6. Effective Dose Ecig | CC:1 Ecig:0.1 Dual:0.55 |
| Note: | Death rates are per million population |

------------------------------------------------------------------------------------------

Results for Age Groups Combined and Years combined, Population and Number deaths adjusted for Ecig scenario,

| Age Group:All | | NULL | Ecig | | | %Drop | | NULL | Ecig | |
|---|---|---|---|---|---|---|---|---|---|---|
| Year | Number Deaths | Attrib Deaths | Attrib Deaths | Drop Deaths | %Drop Deaths | Attrib Deaths | Death Rate | Attrib Rates | Attrib Rates | Drop Rates |
| All Comb | 3093654 | 2244988.113 | 1786344.471 | 458643.6 | 14.825 | 20.430 | 888.629 | 649.875 | 513.114 | 136.761 |

------------------------------------------------------------------------------------------

Results for Age Groups Combined, Population and Number deaths adjusted for Ecig scenario,

| Age Group:All | | NULL | Ecig | | | %Drop | | NULL | Ecig | |
|---|---|---|---|---|---|---|---|---|---|---|
| Year | Number Deaths | Attrib Deaths | Attrib Deaths | Drop Deaths | %Drop Deaths | Attrib Deaths | Death Rate | Attrib Rates | Attrib Rates | Drop Rates |
| 1990 | 78822 | 63385.736 | 63385.736 | 0.000 | 0.000 | 0.000 | 1249.50 | 1004.81 | 1004.81 | 0.000 |
| 1991 | 78462 | 62918.402 | 62872.703 | 45.699 | 0.058 | 0.073 | 1223.41 | 981.049 | 980.337 | 0.713 |
| 1992 | 77988 | 62366.766 | 62245.001 | 121.765 | 0.156 | 0.195 | 1195.35 | 955.930 | 954.057 | 1.872 |
| 1993 | 78544 | 62652.888 | 62414.288 | 238.599 | 0.304 | 0.381 | 1182.40 | 943.195 | 939.581 | 3.614 |
| 1994 | 77443 | 61565.227 | 61190.865 | 374.361 | 0.483 | 0.608 | 1144.02 | 909.520 | 903.941 | 5.579 |
| 1995 | 76915 | 60851.961 | 60309.425 | 542.536 | 0.705 | 0.892 | 1114.40 | 881.759 | 873.809 | 7.949 |
| 1996 | 76249 | 60122.922 | 59369.213 | 753.708 | 0.988 | 1.254 | 1087.51 | 857.643 | 846.751 | 10.892 |
| 1997 | 75499 | 59237.155 | 58258.209 | 978.946 | 1.297 | 1.653 | 1059.17 | 831.234 | 817.293 | 13.941 |
| 1998 | 75419 | 58906.696 | 57647.997 | 1258.699 | 1.669 | 2.137 | 1040.52 | 813.001 | 795.347 | 17.653 |
| 1999 | 73209 | 56930.073 | 55399.259 | 1530.814 | 2.091 | 2.689 | 993.986 | 773.329 | 752.176 | 21.153 |
| 2000 | 73852 | 57137.121 | 55254.434 | 1882.687 | 2.549 | 3.295 | 987.887 | 764.787 | 739.115 | 25.672 |
| 2001 | 73381 | 56481.726 | 54200.315 | 2281.412 | 3.109 | 4.039 | 968.629 | 746.169 | 715.442 | 30.728 |
| 2002 | 72551 | 55588.365 | 52891.169 | 2697.196 | 3.718 | 4.852 | 946.741 | 726.138 | 690.195 | 35.943 |
| 2003 | 72075 | 54945.856 | 51789.900 | 3155.956 | 4.379 | 5.744 | 930.533 | 710.281 | 668.641 | 41.640 |
| 2004 | 71460 | 54203.126 | 50540.282 | 3662.844 | 5.126 | 6.758 | 912.430 | 693.135 | 645.318 | 47.817 |
| 2005 | 71152 | 53744.603 | 49562.246 | 4182.357 | 5.878 | 7.782 | 897.708 | 679.296 | 625.316 | 53.980 |
| 2006 | 69874 | 52535.572 | 47807.259 | 4728.313 | 6.767 | 9.000 | 873.270 | 657.945 | 597.486 | 60.459 |
| 2007 | 68811 | 51492.124 | 46227.326 | 5264.798 | 7.651 | 10.224 | 850.902 | 638.264 | 571.636 | 66.628 |
| 2008 | 68506 | 51030.736 | 45116.926 | 5913.809 | 8.633 | 11.589 | 837.369 | 625.460 | 551.475 | 73.985 |
| 2009 | 67824 | 50276.199 | 43713.686 | 6562.513 | 9.676 | 13.053 | 818.967 | 608.943 | 527.837 | 81.106 |
| 2010 | 67549 | 49840.933 | 42631.525 | 7209.408 | 10.673 | 14.465 | 805.462 | 596.347 | 508.342 | 88.005 |
| 2011 | 66897 | 49072.841 | 41148.281 | 7924.559 | 11.846 | 16.149 | 789.509 | 581.354 | 485.630 | 95.724 |
| 2012 | 66586 | 48539.512 | 39848.528 | 8690.983 | 13.052 | 17.905 | 777.039 | 568.812 | 465.024 | 103.788 |
| 2013 | 65803 | 47675.741 | 38307.790 | 9367.951 | 14.236 | 19.649 | 758.849 | 552.318 | 441.772 | 110.547 |
| 2014 | 65548 | 47194.534 | 37121.017 | 10073.52 | 15.368 | 21.345 | 746.783 | 540.359 | 422.915 | 117.444 |
| 2015 | 64702 | 46305.842 | 35625.329 | 10680.51 | 16.507 | 23.065 | 828.341 | 596.439 | 456.093 | 140.346 |
| 2016 | 62201 | 44208.867 | 33183.682 | 11025.18 | 17.725 | 24.939 | 788.497 | 564.107 | 420.660 | 143.447 |
| 2017 | 60512 | 42702.624 | 31264.576 | 11438.05 | 18.902 | 26.785 | 759.697 | 539.903 | 392.512 | 147.391 |
| 2018 | 63160 | 44321.202 | 31486.136 | 12835.07 | 20.322 | 28.959 | 785.203 | 555.181 | 391.438 | 163.743 |
| 2019 | 63797 | 44493.088 | 30731.668 | 13761.42 | 21.571 | 30.929 | 785.011 | 551.912 | 378.149 | 173.762 |
| 2020 | 64281 | 44529.551 | 29905.614 | 14623.94 | 22.750 | 32.841 | 901.752 | 630.757 | 419.523 | 211.234 |
| 2021 | 57900 | 39852.556 | 25927.563 | 13924.99 | 24.050 | 34.941 | 806.047 | 560.546 | 360.949 | 199.597 |
| 2022 | 58283 | 39853.685 | 25064.576 | 14789.11 | 25.375 | 37.109 | 804.705 | 556.274 | 346.063 | 210.211 |
| 2023 | 58639 | 39847.050 | 24212.251 | 15634.80 | 26.663 | 39.237 | 802.576 | 551.670 | 331.387 | 220.283 |
| 2024 | 58902 | 39726.010 | 23342.914 | 16383.10 | 27.814 | 41.240 | 799.010 | 545.435 | 316.650 | 228.785 |
| 2025 | 58538 | 39207.348 | 22327.571 | 16879.78 | 28.836 | 43.053 | 922.234 | 626.579 | 351.759 | 274.819 |
| 2026 | 52390 | 34803.960 | 19115.631 | 15688.33 | 29.945 | 45.076 | 821.416 | 553.929 | 299.713 | 254.216 |
| 2027 | 52181 | 34484.595 | 18218.552 | 16266.04 | 31.173 | 47.169 | 813.873 | 546.357 | 284.159 | 262.197 |
| 2028 | 51925 | 34100.638 | 17293.857 | 16806.78 | 32.367 | 49.286 | 805.252 | 537.544 | 268.191 | 269.352 |
| 2029 | 51642 | 33669.838 | 16404.842 | 17265.00 | 33.432 | 51.277 | 795.756 | 527.732 | 252.784 | 274.948 |
| 2030 | 50249 | 32557.688 | 15367.469 | 17190.22 | 34.210 | 52.799 | 922.612 | 609.705 | 282.158 | 327.548 |
| 2031 | 44640 | 28646.393 | 13017.570 | 15628.82 | 35.011 | 54.558 | 816.848 | 535.065 | 238.202 | 296.863 |
| 2032 | 44254 | 28227.160 | 12307.227 | 15919.93 | 35.974 | 56.399 | 806.404 | 525.435 | 224.264 | 301.171 |
| 2033 | 43852 | 27786.623 | 11649.857 | 16136.77 | 36.798 | 58.074 | 795.151 | 515.079 | 211.240 | 303.839 |
| 2034 | 43417 | 27348.299 | 11028.913 | 16319.39 | 37.587 | 59.672 | 782.882 | 504.514 | 198.870 | 305.645 |
| 2035 | 40913 | 25570.077 | 10083.707 | 15486.37 | 37.852 | 60.564 | 909.732 | 583.430 | 224.219 | 359.212 |
| 2036 | 35047 | 21717.319 | 8106.466 | 13610.85 | 38.836 | 62.673 | 778.441 | 495.402 | 180.057 | 315.345 |
| 2037 | 34488 | 21256.024 | 7602.310 | 13653.71 | 39.590 | 64.235 | 764.581 | 484.347 | 168.540 | 315.807 |
| 2038 | 33933 | 20747.175 | 7113.643 | 13633.53 | 40.177 | 65.713 | 750.137 | 471.764 | 157.255 | 314.509 |
| 2039 | 33391 | 20327.688 | 6709.166 | 13618.52 | 40.785 | 66.995 | 735.180 | 460.724 | 147.719 | 313.005 |

# Results for PHIM for Run 5 (Sensitivity_Analysis_1.RTF)

************************************************************************************

**Main Results for E-Component for Run: 5, Simulation: 1, COD: IHD, Adjusted**

| Variable Parameter | Value |
|---|---|
| 1. Country | US (United States) |
| 2. Sex | M (Males) |
| 3. Year of start of process | 1990 |
| 4. Cause of Death | IHD |
| 5. Effective Dose NULL | CC:1 |
| 6. Effective Dose Ecig | CC:1 Ecig:0.1 Dual:0.55 |
| Note: | Death rates are per million population |

-----------------------------------------------------------------------------------------

Results for Age Groups Combined and Years combined, Population and Number deaths adjusted for Ecig scenario,

| Age Group:All Year | Number Deaths | NULL Attrib Deaths | Ecig Attrib Deaths | Drop Deaths | %Drop Deaths | %Drop Attrib Deaths | Death Rate | NULL Attrib Rates | Ecig Attrib Rates | Drop Rates |
|---|---|---|---|---|---|---|---|---|---|---|
| All Comb | 6418622 | 1098877.421 | 555151.097 | 543726.3 | 8.471 | 49.480 | 1843.70 | 318.101 | 159.463 | 158.638 |

-----------------------------------------------------------------------------------------

Results for Age Groups Combined, Population and Number deaths adjusted for Ecig scenario,

| Age Group:All Year | Number Deaths | NULL Attrib Deaths | Ecig Attrib Deaths | Drop Deaths | %Drop Deaths | %Drop Attrib Deaths | Death Rate | NULL Attrib Rates | Ecig Attrib Rates | Drop Rates |
|---|---|---|---|---|---|---|---|---|---|---|
| 1990 | 171484 | 38511.725 | 38511.725 | 0.000 | 0.000 | 0.000 | 2718.40 | 610.497 | 610.497 | 0.000 |
| 1991 | 168044 | 37309.838 | 37012.736 | 297.102 | 0.177 | 0.796 | 2620.21 | 581.750 | 577.118 | 4.633 |
| 1992 | 164549 | 36137.488 | 35340.033 | 797.455 | 0.485 | 2.207 | 2522.12 | 553.899 | 541.673 | 12.226 |
| 1993 | 164367 | 35599.588 | 34126.027 | 1473.561 | 0.897 | 4.139 | 2474.37 | 535.926 | 513.731 | 22.195 |
| 1994 | 159877 | 34336.757 | 32092.515 | 2244.242 | 1.404 | 6.536 | 2361.79 | 507.267 | 474.086 | 33.180 |
| 1995 | 158104 | 33455.759 | 30324.459 | 3131.300 | 1.981 | 9.360 | 2290.73 | 484.782 | 439.364 | 45.417 |
| 1996 | 154909 | 32193.824 | 28123.938 | 4069.886 | 2.627 | 12.642 | 2209.38 | 459.239 | 401.117 | 58.123 |
| 1997 | 150020 | 30665.509 | 25688.537 | 4976.972 | 3.318 | 16.230 | 2104.60 | 430.308 | 360.380 | 69.929 |
| 1998 | 145476 | 29188.430 | 23364.458 | 5823.973 | 4.003 | 19.953 | 2007.08 | 402.844 | 322.350 | 80.494 |
| 1999 | 166061 | 33375.386 | 25282.245 | 8093.141 | 4.874 | 24.249 | 2254.66 | 453.366 | 343.266 | 110.100 |
| 2000 | 160401 | 32024.754 | 22975.509 | 9049.245 | 5.642 | 28.257 | 2145.62 | 428.655 | 307.334 | 121.322 |
| 2001 | 154620 | 30682.393 | 20637.837 | 10044.56 | 6.496 | 32.737 | 2040.97 | 405.339 | 272.418 | 132.921 |
| 2002 | 152105 | 30218.370 | 19007.952 | 11210.42 | 7.370 | 37.098 | 1984.87 | 394.736 | 248.041 | 146.694 |
| 2003 | 146993 | 29175.202 | 17108.013 | 12067.19 | 8.209 | 41.361 | 1897.77 | 377.146 | 220.876 | 156.270 |
| 2004 | 138677 | 27488.687 | 14976.462 | 12512.23 | 9.023 | 45.518 | 1770.68 | 351.518 | 191.225 | 160.293 |
| 2005 | 136226 | 27017.439 | 13701.731 | 13315.71 | 9.775 | 49.286 | 1718.73 | 341.482 | 172.872 | 168.610 |
| 2006 | 132314 | 26231.435 | 12233.138 | 13998.30 | 10.580 | 53.365 | 1653.63 | 328.517 | 152.887 | 175.630 |
| 2007 | 126937 | 25103.049 | 10815.967 | 14287.08 | 11.255 | 56.914 | 1569.68 | 311.161 | 133.748 | 177.414 |
| 2008 | 126355 | 24621.740 | 9885.922 | 14735.82 | 11.662 | 59.849 | 1544.47 | 301.777 | 120.838 | 180.939 |
| 2009 | 122977 | 23741.177 | 8858.758 | 14882.42 | 12.102 | 62.686 | 1484.93 | 287.552 | 106.968 | 180.584 |
| 2010 | 121143 | 23189.160 | 8070.045 | 15119.12 | 12.480 | 65.199 | 1444.52 | 277.458 | 96.228 | 181.230 |
| 2011 | 120895 | 22786.927 | 7338.040 | 15448.89 | 12.779 | 67.797 | 1426.80 | 269.951 | 86.603 | 183.348 |
| 2012 | 121681 | 22485.295 | 6741.605 | 15743.69 | 12.938 | 70.018 | 1419.99 | 263.495 | 78.673 | 184.822 |
| 2013 | 123493 | 22251.884 | 6239.836 | 16012.05 | 12.966 | 71.958 | 1424.15 | 257.786 | 71.959 | 185.827 |
| 2014 | 124163 | 21894.593 | 5753.683 | 16140.91 | 13.000 | 73.721 | 1414.58 | 250.685 | 65.551 | 185.134 |
| 2015 | 124805 | 21471.109 | 5325.280 | 16145.83 | 12.937 | 75.198 | 1597.82 | 276.557 | 68.177 | 208.380 |
| 2016 | 127363 | 21411.568 | 4967.329 | 16444.24 | 12.911 | 76.801 | 1614.55 | 273.213 | 62.969 | 210.243 |
| 2017 | 129530 | 21169.399 | 4626.226 | 16543.17 | 12.772 | 78.147 | 1626.19 | 267.651 | 58.080 | 209.571 |
| 2018 | 122710 | 19830.576 | 4065.395 | 15765.18 | 12.848 | 79.499 | 1525.53 | 248.404 | 50.541 | 197.862 |
| 2019 | 123216 | 19431.014 | 3791.779 | 15639.24 | 12.693 | 80.486 | 1516.16 | 241.031 | 46.657 | 194.373 |
| 2020 | 122301 | 18532.612 | 3458.026 | 15074.59 | 12.326 | 81.341 | 1715.66 | 262.513 | 48.510 | 214.003 |
| 2021 | 120970 | 18234.128 | 3213.209 | 15020.92 | 12.417 | 82.378 | 1684.08 | 256.472 | 44.733 | 211.740 |
| 2022 | 121103 | 17812.559 | 2990.413 | 14822.15 | 12.239 | 83.212 | 1672.05 | 248.626 | 41.288 | 207.338 |
| 2023 | 121215 | 17361.668 | 2785.251 | 14576.42 | 12.025 | 83.957 | 1659.04 | 240.367 | 38.121 | 202.246 |
| 2024 | 121181 | 16929.676 | 2618.685 | 14310.99 | 11.810 | 84.532 | 1643.84 | 232.443 | 35.523 | 196.920 |
| 2025 | 117655 | 15501.666 | 2326.813 | 13174.85 | 11.198 | 84.990 | 1853.60 | 247.735 | 36.658 | 211.077 |
| 2026 | 117120 | 15523.814 | 2250.026 | 13273.79 | 11.334 | 85.506 | 1836.31 | 247.072 | 35.278 | 211.794 |
| 2027 | 116123 | 15073.070 | 2126.152 | 12946.92 | 11.149 | 85.894 | 1811.20 | 238.810 | 33.162 | 205.648 |
| 2028 | 115076 | 14636.710 | 2000.279 | 12636.43 | 10.981 | 86.334 | 1784.59 | 230.725 | 31.020 | 199.705 |
| 2029 | 114027 | 14180.166 | 1892.326 | 12287.84 | 10.776 | 86.655 | 1757.06 | 222.256 | 29.159 | 193.097 |
| 2030 | 107203 | 12111.772 | 1587.456 | 10524.32 | 9.817 | 86.893 | 1968.32 | 226.816 | 29.147 | 197.669 |
| 2031 | 107728 | 12417.367 | 1603.446 | 10813.92 | 10.038 | 87.087 | 1971.26 | 231.935 | 29.341 | 202.594 |
| 2032 | 106390 | 12054.064 | 1524.574 | 10529.49 | 9.897 | 87.352 | 1938.65 | 224.381 | 27.781 | 196.600 |
| 2033 | 105066 | 11672.389 | 1441.750 | 10230.64 | 9.737 | 87.648 | 1905.10 | 216.370 | 26.142 | 190.228 |
| 2034 | 103717 | 11365.762 | 1386.592 | 9979.169 | 9.622 | 87.800 | 1870.19 | 209.673 | 25.003 | 184.670 |
| 2035 | 93404 | 8580.360 | 1026.089 | 7554.271 | 8.088 | 88.041 | 2076.89 | 195.777 | 22.816 | 172.962 |
| 2036 | 94789 | 8948.841 | 1053.320 | 7895.521 | 8.330 | 88.230 | 2105.40 | 204.135 | 23.396 | 180.740 |
| 2037 | 93034 | 8624.570 | 1010.677 | 7613.893 | 8.184 | 88.281 | 2062.53 | 196.522 | 22.406 | 174.116 |
| 2038 | 91331 | 8315.017 | 957.185 | 7357.832 | 8.056 | 88.488 | 2018.96 | 189.073 | 21.160 | 167.913 |
| 2039 | 89693 | 8001.132 | 911.648 | 7089.484 | 7.904 | 88.606 | 1974.82 | 181.344 | 20.072 | 161.272 |

# Results for PHIM for Run 5 (Sensitivity_Analysis_1.RTF)

************************************************************************************

**Main Results for E-Component for Run: 5, Simulation: 1, COD: STROKE, Adjusted**

| Variable Parameter | Value |
|---|---|
| 1. Country | US (United States) |
| 2. Sex | M (Males) |
| 3. Year of start of process | 1990 |
| 4. Cause of Death | STROKE |
| 5. Effective Dose NULL | CC:1 |
| 6. Effective Dose Ecig | CC:1 Ecig:0.1 Dual:0.55 |
| Note: | Death rates are per million population |

------------------------------------------------------------------------------------

Results for Age Groups Combined and Years combined, Population and Number deaths adjusted for Ecig scenario,

| Age Group:All | | NULL | Ecig | | | %Drop | | NULL | Ecig | |
|---|---|---|---|---|---|---|---|---|---|---|
| Year | Number | Attrib | Attrib | Drop | %Drop | Attrib | Death | Attrib | Attrib | Drop |
| | Deaths | Deaths | Deaths | Deaths | Deaths | Deaths | Rate | Rates | Rates | Rates |
| All Comb | 1505606 | 149918.462 | 74652.525 | 75265.94 | 4.999 | 50.205 | 432.474 | 43.398 | 21.443 | 21.955 |

------------------------------------------------------------------------------------

Results for Age Groups Combined, Population and Number deaths adjusted for Ecig scenario,

| Age Group:All | | NULL | Ecig | | | %Drop | | NULL | Ecig | |
|---|---|---|---|---|---|---|---|---|---|---|
| Year | Number | Attrib | Attrib | Drop | %Drop | Attrib | Death | Attrib | Attrib | Drop |
| | Deaths | Deaths | Deaths | Deaths | Deaths | Deaths | Rate | Rates | Rates | Rates |
| 1990 | 32862 | 4613.166 | 4613.166 | 0.000 | 0.000 | 0.000 | 520.936 | 73.129 | 73.129 | 0.000 |
| 1991 | 32619 | 4555.566 | 4517.565 | 38.001 | 0.116 | 0.834 | 508.609 | 71.032 | 70.440 | 0.593 |
| 1992 | 32100 | 4453.572 | 4354.179 | 99.393 | 0.310 | 2.232 | 492.015 | 68.262 | 66.738 | 1.524 |
| 1993 | 32897 | 4462.473 | 4274.899 | 187.575 | 0.570 | 4.203 | 495.229 | 67.179 | 64.354 | 2.825 |
| 1994 | 33325 | 4499.513 | 4203.893 | 295.620 | 0.887 | 6.570 | 492.297 | 66.473 | 62.102 | 4.371 |
| 1995 | 33573 | 4472.774 | 4054.067 | 418.707 | 1.247 | 9.361 | 486.435 | 64.812 | 58.738 | 6.073 |
| 1996 | 33877 | 4406.878 | 3852.225 | 554.653 | 1.637 | 12.586 | 483.171 | 62.863 | 54.942 | 7.921 |
| 1997 | 33527 | 4288.158 | 3595.671 | 692.487 | 2.065 | 16.149 | 470.339 | 60.173 | 50.443 | 9.730 |
| 1998 | 32810 | 4120.148 | 3302.009 | 818.139 | 2.494 | 19.857 | 452.666 | 56.864 | 45.557 | 11.308 |
| 1999 | 33396 | 3982.443 | 3039.113 | 943.329 | 2.825 | 23.687 | 453.427 | 54.097 | 41.263 | 12.834 |
| 2000 | 33487 | 4021.411 | 2913.943 | 1107.469 | 3.307 | 27.539 | 447.945 | 53.827 | 38.979 | 14.849 |
| 2001 | 32310 | 3848.092 | 2630.253 | 1217.839 | 3.769 | 31.648 | 426.494 | 50.836 | 34.719 | 16.117 |
| 2002 | 31630 | 3783.081 | 2436.512 | 1346.569 | 4.257 | 35.595 | 412.747 | 49.418 | 31.795 | 17.623 |
| 2003 | 31038 | 3754.415 | 2265.605 | 1488.810 | 4.797 | 39.655 | 400.717 | 48.533 | 29.250 | 19.283 |
| 2004 | 29946 | 3666.274 | 2069.798 | 1596.476 | 5.331 | 43.545 | 382.360 | 46.883 | 26.428 | 20.455 |
| 2005 | 28779 | 3589.688 | 1890.781 | 1698.907 | 5.903 | 47.327 | 363.096 | 45.371 | 23.856 | 21.516 |
| 2006 | 28270 | 3596.985 | 1757.469 | 1839.516 | 6.507 | 51.141 | 353.309 | 45.048 | 21.964 | 23.083 |
| 2007 | 28120 | 3560.798 | 1617.015 | 1943.783 | 6.912 | 54.588 | 347.728 | 44.137 | 19.996 | 24.142 |
| 2008 | 27233 | 3417.001 | 1443.645 | 1973.357 | 7.246 | 57.751 | 332.870 | 41.881 | 17.646 | 24.235 |
| 2009 | 27187 | 3394.390 | 1334.924 | 2059.466 | 7.575 | 60.673 | 328.276 | 41.113 | 16.119 | 24.994 |
| 2010 | 27044 | 3340.137 | 1228.122 | 2112.015 | 7.810 | 63.231 | 322.476 | 39.965 | 14.644 | 25.320 |
| 2011 | 27108 | 3285.435 | 1126.977 | 2158.458 | 7.962 | 65.698 | 319.933 | 38.922 | 13.301 | 25.621 |
| 2012 | 27224 | 3259.209 | 1037.682 | 2221.527 | 8.160 | 68.162 | 317.694 | 38.193 | 12.110 | 26.084 |
| 2013 | 28095 | 3274.703 | 972.394 | 2302.308 | 8.195 | 70.306 | 323.992 | 37.937 | 11.214 | 26.723 |
| 2014 | 29008 | 3298.083 | 912.623 | 2385.460 | 8.223 | 72.329 | 330.487 | 37.762 | 10.397 | 27.364 |
| 2015 | 29805 | 3244.406 | 845.086 | 2399.320 | 8.050 | 73.953 | 381.584 | 41.789 | 10.819 | 30.970 |
| 2016 | 30788 | 3314.836 | 809.286 | 2505.550 | 8.138 | 75.586 | 390.297 | 42.297 | 10.259 | 32.038 |
| 2017 | 31724 | 3333.314 | 763.804 | 2569.510 | 8.100 | 77.086 | 398.283 | 42.144 | 9.589 | 32.555 |
| 2018 | 29663 | 2925.242 | 634.578 | 2290.664 | 7.722 | 78.307 | 368.774 | 36.642 | 7.889 | 28.753 |
| 2019 | 29941 | 2864.628 | 589.409 | 2275.219 | 7.599 | 79.425 | 368.416 | 35.534 | 7.253 | 28.281 |
| 2020 | 29964 | 2730.366 | 535.114 | 2195.252 | 7.326 | 80.401 | 420.342 | 38.675 | 7.507 | 31.169 |
| 2021 | 30503 | 2647.911 | 493.002 | 2154.908 | 7.064 | 81.381 | 424.653 | 37.244 | 6.863 | 30.381 |
| 2022 | 30740 | 2586.165 | 456.864 | 2129.301 | 6.927 | 82.334 | 424.424 | 36.097 | 6.308 | 29.790 |
| 2023 | 30973 | 2517.960 | 424.318 | 2093.642 | 6.760 | 83.148 | 423.922 | 34.860 | 5.808 | 29.053 |
| 2024 | 31160 | 2454.161 | 397.195 | 2056.965 | 6.601 | 83.815 | 422.695 | 33.695 | 5.388 | 28.307 |
| 2025 | 30784 | 2275.364 | 354.831 | 1920.533 | 6.239 | 84.406 | 484.979 | 36.363 | 5.590 | 30.773 |
| 2026 | 31255 | 2183.166 | 328.631 | 1854.535 | 5.933 | 84.947 | 490.052 | 34.747 | 5.153 | 29.594 |
| 2027 | 31160 | 2112.345 | 308.258 | 1804.086 | 5.790 | 85.407 | 486.010 | 33.467 | 4.808 | 28.659 |
| 2028 | 31039 | 2046.677 | 289.354 | 1757.323 | 5.662 | 85.862 | 481.355 | 32.263 | 4.487 | 27.775 |
| 2029 | 30911 | 1977.671 | 272.043 | 1705.628 | 5.518 | 86.244 | 476.313 | 30.997 | 4.192 | 26.806 |
| 2030 | 30010 | 1744.457 | 234.419 | 1510.038 | 5.032 | 86.562 | 551.013 | 32.668 | 4.304 | 28.364 |
| 2031 | 29681 | 1653.738 | 217.493 | 1436.244 | 4.839 | 86.848 | 543.114 | 30.889 | 3.980 | 26.909 |
| 2032 | 29455 | 1603.120 | 204.748 | 1398.372 | 4.747 | 87.228 | 536.732 | 29.841 | 3.731 | 26.110 |
| 2033 | 29220 | 1548.245 | 193.461 | 1354.784 | 4.637 | 87.505 | 529.823 | 28.700 | 3.508 | 25.192 |
| 2034 | 28959 | 1511.985 | 185.270 | 1326.715 | 4.581 | 87.747 | 522.178 | 27.893 | 3.341 | 24.552 |
| 2035 | 27525 | 1239.866 | 149.668 | 1090.198 | 3.961 | 87.929 | 612.039 | 28.290 | 3.328 | 24.962 |
| 2036 | 26373 | 1182.522 | 140.047 | 1042.475 | 3.953 | 88.157 | 585.778 | 26.975 | 3.111 | 23.864 |
| 2037 | 25938 | 1134.483 | 134.191 | 1000.292 | 3.856 | 88.172 | 575.045 | 25.851 | 2.975 | 22.876 |
| 2038 | 25501 | 1093.537 | 126.882 | 966.655 | 3.791 | 88.397 | 563.736 | 24.866 | 2.805 | 22.061 |
| 2039 | 25067 | 1047.905 | 120.043 | 927.862 | 3.702 | 88.544 | 551.910 | 23.751 | 2.643 | 21.108 |

# Results for PHIM for Run 5 (Sensitivity_Analysis_1.RTF)

************************************************************************************

**Main Results for E-Component for Run: 5, Simulation: 1, COD: COPD, Adjusted**

| Variable Parameter | Value |
|---|---|
| 1. Country | US (United States) |
| 2. Sex | M (Males) |
| 3. Year of start of process | 1990 |
| 4. Cause of Death | COPD |
| 5. Effective Dose NULL | CC:1 |
| 6. Effective Dose Ecig | CC:1 Ecig:0.1 Dual:0.55 |
| Note: | Death rates are per million population |

----------------------------------------------------------------------------------------

Results for Age Groups Combined and Years combined, Population and Number deaths adjusted for Ecig scenario,

| Age Group:All Year | Number Deaths | NULL Attrib Deaths | Ecig Attrib Deaths | Drop Deaths | %Drop Deaths | %Drop Attrib Deaths | Death Rate | NULL Attrib Rates | Ecig Attrib Rates | Drop Rates |
|---|---|---|---|---|---|---|---|---|---|---|
| All Comb | 2263604 | 1015606.587 | 706429.182 | 309177.4 | 13.659 | 30.443 | 650.204 | 293.996 | 202.917 | 91.080 |

----------------------------------------------------------------------------------------

Results for Age Groups Combined, Population and Number deaths adjusted for Ecig scenario,

| Age Group:All Year | Number Deaths | NULL Attrib Deaths | Ecig Attrib Deaths | Drop Deaths | %Drop Deaths | %Drop Attrib Deaths | Death Rate | NULL Attrib Rates | Ecig Attrib Rates | Drop Rates |
|---|---|---|---|---|---|---|---|---|---|---|
| 1990 | 33993 | 19262.437 | 19262.437 | 0.000 | 0.000 | 0.000 | 538.865 | 305.353 | 305.353 | 0.000 |
| 1991 | 34092 | 19215.636 | 19194.523 | 21.113 | 0.062 | 0.110 | 531.576 | 299.618 | 299.289 | 0.329 |
| 1992 | 33942 | 19056.189 | 19001.392 | 54.798 | 0.161 | 0.288 | 520.249 | 292.085 | 291.243 | 0.842 |
| 1993 | 35914 | 20095.670 | 19983.809 | 111.861 | 0.311 | 0.557 | 540.648 | 302.526 | 300.835 | 1.691 |
| 1994 | 35076 | 19524.095 | 19353.502 | 170.592 | 0.486 | 0.874 | 518.166 | 288.435 | 285.899 | 2.536 |
| 1995 | 34673 | 19116.002 | 18871.777 | 244.225 | 0.704 | 1.278 | 502.376 | 276.995 | 273.429 | 3.566 |
| 1996 | 34589 | 18975.021 | 18641.691 | 333.330 | 0.964 | 1.757 | 493.329 | 270.675 | 265.876 | 4.799 |
| 1997 | 35278 | 19169.025 | 18726.907 | 442.117 | 1.253 | 2.306 | 494.914 | 268.986 | 262.716 | 6.270 |
| 1998 | 35692 | 19228.937 | 18659.313 | 569.625 | 1.596 | 2.962 | 492.423 | 265.388 | 257.435 | 7.953 |
| 1999 | 38541 | 20589.820 | 19842.544 | 747.276 | 1.939 | 3.629 | 523.281 | 279.689 | 269.410 | 10.279 |
| 2000 | 36314 | 19196.417 | 18352.858 | 843.560 | 2.323 | 4.394 | 485.762 | 256.946 | 245.498 | 11.448 |
| 2001 | 36322 | 19012.731 | 17991.456 | 1021.275 | 2.812 | 5.372 | 479.443 | 251.174 | 237.486 | 13.687 |
| 2002 | 36034 | 18699.357 | 17505.800 | 1193.558 | 3.312 | 6.383 | 470.218 | 244.265 | 228.439 | 15.826 |
| 2003 | 36269 | 18663.550 | 17271.899 | 1391.651 | 3.837 | 7.457 | 468.252 | 241.262 | 222.991 | 18.271 |
| 2004 | 34685 | 17679.164 | 16136.570 | 1542.594 | 4.447 | 8.725 | 442.868 | 226.076 | 206.038 | 20.038 |
| 2005 | 36920 | 18692.832 | 16830.390 | 1862.442 | 5.045 | 9.963 | 465.807 | 236.265 | 212.345 | 23.919 |
| 2006 | 34453 | 17308.564 | 15331.554 | 1977.010 | 5.738 | 11.422 | 430.583 | 216.769 | 191.611 | 25.158 |
| 2007 | 35449 | 17683.181 | 15396.691 | 2286.490 | 6.450 | 12.930 | 438.354 | 219.189 | 190.392 | 28.798 |
| 2008 | 38401 | 19006.374 | 16280.231 | 2726.143 | 7.099 | 14.343 | 469.387 | 232.952 | 198.997 | 33.955 |
| 2009 | 37071 | 18205.573 | 15274.957 | 2930.616 | 7.905 | 16.097 | 447.626 | 220.505 | 184.443 | 36.062 |
| 2010 | 37427 | 18237.758 | 15026.745 | 3211.013 | 8.580 | 17.606 | 446.277 | 218.215 | 179.180 | 39.035 |
| 2011 | 38314 | 18487.916 | 14864.488 | 3623.428 | 9.457 | 19.599 | 452.175 | 219.022 | 175.430 | 43.592 |
| 2012 | 38752 | 18491.158 | 14521.374 | 3969.785 | 10.244 | 21.469 | 452.231 | 216.689 | 169.461 | 47.228 |
| 2013 | 40578 | 19143.452 | 14694.558 | 4448.895 | 10.964 | 23.240 | 467.956 | 221.775 | 169.460 | 52.315 |
| 2014 | 40657 | 18992.005 | 14209.785 | 4782.220 | 11.762 | 25.180 | 463.204 | 217.451 | 161.890 | 55.561 |
| 2015 | 42671 | 19728.885 | 14459.683 | 5269.202 | 12.349 | 26.708 | 546.292 | 254.116 | 185.120 | 68.996 |
| 2016 | 43513 | 19876.724 | 14196.966 | 5679.758 | 13.053 | 28.575 | 551.597 | 253.628 | 179.971 | 73.657 |
| 2017 | 44562 | 20119.790 | 13982.028 | 6137.762 | 13.773 | 30.506 | 559.458 | 254.381 | 175.538 | 78.843 |
| 2018 | 45976 | 20535.123 | 13879.627 | 6655.496 | 14.476 | 32.410 | 571.582 | 257.229 | 172.552 | 84.677 |
| 2019 | 47528 | 21009.029 | 13809.526 | 7199.504 | 15.148 | 34.269 | 584.831 | 260.605 | 169.925 | 90.681 |
| 2020 | 49048 | 21428.512 | 13704.029 | 7724.482 | 15.749 | 36.048 | 688.057 | 303.533 | 192.243 | 111.290 |
| 2021 | 49139 | 21266.697 | 13143.153 | 8123.544 | 16.532 | 38.198 | 684.079 | 299.127 | 182.972 | 116.155 |
| 2022 | 50658 | 21663.568 | 12947.133 | 8716.435 | 17.207 | 40.235 | 699.423 | 302.378 | 178.759 | 123.619 |
| 2023 | 52205 | 22084.131 | 12759.910 | 9324.221 | 17.861 | 42.221 | 714.513 | 305.748 | 174.642 | 131.106 |
| 2024 | 53700 | 22418.456 | 12549.525 | 9868.931 | 18.378 | 44.021 | 728.452 | 307.804 | 170.236 | 137.568 |
| 2025 | 54814 | 22614.834 | 12270.710 | 10344.12 | 18.871 | 45.740 | 863.566 | 361.411 | 193.319 | 168.092 |
| 2026 | 55376 | 22571.522 | 11810.178 | 10761.34 | 19.433 | 47.677 | 868.242 | 359.241 | 185.171 | 174.070 |
| 2027 | 56377 | 22774.416 | 11466.559 | 11307.86 | 20.058 | 49.652 | 879.327 | 360.827 | 178.847 | 181.980 |
| 2028 | 57338 | 22915.561 | 11076.331 | 11839.23 | 20.648 | 51.665 | 889.188 | 361.228 | 171.771 | 189.458 |
| 2029 | 58284 | 22992.027 | 10675.175 | 12316.85 | 21.132 | 53.570 | 898.105 | 360.371 | 164.495 | 195.876 |
| 2030 | 58588 | 22895.360 | 10255.147 | 12640.21 | 21.575 | 55.209 | 1075.72 | 428.760 | 188.292 | 240.468 |
| 2031 | 59015 | 22715.871 | 9809.349 | 12906.52 | 21.870 | 56.817 | 1079.88 | 424.293 | 179.496 | 244.797 |
| 2032 | 59756 | 22759.207 | 9413.236 | 13345.97 | 22.334 | 58.640 | 1088.89 | 423.652 | 171.529 | 252.122 |
| 2033 | 60474 | 22786.258 | 9049.781 | 13736.48 | 22.715 | 60.284 | 1096.55 | 422.387 | 164.095 | 258.293 |
| 2034 | 61134 | 22806.106 | 8675.823 | 14130.28 | 23.114 | 61.958 | 1102.35 | 420.721 | 156.439 | 264.282 |
| 2035 | 60254 | 22262.998 | 8215.030 | 14047.97 | 23.314 | 63.100 | 1339.80 | 507.973 | 182.667 | 325.306 |
| 2036 | 58177 | 21140.051 | 7363.737 | 13776.31 | 23.680 | 65.167 | 1292.21 | 482.234 | 163.560 | 318.674 |
| 2037 | 58365 | 21011.845 | 6950.833 | 14061.01 | 24.092 | 66.919 | 1293.93 | 478.783 | 154.097 | 324.686 |
| 2038 | 58530 | 20787.483 | 6542.926 | 14244.56 | 24.337 | 68.525 | 1293.87 | 472.681 | 144.639 | 328.042 |
| 2039 | 58685 | 20709.267 | 6195.535 | 14513.73 | 24.732 | 70.083 | 1292.09 | 469.372 | 136.410 | 332.962 |

# Results for PHIM for Run 6 (Sensitivity_Analysis_1.RTF)

---

**Control File Details for Run: 6**

| Variable Parameter | Value |
|---|---|
| 1. Name for Reference Scenario | NULL |
| 2. Name for Test Scenario | Ecig |
| 3. Name for Product 1 | CC |
| 4. Name for Product 2 | Ecig |
| 5. Name for Product 3 | - |
| 6. Name for Product 4 | Dual |
| 7. Country | US (United States) |
| 8. Sex | F (Females) |
| 9. Year of start of process | 1990 |
| 10. Number of months of follow-up | 600 |
| 11. Follow-up interval length (in months) | 12 |
| 12. Lower age for risk estimation | 10 |
| 13. Upper age for risk estimation | 79 |
| 14. The effective dose for Product 1 (F1) - Ref | 1 |
| 15. The effective dose for Product 2 (F2) - Ref | - |
| 16. The effective dose for Product 3 (F3) - Ref | - |
| 17. The effective dose for Product 4 (F4) - Ref | - |
| 18. The effective dose for Product 1 (F1) - Test | 1 |
| 19. The effective dose for Product 2 (F2) - Test | 0.1 |
| 20. The effective dose for Product 3 (F3) - Test | - |
| 21. The effective dose for Product 4 (F4) - Test | FROMF2 |
| 22. Number in population to be simulated | 100000 |
| 23. Number of MC simulations | 1 |
| 24. The random number seed for the first simulation | 15975263 |
| 25. Source for the population file (POP) | UN4 |
| 26. Source for the socioeconomic prevalence file (SEP) | - |
| 27. Source for the current smoking prevalence file (CSP) | ISS3 |
| 28. Source for the former smoking prevalence file (FSP) | ISS3 |
| 29. Source for the quit-time distribution file (QTD) | NHIS2006 |
| 30. Source for the death file (MORT) | APR-20 |
| 31. Source for the relative risk file (RR) | PNLEST |
| 32. Source for the half-life file (H) | PNLEST |
| 33. Assumption Set for TTP Factor Reference | F1 |
| 34. Assumption Set for TTP Factor Test | F1 |
| 35. Assumption Set for TTP Reference | ISS3 |
| 36. Assumption Set for TTP Test | ISS3 |
| 37. Assumption Set for TP for socioeconomic group | - |
| 38. Source for Output Choice file (OUTC) | BASIC |
| 39. Output for DTP P1 | N |
| 40. Output for DTP P2 | N |
| 41. Output for Main P Component | Y |
| 42. Output for DTP E1 | N |
| 43. Output for DTP E2 | N |
| 44. Output for DTP E3 | N |
| 45. Output for Main E Component | Y |
| 46. Output file name | Sensitivity_Analysis_1 |
| 47. Run number for P-Component | - |
| 48. Results file for E-Component | - |
| 49. Components required | PE |
| 50. Results folder for P to be used in E-Component | - |
| 51. Fixed age of starting product use | 16 |
| 52. Output absolute risk file? | N |

# Results for PHIM for Run 6 (Sensitivity_Analysis_1.RTF)

************************************************************************************

**TTP values for Run: 6, Scenario: NULL**

| **Variable Parameter** | **Value** |
|---|---|
| 1. Country | US (United States) |
| 2. Sex | F (Females) |
| 3. Assumption Set | ISS3 |
| Note: | Period is in Months |

| Age | Period | **Initiation** Never CC | **Cessation** CC Former | **Re-initiation** Former CC |
|---|---|---|---|---|
| 10-14 | 1-12 | 0.003758 | 0.008742 | 0.000000 |
| 15-19 | 1-12 | 0.003469 | 0.002515 | 0.000000 |
| 20-24 | 1-12 | 0.000276 | 0.000761 | 0.000000 |
| 25-29 | 1-12 | 0.000137 | 0.001375 | 0.000000 |
| 30-34 | 1-12 | 0.000069 | 0.001149 | 0.000000 |
| 35-39 | 1-12 | 0.000000 | 0.000991 | 0.000000 |
| 40-44 | 1-12 | 0.000000 | 0.000833 | 0.000000 |
| 45-49 | 1-12 | 0.000000 | 0.001723 | 0.000000 |
| 50-54 | 1-12 | 0.000000 | 0.002336 | 0.000000 |
| 55-59 | 1-12 | 0.000000 | 0.002233 | 0.000000 |
| 60-64 | 1-12 | 0.000000 | 0.004634 | 0.000000 |
| 65-69 | 1-12 | 0.000000 | 0.003505 | 0.000000 |
| 70-74 | 1-12 | 0.000000 | 0.004329 | 0.000000 |
| 75-79 | 1-12 | 0.000000 | 0.004329 | 0.000000 |
| 10-14 | 13-72 | 0.004794 | 0.008742 | 0.000000 |
| 15-19 | 13-72 | 0.004527 | 0.004082 | 0.000000 |
| 20-24 | 13-72 | 0.000105 | 0.001261 | 0.000000 |
| 25-29 | 13-72 | 0.000053 | 0.000952 | 0.000000 |
| 30-34 | 13-72 | 0.000026 | 0.000733 | 0.000000 |
| 35-39 | 13-72 | 0.000000 | 0.000753 | 0.000000 |
| 40-44 | 13-72 | 0.000000 | 0.001535 | 0.000000 |
| 45-49 | 13-72 | 0.000000 | 0.002998 | 0.000000 |
| 50-54 | 13-72 | 0.000000 | 0.003159 | 0.000000 |
| 55-59 | 13-72 | 0.000000 | 0.002965 | 0.000000 |
| 60-64 | 13-72 | 0.000000 | 0.003633 | 0.000000 |
| 65-69 | 13-72 | 0.000000 | 0.004522 | 0.000000 |
| 70-74 | 13-72 | 0.000000 | 0.005208 | 0.000000 |
| 75-79 | 13-72 | 0.000000 | 0.005208 | 0.000000 |
| 10-14 | 73-132 | 0.003969 | 0.008742 | 0.000000 |
| 15-19 | 73-132 | 0.003056 | 0.003373 | 0.000000 |
| 20-24 | 73-132 | 0.001459 | 0.003881 | 0.000000 |
| 25-29 | 73-132 | 0.000714 | 0.001953 | 0.000000 |
| 30-34 | 73-132 | 0.000111 | 0.001338 | 0.000000 |
| 35-39 | 73-132 | 0.000000 | 0.001507 | 0.000000 |
| 40-44 | 73-132 | 0.000000 | 0.002080 | 0.000000 |
| 45-49 | 73-132 | 0.000000 | 0.003809 | 0.000000 |
| 50-54 | 73-132 | 0.000000 | 0.004871 | 0.000000 |
| 55-59 | 73-132 | 0.000000 | 0.004560 | 0.000000 |
| 60-64 | 73-132 | 0.000000 | 0.007192 | 0.000000 |
| 65-69 | 73-132 | 0.000000 | 0.007033 | 0.000000 |
| 70-74 | 73-132 | 0.000000 | 0.008206 | 0.000000 |
| 75-79 | 73-132 | 0.000000 | 0.008206 | 0.000000 |
| 10-14 | 133-192 | 0.002861 | 0.008742 | 0.000000 |
| 15-19 | 133-192 | 0.002594 | 0.003996 | 0.000000 |
| 20-24 | 133-192 | 0.001247 | 0.002805 | 0.000000 |
| 25-29 | 133-192 | 0.000289 | 0.001574 | 0.000000 |
| 30-34 | 133-192 | 0.000132 | 0.001293 | 0.000000 |
| 35-39 | 133-192 | 0.000000 | 0.001219 | 0.000000 |
| 40-44 | 133-192 | 0.000000 | 0.001657 | 0.000000 |
| 45-49 | 133-192 | 0.000000 | 0.001995 | 0.000000 |
| 50-54 | 133-192 | 0.000000 | 0.002154 | 0.000000 |
| 55-59 | 133-192 | 0.000000 | 0.002072 | 0.000000 |
| 60-64 | 133-192 | 0.000000 | 0.005049 | 0.000000 |
| 65-69 | 133-192 | 0.000000 | 0.003680 | 0.000000 |
| 70-74 | 133-192 | 0.000000 | 0.006144 | 0.000000 |
| 75-79 | 133-192 | 0.000000 | 0.006144 | 0.000000 |
| 10-14 | 193-252 | 0.001859 | 0.008742 | 0.000000 |
| 15-19 | 193-252 | 0.002232 | 0.005770 | 0.000000 |
| 20-24 | 193-252 | 0.001079 | 0.003262 | 0.000000 |
| 25-29 | 193-252 | 0.000531 | 0.002002 | 0.000000 |
| 30-34 | 193-252 | 0.000263 | 0.003240 | 0.000000 |
| 35-39 | 193-252 | 0.000000 | 0.001771 | 0.000000 |
| 40-44 | 193-252 | 0.000000 | 0.002173 | 0.000000 |
| 45-49 | 193-252 | 0.000000 | 0.002075 | 0.000000 |
| 50-54 | 193-252 | 0.000000 | 0.003431 | 0.000000 |

## Results for PHIM for Run 6 (Sensitivity_Analysis_1.RTF)

| | | | | |
|---|---|---|---|---|
| 55-59 | 193-252 | 0.000000 | 0.002906 | 0.000000 |
| 60-64 | 193-252 | 0.000000 | 0.007139 | 0.000000 |
| 65-69 | 193-252 | 0.000000 | 0.004963 | 0.000000 |
| 70-74 | 193-252 | 0.000000 | 0.006269 | 0.000000 |
| 75-79 | 193-252 | 0.000000 | 0.006269 | 0.000000 |
| 10-14 | 253+ | 0.000931 | 0.008742 | 0.000000 |
| 15-19 | 253+ | 0.001798 | 0.009440 | 0.000000 |
| 20-24 | 253+ | 0.000023 | 0.002831 | 0.000000 |
| 25-29 | 253+ | 0.000073 | 0.002655 | 0.000000 |
| 30-34 | 253+ | 0.000037 | 0.004614 | 0.000000 |
| 35-39 | 253+ | 0.000000 | 0.002607 | 0.000000 |
| 40-44 | 253+ | 0.000000 | 0.003115 | 0.000000 |
| 45-49 | 253+ | 0.000000 | 0.002132 | 0.000000 |
| 50-54 | 253+ | 0.000000 | 0.003497 | 0.000000 |
| 55-59 | 253+ | 0.000000 | 0.002942 | 0.000000 |
| 60-64 | 253+ | 0.000000 | 0.005376 | 0.000000 |
| 65-69 | 253+ | 0.000000 | 0.001789 | 0.000000 |
| 70-74 | 253+ | 0.000000 | 0.004077 | 0.000000 |
| 75-79 | 253+ | 0.000000 | 0.004077 | 0.000000 |

# Results for PHIM for Run 6 (Sensitivity_Analysis_1.RTF)

************************************************************************************

**TTP values for Run: 6, Scenario: Ecig**

| **Variable Parameter** | **Value** |
|---|---|
| 1. Country | US (United States) |
| 2. Sex | F (Females) |
| 3. Assumption Set | ISS3 |
| Note: | Period is in Months |

| | | **Initiation** | | | **Cessation** | | | **Re-initiation** | | |
|---|---|---|---|---|---|---|---|---|---|---|
| Age | Period | Never CC | Never Ecig | Never Dual | CC Former | Ecig Former | Dual Former | Former CC | Former Ecig | Former Dual |
| 10-14 | 1-12 | 0.001879 | 0.001879 | 0.000000 | 0.008742 | 0.008742 | 0.000000 | 0.000000 | 0.000000 | 0.000000 |
| 15-19 | 1-12 | 0.001735 | 0.001735 | 0.000000 | 0.002515 | 0.002515 | 0.000000 | 0.000000 | 0.000000 | 0.000000 |
| 20-24 | 1-12 | 0.000138 | 0.000138 | 0.000000 | 0.000761 | 0.000761 | 0.000000 | 0.000000 | 0.000000 | 0.000000 |
| 25-29 | 1-12 | 0.000069 | 0.000069 | 0.000000 | 0.001376 | 0.001376 | 0.000000 | 0.000000 | 0.000000 | 0.000000 |
| 30-34 | 1-12 | 0.000034 | 0.000034 | 0.000000 | 0.001149 | 0.001149 | 0.000000 | 0.000000 | 0.000000 | 0.000000 |
| 35-39 | 1-12 | 0.000000 | 0.000000 | 0.000000 | 0.000991 | 0.000991 | 0.000000 | 0.000000 | 0.000000 | 0.000000 |
| 40-44 | 1-12 | 0.000000 | 0.000000 | 0.000000 | 0.000833 | 0.000833 | 0.000000 | 0.000000 | 0.000000 | 0.000000 |
| 45-49 | 1-12 | 0.000000 | 0.000000 | 0.000000 | 0.001723 | 0.001723 | 0.000000 | 0.000000 | 0.000000 | 0.000000 |
| 50-54 | 1-12 | 0.000000 | 0.000000 | 0.000000 | 0.002336 | 0.002336 | 0.000000 | 0.000000 | 0.000000 | 0.000000 |
| 55-59 | 1-12 | 0.000000 | 0.000000 | 0.000000 | 0.002233 | 0.002233 | 0.000000 | 0.000000 | 0.000000 | 0.000000 |
| 60-64 | 1-12 | 0.000000 | 0.000000 | 0.000000 | 0.004635 | 0.004635 | 0.000000 | 0.000000 | 0.000000 | 0.000000 |
| 65-69 | 1-12 | 0.000000 | 0.000000 | 0.000000 | 0.003505 | 0.003505 | 0.000000 | 0.000000 | 0.000000 | 0.000000 |
| 70-74 | 1-12 | 0.000000 | 0.000000 | 0.000000 | 0.004329 | 0.004329 | 0.000000 | 0.000000 | 0.000000 | 0.000000 |
| 75-79 | 1-12 | 0.000000 | 0.000000 | 0.000000 | 0.004329 | 0.004329 | 0.000000 | 0.000000 | 0.000000 | 0.000000 |
| 10-14 | 13-72 | 0.001918 | 0.002877 | 0.000000 | 0.008742 | 0.008742 | 0.000000 | 0.000000 | 0.000000 | 0.000000 |
| 15-19 | 13-72 | 0.001811 | 0.002716 | 0.000000 | 0.004083 | 0.004083 | 0.000000 | 0.000000 | 0.000000 | 0.000000 |
| 20-24 | 13-72 | 0.000042 | 0.000063 | 0.000000 | 0.001261 | 0.001261 | 0.000000 | 0.000000 | 0.000000 | 0.000000 |
| 25-29 | 13-72 | 0.000021 | 0.000032 | 0.000000 | 0.000952 | 0.000952 | 0.000000 | 0.000000 | 0.000000 | 0.000000 |
| 30-34 | 13-72 | 0.000011 | 0.000016 | 0.000000 | 0.000733 | 0.000733 | 0.000000 | 0.000000 | 0.000000 | 0.000000 |
| 35-39 | 13-72 | 0.000000 | 0.000000 | 0.000000 | 0.000754 | 0.000754 | 0.000000 | 0.000000 | 0.000000 | 0.000000 |
| 40-44 | 13-72 | 0.000000 | 0.000000 | 0.000000 | 0.001535 | 0.001535 | 0.000000 | 0.000000 | 0.000000 | 0.000000 |
| 45-49 | 13-72 | 0.000000 | 0.000000 | 0.000000 | 0.002998 | 0.002998 | 0.000000 | 0.000000 | 0.000000 | 0.000000 |
| 50-54 | 13-72 | 0.000000 | 0.000000 | 0.000000 | 0.003160 | 0.003160 | 0.000000 | 0.000000 | 0.000000 | 0.000000 |
| 55-59 | 13-72 | 0.000000 | 0.000000 | 0.000000 | 0.002965 | 0.002965 | 0.000000 | 0.000000 | 0.000000 | 0.000000 |
| 60-64 | 13-72 | 0.000000 | 0.000000 | 0.000000 | 0.003633 | 0.003633 | 0.000000 | 0.000000 | 0.000000 | 0.000000 |
| 65-69 | 13-72 | 0.000000 | 0.000000 | 0.000000 | 0.004522 | 0.004522 | 0.000000 | 0.000000 | 0.000000 | 0.000000 |
| 70-74 | 13-72 | 0.000000 | 0.000000 | 0.000000 | 0.005208 | 0.005208 | 0.000000 | 0.000000 | 0.000000 | 0.000000 |
| 75-79 | 13-72 | 0.000000 | 0.000000 | 0.000000 | 0.005208 | 0.005208 | 0.000000 | 0.000000 | 0.000000 | 0.000000 |
| 10-14 | 73-132 | 0.000794 | 0.003175 | 0.000000 | 0.008742 | 0.008742 | 0.000000 | 0.000000 | 0.000000 | 0.000000 |
| 15-19 | 73-132 | 0.000611 | 0.002445 | 0.000000 | 0.003373 | 0.003373 | 0.000000 | 0.000000 | 0.000000 | 0.000000 |
| 20-24 | 73-132 | 0.000292 | 0.001167 | 0.000000 | 0.003881 | 0.003881 | 0.000000 | 0.000000 | 0.000000 | 0.000000 |
| 25-29 | 73-132 | 0.000143 | 0.000571 | 0.000000 | 0.001954 | 0.001954 | 0.000000 | 0.000000 | 0.000000 | 0.000000 |
| 30-34 | 73-132 | 0.000022 | 0.000089 | 0.000000 | 0.001338 | 0.001338 | 0.000000 | 0.000000 | 0.000000 | 0.000000 |
| 35-39 | 73-132 | 0.000000 | 0.000000 | 0.000000 | 0.001507 | 0.001507 | 0.000000 | 0.000000 | 0.000000 | 0.000000 |
| 40-44 | 73-132 | 0.000000 | 0.000000 | 0.000000 | 0.002080 | 0.002080 | 0.000000 | 0.000000 | 0.000000 | 0.000000 |
| 45-49 | 73-132 | 0.000000 | 0.000000 | 0.000000 | 0.003809 | 0.003809 | 0.000000 | 0.000000 | 0.000000 | 0.000000 |
| 50-54 | 73-132 | 0.000000 | 0.000000 | 0.000000 | 0.004871 | 0.004871 | 0.000000 | 0.000000 | 0.000000 | 0.000000 |
| 55-59 | 73-132 | 0.000000 | 0.000000 | 0.000000 | 0.004560 | 0.004560 | 0.000000 | 0.000000 | 0.000000 | 0.000000 |
| 60-64 | 73-132 | 0.000000 | 0.000000 | 0.000000 | 0.007192 | 0.007192 | 0.000000 | 0.000000 | 0.000000 | 0.000000 |
| 65-69 | 73-132 | 0.000000 | 0.000000 | 0.000000 | 0.007033 | 0.007033 | 0.000000 | 0.000000 | 0.000000 | 0.000000 |
| 70-74 | 73-132 | 0.000000 | 0.000000 | 0.000000 | 0.008206 | 0.008206 | 0.000000 | 0.000000 | 0.000000 | 0.000000 |
| 75-79 | 73-132 | 0.000000 | 0.000000 | 0.000000 | 0.008206 | 0.008206 | 0.000000 | 0.000000 | 0.000000 | 0.000000 |
| 10-14 | 133-192 | 0.000143 | 0.002718 | 0.000000 | 0.008742 | 0.008742 | 0.000000 | 0.000000 | 0.000000 | 0.000000 |
| 15-19 | 133-192 | 0.000130 | 0.002465 | 0.000000 | 0.003996 | 0.003996 | 0.000000 | 0.000000 | 0.000000 | 0.000000 |
| 20-24 | 133-192 | 0.000062 | 0.001185 | 0.000000 | 0.002805 | 0.002805 | 0.000000 | 0.000000 | 0.000000 | 0.000000 |
| 25-29 | 133-192 | 0.000015 | 0.000275 | 0.000000 | 0.001574 | 0.001574 | 0.000000 | 0.000000 | 0.000000 | 0.000000 |
| 30-34 | 133-192 | 0.000007 | 0.000125 | 0.000000 | 0.001293 | 0.001293 | 0.000000 | 0.000000 | 0.000000 | 0.000000 |
| 35-39 | 133-192 | 0.000000 | 0.000000 | 0.000000 | 0.001219 | 0.001219 | 0.000000 | 0.000000 | 0.000000 | 0.000000 |
| 40-44 | 133-192 | 0.000000 | 0.000000 | 0.000000 | 0.001657 | 0.001657 | 0.000000 | 0.000000 | 0.000000 | 0.000000 |
| 45-49 | 133-192 | 0.000000 | 0.000000 | 0.000000 | 0.001995 | 0.001995 | 0.000000 | 0.000000 | 0.000000 | 0.000000 |
| 50-54 | 133-192 | 0.000000 | 0.000000 | 0.000000 | 0.002154 | 0.002154 | 0.000000 | 0.000000 | 0.000000 | 0.000000 |
| 55-59 | 133-192 | 0.000000 | 0.000000 | 0.000000 | 0.002072 | 0.002072 | 0.000000 | 0.000000 | 0.000000 | 0.000000 |
| 60-64 | 133-192 | 0.000000 | 0.000000 | 0.000000 | 0.005049 | 0.005049 | 0.000000 | 0.000000 | 0.000000 | 0.000000 |
| 65-69 | 133-192 | 0.000000 | 0.000000 | 0.000000 | 0.003681 | 0.003681 | 0.000000 | 0.000000 | 0.000000 | 0.000000 |
| 70-74 | 133-192 | 0.000000 | 0.000000 | 0.000000 | 0.006144 | 0.006144 | 0.000000 | 0.000000 | 0.000000 | 0.000000 |
| 75-79 | 133-192 | 0.000000 | 0.000000 | 0.000000 | 0.006144 | 0.006144 | 0.000000 | 0.000000 | 0.000000 | 0.000000 |
| 10-14 | 193-252 | 0.000093 | 0.001766 | 0.000000 | 0.008742 | 0.008742 | 0.000000 | 0.000000 | 0.000000 | 0.000000 |
| 15-19 | 193-252 | 0.000112 | 0.002120 | 0.000000 | 0.005770 | 0.005770 | 0.000000 | 0.000000 | 0.000000 | 0.000000 |
| 20-24 | 193-252 | 0.000054 | 0.001025 | 0.000000 | 0.003262 | 0.003262 | 0.000000 | 0.000000 | 0.000000 | 0.000000 |
| 25-29 | 193-252 | 0.000027 | 0.000504 | 0.000000 | 0.002002 | 0.002002 | 0.000000 | 0.000000 | 0.000000 | 0.000000 |
| 30-34 | 193-252 | 0.000013 | 0.000250 | 0.000000 | 0.003241 | 0.003241 | 0.000000 | 0.000000 | 0.000000 | 0.000000 |
| 35-39 | 193-252 | 0.000000 | 0.000000 | 0.000000 | 0.001771 | 0.001771 | 0.000000 | 0.000000 | 0.000000 | 0.000000 |
| 40-44 | 193-252 | 0.000000 | 0.000000 | 0.000000 | 0.002173 | 0.002173 | 0.000000 | 0.000000 | 0.000000 | 0.000000 |
| 45-49 | 193-252 | 0.000000 | 0.000000 | 0.000000 | 0.002075 | 0.002075 | 0.000000 | 0.000000 | 0.000000 | 0.000000 |
| 50-54 | 193-252 | 0.000000 | 0.000000 | 0.000000 | 0.003432 | 0.003432 | 0.000000 | 0.000000 | 0.000000 | 0.000000 |

## Results for PHIM for Run 6 (Sensitivity_Analysis_1.RTF)

| | | | | | | | | | | |
|---|---|---|---|---|---|---|---|---|---|---|
| 55-59 | 193-252 | 0.000000 | 0.000000 | 0.000000 | 0.002906 | 0.002906 | 0.000000 | 0.000000 | 0.000000 | 0.000000 |
| 60-64 | 193-252 | 0.000000 | 0.000000 | 0.000000 | 0.007139 | 0.007139 | 0.000000 | 0.000000 | 0.000000 | 0.000000 |
| 65-69 | 193-252 | 0.000000 | 0.000000 | 0.000000 | 0.004963 | 0.004963 | 0.000000 | 0.000000 | 0.000000 | 0.000000 |
| 70-74 | 193-252 | 0.000000 | 0.000000 | 0.000000 | 0.006269 | 0.006269 | 0.000000 | 0.000000 | 0.000000 | 0.000000 |
| 75-79 | 193-252 | 0.000000 | 0.000000 | 0.000000 | 0.006269 | 0.006269 | 0.000000 | 0.000000 | 0.000000 | 0.000000 |
| 10-14 | 253+ | 0.000047 | 0.000884 | 0.000000 | 0.008742 | 0.008742 | 0.000000 | 0.000000 | 0.000000 | 0.000000 |
| 15-19 | 253+ | 0.000090 | 0.001708 | 0.000000 | 0.009440 | 0.009440 | 0.000000 | 0.000000 | 0.000000 | 0.000000 |
| 20-24 | 253+ | 0.000001 | 0.000022 | 0.000000 | 0.002831 | 0.002831 | 0.000000 | 0.000000 | 0.000000 | 0.000000 |
| 25-29 | 253+ | 0.000004 | 0.000070 | 0.000000 | 0.002656 | 0.002656 | 0.000000 | 0.000000 | 0.000000 | 0.000000 |
| 30-34 | 253+ | 0.000002 | 0.000035 | 0.000000 | 0.004614 | 0.004614 | 0.000000 | 0.000000 | 0.000000 | 0.000000 |
| 35-39 | 253+ | 0.000000 | 0.000000 | 0.000000 | 0.002608 | 0.002608 | 0.000000 | 0.000000 | 0.000000 | 0.000000 |
| 40-44 | 253+ | 0.000000 | 0.000000 | 0.000000 | 0.003115 | 0.003115 | 0.000000 | 0.000000 | 0.000000 | 0.000000 |
| 45-49 | 253+ | 0.000000 | 0.000000 | 0.000000 | 0.002132 | 0.002132 | 0.000000 | 0.000000 | 0.000000 | 0.000000 |
| 50-54 | 253+ | 0.000000 | 0.000000 | 0.000000 | 0.003497 | 0.003497 | 0.000000 | 0.000000 | 0.000000 | 0.000000 |
| 55-59 | 253+ | 0.000000 | 0.000000 | 0.000000 | 0.002942 | 0.002942 | 0.000000 | 0.000000 | 0.000000 | 0.000000 |
| 60-64 | 253+ | 0.000000 | 0.000000 | 0.000000 | 0.005376 | 0.005376 | 0.000000 | 0.000000 | 0.000000 | 0.000000 |
| 65-69 | 253+ | 0.000000 | 0.000000 | 0.000000 | 0.001789 | 0.001789 | 0.000000 | 0.000000 | 0.000000 | 0.000000 |
| 70-74 | 253+ | 0.000000 | 0.000000 | 0.000000 | 0.004077 | 0.004077 | 0.000000 | 0.000000 | 0.000000 | 0.000000 |
| 75-79 | 253+ | 0.000000 | 0.000000 | 0.000000 | 0.004077 | 0.004077 | 0.000000 | 0.000000 | 0.000000 | 0.000000 |

## Results for PHIM for Run 6 (Sensitivity_Analysis_1.RTF)

************************************************************************************

| | | Switch from CC | | Switch from Ecig | | Switch from Dual | |
|---|---|---|---|---|---|---|---|
| | | **CC** | **CC** | **Ecig** | **Ecig** | **Dual** | **Dual** |
| Age | Period | Ecig | Dual | CC | Dual | CC | Ecig |
| 10-14 | 1-12 | 0.009055 | 0.000000 | 0.000000 | 0.000000 | 0.000000 | 0.000000 |
| 15-19 | 1-12 | 0.011817 | 0.000000 | 0.000000 | 0.000000 | 0.000000 | 0.000000 |
| 20-24 | 1-12 | 0.013405 | 0.000000 | 0.000000 | 0.000000 | 0.000000 | 0.000000 |
| 25-29 | 1-12 | 0.014127 | 0.000000 | 0.000000 | 0.000000 | 0.000000 | 0.000000 |
| 30-34 | 1-12 | 0.013889 | 0.000000 | 0.000000 | 0.000000 | 0.000000 | 0.000000 |
| 35-39 | 1-12 | 0.012929 | 0.000000 | 0.000000 | 0.000000 | 0.000000 | 0.000000 |
| 40-44 | 1-12 | 0.012785 | 0.000000 | 0.000000 | 0.000000 | 0.000000 | 0.000000 |
| 45-49 | 1-12 | 0.012295 | 0.000000 | 0.000000 | 0.000000 | 0.000000 | 0.000000 |
| 50-54 | 1-12 | 0.011536 | 0.000000 | 0.000000 | 0.000000 | 0.000000 | 0.000000 |
| 55-59 | 1-12 | 0.008988 | 0.000000 | 0.000000 | 0.000000 | 0.000000 | 0.000000 |
| 60-64 | 1-12 | 0.008098 | 0.000000 | 0.000000 | 0.000000 | 0.000000 | 0.000000 |
| 65-69 | 1-12 | 0.006274 | 0.000000 | 0.000000 | 0.000000 | 0.000000 | 0.000000 |
| 70-74 | 1-12 | 0.004141 | 0.000000 | 0.000000 | 0.000000 | 0.000000 | 0.000000 |
| 75-79 | 1-12 | 0.004141 | 0.000000 | 0.000000 | 0.000000 | 0.000000 | 0.000000 |
| 10-14 | 13-72 | 0.009055 | 0.000000 | 0.000000 | 0.000000 | 0.000000 | 0.000000 |
| 15-19 | 13-72 | 0.011817 | 0.000000 | 0.000000 | 0.000000 | 0.000000 | 0.000000 |
| 20-24 | 13-72 | 0.013405 | 0.000000 | 0.000000 | 0.000000 | 0.000000 | 0.000000 |
| 25-29 | 13-72 | 0.014127 | 0.000000 | 0.000000 | 0.000000 | 0.000000 | 0.000000 |
| 30-34 | 13-72 | 0.013889 | 0.000000 | 0.000000 | 0.000000 | 0.000000 | 0.000000 |
| 35-39 | 13-72 | 0.012929 | 0.000000 | 0.000000 | 0.000000 | 0.000000 | 0.000000 |
| 40-44 | 13-72 | 0.012785 | 0.000000 | 0.000000 | 0.000000 | 0.000000 | 0.000000 |
| 45-49 | 13-72 | 0.012295 | 0.000000 | 0.000000 | 0.000000 | 0.000000 | 0.000000 |
| 50-54 | 13-72 | 0.011536 | 0.000000 | 0.000000 | 0.000000 | 0.000000 | 0.000000 |
| 55-59 | 13-72 | 0.008988 | 0.000000 | 0.000000 | 0.000000 | 0.000000 | 0.000000 |
| 60-64 | 13-72 | 0.008098 | 0.000000 | 0.000000 | 0.000000 | 0.000000 | 0.000000 |
| 65-69 | 13-72 | 0.006274 | 0.000000 | 0.000000 | 0.000000 | 0.000000 | 0.000000 |
| 70-74 | 13-72 | 0.004141 | 0.000000 | 0.000000 | 0.000000 | 0.000000 | 0.000000 |
| 75-79 | 13-72 | 0.004141 | 0.000000 | 0.000000 | 0.000000 | 0.000000 | 0.000000 |
| 10-14 | 73-132 | 0.009055 | 0.000000 | 0.000000 | 0.000000 | 0.000000 | 0.000000 |
| 15-19 | 73-132 | 0.011817 | 0.000000 | 0.000000 | 0.000000 | 0.000000 | 0.000000 |
| 20-24 | 73-132 | 0.013405 | 0.000000 | 0.000000 | 0.000000 | 0.000000 | 0.000000 |
| 25-29 | 73-132 | 0.014127 | 0.000000 | 0.000000 | 0.000000 | 0.000000 | 0.000000 |
| 30-34 | 73-132 | 0.013889 | 0.000000 | 0.000000 | 0.000000 | 0.000000 | 0.000000 |
| 35-39 | 73-132 | 0.012929 | 0.000000 | 0.000000 | 0.000000 | 0.000000 | 0.000000 |
| 40-44 | 73-132 | 0.012785 | 0.000000 | 0.000000 | 0.000000 | 0.000000 | 0.000000 |
| 45-49 | 73-132 | 0.012295 | 0.000000 | 0.000000 | 0.000000 | 0.000000 | 0.000000 |
| 50-54 | 73-132 | 0.011536 | 0.000000 | 0.000000 | 0.000000 | 0.000000 | 0.000000 |
| 55-59 | 73-132 | 0.008988 | 0.000000 | 0.000000 | 0.000000 | 0.000000 | 0.000000 |
| 60-64 | 73-132 | 0.008098 | 0.000000 | 0.000000 | 0.000000 | 0.000000 | 0.000000 |
| 65-69 | 73-132 | 0.006274 | 0.000000 | 0.000000 | 0.000000 | 0.000000 | 0.000000 |
| 70-74 | 73-132 | 0.004141 | 0.000000 | 0.000000 | 0.000000 | 0.000000 | 0.000000 |
| 75-79 | 73-132 | 0.004141 | 0.000000 | 0.000000 | 0.000000 | 0.000000 | 0.000000 |
| 10-14 | 133-192 | 0.009055 | 0.000000 | 0.000000 | 0.000000 | 0.000000 | 0.000000 |
| 15-19 | 133-192 | 0.011817 | 0.000000 | 0.000000 | 0.000000 | 0.000000 | 0.000000 |
| 20-24 | 133-192 | 0.013405 | 0.000000 | 0.000000 | 0.000000 | 0.000000 | 0.000000 |
| 25-29 | 133-192 | 0.014127 | 0.000000 | 0.000000 | 0.000000 | 0.000000 | 0.000000 |
| 30-34 | 133-192 | 0.013889 | 0.000000 | 0.000000 | 0.000000 | 0.000000 | 0.000000 |
| 35-39 | 133-192 | 0.012929 | 0.000000 | 0.000000 | 0.000000 | 0.000000 | 0.000000 |
| 40-44 | 133-192 | 0.012785 | 0.000000 | 0.000000 | 0.000000 | 0.000000 | 0.000000 |
| 45-49 | 133-192 | 0.012295 | 0.000000 | 0.000000 | 0.000000 | 0.000000 | 0.000000 |
| 50-54 | 133-192 | 0.011536 | 0.000000 | 0.000000 | 0.000000 | 0.000000 | 0.000000 |
| 55-59 | 133-192 | 0.008988 | 0.000000 | 0.000000 | 0.000000 | 0.000000 | 0.000000 |
| 60-64 | 133-192 | 0.008098 | 0.000000 | 0.000000 | 0.000000 | 0.000000 | 0.000000 |
| 65-69 | 133-192 | 0.006274 | 0.000000 | 0.000000 | 0.000000 | 0.000000 | 0.000000 |
| 70-74 | 133-192 | 0.004141 | 0.000000 | 0.000000 | 0.000000 | 0.000000 | 0.000000 |
| 75-79 | 133-192 | 0.004141 | 0.000000 | 0.000000 | 0.000000 | 0.000000 | 0.000000 |
| 10-14 | 193-252 | 0.009055 | 0.000000 | 0.000000 | 0.000000 | 0.000000 | 0.000000 |
| 15-19 | 193-252 | 0.011817 | 0.000000 | 0.000000 | 0.000000 | 0.000000 | 0.000000 |
| 20-24 | 193-252 | 0.013405 | 0.000000 | 0.000000 | 0.000000 | 0.000000 | 0.000000 |
| 25-29 | 193-252 | 0.014127 | 0.000000 | 0.000000 | 0.000000 | 0.000000 | 0.000000 |
| 30-34 | 193-252 | 0.013889 | 0.000000 | 0.000000 | 0.000000 | 0.000000 | 0.000000 |
| 35-39 | 193-252 | 0.012929 | 0.000000 | 0.000000 | 0.000000 | 0.000000 | 0.000000 |
| 40-44 | 193-252 | 0.012785 | 0.000000 | 0.000000 | 0.000000 | 0.000000 | 0.000000 |
| 45-49 | 193-252 | 0.012295 | 0.000000 | 0.000000 | 0.000000 | 0.000000 | 0.000000 |
| 50-54 | 193-252 | 0.011536 | 0.000000 | 0.000000 | 0.000000 | 0.000000 | 0.000000 |
| 55-59 | 193-252 | 0.008988 | 0.000000 | 0.000000 | 0.000000 | 0.000000 | 0.000000 |
| 60-64 | 193-252 | 0.008098 | 0.000000 | 0.000000 | 0.000000 | 0.000000 | 0.000000 |
| 65-69 | 193-252 | 0.006274 | 0.000000 | 0.000000 | 0.000000 | 0.000000 | 0.000000 |
| 70-74 | 193-252 | 0.004141 | 0.000000 | 0.000000 | 0.000000 | 0.000000 | 0.000000 |
| 75-79 | 193-252 | 0.004141 | 0.000000 | 0.000000 | 0.000000 | 0.000000 | 0.000000 |
| 10-14 | 253+ | 0.009055 | 0.000000 | 0.000000 | 0.000000 | 0.000000 | 0.000000 |
| 15-19 | 253+ | 0.011817 | 0.000000 | 0.000000 | 0.000000 | 0.000000 | 0.000000 |
| 20-24 | 253+ | 0.013405 | 0.000000 | 0.000000 | 0.000000 | 0.000000 | 0.000000 |

## Results for PHIM for Run 6 (Sensitivity_Analysis_1.RTF)

| | | | | | | | |
|---|---|---|---|---|---|---|---|
| 25-29 | 253+ | 0.014127 | 0.000000 | 0.000000 | 0.000000 | 0.000000 | 0.000000 |
| 30-34 | 253+ | 0.013889 | 0.000000 | 0.000000 | 0.000000 | 0.000000 | 0.000000 |
| 35-39 | 253+ | 0.012929 | 0.000000 | 0.000000 | 0.000000 | 0.000000 | 0.000000 |
| 40-44 | 253+ | 0.012785 | 0.000000 | 0.000000 | 0.000000 | 0.000000 | 0.000000 |
| 45-49 | 253+ | 0.012295 | 0.000000 | 0.000000 | 0.000000 | 0.000000 | 0.000000 |
| 50-54 | 253+ | 0.011536 | 0.000000 | 0.000000 | 0.000000 | 0.000000 | 0.000000 |
| 55-59 | 253+ | 0.008988 | 0.000000 | 0.000000 | 0.000000 | 0.000000 | 0.000000 |
| 60-64 | 253+ | 0.008098 | 0.000000 | 0.000000 | 0.000000 | 0.000000 | 0.000000 |
| 65-69 | 253+ | 0.006274 | 0.000000 | 0.000000 | 0.000000 | 0.000000 | 0.000000 |
| 70-74 | 253+ | 0.004141 | 0.000000 | 0.000000 | 0.000000 | 0.000000 | 0.000000 |
| 75-79 | 253+ | 0.004141 | 0.000000 | 0.000000 | 0.000000 | 0.000000 | 0.000000 |

# Results for PHIM for Run 6 (Sensitivity_Analysis_1.RTF)

************************************************************************************

**Main Output for the Tobacco History for Run: 6, Simulation: 1, Scenario: NULL**

| Variable Parameter | Value |
|---|---|
| 1. Country | US (United States) |
| 2. Sex | F (Females) |
| 3. Year of start of process | 1990 |

Year of Follow-up: 2040, Age Group: All Ages

| Period | Age Group | Smoking Group | N | % |
|---|---|---|---|---|
| 50 | All Ages | Never Any | 20670 | 58.80 |
| 50 | All Ages | CC | 2462 | 7.00 |
| 50 | All Ages | Former | 12019 | 34.19 |

Year of Follow-up: 2040, Age Group: 18-19

| Period | Age Group | Smoking Group | N | % |
|---|---|---|---|---|
| 50 | 18-19 | Never Any | 0 | . |
| 50 | 18-19 | CC | 0 | . |
| 50 | 18-19 | Former | 0 | . |

Year of Follow-up: 2040, Age Group: 20-24

| Period | Age Group | Smoking Group | N | % |
|---|---|---|---|---|
| 50 | 20-24 | Never Any | 0 | . |
| 50 | 20-24 | CC | 0 | . |
| 50 | 20-24 | Former | 0 | . |

Year of Follow-up: 2040, Age Group: 25-29

| Period | Age Group | Smoking Group | N | % |
|---|---|---|---|---|
| 50 | 25-29 | Never Any | 0 | . |
| 50 | 25-29 | CC | 0 | . |
| 50 | 25-29 | Former | 0 | . |

Year of Follow-up: 2040, Age Group: 30-34

| Period | Age Group | Smoking Group | N | % |
|---|---|---|---|---|
| 50 | 30-34 | Never Any | 0 | . |
| 50 | 30-34 | CC | 0 | . |
| 50 | 30-34 | Former | 0 | . |

Year of Follow-up: 2040, Age Group: 35-39

| Period | Age Group | Smoking Group | N | % |
|---|---|---|---|---|
| 50 | 35-39 | Never Any | 0 | . |
| 50 | 35-39 | CC | 0 | . |
| 50 | 35-39 | Former | 0 | . |

Year of Follow-up: 2040, Age Group: 40-44

| Period | Age Group | Smoking Group | N | % |
|---|---|---|---|---|
| 50 | 40-44 | Never Any | 0 | . |
| 50 | 40-44 | CC | 0 | . |
| 50 | 40-44 | Former | 0 | . |

Year of Follow-up: 2040, Age Group: 45-49

| Period | Age Group | Smoking Group | N | % |
|---|---|---|---|---|
| 50 | 45-49 | Never Any | 0 | . |
| 50 | 45-49 | CC | 0 | . |
| 50 | 45-49 | Former | 0 | . |

Year of Follow-up: 2040, Age Group: 50-54

| Period | Age Group | Smoking Group | N | % |
|---|---|---|---|---|
| 50 | 50-54 | Never Any | 0 | . |
| 50 | 50-54 | CC | 0 | . |
| 50 | 50-54 | Former | 0 | . |

Year of Follow-up: 2040, Age Group: 55-59

| Period | Age Group | Smoking Group | N | % |
|---|---|---|---|---|
| 50 | 55-59 | Never Any | 0 | . |
| 50 | 55-59 | CC | 0 | . |
| 50 | 55-59 | Former | 0 | . |

Year of Follow-up: 2040, Age Group: 60-64

| Period | Age Group | Smoking Group | N | % |
|---|---|---|---|---|

# Results for PHIM for Run 6 (Sensitivity_Analysis_1.RTF)

| | | | | |
|---|---|---|---|---|
| 50 | 60-64 | Never Any | 4803 | 60.57 |
| 50 | 60-64 | CC | 621 | 7.83 |
| 50 | 60-64 | Former | 2506 | 31.60 |

Year of Follow-up: 2040, Age Group: 65-69

| Period | Age Group | Smoking Group | N | % |
|---|---|---|---|---|
| 50 | 65-69 | Never Any | 5407 | 64.75 |
| 50 | 65-69 | CC | 538 | 6.44 |
| 50 | 65-69 | Former | 2405 | 28.80 |

Year of Follow-up: 2040, Age Group: 70-74

| Period | Age Group | Smoking Group | N | % |
|---|---|---|---|---|
| 50 | 70-74 | Never Any | 5181 | 58.92 |
| 50 | 70-74 | CC | 605 | 6.88 |
| 50 | 70-74 | Former | 3007 | 34.20 |

Year of Follow-up: 2040, Age Group: 75-79

| Period | Age Group | Smoking Group | N | % |
|---|---|---|---|---|
| 50 | 75-79 | Never Any | 5279 | 52.38 |
| 50 | 75-79 | CC | 698 | 6.93 |
| 50 | 75-79 | Former | 4101 | 40.69 |

# Results for PHIM for Run 6 (Sensitivity_Analysis_1.RTF)

**************************************************************************************
**Main Output for the Tobacco History for Run: 6, Simulation: 1, Scenario: Ecig**

| Variable Parameter | Value |
|---|---|
| 1. Country | US (United States) |
| 2. Sex | F (Females) |
| 3. Year of start of process | 1990 |

Year of Follow-up: 1990, Age Group: All Ages

| Period | Age Group | Smoking Group | N | % | Ever Quit (N) | Ever Quit % | Mean Yrs Quit | Short Term (n) | Short Term % |
|---|---|---|---|---|---|---|---|---|---|
| 0 | All Ages | Never Any | 47776 | 54.92 | | | | | |
| 0 | All Ages | CC | 22737 | 26.14 | 0 | 0.00 | | | |
| 0 | All Ages | Ecig | 0 | 0.00 | | | | | |
| 0 | All Ages | Dual | 0 | 0.00 | | | | | |
| 0 | All Ages | Former | 16484 | 18.95 | | | 11.90 | 2815 | 17.08 |

Year of Follow-up: 1990, Age Group: 18-19

| Period | Age Group | Smoking Group | N | % | Ever Quit (N) | Ever Quit % | Mean Yrs Quit | Short Term (n) | Short Term % |
|---|---|---|---|---|---|---|---|---|---|
| 0 | 18-19 | Never Any | 2570 | 78.43 | | | | | |
| 0 | 18-19 | CC | 535 | 16.33 | 0 | 0.00 | | | |
| 0 | 18-19 | Ecig | 0 | 0.00 | | | | | |
| 0 | 18-19 | Dual | 0 | 0.00 | | | | | |
| 0 | 18-19 | Former | 172 | 5.25 | | | 0.20 | 172 | 100.00 |

Year of Follow-up: 1990, Age Group: 20-24

| Period | Age Group | Smoking Group | N | % | Ever Quit (N) | Ever Quit % | Mean Yrs Quit | Short Term (n) | Short Term % |
|---|---|---|---|---|---|---|---|---|---|
| 0 | 20-24 | Never Any | 5354 | 60.89 | | | | | |
| 0 | 20-24 | CC | 2560 | 29.11 | 0 | 0.00 | | | |
| 0 | 20-24 | Ecig | 0 | 0.00 | | | | | |
| 0 | 20-24 | Dual | 0 | 0.00 | | | | | |
| 0 | 20-24 | Former | 879 | 10.00 | | | 1.58 | 420 | 47.78 |

Year of Follow-up: 1990, Age Group: 25-29

| Period | Age Group | Smoking Group | N | % | Ever Quit (N) | Ever Quit % | Mean Yrs Quit | Short Term (n) | Short Term % |
|---|---|---|---|---|---|---|---|---|---|
| 0 | 25-29 | Never Any | 5318 | 52.77 | | | | | |
| 0 | 25-29 | CC | 3335 | 33.09 | 0 | 0.00 | | | |
| 0 | 25-29 | Ecig | 0 | 0.00 | | | | | |
| 0 | 25-29 | Dual | 0 | 0.00 | | | | | |
| 0 | 25-29 | Former | 1425 | 14.14 | | | 3.91 | 404 | 28.35 |

Year of Follow-up: 1990, Age Group: 30-34

| Period | Age Group | Smoking Group | N | % | Ever Quit (N) | Ever Quit % | Mean Yrs Quit | Short Term (n) | Short Term % |
|---|---|---|---|---|---|---|---|---|---|
| 0 | 30-34 | Never Any | 5287 | 50.36 | | | | | |
| 0 | 30-34 | CC | 3326 | 31.68 | 0 | 0.00 | | | |
| 0 | 30-34 | Ecig | 0 | 0.00 | | | | | |
| 0 | 30-34 | Dual | 0 | 0.00 | | | | | |
| 0 | 30-34 | Former | 1886 | 17.96 | | | 4.36 | 566 | 30.01 |

Year of Follow-up: 1990, Age Group: 35-39

| Period | Age Group | Smoking Group | N | % | Ever Quit (N) | Ever Quit % | Mean Yrs Quit | Short Term (n) | Short Term % |
|---|---|---|---|---|---|---|---|---|---|
| 0 | 35-39 | Never Any | 5018 | 52.27 | | | | | |
| 0 | 35-39 | CC | 2653 | 27.64 | 0 | 0.00 | | | |
| 0 | 35-39 | Ecig | 0 | 0.00 | | | | | |
| 0 | 35-39 | Dual | 0 | 0.00 | | | | | |
| 0 | 35-39 | Former | 1929 | 20.09 | | | 7.96 | 324 | 16.80 |

Year of Follow-up: 1990, Age Group: 40-44

| Period | Age Group | Smoking Group | N | % | Ever Quit (N) | Ever Quit % | Mean Yrs Quit | Short Term (n) | Short Term % |
|---|---|---|---|---|---|---|---|---|---|
| 0 | 40-44 | Never Any | 4356 | 51.27 | | | | | |
| 0 | 40-44 | CC | 2397 | 28.21 | 0 | 0.00 | | | |
| 0 | 40-44 | Ecig | 0 | 0.00 | | | | | |
| 0 | 40-44 | Dual | 0 | 0.00 | | | | | |
| 0 | 40-44 | Former | 1744 | 20.52 | | | 7.33 | 339 | 19.44 |

Year of Follow-up: 1990, Age Group: 45-49

| Period | Age Group | Smoking Group | N | % | Ever Quit (N) | Ever Quit % | Mean Yrs Quit | Short Term (n) | Short Term % |
|---|---|---|---|---|---|---|---|---|---|
| 0 | 45-49 | Never Any | 3116 | 47.67 | | | | | |
| 0 | 45-49 | CC | 1875 | 28.69 | 0 | 0.00 | | | |
| 0 | 45-49 | Ecig | 0 | 0.00 | | | | | |
| 0 | 45-49 | Dual | 0 | 0.00 | | | | | |
| 0 | 45-49 | Former | 1545 | 23.64 | | | 12.55 | 194 | 12.56 |

Year of Follow-up: 1990, Age Group: 50-54

| Period | Age Group | Smoking Group | N | % | Ever Quit (N) | Ever Quit % | Mean Yrs Quit | Short Term (n) | Short Term % |
|---|---|---|---|---|---|---|---|---|---|
| 0 | 50-54 | Never Any | 2684 | 48.50 | | | | | |
| 0 | 50-54 | CC | 1572 | 28.41 | 0 | 0.00 | | | |

# Results for PHIM for Run 6 (Sensitivity_Analysis_1.RTF)

| | | | | | | | | | |
|---|---|---|---|---|---|---|---|---|---|
| 0 | 50-54 | Ecig | 0 | 0.00 | | | | | |
| 0 | 50-54 | Dual | 0 | 0.00 | | | | | |
| 0 | 50-54 | Former | 1278 | 23.09 | | | 13.28 | 126 | 9.86 |

Year of Follow-up: 1990, Age Group: 55-59

| Period | Age Group | Smoking Group | N | % | Ever Quit (N) | Ever Quit % | Mean Yrs Quit | Short Term (n) | Short Term % |
|---|---|---|---|---|---|---|---|---|---|
| 0 | 55-59 | Never Any | 2732 | 51.90 | | | | | |
| 0 | 55-59 | CC | 1295 | 24.60 | 0 | 0.00 | | | |
| 0 | 55-59 | Ecig | 0 | 0.00 | | | | | |
| 0 | 55-59 | Dual | 0 | 0.00 | | | | | |
| 0 | 55-59 | Former | 1237 | 23.50 | | | 17.26 | 90 | 7.28 |

Year of Follow-up: 1990, Age Group: 60-64

| Period | Age Group | Smoking Group | N | % | Ever Quit (N) | Ever Quit % | Mean Yrs Quit | Short Term (n) | Short Term % |
|---|---|---|---|---|---|---|---|---|---|
| 0 | 60-64 | Never Any | 3078 | 55.72 | | | | | |
| 0 | 60-64 | CC | 1249 | 22.61 | 0 | 0.00 | | | |
| 0 | 60-64 | Ecig | 0 | 0.00 | | | | | |
| 0 | 60-64 | Dual | 0 | 0.00 | | | | | |
| 0 | 60-64 | Former | 1197 | 21.67 | | | 18.28 | 61 | 5.10 |

Year of Follow-up: 1990, Age Group: 65-69

| Period | Age Group | Smoking Group | N | % | Ever Quit (N) | Ever Quit % | Mean Yrs Quit | Short Term (n) | Short Term % |
|---|---|---|---|---|---|---|---|---|---|
| 0 | 65-69 | Never Any | 3042 | 57.36 | | | | | |
| 0 | 65-69 | CC | 976 | 18.40 | 0 | 0.00 | | | |
| 0 | 65-69 | Ecig | 0 | 0.00 | | | | | |
| 0 | 65-69 | Dual | 0 | 0.00 | | | | | |
| 0 | 65-69 | Former | 1285 | 24.23 | | | 20.65 | 68 | 5.29 |

Year of Follow-up: 1990, Age Group: 70-74

| Period | Age Group | Smoking Group | N | % | Ever Quit (N) | Ever Quit % | Mean Yrs Quit | Short Term (n) | Short Term % |
|---|---|---|---|---|---|---|---|---|---|
| 0 | 70-74 | Never Any | 2738 | 61.05 | | | | | |
| 0 | 70-74 | CC | 610 | 13.60 | 0 | 0.00 | | | |
| 0 | 70-74 | Ecig | 0 | 0.00 | | | | | |
| 0 | 70-74 | Dual | 0 | 0.00 | | | | | |
| 0 | 70-74 | Former | 1137 | 25.35 | | | 24.48 | 27 | 2.37 |

Year of Follow-up: 1990, Age Group: 75-79

| Period | Age Group | Smoking Group | N | % | Ever Quit (N) | Ever Quit % | Mean Yrs Quit | Short Term (n) | Short Term % |
|---|---|---|---|---|---|---|---|---|---|
| 0 | 75-79 | Never Any | 2483 | 68.84 | | | | | |
| 0 | 75-79 | CC | 354 | 9.81 | 0 | 0.00 | | | |
| 0 | 75-79 | Ecig | 0 | 0.00 | | | | | |
| 0 | 75-79 | Dual | 0 | 0.00 | | | | | |
| 0 | 75-79 | Former | 770 | 21.35 | | | 24.48 | 24 | 3.12 |

Year of Follow-up: 1995, Age Group: All Ages

| Period | Age Group | Smoking Group | N | % | Ever Quit (N) | Ever Quit % | Mean Yrs Quit | Short Term (n) | Short Term % |
|---|---|---|---|---|---|---|---|---|---|
| 5 | All Ages | Never Any | 50887 | 55.53 | | | | | |
| 5 | All Ages | CC | 10659 | 11.63 | 0 | 0.00 | | | |
| 5 | All Ages | Ecig | 11697 | 12.76 | | | | | |
| 5 | All Ages | Dual | 0 | 0.00 | | | | | |
| 5 | All Ages | Former | 18396 | 20.07 | | | 14.19 | 1129 | 6.14 |

Year of Follow-up: 1995, Age Group: 18-19

| Period | Age Group | Smoking Group | N | % | Ever Quit (N) | Ever Quit % | Mean Yrs Quit | Short Term (n) | Short Term % |
|---|---|---|---|---|---|---|---|---|---|
| 5 | 18-19 | Never Any | 2434 | 76.64 | | | | | |
| 5 | 18-19 | CC | 233 | 7.34 | 0 | 0.00 | | | |
| 5 | 18-19 | Ecig | 427 | 13.44 | | | | | |
| 5 | 18-19 | Dual | 0 | 0.00 | | | | | |
| 5 | 18-19 | Former | 82 | 2.58 | | | 1.15 | 52 | 63.41 |

Year of Follow-up: 1995, Age Group: 20-24

| Period | Age Group | Smoking Group | N | % | Ever Quit (N) | Ever Quit % | Mean Yrs Quit | Short Term (n) | Short Term % |
|---|---|---|---|---|---|---|---|---|---|
| 5 | 20-24 | Never Any | 5794 | 69.39 | | | | | |
| 5 | 20-24 | CC | 862 | 10.32 | 0 | 0.00 | | | |
| 5 | 20-24 | Ecig | 1250 | 14.97 | | | | | |
| 5 | 20-24 | Dual | 0 | 0.00 | | | | | |
| 5 | 20-24 | Former | 444 | 5.32 | | | 3.23 | 106 | 23.87 |

Year of Follow-up: 1995, Age Group: 25-29

| Period | Age Group | Smoking Group | N | % | Ever Quit (N) | Ever Quit % | Mean Yrs Quit | Short Term (n) | Short Term % |
|---|---|---|---|---|---|---|---|---|---|
| 5 | 25-29 | Never Any | 5313 | 60.42 | | | | | |
| 5 | 25-29 | CC | 1062 | 12.08 | 0 | 0.00 | | | |
| 5 | 25-29 | Ecig | 1395 | 15.86 | | | | | |
| 5 | 25-29 | Dual | 0 | 0.00 | | | | | |
| 5 | 25-29 | Former | 1023 | 11.63 | | | 5.94 | 54 | 5.28 |

Year of Follow-up: 1995, Age Group: 30-34

| Period | Age Group | Smoking Group | N | % | Ever Quit (N) | Ever Quit % | Mean Yrs Quit | Short Term (n) | Short Term % |
|---|---|---|---|---|---|---|---|---|---|

# Results for PHIM for Run 6 (Sensitivity_Analysis_1.RTF)

| | | | | | | | | | |
|---|---|---|---|---|---|---|---|---|---|
| 5 | 30-34 | Never Any | 5304 | 52.63 | | | | | |
| 5 | 30-34 | CC | 1373 | 13.62 | 0 | 0.00 | | | |
| 5 | 30-34 | Ecig | 1802 | 17.88 | | | | | |
| 5 | 30-34 | Dual | 0 | 0.00 | | | | | |
| 5 | 30-34 | Former | 1599 | 15.87 | | | 8.16 | 67 | 4.19 |

Year of Follow-up: 1995, Age Group: 35-39

| Period | Age Group | Smoking Group | N | % | Ever Quit (N) | Ever Quit % | Mean Yrs Quit | Short Term (n) | Short Term % |
|---|---|---|---|---|---|---|---|---|---|
| 5 | 35-39 | Never Any | 5278 | 50.27 | | | | | |
| 5 | 35-39 | CC | 1410 | 13.43 | 0 | 0.00 | | | |
| 5 | 35-39 | Ecig | 1746 | 16.63 | | | | | |
| 5 | 35-39 | Dual | 0 | 0.00 | | | | | |
| 5 | 35-39 | Former | 2065 | 19.67 | | | 8.75 | 62 | 3.00 |

Year of Follow-up: 1995, Age Group: 40-44

| Period | Age Group | Smoking Group | N | % | Ever Quit (N) | Ever Quit % | Mean Yrs Quit | Short Term (n) | Short Term % |
|---|---|---|---|---|---|---|---|---|---|
| 5 | 40-44 | Never Any | 5018 | 52.27 | | | | | |
| 5 | 40-44 | CC | 1135 | 11.82 | 0 | 0.00 | | | |
| 5 | 40-44 | Ecig | 1356 | 14.13 | | | | | |
| 5 | 40-44 | Dual | 0 | 0.00 | | | | | |
| 5 | 40-44 | Former | 2091 | 21.78 | | | 12.09 | 81 | 3.87 |

Year of Follow-up: 1995, Age Group: 45-49

| Period | Age Group | Smoking Group | N | % | Ever Quit (N) | Ever Quit % | Mean Yrs Quit | Short Term (n) | Short Term % |
|---|---|---|---|---|---|---|---|---|---|
| 5 | 45-49 | Never Any | 4356 | 51.27 | | | | | |
| 5 | 45-49 | CC | 974 | 11.46 | 0 | 0.00 | | | |
| 5 | 45-49 | Ecig | 1161 | 13.66 | | | | | |
| 5 | 45-49 | Dual | 0 | 0.00 | | | | | |
| 5 | 45-49 | Former | 2006 | 23.61 | | | 10.93 | 133 | 6.63 |

Year of Follow-up: 1995, Age Group: 50-54

| Period | Age Group | Smoking Group | N | % | Ever Quit (N) | Ever Quit % | Mean Yrs Quit | Short Term (n) | Short Term % |
|---|---|---|---|---|---|---|---|---|---|
| 5 | 50-54 | Never Any | 3116 | 47.67 | | | | | |
| 5 | 50-54 | CC | 739 | 11.31 | 0 | 0.00 | | | |
| 5 | 50-54 | Ecig | 826 | 12.64 | | | | | |
| 5 | 50-54 | Dual | 0 | 0.00 | | | | | |
| 5 | 50-54 | Former | 1855 | 28.38 | | | 14.92 | 140 | 7.55 |

Year of Follow-up: 1995, Age Group: 55-59

| Period | Age Group | Smoking Group | N | % | Ever Quit (N) | Ever Quit % | Mean Yrs Quit | Short Term (n) | Short Term % |
|---|---|---|---|---|---|---|---|---|---|
| 5 | 55-59 | Never Any | 2684 | 48.50 | | | | | |
| 5 | 55-59 | CC | 722 | 13.05 | 0 | 0.00 | | | |
| 5 | 55-59 | Ecig | 627 | 11.33 | | | | | |
| 5 | 55-59 | Dual | 0 | 0.00 | | | | | |
| 5 | 55-59 | Former | 1501 | 27.12 | | | 15.84 | 93 | 6.20 |

Year of Follow-up: 1995, Age Group: 60-64

| Period | Age Group | Smoking Group | N | % | Ever Quit (N) | Ever Quit % | Mean Yrs Quit | Short Term (n) | Short Term % |
|---|---|---|---|---|---|---|---|---|---|
| 5 | 60-64 | Never Any | 2732 | 51.90 | | | | | |
| 5 | 60-64 | CC | 633 | 12.03 | 0 | 0.00 | | | |
| 5 | 60-64 | Ecig | 444 | 8.43 | | | | | |
| 5 | 60-64 | Dual | 0 | 0.00 | | | | | |
| 5 | 60-64 | Former | 1455 | 27.64 | | | 19.22 | 87 | 5.98 |

Year of Follow-up: 1995, Age Group: 65-69

| Period | Age Group | Smoking Group | N | % | Ever Quit (N) | Ever Quit % | Mean Yrs Quit | Short Term (n) | Short Term % |
|---|---|---|---|---|---|---|---|---|---|
| 5 | 65-69 | Never Any | 3078 | 55.72 | | | | | |
| 5 | 65-69 | CC | 602 | 10.90 | 0 | 0.00 | | | |
| 5 | 65-69 | Ecig | 374 | 6.77 | | | | | |
| 5 | 65-69 | Dual | 0 | 0.00 | | | | | |
| 5 | 65-69 | Former | 1470 | 26.61 | | | 19.34 | 107 | 7.28 |

Year of Follow-up: 1995, Age Group: 70-74

| Period | Age Group | Smoking Group | N | % | Ever Quit (N) | Ever Quit % | Mean Yrs Quit | Short Term (n) | Short Term % |
|---|---|---|---|---|---|---|---|---|---|
| 5 | 70-74 | Never Any | 3042 | 57.36 | | | | | |
| 5 | 70-74 | CC | 577 | 10.88 | 0 | 0.00 | | | |
| 5 | 70-74 | Ecig | 188 | 3.55 | | | | | |
| 5 | 70-74 | Dual | 0 | 0.00 | | | | | |
| 5 | 70-74 | Former | 1496 | 28.21 | | | 22.32 | 77 | 5.15 |

Year of Follow-up: 1995, Age Group: 75-79

| Period | Age Group | Smoking Group | N | % | Ever Quit (N) | Ever Quit % | Mean Yrs Quit | Short Term (n) | Short Term % |
|---|---|---|---|---|---|---|---|---|---|
| 5 | 75-79 | Never Any | 2738 | 61.05 | | | | | |
| 5 | 75-79 | CC | 337 | 7.51 | 0 | 0.00 | | | |
| 5 | 75-79 | Ecig | 101 | 2.25 | | | | | |
| 5 | 75-79 | Dual | 0 | 0.00 | | | | | |
| 5 | 75-79 | Former | 1309 | 29.19 | | | 25.87 | 70 | 5.35 |

Year of Follow-up: 2000, Age Group: All Ages

# Results for PHIM for Run 6 (Sensitivity_Analysis_1.RTF)

| Period | Age Group | Smoking Group | N | % | Ever Quit (N) | Ever Quit % | Mean Yrs Quit | Short Term (n) | Short Term % |
|---|---|---|---|---|---|---|---|---|---|
| 10 | All Ages | Never Any | 50423 | 54.86 | | | | | |
| 10 | All Ages | CC | 4595 | 5.00 | 0 | 0.00 | | | |
| 10 | All Ages | Ecig | 15644 | 17.02 | | | | | |
| 10 | All Ages | Dual | 0 | 0.00 | | | | | |
| 10 | All Ages | Former | 21246 | 23.12 | | | 15.11 | 1717 | 8.08 |

Year of Follow-up: 2000, Age Group: 18-19

| Period | Age Group | Smoking Group | N | % | Ever Quit (N) | Ever Quit % | Mean Yrs Quit | Short Term (n) | Short Term % |
|---|---|---|---|---|---|---|---|---|---|
| 10 | 18-19 | Never Any | 0 | . | | | | | |
| 10 | 18-19 | CC | 0 | . | 0 | . | | | |
| 10 | 18-19 | Ecig | 0 | . | | | | | |
| 10 | 18-19 | Dual | 0 | . | | | | | |
| 10 | 18-19 | Former | 0 | . | | | . | 0 | . |

Year of Follow-up: 2000, Age Group: 20-24

| Period | Age Group | Smoking Group | N | % | Ever Quit (N) | Ever Quit % | Mean Yrs Quit | Short Term (n) | Short Term % |
|---|---|---|---|---|---|---|---|---|---|
| 10 | 20-24 | Never Any | 5128 | 64.67 | | | | | |
| 10 | 20-24 | CC | 370 | 4.67 | 0 | 0.00 | | | |
| 10 | 20-24 | Ecig | 1780 | 22.45 | | | | | |
| 10 | 20-24 | Dual | 0 | 0.00 | | | | | |
| 10 | 20-24 | Former | 652 | 8.22 | | | 3.55 | 169 | 25.92 |

Year of Follow-up: 2000, Age Group: 25-29

| Period | Age Group | Smoking Group | N | % | Ever Quit (N) | Ever Quit % | Mean Yrs Quit | Short Term (n) | Short Term % |
|---|---|---|---|---|---|---|---|---|---|
| 10 | 25-29 | Never Any | 5493 | 65.78 | | | | | |
| 10 | 25-29 | CC | 400 | 4.79 | 0 | 0.00 | | | |
| 10 | 25-29 | Ecig | 1690 | 20.24 | | | | | |
| 10 | 25-29 | Dual | 0 | 0.00 | | | | | |
| 10 | 25-29 | Former | 767 | 9.19 | | | 5.59 | 126 | 16.43 |

Year of Follow-up: 2000, Age Group: 30-34

| Period | Age Group | Smoking Group | N | % | Ever Quit (N) | Ever Quit % | Mean Yrs Quit | Short Term (n) | Short Term % |
|---|---|---|---|---|---|---|---|---|---|
| 10 | 30-34 | Never Any | 5214 | 59.30 | | | | | |
| 10 | 30-34 | CC | 417 | 4.74 | 0 | 0.00 | | | |
| 10 | 30-34 | Ecig | 1908 | 21.70 | | | | | |
| 10 | 30-34 | Dual | 0 | 0.00 | | | | | |
| 10 | 30-34 | Former | 1254 | 14.26 | | | 9.28 | 91 | 7.26 |

Year of Follow-up: 2000, Age Group: 35-39

| Period | Age Group | Smoking Group | N | % | Ever Quit (N) | Ever Quit % | Mean Yrs Quit | Short Term (n) | Short Term % |
|---|---|---|---|---|---|---|---|---|---|
| 10 | 35-39 | Never Any | 5284 | 52.43 | | | | | |
| 10 | 35-39 | CC | 561 | 5.57 | 0 | 0.00 | | | |
| 10 | 35-39 | Ecig | 2413 | 23.94 | | | | | |
| 10 | 35-39 | Dual | 0 | 0.00 | | | | | |
| 10 | 35-39 | Former | 1820 | 18.06 | | | 11.77 | 105 | 5.77 |

Year of Follow-up: 2000, Age Group: 40-44

| Period | Age Group | Smoking Group | N | % | Ever Quit (N) | Ever Quit % | Mean Yrs Quit | Short Term (n) | Short Term % |
|---|---|---|---|---|---|---|---|---|---|
| 10 | 40-44 | Never Any | 5278 | 50.27 | | | | | |
| 10 | 40-44 | CC | 584 | 5.56 | 0 | 0.00 | | | |
| 10 | 40-44 | Ecig | 2292 | 21.83 | | | | | |
| 10 | 40-44 | Dual | 0 | 0.00 | | | | | |
| 10 | 40-44 | Former | 2345 | 22.34 | | | 12.30 | 143 | 6.10 |

Year of Follow-up: 2000, Age Group: 45-49

| Period | Age Group | Smoking Group | N | % | Ever Quit (N) | Ever Quit % | Mean Yrs Quit | Short Term (n) | Short Term % |
|---|---|---|---|---|---|---|---|---|---|
| 10 | 45-49 | Never Any | 5018 | 52.27 | | | | | |
| 10 | 45-49 | CC | 446 | 4.65 | 0 | 0.00 | | | |
| 10 | 45-49 | Ecig | 1673 | 17.43 | | | | | |
| 10 | 45-49 | Dual | 0 | 0.00 | | | | | |
| 10 | 45-49 | Former | 2463 | 25.66 | | | 14.76 | 181 | 7.35 |

Year of Follow-up: 2000, Age Group: 50-54

| Period | Age Group | Smoking Group | N | % | Ever Quit (N) | Ever Quit % | Mean Yrs Quit | Short Term (n) | Short Term % |
|---|---|---|---|---|---|---|---|---|---|
| 10 | 50-54 | Never Any | 4356 | 51.27 | | | | | |
| 10 | 50-54 | CC | 378 | 4.45 | 0 | 0.00 | | | |
| 10 | 50-54 | Ecig | 1299 | 15.29 | | | | | |
| 10 | 50-54 | Dual | 0 | 0.00 | | | | | |
| 10 | 50-54 | Former | 2464 | 29.00 | | | 13.32 | 188 | 7.63 |

Year of Follow-up: 2000, Age Group: 55-59

| Period | Age Group | Smoking Group | N | % | Ever Quit (N) | Ever Quit % | Mean Yrs Quit | Short Term (n) | Short Term % |
|---|---|---|---|---|---|---|---|---|---|
| 10 | 55-59 | Never Any | 3116 | 47.67 | | | | | |
| 10 | 55-59 | CC | 283 | 4.33 | 0 | 0.00 | | | |
| 10 | 55-59 | Ecig | 912 | 13.95 | | | | | |
| 10 | 55-59 | Dual | 0 | 0.00 | | | | | |

# Results for PHIM for Run 6 (Sensitivity_Analysis_1.RTF)

| | | | | | | | | | |
|---|---|---|---|---|---|---|---|---|---|
| 10 | 55-59 | Former | 2225 | 34.04 | | | 16.92 | 157 | 7.06 |

Year of Follow-up: 2000, Age Group: 60-64

| Period | Age Group | Smoking Group | N | % | Ever Quit (N) | Ever Quit % | Mean Yrs Quit | Short Term (n) | Short Term % |
|---|---|---|---|---|---|---|---|---|---|
| 10 | 60-64 | Never Any | 2684 | 48.50 | | | | | |
| 10 | 60-64 | CC | 334 | 6.04 | 0 | 0.00 | | | |
| 10 | 60-64 | Ecig | 663 | 11.98 | | | | | |
| 10 | 60-64 | Dual | 0 | 0.00 | | | | | |
| 10 | 60-64 | Former | 1853 | 33.48 | | | 17.23 | 149 | 8.04 |

Year of Follow-up: 2000, Age Group: 65-69

| Period | Age Group | Smoking Group | N | % | Ever Quit (N) | Ever Quit % | Mean Yrs Quit | Short Term (n) | Short Term % |
|---|---|---|---|---|---|---|---|---|---|
| 10 | 65-69 | Never Any | 2732 | 51.90 | | | | | |
| 10 | 65-69 | CC | 280 | 5.32 | 0 | 0.00 | | | |
| 10 | 65-69 | Ecig | 443 | 8.42 | | | | | |
| 10 | 65-69 | Dual | 0 | 0.00 | | | | | |
| 10 | 65-69 | Former | 1809 | 34.37 | | | 19.85 | 146 | 8.07 |

Year of Follow-up: 2000, Age Group: 70-74

| Period | Age Group | Smoking Group | N | % | Ever Quit (N) | Ever Quit % | Mean Yrs Quit | Short Term (n) | Short Term % |
|---|---|---|---|---|---|---|---|---|---|
| 10 | 70-74 | Never Any | 3078 | 55.72 | | | | | |
| 10 | 70-74 | CC | 274 | 4.96 | 0 | 0.00 | | | |
| 10 | 70-74 | Ecig | 368 | 6.66 | | | | | |
| 10 | 70-74 | Dual | 0 | 0.00 | | | | | |
| 10 | 70-74 | Former | 1804 | 32.66 | | | 20.18 | 154 | 8.54 |

Year of Follow-up: 2000, Age Group: 75-79

| Period | Age Group | Smoking Group | N | % | Ever Quit (N) | Ever Quit % | Mean Yrs Quit | Short Term (n) | Short Term % |
|---|---|---|---|---|---|---|---|---|---|
| 10 | 75-79 | Never Any | 3042 | 57.36 | | | | | |
| 10 | 75-79 | CC | 268 | 5.05 | 0 | 0.00 | | | |
| 10 | 75-79 | Ecig | 203 | 3.83 | | | | | |
| 10 | 75-79 | Dual | 0 | 0.00 | | | | | |
| 10 | 75-79 | Former | 1790 | 33.75 | | | 23.17 | 108 | 6.03 |

Year of Follow-up: 2005, Age Group: All Ages

| Period | Age Group | Smoking Group | N | % | Ever Quit (N) | Ever Quit % | Mean Yrs Quit | Short Term (n) | Short Term % |
|---|---|---|---|---|---|---|---|---|---|
| 15 | All Ages | Never Any | 47002 | 54.27 | | | | | |
| 15 | All Ages | CC | 1890 | 2.18 | 0 | 0.00 | | | |
| 15 | All Ages | Ecig | 15617 | 18.03 | | | | | |
| 15 | All Ages | Dual | 0 | 0.00 | | | | | |
| 15 | All Ages | Former | 22096 | 25.51 | | | 17.32 | 941 | 4.26 |

Year of Follow-up: 2005, Age Group: 18-19

| Period | Age Group | Smoking Group | N | % | Ever Quit (N) | Ever Quit % | Mean Yrs Quit | Short Term (n) | Short Term % |
|---|---|---|---|---|---|---|---|---|---|
| 15 | 18-19 | Never Any | 0 | . | | | | | |
| 15 | 18-19 | CC | 0 | . | 0 | . | | | |
| 15 | 18-19 | Ecig | 0 | . | | | | | |
| 15 | 18-19 | Dual | 0 | . | | | | | |
| 15 | 18-19 | Former | 0 | . | | | . | 0 | . |

Year of Follow-up: 2005, Age Group: 20-24

| Period | Age Group | Smoking Group | N | % | Ever Quit (N) | Ever Quit % | Mean Yrs Quit | Short Term (n) | Short Term % |
|---|---|---|---|---|---|---|---|---|---|
| 15 | 20-24 | Never Any | 0 | . | | | | | |
| 15 | 20-24 | CC | 0 | . | 0 | . | | | |
| 15 | 20-24 | Ecig | 0 | . | | | | | |
| 15 | 20-24 | Dual | 0 | . | | | | | |
| 15 | 20-24 | Former | 0 | . | | | . | 0 | . |

Year of Follow-up: 2005, Age Group: 25-29

| Period | Age Group | Smoking Group | N | % | Ever Quit (N) | Ever Quit % | Mean Yrs Quit | Short Term (n) | Short Term % |
|---|---|---|---|---|---|---|---|---|---|
| 15 | 25-29 | Never Any | 4860 | 61.29 | | | | | |
| 15 | 25-29 | CC | 166 | 2.09 | 0 | 0.00 | | | |
| 15 | 25-29 | Ecig | 1941 | 24.48 | | | | | |
| 15 | 25-29 | Dual | 0 | 0.00 | | | | | |
| 15 | 25-29 | Former | 963 | 12.14 | | | 6.57 | 96 | 9.97 |

Year of Follow-up: 2005, Age Group: 30-34

| Period | Age Group | Smoking Group | N | % | Ever Quit (N) | Ever Quit % | Mean Yrs Quit | Short Term (n) | Short Term % |
|---|---|---|---|---|---|---|---|---|---|
| 15 | 30-34 | Never Any | 5407 | 64.75 | | | | | |
| 15 | 30-34 | CC | 149 | 1.78 | 0 | 0.00 | | | |
| 15 | 30-34 | Ecig | 1822 | 21.82 | | | | | |
| 15 | 30-34 | Dual | 0 | 0.00 | | | | | |
| 15 | 30-34 | Former | 972 | 11.64 | | | 8.77 | 80 | 8.23 |

Year of Follow-up: 2005, Age Group: 35-39

| Period | Age Group | Smoking Group | N | % | Ever Quit (N) | Ever Quit % | Mean Yrs Quit | Short Term (n) | Short Term % |
|---|---|---|---|---|---|---|---|---|---|
| 15 | 35-39 | Never Any | 5189 | 59.01 | | | | | |
| 15 | 35-39 | CC | 167 | 1.90 | 0 | 0.00 | | | |

# Results for PHIM for Run 6 (Sensitivity_Analysis_1.RTF)

| | | | | | | | | | |
|---|---|---|---|---|---|---|---|---|---|
| 15 | 35-39 | Ecig | 2002 | 22.77 | | | | | |
| 15 | 35-39 | Dual | 0 | 0.00 | | | | | |
| 15 | 35-39 | Former | 1435 | 16.32 | | | 12.75 | 66 | 4.60 |

Year of Follow-up: 2005, Age Group: 40-44

| Period | Age Group | Smoking Group | N | % | Ever Quit (N) | Ever Quit % | Mean Yrs Quit | Short Term (n) | Short Term % |
|---|---|---|---|---|---|---|---|---|---|
| 15 | 40-44 | Never Any | 5284 | 52.43 | | | | | |
| 15 | 40-44 | CC | 238 | 2.36 | 0 | 0.00 | | | |
| 15 | 40-44 | Ecig | 2500 | 24.81 | | | | | |
| 15 | 40-44 | Dual | 0 | 0.00 | | | | | |
| 15 | 40-44 | Former | 2056 | 20.40 | | | 15.08 | 101 | 4.91 |

Year of Follow-up: 2005, Age Group: 45-49

| Period | Age Group | Smoking Group | N | % | Ever Quit (N) | Ever Quit % | Mean Yrs Quit | Short Term (n) | Short Term % |
|---|---|---|---|---|---|---|---|---|---|
| 15 | 45-49 | Never Any | 5278 | 50.27 | | | | | |
| 15 | 45-49 | CC | 237 | 2.26 | 0 | 0.00 | | | |
| 15 | 45-49 | Ecig | 2318 | 22.08 | | | | | |
| 15 | 45-49 | Dual | 0 | 0.00 | | | | | |
| 15 | 45-49 | Former | 2666 | 25.39 | | | 15.45 | 135 | 5.06 |

Year of Follow-up: 2005, Age Group: 50-54

| Period | Age Group | Smoking Group | N | % | Ever Quit (N) | Ever Quit % | Mean Yrs Quit | Short Term (n) | Short Term % |
|---|---|---|---|---|---|---|---|---|---|
| 15 | 50-54 | Never Any | 5018 | 52.27 | | | | | |
| 15 | 50-54 | CC | 191 | 1.99 | 0 | 0.00 | | | |
| 15 | 50-54 | Ecig | 1622 | 16.90 | | | | | |
| 15 | 50-54 | Dual | 0 | 0.00 | | | | | |
| 15 | 50-54 | Former | 2769 | 28.84 | | | 17.84 | 99 | 3.58 |

Year of Follow-up: 2005, Age Group: 55-59

| Period | Age Group | Smoking Group | N | % | Ever Quit (N) | Ever Quit % | Mean Yrs Quit | Short Term (n) | Short Term % |
|---|---|---|---|---|---|---|---|---|---|
| 15 | 55-59 | Never Any | 4356 | 51.27 | | | | | |
| 15 | 55-59 | CC | 166 | 1.95 | 0 | 0.00 | | | |
| 15 | 55-59 | Ecig | 1281 | 15.08 | | | | | |
| 15 | 55-59 | Dual | 0 | 0.00 | | | | | |
| 15 | 55-59 | Former | 2694 | 31.71 | | | 16.95 | 78 | 2.90 |

Year of Follow-up: 2005, Age Group: 60-64

| Period | Age Group | Smoking Group | N | % | Ever Quit (N) | Ever Quit % | Mean Yrs Quit | Short Term (n) | Short Term % |
|---|---|---|---|---|---|---|---|---|---|
| 15 | 60-64 | Never Any | 3116 | 47.67 | | | | | |
| 15 | 60-64 | CC | 131 | 2.00 | 0 | 0.00 | | | |
| 15 | 60-64 | Ecig | 852 | 13.04 | | | | | |
| 15 | 60-64 | Dual | 0 | 0.00 | | | | | |
| 15 | 60-64 | Former | 2437 | 37.29 | | | 20.18 | 93 | 3.82 |

Year of Follow-up: 2005, Age Group: 65-69

| Period | Age Group | Smoking Group | N | % | Ever Quit (N) | Ever Quit % | Mean Yrs Quit | Short Term (n) | Short Term % |
|---|---|---|---|---|---|---|---|---|---|
| 15 | 65-69 | Never Any | 2684 | 48.50 | | | | | |
| 15 | 65-69 | CC | 160 | 2.89 | 0 | 0.00 | | | |
| 15 | 65-69 | Ecig | 591 | 10.68 | | | | | |
| 15 | 65-69 | Dual | 0 | 0.00 | | | | | |
| 15 | 65-69 | Former | 2099 | 37.93 | | | 19.92 | 68 | 3.24 |

Year of Follow-up: 2005, Age Group: 70-74

| Period | Age Group | Smoking Group | N | % | Ever Quit (N) | Ever Quit % | Mean Yrs Quit | Short Term (n) | Short Term % |
|---|---|---|---|---|---|---|---|---|---|
| 15 | 70-74 | Never Any | 2732 | 51.90 | | | | | |
| 15 | 70-74 | CC | 139 | 2.64 | 0 | 0.00 | | | |
| 15 | 70-74 | Ecig | 391 | 7.43 | | | | | |
| 15 | 70-74 | Dual | 0 | 0.00 | | | | | |
| 15 | 70-74 | Former | 2002 | 38.03 | | | 22.67 | 69 | 3.45 |

Year of Follow-up: 2005, Age Group: 75-79

| Period | Age Group | Smoking Group | N | % | Ever Quit (N) | Ever Quit % | Mean Yrs Quit | Short Term (n) | Short Term % |
|---|---|---|---|---|---|---|---|---|---|
| 15 | 75-79 | Never Any | 3078 | 55.72 | | | | | |
| 15 | 75-79 | CC | 146 | 2.64 | 0 | 0.00 | | | |
| 15 | 75-79 | Ecig | 297 | 5.38 | | | | | |
| 15 | 75-79 | Dual | 0 | 0.00 | | | | | |
| 15 | 75-79 | Former | 2003 | 36.26 | | | 22.91 | 56 | 2.80 |

Year of Follow-up: 2010, Age Group: All Ages

| Period | Age Group | Smoking Group | N | % | Ever Quit (N) | Ever Quit % | Mean Yrs Quit | Short Term (n) | Short Term % |
|---|---|---|---|---|---|---|---|---|---|
| 20 | All Ages | Never Any | 43759 | 53.97 | | | | | |
| 20 | All Ages | CC | 765 | 0.94 | 0 | 0.00 | | | |
| 20 | All Ages | Ecig | 13785 | 17.00 | | | | | |
| 20 | All Ages | Dual | 0 | 0.00 | | | | | |
| 20 | All Ages | Former | 22772 | 28.09 | | | 19.42 | 1117 | 4.91 |

Year of Follow-up: 2010, Age Group: 18-19

| Period | Age Group | Smoking Group | N | % | Ever Quit (N) | Ever Quit % | Mean Yrs Quit | Short Term (n) | Short Term % |
|---|---|---|---|---|---|---|---|---|---|

## Results for PHIM for Run 6 (Sensitivity_Analysis_1.RTF)

| | | | | | | | | | |
|---|---|---|---|---|---|---|---|---|---|
| 20 | 18-19 | Never Any | 0 | . | | | | | |
| 20 | 18-19 | CC | 0 | . | 0 | . | | | |
| 20 | 18-19 | Ecig | 0 | . | | | | | |
| 20 | 18-19 | Dual | 0 | . | | | | | |
| 20 | 18-19 | Former | 0 | . | | | . | 0 | . |

Year of Follow-up: 2010, Age Group: 20-24

| Period | Age Group | Smoking Group | N | % | Ever Quit (N) | Ever Quit % | Mean Yrs Quit | Short Term (n) | Short Term % |
|---|---|---|---|---|---|---|---|---|---|
| 20 | 20-24 | Never Any | 0 | . | | | | | |
| 20 | 20-24 | CC | 0 | . | 0 | . | | | |
| 20 | 20-24 | Ecig | 0 | . | | | | | |
| 20 | 20-24 | Dual | 0 | . | | | | | |
| 20 | 20-24 | Former | 0 | . | | | . | 0 | . |

Year of Follow-up: 2010, Age Group: 25-29

| Period | Age Group | Smoking Group | N | % | Ever Quit (N) | Ever Quit % | Mean Yrs Quit | Short Term (n) | Short Term % |
|---|---|---|---|---|---|---|---|---|---|
| 20 | 25-29 | Never Any | 0 | . | | | | | |
| 20 | 25-29 | CC | 0 | . | 0 | . | | | |
| 20 | 25-29 | Ecig | 0 | . | | | | | |
| 20 | 25-29 | Dual | 0 | . | | | | | |
| 20 | 25-29 | Former | 0 | . | | | . | 0 | . |

Year of Follow-up: 2010, Age Group: 30-34

| Period | Age Group | Smoking Group | N | % | Ever Quit (N) | Ever Quit % | Mean Yrs Quit | Short Term (n) | Short Term % |
|---|---|---|---|---|---|---|---|---|---|
| 20 | 30-34 | Never Any | 4743 | 59.81 | | | | | |
| 20 | 30-34 | CC | 65 | 0.82 | 0 | 0.00 | | | |
| 20 | 30-34 | Ecig | 1881 | 23.72 | | | | | |
| 20 | 30-34 | Dual | 0 | 0.00 | | | | | |
| 20 | 30-34 | Former | 1241 | 15.65 | | | 9.36 | 130 | 10.48 |

Year of Follow-up: 2010, Age Group: 35-39

| Period | Age Group | Smoking Group | N | % | Ever Quit (N) | Ever Quit % | Mean Yrs Quit | Short Term (n) | Short Term % |
|---|---|---|---|---|---|---|---|---|---|
| 20 | 35-39 | Never Any | 5359 | 64.18 | | | | | |
| 20 | 35-39 | CC | 58 | 0.69 | 0 | 0.00 | | | |
| 20 | 35-39 | Ecig | 1717 | 20.56 | | | | | |
| 20 | 35-39 | Dual | 0 | 0.00 | | | | | |
| 20 | 35-39 | Former | 1216 | 14.56 | | | 11.38 | 98 | 8.06 |

Year of Follow-up: 2010, Age Group: 40-44

| Period | Age Group | Smoking Group | N | % | Ever Quit (N) | Ever Quit % | Mean Yrs Quit | Short Term (n) | Short Term % |
|---|---|---|---|---|---|---|---|---|---|
| 20 | 40-44 | Never Any | 5189 | 59.01 | | | | | |
| 20 | 40-44 | CC | 64 | 0.73 | 0 | 0.00 | | | |
| 20 | 40-44 | Ecig | 1849 | 21.03 | | | | | |
| 20 | 40-44 | Dual | 0 | 0.00 | | | | | |
| 20 | 40-44 | Former | 1691 | 19.23 | | | 15.34 | 112 | 6.62 |

Year of Follow-up: 2010, Age Group: 45-49

| Period | Age Group | Smoking Group | N | % | Ever Quit (N) | Ever Quit % | Mean Yrs Quit | Short Term (n) | Short Term % |
|---|---|---|---|---|---|---|---|---|---|
| 20 | 45-49 | Never Any | 5284 | 52.43 | | | | | |
| 20 | 45-49 | CC | 105 | 1.04 | 0 | 0.00 | | | |
| 20 | 45-49 | Ecig | 2327 | 23.09 | | | | | |
| 20 | 45-49 | Dual | 0 | 0.00 | | | | | |
| 20 | 45-49 | Former | 2362 | 23.44 | | | 17.71 | 131 | 5.55 |

Year of Follow-up: 2010, Age Group: 50-54

| Period | Age Group | Smoking Group | N | % | Ever Quit (N) | Ever Quit % | Mean Yrs Quit | Short Term (n) | Short Term % |
|---|---|---|---|---|---|---|---|---|---|
| 20 | 50-54 | Never Any | 5278 | 50.27 | | | | | |
| 20 | 50-54 | CC | 101 | 0.96 | 0 | 0.00 | | | |
| 20 | 50-54 | Ecig | 2101 | 20.01 | | | | | |
| 20 | 50-54 | Dual | 0 | 0.00 | | | | | |
| 20 | 50-54 | Former | 3019 | 28.76 | | | 18.27 | 163 | 5.40 |

Year of Follow-up: 2010, Age Group: 55-59

| Period | Age Group | Smoking Group | N | % | Ever Quit (N) | Ever Quit % | Mean Yrs Quit | Short Term (n) | Short Term % |
|---|---|---|---|---|---|---|---|---|---|
| 20 | 55-59 | Never Any | 5018 | 52.27 | | | | | |
| 20 | 55-59 | CC | 91 | 0.95 | 0 | 0.00 | | | |
| 20 | 55-59 | Ecig | 1433 | 14.93 | | | | | |
| 20 | 55-59 | Dual | 0 | 0.00 | | | | | |
| 20 | 55-59 | Former | 3058 | 31.85 | | | 20.88 | 103 | 3.37 |

Year of Follow-up: 2010, Age Group: 60-64

| Period | Age Group | Smoking Group | N | % | Ever Quit (N) | Ever Quit % | Mean Yrs Quit | Short Term (n) | Short Term % |
|---|---|---|---|---|---|---|---|---|---|
| 20 | 60-64 | Never Any | 4356 | 51.27 | | | | | |
| 20 | 60-64 | CC | 71 | 0.84 | 0 | 0.00 | | | |
| 20 | 60-64 | Ecig | 1079 | 12.70 | | | | | |
| 20 | 60-64 | Dual | 0 | 0.00 | | | | | |
| 20 | 60-64 | Former | 2991 | 35.20 | | | 19.94 | 144 | 4.81 |

Year of Follow-up: 2010, Age Group: 65-69

# Results for PHIM for Run 6 (Sensitivity_Analysis_1.RTF)

| Period | Age Group | Smoking Group | N | % | Ever Quit (N) | Ever Quit % | Mean Yrs Quit | Short Term (n) | Short Term % |
|---|---|---|---|---|---|---|---|---|---|
| 20 | 65-69 | Never Any | 3116 | 47.67 | | | | | |
| 20 | 65-69 | CC | 47 | 0.72 | 0 | 0.00 | | | |
| 20 | 65-69 | Ecig | 645 | 9.87 | | | | | |
| 20 | 65-69 | Dual | 0 | 0.00 | | | | | |
| 20 | 65-69 | Former | 2728 | 41.74 | | | 22.72 | 95 | 3.48 |

Year of Follow-up: 2010, Age Group: 70-74

| Period | Age Group | Smoking Group | N | % | Ever Quit (N) | Ever Quit % | Mean Yrs Quit | Short Term (n) | Short Term % |
|---|---|---|---|---|---|---|---|---|---|
| 20 | 70-74 | Never Any | 2684 | 48.50 | | | | | |
| 20 | 70-74 | CC | 88 | 1.59 | 0 | 0.00 | | | |
| 20 | 70-74 | Ecig | 477 | 8.62 | | | | | |
| 20 | 70-74 | Dual | 0 | 0.00 | | | | | |
| 20 | 70-74 | Former | 2285 | 41.29 | | | 23.05 | 69 | 3.02 |

Year of Follow-up: 2010, Age Group: 75-79

| Period | Age Group | Smoking Group | N | % | Ever Quit (N) | Ever Quit % | Mean Yrs Quit | Short Term (n) | Short Term % |
|---|---|---|---|---|---|---|---|---|---|
| 20 | 75-79 | Never Any | 2732 | 51.90 | | | | | |
| 20 | 75-79 | CC | 75 | 1.42 | 0 | 0.00 | | | |
| 20 | 75-79 | Ecig | 276 | 5.24 | | | | | |
| 20 | 75-79 | Dual | 0 | 0.00 | | | | | |
| 20 | 75-79 | Former | 2181 | 41.43 | | | 25.56 | 72 | 3.30 |

Year of Follow-up: 2015, Age Group: All Ages

| Period | Age Group | Smoking Group | N | % | Ever Quit (N) | Ever Quit % | Mean Yrs Quit | Short Term (n) | Short Term % |
|---|---|---|---|---|---|---|---|---|---|
| 25 | All Ages | Never Any | 41010 | 54.09 | | | | | |
| 25 | All Ages | CC | 340 | 0.45 | 0 | 0.00 | | | |
| 25 | All Ages | Ecig | 11438 | 15.09 | | | | | |
| 25 | All Ages | Dual | 0 | 0.00 | | | | | |
| 25 | All Ages | Former | 23029 | 30.37 | | | 21.48 | 914 | 3.97 |

Year of Follow-up: 2015, Age Group: 18-19

| Period | Age Group | Smoking Group | N | % | Ever Quit (N) | Ever Quit % | Mean Yrs Quit | Short Term (n) | Short Term % |
|---|---|---|---|---|---|---|---|---|---|
| 25 | 18-19 | Never Any | 0 | . | | | | | |
| 25 | 18-19 | CC | 0 | . | 0 | . | | | |
| 25 | 18-19 | Ecig | 0 | . | | | | | |
| 25 | 18-19 | Dual | 0 | . | | | | | |
| 25 | 18-19 | Former | 0 | . | | | . | 0 | . |

Year of Follow-up: 2015, Age Group: 20-24

| Period | Age Group | Smoking Group | N | % | Ever Quit (N) | Ever Quit % | Mean Yrs Quit | Short Term (n) | Short Term % |
|---|---|---|---|---|---|---|---|---|---|
| 25 | 20-24 | Never Any | 0 | . | | | | | |
| 25 | 20-24 | CC | 0 | . | 0 | . | | | |
| 25 | 20-24 | Ecig | 0 | . | | | | | |
| 25 | 20-24 | Dual | 0 | . | | | | | |
| 25 | 20-24 | Former | 0 | . | | | . | 0 | . |

Year of Follow-up: 2015, Age Group: 25-29

| Period | Age Group | Smoking Group | N | % | Ever Quit (N) | Ever Quit % | Mean Yrs Quit | Short Term (n) | Short Term % |
|---|---|---|---|---|---|---|---|---|---|
| 25 | 25-29 | Never Any | 0 | . | | | | | |
| 25 | 25-29 | CC | 0 | . | 0 | . | | | |
| 25 | 25-29 | Ecig | 0 | . | | | | | |
| 25 | 25-29 | Dual | 0 | . | | | | | |
| 25 | 25-29 | Former | 0 | . | | | . | 0 | . |

Year of Follow-up: 2015, Age Group: 30-34

| Period | Age Group | Smoking Group | N | % | Ever Quit (N) | Ever Quit % | Mean Yrs Quit | Short Term (n) | Short Term % |
|---|---|---|---|---|---|---|---|---|---|
| 25 | 30-34 | Never Any | 0 | . | | | | | |
| 25 | 30-34 | CC | 0 | . | 0 | . | | | |
| 25 | 30-34 | Ecig | 0 | . | | | | | |
| 25 | 30-34 | Dual | 0 | . | | | | | |
| 25 | 30-34 | Former | 0 | . | | | . | 0 | . |

Year of Follow-up: 2015, Age Group: 35-39

| Period | Age Group | Smoking Group | N | % | Ever Quit (N) | Ever Quit % | Mean Yrs Quit | Short Term (n) | Short Term % |
|---|---|---|---|---|---|---|---|---|---|
| 25 | 35-39 | Never Any | 4726 | 59.60 | | | | | |
| 25 | 35-39 | CC | 20 | 0.25 | 0 | 0.00 | | | |
| 25 | 35-39 | Ecig | 1567 | 19.76 | | | | | |
| 25 | 35-39 | Dual | 0 | 0.00 | | | | | |
| 25 | 35-39 | Former | 1617 | 20.39 | | | 11.52 | 129 | 7.98 |

Year of Follow-up: 2015, Age Group: 40-44

| Period | Age Group | Smoking Group | N | % | Ever Quit (N) | Ever Quit % | Mean Yrs Quit | Short Term (n) | Short Term % |
|---|---|---|---|---|---|---|---|---|---|
| 25 | 40-44 | Never Any | 5359 | 64.18 | | | | | |
| 25 | 40-44 | CC | 33 | 0.40 | 0 | 0.00 | | | |
| 25 | 40-44 | Ecig | 1502 | 17.99 | | | | | |
| 25 | 40-44 | Dual | 0 | 0.00 | | | | | |

# Results for PHIM for Run 6 (Sensitivity_Analysis_1.RTF)

| | | | | | | | | | |
|---|---|---|---|---|---|---|---|---|---|
| 25 | 40-44 | Former | 1456 | 17.44 | | | 14.00 | 103 | 7.07 |

Year of Follow-up: 2015, Age Group: 45-49

| Period | Age Group | Smoking Group | N | % | Ever Quit (N) | Ever Quit % | Mean Yrs Quit | Short Term (n) | Short Term % |
|---|---|---|---|---|---|---|---|---|---|
| 25 | 45-49 | Never Any | 5189 | 59.01 | | | | | |
| 25 | 45-49 | CC | 32 | 0.36 | 0 | 0.00 | | | |
| 25 | 45-49 | Ecig | 1627 | 18.50 | | | | | |
| 25 | 45-49 | Dual | 0 | 0.00 | | | | | |
| 25 | 45-49 | Former | 1945 | 22.12 | | | 17.95 | 96 | 4.94 |

Year of Follow-up: 2015, Age Group: 50-54

| Period | Age Group | Smoking Group | N | % | Ever Quit (N) | Ever Quit % | Mean Yrs Quit | Short Term (n) | Short Term % |
|---|---|---|---|---|---|---|---|---|---|
| 25 | 50-54 | Never Any | 5284 | 52.43 | | | | | |
| 25 | 50-54 | CC | 45 | 0.45 | 0 | 0.00 | | | |
| 25 | 50-54 | Ecig | 2059 | 20.43 | | | | | |
| 25 | 50-54 | Dual | 0 | 0.00 | | | | | |
| 25 | 50-54 | Former | 2690 | 26.69 | | | 20.17 | 150 | 5.58 |

Year of Follow-up: 2015, Age Group: 55-59

| Period | Age Group | Smoking Group | N | % | Ever Quit (N) | Ever Quit % | Mean Yrs Quit | Short Term (n) | Short Term % |
|---|---|---|---|---|---|---|---|---|---|
| 25 | 55-59 | Never Any | 5278 | 50.27 | | | | | |
| 25 | 55-59 | CC | 37 | 0.35 | 0 | 0.00 | | | |
| 25 | 55-59 | Ecig | 1773 | 16.89 | | | | | |
| 25 | 55-59 | Dual | 0 | 0.00 | | | | | |
| 25 | 55-59 | Former | 3411 | 32.49 | | | 20.84 | 140 | 4.10 |

Year of Follow-up: 2015, Age Group: 60-64

| Period | Age Group | Smoking Group | N | % | Ever Quit (N) | Ever Quit % | Mean Yrs Quit | Short Term (n) | Short Term % |
|---|---|---|---|---|---|---|---|---|---|
| 25 | 60-64 | Never Any | 5018 | 52.27 | | | | | |
| 25 | 60-64 | CC | 42 | 0.44 | 0 | 0.00 | | | |
| 25 | 60-64 | Ecig | 1154 | 12.02 | | | | | |
| 25 | 60-64 | Dual | 0 | 0.00 | | | | | |
| 25 | 60-64 | Former | 3386 | 35.27 | | | 23.56 | 144 | 4.25 |

Year of Follow-up: 2015, Age Group: 65-69

| Period | Age Group | Smoking Group | N | % | Ever Quit (N) | Ever Quit % | Mean Yrs Quit | Short Term (n) | Short Term % |
|---|---|---|---|---|---|---|---|---|---|
| 25 | 65-69 | Never Any | 4356 | 51.27 | | | | | |
| 25 | 65-69 | CC | 41 | 0.48 | 0 | 0.00 | | | |
| 25 | 65-69 | Ecig | 844 | 9.93 | | | | | |
| 25 | 65-69 | Dual | 0 | 0.00 | | | | | |
| 25 | 65-69 | Former | 3256 | 38.32 | | | 23.12 | 70 | 2.15 |

Year of Follow-up: 2015, Age Group: 70-74

| Period | Age Group | Smoking Group | N | % | Ever Quit (N) | Ever Quit % | Mean Yrs Quit | Short Term (n) | Short Term % |
|---|---|---|---|---|---|---|---|---|---|
| 25 | 70-74 | Never Any | 3116 | 47.67 | | | | | |
| 25 | 70-74 | CC | 31 | 0.47 | 0 | 0.00 | | | |
| 25 | 70-74 | Ecig | 527 | 8.06 | | | | | |
| 25 | 70-74 | Dual | 0 | 0.00 | | | | | |
| 25 | 70-74 | Former | 2862 | 43.79 | | | 26.54 | 42 | 1.47 |

Year of Follow-up: 2015, Age Group: 75-79

| Period | Age Group | Smoking Group | N | % | Ever Quit (N) | Ever Quit % | Mean Yrs Quit | Short Term (n) | Short Term % |
|---|---|---|---|---|---|---|---|---|---|
| 25 | 75-79 | Never Any | 2684 | 48.50 | | | | | |
| 25 | 75-79 | CC | 59 | 1.07 | 0 | 0.00 | | | |
| 25 | 75-79 | Ecig | 385 | 6.96 | | | | | |
| 25 | 75-79 | Dual | 0 | 0.00 | | | | | |
| 25 | 75-79 | Former | 2406 | 43.48 | | | 26.76 | 40 | 1.66 |

Year of Follow-up: 2020, Age Group: All Ages

| Period | Age Group | Smoking Group | N | % | Ever Quit (N) | Ever Quit % | Mean Yrs Quit | Short Term (n) | Short Term % |
|---|---|---|---|---|---|---|---|---|---|
| 30 | All Ages | Never Any | 38326 | 54.53 | | | | | |
| 30 | All Ages | CC | 115 | 0.16 | 0 | 0.00 | | | |
| 30 | All Ages | Ecig | 9272 | 13.19 | | | | | |
| 30 | All Ages | Dual | 0 | 0.00 | | | | | |
| 30 | All Ages | Former | 22570 | 32.11 | | | 23.81 | 756 | 3.35 |

Year of Follow-up: 2020, Age Group: 18-19

| Period | Age Group | Smoking Group | N | % | Ever Quit (N) | Ever Quit % | Mean Yrs Quit | Short Term (n) | Short Term % |
|---|---|---|---|---|---|---|---|---|---|
| 30 | 18-19 | Never Any | 0 | . | | | | | |
| 30 | 18-19 | CC | 0 | . | 0 | . | | | |
| 30 | 18-19 | Ecig | 0 | . | | | | | |
| 30 | 18-19 | Dual | 0 | . | | | | | |
| 30 | 18-19 | Former | 0 | . | | | . | 0 | . |

Year of Follow-up: 2020, Age Group: 20-24

| Period | Age Group | Smoking Group | N | % | Ever Quit (N) | Ever Quit % | Mean Yrs Quit | Short Term (n) | Short Term % |
|---|---|---|---|---|---|---|---|---|---|
| 30 | 20-24 | Never Any | 0 | . | | | | | |
| 30 | 20-24 | CC | 0 | . | 0 | . | | | |

# Results for PHIM for Run 6 (Sensitivity_Analysis_1.RTF)

| | | | | | | | | | |
|---|---|---|---|---|---|---|---|---|---|
| 30 | 20-24 | Ecig | 0 | . | | | | | |
| 30 | 20-24 | Dual | 0 | . | | | | | |
| 30 | 20-24 | Former | 0 | . | | | . | 0 | . |

Year of Follow-up: 2020, Age Group: 25-29

| Period | Age Group | Smoking Group | N | % | Ever Quit (N) | Ever Quit % | Mean Yrs Quit | Short Term (n) | Short Term % |
|---|---|---|---|---|---|---|---|---|---|
| 30 | 25-29 | Never Any | 0 | . | | | | | |
| 30 | 25-29 | CC | 0 | . | 0 | . | | | |
| 30 | 25-29 | Ecig | 0 | . | | | | | |
| 30 | 25-29 | Dual | 0 | . | | | | | |
| 30 | 25-29 | Former | 0 | . | | | . | 0 | . |

Year of Follow-up: 2020, Age Group: 30-34

| Period | Age Group | Smoking Group | N | % | Ever Quit (N) | Ever Quit % | Mean Yrs Quit | Short Term (n) | Short Term % |
|---|---|---|---|---|---|---|---|---|---|
| 30 | 30-34 | Never Any | 0 | . | | | | | |
| 30 | 30-34 | CC | 0 | . | 0 | . | | | |
| 30 | 30-34 | Ecig | 0 | . | | | | | |
| 30 | 30-34 | Dual | 0 | . | | | | | |
| 30 | 30-34 | Former | 0 | . | | | . | 0 | . |

Year of Follow-up: 2020, Age Group: 35-39

| Period | Age Group | Smoking Group | N | % | Ever Quit (N) | Ever Quit % | Mean Yrs Quit | Short Term (n) | Short Term % |
|---|---|---|---|---|---|---|---|---|---|
| 30 | 35-39 | Never Any | 0 | . | | | | | |
| 30 | 35-39 | CC | 0 | . | 0 | . | | | |
| 30 | 35-39 | Ecig | 0 | . | | | | | |
| 30 | 35-39 | Dual | 0 | . | | | | | |
| 30 | 35-39 | Former | 0 | . | | | . | 0 | . |

Year of Follow-up: 2020, Age Group: 40-44

| Period | Age Group | Smoking Group | N | % | Ever Quit (N) | Ever Quit % | Mean Yrs Quit | Short Term (n) | Short Term % |
|---|---|---|---|---|---|---|---|---|---|
| 30 | 40-44 | Never Any | 4726 | 59.60 | | | | | |
| 30 | 40-44 | CC | 6 | 0.08 | 0 | 0.00 | | | |
| 30 | 40-44 | Ecig | 1345 | 16.96 | | | | | |
| 30 | 40-44 | Dual | 0 | 0.00 | | | | | |
| 30 | 40-44 | Former | 1853 | 23.37 | | | 14.68 | 82 | 4.43 |

Year of Follow-up: 2020, Age Group: 45-49

| Period | Age Group | Smoking Group | N | % | Ever Quit (N) | Ever Quit % | Mean Yrs Quit | Short Term (n) | Short Term % |
|---|---|---|---|---|---|---|---|---|---|
| 30 | 45-49 | Never Any | 5359 | 64.18 | | | | | |
| 30 | 45-49 | CC | 13 | 0.16 | 0 | 0.00 | | | |
| 30 | 45-49 | Ecig | 1317 | 15.77 | | | | | |
| 30 | 45-49 | Dual | 0 | 0.00 | | | | | |
| 30 | 45-49 | Former | 1661 | 19.89 | | | 16.93 | 71 | 4.27 |

Year of Follow-up: 2020, Age Group: 50-54

| Period | Age Group | Smoking Group | N | % | Ever Quit (N) | Ever Quit % | Mean Yrs Quit | Short Term (n) | Short Term % |
|---|---|---|---|---|---|---|---|---|---|
| 30 | 50-54 | Never Any | 5189 | 59.01 | | | | | |
| 30 | 50-54 | CC | 9 | 0.10 | 0 | 0.00 | | | |
| 30 | 50-54 | Ecig | 1395 | 15.86 | | | | | |
| 30 | 50-54 | Dual | 0 | 0.00 | | | | | |
| 30 | 50-54 | Former | 2200 | 25.02 | | | 20.50 | 121 | 5.50 |

Year of Follow-up: 2020, Age Group: 55-59

| Period | Age Group | Smoking Group | N | % | Ever Quit (N) | Ever Quit % | Mean Yrs Quit | Short Term (n) | Short Term % |
|---|---|---|---|---|---|---|---|---|---|
| 30 | 55-59 | Never Any | 5284 | 52.43 | | | | | |
| 30 | 55-59 | CC | 14 | 0.14 | 0 | 0.00 | | | |
| 30 | 55-59 | Ecig | 1708 | 16.95 | | | | | |
| 30 | 55-59 | Dual | 0 | 0.00 | | | | | |
| 30 | 55-59 | Former | 3072 | 30.48 | | | 22.30 | 140 | 4.56 |

Year of Follow-up: 2020, Age Group: 60-64

| Period | Age Group | Smoking Group | N | % | Ever Quit (N) | Ever Quit % | Mean Yrs Quit | Short Term (n) | Short Term % |
|---|---|---|---|---|---|---|---|---|---|
| 30 | 60-64 | Never Any | 5278 | 50.27 | | | | | |
| 30 | 60-64 | CC | 15 | 0.14 | 0 | 0.00 | | | |
| 30 | 60-64 | Ecig | 1428 | 13.60 | | | | | |
| 30 | 60-64 | Dual | 0 | 0.00 | | | | | |
| 30 | 60-64 | Former | 3778 | 35.98 | | | 23.52 | 153 | 4.05 |

Year of Follow-up: 2020, Age Group: 65-69

| Period | Age Group | Smoking Group | N | % | Ever Quit (N) | Ever Quit % | Mean Yrs Quit | Short Term (n) | Short Term % |
|---|---|---|---|---|---|---|---|---|---|
| 30 | 65-69 | Never Any | 5018 | 52.27 | | | | | |
| 30 | 65-69 | CC | 18 | 0.19 | 0 | 0.00 | | | |
| 30 | 65-69 | Ecig | 917 | 9.55 | | | | | |
| 30 | 65-69 | Dual | 0 | 0.00 | | | | | |
| 30 | 65-69 | Former | 3647 | 37.99 | | | 26.68 | 75 | 2.06 |

Year of Follow-up: 2020, Age Group: 70-74

| Period | Age Group | Smoking Group | N | % | Ever Quit (N) | Ever Quit % | Mean Yrs Quit | Short Term (n) | Short Term % |
|---|---|---|---|---|---|---|---|---|---|

# Results for PHIM for Run 6 (Sensitivity_Analysis_1.RTF)

| | | | | | | | | | |
|---|---|---|---|---|---|---|---|---|---|
| 30 | 70-74 | Never Any | 4356 | 51.27 | | | | | |
| 30 | 70-74 | CC | 22 | 0.26 | 0 | 0.00 | | | |
| 30 | 70-74 | Ecig | 727 | 8.56 | | | | | |
| 30 | 70-74 | Dual | 0 | 0.00 | | | | | |
| 30 | 70-74 | Former | 3392 | 39.92 | | | 27.06 | 66 | 1.95 |

Year of Follow-up: 2020, Age Group: 75-79

| Period | Age Group | Smoking Group | N | % | Ever Quit (N) | Ever Quit % | Mean Yrs Quit | Short Term (n) | Short Term % |
|---|---|---|---|---|---|---|---|---|---|
| 30 | 75-79 | Never Any | 3116 | 47.67 | | | | | |
| 30 | 75-79 | CC | 18 | 0.28 | 0 | 0.00 | | | |
| 30 | 75-79 | Ecig | 435 | 6.66 | | | | | |
| 30 | 75-79 | Dual | 0 | 0.00 | | | | | |
| 30 | 75-79 | Former | 2967 | 45.39 | | | 30.49 | 48 | 1.62 |

Year of Follow-up: 2025, Age Group: All Ages

| Period | Age Group | Smoking Group | N | % | Ever Quit (N) | Ever Quit % | Mean Yrs Quit | Short Term (n) | Short Term % |
|---|---|---|---|---|---|---|---|---|---|
| 35 | All Ages | Never Any | 35210 | 55.23 | | | | | |
| 35 | All Ages | CC | 47 | 0.07 | 0 | 0.00 | | | |
| 35 | All Ages | Ecig | 7305 | 11.46 | | | | | |
| 35 | All Ages | Dual | 0 | 0.00 | | | | | |
| 35 | All Ages | Former | 21185 | 33.23 | | | 25.87 | 616 | 2.91 |

Year of Follow-up: 2025, Age Group: 18-19

| Period | Age Group | Smoking Group | N | % | Ever Quit (N) | Ever Quit % | Mean Yrs Quit | Short Term (n) | Short Term % |
|---|---|---|---|---|---|---|---|---|---|
| 35 | 18-19 | Never Any | 0 | . | | | | | |
| 35 | 18-19 | CC | 0 | . | 0 | . | | | |
| 35 | 18-19 | Ecig | 0 | . | | | | | |
| 35 | 18-19 | Dual | 0 | . | | | | | |
| 35 | 18-19 | Former | 0 | . | | | . | 0 | . |

Year of Follow-up: 2025, Age Group: 20-24

| Period | Age Group | Smoking Group | N | % | Ever Quit (N) | Ever Quit % | Mean Yrs Quit | Short Term (n) | Short Term % |
|---|---|---|---|---|---|---|---|---|---|
| 35 | 20-24 | Never Any | 0 | . | | | | | |
| 35 | 20-24 | CC | 0 | . | 0 | . | | | |
| 35 | 20-24 | Ecig | 0 | . | | | | | |
| 35 | 20-24 | Dual | 0 | . | | | | | |
| 35 | 20-24 | Former | 0 | . | | | . | 0 | . |

Year of Follow-up: 2025, Age Group: 25-29

| Period | Age Group | Smoking Group | N | % | Ever Quit (N) | Ever Quit % | Mean Yrs Quit | Short Term (n) | Short Term % |
|---|---|---|---|---|---|---|---|---|---|
| 35 | 25-29 | Never Any | 0 | . | | | | | |
| 35 | 25-29 | CC | 0 | . | 0 | . | | | |
| 35 | 25-29 | Ecig | 0 | . | | | | | |
| 35 | 25-29 | Dual | 0 | . | | | | | |
| 35 | 25-29 | Former | 0 | . | | | . | 0 | . |

Year of Follow-up: 2025, Age Group: 30-34

| Period | Age Group | Smoking Group | N | % | Ever Quit (N) | Ever Quit % | Mean Yrs Quit | Short Term (n) | Short Term % |
|---|---|---|---|---|---|---|---|---|---|
| 35 | 30-34 | Never Any | 0 | . | | | | | |
| 35 | 30-34 | CC | 0 | . | 0 | . | | | |
| 35 | 30-34 | Ecig | 0 | . | | | | | |
| 35 | 30-34 | Dual | 0 | . | | | | | |
| 35 | 30-34 | Former | 0 | . | | | . | 0 | . |

Year of Follow-up: 2025, Age Group: 35-39

| Period | Age Group | Smoking Group | N | % | Ever Quit (N) | Ever Quit % | Mean Yrs Quit | Short Term (n) | Short Term % |
|---|---|---|---|---|---|---|---|---|---|
| 35 | 35-39 | Never Any | 0 | . | | | | | |
| 35 | 35-39 | CC | 0 | . | 0 | . | | | |
| 35 | 35-39 | Ecig | 0 | . | | | | | |
| 35 | 35-39 | Dual | 0 | . | | | | | |
| 35 | 35-39 | Former | 0 | . | | | . | 0 | . |

Year of Follow-up: 2025, Age Group: 40-44

| Period | Age Group | Smoking Group | N | % | Ever Quit (N) | Ever Quit % | Mean Yrs Quit | Short Term (n) | Short Term % |
|---|---|---|---|---|---|---|---|---|---|
| 35 | 40-44 | Never Any | 0 | . | | | | | |
| 35 | 40-44 | CC | 0 | . | 0 | . | | | |
| 35 | 40-44 | Ecig | 0 | . | | | | | |
| 35 | 40-44 | Dual | 0 | . | | | | | |
| 35 | 40-44 | Former | 0 | . | | | . | 0 | . |

Year of Follow-up: 2025, Age Group: 45-49

| Period | Age Group | Smoking Group | N | % | Ever Quit (N) | Ever Quit % | Mean Yrs Quit | Short Term (n) | Short Term % |
|---|---|---|---|---|---|---|---|---|---|
| 35 | 45-49 | Never Any | 4726 | 59.60 | | | | | |
| 35 | 45-49 | CC | 1 | 0.01 | 0 | 0.00 | | | |
| 35 | 45-49 | Ecig | 1146 | 14.45 | | | | | |
| 35 | 45-49 | Dual | 0 | 0.00 | | | | | |
| 35 | 45-49 | Former | 2057 | 25.94 | | | 17.95 | 74 | 3.60 |

Year of Follow-up: 2025, Age Group: 50-54

# Results for PHIM for Run 6 (Sensitivity_Analysis_1.RTF)

| Period | Age Group | Smoking Group | N | % | Ever Quit (N) | Ever Quit % | Mean Yrs Quit | Short Term (n) | Short Term % |
|---|---|---|---|---|---|---|---|---|---|
| 35 | 50-54 | Never Any | 5359 | 64.18 | | | | | |
| 35 | 50-54 | CC | 3 | 0.04 | 0 | 0.00 | | | |
| 35 | 50-54 | Ecig | 1112 | 13.32 | | | | | |
| 35 | 50-54 | Dual | 0 | 0.00 | | | | | |
| 35 | 50-54 | Former | 1876 | 22.47 | | | 19.62 | 104 | 5.54 |

Year of Follow-up: 2025, Age Group: 55-59

| Period | Age Group | Smoking Group | N | % | Ever Quit (N) | Ever Quit % | Mean Yrs Quit | Short Term (n) | Short Term % |
|---|---|---|---|---|---|---|---|---|---|
| 35 | 55-59 | Never Any | 5189 | 59.01 | | | | | |
| 35 | 55-59 | CC | 4 | 0.05 | 0 | 0.00 | | | |
| 35 | 55-59 | Ecig | 1153 | 13.11 | | | | | |
| 35 | 55-59 | Dual | 0 | 0.00 | | | | | |
| 35 | 55-59 | Former | 2447 | 27.83 | | | 23.13 | 98 | 4.00 |

Year of Follow-up: 2025, Age Group: 60-64

| Period | Age Group | Smoking Group | N | % | Ever Quit (N) | Ever Quit % | Mean Yrs Quit | Short Term (n) | Short Term % |
|---|---|---|---|---|---|---|---|---|---|
| 35 | 60-64 | Never Any | 5284 | 52.43 | | | | | |
| 35 | 60-64 | CC | 7 | 0.07 | 0 | 0.00 | | | |
| 35 | 60-64 | Ecig | 1378 | 13.67 | | | | | |
| 35 | 60-64 | Dual | 0 | 0.00 | | | | | |
| 35 | 60-64 | Former | 3409 | 33.83 | | | 24.79 | 139 | 4.08 |

Year of Follow-up: 2025, Age Group: 65-69

| Period | Age Group | Smoking Group | N | % | Ever Quit (N) | Ever Quit % | Mean Yrs Quit | Short Term (n) | Short Term % |
|---|---|---|---|---|---|---|---|---|---|
| 35 | 65-69 | Never Any | 5278 | 50.27 | | | | | |
| 35 | 65-69 | CC | 7 | 0.07 | 0 | 0.00 | | | |
| 35 | 65-69 | Ecig | 1139 | 10.85 | | | | | |
| 35 | 65-69 | Dual | 0 | 0.00 | | | | | |
| 35 | 65-69 | Former | 4075 | 38.81 | | | 26.61 | 83 | 2.04 |

Year of Follow-up: 2025, Age Group: 70-74

| Period | Age Group | Smoking Group | N | % | Ever Quit (N) | Ever Quit % | Mean Yrs Quit | Short Term (n) | Short Term % |
|---|---|---|---|---|---|---|---|---|---|
| 35 | 70-74 | Never Any | 5018 | 52.27 | | | | | |
| 35 | 70-74 | CC | 11 | 0.11 | 0 | 0.00 | | | |
| 35 | 70-74 | Ecig | 789 | 8.22 | | | | | |
| 35 | 70-74 | Dual | 0 | 0.00 | | | | | |
| 35 | 70-74 | Former | 3782 | 39.40 | | | 30.61 | 65 | 1.72 |

Year of Follow-up: 2025, Age Group: 75-79

| Period | Age Group | Smoking Group | N | % | Ever Quit (N) | Ever Quit % | Mean Yrs Quit | Short Term (n) | Short Term % |
|---|---|---|---|---|---|---|---|---|---|
| 35 | 75-79 | Never Any | 4356 | 51.27 | | | | | |
| 35 | 75-79 | CC | 14 | 0.16 | 0 | 0.00 | | | |
| 35 | 75-79 | Ecig | 588 | 6.92 | | | | | |
| 35 | 75-79 | Dual | 0 | 0.00 | | | | | |
| 35 | 75-79 | Former | 3539 | 41.65 | | | 30.81 | 53 | 1.50 |

Year of Follow-up: 2030, Age Group: All Ages

| Period | Age Group | Smoking Group | N | % | Ever Quit (N) | Ever Quit % | Mean Yrs Quit | Short Term (n) | Short Term % |
|---|---|---|---|---|---|---|---|---|---|
| 40 | All Ages | Never Any | 30854 | 55.84 | | | | | |
| 40 | All Ages | CC | 14 | 0.03 | 0 | 0.00 | | | |
| 40 | All Ages | Ecig | 5391 | 9.76 | | | | | |
| 40 | All Ages | Dual | 0 | 0.00 | | | | | |
| 40 | All Ages | Former | 18991 | 34.37 | | | 27.90 | 543 | 2.86 |

Year of Follow-up: 2030, Age Group: 18-19

| Period | Age Group | Smoking Group | N | % | Ever Quit (N) | Ever Quit % | Mean Yrs Quit | Short Term (n) | Short Term % |
|---|---|---|---|---|---|---|---|---|---|
| 40 | 18-19 | Never Any | 0 | . | | | | | |
| 40 | 18-19 | CC | 0 | . | 0 | . | | | |
| 40 | 18-19 | Ecig | 0 | . | | | | | |
| 40 | 18-19 | Dual | 0 | . | | | | | |
| 40 | 18-19 | Former | 0 | . | | | . | 0 | . |

Year of Follow-up: 2030, Age Group: 20-24

| Period | Age Group | Smoking Group | N | % | Ever Quit (N) | Ever Quit % | Mean Yrs Quit | Short Term (n) | Short Term % |
|---|---|---|---|---|---|---|---|---|---|
| 40 | 20-24 | Never Any | 0 | . | | | | | |
| 40 | 20-24 | CC | 0 | . | 0 | . | | | |
| 40 | 20-24 | Ecig | 0 | . | | | | | |
| 40 | 20-24 | Dual | 0 | . | | | | | |
| 40 | 20-24 | Former | 0 | . | | | . | 0 | . |

Year of Follow-up: 2030, Age Group: 25-29

| Period | Age Group | Smoking Group | N | % | Ever Quit (N) | Ever Quit % | Mean Yrs Quit | Short Term (n) | Short Term % |
|---|---|---|---|---|---|---|---|---|---|
| 40 | 25-29 | Never Any | 0 | . | | | | | |
| 40 | 25-29 | CC | 0 | . | 0 | . | | | |
| 40 | 25-29 | Ecig | 0 | . | | | | | |
| 40 | 25-29 | Dual | 0 | . | | | | | |

# Results for PHIM for Run 6 (Sensitivity_Analysis_1.RTF)

| | | | | | | | | | |
|---|---|---|---|---|---|---|---|---|---|
| 40 | 25-29 | Former | 0 | . | | | . | 0 | . |

Year of Follow-up: 2030, Age Group: 30-34

| Period | Age Group | Smoking Group | N | % | Ever Quit (N) | Ever Quit % | Mean Yrs Quit | Short Term (n) | Short Term % |
|---|---|---|---|---|---|---|---|---|---|
| 40 | 30-34 | Never Any | 0 | . | | | | | |
| 40 | 30-34 | CC | 0 | . | 0 | . | | | |
| 40 | 30-34 | Ecig | 0 | . | | | | | |
| 40 | 30-34 | Dual | 0 | . | | | | | |
| 40 | 30-34 | Former | 0 | . | | | . | 0 | . |

Year of Follow-up: 2030, Age Group: 35-39

| Period | Age Group | Smoking Group | N | % | Ever Quit (N) | Ever Quit % | Mean Yrs Quit | Short Term (n) | Short Term % |
|---|---|---|---|---|---|---|---|---|---|
| 40 | 35-39 | Never Any | 0 | . | | | | | |
| 40 | 35-39 | CC | 0 | . | 0 | . | | | |
| 40 | 35-39 | Ecig | 0 | . | | | | | |
| 40 | 35-39 | Dual | 0 | . | | | | | |
| 40 | 35-39 | Former | 0 | . | | | . | 0 | . |

Year of Follow-up: 2030, Age Group: 40-44

| Period | Age Group | Smoking Group | N | % | Ever Quit (N) | Ever Quit % | Mean Yrs Quit | Short Term (n) | Short Term % |
|---|---|---|---|---|---|---|---|---|---|
| 40 | 40-44 | Never Any | 0 | . | | | | | |
| 40 | 40-44 | CC | 0 | . | 0 | . | | | |
| 40 | 40-44 | Ecig | 0 | . | | | | | |
| 40 | 40-44 | Dual | 0 | . | | | | | |
| 40 | 40-44 | Former | 0 | . | | | . | 0 | . |

Year of Follow-up: 2030, Age Group: 45-49

| Period | Age Group | Smoking Group | N | % | Ever Quit (N) | Ever Quit % | Mean Yrs Quit | Short Term (n) | Short Term % |
|---|---|---|---|---|---|---|---|---|---|
| 40 | 45-49 | Never Any | 0 | . | | | | | |
| 40 | 45-49 | CC | 0 | . | 0 | . | | | |
| 40 | 45-49 | Ecig | 0 | . | | | | | |
| 40 | 45-49 | Dual | 0 | . | | | | | |
| 40 | 45-49 | Former | 0 | . | | | . | 0 | . |

Year of Follow-up: 2030, Age Group: 50-54

| Period | Age Group | Smoking Group | N | % | Ever Quit (N) | Ever Quit % | Mean Yrs Quit | Short Term (n) | Short Term % |
|---|---|---|---|---|---|---|---|---|---|
| 40 | 50-54 | Never Any | 4726 | 59.60 | | | | | |
| 40 | 50-54 | CC | 0 | 0.00 | 0 | 0.00 | | | |
| 40 | 50-54 | Ecig | 959 | 12.09 | | | | | |
| 40 | 50-54 | Dual | 0 | 0.00 | | | | | |
| 40 | 50-54 | Former | 2245 | 28.31 | | | 21.16 | 100 | 4.45 |

Year of Follow-up: 2030, Age Group: 55-59

| Period | Age Group | Smoking Group | N | % | Ever Quit (N) | Ever Quit % | Mean Yrs Quit | Short Term (n) | Short Term % |
|---|---|---|---|---|---|---|---|---|---|
| 40 | 55-59 | Never Any | 5359 | 64.18 | | | | | |
| 40 | 55-59 | CC | 0 | 0.00 | 0 | 0.00 | | | |
| 40 | 55-59 | Ecig | 897 | 10.74 | | | | | |
| 40 | 55-59 | Dual | 0 | 0.00 | | | | | |
| 40 | 55-59 | Former | 2094 | 25.08 | | | 22.27 | 80 | 3.82 |

Year of Follow-up: 2030, Age Group: 60-64

| Period | Age Group | Smoking Group | N | % | Ever Quit (N) | Ever Quit % | Mean Yrs Quit | Short Term (n) | Short Term % |
|---|---|---|---|---|---|---|---|---|---|
| 40 | 60-64 | Never Any | 5189 | 59.01 | | | | | |
| 40 | 60-64 | CC | 3 | 0.03 | 0 | 0.00 | | | |
| 40 | 60-64 | Ecig | 886 | 10.08 | | | | | |
| 40 | 60-64 | Dual | 0 | 0.00 | | | | | |
| 40 | 60-64 | Former | 2715 | 30.88 | | | 25.52 | 128 | 4.71 |

Year of Follow-up: 2030, Age Group: 65-69

| Period | Age Group | Smoking Group | N | % | Ever Quit (N) | Ever Quit % | Mean Yrs Quit | Short Term (n) | Short Term % |
|---|---|---|---|---|---|---|---|---|---|
| 40 | 65-69 | Never Any | 5284 | 52.43 | | | | | |
| 40 | 65-69 | CC | 3 | 0.03 | 0 | 0.00 | | | |
| 40 | 65-69 | Ecig | 1080 | 10.72 | | | | | |
| 40 | 65-69 | Dual | 0 | 0.00 | | | | | |
| 40 | 65-69 | Former | 3711 | 36.82 | | | 27.57 | 73 | 1.97 |

Year of Follow-up: 2030, Age Group: 70-74

| Period | Age Group | Smoking Group | N | % | Ever Quit (N) | Ever Quit % | Mean Yrs Quit | Short Term (n) | Short Term % |
|---|---|---|---|---|---|---|---|---|---|
| 40 | 70-74 | Never Any | 5278 | 50.27 | | | | | |
| 40 | 70-74 | CC | 4 | 0.04 | 0 | 0.00 | | | |
| 40 | 70-74 | Ecig | 957 | 9.12 | | | | | |
| 40 | 70-74 | Dual | 0 | 0.00 | | | | | |
| 40 | 70-74 | Former | 4260 | 40.58 | | | 30.32 | 86 | 2.02 |

Year of Follow-up: 2030, Age Group: 75-79

| Period | Age Group | Smoking Group | N | % | Ever Quit (N) | Ever Quit % | Mean Yrs Quit | Short Term (n) | Short Term % |
|---|---|---|---|---|---|---|---|---|---|
| 40 | 75-79 | Never Any | 5018 | 52.27 | | | | | |
| 40 | 75-79 | CC | 4 | 0.04 | 0 | 0.00 | | | |

# Results for PHIM for Run 6 (Sensitivity_Analysis_1.RTF)

| | | | | | | | | | |
|---|---|---|---|---|---|---|---|---|---|
| 40 | 75-79 | Ecig | 612 | 6.38 | | | | | |
| 40 | 75-79 | Dual | 0 | 0.00 | | | | | |
| 40 | 75-79 | Former | 3966 | 41.31 | | | 34.05 | 76 | 1.92 |

Year of Follow-up: 2035, Age Group: All Ages

| Period | Age Group | Smoking Group | N | % | Ever Quit (N) | Ever Quit % | Mean Yrs Quit | Short Term (n) | Short Term % |
|---|---|---|---|---|---|---|---|---|---|
| 45 | All Ages | Never Any | 25836 | 56.60 | | | | | |
| 45 | All Ages | CC | 5 | 0.01 | 0 | 0.00 | | | |
| 45 | All Ages | Ecig | 3886 | 8.51 | | | | | |
| 45 | All Ages | Dual | 0 | 0.00 | | | | | |
| 45 | All Ages | Former | 15923 | 34.88 | | | 29.63 | 351 | 2.20 |

Year of Follow-up: 2035, Age Group: 18-19

| Period | Age Group | Smoking Group | N | % | Ever Quit (N) | Ever Quit % | Mean Yrs Quit | Short Term (n) | Short Term % |
|---|---|---|---|---|---|---|---|---|---|
| 45 | 18-19 | Never Any | 0 | . | | | | | |
| 45 | 18-19 | CC | 0 | . | 0 | . | | | |
| 45 | 18-19 | Ecig | 0 | . | | | | | |
| 45 | 18-19 | Dual | 0 | . | | | | | |
| 45 | 18-19 | Former | 0 | . | | | . | 0 | . |

Year of Follow-up: 2035, Age Group: 20-24

| Period | Age Group | Smoking Group | N | % | Ever Quit (N) | Ever Quit % | Mean Yrs Quit | Short Term (n) | Short Term % |
|---|---|---|---|---|---|---|---|---|---|
| 45 | 20-24 | Never Any | 0 | . | | | | | |
| 45 | 20-24 | CC | 0 | . | 0 | . | | | |
| 45 | 20-24 | Ecig | 0 | . | | | | | |
| 45 | 20-24 | Dual | 0 | . | | | | | |
| 45 | 20-24 | Former | 0 | . | | | . | 0 | . |

Year of Follow-up: 2035, Age Group: 25-29

| Period | Age Group | Smoking Group | N | % | Ever Quit (N) | Ever Quit % | Mean Yrs Quit | Short Term (n) | Short Term % |
|---|---|---|---|---|---|---|---|---|---|
| 45 | 25-29 | Never Any | 0 | . | | | | | |
| 45 | 25-29 | CC | 0 | . | 0 | . | | | |
| 45 | 25-29 | Ecig | 0 | . | | | | | |
| 45 | 25-29 | Dual | 0 | . | | | | | |
| 45 | 25-29 | Former | 0 | . | | | . | 0 | . |

Year of Follow-up: 2035, Age Group: 30-34

| Period | Age Group | Smoking Group | N | % | Ever Quit (N) | Ever Quit % | Mean Yrs Quit | Short Term (n) | Short Term % |
|---|---|---|---|---|---|---|---|---|---|
| 45 | 30-34 | Never Any | 0 | . | | | | | |
| 45 | 30-34 | CC | 0 | . | 0 | . | | | |
| 45 | 30-34 | Ecig | 0 | . | | | | | |
| 45 | 30-34 | Dual | 0 | . | | | | | |
| 45 | 30-34 | Former | 0 | . | | | . | 0 | . |

Year of Follow-up: 2035, Age Group: 35-39

| Period | Age Group | Smoking Group | N | % | Ever Quit (N) | Ever Quit % | Mean Yrs Quit | Short Term (n) | Short Term % |
|---|---|---|---|---|---|---|---|---|---|
| 45 | 35-39 | Never Any | 0 | . | | | | | |
| 45 | 35-39 | CC | 0 | . | 0 | . | | | |
| 45 | 35-39 | Ecig | 0 | . | | | | | |
| 45 | 35-39 | Dual | 0 | . | | | | | |
| 45 | 35-39 | Former | 0 | . | | | . | 0 | . |

Year of Follow-up: 2035, Age Group: 40-44

| Period | Age Group | Smoking Group | N | % | Ever Quit (N) | Ever Quit % | Mean Yrs Quit | Short Term (n) | Short Term % |
|---|---|---|---|---|---|---|---|---|---|
| 45 | 40-44 | Never Any | 0 | . | | | | | |
| 45 | 40-44 | CC | 0 | . | 0 | . | | | |
| 45 | 40-44 | Ecig | 0 | . | | | | | |
| 45 | 40-44 | Dual | 0 | . | | | | | |
| 45 | 40-44 | Former | 0 | . | | | . | 0 | . |

Year of Follow-up: 2035, Age Group: 45-49

| Period | Age Group | Smoking Group | N | % | Ever Quit (N) | Ever Quit % | Mean Yrs Quit | Short Term (n) | Short Term % |
|---|---|---|---|---|---|---|---|---|---|
| 45 | 45-49 | Never Any | 0 | . | | | | | |
| 45 | 45-49 | CC | 0 | . | 0 | . | | | |
| 45 | 45-49 | Ecig | 0 | . | | | | | |
| 45 | 45-49 | Dual | 0 | . | | | | | |
| 45 | 45-49 | Former | 0 | . | | | . | 0 | . |

Year of Follow-up: 2035, Age Group: 50-54

| Period | Age Group | Smoking Group | N | % | Ever Quit (N) | Ever Quit % | Mean Yrs Quit | Short Term (n) | Short Term % |
|---|---|---|---|---|---|---|---|---|---|
| 45 | 50-54 | Never Any | 0 | . | | | | | |
| 45 | 50-54 | CC | 0 | . | 0 | . | | | |
| 45 | 50-54 | Ecig | 0 | . | | | | | |
| 45 | 50-54 | Dual | 0 | . | | | | | |
| 45 | 50-54 | Former | 0 | . | | | . | 0 | . |

Year of Follow-up: 2035, Age Group: 55-59

| Period | Age Group | Smoking Group | N | % | Ever Quit (N) | Ever Quit % | Mean Yrs Quit | Short Term (n) | Short Term % |
|---|---|---|---|---|---|---|---|---|---|

# Results for PHIM for Run 6 (Sensitivity_Analysis_1.RTF)

| | | | | | | | | | |
|---|---|---|---|---|---|---|---|---|---|
| 45 | 55-59 | Never Any | 4726 | 59.60 | | | | | |
| 45 | 55-59 | CC | 0 | 0.00 | 0 | 0.00 | | | |
| 45 | 55-59 | Ecig | 792 | 9.99 | | | | | |
| 45 | 55-59 | Dual | 0 | 0.00 | | | | | |
| 45 | 55-59 | Former | 2412 | 30.42 | | | 24.49 | 64 | 2.65 |

Year of Follow-up: 2035, Age Group: 60-64

| Period | Age Group | Smoking Group | N | % | Ever Quit (N) | Ever Quit % | Mean Yrs Quit | Short Term (n) | Short Term % |
|---|---|---|---|---|---|---|---|---|---|
| 45 | 60-64 | Never Any | 5359 | 64.18 | | | | | |
| 45 | 60-64 | CC | 0 | 0.00 | 0 | 0.00 | | | |
| 45 | 60-64 | Ecig | 719 | 8.61 | | | | | |
| 45 | 60-64 | Dual | 0 | 0.00 | | | | | |
| 45 | 60-64 | Former | 2272 | 27.21 | | | 25.28 | 77 | 3.39 |

Year of Follow-up: 2035, Age Group: 65-69

| Period | Age Group | Smoking Group | N | % | Ever Quit (N) | Ever Quit % | Mean Yrs Quit | Short Term (n) | Short Term % |
|---|---|---|---|---|---|---|---|---|---|
| 45 | 65-69 | Never Any | 5189 | 59.01 | | | | | |
| 45 | 65-69 | CC | 3 | 0.03 | 0 | 0.00 | | | |
| 45 | 65-69 | Ecig | 694 | 7.89 | | | | | |
| 45 | 65-69 | Dual | 0 | 0.00 | | | | | |
| 45 | 65-69 | Former | 2907 | 33.06 | | | 28.66 | 55 | 1.89 |

Year of Follow-up: 2035, Age Group: 70-74

| Period | Age Group | Smoking Group | N | % | Ever Quit (N) | Ever Quit % | Mean Yrs Quit | Short Term (n) | Short Term % |
|---|---|---|---|---|---|---|---|---|---|
| 45 | 70-74 | Never Any | 5284 | 52.43 | | | | | |
| 45 | 70-74 | CC | 1 | 0.01 | 0 | 0.00 | | | |
| 45 | 70-74 | Ecig | 934 | 9.27 | | | | | |
| 45 | 70-74 | Dual | 0 | 0.00 | | | | | |
| 45 | 70-74 | Former | 3859 | 38.29 | | | 31.38 | 76 | 1.97 |

Year of Follow-up: 2035, Age Group: 75-79

| Period | Age Group | Smoking Group | N | % | Ever Quit (N) | Ever Quit % | Mean Yrs Quit | Short Term (n) | Short Term % |
|---|---|---|---|---|---|---|---|---|---|
| 45 | 75-79 | Never Any | 5278 | 50.27 | | | | | |
| 45 | 75-79 | CC | 1 | 0.01 | 0 | 0.00 | | | |
| 45 | 75-79 | Ecig | 747 | 7.11 | | | | | |
| 45 | 75-79 | Dual | 0 | 0.00 | | | | | |
| 45 | 75-79 | Former | 4473 | 42.60 | | | 33.73 | 79 | 1.77 |

Year of Follow-up: 2040, Age Group: All Ages

| Period | Age Group | Smoking Group | N | % | Ever Quit (N) | Ever Quit % | Mean Yrs Quit | Short Term (n) | Short Term % |
|---|---|---|---|---|---|---|---|---|---|
| 50 | All Ages | Never Any | 20558 | 58.48 | | | | | |
| 50 | All Ages | CC | 4 | 0.01 | 0 | 0.00 | | | |
| 50 | All Ages | Ecig | 2497 | 7.10 | | | | | |
| 50 | All Ages | Dual | 0 | 0.00 | | | | | |
| 50 | All Ages | Former | 12092 | 34.40 | | | 31.39 | 238 | 1.97 |

Year of Follow-up: 2040, Age Group: 18-19

| Period | Age Group | Smoking Group | N | % | Ever Quit (N) | Ever Quit % | Mean Yrs Quit | Short Term (n) | Short Term % |
|---|---|---|---|---|---|---|---|---|---|
| 50 | 18-19 | Never Any | 0 | . | | | | | |
| 50 | 18-19 | CC | 0 | . | 0 | . | | | |
| 50 | 18-19 | Ecig | 0 | . | | | | | |
| 50 | 18-19 | Dual | 0 | . | | | | | |
| 50 | 18-19 | Former | 0 | . | | | . | 0 | . |

Year of Follow-up: 2040, Age Group: 20-24

| Period | Age Group | Smoking Group | N | % | Ever Quit (N) | Ever Quit % | Mean Yrs Quit | Short Term (n) | Short Term % |
|---|---|---|---|---|---|---|---|---|---|
| 50 | 20-24 | Never Any | 0 | . | | | | | |
| 50 | 20-24 | CC | 0 | . | 0 | . | | | |
| 50 | 20-24 | Ecig | 0 | . | | | | | |
| 50 | 20-24 | Dual | 0 | . | | | | | |
| 50 | 20-24 | Former | 0 | . | | | . | 0 | . |

Year of Follow-up: 2040, Age Group: 25-29

| Period | Age Group | Smoking Group | N | % | Ever Quit (N) | Ever Quit % | Mean Yrs Quit | Short Term (n) | Short Term % |
|---|---|---|---|---|---|---|---|---|---|
| 50 | 25-29 | Never Any | 0 | . | | | | | |
| 50 | 25-29 | CC | 0 | . | 0 | . | | | |
| 50 | 25-29 | Ecig | 0 | . | | | | | |
| 50 | 25-29 | Dual | 0 | . | | | | | |
| 50 | 25-29 | Former | 0 | . | | | . | 0 | . |

Year of Follow-up: 2040, Age Group: 30-34

| Period | Age Group | Smoking Group | N | % | Ever Quit (N) | Ever Quit % | Mean Yrs Quit | Short Term (n) | Short Term % |
|---|---|---|---|---|---|---|---|---|---|
| 50 | 30-34 | Never Any | 0 | . | | | | | |
| 50 | 30-34 | CC | 0 | . | 0 | . | | | |
| 50 | 30-34 | Ecig | 0 | . | | | | | |
| 50 | 30-34 | Dual | 0 | . | | | | | |
| 50 | 30-34 | Former | 0 | . | | | . | 0 | . |

Year of Follow-up: 2040, Age Group: 35-39

# Results for PHIM for Run 6 (Sensitivity_Analysis_1.RTF)

| Period | Age Group | Smoking Group | N | % | Ever Quit (N) | Ever Quit % | Mean Yrs Quit | Short Term (n) | Short Term % |
|---|---|---|---|---|---|---|---|---|---|
| 50 | 35-39 | Never Any | 0 | . | | | | | |
| 50 | 35-39 | CC | 0 | . | 0 | . | | | |
| 50 | 35-39 | Ecig | 0 | . | | | | | |
| 50 | 35-39 | Dual | 0 | . | | | | | |
| 50 | 35-39 | Former | 0 | . | | | . | 0 | . |

Year of Follow-up: 2040, Age Group: 40-44

| Period | Age Group | Smoking Group | N | % | Ever Quit (N) | Ever Quit % | Mean Yrs Quit | Short Term (n) | Short Term % |
|---|---|---|---|---|---|---|---|---|---|
| 50 | 40-44 | Never Any | 0 | . | | | | | |
| 50 | 40-44 | CC | 0 | . | 0 | . | | | |
| 50 | 40-44 | Ecig | 0 | . | | | | | |
| 50 | 40-44 | Dual | 0 | . | | | | | |
| 50 | 40-44 | Former | 0 | . | | | . | 0 | . |

Year of Follow-up: 2040, Age Group: 45-49

| Period | Age Group | Smoking Group | N | % | Ever Quit (N) | Ever Quit % | Mean Yrs Quit | Short Term (n) | Short Term % |
|---|---|---|---|---|---|---|---|---|---|
| 50 | 45-49 | Never Any | 0 | . | | | | | |
| 50 | 45-49 | CC | 0 | . | 0 | . | | | |
| 50 | 45-49 | Ecig | 0 | . | | | | | |
| 50 | 45-49 | Dual | 0 | . | | | | | |
| 50 | 45-49 | Former | 0 | . | | | . | 0 | . |

Year of Follow-up: 2040, Age Group: 50-54

| Period | Age Group | Smoking Group | N | % | Ever Quit (N) | Ever Quit % | Mean Yrs Quit | Short Term (n) | Short Term % |
|---|---|---|---|---|---|---|---|---|---|
| 50 | 50-54 | Never Any | 0 | . | | | | | |
| 50 | 50-54 | CC | 0 | . | 0 | . | | | |
| 50 | 50-54 | Ecig | 0 | . | | | | | |
| 50 | 50-54 | Dual | 0 | . | | | | | |
| 50 | 50-54 | Former | 0 | . | | | . | 0 | . |

Year of Follow-up: 2040, Age Group: 55-59

| Period | Age Group | Smoking Group | N | % | Ever Quit (N) | Ever Quit % | Mean Yrs Quit | Short Term (n) | Short Term % |
|---|---|---|---|---|---|---|---|---|---|
| 50 | 55-59 | Never Any | 0 | . | | | | | |
| 50 | 55-59 | CC | 0 | . | 0 | . | | | |
| 50 | 55-59 | Ecig | 0 | . | | | | | |
| 50 | 55-59 | Dual | 0 | . | | | | | |
| 50 | 55-59 | Former | 0 | . | | | . | 0 | . |

Year of Follow-up: 2040, Age Group: 60-64

| Period | Age Group | Smoking Group | N | % | Ever Quit (N) | Ever Quit % | Mean Yrs Quit | Short Term (n) | Short Term % |
|---|---|---|---|---|---|---|---|---|---|
| 50 | 60-64 | Never Any | 4726 | 59.60 | | | | | |
| 50 | 60-64 | CC | 0 | 0.00 | 0 | 0.00 | | | |
| 50 | 60-64 | Ecig | 613 | 7.73 | | | | | |
| 50 | 60-64 | Dual | 0 | 0.00 | | | | | |
| 50 | 60-64 | Former | 2591 | 32.67 | | | 27.58 | 77 | 2.97 |

Year of Follow-up: 2040, Age Group: 65-69

| Period | Age Group | Smoking Group | N | % | Ever Quit (N) | Ever Quit % | Mean Yrs Quit | Short Term (n) | Short Term % |
|---|---|---|---|---|---|---|---|---|---|
| 50 | 65-69 | Never Any | 5359 | 64.18 | | | | | |
| 50 | 65-69 | CC | 0 | 0.00 | 0 | 0.00 | | | |
| 50 | 65-69 | Ecig | 578 | 6.92 | | | | | |
| 50 | 65-69 | Dual | 0 | 0.00 | | | | | |
| 50 | 65-69 | Former | 2413 | 28.90 | | | 28.67 | 34 | 1.41 |

Year of Follow-up: 2040, Age Group: 70-74

| Period | Age Group | Smoking Group | N | % | Ever Quit (N) | Ever Quit % | Mean Yrs Quit | Short Term (n) | Short Term % |
|---|---|---|---|---|---|---|---|---|---|
| 50 | 70-74 | Never Any | 5189 | 59.01 | | | | | |
| 50 | 70-74 | CC | 3 | 0.03 | 0 | 0.00 | | | |
| 50 | 70-74 | Ecig | 583 | 6.63 | | | | | |
| 50 | 70-74 | Dual | 0 | 0.00 | | | | | |
| 50 | 70-74 | Former | 3018 | 34.32 | | | 32.48 | 56 | 1.86 |

Year of Follow-up: 2040, Age Group: 75-79

| Period | Age Group | Smoking Group | N | % | Ever Quit (N) | Ever Quit % | Mean Yrs Quit | Short Term (n) | Short Term % |
|---|---|---|---|---|---|---|---|---|---|
| 50 | 75-79 | Never Any | 5284 | 52.43 | | | | | |
| 50 | 75-79 | CC | 1 | 0.01 | 0 | 0.00 | | | |
| 50 | 75-79 | Ecig | 723 | 7.17 | | | | | |
| 50 | 75-79 | Dual | 0 | 0.00 | | | | | |
| 50 | 75-79 | Former | 4070 | 40.38 | | | 34.61 | 71 | 1.74 |

# Results for PHIM for Run 6 (Sensitivity_Analysis_1.RTF)

************************************************************************************

**Main Results for E-Component for Run: 6, Simulation: 1, COD: Four Diseases Combined**

| Variable Parameter | Value |
|---|---|
| 1. Country | US (United States) |
| 2. Sex | F (Females) |
| 3. Year of start of process | 1990 |
| 4. Cause of Death | Four Diseases Combined |
| 5. Effective Dose NULL | CC:1 |
| 6. Effective Dose Ecig | CC:1 Ecig:0.1 Dual:0.55 |
| Note: | Death rates are per million population |

------------------------------------------------------------------------------------------

Results for Age Groups Combined and Years combined, Population unadjusted for deaths in Ecig scenario,

| Age Group:All Year | Number Deaths | NULL Attrib Deaths | Ecig Attrib Deaths | Drop Deaths | %Drop Deaths | %Drop Attrib Deaths | Death Rate | NULL Attrib Rates | Ecig Attrib Rates | Drop Rates |
|---|---|---|---|---|---|---|---|---|---|---|
| All Comb | 9082078 | 2946250.006 | 2025402.456 | 920847.5 | 10.139 | 31.255 | 2493.45 | 808.881 | 556.066 | 252.815 |

------------------------------------------------------------------------------------------

Results for Age Groups Combined, Population unadjusted for deaths in Ecig scenario,

| Age Group:All Year | Number Deaths | NULL Attrib Deaths | Ecig Attrib Deaths | Drop Deaths | %Drop Deaths | %Drop Attrib Deaths | Death Rate | NULL Attrib Rates | Ecig Attrib Rates | Drop Rates |
|---|---|---|---|---|---|---|---|---|---|---|
| 1990 | 199848 | 57524.822 | 57524.822 | 0.000 | 0.000 | 0.000 | 2926.84 | 842.471 | 842.471 | 0.000 |
| 1991 | 199162 | 59078.486 | 58918.742 | 159.743 | 0.080 | 0.270 | 2877.87 | 853.678 | 851.370 | 2.308 |
| 1992 | 198532 | 60494.150 | 60047.677 | 446.472 | 0.225 | 0.738 | 2829.84 | 862.271 | 855.907 | 6.364 |
| 1993 | 202512 | 62694.848 | 61826.443 | 868.404 | 0.429 | 1.385 | 2845.85 | 881.034 | 868.830 | 12.203 |
| 1994 | 200658 | 63202.598 | 61831.711 | 1370.887 | 0.683 | 2.169 | 2777.87 | 874.966 | 855.987 | 18.978 |
| 1995 | 201023 | 63766.862 | 61787.276 | 1979.586 | 0.985 | 3.104 | 2739.80 | 869.097 | 842.117 | 26.980 |
| 1996 | 201180 | 64591.163 | 61880.925 | 2710.238 | 1.347 | 4.196 | 2706.36 | 868.908 | 832.449 | 36.459 |
| 1997 | 198639 | 64686.912 | 61295.660 | 3391.252 | 1.707 | 5.243 | 2635.18 | 858.147 | 813.158 | 44.989 |
| 1998 | 197573 | 65295.961 | 61115.834 | 4180.127 | 2.116 | 6.402 | 2584.18 | 854.046 | 799.372 | 54.674 |
| 1999 | 211329 | 67327.521 | 61933.766 | 5393.755 | 2.552 | 8.011 | 2726.91 | 868.771 | 799.171 | 69.599 |
| 2000 | 208496 | 67596.897 | 61316.457 | 6280.439 | 3.012 | 9.291 | 2656.96 | 861.419 | 781.385 | 80.035 |
| 2001 | 203606 | 67008.811 | 59793.938 | 7214.873 | 3.544 | 10.767 | 2565.88 | 844.458 | 753.535 | 90.923 |
| 2002 | 200828 | 67067.744 | 58810.715 | 8257.028 | 4.111 | 12.311 | 2506.09 | 836.925 | 733.887 | 103.038 |
| 2003 | 196352 | 66756.217 | 57559.978 | 9196.239 | 4.684 | 13.776 | 2427.31 | 825.244 | 711.560 | 113.684 |
| 2004 | 188160 | 65058.725 | 55072.987 | 9985.738 | 5.307 | 15.349 | 2302.83 | 796.234 | 674.022 | 122.212 |
| 2005 | 186416 | 65214.724 | 54164.690 | 11050.03 | 5.928 | 16.944 | 2256.27 | 789.321 | 655.578 | 133.743 |
| 2006 | 180088 | 63771.146 | 51919.709 | 11851.44 | 6.581 | 18.584 | 2160.67 | 765.117 | 622.925 | 142.192 |
| 2007 | 175644 | 63110.718 | 50438.489 | 12672.23 | 7.215 | 20.079 | 2086.36 | 749.650 | 599.125 | 150.525 |
| 2008 | 177570 | 63718.624 | 49853.115 | 13865.51 | 7.808 | 21.761 | 2086.39 | 748.674 | 585.758 | 162.915 |
| 2009 | 172806 | 62794.698 | 48168.666 | 14626.03 | 8.464 | 23.292 | 2007.57 | 729.515 | 559.598 | 169.917 |
| 2010 | 170489 | 61917.074 | 46496.886 | 15420.19 | 9.045 | 24.905 | 1957.97 | 711.083 | 533.991 | 177.092 |
| 2011 | 170305 | 61680.114 | 45184.631 | 16495.48 | 9.686 | 26.744 | 1937.46 | 701.700 | 514.040 | 187.660 |
| 2012 | 171421 | 62023.127 | 44341.940 | 17681.19 | 10.314 | 28.507 | 1930.22 | 698.386 | 499.294 | 199.092 |
| 2013 | 172699 | 62037.765 | 43285.199 | 18752.57 | 10.859 | 30.228 | 1923.75 | 691.059 | 482.168 | 208.891 |
| 2014 | 174169 | 61921.017 | 41996.682 | 19924.34 | 11.440 | 32.177 | 1918.85 | 682.195 | 462.685 | 219.510 |
| 2015 | 177354 | 61856.204 | 40938.560 | 20917.64 | 11.794 | 33.817 | 2184.51 | 761.899 | 504.251 | 257.648 |
| 2016 | 176853 | 60464.119 | 38751.917 | 21712.20 | 12.277 | 35.909 | 2157.59 | 737.655 | 472.769 | 264.886 |
| 2017 | 179309 | 60243.839 | 37456.732 | 22787.11 | 12.708 | 37.825 | 2166.86 | 728.017 | 452.646 | 275.371 |
| 2018 | 177157 | 60713.327 | 36970.863 | 23742.46 | 13.402 | 39.106 | 2120.51 | 726.721 | 442.530 | 284.190 |
| 2019 | 179227 | 61148.172 | 36165.541 | 24982.63 | 13.939 | 40.856 | 2124.53 | 724.841 | 428.701 | 296.140 |
| 2020 | 180467 | 61149.317 | 35326.252 | 25823.06 | 14.309 | 42.230 | 2424.25 | 821.432 | 474.545 | 346.887 |
| 2021 | 178116 | 58727.786 | 32539.274 | 26188.51 | 14.703 | 44.593 | 2375.42 | 783.216 | 433.956 | 349.260 |
| 2022 | 179408 | 58848.802 | 31630.884 | 27217.92 | 15.171 | 46.251 | 2374.52 | 778.881 | 418.644 | 360.237 |
| 2023 | 180640 | 58816.149 | 30698.802 | 28117.35 | 15.565 | 47.805 | 2372.26 | 772.407 | 403.154 | 369.253 |
| 2024 | 181610 | 58955.232 | 29791.167 | 29164.06 | 16.059 | 49.468 | 2366.77 | 768.316 | 388.244 | 380.072 |
| 2025 | 180418 | 58297.744 | 28705.640 | 29592.10 | 16.402 | 50.760 | 2713.69 | 876.861 | 431.764 | 445.097 |
| 2026 | 180313 | 56469.971 | 26674.228 | 29795.74 | 16.524 | 52.764 | 2701.83 | 846.150 | 399.688 | 446.461 |
| 2027 | 179619 | 56209.427 | 25625.497 | 30583.93 | 17.027 | 54.411 | 2680.68 | 838.883 | 382.441 | 456.442 |
| 2028 | 178762 | 55733.105 | 24516.879 | 31216.23 | 17.462 | 56.010 | 2656.74 | 828.298 | 364.367 | 463.931 |
| 2029 | 177808 | 55145.838 | 23377.371 | 31768.47 | 17.867 | 57.608 | 2630.89 | 815.951 | 345.897 | 470.054 |
| 2030 | 173587 | 53615.097 | 22112.847 | 31502.25 | 18.148 | 58.756 | 3040.33 | 939.056 | 387.301 | 551.755 |
| 2031 | 174985 | 52046.561 | 20843.241 | 31203.32 | 17.832 | 59.953 | 3060.73 | 910.364 | 364.576 | 545.788 |
| 2032 | 173400 | 51582.636 | 19919.411 | 31663.22 | 18.260 | 61.383 | 3027.10 | 900.496 | 347.740 | 552.756 |
| 2033 | 171756 | 50839.758 | 18966.858 | 31872.90 | 18.557 | 62.693 | 2991.14 | 885.375 | 330.308 | 555.067 |
| 2034 | 169973 | 50215.451 | 18047.119 | 32168.33 | 18.926 | 64.061 | 2951.96 | 872.104 | 313.429 | 558.675 |
| 2035 | 162644 | 47773.075 | 16809.969 | 30963.11 | 19.037 | 64.813 | 3459.14 | 1016.05 | 357.517 | 658.528 |
| 2036 | 156254 | 43298.709 | 14572.432 | 28726.28 | 18.384 | 66.344 | 3328.25 | 922.274 | 310.397 | 611.877 |
| 2037 | 153593 | 42241.563 | 13606.907 | 28634.66 | 18.643 | 67.788 | 3274.10 | 900.450 | 290.054 | 610.396 |
| 2038 | 150947 | 41356.353 | 12837.243 | 28519.11 | 18.893 | 68.959 | 3217.50 | 881.531 | 273.632 | 607.899 |
| 2039 | 148373 | 40161.050 | 11949.885 | 28211.16 | 19.014 | 70.245 | 3159.42 | 855.178 | 254.457 | 600.720 |

# Results for PHIM for Run 6 (Sensitivity_Analysis_1.RTF)

***********************************************************************************
**Main Results for E-Component for Run: 6, Simulation: 1, COD: LUNG CANCER**

| Variable Parameter | Value |
|---|---|
| 1. Country | US (United States) |
| 2. Sex | F (Females) |
| 3. Year of start of process | 1990 |
| 4. Cause of Death | LUNG CANCER |
| 5. Effective Dose NULL | CC:1 |
| 6. Effective Dose Ecig | CC:1 Ecig:0.1 Dual:0.55 |
| Note: | Death rates are per million population |

--------------------------------------------------------------------------------------
Results for Age Groups Combined and Years combined, Population unadjusted for deaths in Ecig scenario,

| Age Group:All Year | Number Deaths | NULL Attrib Deaths | Ecig Attrib Deaths | Drop Deaths | %Drop Deaths | %Drop Attrib Deaths | Death Rate | NULL Attrib Rates | Ecig Attrib Rates | Drop Rates |
|---|---|---|---|---|---|---|---|---|---|---|
| All Comb | 2327764 | 1580465.217 | 1185643.707 | 394821.5 | 16.961 | 24.981 | 639.078 | 433.910 | 325.514 | 108.397 |

--------------------------------------------------------------------------------------
Results for Age Groups Combined, Population unadjusted for deaths in Ecig scenario,

| Age Group:All Year | Number Deaths | NULL Attrib Deaths | Ecig Attrib Deaths | Drop Deaths | %Drop Deaths | %Drop Attrib Deaths | Death Rate | NULL Attrib Rates | Ecig Attrib Rates | Drop Rates |
|---|---|---|---|---|---|---|---|---|---|---|
| 1990 | 42690 | 30614.201 | 30614.201 | 0.000 | 0.000 | 0.000 | 625.210 | 448.355 | 448.355 | 0.000 |
| 1991 | 43692 | 31412.010 | 31381.330 | 30.680 | 0.070 | 0.098 | 631.345 | 453.901 | 453.457 | 0.443 |
| 1992 | 45423 | 32782.407 | 32693.404 | 89.003 | 0.196 | 0.271 | 647.450 | 467.274 | 466.005 | 1.269 |
| 1993 | 46291 | 33491.183 | 33315.253 | 175.931 | 0.380 | 0.525 | 650.515 | 470.643 | 468.170 | 2.472 |
| 1994 | 47017 | 34074.347 | 33789.310 | 285.037 | 0.606 | 0.837 | 650.895 | 471.719 | 467.773 | 3.946 |
| 1995 | 47840 | 34694.454 | 34264.615 | 429.839 | 0.898 | 1.239 | 652.025 | 472.861 | 467.002 | 5.858 |
| 1996 | 48244 | 35027.269 | 34432.860 | 594.408 | 1.232 | 1.697 | 648.999 | 471.202 | 463.206 | 7.996 |
| 1997 | 49035 | 35590.763 | 34800.607 | 790.156 | 1.611 | 2.220 | 650.506 | 472.153 | 461.670 | 10.482 |
| 1998 | 49686 | 36100.785 | 35079.413 | 1021.371 | 2.056 | 2.829 | 649.874 | 472.184 | 458.825 | 13.359 |
| 1999 | 48883 | 35451.030 | 34221.965 | 1229.064 | 2.514 | 3.467 | 630.769 | 457.448 | 441.588 | 15.859 |
| 2000 | 50331 | 36447.986 | 34910.251 | 1537.735 | 3.055 | 4.219 | 641.392 | 464.474 | 444.878 | 19.596 |
| 2001 | 50389 | 36411.326 | 34561.884 | 1849.443 | 3.670 | 5.079 | 635.012 | 458.863 | 435.556 | 23.307 |
| 2002 | 51445 | 37078.545 | 34842.310 | 2236.235 | 4.347 | 6.031 | 641.972 | 462.696 | 434.790 | 27.906 |
| 2003 | 51408 | 36999.876 | 34395.541 | 2604.335 | 5.066 | 7.039 | 635.509 | 457.395 | 425.200 | 32.195 |
| 2004 | 51399 | 36861.555 | 33850.860 | 3010.695 | 5.857 | 8.168 | 629.057 | 451.138 | 414.291 | 36.847 |
| 2005 | 51421 | 36689.529 | 33250.555 | 3438.974 | 6.688 | 9.373 | 622.370 | 444.069 | 402.445 | 41.623 |
| 2006 | 51355 | 36504.774 | 32617.934 | 3886.840 | 7.569 | 10.647 | 616.150 | 437.979 | 391.345 | 46.634 |
| 2007 | 51839 | 36663.202 | 32243.482 | 4419.720 | 8.526 | 12.055 | 615.761 | 435.498 | 382.999 | 52.499 |
| 2008 | 50976 | 35898.803 | 31082.458 | 4816.346 | 9.448 | 13.416 | 598.952 | 421.800 | 365.209 | 56.591 |
| 2009 | 51193 | 35934.525 | 30550.082 | 5384.443 | 10.518 | 14.984 | 594.733 | 417.468 | 354.914 | 62.554 |
| 2010 | 50853 | 35502.829 | 29657.737 | 5845.093 | 11.494 | 16.464 | 584.018 | 407.730 | 340.602 | 67.128 |
| 2011 | 50509 | 35136.342 | 28754.605 | 6381.737 | 12.635 | 18.163 | 574.613 | 399.726 | 327.125 | 72.601 |
| 2012 | 51245 | 35450.201 | 28402.167 | 7048.034 | 13.754 | 19.882 | 577.023 | 399.173 | 319.811 | 79.362 |
| 2013 | 51069 | 35123.224 | 27490.871 | 7632.353 | 14.945 | 21.730 | 568.874 | 391.249 | 306.230 | 85.019 |
| 2014 | 51251 | 35079.370 | 26824.198 | 8255.172 | 16.107 | 23.533 | 564.641 | 386.476 | 295.527 | 90.949 |
| 2015 | 50443 | 34324.171 | 25670.086 | 8654.085 | 17.156 | 25.213 | 621.319 | 422.780 | 316.185 | 106.595 |
| 2016 | 48854 | 33054.753 | 24055.255 | 8999.498 | 18.421 | 27.226 | 596.013 | 403.264 | 293.471 | 109.793 |
| 2017 | 48325 | 32471.234 | 22965.940 | 9505.294 | 19.670 | 29.273 | 583.983 | 392.399 | 277.532 | 114.867 |
| 2018 | 50465 | 33657.595 | 23055.181 | 10602.41 | 21.009 | 31.501 | 604.052 | 402.871 | 275.964 | 126.908 |
| 2019 | 51036 | 33863.424 | 22489.194 | 11374.23 | 22.287 | 33.589 | 604.975 | 401.412 | 266.583 | 134.828 |
| 2020 | 51444 | 33910.830 | 21903.585 | 12007.24 | 23.341 | 35.408 | 691.053 | 455.532 | 294.236 | 161.296 |
| 2021 | 48037 | 31531.880 | 19675.090 | 11856.79 | 24.683 | 37.603 | 640.639 | 420.521 | 262.394 | 158.127 |
| 2022 | 48335 | 31560.212 | 19041.377 | 12518.84 | 25.900 | 39.667 | 639.727 | 417.708 | 252.018 | 165.690 |
| 2023 | 48599 | 31508.781 | 18389.410 | 13119.37 | 26.995 | 41.637 | 638.227 | 413.791 | 241.500 | 172.291 |
| 2024 | 48784 | 31517.293 | 17743.687 | 13773.61 | 28.234 | 43.702 | 635.757 | 410.740 | 231.239 | 179.500 |
| 2025 | 48477 | 31175.389 | 17002.511 | 14172.88 | 29.237 | 45.462 | 729.141 | 468.912 | 255.736 | 213.175 |
| 2026 | 45324 | 29044.598 | 15268.331 | 13776.27 | 30.395 | 47.431 | 679.138 | 435.206 | 228.782 | 206.425 |
| 2027 | 45107 | 28825.765 | 14577.285 | 14248.48 | 31.588 | 49.430 | 673.191 | 430.203 | 217.555 | 212.648 |
| 2028 | 44842 | 28513.846 | 13866.123 | 14647.72 | 32.665 | 51.371 | 666.430 | 423.769 | 206.077 | 217.693 |
| 2029 | 44539 | 28148.109 | 13141.126 | 15006.98 | 33.694 | 53.314 | 659.013 | 416.486 | 194.439 | 222.047 |
| 2030 | 43393 | 27314.475 | 12341.048 | 14973.43 | 34.507 | 54.819 | 760.018 | 478.407 | 216.151 | 262.256 |
| 2031 | 40748 | 25423.102 | 11176.374 | 14246.73 | 34.963 | 56.039 | 712.731 | 444.684 | 195.490 | 249.194 |
| 2032 | 40325 | 25085.902 | 10601.994 | 14483.91 | 35.918 | 57.737 | 703.969 | 437.933 | 185.083 | 252.851 |
| 2033 | 39882 | 24645.067 | 10031.091 | 14613.98 | 36.643 | 59.298 | 694.543 | 429.194 | 174.691 | 254.502 |
| 2034 | 39404 | 24239.126 | 9483.000 | 14756.13 | 37.448 | 60.877 | 684.347 | 420.967 | 164.694 | 256.273 |
| 2035 | 37454 | 22944.147 | 8738.957 | 14205.19 | 37.927 | 61.912 | 796.588 | 487.980 | 185.862 | 302.118 |
| 2036 | 32988 | 19950.581 | 7298.440 | 12652.14 | 38.353 | 63.417 | 702.663 | 424.953 | 155.459 | 269.494 |
| 2037 | 32403 | 19415.334 | 6788.054 | 12627.28 | 38.969 | 65.038 | 690.731 | 413.871 | 144.699 | 269.172 |
| 2038 | 31820 | 18939.317 | 6383.291 | 12556.03 | 39.460 | 66.296 | 678.257 | 403.701 | 136.063 | 267.638 |
| 2039 | 31251 | 18379.752 | 5929.386 | 12450.37 | 39.839 | 67.740 | 665.457 | 391.373 | 126.259 | 265.114 |

# Results for PHIM for Run 6 (Sensitivity_Analysis_1.RTF)

************************************************************************************
**Main Results for E-Component for Run: 6, Simulation: 1, COD: IHD**

| Variable Parameter | Value |
|---|---|
| 1. Country | US (United States) |
| 2. Sex | F (Females) |
| 3. Year of start of process | 1990 |
| 4. Cause of Death | IHD |
| 5. Effective Dose NULL | CC:1 |
| 6. Effective Dose Ecig | CC:1 Ecig:0.1 Dual:0.55 |
| Note: | Death rates are per million population |

-----------------------------------------------------------------------------------------
Results for Age Groups Combined and Years combined, Population unadjusted for deaths in Ecig scenario,

| Age Group:All | | NULL | Ecig | | | %Drop | | NULL | Ecig | |
|---|---|---|---|---|---|---|---|---|---|---|
| Year | Number Deaths | Attrib Deaths | Attrib Deaths | Drop Deaths | %Drop Deaths | Attrib Deaths | Death Rate | Attrib Rates | Attrib Rates | Drop Rates |
| All Comb | 3242167 | 398833.796 | 212062.316 | 186771.5 | 5.761 | 46.829 | 890.124 | 109.498 | 58.221 | 51.277 |

-----------------------------------------------------------------------------------------
Results for Age Groups Combined, Population unadjusted for deaths in Ecig scenario,

| Age Group:All | | NULL | Ecig | | | %Drop | | NULL | Ecig | |
|---|---|---|---|---|---|---|---|---|---|---|
| Year | Number Deaths | Attrib Deaths | Attrib Deaths | Drop Deaths | %Drop Deaths | Attrib Deaths | Death Rate | Attrib Rates | Attrib Rates | Drop Rates |
| 1990 | 98916 | 12922.892 | 12922.892 | 0.000 | 0.000 | 0.000 | 1448.66 | 189.260 | 189.260 | 0.000 |
| 1991 | 96403 | 12856.479 | 12770.826 | 85.653 | 0.089 | 0.666 | 1393.01 | 185.775 | 184.537 | 1.238 |
| 1992 | 93721 | 12662.244 | 12425.222 | 237.021 | 0.253 | 1.872 | 1335.88 | 180.485 | 177.107 | 3.378 |
| 1993 | 93679 | 12730.613 | 12278.638 | 451.975 | 0.482 | 3.550 | 1316.45 | 178.900 | 172.548 | 6.351 |
| 1994 | 90832 | 12495.323 | 11792.090 | 703.233 | 0.774 | 5.628 | 1257.46 | 172.983 | 163.248 | 9.735 |
| 1995 | 89698 | 12365.590 | 11372.529 | 993.061 | 1.107 | 8.031 | 1222.52 | 168.534 | 155.000 | 13.535 |
| 1996 | 88286 | 12290.551 | 10937.948 | 1352.603 | 1.532 | 11.005 | 1187.66 | 165.338 | 147.142 | 18.196 |
| 1997 | 84777 | 11719.531 | 10091.960 | 1627.571 | 1.920 | 13.888 | 1124.67 | 155.473 | 133.882 | 21.592 |
| 1998 | 82517 | 11416.794 | 9469.837 | 1946.958 | 2.359 | 17.053 | 1079.29 | 149.327 | 123.862 | 25.465 |
| 1999 | 93939 | 13028.038 | 10350.205 | 2677.833 | 2.851 | 20.554 | 1212.16 | 168.109 | 133.555 | 34.554 |
| 2000 | 90039 | 12426.299 | 9437.910 | 2988.389 | 3.319 | 24.049 | 1147.41 | 158.354 | 120.272 | 38.082 |
| 2001 | 86119 | 11939.281 | 8574.080 | 3365.202 | 3.908 | 28.186 | 1085.29 | 150.461 | 108.052 | 42.409 |
| 2002 | 83014 | 11528.794 | 7796.134 | 3732.660 | 4.496 | 32.377 | 1035.91 | 143.865 | 97.286 | 46.579 |
| 2003 | 79302 | 11048.407 | 7038.629 | 4009.778 | 5.056 | 36.293 | 980.336 | 136.581 | 87.012 | 49.569 |
| 2004 | 73861 | 10375.132 | 6179.200 | 4195.931 | 5.681 | 40.442 | 903.963 | 126.978 | 75.625 | 51.353 |
| 2005 | 71649 | 10051.029 | 5601.937 | 4449.092 | 6.210 | 44.265 | 867.197 | 121.652 | 67.803 | 53.849 |
| 2006 | 67551 | 9542.523 | 4932.261 | 4610.262 | 6.825 | 48.313 | 810.467 | 114.490 | 59.177 | 55.313 |
| 2007 | 63503 | 8936.533 | 4295.643 | 4640.890 | 7.308 | 51.932 | 754.310 | 106.151 | 51.025 | 55.126 |
| 2008 | 63171 | 8944.180 | 3993.003 | 4951.178 | 7.838 | 55.356 | 742.239 | 105.091 | 46.917 | 58.175 |
| 2009 | 59747 | 8390.033 | 3500.327 | 4889.707 | 8.184 | 58.280 | 694.108 | 97.471 | 40.665 | 56.806 |
| 2010 | 58008 | 8157.570 | 3170.297 | 4987.272 | 8.598 | 61.137 | 666.189 | 93.685 | 36.409 | 57.276 |
| 2011 | 57368 | 8035.356 | 2889.574 | 5145.782 | 8.970 | 64.039 | 652.644 | 91.414 | 32.873 | 58.541 |
| 2012 | 57411 | 7970.766 | 2647.005 | 5323.761 | 9.273 | 66.791 | 646.453 | 89.752 | 29.806 | 59.946 |
| 2013 | 57067 | 7743.216 | 2402.764 | 5340.452 | 9.358 | 68.969 | 635.688 | 86.254 | 26.765 | 59.489 |
| 2014 | 57538 | 7774.935 | 2238.053 | 5536.882 | 9.623 | 71.215 | 633.906 | 85.658 | 24.657 | 61.001 |
| 2015 | 58395 | 7722.535 | 2098.695 | 5623.840 | 9.631 | 72.824 | 719.266 | 95.120 | 25.850 | 69.270 |
| 2016 | 58658 | 7600.353 | 1921.489 | 5678.865 | 9.681 | 74.718 | 715.621 | 92.723 | 23.442 | 69.281 |
| 2017 | 59004 | 7470.266 | 1768.015 | 5702.252 | 9.664 | 76.333 | 713.034 | 90.274 | 21.366 | 68.909 |
| 2018 | 56077 | 6907.050 | 1531.832 | 5375.218 | 9.585 | 77.822 | 671.224 | 82.675 | 18.336 | 64.340 |
| 2019 | 55886 | 6732.046 | 1408.584 | 5323.462 | 9.526 | 79.076 | 662.462 | 79.801 | 16.697 | 63.104 |
| 2020 | 55313 | 6429.836 | 1282.207 | 5147.629 | 9.306 | 80.058 | 743.037 | 86.373 | 17.224 | 69.149 |
| 2021 | 56292 | 6562.016 | 1221.243 | 5340.772 | 9.488 | 81.389 | 750.730 | 87.513 | 16.287 | 71.227 |
| 2022 | 55827 | 6352.366 | 1124.854 | 5227.511 | 9.364 | 82.292 | 738.885 | 84.075 | 14.888 | 69.188 |
| 2023 | 55338 | 6134.123 | 1041.960 | 5092.163 | 9.202 | 83.014 | 726.728 | 80.557 | 13.684 | 66.873 |
| 2024 | 54770 | 5922.193 | 963.654 | 4958.538 | 9.053 | 83.728 | 713.771 | 77.179 | 12.559 | 64.621 |
| 2025 | 53295 | 5504.188 | 866.549 | 4637.638 | 8.702 | 84.257 | 801.612 | 82.789 | 13.034 | 69.755 |
| 2026 | 55278 | 5641.075 | 853.981 | 4787.095 | 8.660 | 84.861 | 828.283 | 84.526 | 12.796 | 71.730 |
| 2027 | 54199 | 5426.216 | 795.542 | 4630.674 | 8.544 | 85.339 | 808.875 | 80.982 | 11.873 | 69.109 |
| 2028 | 53090 | 5197.830 | 739.597 | 4458.233 | 8.397 | 85.771 | 789.020 | 77.249 | 10.992 | 66.258 |
| 2029 | 51974 | 4973.547 | 689.428 | 4284.119 | 8.243 | 86.138 | 769.023 | 73.590 | 10.201 | 63.389 |
| 2030 | 49479 | 4450.618 | 599.216 | 3851.402 | 7.784 | 86.536 | 866.605 | 77.952 | 10.495 | 67.456 |
| 2031 | 51987 | 4555.052 | 603.094 | 3951.958 | 7.602 | 86.760 | 909.317 | 79.674 | 10.549 | 69.125 |
| 2032 | 50677 | 4349.999 | 564.220 | 3785.780 | 7.470 | 87.029 | 884.689 | 75.939 | 9.850 | 66.090 |
| 2033 | 49380 | 4147.948 | 531.717 | 3616.231 | 7.323 | 87.181 | 859.955 | 72.237 | 9.260 | 62.977 |
| 2034 | 48076 | 3966.133 | 500.523 | 3465.610 | 7.209 | 87.380 | 834.943 | 68.881 | 8.693 | 60.188 |
| 2035 | 44518 | 3289.064 | 404.021 | 2885.044 | 6.481 | 87.716 | 946.825 | 69.952 | 8.593 | 61.360 |
| 2036 | 46347 | 3313.192 | 399.000 | 2914.193 | 6.288 | 87.957 | 987.211 | 70.572 | 8.499 | 62.073 |
| 2037 | 44841 | 3117.951 | 370.446 | 2747.505 | 6.127 | 88.119 | 955.859 | 66.464 | 7.897 | 58.568 |
| 2038 | 43378 | 2949.775 | 348.059 | 2601.716 | 5.998 | 88.200 | 924.621 | 62.876 | 7.419 | 55.457 |
| 2039 | 41973 | 2766.312 | 325.424 | 2440.888 | 5.815 | 88.236 | 893.753 | 58.905 | 6.929 | 51.976 |

# Results for PHIM for Run 6 (Sensitivity_Analysis_1.RTF)

************************************************************************************

**Main Results for E-Component for Run: 6, Simulation: 1, COD: STROKE**

| Variable Parameter | Value |
|---|---|
| 1. Country | US (United States) |
| 2. Sex | F (Females) |
| 3. Year of start of process | 1990 |
| 4. Cause of Death | STROKE |
| 5. Effective Dose NULL | CC:1 |
| 6. Effective Dose Ecig | CC:1 Ecig:0.1 Dual:0.55 |
| Note: | Death rates are per million population |

-----------------------------------------------------------------------------------------

Results for Age Groups Combined and Years combined, Population unadjusted for deaths in Ecig scenario,

| Age Group:All | | NULL | Ecig | | | %Drop | | NULL | Ecig | |
|---|---|---|---|---|---|---|---|---|---|---|
| Year | Number | Attrib | Attrib | Drop | %Drop | Attrib | Death | Attrib | Attrib | Drop |
| | Deaths | Deaths | Deaths | Deaths | Deaths | Deaths | Rate | Rates | Rates | Rates |
| All Comb | 1352207 | 104899.750 | 54615.614 | 50284.14 | 3.719 | 47.935 | 371.243 | 28.800 | 14.995 | 13.805 |

-----------------------------------------------------------------------------------------

Results for Age Groups Combined, Population unadjusted for deaths in Ecig scenario,

| Age Group:All | | NULL | Ecig | | | %Drop | | NULL | Ecig | |
|---|---|---|---|---|---|---|---|---|---|---|
| Year | Number | Attrib | Attrib | Drop | %Drop | Attrib | Death | Attrib | Attrib | Drop |
| | Deaths | Deaths | Deaths | Deaths | Deaths | Deaths | Rate | Rates | Rates | Rates |
| 1990 | 33504 | 3293.017 | 3293.017 | 0.000 | 0.000 | 0.000 | 490.678 | 48.227 | 48.227 | 0.000 |
| 1991 | 32505 | 3207.063 | 3180.986 | 26.077 | 0.080 | 0.813 | 469.694 | 46.342 | 45.965 | 0.377 |
| 1992 | 32560 | 3218.125 | 3145.290 | 72.835 | 0.224 | 2.263 | 464.104 | 45.871 | 44.832 | 1.038 |
| 1993 | 32768 | 3226.680 | 3088.849 | 137.831 | 0.421 | 4.272 | 460.480 | 45.344 | 43.407 | 1.937 |
| 1994 | 33005 | 3277.266 | 3057.660 | 219.606 | 0.665 | 6.701 | 456.915 | 45.370 | 42.330 | 3.040 |
| 1995 | 33730 | 3309.378 | 2994.867 | 314.511 | 0.932 | 9.504 | 459.716 | 45.104 | 40.818 | 4.287 |
| 1996 | 33750 | 3312.987 | 2887.919 | 425.068 | 1.259 | 12.830 | 454.019 | 44.568 | 38.850 | 5.718 |
| 1997 | 33699 | 3267.066 | 2735.153 | 531.913 | 1.578 | 16.281 | 447.056 | 43.341 | 36.285 | 7.056 |
| 1998 | 33274 | 3178.725 | 2544.811 | 633.913 | 1.905 | 19.942 | 435.211 | 41.577 | 33.285 | 8.291 |
| 1999 | 33891 | 3120.351 | 2382.803 | 737.548 | 2.176 | 23.637 | 437.317 | 40.264 | 30.747 | 9.517 |
| 2000 | 33827 | 3142.808 | 2280.569 | 862.239 | 2.549 | 27.435 | 431.074 | 40.050 | 29.062 | 10.988 |
| 2001 | 32506 | 2961.588 | 2036.209 | 925.379 | 2.847 | 31.246 | 409.647 | 37.323 | 25.661 | 11.662 |
| 2002 | 32078 | 2940.627 | 1900.619 | 1040.008 | 3.242 | 35.367 | 400.295 | 36.695 | 23.717 | 12.978 |
| 2003 | 30568 | 2819.545 | 1712.872 | 1106.673 | 3.620 | 39.250 | 377.883 | 34.855 | 21.175 | 13.681 |
| 2004 | 29388 | 2730.466 | 1552.683 | 1177.783 | 4.008 | 43.135 | 359.671 | 33.417 | 19.003 | 14.415 |
| 2005 | 27954 | 2670.510 | 1421.385 | 1249.125 | 4.469 | 46.775 | 338.339 | 32.322 | 17.204 | 15.119 |
| 2006 | 27201 | 2623.285 | 1304.912 | 1318.373 | 4.847 | 50.257 | 326.354 | 31.474 | 15.656 | 15.818 |
| 2007 | 26302 | 2536.599 | 1176.902 | 1359.697 | 5.170 | 53.603 | 312.424 | 30.131 | 13.980 | 16.151 |
| 2008 | 25998 | 2469.443 | 1071.119 | 1398.324 | 5.379 | 56.625 | 305.468 | 29.015 | 12.585 | 16.430 |
| 2009 | 25040 | 2386.945 | 966.653 | 1420.292 | 5.672 | 59.503 | 290.901 | 27.730 | 11.230 | 16.500 |
| 2010 | 24840 | 2329.274 | 881.552 | 1447.723 | 5.828 | 62.153 | 285.273 | 26.750 | 10.124 | 16.626 |
| 2011 | 24792 | 2286.581 | 804.618 | 1481.963 | 5.978 | 64.811 | 282.045 | 26.013 | 9.154 | 16.859 |
| 2012 | 24500 | 2243.311 | 734.371 | 1508.940 | 6.159 | 67.264 | 275.872 | 25.260 | 8.269 | 16.991 |
| 2013 | 24451 | 2176.503 | 663.640 | 1512.863 | 6.187 | 69.509 | 272.368 | 24.245 | 7.393 | 16.852 |
| 2014 | 25296 | 2211.619 | 629.778 | 1581.841 | 6.253 | 71.524 | 278.691 | 24.366 | 6.938 | 17.427 |
| 2015 | 26316 | 2237.016 | 601.422 | 1635.594 | 6.215 | 73.115 | 324.141 | 27.554 | 7.408 | 20.146 |
| 2016 | 26735 | 2229.057 | 563.086 | 1665.971 | 6.231 | 74.739 | 326.164 | 27.194 | 6.870 | 20.325 |
| 2017 | 27616 | 2221.649 | 528.893 | 1692.756 | 6.130 | 76.194 | 333.726 | 26.848 | 6.391 | 20.456 |
| 2018 | 25256 | 1939.420 | 437.383 | 1502.037 | 5.947 | 77.448 | 302.304 | 23.214 | 5.235 | 17.979 |
| 2019 | 25429 | 1904.400 | 404.859 | 1499.540 | 5.897 | 78.741 | 301.434 | 22.574 | 4.799 | 17.775 |
| 2020 | 25379 | 1806.054 | 365.705 | 1440.349 | 5.675 | 79.751 | 340.922 | 24.261 | 4.913 | 19.349 |
| 2021 | 25448 | 1748.172 | 334.869 | 1413.304 | 5.554 | 80.845 | 339.386 | 23.314 | 4.466 | 18.848 |
| 2022 | 25539 | 1701.148 | 310.226 | 1390.922 | 5.446 | 81.764 | 338.015 | 22.515 | 4.106 | 18.409 |
| 2023 | 25620 | 1652.087 | 288.951 | 1363.136 | 5.321 | 82.510 | 336.461 | 21.696 | 3.795 | 17.901 |
| 2024 | 25659 | 1597.127 | 267.248 | 1329.878 | 5.183 | 83.267 | 334.391 | 20.814 | 3.483 | 17.331 |
| 2025 | 25260 | 1467.622 | 236.928 | 1230.694 | 4.872 | 83.856 | 379.931 | 22.075 | 3.564 | 18.511 |
| 2026 | 25480 | 1390.505 | 216.692 | 1173.814 | 4.607 | 84.416 | 381.790 | 20.835 | 3.247 | 17.589 |
| 2027 | 25264 | 1342.095 | 201.865 | 1140.231 | 4.513 | 84.959 | 377.042 | 20.030 | 3.013 | 17.017 |
| 2028 | 25022 | 1289.536 | 188.176 | 1101.360 | 4.402 | 85.407 | 371.870 | 19.165 | 2.797 | 16.368 |
| 2029 | 24767 | 1242.216 | 176.023 | 1066.193 | 4.305 | 85.830 | 366.459 | 18.380 | 2.604 | 15.776 |
| 2030 | 23959 | 1096.803 | 150.322 | 946.481 | 3.950 | 86.295 | 419.641 | 19.210 | 2.633 | 16.577 |
| 2031 | 23963 | 1019.991 | 137.038 | 882.953 | 3.685 | 86.565 | 419.142 | 17.841 | 2.397 | 15.444 |
| 2032 | 23615 | 977.406 | 128.334 | 849.072 | 3.595 | 86.870 | 412.259 | 17.063 | 2.240 | 14.823 |
| 2033 | 23259 | 937.450 | 121.330 | 816.120 | 3.509 | 87.057 | 405.051 | 16.326 | 2.113 | 14.213 |
| 2034 | 22879 | 900.763 | 114.446 | 786.317 | 3.437 | 87.295 | 397.339 | 15.644 | 1.988 | 13.656 |
| 2035 | 21733 | 745.023 | 92.421 | 652.603 | 3.003 | 87.595 | 462.224 | 15.845 | 1.966 | 13.880 |
| 2036 | 20904 | 680.360 | 82.347 | 598.013 | 2.861 | 87.897 | 445.263 | 14.492 | 1.754 | 12.738 |
| 2037 | 20397 | 644.109 | 76.989 | 567.121 | 2.780 | 88.047 | 434.791 | 13.730 | 1.641 | 12.089 |
| 2038 | 19890 | 612.448 | 72.589 | 539.859 | 2.714 | 88.148 | 423.966 | 13.055 | 1.547 | 11.507 |
| 2039 | 19392 | 577.529 | 68.235 | 509.294 | 2.626 | 88.185 | 412.930 | 12.298 | 1.453 | 10.845 |

# Results for PHIM for Run 6 (Sensitivity_Analysis_1.RTF)

********************************************************************************

**Main Results for E-Component for Run: 6, Simulation: 1, COD: COPD**

| Variable Parameter | Value |
|---|---|
| 1. Country | US (United States) |
| 2. Sex | F (Females) |
| 3. Year of start of process | 1990 |
| 4. Cause of Death | COPD |
| 5. Effective Dose NULL | CC:1 |
| 6. Effective Dose Ecig | CC:1 Ecig:0.1 Dual:0.55 |
| Note: | Death rates are per million population |

---------------------------------------------------------------------------------------

Results for Age Groups Combined and Years combined, Population unadjusted for deaths in Ecig scenario,

| Age Group:All | | NULL | Ecig | | | %Drop | | NULL | Ecig | |
|---|---|---|---|---|---|---|---|---|---|---|
| Year | Number | Attrib | Attrib | Drop | %Drop | Attrib | Death | Attrib | Attrib | Drop |
| | Deaths | Deaths | Deaths | Deaths | Deaths | Deaths | Rate | Rates | Rates | Rates |
| All Comb | 2159941 | 862051.244 | 573080.819 | 288970.4 | 13.379 | 33.521 | 593.003 | 236.673 | 157.337 | 79.336 |

---------------------------------------------------------------------------------------

Results for Age Groups Combined, Population unadjusted for deaths in Ecig scenario,

| Age Group:All | | NULL | Ecig | | | %Drop | | NULL | Ecig | |
|---|---|---|---|---|---|---|---|---|---|---|
| Year | Number | Attrib | Attrib | Drop | %Drop | Attrib | Death | Attrib | Attrib | Drop |
| | Deaths | Deaths | Deaths | Deaths | Deaths | Deaths | Rate | Rates | Rates | Rates |
| 1990 | 24738 | 10694.712 | 10694.712 | 0.000 | 0.000 | 0.000 | 362.296 | 156.628 | 156.628 | 0.000 |
| 1991 | 26562 | 11602.933 | 11585.600 | 17.333 | 0.065 | 0.149 | 383.818 | 167.661 | 167.411 | 0.250 |
| 1992 | 26828 | 11831.375 | 11783.762 | 47.613 | 0.177 | 0.402 | 382.401 | 168.642 | 167.963 | 0.679 |
| 1993 | 29774 | 13246.371 | 13143.703 | 102.668 | 0.345 | 0.775 | 418.406 | 186.148 | 184.705 | 1.443 |
| 1994 | 29804 | 13355.662 | 13192.651 | 163.011 | 0.547 | 1.221 | 412.601 | 184.893 | 182.637 | 2.257 |
| 1995 | 29755 | 13397.439 | 13155.264 | 242.175 | 0.814 | 1.808 | 405.540 | 182.598 | 179.297 | 3.301 |
| 1996 | 30900 | 13960.356 | 13622.197 | 338.158 | 1.094 | 2.422 | 415.680 | 187.801 | 183.252 | 4.549 |
| 1997 | 31128 | 14109.552 | 13667.941 | 441.612 | 1.419 | 3.130 | 412.949 | 187.180 | 181.321 | 5.858 |
| 1998 | 32096 | 14599.657 | 14021.772 | 577.885 | 1.800 | 3.958 | 419.803 | 190.958 | 183.399 | 7.559 |
| 1999 | 34616 | 15728.102 | 14978.792 | 749.310 | 2.165 | 4.764 | 446.673 | 202.950 | 193.281 | 9.669 |
| 2000 | 34299 | 15579.803 | 14687.727 | 892.076 | 2.601 | 5.726 | 437.088 | 198.541 | 187.173 | 11.368 |
| 2001 | 34592 | 15696.614 | 14621.765 | 1074.850 | 3.107 | 6.848 | 435.935 | 197.812 | 184.266 | 13.545 |
| 2002 | 34291 | 15519.777 | 14271.652 | 1248.125 | 3.640 | 8.042 | 427.910 | 193.668 | 178.093 | 15.575 |
| 2003 | 35074 | 15888.389 | 14412.936 | 1475.453 | 4.207 | 9.286 | 433.587 | 196.413 | 178.174 | 18.240 |
| 2004 | 33512 | 15091.573 | 13490.243 | 1601.330 | 4.778 | 10.611 | 410.143 | 184.701 | 165.103 | 19.598 |
| 2005 | 35392 | 15803.656 | 13890.812 | 1912.843 | 5.405 | 12.104 | 428.364 | 191.278 | 168.126 | 23.152 |
| 2006 | 33981 | 15100.563 | 13064.602 | 2035.962 | 5.991 | 13.483 | 407.699 | 181.174 | 156.747 | 24.427 |
| 2007 | 34000 | 14974.384 | 12722.462 | 2251.922 | 6.623 | 15.038 | 403.863 | 177.871 | 151.122 | 26.749 |
| 2008 | 37425 | 16406.197 | 13706.536 | 2699.662 | 7.214 | 16.455 | 439.732 | 192.768 | 161.047 | 31.720 |
| 2009 | 36826 | 16083.194 | 13151.604 | 2931.590 | 7.961 | 18.228 | 427.825 | 186.846 | 152.788 | 34.058 |
| 2010 | 36788 | 15927.400 | 12787.300 | 3140.100 | 8.536 | 19.715 | 422.489 | 182.917 | 146.855 | 36.062 |
| 2011 | 37636 | 16221.836 | 12735.835 | 3486.000 | 9.262 | 21.490 | 428.164 | 184.547 | 144.888 | 39.658 |
| 2012 | 38265 | 16358.849 | 12558.396 | 3800.453 | 9.932 | 23.232 | 430.867 | 184.202 | 141.409 | 42.793 |
| 2013 | 40112 | 16994.822 | 12727.924 | 4266.899 | 10.637 | 25.107 | 446.821 | 189.311 | 141.780 | 47.530 |
| 2014 | 40084 | 16855.093 | 12304.653 | 4550.441 | 11.352 | 26.997 | 441.613 | 185.696 | 135.563 | 50.133 |
| 2015 | 42200 | 17572.483 | 12568.358 | 5004.125 | 11.858 | 28.477 | 519.788 | 216.445 | 154.808 | 61.637 |
| 2016 | 42606 | 17579.955 | 12212.087 | 5367.868 | 12.599 | 30.534 | 519.788 | 214.473 | 148.986 | 65.487 |
| 2017 | 44364 | 18080.688 | 12193.884 | 5886.805 | 13.269 | 32.559 | 536.117 | 218.496 | 147.357 | 71.139 |
| 2018 | 45359 | 18209.262 | 11946.467 | 6262.795 | 13.807 | 34.393 | 542.932 | 217.960 | 142.996 | 74.964 |
| 2019 | 46876 | 18648.302 | 11862.904 | 6785.398 | 14.475 | 36.386 | 555.657 | 221.054 | 140.621 | 80.433 |
| 2020 | 48331 | 19002.597 | 11774.754 | 7227.843 | 14.955 | 38.036 | 649.239 | 255.266 | 158.173 | 97.093 |
| 2021 | 48338 | 18885.717 | 11308.072 | 7577.645 | 15.676 | 40.124 | 644.660 | 251.867 | 150.809 | 101.058 |
| 2022 | 49708 | 19235.076 | 11154.426 | 8080.650 | 16.256 | 42.010 | 657.895 | 254.582 | 147.632 | 106.950 |
| 2023 | 51083 | 19521.158 | 10978.481 | 8542.677 | 16.723 | 43.761 | 670.849 | 256.363 | 144.176 | 112.187 |
| 2024 | 52397 | 19918.620 | 10816.578 | 9102.042 | 17.371 | 45.696 | 682.854 | 259.583 | 140.964 | 118.620 |
| 2025 | 53387 | 20150.545 | 10599.651 | 9550.894 | 17.890 | 47.398 | 803.004 | 303.086 | 159.430 | 143.656 |
| 2026 | 54232 | 20393.792 | 10335.225 | 10058.57 | 18.547 | 49.322 | 812.615 | 305.582 | 154.864 | 150.718 |
| 2027 | 55049 | 20615.351 | 10050.805 | 10564.55 | 19.191 | 51.246 | 821.568 | 307.668 | 150.001 | 157.668 |
| 2028 | 55809 | 20731.894 | 9722.983 | 11008.91 | 19.726 | 53.101 | 829.419 | 308.115 | 144.502 | 163.613 |
| 2029 | 56527 | 20781.967 | 9370.794 | 11411.17 | 20.187 | 54.909 | 836.392 | 307.495 | 138.652 | 168.842 |
| 2030 | 56756 | 20753.201 | 9022.261 | 11730.94 | 20.669 | 56.526 | 994.067 | 363.487 | 158.023 | 205.465 |
| 2031 | 58288 | 21048.417 | 8926.736 | 12121.68 | 20.796 | 57.590 | 1019.54 | 368.165 | 156.140 | 212.024 |
| 2032 | 58782 | 21169.329 | 8624.865 | 12544.46 | 21.341 | 59.258 | 1026.18 | 369.560 | 150.567 | 218.993 |
| 2033 | 59236 | 21109.293 | 8282.720 | 12826.57 | 21.654 | 60.763 | 1031.59 | 367.618 | 144.244 | 223.375 |
| 2034 | 59614 | 21109.429 | 7949.149 | 13160.28 | 22.076 | 62.343 | 1035.33 | 366.613 | 138.055 | 228.558 |
| 2035 | 58938 | 20794.840 | 7574.571 | 13220.27 | 22.431 | 63.575 | 1253.51 | 442.268 | 161.097 | 281.171 |
| 2036 | 56014 | 19354.577 | 6792.646 | 12561.93 | 22.426 | 64.904 | 1193.11 | 412.258 | 144.685 | 267.573 |
| 2037 | 55953 | 19064.169 | 6371.418 | 12692.75 | 22.685 | 66.579 | 1192.72 | 406.385 | 135.818 | 270.567 |
| 2038 | 55859 | 18854.812 | 6033.303 | 12821.51 | 22.953 | 68.001 | 1190.66 | 401.900 | 128.603 | 273.297 |
| 2039 | 55757 | 18437.457 | 5626.839 | 12810.62 | 22.976 | 69.481 | 1187.28 | 392.602 | 119.816 | 272.786 |

# Results for PHIM for Run 6 (Sensitivity_Analysis_1.RTF)

************************************************************************************

**Main Results for E-Component for Run: 6, Simulation: 1, COD: Four Diseases Combined, Adjusted**

| Variable Parameter | Value |
|---|---|
| 1. Country | US (United States) |
| 2. Sex | F (Females) |
| 3. Year of start of process | 1990 |
| 4. Cause of Death | Four Diseases Combined |
| 5. Effective Dose NULL | CC:1 |
| 6. Effective Dose Ecig | CC:1 Ecig:0.1 Dual:0.55 |
| Note: | Death rates are per million population |

-----------------------------------------------------------------------------------------

Results for Age Groups Combined and Years combined, Population and Number deaths adjusted for Ecig scenario,

| Age Group:All | | NULL | Ecig | | | %Drop | | NULL | Ecig | |
|---|---|---|---|---|---|---|---|---|---|---|
| Year | Number Deaths | Attrib Deaths | Attrib Deaths | Drop Deaths | %Drop Deaths | Attrib Deaths | Death Rate | Attrib Rates | Attrib Rates | Drop Rates |
| All Comb | 9143821 | 2946250.006 | 2034522.086 | 911727.9 | 9.971 | 30.945 | 2499.23 | 808.881 | 556.084 | 252.798 |

-----------------------------------------------------------------------------------------

Results for Age Groups Combined, Population and Number deaths adjusted for Ecig scenario,

| Age Group:All | | NULL | Ecig | | | %Drop | | NULL | Ecig | |
|---|---|---|---|---|---|---|---|---|---|---|
| Year | Number Deaths | Attrib Deaths | Attrib Deaths | Drop Deaths | %Drop Deaths | Attrib Deaths | Death Rate | Attrib Rates | Attrib Rates | Drop Rates |
| 1990 | 199848 | 57524.822 | 57524.822 | 0.000 | 0.000 | 0.000 | 2926.84 | 842.471 | 842.471 | 0.000 |
| 1991 | 199162 | 59078.486 | 58918.742 | 159.743 | 0.080 | 0.270 | 2877.87 | 853.678 | 851.370 | 2.308 |
| 1992 | 198533 | 60494.150 | 60047.891 | 446.258 | 0.225 | 0.738 | 2829.84 | 862.271 | 855.909 | 6.363 |
| 1993 | 202515 | 62694.848 | 61827.277 | 867.571 | 0.428 | 1.384 | 2845.86 | 881.034 | 868.834 | 12.199 |
| 1994 | 200664 | 63202.598 | 61833.757 | 1368.841 | 0.682 | 2.166 | 2777.91 | 874.966 | 855.998 | 18.968 |
| 1995 | 201035 | 63766.862 | 61791.203 | 1975.659 | 0.983 | 3.098 | 2739.86 | 869.097 | 842.137 | 26.961 |
| 1996 | 201201 | 64591.163 | 61887.611 | 2703.551 | 1.344 | 4.186 | 2706.46 | 868.908 | 832.482 | 36.426 |
| 1997 | 198672 | 64686.912 | 61306.029 | 3380.883 | 1.702 | 5.227 | 2635.33 | 858.147 | 813.210 | 44.937 |
| 1998 | 197620 | 65295.961 | 61130.977 | 4164.984 | 2.108 | 6.379 | 2584.41 | 854.046 | 799.449 | 54.597 |
| 1999 | 211400 | 67327.521 | 61955.149 | 5372.372 | 2.541 | 7.979 | 2727.26 | 868.771 | 799.282 | 69.489 |
| 2000 | 208591 | 67596.897 | 61345.201 | 6251.696 | 2.997 | 9.248 | 2657.43 | 861.419 | 781.533 | 79.886 |
| 2001 | 203727 | 67008.811 | 59830.638 | 7178.173 | 3.523 | 10.712 | 2566.48 | 844.458 | 753.725 | 90.733 |
| 2002 | 200981 | 67067.744 | 58856.557 | 8211.186 | 4.086 | 12.243 | 2506.86 | 836.925 | 734.124 | 102.801 |
| 2003 | 196539 | 66756.217 | 57616.052 | 9140.165 | 4.651 | 13.692 | 2428.26 | 825.244 | 711.851 | 113.393 |
| 2004 | 188381 | 65058.725 | 55138.743 | 9919.982 | 5.266 | 15.248 | 2303.96 | 796.234 | 674.365 | 121.869 |
| 2005 | 186678 | 65214.724 | 54242.012 | 10972.71 | 5.878 | 16.826 | 2257.60 | 789.321 | 655.979 | 133.342 |
| 2006 | 180388 | 63771.146 | 52007.445 | 11763.70 | 6.521 | 18.447 | 2162.20 | 765.117 | 623.381 | 141.736 |
| 2007 | 175985 | 63110.718 | 50537.935 | 12572.78 | 7.144 | 19.922 | 2088.09 | 749.650 | 599.640 | 150.010 |
| 2008 | 177969 | 63718.624 | 49966.749 | 13751.88 | 7.727 | 21.582 | 2088.41 | 748.674 | 586.346 | 162.328 |
| 2009 | 173250 | 62794.698 | 48294.270 | 14500.43 | 8.370 | 23.092 | 2009.81 | 729.515 | 560.243 | 169.272 |
| 2010 | 170985 | 61917.074 | 46634.170 | 15282.90 | 8.938 | 24.683 | 1960.45 | 711.083 | 534.690 | 176.393 |
| 2011 | 170861 | 61680.114 | 45334.248 | 16345.87 | 9.567 | 26.501 | 1940.23 | 701.700 | 514.798 | 186.902 |
| 2012 | 172045 | 62023.127 | 44505.822 | 17517.31 | 10.182 | 28.243 | 1933.29 | 698.386 | 500.119 | 198.267 |
| 2013 | 173398 | 62037.765 | 43463.061 | 18574.70 | 10.712 | 29.941 | 1927.18 | 691.059 | 483.057 | 208.002 |
| 2014 | 174948 | 61921.017 | 42187.471 | 19733.55 | 11.280 | 31.869 | 1922.64 | 682.195 | 463.631 | 218.564 |
| 2015 | 178230 | 61856.204 | 41143.939 | 20712.26 | 11.621 | 33.485 | 2188.59 | 761.899 | 505.230 | 256.669 |
| 2016 | 177809 | 60464.119 | 38964.801 | 21499.32 | 12.091 | 35.557 | 2162.00 | 737.655 | 473.777 | 263.879 |
| 2017 | 180369 | 60243.839 | 37681.959 | 22561.88 | 12.509 | 37.451 | 2171.75 | 728.017 | 453.712 | 274.305 |
| 2018 | 178300 | 60713.327 | 37213.304 | 23500.02 | 13.180 | 38.707 | 2125.79 | 726.721 | 443.677 | 283.044 |
| 2019 | 180485 | 61148.172 | 36423.813 | 24724.36 | 13.699 | 40.434 | 2130.34 | 724.841 | 429.926 | 294.916 |
| 2020 | 181848 | 61149.317 | 35600.730 | 25548.59 | 14.049 | 41.781 | 2430.19 | 821.432 | 475.764 | 345.668 |
| 2021 | 179584 | 58727.786 | 32812.004 | 25915.78 | 14.431 | 44.129 | 2381.74 | 783.216 | 435.170 | 348.046 |
| 2022 | 180998 | 58848.802 | 31915.986 | 26932.82 | 14.880 | 45.766 | 2381.39 | 778.881 | 419.918 | 358.963 |
| 2023 | 182357 | 58816.149 | 30995.692 | 27820.46 | 15.256 | 47.301 | 2379.71 | 772.407 | 404.487 | 367.920 |
| 2024 | 183455 | 58955.232 | 30099.607 | 28855.62 | 15.729 | 48.945 | 2374.82 | 768.316 | 389.637 | 378.679 |
| 2025 | 182393 | 58297.744 | 29024.613 | 29273.13 | 16.049 | 50.213 | 2721.39 | 876.861 | 433.061 | 443.800 |
| 2026 | 182425 | 56469.971 | 26992.886 | 29477.08 | 16.158 | 52.200 | 2710.29 | 846.150 | 401.033 | 445.117 |
| 2027 | 181854 | 56209.427 | 25951.556 | 30257.87 | 16.639 | 53.831 | 2689.78 | 838.883 | 383.845 | 455.038 |
| 2028 | 181123 | 55733.105 | 24848.121 | 30884.98 | 17.052 | 55.416 | 2666.51 | 828.298 | 365.817 | 462.481 |
| 2029 | 180295 | 55145.838 | 23712.410 | 31433.43 | 17.434 | 57.001 | 2641.35 | 815.951 | 347.392 | 468.559 |
| 2030 | 176190 | 53615.097 | 22451.445 | 31163.65 | 17.688 | 58.125 | 3049.83 | 939.056 | 388.633 | 550.423 |
| 2031 | 177783 | 52046.561 | 21185.589 | 30860.97 | 17.359 | 59.295 | 3071.59 | 910.364 | 366.027 | 544.336 |
| 2032 | 176324 | 51582.636 | 20265.333 | 31317.30 | 17.761 | 60.713 | 3038.80 | 900.496 | 349.257 | 551.238 |
| 2033 | 174806 | 50839.758 | 19314.401 | 31525.36 | 18.034 | 62.009 | 3003.71 | 885.375 | 331.881 | 553.494 |
| 2034 | 173144 | 50215.451 | 18395.407 | 31820.04 | 18.378 | 63.367 | 2965.39 | 872.104 | 315.053 | 557.051 |
| 2035 | 165899 | 47773.075 | 17156.808 | 30616.27 | 18.455 | 64.087 | 3471.07 | 1016.05 | 358.969 | 657.077 |
| 2036 | 159533 | 43298.709 | 14888.444 | 28410.27 | 17.808 | 65.615 | 3340.82 | 922.274 | 311.783 | 610.491 |
| 2037 | 156957 | 42241.563 | 13915.028 | 28326.54 | 18.047 | 67.058 | 3287.53 | 900.450 | 291.457 | 608.994 |
| 2038 | 154390 | 41356.353 | 13140.695 | 28215.66 | 18.276 | 68.226 | 3231.78 | 881.531 | 275.069 | 606.462 |
| 2039 | 151894 | 40161.050 | 12243.680 | 27917.37 | 18.380 | 69.514 | 3174.53 | 855.178 | 255.889 | 599.288 |

# Results for PHIM for Run 6 (Sensitivity_Analysis_1.RTF)

*************************************************************************************
**Main Results for E-Component for Run: 6, Simulation: 1, COD: LUNG CANCER, Adjusted**

| Variable Parameter | Value |
|---|---|
| 1. Country | US (United States) |
| 2. Sex | F (Females) |
| 3. Year of start of process | 1990 |
| 4. Cause of Death | LUNG CANCER |
| 5. Effective Dose NULL | CC:1 |
| 6. Effective Dose Ecig | CC:1 Ecig:0.1 Dual:0.55 |
| Note: | Death rates are per million population |

-----------------------------------------------------------------------------------------
Results for Age Groups Combined and Years combined, Population and Number deaths adjusted for Ecig scenario,

| Age Group:All Year | Number Deaths | NULL Attrib Deaths | Ecig Attrib Deaths | Drop Deaths | %Drop Deaths | %Drop Attrib Deaths | Death Rate | NULL Attrib Rates | Ecig Attrib Rates | Drop Rates |
|---|---|---|---|---|---|---|---|---|---|---|
| All Comb | 2342820 | 1580465.217 | 1190837.525 | 389627.7 | 16.631 | 24.653 | 640.349 | 433.910 | 325.485 | 108.426 |

-----------------------------------------------------------------------------------------
Results for Age Groups Combined, Population and Number deaths adjusted for Ecig scenario,

| Age Group:All Year | Number Deaths | NULL Attrib Deaths | Ecig Attrib Deaths | Drop Deaths | %Drop Deaths | %Drop Attrib Deaths | Death Rate | NULL Attrib Rates | Ecig Attrib Rates | Drop Rates |
|---|---|---|---|---|---|---|---|---|---|---|
| 1990 | 42690 | 30614.201 | 30614.201 | 0.000 | 0.000 | 0.000 | 625.210 | 448.355 | 448.355 | 0.000 |
| 1991 | 43692 | 31412.010 | 31381.330 | 30.680 | 0.070 | 0.098 | 631.345 | 453.901 | 453.457 | 0.443 |
| 1992 | 45423 | 32782.407 | 32693.521 | 88.886 | 0.196 | 0.271 | 647.451 | 467.274 | 466.006 | 1.268 |
| 1993 | 46292 | 33491.183 | 33315.705 | 175.478 | 0.379 | 0.524 | 650.518 | 470.643 | 468.173 | 2.470 |
| 1994 | 47019 | 34074.347 | 33790.439 | 283.909 | 0.604 | 0.833 | 650.903 | 471.719 | 467.779 | 3.940 |
| 1995 | 47843 | 34694.454 | 34266.814 | 427.640 | 0.894 | 1.233 | 652.041 | 472.861 | 467.014 | 5.847 |
| 1996 | 48249 | 35027.269 | 34436.621 | 590.647 | 1.224 | 1.686 | 649.026 | 471.202 | 463.225 | 7.977 |
| 1997 | 49043 | 35590.763 | 34806.538 | 784.225 | 1.599 | 2.203 | 650.548 | 472.153 | 461.701 | 10.452 |
| 1998 | 49698 | 36100.785 | 35088.162 | 1012.623 | 2.038 | 2.805 | 649.937 | 472.184 | 458.870 | 13.314 |
| 1999 | 48900 | 35451.030 | 34233.832 | 1217.197 | 2.489 | 3.433 | 630.856 | 457.448 | 441.650 | 15.798 |
| 2000 | 50355 | 36447.986 | 34926.687 | 1521.299 | 3.021 | 4.174 | 641.514 | 464.474 | 444.963 | 19.511 |
| 2001 | 50420 | 36411.326 | 34583.147 | 1828.180 | 3.626 | 5.021 | 635.171 | 458.863 | 435.666 | 23.197 |
| 2002 | 51485 | 37078.545 | 34869.547 | 2208.998 | 4.291 | 5.958 | 642.179 | 462.696 | 434.931 | 27.764 |
| 2003 | 51458 | 36999.876 | 34429.117 | 2570.759 | 4.996 | 6.948 | 635.768 | 457.395 | 425.375 | 32.020 |
| 2004 | 51460 | 36861.555 | 33891.317 | 2970.238 | 5.772 | 8.058 | 629.374 | 451.138 | 414.502 | 36.636 |
| 2005 | 51494 | 36689.529 | 33298.003 | 3391.525 | 6.586 | 9.244 | 622.745 | 444.069 | 402.691 | 41.377 |
| 2006 | 51441 | 36504.774 | 32672.980 | 3831.794 | 7.449 | 10.497 | 616.591 | 437.979 | 391.631 | 46.348 |
| 2007 | 51940 | 36663.202 | 32306.942 | 4356.260 | 8.387 | 11.882 | 616.277 | 435.498 | 383.327 | 52.171 |
| 2008 | 51091 | 35898.803 | 31153.063 | 4745.740 | 9.289 | 13.220 | 599.534 | 421.800 | 365.572 | 56.227 |
| 2009 | 51324 | 35934.525 | 30629.503 | 5305.022 | 10.336 | 14.763 | 595.395 | 417.468 | 355.321 | 62.147 |
| 2010 | 51001 | 35502.829 | 29744.979 | 5757.850 | 11.290 | 16.218 | 584.754 | 407.730 | 341.045 | 66.685 |
| 2011 | 50673 | 35136.342 | 28849.438 | 6286.903 | 12.407 | 17.893 | 575.425 | 399.726 | 327.603 | 72.124 |
| 2012 | 51431 | 35450.201 | 28506.749 | 6943.452 | 13.501 | 19.586 | 577.936 | 399.173 | 320.335 | 78.838 |
| 2013 | 51275 | 35123.224 | 27603.343 | 7519.881 | 14.666 | 21.410 | 569.876 | 391.249 | 306.789 | 84.460 |
| 2014 | 51479 | 35079.370 | 26945.630 | 8133.740 | 15.800 | 23.187 | 565.745 | 386.476 | 296.127 | 90.349 |
| 2015 | 50691 | 34324.171 | 25798.337 | 8525.833 | 16.819 | 24.839 | 622.461 | 422.780 | 316.792 | 105.987 |
| 2016 | 49117 | 33054.753 | 24186.953 | 8867.799 | 18.055 | 26.828 | 597.214 | 403.264 | 294.091 | 109.173 |
| 2017 | 48609 | 32471.234 | 23103.575 | 9367.660 | 19.271 | 28.849 | 585.283 | 392.399 | 278.180 | 114.219 |
| 2018 | 50787 | 33657.595 | 23205.359 | 10452.24 | 20.580 | 31.055 | 605.513 | 402.871 | 276.667 | 126.205 |
| 2019 | 51390 | 33863.424 | 22648.634 | 11214.79 | 21.823 | 33.118 | 606.578 | 401.412 | 267.331 | 134.081 |
| 2020 | 51831 | 33910.830 | 22072.375 | 11838.45 | 22.840 | 34.911 | 692.666 | 455.532 | 294.973 | 160.559 |
| 2021 | 48428 | 31531.880 | 19838.760 | 11693.12 | 24.145 | 37.083 | 642.275 | 420.521 | 263.112 | 157.409 |
| 2022 | 48757 | 31560.212 | 19211.675 | 12348.54 | 25.326 | 39.127 | 641.500 | 417.708 | 252.768 | 164.941 |
| 2023 | 49054 | 31508.781 | 18565.828 | 12942.95 | 26.385 | 41.077 | 640.145 | 413.791 | 242.280 | 171.511 |
| 2024 | 49272 | 31517.293 | 17925.864 | 13591.43 | 27.584 | 43.124 | 637.822 | 410.740 | 232.049 | 178.691 |
| 2025 | 48998 | 31175.389 | 17189.776 | 13985.61 | 28.543 | 44.861 | 731.074 | 468.912 | 256.480 | 212.432 |
| 2026 | 45848 | 29044.598 | 15449.233 | 13595.36 | 29.653 | 46.809 | 681.164 | 435.206 | 229.529 | 205.677 |
| 2027 | 45661 | 28825.765 | 14761.151 | 14064.61 | 30.802 | 48.792 | 675.367 | 430.203 | 218.330 | 211.873 |
| 2028 | 45426 | 28513.846 | 14051.731 | 14462.11 | 31.837 | 50.720 | 668.761 | 423.769 | 206.871 | 216.898 |
| 2029 | 45153 | 28148.109 | 13327.605 | 14820.50 | 32.823 | 52.652 | 661.503 | 416.486 | 195.252 | 221.234 |
| 2030 | 44034 | 27314.475 | 12528.172 | 14786.30 | 33.579 | 54.134 | 762.225 | 478.407 | 216.862 | 261.545 |
| 2031 | 41393 | 25423.102 | 11358.255 | 14064.85 | 33.979 | 55.323 | 715.150 | 444.684 | 196.239 | 248.445 |
| 2032 | 40998 | 25085.902 | 10784.230 | 14301.67 | 34.884 | 57.011 | 706.563 | 437.933 | 185.858 | 252.075 |
| 2033 | 40582 | 24645.067 | 10212.852 | 14432.22 | 35.563 | 58.560 | 697.319 | 429.194 | 175.488 | 253.706 |
| 2034 | 40130 | 24239.126 | 9663.790 | 14575.34 | 36.320 | 60.131 | 687.301 | 420.967 | 165.509 | 255.458 |
| 2035 | 38196 | 22944.147 | 8917.356 | 14026.79 | 36.723 | 61.135 | 799.169 | 487.980 | 186.576 | 301.404 |
| 2036 | 33672 | 19950.581 | 7454.614 | 12495.97 | 37.111 | 62.635 | 705.125 | 424.953 | 156.109 | 268.844 |
| 2037 | 33103 | 19415.334 | 6939.485 | 12475.85 | 37.688 | 64.258 | 693.350 | 413.871 | 145.351 | 268.520 |
| 2038 | 32535 | 18939.317 | 6531.736 | 12407.58 | 38.137 | 65.512 | 681.032 | 403.701 | 136.726 | 266.975 |
| 2039 | 31981 | 18379.752 | 6072.567 | 12307.18 | 38.483 | 66.961 | 668.384 | 391.373 | 126.915 | 264.458 |

## Results for PHIM for Run 6 (Sensitivity_Analysis_1.RTF)

************************************************************************************

**Main Results for E-Component for Run: 6, Simulation: 1, COD: IHD, Adjusted**

| Variable Parameter | Value |
|---|---|
| 1. Country | US (United States) |
| 2. Sex | F (Females) |
| 3. Year of start of process | 1990 |
| 4. Cause of Death | IHD |
| 5. Effective Dose NULL | CC:1 |
| 6. Effective Dose Ecig | CC:1 Ecig:0.1 Dual:0.55 |
| Note: | Death rates are per million population |

------------------------------------------------------------------------------------------

Results for Age Groups Combined and Years combined, Population and Number deaths adjusted for Ecig scenario,

| Age Group:All | | NULL | Ecig | | | %Drop | | NULL | Ecig | |
|---|---|---|---|---|---|---|---|---|---|---|
| Year | Number Deaths | Attrib Deaths | Attrib Deaths | Drop Deaths | %Drop Deaths | Attrib Deaths | Death Rate | Attrib Rates | Attrib Rates | Drop Rates |
| All Comb | 3260296 | 398833.796 | 212396.898 | 186436.9 | 5.718 | 46.746 | 891.117 | 109.498 | 58.053 | 51.445 |

------------------------------------------------------------------------------------------

Results for Age Groups Combined, Population and Number deaths adjusted for Ecig scenario,

| Age Group:All | | NULL | Ecig | | | %Drop | | NULL | Ecig | |
|---|---|---|---|---|---|---|---|---|---|---|
| Year | Number Deaths | Attrib Deaths | Attrib Deaths | Drop Deaths | %Drop Deaths | Attrib Deaths | Death Rate | Attrib Rates | Attrib Rates | Drop Rates |
| 1990 | 98916 | 12922.892 | 12922.892 | 0.000 | 0.000 | 0.000 | 1448.66 | 189.260 | 189.260 | 0.000 |
| 1991 | 96403 | 12856.479 | 12770.826 | 85.653 | 0.089 | 0.666 | 1393.01 | 185.775 | 184.537 | 1.238 |
| 1992 | 93721 | 12662.244 | 12425.266 | 236.977 | 0.253 | 1.872 | 1335.88 | 180.485 | 177.107 | 3.378 |
| 1993 | 93680 | 12730.613 | 12278.803 | 451.809 | 0.482 | 3.549 | 1316.45 | 178.900 | 172.549 | 6.351 |
| 1994 | 90835 | 12495.323 | 11792.480 | 702.843 | 0.774 | 5.625 | 1257.48 | 172.983 | 163.250 | 9.733 |
| 1995 | 89703 | 12365.590 | 11373.253 | 992.338 | 1.106 | 8.025 | 1222.55 | 168.534 | 155.003 | 13.531 |
| 1996 | 88295 | 12290.551 | 10939.128 | 1351.424 | 1.531 | 10.996 | 1187.70 | 165.338 | 147.148 | 18.190 |
| 1997 | 84791 | 11719.531 | 10093.676 | 1625.855 | 1.917 | 13.873 | 1124.73 | 155.473 | 133.890 | 21.583 |
| 1998 | 82537 | 11416.794 | 9472.200 | 1944.594 | 2.356 | 17.033 | 1079.38 | 149.327 | 123.874 | 25.453 |
| 1999 | 93970 | 13028.038 | 10353.814 | 2674.224 | 2.846 | 20.527 | 1212.31 | 168.109 | 133.574 | 34.535 |
| 2000 | 90080 | 12426.299 | 9442.378 | 2983.921 | 3.313 | 24.013 | 1147.61 | 158.354 | 120.295 | 38.059 |
| 2001 | 86170 | 11939.281 | 8579.381 | 3359.900 | 3.899 | 28.142 | 1085.54 | 150.461 | 108.080 | 42.381 |
| 2002 | 83077 | 11528.794 | 7802.226 | 3726.568 | 4.486 | 32.324 | 1036.23 | 143.865 | 97.318 | 46.548 |
| 2003 | 79377 | 11048.407 | 7045.475 | 4002.932 | 5.043 | 36.231 | 980.711 | 136.581 | 87.047 | 49.534 |
| 2004 | 73947 | 10375.132 | 6186.524 | 4188.608 | 5.664 | 40.372 | 904.397 | 126.978 | 75.663 | 51.315 |
| 2005 | 71749 | 10051.029 | 5609.852 | 4441.177 | 6.190 | 44.186 | 867.703 | 121.652 | 67.843 | 53.809 |
| 2006 | 67663 | 9542.523 | 4940.450 | 4602.073 | 6.801 | 48.227 | 811.033 | 114.490 | 59.218 | 55.272 |
| 2007 | 63626 | 8936.533 | 4303.910 | 4632.623 | 7.281 | 51.839 | 754.927 | 106.151 | 51.067 | 55.085 |
| 2008 | 63312 | 8944.180 | 4001.797 | 4942.383 | 7.806 | 55.258 | 742.943 | 105.091 | 46.960 | 58.131 |
| 2009 | 59899 | 8390.033 | 3509.118 | 4880.916 | 8.149 | 58.175 | 694.867 | 97.471 | 40.708 | 56.763 |
| 2010 | 58175 | 8157.570 | 3179.247 | 4978.322 | 8.557 | 61.027 | 667.014 | 93.685 | 36.452 | 57.233 |
| 2011 | 57553 | 8035.356 | 2898.661 | 5136.695 | 8.925 | 63.926 | 653.553 | 91.414 | 32.916 | 58.498 |
| 2012 | 57617 | 7970.766 | 2656.163 | 5314.603 | 9.224 | 66.676 | 647.453 | 89.752 | 29.848 | 59.904 |
| 2013 | 57295 | 7743.216 | 2411.941 | 5331.275 | 9.305 | 68.851 | 636.792 | 86.254 | 26.807 | 59.447 |
| 2014 | 57792 | 7774.935 | 2247.402 | 5527.533 | 9.565 | 71.094 | 635.119 | 85.658 | 24.698 | 60.959 |
| 2015 | 58679 | 7722.535 | 2108.338 | 5614.197 | 9.568 | 72.699 | 720.555 | 95.120 | 25.889 | 69.231 |
| 2016 | 58971 | 7600.353 | 1931.090 | 5669.263 | 9.614 | 74.592 | 717.029 | 92.723 | 23.480 | 69.243 |
| 2017 | 59347 | 7470.266 | 1777.590 | 5692.676 | 9.592 | 76.204 | 714.575 | 90.274 | 21.403 | 68.871 |
| 2018 | 56432 | 6907.050 | 1540.766 | 5366.284 | 9.509 | 77.693 | 672.813 | 82.675 | 18.370 | 64.306 |
| 2019 | 56271 | 6732.046 | 1417.449 | 5314.597 | 9.445 | 78.945 | 664.188 | 79.801 | 16.731 | 63.070 |
| 2020 | 55729 | 6429.836 | 1291.000 | 5138.835 | 9.221 | 79.922 | 744.762 | 86.373 | 17.253 | 69.121 |
| 2021 | 56746 | 6562.016 | 1230.118 | 5331.897 | 9.396 | 81.254 | 752.588 | 87.513 | 16.314 | 71.199 |
| 2022 | 56310 | 6352.366 | 1133.574 | 5218.792 | 9.268 | 82.155 | 740.874 | 84.075 | 14.914 | 69.161 |
| 2023 | 55852 | 6134.123 | 1050.552 | 5083.571 | 9.102 | 82.874 | 728.852 | 80.557 | 13.709 | 66.847 |
| 2024 | 55314 | 5922.193 | 972.079 | 4950.114 | 8.949 | 83.586 | 716.029 | 77.179 | 12.583 | 64.596 |
| 2025 | 53867 | 5504.188 | 874.823 | 4629.365 | 8.594 | 84.106 | 803.720 | 82.789 | 13.053 | 69.736 |
| 2026 | 55907 | 5641.075 | 862.594 | 4778.481 | 8.547 | 84.709 | 830.612 | 84.526 | 12.816 | 71.711 |
| 2027 | 54854 | 5426.216 | 804.025 | 4622.190 | 8.426 | 85.183 | 811.339 | 80.982 | 11.892 | 69.090 |
| 2028 | 53771 | 5197.830 | 747.889 | 4449.941 | 8.276 | 85.612 | 791.623 | 77.249 | 11.011 | 66.239 |
| 2029 | 52679 | 4973.547 | 697.550 | 4275.997 | 8.117 | 85.975 | 771.765 | 73.590 | 10.219 | 63.371 |
| 2030 | 50203 | 4450.618 | 607.055 | 3843.563 | 7.656 | 86.360 | 869.010 | 77.952 | 10.508 | 67.443 |
| 2031 | 52790 | 4555.052 | 611.350 | 3943.702 | 7.470 | 86.579 | 912.071 | 79.674 | 10.562 | 69.112 |
| 2032 | 51502 | 4349.999 | 572.289 | 3777.710 | 7.335 | 86.844 | 887.603 | 75.939 | 9.863 | 66.076 |
| 2033 | 50226 | 4147.948 | 539.665 | 3608.283 | 7.184 | 86.990 | 863.030 | 72.237 | 9.273 | 62.963 |
| 2034 | 48939 | 3966.133 | 508.308 | 3457.826 | 7.066 | 87.184 | 838.171 | 68.881 | 8.706 | 60.175 |
| 2035 | 45384 | 3289.064 | 411.266 | 2877.798 | 6.341 | 87.496 | 949.564 | 69.952 | 8.605 | 61.348 |
| 2036 | 47288 | 3313.192 | 406.359 | 2906.833 | 6.147 | 87.735 | 990.274 | 70.572 | 8.510 | 62.062 |
| 2037 | 45789 | 3117.951 | 377.481 | 2740.470 | 5.985 | 87.893 | 959.069 | 66.464 | 7.907 | 58.558 |
| 2038 | 44331 | 2949.775 | 354.870 | 2594.905 | 5.853 | 87.970 | 927.970 | 62.876 | 7.428 | 55.448 |
| 2039 | 42930 | 2766.312 | 331.953 | 2434.359 | 5.670 | 88.000 | 897.232 | 58.905 | 6.938 | 51.967 |

# Results for PHIM for Run 6 (Sensitivity_Analysis_1.RTF)

************************************************************************************
**Main Results for E-Component for Run: 6, Simulation: 1, COD: STROKE, Adjusted**

| Variable Parameter | Value |
|---|---|
| 1. Country | US (United States) |
| 2. Sex | F (Females) |
| 3. Year of start of process | 1990 |
| 4. Cause of Death | STROKE |
| 5. Effective Dose NULL | CC:1 |
| 6. Effective Dose Ecig | CC:1 Ecig:0.1 Dual:0.55 |
| Note: | Death rates are per million population |

------------------------------------------------------------------------------------------
Results for Age Groups Combined and Years combined, Population and Number deaths adjusted for Ecig scenario,

| Age Group:All Year | Number Deaths | NULL Attrib Deaths | Ecig Attrib Deaths | Drop Deaths | %Drop Deaths | %Drop Attrib Deaths | Death Rate | NULL Attrib Rates | Ecig Attrib Rates | Drop Rates |
|---|---|---|---|---|---|---|---|---|---|---|
| All Comb | 1360845 | 104899.750 | 54692.466 | 50207.28 | 3.689 | 47.862 | 371.952 | 28.800 | 14.949 | 13.851 |

------------------------------------------------------------------------------------------
Results for Age Groups Combined, Population and Number deaths adjusted for Ecig scenario,

| Age Group:All Year | Number Deaths | NULL Attrib Deaths | Ecig Attrib Deaths | Drop Deaths | %Drop Deaths | %Drop Attrib Deaths | Death Rate | NULL Attrib Rates | Ecig Attrib Rates | Drop Rates |
|---|---|---|---|---|---|---|---|---|---|---|
| 1990 | 33504 | 3293.017 | 3293.017 | 0.000 | 0.000 | 0.000 | 490.678 | 48.227 | 48.227 | 0.000 |
| 1991 | 32505 | 3207.063 | 3180.986 | 26.077 | 0.080 | 0.813 | 469.694 | 46.342 | 45.965 | 0.377 |
| 1992 | 32560 | 3218.125 | 3145.300 | 72.825 | 0.224 | 2.263 | 464.104 | 45.871 | 44.832 | 1.038 |
| 1993 | 32768 | 3226.680 | 3088.888 | 137.793 | 0.421 | 4.270 | 460.482 | 45.344 | 43.407 | 1.937 |
| 1994 | 33006 | 3277.266 | 3057.752 | 219.514 | 0.665 | 6.698 | 456.920 | 45.370 | 42.330 | 3.040 |
| 1995 | 33732 | 3309.378 | 2995.041 | 314.337 | 0.932 | 9.498 | 459.725 | 45.104 | 40.819 | 4.286 |
| 1996 | 33753 | 3312.987 | 2888.201 | 424.786 | 1.258 | 12.822 | 454.034 | 44.568 | 38.851 | 5.717 |
| 1997 | 33704 | 3267.066 | 2735.571 | 531.495 | 1.577 | 16.268 | 447.079 | 43.341 | 36.287 | 7.055 |
| 1998 | 33282 | 3178.725 | 2545.381 | 633.344 | 1.903 | 19.924 | 435.244 | 41.577 | 33.288 | 8.289 |
| 1999 | 33902 | 3120.351 | 2383.543 | 736.808 | 2.173 | 23.613 | 437.366 | 40.264 | 30.750 | 9.514 |
| 2000 | 33842 | 3142.808 | 2281.523 | 861.285 | 2.545 | 27.405 | 431.139 | 40.050 | 29.066 | 10.984 |
| 2001 | 32524 | 2961.588 | 2037.317 | 924.272 | 2.842 | 31.209 | 409.731 | 37.323 | 25.665 | 11.657 |
| 2002 | 32101 | 2940.627 | 1901.910 | 1038.718 | 3.236 | 35.323 | 400.403 | 36.695 | 23.723 | 12.973 |
| 2003 | 30596 | 2819.545 | 1714.303 | 1105.242 | 3.612 | 39.199 | 378.014 | 34.855 | 21.180 | 13.675 |
| 2004 | 29421 | 2730.466 | 1554.245 | 1176.221 | 3.998 | 43.078 | 359.829 | 33.417 | 19.009 | 14.408 |
| 2005 | 27992 | 2670.510 | 1423.058 | 1247.452 | 4.457 | 46.712 | 338.519 | 32.322 | 17.210 | 15.112 |
| 2006 | 27245 | 2623.285 | 1306.720 | 1316.565 | 4.832 | 50.188 | 326.566 | 31.474 | 15.663 | 15.811 |
| 2007 | 26351 | 2536.599 | 1178.778 | 1357.821 | 5.153 | 53.529 | 312.662 | 30.131 | 13.986 | 16.144 |
| 2008 | 26054 | 2469.443 | 1073.073 | 1396.370 | 5.359 | 56.546 | 305.742 | 29.015 | 12.592 | 16.423 |
| 2009 | 25102 | 2386.945 | 968.655 | 1418.290 | 5.650 | 59.419 | 291.204 | 27.730 | 11.237 | 16.493 |
| 2010 | 24910 | 2329.274 | 883.608 | 1445.666 | 5.803 | 62.065 | 285.614 | 26.750 | 10.131 | 16.619 |
| 2011 | 24871 | 2286.581 | 806.711 | 1479.870 | 5.950 | 64.720 | 282.426 | 26.013 | 9.161 | 16.852 |
| 2012 | 24587 | 2243.311 | 736.472 | 1506.839 | 6.129 | 67.170 | 276.292 | 25.260 | 8.276 | 16.984 |
| 2013 | 24548 | 2176.503 | 665.738 | 1510.766 | 6.154 | 69.413 | 272.833 | 24.245 | 7.399 | 16.846 |
| 2014 | 25407 | 2211.619 | 631.970 | 1579.649 | 6.217 | 71.425 | 279.223 | 24.366 | 6.945 | 17.421 |
| 2015 | 26444 | 2237.016 | 603.738 | 1633.278 | 6.176 | 73.011 | 324.726 | 27.554 | 7.414 | 20.140 |
| 2016 | 26878 | 2229.057 | 565.456 | 1663.601 | 6.189 | 74.632 | 326.811 | 27.194 | 6.875 | 20.319 |
| 2017 | 27778 | 2221.649 | 531.341 | 1690.308 | 6.085 | 76.083 | 334.462 | 26.848 | 6.398 | 20.450 |
| 2018 | 25418 | 1939.420 | 439.600 | 1499.820 | 5.901 | 77.333 | 303.046 | 23.214 | 5.241 | 17.973 |
| 2019 | 25607 | 1904.400 | 407.078 | 1497.321 | 5.847 | 78.624 | 302.251 | 22.574 | 4.805 | 17.770 |
| 2020 | 25574 | 1806.054 | 367.916 | 1438.139 | 5.624 | 79.629 | 341.762 | 24.261 | 4.917 | 19.344 |
| 2021 | 25660 | 1748.172 | 337.064 | 1411.108 | 5.499 | 80.719 | 340.312 | 23.314 | 4.470 | 18.844 |
| 2022 | 25767 | 1701.148 | 312.404 | 1388.744 | 5.390 | 81.636 | 339.018 | 22.515 | 4.110 | 18.405 |
| 2023 | 25866 | 1652.087 | 291.115 | 1360.971 | 5.262 | 82.379 | 337.544 | 21.696 | 3.799 | 17.897 |
| 2024 | 25922 | 1597.127 | 269.373 | 1327.754 | 5.122 | 83.134 | 335.556 | 20.814 | 3.487 | 17.327 |
| 2025 | 25540 | 1467.622 | 239.013 | 1228.609 | 4.811 | 83.714 | 381.062 | 22.075 | 3.566 | 18.508 |
| 2026 | 25782 | 1390.505 | 218.737 | 1171.768 | 4.545 | 84.269 | 383.049 | 20.835 | 3.250 | 17.586 |
| 2027 | 25583 | 1342.095 | 203.879 | 1138.216 | 4.449 | 84.809 | 378.388 | 20.030 | 3.016 | 17.014 |
| 2028 | 25357 | 1289.536 | 190.152 | 1099.384 | 4.336 | 85.254 | 373.306 | 19.165 | 2.799 | 16.365 |
| 2029 | 25118 | 1242.216 | 177.966 | 1064.250 | 4.237 | 85.674 | 367.988 | 18.380 | 2.607 | 15.773 |
| 2030 | 24325 | 1096.803 | 152.187 | 944.616 | 3.883 | 86.124 | 421.063 | 19.210 | 2.634 | 16.576 |
| 2031 | 24352 | 1019.991 | 138.827 | 881.164 | 3.619 | 86.389 | 420.725 | 17.841 | 2.399 | 15.442 |
| 2032 | 24019 | 977.406 | 130.082 | 847.324 | 3.528 | 86.691 | 413.957 | 17.063 | 2.242 | 14.821 |
| 2033 | 23678 | 937.450 | 123.057 | 814.392 | 3.439 | 86.873 | 406.865 | 16.326 | 2.114 | 14.211 |
| 2034 | 23312 | 900.763 | 116.139 | 784.624 | 3.366 | 87.107 | 399.266 | 15.644 | 1.989 | 13.655 |
| 2035 | 22176 | 745.023 | 93.994 | 651.029 | 2.936 | 87.384 | 463.978 | 15.845 | 1.967 | 13.879 |
| 2036 | 21351 | 680.360 | 83.784 | 596.575 | 2.794 | 87.685 | 447.121 | 14.492 | 1.755 | 12.737 |
| 2037 | 20852 | 644.109 | 78.367 | 565.742 | 2.713 | 87.833 | 436.763 | 13.730 | 1.641 | 12.089 |
| 2038 | 20353 | 612.448 | 73.923 | 538.525 | 2.646 | 87.930 | 426.050 | 13.055 | 1.547 | 11.507 |
| 2039 | 19862 | 577.529 | 69.514 | 508.015 | 2.558 | 87.964 | 415.120 | 12.298 | 1.453 | 10.845 |

# Results for PHIM for Run 6 (Sensitivity_Analysis_1.RTF)

*********************************************************************************
**Main Results for E-Component for Run: 6, Simulation: 1, COD: COPD, Adjusted**

| Variable Parameter | Value |
|---|---|
| 1. Country | US (United States) |
| 2. Sex | F (Females) |
| 3. Year of start of process | 1990 |
| 4. Cause of Death | COPD |
| 5. Effective Dose NULL | CC:1 |
| 6. Effective Dose Ecig | CC:1 Ecig:0.1 Dual:0.55 |
| Note: | Death rates are per million population |

-----------------------------------------------------------------------------------------
Results for Age Groups Combined and Years combined, Population and Number deaths adjusted for Ecig scenario,

| Age Group:All | | NULL | Ecig | | | %Drop | | NULL | Ecig | |
|---|---|---|---|---|---|---|---|---|---|---|
| Year | Number | Attrib | Attrib | Drop | %Drop | Attrib | Death | Attrib | Attrib | Drop |
| | Deaths | Deaths | Deaths | Deaths | Deaths | Deaths | Rate | Rates | Rates | Rates |
| All Comb | 2179861 | 862051.244 | 576595.197 | 285456.0 | 13.095 | 33.114 | 595.808 | 236.673 | 157.597 | 79.075 |

-----------------------------------------------------------------------------------------
Results for Age Groups Combined, Population and Number deaths adjusted for Ecig scenario,

| Age Group:All | | NULL | Ecig | | | %Drop | | NULL | Ecig | |
|---|---|---|---|---|---|---|---|---|---|---|
| Year | Number | Attrib | Attrib | Drop | %Drop | Attrib | Death | Attrib | Attrib | Drop |
| | Deaths | Deaths | Deaths | Deaths | Deaths | Deaths | Rate | Rates | Rates | Rates |
| 1990 | 24738 | 10694.712 | 10694.712 | 0.000 | 0.000 | 0.000 | 362.296 | 156.628 | 156.628 | 0.000 |
| 1991 | 26562 | 11602.933 | 11585.600 | 17.333 | 0.065 | 0.149 | 383.818 | 167.661 | 167.411 | 0.250 |
| 1992 | 26828 | 11831.375 | 11783.804 | 47.571 | 0.177 | 0.402 | 382.401 | 168.642 | 167.964 | 0.678 |
| 1993 | 29774 | 13246.371 | 13143.880 | 102.491 | 0.344 | 0.774 | 418.408 | 186.148 | 184.706 | 1.442 |
| 1994 | 29805 | 13355.662 | 13193.087 | 162.575 | 0.545 | 1.217 | 412.606 | 184.893 | 182.639 | 2.255 |
| 1995 | 29757 | 13397.439 | 13156.096 | 241.344 | 0.811 | 1.801 | 405.549 | 182.598 | 179.301 | 3.297 |
| 1996 | 30903 | 13960.356 | 13623.662 | 336.694 | 1.090 | 2.412 | 415.696 | 187.801 | 183.259 | 4.542 |
| 1997 | 31133 | 14109.552 | 13670.245 | 439.308 | 1.411 | 3.114 | 412.975 | 187.180 | 181.333 | 5.847 |
| 1998 | 32104 | 14599.657 | 14025.234 | 574.423 | 1.789 | 3.934 | 419.842 | 190.958 | 183.417 | 7.541 |
| 1999 | 34628 | 15728.102 | 14983.960 | 744.142 | 2.149 | 4.731 | 446.732 | 202.950 | 193.308 | 9.642 |
| 2000 | 34315 | 15579.803 | 14694.613 | 885.191 | 2.580 | 5.682 | 437.170 | 198.541 | 187.208 | 11.333 |
| 2001 | 34613 | 15696.614 | 14630.793 | 1065.821 | 3.079 | 6.790 | 436.044 | 197.812 | 184.313 | 13.498 |
| 2002 | 34318 | 15519.777 | 14282.875 | 1236.902 | 3.604 | 7.970 | 428.049 | 193.668 | 178.152 | 15.516 |
| 2003 | 35108 | 15888.389 | 14427.156 | 1461.233 | 4.162 | 9.197 | 433.767 | 196.413 | 178.249 | 18.164 |
| 2004 | 33552 | 15091.573 | 13506.657 | 1584.916 | 4.724 | 10.502 | 410.358 | 184.701 | 165.191 | 19.510 |
| 2005 | 35443 | 15803.656 | 13911.098 | 1892.557 | 5.340 | 11.975 | 428.635 | 191.278 | 168.235 | 23.044 |
| 2006 | 34040 | 15100.563 | 13087.295 | 2013.268 | 5.915 | 13.332 | 408.011 | 181.174 | 156.869 | 24.305 |
| 2007 | 34068 | 14974.384 | 12748.304 | 2226.079 | 6.534 | 14.866 | 404.227 | 177.871 | 151.261 | 26.610 |
| 2008 | 37512 | 16406.197 | 13738.816 | 2667.382 | 7.111 | 16.258 | 440.196 | 192.768 | 161.221 | 31.546 |
| 2009 | 36924 | 16083.194 | 13186.995 | 2896.199 | 7.844 | 18.008 | 428.340 | 186.846 | 152.977 | 33.869 |
| 2010 | 36899 | 15927.400 | 12826.335 | 3101.065 | 8.404 | 19.470 | 423.069 | 182.917 | 147.062 | 35.855 |
| 2011 | 37763 | 16221.836 | 12779.437 | 3442.398 | 9.116 | 21.221 | 428.823 | 184.547 | 145.118 | 39.428 |
| 2012 | 38409 | 16358.849 | 12606.438 | 3752.412 | 9.770 | 22.938 | 431.613 | 184.202 | 141.661 | 42.542 |
| 2013 | 40280 | 16994.822 | 12782.039 | 4212.783 | 10.459 | 24.789 | 447.683 | 189.311 | 142.062 | 47.249 |
| 2014 | 40270 | 16855.093 | 12362.469 | 4492.624 | 11.156 | 26.654 | 442.555 | 185.696 | 135.861 | 49.835 |
| 2015 | 42416 | 17572.483 | 12633.526 | 4938.957 | 11.644 | 28.106 | 520.850 | 216.445 | 155.134 | 61.311 |
| 2016 | 42844 | 17579.955 | 12281.301 | 5298.654 | 12.367 | 30.140 | 520.945 | 214.473 | 149.329 | 65.144 |
| 2017 | 44635 | 18080.688 | 12269.452 | 5811.236 | 13.020 | 32.141 | 537.428 | 218.496 | 147.731 | 70.765 |
| 2018 | 45663 | 18209.262 | 12027.579 | 6181.683 | 13.538 | 33.948 | 544.414 | 217.960 | 143.399 | 74.560 |
| 2019 | 47217 | 18648.302 | 11950.652 | 6697.650 | 14.185 | 35.916 | 557.322 | 221.054 | 141.059 | 79.996 |
| 2020 | 48714 | 19002.597 | 11869.439 | 7133.158 | 14.643 | 37.538 | 651.002 | 255.266 | 158.622 | 96.644 |
| 2021 | 48751 | 18885.717 | 11406.061 | 7479.656 | 15.343 | 39.605 | 646.561 | 251.867 | 151.273 | 100.594 |
| 2022 | 50163 | 19235.076 | 11258.333 | 7976.743 | 15.901 | 41.470 | 660.000 | 254.582 | 148.126 | 106.456 |
| 2023 | 51585 | 19521.158 | 11088.196 | 8432.962 | 16.348 | 43.199 | 673.173 | 256.363 | 144.699 | 111.664 |
| 2024 | 52948 | 19918.620 | 10932.292 | 8986.328 | 16.972 | 45.115 | 685.410 | 259.583 | 141.518 | 118.066 |
| 2025 | 53988 | 20150.545 | 10721.002 | 9429.543 | 17.466 | 46.795 | 805.534 | 303.086 | 159.962 | 143.124 |
| 2026 | 54887 | 20393.792 | 10462.322 | 9931.470 | 18.094 | 48.698 | 815.463 | 305.582 | 155.439 | 150.143 |
| 2027 | 55756 | 20615.351 | 10182.501 | 10432.85 | 18.711 | 50.607 | 824.684 | 307.668 | 150.608 | 157.061 |
| 2028 | 56569 | 20731.894 | 9858.350 | 10873.54 | 19.222 | 52.448 | 832.821 | 308.115 | 145.136 | 162.979 |
| 2029 | 57344 | 20781.967 | 9509.289 | 11272.68 | 19.658 | 54.243 | 840.096 | 307.495 | 139.313 | 168.182 |
| 2030 | 57628 | 20753.201 | 9164.031 | 11589.17 | 20.110 | 55.843 | 997.534 | 363.487 | 158.629 | 204.859 |
| 2031 | 59248 | 21048.417 | 9077.157 | 11971.26 | 20.205 | 56.875 | 1023.64 | 368.165 | 156.828 | 211.337 |
| 2032 | 59804 | 21169.329 | 8778.731 | 12390.60 | 20.719 | 58.531 | 1030.68 | 369.560 | 151.295 | 218.266 |
| 2033 | 60321 | 21109.293 | 8438.827 | 12670.47 | 21.005 | 60.023 | 1036.49 | 367.618 | 145.005 | 222.613 |
| 2034 | 60762 | 21109.429 | 8107.170 | 13002.26 | 21.399 | 61.595 | 1040.65 | 366.613 | 138.849 | 227.763 |
| 2035 | 60143 | 20794.840 | 7734.192 | 13060.65 | 21.716 | 62.807 | 1258.36 | 442.268 | 161.821 | 280.447 |
| 2036 | 57222 | 19354.577 | 6943.687 | 12410.89 | 21.689 | 64.124 | 1198.30 | 412.258 | 145.410 | 266.848 |
| 2037 | 57213 | 19064.169 | 6519.695 | 12544.47 | 21.926 | 65.801 | 1198.35 | 406.385 | 136.558 | 269.827 |
| 2038 | 57171 | 18854.812 | 6180.166 | 12674.65 | 22.170 | 67.222 | 1196.73 | 401.900 | 129.367 | 272.533 |
| 2039 | 57120 | 18437.457 | 5769.646 | 12667.81 | 22.177 | 68.707 | 1193.80 | 392.602 | 120.584 | 272.018 |

# Results for PHIM for Run 7 (Sensitivity_Analysis_1.RTF)

---

-----------------------------------------------------------------------------------------

**Control File Details for Run: 7**

| Variable Parameter | Value |
|---|---|
| 1. Name for Reference Scenario | NULL |
| 2. Name for Test Scenario | Ecig |
| 3. Name for Product 1 | CC |
| 4. Name for Product 2 | Ecig |
| 5. Name for Product 3 | - |
| 6. Name for Product 4 | Dual |
| 7. Country | US (United States) |
| 8. Sex | M (Males) |
| 9. Year of start of process | 1990 |
| 10. Number of months of follow-up | 600 |
| 11. Follow-up interval length (in months) | 12 |
| 12. Lower age for risk estimation | 10 |
| 13. Upper age for risk estimation | 79 |
| 14. The effective dose for Product 1 (F1) - Ref | 1 |
| 15. The effective dose for Product 2 (F2) - Ref | - |
| 16. The effective dose for Product 3 (F3) - Ref | - |
| 17. The effective dose for Product 4 (F4) - Ref | - |
| 18. The effective dose for Product 1 (F1) - Test | 1 |
| 19. The effective dose for Product 2 (F2) - Test | 0.2 |
| 20. The effective dose for Product 3 (F3) - Test | - |
| 21. The effective dose for Product 4 (F4) - Test | FROMF2 |
| 22. Number in population to be simulated | 100000 |
| 23. Number of MC simulations | 1 |
| 24. The random number seed for the first simulation | 15975263 |
| 25. Source for the population file (POP) | UN4 |
| 26. Source for the socioeconomic prevalence file (SEP) | - |
| 27. Source for the current smoking prevalence file (CSP) | ISS3 |
| 28. Source for the former smoking prevalence file (FSP) | ISS3 |
| 29. Source for the quit-time distribution file (QTD) | NHIS2006 |
| 30. Source for the death file (MORT) | APR-20 |
| 31. Source for the relative risk file (RR) | PNLEST |
| 32. Source for the half-life file (H) | PNLEST |
| 33. Assumption Set for TTP Factor Reference | F1 |
| 34. Assumption Set for TTP Factor Test | F1 |
| 35. Assumption Set for TTP Reference | ISS3 |
| 36. Assumption Set for TTP Test | ISS3 |
| 37. Assumption Set for TP for socioeconomic group | - |
| 38. Source for Output Choice file (OUTC) | BASIC |
| 39. Output for DTP P1 | N |
| 40. Output for DTP P2 | N |
| 41. Output for Main P Component | Y |
| 42. Output for DTP E1 | N |
| 43. Output for DTP E2 | N |
| 44. Output for DTP E3 | N |
| 45. Output for Main E Component | Y |
| 46. Output file name | Sensitivity_Analysis_1 |
| 47. Run number for P-Component | - |
| 48. Results file for E-Component | - |
| 49. Components required | PE |
| 50. Results folder for P to be used in E-Component | - |
| 51. Fixed age of starting product use | 16 |
| 52. Output absolute risk file? | N |

# Results for PHIM for Run 7 (Sensitivity_Analysis_1.RTF)

************************************************************************************
**TTP values for Run: 7, Scenario: NULL**

| **Variable Parameter** | **Value** |
|---|---|
| 1. Country | US (United States) |
| 2. Sex | M (Males) |
| 3. Assumption Set | ISS3 |
| Note: | Period is in Months |

| Age | Period | **Initiation** Never CC | **Cessation** CC Former | **Re-initiation** Former CC |
|---|---|---|---|---|
| 10-14 | 1-12 | 0.003842 | 0.008742 | 0.000000 |
| 15-19 | 1-12 | 0.004720 | 0.003614 | 0.000000 |
| 20-24 | 1-12 | 0.000376 | 0.000943 | 0.000000 |
| 25-29 | 1-12 | 0.000187 | 0.001143 | 0.000000 |
| 30-34 | 1-12 | 0.000093 | 0.000871 | 0.000000 |
| 35-39 | 1-12 | 0.000000 | 0.000867 | 0.000000 |
| 40-44 | 1-12 | 0.000000 | 0.001399 | 0.000000 |
| 45-49 | 1-12 | 0.000000 | 0.002033 | 0.000000 |
| 50-54 | 1-12 | 0.000000 | 0.003209 | 0.000000 |
| 55-59 | 1-12 | 0.000000 | 0.003574 | 0.000000 |
| 60-64 | 1-12 | 0.000000 | 0.003460 | 0.000000 |
| 65-69 | 1-12 | 0.000000 | 0.004136 | 0.000000 |
| 70-74 | 1-12 | 0.000000 | 0.005592 | 0.000000 |
| 75-79 | 1-12 | 0.000000 | 0.005592 | 0.000000 |
| 10-14 | 13-72 | 0.004906 | 0.008742 | 0.000000 |
| 15-19 | 13-72 | 0.006989 | 0.004426 | 0.000000 |
| 20-24 | 13-72 | 0.000687 | 0.001527 | 0.000000 |
| 25-29 | 13-72 | 0.000962 | 0.001515 | 0.000000 |
| 30-34 | 13-72 | 0.000364 | 0.001227 | 0.000000 |
| 35-39 | 13-72 | 0.000000 | 0.001630 | 0.000000 |
| 40-44 | 13-72 | 0.000000 | 0.001852 | 0.000000 |
| 45-49 | 13-72 | 0.000000 | 0.002667 | 0.000000 |
| 50-54 | 13-72 | 0.000000 | 0.003208 | 0.000000 |
| 55-59 | 13-72 | 0.000000 | 0.003978 | 0.000000 |
| 60-64 | 13-72 | 0.000000 | 0.005771 | 0.000000 |
| 65-69 | 13-72 | 0.000000 | 0.010432 | 0.000000 |
| 70-74 | 13-72 | 0.000000 | 0.007079 | 0.000000 |
| 75-79 | 13-72 | 0.000000 | 0.007079 | 0.000000 |
| 10-14 | 73-132 | 0.004312 | 0.008742 | 0.000000 |
| 15-19 | 73-132 | 0.005330 | 0.003289 | 0.000000 |
| 20-24 | 73-132 | 0.002456 | 0.003844 | 0.000000 |
| 25-29 | 73-132 | 0.000839 | 0.002006 | 0.000000 |
| 30-34 | 73-132 | 0.000414 | 0.001329 | 0.000000 |
| 35-39 | 73-132 | 0.000000 | 0.001278 | 0.000000 |
| 40-44 | 73-132 | 0.000000 | 0.001691 | 0.000000 |
| 45-49 | 73-132 | 0.000000 | 0.002904 | 0.000000 |
| 50-54 | 73-132 | 0.000000 | 0.003319 | 0.000000 |
| 55-59 | 73-132 | 0.000000 | 0.003976 | 0.000000 |
| 60-64 | 73-132 | 0.000000 | 0.005235 | 0.000000 |
| 65-69 | 73-132 | 0.000000 | 0.005901 | 0.000000 |
| 70-74 | 73-132 | 0.000000 | 0.005615 | 0.000000 |
| 75-79 | 73-132 | 0.000000 | 0.005615 | 0.000000 |
| 10-14 | 133-192 | 0.003468 | 0.008742 | 0.000000 |
| 15-19 | 133-192 | 0.004675 | 0.003732 | 0.000000 |
| 20-24 | 133-192 | 0.000062 | 0.002260 | 0.000000 |
| 25-29 | 133-192 | 0.001422 | 0.002822 | 0.000000 |
| 30-34 | 133-192 | 0.000696 | 0.002610 | 0.000000 |
| 35-39 | 133-192 | 0.000000 | 0.002058 | 0.000000 |
| 40-44 | 133-192 | 0.000000 | 0.002310 | 0.000000 |
| 45-49 | 133-192 | 0.000000 | 0.002256 | 0.000000 |
| 50-54 | 133-192 | 0.000000 | 0.002663 | 0.000000 |
| 55-59 | 133-192 | 0.000000 | 0.001746 | 0.000000 |
| 60-64 | 133-192 | 0.000000 | 0.005809 | 0.000000 |
| 65-69 | 133-192 | 0.000000 | 0.005896 | 0.000000 |
| 70-74 | 133-192 | 0.000000 | 0.007323 | 0.000000 |
| 75-79 | 133-192 | 0.000000 | 0.007323 | 0.000000 |
| 10-14 | 193-252 | 0.002314 | 0.008742 | 0.000000 |
| 15-19 | 193-252 | 0.003987 | 0.005036 | 0.000000 |
| 20-24 | 193-252 | 0.000320 | 0.002857 | 0.000000 |
| 25-29 | 193-252 | 0.000251 | 0.002314 | 0.000000 |
| 30-34 | 193-252 | 0.000125 | 0.004343 | 0.000000 |
| 35-39 | 193-252 | 0.000000 | 0.002092 | 0.000000 |
| 40-44 | 193-252 | 0.000000 | 0.002566 | 0.000000 |
| 45-49 | 193-252 | 0.000000 | 0.002311 | 0.000000 |
| 50-54 | 193-252 | 0.000000 | 0.003209 | 0.000000 |

## Results for PHIM for Run 7 (Sensitivity_Analysis_1.RTF)

| | | | | |
|---|---|---|---|---|
| 55-59 | 193-252 | 0.000000 | 0.004057 | 0.000000 |
| 60-64 | 193-252 | 0.000000 | 0.007166 | 0.000000 |
| 65-69 | 193-252 | 0.000000 | 0.004281 | 0.000000 |
| 70-74 | 193-252 | 0.000000 | 0.004580 | 0.000000 |
| 75-79 | 193-252 | 0.000000 | 0.004580 | 0.000000 |
| 10-14 | 253+ | 0.001325 | 0.008742 | 0.000000 |
| 15-19 | 253+ | 0.002984 | 0.010602 | 0.000000 |
| 20-24 | 253+ | 0.000452 | 0.004314 | 0.000000 |
| 25-29 | 253+ | 0.000225 | 0.003645 | 0.000000 |
| 30-34 | 253+ | 0.000112 | 0.004512 | 0.000000 |
| 35-39 | 253+ | 0.000000 | 0.002147 | 0.000000 |
| 40-44 | 253+ | 0.000000 | 0.002636 | 0.000000 |
| 45-49 | 253+ | 0.000000 | 0.001051 | 0.000000 |
| 50-54 | 253+ | 0.000000 | 0.002519 | 0.000000 |
| 55-59 | 253+ | 0.000000 | 0.003693 | 0.000000 |
| 60-64 | 253+ | 0.000000 | 0.005888 | 0.000000 |
| 65-69 | 253+ | 0.000000 | 0.004823 | 0.000000 |
| 70-74 | 253+ | 0.000000 | 0.004733 | 0.000000 |
| 75-79 | 253+ | 0.000000 | 0.004733 | 0.000000 |

# Results for PHIM for Run 7 (Sensitivity_Analysis_1.RTF)

************************************************************************************
**TTP values for Run: 7, Scenario: Ecig**

| **Variable Parameter** | **Value** |
|---|---|
[truncated: 429,948 more chars]
